# Uncovering metabolite–inflammatory protein interactions in gout pathogenesis: A 2-sample bidirectional Mendelian randomization study

**DOI:** 10.1097/MD.0000000000051276

**Published:** 2026-10-30

**Authors:** Shuai Chen, Yudong Gan, Yan Jin, Feng Wang, Zhongxiong Wu, Zenghui Liu, Zixiao Yan, Cheng Li, Shenglong Duan, Zhongzi Zhang

**Affiliations:** a Department of Orthopedics, The Affiliated Yan’an Hospital of Kunming Medical University, Kunming, Yunnan, China; b Department of Zoology, Kunming Medical University, Kunming, Yunnan, China; c School of Basic Medical Sciences, Mudanjiang Medical University, Mudanjiang, Heilongjiang, China; d Department of Orthopedics, Tengchong People’s Hospital, Tengchong, Yunnan, China.

**Keywords:** FGF19, gout, inflammatory proteins, mediation analysis, Mendelian randomization, metabolomics

## Abstract

Gout is a widespread inflammatory arthritis whose current treatments focus mainly on symptom relief and urate lowering. Recent work suggests that circulating metabolites and inflammatory proteins interact in complex ways to influence disease risk, with fibroblast growth factor 19 (FGF19) standing out as a potential regulatory node linking metabolism and inflammation. We performed bidirectional 2-sample Mendelian randomization using genome-wide association study summary statistics for 1440 serum metabolites and 91 inflammatory proteins, with gout outcomes drawn from FinnGen R12. Mediation analyses were then applied to examine whether FGF19 acts as an intermediary in selected metabolite–gout pathways. Genetically predicted levels of vanillactate and histidine betaine were associated with higher gout risk, whereas sphingomyelin species and taurolithocholate 3-sulfate showed protective effects. Among inflammatory proteins, FGF19, adenosine deaminase and FGF5 appeared protective, while cluster of differentiation 244 increased risk. Bidirectional analyses revealed reciprocal relationships, including a negative causal effect of propionylcarnitine on FGF19. Mediation testing further showed that FGF19 explained 7.88% of the total effect of lipid-related metabolites on gout via glycodeoxycholate 3-sulfate and 9.58% via cis-3,4-methyleneheptanoylglycine. These findings indicate that FGF19 may exert protective effects against gout partly by modulating bile acid and fatty acid metabolism. The results offer genetic evidence for metabolic–immune crosstalk in gout pathogenesis and highlight FGF19-related pathways as possible targets for more precise therapeutic strategies.

## 1. Introduction

Gout is a common chronic inflammatory arthropathy caused by the deposition of sodium urate crystals secondary to hyperuricemia. Global estimates suggest that the number of affected individuals could reach approximately 95.8 million by 2050, with particularly rapid increases in developed countries and the Pacific region.^[1]^ The disease significantly impairs quality of life and is associated with higher risks of cardiovascular disease, renal impairment, and metabolic syndrome.^[2]^ Clinically, it presents with recurrent acute attacks of severe joint pain, swelling, and erythema, and may progress to chronic tophaceous arthritis with joint destruction. Current treatment mainly relies on anti-inflammatory agents such as non-steroidal anti-inflammatory drugs, colchicine, and corticosteroids during flares, while long-term management depends on urate-lowering therapies.^[3]^ Although these approaches can reduce serum uric acid and relieve symptoms, many patients still experience recurrent attacks or develop drug resistance, indicating that the mechanisms sustaining chronic inflammation are not fully understood.

Serum metabolites are closely linked to gout. Hyperuricemia is frequently accompanied by dysregulation of other metabolites, including lipids and amino acids.^[4]^ These molecules can influence uric acid crystal formation and clearance while also activating inflammatory pathways.^[5]^ Certain gut-derived metabolites have been shown to affect renal urate transporters such as urate transporter 1 and glucose transporter, and some may act as danger signals that directly stimulate innate immune responses.^[6]^ These observations suggest that metabolic changes may contribute to gout through both direct effects on urate handling and indirect effects on inflammatory processes.

In the inflammatory cascade of gout, urate crystal recognition by immune cells triggers the rapid release of pro-inflammatory cytokines, including interleukin-1 beta, tumor necrosis factor-alpha, and IL-6, leading to sustained inflammation. Recent evidence indicates that dysregulated bile acid metabolism also plays an important role in this process. Altered bile acid profiles in gout patients have been associated with increased hepatic xanthine oxidase (XOD) activity, impaired uric acid excretion, and enhanced inflammatory responses mediated by NOD-like receptor family pyrin domain-containing 3 inflammasome activation.^[7]^ Fibroblast growth factor 19 (FGF19), an enterohepatic hormone that regulates bile acid synthesis through the farnesoid X receptor–FGF19–fibroblast growth factor receptor 4 axis, may therefore influence both urate metabolism and the inflammatory microenvironment in gout.^[8]^ These findings suggest that certain serum metabolites could affect gout risk partly by modulating inflammatory proteins involved in bile acid homeostasis and immune regulation.

We hypothesized that specific metabolites may influence gout susceptibility through effects on inflammatory intermediates, including FGF19. Although associations between metabolic disorders and immune activation in gout have been observed, causal pathways and mediating mechanisms remain insufficiently defined. Most existing studies have been limited to observational associations without establishing causal relationships.^[9]^ A clearer understanding of the pathways linking metabolites, inflammatory proteins, and gout is therefore needed.

The present study used Mendelian randomization (MR) to investigate potential causal relationships from serum metabolites to inflammatory proteins and subsequently to gout. This genetic approach was employed to reduce confounding and reverse causation that commonly affect observational research. The findings are expected to provide evidence for metabolic–inflammatory interactions in gout pathogenesis.

## 2. Methods and materials

### 2.1. Methods

This study leveraged publicly accessible databases alongside published genome-wide association study (GWAS) datasets, which included 1440 categories of serum metabolites and 91 inflammatory proteins, in addition to GWAS information related to gout. The research adhered to the Strengthening the Reporting of Observational Studies in Epidemiology-MR guidelines for MR, which stipulate that genetic variants used as instrumental variables (IVs) must fulfill 3 essential criteria: a robust association with both serum metabolites and gout; independence from potential confounding factors; and an influence on the outcome exclusively through indirect pathways associated with exposure, without any direct effects. Data regarding all exposures and outcomes were acquired from separate GWAS studies to bolster the study’s validity. We implemented 2-sample bidirectional MR to investigate causal relationships among serum metabolites, inflammatory cytokines, and gout, while also conducting mediation analyses to evaluate the roles of inflammatory proteins (FGF19, adenosine deaminase [ADA], FGF5, and cluster of differentiation 244 [CD244]) as potential intermediaries in the pathways from serum metabolites to gout. The overall design of the study is depicted in Figure 1.

**Figure 1.**
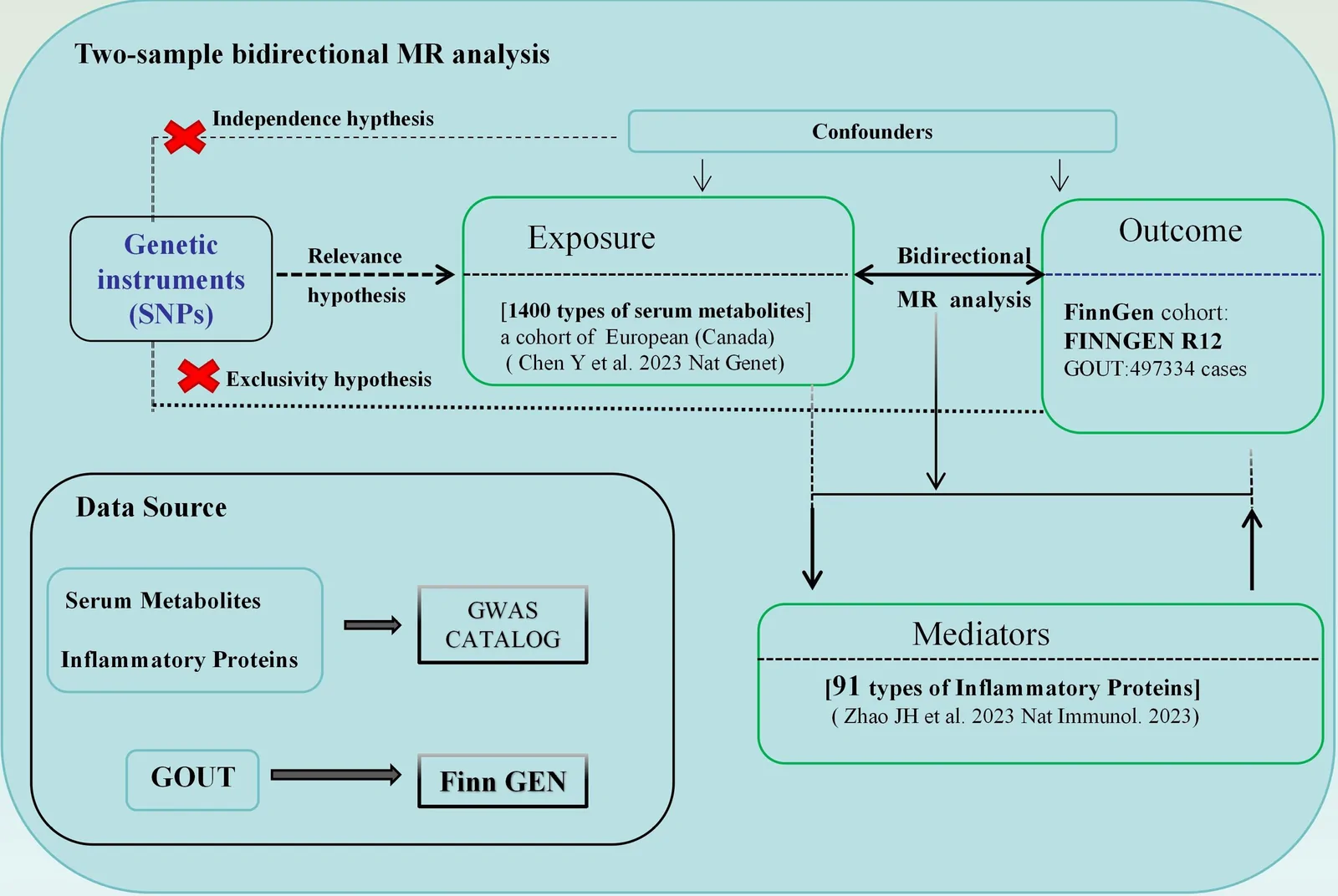
Research plan flowchart. Flowchart illustrating the 2-sample bidirectional Mendelian randomization (MR) analysis framework using serum metabolite traits as exposure, gout as outcome, and inflammatory proteins as potential mediators, based on IEU-GWAS, GWAS catalog, and FinnGen datasets. GWAS = genome-wide association study, IEU = integrative epidemiology unit.

### 2.2. Data sources and IVs

The data utilized for this study were derived from several key sources. Exposure variables: serum metabolite datasets were procured from the GWAS catalog (ranging from GCST90199621 to GCST90201020). Mediator variables: data pertaining to inflammatory proteins were sourced from the GWAS catalog (IDs GCST90274758 to GCST90274848). Outcome variables: information on gout was obtained from the FinnGen R12 version (https://www.finngen.fi/en) (File S1, Supplemental Digital Content 1).

In our MR analyses, linkage disequilibrium (LD) clumping of exposure data was performed with the following parameters: *r*^2^ < 0.001, a 10 Mb clumping window, and the 1000 Genomes European reference panel. For the serum metabolites and inflammatory proteins, an initial screening threshold for single nucleotide polymorphisms (SNPs) was set at *P* values <1E−08; however, this was modified to <5E−08 due to a limited number of IVs. For gout, the SNP screening criterion was *P* < 5E−07. This approach was adopted to mitigate LD, thereby ensuring the independence of SNPs. Alleles were coordinated, and ambiguous SNPs were excluded. SNPs were selected based on having an F-statistic exceeding 10 and a minor allele frequency >0.01, designating them as robust IVs.

Quantitative evaluation of SNP *P* value thresholds showed that 68% of metabolite-related traits and 42% of inflammatory protein-related traits required threshold relaxation to achieve adequate instruments. On average, 4.2 to 5.1 SNPs were retained per trait, with a mean *F* statistic of 28.4. Sensitivity analyses restricted to a stricter *P* < 5 × 10^−8^ threshold yielded consistent effect directions. Analyses were conducted in European-ancestry samples, using exposures and mediators from non-Finnish European datasets and outcomes from Finnish datasets. This strategy maintained a consistent genetic background across distinct geographic regions, minimized sample overlap, and strengthened the reliability of causal inference.

Two-sample MR analyses were performed in R (version 4.5.1; R Foundation for Statistical Computing, Vienna, Austria) using the TwoSampleMR and MendelianRandomization packages. Instrument selection, harmonization, primary inverse-variance weighted (IVW) estimates, and sensitivity analyses (MR-Egger, weighted median, Mendelian Randomization Pleiotropy RESidual Sum and Outlier (MR-PRESSO), leave-one-out) followed the procedures detailed in the File S2, Supplemental Digital Content 2.

### 2.3. Statistical analysis strategy

We used several MR approaches to examine the relationships of interest. The main results came from IVW analysis under a random-effects model. When only 1 valid IV remained after clumping and harmonization, we calculated causal estimates using the Wald ratio method. In cases with multiple instruments, we also ran MR-Egger regression to check for directional pleiotropy, along with weighted median and weighted mode estimators. MR-PRESSO helped identify and correct for potential outliers, and we additionally applied the robust adjusted profile score approach. This combination of methods allowed us to assess the consistency of our findings across different assumptions. To evaluate heterogeneity among the genetic instruments, we applied Cochran *Q* statistic. We also examined the MR-Egger intercept as an indicator of directional pleiotropy, using a *P* value threshold of .05. Because metabolites are biologically related, some heterogeneity is not unexpected. For this reason, we considered results to be reliable when the IVW estimate showed the same direction and reached statistical significance in at least 2 of the sensitivity analyses (particularly the weighted median and MR-PRESSO methods), and when there was little evidence of directional pleiotropy from the MR-Egger test.

We further conducted a 2-step mediation analysis to explore whether certain inflammatory proteins might lie on the causal pathway between serum metabolites and gout. In the 1st step, we tested which inflammatory proteins had a causal effect on gout. In the 2nd step, we examined the effects of metabolites on these proteins and of the proteins on gout. To reduce the chance that reverse causation would distort the mediation estimates, we removed from the main analysis any proteins that showed a significant effect in the reverse direction (i.e., from gout back to the protein). The indirect effect was estimated using the product-of-coefficients method (β*a* × β*b*)/β*c*, where β*a* represents the metabolite-to-protein effect, β*b* the protein-to-gout effect, and β*c* the total metabolite-to-gout effect. We obtained confidence intervals via the delta method and regarded associations with a 2-sided *P* value below .05 as statistically significant. Given the very large number of tests performed across roughly 1440 metabolites and 91 inflammatory proteins in both directions, plus the mediation analyses, we did not apply a strict multiple-testing correction such as Bonferroni. The analysis was exploratory in nature, and many metabolites are correlated, which would make such a correction overly conservative. We therefore report nominal *P* values and note that some of the observed associations could be false positives. All analyses were carried out in R version 4.5.1 with the TwoSampleMR package.

## 3. Results

This study systematically evaluated causal associations between serum metabolites, inflammatory proteins, and gout using 2-sample bidirectional MR and mediation analysis. By integrating genetic IVs, we separately analyzed the effects of serum metabolites and inflammatory proteins on gout, the bidirectional causal effects between serum metabolites and inflammatory proteins, and mediation analysis. This study aims to reveal potential biological pathways through which metabolites and inflammatory proteins influence gout pathogenesis from a genetic perspective, providing new theoretical foundations for understanding the etiological mechanisms of gout and enabling early intervention strategies.

### 3.1. Results of 2-sample bidirectional MR analysis

#### 3.1.1. Causal associations between serum metabolites and gout

Significant causal relationships exist between serum metabolite levels and gout risk. Bidirectional MR analysis of serum metabolite levels and gout revealed metabolites significantly positively associated with gout risk, including: vanillactate levels (odds ratio [OR] = 1.73, 95% confidence interval [CI]: 1.32–2.28, *P* = .0001), histidine betaine (hercynine) levels (OR = 1.42, 95% CI: 1.16–1.74, *P* = .0005), glycolithocholate to glycolithocholate sulfate ratio (OR = 1.25, 95% CI: 1.10–1.42, *P* = .0007), N-acetyl-aspartyl-glutamate (NAAG) levels (OR = 1.11, 95% CI: 1.04–1.20, *P* = .0034), and undecenoylcarnitine (C11:1) levels (OR = 1.15, 95% CI: 1.05–1.27, *P* = .0036) (Fig. 2) (Files S3 and S4, Supplemental Digital Content 3 and 4). These positive correlations indicate that these metabolites, primarily involving amino acid derivatives, bile acid metabolites, and acylcarnitines, may increase gout risk by promoting inflammatory responses or disrupting uric acid metabolism. The most significant negative correlations included: X-10458 levels (OR = 0.78, 95% CI: 0.63–0.96, *P* = .0191), sphingomyelin (d18:2/21:0, d16:2/23:0) (OR = 0.80, 95% CI: 0.67–0.95, *P* = .0094), taurolithocholate 3-sulfate (OR = 0.80, 95% CI: 0.68–0.95, *P* = .0086), N-oleoylserine (OR = 0.83, 95% CI: 0.73–0.94, *P* = .0032), and glycolithocholate sulfate (OR = 0.85, 95% CI: 0.73–0.98, *P* = .0278) (Fig. 2) (Files S3 and S4, Supplemental Digital Content 3 and 4). These metabolites, predominantly sphingolipids and bile acid sulfates, may exert protective effects against gout by inhibiting crystal deposition and joint damage through anti-inflammatory or antioxidant mechanisms. The bidirectional causal pattern of serum metabolites in gout highlights their central role in the disease pathogenesis.

**Figure 2.**
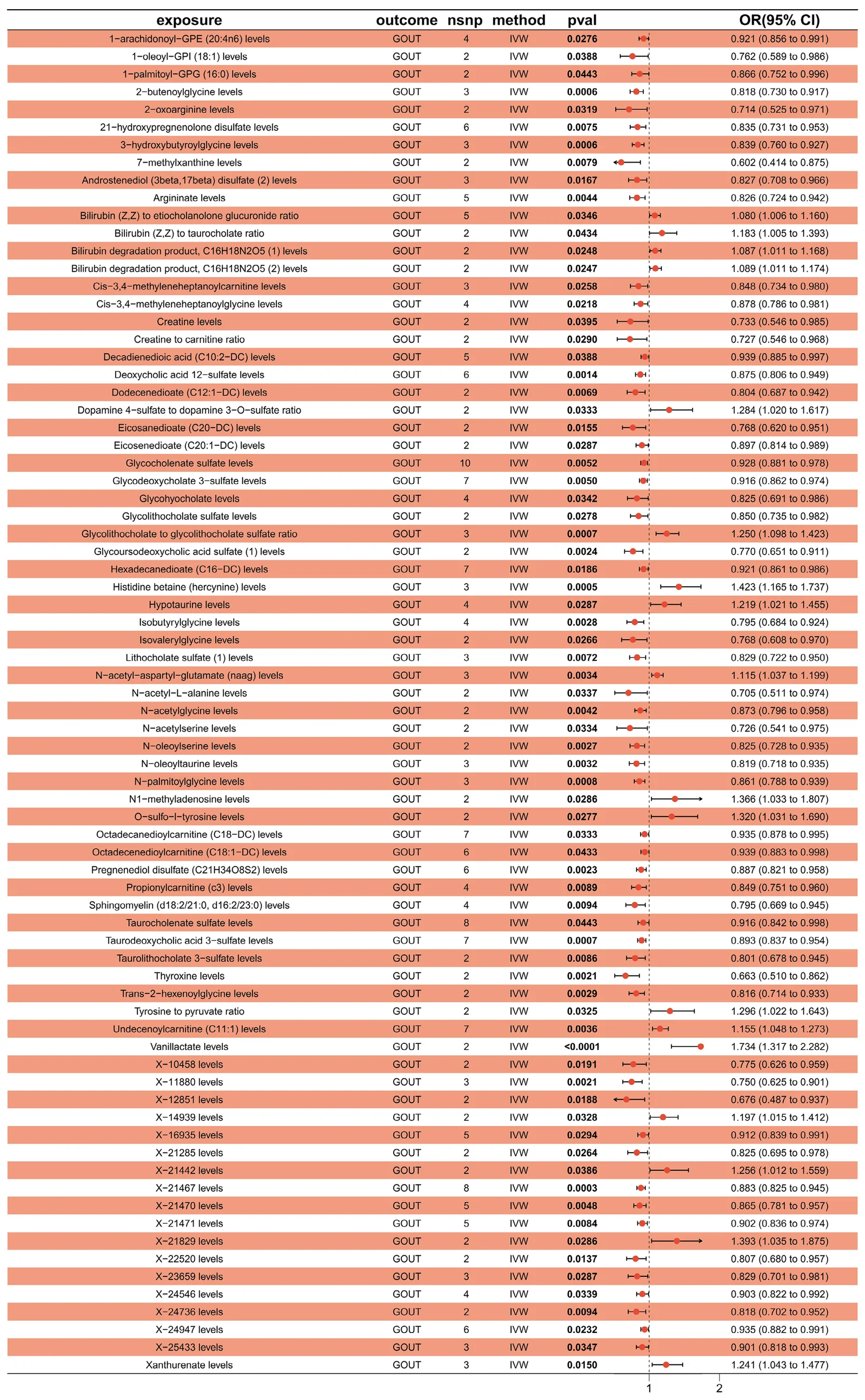
Illustration of the impact of serum metabolites on gout via bidirectional Mendelian randomization. Forest plots showing significant bidirectional Mendelian randomization associations between serum metabolite traits (exposure) and gout outcomes (*a*, *b*, and *c*), with odds ratios (OR), 95% confidence intervals (CIs), and *P* values from the inverse-variance weighted (IVW) method.

#### 3.1.2. Causal associations between inflammatory proteins and gout

Inflammatory proteins demonstrate substantial causal relationships with the risk of developing gout. A bidirectional MR analysis identified causally associated inflammatory proteins, which included a positive correlation for CD244 (OR = 1.17, 95% CI: 1.03–1.32, *P* = .0154). In contrast, negative associations were noted for FGF19 (OR = 0.82, 95% CI: 0.69–0.97, *P* = .0203), ADA (OR = 0.90, 95% CI: 0.84–0.97, *P* = .0073), and FGF5 (OR = 0.93, 95% CI: 0.87–0.99, *P* = .028), with statistical significance as illustrated in Figure 3 (Files S5 and S6, Supplemental Digital Content 5 and 6). The upregulation of CD244 may lead to heightened immune activation and disrupt the regulation of uric acid, increasing the likelihood of gout development. Conversely, FGF19, FGF5, and ADA may offer protective benefits against gout through the modulation of purine metabolism, the attenuation of inflammatory signaling, or the enhancement of metabolic homeostasis.

**Figure 3.**
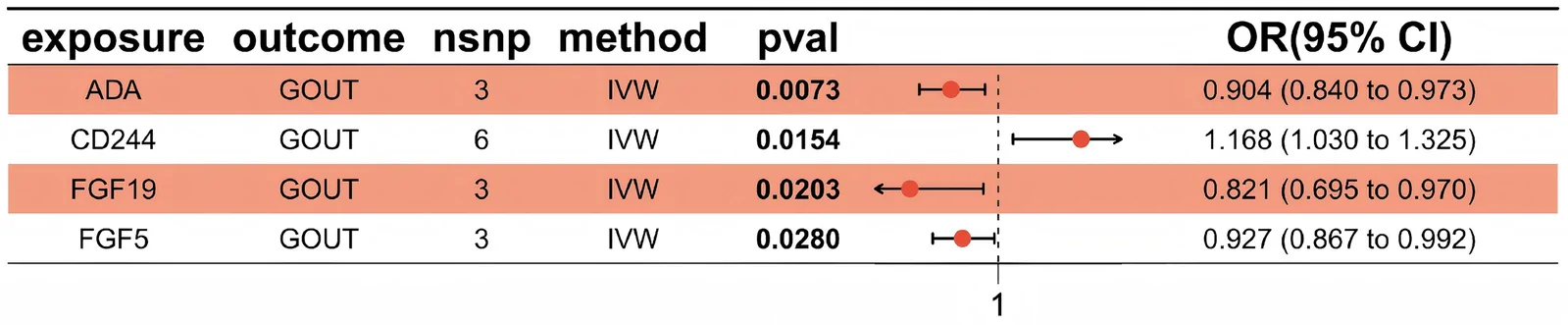
Illustration of the impact of inflammatory proteins on gout via bidirectional Mendelian randomization. Forest plot showing significant bidirectional Mendelian randomization results for inflammatory proteins (exposure) on gout (outcome), including odds ratios (OR), 95% CI, and IVW *P* values. ADA = adenosine deaminase, CD244 = cluster of differentiation 244, CI = confidence interval, FGF = fibroblast growth factor, IVW = inverse-variance weighted.

#### 3.1.3. Causal associations between serum metabolites and inflammatory proteins in gout

Furthermore, serum metabolite levels were found to have significant causal relationships with inflammatory proteins in relation to gout risk. A 2-sample MR analysis revealed positive associations with several metabolites, including X-21471 levels correlating with ADA (OR = 1.10, 95% CI: 1.05–1.15, *P* = .0001), androstenediol (3beta, 17beta) disulfate (2) levels with ADA (OR = 1.19, 95% CI: 1.07–1.32, *P* = .001), and lithocholate sulfate (1) levels with ADA (OR = 1.12, 95% CI: 1.03–1.22, *P* = .0098). Other significant associations included X-24546 levels with ADA (OR = 1.11, 95% CI: 1.03–1.19, *P* = .0081) and glycocholenate sulfate levels with ADA (OR = 1.06, 95% CI: 1.01–1.11, *P* = .0315), as shown in Figure 4 (Files S7 and S8, Supplemental Digital Content 7 and 8). The elevation of these metabolites may activate ADA-related inflammatory signaling pathways, thereby impacting the pathogenesis of gout. This observation sheds light on the potential mechanism by which ADA operates within the metabolism–inflammation axis relevant to gout. Negative correlation analysis indicated that levels of propionylcarnitine (c3) were inversely associated with FGF19 (OR = 0.91, 95% CI: 0.85–0.98, *P* = .0166), while NAAG levels were also negatively correlated with FGF19 (OR = 0.95, 95% CI: 0.91–0.99, *P* = .0254). Additionally, X-25433 levels exhibited a negative correlation with FGF5 (OR = 0.92, 95% CI: 0.86–1.00, *P* = .0214), as did taurocholenate sulfate levels with FGF5 (OR = 0.94, 95% CI: 0.88–0.99, *P* = .0296) (Fig. 4) (Files S7 and S8, Supplemental Digital Content 7 and 8). These findings suggest that metabolites may influence gout-associated signaling pathways through inflammatory proteins ADA, FGF19, and FGF5, proposing a potential metabolic–inflammatory axis mechanism.

**Figure 4.**
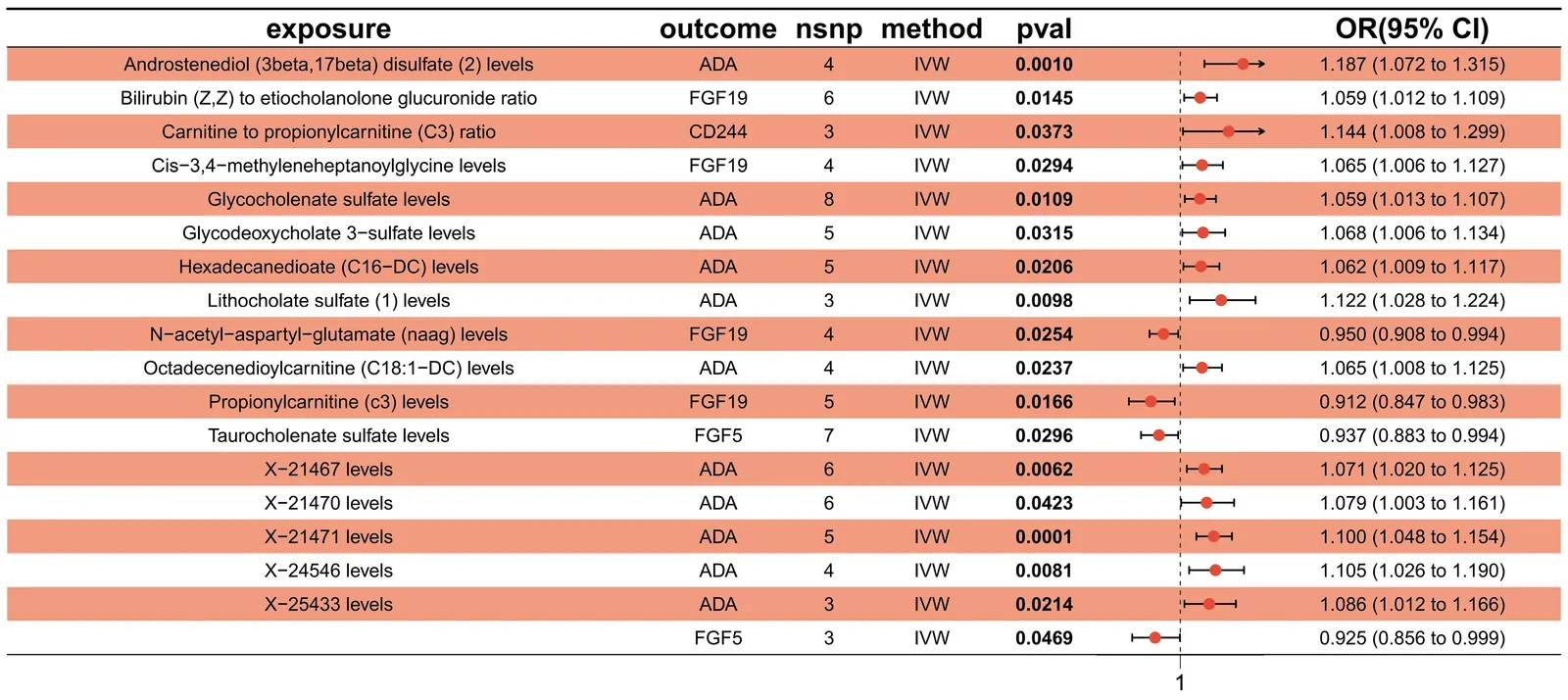
Results of serum metabolites affecting inflammatory proteins via bidirectional Mendelian randomization in gout. Forest plot displaying significant bidirectional Mendelian randomization associations from serum metabolites (exposure) to inflammatory proteins (outcome) in gout, with IVW odds ratios, 95% CI, and *P* values. ADA = adenosine deaminase, CD244 = cluster of differentiation 244, CI = confidence interval, FGF = fibroblast growth factor, IVW = inverse-variance weighted.

Significant causal associations have been identified between inflammatory proteins and serum metabolite concentrations in relation to the risk of gout, as evidenced by a 2-sample MR analysis. This analysis highlighted positive correlations involving FGF19 with glycodeoxycholate 3-sulfate (OR = 1.19, 95% CI: 1.03–1.39, *P* = .0209), FGF5 with glycodeoxycholate 3-sulfate (OR = 1.07, 95% CI: 1.00–1.13, *P* = .0419), and X-21471 levels (OR = 1.07, 95% CI: 1.00–1.13, *P* = .043). Additionally, ADA was positively associated with 2-butenoylglycine (OR = 1.09, 95% CI: 1.02–1.16, *P* = .0161) and 3-hydroxybutyroylglycine (OR = 1.06, 95% CI: 1.00–1.13, *P* = .0388), with the latter also demonstrating a significant association with gout (Fig. 5) (Files S9 and S10, Supplemental Digital Content 9 and 10). The upregulation of the inflammatory proteins FGF19, FGF5, and ADA may facilitate metabolite transformation and the crystallization of gout. This observation underscores the potential significance of the interplay between inflammation and metabolism as therapeutic targets in the prevention of gout. Conversely, significant negative correlations were observed, with CD244 exhibiting a notable negative association with taurodeoxycholic acid 3-sulfate levels (OR = 0.85, 95% CI: 0.75–0.95, *P* = .0056) (Fig. 5) (Files S9 and S10, Supplemental Digital Content 9 and 10). The inflammatory protein CD244, linked to joint inflammation pathways and the formation of gout crystals, may be pivotal in the progression of gout.

**Figure 5.**
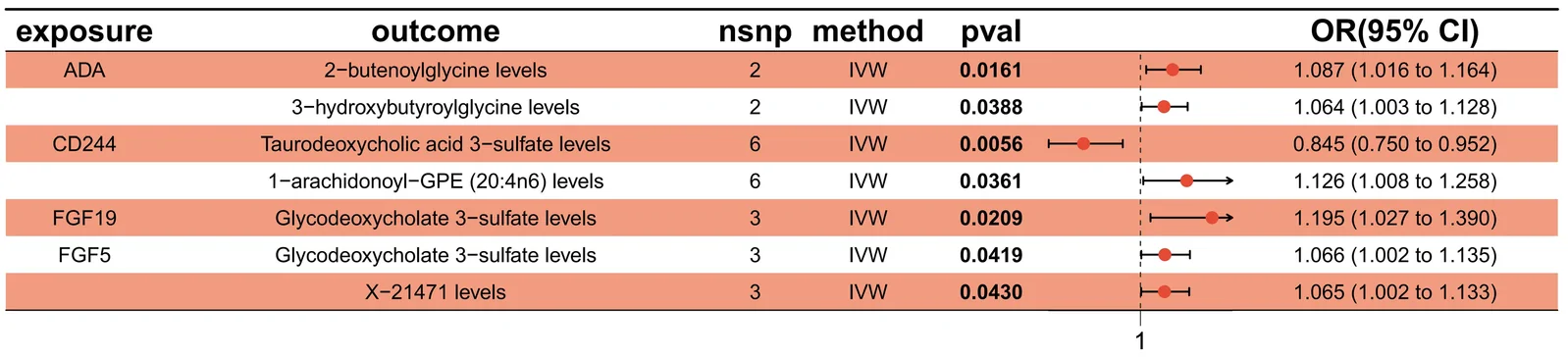
Results of inflammatory proteins affecting serum metabolites via bidirectional Mendelian randomization in gout. Forest plot showing significant bidirectional Mendelian randomization results of inflammatory protein traits (exposure) on serum metabolites (outcome) specifically in gout, with IVW odds ratios, 95% CI, and *P* values. ADA = adenosine deaminase, CD244 = cluster of differentiation 244, CI = confidence interval, FGF = fibroblast growth factor, IVW = inverse-variance weighted.

These findings elucidate the causal relationships between various metabolites and inflammatory proteins in the context of gout. Importantly, several inflammatory proteins display negative effects across multiple causal pathways, suggesting their potential role as protective factors against the progression of gout.

### 3.2. Mediator analysis results

In the mediation analysis, 2 primary pathways related to the pathogenesis of gout were identified, particularly emphasizing the interaction between metabolites associated with lipid metabolism and FGF19 (Table 1). These pathways underscore the possible involvement of dysregulation in bile acid and fatty acid metabolism in the progression of gout. Specifically, in the pathway FGF19 → glycodeoxycholate 3-sulfate levels → gout, the total effect value was calculated to be −0.1818, which included a mediation effect of −0.0155 (accounting for 7.88% of the total effect) and a direct effect of −0.1663. In the pathway cis-3,4-methyleneheptanoylglycine levels → FGF19 → gout, the total effect value was −0.1175, with a mediation effect value of −0.0124 (representing 9.58% of the total effect) and a direct effect value of −0.1051. From the perspective of metabolite classification, both metabolites are related to lipid metabolism: glycodeoxycholate 3-sulfate pertains to bile acid metabolic pathways, while cis-3,4-methyleneheptanoylglycine is associated with branched-chain fatty acid metabolic pathways. Both pathways fall under the broader category of lipid metabolism, with the former focusing on bile acid sulfation and the latter on fatty acid acylation. Both pathways exhibit negative mediating effects, which may indicate protective mechanisms, though their low proportions (<10%) suggest that other non-mediated factors predominantly influence the overall effect. The mediating effect values for both pathways correspond with their respective direct effect values, indicating the potential for synergistic interactions.

**Table 1:** Mediation analysis for gout.

Exposure	Mediator	Outcome	Mediated effect	Proportion	Beta direct
FGF19	Glycodeoxycholate 3-sulfate levels	Gout	−0.0155	7.88%	−0.1818
Cis-3,4-methyleneheptanoylglycine levels	FGF19	Gout	−0.0124	9.58%	−0.1175

Significant mediation effects of inflammatory proteins on the causal associations between serum metabolite traits and gout outcome.

FGF19 = fibroblast growth factor 19.

## 4. Discussion

### 4.1. Key findings

Using bidirectional MR and mediation analysis, we identified several causal links between circulating metabolites and inflammatory proteins in gout. A number of metabolites showed direct effects on gout risk, notably glycodeoxycholate 3-sulfate, 2-butenoylglycine, 3-hydroxybutyroylglycine, and taurodeoxycholic acid 3-sulfate. In the opposite direction, gout itself appeared to alter levels of certain metabolites, pointing to reciprocal regulation between metabolic and inflammatory pathways. Mediation analyses suggested that some inflammatory proteins may partly explain how metabolites influence gout, although the proportions explained were generally modest. Taken together, the results support the idea that the immune–metabolic axis plays a meaningful role in gout development and highlight bile acid metabolism and branched-chain amino acid derivatives as pathways worth further investigation.

### 4.2. Bidirectional relationships between metabolites and inflammatory proteins

Gout is classified as a metabolic inflammatory condition characterized by the deposition of urate crystals in the joints and adjacent tissues. The pathophysiology of gout involves several mechanisms, including disturbances in uric acid metabolism, activation of inflammatory responses, and genetic predispositions.^[10]^ Our bidirectional MR and mediation analyses explored the causal relationships between metabolites and inflammatory proteins in gout. The results revealed reciprocal interactions between inflammatory proteins and metabolites, suggesting potential biological pathways involved in gout pathogenesis.

Bidirectional MR analyses showed that individuals with gout had elevated levels of several serum metabolites, including glycodeoxycholate 3-sulfate, which may worsen crystal deposition by disrupting uric acid transport mechanisms.^[11]^ Bile acid metabolites have been shown to modulate inflammation through the gut–liver axis, with observational studies supporting that their dysregulation may induce oxidative stress, thereby triggering gout flare-ups.^[12]^ Furthermore, derivatives of branched-chain amino acids, such as 2-butenoylglycine, are associated with inflammatory processes, and their increased concentrations could potentially enhance metabolic reprogramming within immune cells.^[13]^ Fluctuations in levels of 3-hydroxybutyroylglycine are indicative of mitochondrial dysfunction, with clinical cohort studies demonstrating a positive association between these levels and the extent of joint damage in gout patients.^[14]^ Additionally, taurodeoxycholic acid 3-sulfate is implicated in lipid metabolism; studies using animal models suggest that its aberrations elevate the expression of inflammatory mediators.^[15]^ The interplay between immune phenotypes and serum metabolites is central to the pathology of gout. ADA modulates adenosine signaling,^[16]^ which in turn influences the synthesis of metabolites such as NAAG, exacerbating joint inflammation.^[17]^ FGF19 has been shown to inhibit the activity of metabolic enzymes through feedback mechanisms from the gut.^[18]^ The role of FGF5 in fibrosis may also indirectly affect metabolite concentrations.^[19]^ These interactions highlight the critical importance of the immune-metabolic axis in the context of gout. Further investigations indicate that serum metabolites can reciprocally influence immune phenotypes. For instance, 2-butenoylglycine affects the functionality of natural killer and T cells via reactive oxygen species-mediated signaling pathways, such as mechanistic target of rapamycin or Janus kinase/signal transducer and activator of transcription, potentially resulting in immune exhaustion or heightened inflammatory reactions.^[20]^ Observational evidence also corroborates that elevated levels of taurodeoxycholic acid 3-sulfate are associated with dysfunctional immune cells.^[21]^ Such bidirectional regulatory mechanisms elucidate the chronic inflammatory nature characteristic of gout.

Our analyses identified causal relationships between specific inflammatory proteins and metabolites relevant to gout progression. One inflammatory protein in particular showed both positive and negative associations with several metabolites, largely through hepaticoenteric signaling pathways.^[18]^ In forward MR, higher levels of this protein together with cis-3,4-methylenheptanoylglycine were associated with lower gout risk, suggesting a potential protective effect. In investigations focused on positive correlations, levels of FGF19 and cis-3,4-methylenheptanoylglycine displayed negative causal connections with gout, functioning as protective elements. Conversely, the concentration of cis-3,4-methylenheptanoylglycine is identified as a risk factor for FGF19, demonstrating a positive relationship with its expression. These observations suggest that elevated levels of cis-3,4-methylenheptanoylglycine – an intermediate in the process of fatty acid β-oxidation – are indicative of metabolic dysfunction during fatty acid oxidation. While the precise mechanism connecting cis-3,4-methylenheptanoylglycine to gout remains elusive, it is evident that fatty acid metabolism plays a significant role in the pathogenesis of gout through the modulation of XOD activity, which subsequently influences uric acid production.^[22]^ Our results support the view that disruption of metabolic homeostasis is an important factor in gout development.

In reverse MR analyses, the same inflammatory protein was positively associated with glycodeoxycholate 3-sulfate, showcasing a positive correlation with its metabolic concentrations. This reverse interaction suggests a feedback regulatory mechanism where metabolites influence FGF19. As a principal component of bile acids, glycodeoxycholate 3-sulfate may exert predominant effects akin to those observed with other bile acids. The action of FGF19 leads to a decrease in bile acid synthesis by activating the hepatocyte fibroblast growth factor receptor 4-βKlotho receptor complex.^[23]^ Previous studies have demonstrated that bile acids can suppress the expression and activity of XOD via peroxisome proliferator-activated receptor alpha, thereby inducing circadian variations in uric acid production and enhancing uric acid synthesis.^[10]^ Additionally, bile acids may promote the release of pro-inflammatory factors (such as IL-6 and tumor necrosis factor-alpha) from intestinal epithelial cells, resulting in dysfunction of the intestinal barrier that hinders uric acid excretion. This accumulation contributes to hyperuricemia.^[24]^ These observations are consistent with a possible feedback loop in which bile acid metabolites and inflammatory proteins jointly influence uric acid metabolism.

Mediation analysis suggested that this inflammatory protein partly mediated the effect of glycodeoxycholate 3-sulfate on gout (mediation effect −0.0155 and proportion mediated 7.88%) in the development of gout. The findings indicated that FGF19 mediated gout through the levels of glycodeoxycholate 3-sulfate, resulting in a mediation effect quantified at −0.0155 and a mediation proportion of 7.88%, alongside a direct effect measured at −0.1818. This suggests that FGF19 predominantly exerts a direct suppressive effect on gout while also offering partial protection by diminishing this specific metabolite. Furthermore, the study identified that cis-3,4-methyleneheptanoylglycine levels mediated gout through FGF19, with a mediation effect of −0.0124 and a mediation proportion of 9.58%, coupled with a direct effect of −0.1175. This indicates that this metabolite is capable of modulating FGF19 expression, thereby exerting an indirect influence on the risk of developing gout. The presence of multiple mediating pathways illustrates that FGF19’s mediation mechanism entails the inhibition of uric acid production via the reduction of bile acids. Clinically, elevated levels of FGF19 have been recorded alongside diminished uric acid levels in patients with diabetes.^[25]^ Both mediation proportions were modest, indicating that other pathways likely contribute to the observed relationships. By leveraging the Finnish database, this mediation analysis effectively controlled for genetic confounding factors, thereby providing substantial evidence. However, the study has limitations, including a low mediation proportion, which implies the existence of alternative pathways. While analogues of this protein have shown benefits in metabolic disorders through enterohepatic mechanisms,^[23]^ the modest mediation proportions in our data suggest that any potential therapeutic relevance to gout would require further investigation.

Comprehensive sensitivity analyses supported the robustness of both the bidirectional and mediation findings, confirming both positive and negative interactions, as well as the mediation analysis, while circumventing horizontal pleiotropy and IV bias. The bidirectional MR, combined with mediation analysis, provides a clearer view of directionality than unidirectional approaches, examining factors that affect the progression of gout from both metabolomic and inflammatory protein perspectives. Collectively, these associations between metabolites and FGF19 shed light on gout’s metabolic network, proposing innovative intervention strategies – such as dietary modifications aimed at balancing bile acids to mitigate gout recurrence. Further cohort studies are warranted to validate the consistency of these causal relationships across diverse populations.

In comparison to other similar publications in SCI journals, these methodological features strengthen confidence in the overall pattern of results. We highlight the previously neglected sulfated bile acid pathway, thereby providing novel mechanistic insights. Methodologically, our application of bidirectional MR, combined with mediation analysis, surpasses the effectiveness of unidirectional MR by minimizing reverse causality bias and improving the precision of causal inferences. The selection of the Finnish R12 dataset encompasses a wider genetic variety and offers enhanced representation of European populations compared to earlier UK Biobank data, thereby bolstering the robustness of our results. Finally, the considerable sample size and the application of the stringent gout-serous tubal intraepithelial carcinoma definition for gout outcomes have effectively mitigated the risk of diagnostic misclassification.

### 4.3. Multiple testing burden

With 1400 metabolites and 91 inflammatory proteins tested, over 6000 tests were conducted. The sheer number of statistical tests raises legitimate concerns about false positives. To manage this, we performed LD clumping using a strict *r*^2^ threshold of 0.001 within a 10 Mb window and the 1000 Genomes European reference panel. Even so, many exposures still had too few genome-wide significant SNPs to serve as instruments. For 68% of the metabolite exposures and 42% of the inflammatory protein exposures, we had to relax the *P* value threshold to retain enough variants. After clumping, we kept on average only 4.2 to 5.1 SNPs per exposure, and the mean F-statistic across these instruments was 28.4. Although this exceeds the usual rule-of-thumb threshold of 10, the relatively small number of instruments for many exposures means power is limited and weak-instrument bias cannot be entirely ruled out. We therefore placed more weight on findings that survived multiple sensitivity methods and that aligned with existing biological knowledge. Still, we recognize that some of the nominally significant results may not hold up under stricter multiple-testing correction, and independent replication will be important.

## 5. Limitations

Our study has several limitations that should be kept in mind. First, the need to relax instrument-selection thresholds for a large fraction of exposures means that many of our instruments rest on relatively few SNPs. With an average of only 4 to 5 independent variants per exposure and a mean *F* statistic of 28.4, weak-instrument bias remains a possibility, especially for those exposures with the smallest number of SNPs. Second, although we deliberately drew exposure and mediator data from non-Finnish European cohorts and outcome data from the Finnish biobank to reduce sample overlap, some degree of overlap may still exist in these large datasets and could bias causal estimates toward the null or inflate type I error. Third, all participants were of European ancestry. While splitting the European data into non-Finnish and Finnish subsets helped limit population stratification while preserving a broadly similar genetic background, our findings may not apply to other ancestral groups. Larger multi-ancestry MR studies will be needed before these relationships can be considered generalizable.

On the positive side, we conducted extensive sensitivity analyses to check for horizontal pleiotropy and inconsistent results across MR methods. Compared with earlier unidirectional MR studies, the bidirectional plus mediation design gives a clearer picture of directionality and potential mediating mechanisms. The Finnish R12 release also offers broader European coverage than some older UK Biobank releases. In addition, the large sample size and the use of a strict gout definition (gout-serous tubal intraepithelial carcinoma) should have reduced misclassification. These strengths do not eliminate the limitations above, but they do increase confidence in the overall pattern of results.

This investigation utilizes MR methodologies to elucidate the potential role of FGF19 in the context of gout; however, it is not without certain constraints. Firstly, the dependence on the Finnish Genome Database version R12 may lead to population-specific biases, particularly given the genetic disparities between Nordic and Asian cohorts, which could restrict the generalizability of the findings. Secondly, while MR presumes the absence of confounding by tool variables, the potential for multivariate confounding remains unaddressed, potentially introducing bias into the results. Thirdly, the observational nature of the study design lacks the validation provided by randomized controlled trials, which are essential for establishing causality. Moreover, despite the thorough screening of 91 inflammatory proteins and 1400 metabolites, the omission of environmental determinants such as dietary factors may have resulted in an underestimation of interaction effects. Future studies could help address these limitations by incorporating multi-omics approaches, such as epigenetic data, and by following participants prospectively across more diverse populations. These efforts would be valuable for confirming the causal relationships observed in this study and for assessing whether the findings extend beyond European ancestry groups.

## 6. Conclusion

This study used bidirectional MR and mediation analysis to investigate causal relationships between serum metabolites and inflammatory proteins in gout. We identified multiple metabolites with causal effects on gout risk and found evidence of reciprocal relationships, particularly involving bile acid and fatty acid metabolism pathways. While 1 inflammatory protein showed modest mediating effects through specific metabolites, the mediation proportions were relatively small, suggesting that other pathways are also involved. The findings provide genetic support for the role of metabolic–inflammatory interactions in gout pathogenesis and demonstrate the value of combining bidirectional MR with mediation analysis. Although limited to European populations and subject to the usual assumptions of MR, this work offers a more comprehensive view of potential causal mechanisms than previous studies. Further research in independent cohorts and functional validation will be needed to confirm these relationships and explore their therapeutic implications.

## Acknowledgments

We thank our colleagues for their assistance with this study.

## Author contributions

**Conceptualization:** Shuai Chen, Zixiao Yan.

**Data curation:** Shuai Chen, Yudong Gan.

**Formal analysis:** Shuai Chen, Zenghui Liu.

**Methodology:** Shuai Chen, Yan Jin, Feng Wang.

**Supervision:** Shenglong Duan, Zhongzi Zhang.

**Writing – original draft:** Shuai Chen, Zhongxiong Wu.

**Writing – review & editing:** Shuai Chen, Cheng Li.




















