# Supplementary material for: Uncovering metabolite–inflammatory protein interactions in gout pathogenesis: A 2-sample bidirectional Mendelian randomization study
Source: Medicine (Baltimore). 2026 Oct 30;105(44):e51276. doi: 10.1097/MD.0000000000051276 (PMC13727226; doi:10.1097/MD.0000000000051276)
Supplement: Supplementary file 1 [file medi-105-e51276-s004.pdf]

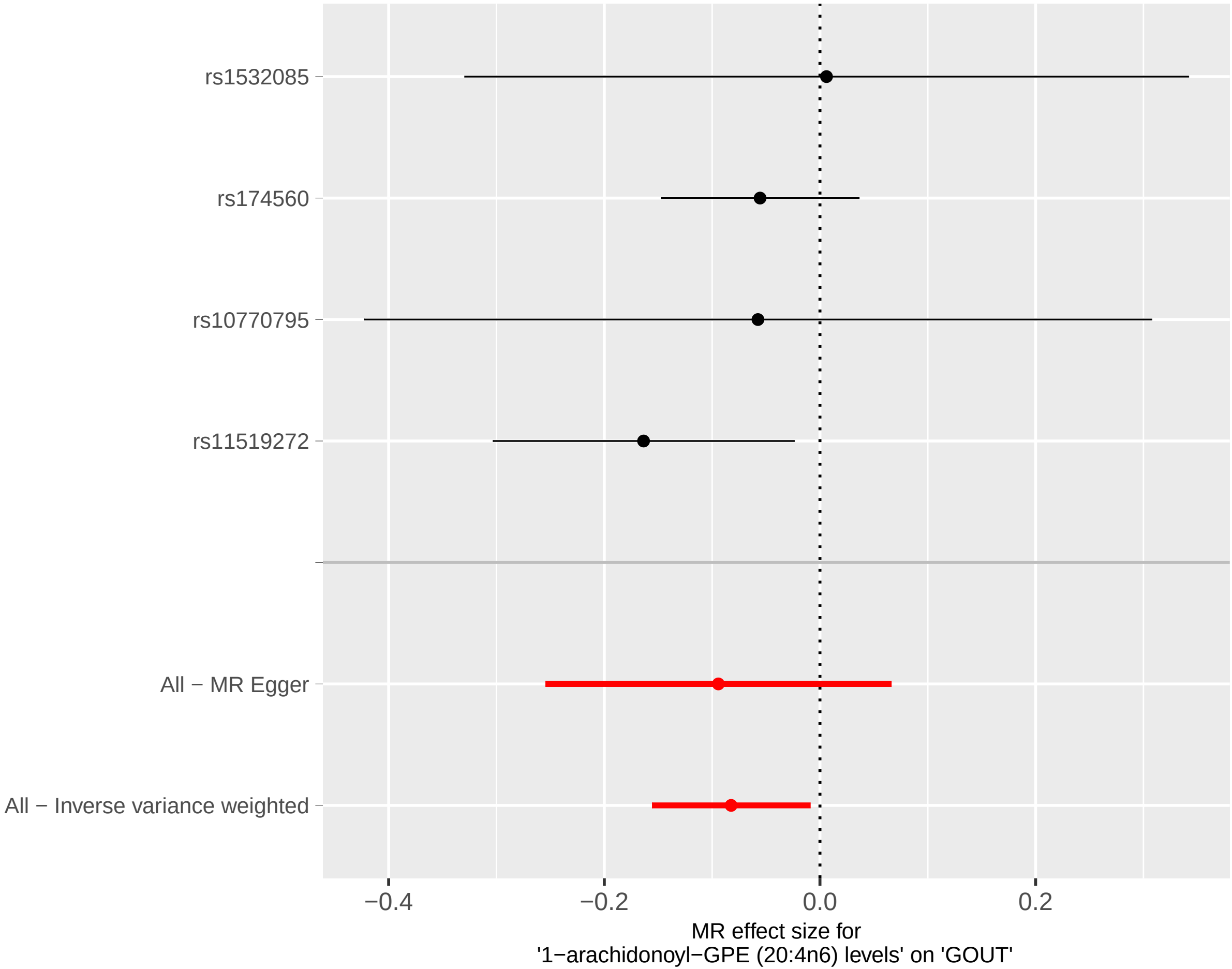
rs1532085
rs174560
rs10770795
rs11519272
All − MR Egger
All − Inverse variance weighted
−0.4
−0.2
0.0
0.2
MR effect size for
'1−arachidonoyl−GPE (20:4n6) levels' on 'GOUT'

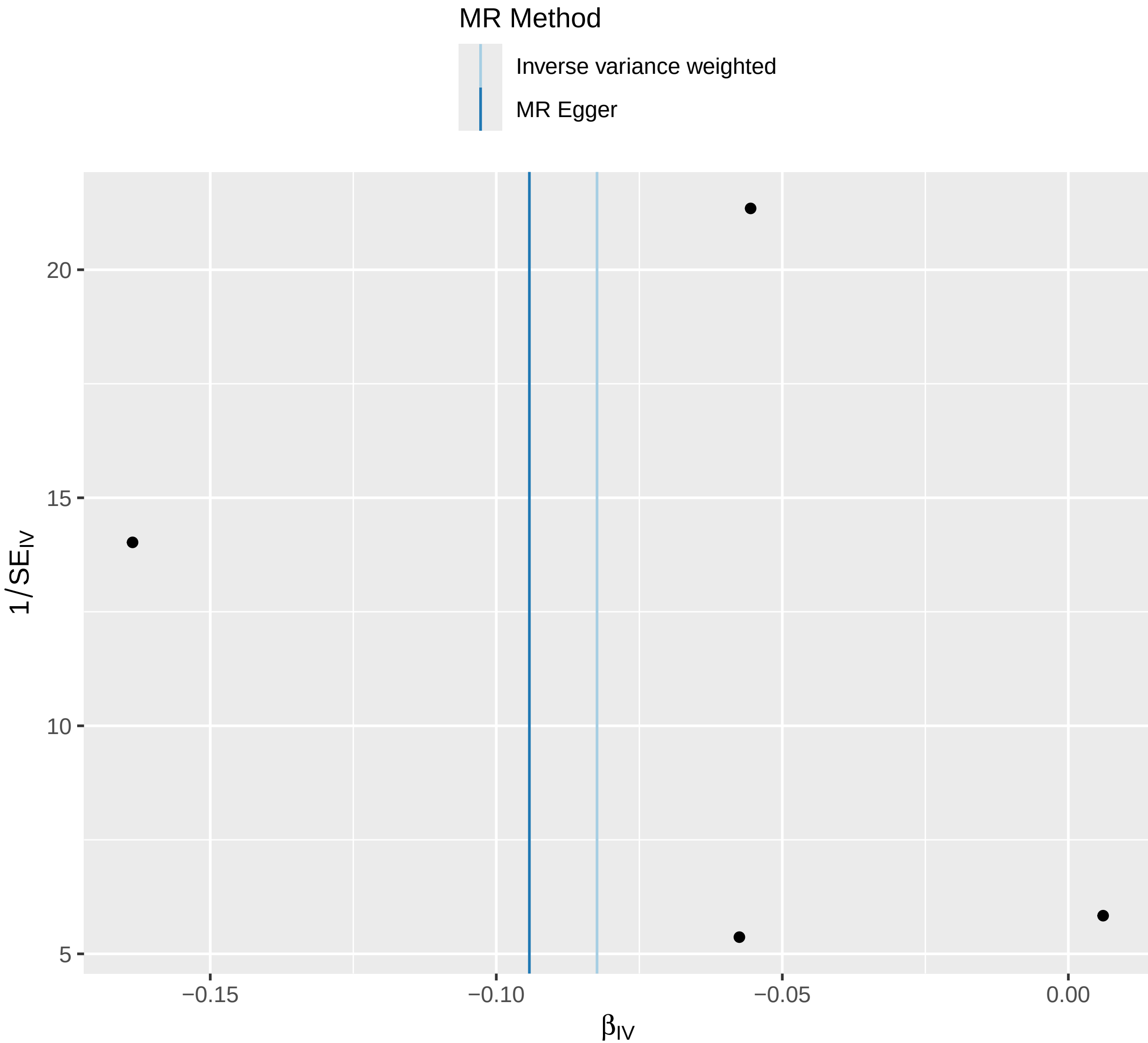
MR Method
Inverse variance weighted
MR Egger
20
15
10
5
1/SE_IV
−0.15
−0.10
−0.05
0.00
β_IV

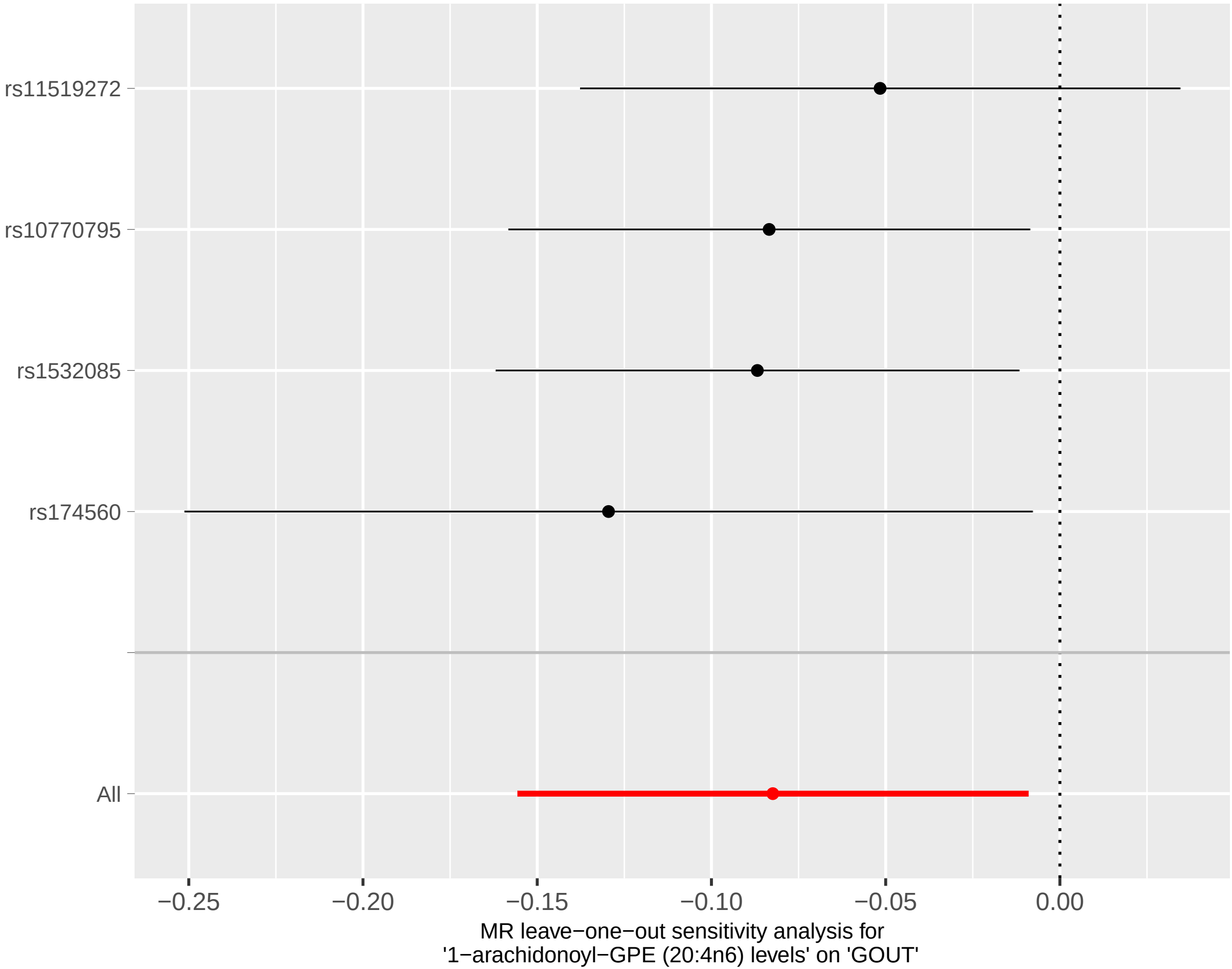
rs11519272
rs10770795
rs1532085
rs174560
All
−0.25
−0.20
−0.15
−0.10
−0.05
0.00
MR leave−one−out sensitivity analysis for
'1−arachidonoyl−GPE (20:4n6) levels' on 'GOUT'

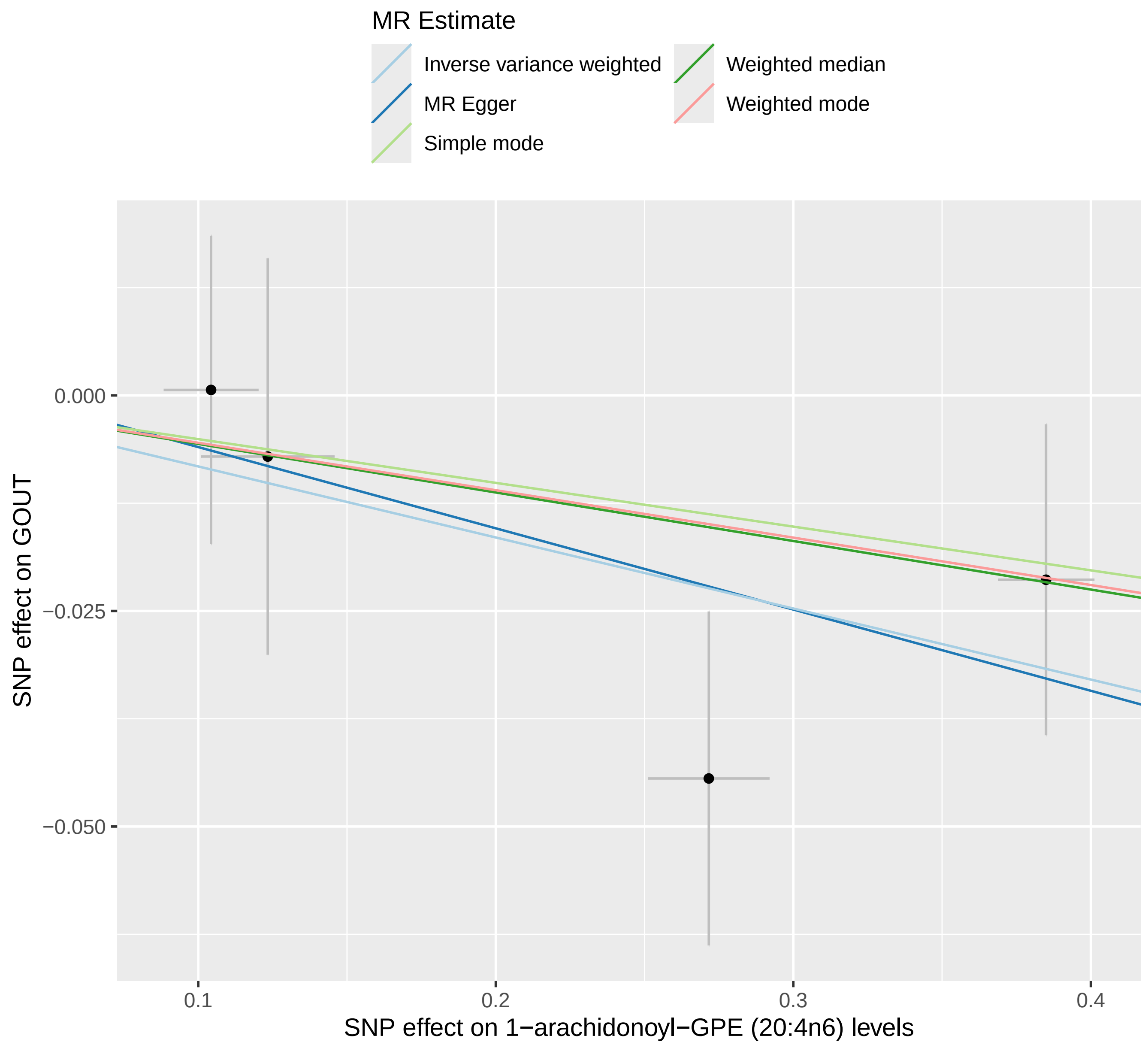
MR Estimate
Inverse variance weighted
MR Egger
Simple mode
Weighted median
Weighted mode
0.000
−0.025
−0.050
0.1
0.2
0.3
0.4
SNP effect on GOUT
SNP effect on 1−arachidonoyl−GPE (20:4n6) levels

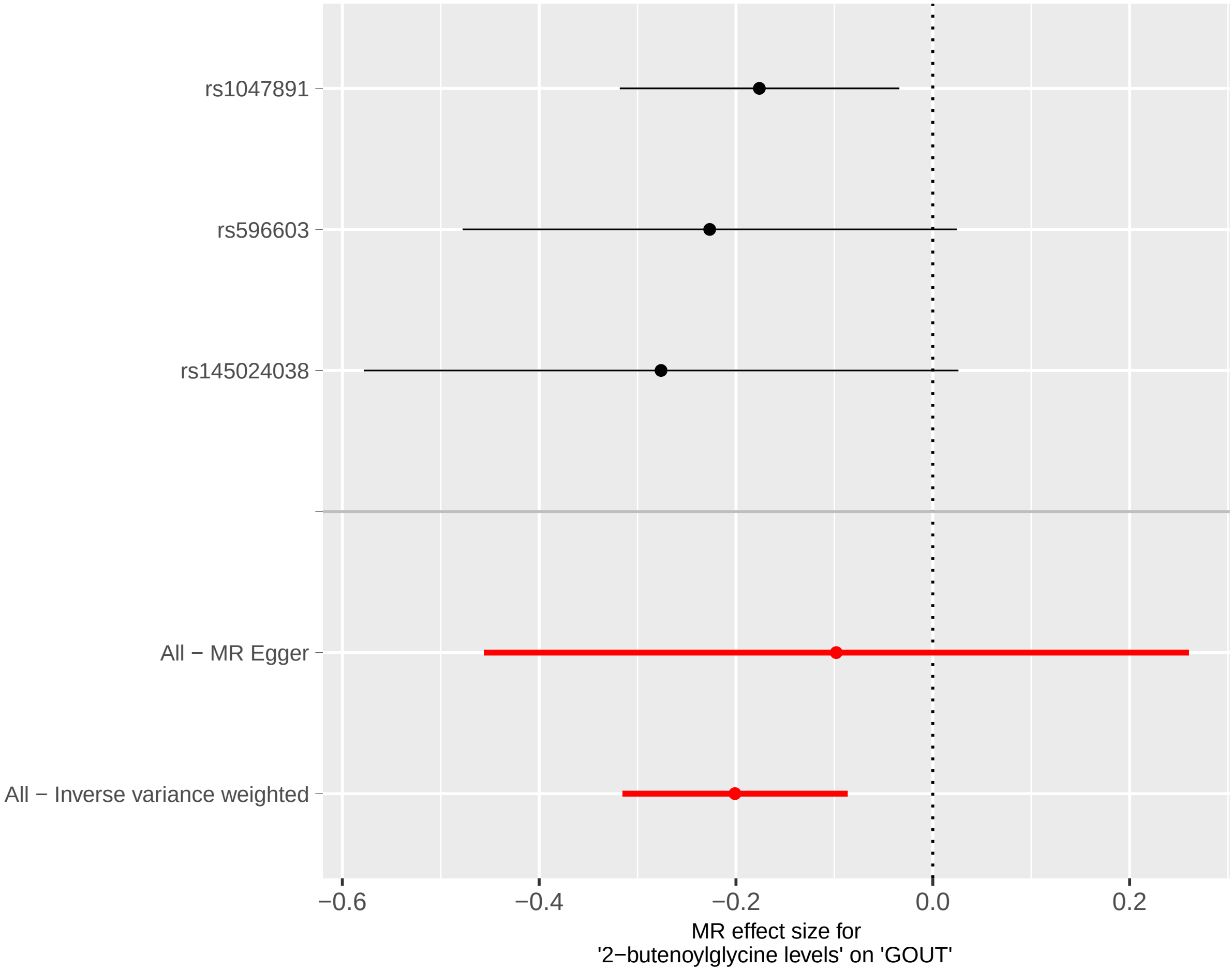
rs1047891
rs596603
rs145024038
All − MR Egger
All − Inverse variance weighted
−0.6
−0.4
−0.2
0.0
0.2
MR effect size for
'2−butenoylglycine levels' on 'GOUT'

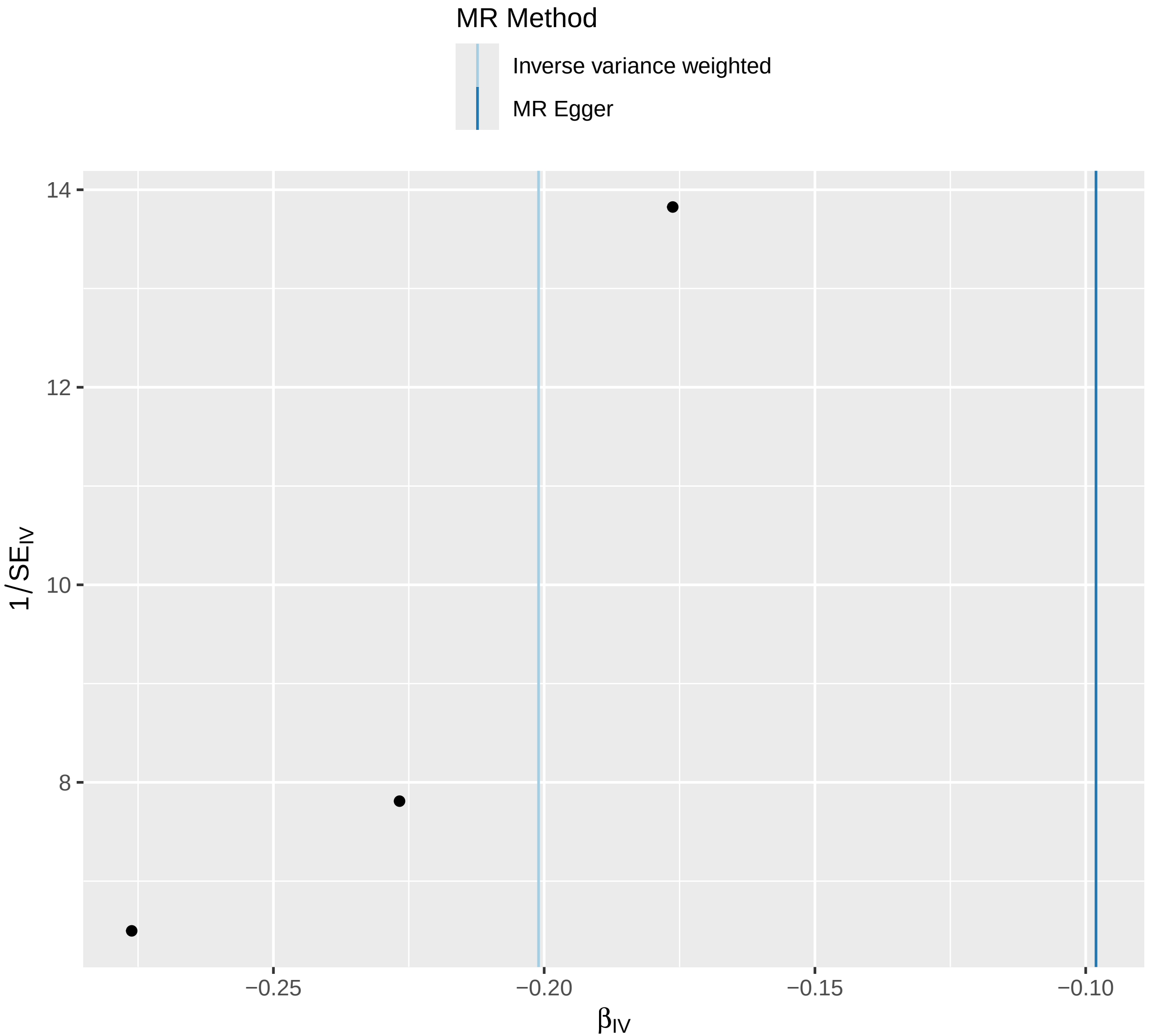
MR Method
Inverse variance weighted
MR Egger
14
12
10
8
1/SE_IV
−0.25
−0.20
−0.15
−0.10
β_IV

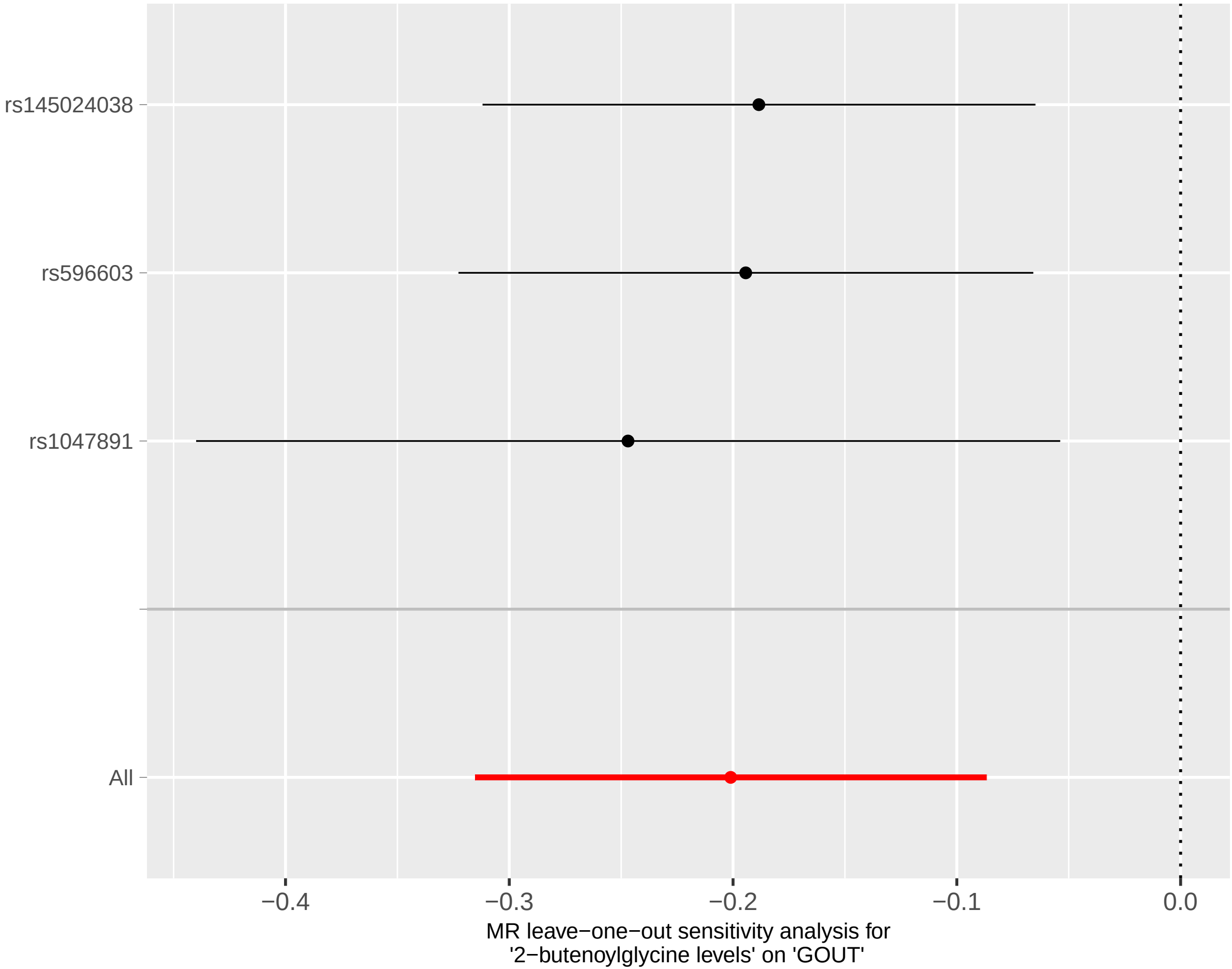
rs145024038
rs596603
rs1047891
All
−0.4
−0.3
−0.2
−0.1
0.0
MR leave−one−out sensitivity analysis for
'2−butenoylglycine levels' on 'GOUT'

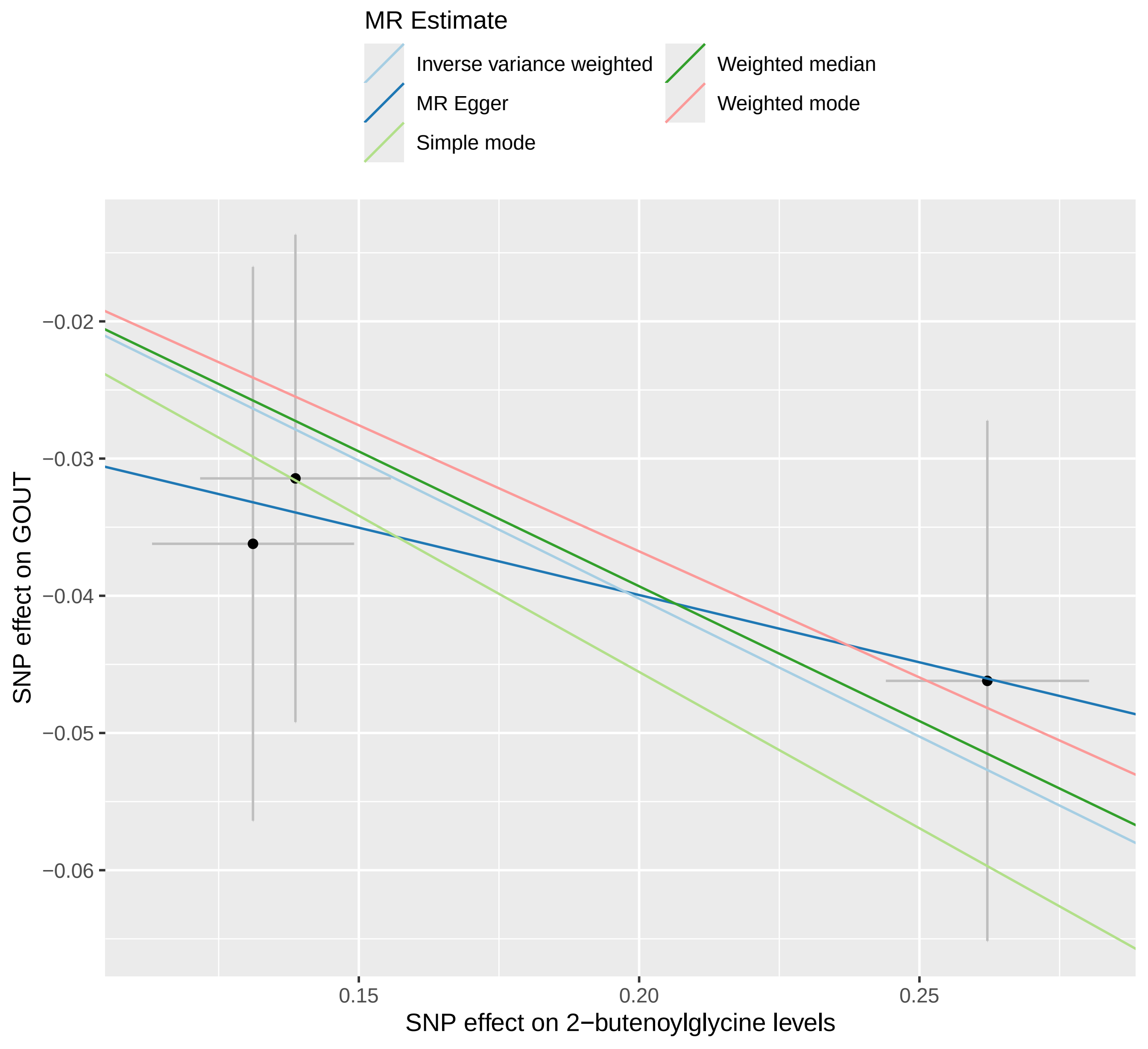
MR Estimate
Inverse variance weighted
MR Egger
Simple mode
Weighted median
Weighted mode
SNP effect on GOUT
−0.02
−0.03
−0.04
−0.05
−0.06
0.15
0.20
0.25
SNP effect on 2−butenoylglycine levels

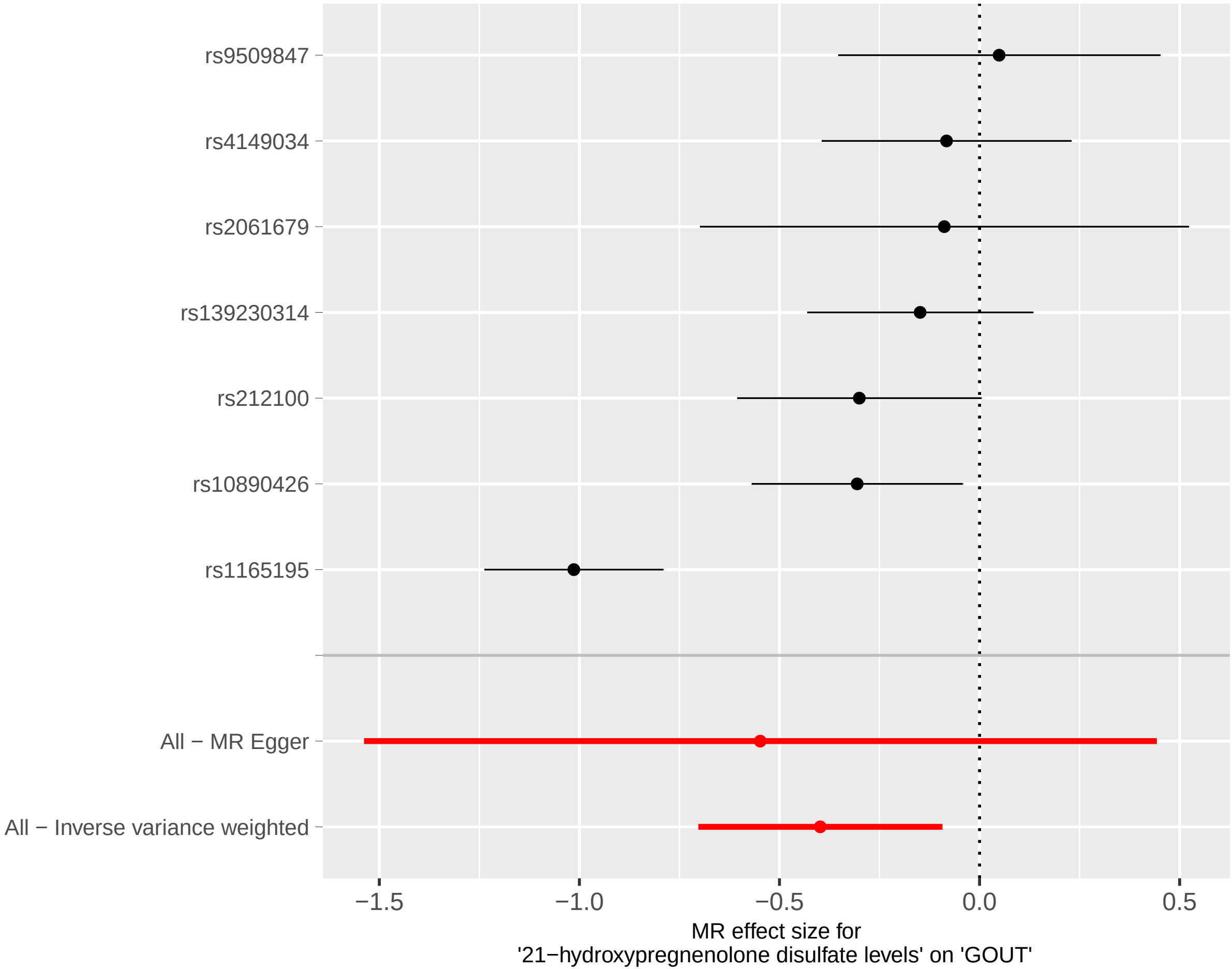
rs9509847
rs4149034
rs2061679
rs139230314
rs212100
rs10890426
rs1165195
All − MR Egger
All − Inverse variance weighted
−1.5
−1.0
−0.5
0.0
0.5
MR effect size for
'21−hydroxypregnenolone disulfate levels' on 'GOUT'

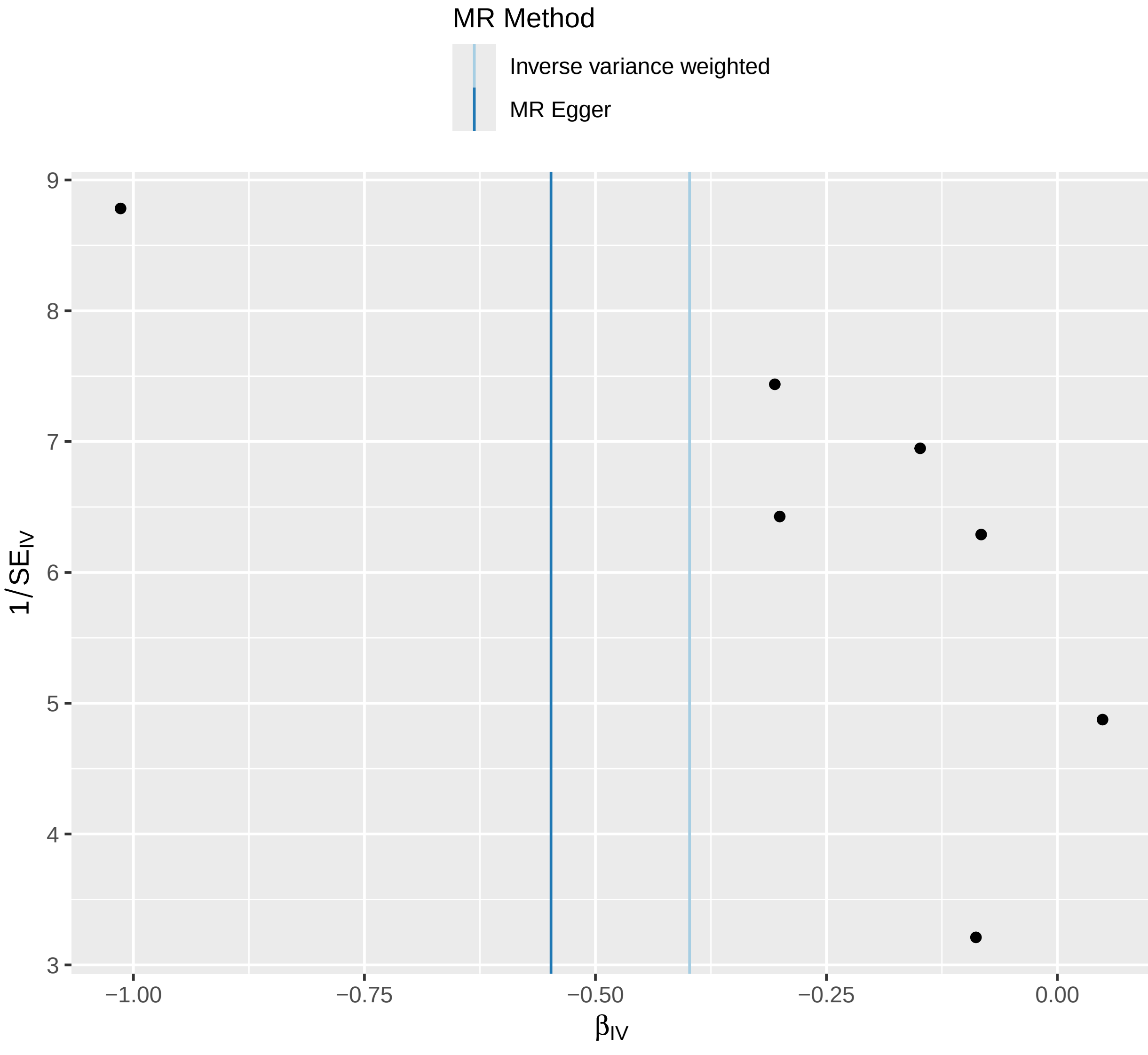
MR Method
Inverse variance weighted
MR Egger
9
8
7
6
5
4
3
$1/SE_{IV}$
−1.00
−0.75
−0.50
−0.25
0.00
β_{IV}

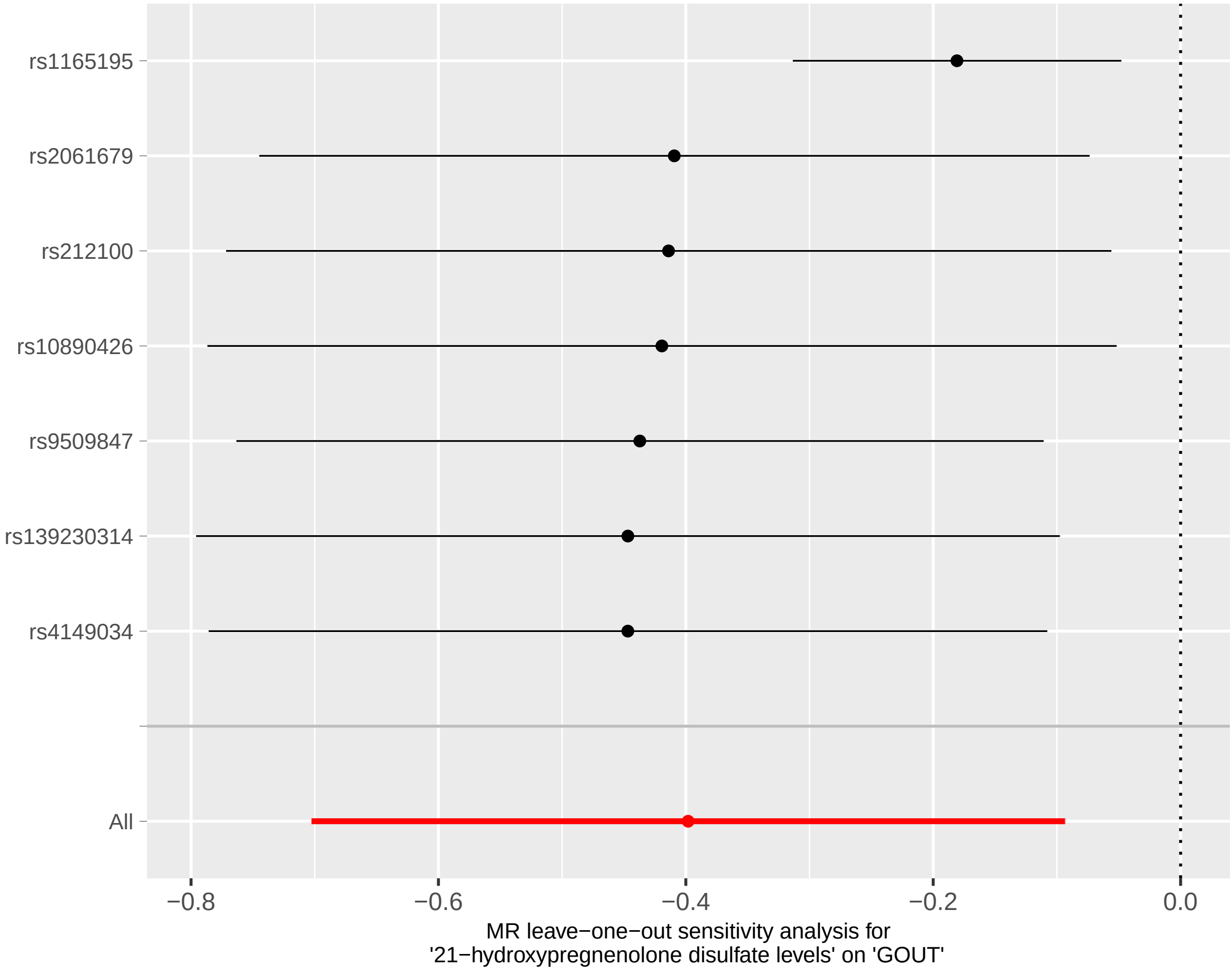
rs1165195
rs2061679
rs212100
rs10890426
rs9509847
rs139230314
rs4149034
All
−0.8
−0.6
−0.4
−0.2
0.0
MR leave−one−out sensitivity analysis for
'21−hydroxypregnenolone disulfate levels' on 'GOUT'

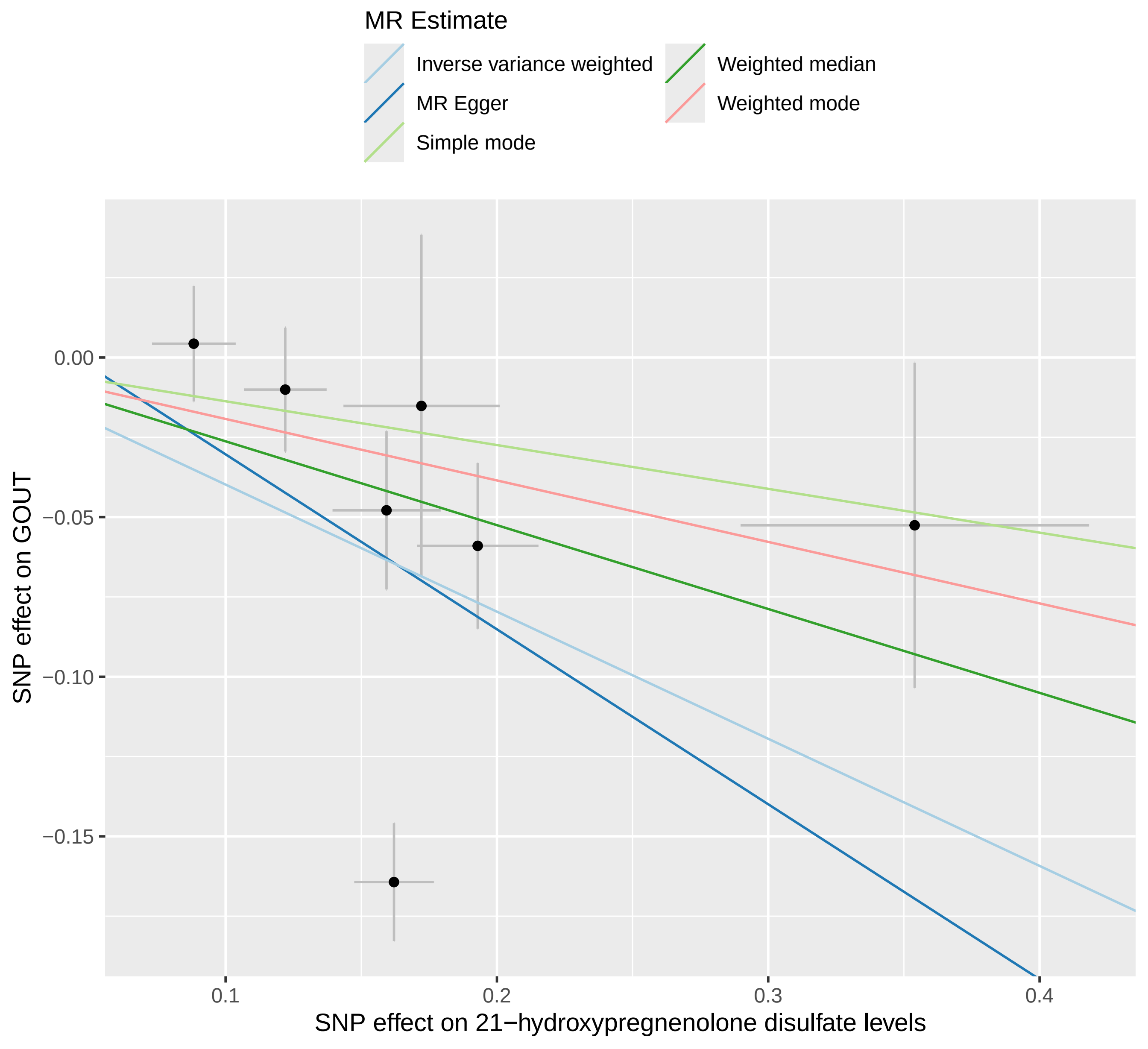
MR Estimate
Inverse variance weighted
MR Egger
Simple mode
Weighted median
Weighted mode
0.00
−0.05
−0.10
−0.15
SNP effect on GOUT
0.1
0.2
0.3
0.4
SNP effect on 21−hydroxypregnenolone disulfate levels

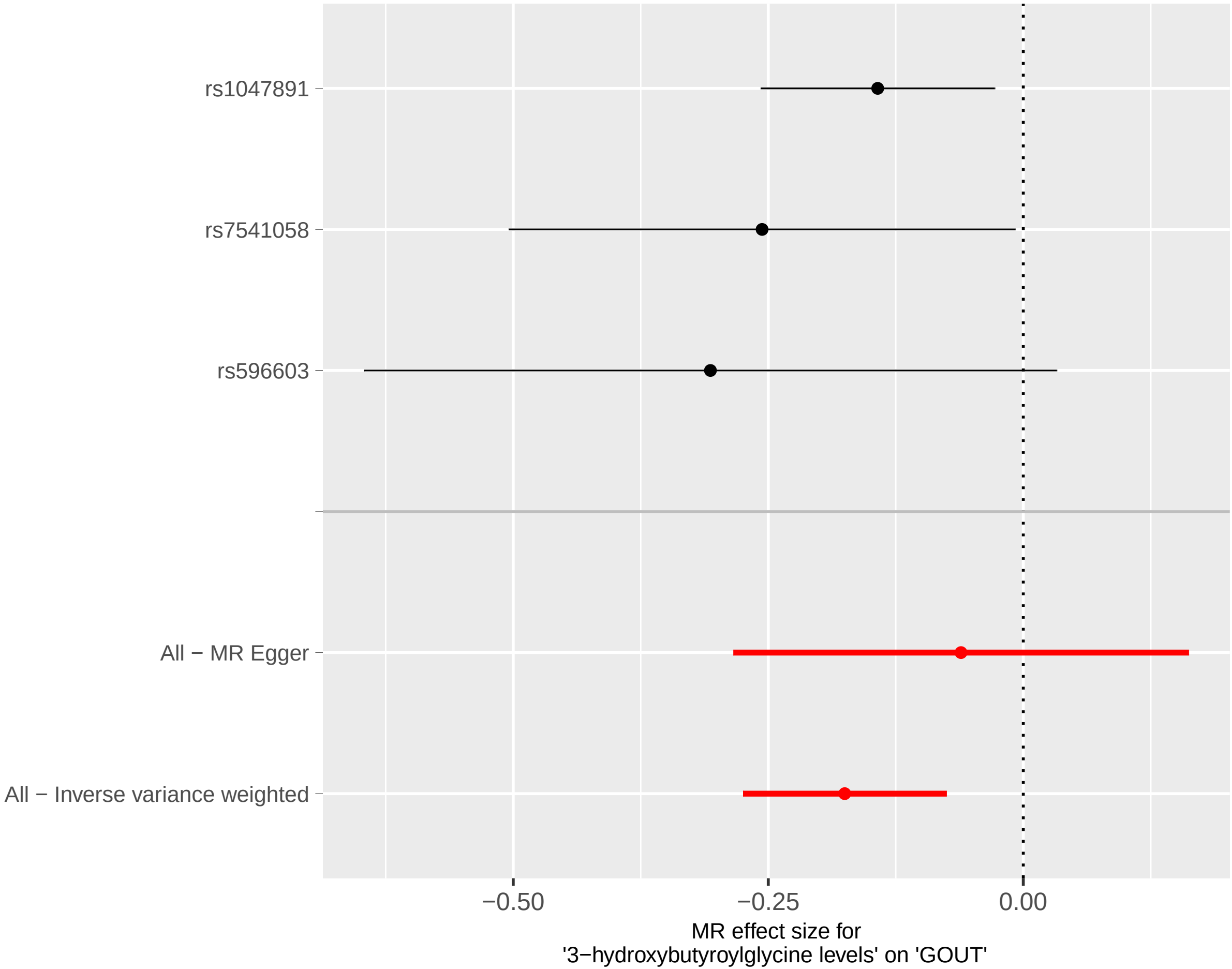
rs1047891
rs7541058
rs596603
All − MR Egger
All − Inverse variance weighted
−0.50
−0.25
0.00
MR effect size for
'3−hydroxybutyroylglycine levels' on 'GOUT'

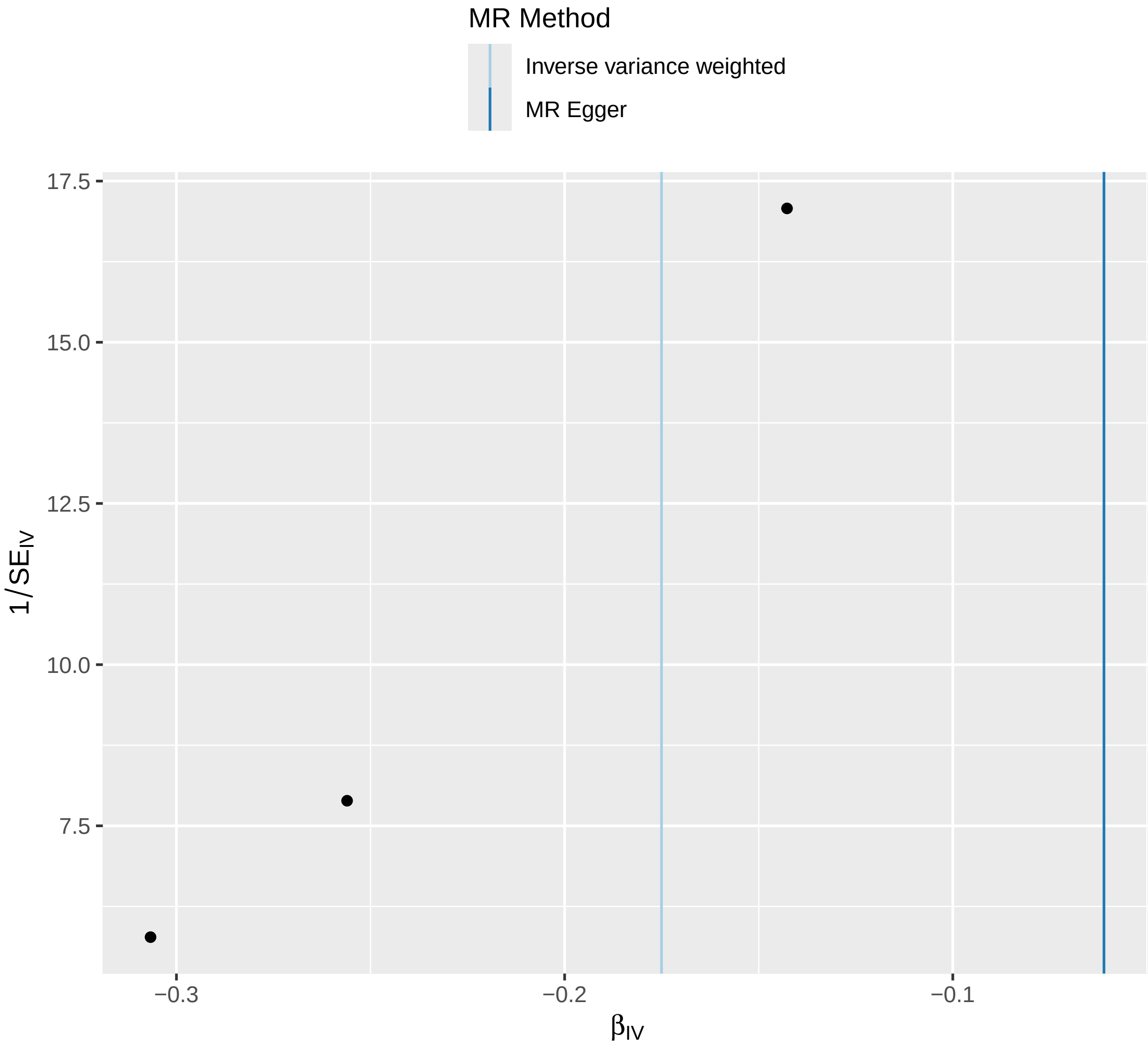

MR Method
Inverse variance weighted
MR Egger
17.5
15.0
12.5
10.0
7.5
$1/SE_{IV}$
−0.3
−0.2
−0.1
β_{IV}

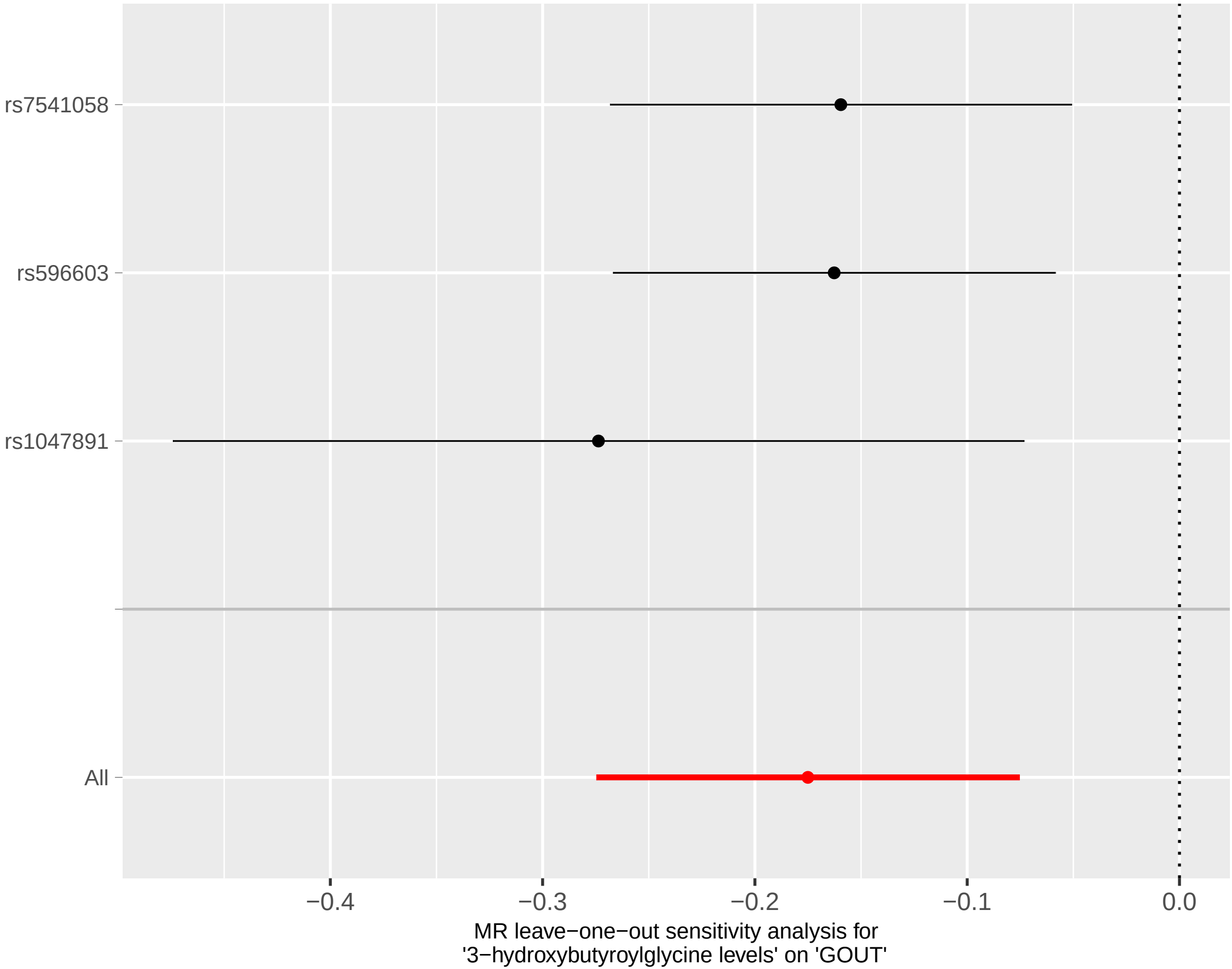
rs7541058
rs596603
rs1047891
All
−0.4
−0.3
−0.2
−0.1
0.0
MR leave−one−out sensitivity analysis for
'3−hydroxybutyroylglycine levels' on 'GOUT'

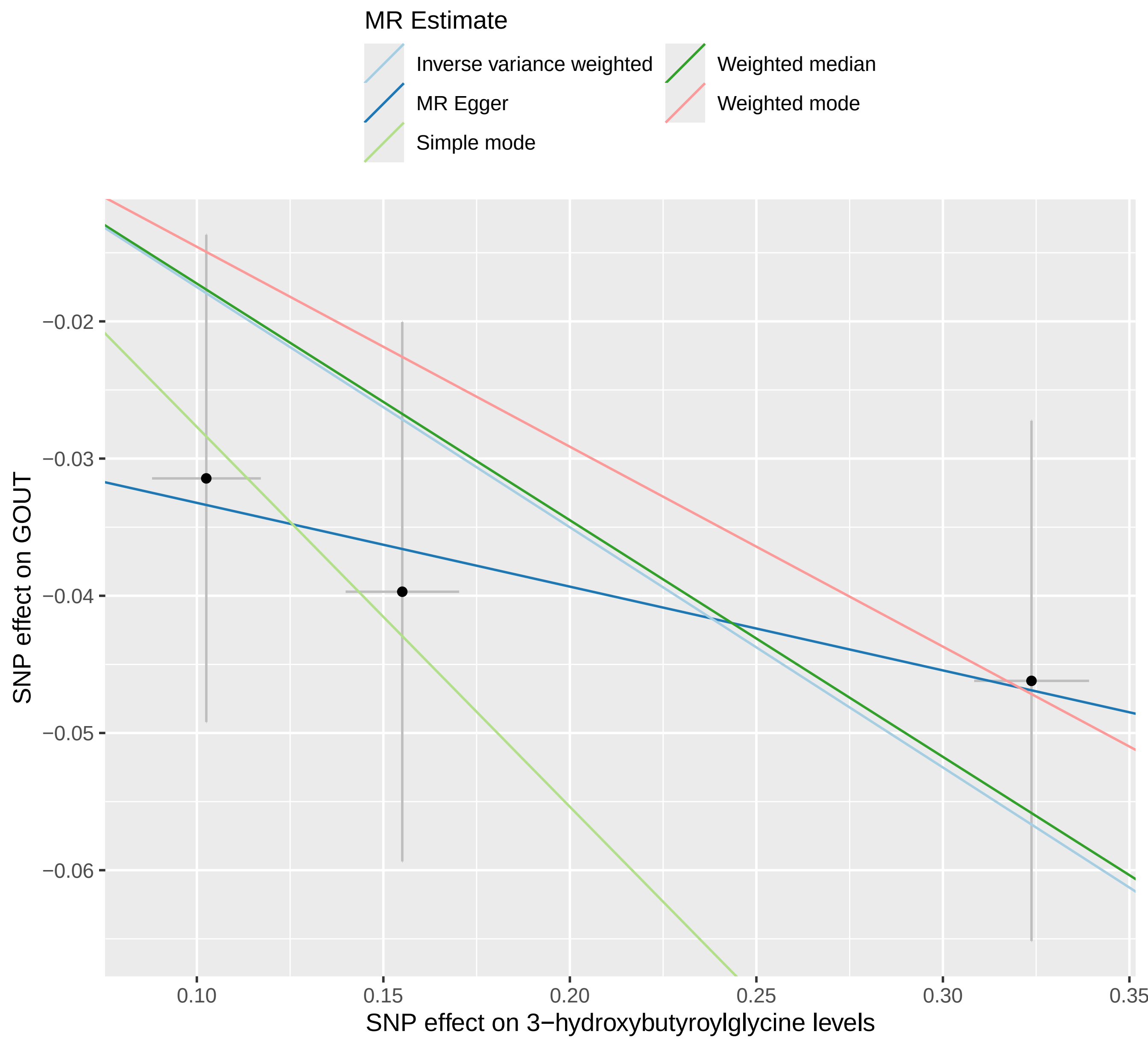
MR Estimate
Inverse variance weighted
MR Egger
Simple mode
Weighted median
Weighted mode
SNP effect on GOUT
−0.02
−0.03
−0.04
−0.05
−0.06
0.10
0.15
0.20
0.25
0.30
0.35
SNP effect on 3−hydroxybutyroylglycine levels

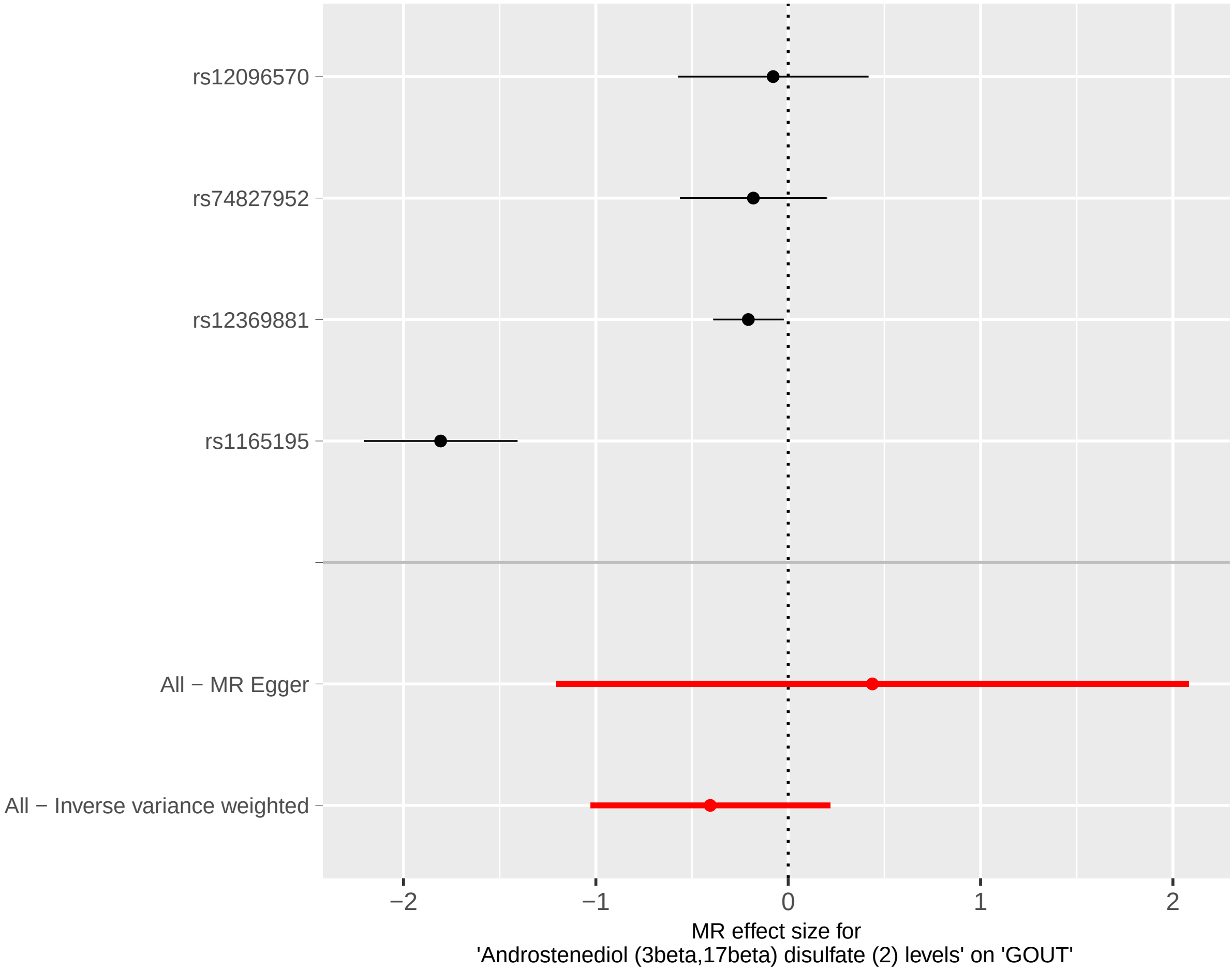

rs12096570
rs74827952
rs12369881
rs1165195
All − MR Egger
All − Inverse variance weighted
−2
−1
0
1
2
MR effect size for
'Androstenediol (3beta,17beta) disulfate (2) levels' on 'GOUT'

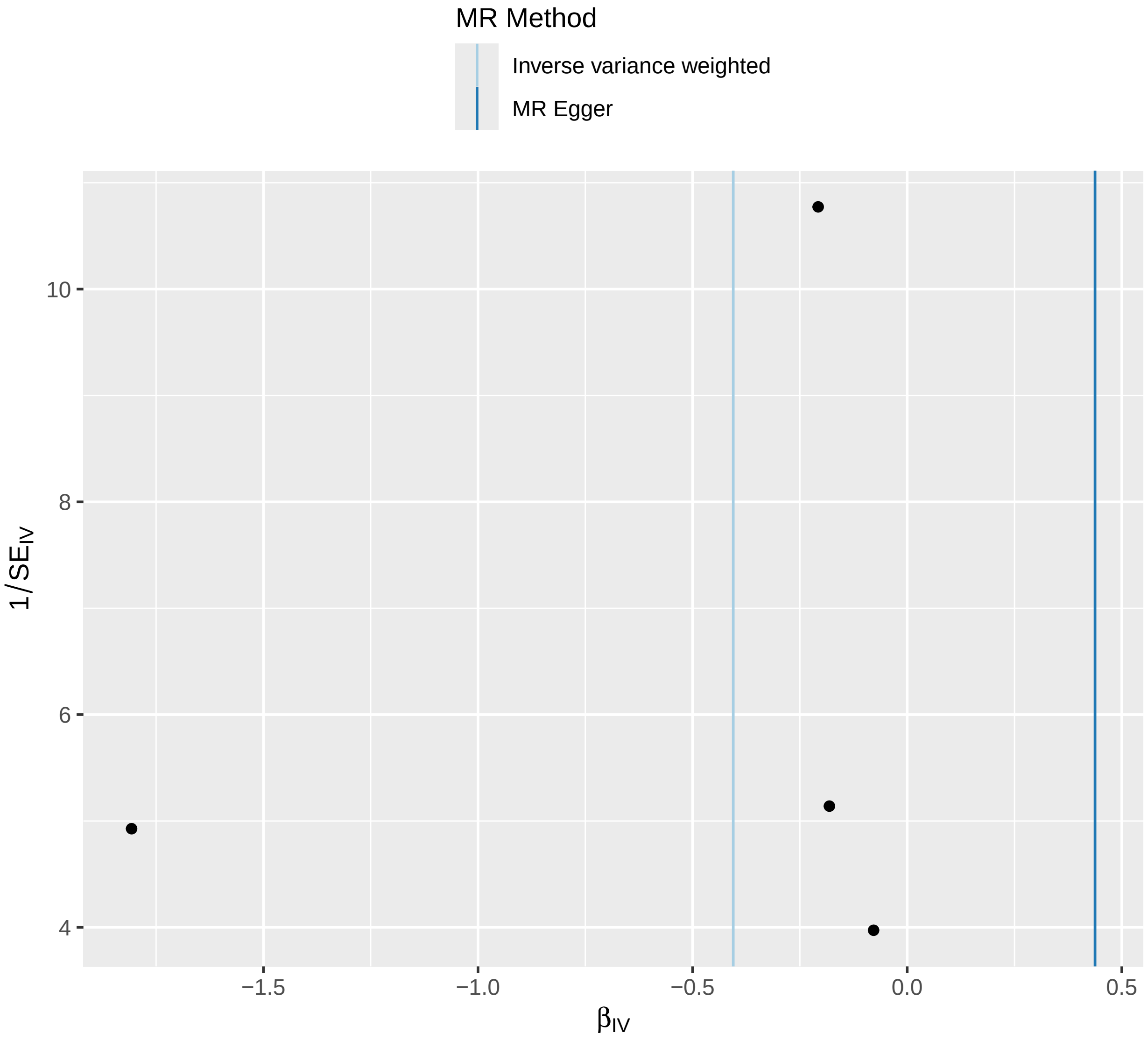

MR Method
Inverse variance weighted
MR Egger
10
8
6
4
1/SE$_{IV}$
−1.5
−1.0
−0.5
0.0
0.5
β$_{IV}$

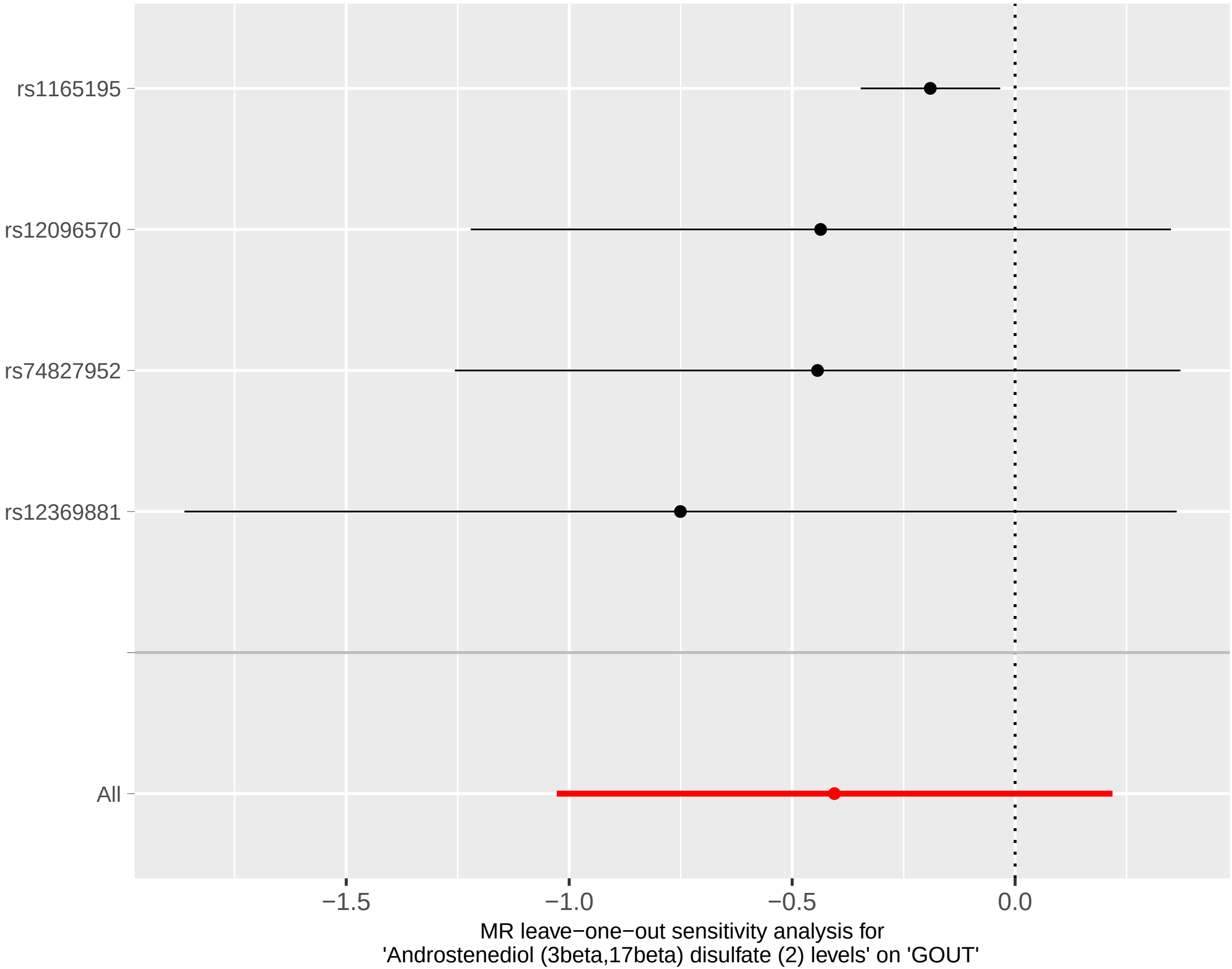

rs1165195
rs12096570
rs74827952
rs12369881
All
−1.5
−1.0
−0.5
0.0
MR leave−one−out sensitivity analysis for
'Androstenediol (3beta,17beta) disulfate (2) levels' on 'GOUT'

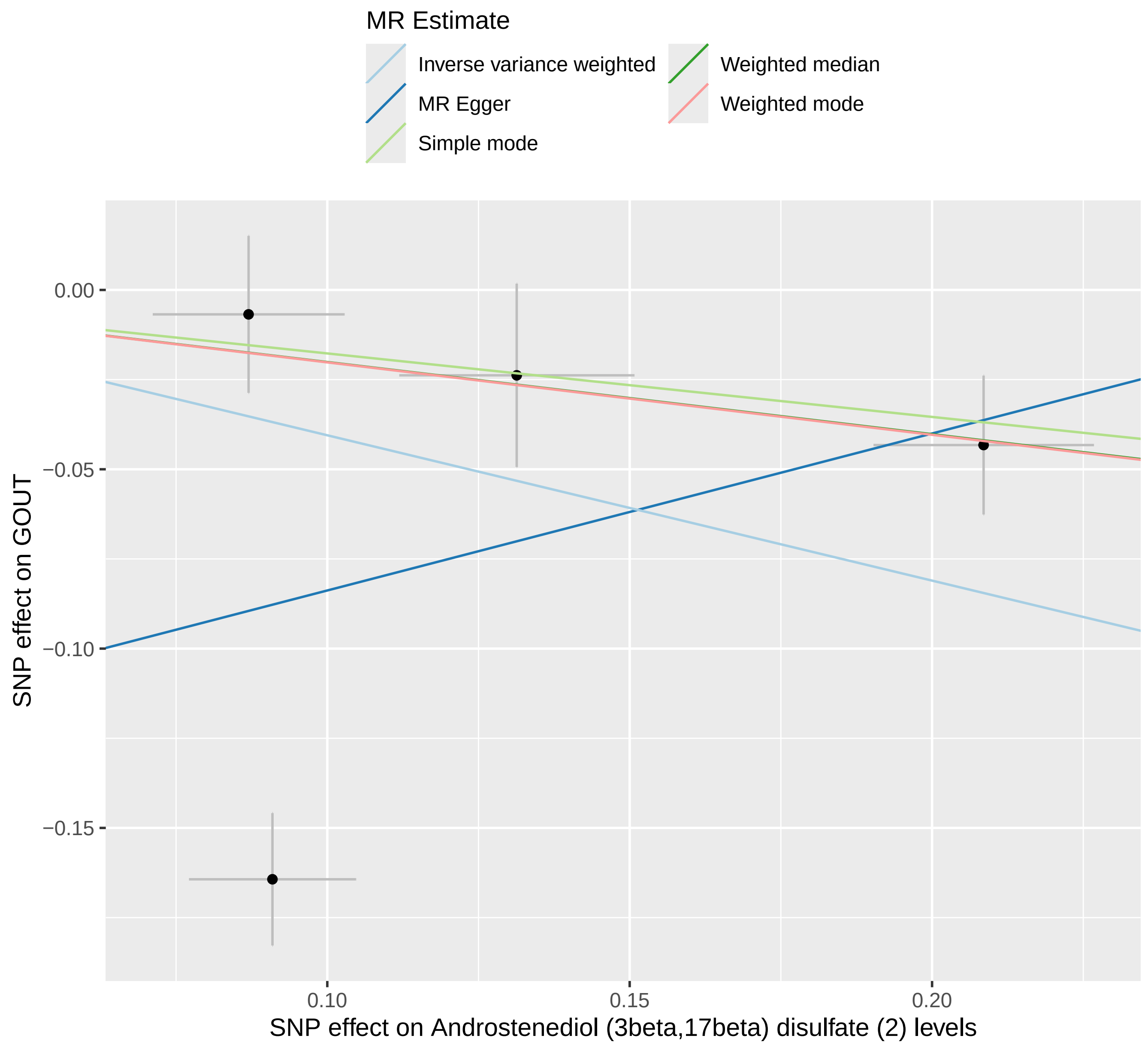
MR Estimate
Inverse variance weighted
MR Egger
Simple mode
Weighted median
Weighted mode
0.00
−0.05
−0.10
−0.15
SNP effect on GOUT
0.10
0.15
0.20
SNP effect on Androstenediol (3beta,17beta) disulfate (2) levels

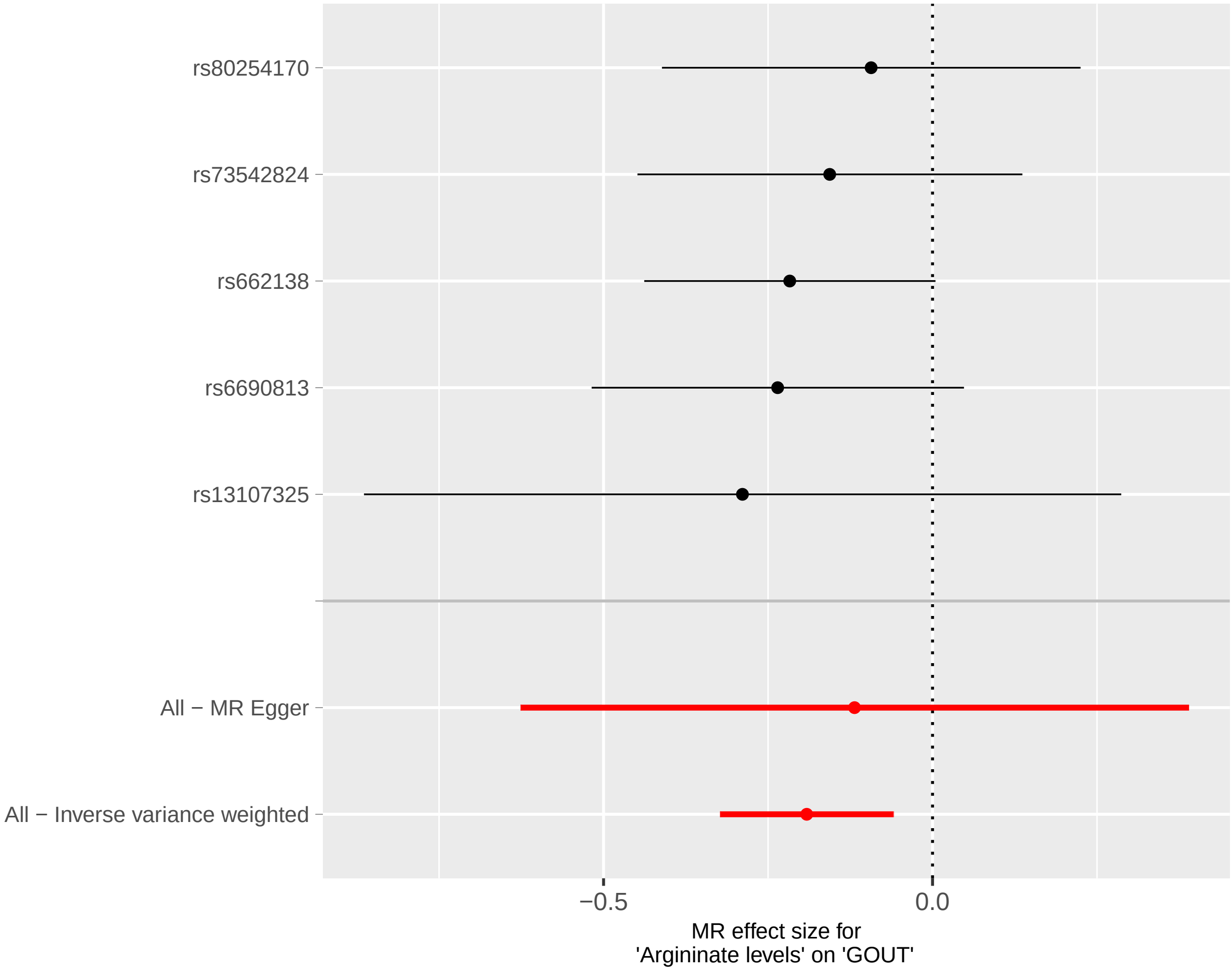
rs80254170
rs73542824
rs662138
rs6690813
rs13107325
All − MR Egger
All − Inverse variance weighted
−0.5
0.0
MR effect size for
'Argininate levels' on 'GOUT'

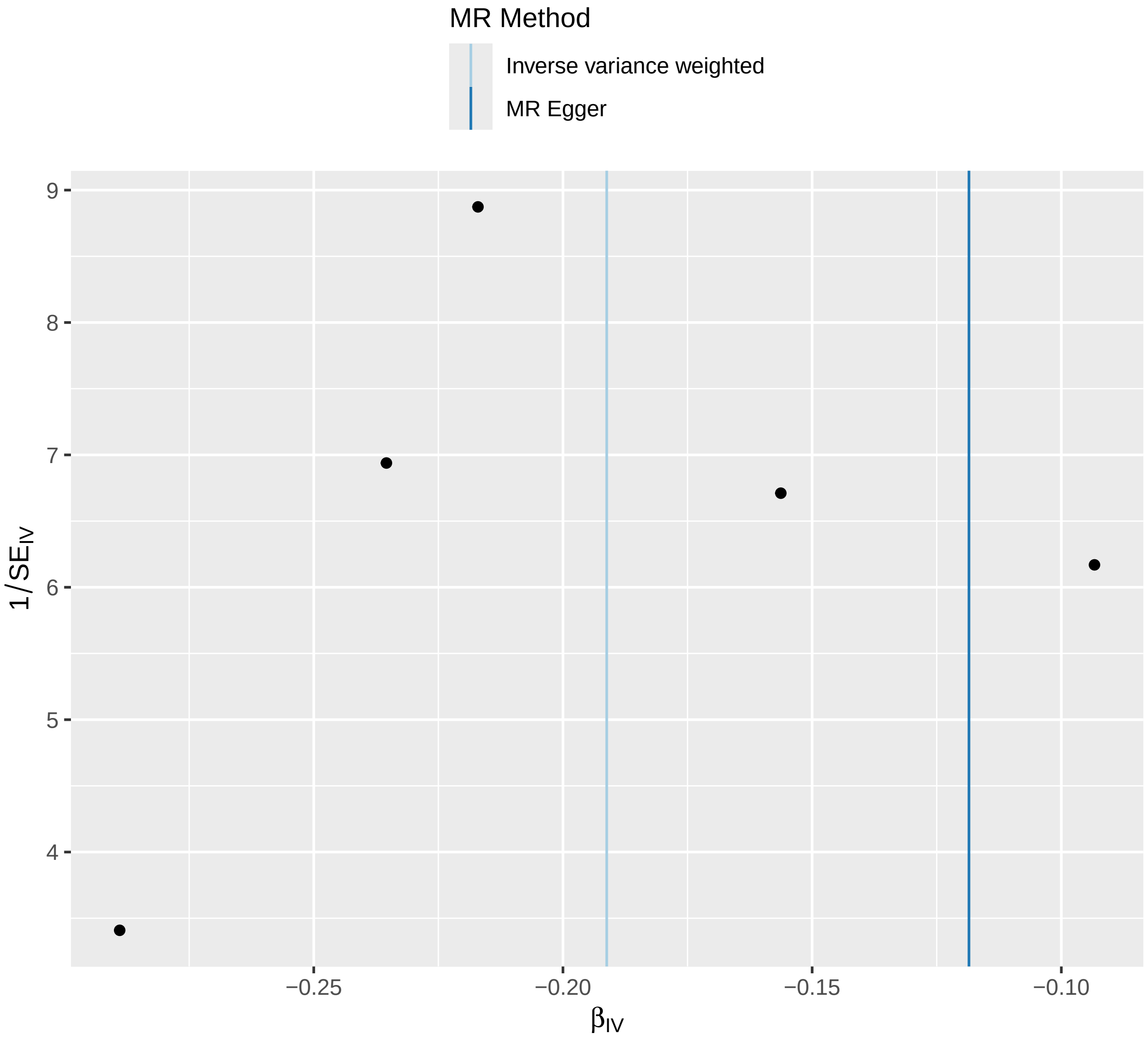

MR Method
Inverse variance weighted
MR Egger
9
8
7
6
5
4
1/SE_IV
−0.25
−0.20
−0.15
−0.10
β_IV

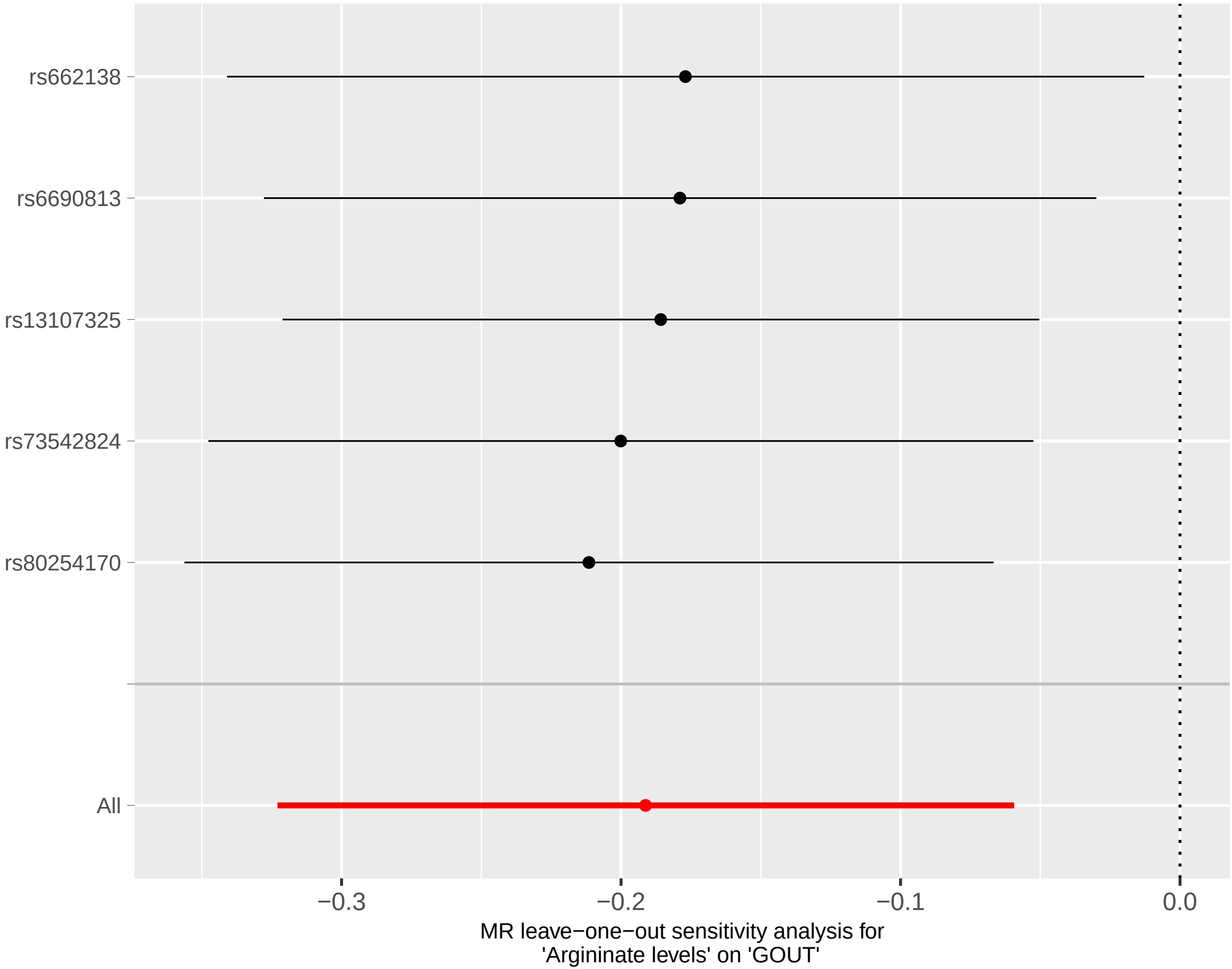

rs662138
rs6690813
rs13107325
rs73542824
rs80254170
All
−0.3
−0.2
−0.1
0.0
MR leave−one−out sensitivity analysis for
'Argininate levels' on 'GOUT'

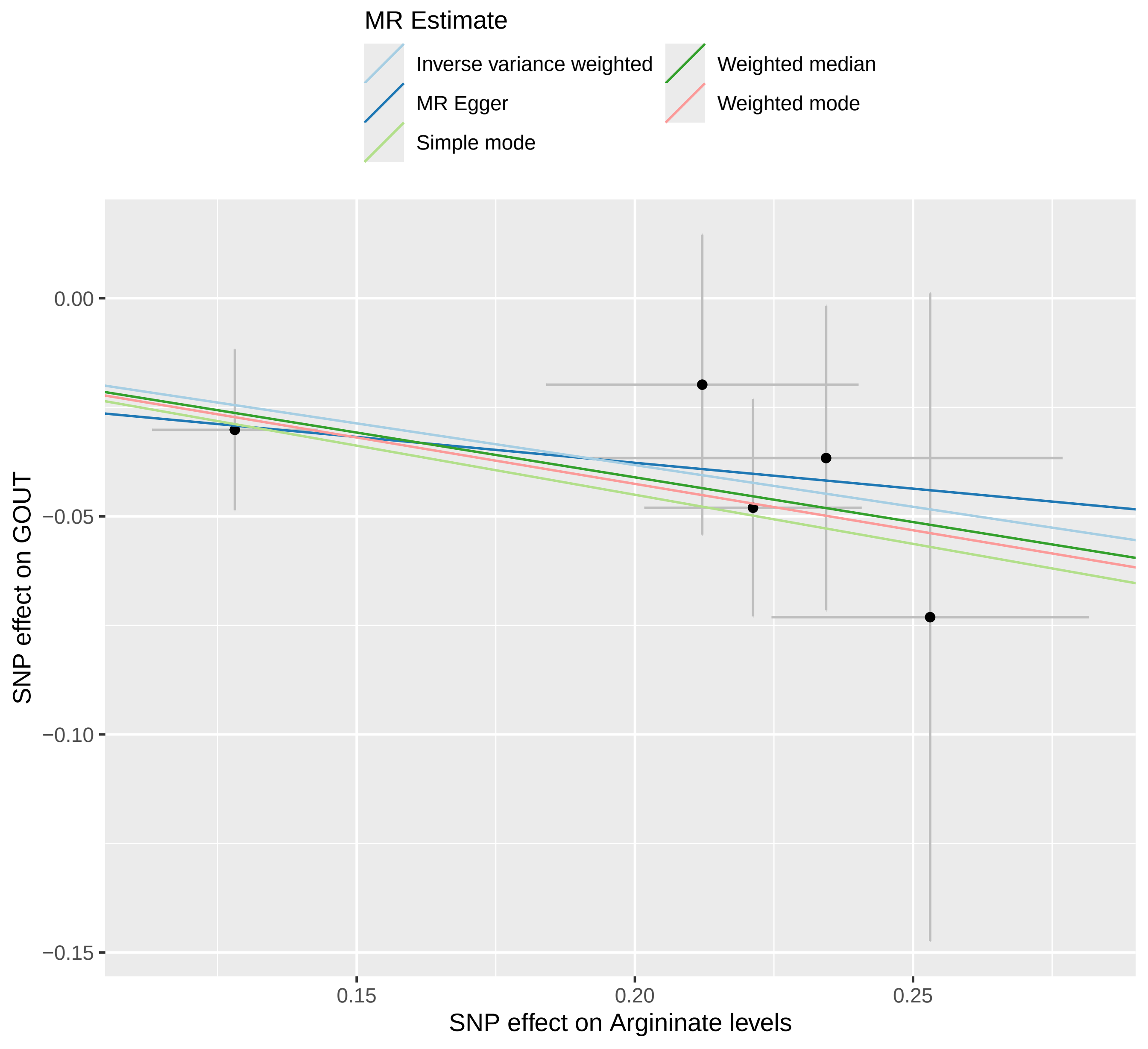
MR Estimate
Inverse variance weighted
MR Egger
Simple mode
Weighted median
Weighted mode
0.00
−0.05
−0.10
−0.15
SNP effect on GOUT
0.15
0.20
0.25
SNP effect on Argininate levels

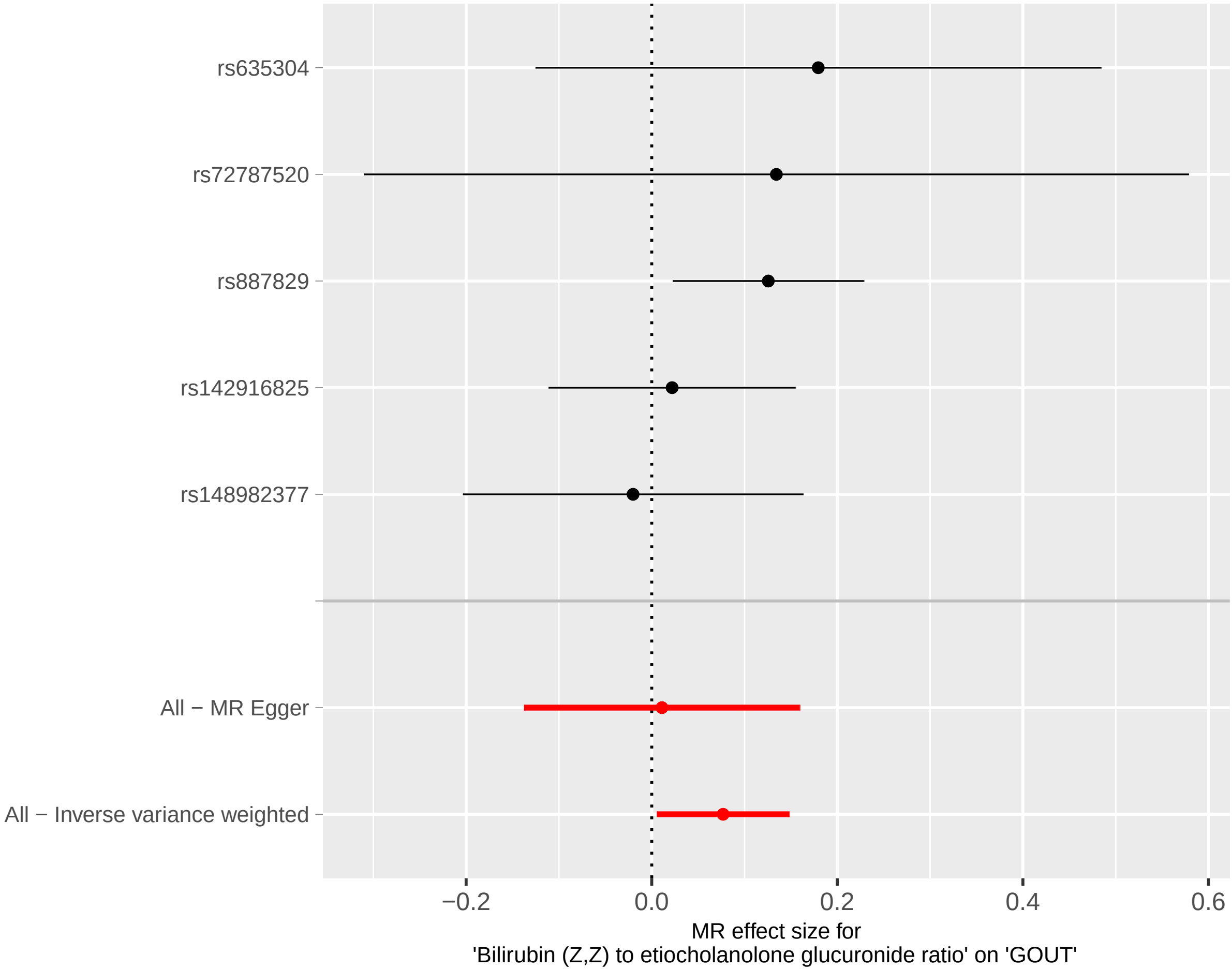
rs635304
rs72787520
rs887829
rs142916825
rs148982377
All − MR Egger
All − Inverse variance weighted
−0.2
0.0
0.2
0.4
0.6
MR effect size for
'Bilirubin (Z,Z) to etiocholanolone glucuronide ratio' on 'GOUT'

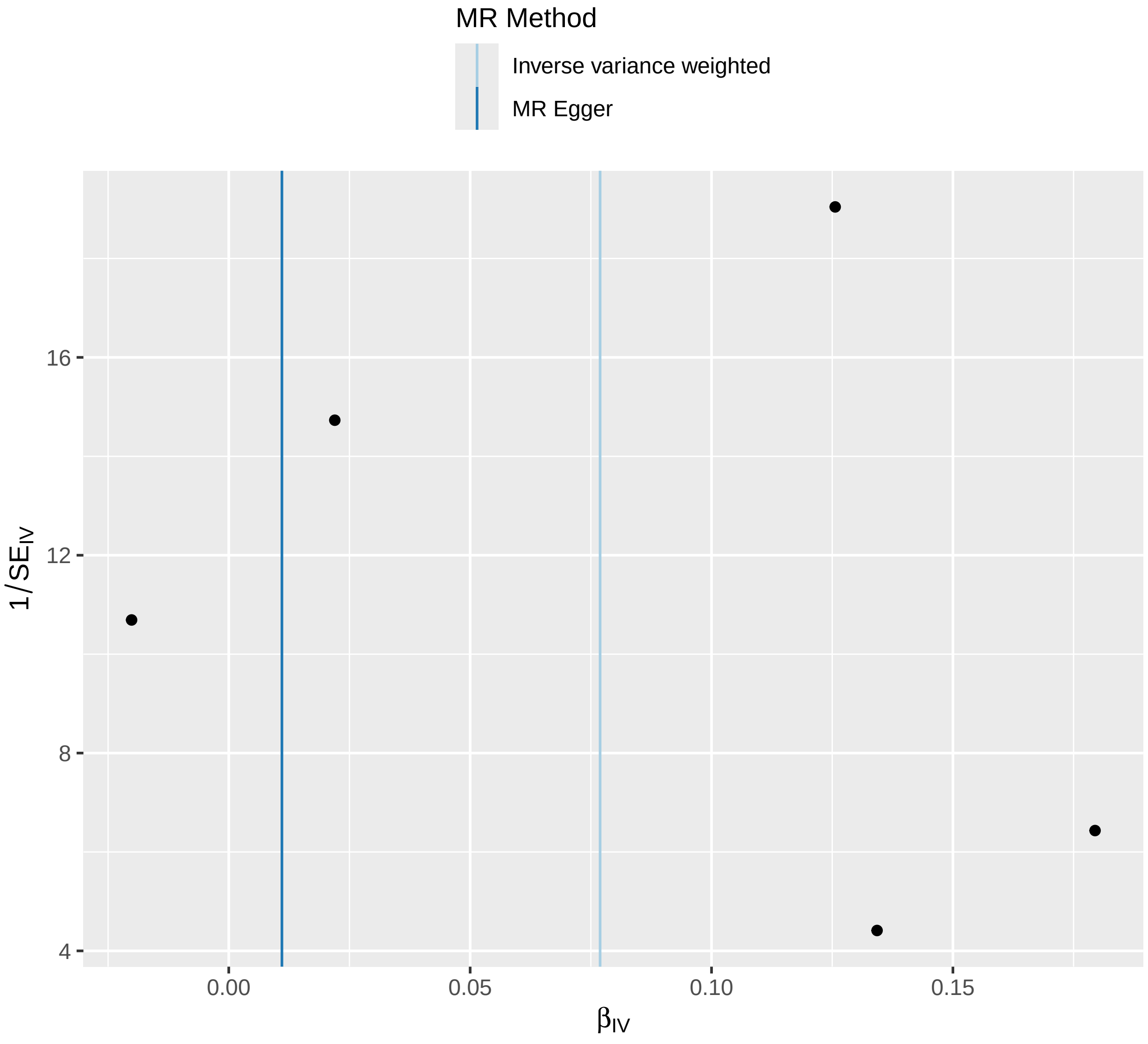

MR Method
Inverse variance weighted
MR Egger
16
12
8
4
1/SE_IV
0.00
0.05
0.10
0.15
β_IV

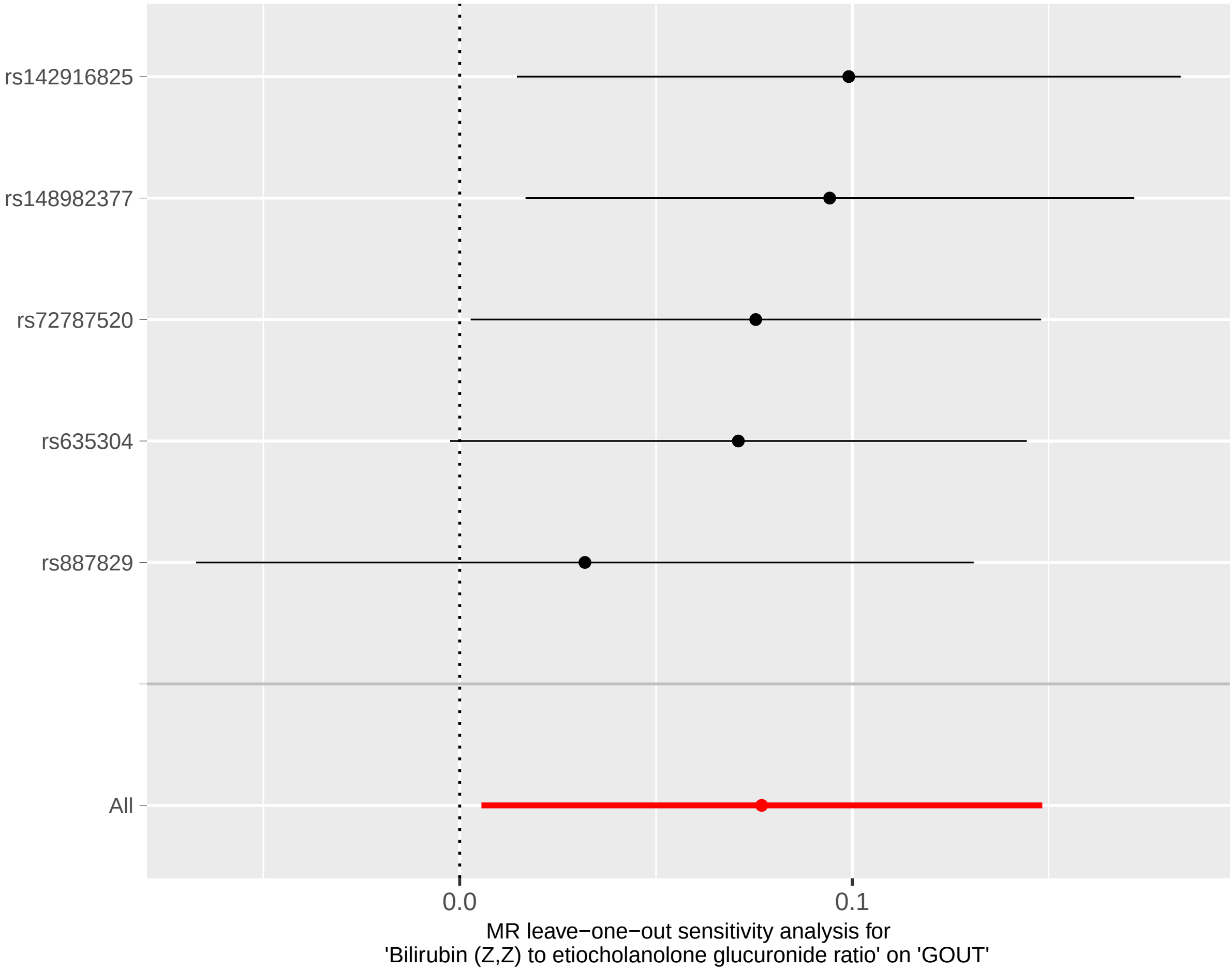
rs142916825
rs148982377
rs72787520
rs635304
rs887829
All
0.0
0.1
MR leave−one−out sensitivity analysis for
'Bilirubin (Z,Z) to etiocholanolone glucuronide ratio' on 'GOUT'

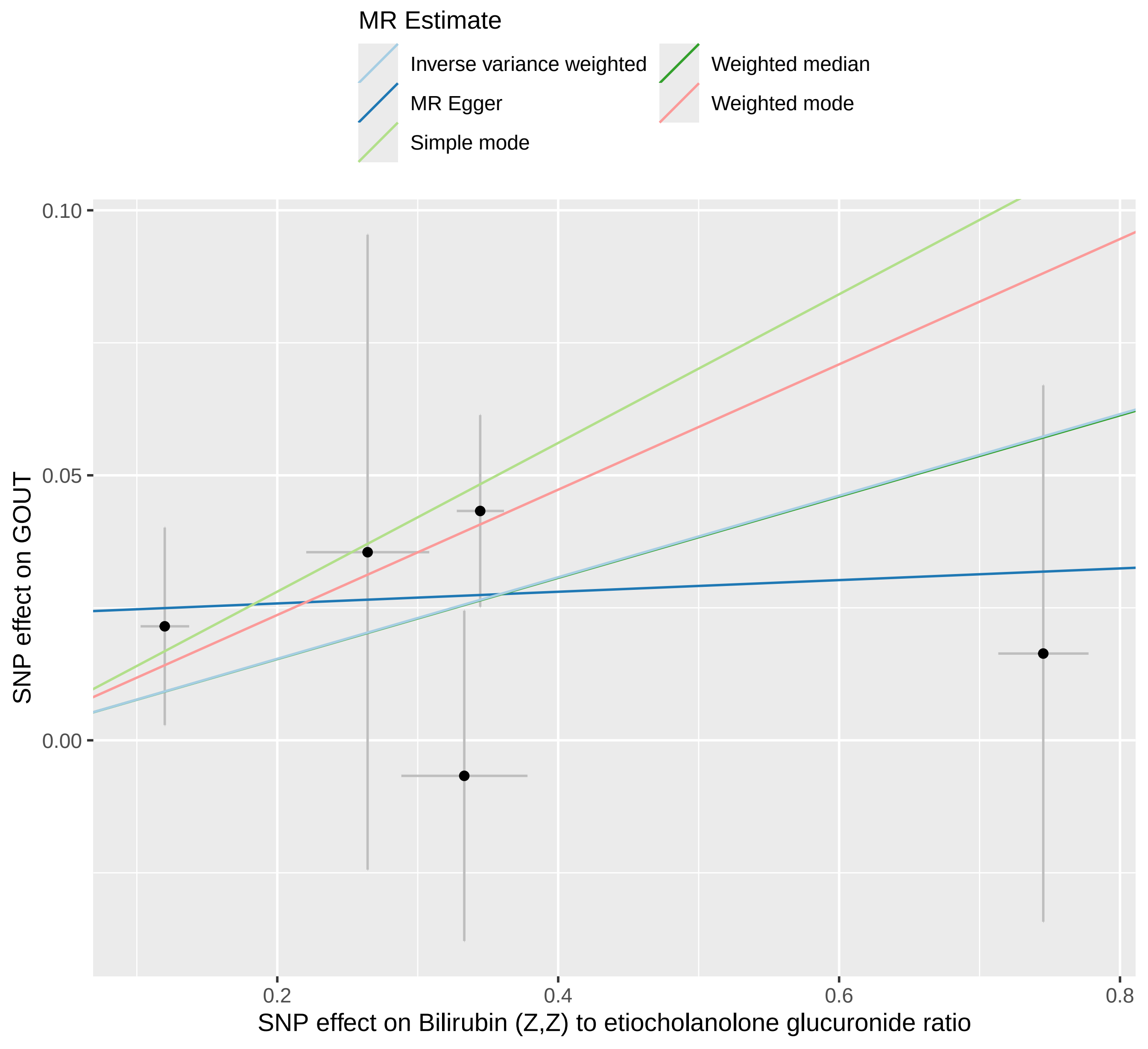
MR Estimate
Inverse variance weighted
MR Egger
Simple mode
Weighted median
Weighted mode
0.10
0.05
0.00
0.2
0.4
0.6
0.8
SNP effect on GOUT
SNP effect on Bilirubin (Z,Z) to etiocholanolone glucuronide ratio

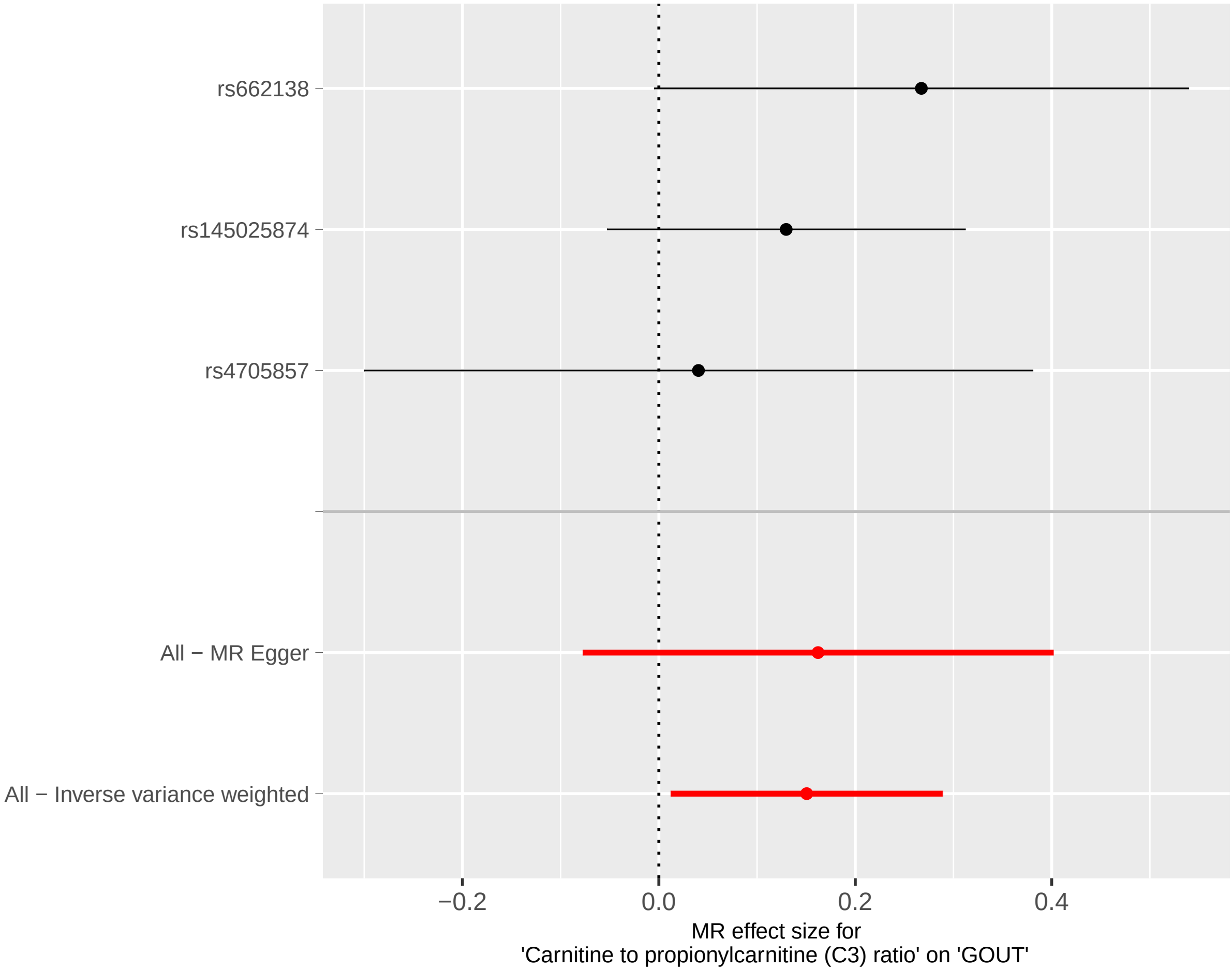

rs662138
rs145025874
rs4705857
All − MR Egger
All − Inverse variance weighted
−0.2
0.0
0.2
0.4
MR effect size for
'Carnitine to propionylcarnitine (C3) ratio' on 'GOUT'

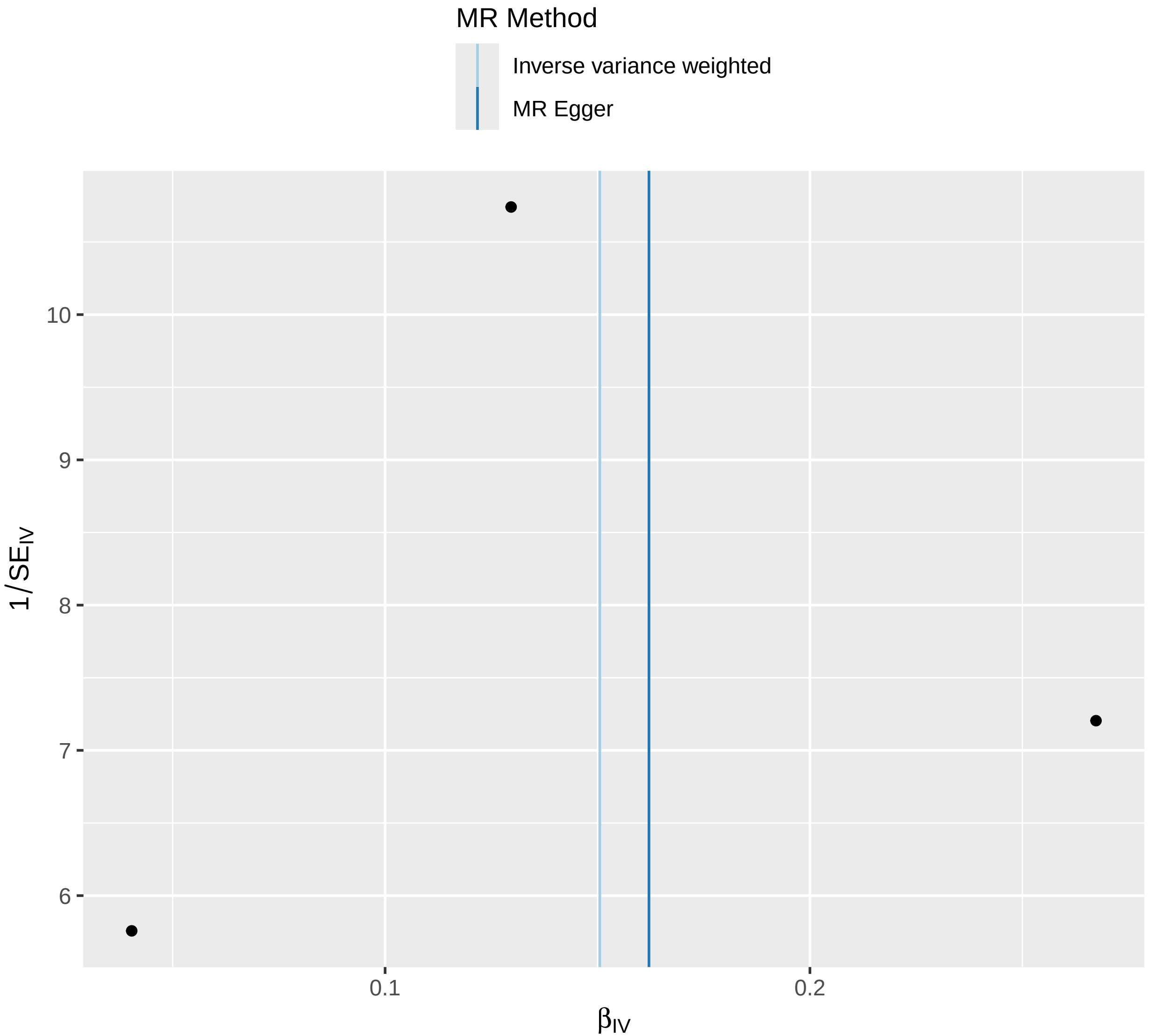
MR Method
Inverse variance weighted
MR Egger
10
9
8
7
6
1/SE_IV
0.1
0.2
β_IV

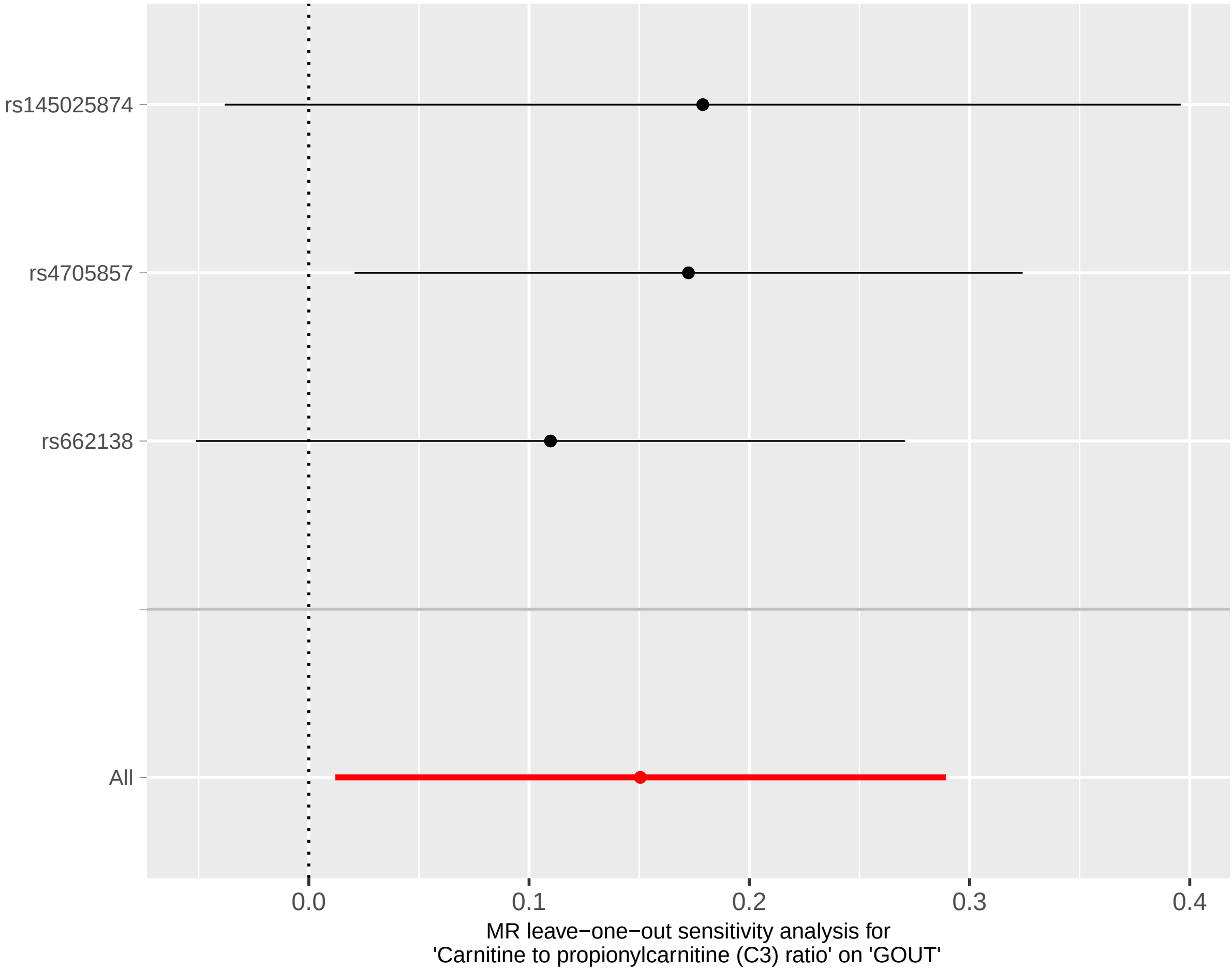
rs145025874
rs4705857
rs662138
All
0.0
0.1
0.2
0.3
0.4
MR leave−one−out sensitivity analysis for
'Carnitine to propionylcarnitine (C3) ratio' on 'GOUT'

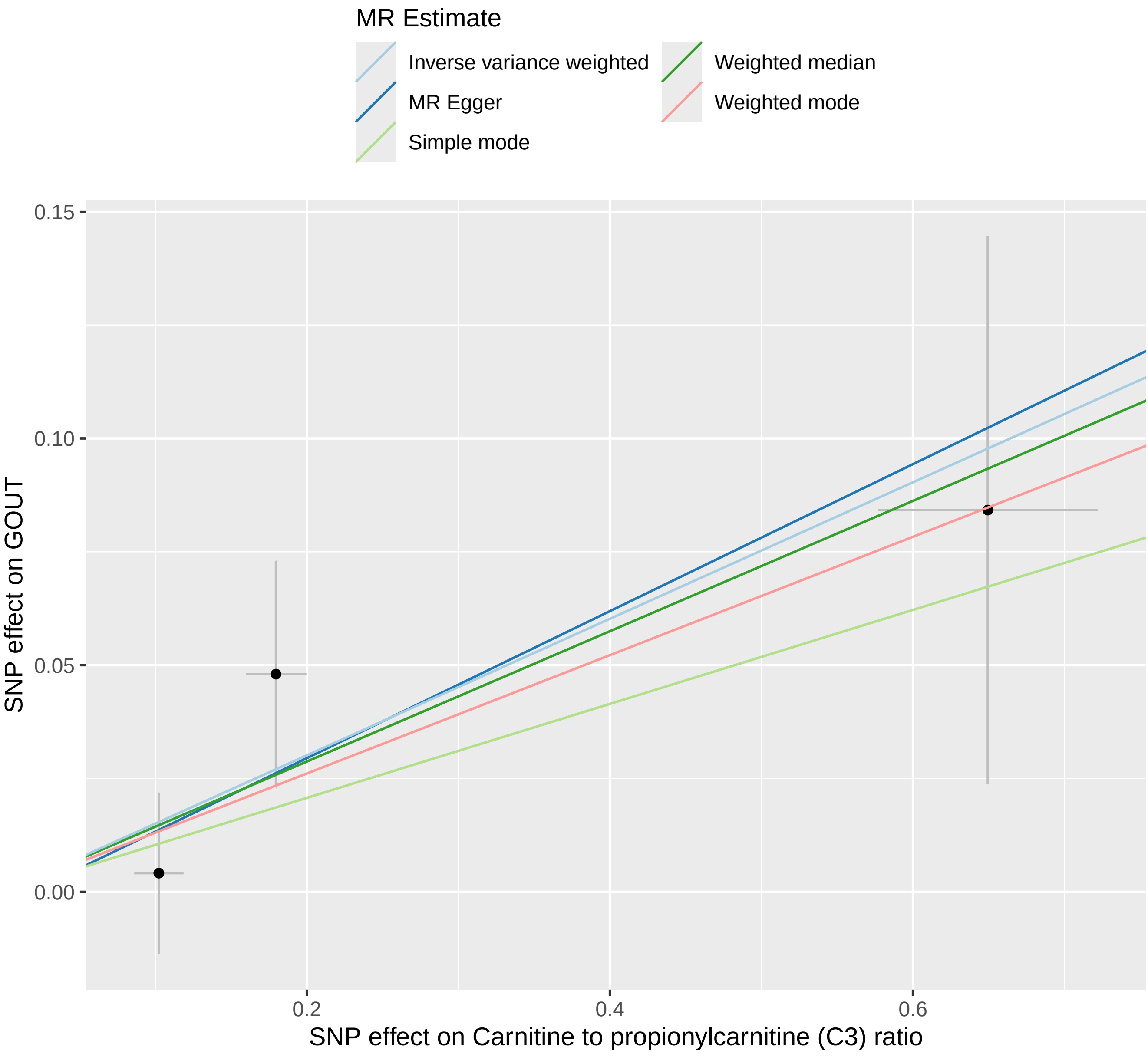
MR Estimate
Inverse variance weighted
MR Egger
Simple mode
Weighted median
Weighted mode
0.15
0.10
0.05
0.00
0.2
0.4
0.6
SNP effect on GOUT
SNP effect on Carnitine to propionylcarnitine (C3) ratio

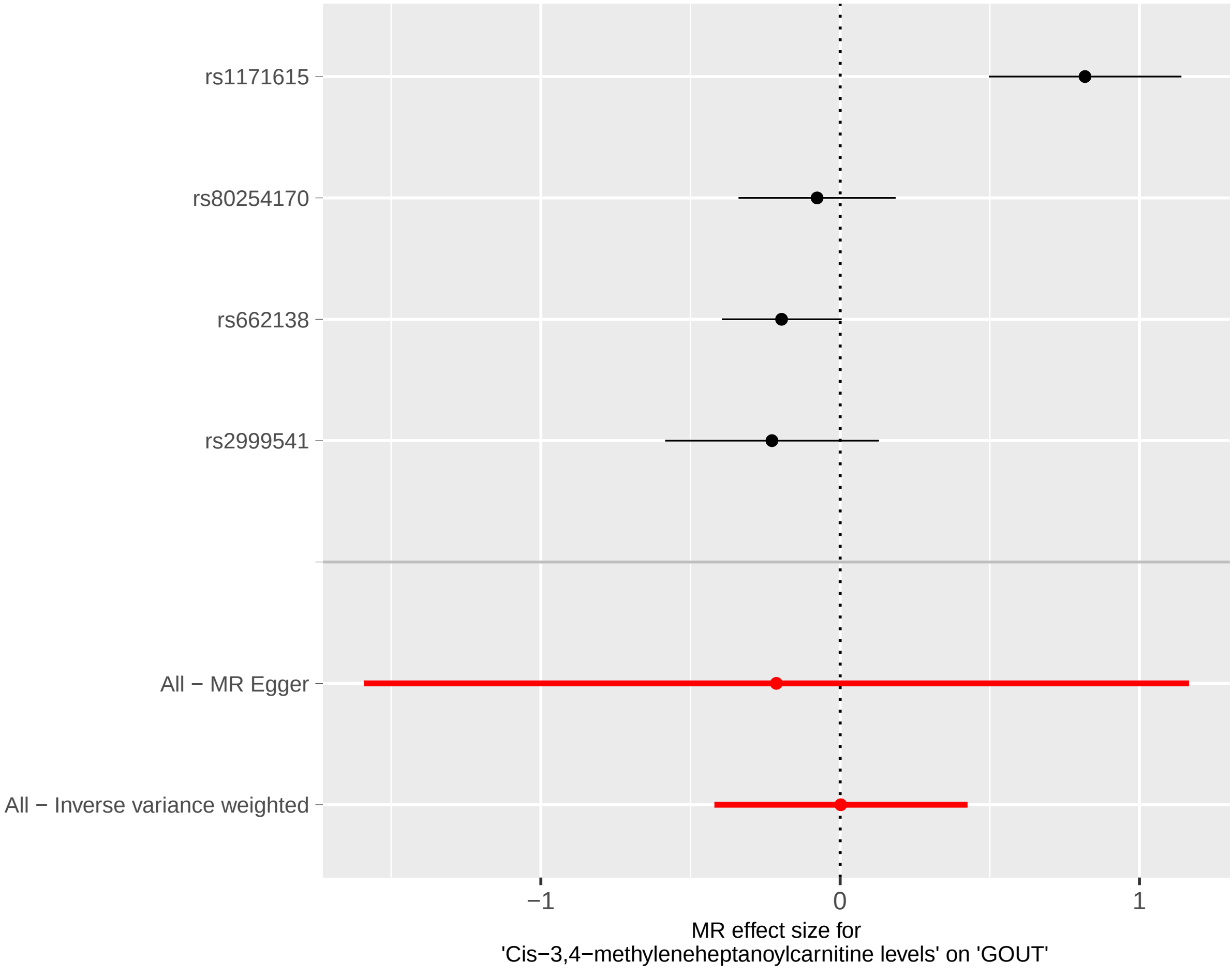

rs1171615
rs80254170
rs662138
rs2999541
All − MR Egger
All − Inverse variance weighted
−1
0
1
MR effect size for
'Cis−3,4−methyleneheptanoylcarnitine levels' on 'GOUT'

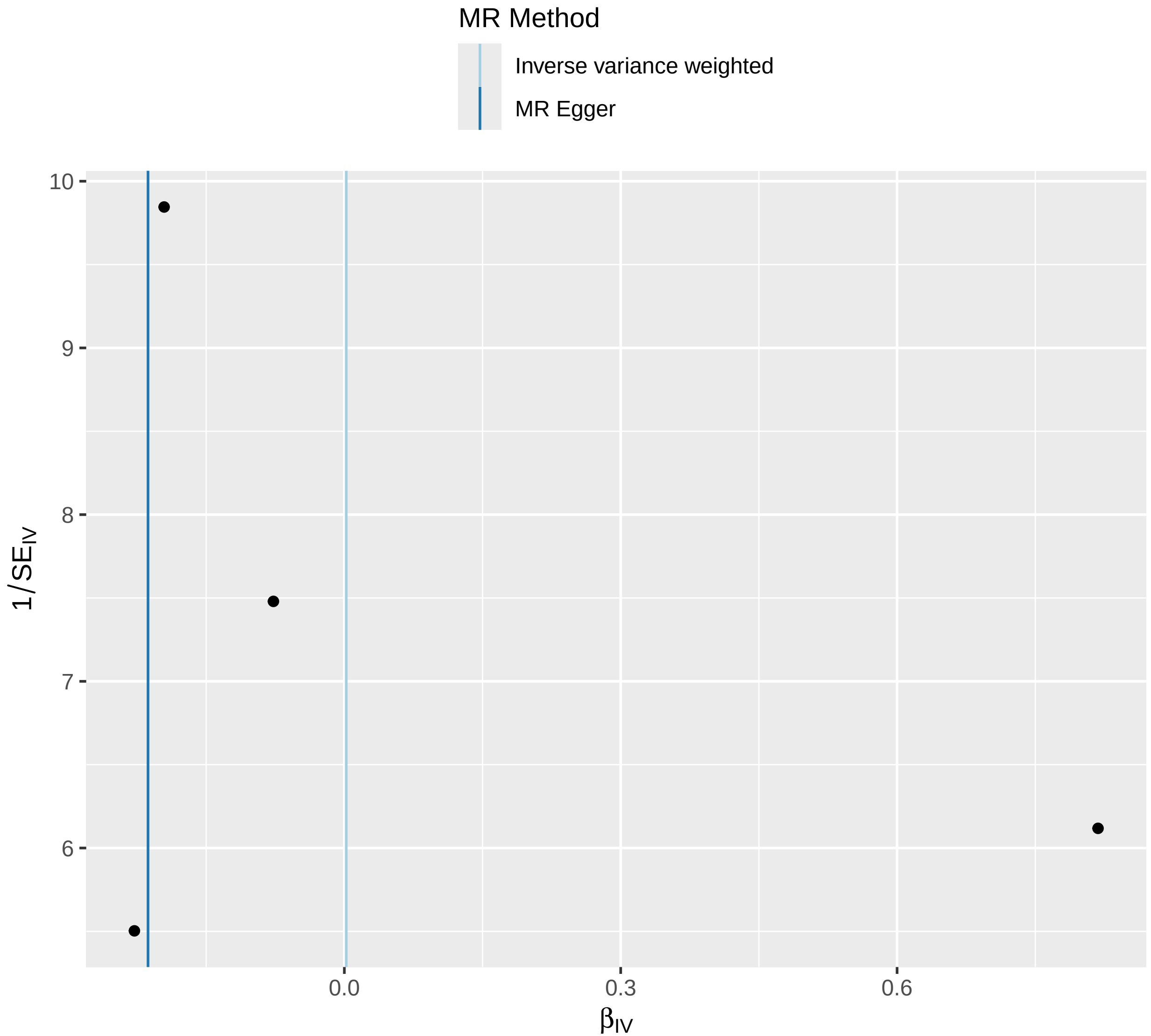

MR Method
Inverse variance weighted
MR Egger
10
9
8
7
6
$1/SE_{IV}$
0.0
0.3
0.6
β_{IV}

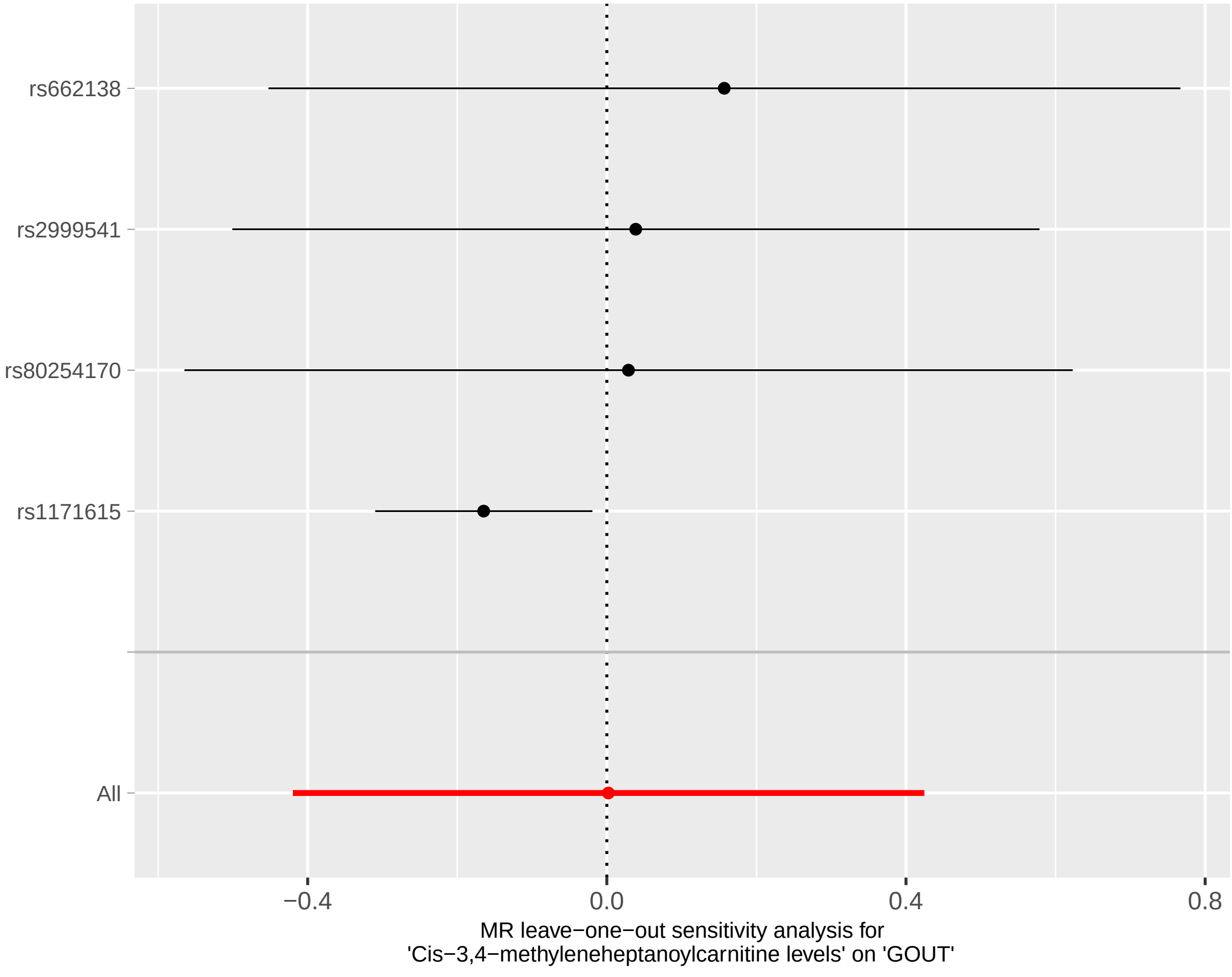
rs662138
rs2999541
rs80254170
rs1171615
All
−0.4
0.0
0.4
0.8
MR leave−one−out sensitivity analysis for
'Cis−3,4−methyleneheptanoylcarnitine levels' on 'GOUT'

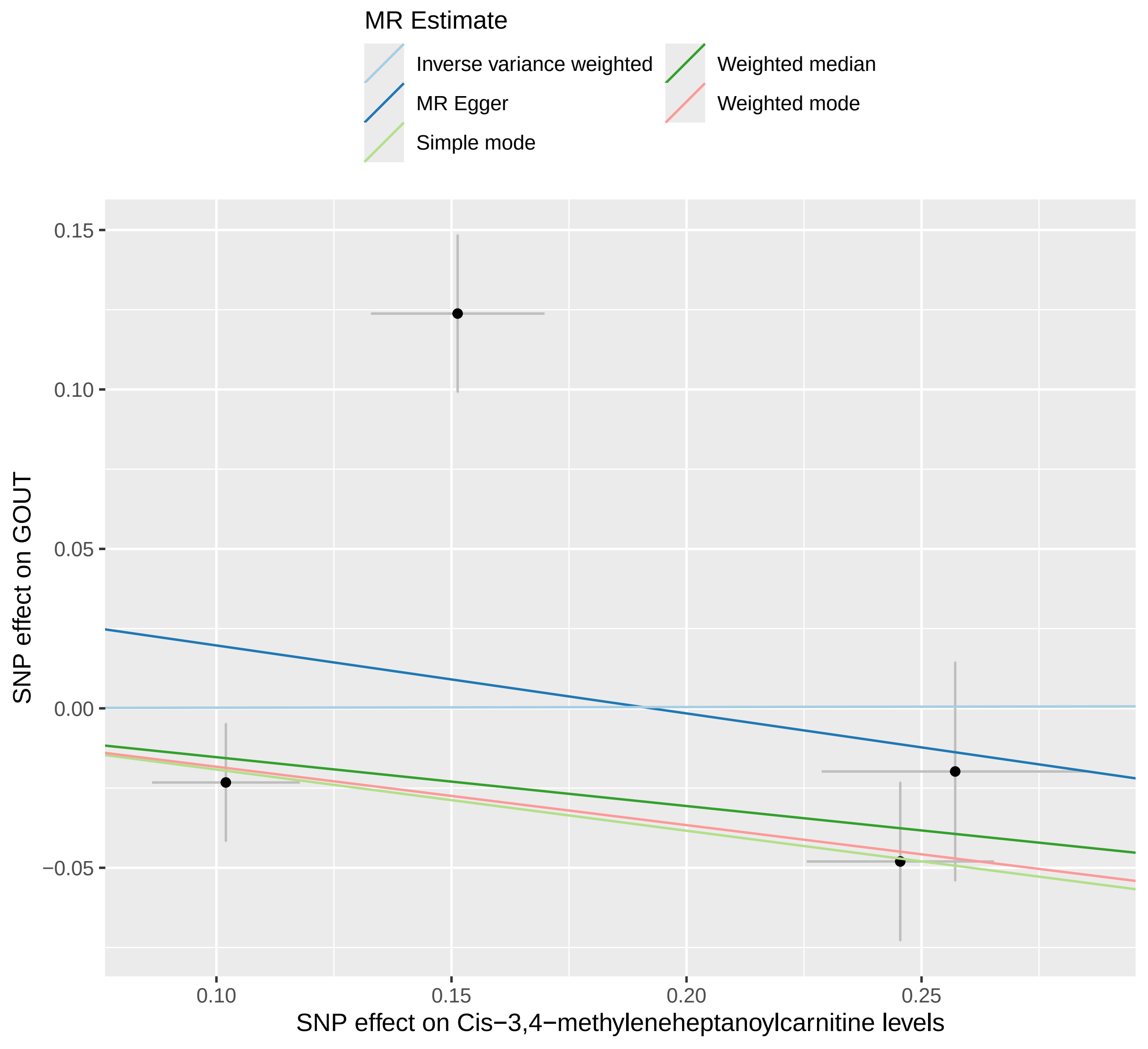
MR Estimate
Inverse variance weighted
MR Egger
Simple mode
Weighted median
Weighted mode
0.15
0.10
0.05
0.00
−0.05
SNP effect on GOUT
0.10
0.15
0.20
0.25
SNP effect on Cis−3,4−methyleneheptanoylcarnitine levels

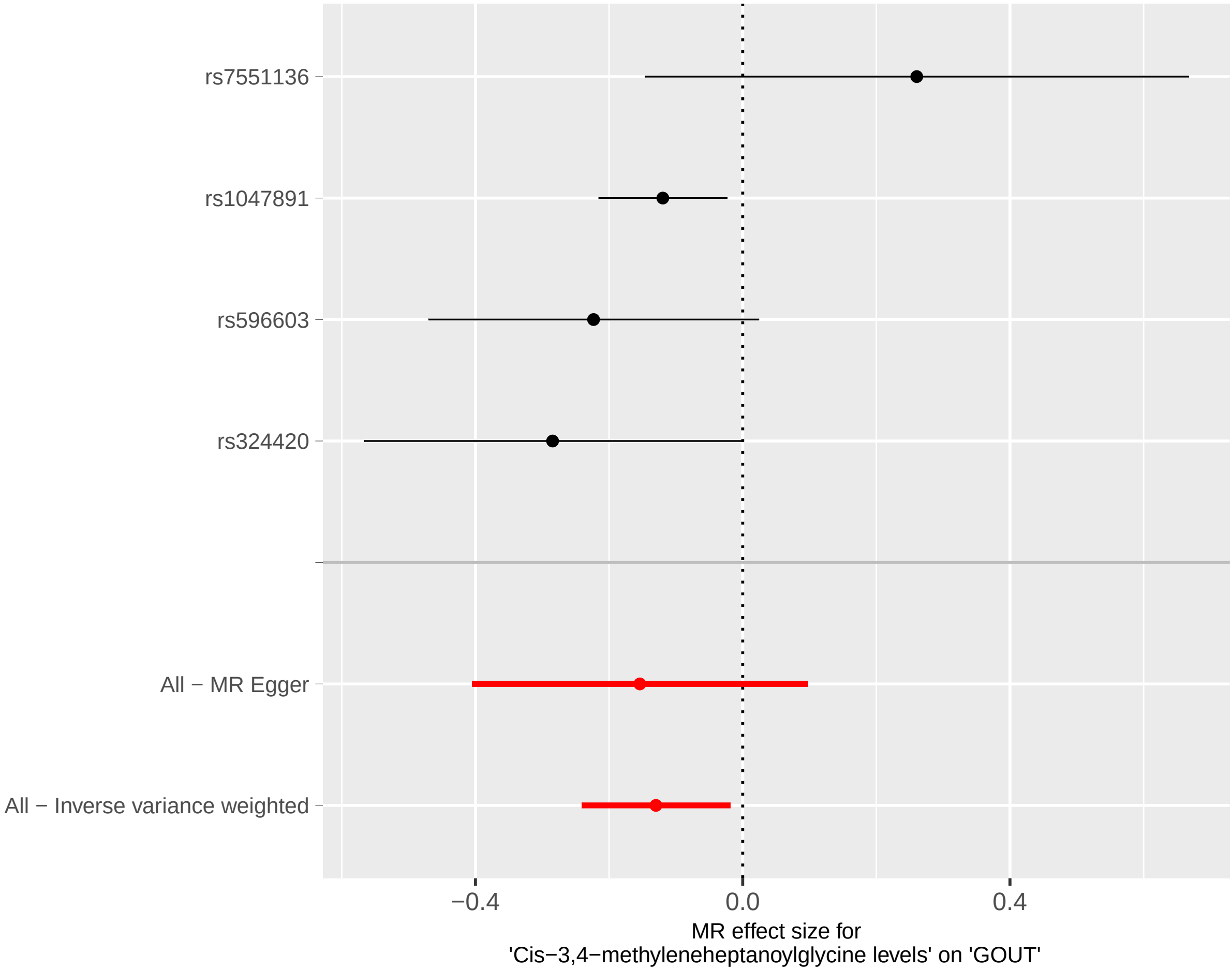
rs7551136
rs1047891
rs596603
rs324420
All − MR Egger
All − Inverse variance weighted
−0.4
0.0
0.4
MR effect size for
'Cis−3,4−methyleneheptanoylglycine levels' on 'GOUT'

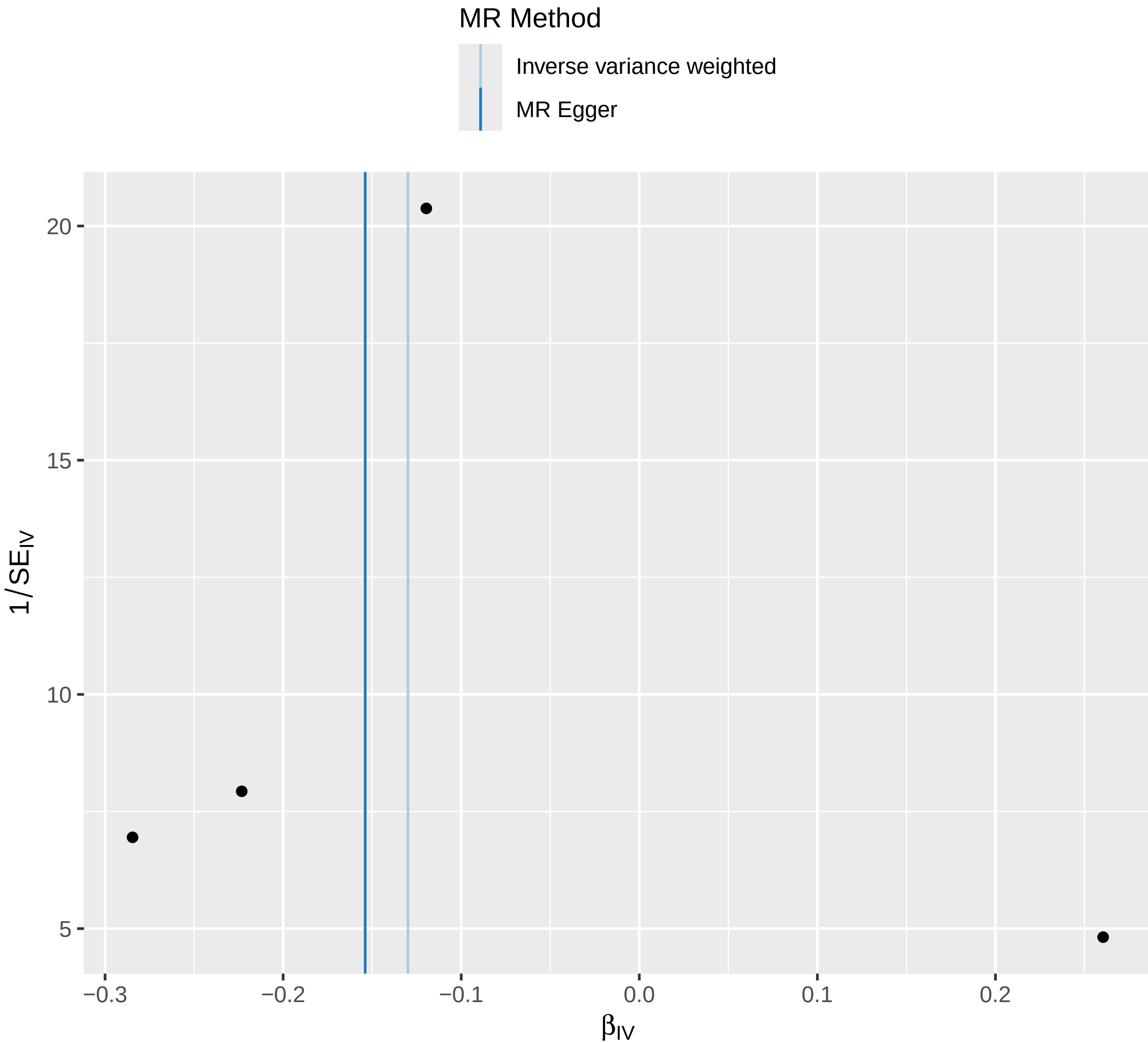
MR Method
Inverse variance weighted
MR Egger
20
15
10
5
1/SE_IV
−0.3
−0.2
−0.1
0.0
0.1
0.2
β_IV

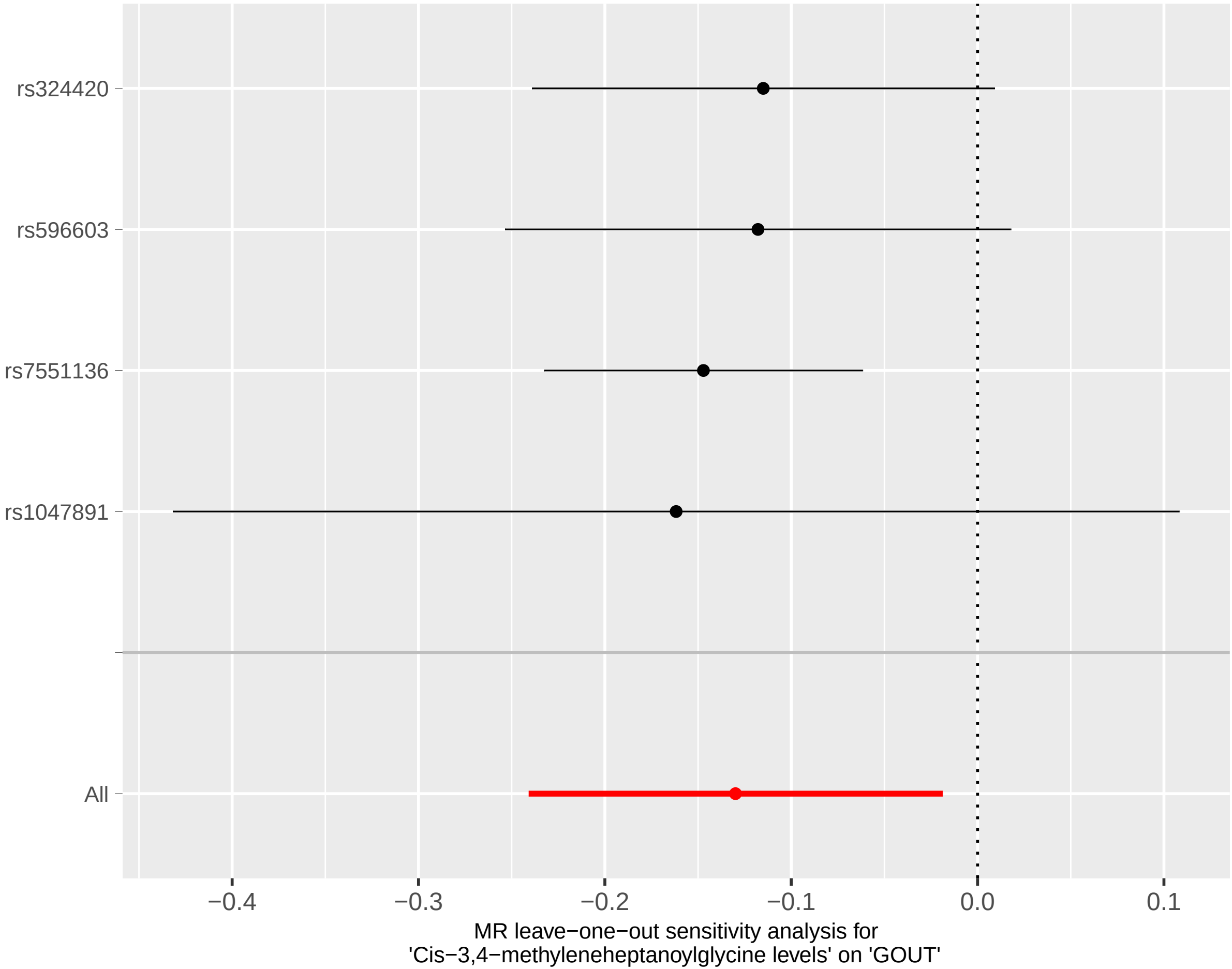

rs324420
rs596603
rs7551136
rs1047891
All
−0.4
−0.3
−0.2
−0.1
0.0
0.1
MR leave−one−out sensitivity analysis for
'Cis−3,4−methyleneheptanoylglycine levels' on 'GOUT'

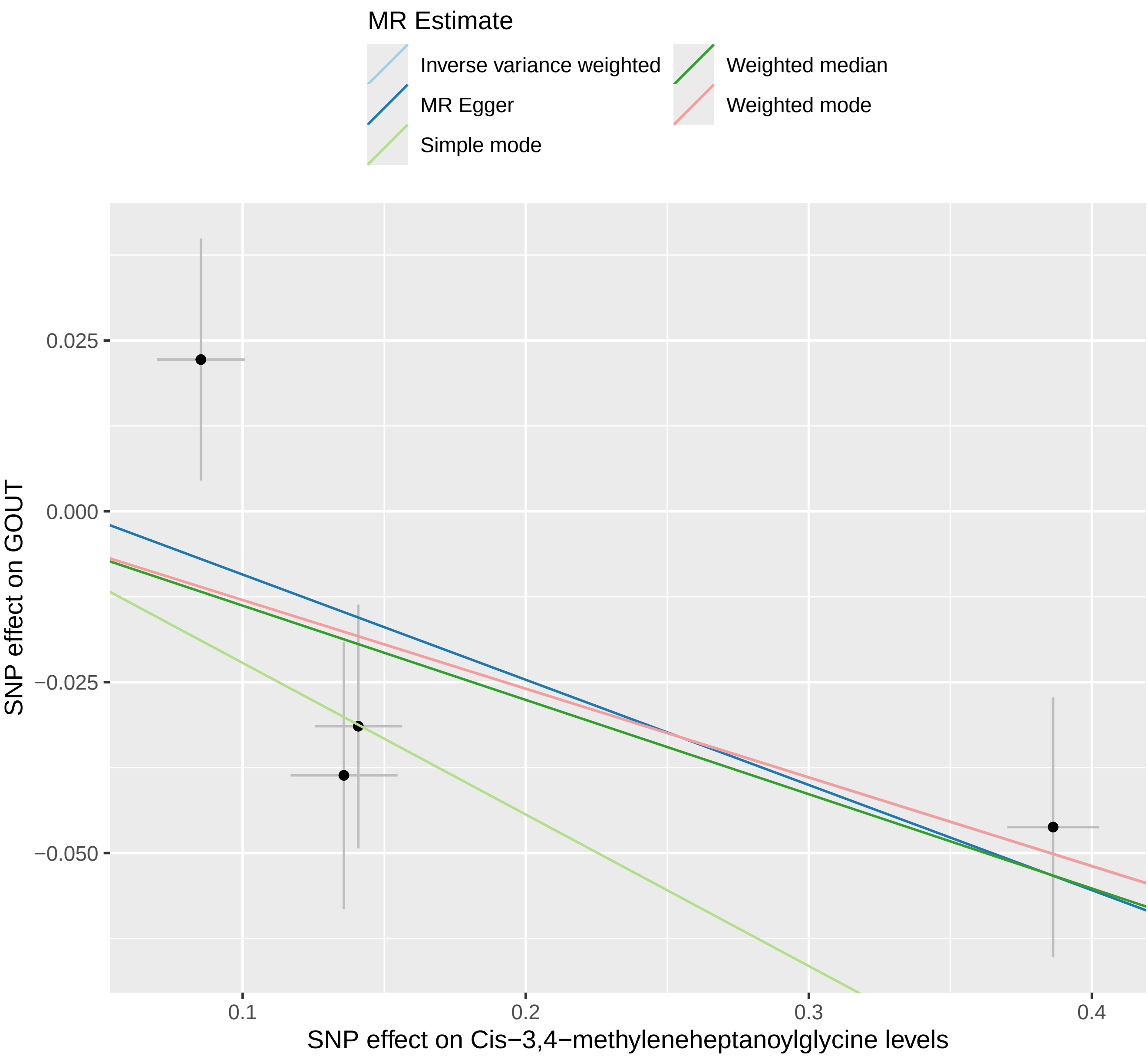
MR Estimate
Inverse variance weighted
MR Egger
Simple mode
Weighted median
Weighted mode
0.025
0.000
−0.025
−0.050
0.1
0.2
0.3
0.4
SNP effect on GOUT
SNP effect on Cis−3,4−methyleneheptanoylglycine levels

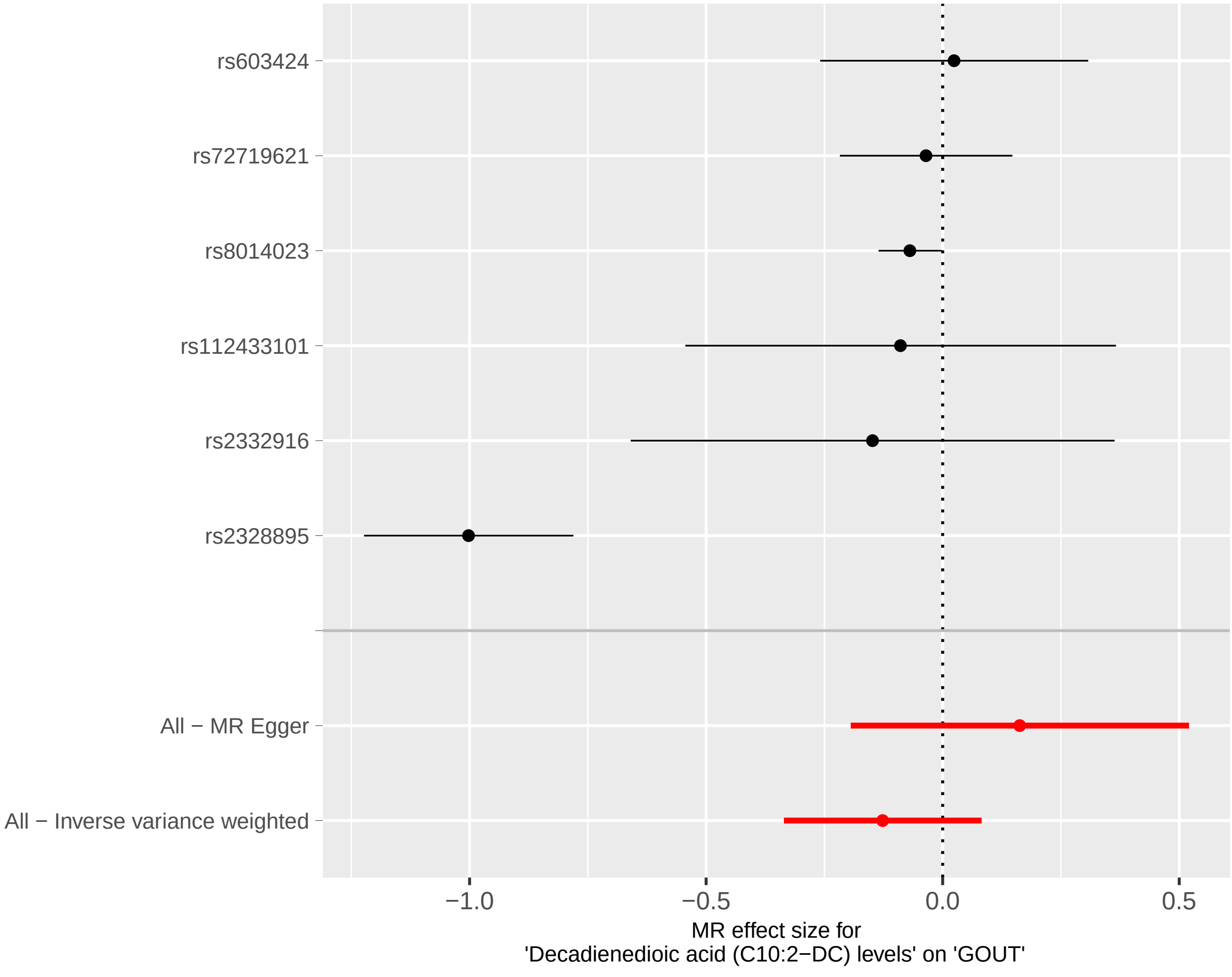
rs603424
rs72719621
rs8014023
rs112433101
rs2332916
rs2328895
All − MR Egger
All − Inverse variance weighted
−1.0
−0.5
0.0
0.5
MR effect size for
'Decadienedioic acid (C10:2−DC) levels' on 'GOUT'

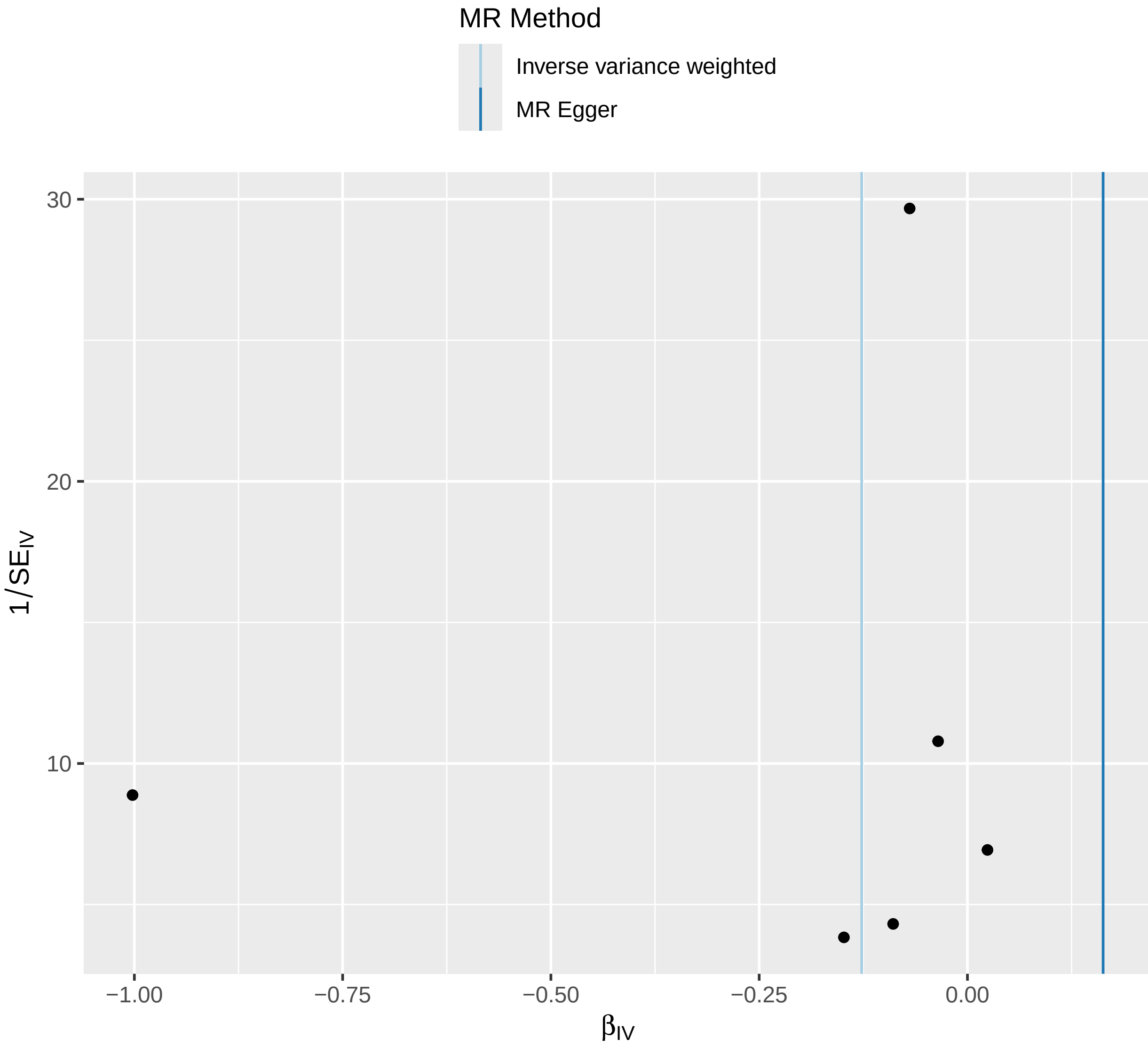
MR Method
Inverse variance weighted
MR Egger
30
20
10
1/SE_IV
−1.00
−0.75
−0.50
−0.25
0.00
β_IV

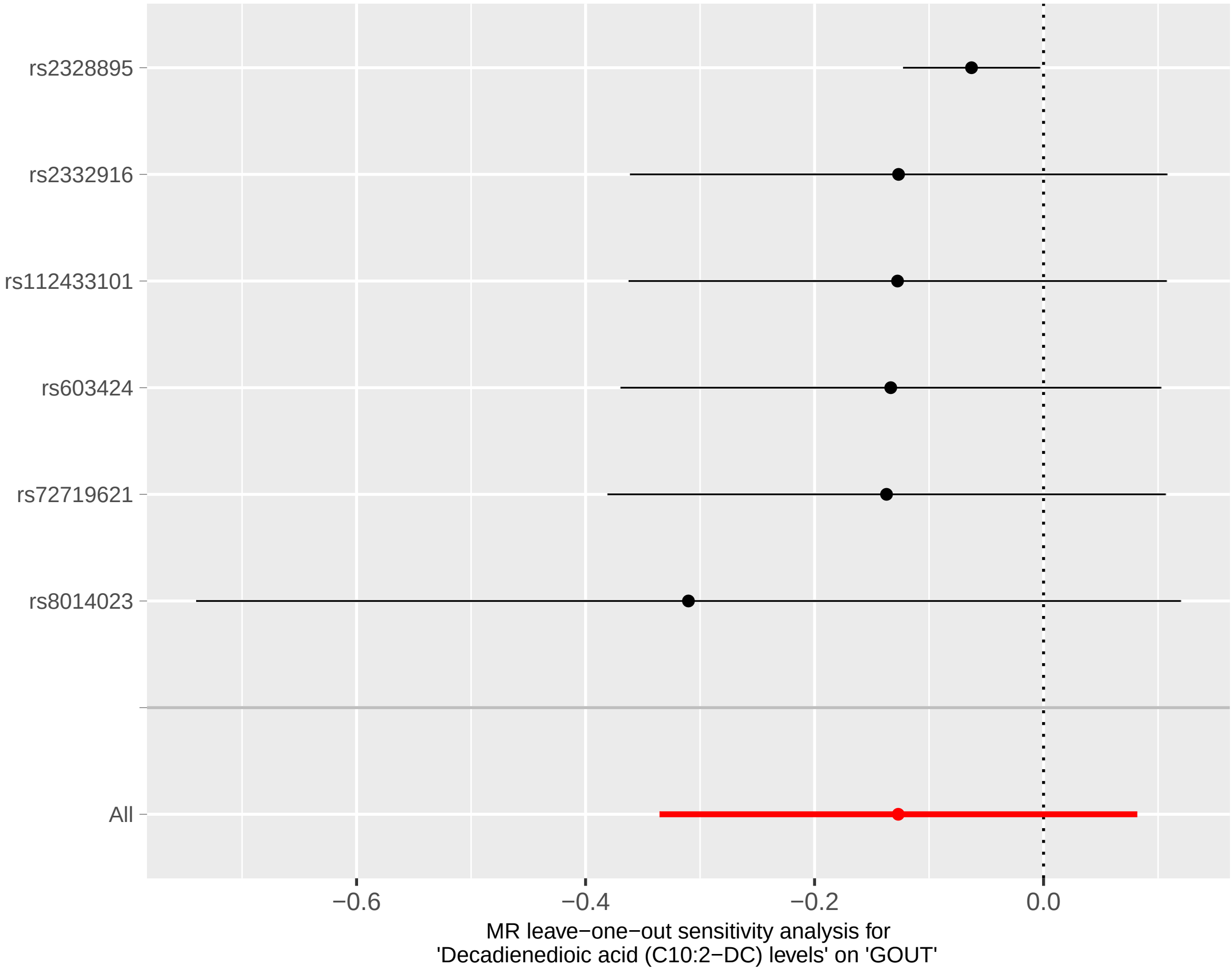
rs2328895
rs2332916
rs112433101
rs603424
rs72719621
rs8014023
All
−0.6
−0.4
−0.2
0.0
MR leave−one−out sensitivity analysis for
'Decadienedioic acid (C10:2−DC) levels' on 'GOUT'

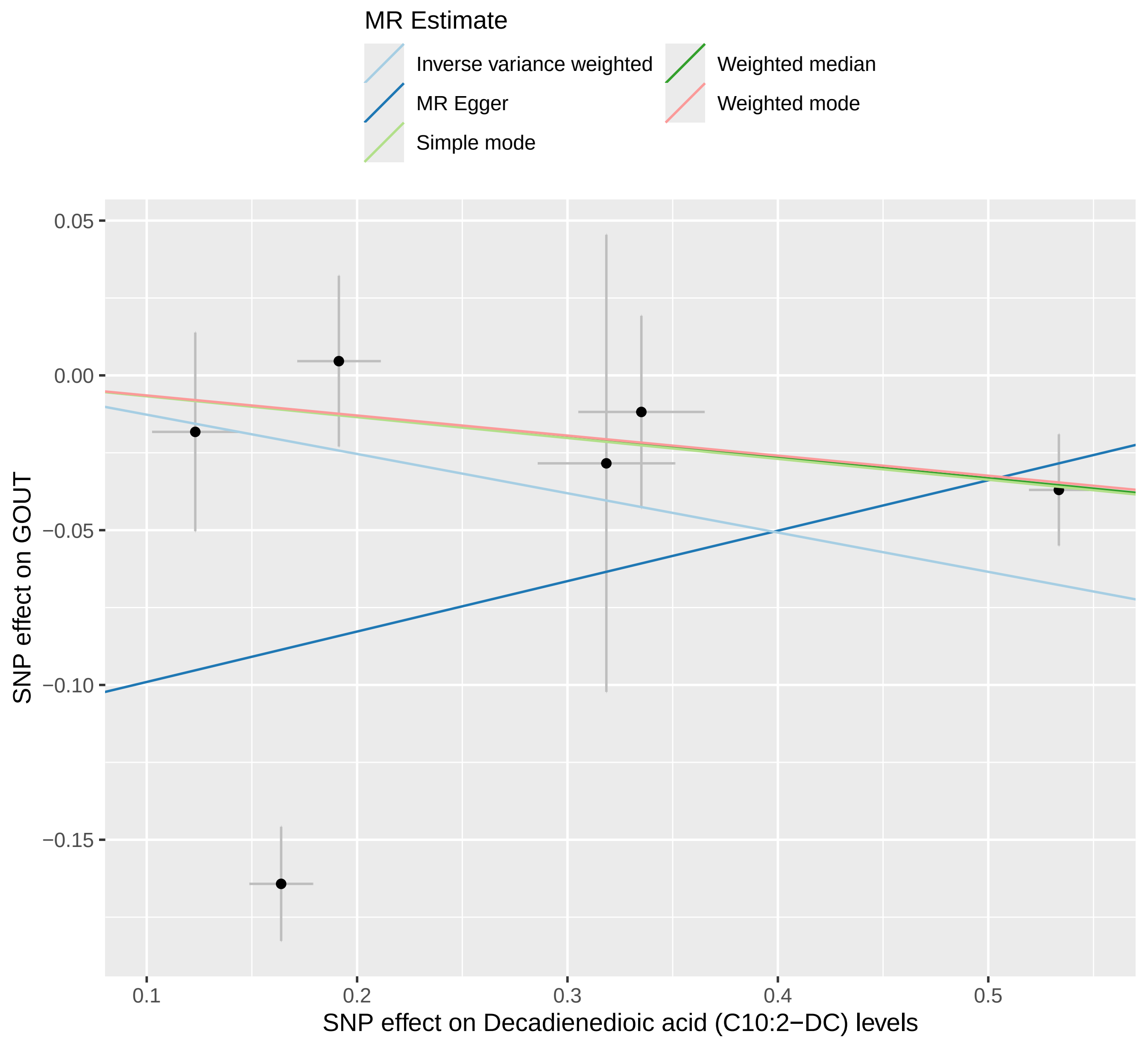
MR Estimate
Inverse variance weighted
MR Egger
Simple mode
Weighted median
Weighted mode
0.05
0.00
−0.05
−0.10
−0.15
SNP effect on GOUT
0.1
0.2
0.3
0.4
0.5
SNP effect on Decadienedioic acid (C10:2−DC) levels

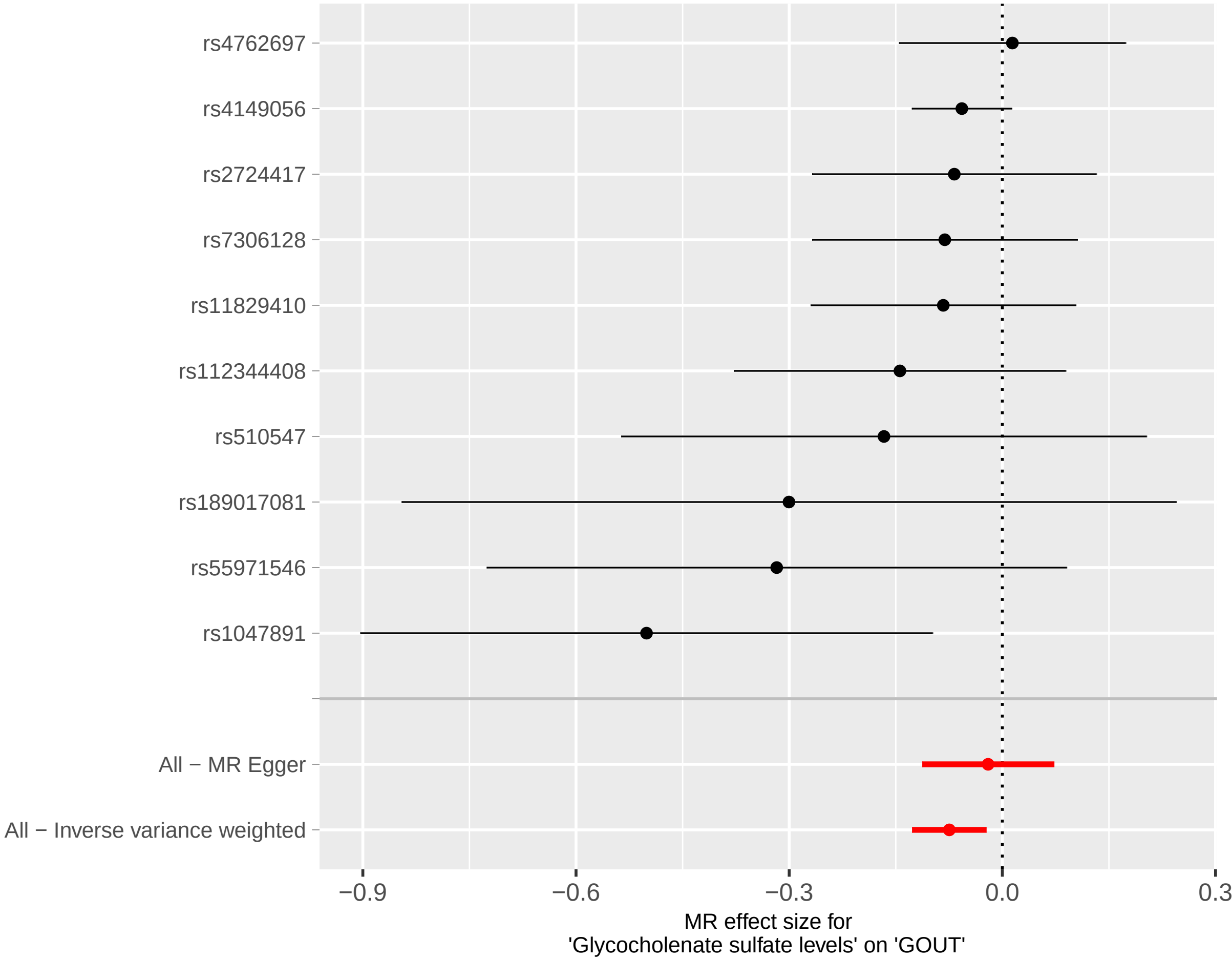
rs4762697
rs4149056
rs2724417
rs7306128
rs11829410
rs112344408
rs510547
rs189017081
rs55971546
rs1047891
All − MR Egger
All − Inverse variance weighted
−0.9
−0.6
−0.3
0.0
0.3
MR effect size for
'Glycocholenate sulfate levels' on 'GOUT'

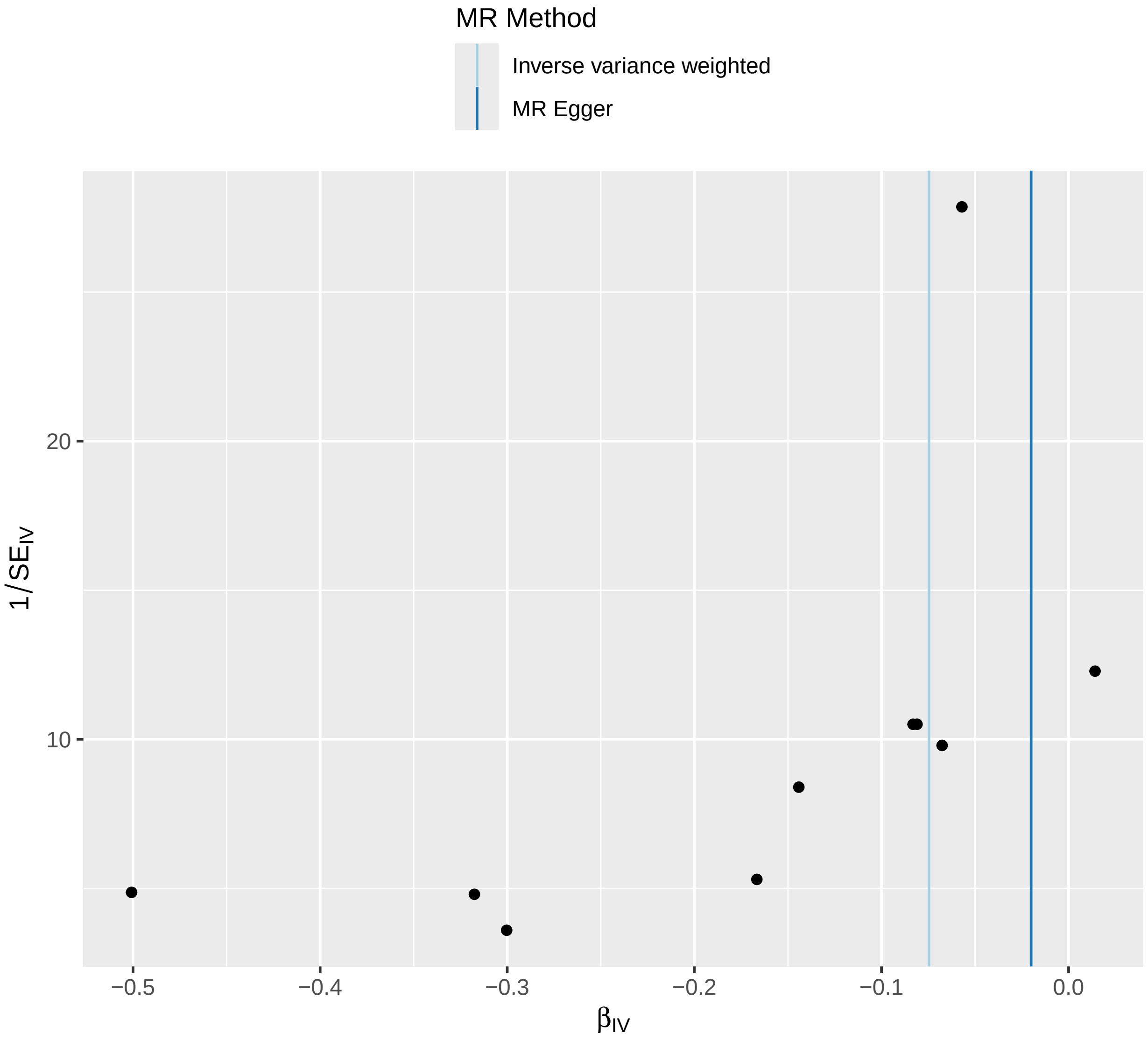
MR Method
Inverse variance weighted
MR Egger
20
10
1/SE_IV
−0.5
−0.4
−0.3
−0.2
−0.1
0.0
β_IV

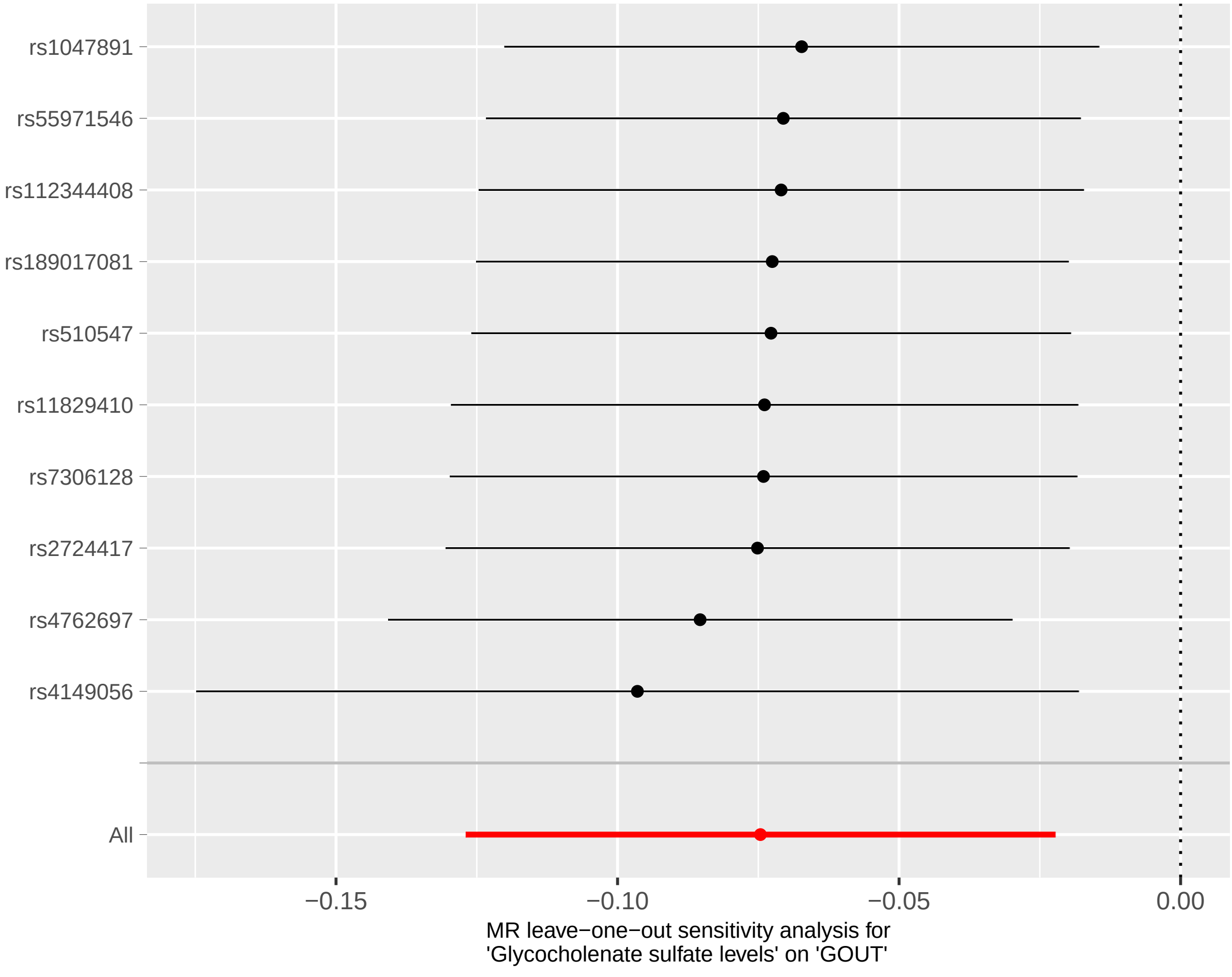
rs1047891
rs55971546
rs112344408
rs189017081
rs510547
rs11829410
rs7306128
rs2724417
rs4762697
rs4149056
All
−0.15
−0.10
−0.05
0.00
MR leave−one−out sensitivity analysis for
'Glycocholenate sulfate levels' on 'GOUT'

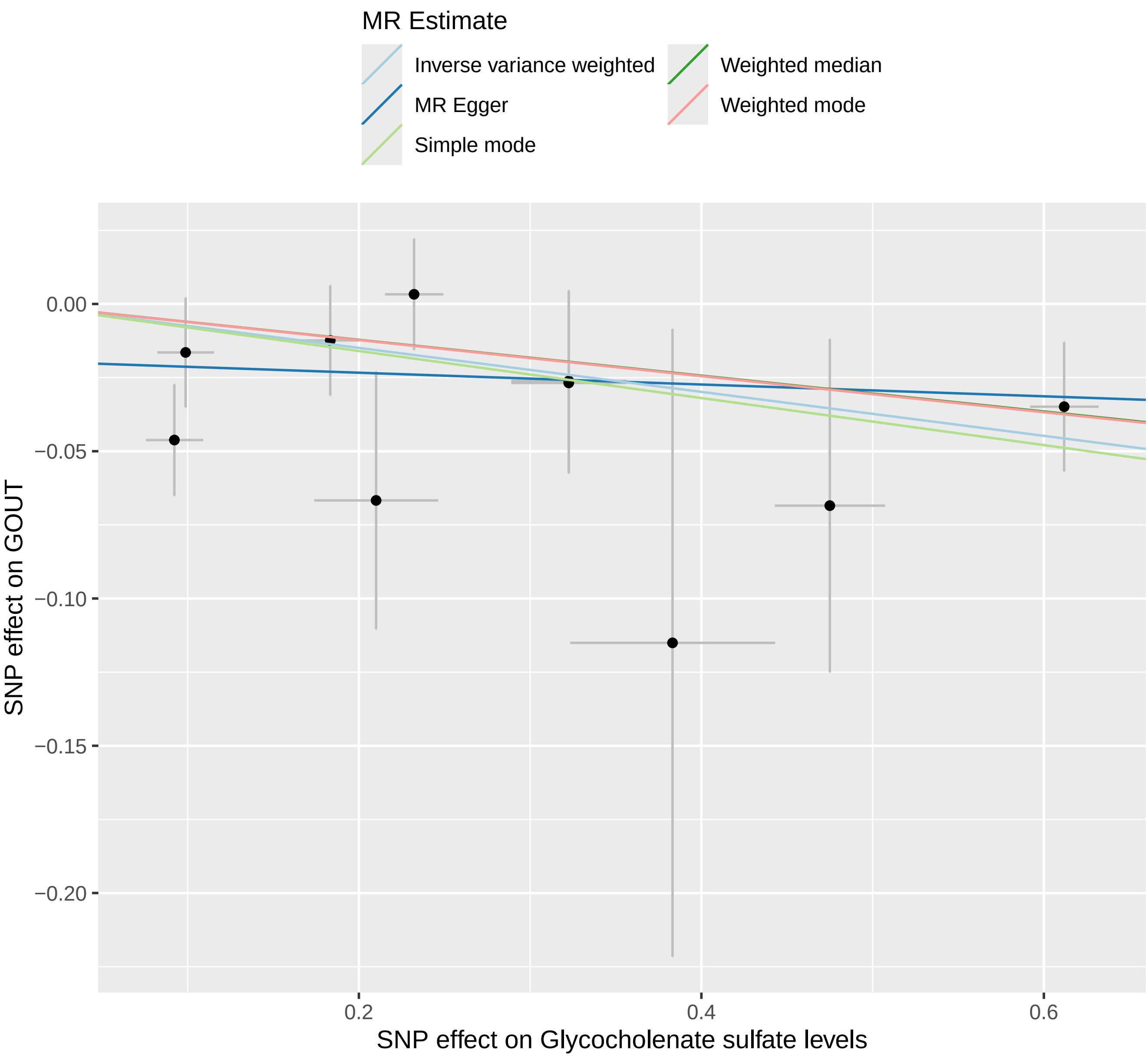

MR Estimate
Inverse variance weighted
MR Egger
Simple mode
Weighted median
Weighted mode
0.00
−0.05
−0.10
−0.15
−0.20
SNP effect on GOUT
0.2
0.4
0.6
SNP effect on Glycocholenate sulfate levels

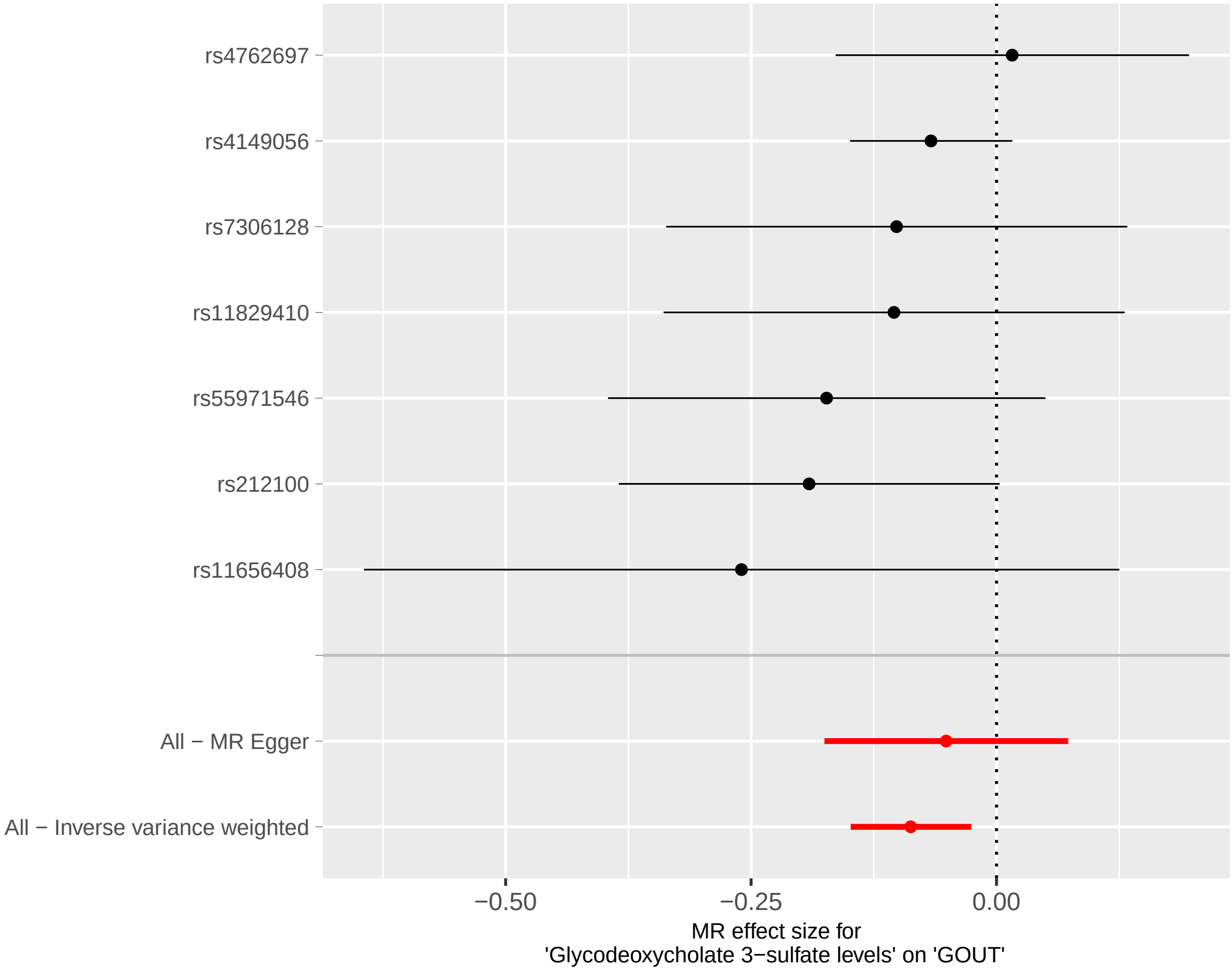
rs4762697
rs4149056
rs7306128
rs11829410
rs55971546
rs212100
rs11656408
All − MR Egger
All − Inverse variance weighted
−0.50
−0.25
0.00
MR effect size for
'Glycodeoxycholate 3−sulfate levels' on 'GOUT'

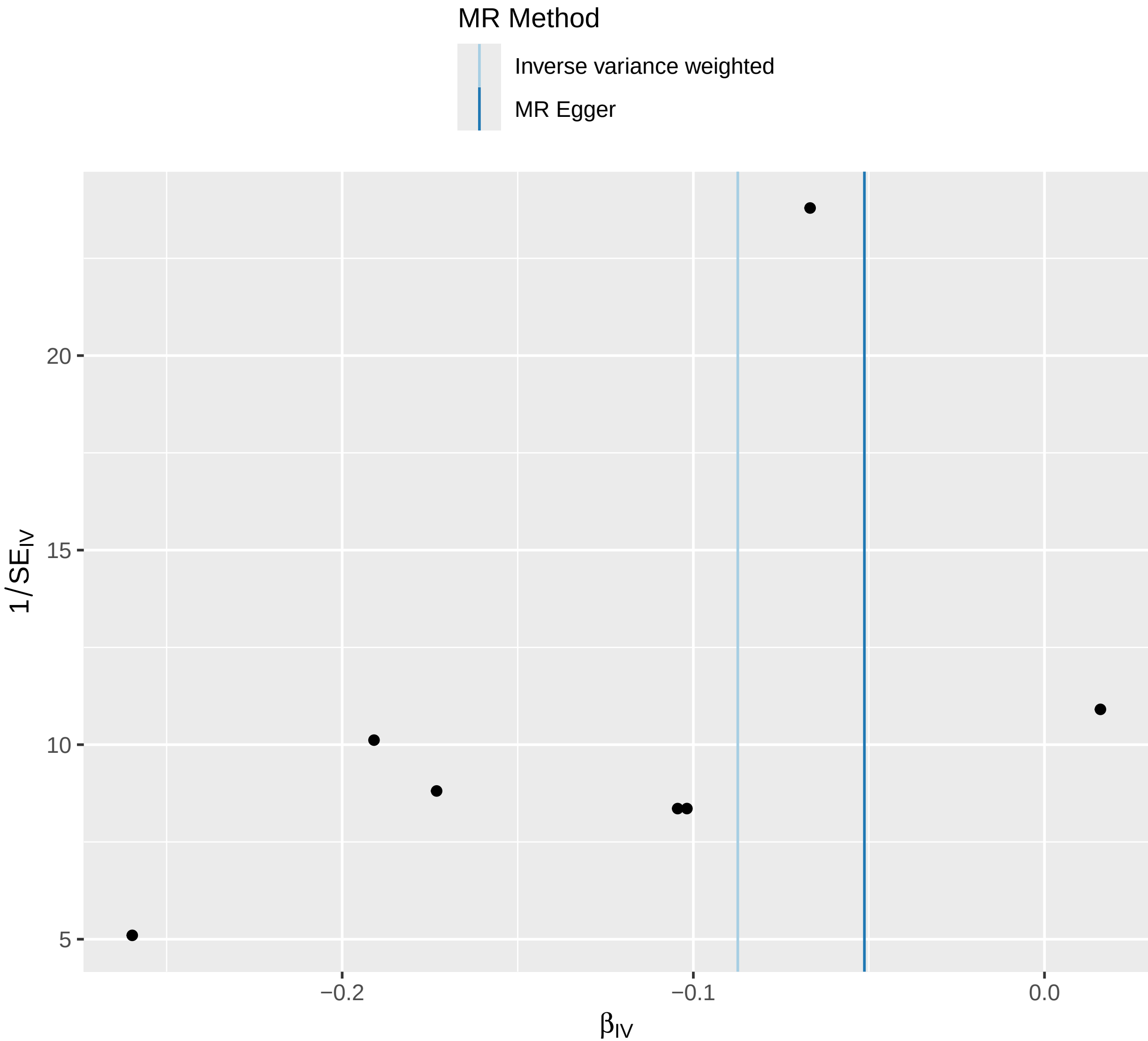

MR Method
Inverse variance weighted
MR Egger
20
15
10
5
1/SE_IV
−0.2
−0.1
0.0
β_IV

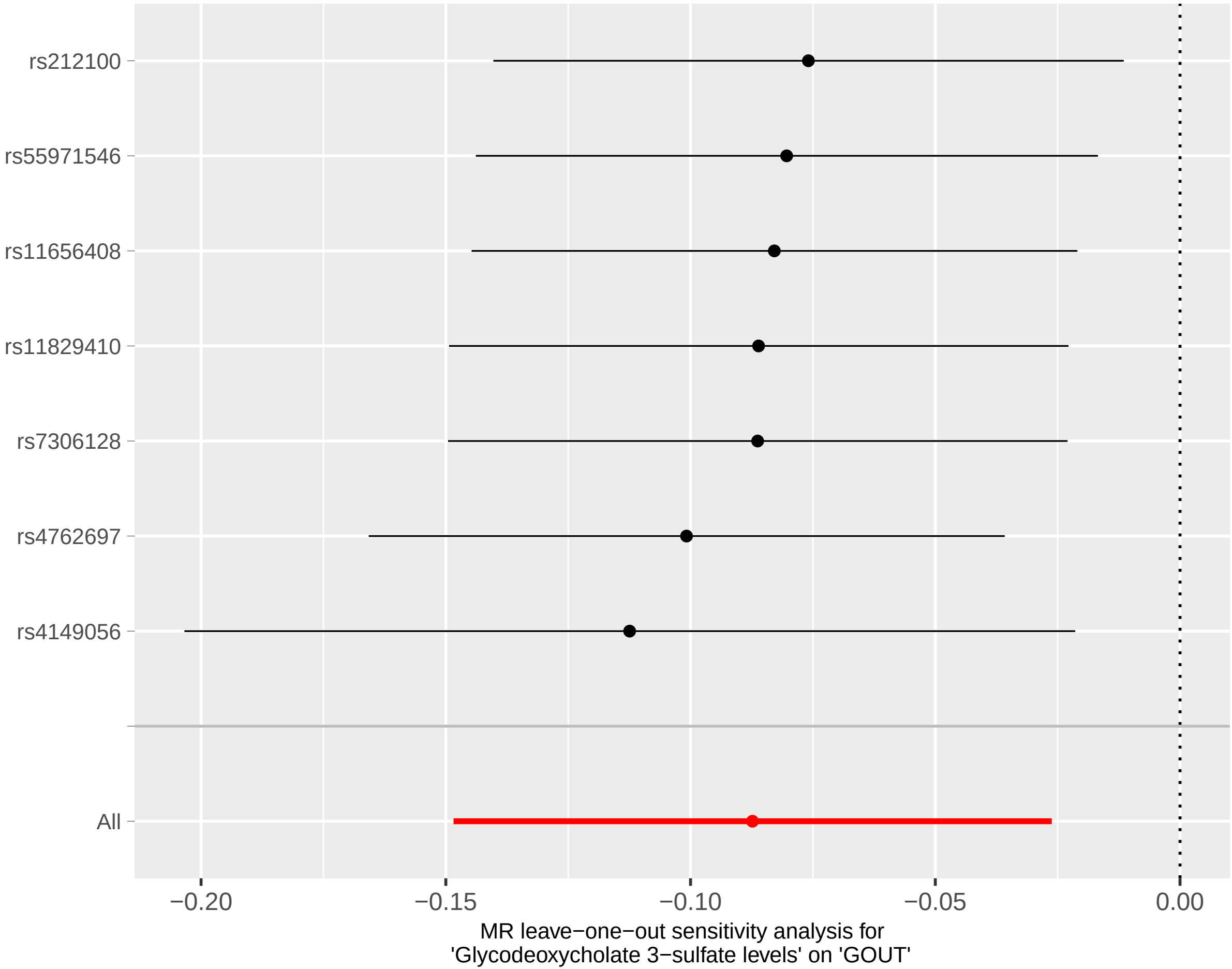
rs212100
rs55971546
rs11656408
rs11829410
rs7306128
rs4762697
rs4149056
All
−0.20
−0.15
−0.10
−0.05
0.00
MR leave−one−out sensitivity analysis for
'Glycodeoxycholate 3−sulfate levels' on 'GOUT'

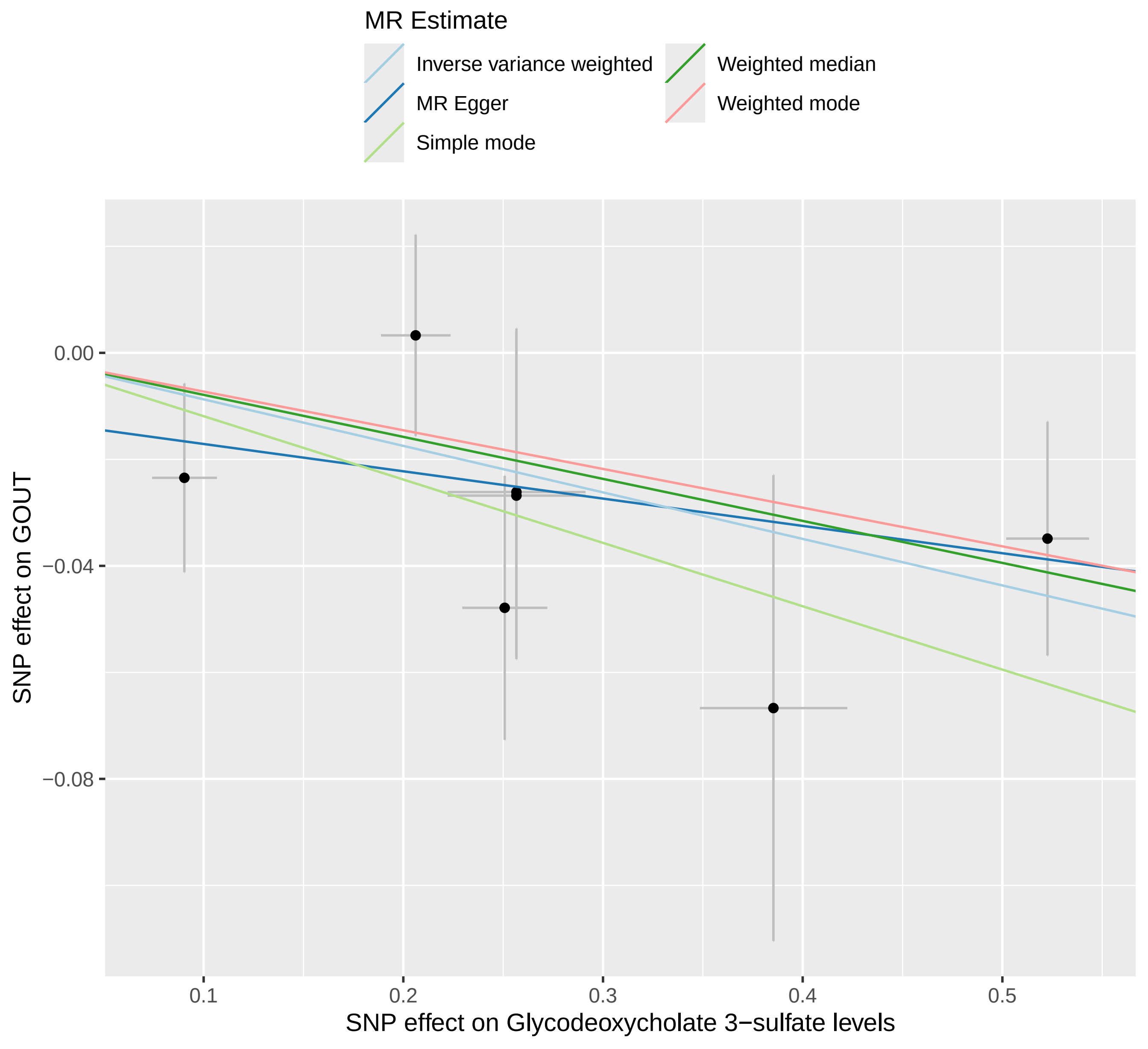
MR Estimate
Inverse variance weighted
MR Egger
Simple mode
Weighted median
Weighted mode
0.00
−0.04
−0.08
SNP effect on GOUT
0.1
0.2
0.3
0.4
0.5
SNP effect on Glycodeoxycholate 3−sulfate levels

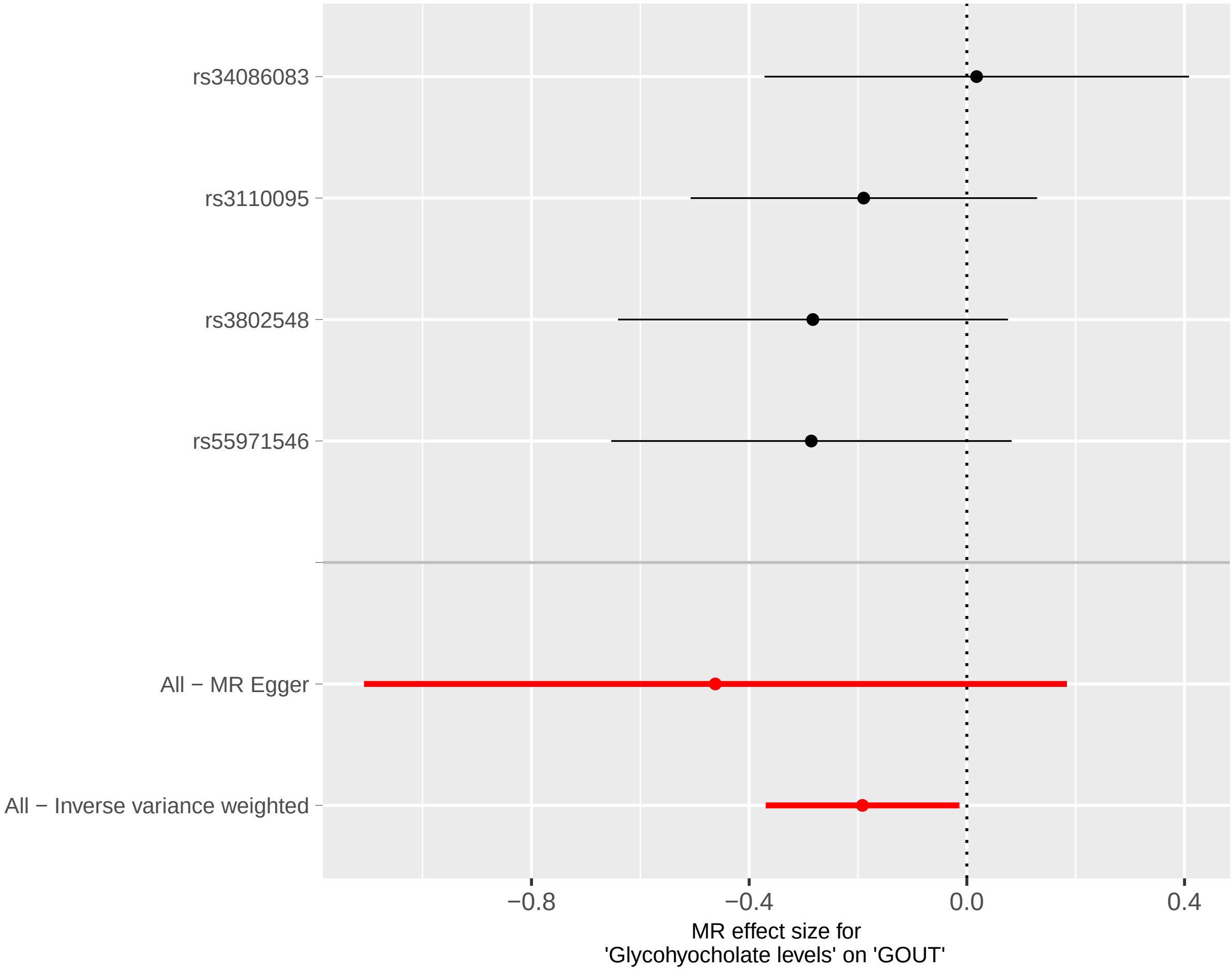
rs34086083
rs3110095
rs3802548
rs55971546
All − MR Egger
All − Inverse variance weighted
−0.8
−0.4
0.0
0.4
MR effect size for
'Glycohyocholate levels' on 'GOUT'

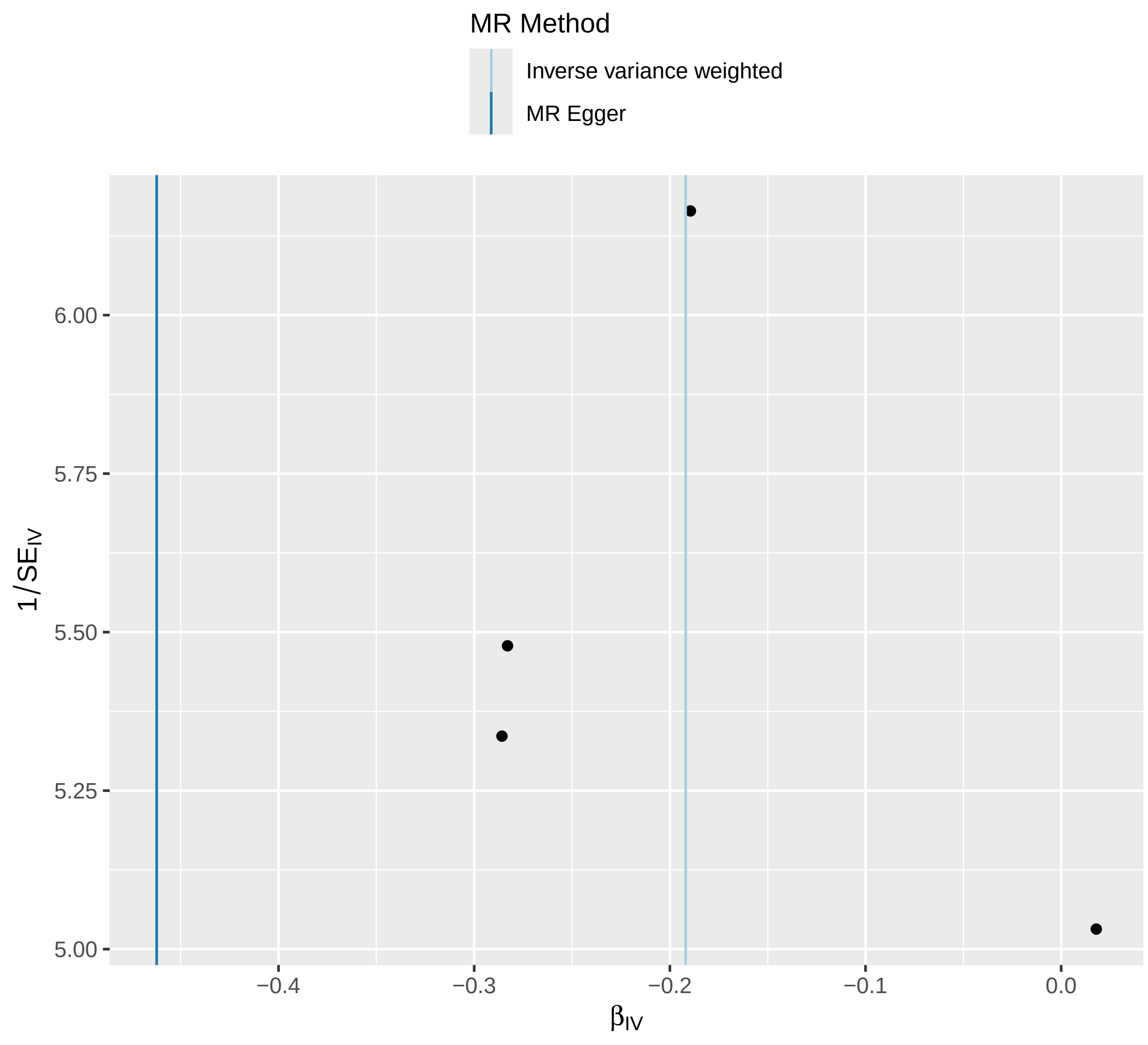

MR Method
Inverse variance weighted
MR Egger
6.00
5.75
5.50
5.25
5.00
1/SE$_{IV}$
−0.4
−0.3
−0.2
−0.1
0.0
β$_{IV}$

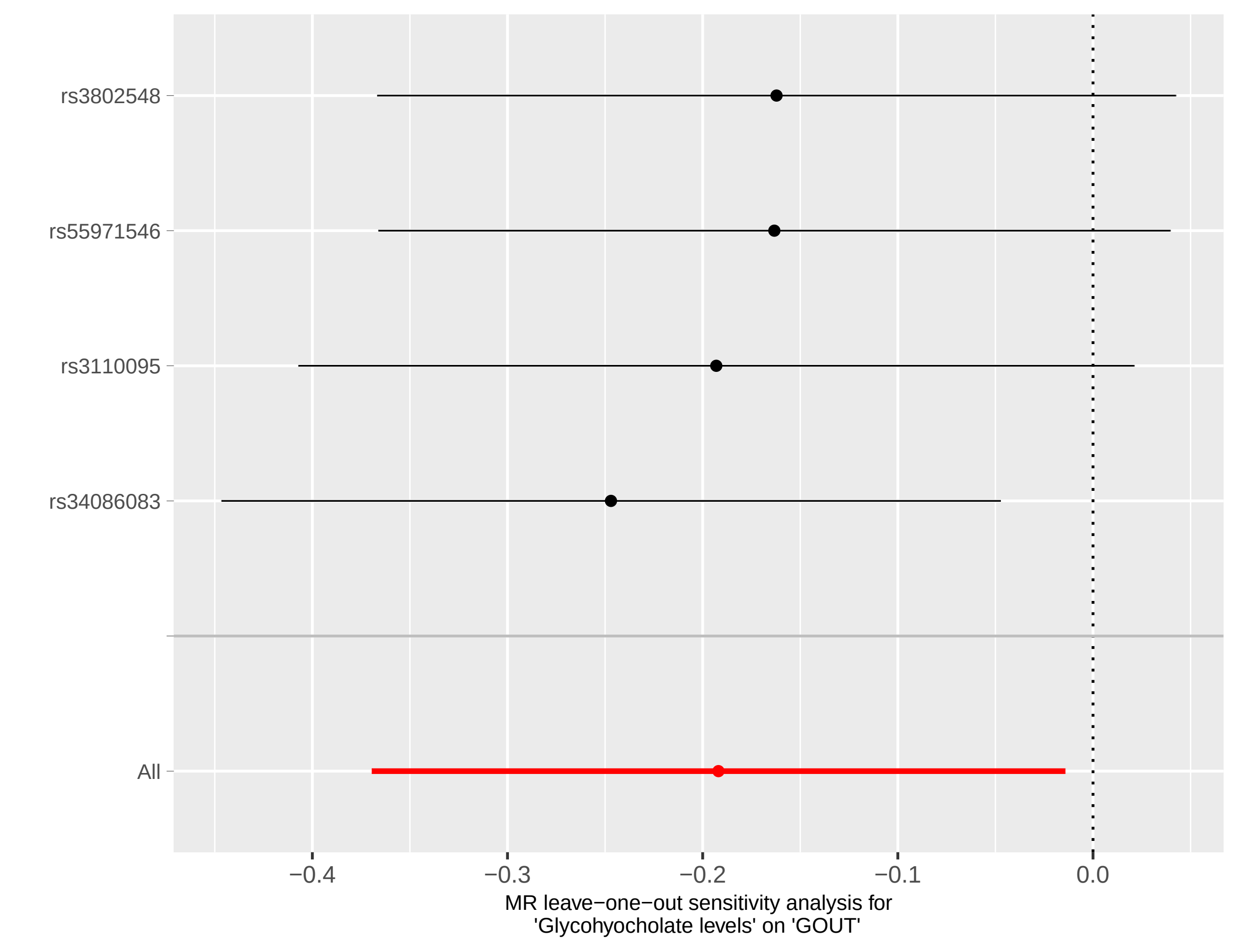
rs3802548
rs55971546
rs3110095
rs34086083
All
−0.4
−0.3
−0.2
−0.1
0.0
MR leave−one−out sensitivity analysis for
'Glycohyocholate levels' on 'GOUT'

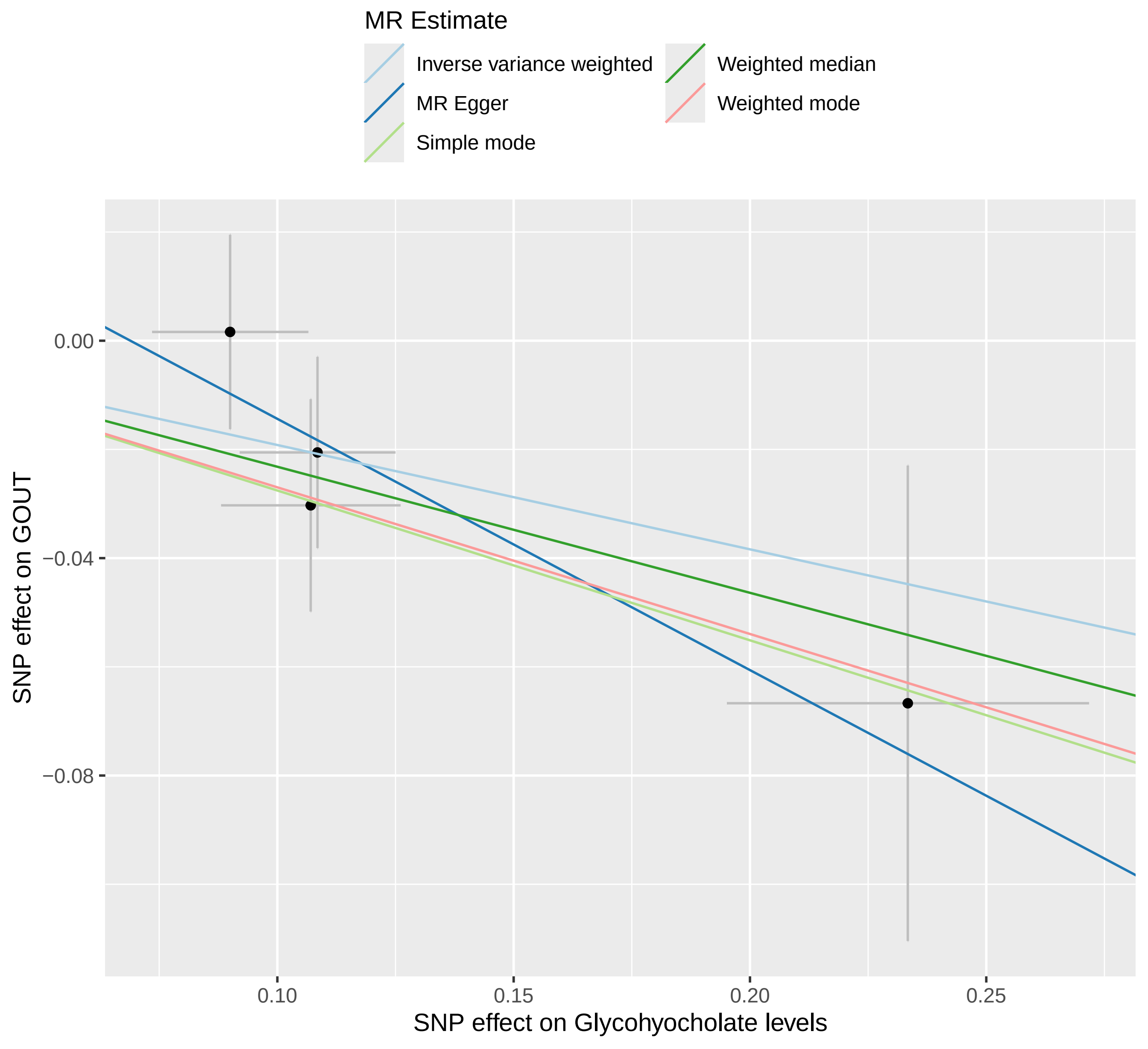
MR Estimate
Inverse variance weighted
MR Egger
Simple mode
Weighted median
Weighted mode
0.00
−0.04
−0.08
SNP effect on GOUT
0.10
0.15
0.20
0.25
SNP effect on Glycohyocholate levels

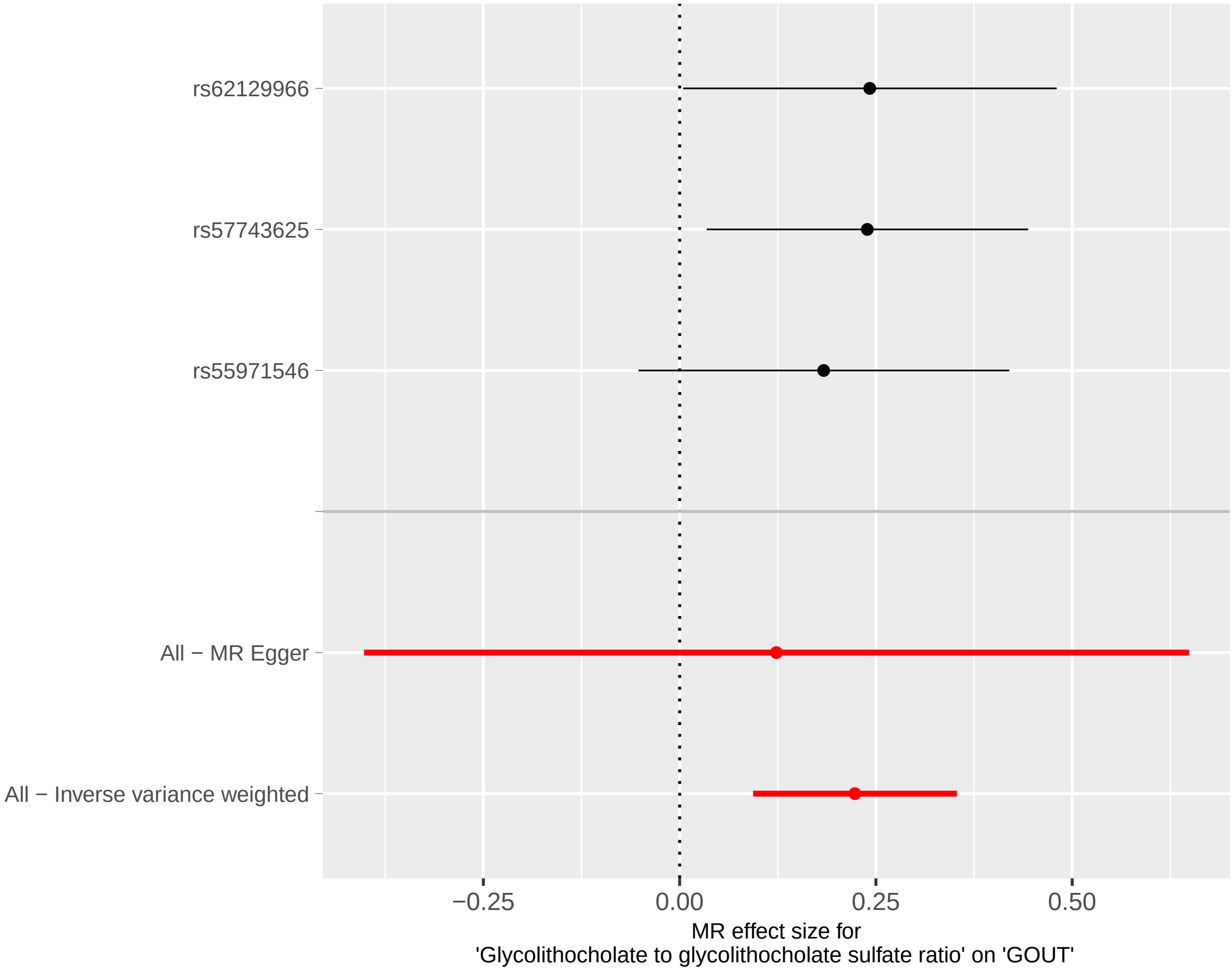
rs62129966
rs57743625
rs55971546
All − MR Egger
All − Inverse variance weighted
−0.25
0.00
0.25
0.50
MR effect size for
'Glycolithocholate to glycolithocholate sulfate ratio' on 'GOUT'

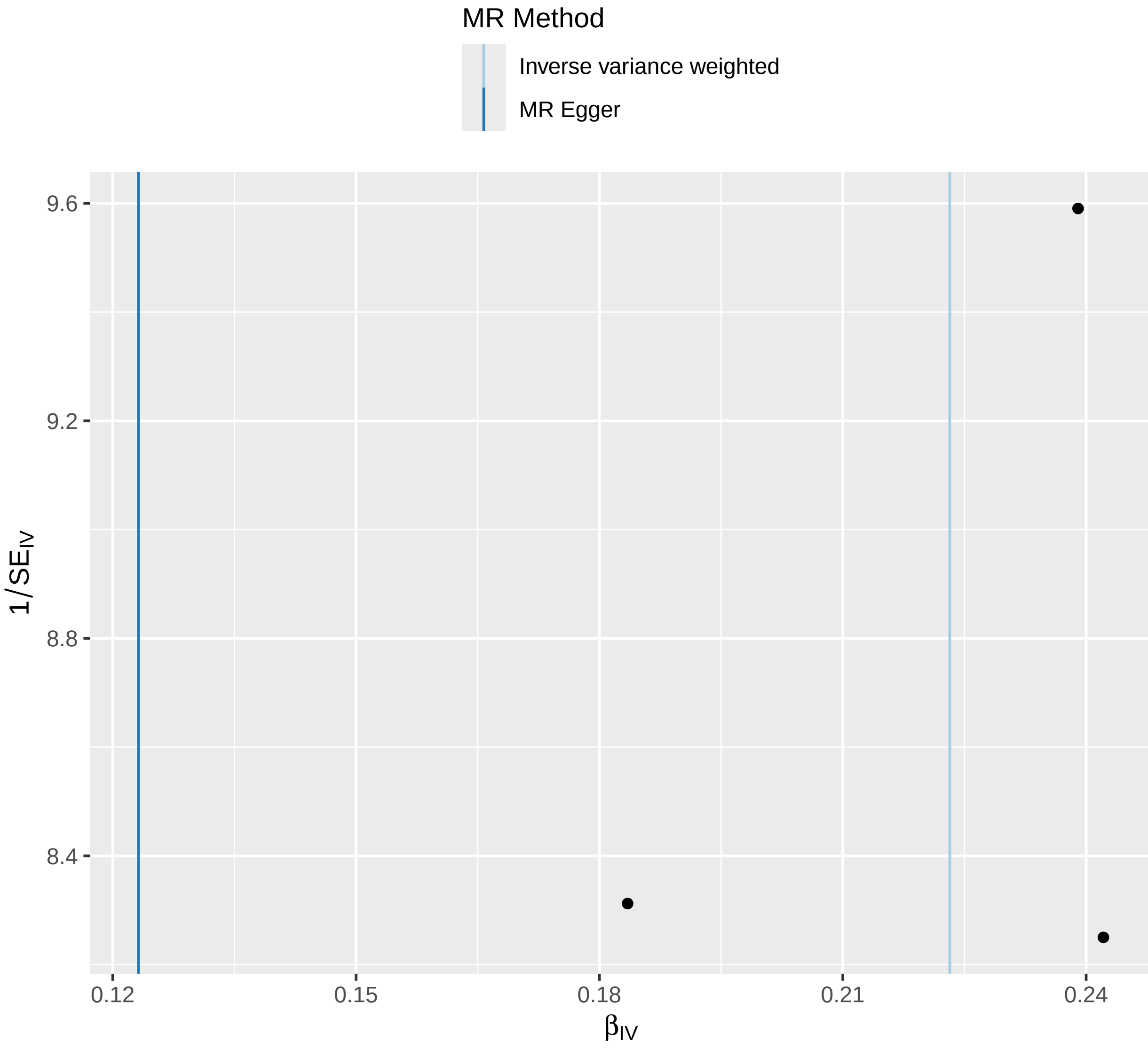
MR Method
Inverse variance weighted
MR Egger
9.6
9.2
8.8
8.4
1/SE_IV
0.12
0.15
0.18
0.21
0.24
β_IV

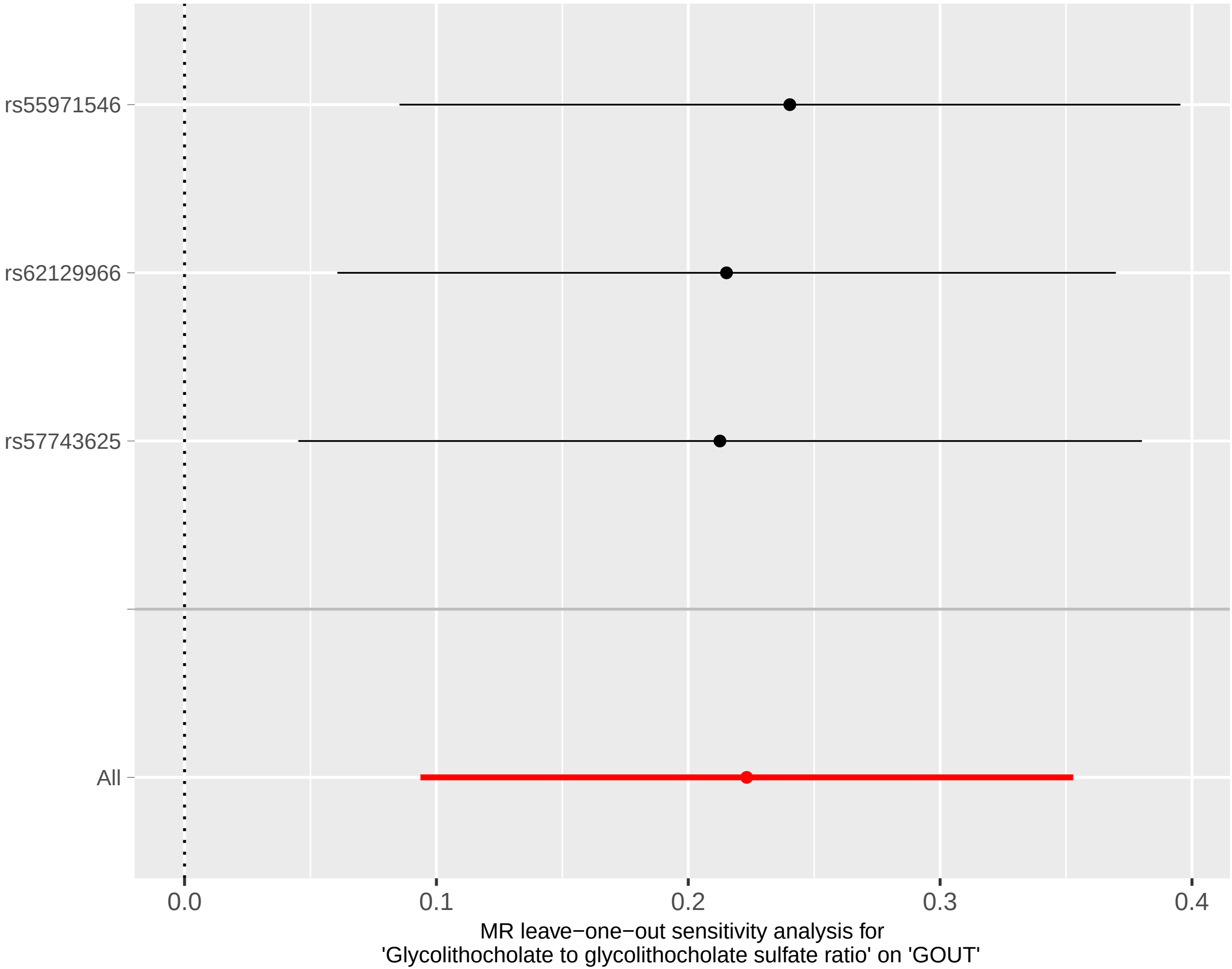
rs55971546
rs62129966
rs57743625
All
0.0
0.1
0.2
0.3
0.4
MR leave−one−out sensitivity analysis for
'Glycolithocholate to glycolithocholate sulfate ratio' on 'GOUT'

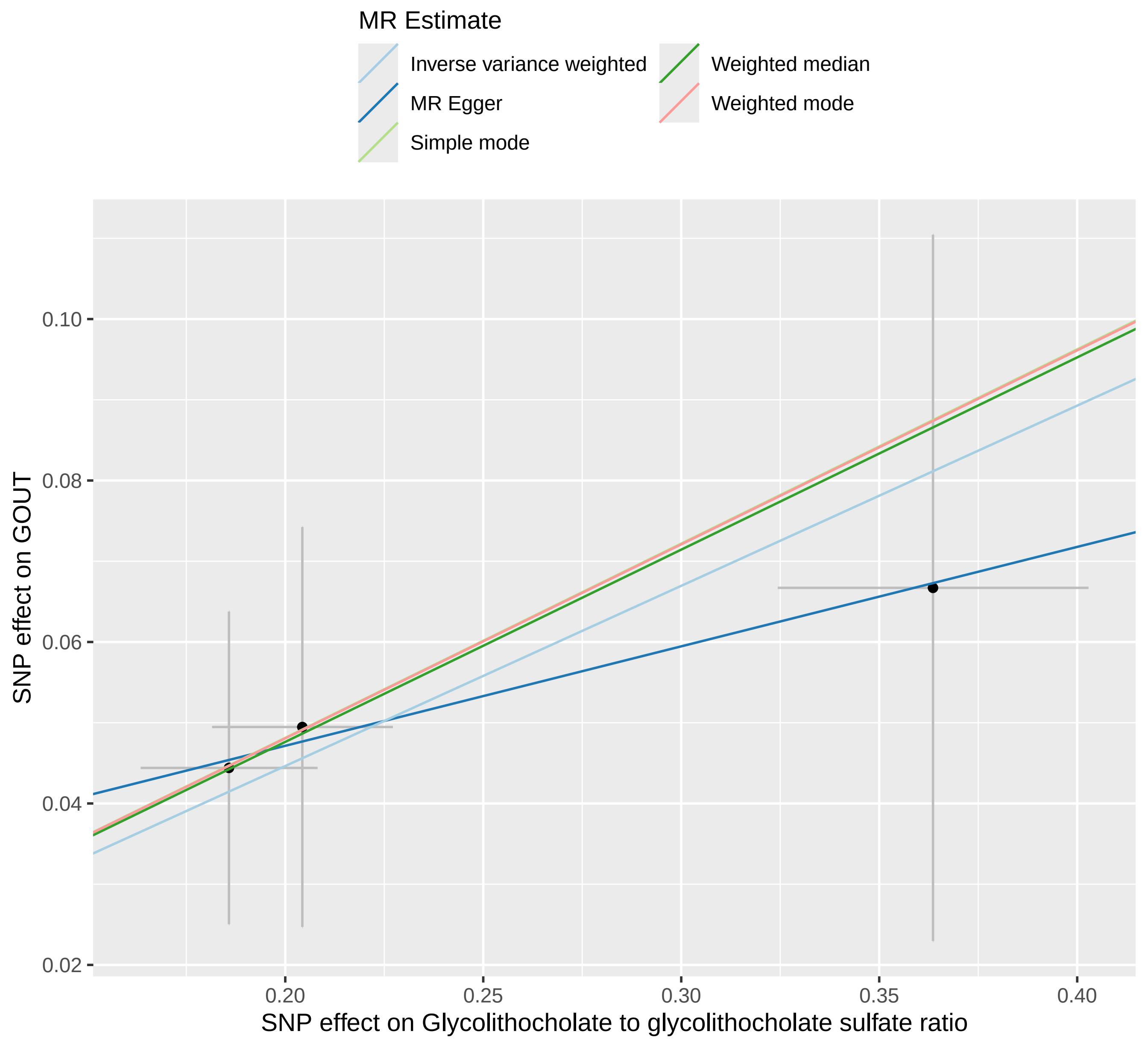
MR Estimate
Inverse variance weighted
MR Egger
Simple mode
Weighted median
Weighted mode
0.10
0.08
0.06
0.04
0.02
0.20
0.25
0.30
0.35
0.40
SNP effect on GOUT
SNP effect on Glycolithocholate to glycolithocholate sulfate ratio

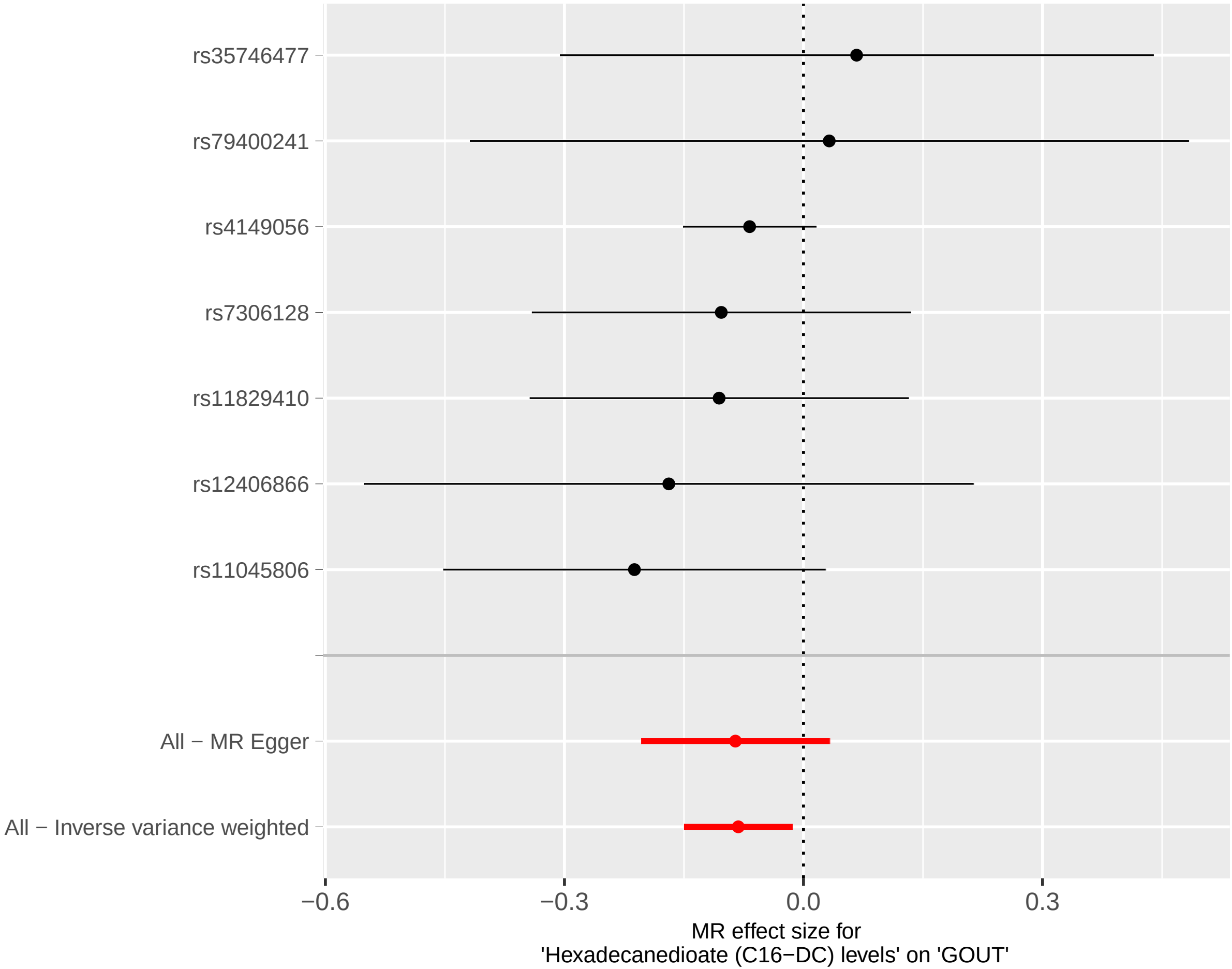

rs35746477
rs79400241
rs4149056
rs7306128
rs11829410
rs12406866
rs11045806
All − MR Egger
All − Inverse variance weighted
−0.6
−0.3
0.0
0.3
MR effect size for
'Hexadecanedioate (C16−DC) levels' on 'GOUT'

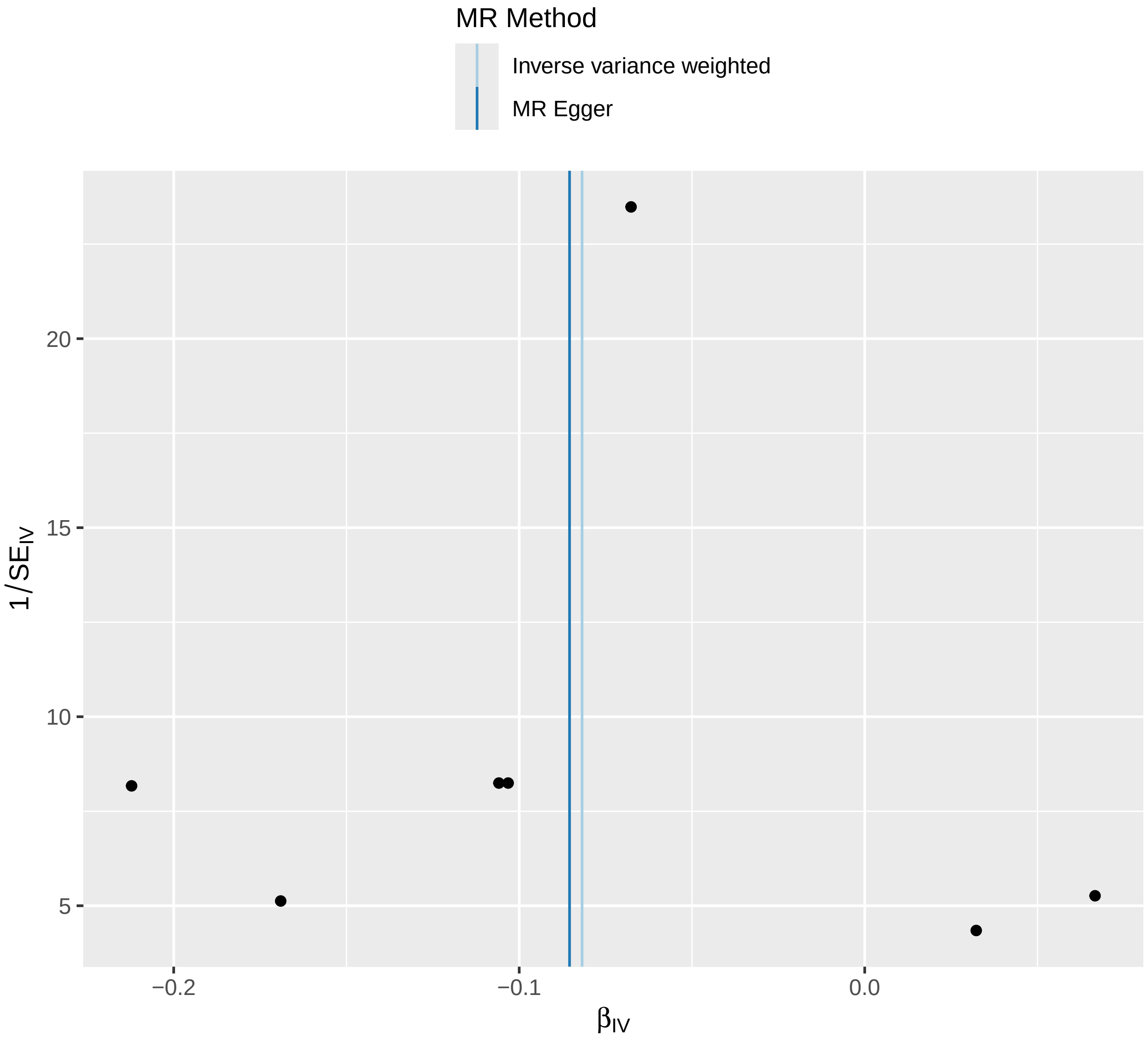
MR Method
Inverse variance weighted
MR Egger
20
15
10
5
$1/SE_{IV}$
−0.2
−0.1
0.0
β_{IV}

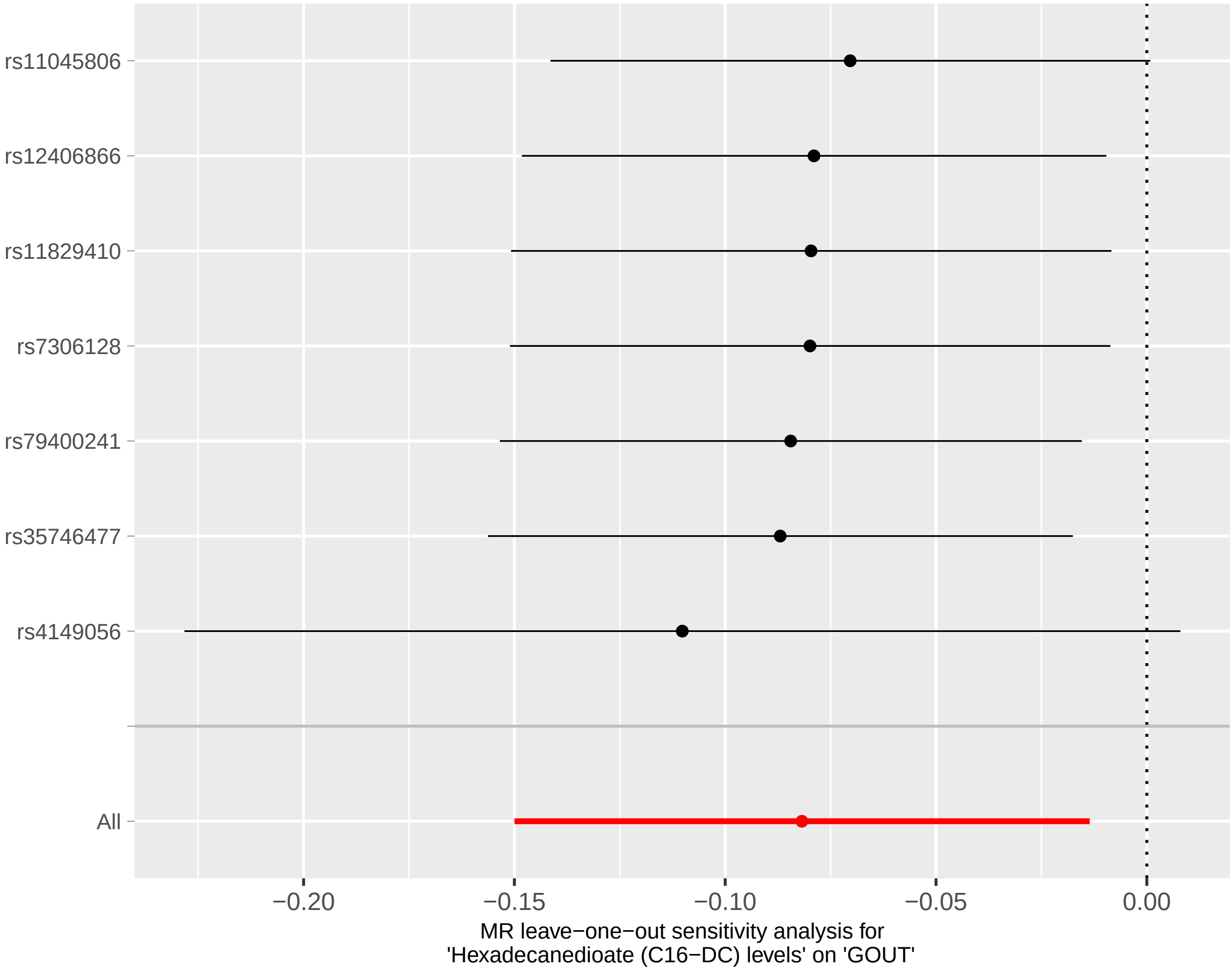
rs11045806
rs12406866
rs11829410
rs7306128
rs79400241
rs35746477
rs4149056
All
−0.20
−0.15
−0.10
−0.05
0.00
MR leave−one−out sensitivity analysis for
'Hexadecanedioate (C16−DC) levels' on 'GOUT'

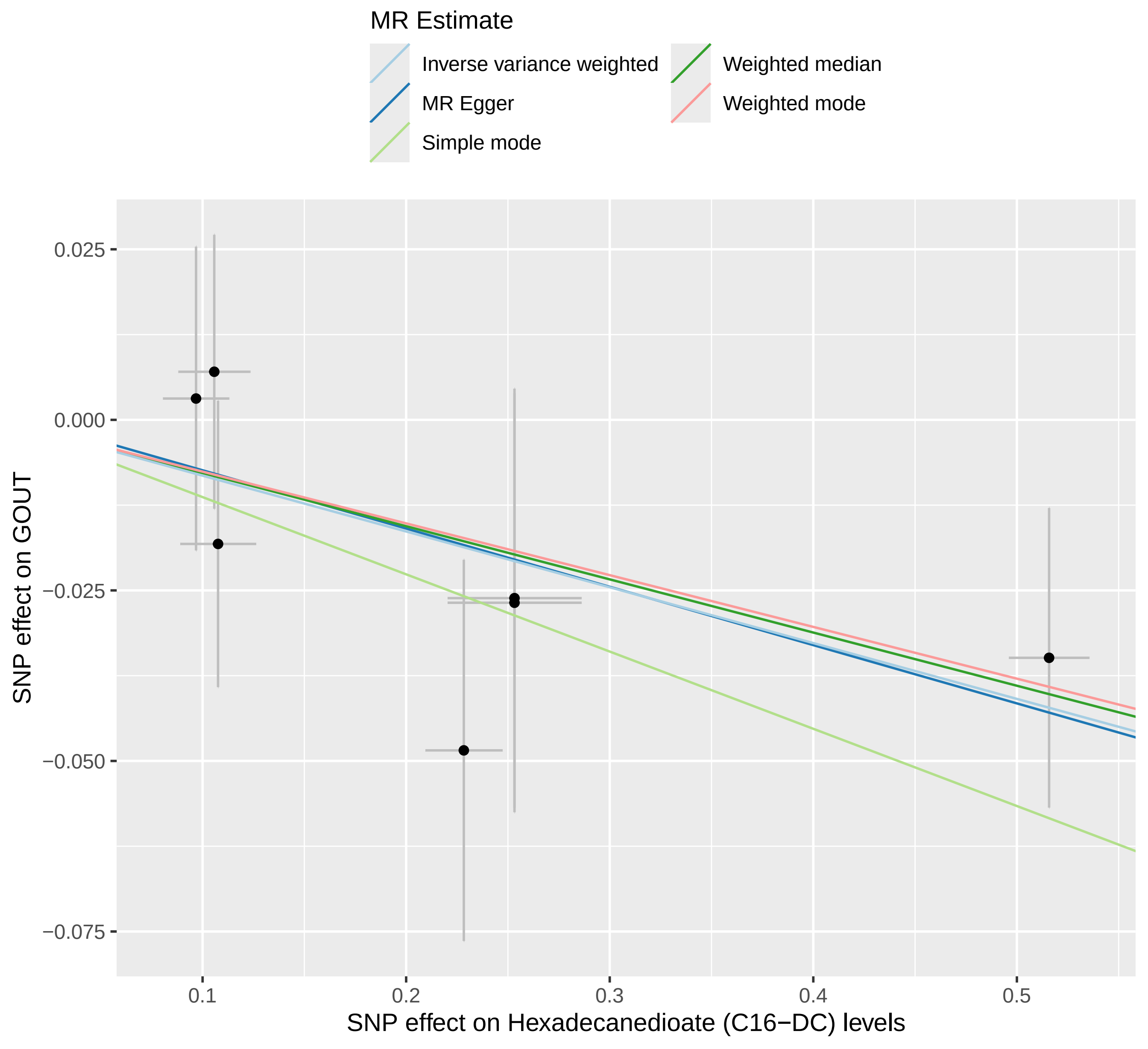
MR Estimate
Inverse variance weighted
MR Egger
Simple mode
Weighted median
Weighted mode
0.025
0.000
−0.025
−0.050
−0.075
0.1
0.2
0.3
0.4
0.5
SNP effect on GOUT
SNP effect on Hexadecanedioate (C16−DC) levels

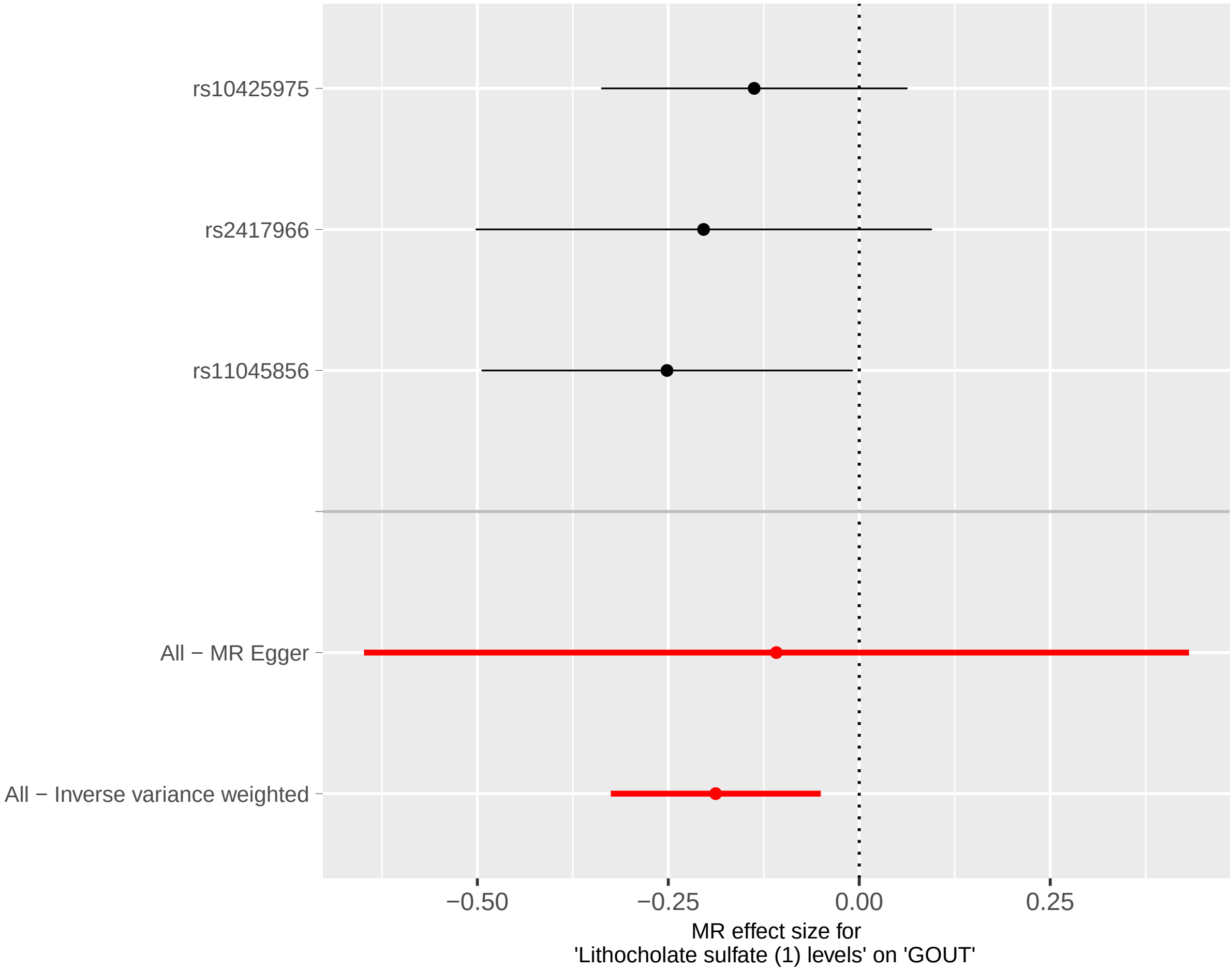

rs10425975
rs2417966
rs11045856
All − MR Egger
All − Inverse variance weighted
−0.50
−0.25
0.00
0.25
MR effect size for
'Lithocholate sulfate (1) levels' on 'GOUT'

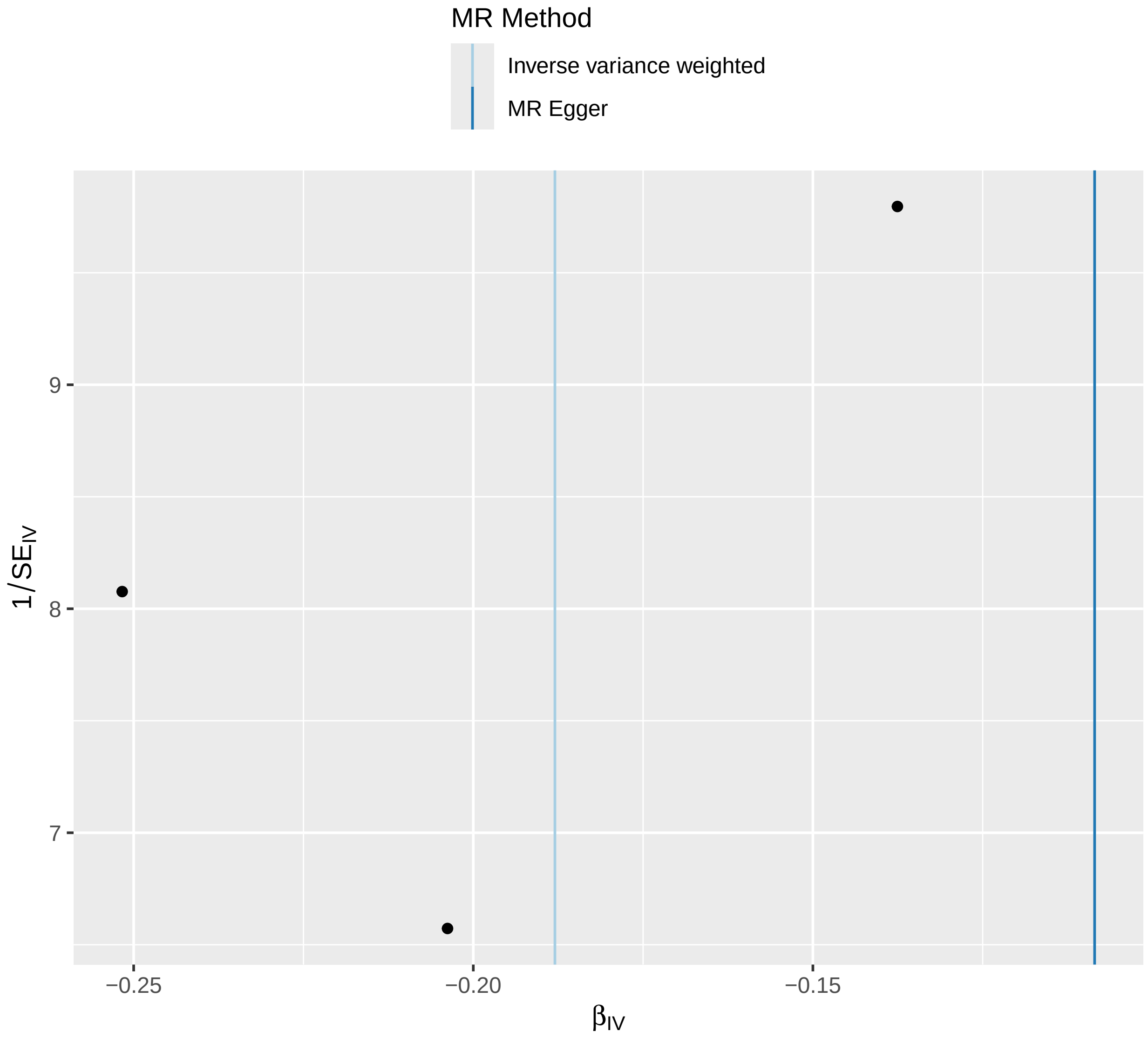
MR Method
Inverse variance weighted
MR Egger
9
8
7
1/SE$_{IV}$
−0.25
−0.20
−0.15
β$_{IV}$

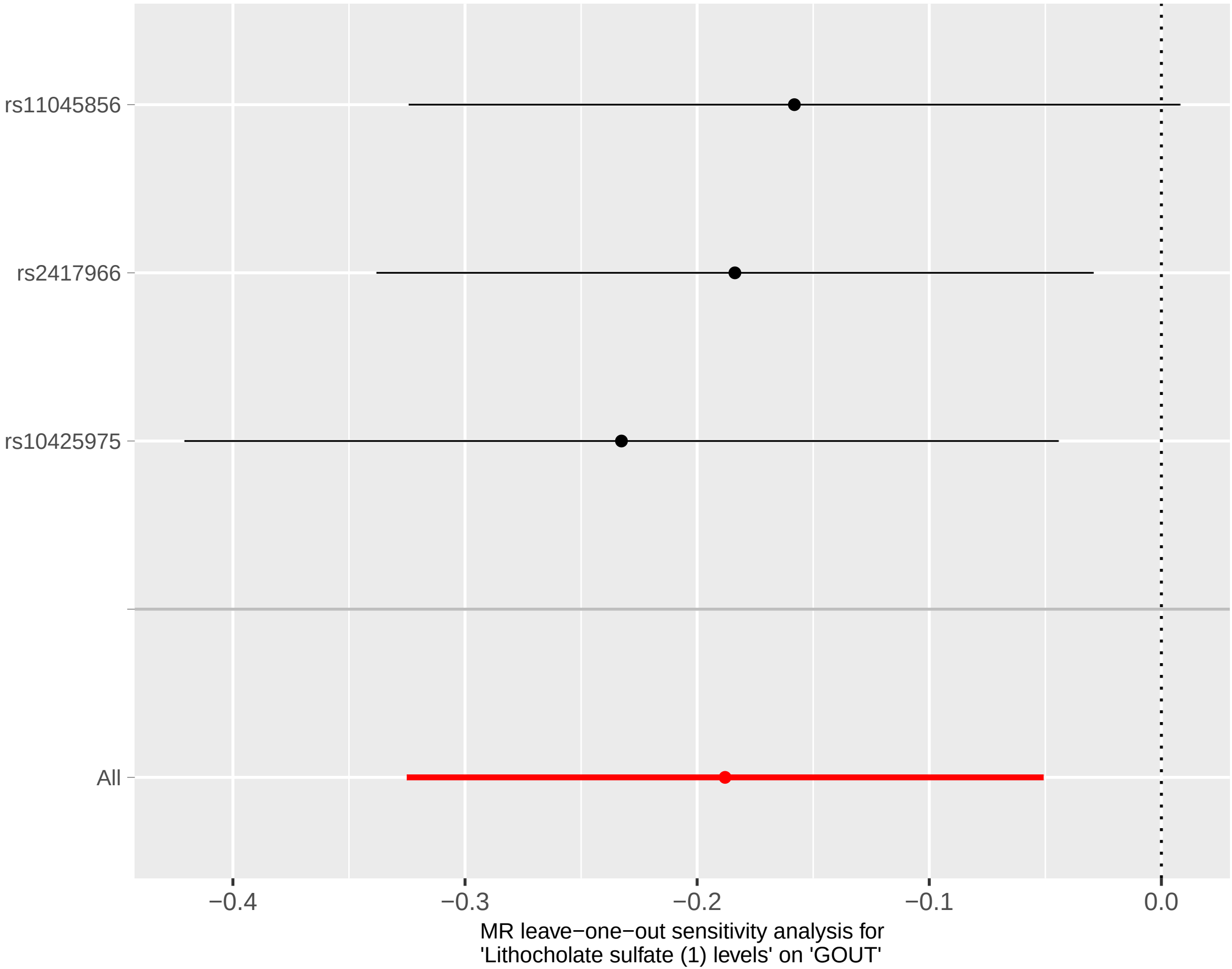
rs11045856
rs2417966
rs10425975
All
−0.4
−0.3
−0.2
−0.1
0.0
MR leave−one−out sensitivity analysis for
'Lithocholate sulfate (1) levels' on 'GOUT'

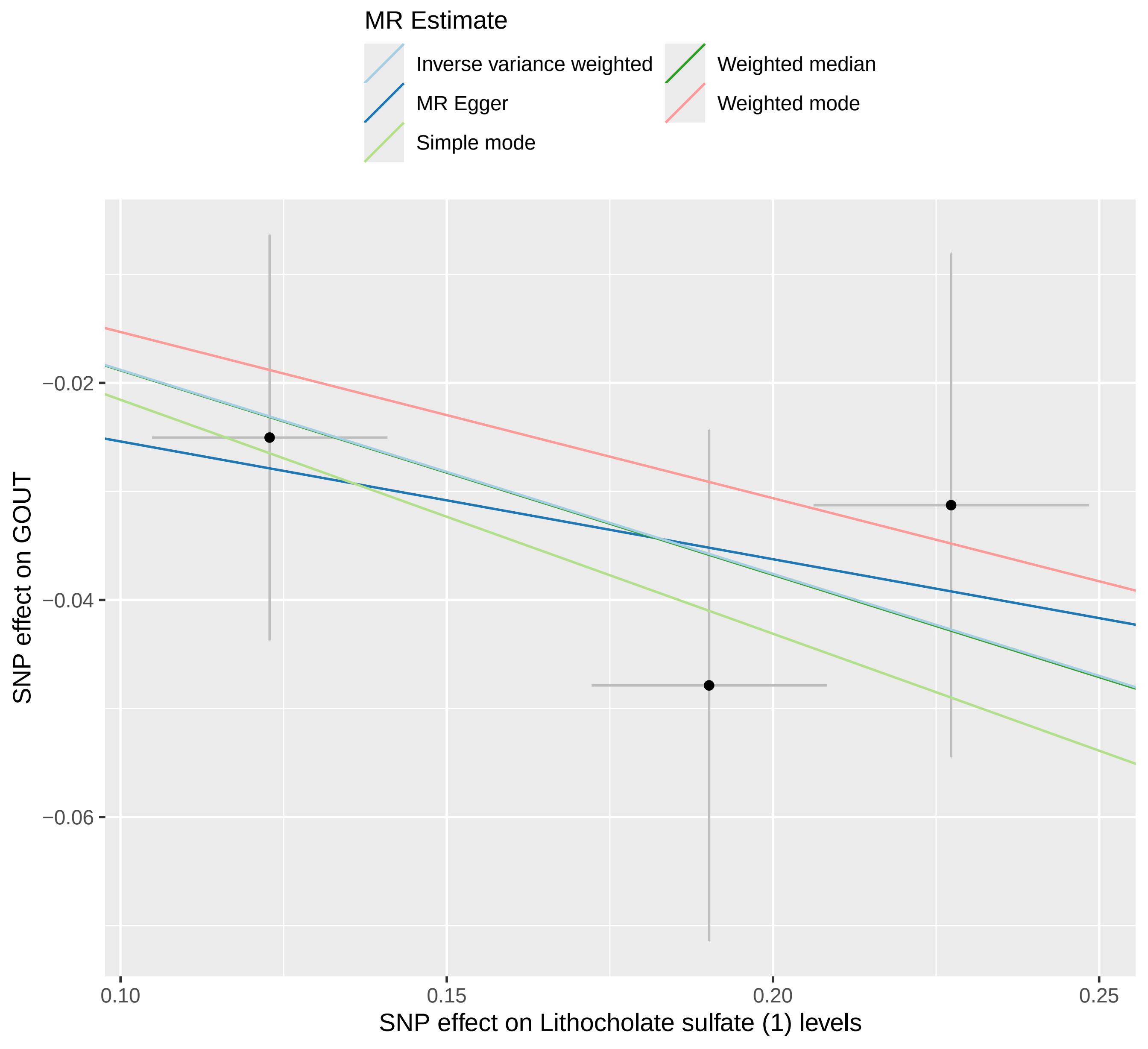
MR Estimate
Inverse variance weighted
MR Egger
Simple mode
Weighted median
Weighted mode
−0.02
−0.04
−0.06
SNP effect on GOUT
0.10
0.15
0.20
0.25
SNP effect on Lithocholate sulfate (1) levels

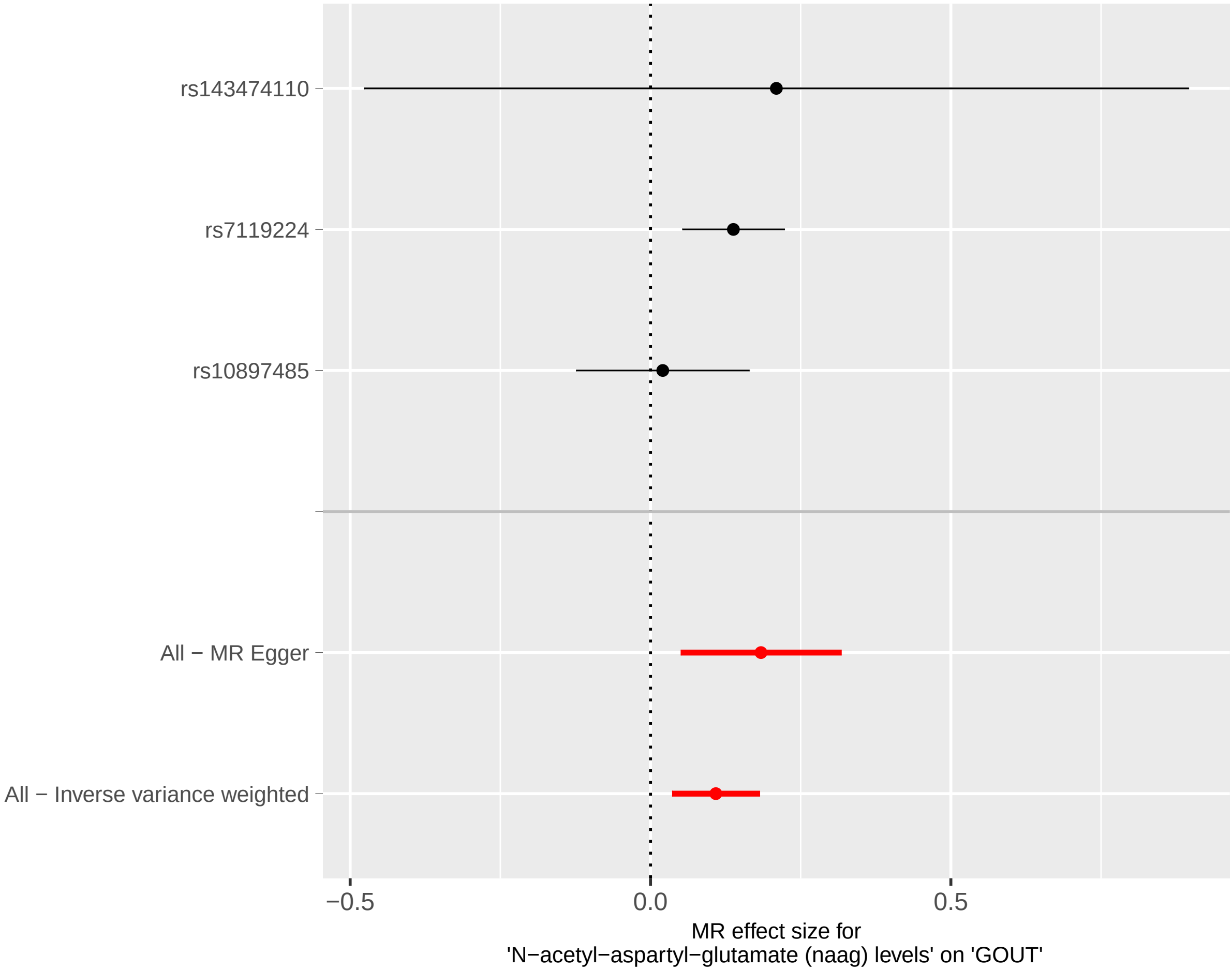

rs143474110
rs7119224
rs10897485
All − MR Egger
All − Inverse variance weighted
−0.5
0.0
0.5
MR effect size for
'N−acetyl−aspartyl−glutamate (naag) levels' on 'GOUT'

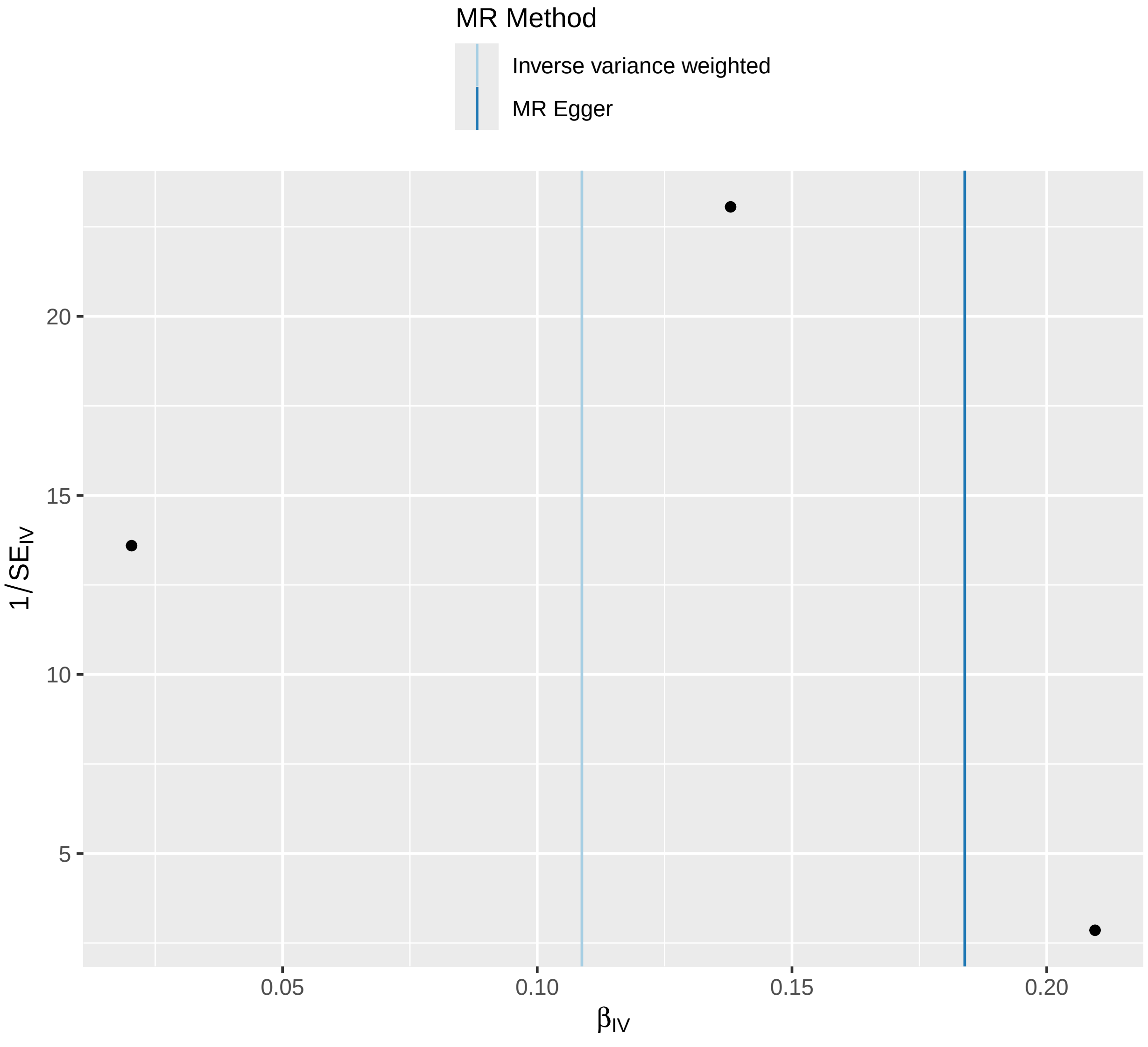
MR Method
Inverse variance weighted
MR Egger
20
15
10
5
$1/SE_{IV}$
0.05
0.10
0.15
0.20
β_{IV}

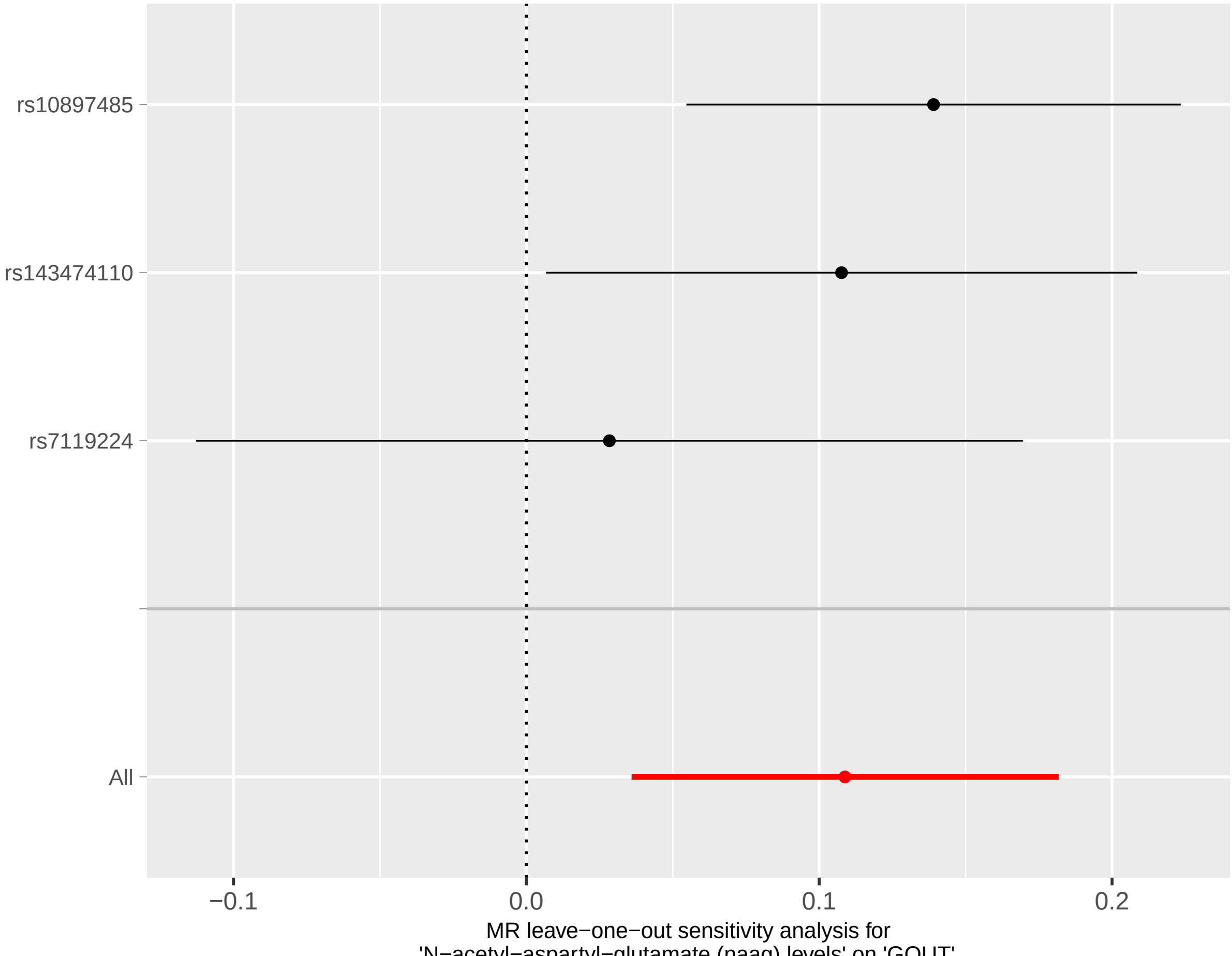
rs10897485
rs143474110
rs7119224
All
−0.1
0.0
0.1
0.2
MR leave−one−out sensitivity analysis for
'N−acetyl−aspartyl−glutamate (naag) levels' on 'GOUT'

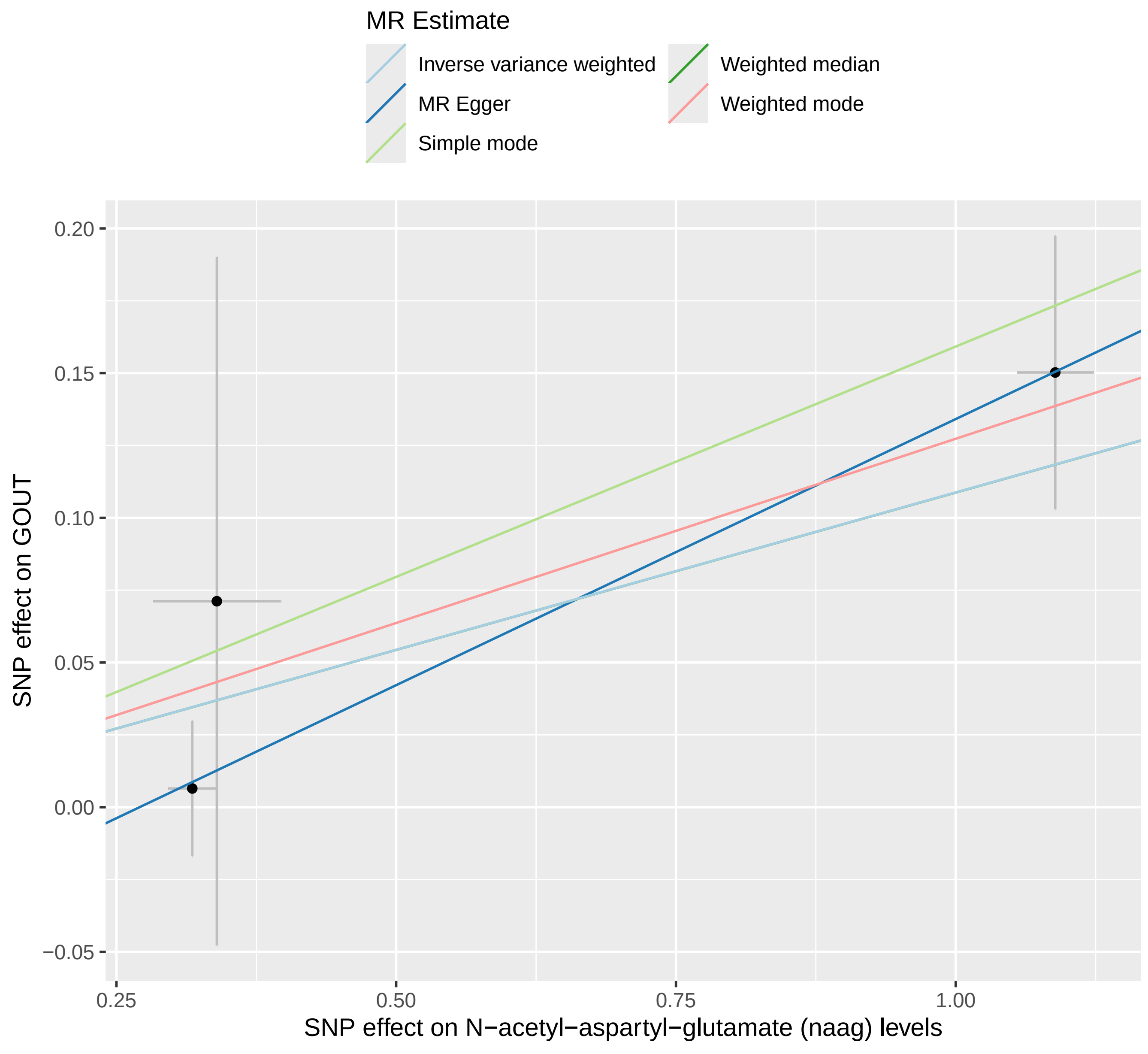
MR Estimate
Inverse variance weighted
MR Egger
Simple mode
Weighted median
Weighted mode
0.20
0.15
0.10
0.05
0.00
−0.05
SNP effect on GOUT
0.25
0.50
0.75
1.00
SNP effect on N−acetyl−aspartyl−glutamate (naag) levels

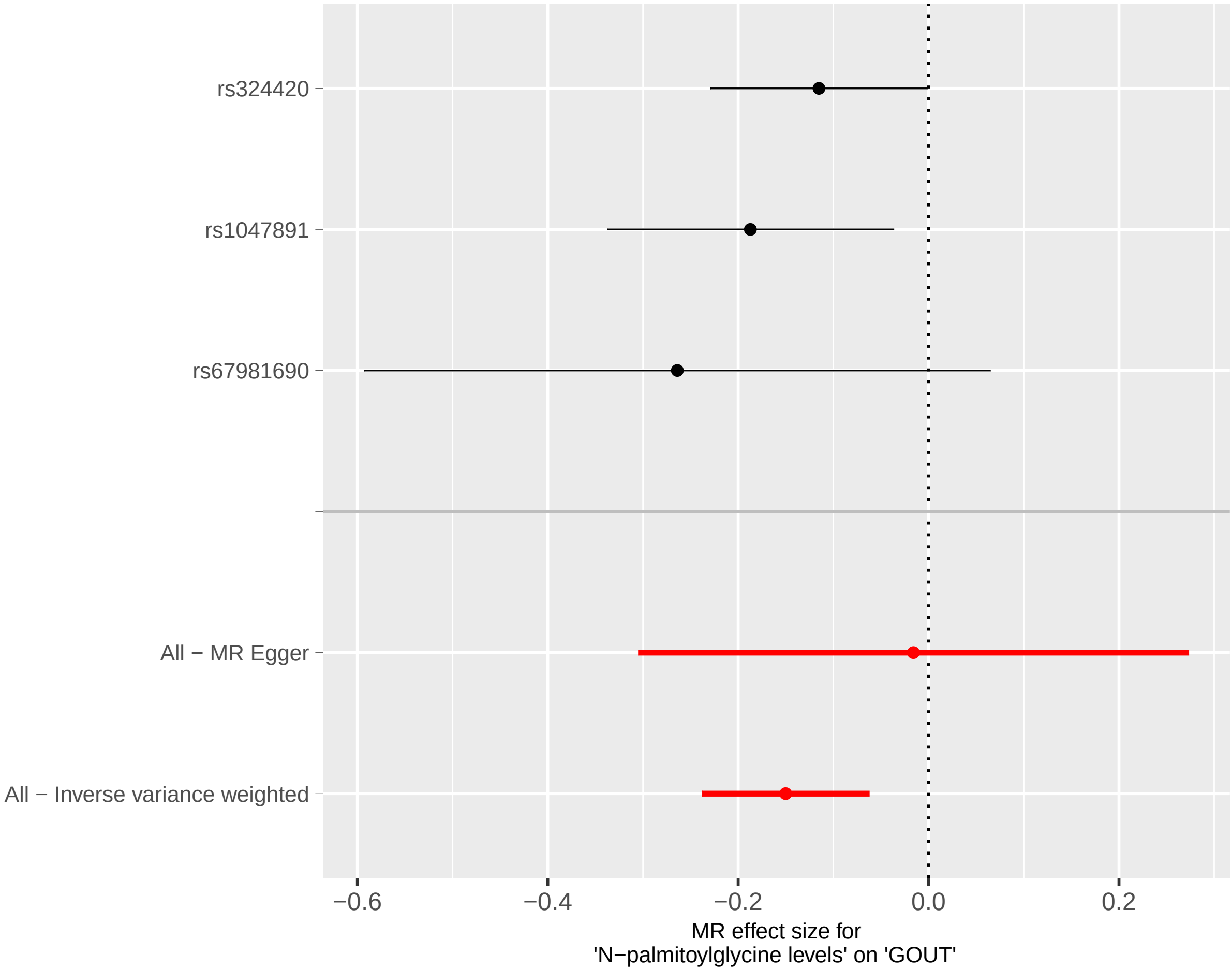

rs324420
rs1047891
rs67981690
All − MR Egger
All − Inverse variance weighted
−0.6
−0.4
−0.2
0.0
0.2
MR effect size for
'N−palmitoylglycine levels' on 'GOUT'

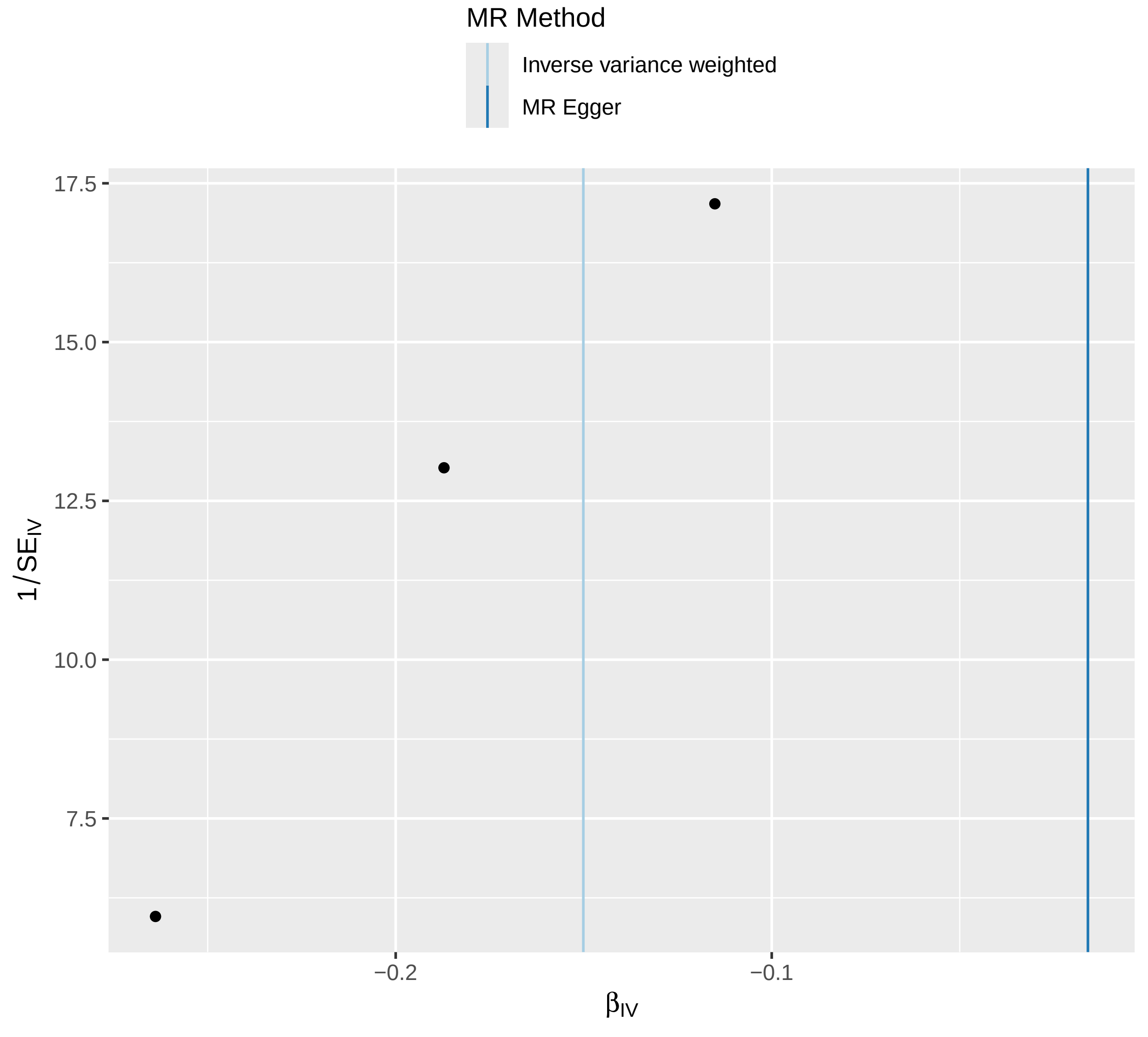
MR Method
Inverse variance weighted
MR Egger
17.5
15.0
12.5
10.0
7.5
1/SE_IV
−0.2
−0.1
β_IV

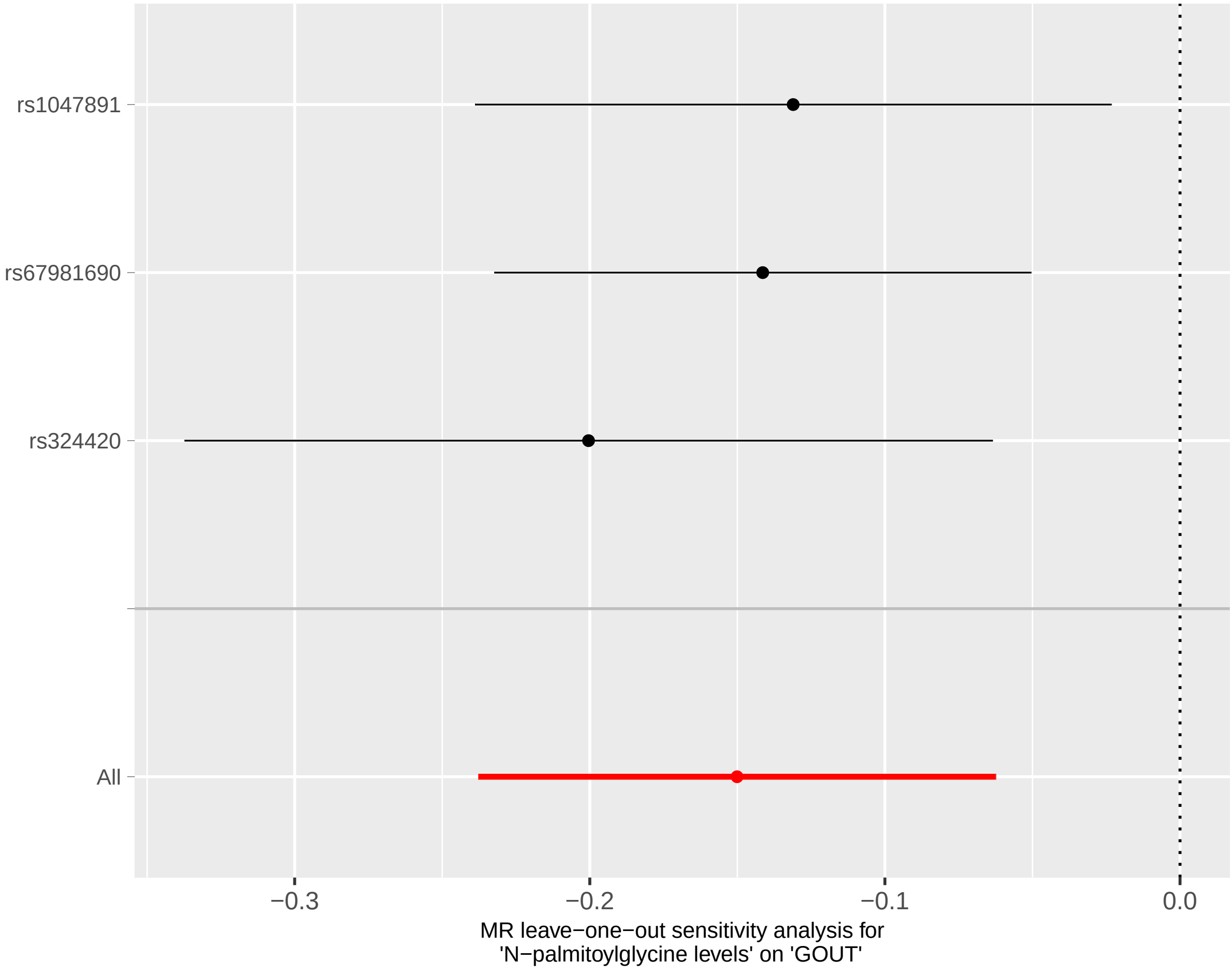
rs1047891
rs67981690
rs324420
All
−0.3
−0.2
−0.1
0.0
MR leave−one−out sensitivity analysis for
'N−palmitoylglycine levels' on 'GOUT'

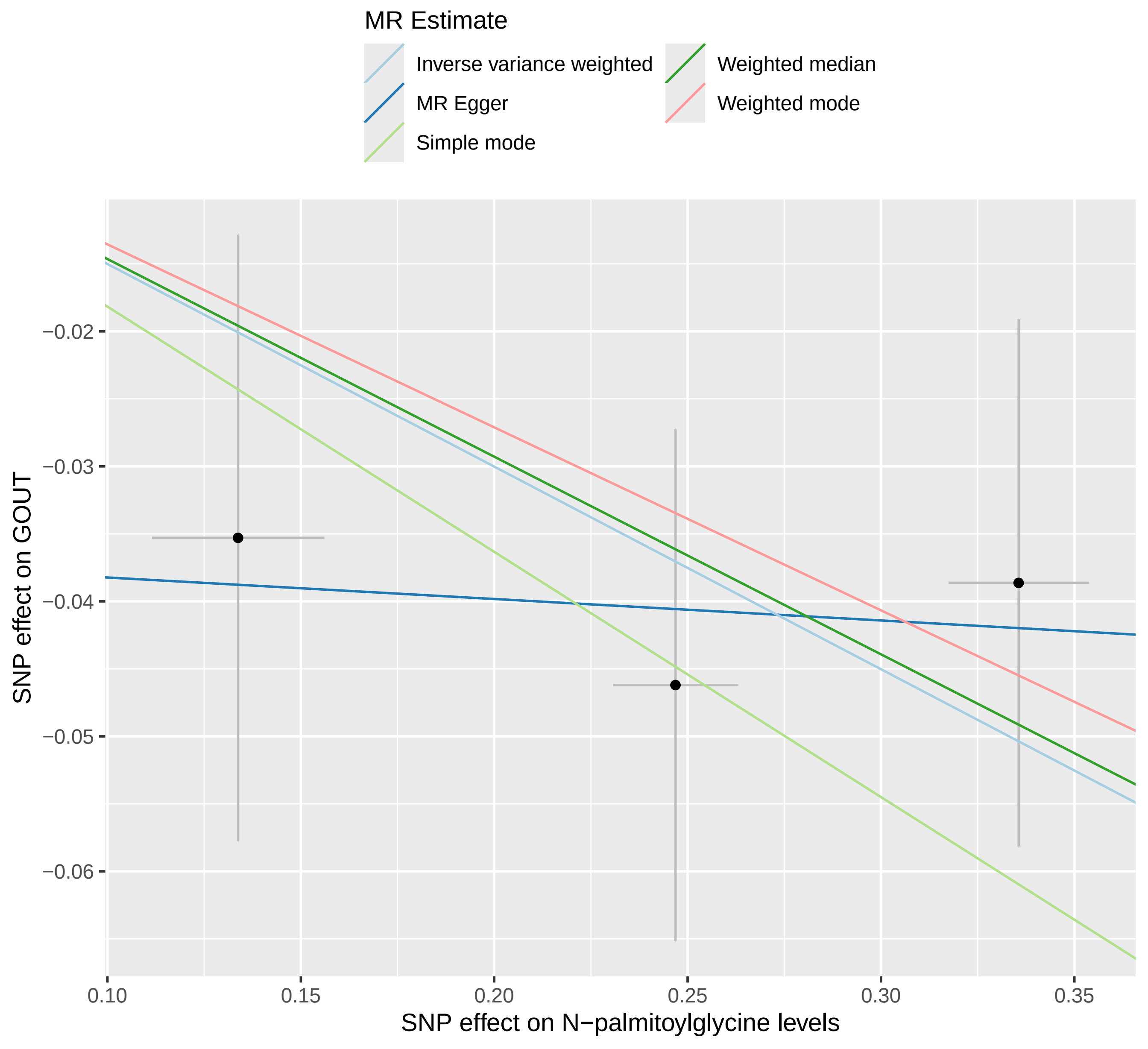
MR Estimate
Inverse variance weighted
MR Egger
Simple mode
Weighted median
Weighted mode
−0.02
−0.03
−0.04
−0.05
−0.06
SNP effect on GOUT
0.10
0.15
0.20
0.25
0.30
0.35
SNP effect on N−palmitoylglycine levels

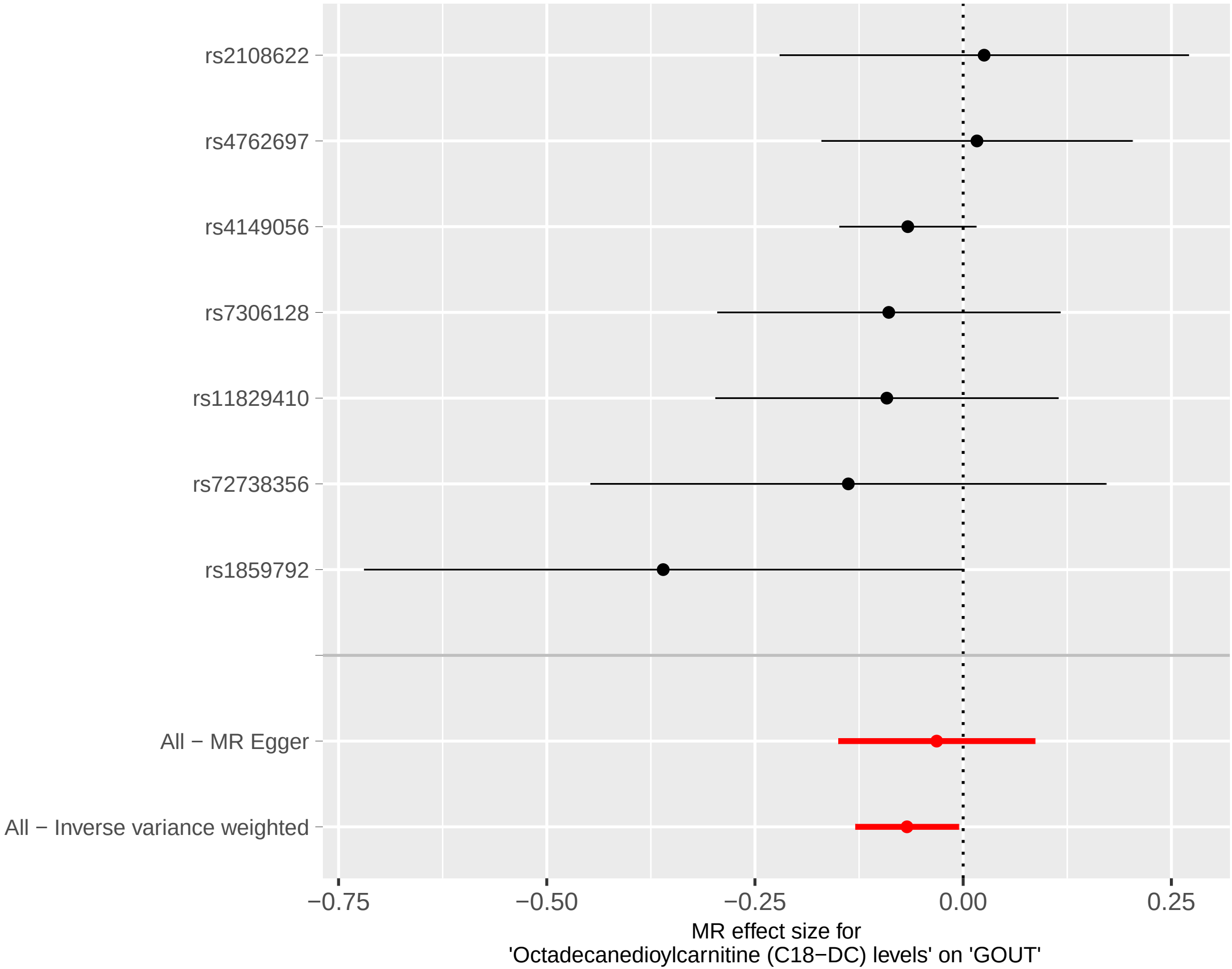

rs2108622
rs4762697
rs4149056
rs7306128
rs11829410
rs72738356
rs1859792
All − MR Egger
All − Inverse variance weighted
−0.75
−0.50
−0.25
0.00
0.25
MR effect size for
'Octadecanedioylcarnitine (C18−DC) levels' on 'GOUT'

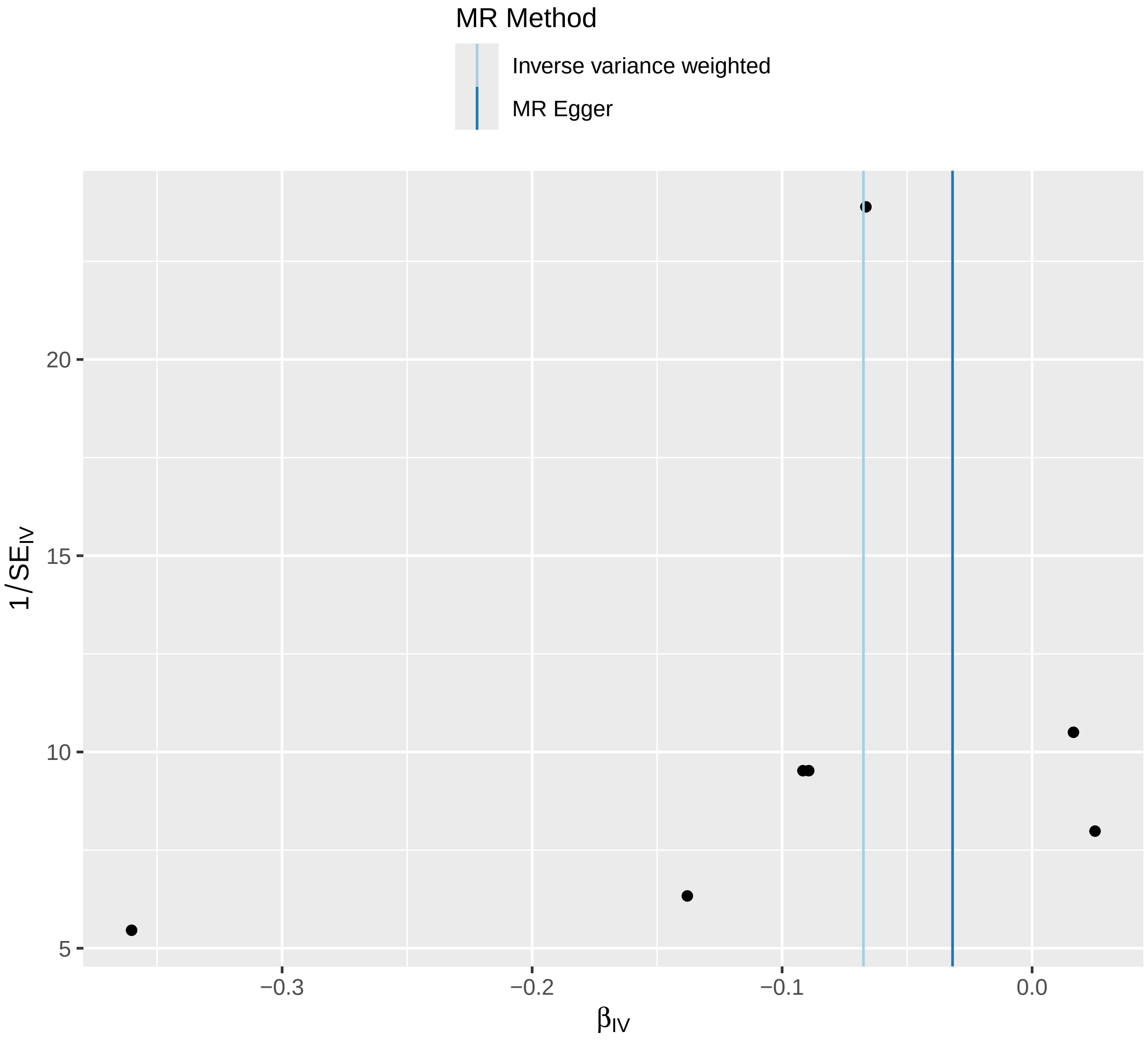
MR Method
Inverse variance weighted
MR Egger
20
15
10
5
1/SE_IV
−0.3
−0.2
−0.1
0.0
β_IV

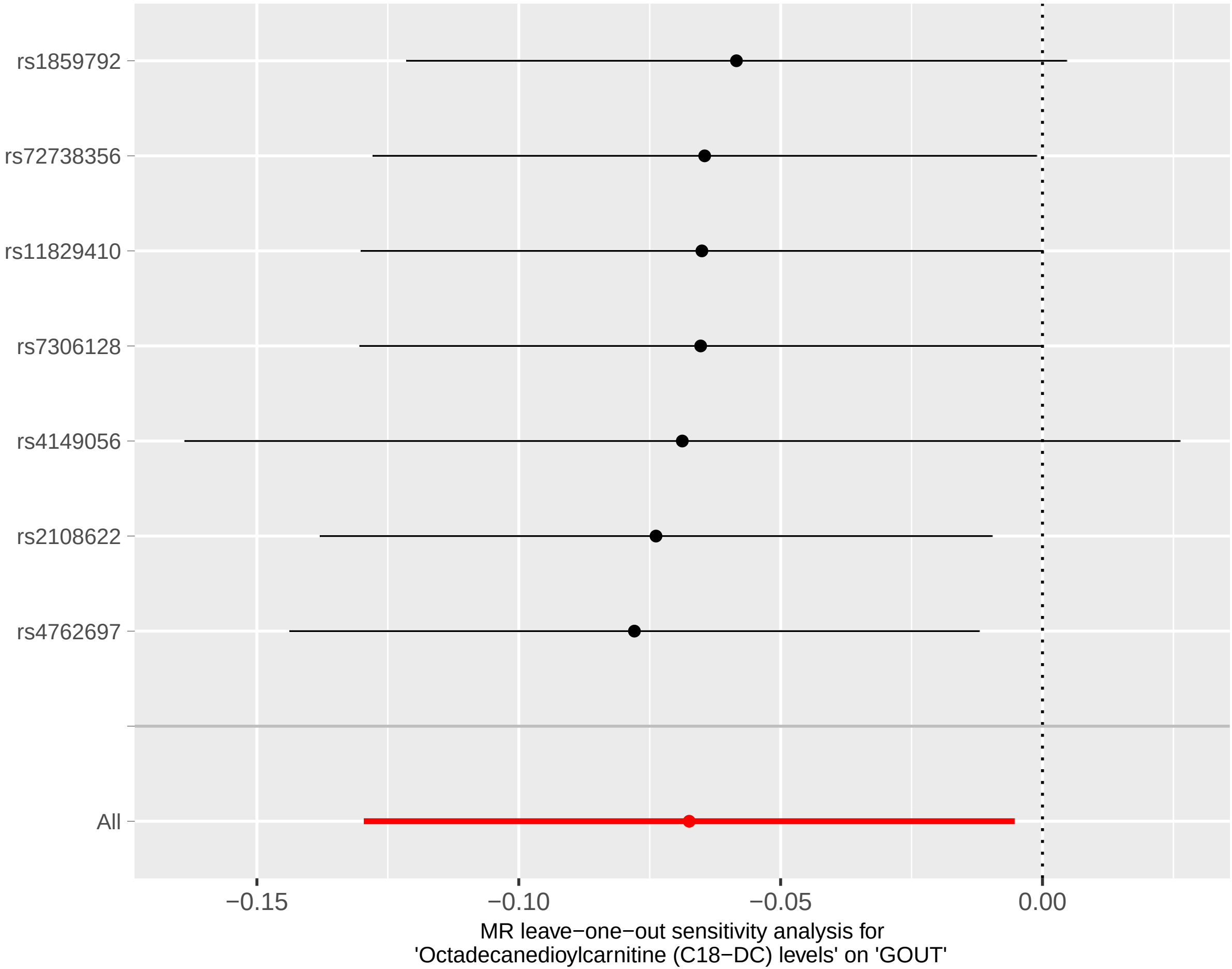
rs1859792
rs72738356
rs11829410
rs7306128
rs4149056
rs2108622
rs4762697
All
−0.15
−0.10
−0.05
0.00
MR leave−one−out sensitivity analysis for
'Octadecanedioylcarnitine (C18−DC) levels' on 'GOUT'

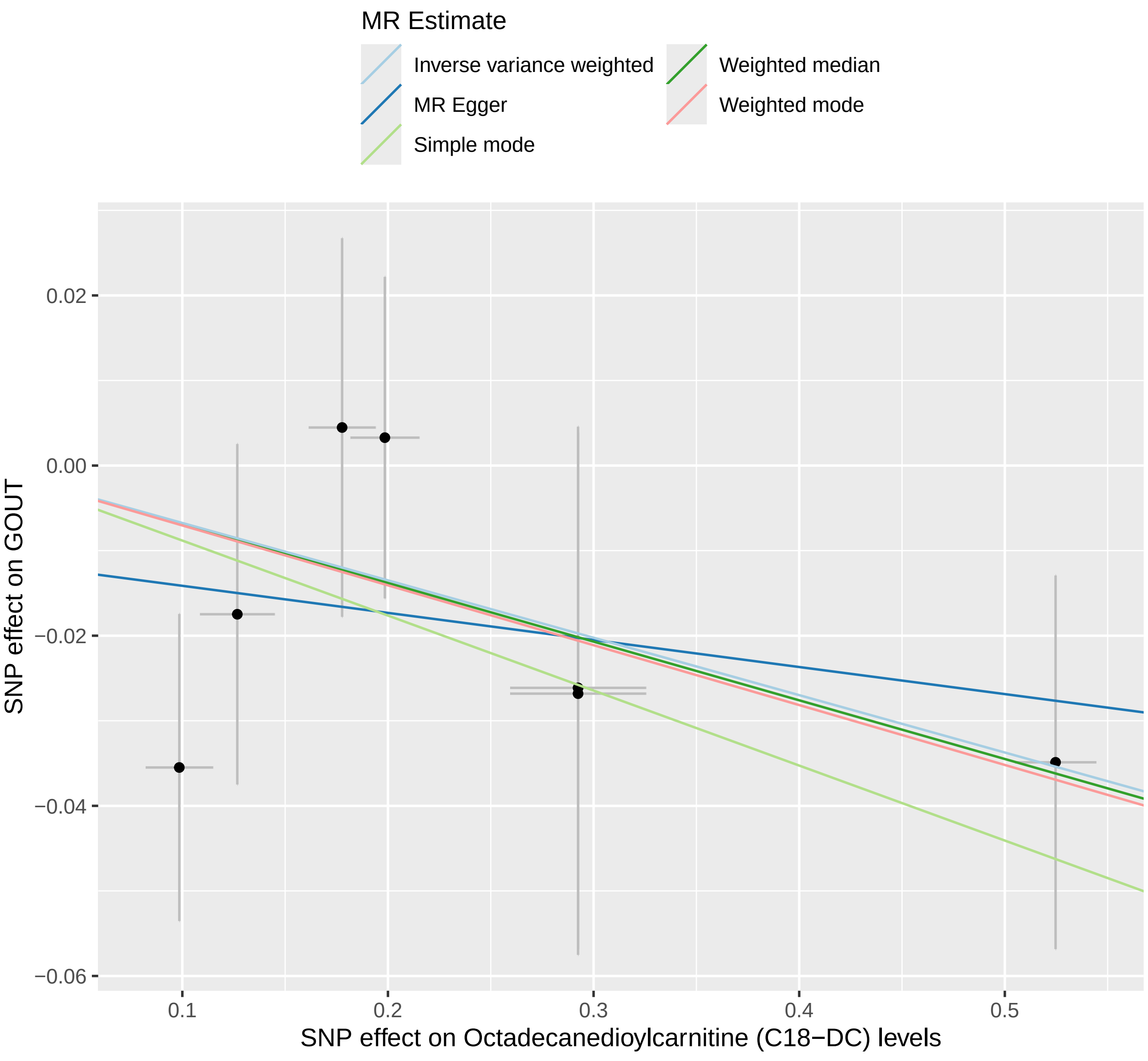
MR Estimate
Inverse variance weighted
MR Egger
Simple mode
Weighted median
Weighted mode
0.02
0.00
−0.02
−0.04
−0.06
0.1
0.2
0.3
0.4
0.5
SNP effect on GOUT
SNP effect on Octadecanedioylcarnitine (C18−DC) levels

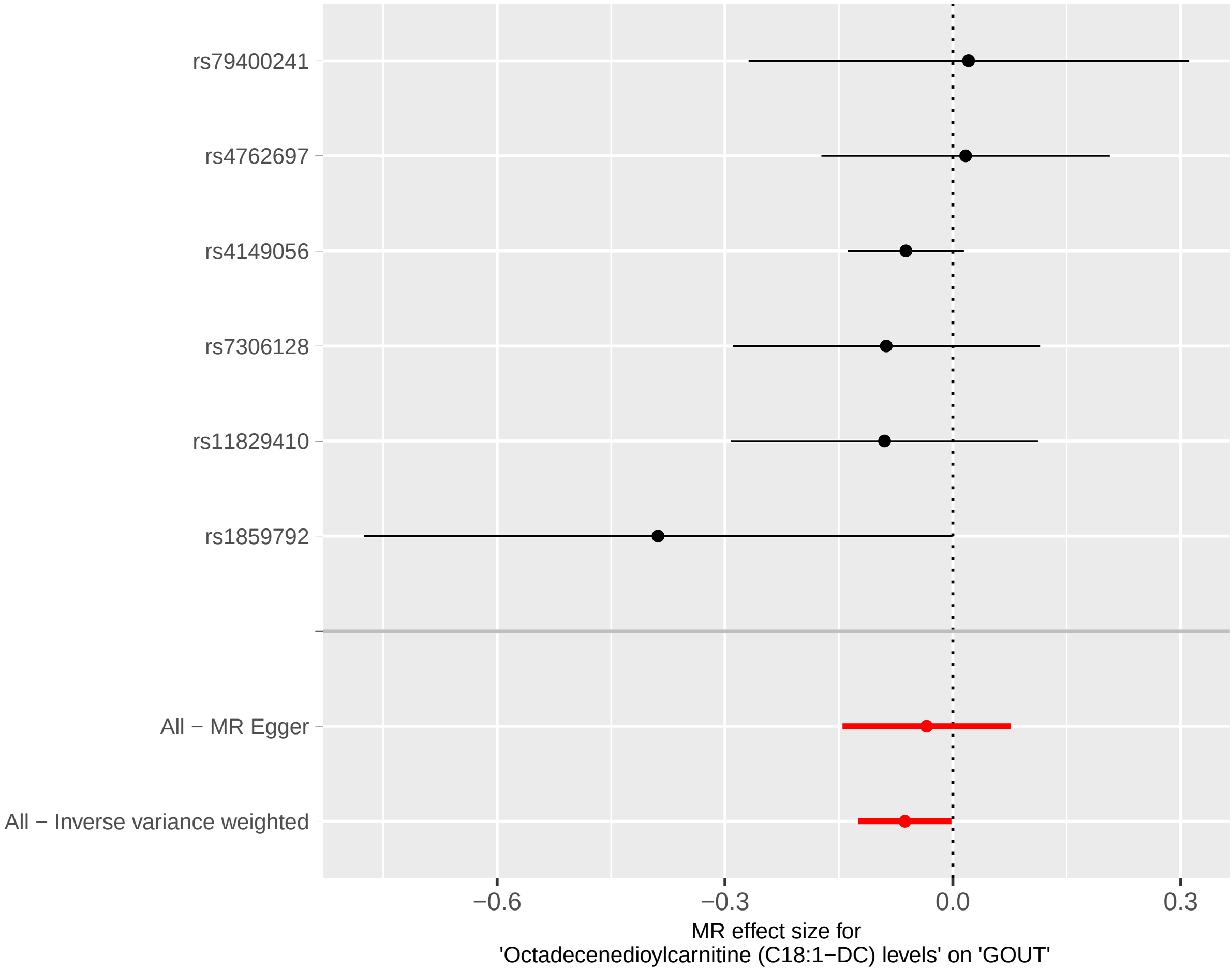

rs79400241
rs4762697
rs4149056
rs7306128
rs11829410
rs1859792
All − MR Egger
All − Inverse variance weighted
−0.6
−0.3
0.0
0.3
MR effect size for
'Octadecenedioylcarnitine (C18:1−DC) levels' on 'GOUT'

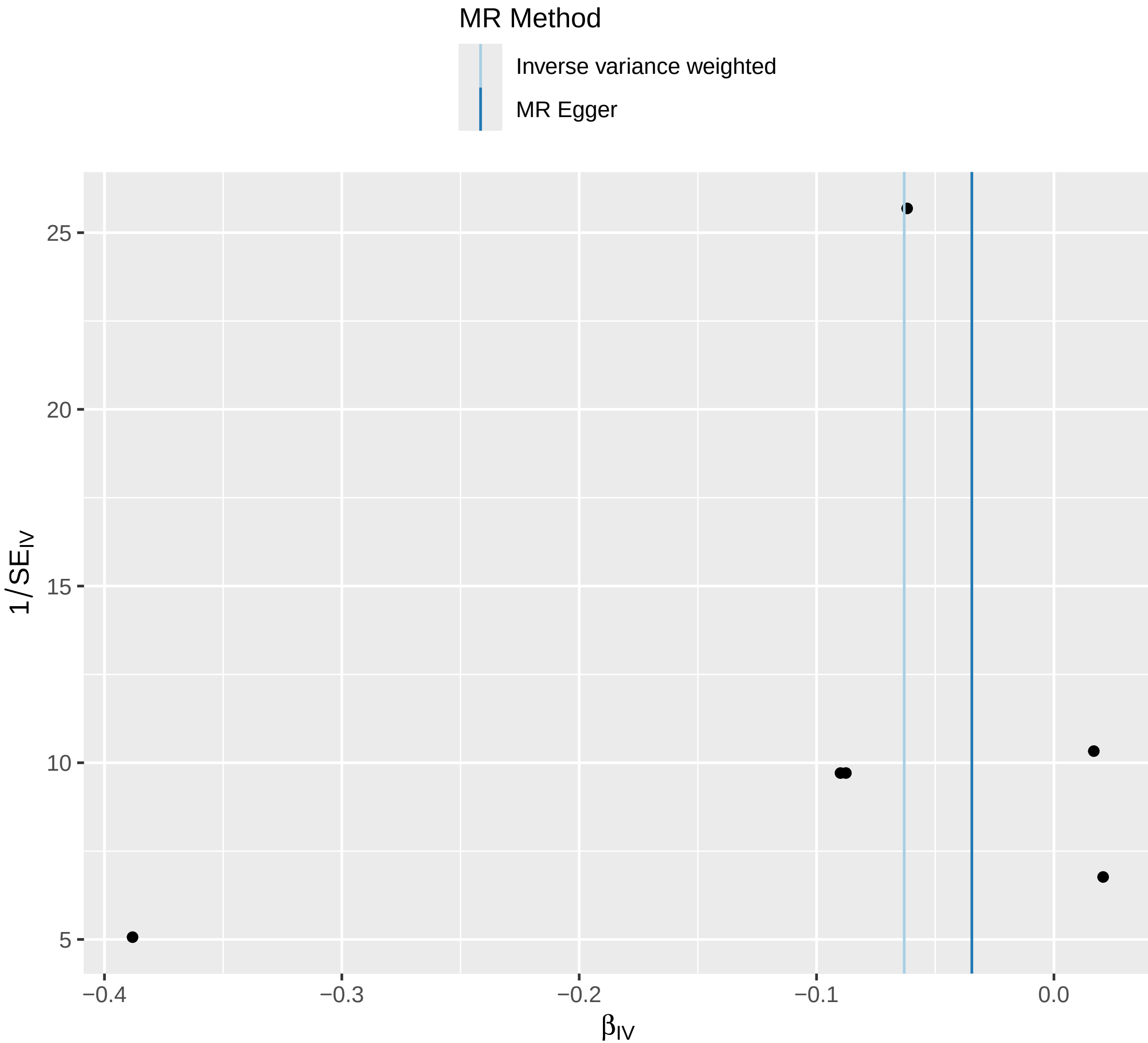
MR Method
Inverse variance weighted
MR Egger
25
20
15
10
5
1/SE_IV
−0.4
−0.3
−0.2
−0.1
0.0
β_IV

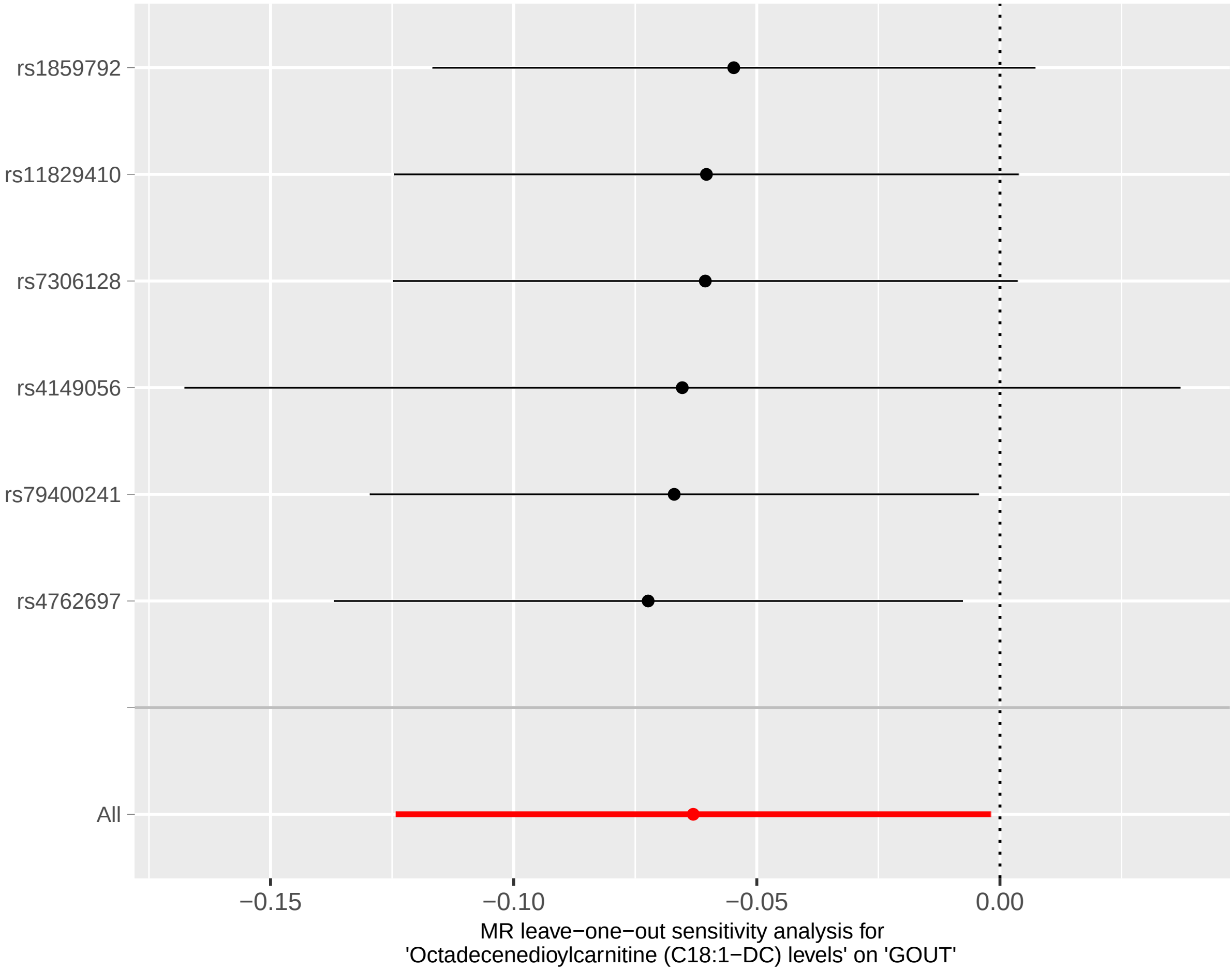
rs1859792
rs11829410
rs7306128
rs4149056
rs79400241
rs4762697
All
−0.15
−0.10
−0.05
0.00
MR leave−one−out sensitivity analysis for
'Octadecenedioylcarnitine (C18:1−DC) levels' on 'GOUT'

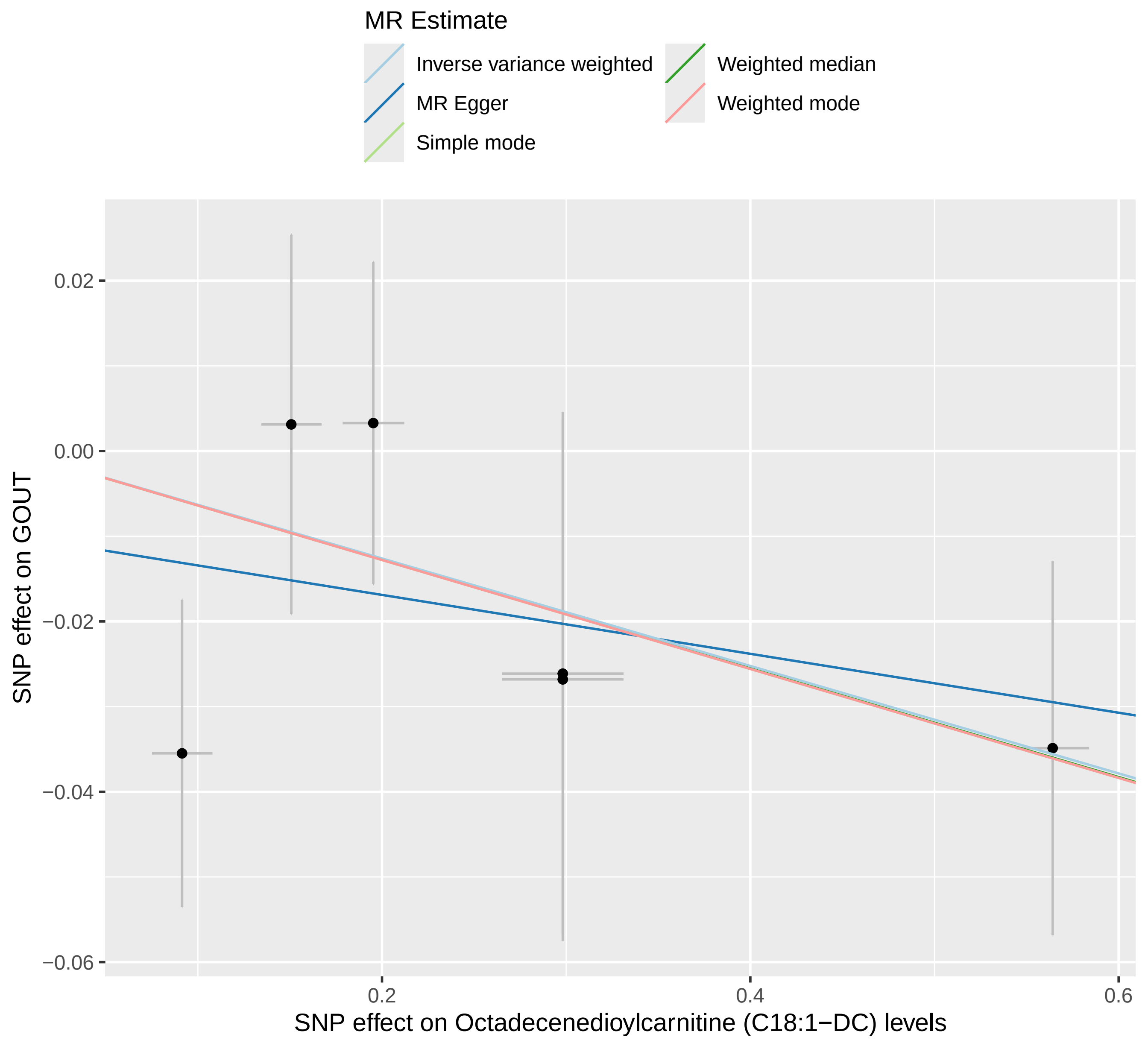

MR Estimate
Inverse variance weighted
MR Egger
Simple mode
Weighted median
Weighted mode
0.02
0.00
−0.02
−0.04
−0.06
SNP effect on GOUT
0.2
0.4
0.6
SNP effect on Octadecenedioylcarnitine (C18:1−DC) levels

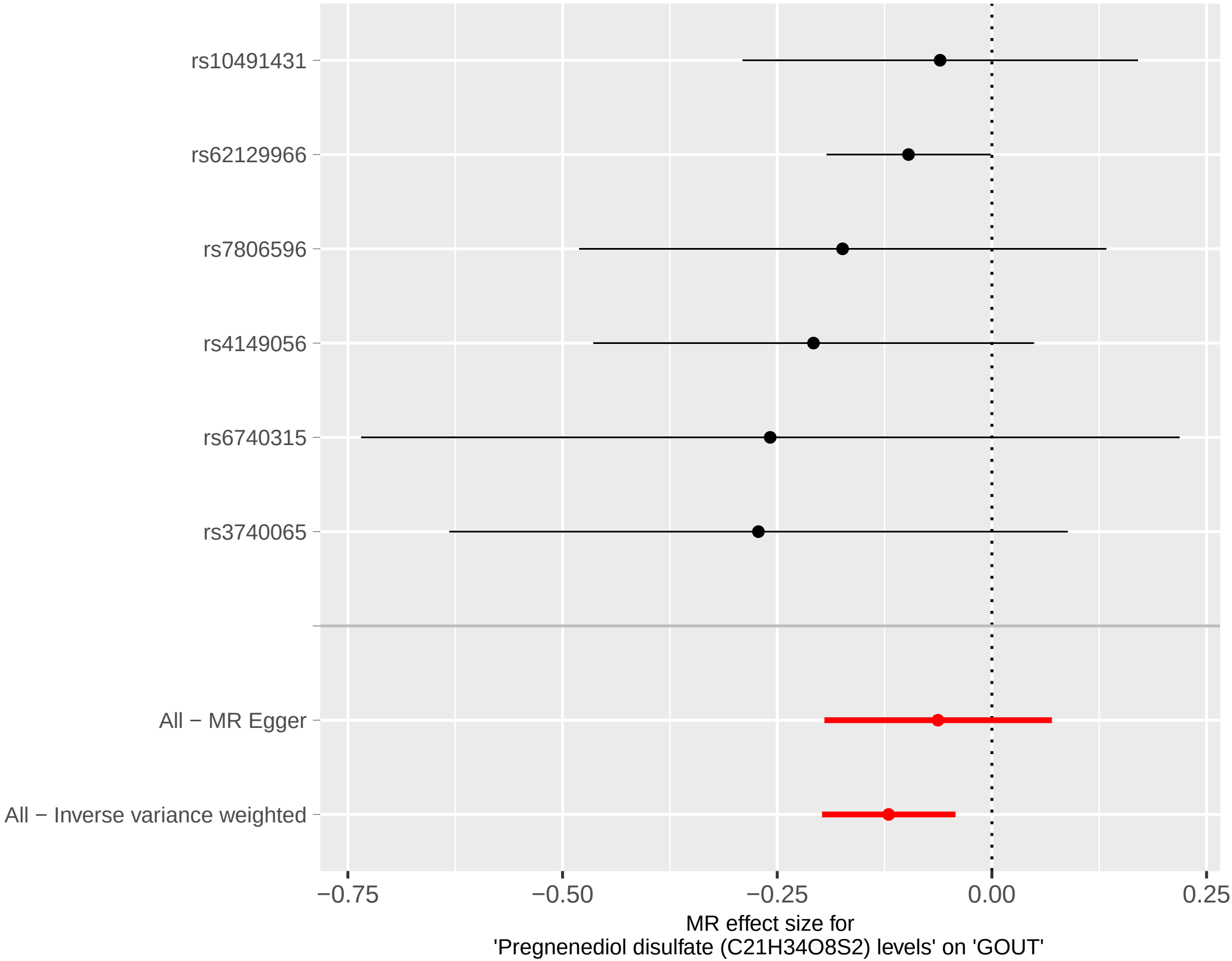

rs10491431
rs62129966
rs7806596
rs4149056
rs6740315
rs3740065
All − MR Egger
All − Inverse variance weighted
−0.75
−0.50
−0.25
0.00
0.25
MR effect size for
'Pregnenediol disulfate (C21H34O8S2) levels' on 'GOUT'

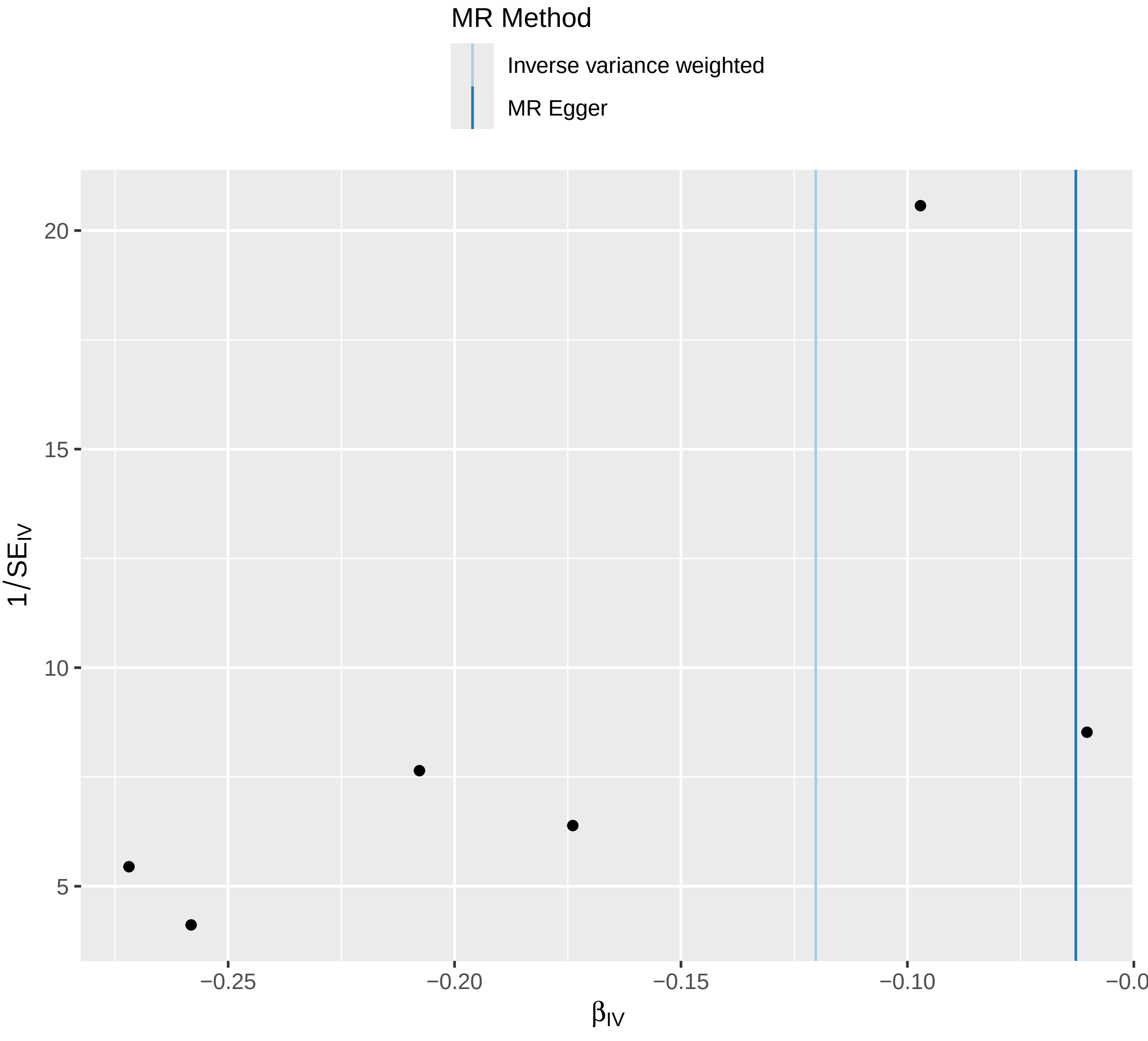
MR Method
Inverse variance weighted
MR Egger
20
15
10
5
1/SE$_{IV}$
−0.25
−0.20
−0.15
−0.10
−0.0
β$_{IV}$

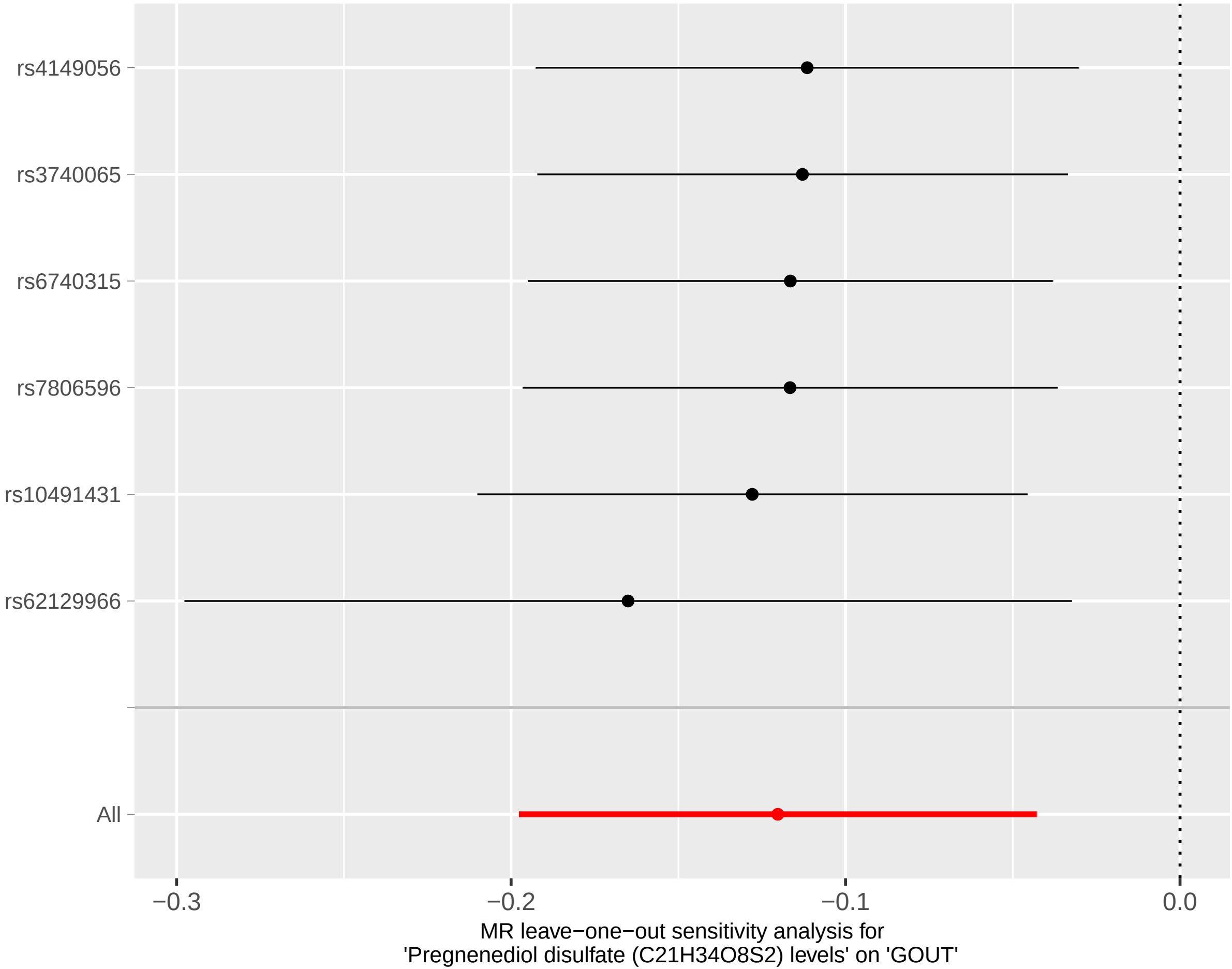
rs4149056
rs3740065
rs6740315
rs7806596
rs10491431
rs62129966
All
−0.3
−0.2
−0.1
0.0
MR leave−one−out sensitivity analysis for
'Pregnenediol disulfate (C21H34O8S2) levels' on 'GOUT'

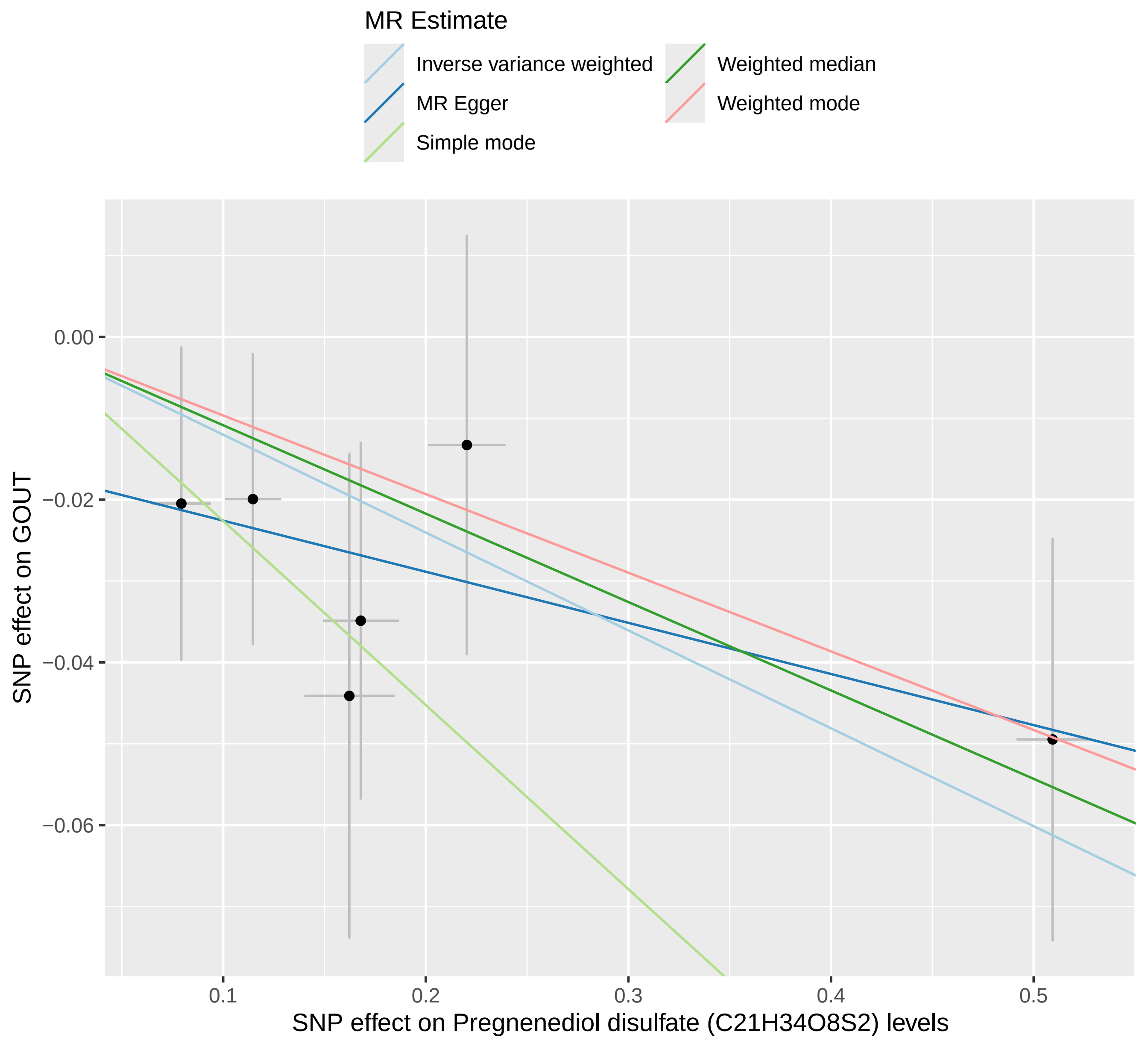
MR Estimate
Inverse variance weighted
MR Egger
Simple mode
Weighted median
Weighted mode
0.00
−0.02
−0.04
−0.06
SNP effect on GOUT
0.1
0.2
0.3
0.4
0.5
SNP effect on Pregnenediol disulfate (C21H34O8S2) levels

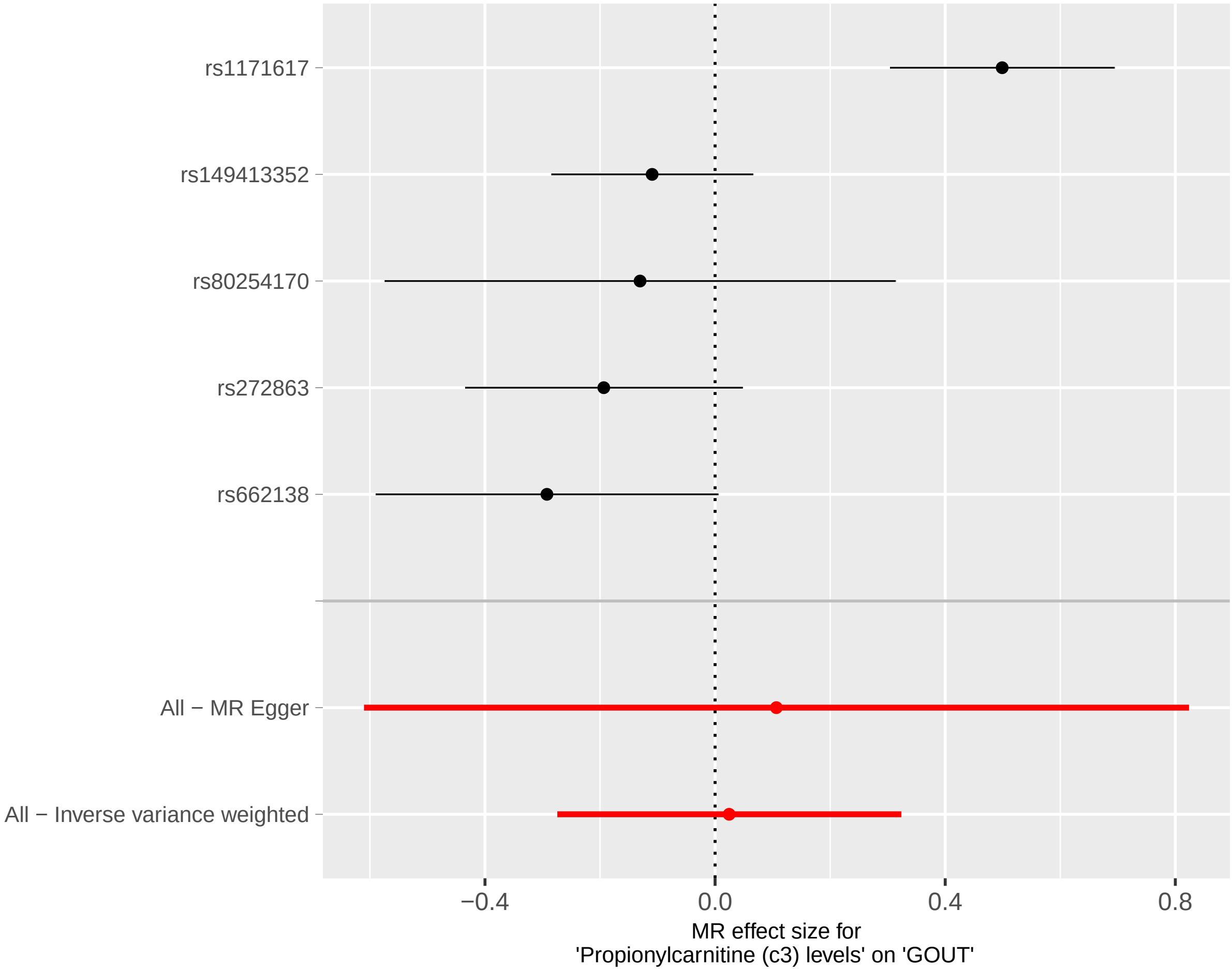
rs1171617
rs149413352
rs80254170
rs272863
rs662138
All − MR Egger
All − Inverse variance weighted
−0.4
0.0
0.4
0.8
MR effect size for
'Propionylcarnitine (c3) levels' on 'GOUT'

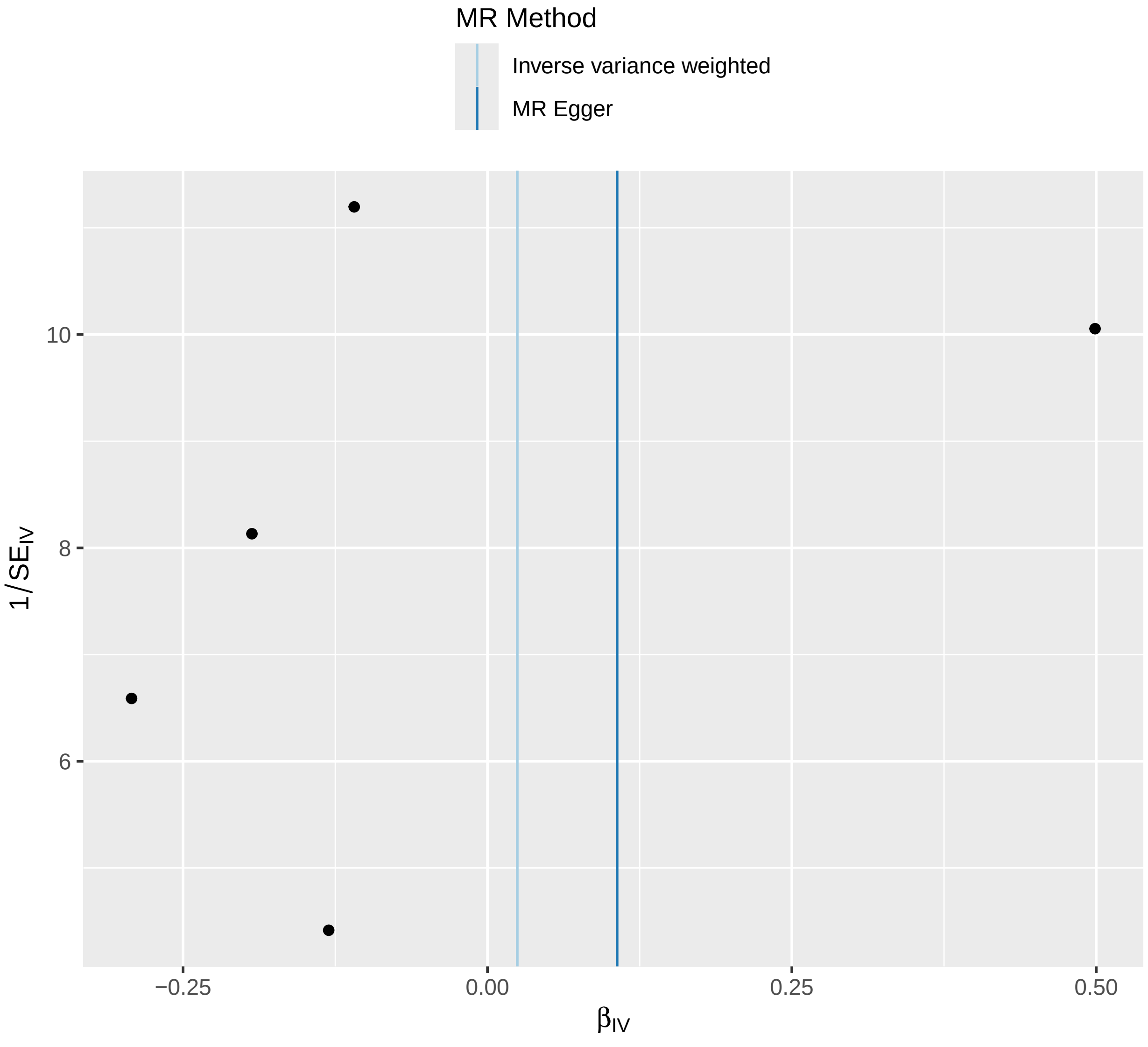
MR Method
Inverse variance weighted
MR Egger
10
8
6
1/SE$_{IV}$
−0.25
0.00
0.25
0.50
β$_{IV}$

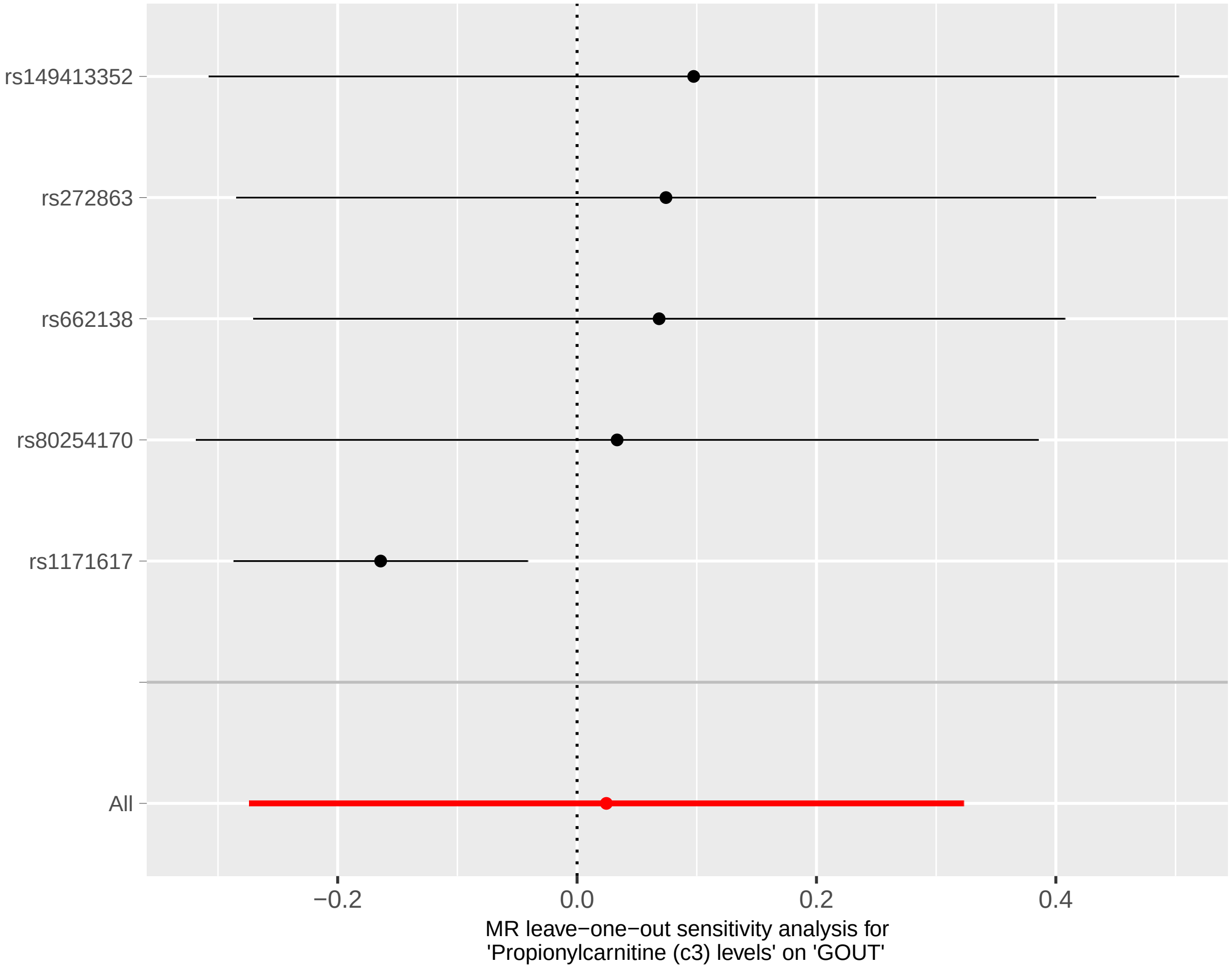
rs149413352
rs272863
rs662138
rs80254170
rs1171617
All
−0.2
0.0
0.2
0.4
MR leave−one−out sensitivity analysis for
'Propionylcarnitine (c3) levels' on 'GOUT'

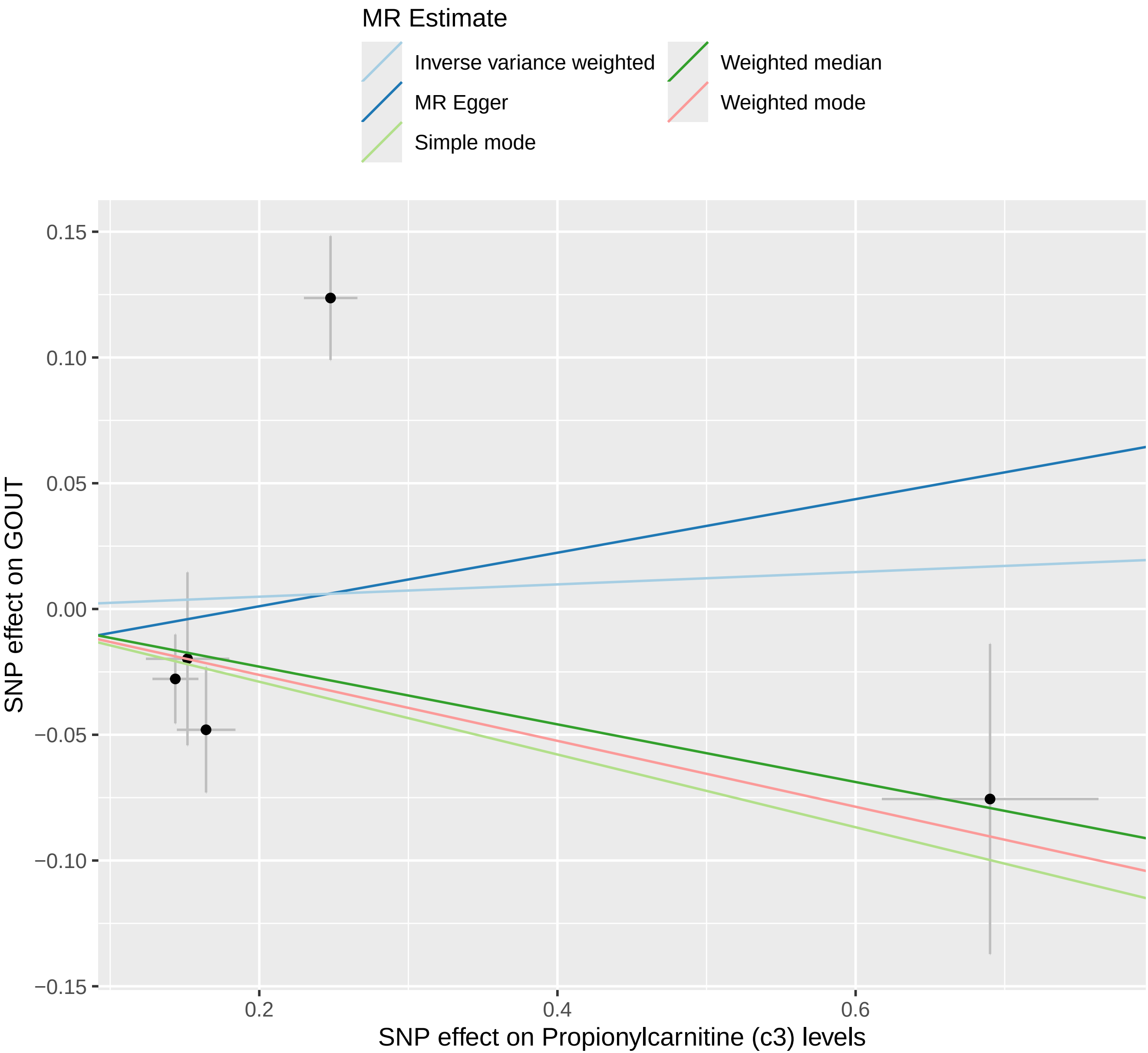
MR Estimate
Inverse variance weighted
MR Egger
Simple mode
Weighted median
Weighted mode
0.15
0.10
0.05
0.00
−0.05
−0.10
−0.15
SNP effect on GOUT
0.2
0.4
0.6
SNP effect on Propionylcarnitine (c3) levels

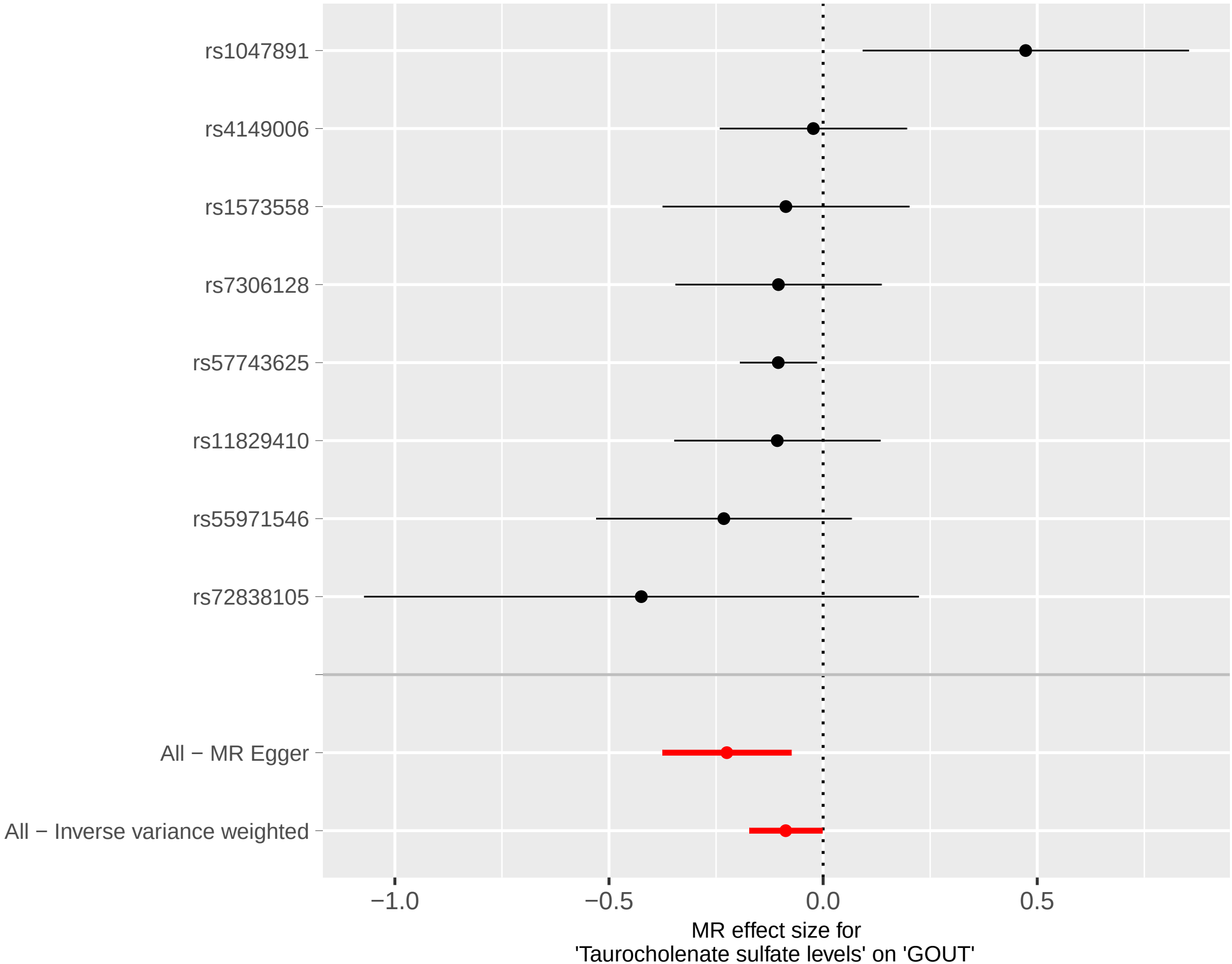
rs1047891
rs4149006
rs1573558
rs7306128
rs57743625
rs11829410
rs55971546
rs72838105
All − MR Egger
All − Inverse variance weighted
−1.0
−0.5
0.0
0.5
MR effect size for
'Taurocholenate sulfate levels' on 'GOUT'

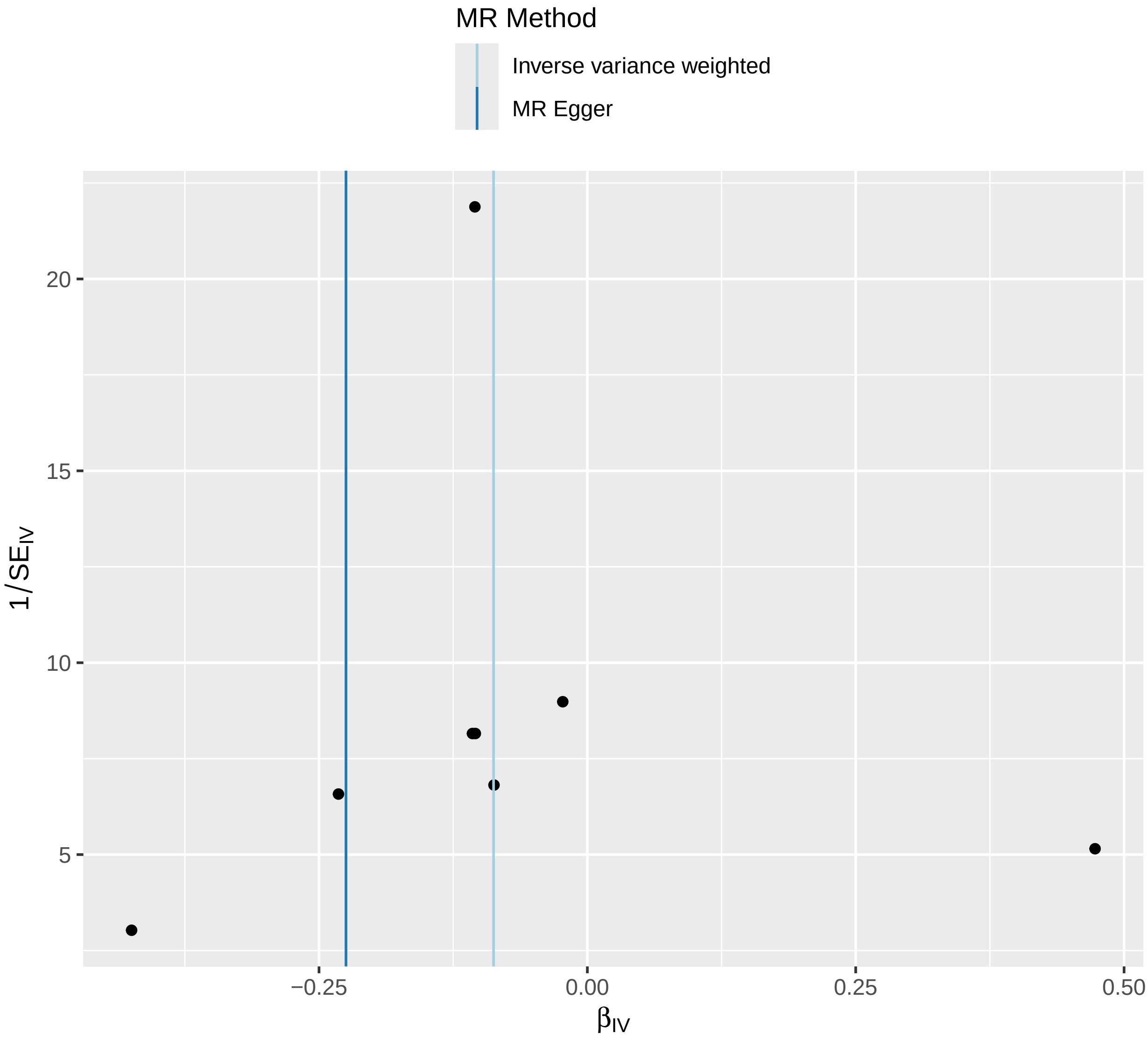

MR Method
Inverse variance weighted
MR Egger
20
15
10
5
1/SE_{IV}
−0.25
0.00
0.25
0.50
β_{IV}

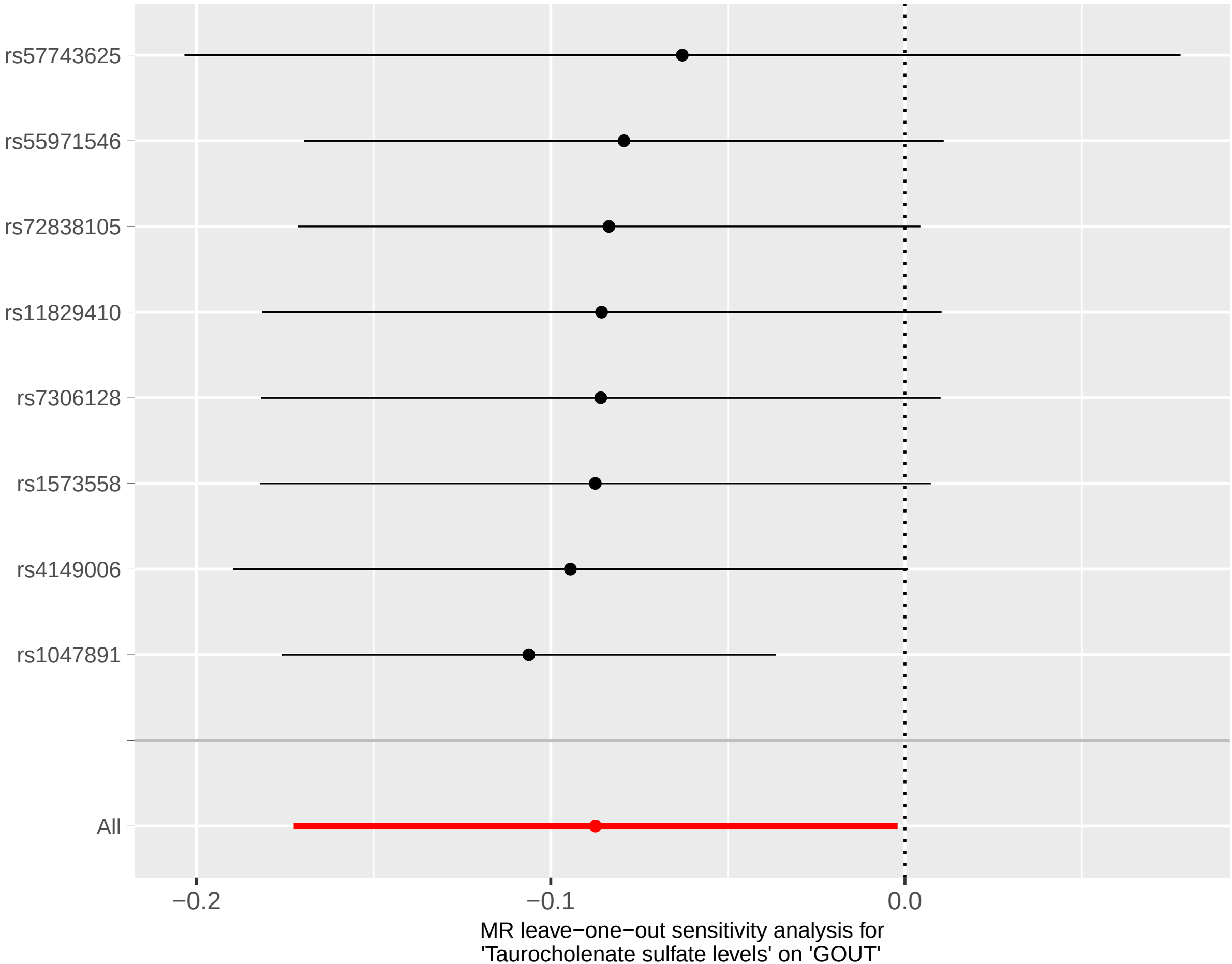
rs57743625
rs55971546
rs72838105
rs11829410
rs7306128
rs1573558
rs4149006
rs1047891
All
−0.2
−0.1
0.0
MR leave−one−out sensitivity analysis for
'Taurocholenate sulfate levels' on 'GOUT'

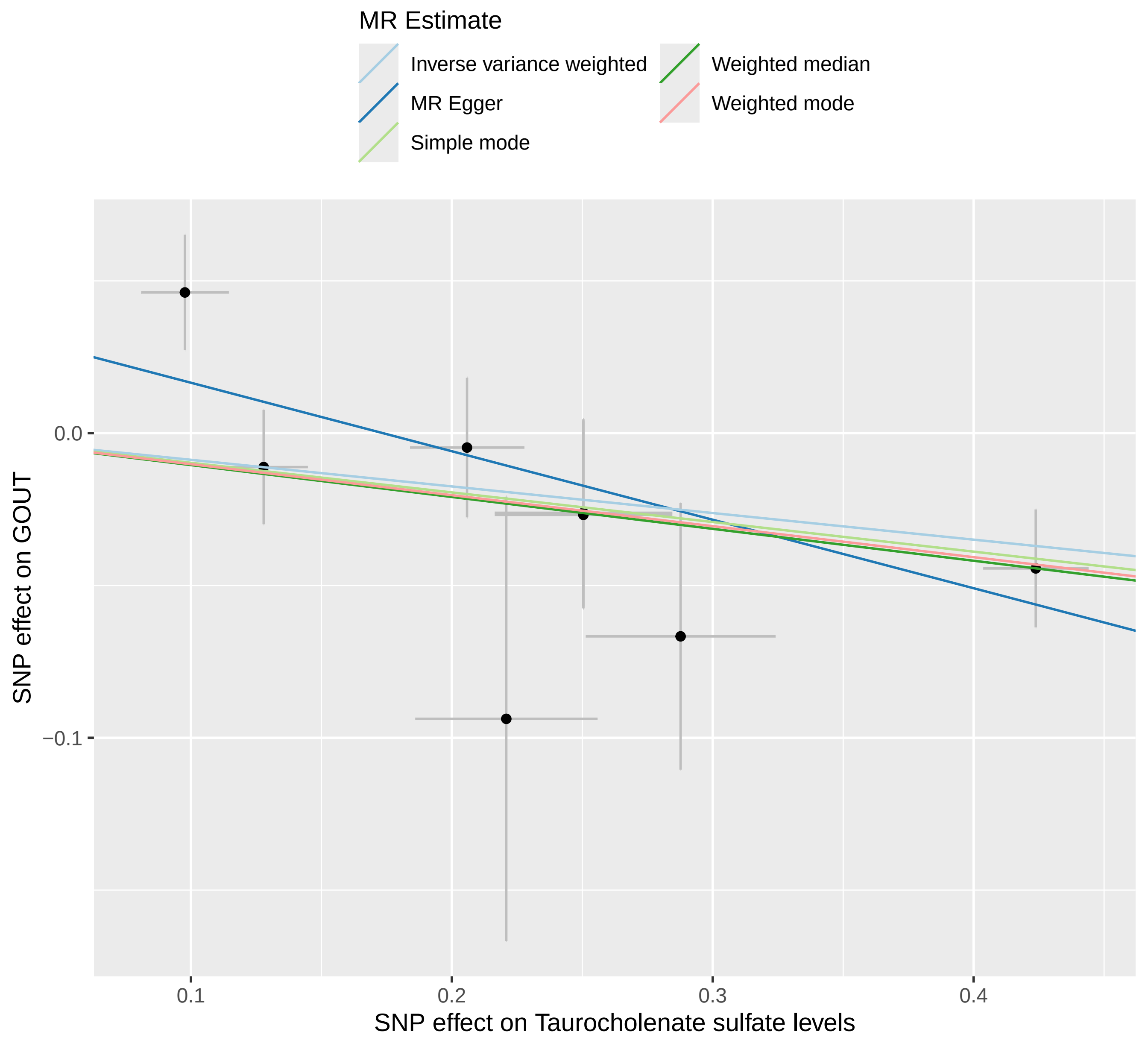
MR Estimate
Inverse variance weighted
MR Egger
Simple mode
Weighted median
Weighted mode
0.0
−0.1
0.1
0.2
0.3
0.4
SNP effect on GOUT
SNP effect on Taurocholenate sulfate levels

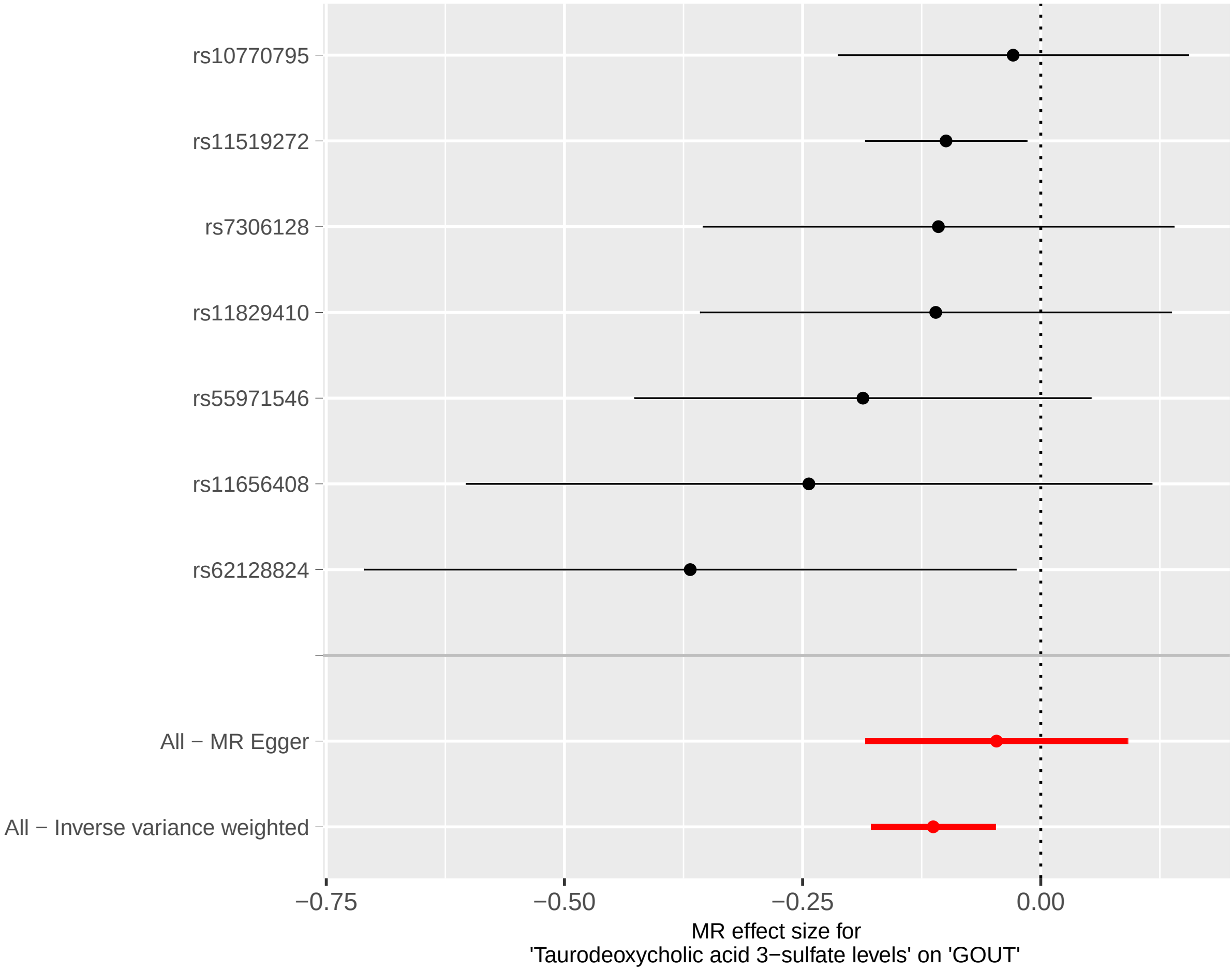
rs10770795
rs11519272
rs7306128
rs11829410
rs55971546
rs11656408
rs62128824
All − MR Egger
All − Inverse variance weighted
−0.75
−0.50
−0.25
0.00
MR effect size for
'Taurodeoxycholic acid 3−sulfate levels' on 'GOUT'

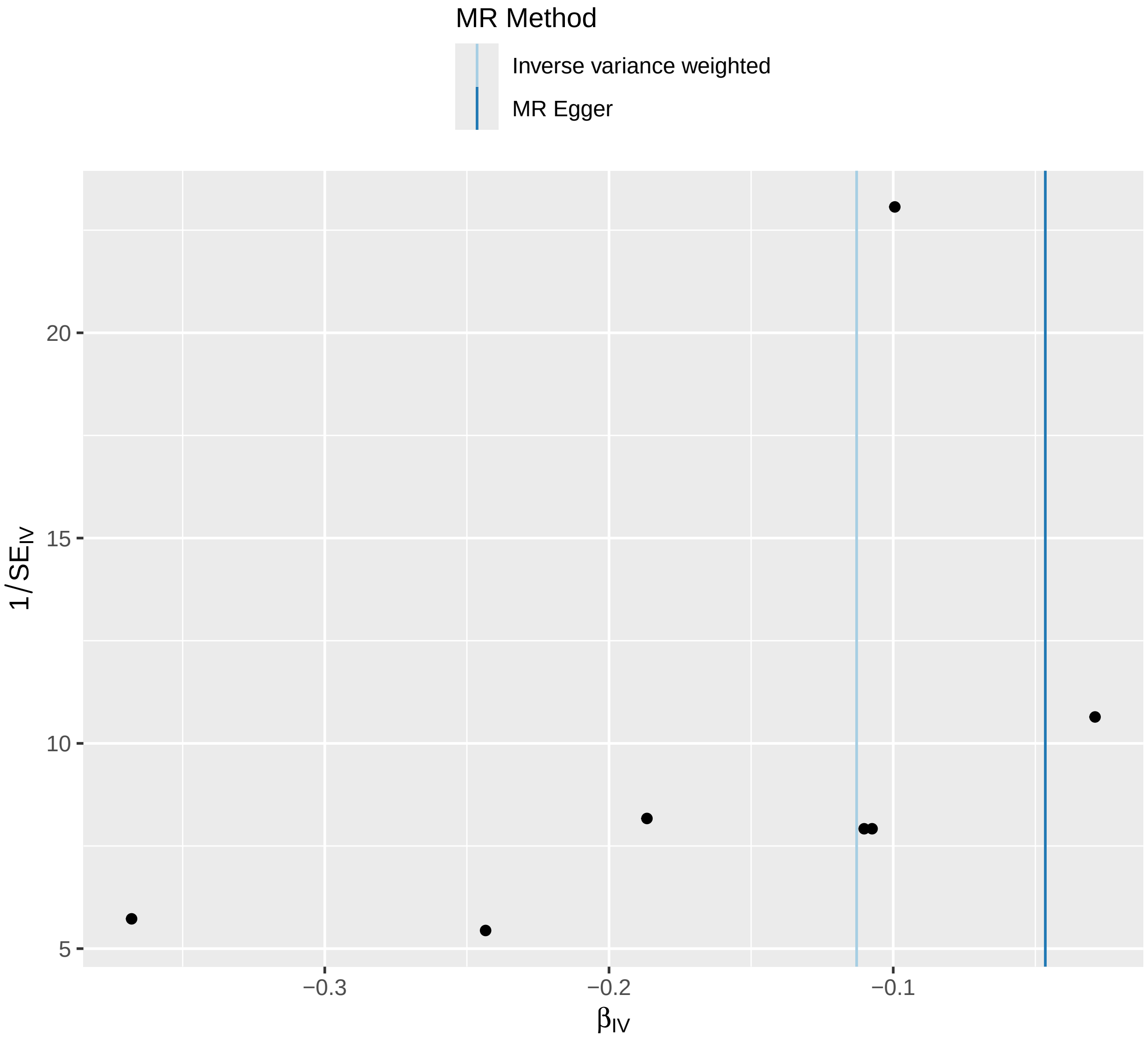
MR Method
Inverse variance weighted
MR Egger
20
15
10
5
1/SE$_{IV}$
−0.3
−0.2
−0.1
β$_{IV}$

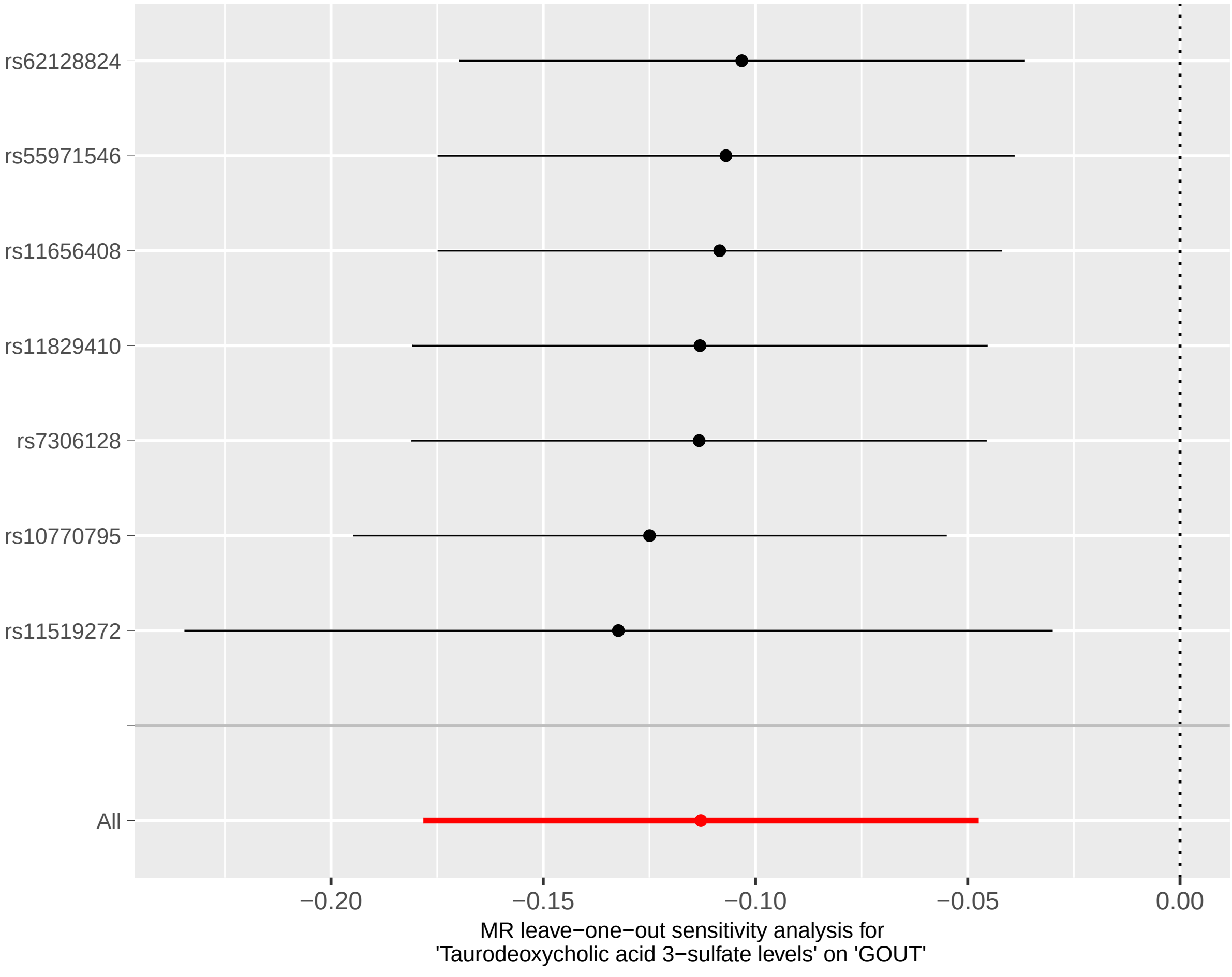

rs62128824
rs55971546
rs11656408
rs11829410
rs7306128
rs10770795
rs11519272
All
−0.20
−0.15
−0.10
−0.05
0.00
MR leave−one−out sensitivity analysis for
'Taurodeoxycholic acid 3−sulfate levels' on 'GOUT'

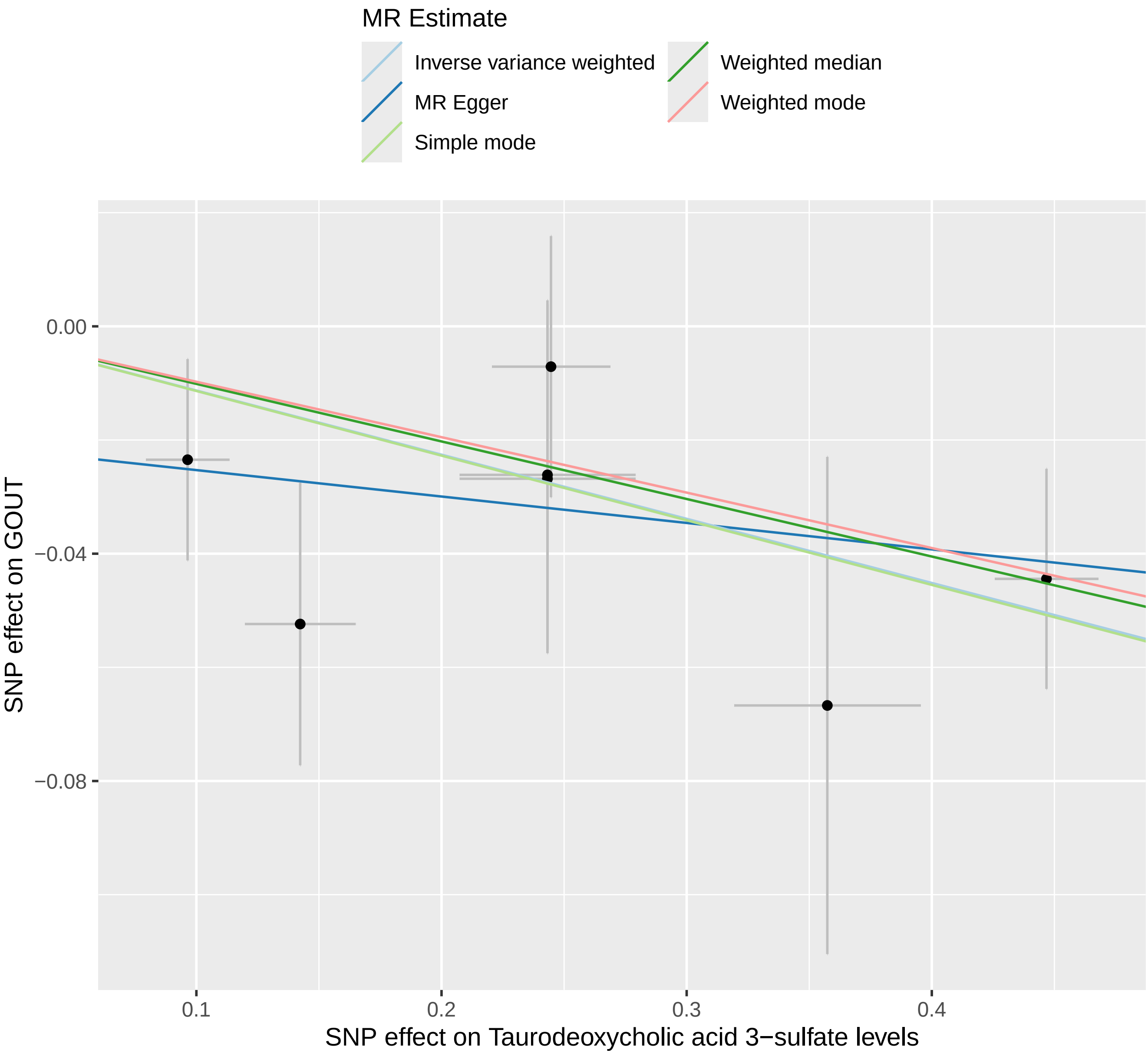
MR Estimate
Inverse variance weighted
MR Egger
Simple mode
Weighted median
Weighted mode
0.00
−0.04
−0.08
SNP effect on GOUT
0.1
0.2
0.3
0.4
SNP effect on Taurodeoxycholic acid 3−sulfate levels

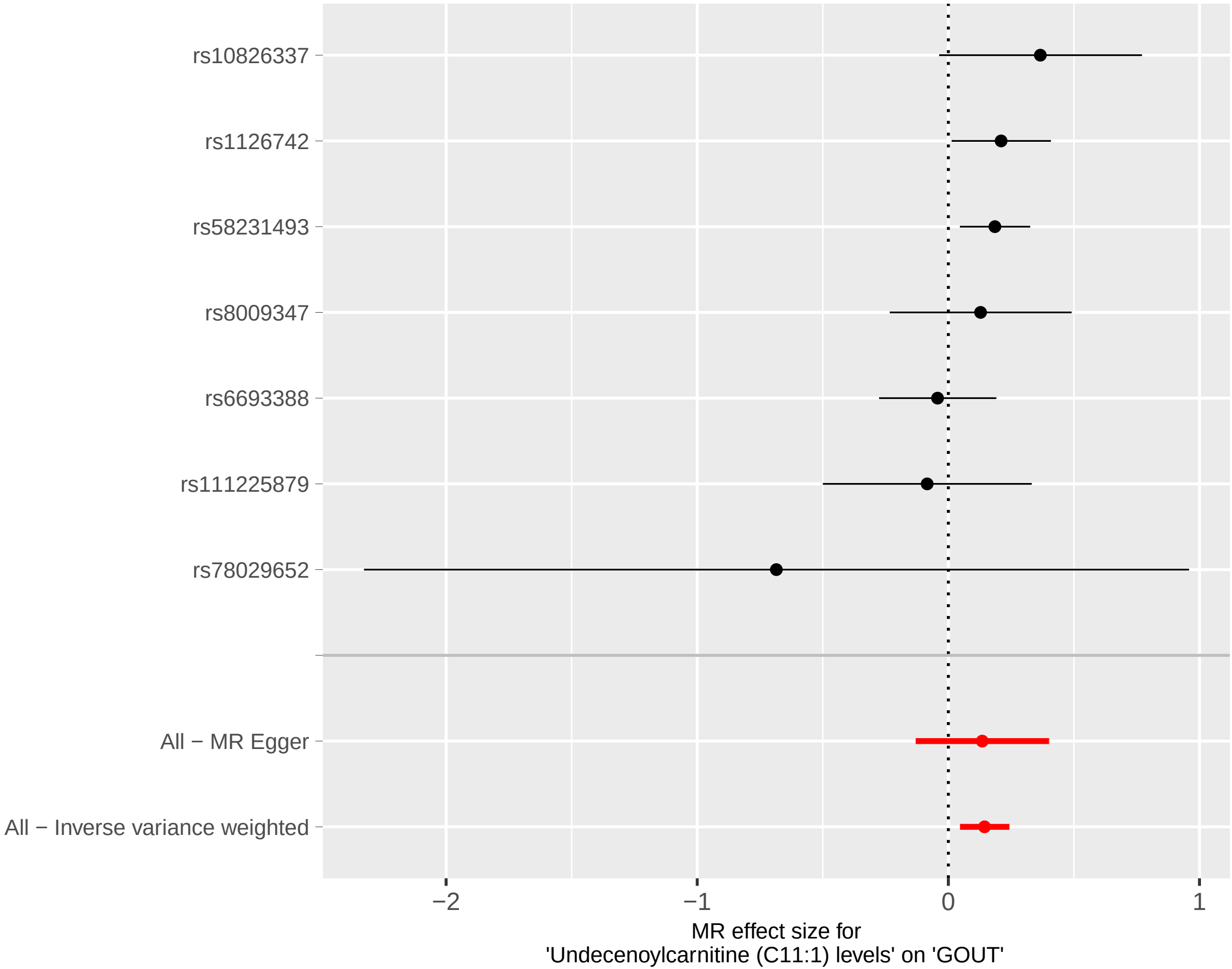
rs10826337
rs1126742
rs58231493
rs8009347
rs6693388
rs111225879
rs78029652
All − MR Egger
All − Inverse variance weighted
−2
−1
0
1
MR effect size for
'Undecenoylcarnitine (C11:1) levels' on 'GOUT'

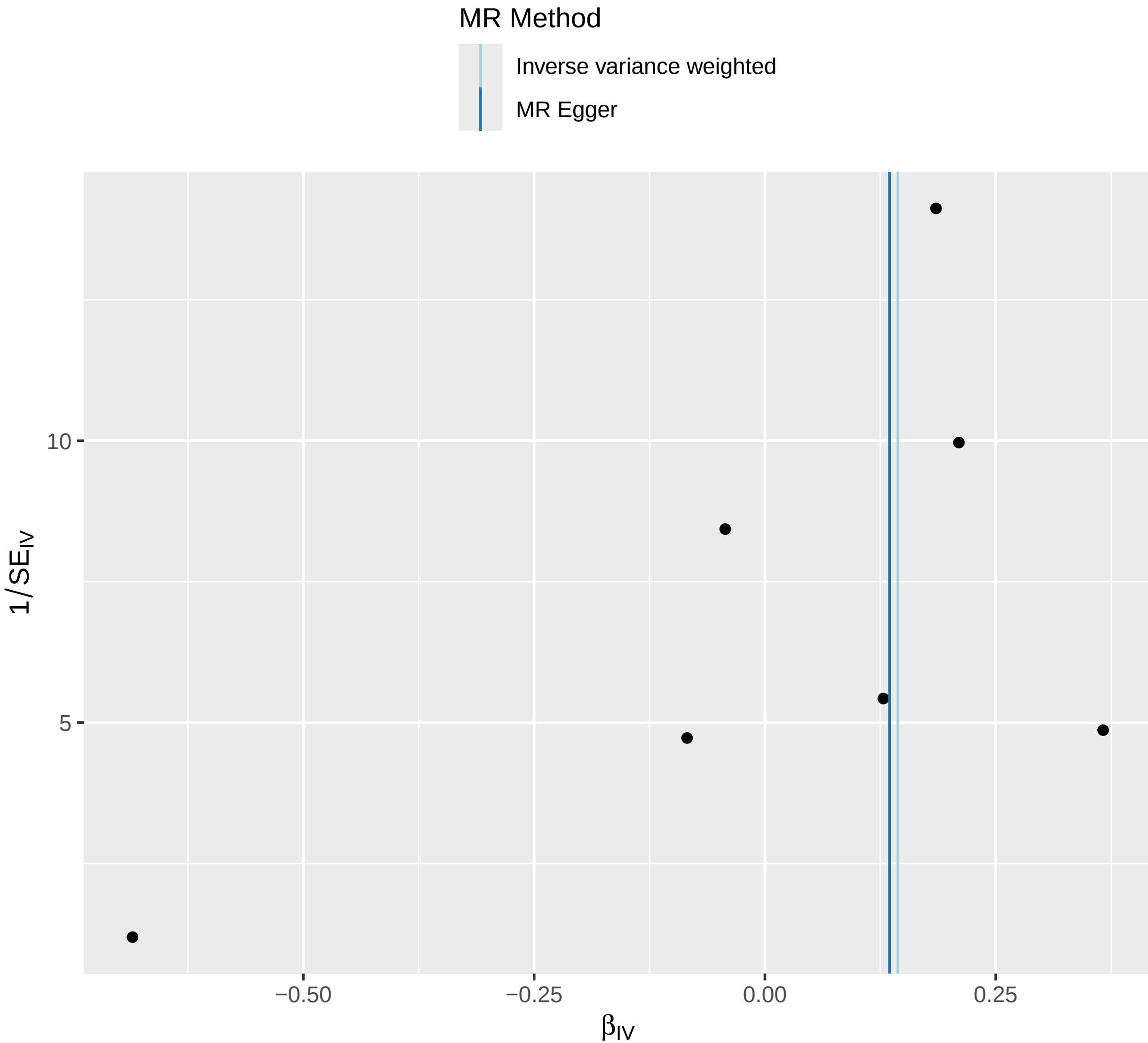

MR Method
Inverse variance weighted
MR Egger
10
5
1/SE$_{IV}$
−0.50
−0.25
0.00
0.25
β$_{IV}$

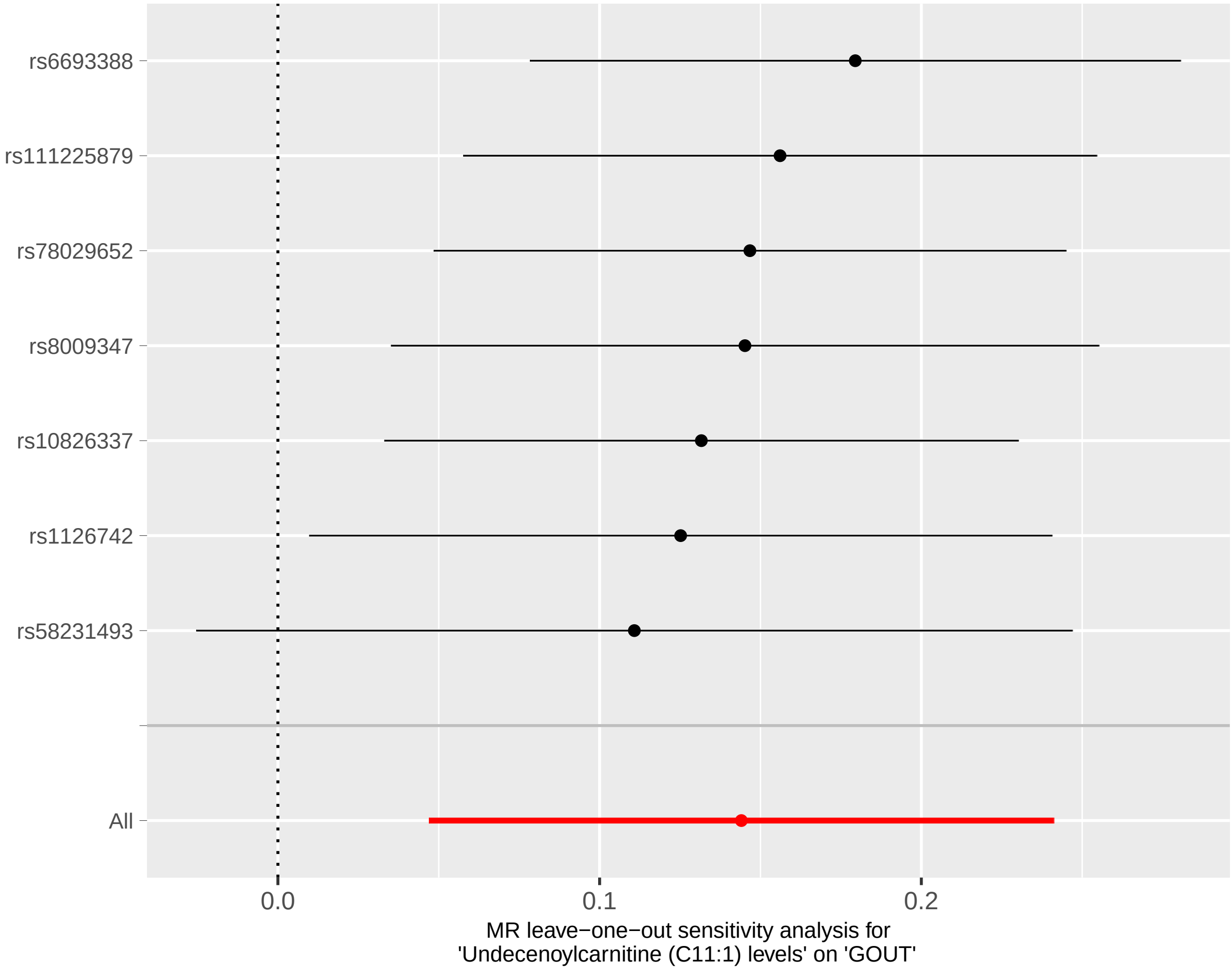
rs6693388
rs111225879
rs78029652
rs8009347
rs10826337
rs1126742
rs58231493
All
0.0
0.1
0.2
MR leave−one−out sensitivity analysis for
'Undecenoylcarnitine (C11:1) levels' on 'GOUT'

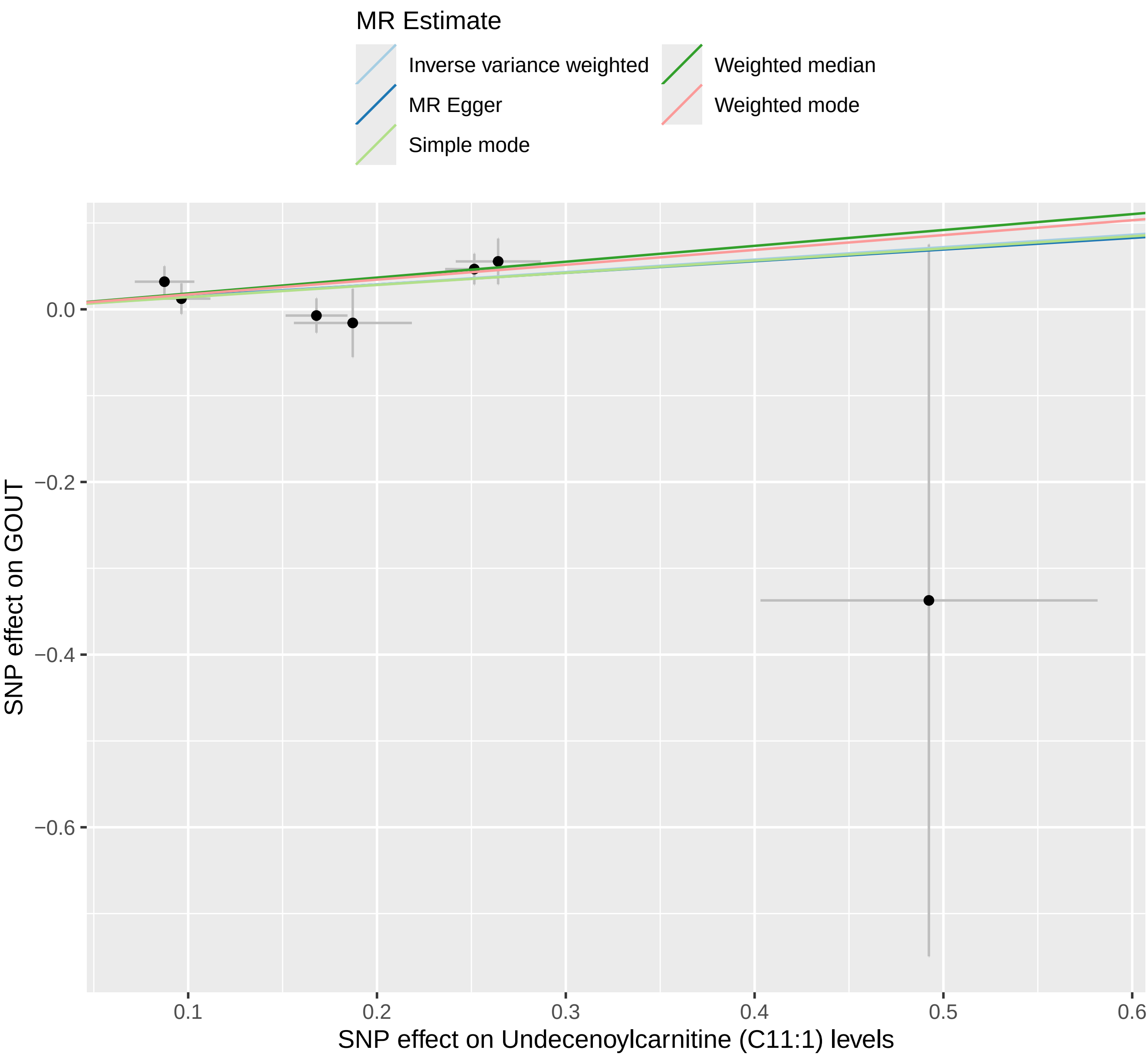
MR Estimate
Inverse variance weighted
MR Egger
Simple mode
Weighted median
Weighted mode
0.0
−0.2
−0.4
−0.6
0.1
0.2
0.3
0.4
0.5
0.6
SNP effect on GOUT
SNP effect on Undecenoylcarnitine (C11:1) levels

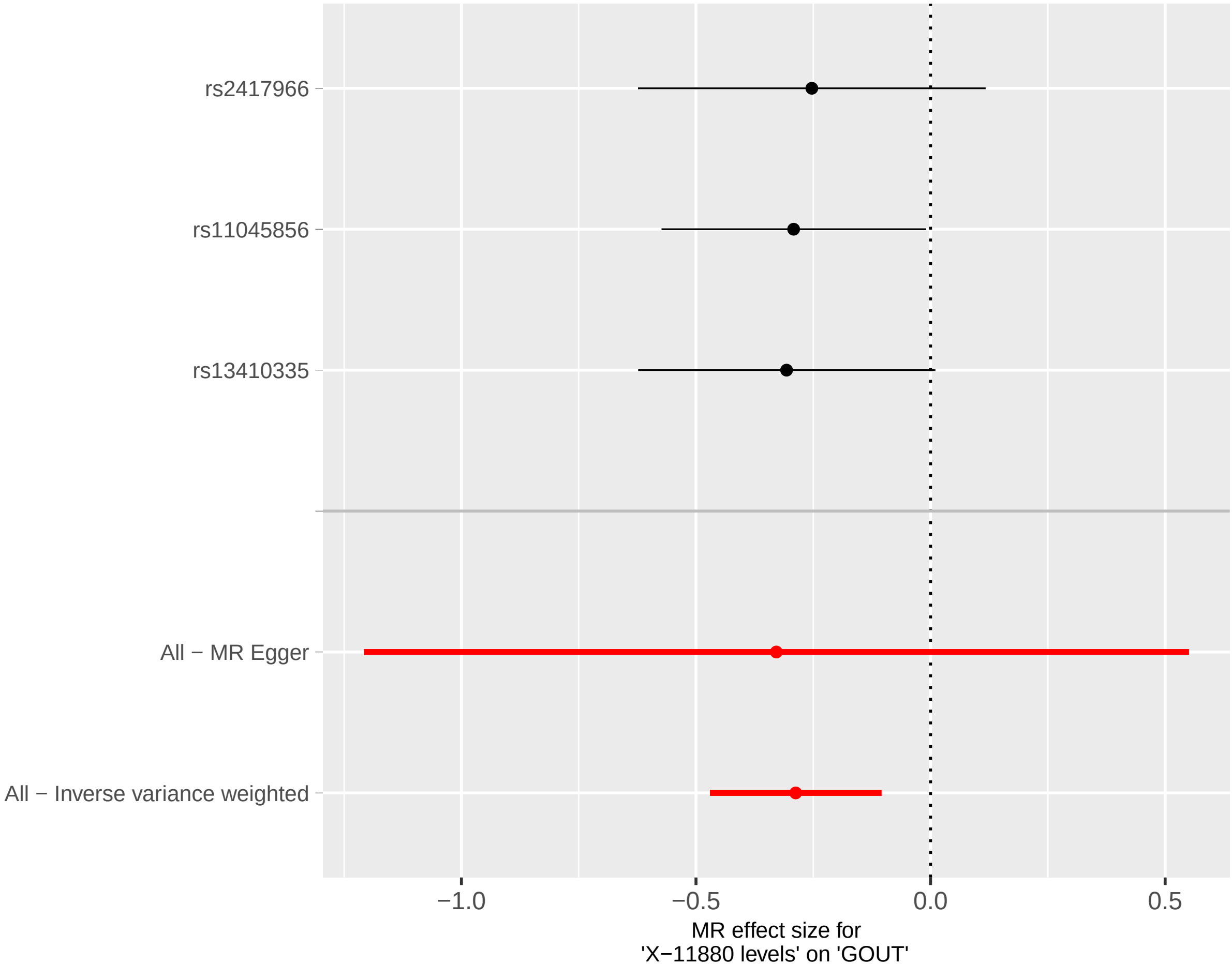

rs2417966
rs11045856
rs13410335
All − MR Egger
All − Inverse variance weighted
−1.0
−0.5
0.0
0.5
MR effect size for
'X−11880 levels' on 'GOUT'

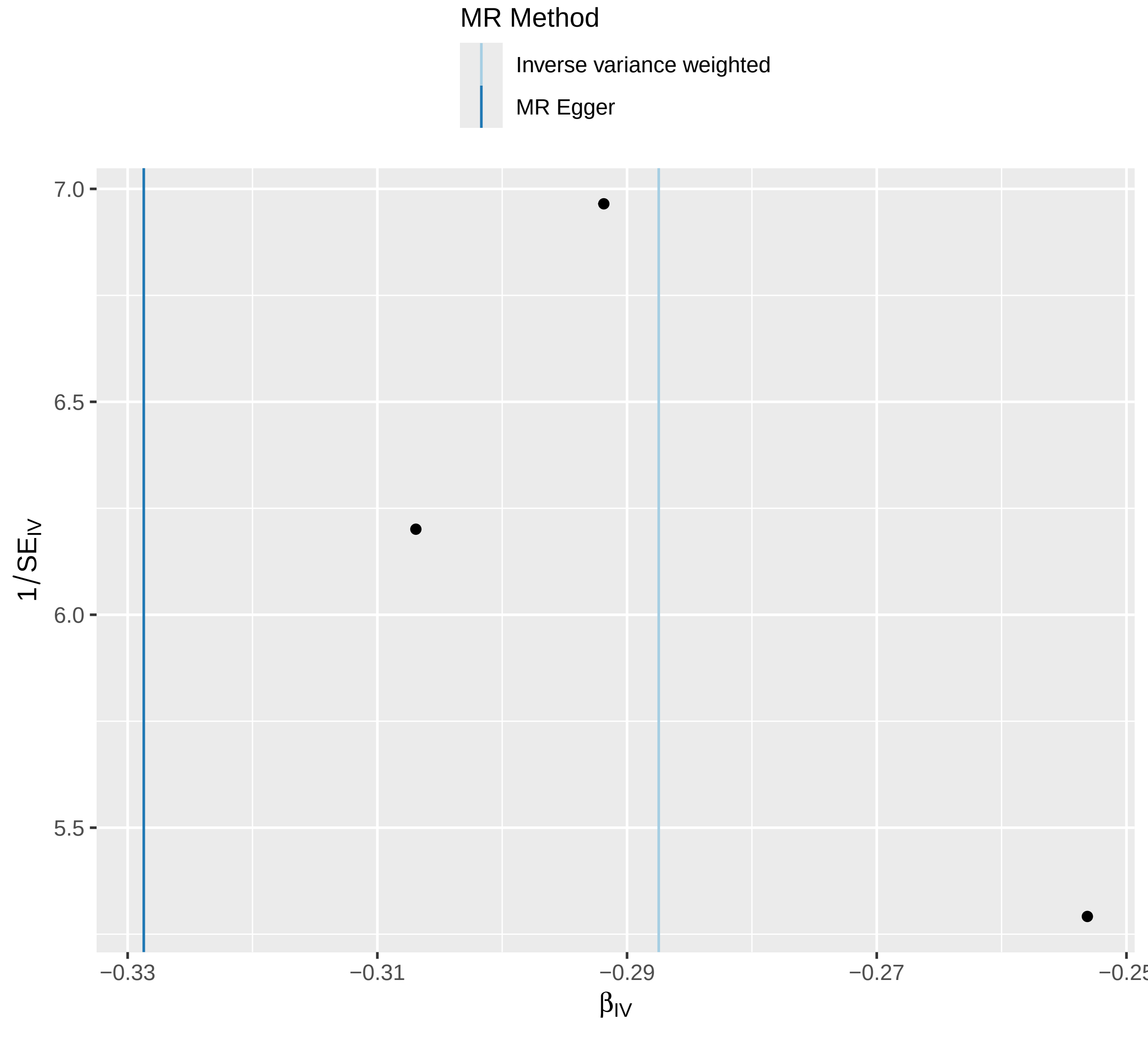
MR Method
Inverse variance weighted
MR Egger
7.0
6.5
6.0
5.5
$1/SE_{IV}$
−0.33
−0.31
−0.29
−0.27
−0.25
β_{IV}

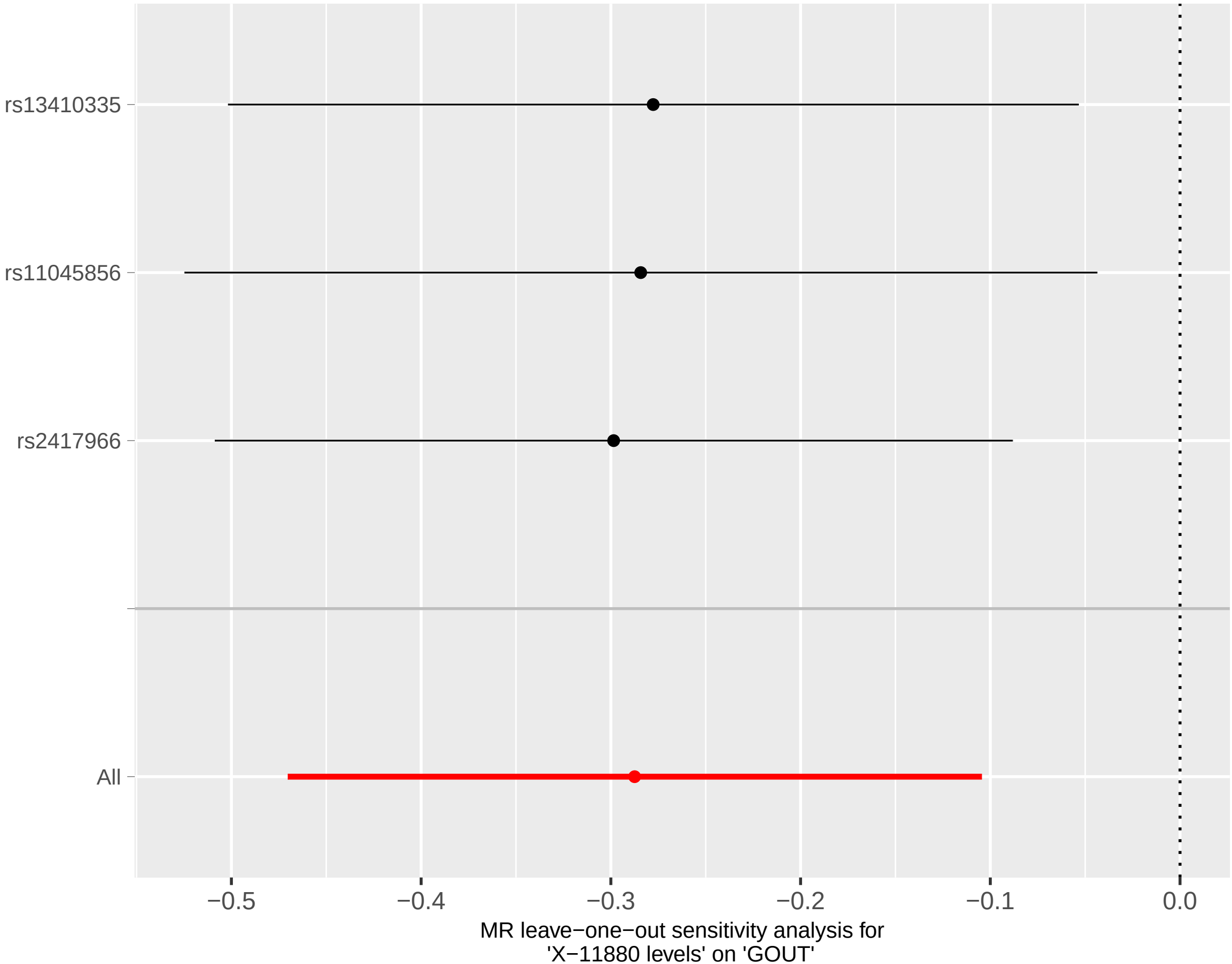
rs13410335
rs11045856
rs2417966
All
−0.5
−0.4
−0.3
−0.2
−0.1
0.0
MR leave−one−out sensitivity analysis for
'X−11880 levels' on 'GOUT'

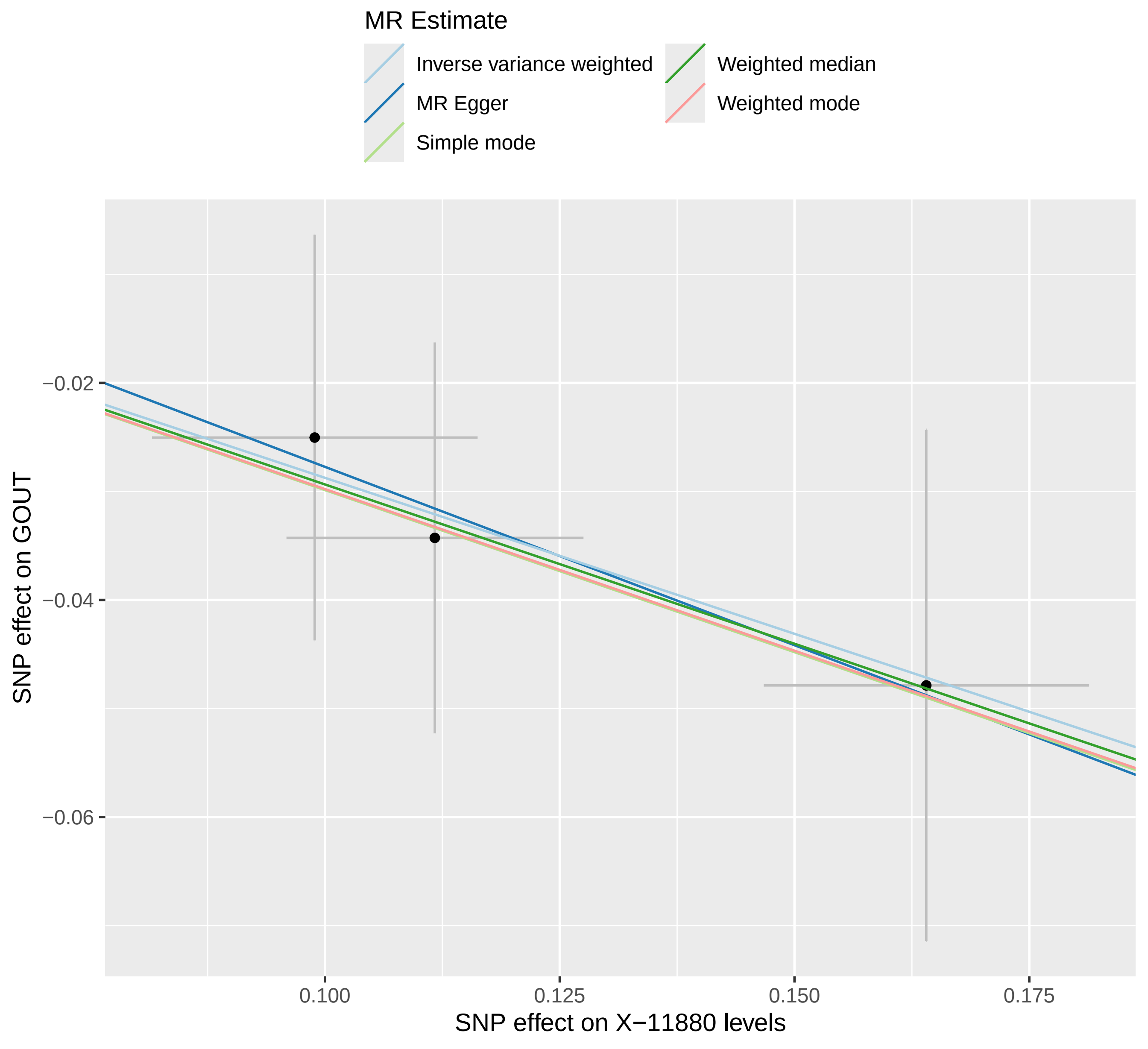
MR Estimate
Inverse variance weighted
MR Egger
Simple mode
Weighted median
Weighted mode
−0.02
−0.04
−0.06
SNP effect on GOUT
0.100
0.125
0.150
0.175
SNP effect on X−11880 levels

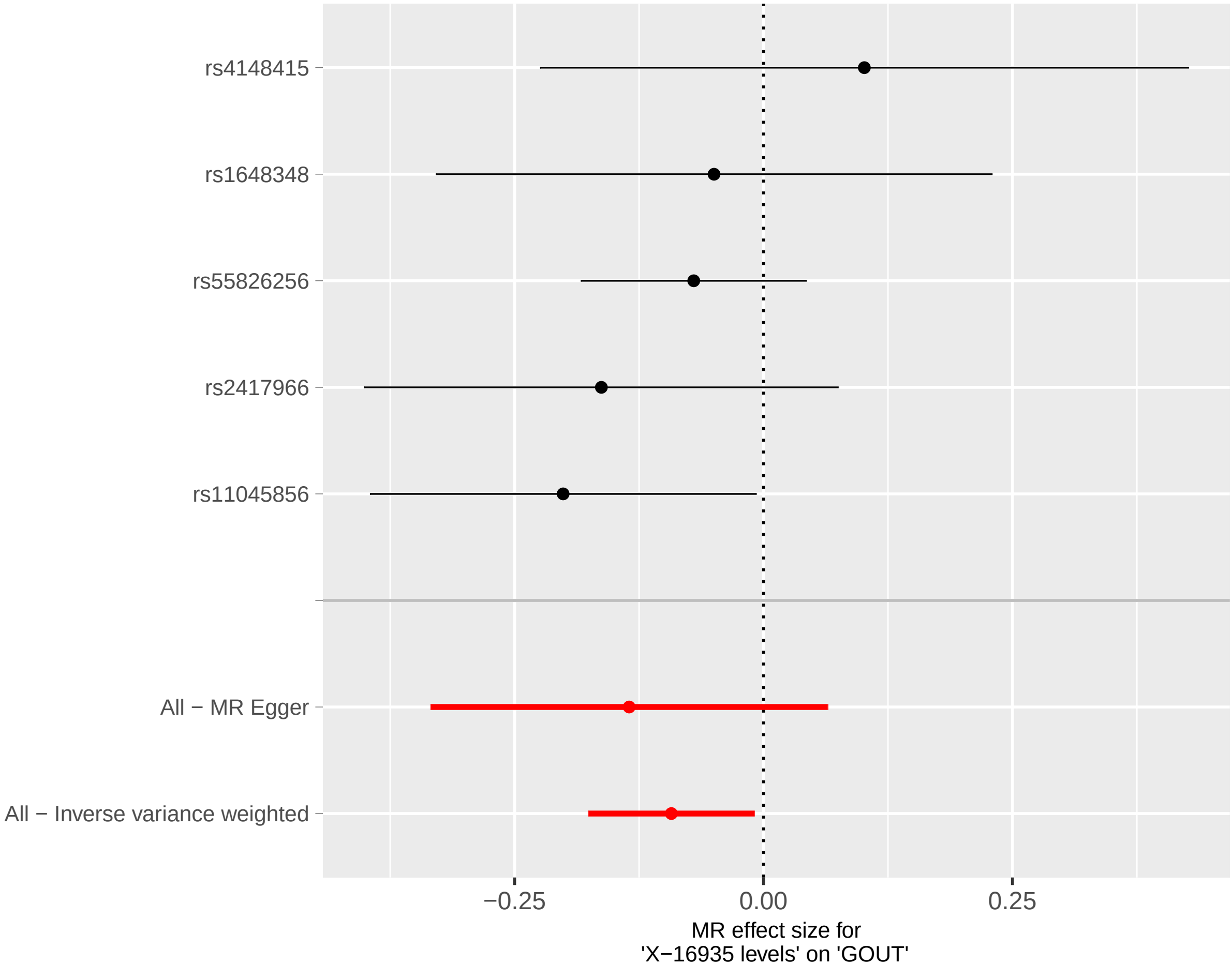
rs4148415
rs1648348
rs55826256
rs2417966
rs11045856
All − MR Egger
All − Inverse variance weighted
−0.25
0.00
0.25
MR effect size for
'X−16935 levels' on 'GOUT'

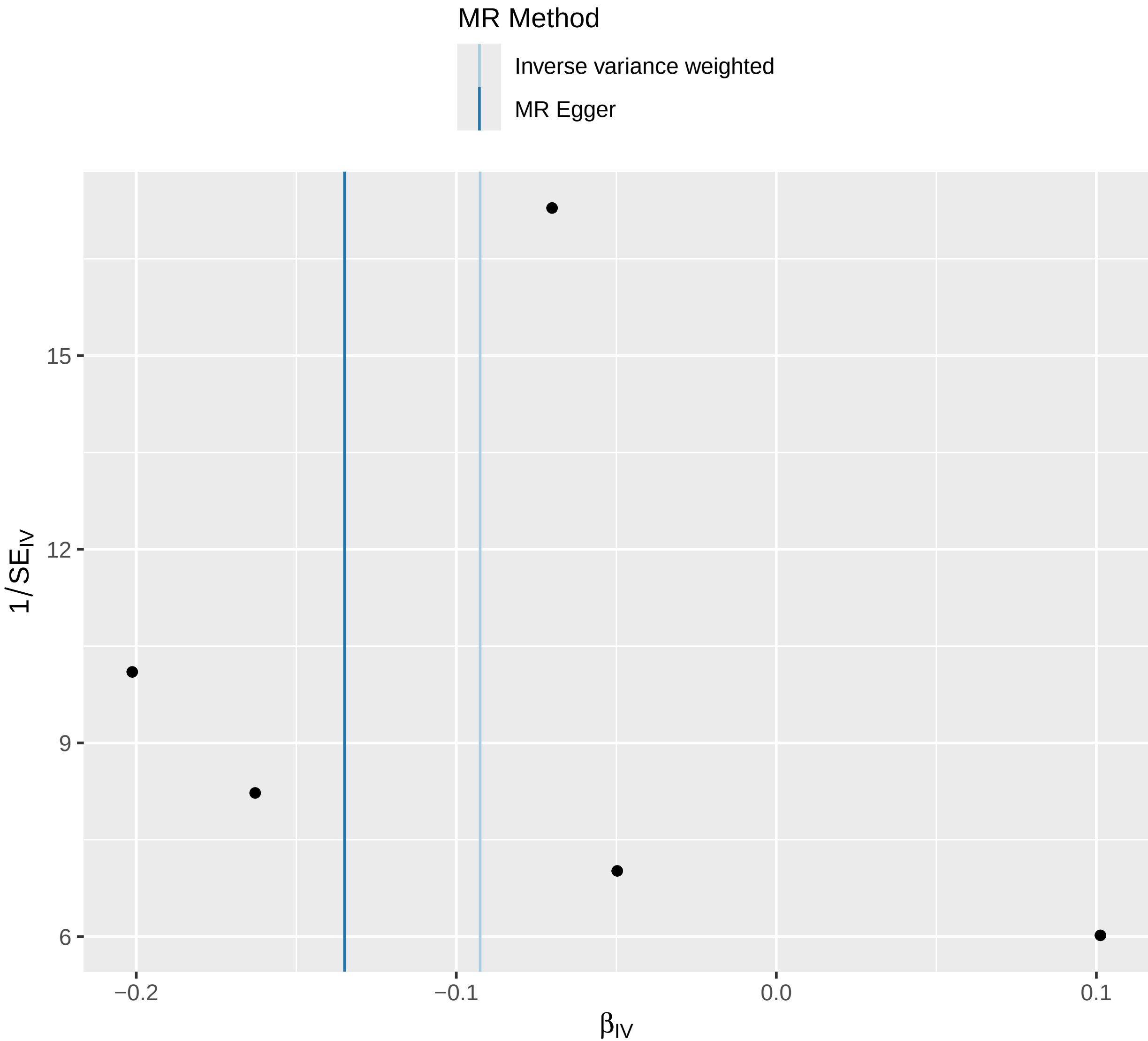
MR Method
Inverse variance weighted
MR Egger
15
12
9
6
1/SE_IV
−0.2
−0.1
0.0
0.1
β_IV

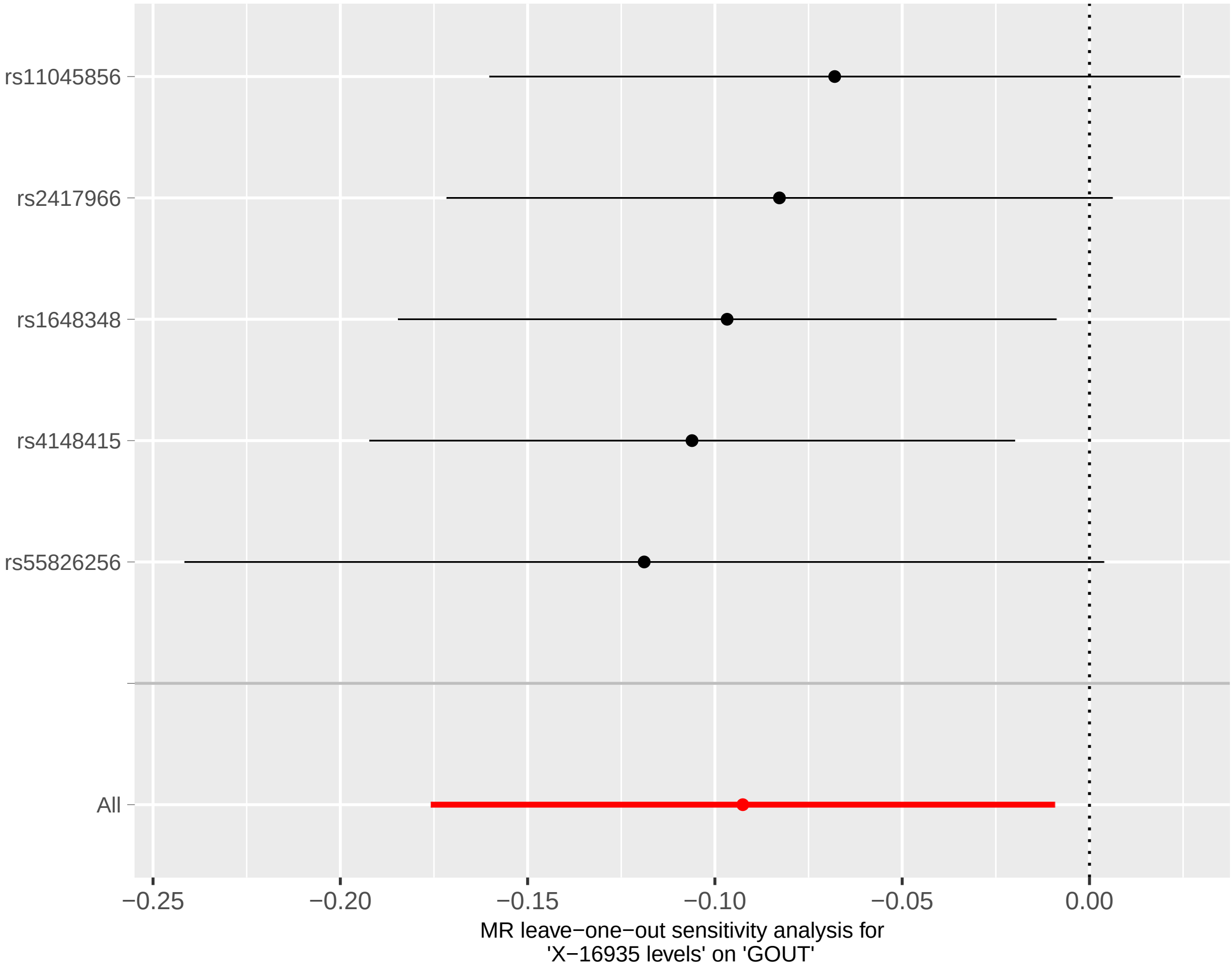
rs11045856
rs2417966
rs1648348
rs4148415
rs55826256
All
−0.25
−0.20
−0.15
−0.10
−0.05
0.00
MR leave−one−out sensitivity analysis for
'X−16935 levels' on 'GOUT'

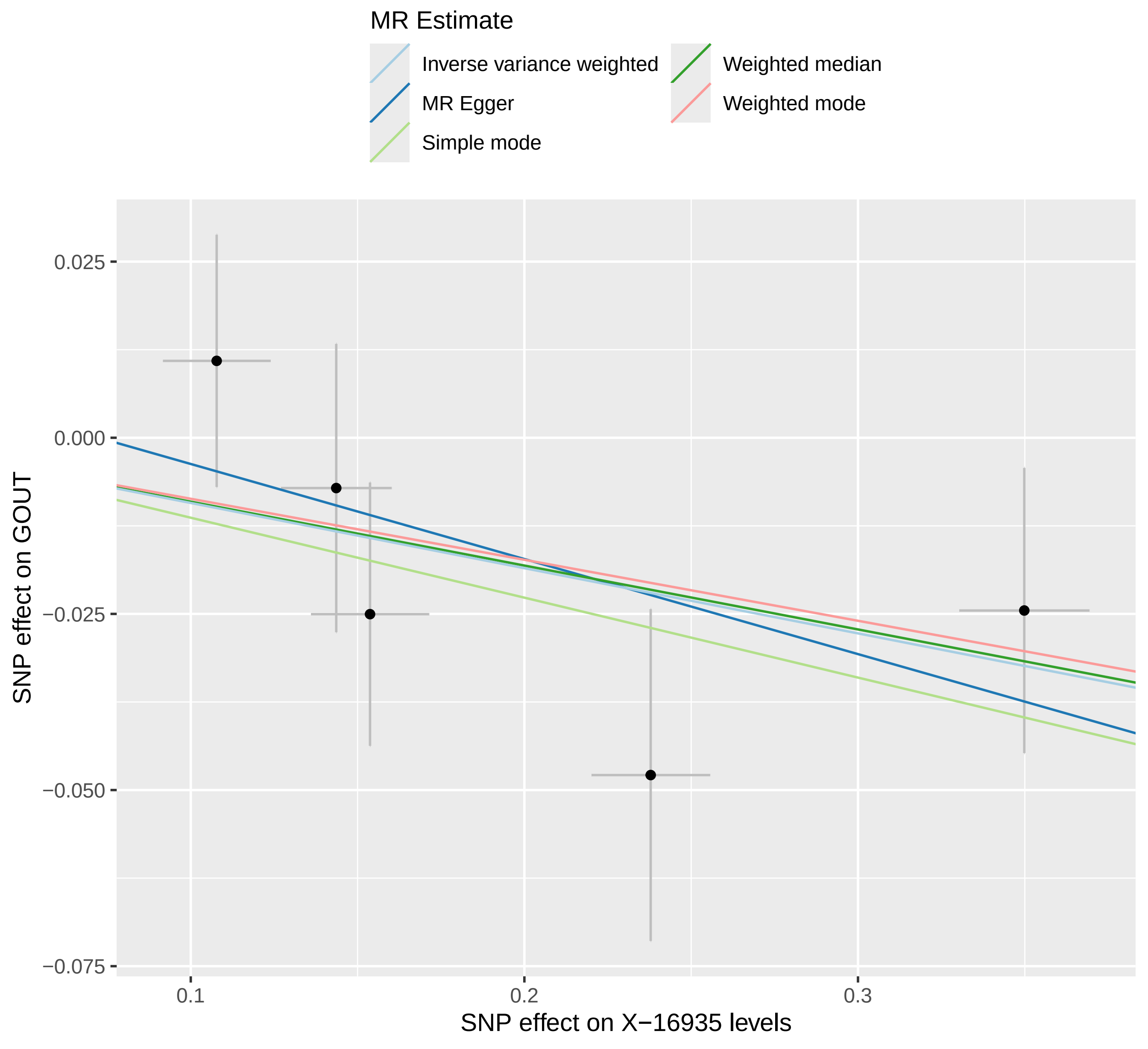
MR Estimate
Inverse variance weighted
MR Egger
Simple mode
Weighted median
Weighted mode
0.025
0.000
−0.025
−0.050
−0.075
SNP effect on GOUT
0.1
0.2
0.3
SNP effect on X−16935 levels

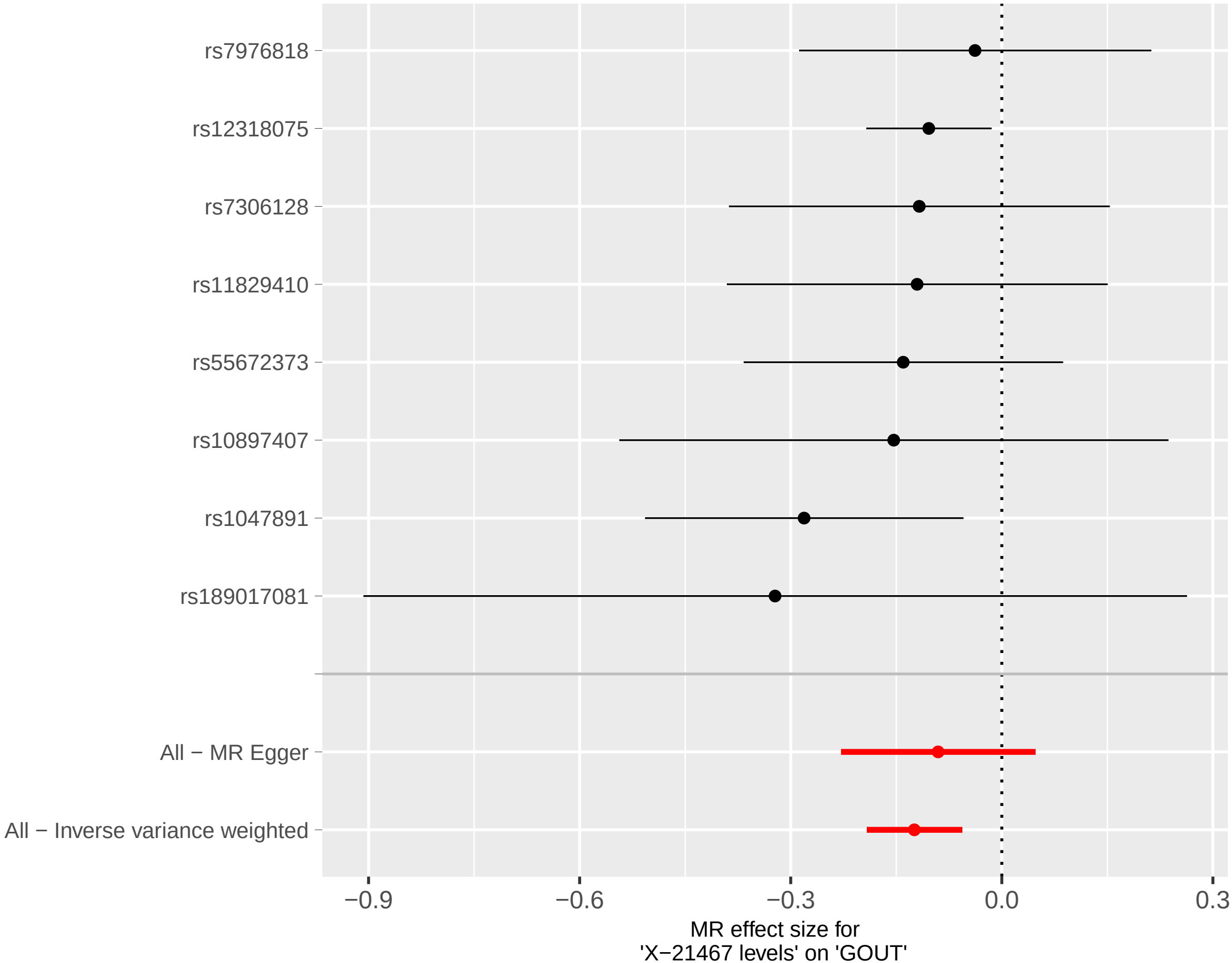
rs7976818
rs12318075
rs7306128
rs11829410
rs55672373
rs10897407
rs1047891
rs189017081
All − MR Egger
All − Inverse variance weighted
−0.9
−0.6
−0.3
0.0
0.3
MR effect size for
'X−21467 levels' on 'GOUT'

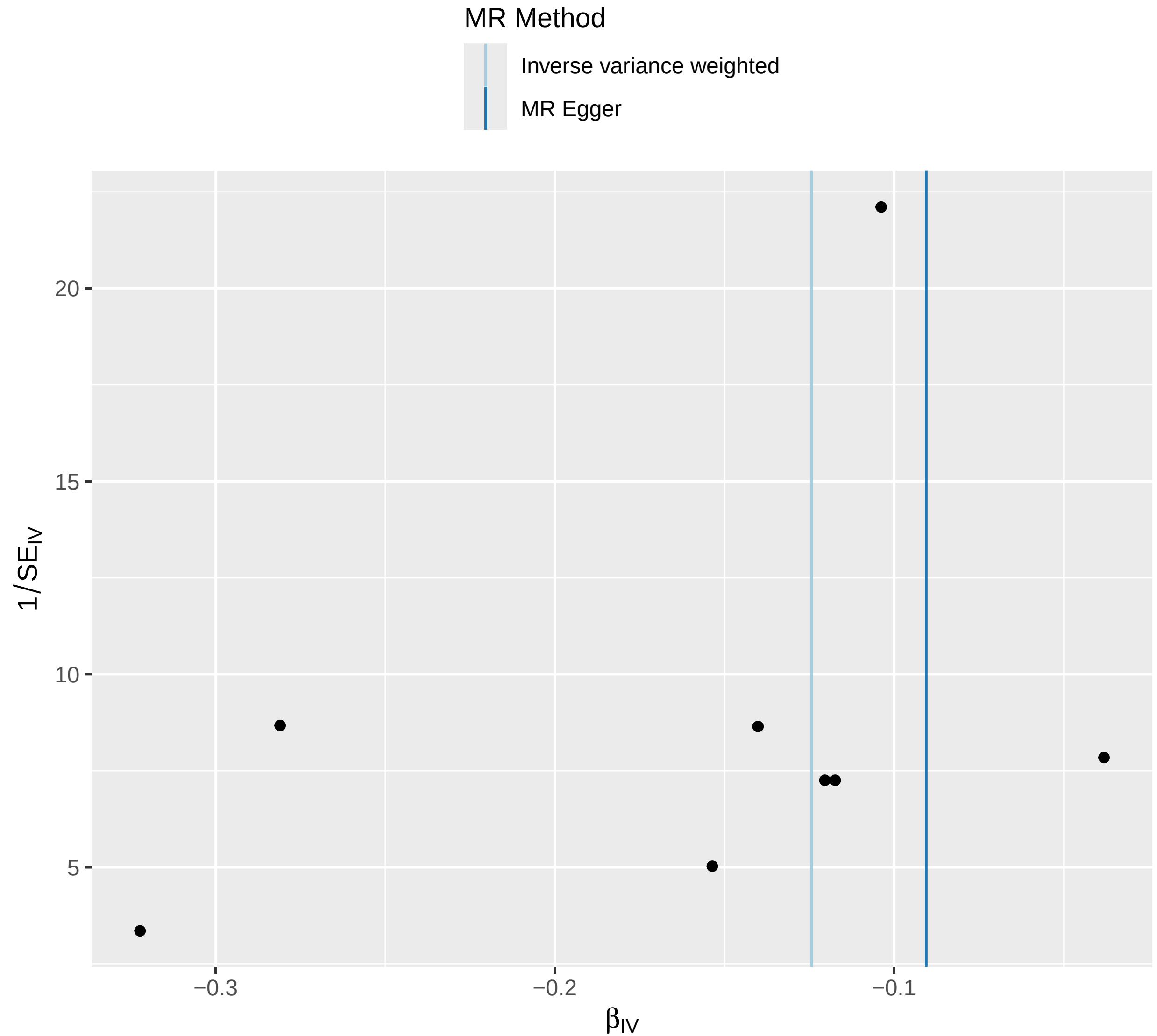
MR Method
Inverse variance weighted
MR Egger
20
15
10
5
1/SE_IV
−0.3
−0.2
−0.1
β_IV

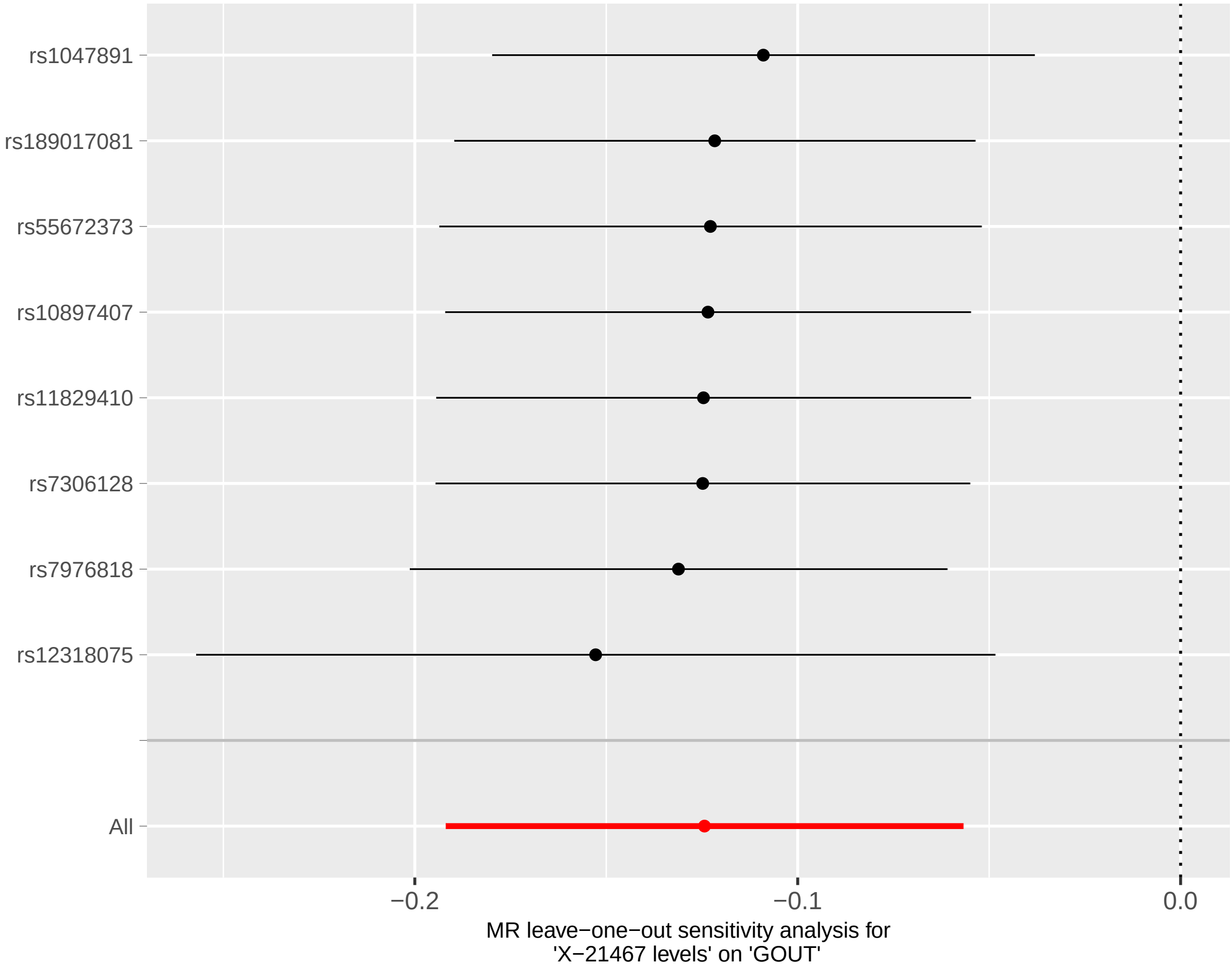
rs1047891
rs189017081
rs55672373
rs10897407
rs11829410
rs7306128
rs7976818
rs12318075
All
−0.2
−0.1
0.0
MR leave−one−out sensitivity analysis for
'X−21467 levels' on 'GOUT'

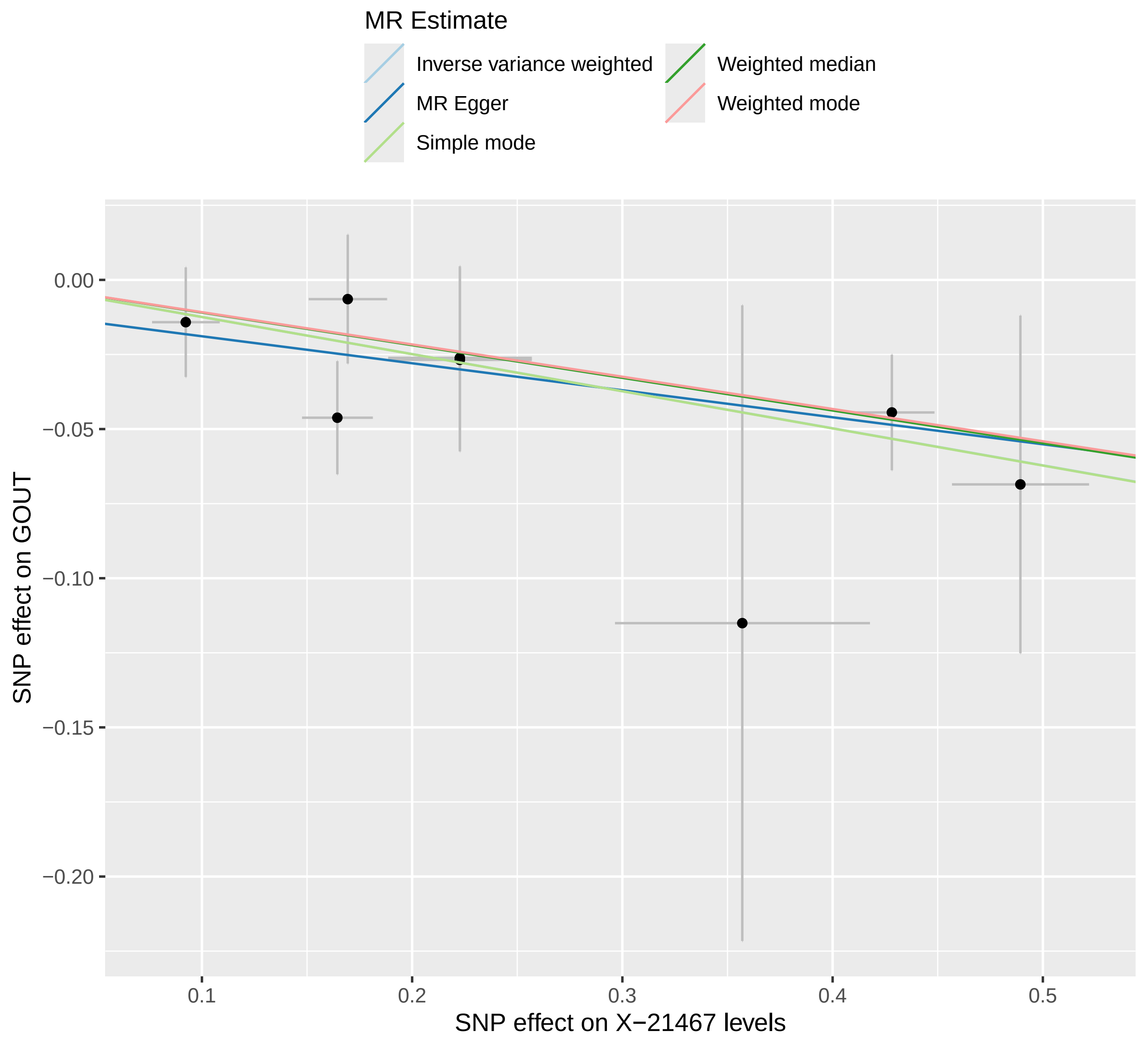

MR Estimate
Inverse variance weighted
MR Egger
Simple mode
Weighted median
Weighted mode
0.00
−0.05
−0.10
−0.15
−0.20
0.1
0.2
0.3
0.4
0.5
SNP effect on GOUT
SNP effect on X−21467 levels

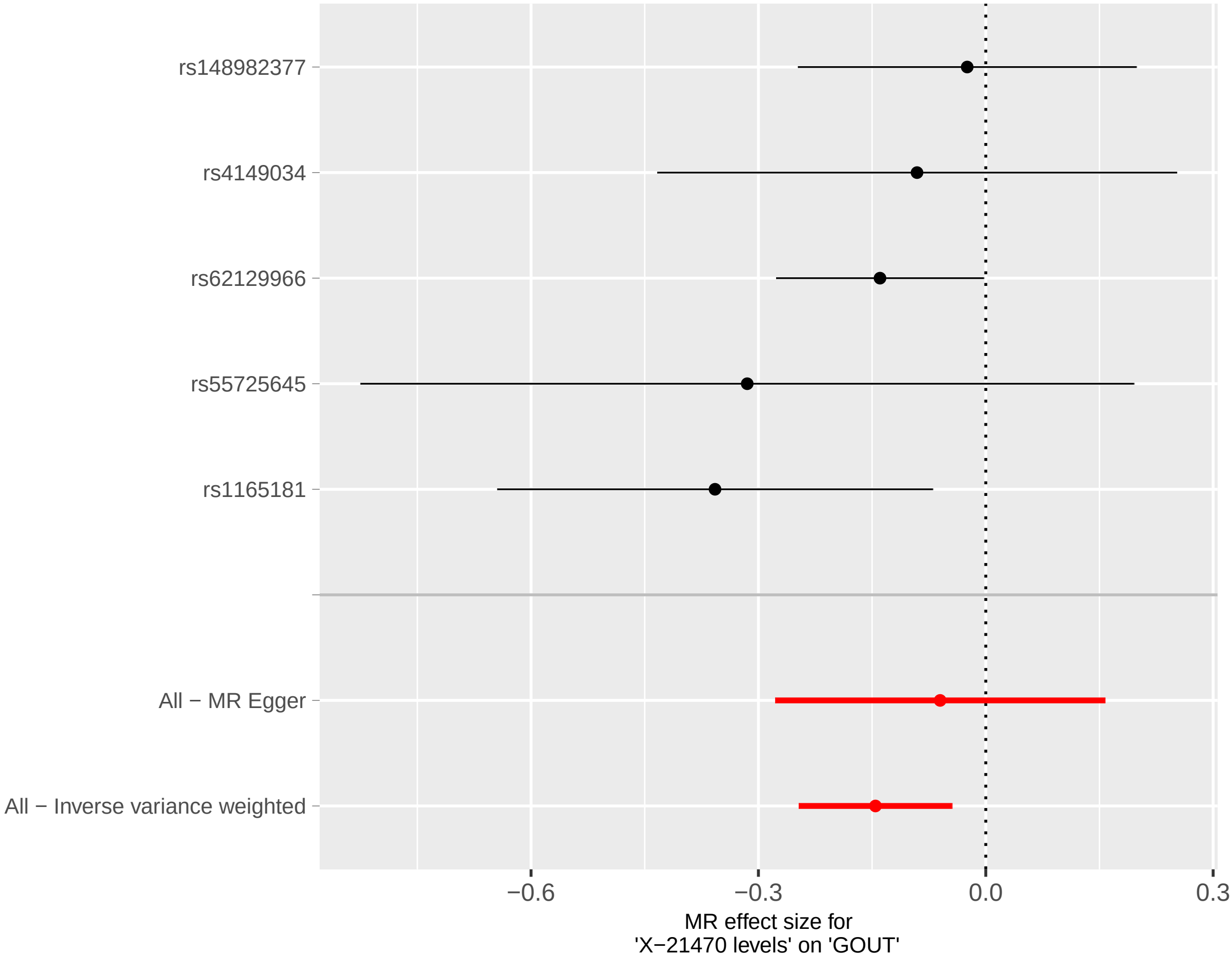
rs148982377
rs4149034
rs62129966
rs55725645
rs1165181
All − MR Egger
All − Inverse variance weighted
−0.6
−0.3
0.0
0.3
MR effect size for
'X−21470 levels' on 'GOUT'

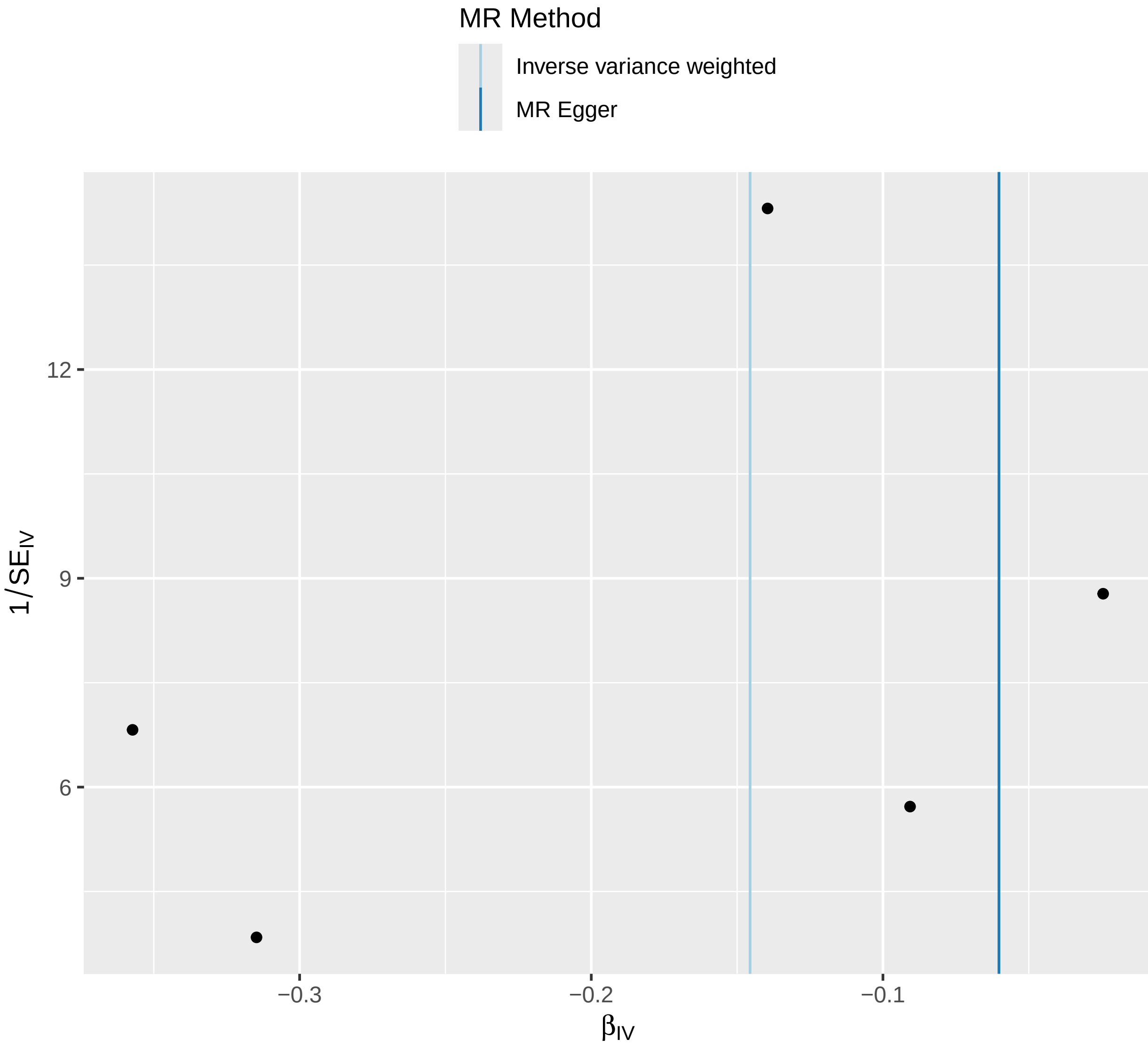
MR Method
Inverse variance weighted
MR Egger
12
9
6
−0.3
−0.2
−0.1
1/SE_IV
β_IV

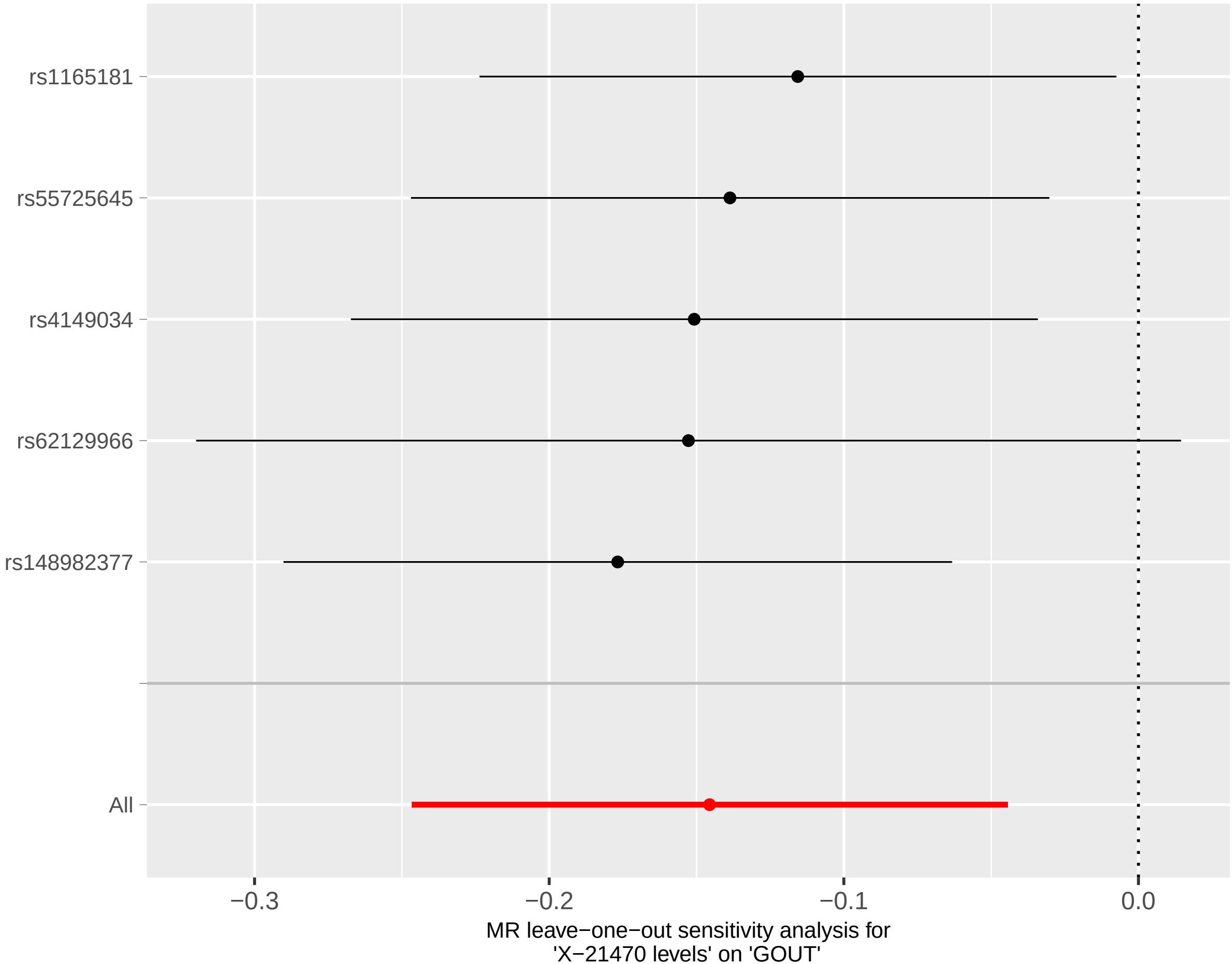
rs1165181
rs55725645
rs4149034
rs62129966
rs148982377
All
−0.3
−0.2
−0.1
0.0
MR leave−one−out sensitivity analysis for
'X−21470 levels' on 'GOUT'

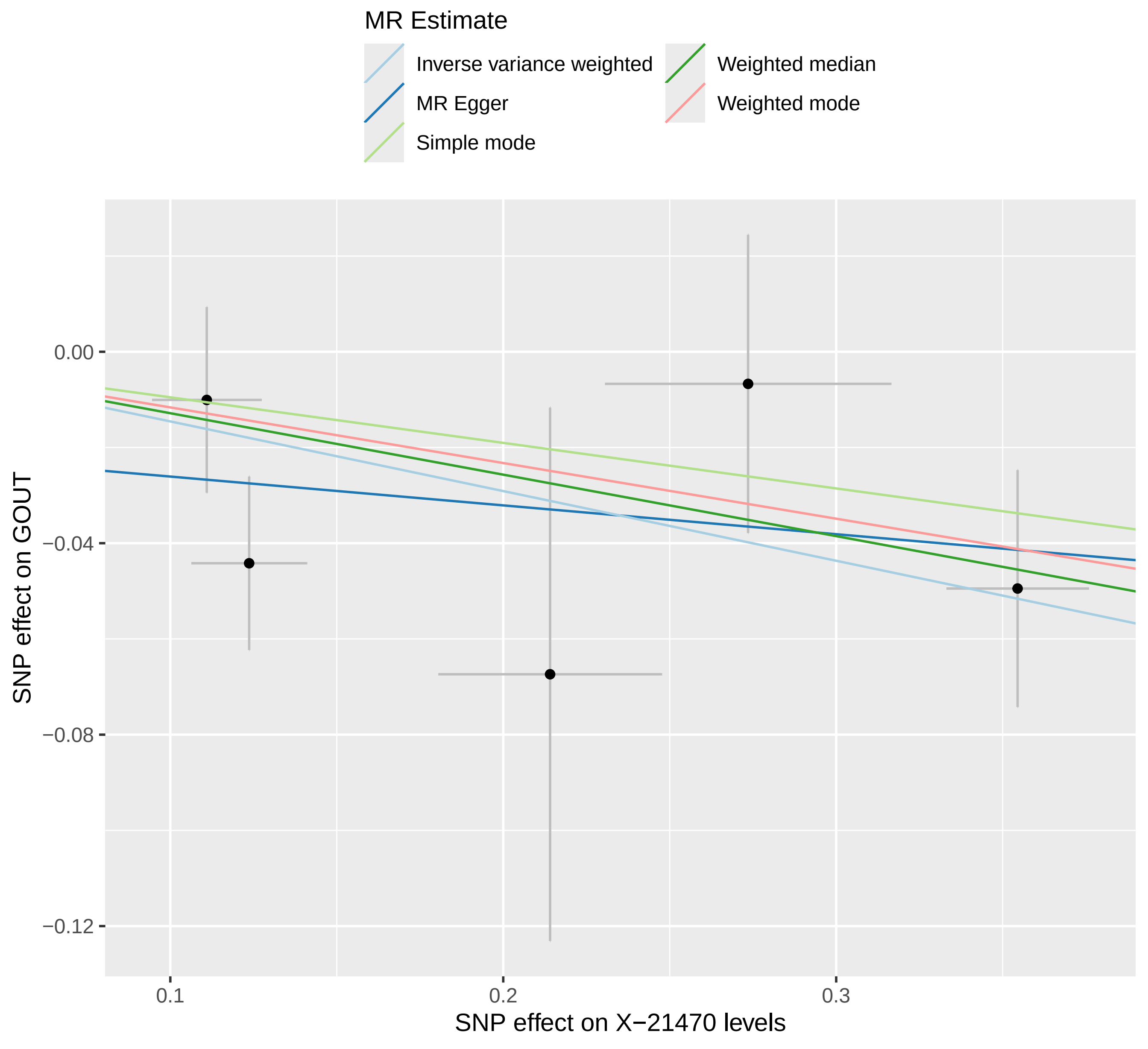
MR Estimate
Inverse variance weighted
MR Egger
Simple mode
Weighted median
Weighted mode
0.00
−0.04
−0.08
−0.12
SNP effect on GOUT
0.1
0.2
0.3
SNP effect on X−21470 levels

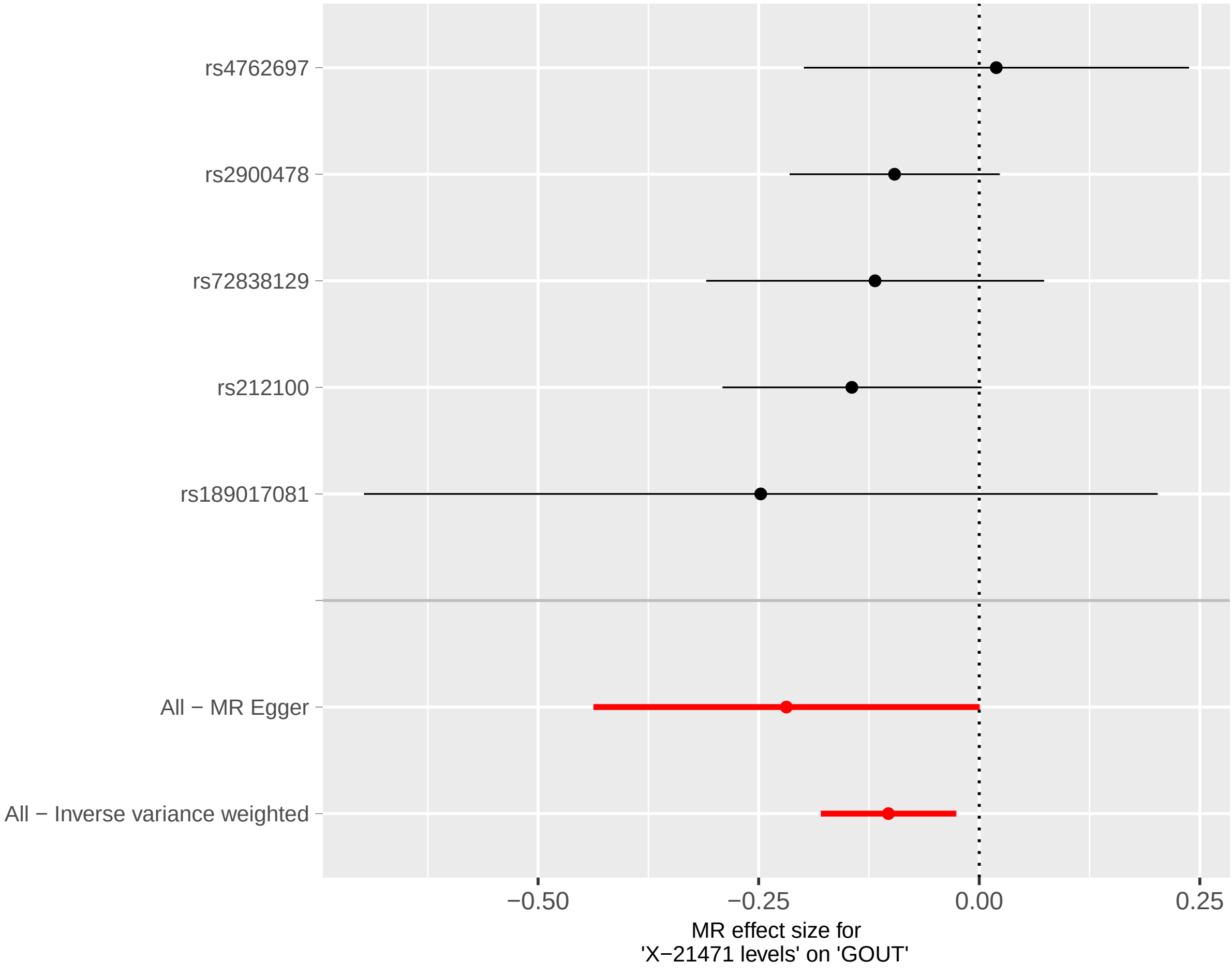
rs4762697
rs2900478
rs72838129
rs212100
rs189017081
All − MR Egger
All − Inverse variance weighted
−0.50
−0.25
0.00
0.25
MR effect size for
'X−21471 levels' on 'GOUT'

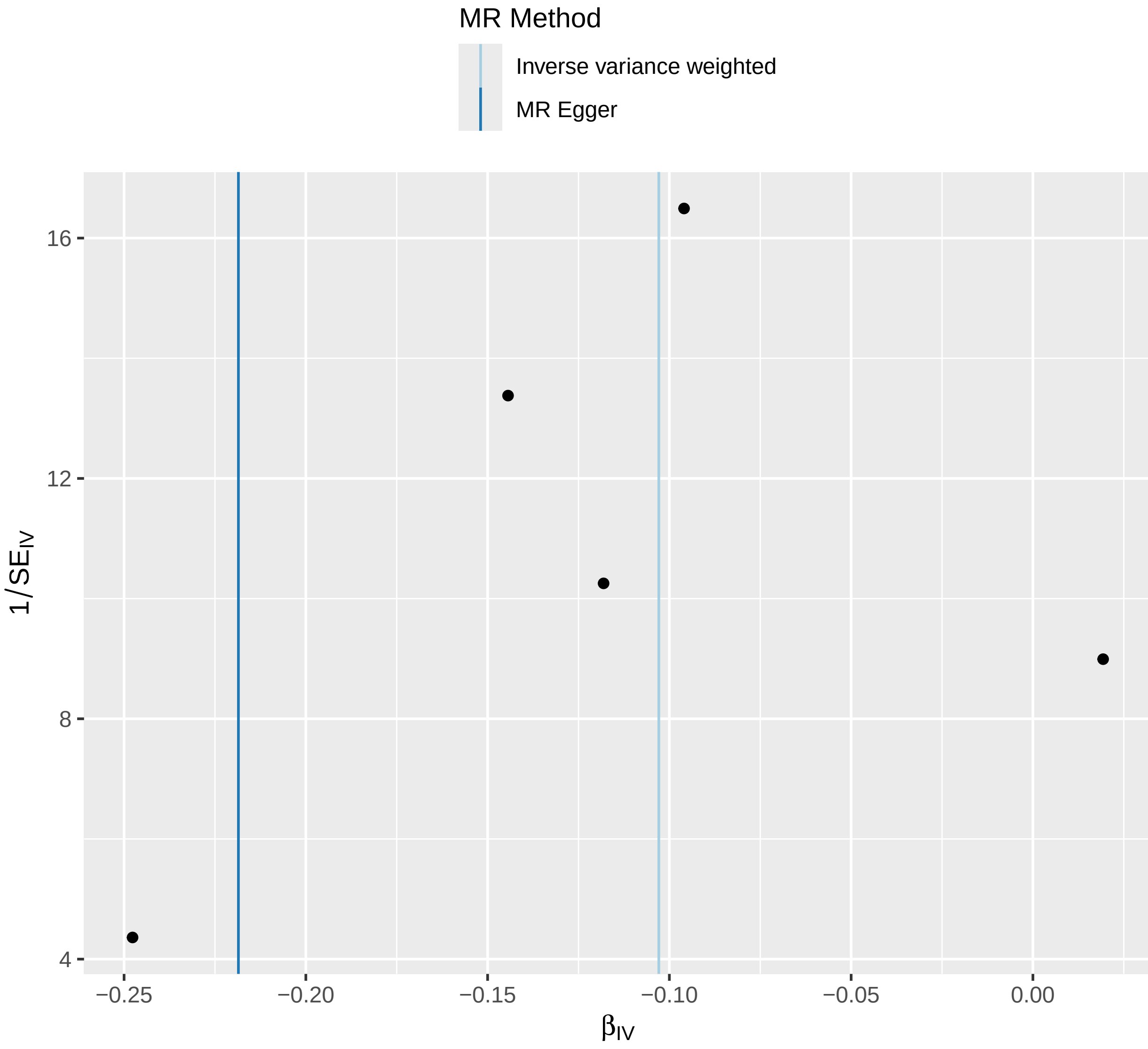
MR Method
Inverse variance weighted
MR Egger
16
12
8
4
1/SE_IV
−0.25
−0.20
−0.15
−0.10
−0.05
0.00
β_IV

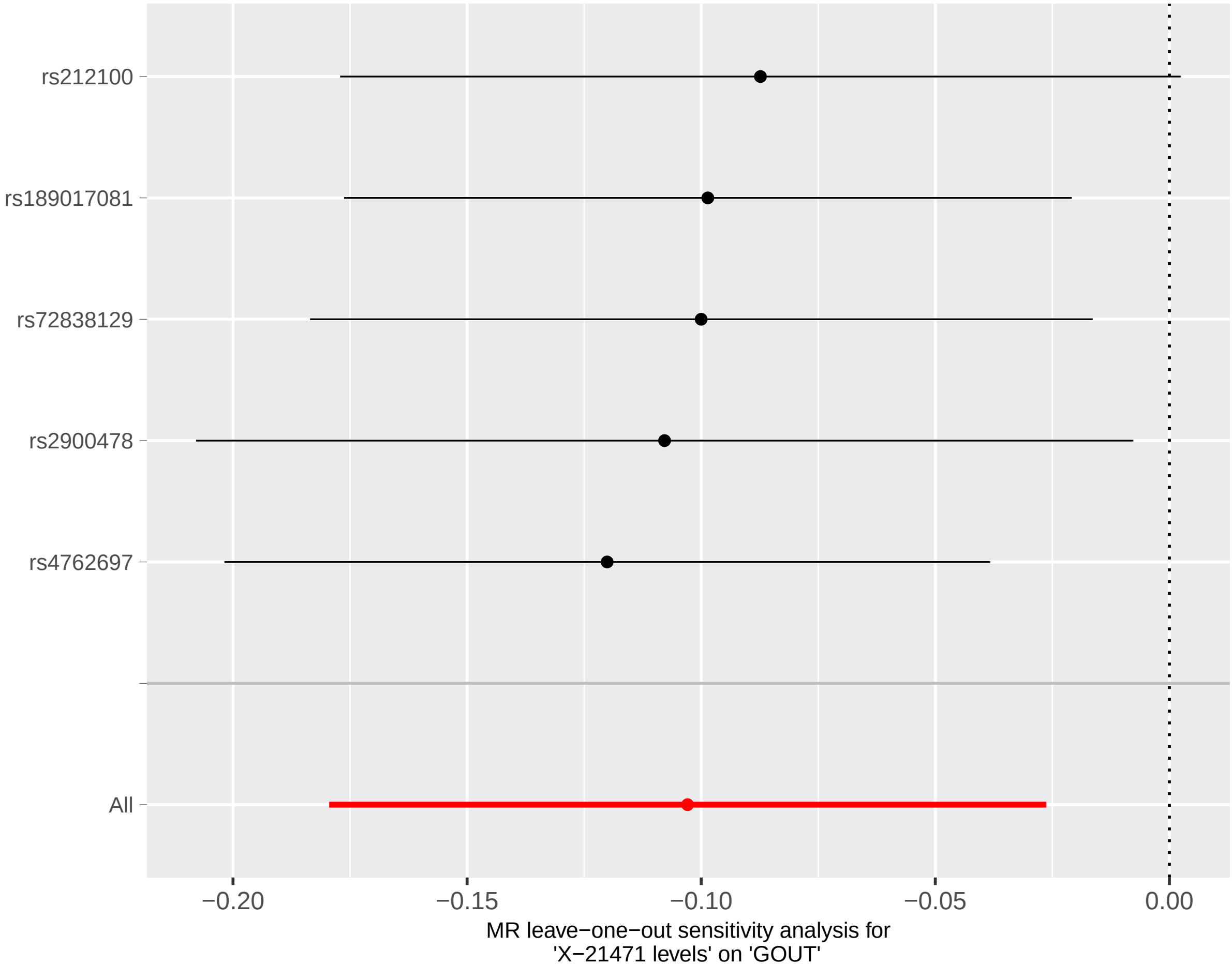
rs212100
rs189017081
rs72838129
rs2900478
rs4762697
All
−0.20
−0.15
−0.10
−0.05
0.00
MR leave−one−out sensitivity analysis for
'X−21471 levels' on 'GOUT'

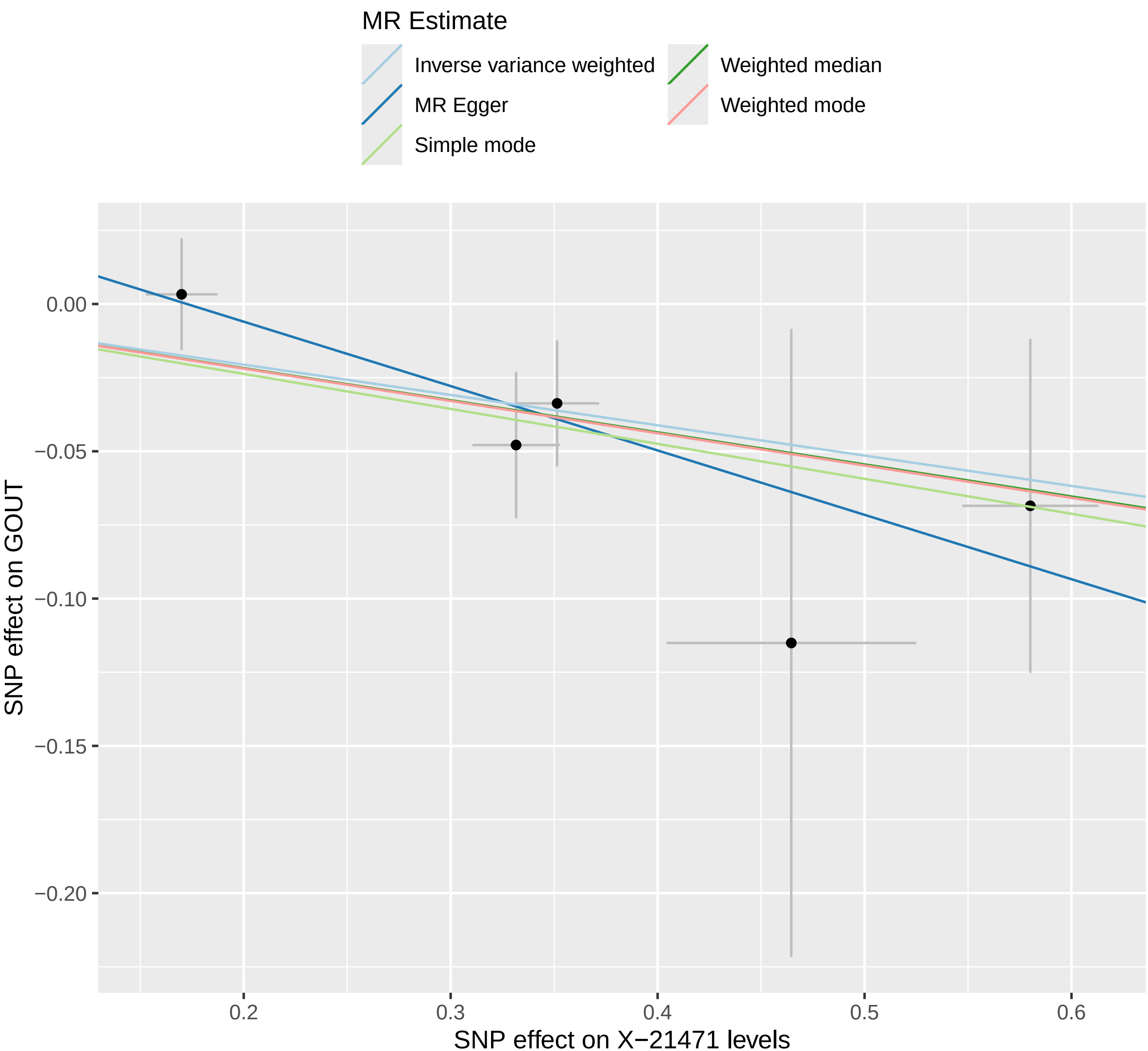
MR Estimate
Inverse variance weighted
MR Egger
Simple mode
Weighted median
Weighted mode
0.00
−0.05
−0.10
−0.15
−0.20
SNP effect on GOUT
0.2
0.3
0.4
0.5
0.6
SNP effect on X−21471 levels

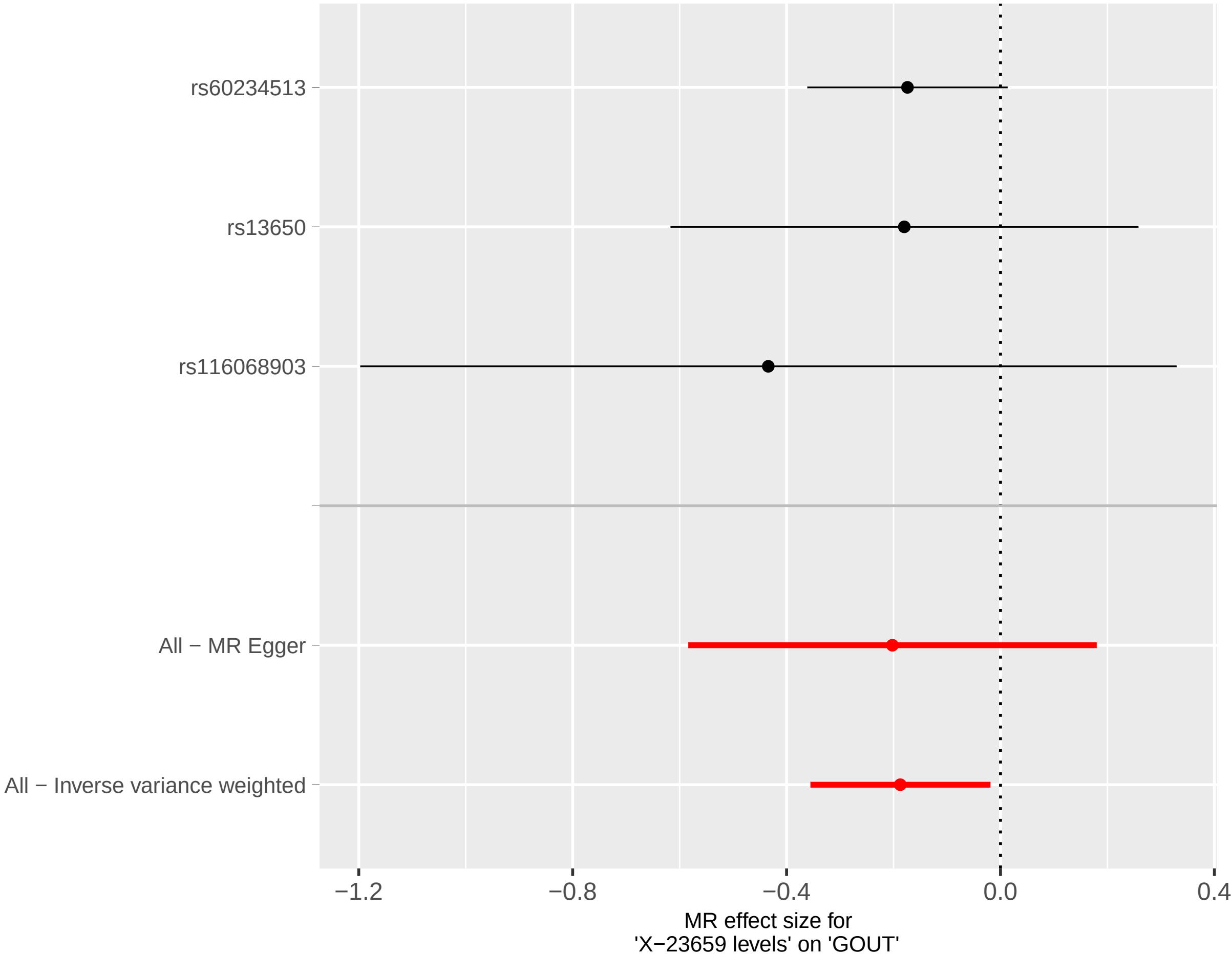
rs60234513
rs13650
rs116068903
All − MR Egger
All − Inverse variance weighted
−1.2
−0.8
−0.4
0.0
0.4
MR effect size for
'X−23659 levels' on 'GOUT'

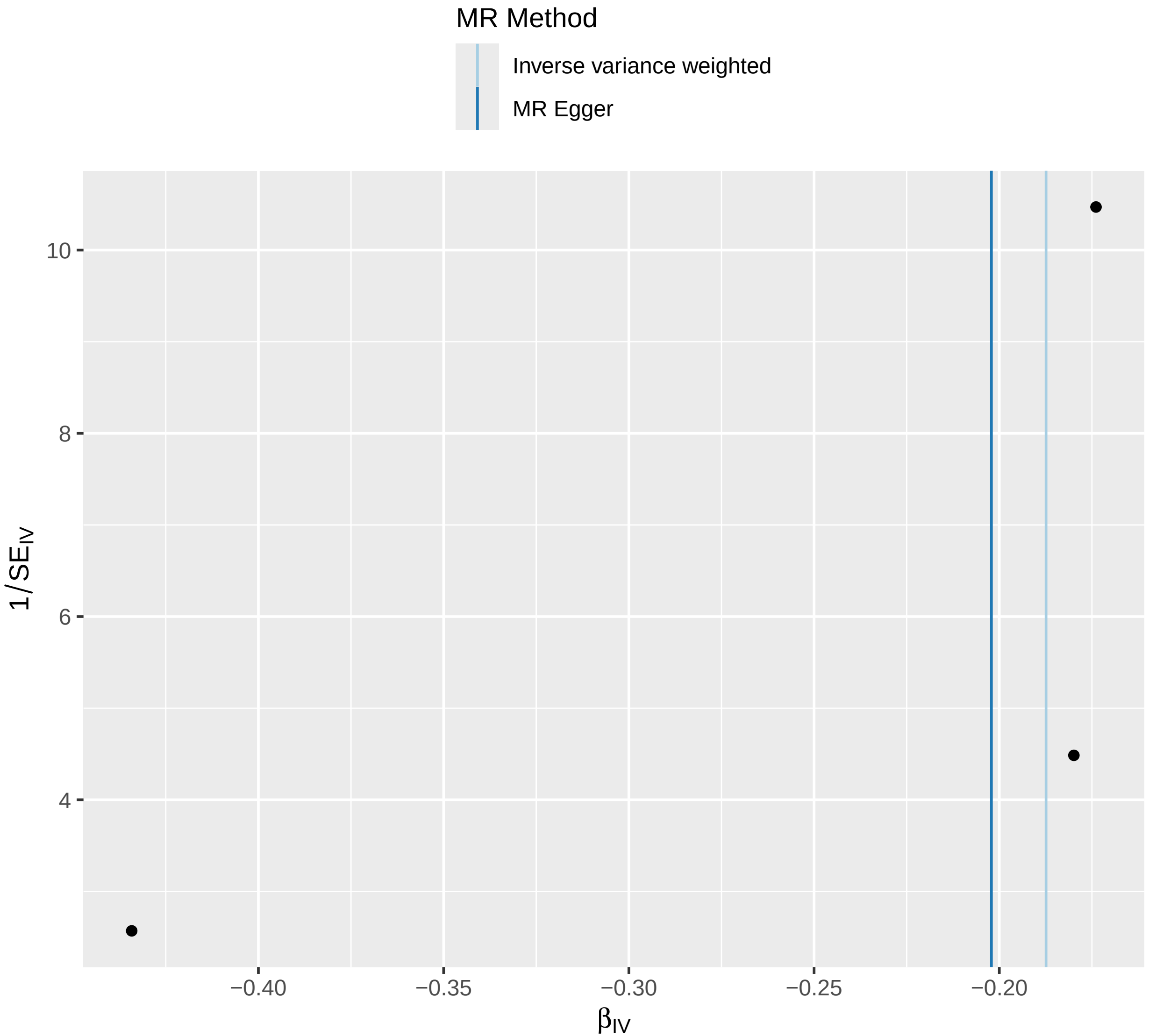
MR Method
Inverse variance weighted
MR Egger
10
8
6
4
1/SE_IV
−0.40
−0.35
−0.30
−0.25
−0.20
β_IV

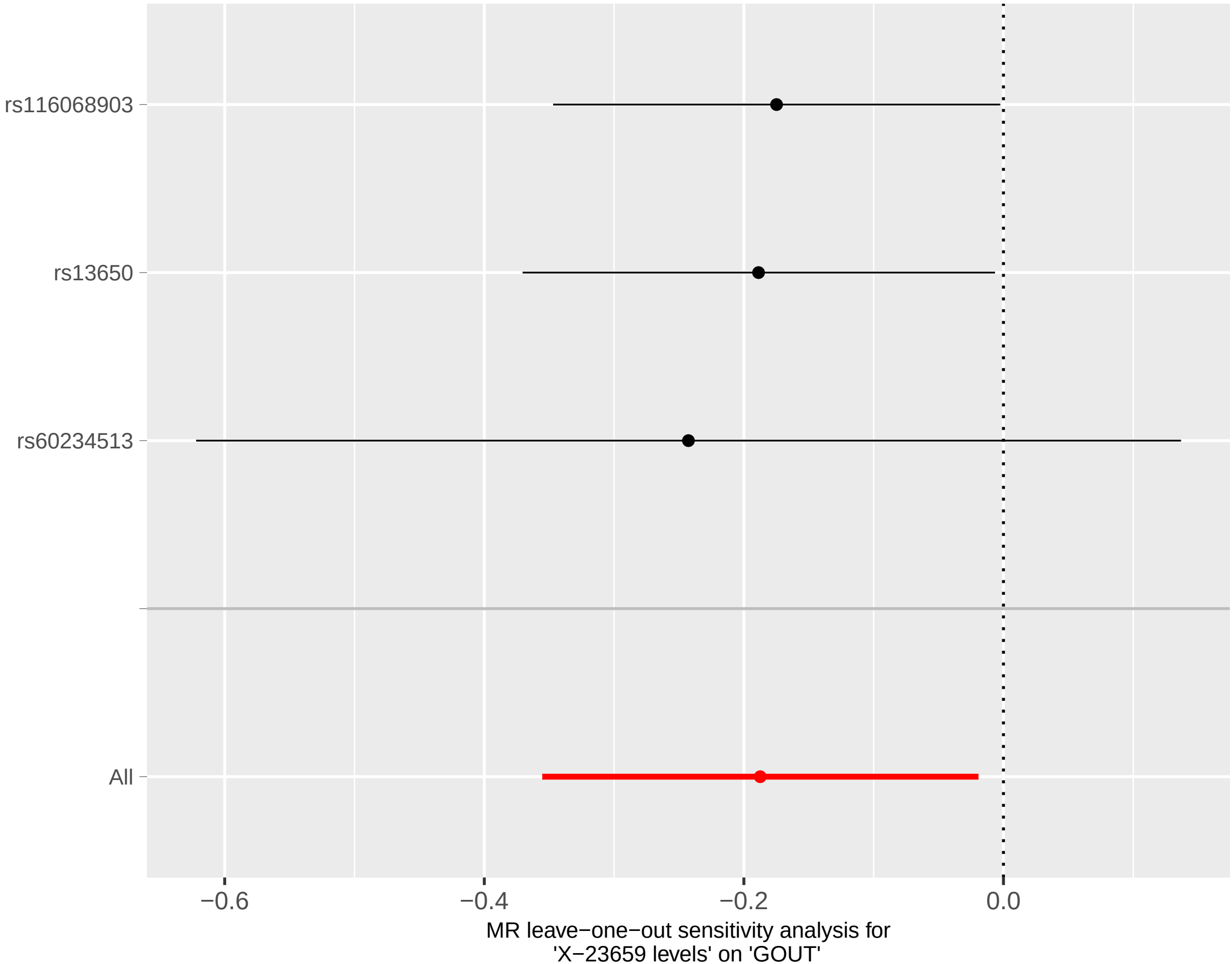
rs116068903
rs13650
rs60234513
All
−0.6
−0.4
−0.2
0.0
MR leave−one−out sensitivity analysis for
'X−23659 levels' on 'GOUT'

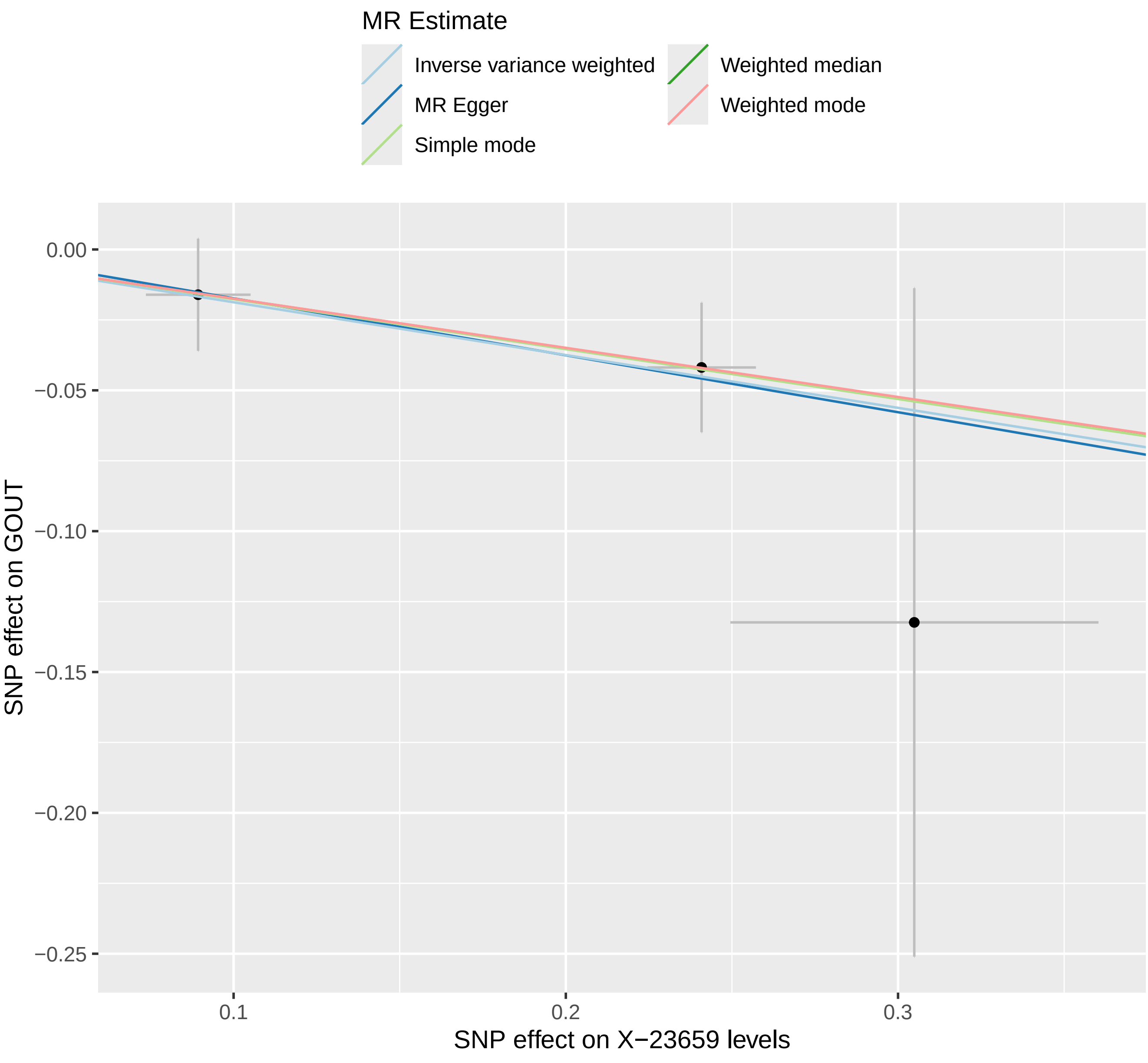
MR Estimate
Inverse variance weighted
MR Egger
Simple mode
Weighted median
Weighted mode
0.00
−0.05
−0.10
−0.15
−0.20
−0.25
SNP effect on GOUT
0.1
0.2
0.3
SNP effect on X−23659 levels

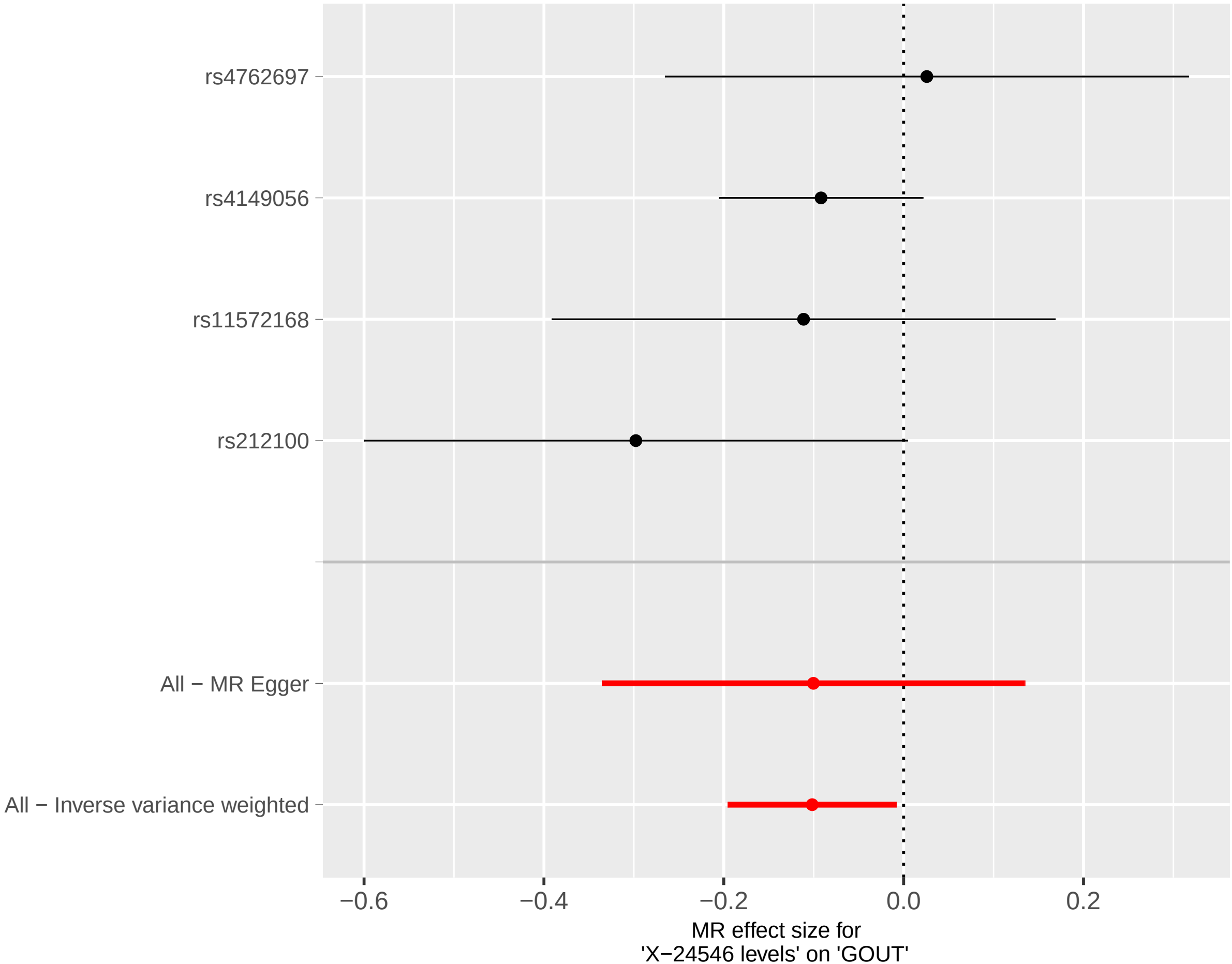
rs4762697
rs4149056
rs11572168
rs212100
All − MR Egger
All − Inverse variance weighted
−0.6
−0.4
−0.2
0.0
0.2
MR effect size for
'X−24546 levels' on 'GOUT'

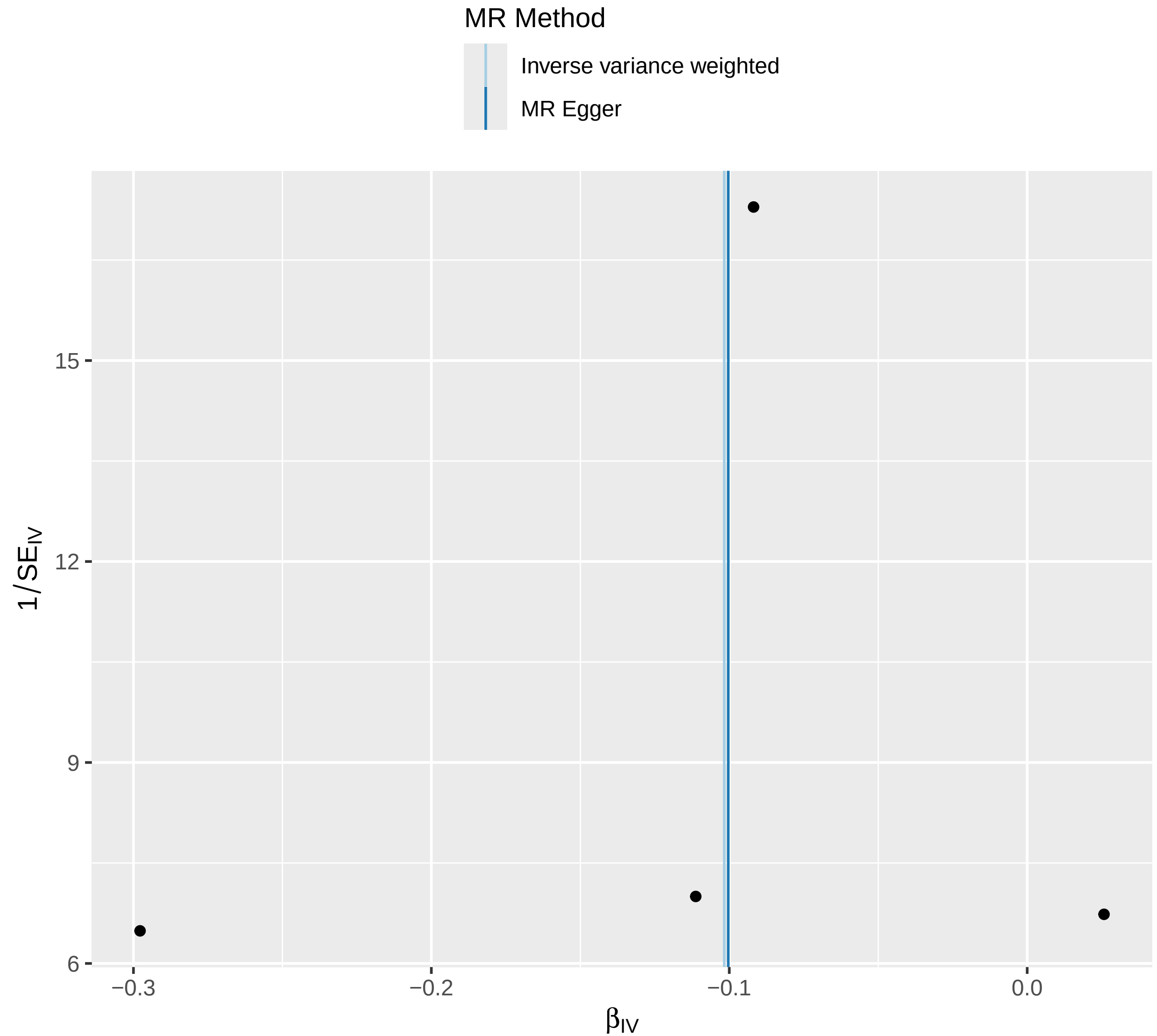
MR Method
Inverse variance weighted
MR Egger
15
12
9
6
1/SE$_{IV}$
−0.3
−0.2
−0.1
0.0
β_{IV}

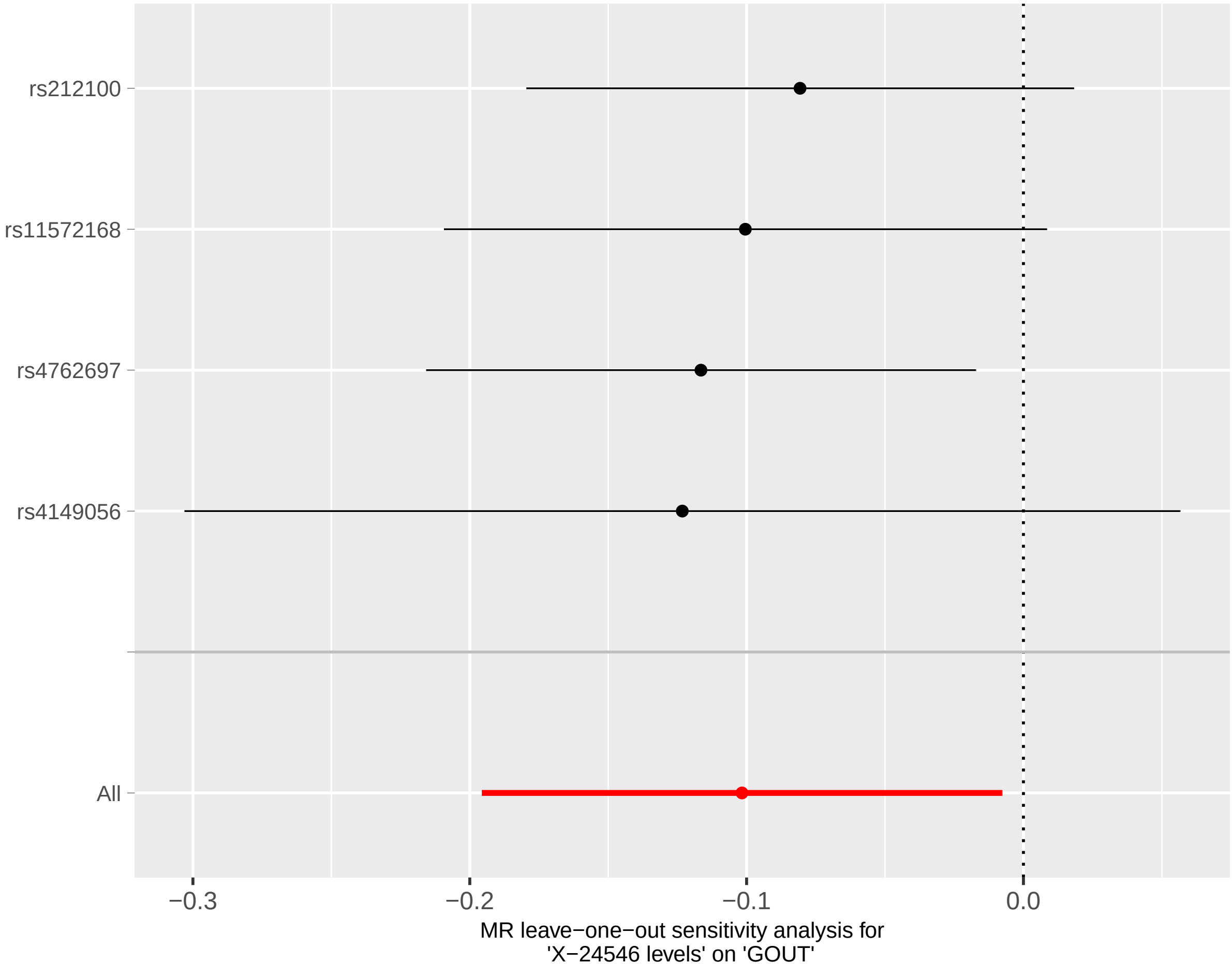
rs212100
rs11572168
rs4762697
rs4149056
All
−0.3
−0.2
−0.1
0.0
MR leave−one−out sensitivity analysis for
'X−24546 levels' on 'GOUT'

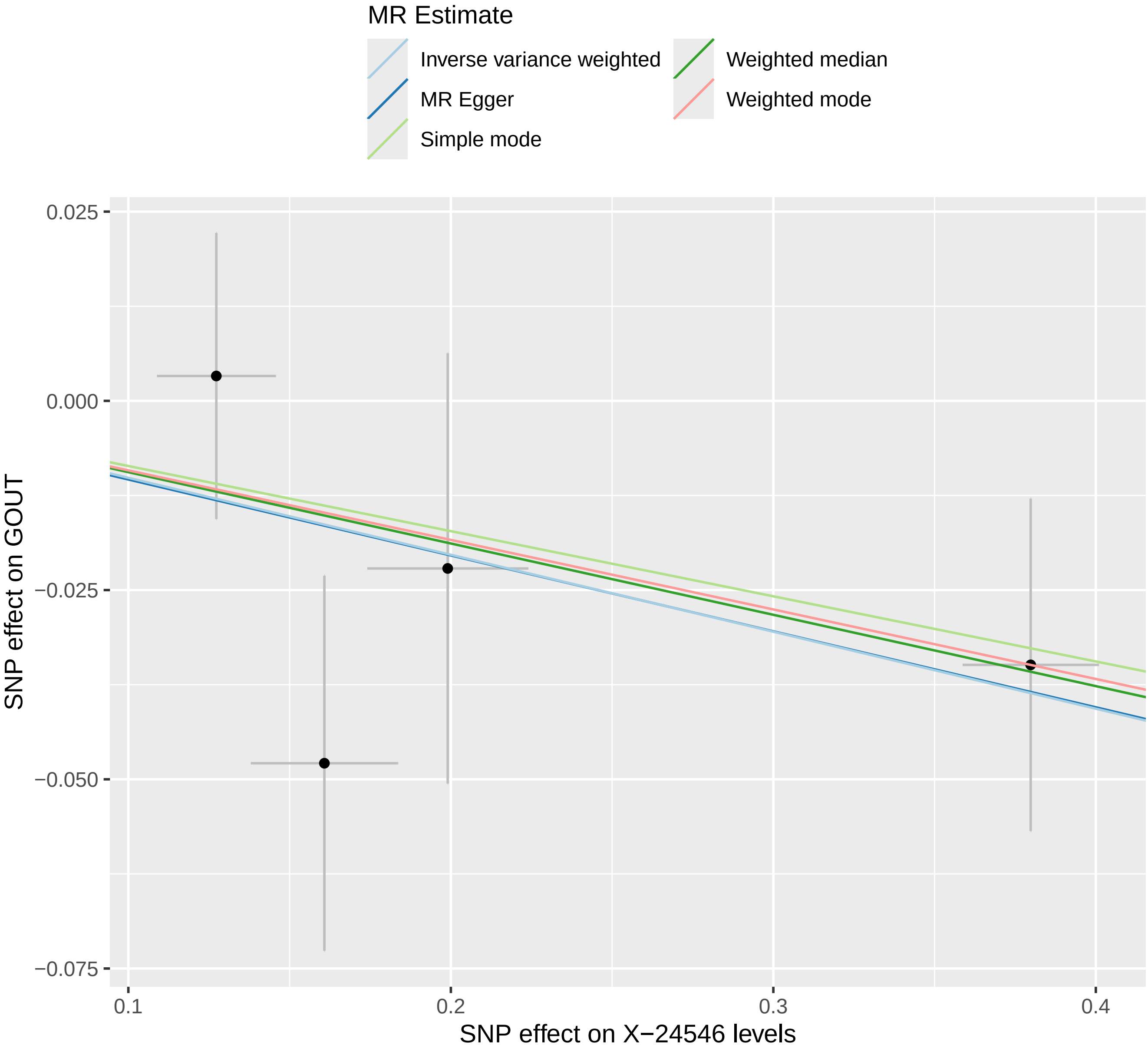
MR Estimate
Inverse variance weighted
MR Egger
Simple mode
Weighted median
Weighted mode
0.025
0.000
−0.025
−0.050
−0.075
0.1
0.2
0.3
0.4
SNP effect on GOUT
SNP effect on X−24546 levels

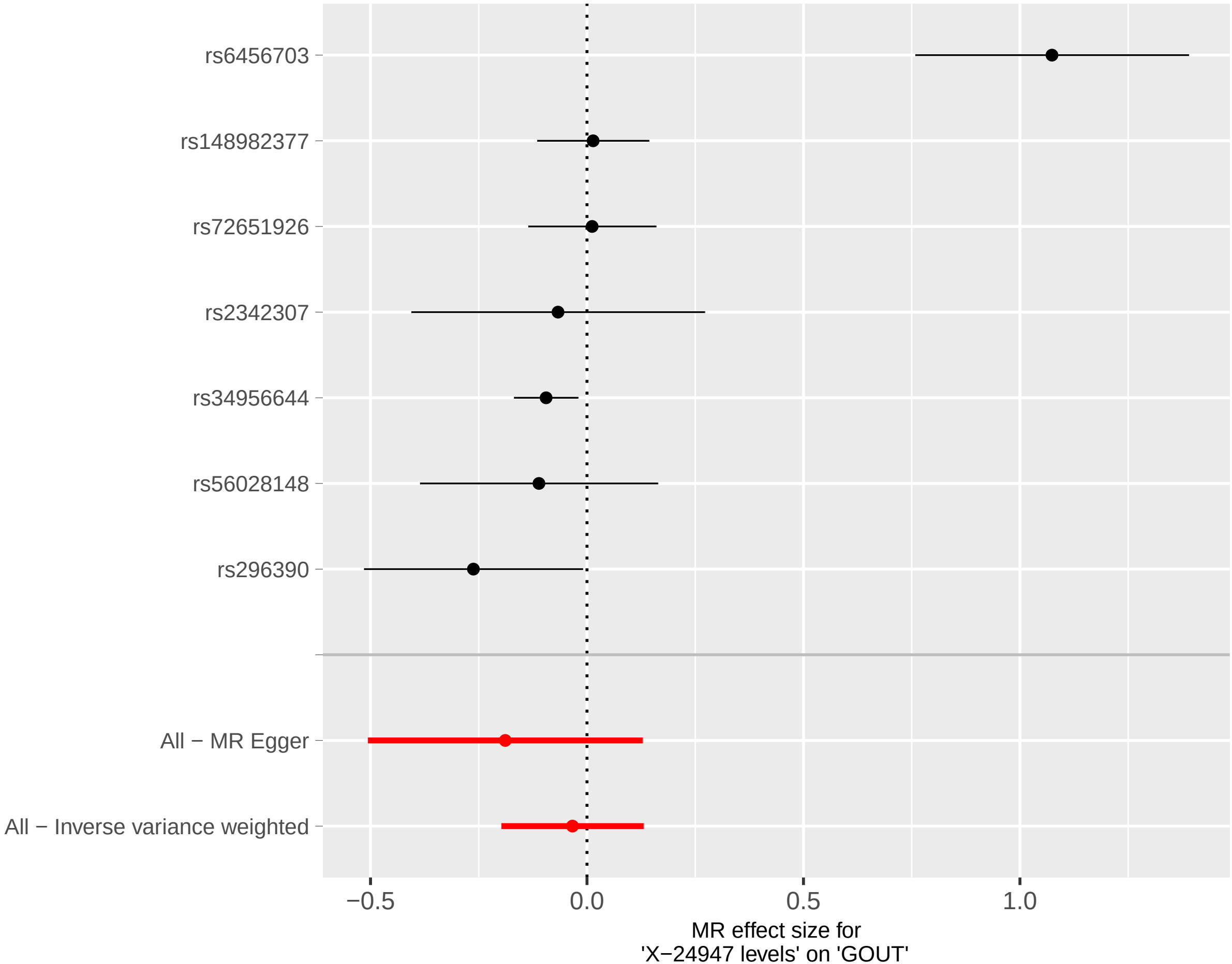
rs6456703
rs148982377
rs72651926
rs2342307
rs34956644
rs56028148
rs296390
All − MR Egger
All − Inverse variance weighted
−0.5
0.0
0.5
1.0
MR effect size for
'X−24947 levels' on 'GOUT'

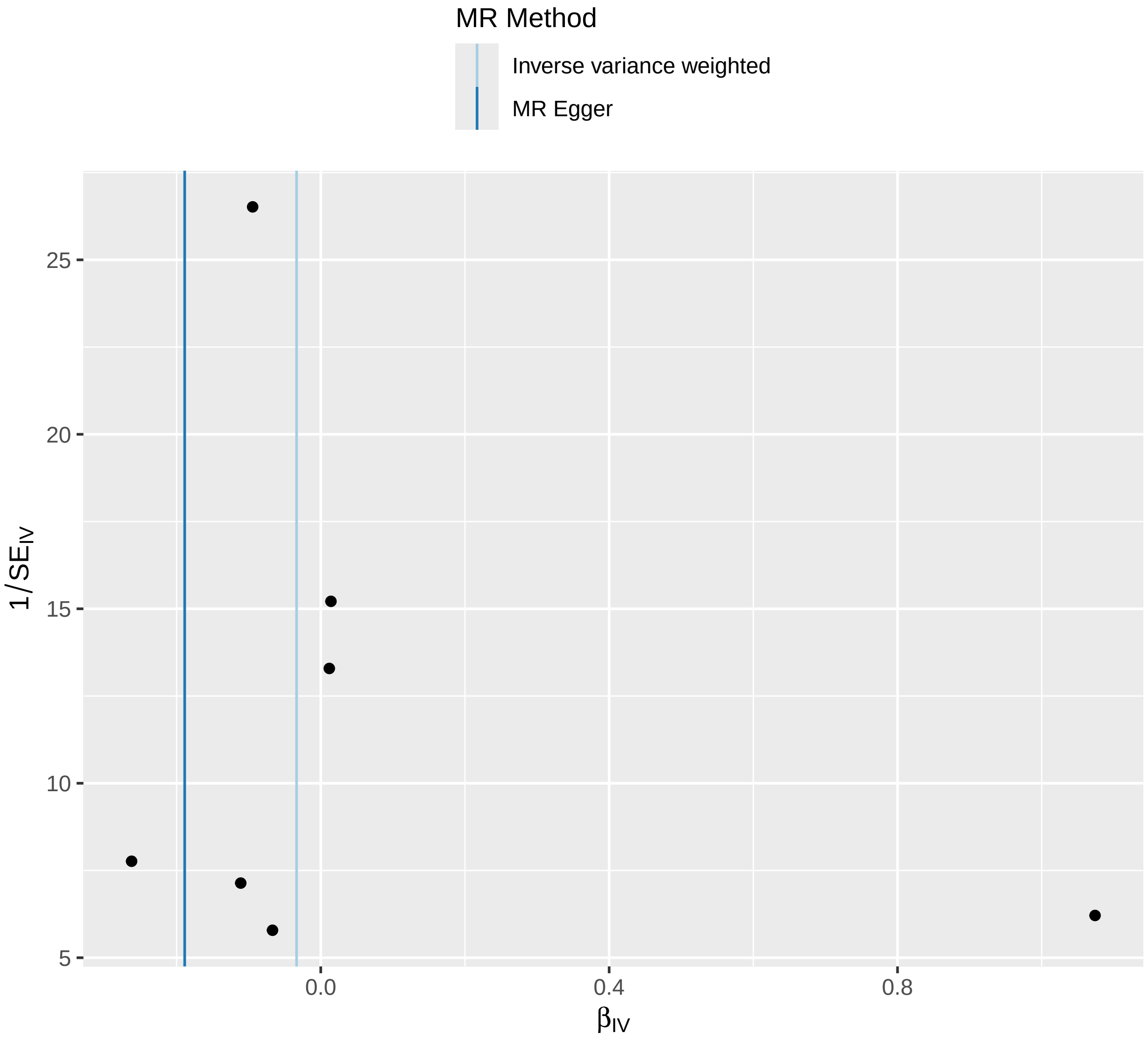
MR Method
Inverse variance weighted
MR Egger
25
20
15
10
5
1/SE_IV
0.0
0.4
0.8
β_IV

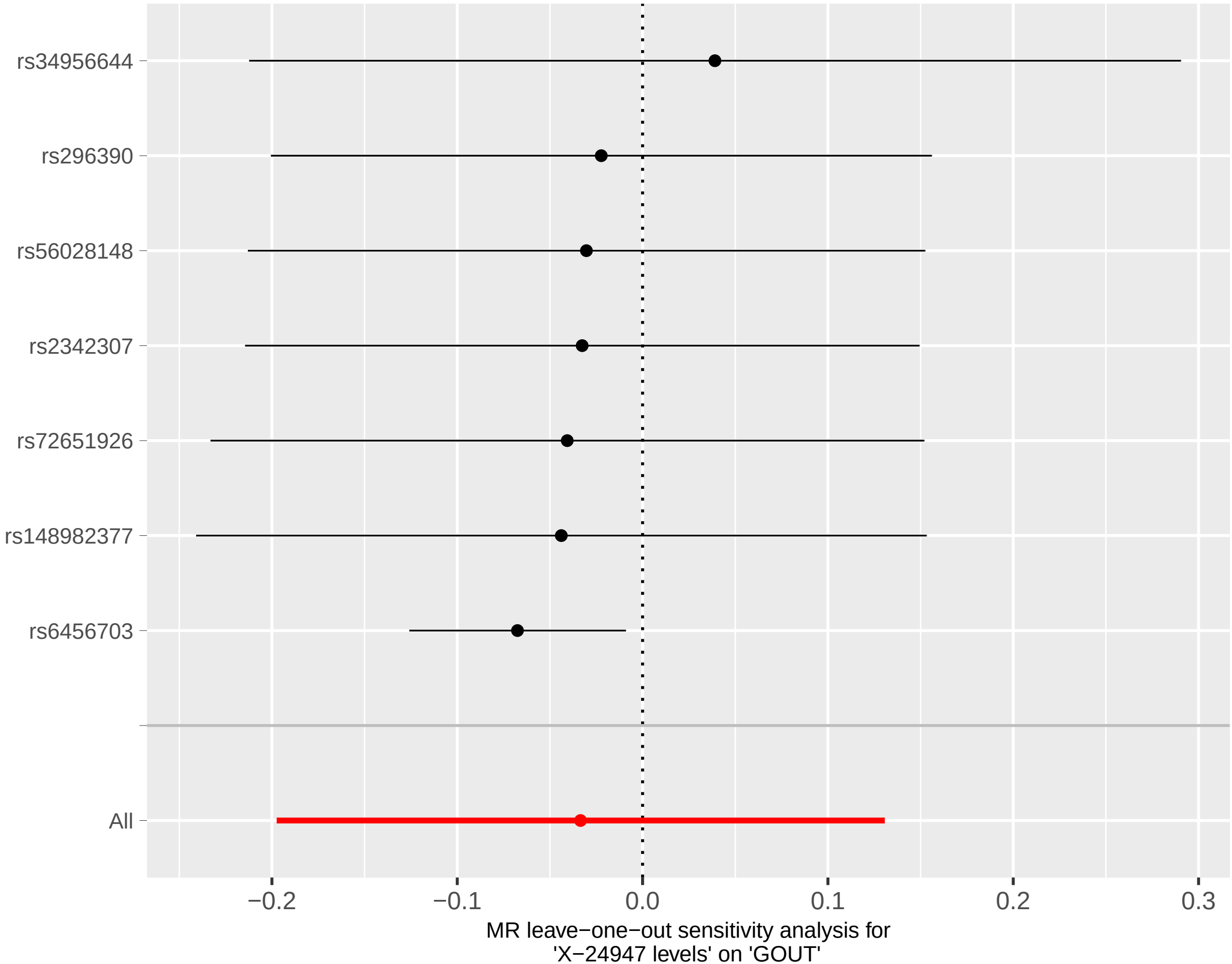
rs34956644
rs296390
rs56028148
rs2342307
rs72651926
rs148982377
rs6456703
All
−0.2
−0.1
0.0
0.1
0.2
0.3
MR leave−one−out sensitivity analysis for
'X−24947 levels' on 'GOUT'

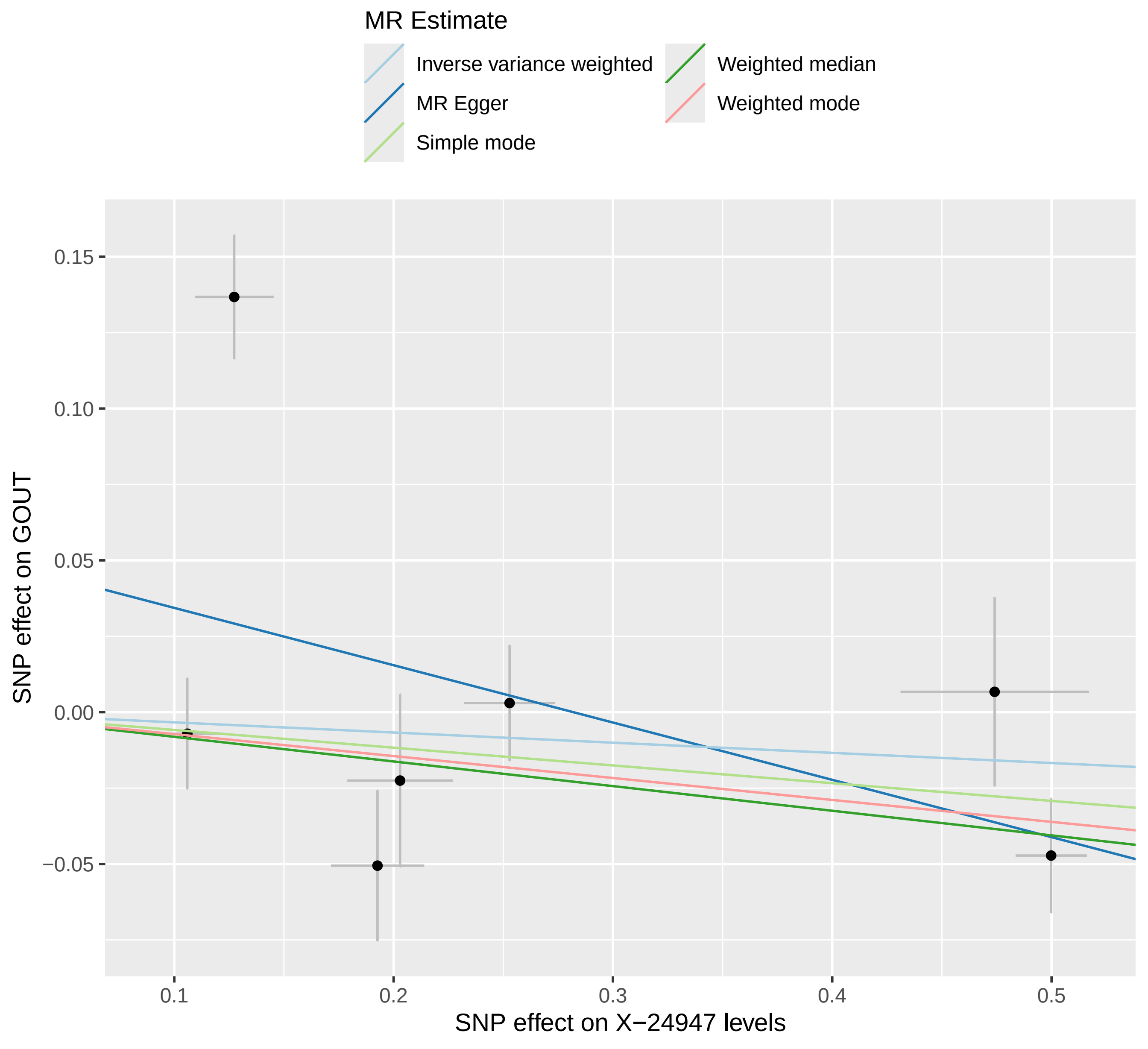
MR Estimate
Inverse variance weighted
MR Egger
Simple mode
Weighted median
Weighted mode
0.15
0.10
0.05
0.00
−0.05
0.1
0.2
0.3
0.4
0.5
SNP effect on GOUT
SNP effect on X−24947 levels

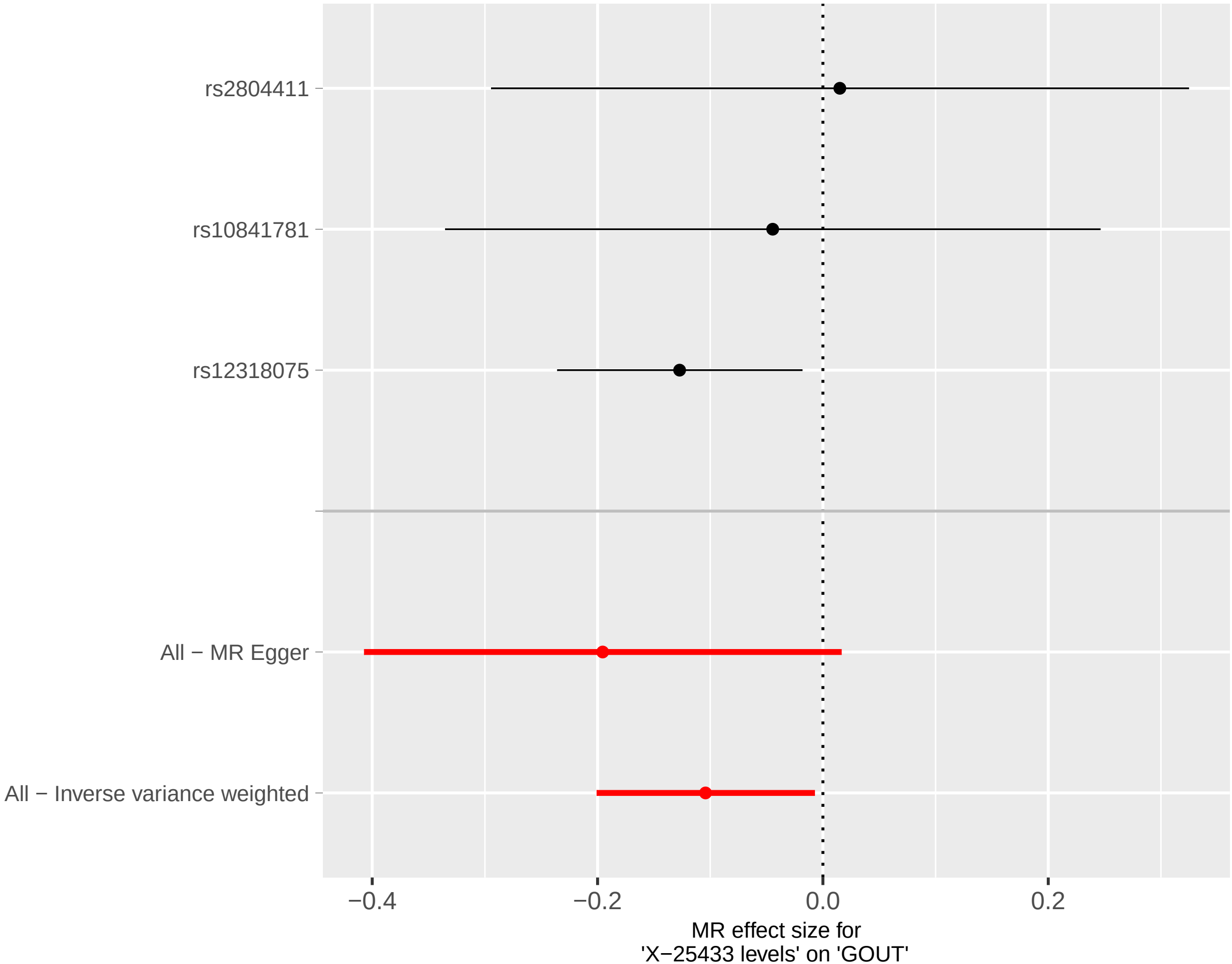
rs2804411
rs10841781
rs12318075
All − MR Egger
All − Inverse variance weighted
−0.4
−0.2
0.0
0.2
MR effect size for
'X−25433 levels' on 'GOUT'

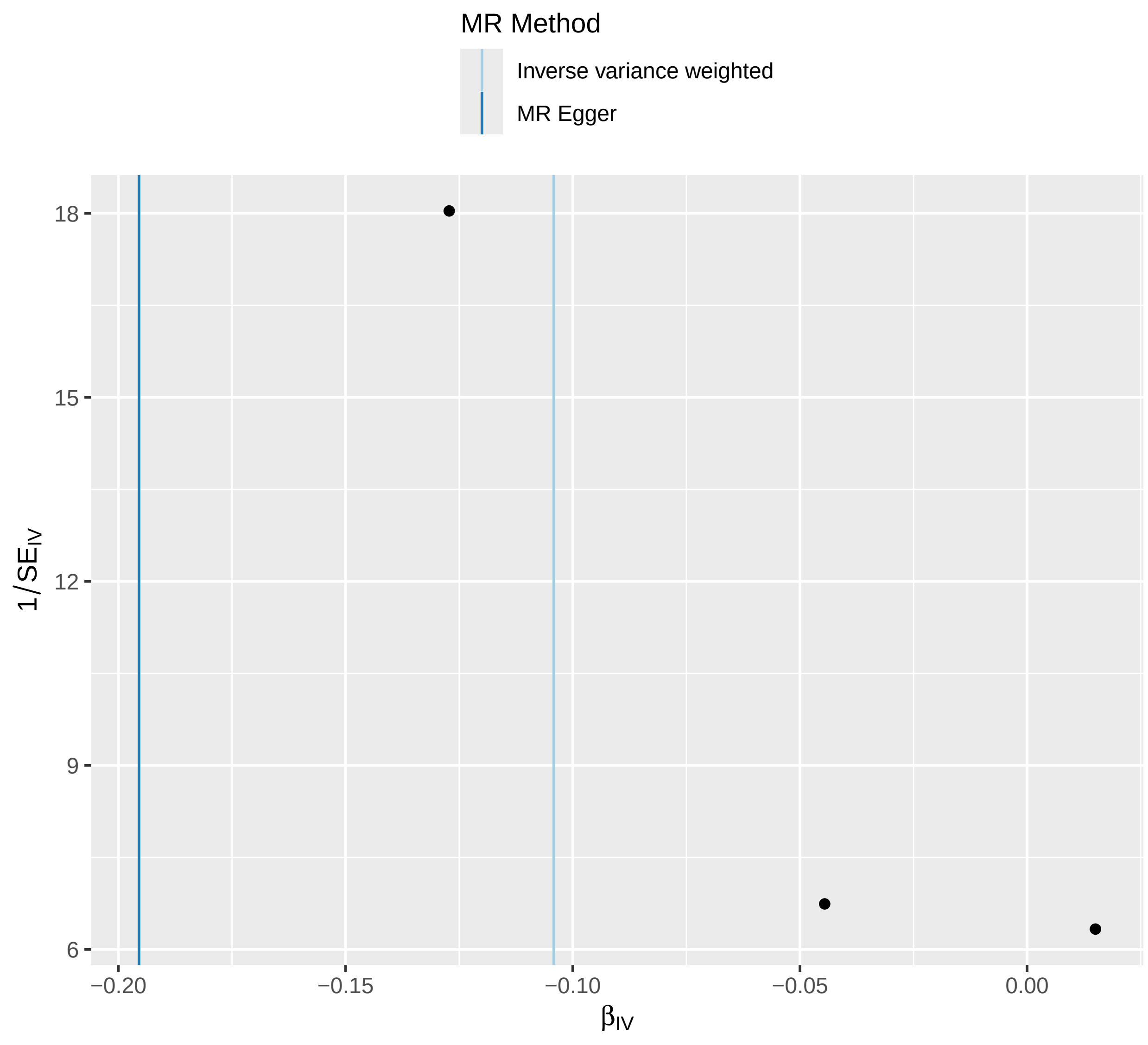

MR Method
Inverse variance weighted
MR Egger
18
15
12
9
6
1/SE$_{IV}$
−0.20
−0.15
−0.10
−0.05
0.00
β$_{IV}$

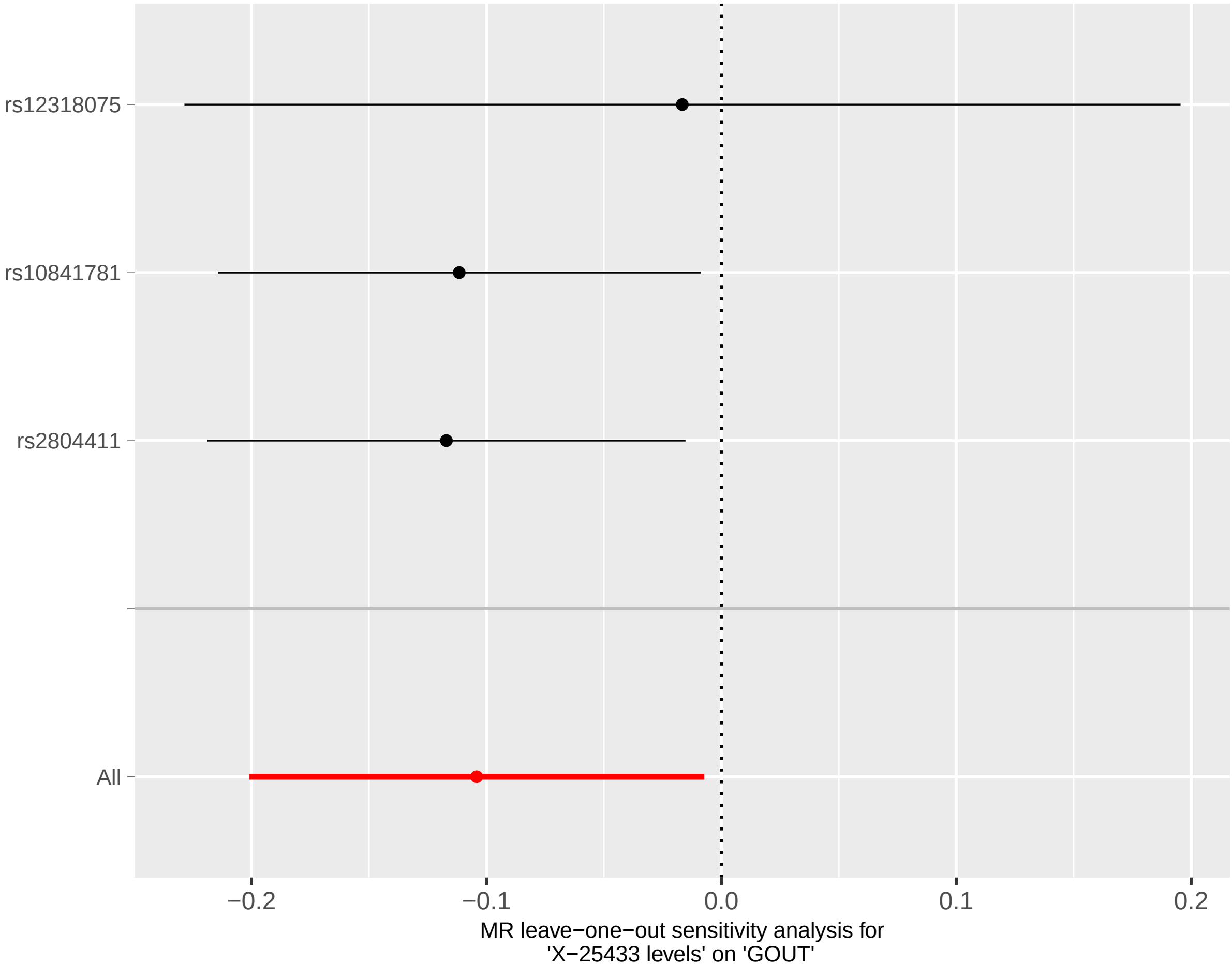

rs12318075
rs10841781
rs2804411
All
−0.2
−0.1
0.0
0.1
0.2
MR leave−one−out sensitivity analysis for
'X−25433 levels' on 'GOUT'

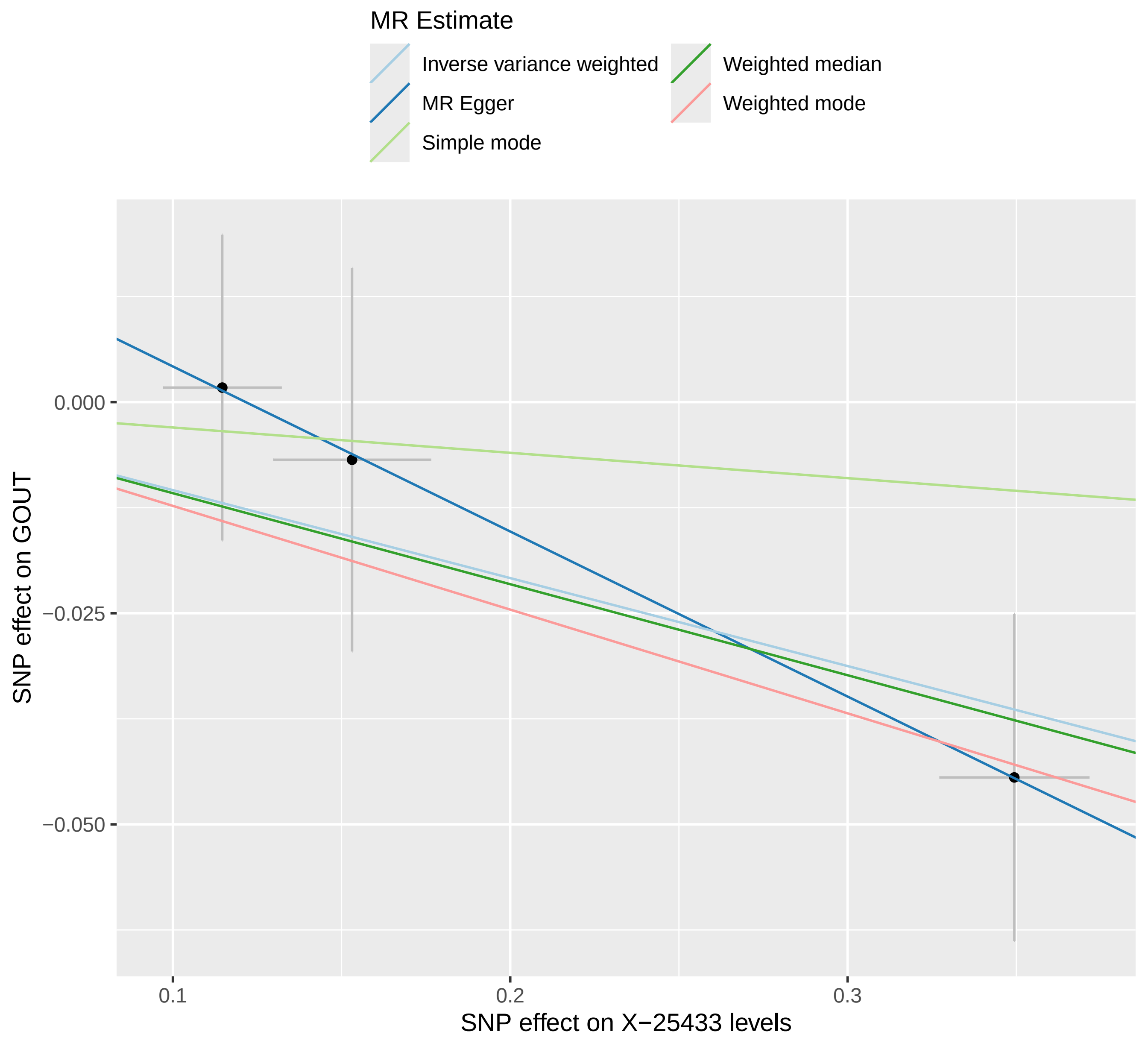

MR Estimate
Inverse variance weighted
MR Egger
Simple mode
Weighted median
Weighted mode
0.000
−0.025
−0.050
SNP effect on GOUT
0.1
0.2
0.3
SNP effect on X−25433 levels

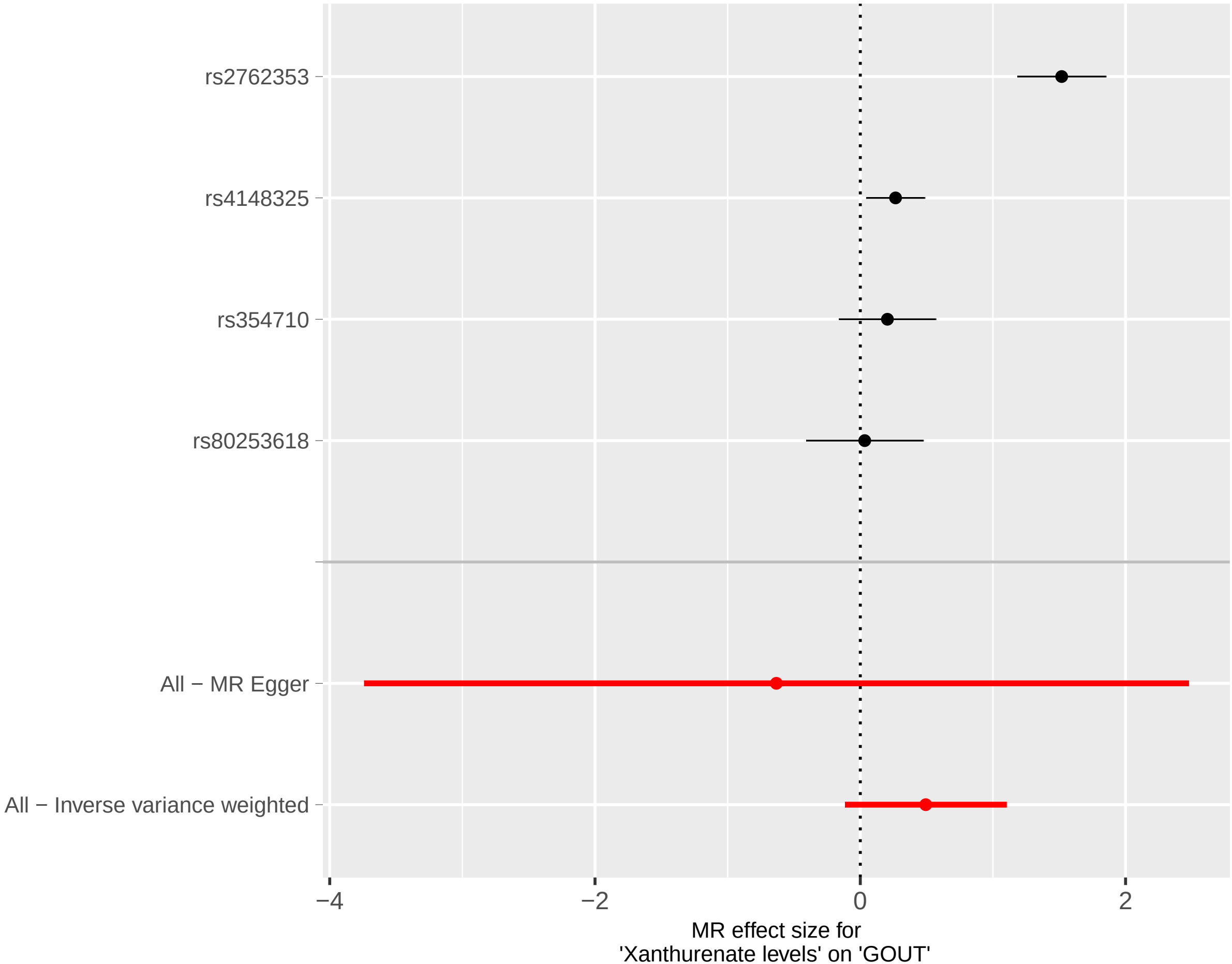
rs2762353
rs4148325
rs354710
rs80253618
All − MR Egger
All − Inverse variance weighted
−4
−2
0
2
MR effect size for
'Xanthurenate levels' on 'GOUT'

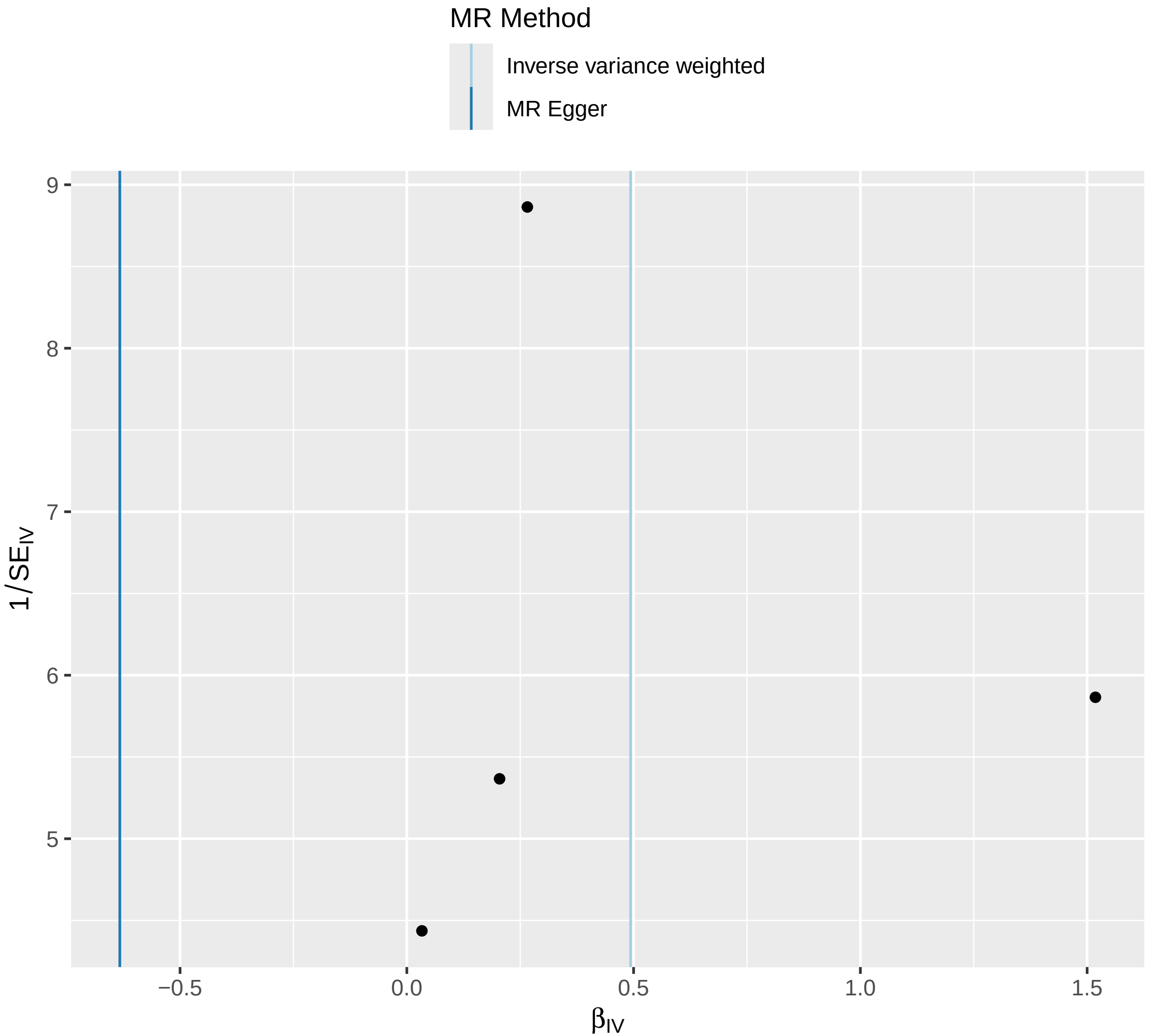
MR Method
Inverse variance weighted
MR Egger
9
8
7
6
5
1/SE_IV
−0.5
0.0
0.5
1.0
1.5
β_IV

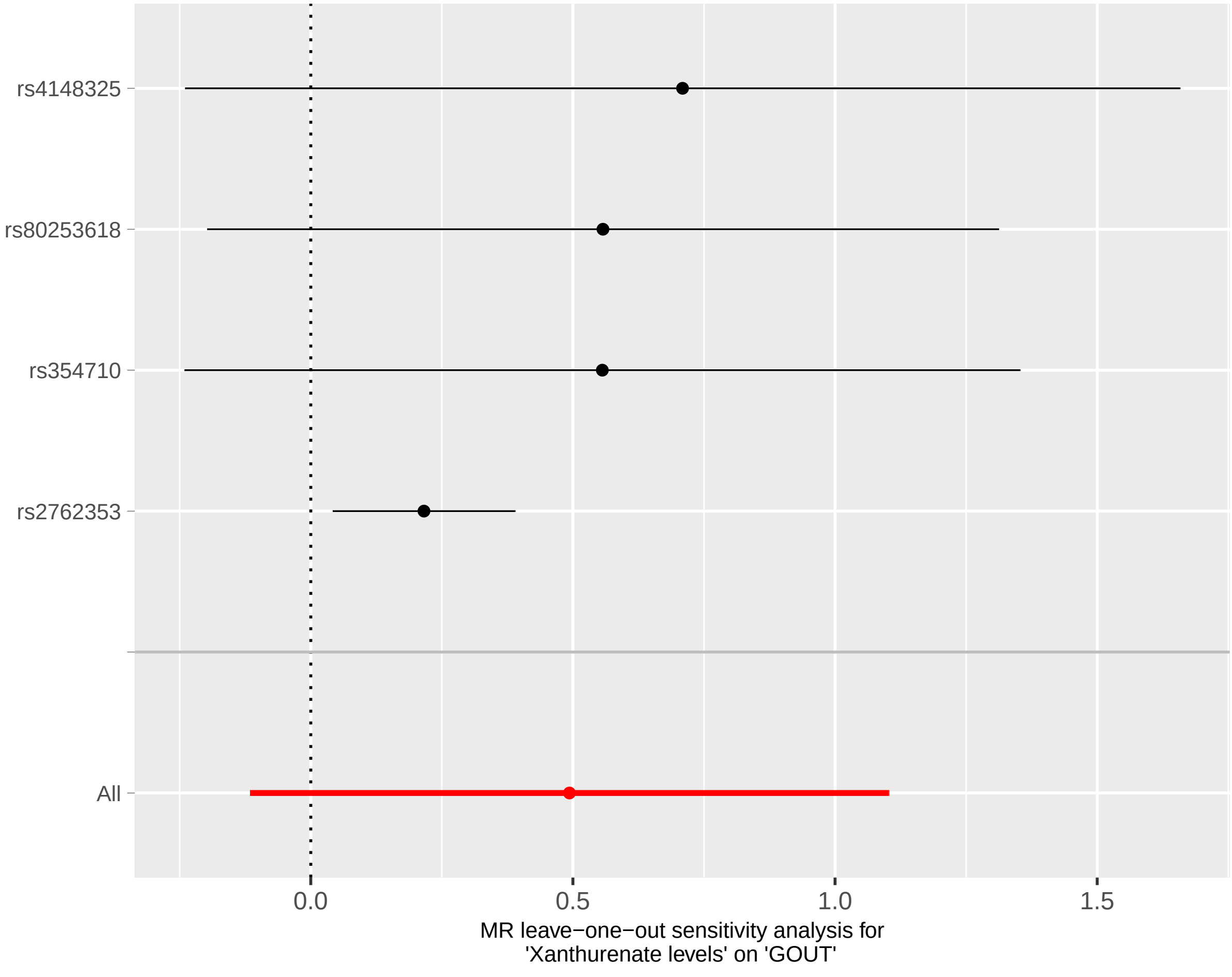
rs4148325
rs80253618
rs354710
rs2762353
All
0.0
0.5
1.0
1.5
MR leave−one−out sensitivity analysis for
'Xanthurenate levels' on 'GOUT'

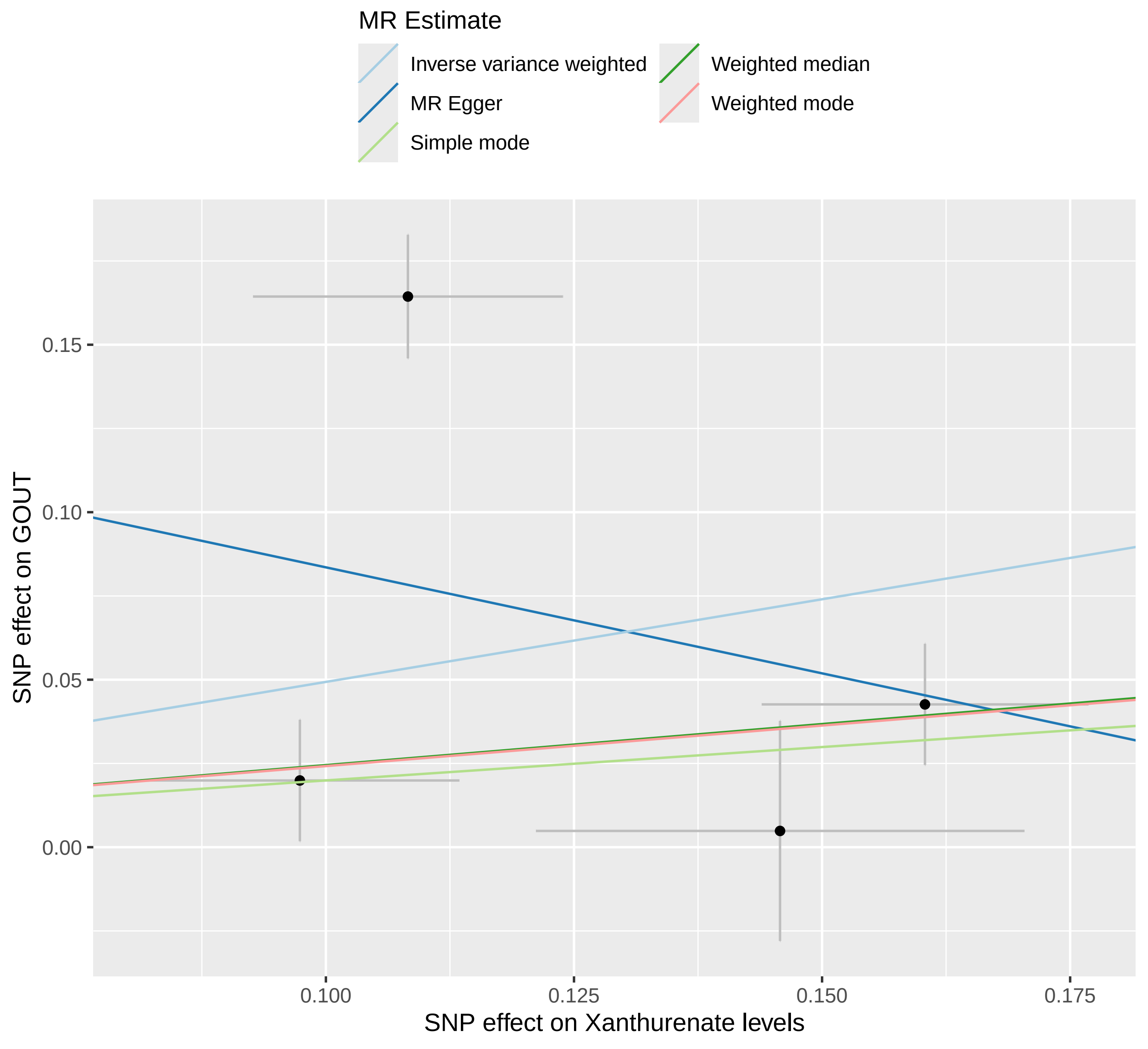

MR Estimate
Inverse variance weighted
MR Egger
Simple mode
Weighted median
Weighted mode
0.15
0.10
0.05
0.00
SNP effect on GOUT
0.100
0.125
0.150
0.175
SNP effect on Xanthurenate levels
