# Supplementary material for: Uncovering metabolite–inflammatory protein interactions in gout pathogenesis: A 2-sample bidirectional Mendelian randomization study
Source: Medicine (Baltimore). 2026 Oct 30;105(44):e51276. doi: 10.1097/MD.0000000000051276 (PMC13727226; doi:10.1097/MD.0000000000051276)
Supplement: Supplementary file 2 [file medi-105-e51276-s006.pdf]

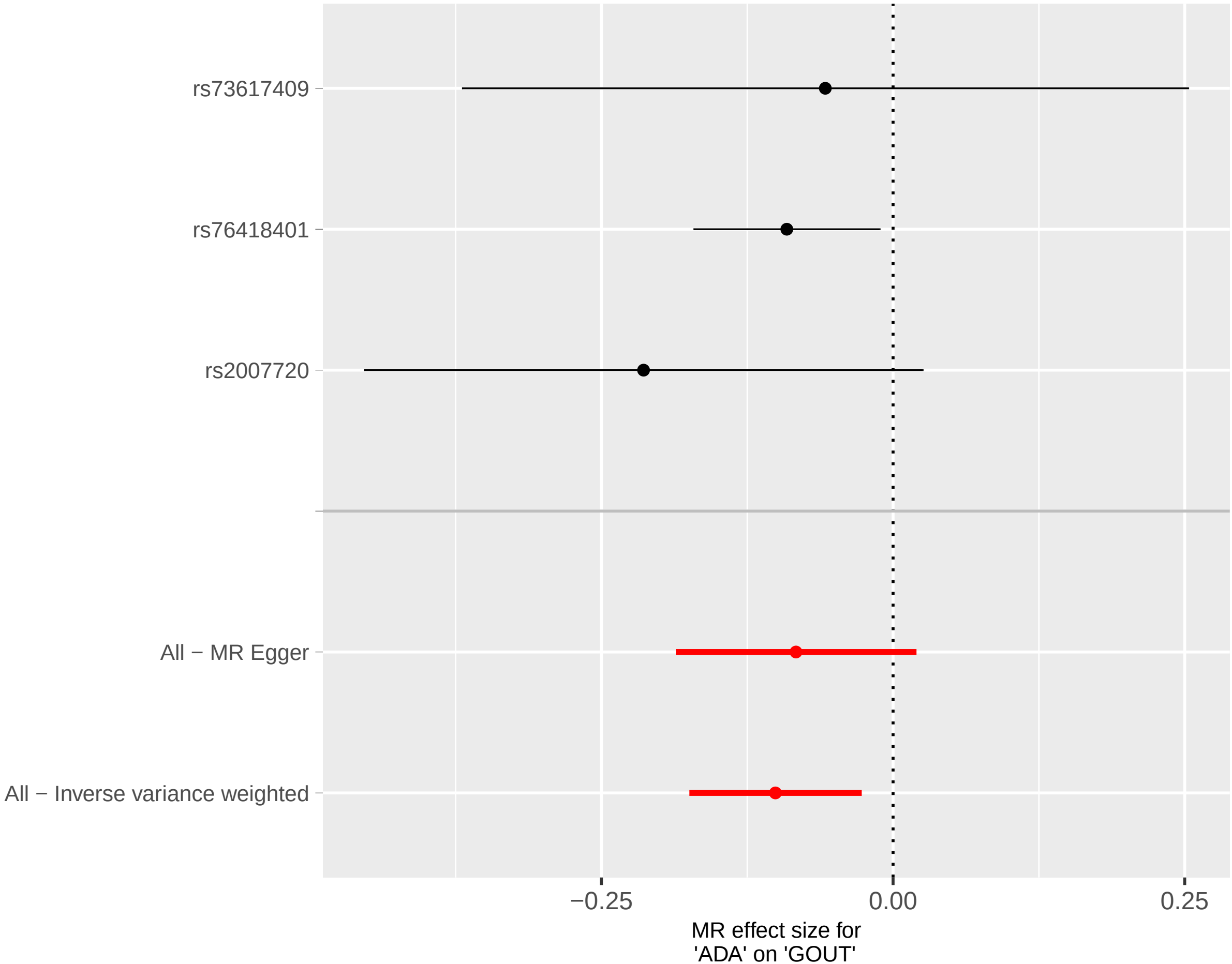
rs73617409
rs76418401
rs2007720
All − MR Egger
All − Inverse variance weighted
−0.25
0.00
0.25
MR effect size for
'ADA' on 'GOUT'

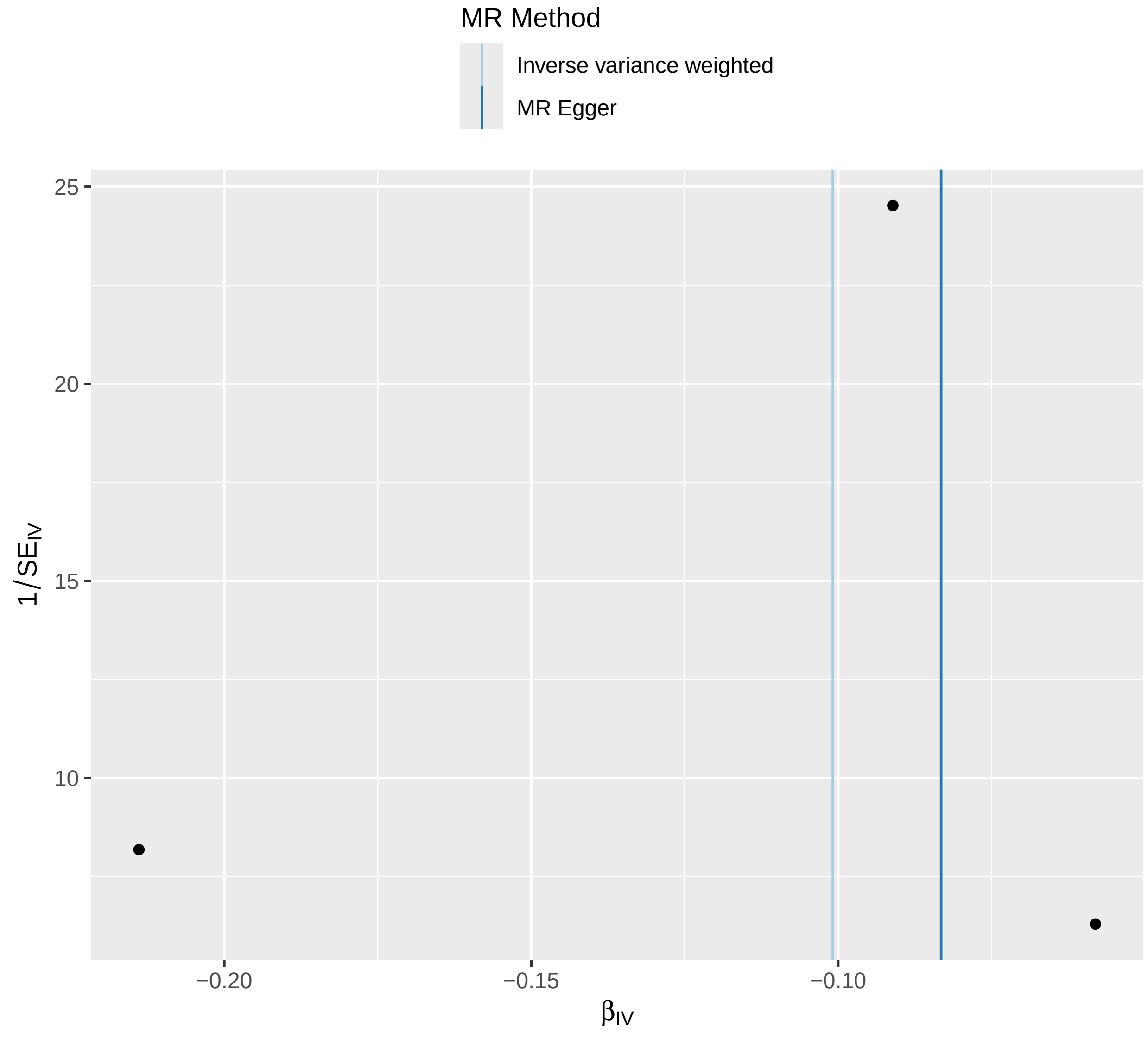

MR Method
Inverse variance weighted
MR Egger
25
20
15
10
$1/SE_{IV}$
−0.20
−0.15
−0.10
β_{IV}

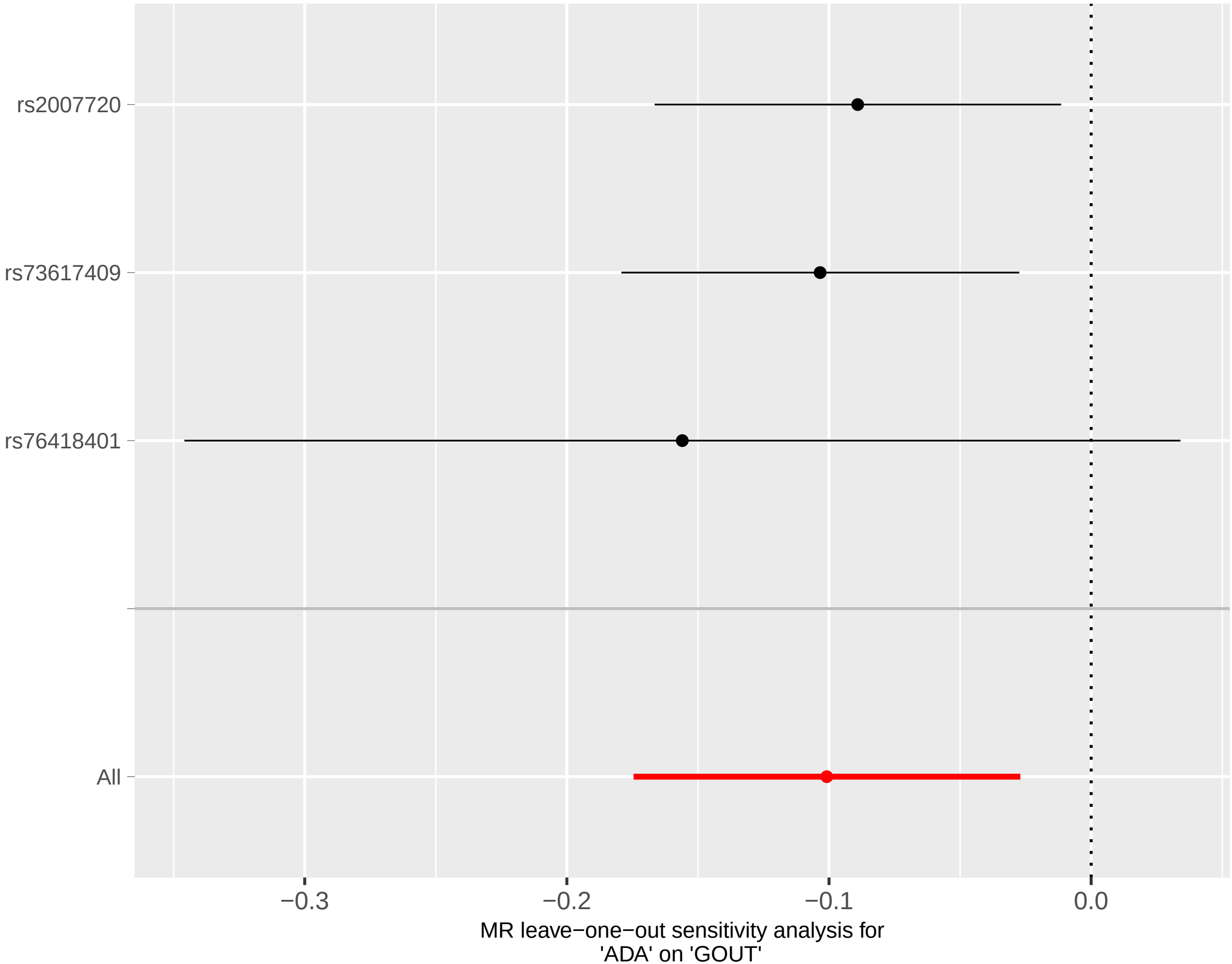
rs2007720
rs73617409
rs76418401
All
−0.3
−0.2
−0.1
0.0
MR leave−one−out sensitivity analysis for
'ADA' on 'GOUT'

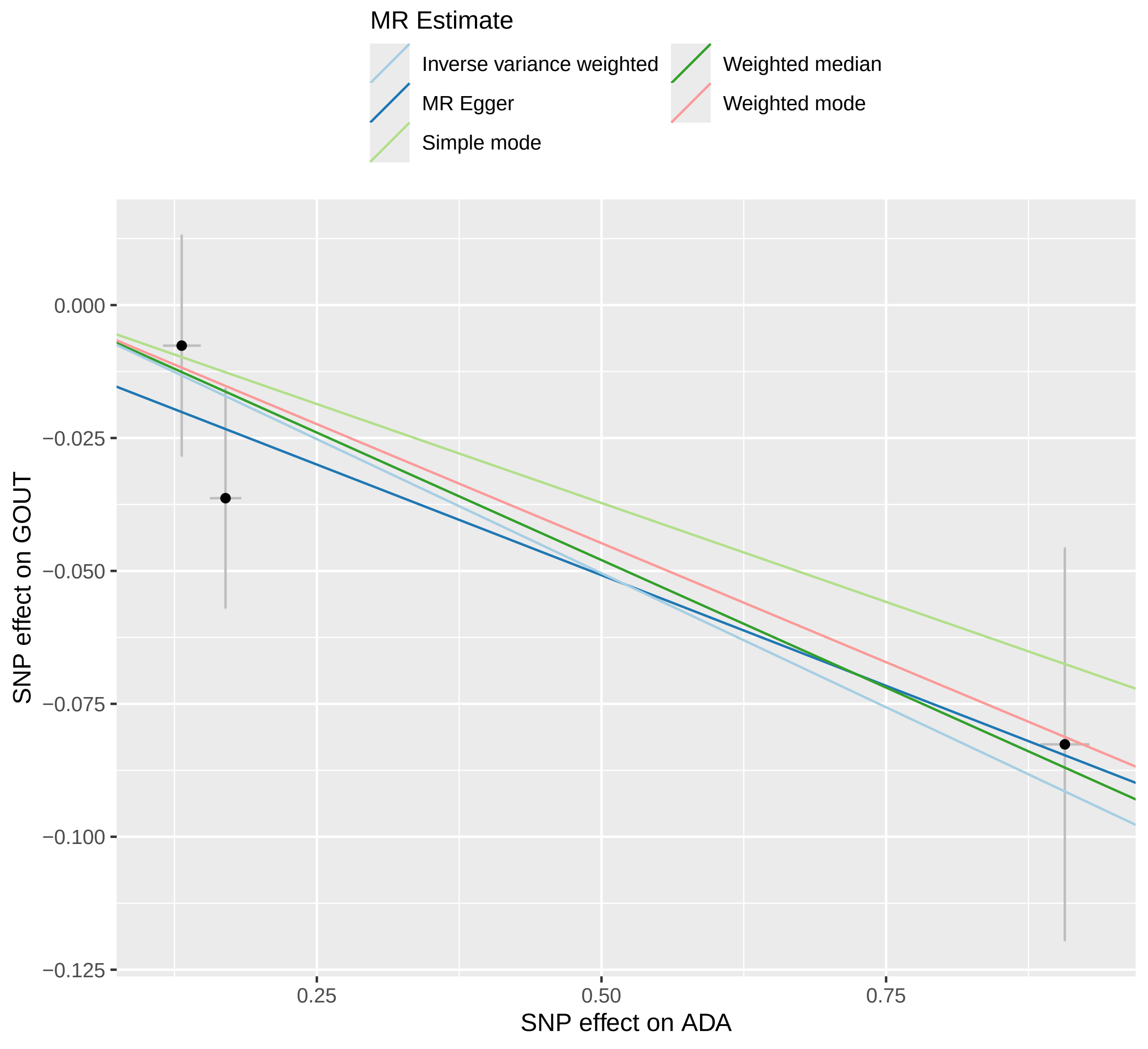
MR Estimate
Inverse variance weighted
MR Egger
Simple mode
Weighted median
Weighted mode
SNP effect on GOUT
0.000
−0.025
−0.050
−0.075
−0.100
−0.125
0.25
0.50
0.75
SNP effect on ADA

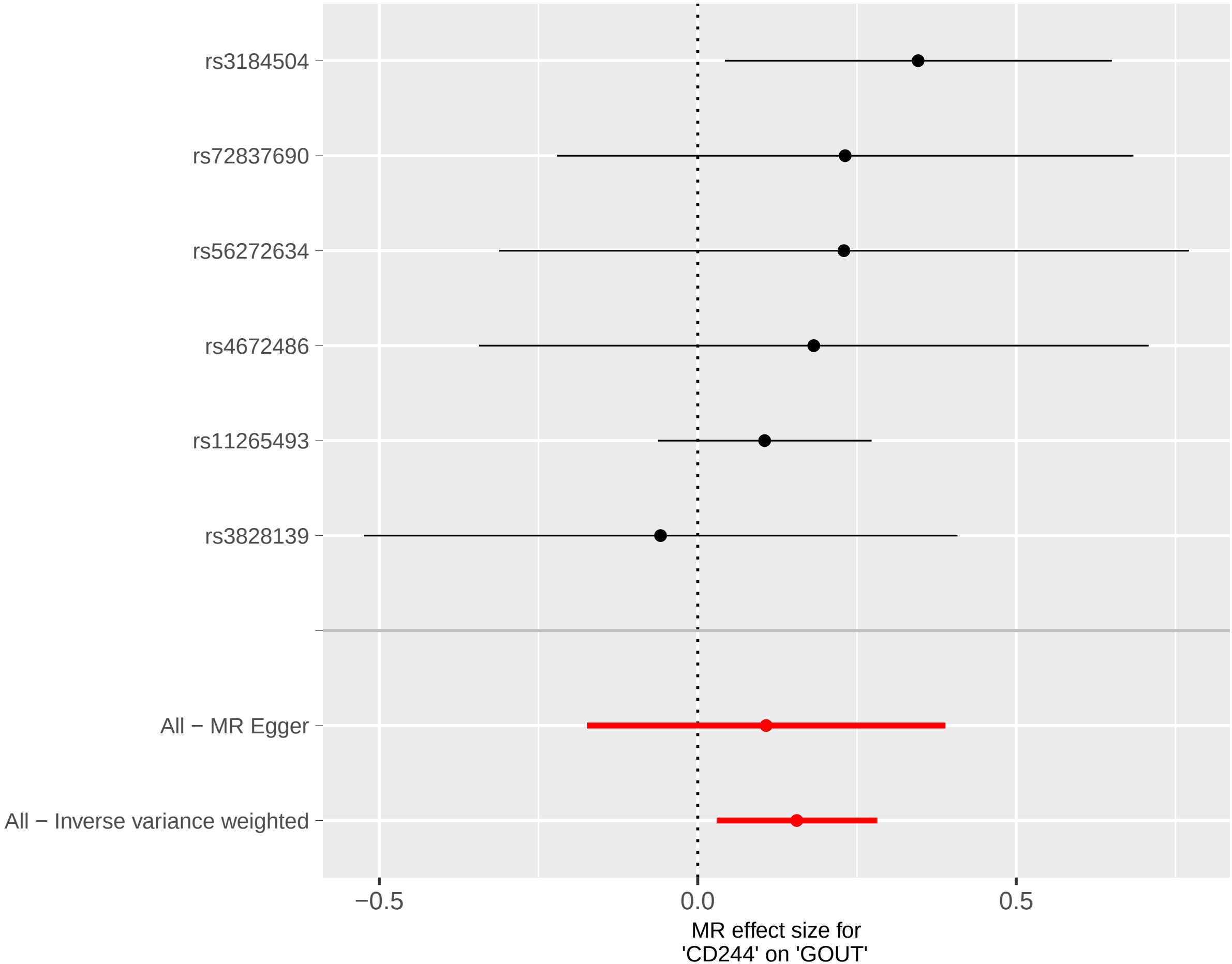
rs3184504
rs72837690
rs56272634
rs4672486
rs11265493
rs3828139
All − MR Egger
All − Inverse variance weighted
−0.5
0.0
0.5
MR effect size for
'CD244' on 'GOUT'

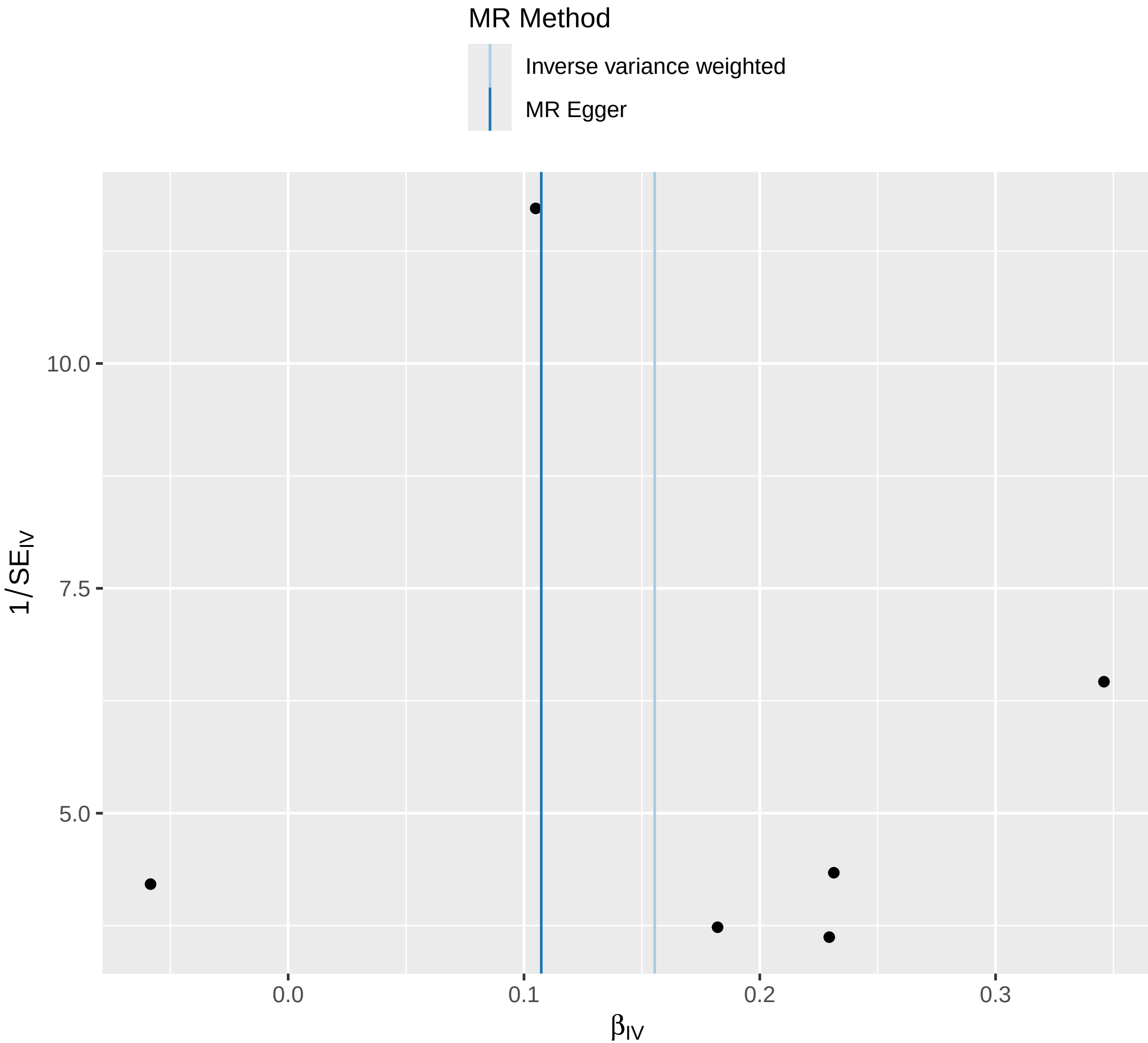

MR Method
Inverse variance weighted
MR Egger
10.0
7.5
5.0
1/SE_IV
0.0
0.1
0.2
0.3
β_IV

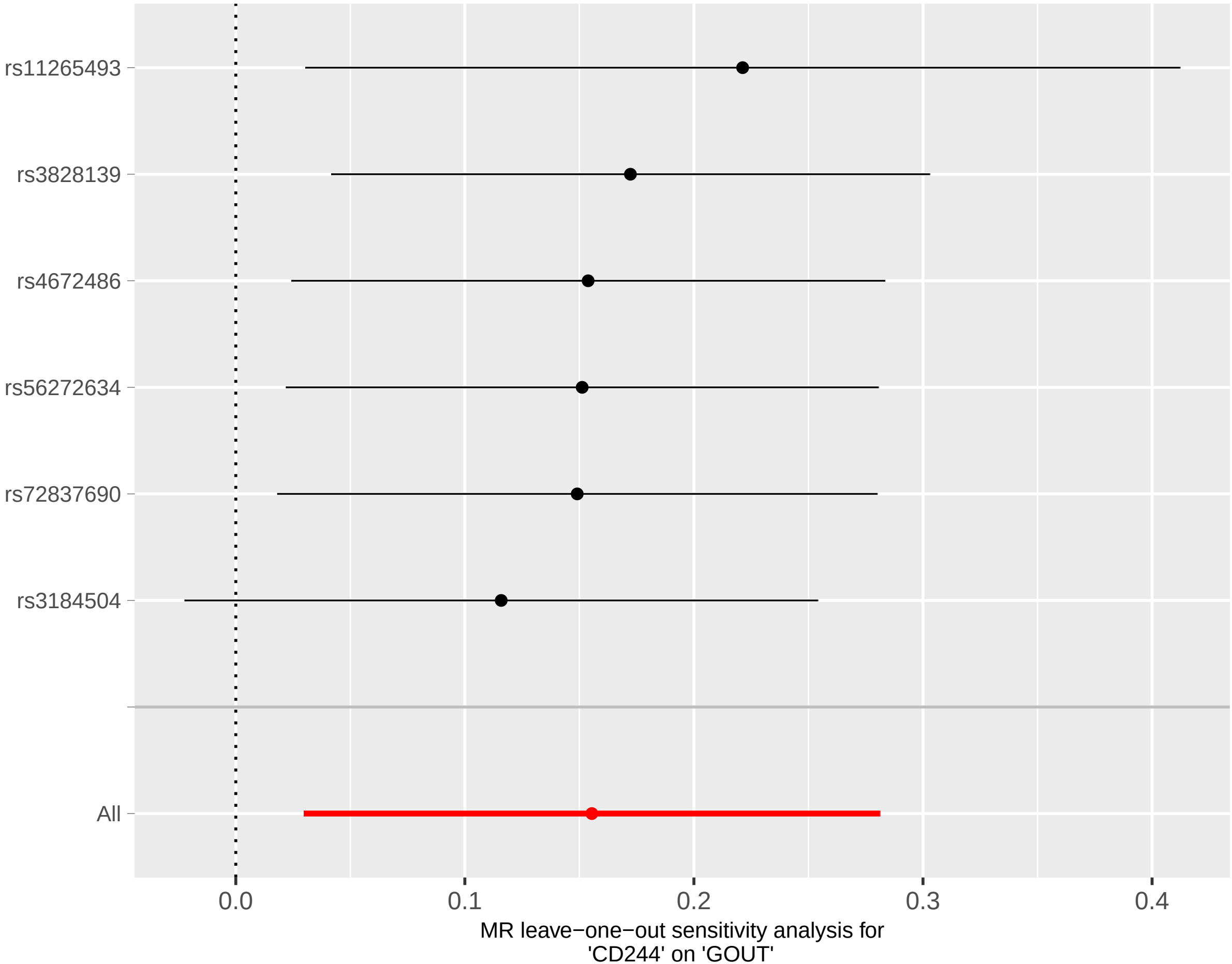
rs11265493
rs3828139
rs4672486
rs56272634
rs72837690
rs3184504
All
0.0
0.1
0.2
0.3
0.4
MR leave−one−out sensitivity analysis for
'CD244' on 'GOUT'

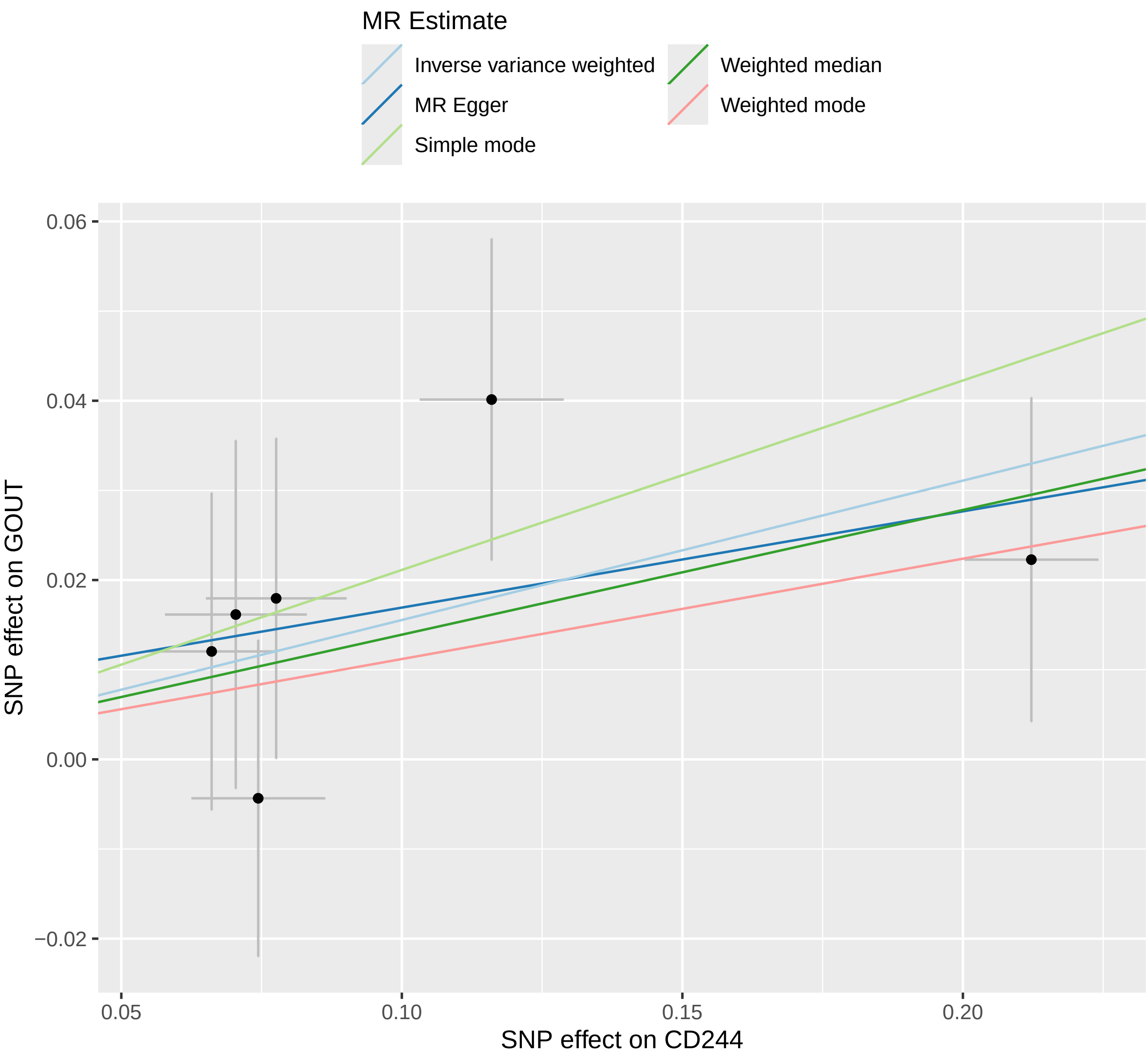
MR Estimate
Inverse variance weighted
MR Egger
Simple mode
Weighted median
Weighted mode
0.06
0.04
0.02
0.00
−0.02
SNP effect on GOUT
0.05
0.10
0.15
0.20
SNP effect on CD244

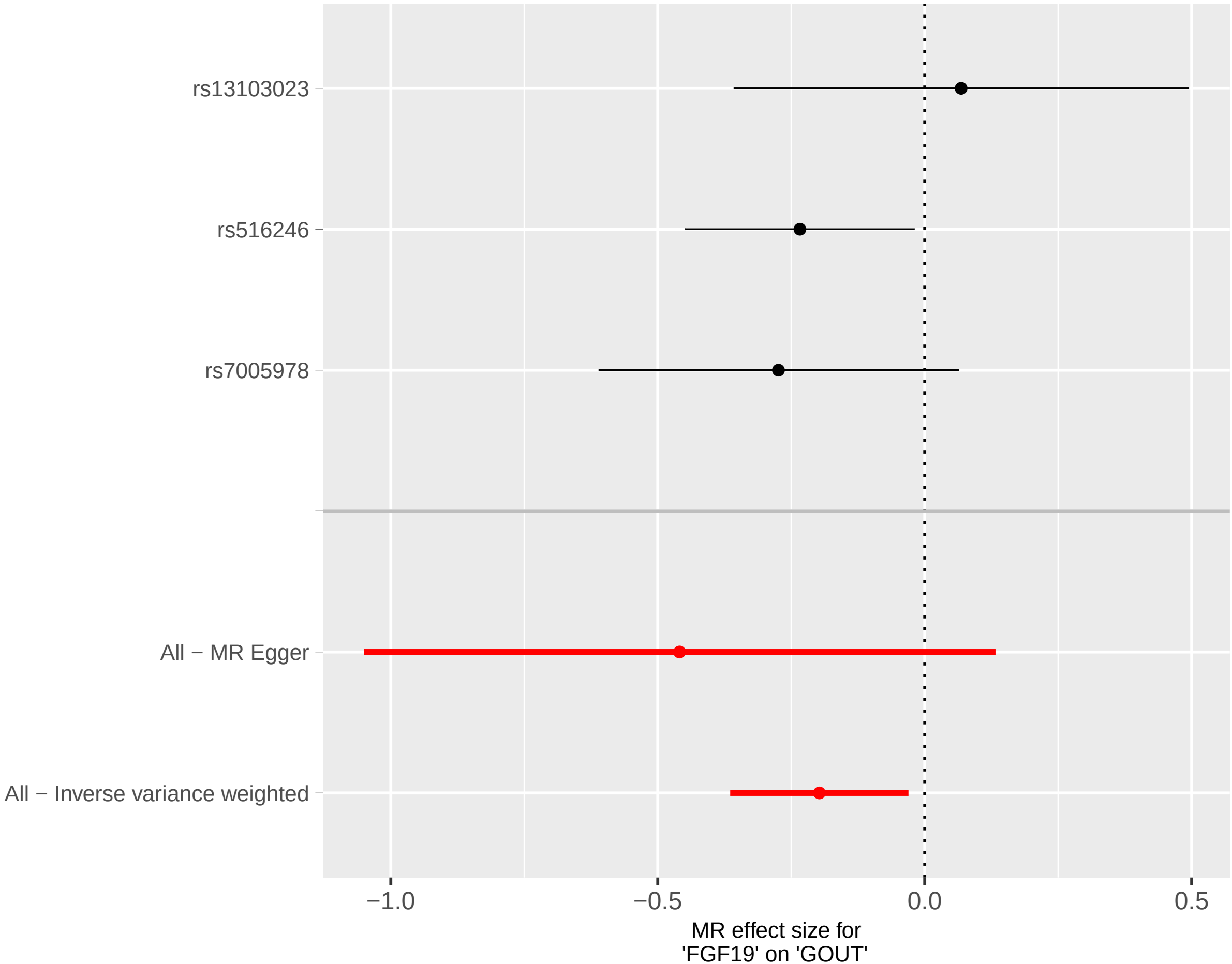
rs13103023
rs516246
rs7005978
All − MR Egger
All − Inverse variance weighted
−1.0
−0.5
0.0
0.5
MR effect size for
'FGF19' on 'GOUT'

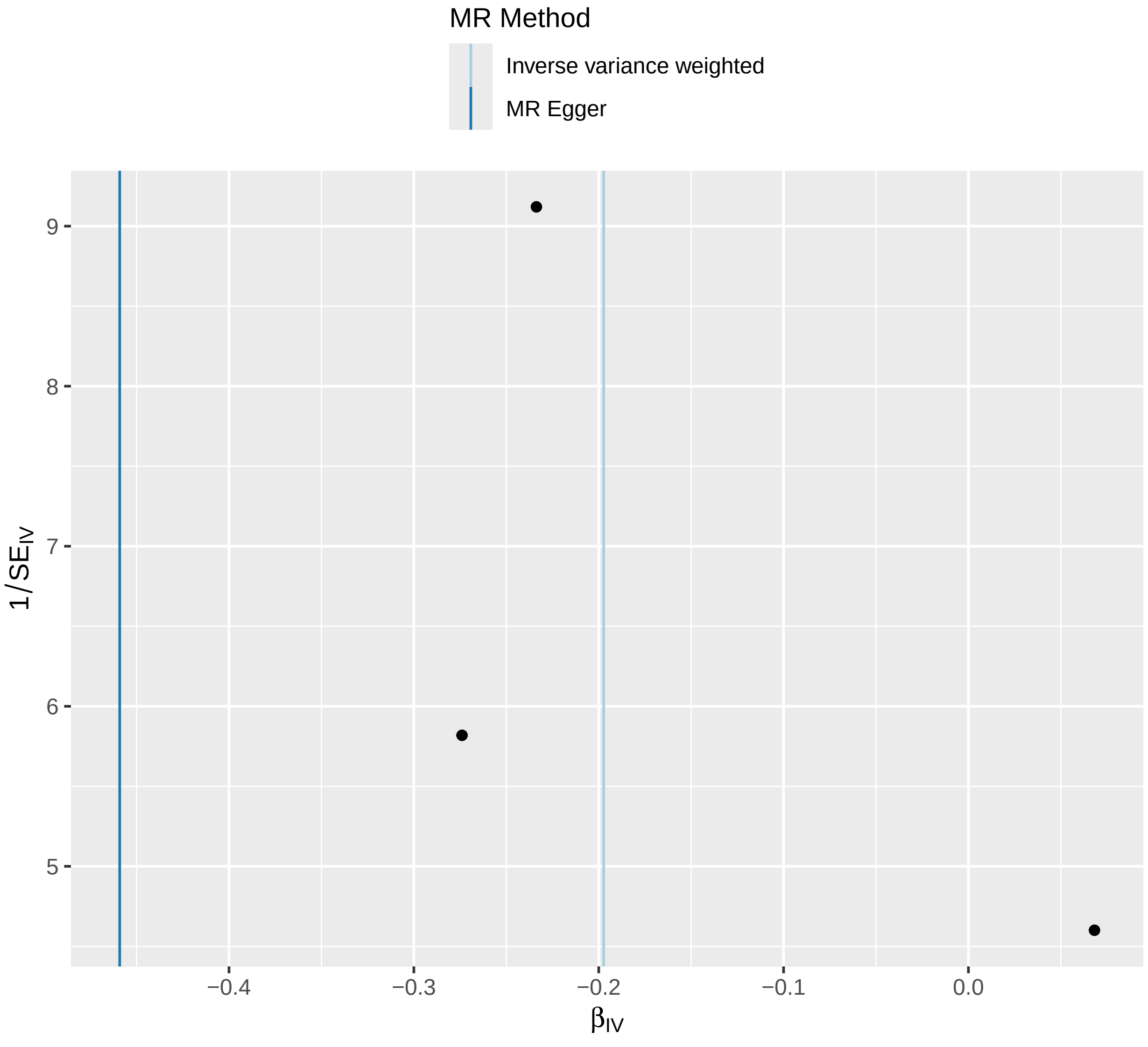
MR Method
Inverse variance weighted
MR Egger
1/SE_IV
9
8
7
6
5
−0.4
−0.3
−0.2
−0.1
0.0
β_IV

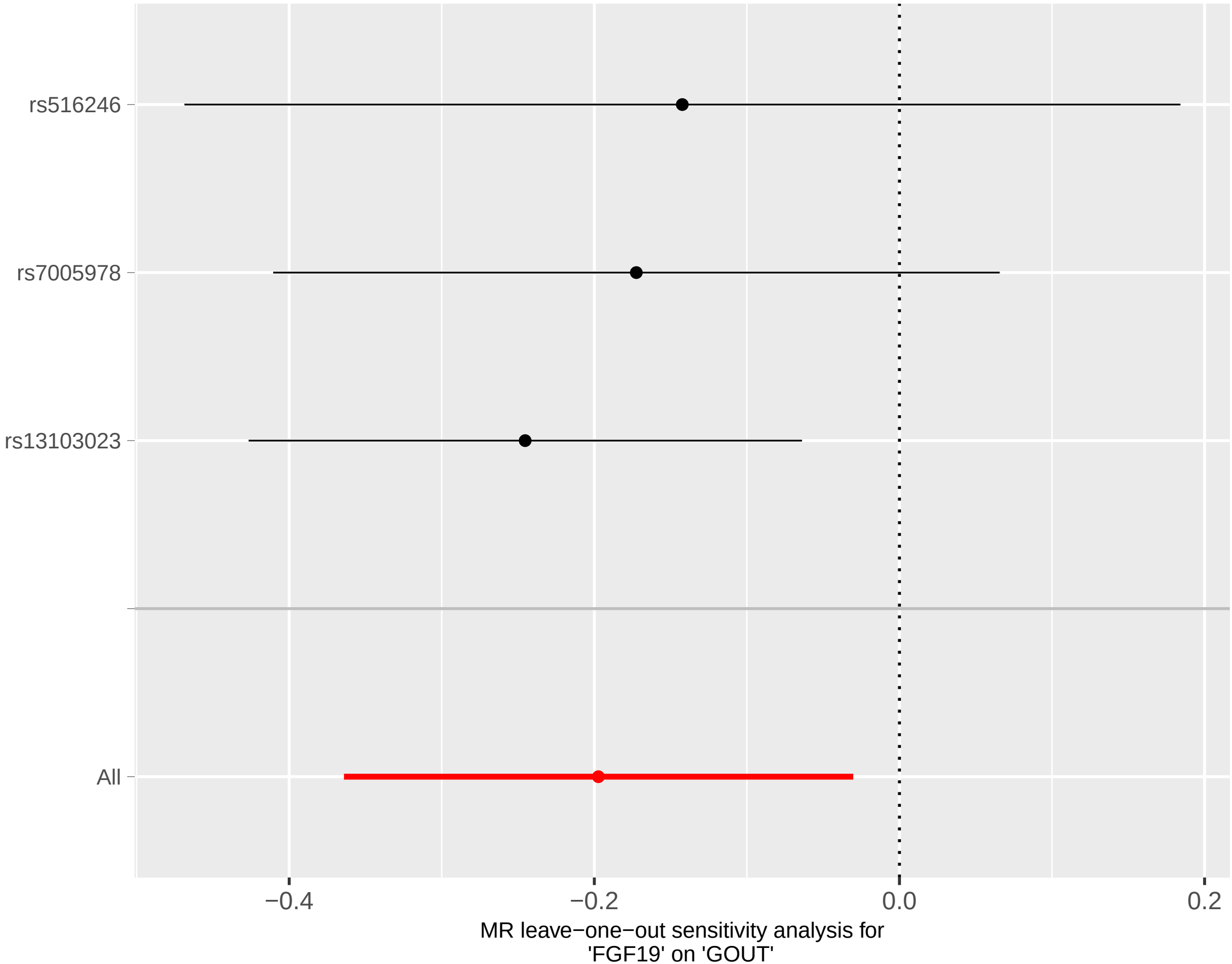
rs516246
rs7005978
rs13103023
All
−0.4
−0.2
0.0
0.2
MR leave−one−out sensitivity analysis for
'FGF19' on 'GOUT'

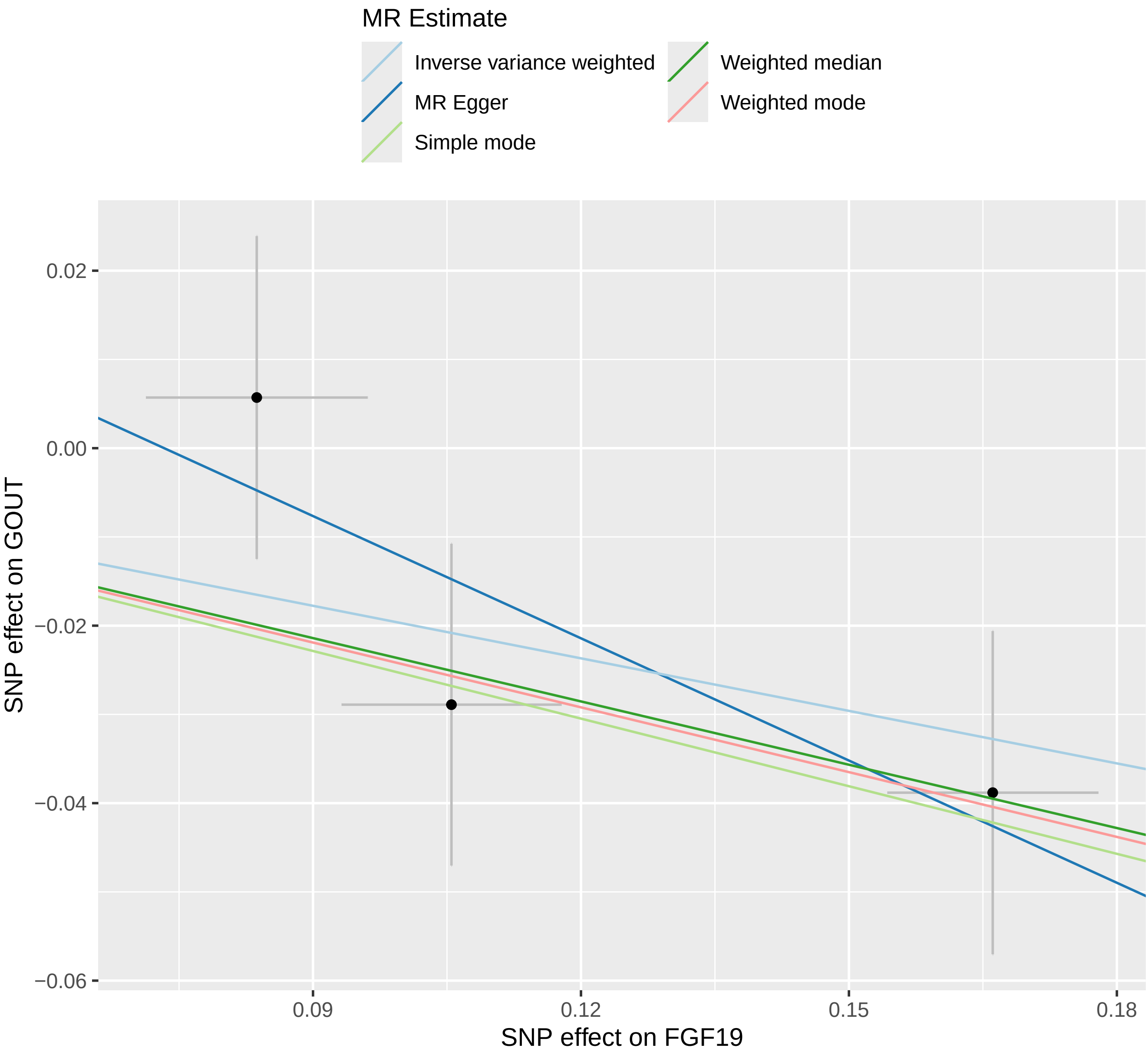
MR Estimate
Inverse variance weighted
MR Egger
Simple mode
Weighted median
Weighted mode
0.02
0.00
−0.02
−0.04
−0.06
0.09
0.12
0.15
0.18
SNP effect on GOUT
SNP effect on FGF19

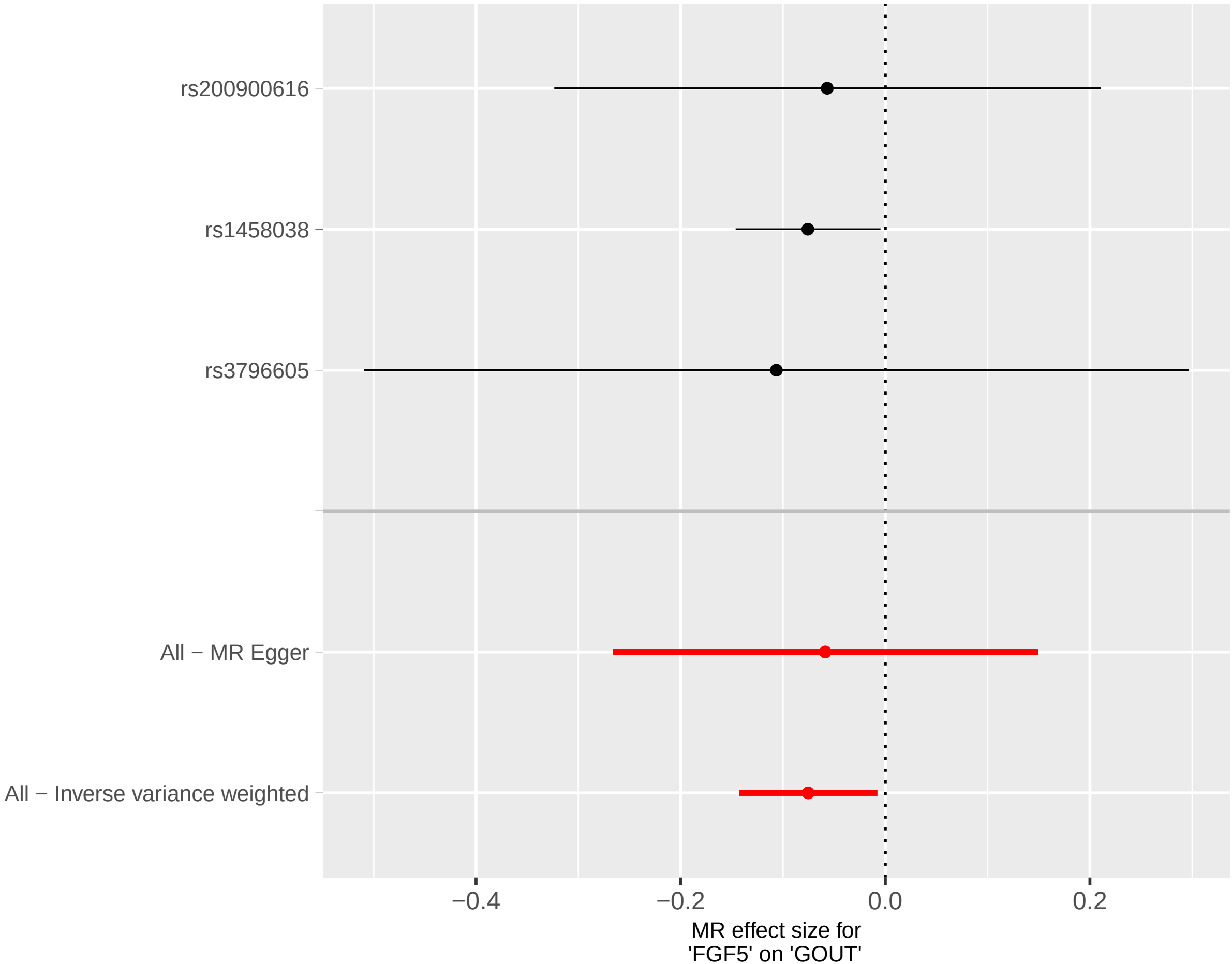
rs200900616
rs1458038
rs3796605
All − MR Egger
All − Inverse variance weighted
−0.4
−0.2
0.0
0.2
MR effect size for
'FGF5' on 'GOUT'

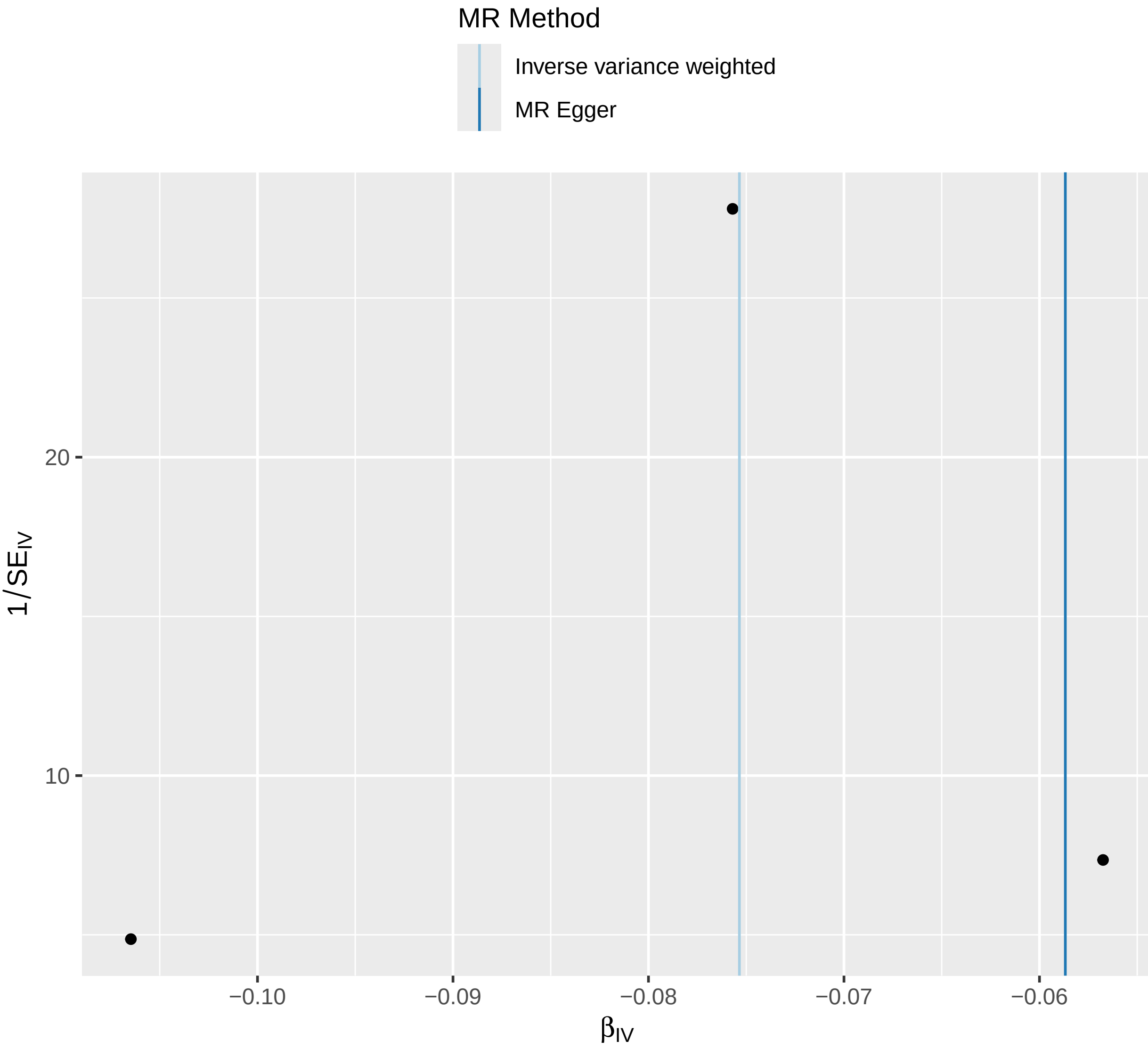
MR Method
Inverse variance weighted
MR Egger
20
10
1/SE_IV
−0.10
−0.09
−0.08
−0.07
−0.06
β_IV

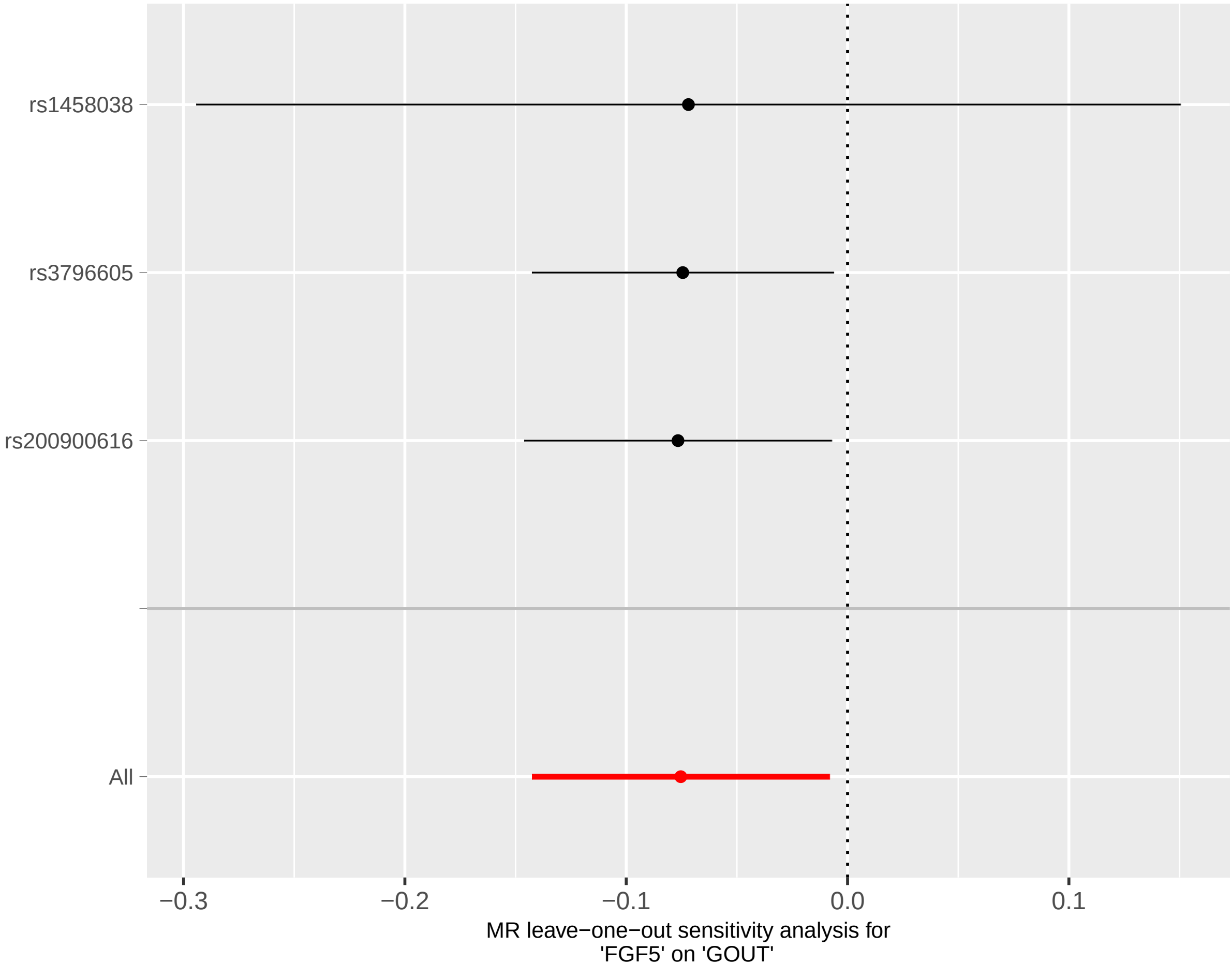
rs1458038
rs3796605
rs200900616
All
−0.3
−0.2
−0.1
0.0
0.1
MR leave−one−out sensitivity analysis for
'FGF5' on 'GOUT'

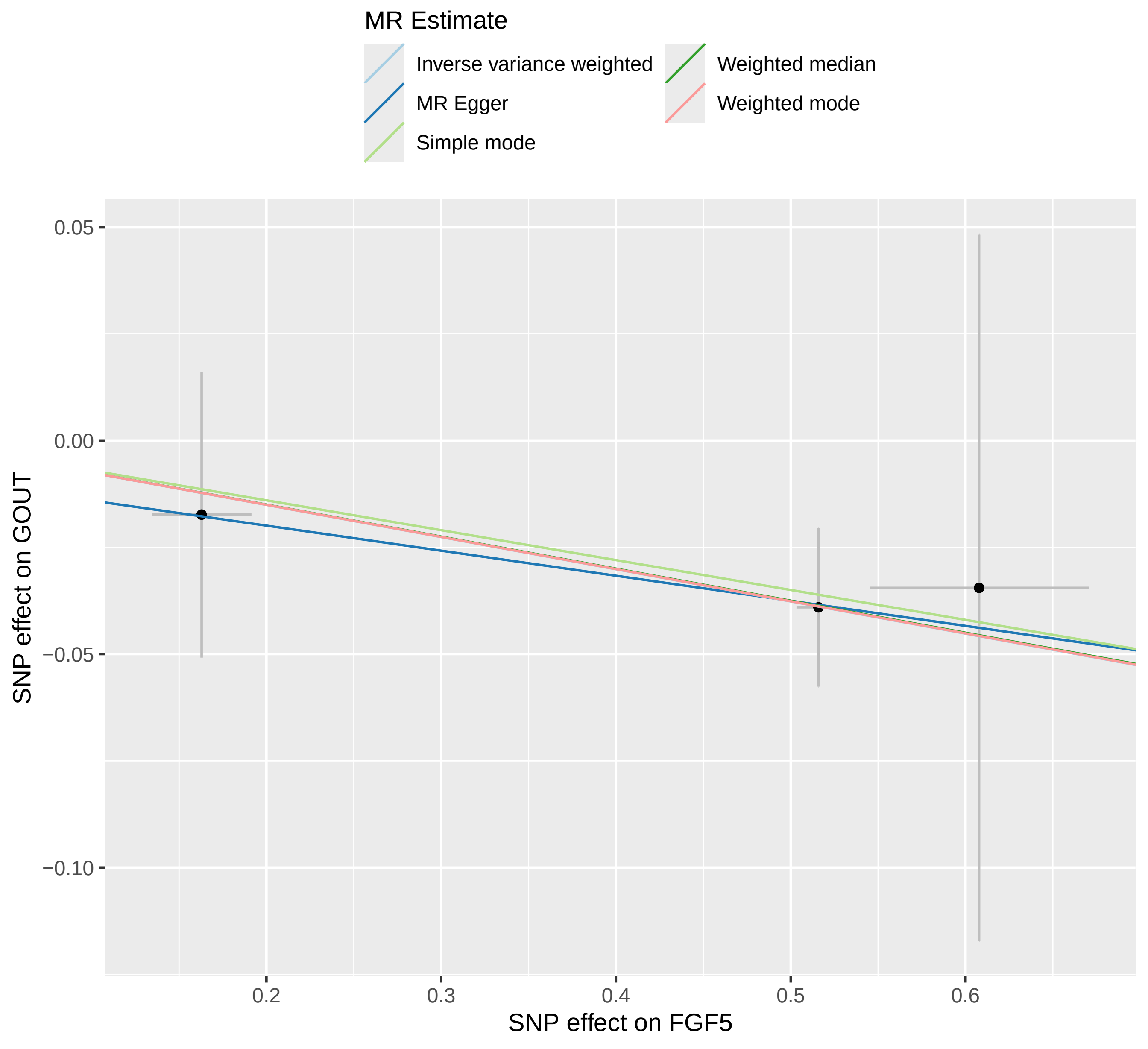

MR Estimate
Inverse variance weighted
MR Egger
Simple mode
Weighted median
Weighted mode
0.05
0.00
−0.05
−0.10
0.2
0.3
0.4
0.5
0.6
SNP effect on GOUT
SNP effect on FGF5
