# Supplementary material for: Uncovering metabolite–inflammatory protein interactions in gout pathogenesis: A 2-sample bidirectional Mendelian randomization study
Source: Medicine (Baltimore). 2026 Oct 30;105(44):e51276. doi: 10.1097/MD.0000000000051276 (PMC13727226; doi:10.1097/MD.0000000000051276)
Supplement: Supplementary file 3 [file medi-105-e51276-s008.pdf]

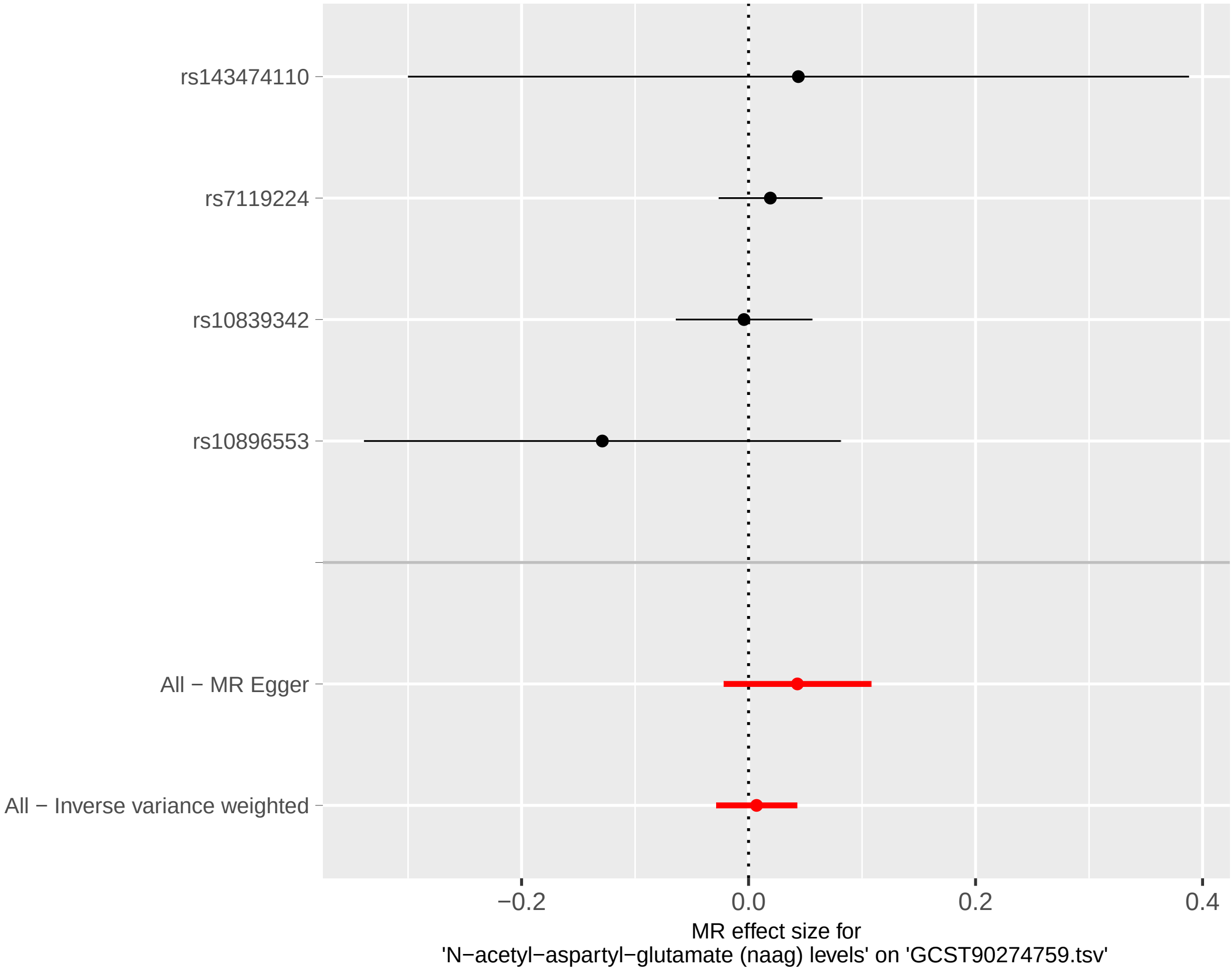
rs143474110
rs7119224
rs10839342
rs10896553
All − MR Egger
All − Inverse variance weighted
−0.2
0.0
0.2
0.4
MR effect size for
'N−acetyl−aspartyl−glutamate (naag) levels' on 'GCST90274759.tsv'

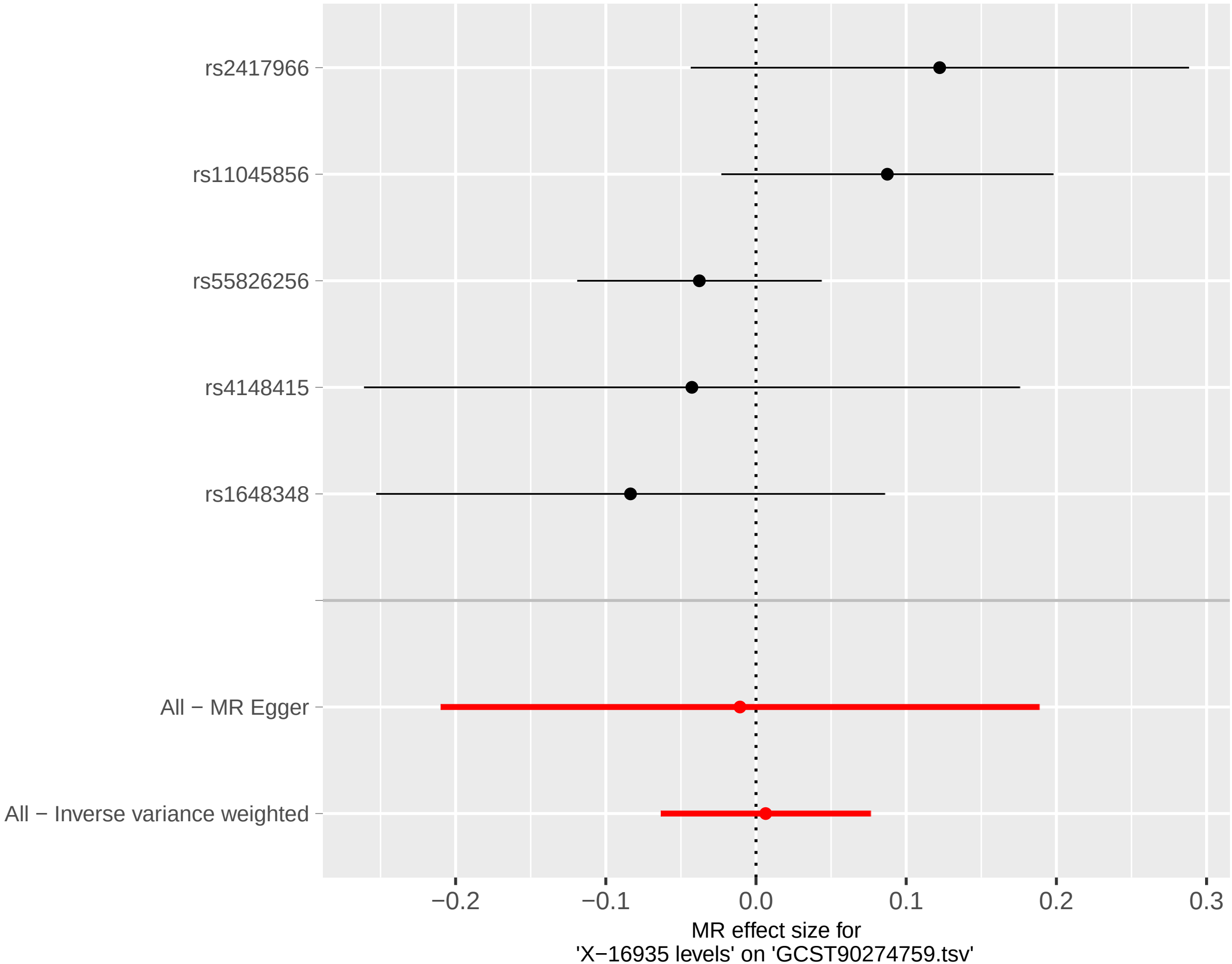
rs2417966
rs11045856
rs55826256
rs4148415
rs1648348
All − MR Egger
All − Inverse variance weighted
−0.2
−0.1
0.0
0.1
0.2
0.3
MR effect size for
'X−16935 levels' on 'GCST90274759.tsv'

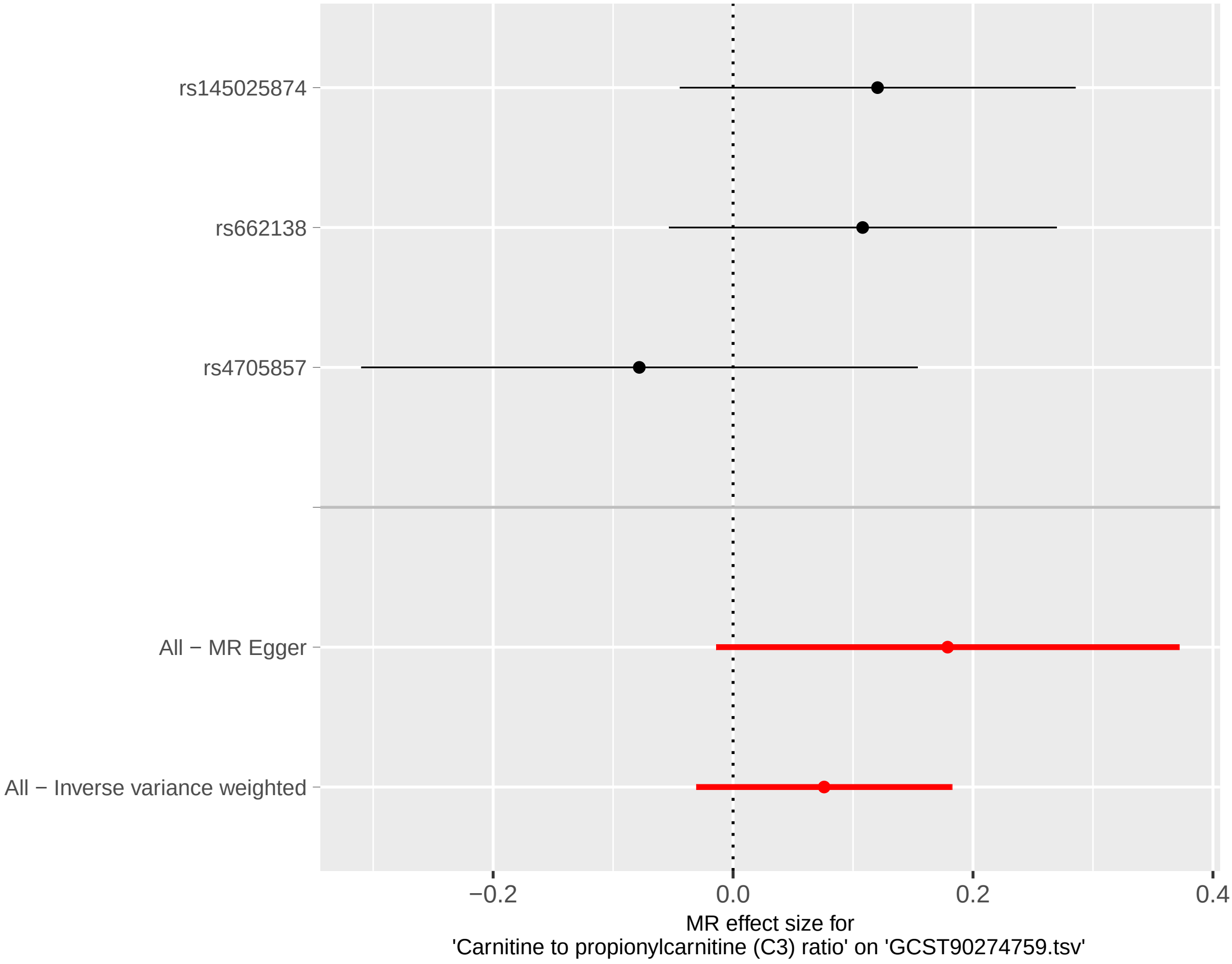
rs145025874
rs662138
rs4705857
All − MR Egger
All − Inverse variance weighted
−0.2
0.0
0.2
0.4
MR effect size for
'Carnitine to propionylcarnitine (C3) ratio' on 'GCST90274759.tsv'

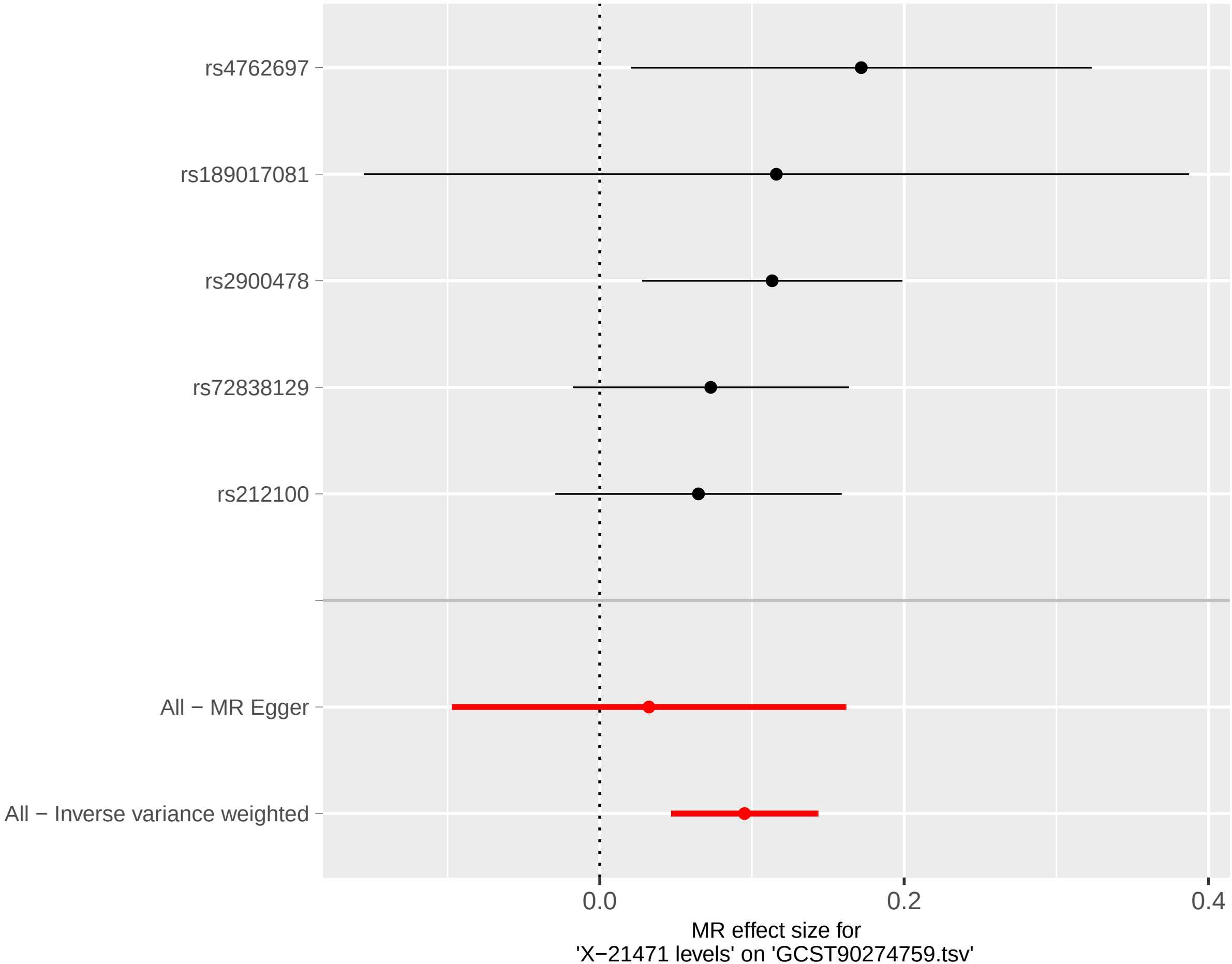
rs4762697
rs189017081
rs2900478
rs72838129
rs212100
All − MR Egger
All − Inverse variance weighted
0.0
0.2
0.4
MR effect size for
'X−21471 levels' on 'GCST90274759.tsv'

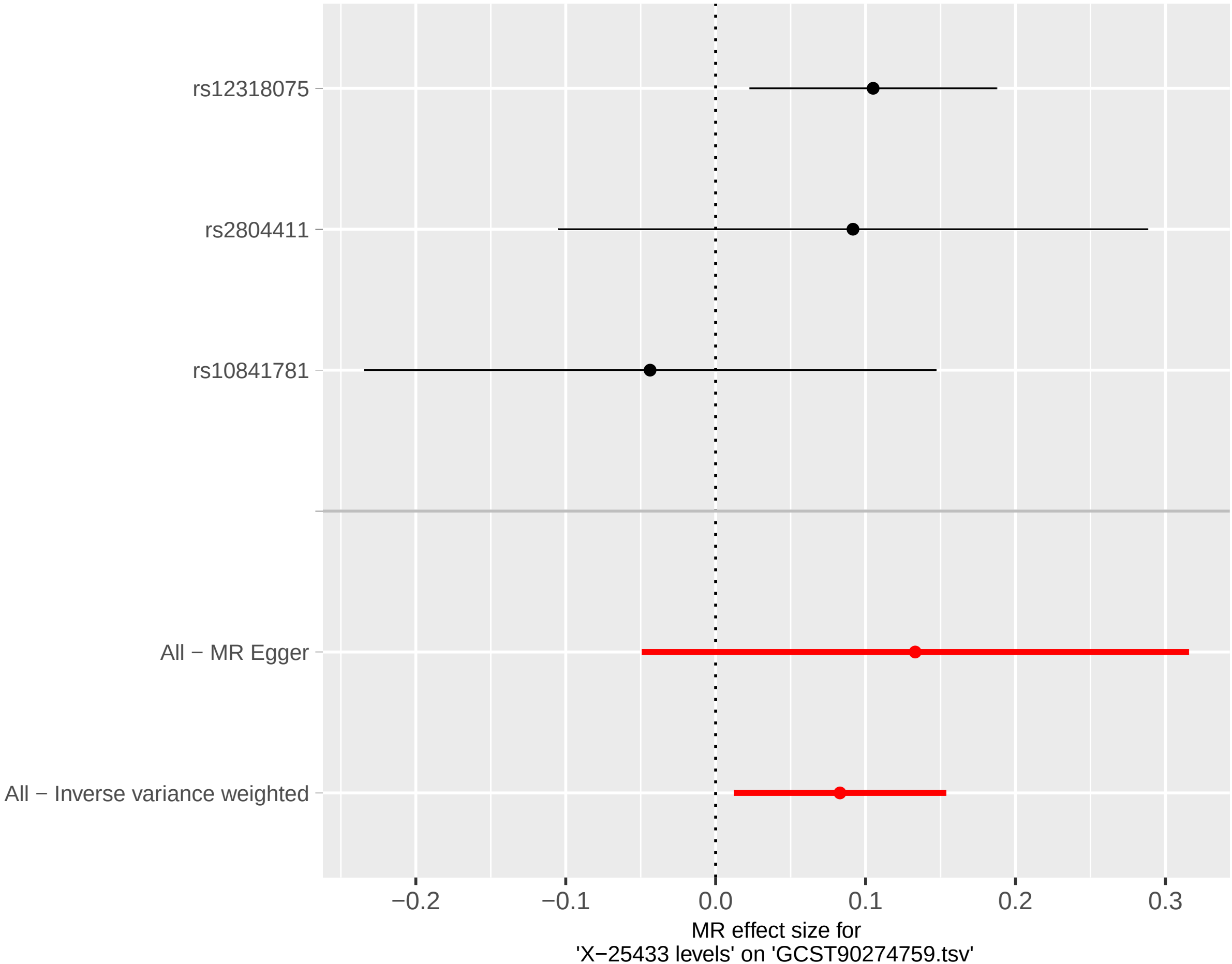
rs12318075
rs2804411
rs10841781
All − MR Egger
All − Inverse variance weighted
−0.2
−0.1
0.0
0.1
0.2
0.3
MR effect size for
'X−25433 levels' on 'GCST90274759.tsv'

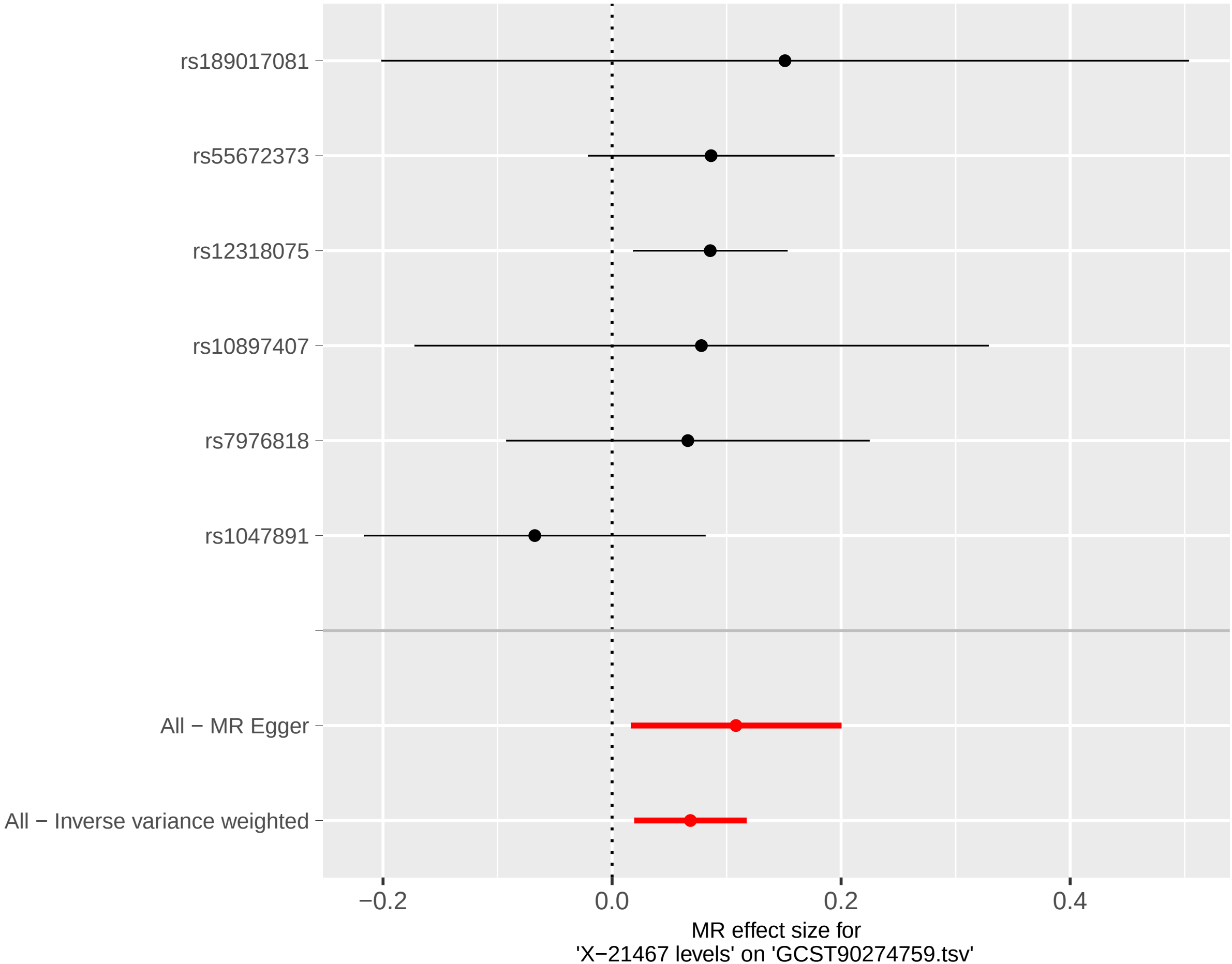
rs189017081
rs55672373
rs12318075
rs10897407
rs7976818
rs1047891
All − MR Egger
All − Inverse variance weighted
−0.2
0.0
0.2
0.4
MR effect size for
'X−21467 levels' on 'GCST90274759.tsv'

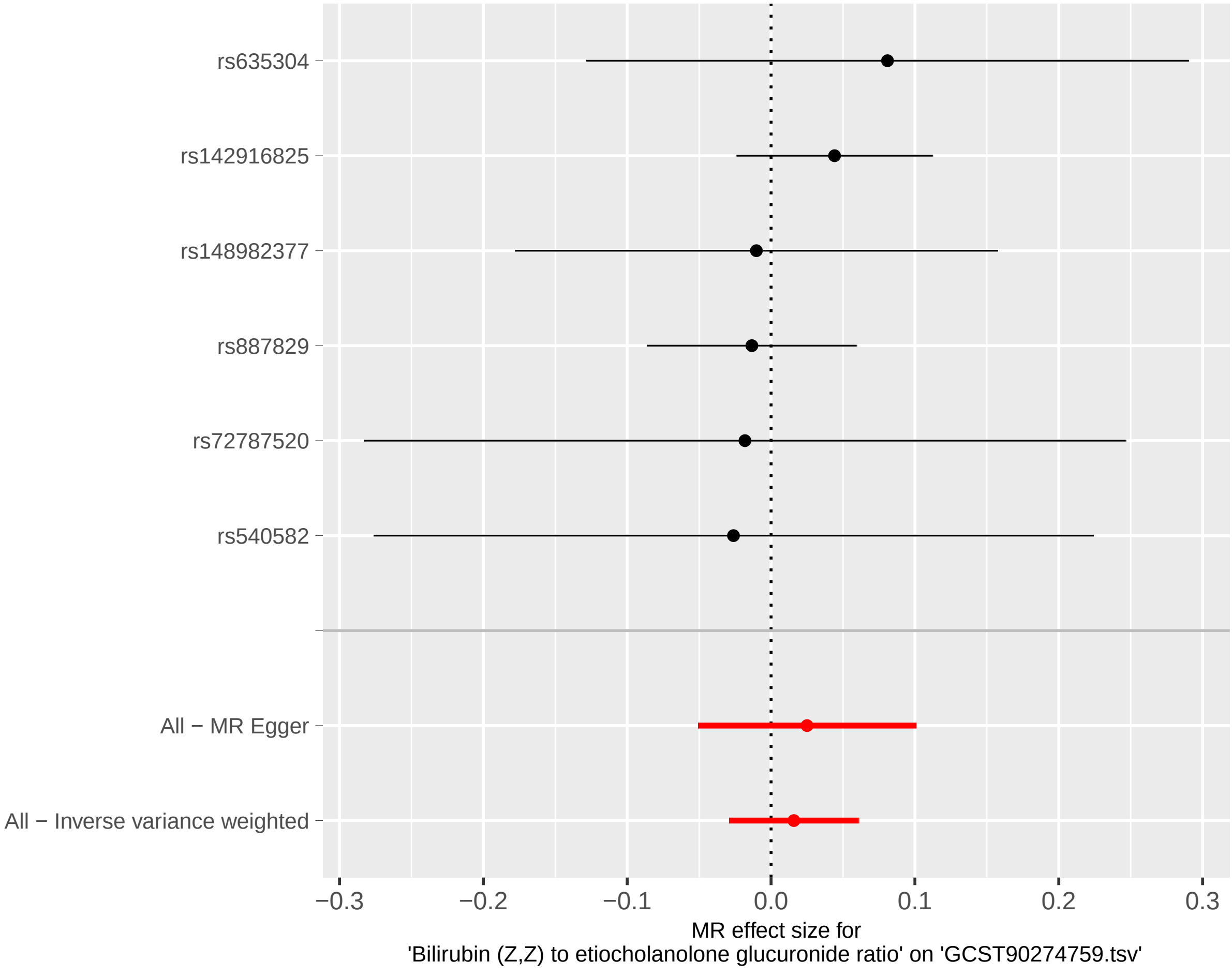

rs635304
rs142916825
rs148982377
rs887829
rs72787520
rs540582
All − MR Egger
All − Inverse variance weighted
−0.3
−0.2
−0.1
0.0
0.1
0.2
0.3
MR effect size for
'Bilirubin (Z,Z) to etiocholanolone glucuronide ratio' on 'GCST90274759.tsv'

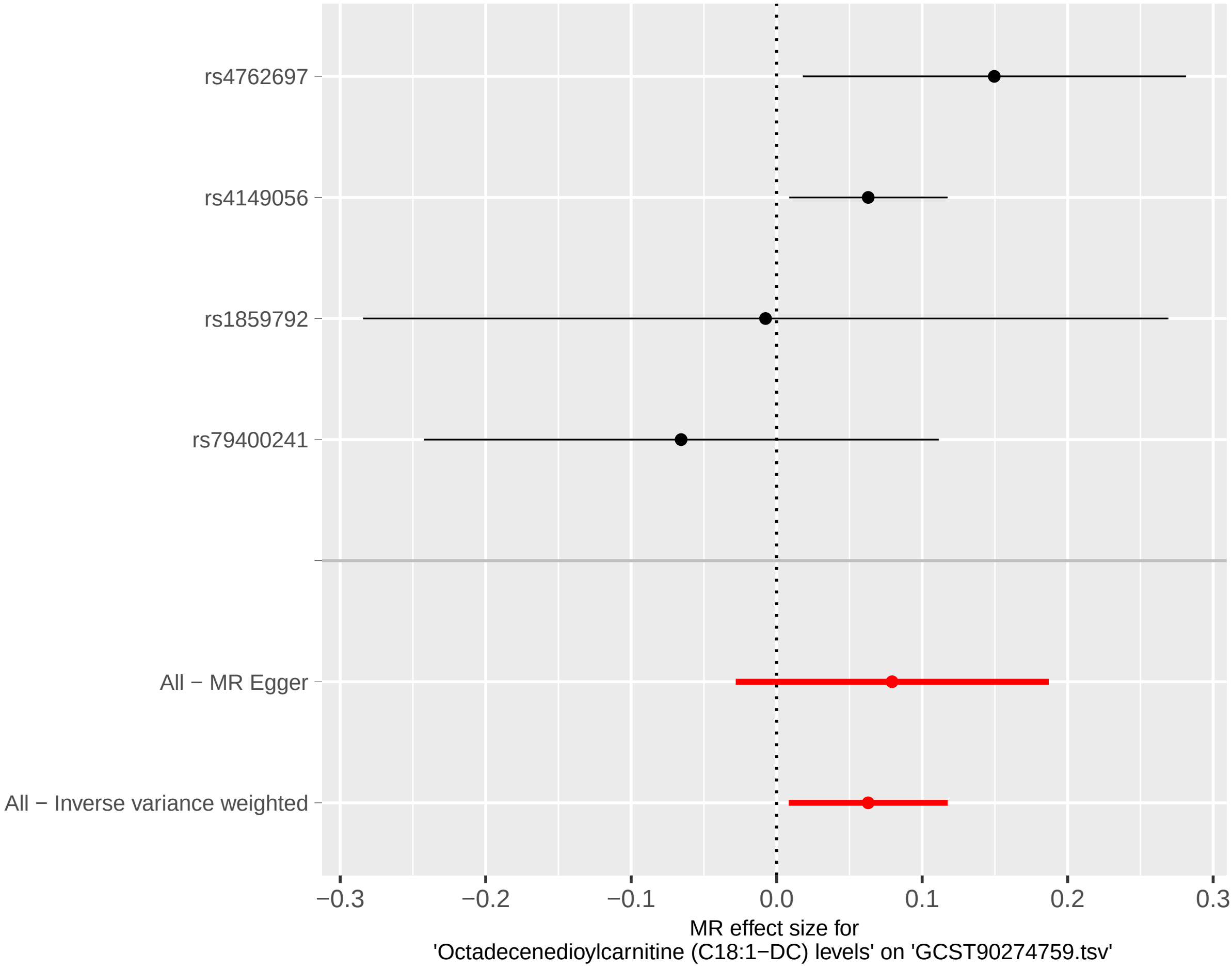
rs4762697
rs4149056
rs1859792
rs79400241
All − MR Egger
All − Inverse variance weighted
−0.3
−0.2
−0.1
0.0
0.1
0.2
0.3
MR effect size for
'Octadecenedioylcarnitine (C18:1−DC) levels' on 'GCST90274759.tsv'

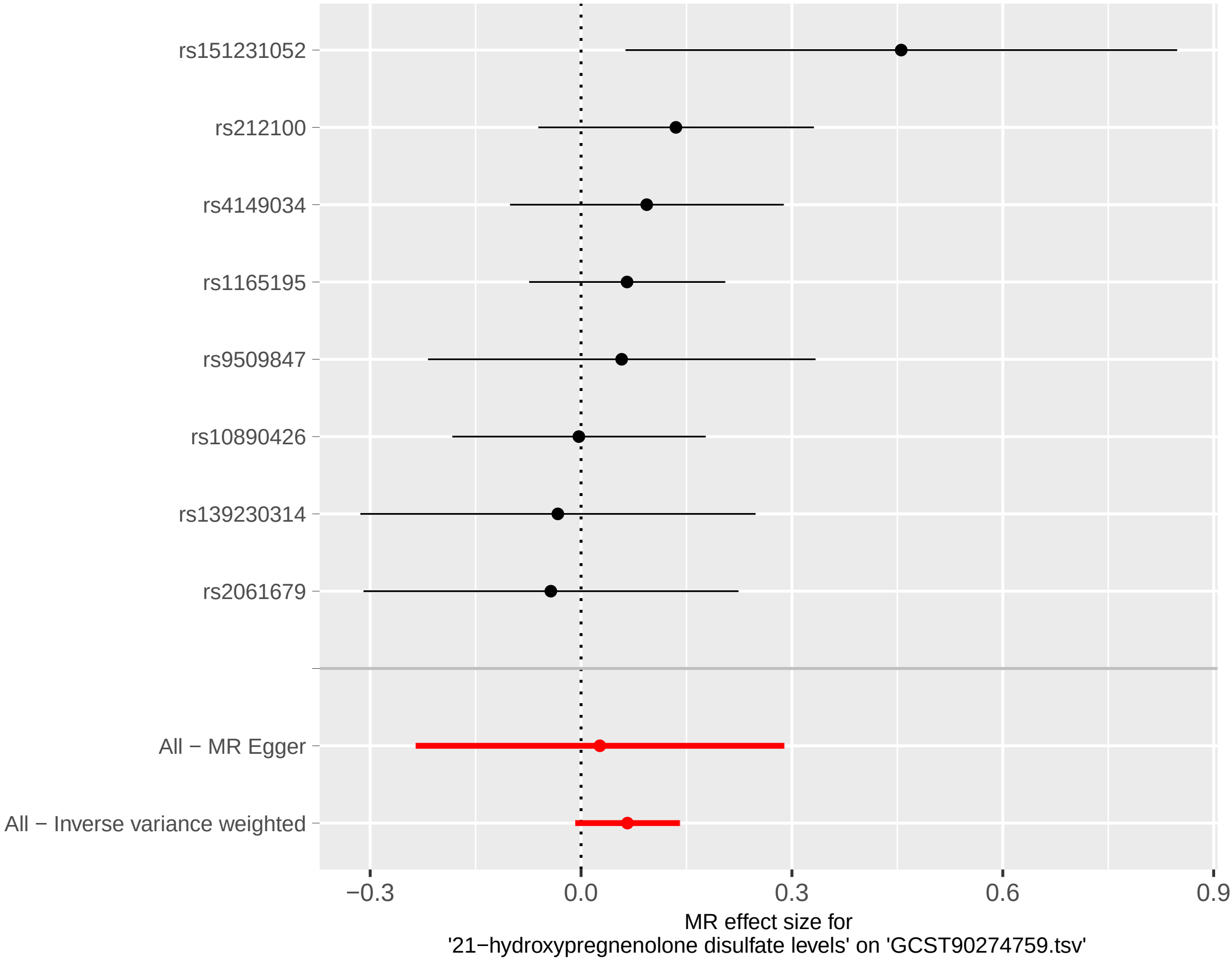
rs151231052
rs212100
rs4149034
rs1165195
rs9509847
rs10890426
rs139230314
rs2061679
All − MR Egger
All − Inverse variance weighted
−0.3
0.0
0.3
0.6
0.9
MR effect size for
'21−hydroxypregnenolone disulfate levels' on 'GCST90274759.tsv'

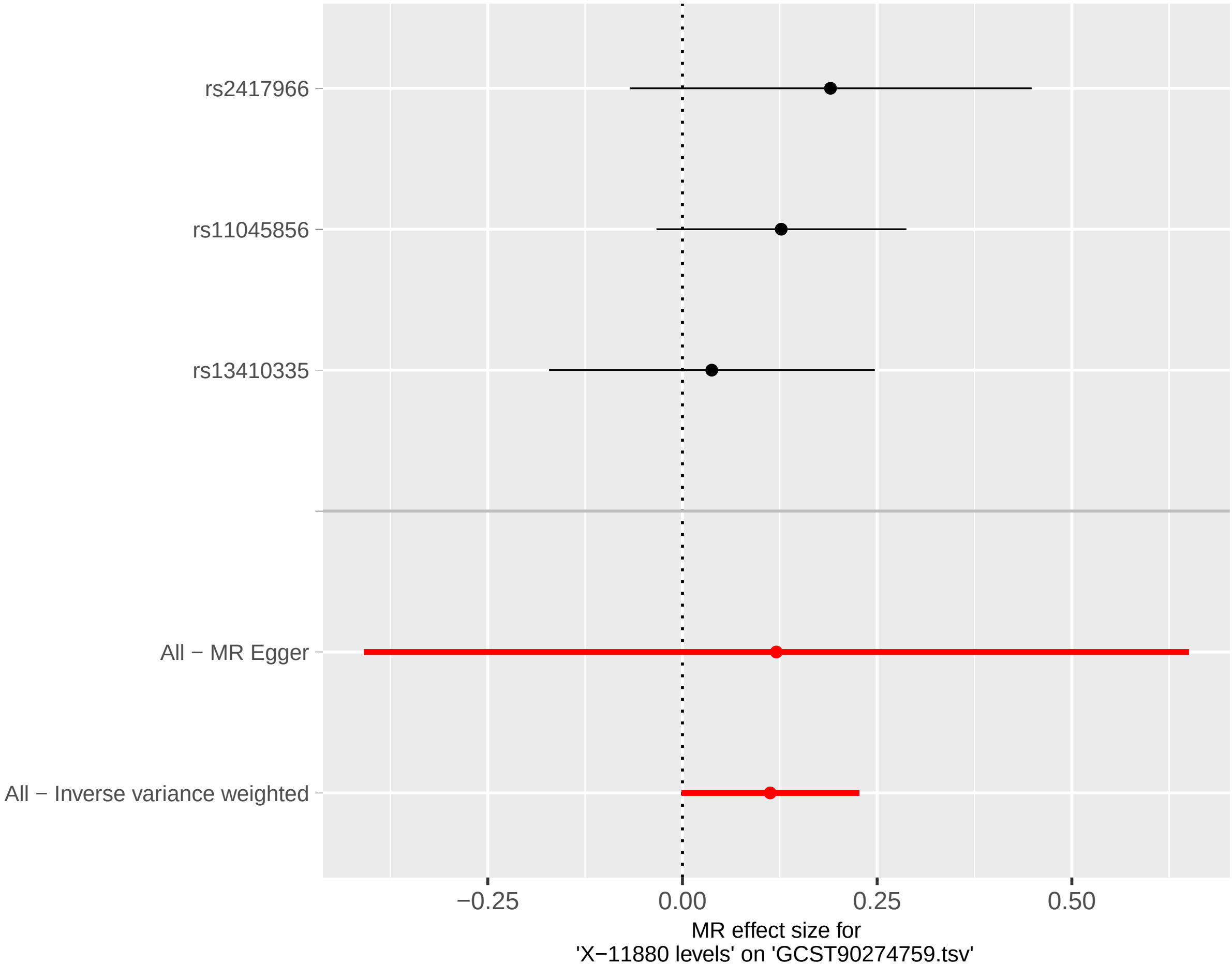
rs2417966
rs11045856
rs13410335
All − MR Egger
All − Inverse variance weighted
−0.25
0.00
0.25
0.50
MR effect size for
'X−11880 levels' on 'GCST90274759.tsv'

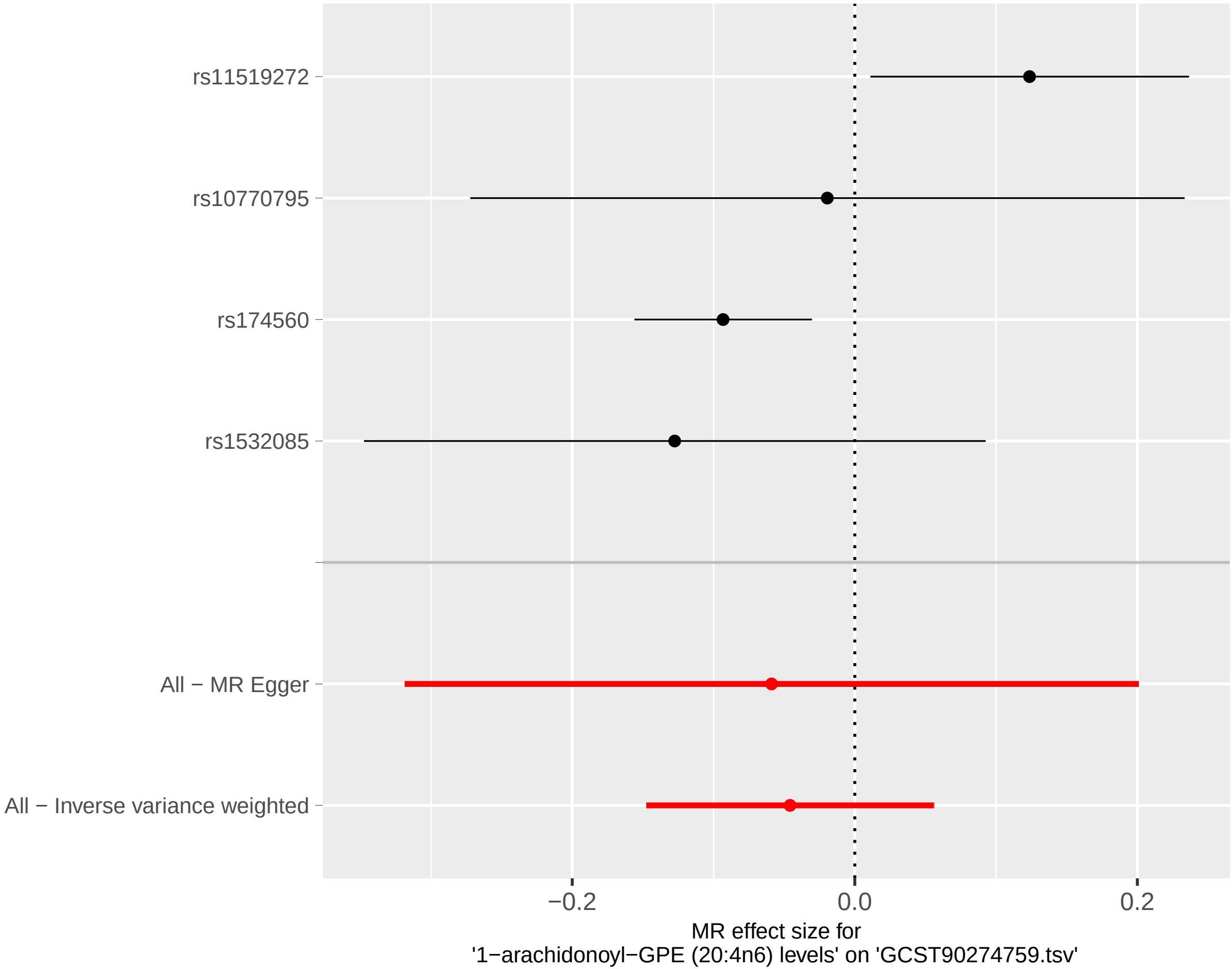
rs11519272
rs10770795
rs174560
rs1532085
All − MR Egger
All − Inverse variance weighted
−0.2
0.0
0.2
MR effect size for
'1−arachidonoyl−GPE (20:4n6) levels' on 'GCST90274759.tsv'

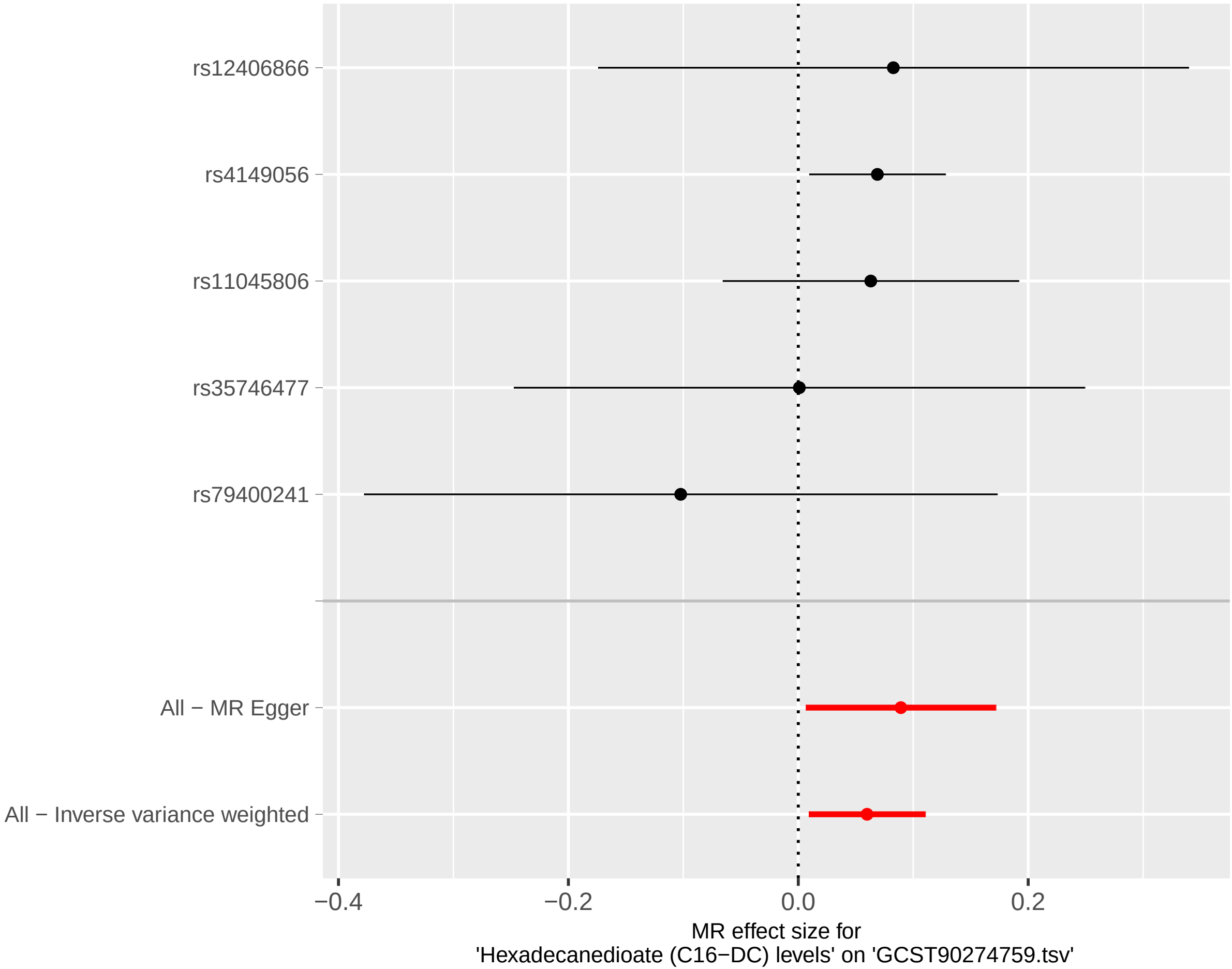

rs12406866
rs4149056
rs11045806
rs35746477
rs79400241
All − MR Egger
All − Inverse variance weighted
−0.4
−0.2
0.0
0.2
MR effect size for
'Hexadecanedioate (C16−DC) levels' on 'GCST90274759.tsv'

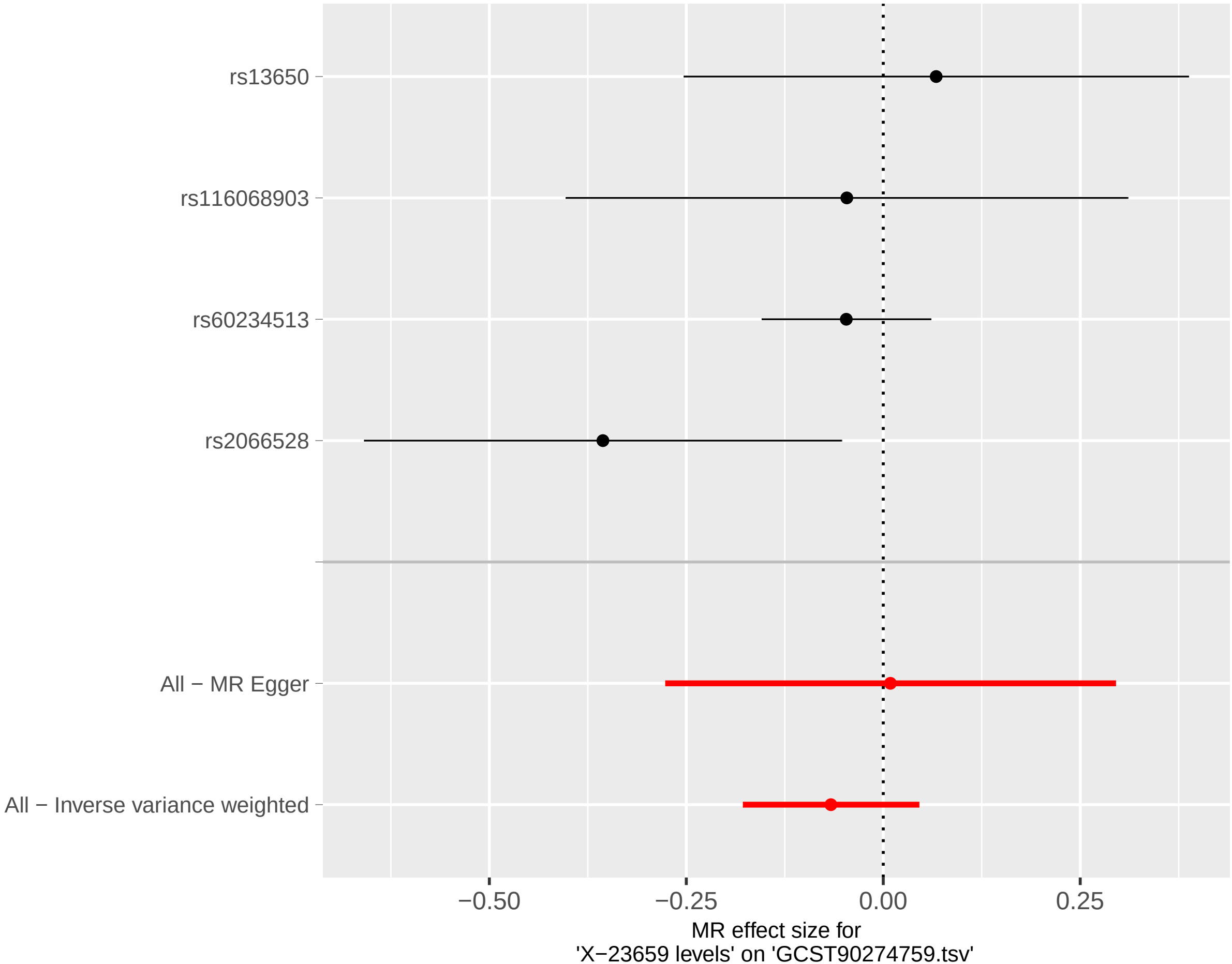

rs13650
rs116068903
rs60234513
rs2066528
All − MR Egger
All − Inverse variance weighted
−0.50
−0.25
0.00
0.25
MR effect size for
'X−23659 levels' on 'GCST90274759.tsv'

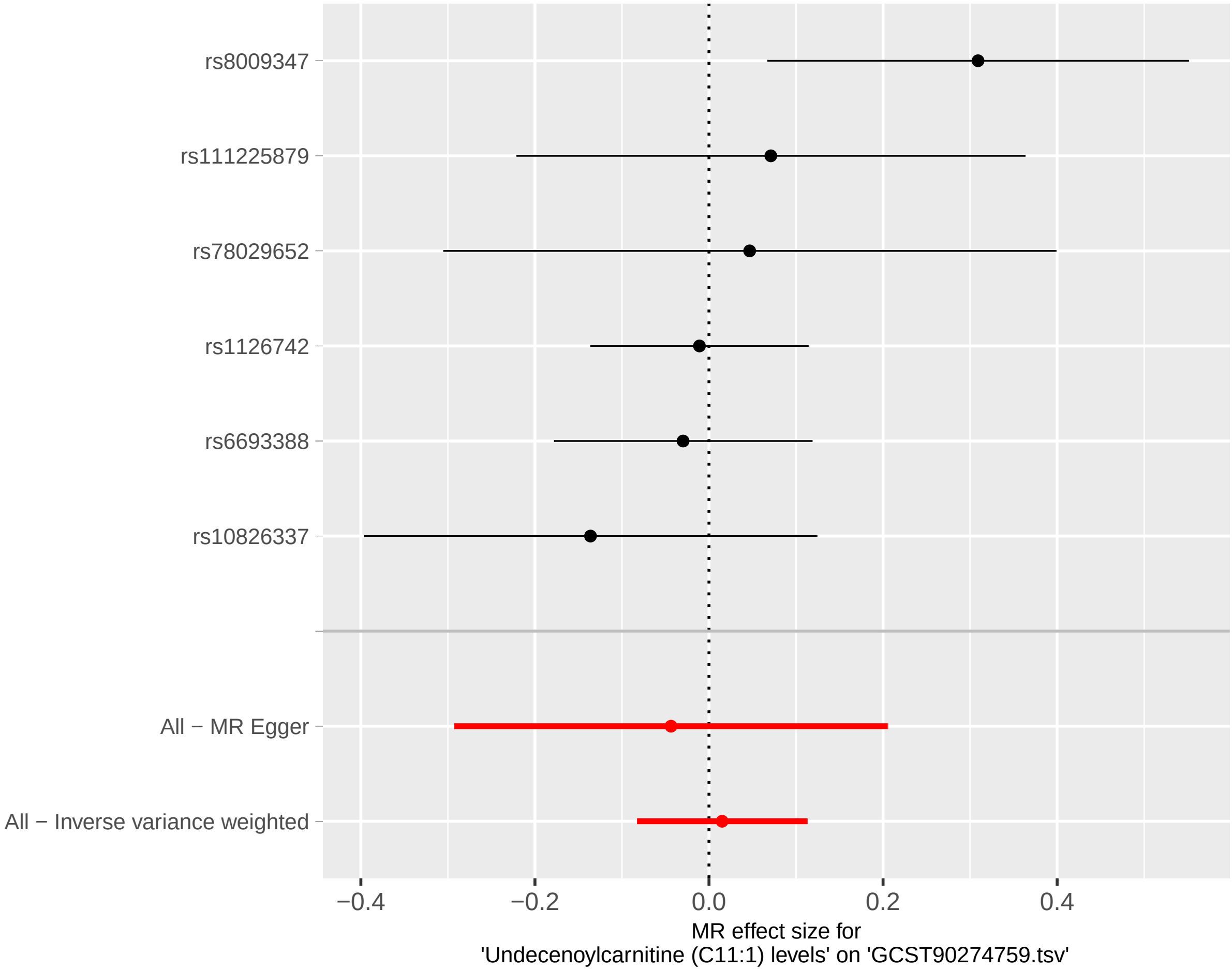
rs8009347
rs111225879
rs78029652
rs1126742
rs6693388
rs10826337
All − MR Egger
All − Inverse variance weighted
−0.4
−0.2
0.0
0.2
0.4
MR effect size for
'Undecenoylcarnitine (C11:1) levels' on 'GCST90274759.tsv'

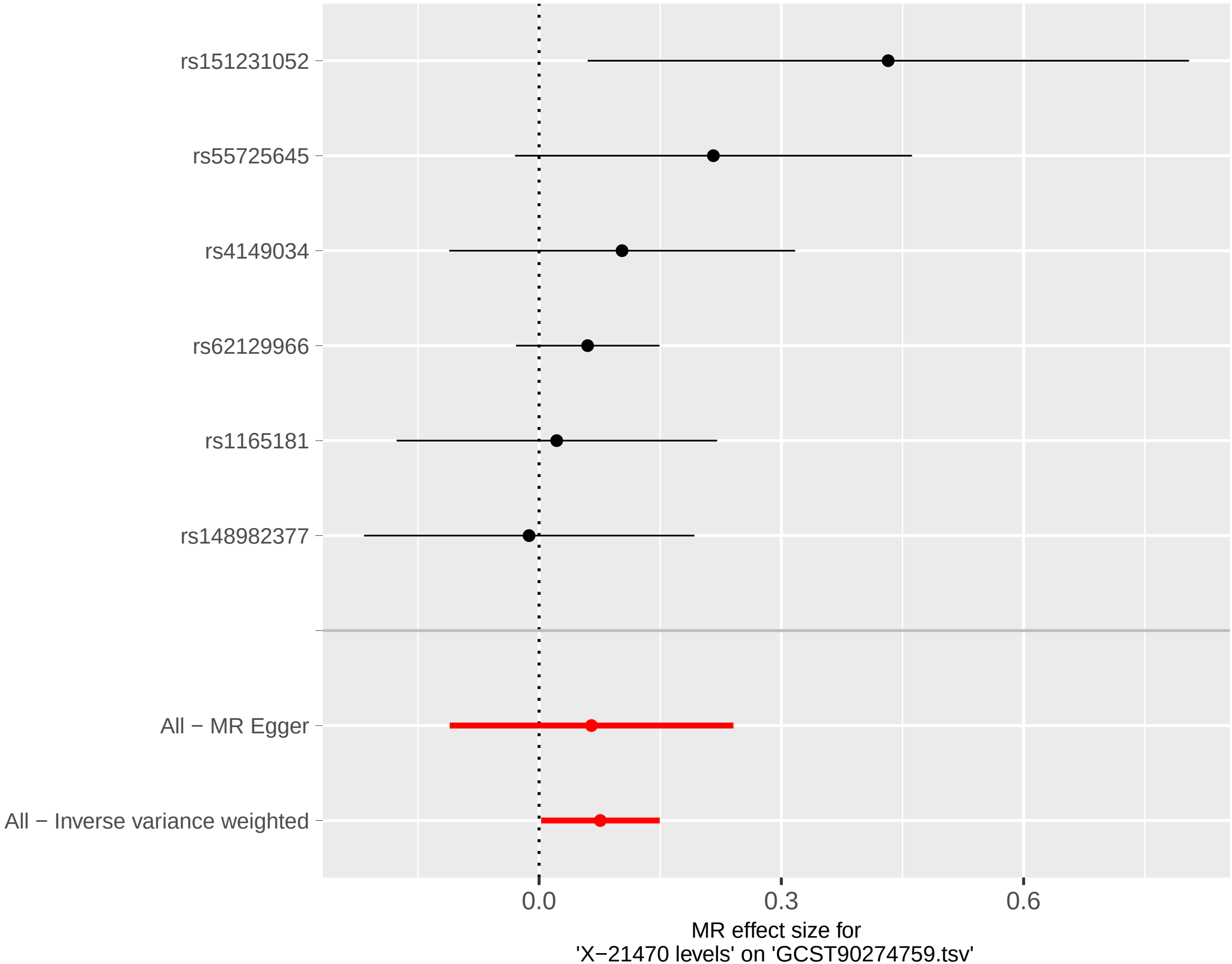

rs151231052
rs55725645
rs4149034
rs62129966
rs1165181
rs148982377
All − MR Egger
All − Inverse variance weighted
0.0
0.3
0.6
MR effect size for
'X−21470 levels' on 'GCST90274759.tsv'

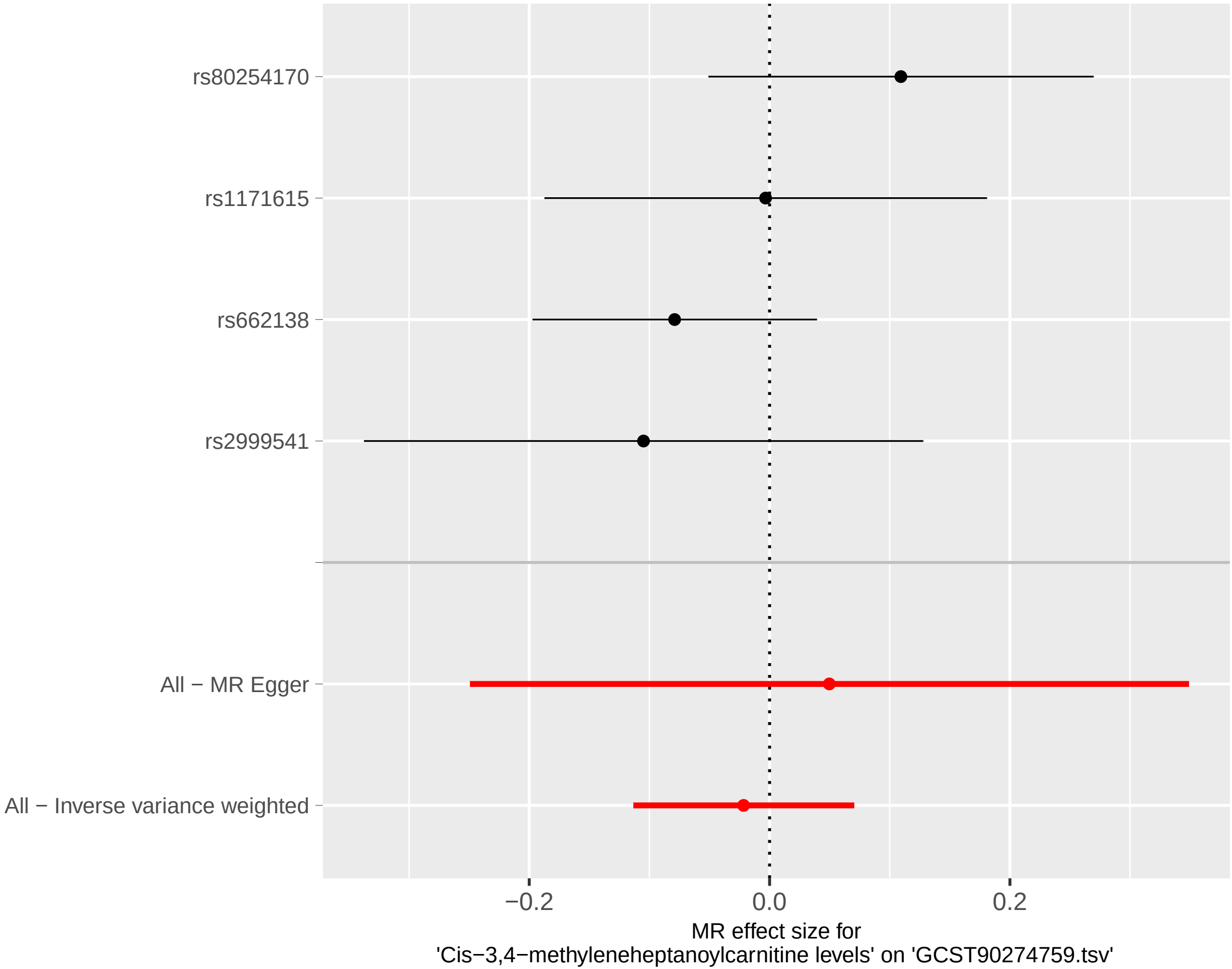
rs80254170
rs1171615
rs662138
rs2999541
All − MR Egger
All − Inverse variance weighted
−0.2
0.0
0.2
MR effect size for
'Cis−3,4−methyleneheptanoylcarnitine levels' on 'GCST90274759.tsv'

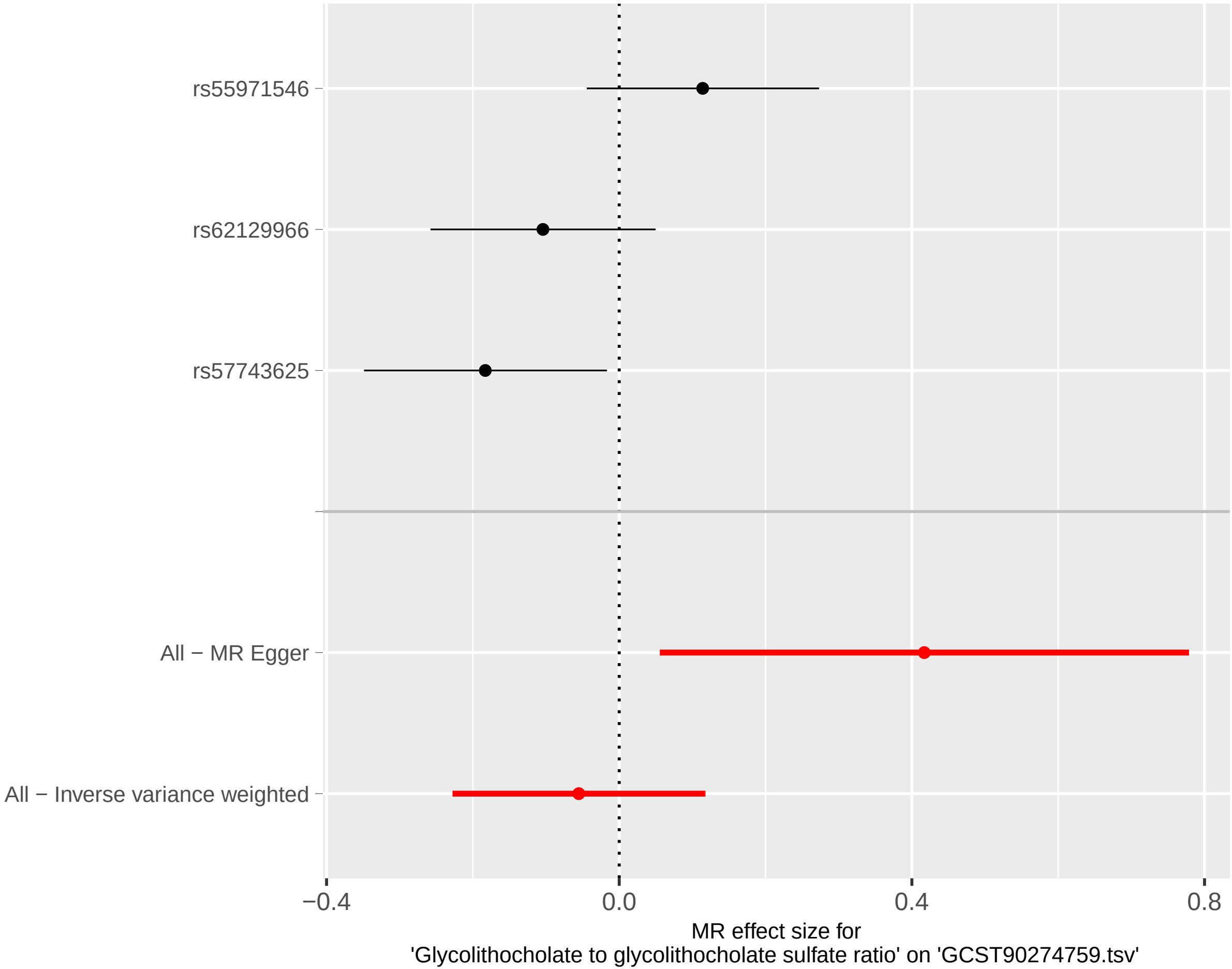
rs55971546
rs62129966
rs57743625
All − MR Egger
All − Inverse variance weighted
−0.4
0.0
0.4
0.8
MR effect size for
'Glycolithocholate to glycolithocholate sulfate ratio' on 'GCST90274759.tsv'

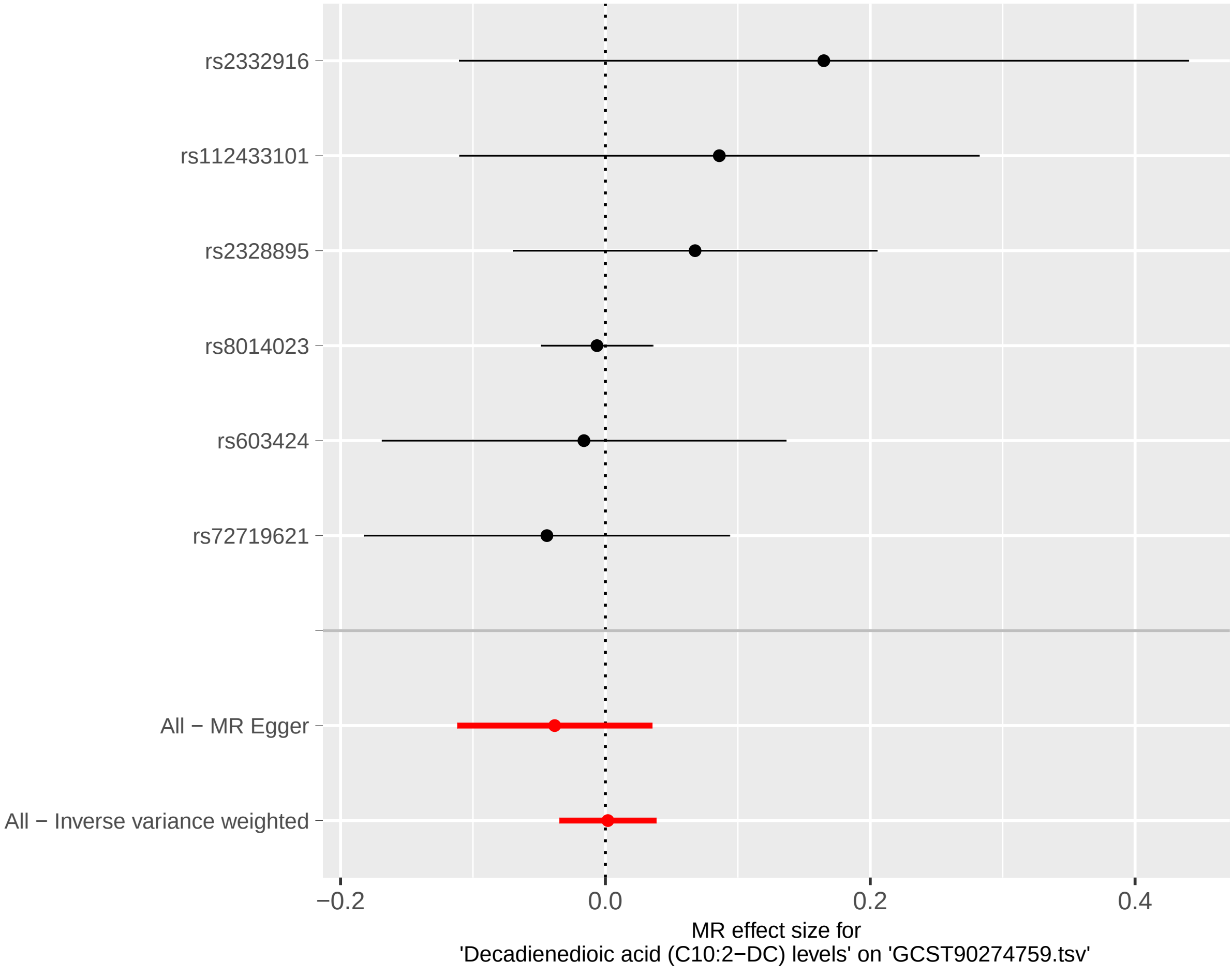
rs2332916
rs112433101
rs2328895
rs8014023
rs603424
rs72719621
All − MR Egger
All − Inverse variance weighted
−0.2
0.0
0.2
0.4
MR effect size for
'Decadienedioic acid (C10:2−DC) levels' on 'GCST90274759.tsv'

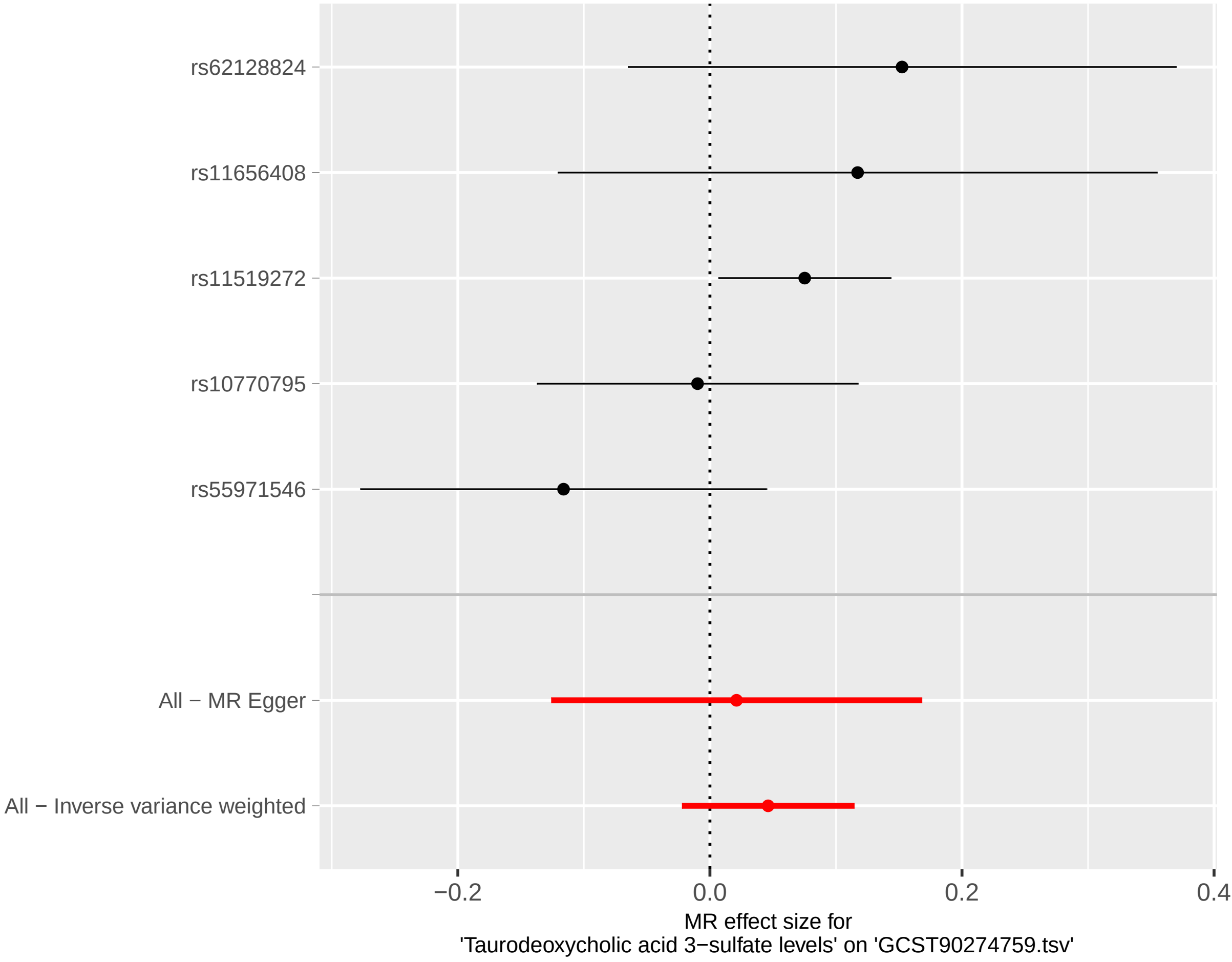

rs62128824
rs11656408
rs11519272
rs10770795
rs55971546
All − MR Egger
All − Inverse variance weighted
−0.2
0.0
0.2
0.4
MR effect size for
'Taurodeoxycholic acid 3−sulfate levels' on 'GCST90274759.tsv'

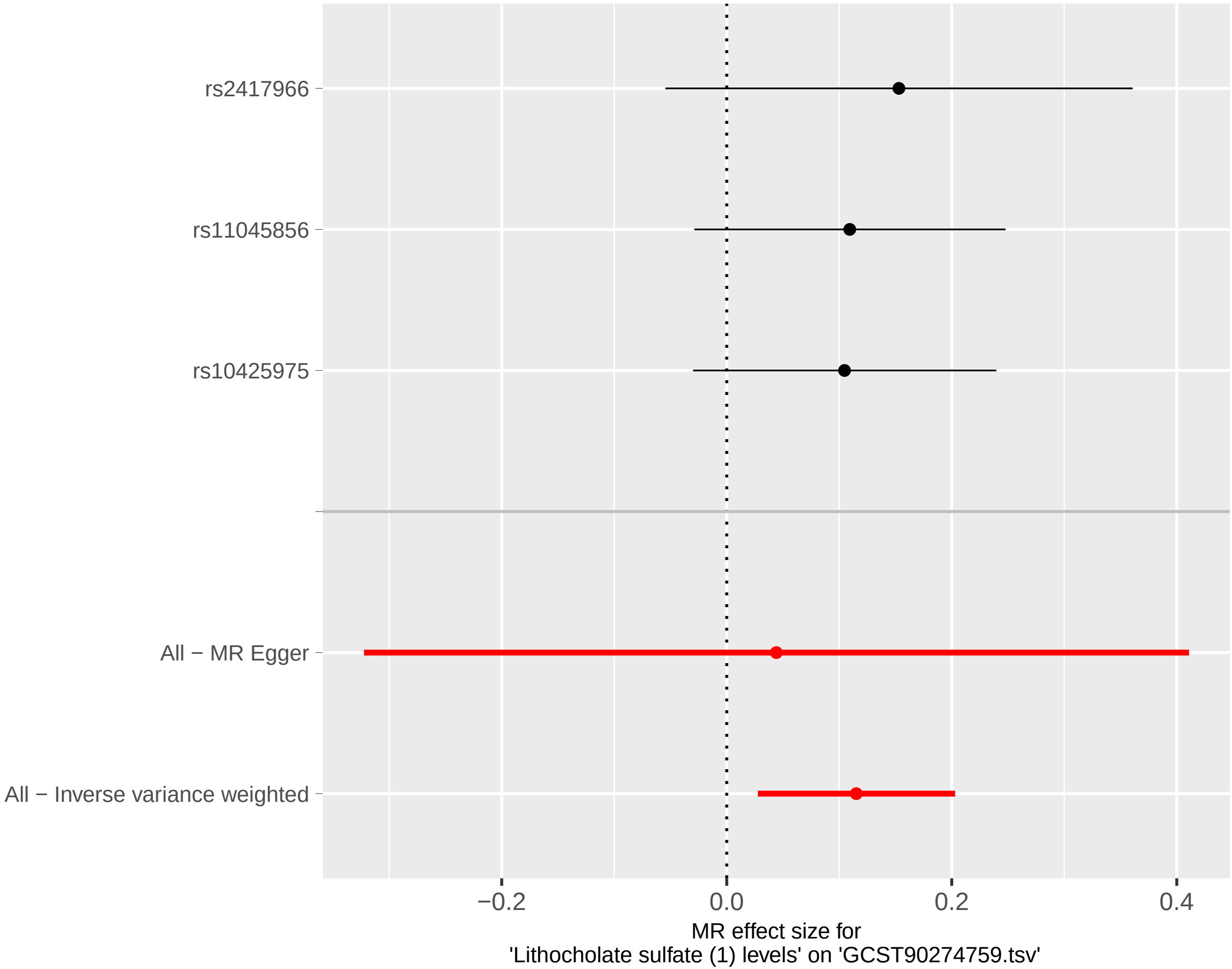

rs2417966
rs11045856
rs10425975
All − MR Egger
All − Inverse variance weighted
−0.2
0.0
0.2
0.4
MR effect size for
'Lithocholate sulfate (1) levels' on 'GCST90274759.tsv'

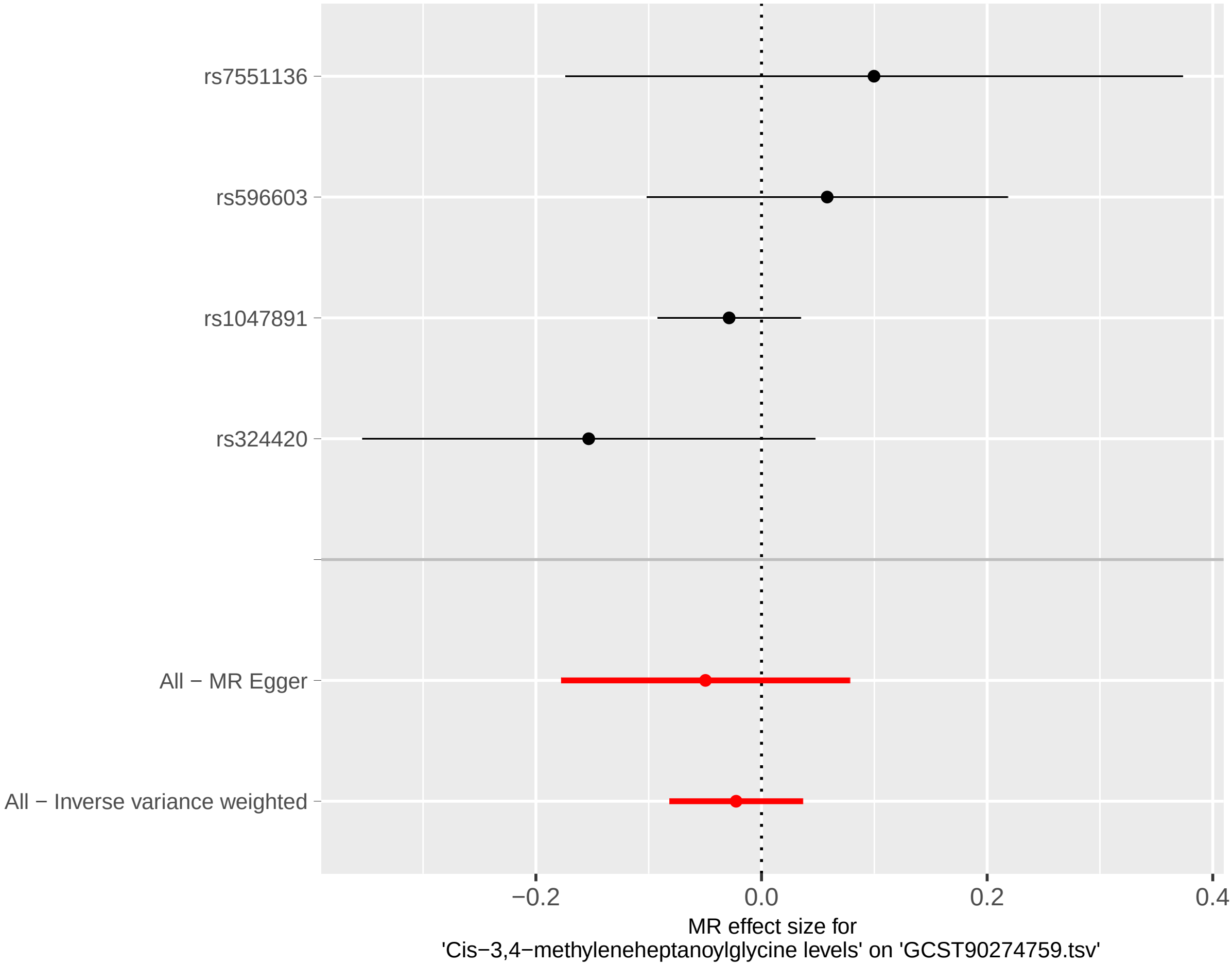
rs7551136
rs596603
rs1047891
rs324420
All − MR Egger
All − Inverse variance weighted
−0.2
0.0
0.2
0.4
MR effect size for
'Cis−3,4−methyleneheptanoylglycine levels' on 'GCST90274759.tsv'

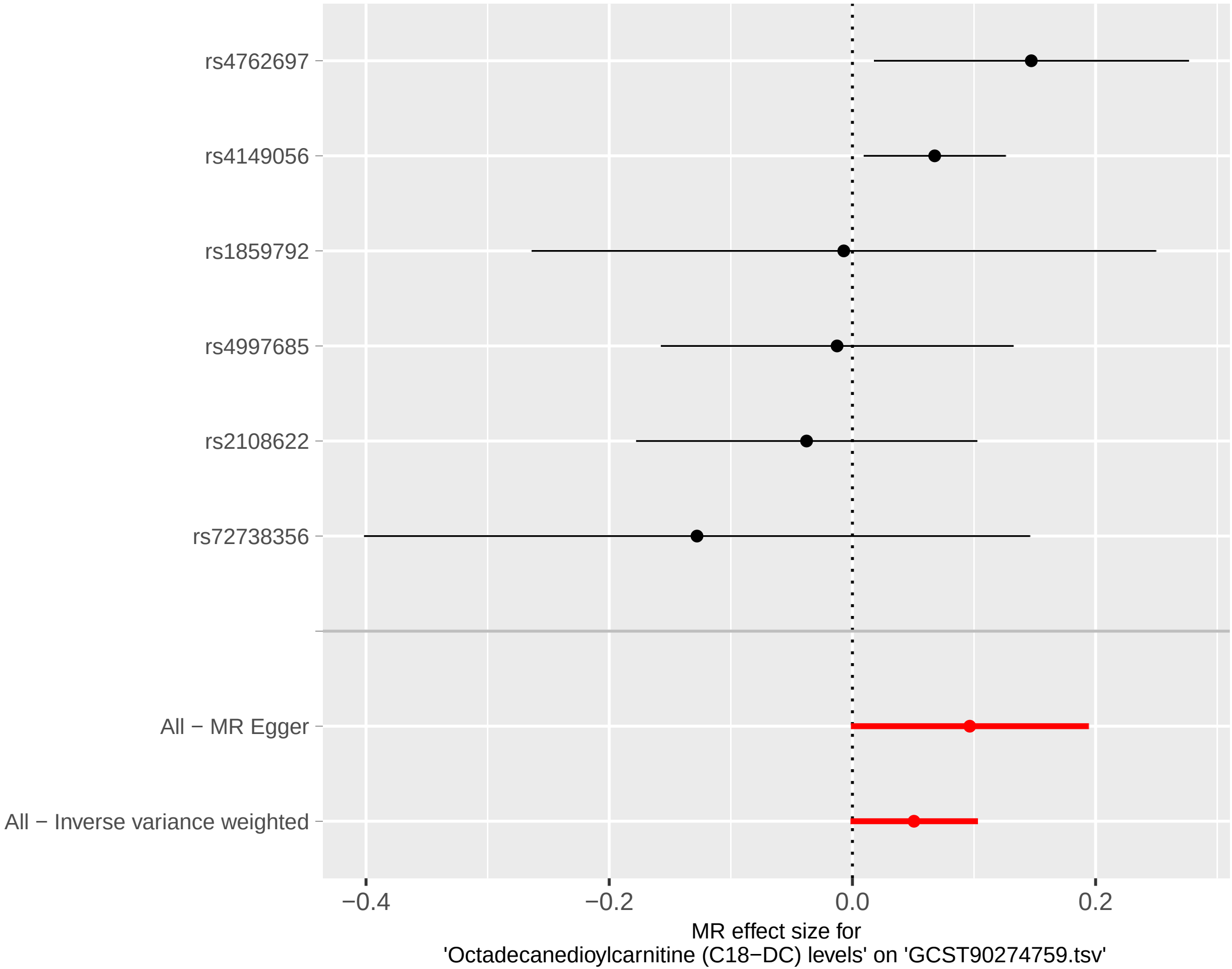
rs4762697
rs4149056
rs1859792
rs4997685
rs2108622
rs72738356
All − MR Egger
All − Inverse variance weighted
−0.4
−0.2
0.0
0.2
MR effect size for
'Octadecanedioylcarnitine (C18−DC) levels' on 'GCST90274759.tsv'

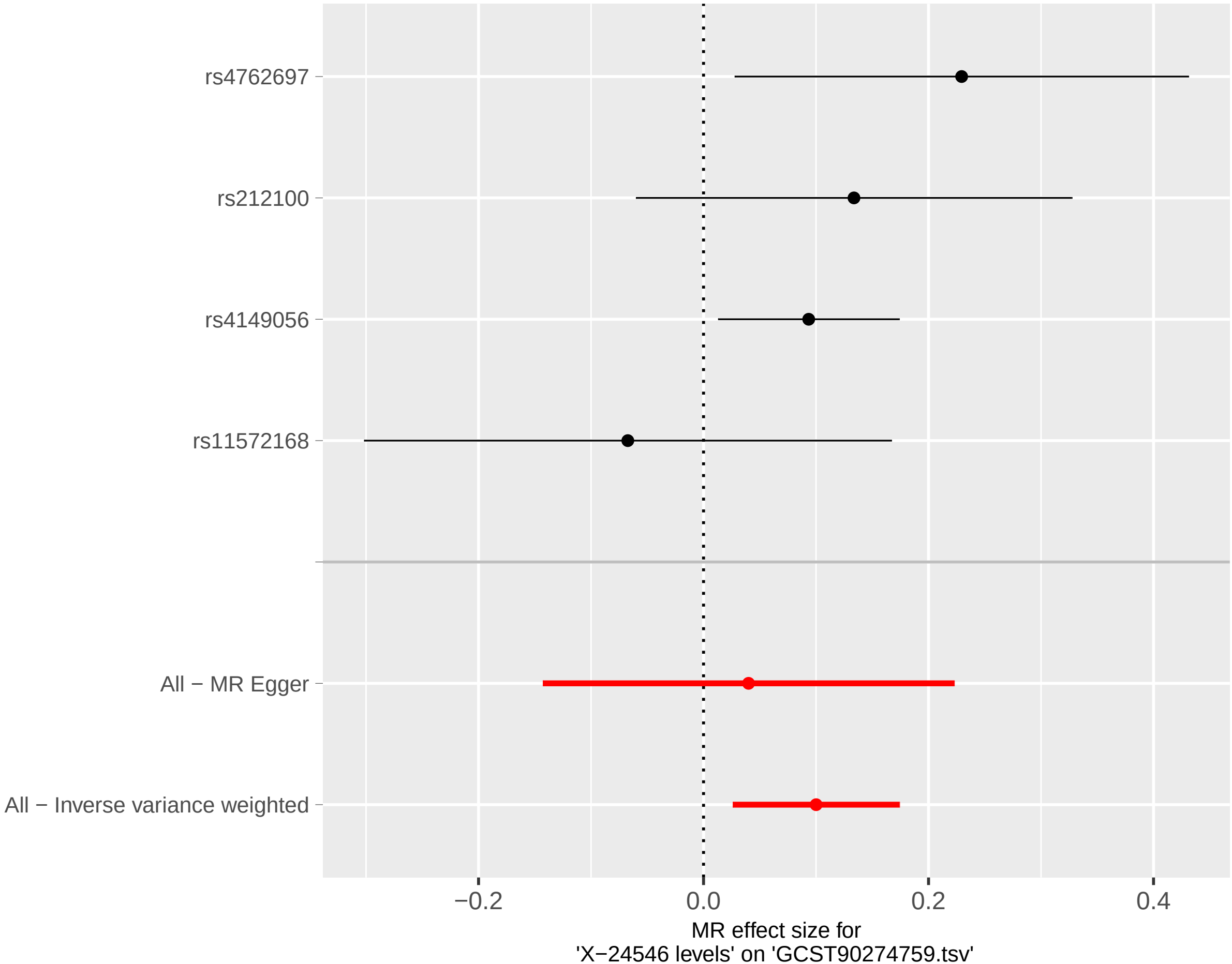
rs4762697
rs212100
rs4149056
rs11572168
All − MR Egger
All − Inverse variance weighted
−0.2
0.0
0.2
0.4
MR effect size for
'X−24546 levels' on 'GCST90274759.tsv'

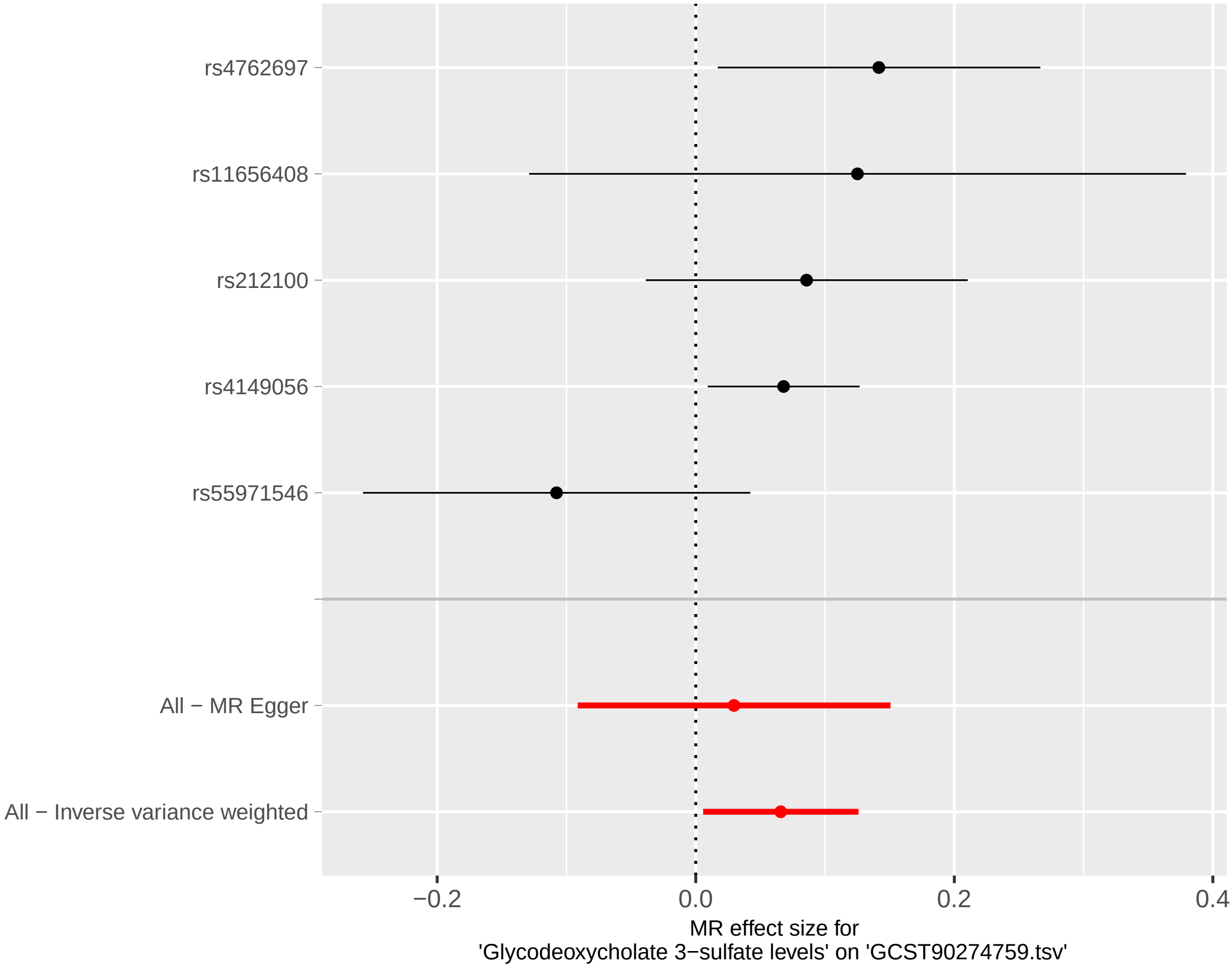
rs4762697
rs11656408
rs212100
rs4149056
rs55971546
All − MR Egger
All − Inverse variance weighted
−0.2
0.0
0.2
0.4
MR effect size for
'Glycodeoxycholate 3−sulfate levels' on 'GCST90274759.tsv'

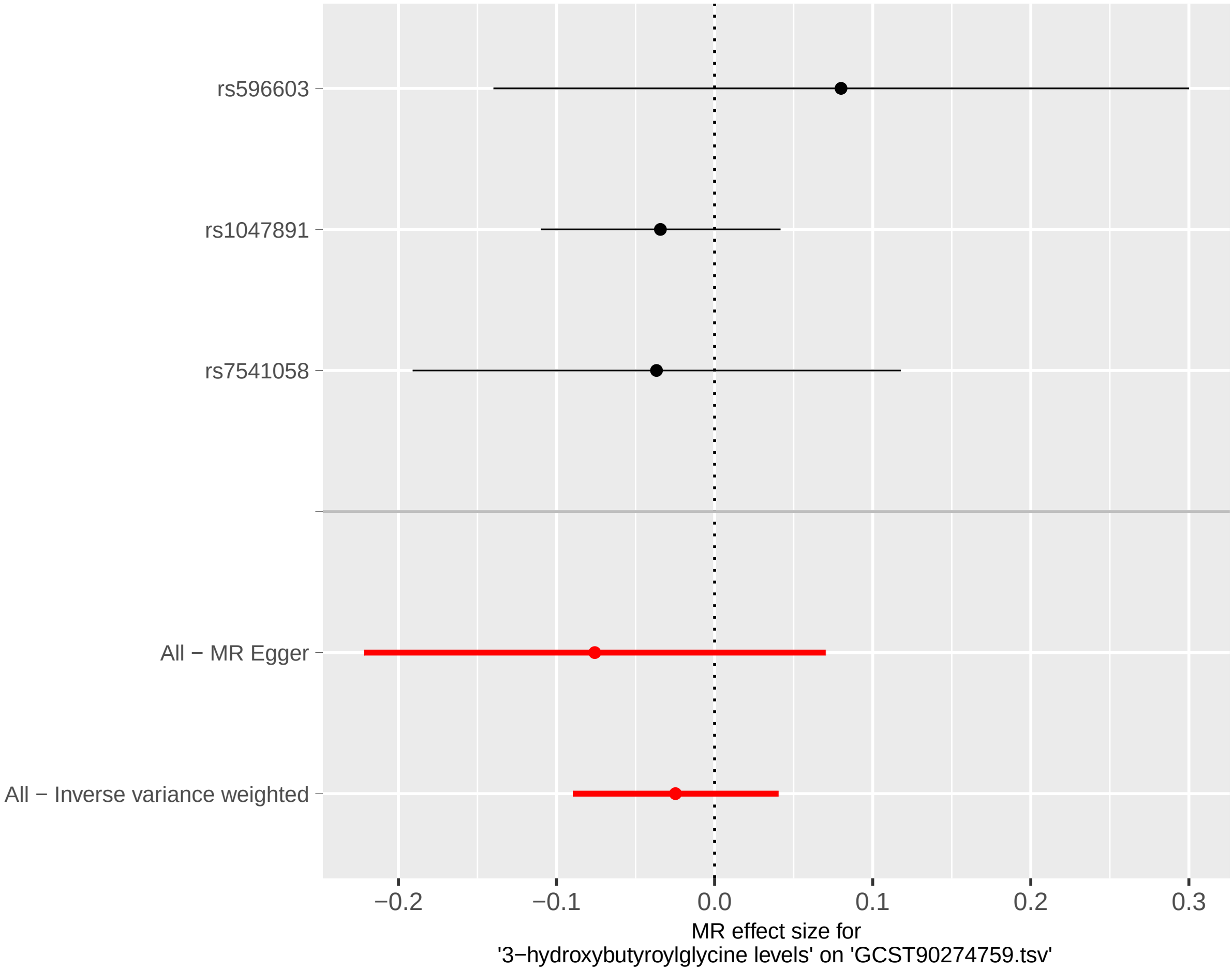
rs596603
rs1047891
rs7541058
All − MR Egger
All − Inverse variance weighted
−0.2
−0.1
0.0
0.1
0.2
0.3
MR effect size for
'3−hydroxybutyroylglycine levels' on 'GCST90274759.tsv'

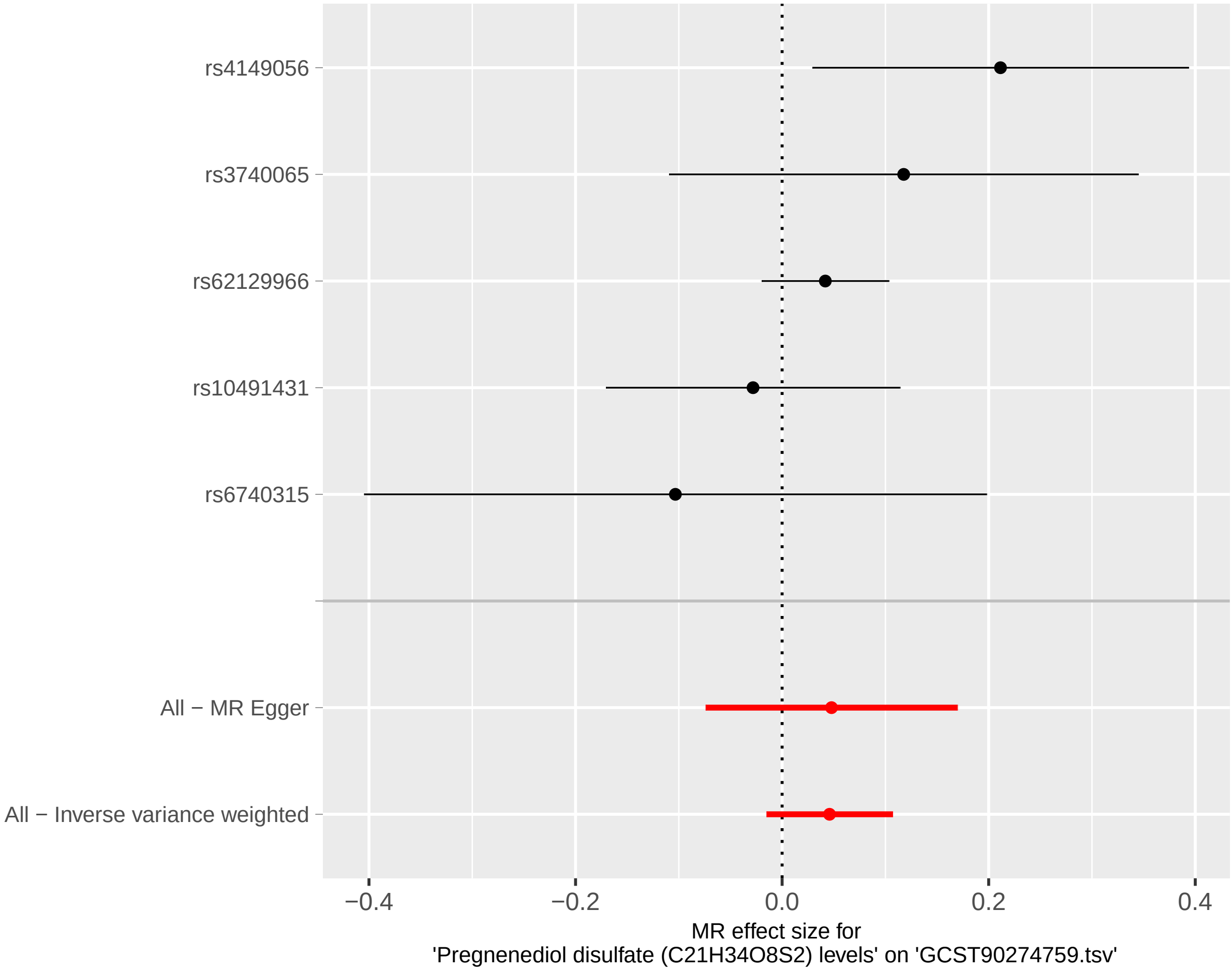
rs4149056
rs3740065
rs62129966
rs10491431
rs6740315
All − MR Egger
All − Inverse variance weighted
−0.4
−0.2
0.0
0.2
0.4
MR effect size for
'Pregnenediol disulfate (C21H34O8S2) levels' on 'GCST90274759.tsv'

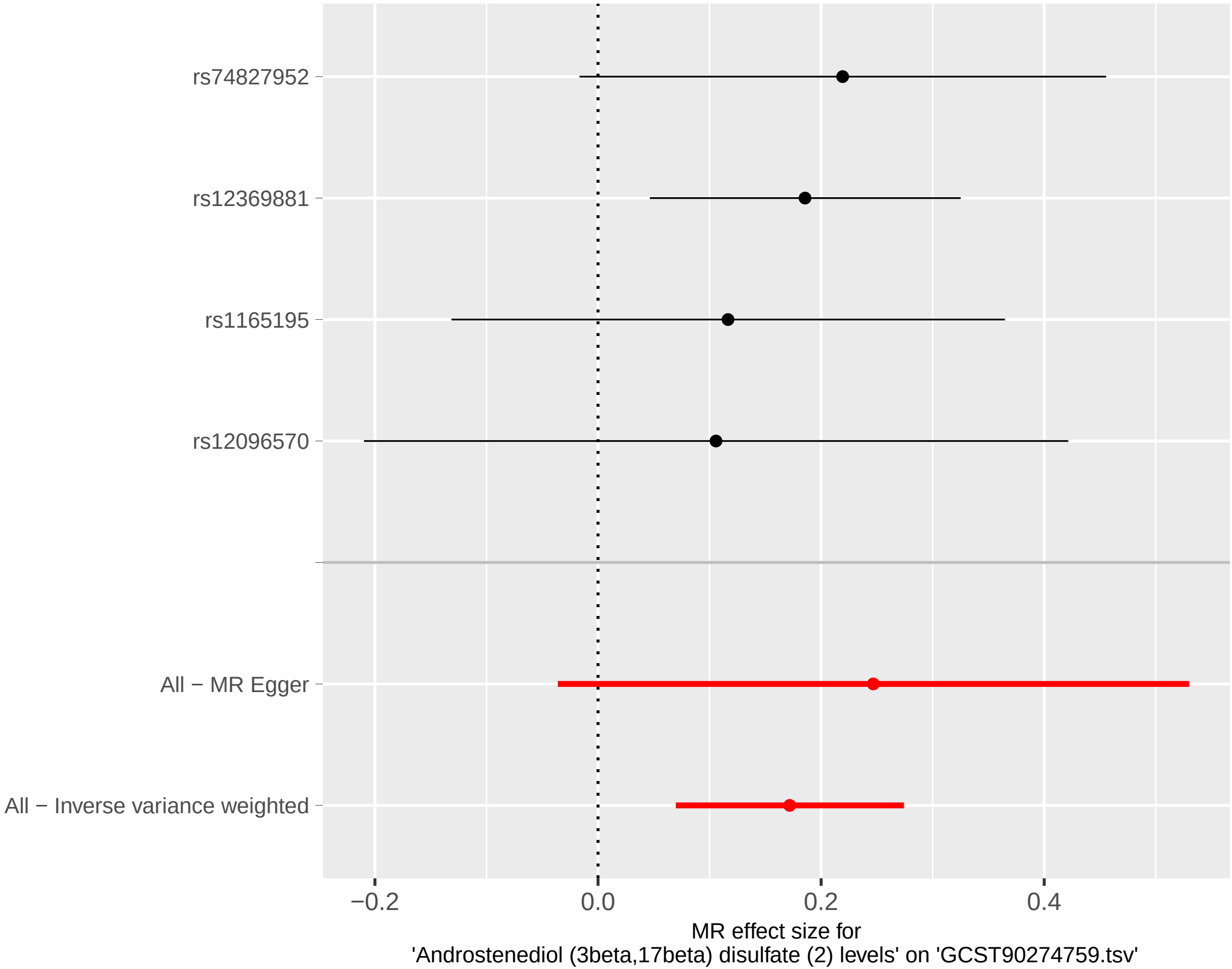
rs74827952
rs12369881
rs1165195
rs12096570
All − MR Egger
All − Inverse variance weighted
−0.2
0.0
0.2
0.4
MR effect size for
'Androstenediol (3beta,17beta) disulfate (2) levels' on 'GCST90274759.tsv'

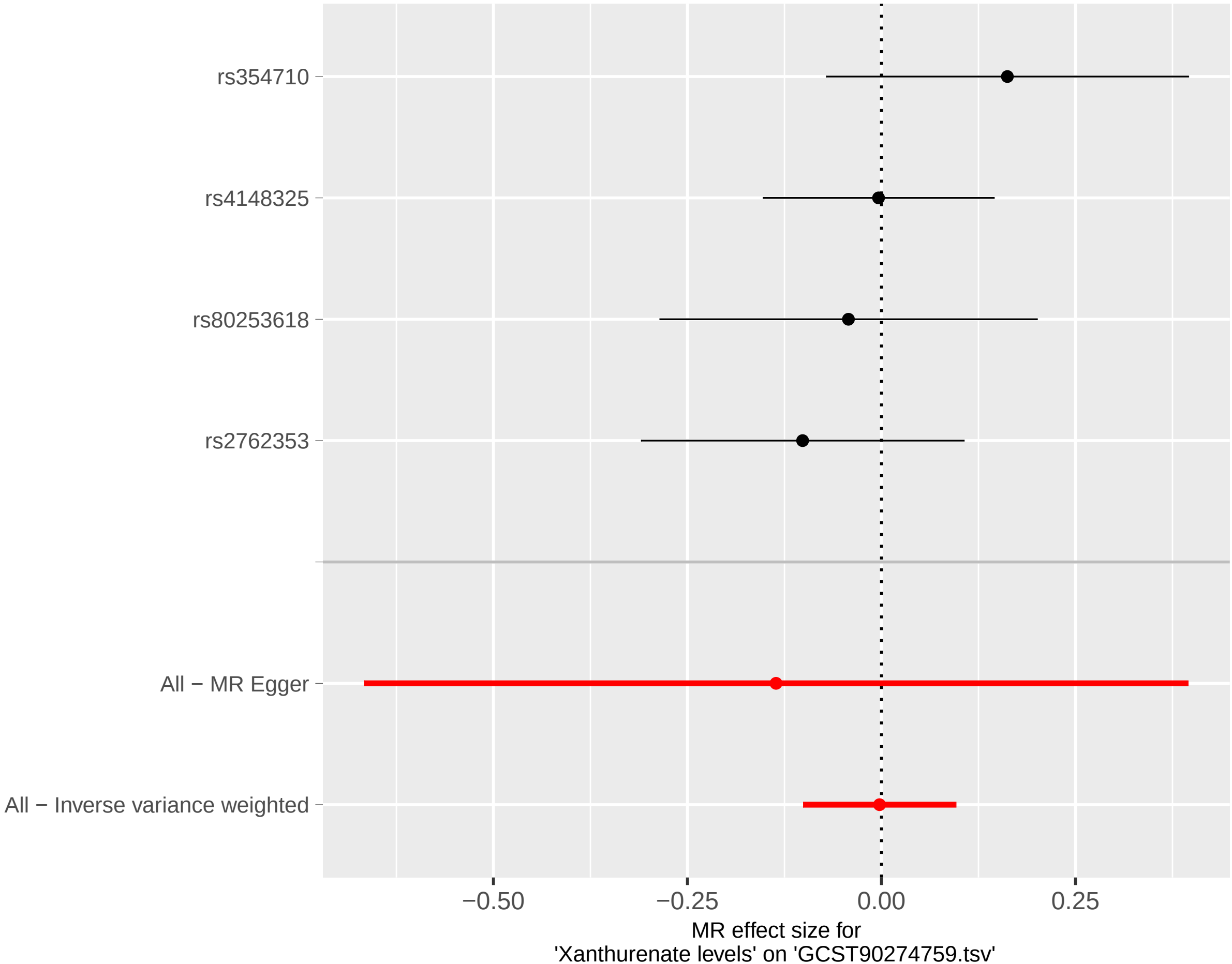
rs354710
rs4148325
rs80253618
rs2762353
All − MR Egger
All − Inverse variance weighted
−0.50
−0.25
0.00
0.25
MR effect size for
'Xanthurenate levels' on 'GCST90274759.tsv'

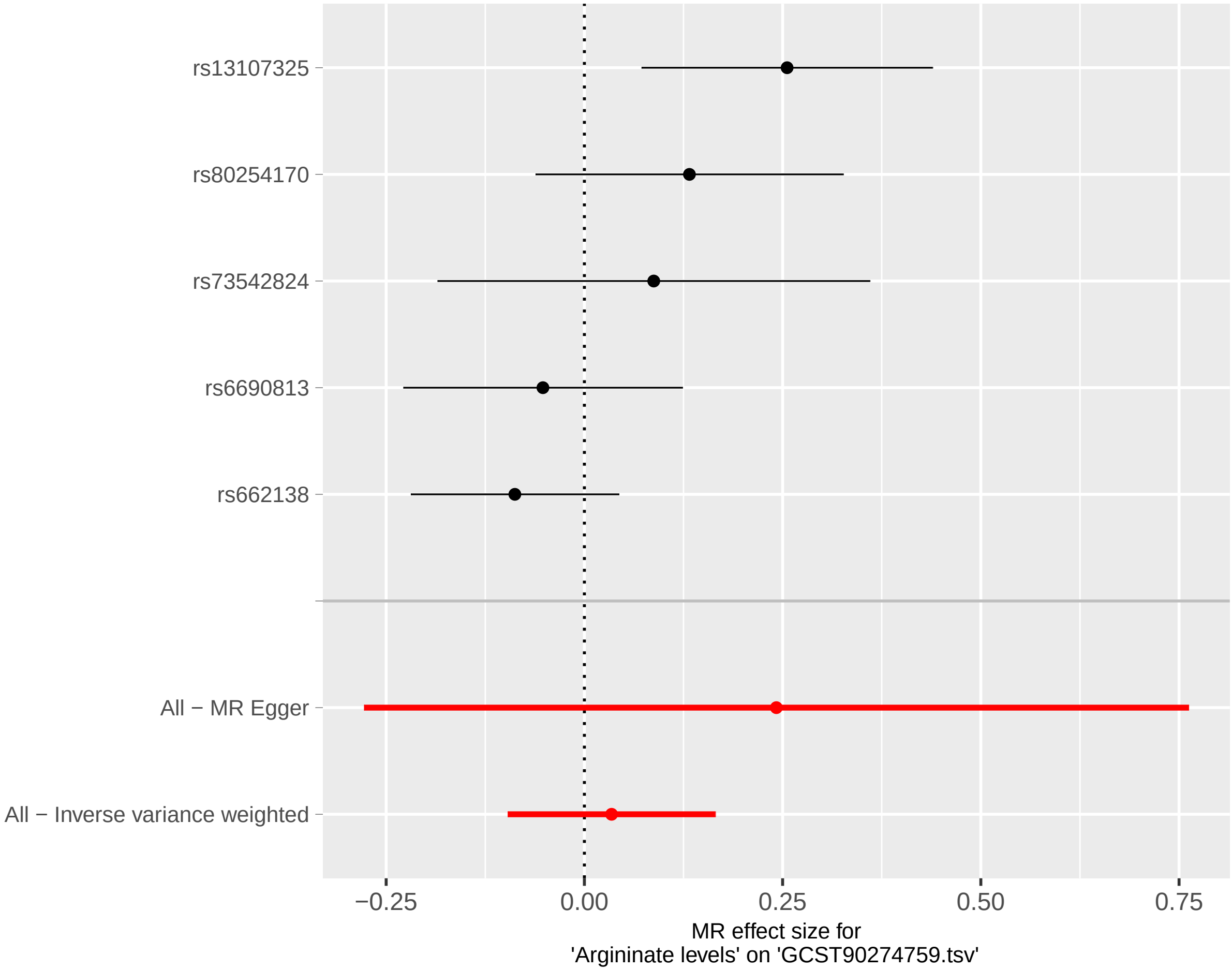
rs13107325
rs80254170
rs73542824
rs6690813
rs662138
All − MR Egger
All − Inverse variance weighted
−0.25
0.00
0.25
0.50
0.75
MR effect size for
'Argininate levels' on 'GCST90274759.tsv'

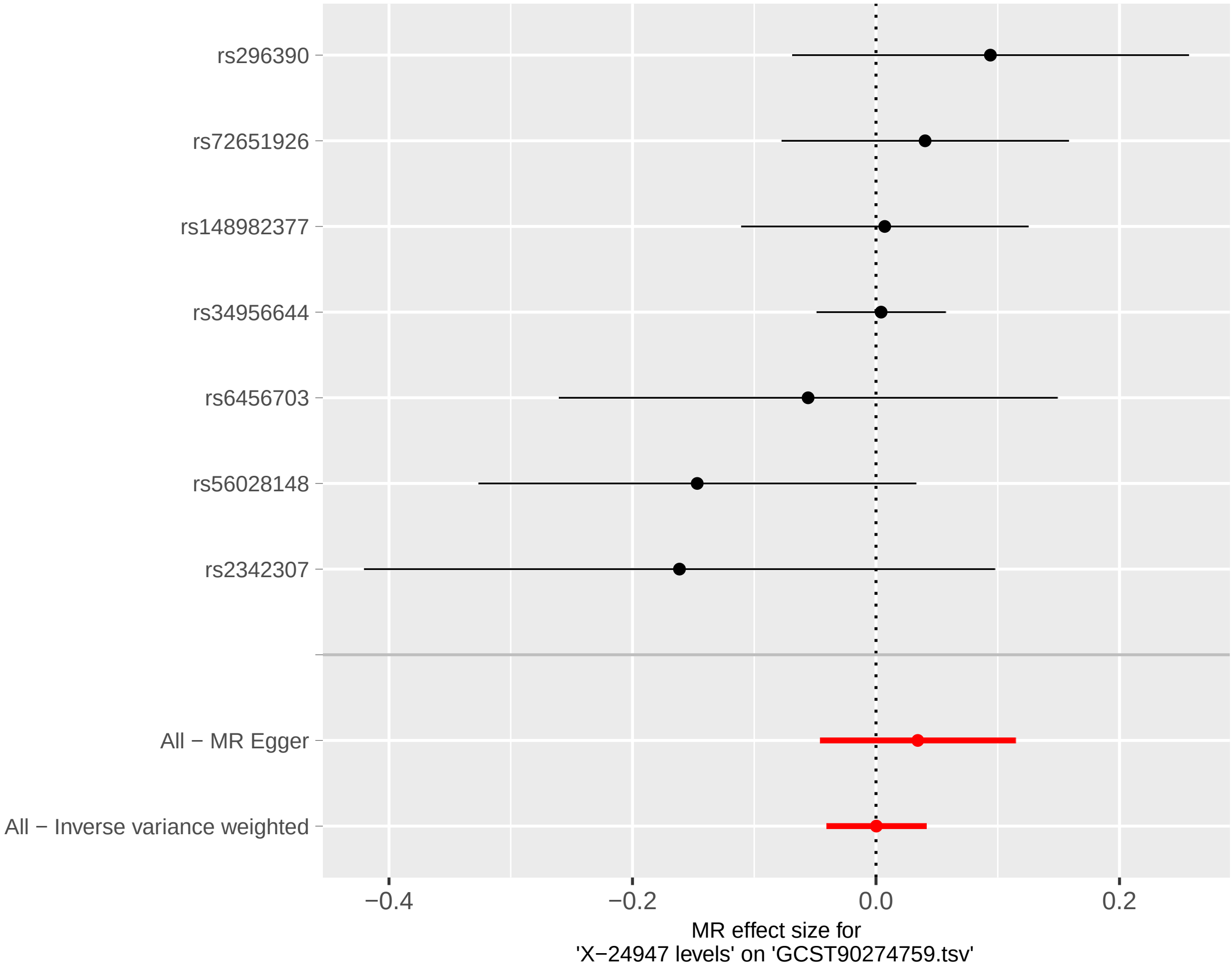
rs296390
rs72651926
rs148982377
rs34956644
rs6456703
rs56028148
rs2342307
All − MR Egger
All − Inverse variance weighted
−0.4
−0.2
0.0
0.2
MR effect size for
'X−24947 levels' on 'GCST90274759.tsv'

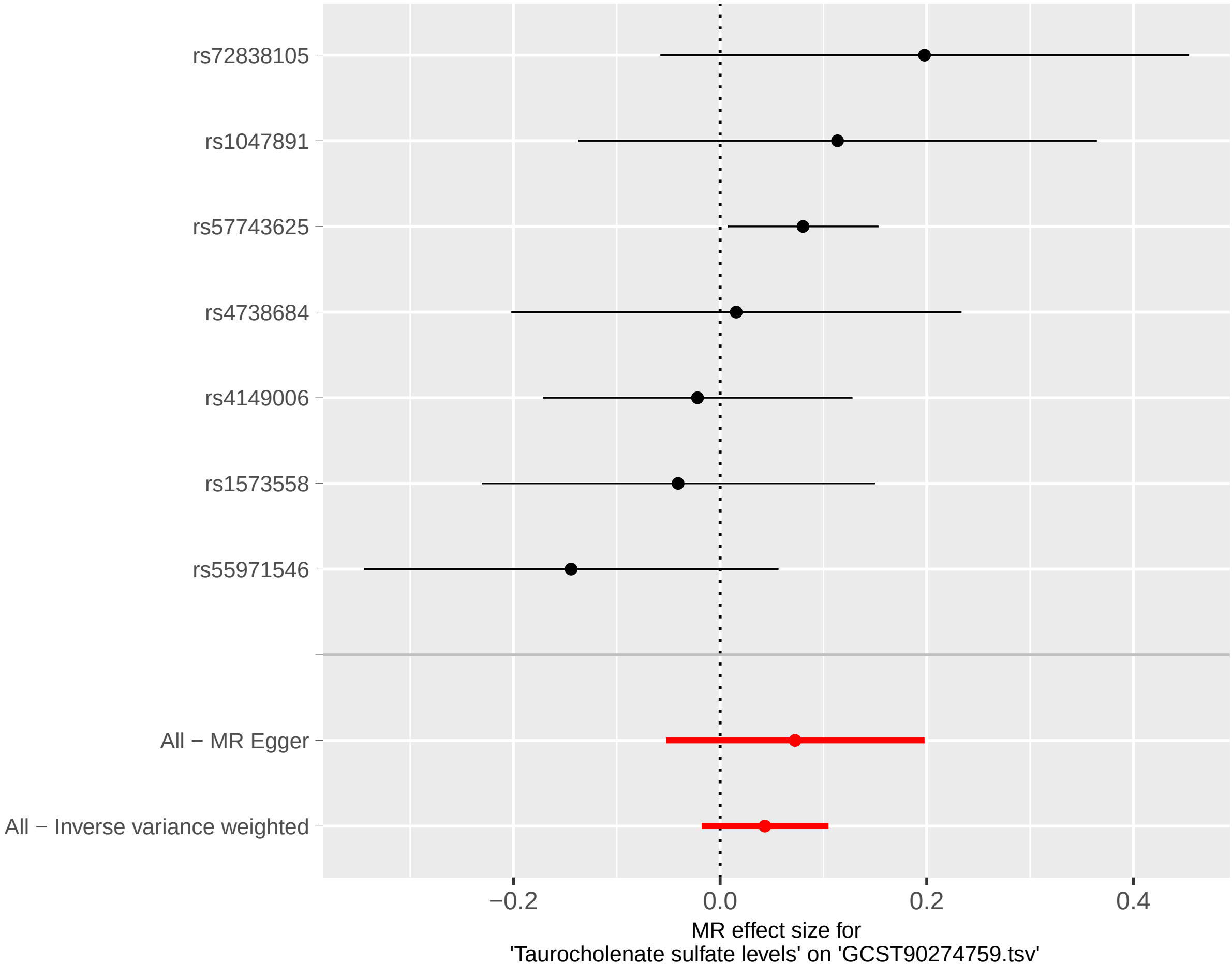

rs72838105
rs1047891
rs57743625
rs4738684
rs4149006
rs1573558
rs55971546
All − MR Egger
All − Inverse variance weighted
−0.2
0.0
0.2
0.4
MR effect size for
'Taurocholenate sulfate levels' on 'GCST90274759.tsv'

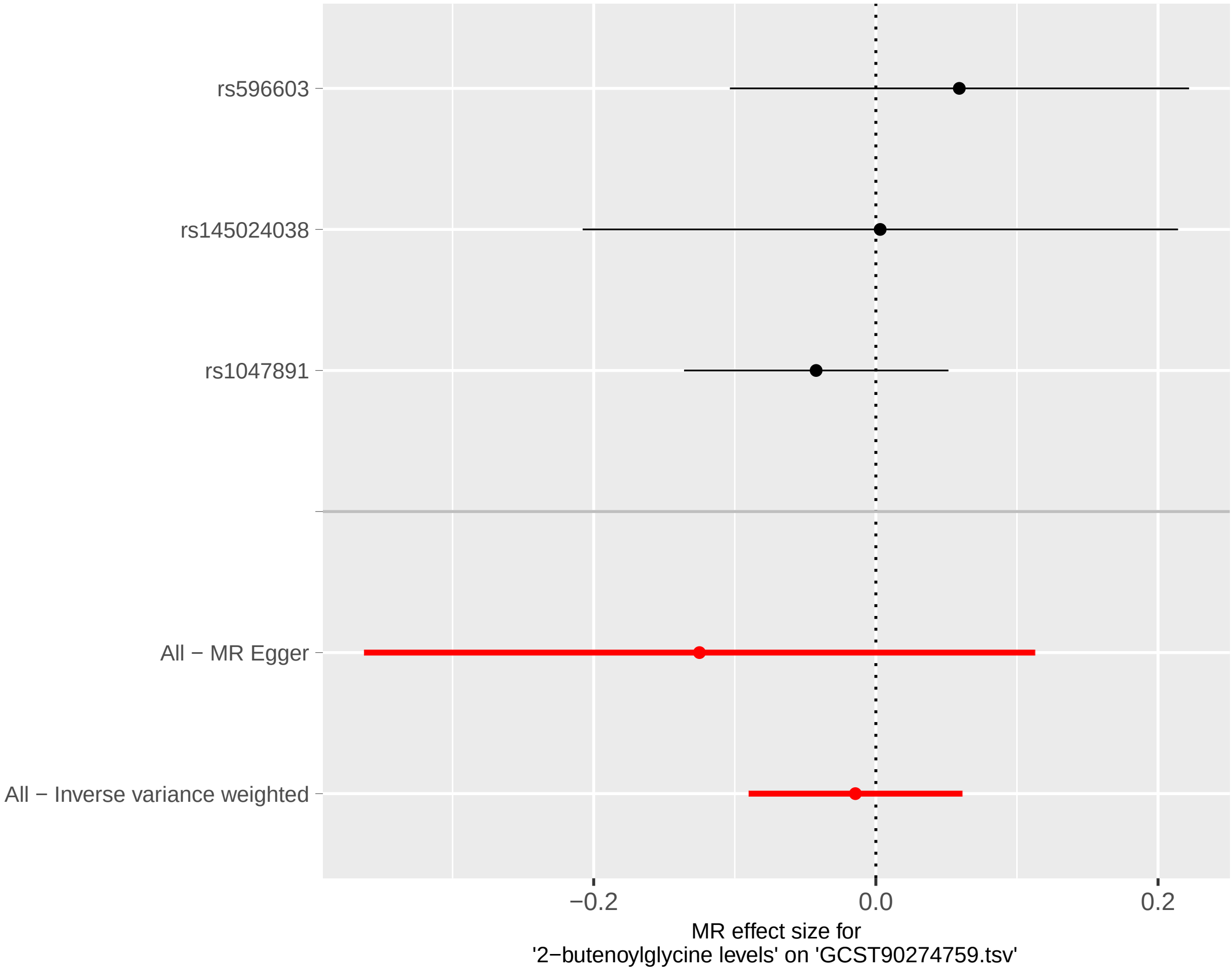
rs596603
rs145024038
rs1047891
All − MR Egger
All − Inverse variance weighted
−0.2
0.0
0.2
MR effect size for
'2−butenoylglycine levels' on 'GCST90274759.tsv'

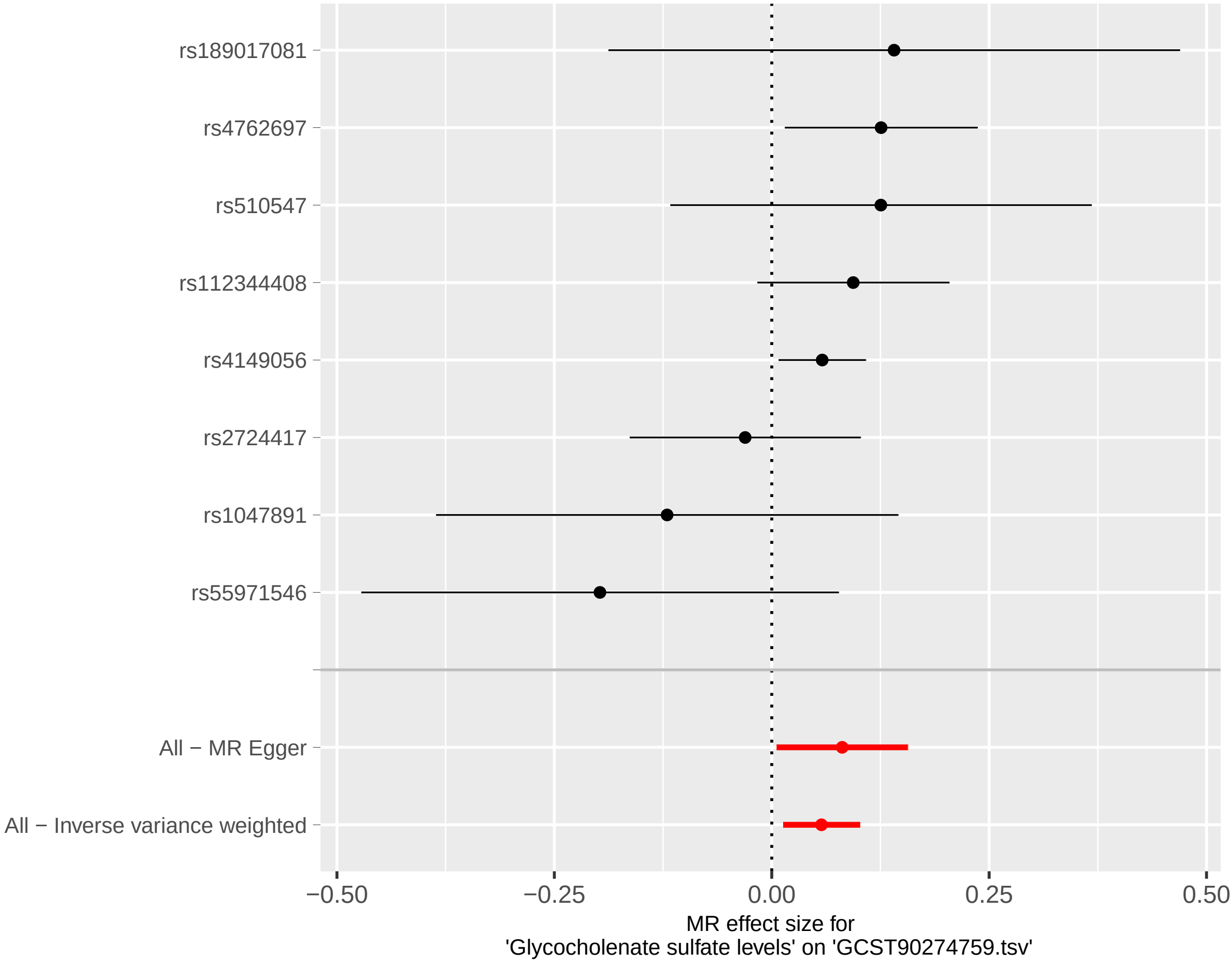
rs189017081
rs4762697
rs510547
rs112344408
rs4149056
rs2724417
rs1047891
rs55971546
All − MR Egger
All − Inverse variance weighted
−0.50
−0.25
0.00
0.25
0.50
MR effect size for
'Glycocholenate sulfate levels' on 'GCST90274759.tsv'

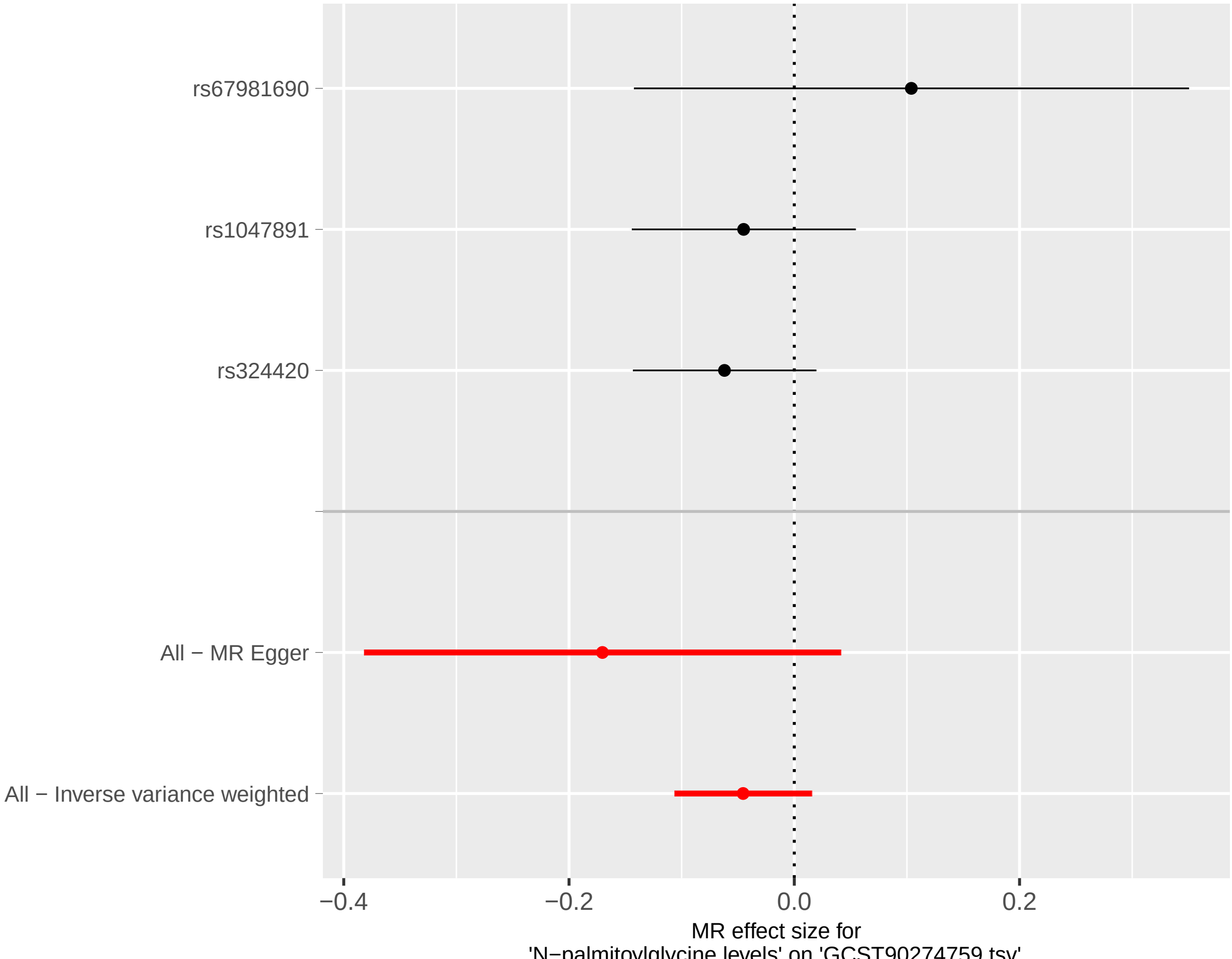
rs67981690
rs1047891
rs324420
All − MR Egger
All − Inverse variance weighted
−0.4
−0.2
0.0
0.2
MR effect size for
'N−palmitoylglycine levels' on 'GCST90274759.tsv'

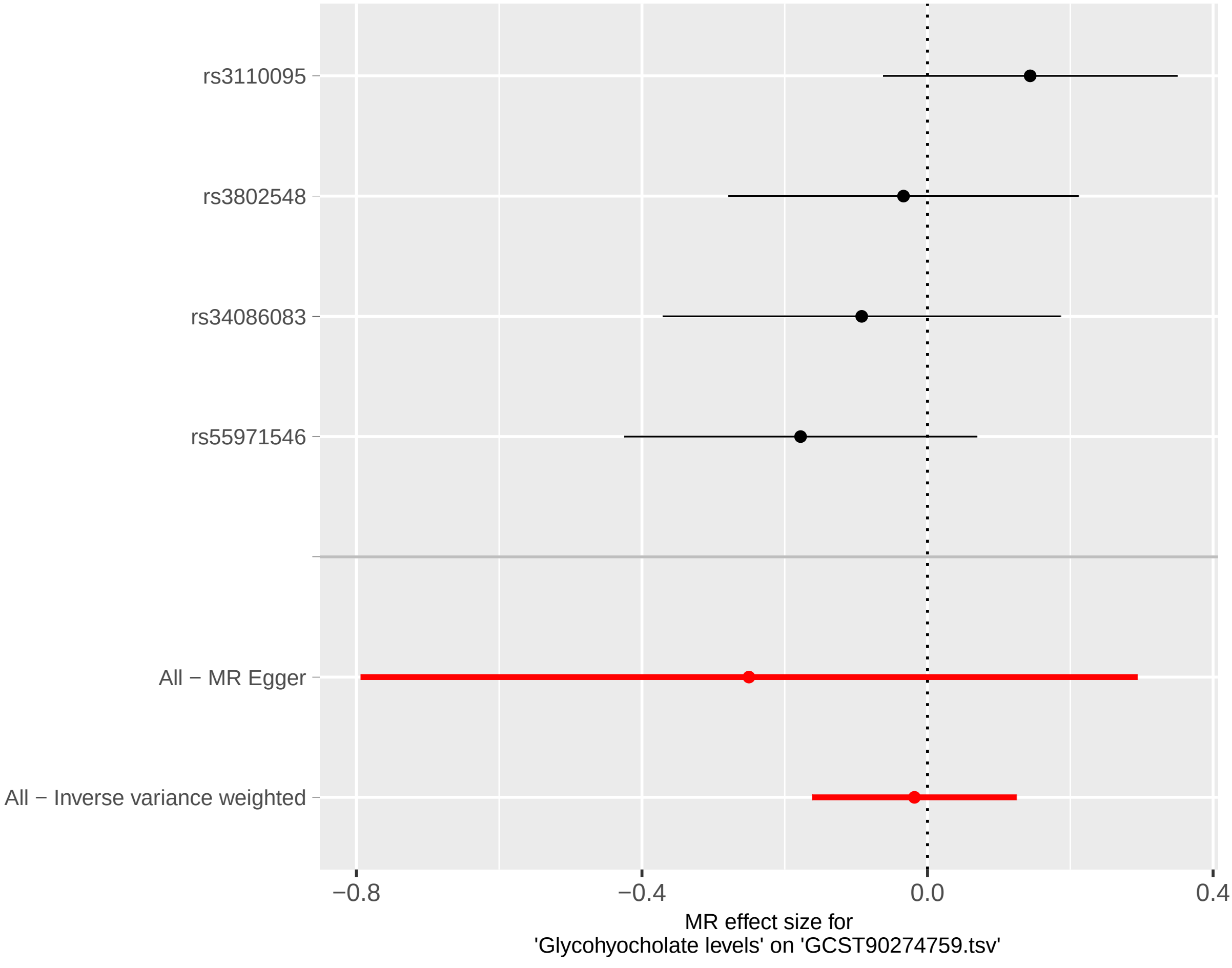
rs3110095
rs3802548
rs34086083
rs55971546
All − MR Egger
All − Inverse variance weighted
−0.8
−0.4
0.0
0.4
MR effect size for
'Glycohyocholate levels' on 'GCST90274759.tsv'

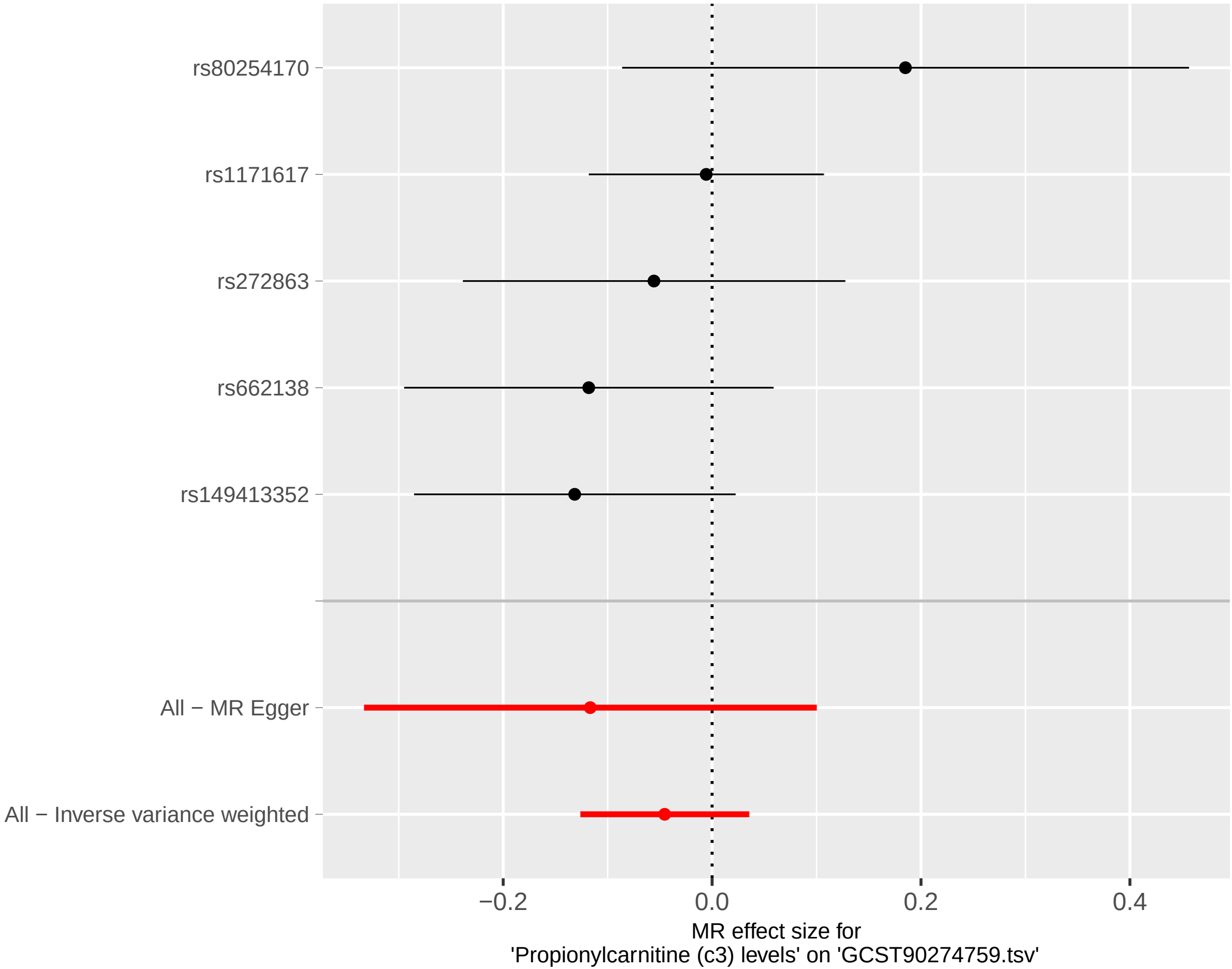
rs80254170
rs1171617
rs272863
rs662138
rs149413352
All − MR Egger
All − Inverse variance weighted
−0.2
0.0
0.2
0.4
MR effect size for
'Propionylcarnitine (c3) levels' on 'GCST90274759.tsv'

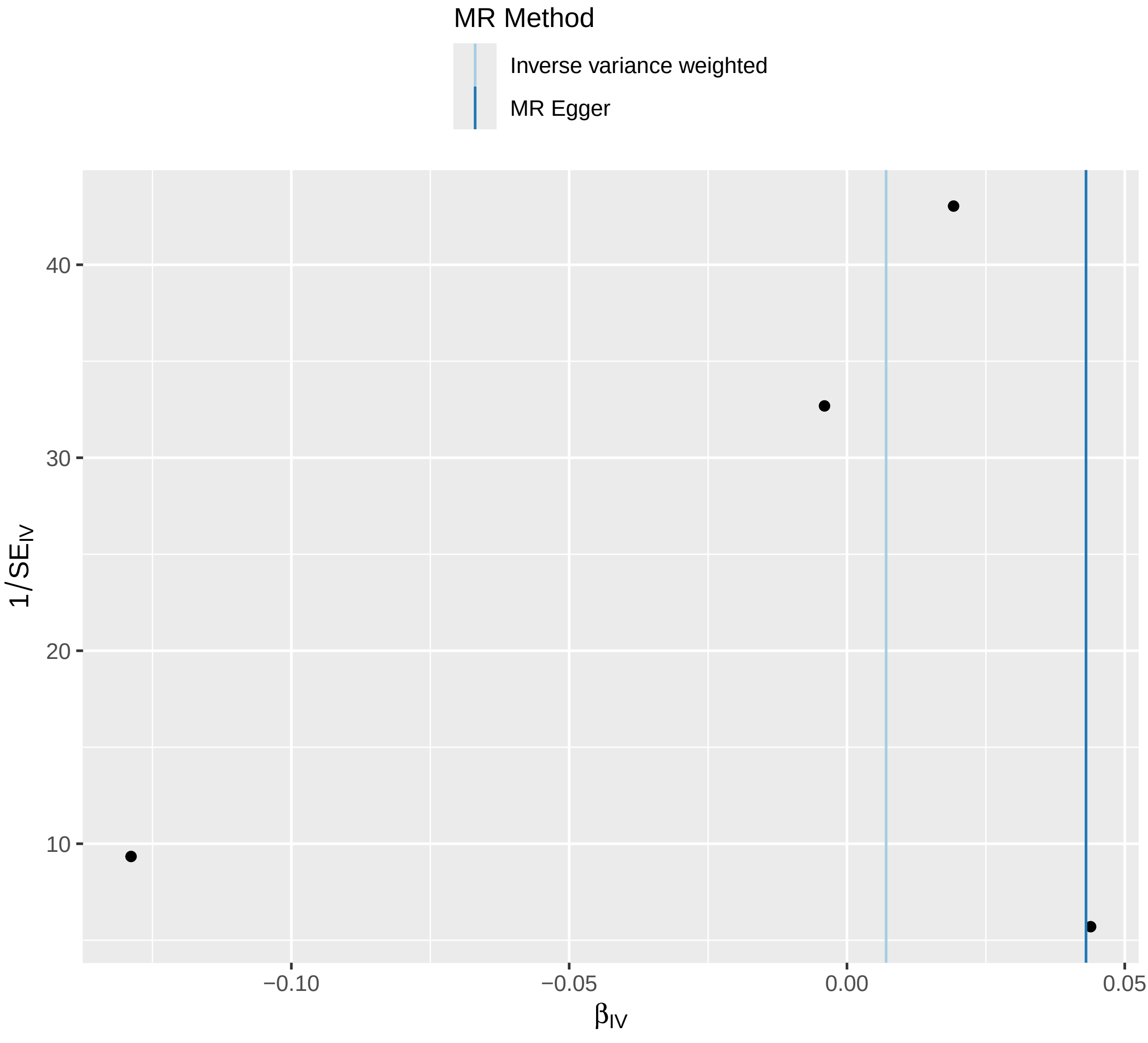
MR Method
Inverse variance weighted
MR Egger
40
30
20
10
1/SE_IV
−0.10
−0.05
0.00
0.05
β_IV

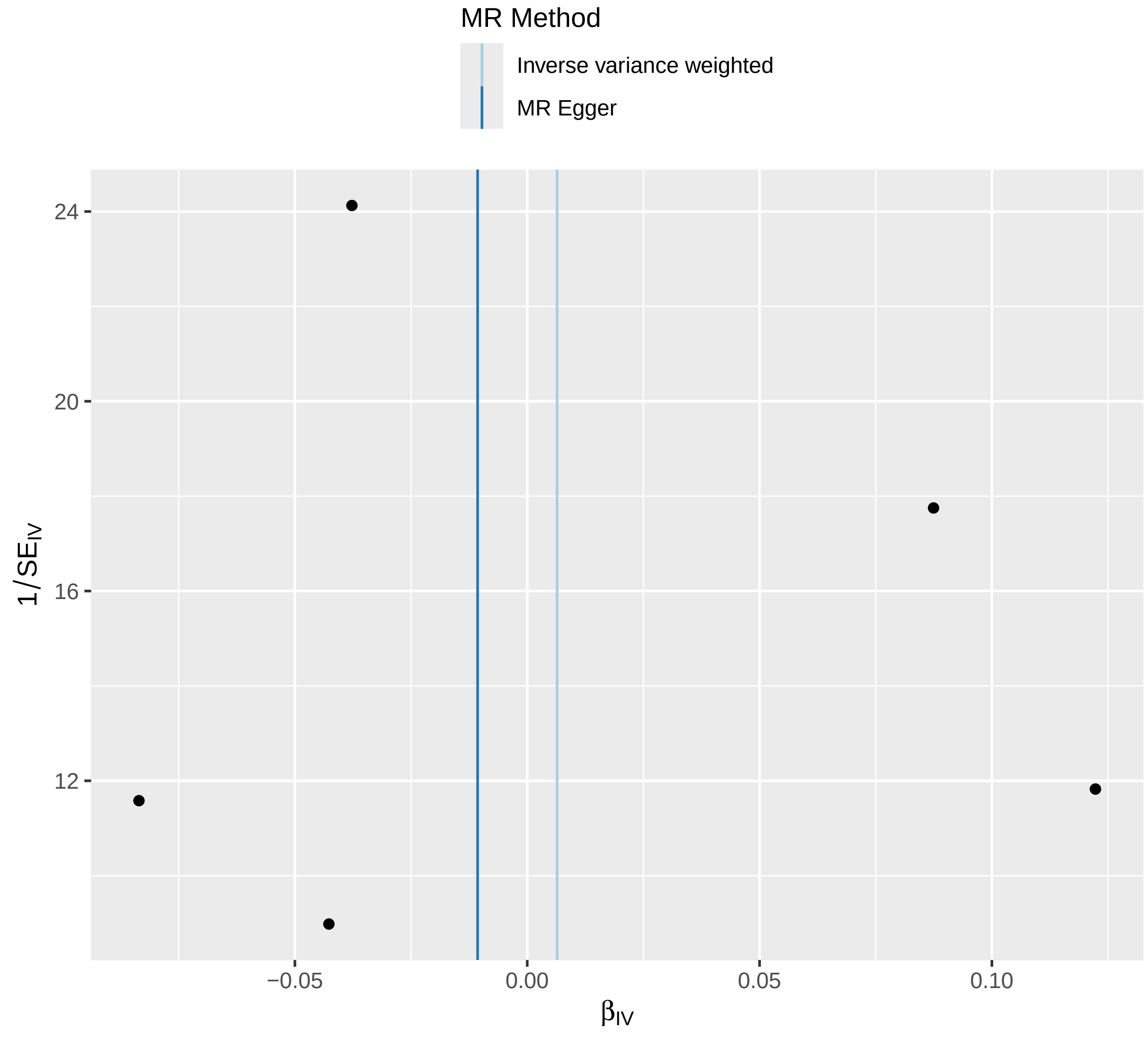
MR Method
Inverse variance weighted
MR Egger
24
20
16
12
1/SE_IV
−0.05
0.00
0.05
0.10
β_IV

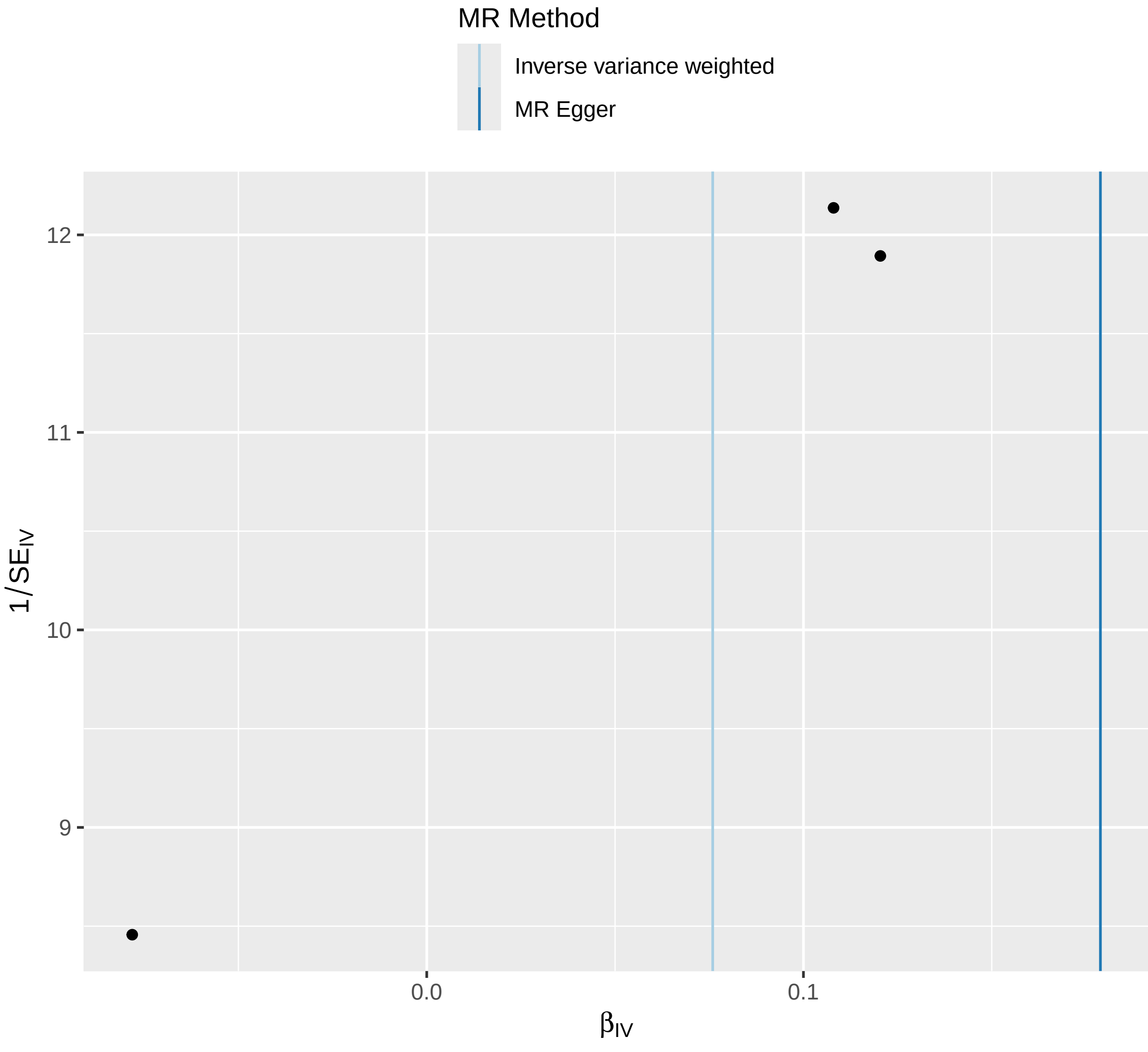
MR Method
Inverse variance weighted
MR Egger
12
11
10
9
1/SE$_{IV}$
0.0
0.1
β$_{IV}$

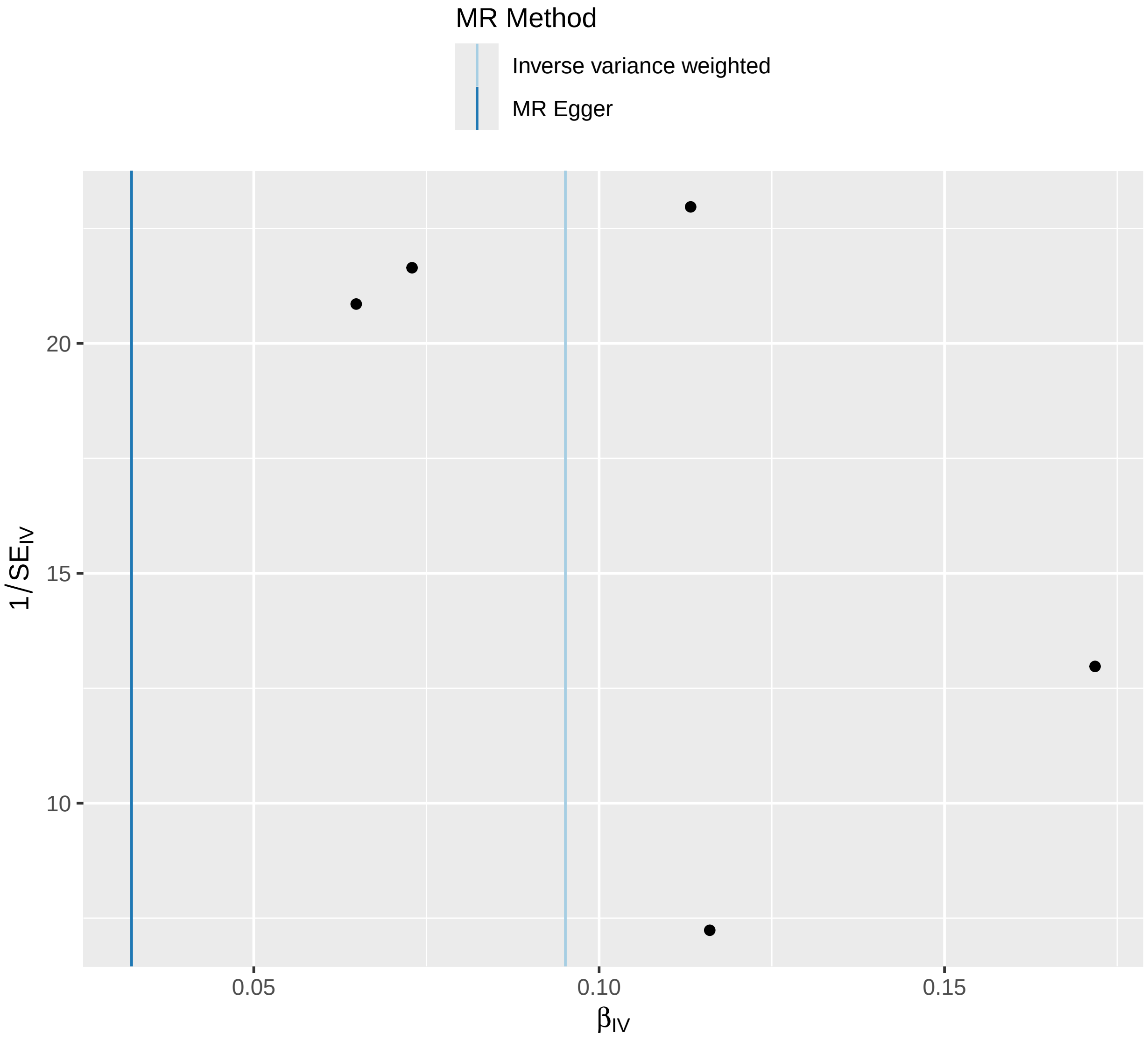

MR Method
Inverse variance weighted
MR Egger
20
15
10
0.05
0.10
0.15
1/SE_IV
β_IV

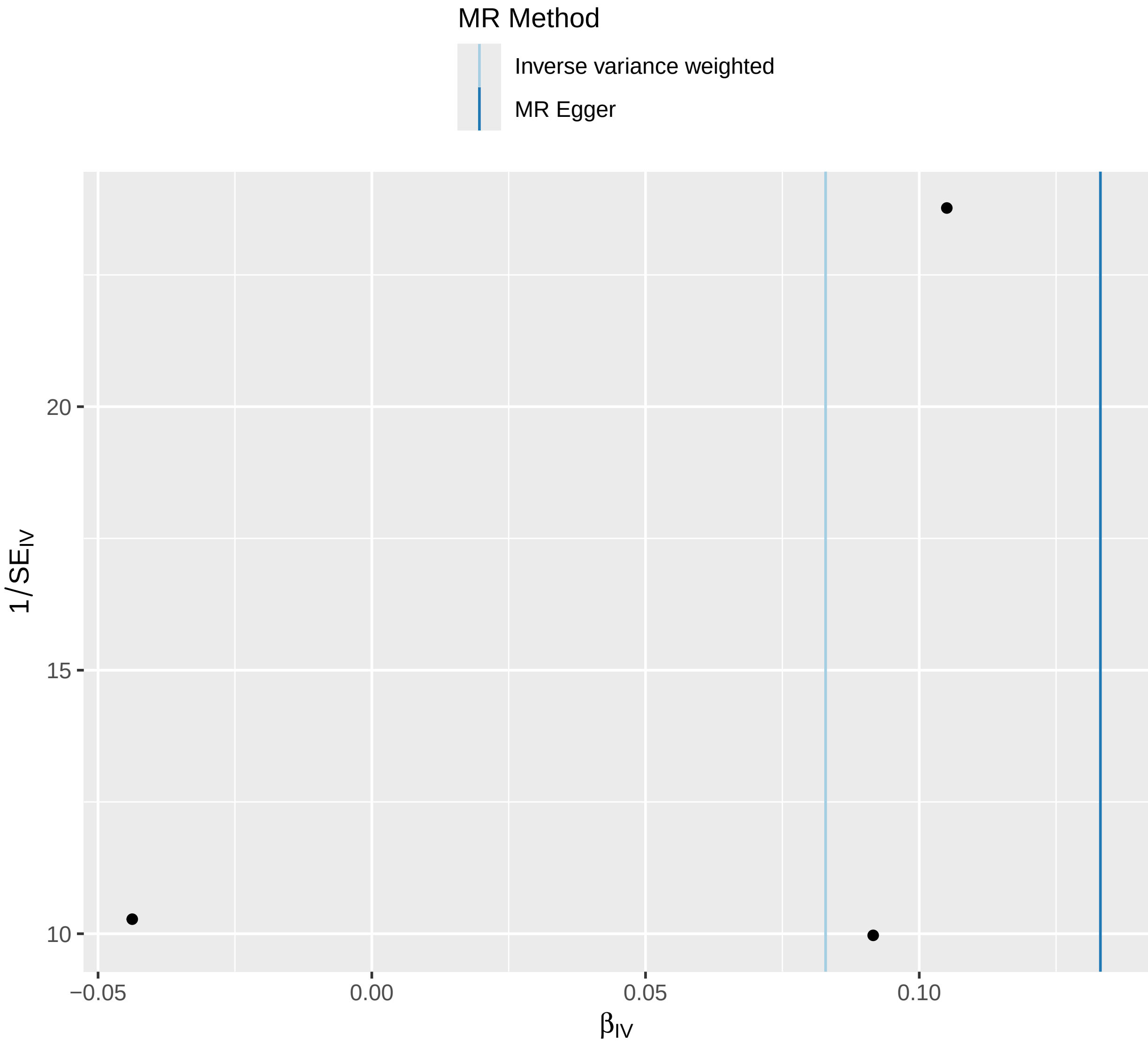
MR Method
Inverse variance weighted
MR Egger
20
15
10
$1/SE_{IV}$
−0.05
0.00
0.05
0.10
β_{IV}

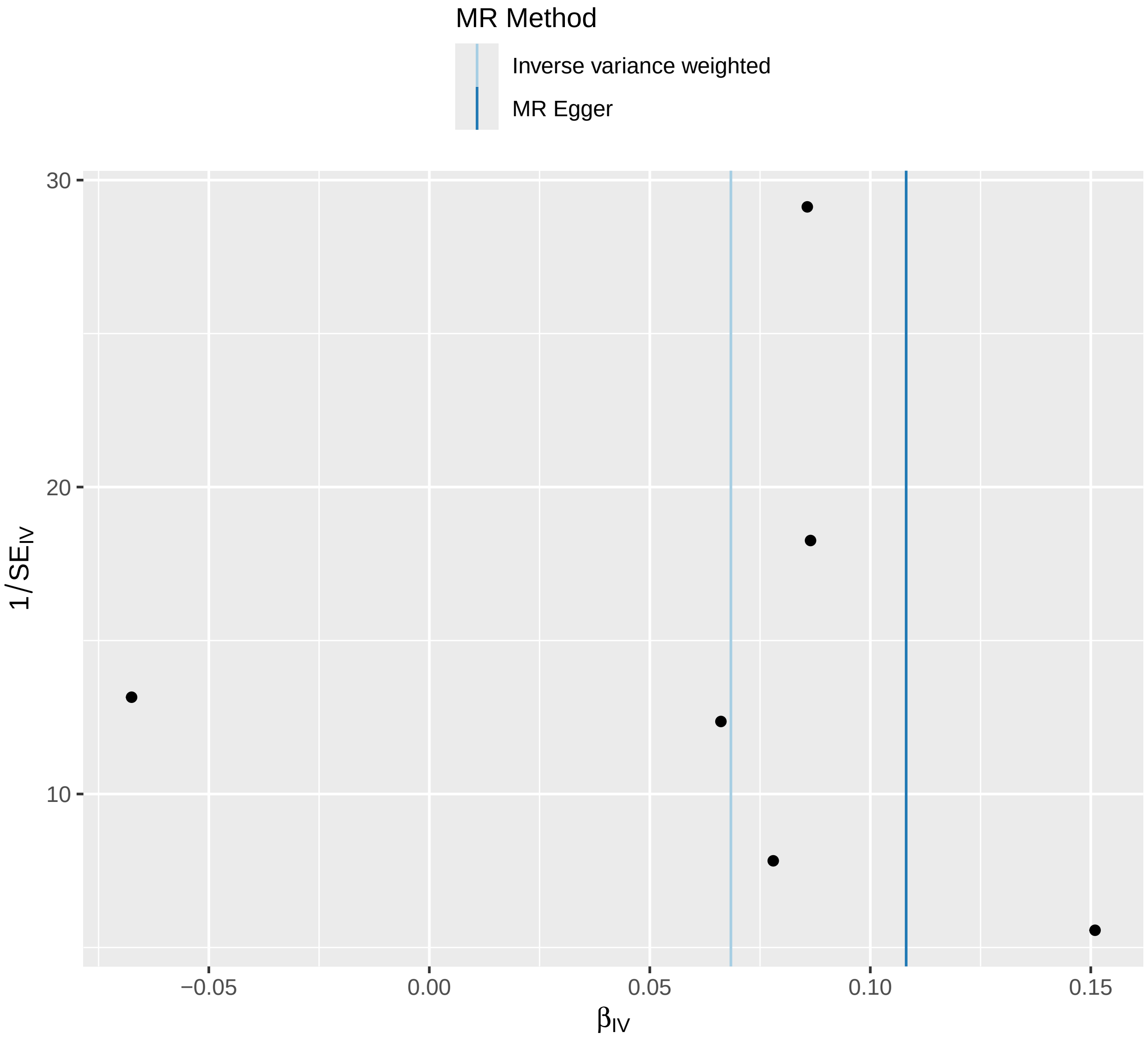
MR Method
Inverse variance weighted
MR Egger
30
20
10
1/SE_IV
−0.05
0.00
0.05
0.10
0.15
β_IV

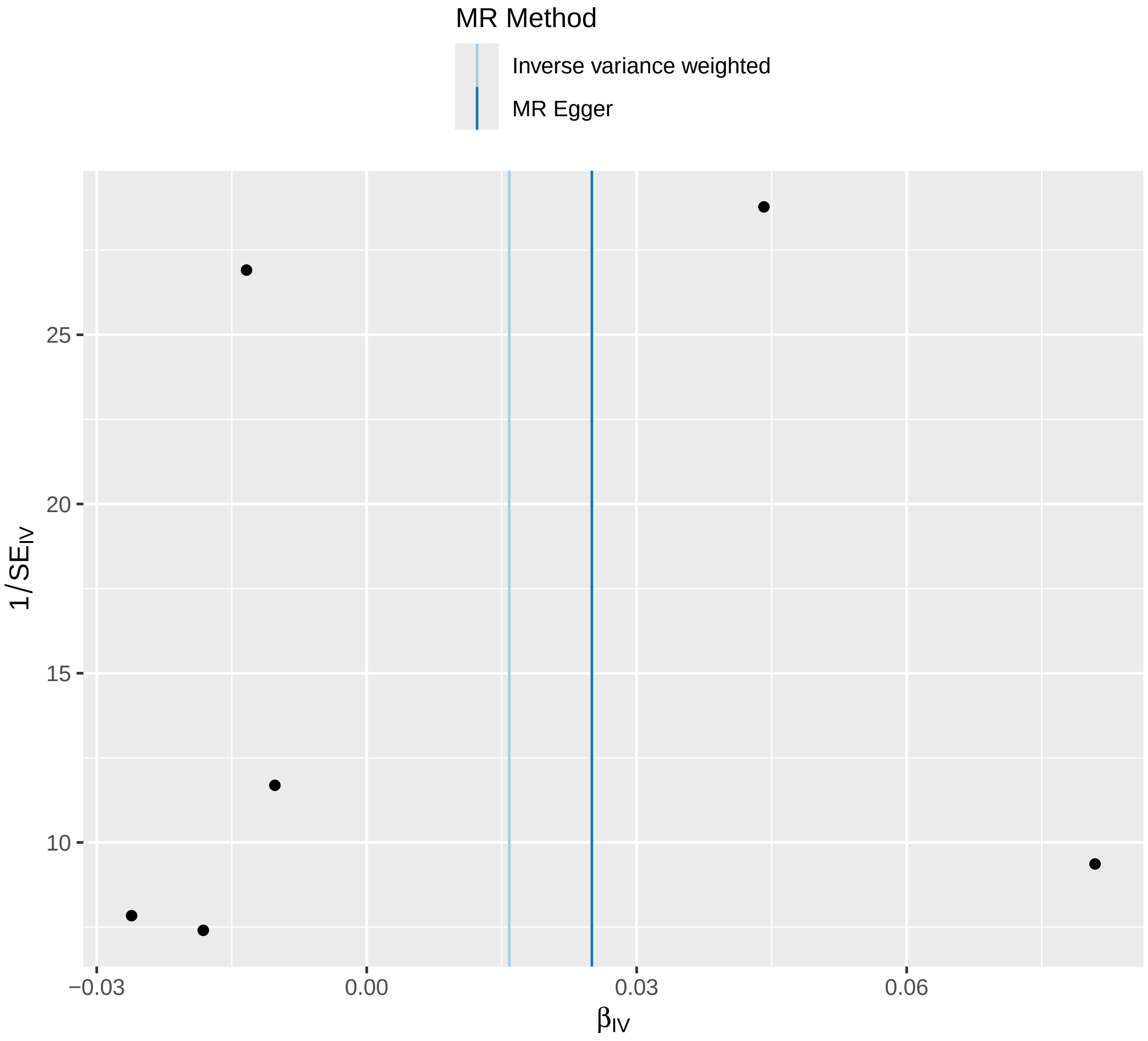

MR Method
Inverse variance weighted
MR Egger
25
20
15
10
1/SE$_{IV}$
−0.03
0.00
0.03
0.06
β_{IV}

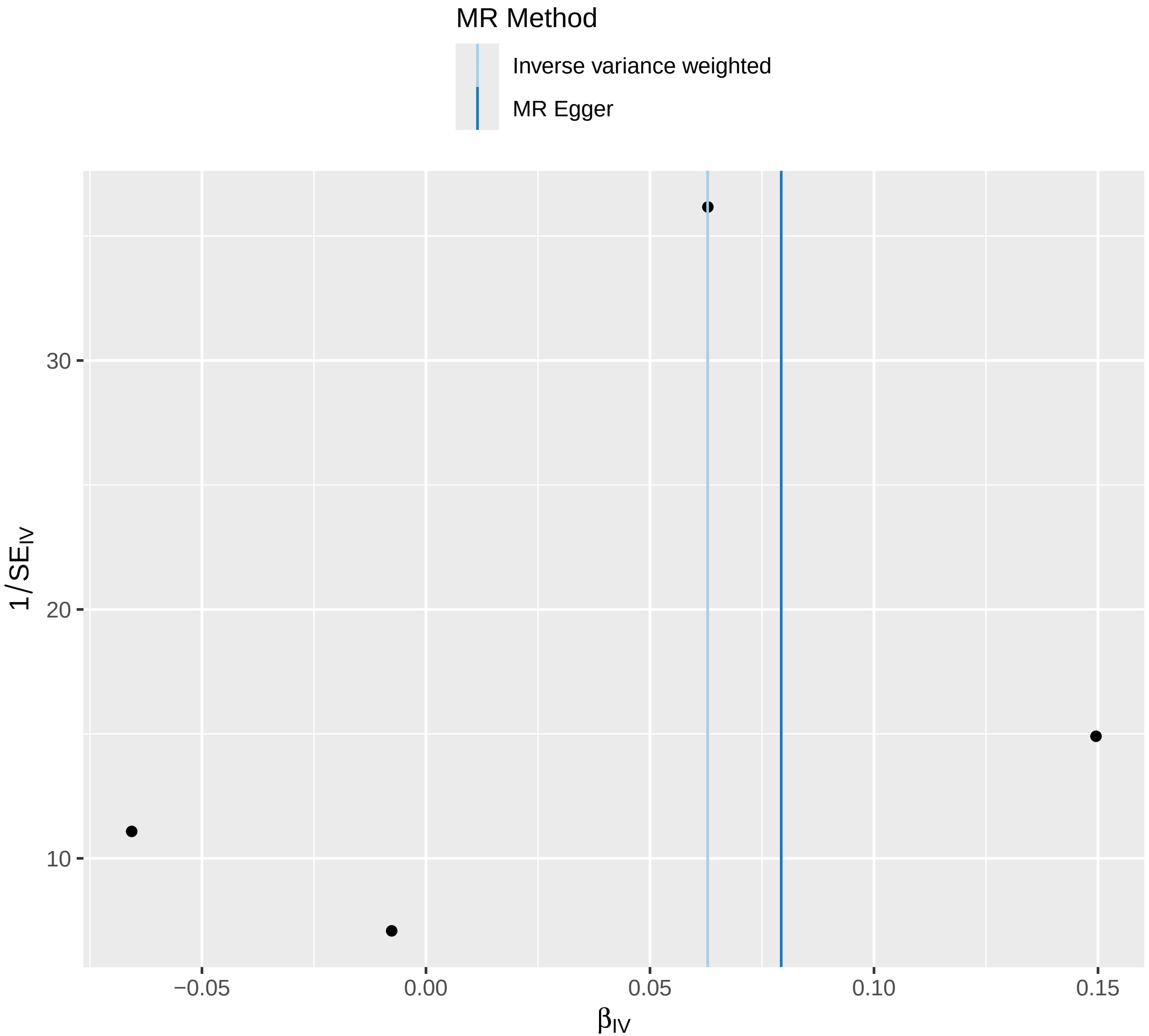

MR Method
Inverse variance weighted
MR Egger
1/SE_IV
30
20
10
−0.05
0.00
0.05
0.10
0.15
β_IV

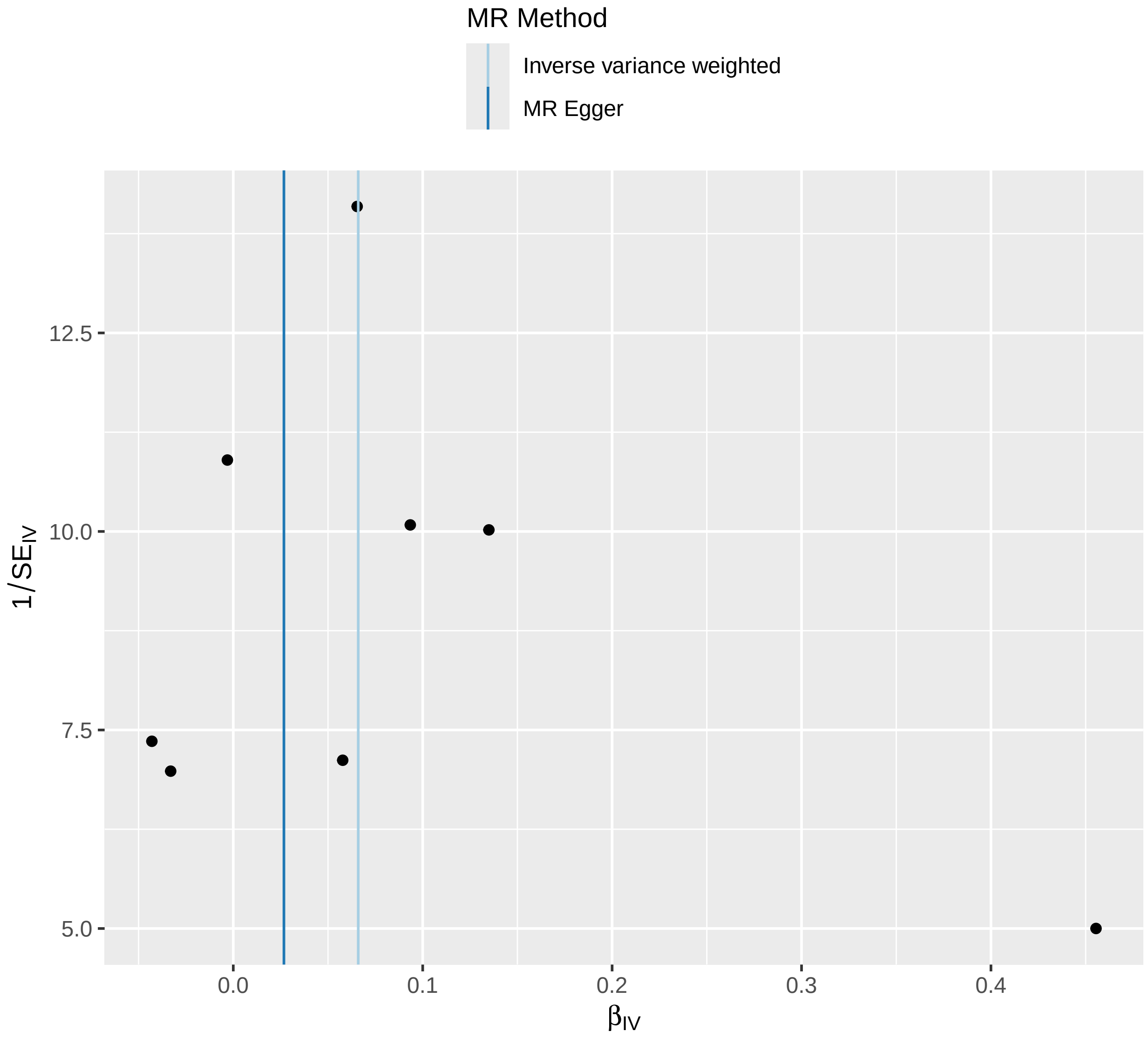
MR Method
Inverse variance weighted
MR Egger
12.5
10.0
7.5
5.0
1/SE_IV
0.0
0.1
0.2
0.3
0.4
β_IV

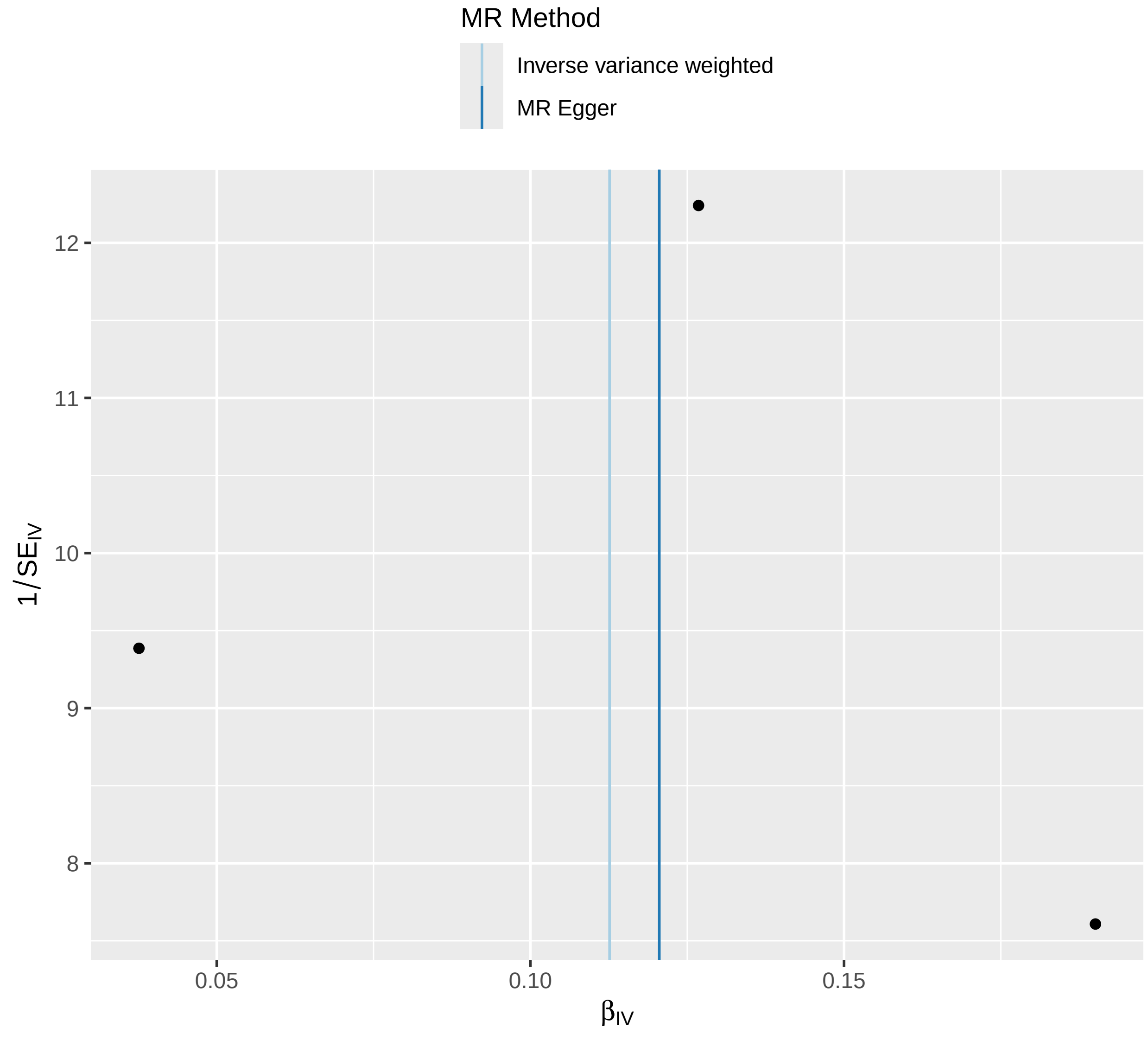

MR Method
Inverse variance weighted
MR Egger
12
11
10
9
8
$1/SE_{IV}$
0.05
0.10
0.15
β_{IV}

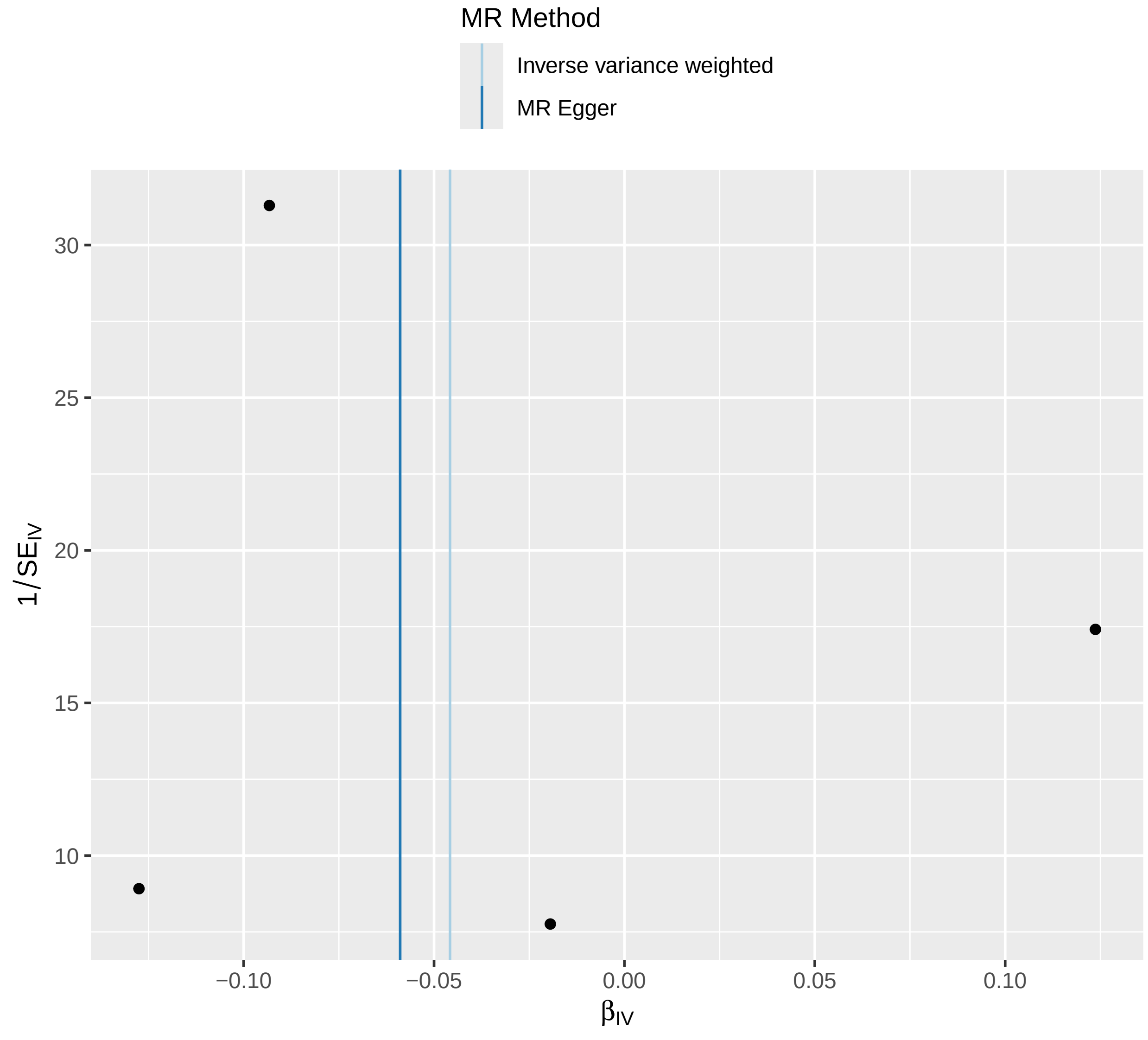
MR Method
Inverse variance weighted
MR Egger
30
25
20
15
10
1/SE_IV
−0.10
−0.05
0.00
0.05
0.10
β_IV

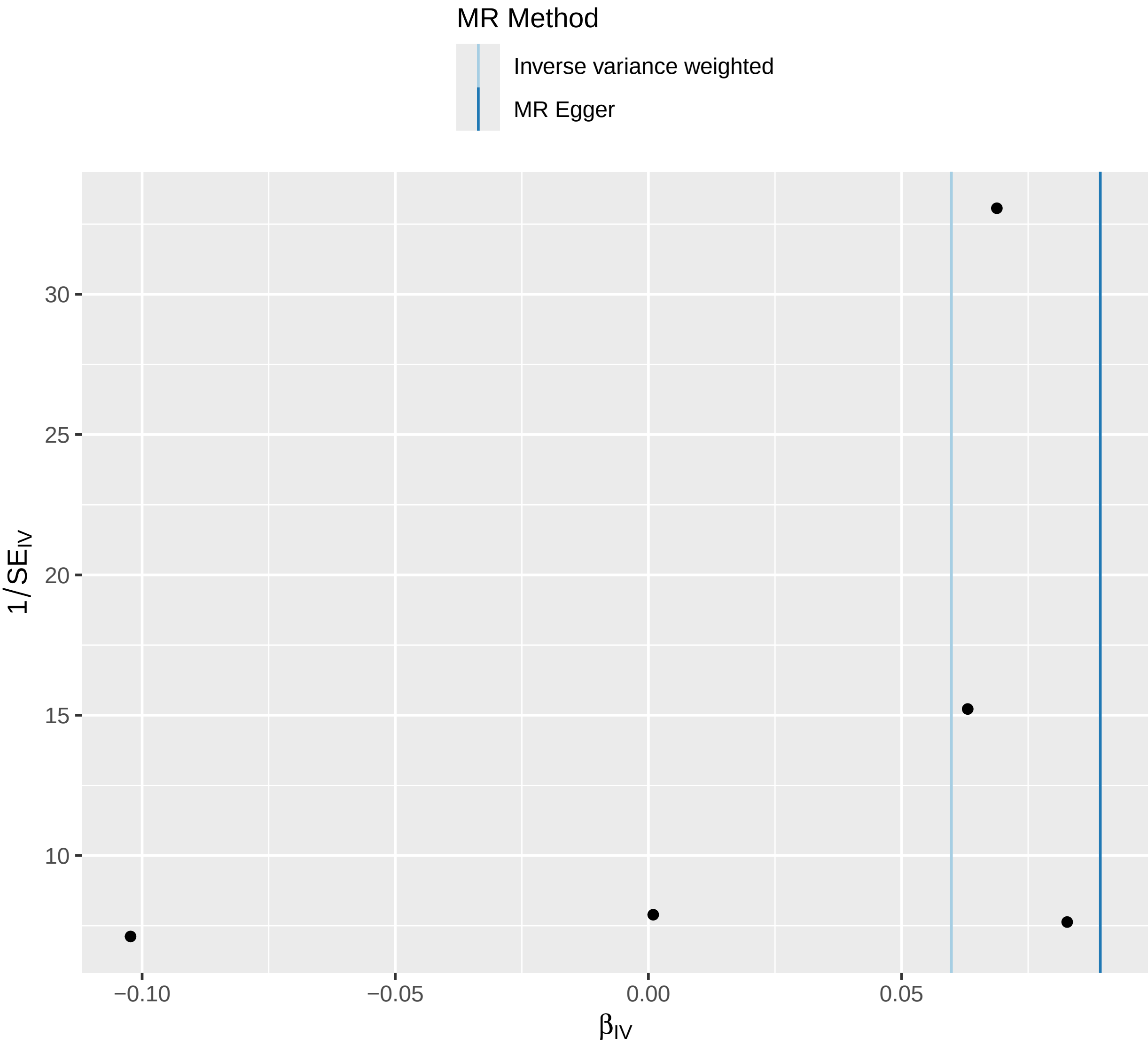
MR Method
Inverse variance weighted
MR Egger
30
25
20
15
10
1/SE_IV
−0.10
−0.05
0.00
0.05
β_IV

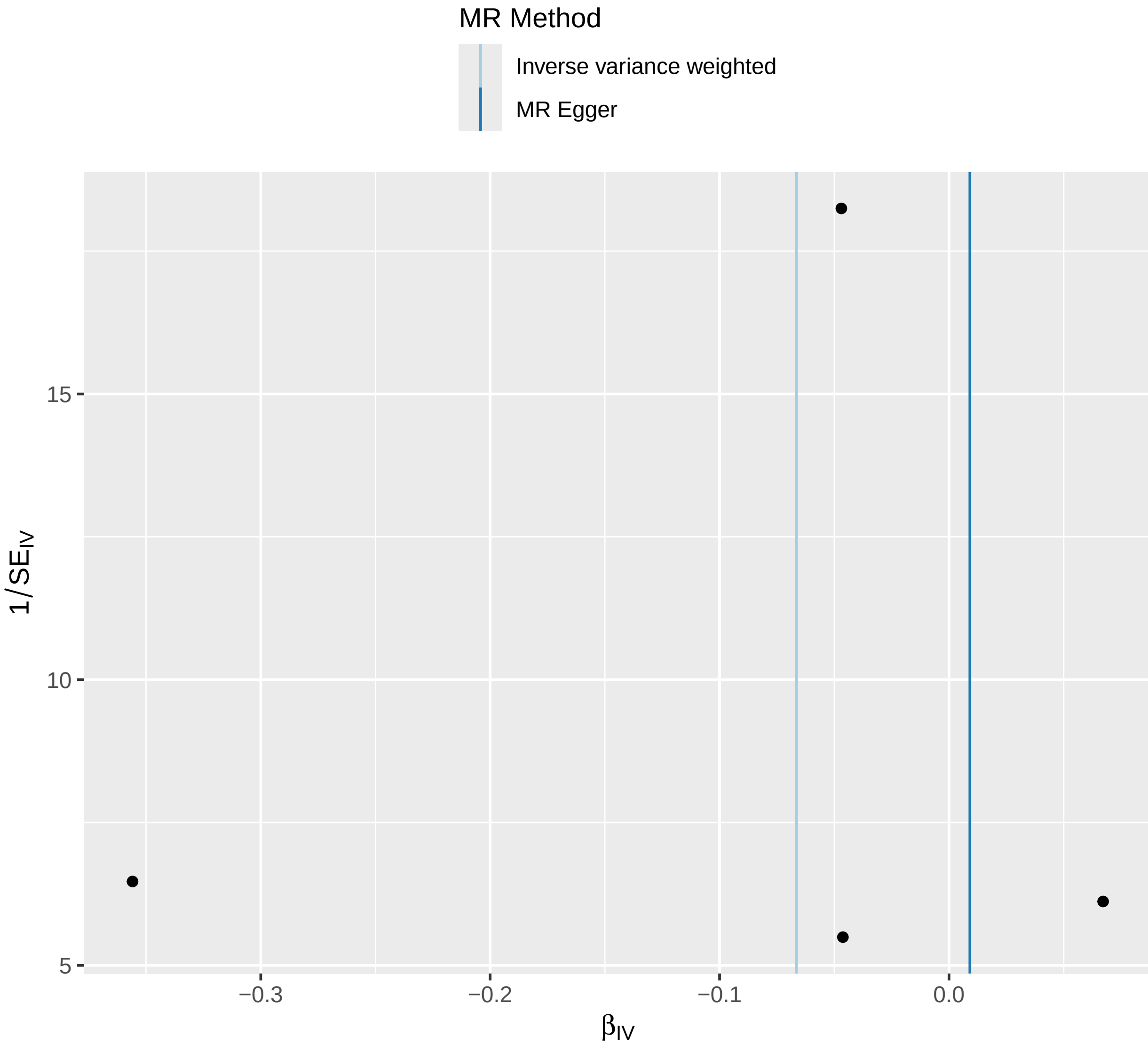
MR Method
Inverse variance weighted
MR Egger
15
10
5
−0.3
−0.2
−0.1
0.0
$1/SE_{IV}$
β_{IV}

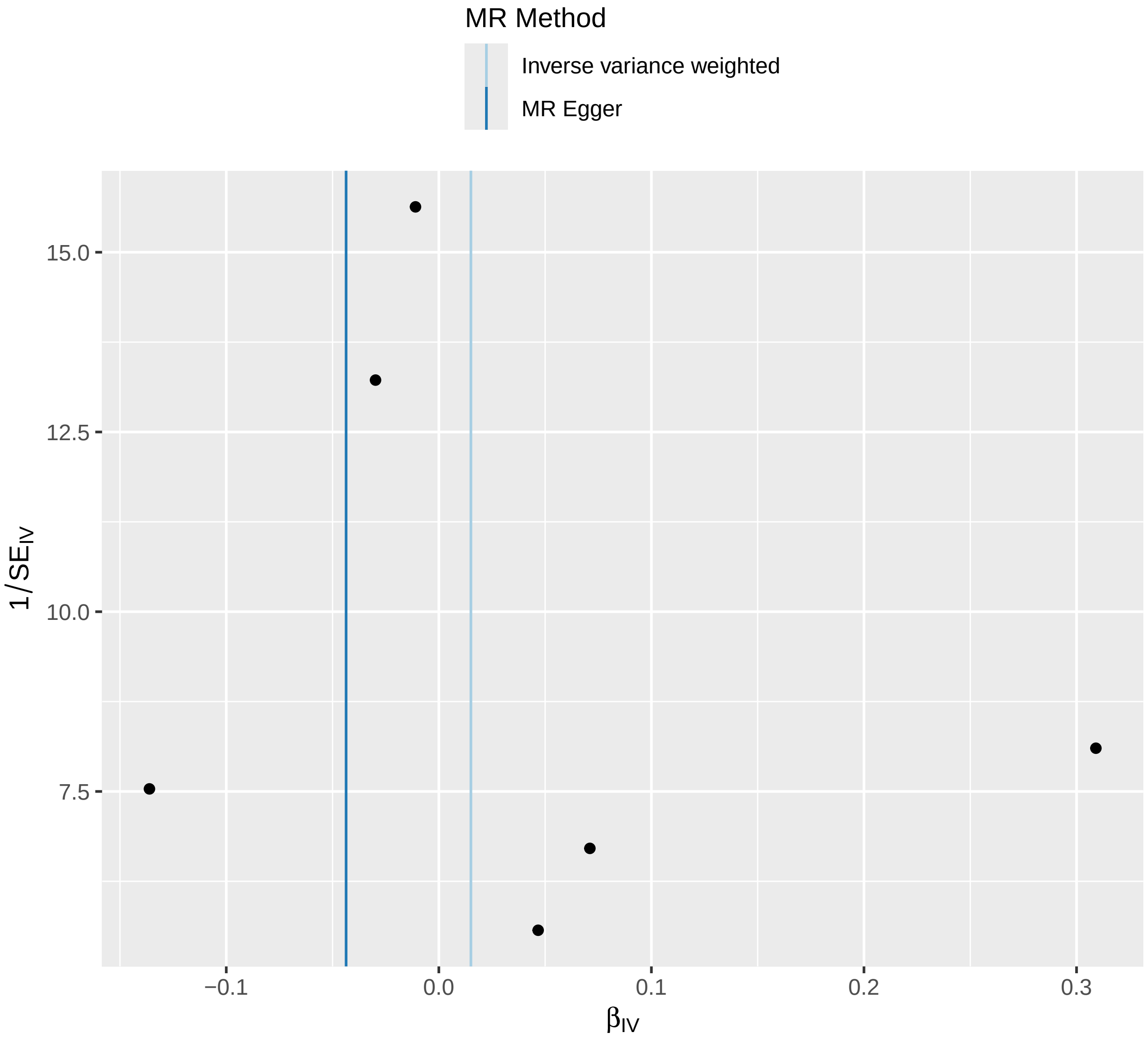
MR Method
Inverse variance weighted
MR Egger
15.0
12.5
10.0
7.5
1/SE$_{IV}$
−0.1
0.0
0.1
0.2
0.3
β_{IV}

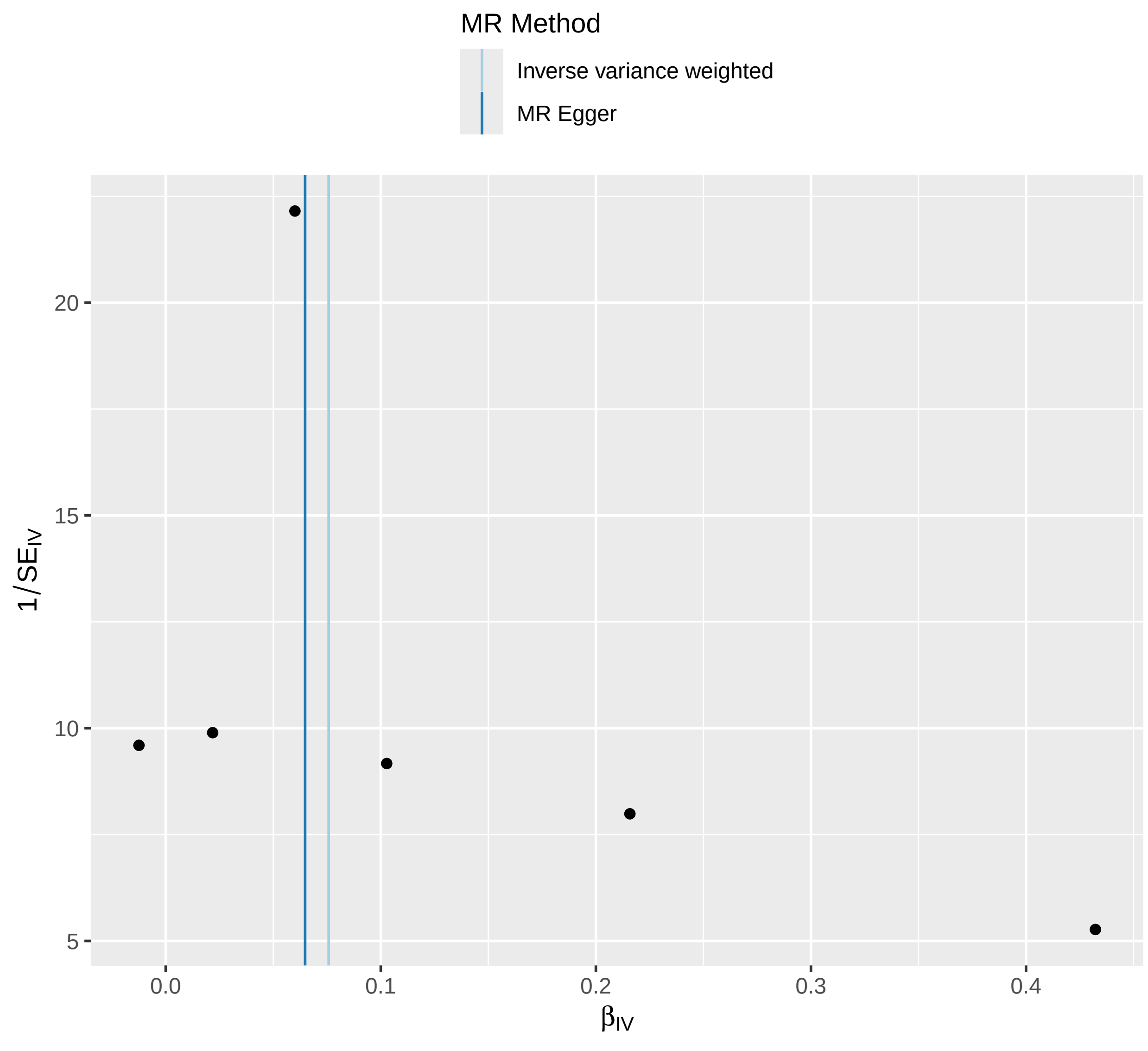

MR Method
Inverse variance weighted
MR Egger
20
15
10
5
1/SE_IV
0.0
0.1
0.2
0.3
0.4
β_IV

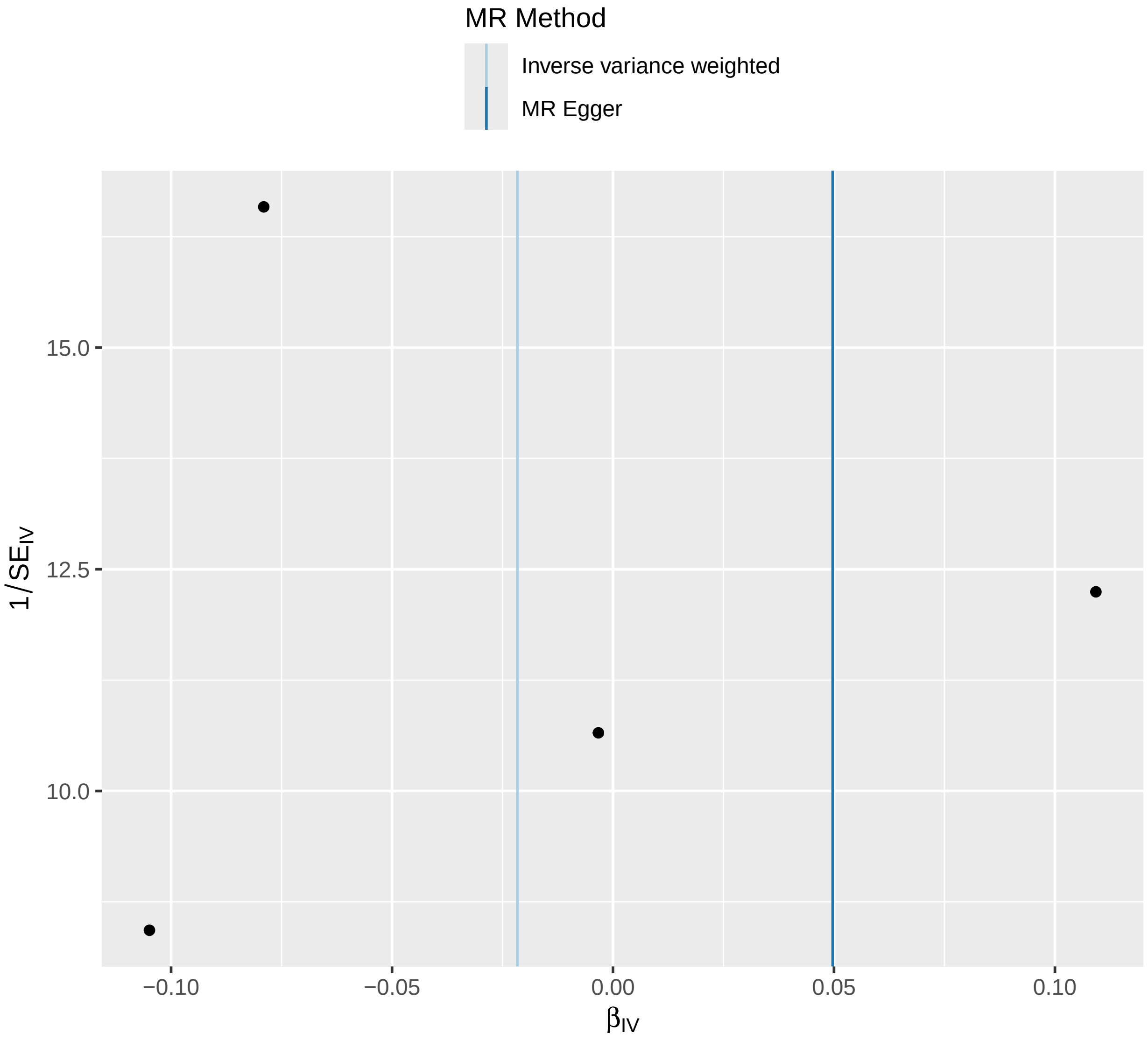
MR Method
Inverse variance weighted
MR Egger
15.0
12.5
10.0
$1/SE_{IV}$
−0.10
−0.05
0.00
0.05
0.10
β_{IV}

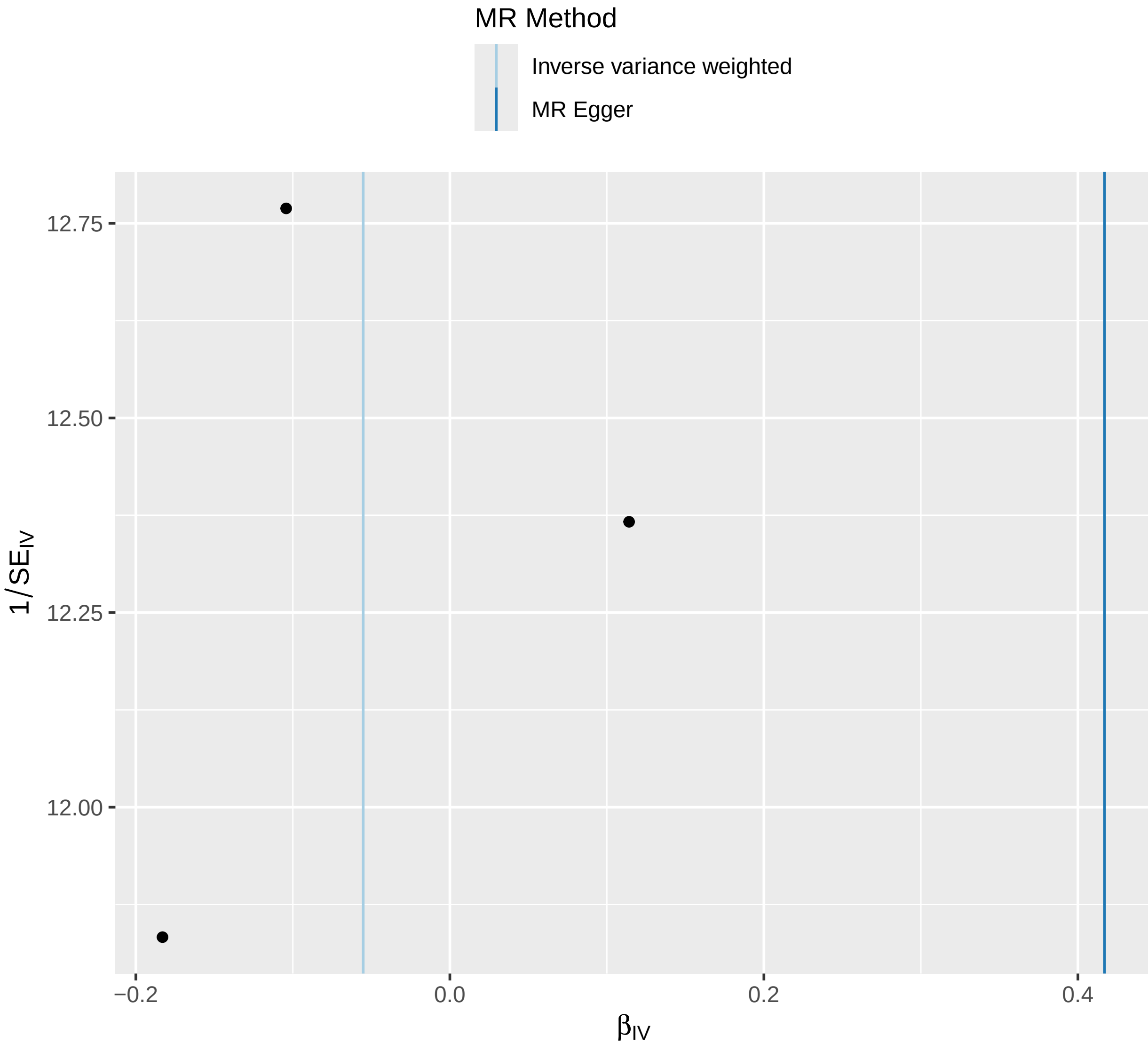
MR Method
Inverse variance weighted
MR Egger
12.75
12.50
12.25
12.00
1/SE_IV
−0.2
0.0
0.2
0.4
β_IV

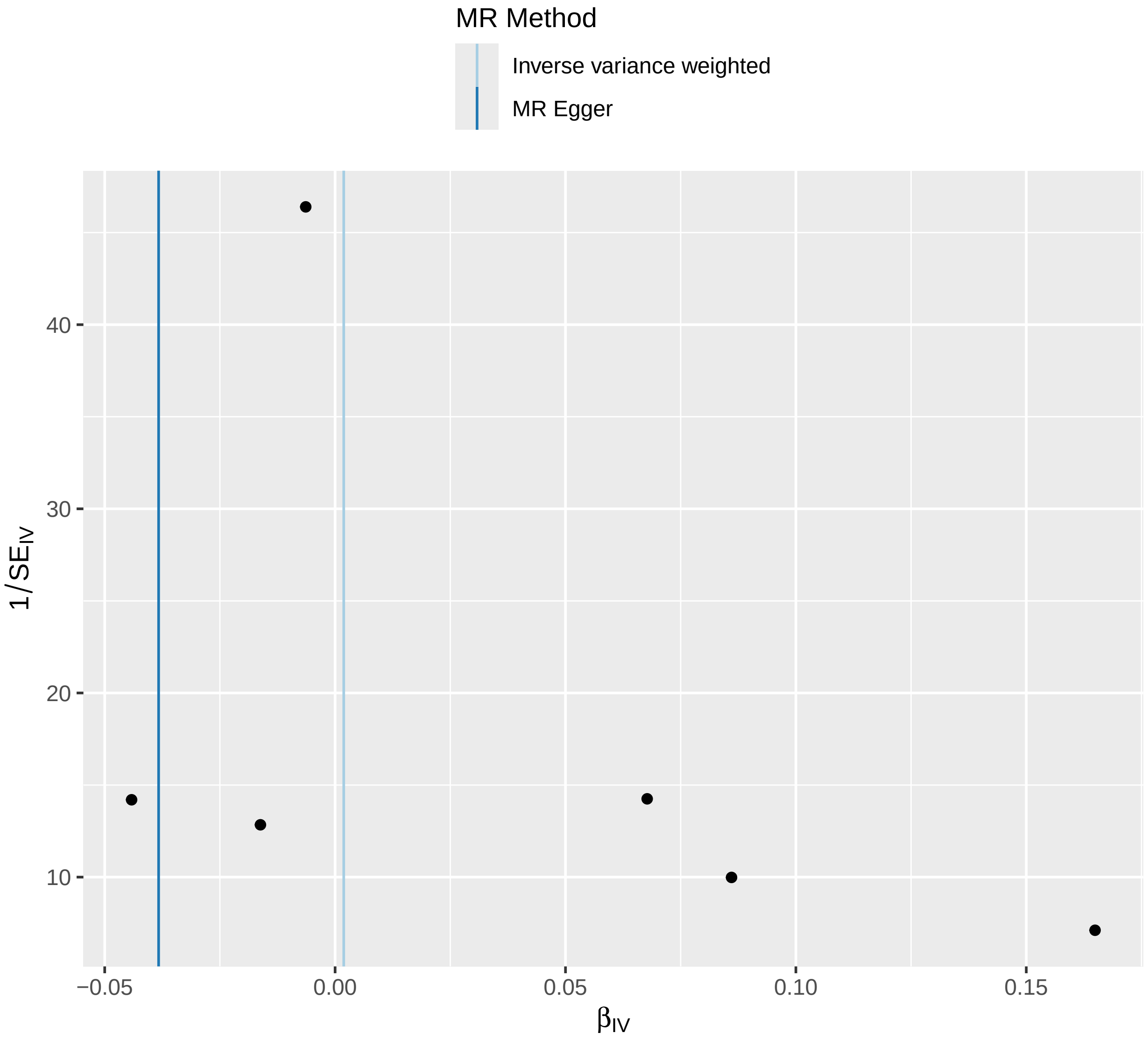

MR Method
Inverse variance weighted
MR Egger
40
30
20
10
1/SE_IV
−0.05
0.00
0.05
0.10
0.15
β_IV

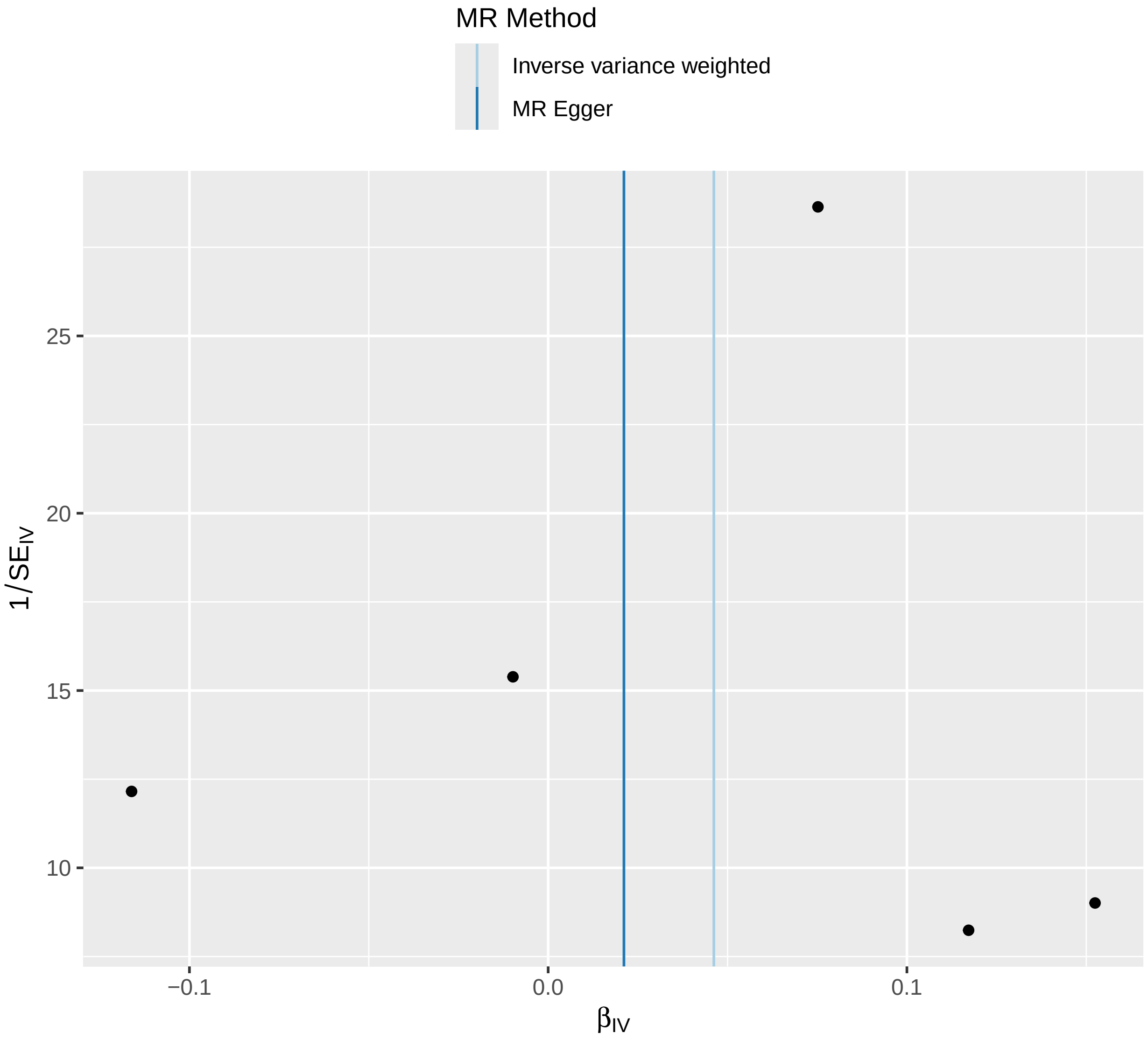

MR Method
Inverse variance weighted
MR Egger
25
20
15
10
1/SE_IV
−0.1
0.0
0.1
β_IV

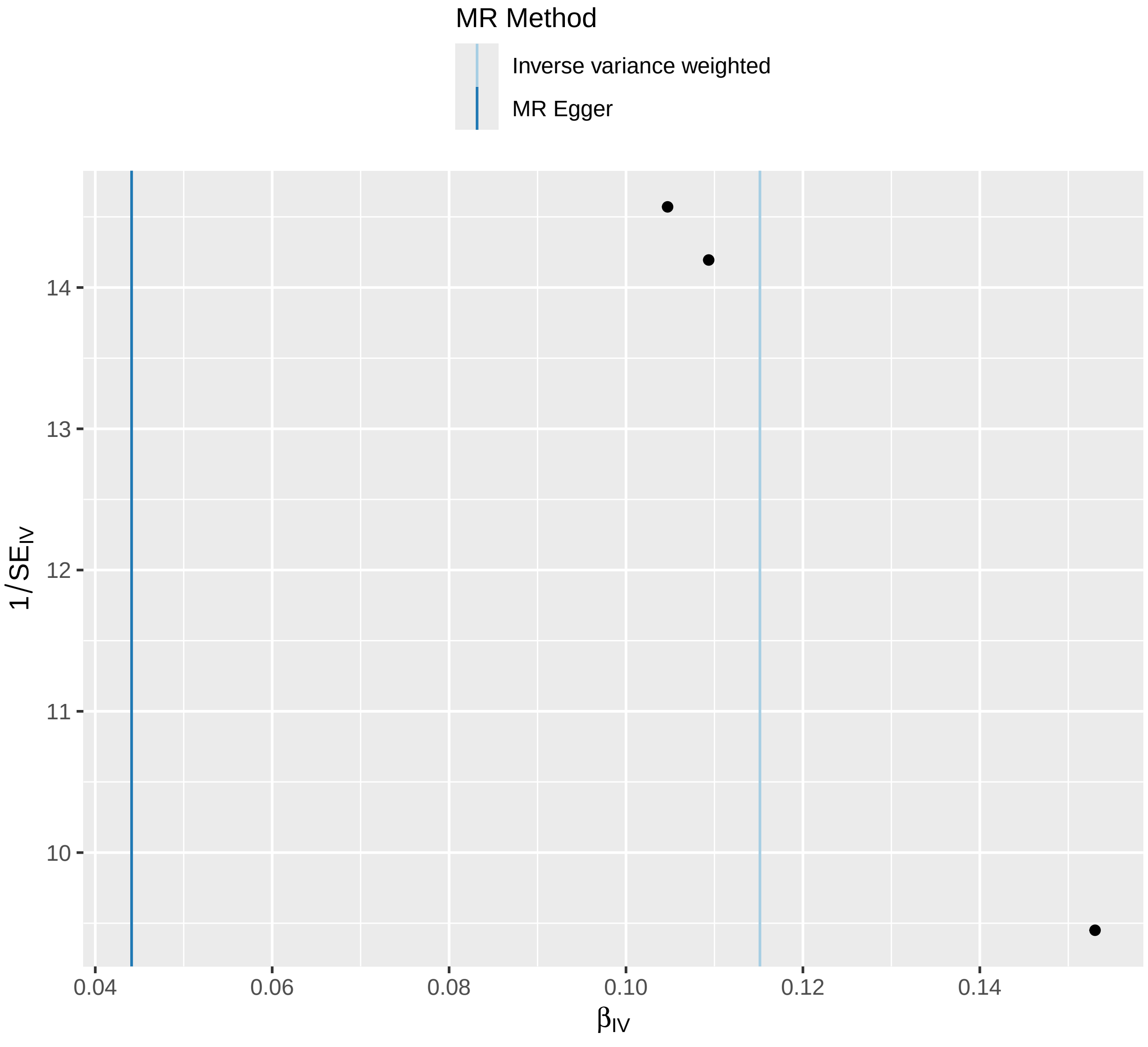

MR Method
Inverse variance weighted
MR Egger
1/SE_IV
14
13
12
11
10
0.04
0.06
0.08
0.10
0.12
0.14
β_IV

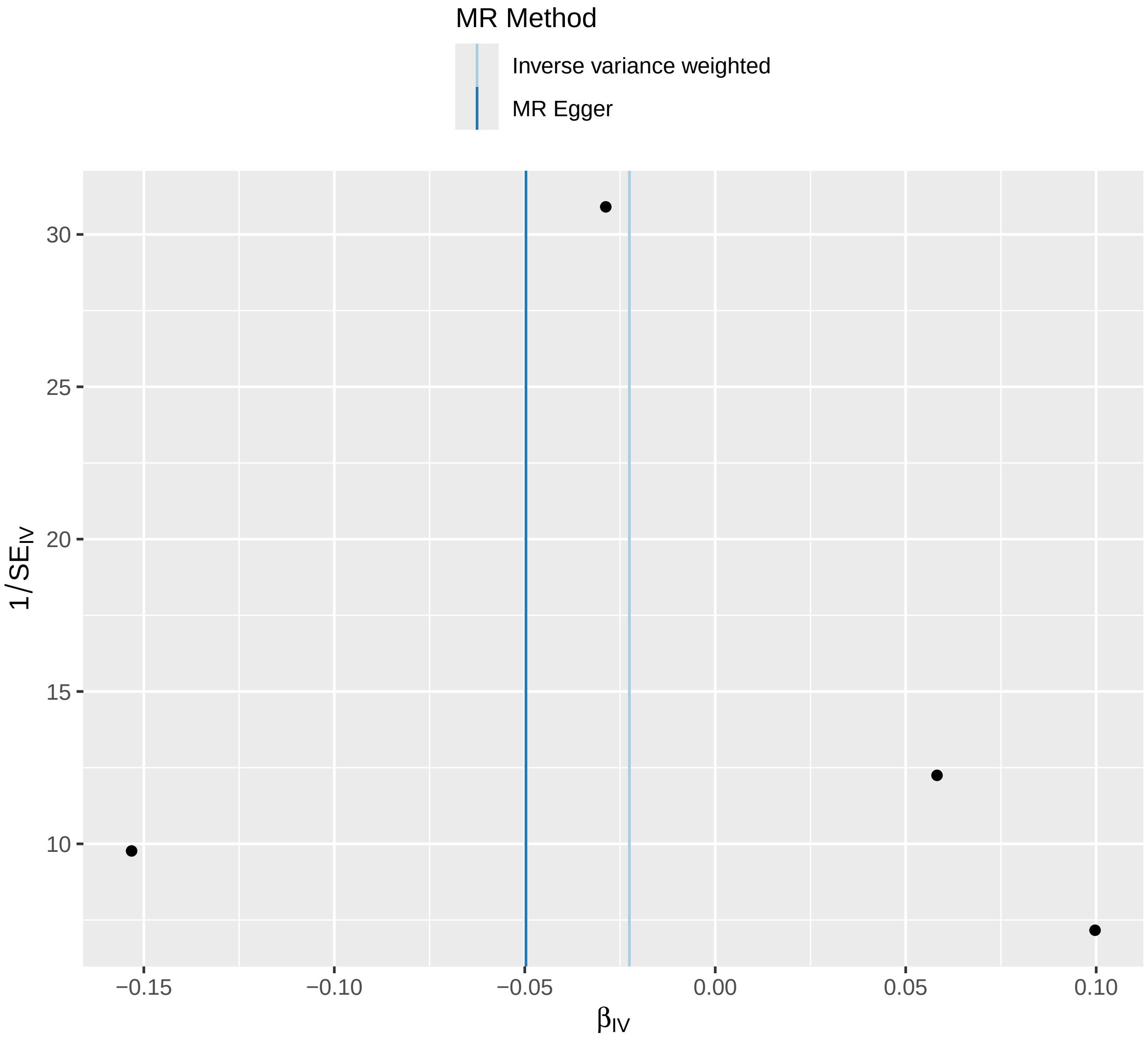
MR Method
Inverse variance weighted
MR Egger
30
25
20
15
10
1/SE_IV
−0.15
−0.10
−0.05
0.00
0.05
0.10
β_IV

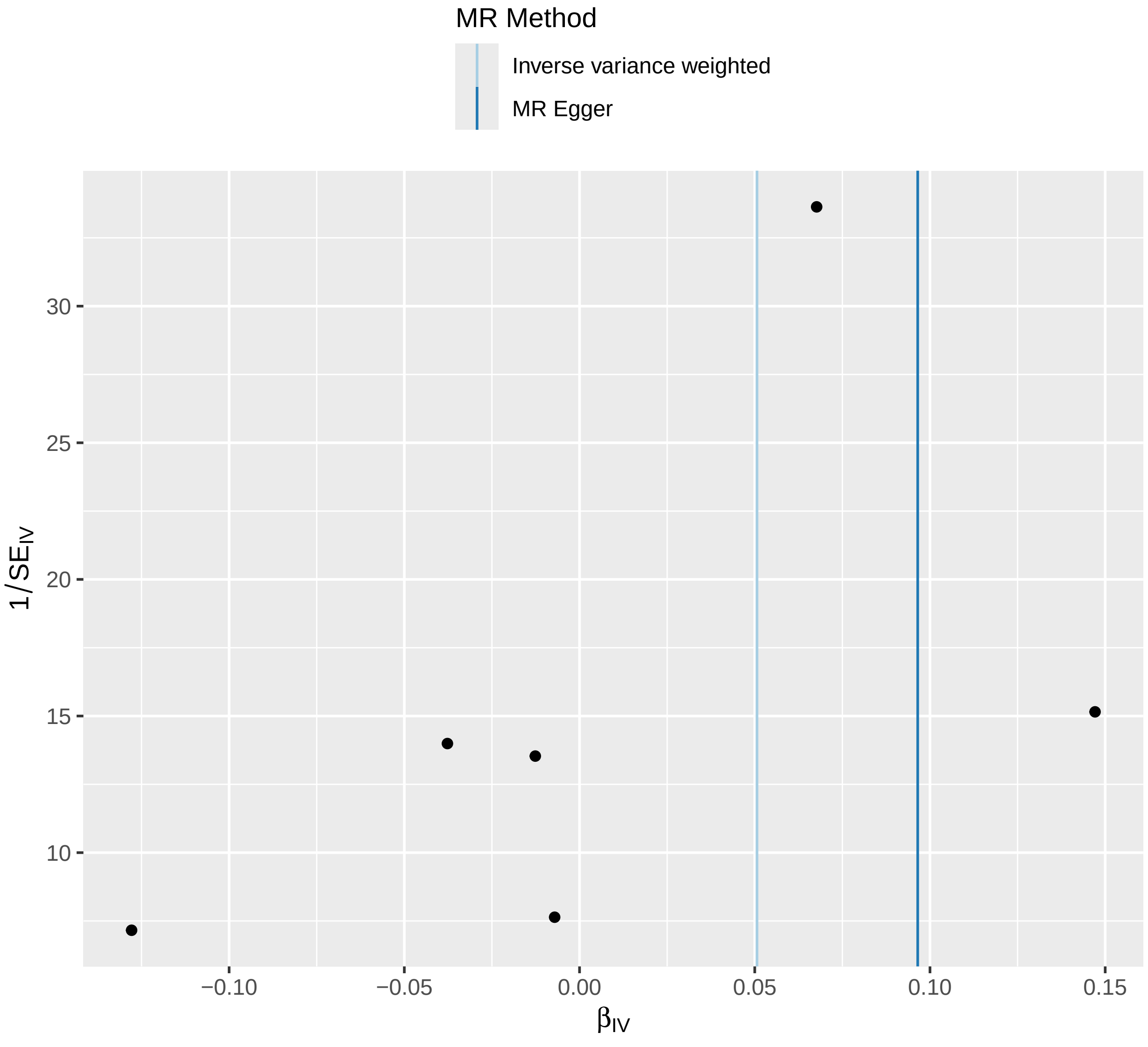
MR Method
Inverse variance weighted
MR Egger
30
25
20
15
10
1/SE$_{IV}$
−0.10
−0.05
0.00
0.05
0.10
0.15
β$_{IV}$

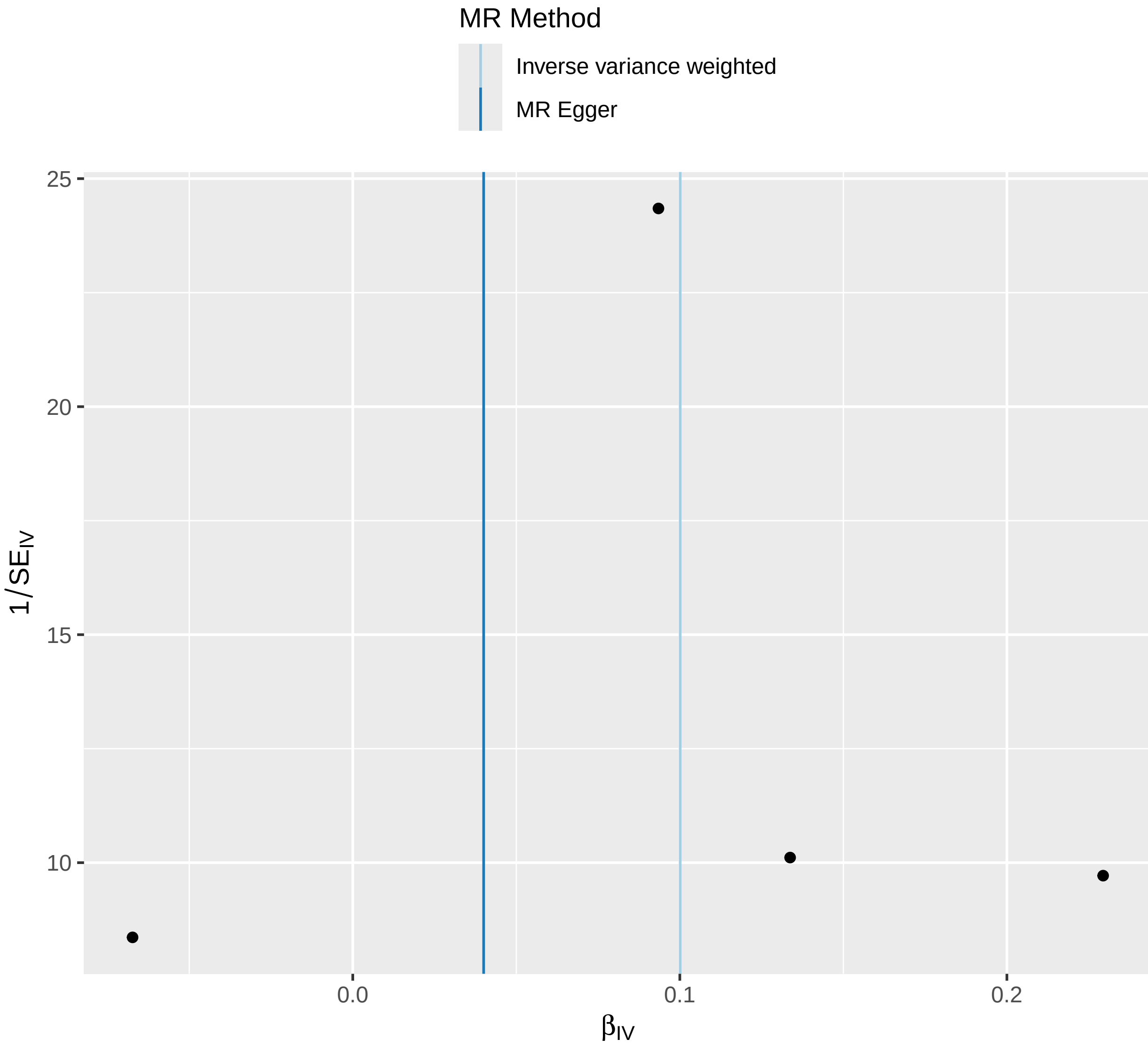
MR Method
Inverse variance weighted
MR Egger
25
20
15
10
1/SE$_{IV}$
0.0
0.1
0.2
β_{IV}

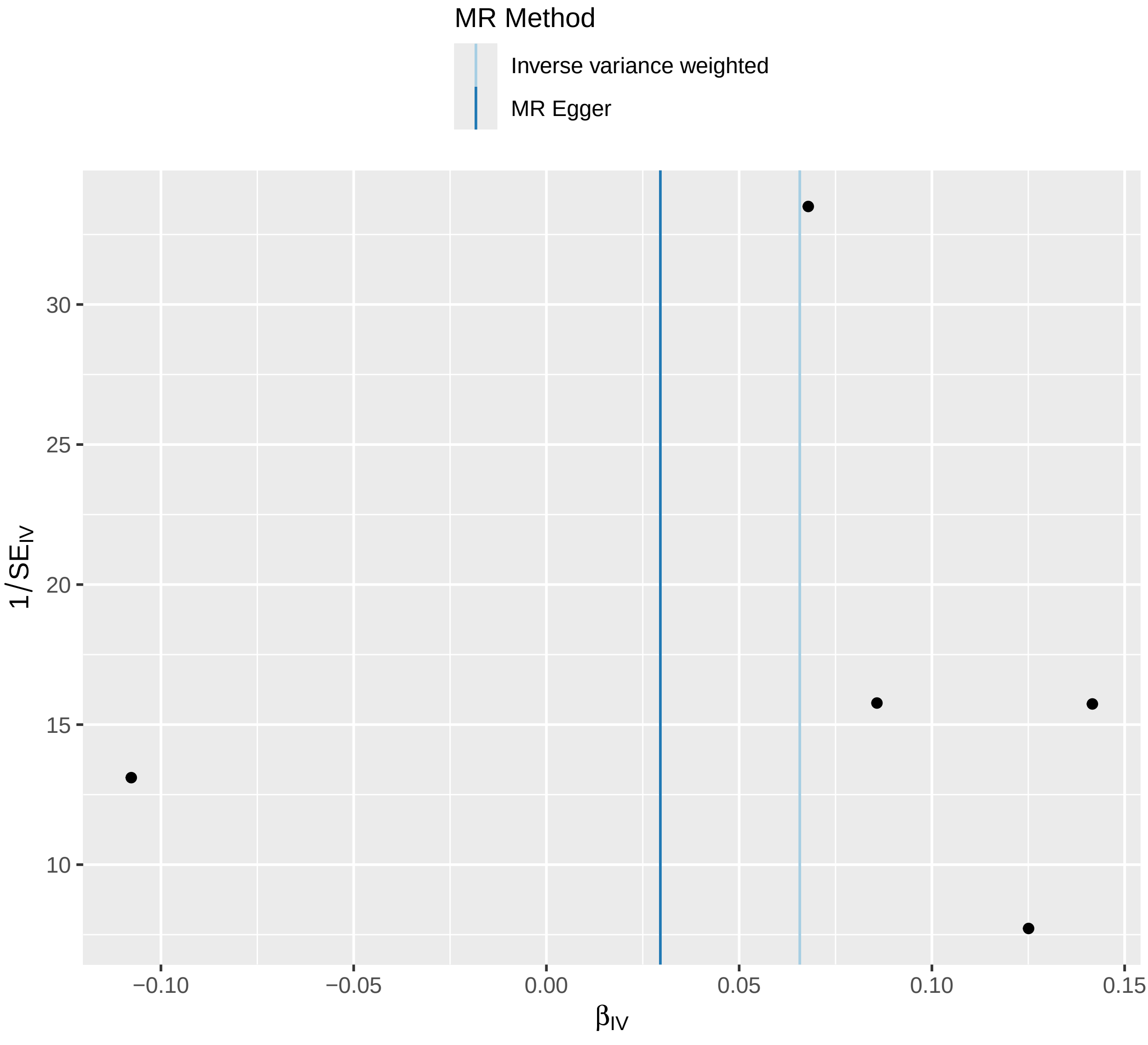

MR Method
Inverse variance weighted
MR Egger
30
25
20
15
10
$1/SE_{IV}$
−0.10
−0.05
0.00
0.05
0.10
0.15
β_{IV}

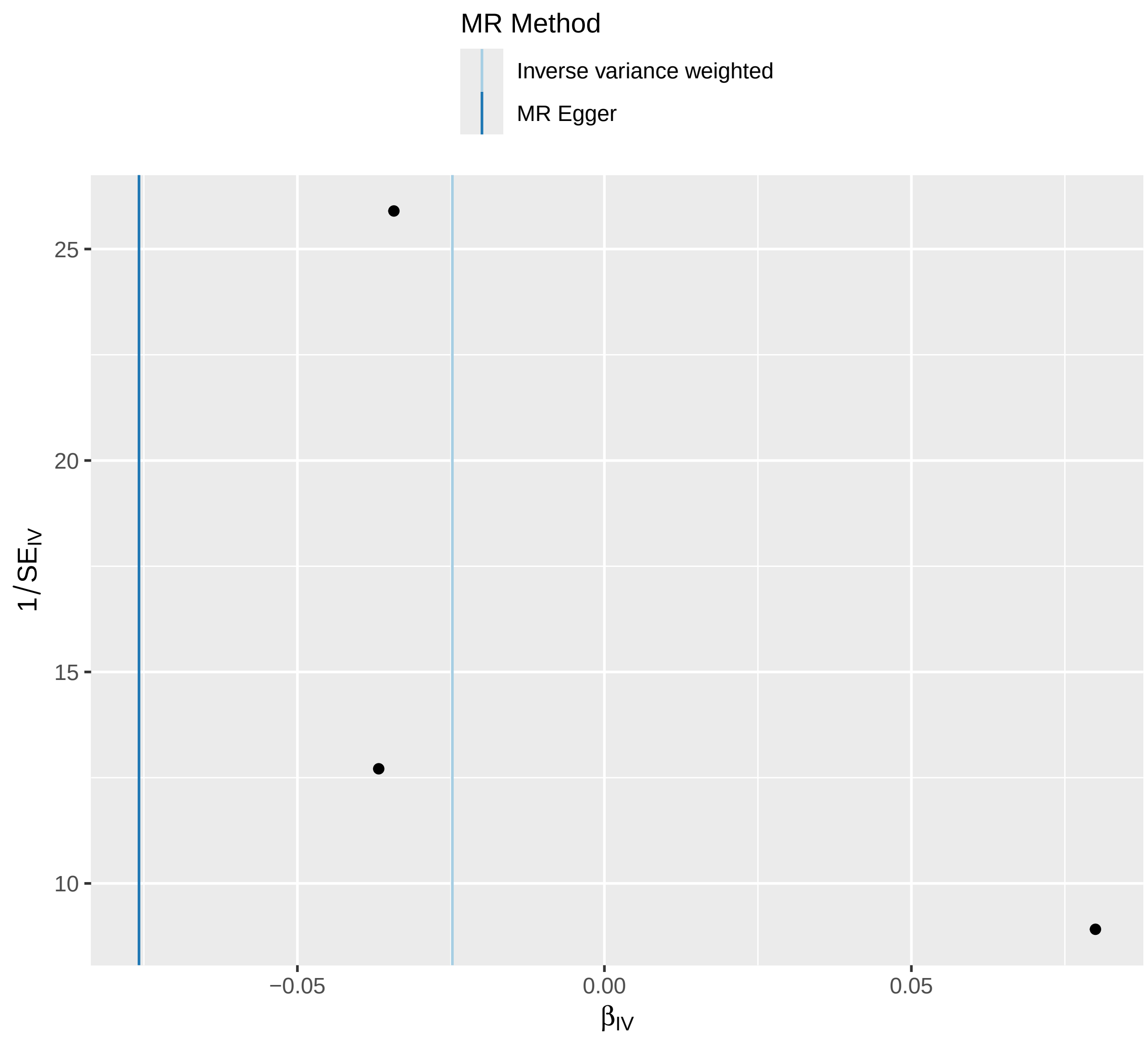

MR Method
Inverse variance weighted
MR Egger
25
20
15
10
−0.05
0.00
0.05
1/SE_IV
β_IV

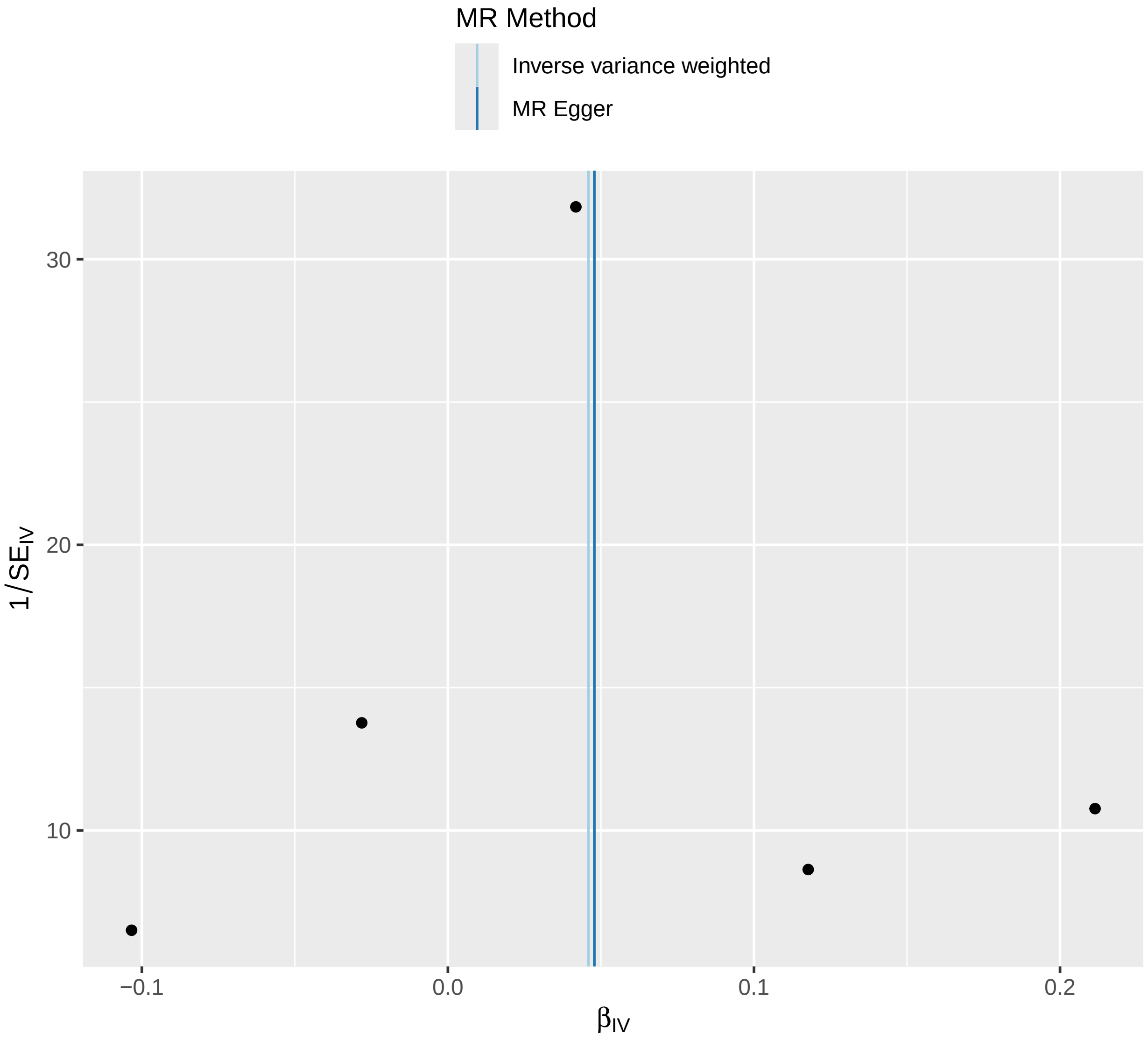
MR Method
Inverse variance weighted
MR Egger
30
20
10
1/SE$_{IV}$
−0.1
0.0
0.1
0.2
β$_{IV}$

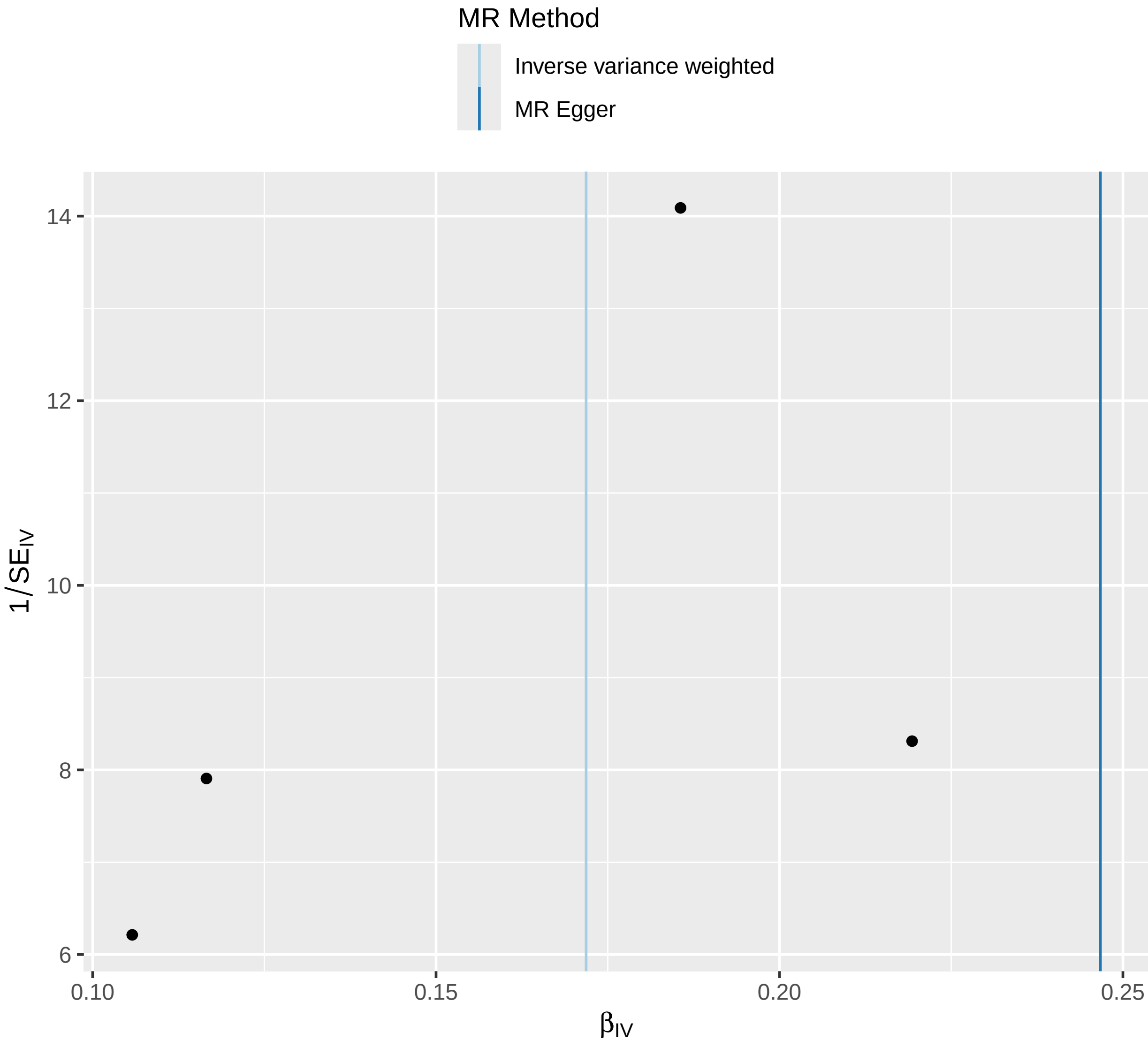

MR Method
Inverse variance weighted
MR Egger
14
12
10
8
6
1/SE_IV
0.10
0.15
0.20
0.25
β_IV

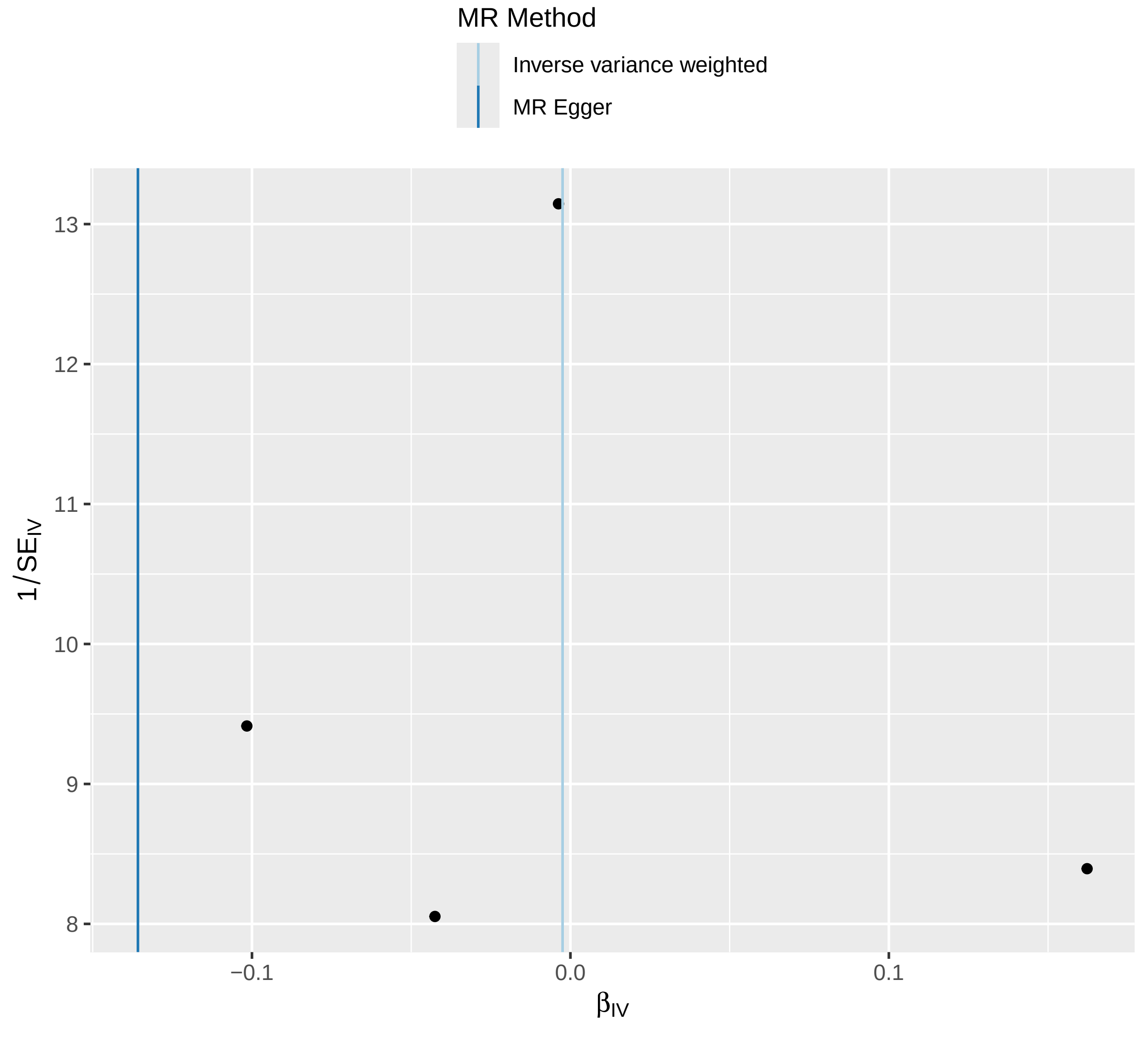
MR Method
Inverse variance weighted
MR Egger
13
12
11
10
9
8
1/SE_IV
−0.1
0.0
0.1
β_IV

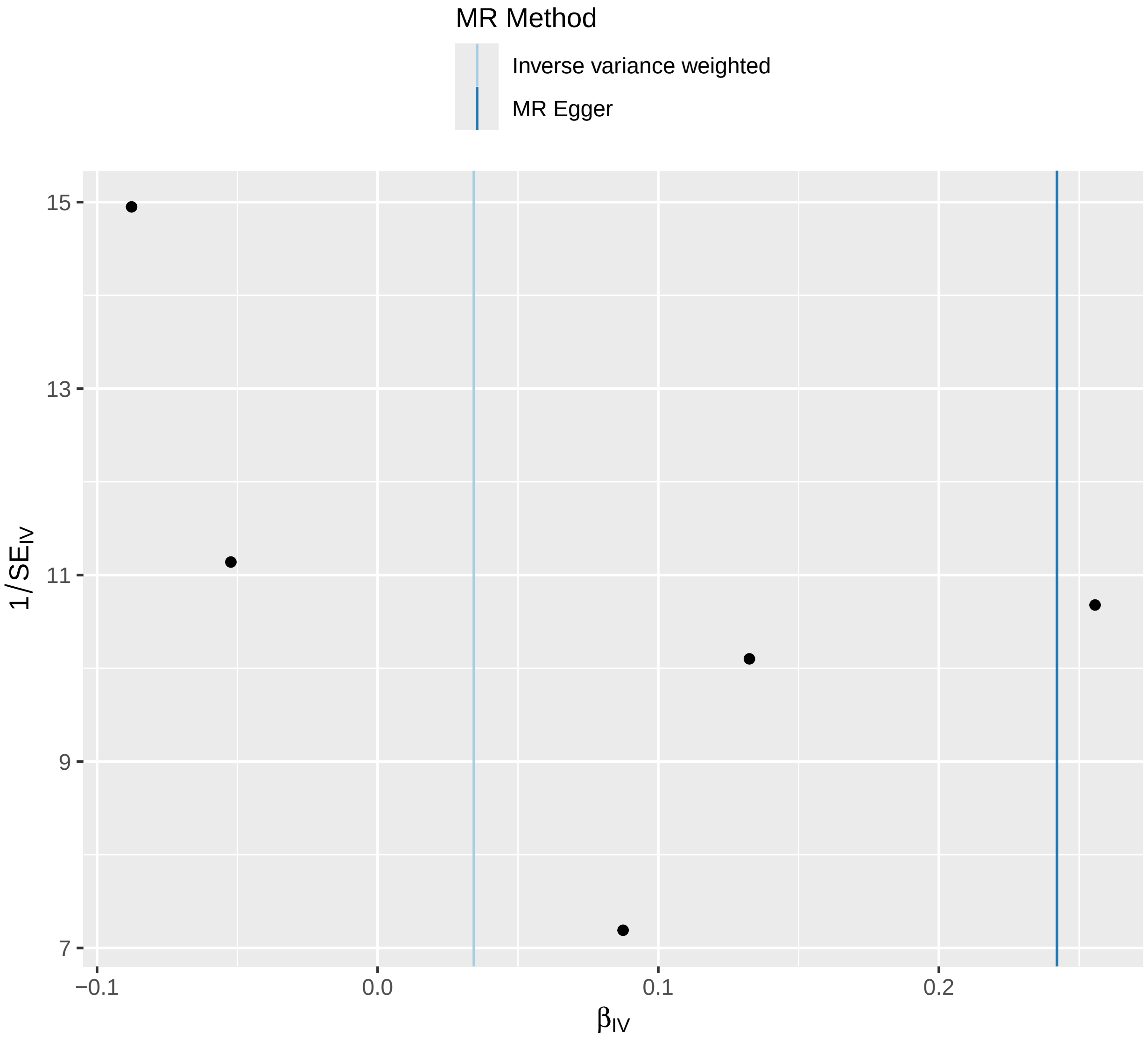

MR Method
Inverse variance weighted
MR Egger
15
13
11
9
7
1/SE_IV
−0.1
0.0
0.1
0.2
β_IV

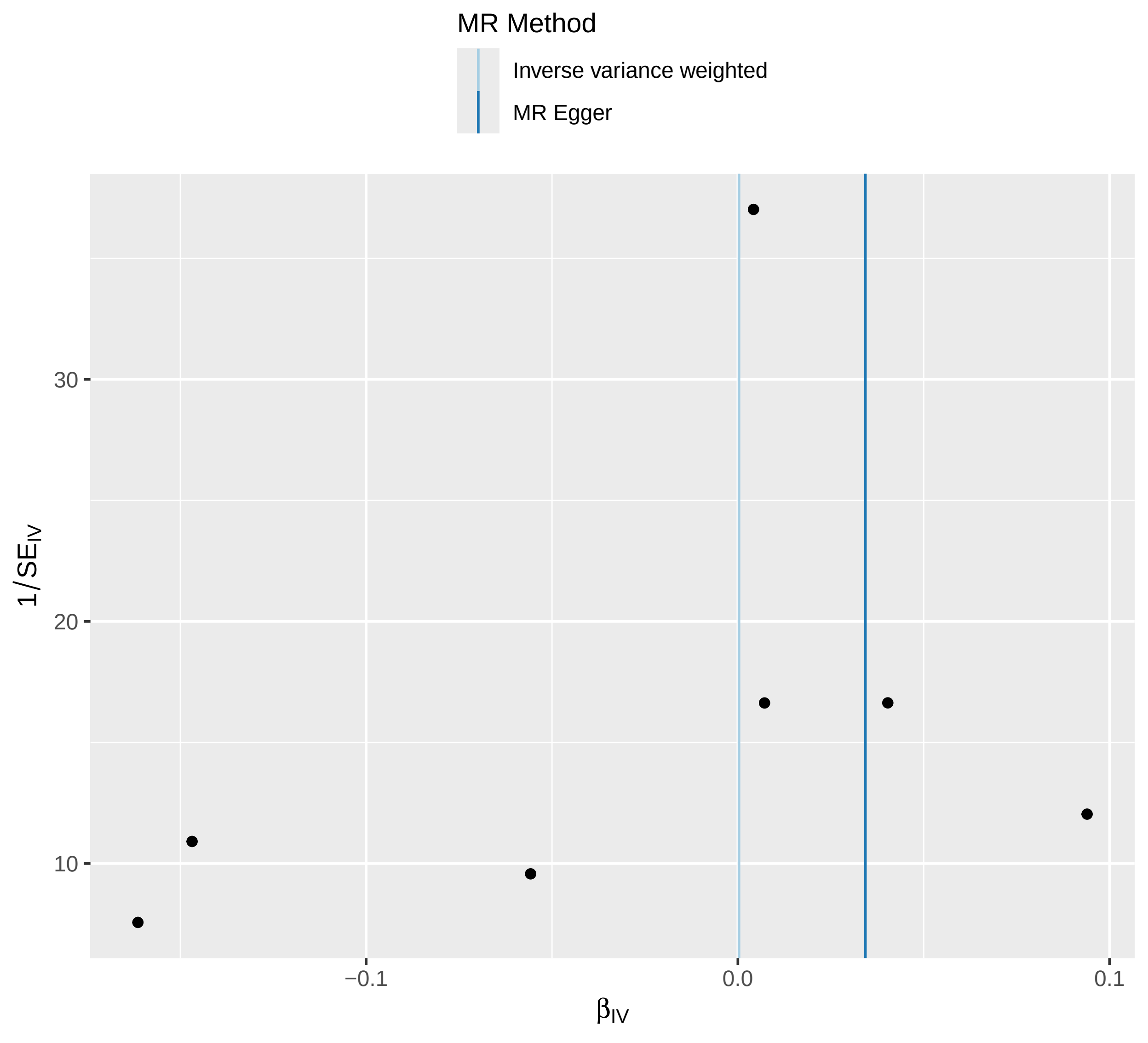

MR Method
Inverse variance weighted
MR Egger
30
20
10
1/SE_IV
−0.1
0.0
0.1
β_IV

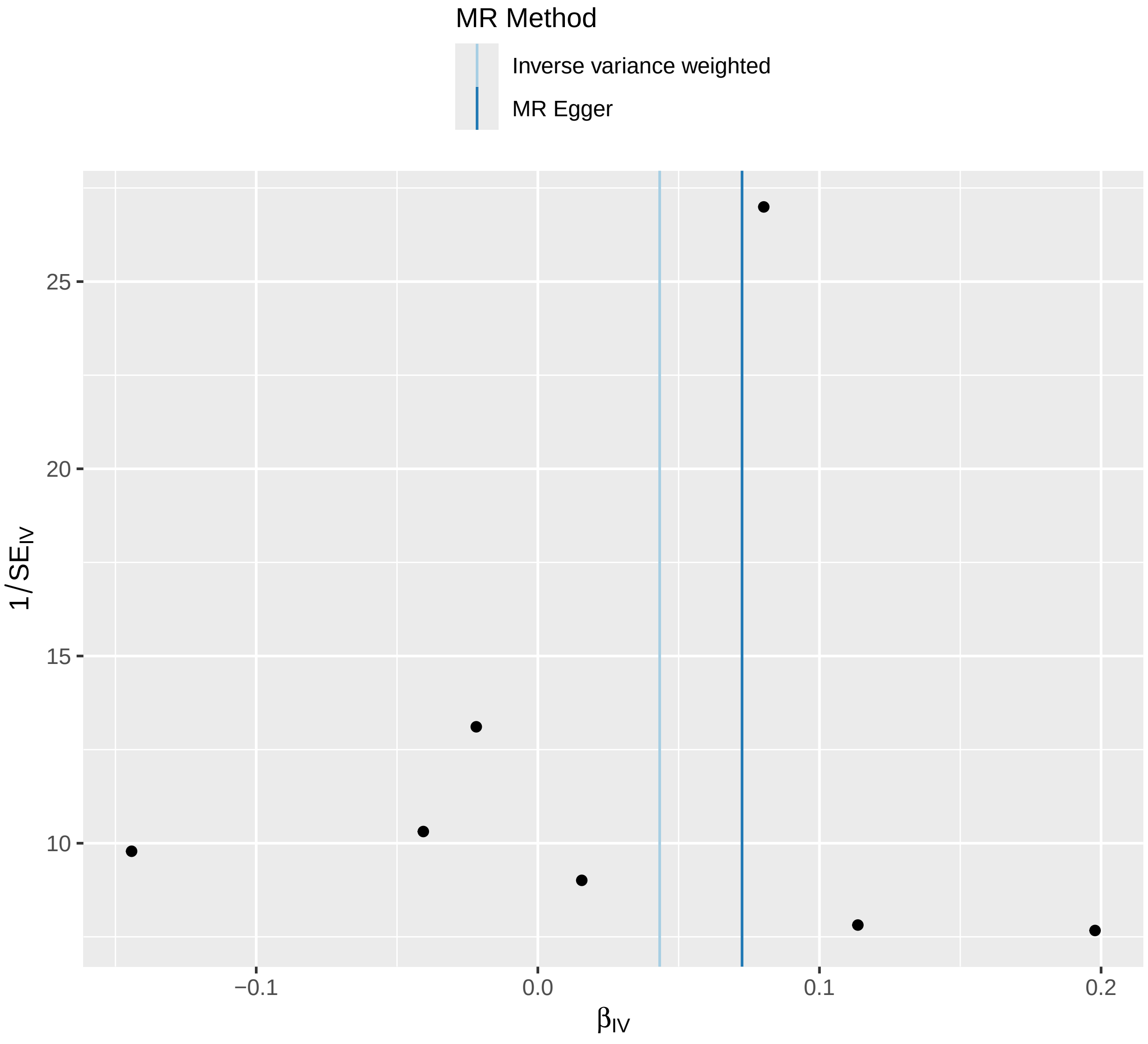
MR Method
Inverse variance weighted
MR Egger
25
20
15
10
1/SE_IV
−0.1
0.0
0.1
0.2
β_IV

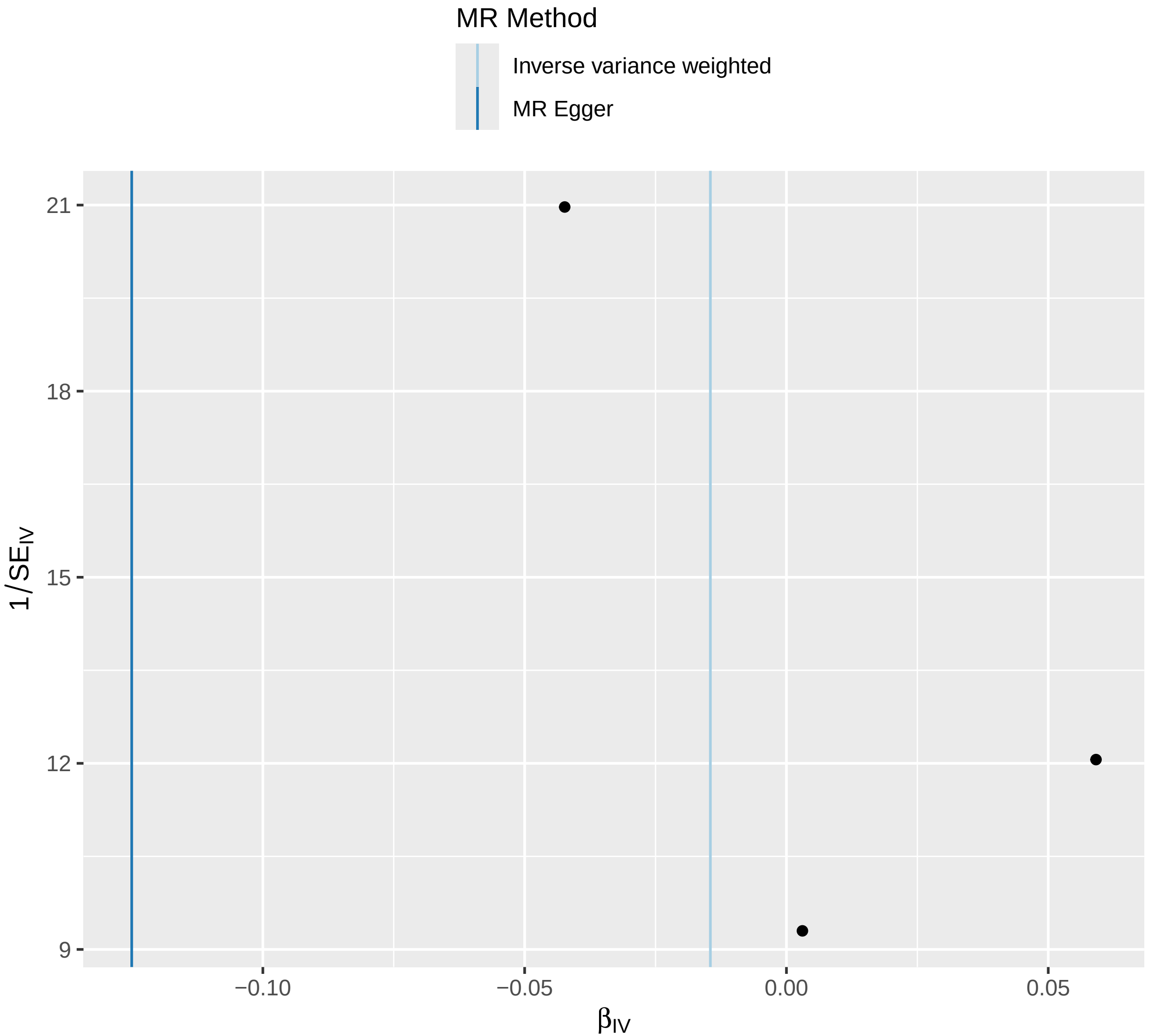

MR Method
Inverse variance weighted
MR Egger
21
18
15
12
9
1/SE_IV
−0.10
−0.05
0.00
0.05
β_IV

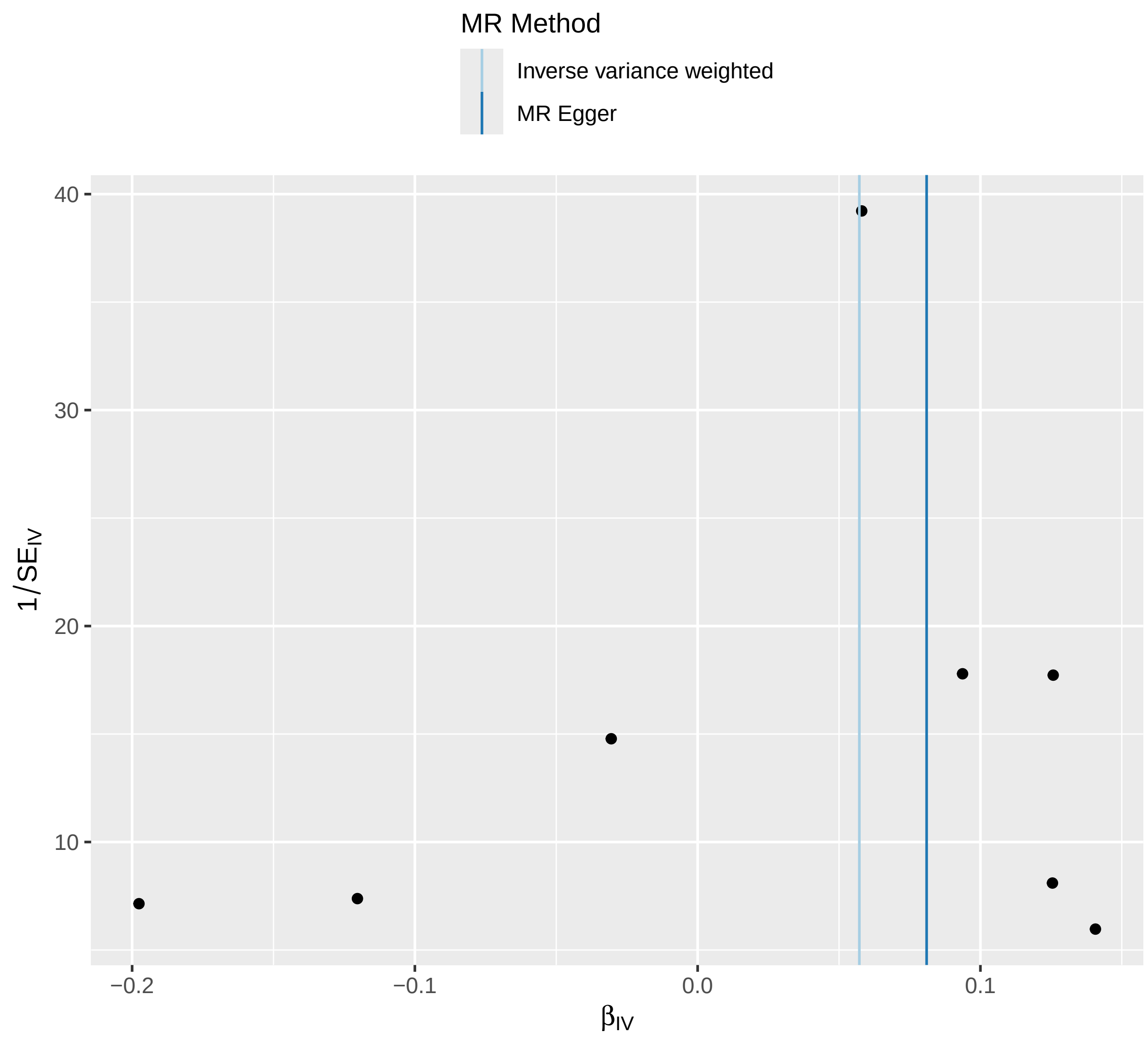

MR Method
Inverse variance weighted
MR Egger
40
30
20
10
1/SE_IV
−0.2
−0.1
0.0
0.1
β_IV

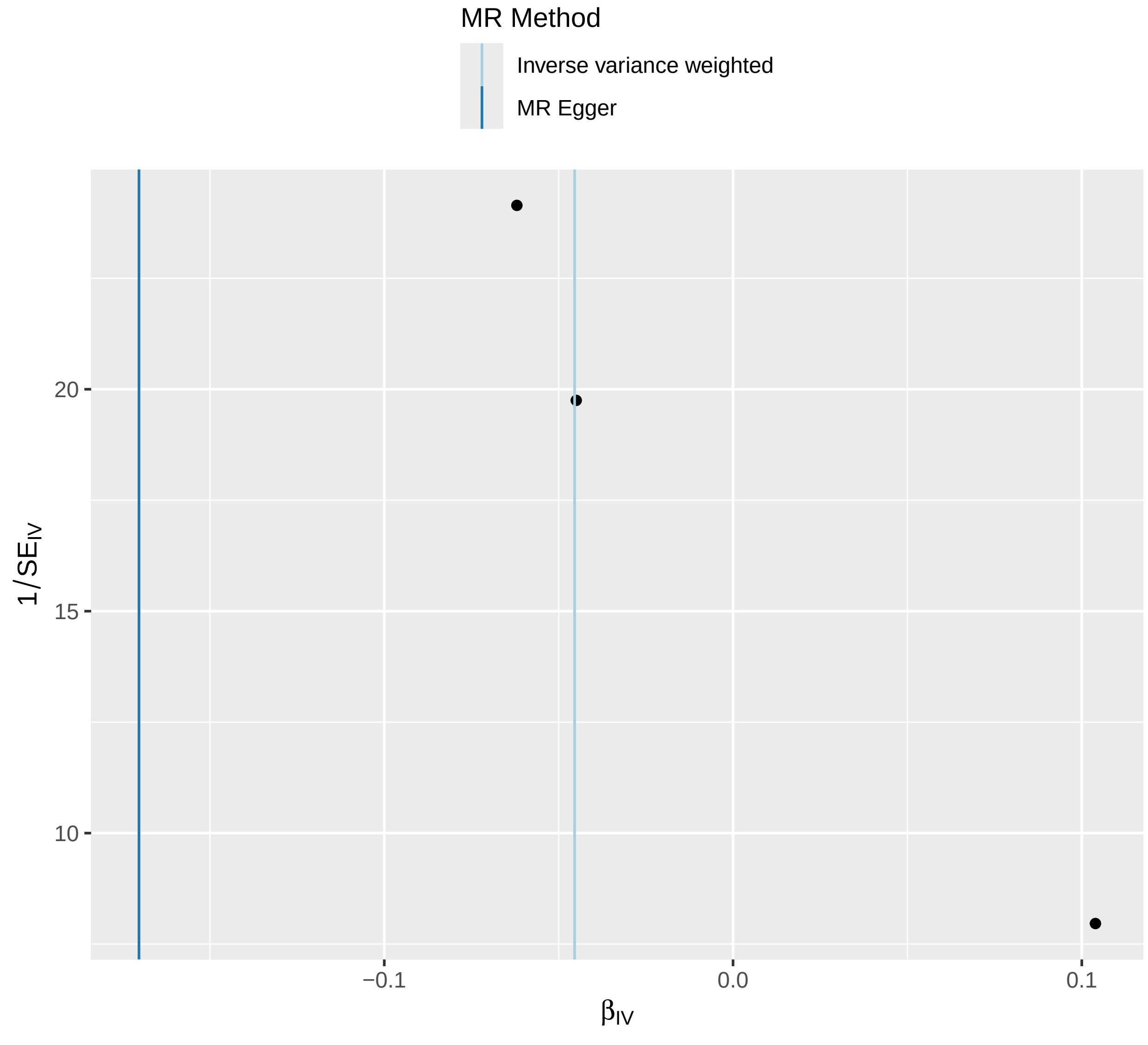

MR Method
Inverse variance weighted
MR Egger
20
15
10
$1/SE_{IV}$
−0.1
0.0
0.1
β_{IV}

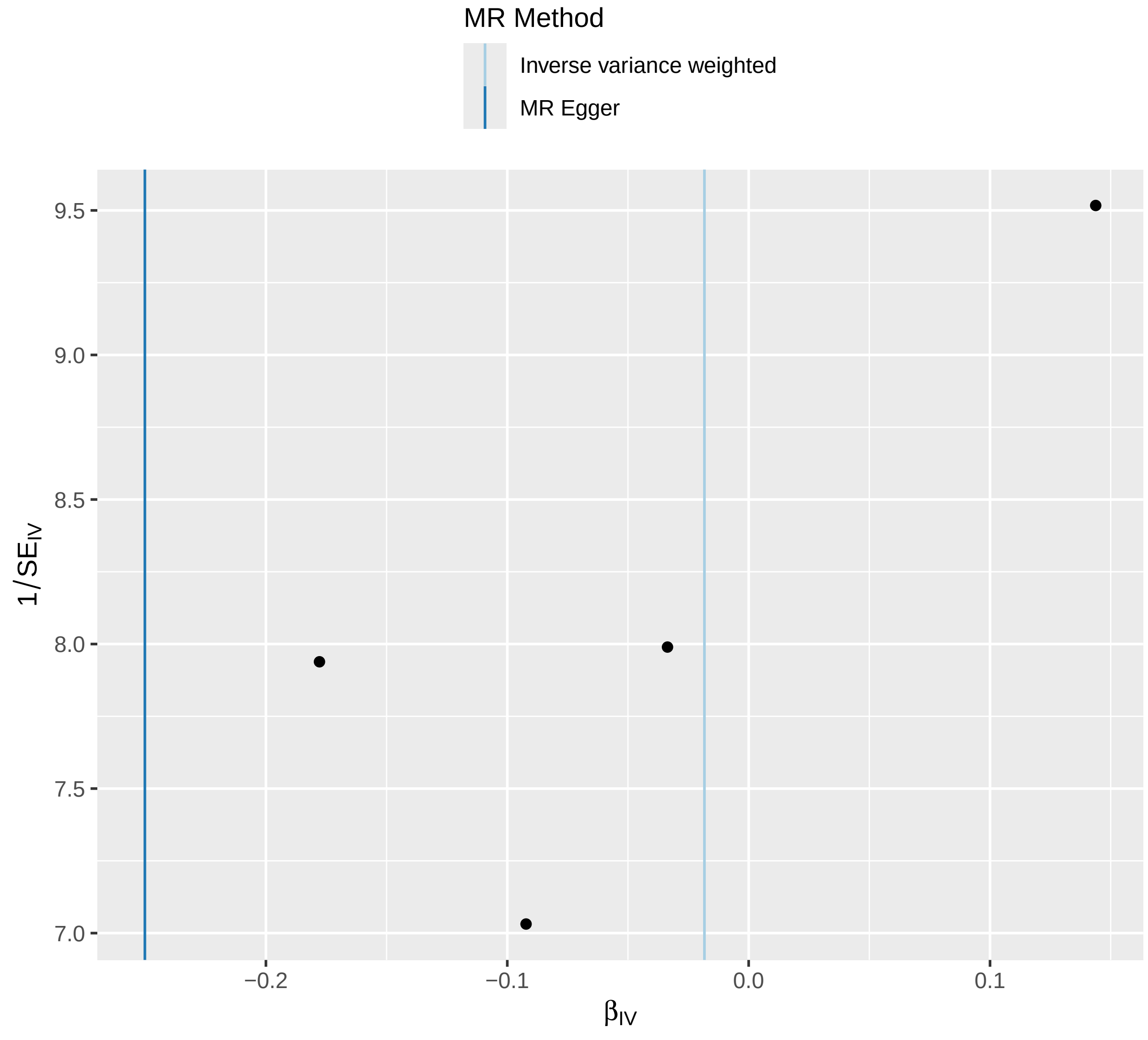
MR Method
Inverse variance weighted
MR Egger
9.5
9.0
8.5
8.0
7.5
7.0
1/SE_IV
−0.2
−0.1
0.0
0.1
β_IV

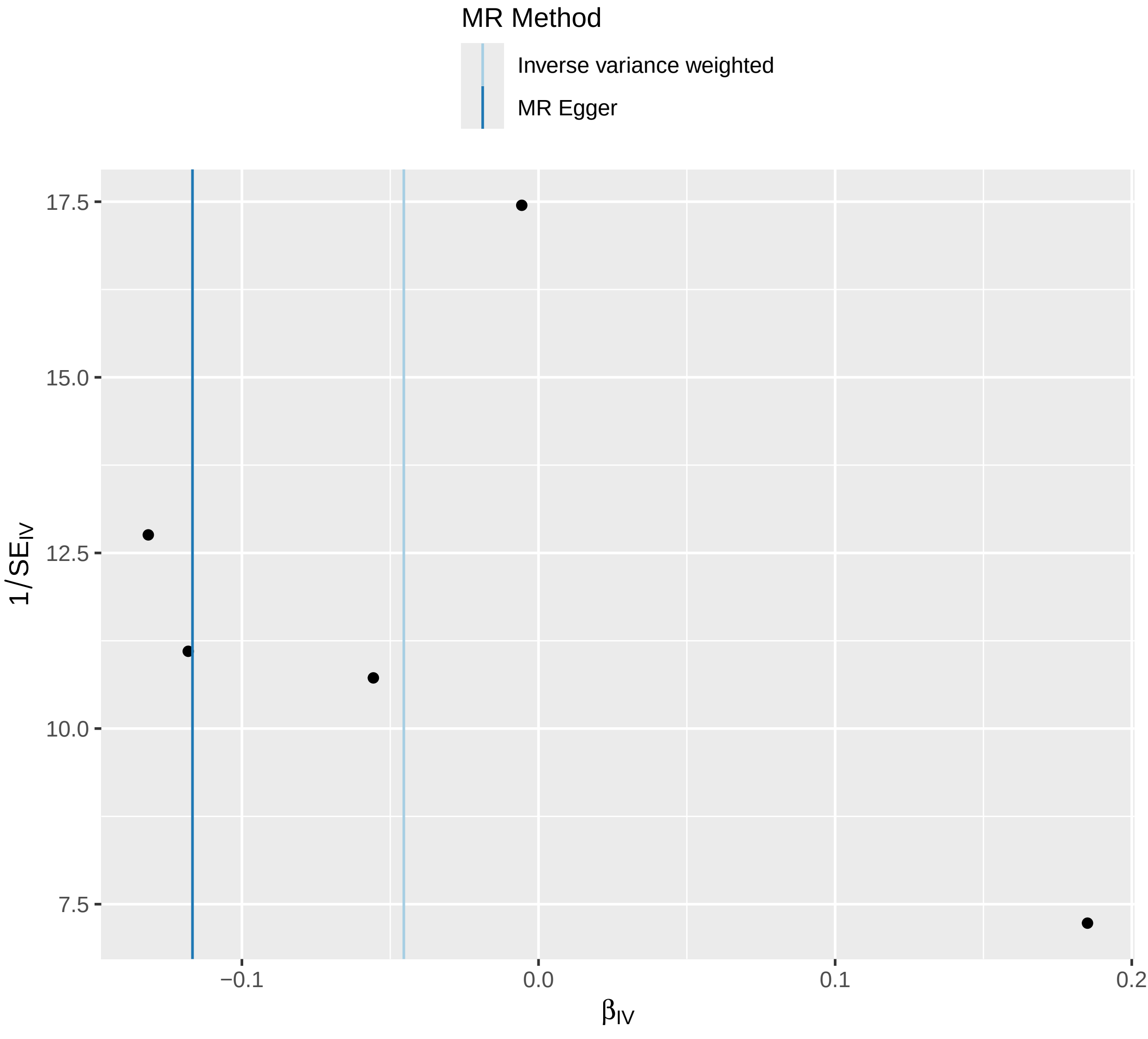

MR Method
Inverse variance weighted
MR Egger
17.5
15.0
12.5
10.0
7.5
1/SE_IV
−0.1
0.0
0.1
0.2
β_IV

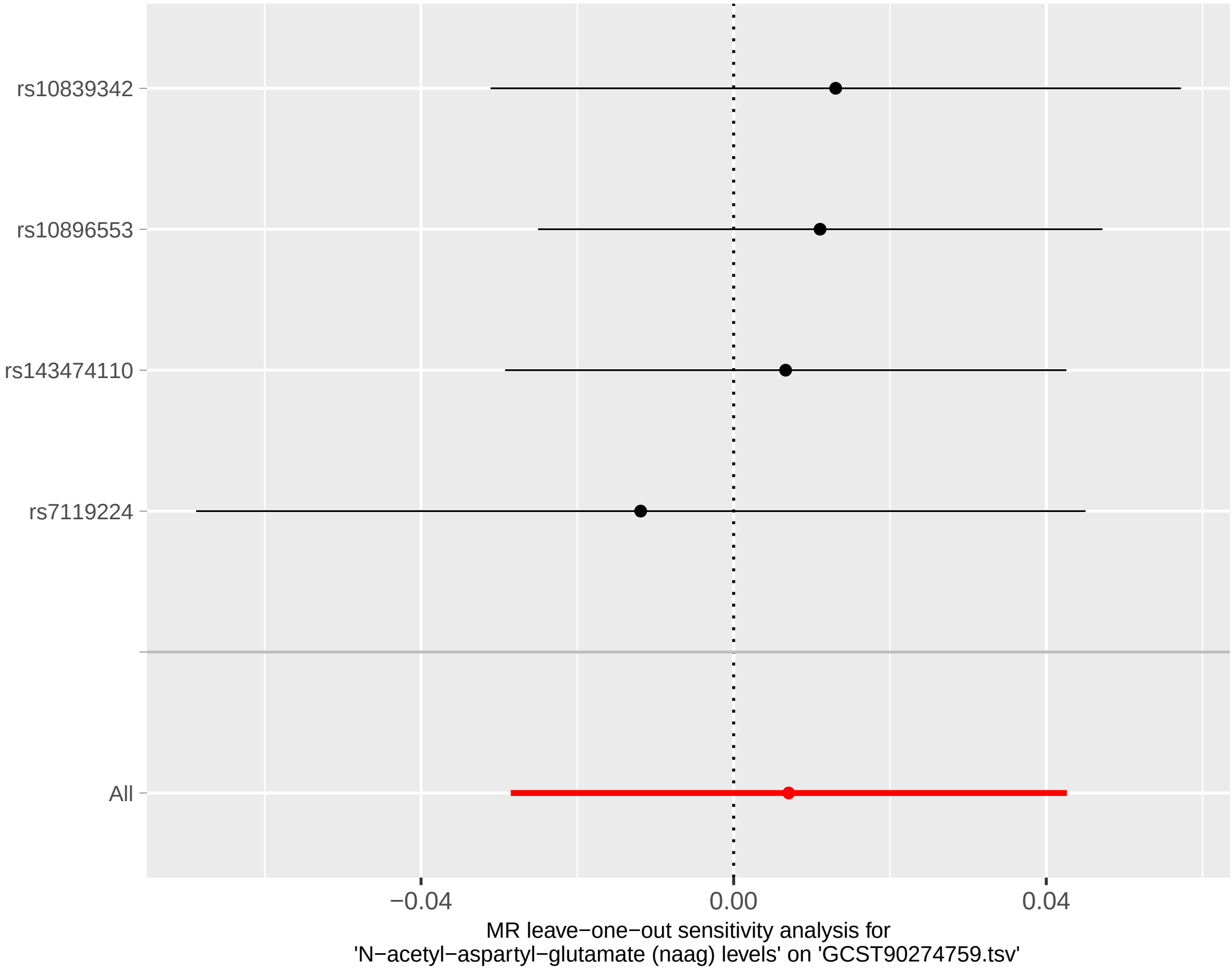
rs10839342
rs10896553
rs143474110
rs7119224
All
−0.04
0.00
0.04
MR leave−one−out sensitivity analysis for
'N−acetyl−aspartyl−glutamate (naag) levels' on 'GCST90274759.tsv'

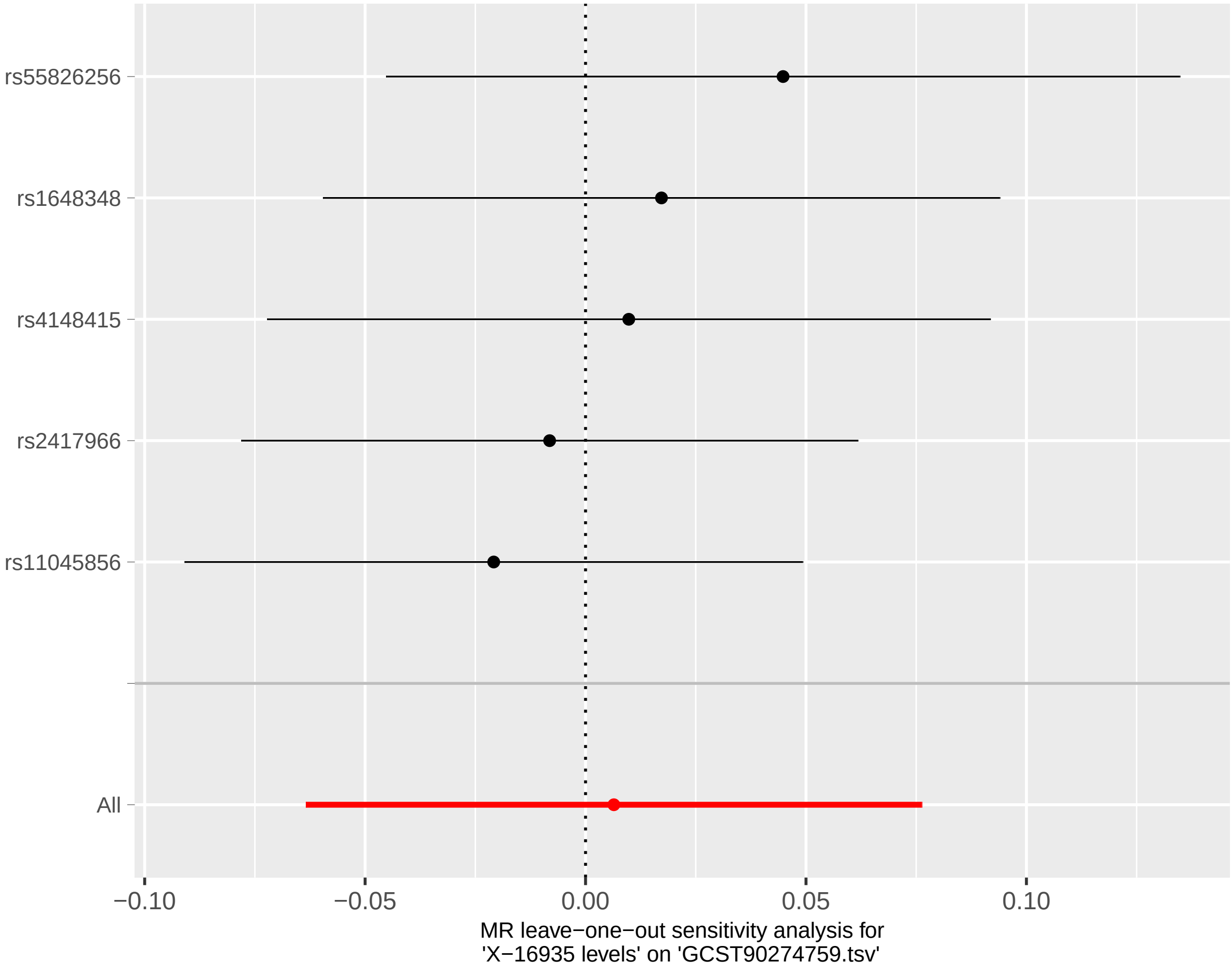
rs55826256
rs1648348
rs4148415
rs2417966
rs11045856
All
−0.10
−0.05
0.00
0.05
0.10
MR leave−one−out sensitivity analysis for
'X−16935 levels' on 'GCST90274759.tsv'

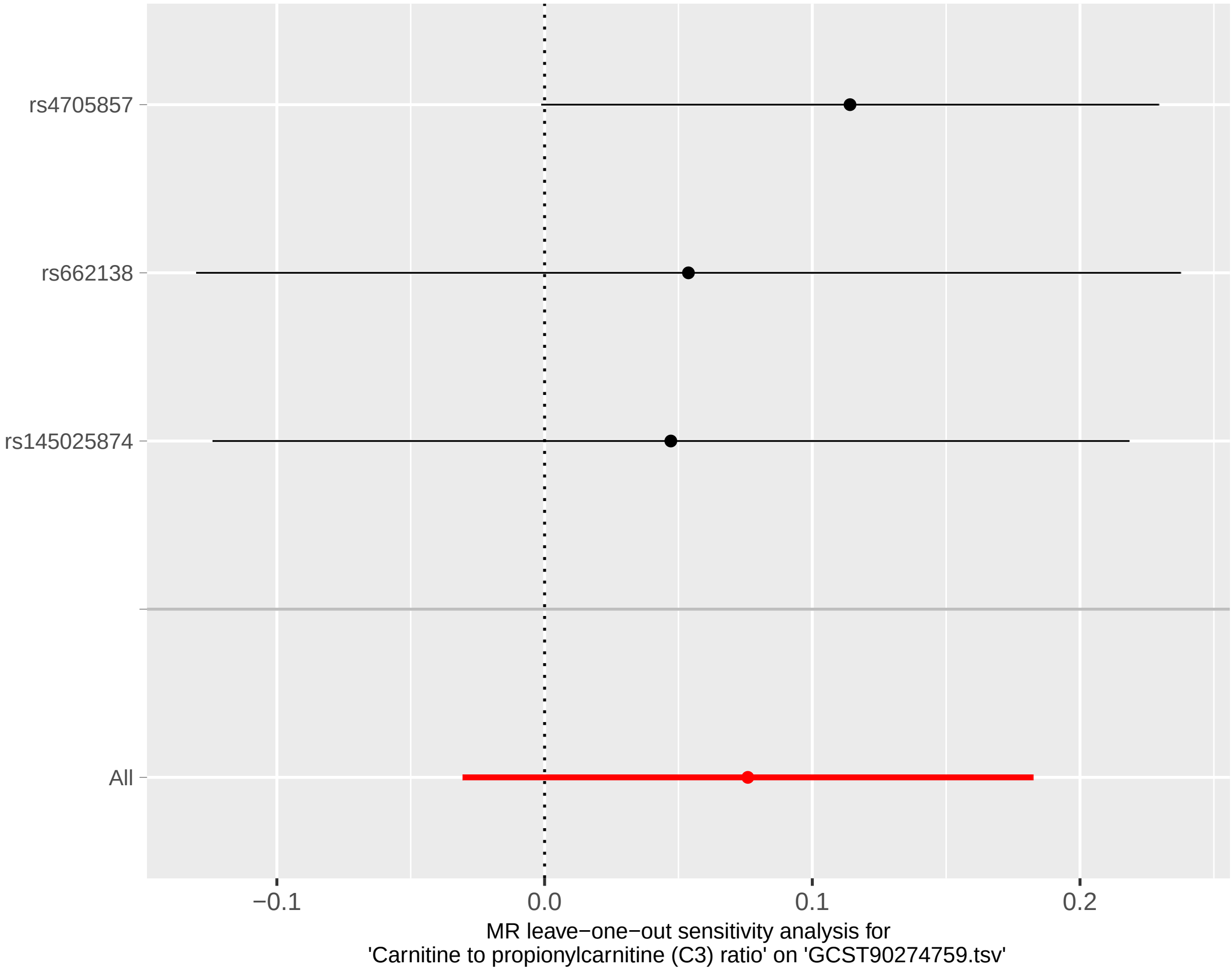
rs4705857
rs662138
rs145025874
All
−0.1
0.0
0.1
0.2
MR leave−one−out sensitivity analysis for
'Carnitine to propionylcarnitine (C3) ratio' on 'GCST90274759.tsv'

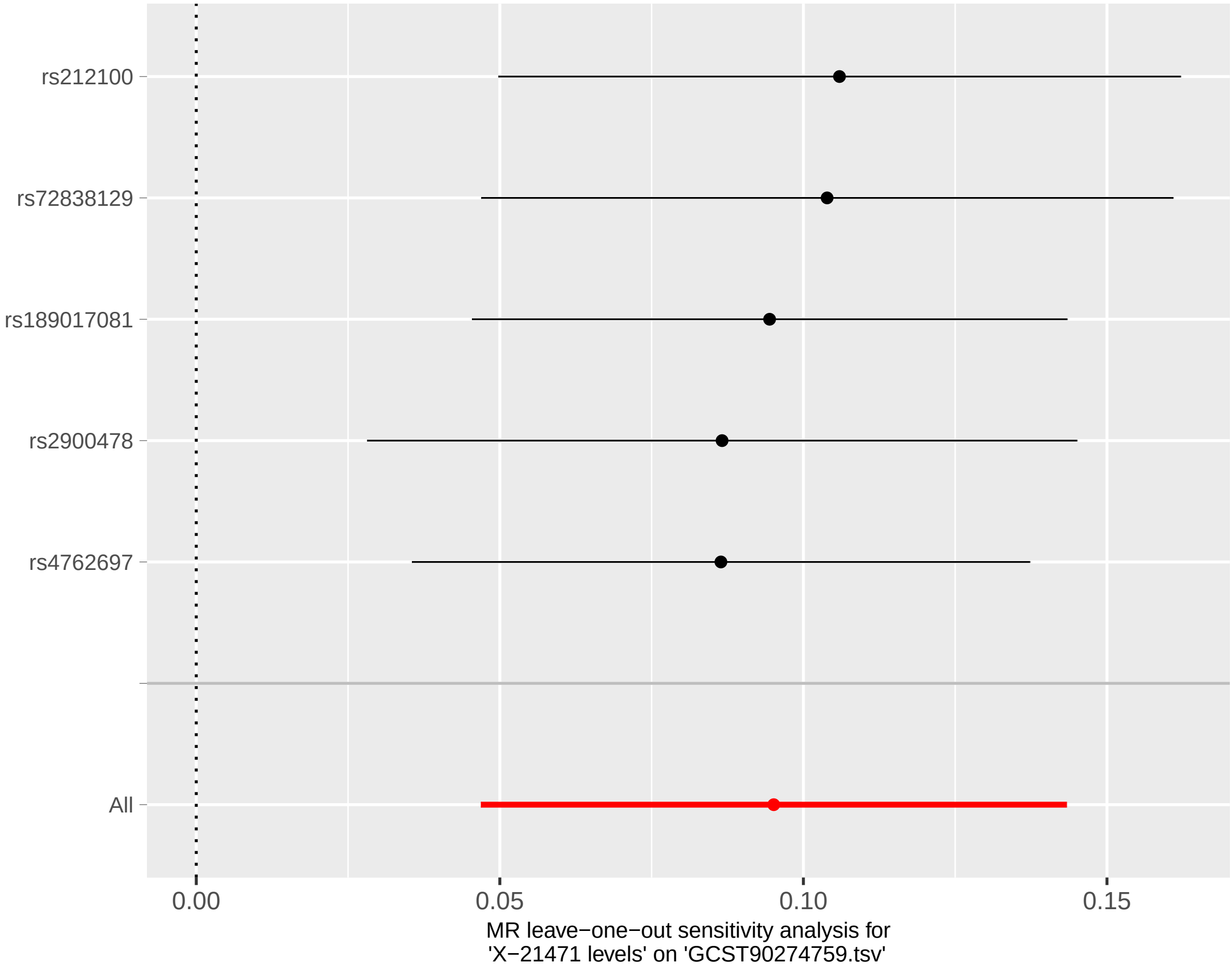
rs212100
rs72838129
rs189017081
rs2900478
rs4762697
All
0.00
0.05
0.10
0.15
MR leave−one−out sensitivity analysis for
'X−21471 levels' on 'GCST90274759.tsv'

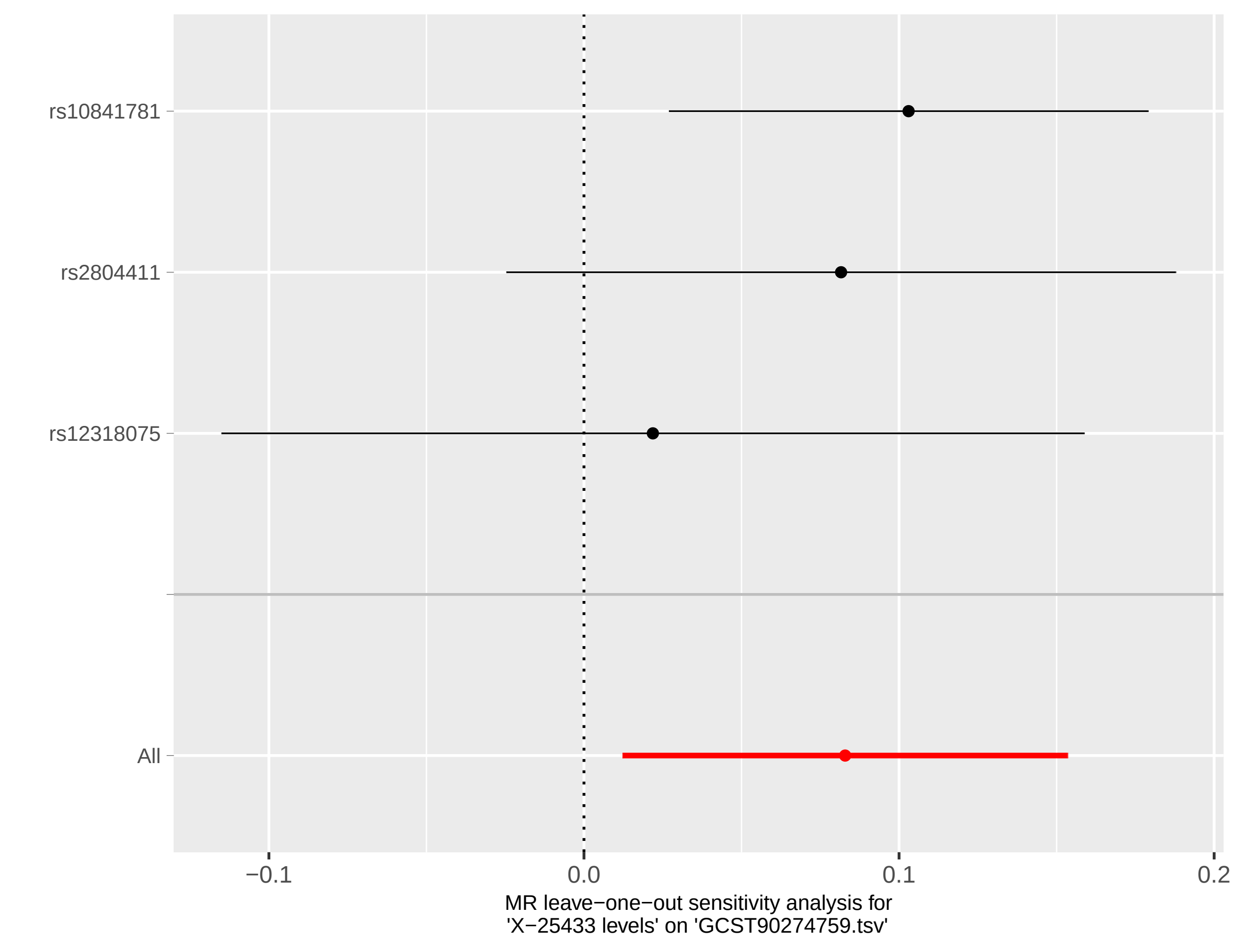
rs10841781
rs2804411
rs12318075
All
−0.1
0.0
0.1
0.2
MR leave−one−out sensitivity analysis for
'X−25433 levels' on 'GCST90274759.tsv'

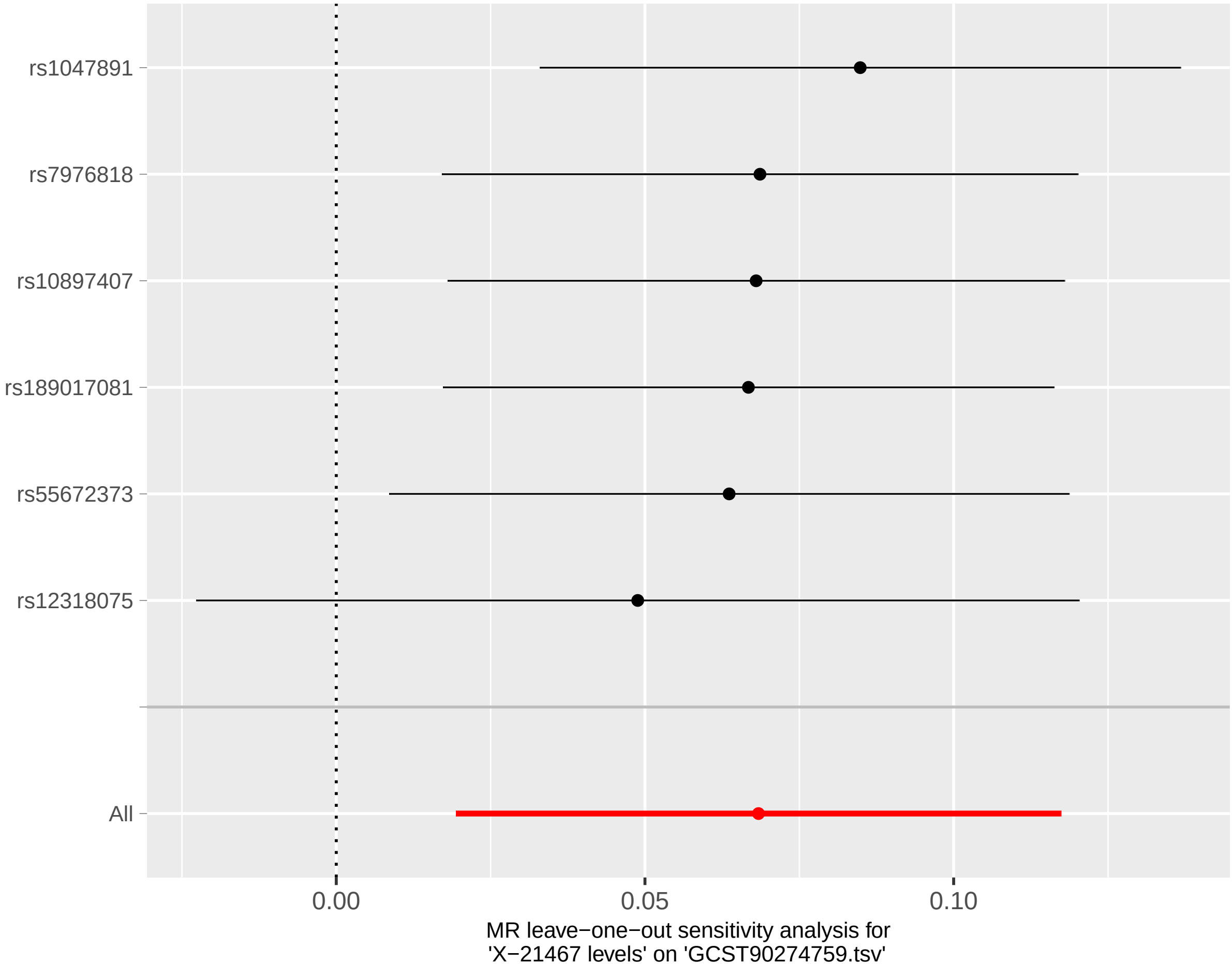
rs1047891
rs7976818
rs10897407
rs189017081
rs55672373
rs12318075
All
0.00
0.05
0.10
MR leave−one−out sensitivity analysis for
'X−21467 levels' on 'GCST90274759.tsv'

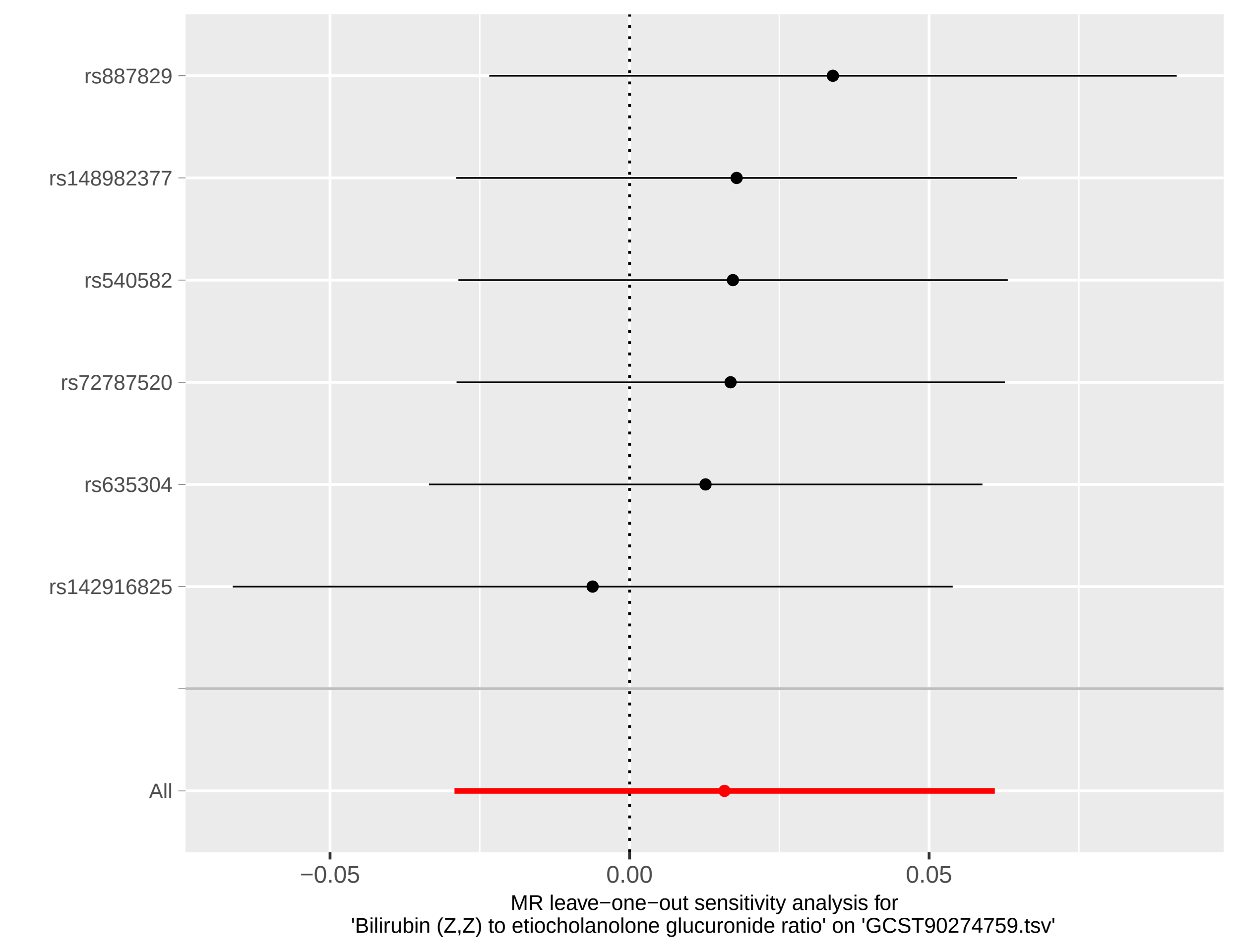
rs887829
rs148982377
rs540582
rs72787520
rs635304
rs142916825
All
−0.05
0.00
0.05
MR leave−one−out sensitivity analysis for
'Bilirubin (Z,Z) to etiocholanolone glucuronide ratio' on 'GCST90274759.tsv'

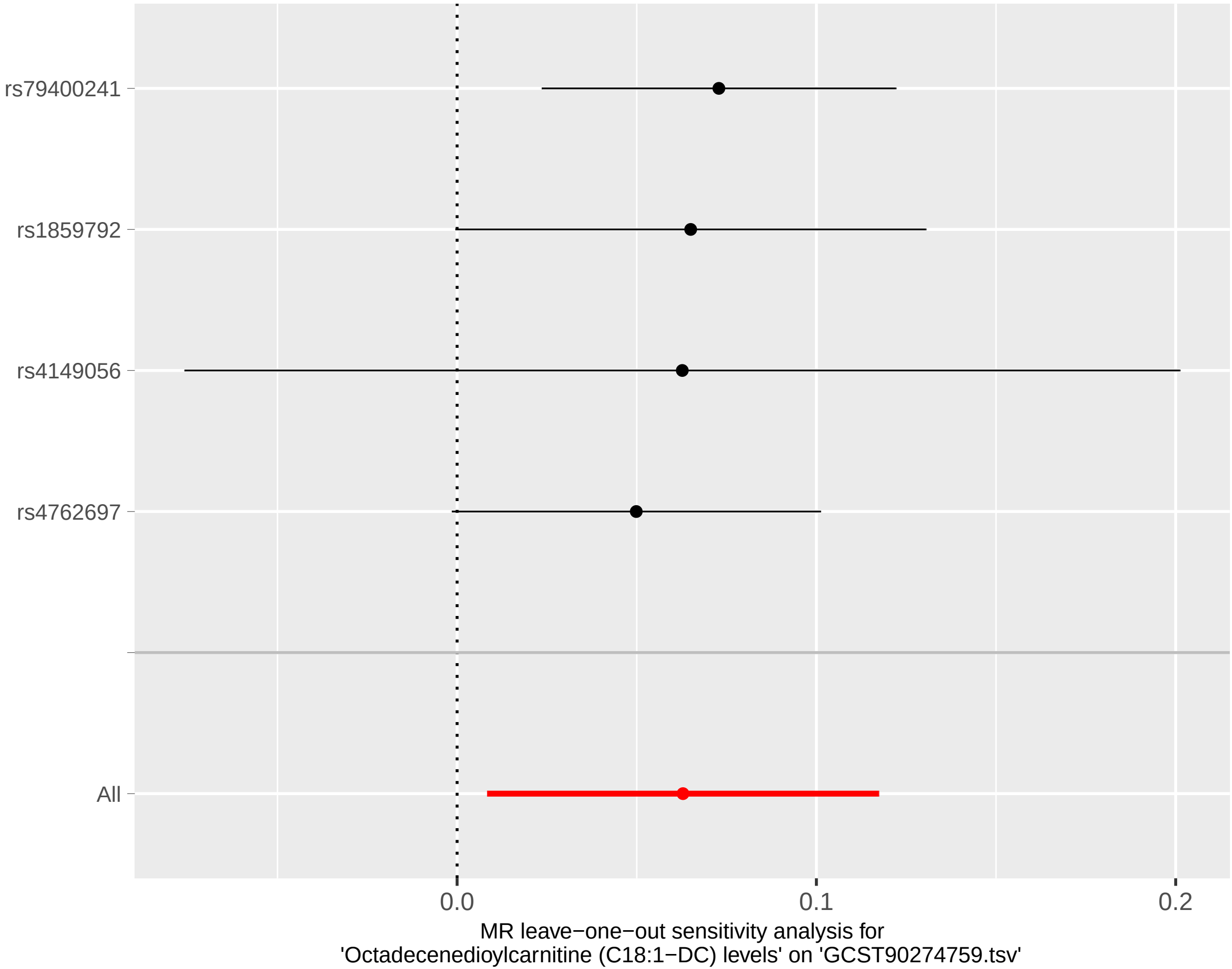
rs79400241
rs1859792
rs4149056
rs4762697
All
0.0
0.1
0.2
MR leave−one−out sensitivity analysis for
'Octadecenedioylcarnitine (C18:1−DC) levels' on 'GCST90274759.tsv'

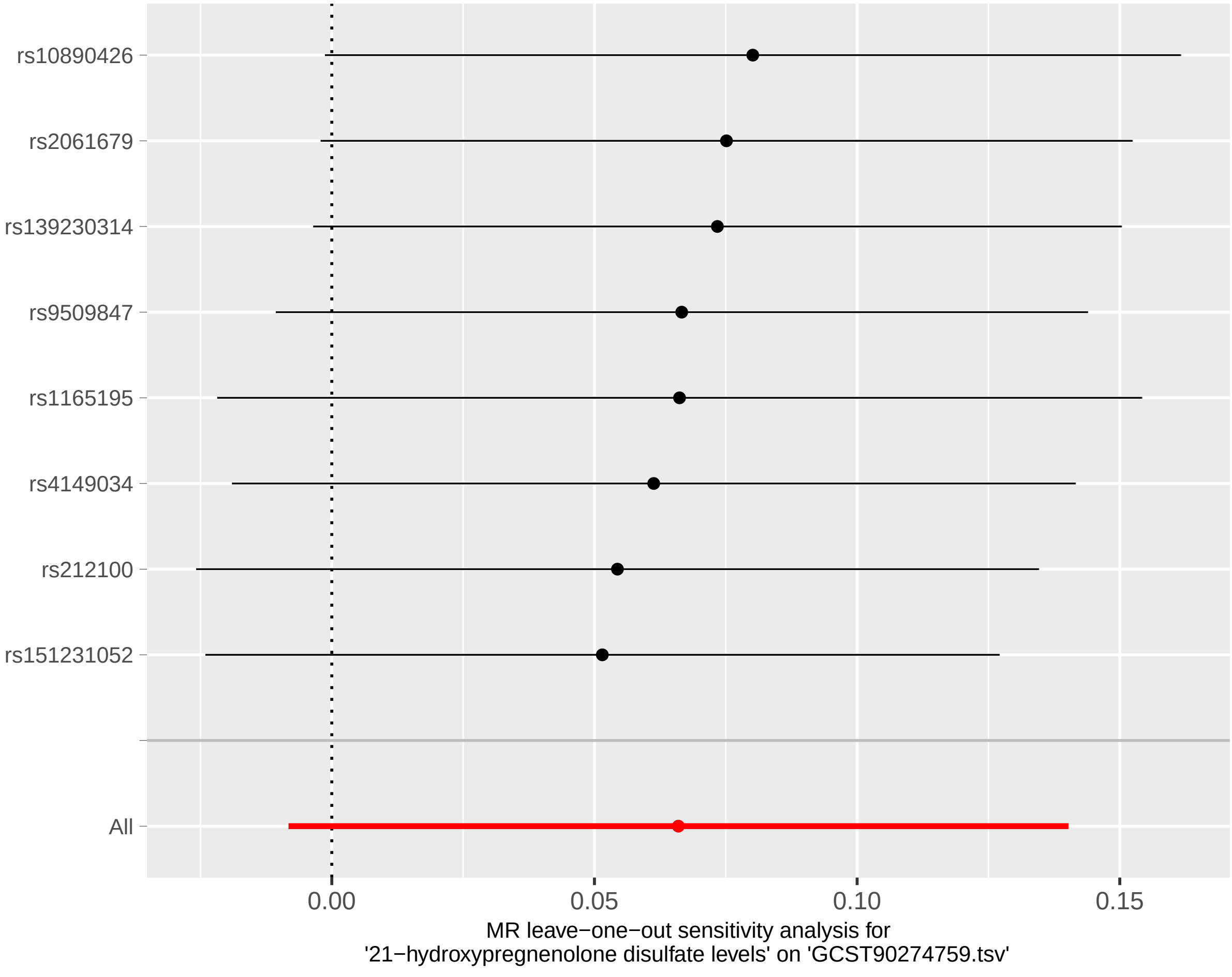

rs10890426
rs2061679
rs139230314
rs9509847
rs1165195
rs4149034
rs212100
rs151231052
All
0.00
0.05
0.10
0.15
MR leave−one−out sensitivity analysis for
'21−hydroxypregnenolone disulfate levels' on 'GCST90274759.tsv'

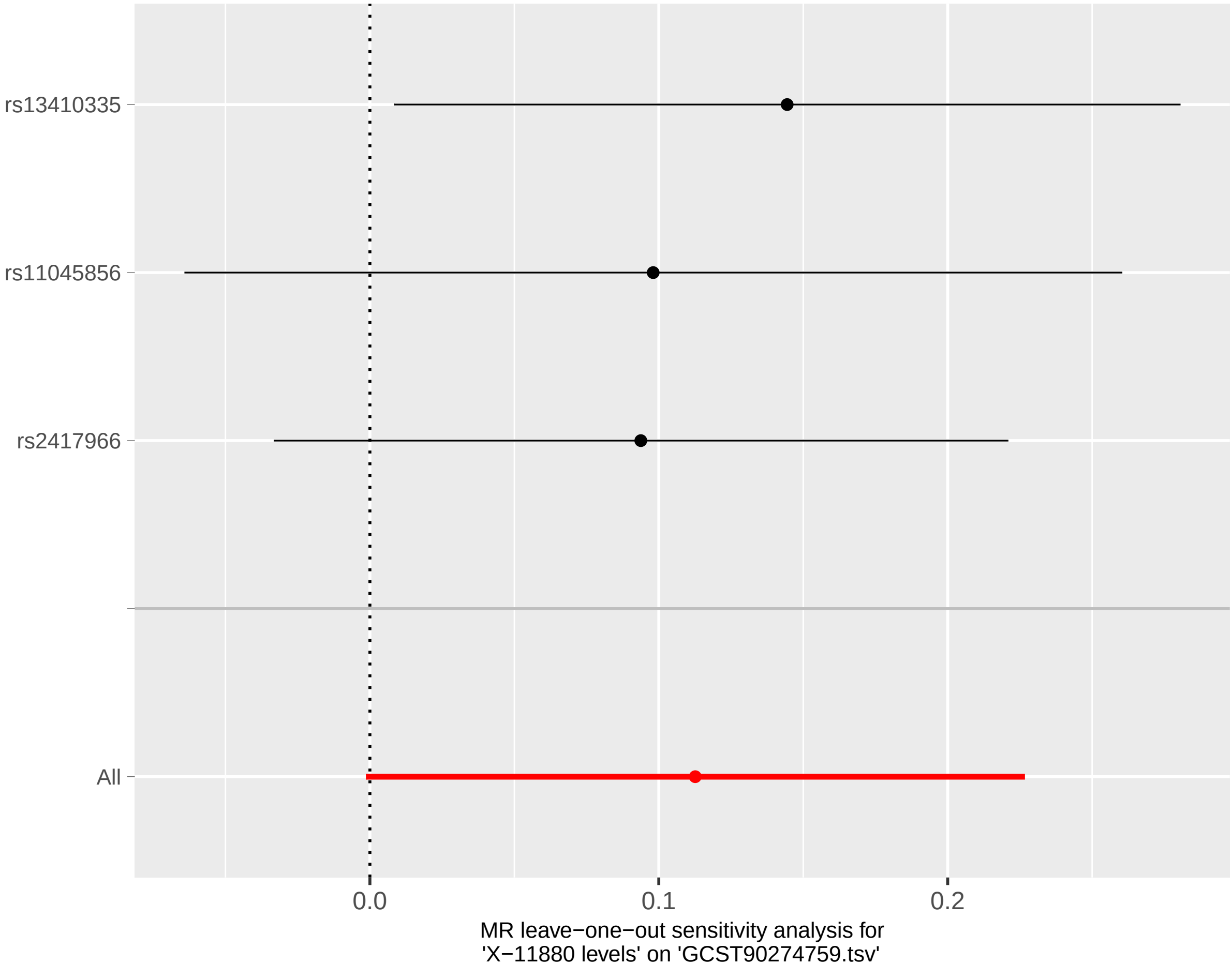
rs13410335
rs11045856
rs2417966
All
0.0
0.1
0.2
MR leave−one−out sensitivity analysis for
'X−11880 levels' on 'GCST90274759.tsv'

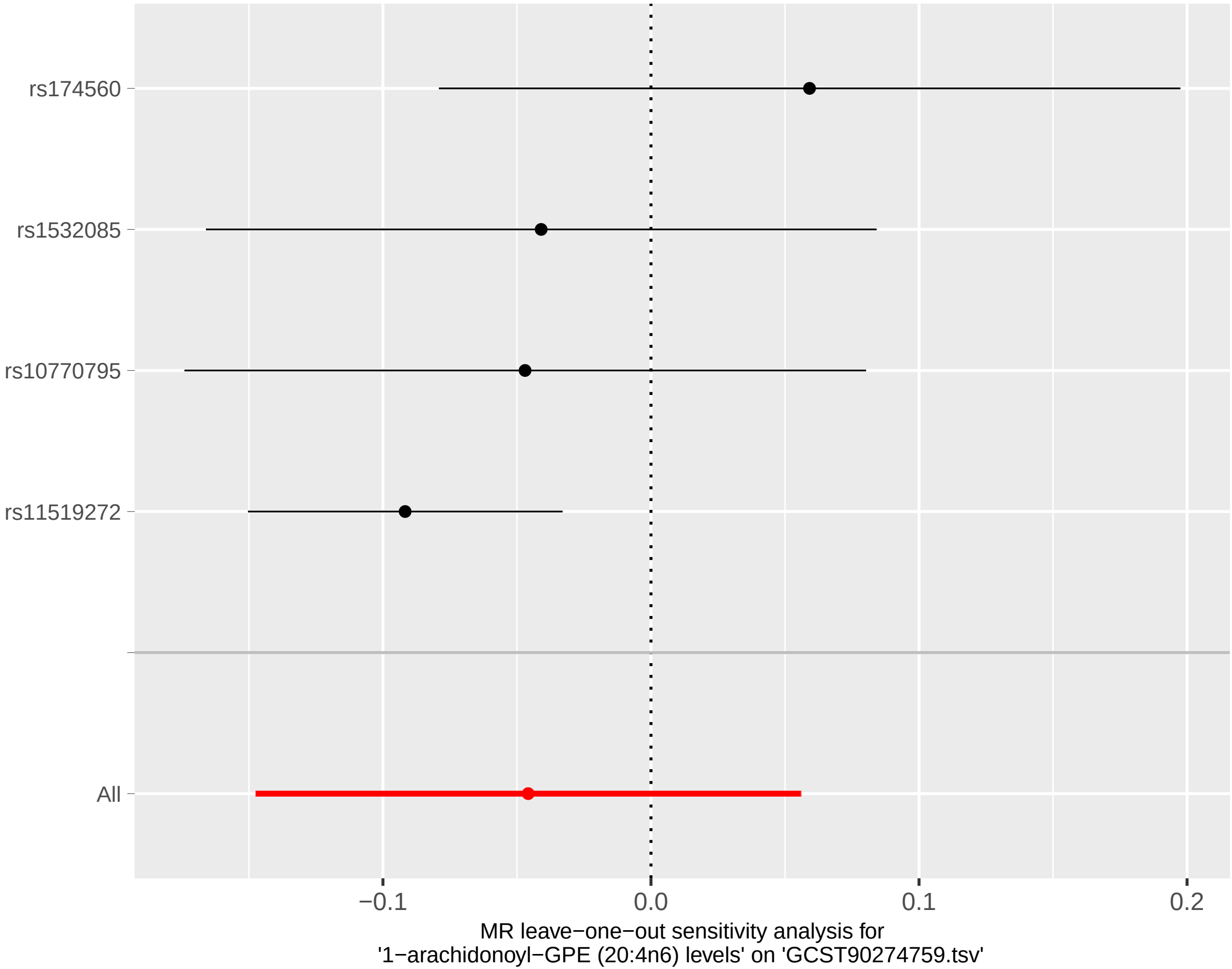
rs174560
rs1532085
rs10770795
rs11519272
All
−0.1
0.0
0.1
0.2
MR leave−one−out sensitivity analysis for
'1−arachidonoyl−GPE (20:4n6) levels' on 'GCST90274759.tsv'

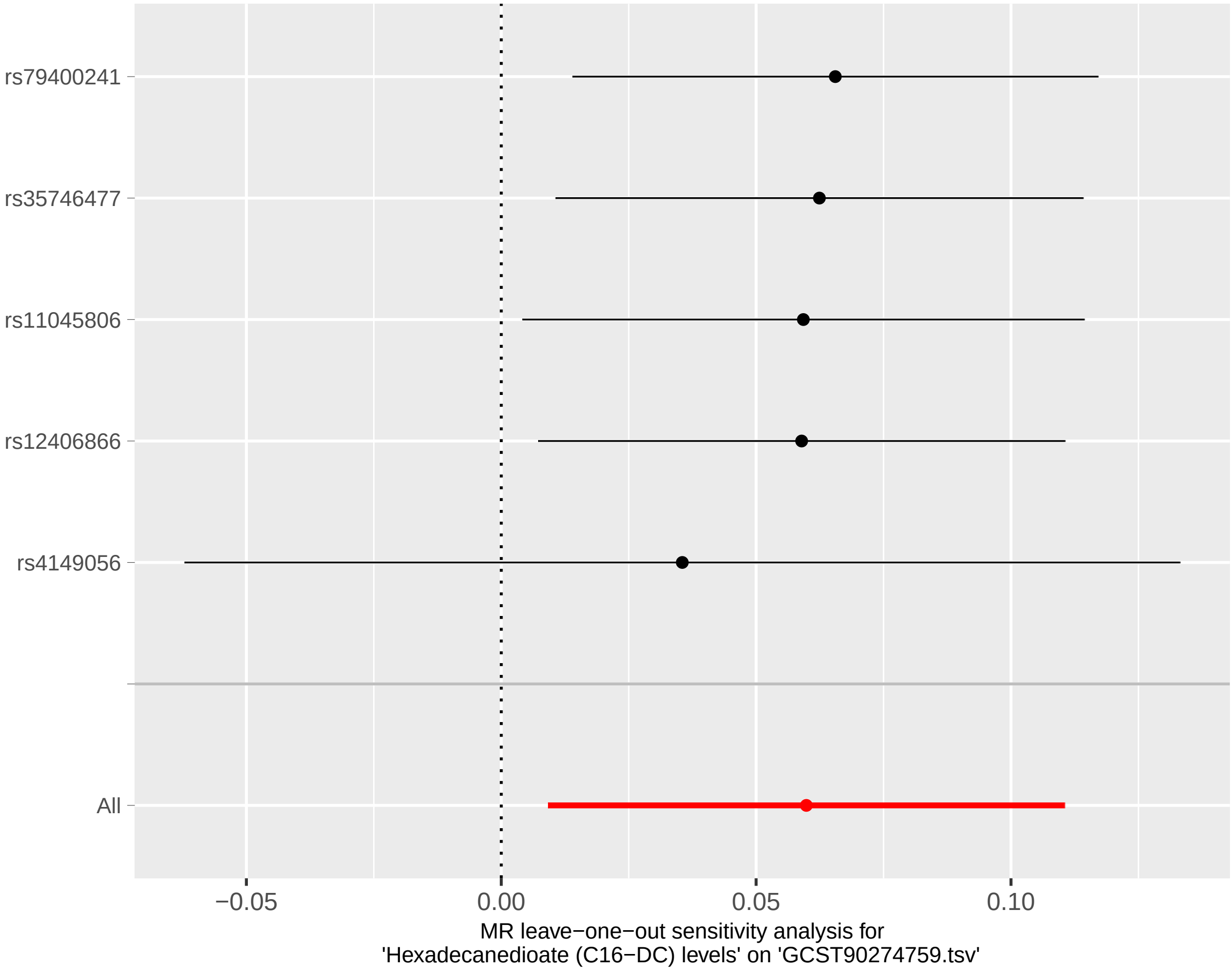
rs79400241
rs35746477
rs11045806
rs12406866
rs4149056
All
−0.05
0.00
0.05
0.10
MR leave−one−out sensitivity analysis for
'Hexadecanedioate (C16−DC) levels' on 'GCST90274759.tsv'

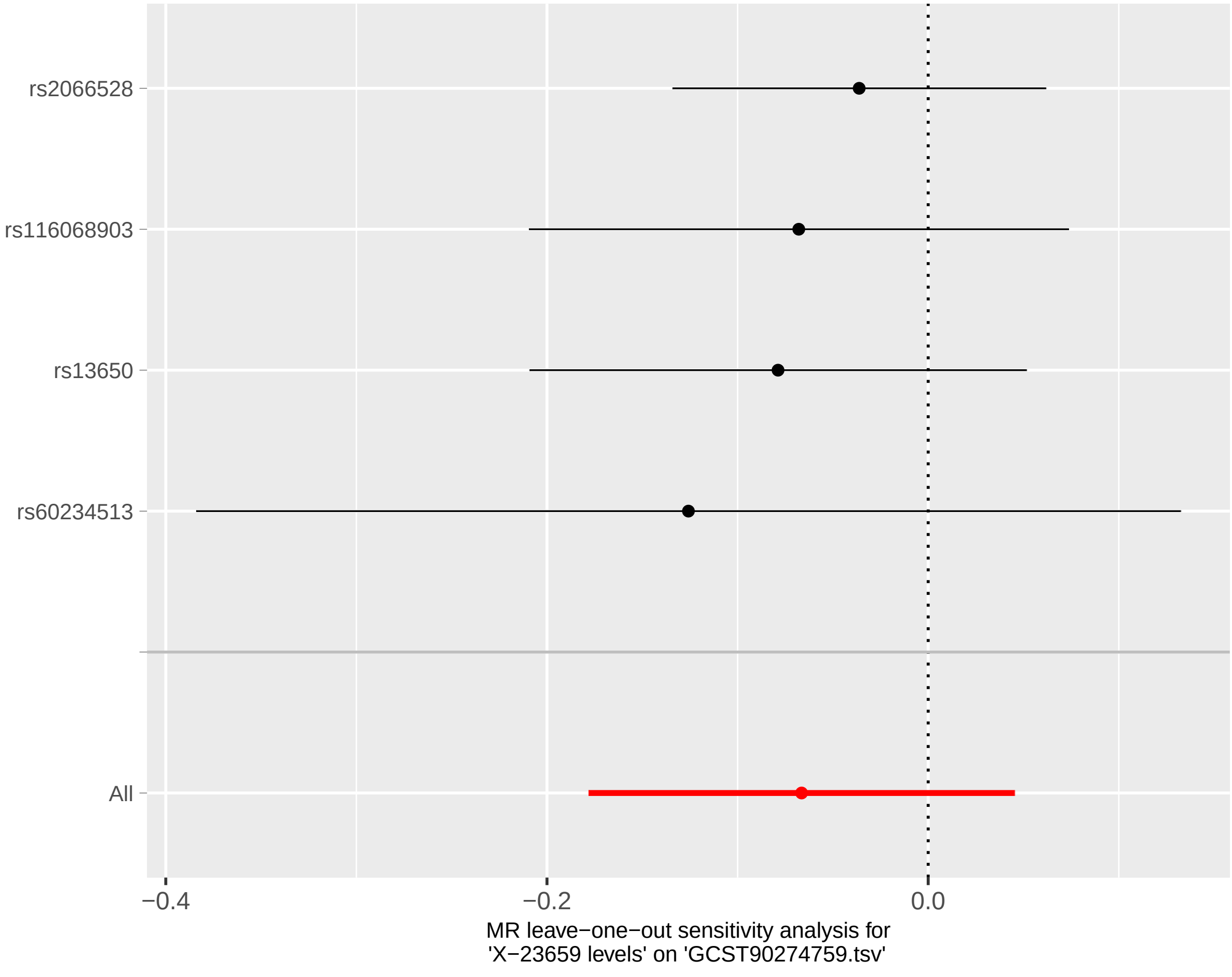
rs2066528
rs116068903
rs13650
rs60234513
All
−0.4
−0.2
0.0
MR leave−one−out sensitivity analysis for
'X−23659 levels' on 'GCST90274759.tsv'

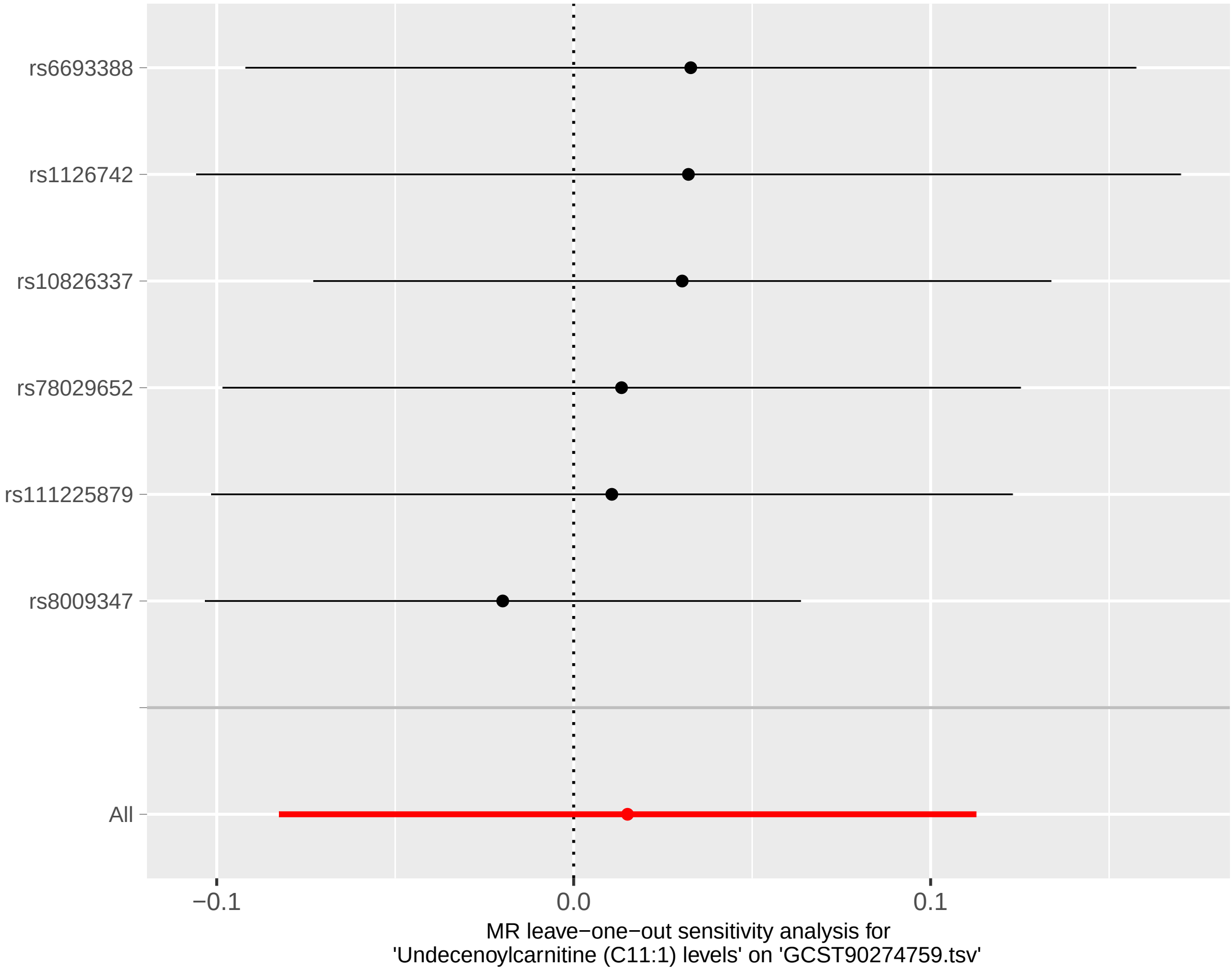
rs6693388
rs1126742
rs10826337
rs78029652
rs111225879
rs8009347
All
−0.1
0.0
0.1
MR leave−one−out sensitivity analysis for
'Undecenoylcarnitine (C11:1) levels' on 'GCST90274759.tsv'

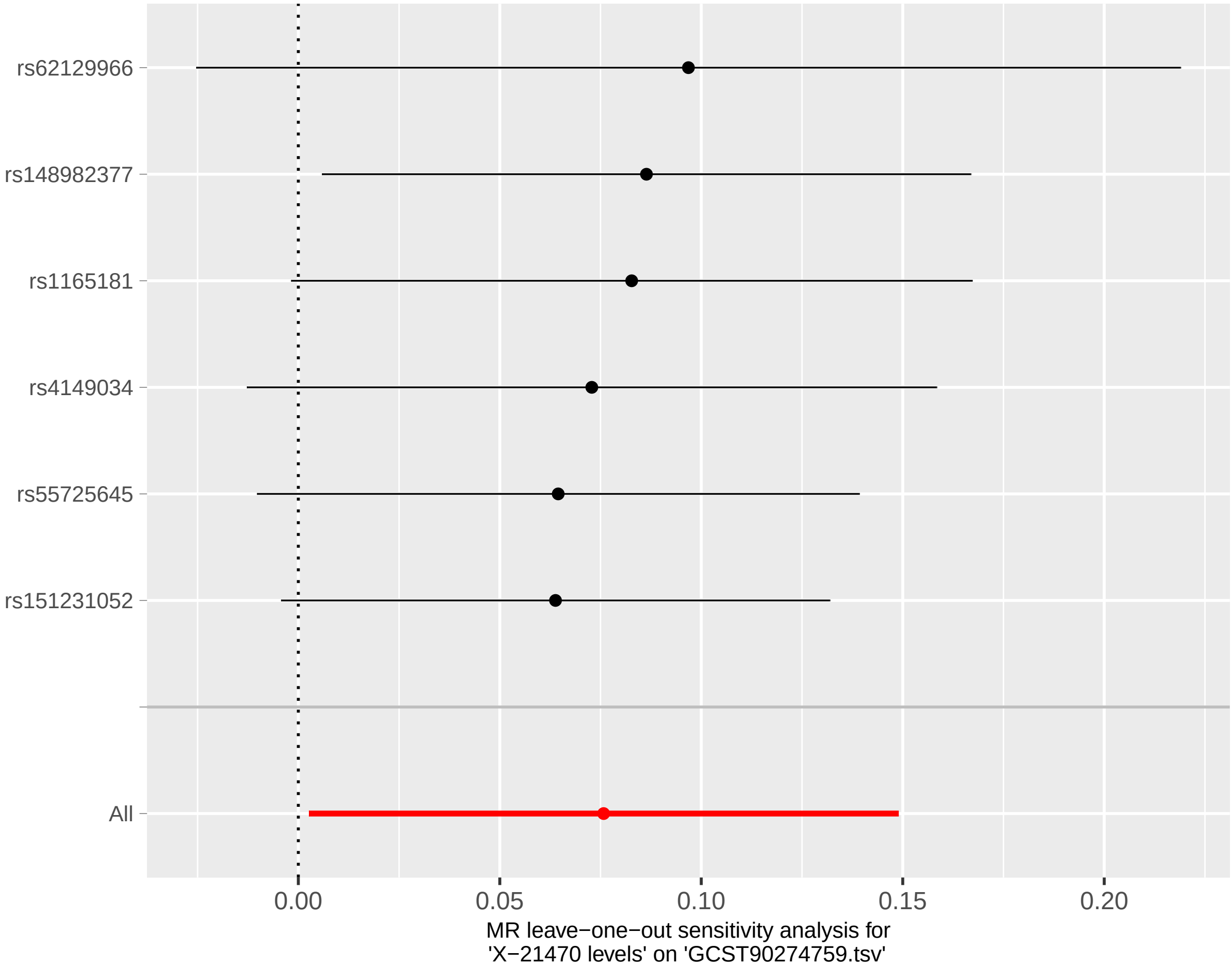
rs62129966
rs148982377
rs1165181
rs4149034
rs55725645
rs151231052
All
0.00
0.05
0.10
0.15
0.20
MR leave−one−out sensitivity analysis for
'X−21470 levels' on 'GCST90274759.tsv'

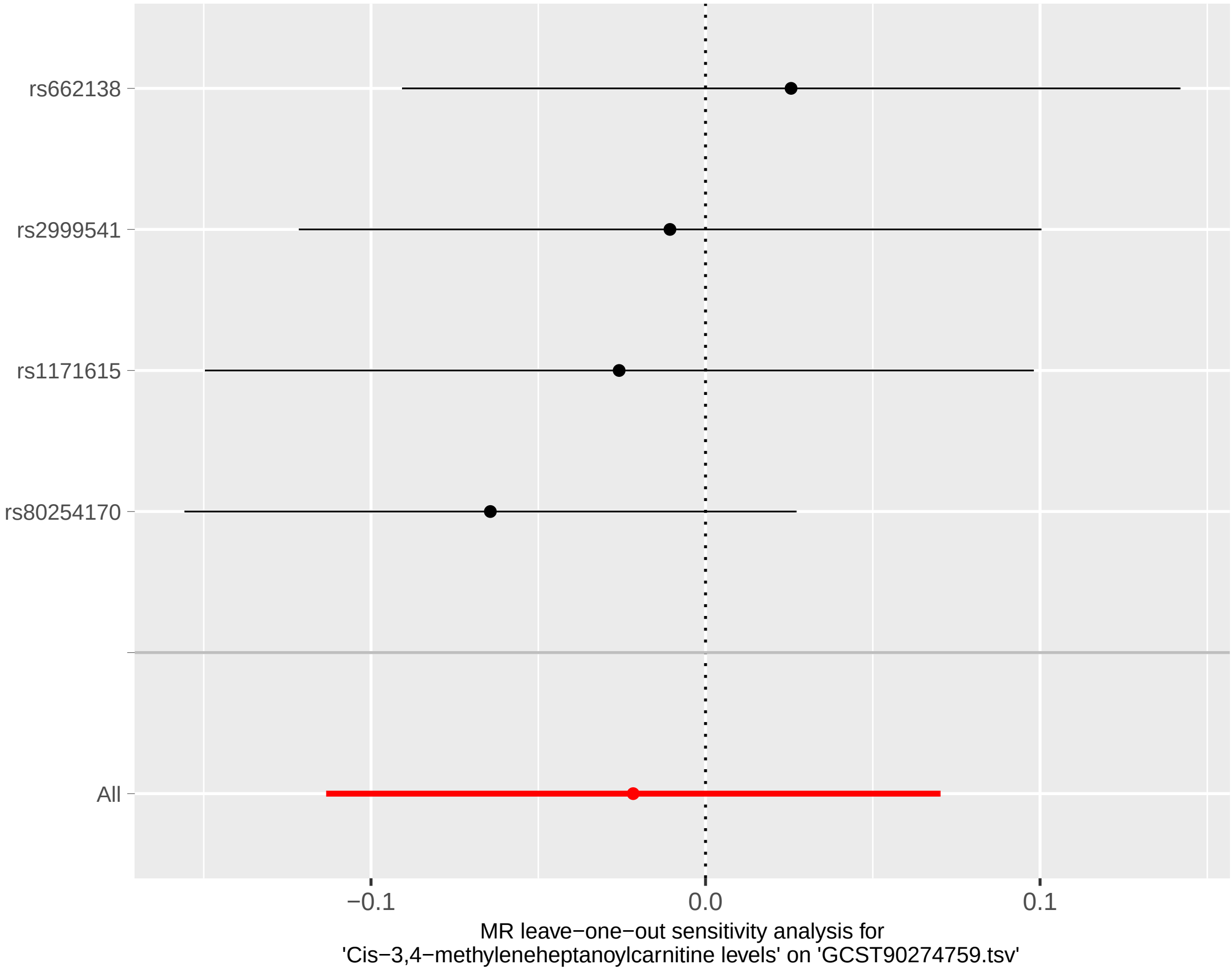
rs662138
rs2999541
rs1171615
rs80254170
All
−0.1
0.0
0.1
MR leave−one−out sensitivity analysis for
'Cis−3,4−methyleneheptanoylcarnitine levels' on 'GCST90274759.tsv'

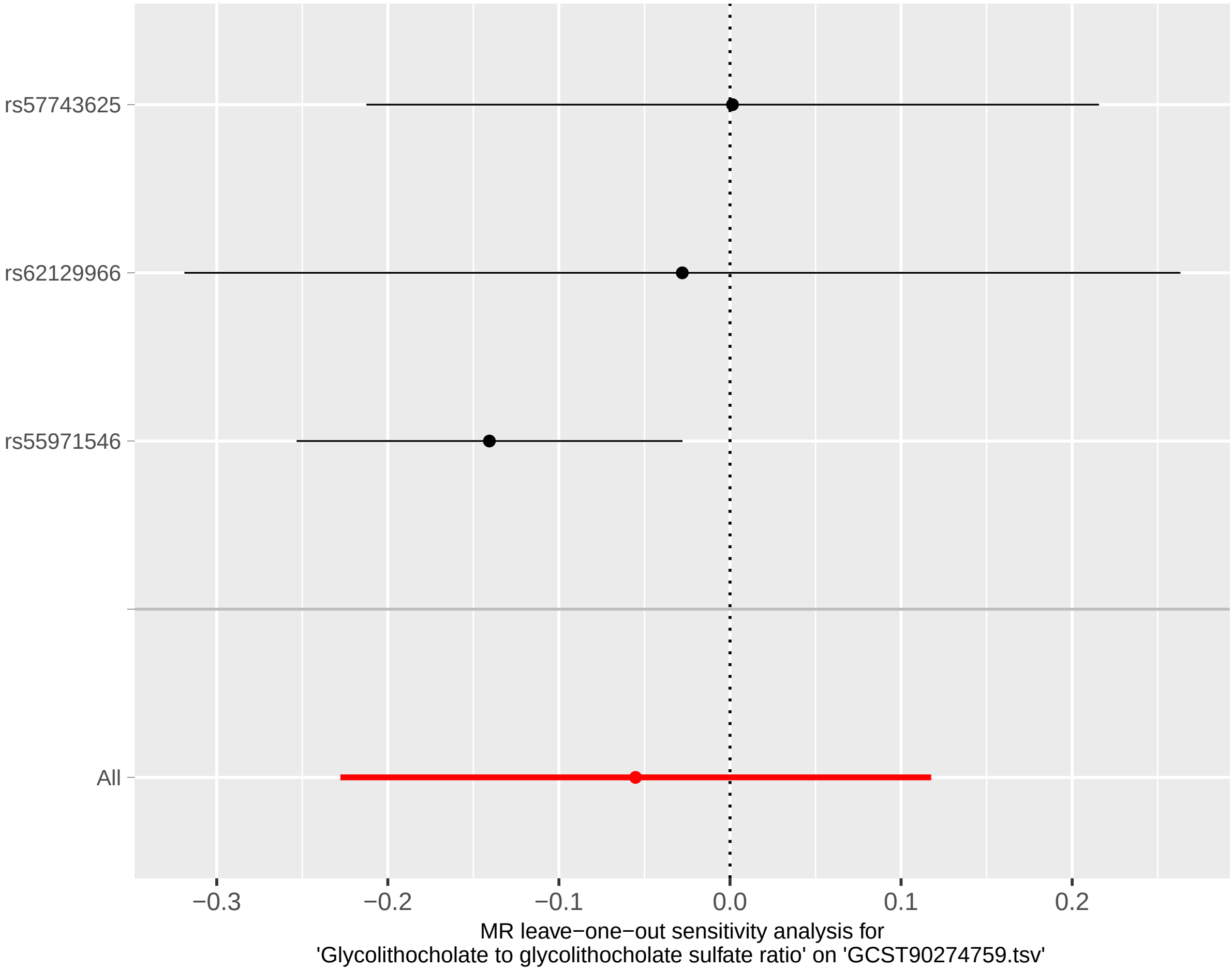

rs57743625
rs62129966
rs55971546
All
−0.3
−0.2
−0.1
0.0
0.1
0.2
MR leave−one−out sensitivity analysis for
'Glycolithocholate to glycolithocholate sulfate ratio' on 'GCST90274759.tsv'

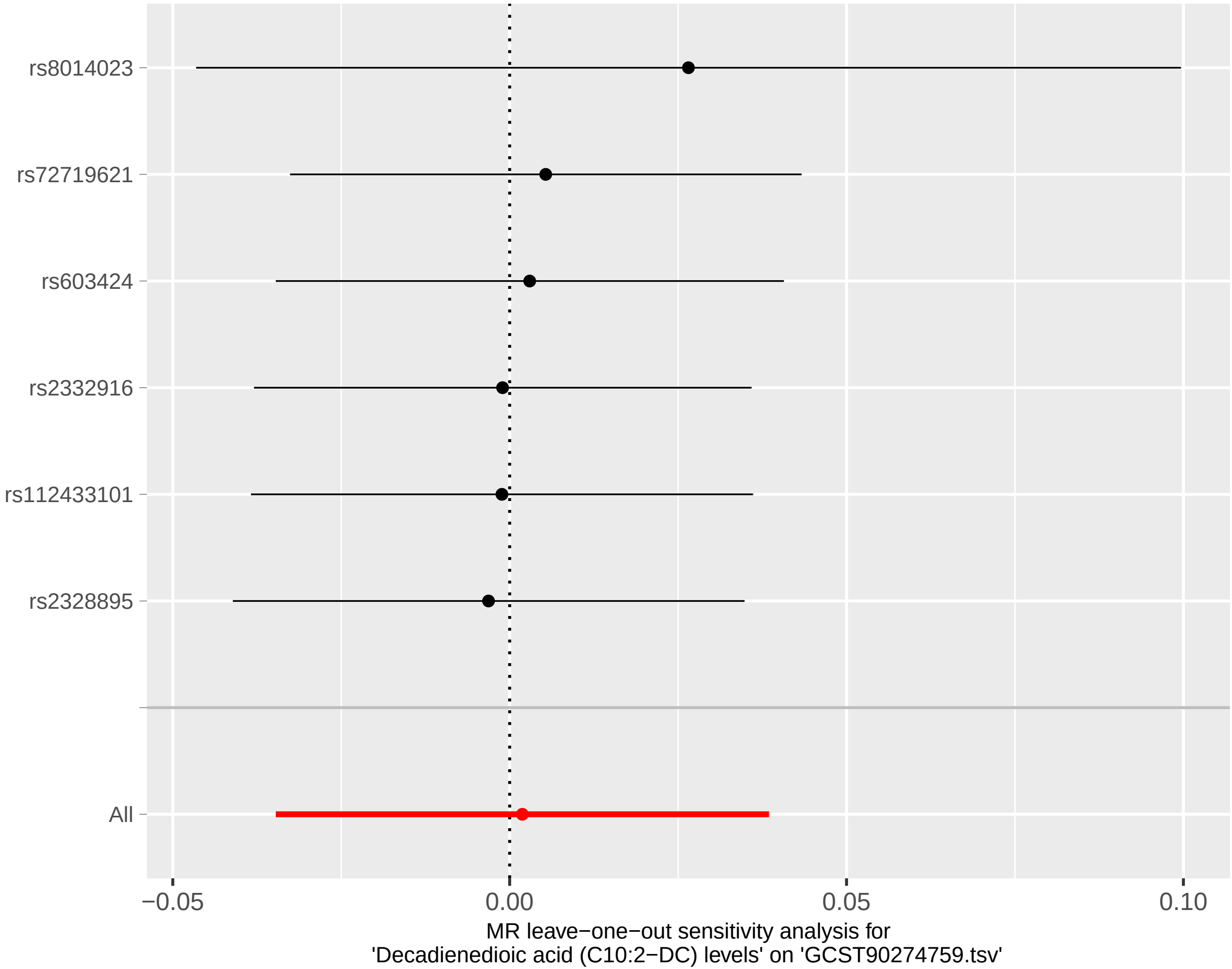
rs8014023
rs72719621
rs603424
rs2332916
rs112433101
rs2328895
All
−0.05
0.00
0.05
0.10
MR leave−one−out sensitivity analysis for
'Decadienedioic acid (C10:2−DC) levels' on 'GCST90274759.tsv'

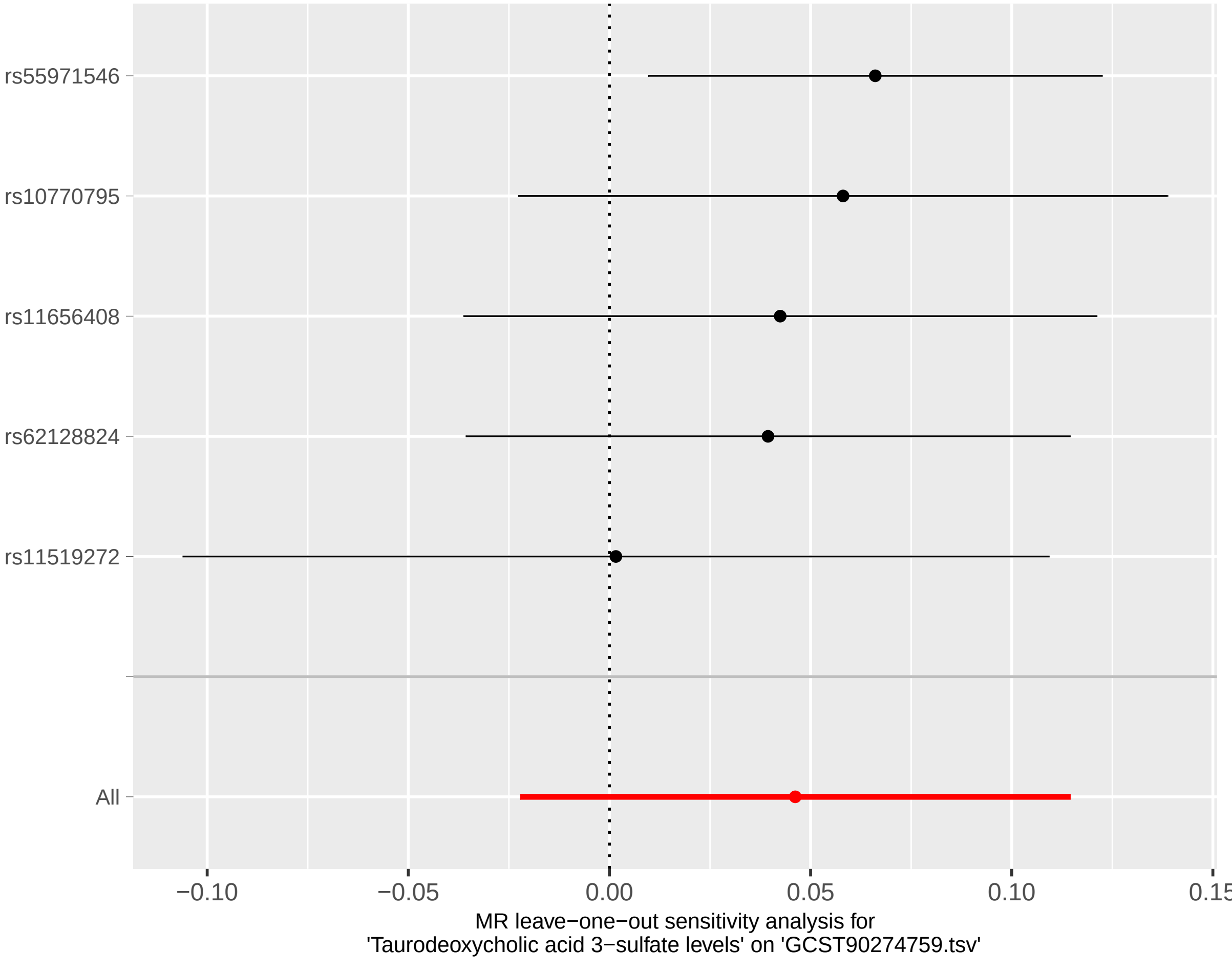

rs55971546
rs10770795
rs11656408
rs62128824
rs11519272
All
−0.10
−0.05
0.00
0.05
0.10
0.15
MR leave−one−out sensitivity analysis for
'Taurodeoxycholic acid 3−sulfate levels' on 'GCST90274759.tsv'

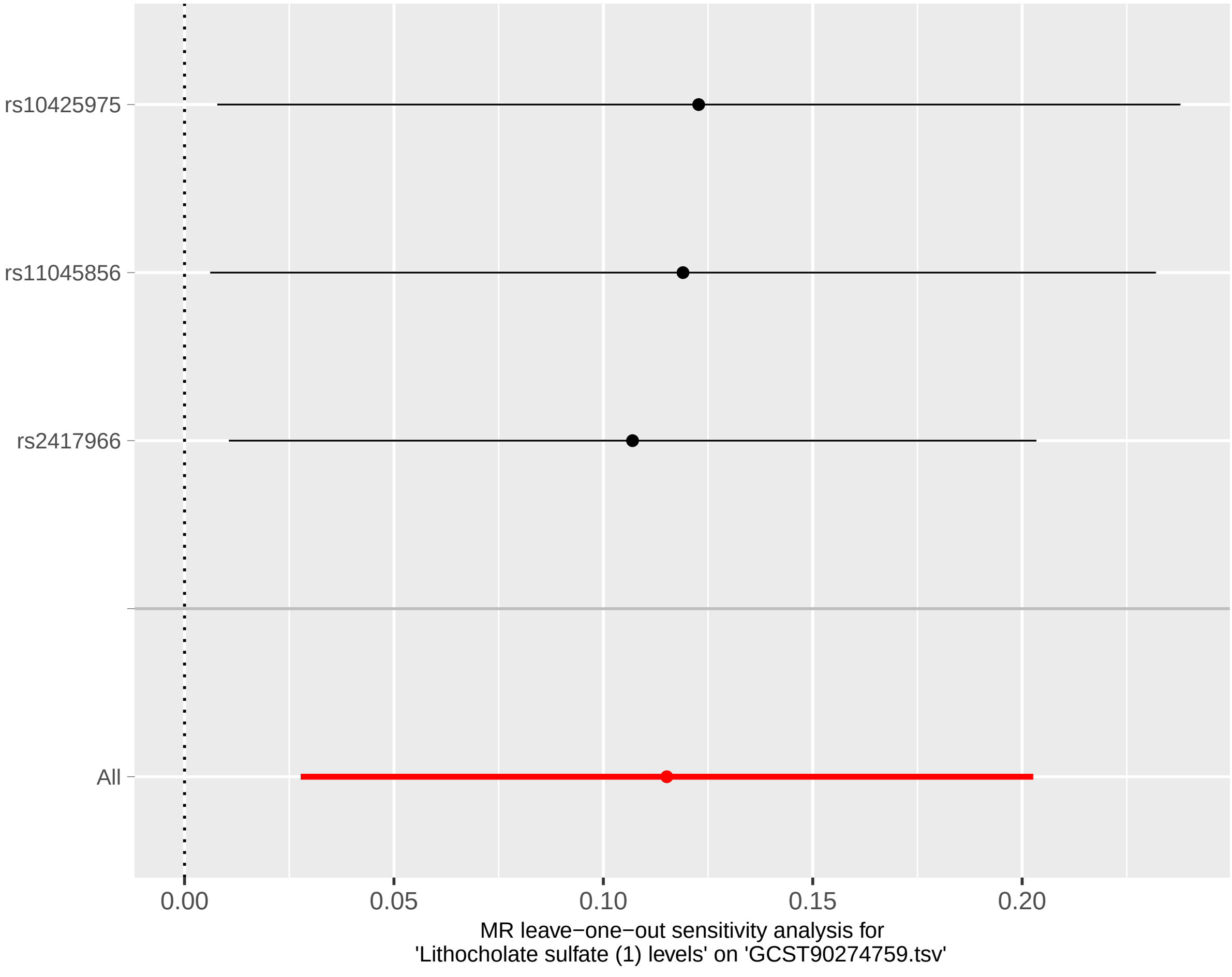

rs10425975
rs11045856
rs2417966
All
0.00
0.05
0.10
0.15
0.20
MR leave−one−out sensitivity analysis for
'Lithocholate sulfate (1) levels' on 'GCST90274759.tsv'

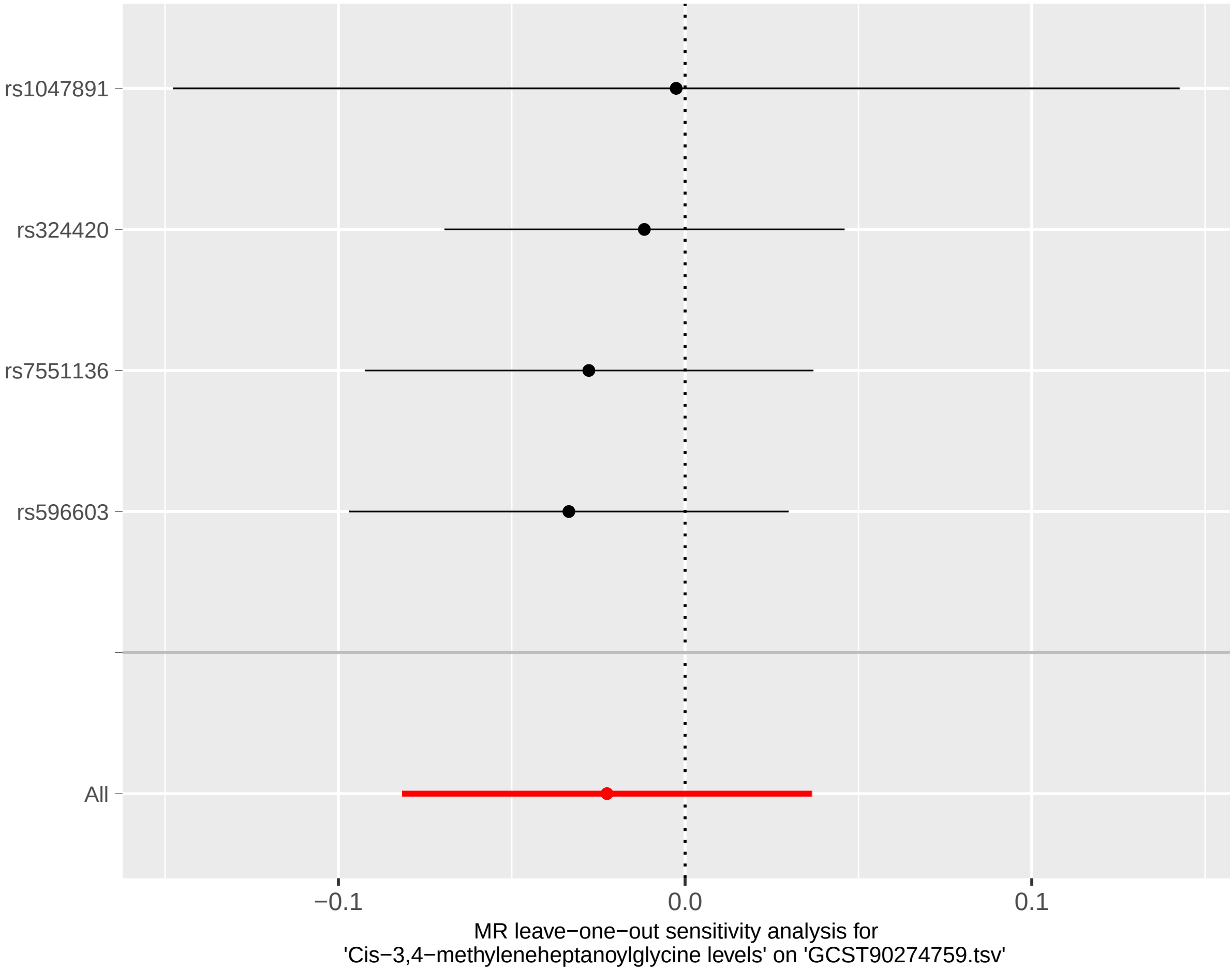

rs1047891
rs324420
rs7551136
rs596603
All
−0.1
0.0
0.1
MR leave−one−out sensitivity analysis for
'Cis−3,4−methyleneheptanoylglycine levels' on 'GCST90274759.tsv'

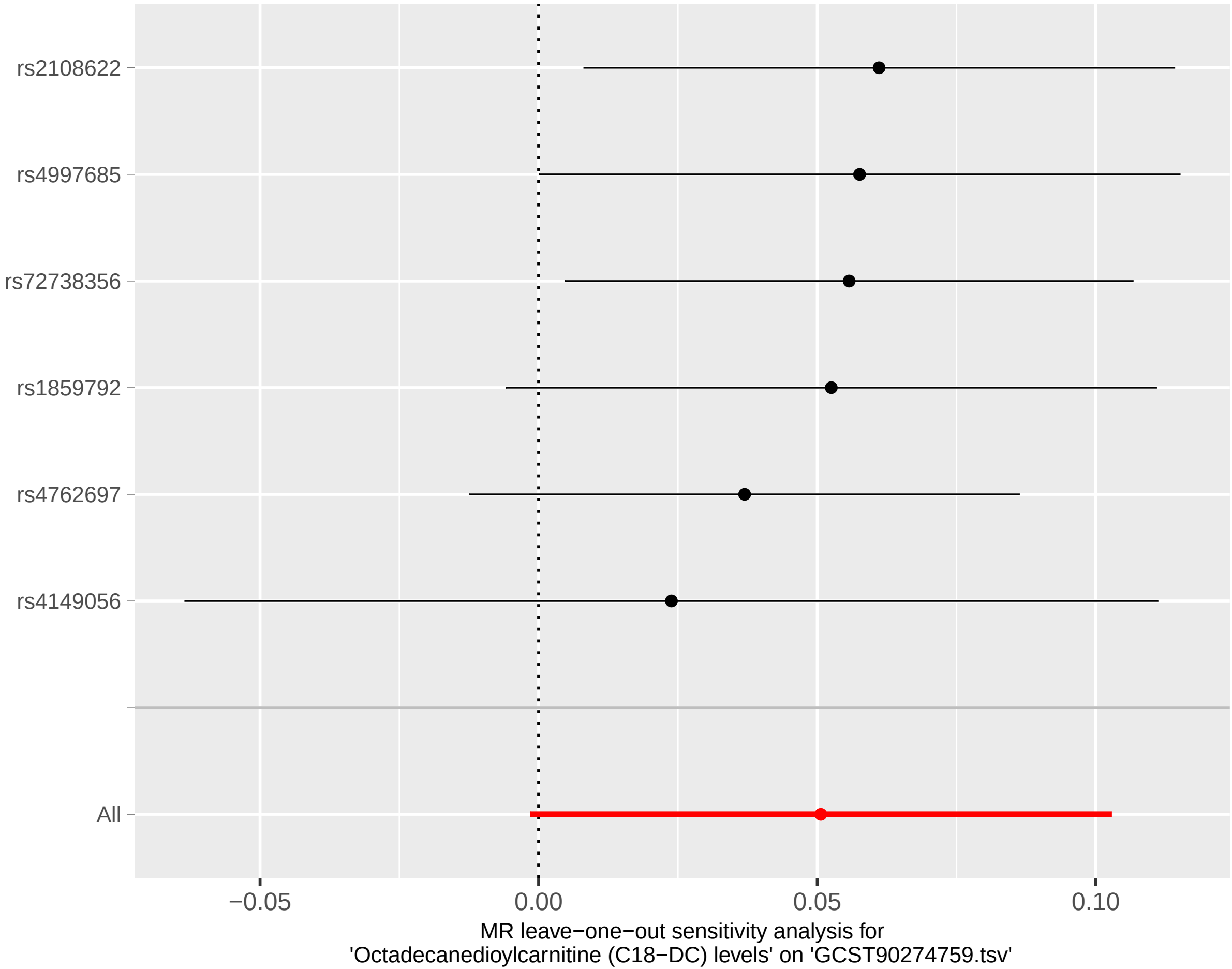
rs2108622
rs4997685
rs72738356
rs1859792
rs4762697
rs4149056
All
−0.05
0.00
0.05
0.10
MR leave−one−out sensitivity analysis for
'Octadecanedioylcarnitine (C18−DC) levels' on 'GCST90274759.tsv'

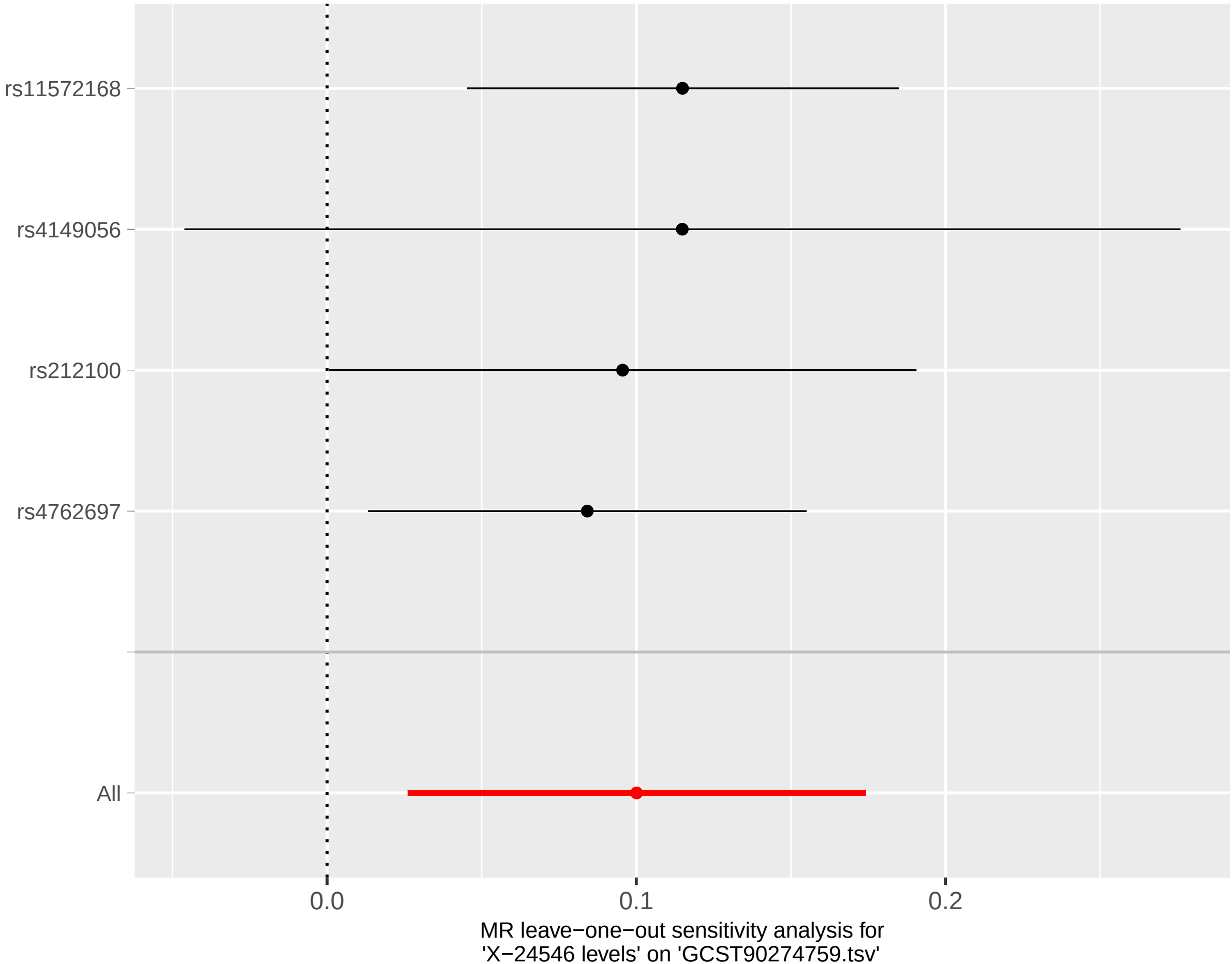
rs11572168
rs4149056
rs212100
rs4762697
All
0.0
0.1
0.2
MR leave−one−out sensitivity analysis for
'X−24546 levels' on 'GCST90274759.tsv'

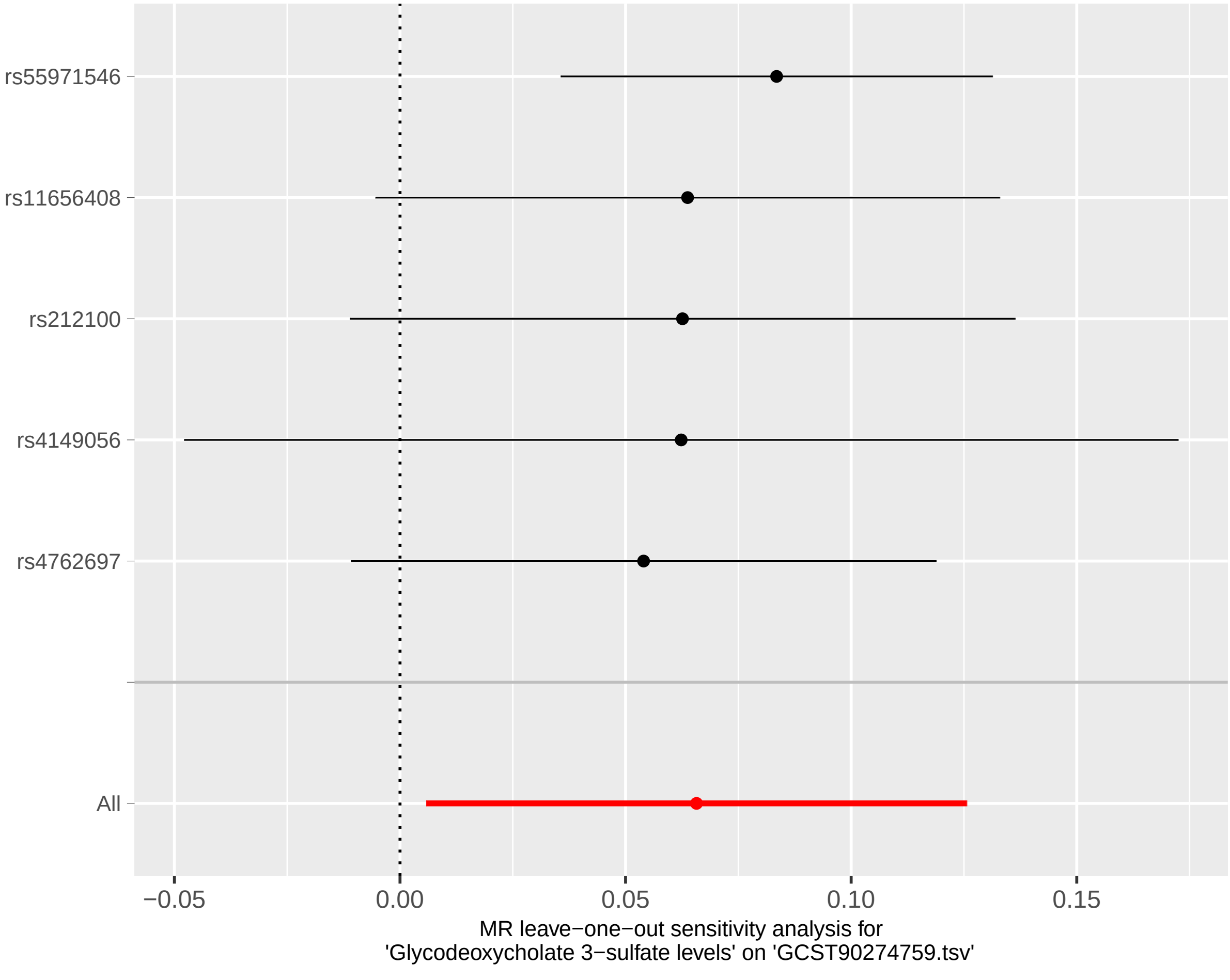
rs55971546
rs11656408
rs212100
rs4149056
rs4762697
All
−0.05
0.00
0.05
0.10
0.15
MR leave−one−out sensitivity analysis for
'Glycodeoxycholate 3−sulfate levels' on 'GCST90274759.tsv'

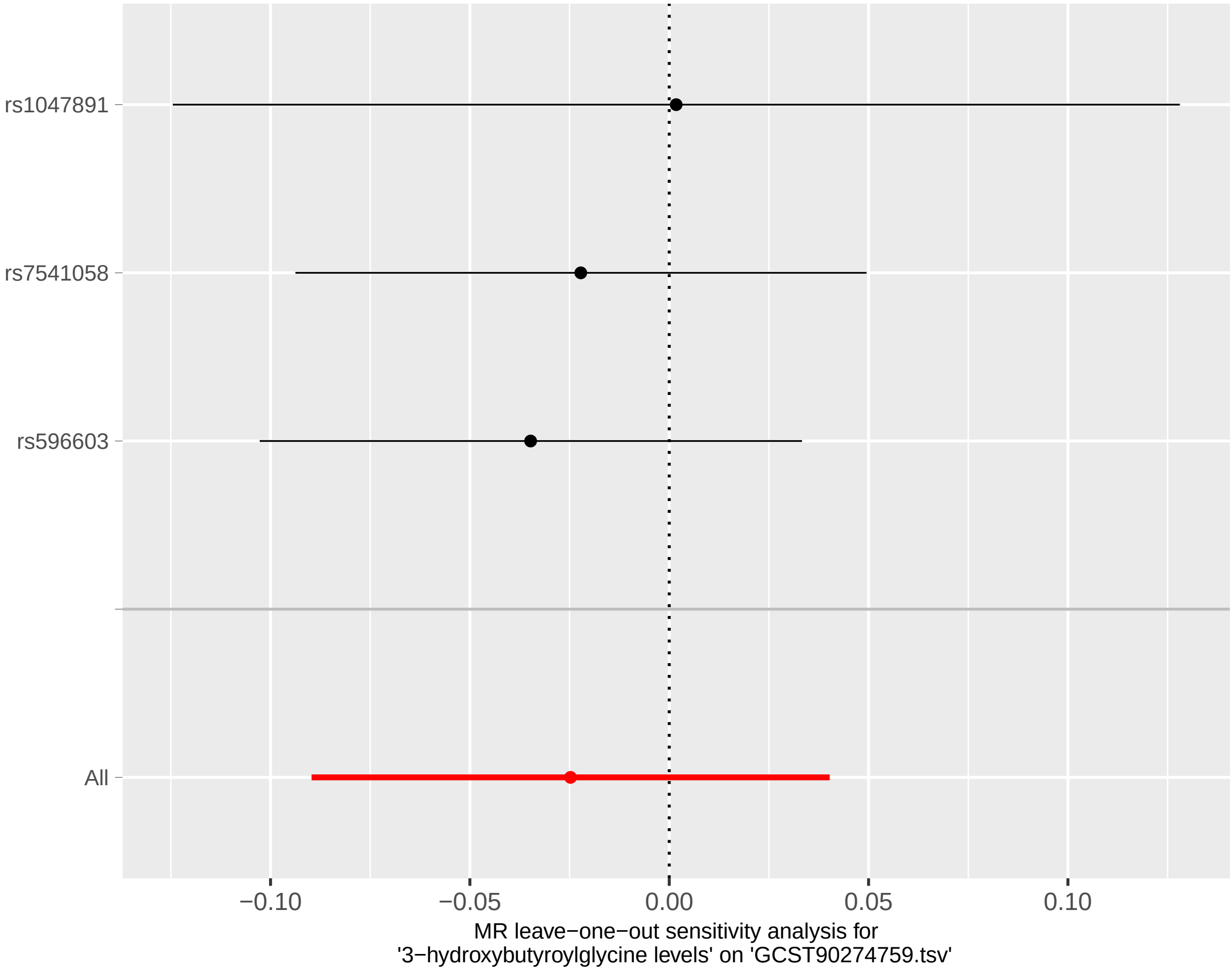
rs1047891
rs7541058
rs596603
All
−0.10
−0.05
0.00
0.05
0.10
MR leave−one−out sensitivity analysis for
'3−hydroxybutyroylglycine levels' on 'GCST90274759.tsv'

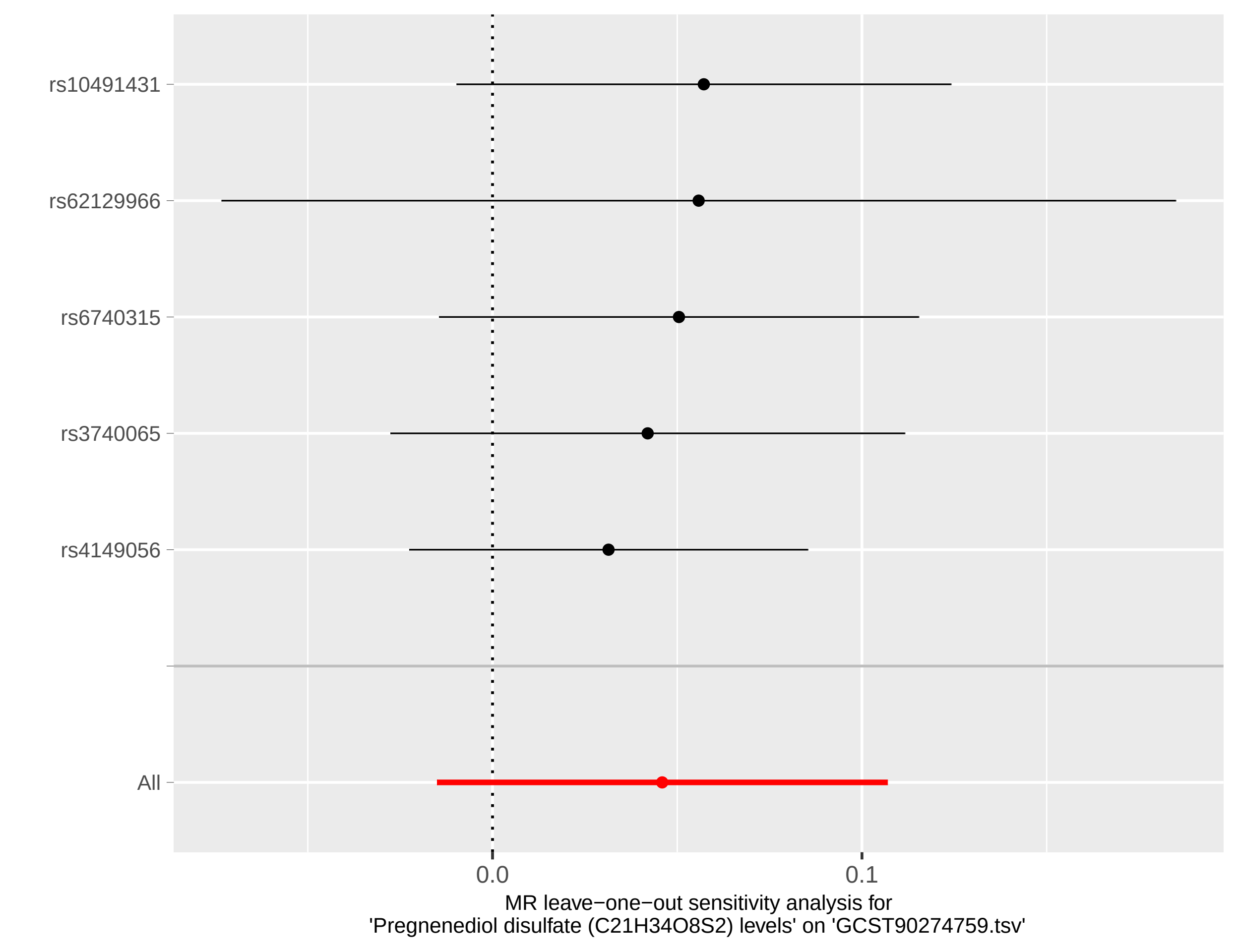
rs10491431
rs62129966
rs6740315
rs3740065
rs4149056
All
0.0
0.1
MR leave−one−out sensitivity analysis for
'Pregnenediol disulfate (C21H34O8S2) levels' on 'GCST90274759.tsv'

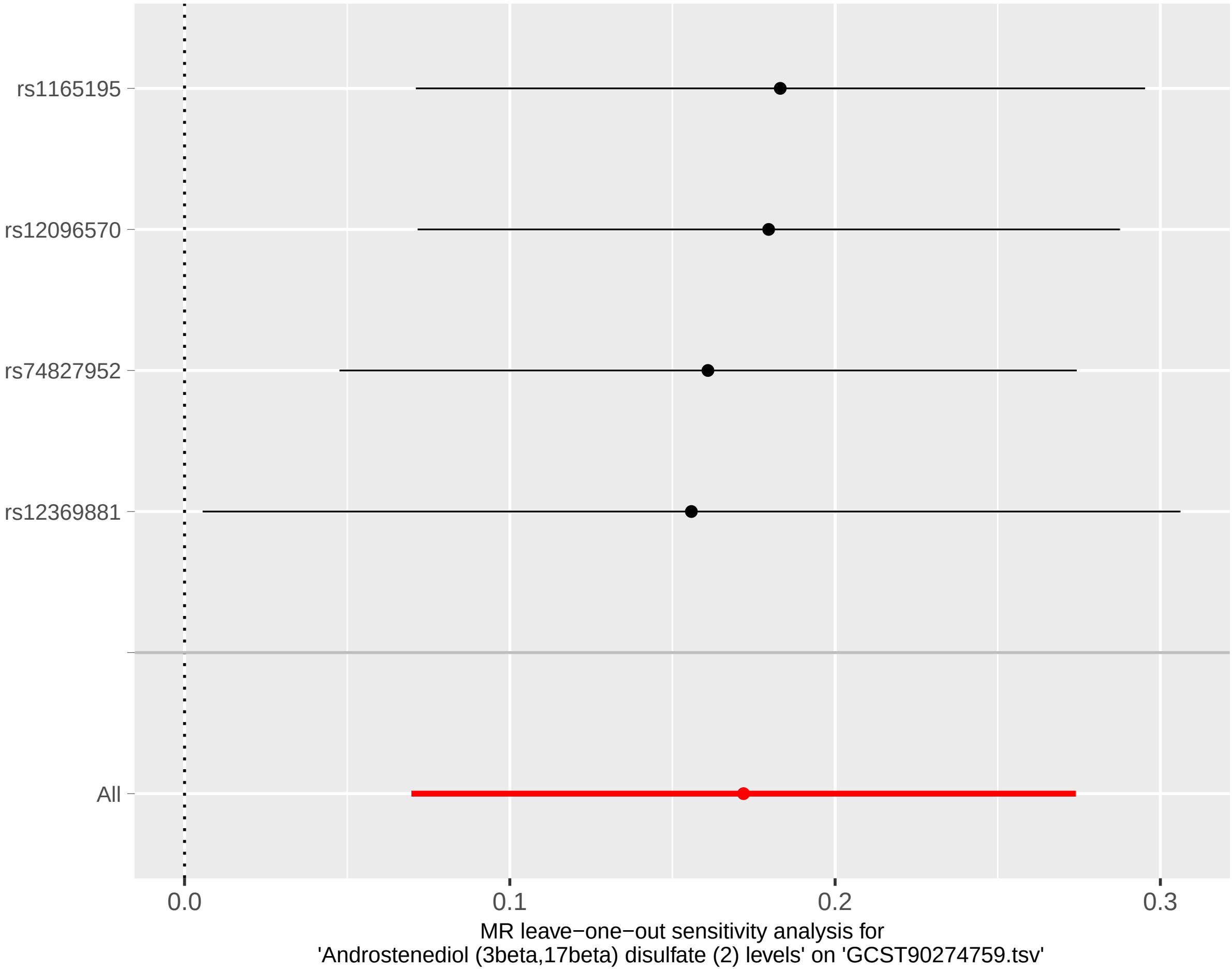

rs1165195
rs12096570
rs74827952
rs12369881
All
0.0
0.1
0.2
0.3
MR leave−one−out sensitivity analysis for
'Androstenediol (3beta,17beta) disulfate (2) levels' on 'GCST90274759.tsv'

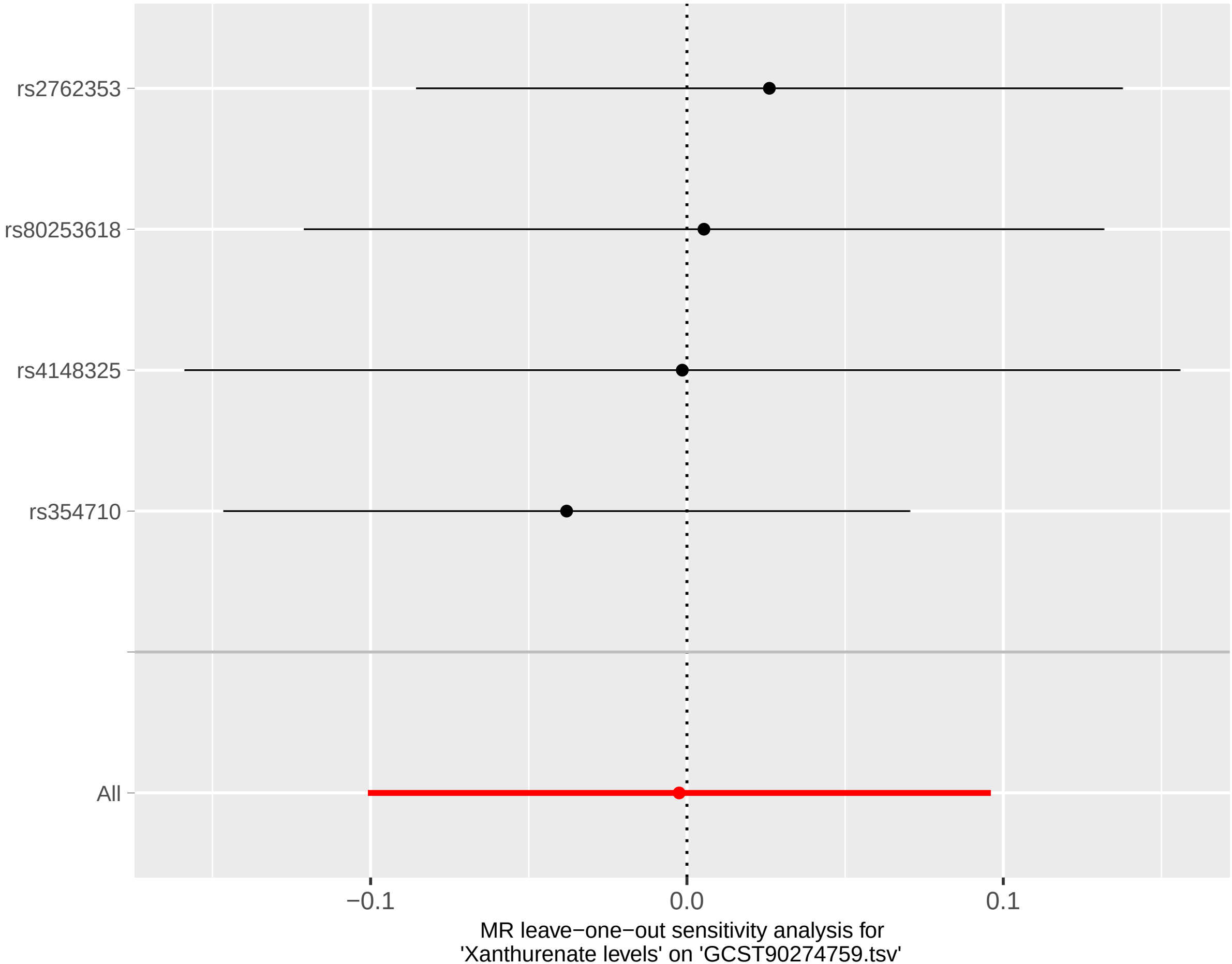
rs2762353
rs80253618
rs4148325
rs354710
All
−0.1
0.0
0.1
MR leave−one−out sensitivity analysis for
'Xanthurenate levels' on 'GCST90274759.tsv'

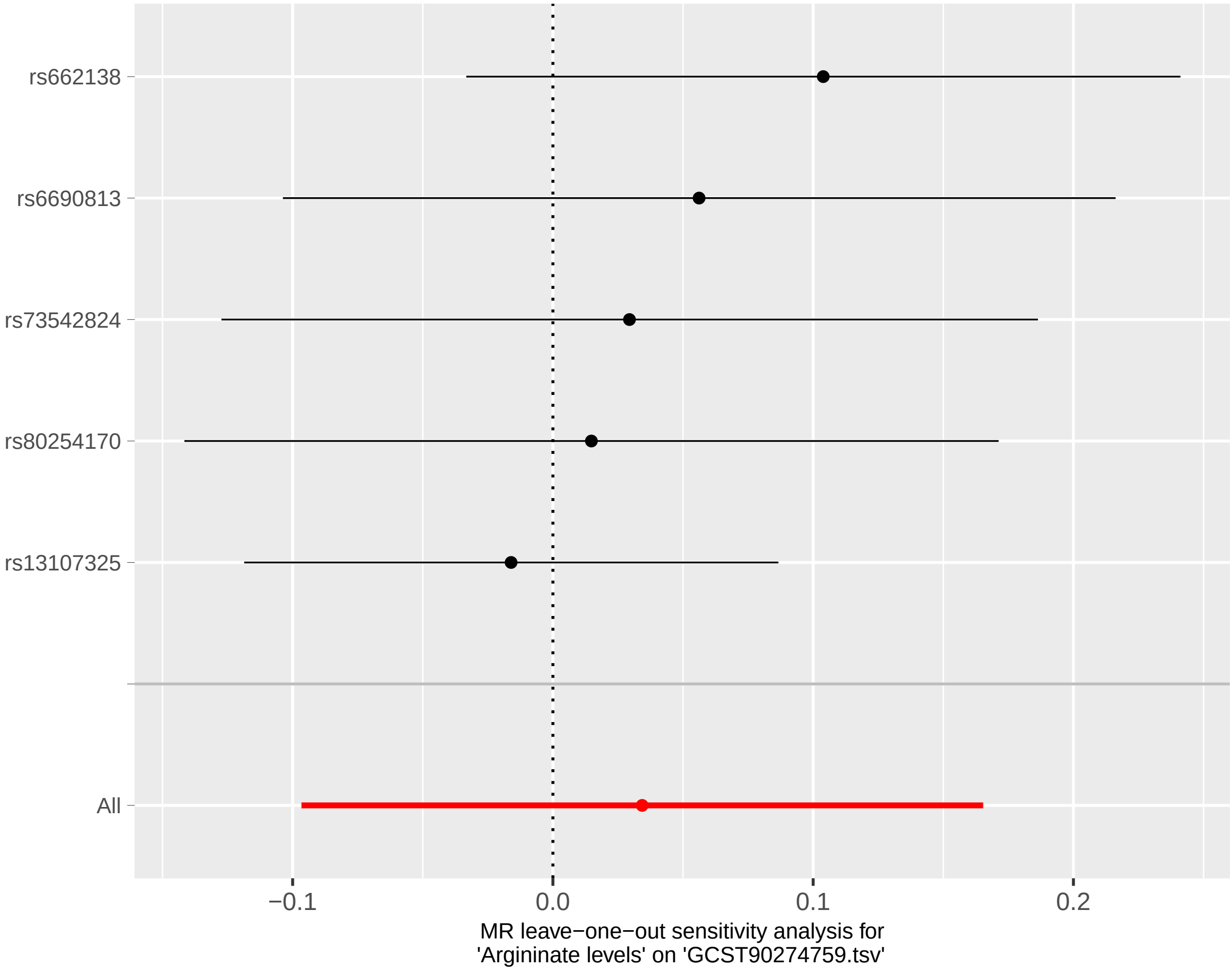
rs662138
rs6690813
rs73542824
rs80254170
rs13107325
All
−0.1
0.0
0.1
0.2
MR leave−one−out sensitivity analysis for
'Argininate levels' on 'GCST90274759.tsv'

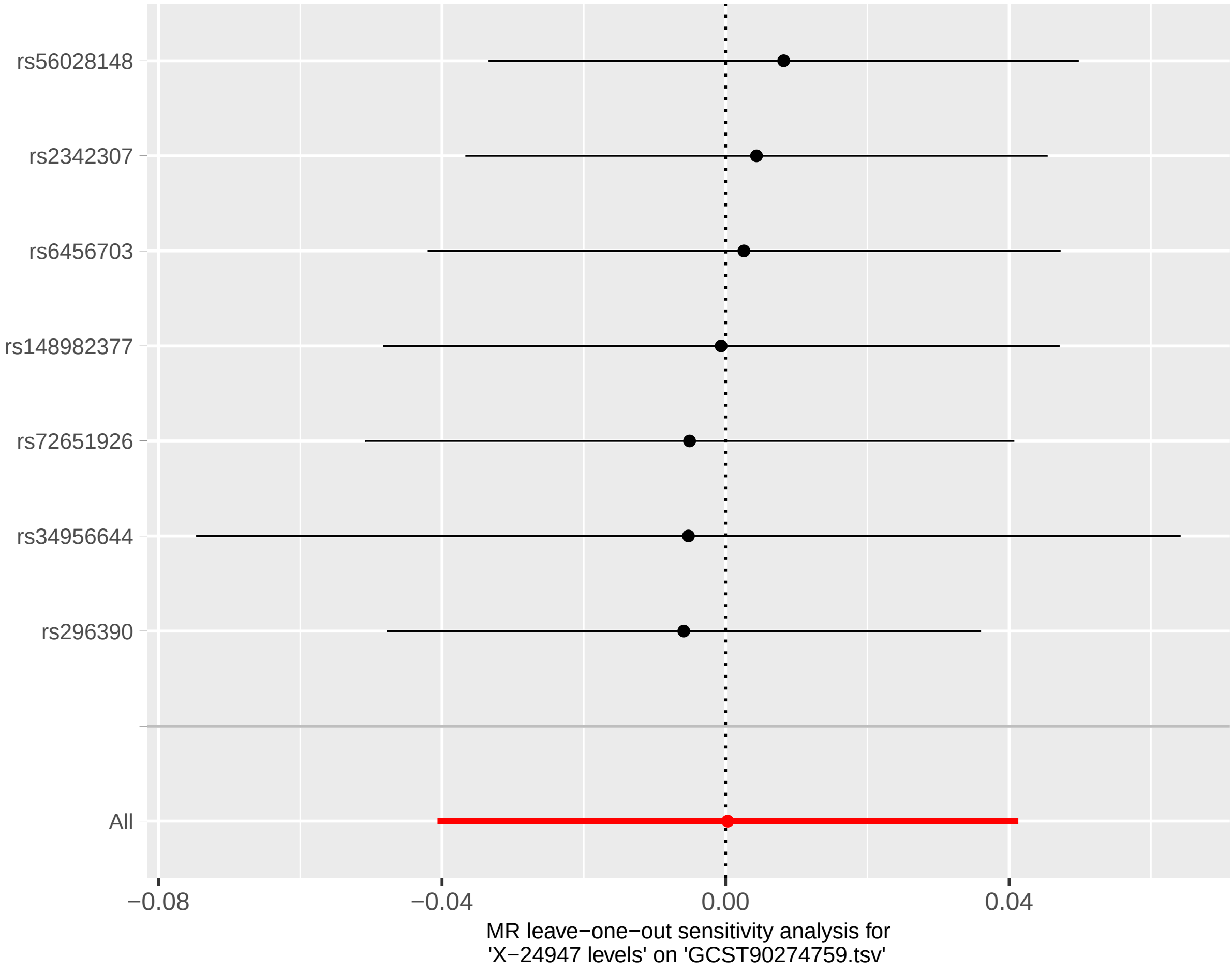
rs56028148
rs2342307
rs6456703
rs148982377
rs72651926
rs34956644
rs296390
All
−0.08
−0.04
0.00
0.04
MR leave−one−out sensitivity analysis for
'X−24947 levels' on 'GCST90274759.tsv'

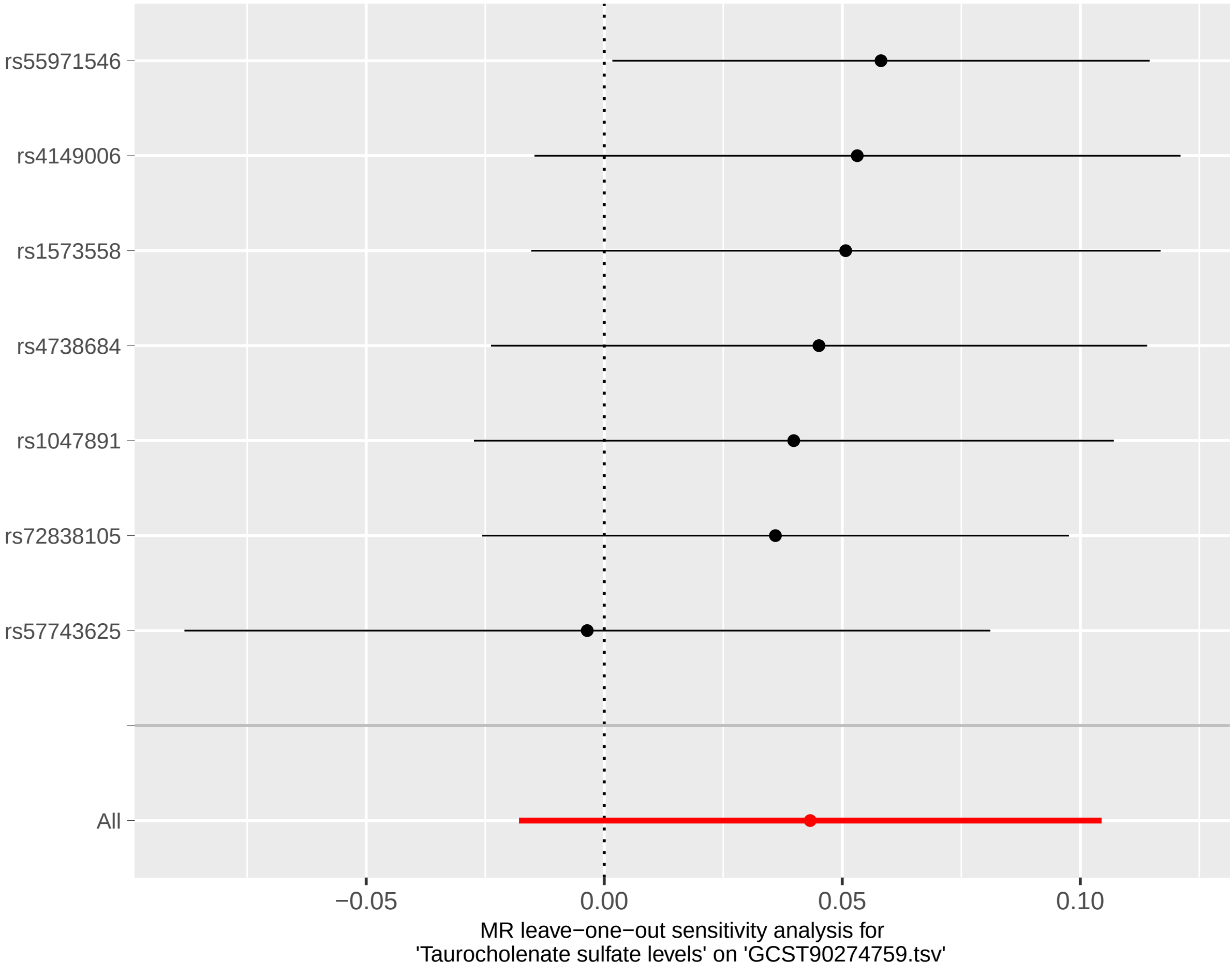

rs55971546
rs4149006
rs1573558
rs4738684
rs1047891
rs72838105
rs57743625
All
−0.05
0.00
0.05
0.10
MR leave−one−out sensitivity analysis for
'Taurocholenate sulfate levels' on 'GCST90274759.tsv'

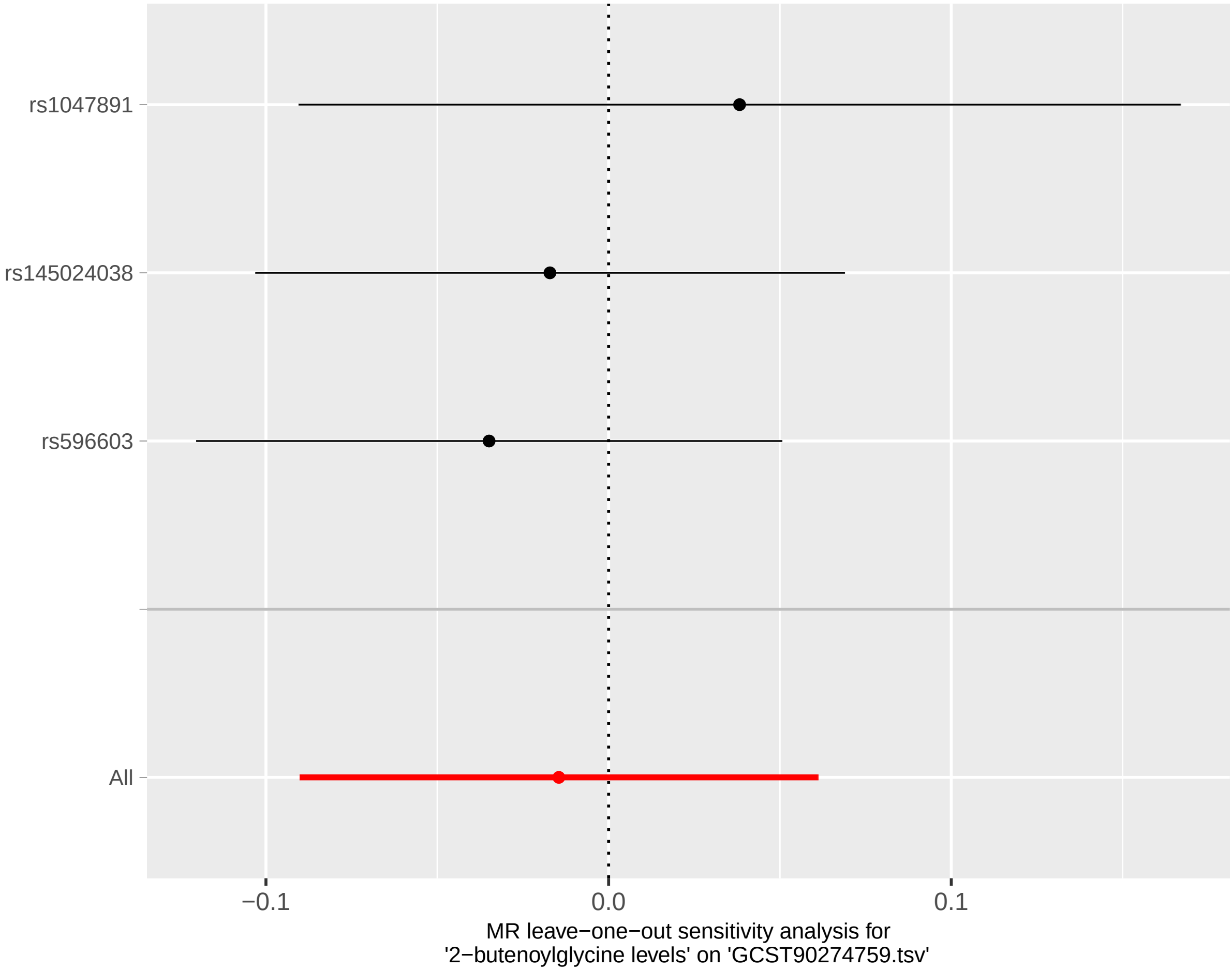
rs1047891
rs145024038
rs596603
All
−0.1
0.0
0.1
MR leave−one−out sensitivity analysis for
'2−butenoylglycine levels' on 'GCST90274759.tsv'

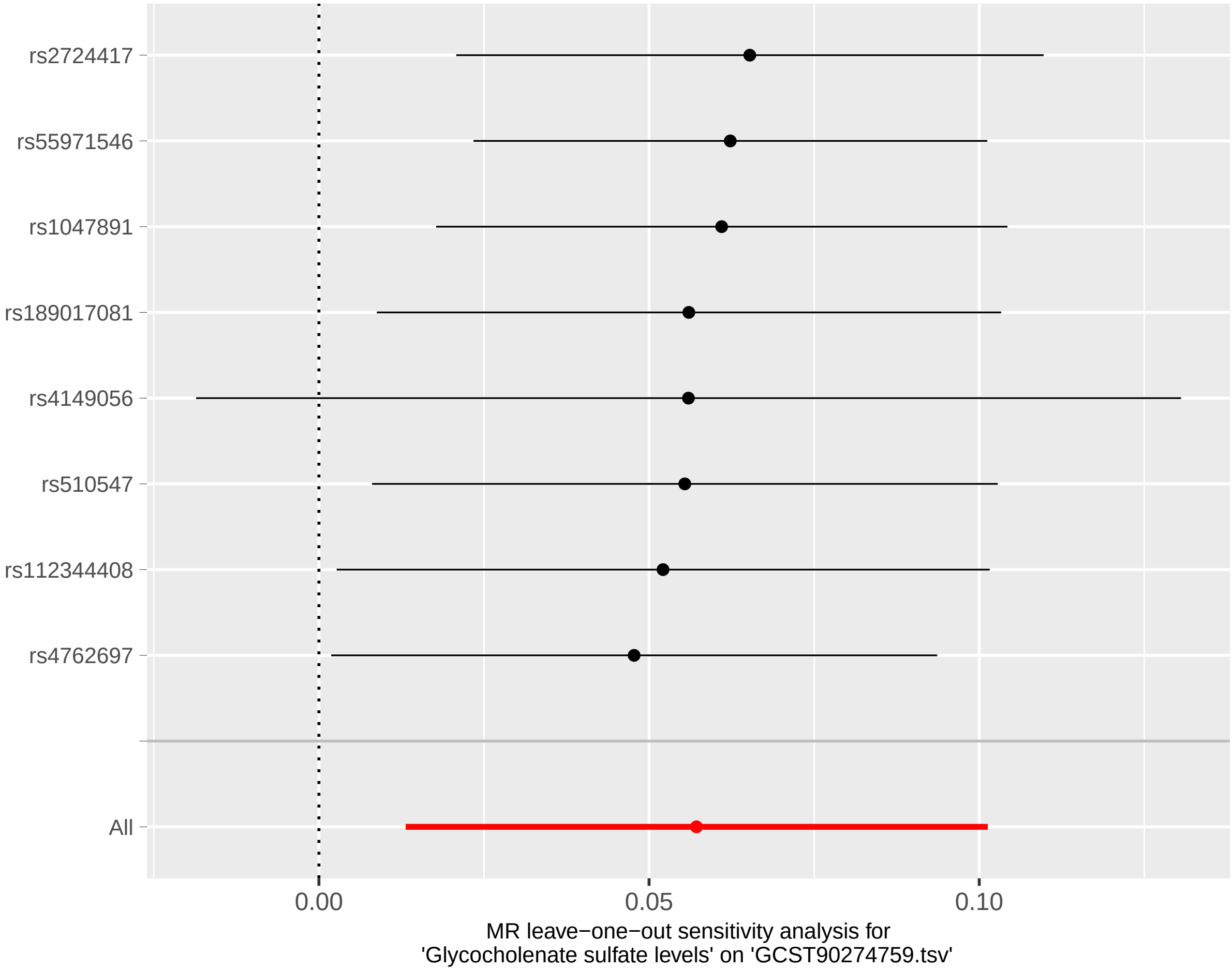
rs2724417
rs55971546
rs1047891
rs189017081
rs4149056
rs510547
rs112344408
rs4762697
All
0.00
0.05
0.10
MR leave−one−out sensitivity analysis for
'Glycocholenate sulfate levels' on 'GCST90274759.tsv'

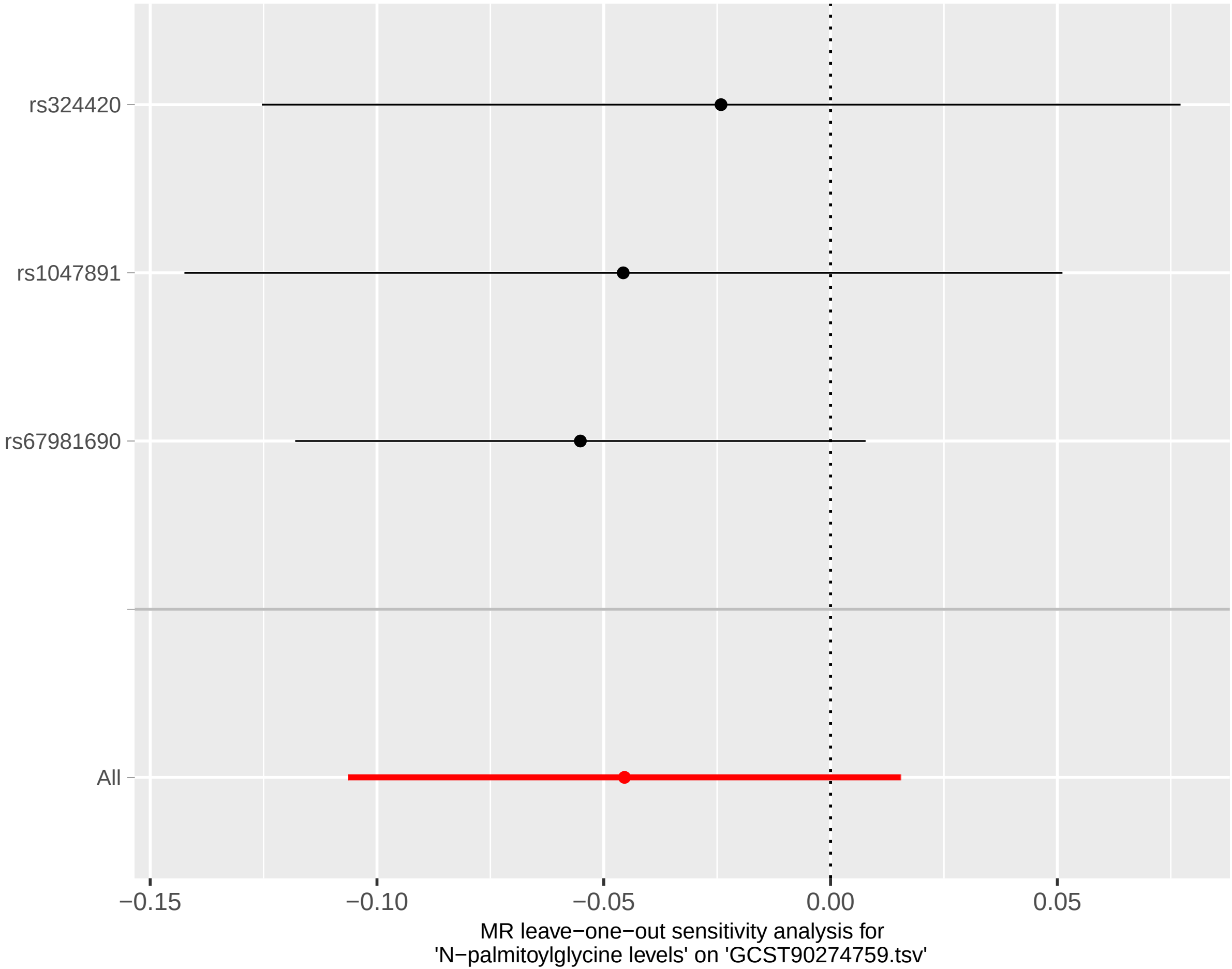
rs324420
rs1047891
rs67981690
All
−0.15
−0.10
−0.05
0.00
0.05
MR leave−one−out sensitivity analysis for
'N−palmitoylglycine levels' on 'GCST90274759.tsv'

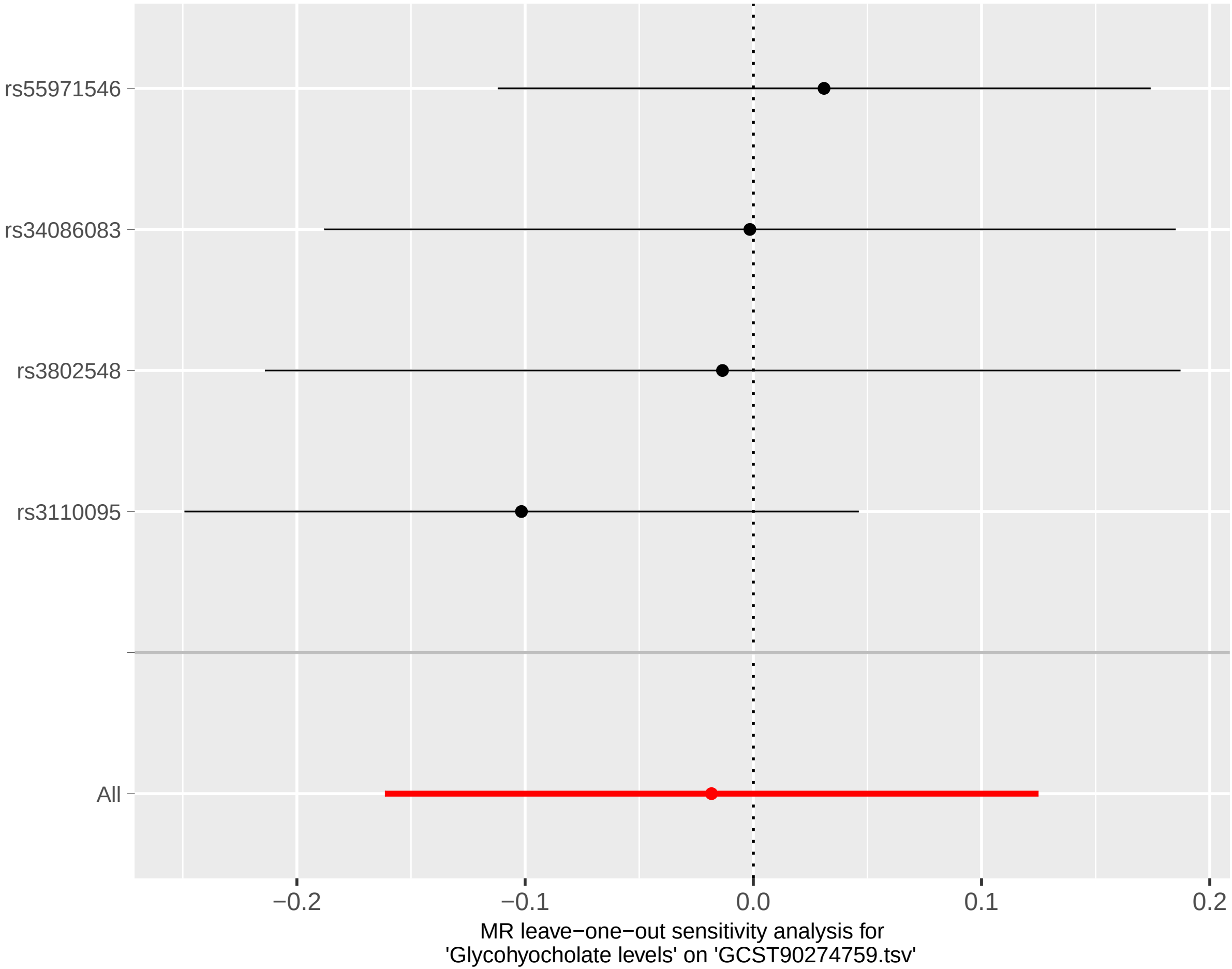
rs55971546
rs34086083
rs3802548
rs3110095
All
−0.2
−0.1
0.0
0.1
0.2
MR leave−one−out sensitivity analysis for
'Glycohyocholate levels' on 'GCST90274759.tsv'

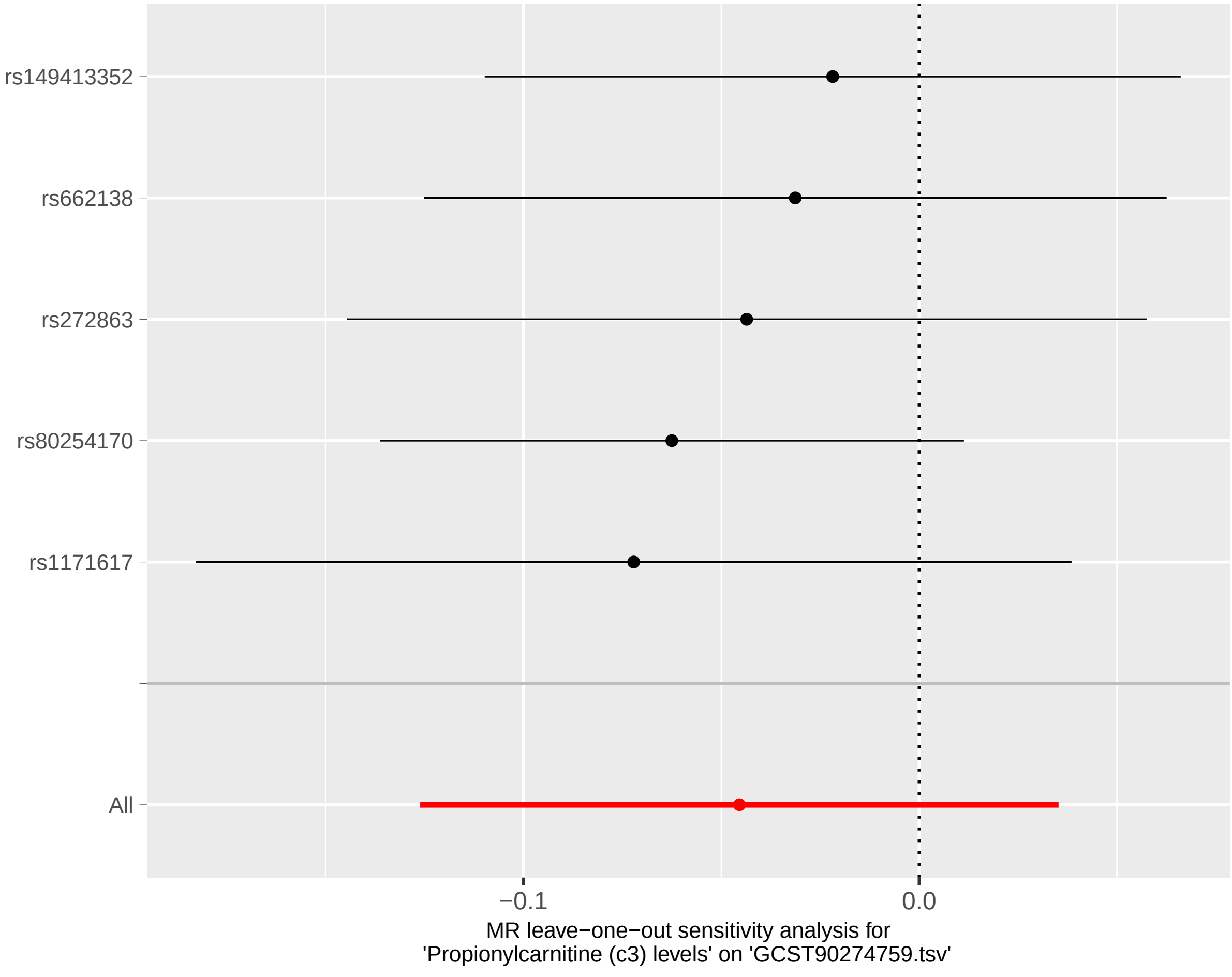
rs149413352
rs662138
rs272863
rs80254170
rs1171617
All
−0.1
0.0
MR leave−one−out sensitivity analysis for
'Propionylcarnitine (c3) levels' on 'GCST90274759.tsv'

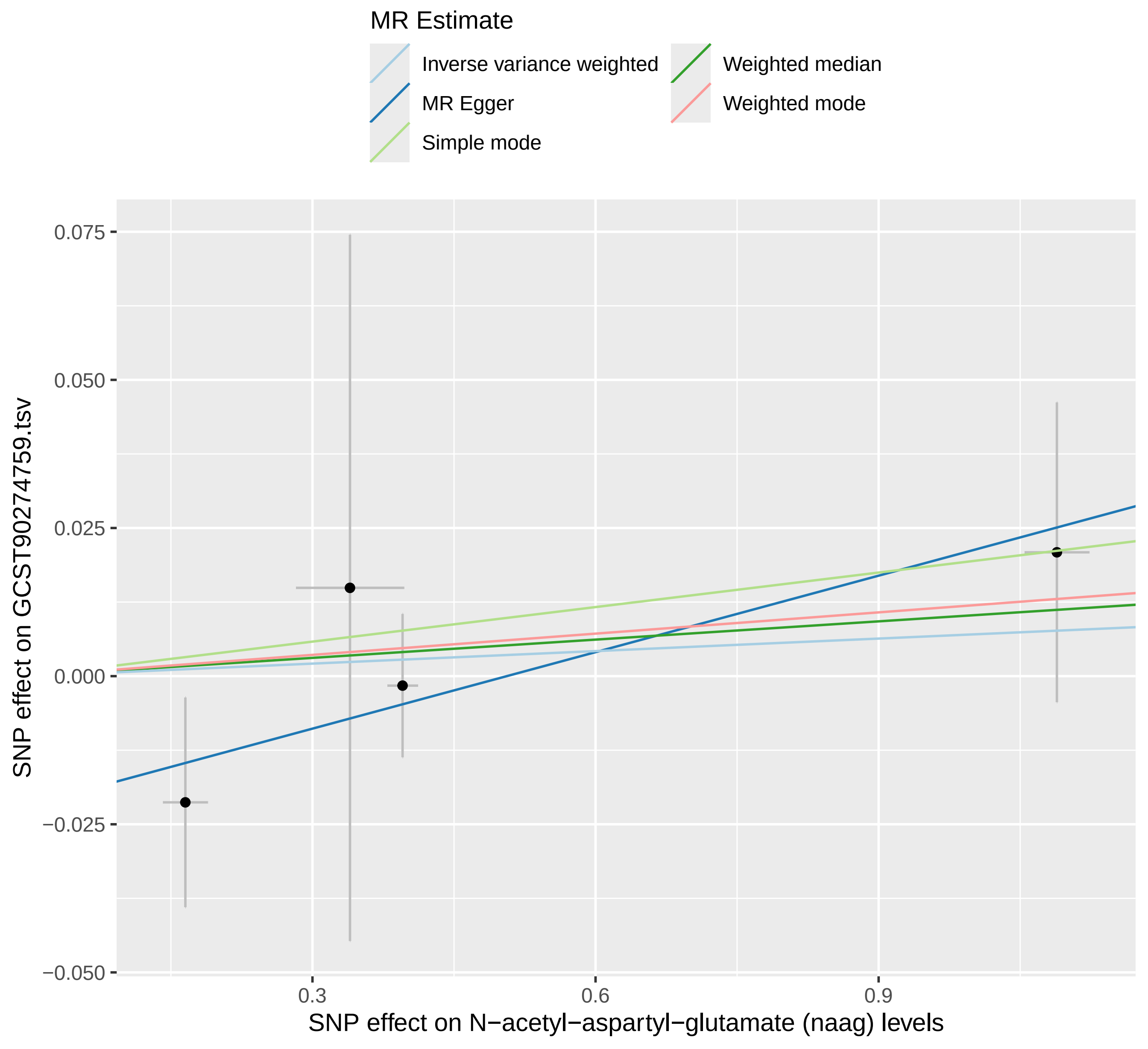
MR Estimate
Inverse variance weighted
MR Egger
Simple mode
Weighted median
Weighted mode
0.075
0.050
0.025
0.000
−0.025
−0.050
SNP effect on GCST90274759.tsv
0.3
0.6
0.9
SNP effect on N−acetyl−aspartyl−glutamate (naag) levels

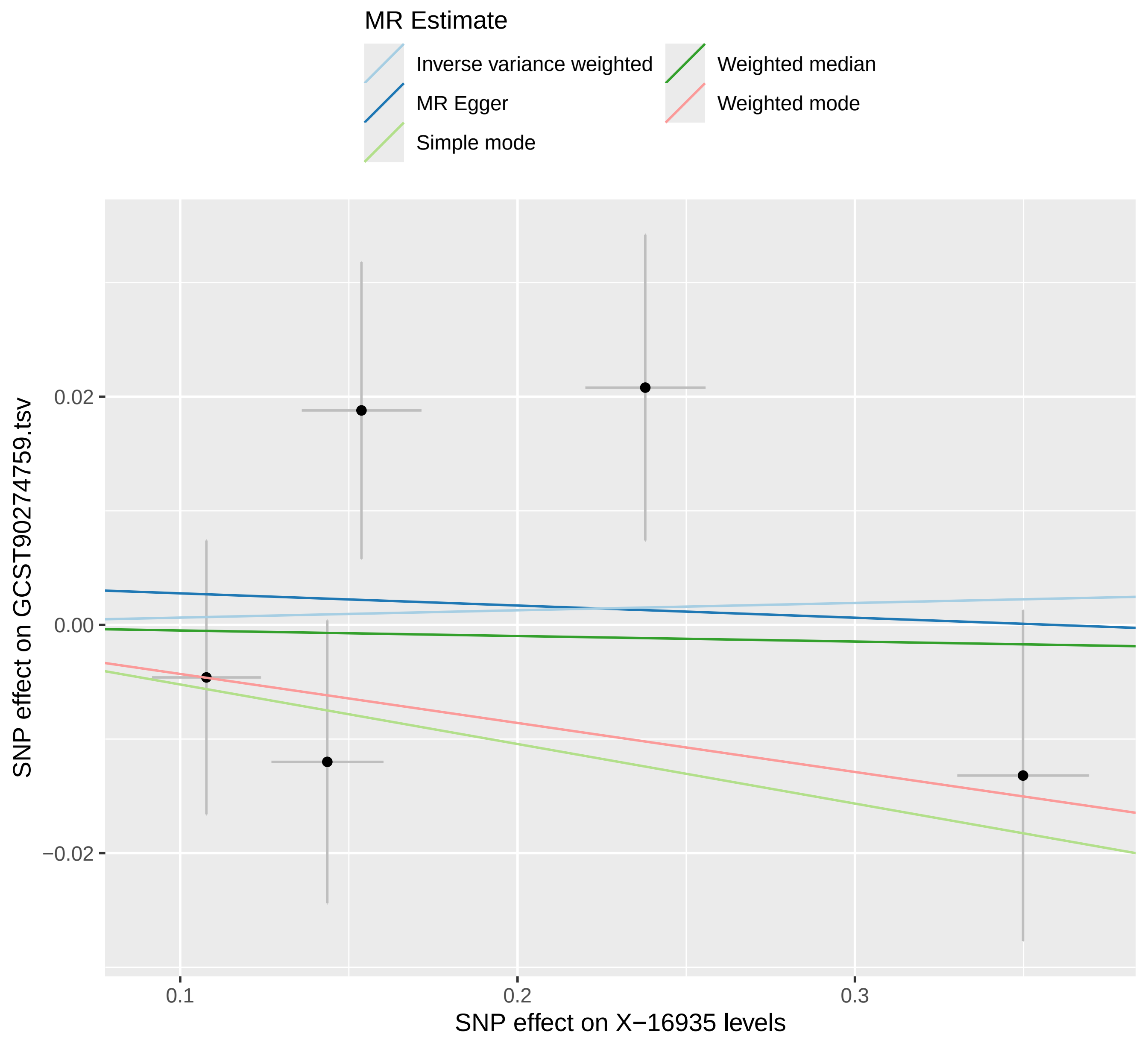
MR Estimate
Inverse variance weighted
MR Egger
Simple mode
Weighted median
Weighted mode
0.02
0.00
−0.02
0.1
0.2
0.3
SNP effect on GCST90274759.tsv
SNP effect on X−16935 levels

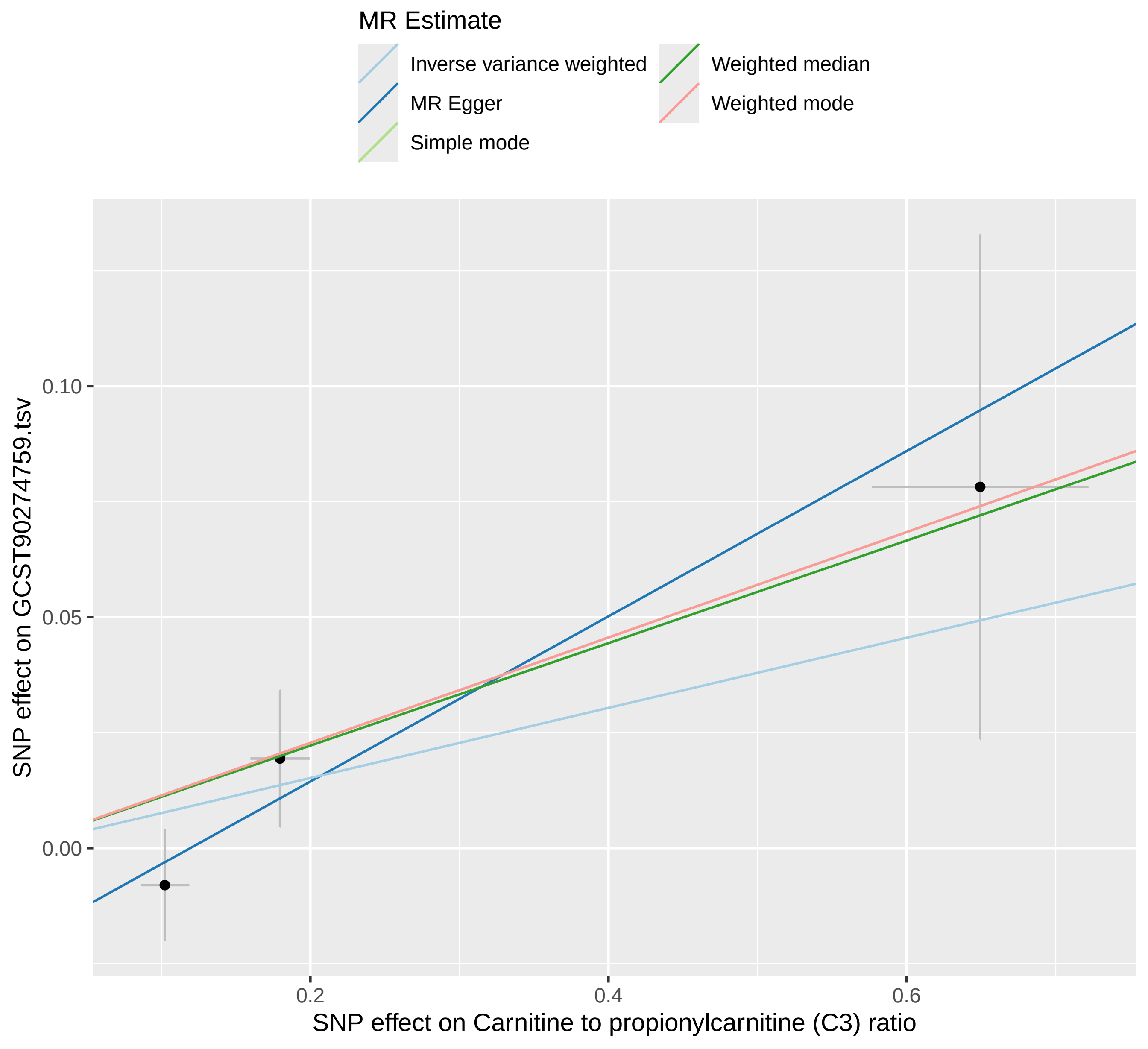
MR Estimate
Inverse variance weighted
MR Egger
Simple mode
Weighted median
Weighted mode
0.10
0.05
0.00
SNP effect on GCST90274759.tsv
0.2
0.4
0.6
SNP effect on Carnitine to propionylcarnitine (C3) ratio

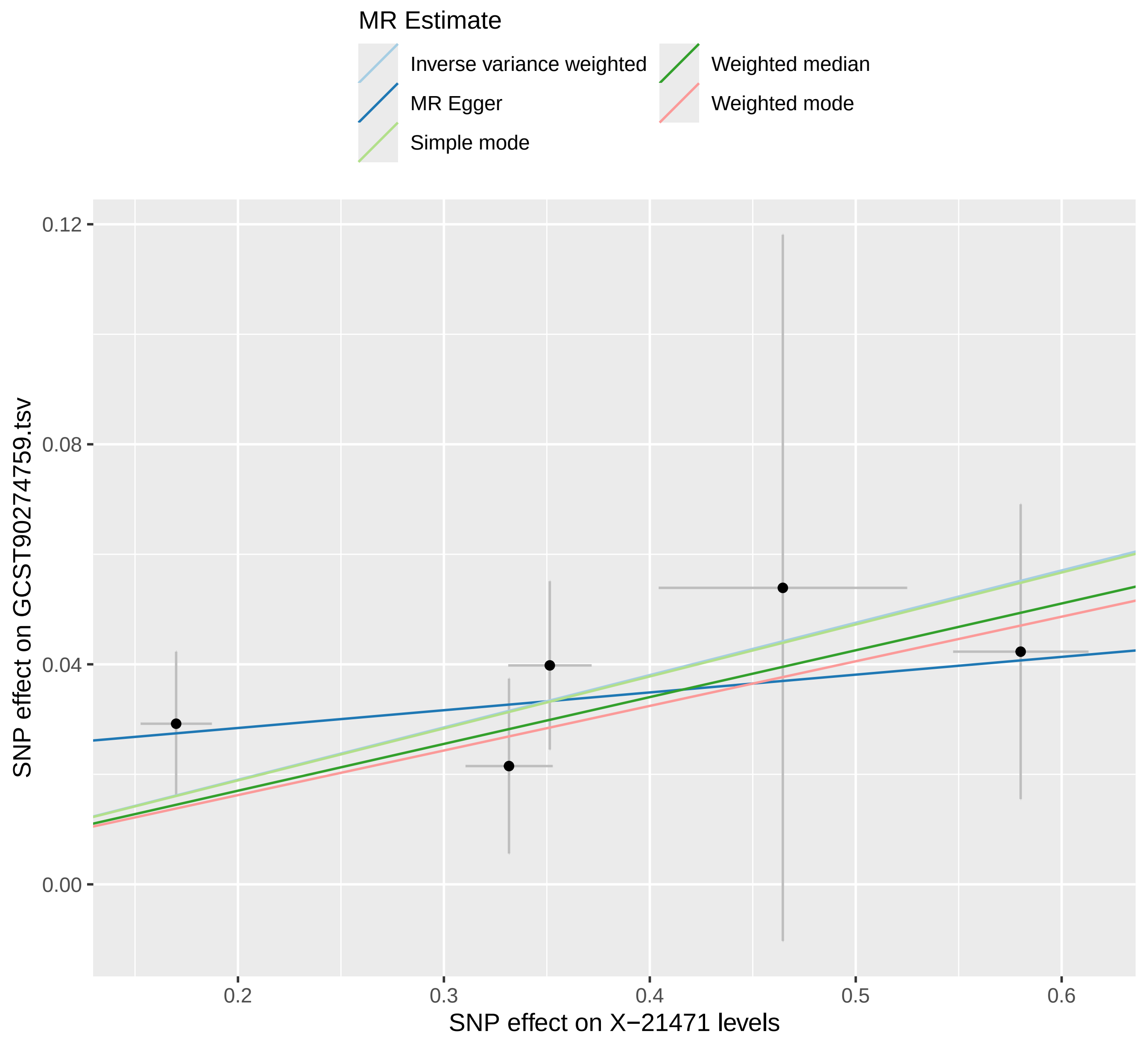

MR Estimate
Inverse variance weighted
MR Egger
Simple mode
Weighted median
Weighted mode
0.12
0.08
0.04
0.00
0.2
0.3
0.4
0.5
0.6
SNP effect on X−21471 levels
SNP effect on GCST90274759.tsv

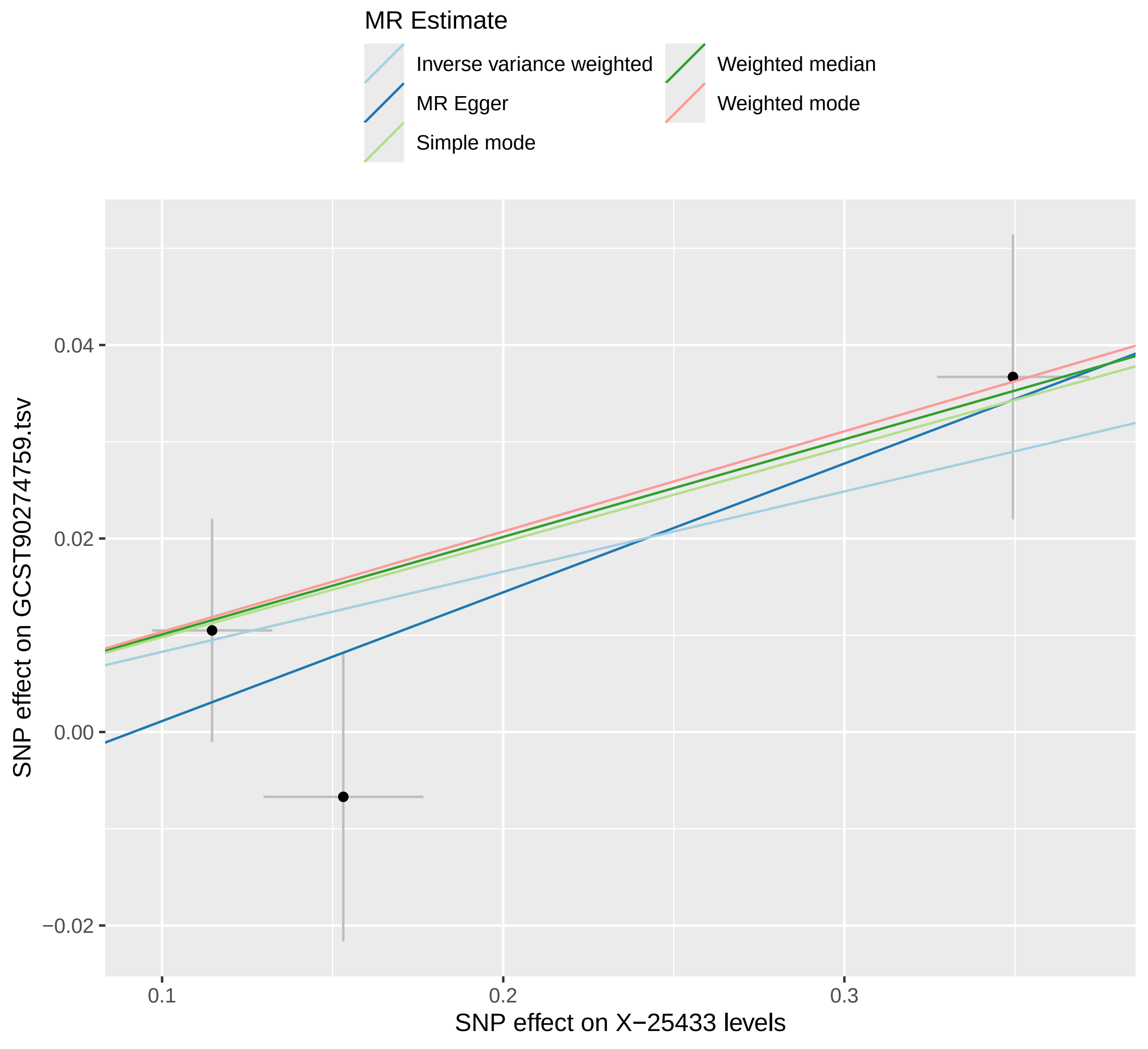
MR Estimate
Inverse variance weighted
MR Egger
Simple mode
Weighted median
Weighted mode
SNP effect on GCST90274759.tsv
0.04
0.02
0.00
−0.02
0.1
0.2
0.3
SNP effect on X−25433 levels

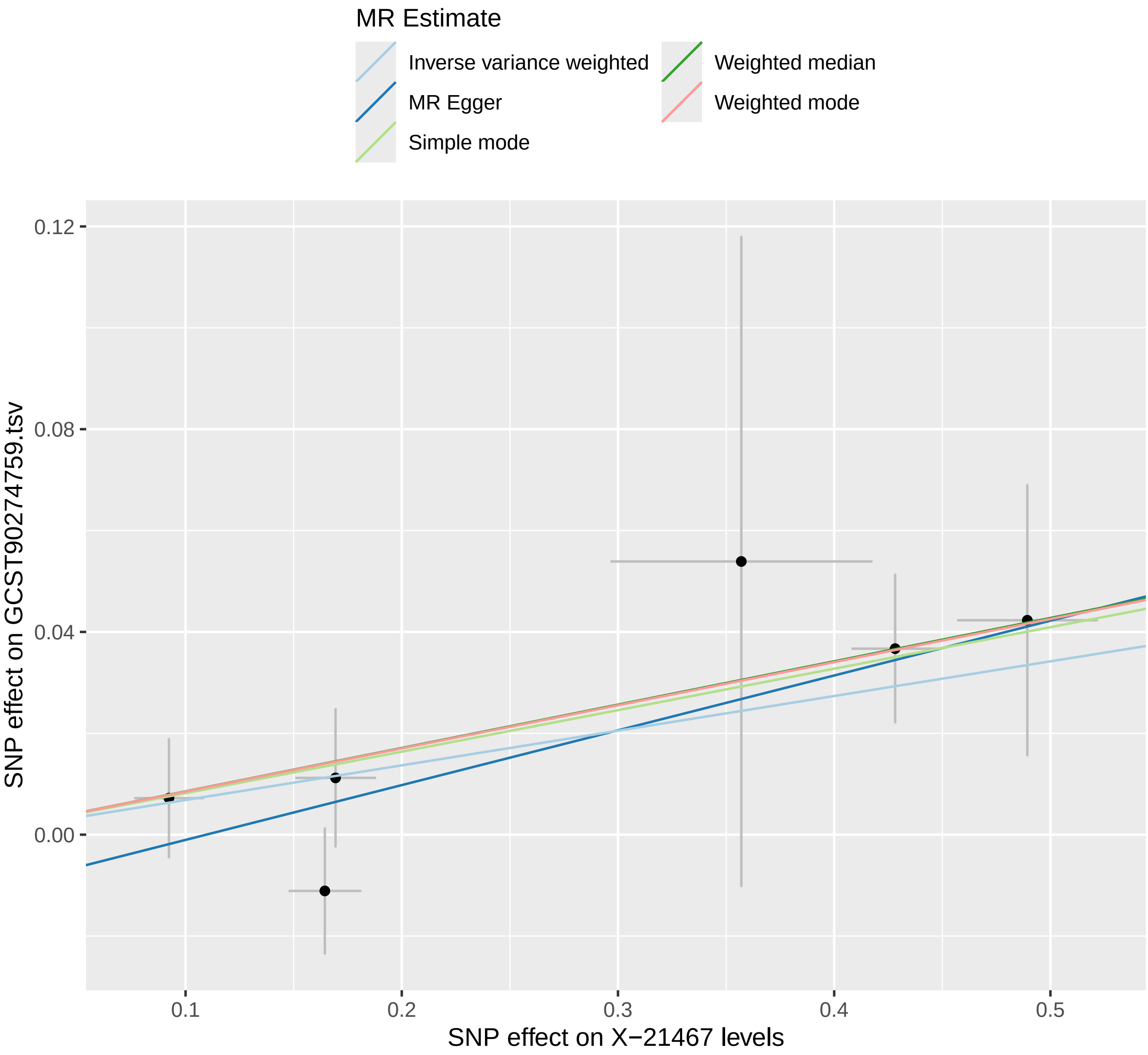

MR Estimate
Inverse variance weighted
MR Egger
Simple mode
Weighted median
Weighted mode
0.12
0.08
0.04
0.00
0.1
0.2
0.3
0.4
0.5
SNP effect on GCST90274759.tsv
SNP effect on X−21467 levels

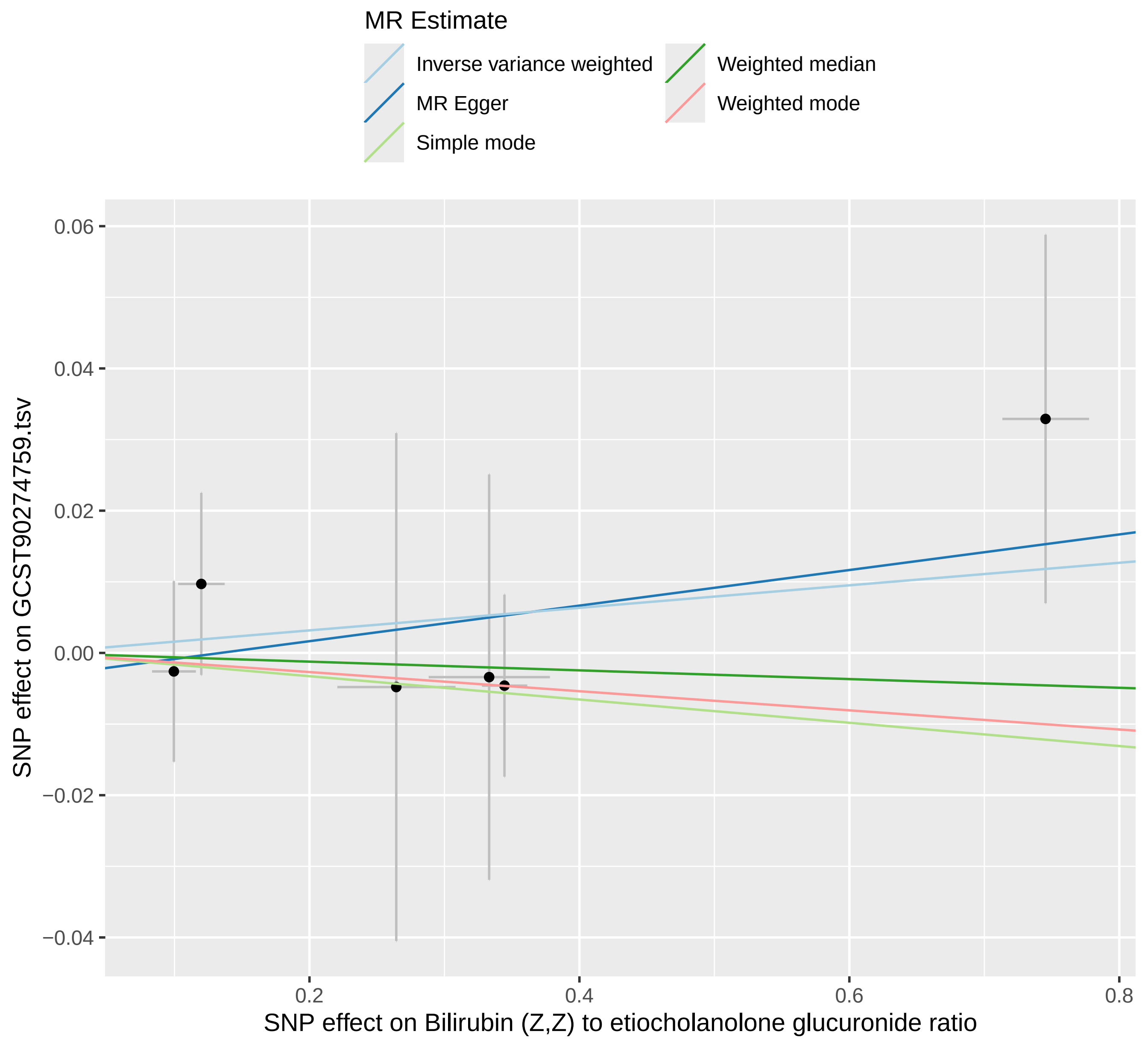
MR Estimate
Inverse variance weighted
MR Egger
Simple mode
Weighted median
Weighted mode
0.06
0.04
0.02
0.00
−0.02
−0.04
0.2
0.4
0.6
0.8
SNP effect on GCST90274759.tsv
SNP effect on Bilirubin (Z,Z) to etiocholanolone glucuronide ratio

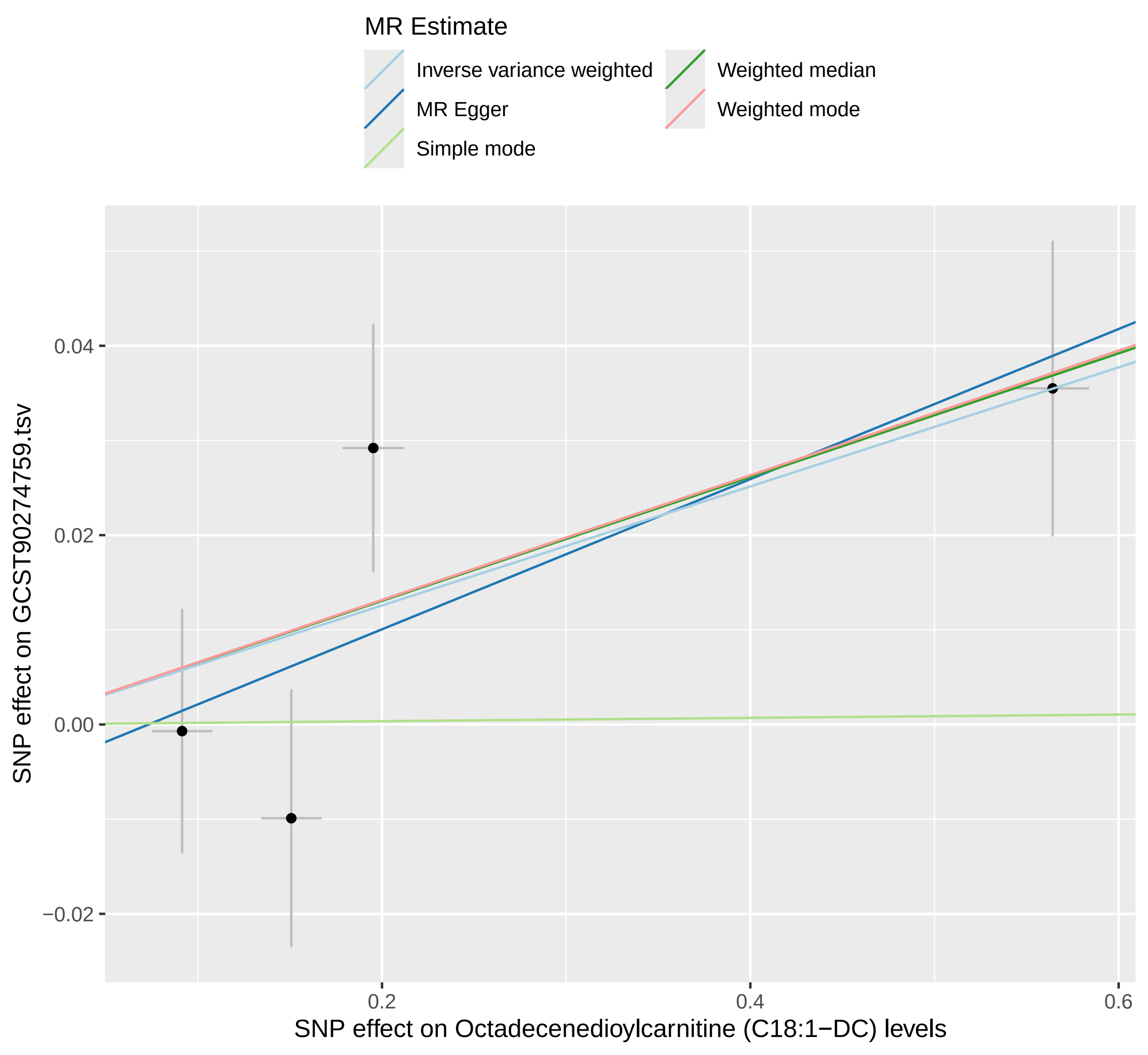

MR Estimate
Inverse variance weighted
MR Egger
Simple mode
Weighted median
Weighted mode
SNP effect on GCST90274759.tsv
0.04
0.02
0.00
−0.02
0.2
0.4
0.6
SNP effect on Octadecenedioylcarnitine (C18:1−DC) levels

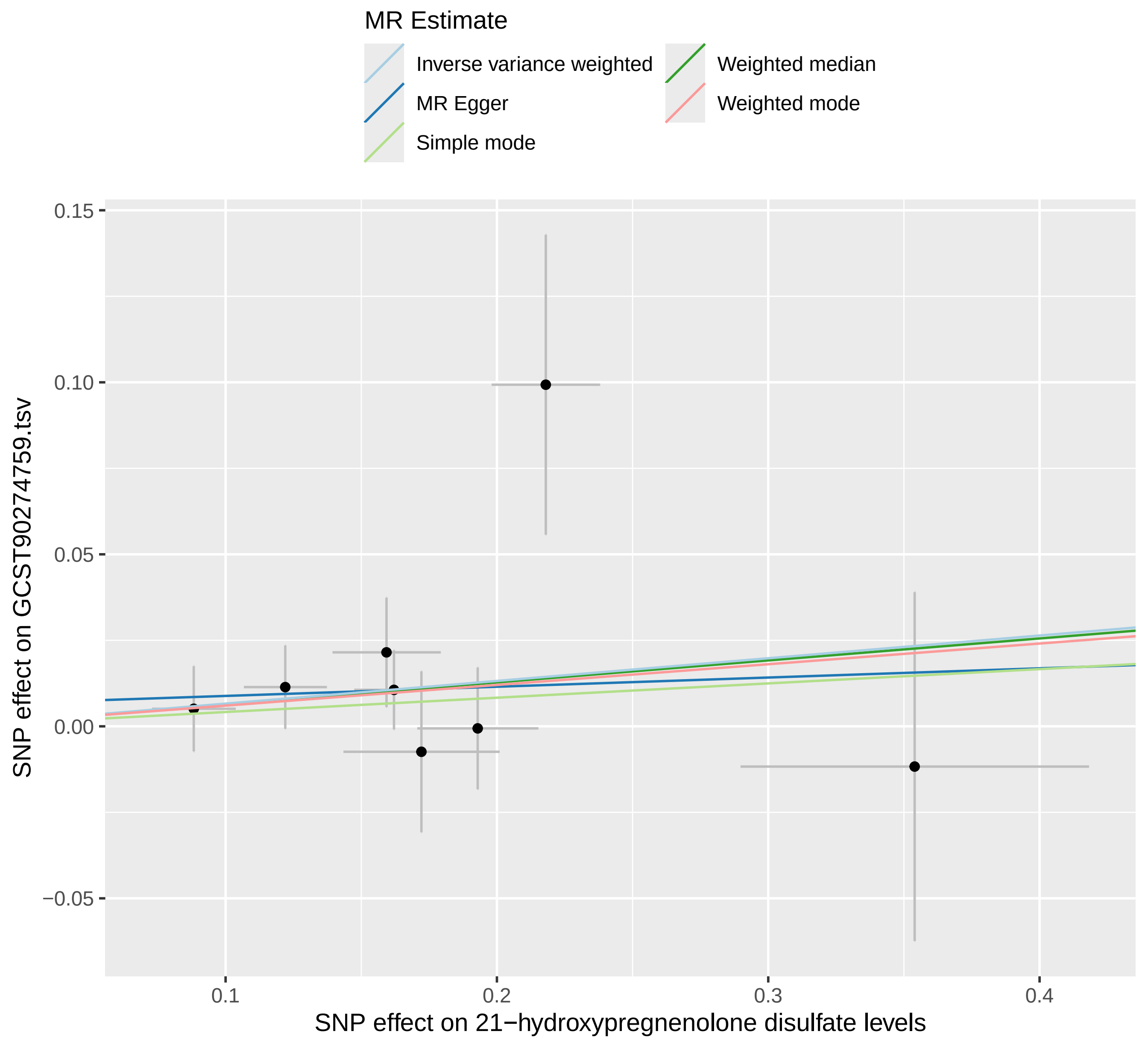

MR Estimate
Inverse variance weighted
MR Egger
Simple mode
Weighted median
Weighted mode
0.15
0.10
0.05
0.00
−0.05
0.1
0.2
0.3
0.4
SNP effect on GCST90274759.tsv
SNP effect on 21−hydroxypregnenolone disulfate levels

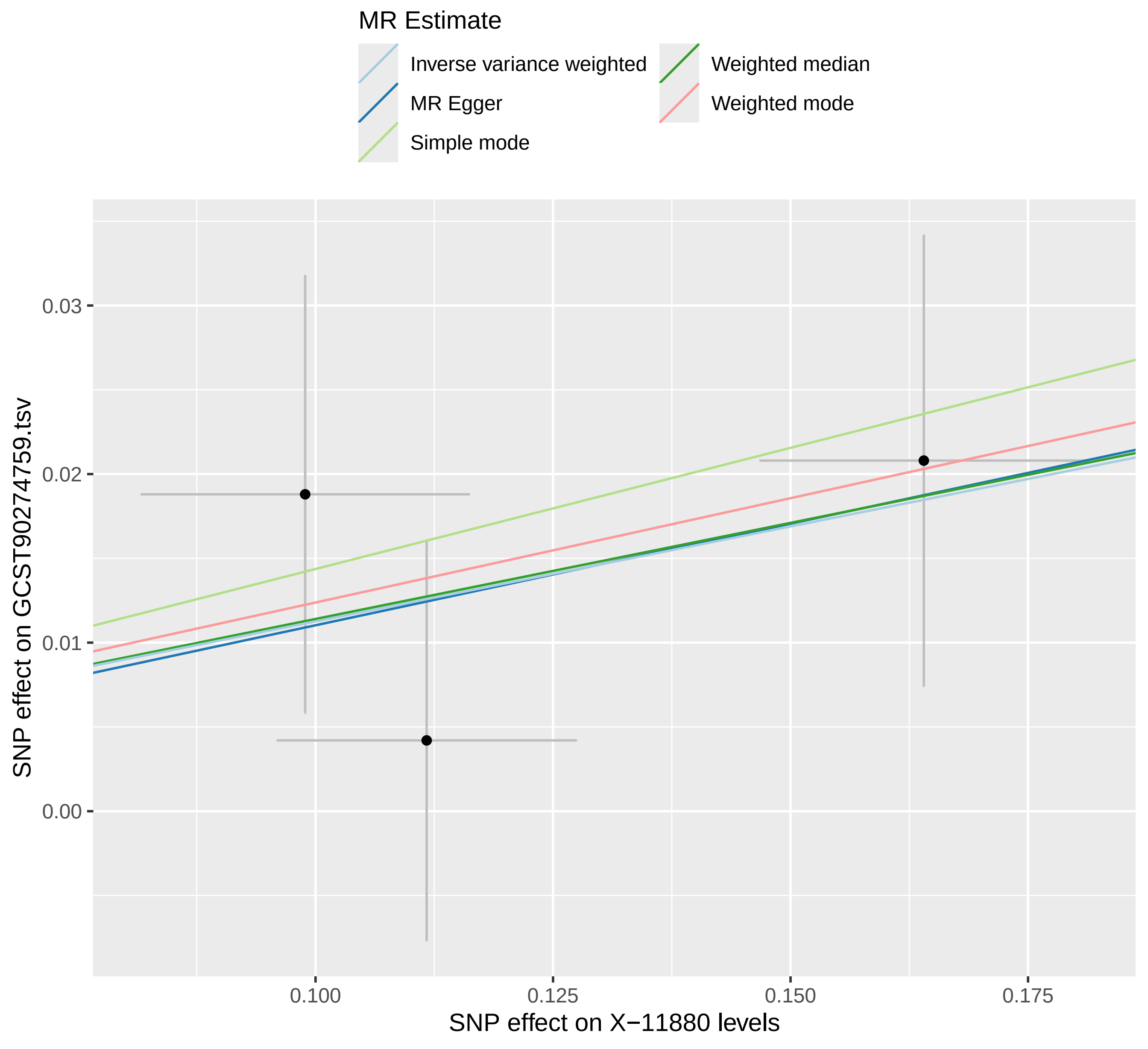
MR Estimate
Inverse variance weighted
MR Egger
Simple mode
Weighted median
Weighted mode
0.03
0.02
0.01
0.00
0.100
0.125
0.150
0.175
SNP effect on GCST90274759.tsv
SNP effect on X−11880 levels

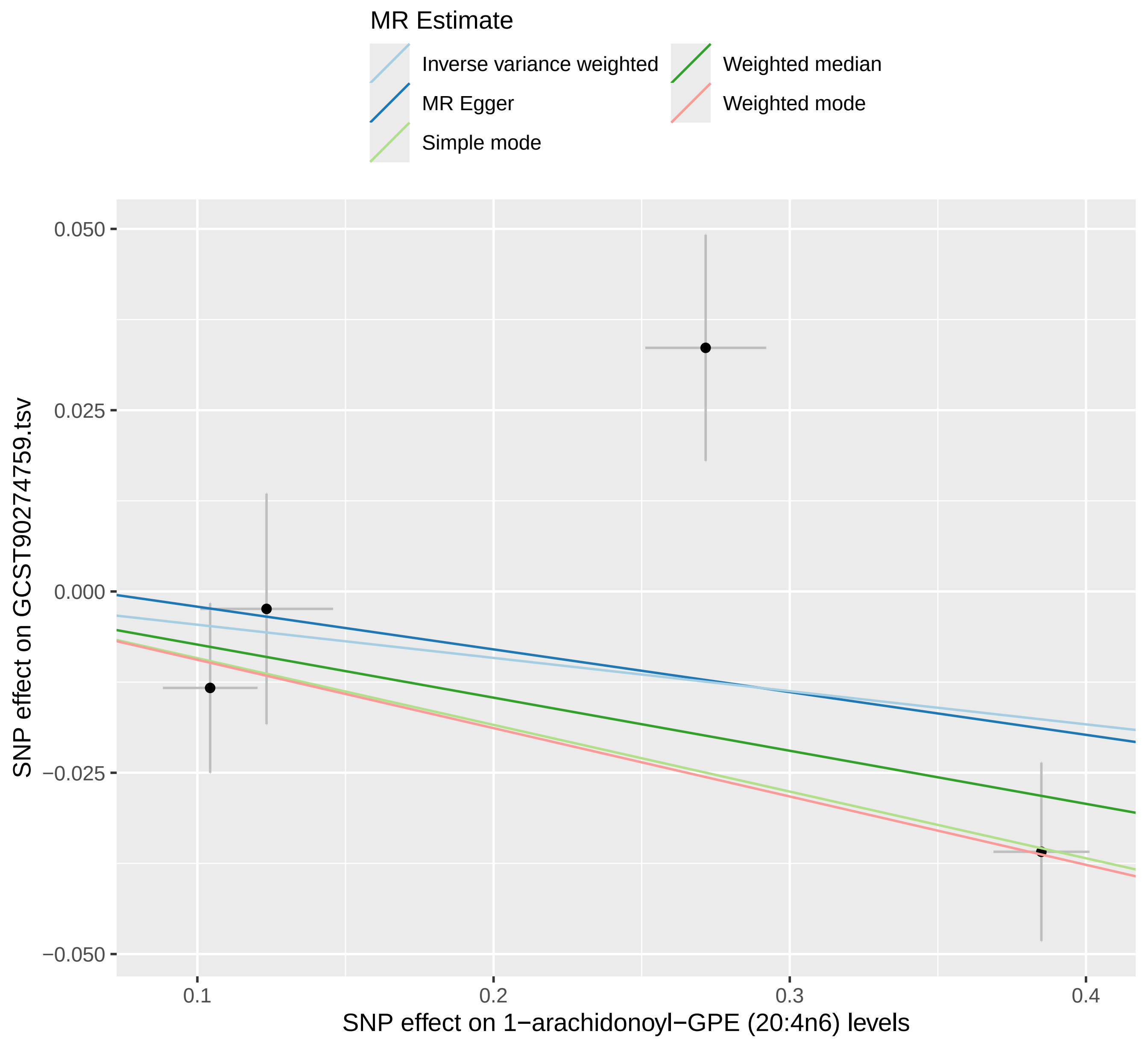
MR Estimate
Inverse variance weighted
MR Egger
Simple mode
Weighted median
Weighted mode
0.050
0.025
0.000
−0.025
−0.050
SNP effect on GCST90274759.tsv
0.1
0.2
0.3
0.4
SNP effect on 1−arachidonoyl−GPE (20:4n6) levels

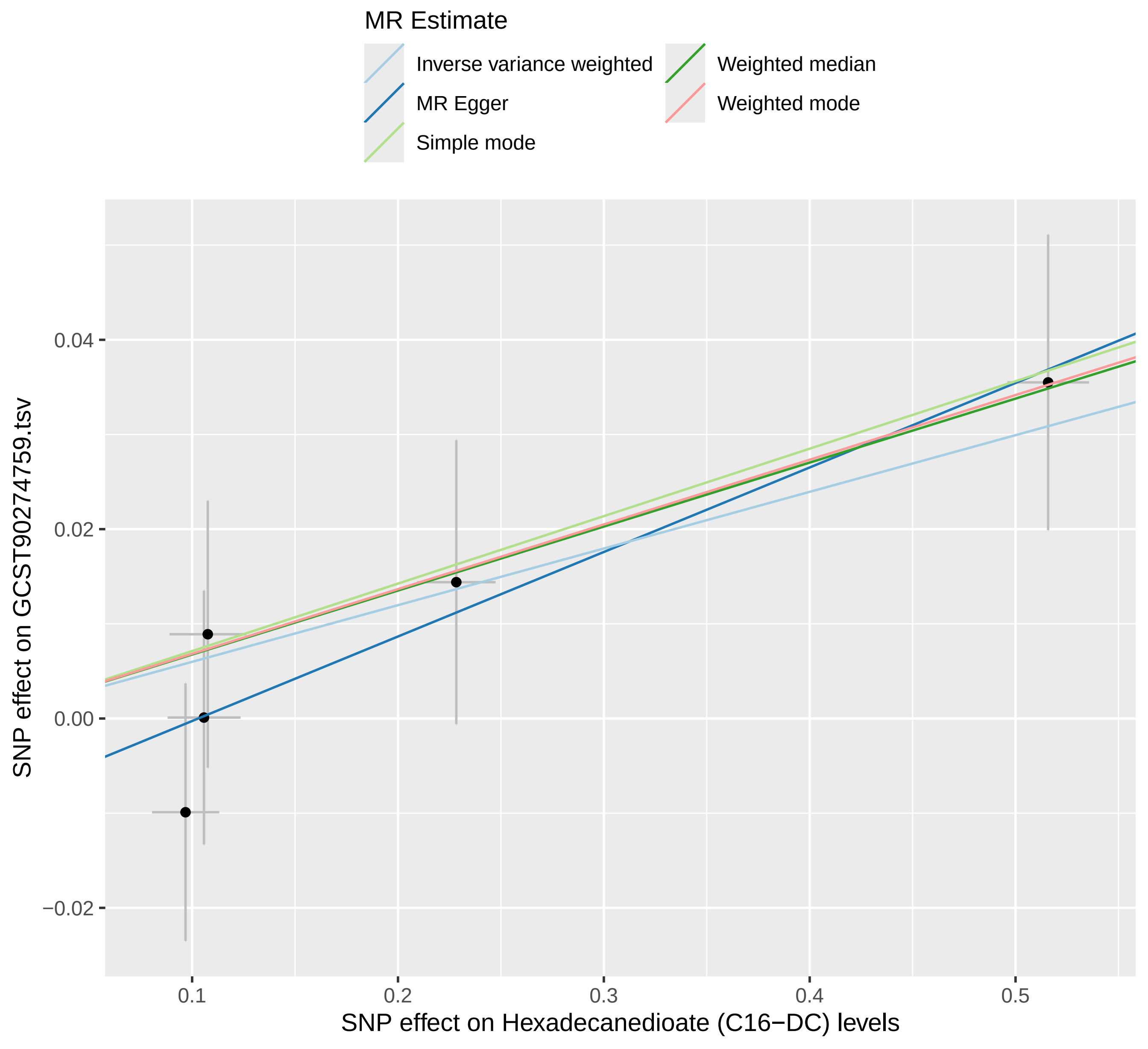
MR Estimate
Inverse variance weighted
MR Egger
Simple mode
Weighted median
Weighted mode
0.04
0.02
0.00
−0.02
0.1
0.2
0.3
0.4
0.5
SNP effect on GCST90274759.tsv
SNP effect on Hexadecanedioate (C16−DC) levels

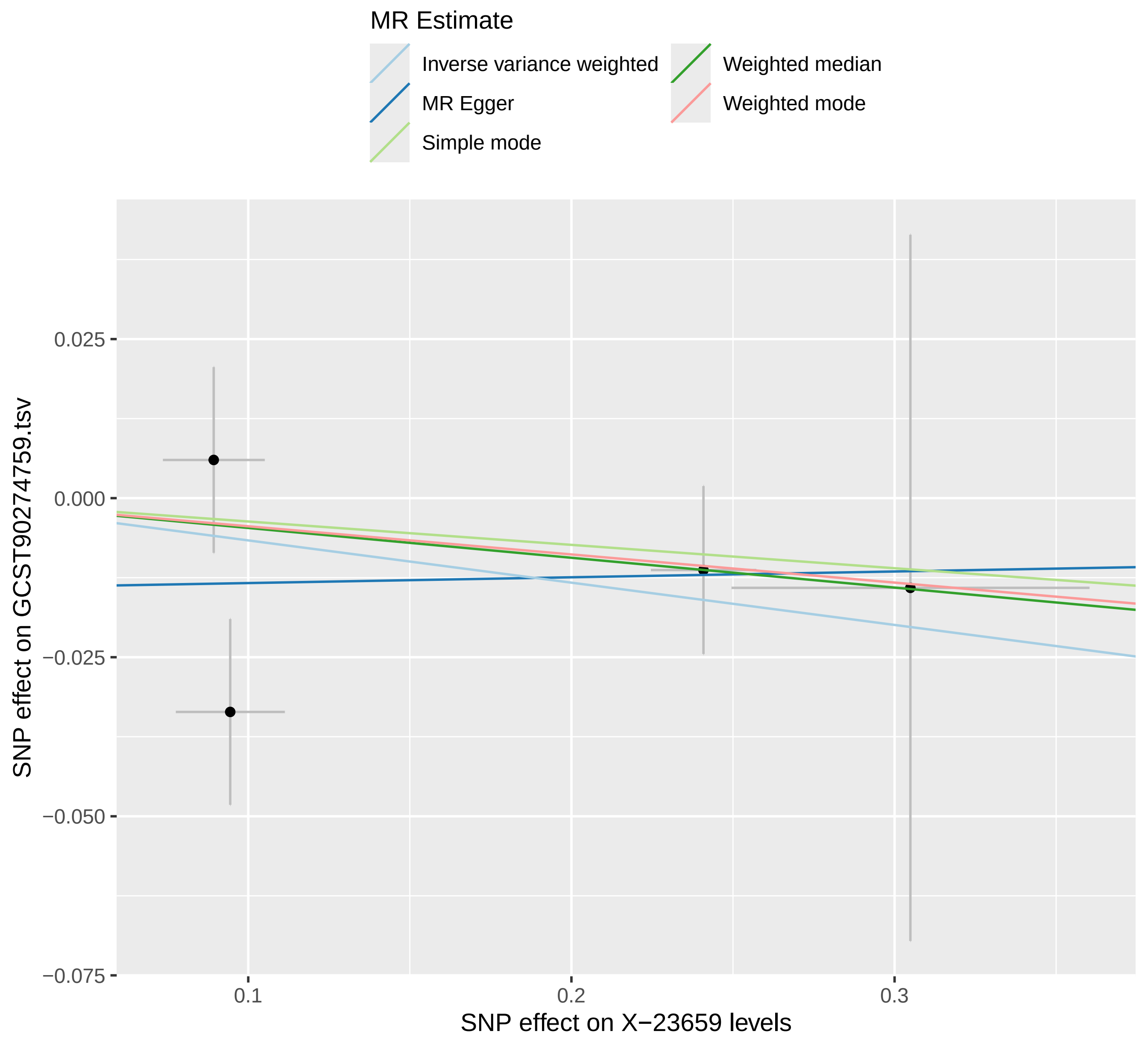
MR Estimate
Inverse variance weighted
MR Egger
Simple mode
Weighted median
Weighted mode
0.025
0.000
−0.025
−0.050
−0.075
0.1
0.2
0.3
SNP effect on GCST90274759.tsv
SNP effect on X−23659 levels

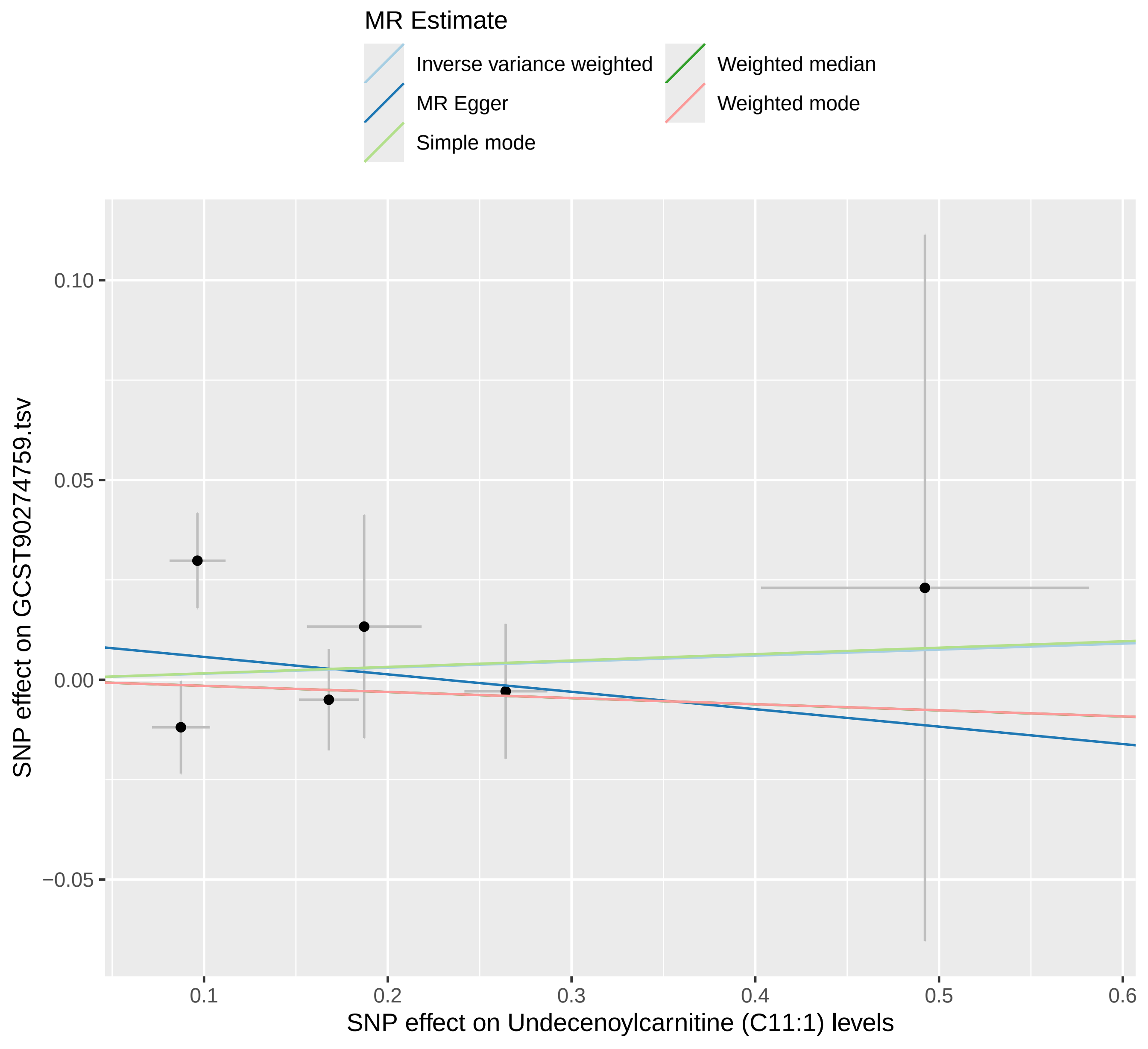
MR Estimate
Inverse variance weighted
MR Egger
Simple mode
Weighted median
Weighted mode
0.10
0.05
0.00
−0.05
0.1
0.2
0.3
0.4
0.5
0.6
SNP effect on GCST90274759.tsv
SNP effect on Undecenoylcarnitine (C11:1) levels

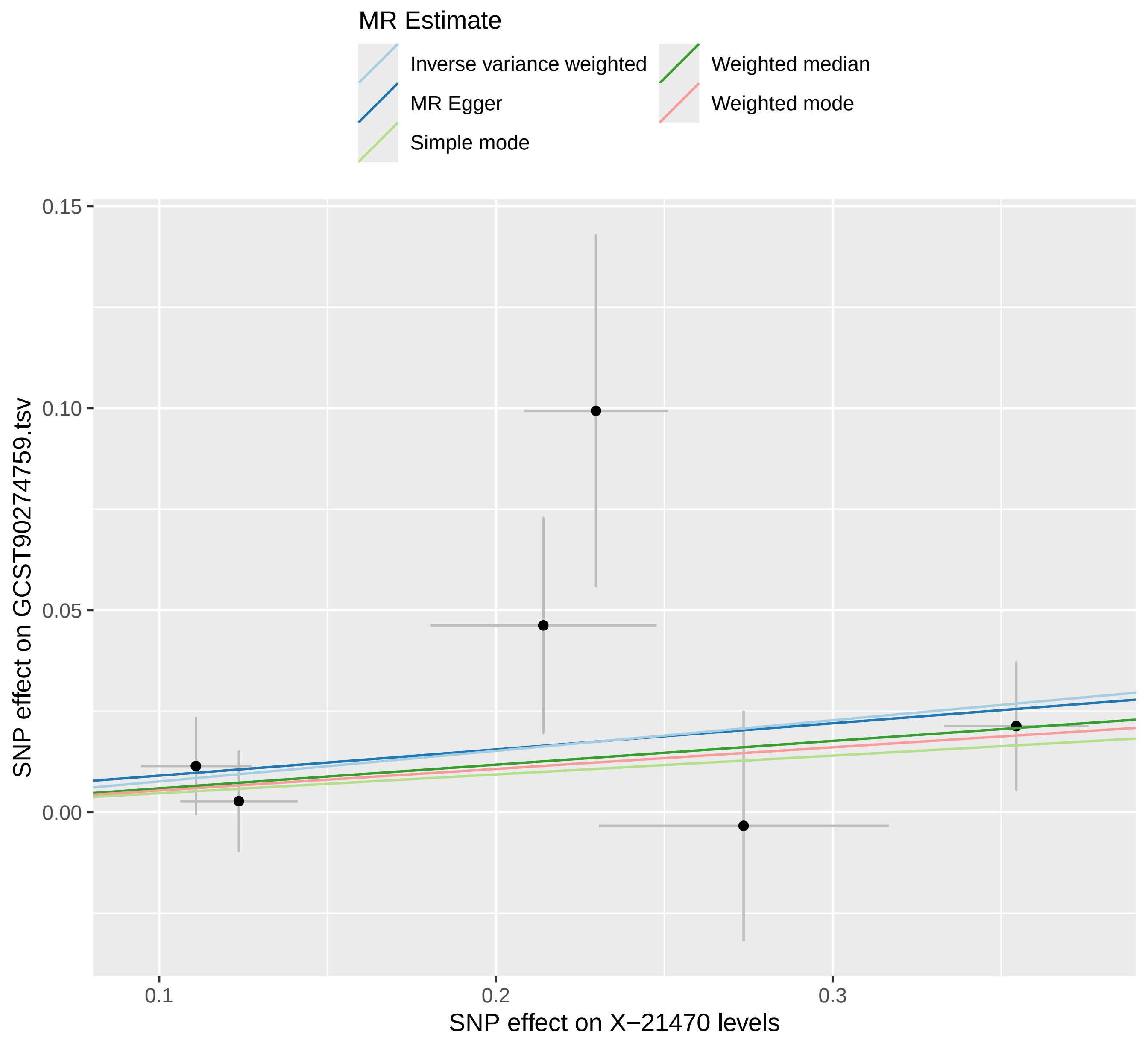
MR Estimate
Inverse variance weighted
MR Egger
Simple mode
Weighted median
Weighted mode
0.15
0.10
0.05
0.00
0.1
0.2
0.3
SNP effect on GCST90274759.tsv
SNP effect on X−21470 levels

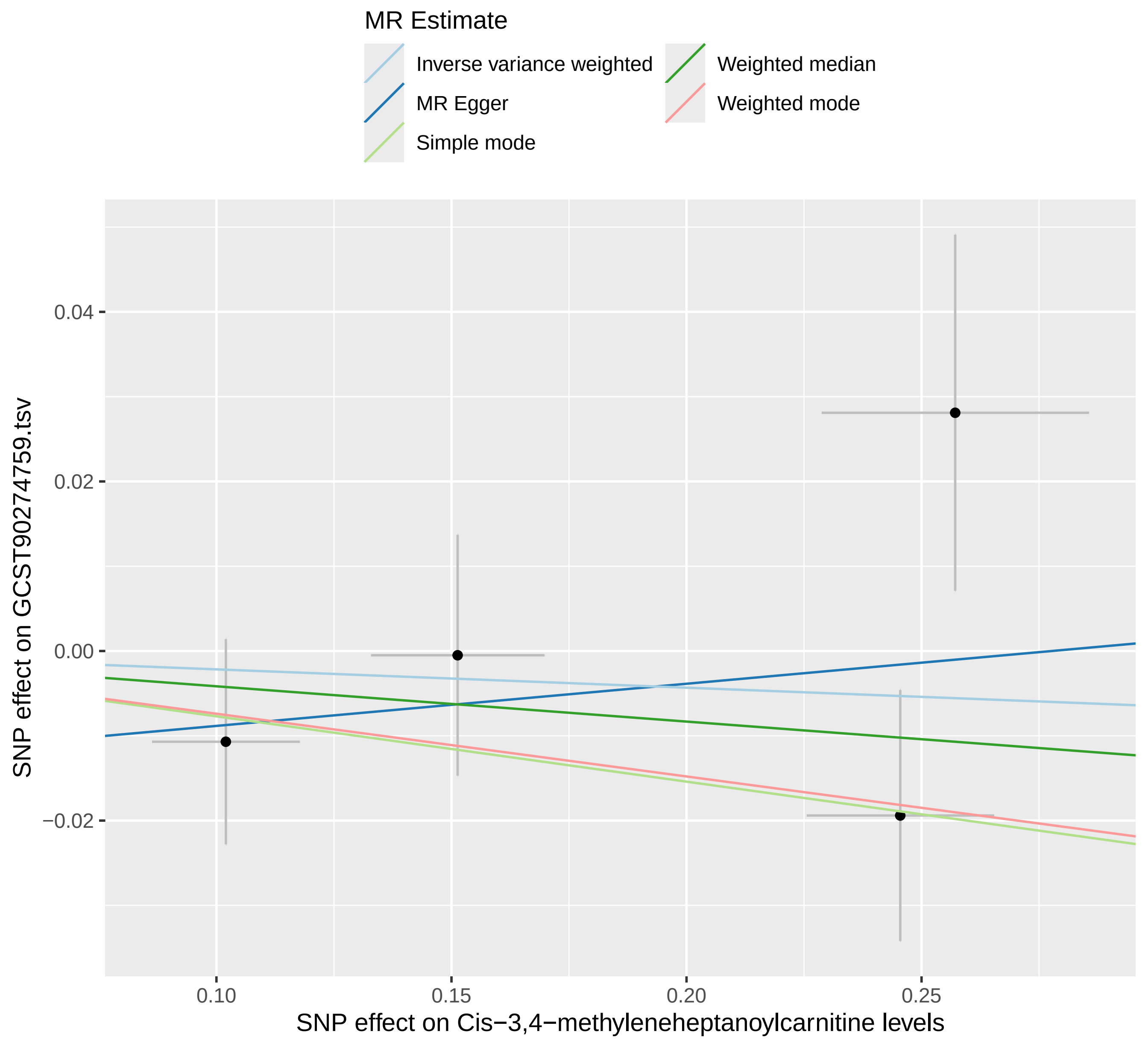
MR Estimate
Inverse variance weighted
MR Egger
Simple mode
Weighted median
Weighted mode
0.04
0.02
0.00
−0.02
0.10
0.15
0.20
0.25
SNP effect on GCST90274759.tsv
SNP effect on Cis−3,4−methyleneheptanoylcarnitine levels

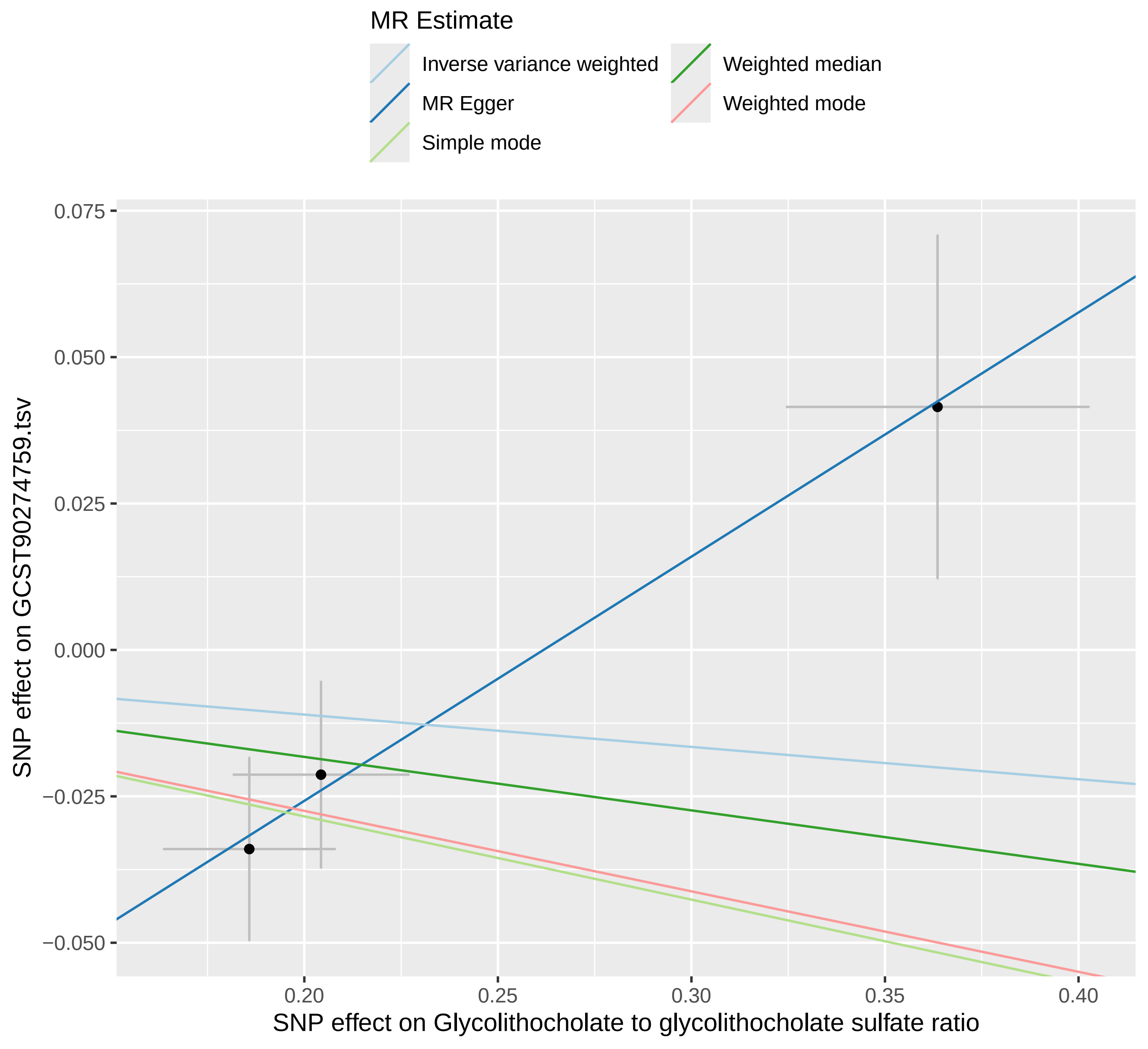
MR Estimate
Inverse variance weighted
MR Egger
Simple mode
Weighted median
Weighted mode
0.075
0.050
0.025
0.000
−0.025
−0.050
0.20
0.25
0.30
0.35
0.40
SNP effect on GCST90274759.tsv
SNP effect on Glycolithocholate to glycolithocholate sulfate ratio

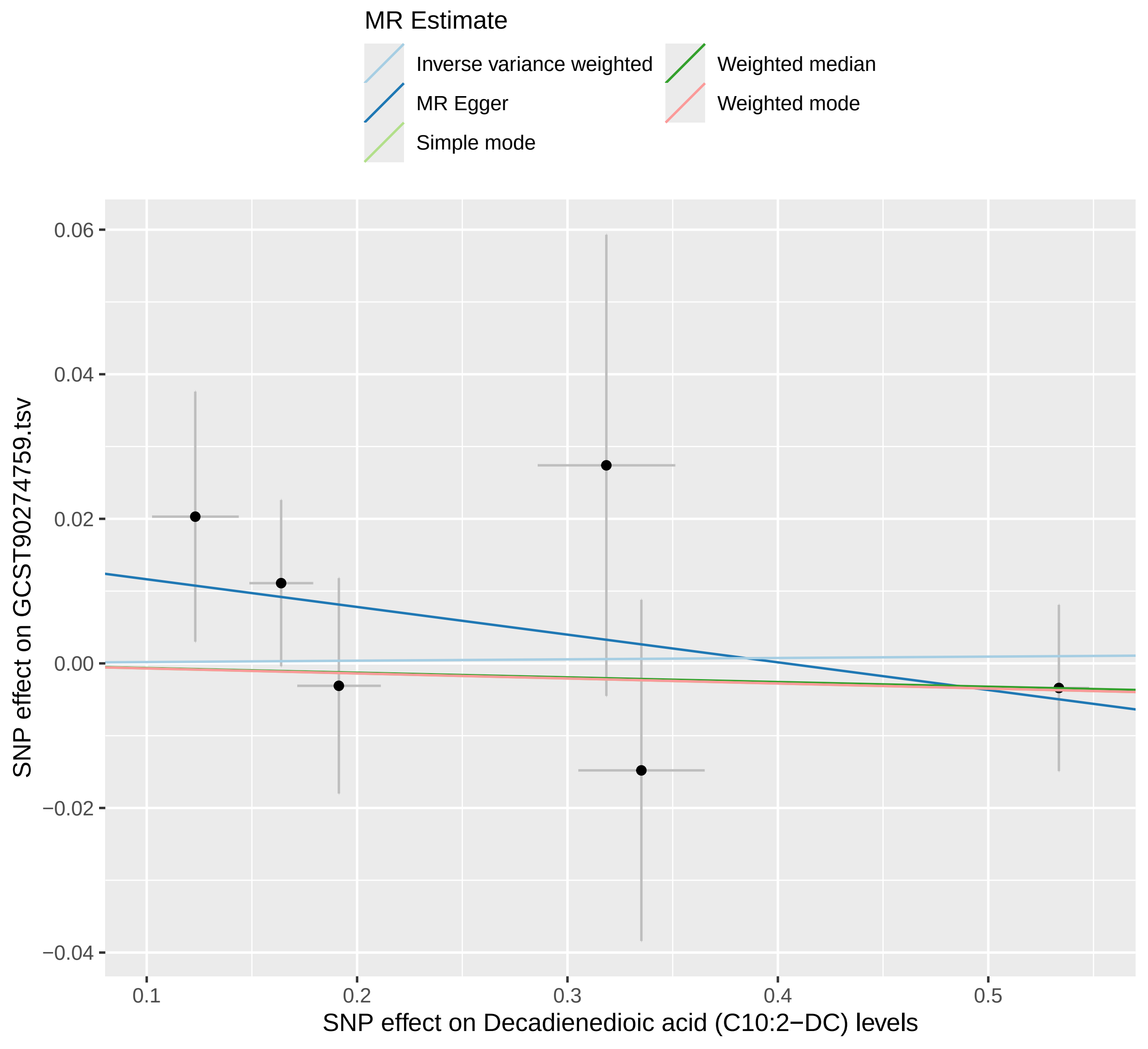

MR Estimate
Inverse variance weighted
MR Egger
Simple mode
Weighted median
Weighted mode
0.06
0.04
0.02
0.00
−0.02
−0.04
0.1
0.2
0.3
0.4
0.5
SNP effect on GCST90274759.tsv
SNP effect on Decadienedioic acid (C10:2−DC) levels

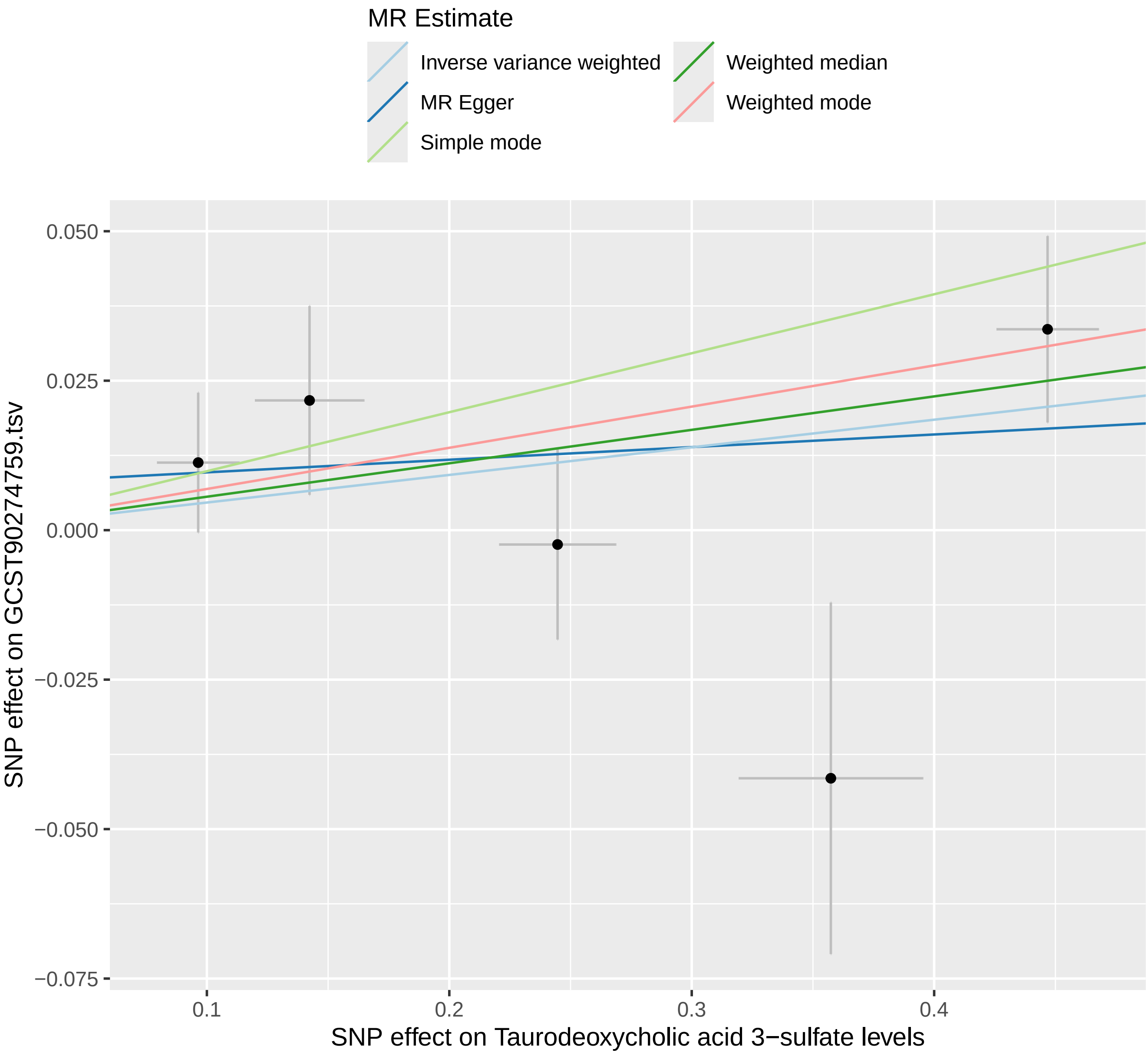

MR Estimate
Inverse variance weighted
MR Egger
Simple mode
Weighted median
Weighted mode
0.050
0.025
0.000
−0.025
−0.050
−0.075
0.1
0.2
0.3
0.4
SNP effect on GCST90274759.tsv
SNP effect on Taurodeoxycholic acid 3−sulfate levels

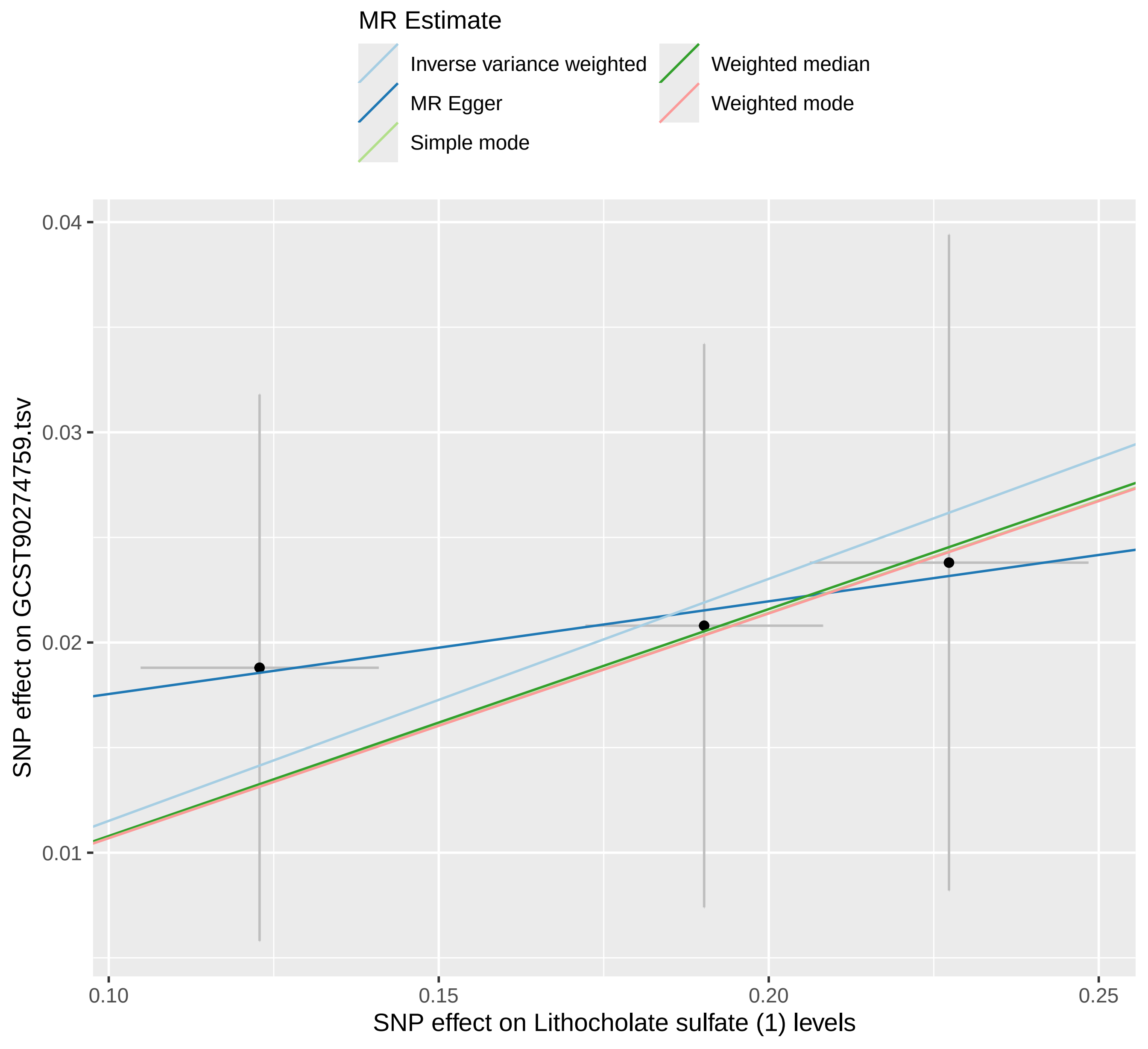
MR Estimate
Inverse variance weighted
MR Egger
Simple mode
Weighted median
Weighted mode
0.04
0.03
0.02
0.01
0.10
0.15
0.20
0.25
SNP effect on GCST90274759.tsv
SNP effect on Lithocholate sulfate (1) levels

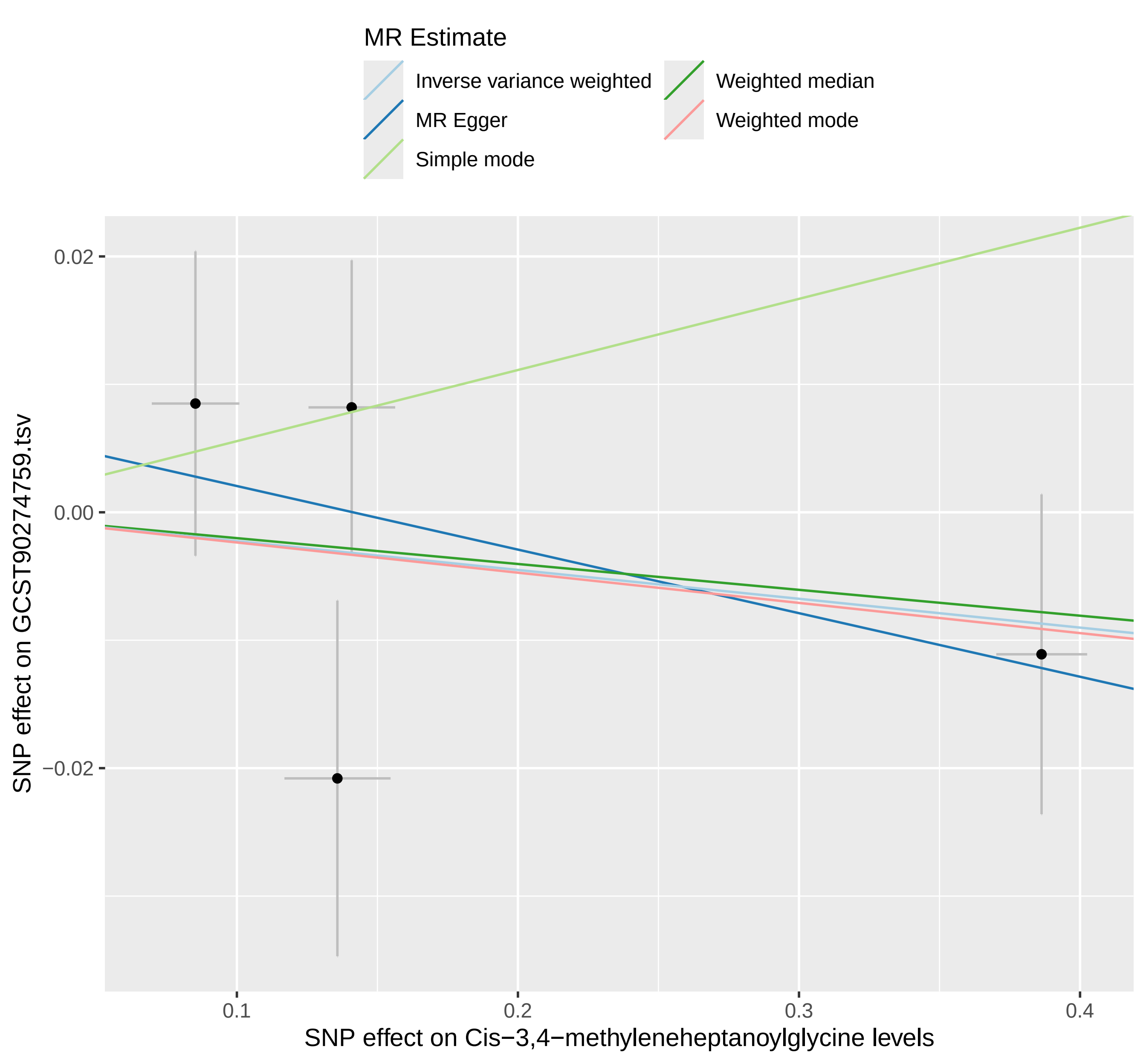
MR Estimate
Inverse variance weighted
MR Egger
Simple mode
Weighted median
Weighted mode
0.02
0.00
−0.02
0.1
0.2
0.3
0.4
SNP effect on GCST90274759.tsv
SNP effect on Cis−3,4−methyleneheptanoylglycine levels

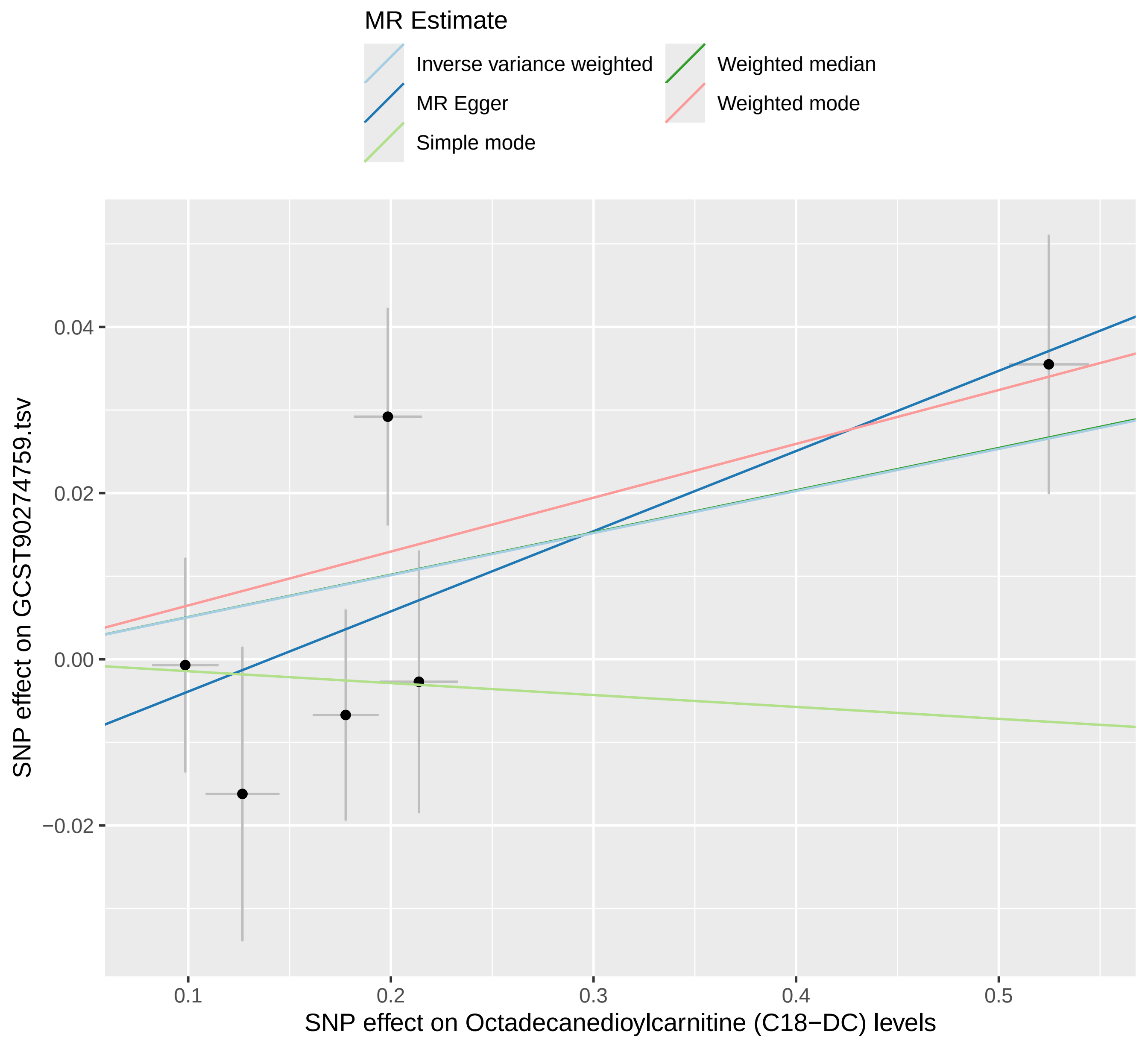

MR Estimate
Inverse variance weighted
MR Egger
Simple mode
Weighted median
Weighted mode
0.04
0.02
0.00
−0.02
0.1
0.2
0.3
0.4
0.5
SNP effect on GCST90274759.tsv
SNP effect on Octadecanedioylcarnitine (C18−DC) levels

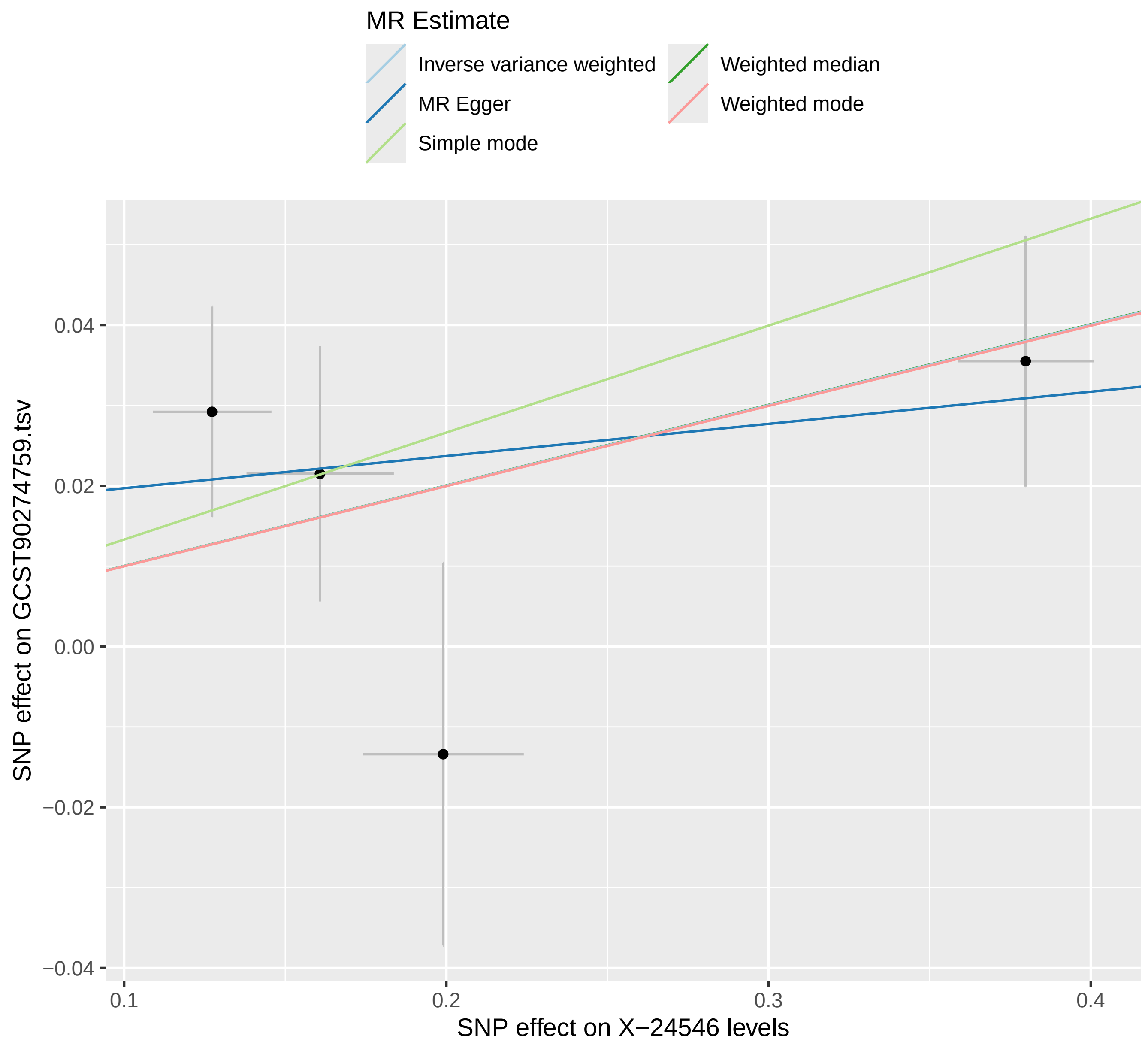
MR Estimate
Inverse variance weighted
MR Egger
Simple mode
Weighted median
Weighted mode
SNP effect on GCST90274759.tsv
0.04
0.02
0.00
−0.02
−0.04
0.1
0.2
0.3
0.4
SNP effect on X−24546 levels

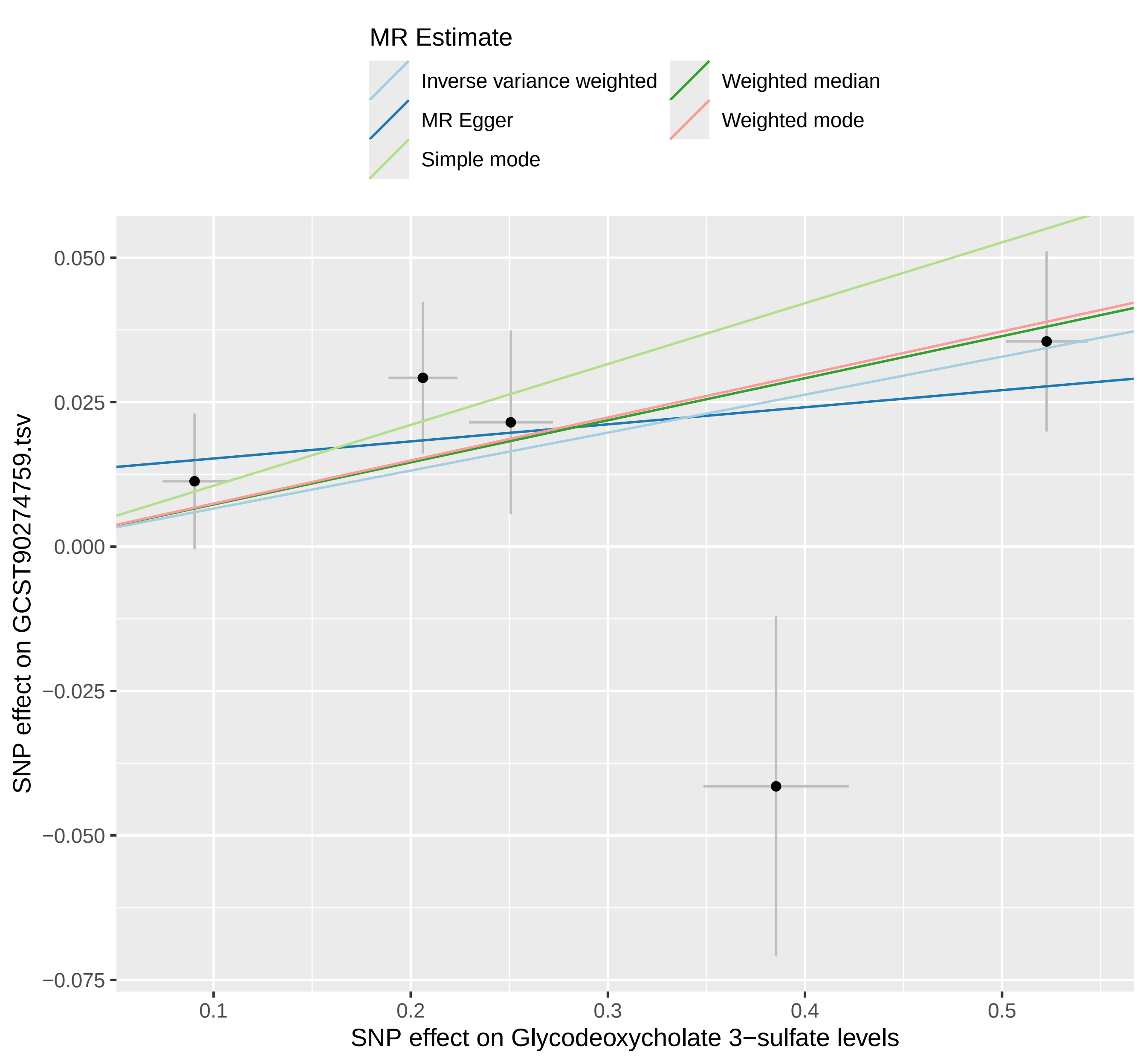
MR Estimate
Inverse variance weighted
MR Egger
Simple mode
Weighted median
Weighted mode
0.050
0.025
0.000
−0.025
−0.050
−0.075
0.1
0.2
0.3
0.4
0.5
SNP effect on GCST90274759.tsv
SNP effect on Glycodeoxycholate 3−sulfate levels

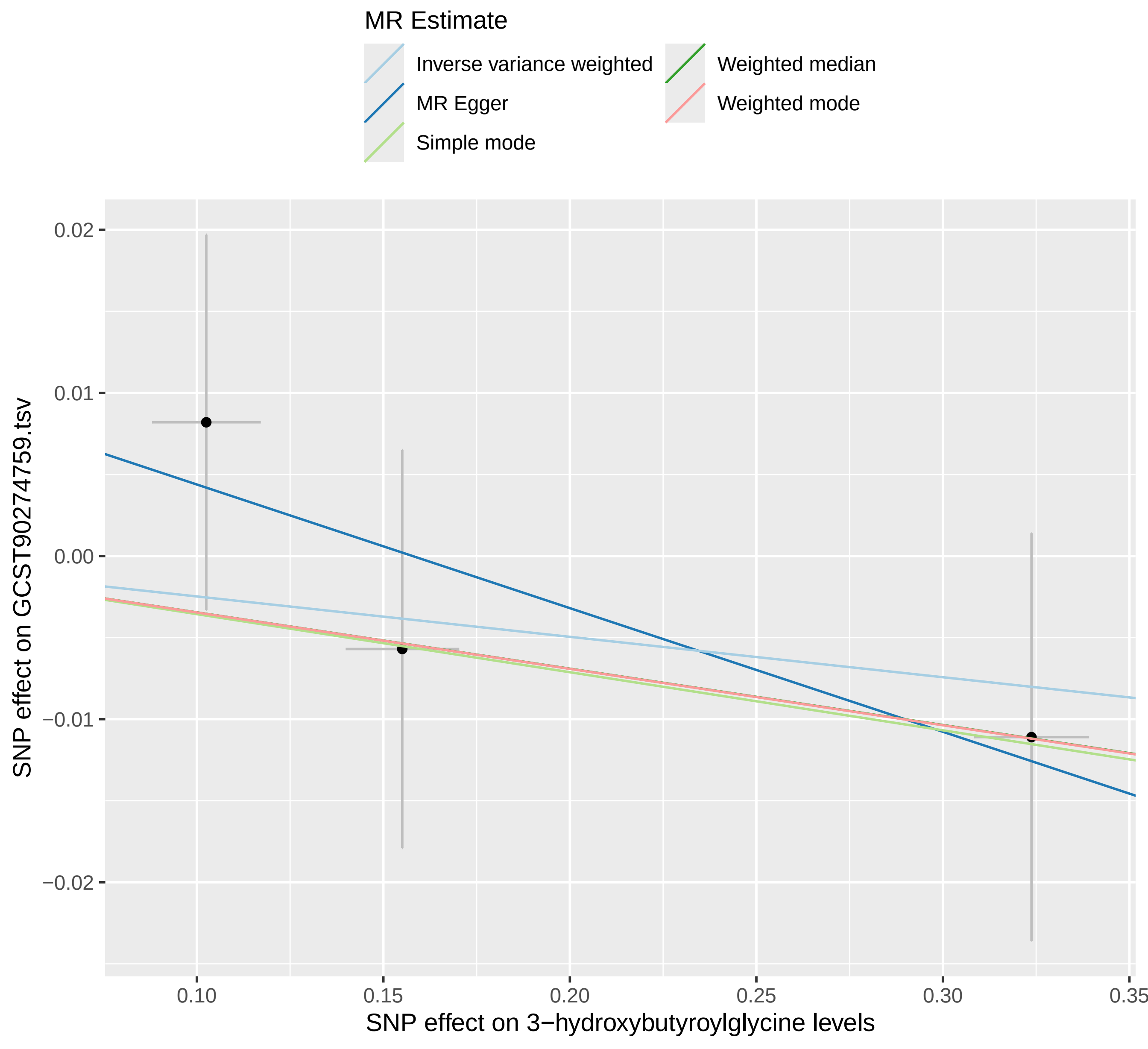
MR Estimate
Inverse variance weighted
MR Egger
Simple mode
Weighted median
Weighted mode
0.02
0.01
0.00
−0.01
−0.02
SNP effect on GCST90274759.tsv
0.10
0.15
0.20
0.25
0.30
0.35
SNP effect on 3−hydroxybutyroylglycine levels

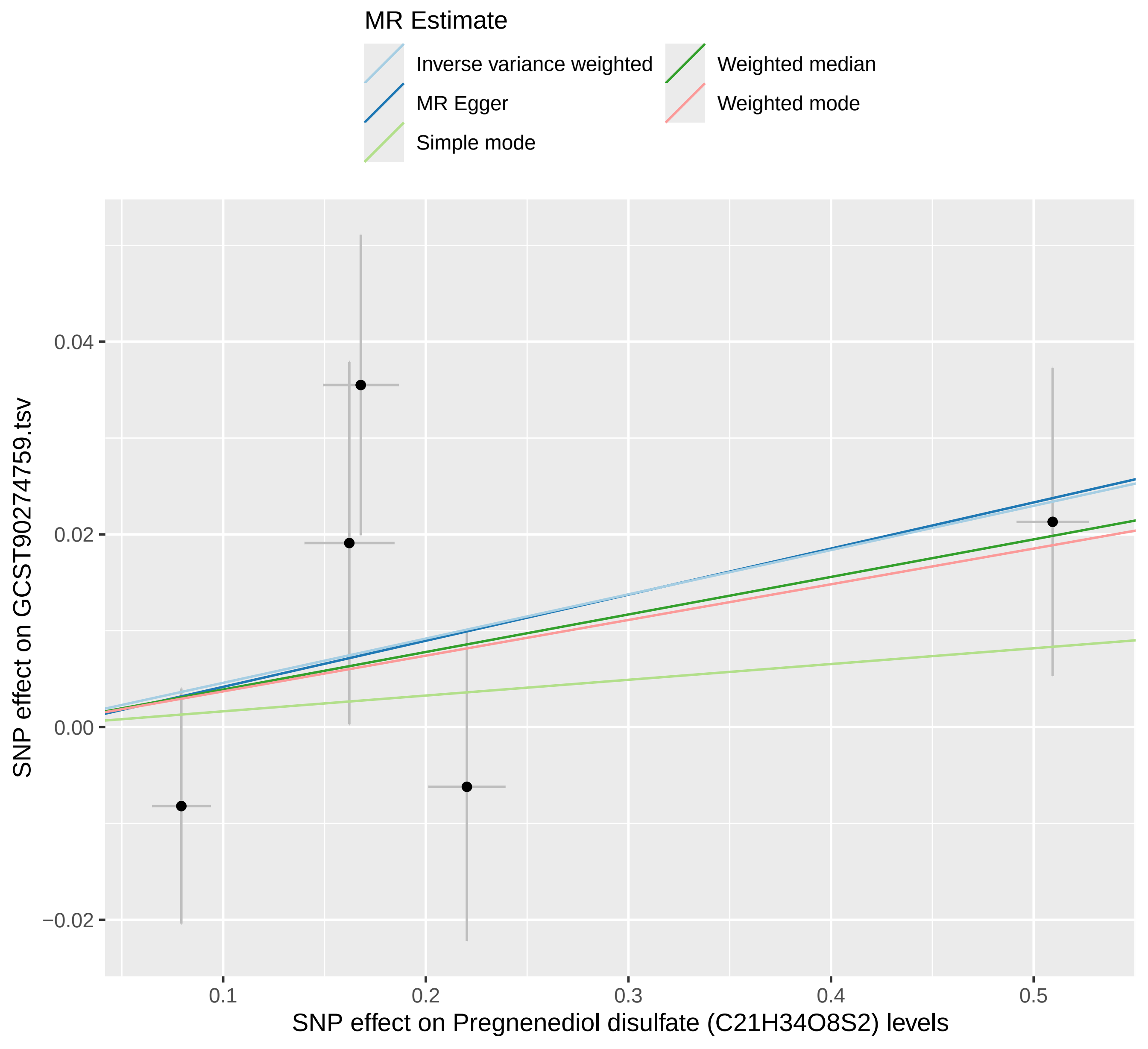

MR Estimate
Inverse variance weighted
MR Egger
Simple mode
Weighted median
Weighted mode
0.04
0.02
0.00
−0.02
SNP effect on GCST90274759.tsv
0.1
0.2
0.3
0.4
0.5
SNP effect on Pregnenediol disulfate (C21H34O8S2) levels

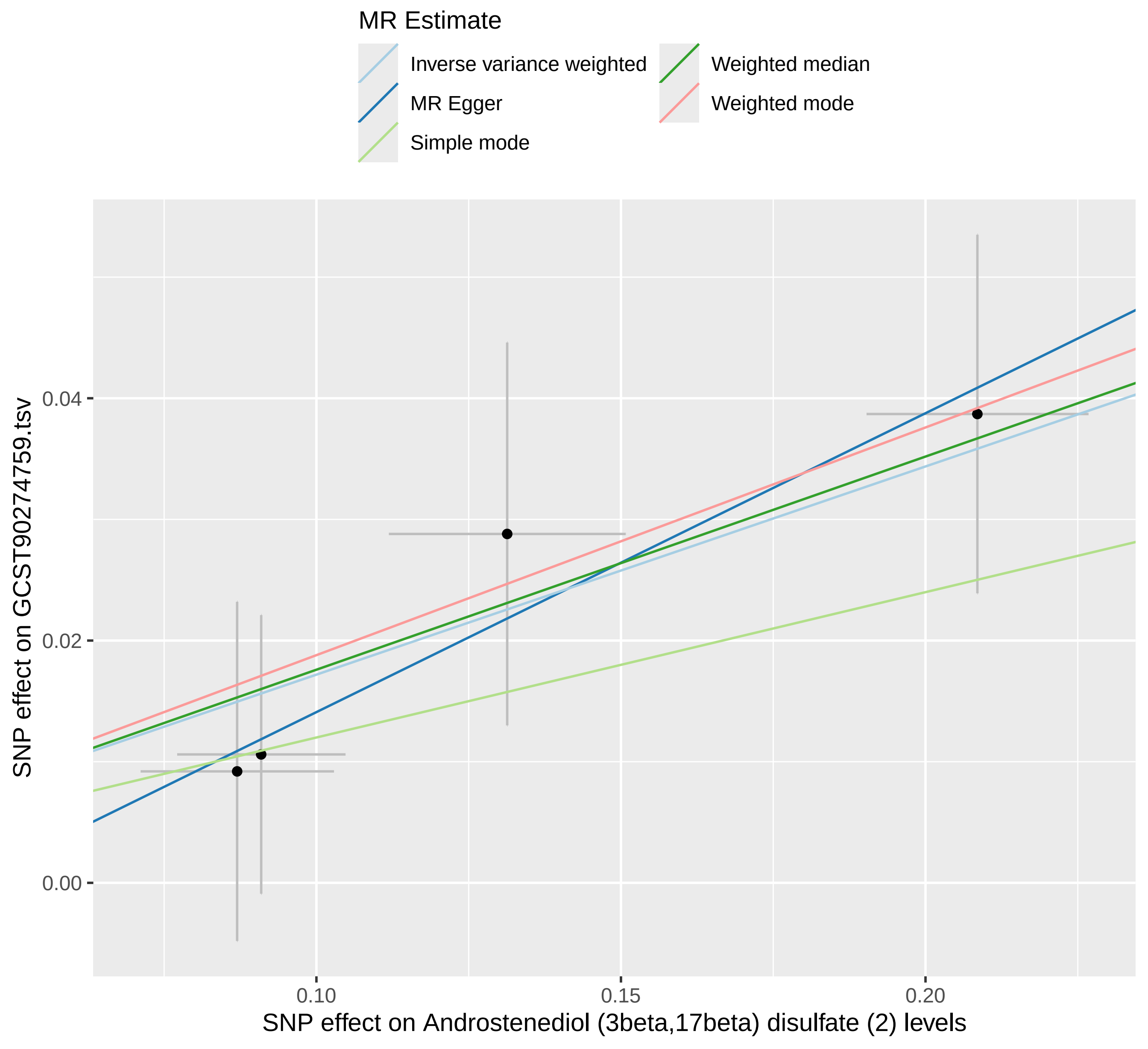
MR Estimate
Inverse variance weighted
MR Egger
Simple mode
Weighted median
Weighted mode
0.00
0.02
0.04
0.10
0.15
0.20
SNP effect on GCST90274759.tsv
SNP effect on Androstenediol (3beta,17beta) disulfate (2) levels

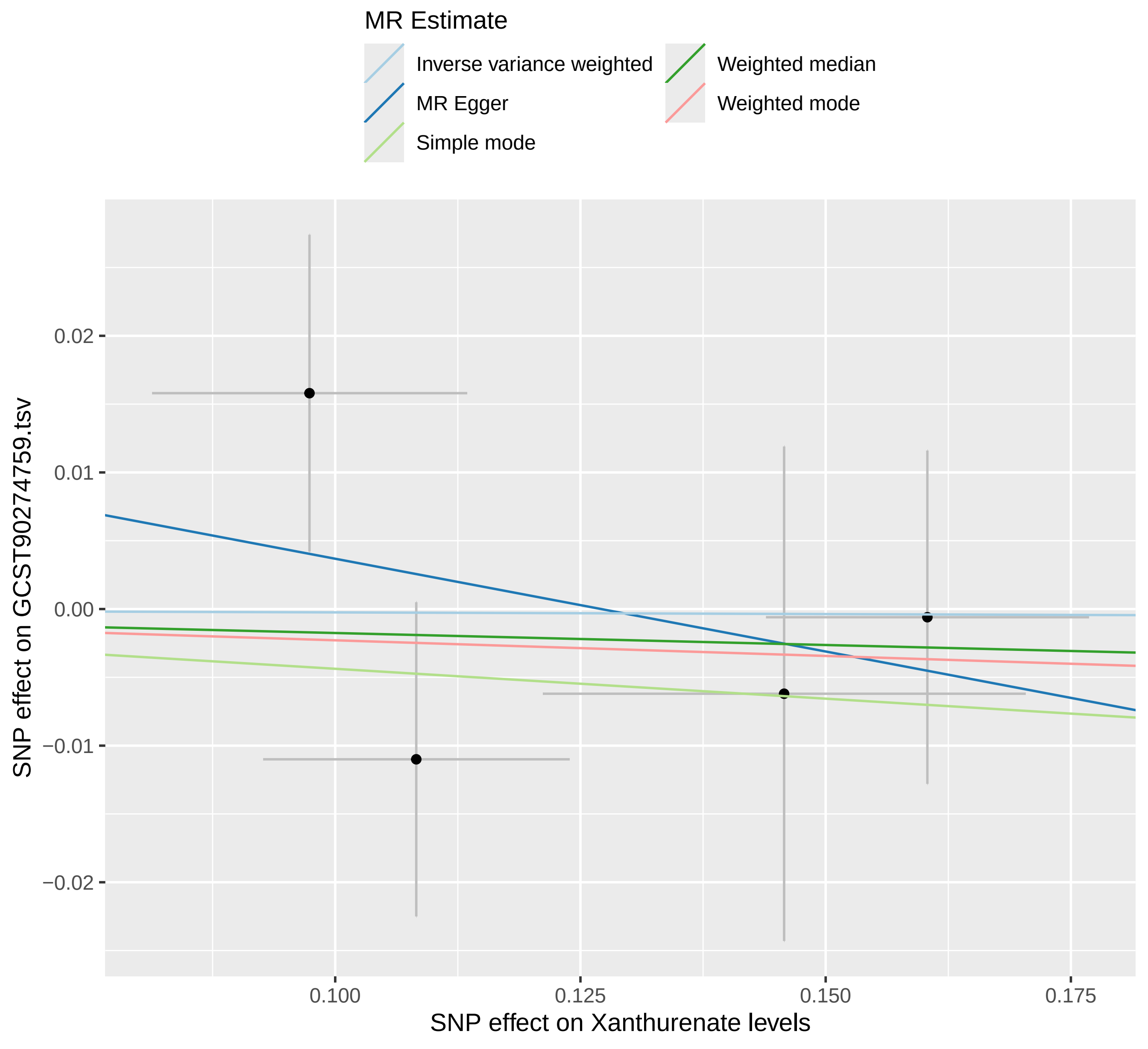
MR Estimate
Inverse variance weighted
MR Egger
Simple mode
Weighted median
Weighted mode
0.02
0.01
0.00
−0.01
−0.02
SNP effect on GCST90274759.tsv
0.100
0.125
0.150
0.175
SNP effect on Xanthurenate levels

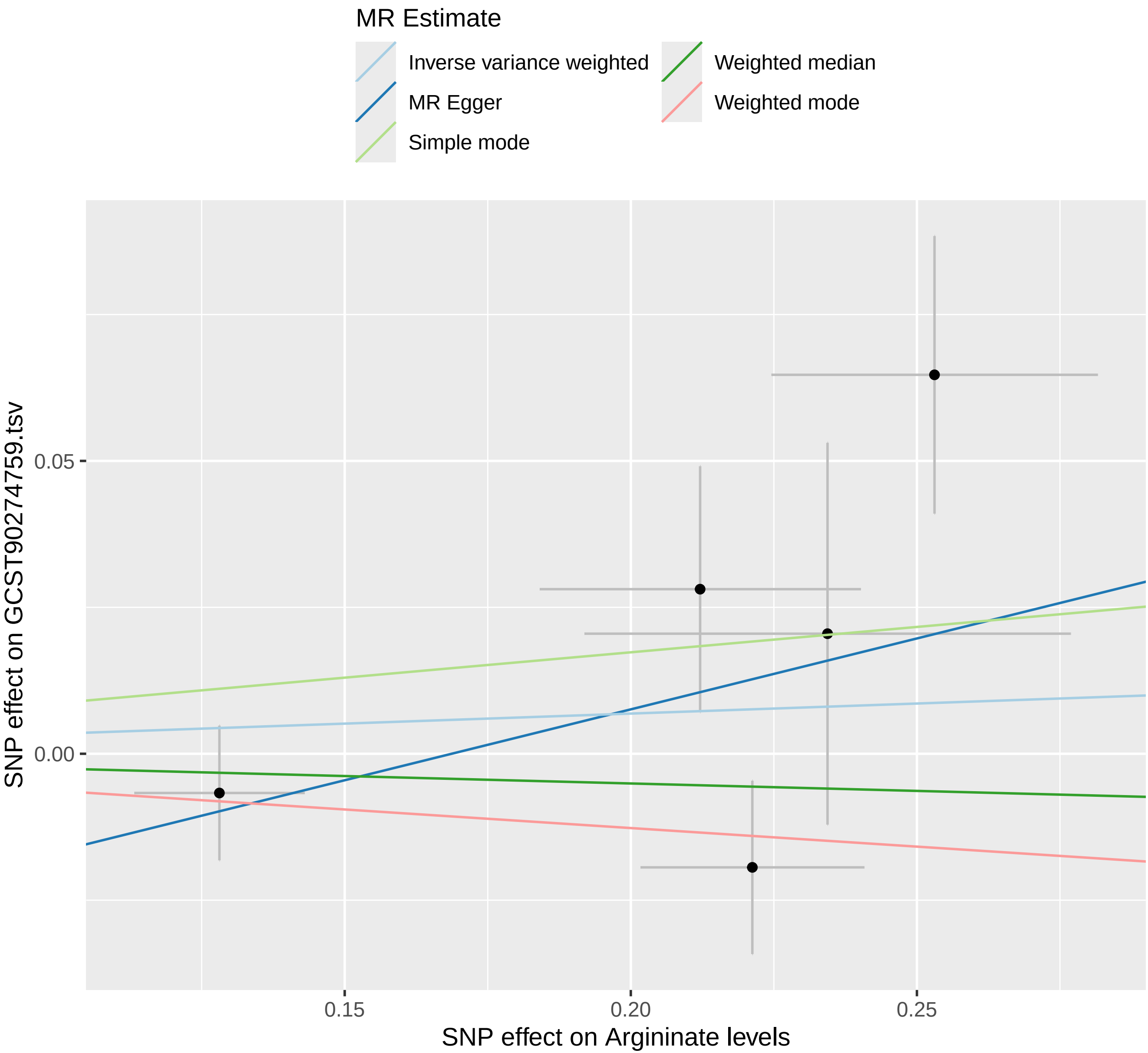

MR Estimate
Inverse variance weighted
MR Egger
Simple mode
Weighted median
Weighted mode
SNP effect on GCST90274759.tsv
0.05
0.00
0.15
0.20
0.25
SNP effect on Argininate levels

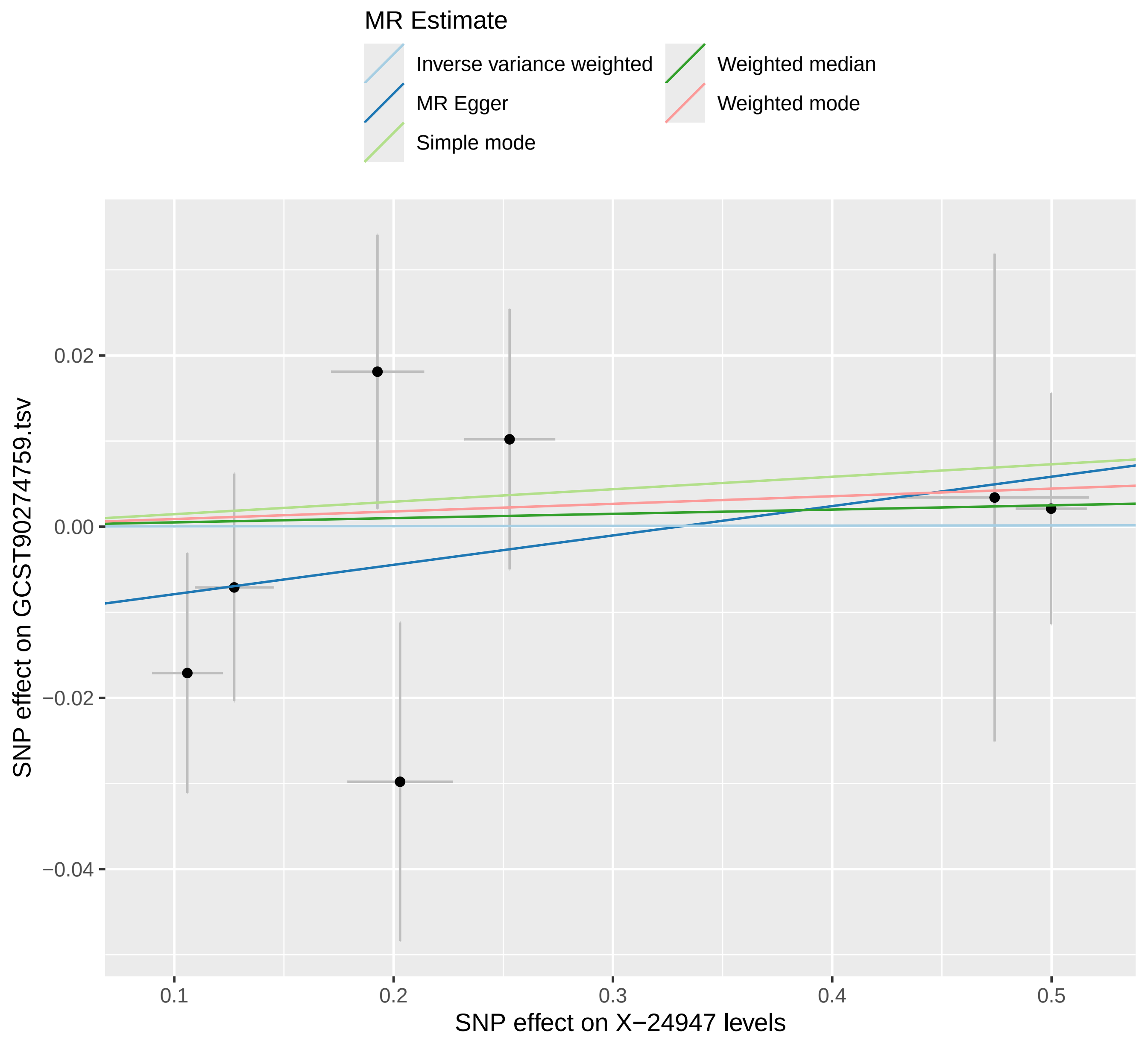

MR Estimate
Inverse variance weighted
MR Egger
Simple mode
Weighted median
Weighted mode
0.02
0.00
−0.02
−0.04
SNP effect on GCST90274759.tsv
0.1
0.2
0.3
0.4
0.5
SNP effect on X−24947 levels

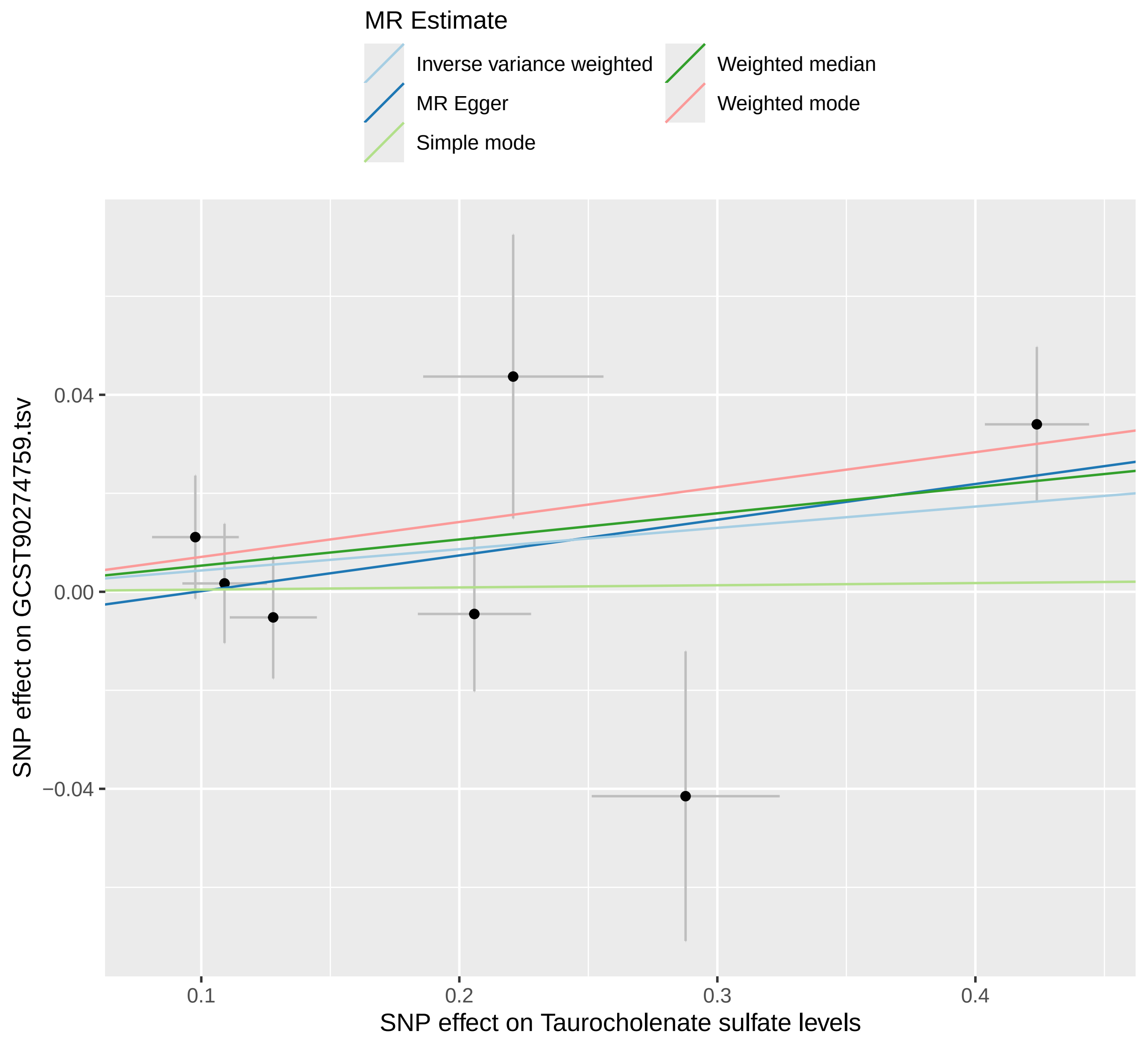
MR Estimate
Inverse variance weighted
MR Egger
Simple mode
Weighted median
Weighted mode
0.04
0.00
−0.04
0.1
0.2
0.3
0.4
SNP effect on GCST90274759.tsv
SNP effect on Taurocholenate sulfate levels

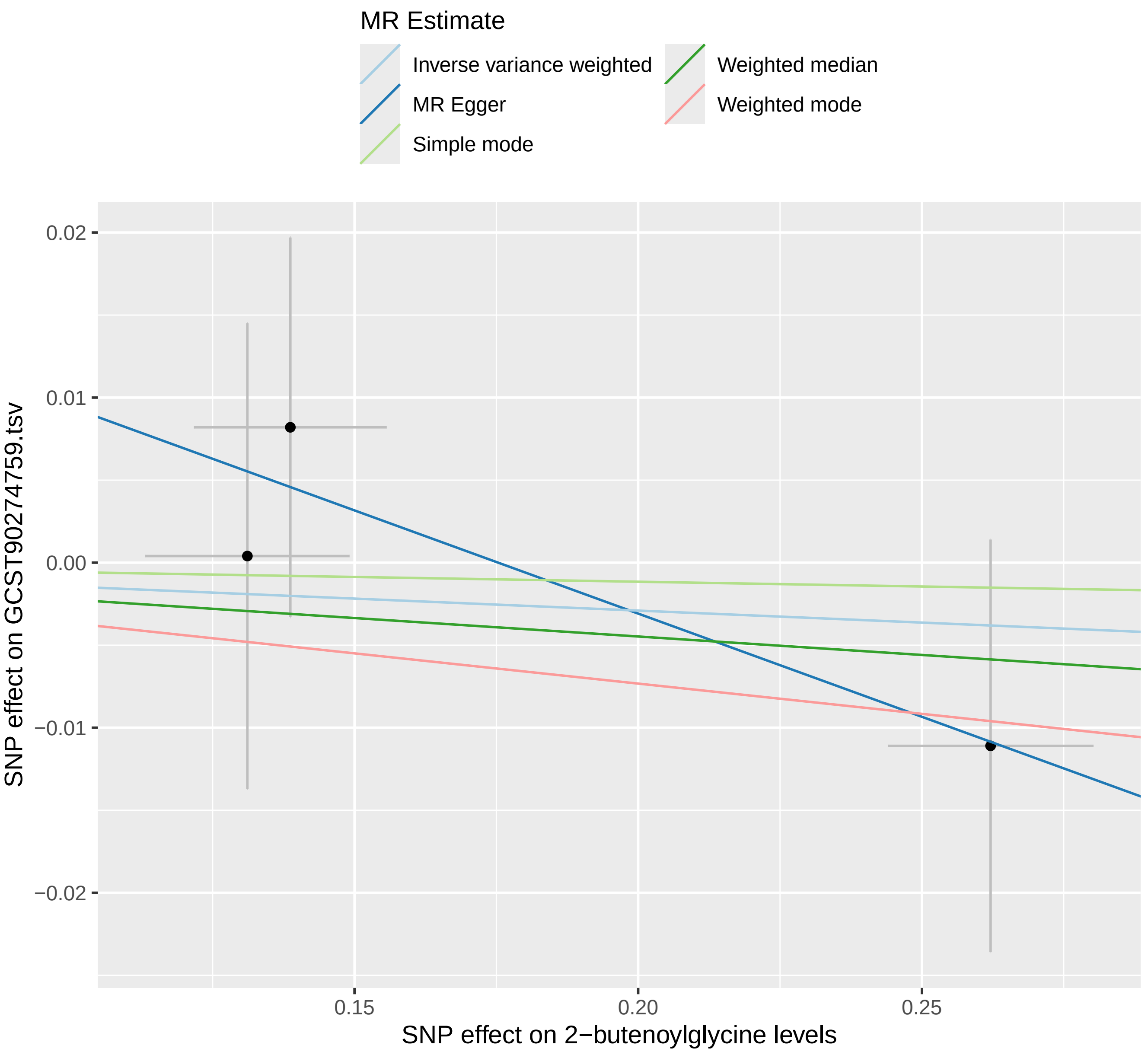
MR Estimate
Inverse variance weighted
MR Egger
Simple mode
Weighted median
Weighted mode
0.02
0.01
0.00
−0.01
−0.02
SNP effect on GCST90274759.tsv
0.15
0.20
0.25
SNP effect on 2−butenoylglycine levels

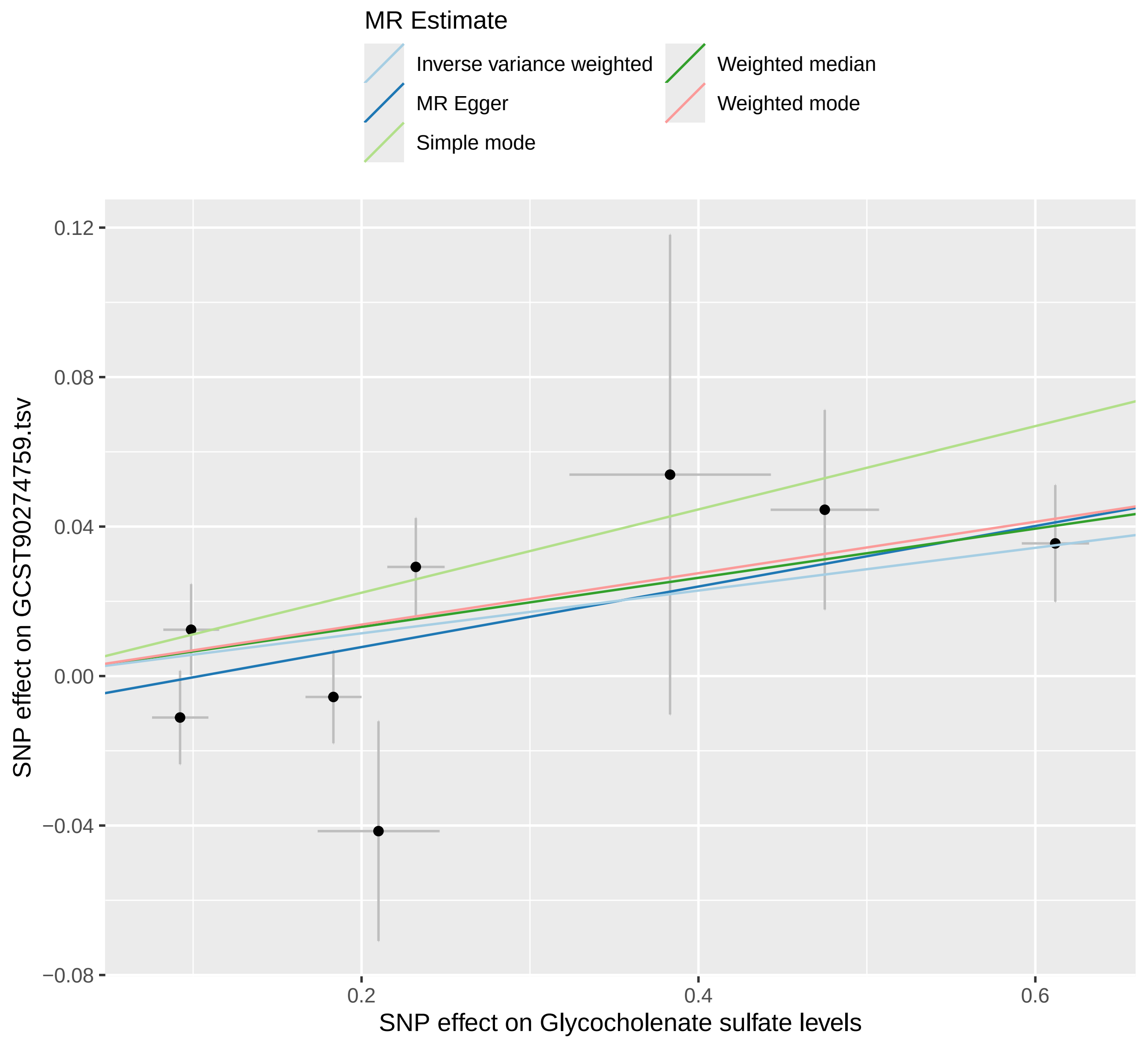
MR Estimate
Inverse variance weighted
MR Egger
Simple mode
Weighted median
Weighted mode
0.12
0.08
0.04
0.00
−0.04
−0.08
0.2
0.4
0.6
SNP effect on GCST90274759.tsv
SNP effect on Glycocholenate sulfate levels

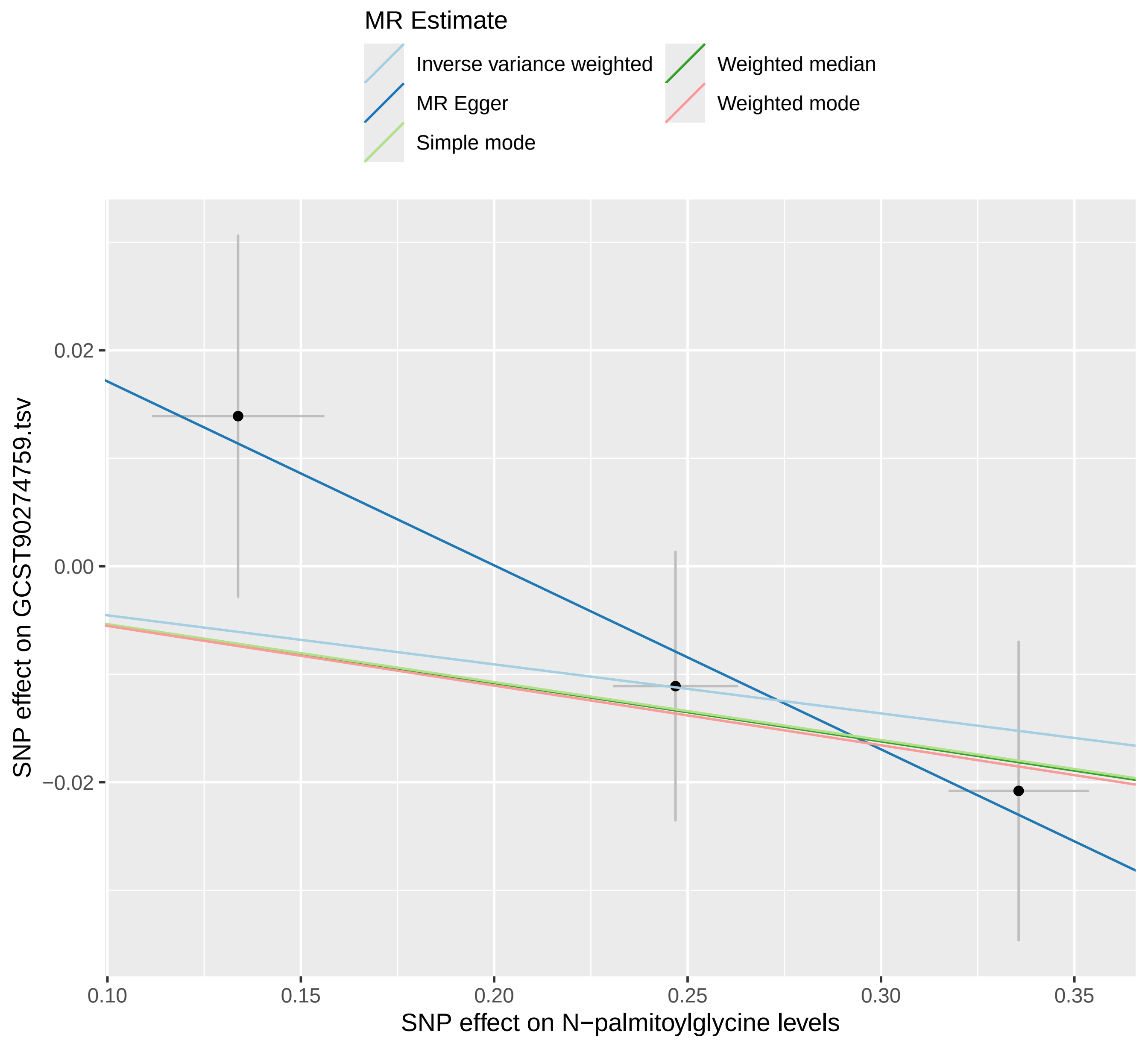

MR Estimate
Inverse variance weighted
MR Egger
Simple mode
Weighted median
Weighted mode
0.02
0.00
−0.02
SNP effect on GCST90274759.tsv
0.10
0.15
0.20
0.25
0.30
0.35
SNP effect on N−palmitoylglycine levels

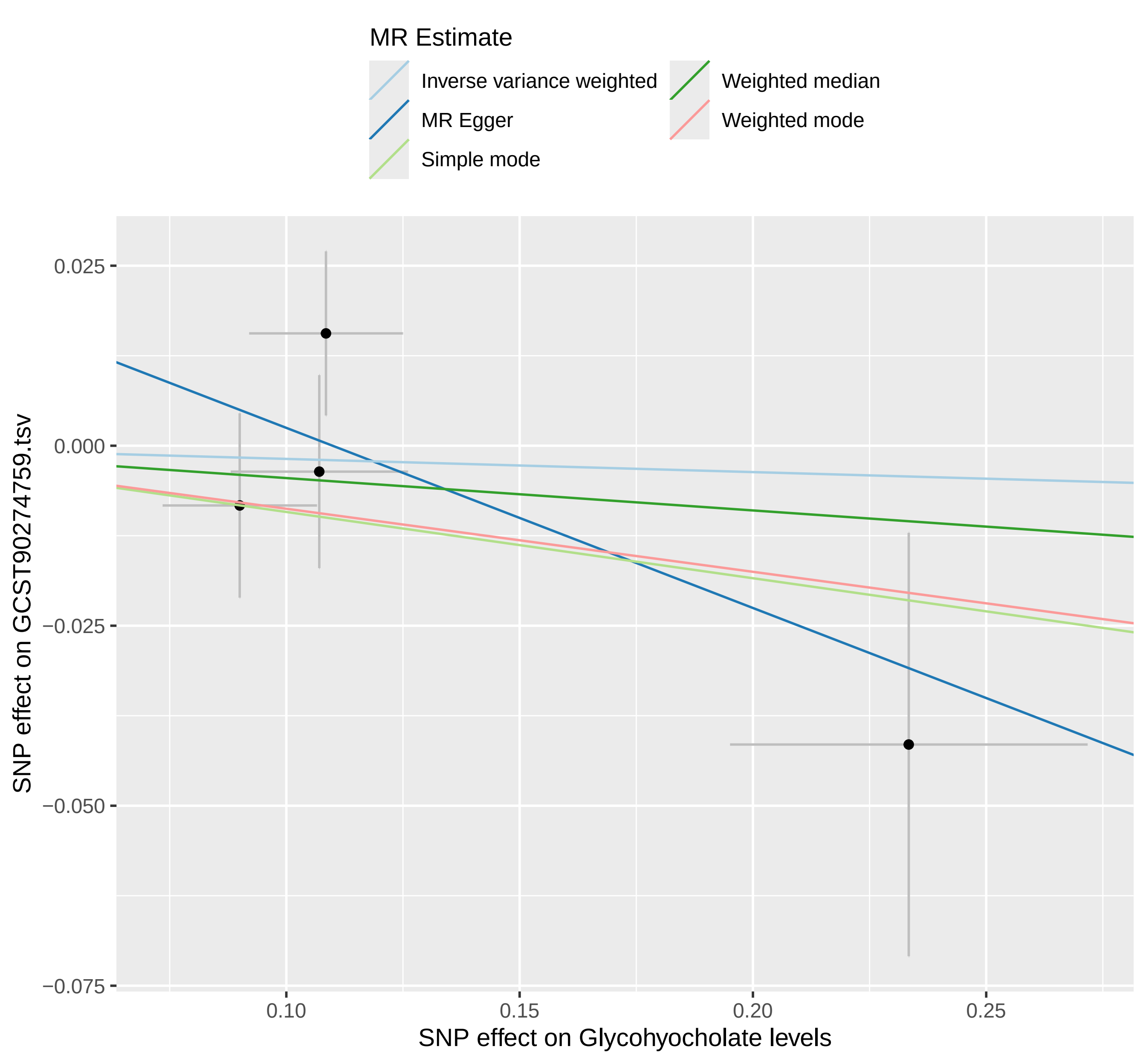

MR Estimate
Inverse variance weighted
MR Egger
Simple mode
Weighted median
Weighted mode
0.025
0.000
−0.025
−0.050
−0.075
0.10
0.15
0.20
0.25
SNP effect on GCST90274759.tsv
SNP effect on Glycohyocholate levels

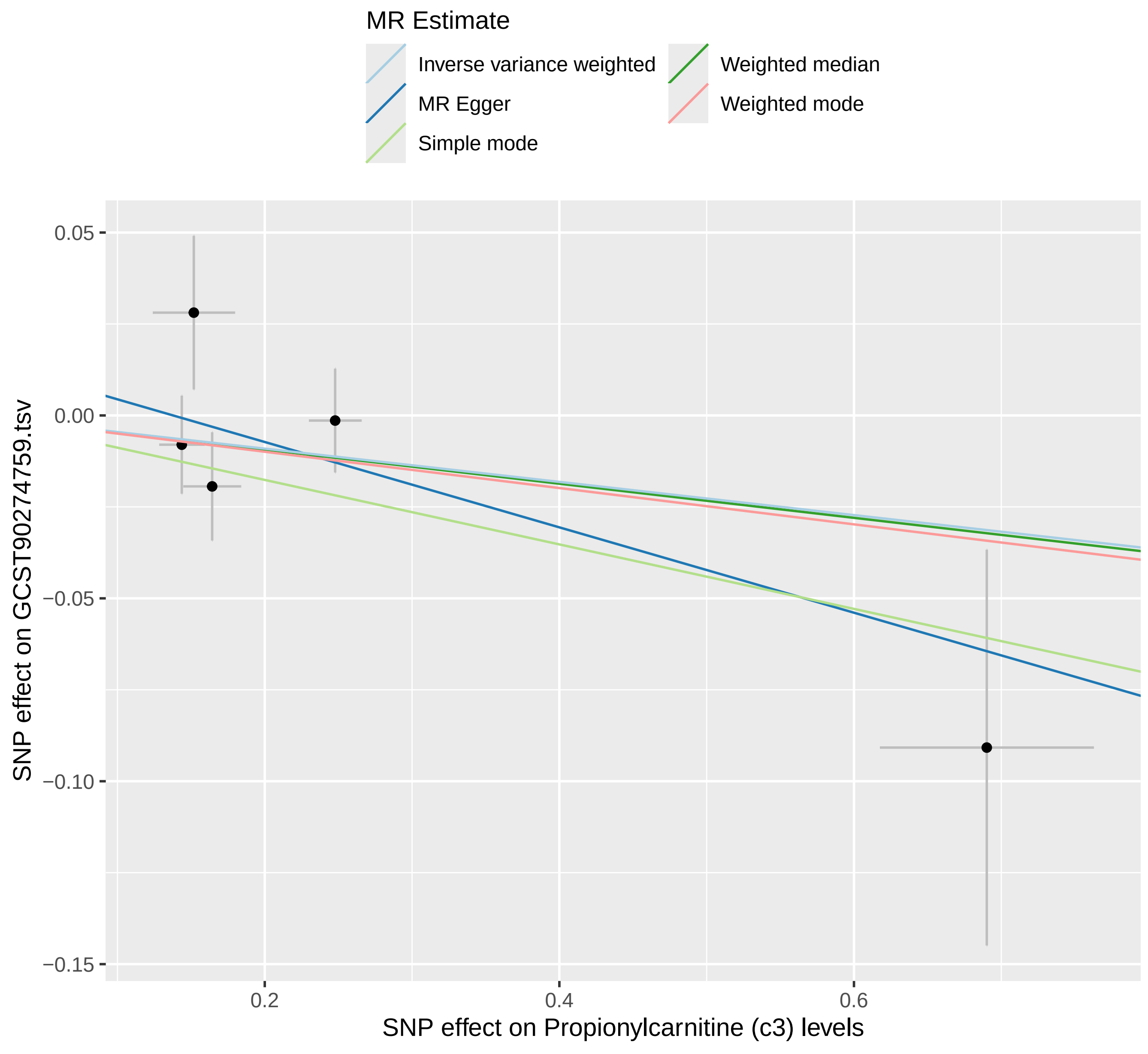

MR Estimate
Inverse variance weighted
MR Egger
Simple mode
Weighted median
Weighted mode
0.05
0.00
−0.05
−0.10
−0.15
0.2
0.4
0.6
SNP effect on GCST90274759.tsv
SNP effect on Propionylcarnitine (c3) levels

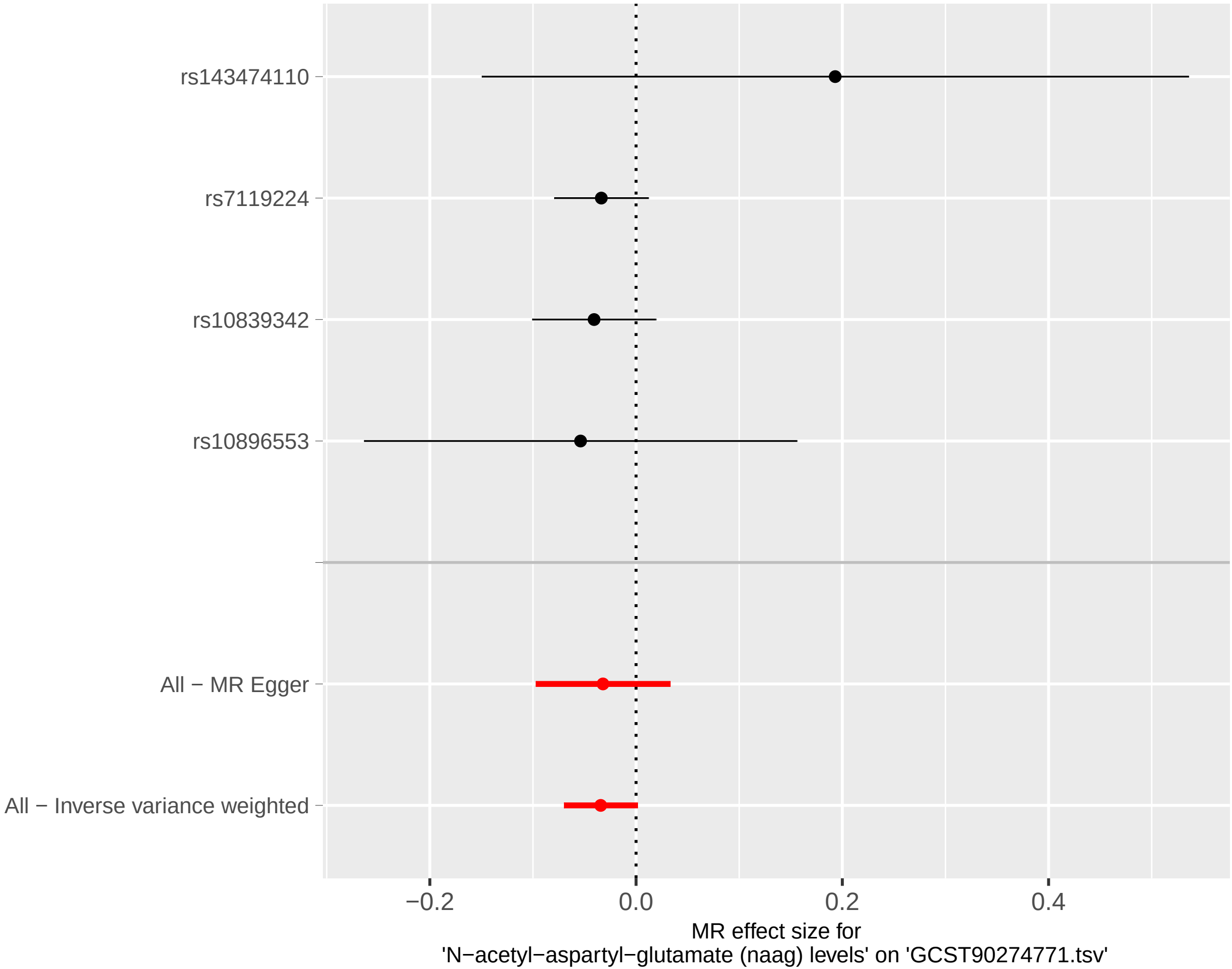
rs143474110
rs7119224
rs10839342
rs10896553
All − MR Egger
All − Inverse variance weighted
−0.2
0.0
0.2
0.4
MR effect size for
'N−acetyl−aspartyl−glutamate (naag) levels' on 'GCST90274771.tsv'

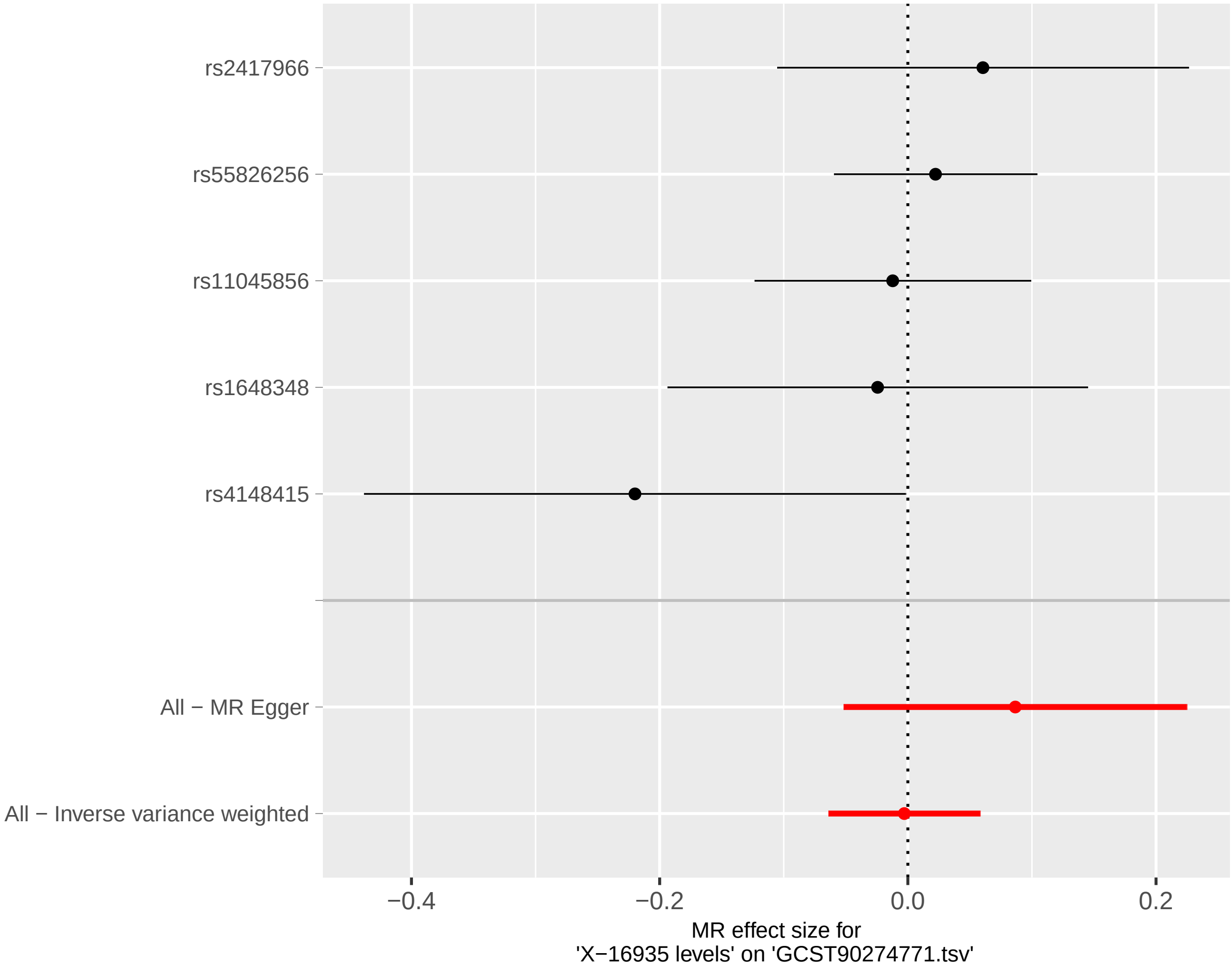
rs2417966
rs55826256
rs11045856
rs1648348
rs4148415
All − MR Egger
All − Inverse variance weighted
−0.4
−0.2
0.0
0.2
MR effect size for
'X−16935 levels' on 'GCST90274771.tsv'

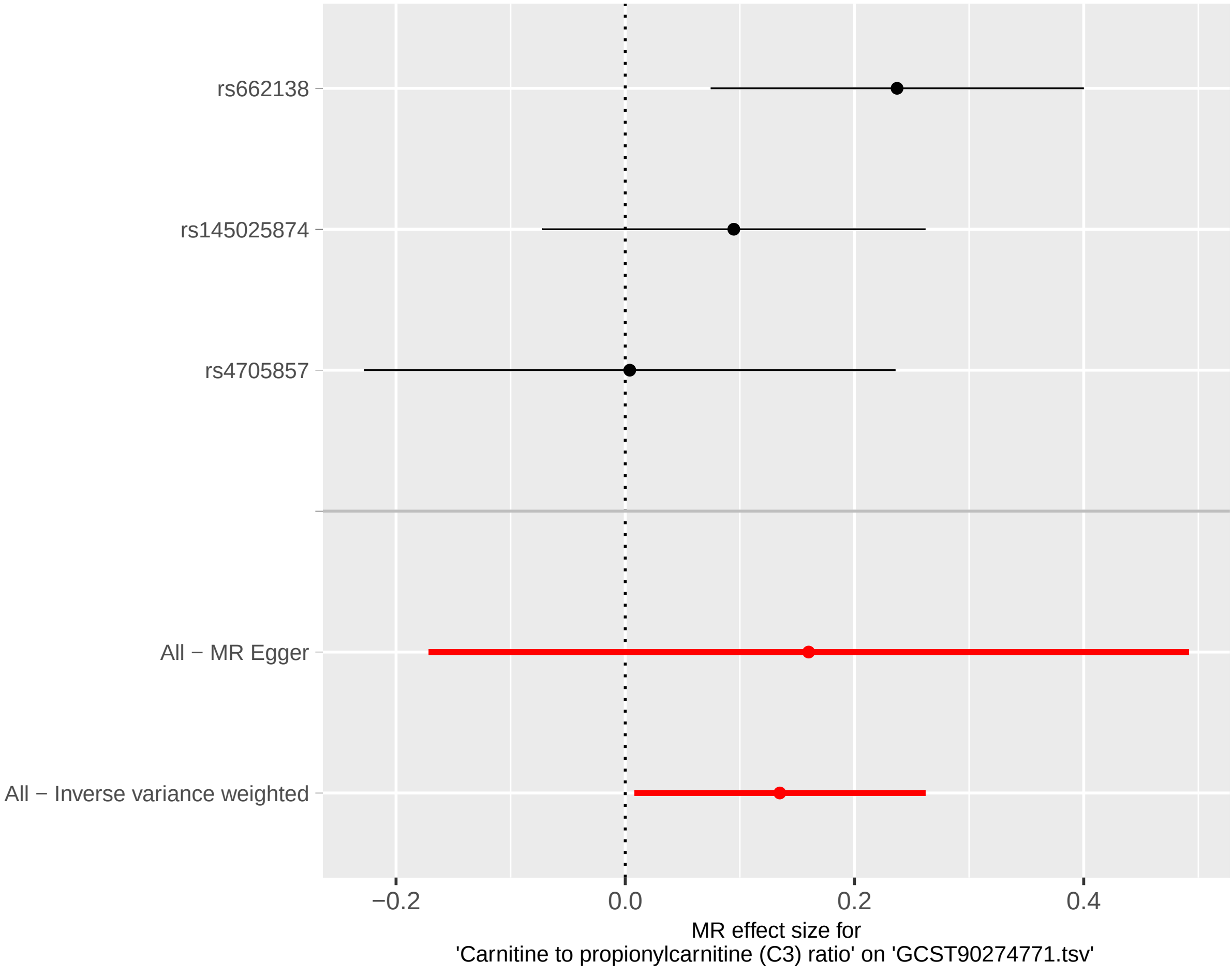
rs662138
rs145025874
rs4705857
All − MR Egger
All − Inverse variance weighted
−0.2
0.0
0.2
0.4
MR effect size for
'Carnitine to propionylcarnitine (C3) ratio' on 'GCST90274771.tsv'

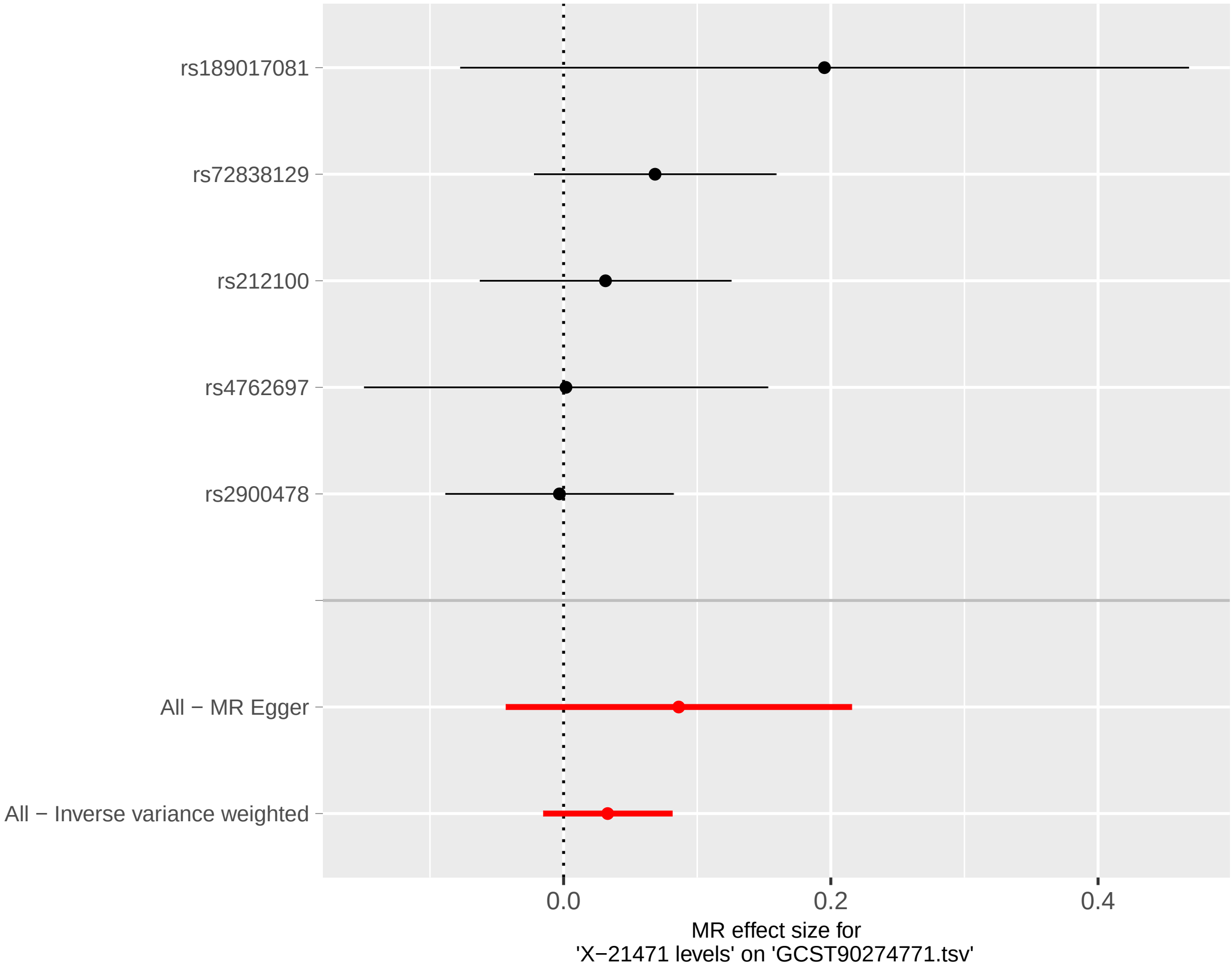
rs189017081
rs72838129
rs212100
rs4762697
rs2900478
All − MR Egger
All − Inverse variance weighted
0.0
0.2
0.4
MR effect size for
'X−21471 levels' on 'GCST90274771.tsv'

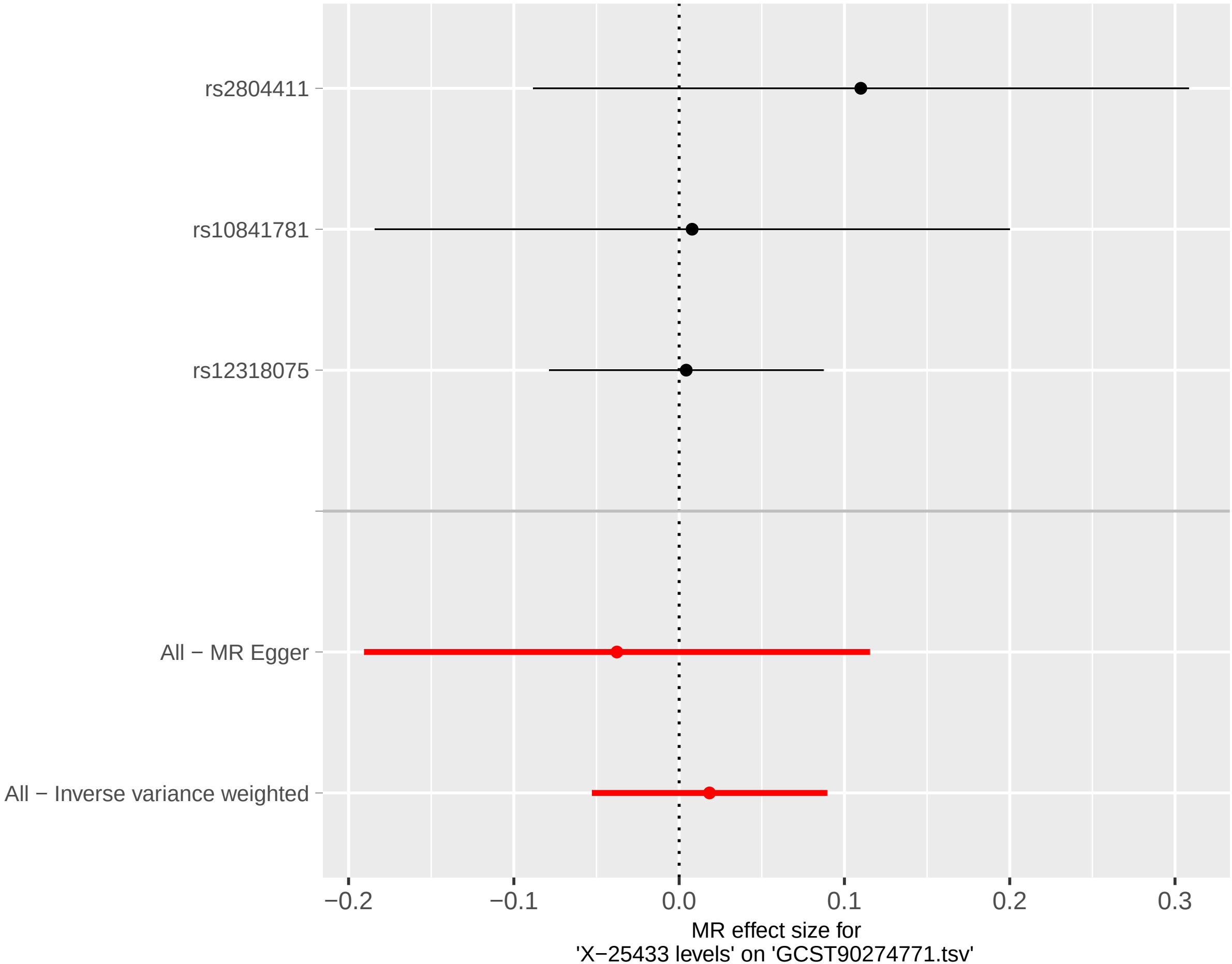
rs2804411
rs10841781
rs12318075
All − MR Egger
All − Inverse variance weighted
−0.2
−0.1
0.0
0.1
0.2
0.3
MR effect size for
'X−25433 levels' on 'GCST90274771.tsv'

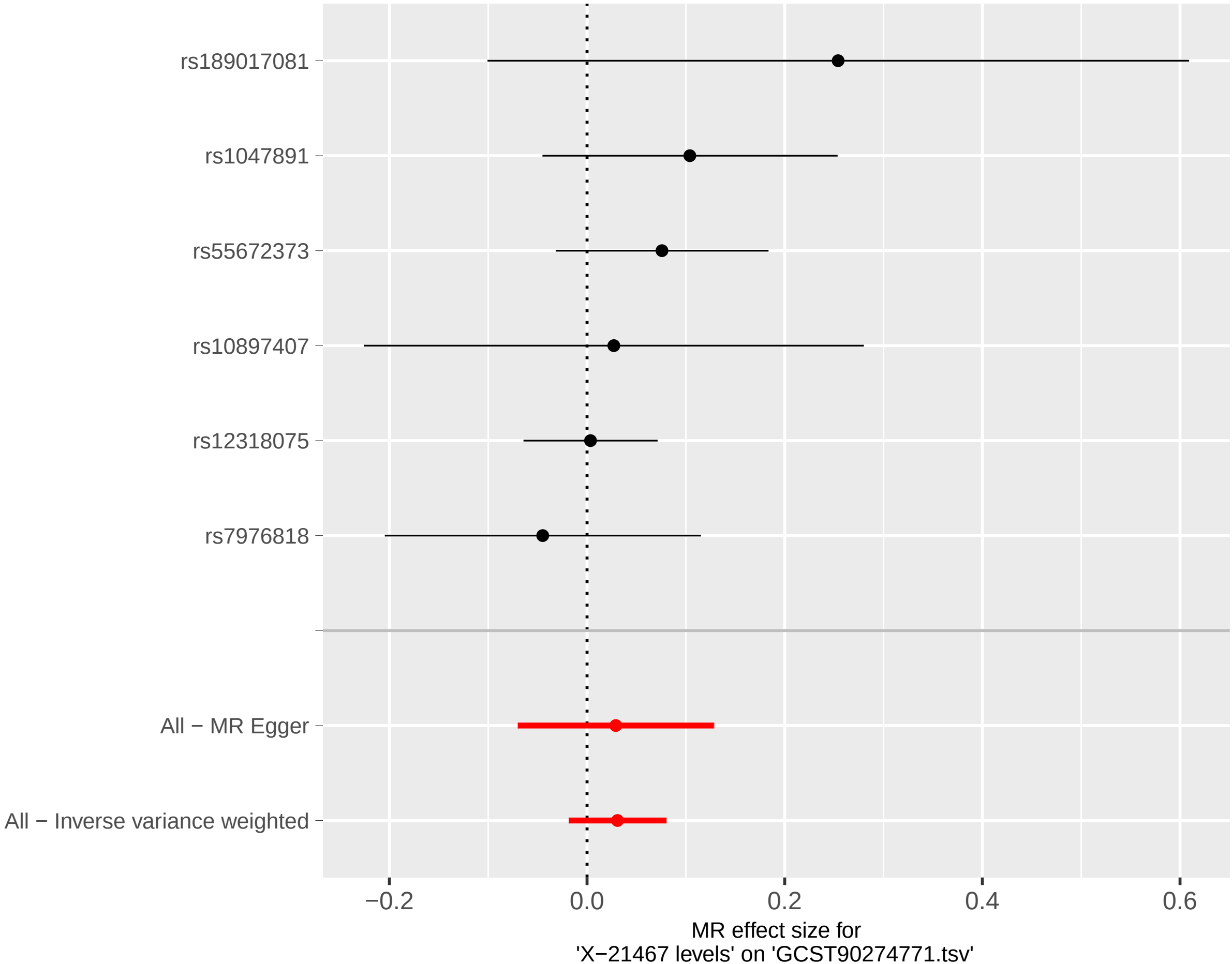
rs189017081
rs1047891
rs55672373
rs10897407
rs12318075
rs7976818
All − MR Egger
All − Inverse variance weighted
−0.2
0.0
0.2
0.4
0.6
MR effect size for
'X−21467 levels' on 'GCST90274771.tsv'

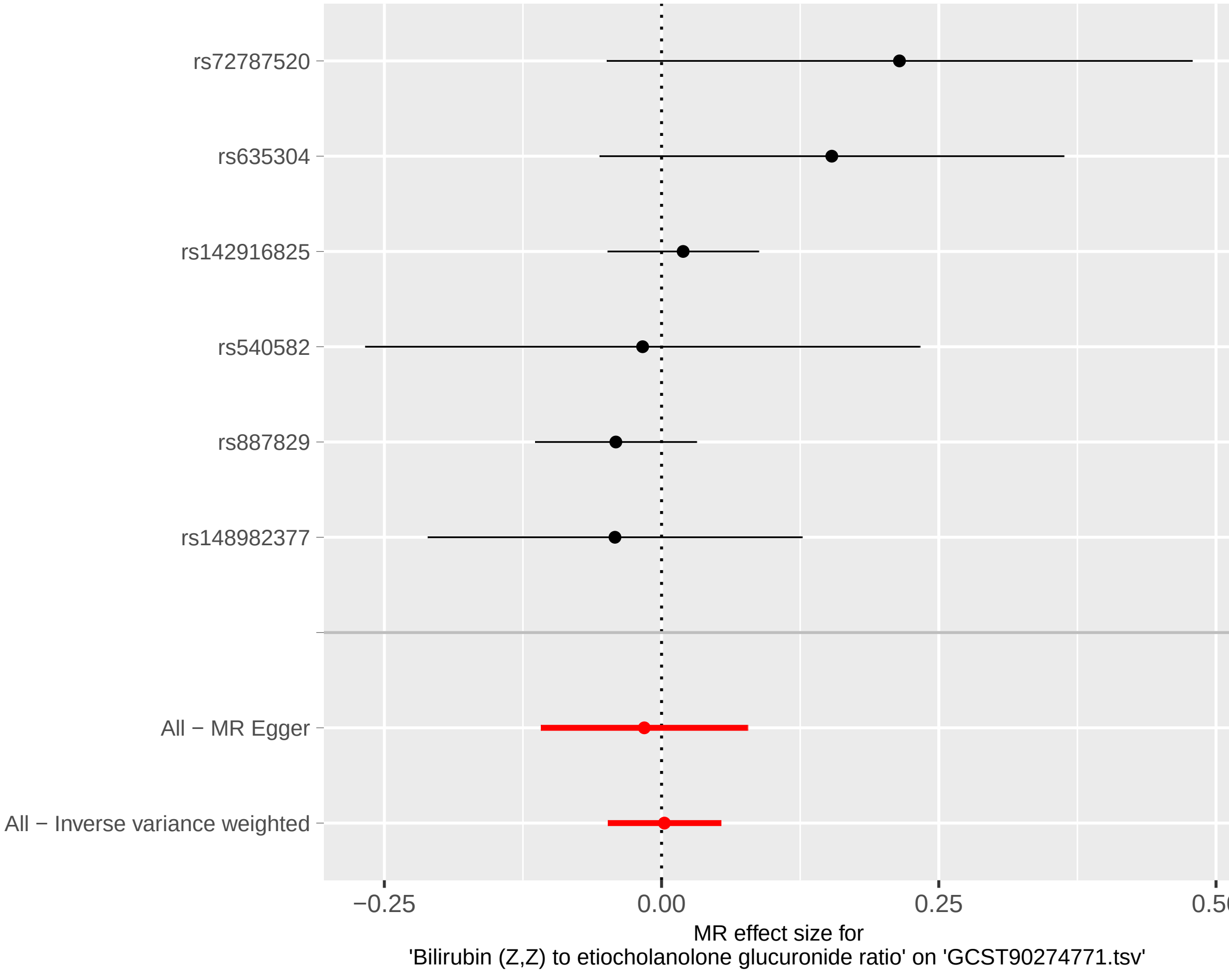

rs72787520
rs635304
rs142916825
rs540582
rs887829
rs148982377
All − MR Egger
All − Inverse variance weighted
−0.25
0.00
0.25
0.50
MR effect size for
'Bilirubin (Z,Z) to etiocholanolone glucuronide ratio' on 'GCST90274771.tsv'

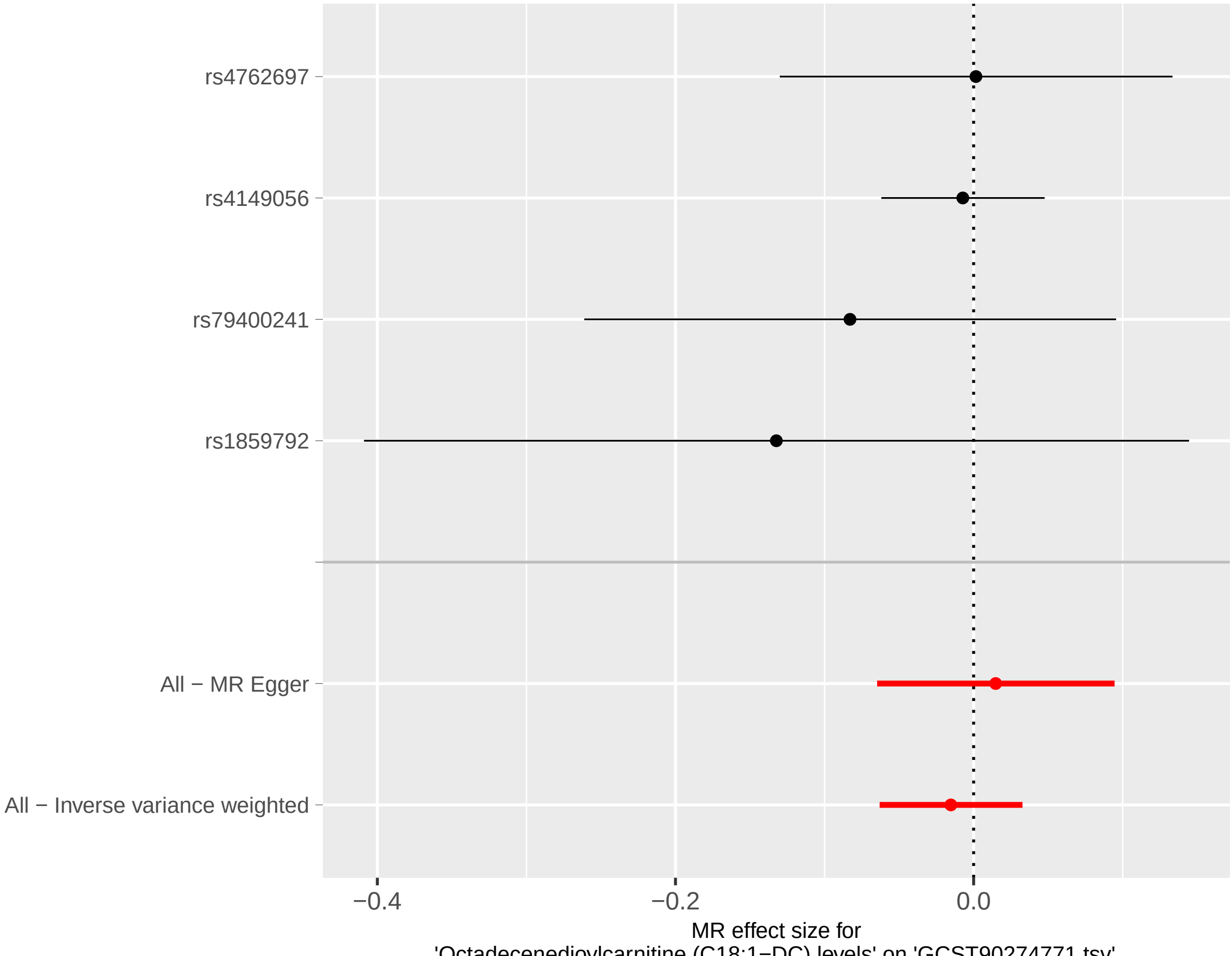

rs4762697
rs4149056
rs79400241
rs1859792
All − MR Egger
All − Inverse variance weighted
−0.4
−0.2
0.0
MR effect size for
'Octadecenedioylcarnitine (C18:1−DC) levels' on 'GCST90274771.tsv'

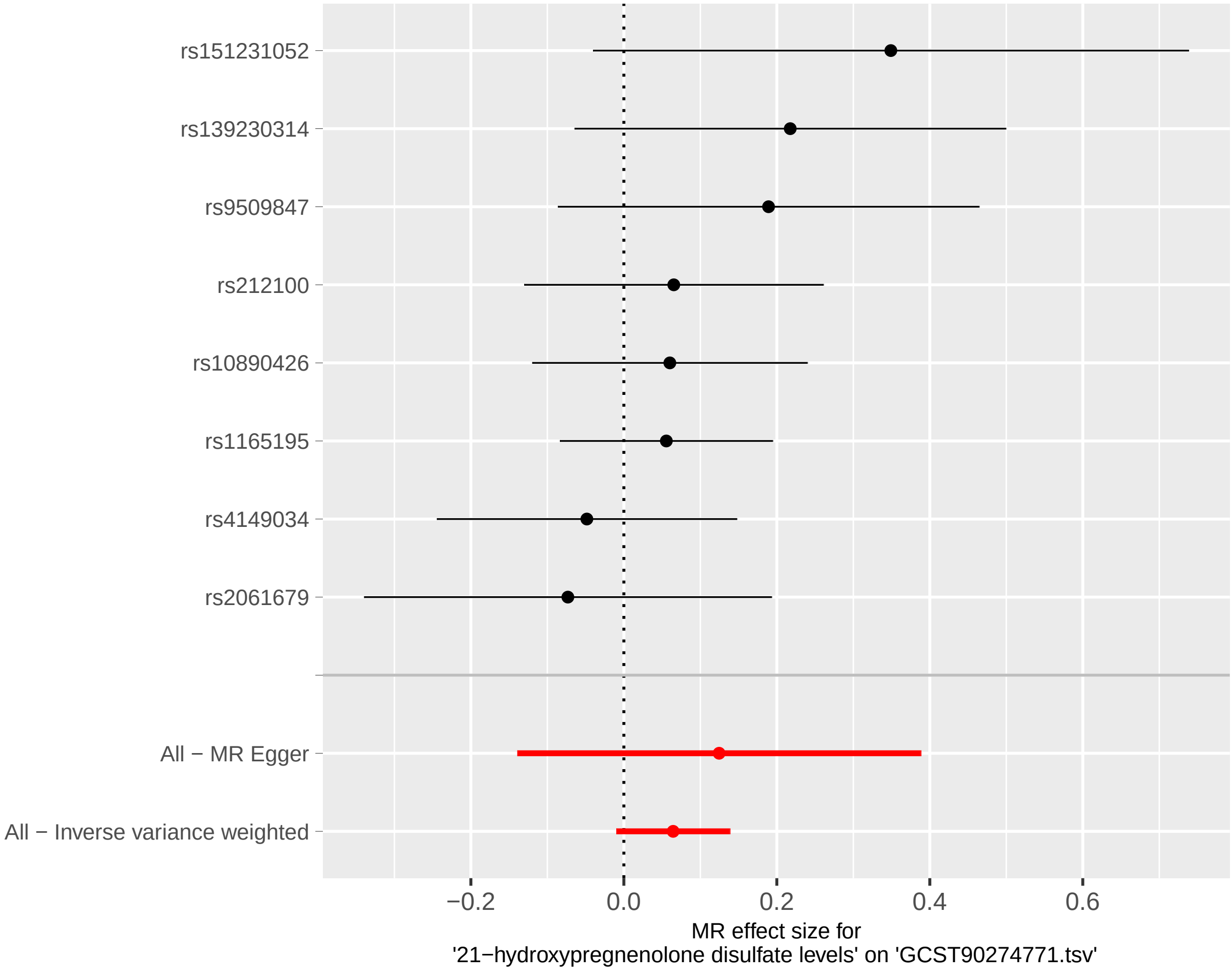
rs151231052
rs139230314
rs9509847
rs212100
rs10890426
rs1165195
rs4149034
rs2061679
All − MR Egger
All − Inverse variance weighted
−0.2
0.0
0.2
0.4
0.6
MR effect size for
'21−hydroxypregnenolone disulfate levels' on 'GCST90274771.tsv'

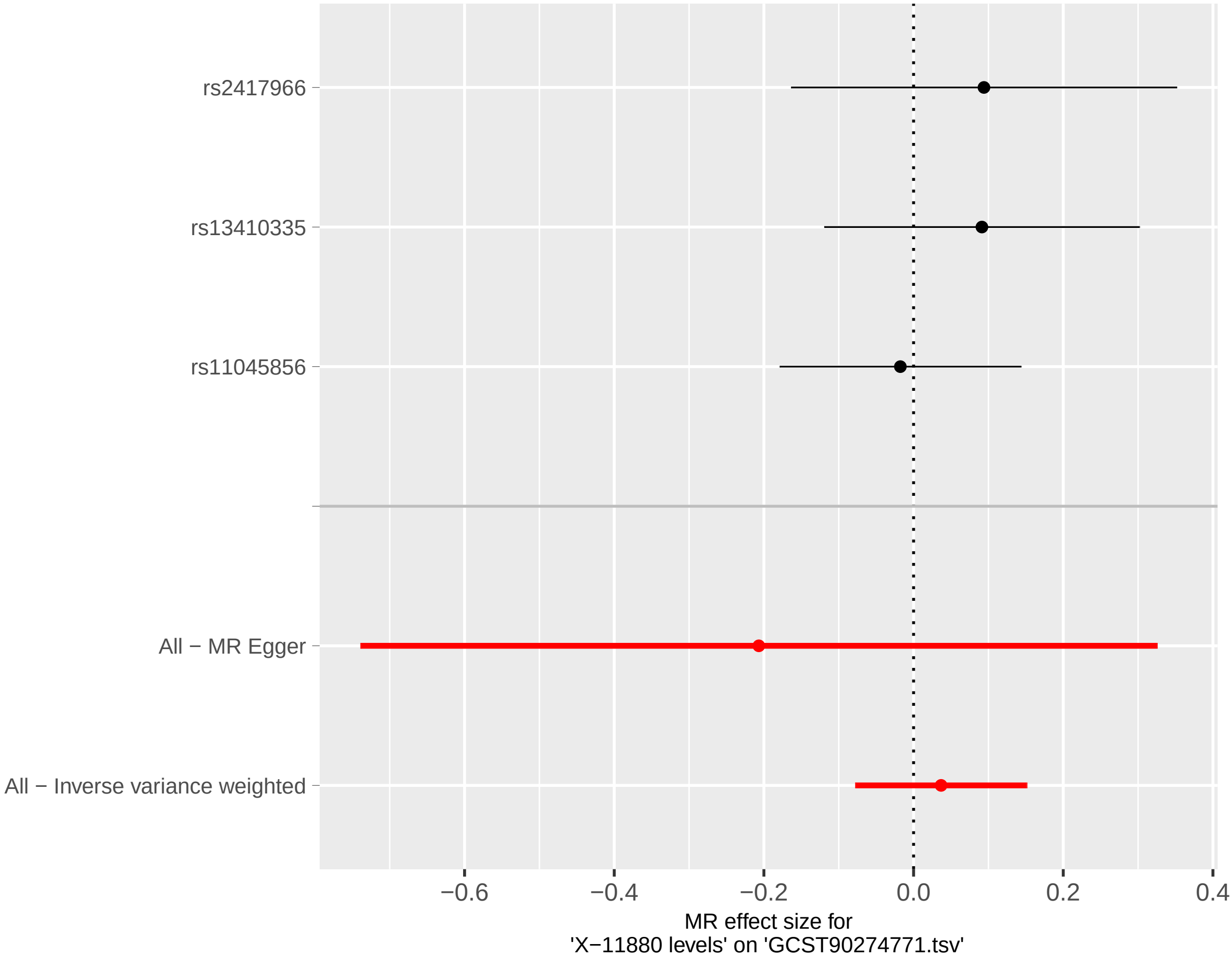

rs2417966
rs13410335
rs11045856
All − MR Egger
All − Inverse variance weighted
−0.6
−0.4
−0.2
0.0
0.2
0.4
MR effect size for
'X−11880 levels' on 'GCST90274771.tsv'

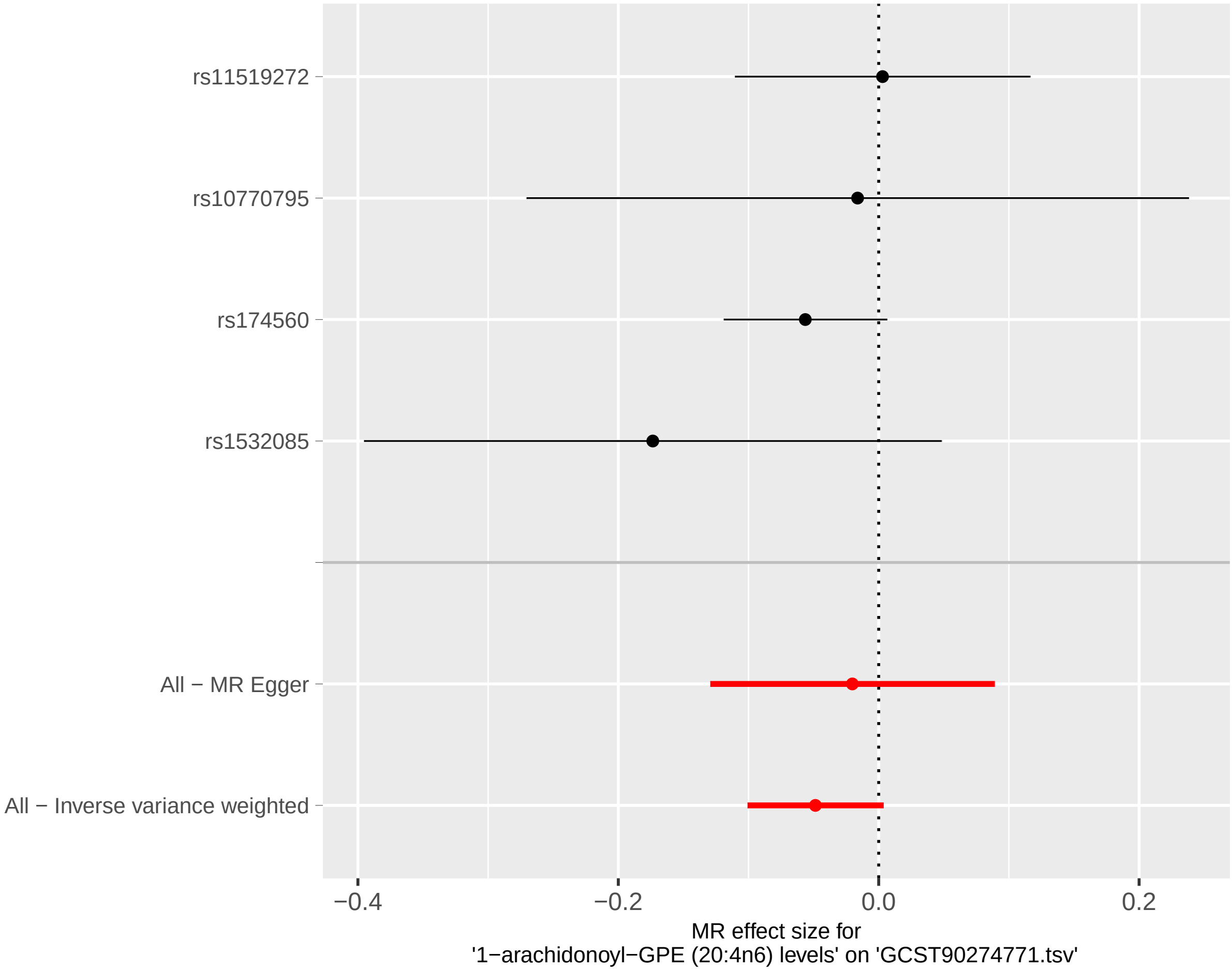
rs11519272
rs10770795
rs174560
rs1532085
All − MR Egger
All − Inverse variance weighted
−0.4
−0.2
0.0
0.2
MR effect size for
'1−arachidonoyl−GPE (20:4n6) levels' on 'GCST90274771.tsv'

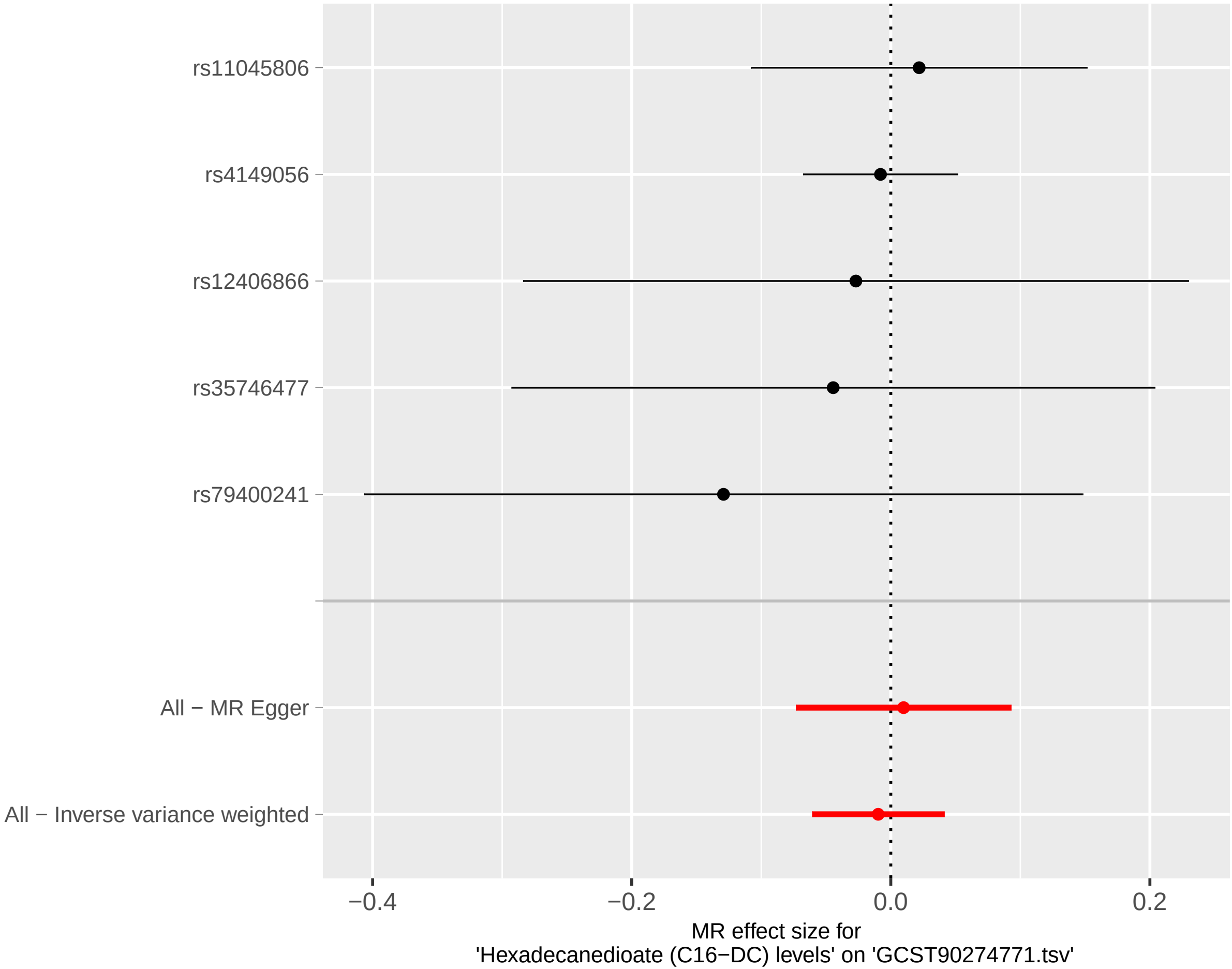
rs11045806
rs4149056
rs12406866
rs35746477
rs79400241
All − MR Egger
All − Inverse variance weighted
−0.4
−0.2
0.0
0.2
MR effect size for
'Hexadecanedioate (C16−DC) levels' on 'GCST90274771.tsv'

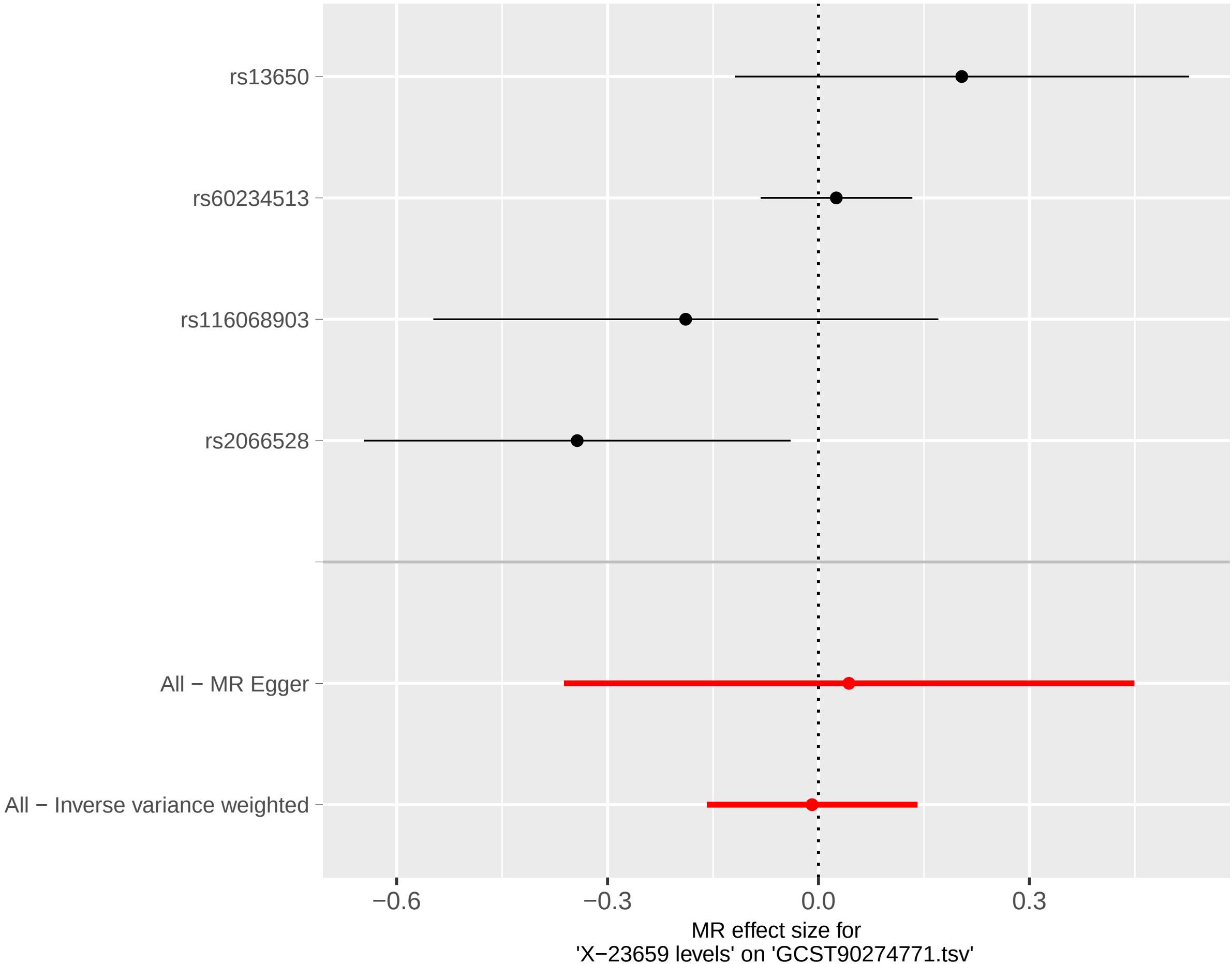
rs13650
rs60234513
rs116068903
rs2066528
All − MR Egger
All − Inverse variance weighted
−0.6
−0.3
0.0
0.3
MR effect size for
'X−23659 levels' on 'GCST90274771.tsv'

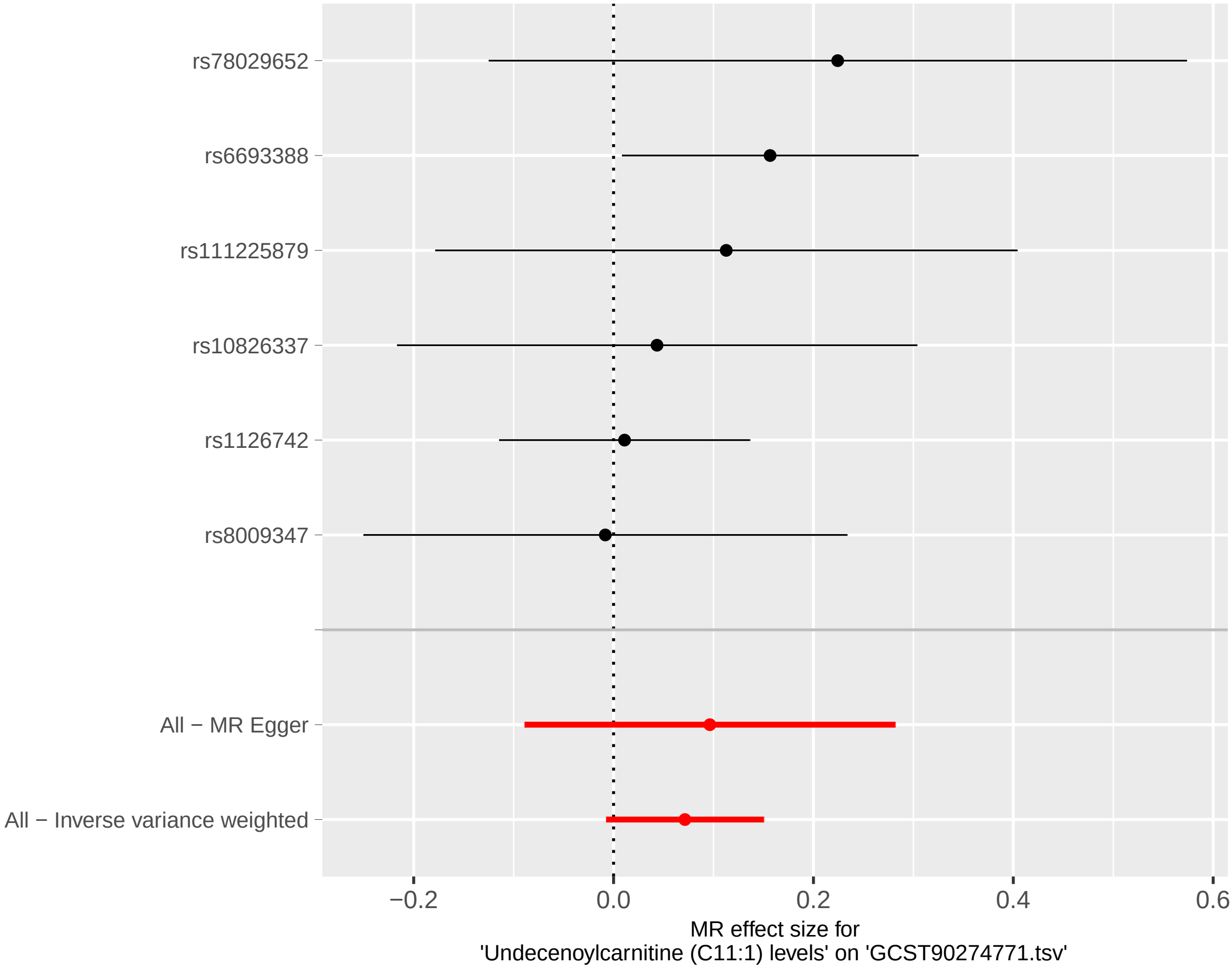

rs78029652
rs6693388
rs111225879
rs10826337
rs1126742
rs8009347
All − MR Egger
All − Inverse variance weighted
−0.2
0.0
0.2
0.4
0.6
MR effect size for
'Undecenoylcarnitine (C11:1) levels' on 'GCST90274771.tsv'

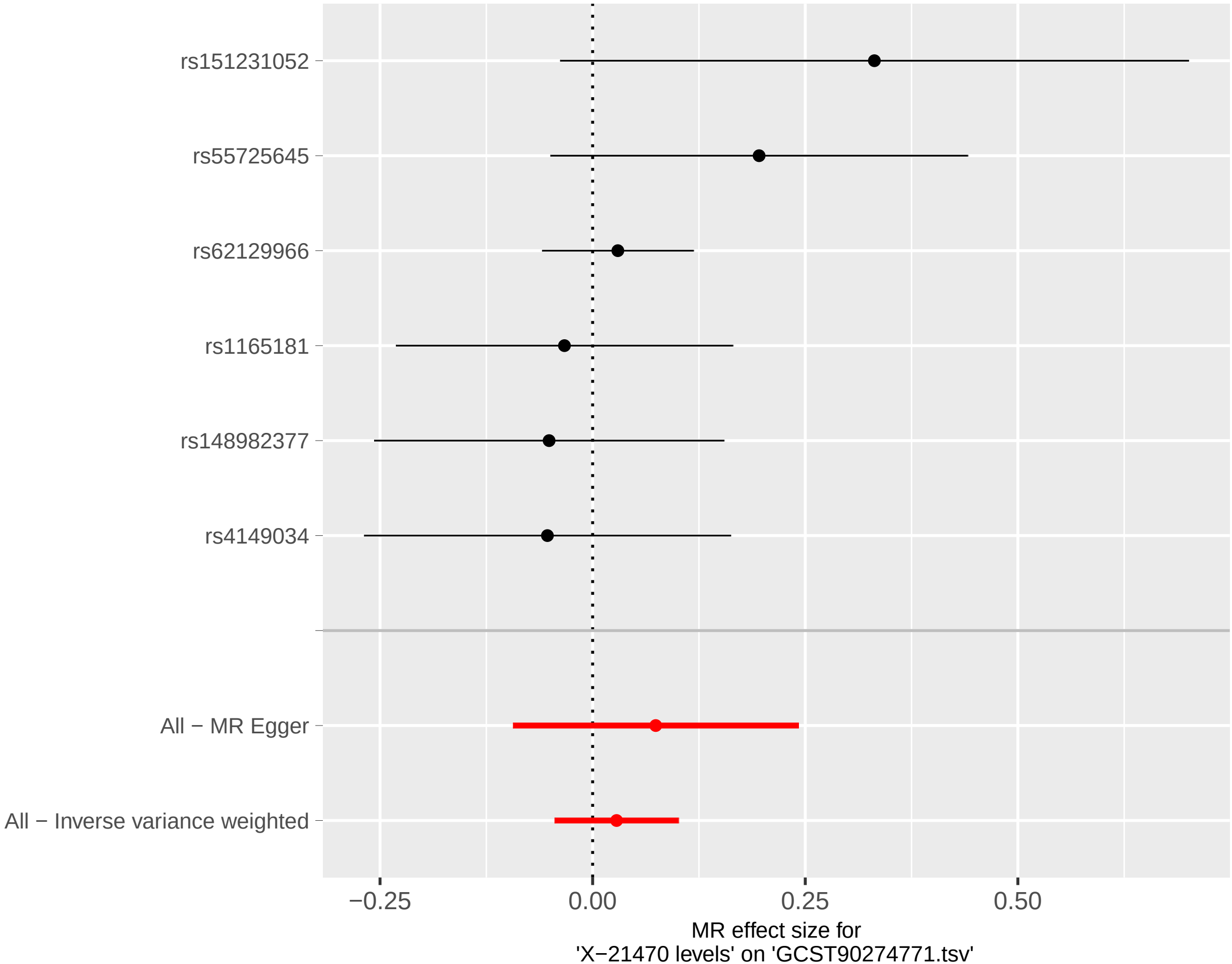
rs151231052
rs55725645
rs62129966
rs1165181
rs148982377
rs4149034
All − MR Egger
All − Inverse variance weighted
−0.25
0.00
0.25
0.50
MR effect size for
'X−21470 levels' on 'GCST90274771.tsv'

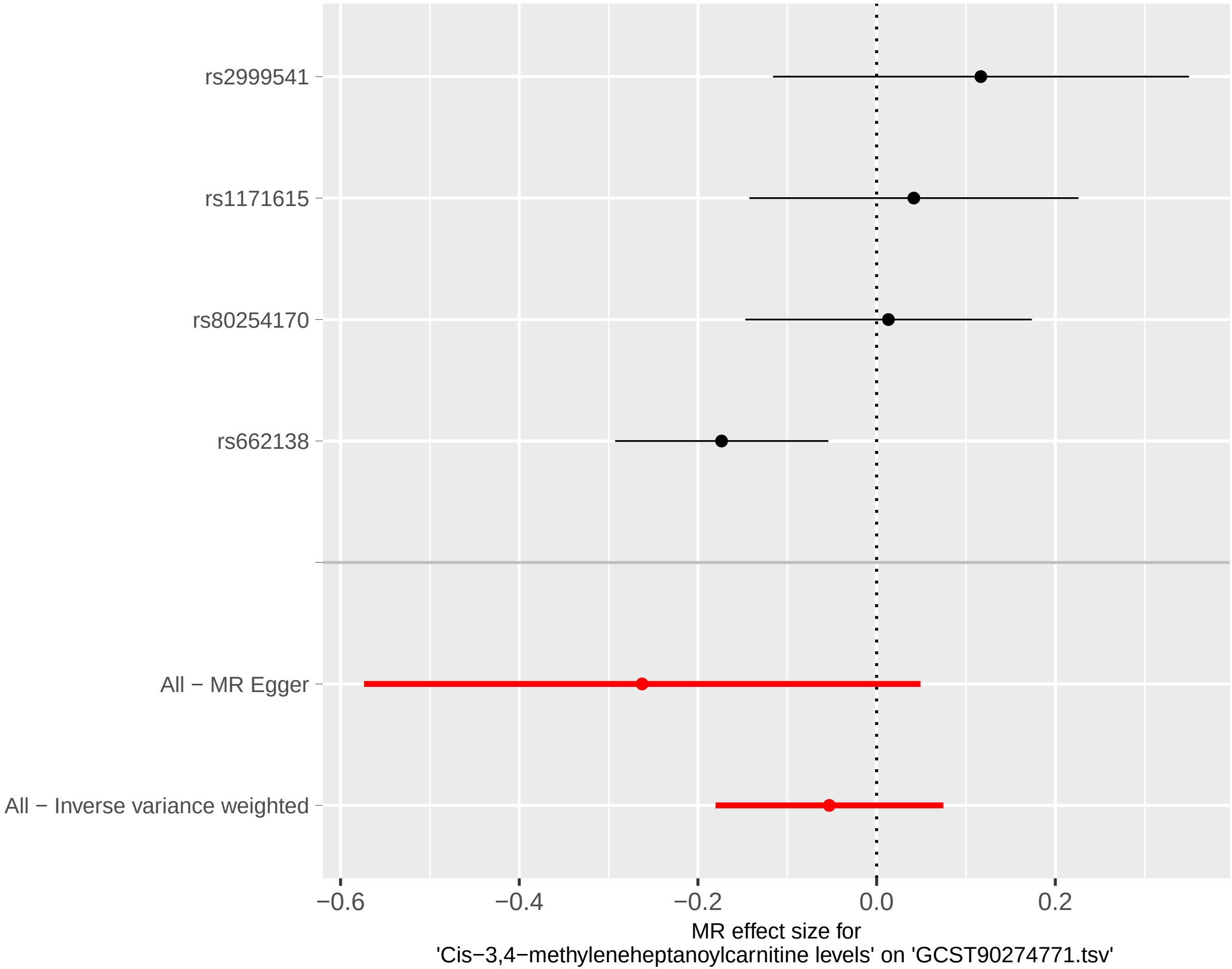
rs2999541
rs1171615
rs80254170
rs662138
All − MR Egger
All − Inverse variance weighted
−0.6
−0.4
−0.2
0.0
0.2
MR effect size for
'Cis−3,4−methyleneheptanoylcarnitine levels' on 'GCST90274771.tsv'

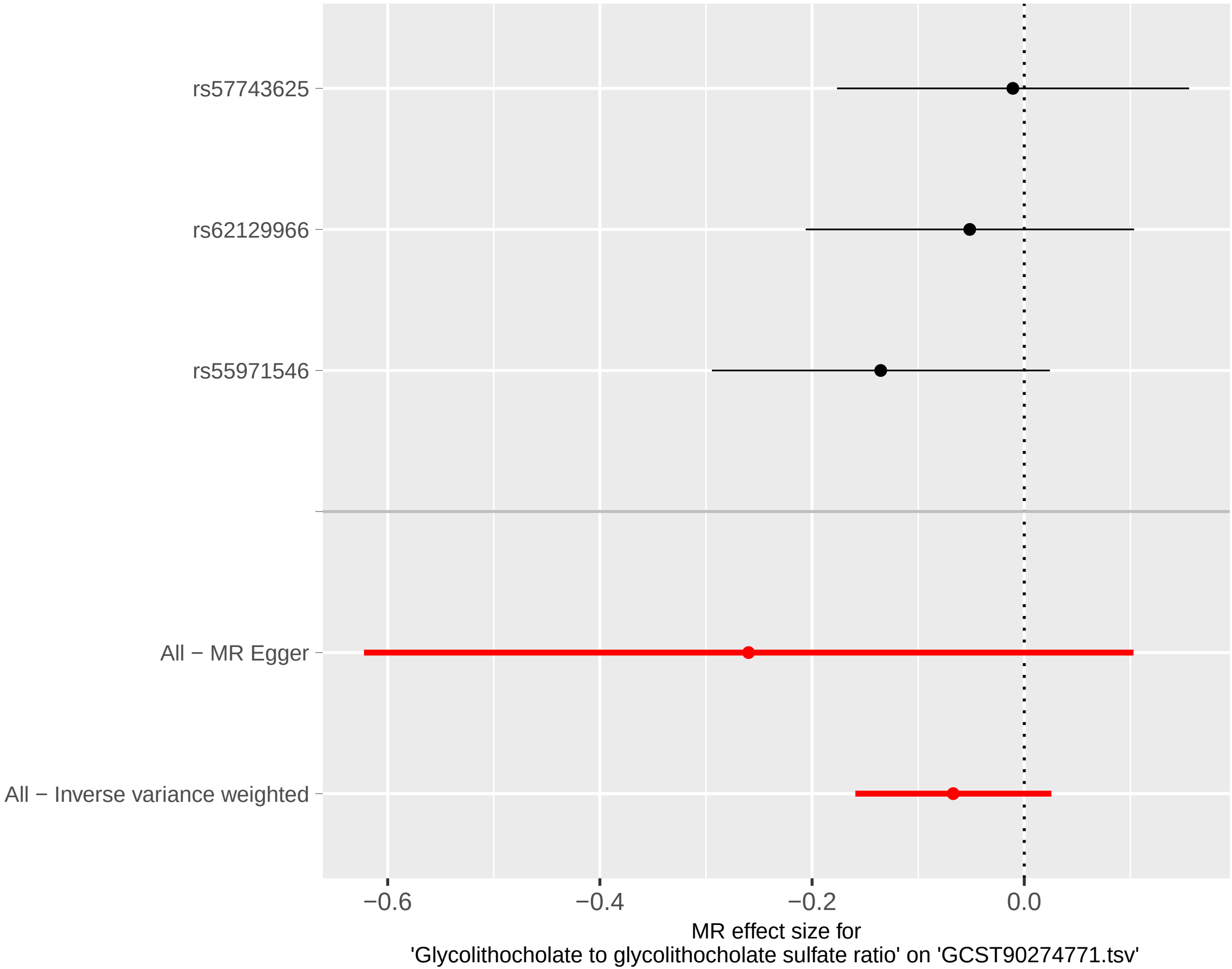

rs57743625
rs62129966
rs55971546
All − MR Egger
All − Inverse variance weighted
−0.6
−0.4
−0.2
0.0
MR effect size for
'Glycolithocholate to glycolithocholate sulfate ratio' on 'GCST90274771.tsv'

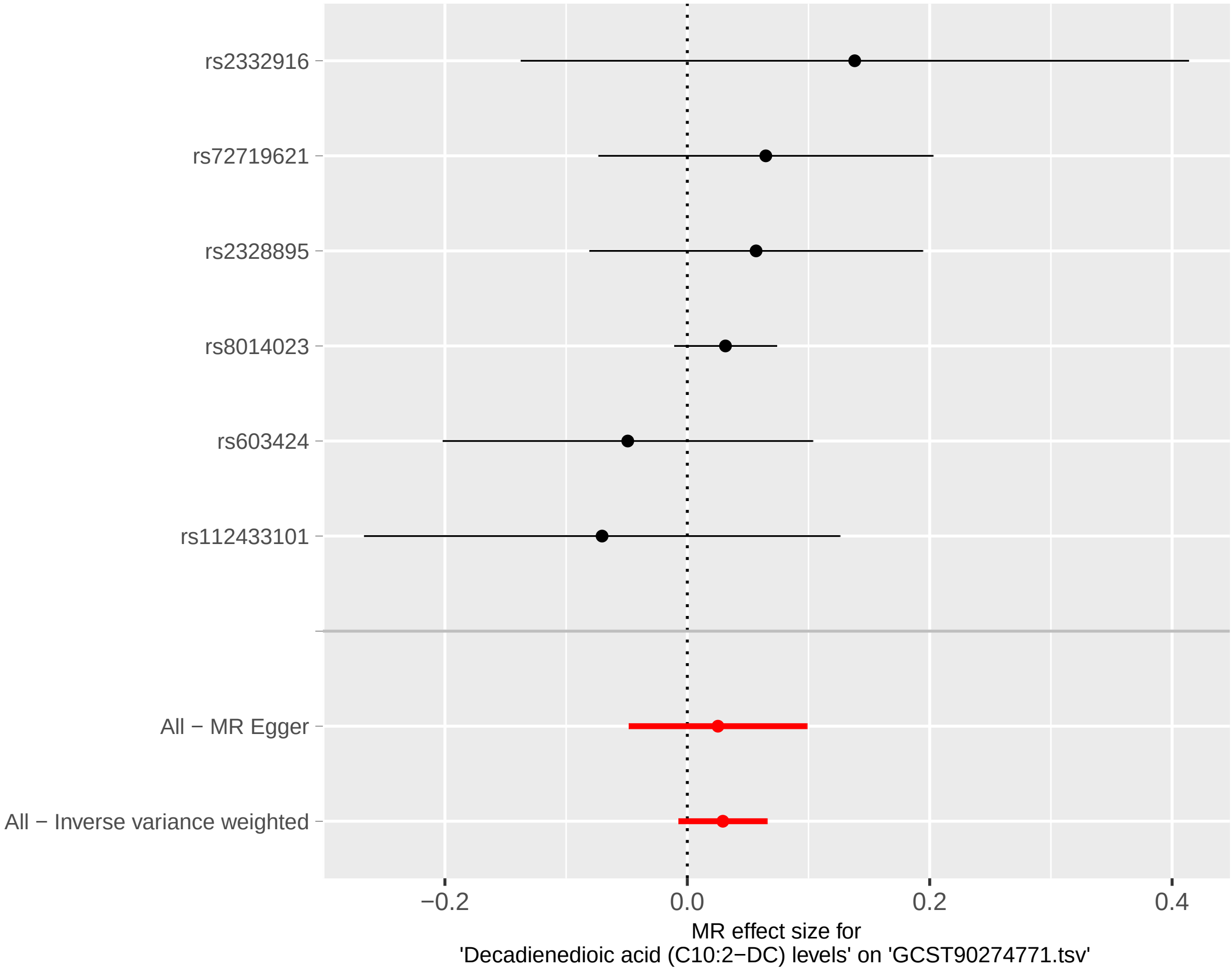

rs2332916
rs72719621
rs2328895
rs8014023
rs603424
rs112433101
All − MR Egger
All − Inverse variance weighted
−0.2
0.0
0.2
0.4
MR effect size for
'Decadienedioic acid (C10:2−DC) levels' on 'GCST90274771.tsv'

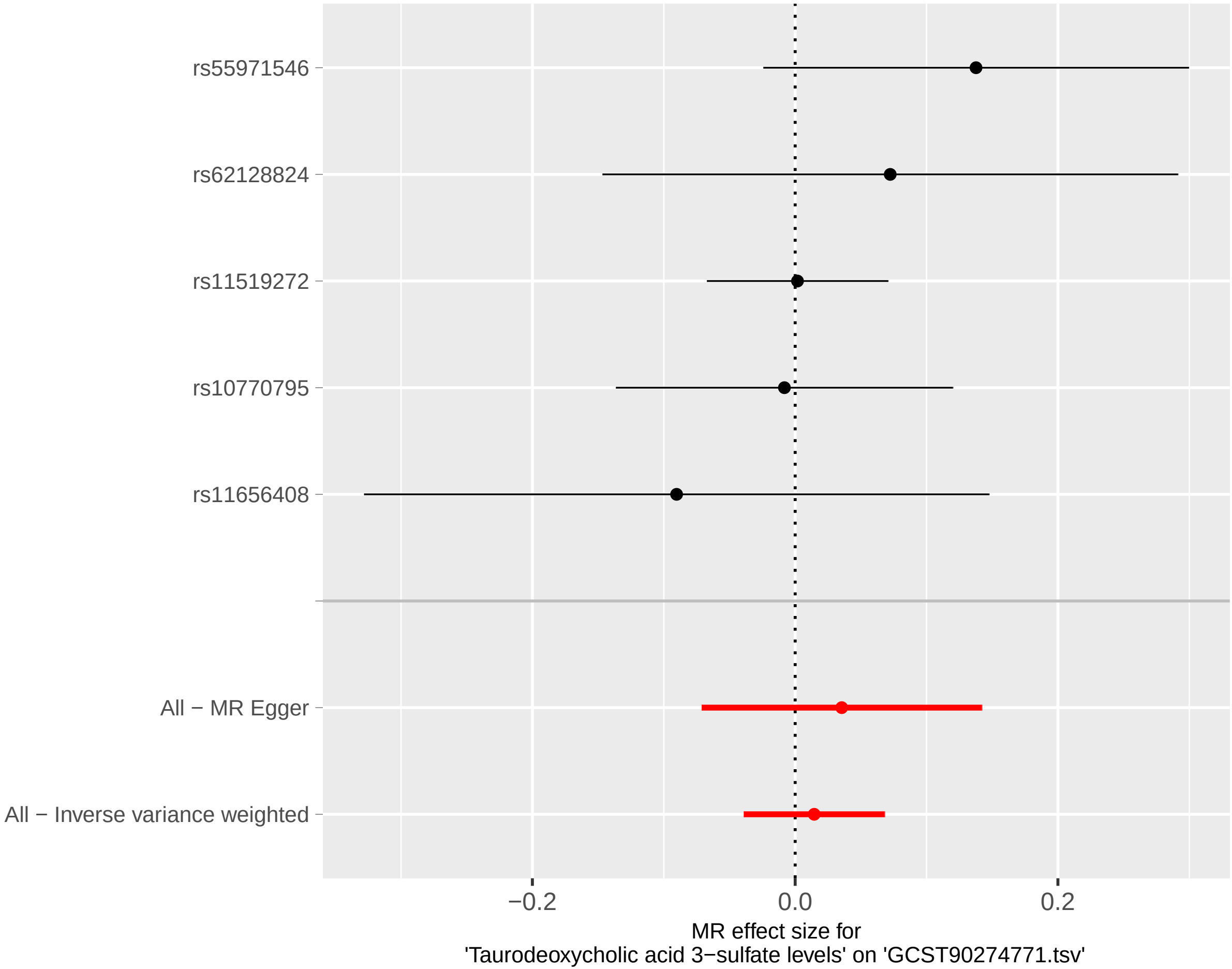
rs55971546
rs62128824
rs11519272
rs10770795
rs11656408
All − MR Egger
All − Inverse variance weighted
−0.2
0.0
0.2
MR effect size for
'Taurodeoxycholic acid 3−sulfate levels' on 'GCST90274771.tsv'

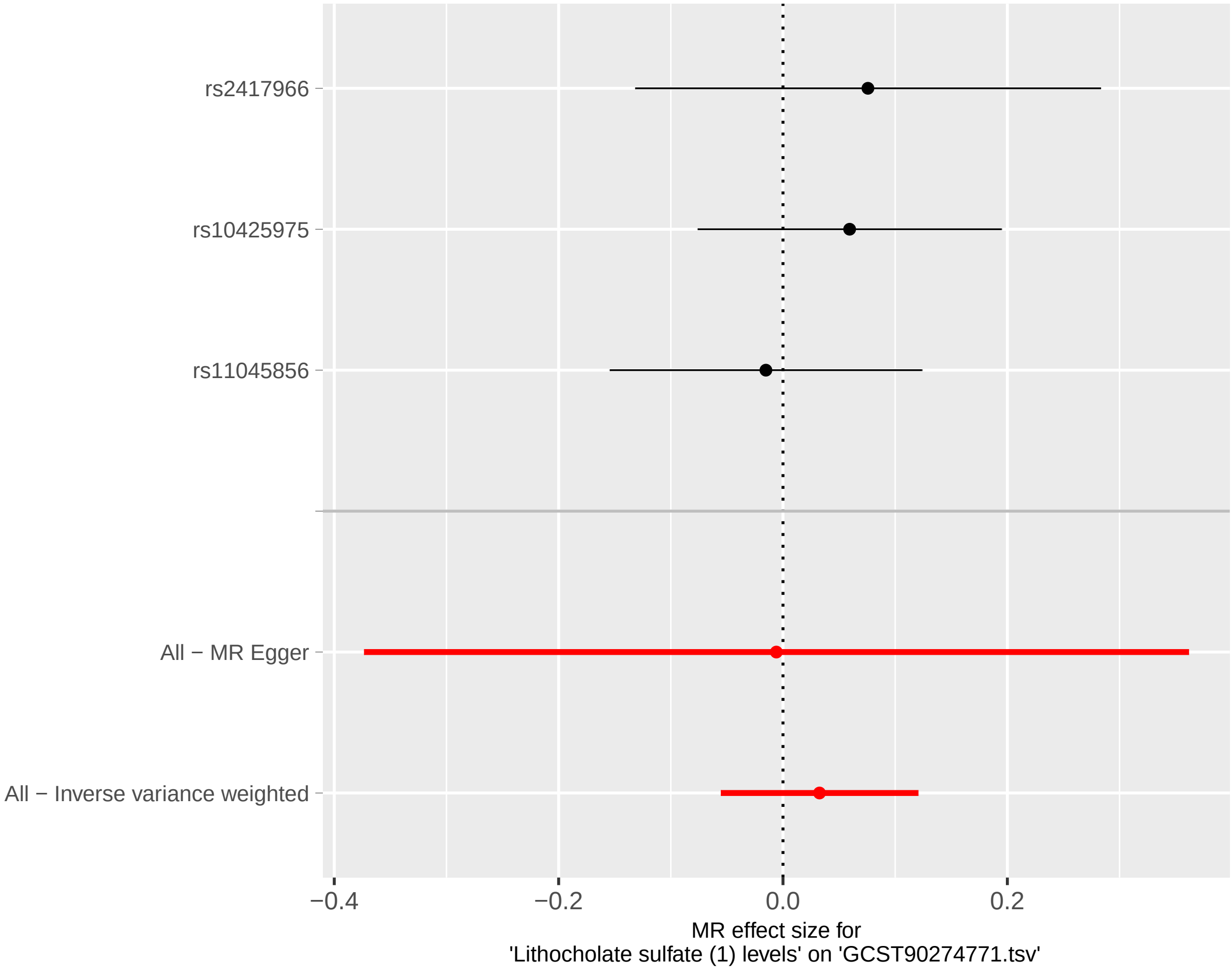
rs2417966
rs10425975
rs11045856
All − MR Egger
All − Inverse variance weighted
−0.4
−0.2
0.0
0.2
MR effect size for
'Lithocholate sulfate (1) levels' on 'GCST90274771.tsv'

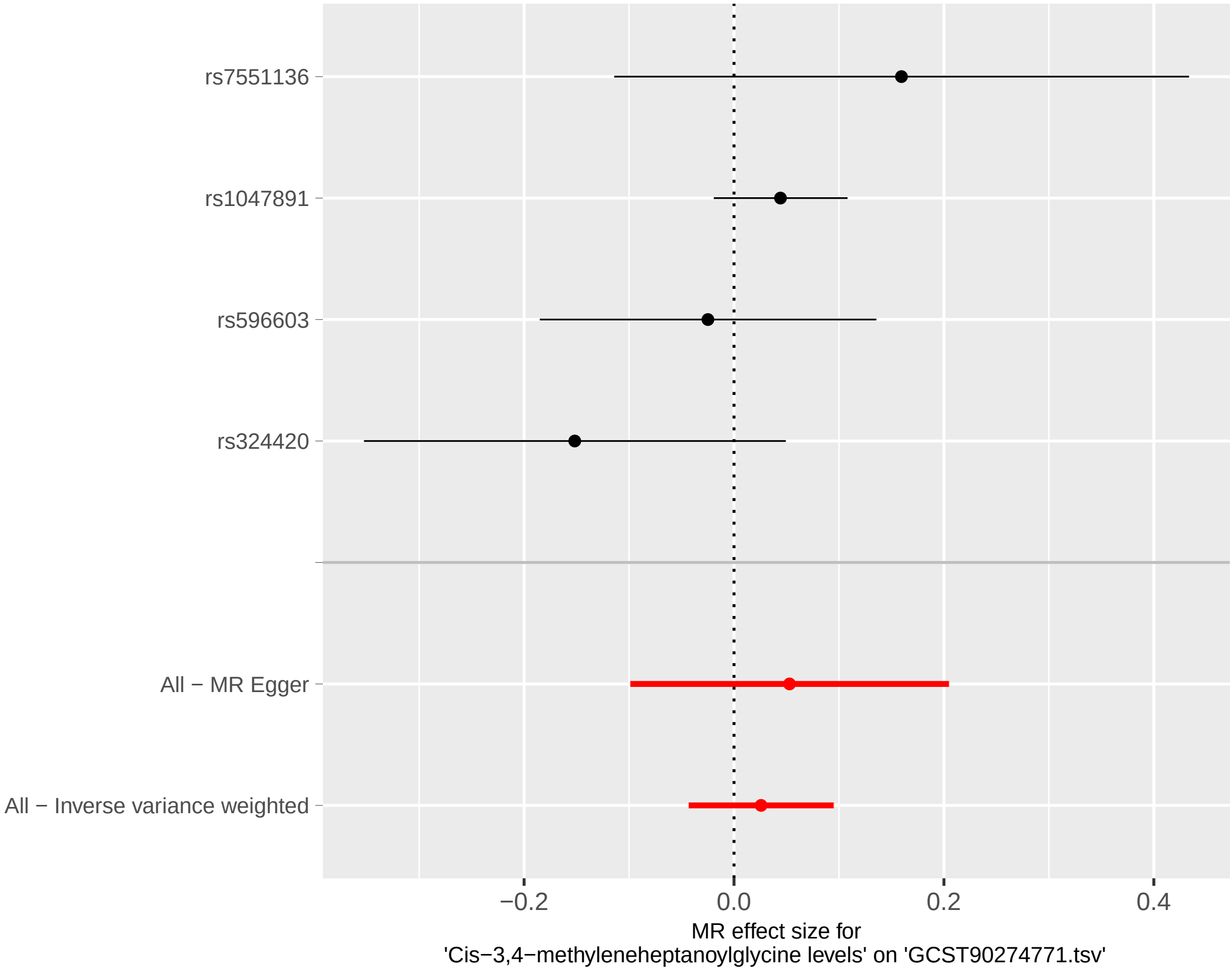
rs7551136
rs1047891
rs596603
rs324420
All − MR Egger
All − Inverse variance weighted
−0.2
0.0
0.2
0.4
MR effect size for
'Cis−3,4−methyleneheptanoylglycine levels' on 'GCST90274771.tsv'

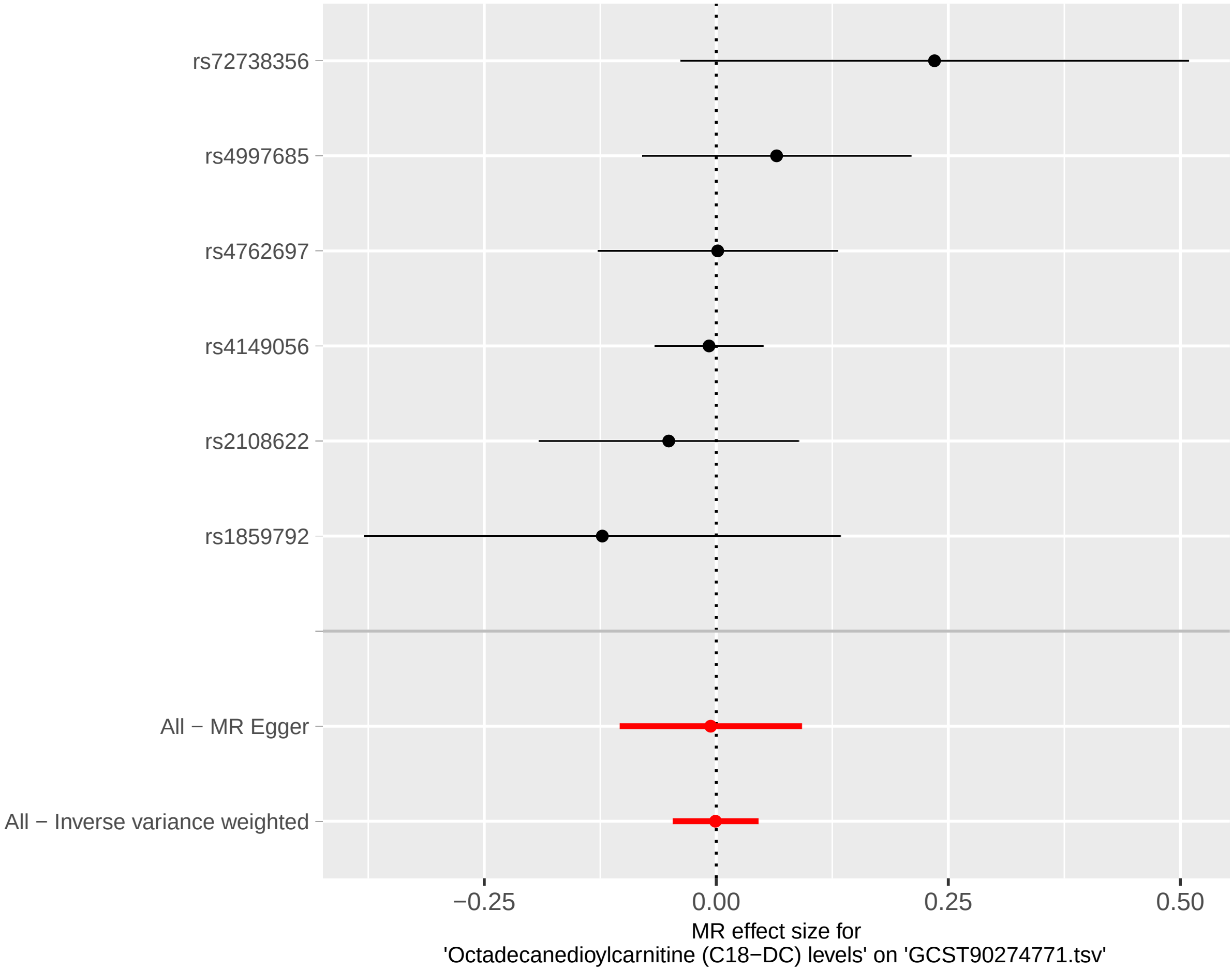
rs72738356
rs4997685
rs4762697
rs4149056
rs2108622
rs1859792
All − MR Egger
All − Inverse variance weighted
−0.25
0.00
0.25
0.50
MR effect size for
'Octadecanedioylcarnitine (C18−DC) levels' on 'GCST90274771.tsv'

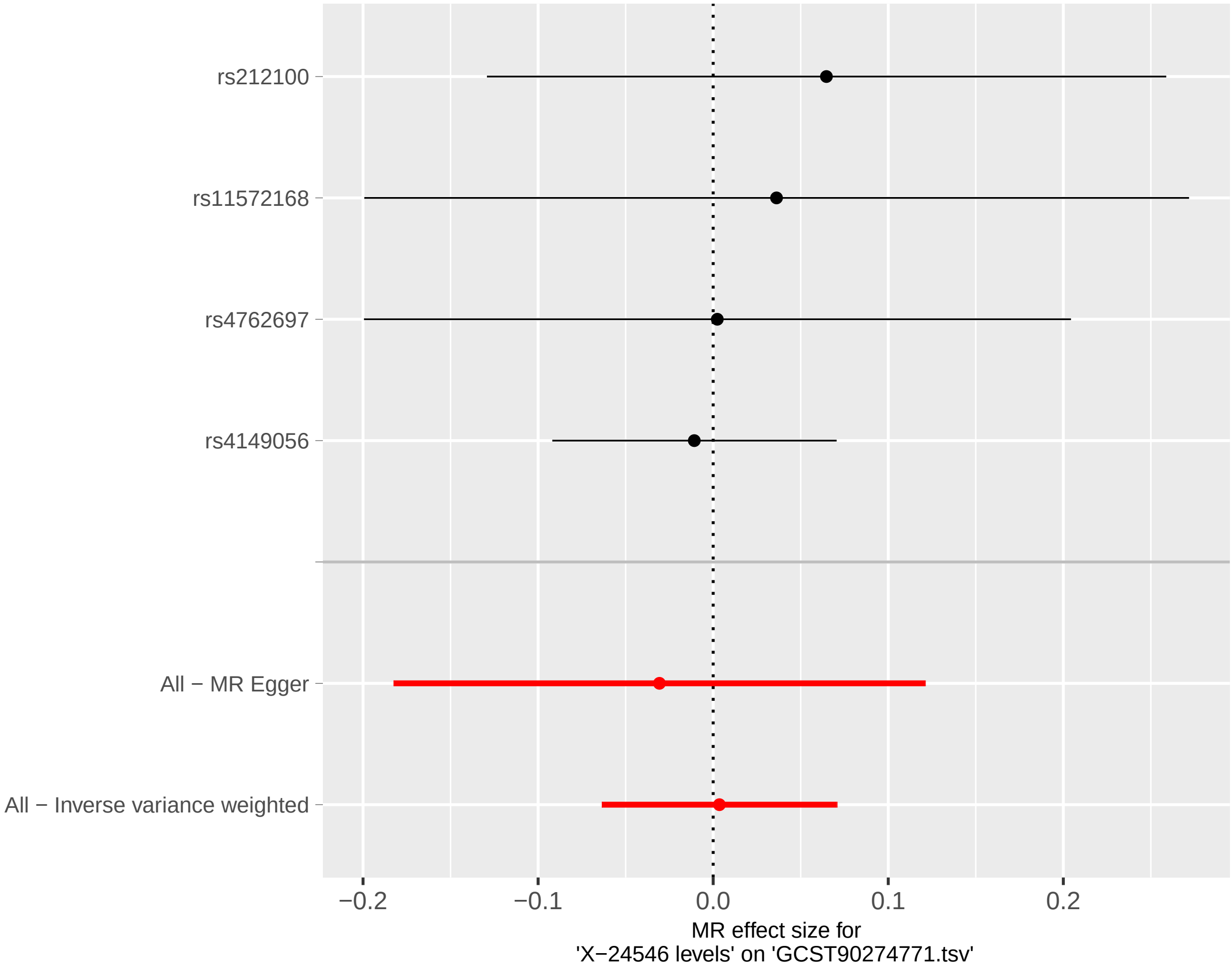

rs212100
rs11572168
rs4762697
rs4149056
All − MR Egger
All − Inverse variance weighted
−0.2
−0.1
0.0
0.1
0.2
MR effect size for
'X−24546 levels' on 'GCST90274771.tsv'

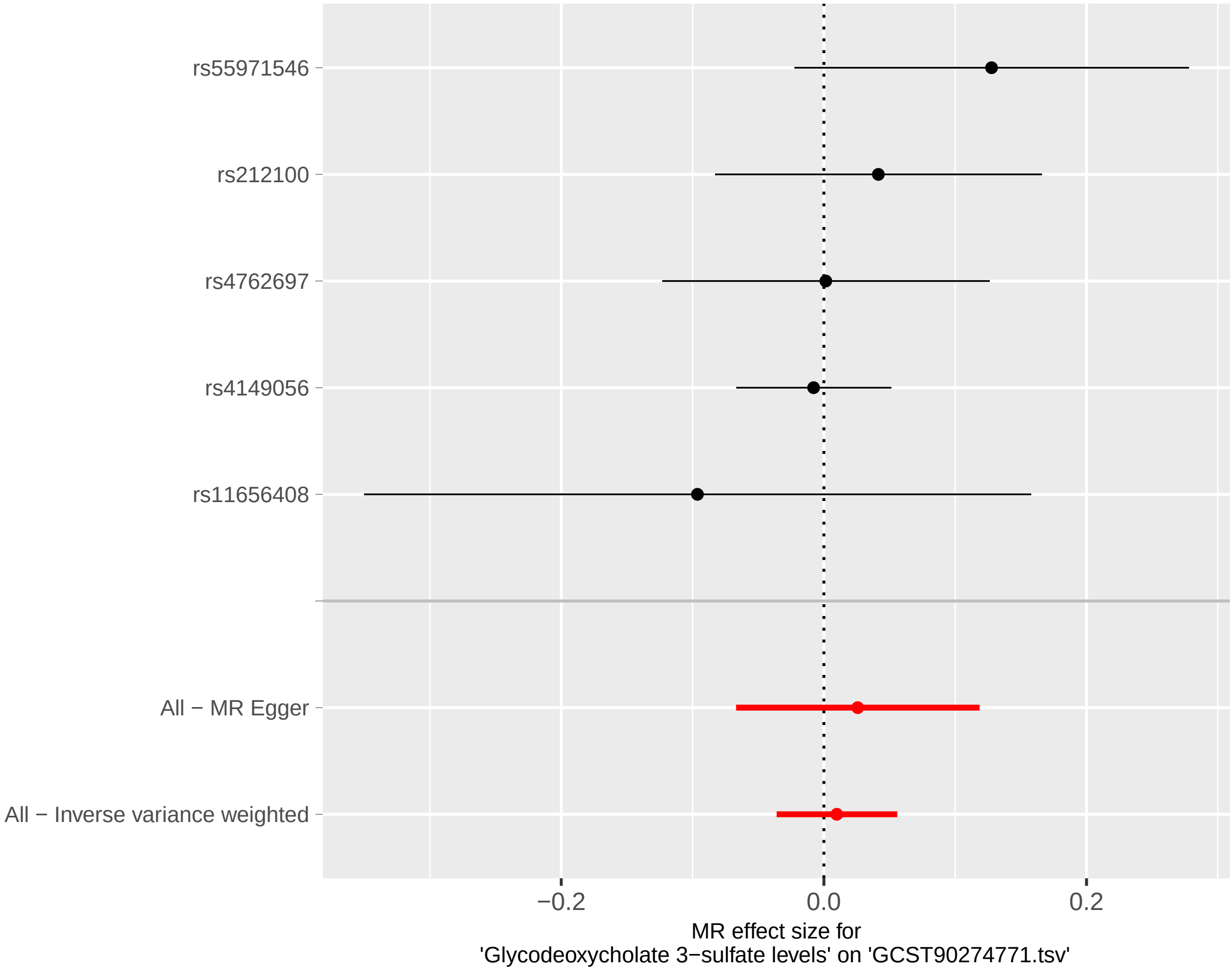

rs55971546
rs212100
rs4762697
rs4149056
rs11656408
All − MR Egger
All − Inverse variance weighted
−0.2
0.0
0.2
MR effect size for
'Glycodeoxycholate 3−sulfate levels' on 'GCST90274771.tsv'

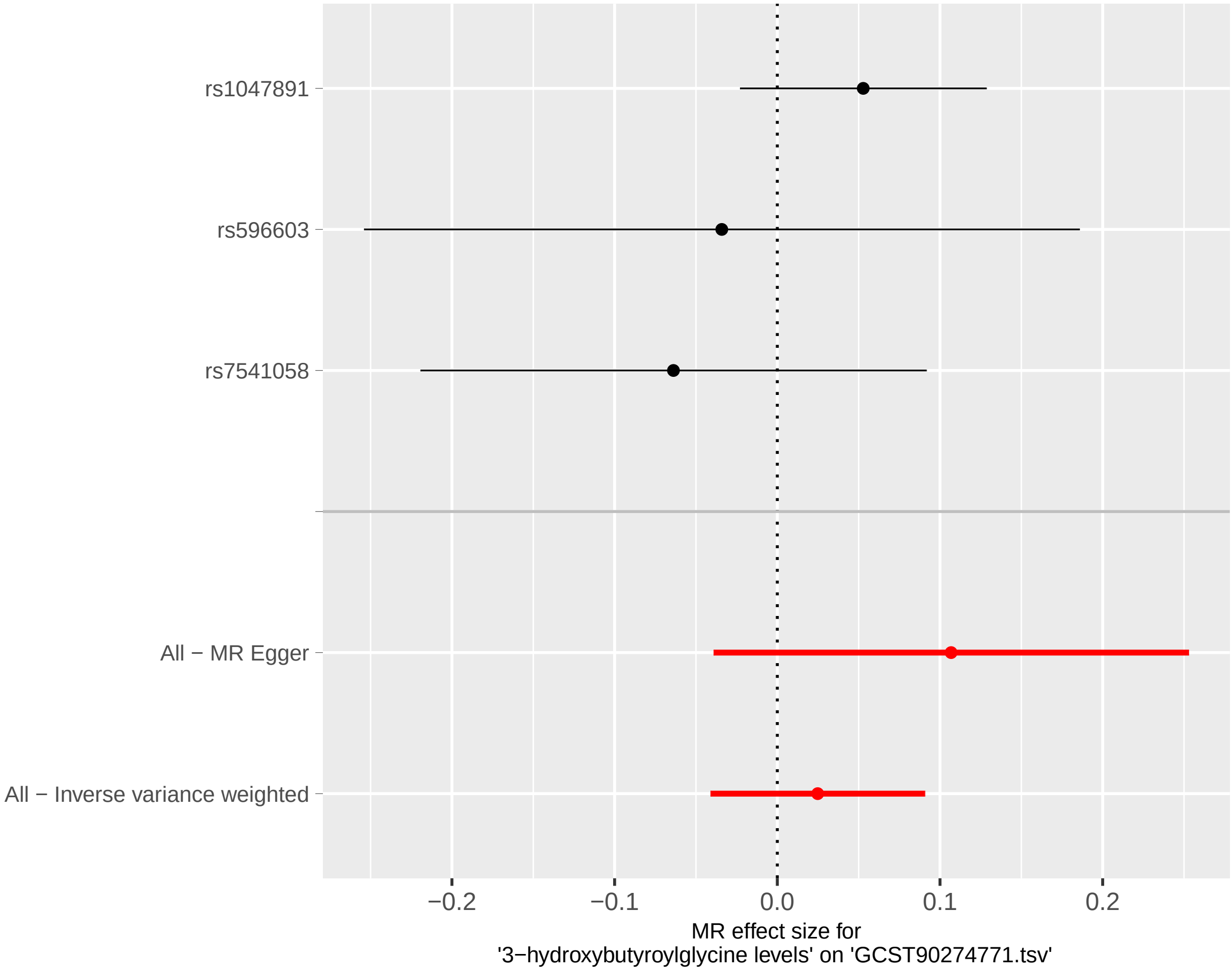
rs1047891
rs596603
rs7541058
All − MR Egger
All − Inverse variance weighted
−0.2
−0.1
0.0
0.1
0.2
MR effect size for
'3−hydroxybutyroylglycine levels' on 'GCST90274771.tsv'

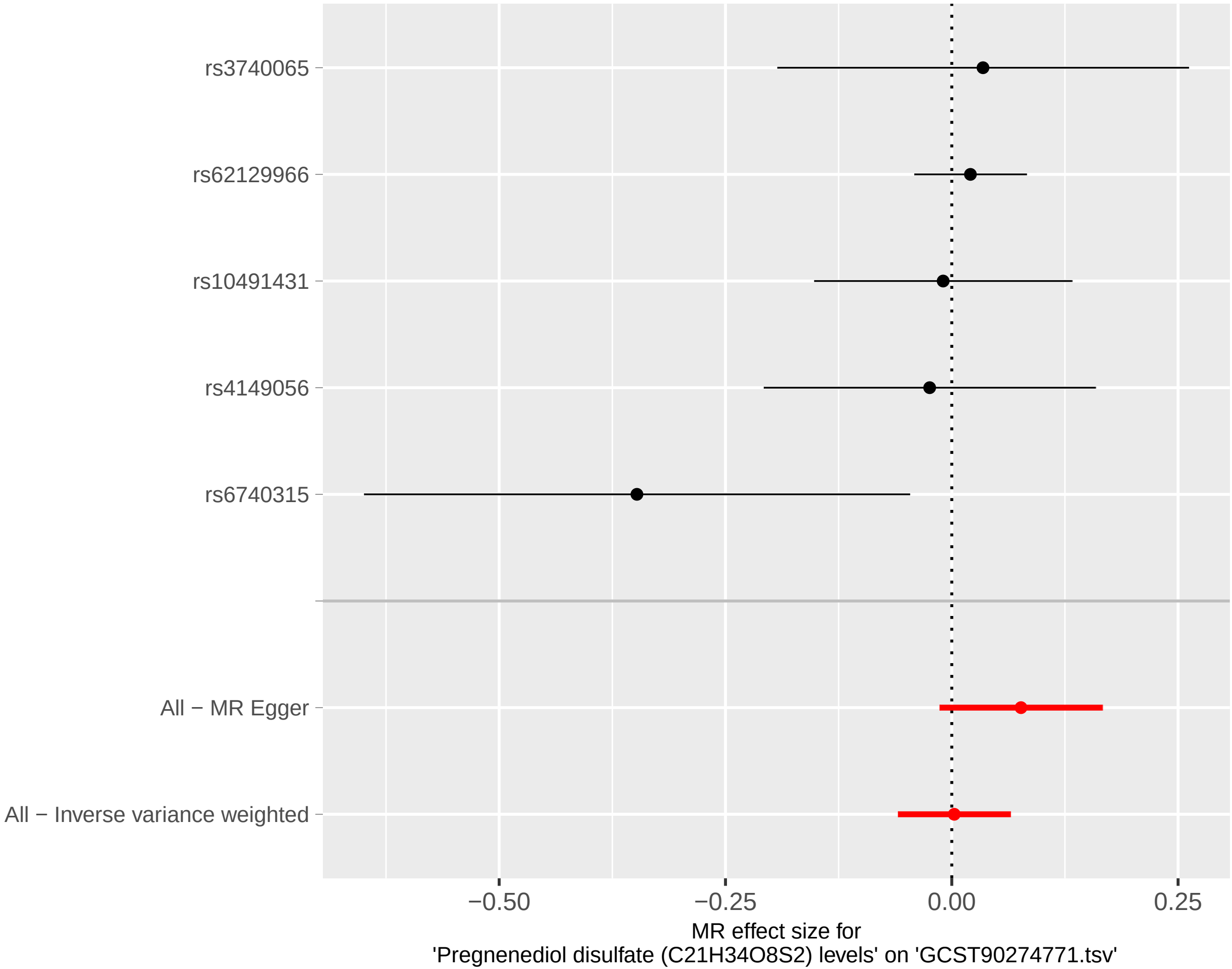
rs3740065
rs62129966
rs10491431
rs4149056
rs6740315
All − MR Egger
All − Inverse variance weighted
−0.50
−0.25
0.00
0.25
MR effect size for
'Pregnenediol disulfate (C21H34O8S2) levels' on 'GCST90274771.tsv'

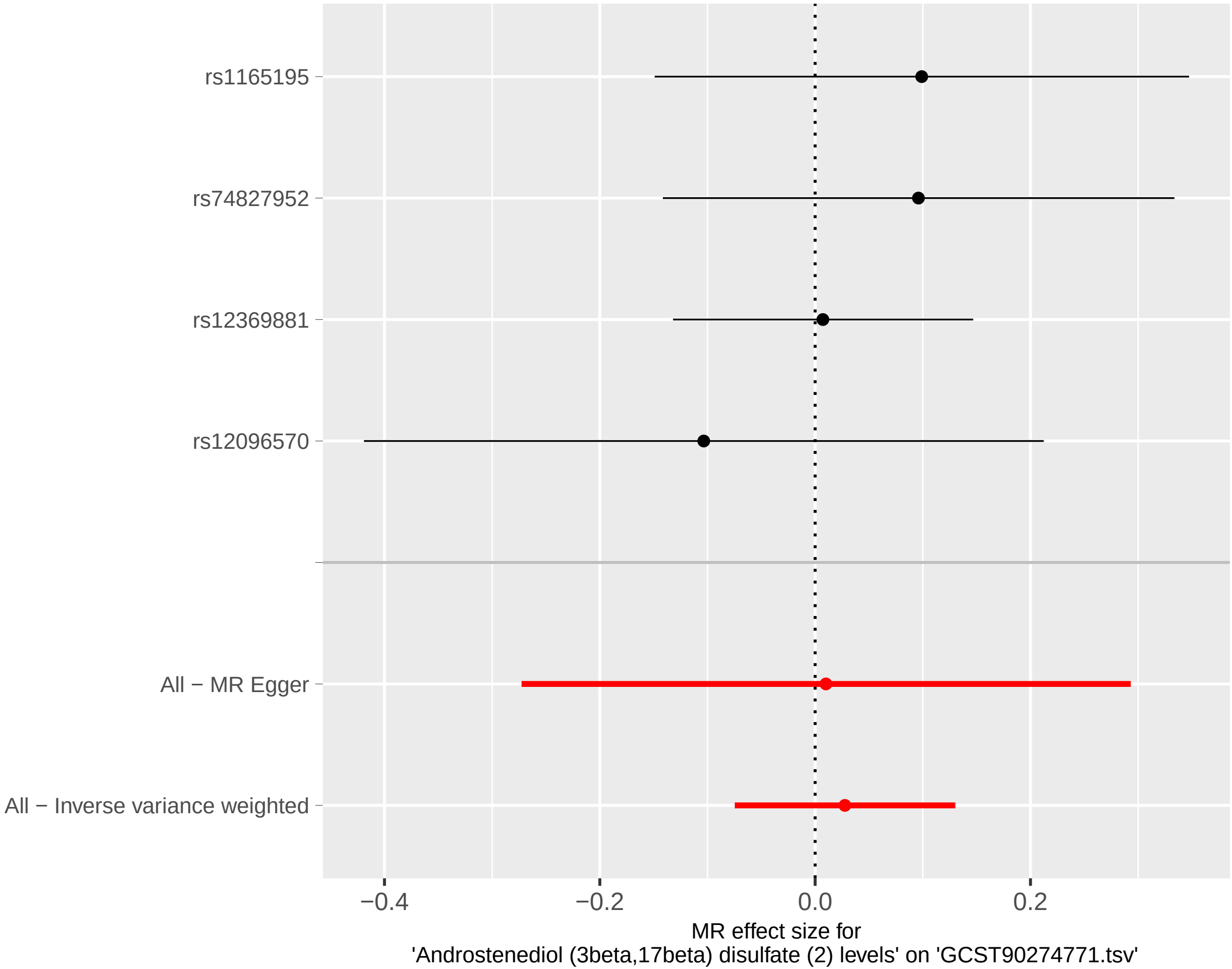
rs1165195
rs74827952
rs12369881
rs12096570
All − MR Egger
All − Inverse variance weighted
−0.4
−0.2
0.0
0.2
MR effect size for
'Androstenediol (3beta,17beta) disulfate (2) levels' on 'GCST90274771.tsv'

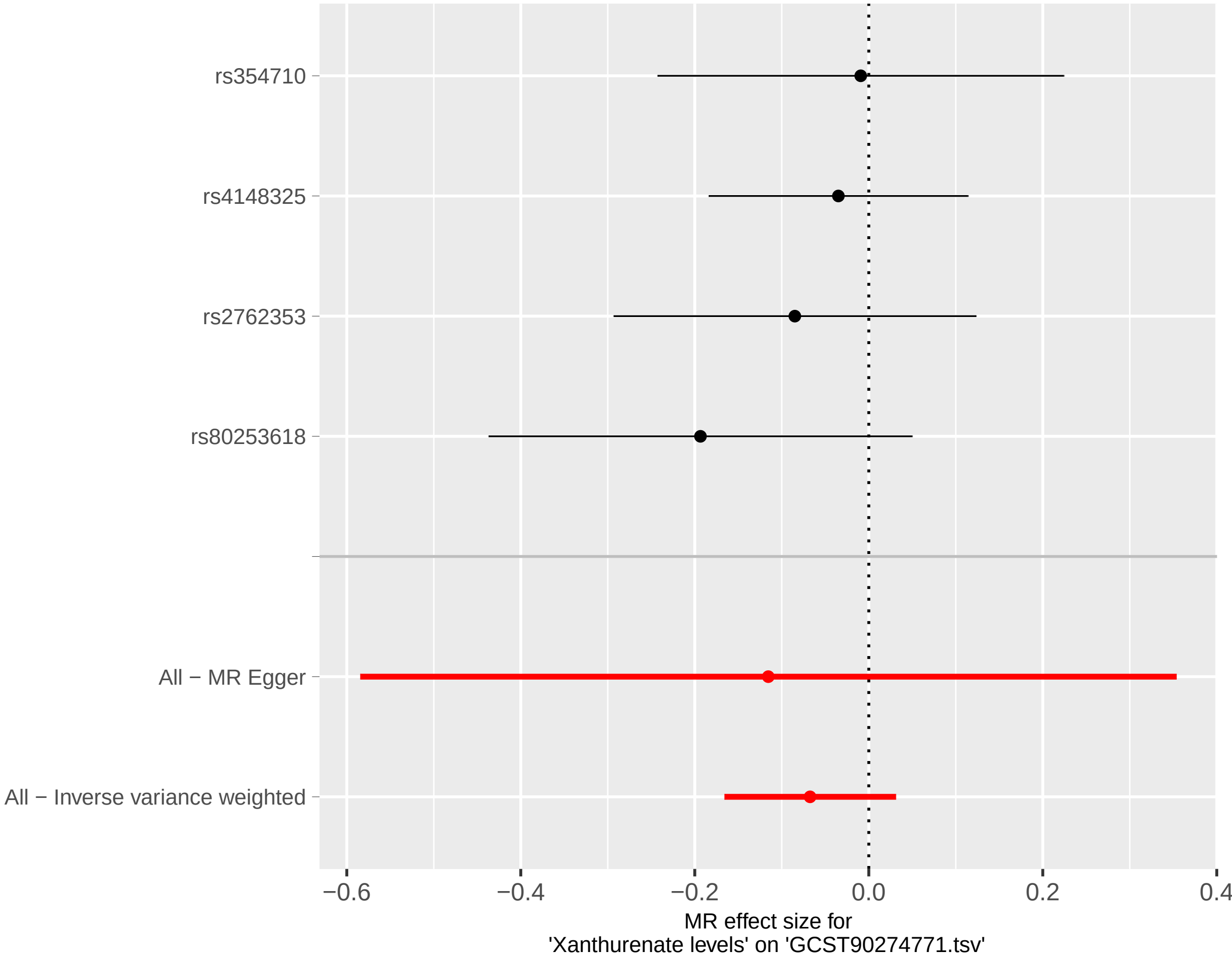

rs354710
rs4148325
rs2762353
rs80253618
All − MR Egger
All − Inverse variance weighted
−0.6
−0.4
−0.2
0.0
0.2
0.4
MR effect size for
'Xanthurenate levels' on 'GCST90274771.tsv'

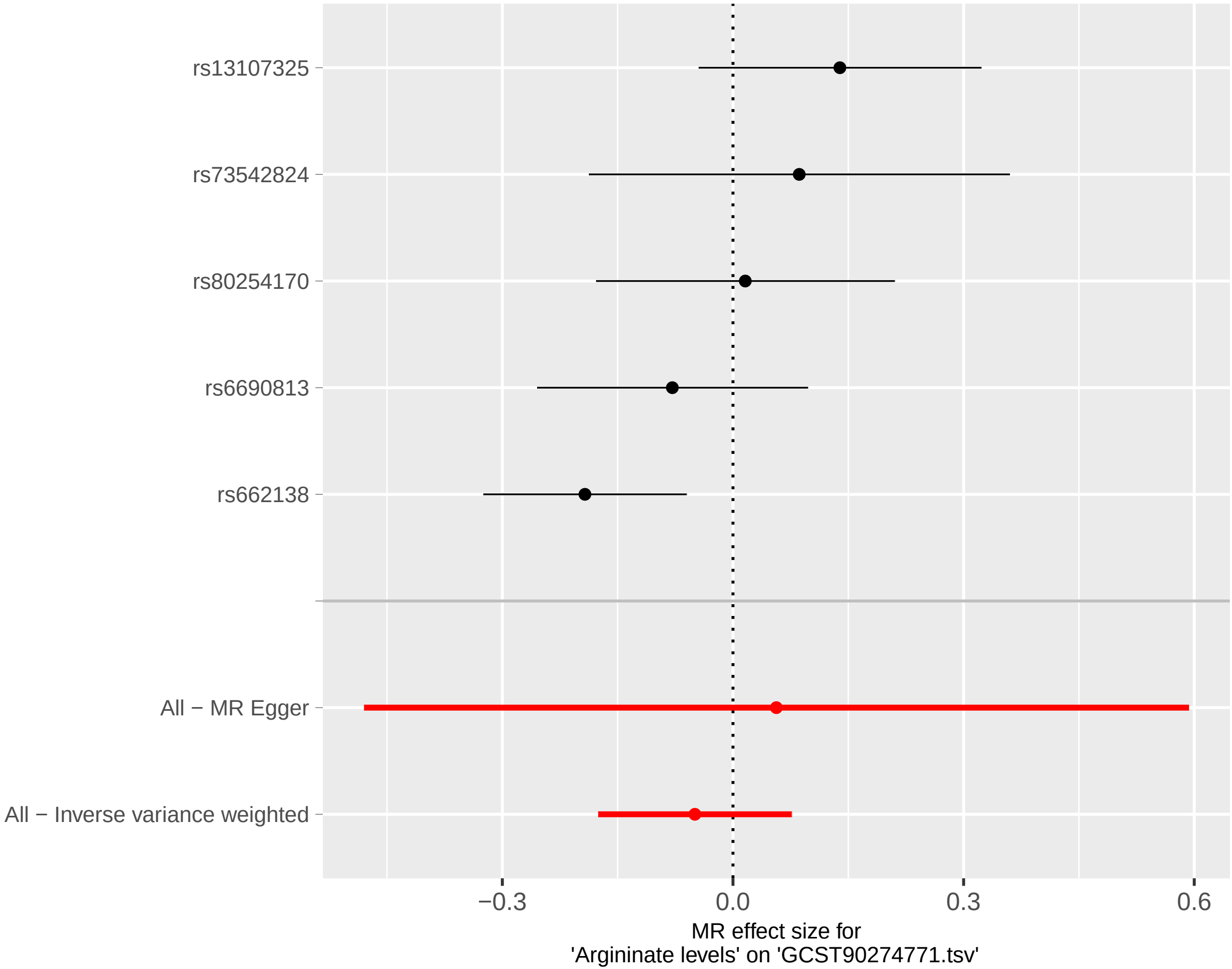
rs13107325
rs73542824
rs80254170
rs6690813
rs662138
All − MR Egger
All − Inverse variance weighted
−0.3
0.0
0.3
0.6
MR effect size for
'Argininate levels' on 'GCST90274771.tsv'

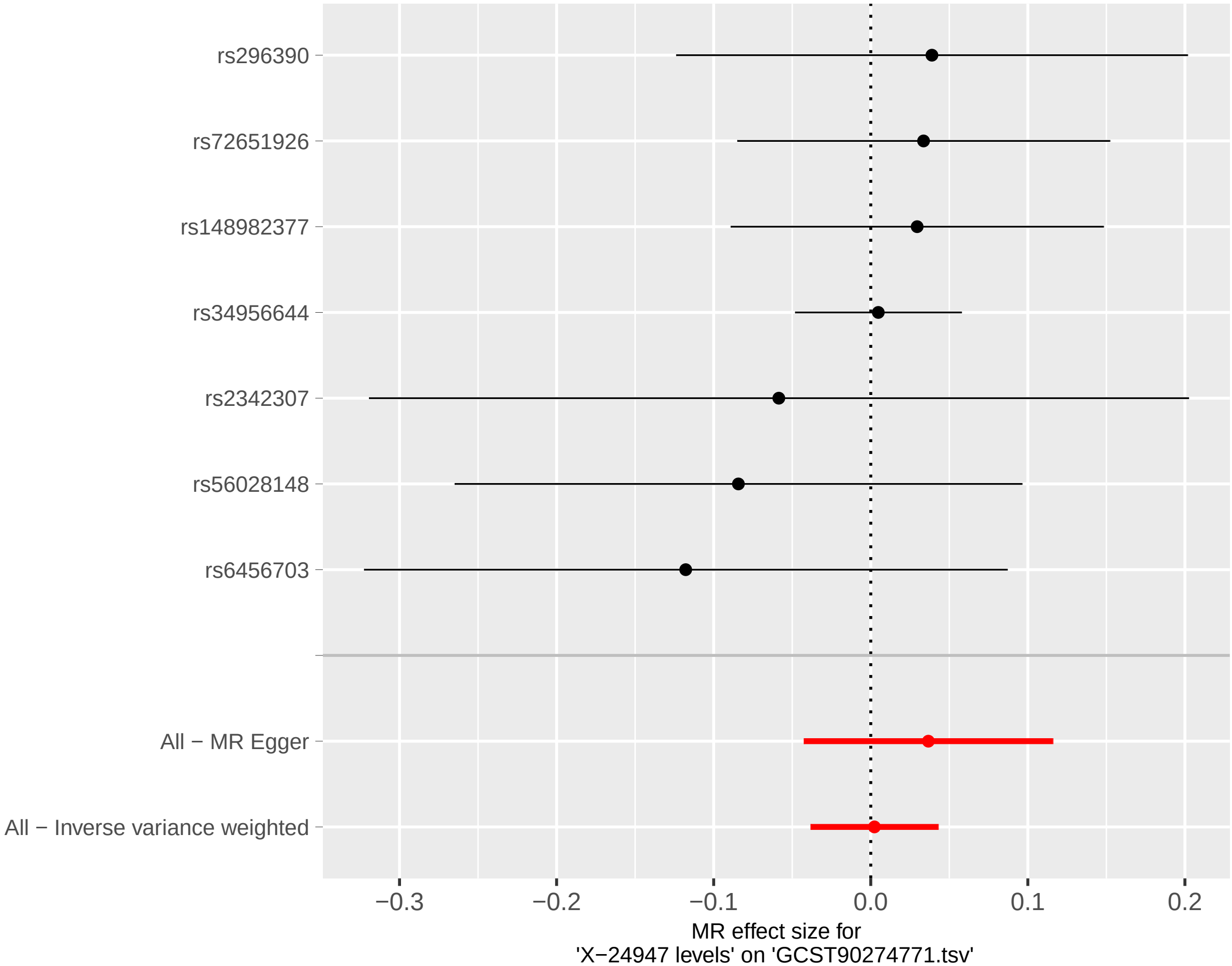
rs296390
rs72651926
rs148982377
rs34956644
rs2342307
rs56028148
rs6456703
All − MR Egger
All − Inverse variance weighted
−0.3
−0.2
−0.1
0.0
0.1
0.2
MR effect size for
'X−24947 levels' on 'GCST90274771.tsv'

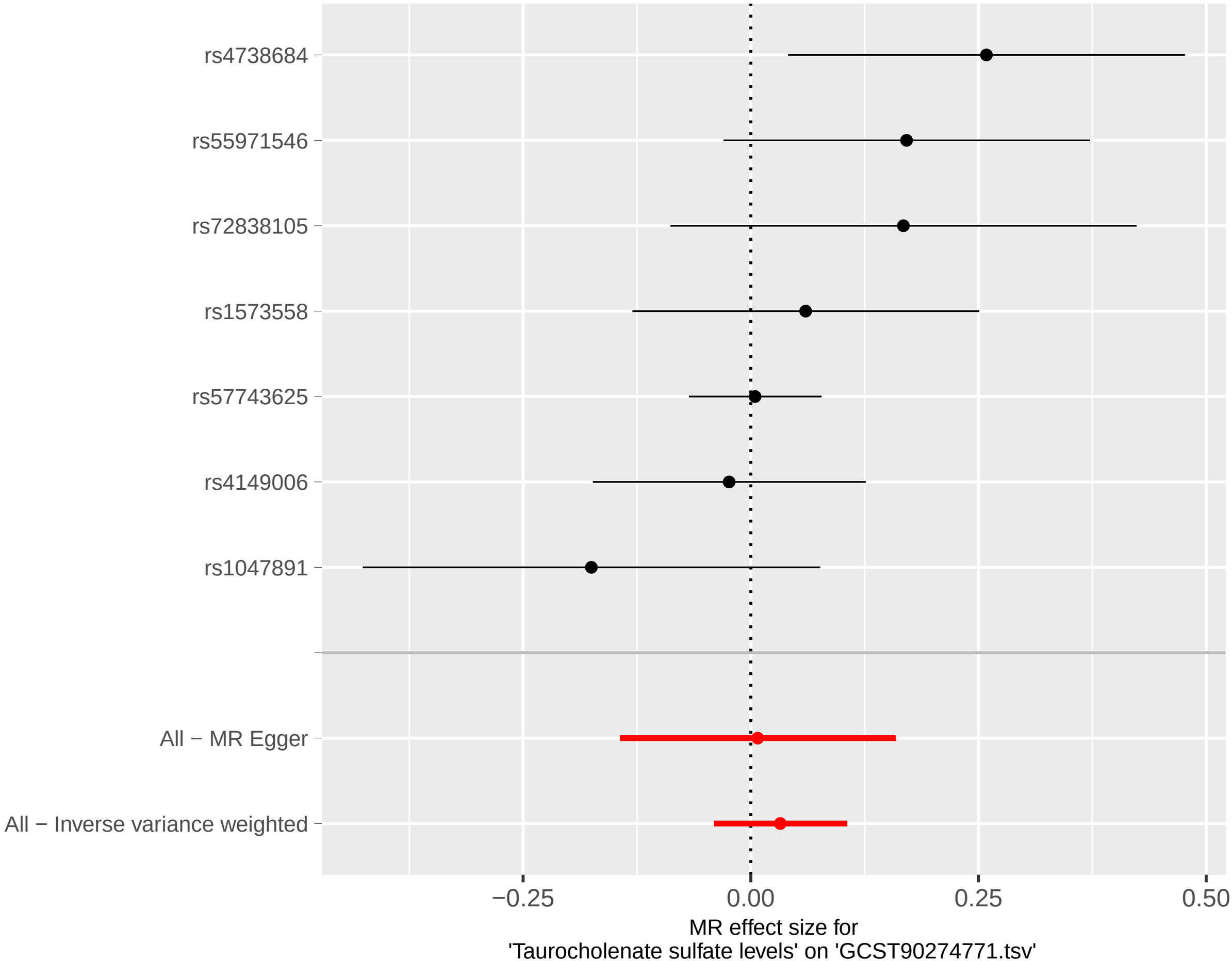

rs4738684
rs55971546
rs72838105
rs1573558
rs57743625
rs4149006
rs1047891
All − MR Egger
All − Inverse variance weighted
−0.25
0.00
0.25
0.50
MR effect size for
'Taurocholenate sulfate levels' on 'GCST90274771.tsv'

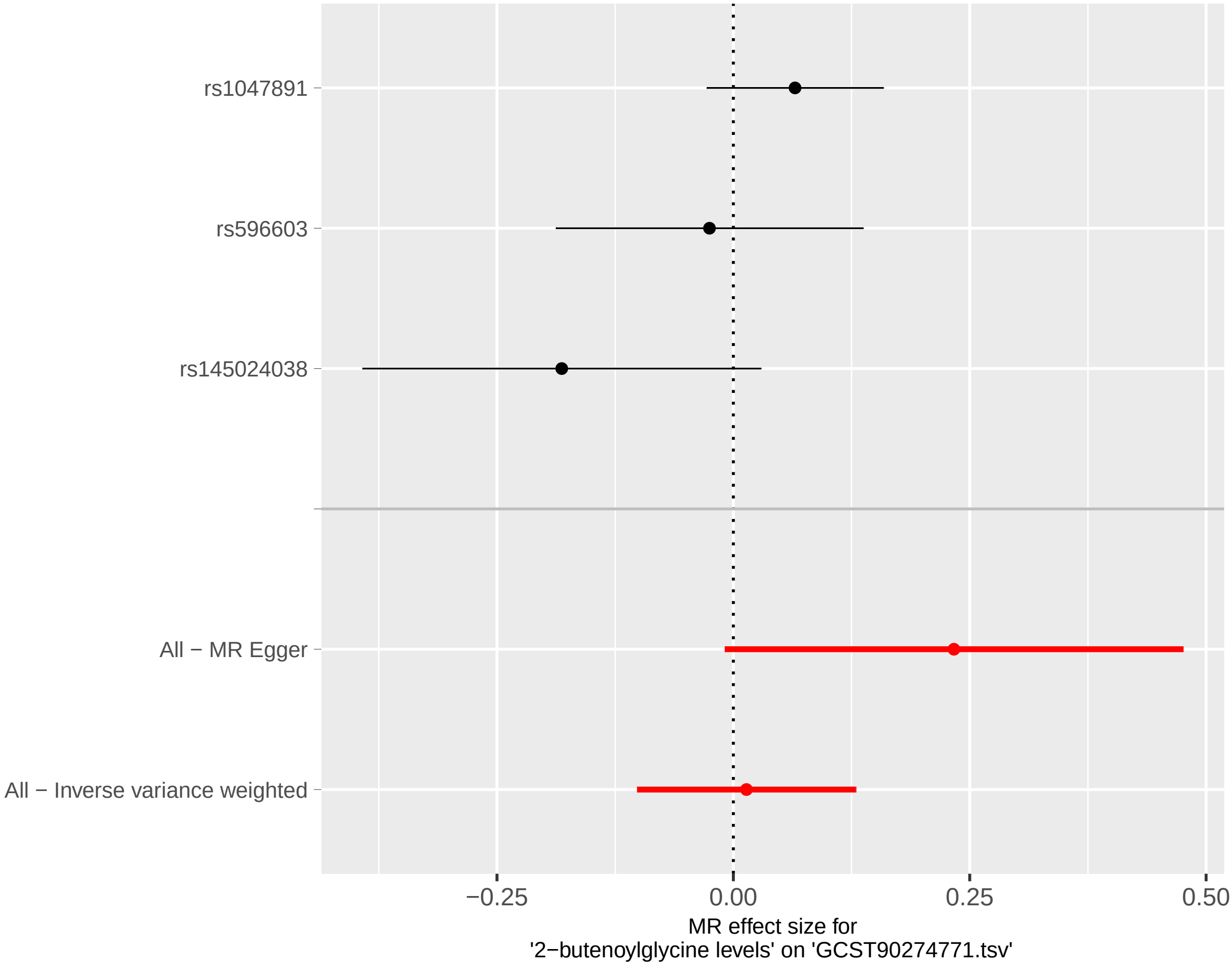
rs1047891
rs596603
rs145024038
All − MR Egger
All − Inverse variance weighted
−0.25
0.00
0.25
0.50
MR effect size for
'2−butenoylglycine levels' on 'GCST90274771.tsv'

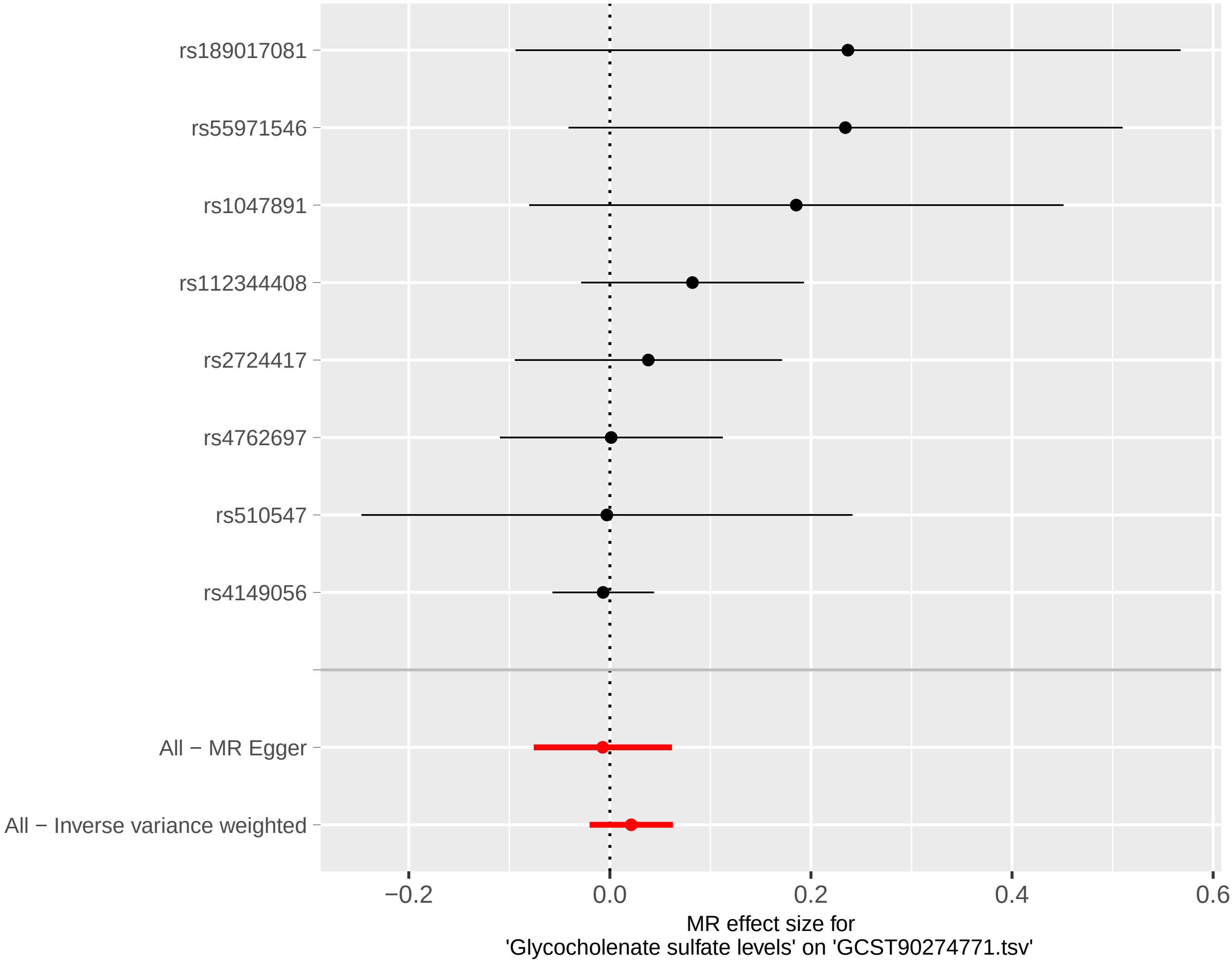
rs189017081
rs55971546
rs1047891
rs112344408
rs2724417
rs4762697
rs510547
rs4149056
All − MR Egger
All − Inverse variance weighted
−0.2
0.0
0.2
0.4
0.6
MR effect size for
'Glycocholenate sulfate levels' on 'GCST90274771.tsv'

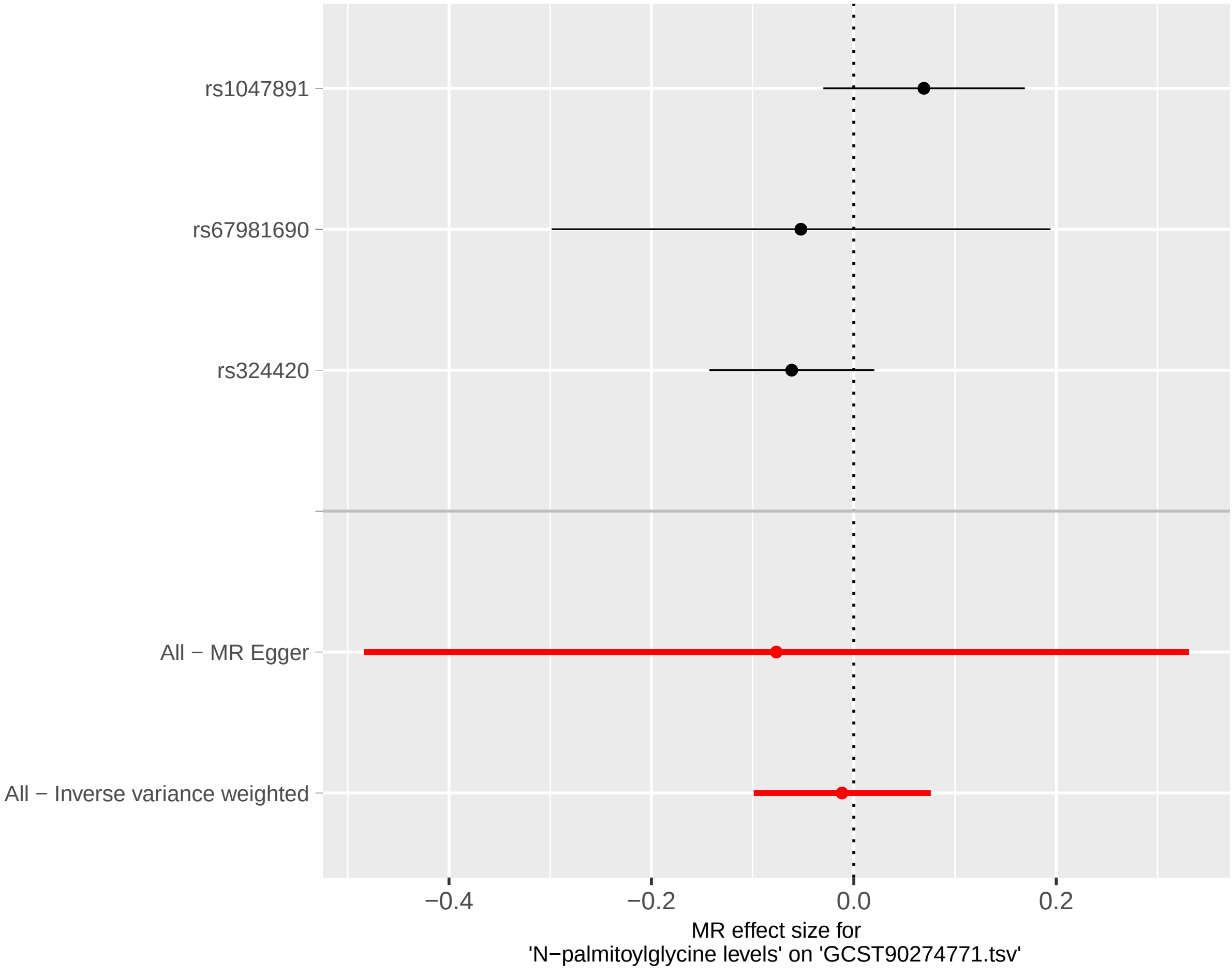
rs1047891
rs67981690
rs324420
All − MR Egger
All − Inverse variance weighted
−0.4
−0.2
0.0
0.2
MR effect size for
'N−palmitoylglycine levels' on 'GCST90274771.tsv'

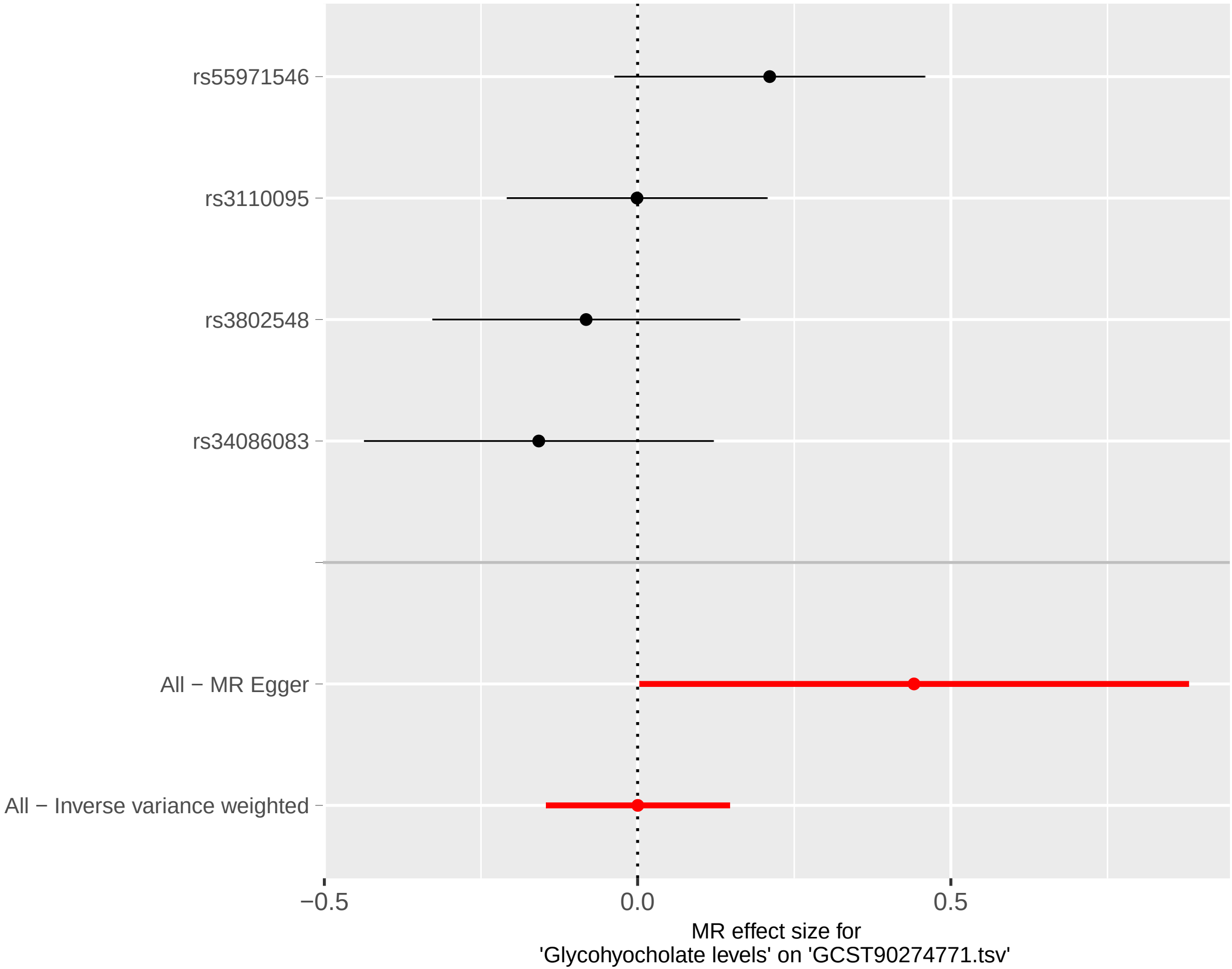
rs55971546
rs3110095
rs3802548
rs34086083
All − MR Egger
All − Inverse variance weighted
−0.5
0.0
0.5
MR effect size for
'Glycohyocholate levels' on 'GCST90274771.tsv'

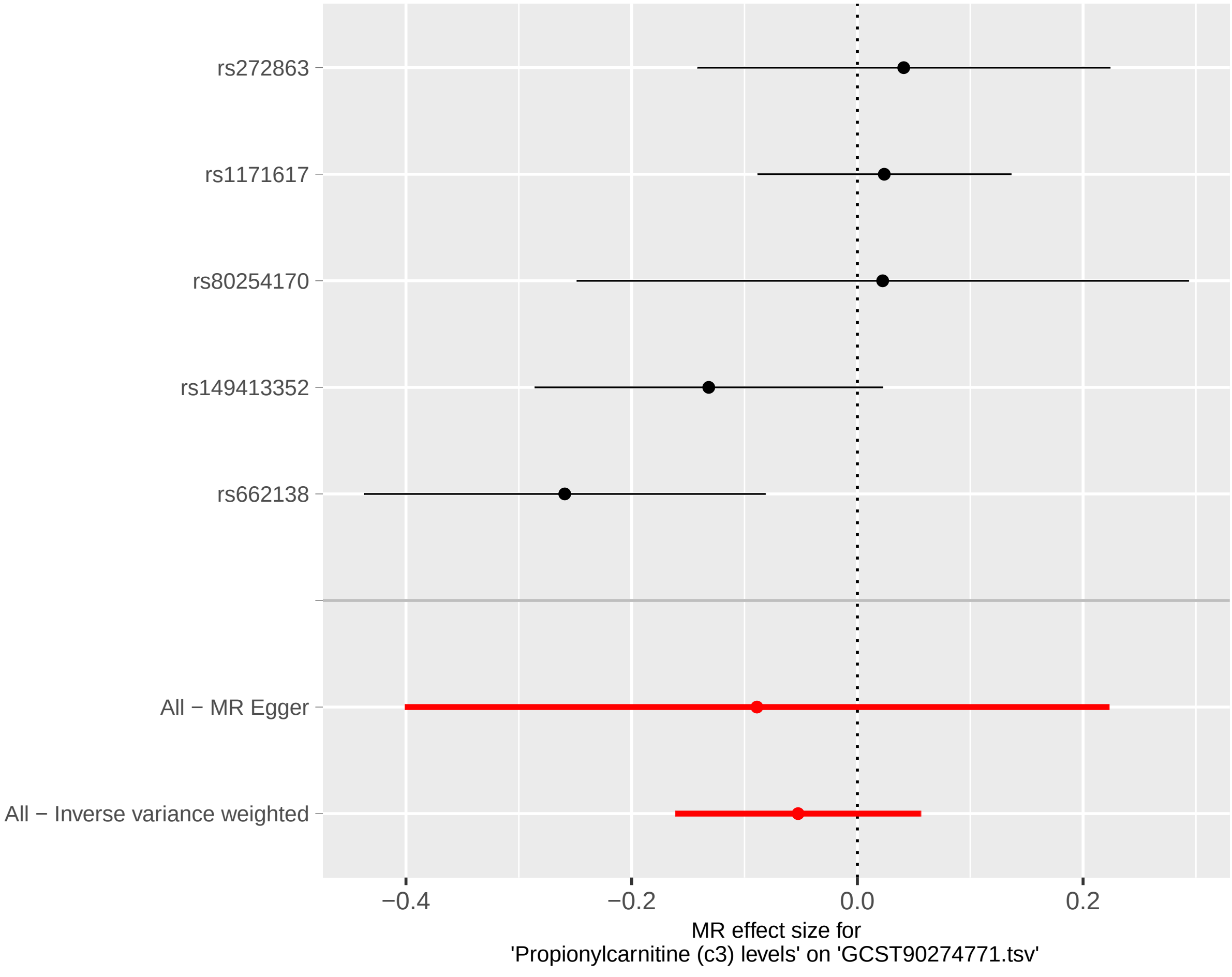
rs272863
rs1171617
rs80254170
rs149413352
rs662138
All − MR Egger
All − Inverse variance weighted
−0.4
−0.2
0.0
0.2
MR effect size for
'Propionylcarnitine (c3) levels' on 'GCST90274771.tsv'

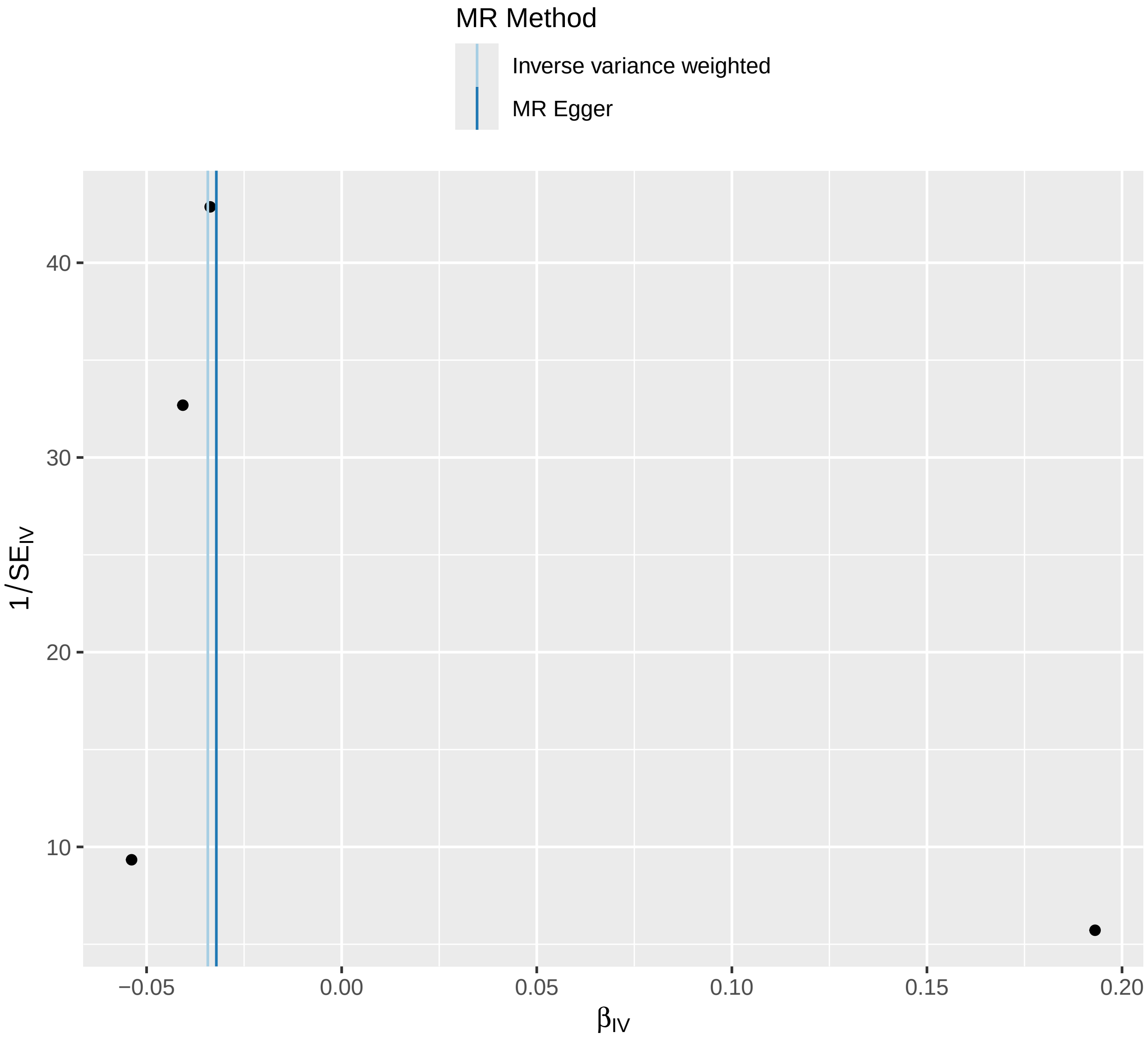
MR Method
Inverse variance weighted
MR Egger
40
30
20
10
1/SE$_{IV}$
−0.05
0.00
0.05
0.10
0.15
0.20
β$_{IV}$

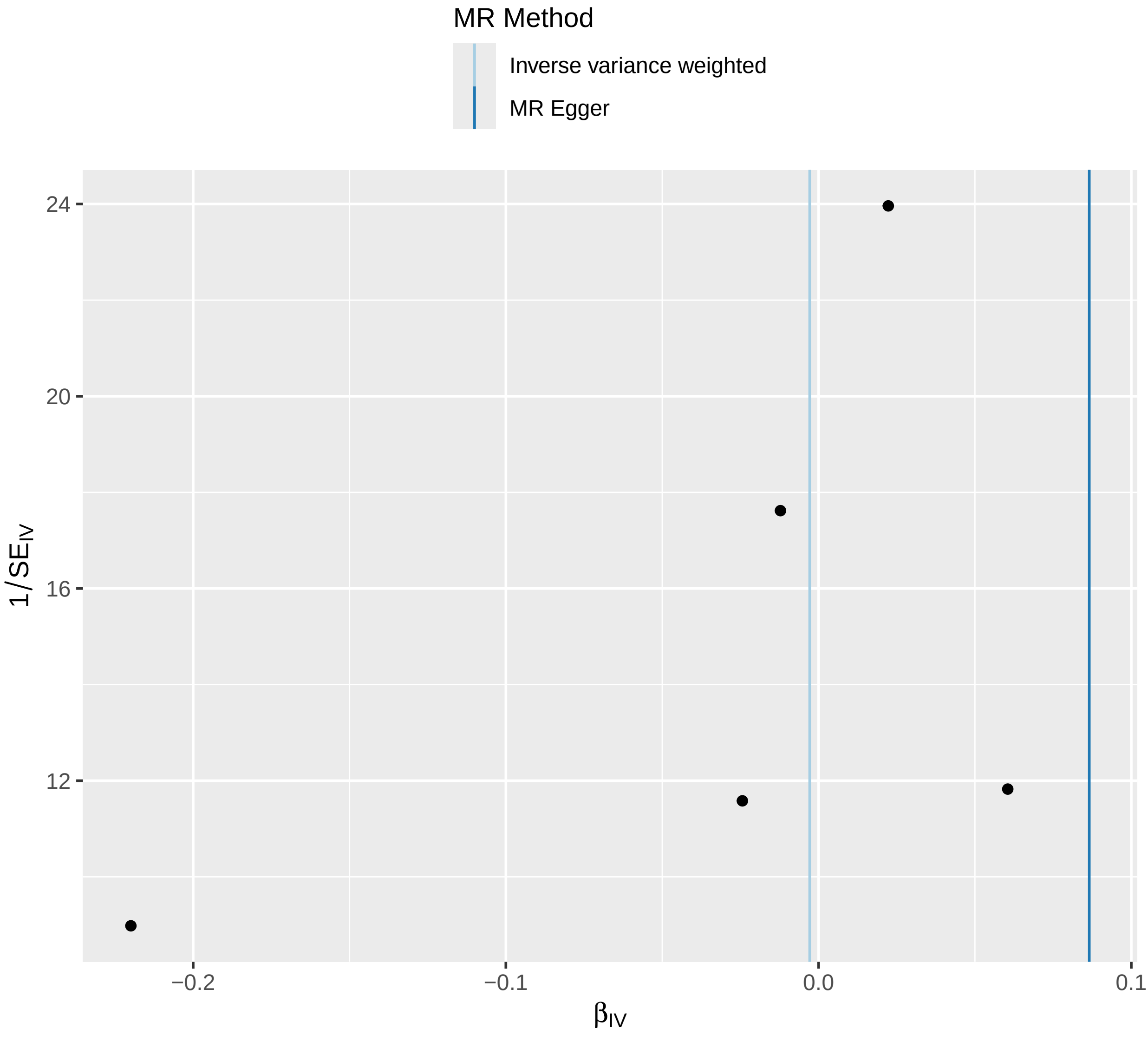
MR Method
Inverse variance weighted
MR Egger
24
20
16
12
1/SE_IV
−0.2
−0.1
0.0
0.1
β_IV

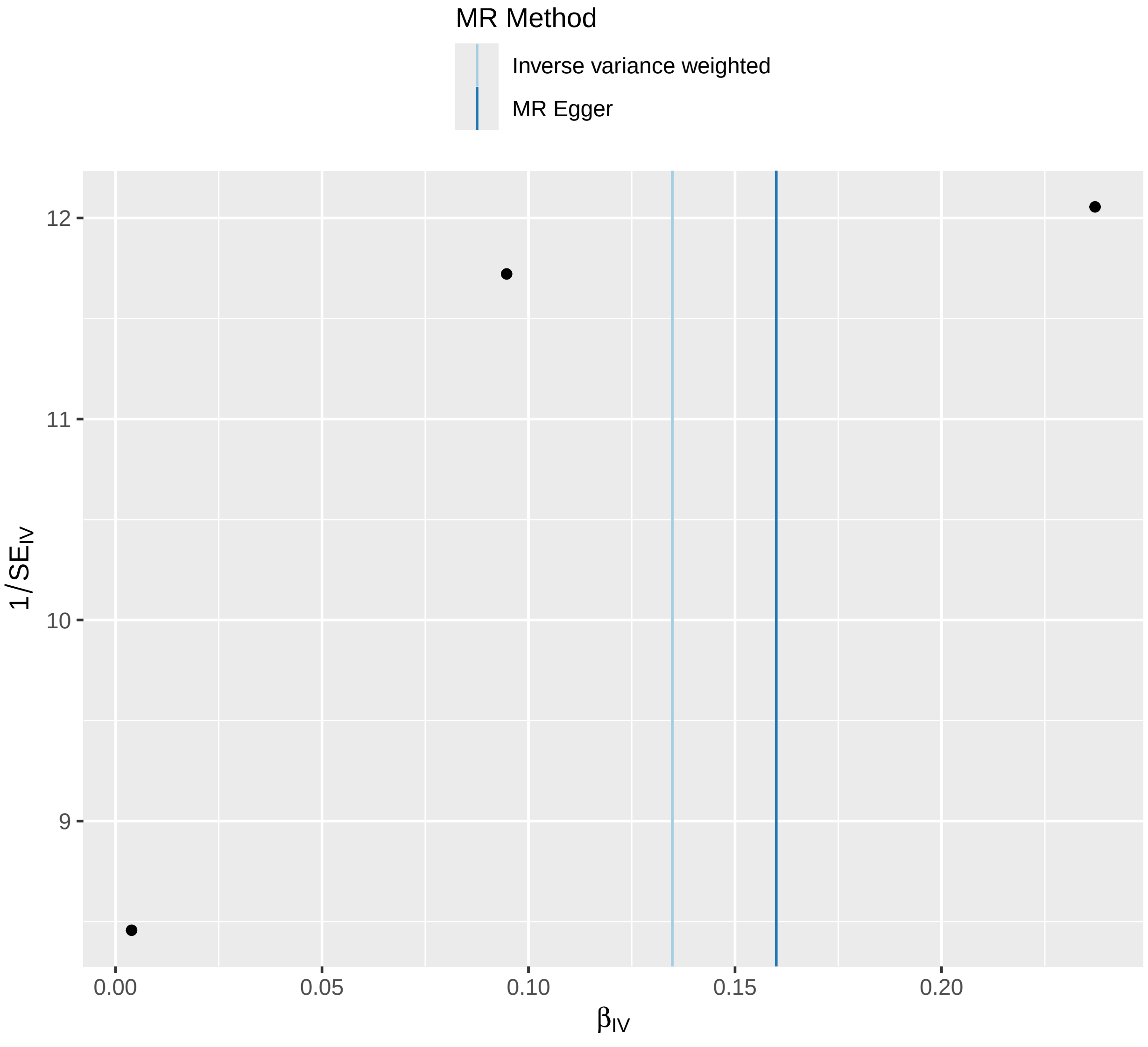

MR Method
Inverse variance weighted
MR Egger
12
11
10
9
1/SE_IV
0.00
0.05
0.10
0.15
0.20
β_IV

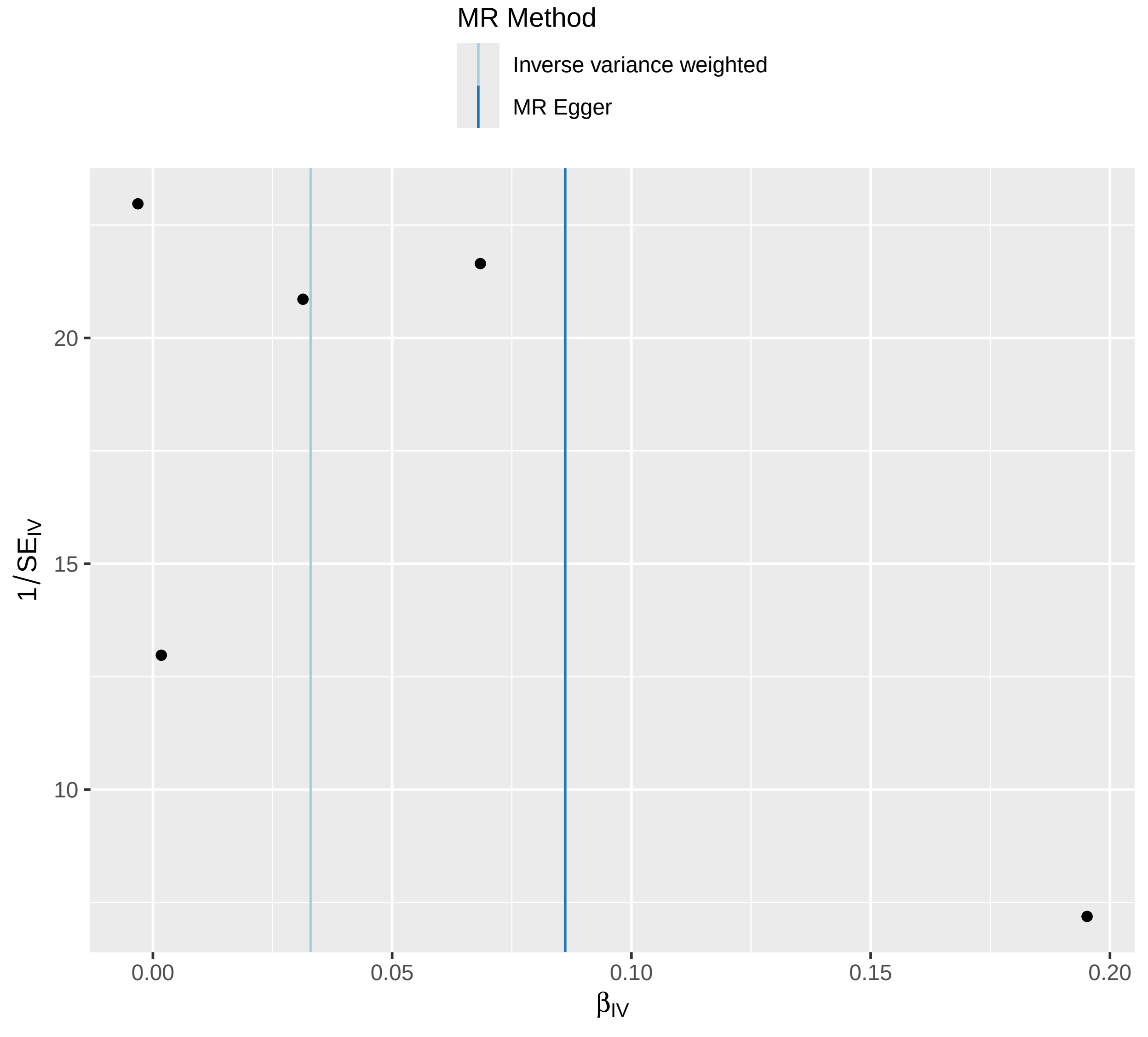
MR Method
Inverse variance weighted
MR Egger
20
15
10
1/SE$_{IV}$
0.00
0.05
0.10
0.15
0.20
β_{IV}

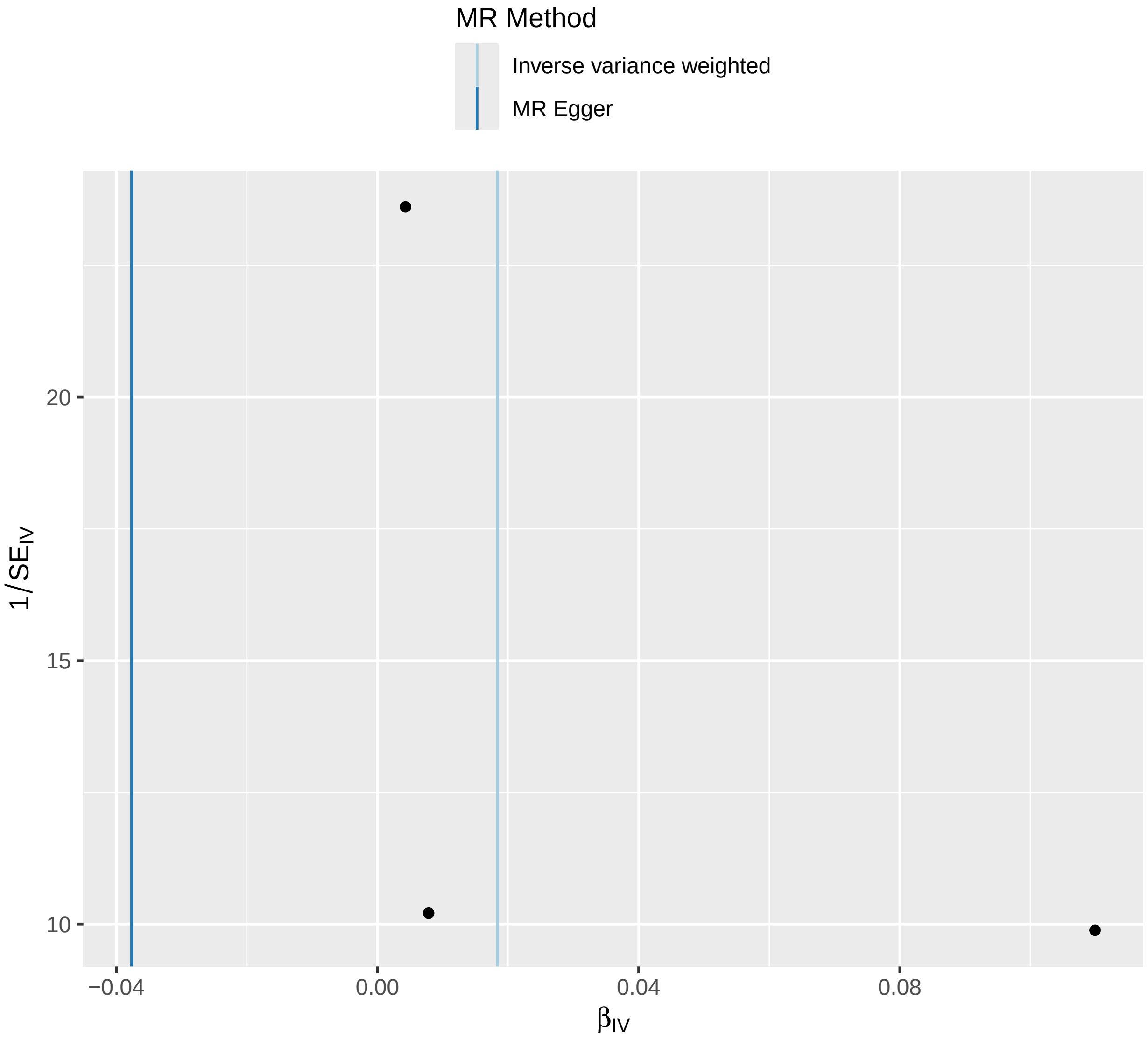

MR Method
Inverse variance weighted
MR Egger
20
15
10
1/SE_IV
−0.04
0.00
0.04
0.08
β_IV

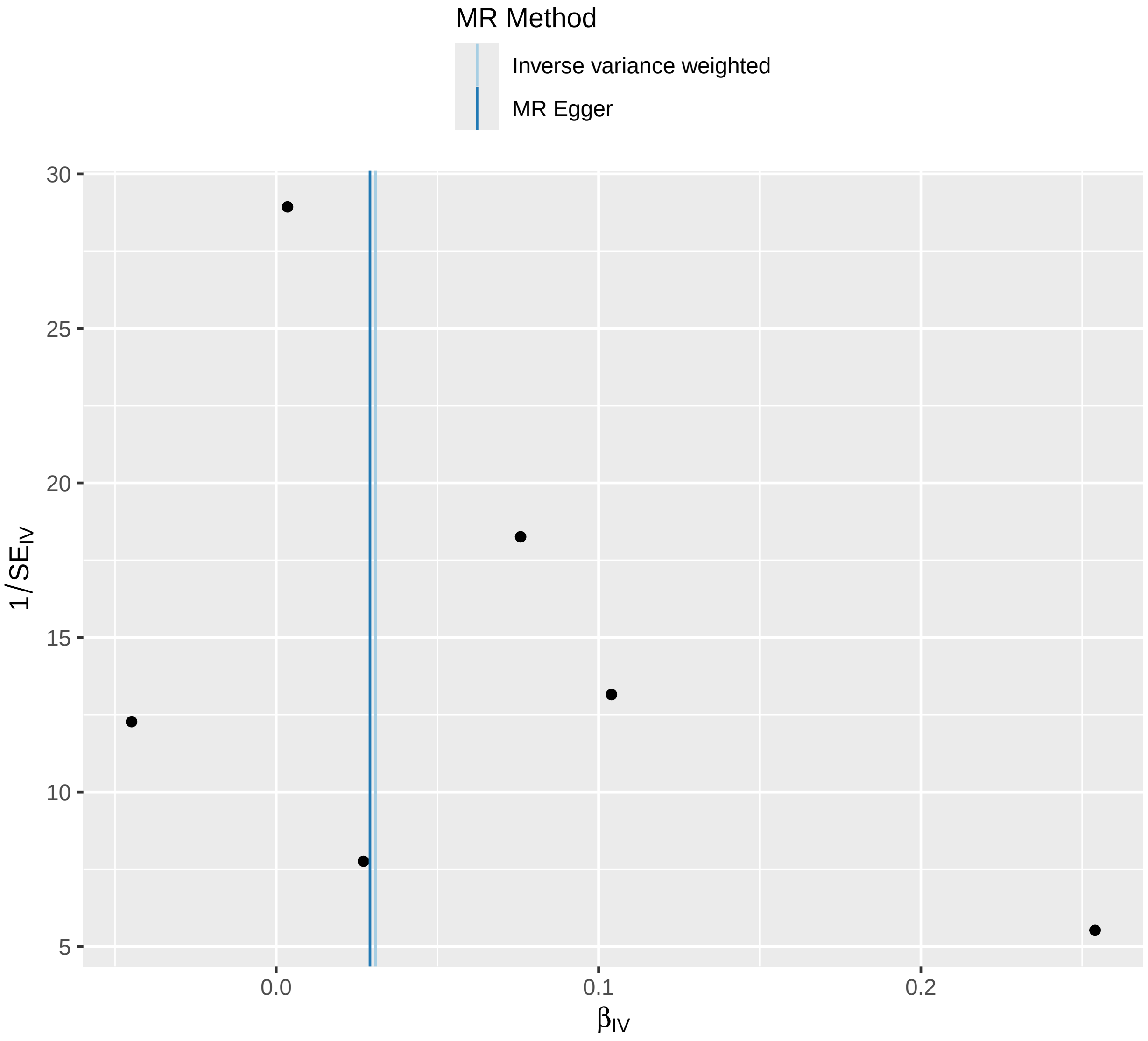
MR Method
Inverse variance weighted
MR Egger
30
25
20
15
10
5
$1/SE_{IV}$
0.0
0.1
0.2
β_{IV}

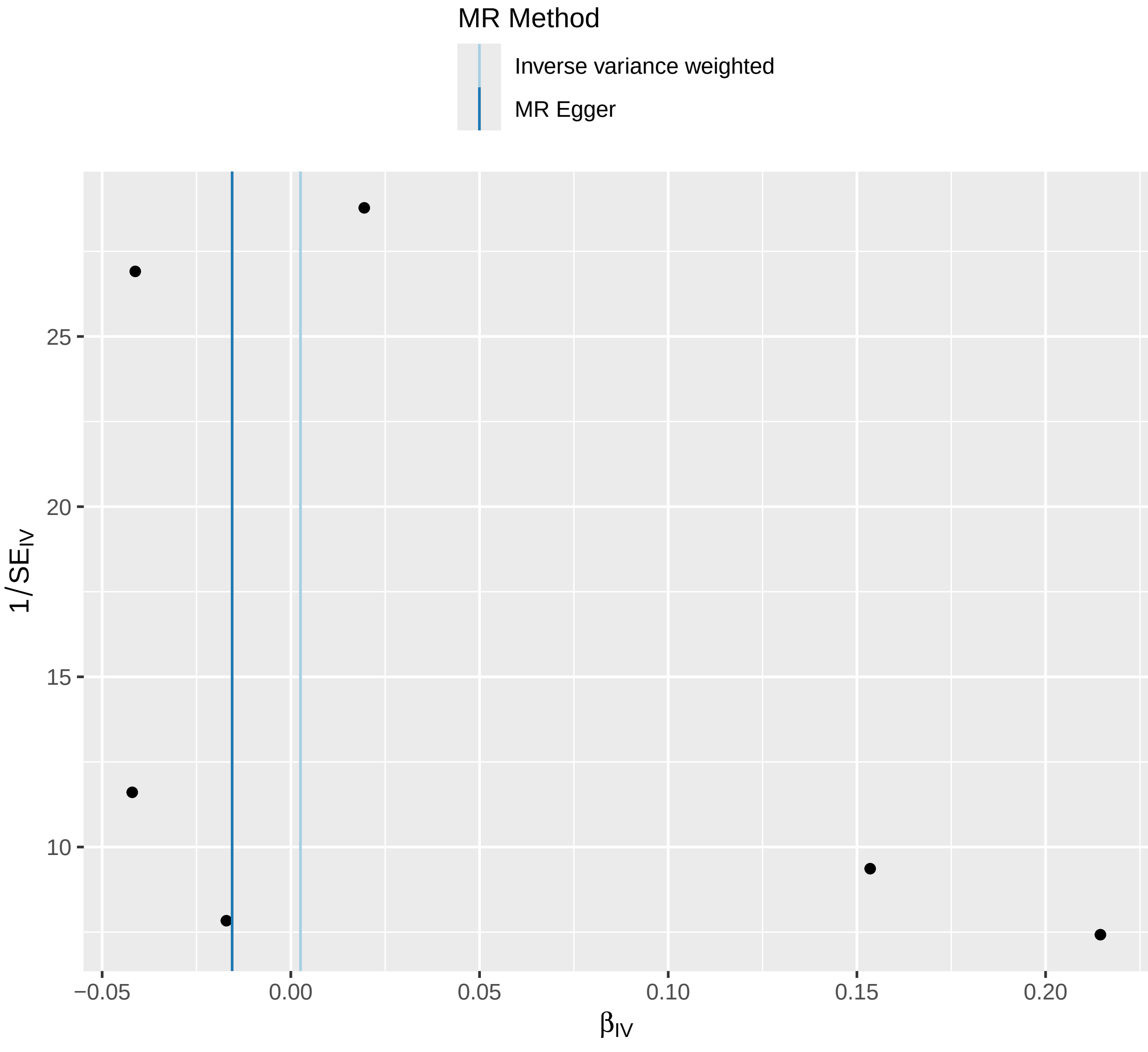
MR Method
Inverse variance weighted
MR Egger
25
20
15
10
−0.05
0.00
0.05
0.10
0.15
0.20
β_{IV}
$1/SE_{IV}$

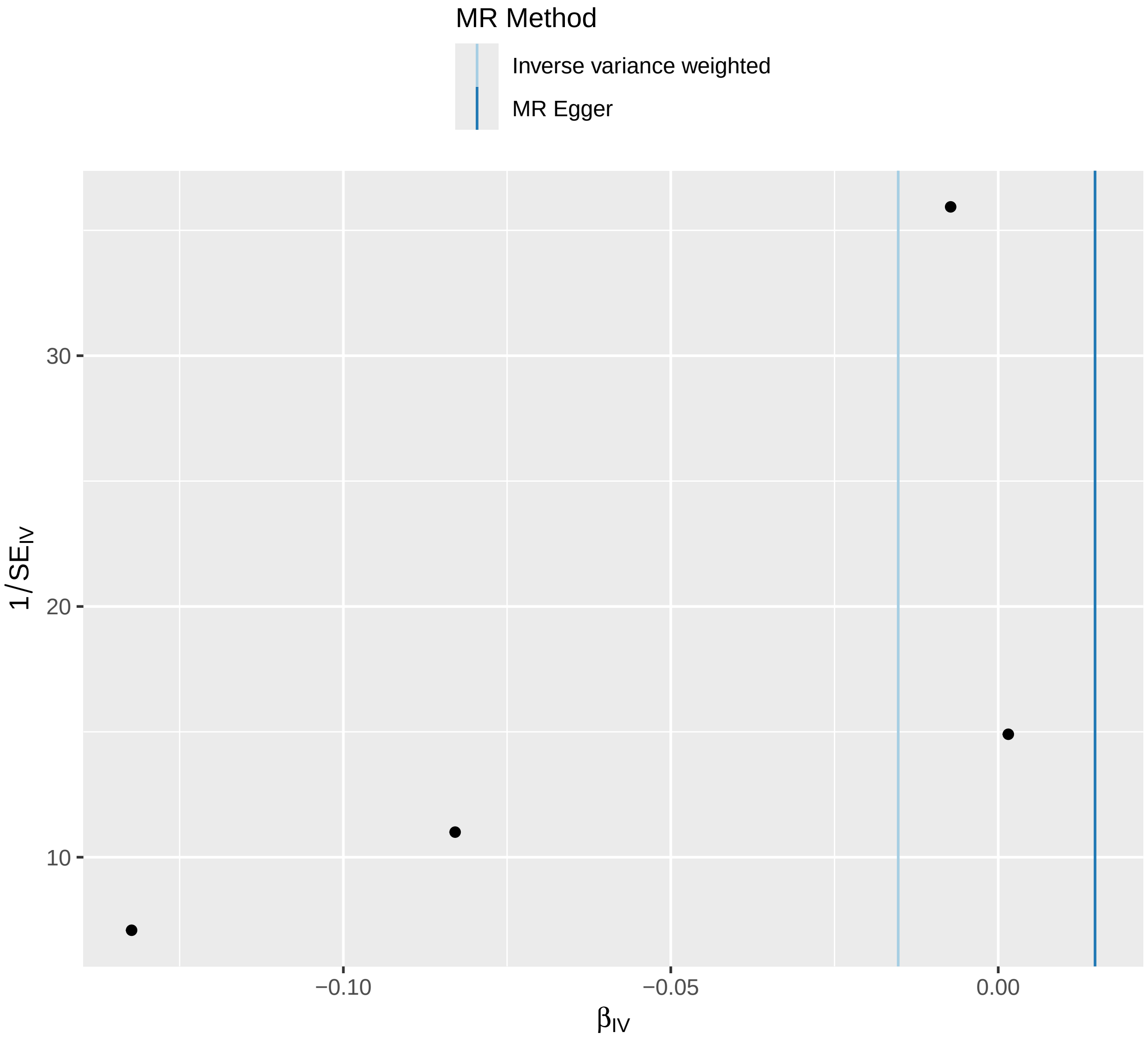
MR Method
Inverse variance weighted
MR Egger
30
20
10
1/SE_{IV}
−0.10
−0.05
0.00
β_{IV}

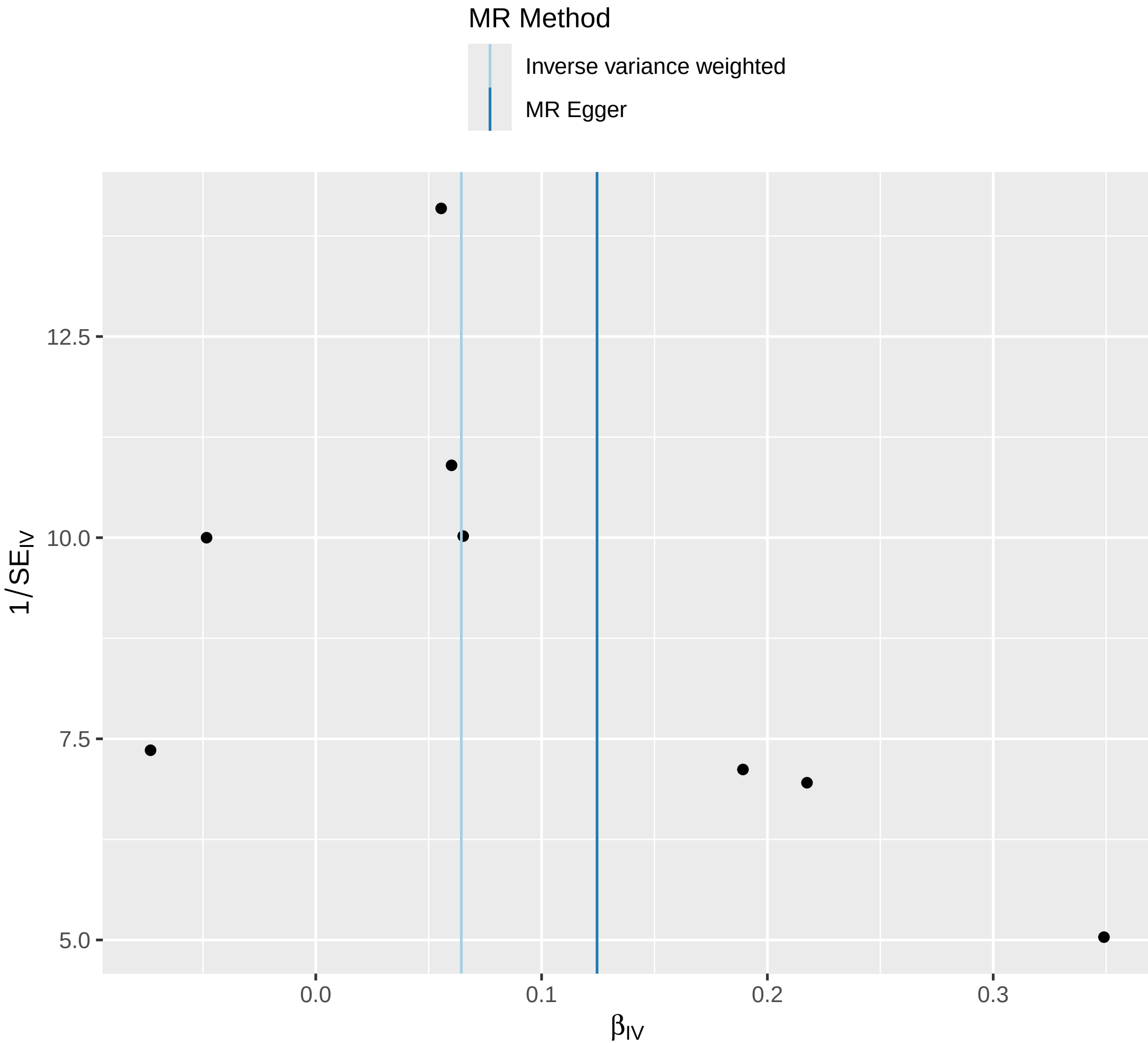

MR Method
Inverse variance weighted
MR Egger
12.5
10.0
7.5
5.0
1/SE_IV
0.0
0.1
0.2
0.3
β_IV

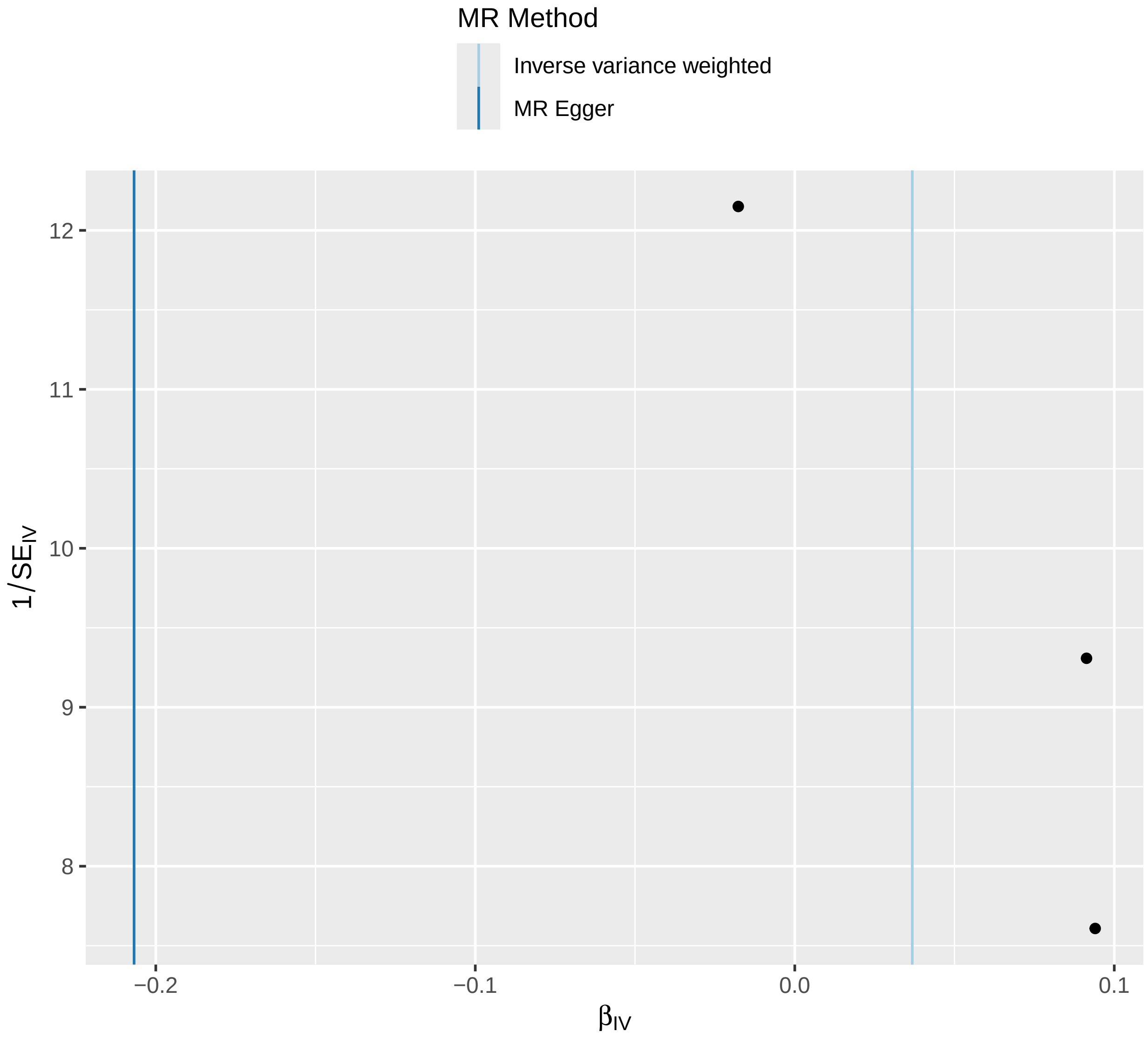

MR Method
Inverse variance weighted
MR Egger
12
11
10
9
8
1/SE_IV
−0.2
−0.1
0.0
0.1
β_IV

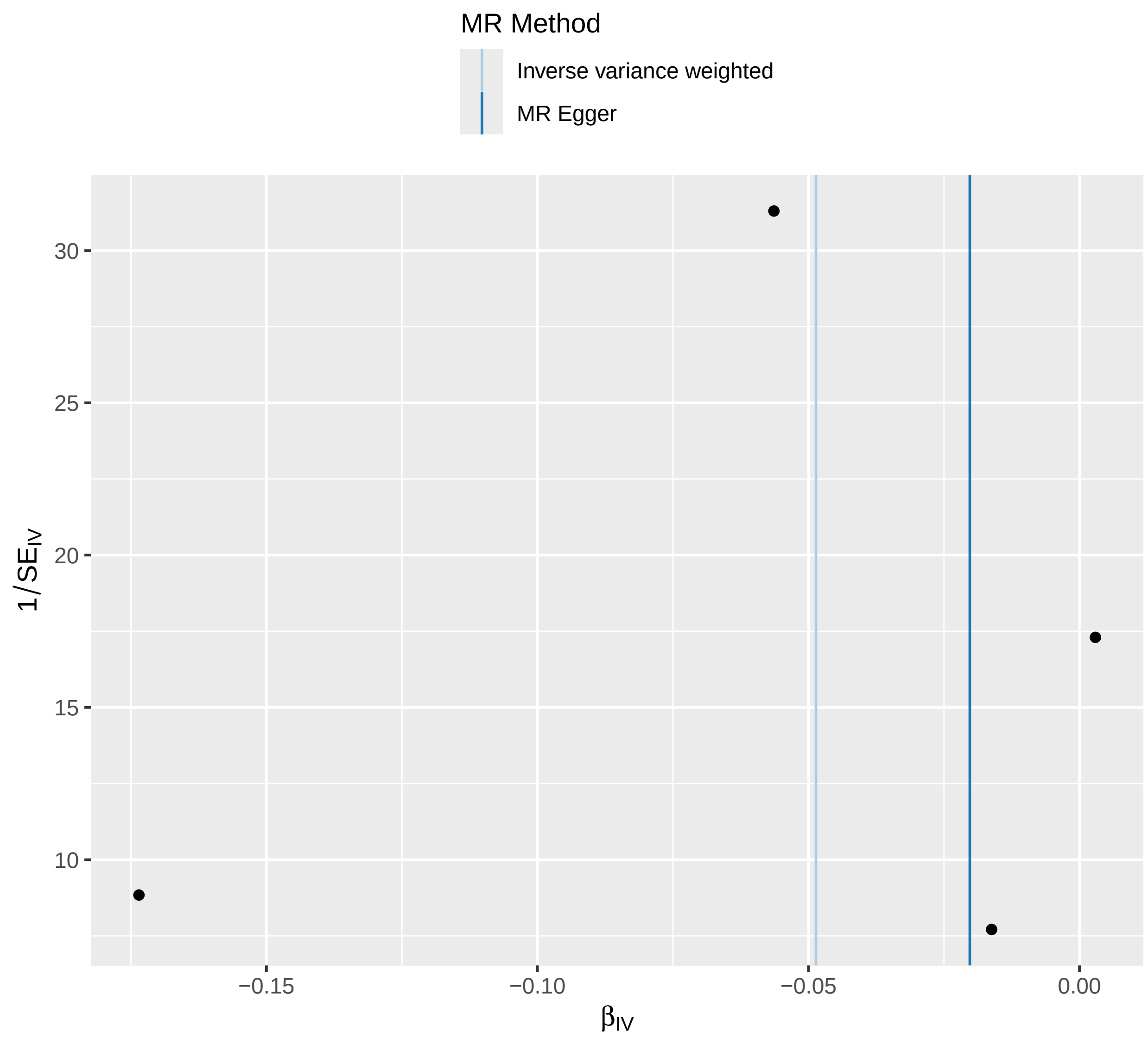

MR Method
Inverse variance weighted
MR Egger
30
25
20
15
10
$1/SE_{IV}$
−0.15
−0.10
−0.05
0.00
β_{IV}

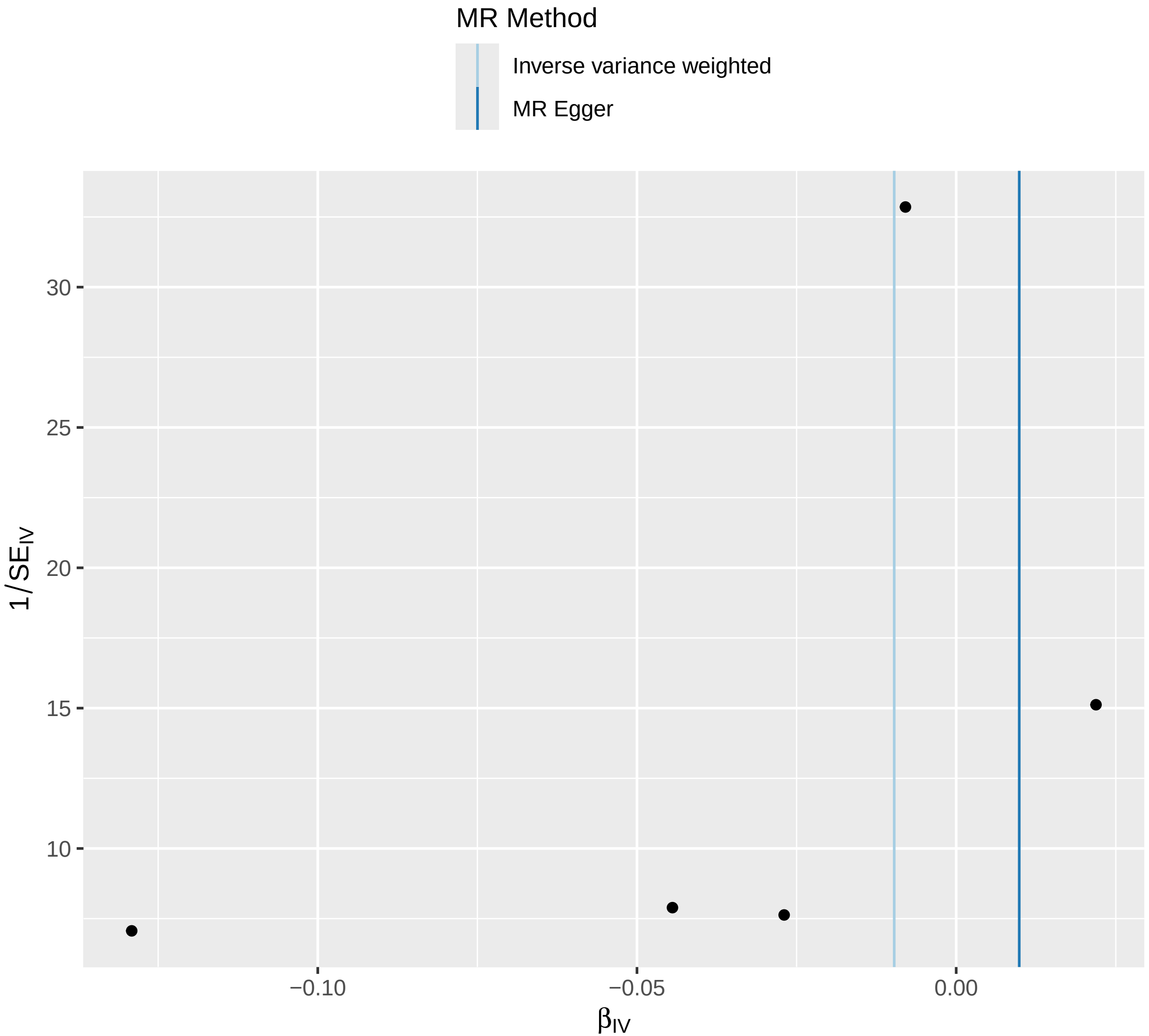
MR Method
Inverse variance weighted
MR Egger
30
25
20
15
10
1/SE$_{IV}$
−0.10
−0.05
0.00
β$_{IV}$

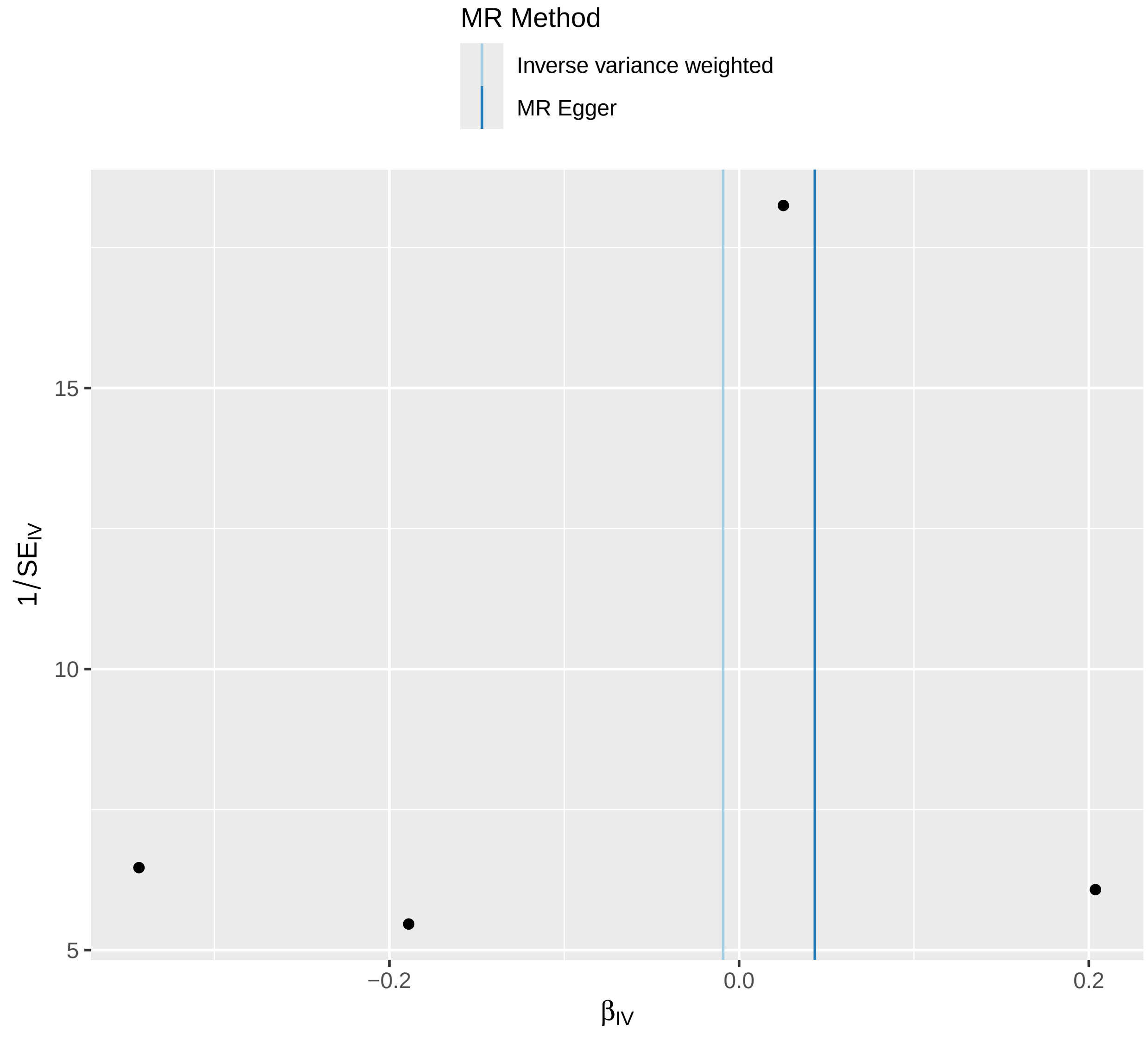
MR Method
Inverse variance weighted
MR Egger
15
10
5
1/SE_IV
−0.2
0.0
0.2
β_IV

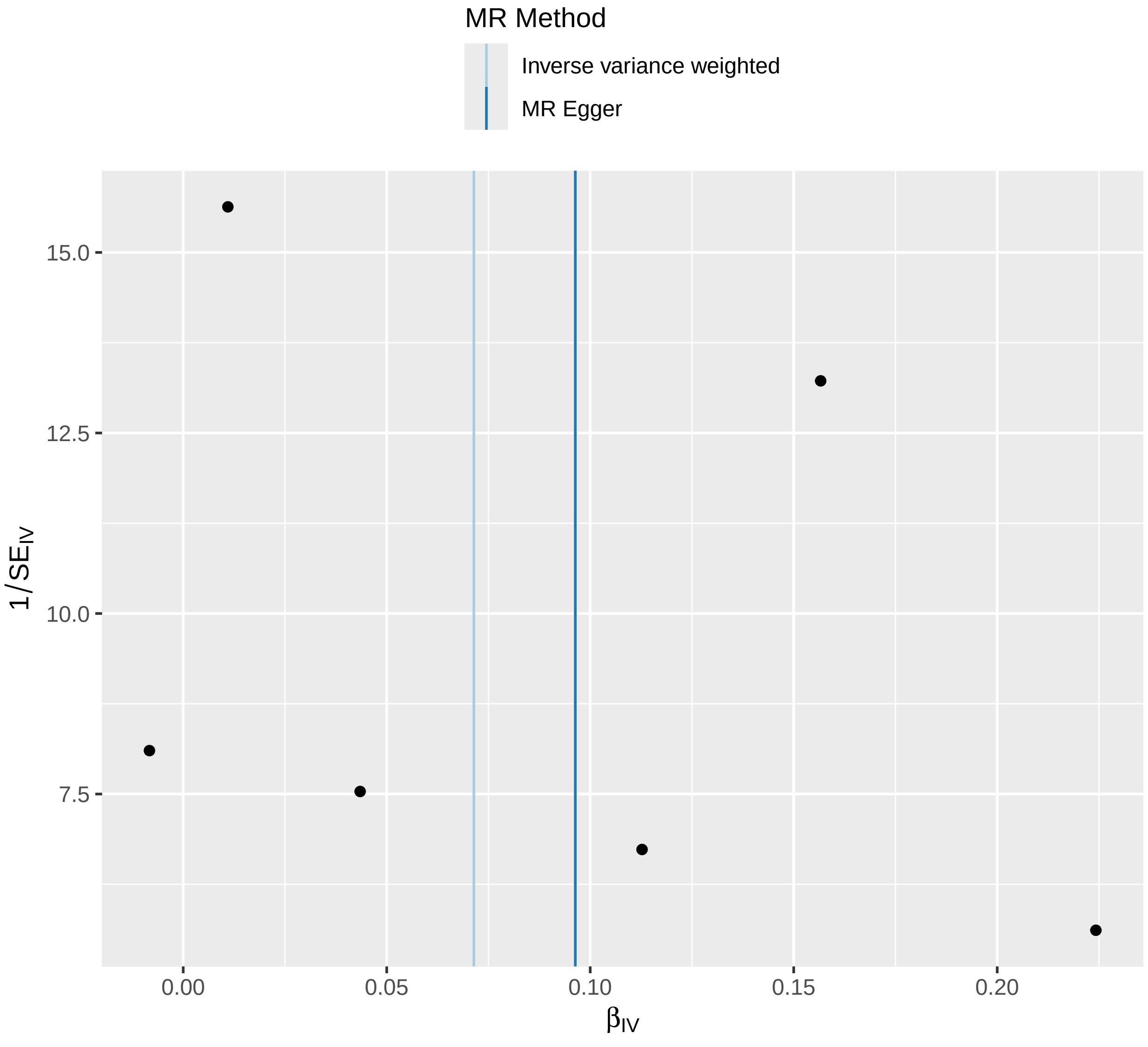

MR Method
Inverse variance weighted
MR Egger
15.0
12.5
10.0
7.5
$1/SE_{IV}$
0.00
0.05
0.10
0.15
0.20
β_{IV}

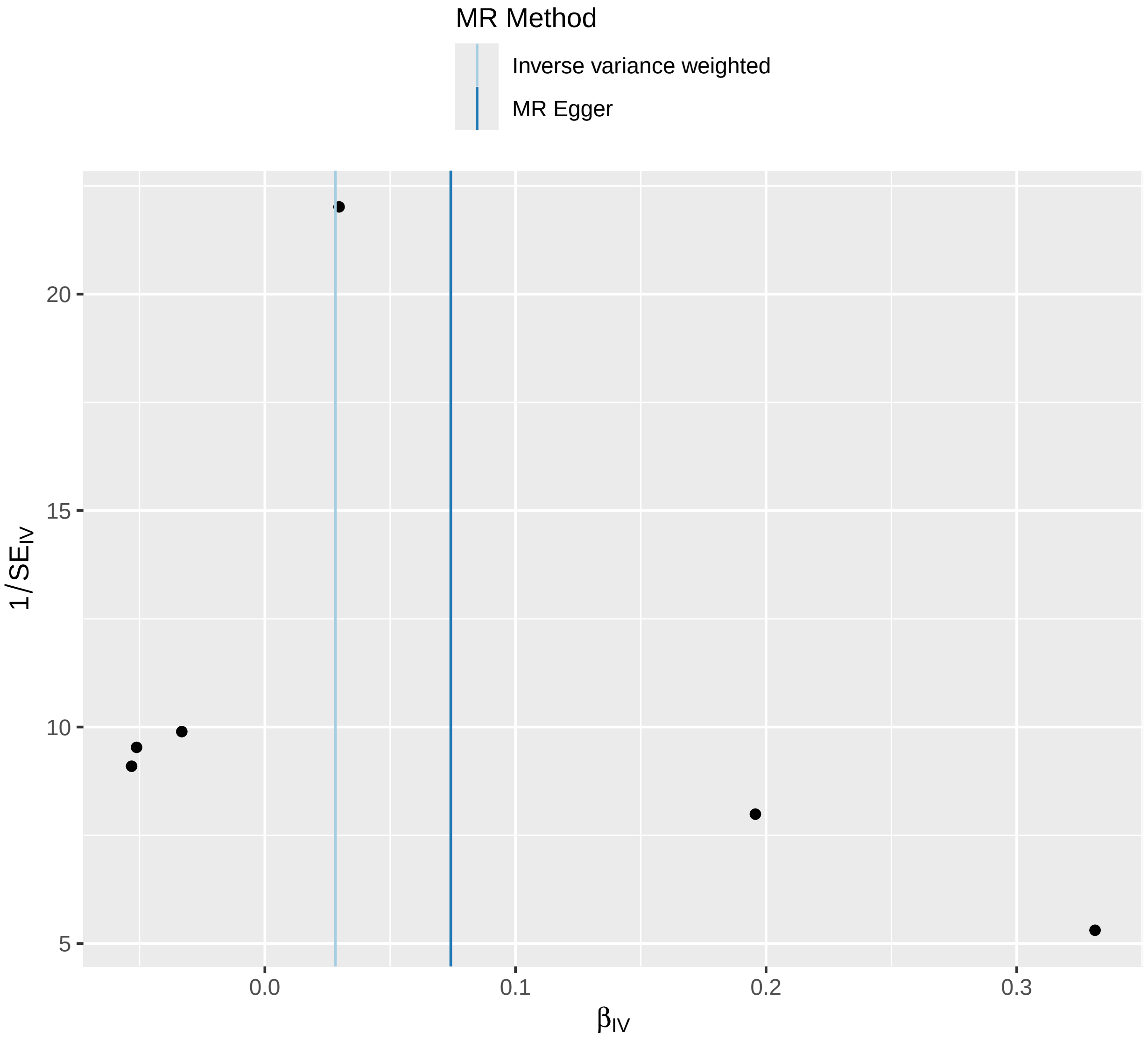

MR Method
Inverse variance weighted
MR Egger
20
15
10
5
$1/SE_{IV}$
0.0
0.1
0.2
0.3
β_{IV}

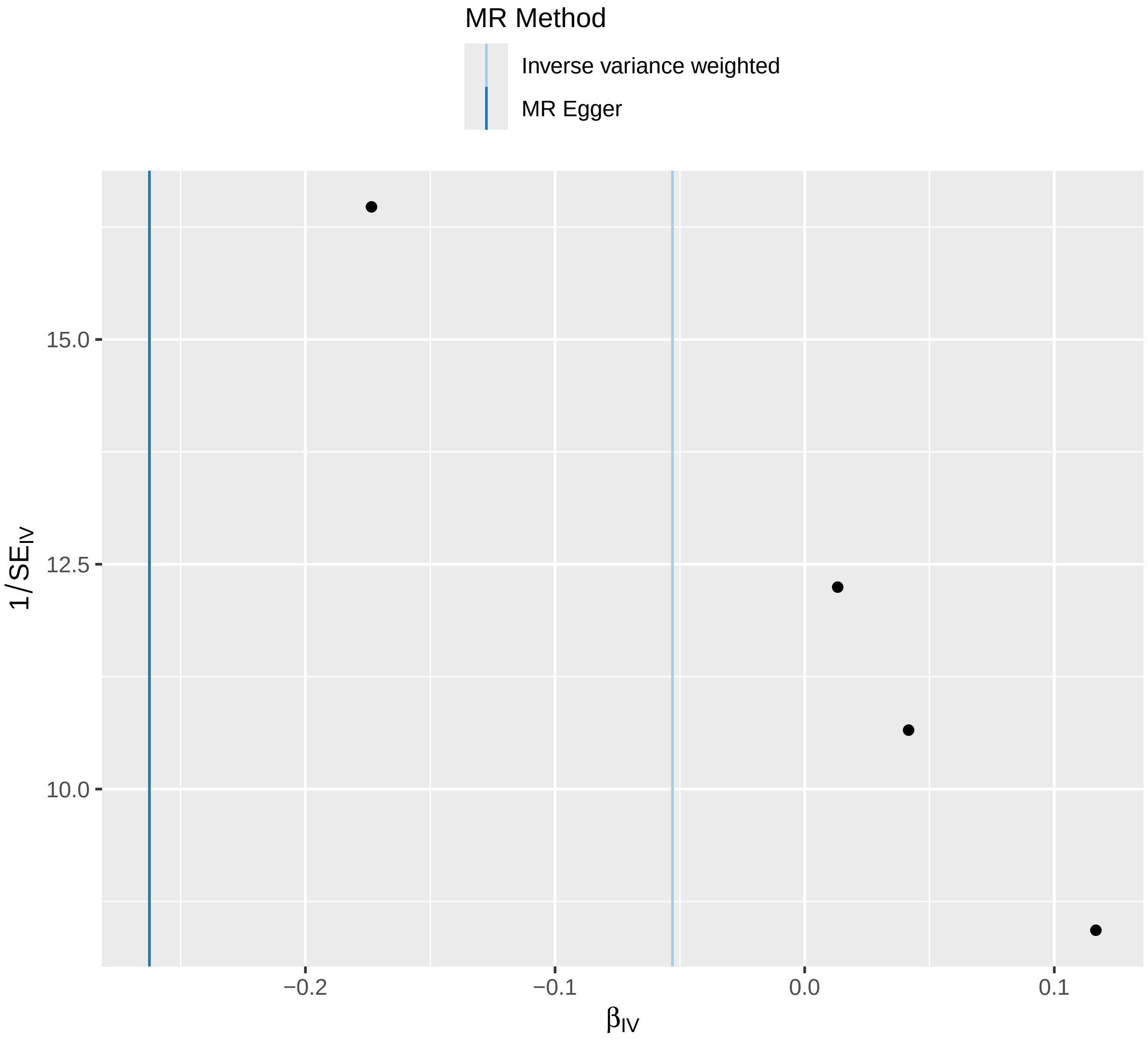
MR Method
Inverse variance weighted
MR Egger
15.0
12.5
10.0
1/SE$_{IV}$
−0.2
−0.1
0.0
0.1
β$_{IV}$

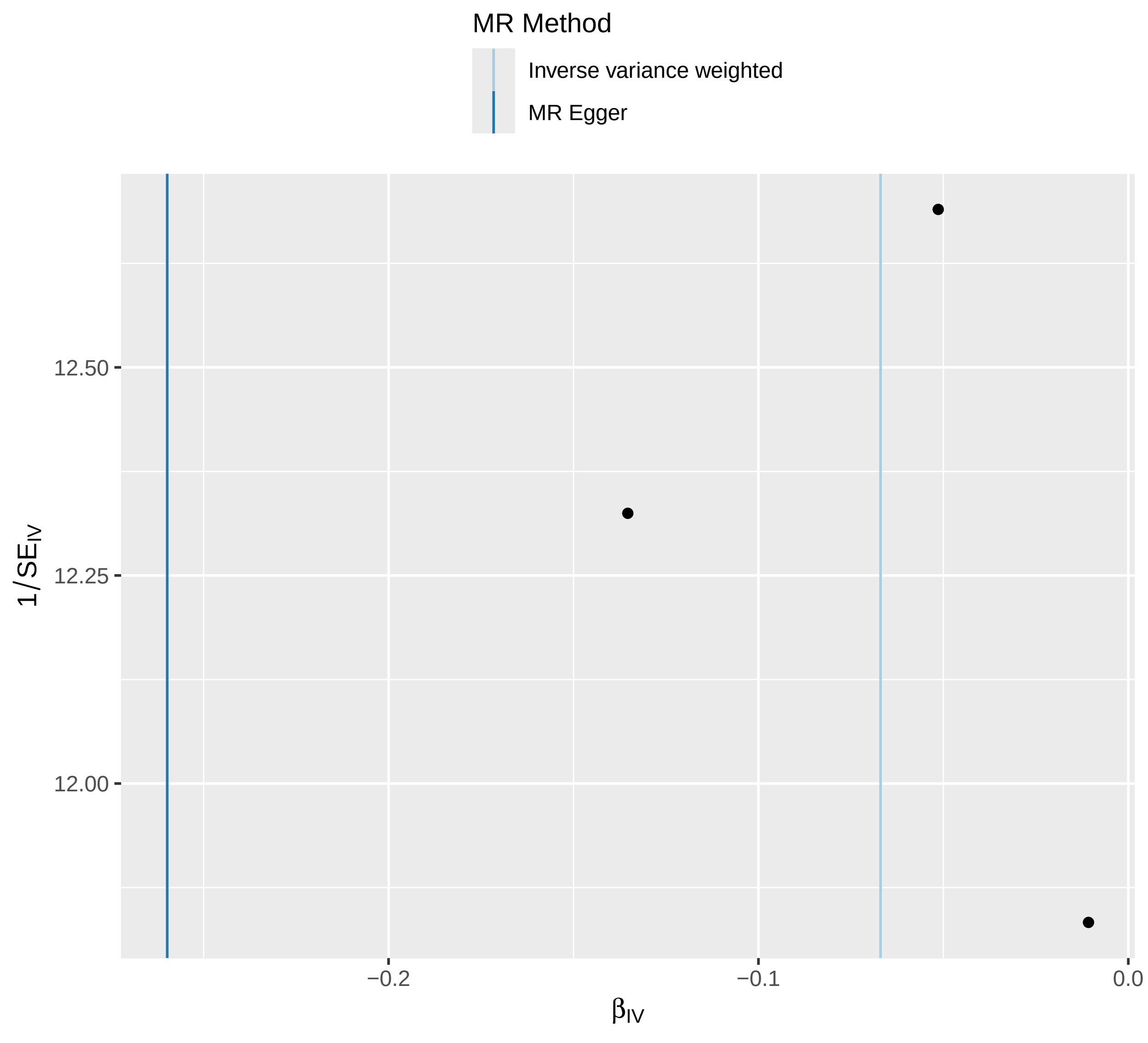
MR Method
Inverse variance weighted
MR Egger
12.50
12.25
12.00
1/SE$_{IV}$
−0.2
−0.1
0.0
β$_{IV}$

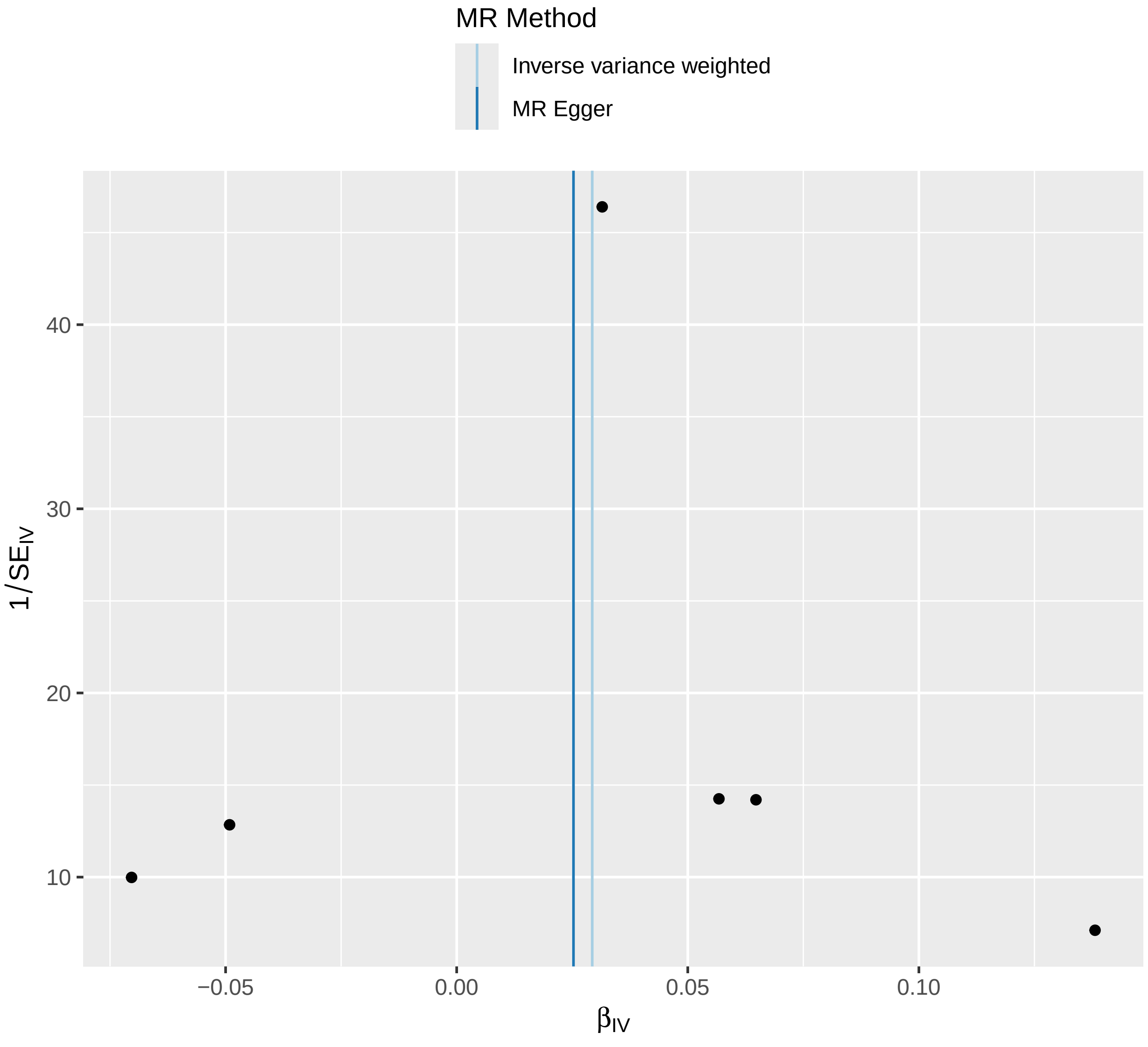

MR Method
Inverse variance weighted
MR Egger
40
30
20
10
1/SE_IV
−0.05
0.00
0.05
0.10
β_IV

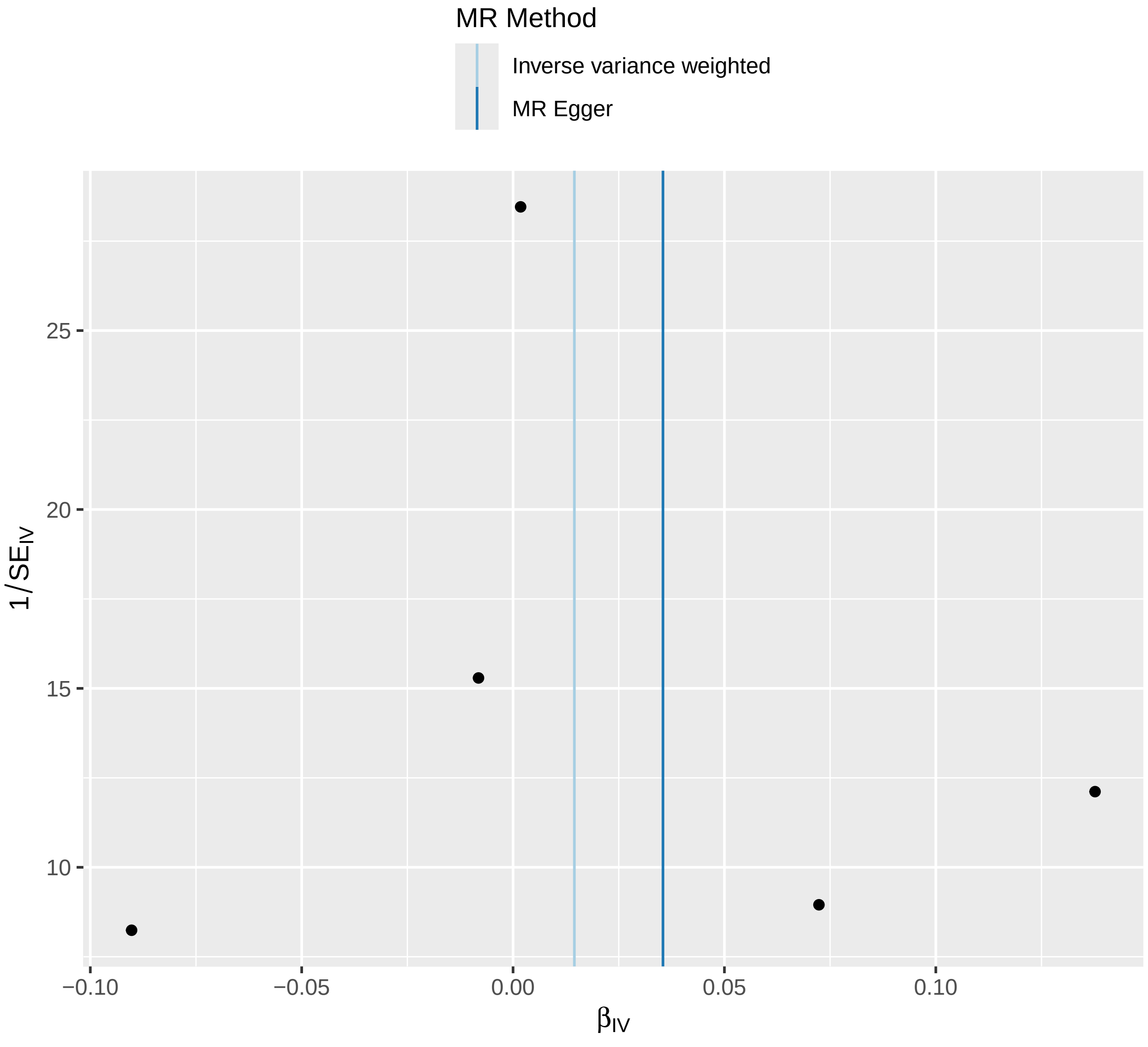
MR Method
Inverse variance weighted
MR Egger
25
20
15
10
1/SE_IV
−0.10
−0.05
0.00
0.05
0.10
β_IV

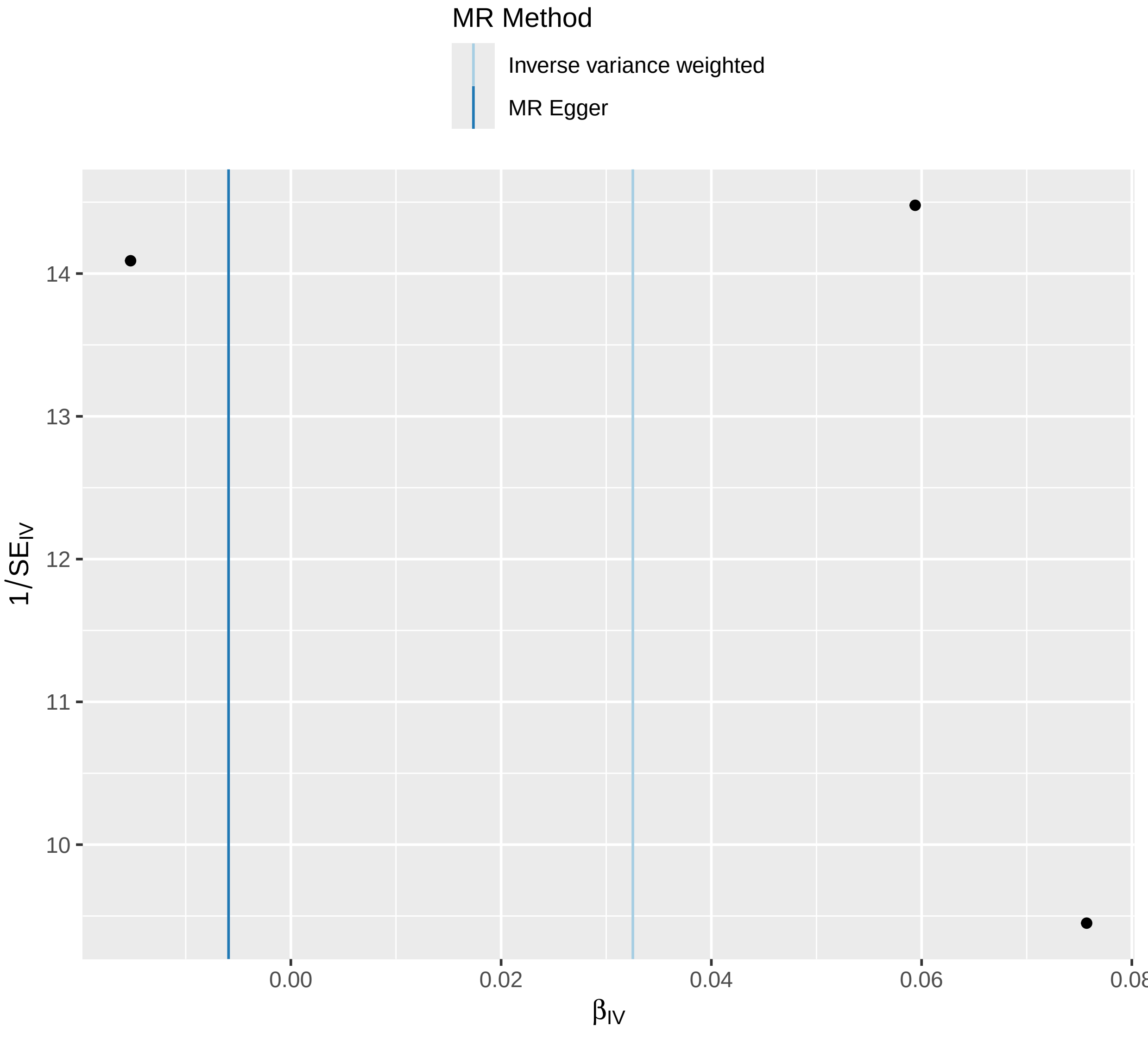
MR Method
Inverse variance weighted
MR Egger
14
13
12
11
10
1/SE_IV
0.00
0.02
0.04
0.06
0.08
β_IV

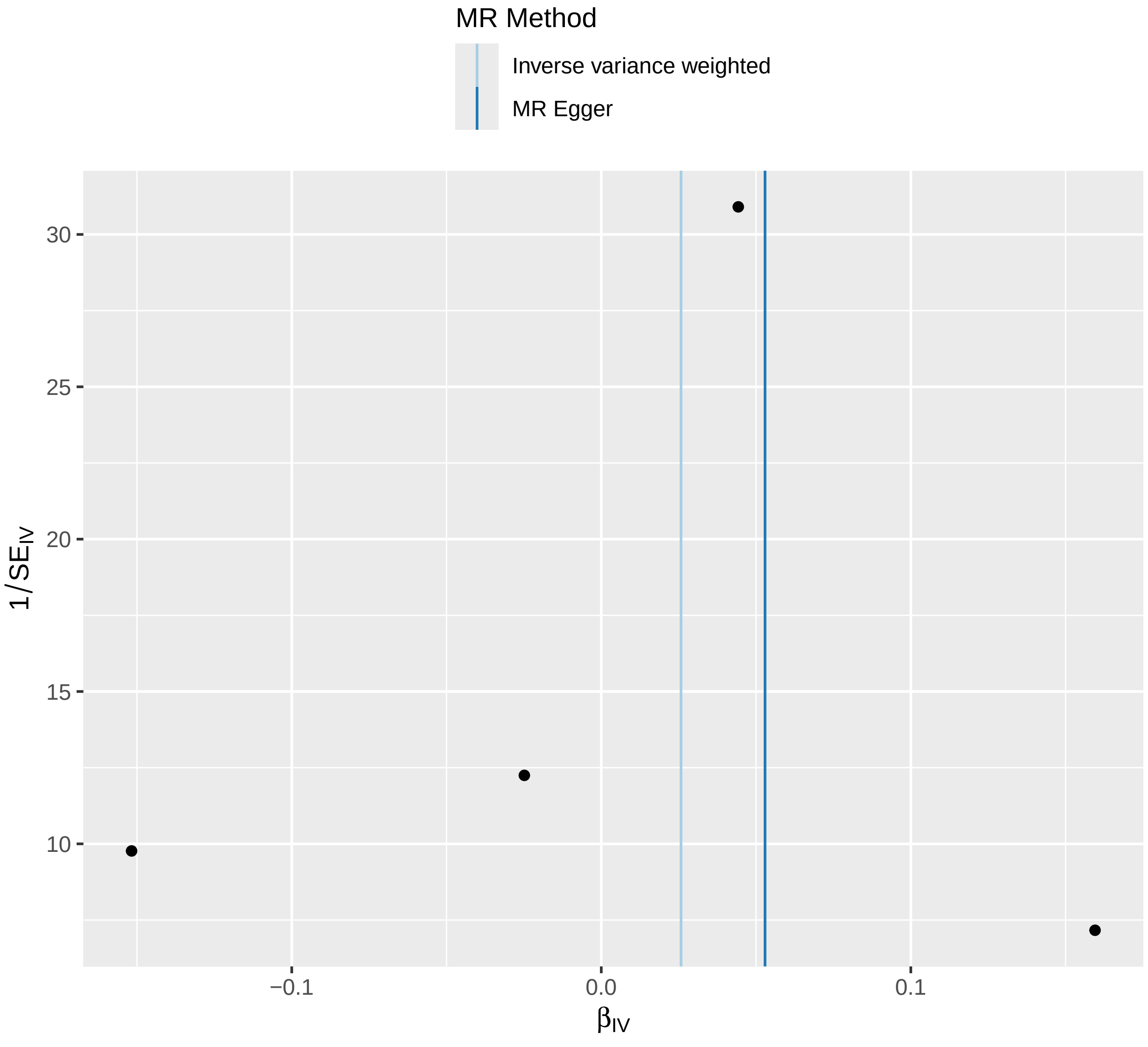
MR Method
Inverse variance weighted
MR Egger
30
25
20
15
10
1/SE$_{IV}$
−0.1
0.0
0.1
β$_{IV}$

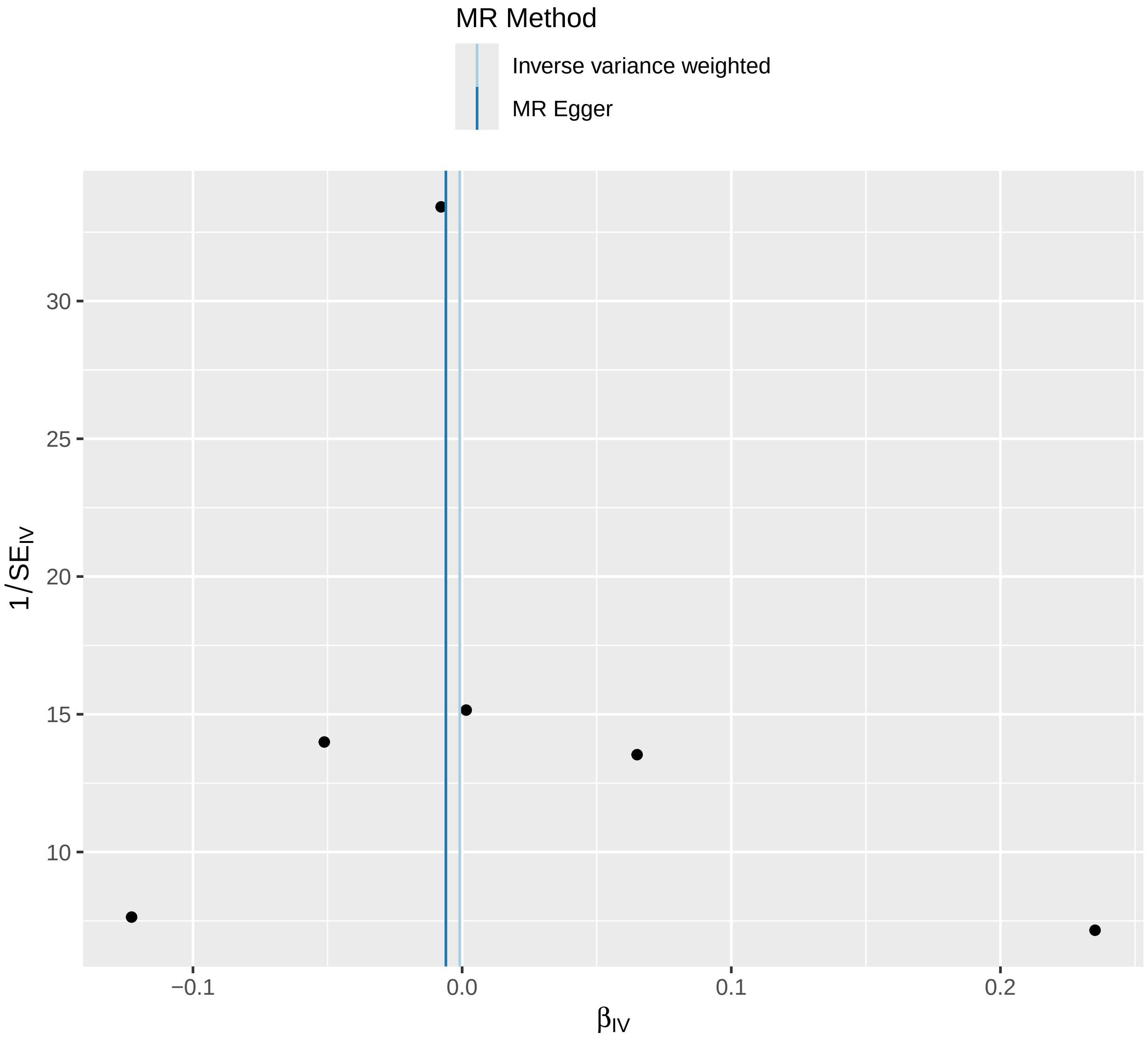
MR Method
Inverse variance weighted
MR Egger
1/SE_IV
30
25
20
15
10
−0.1
0.0
0.1
0.2
β_IV

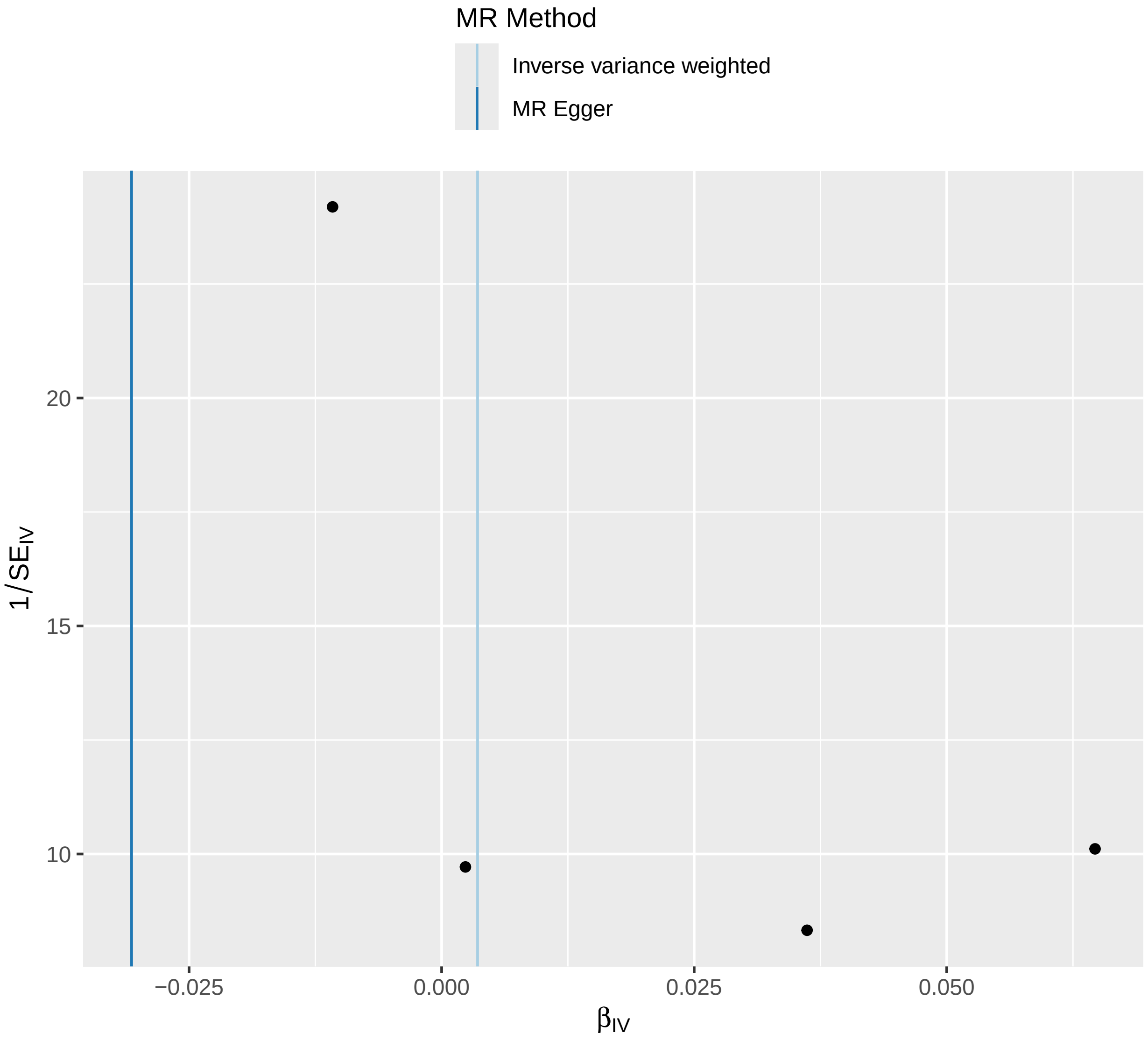
MR Method
Inverse variance weighted
MR Egger
20
15
10
1/SE_IV
−0.025
0.000
0.025
0.050
β_IV

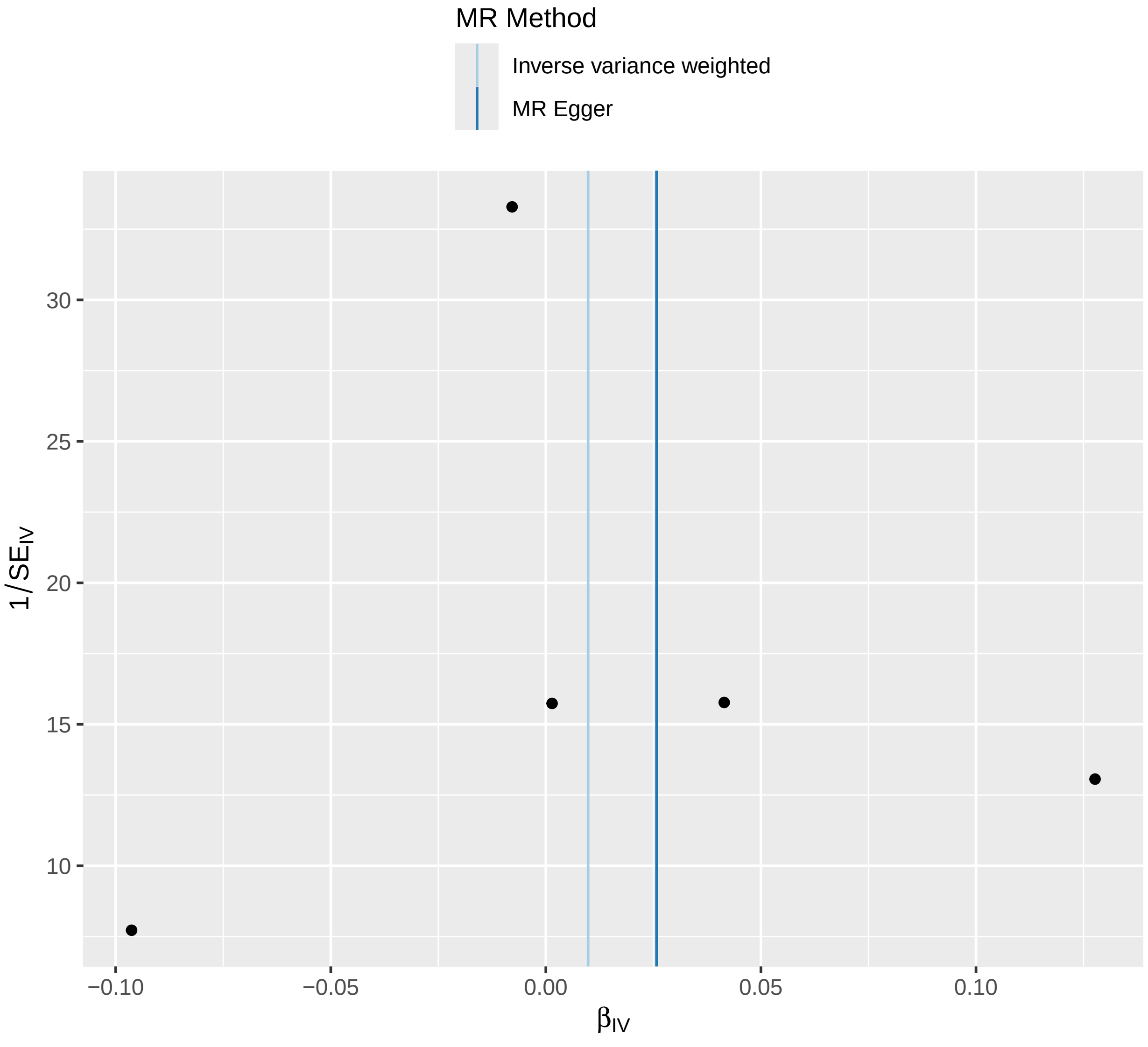
MR Method
Inverse variance weighted
MR Egger
30
25
20
15
10
1/SE_IV
−0.10
−0.05
0.00
0.05
0.10
β_IV

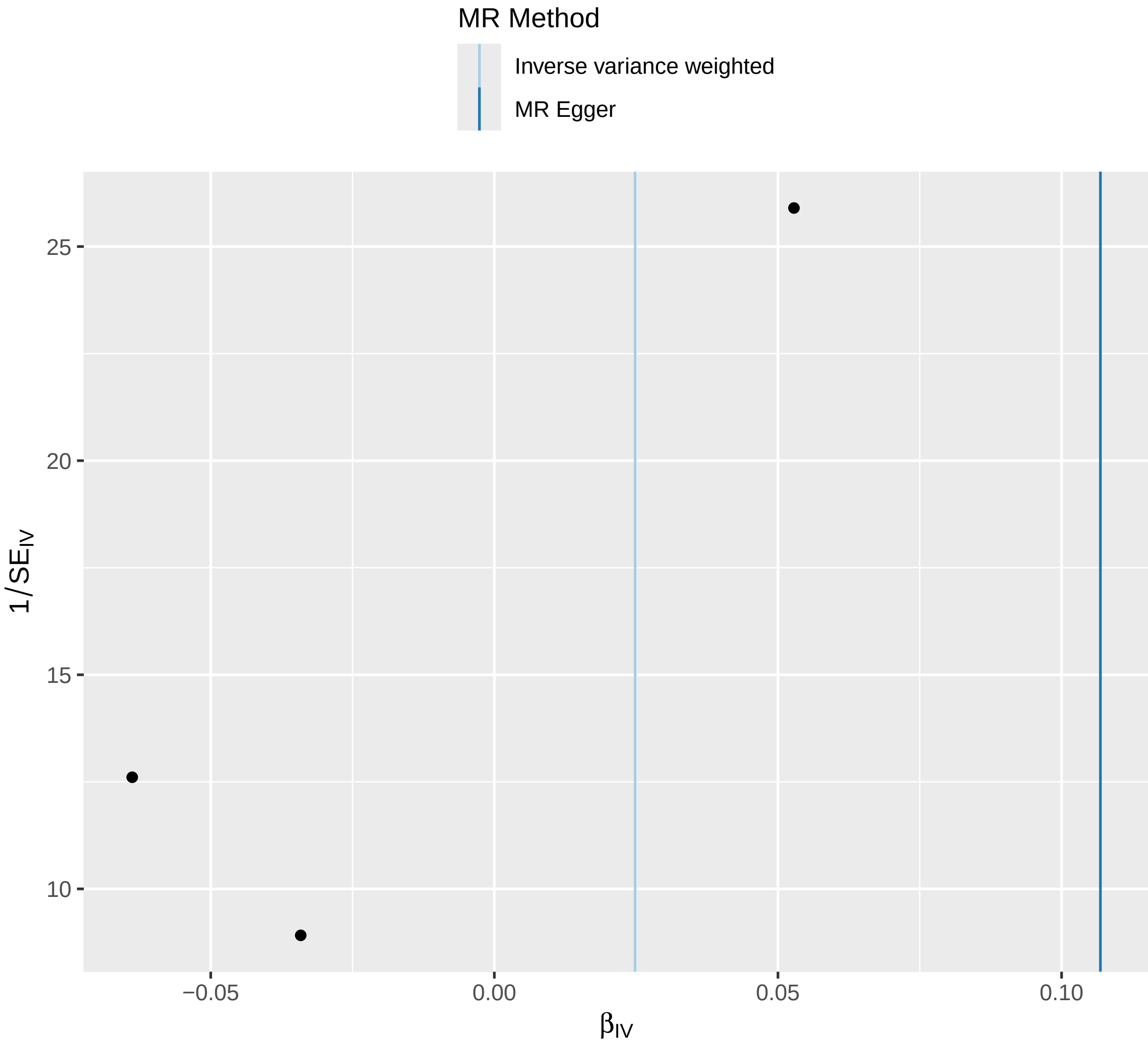

MR Method
Inverse variance weighted
MR Egger
25
20
15
10
1/SE_IV
−0.05
0.00
0.05
0.10
β_IV

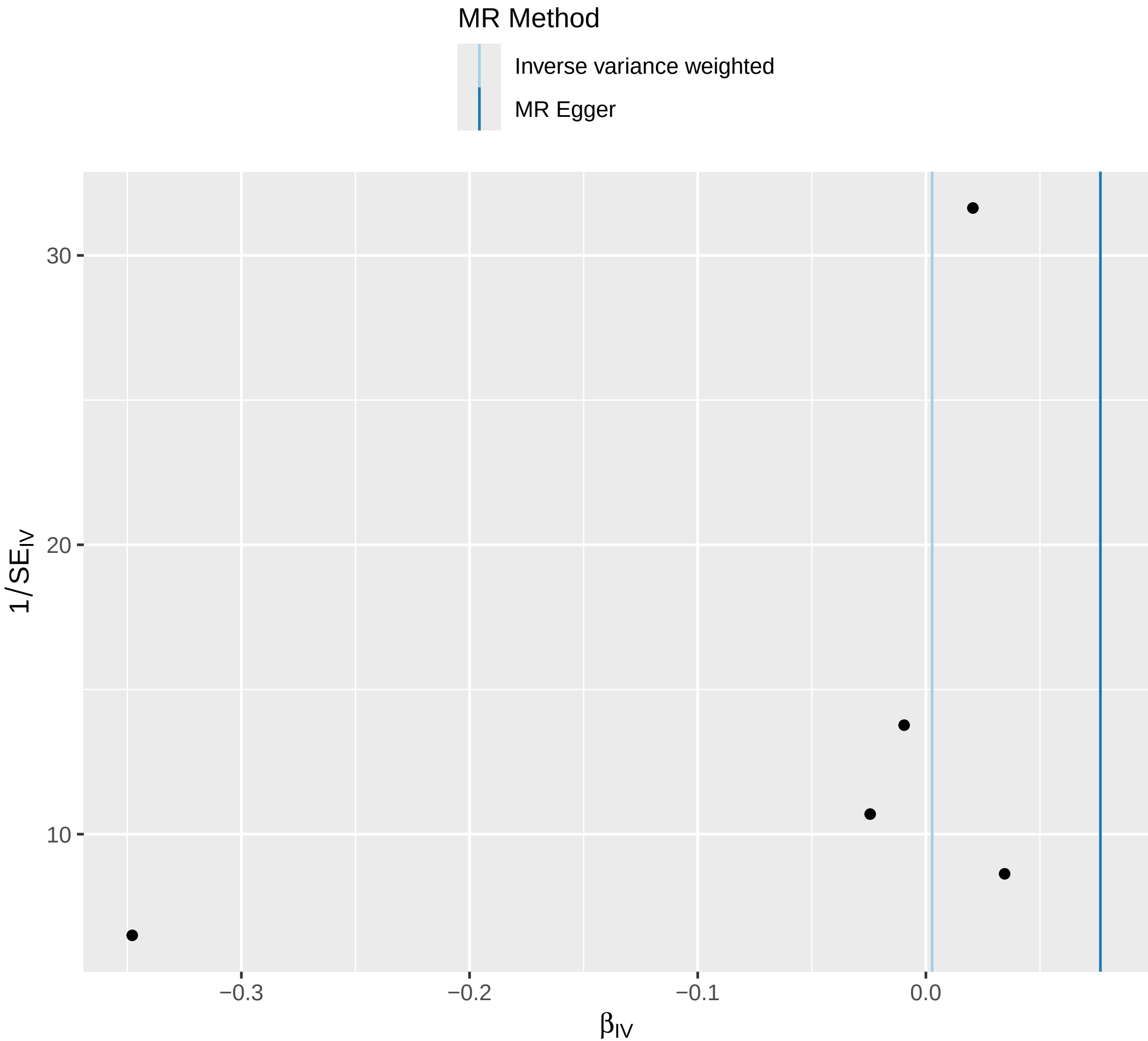
MR Method
Inverse variance weighted
MR Egger
30
20
10
1/SE_IV
−0.3
−0.2
−0.1
0.0
β_IV

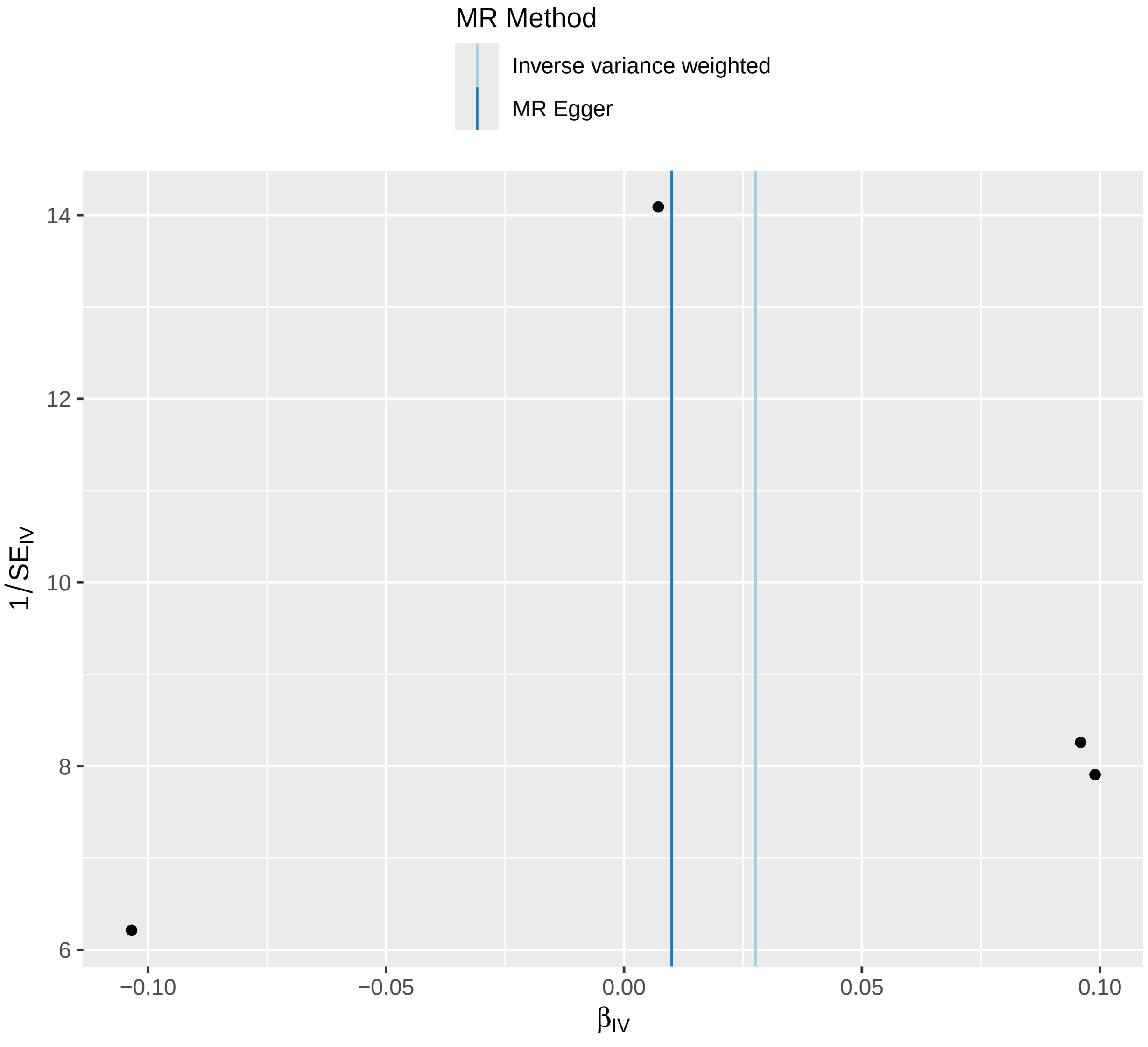
MR Method
Inverse variance weighted
MR Egger
14
12
10
8
6
1/SE$_{IV}$
−0.10
−0.05
0.00
0.05
0.10
β$_{IV}$

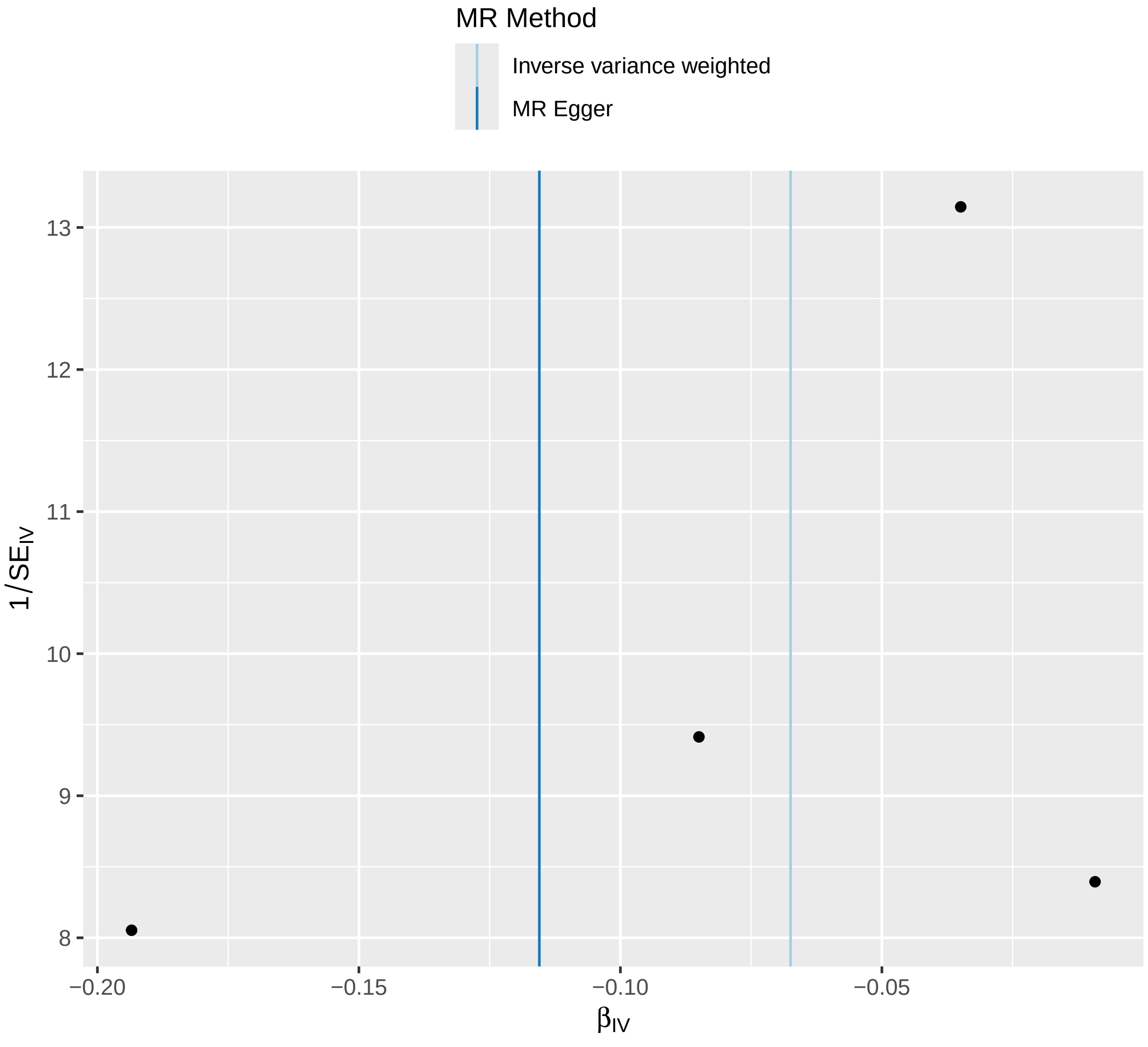

MR Method
Inverse variance weighted
MR Egger
13
12
11
10
9
8
1/SE$_{IV}$
−0.20
−0.15
−0.10
−0.05
β$_{IV}$

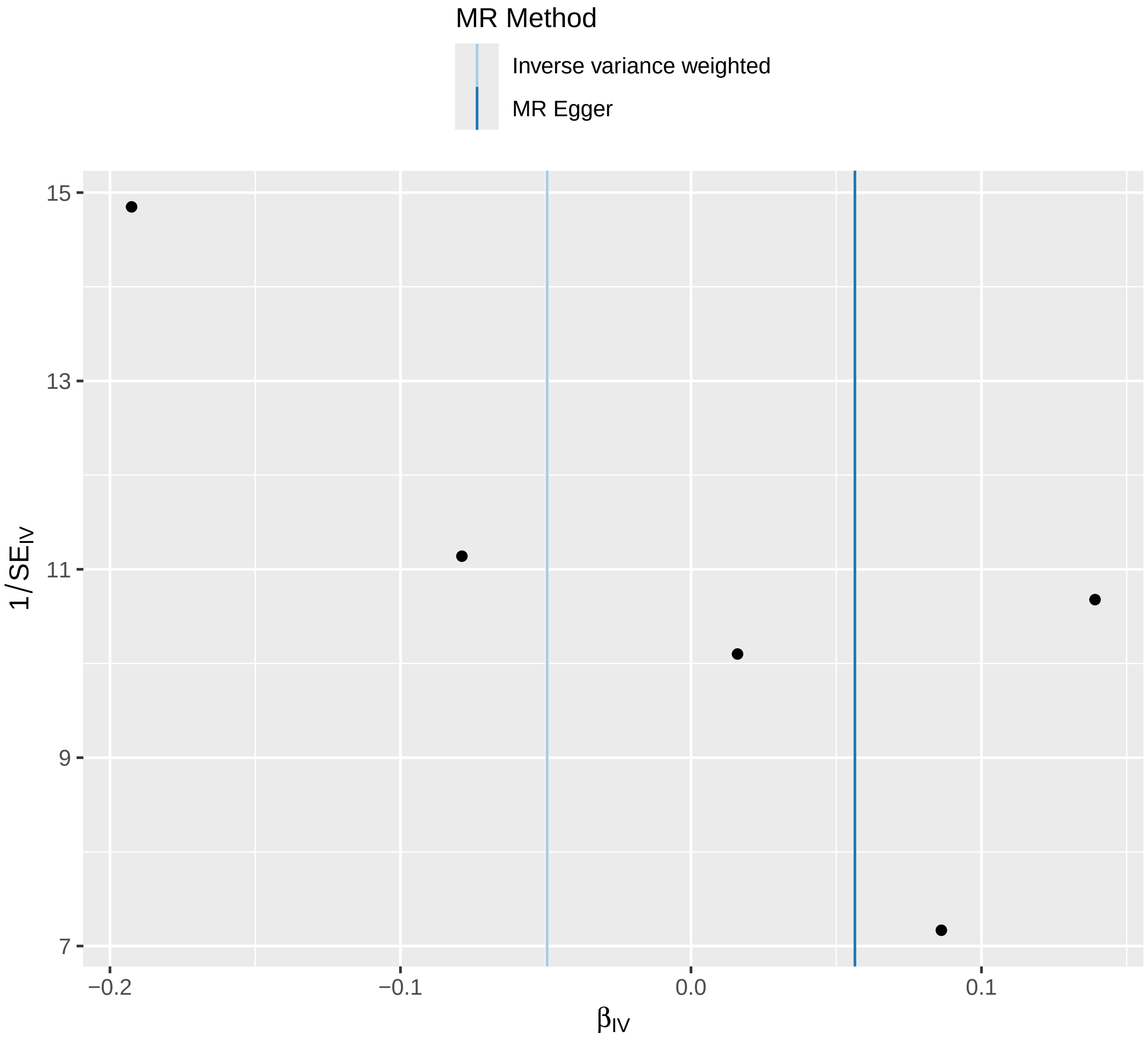
MR Method
Inverse variance weighted
MR Egger
15
13
11
9
7
1/SE_IV
−0.2
−0.1
0.0
0.1
β_IV

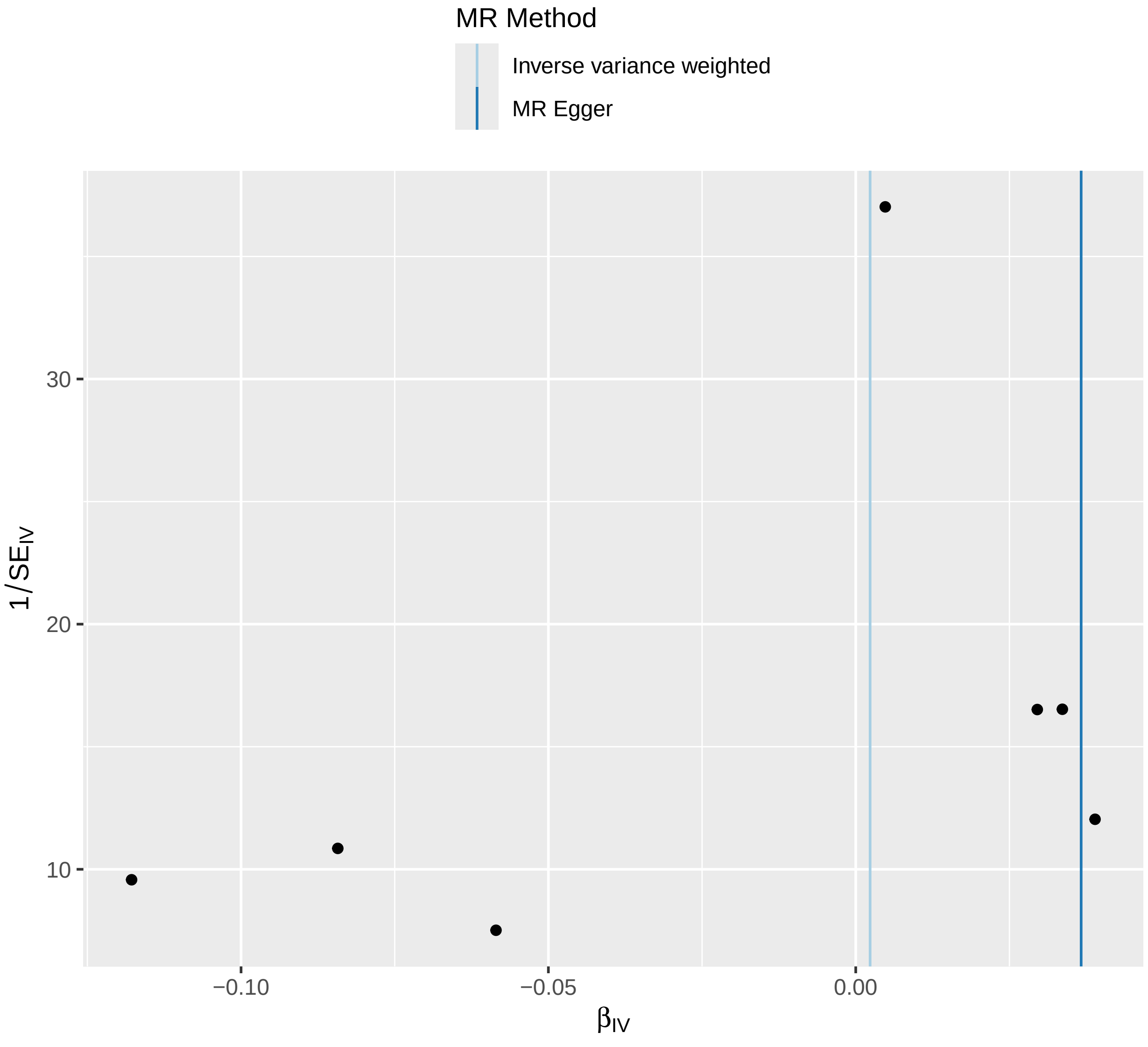
MR Method
Inverse variance weighted
MR Egger
30
20
10
1/SE_IV
−0.10
−0.05
0.00
β_IV

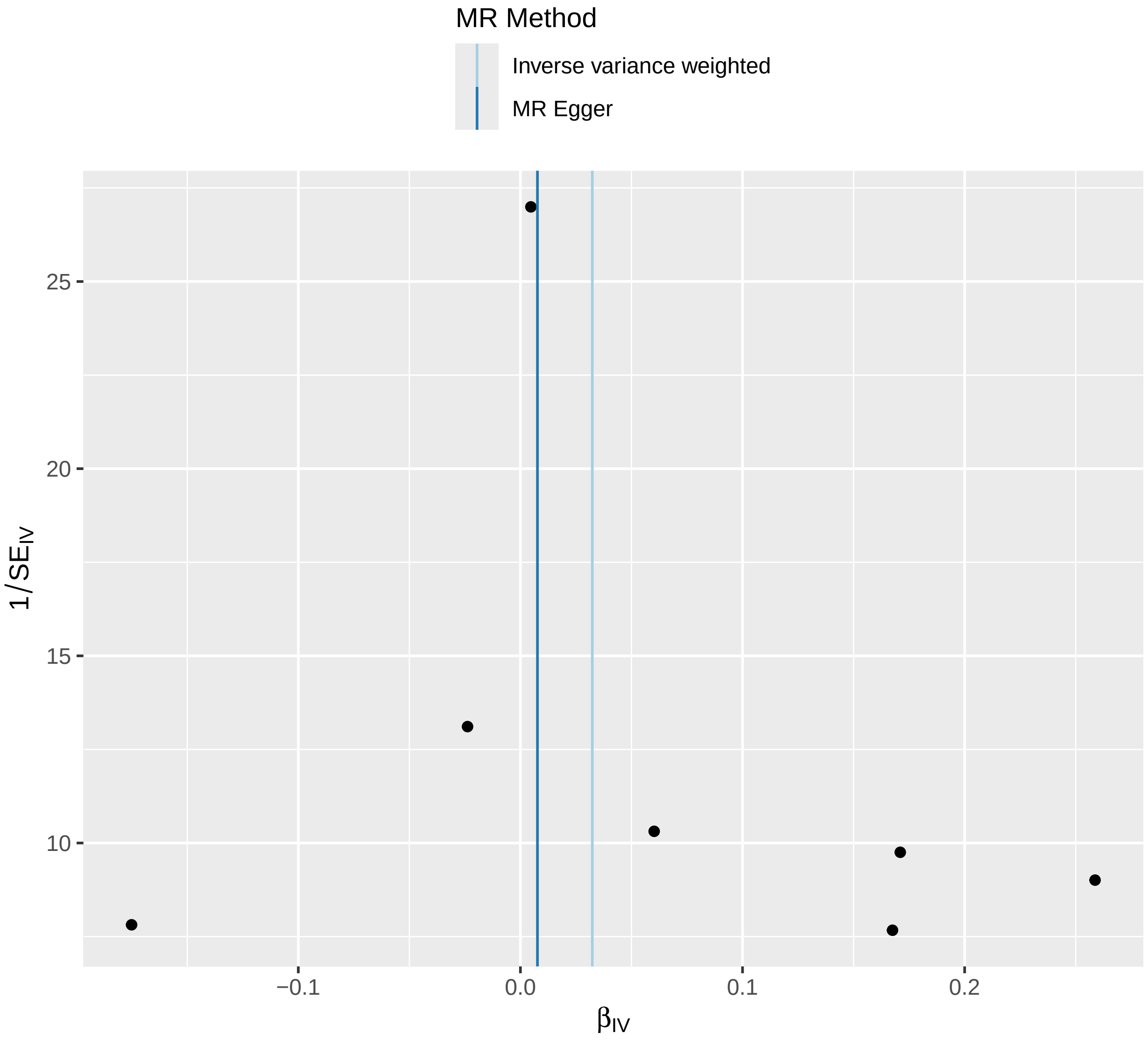
MR Method
Inverse variance weighted
MR Egger
25
20
15
10
1/SE$_{IV}$
−0.1
0.0
0.1
0.2
β_{IV}

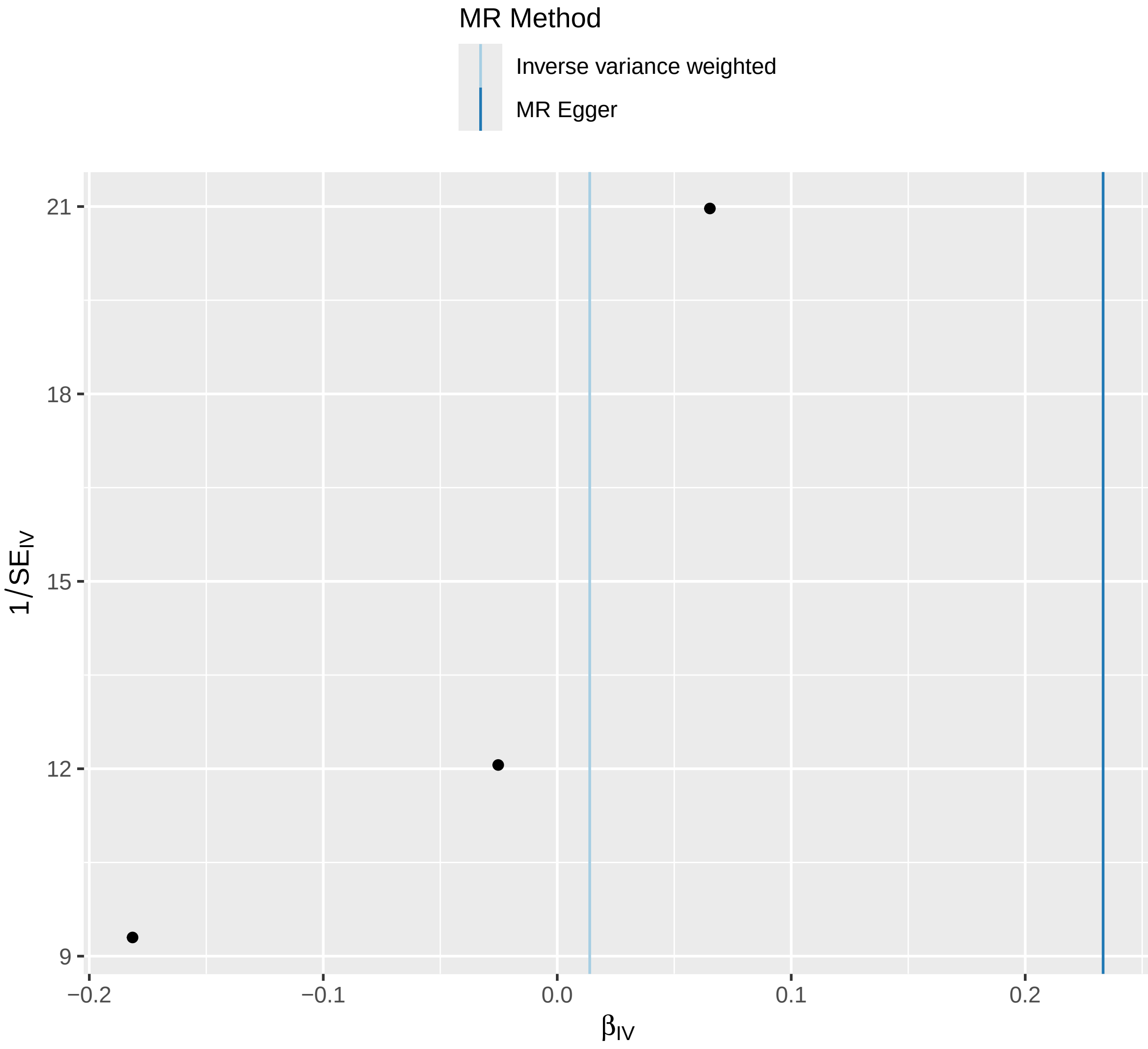

MR Method
Inverse variance weighted
MR Egger
21
18
15
12
9
1/SE_IV
−0.2
−0.1
0.0
0.1
0.2
β_IV

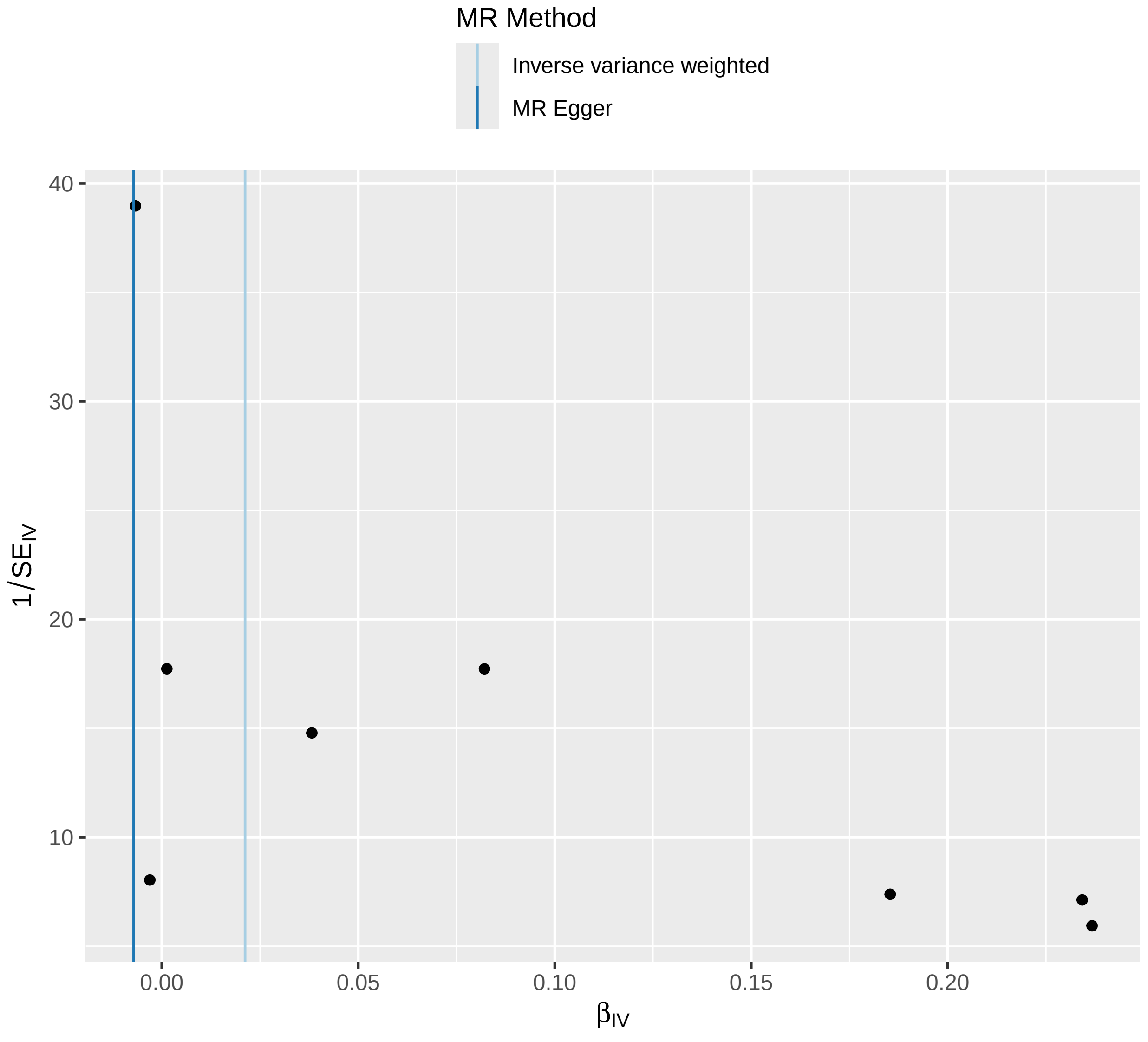

MR Method
Inverse variance weighted
MR Egger
40
30
20
10
1/SE_IV
0.00
0.05
0.10
0.15
0.20
β_IV

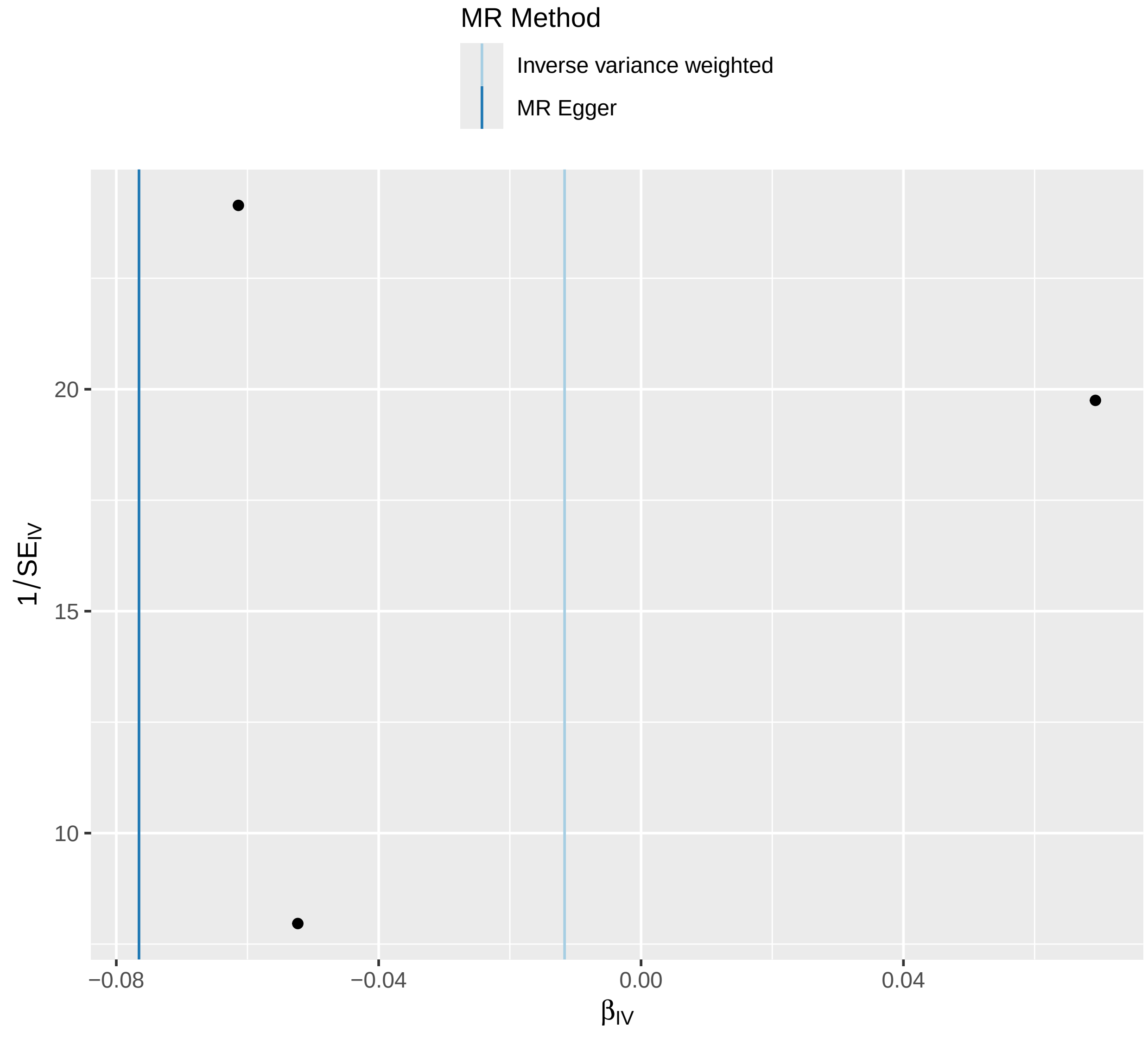
MR Method
Inverse variance weighted
MR Egger
20
15
10
$1/SE_{IV}$
−0.08
−0.04
0.00
0.04
β_{IV}

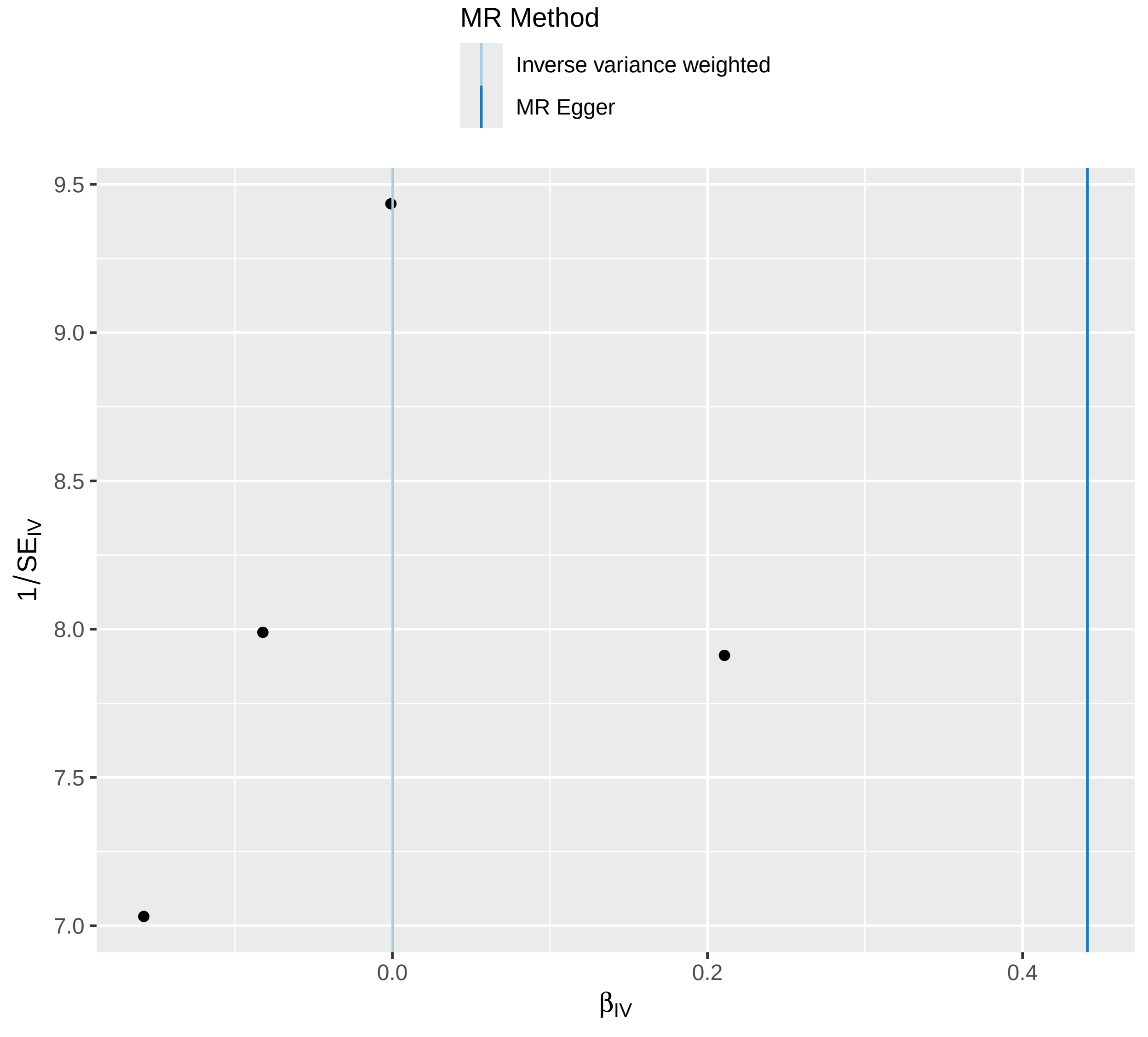

MR Method
Inverse variance weighted
MR Egger
9.5
9.0
8.5
8.0
7.5
7.0
1/SE_IV
0.0
0.2
0.4
β_IV

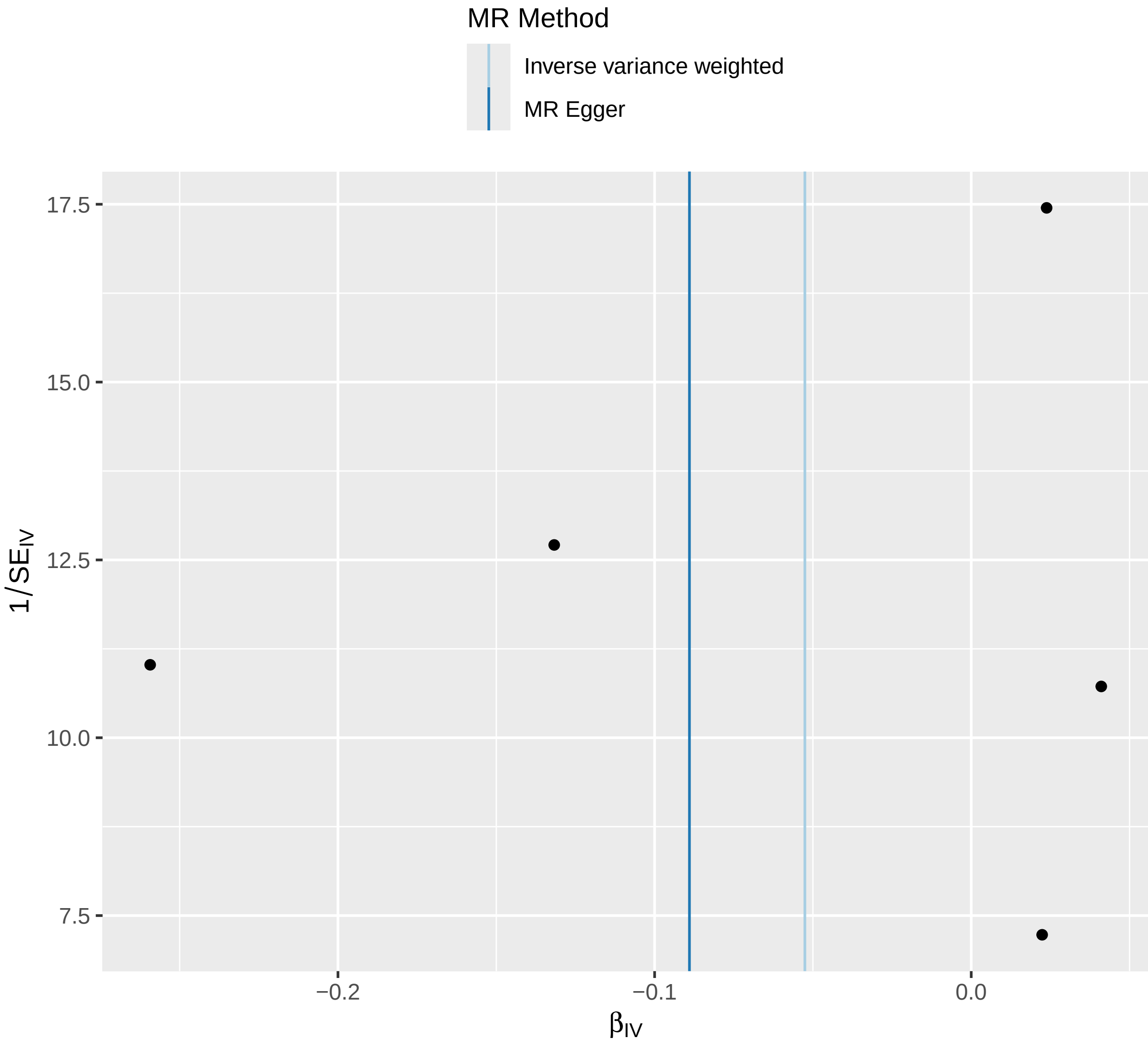
MR Method
Inverse variance weighted
MR Egger
17.5
15.0
12.5
10.0
7.5
1/SE_IV
−0.2
−0.1
0.0
β_IV

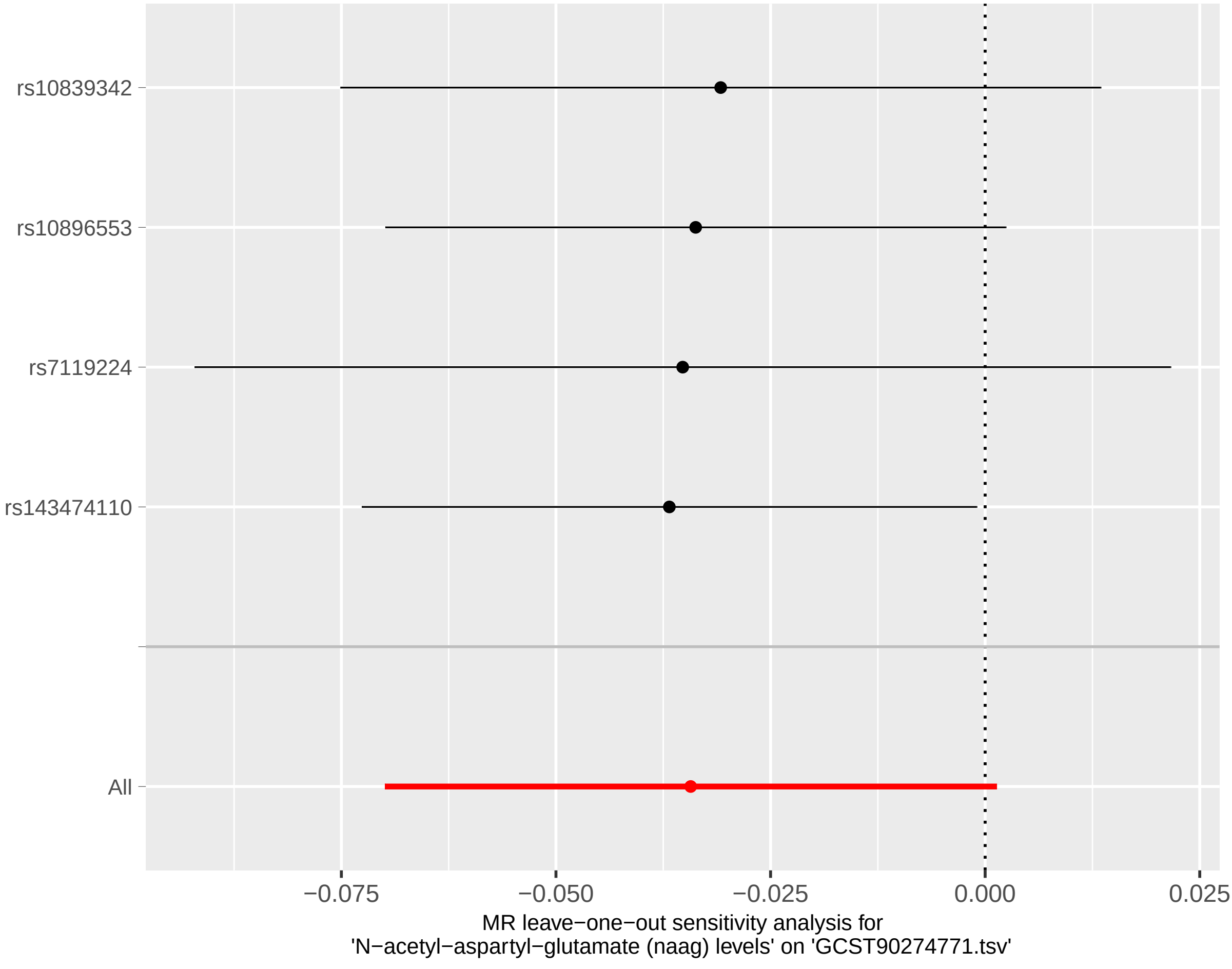
rs10839342
rs10896553
rs7119224
rs143474110
All
−0.075
−0.050
−0.025
0.000
0.025
MR leave−one−out sensitivity analysis for
'N−acetyl−aspartyl−glutamate (naag) levels' on 'GCST90274771.tsv'

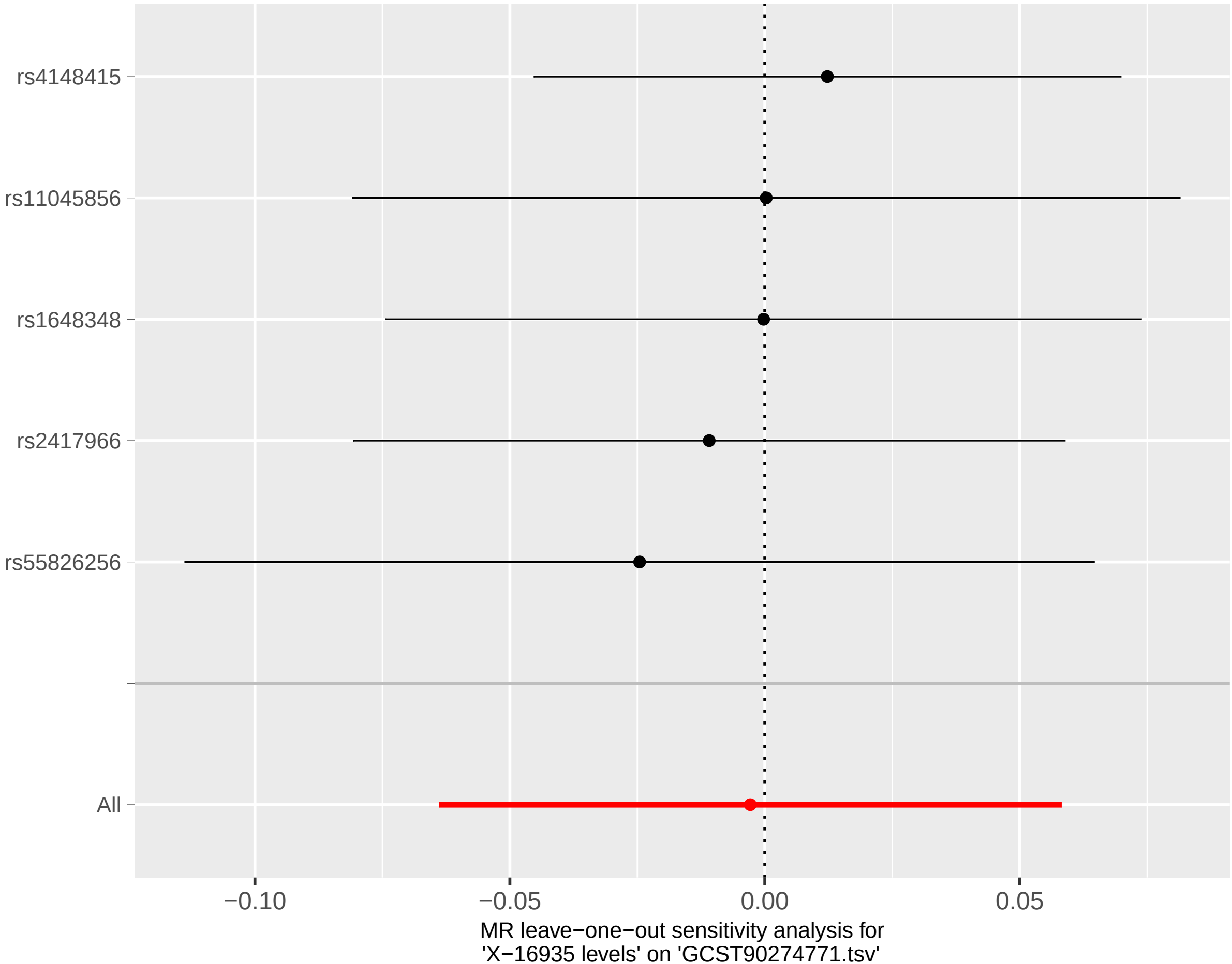
rs4148415
rs11045856
rs1648348
rs2417966
rs55826256
All
−0.10
−0.05
0.00
0.05
MR leave−one−out sensitivity analysis for
'X−16935 levels' on 'GCST90274771.tsv'

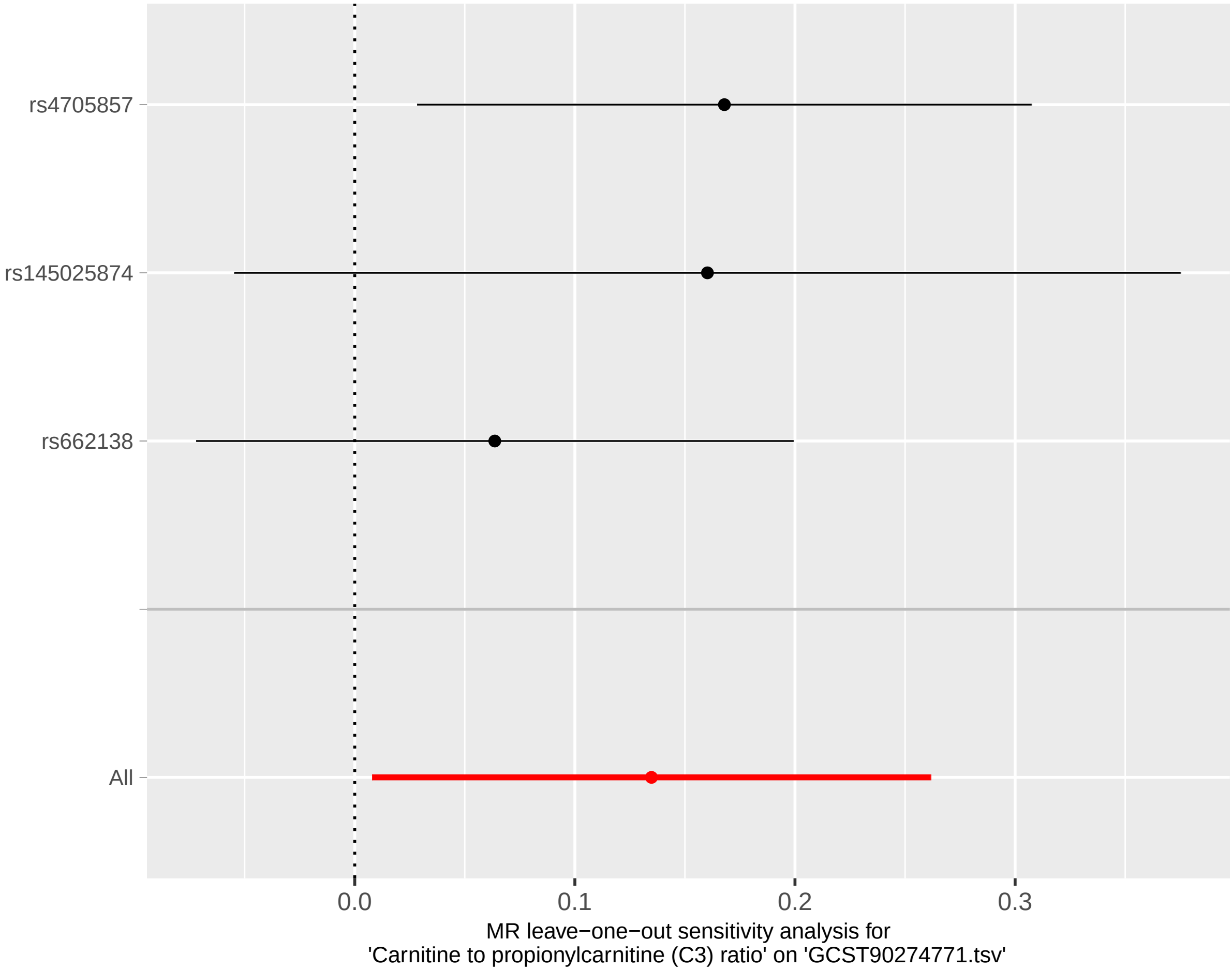
rs4705857
rs145025874
rs662138
All
0.0
0.1
0.2
0.3
MR leave−one−out sensitivity analysis for
'Carnitine to propionylcarnitine (C3) ratio' on 'GCST90274771.tsv'

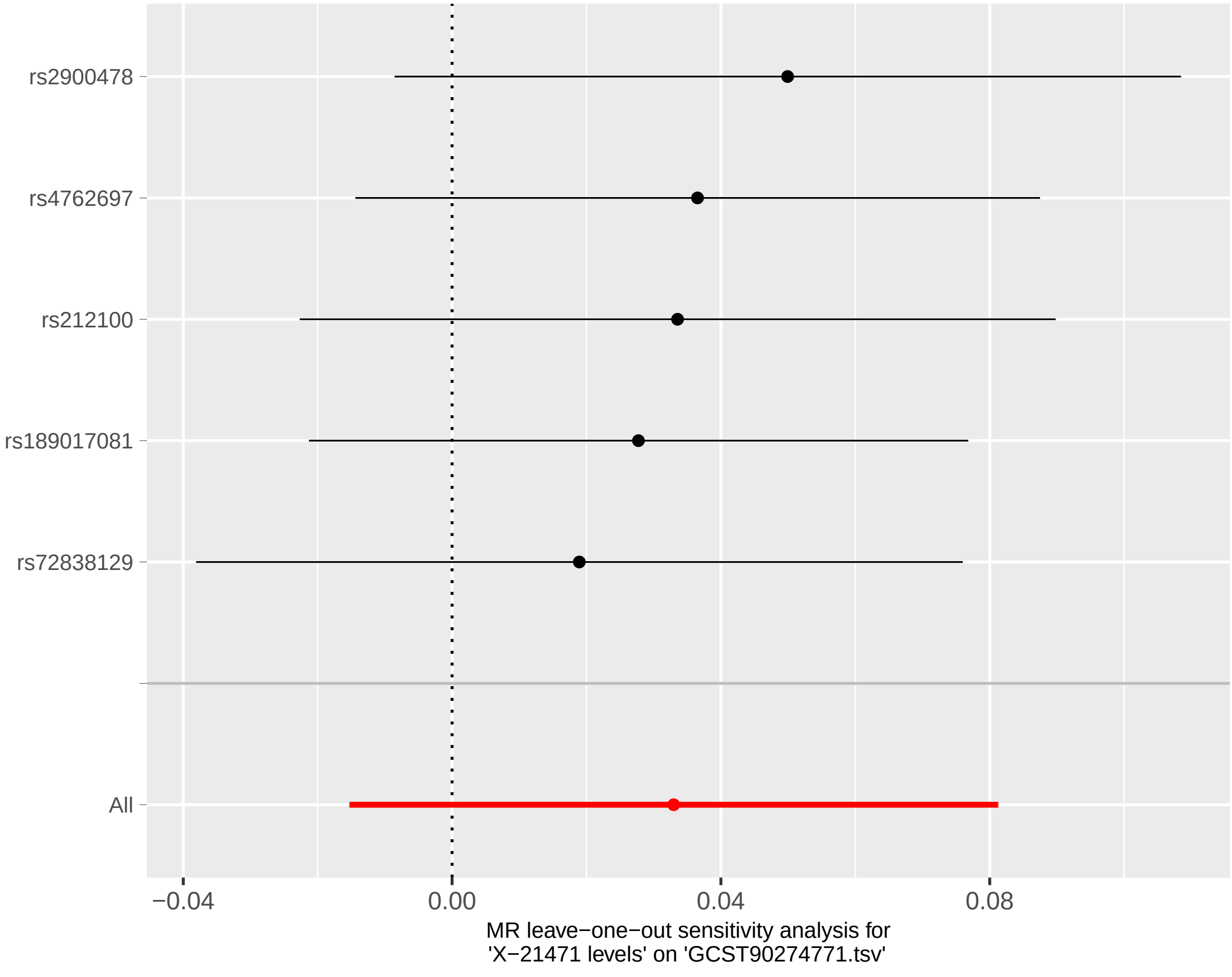
rs2900478
rs4762697
rs212100
rs189017081
rs72838129
All
−0.04
0.00
0.04
0.08
MR leave−one−out sensitivity analysis for
'X−21471 levels' on 'GCST90274771.tsv'

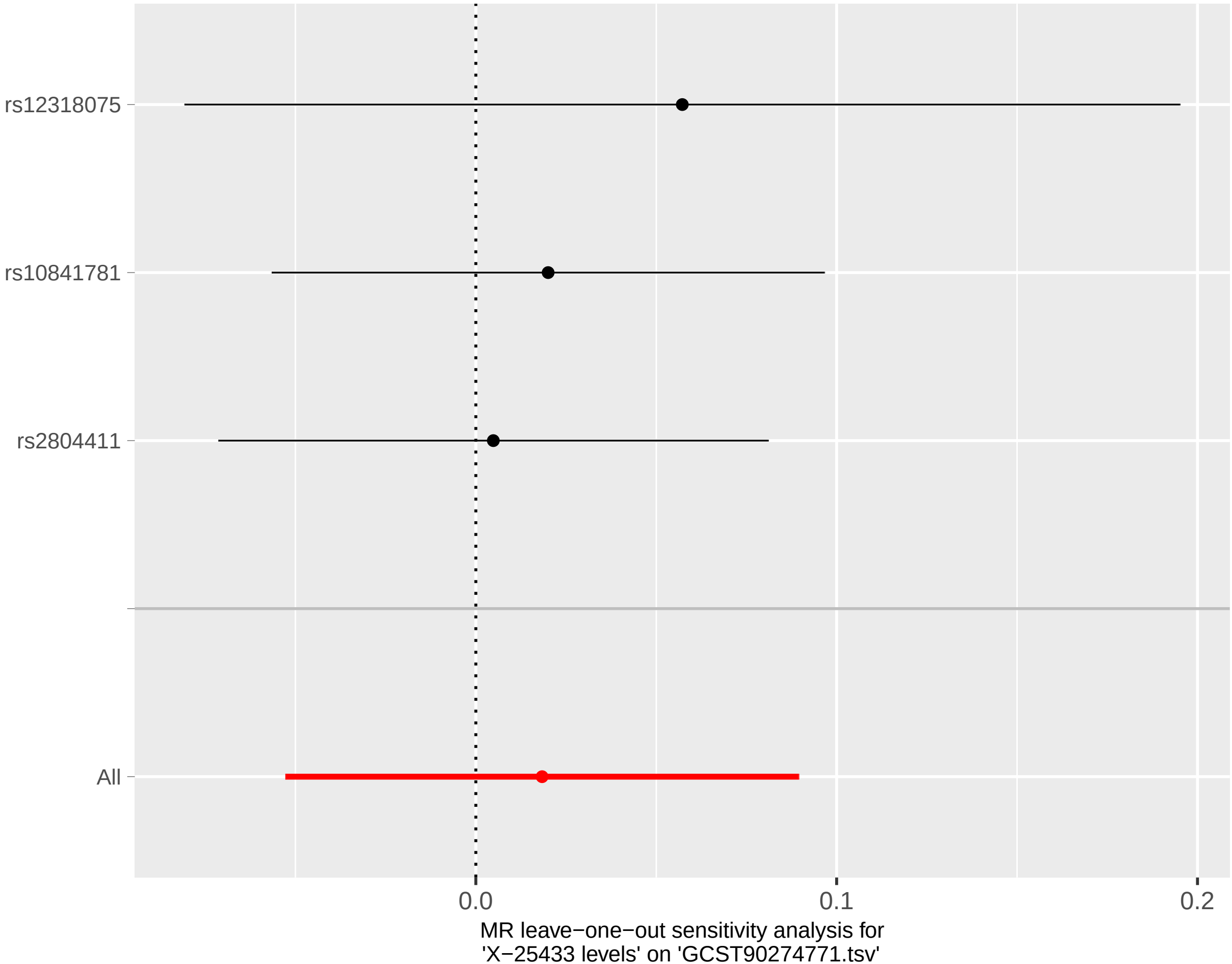

rs12318075
rs10841781
rs2804411
All
0.0
0.1
0.2
MR leave−one−out sensitivity analysis for
'X−25433 levels' on 'GCST90274771.tsv'

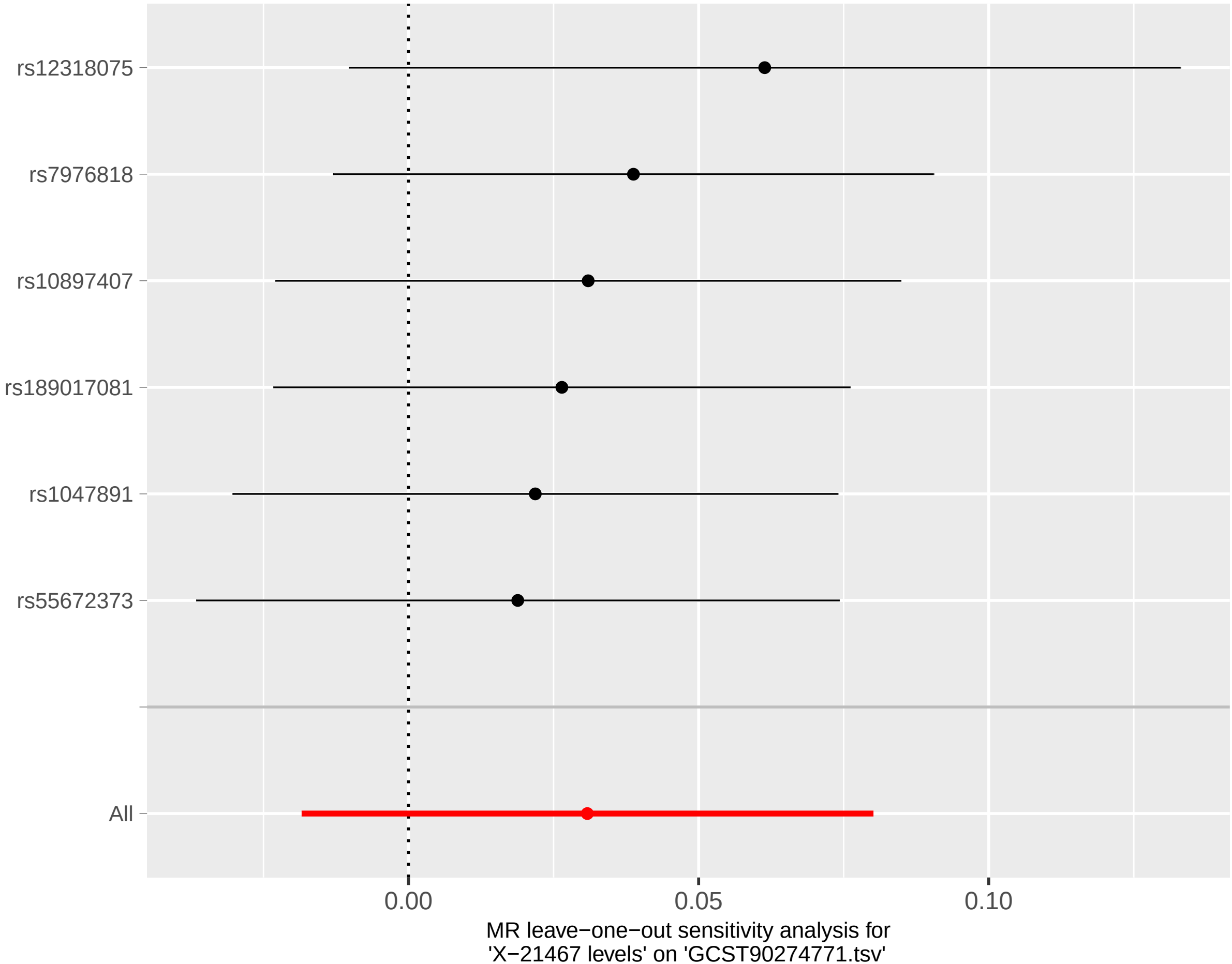
rs12318075
rs7976818
rs10897407
rs189017081
rs1047891
rs55672373
All
0.00
0.05
0.10
MR leave−one−out sensitivity analysis for
'X−21467 levels' on 'GCST90274771.tsv'

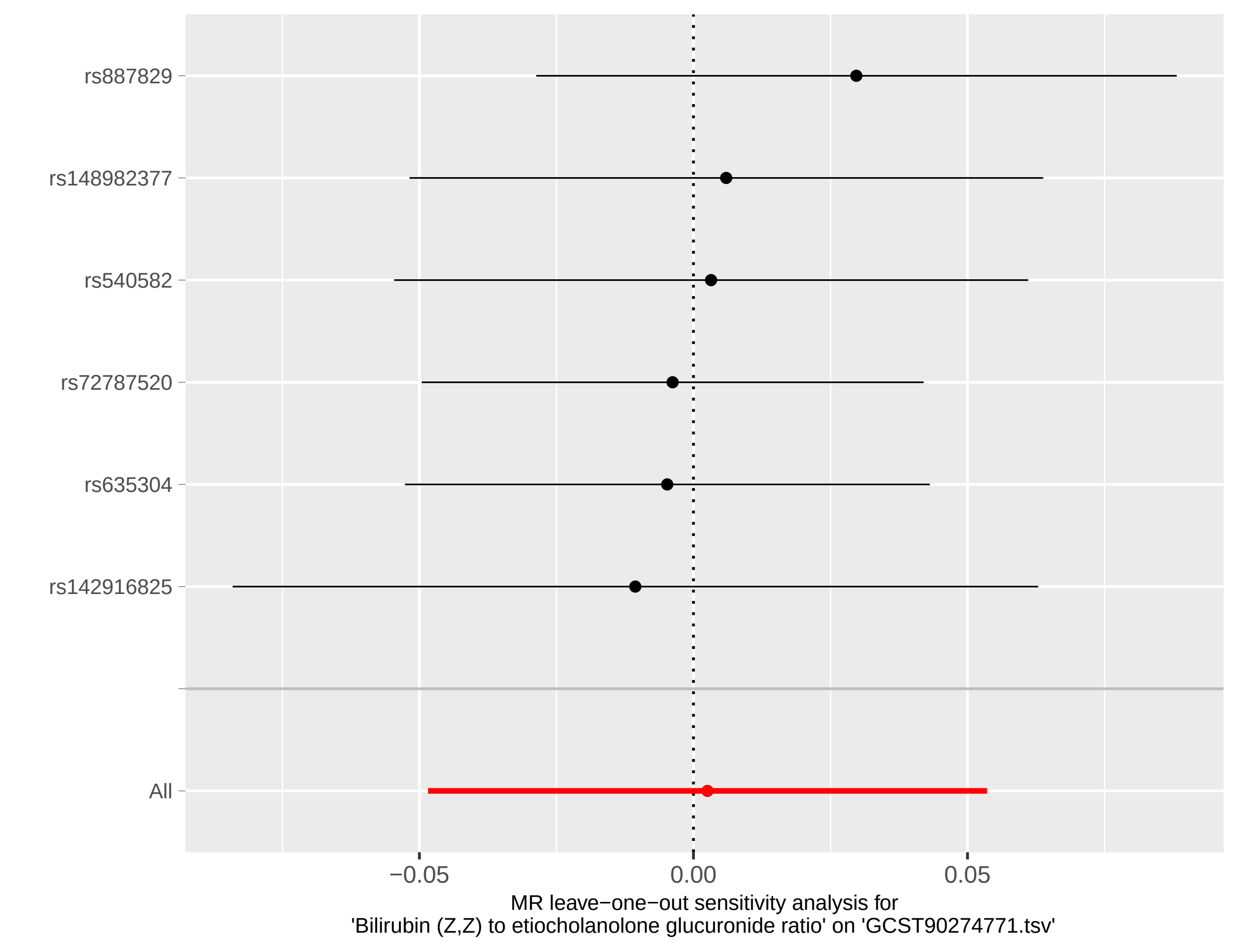
rs887829
rs148982377
rs540582
rs72787520
rs635304
rs142916825
All
−0.05
0.00
0.05
MR leave−one−out sensitivity analysis for
'Bilirubin (Z,Z) to etiocholanolone glucuronide ratio' on 'GCST90274771.tsv'

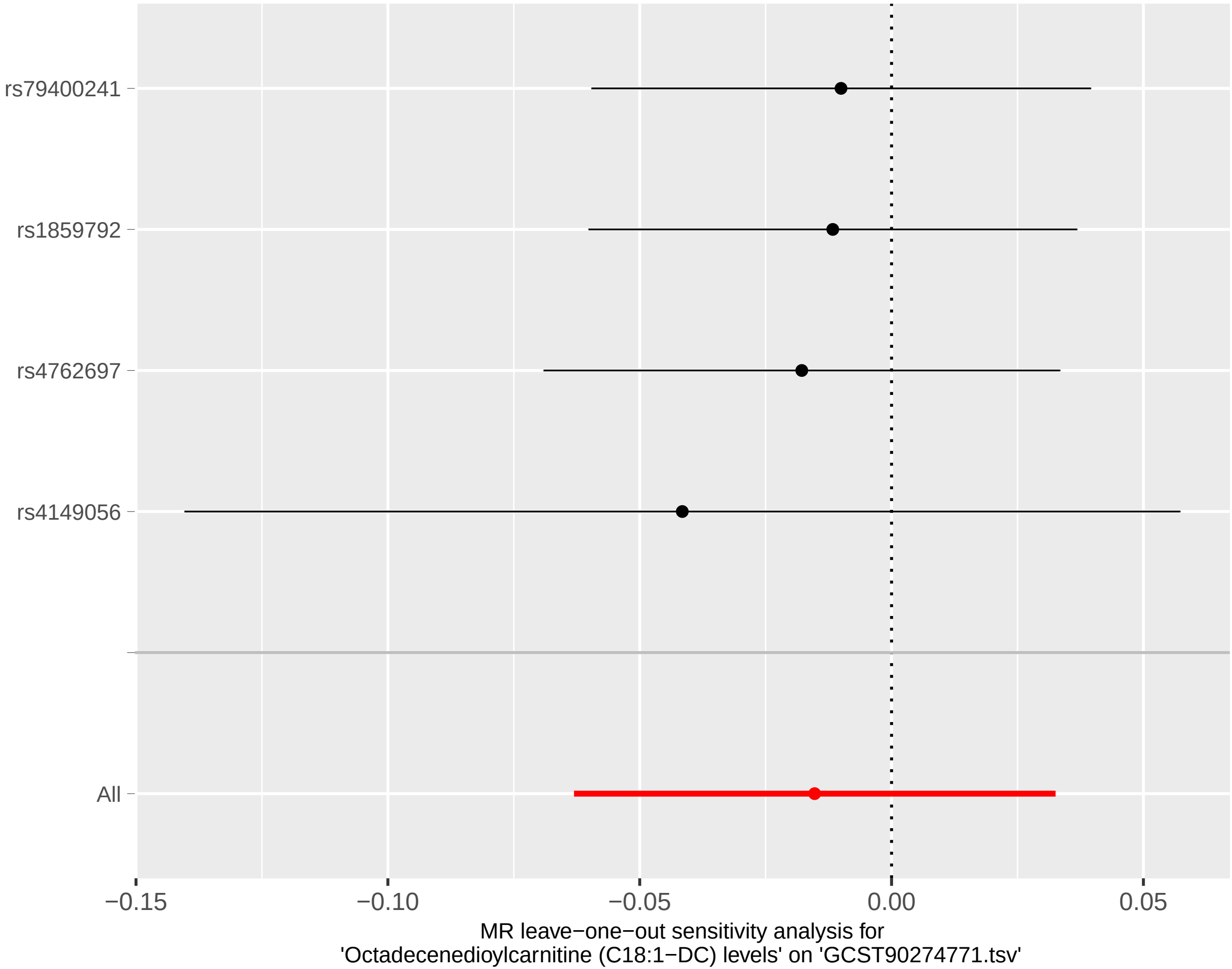
rs79400241
rs1859792
rs4762697
rs4149056
All
−0.15
−0.10
−0.05
0.00
0.05
MR leave−one−out sensitivity analysis for
'Octadecenedioylcarnitine (C18:1−DC) levels' on 'GCST90274771.tsv'

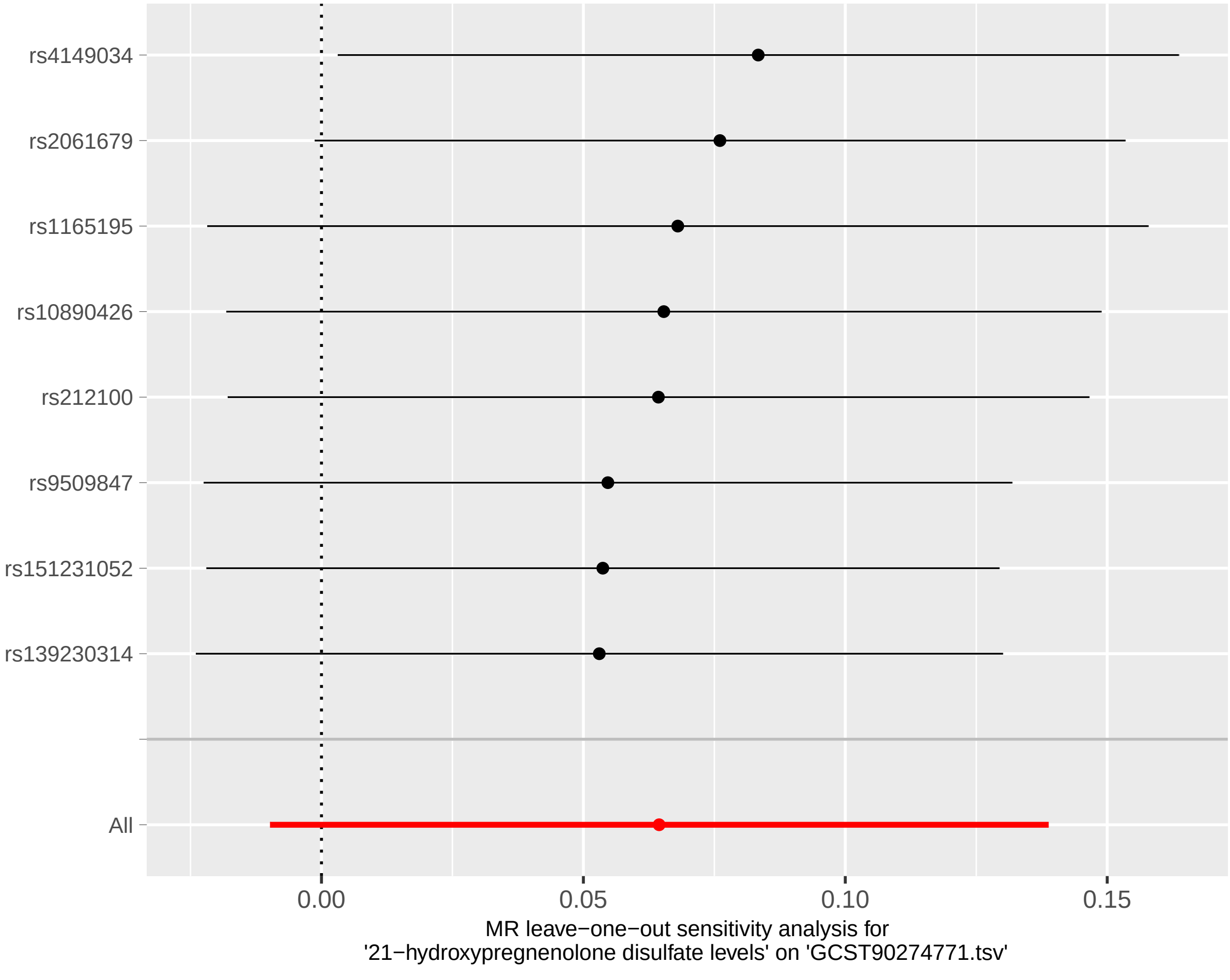
rs4149034
rs2061679
rs1165195
rs10890426
rs212100
rs9509847
rs151231052
rs139230314
All
0.00
0.05
0.10
0.15
MR leave−one−out sensitivity analysis for
'21−hydroxypregnenolone disulfate levels' on 'GCST90274771.tsv'

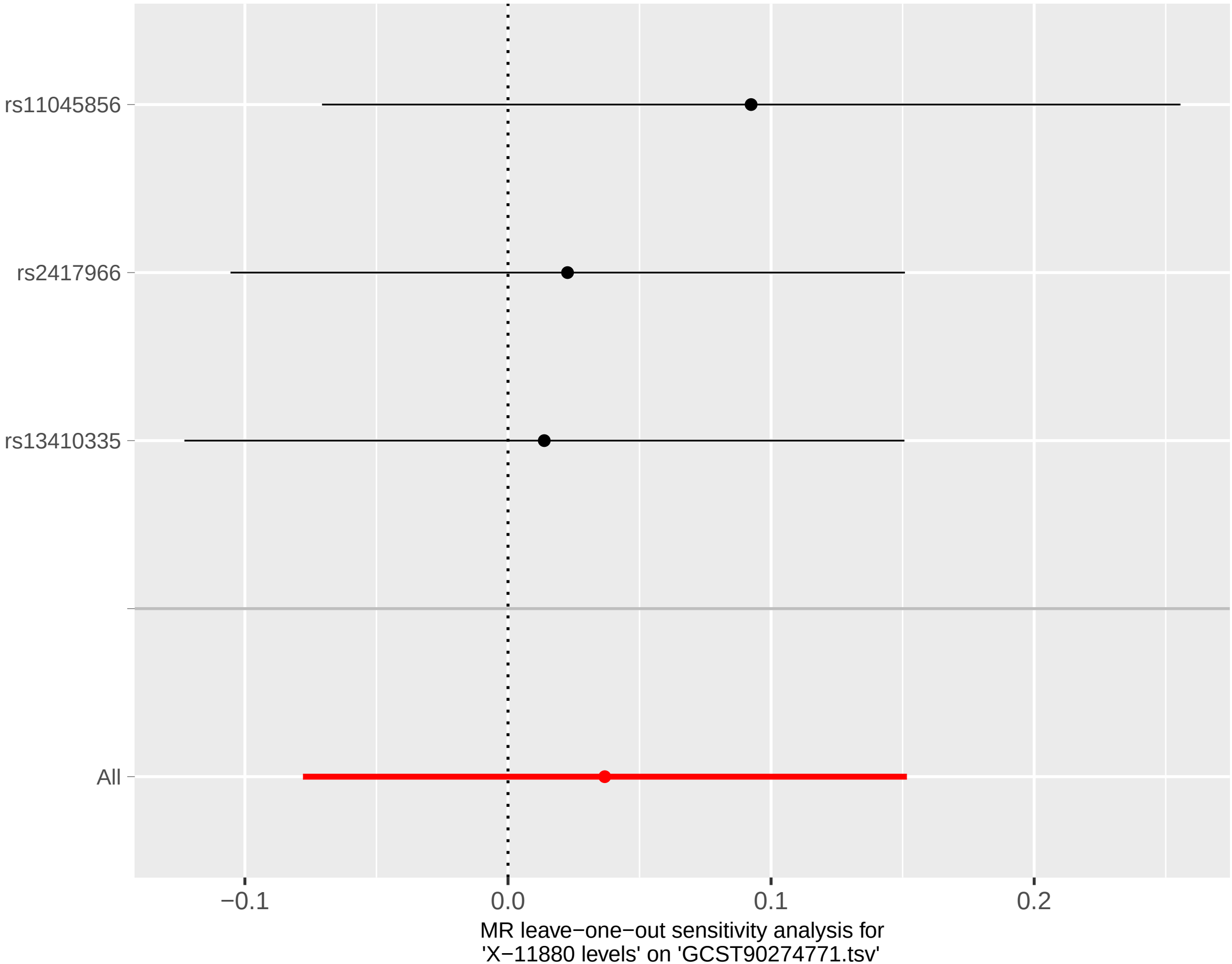
rs11045856
rs2417966
rs13410335
All
−0.1
0.0
0.1
0.2
MR leave−one−out sensitivity analysis for
'X−11880 levels' on 'GCST90274771.tsv'

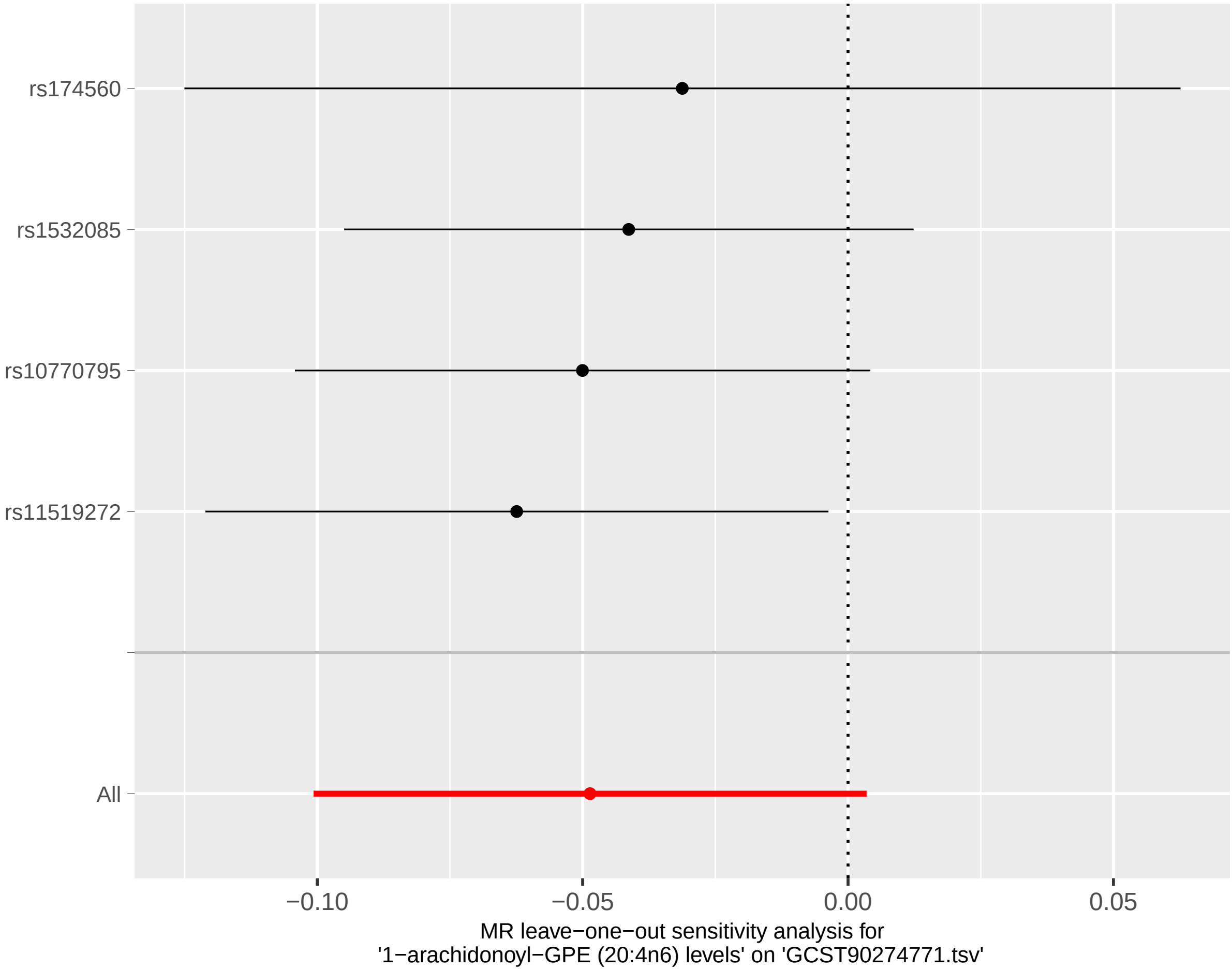
rs174560
rs1532085
rs10770795
rs11519272
All
−0.10
−0.05
0.00
0.05
MR leave−one−out sensitivity analysis for
'1−arachidonoyl−GPE (20:4n6) levels' on 'GCST90274771.tsv'

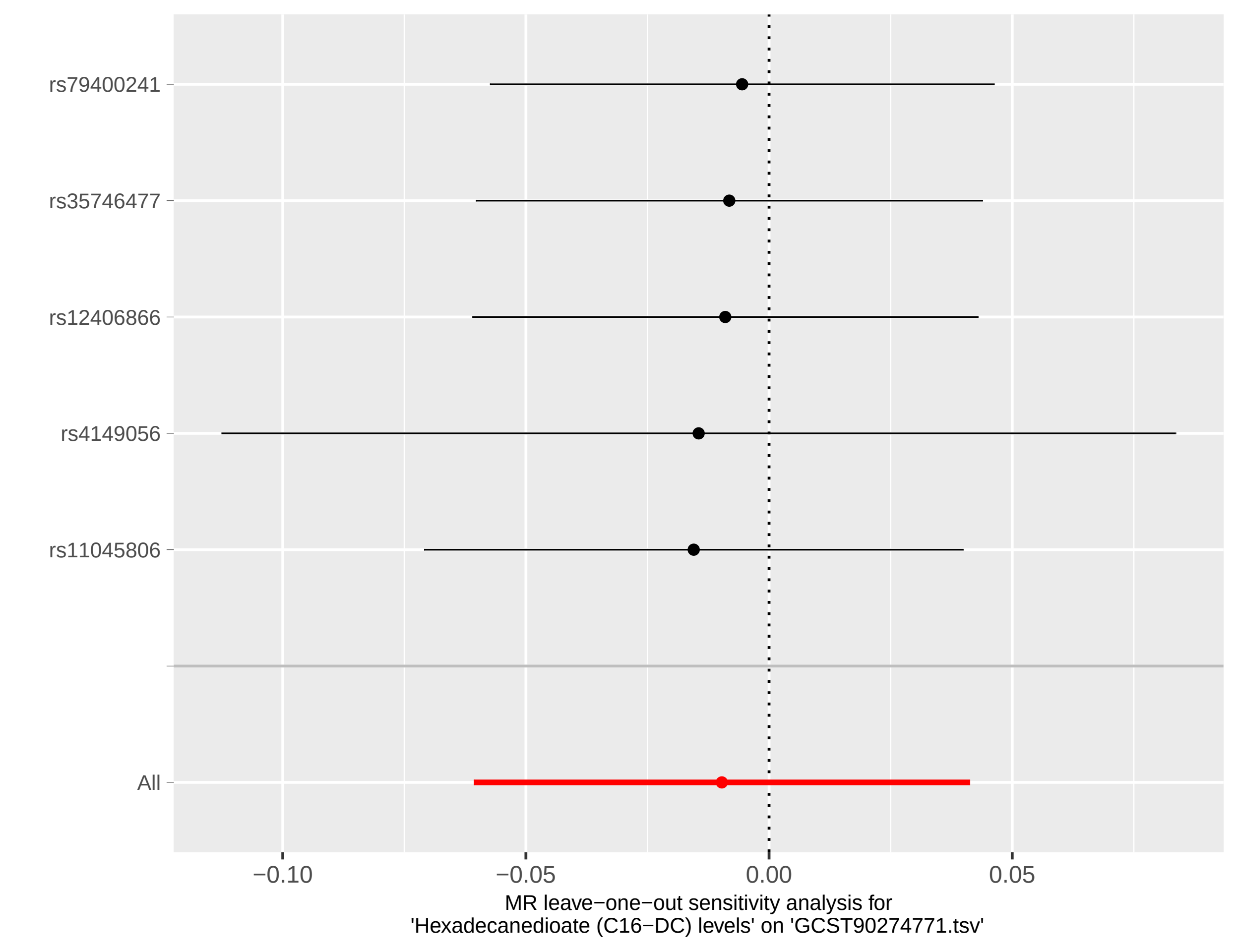
rs79400241
rs35746477
rs12406866
rs4149056
rs11045806
All
−0.10
−0.05
0.00
0.05
MR leave−one−out sensitivity analysis for
'Hexadecanedioate (C16−DC) levels' on 'GCST90274771.tsv'

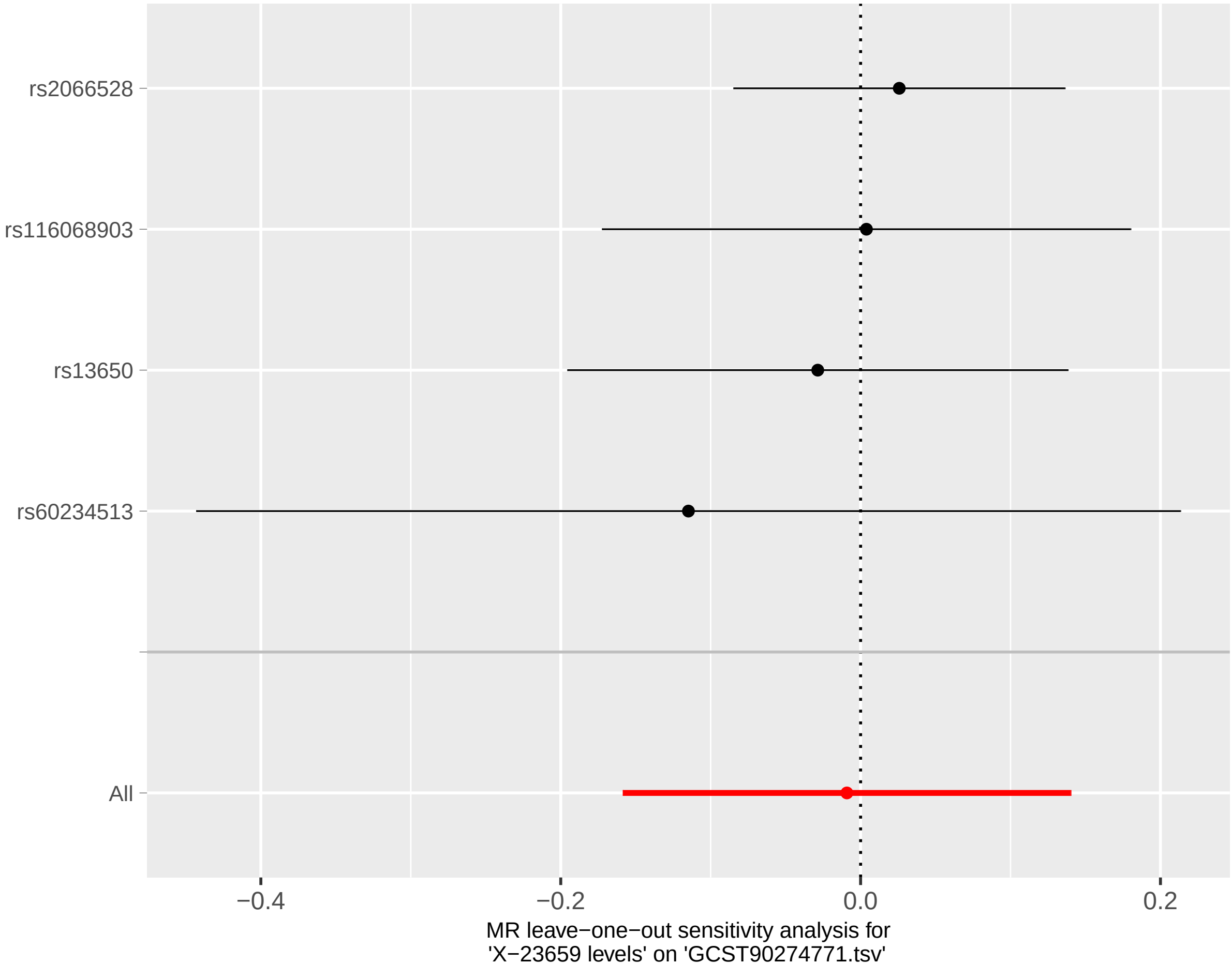
rs2066528
rs116068903
rs13650
rs60234513
All
−0.4
−0.2
0.0
0.2
MR leave−one−out sensitivity analysis for
'X−23659 levels' on 'GCST90274771.tsv'

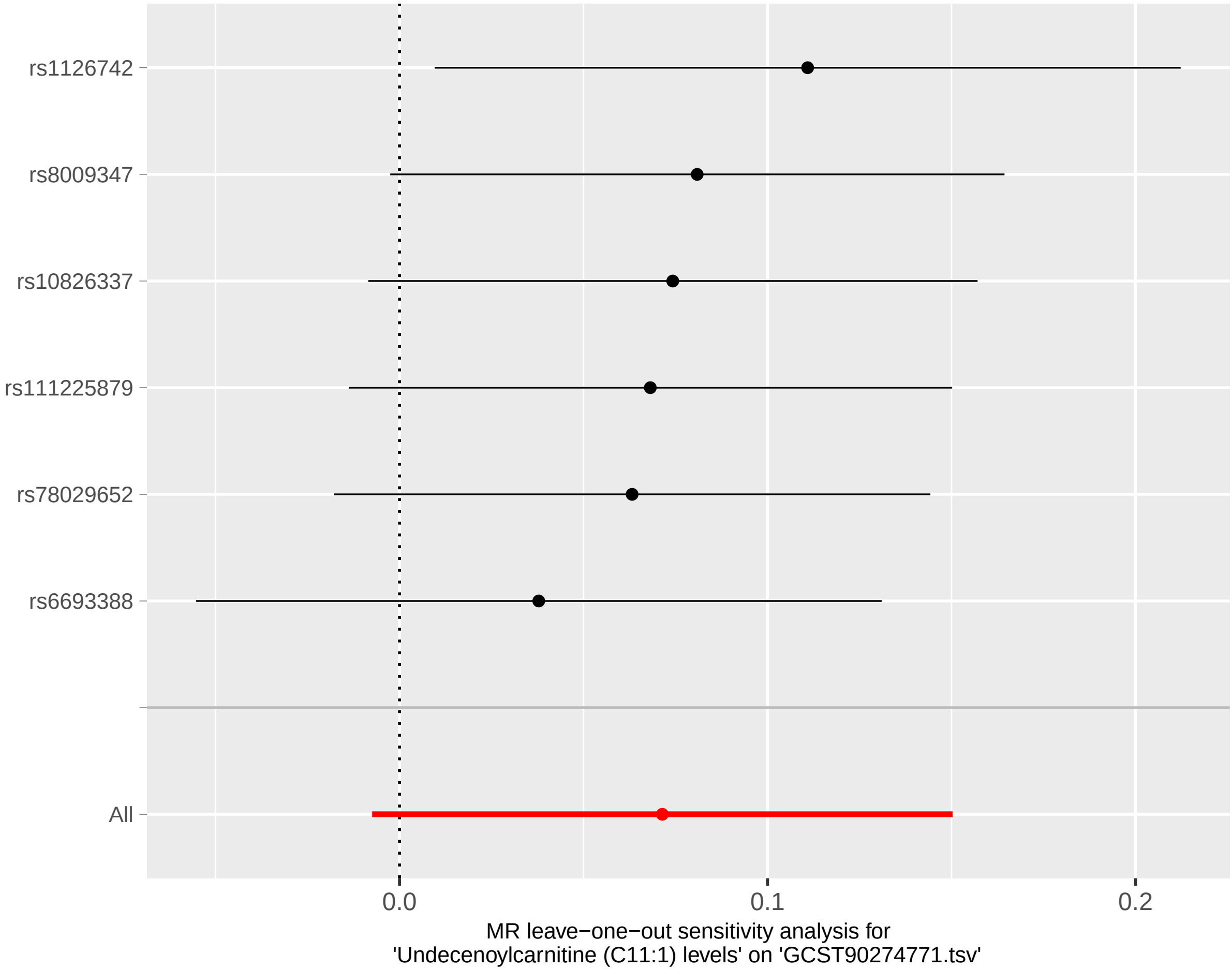
rs1126742
rs8009347
rs10826337
rs111225879
rs78029652
rs6693388
All
0.0
0.1
0.2
MR leave−one−out sensitivity analysis for
'Undecenoylcarnitine (C11:1) levels' on 'GCST90274771.tsv'

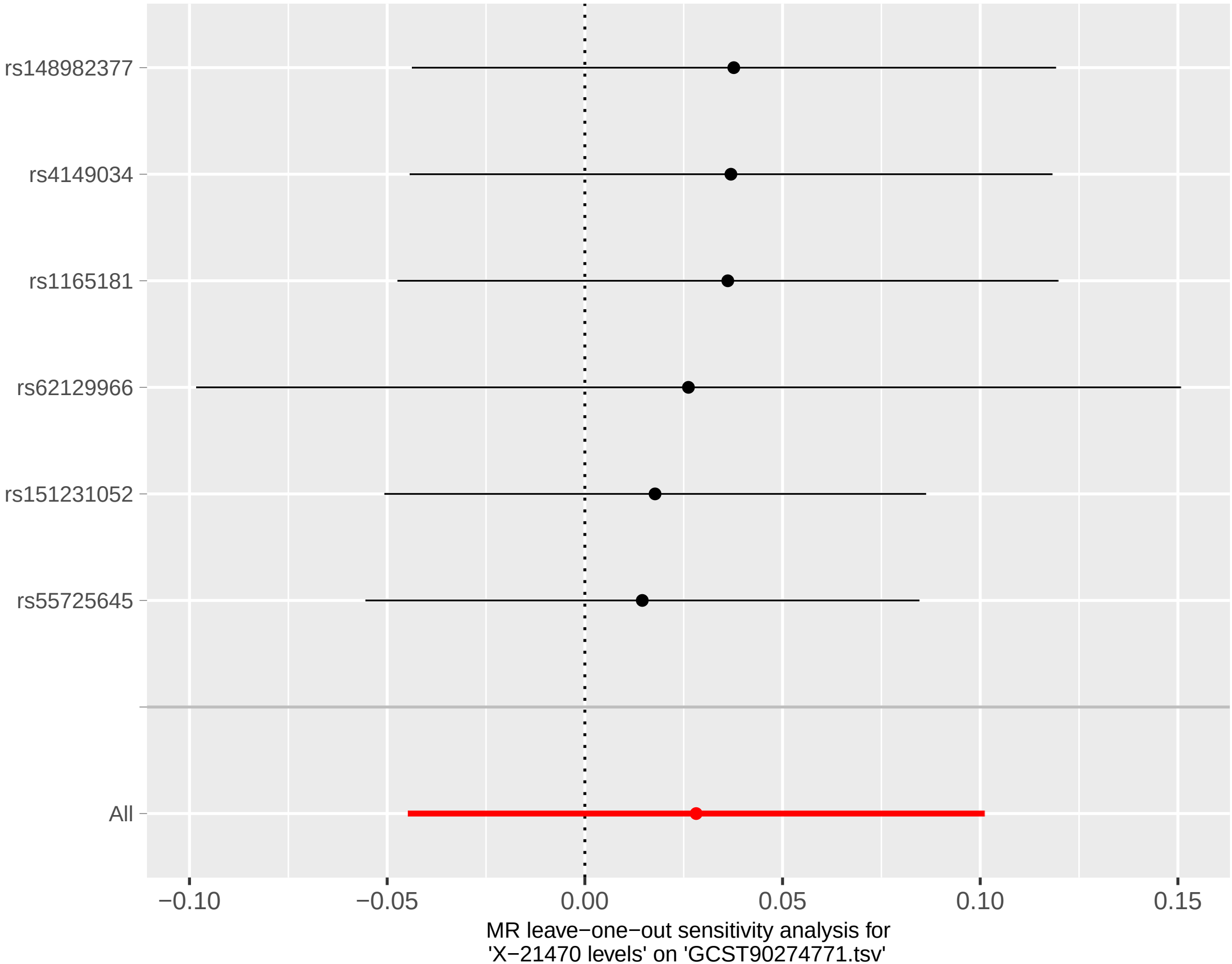
rs148982377
rs4149034
rs1165181
rs62129966
rs151231052
rs55725645
All
−0.10
−0.05
0.00
0.05
0.10
0.15
MR leave−one−out sensitivity analysis for
'X−21470 levels' on 'GCST90274771.tsv'

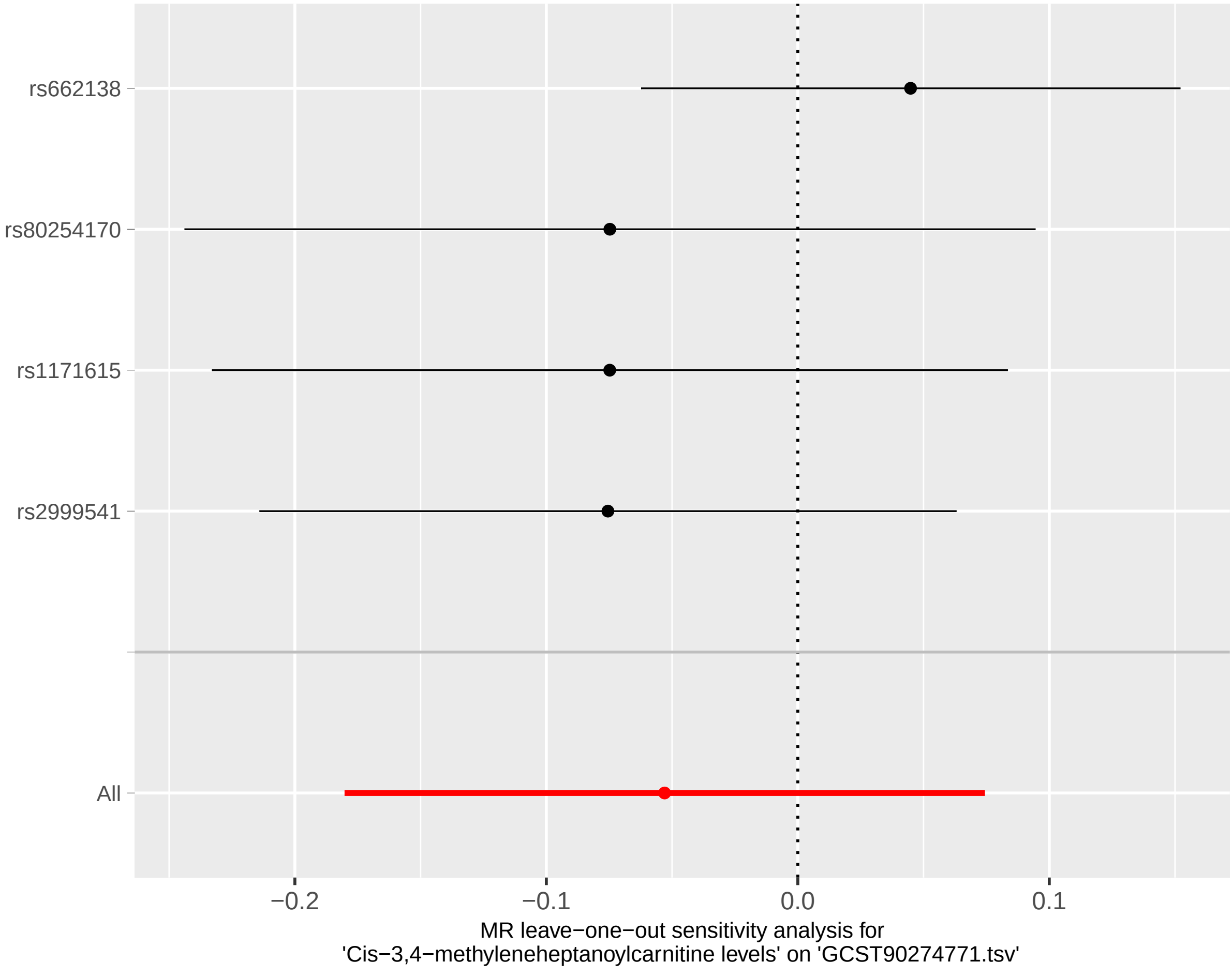
rs662138
rs80254170
rs1171615
rs2999541
All
−0.2
−0.1
0.0
0.1
MR leave−one−out sensitivity analysis for
'Cis−3,4−methyleneheptanoylcarnitine levels' on 'GCST90274771.tsv'

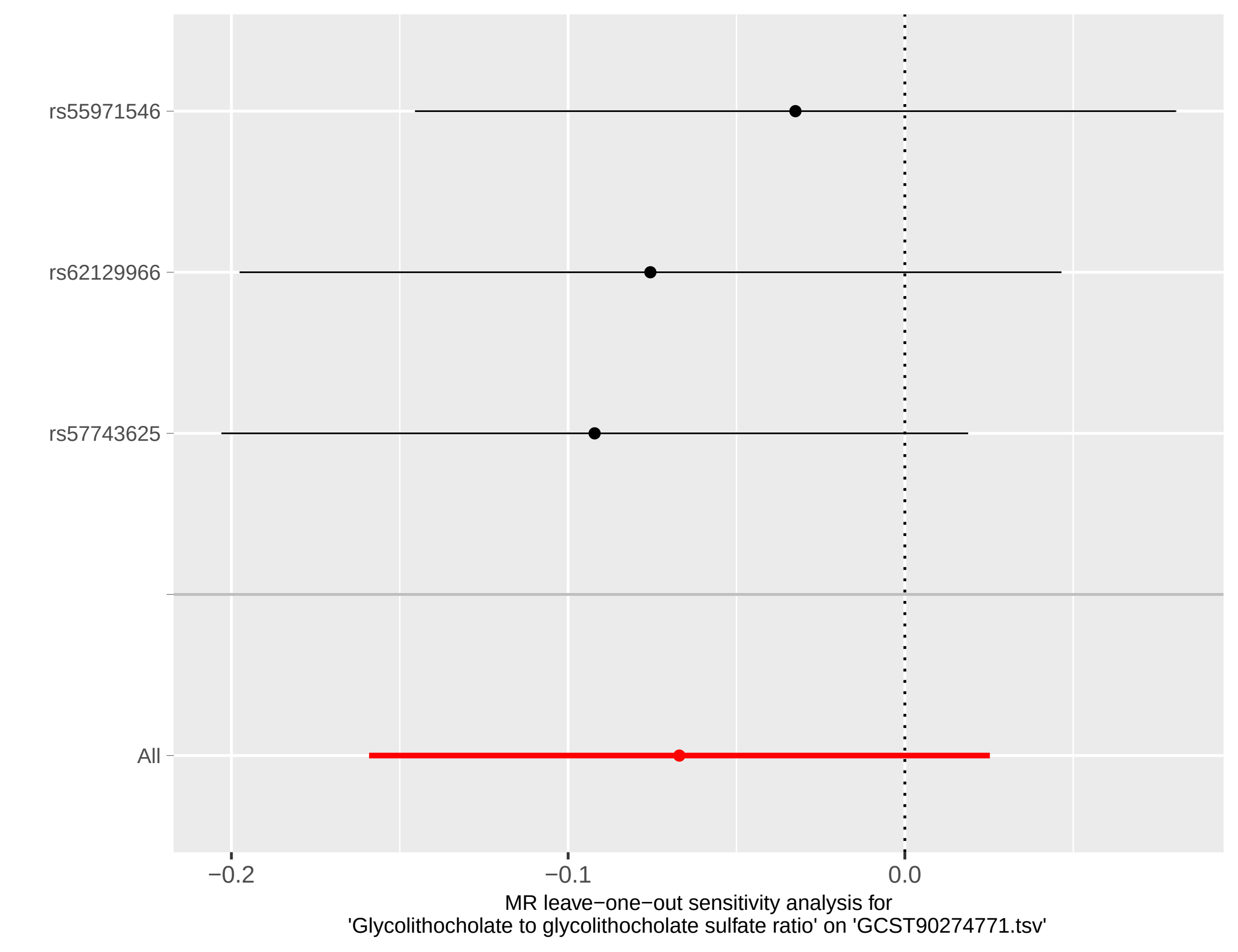

rs55971546
rs62129966
rs57743625
All
−0.2
−0.1
0.0
MR leave−one−out sensitivity analysis for
'Glycolithocholate to glycolithocholate sulfate ratio' on 'GCST90274771.tsv'

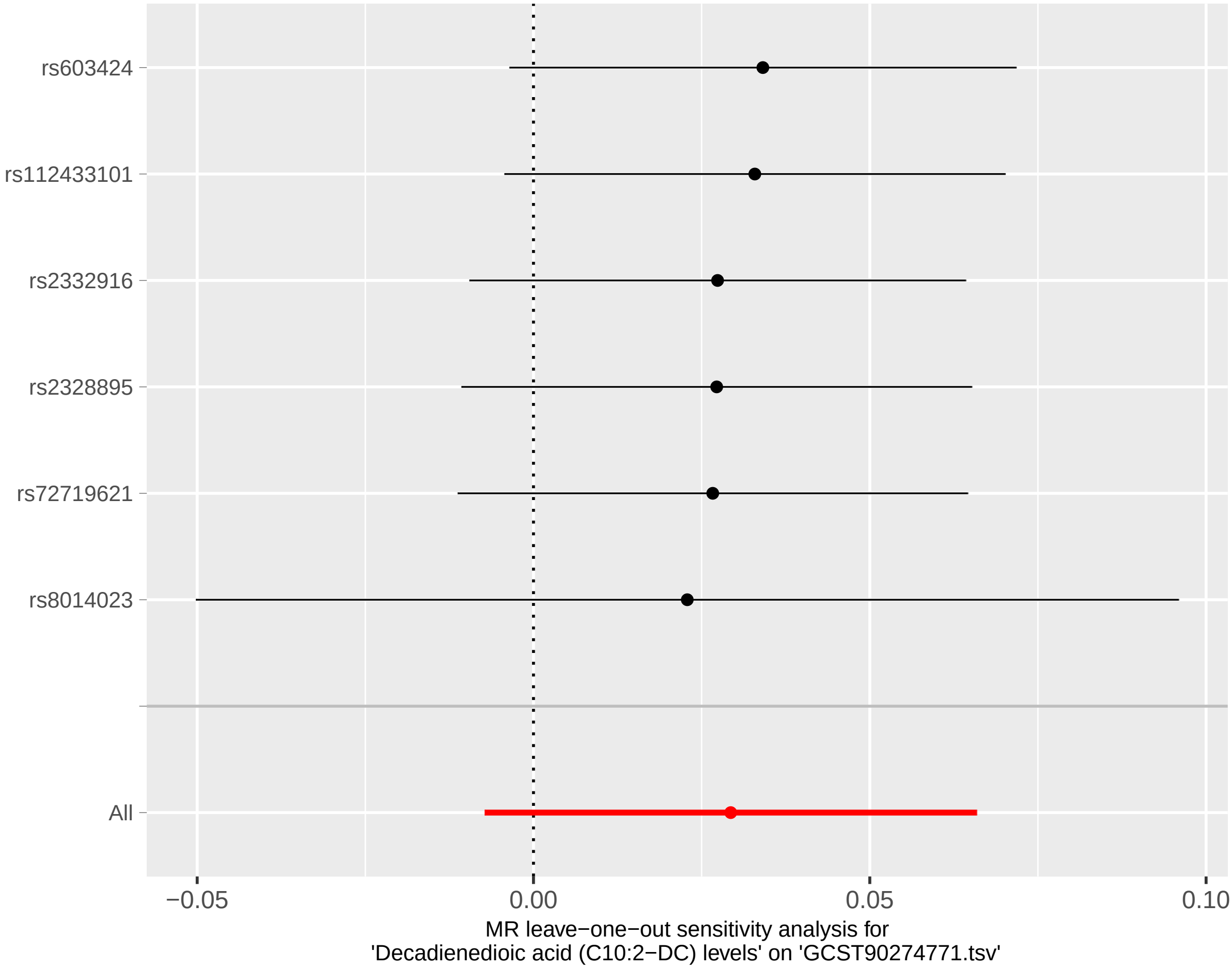
rs603424
rs112433101
rs2332916
rs2328895
rs72719621
rs8014023
All
−0.05
0.00
0.05
0.10
MR leave−one−out sensitivity analysis for
'Decadienedioic acid (C10:2−DC) levels' on 'GCST90274771.tsv'

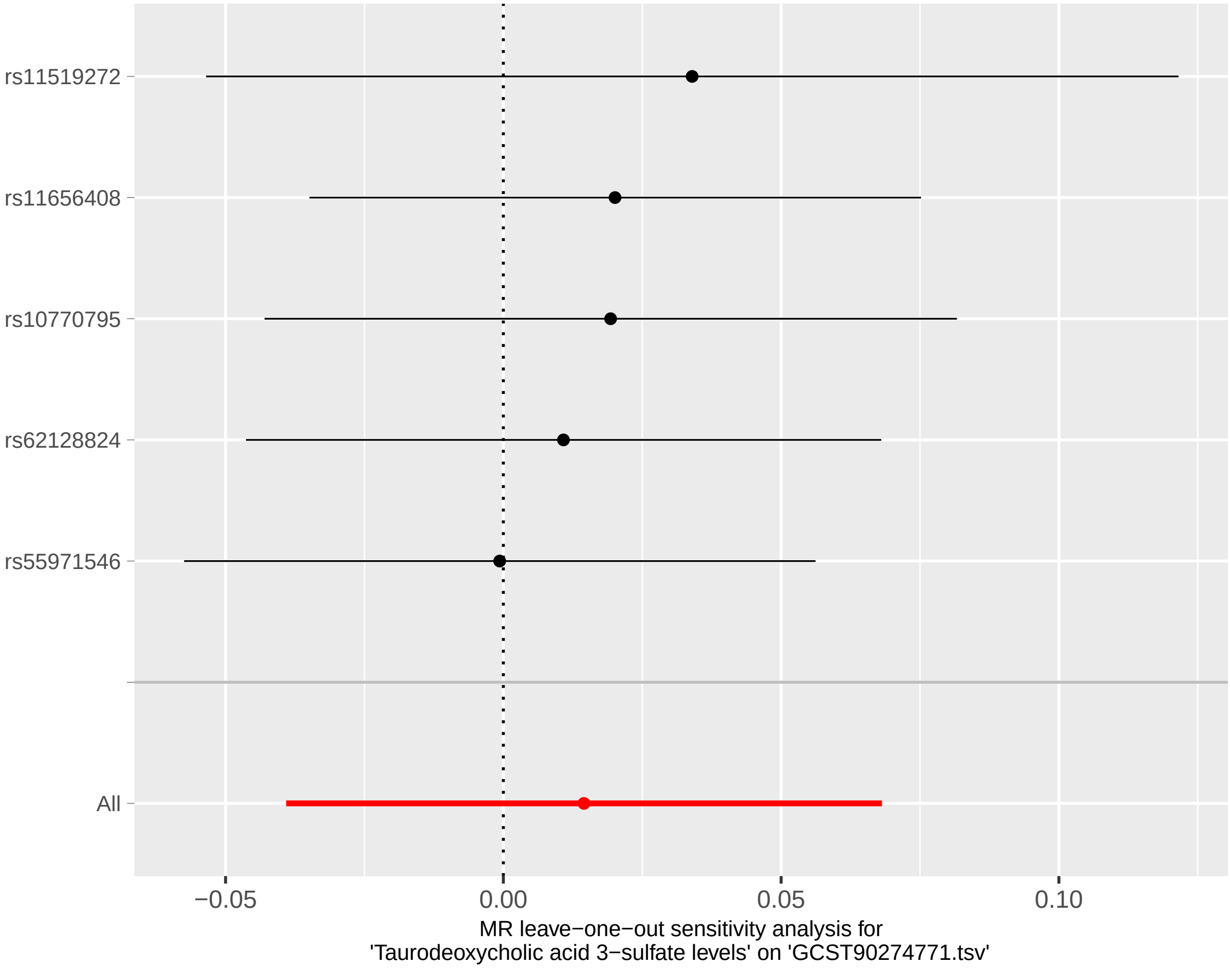
rs11519272
rs11656408
rs10770795
rs62128824
rs55971546
All
−0.05
0.00
0.05
0.10
MR leave−one−out sensitivity analysis for
'Taurodeoxycholic acid 3−sulfate levels' on 'GCST90274771.tsv'

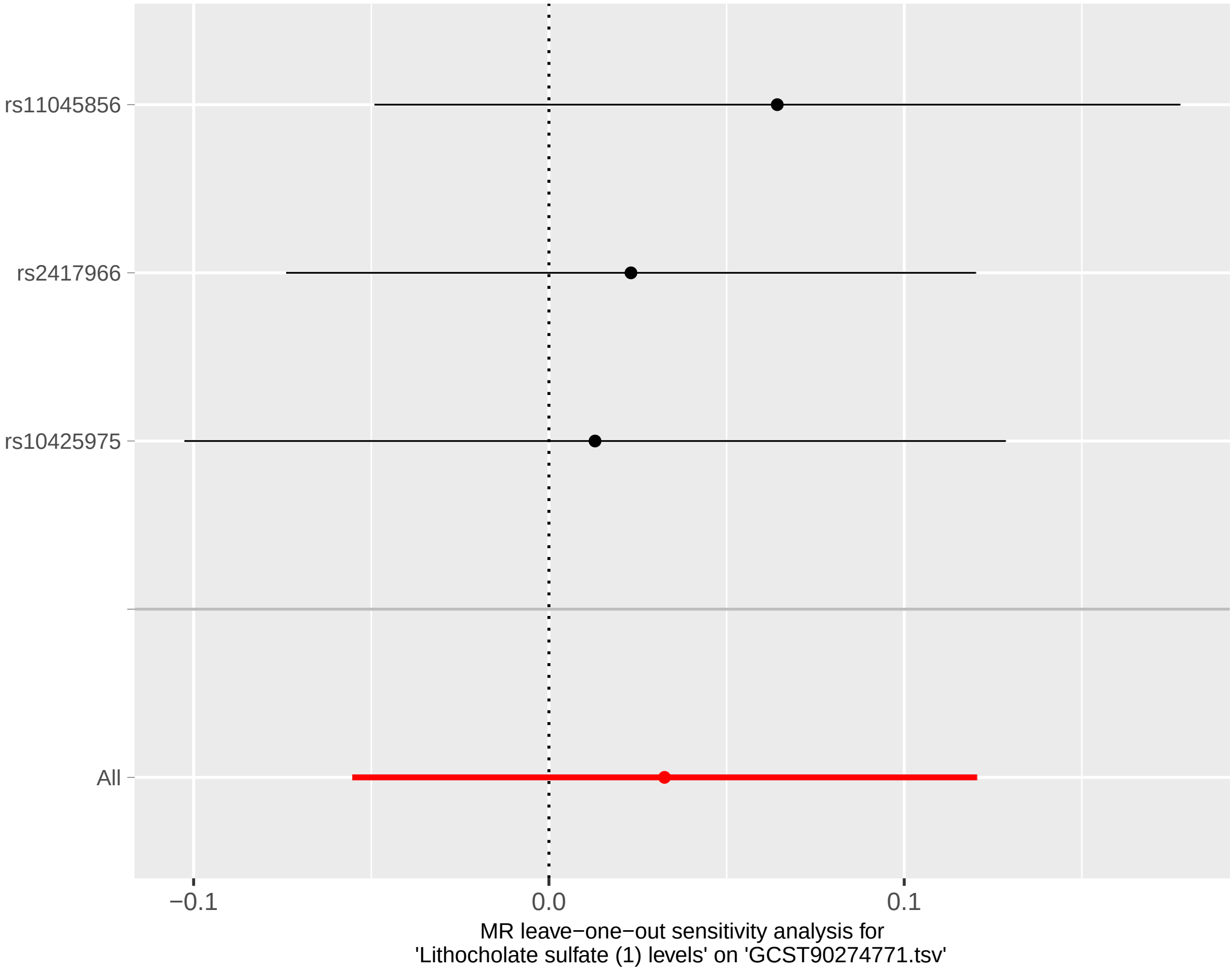
rs11045856
rs2417966
rs10425975
All
−0.1
0.0
0.1
MR leave−one−out sensitivity analysis for
'Lithocholate sulfate (1) levels' on 'GCST90274771.tsv'

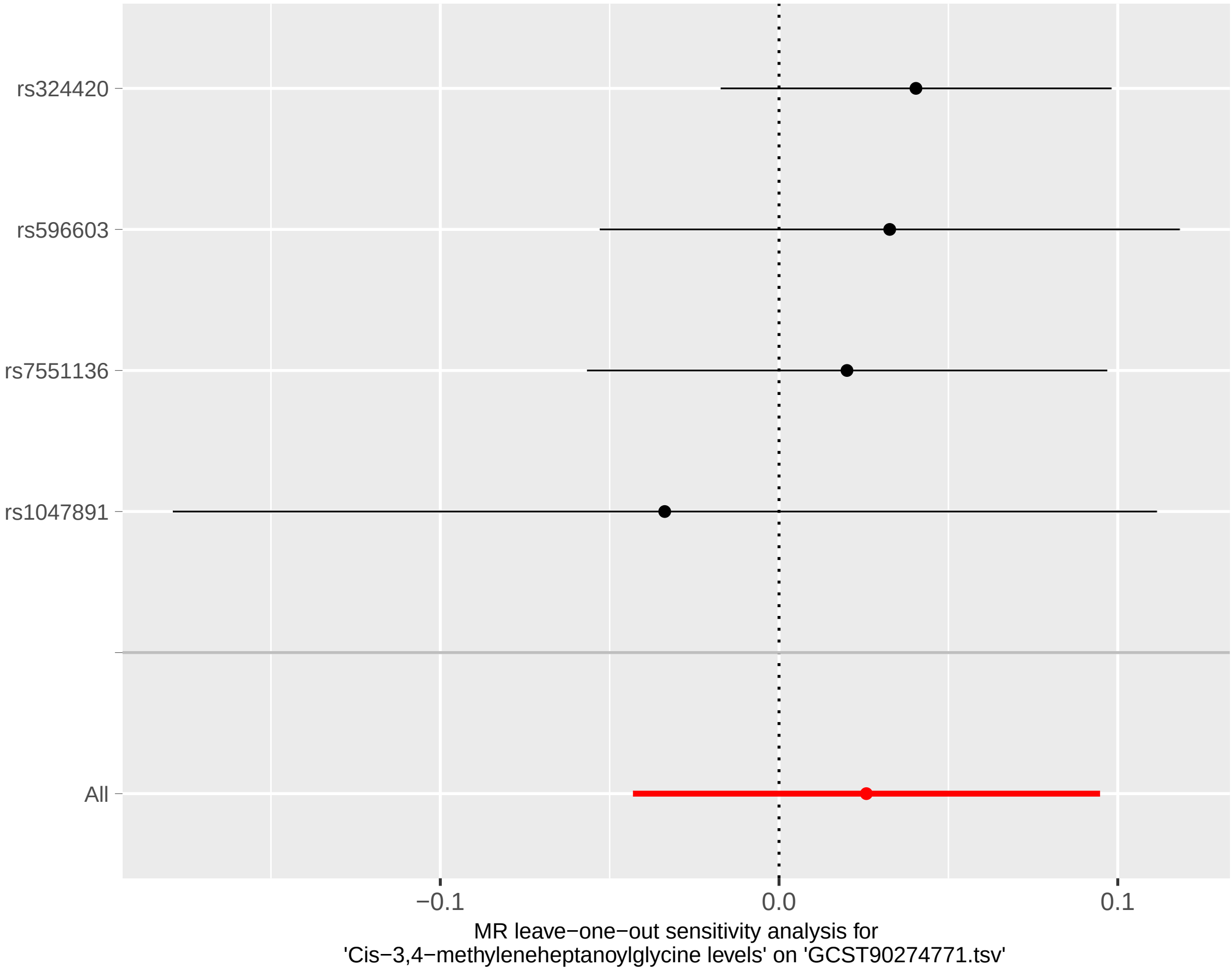
rs324420
rs596603
rs7551136
rs1047891
All
−0.1
0.0
0.1
MR leave−one−out sensitivity analysis for
'Cis−3,4−methyleneheptanoylglycine levels' on 'GCST90274771.tsv'

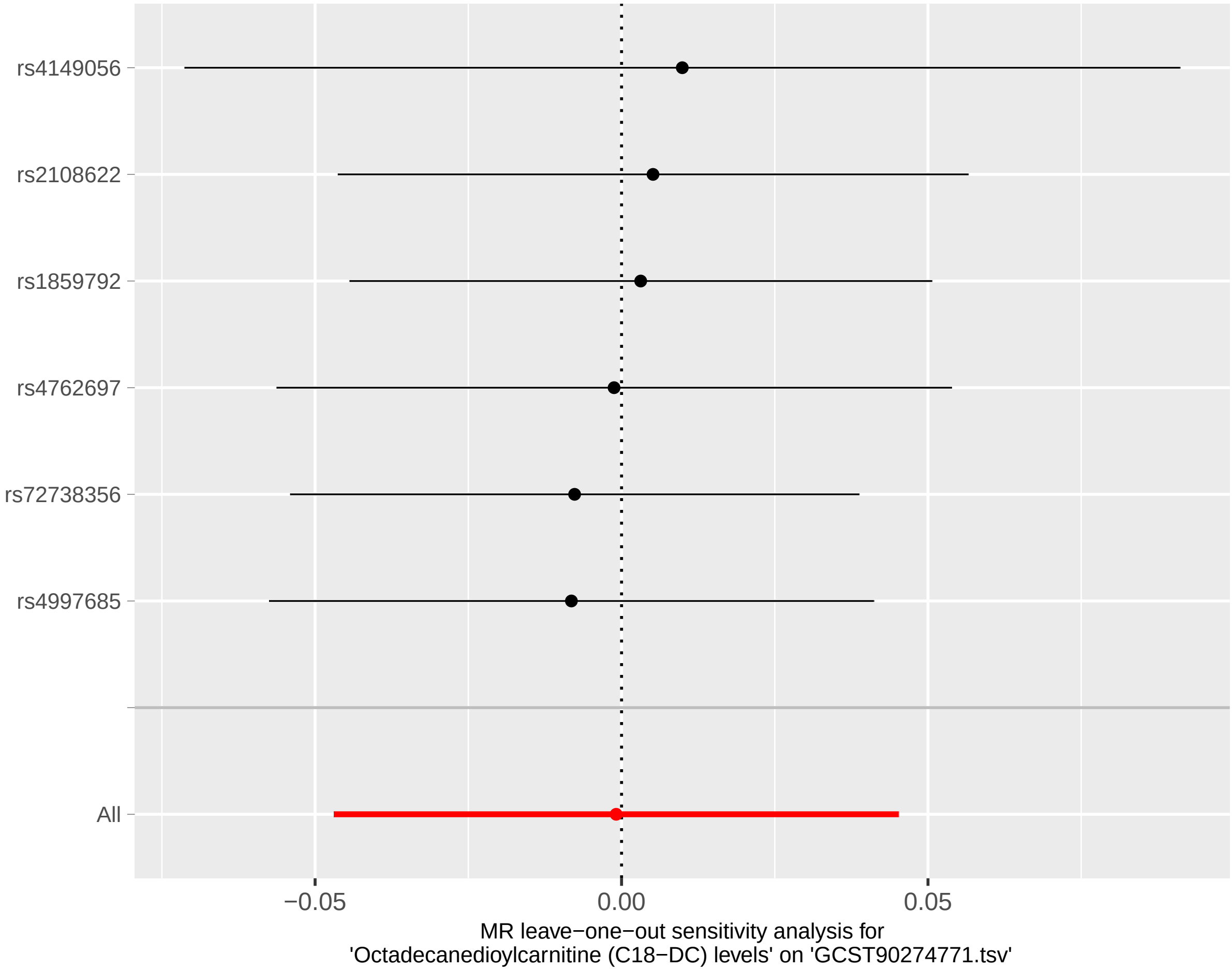
rs4149056
rs2108622
rs1859792
rs4762697
rs72738356
rs4997685
All
−0.05
0.00
0.05
MR leave−one−out sensitivity analysis for
'Octadecanedioylcarnitine (C18−DC) levels' on 'GCST90274771.tsv'

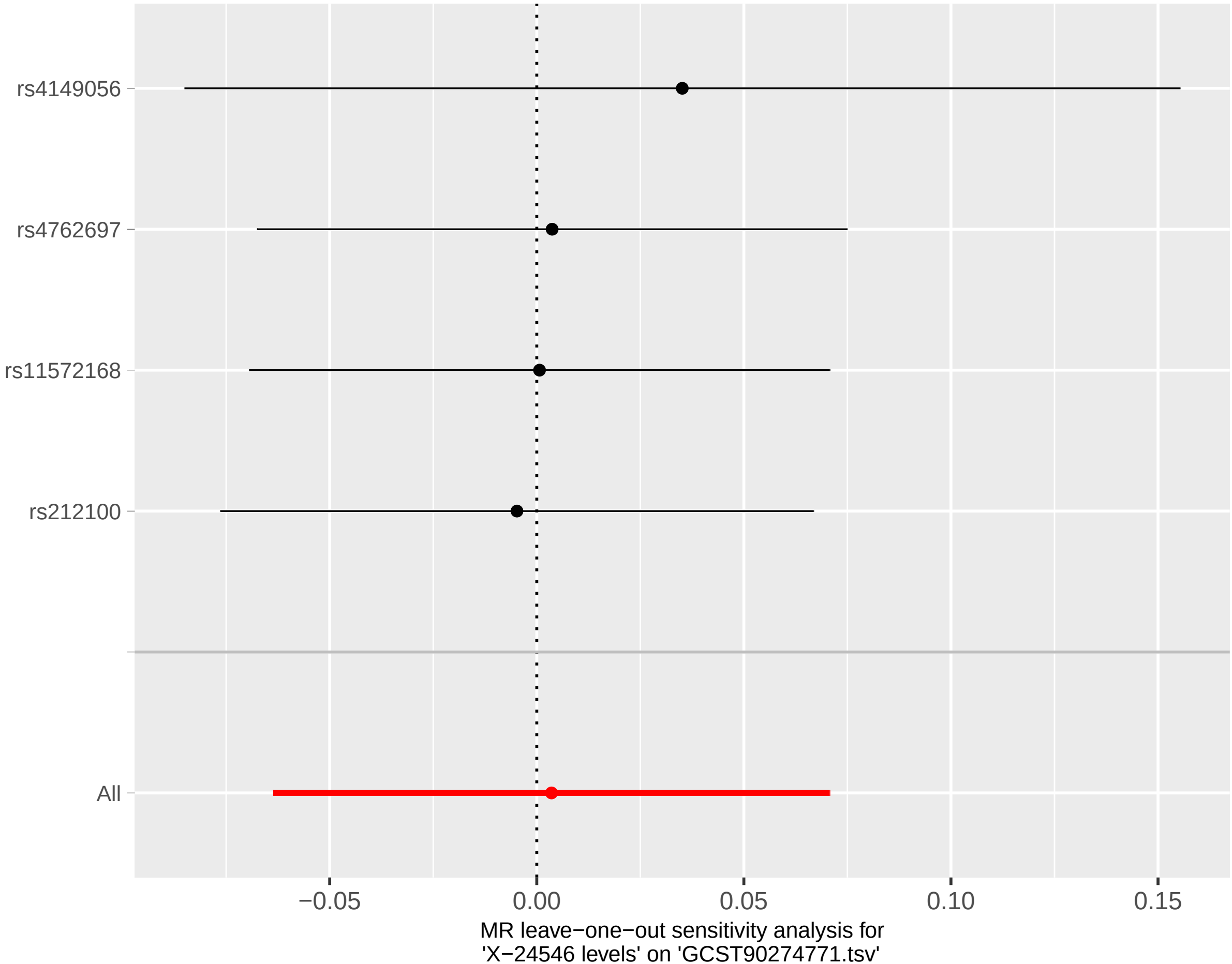
rs4149056
rs4762697
rs11572168
rs212100
All
−0.05
0.00
0.05
0.10
0.15
MR leave−one−out sensitivity analysis for
'X−24546 levels' on 'GCST90274771.tsv'

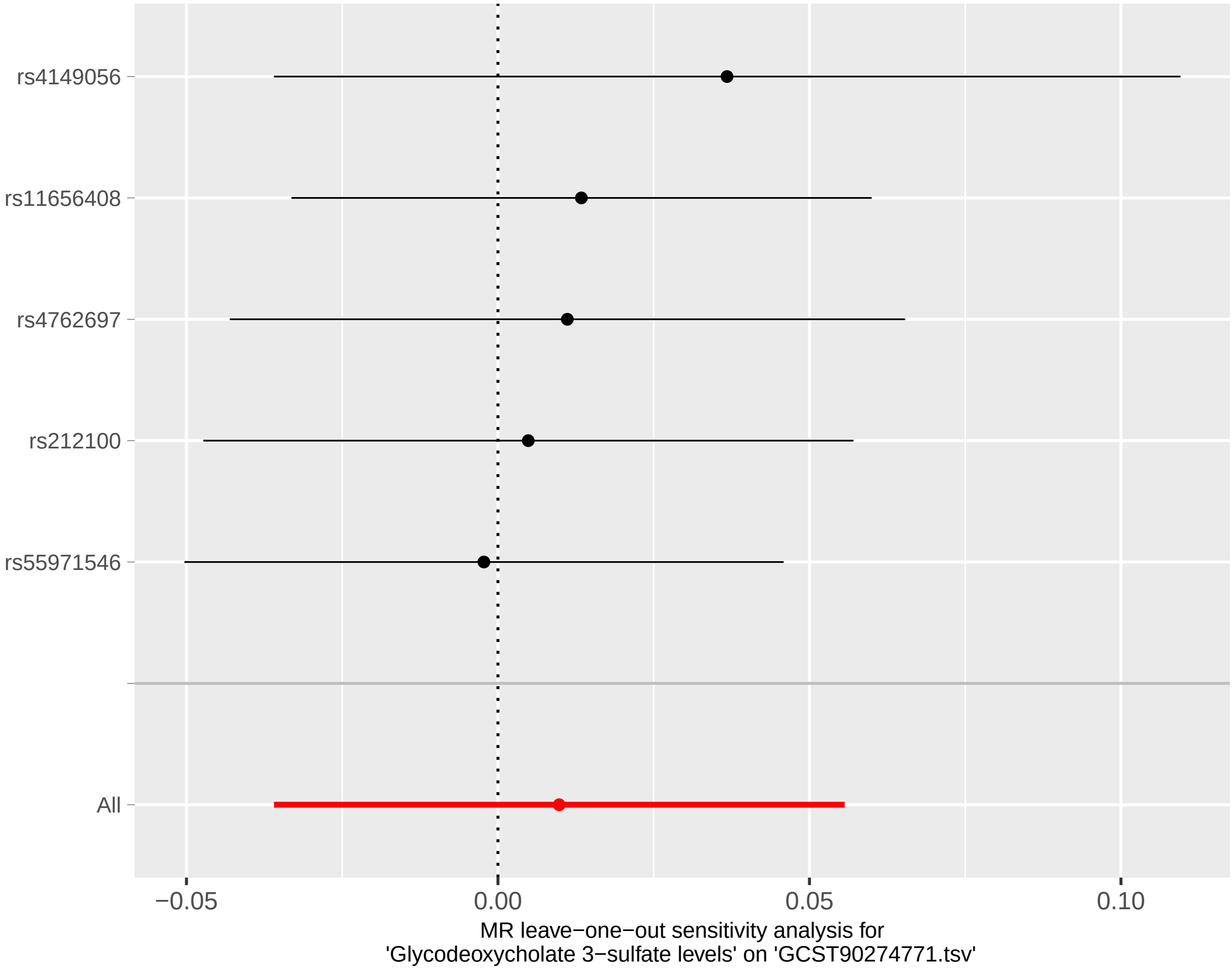

rs4149056
rs11656408
rs4762697
rs212100
rs55971546
All
−0.05
0.00
0.05
0.10
MR leave−one−out sensitivity analysis for
'Glycodeoxycholate 3−sulfate levels' on 'GCST90274771.tsv'

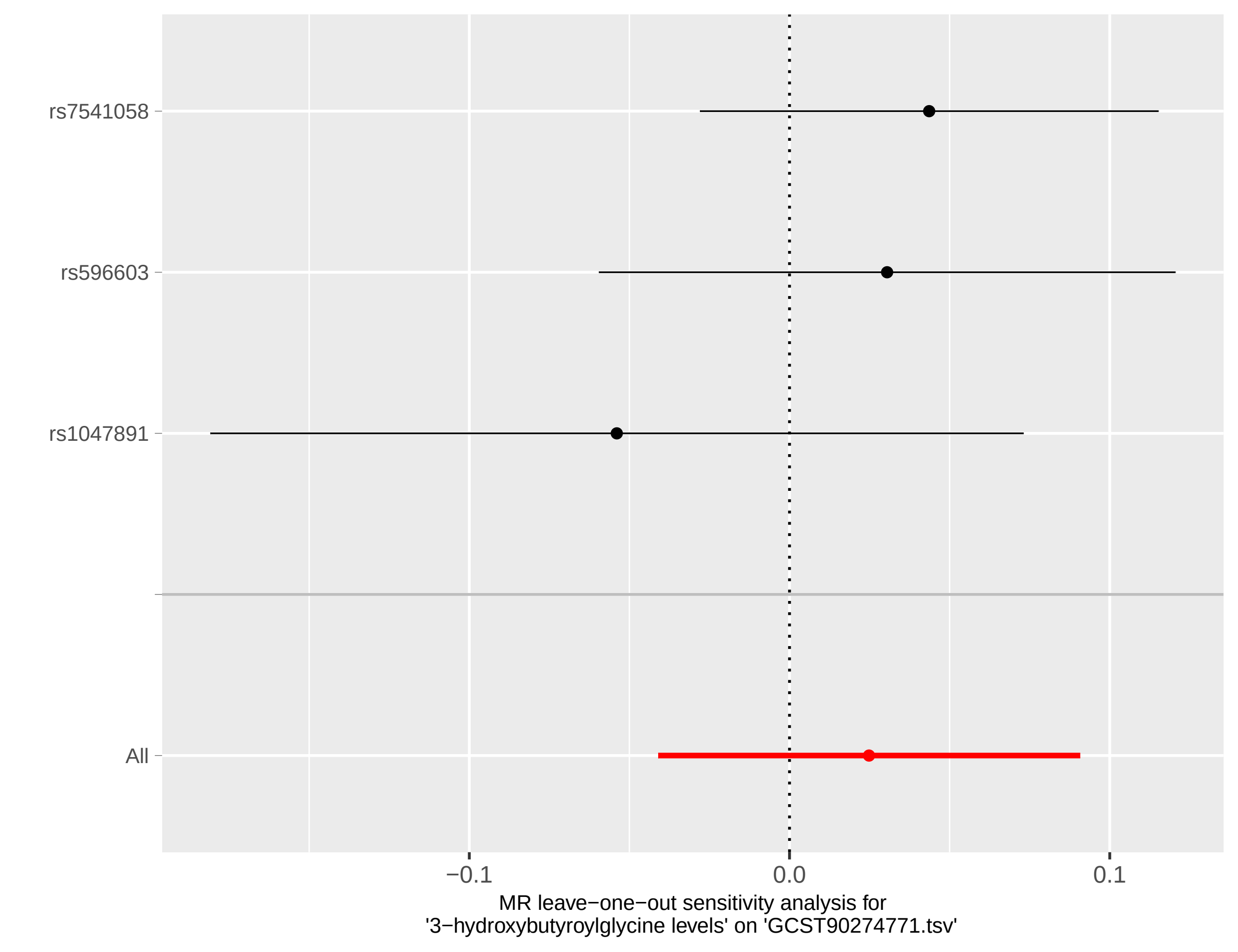
rs7541058
rs596603
rs1047891
All
−0.1
0.0
0.1
MR leave−one−out sensitivity analysis for
'3−hydroxybutyroylglycine levels' on 'GCST90274771.tsv'

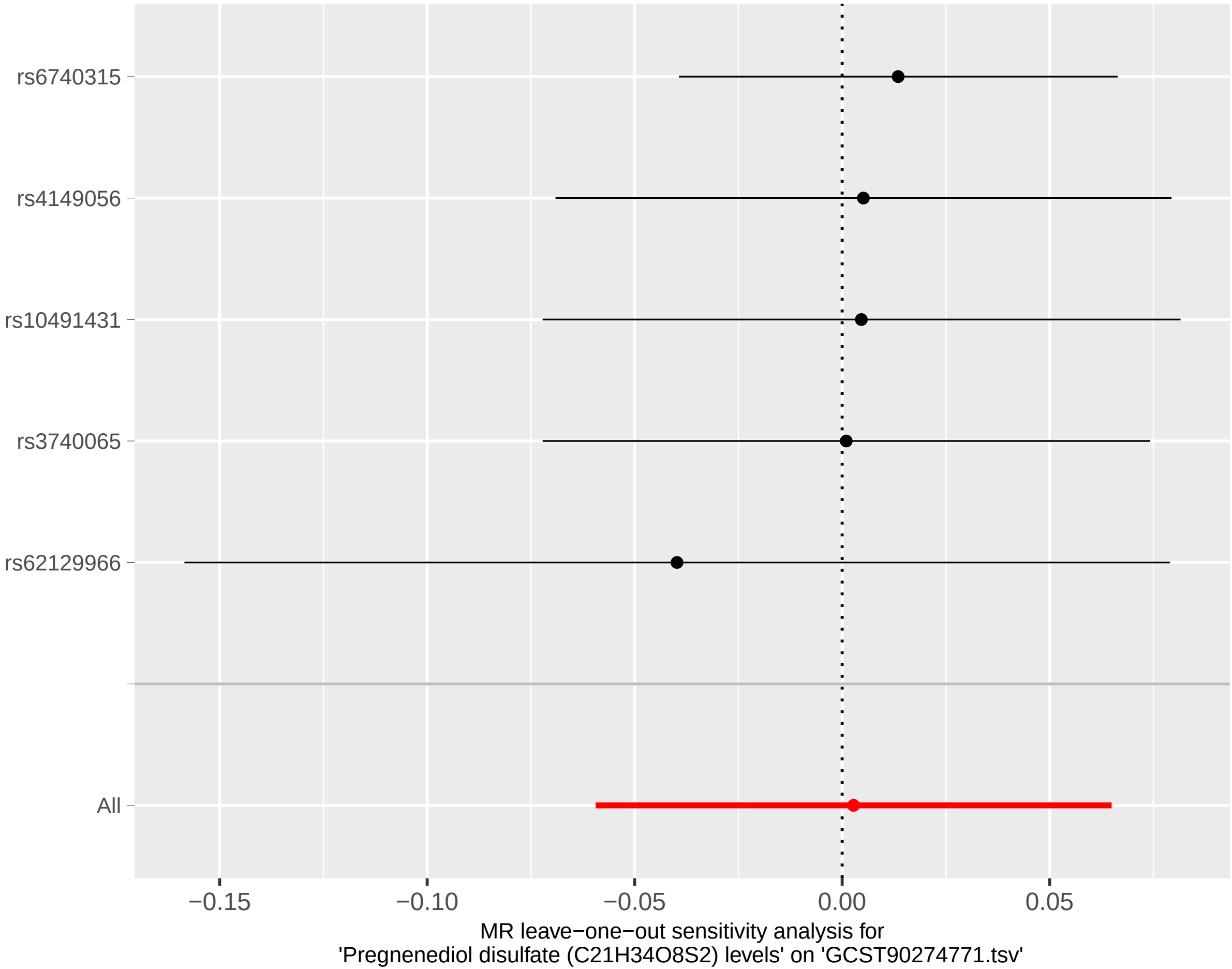

rs6740315
rs4149056
rs10491431
rs3740065
rs62129966
All
−0.15
−0.10
−0.05
0.00
0.05
MR leave−one−out sensitivity analysis for
'Pregnenediol disulfate (C21H34O8S2) levels' on 'GCST90274771.tsv'

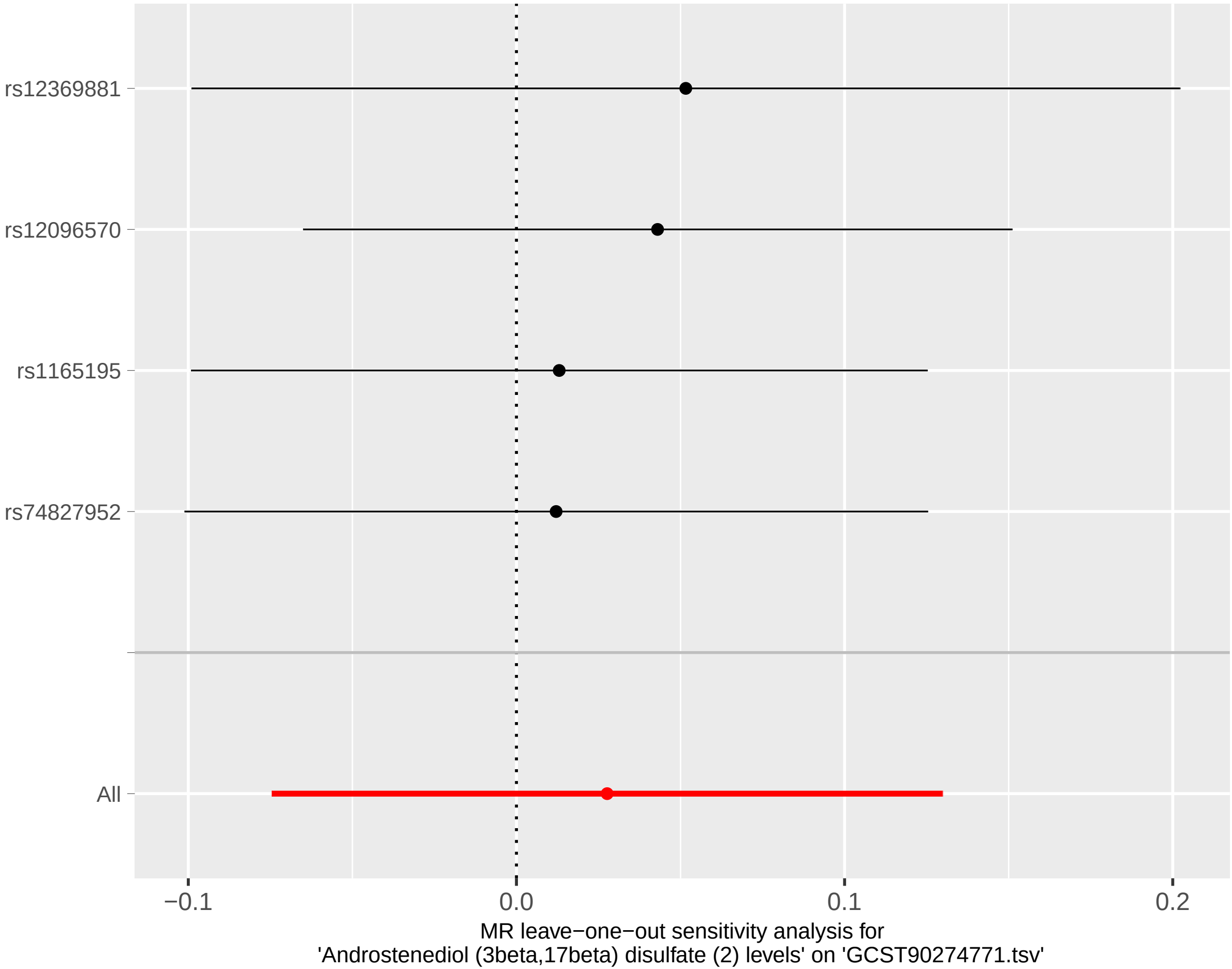
rs12369881
rs12096570
rs1165195
rs74827952
All
−0.1
0.0
0.1
0.2
MR leave−one−out sensitivity analysis for
'Androstenediol (3beta,17beta) disulfate (2) levels' on 'GCST90274771.tsv'

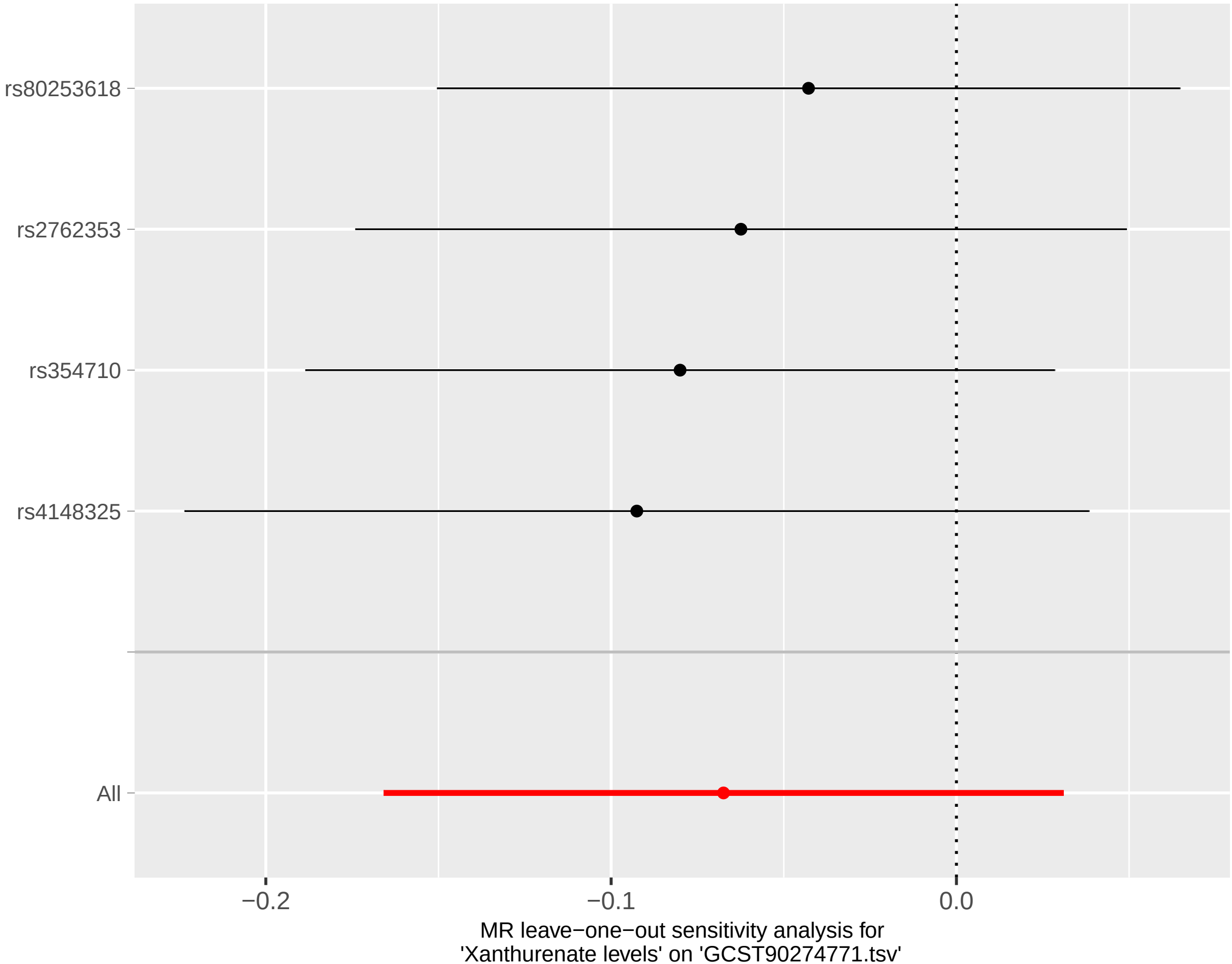
rs80253618
rs2762353
rs354710
rs4148325
All
−0.2
−0.1
0.0
MR leave−one−out sensitivity analysis for
'Xanthurenate levels' on 'GCST90274771.tsv'

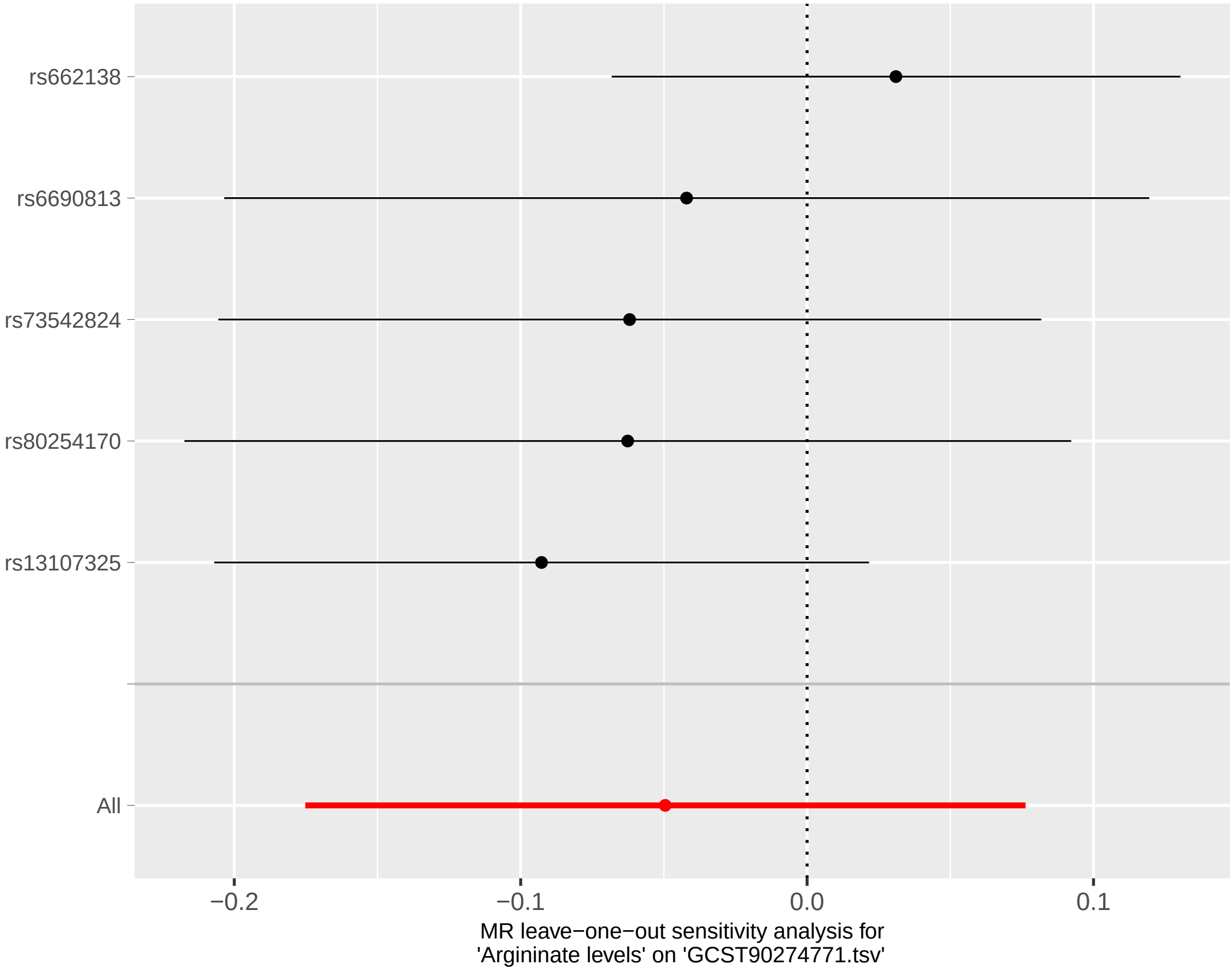
rs662138
rs6690813
rs73542824
rs80254170
rs13107325
All
−0.2
−0.1
0.0
0.1
MR leave−one−out sensitivity analysis for
'Argininate levels' on 'GCST90274771.tsv'

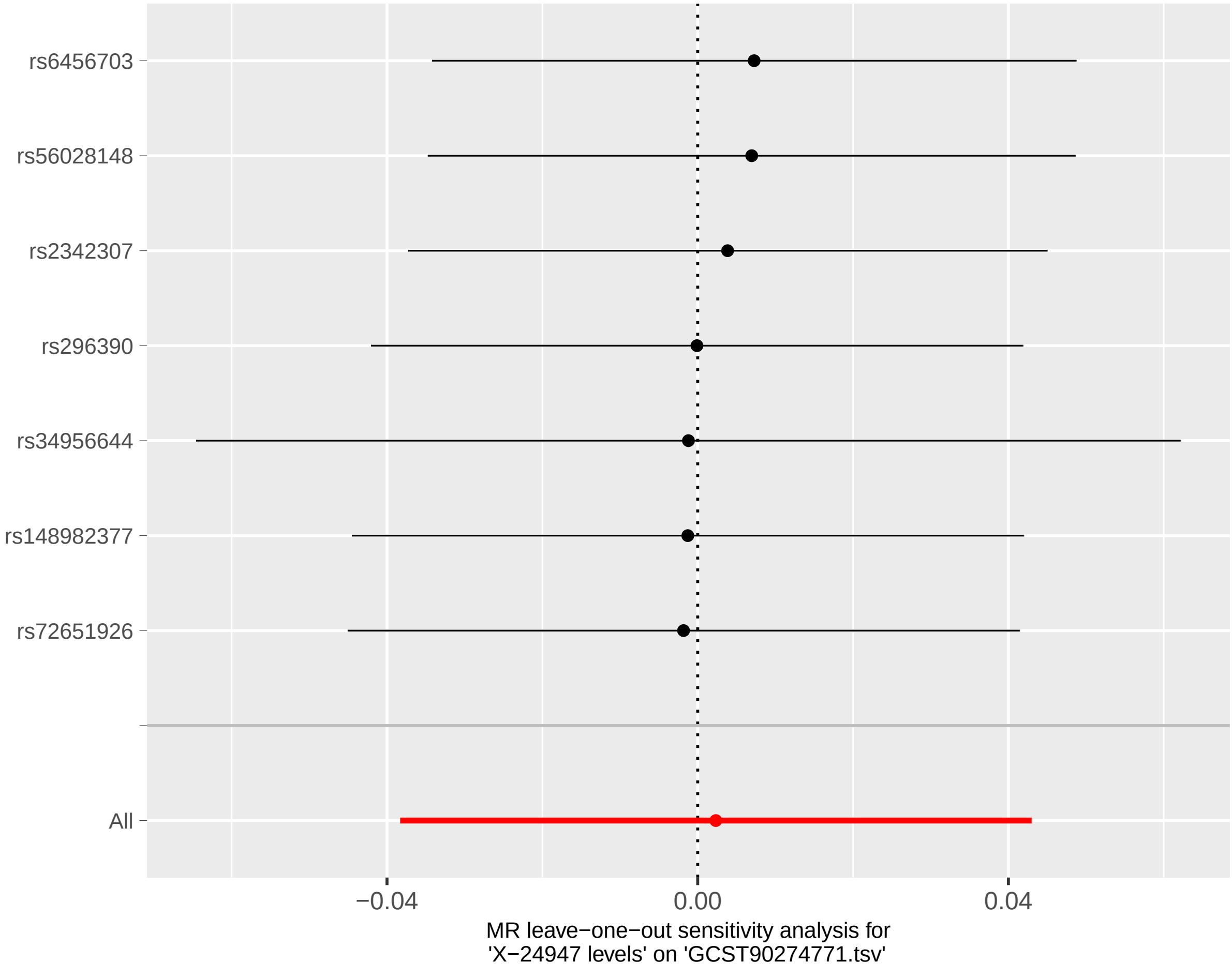

rs6456703
rs56028148
rs2342307
rs296390
rs34956644
rs148982377
rs72651926
All
−0.04
0.00
0.04
MR leave−one−out sensitivity analysis for
'X−24947 levels' on 'GCST90274771.tsv'

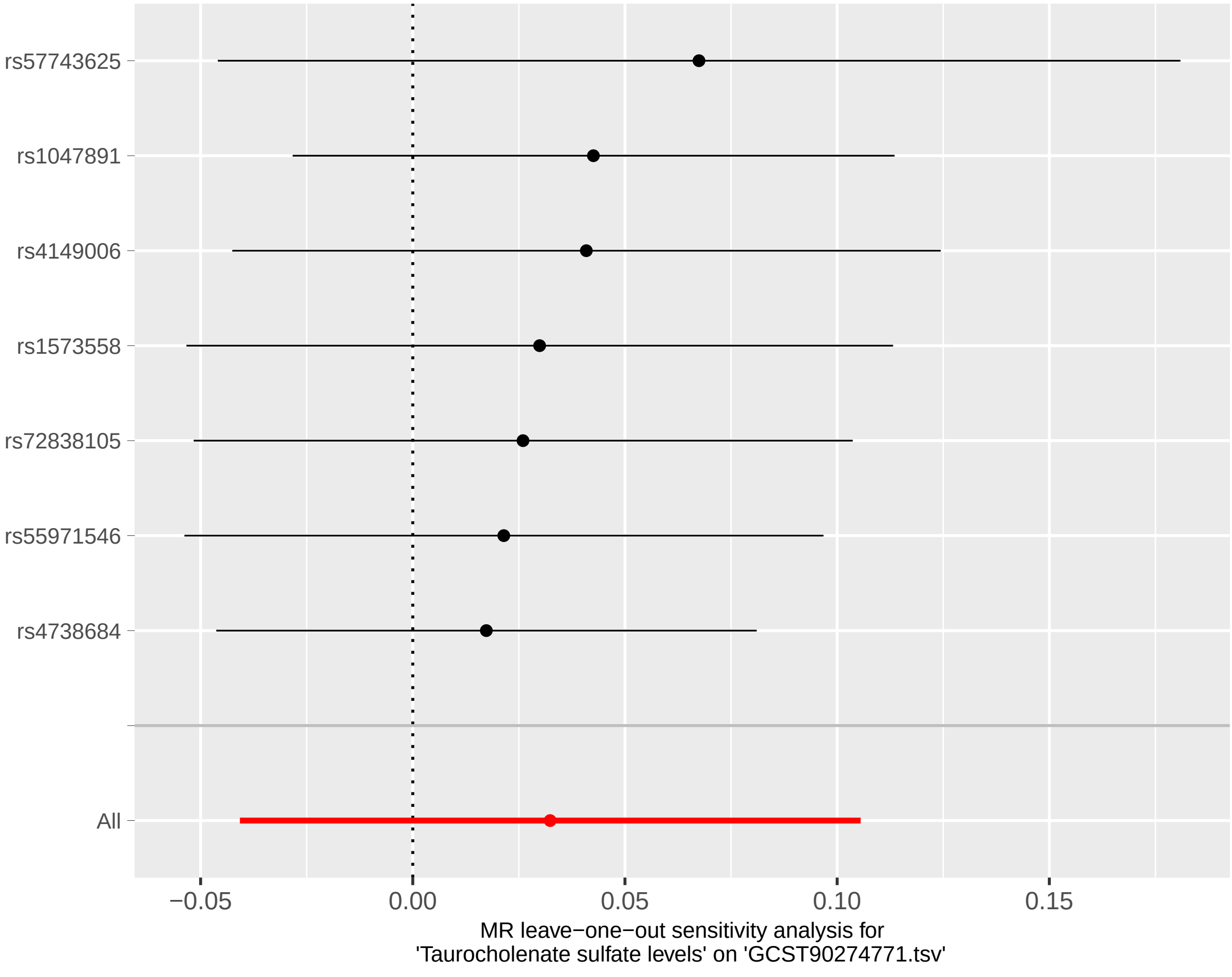
rs57743625
rs1047891
rs4149006
rs1573558
rs72838105
rs55971546
rs4738684
All
−0.05
0.00
0.05
0.10
0.15
MR leave−one−out sensitivity analysis for
'Taurocholenate sulfate levels' on 'GCST90274771.tsv'

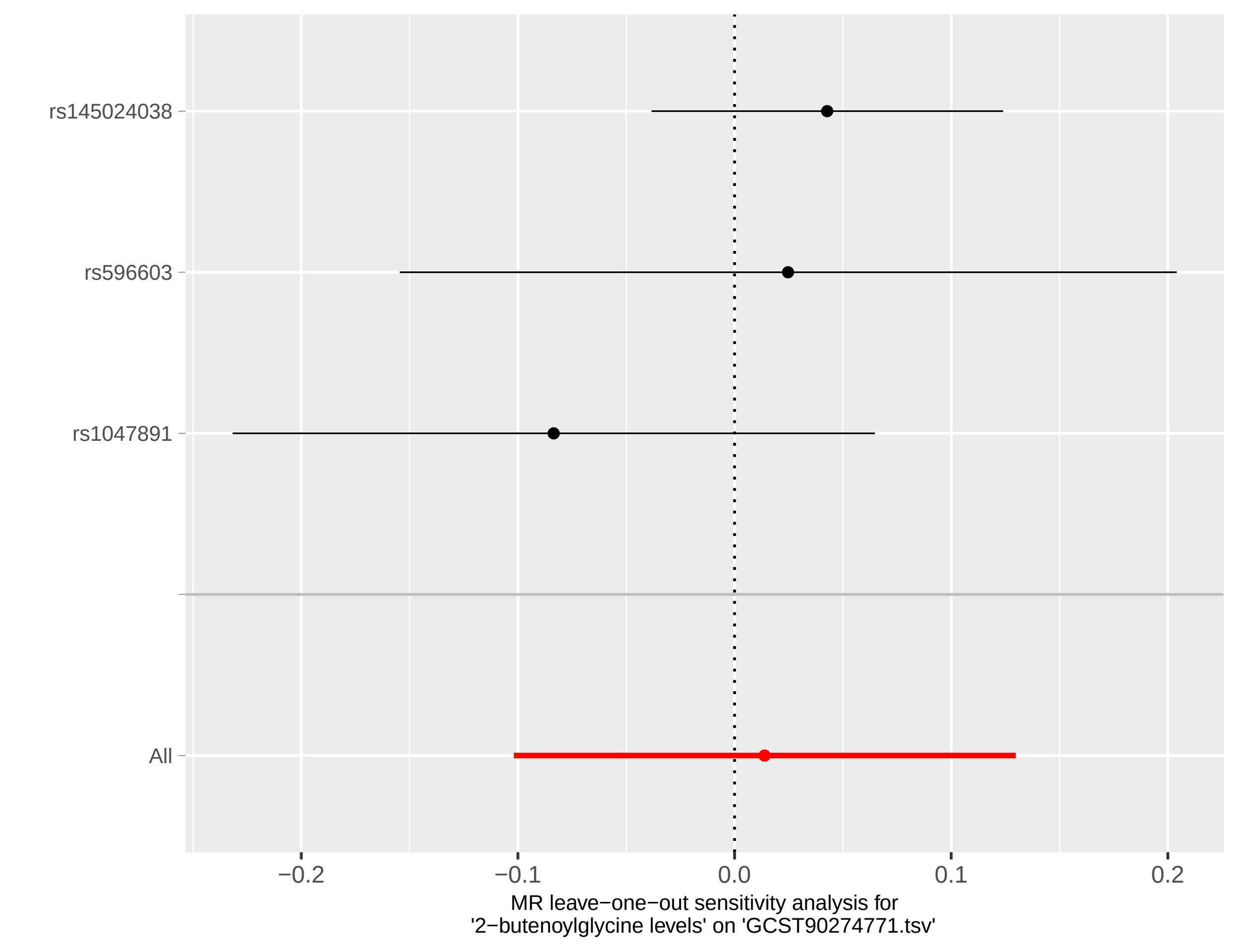
rs145024038
rs596603
rs1047891
All
−0.2
−0.1
0.0
0.1
0.2
MR leave−one−out sensitivity analysis for
'2−butenoylglycine levels' on 'GCST90274771.tsv'

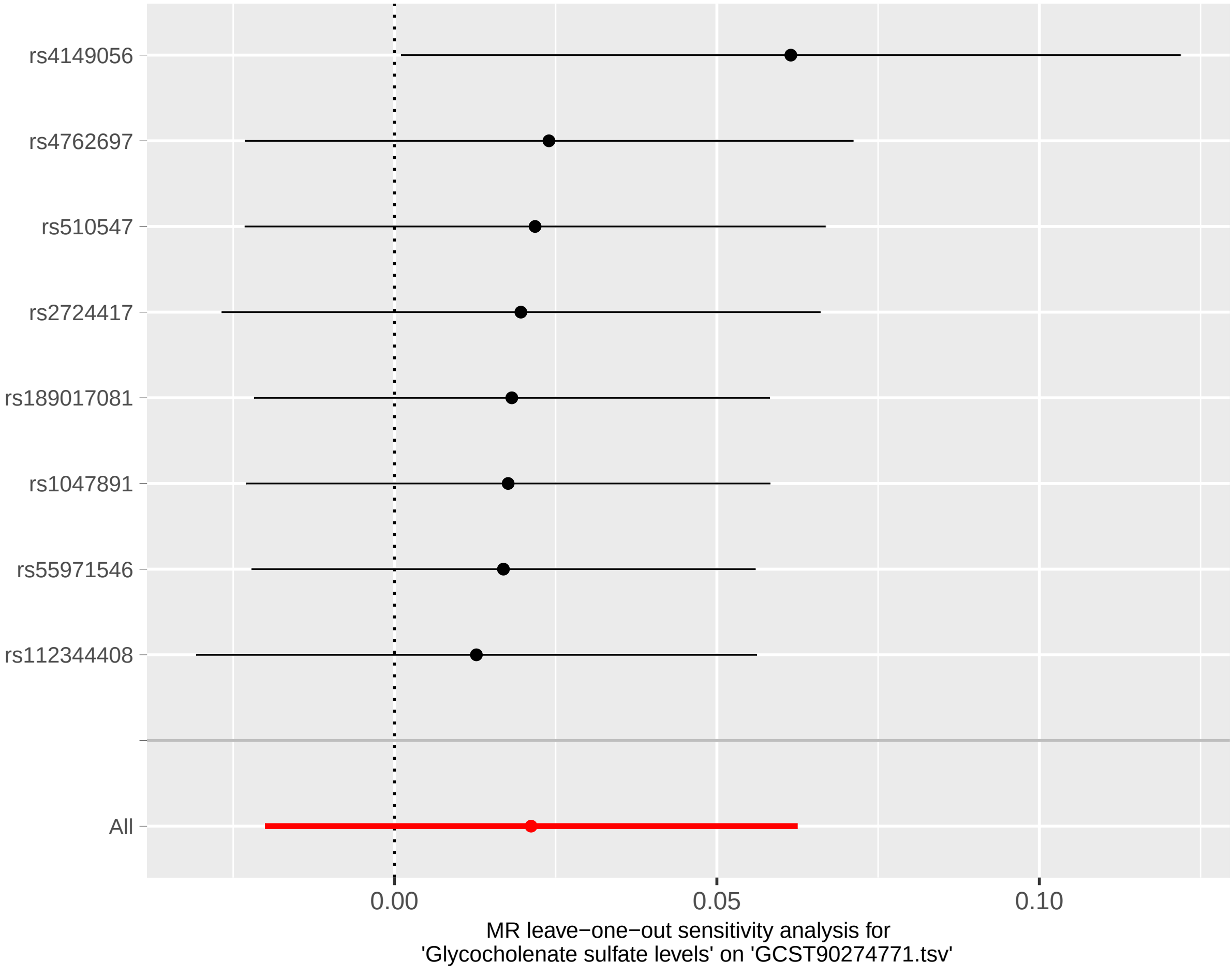
rs4149056
rs4762697
rs510547
rs2724417
rs189017081
rs1047891
rs55971546
rs112344408
All
0.00
0.05
0.10
MR leave−one−out sensitivity analysis for
'Glycocholenate sulfate levels' on 'GCST90274771.tsv'

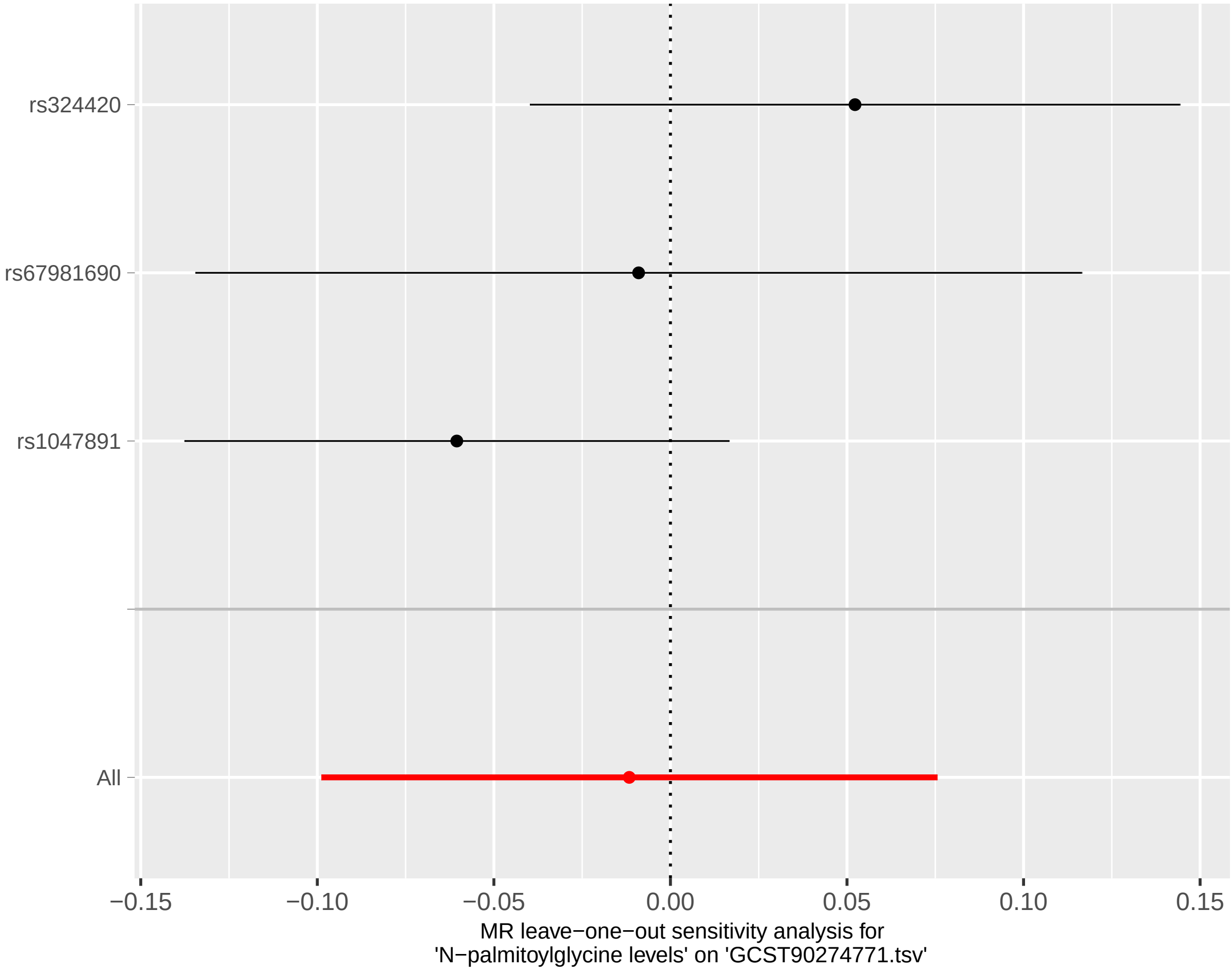
rs324420
rs67981690
rs1047891
All
−0.15
−0.10
−0.05
0.00
0.05
0.10
0.15
MR leave−one−out sensitivity analysis for
'N−palmitoylglycine levels' on 'GCST90274771.tsv'

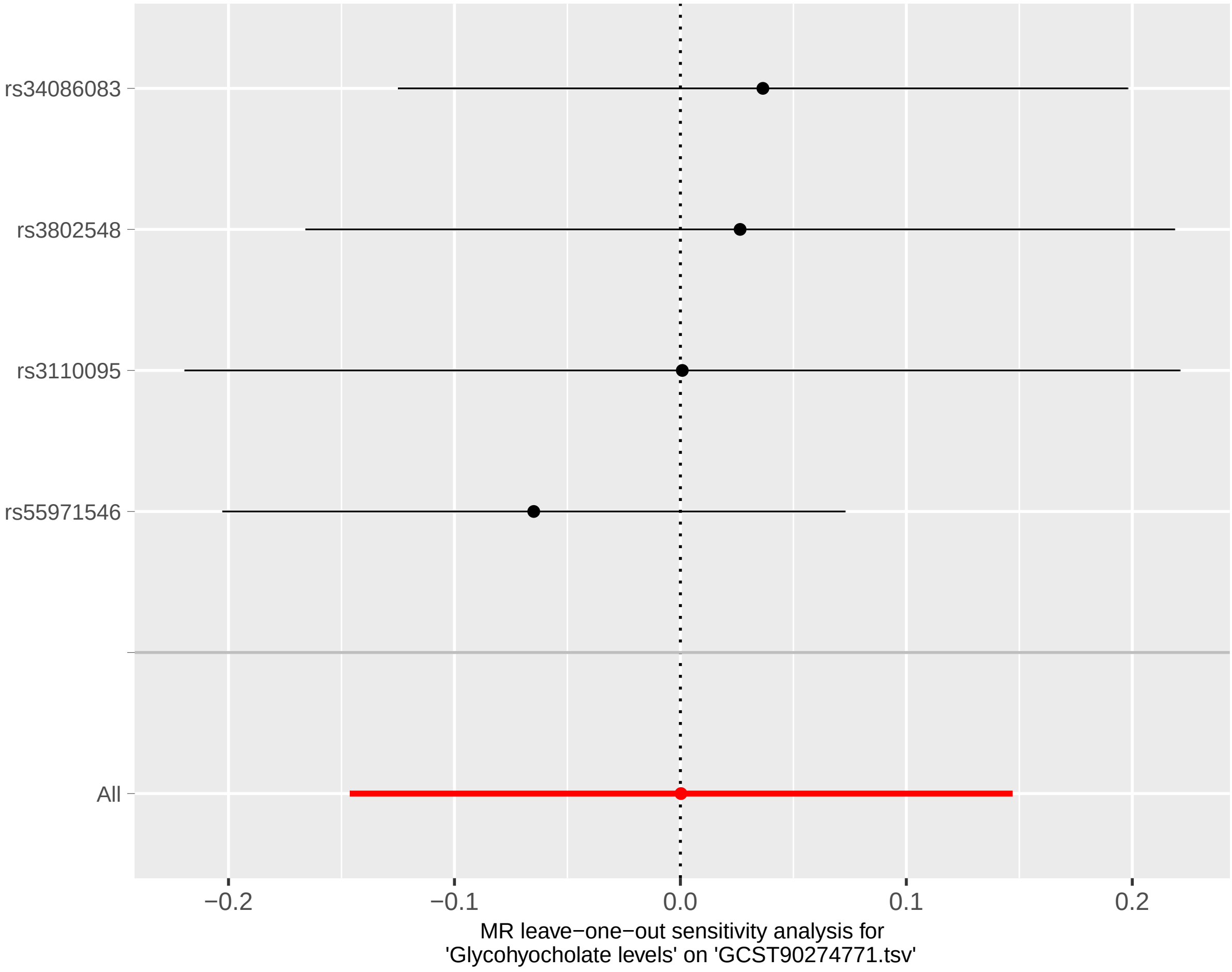

rs34086083
rs3802548
rs3110095
rs55971546
All
−0.2
−0.1
0.0
0.1
0.2
MR leave−one−out sensitivity analysis for
'Glycohyocholate levels' on 'GCST90274771.tsv'

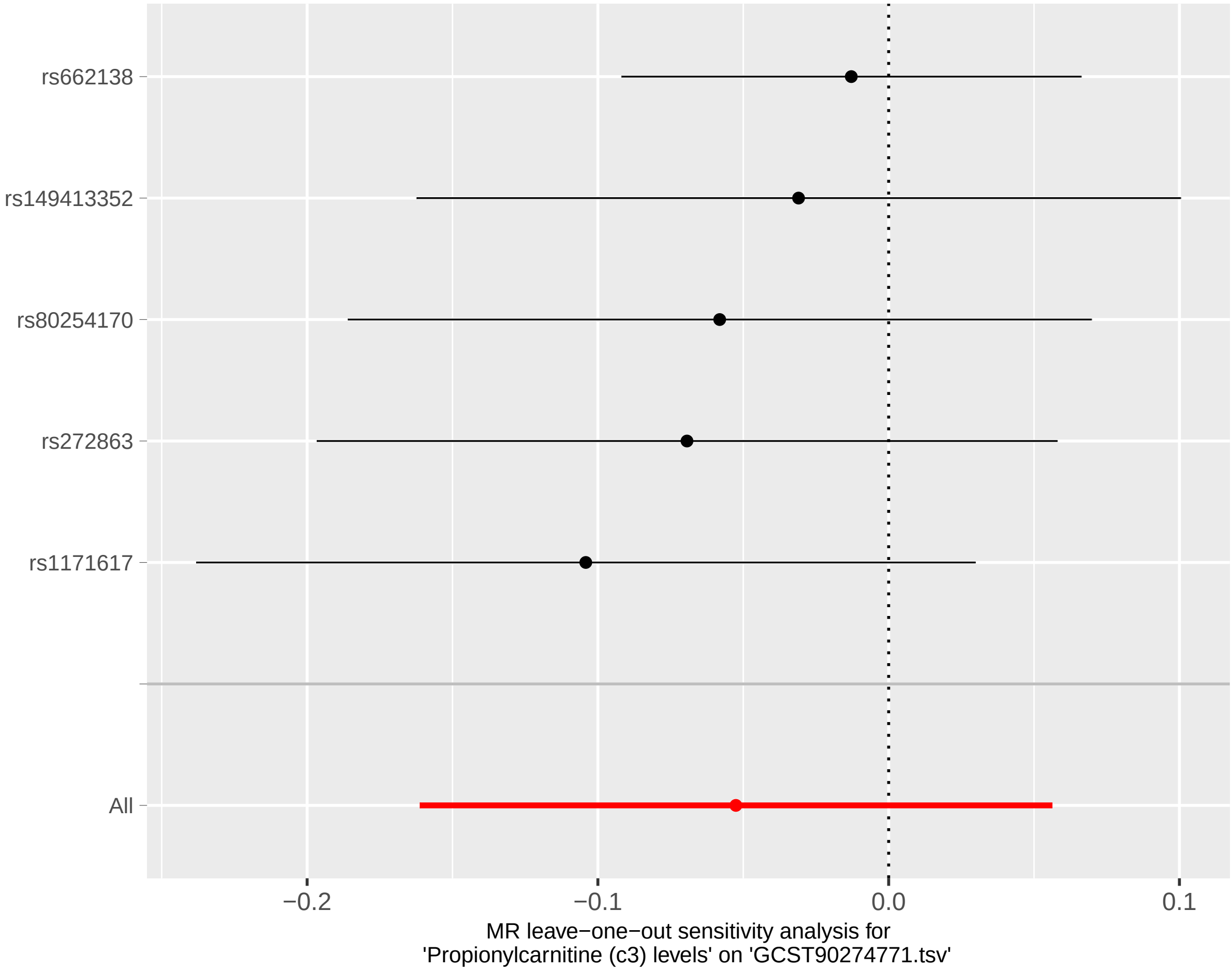
rs662138
rs149413352
rs80254170
rs272863
rs1171617
All
−0.2
−0.1
0.0
0.1
MR leave−one−out sensitivity analysis for
'Propionylcarnitine (c3) levels' on 'GCST90274771.tsv'

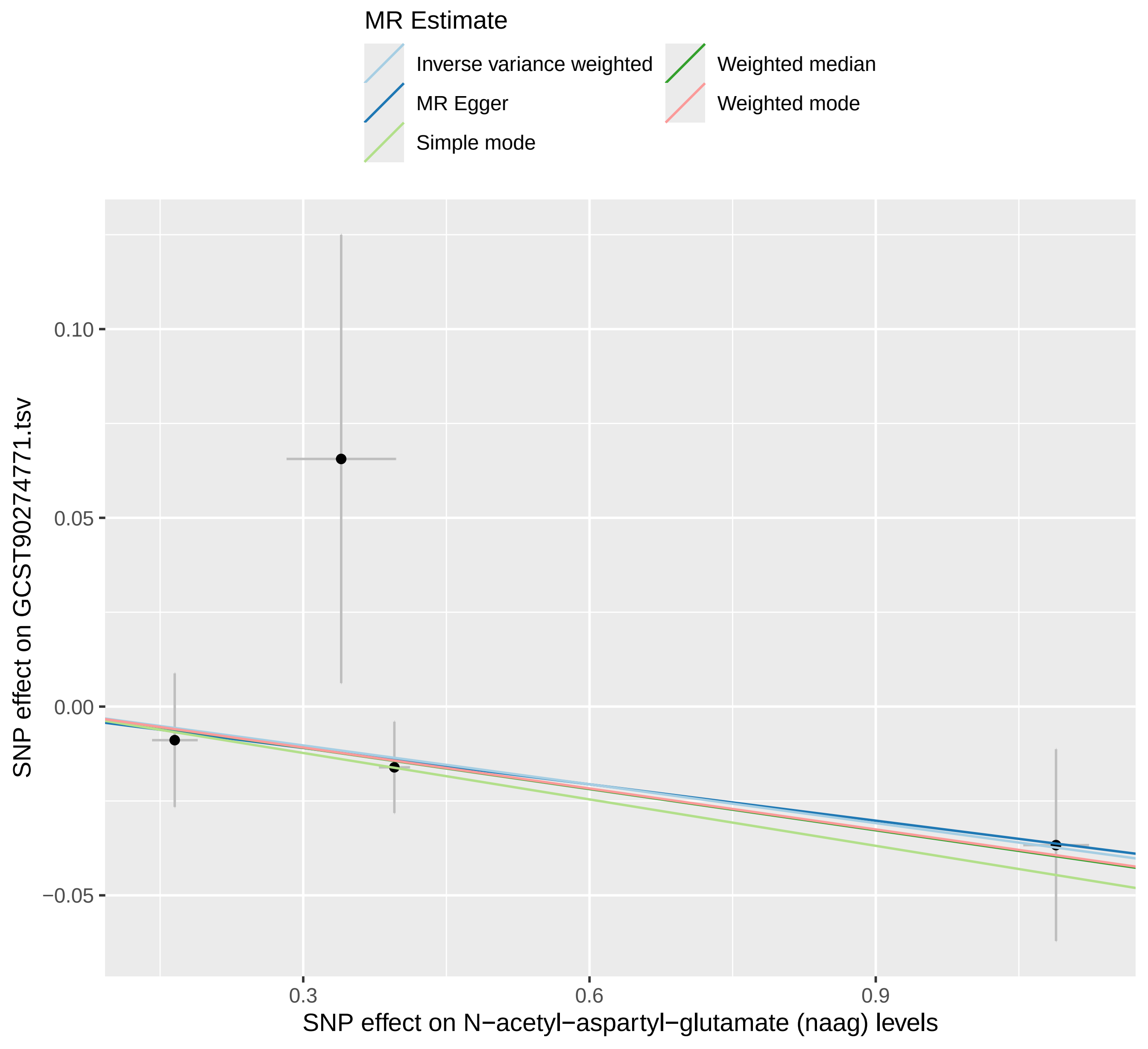

MR Estimate
Inverse variance weighted
MR Egger
Simple mode
Weighted median
Weighted mode
0.10
0.05
0.00
−0.05
0.3
0.6
0.9
SNP effect on GCST90274771.tsv
SNP effect on N−acetyl−aspartyl−glutamate (naag) levels

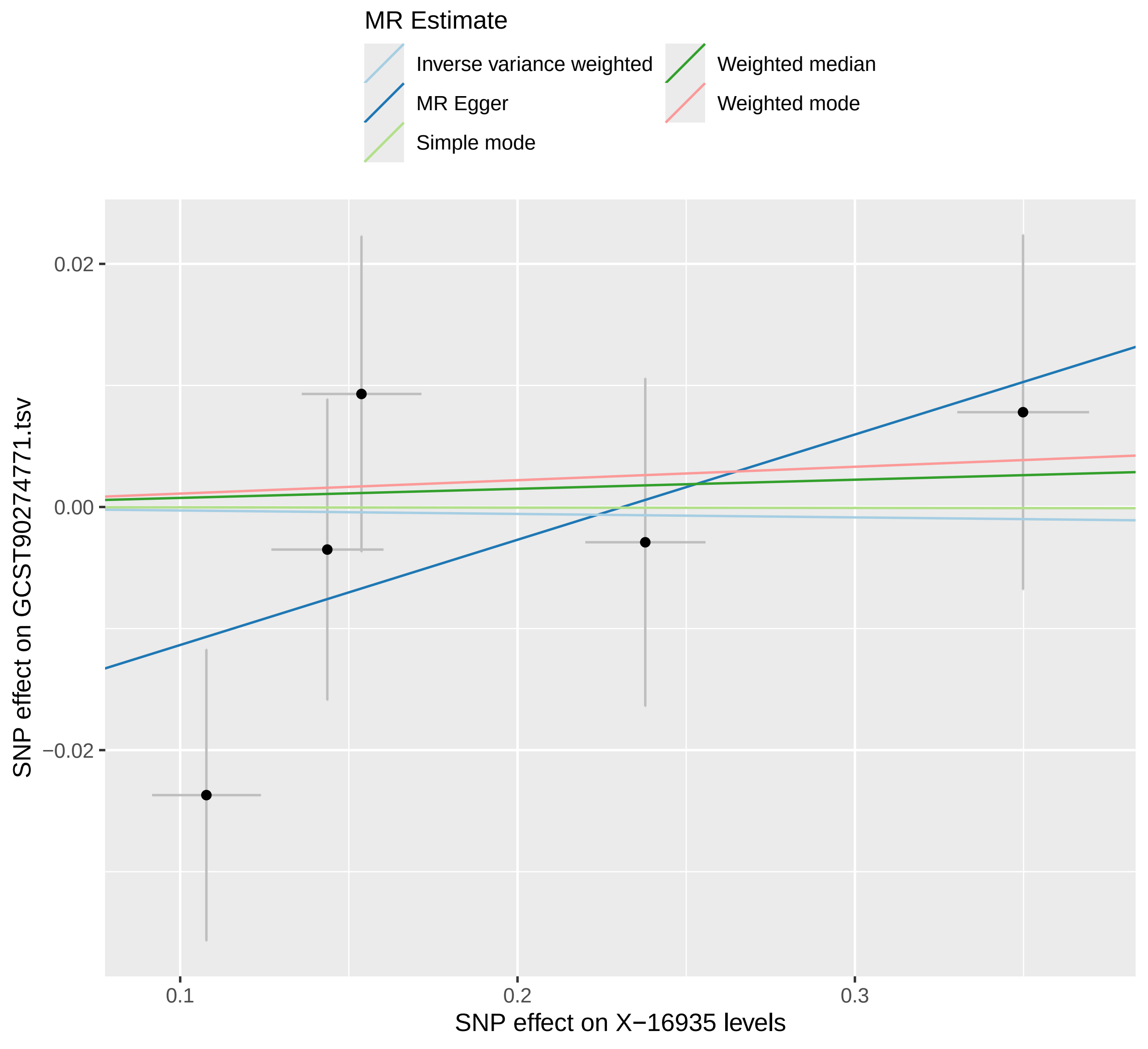
MR Estimate
Inverse variance weighted
MR Egger
Simple mode
Weighted median
Weighted mode
0.02
0.00
−0.02
0.1
0.2
0.3
SNP effect on GCST90274771.tsv
SNP effect on X−16935 levels

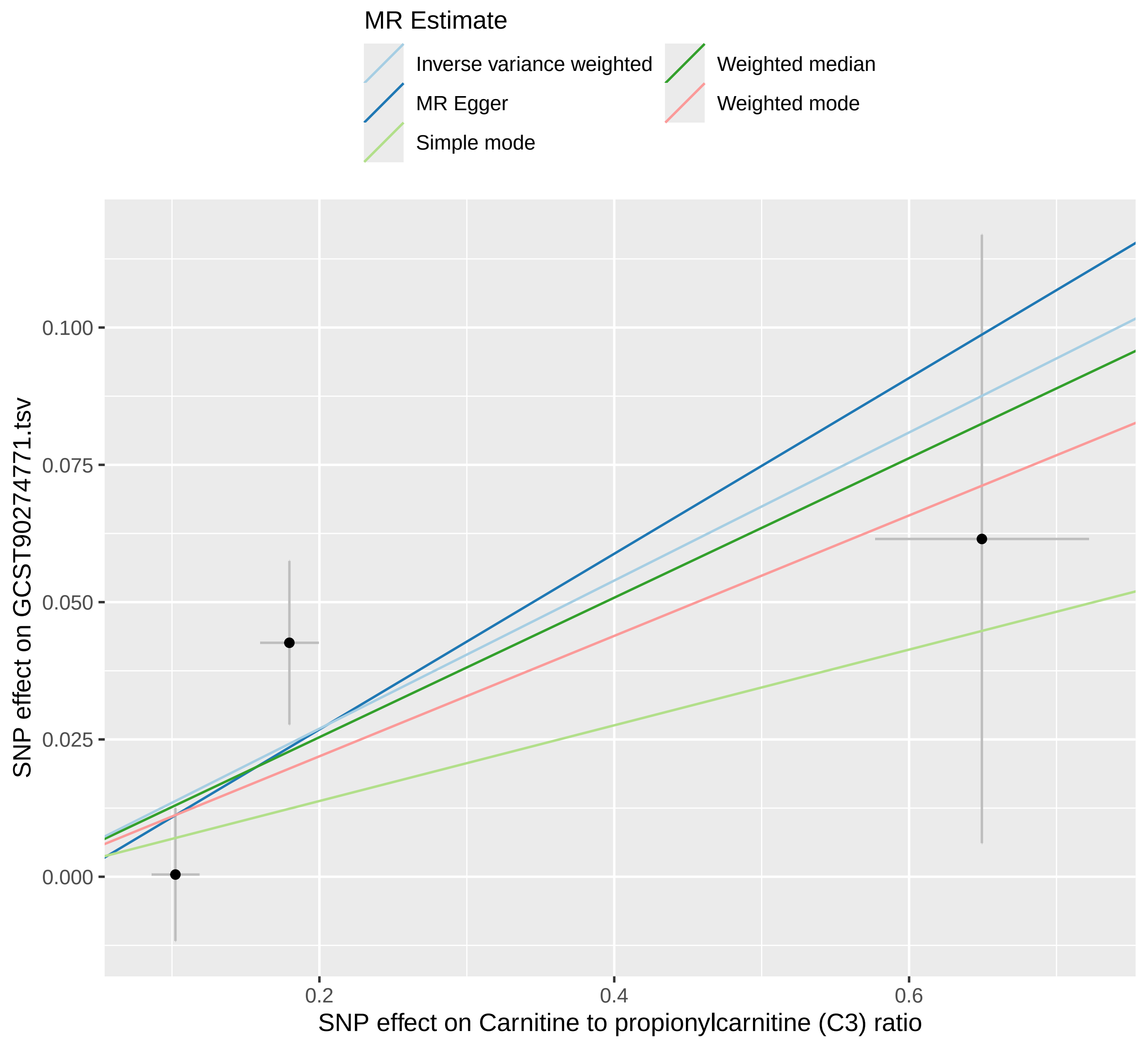
MR Estimate
Inverse variance weighted
MR Egger
Simple mode
Weighted median
Weighted mode
0.100
0.075
0.050
0.025
0.000
0.2
0.4
0.6
SNP effect on GCST90274771.tsv
SNP effect on Carnitine to propionylcarnitine (C3) ratio

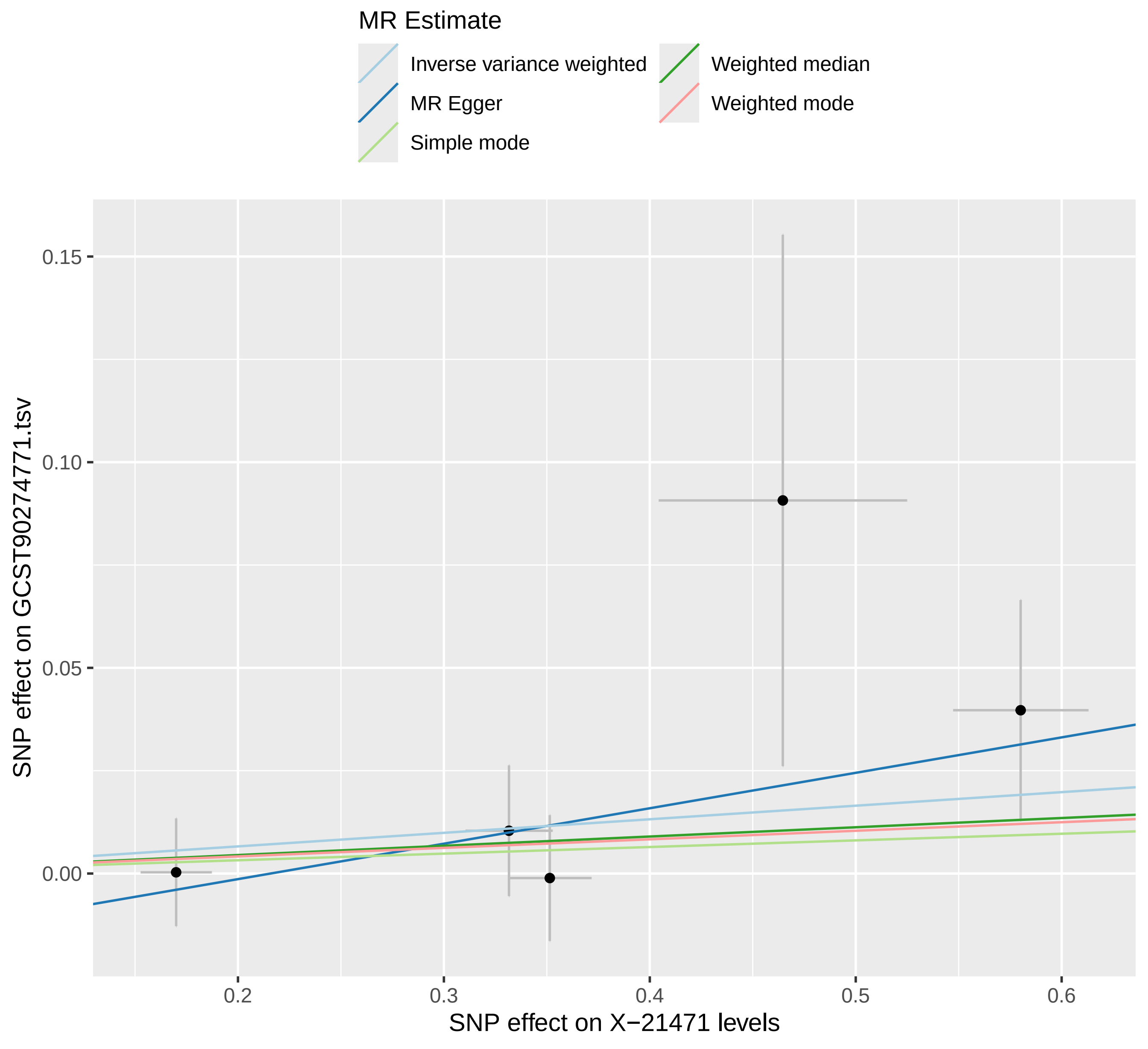
MR Estimate
Inverse variance weighted
MR Egger
Simple mode
Weighted median
Weighted mode
SNP effect on GCST90274771.tsv
SNP effect on X−21471 levels
0.15
0.10
0.05
0.00
0.2
0.3
0.4
0.5
0.6

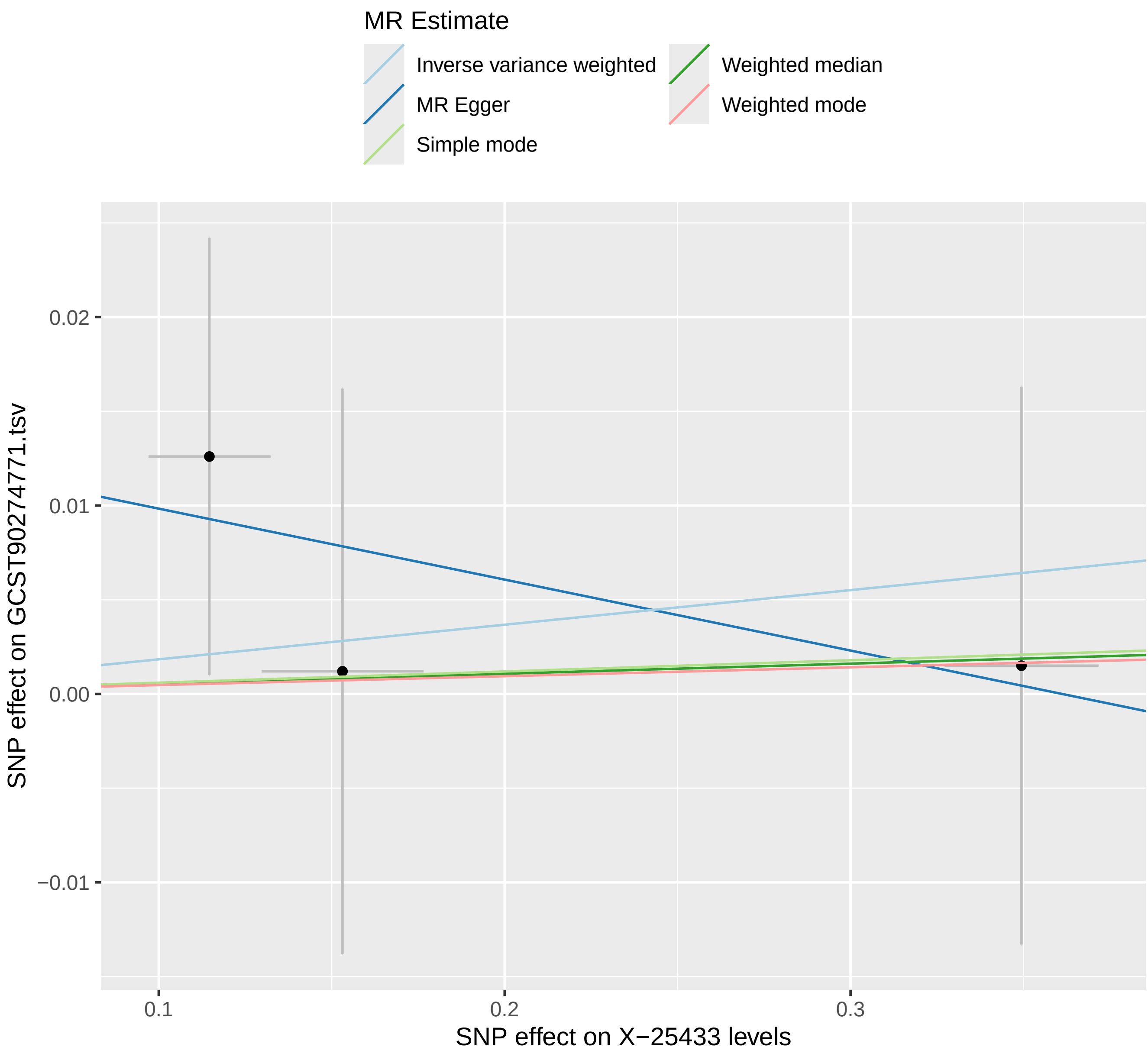
MR Estimate
Inverse variance weighted
MR Egger
Simple mode
Weighted median
Weighted mode
0.02
0.01
0.00
−0.01
0.1
0.2
0.3
SNP effect on GCST90274771.tsv
SNP effect on X−25433 levels

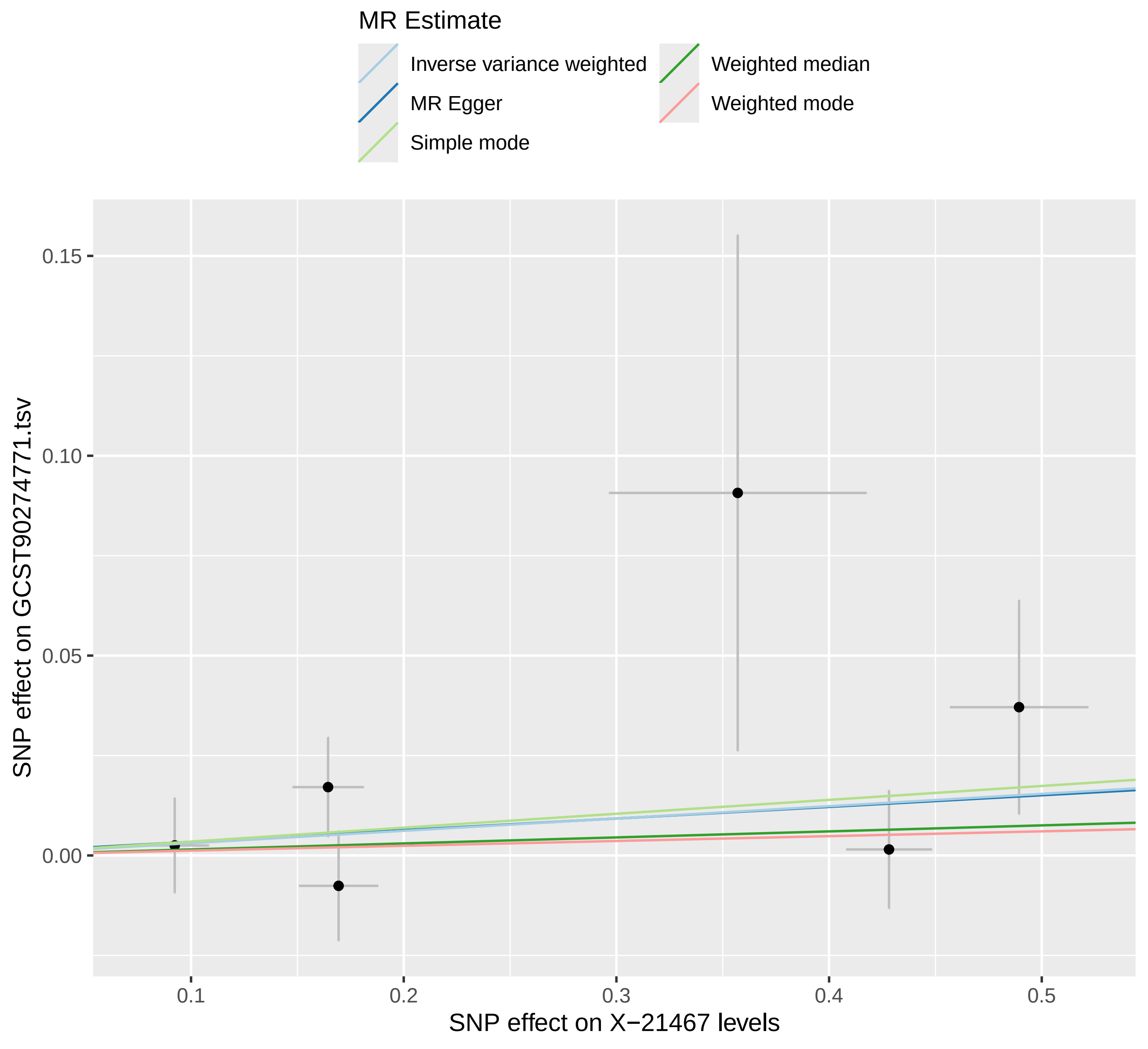
MR Estimate
Inverse variance weighted
MR Egger
Simple mode
Weighted median
Weighted mode
0.15
0.10
0.05
0.00
0.1
0.2
0.3
0.4
0.5
SNP effect on GCST90274771.tsv
SNP effect on X−21467 levels

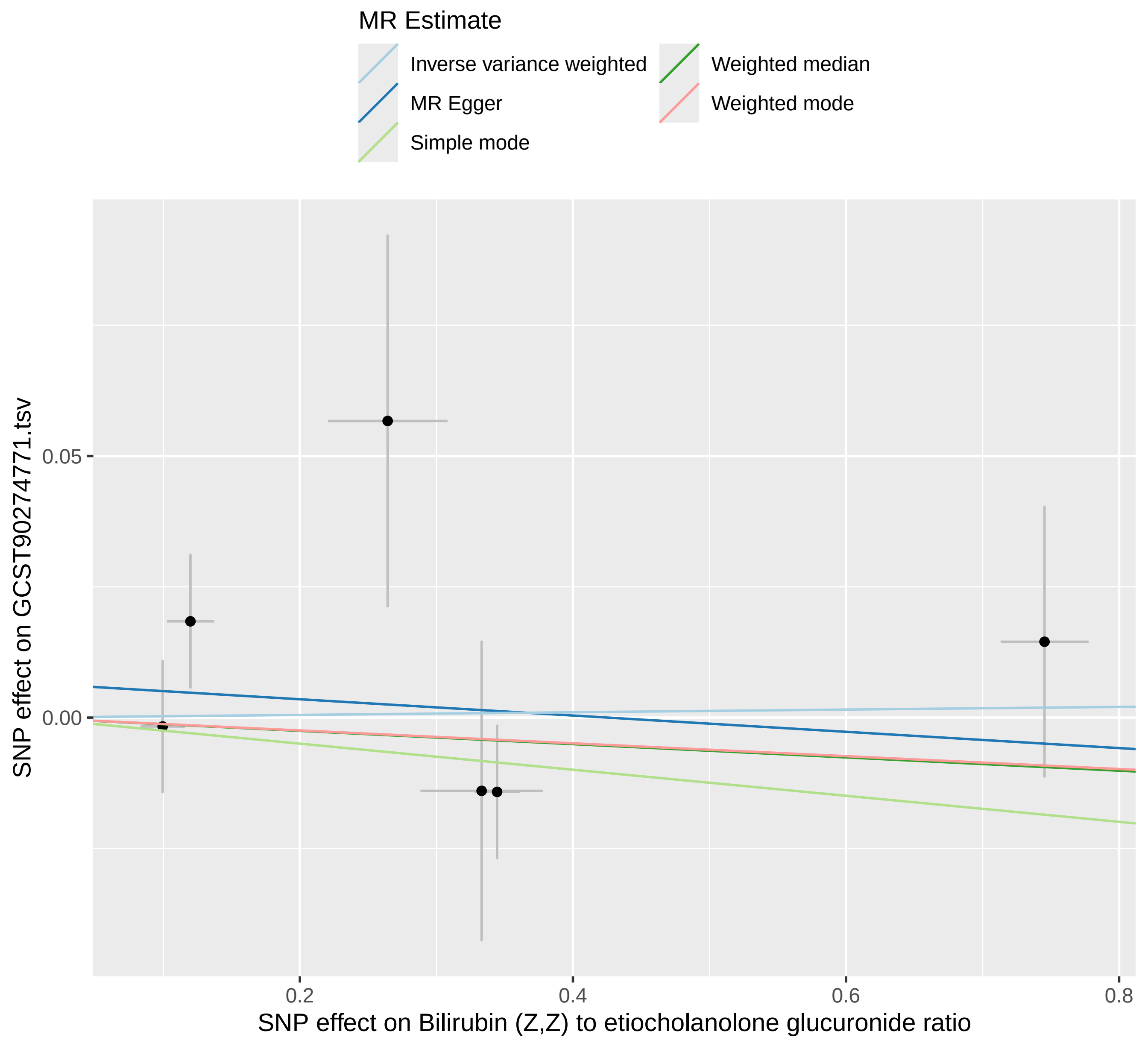

MR Estimate
Inverse variance weighted
MR Egger
Simple mode
Weighted median
Weighted mode
SNP effect on GCST90274771.tsv
0.05
0.00
0.2
0.4
0.6
0.8
SNP effect on Bilirubin (Z,Z) to etiocholanolone glucuronide ratio

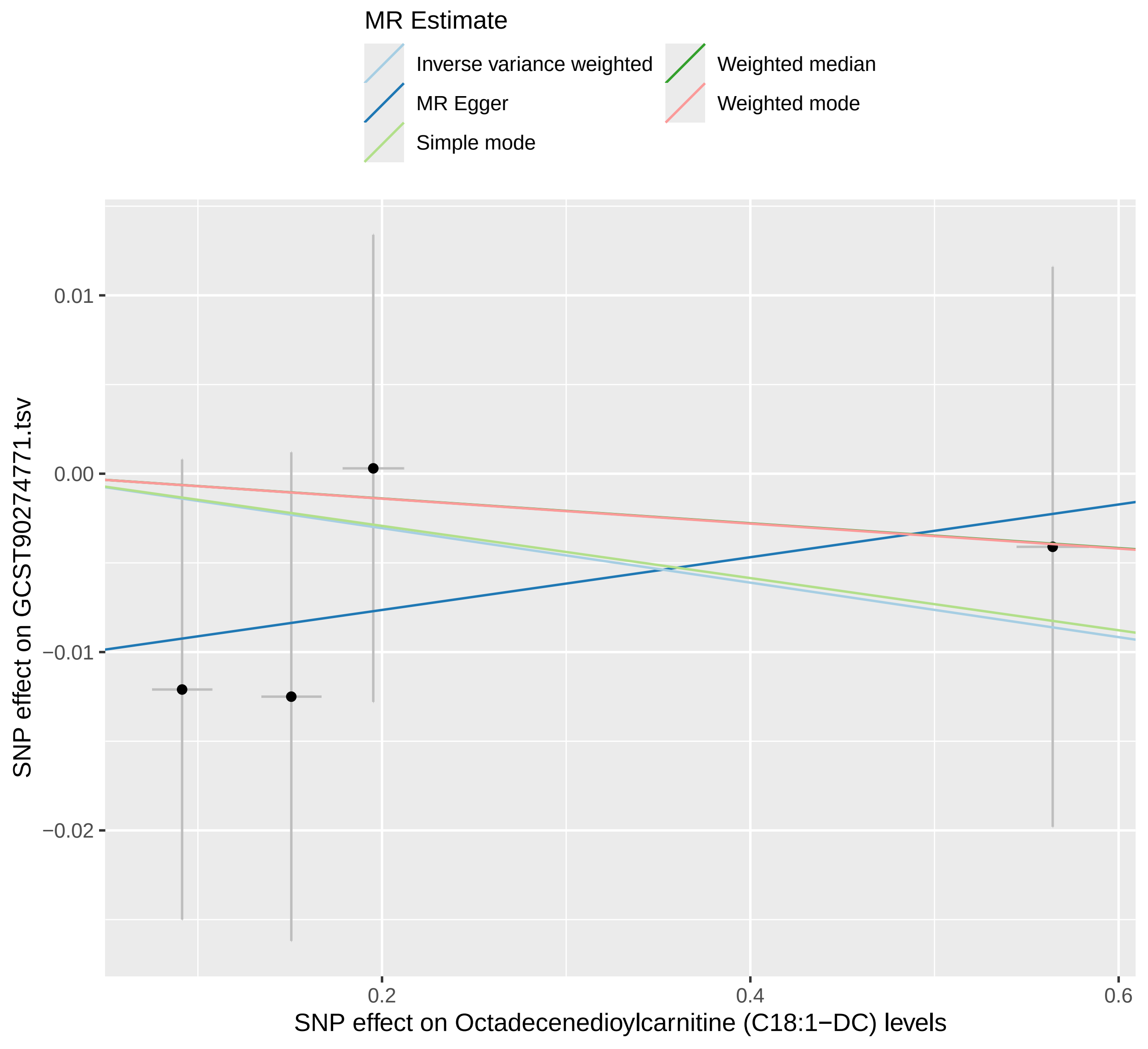

MR Estimate
Inverse variance weighted
MR Egger
Simple mode
Weighted median
Weighted mode
0.01
0.00
−0.01
−0.02
0.2
0.4
0.6
SNP effect on GCST90274771.tsv
SNP effect on Octadecenedioylcarnitine (C18:1−DC) levels

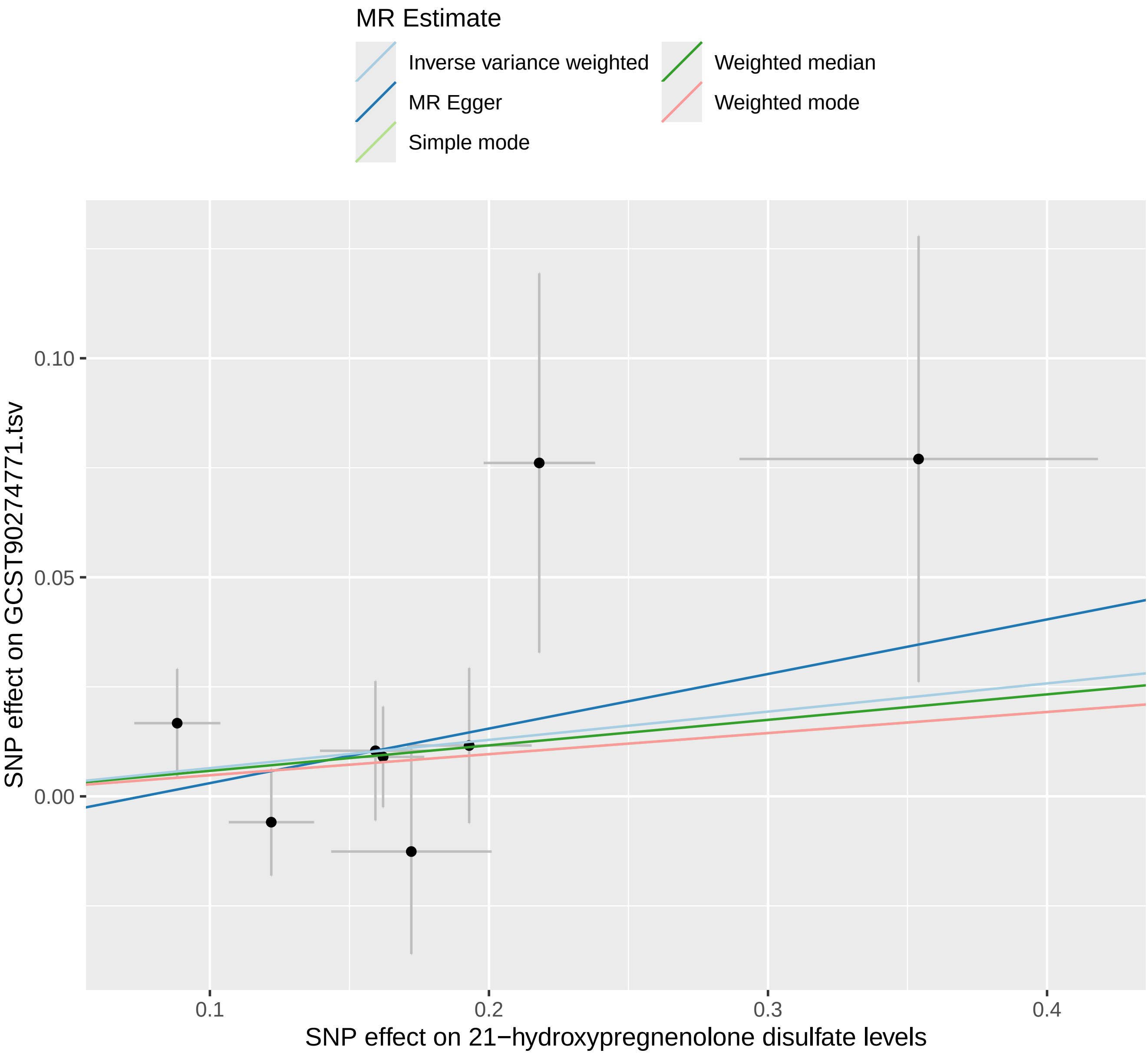
MR Estimate
Inverse variance weighted
MR Egger
Simple mode
Weighted median
Weighted mode
0.10
0.05
0.00
0.1
0.2
0.3
0.4
SNP effect on GCST90274771.tsv
SNP effect on 21−hydroxypregnenolone disulfate levels

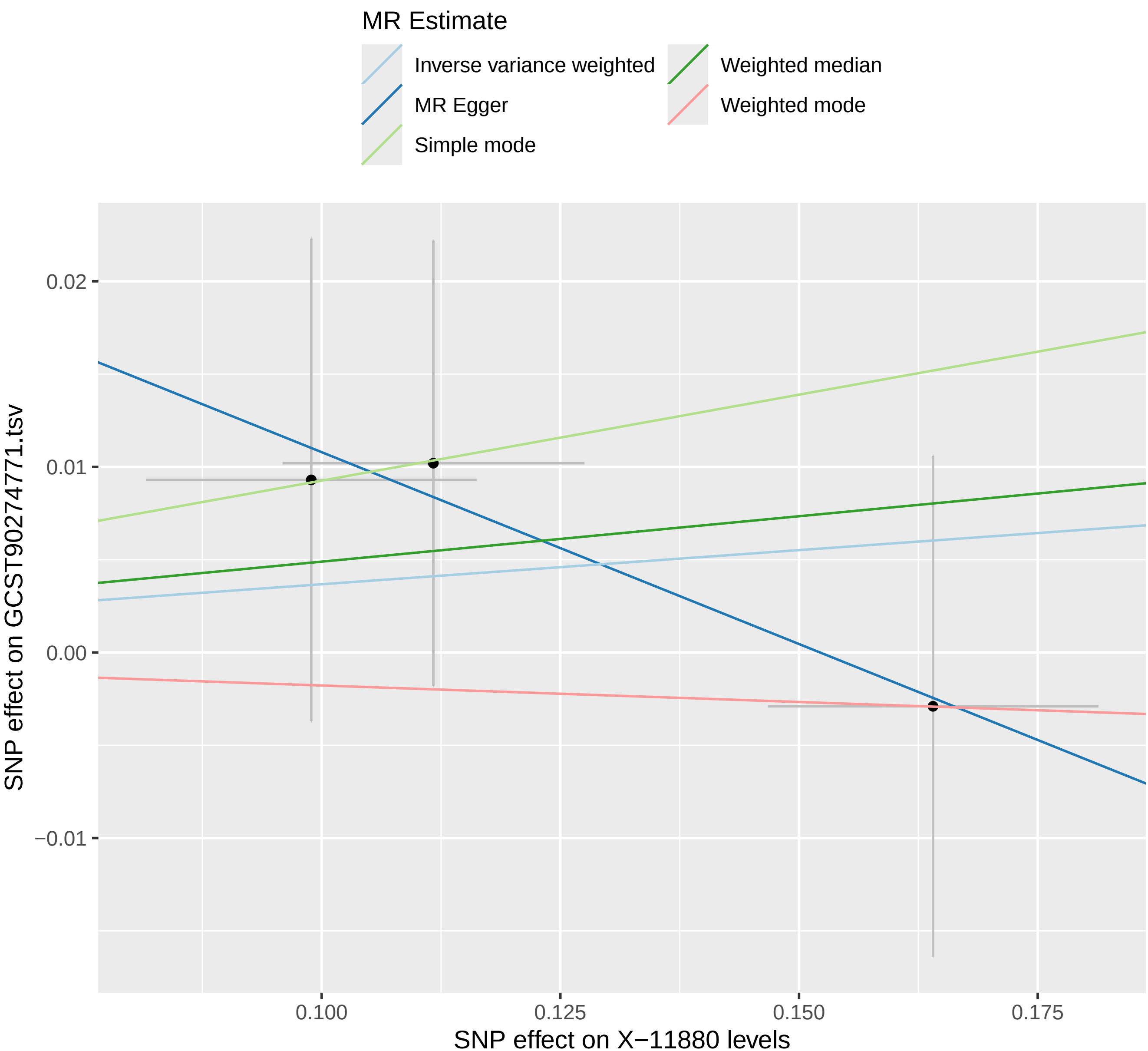
MR Estimate
Inverse variance weighted
MR Egger
Simple mode
Weighted median
Weighted mode
0.02
0.01
0.00
−0.01
0.100
0.125
0.150
0.175
SNP effect on GCST90274771.tsv
SNP effect on X−11880 levels

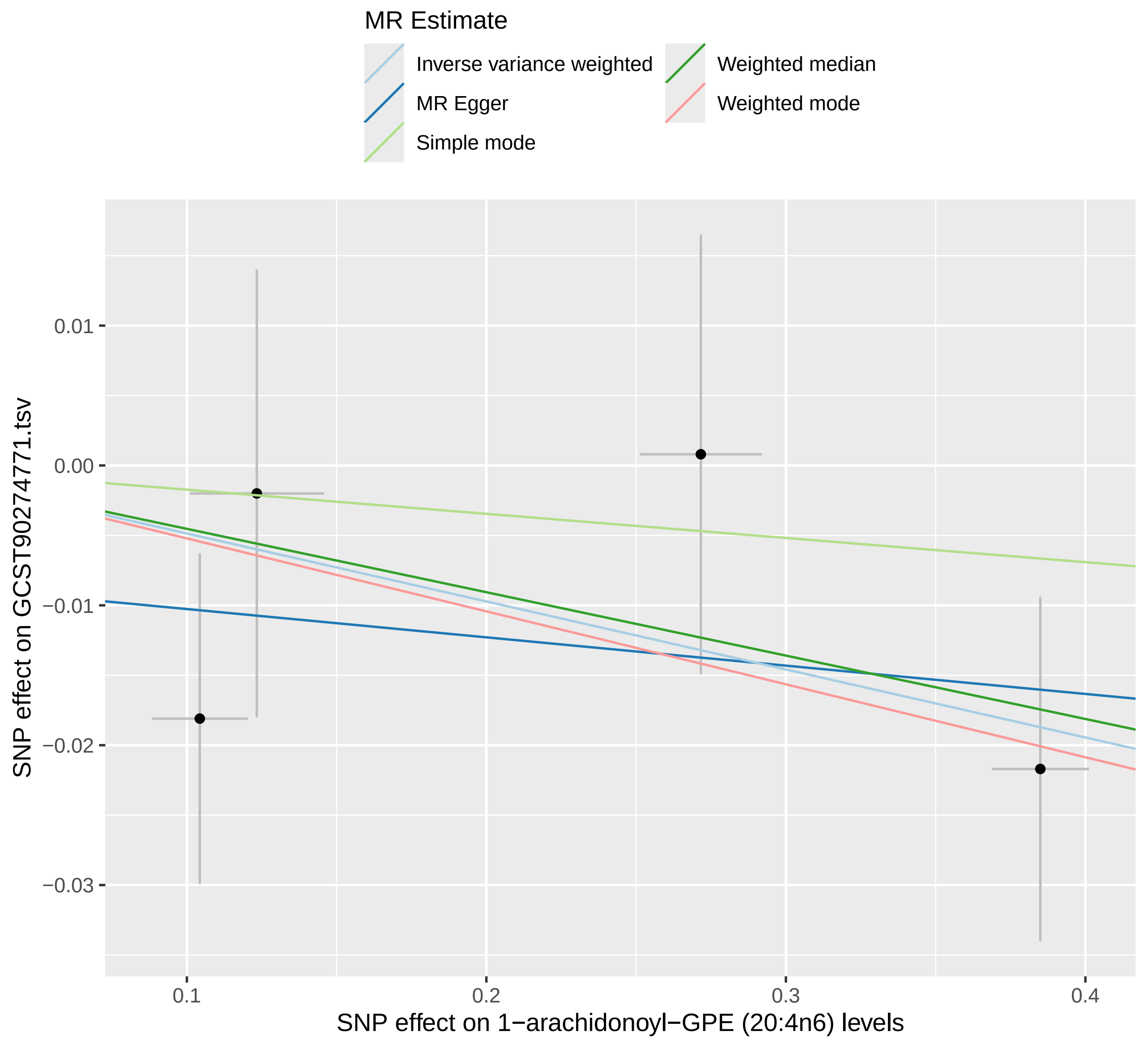

MR Estimate
Inverse variance weighted
MR Egger
Simple mode
Weighted median
Weighted mode
0.01
0.00
−0.01
−0.02
−0.03
0.1
0.2
0.3
0.4
SNP effect on GCST90274771.tsv
SNP effect on 1−arachidonoyl−GPE (20:4n6) levels

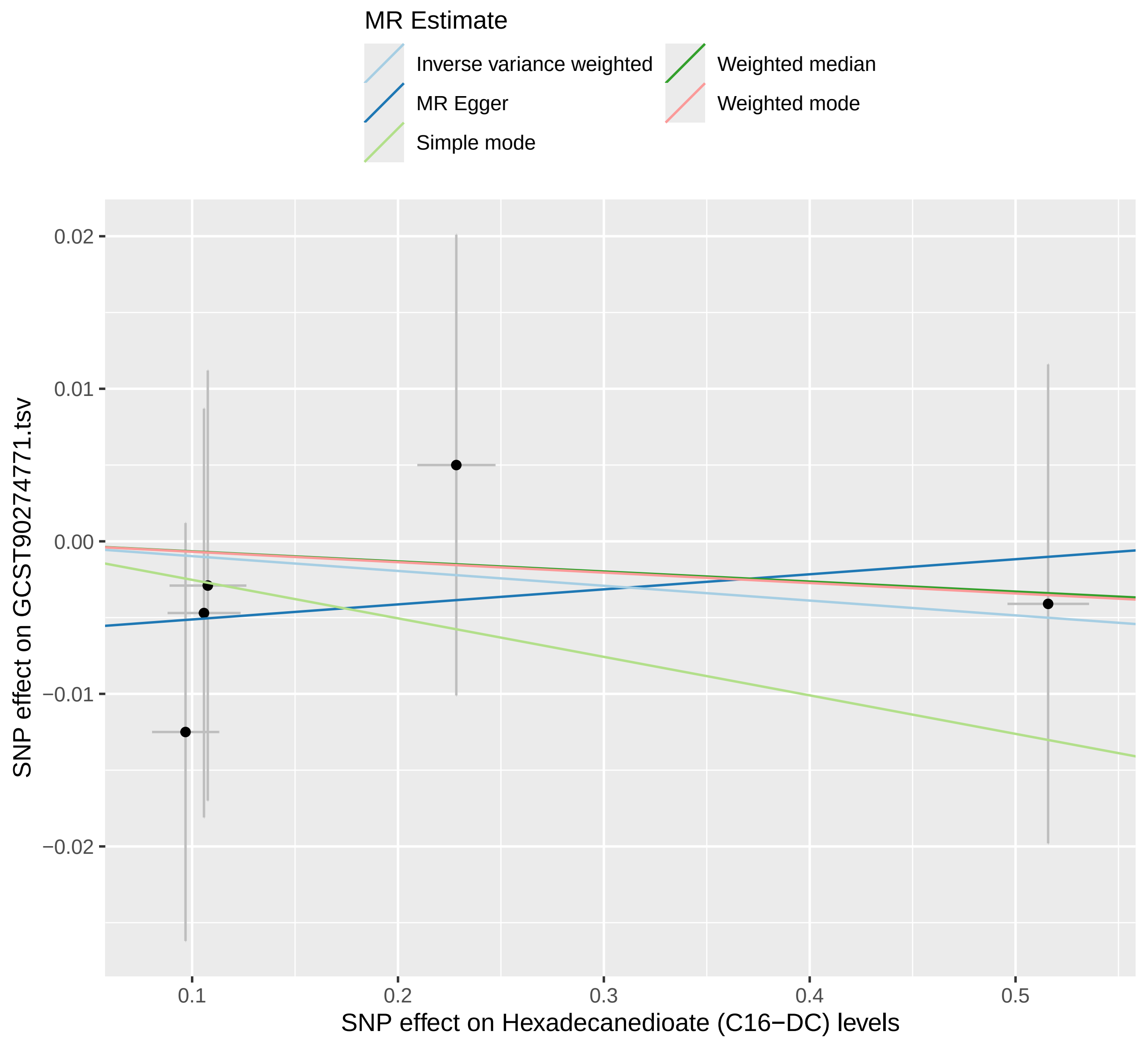

MR Estimate
Inverse variance weighted
MR Egger
Simple mode
Weighted median
Weighted mode
0.02
0.01
0.00
−0.01
−0.02
0.1
0.2
0.3
0.4
0.5
SNP effect on GCST90274771.tsv
SNP effect on Hexadecanedioate (C16−DC) levels

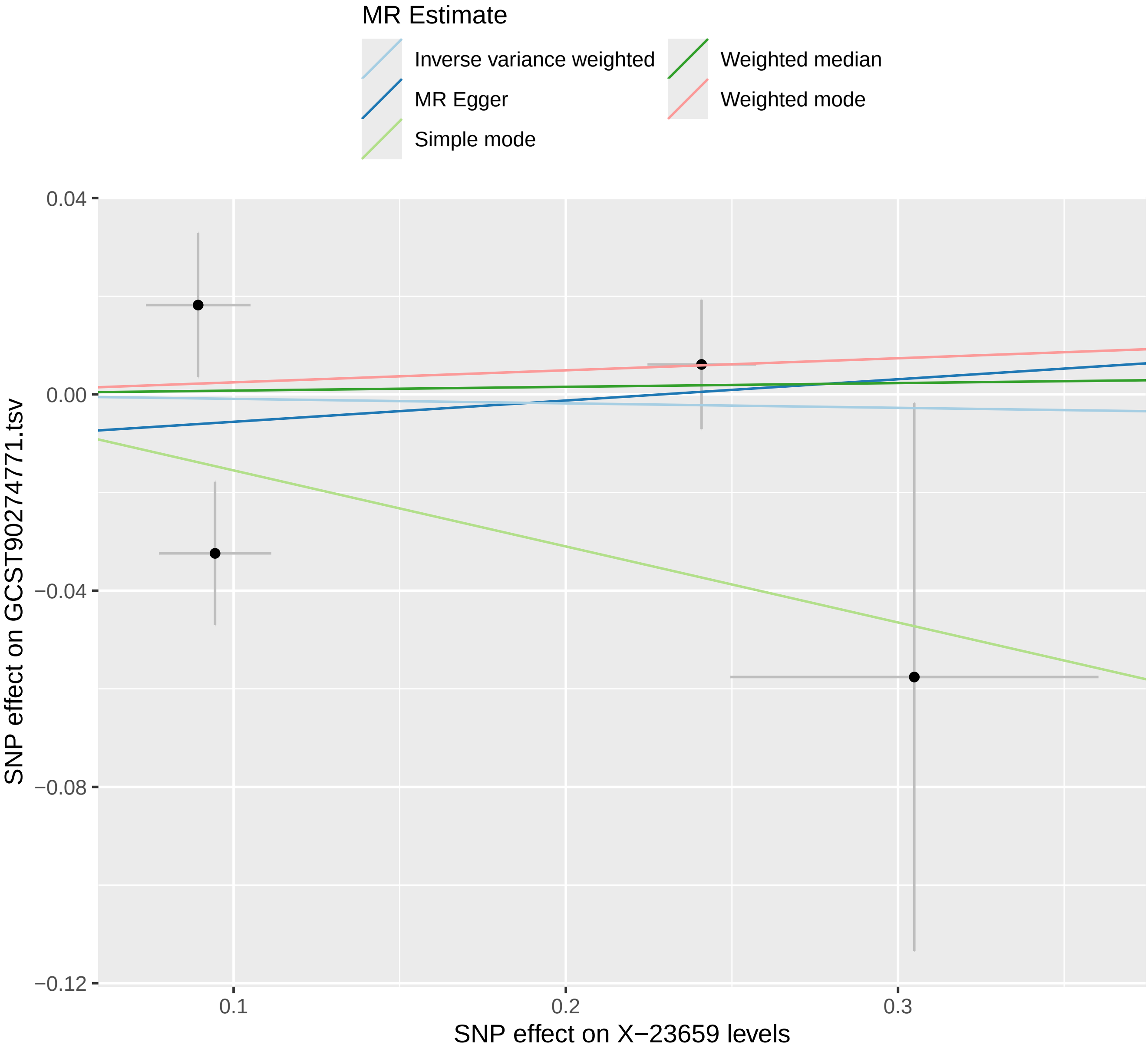
MR Estimate
Inverse variance weighted
MR Egger
Simple mode
Weighted median
Weighted mode
0.04
0.00
−0.04
−0.08
−0.12
0.1
0.2
0.3
SNP effect on GCST90274771.tsv
SNP effect on X−23659 levels

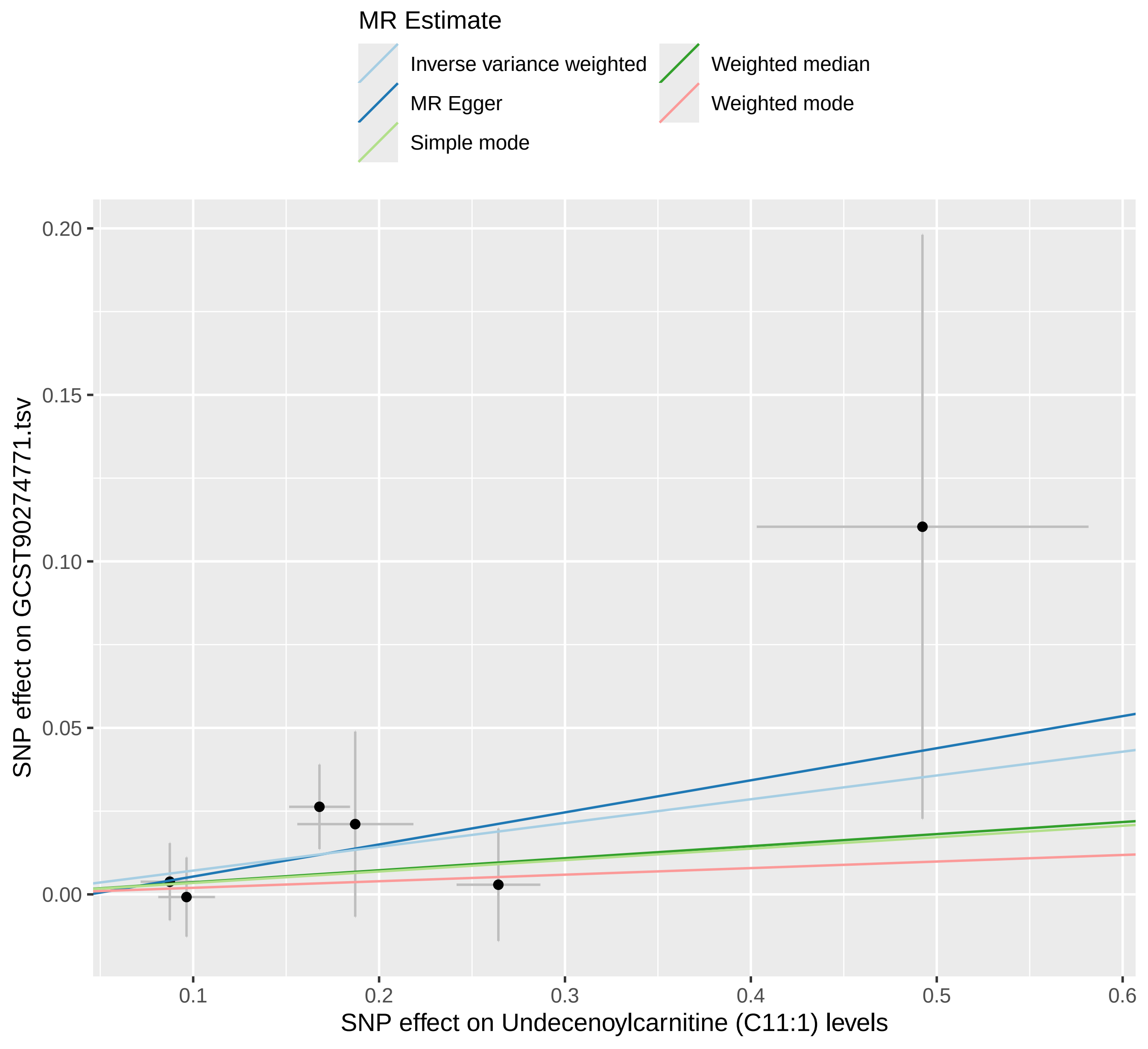

MR Estimate
Inverse variance weighted
MR Egger
Simple mode
Weighted median
Weighted mode
0.20
0.15
0.10
0.05
0.00
SNP effect on GCST90274771.tsv
0.1
0.2
0.3
0.4
0.5
0.6
SNP effect on Undecenoylcarnitine (C11:1) levels

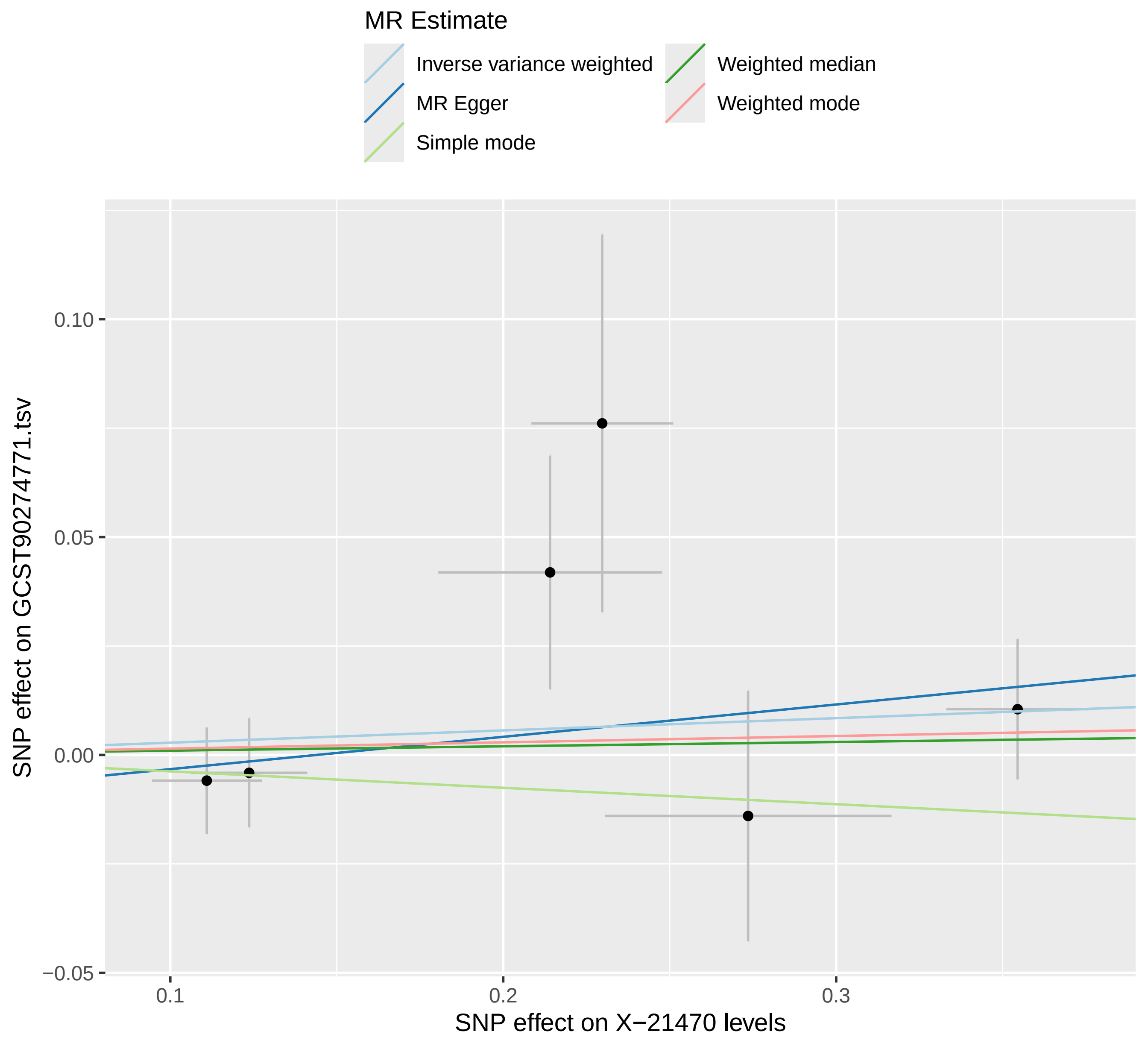
MR Estimate
Inverse variance weighted
MR Egger
Simple mode
Weighted median
Weighted mode
SNP effect on GCST90274771.tsv
0.10
0.05
0.00
−0.05
0.1
0.2
0.3
SNP effect on X−21470 levels

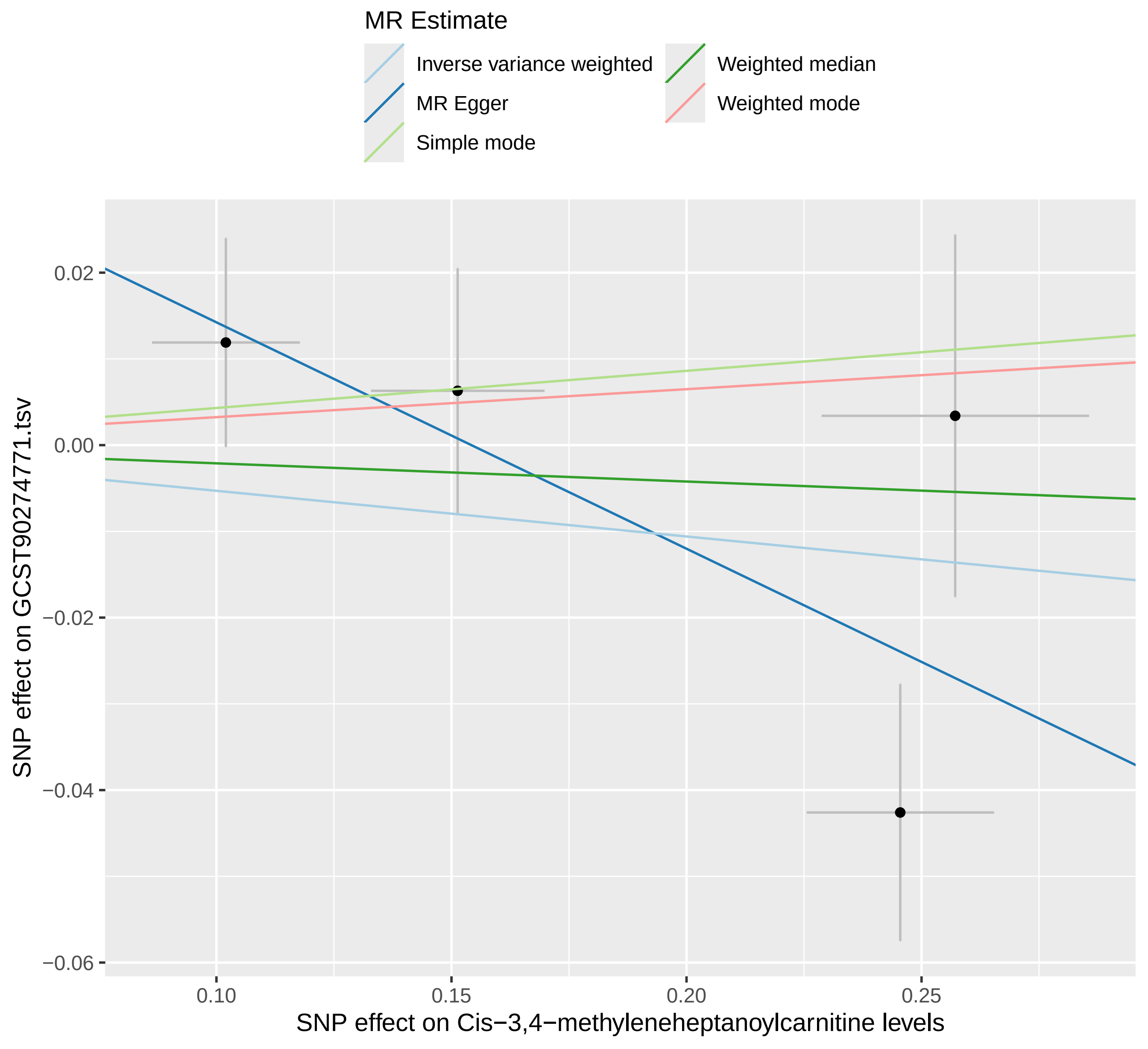
MR Estimate
Inverse variance weighted
MR Egger
Simple mode
Weighted median
Weighted mode
0.02
0.00
−0.02
−0.04
−0.06
SNP effect on GCST90274771.tsv
0.10
0.15
0.20
0.25
SNP effect on Cis−3,4−methyleneheptanoylcarnitine levels

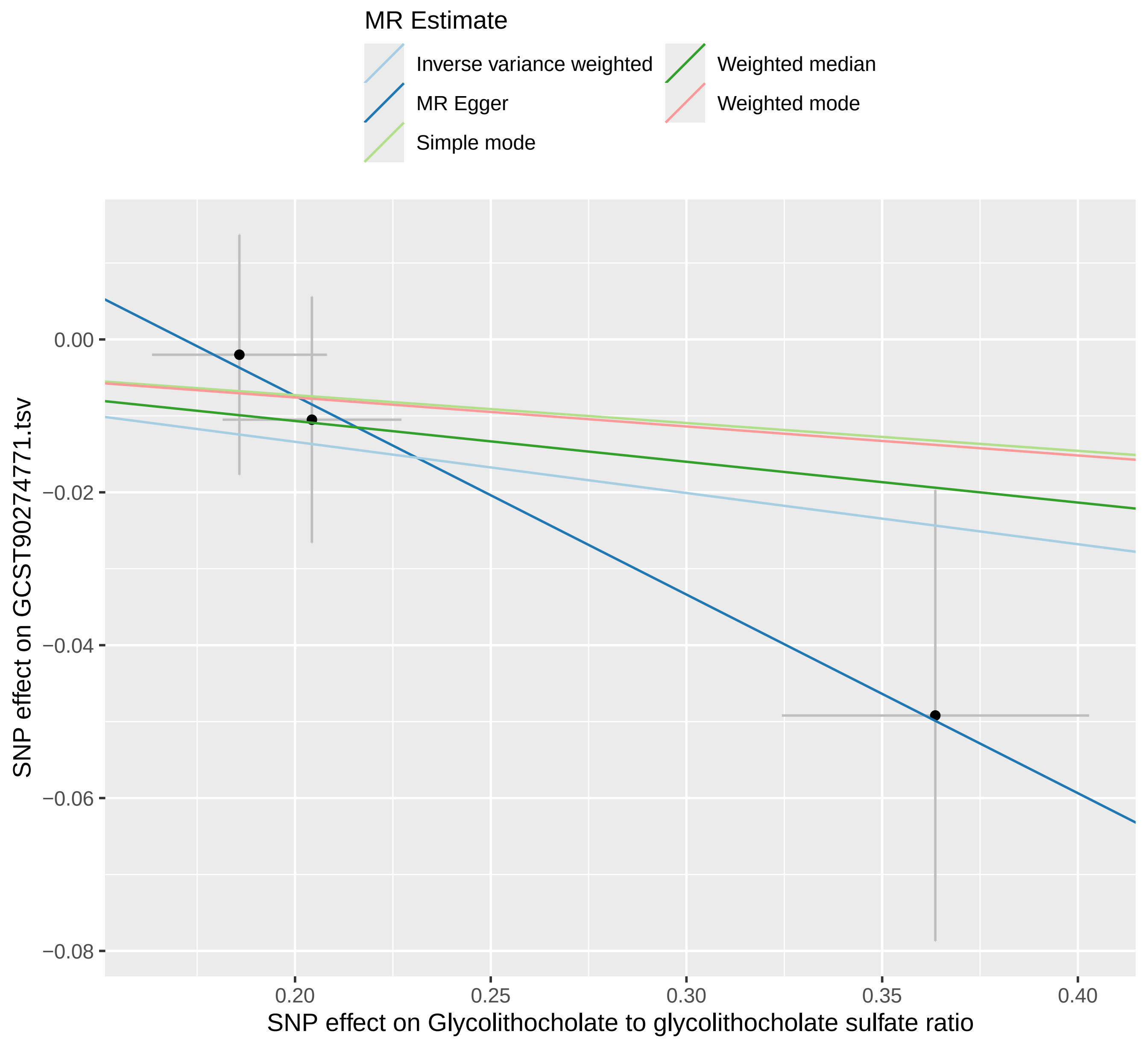

MR Estimate
Inverse variance weighted
MR Egger
Simple mode
Weighted median
Weighted mode
0.00
−0.02
−0.04
−0.06
−0.08
0.20
0.25
0.30
0.35
0.40
SNP effect on GCST90274771.tsv
SNP effect on Glycolithocholate to glycolithocholate sulfate ratio

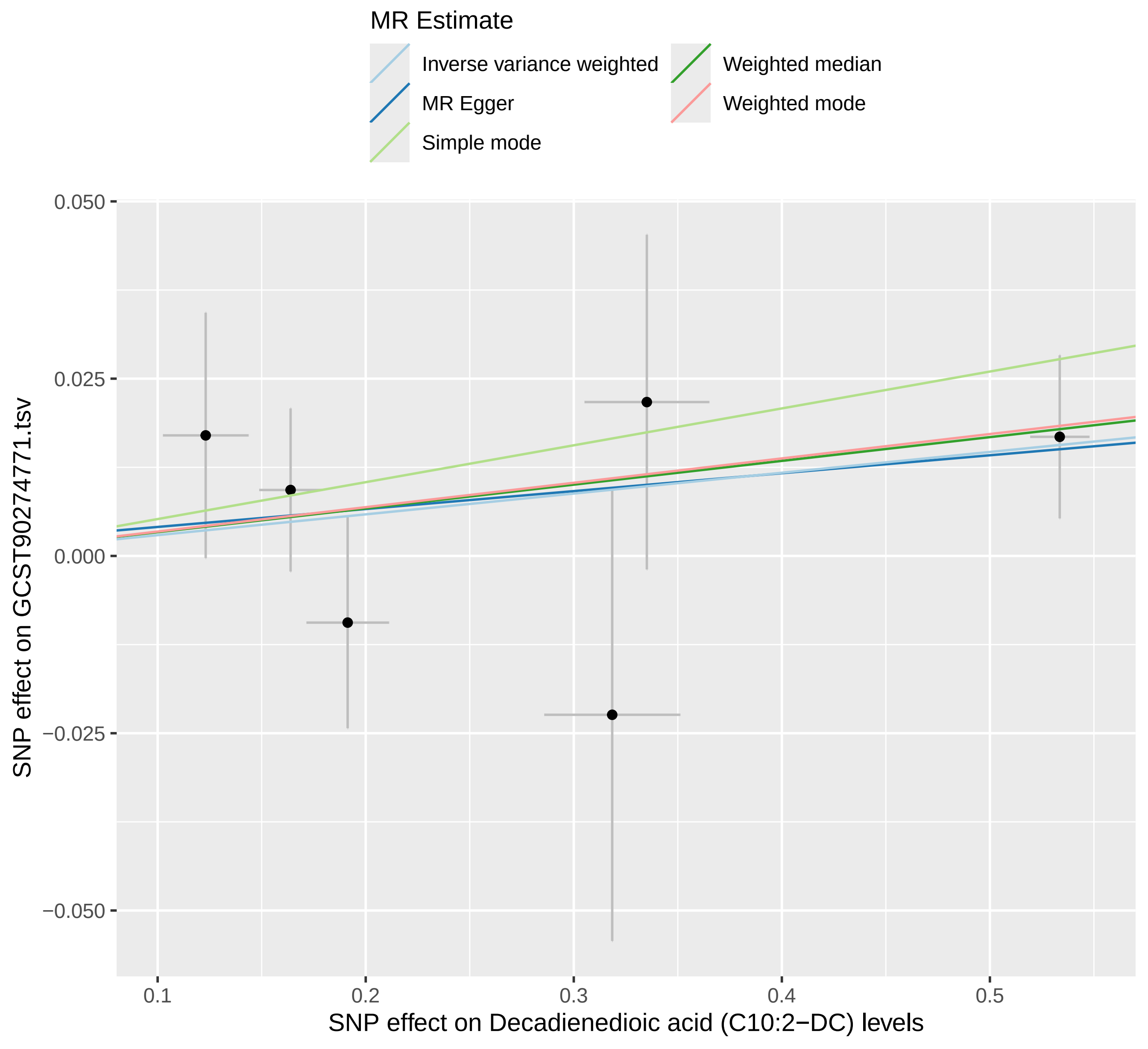

MR Estimate
Inverse variance weighted
MR Egger
Simple mode
Weighted median
Weighted mode
0.050
0.025
0.000
−0.025
−0.050
0.1
0.2
0.3
0.4
0.5
SNP effect on GCST90274771.tsv
SNP effect on Decadienedioic acid (C10:2−DC) levels

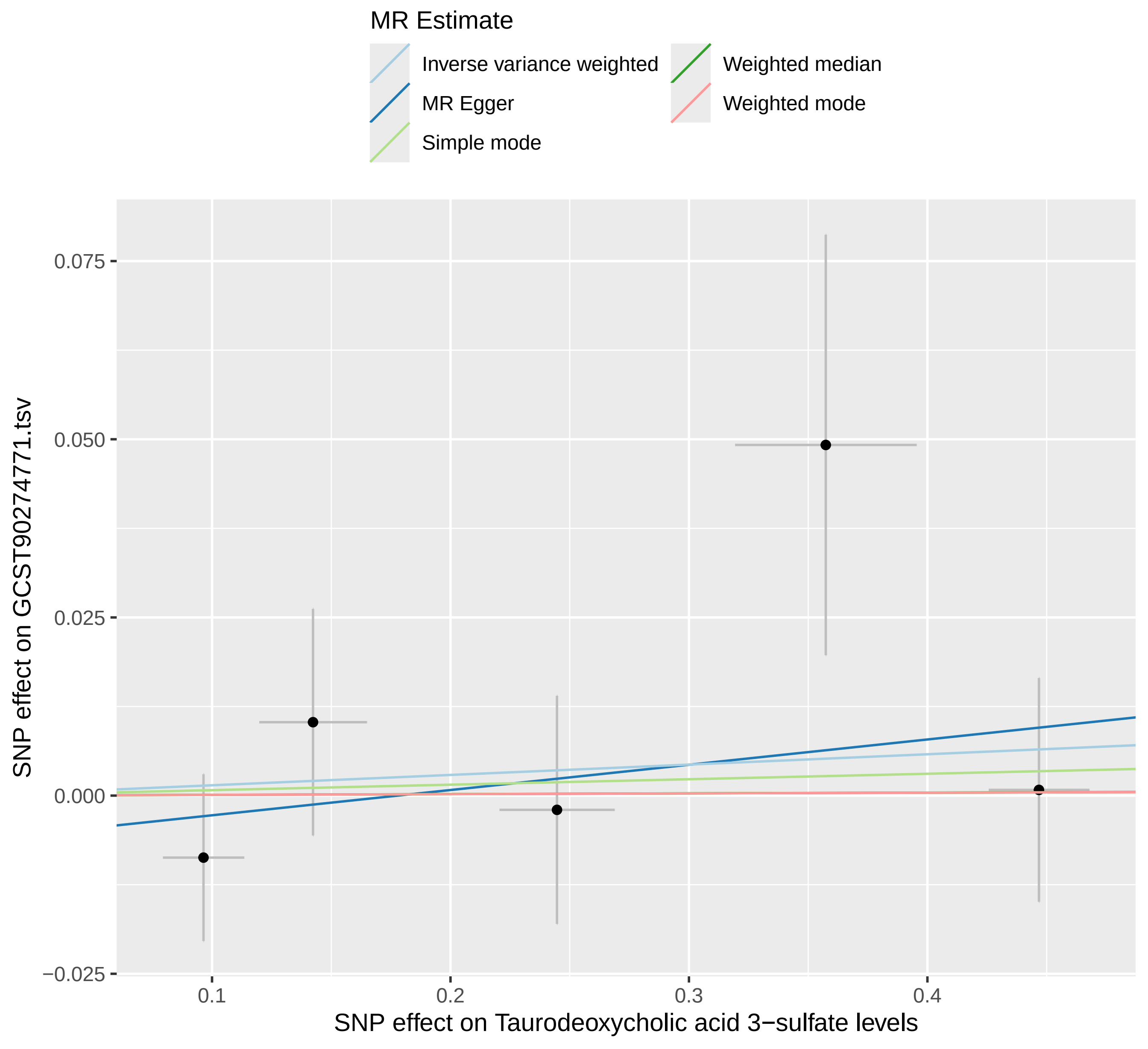
MR Estimate
Inverse variance weighted
MR Egger
Simple mode
Weighted median
Weighted mode
0.075
0.050
0.025
0.000
−0.025
0.1
0.2
0.3
0.4
SNP effect on GCST90274771.tsv
SNP effect on Taurodeoxycholic acid 3−sulfate levels

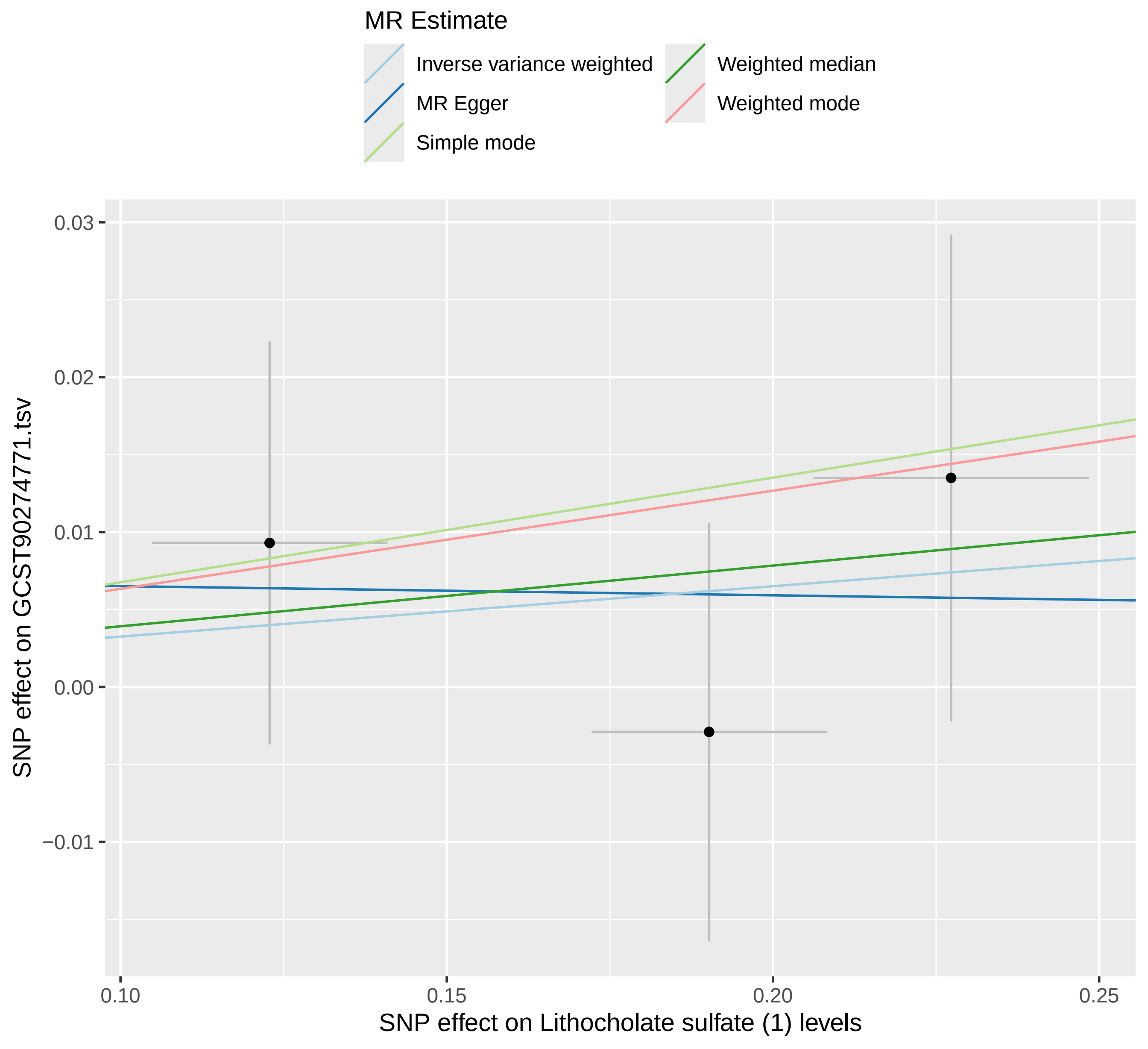
MR Estimate
Inverse variance weighted
MR Egger
Simple mode
Weighted median
Weighted mode
0.03
0.02
0.01
0.00
−0.01
0.10
0.15
0.20
0.25
SNP effect on GCST90274771.tsv
SNP effect on Lithocholate sulfate (1) levels

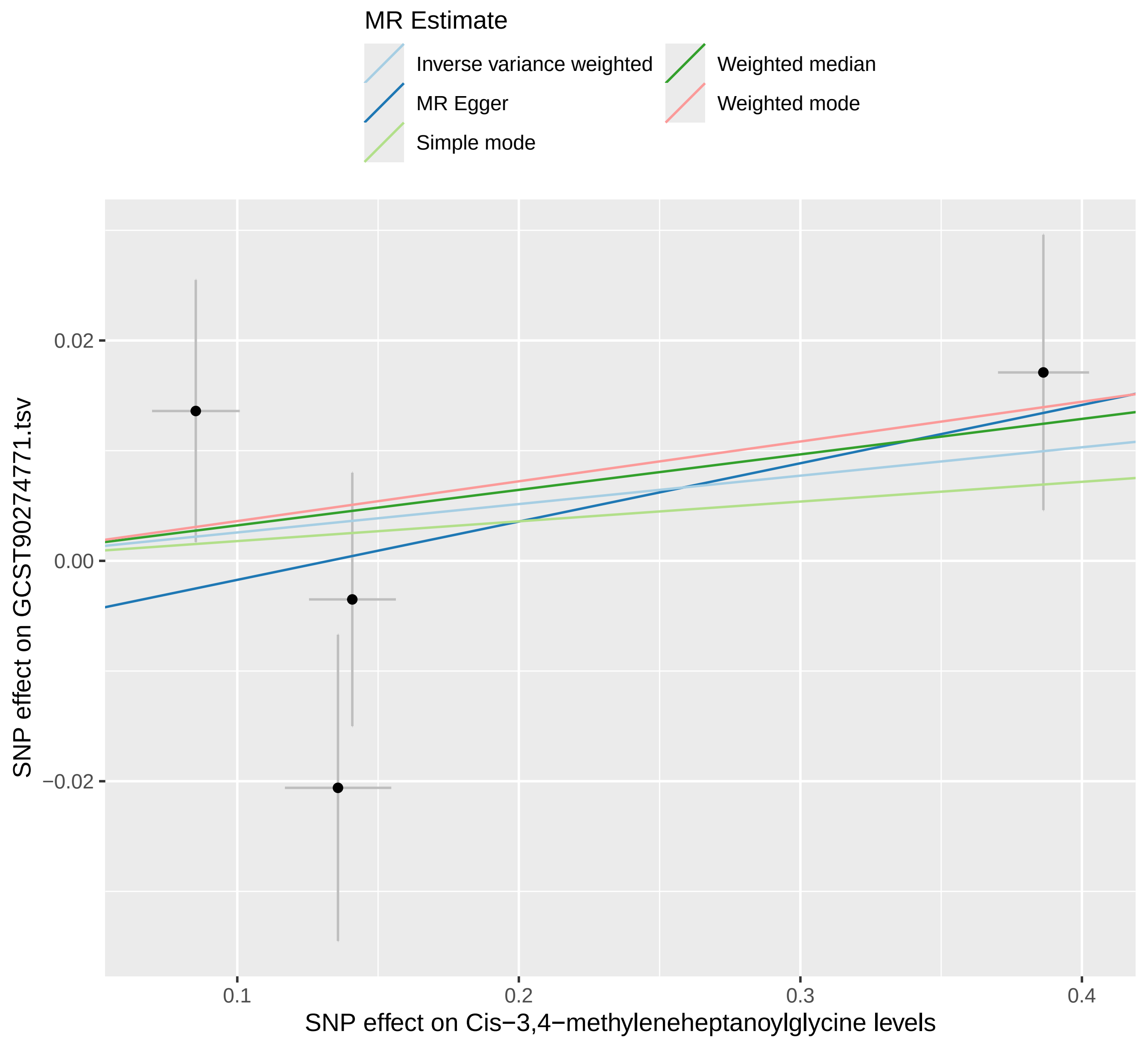
MR Estimate
Inverse variance weighted
MR Egger
Simple mode
Weighted median
Weighted mode
0.02
0.00
−0.02
0.1
0.2
0.3
0.4
SNP effect on GCST90274771.tsv
SNP effect on Cis−3,4−methyleneheptanoylglycine levels

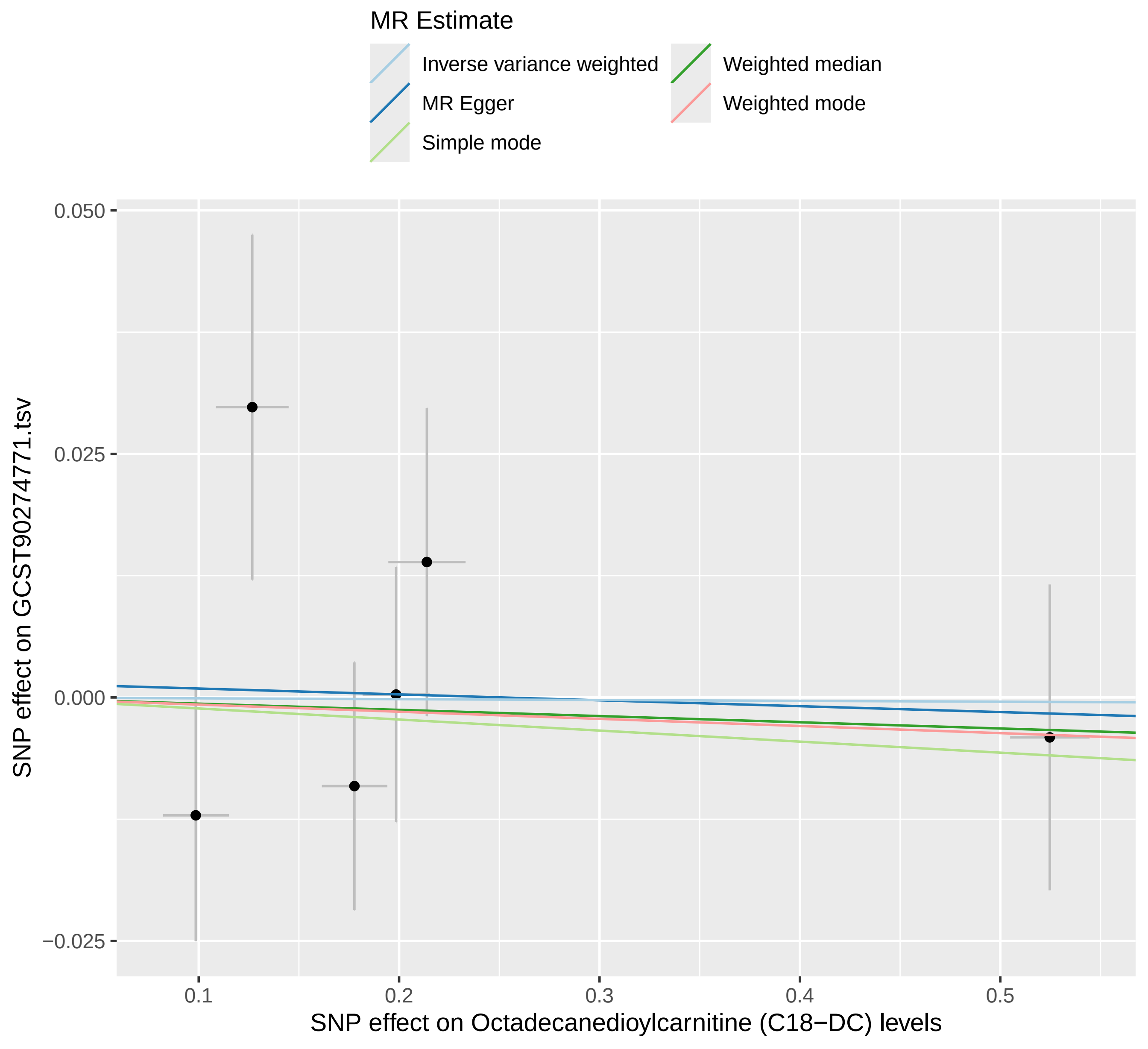

MR Estimate
Inverse variance weighted
MR Egger
Simple mode
Weighted median
Weighted mode
0.050
0.025
0.000
−0.025
0.1
0.2
0.3
0.4
0.5
SNP effect on GCST90274771.tsv
SNP effect on Octadecanedioylcarnitine (C18−DC) levels

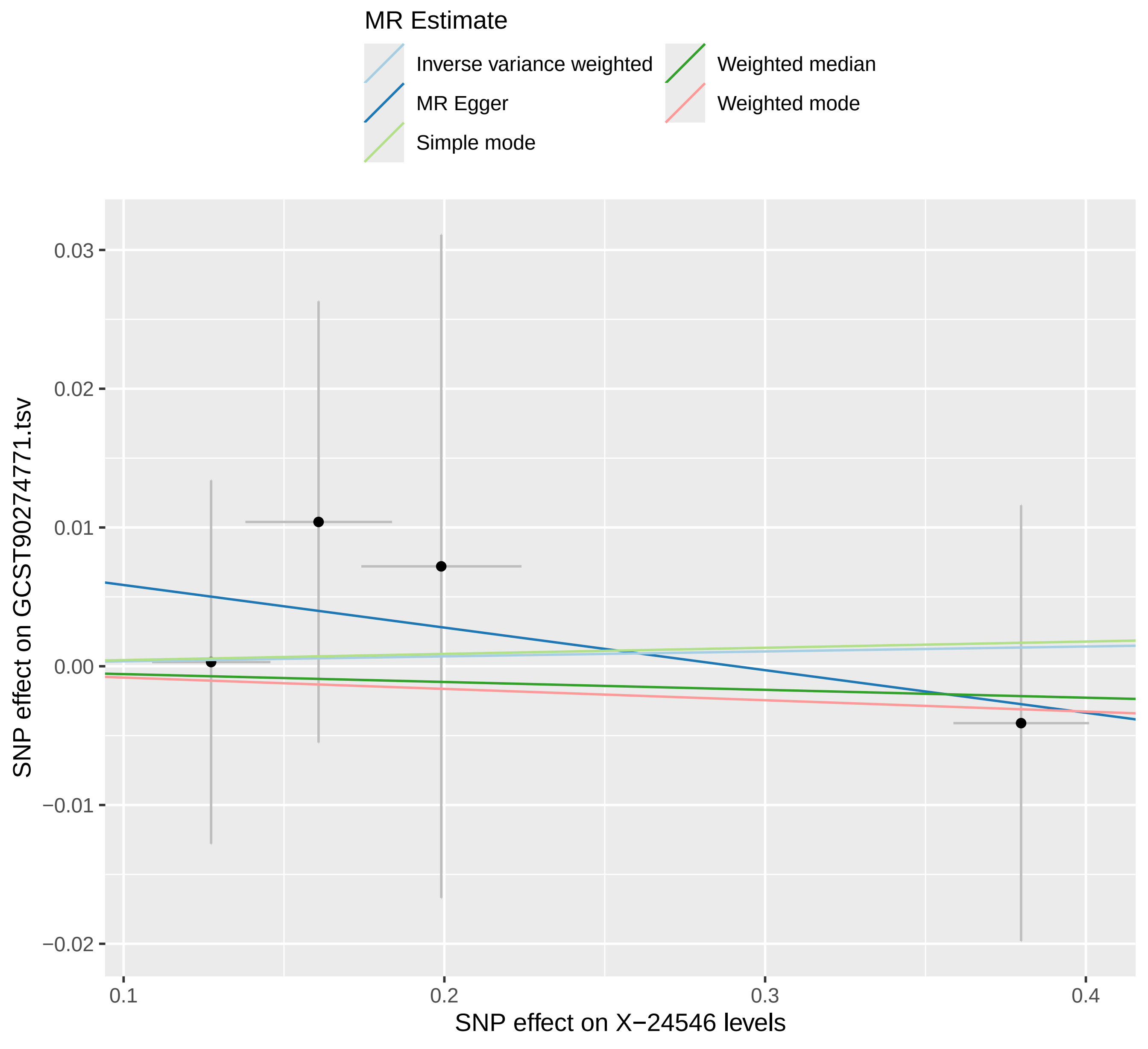

MR Estimate
Inverse variance weighted
MR Egger
Simple mode
Weighted median
Weighted mode
0.03
0.02
0.01
0.00
−0.01
−0.02
SNP effect on GCST90274771.tsv
0.1
0.2
0.3
0.4
SNP effect on X−24546 levels

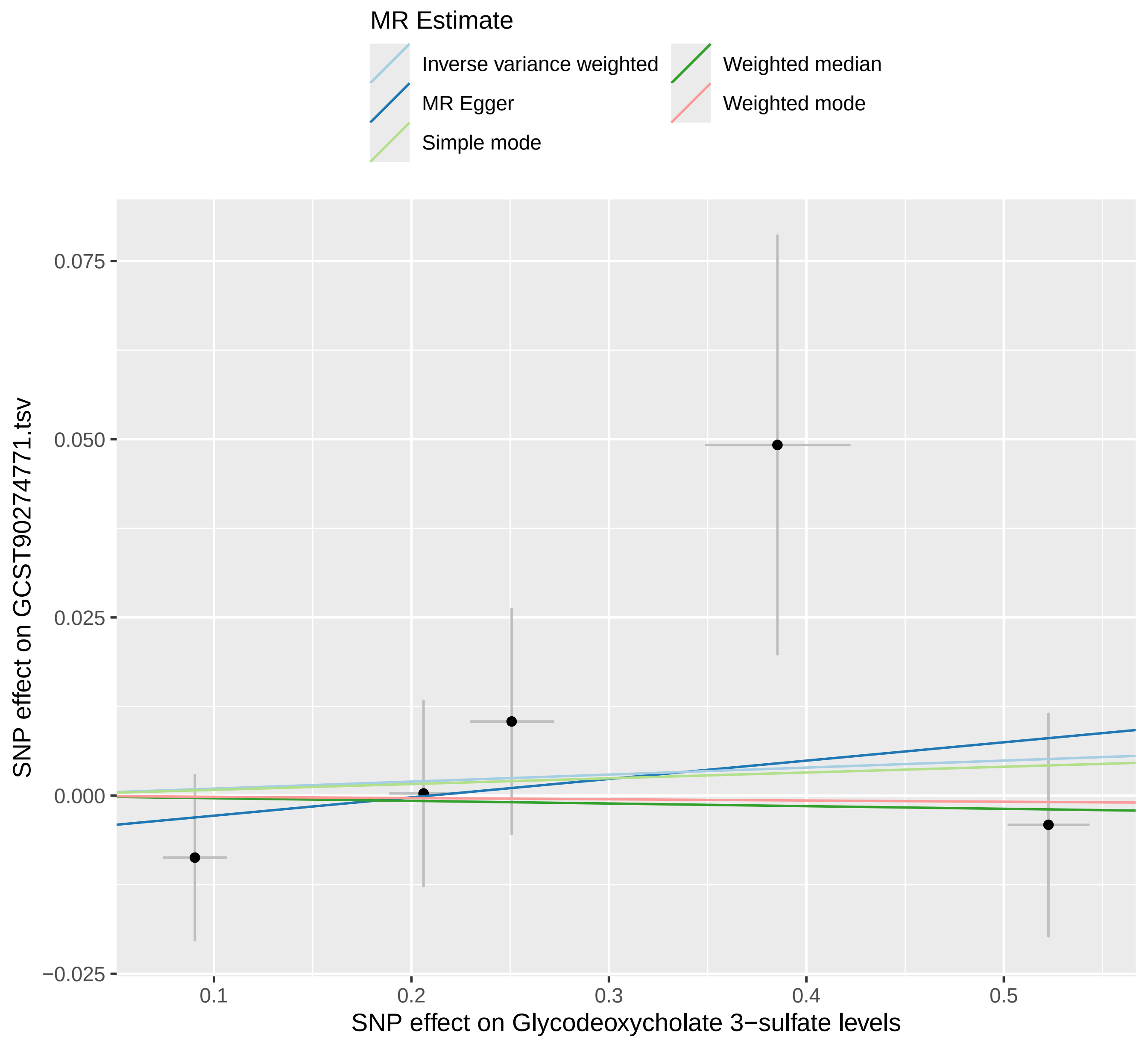
MR Estimate
Inverse variance weighted
MR Egger
Simple mode
Weighted median
Weighted mode
0.075
0.050
0.025
0.000
−0.025
0.1
0.2
0.3
0.4
0.5
SNP effect on GCST90274771.tsv
SNP effect on Glycodeoxycholate 3−sulfate levels

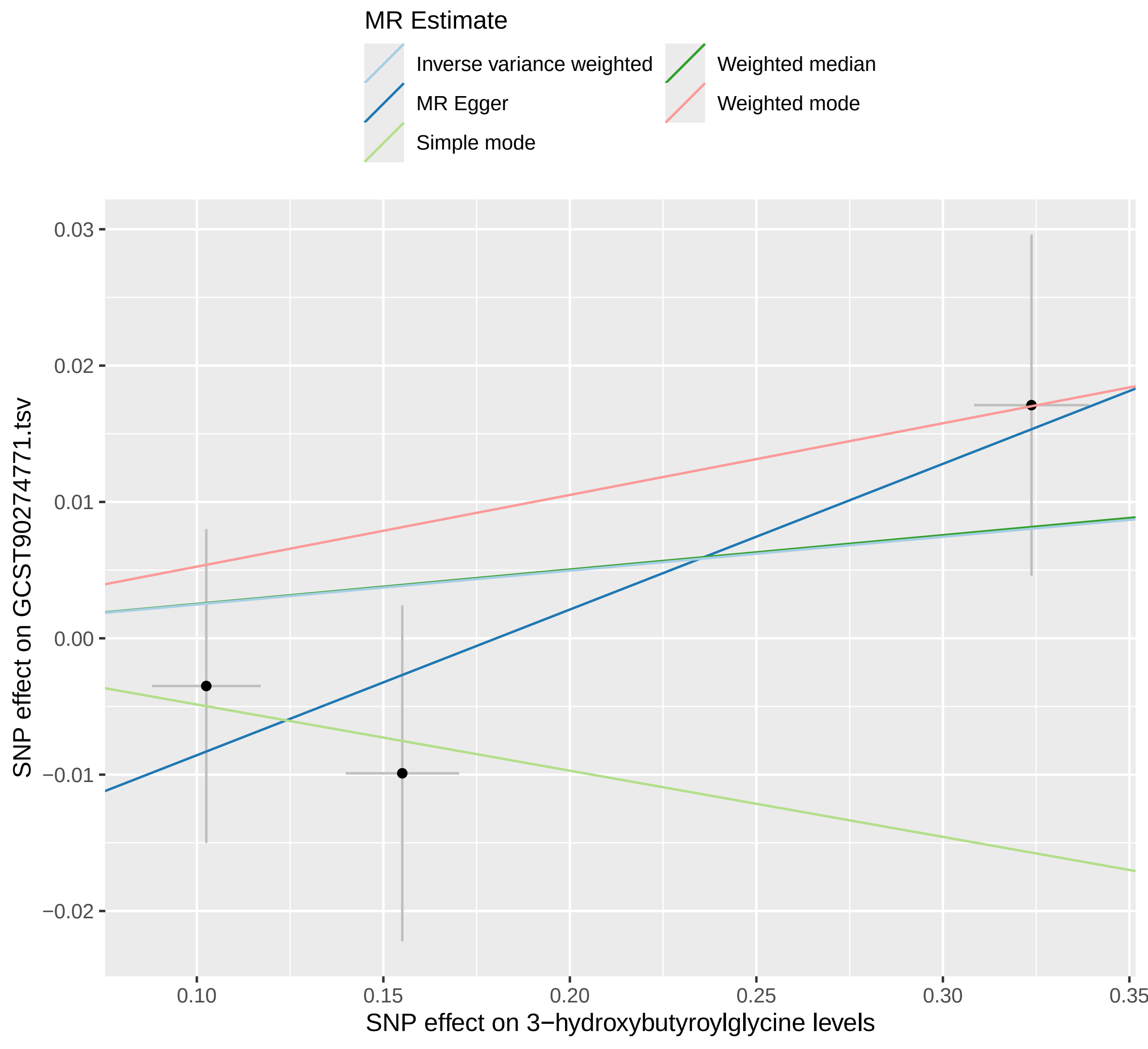
MR Estimate
Inverse variance weighted
MR Egger
Simple mode
Weighted median
Weighted mode
0.03
0.02
0.01
0.00
−0.01
−0.02
SNP effect on GCST90274771.tsv
0.10
0.15
0.20
0.25
0.30
0.35
SNP effect on 3−hydroxybutyroylglycine levels

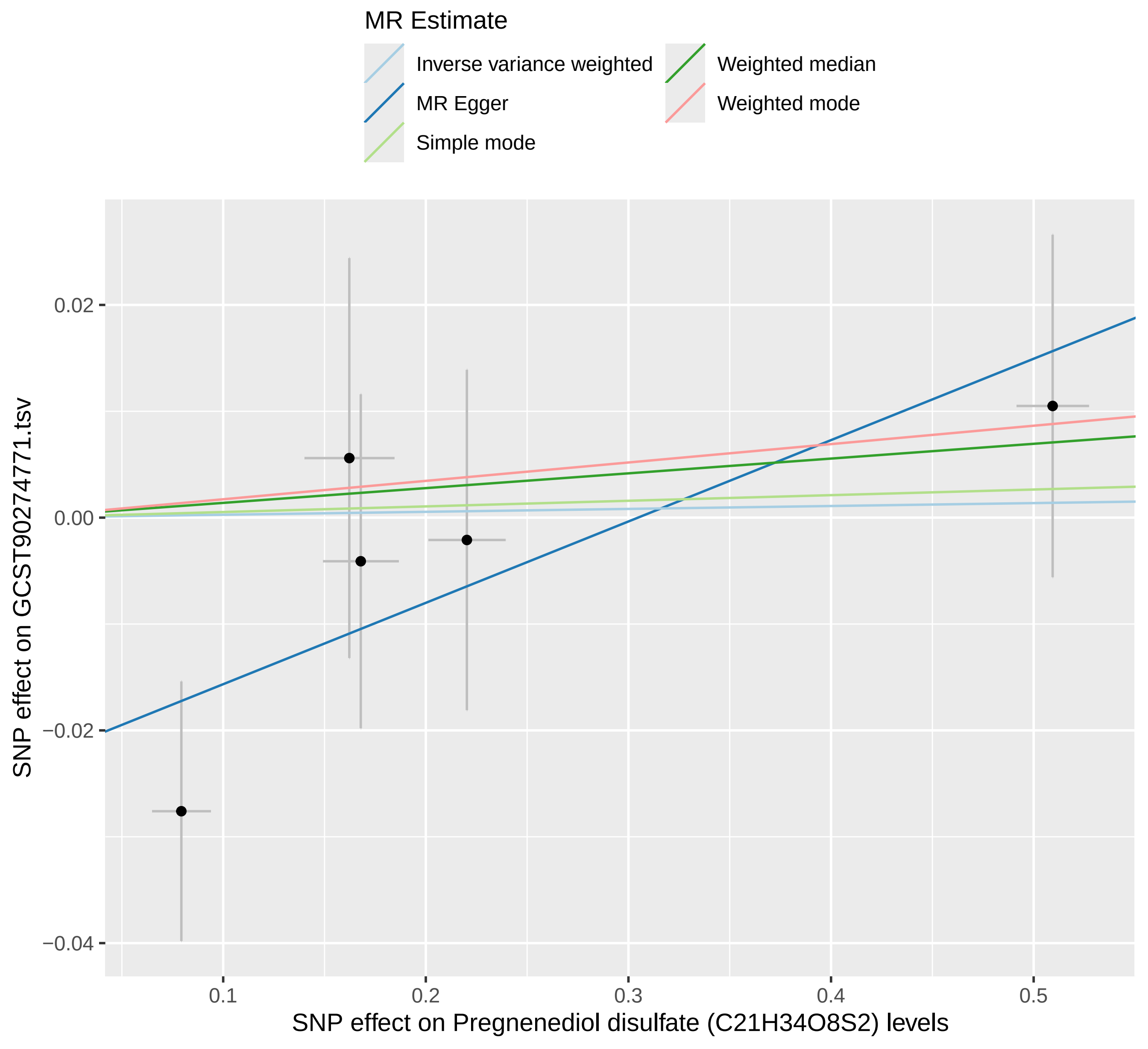

MR Estimate
Inverse variance weighted
MR Egger
Simple mode
Weighted median
Weighted mode
0.02
0.00
−0.02
−0.04
0.1
0.2
0.3
0.4
0.5
SNP effect on GCST90274771.tsv
SNP effect on Pregnenediol disulfate (C21H34O8S2) levels

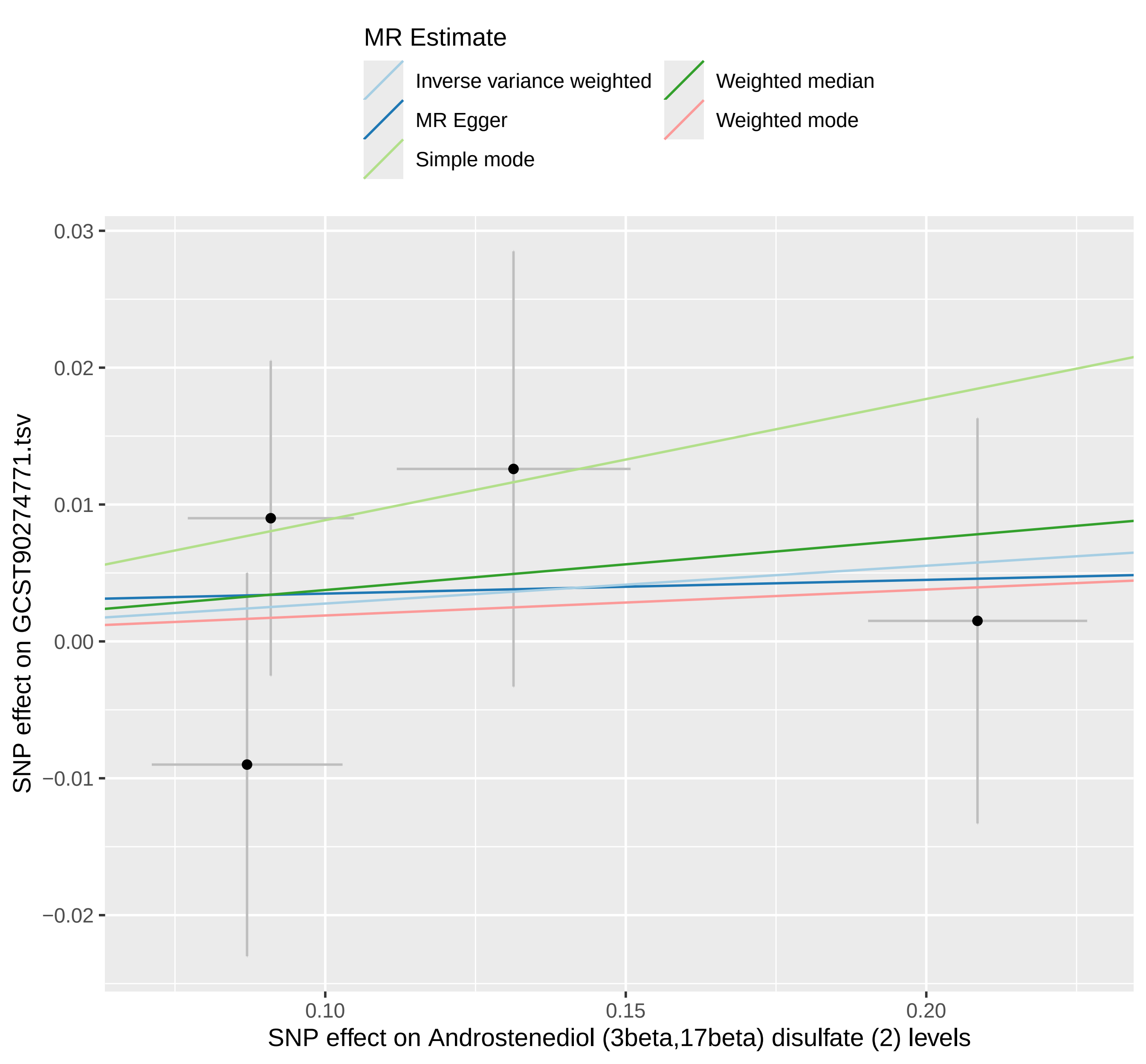
MR Estimate
Inverse variance weighted
MR Egger
Simple mode
Weighted median
Weighted mode
0.03
0.02
0.01
0.00
−0.01
−0.02
0.10
0.15
0.20
SNP effect on GCST90274771.tsv
SNP effect on Androstenediol (3beta,17beta) disulfate (2) levels

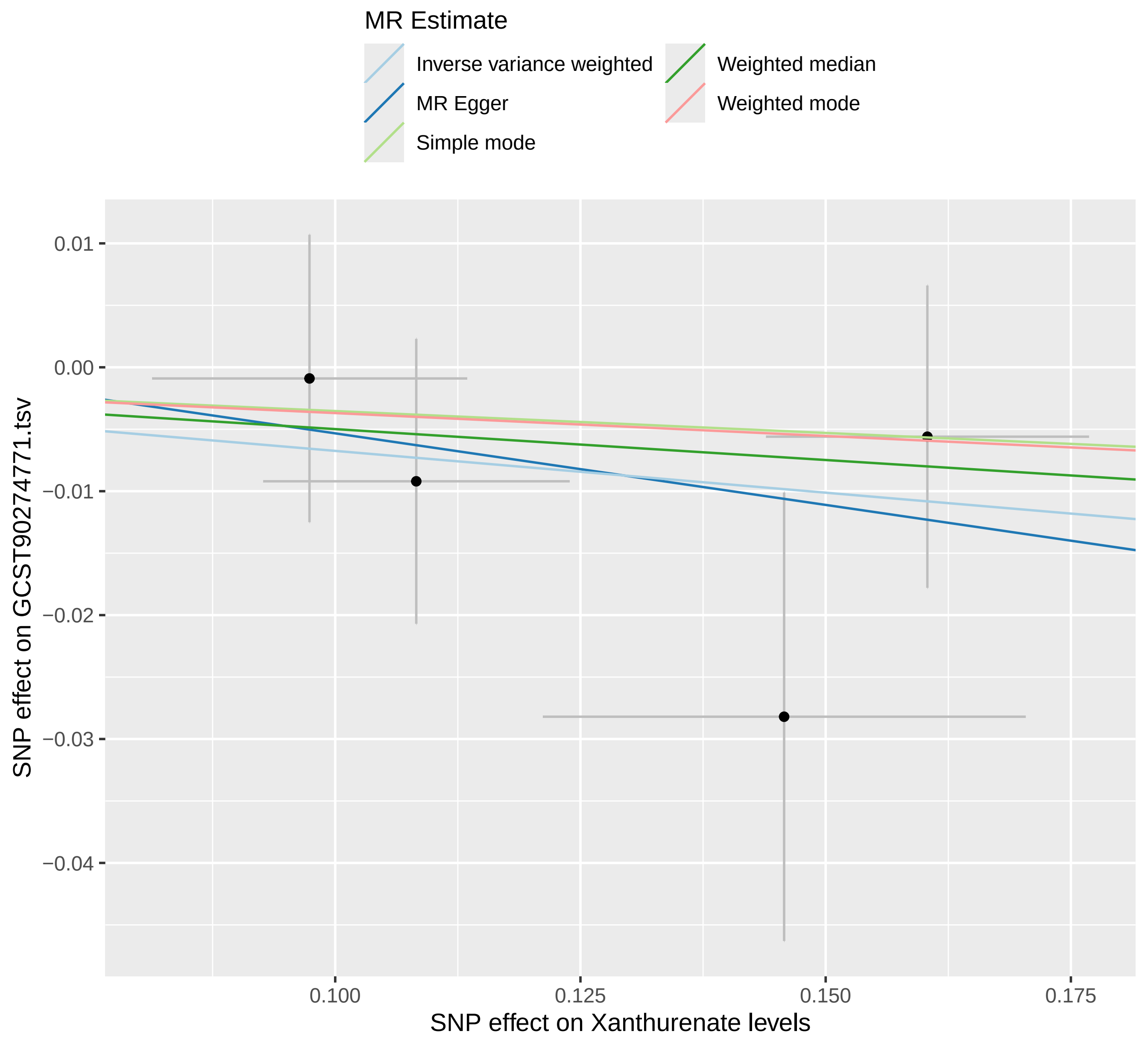

MR Estimate
Inverse variance weighted
MR Egger
Simple mode
Weighted median
Weighted mode
0.01
0.00
−0.01
−0.02
−0.03
−0.04
SNP effect on GCST90274771.tsv
0.100
0.125
0.150
0.175
SNP effect on Xanthurenate levels

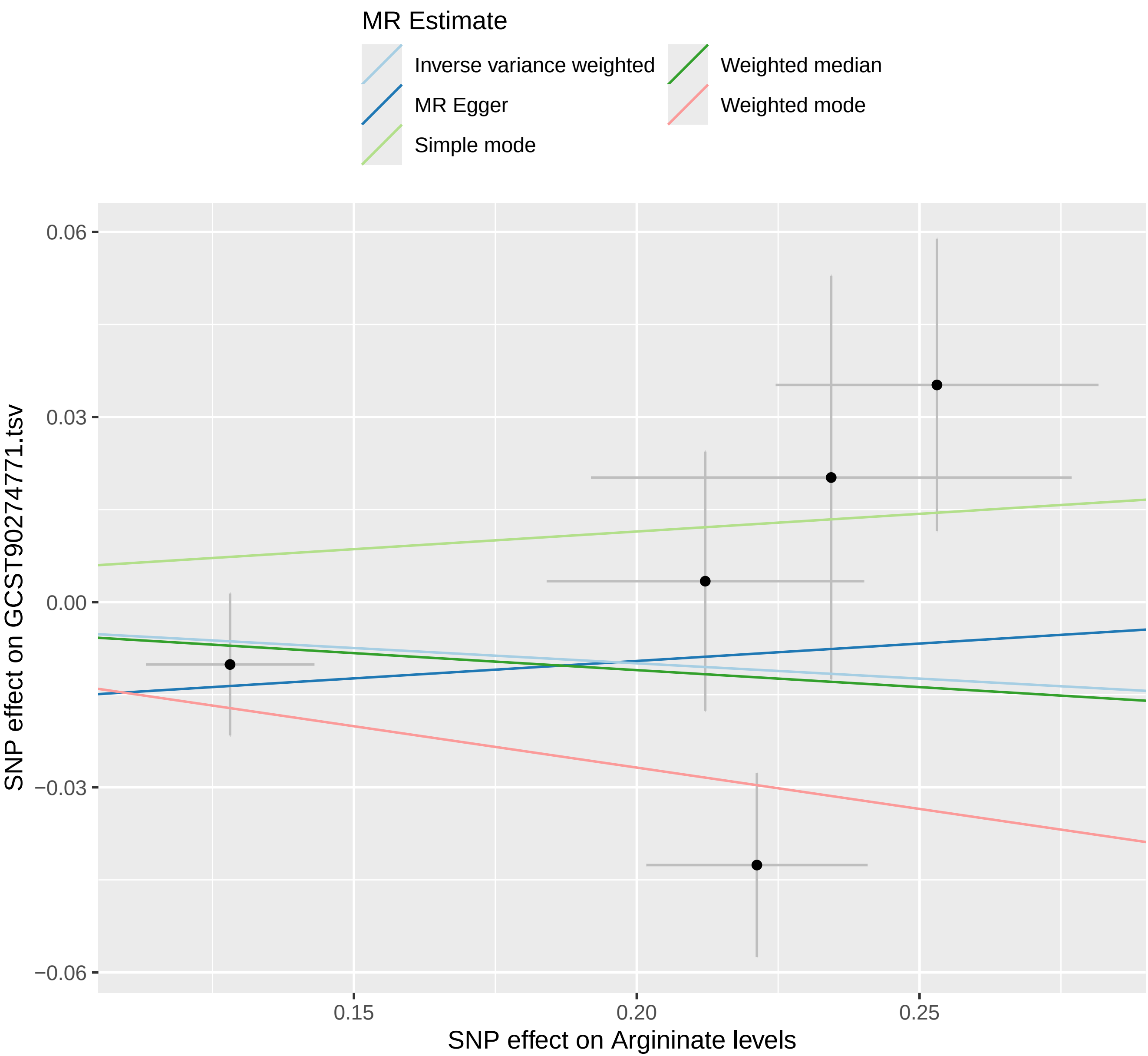
MR Estimate
Inverse variance weighted
MR Egger
Simple mode
Weighted median
Weighted mode
0.06
0.03
0.00
−0.03
−0.06
0.15
0.20
0.25
SNP effect on GCST90274771.tsv
SNP effect on Argininate levels

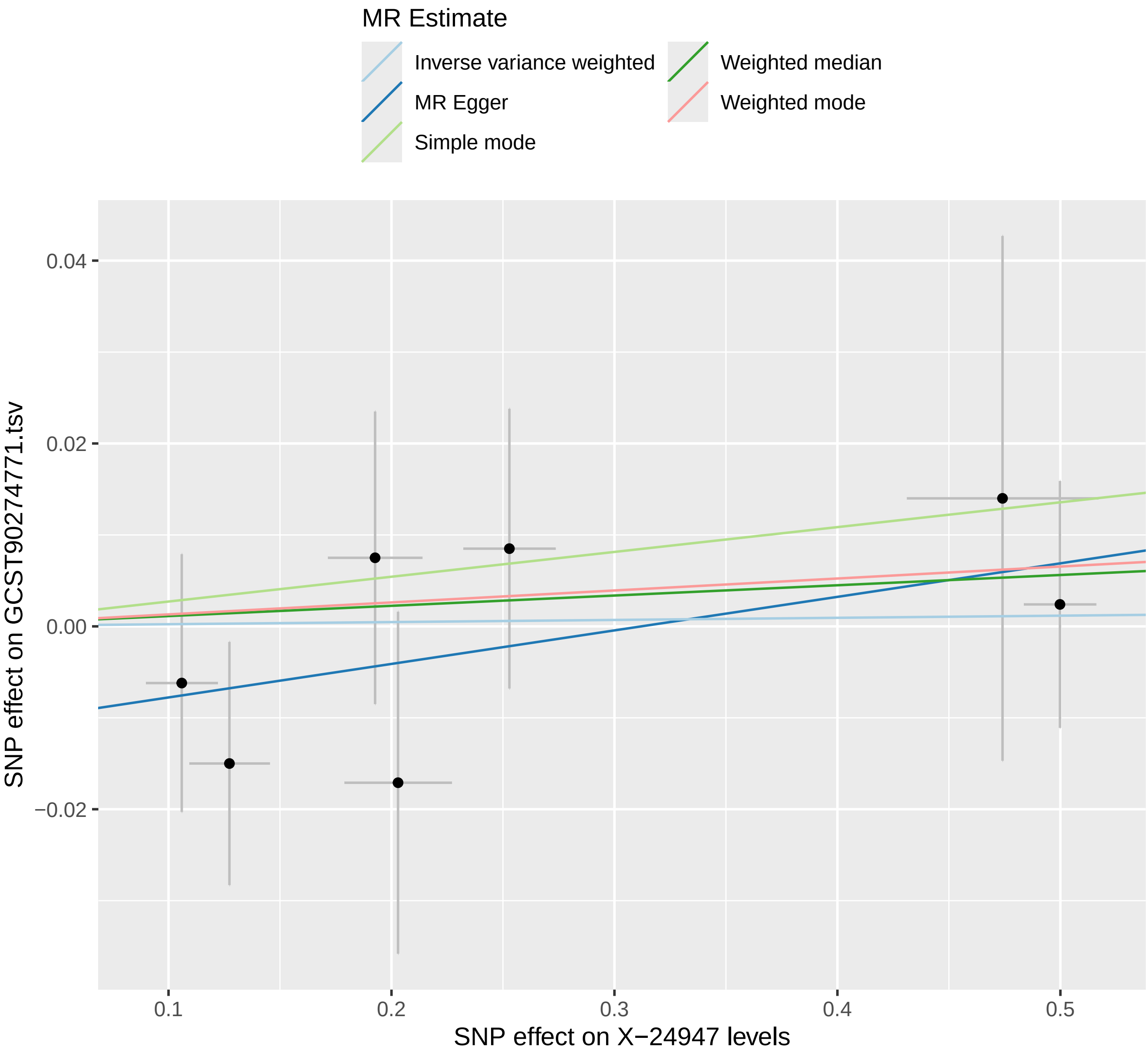
MR Estimate
Inverse variance weighted
MR Egger
Simple mode
Weighted median
Weighted mode
0.04
0.02
0.00
−0.02
0.1
0.2
0.3
0.4
0.5
SNP effect on GCST90274771.tsv
SNP effect on X−24947 levels

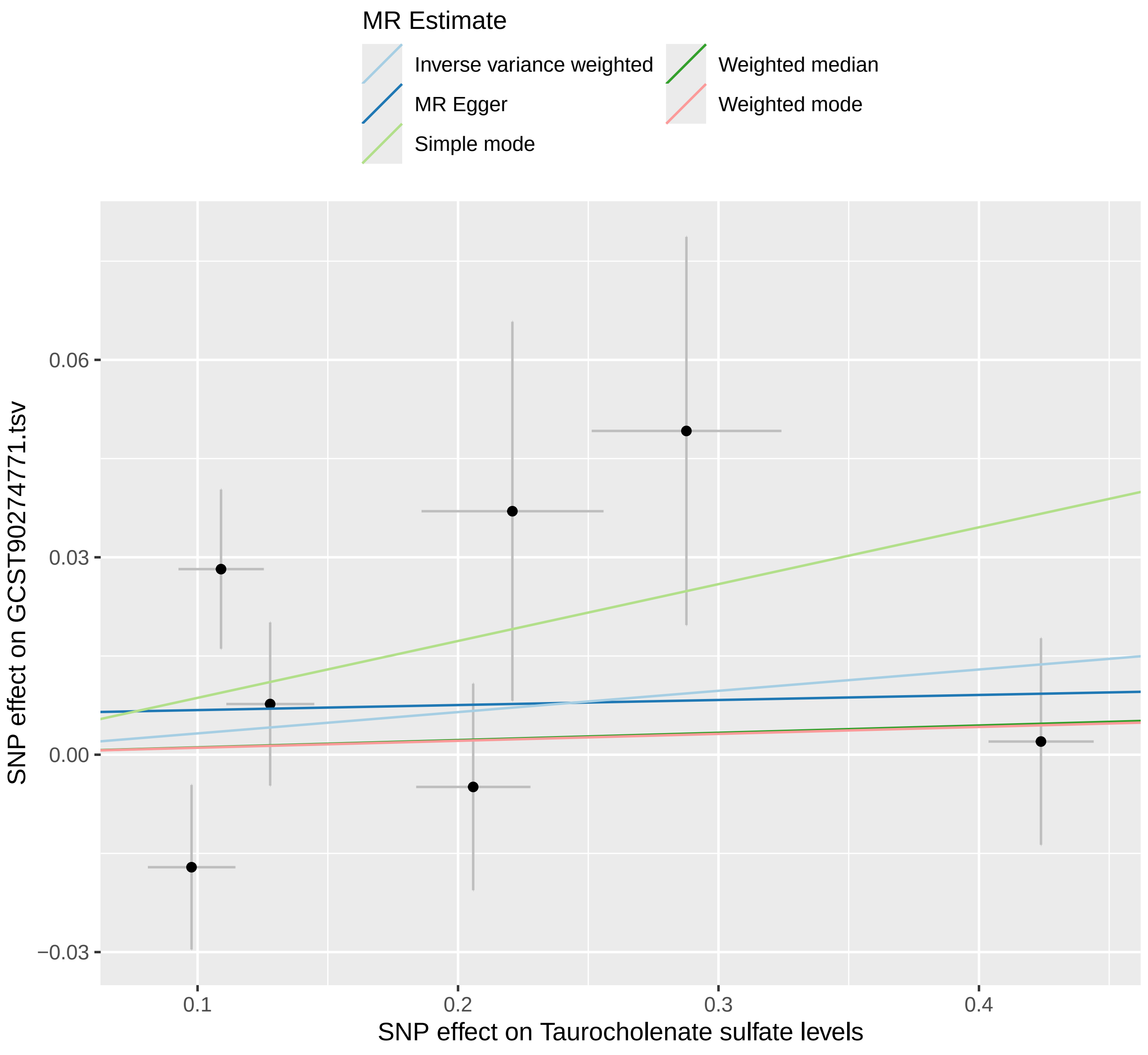
MR Estimate
Inverse variance weighted
MR Egger
Simple mode
Weighted median
Weighted mode
0.06
0.03
0.00
−0.03
0.1
0.2
0.3
0.4
SNP effect on GCST90274771.tsv
SNP effect on Taurocholenate sulfate levels

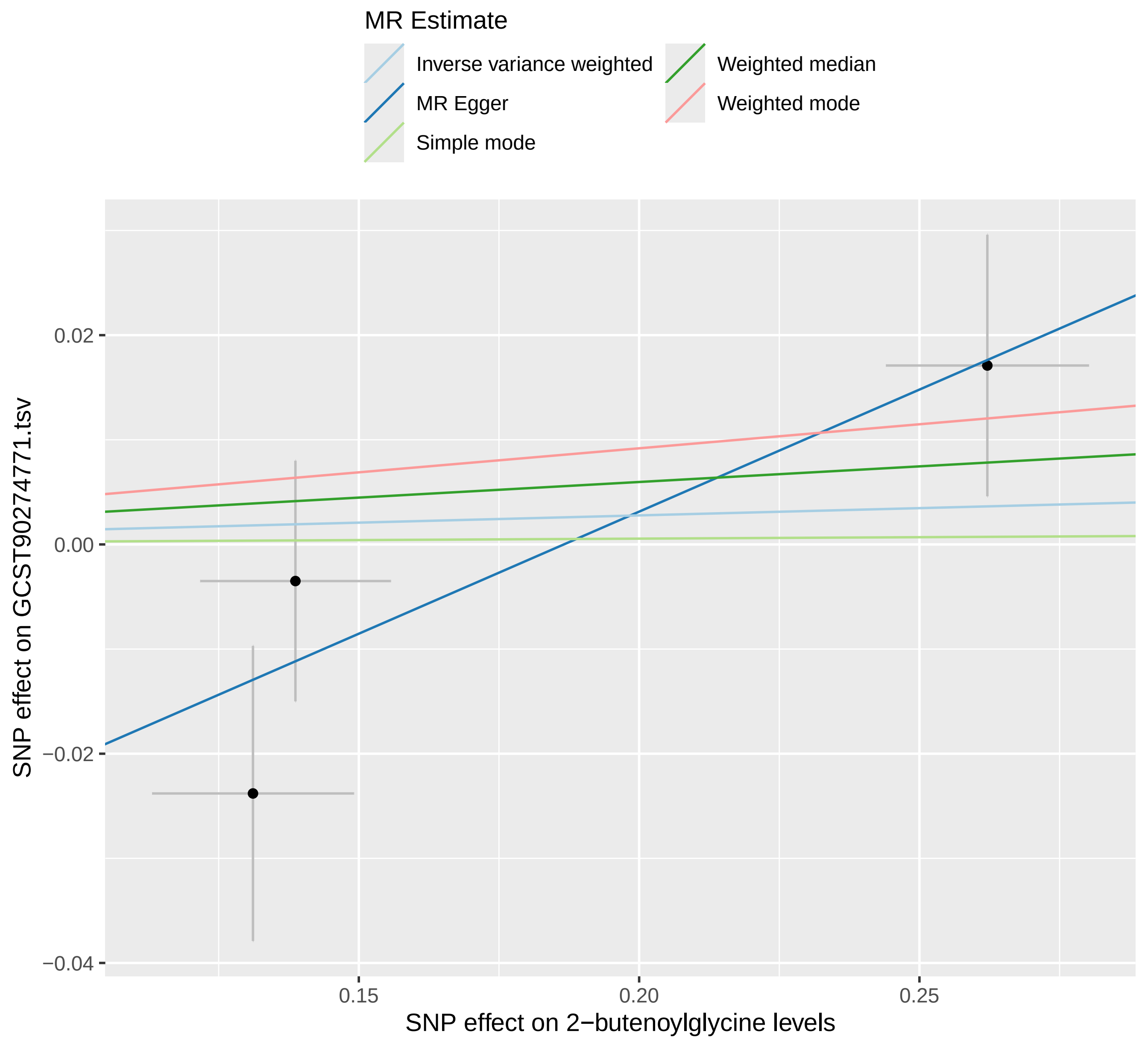

MR Estimate
Inverse variance weighted
MR Egger
Simple mode
Weighted median
Weighted mode
SNP effect on GCST90274771.tsv
0.02
0.00
−0.02
−0.04
0.15
0.20
0.25
SNP effect on 2−butenoylglycine levels

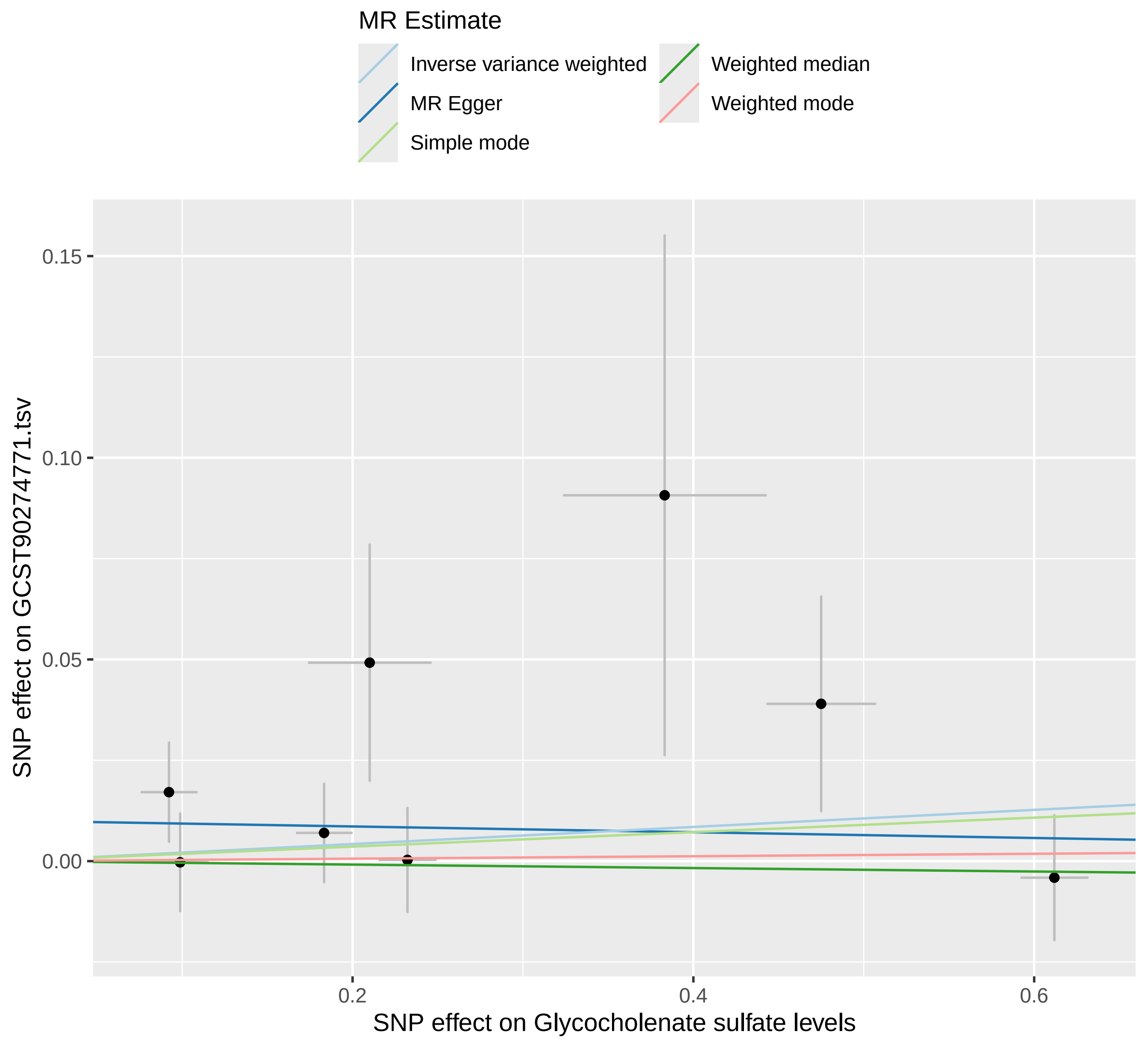
MR Estimate
Inverse variance weighted
MR Egger
Simple mode
Weighted median
Weighted mode
0.15
0.10
0.05
0.00
SNP effect on GCST90274771.tsv
0.2
0.4
0.6
SNP effect on Glycocholenate sulfate levels

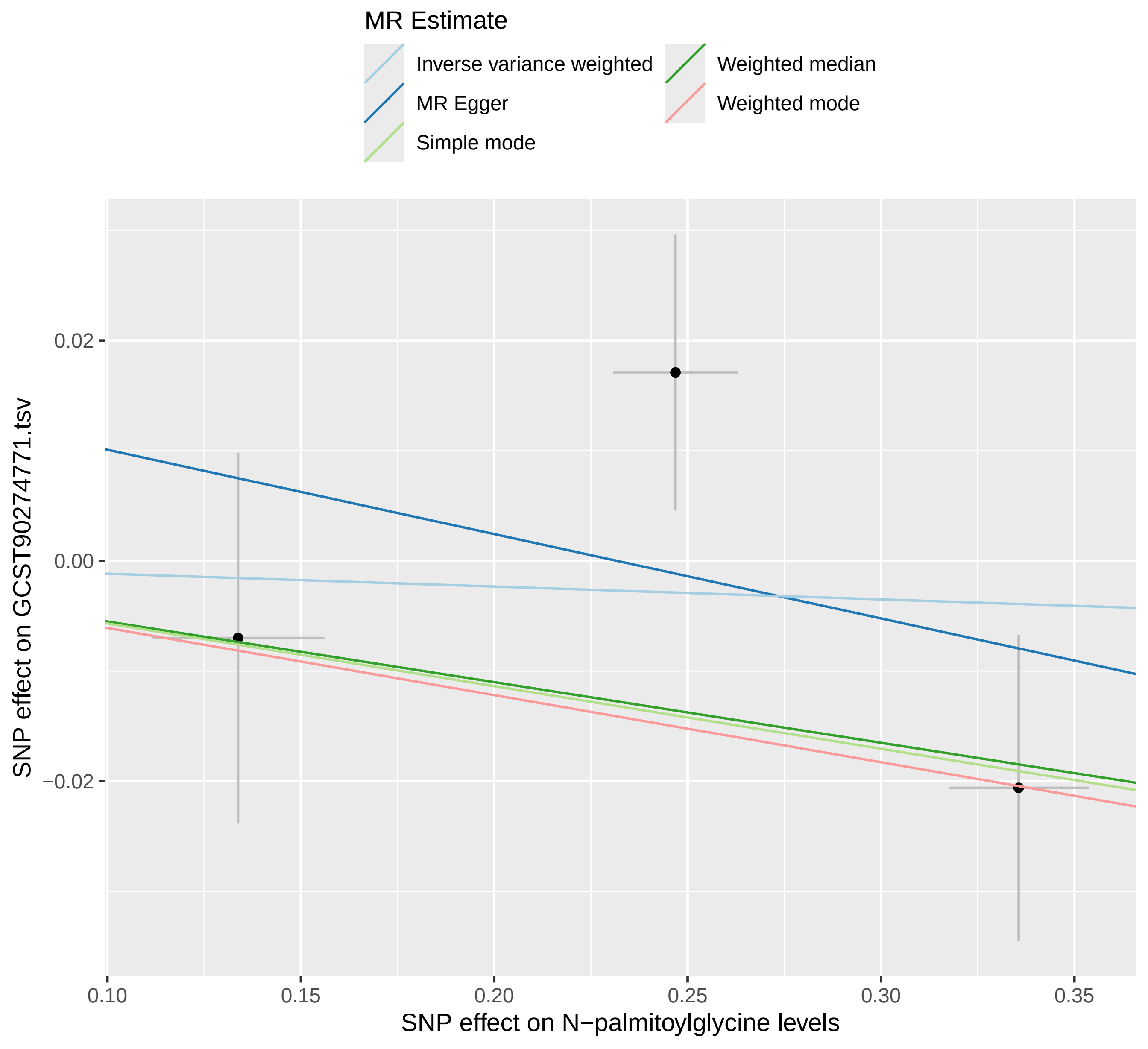
MR Estimate
Inverse variance weighted
MR Egger
Simple mode
Weighted median
Weighted mode
0.02
0.00
−0.02
SNP effect on GCST90274771.tsv
0.10
0.15
0.20
0.25
0.30
0.35
SNP effect on N−palmitoylglycine levels

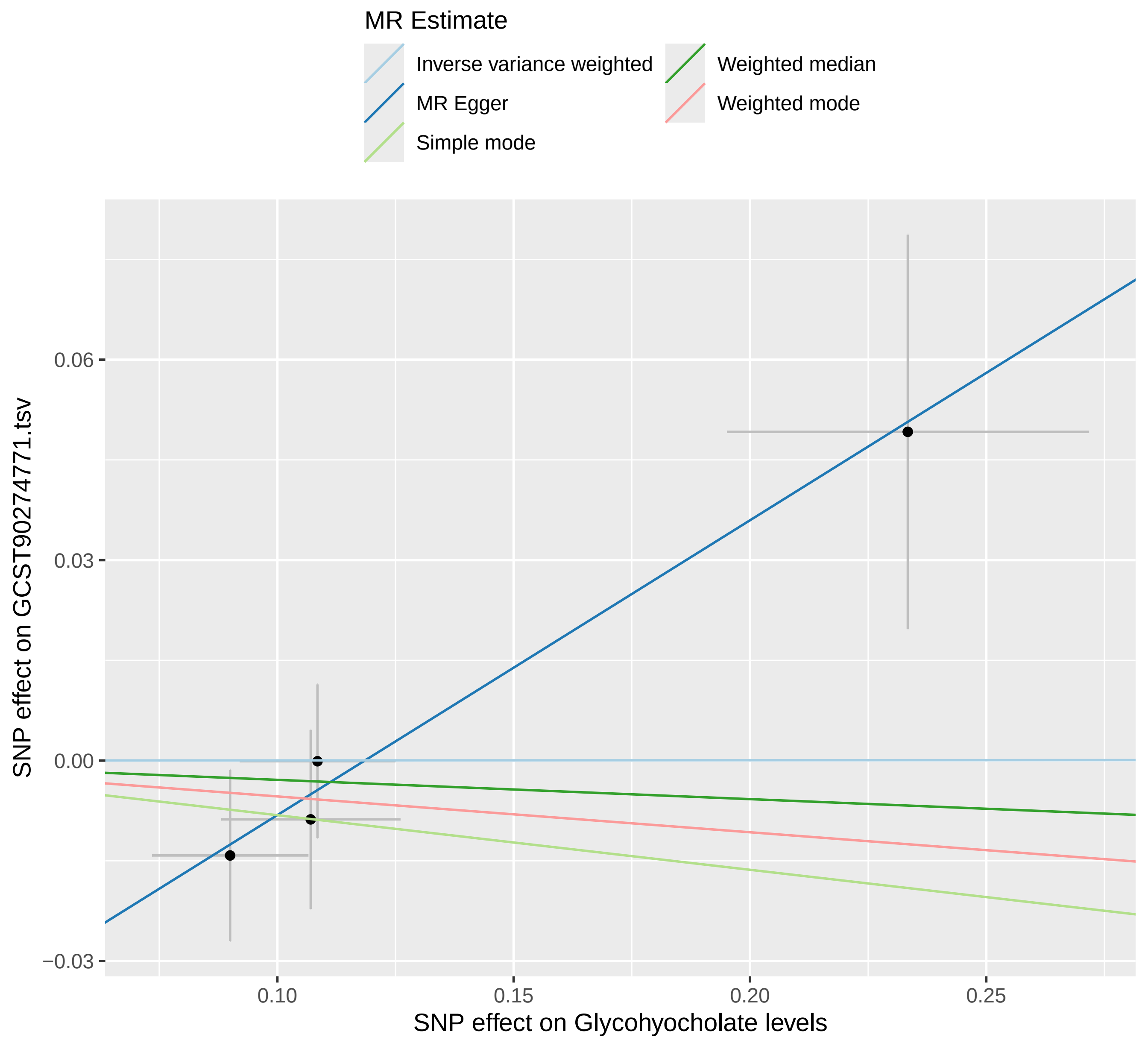
MR Estimate
Inverse variance weighted
MR Egger
Simple mode
Weighted median
Weighted mode
0.06
0.03
0.00
−0.03
0.10
0.15
0.20
0.25
SNP effect on GCST90274771.tsv
SNP effect on Glycohyocholate levels

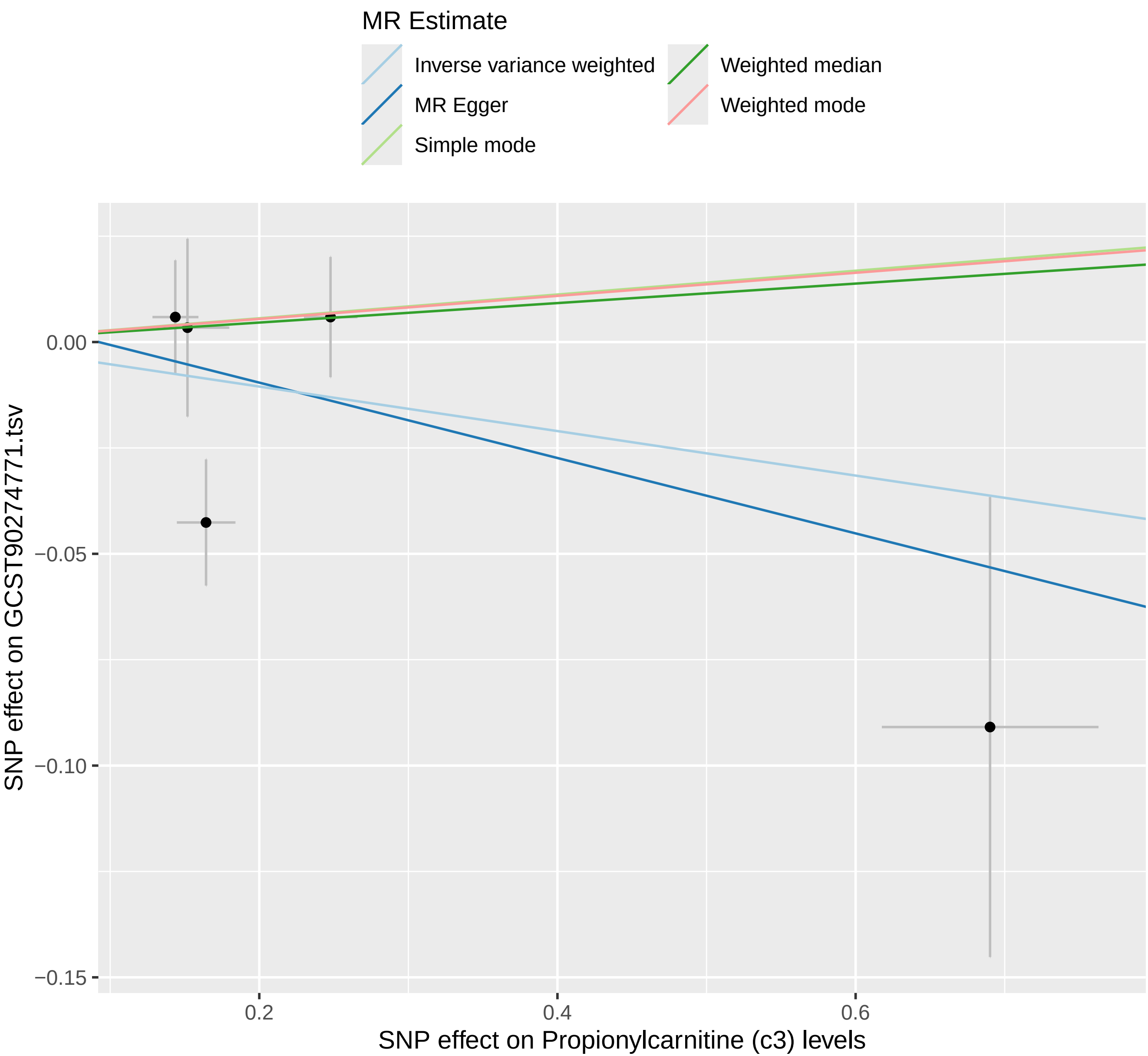
MR Estimate
Inverse variance weighted
MR Egger
Simple mode
Weighted median
Weighted mode
0.00
−0.05
−0.10
−0.15
0.2
0.4
0.6
SNP effect on GCST90274771.tsv
SNP effect on Propionylcarnitine (c3) levels

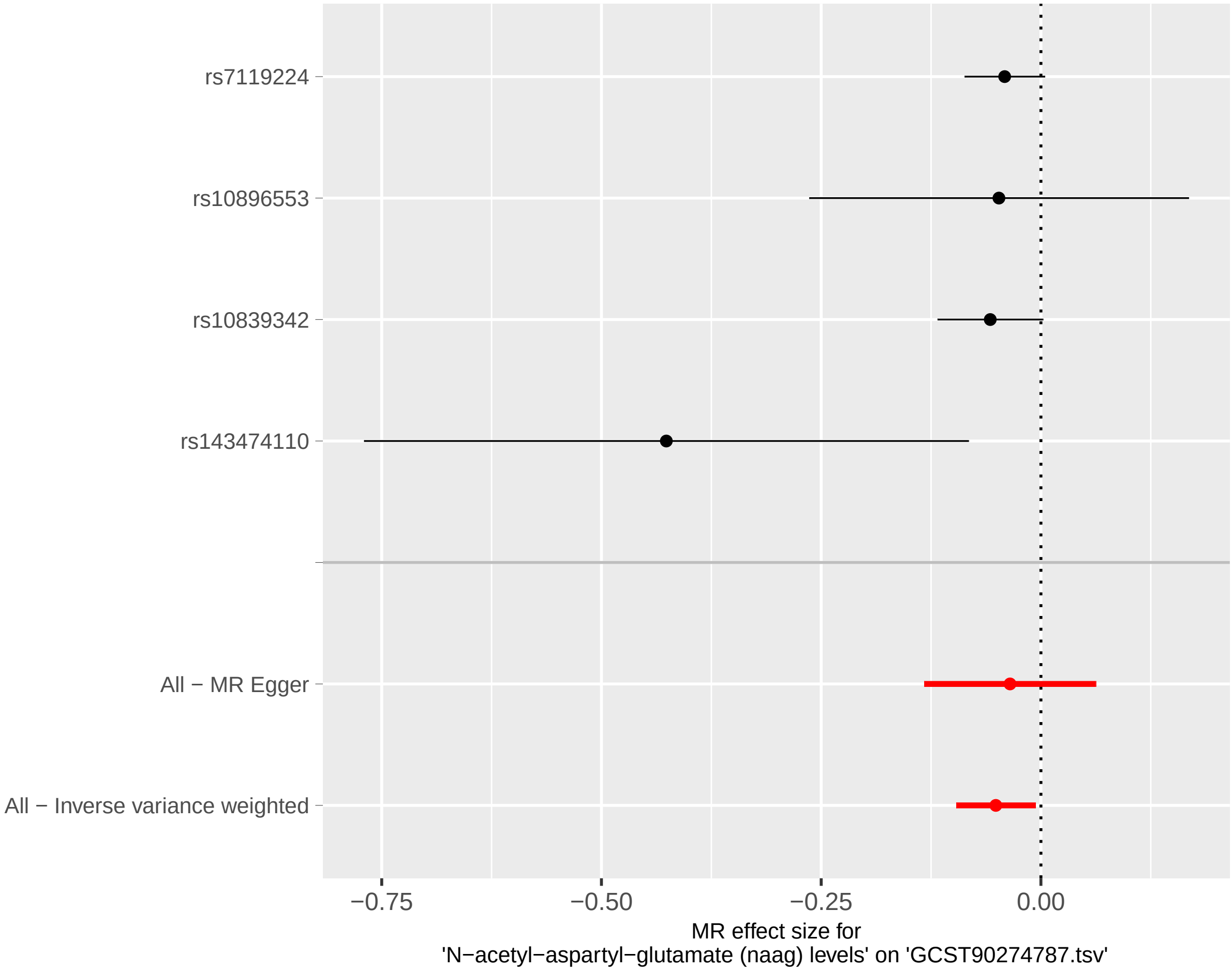

rs7119224
rs10896553
rs10839342
rs143474110
All − MR Egger
All − Inverse variance weighted
−0.75
−0.50
−0.25
0.00
MR effect size for
'N−acetyl−aspartyl−glutamate (naag) levels' on 'GCST90274787.tsv'

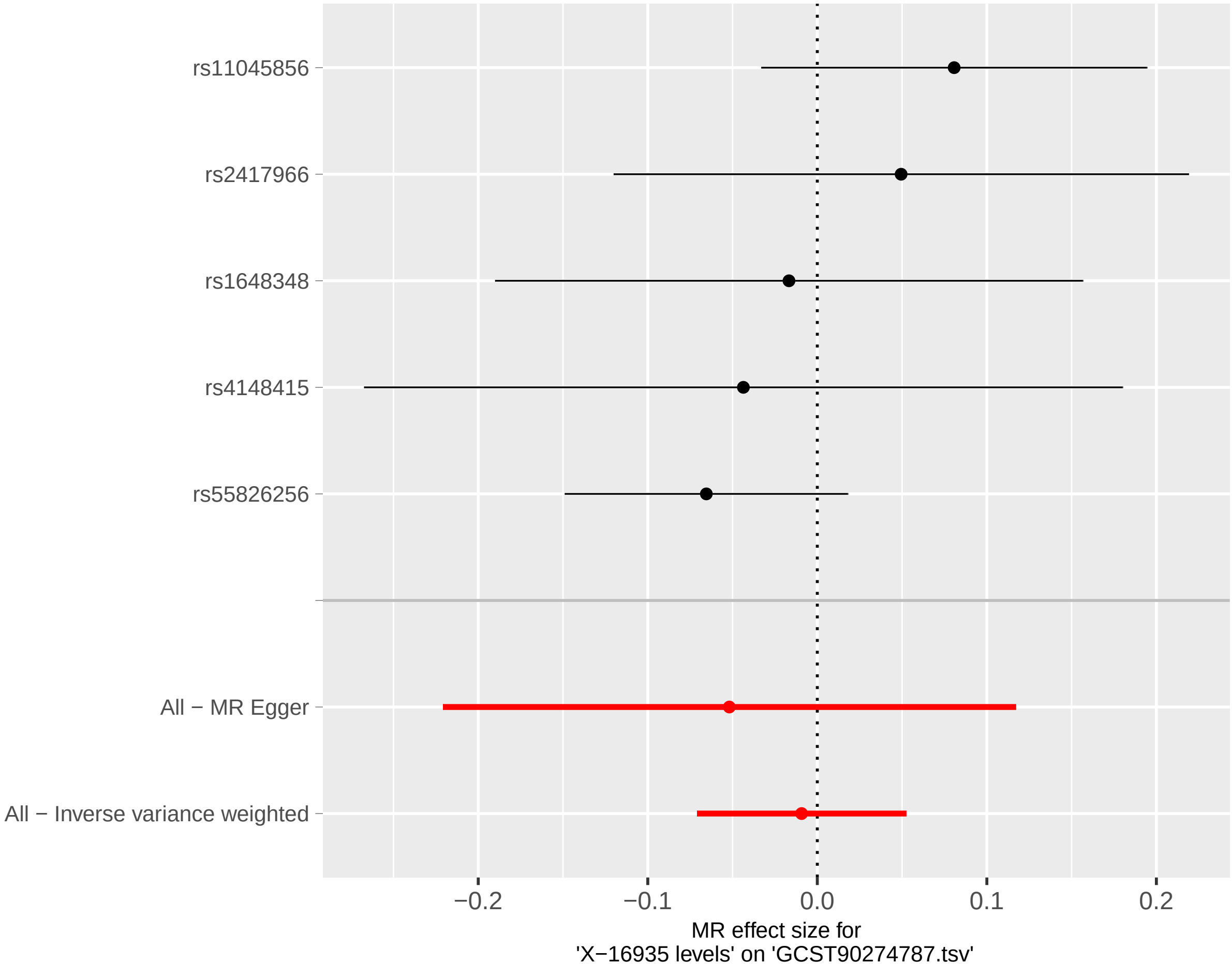

rs11045856
rs2417966
rs1648348
rs4148415
rs55826256
All − MR Egger
All − Inverse variance weighted
−0.2
−0.1
0.0
0.1
0.2
MR effect size for
'X−16935 levels' on 'GCST90274787.tsv'

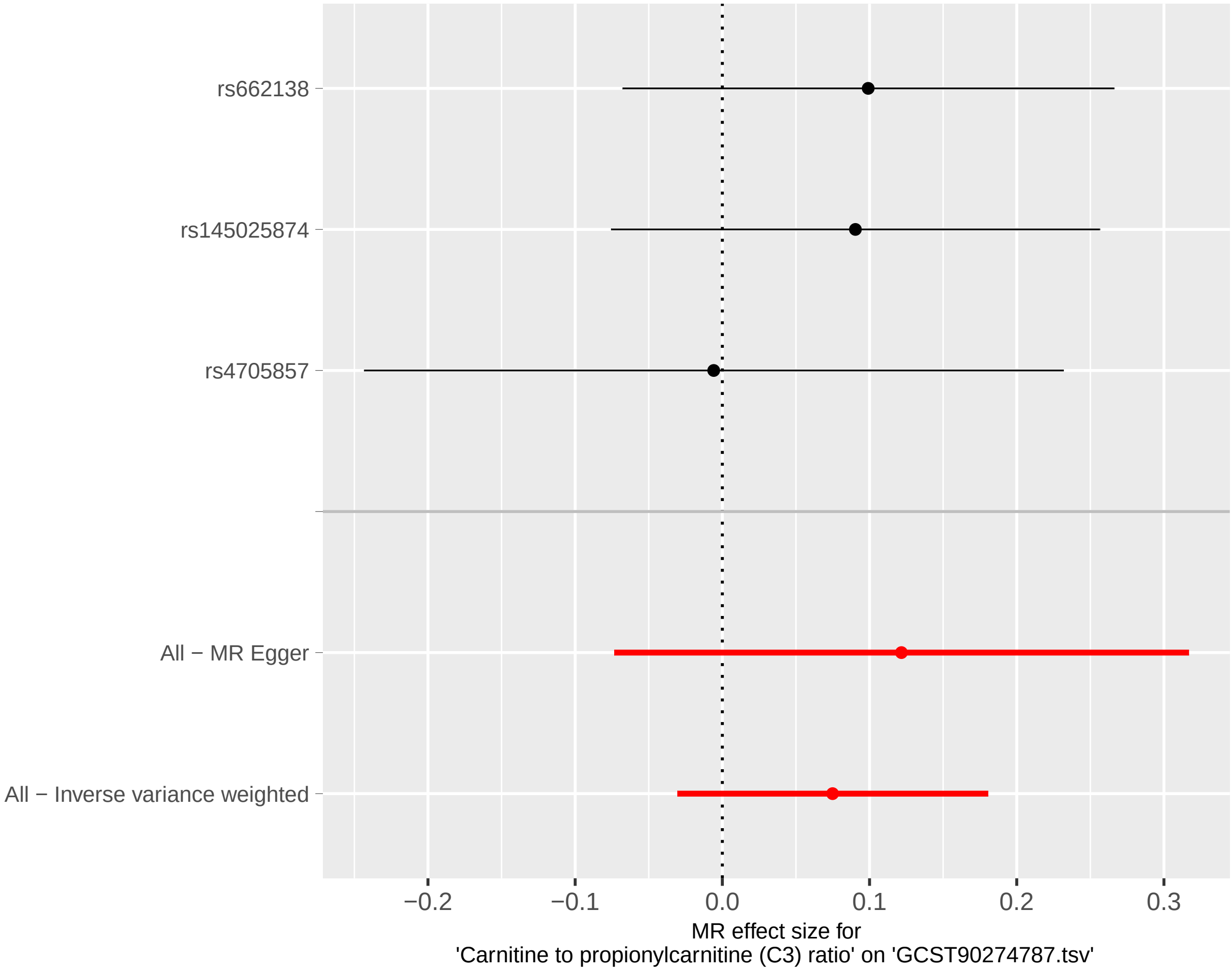

rs662138
rs145025874
rs4705857
All − MR Egger
All − Inverse variance weighted
−0.2
−0.1
0.0
0.1
0.2
0.3
MR effect size for
'Carnitine to propionylcarnitine (C3) ratio' on 'GCST90274787.tsv'

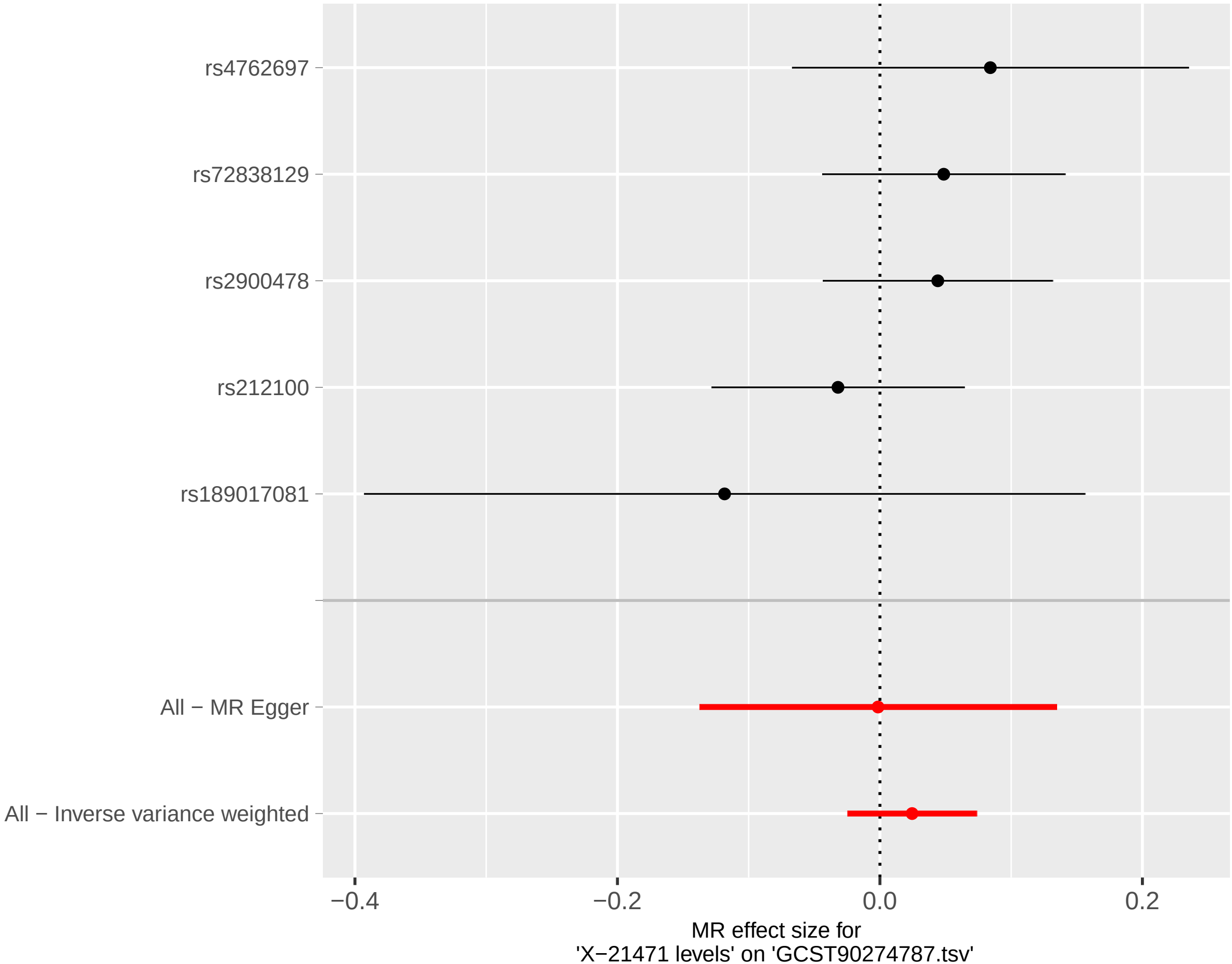
rs4762697
rs72838129
rs2900478
rs212100
rs189017081
All − MR Egger
All − Inverse variance weighted
−0.4
−0.2
0.0
0.2
MR effect size for
'X−21471 levels' on 'GCST90274787.tsv'

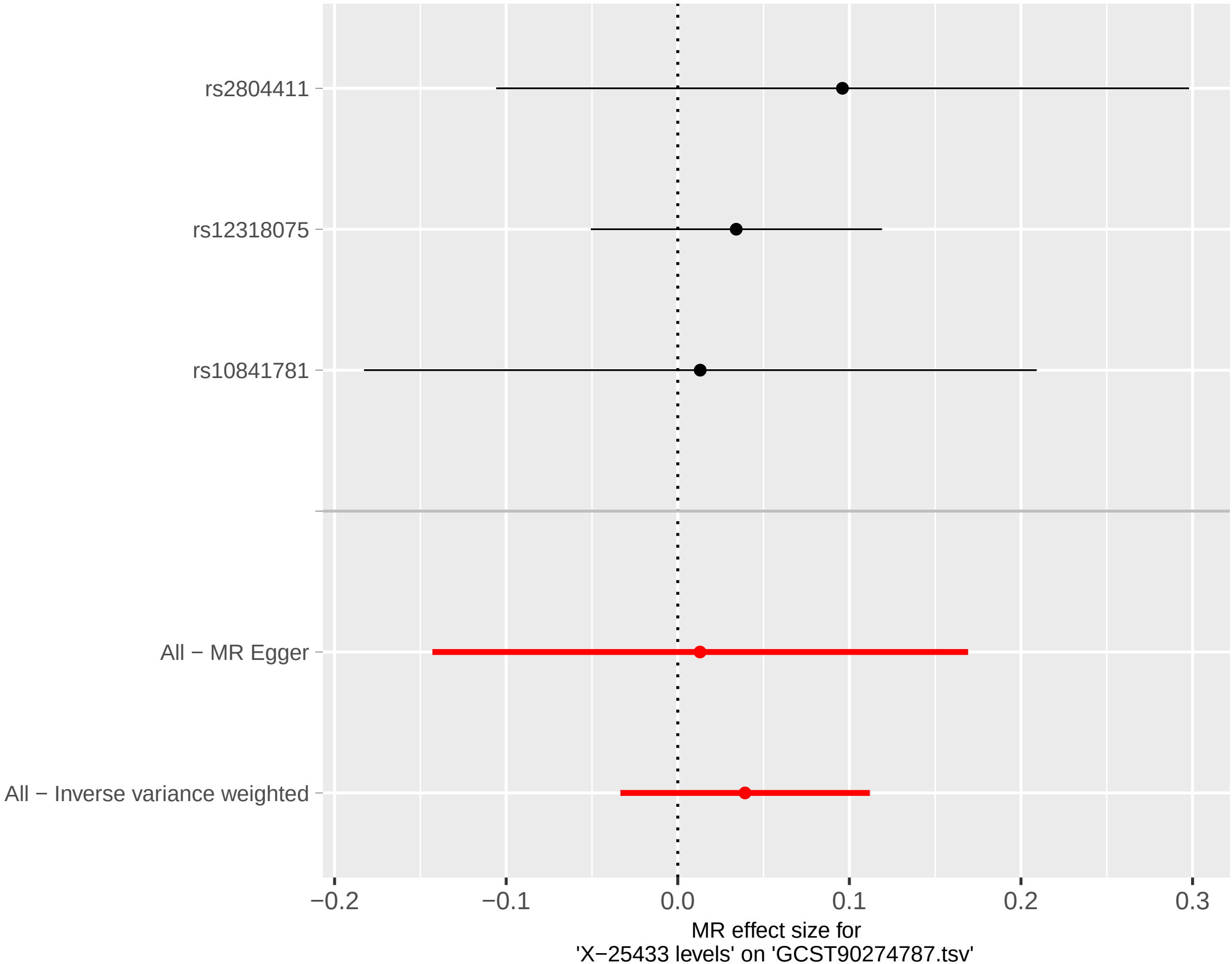
rs2804411
rs12318075
rs10841781
All − MR Egger
All − Inverse variance weighted
−0.2
−0.1
0.0
0.1
0.2
0.3
MR effect size for
'X−25433 levels' on 'GCST90274787.tsv'

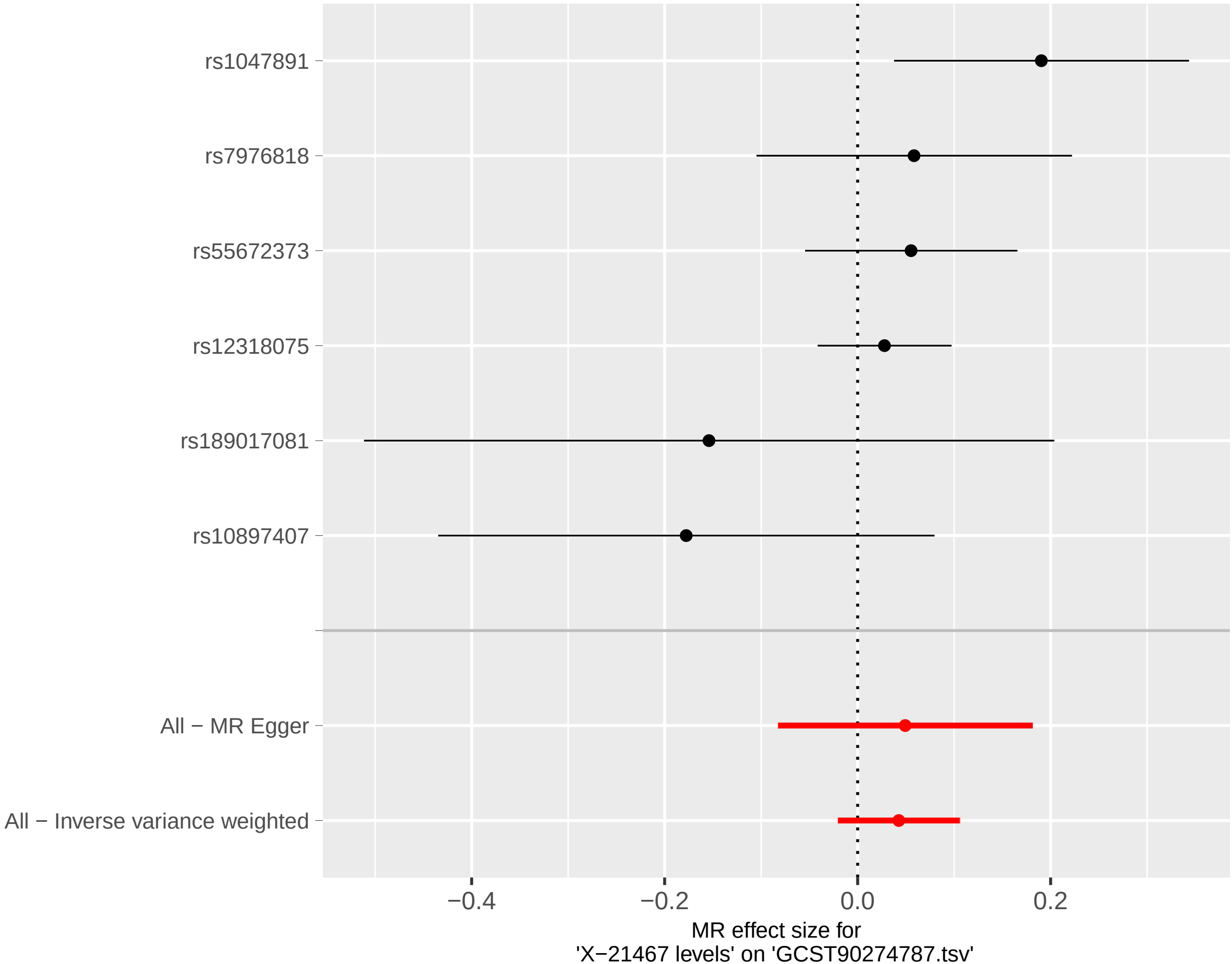
rs1047891
rs7976818
rs55672373
rs12318075
rs189017081
rs10897407
All − MR Egger
All − Inverse variance weighted
−0.4
−0.2
0.0
0.2
MR effect size for
'X−21467 levels' on 'GCST90274787.tsv'

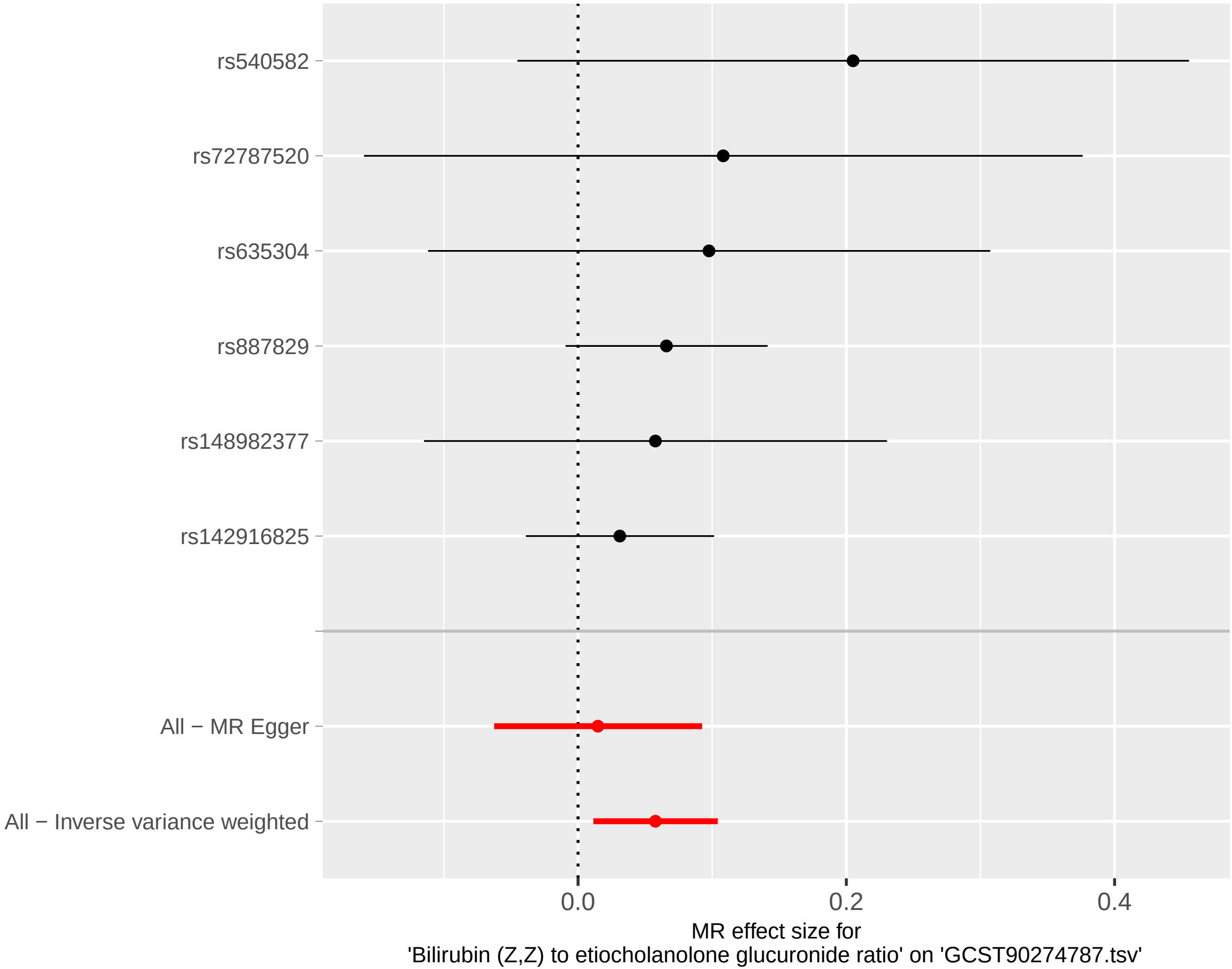
rs540582
rs72787520
rs635304
rs887829
rs148982377
rs142916825
All − MR Egger
All − Inverse variance weighted
0.0
0.2
0.4
MR effect size for
'Bilirubin (Z,Z) to etiocholanolone glucuronide ratio' on 'GCST90274787.tsv'

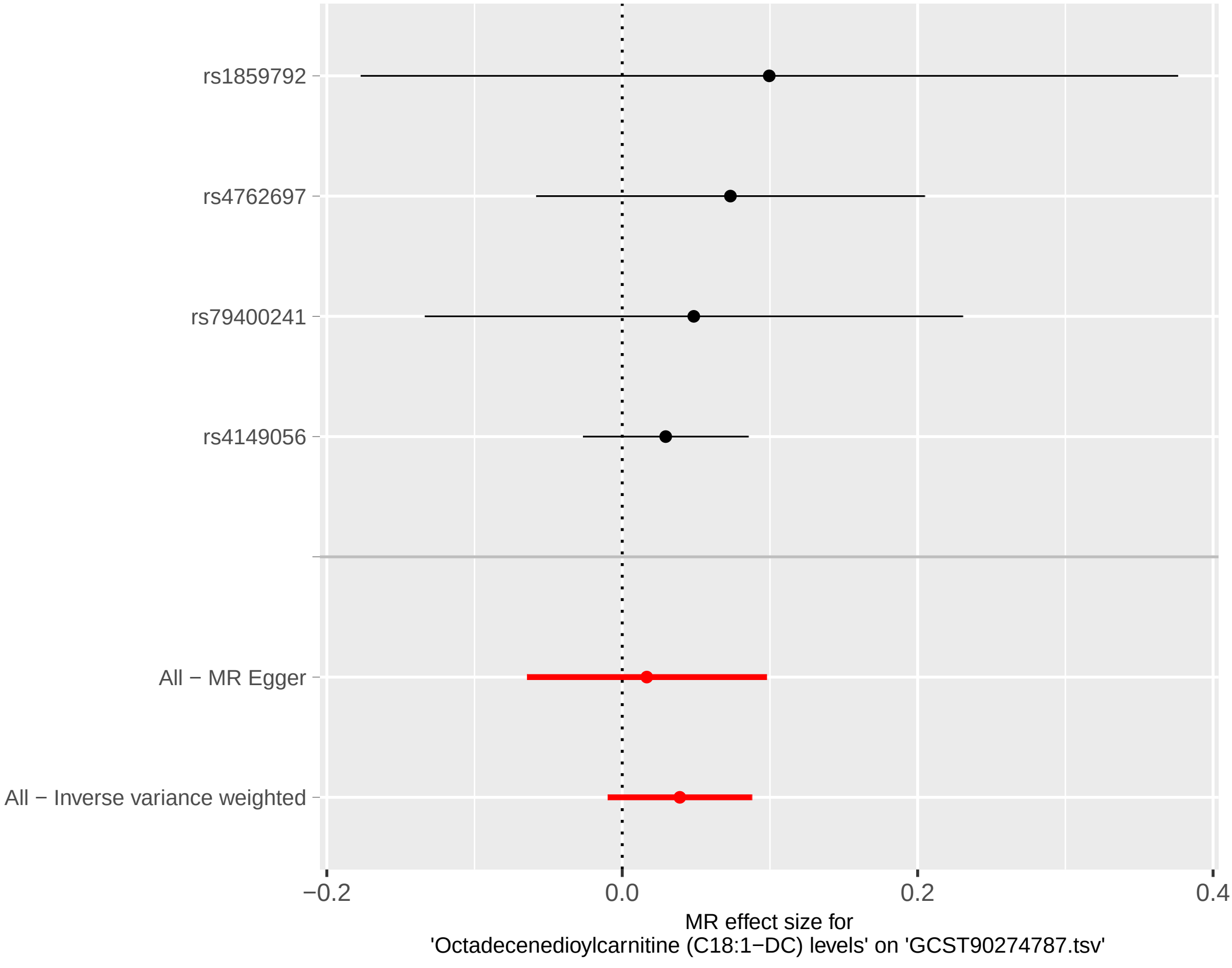

rs1859792
rs4762697
rs79400241
rs4149056
All − MR Egger
All − Inverse variance weighted
−0.2
0.0
0.2
0.4
MR effect size for
'Octadecenedioylcarnitine (C18:1−DC) levels' on 'GCST90274787.tsv'

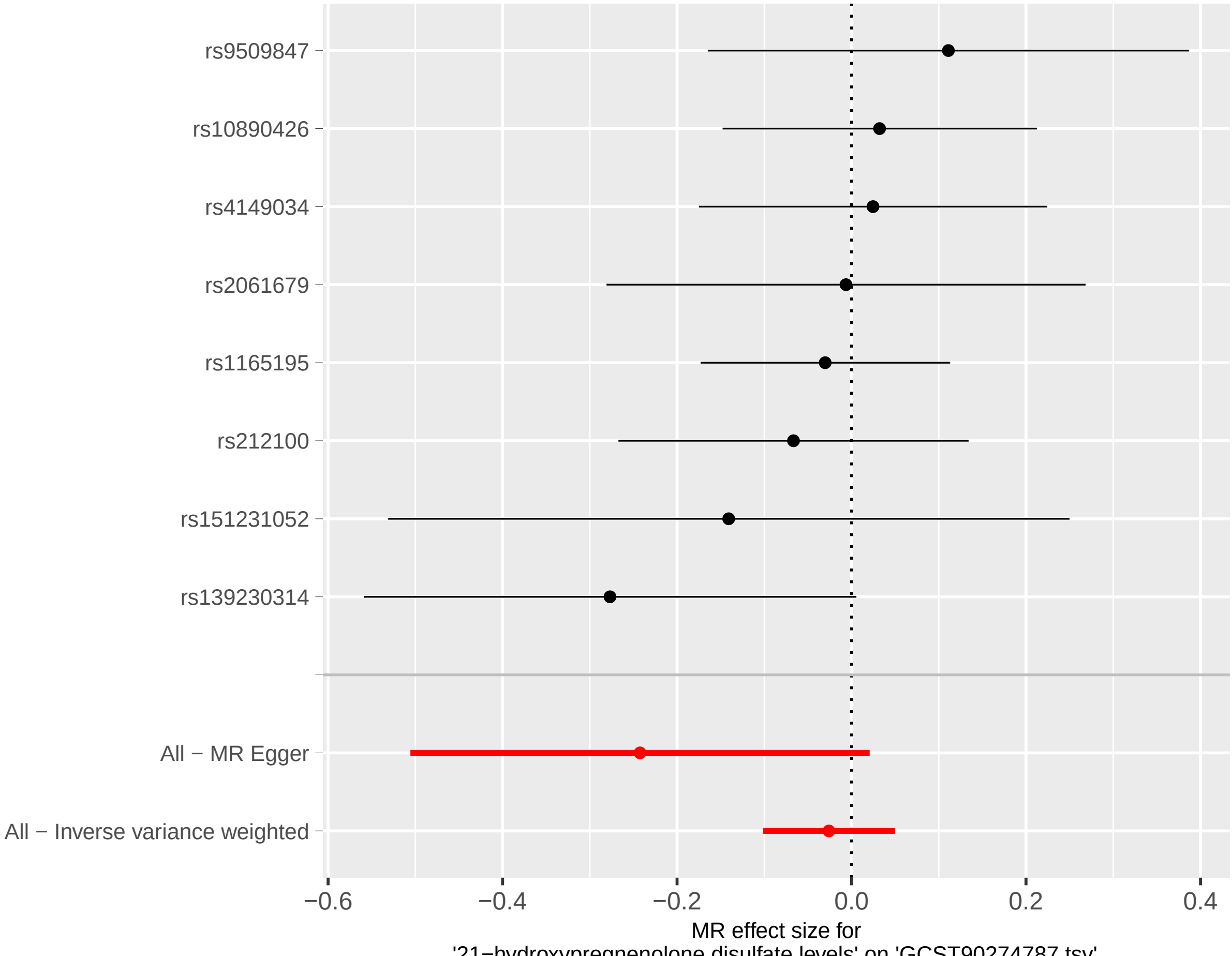

rs9509847
rs10890426
rs4149034
rs2061679
rs1165195
rs212100
rs151231052
rs139230314
All − MR Egger
All − Inverse variance weighted
−0.6
−0.4
−0.2
0.0
0.2
0.4
MR effect size for
'21−hydroxypregnenolone disulfate levels' on 'GCST90274787.tsv'

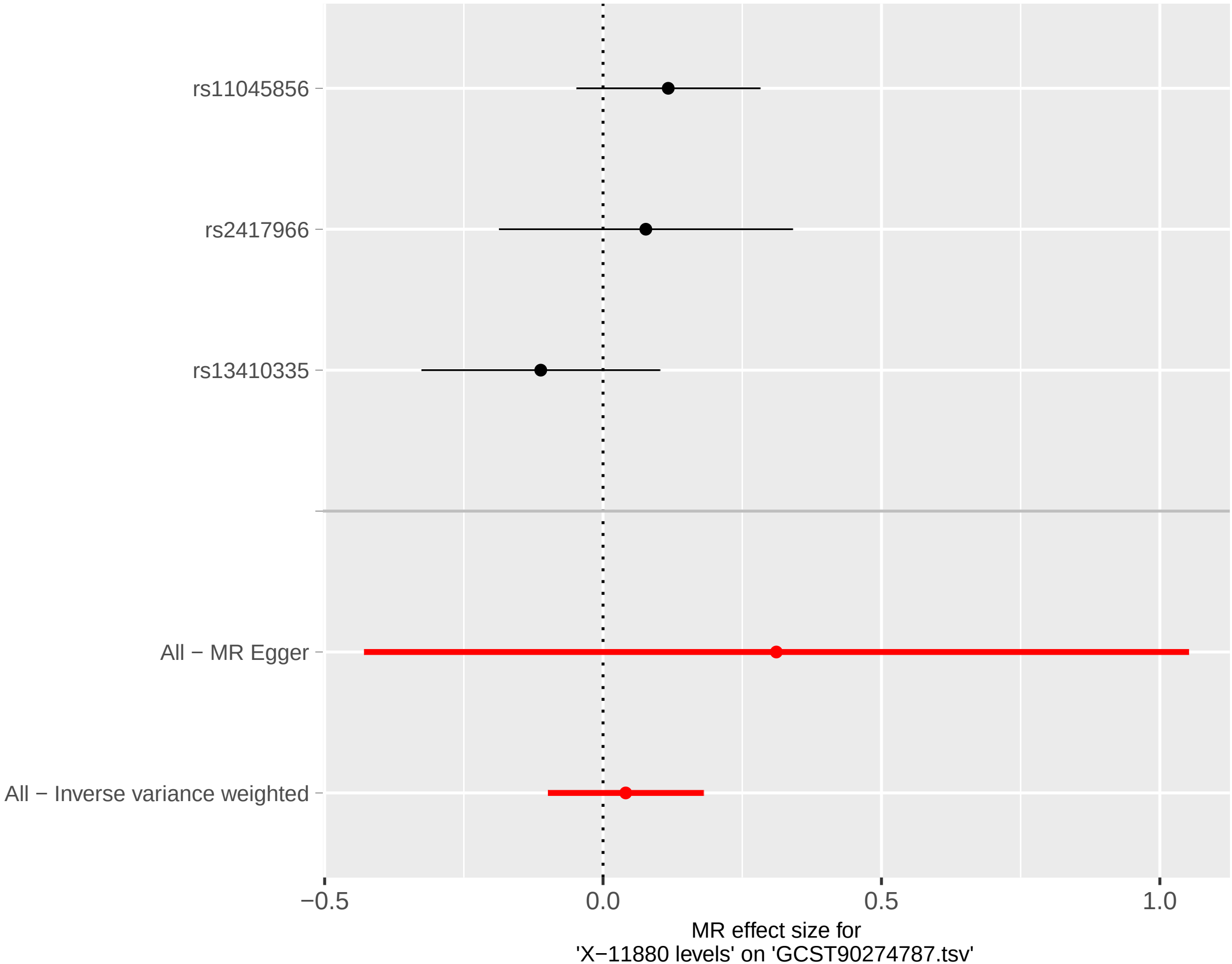
rs11045856
rs2417966
rs13410335
All − MR Egger
All − Inverse variance weighted
−0.5
0.0
0.5
1.0
MR effect size for
'X−11880 levels' on 'GCST90274787.tsv'

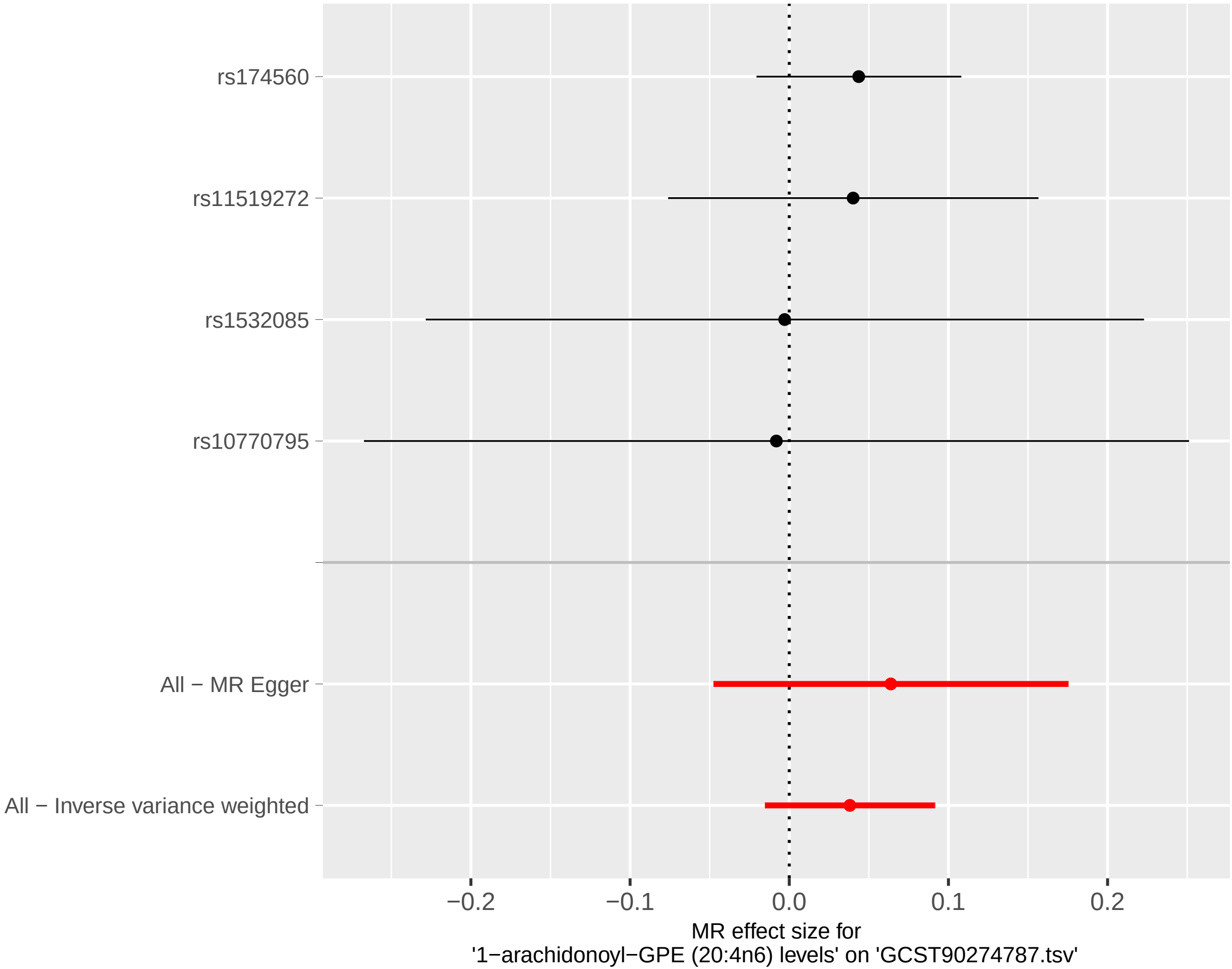
rs174560
rs11519272
rs1532085
rs10770795
All − MR Egger
All − Inverse variance weighted
−0.2
−0.1
0.0
0.1
0.2
MR effect size for
'1−arachidonoyl−GPE (20:4n6) levels' on 'GCST90274787.tsv'

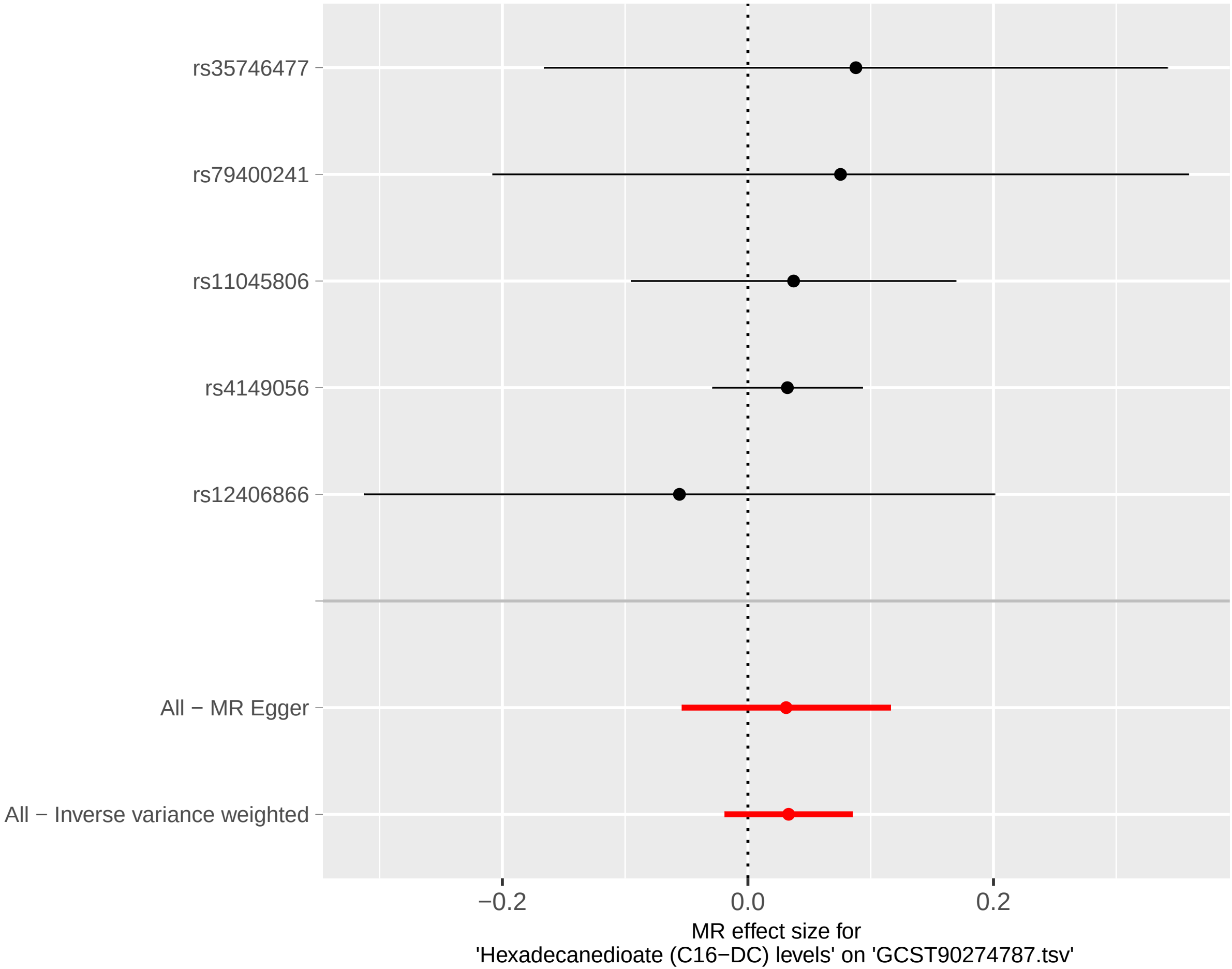

rs35746477
rs79400241
rs11045806
rs4149056
rs12406866
All − MR Egger
All − Inverse variance weighted
−0.2
0.0
0.2
MR effect size for
'Hexadecanedioate (C16−DC) levels' on 'GCST90274787.tsv'

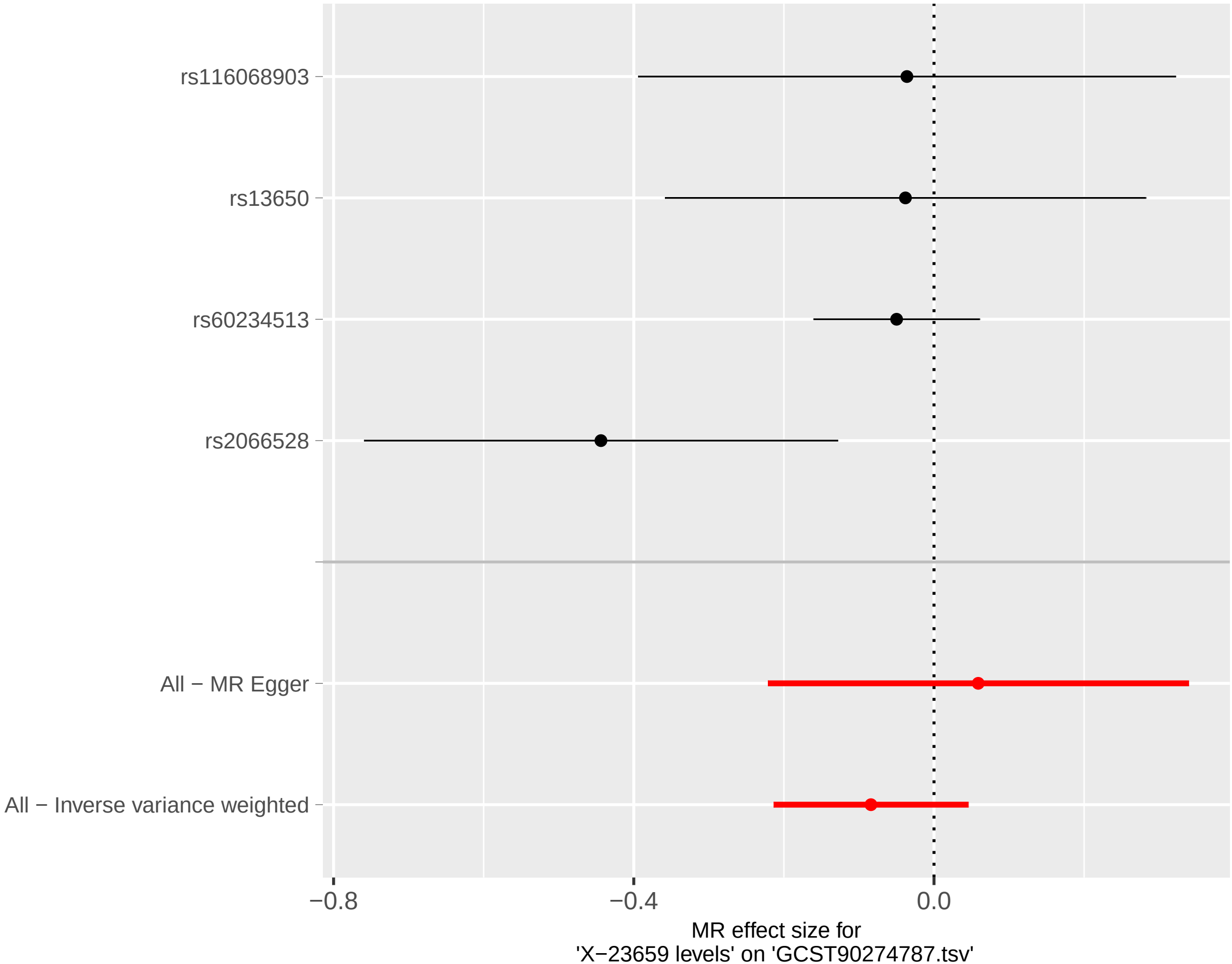
rs116068903
rs13650
rs60234513
rs2066528
All − MR Egger
All − Inverse variance weighted
−0.8
−0.4
0.0
MR effect size for
'X−23659 levels' on 'GCST90274787.tsv'

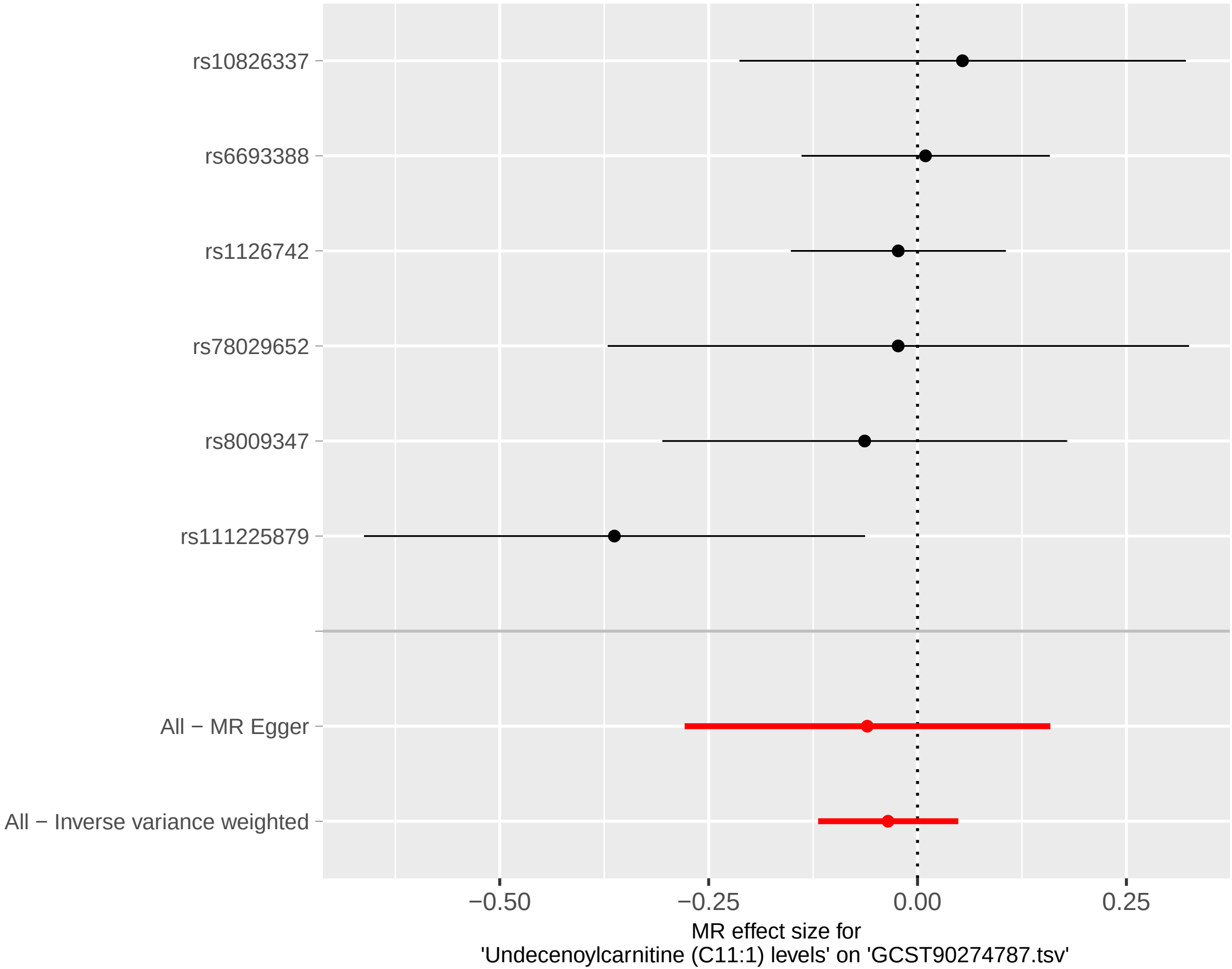

rs10826337
rs6693388
rs1126742
rs78029652
rs8009347
rs111225879
All − MR Egger
All − Inverse variance weighted
−0.50
−0.25
0.00
0.25
MR effect size for
'Undecenoylcarnitine (C11:1) levels' on 'GCST90274787.tsv'

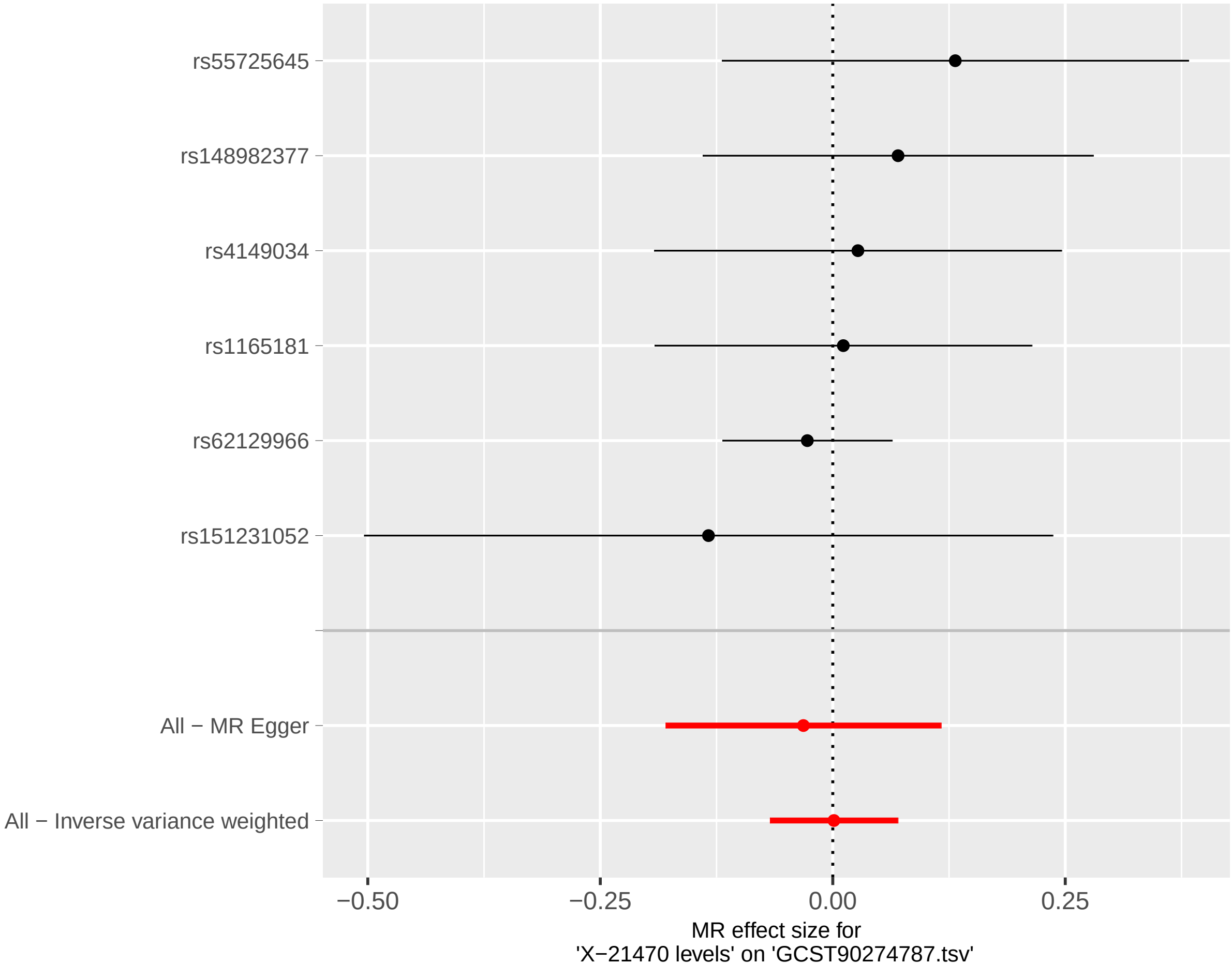
rs55725645
rs148982377
rs4149034
rs1165181
rs62129966
rs151231052
All − MR Egger
All − Inverse variance weighted
−0.50
−0.25
0.00
0.25
MR effect size for
'X−21470 levels' on 'GCST90274787.tsv'

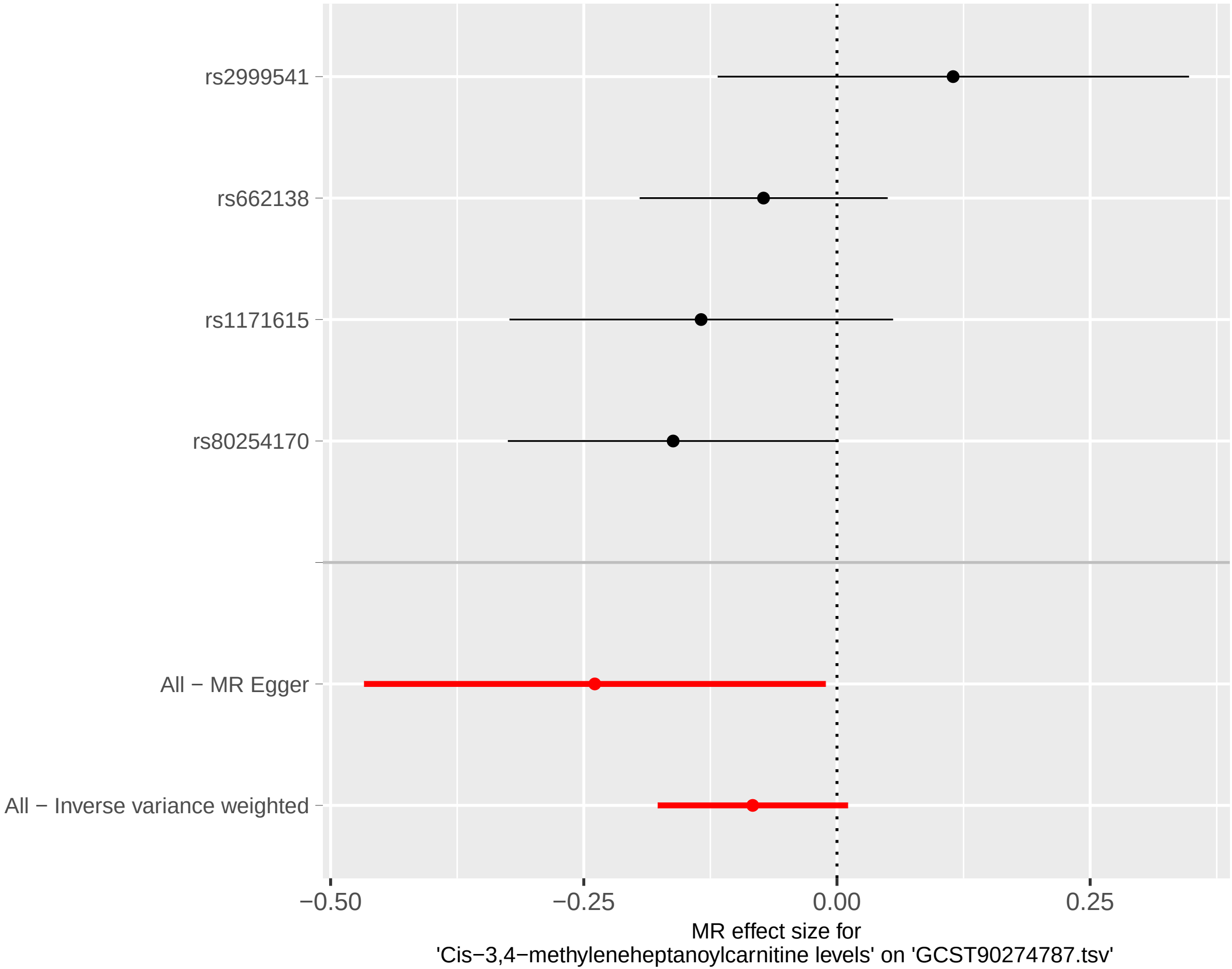

rs2999541
rs662138
rs1171615
rs80254170
All − MR Egger
All − Inverse variance weighted
−0.50
−0.25
0.00
0.25
MR effect size for
'Cis−3,4−methyleneheptanoylcarnitine levels' on 'GCST90274787.tsv'

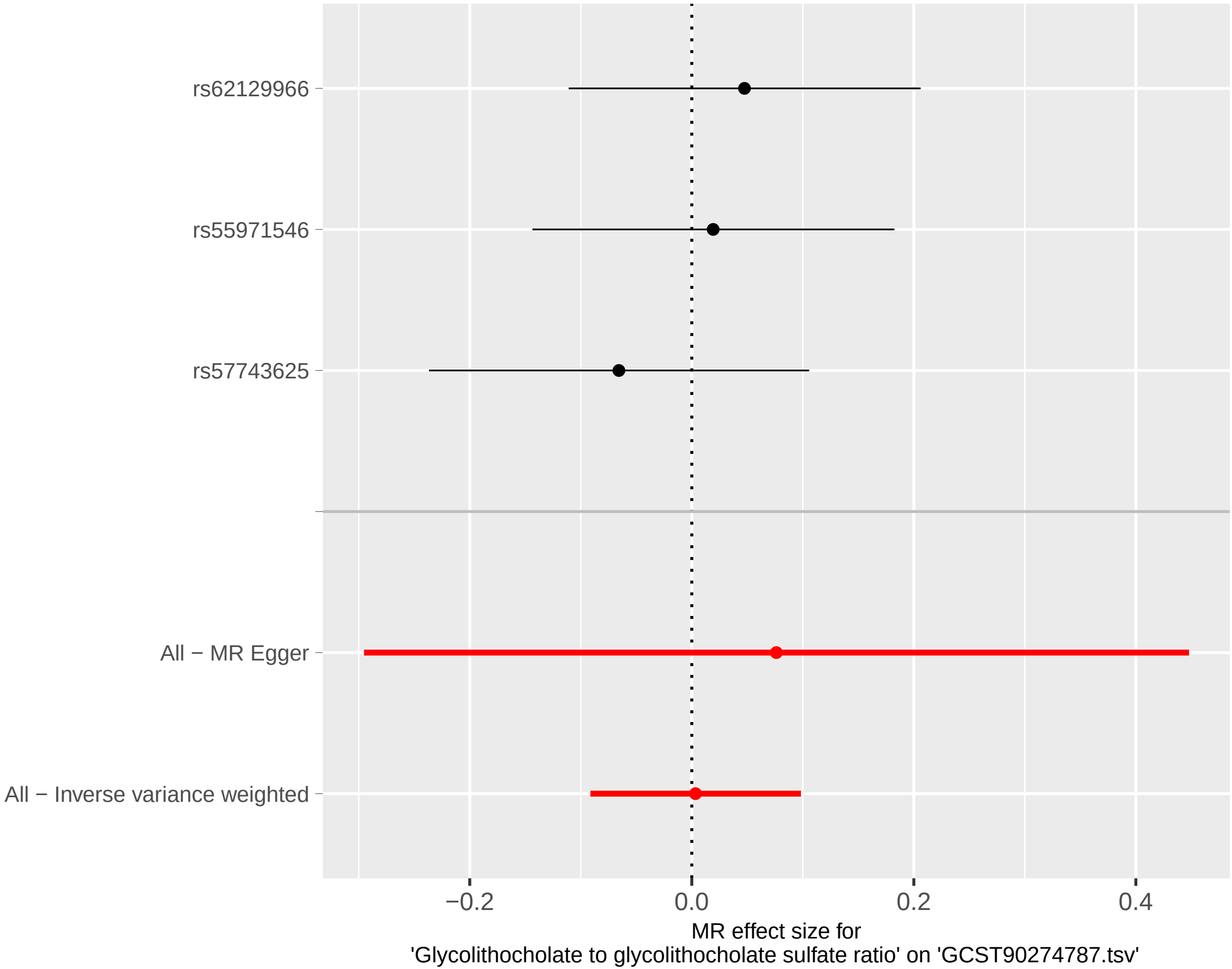
rs62129966
rs55971546
rs57743625
All − MR Egger
All − Inverse variance weighted
−0.2
0.0
0.2
0.4
MR effect size for
'Glycolithocholate to glycolithocholate sulfate ratio' on 'GCST90274787.tsv'

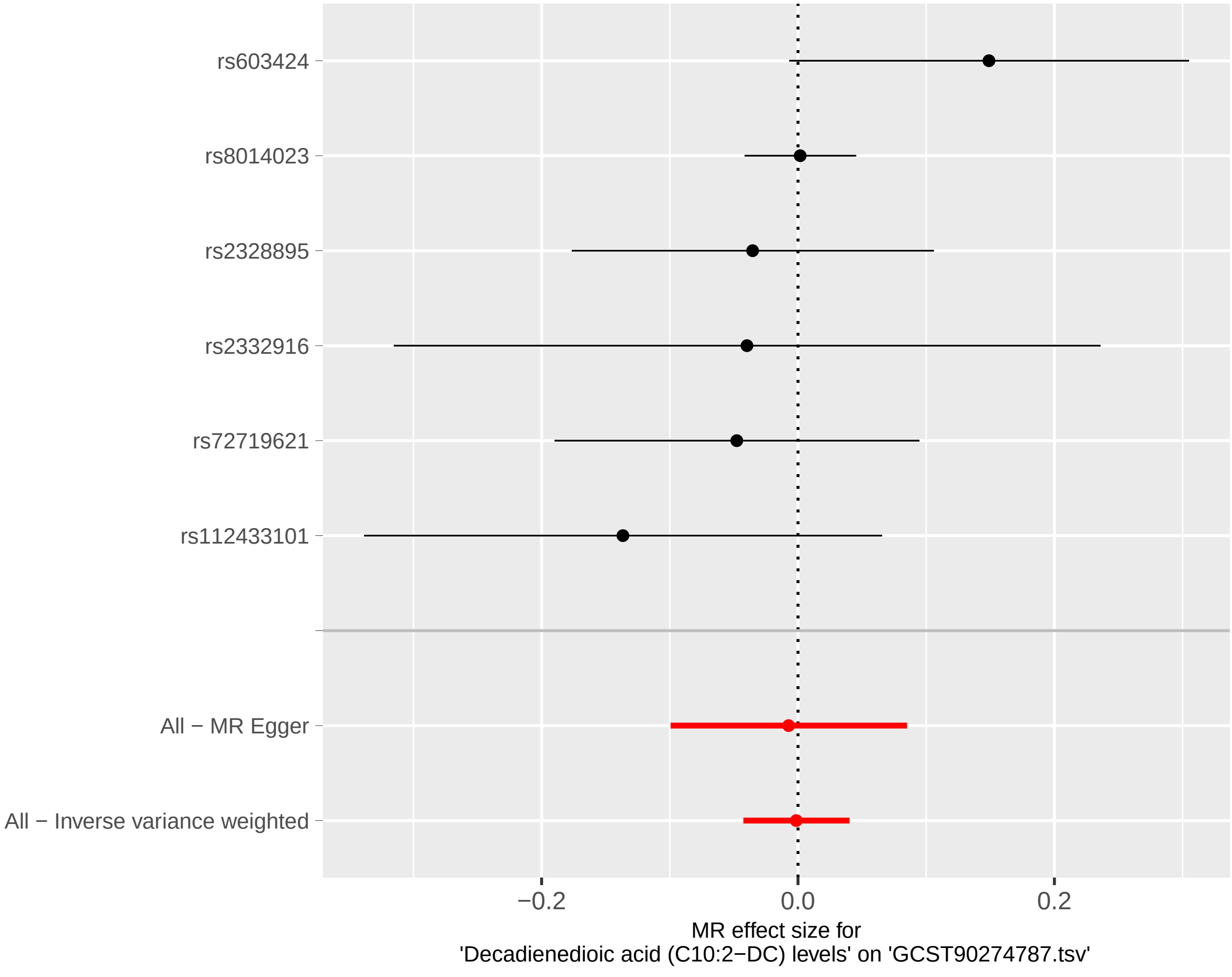

rs603424
rs8014023
rs2328895
rs2332916
rs72719621
rs112433101
All − MR Egger
All − Inverse variance weighted
−0.2
0.0
0.2
MR effect size for
'Decadienedioic acid (C10:2−DC) levels' on 'GCST90274787.tsv'

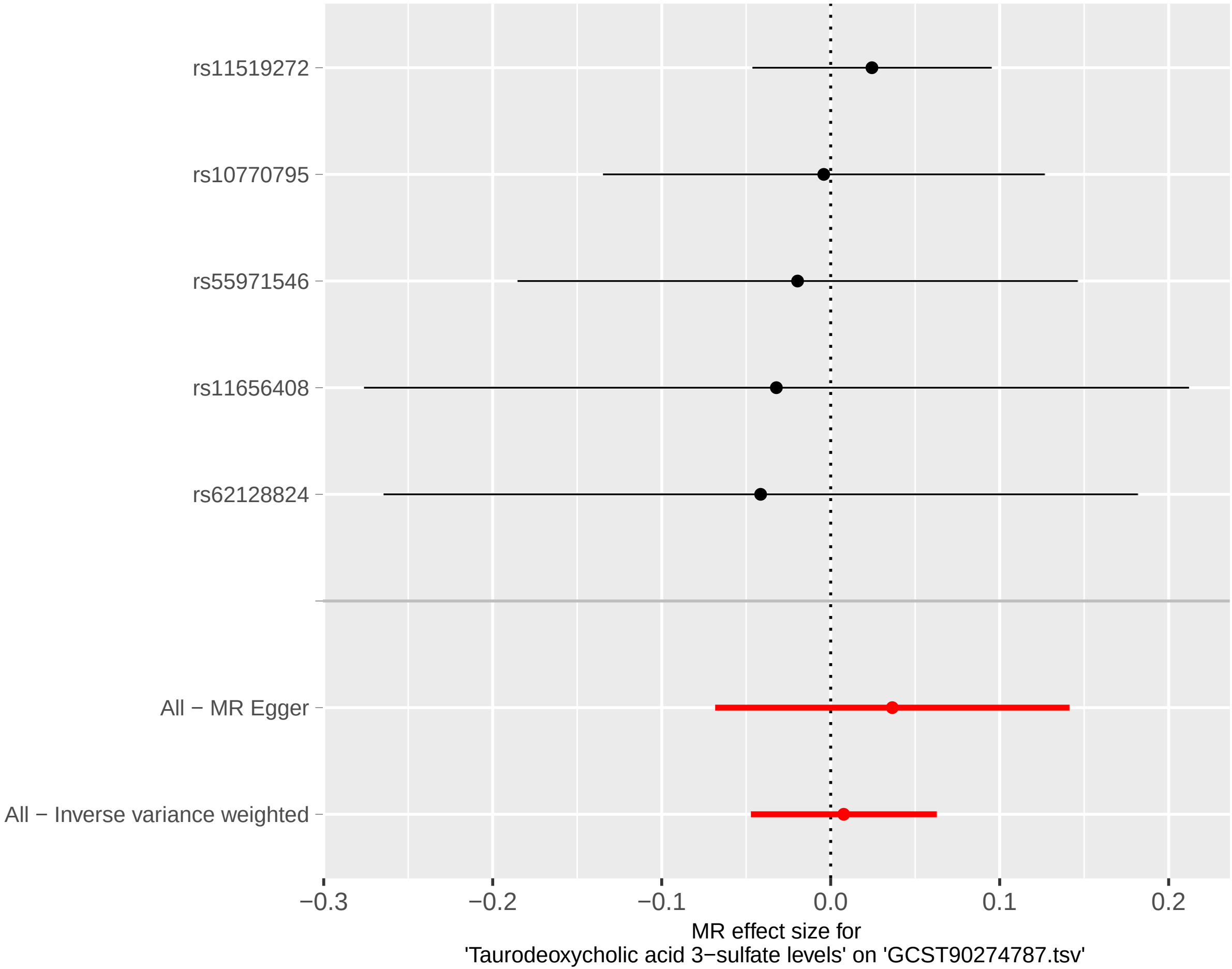
rs11519272
rs10770795
rs55971546
rs11656408
rs62128824
All − MR Egger
All − Inverse variance weighted
−0.3
−0.2
−0.1
0.0
0.1
0.2
MR effect size for
'Taurodeoxycholic acid 3−sulfate levels' on 'GCST90274787.tsv'

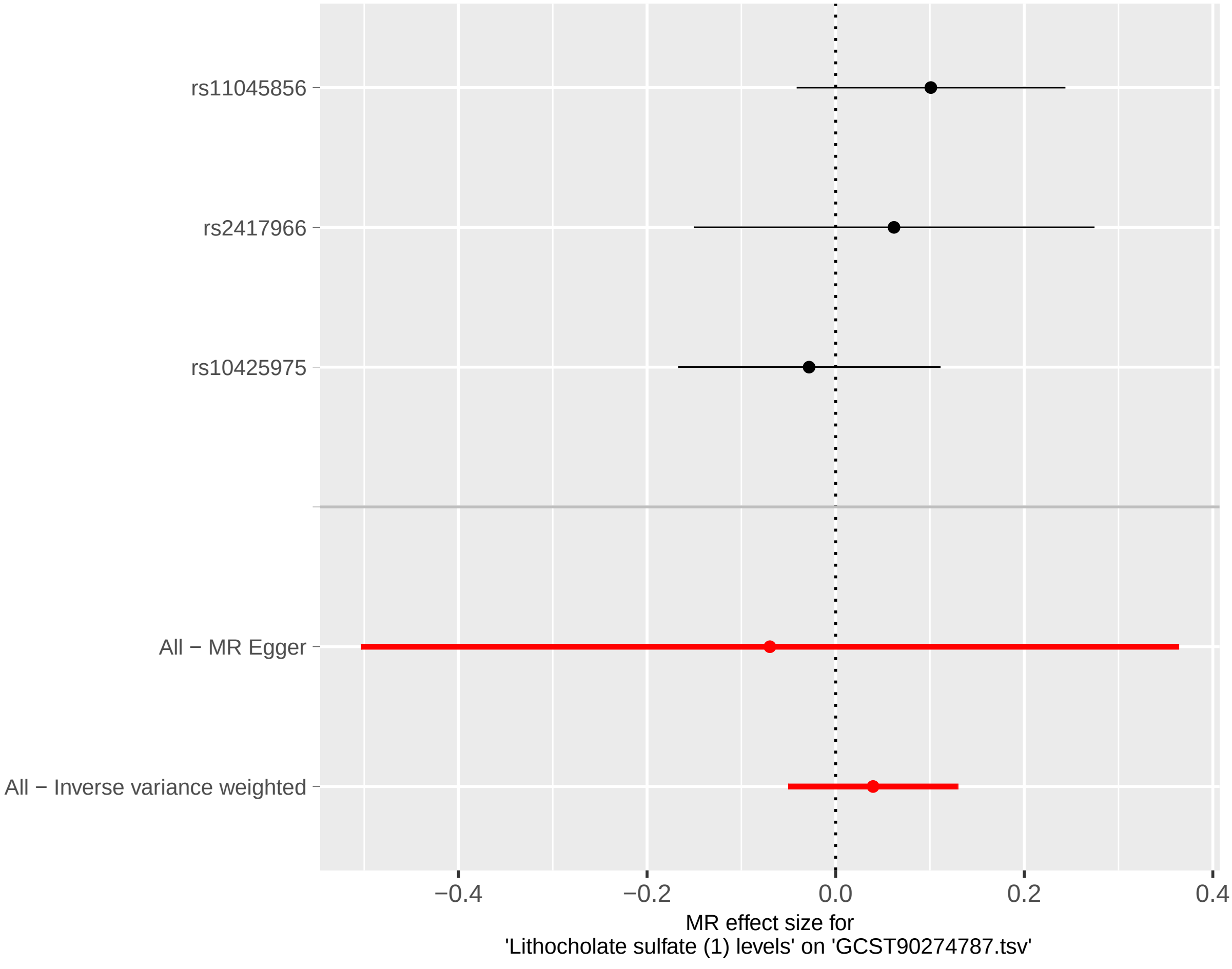
rs11045856
rs2417966
rs10425975
All − MR Egger
All − Inverse variance weighted
−0.4
−0.2
0.0
0.2
0.4
MR effect size for
'Lithocholate sulfate (1) levels' on 'GCST90274787.tsv'

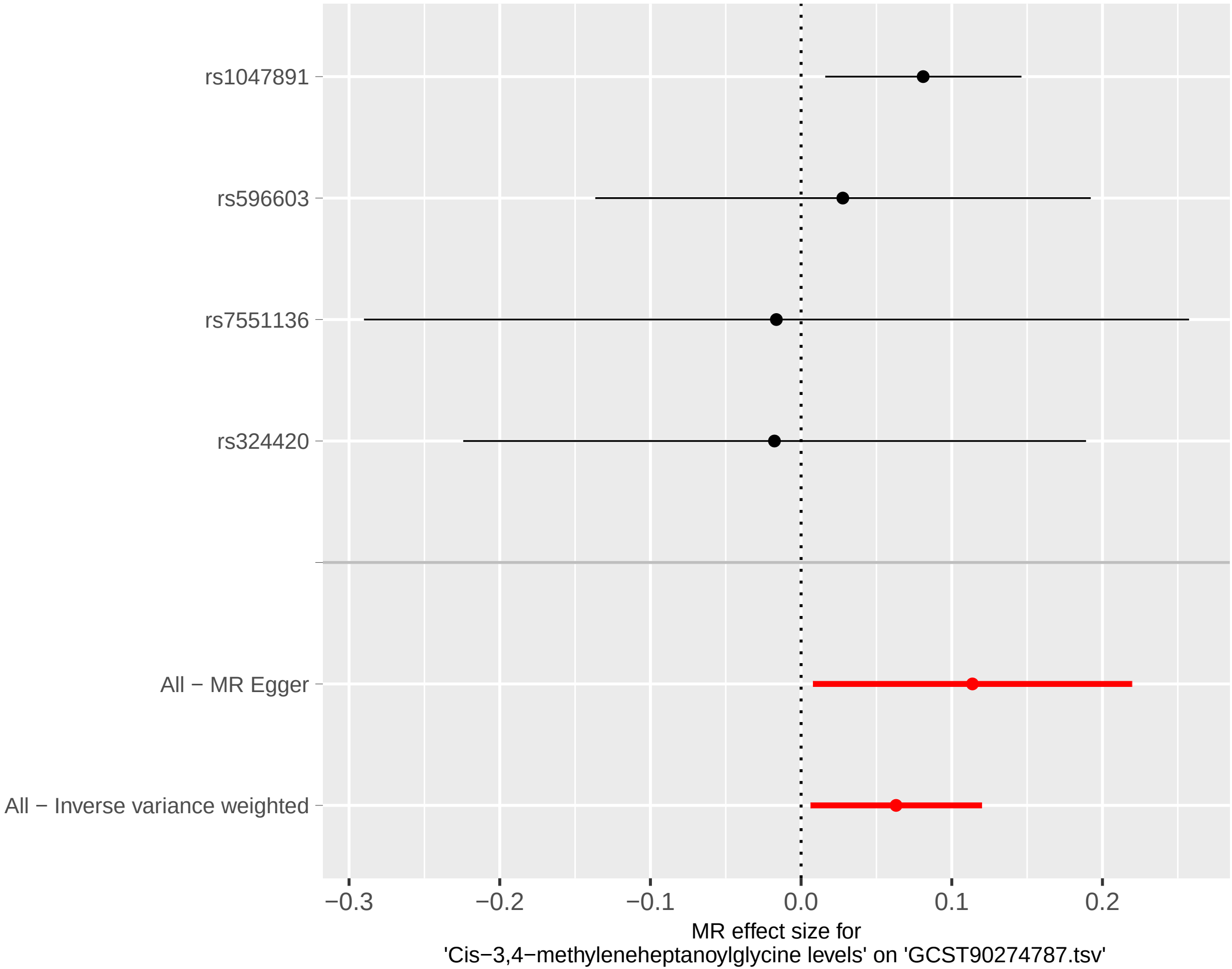
rs1047891
rs596603
rs7551136
rs324420
All − MR Egger
All − Inverse variance weighted
−0.3
−0.2
−0.1
0.0
0.1
0.2
MR effect size for
'Cis−3,4−methyleneheptanoylglycine levels' on 'GCST90274787.tsv'

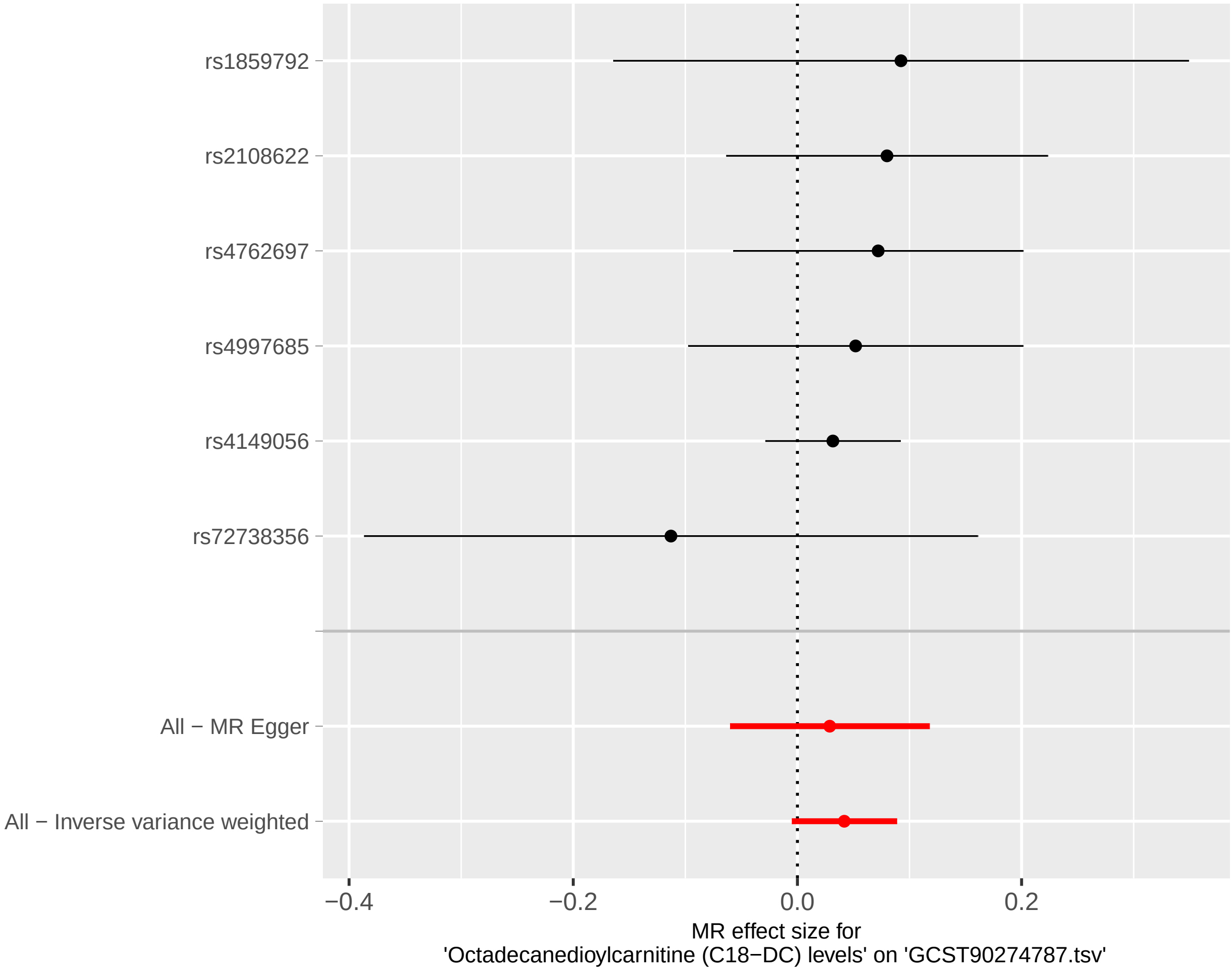

rs1859792
rs2108622
rs4762697
rs4997685
rs4149056
rs72738356
All − MR Egger
All − Inverse variance weighted
−0.4
−0.2
0.0
0.2
MR effect size for
'Octadecanedioylcarnitine (C18−DC) levels' on 'GCST90274787.tsv'

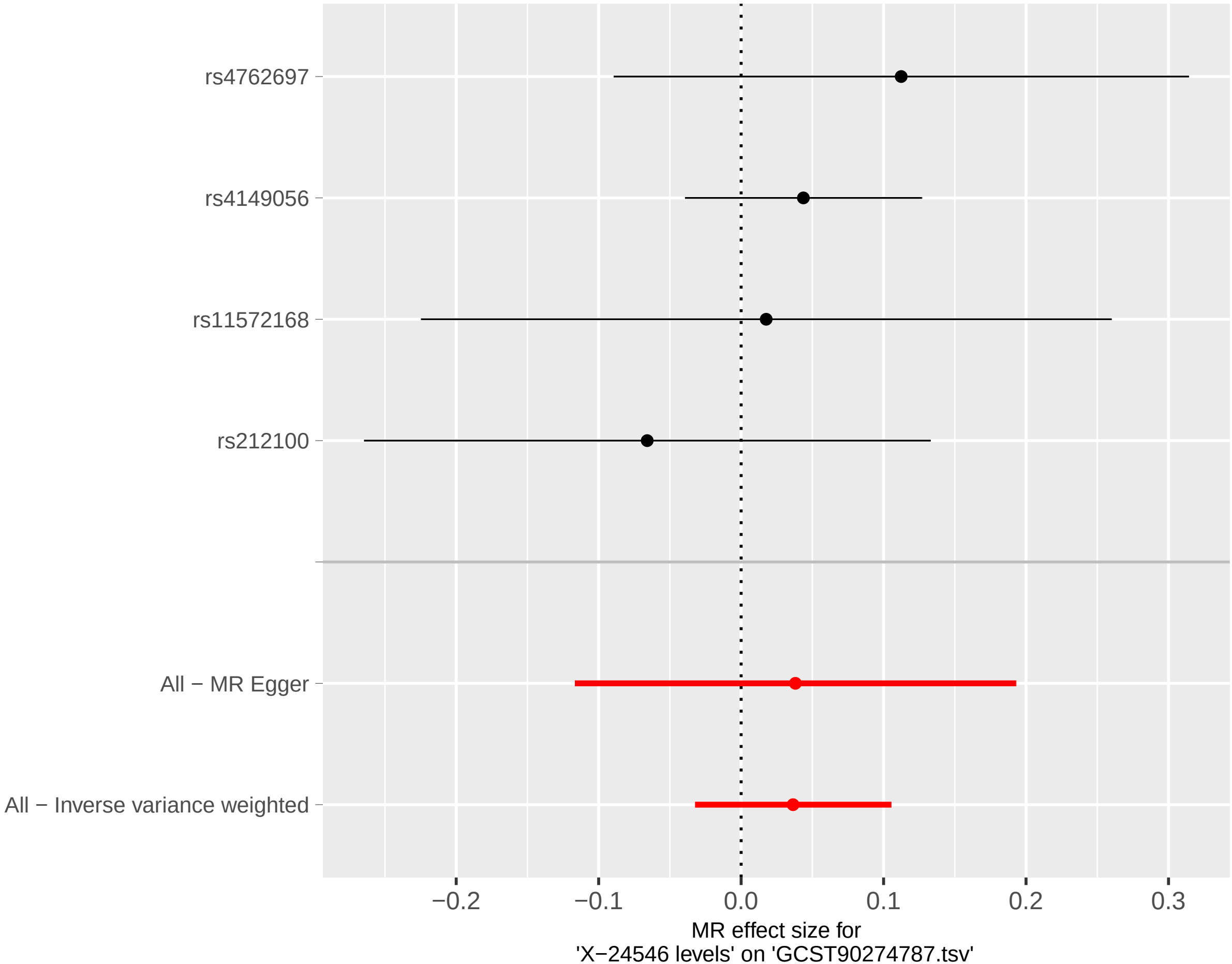
rs4762697
rs4149056
rs11572168
rs212100
All − MR Egger
All − Inverse variance weighted
−0.2
−0.1
0.0
0.1
0.2
0.3
MR effect size for
'X−24546 levels' on 'GCST90274787.tsv'

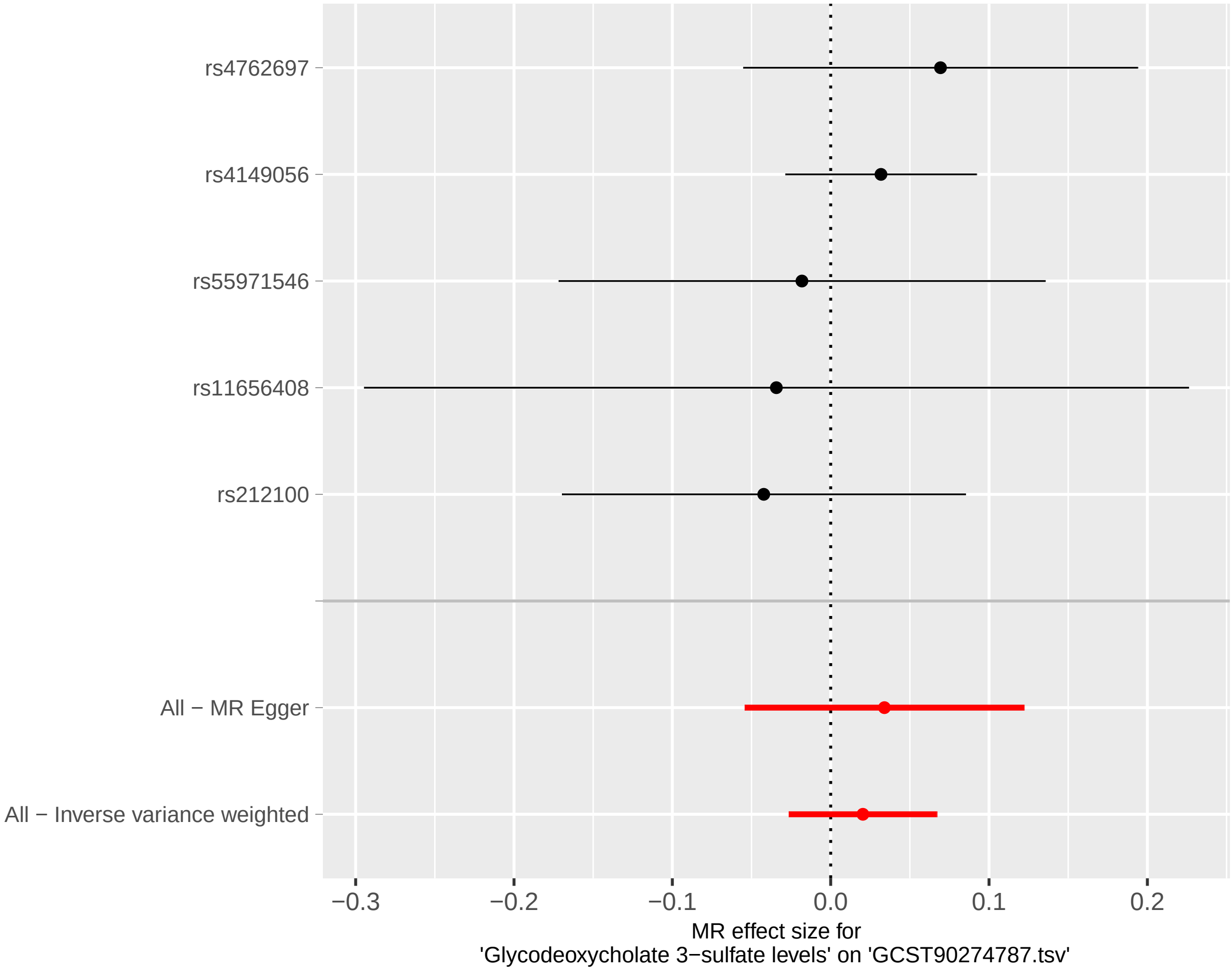
rs4762697
rs4149056
rs55971546
rs11656408
rs212100
All − MR Egger
All − Inverse variance weighted
−0.3
−0.2
−0.1
0.0
0.1
0.2
MR effect size for
'Glycodeoxycholate 3−sulfate levels' on 'GCST90274787.tsv'

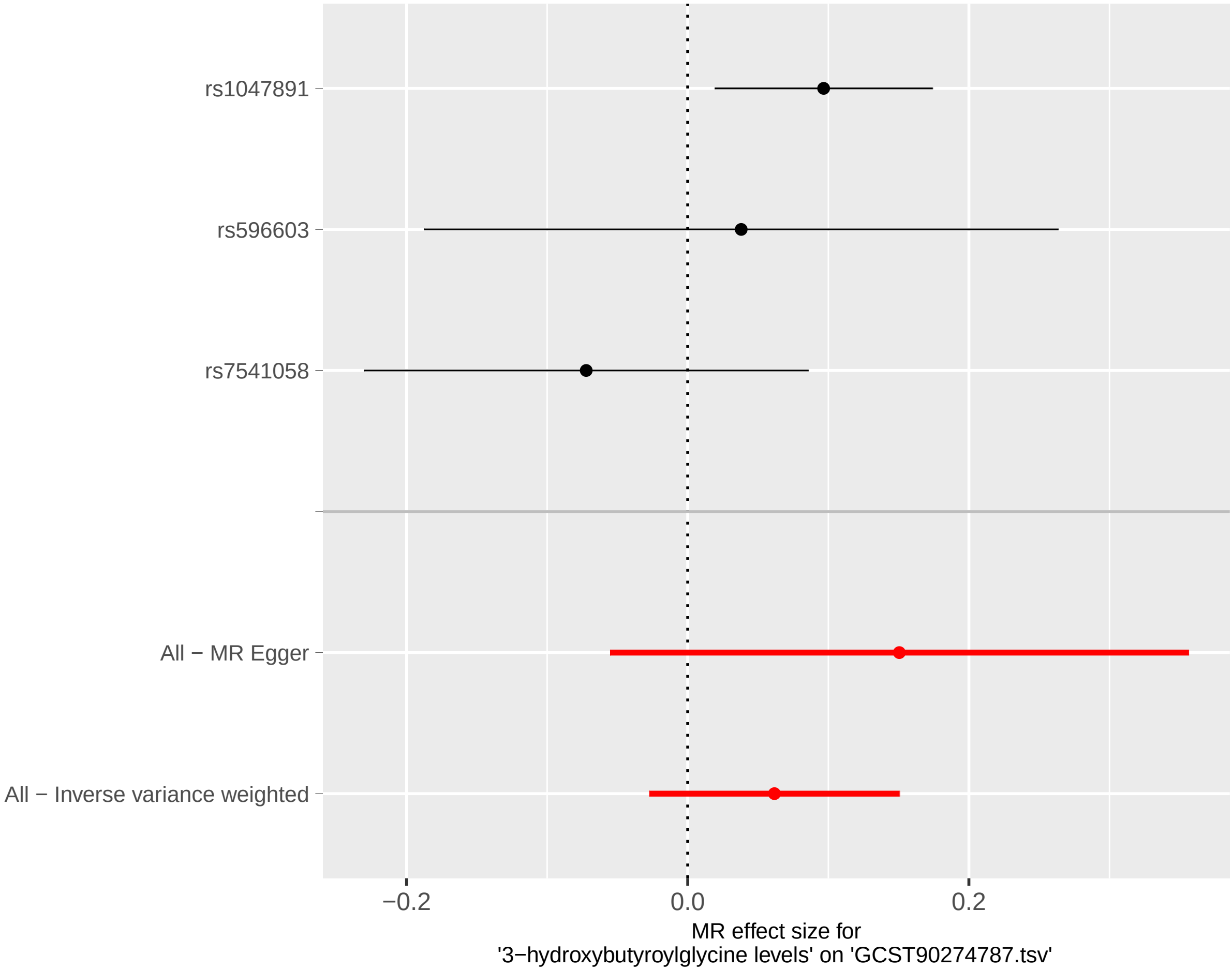
rs1047891
rs596603
rs7541058
All − MR Egger
All − Inverse variance weighted
−0.2
0.0
0.2
MR effect size for
'3−hydroxybutyroylglycine levels' on 'GCST90274787.tsv'

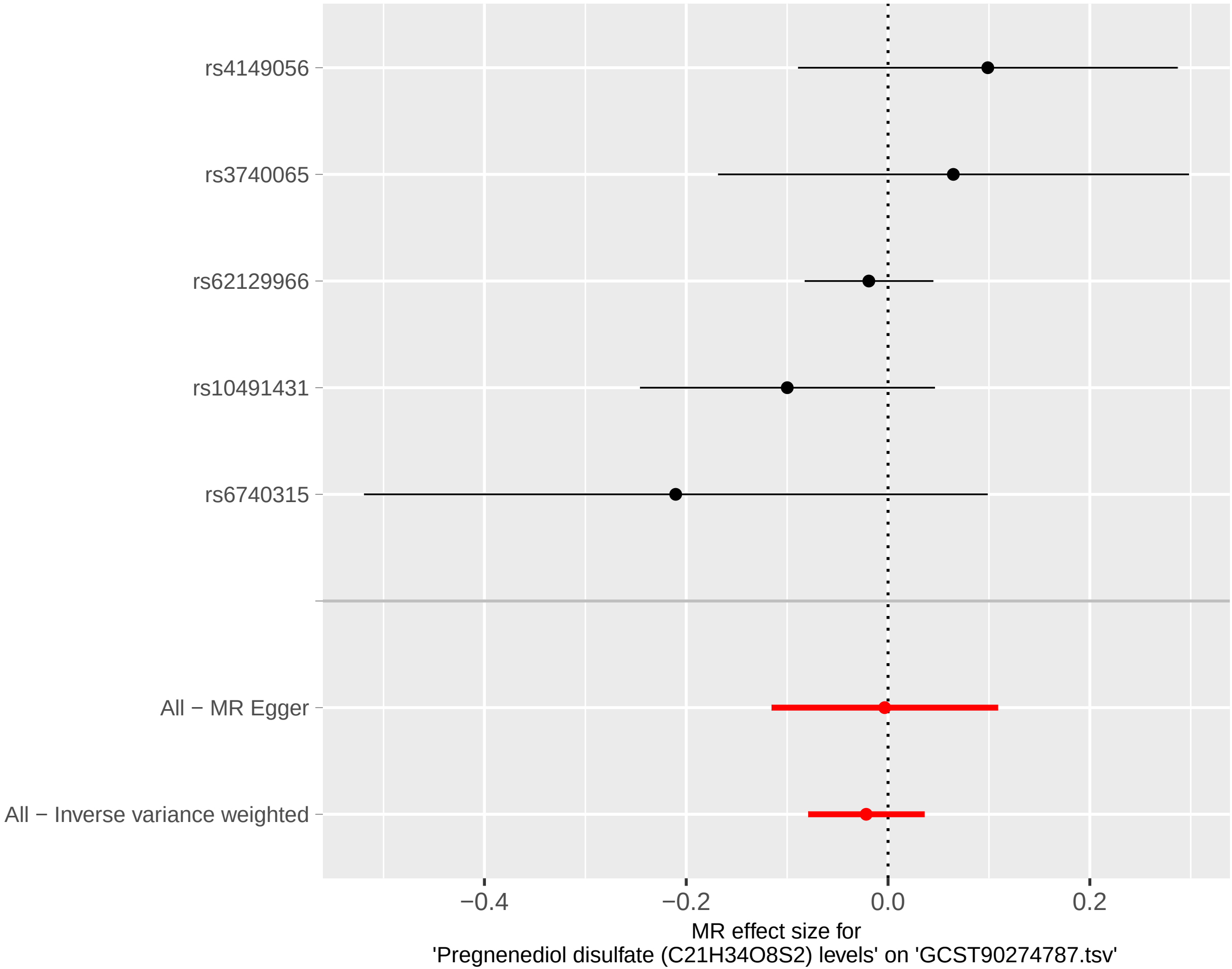

rs4149056
rs3740065
rs62129966
rs10491431
rs6740315
All − MR Egger
All − Inverse variance weighted
−0.4
−0.2
0.0
0.2
MR effect size for
'Pregnenediol disulfate (C21H34O8S2) levels' on 'GCST90274787.tsv'

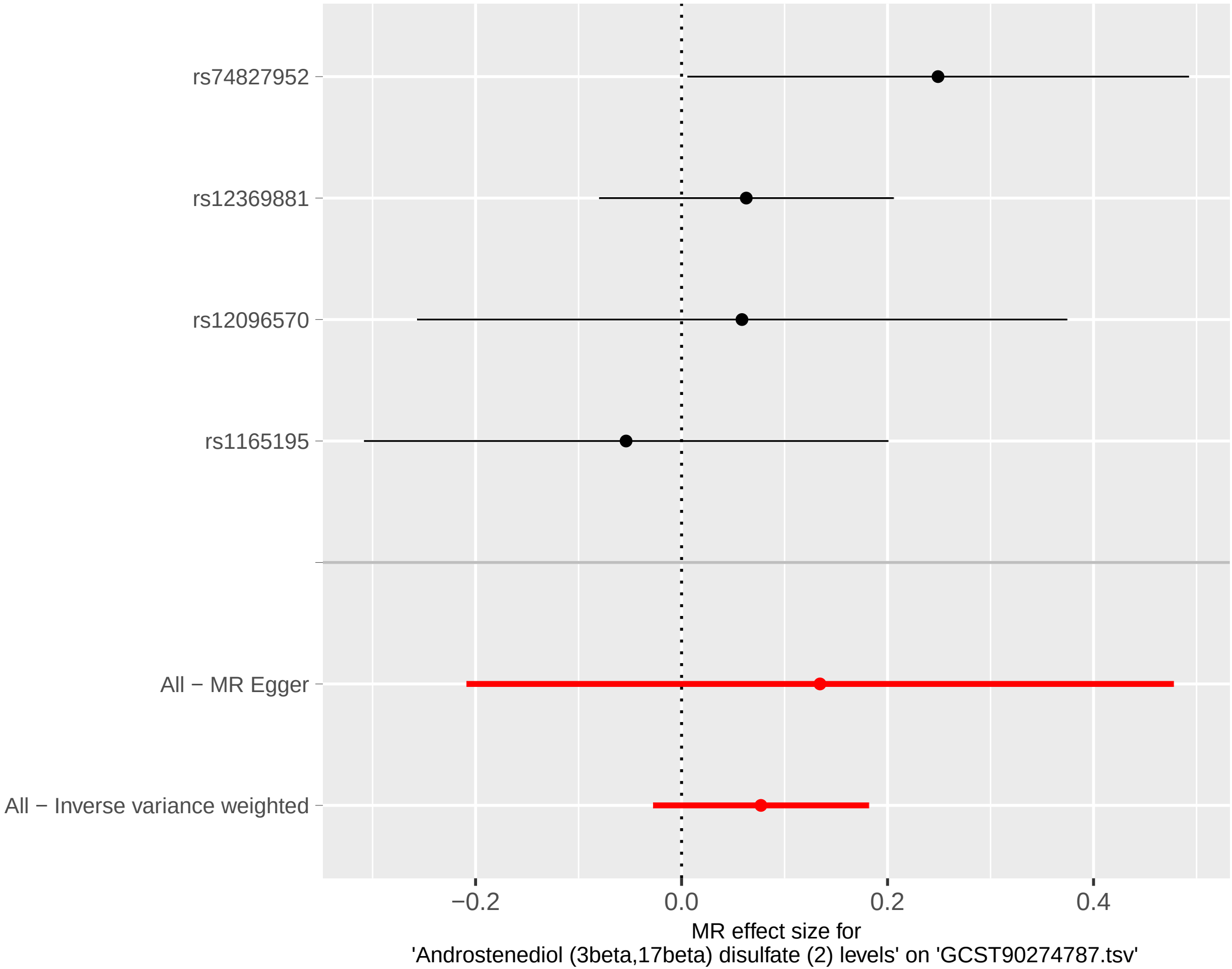
rs74827952
rs12369881
rs12096570
rs1165195
All − MR Egger
All − Inverse variance weighted
−0.2
0.0
0.2
0.4
MR effect size for
'Androstenediol (3beta,17beta) disulfate (2) levels' on 'GCST90274787.tsv'

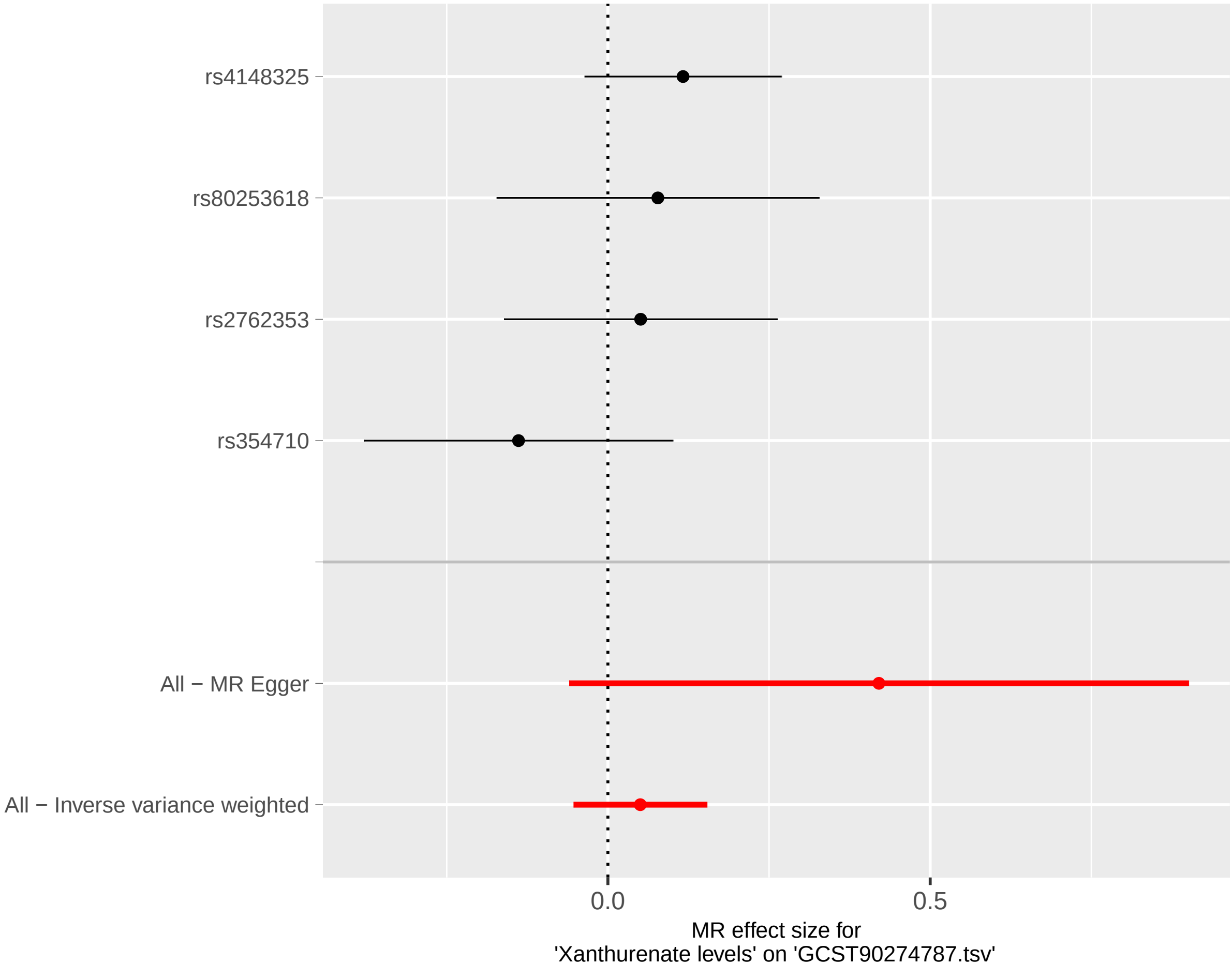
rs4148325
rs80253618
rs2762353
rs354710
All − MR Egger
All − Inverse variance weighted
0.0
0.5
MR effect size for
'Xanthurenate levels' on 'GCST90274787.tsv'

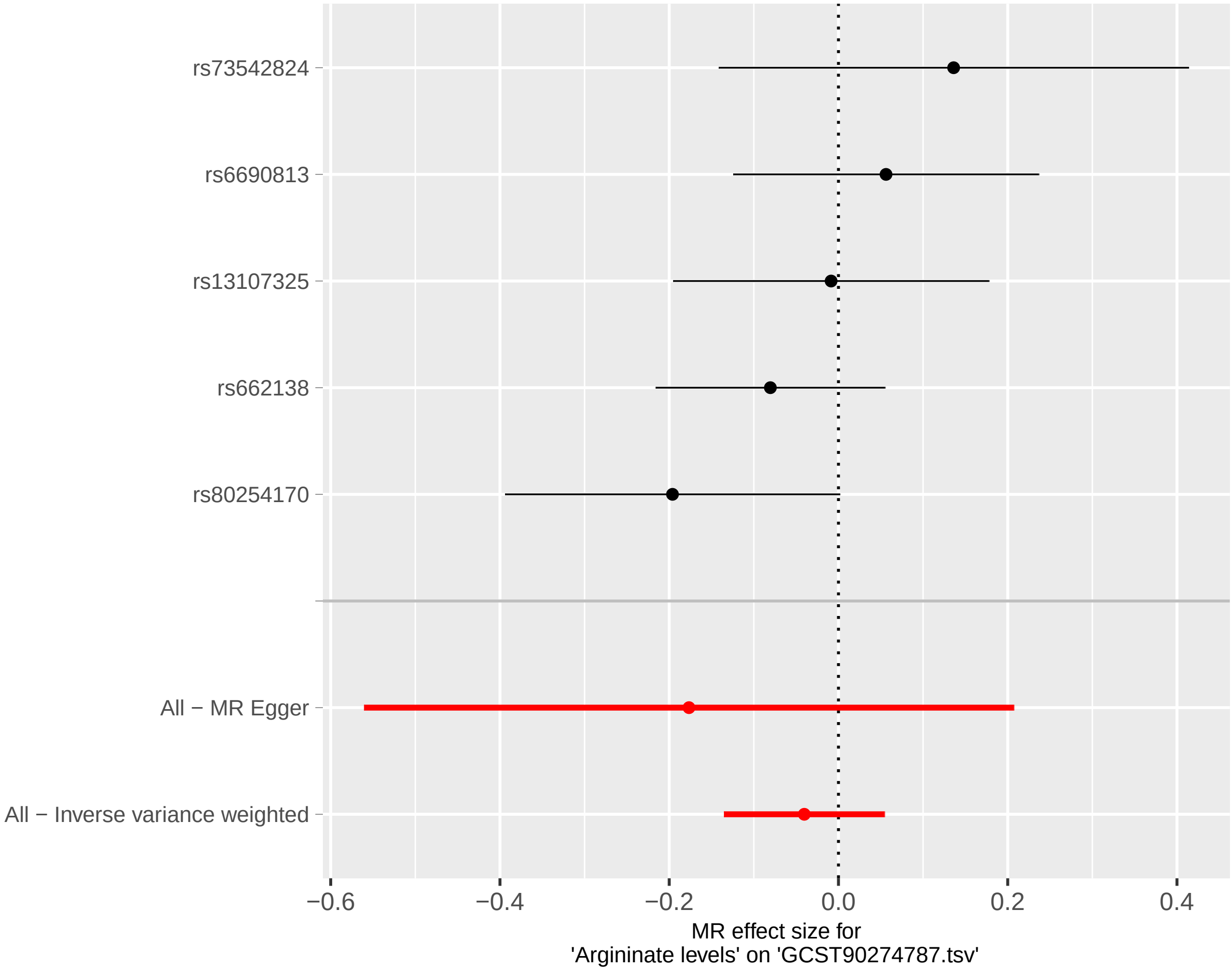

rs73542824
rs6690813
rs13107325
rs662138
rs80254170
All − MR Egger
All − Inverse variance weighted
−0.6
−0.4
−0.2
0.0
0.2
0.4
MR effect size for
'Argininate levels' on 'GCST90274787.tsv'

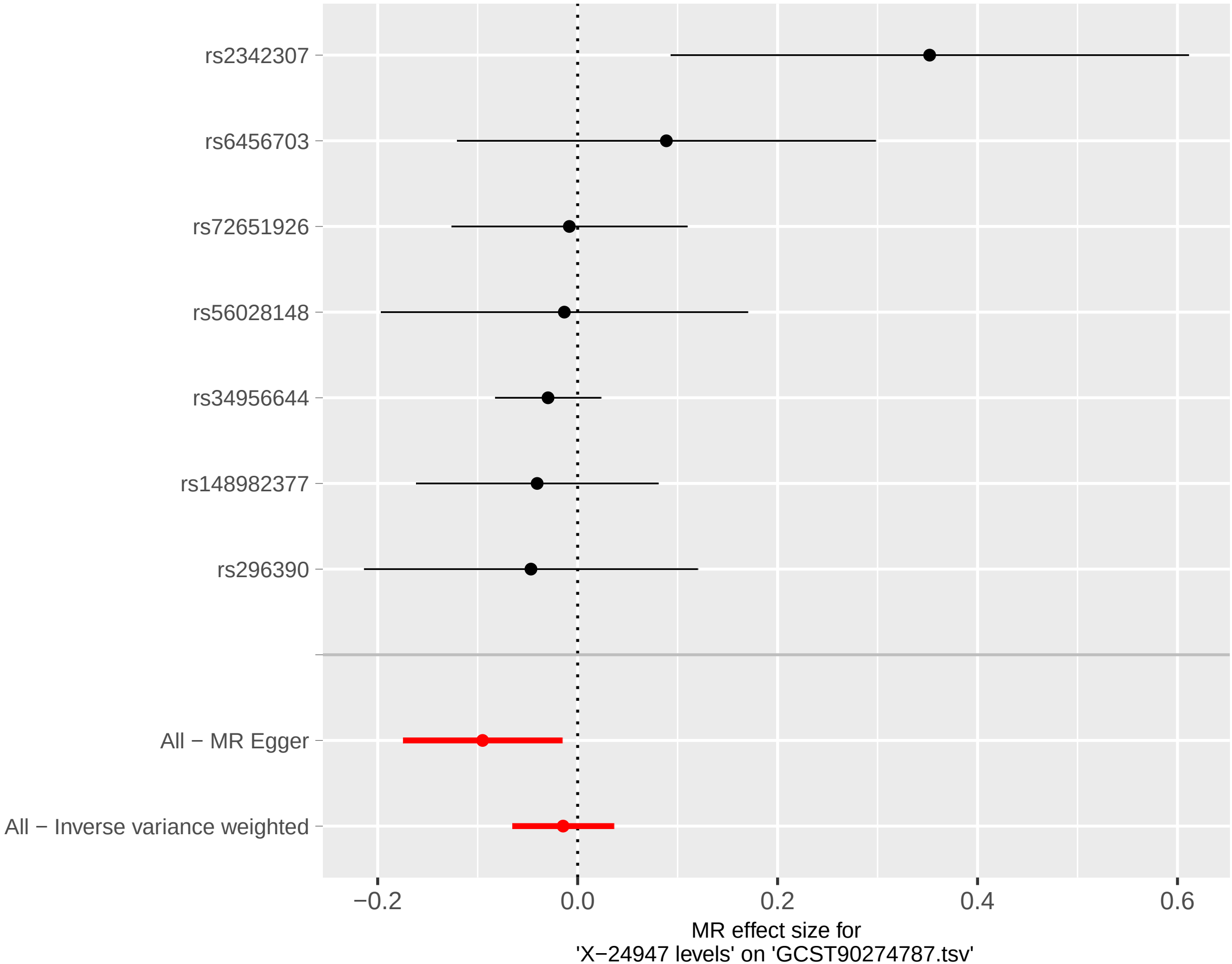
rs2342307
rs6456703
rs72651926
rs56028148
rs34956644
rs148982377
rs296390
All − MR Egger
All − Inverse variance weighted
−0.2
0.0
0.2
0.4
0.6
MR effect size for
'X−24947 levels' on 'GCST90274787.tsv'

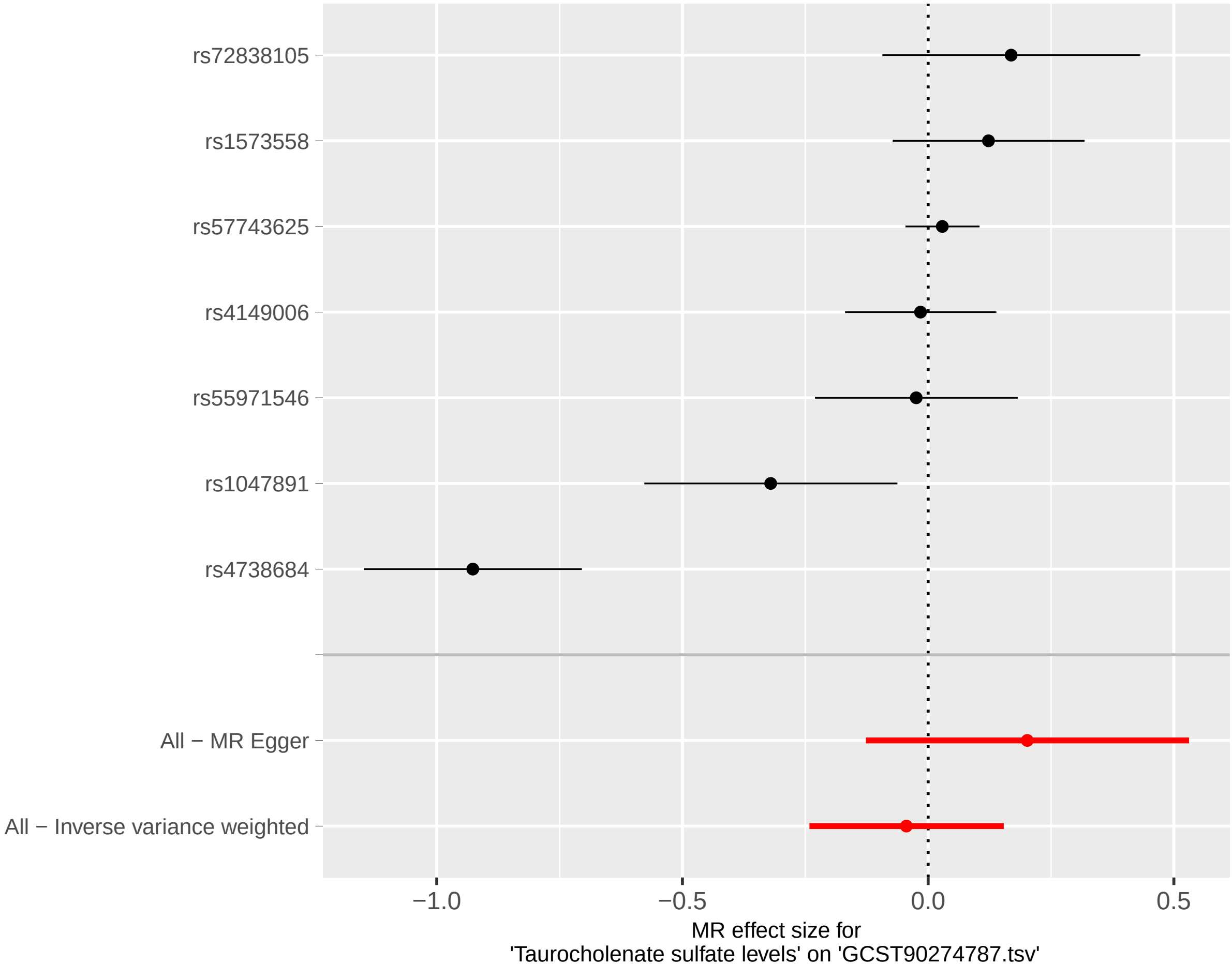
rs72838105
rs1573558
rs57743625
rs4149006
rs55971546
rs1047891
rs4738684
All − MR Egger
All − Inverse variance weighted
−1.0
−0.5
0.0
0.5
MR effect size for
'Taurocholenate sulfate levels' on 'GCST90274787.tsv'

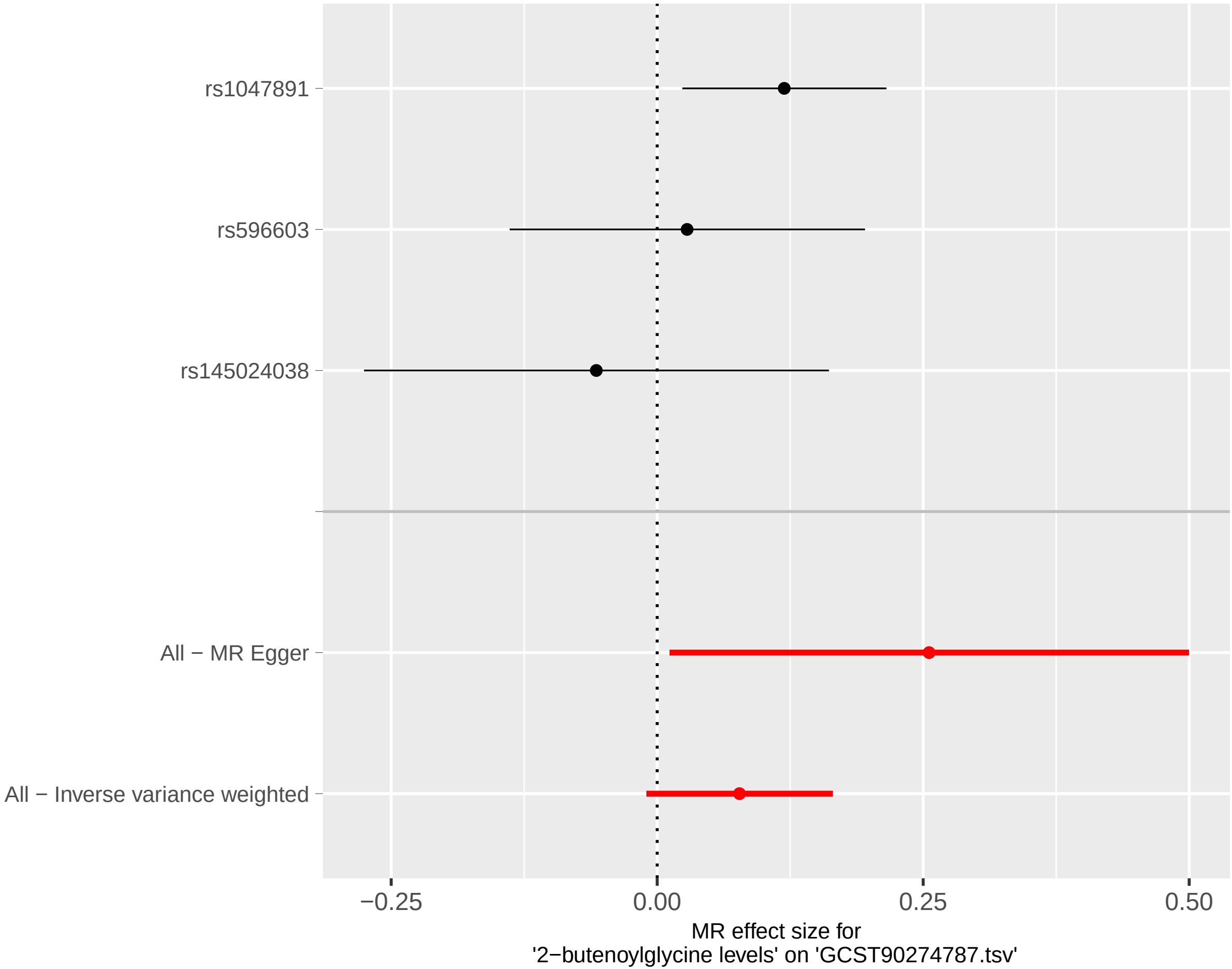
rs1047891
rs596603
rs145024038
All − MR Egger
All − Inverse variance weighted
−0.25
0.00
0.25
0.50
MR effect size for
'2−butenoylglycine levels' on 'GCST90274787.tsv'

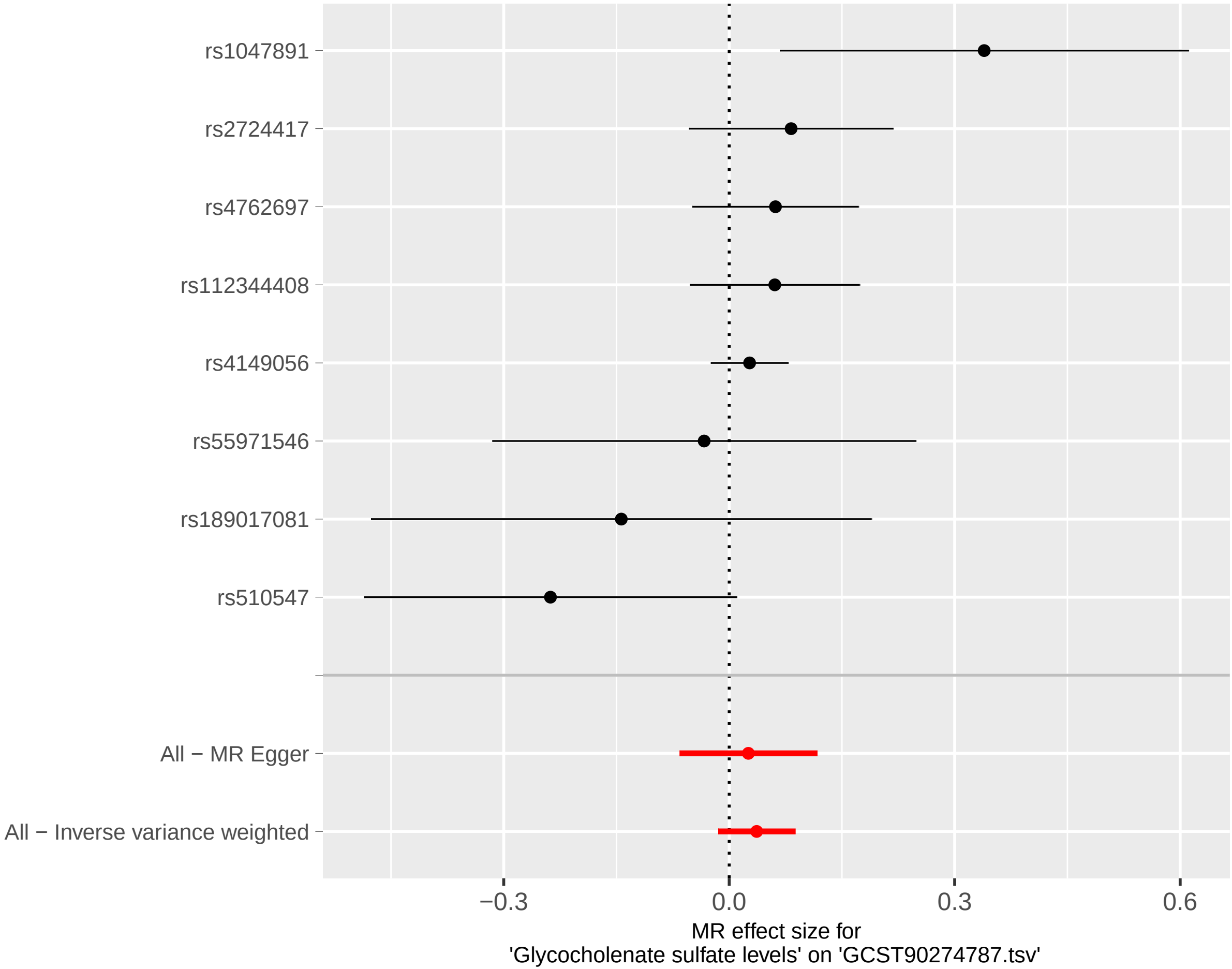

rs1047891
rs2724417
rs4762697
rs112344408
rs4149056
rs55971546
rs189017081
rs510547
All − MR Egger
All − Inverse variance weighted
−0.3
0.0
0.3
0.6
MR effect size for
'Glycocholenate sulfate levels' on 'GCST90274787.tsv'

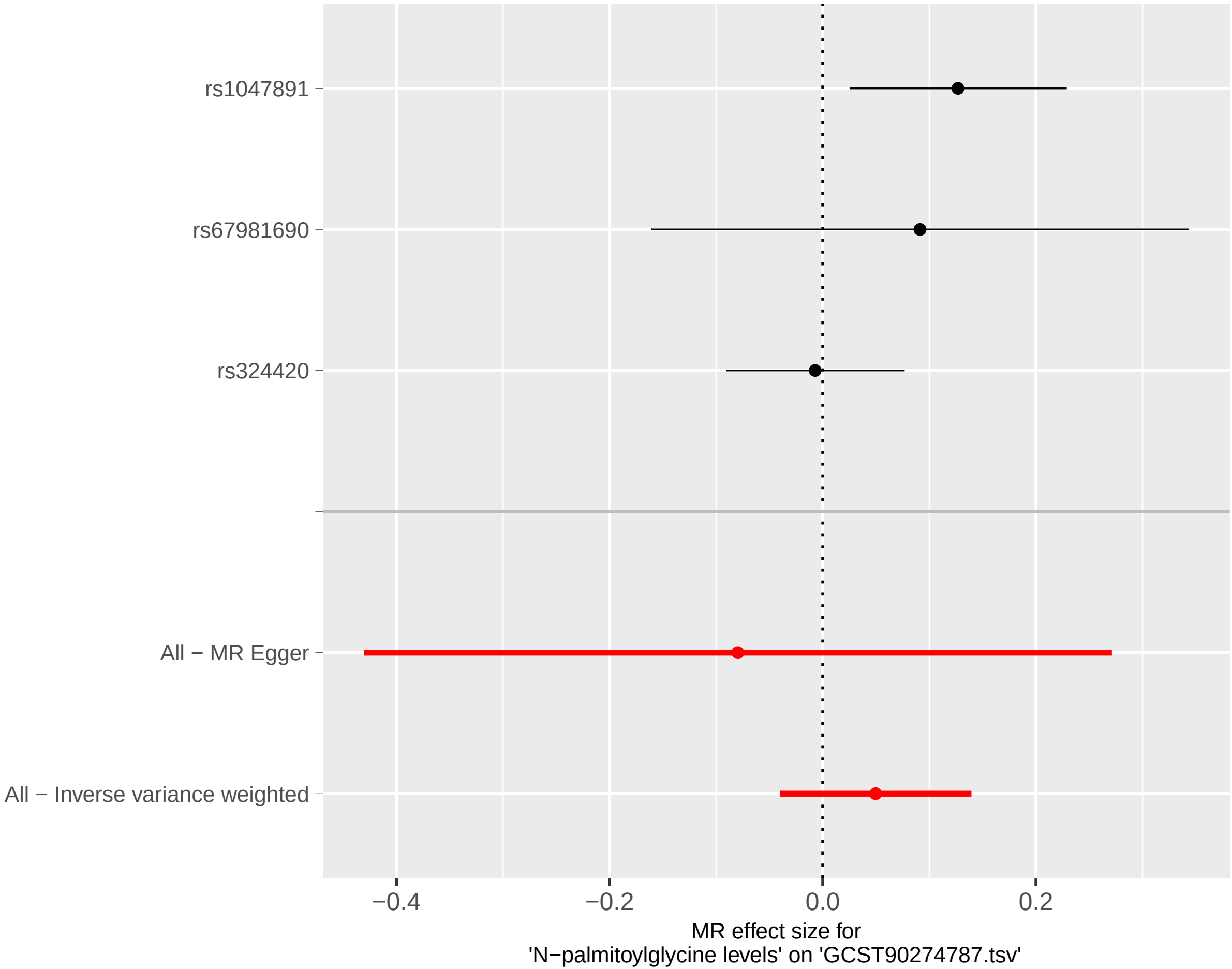
rs1047891
rs67981690
rs324420
All − MR Egger
All − Inverse variance weighted
−0.4
−0.2
0.0
0.2
MR effect size for
'N−palmitoylglycine levels' on 'GCST90274787.tsv'

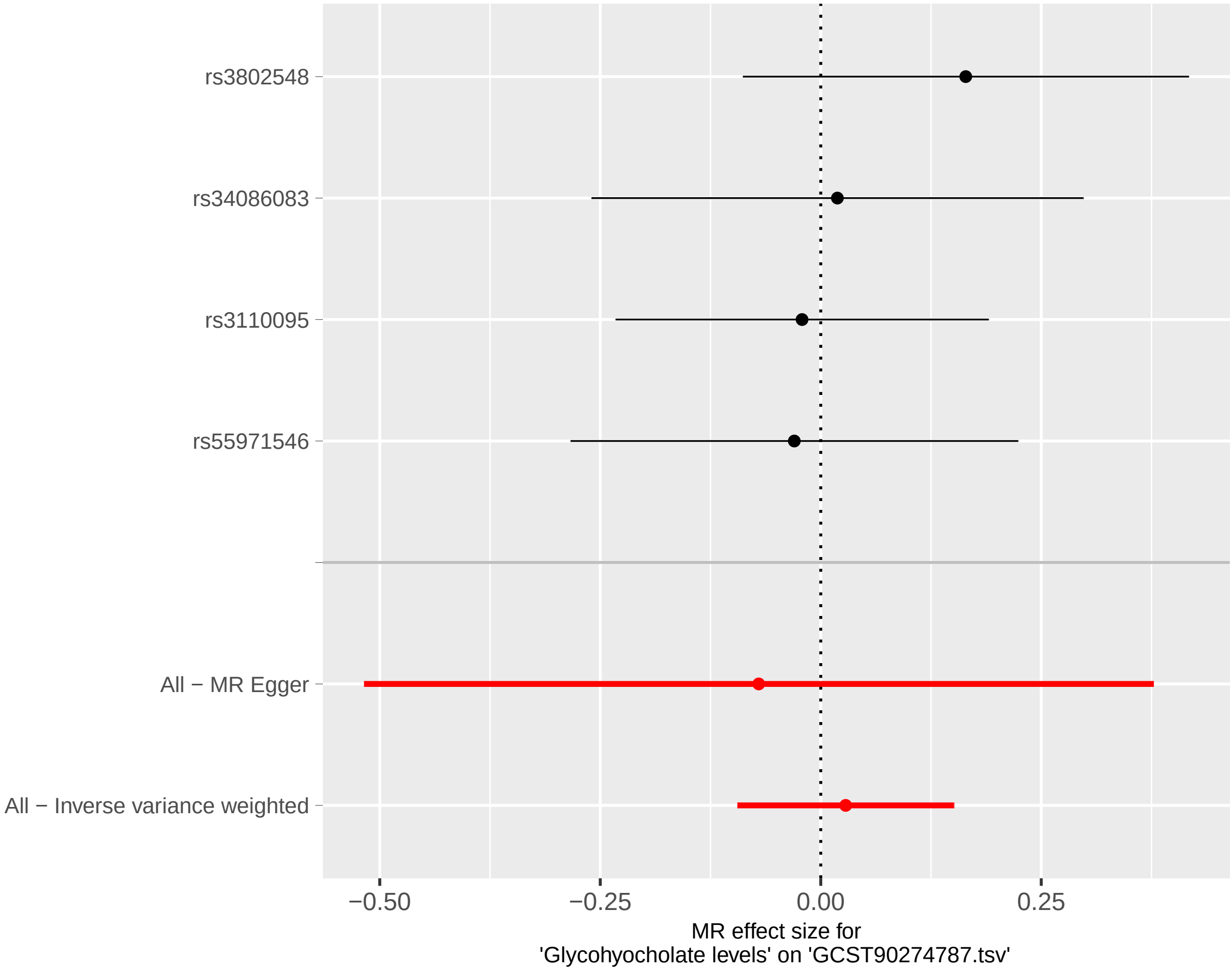
rs3802548
rs34086083
rs3110095
rs55971546
All − MR Egger
All − Inverse variance weighted
−0.50
−0.25
0.00
0.25
MR effect size for
'Glycohyocholate levels' on 'GCST90274787.tsv'

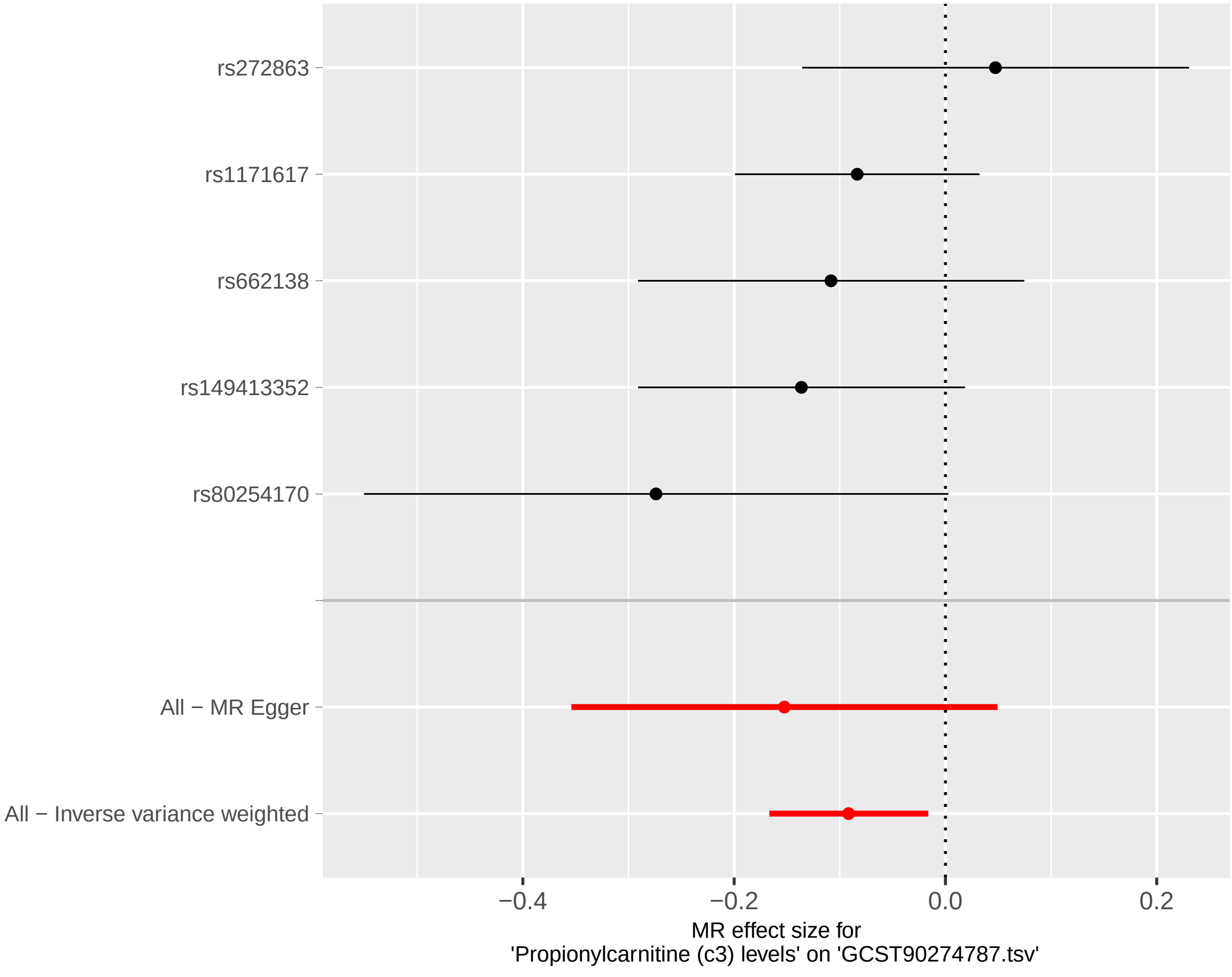
rs272863
rs1171617
rs662138
rs149413352
rs80254170
All − MR Egger
All − Inverse variance weighted
−0.4
−0.2
0.0
0.2
MR effect size for
'Propionylcarnitine (c3) levels' on 'GCST90274787.tsv'

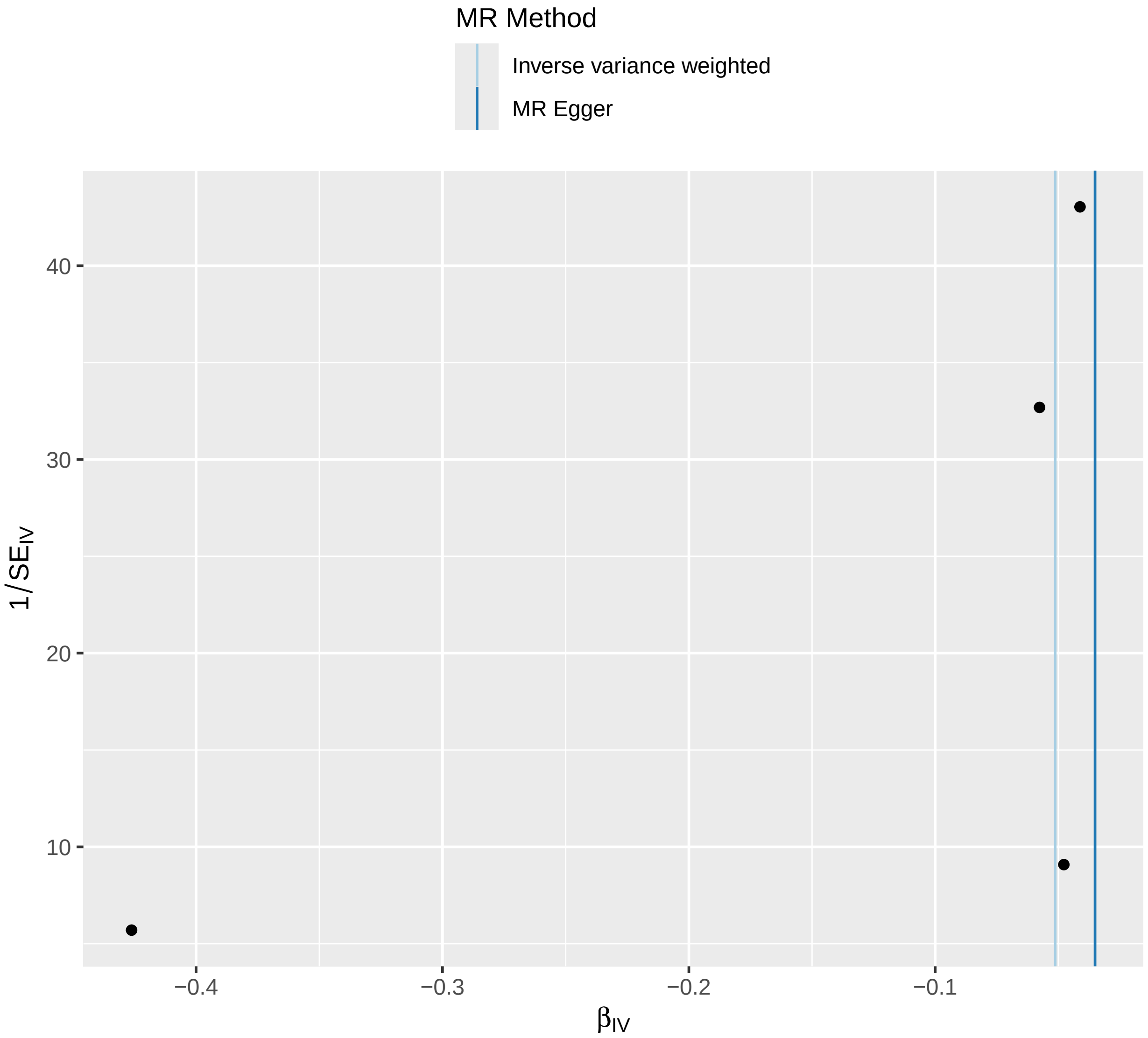
MR Method
Inverse variance weighted
MR Egger
40
30
20
10
1/SE$_{IV}$
−0.4
−0.3
−0.2
−0.1
β$_{IV}$

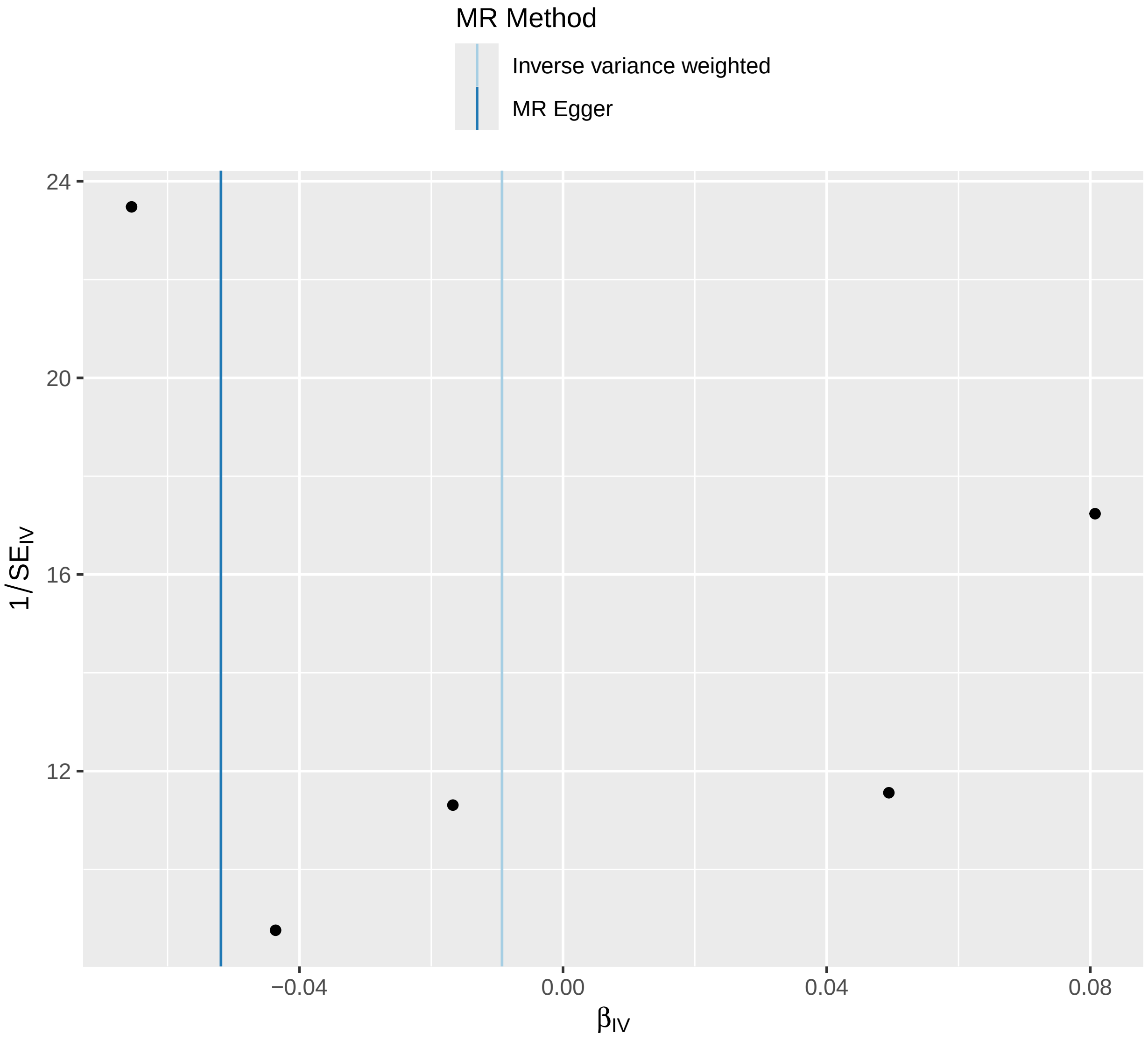

MR Method
Inverse variance weighted
MR Egger
24
20
16
12
1/SE_IV
−0.04
0.00
0.04
0.08
β_IV

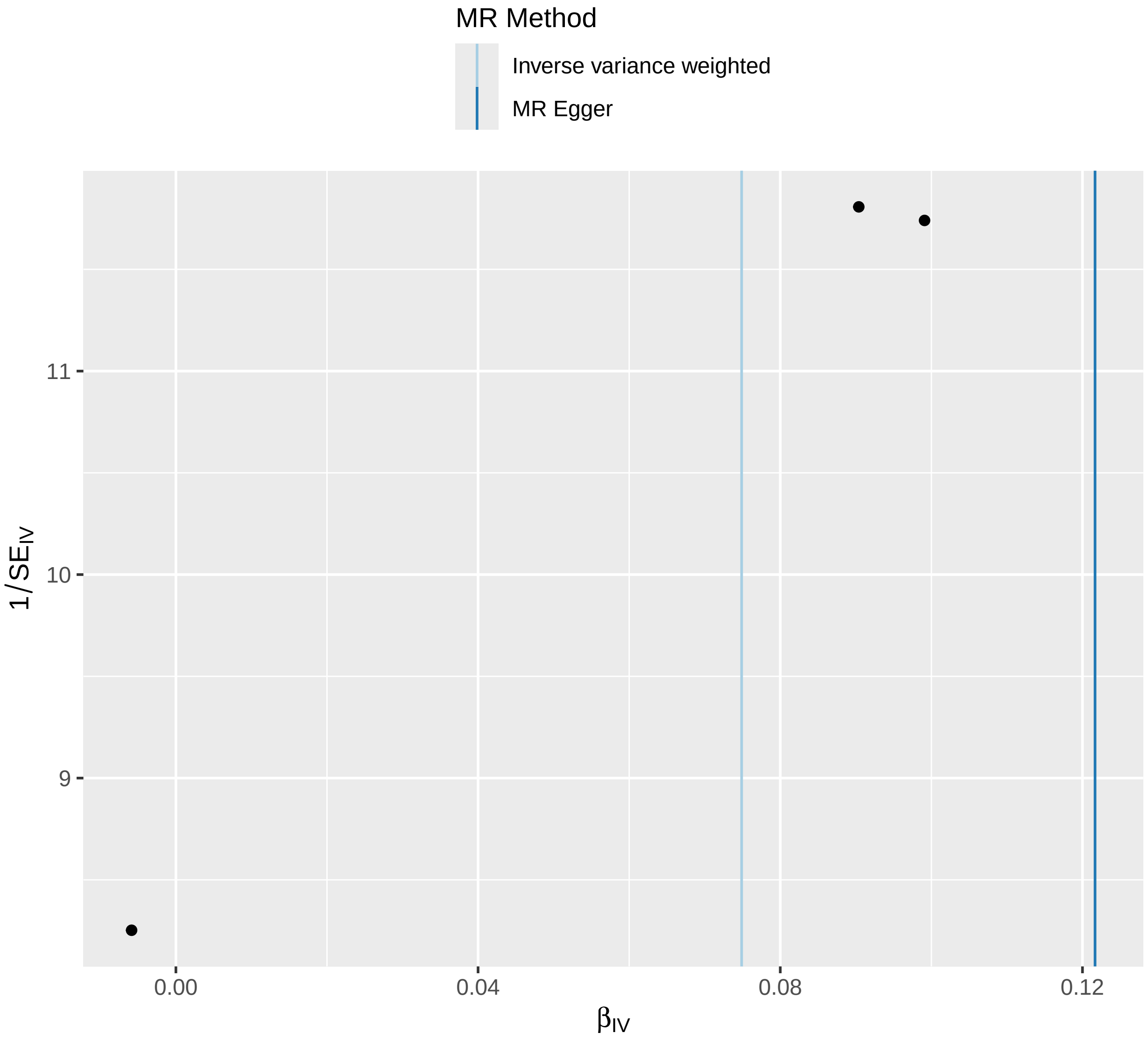
MR Method
Inverse variance weighted
MR Egger
1/SE$_{IV}$
11
10
9
0.00
0.04
0.08
0.12
β$_{IV}$

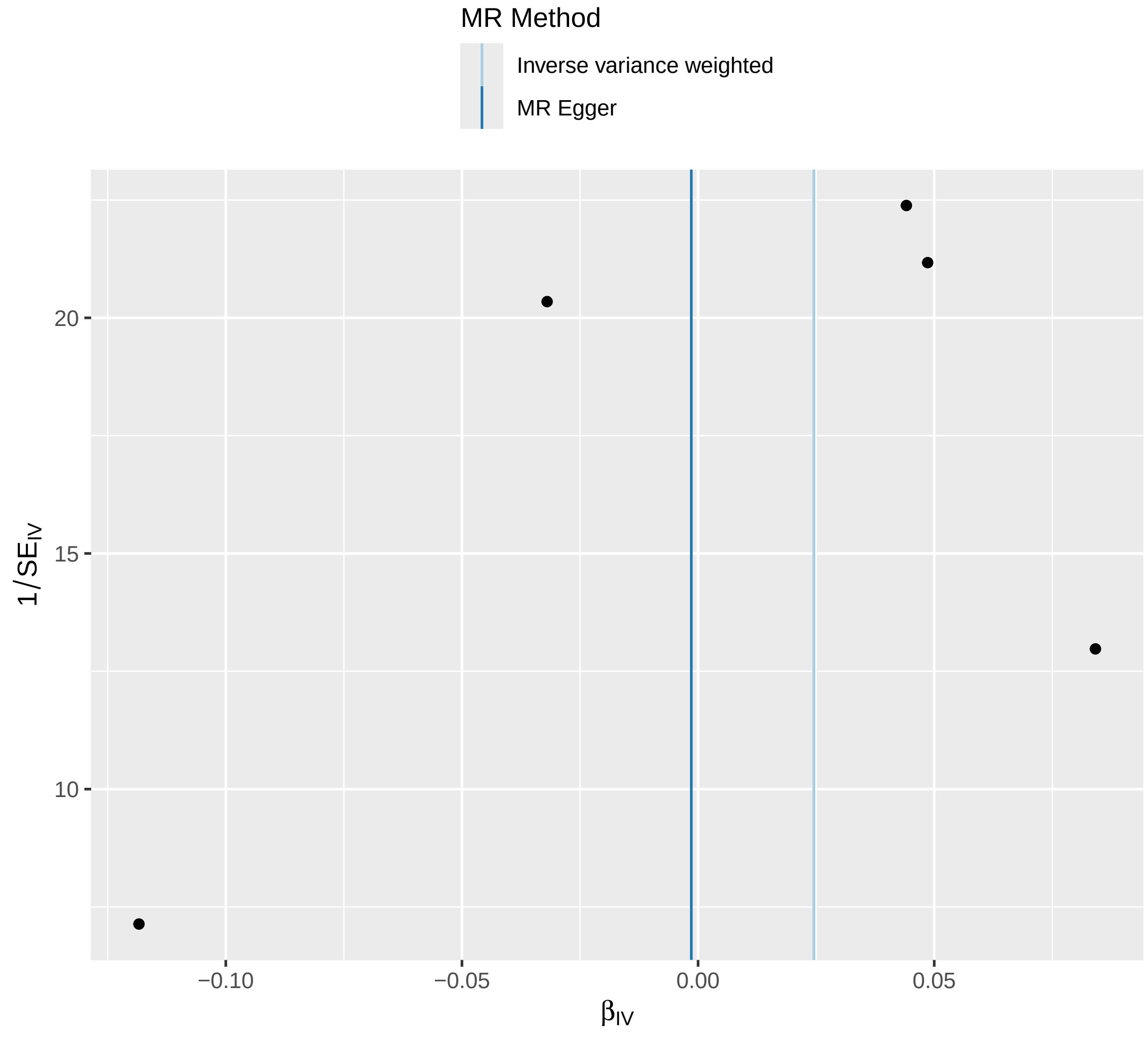
MR Method
Inverse variance weighted
MR Egger
1/SE_IV
20
15
10
−0.10
−0.05
0.00
0.05
β_IV

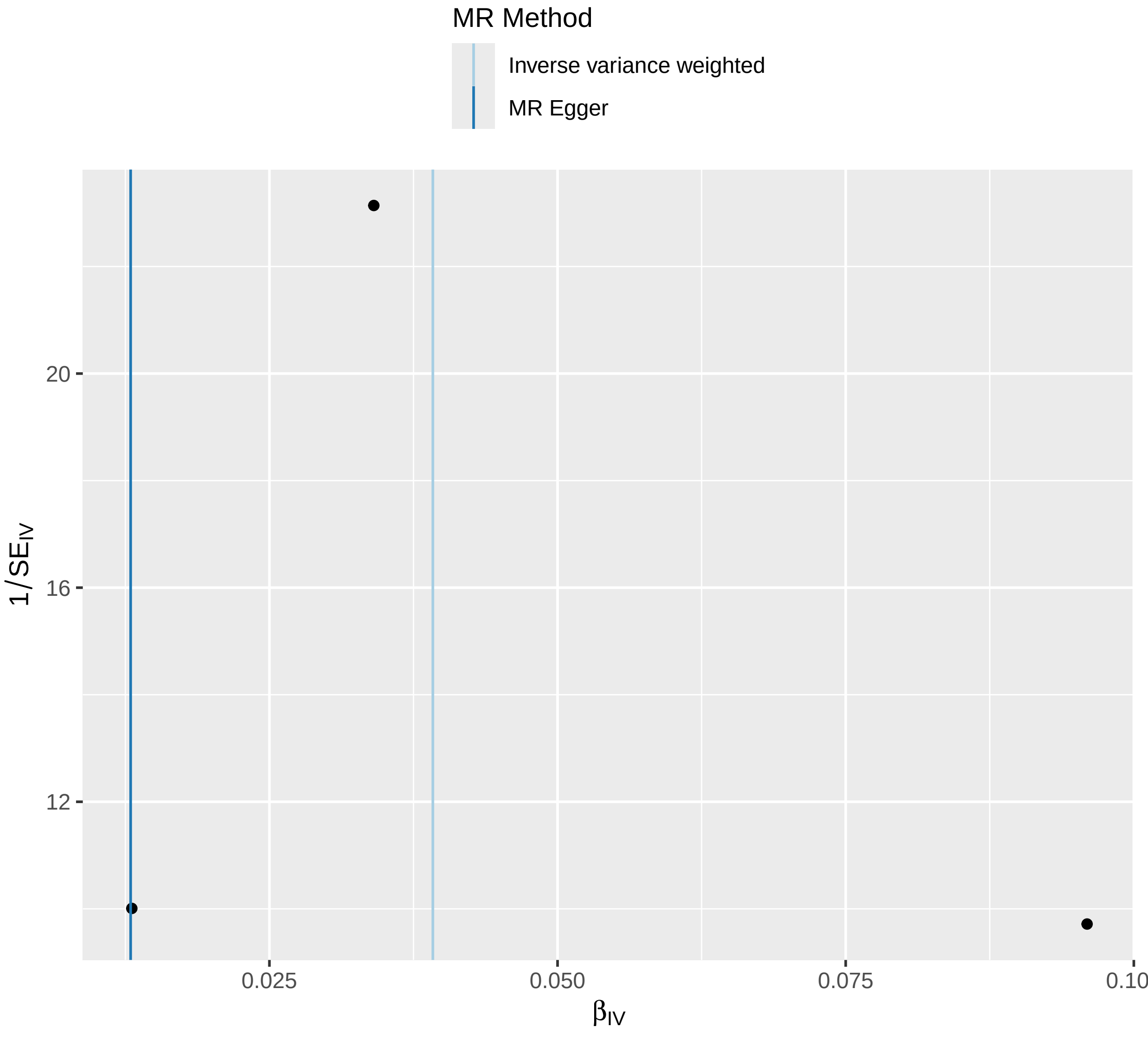

MR Method
Inverse variance weighted
MR Egger
20
16
12
$1/SE_{IV}$
0.025
0.050
0.075
0.100
β_{IV}

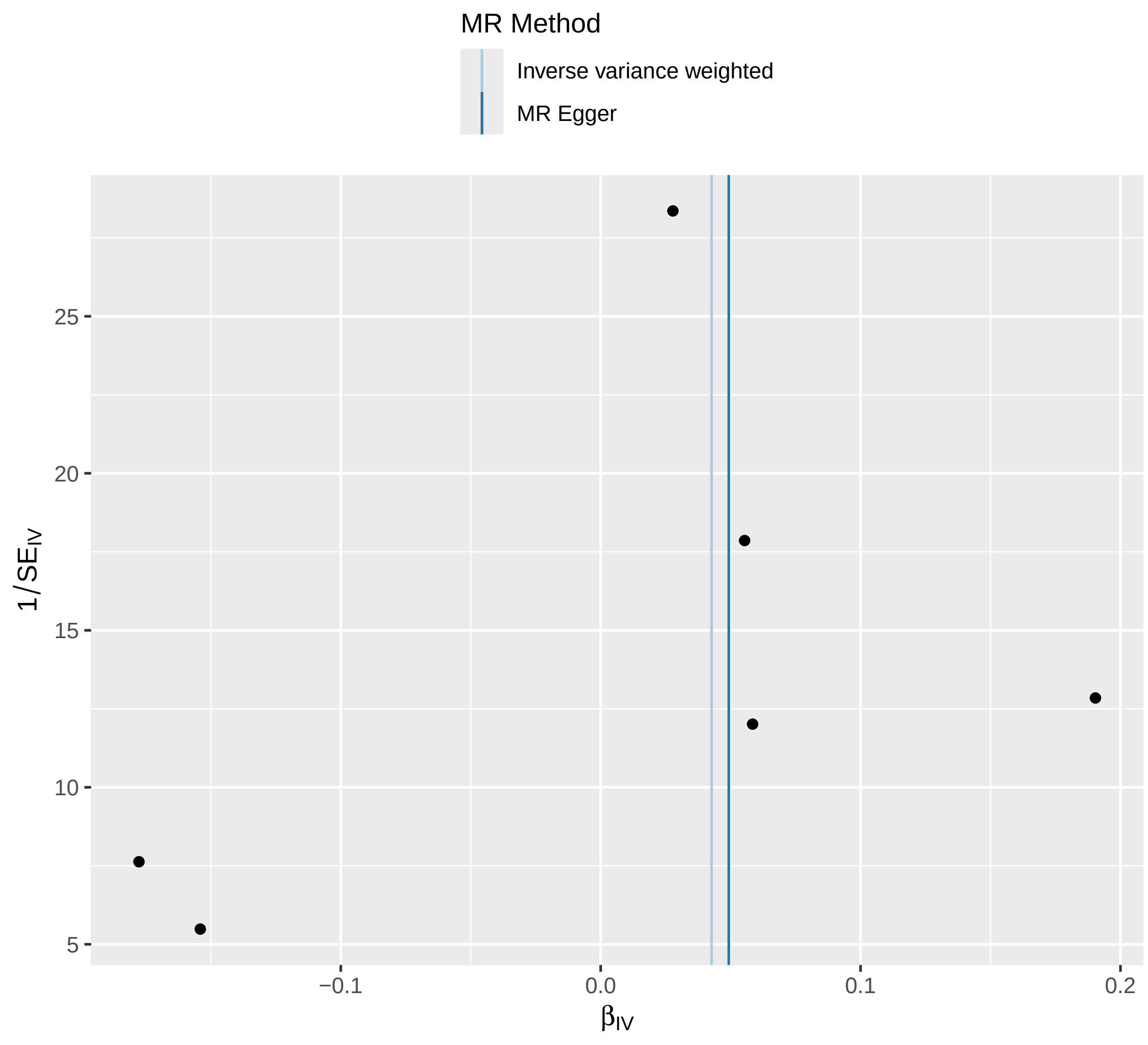
MR Method
Inverse variance weighted
MR Egger
25
20
15
10
5
1/SE_IV
−0.1
0.0
0.1
0.2
β_IV

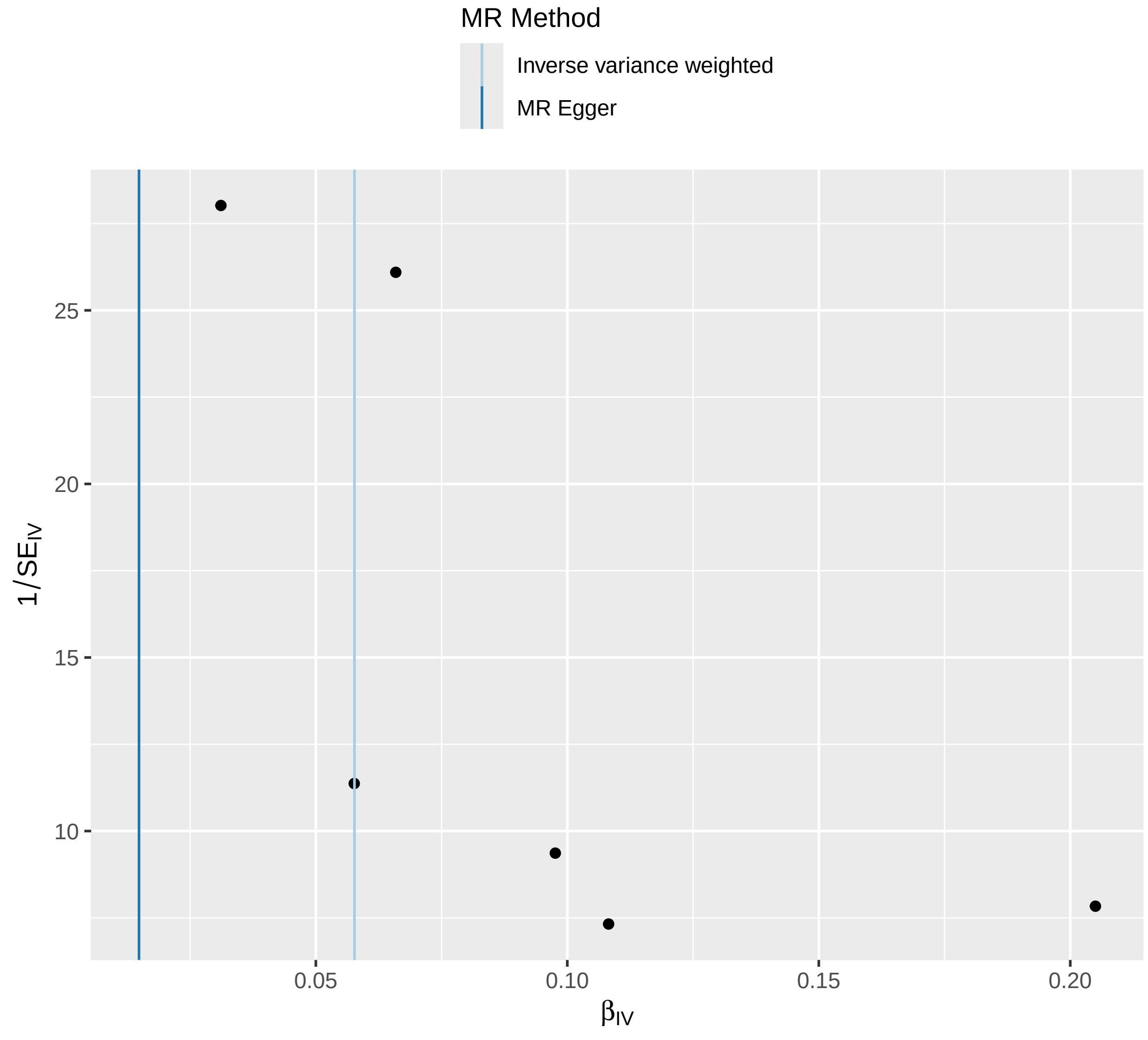

MR Method
Inverse variance weighted
MR Egger
25
20
15
10
1/SE$_{IV}$
0.05
0.10
0.15
0.20
β_{IV}

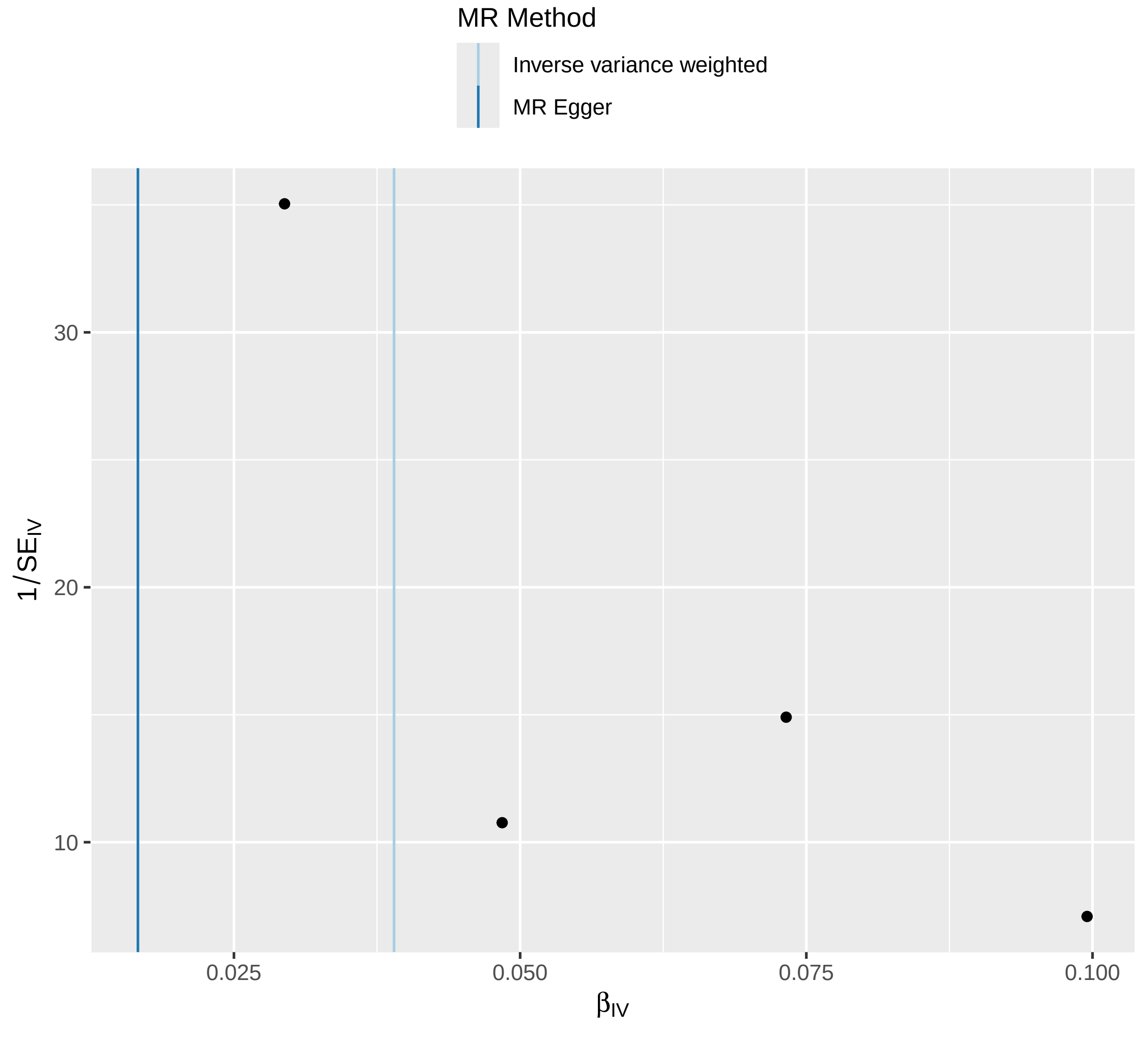

MR Method
Inverse variance weighted
MR Egger
30
20
10
0.025
0.050
0.075
0.100
1/SE_IV
β_IV

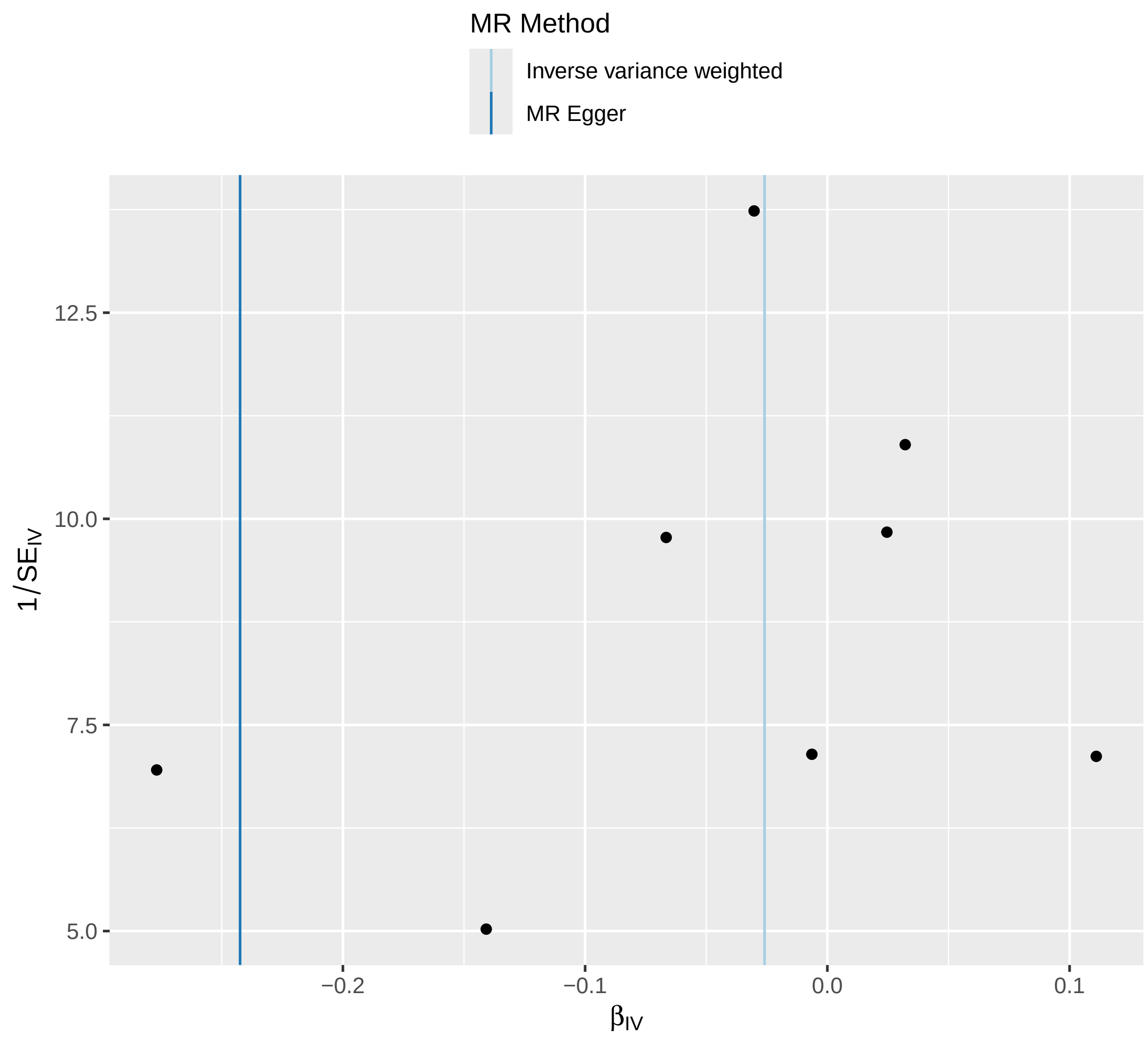

MR Method
Inverse variance weighted
MR Egger
12.5
10.0
7.5
5.0
1/SE_IV
−0.2
−0.1
0.0
0.1
β_IV

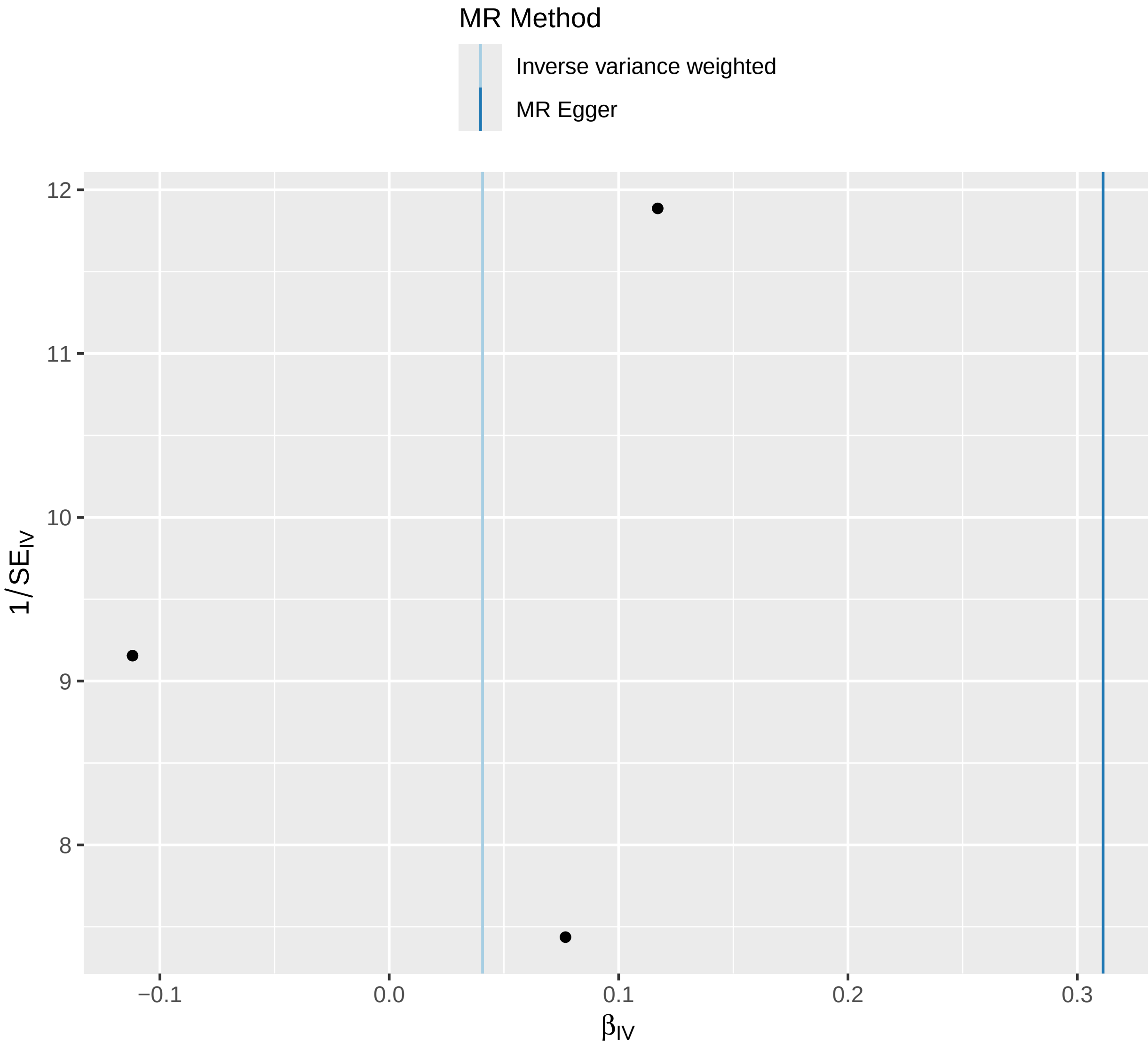
MR Method
Inverse variance weighted
MR Egger
12
11
10
9
8
$1/SE_{IV}$
−0.1
0.0
0.1
0.2
0.3
β_{IV}

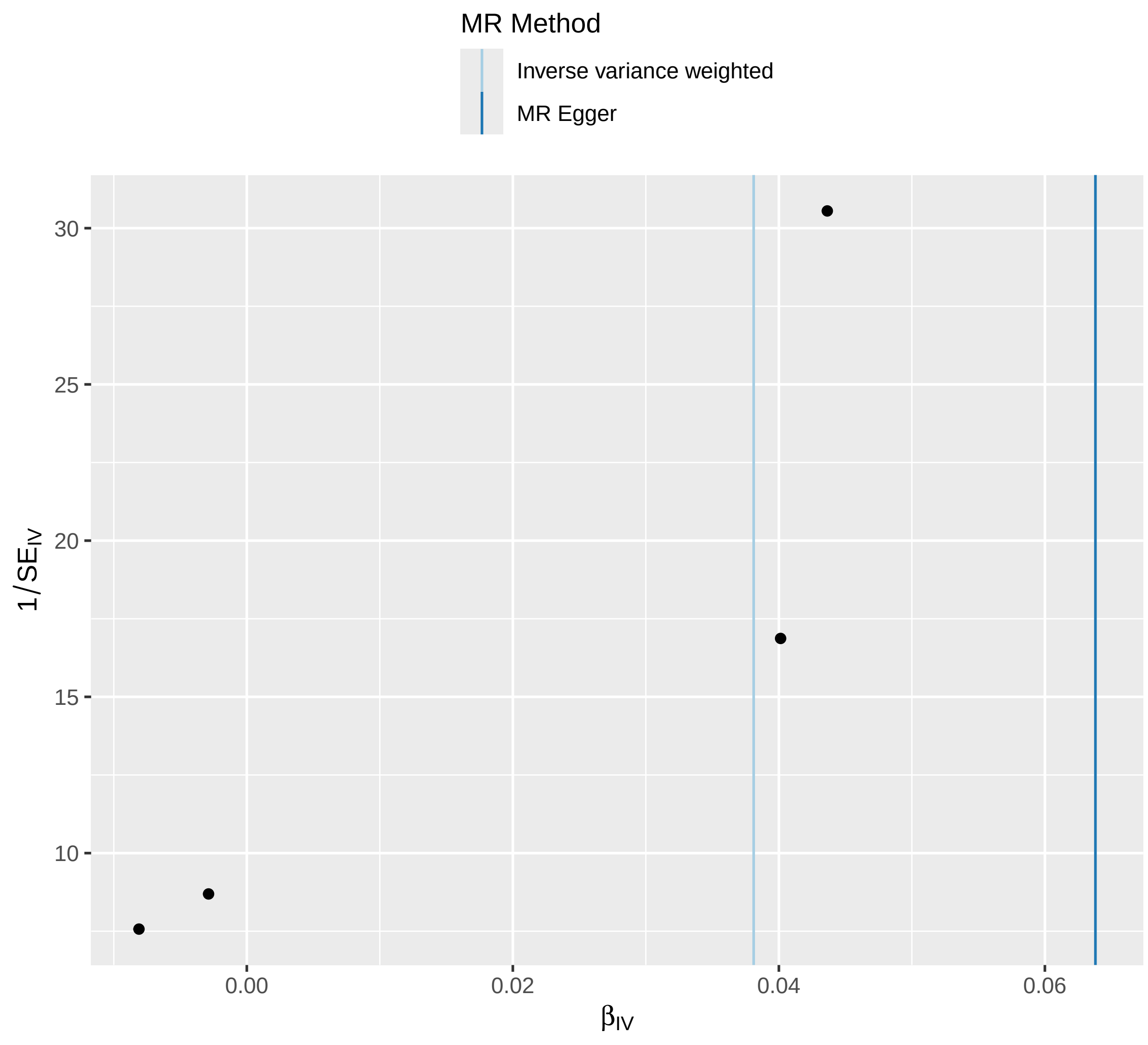
MR Method
Inverse variance weighted
MR Egger
30
25
20
15
10
1/SE_IV
0.00
0.02
0.04
0.06
β_IV

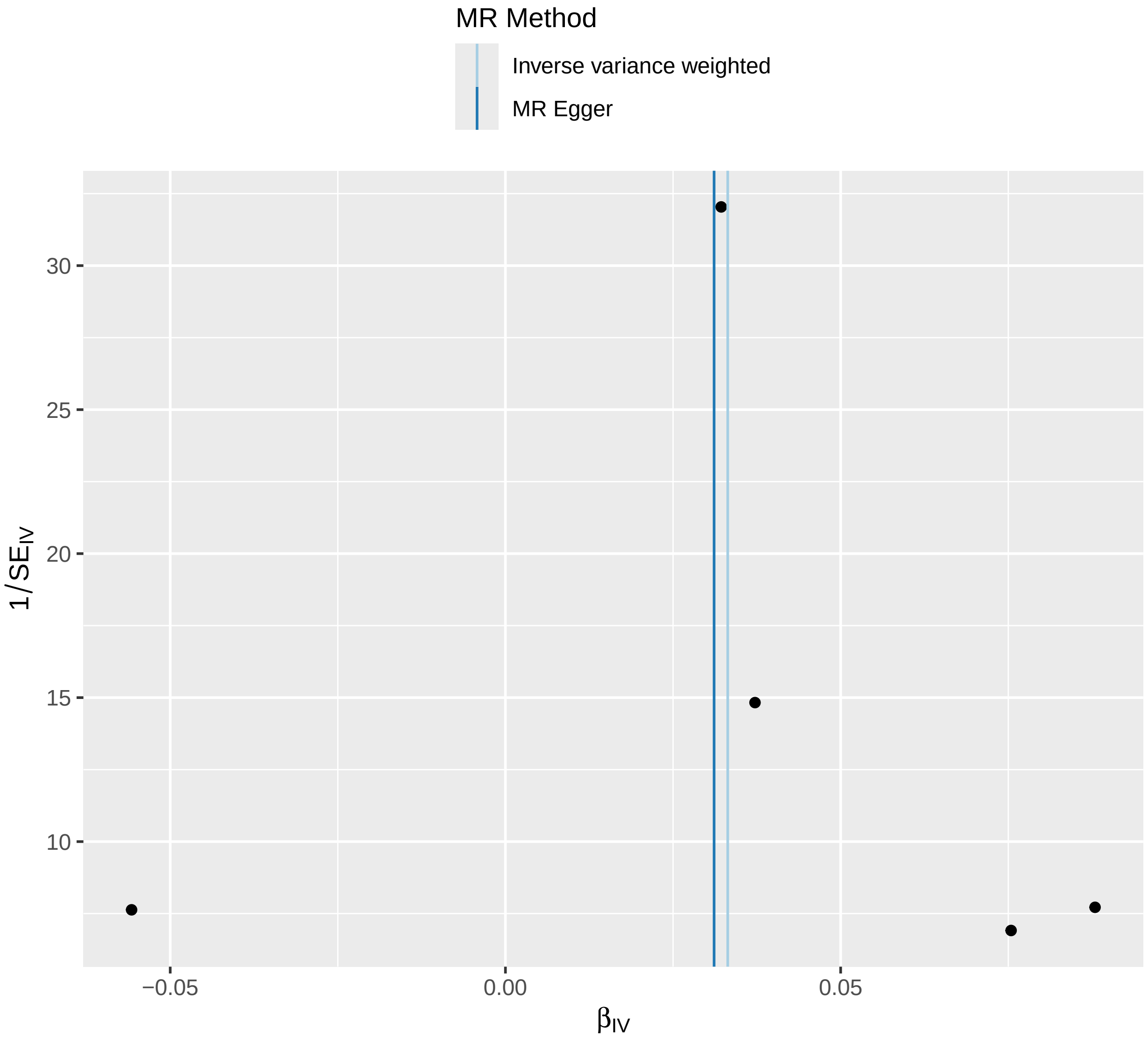

MR Method
Inverse variance weighted
MR Egger
30
25
20
15
10
1/SE_IV
−0.05
0.00
0.05
β_IV

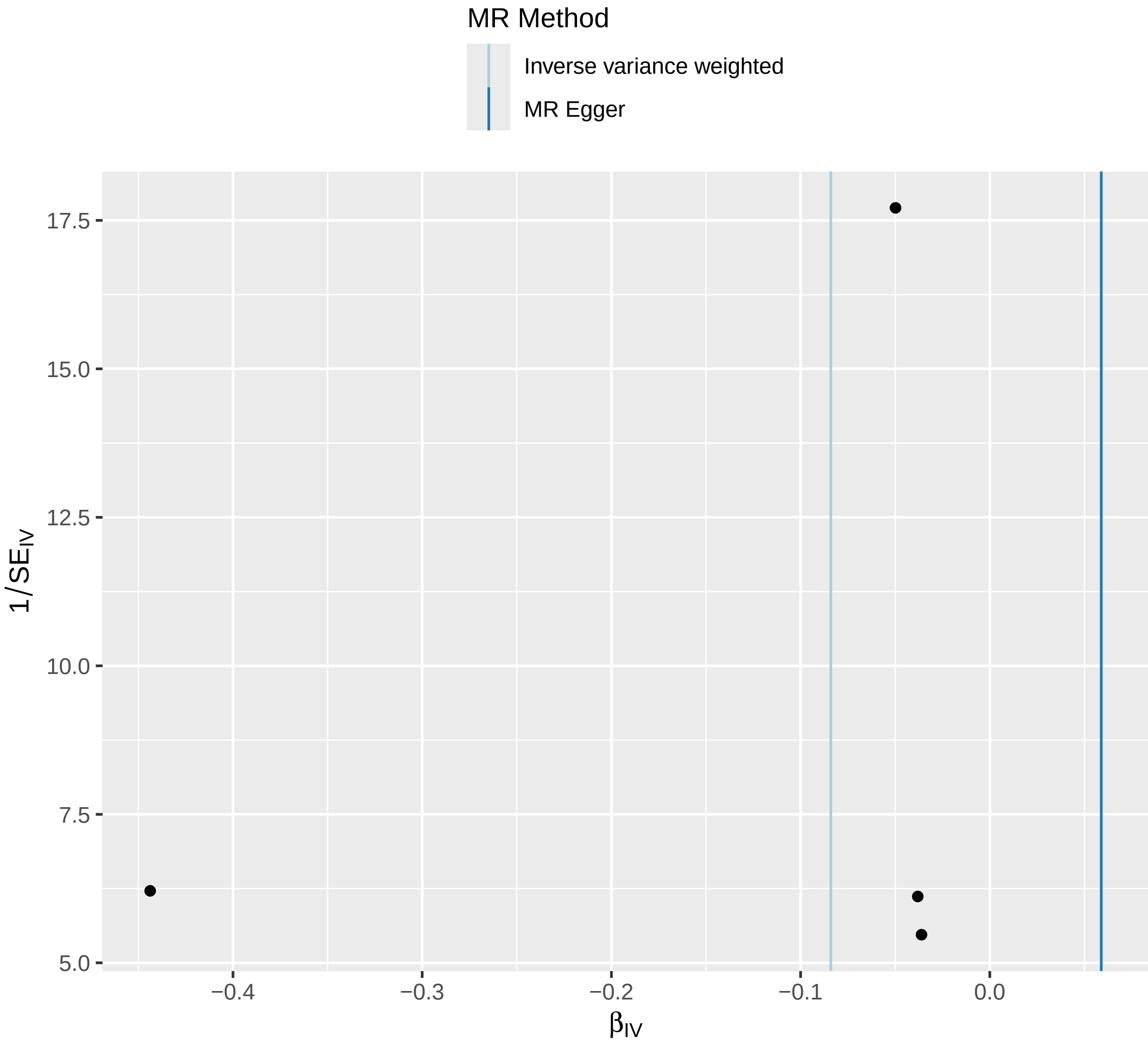
MR Method
Inverse variance weighted
MR Egger
17.5
15.0
12.5
10.0
7.5
5.0
1/SE_IV
−0.4
−0.3
−0.2
−0.1
0.0
β_IV

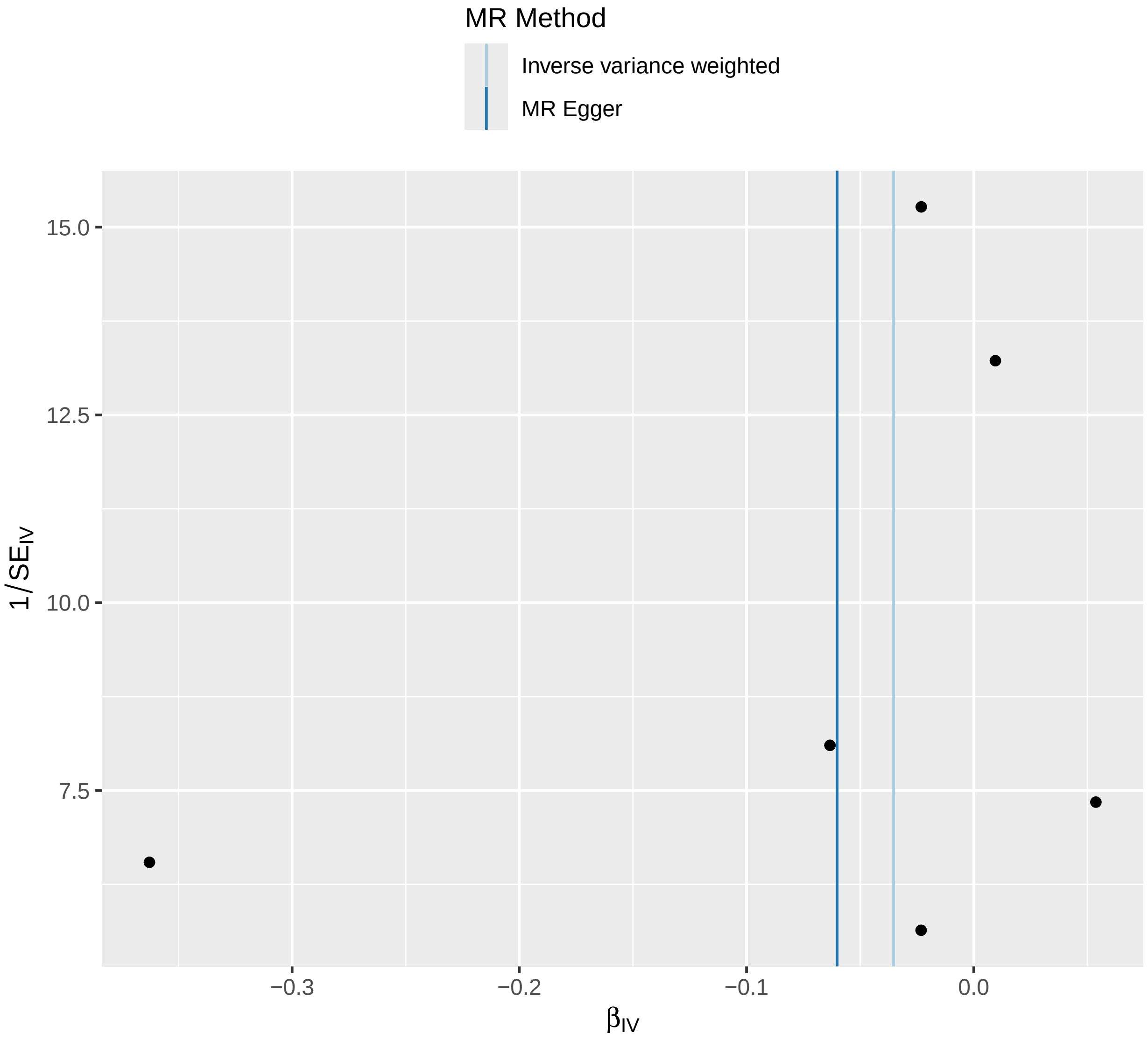

MR Method
Inverse variance weighted
MR Egger
15.0
12.5
10.0
7.5
1/SE_IV
−0.3
−0.2
−0.1
0.0
β_IV

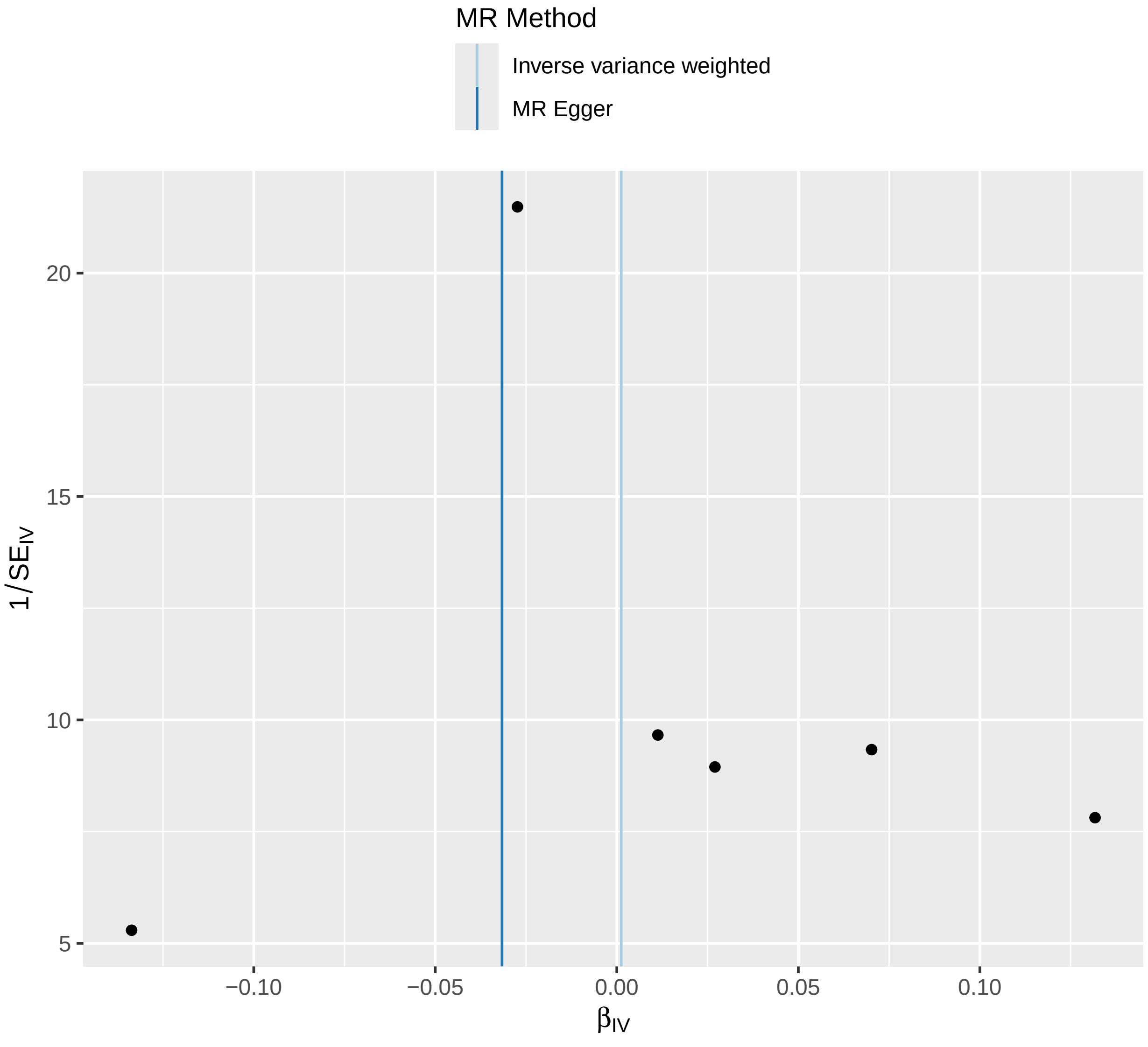

MR Method
Inverse variance weighted
MR Egger
20
15
10
5
1/SE$_{IV}$
−0.10
−0.05
0.00
0.05
0.10
β$_{IV}$

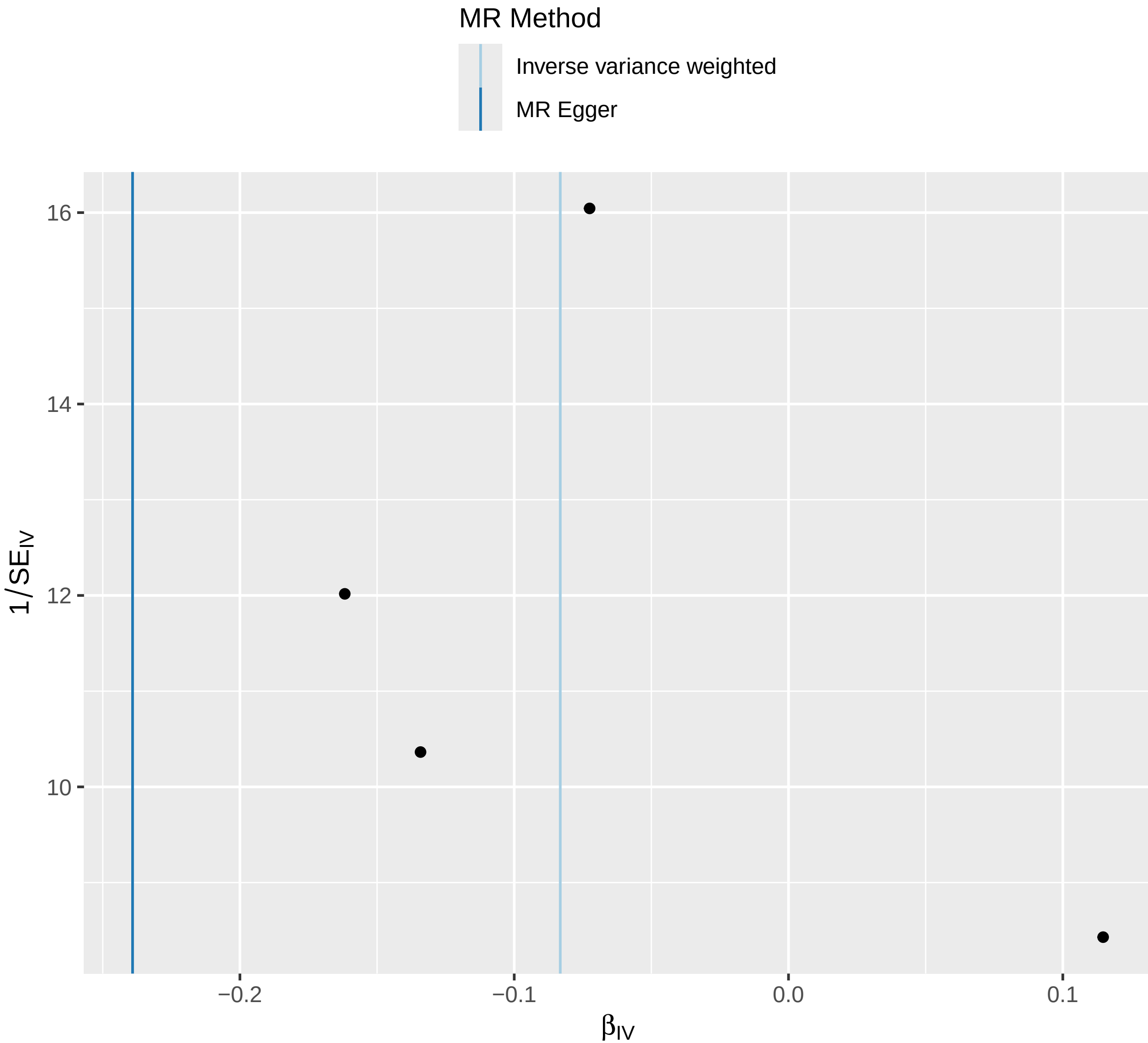
MR Method
Inverse variance weighted
MR Egger
16
14
12
10
$1/SE_{IV}$
−0.2
−0.1
0.0
0.1
β_{IV}

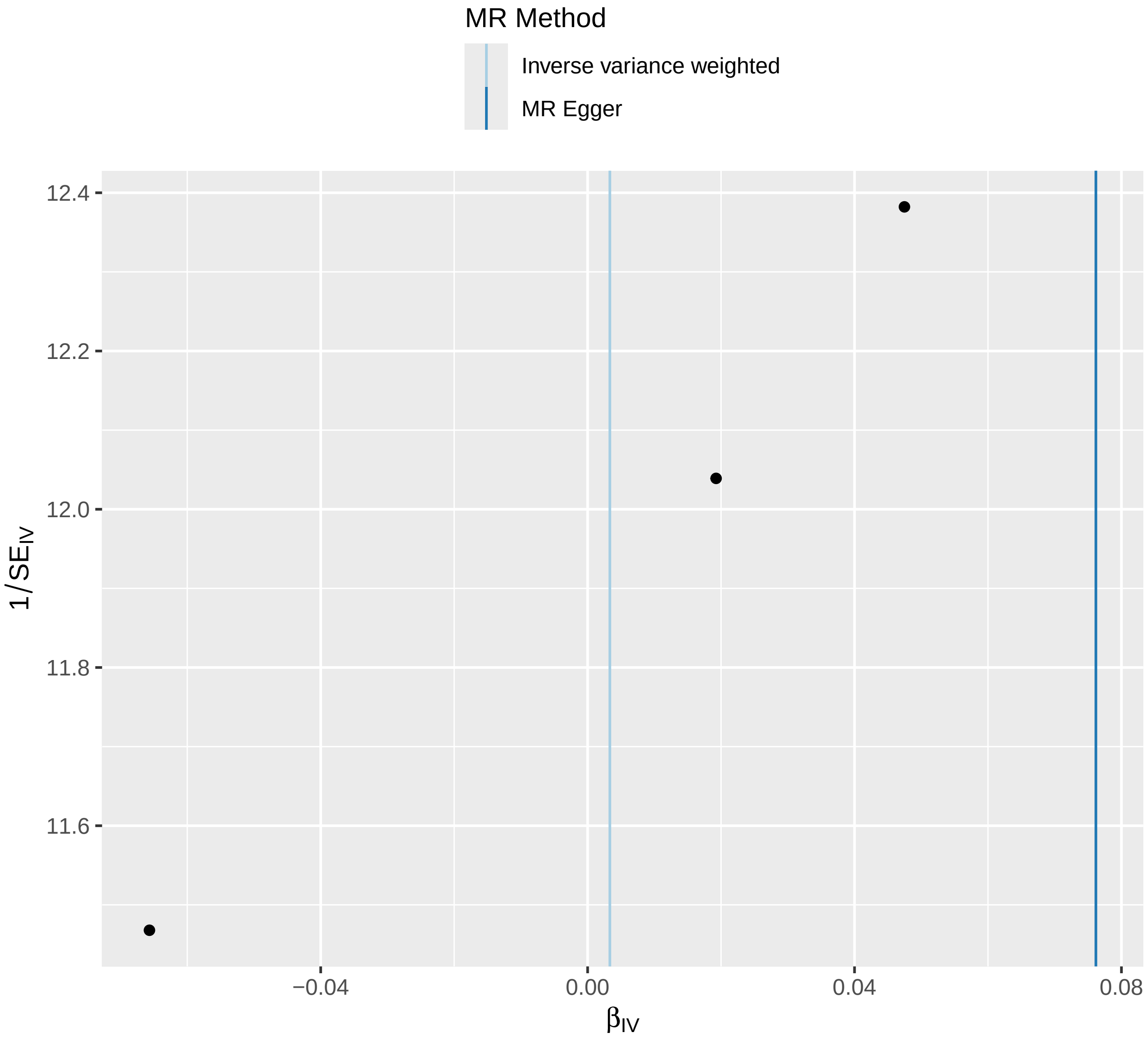
MR Method
Inverse variance weighted
MR Egger
12.4
12.2
12.0
11.8
11.6
1/SE_IV
−0.04
0.00
0.04
0.08
β_IV

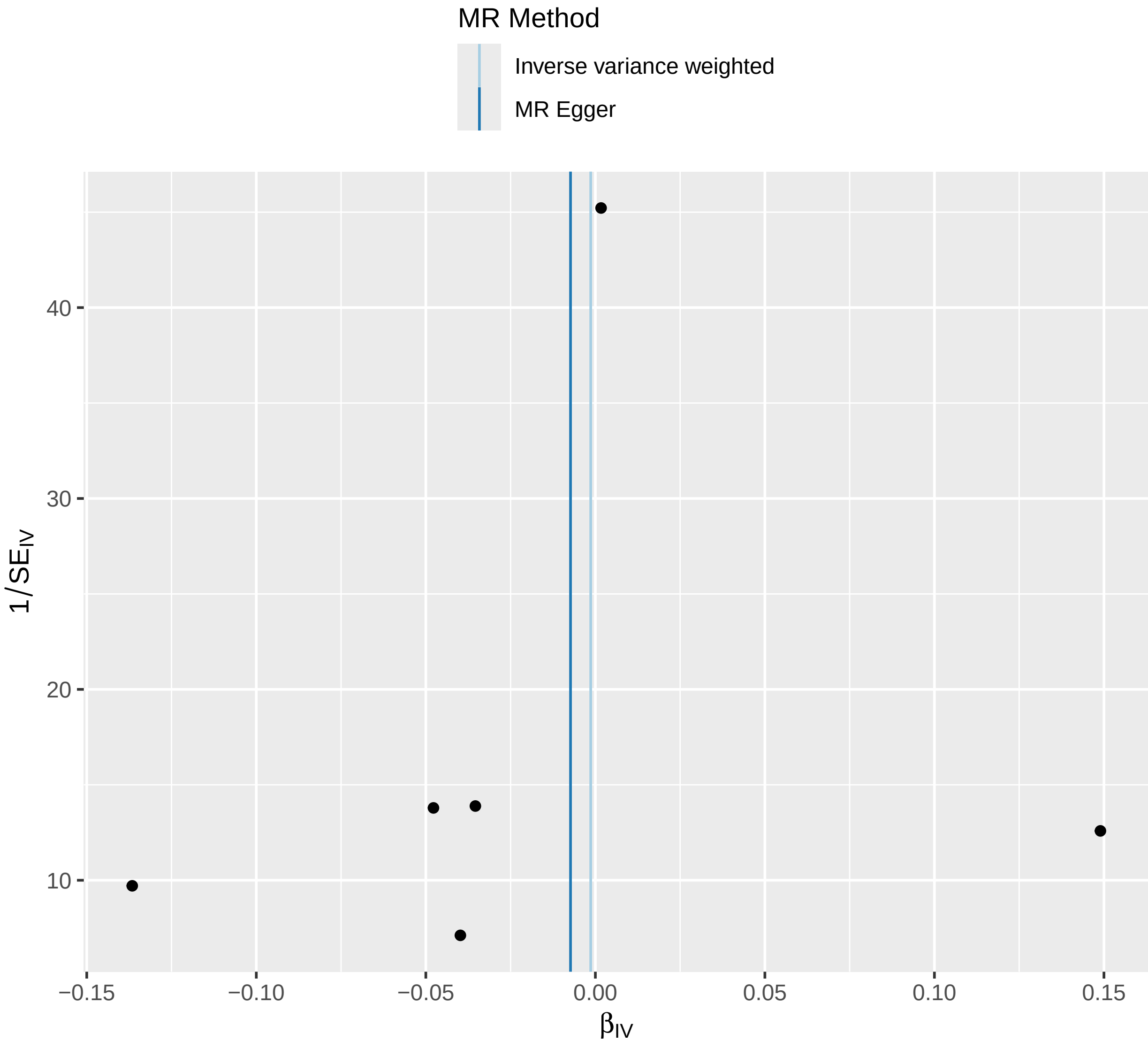
MR Method
Inverse variance weighted
MR Egger
40
30
20
10
1/SE$_{IV}$
−0.15
−0.10
−0.05
0.00
0.05
0.10
0.15
β$_{IV}$

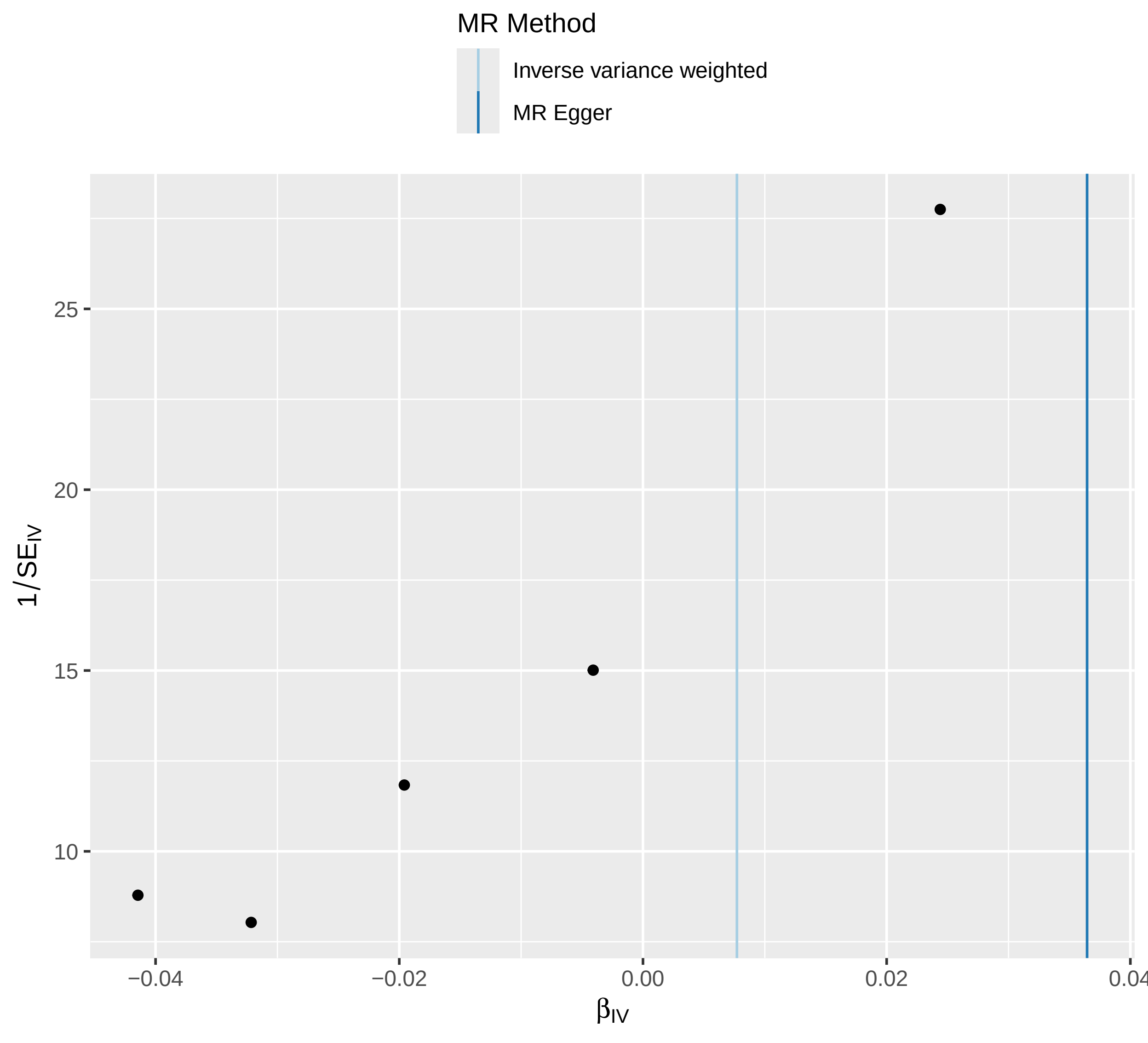

MR Method
Inverse variance weighted
MR Egger
25
20
15
10
1/SE_IV
−0.04
−0.02
0.00
0.02
0.04
β_IV

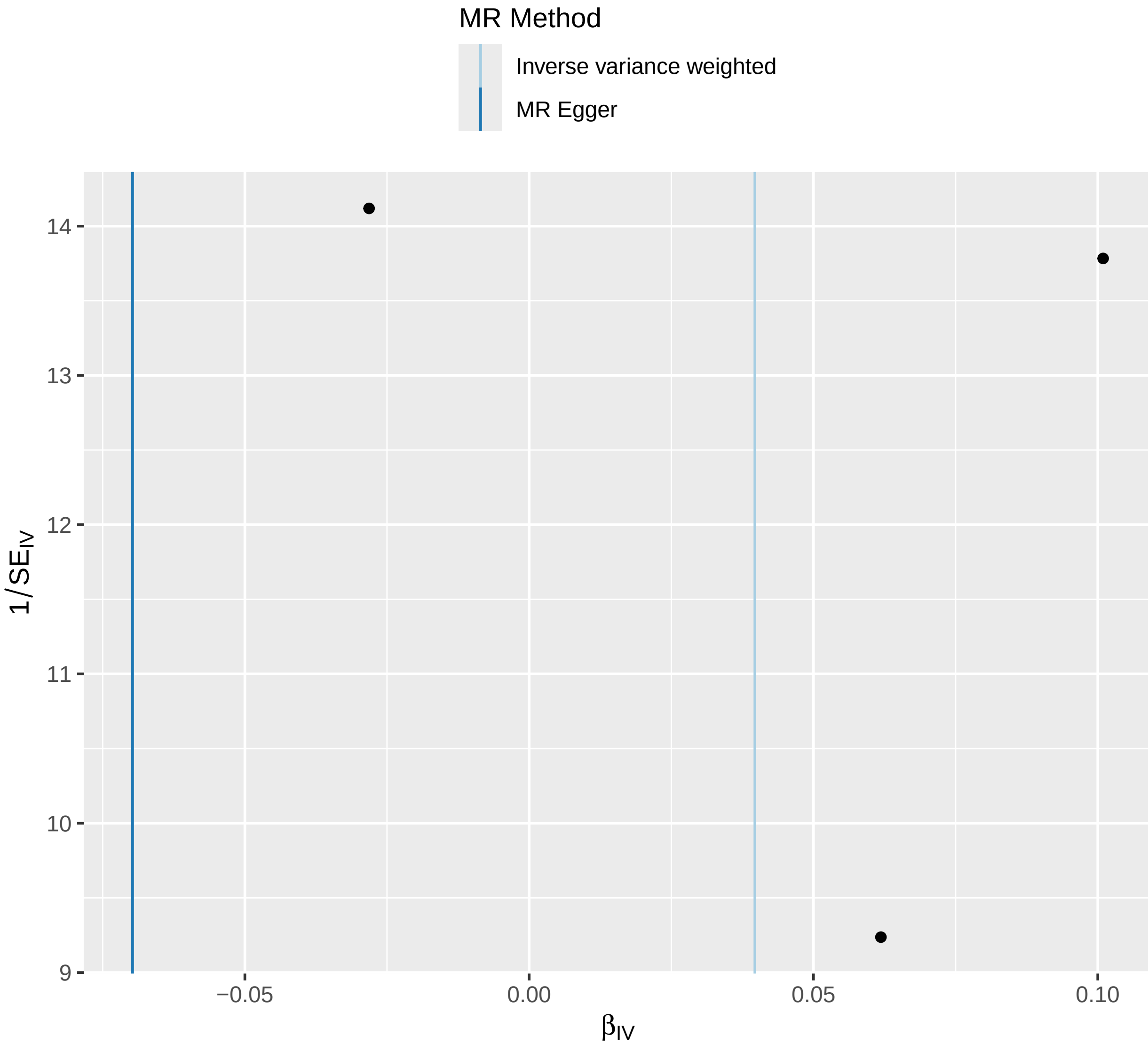
MR Method
Inverse variance weighted
MR Egger
14
13
12
11
10
9
1/SE_IV
−0.05
0.00
0.05
0.10
β_IV

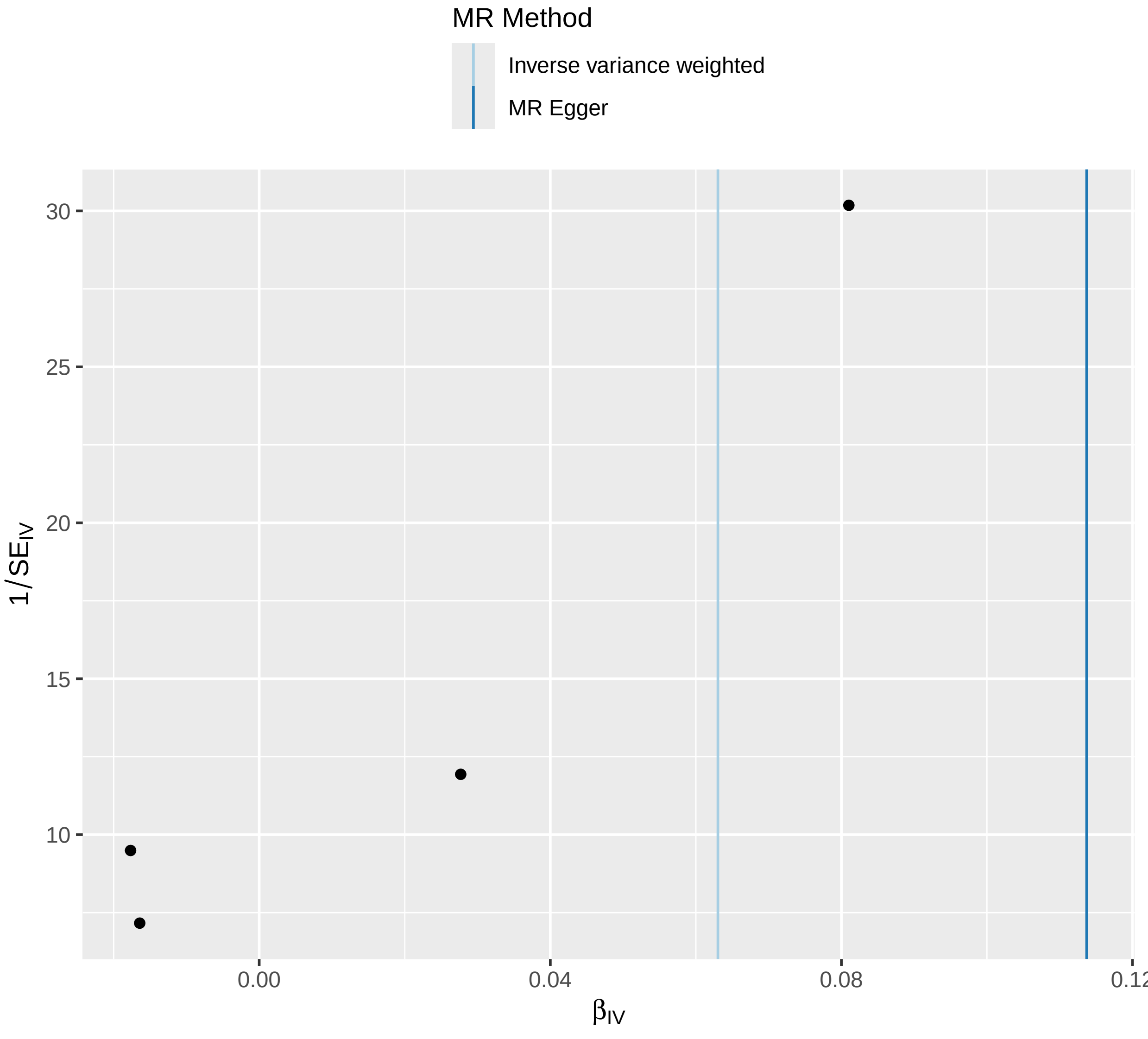

MR Method
Inverse variance weighted
MR Egger
30
25
20
15
10
$1/SE_{IV}$
0.00
0.04
0.08
0.12
β_{IV}

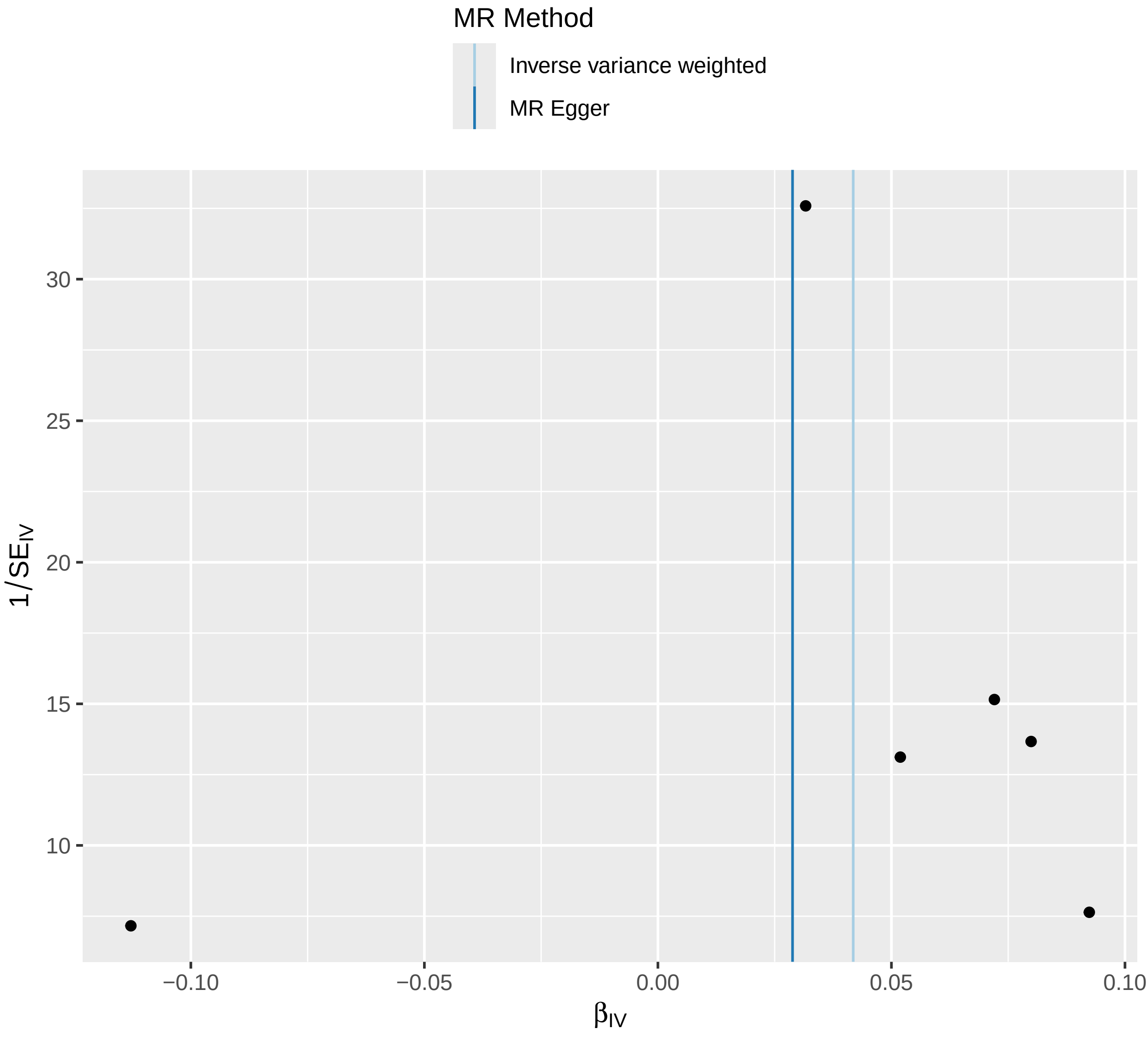

MR Method
Inverse variance weighted
MR Egger
30
25
20
15
10
1/SE_IV
−0.10
−0.05
0.00
0.05
0.10
β_IV

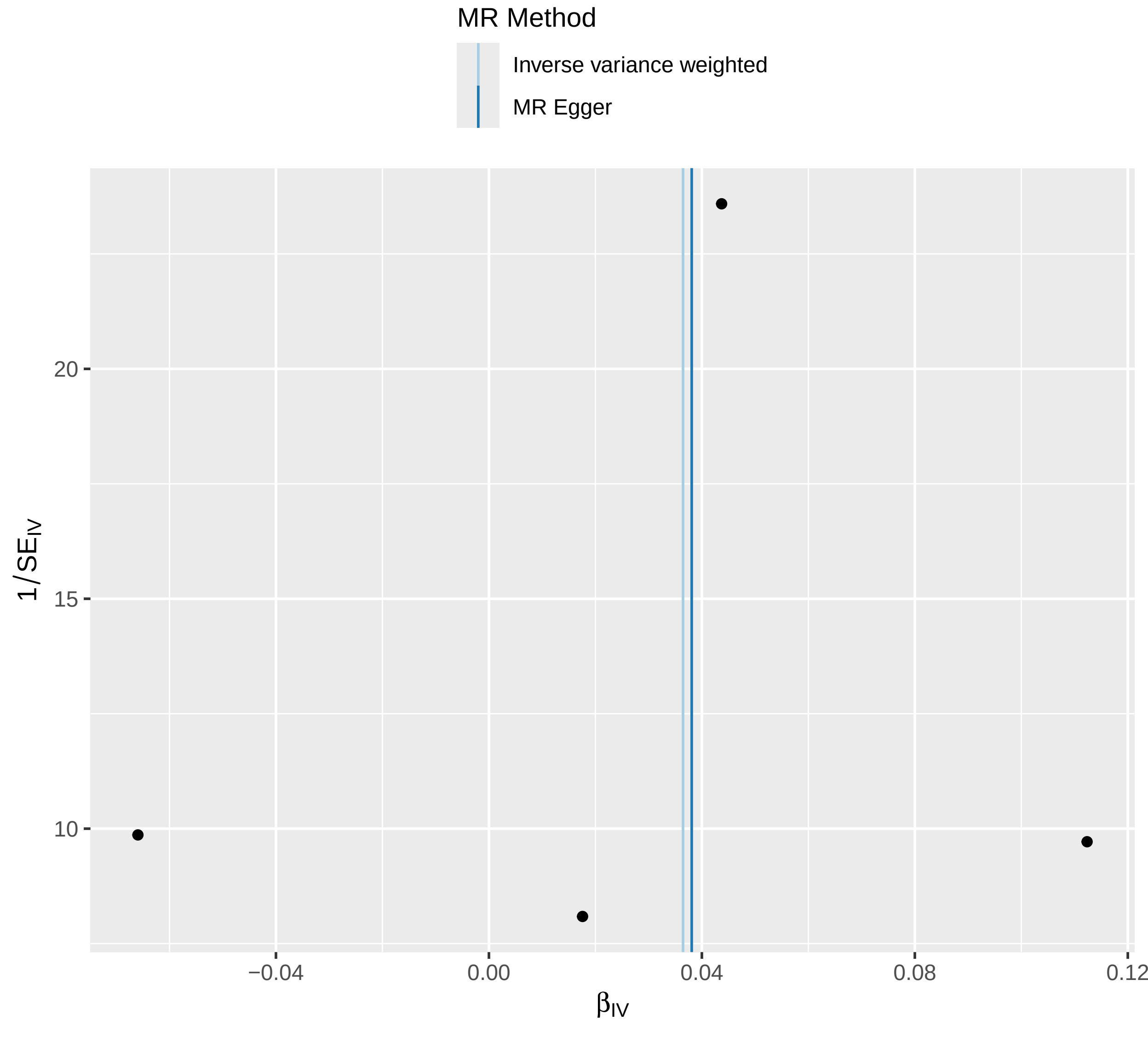
MR Method
Inverse variance weighted
MR Egger
20
15
10
1/SE_IV
−0.04
0.00
0.04
0.08
0.12
β_IV

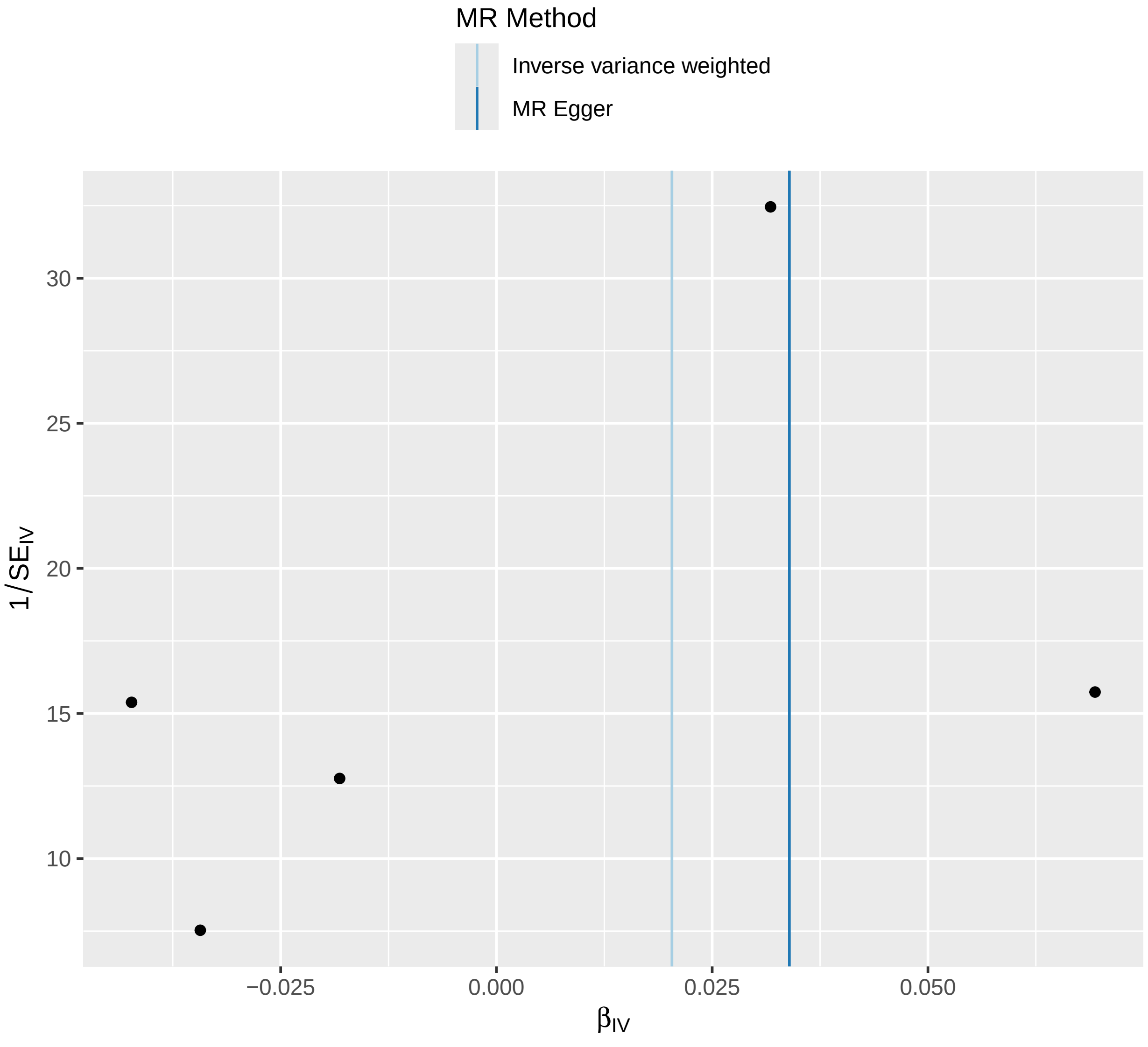
MR Method
Inverse variance weighted
MR Egger
30
25
20
15
10
1/SE_IV
−0.025
0.000
0.025
0.050
β_IV

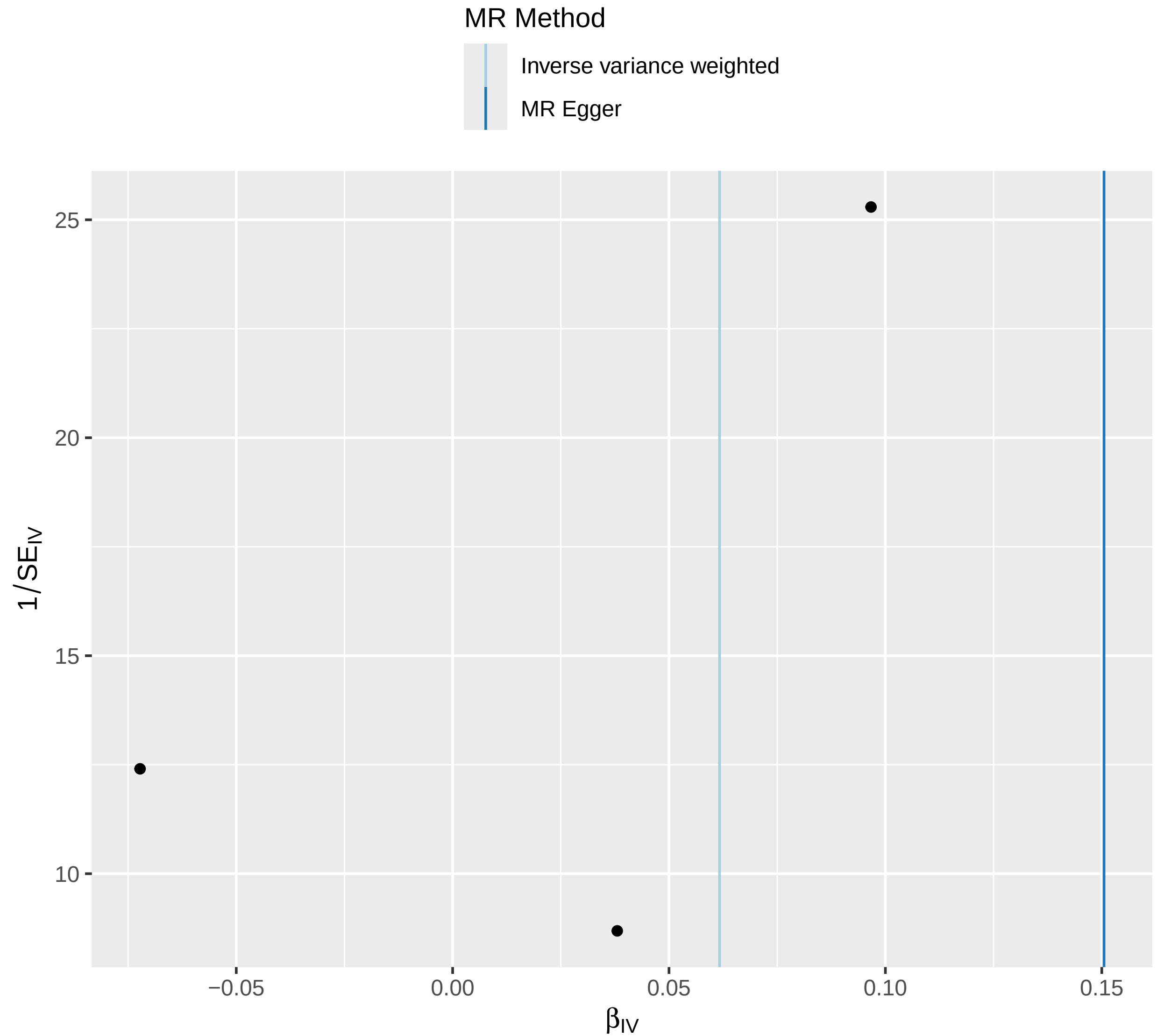

MR Method
Inverse variance weighted
MR Egger
25
20
15
10
1/SE_IV
−0.05
0.00
0.05
0.10
0.15
β_IV

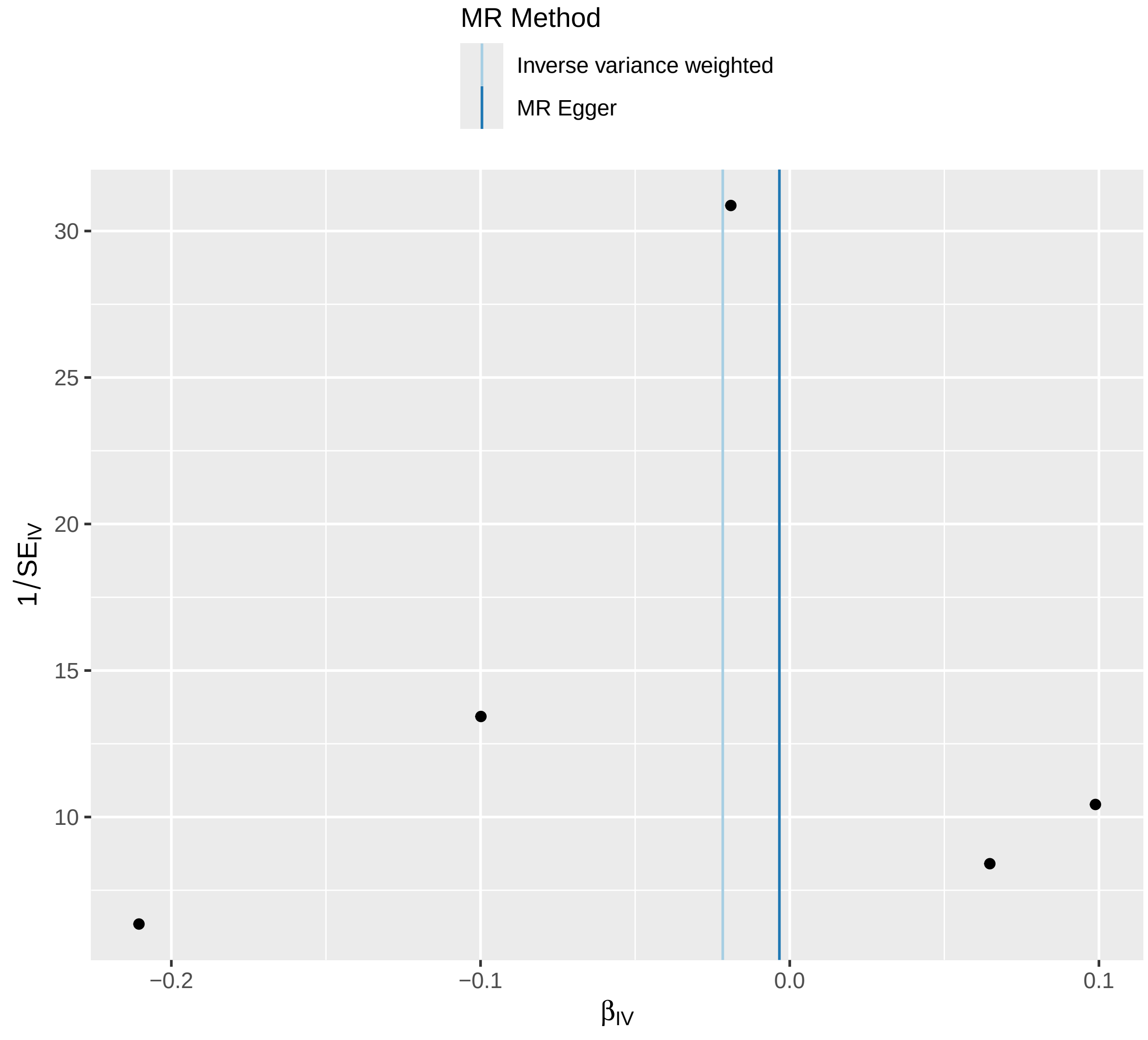
MR Method
Inverse variance weighted
MR Egger
30
25
20
15
10
1/SE_IV
−0.2
−0.1
0.0
0.1
β_IV

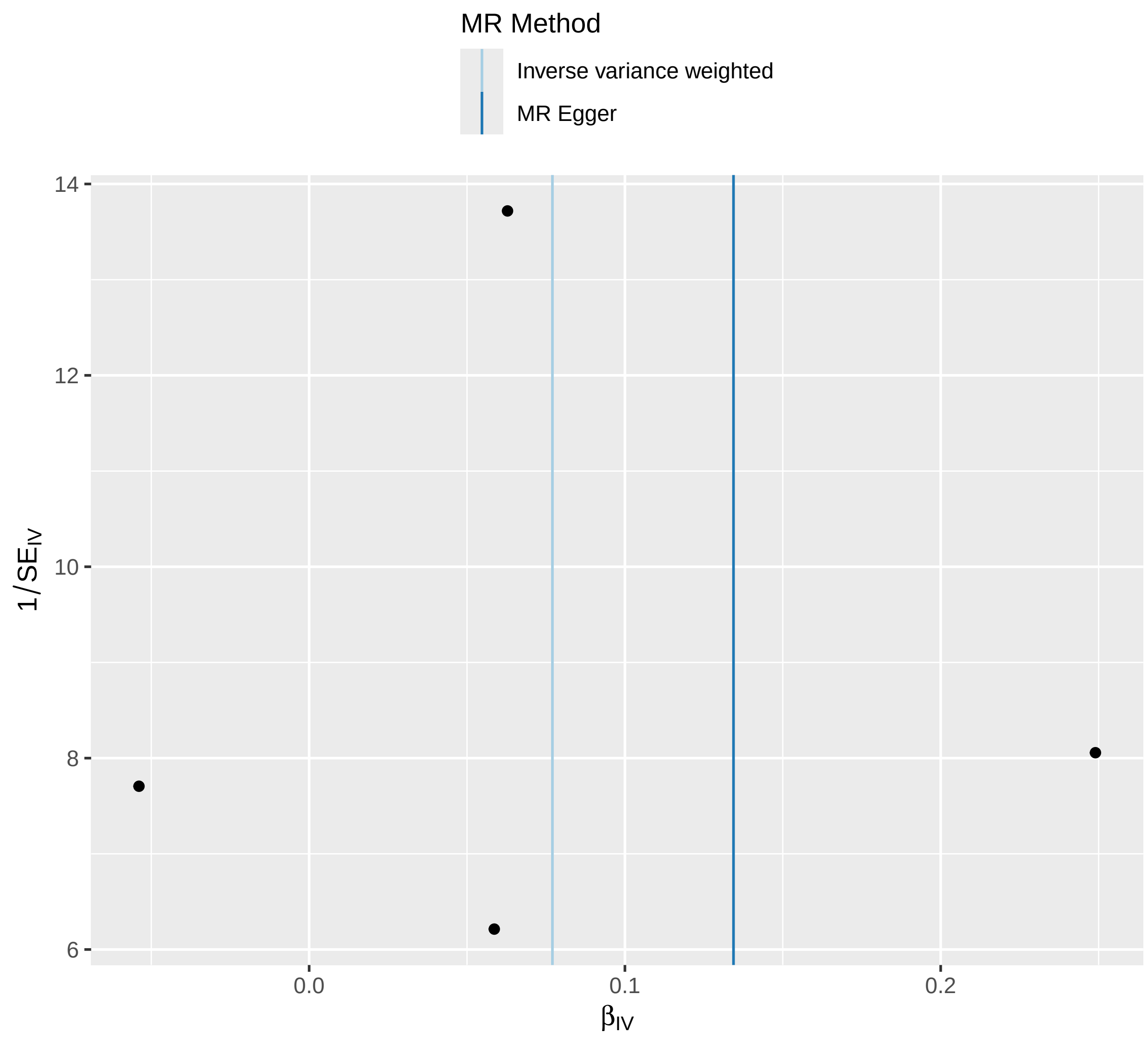

MR Method
Inverse variance weighted
MR Egger
14
12
10
8
6
1/SE$_{IV}$
0.0
0.1
0.2
β_{IV}

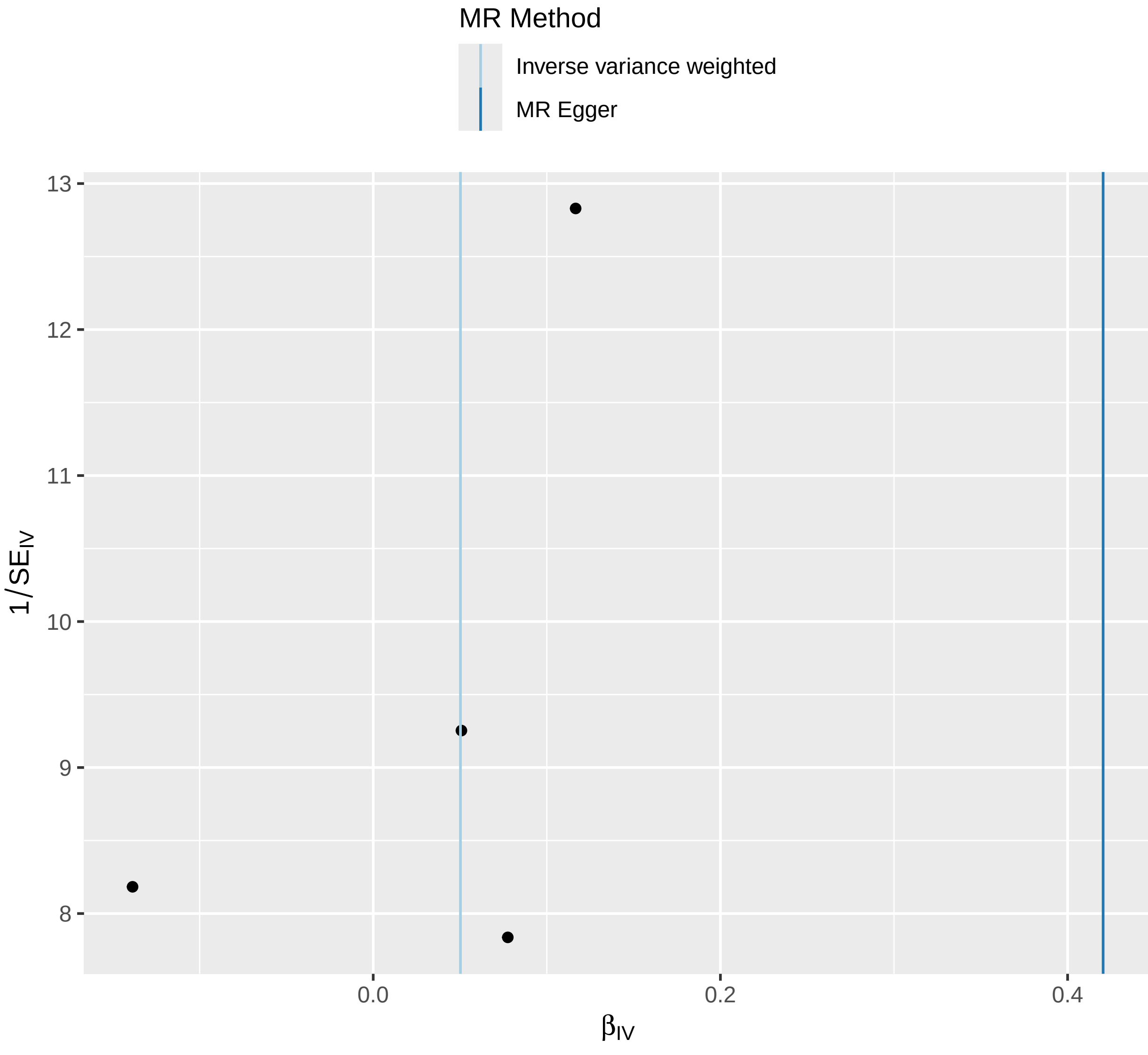
MR Method
Inverse variance weighted
MR Egger
13
12
11
10
9
8
0.0
0.2
0.4
$1/SE_{IV}$
β_{IV}

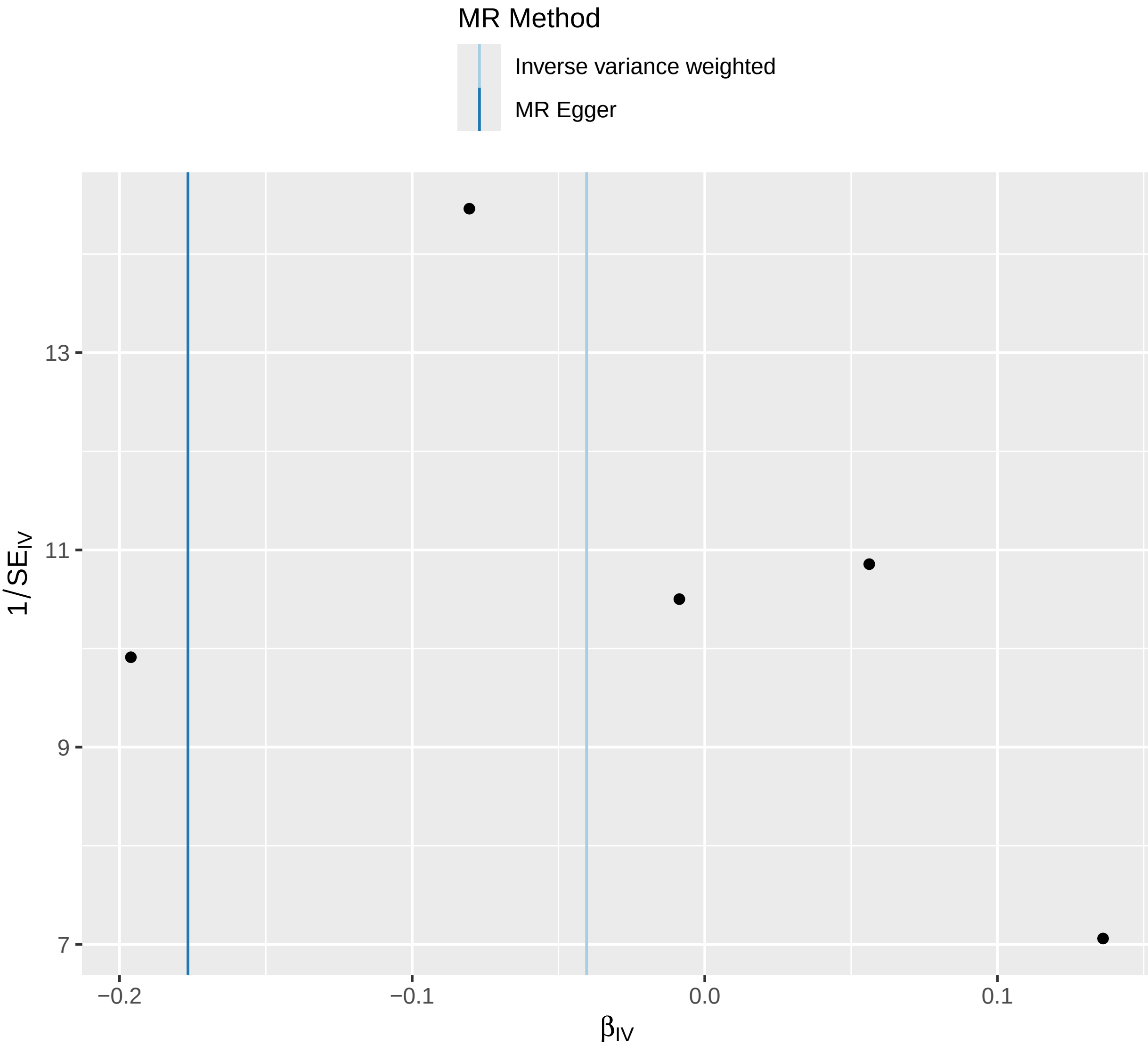
MR Method
Inverse variance weighted
MR Egger
13
11
9
7
1/SE_IV
−0.2
−0.1
0.0
0.1
β_IV

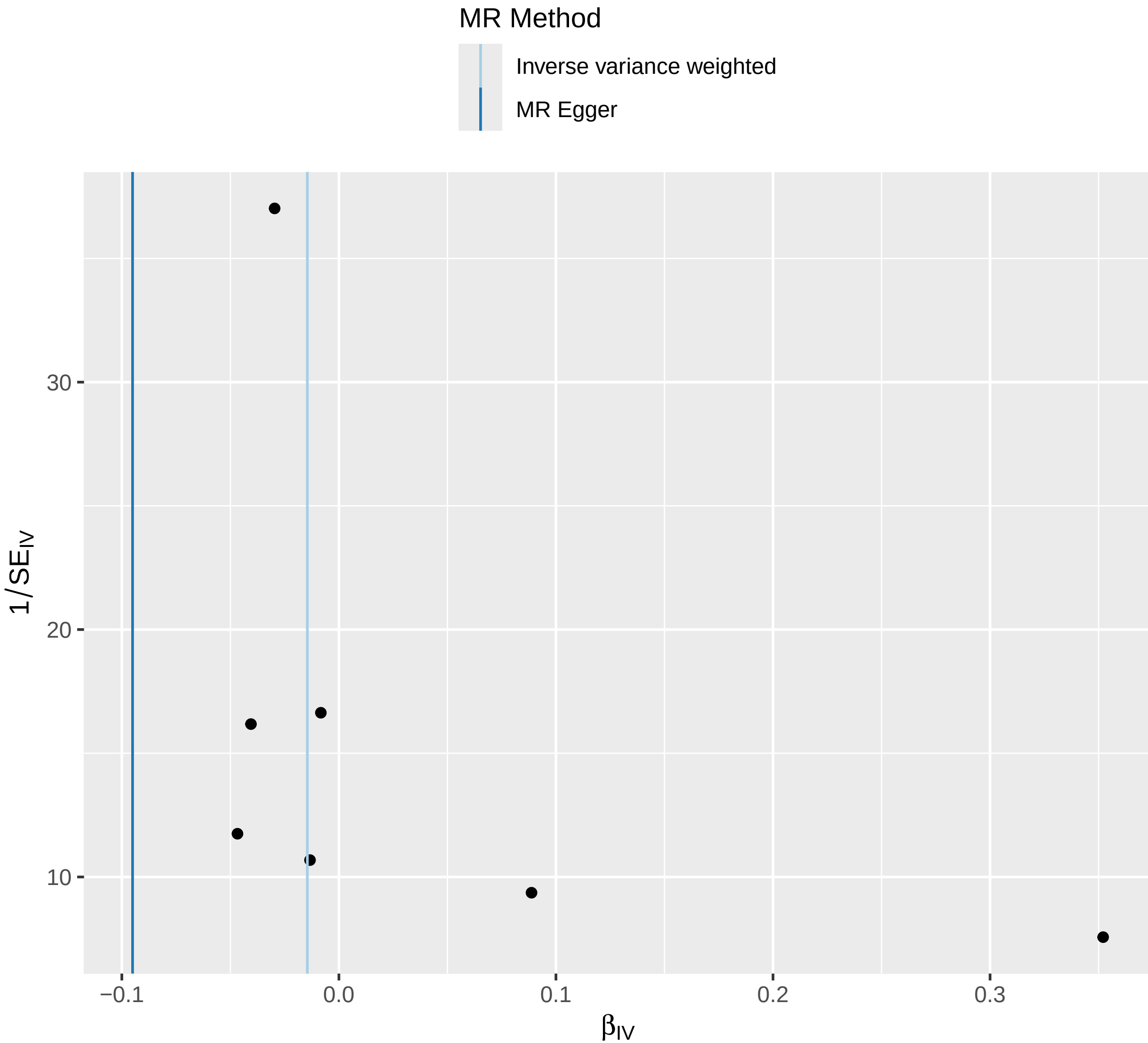

MR Method
Inverse variance weighted
MR Egger
30
20
10
1/SE$_{IV}$
−0.1
0.0
0.1
0.2
0.3
β$_{IV}$

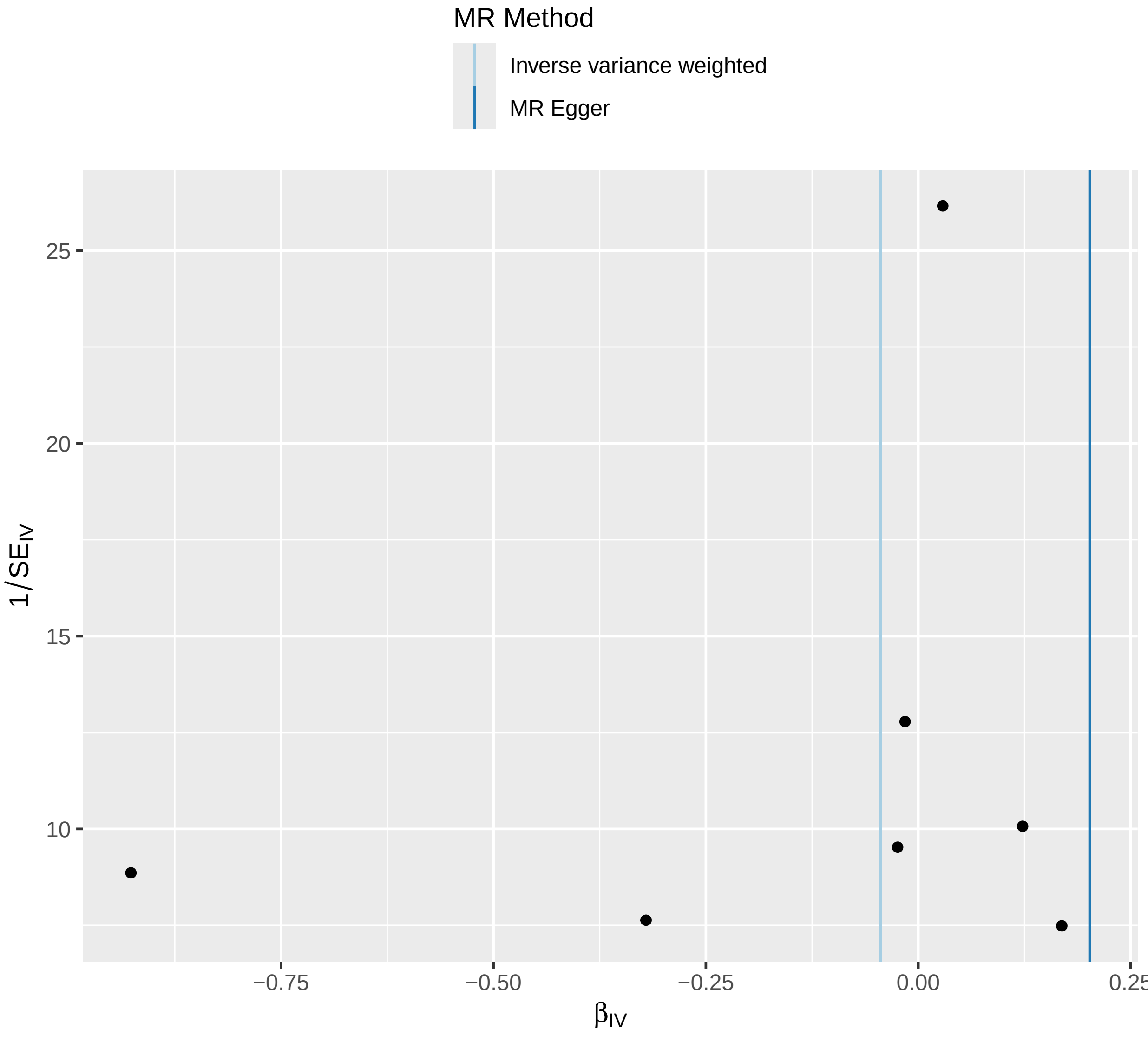
MR Method
Inverse variance weighted
MR Egger
25
20
15
10
$1/SE_{IV}$
−0.75
−0.50
−0.25
0.00
0.25
β_{IV}

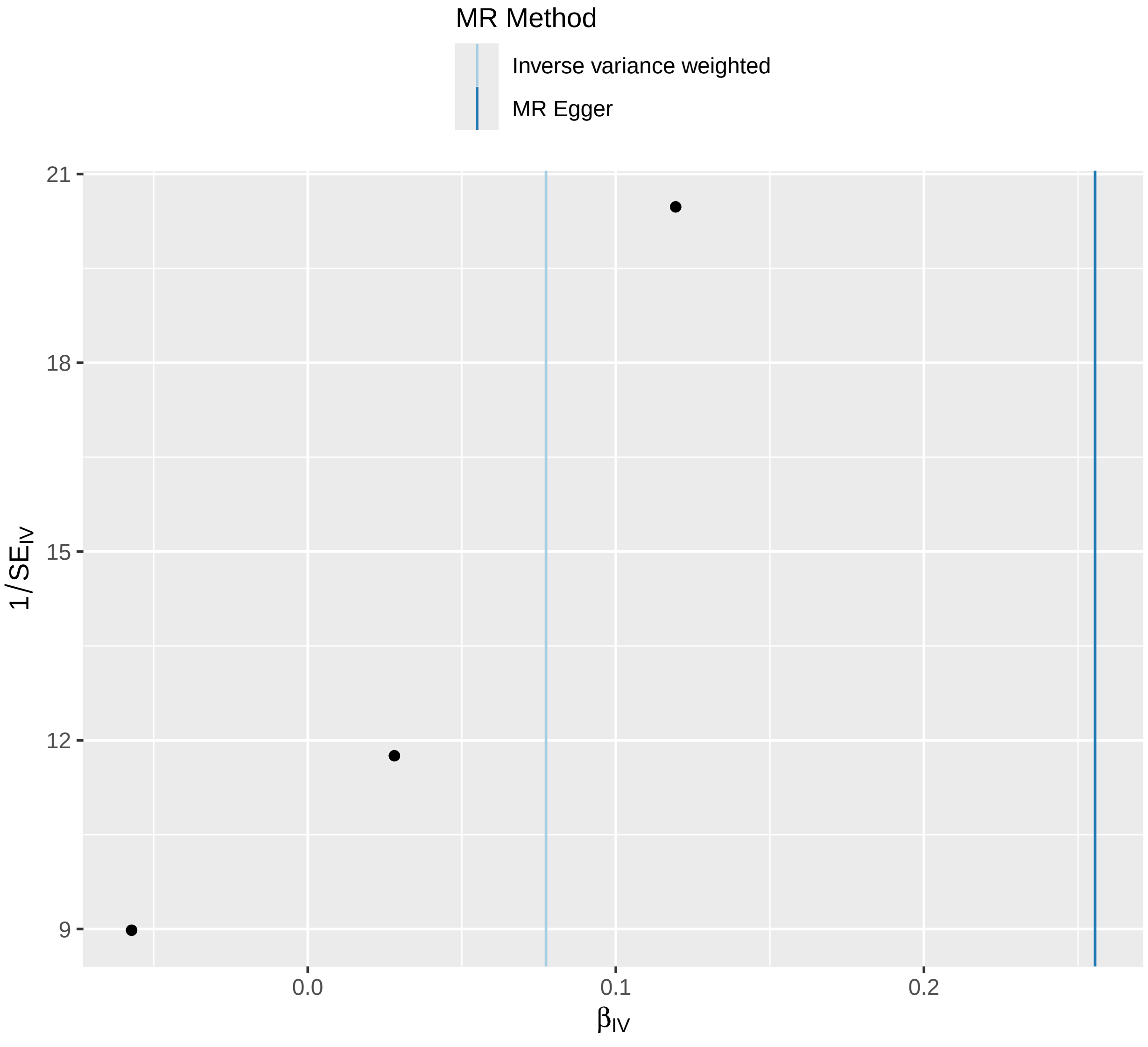
MR Method
Inverse variance weighted
MR Egger
21
18
15
12
9
1/SE_IV
0.0
0.1
0.2
β_IV

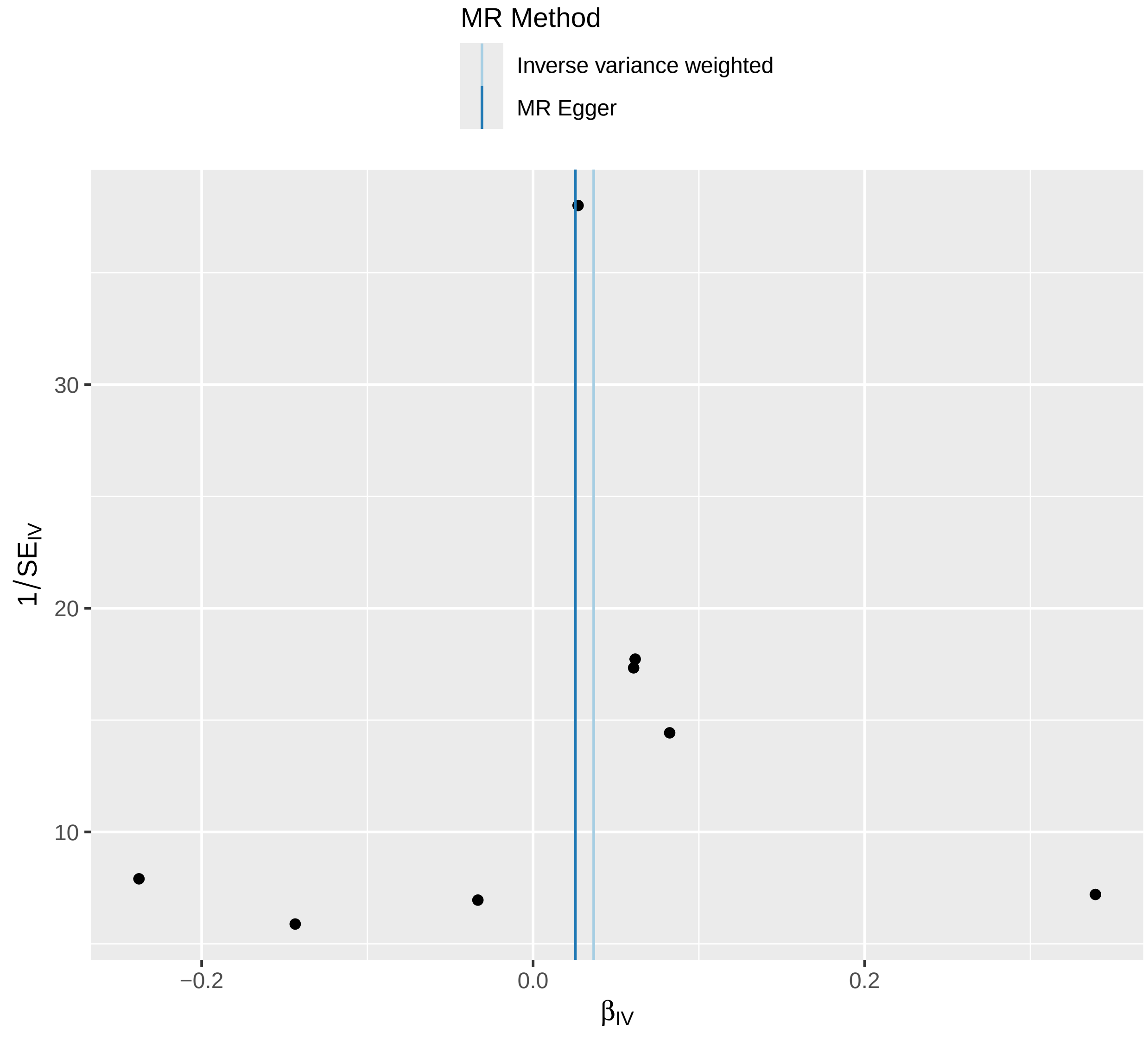

MR Method
Inverse variance weighted
MR Egger
30
20
10
$1/SE_{IV}$
−0.2
0.0
0.2
β_{IV}

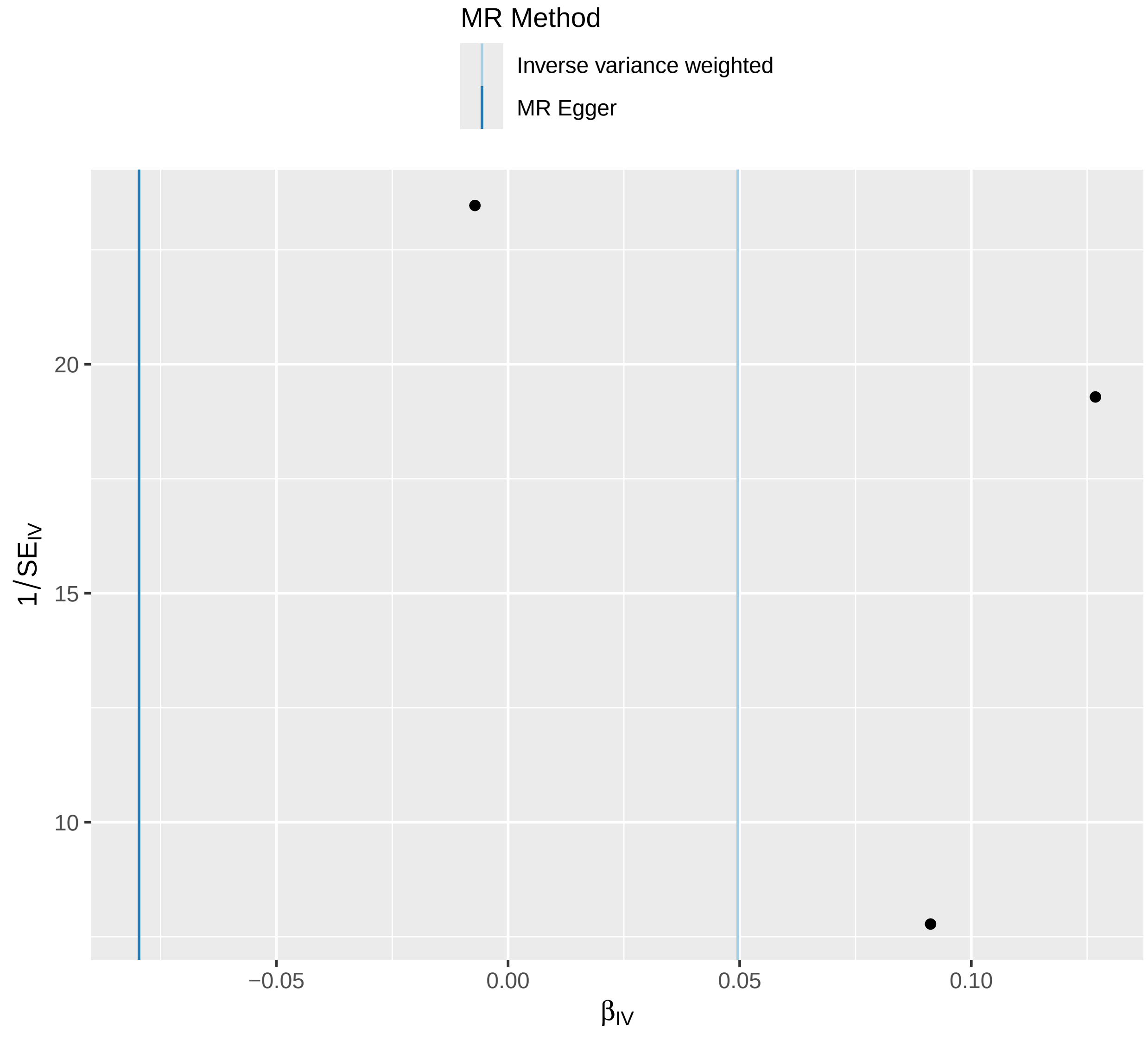
MR Method
Inverse variance weighted
MR Egger
20
15
10
−0.05
0.00
0.05
0.10
$1/SE_{IV}$
β_{IV}

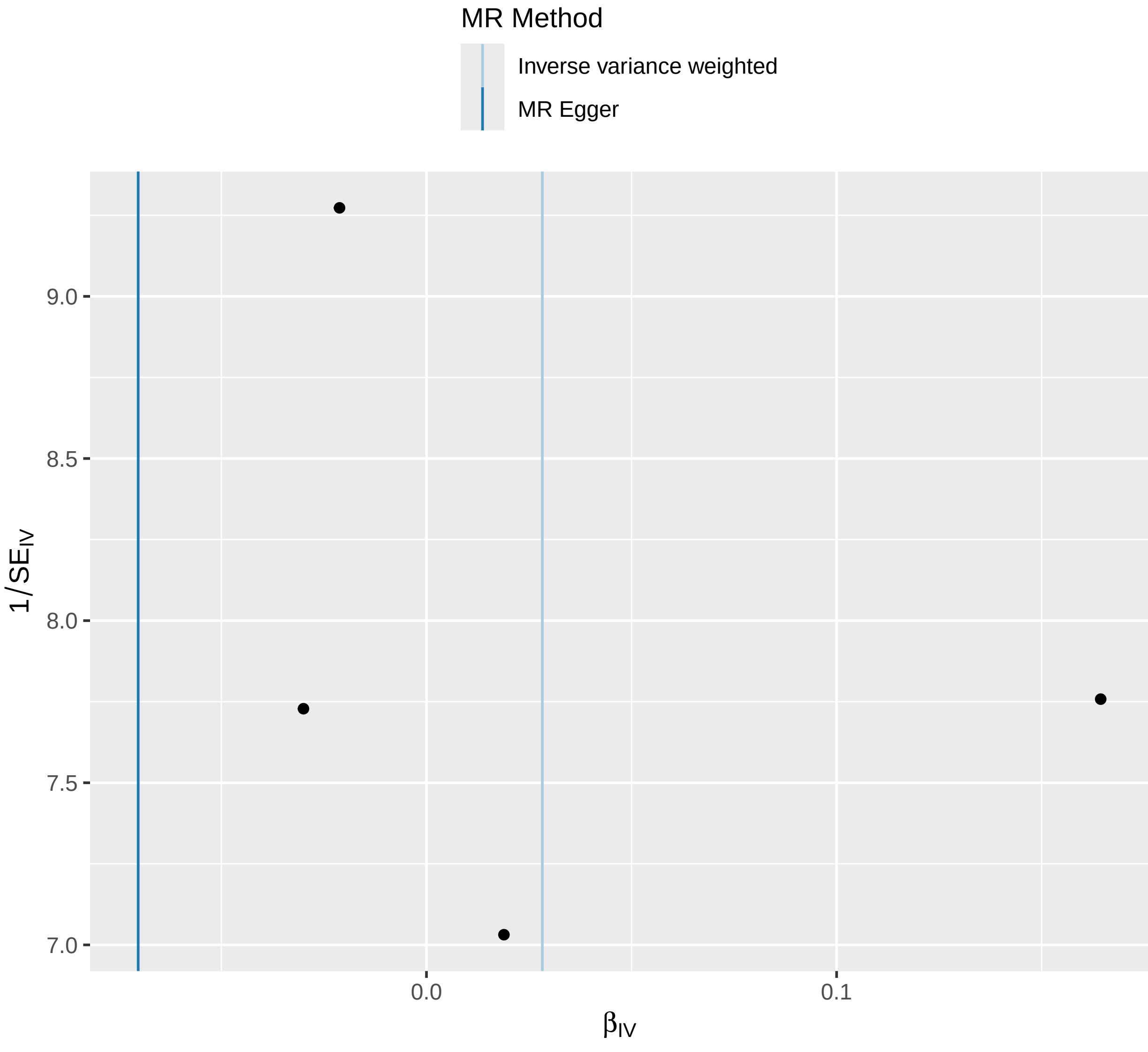
MR Method
Inverse variance weighted
MR Egger
9.0
8.5
8.0
7.5
7.0
1/SE_IV
0.0
0.1
β_IV

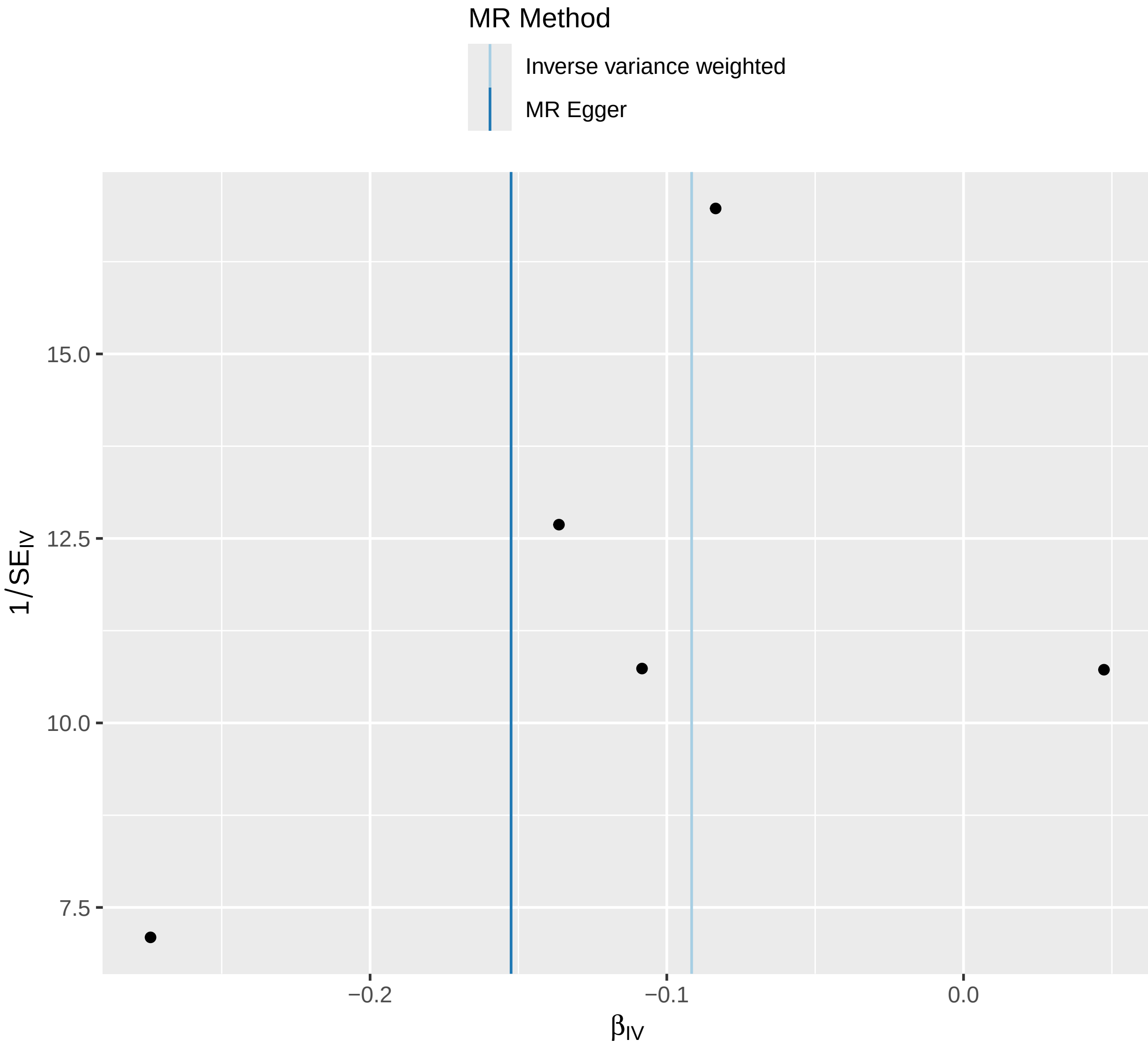
MR Method
Inverse variance weighted
MR Egger
15.0
12.5
10.0
7.5
$1/SE_{IV}$
−0.2
−0.1
0.0
β_{IV}

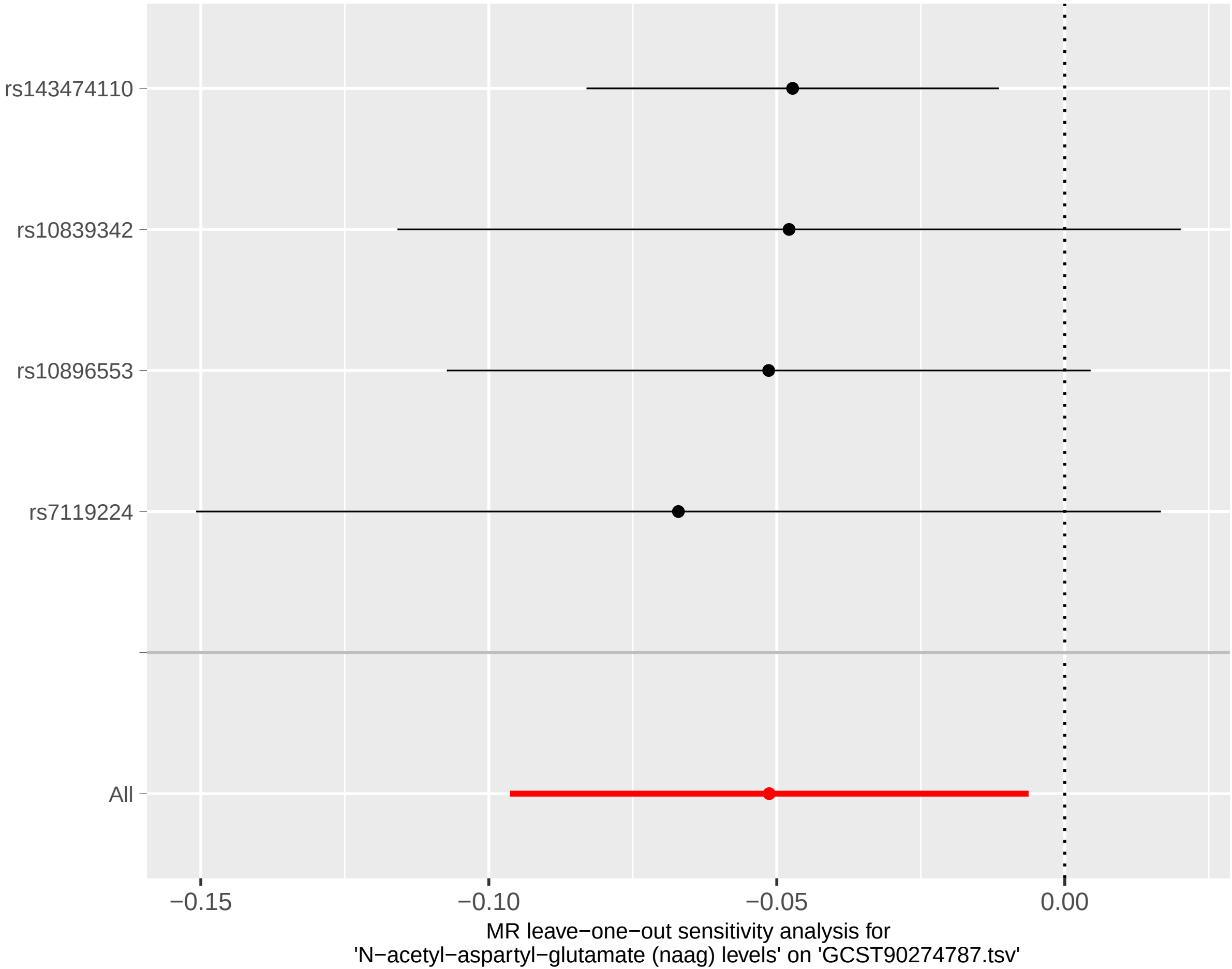

rs143474110
rs10839342
rs10896553
rs7119224
All
−0.15
−0.10
−0.05
0.00
MR leave−one−out sensitivity analysis for
'N−acetyl−aspartyl−glutamate (naag) levels' on 'GCST90274787.tsv'

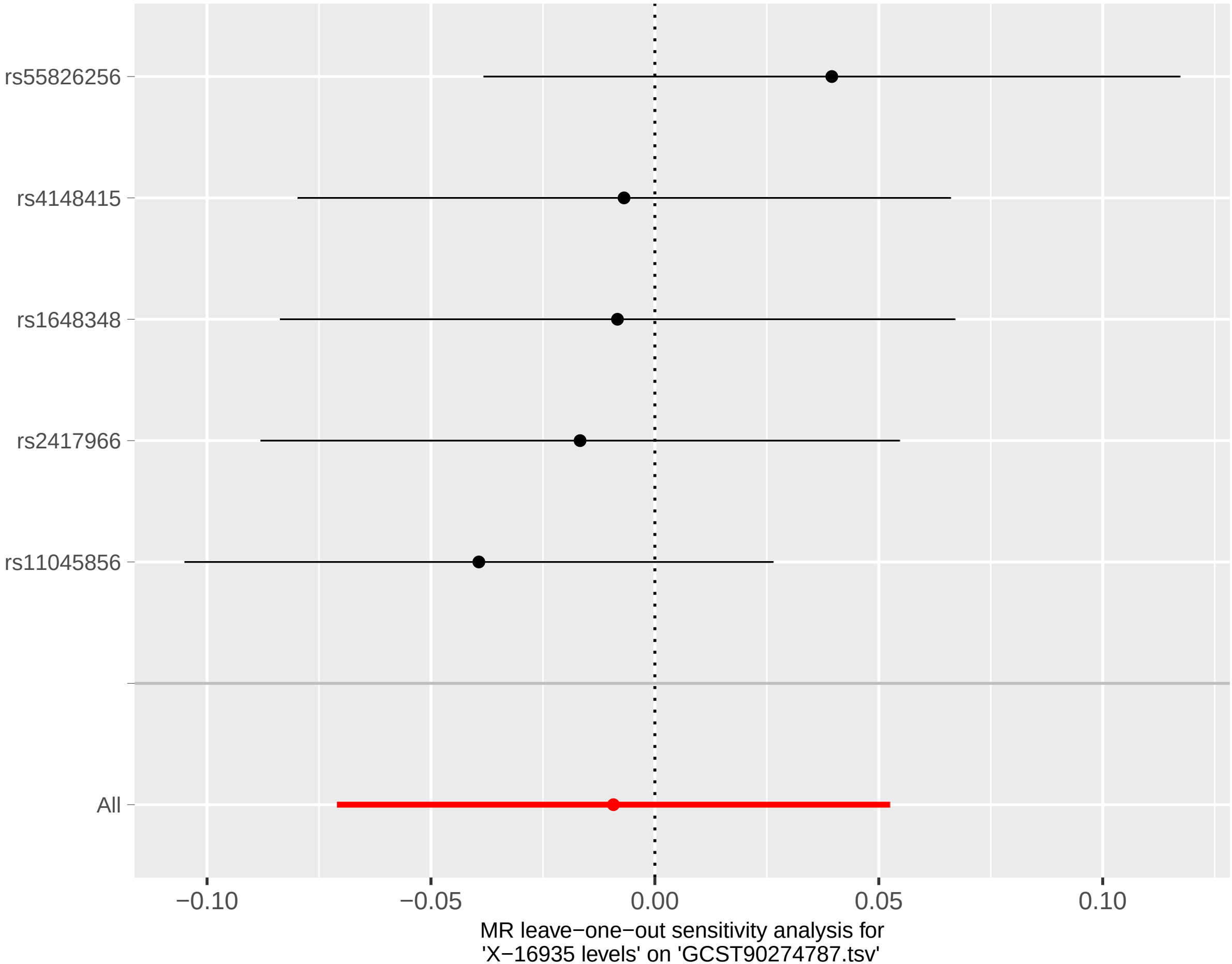
rs55826256
rs4148415
rs1648348
rs2417966
rs11045856
All
−0.10
−0.05
0.00
0.05
0.10
MR leave−one−out sensitivity analysis for
'X−16935 levels' on 'GCST90274787.tsv'

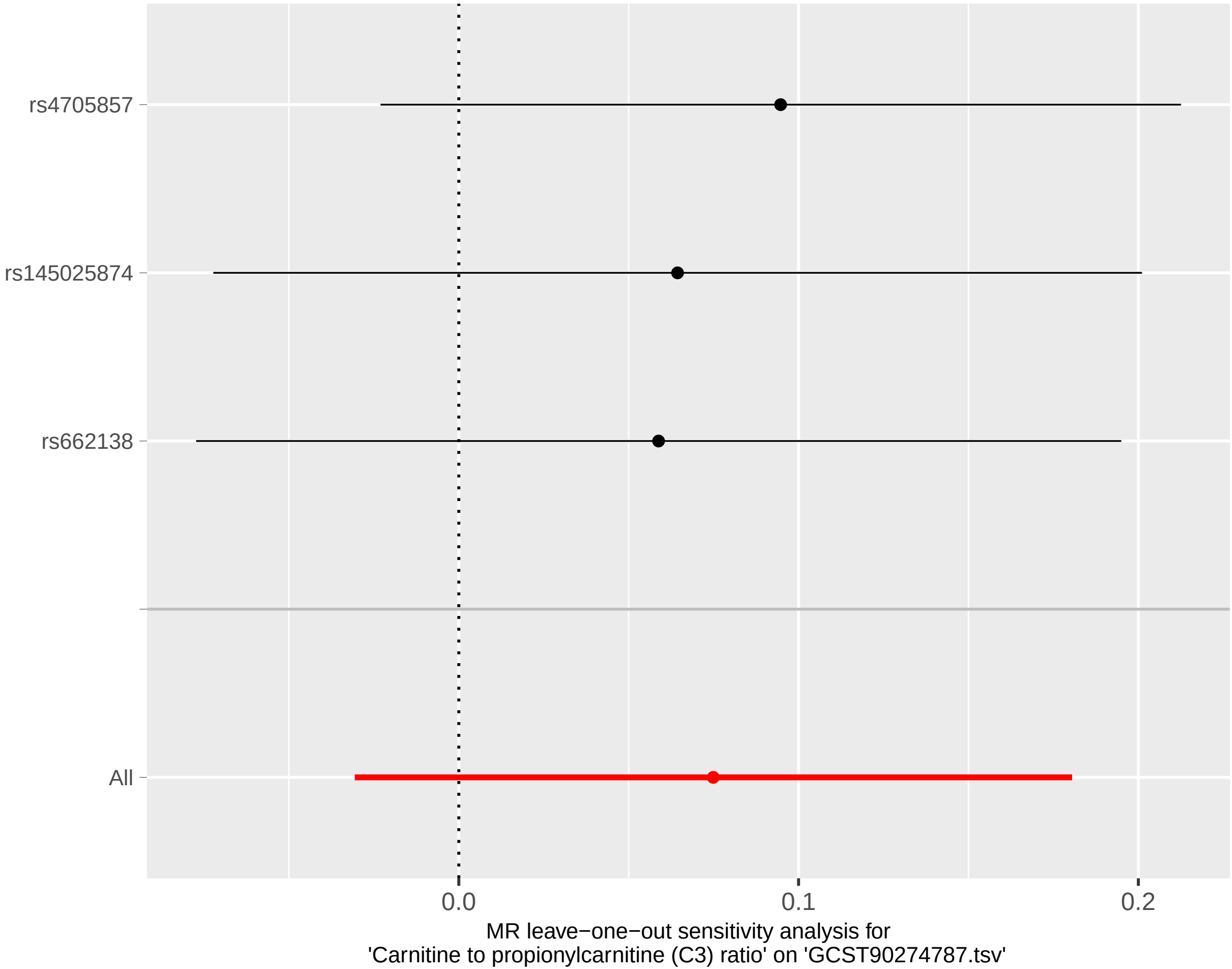
rs4705857
rs145025874
rs662138
All
0.0
0.1
0.2
MR leave−one−out sensitivity analysis for
'Carnitine to propionylcarnitine (C3) ratio' on 'GCST90274787.tsv'

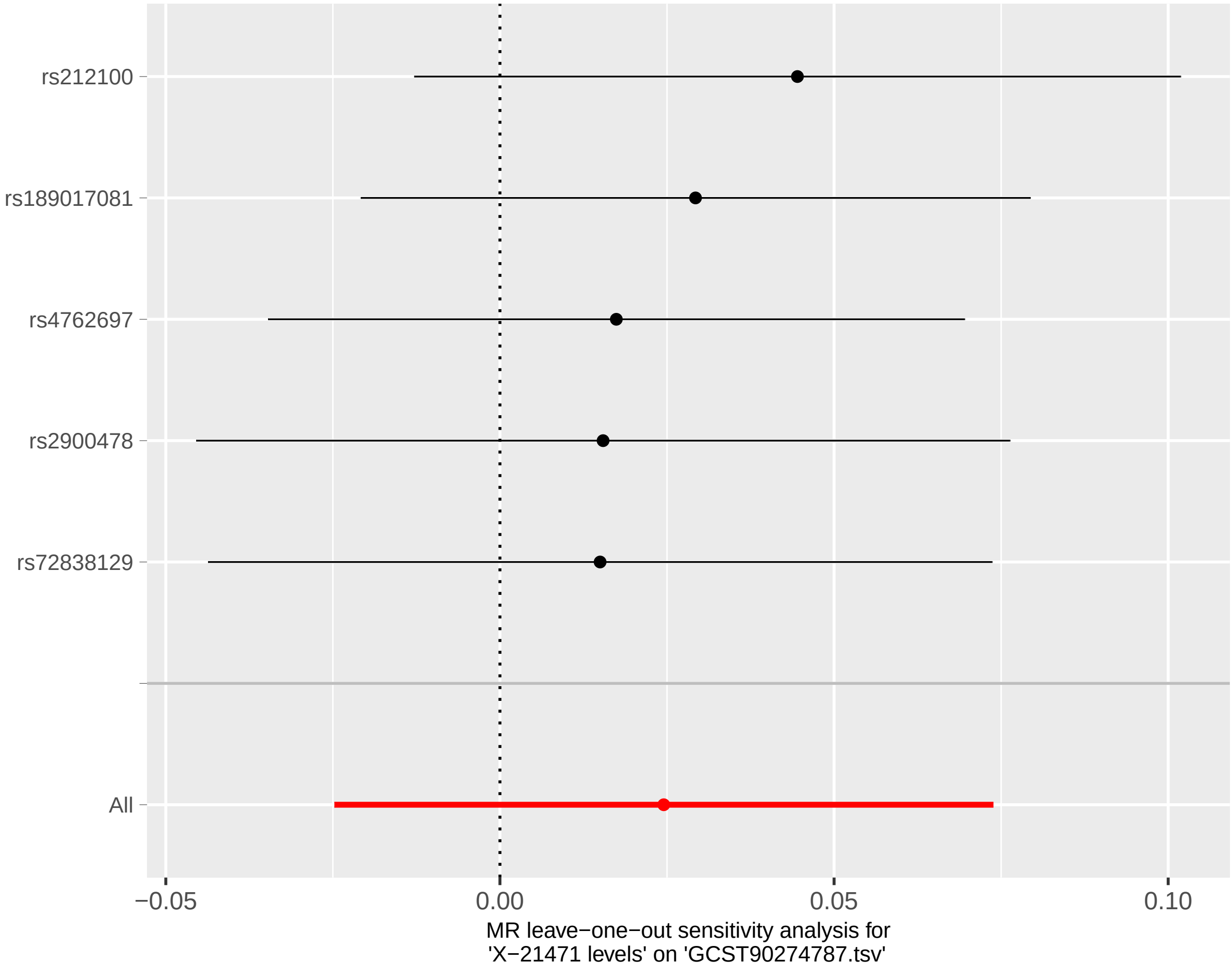
rs212100
rs189017081
rs4762697
rs2900478
rs72838129
All
−0.05
0.00
0.05
0.10
MR leave−one−out sensitivity analysis for
'X−21471 levels' on 'GCST90274787.tsv'

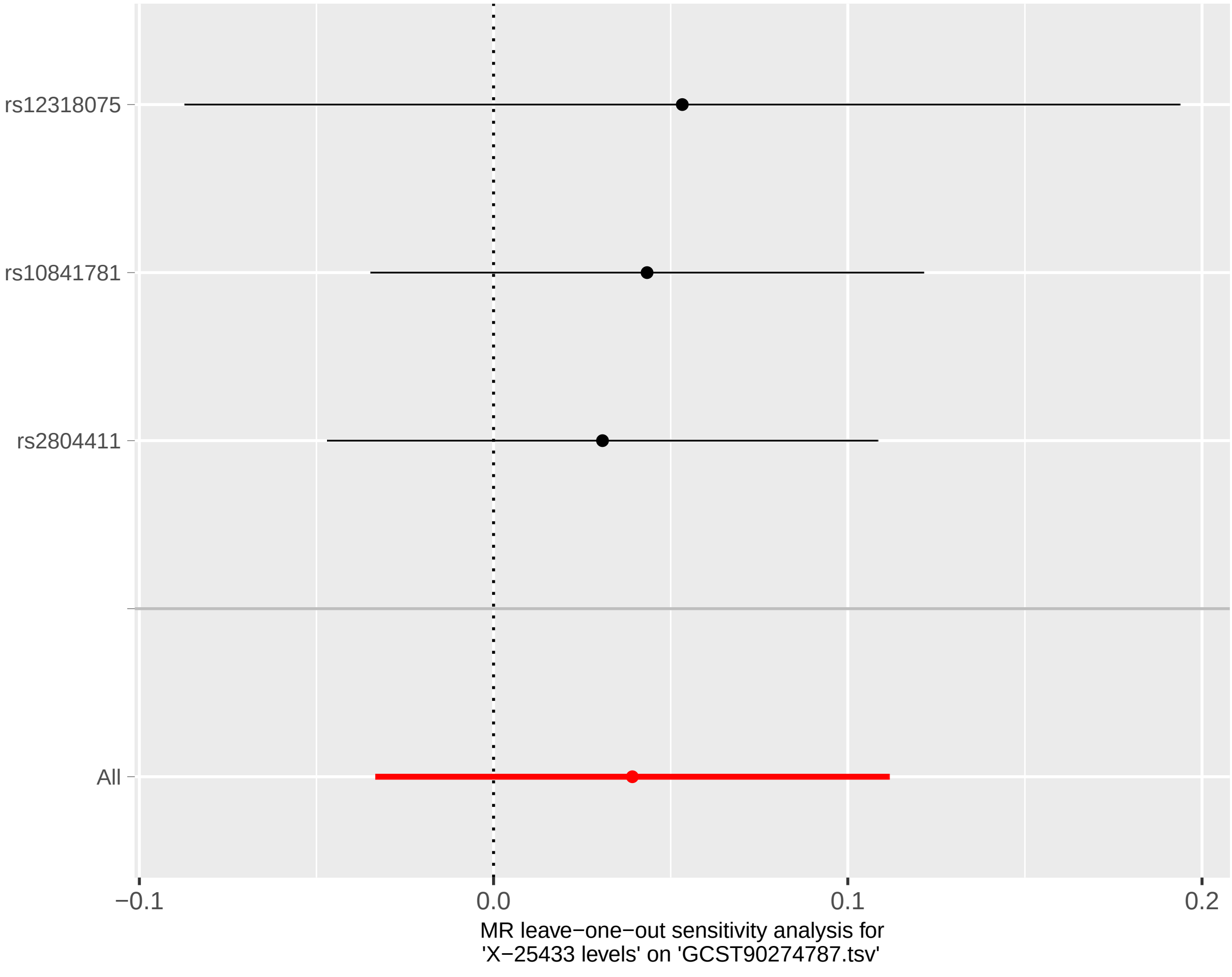
rs12318075
rs10841781
rs2804411
All
−0.1
0.0
0.1
0.2
MR leave−one−out sensitivity analysis for
'X−25433 levels' on 'GCST90274787.tsv'

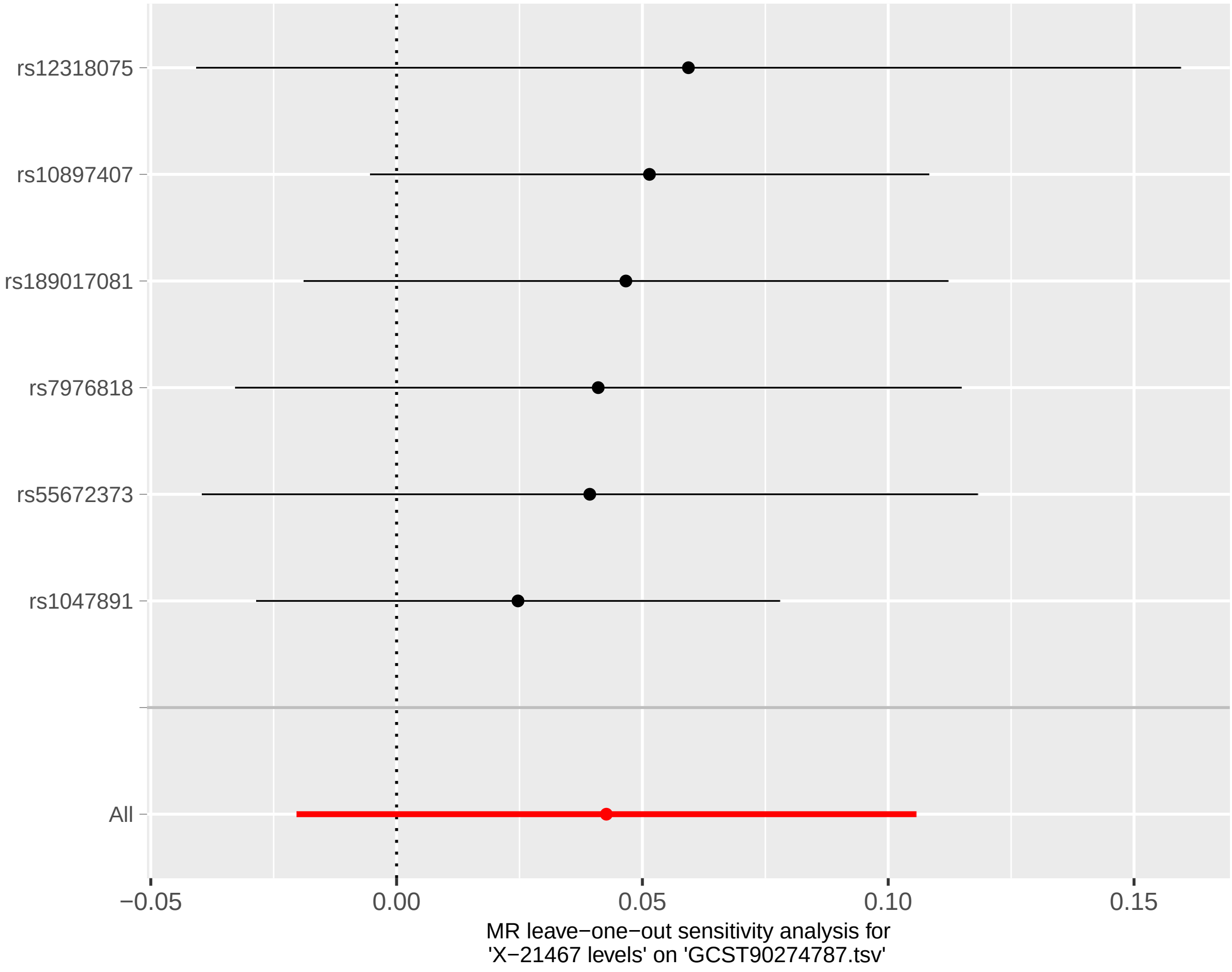
rs12318075
rs10897407
rs189017081
rs7976818
rs55672373
rs1047891
All
−0.05
0.00
0.05
0.10
0.15
MR leave−one−out sensitivity analysis for
'X−21467 levels' on 'GCST90274787.tsv'

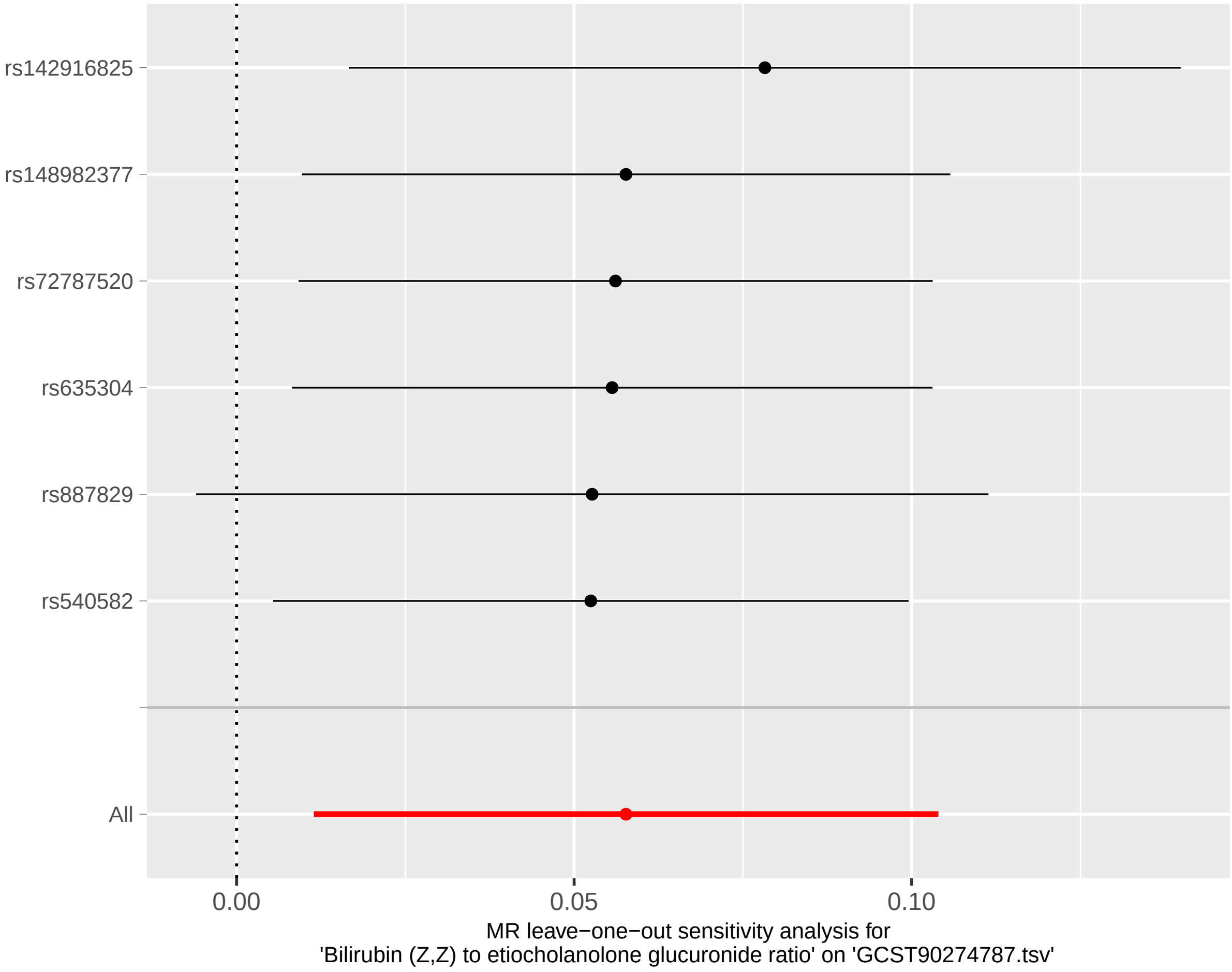
rs142916825
rs148982377
rs72787520
rs635304
rs887829
rs540582
All
0.00
0.05
0.10
MR leave−one−out sensitivity analysis for
'Bilirubin (Z,Z) to etiocholanolone glucuronide ratio' on 'GCST90274787.tsv'

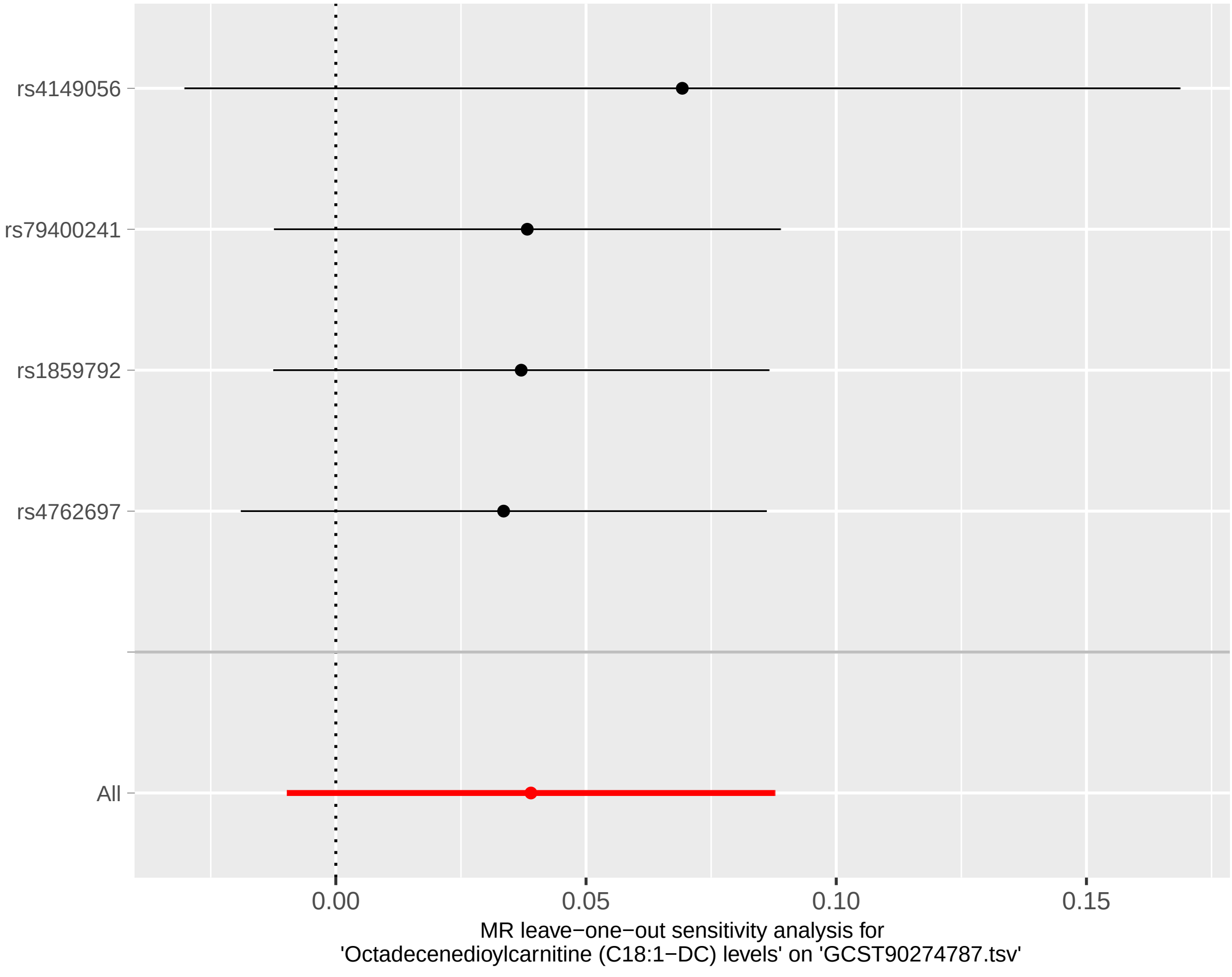
rs4149056
rs79400241
rs1859792
rs4762697
All
0.00
0.05
0.10
0.15
MR leave−one−out sensitivity analysis for
'Octadecenedioylcarnitine (C18:1−DC) levels' on 'GCST90274787.tsv'

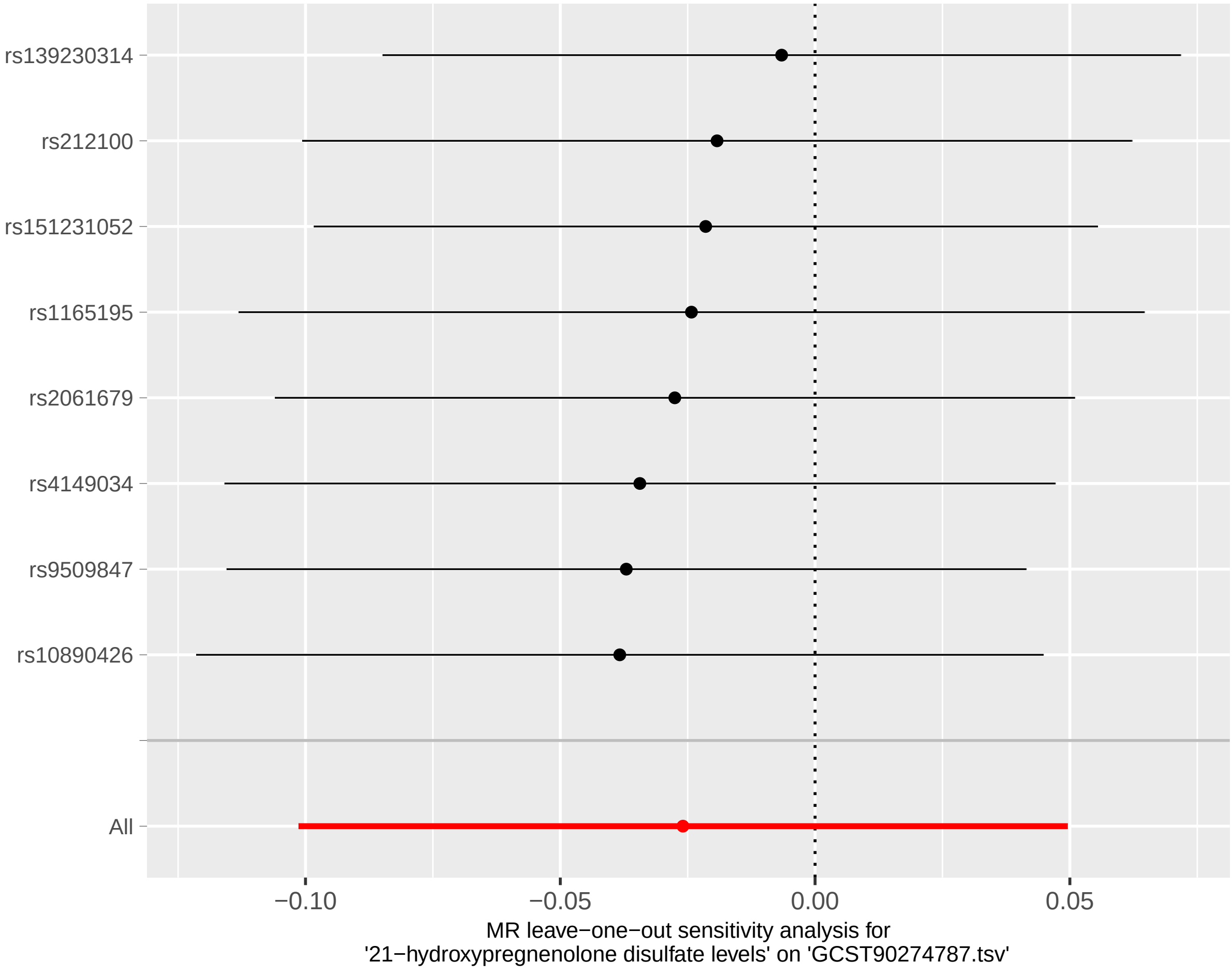
rs139230314
rs212100
rs151231052
rs1165195
rs2061679
rs4149034
rs9509847
rs10890426
All
−0.10
−0.05
0.00
0.05
MR leave−one−out sensitivity analysis for
'21−hydroxypregnenolone disulfate levels' on 'GCST90274787.tsv'

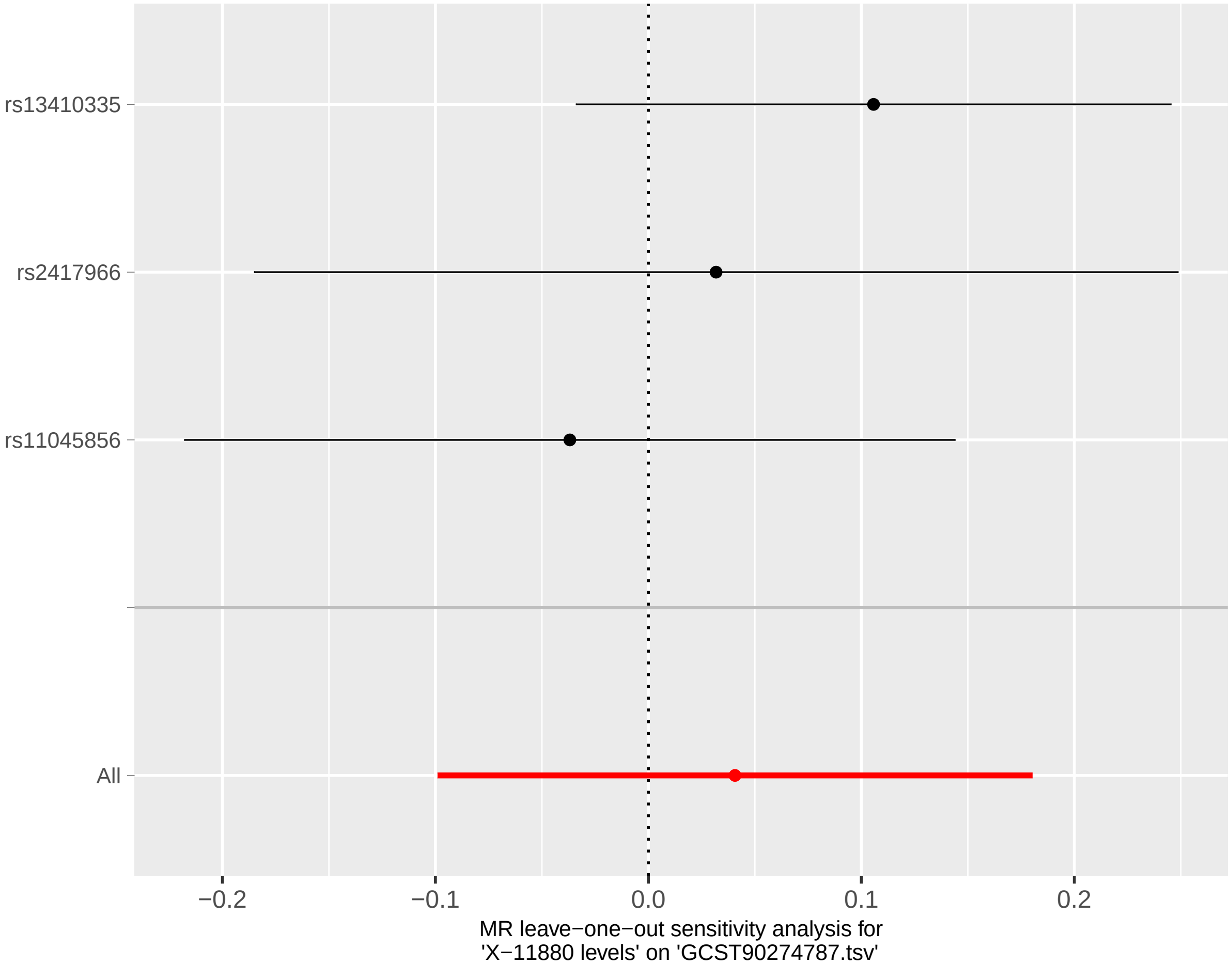
rs13410335
rs2417966
rs11045856
All
−0.2
−0.1
0.0
0.1
0.2
MR leave−one−out sensitivity analysis for
'X−11880 levels' on 'GCST90274787.tsv'

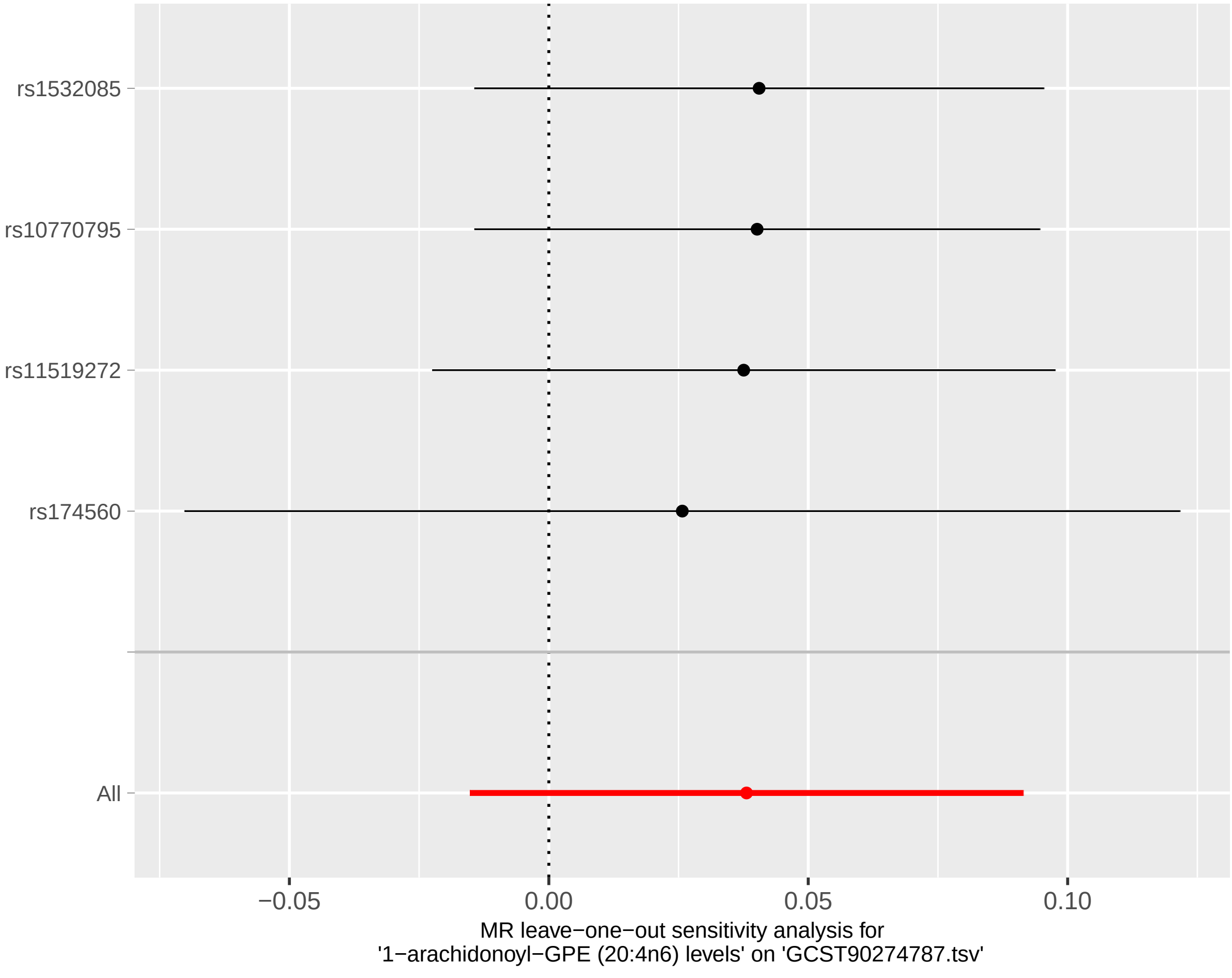
rs1532085
rs10770795
rs11519272
rs174560
All
−0.05
0.00
0.05
0.10
MR leave−one−out sensitivity analysis for
'1−arachidonoyl−GPE (20:4n6) levels' on 'GCST90274787.tsv'

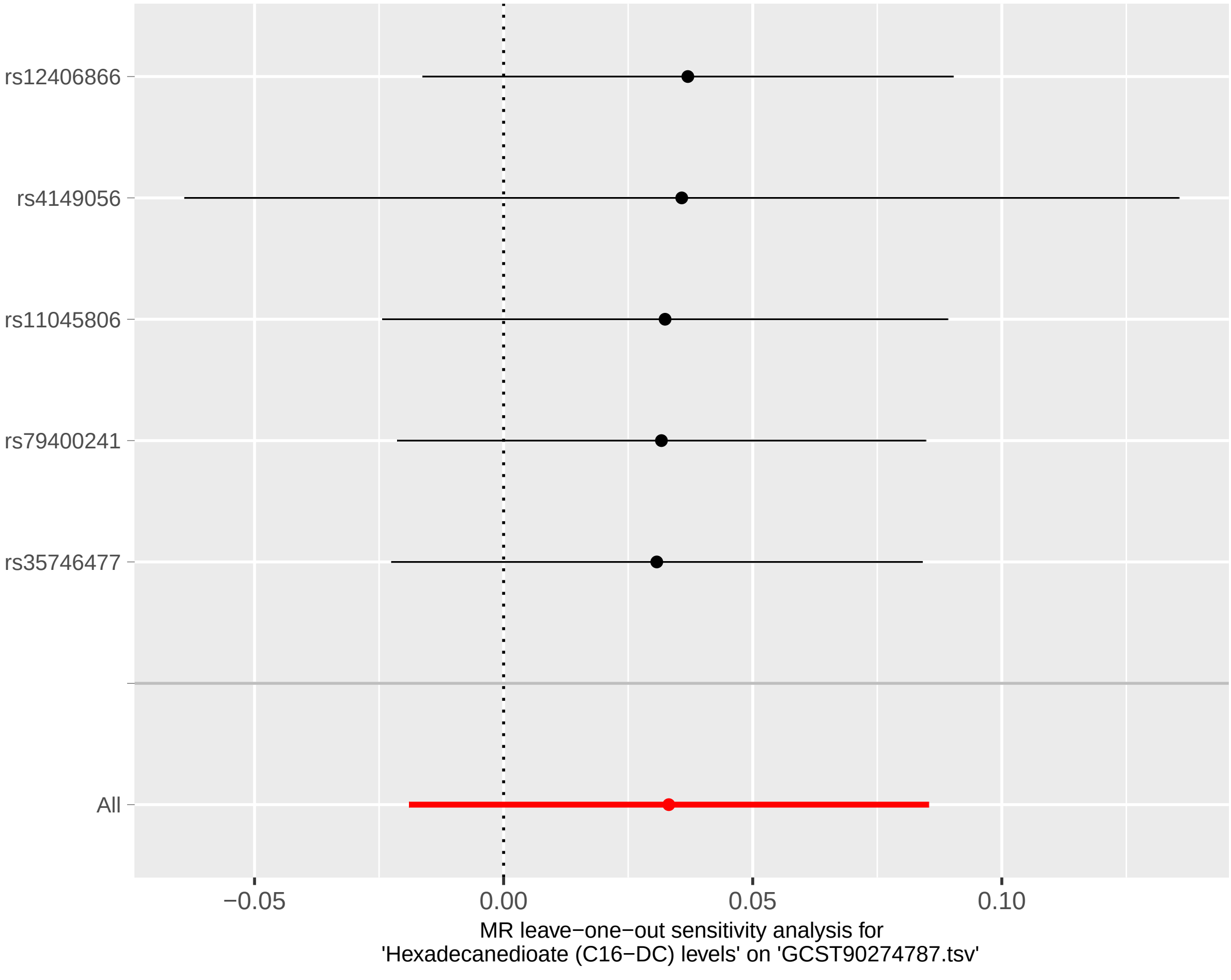
rs12406866
rs4149056
rs11045806
rs79400241
rs35746477
All
−0.05
0.00
0.05
0.10
MR leave−one−out sensitivity analysis for
'Hexadecanedioate (C16−DC) levels' on 'GCST90274787.tsv'

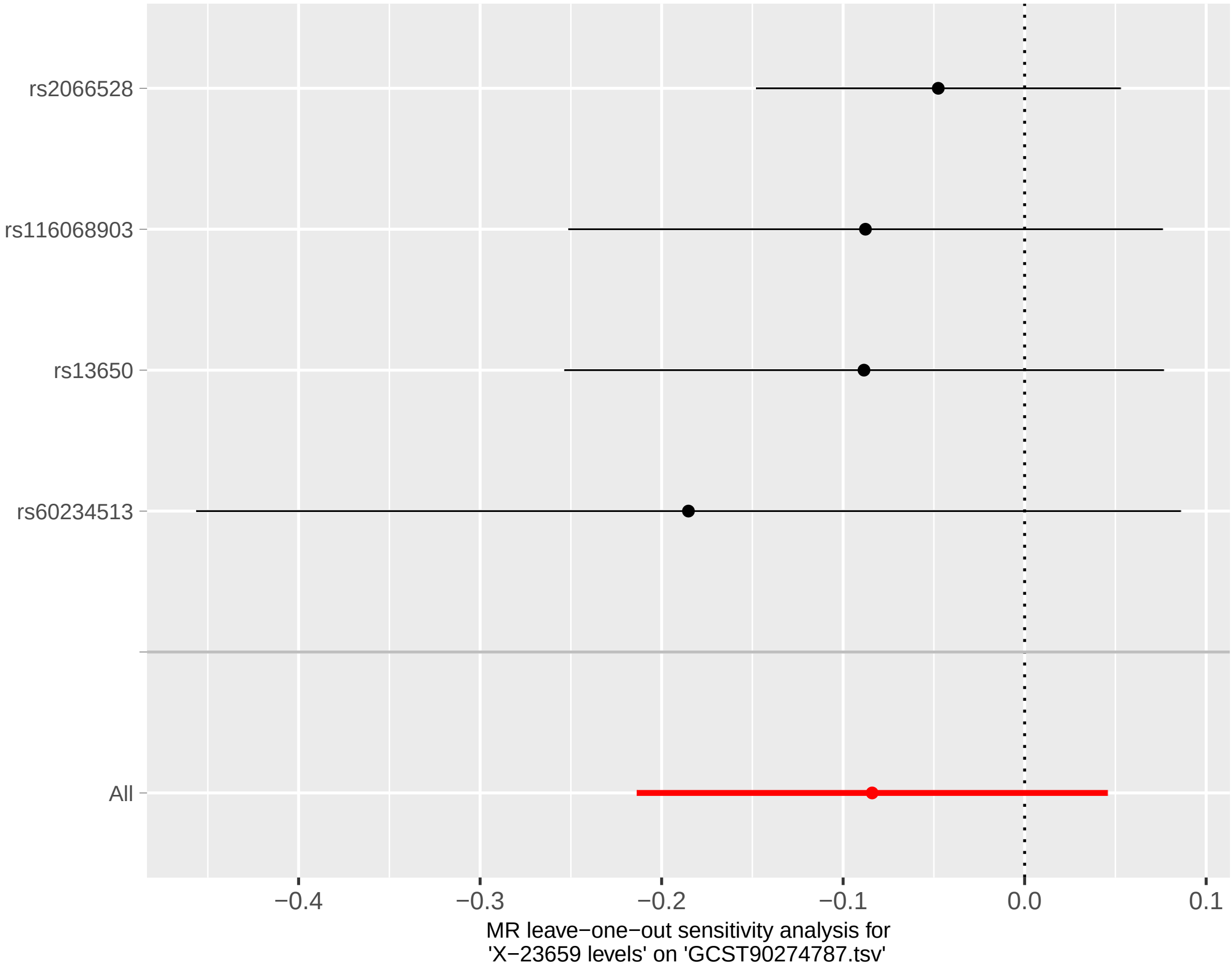
rs2066528
rs116068903
rs13650
rs60234513
All
−0.4
−0.3
−0.2
−0.1
0.0
0.1
MR leave−one−out sensitivity analysis for
'X−23659 levels' on 'GCST90274787.tsv'

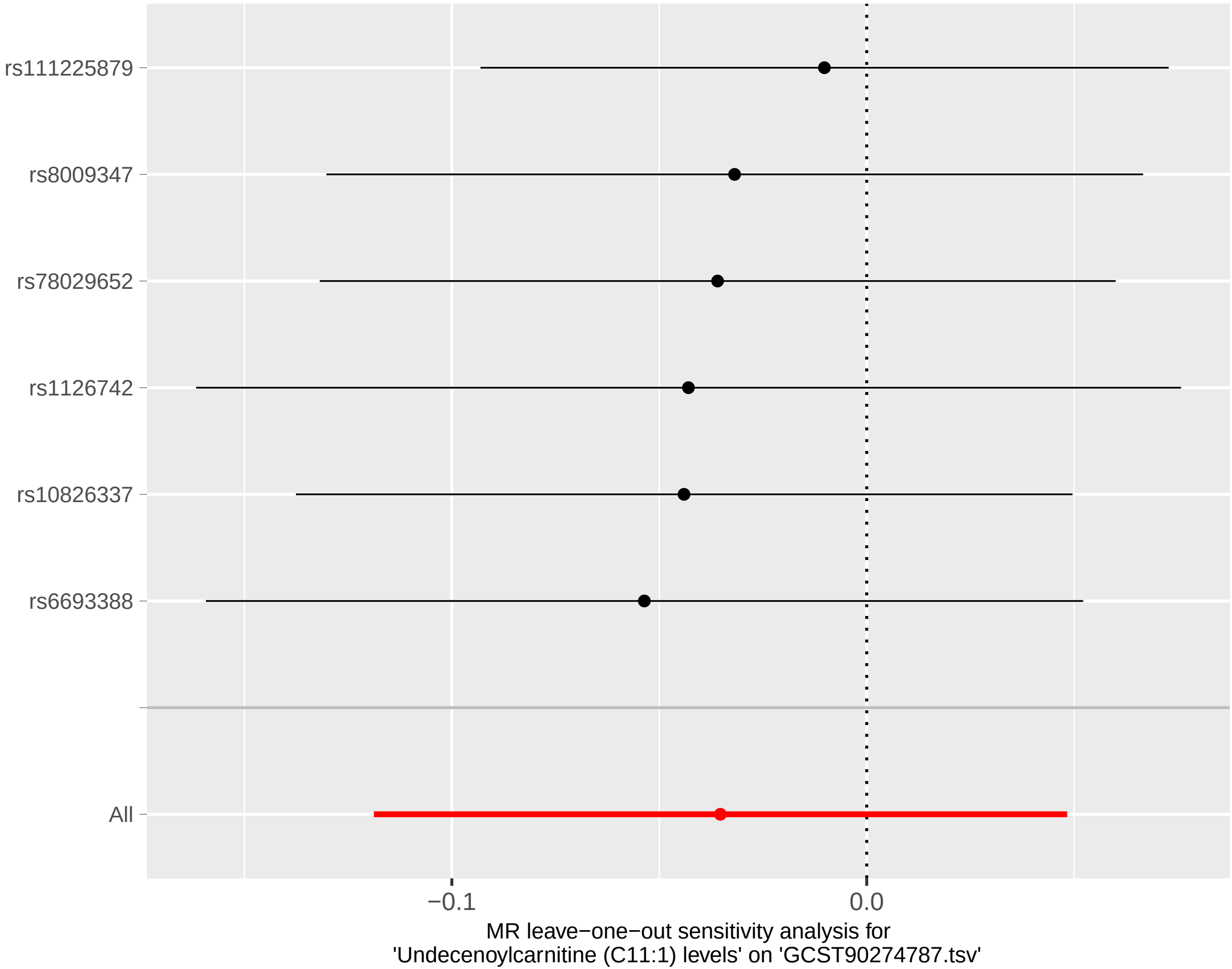
rs111225879
rs8009347
rs78029652
rs1126742
rs10826337
rs6693388
All
−0.1
0.0
MR leave−one−out sensitivity analysis for
'Undecenoylcarnitine (C11:1) levels' on 'GCST90274787.tsv'

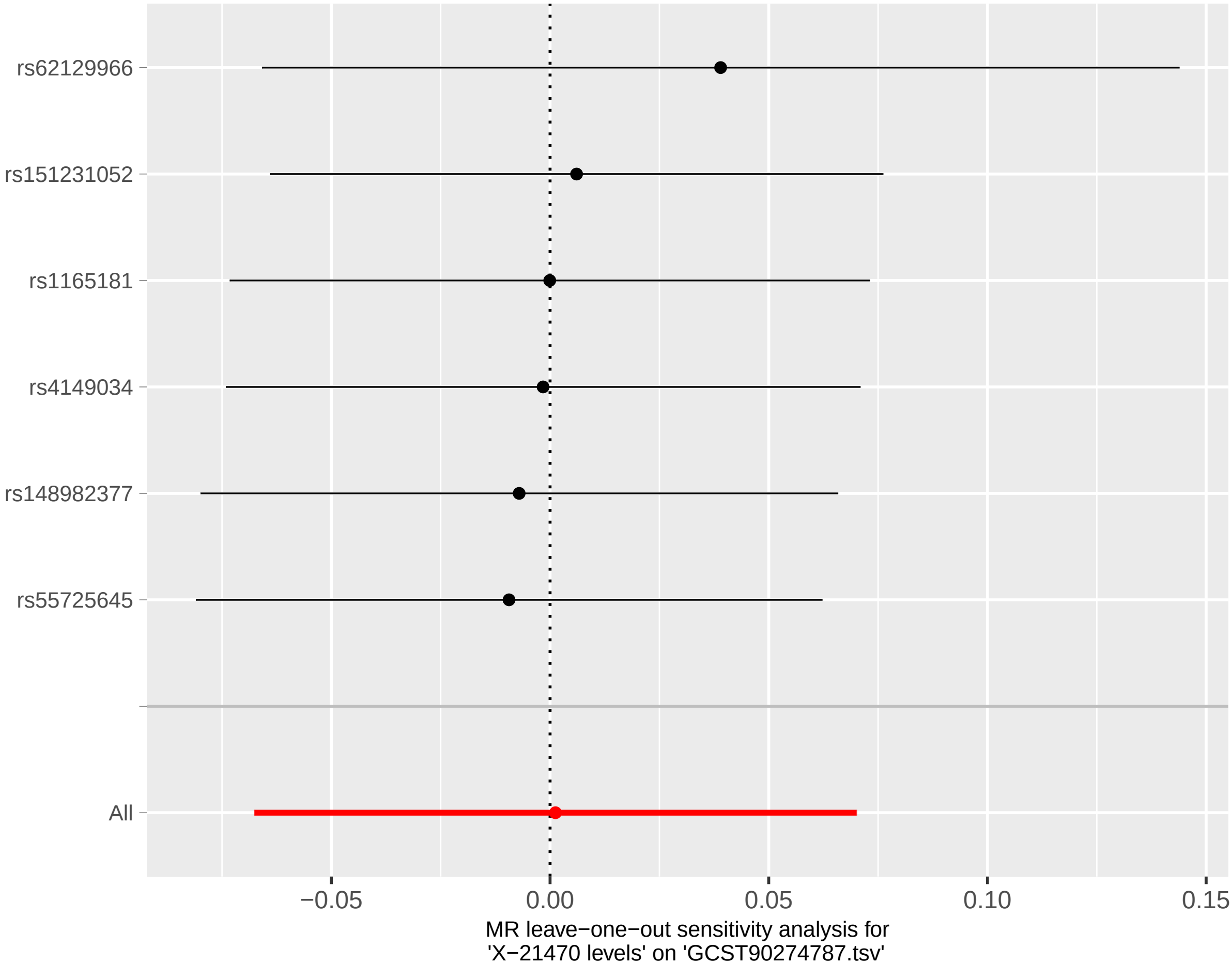

rs62129966
rs151231052
rs1165181
rs4149034
rs148982377
rs55725645
All
−0.05
0.00
0.05
0.10
0.15
MR leave−one−out sensitivity analysis for
'X−21470 levels' on 'GCST90274787.tsv'

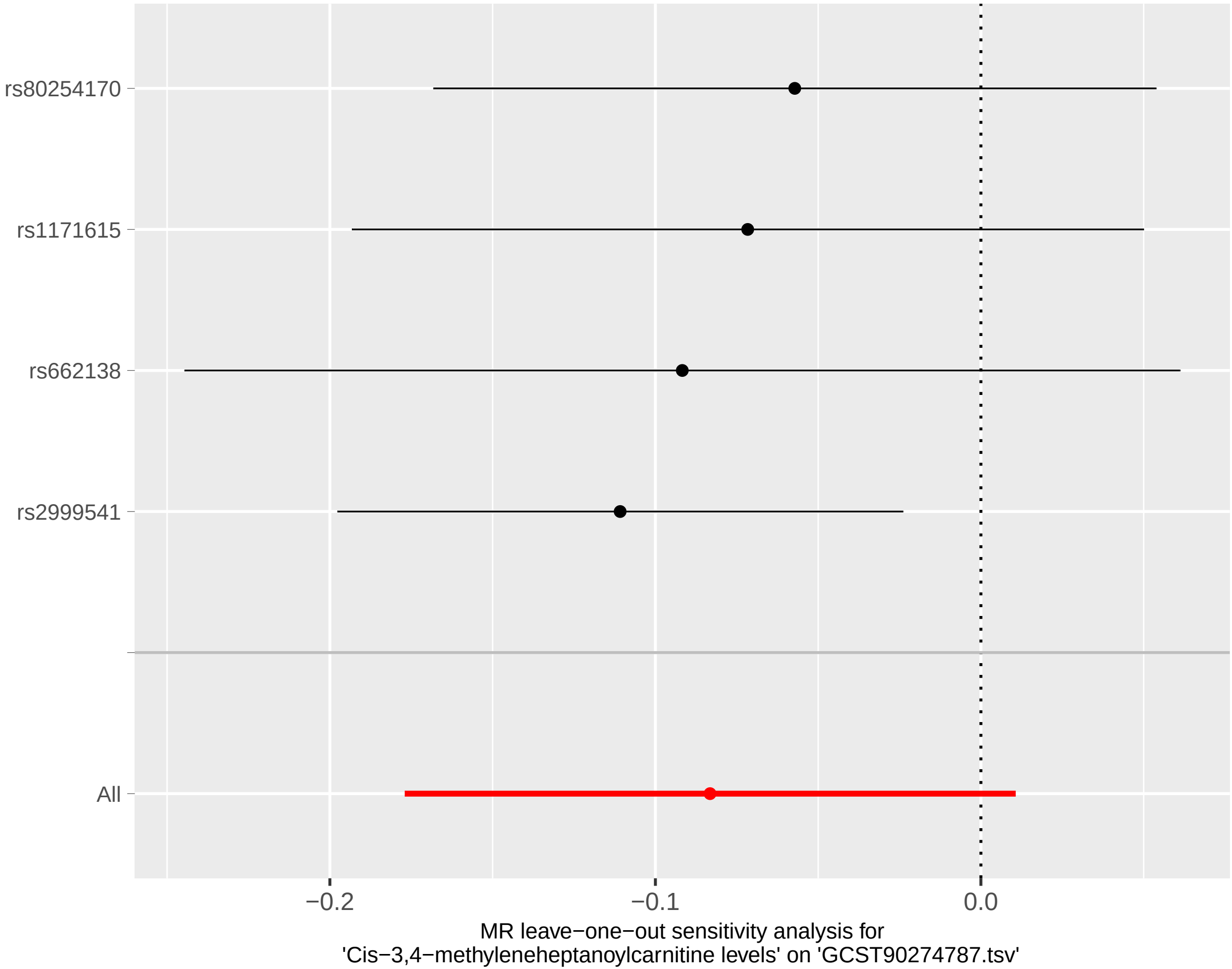

rs80254170
rs1171615
rs662138
rs2999541
All
−0.2
−0.1
0.0
MR leave−one−out sensitivity analysis for
'Cis−3,4−methyleneheptanoylcarnitine levels' on 'GCST90274787.tsv'

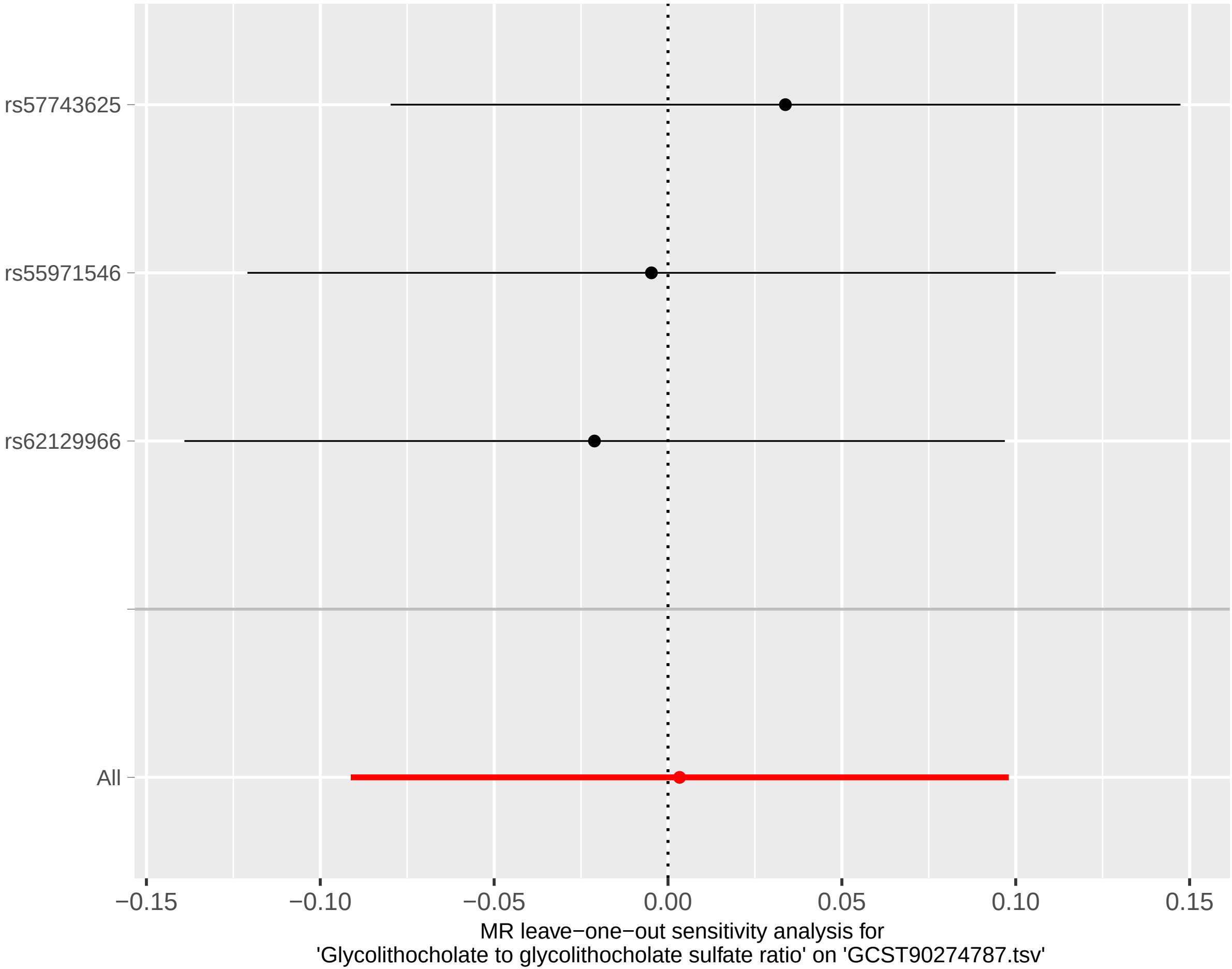
rs57743625
rs55971546
rs62129966
All
−0.15
−0.10
−0.05
0.00
0.05
0.10
0.15
MR leave−one−out sensitivity analysis for
'Glycolithocholate to glycolithocholate sulfate ratio' on 'GCST90274787.tsv'

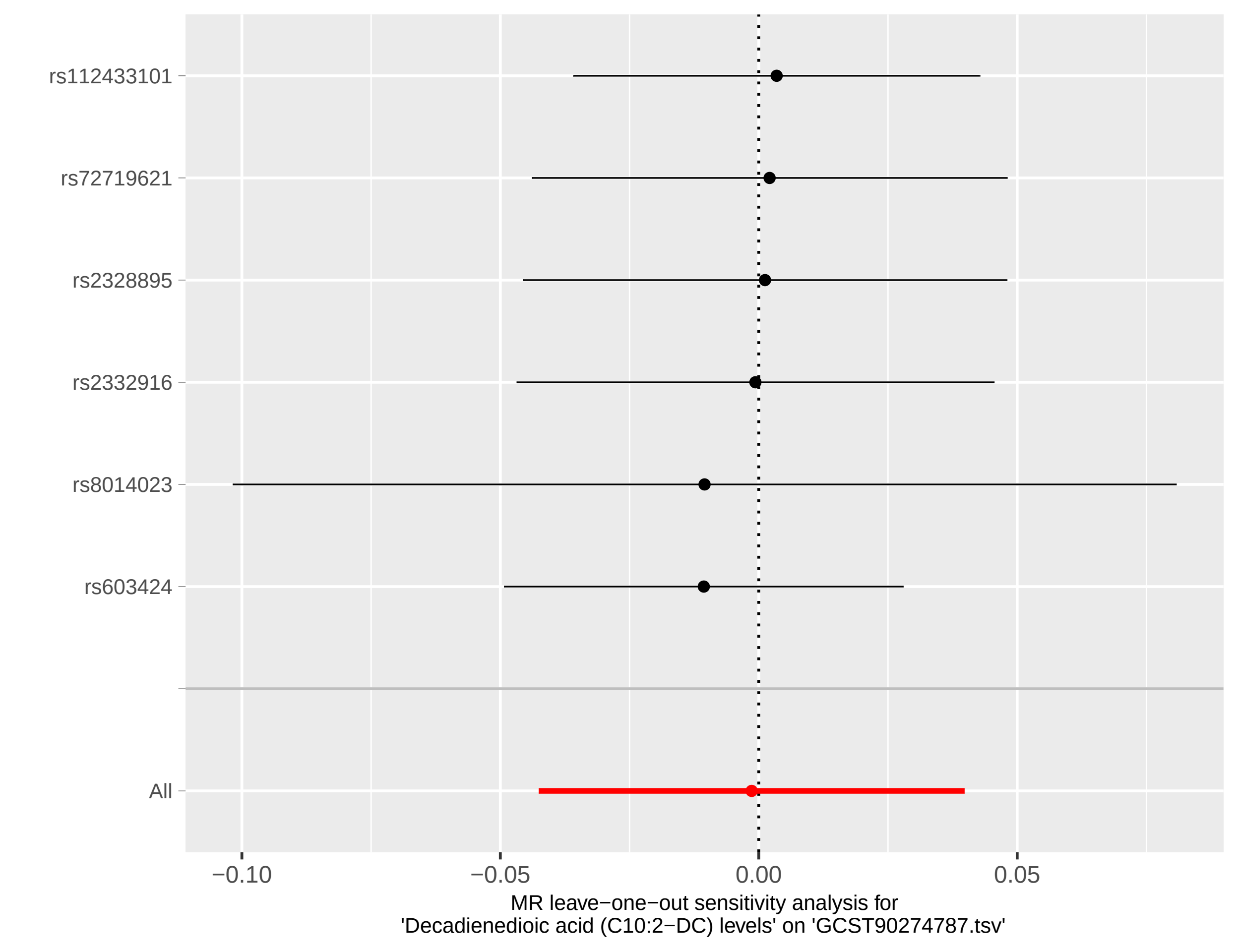

rs112433101
rs72719621
rs2328895
rs2332916
rs8014023
rs603424
All
−0.10
−0.05
0.00
0.05
MR leave−one−out sensitivity analysis for
'Decadienedioic acid (C10:2−DC) levels' on 'GCST90274787.tsv'

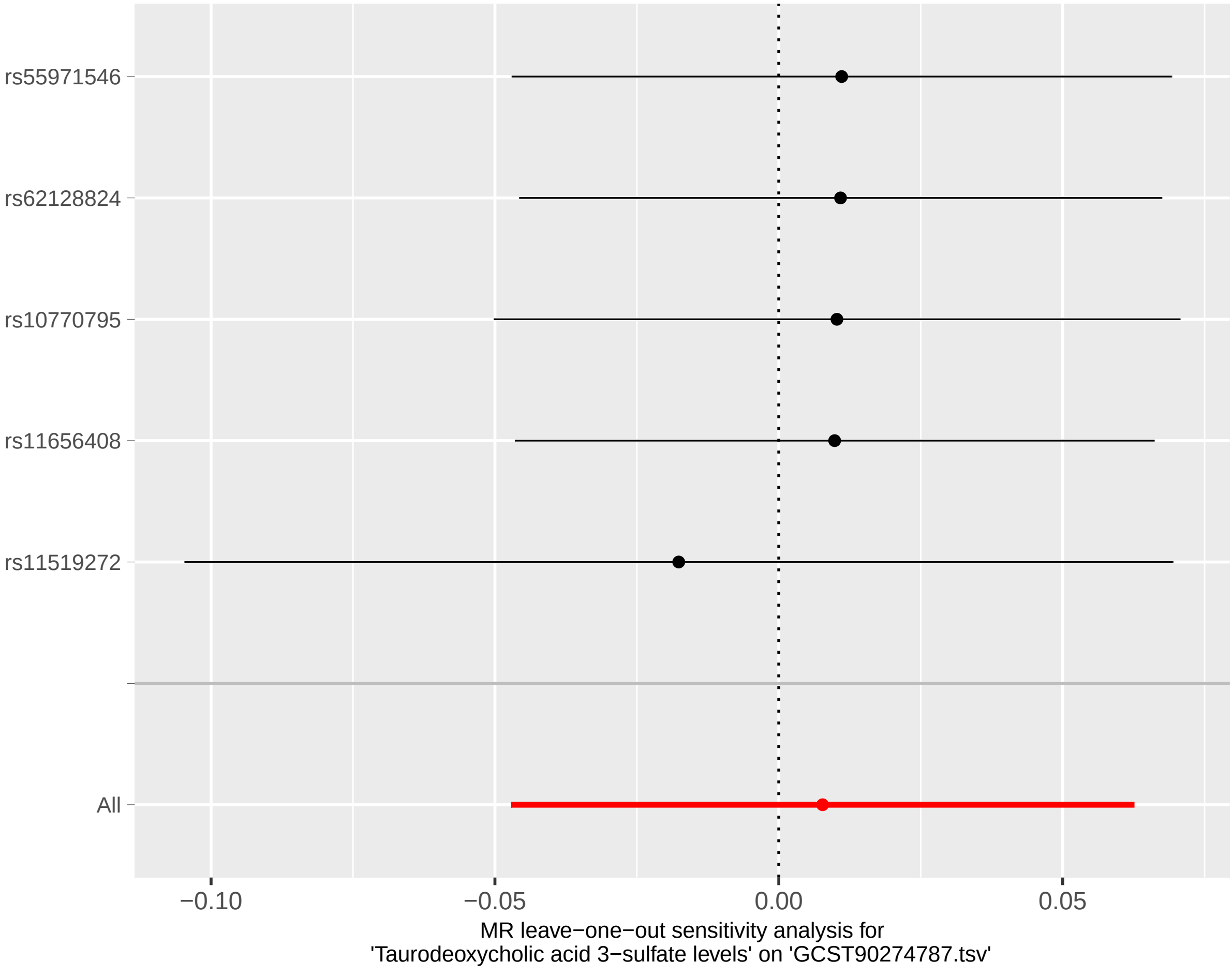
rs55971546
rs62128824
rs10770795
rs11656408
rs11519272
All
−0.10
−0.05
0.00
0.05
MR leave−one−out sensitivity analysis for
'Taurodeoxycholic acid 3−sulfate levels' on 'GCST90274787.tsv'

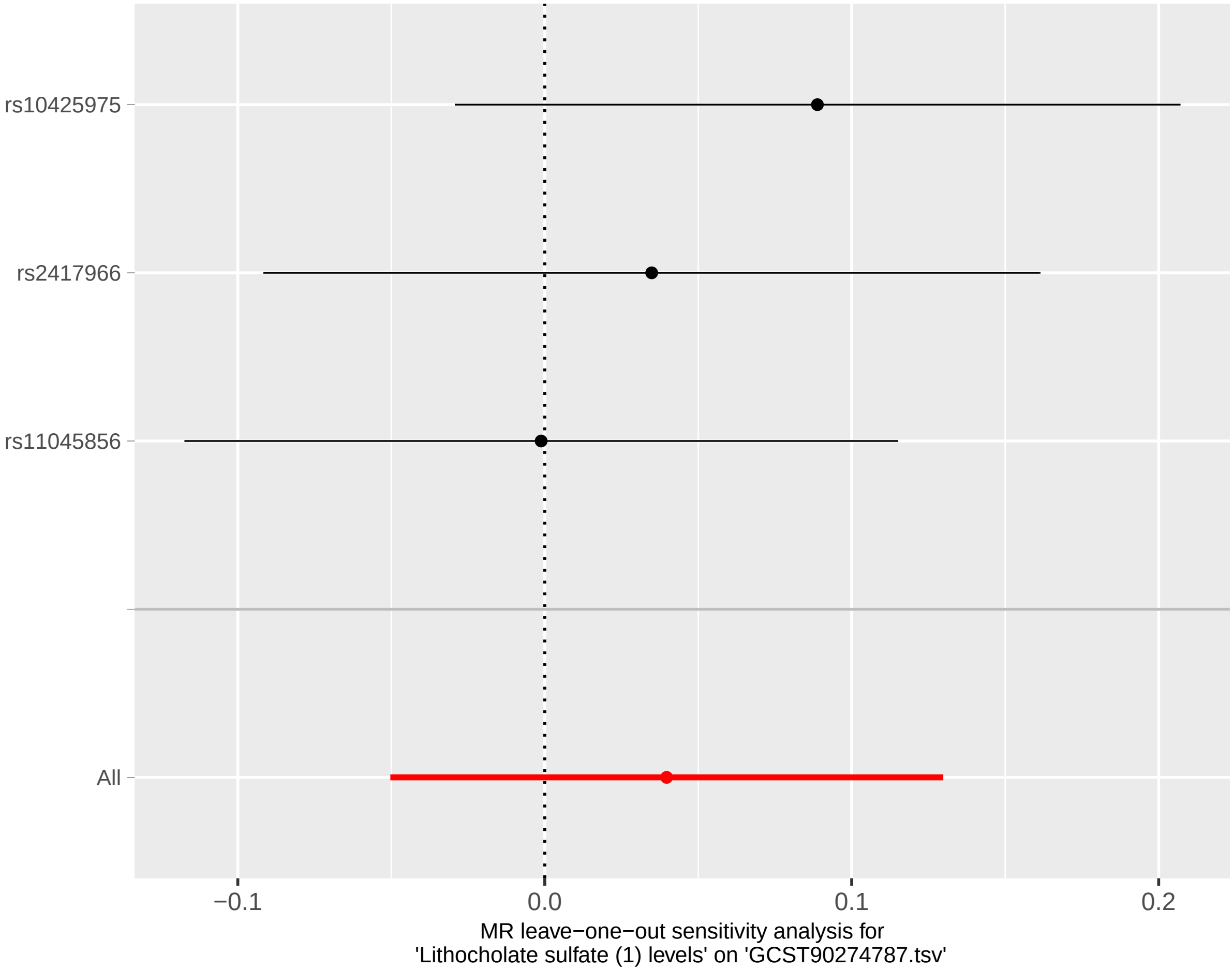
rs10425975
rs2417966
rs11045856
All
−0.1
0.0
0.1
0.2
MR leave−one−out sensitivity analysis for
'Lithocholate sulfate (1) levels' on 'GCST90274787.tsv'

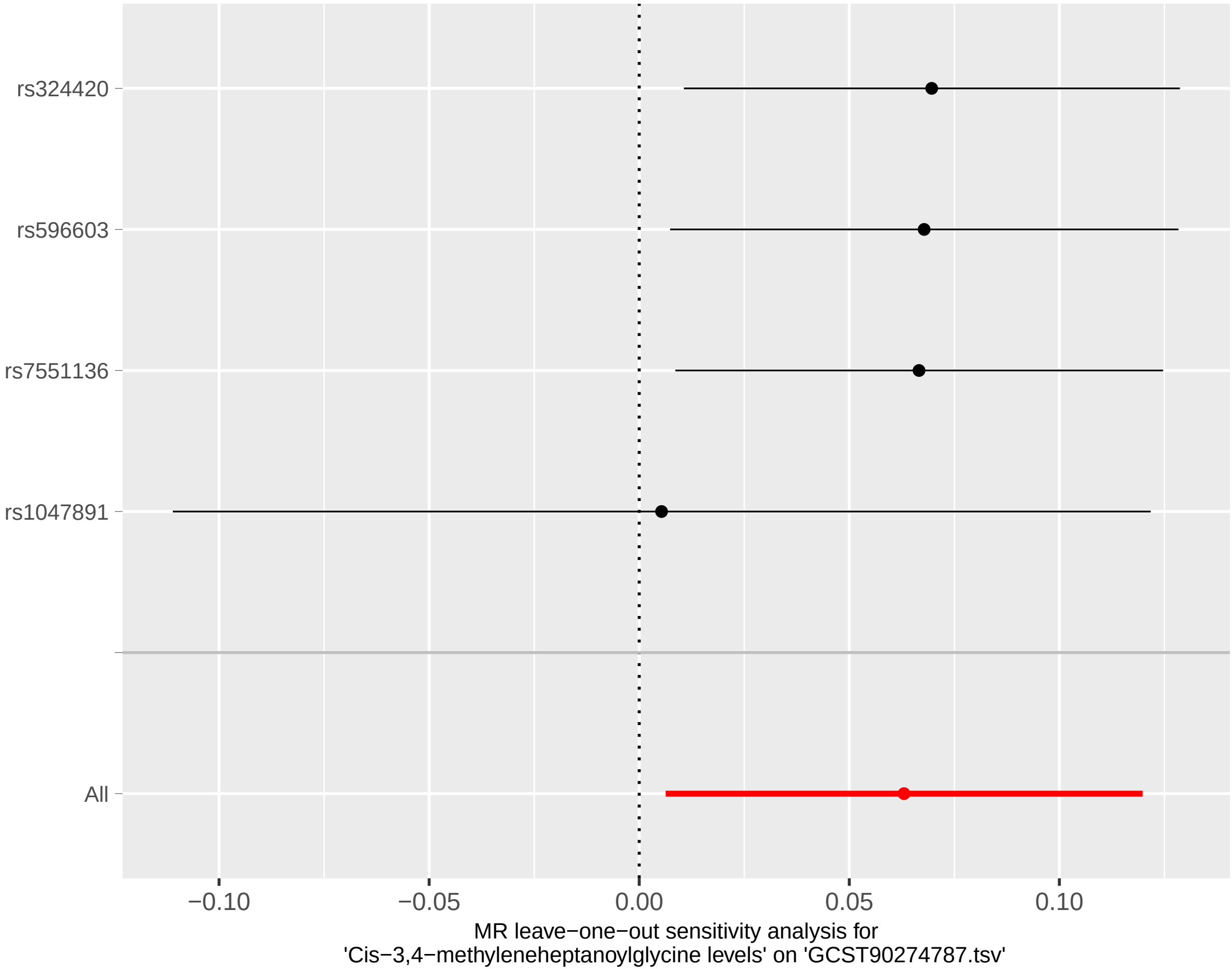
rs324420
rs596603
rs7551136
rs1047891
All
−0.10
−0.05
0.00
0.05
0.10
MR leave−one−out sensitivity analysis for
'Cis−3,4−methyleneheptanoylglycine levels' on 'GCST90274787.tsv'

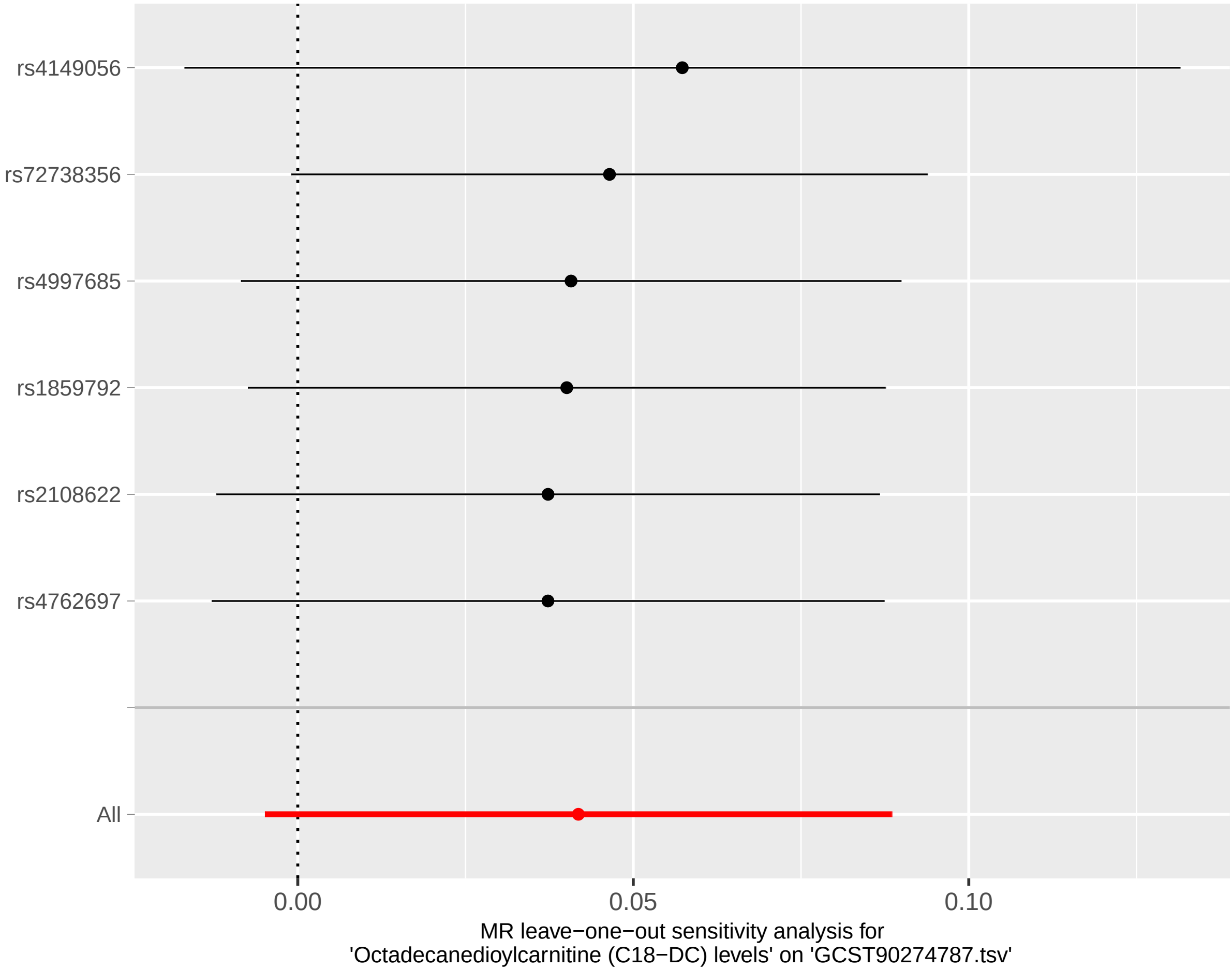
rs4149056
rs72738356
rs4997685
rs1859792
rs2108622
rs4762697
All
0.00
0.05
0.10
MR leave−one−out sensitivity analysis for
'Octadecanedioylcarnitine (C18−DC) levels' on 'GCST90274787.tsv'

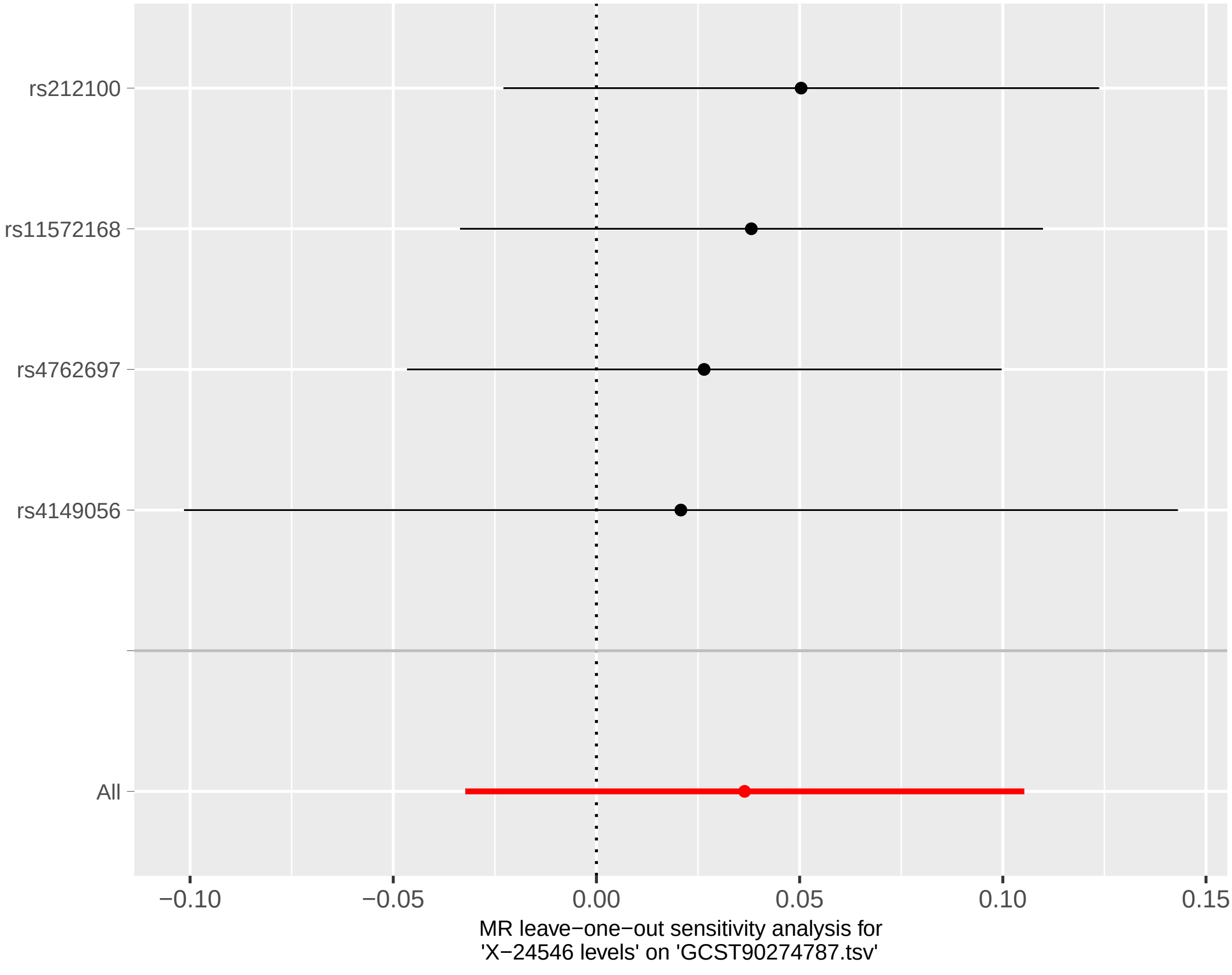

rs212100
rs11572168
rs4762697
rs4149056
All
−0.10
−0.05
0.00
0.05
0.10
0.15
MR leave−one−out sensitivity analysis for
'X−24546 levels' on 'GCST90274787.tsv'

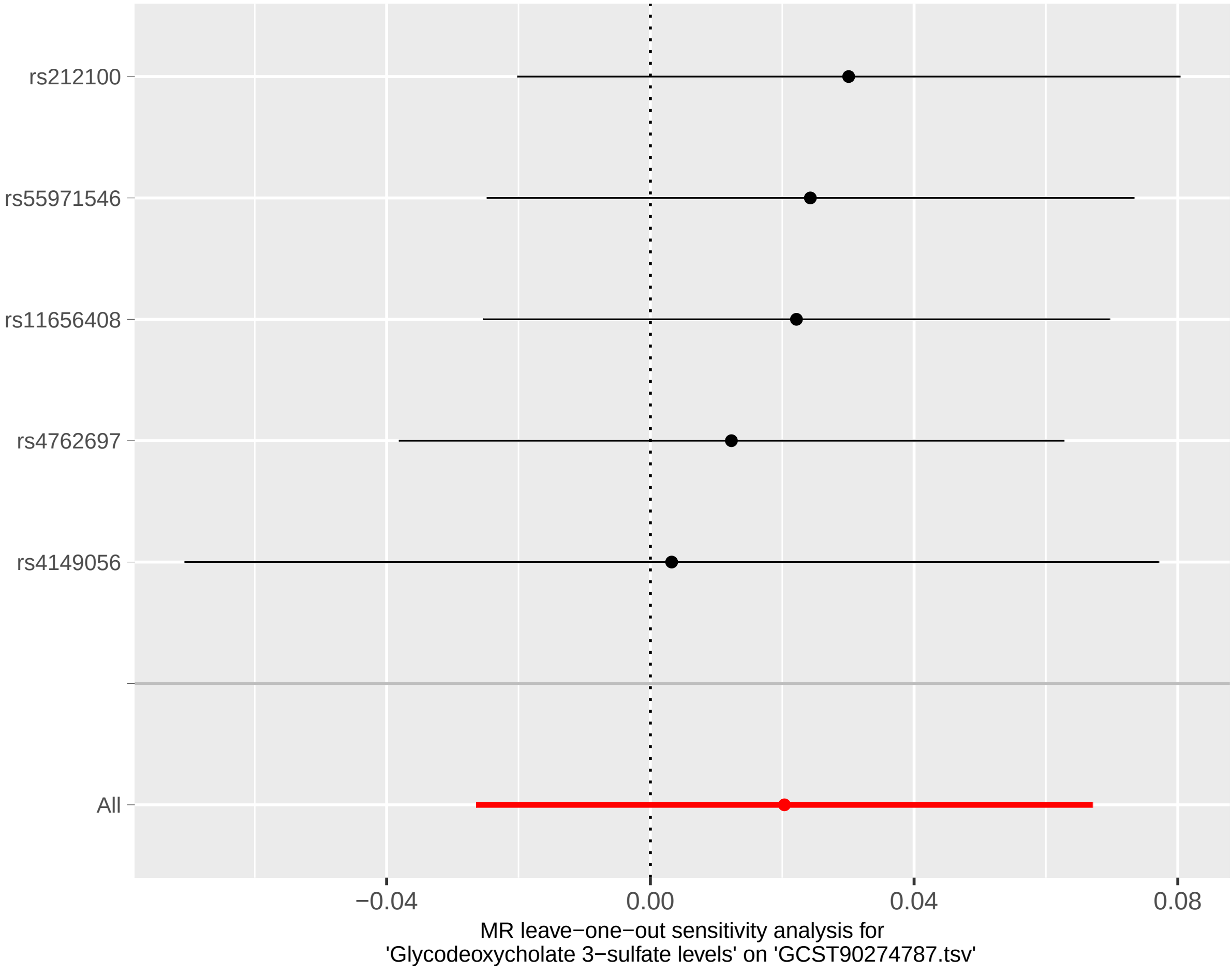

rs212100
rs55971546
rs11656408
rs4762697
rs4149056
All
−0.04
0.00
0.04
0.08
MR leave−one−out sensitivity analysis for
'Glycodeoxycholate 3−sulfate levels' on 'GCST90274787.tsv'

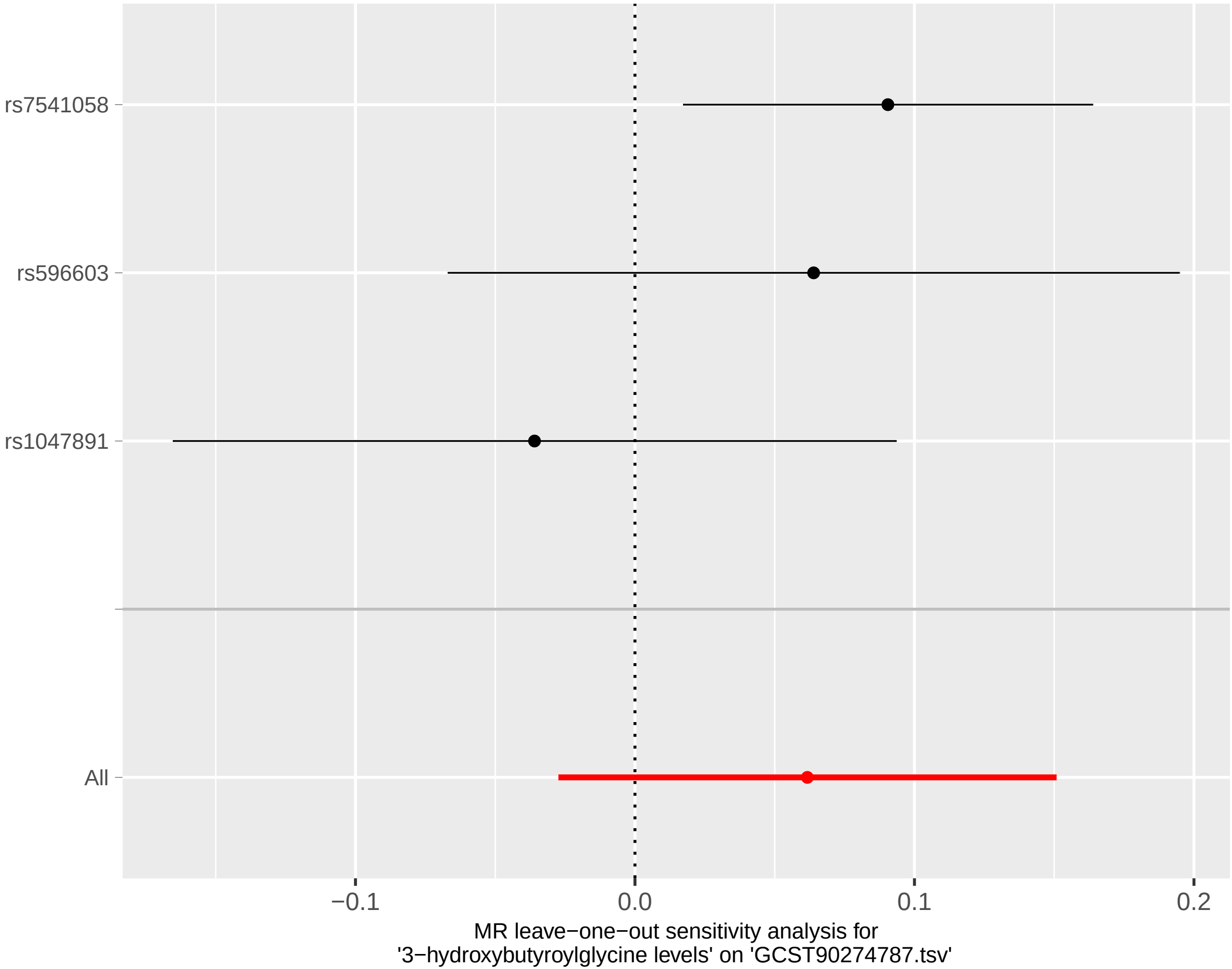
rs7541058
rs596603
rs1047891
All
−0.1
0.0
0.1
0.2
MR leave−one−out sensitivity analysis for
'3−hydroxybutyroylglycine levels' on 'GCST90274787.tsv'

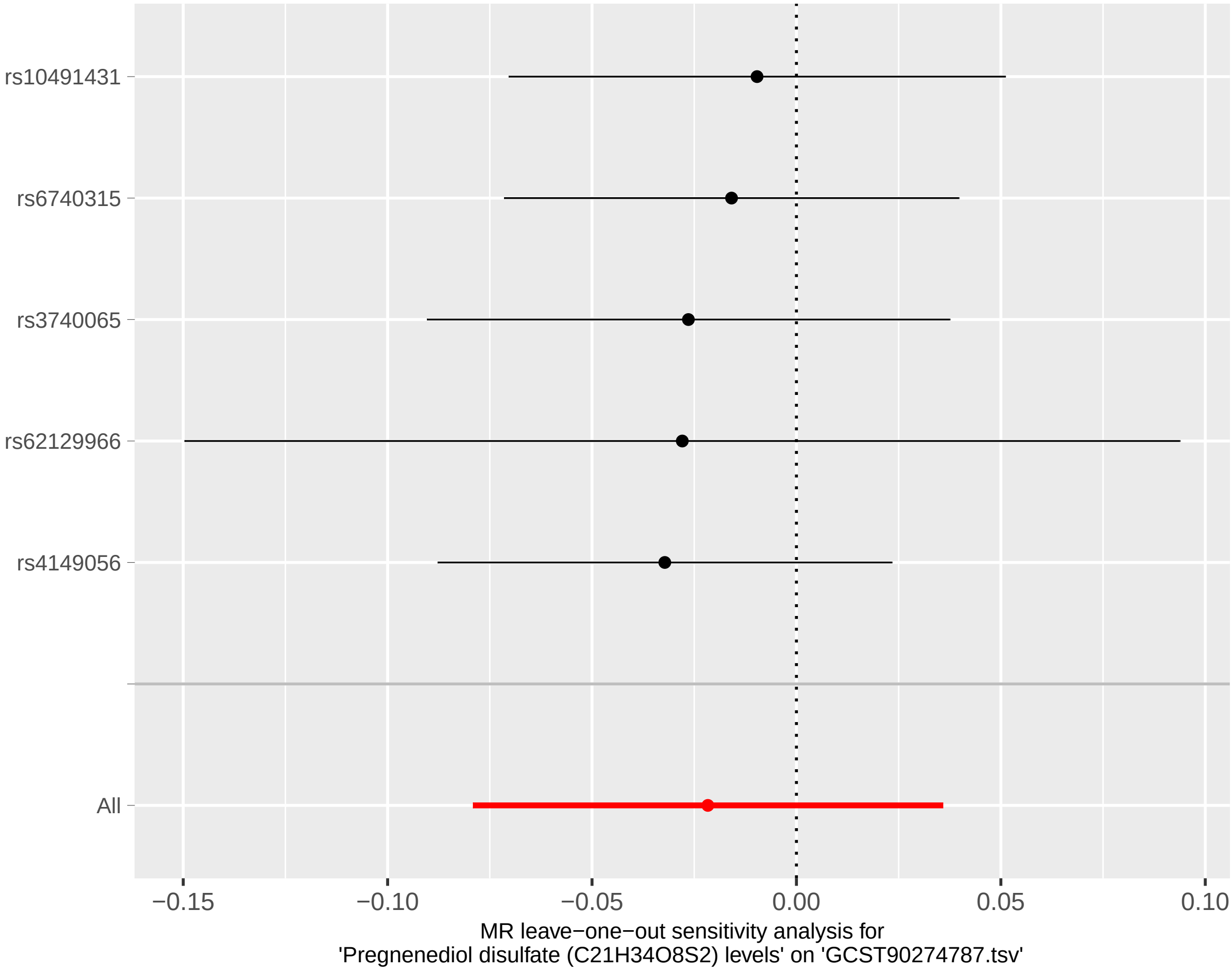

rs10491431
rs6740315
rs3740065
rs62129966
rs4149056
All
−0.15
−0.10
−0.05
0.00
0.05
0.10
MR leave−one−out sensitivity analysis for
'Pregnenediol disulfate (C21H34O8S2) levels' on 'GCST90274787.tsv'

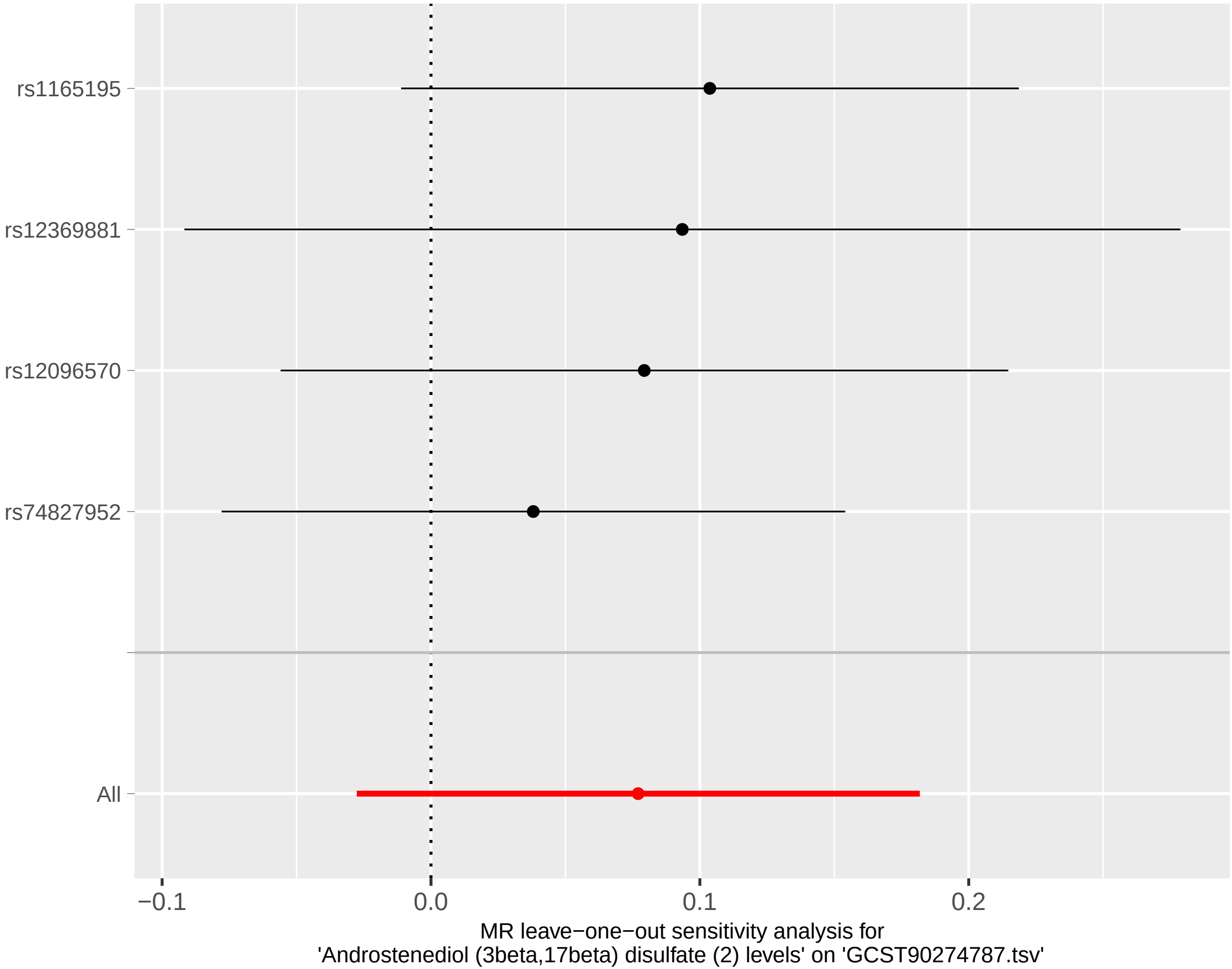
rs1165195
rs12369881
rs12096570
rs74827952
All
−0.1
0.0
0.1
0.2
MR leave−one−out sensitivity analysis for
'Androstenediol (3beta,17beta) disulfate (2) levels' on 'GCST90274787.tsv'

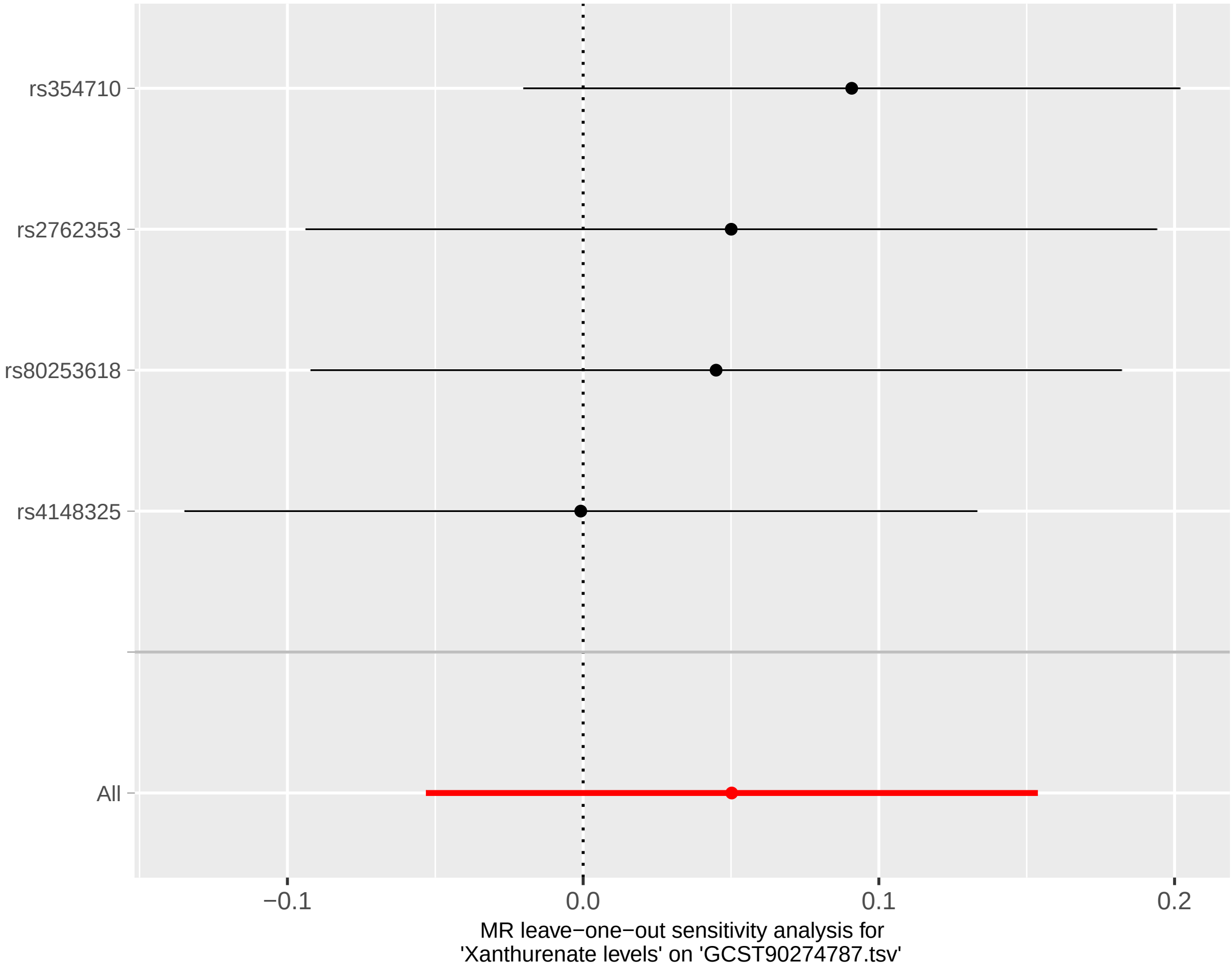
rs354710
rs2762353
rs80253618
rs4148325
All
−0.1
0.0
0.1
0.2
MR leave−one−out sensitivity analysis for
'Xanthurenate levels' on 'GCST90274787.tsv'

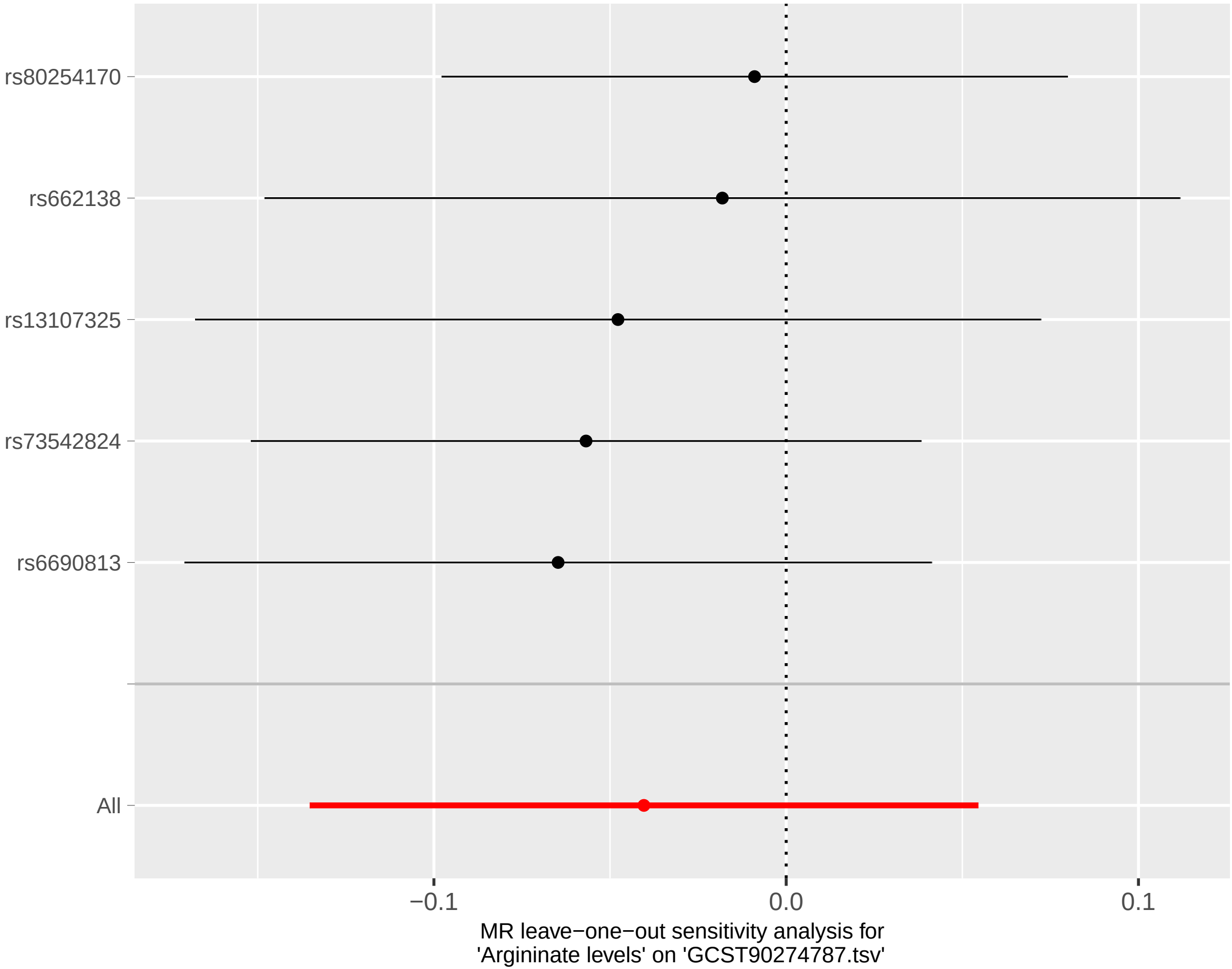
rs80254170
rs662138
rs13107325
rs73542824
rs6690813
All
−0.1
0.0
0.1
MR leave−one−out sensitivity analysis for
'Argininate levels' on 'GCST90274787.tsv'

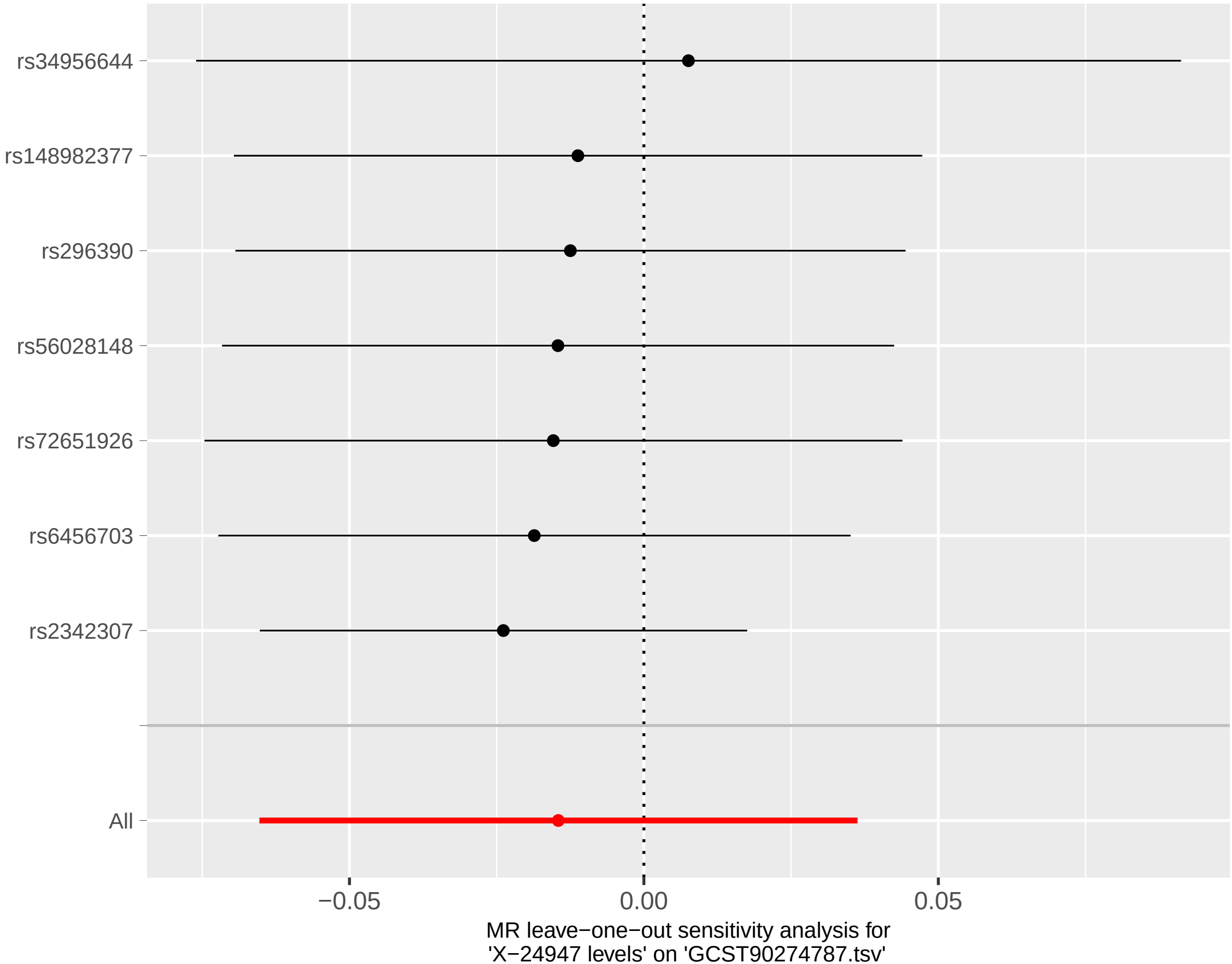
rs34956644
rs148982377
rs296390
rs56028148
rs72651926
rs6456703
rs2342307
All
−0.05
0.00
0.05
MR leave−one−out sensitivity analysis for
'X−24947 levels' on 'GCST90274787.tsv'

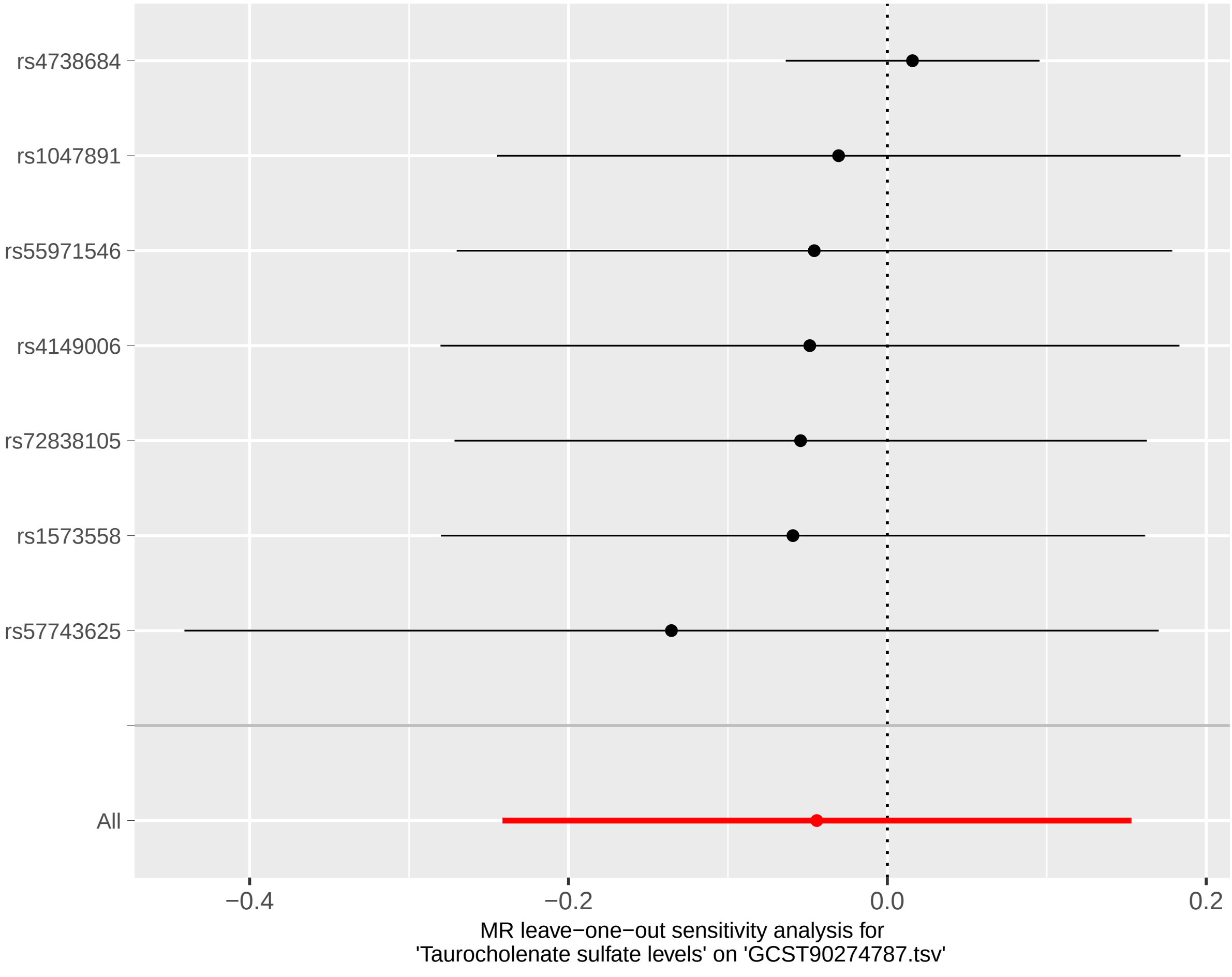
rs4738684
rs1047891
rs55971546
rs4149006
rs72838105
rs1573558
rs57743625
All
−0.4
−0.2
0.0
0.2
MR leave−one−out sensitivity analysis for
'Taurocholenate sulfate levels' on 'GCST90274787.tsv'

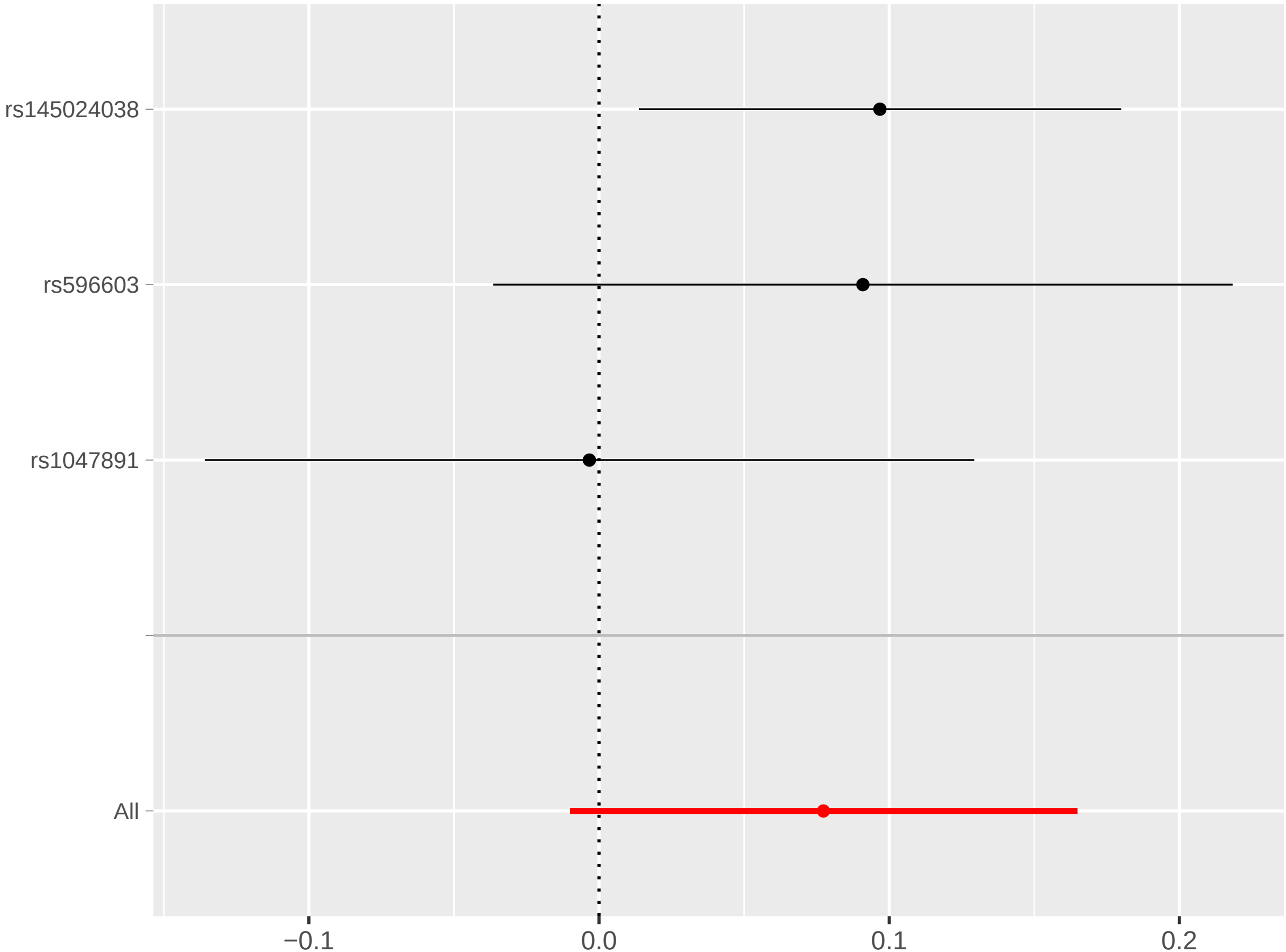

MR leave−one−out sensitivity analysis for
'2−butenoylglycine levels' on 'GCST90274787.tsv'

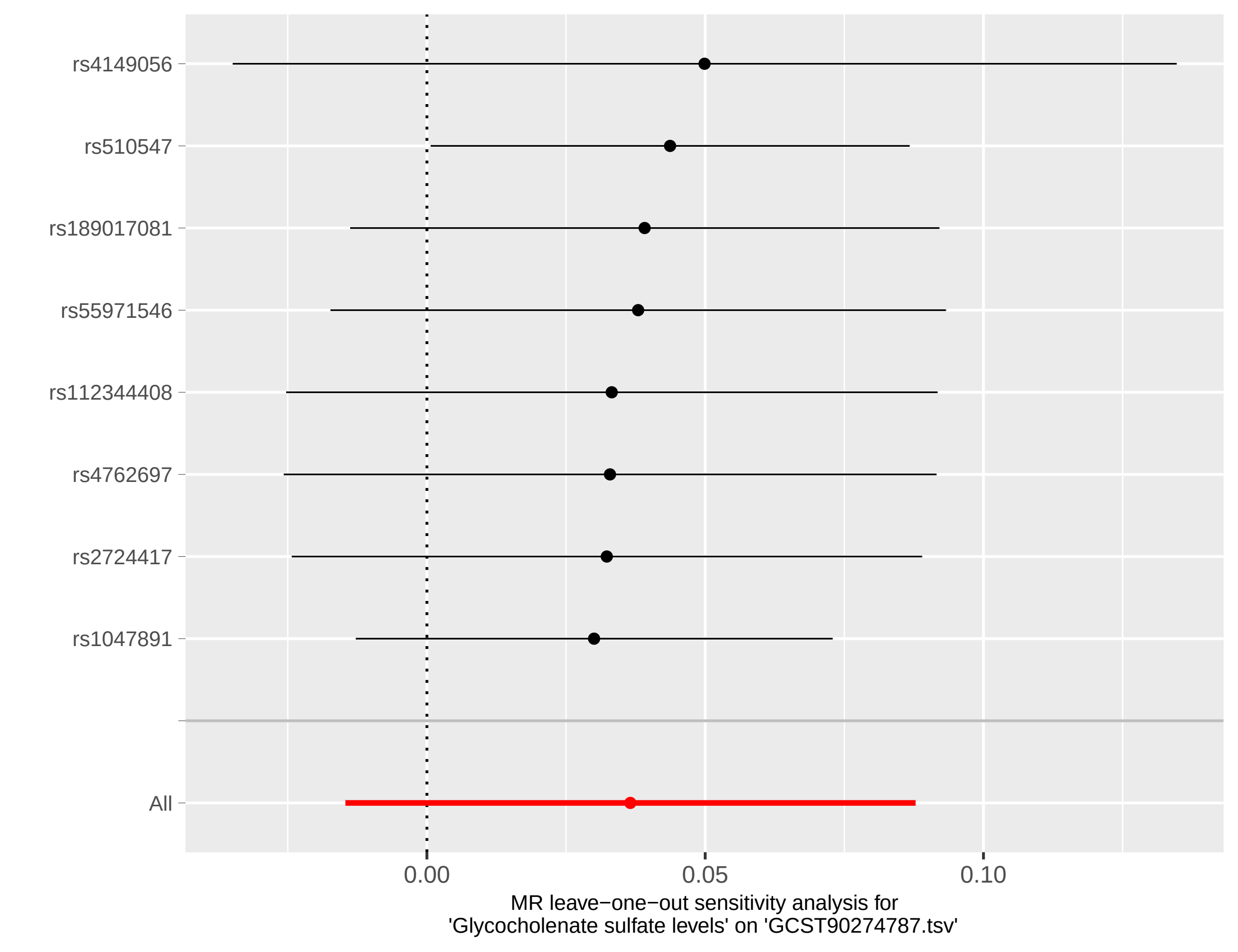
rs4149056
rs510547
rs189017081
rs55971546
rs112344408
rs4762697
rs2724417
rs1047891
All
0.00
0.05
0.10
MR leave−one−out sensitivity analysis for
'Glycocholenate sulfate levels' on 'GCST90274787.tsv'

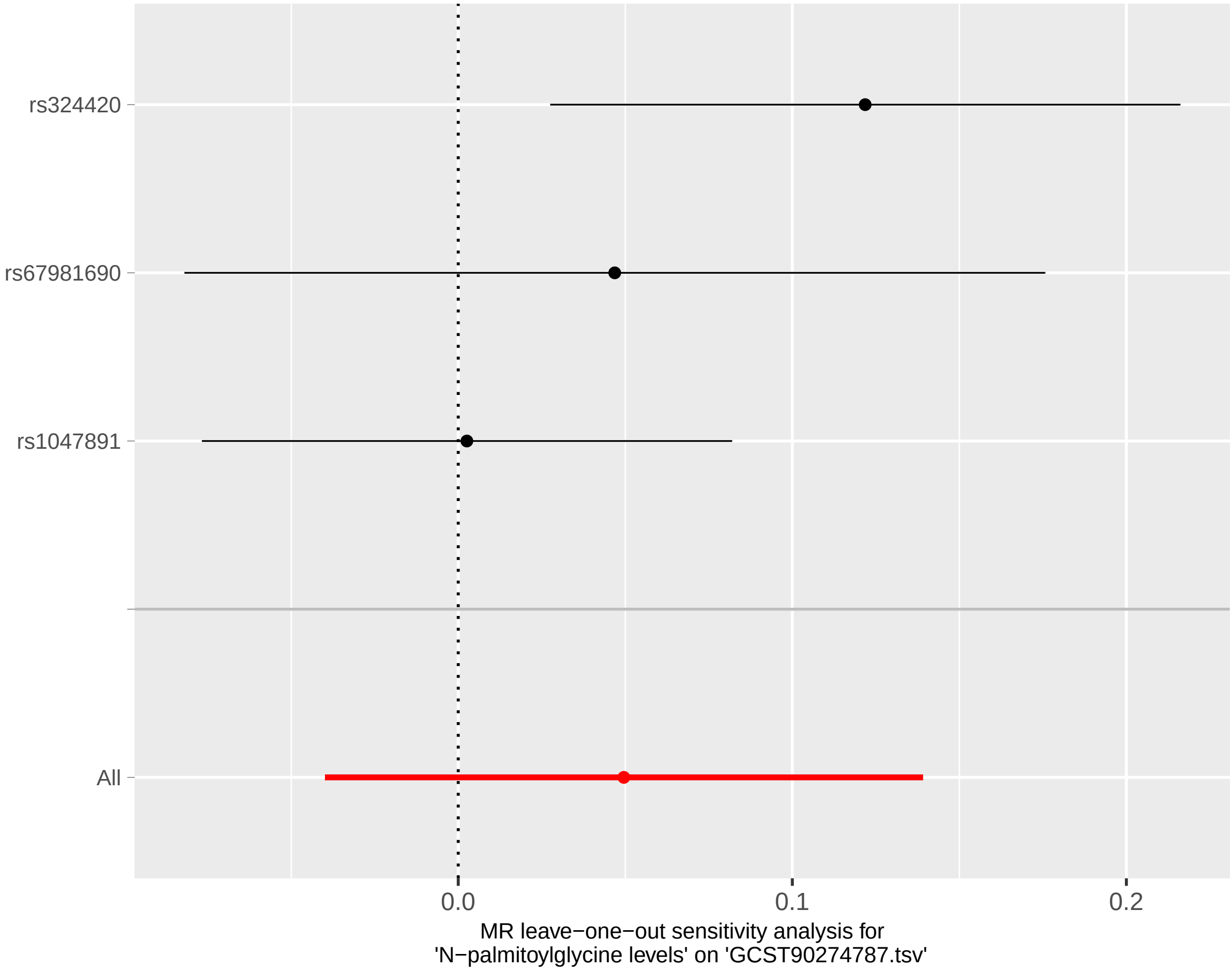
rs324420
rs67981690
rs1047891
All
0.0
0.1
0.2
MR leave−one−out sensitivity analysis for
'N−palmitoylglycine levels' on 'GCST90274787.tsv'

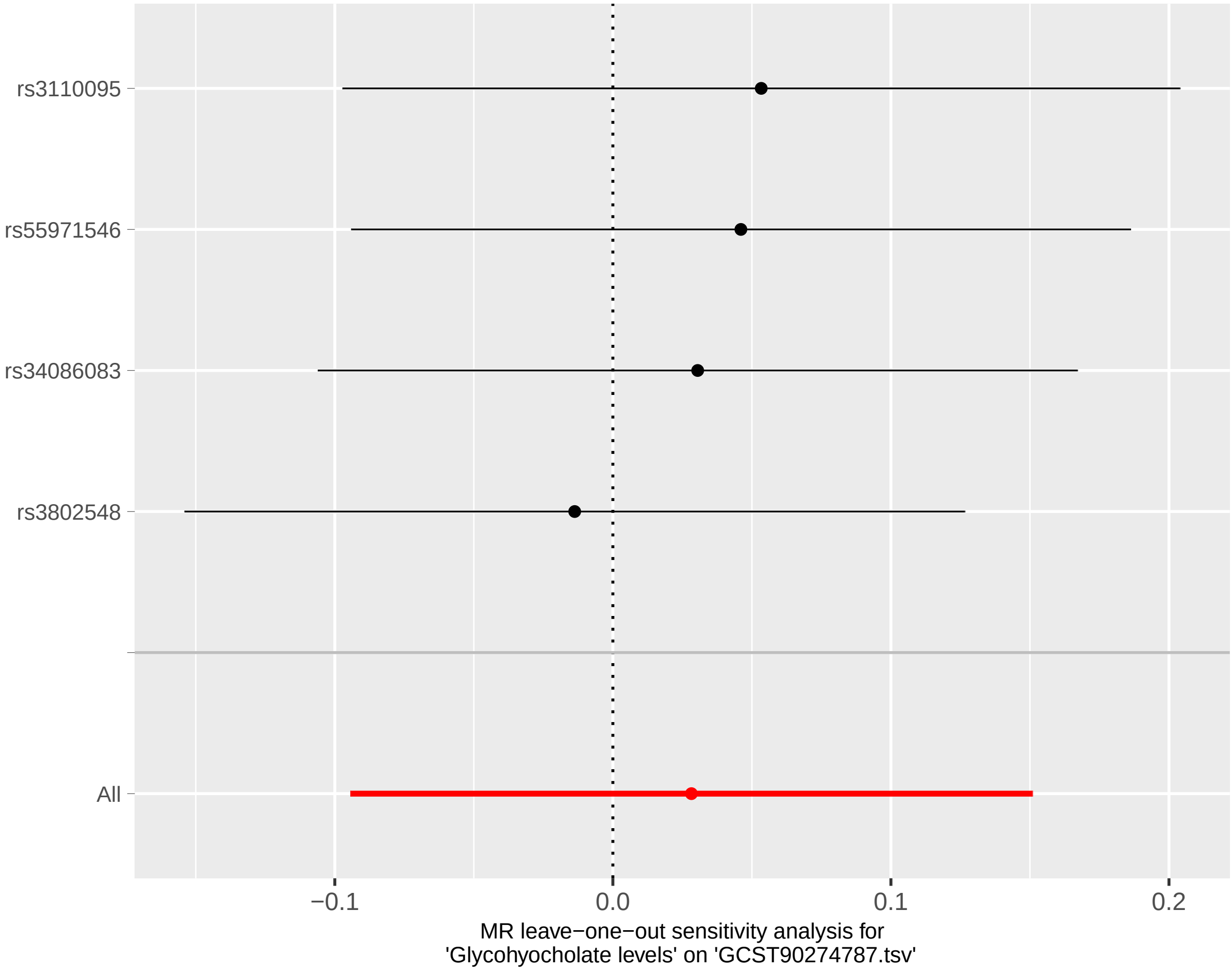
rs3110095
rs55971546
rs34086083
rs3802548
All
−0.1
0.0
0.1
0.2
MR leave−one−out sensitivity analysis for
'Glycohyocholate levels' on 'GCST90274787.tsv'

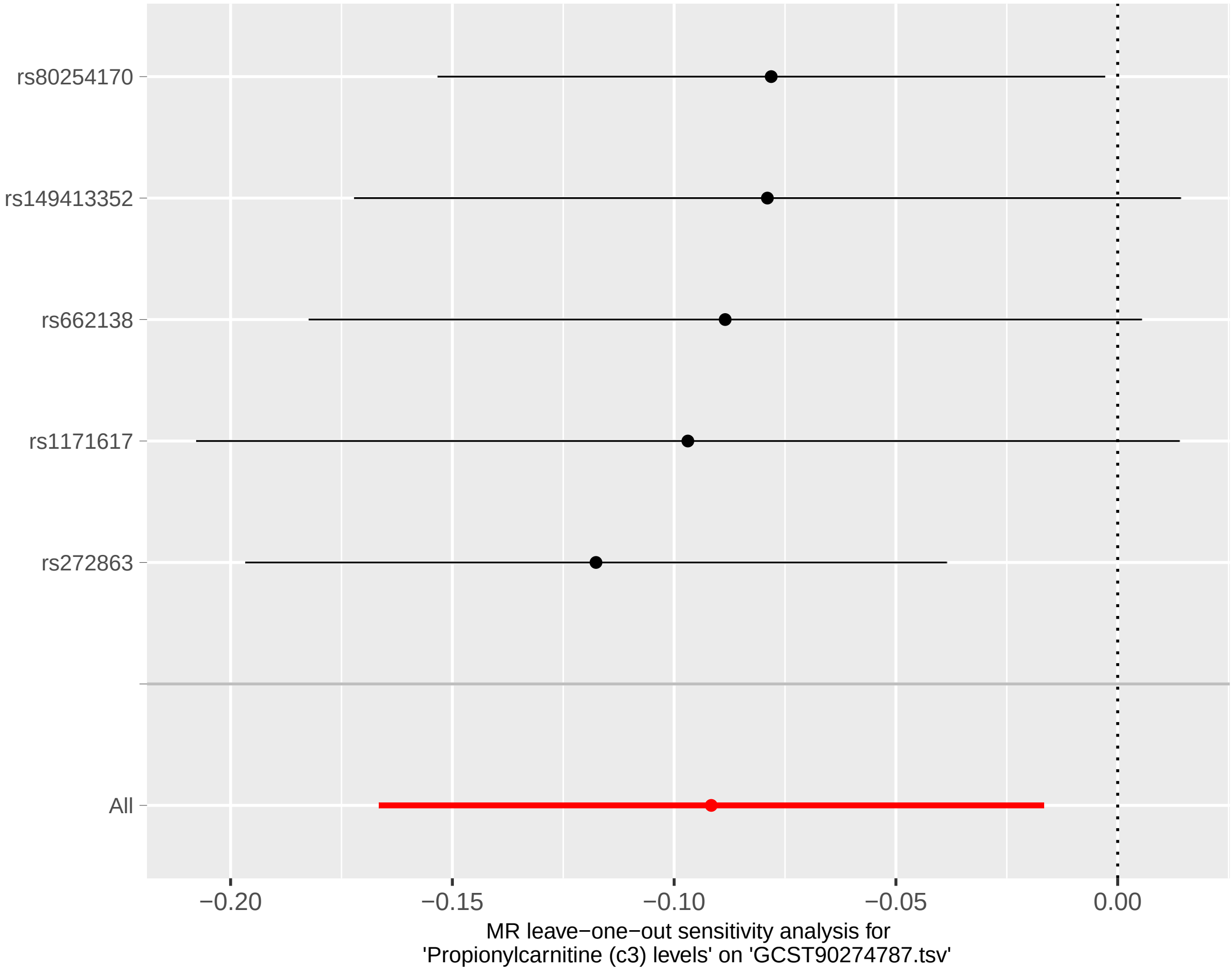
rs80254170
rs149413352
rs662138
rs1171617
rs272863
All
−0.20
−0.15
−0.10
−0.05
0.00
MR leave−one−out sensitivity analysis for
'Propionylcarnitine (c3) levels' on 'GCST90274787.tsv'

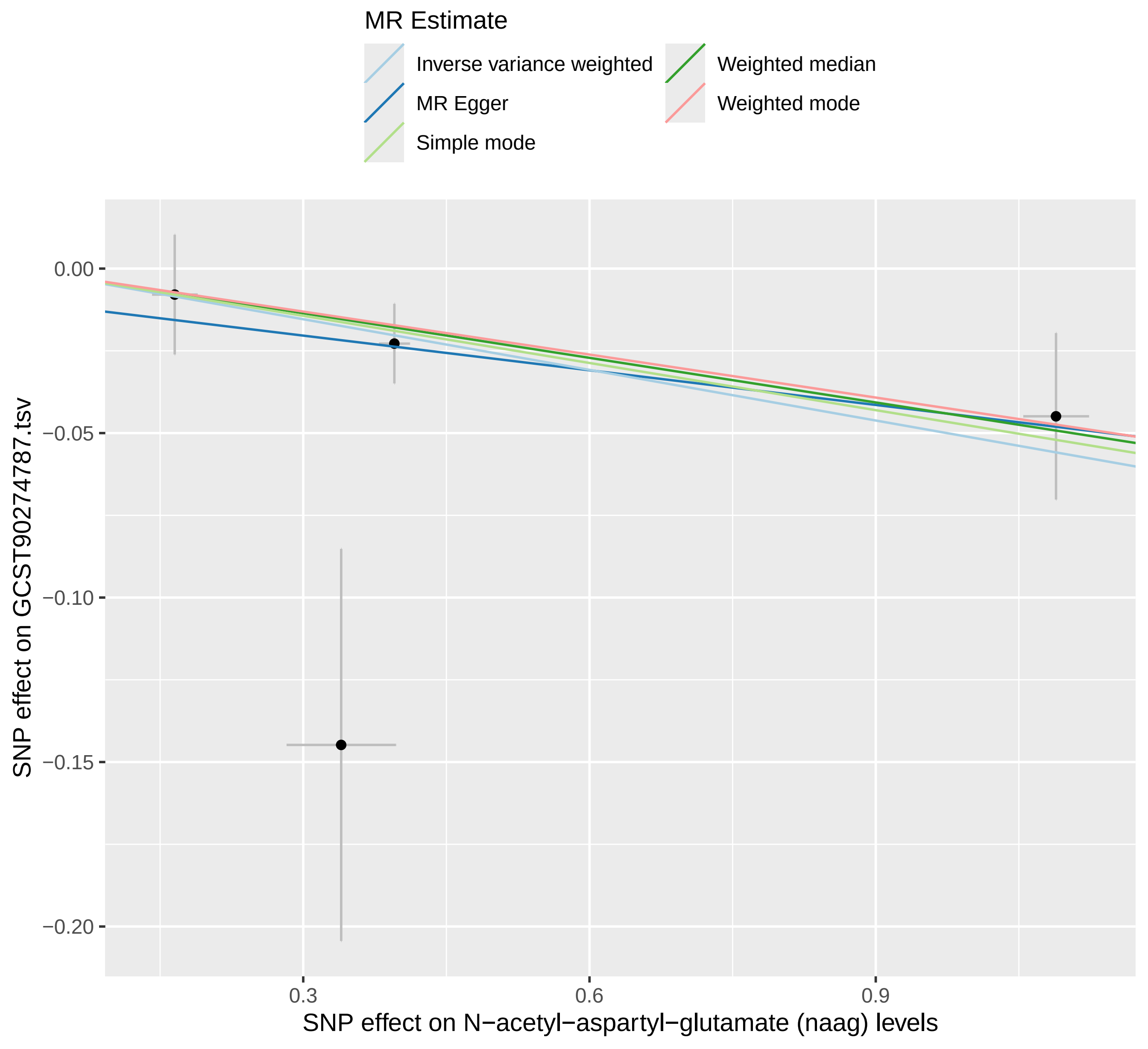

MR Estimate
Inverse variance weighted
MR Egger
Simple mode
Weighted median
Weighted mode
0.00
−0.05
−0.10
−0.15
−0.20
0.3
0.6
0.9
SNP effect on GCST90274787.tsv
SNP effect on N−acetyl−aspartyl−glutamate (naag) levels

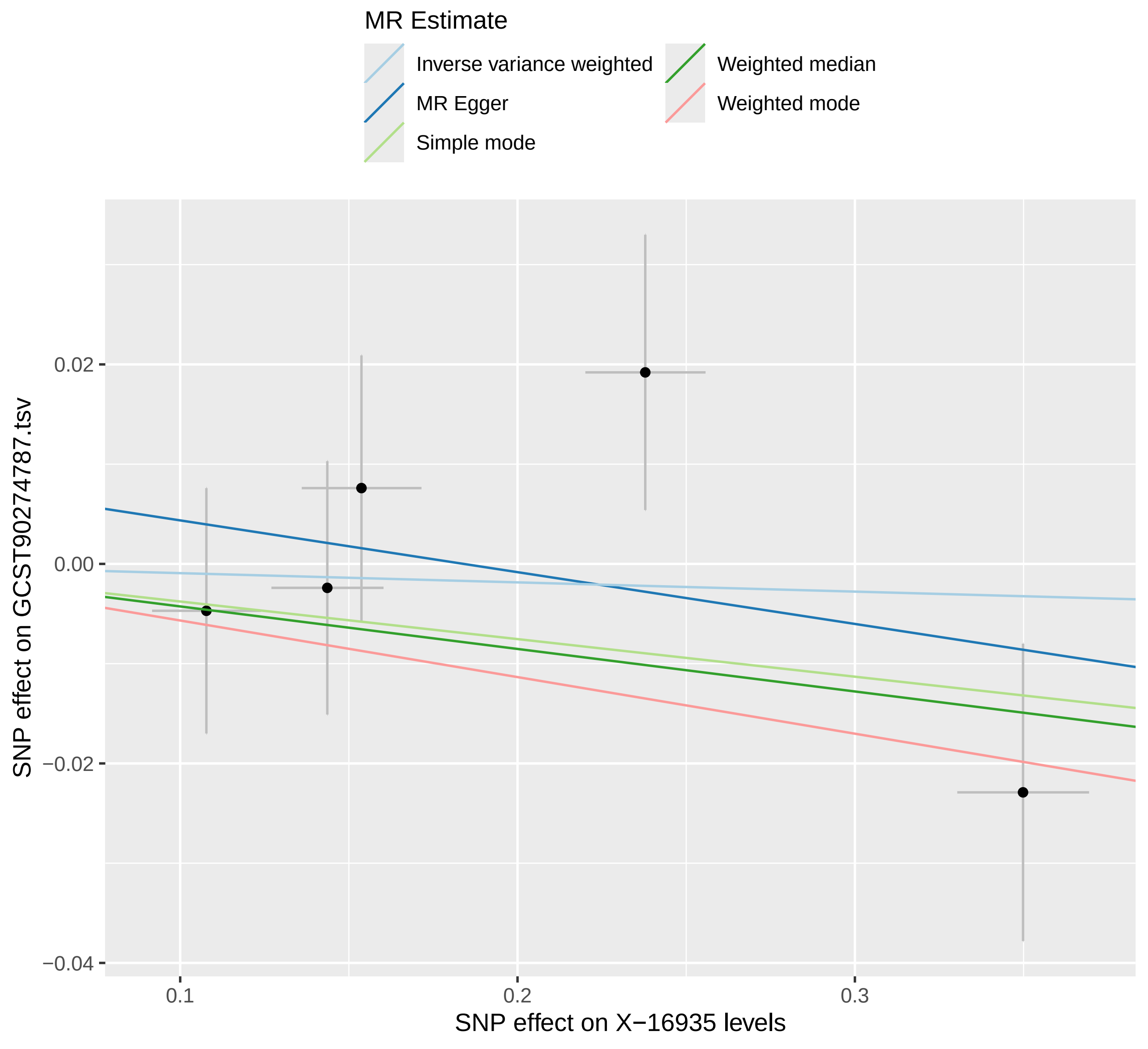
MR Estimate
Inverse variance weighted
MR Egger
Simple mode
Weighted median
Weighted mode
0.02
0.00
−0.02
−0.04
0.1
0.2
0.3
SNP effect on GCST90274787.tsv
SNP effect on X−16935 levels

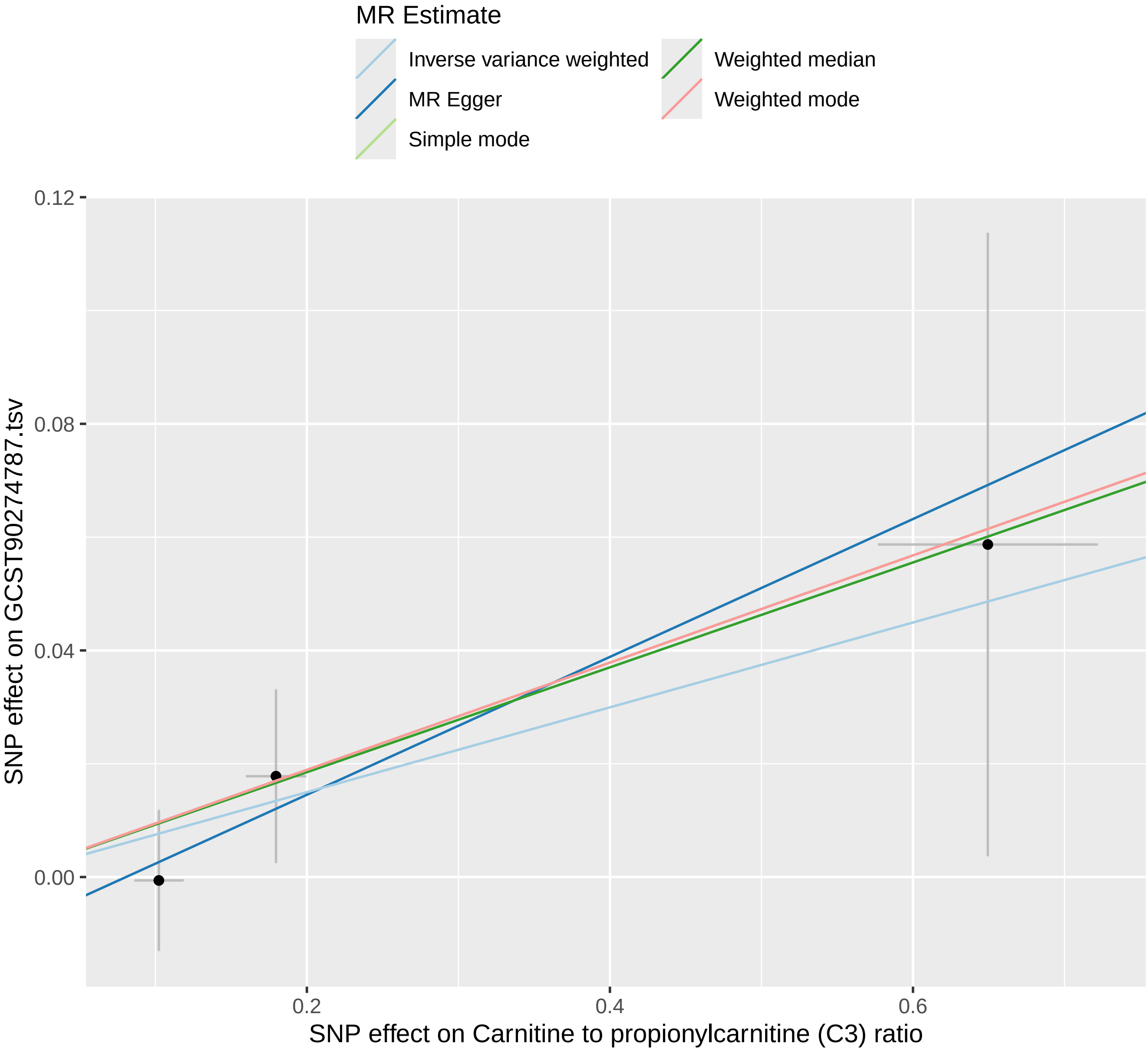
MR Estimate
Inverse variance weighted
MR Egger
Simple mode
Weighted median
Weighted mode
0.12
0.08
0.04
0.00
0.2
0.4
0.6
SNP effect on GCST90274787.tsv
SNP effect on Carnitine to propionylcarnitine (C3) ratio

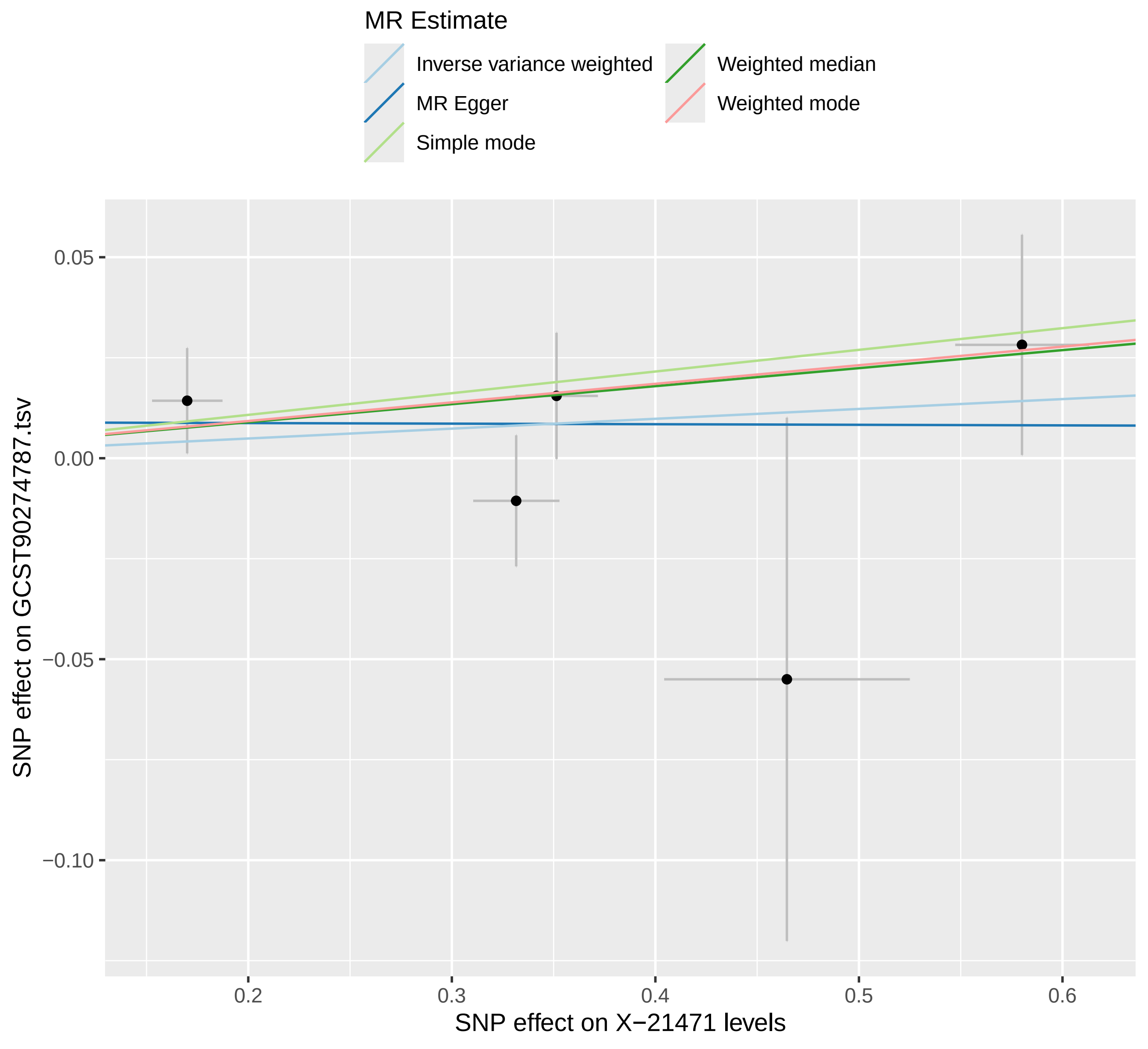
MR Estimate
Inverse variance weighted
MR Egger
Simple mode
Weighted median
Weighted mode
0.05
0.00
−0.05
−0.10
0.2
0.3
0.4
0.5
0.6
SNP effect on GCST90274787.tsv
SNP effect on X−21471 levels

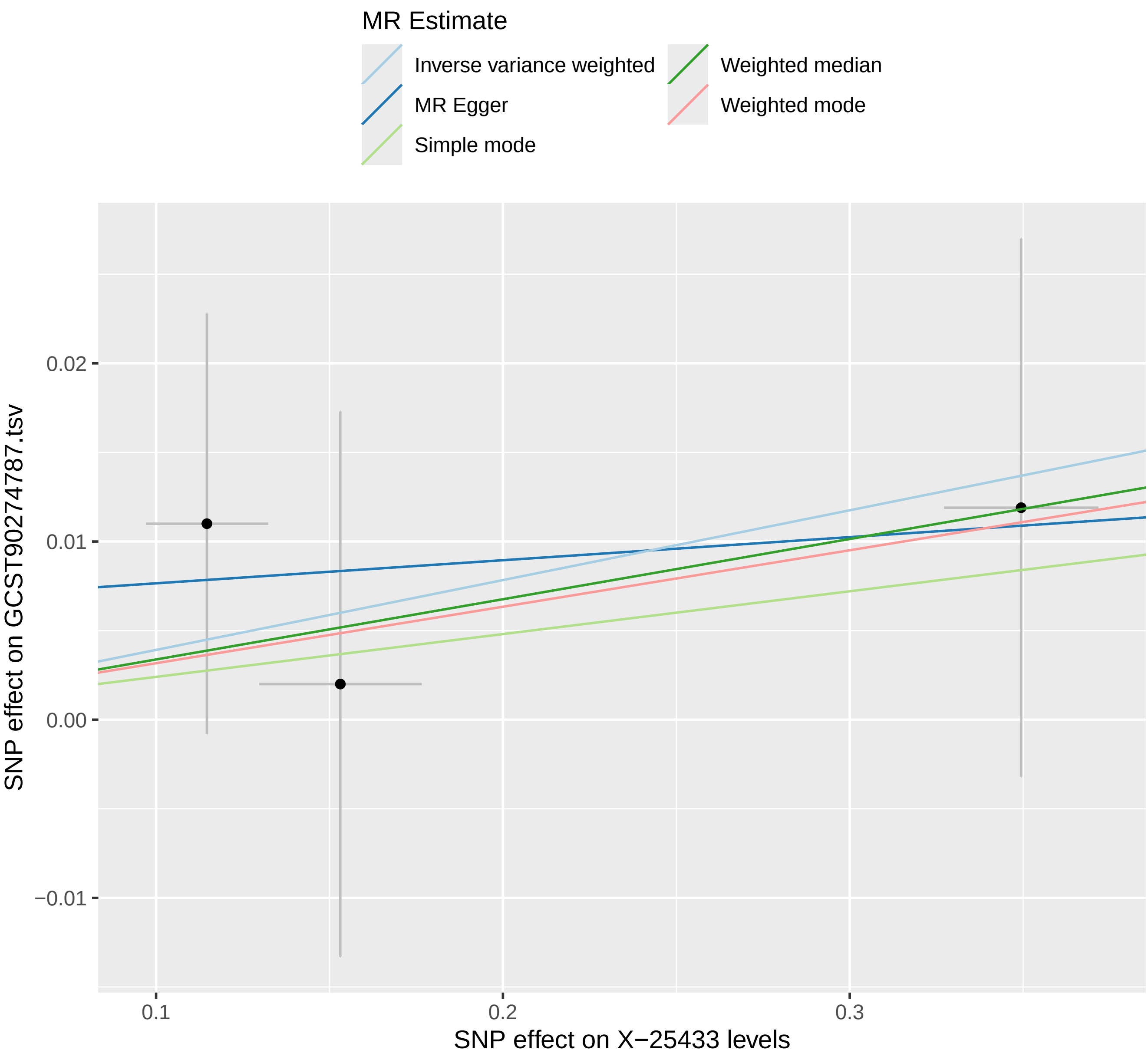
MR Estimate
Inverse variance weighted
MR Egger
Simple mode
Weighted median
Weighted mode
SNP effect on GCST90274787.tsv
0.02
0.01
0.00
−0.01
0.1
0.2
0.3
SNP effect on X−25433 levels

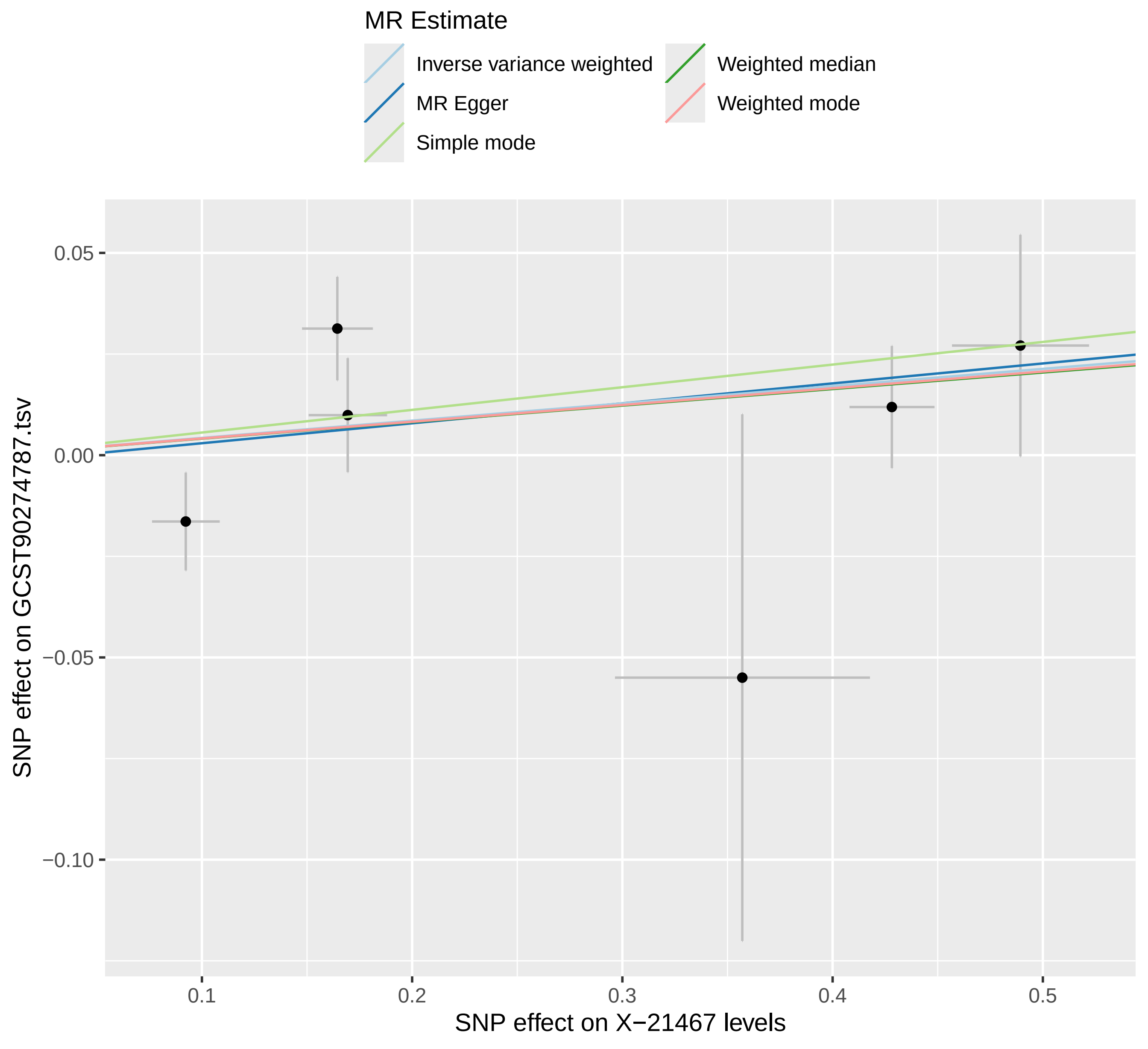

MR Estimate
Inverse variance weighted
MR Egger
Simple mode
Weighted median
Weighted mode
0.05
0.00
−0.05
−0.10
0.1
0.2
0.3
0.4
0.5
SNP effect on GCST90274787.tsv
SNP effect on X−21467 levels

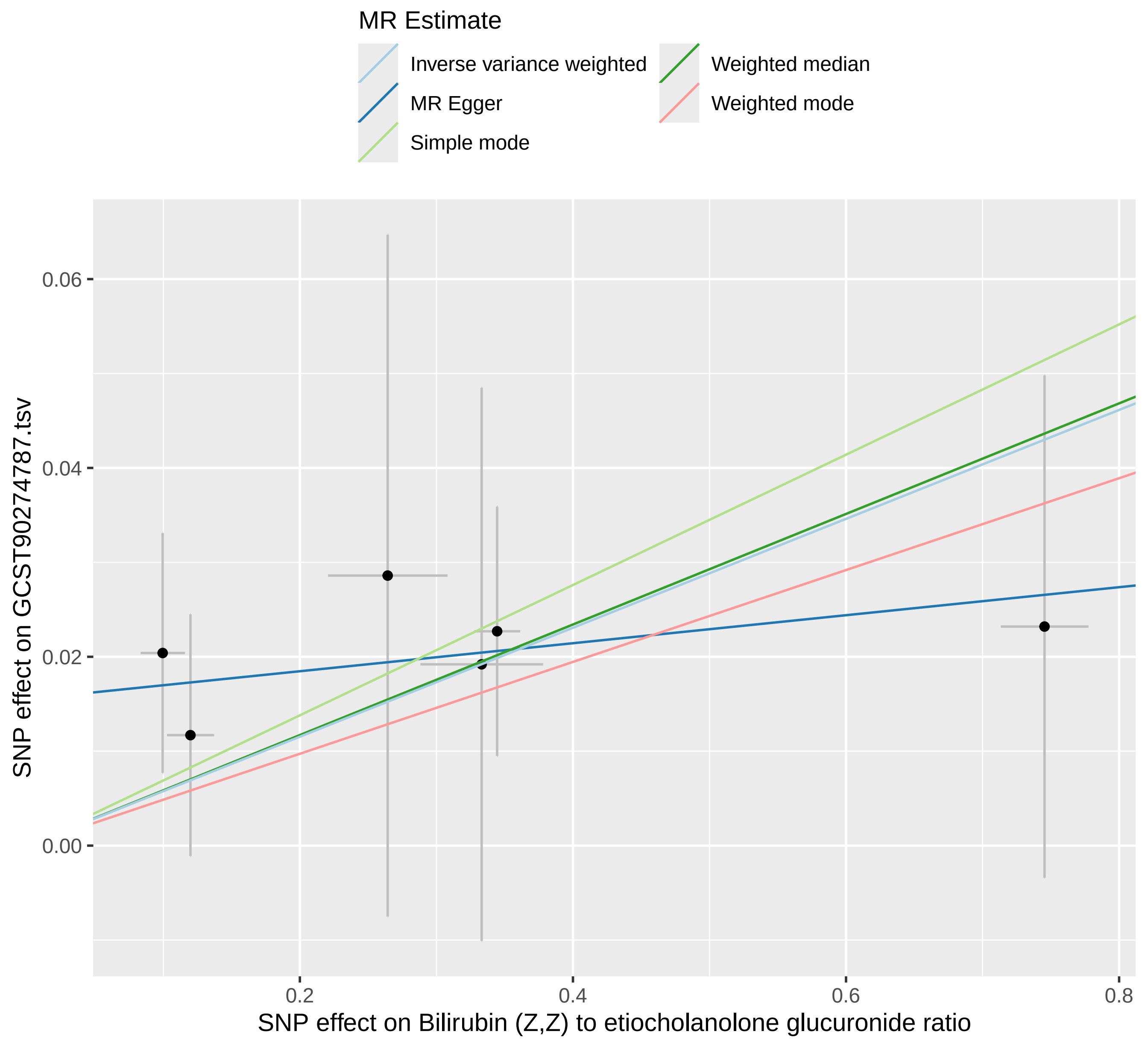
MR Estimate
Inverse variance weighted
MR Egger
Simple mode
Weighted median
Weighted mode
0.06
0.04
0.02
0.00
0.2
0.4
0.6
0.8
SNP effect on GCST90274787.tsv
SNP effect on Bilirubin (Z,Z) to etiocholanolone glucuronide ratio

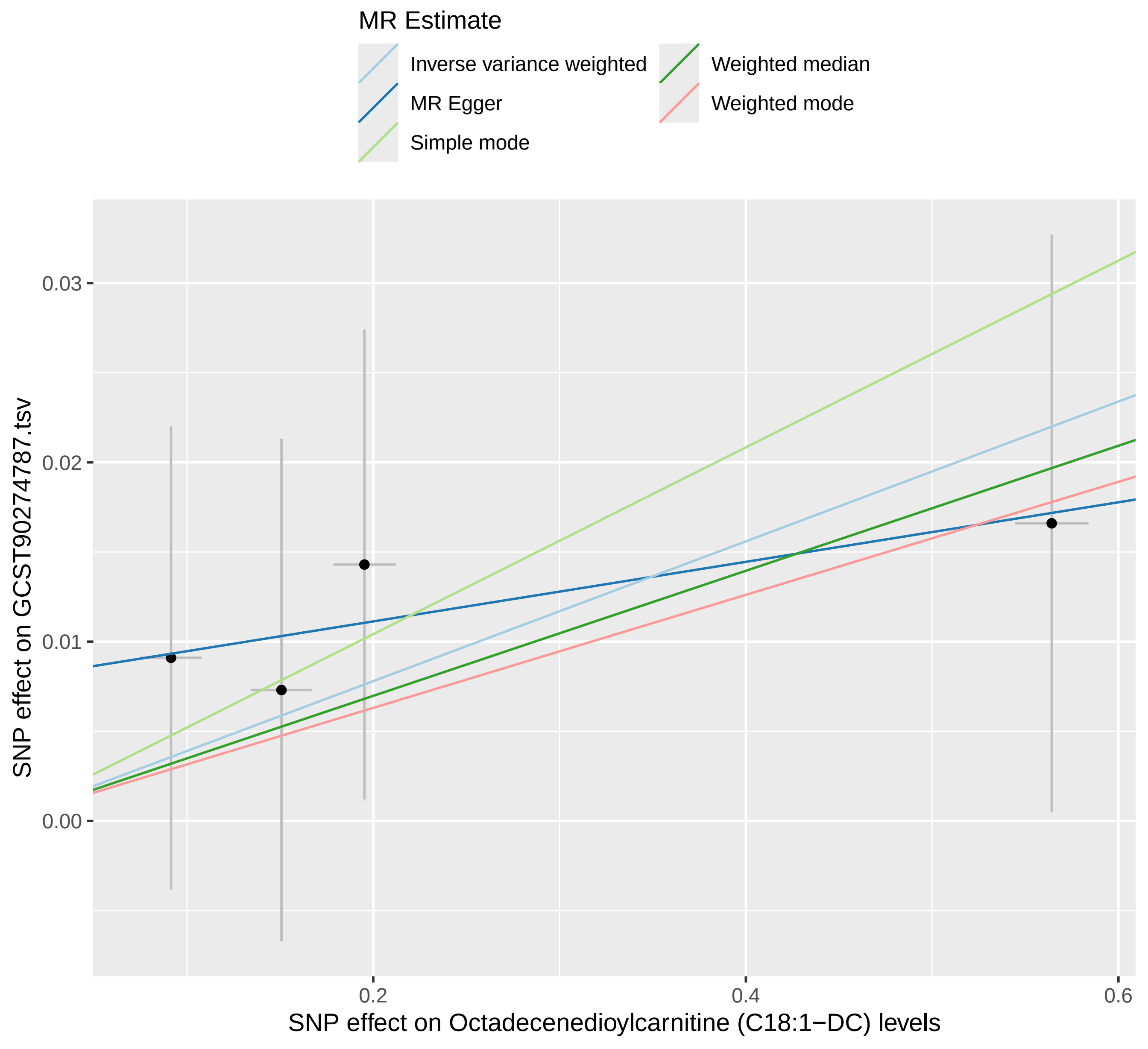
MR Estimate
Inverse variance weighted
MR Egger
Simple mode
Weighted median
Weighted mode
0.03
0.02
0.01
0.00
0.2
0.4
0.6
SNP effect on GCST90274787.tsv
SNP effect on Octadecenedioylcarnitine (C18:1−DC) levels

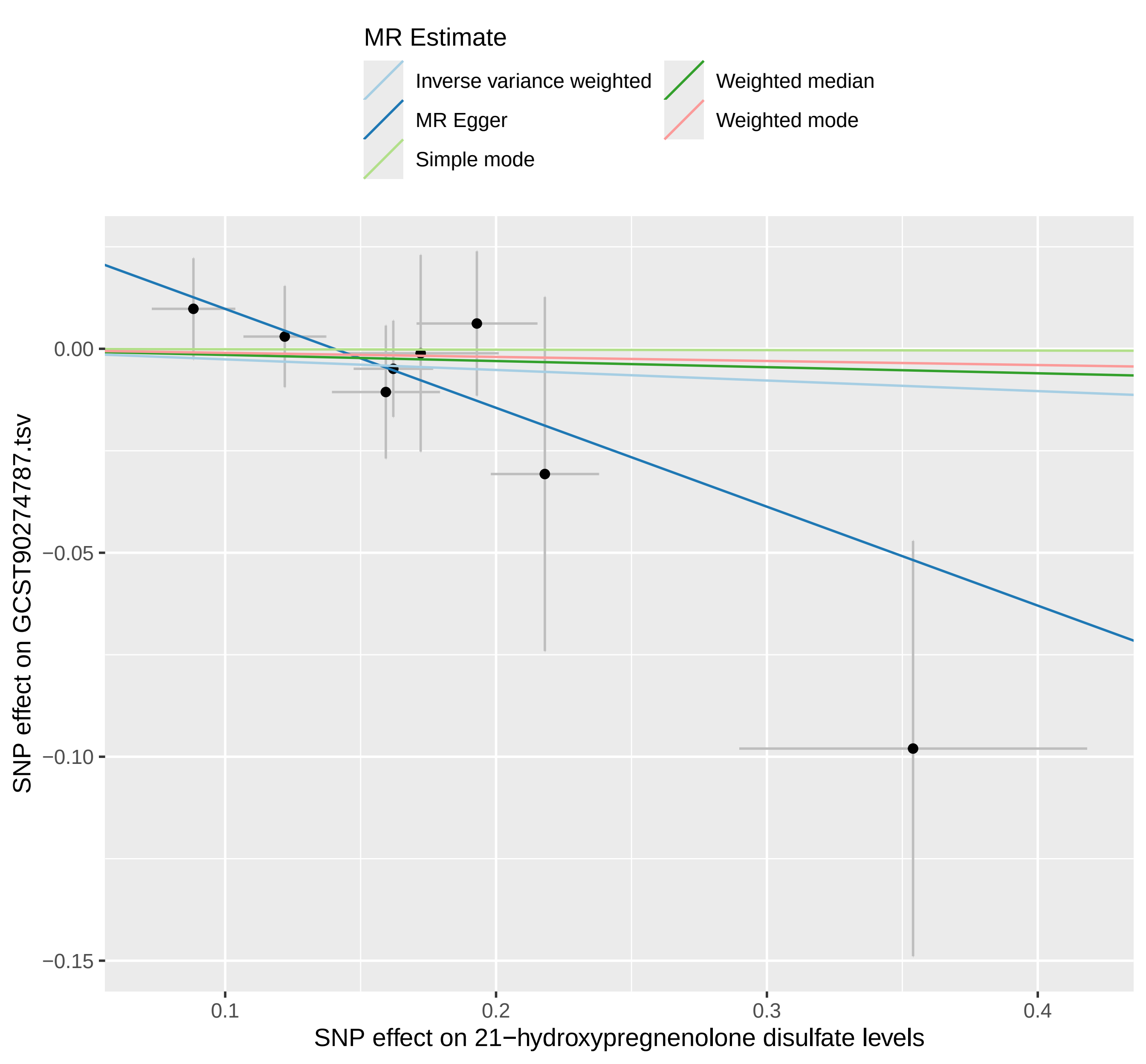
MR Estimate
Inverse variance weighted
MR Egger
Simple mode
Weighted median
Weighted mode
SNP effect on GCST90274787.tsv
0.00
−0.05
−0.10
−0.15
0.1
0.2
0.3
0.4
SNP effect on 21−hydroxypregnenolone disulfate levels

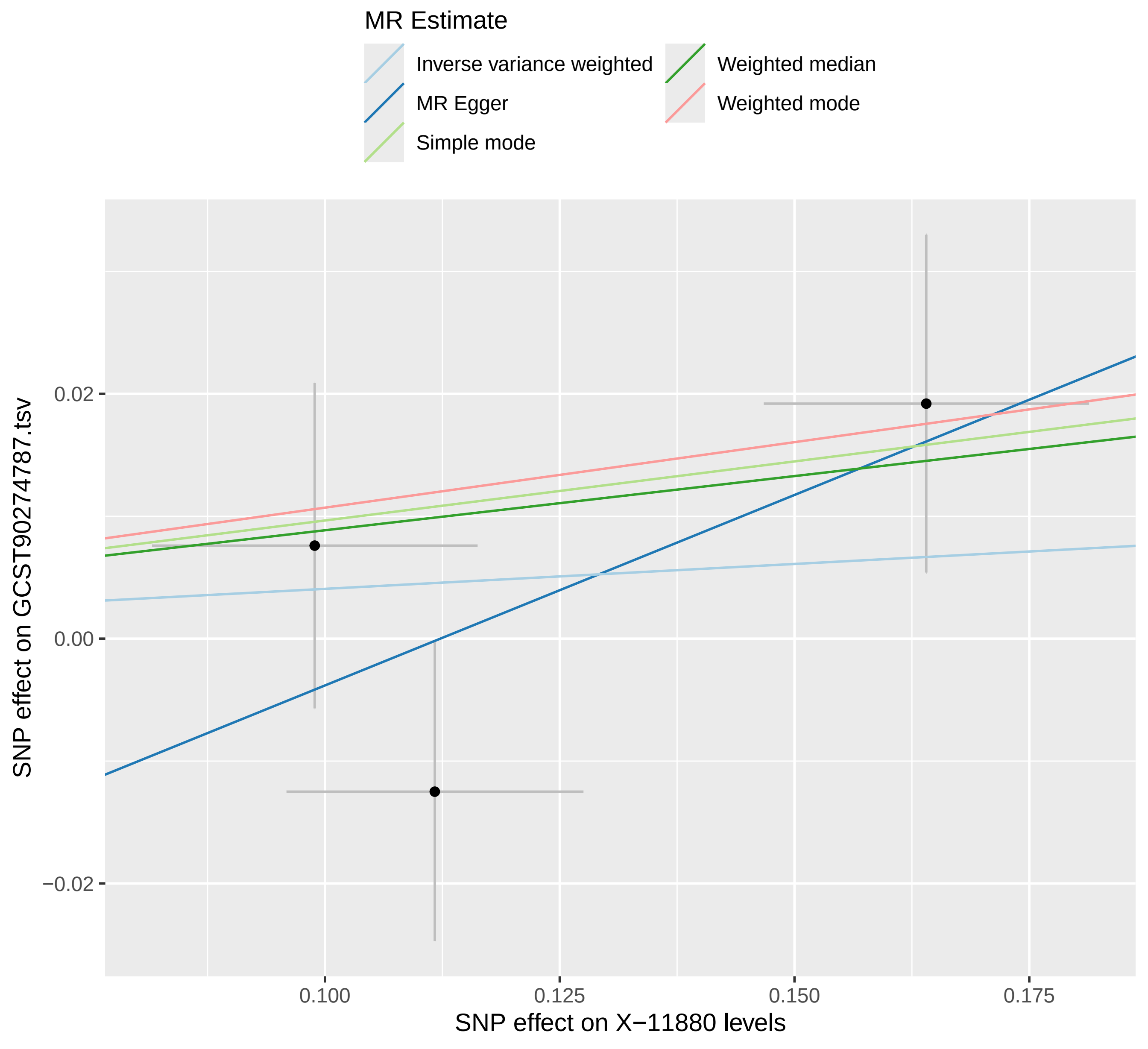
MR Estimate
Inverse variance weighted
MR Egger
Simple mode
Weighted median
Weighted mode
SNP effect on GCST90274787.tsv
0.02
0.00
−0.02
0.100
0.125
0.150
0.175
SNP effect on X−11880 levels

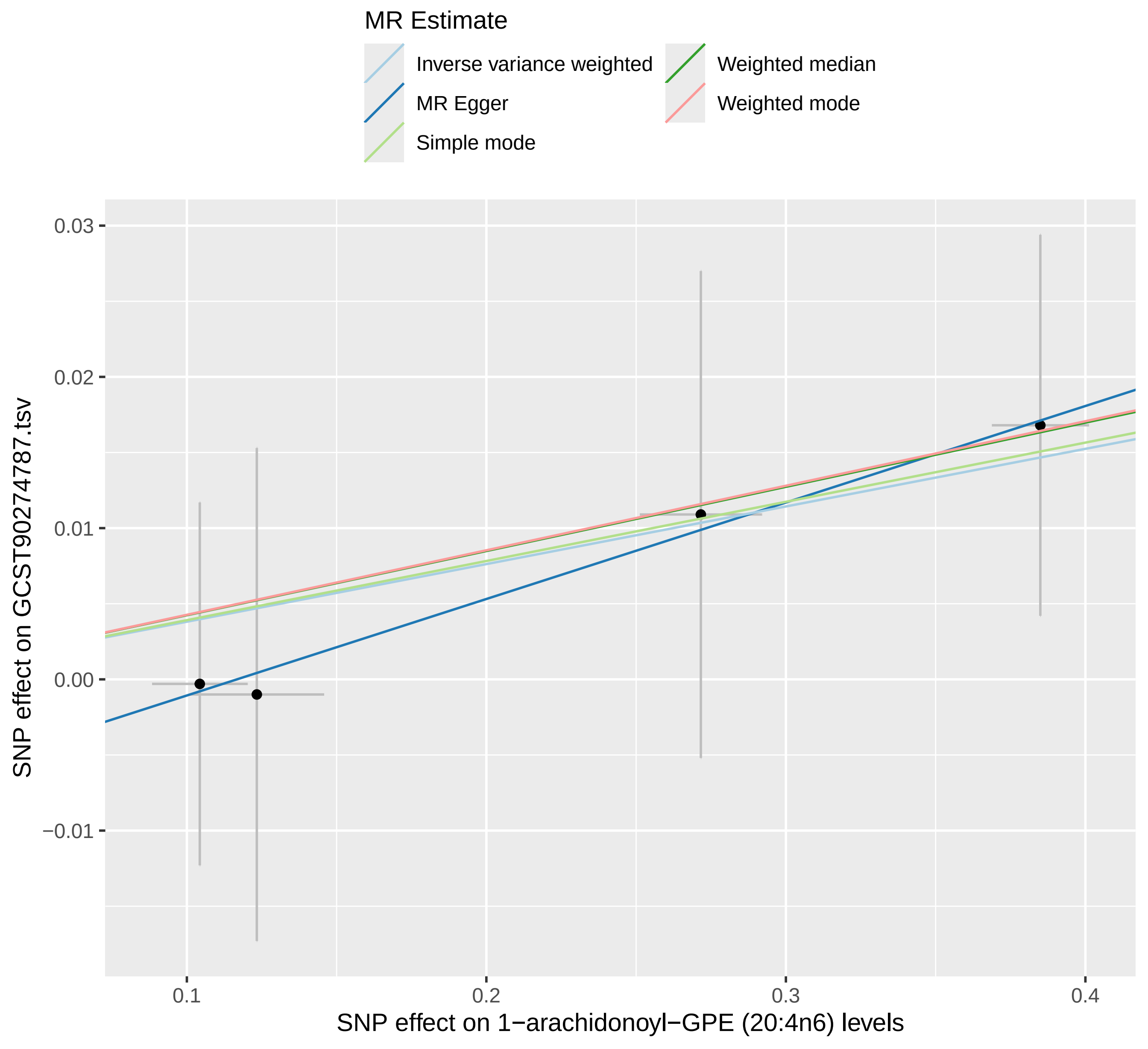

MR Estimate
Inverse variance weighted
MR Egger
Simple mode
Weighted median
Weighted mode
SNP effect on GCST90274787.tsv
0.03
0.02
0.01
0.00
−0.01
0.1
0.2
0.3
0.4
SNP effect on 1−arachidonoyl−GPE (20:4n6) levels

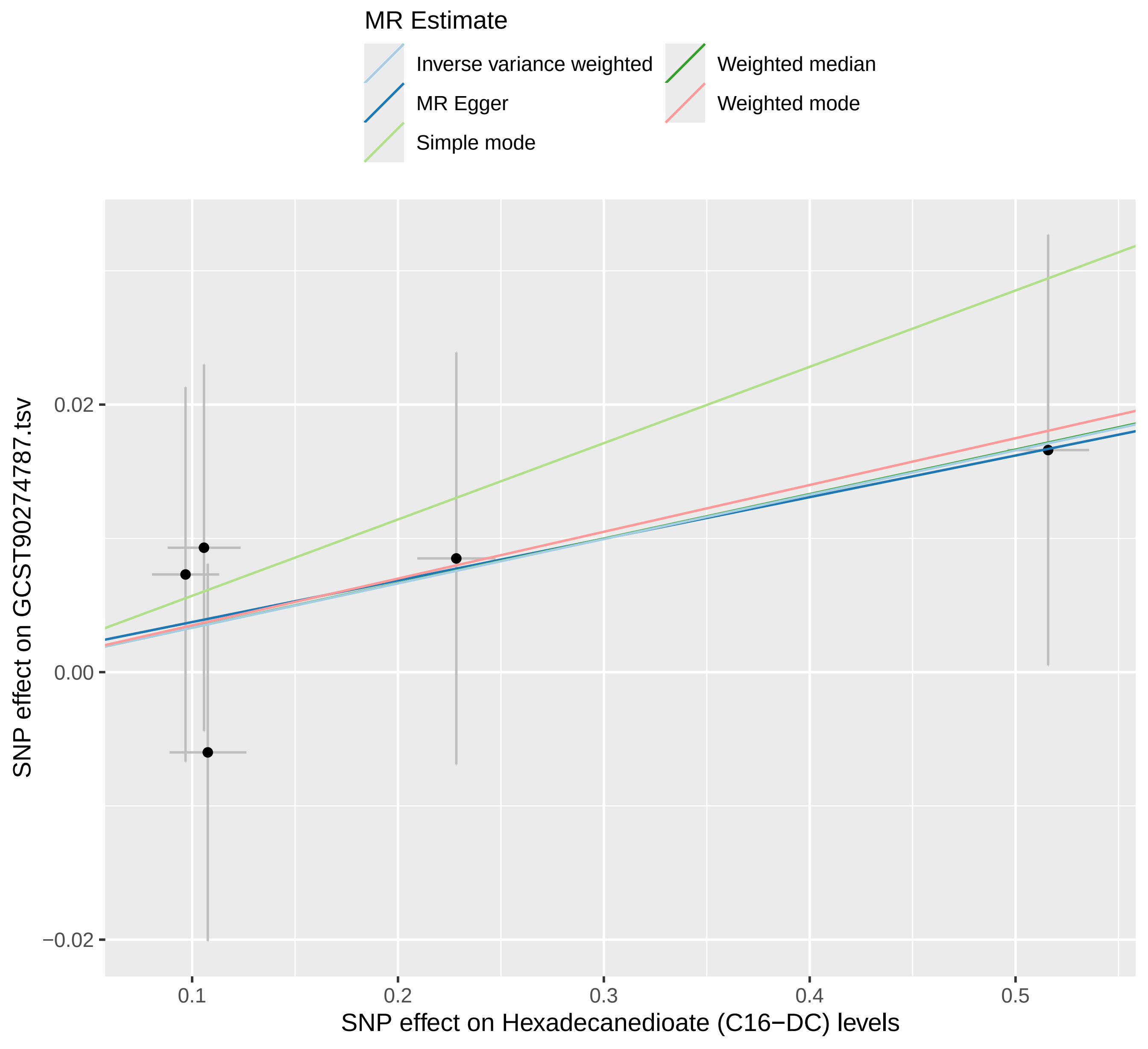
MR Estimate
Inverse variance weighted
MR Egger
Simple mode
Weighted median
Weighted mode
SNP effect on GCST90274787.tsv
0.02
0.00
−0.02
0.1
0.2
0.3
0.4
0.5
SNP effect on Hexadecanedioate (C16−DC) levels

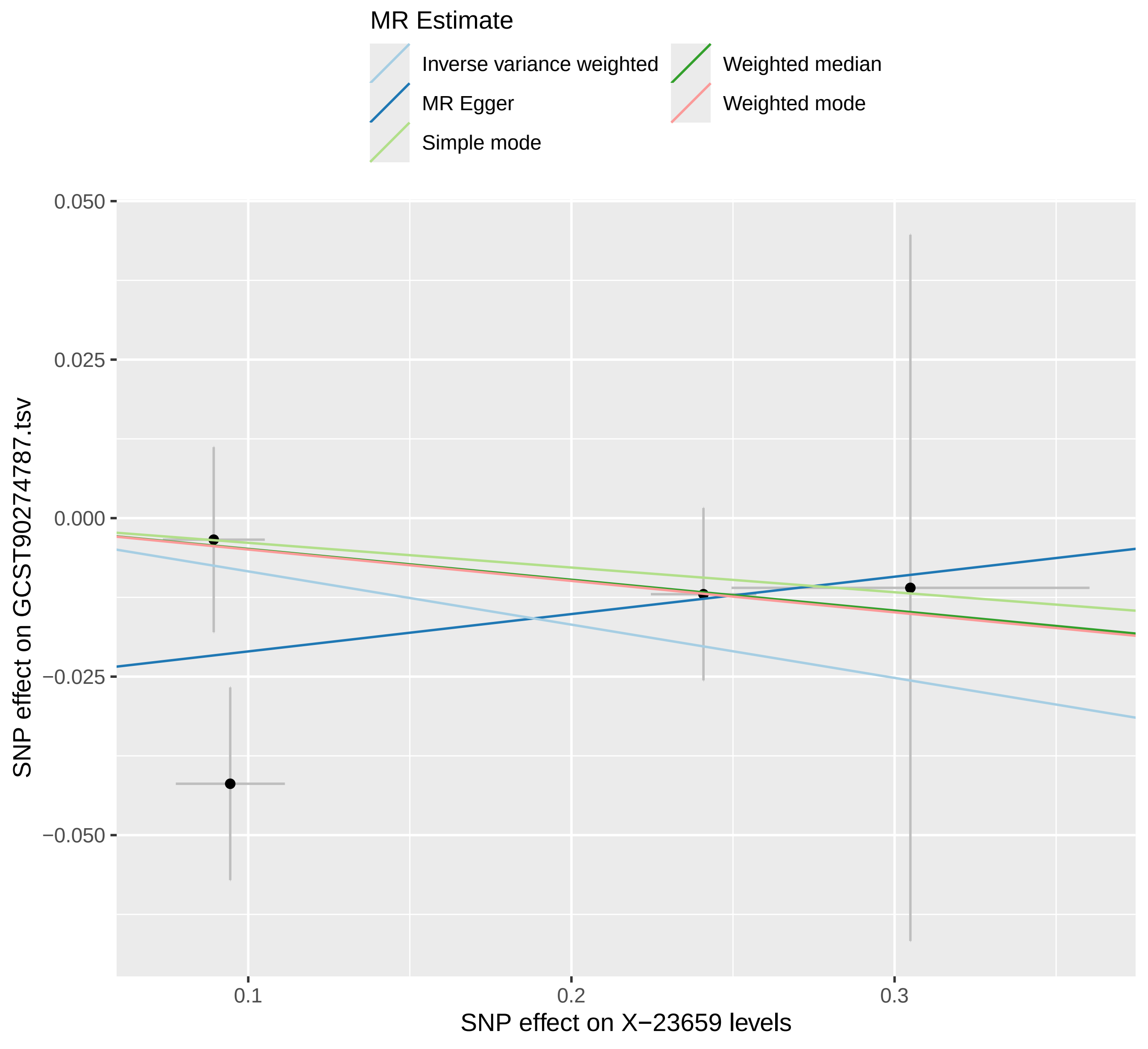
MR Estimate
Inverse variance weighted
MR Egger
Simple mode
Weighted median
Weighted mode
0.050
0.025
0.000
−0.025
−0.050
SNP effect on GCST90274787.tsv
0.1
0.2
0.3
SNP effect on X−23659 levels

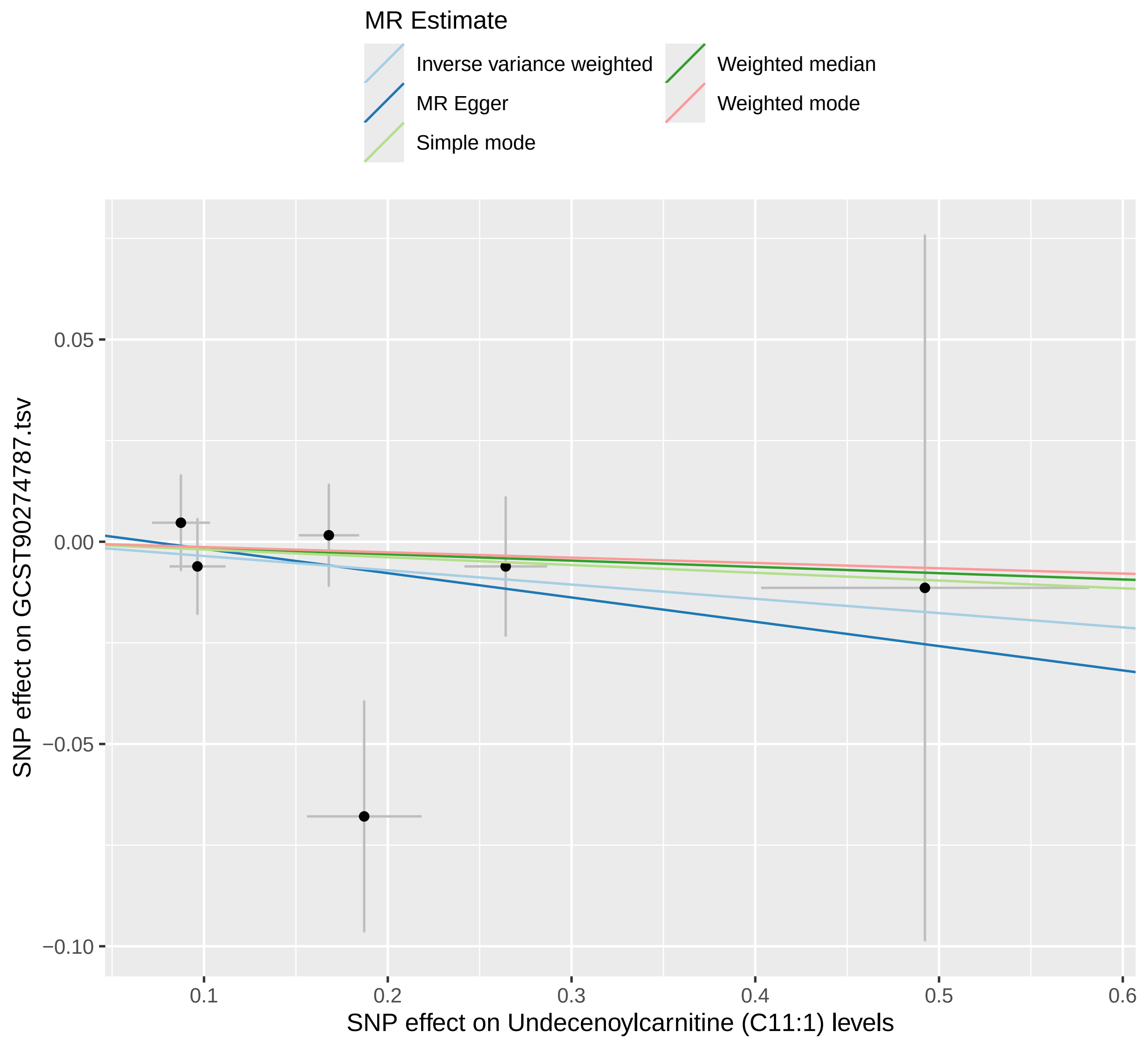
MR Estimate
Inverse variance weighted
MR Egger
Simple mode
Weighted median
Weighted mode
0.05
0.00
−0.05
−0.10
0.1
0.2
0.3
0.4
0.5
0.6
SNP effect on GCST90274787.tsv
SNP effect on Undecenoylcarnitine (C11:1) levels

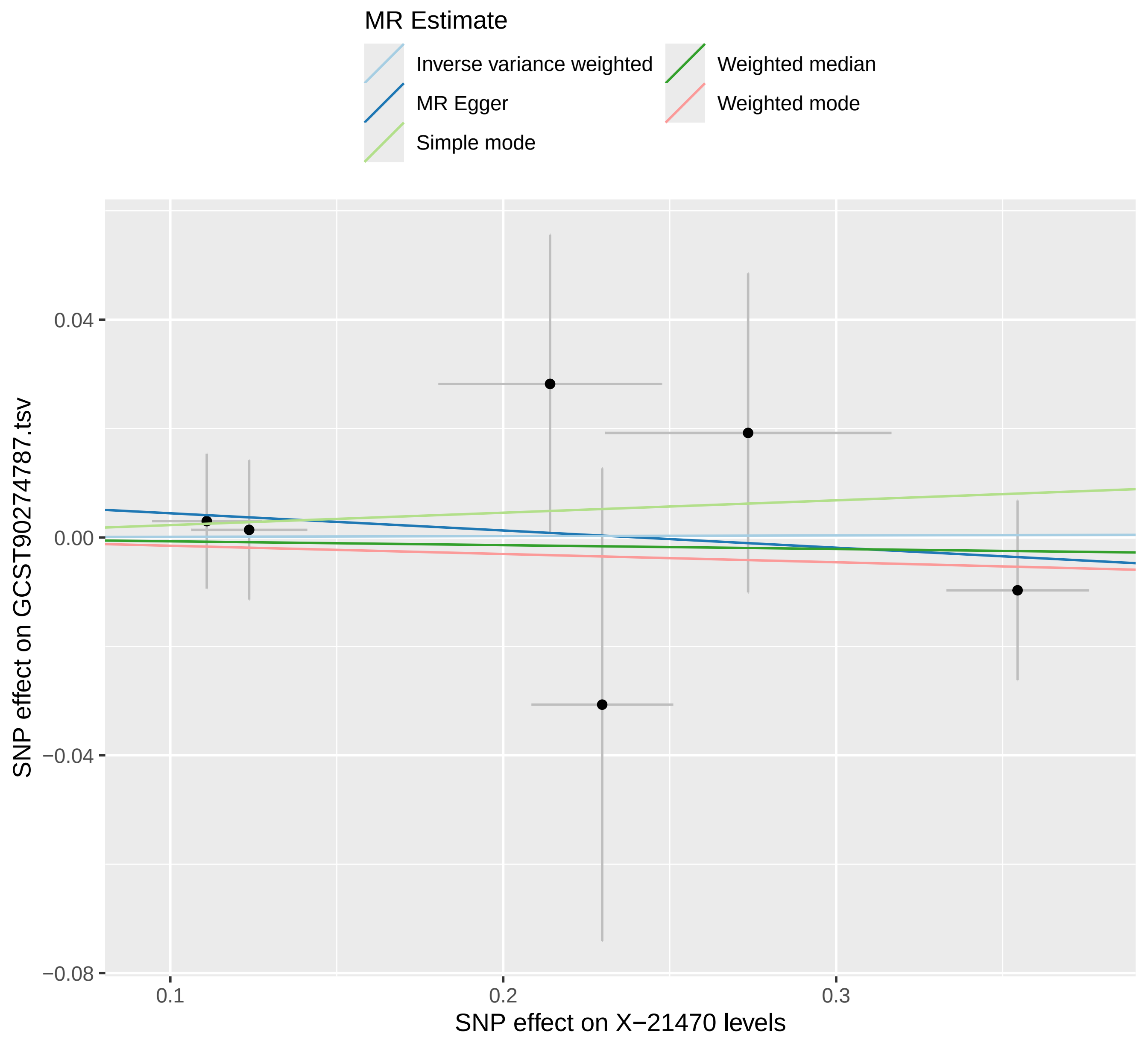
MR Estimate
Inverse variance weighted
MR Egger
Simple mode
Weighted median
Weighted mode
0.04
0.00
−0.04
−0.08
0.1
0.2
0.3
SNP effect on GCST90274787.tsv
SNP effect on X−21470 levels

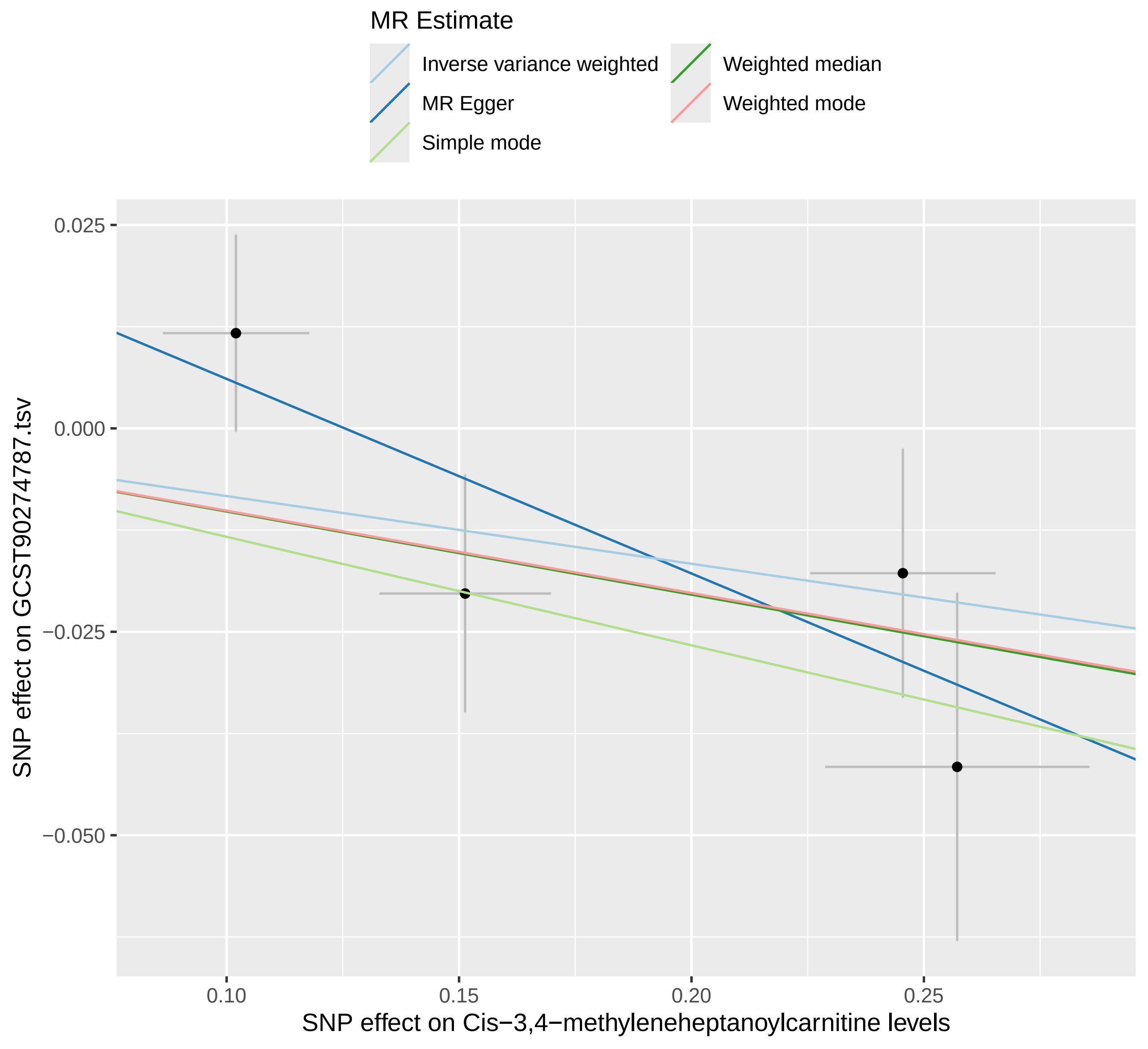

MR Estimate
Inverse variance weighted
MR Egger
Simple mode
Weighted median
Weighted mode
0.025
0.000
−0.025
−0.050
0.10
0.15
0.20
0.25
SNP effect on GCST90274787.tsv
SNP effect on Cis−3,4−methyleneheptanoylcarnitine levels

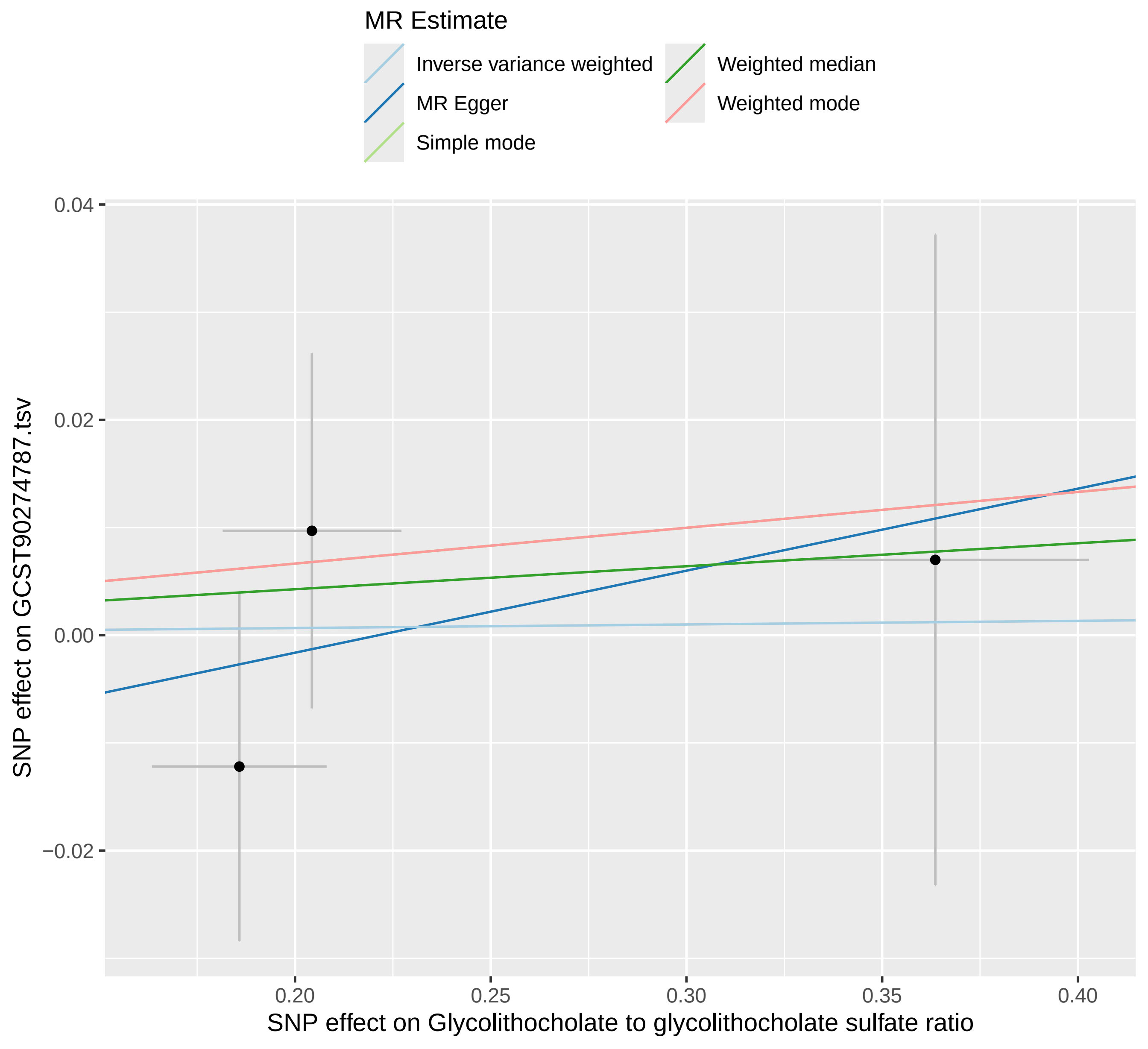
MR Estimate
Inverse variance weighted
MR Egger
Simple mode
Weighted median
Weighted mode
0.04
0.02
0.00
−0.02
0.20
0.25
0.30
0.35
0.40
SNP effect on GCST90274787.tsv
SNP effect on Glycolithocholate to glycolithocholate sulfate ratio

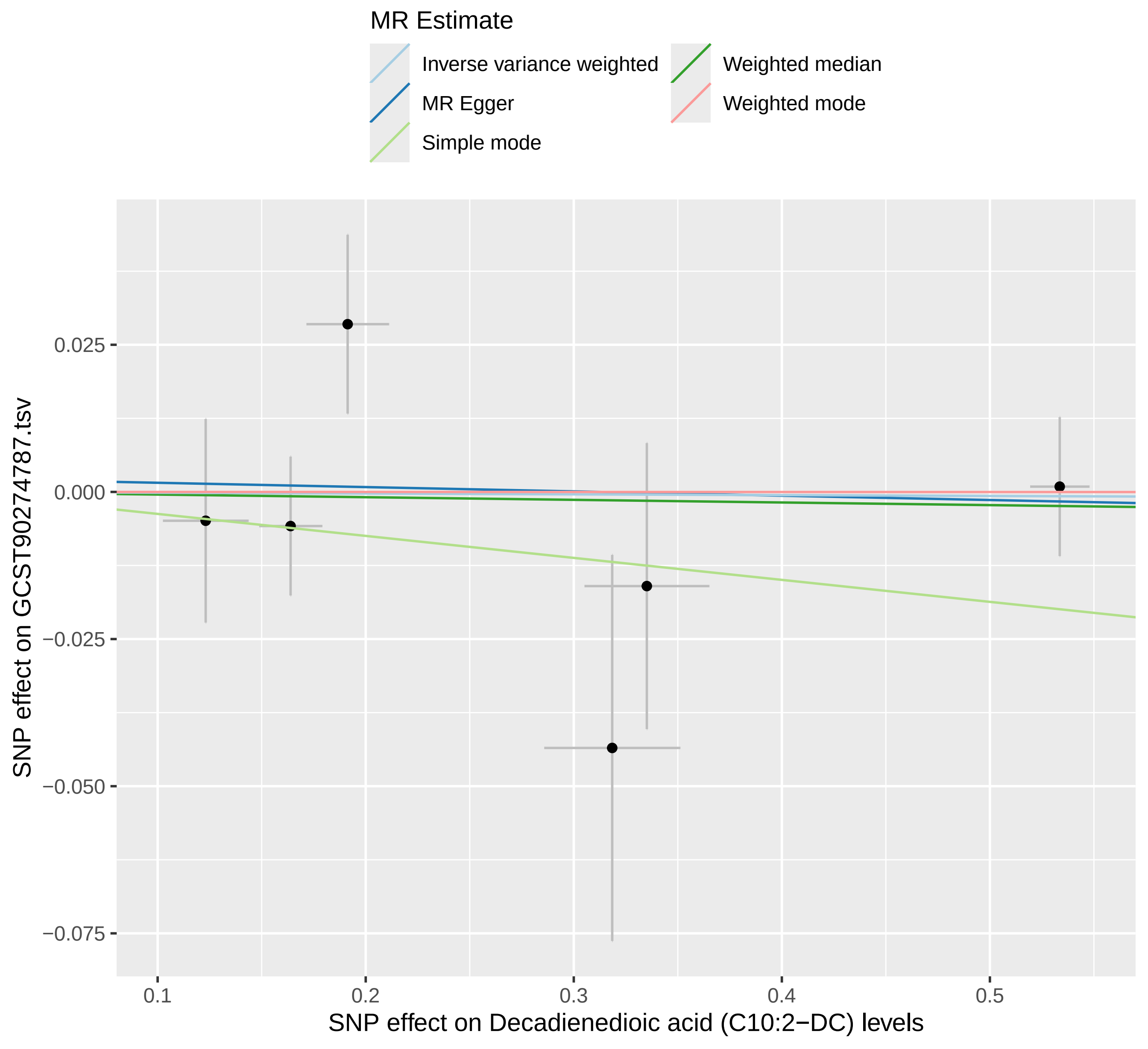
MR Estimate
Inverse variance weighted
MR Egger
Simple mode
Weighted median
Weighted mode
0.025
0.000
−0.025
−0.050
−0.075
SNP effect on GCST90274787.tsv
0.1
0.2
0.3
0.4
0.5
SNP effect on Decadienedioic acid (C10:2−DC) levels

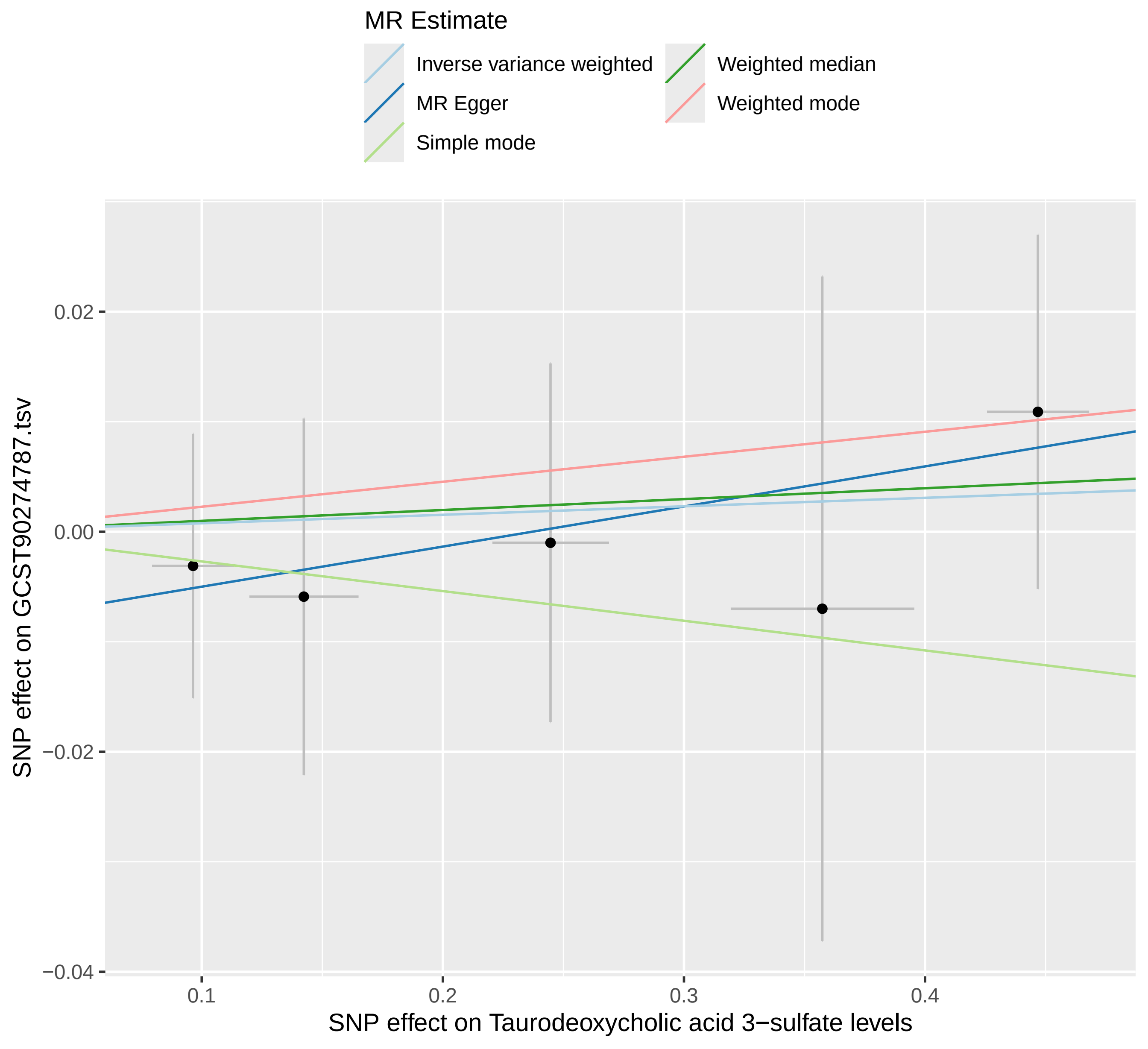
MR Estimate
Inverse variance weighted
MR Egger
Simple mode
Weighted median
Weighted mode
0.02
0.00
−0.02
−0.04
0.1
0.2
0.3
0.4
SNP effect on GCST90274787.tsv
SNP effect on Taurodeoxycholic acid 3−sulfate levels

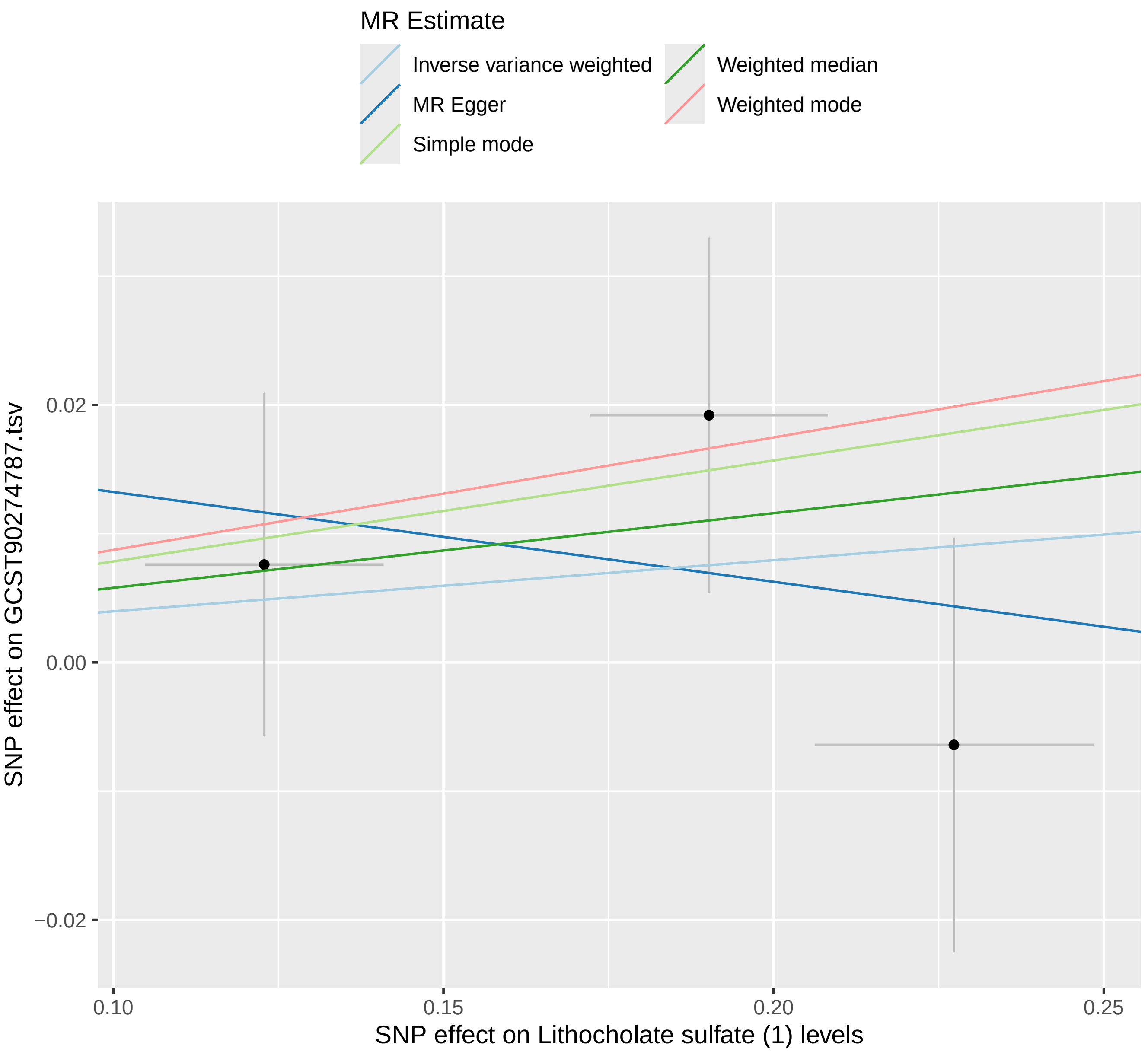
MR Estimate
Inverse variance weighted
MR Egger
Simple mode
Weighted median
Weighted mode
0.02
0.00
−0.02
0.10
0.15
0.20
0.25
SNP effect on GCST90274787.tsv
SNP effect on Lithocholate sulfate (1) levels

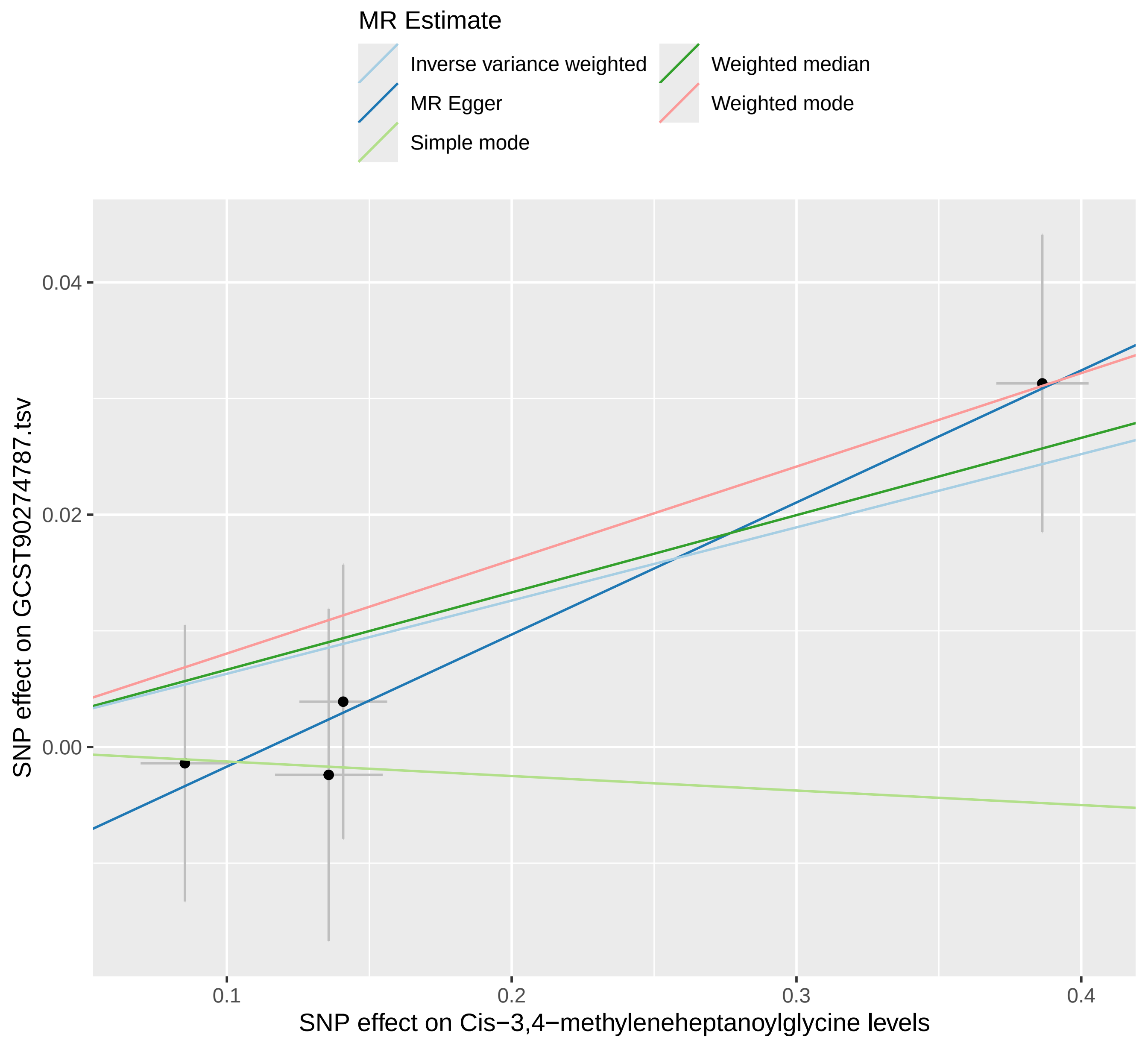
MR Estimate
Inverse variance weighted
MR Egger
Simple mode
Weighted median
Weighted mode
0.04
0.02
0.00
0.1
0.2
0.3
0.4
SNP effect on GCST90274787.tsv
SNP effect on Cis−3,4−methyleneheptanoylglycine levels

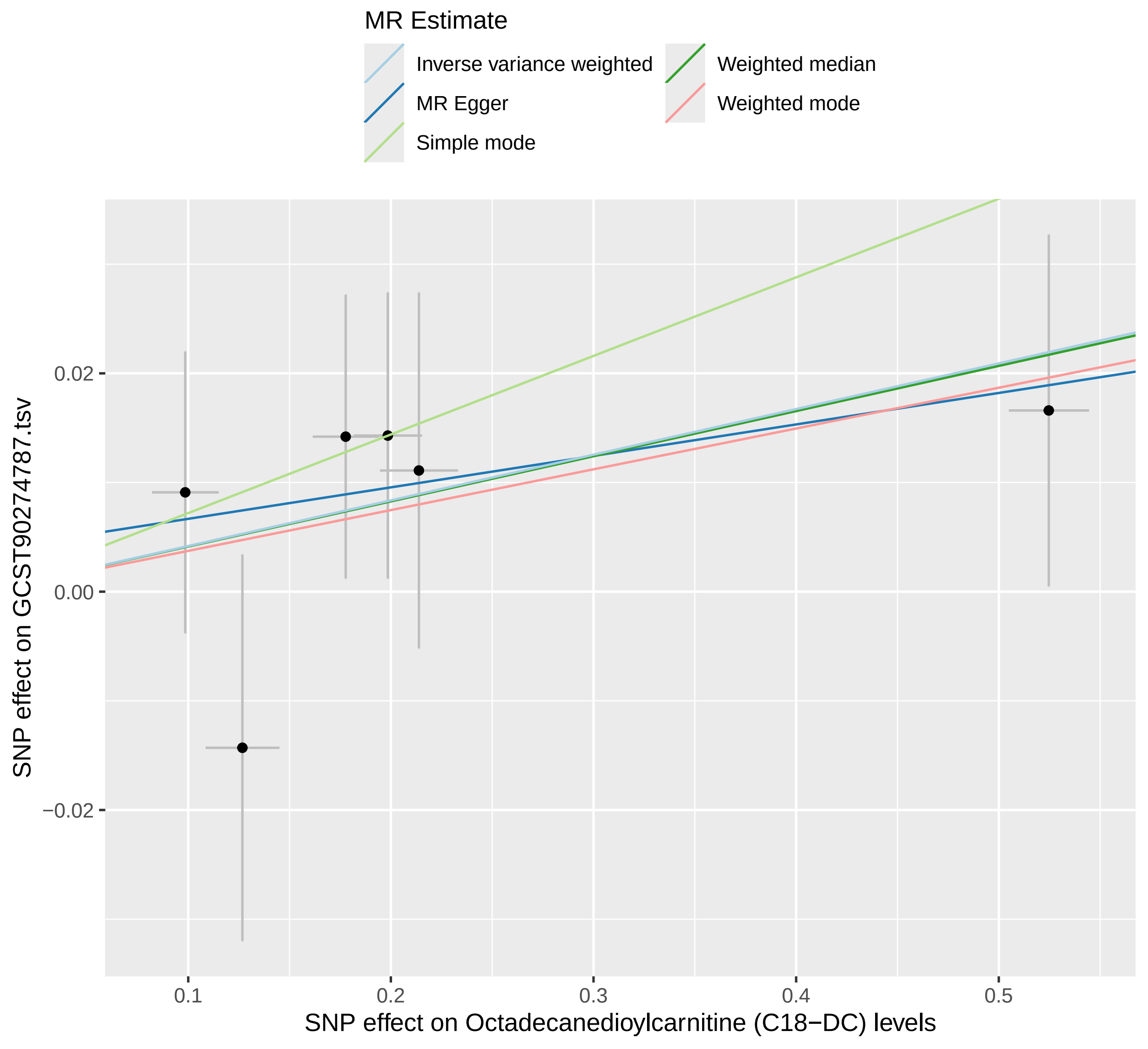
MR Estimate
Inverse variance weighted
MR Egger
Simple mode
Weighted median
Weighted mode
0.02
0.00
−0.02
0.1
0.2
0.3
0.4
0.5
SNP effect on GCST90274787.tsv
SNP effect on Octadecanedioylcarnitine (C18−DC) levels

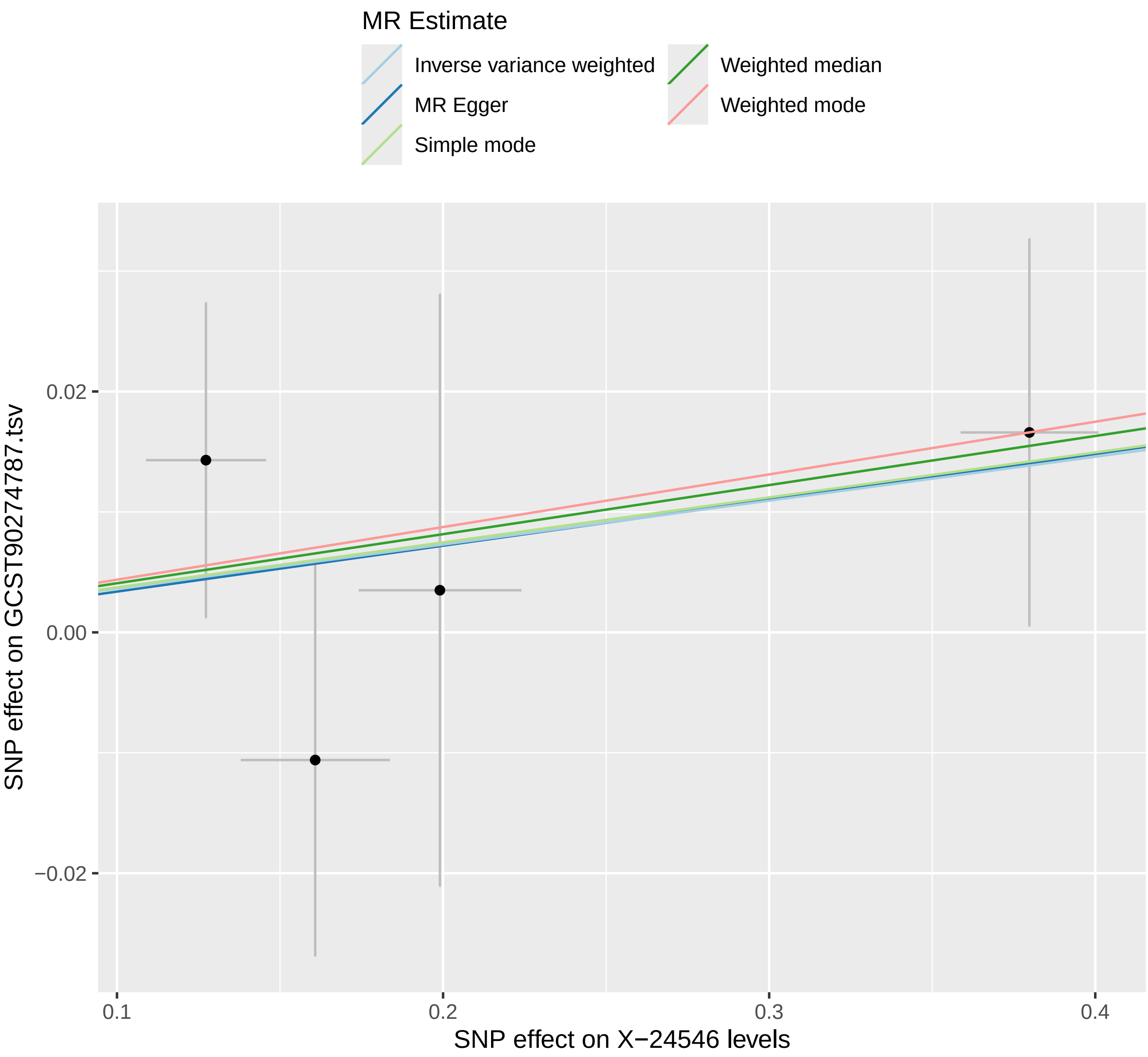
MR Estimate
Inverse variance weighted
MR Egger
Simple mode
Weighted median
Weighted mode
0.02
0.00
−0.02
SNP effect on GCST90274787.tsv
0.1
0.2
0.3
0.4
SNP effect on X−24546 levels

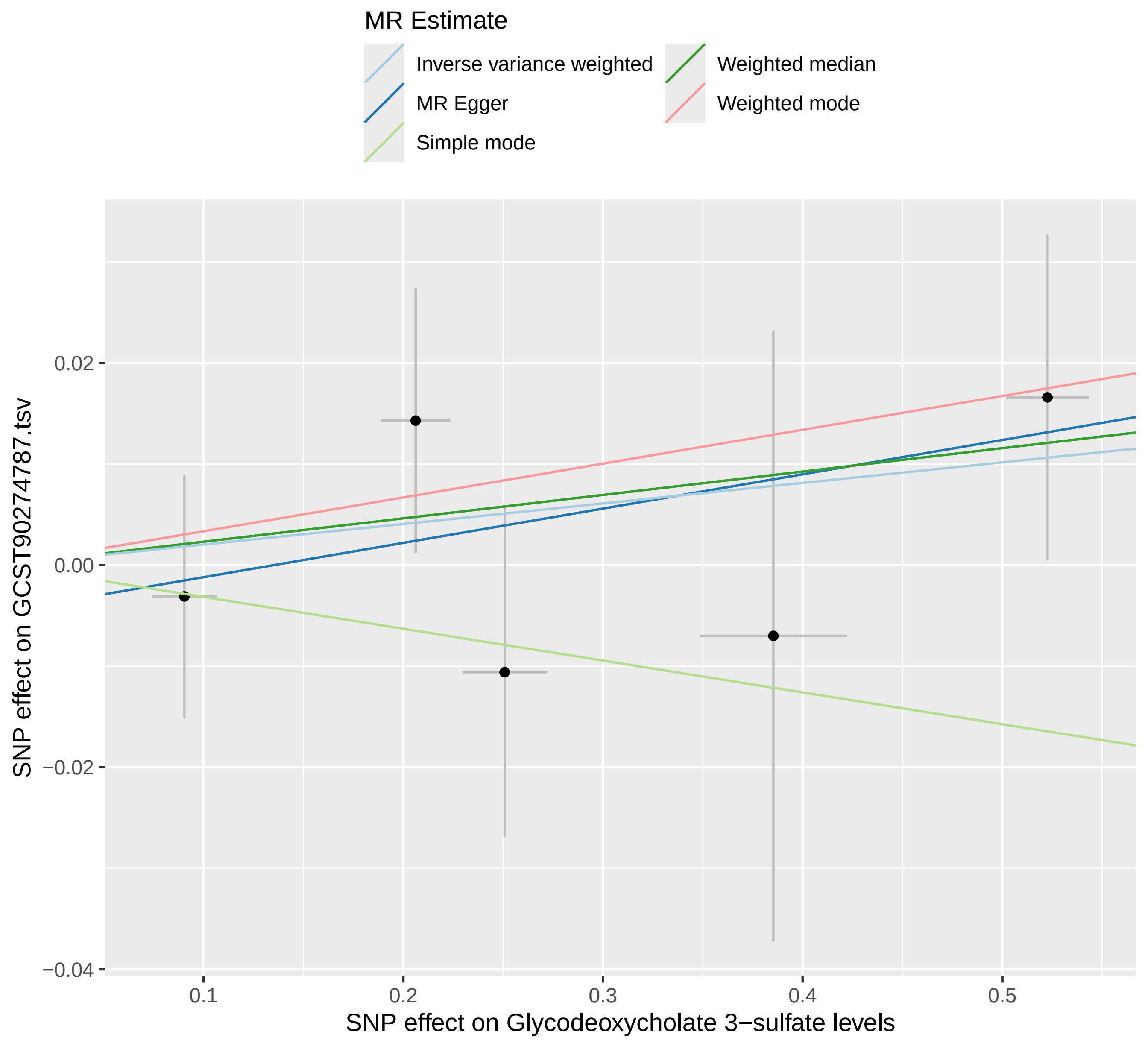
MR Estimate
Inverse variance weighted
MR Egger
Simple mode
Weighted median
Weighted mode
0.02
0.00
−0.02
−0.04
0.1
0.2
0.3
0.4
0.5
SNP effect on GCST90274787.tsv
SNP effect on Glycodeoxycholate 3−sulfate levels

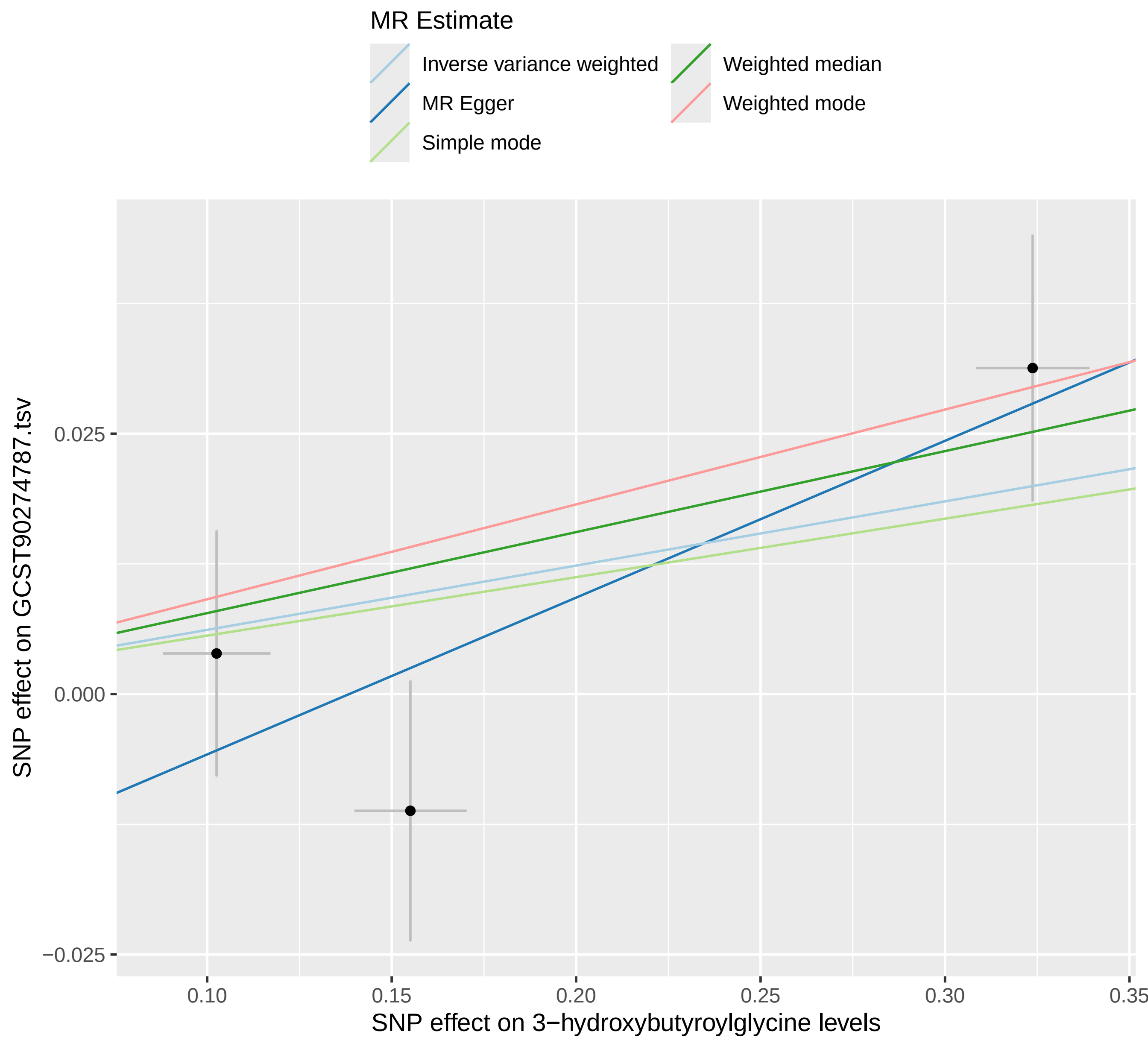
MR Estimate
Inverse variance weighted
MR Egger
Simple mode
Weighted median
Weighted mode
SNP effect on GCST90274787.tsv
0.025
0.000
−0.025
0.10
0.15
0.20
0.25
0.30
0.35
SNP effect on 3−hydroxybutyroylglycine levels

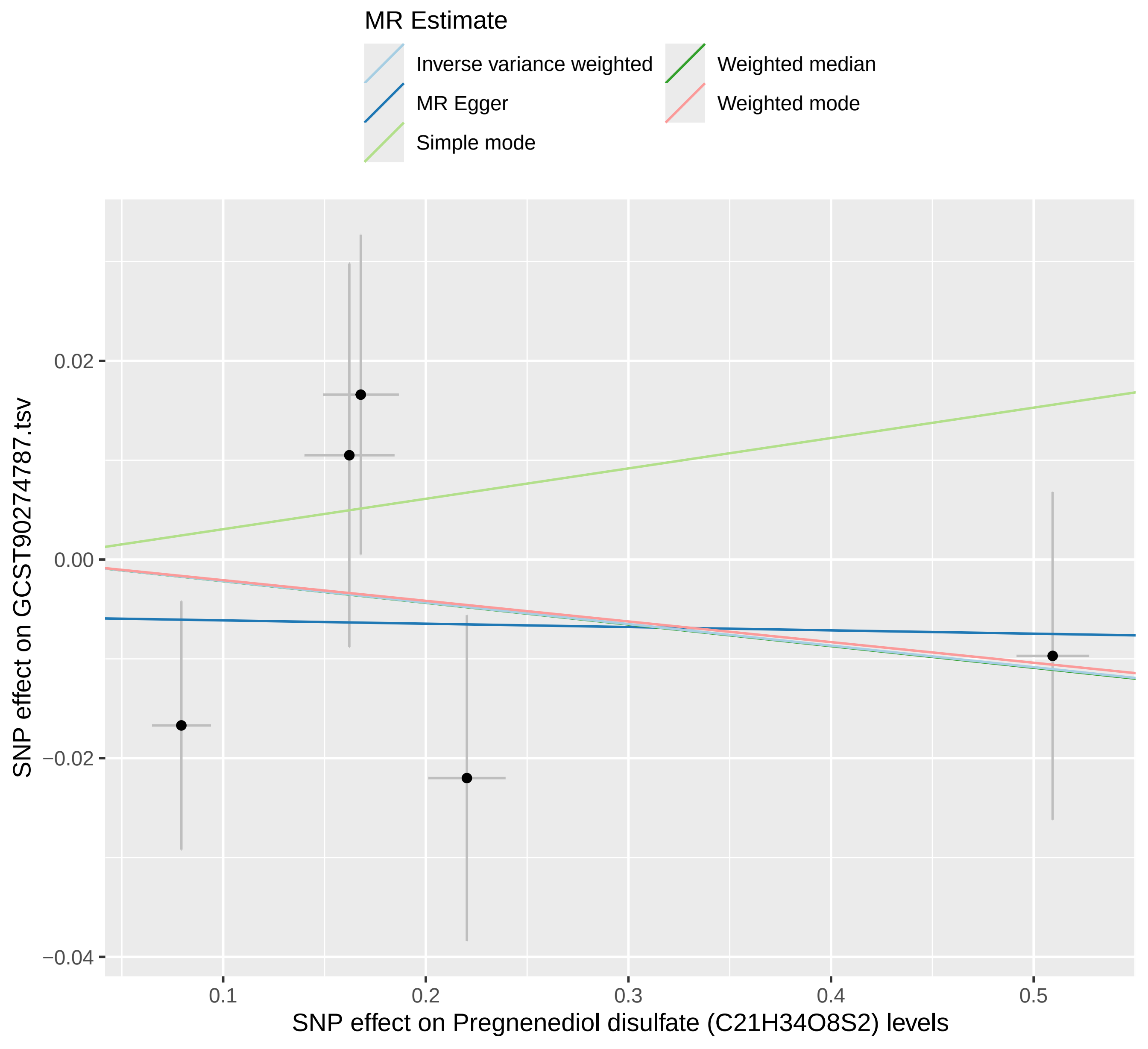

MR Estimate
Inverse variance weighted
MR Egger
Simple mode
Weighted median
Weighted mode
0.02
0.00
−0.02
−0.04
0.1
0.2
0.3
0.4
0.5
SNP effect on GCST90274787.tsv
SNP effect on Pregnenediol disulfate (C21H34O8S2) levels

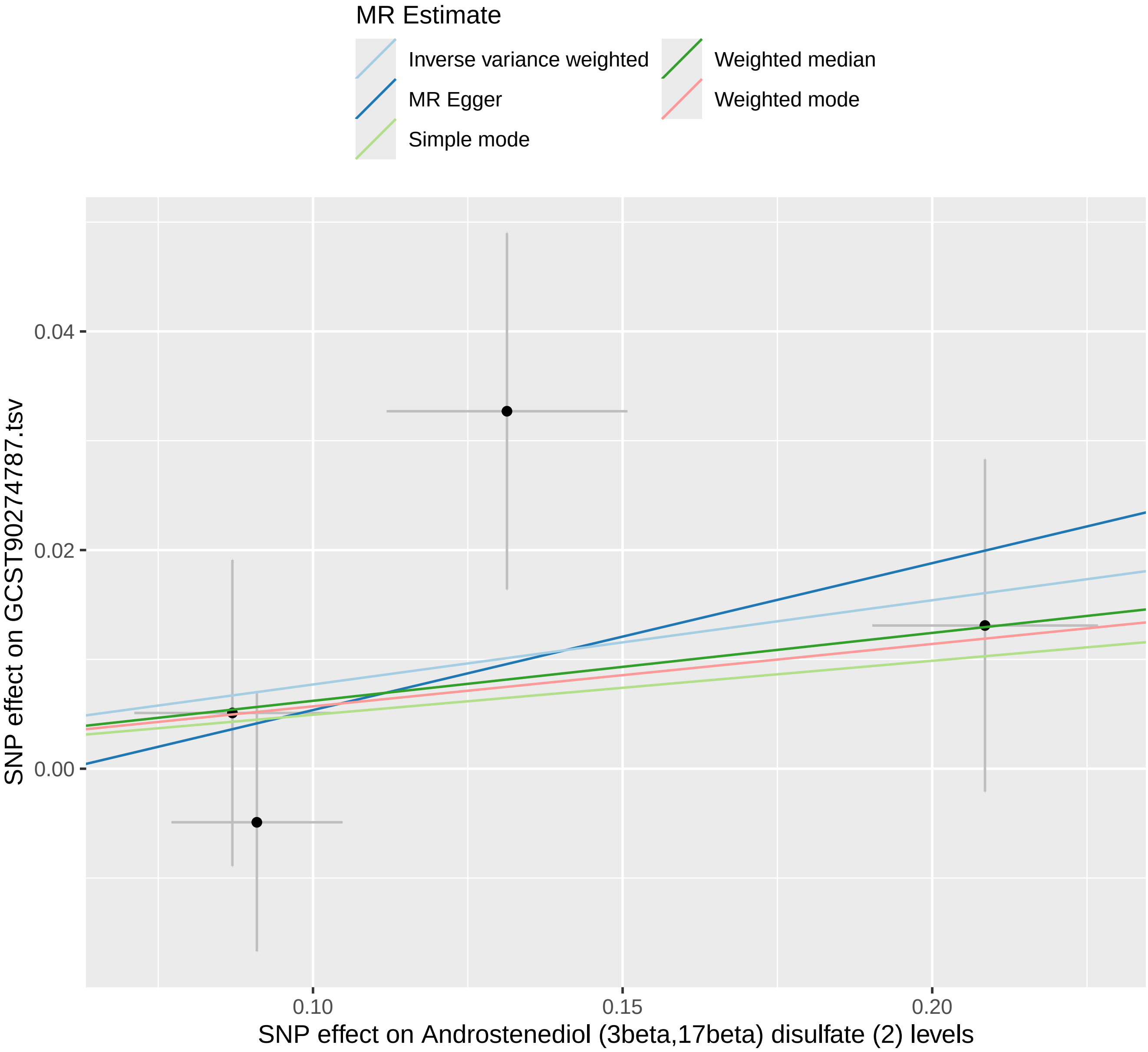

MR Estimate
Inverse variance weighted
MR Egger
Simple mode
Weighted median
Weighted mode
0.04
0.02
0.00
0.10
0.15
0.20
SNP effect on GCST90274787.tsv
SNP effect on Androstenediol (3beta,17beta) disulfate (2) levels

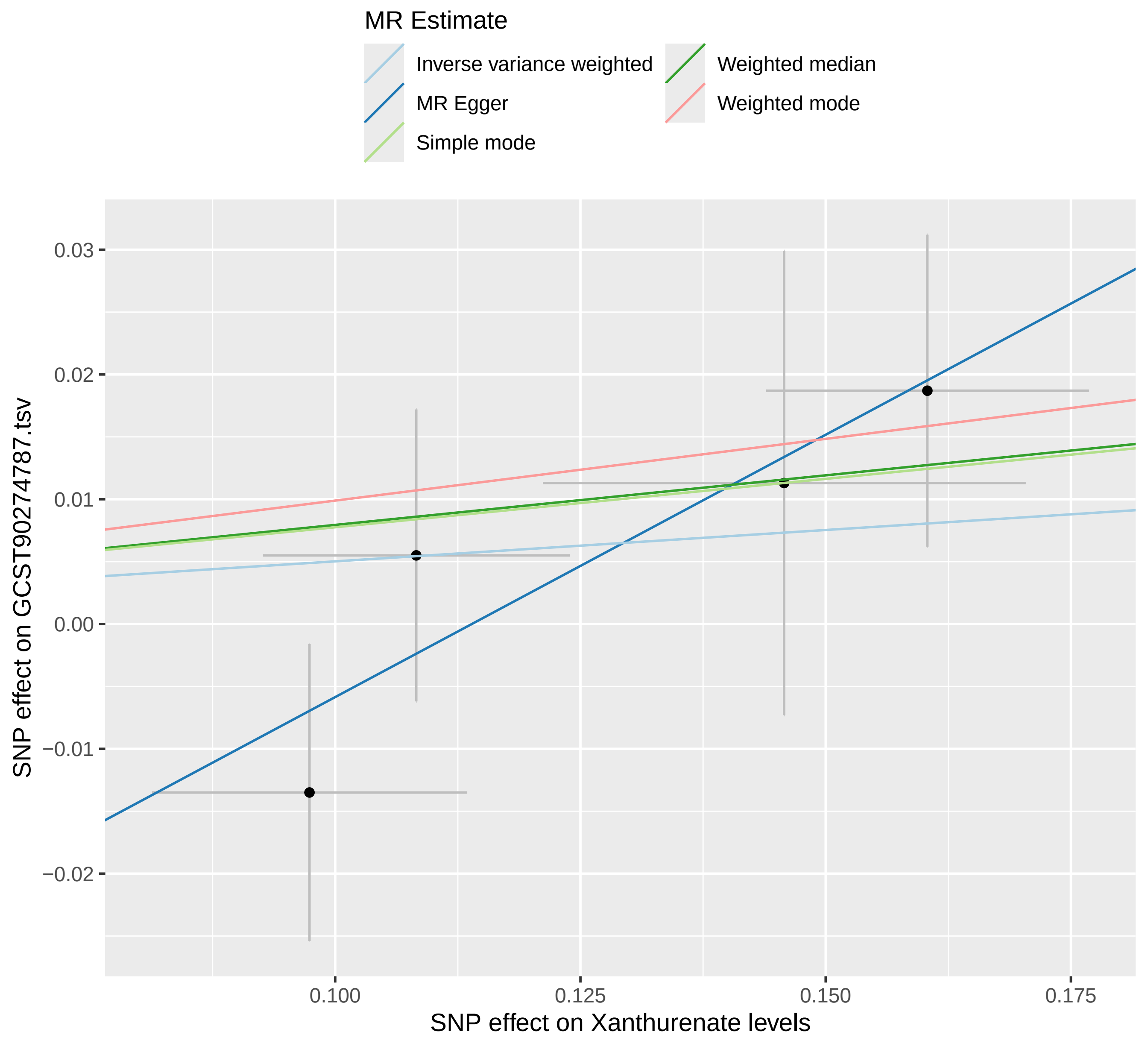

MR Estimate
Inverse variance weighted
MR Egger
Simple mode
Weighted median
Weighted mode
0.03
0.02
0.01
0.00
−0.01
−0.02
SNP effect on GCST90274787.tsv
0.100
0.125
0.150
0.175
SNP effect on Xanthurenate levels

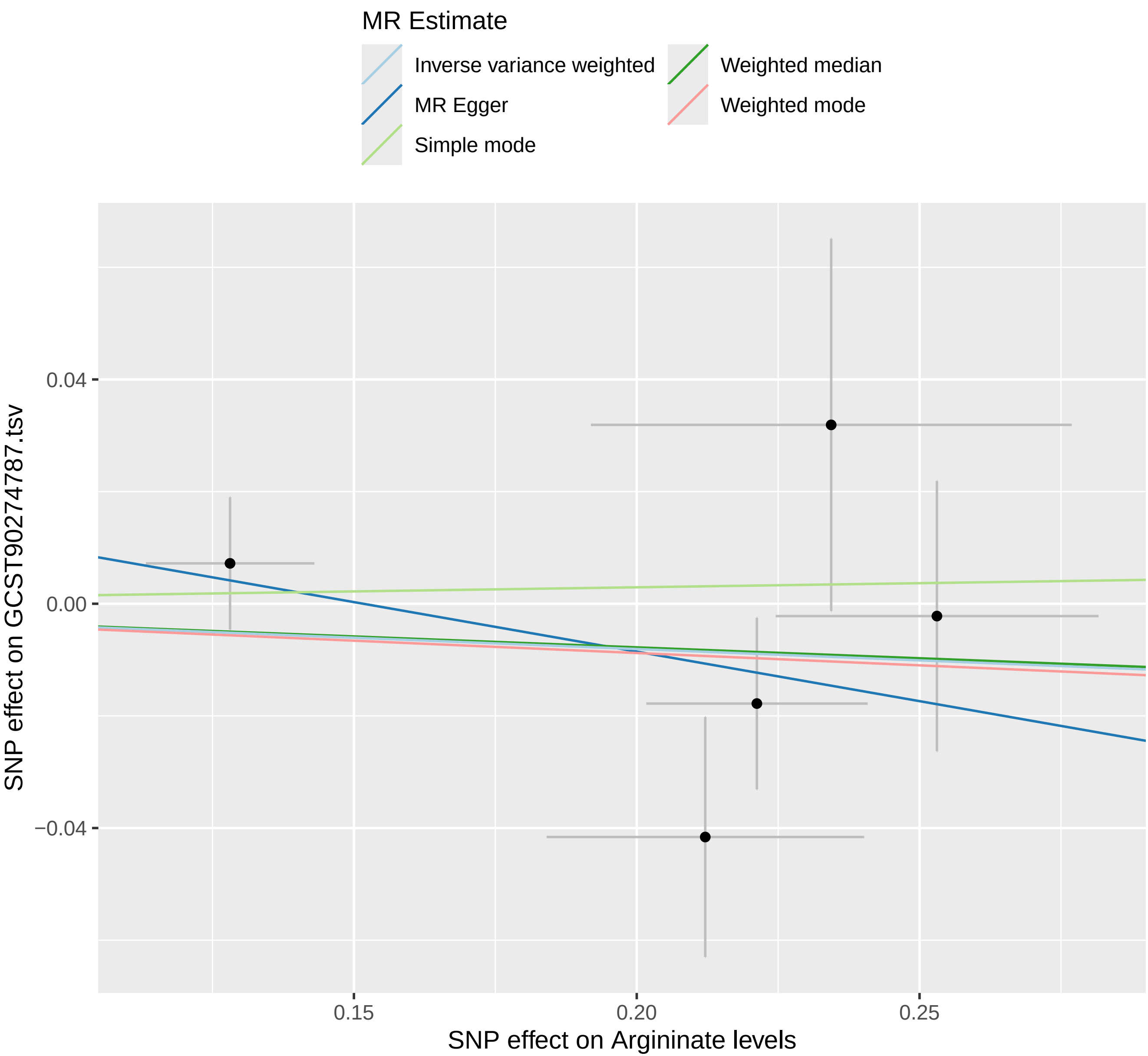
MR Estimate
Inverse variance weighted
MR Egger
Simple mode
Weighted median
Weighted mode
0.04
0.00
−0.04
SNP effect on GCST90274787.tsv
0.15
0.20
0.25
SNP effect on Argininate levels

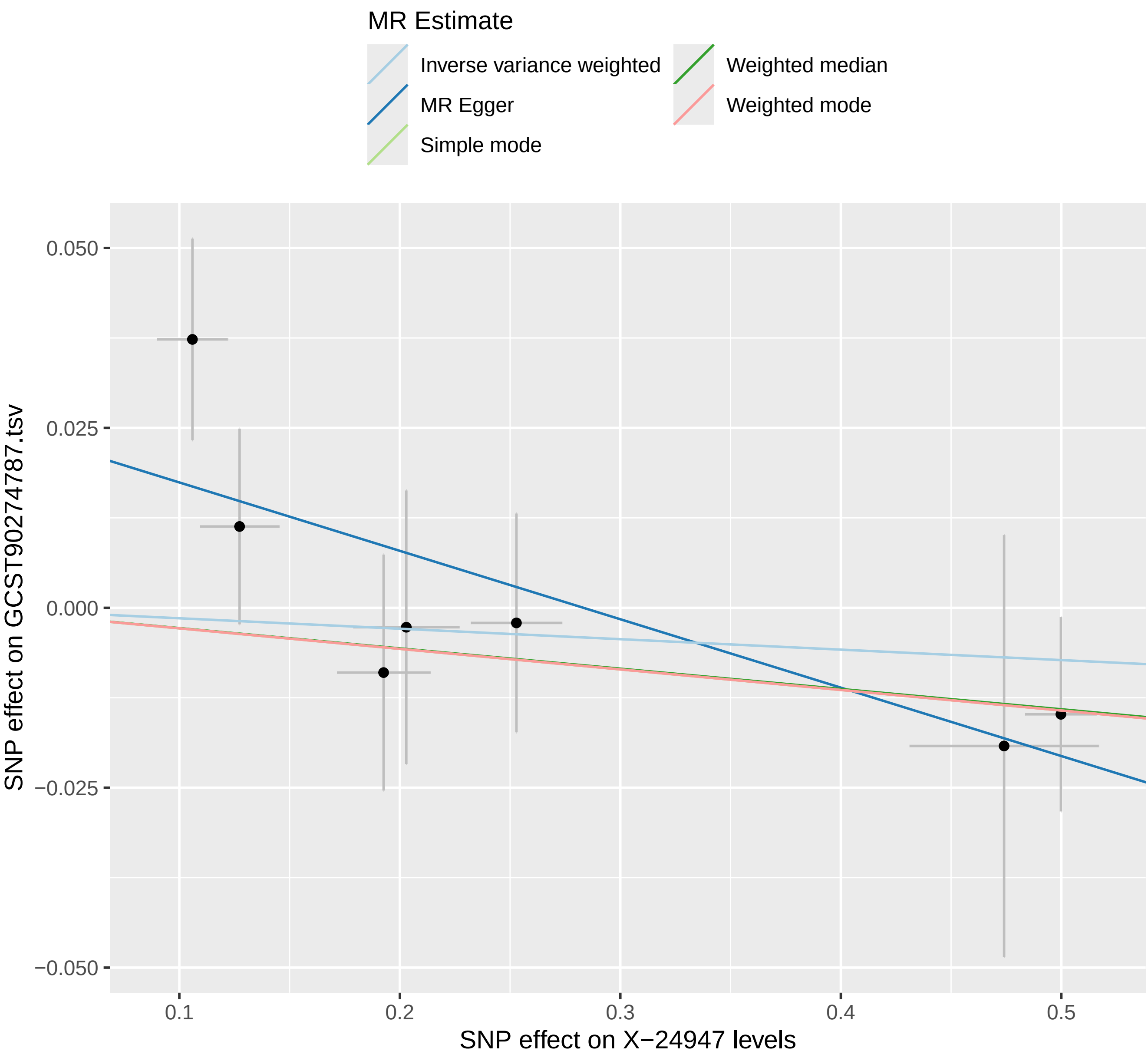

MR Estimate
Inverse variance weighted
MR Egger
Simple mode
Weighted median
Weighted mode
0.050
0.025
0.000
−0.025
−0.050
SNP effect on GCST90274787.tsv
0.1
0.2
0.3
0.4
0.5
SNP effect on X−24947 levels

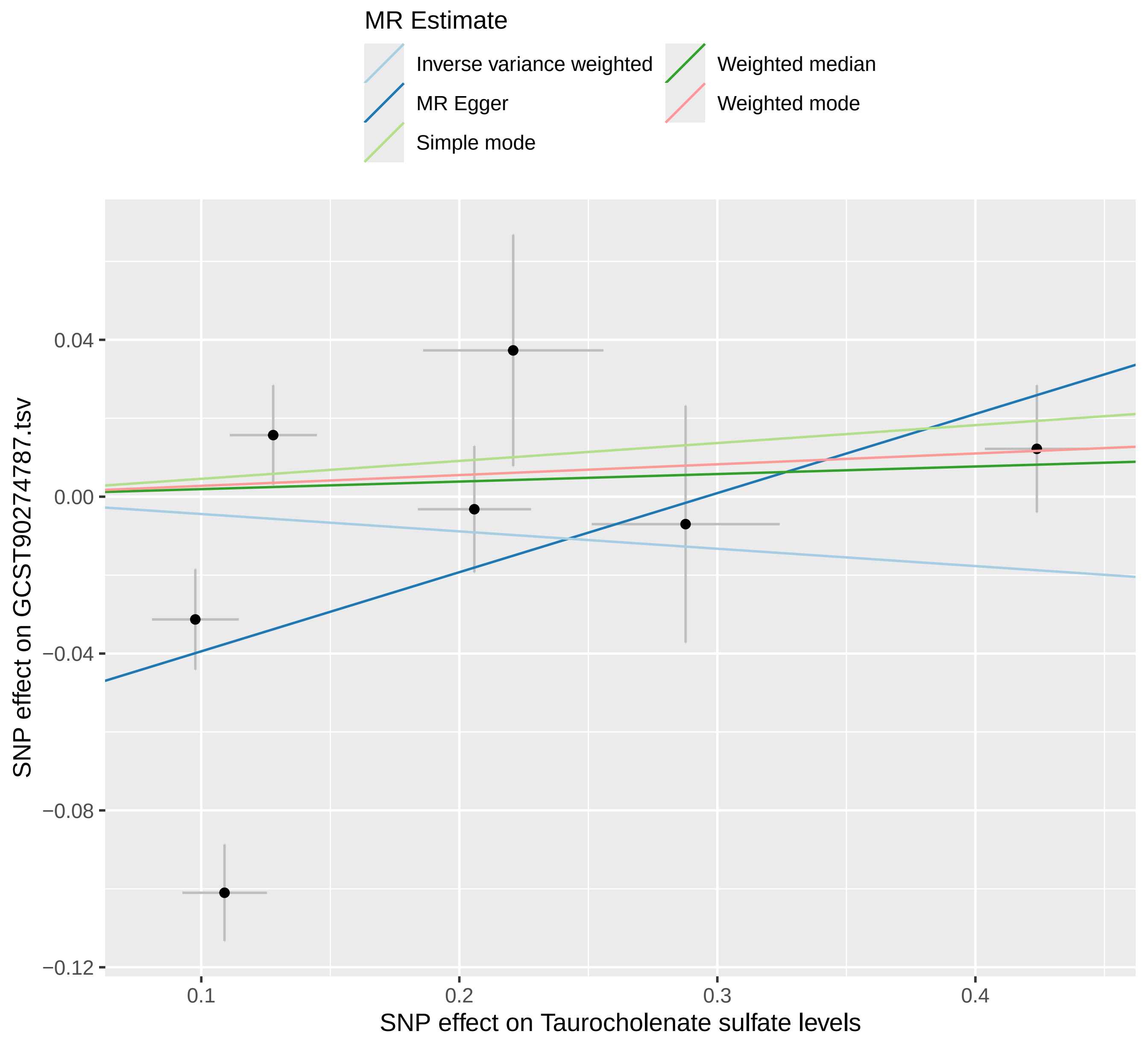
MR Estimate
Inverse variance weighted
MR Egger
Simple mode
Weighted median
Weighted mode
SNP effect on GCST90274787.tsv
0.04
0.00
−0.04
−0.08
−0.12
0.1
0.2
0.3
0.4
SNP effect on Taurocholenate sulfate levels

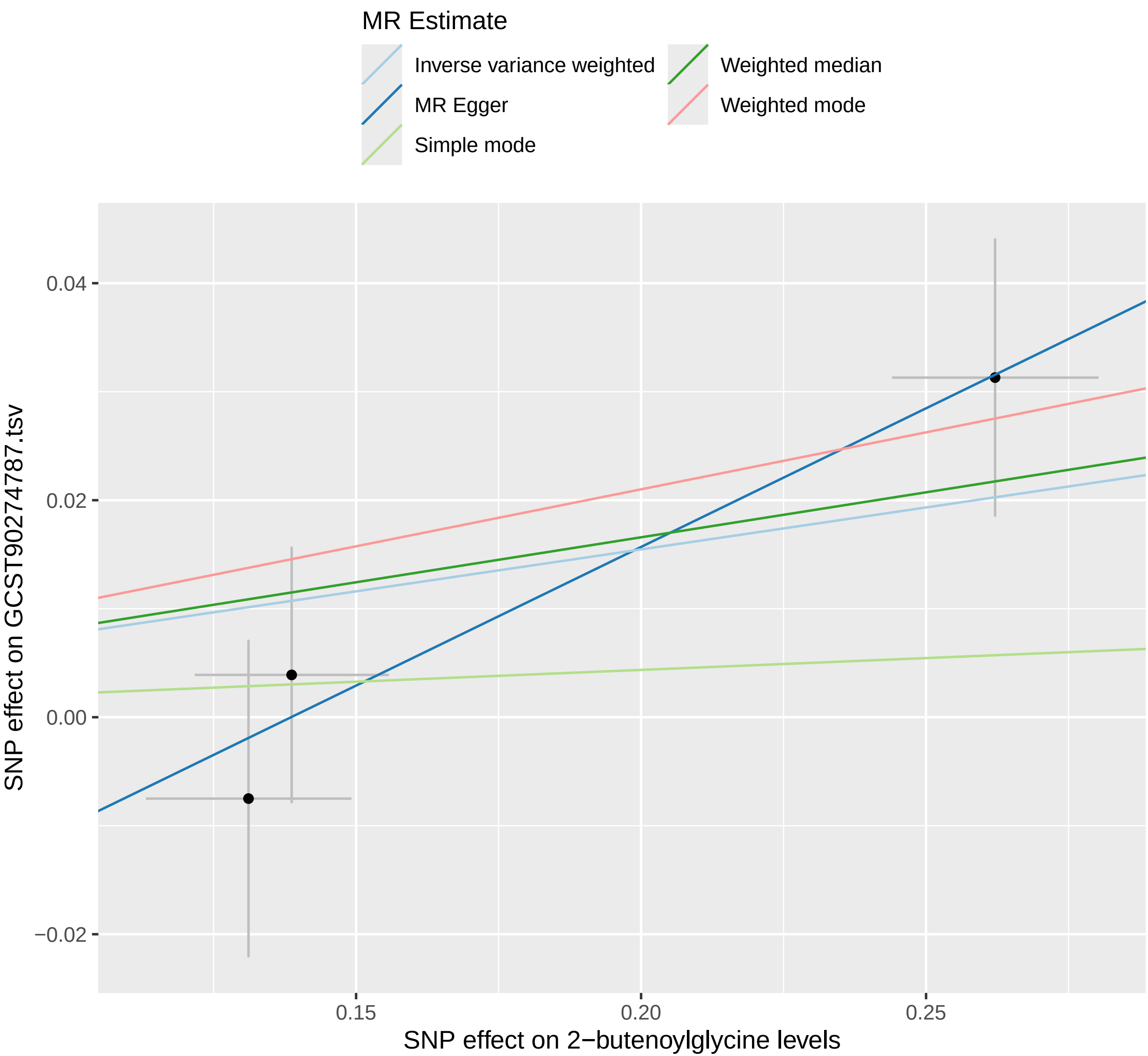
MR Estimate
Inverse variance weighted
MR Egger
Simple mode
Weighted median
Weighted mode
0.04
0.02
0.00
−0.02
0.15
0.20
0.25
SNP effect on GCST90274787.tsv
SNP effect on 2−butenoylglycine levels

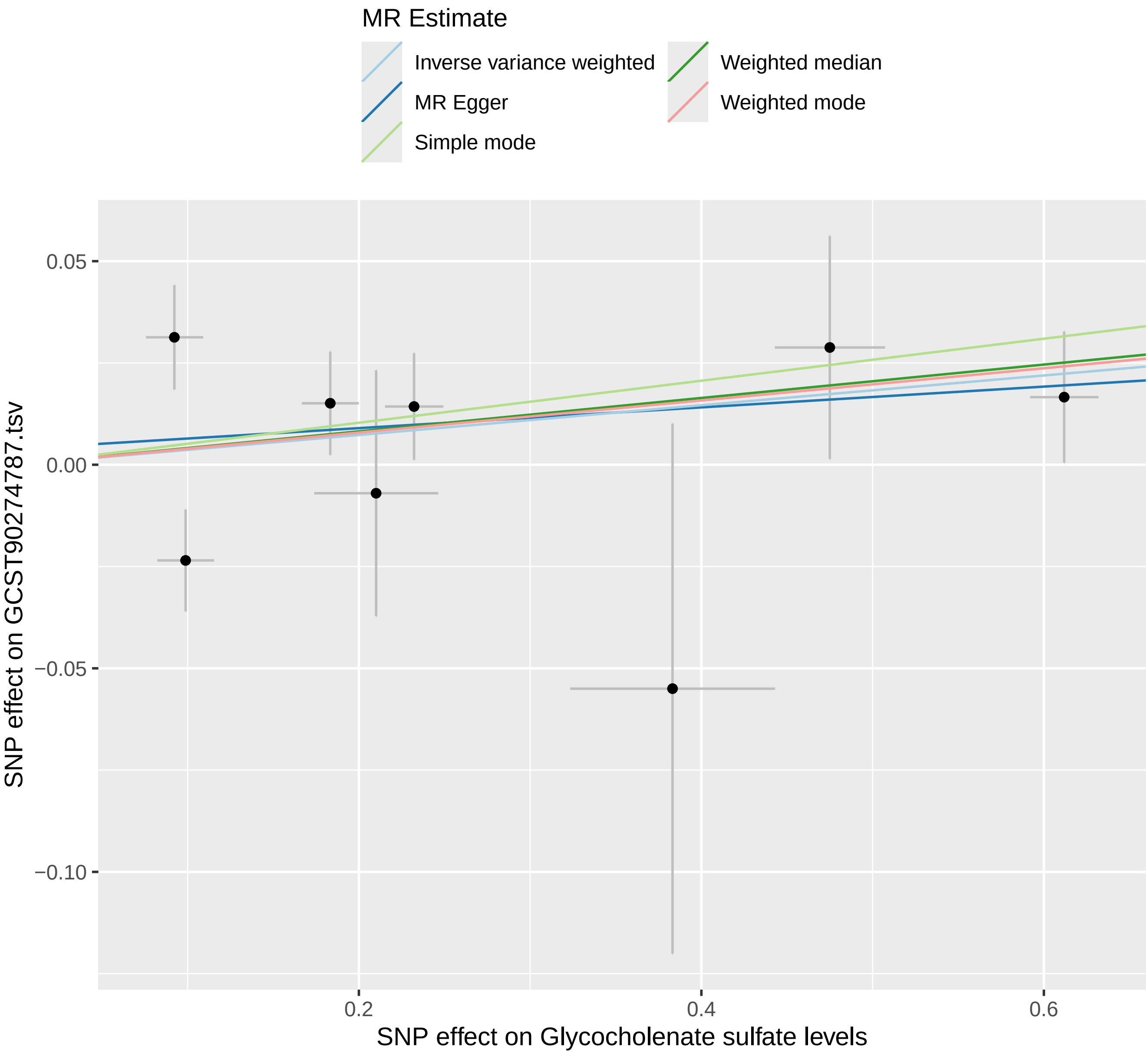
MR Estimate
Inverse variance weighted
MR Egger
Simple mode
Weighted median
Weighted mode
0.05
0.00
−0.05
−0.10
0.2
0.4
0.6
SNP effect on GCST90274787.tsv
SNP effect on Glycocholenate sulfate levels

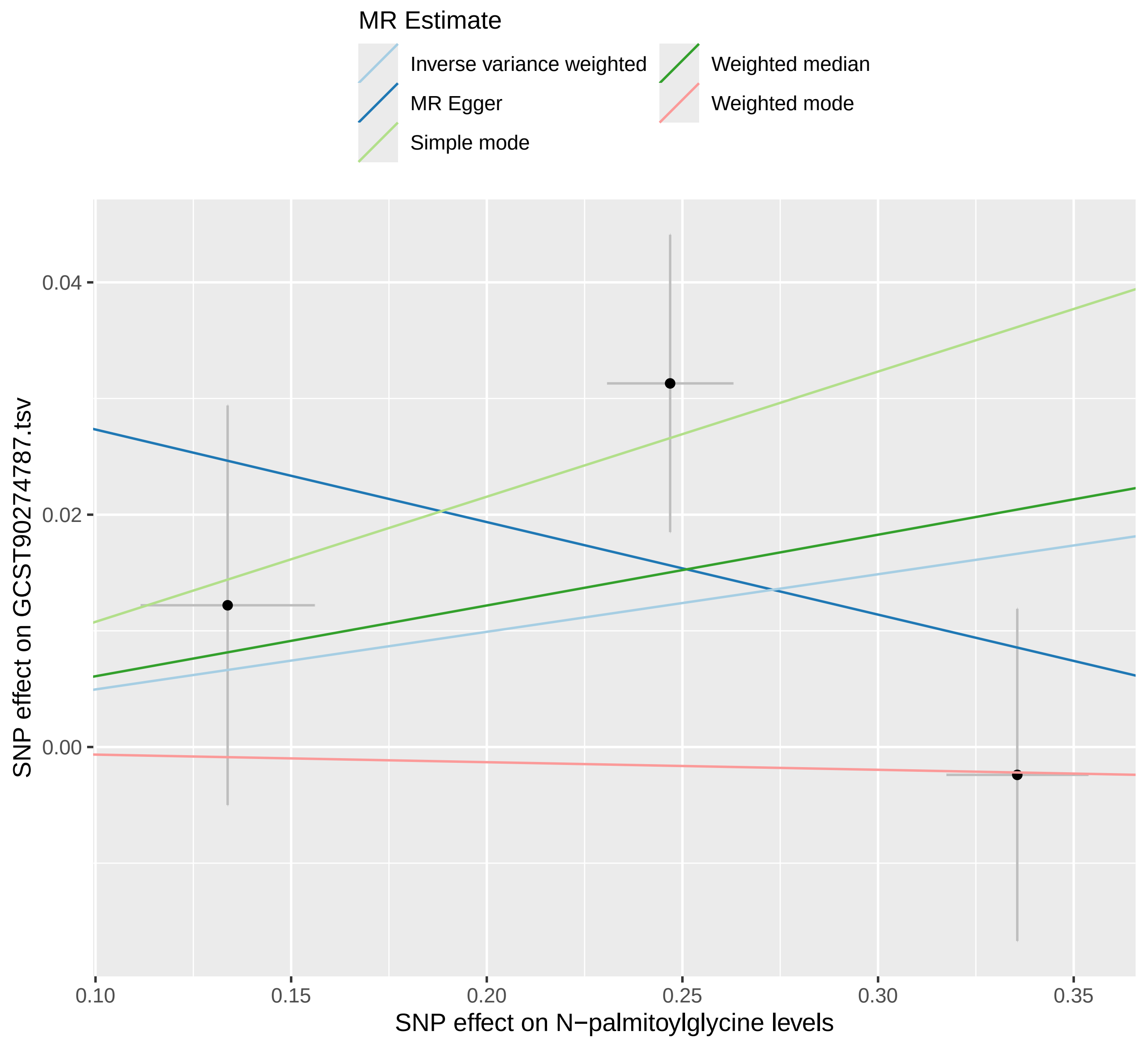
MR Estimate
Inverse variance weighted
MR Egger
Simple mode
Weighted median
Weighted mode
0.04
0.02
0.00
0.10
0.15
0.20
0.25
0.30
0.35
SNP effect on GCST90274787.tsv
SNP effect on N−palmitoylglycine levels

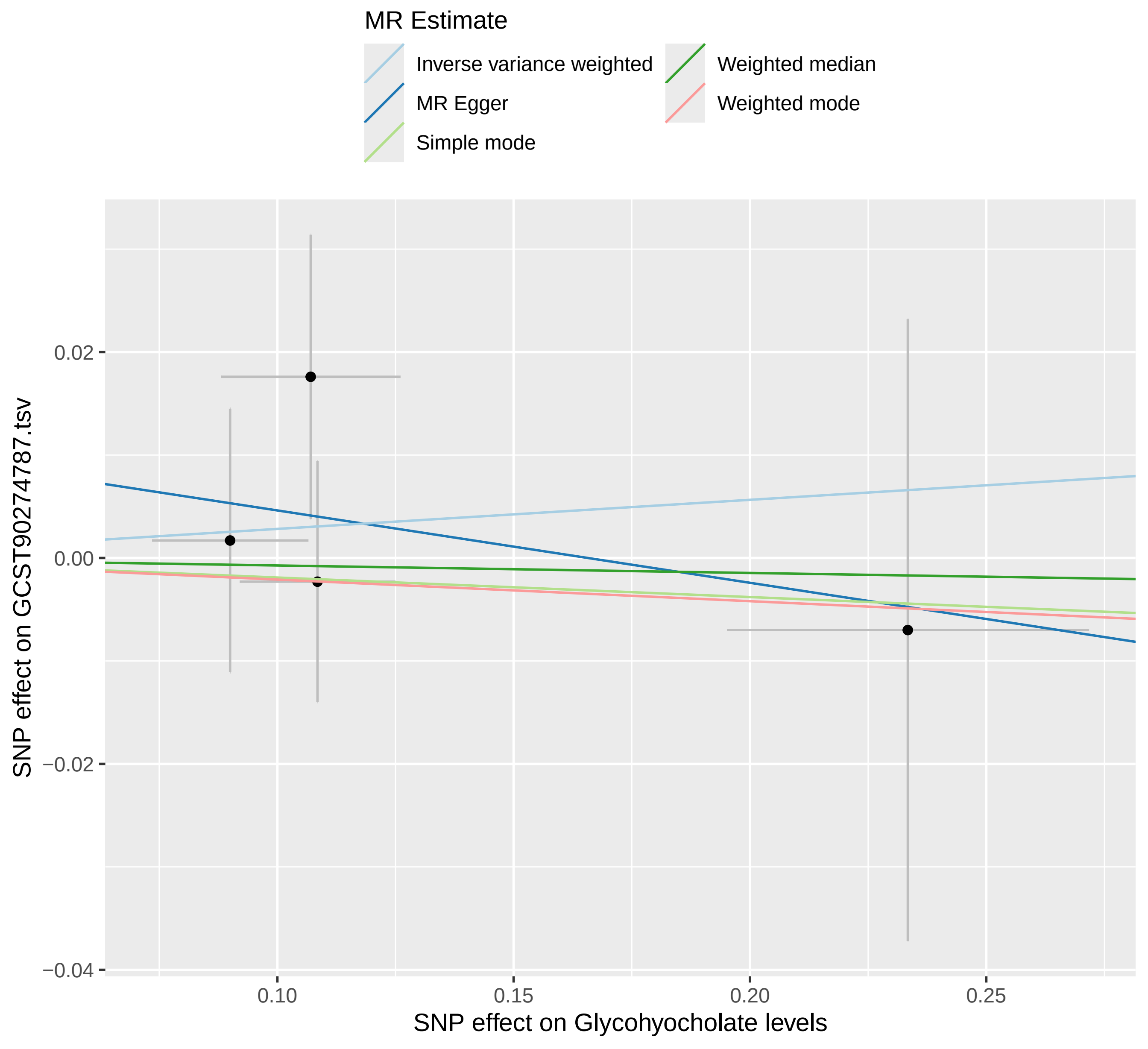
MR Estimate
Inverse variance weighted
MR Egger
Simple mode
Weighted median
Weighted mode
0.02
0.00
−0.02
−0.04
0.10
0.15
0.20
0.25
SNP effect on GCST90274787.tsv
SNP effect on Glycohyocholate levels

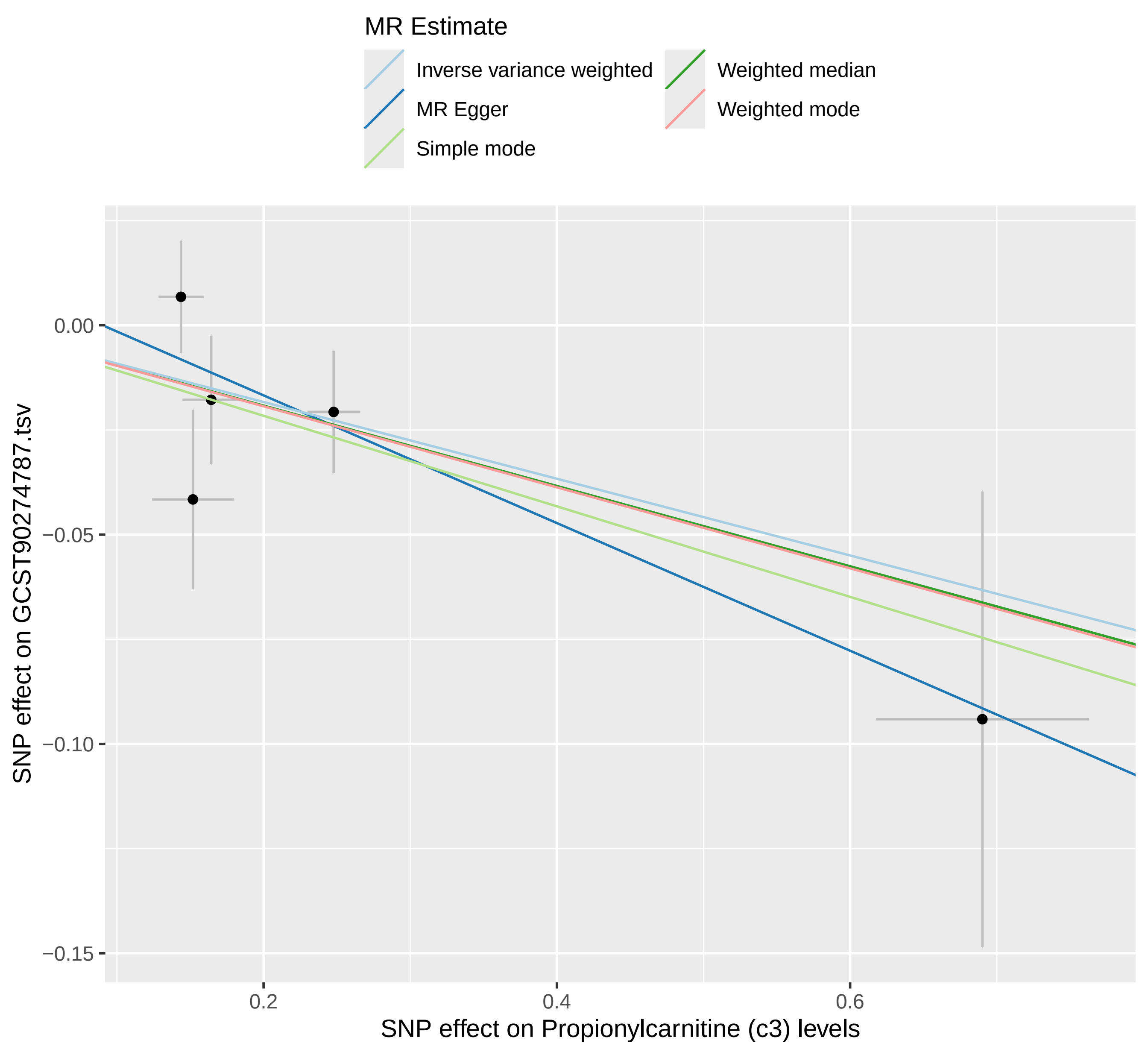
MR Estimate
Inverse variance weighted
MR Egger
Simple mode
Weighted median
Weighted mode
SNP effect on GCST90274787.tsv
0.00
−0.05
−0.10
−0.15
0.2
0.4
0.6
SNP effect on Propionylcarnitine (c3) levels

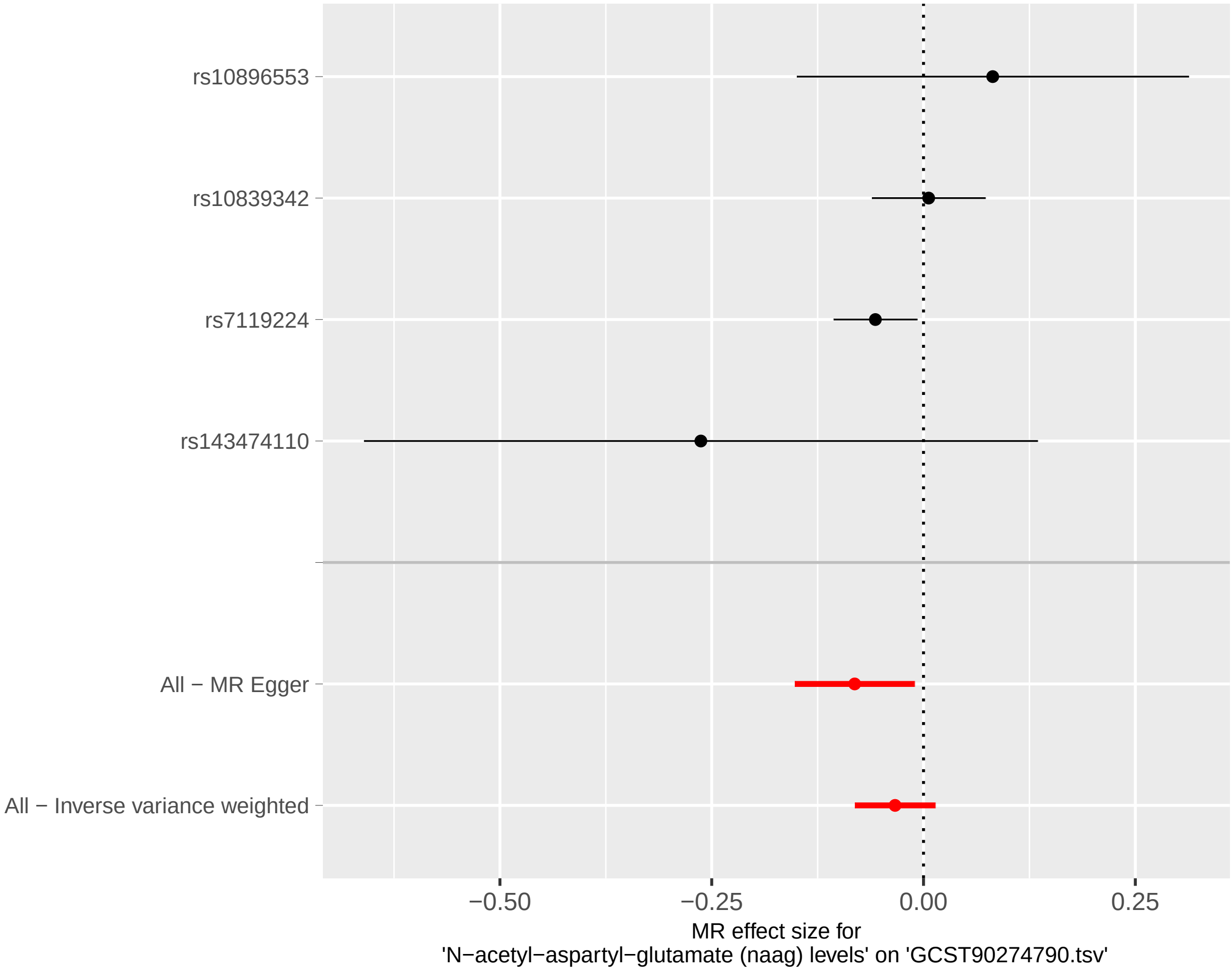
rs10896553
rs10839342
rs7119224
rs143474110
All − MR Egger
All − Inverse variance weighted
−0.50
−0.25
0.00
0.25
MR effect size for
'N−acetyl−aspartyl−glutamate (naag) levels' on 'GCST90274790.tsv'

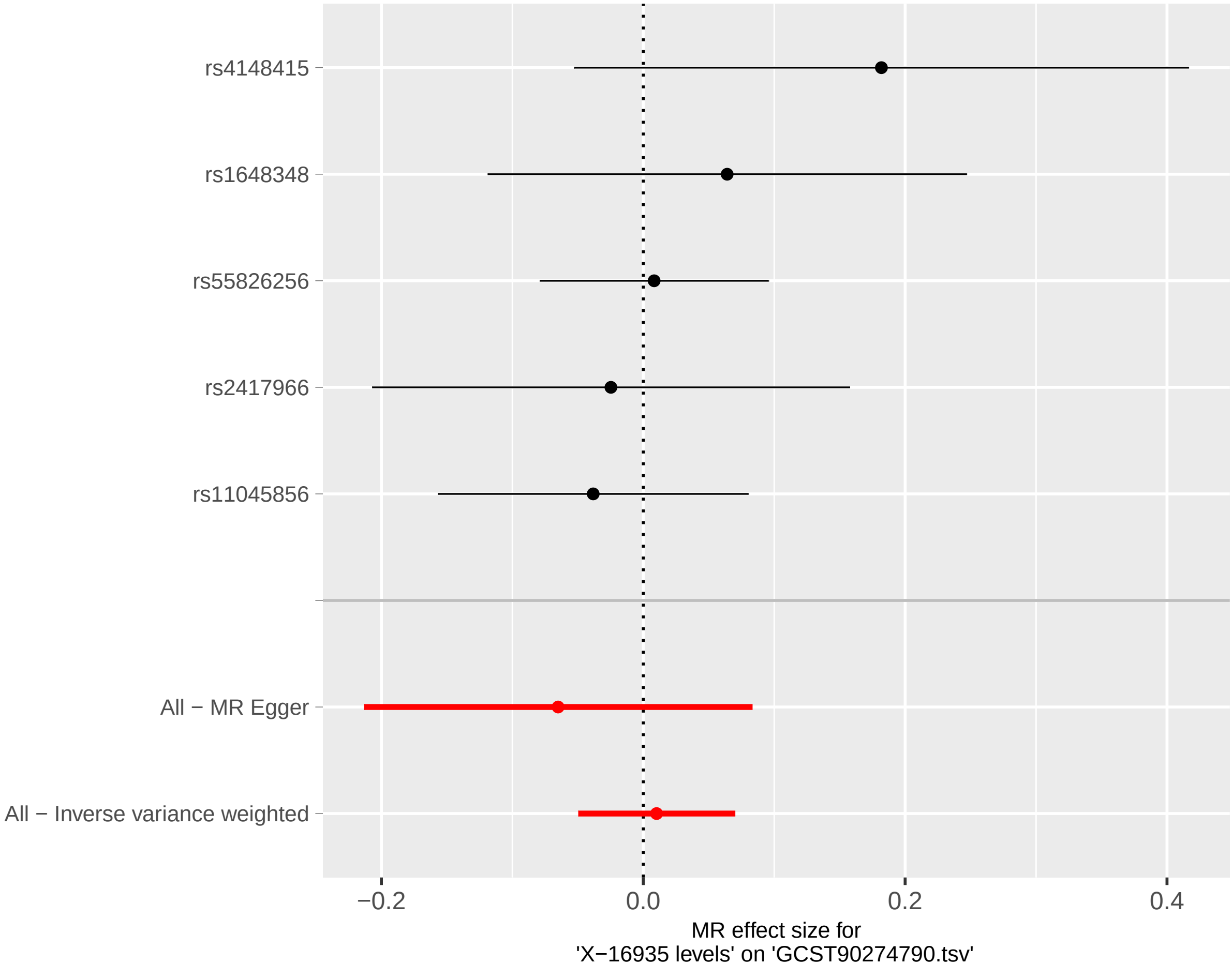
rs4148415
rs1648348
rs55826256
rs2417966
rs11045856
All − MR Egger
All − Inverse variance weighted
−0.2
0.0
0.2
0.4
MR effect size for
'X−16935 levels' on 'GCST90274790.tsv'

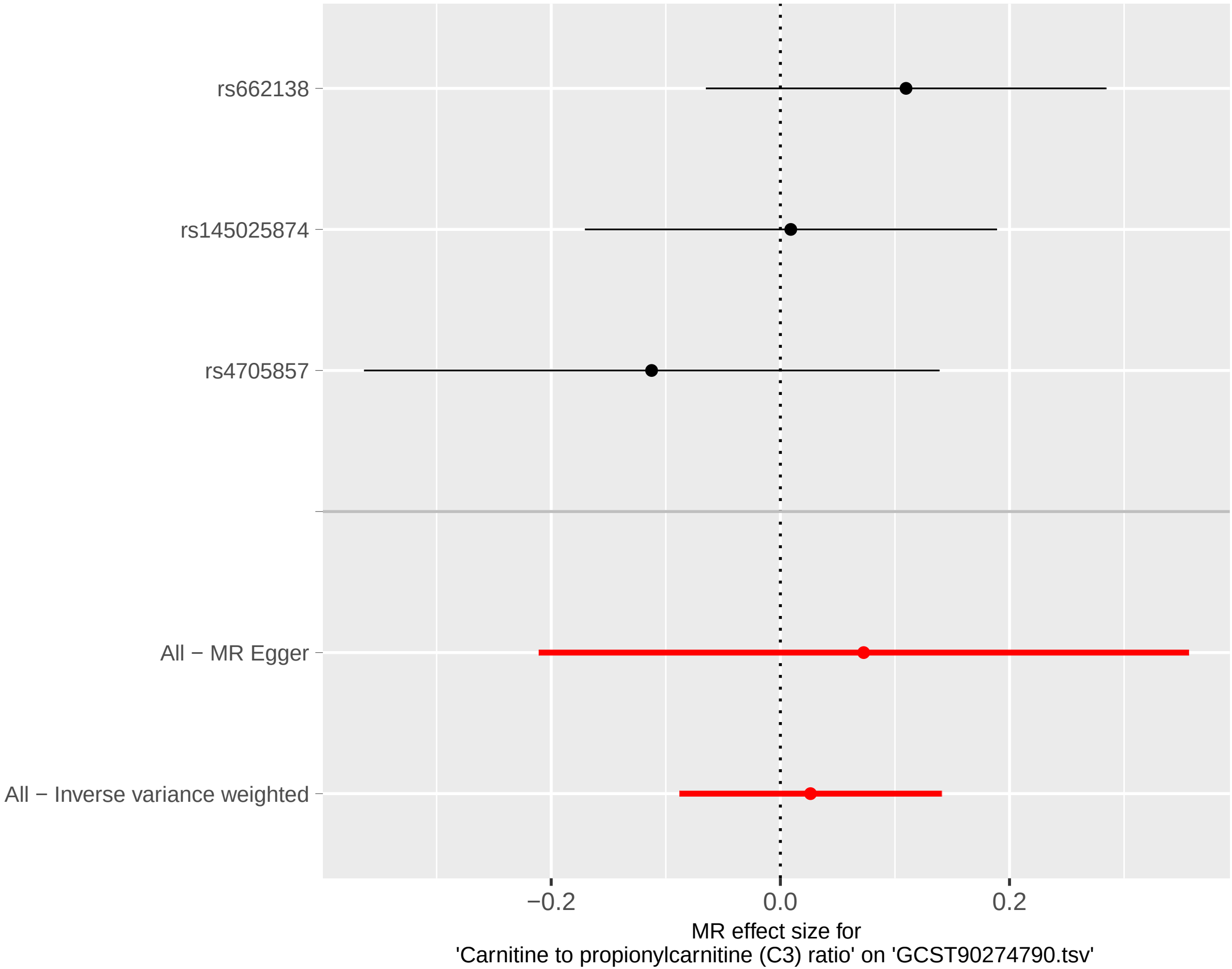

rs662138
rs145025874
rs4705857
All − MR Egger
All − Inverse variance weighted
−0.2
0.0
0.2
MR effect size for
'Carnitine to propionylcarnitine (C3) ratio' on 'GCST90274790.tsv'

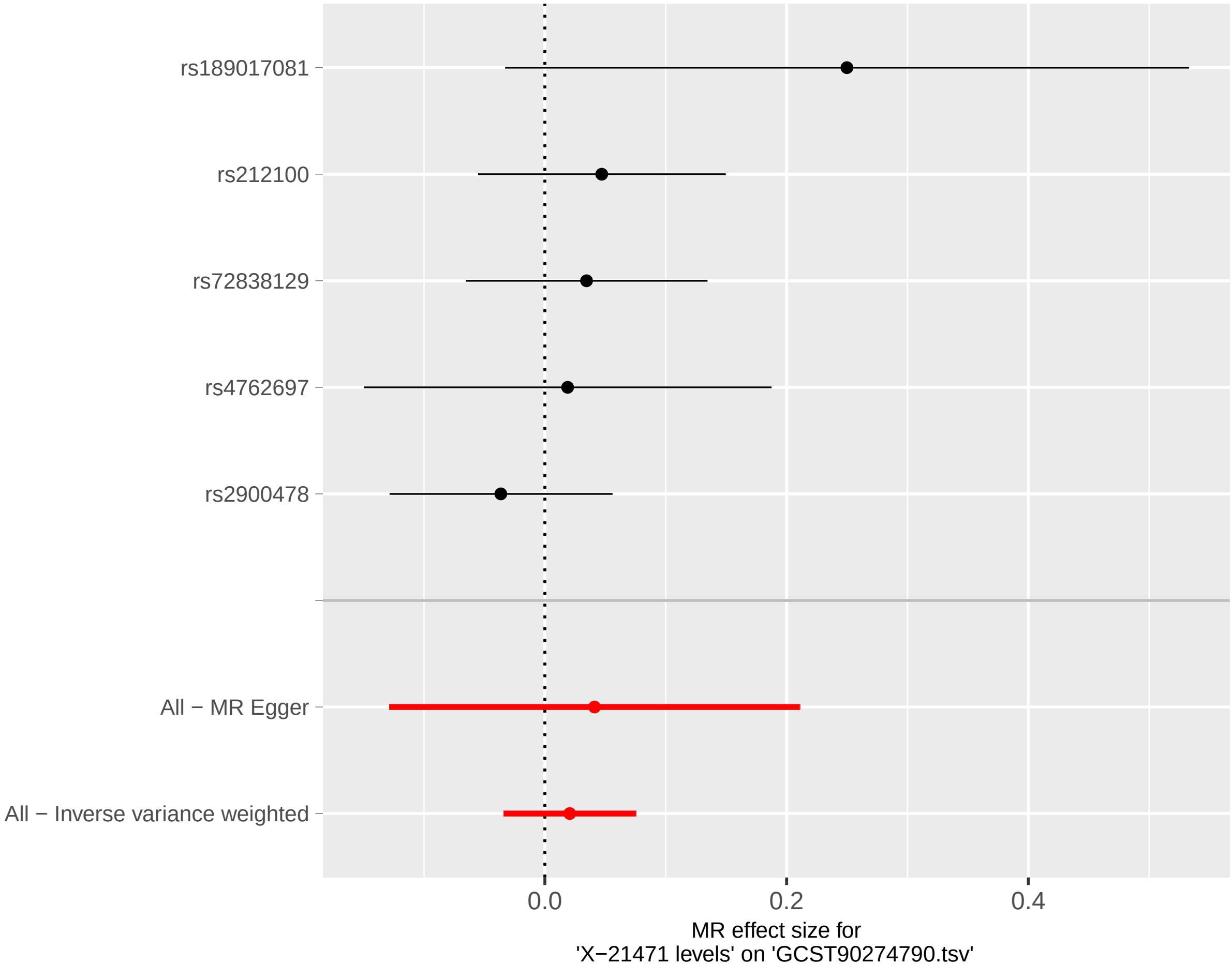

rs189017081
rs212100
rs72838129
rs4762697
rs2900478
All − MR Egger
All − Inverse variance weighted
0.0
0.2
0.4
MR effect size for
'X−21471 levels' on 'GCST90274790.tsv'

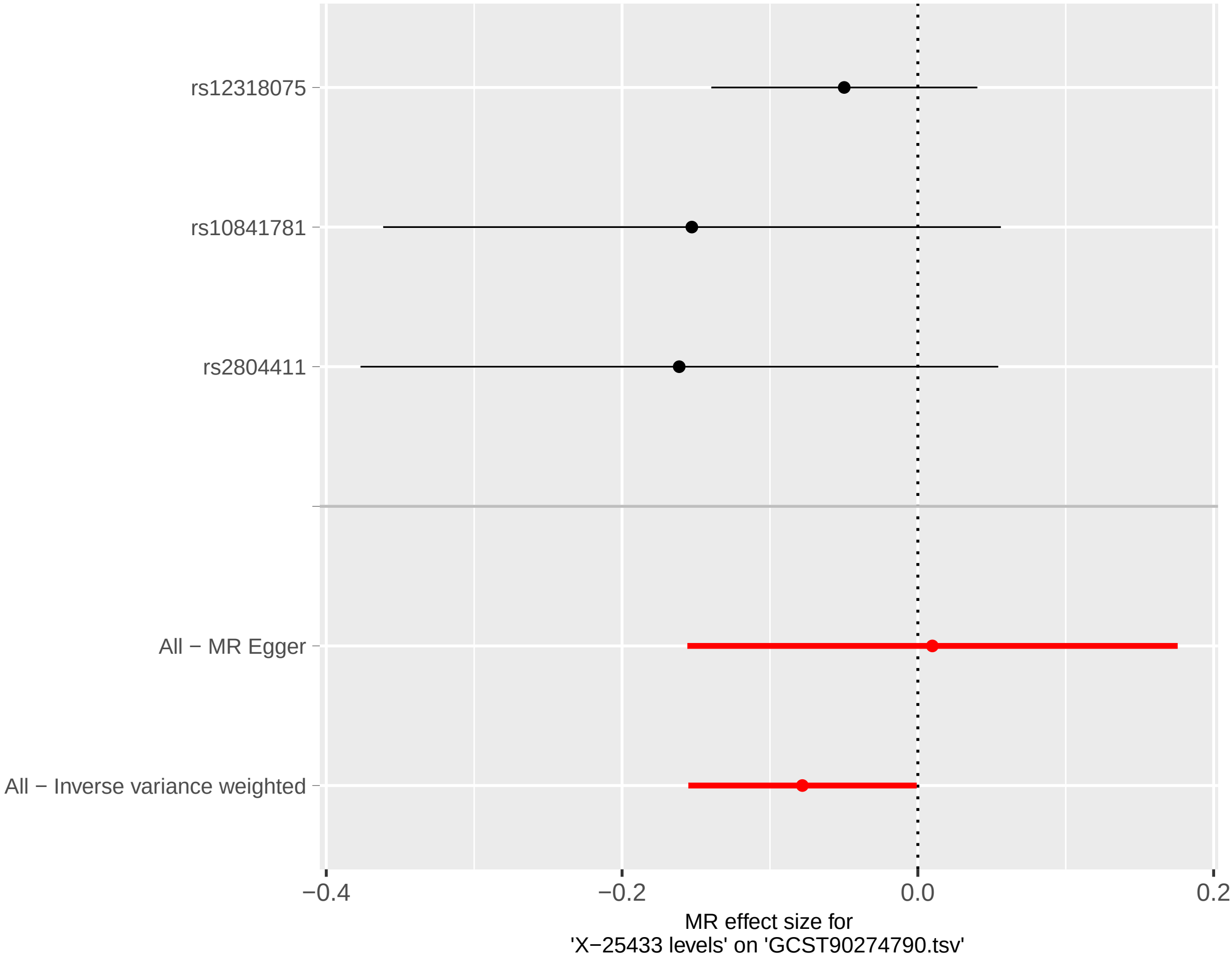
rs12318075
rs10841781
rs2804411
All − MR Egger
All − Inverse variance weighted
−0.4
−0.2
0.0
0.2
MR effect size for
'X−25433 levels' on 'GCST90274790.tsv'

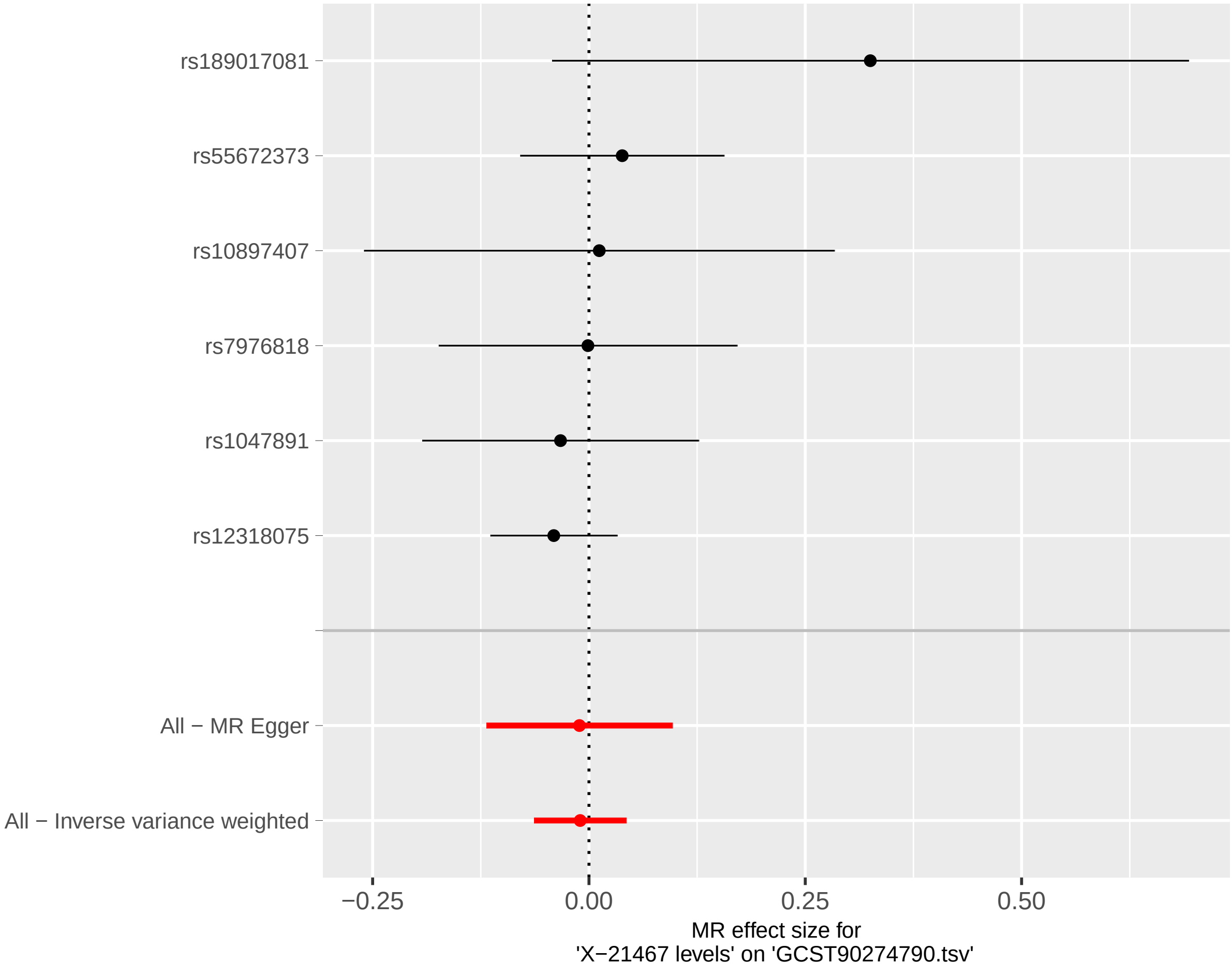

rs189017081
rs55672373
rs10897407
rs7976818
rs1047891
rs12318075
All − MR Egger
All − Inverse variance weighted
−0.25
0.00
0.25
0.50
MR effect size for
'X−21467 levels' on 'GCST90274790.tsv'

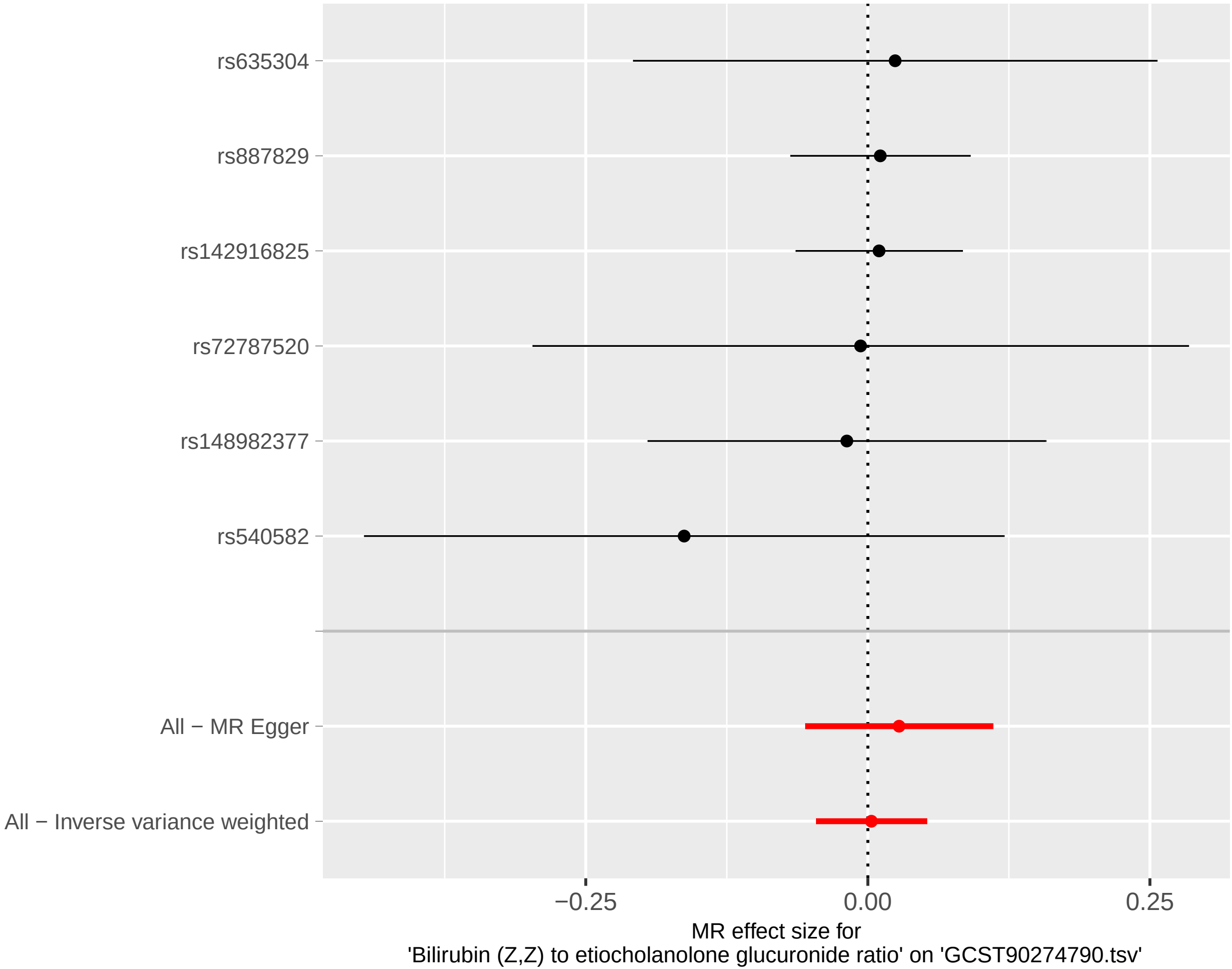
rs635304
rs887829
rs142916825
rs72787520
rs148982377
rs540582
All − MR Egger
All − Inverse variance weighted
−0.25
0.00
0.25
MR effect size for
'Bilirubin (Z,Z) to etiocholanolone glucuronide ratio' on 'GCST90274790.tsv'

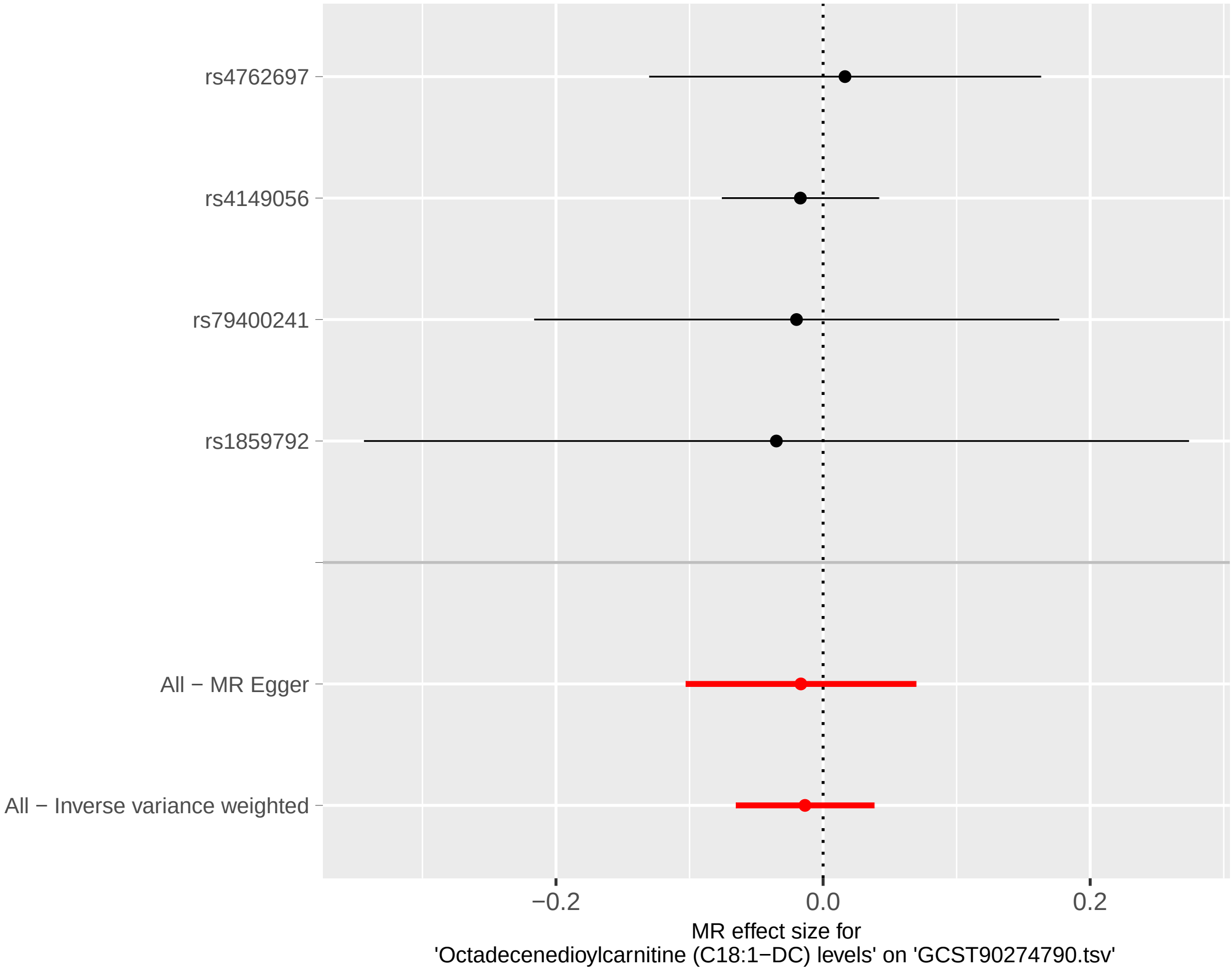
rs4762697
rs4149056
rs79400241
rs1859792
All − MR Egger
All − Inverse variance weighted
−0.2
0.0
0.2
MR effect size for
'Octadecenedioylcarnitine (C18:1−DC) levels' on 'GCST90274790.tsv'

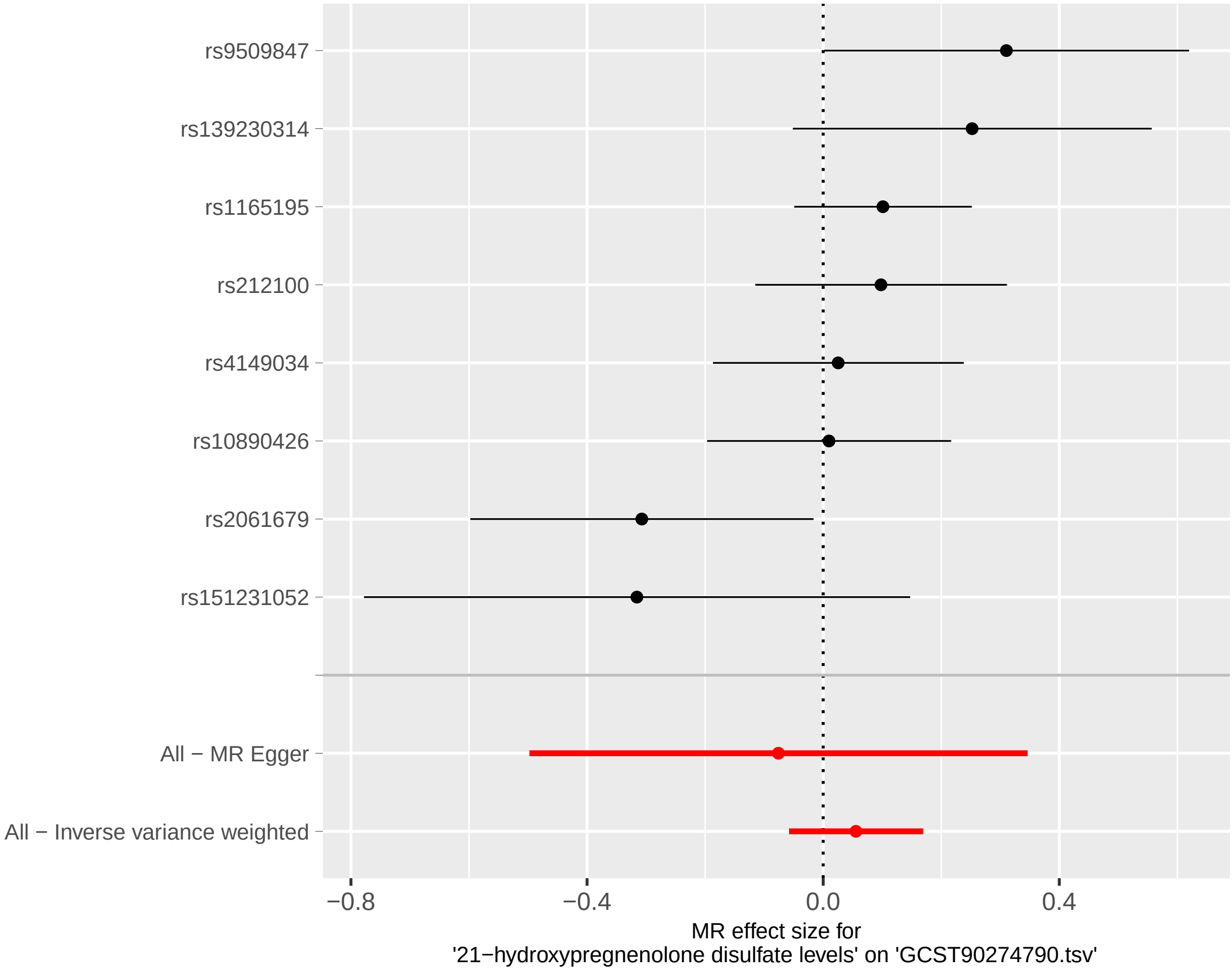
rs9509847
rs139230314
rs1165195
rs212100
rs4149034
rs10890426
rs2061679
rs151231052
All − MR Egger
All − Inverse variance weighted
−0.8
−0.4
0.0
0.4
MR effect size for
'21−hydroxypregnenolone disulfate levels' on 'GCST90274790.tsv'

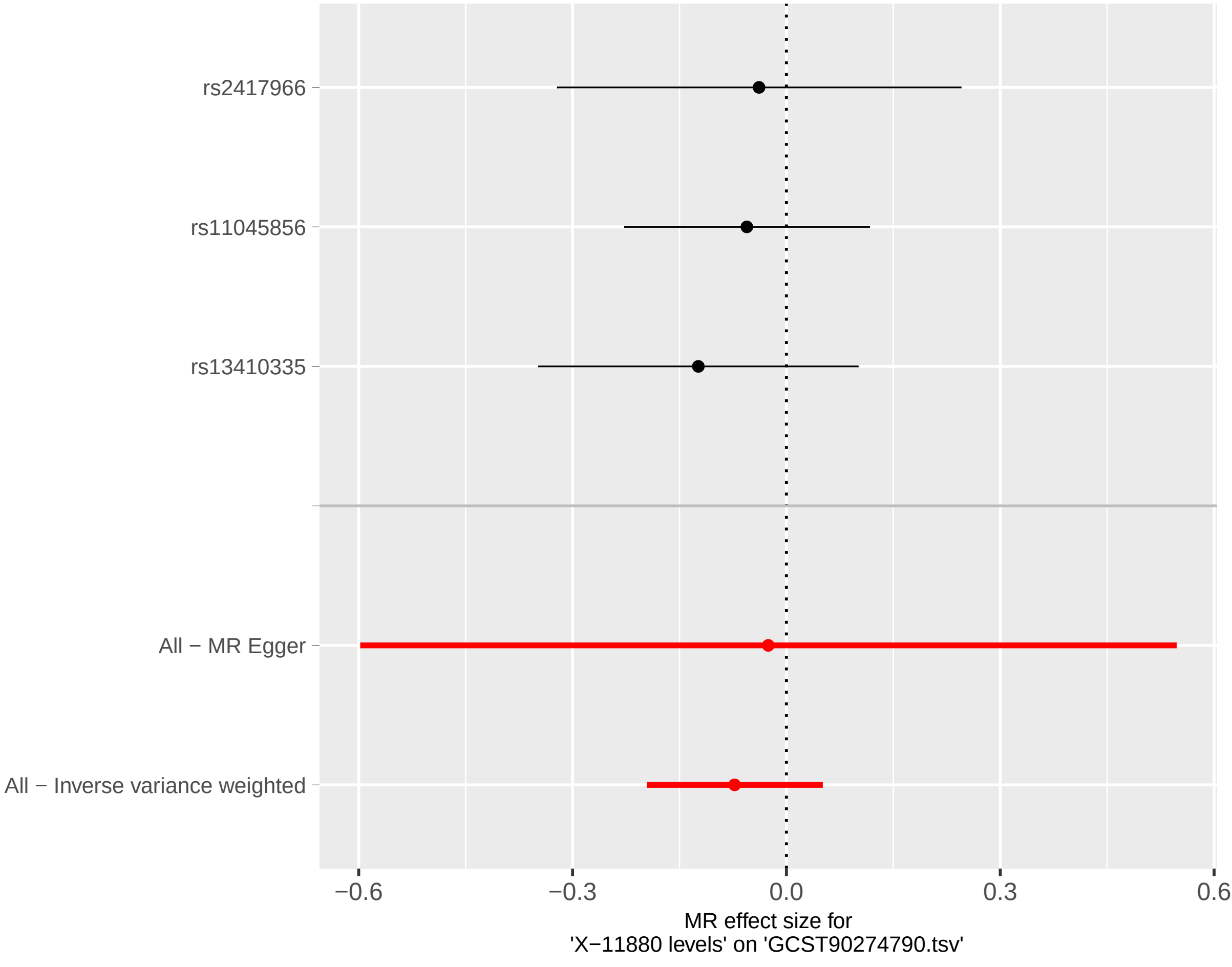
rs2417966
rs11045856
rs13410335
All − MR Egger
All − Inverse variance weighted
−0.6
−0.3
0.0
0.3
0.6
MR effect size for
'X−11880 levels' on 'GCST90274790.tsv'

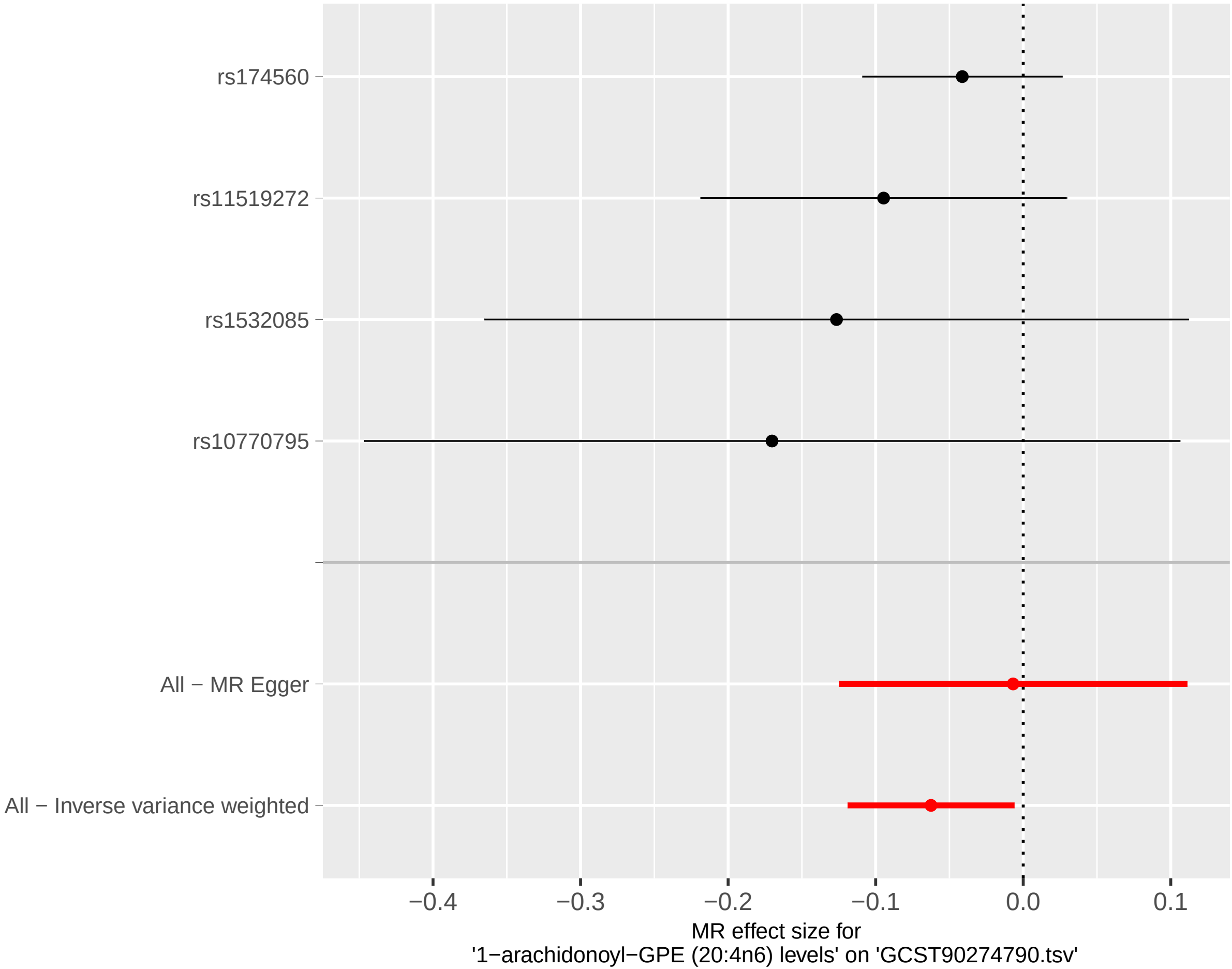
rs174560
rs11519272
rs1532085
rs10770795
All − MR Egger
All − Inverse variance weighted
−0.4
−0.3
−0.2
−0.1
0.0
0.1
MR effect size for
'1−arachidonoyl−GPE (20:4n6) levels' on 'GCST90274790.tsv'

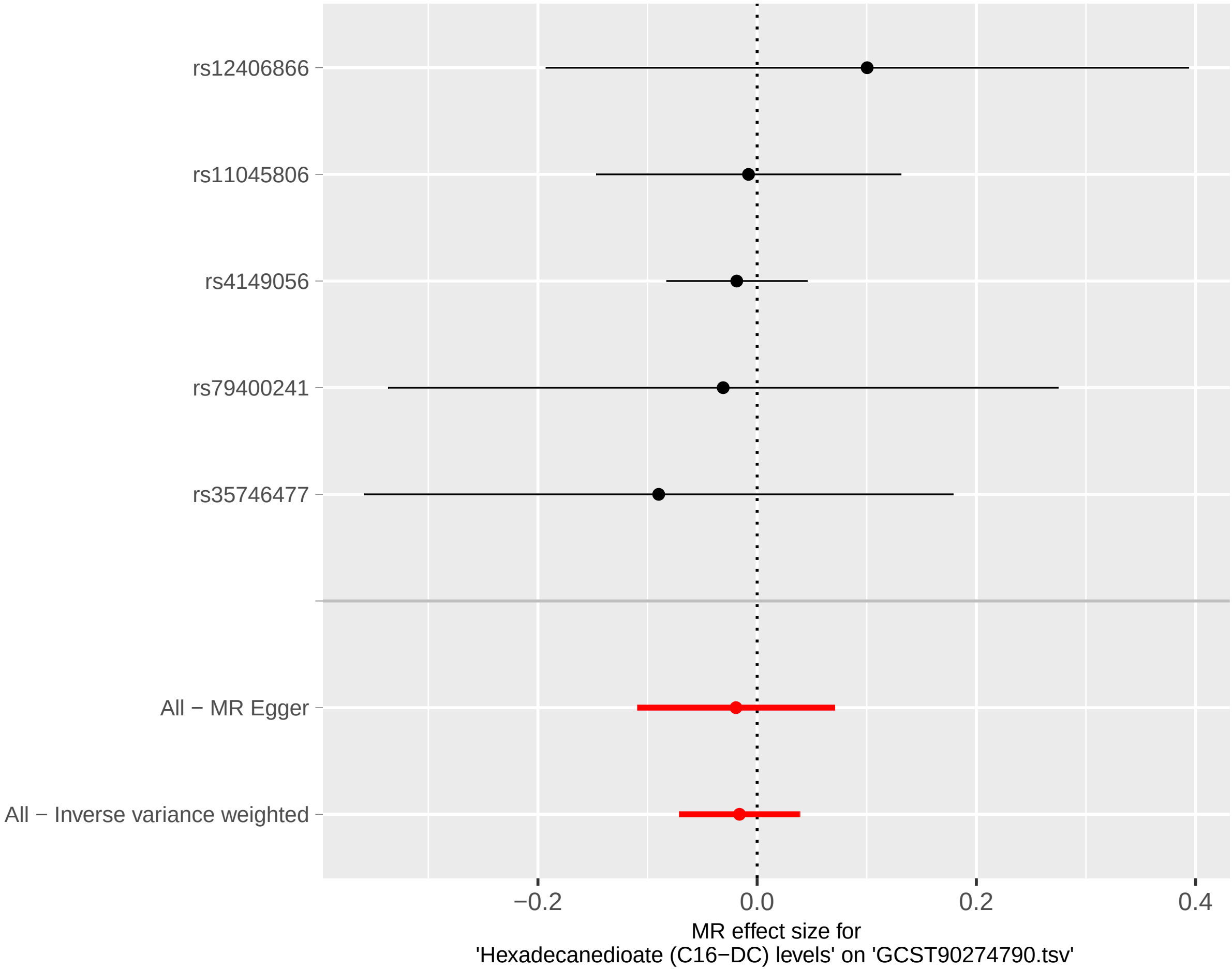
rs12406866
rs11045806
rs4149056
rs79400241
rs35746477
All − MR Egger
All − Inverse variance weighted
−0.2
0.0
0.2
0.4
MR effect size for
'Hexadecanedioate (C16−DC) levels' on 'GCST90274790.tsv'

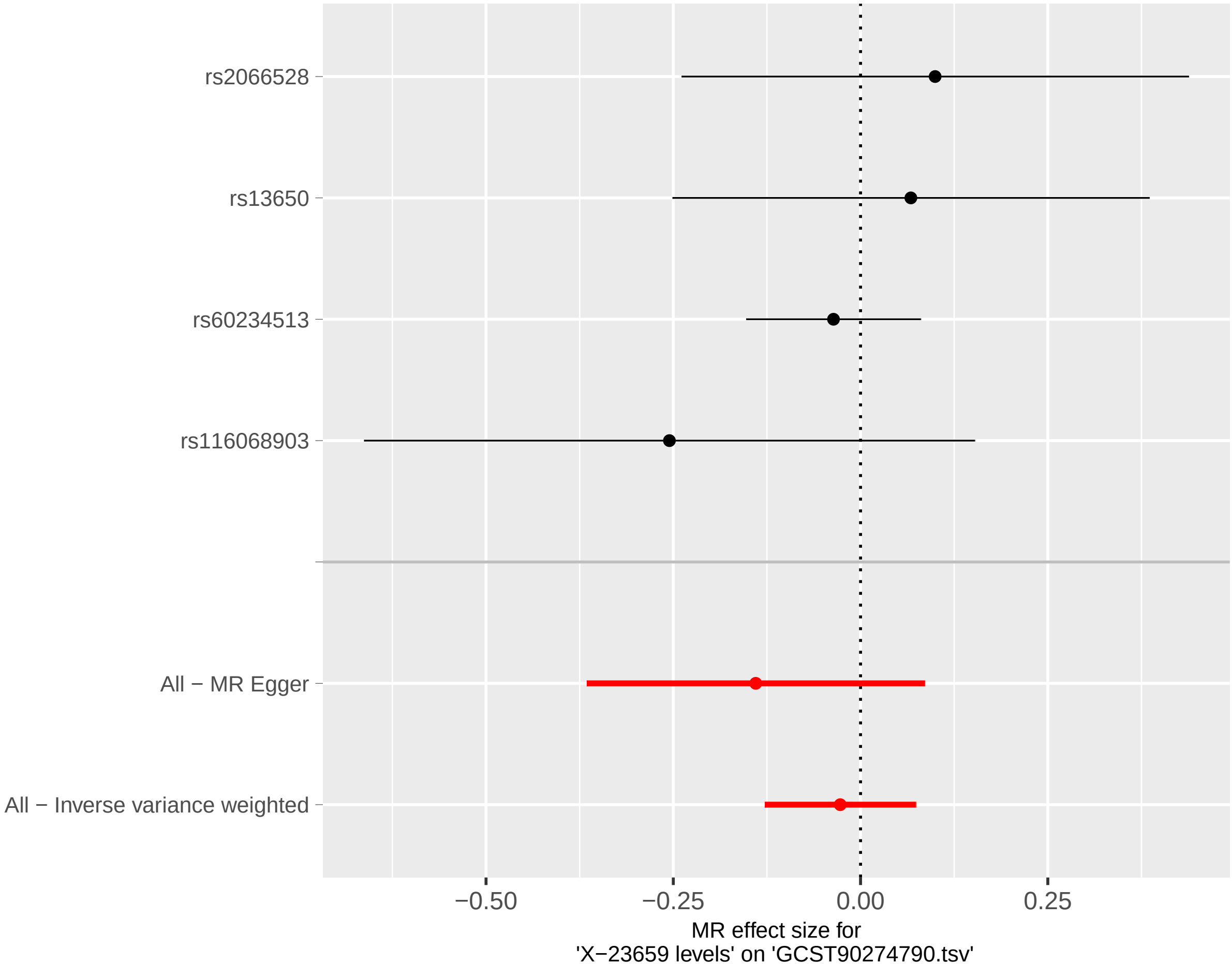
rs2066528
rs13650
rs60234513
rs116068903
All − MR Egger
All − Inverse variance weighted
−0.50
−0.25
0.00
0.25
MR effect size for
'X−23659 levels' on 'GCST90274790.tsv'

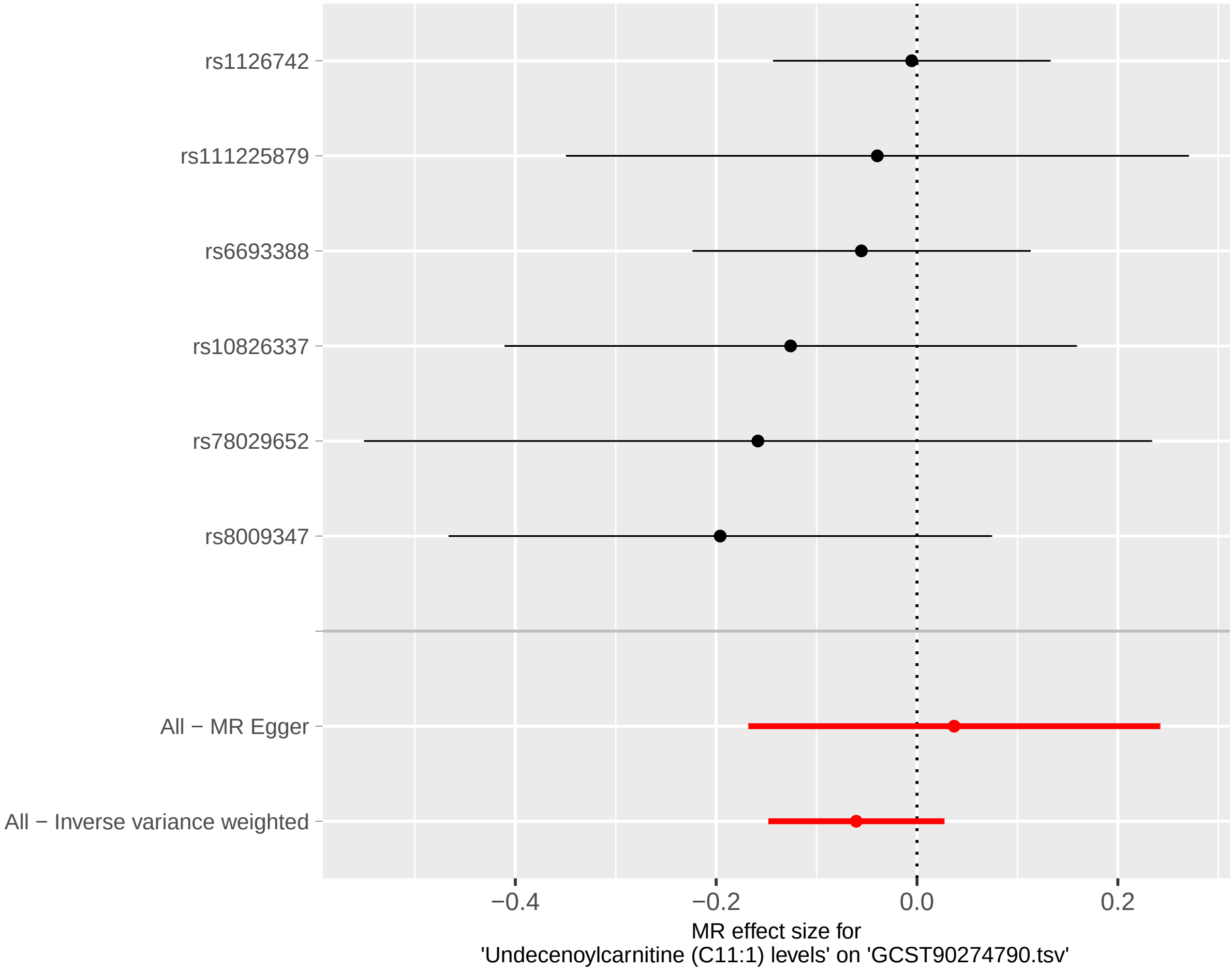
rs1126742
rs111225879
rs6693388
rs10826337
rs78029652
rs8009347
All − MR Egger
All − Inverse variance weighted
−0.4
−0.2
0.0
0.2
MR effect size for
'Undecenoylcarnitine (C11:1) levels' on 'GCST90274790.tsv'

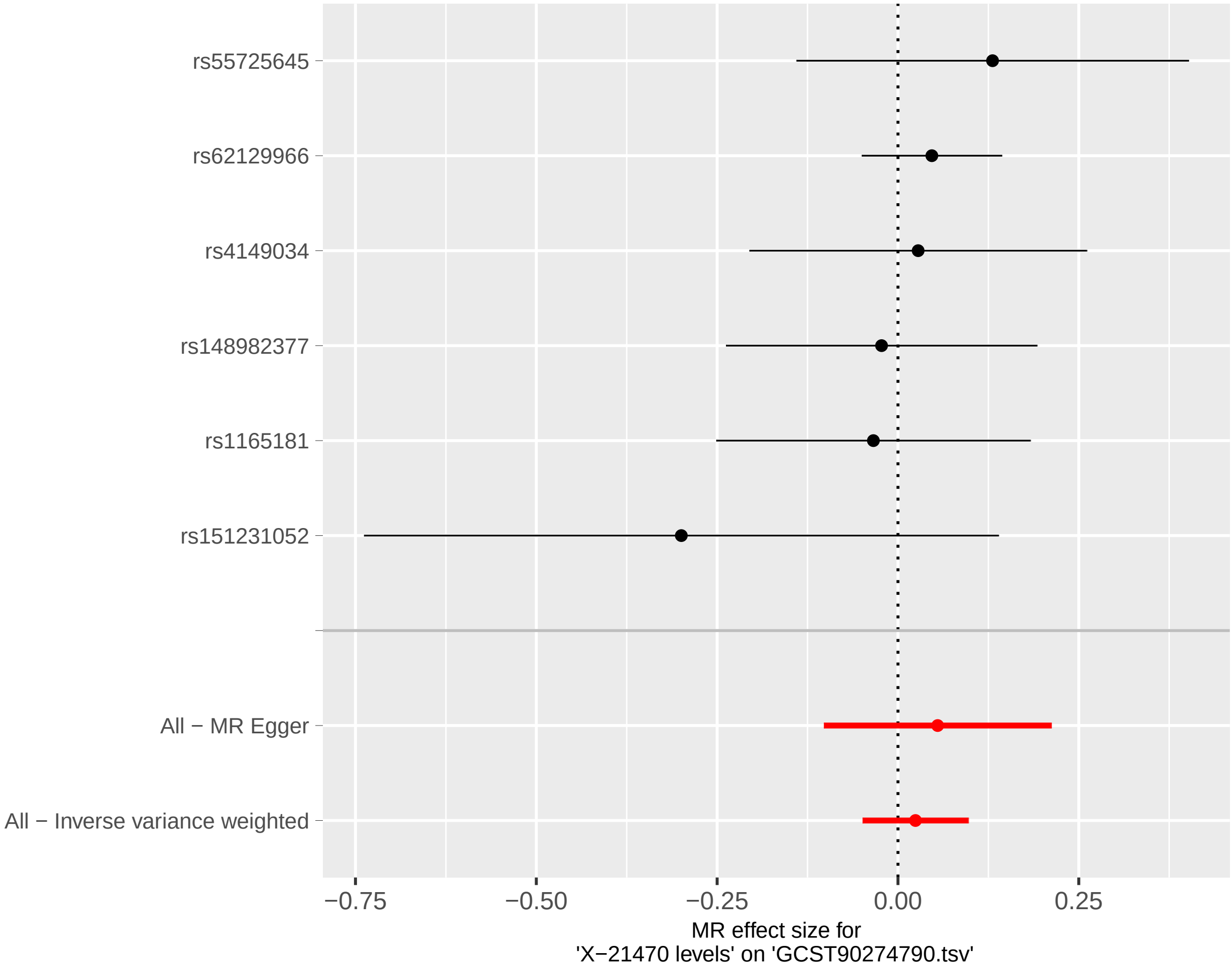
rs55725645
rs62129966
rs4149034
rs148982377
rs1165181
rs151231052
All − MR Egger
All − Inverse variance weighted
−0.75
−0.50
−0.25
0.00
0.25
MR effect size for
'X−21470 levels' on 'GCST90274790.tsv'

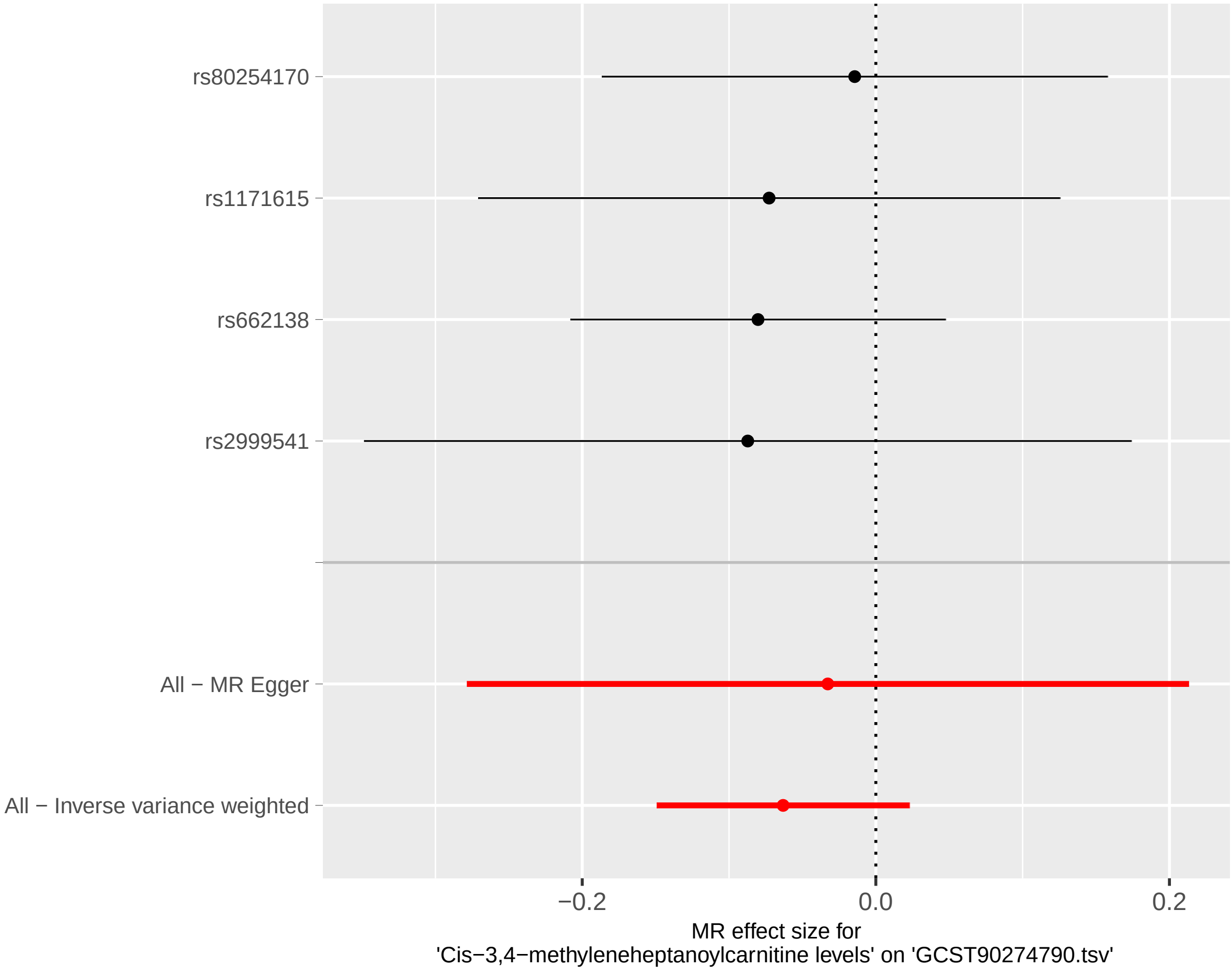
rs80254170
rs1171615
rs662138
rs2999541
All − MR Egger
All − Inverse variance weighted
−0.2
0.0
0.2
MR effect size for
'Cis−3,4−methyleneheptanoylcarnitine levels' on 'GCST90274790.tsv'

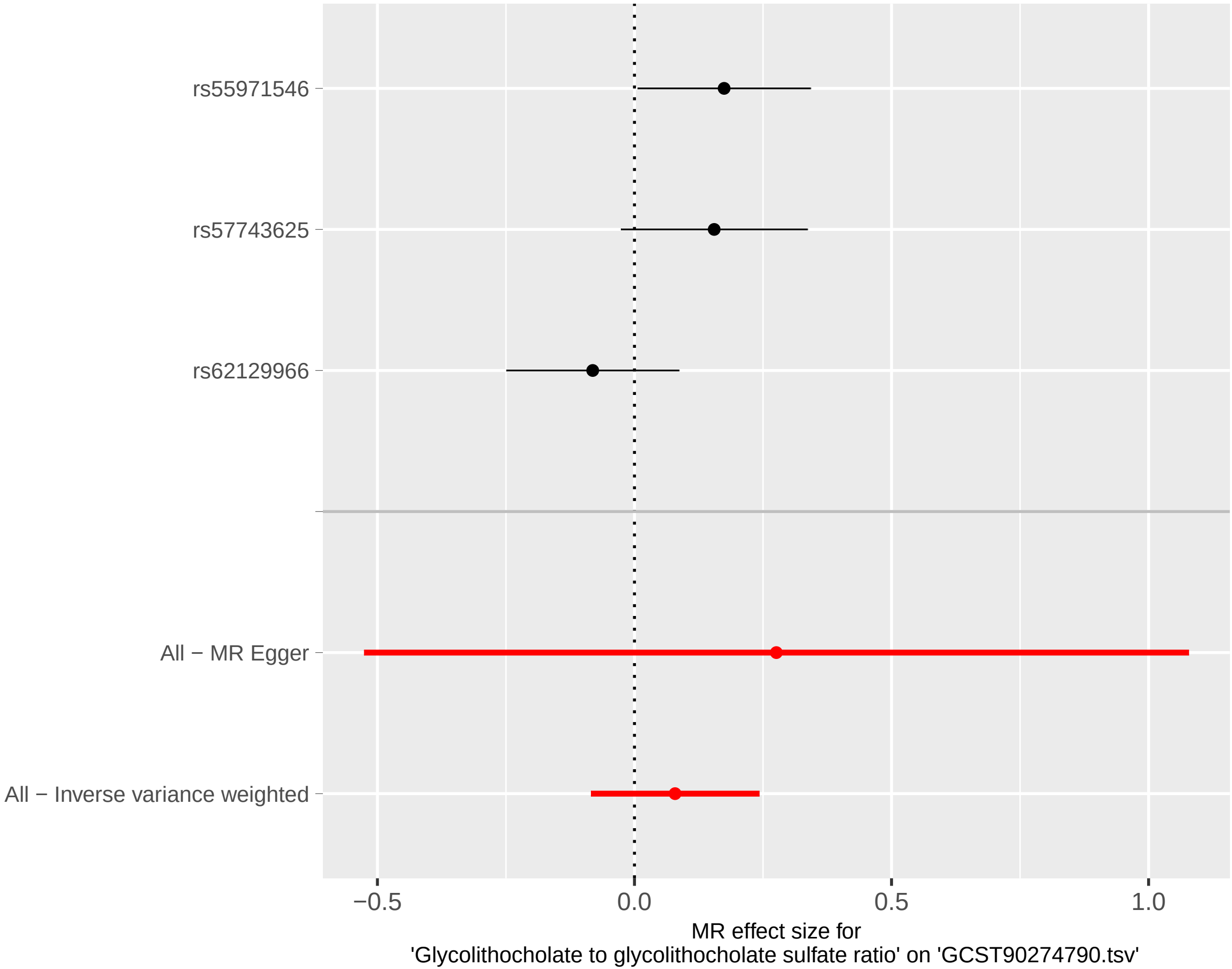
rs55971546
rs57743625
rs62129966
All − MR Egger
All − Inverse variance weighted
−0.5
0.0
0.5
1.0
MR effect size for
'Glycolithocholate to glycolithocholate sulfate ratio' on 'GCST90274790.tsv'

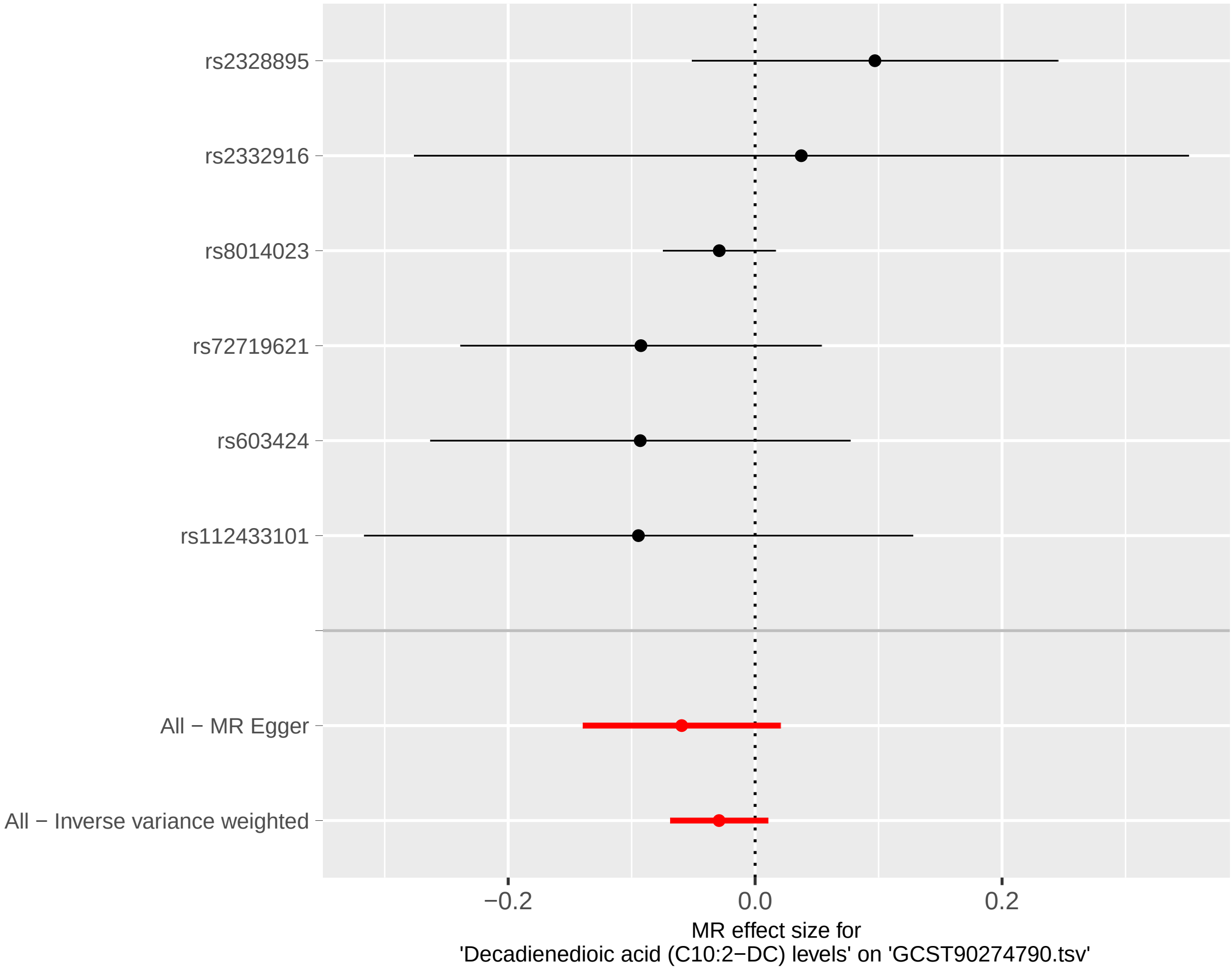

rs2328895
rs2332916
rs8014023
rs72719621
rs603424
rs112433101
All − MR Egger
All − Inverse variance weighted
−0.2
0.0
0.2
MR effect size for
'Decadienedioic acid (C10:2−DC) levels' on 'GCST90274790.tsv'

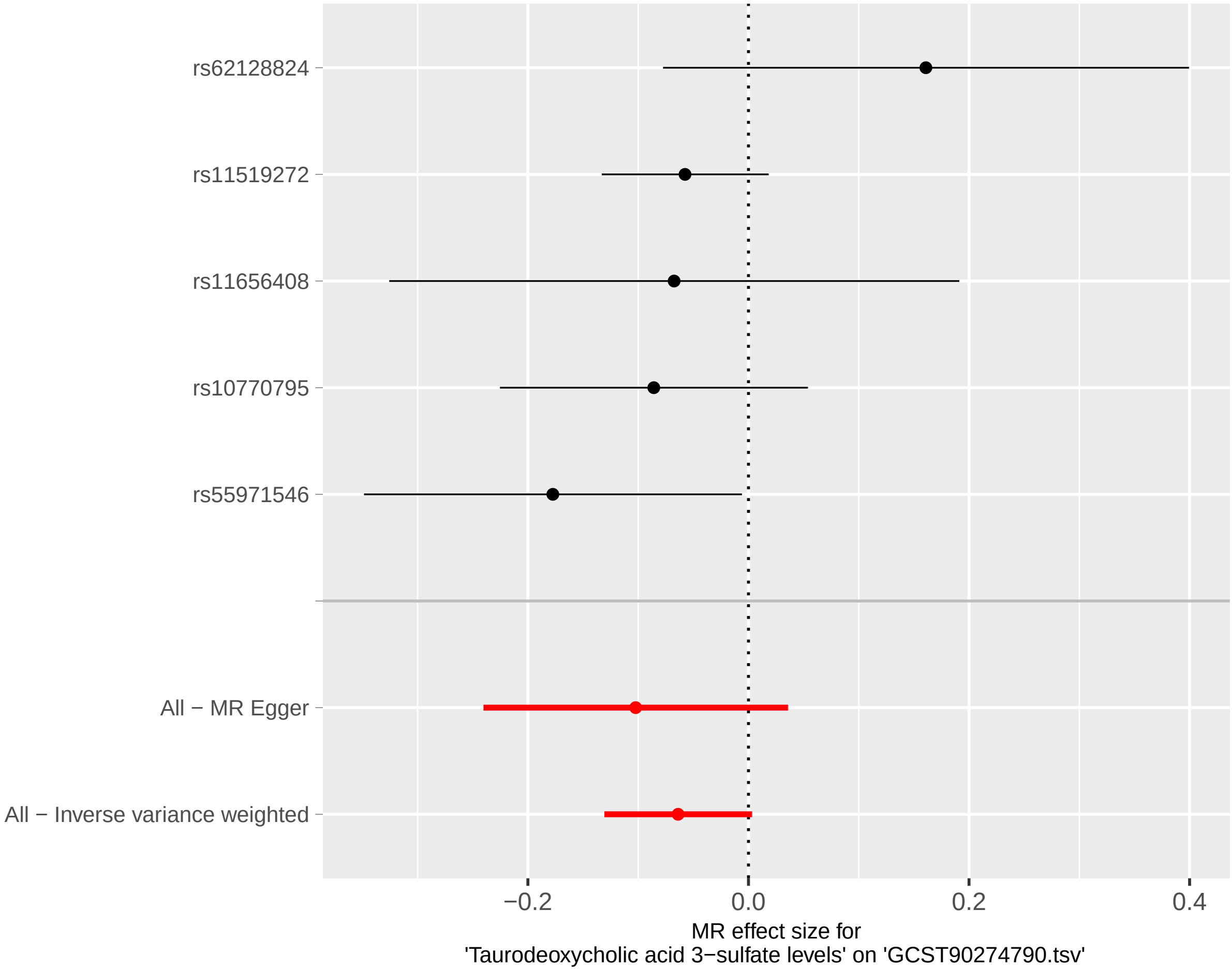
rs62128824
rs11519272
rs11656408
rs10770795
rs55971546
All − MR Egger
All − Inverse variance weighted
−0.2
0.0
0.2
0.4
MR effect size for
'Taurodeoxycholic acid 3−sulfate levels' on 'GCST90274790.tsv'

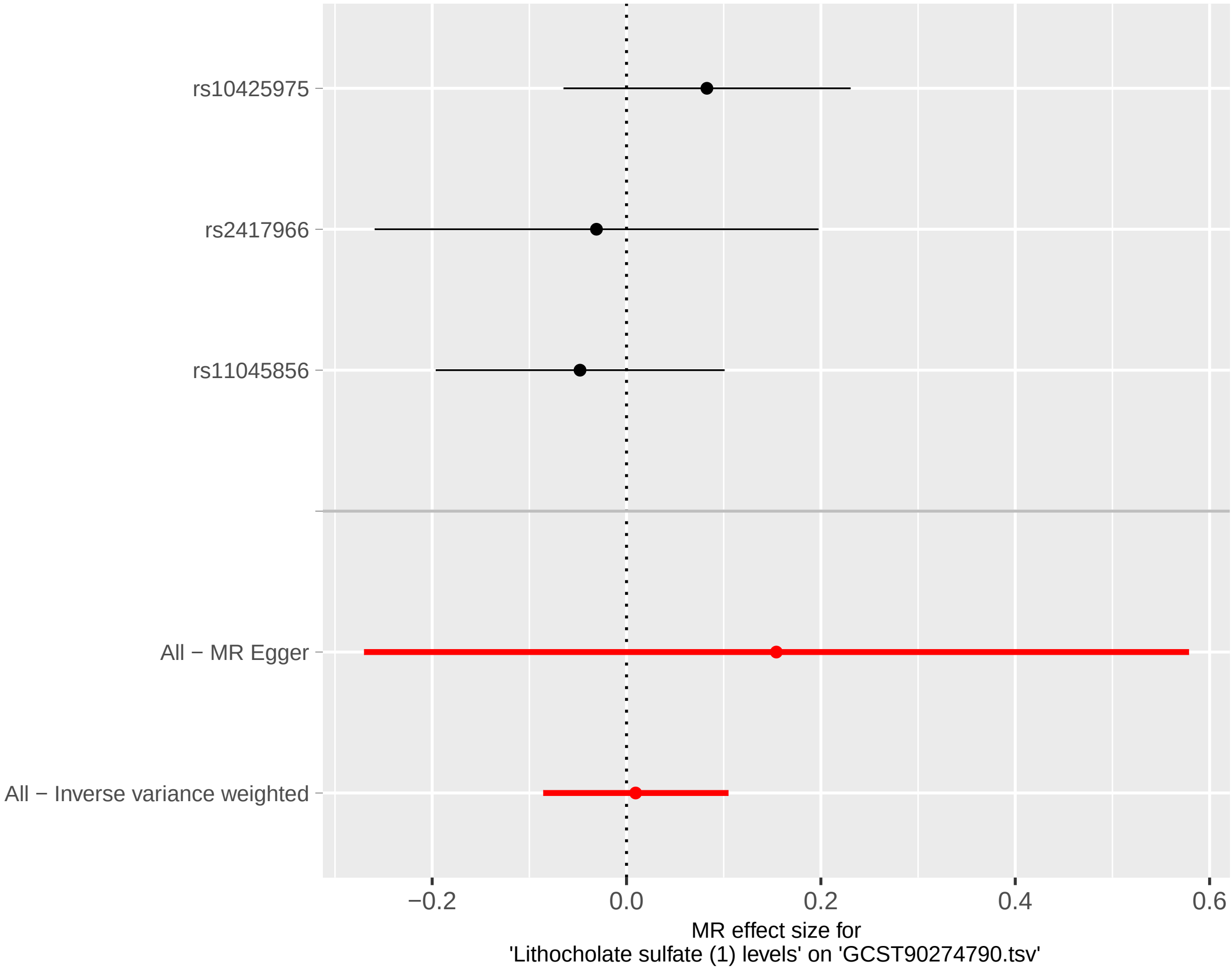

rs10425975
rs2417966
rs11045856
All − MR Egger
All − Inverse variance weighted
−0.2
0.0
0.2
0.4
0.6
MR effect size for
'Lithocholate sulfate (1) levels' on 'GCST90274790.tsv'

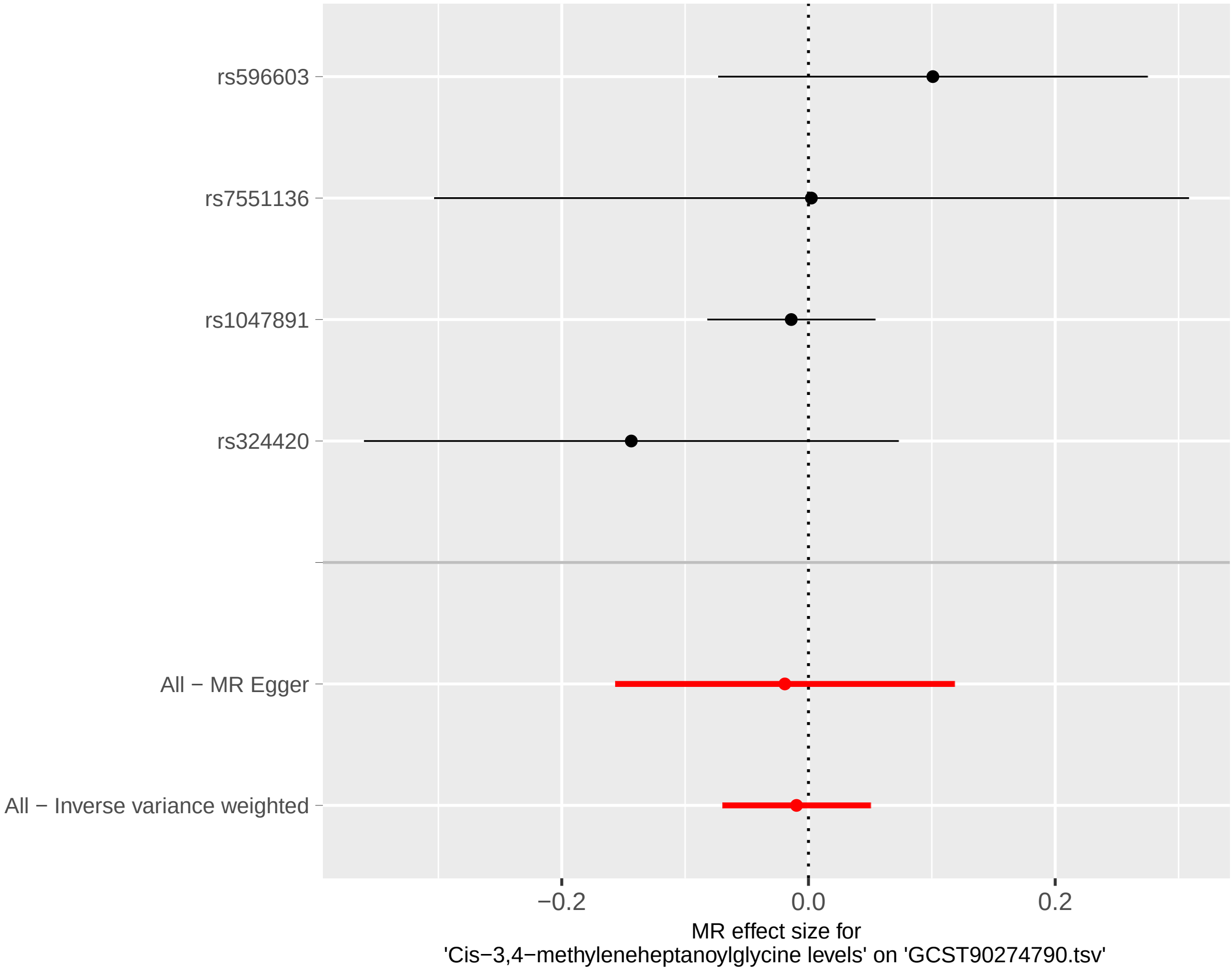
rs596603
rs7551136
rs1047891
rs324420
All − MR Egger
All − Inverse variance weighted
−0.2
0.0
0.2
MR effect size for
'Cis−3,4−methyleneheptanoylglycine levels' on 'GCST90274790.tsv'

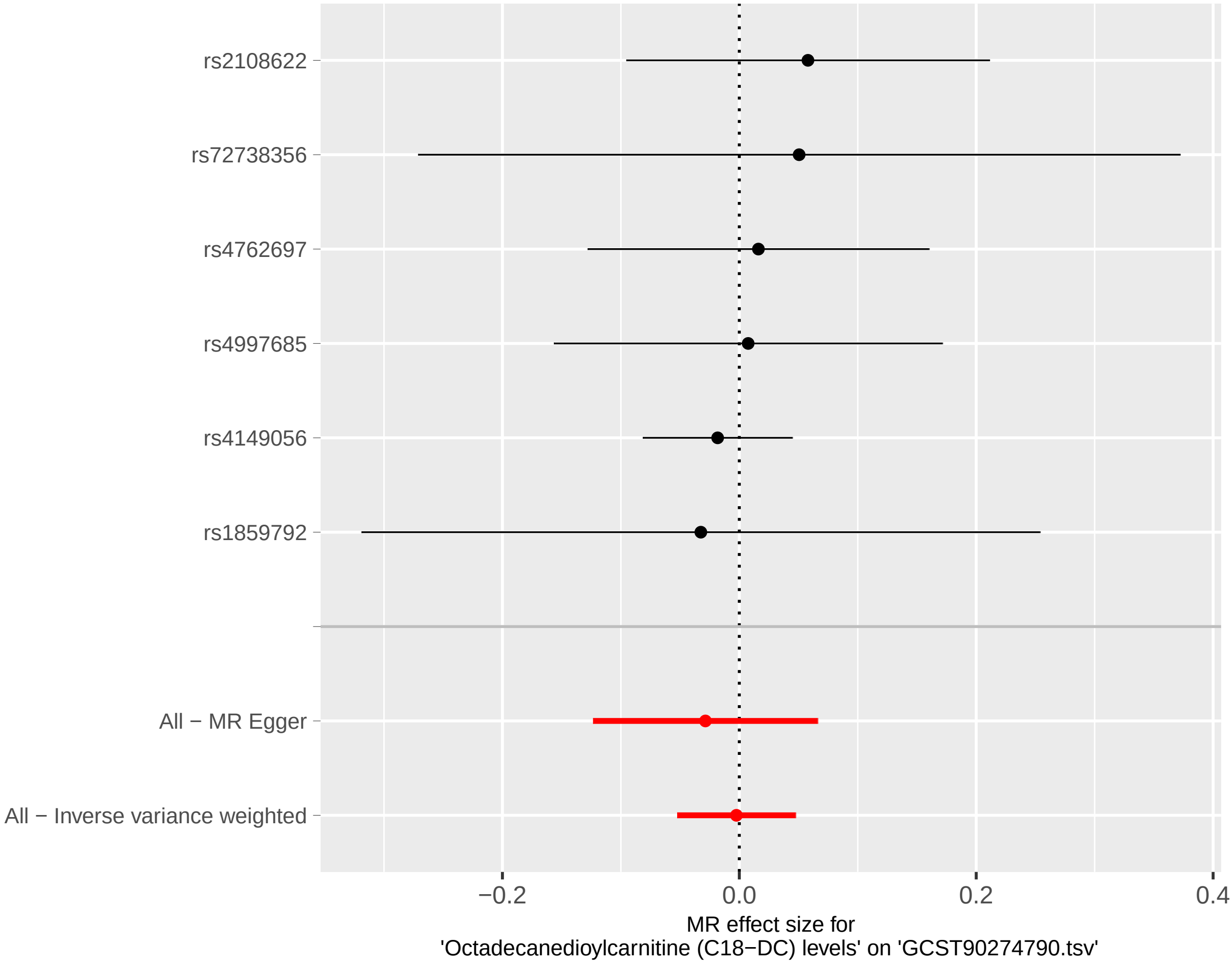
rs2108622
rs72738356
rs4762697
rs4997685
rs4149056
rs1859792
All − MR Egger
All − Inverse variance weighted
−0.2
0.0
0.2
0.4
MR effect size for
'Octadecanedioylcarnitine (C18−DC) levels' on 'GCST90274790.tsv'

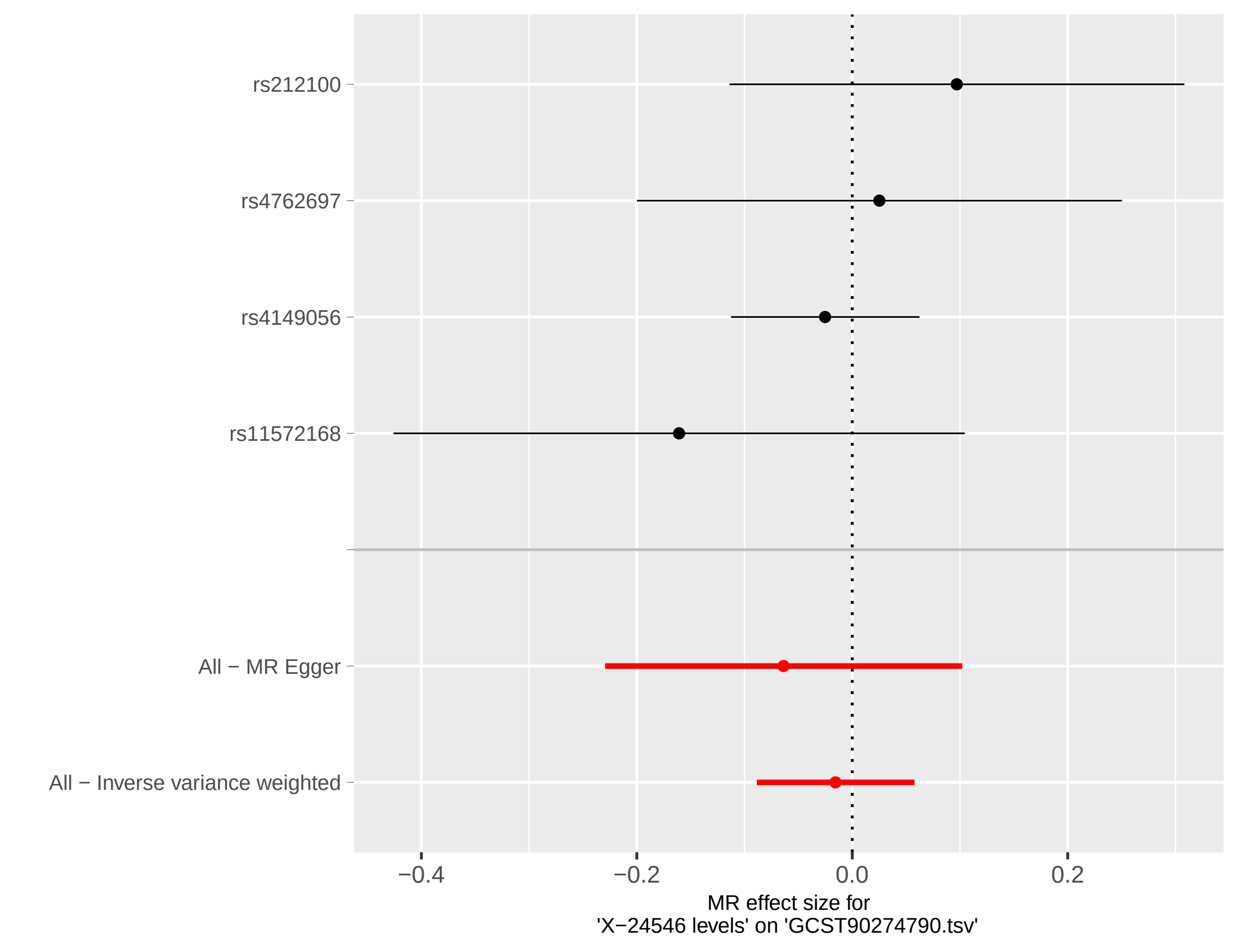
rs212100
rs4762697
rs4149056
rs11572168
All − MR Egger
All − Inverse variance weighted
−0.4
−0.2
0.0
0.2
MR effect size for
'X−24546 levels' on 'GCST90274790.tsv'

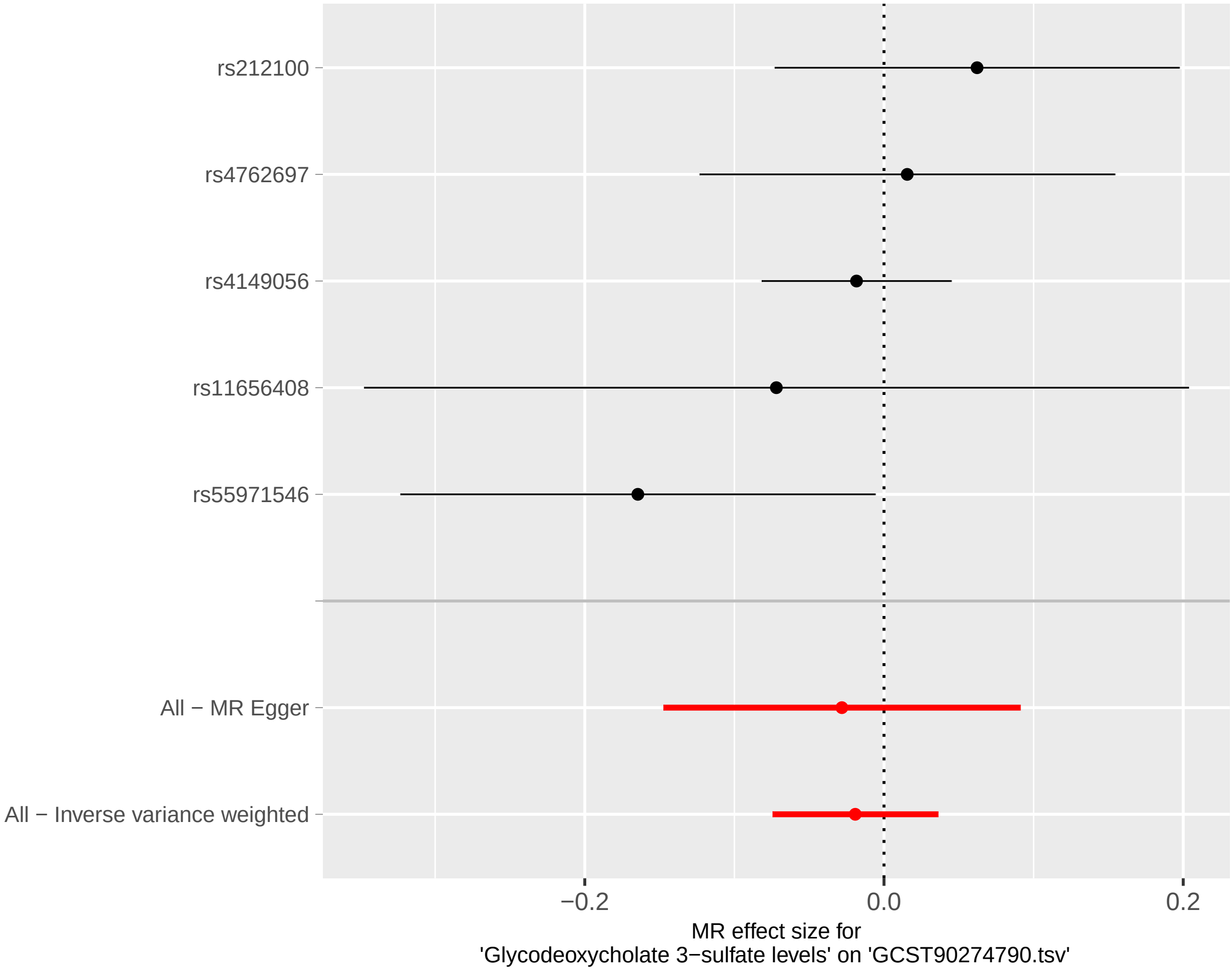
rs212100
rs4762697
rs4149056
rs11656408
rs55971546
All − MR Egger
All − Inverse variance weighted
−0.2
0.0
0.2
MR effect size for
'Glycodeoxycholate 3−sulfate levels' on 'GCST90274790.tsv'

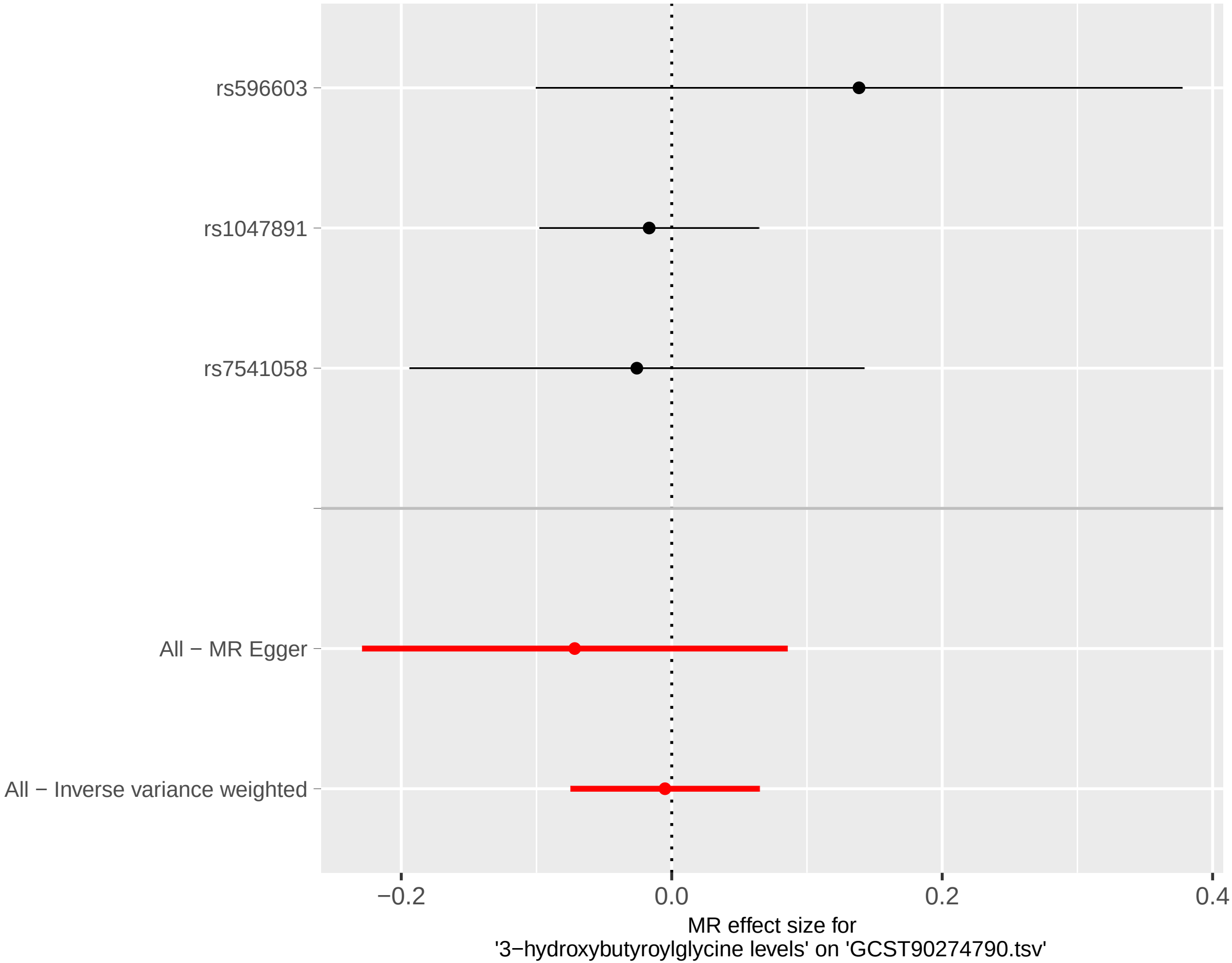
rs596603
rs1047891
rs7541058
All − MR Egger
All − Inverse variance weighted
−0.2
0.0
0.2
0.4
MR effect size for
'3−hydroxybutyroylglycine levels' on 'GCST90274790.tsv'

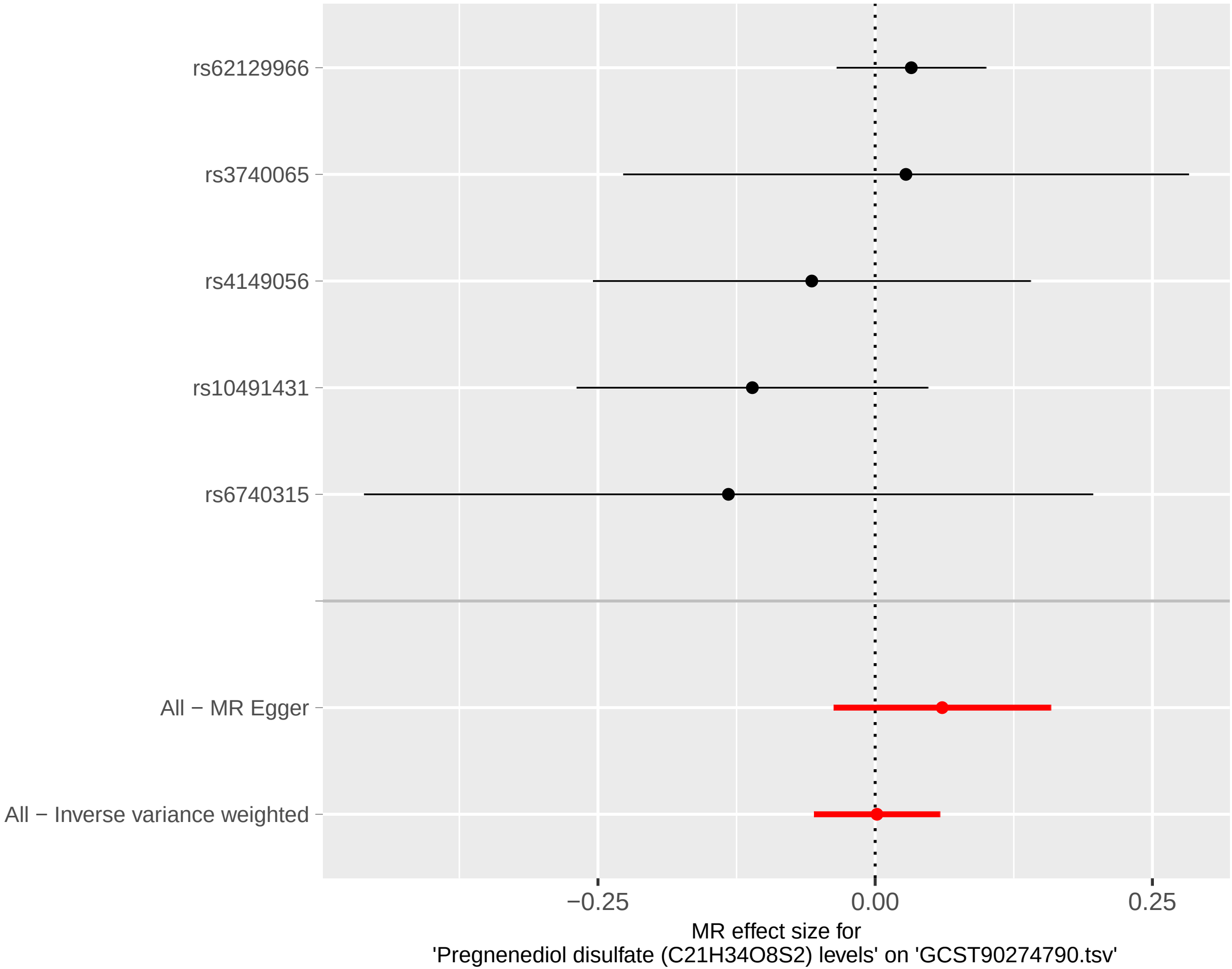

rs62129966
rs3740065
rs4149056
rs10491431
rs6740315
All − MR Egger
All − Inverse variance weighted
−0.25
0.00
0.25
MR effect size for
'Pregnenediol disulfate (C21H34O8S2) levels' on 'GCST90274790.tsv'

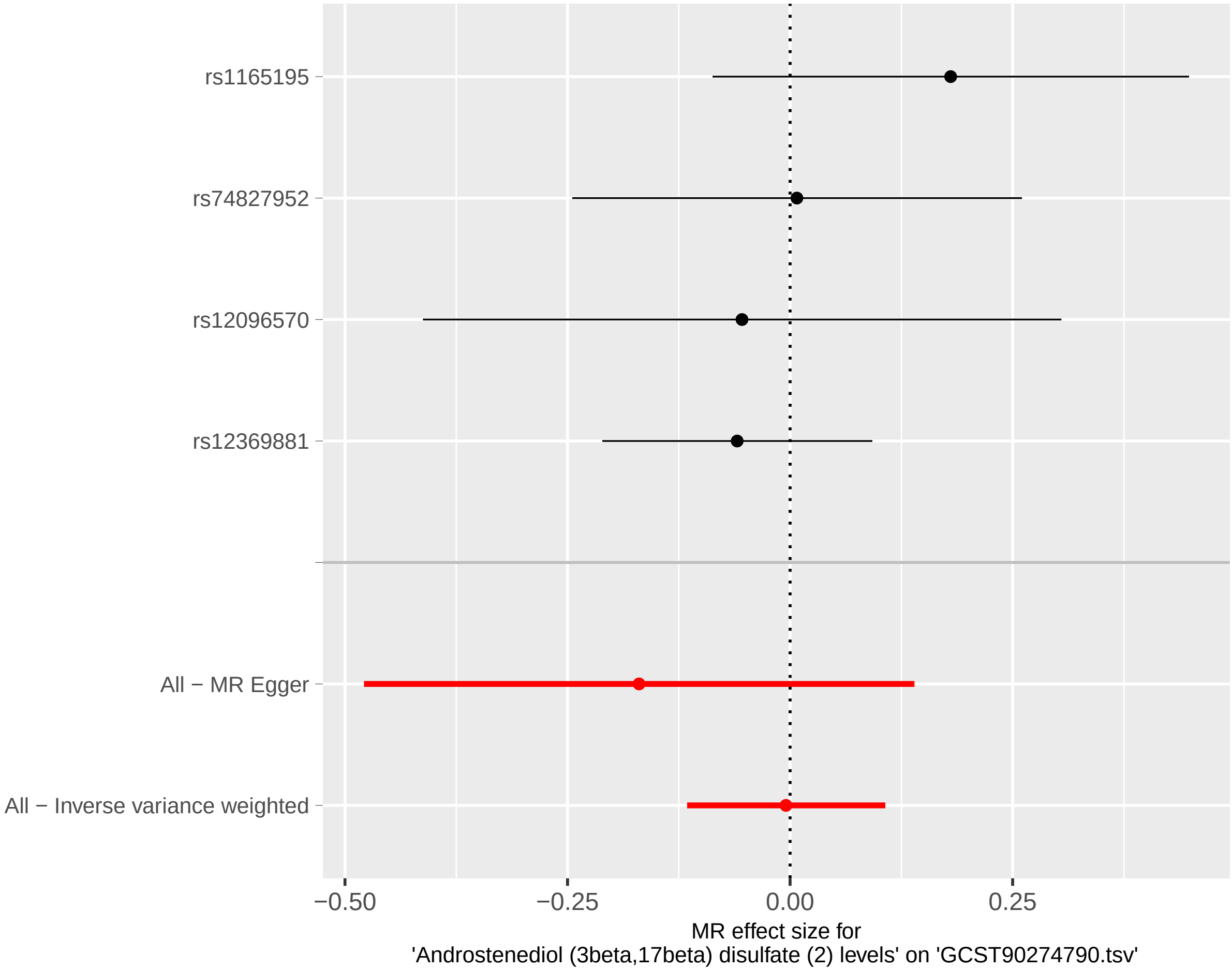
rs1165195
rs74827952
rs12096570
rs12369881
All − MR Egger
All − Inverse variance weighted
−0.50
−0.25
0.00
0.25
MR effect size for
'Androstenediol (3beta,17beta) disulfate (2) levels' on 'GCST90274790.tsv'

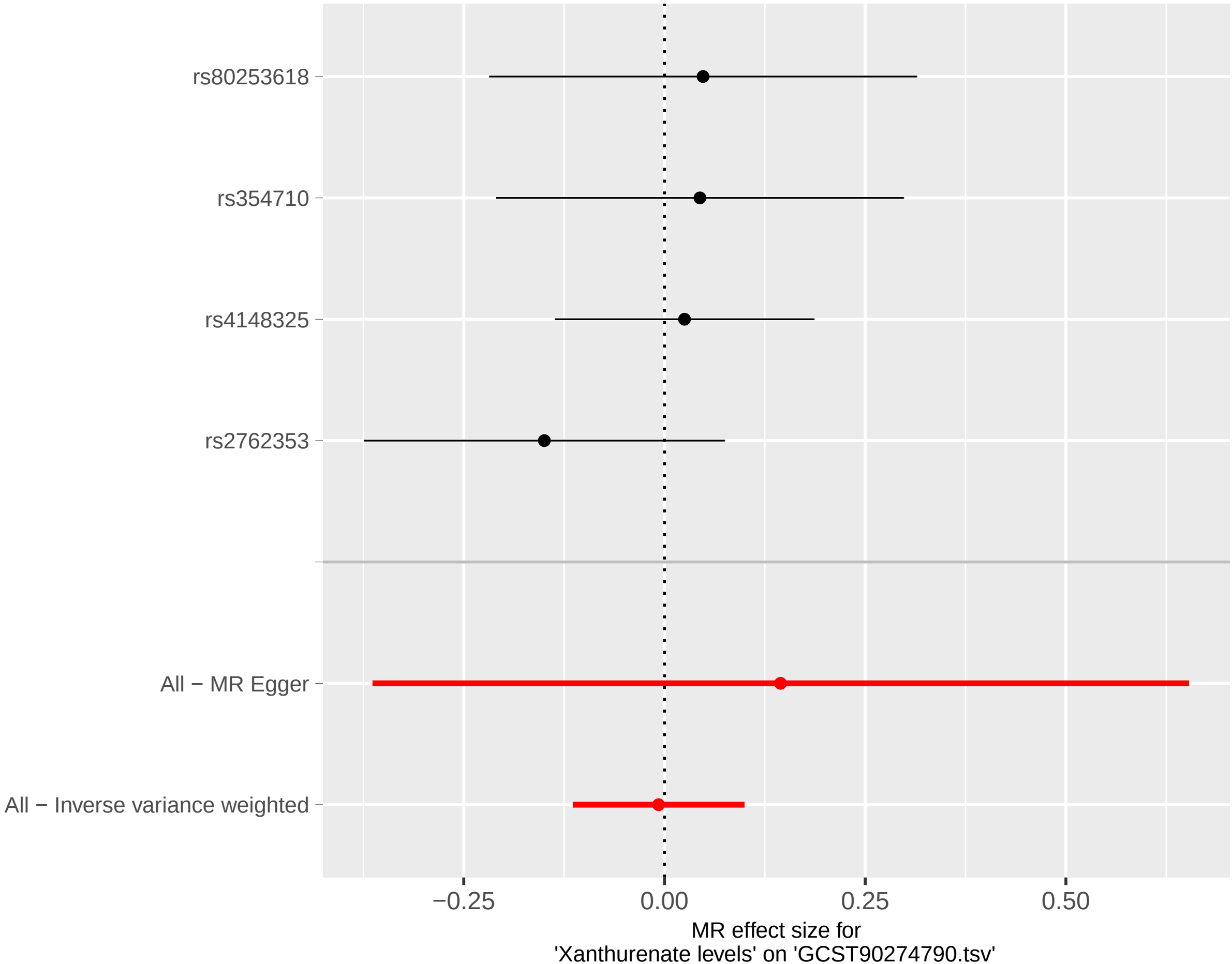

rs80253618
rs354710
rs4148325
rs2762353
All − MR Egger
All − Inverse variance weighted
−0.25
0.00
0.25
0.50
MR effect size for
'Xanthurenate levels' on 'GCST90274790.tsv'

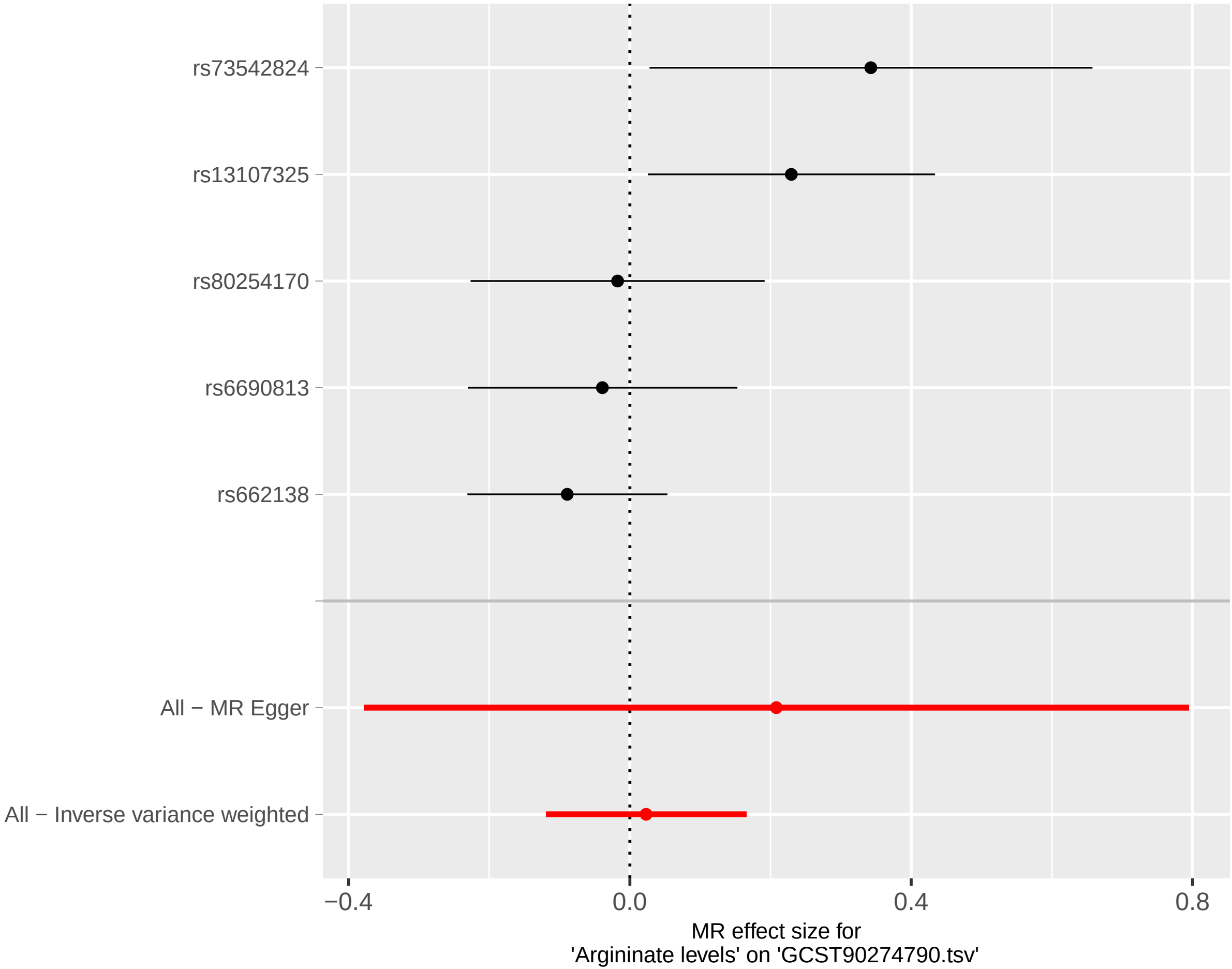

rs73542824
rs13107325
rs80254170
rs6690813
rs662138
All − MR Egger
All − Inverse variance weighted
−0.4
0.0
0.4
0.8
MR effect size for
'Argininate levels' on 'GCST90274790.tsv'

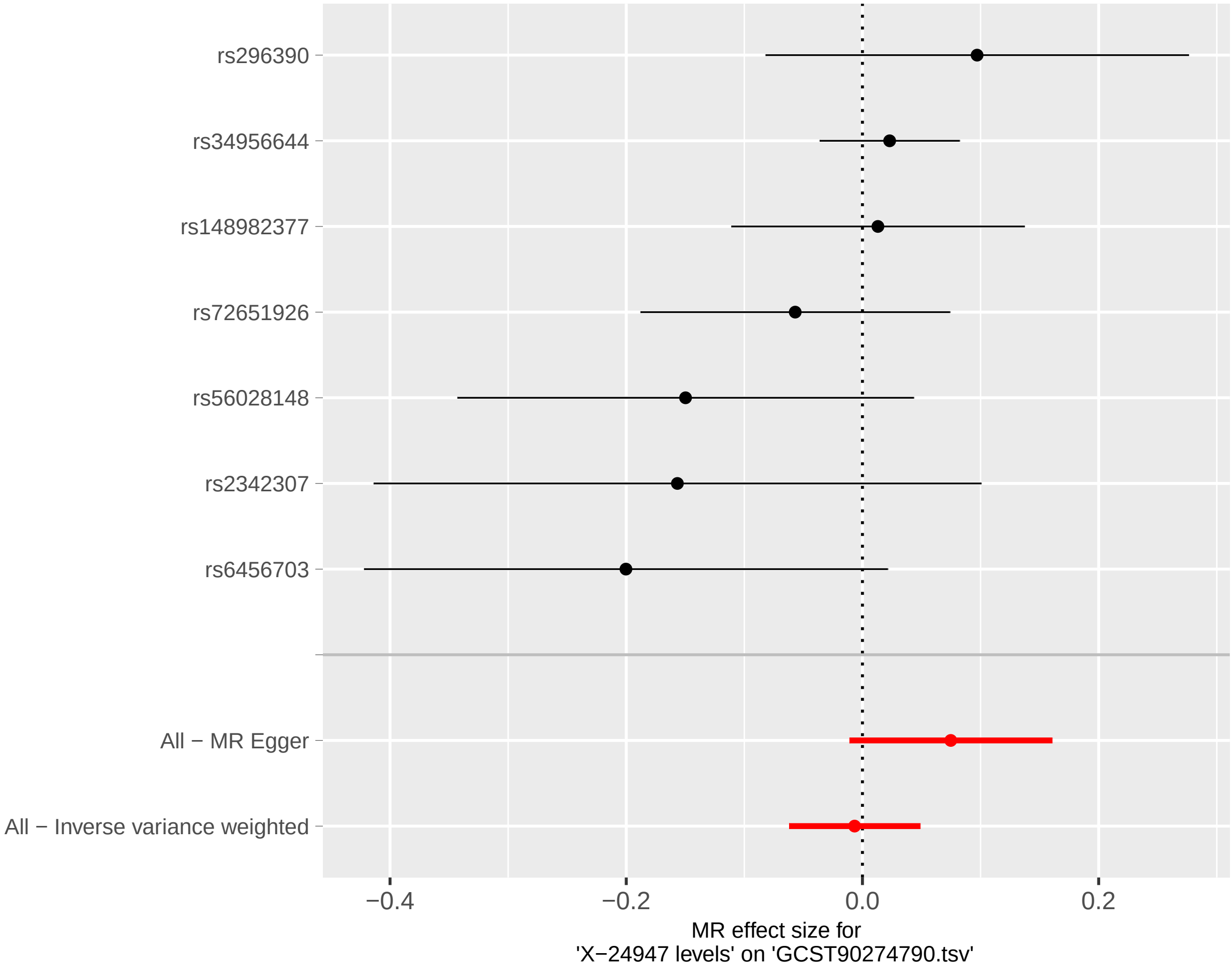
rs296390
rs34956644
rs148982377
rs72651926
rs56028148
rs2342307
rs6456703
All − MR Egger
All − Inverse variance weighted
−0.4
−0.2
0.0
0.2
MR effect size for
'X−24947 levels' on 'GCST90274790.tsv'

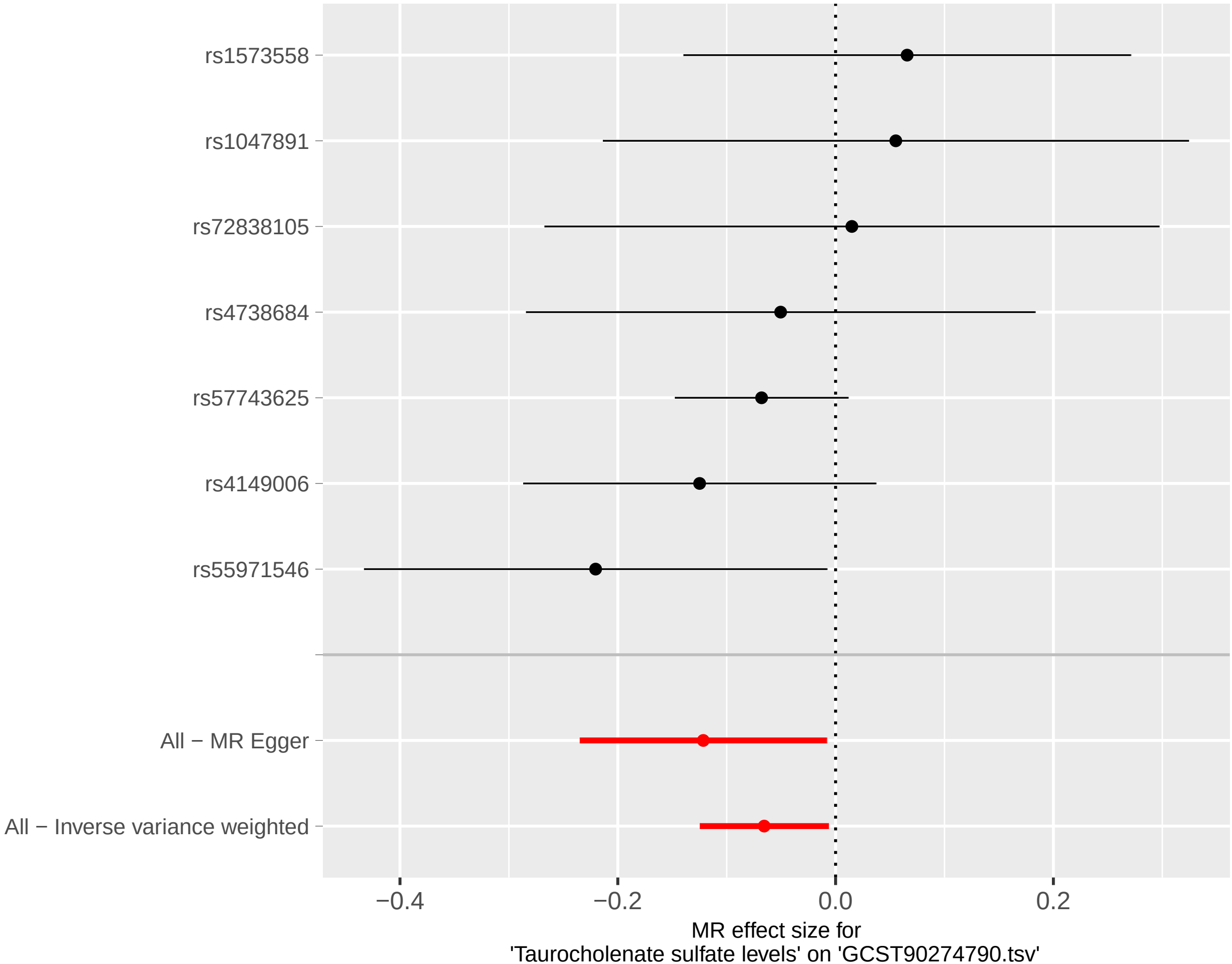
rs1573558
rs1047891
rs72838105
rs4738684
rs57743625
rs4149006
rs55971546
All − MR Egger
All − Inverse variance weighted
−0.4
−0.2
0.0
0.2
MR effect size for
'Taurocholenate sulfate levels' on 'GCST90274790.tsv'

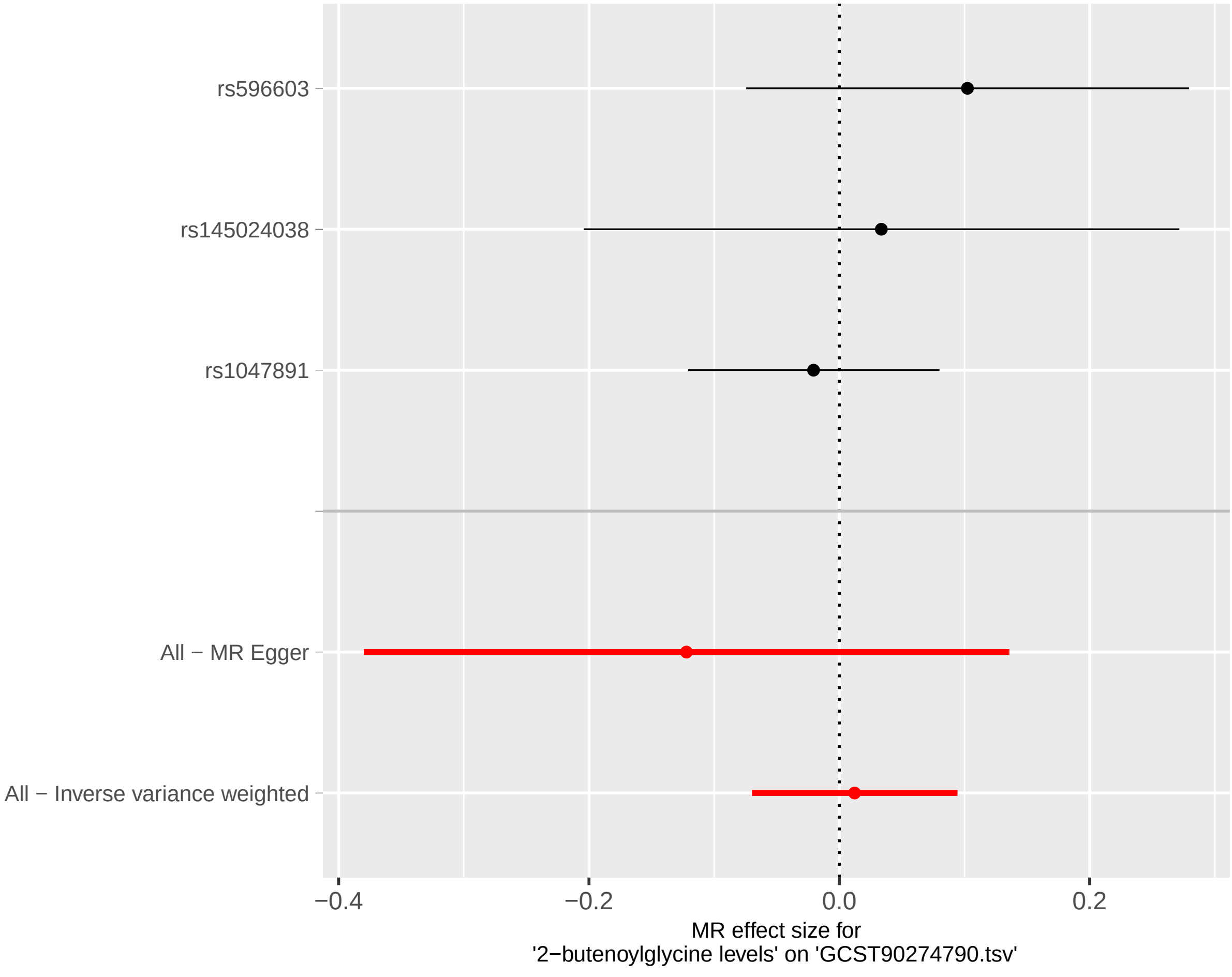

rs596603
rs145024038
rs1047891
All − MR Egger
All − Inverse variance weighted
−0.4
−0.2
0.0
0.2
MR effect size for
'2−butenoylglycine levels' on 'GCST90274790.tsv'

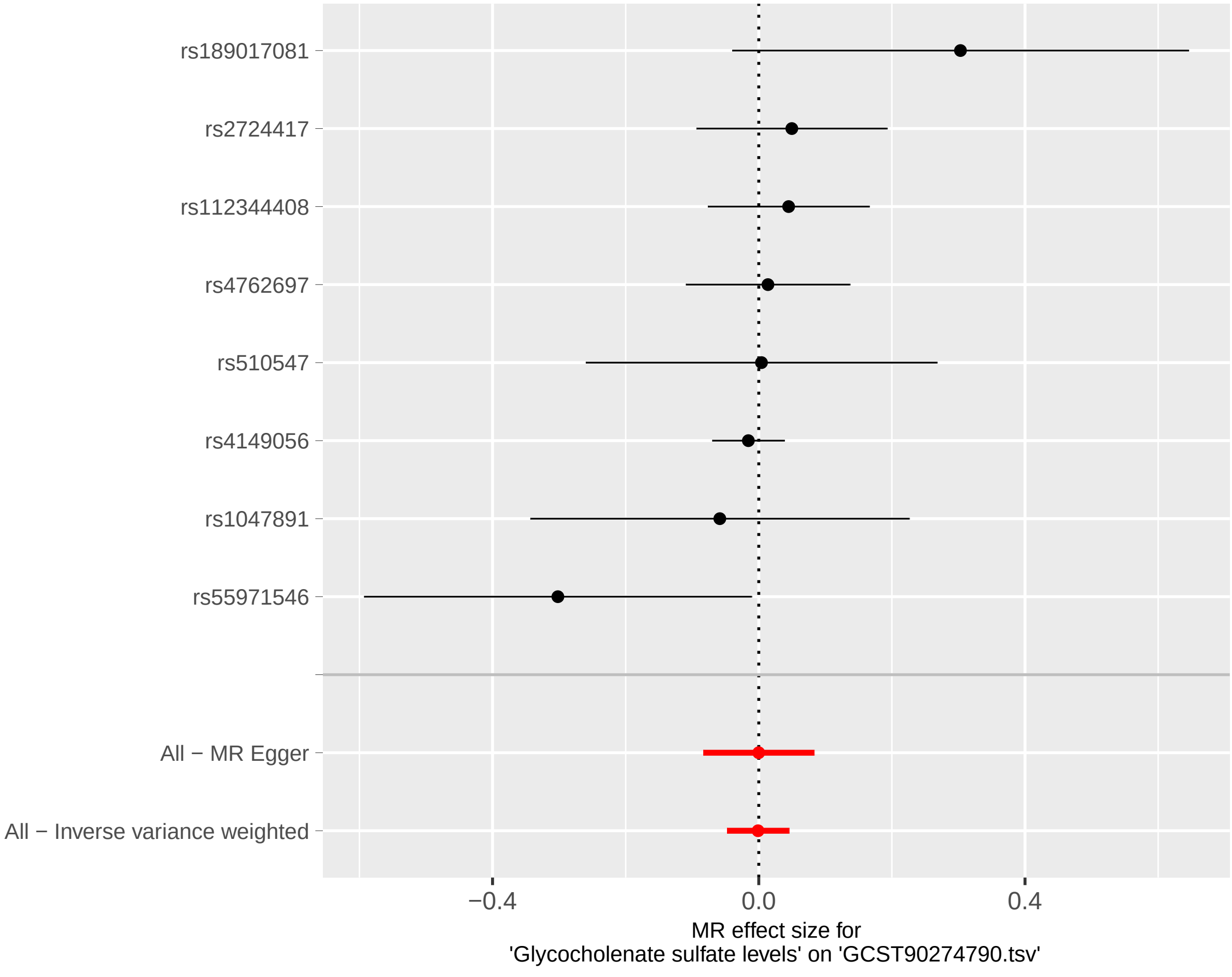
rs189017081
rs2724417
rs112344408
rs4762697
rs510547
rs4149056
rs1047891
rs55971546
All − MR Egger
All − Inverse variance weighted
−0.4
0.0
0.4
MR effect size for
'Glycocholenate sulfate levels' on 'GCST90274790.tsv'

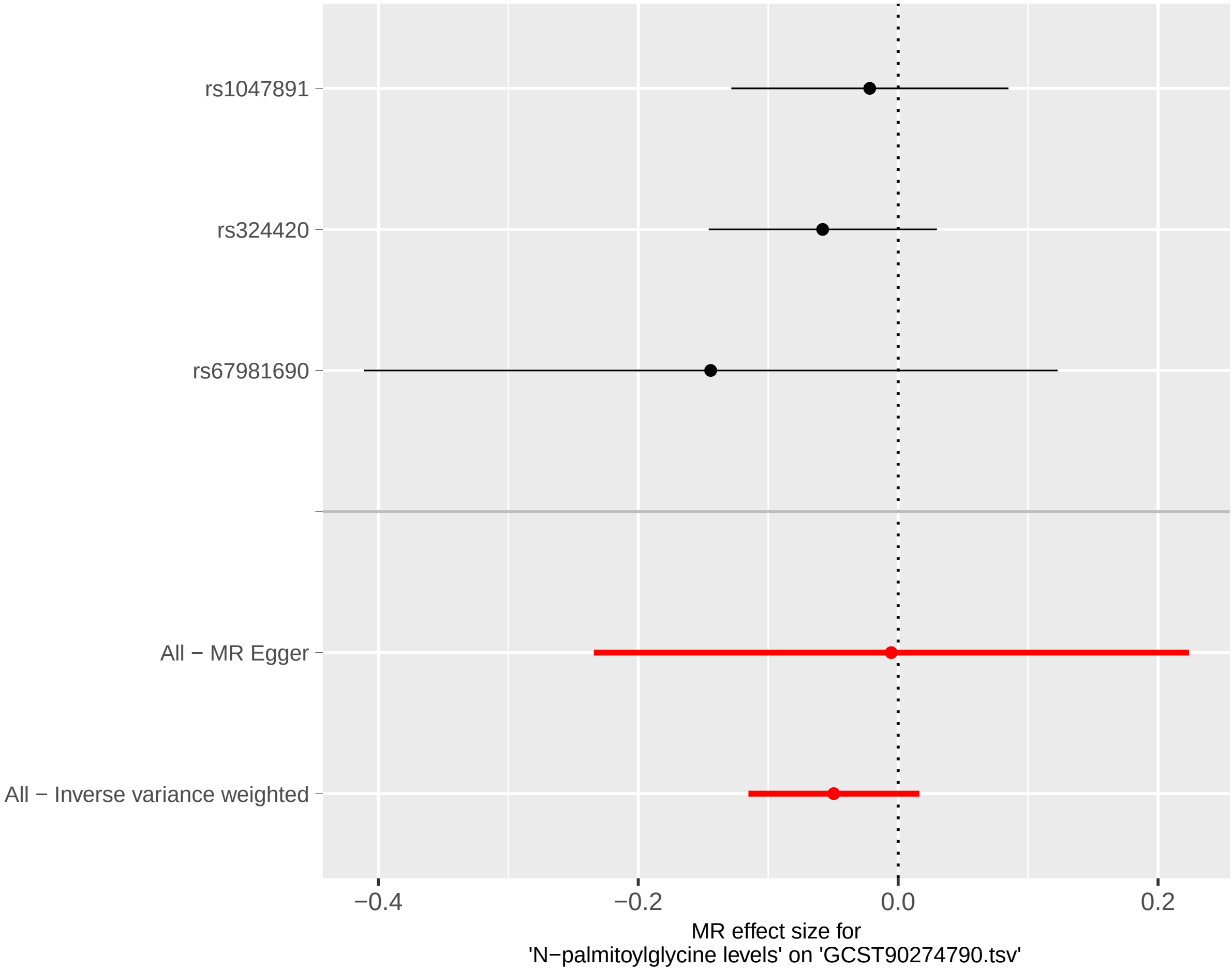
rs1047891
rs324420
rs67981690
All − MR Egger
All − Inverse variance weighted
−0.4
−0.2
0.0
0.2
MR effect size for
'N−palmitoylglycine levels' on 'GCST90274790.tsv'

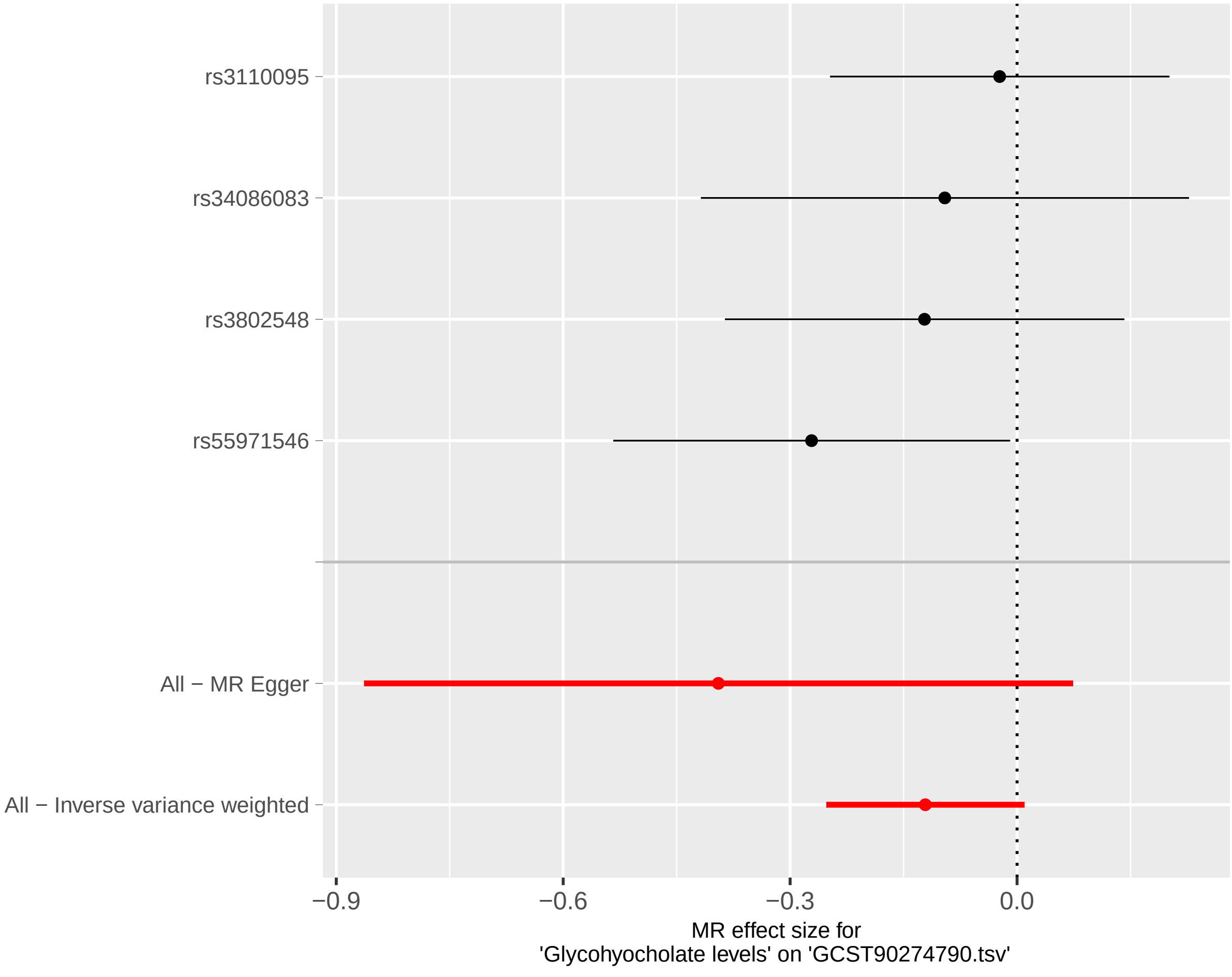
rs3110095
rs34086083
rs3802548
rs55971546
All − MR Egger
All − Inverse variance weighted
−0.9
−0.6
−0.3
0.0
MR effect size for
'Glycohyocholate levels' on 'GCST90274790.tsv'

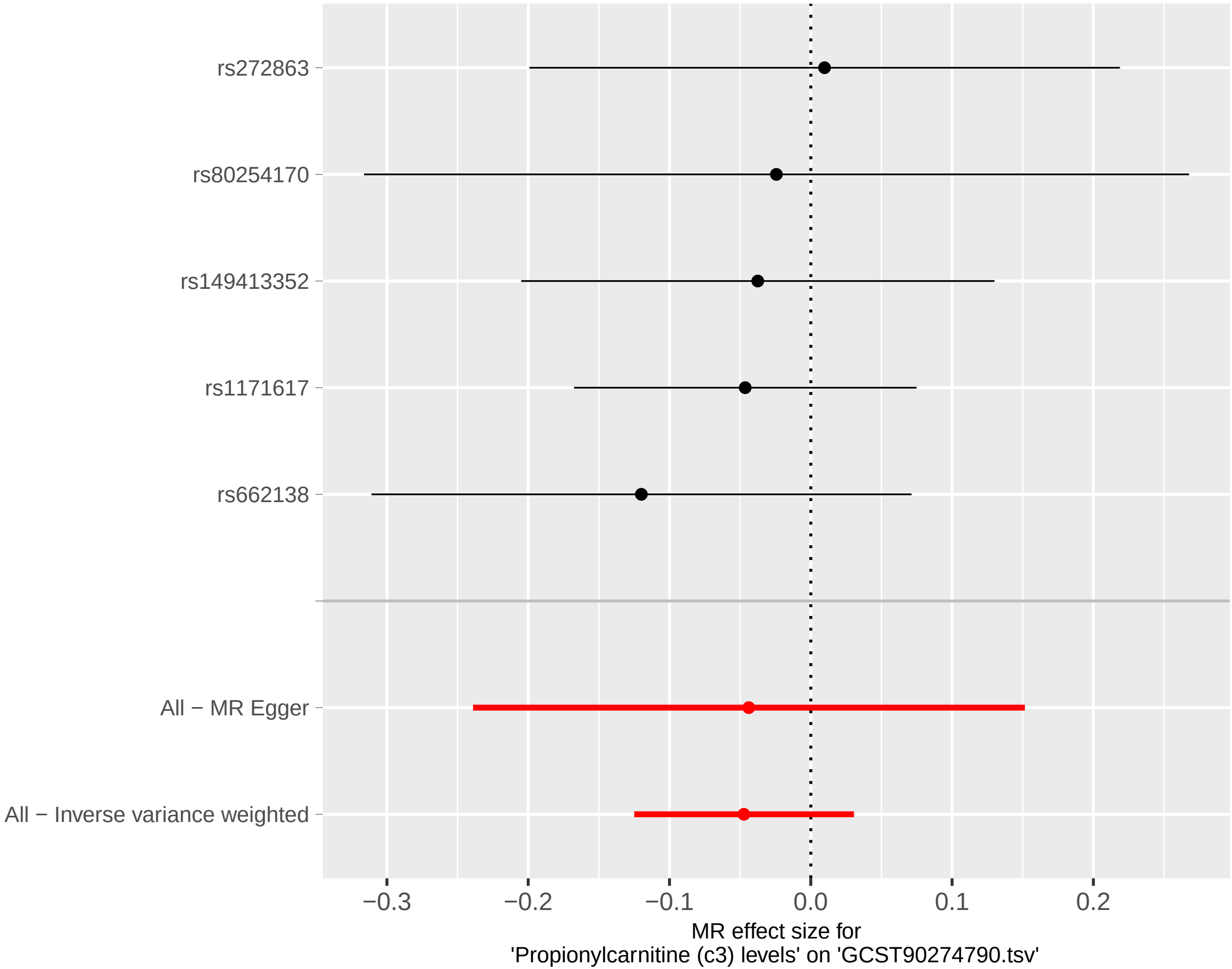
rs272863
rs80254170
rs149413352
rs1171617
rs662138
All − MR Egger
All − Inverse variance weighted
−0.3
−0.2
−0.1
0.0
0.1
0.2
MR effect size for
'Propionylcarnitine (c3) levels' on 'GCST90274790.tsv'

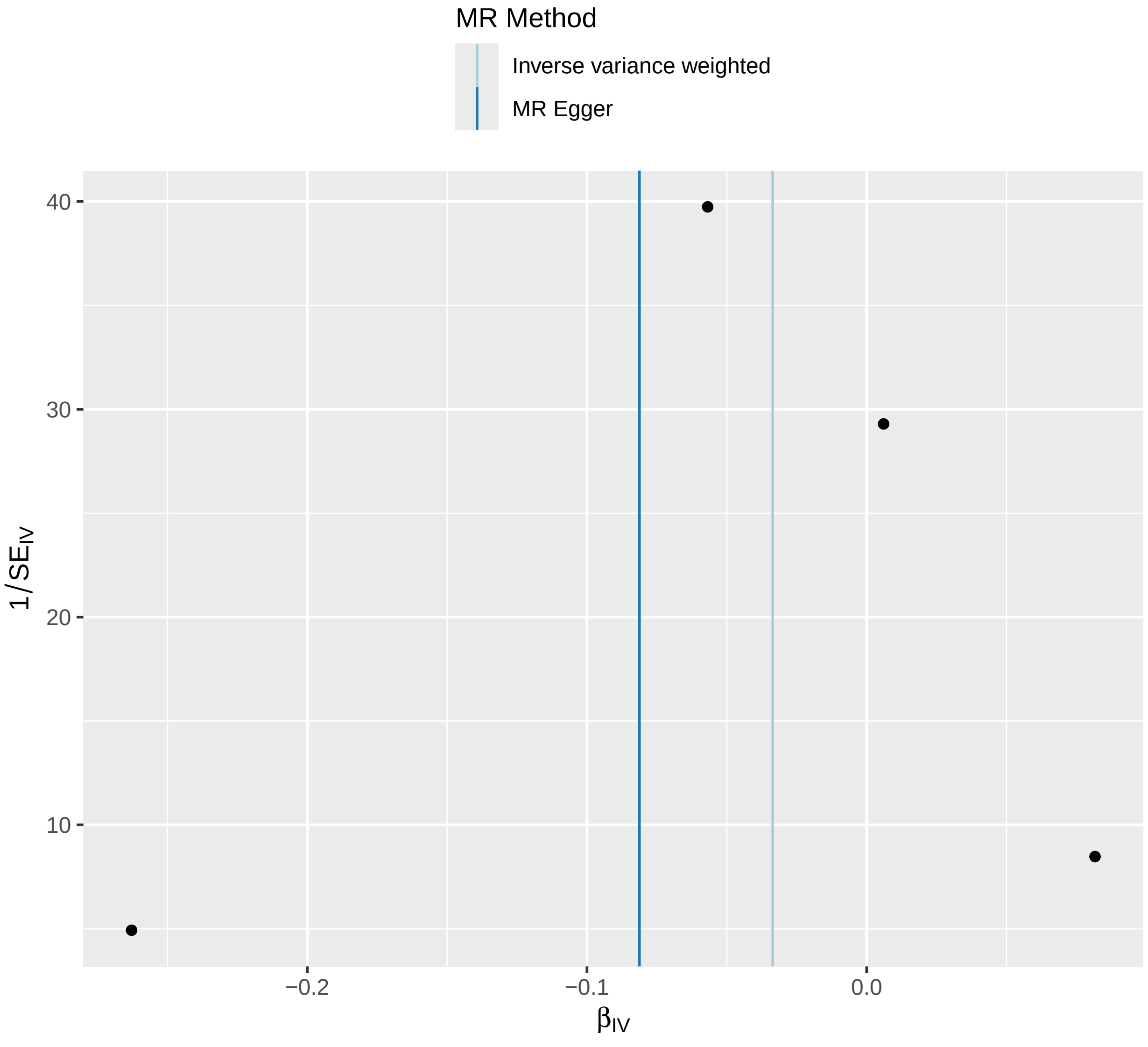
MR Method
Inverse variance weighted
MR Egger
40
30
20
10
1/SE$_{IV}$
−0.2
−0.1
0.0
β$_{IV}$

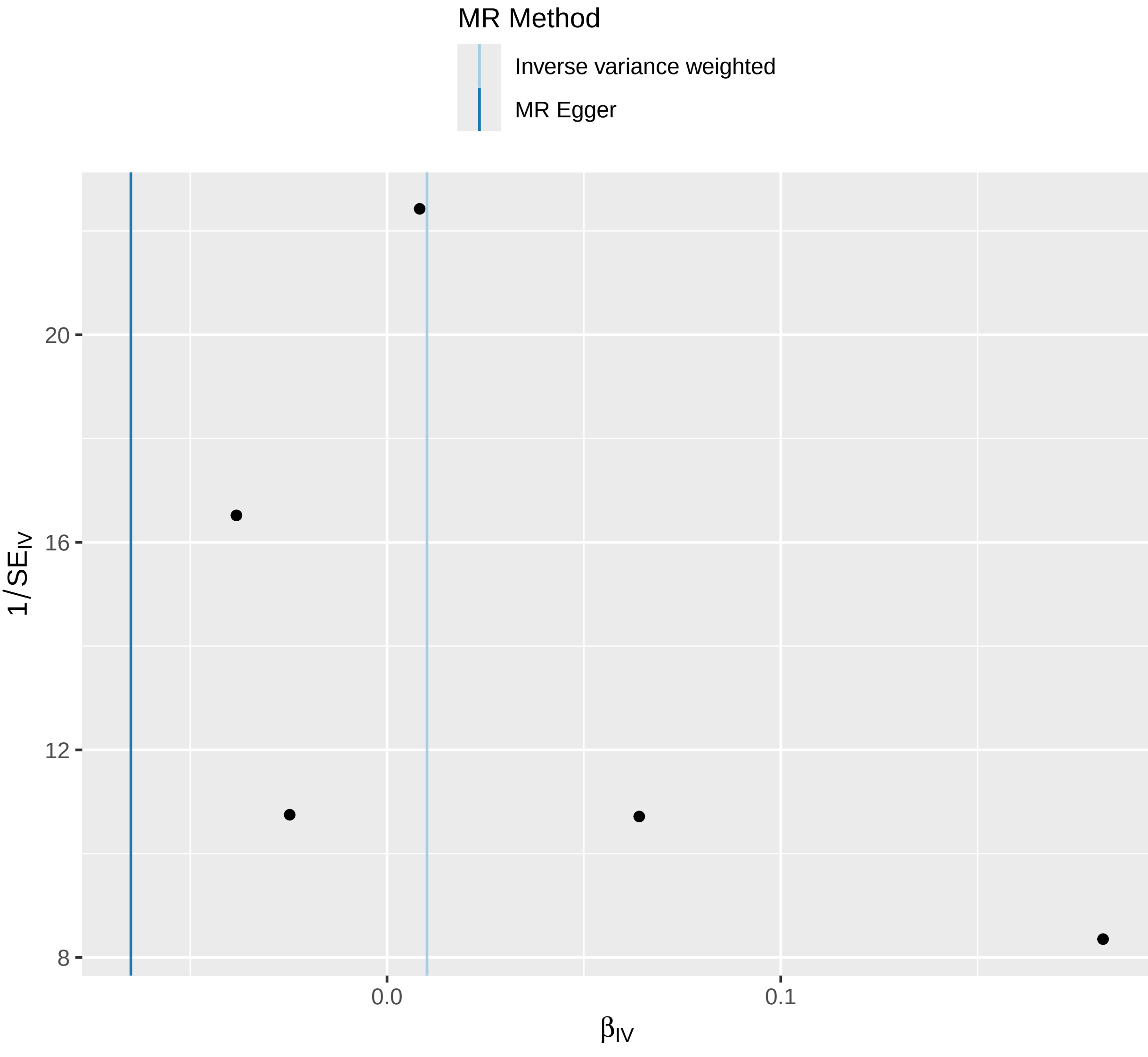

MR Method
Inverse variance weighted
MR Egger
20
16
12
8
1/SE$_{IV}$
0.0
0.1
β$_{IV}$

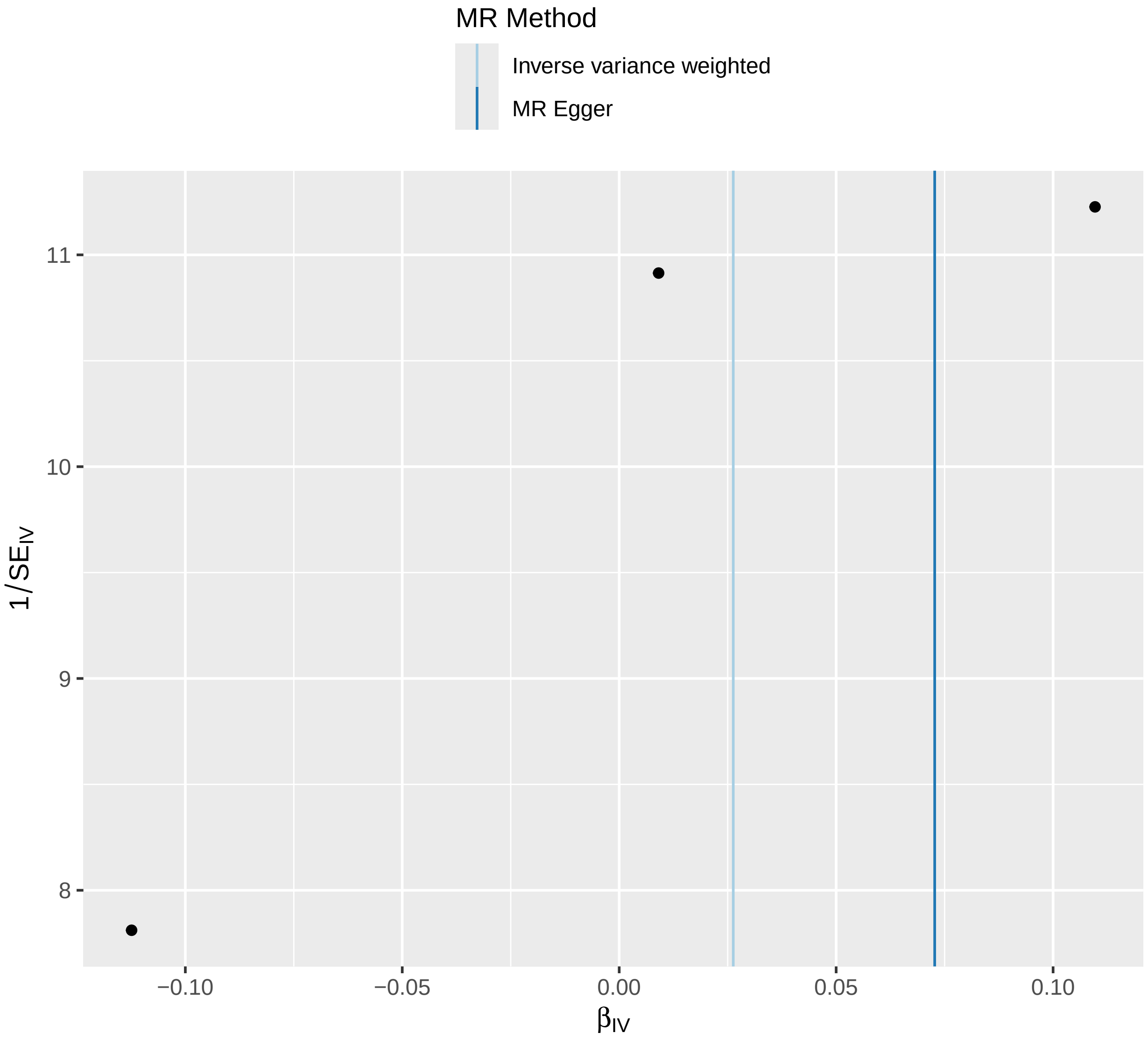
MR Method
Inverse variance weighted
MR Egger
11
10
9
8
1/SE_IV
−0.10
−0.05
0.00
0.05
0.10
β_IV

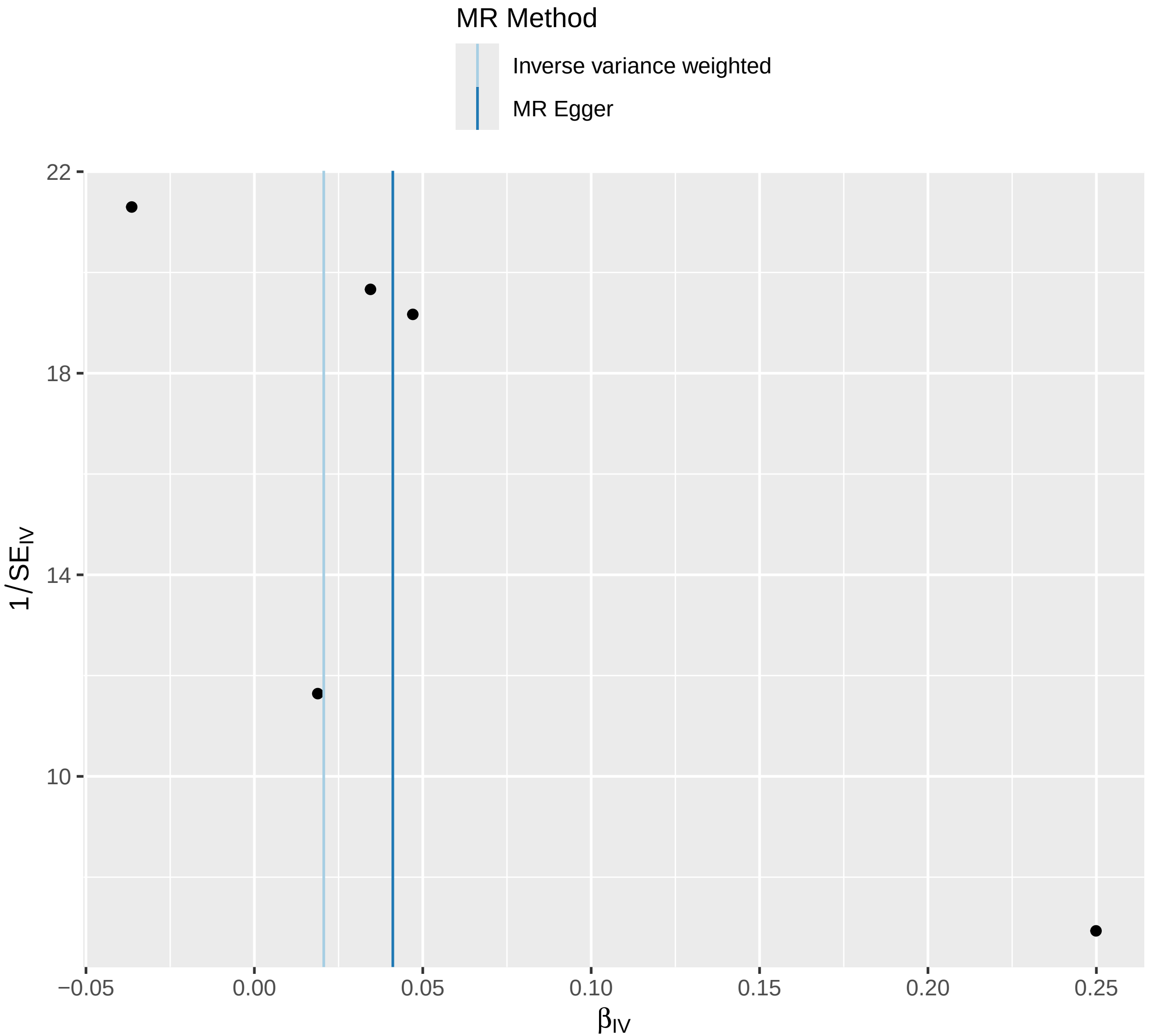
MR Method
Inverse variance weighted
MR Egger
22
18
14
10
1/SE_IV
−0.05
0.00
0.05
0.10
0.15
0.20
0.25
β_IV

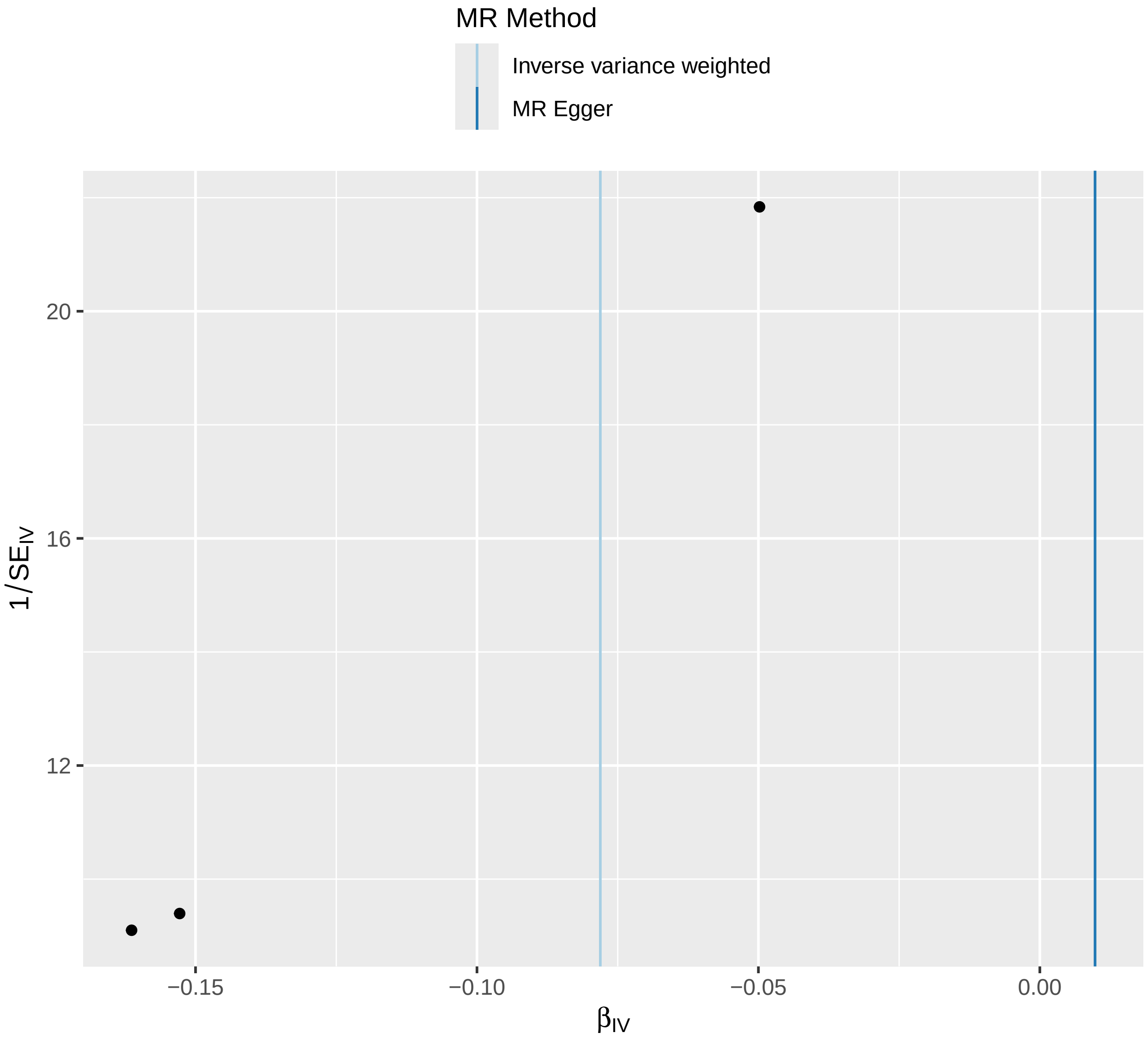

MR Method
Inverse variance weighted
MR Egger
20
16
12
1/SE_IV
−0.15
−0.10
−0.05
0.00
β_IV

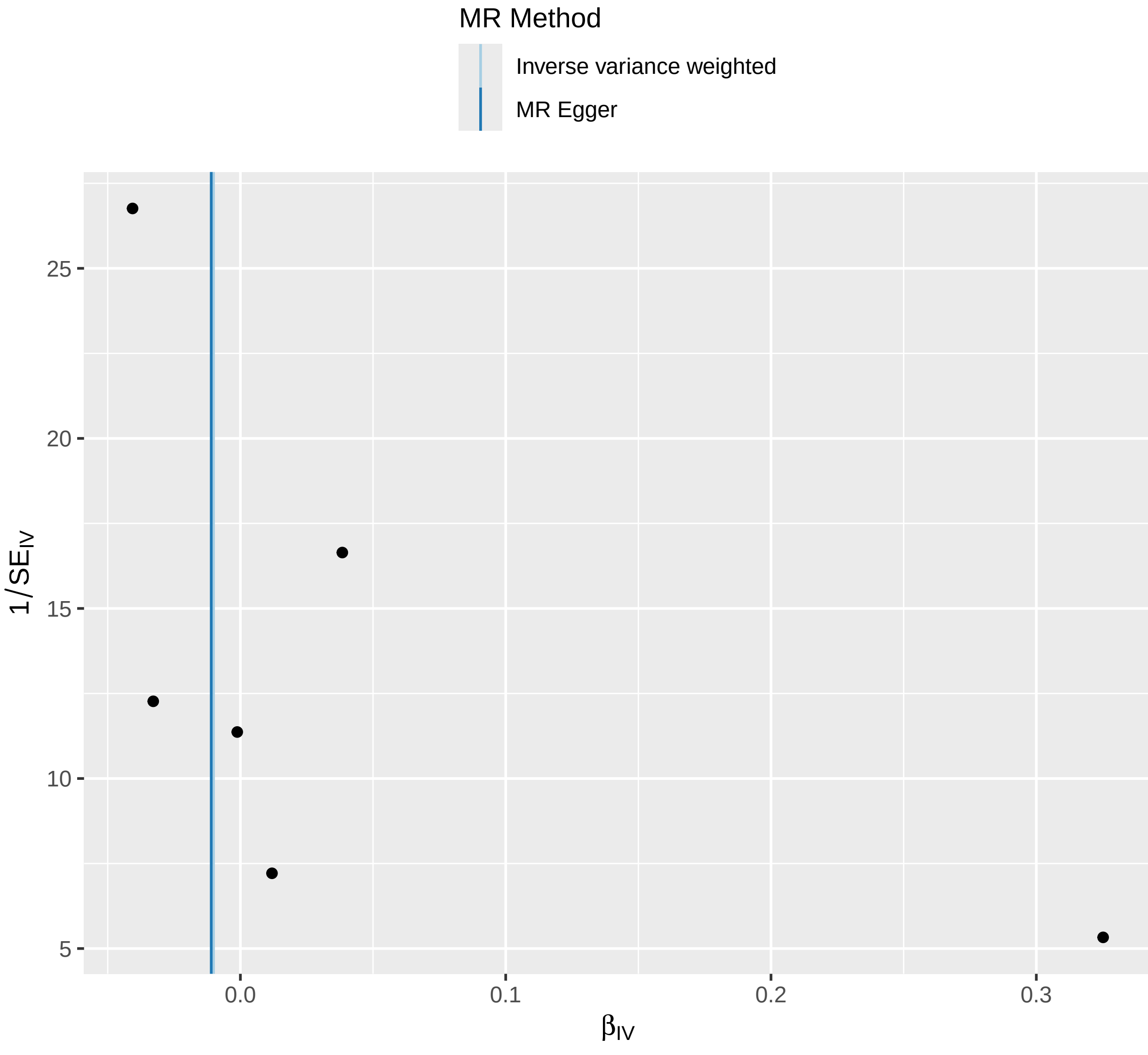
MR Method
Inverse variance weighted
MR Egger
25
20
15
10
5
0.0
0.1
0.2
0.3
$1/SE_{IV}$
β_{IV}

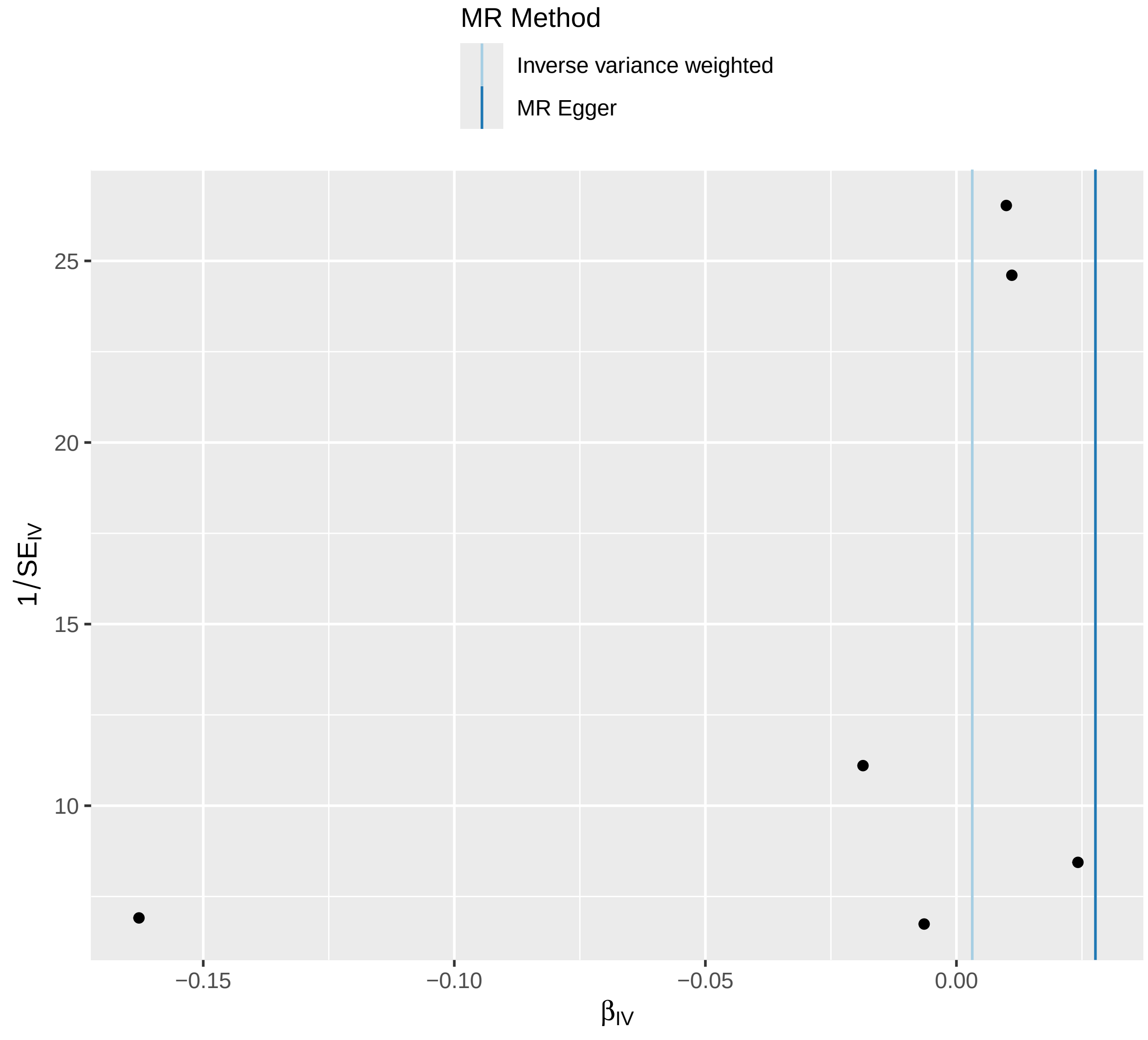
MR Method
Inverse variance weighted
MR Egger
25
20
15
10
1/SE_IV
−0.15
−0.10
−0.05
0.00
β_IV

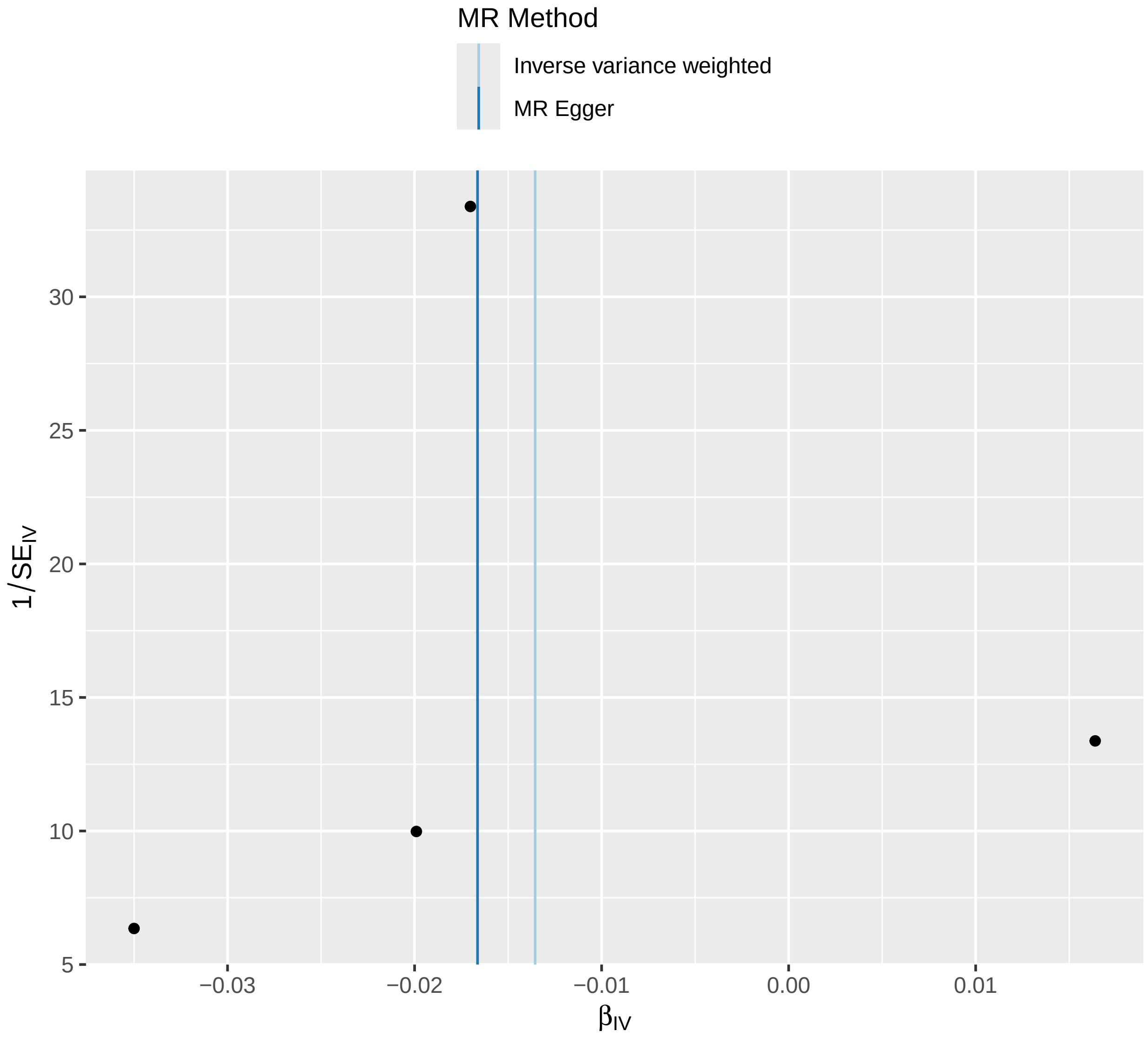
MR Method
Inverse variance weighted
MR Egger
1/SE_IV
30
25
20
15
10
5
−0.03
−0.02
−0.01
0.00
0.01
β_IV

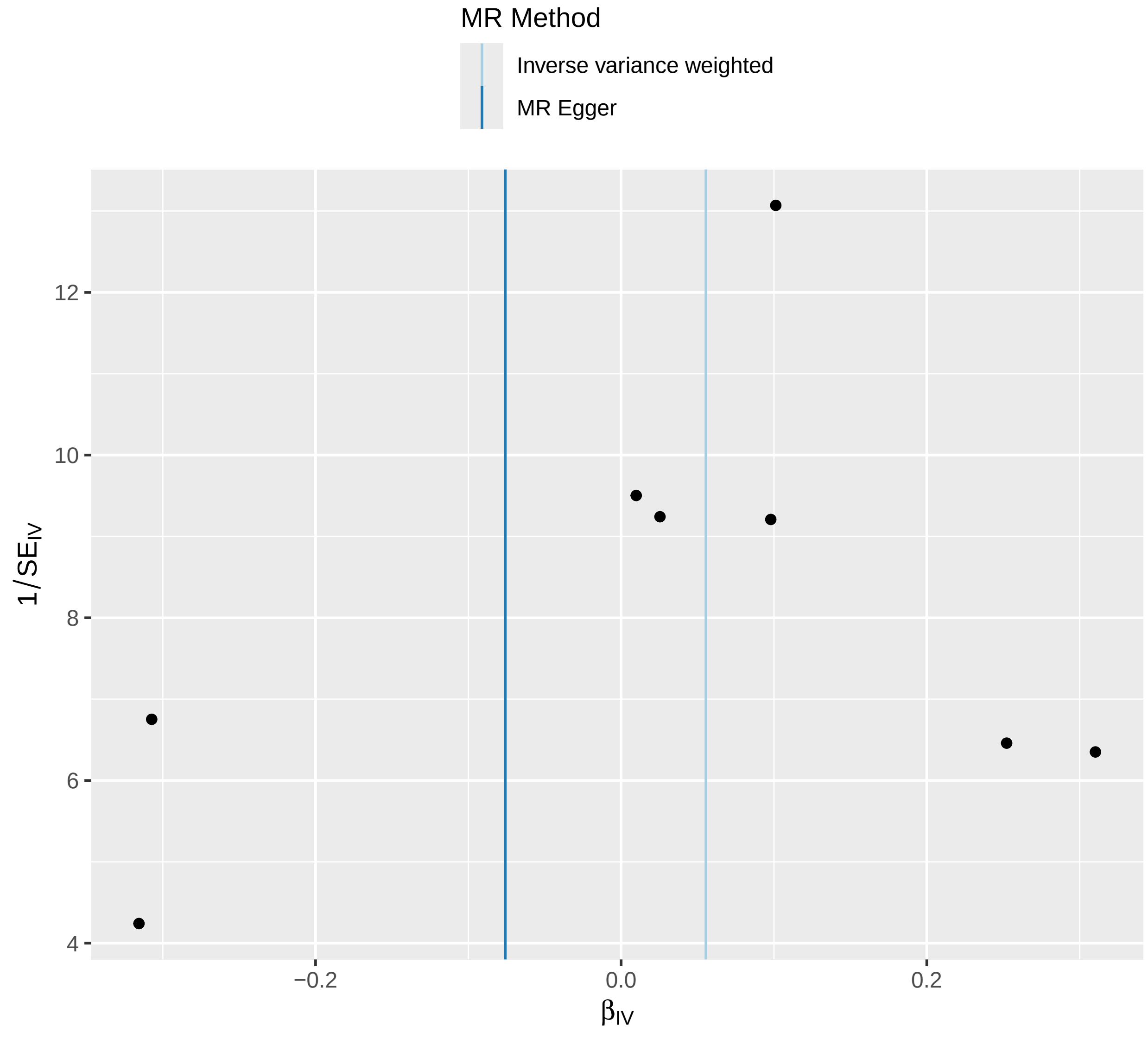
MR Method
Inverse variance weighted
MR Egger
12
10
8
6
4
−0.2
0.0
0.2
$1/SE_{IV}$
β_{IV}

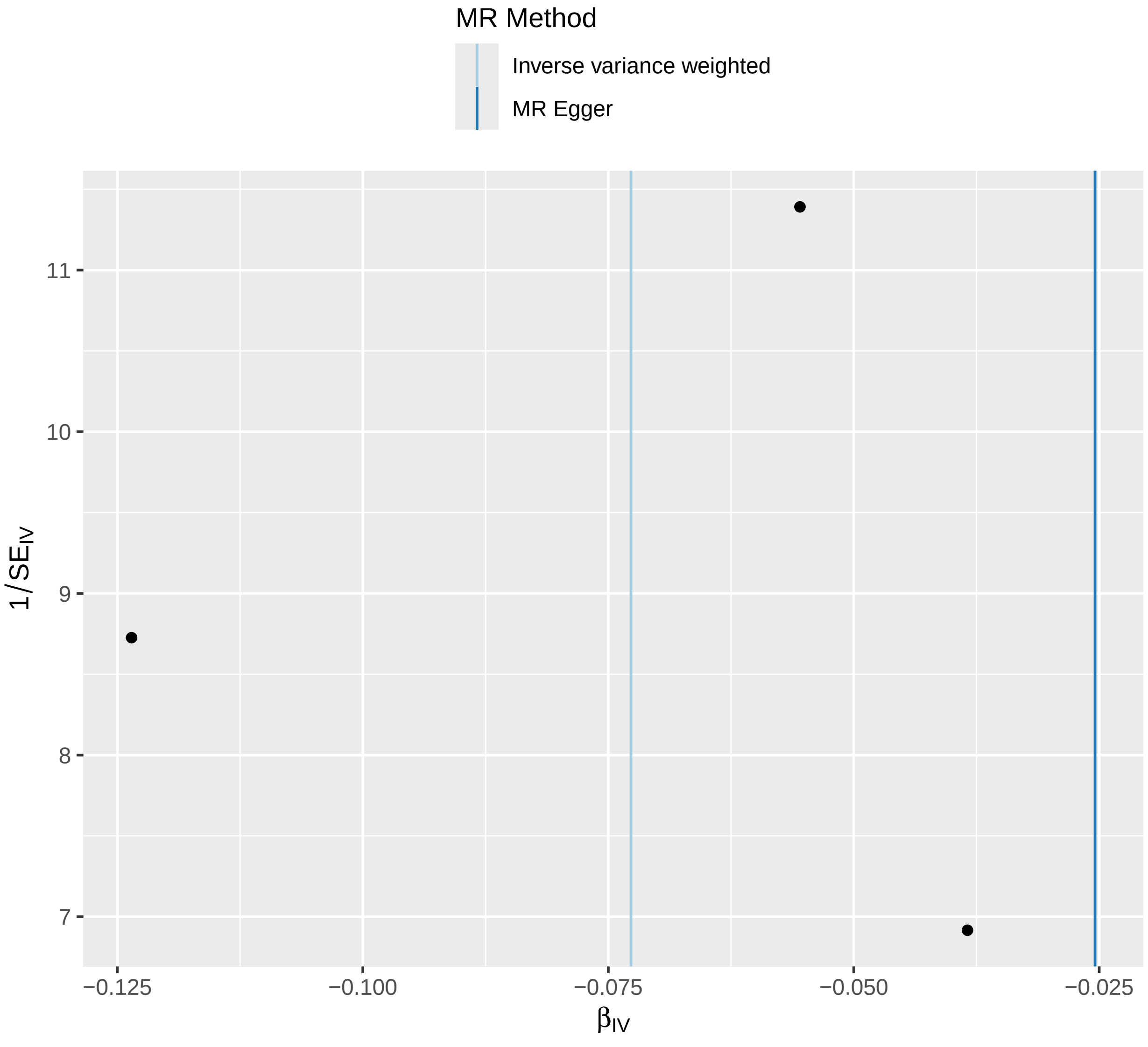

MR Method
Inverse variance weighted
MR Egger
11
10
9
8
7
1/SE_IV
−0.125
−0.100
−0.075
−0.050
−0.025
β_IV

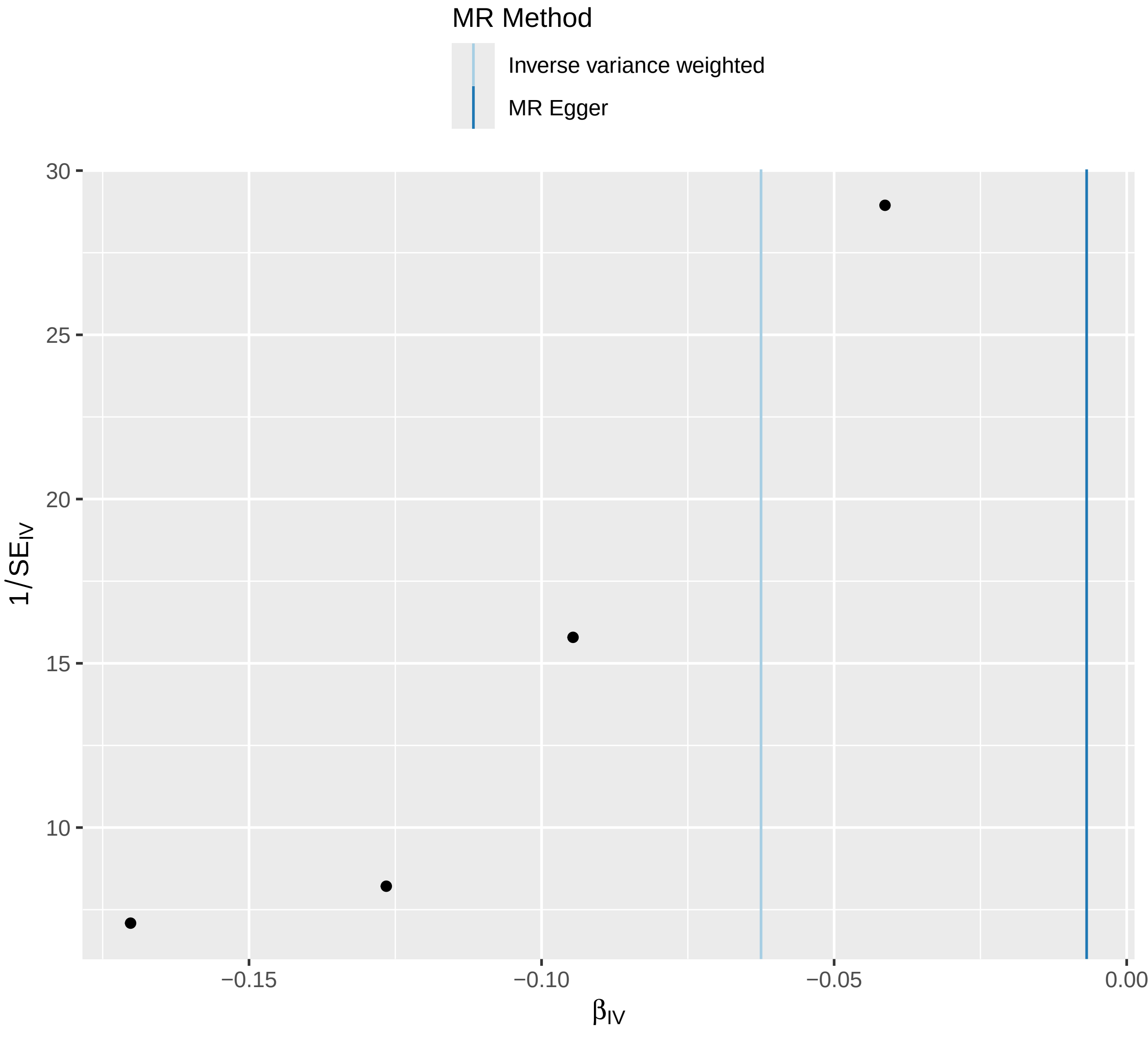
MR Method
Inverse variance weighted
MR Egger
30
25
20
15
10
1/SE_IV
−0.15
−0.10
−0.05
0.00
β_IV

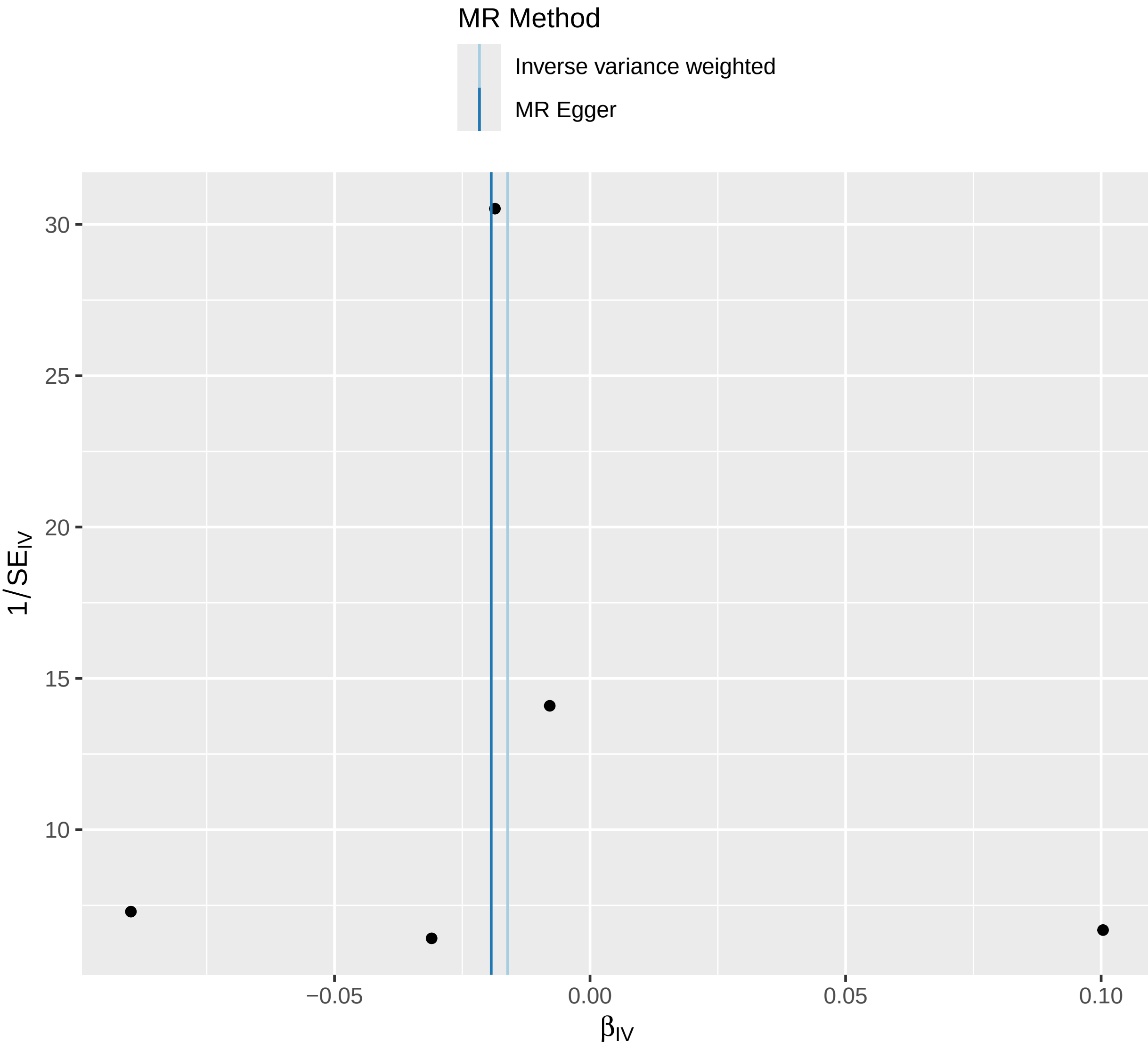
MR Method
Inverse variance weighted
MR Egger
30
25
20
15
10
1/SE_IV
−0.05
0.00
0.05
0.10
β_IV

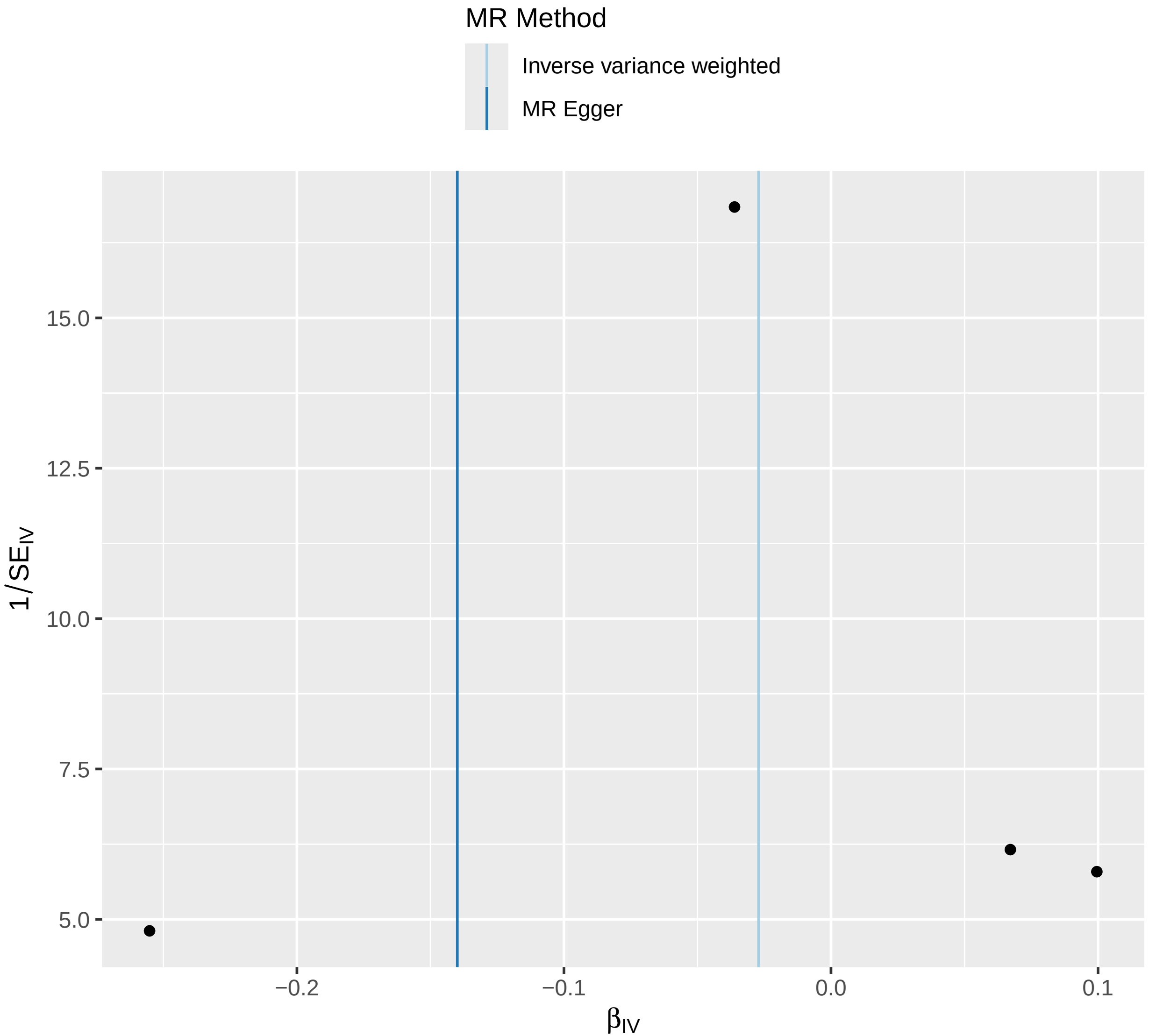
MR Method
Inverse variance weighted
MR Egger
15.0
12.5
10.0
7.5
5.0
1/SE_IV
−0.2
−0.1
0.0
0.1
β_IV

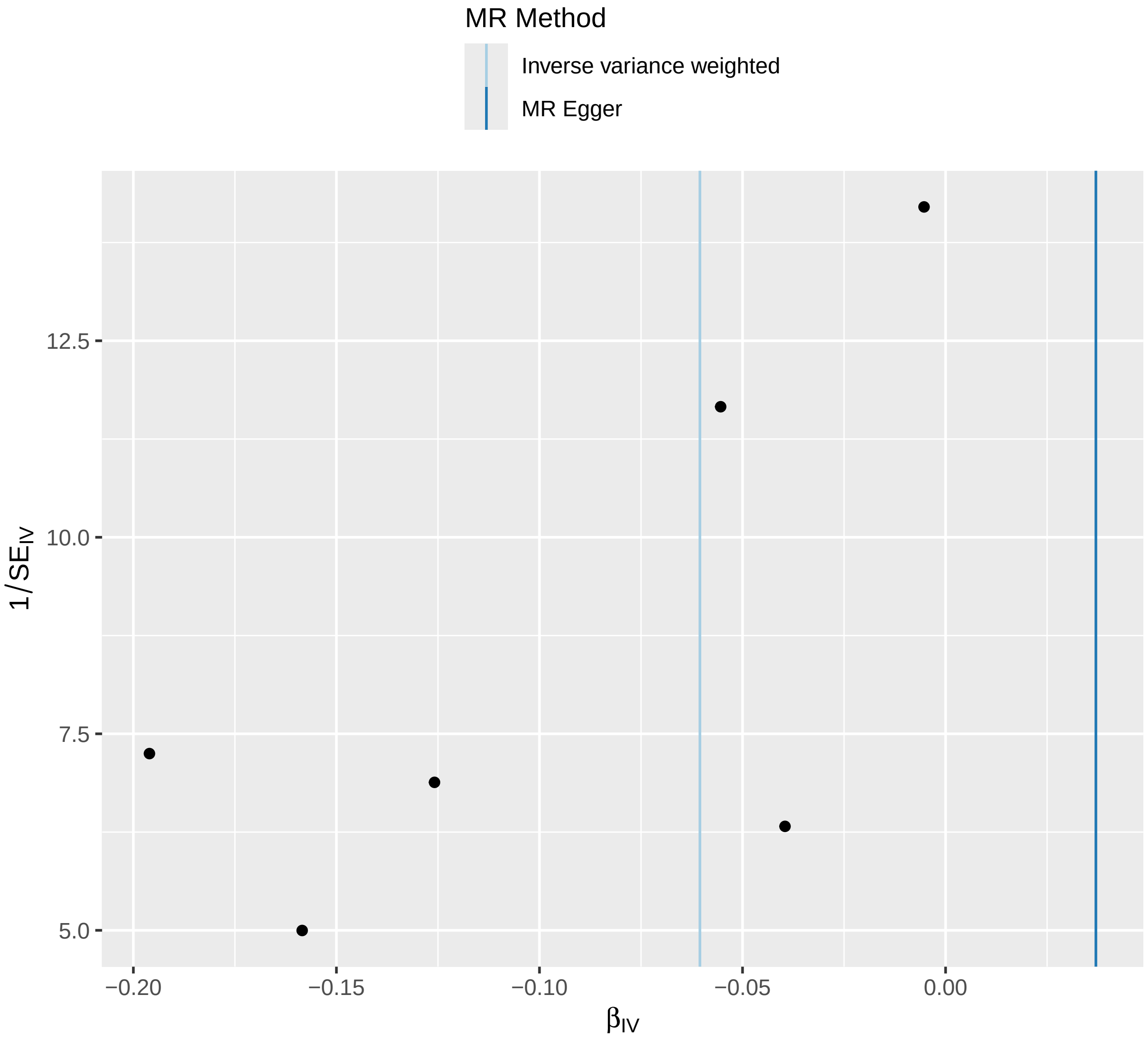

MR Method
Inverse variance weighted
MR Egger
12.5
10.0
7.5
5.0
$1/SE_{IV}$
−0.20
−0.15
−0.10
−0.05
0.00
β_{IV}

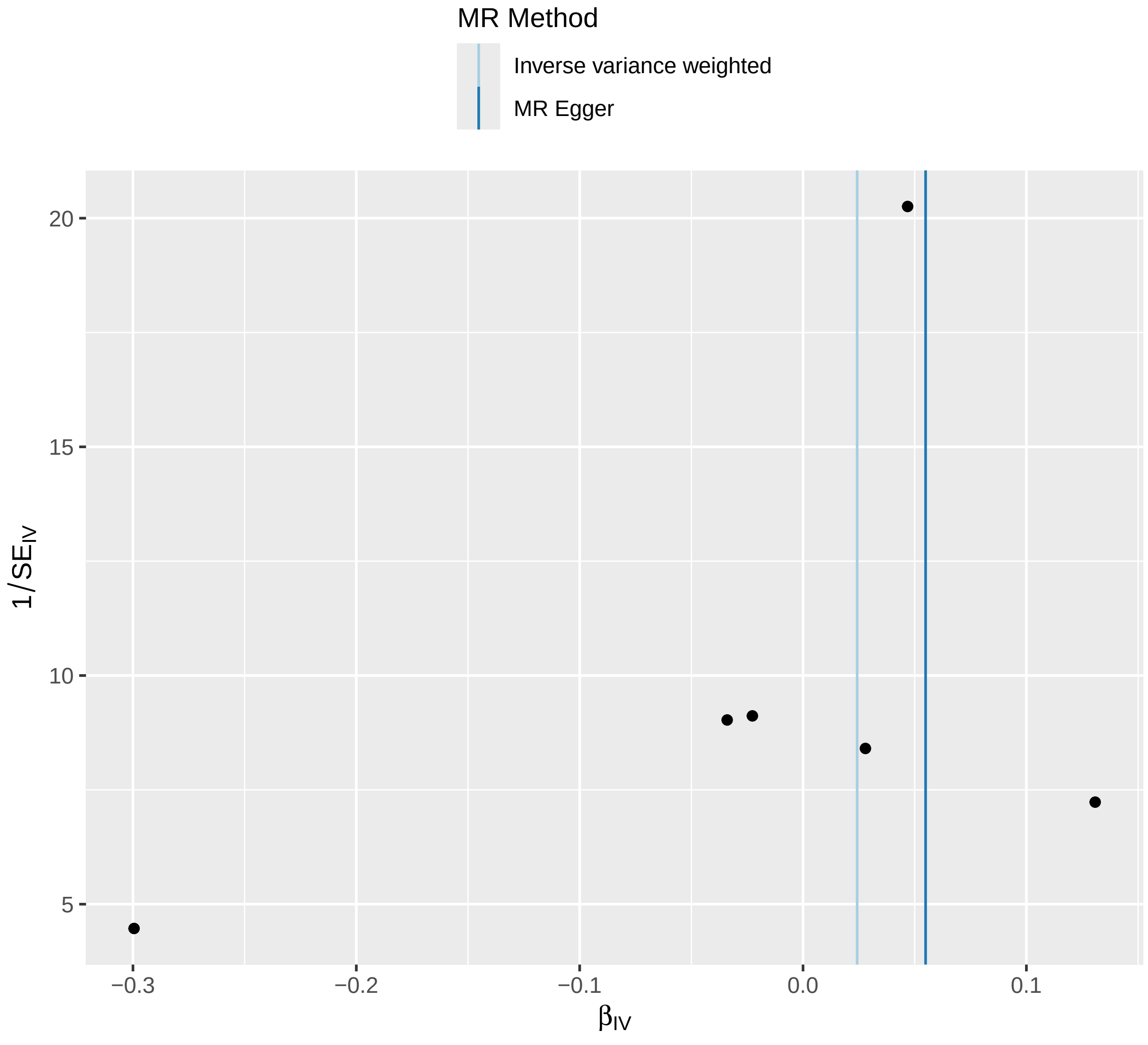
MR Method
Inverse variance weighted
MR Egger
20
15
10
5
1/SE_IV
−0.3
−0.2
−0.1
0.0
0.1
β_IV

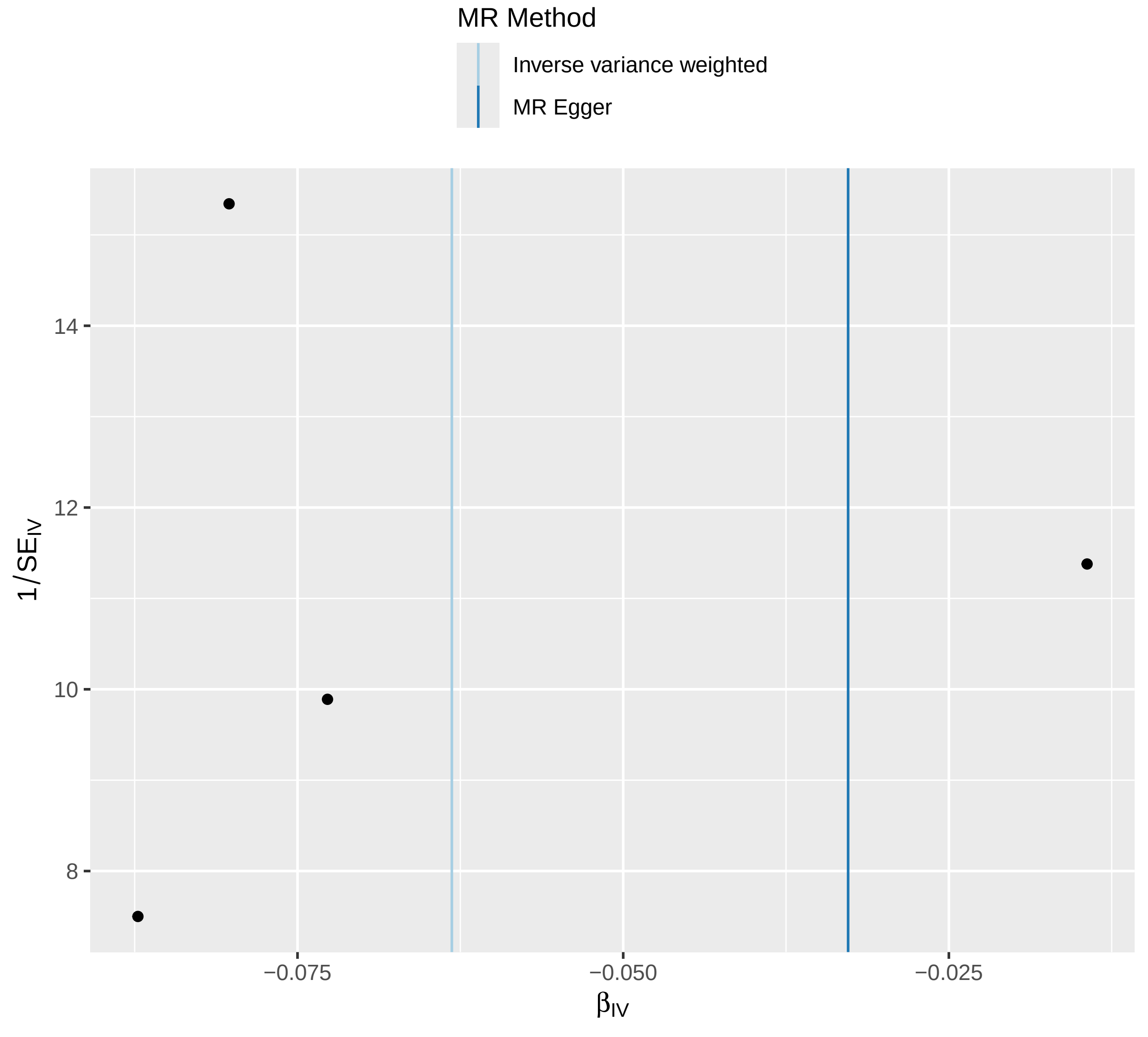
MR Method
Inverse variance weighted
MR Egger
14
12
10
8
1/SE_IV
−0.075
−0.050
−0.025
β_IV

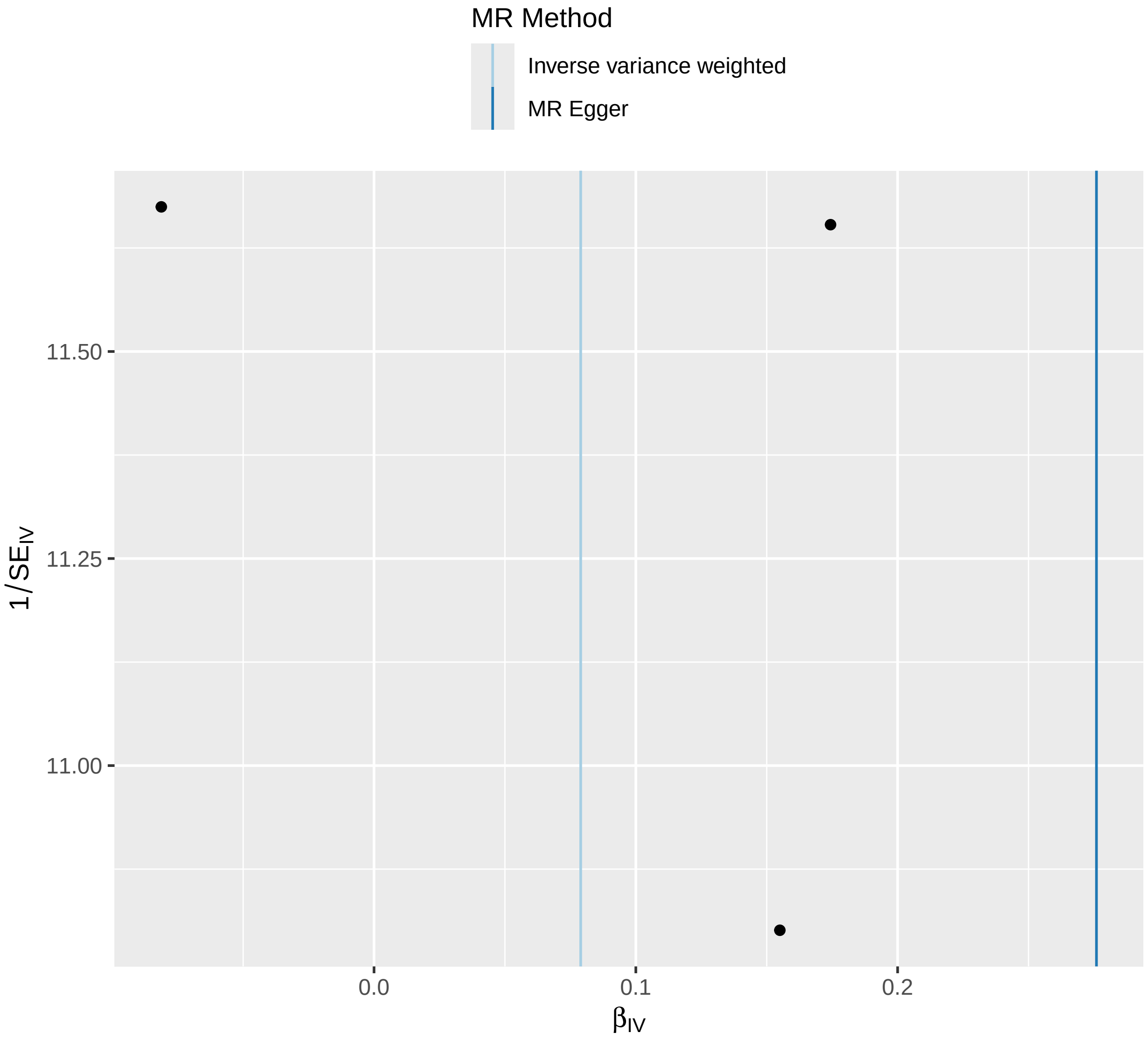
MR Method
Inverse variance weighted
MR Egger
11.50
11.25
11.00
1/SE_{IV}
0.0
0.1
0.2
β_{IV}

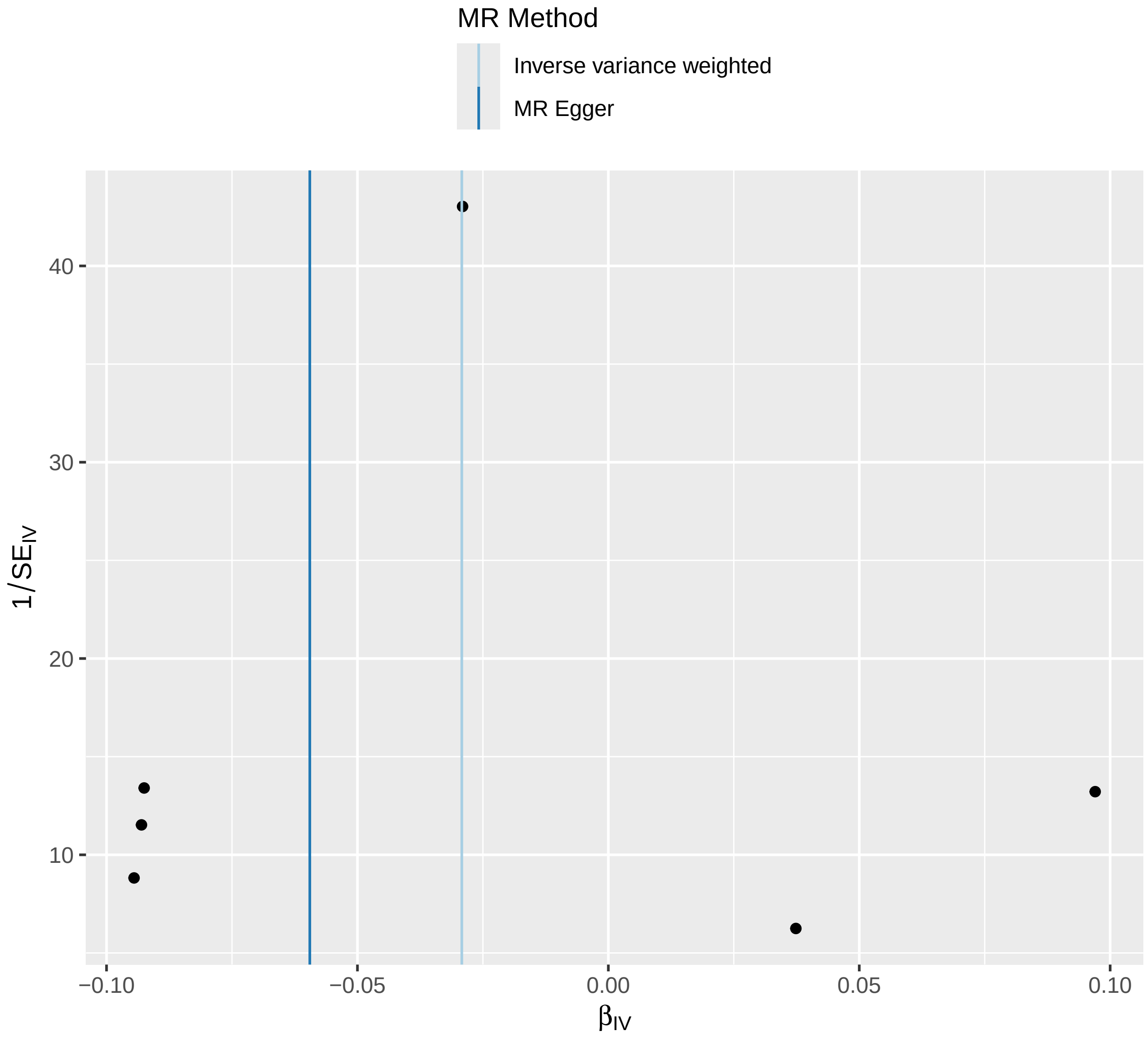
MR Method
Inverse variance weighted
MR Egger
40
30
20
10
1/SE_IV
−0.10
−0.05
0.00
0.05
0.10
β_IV

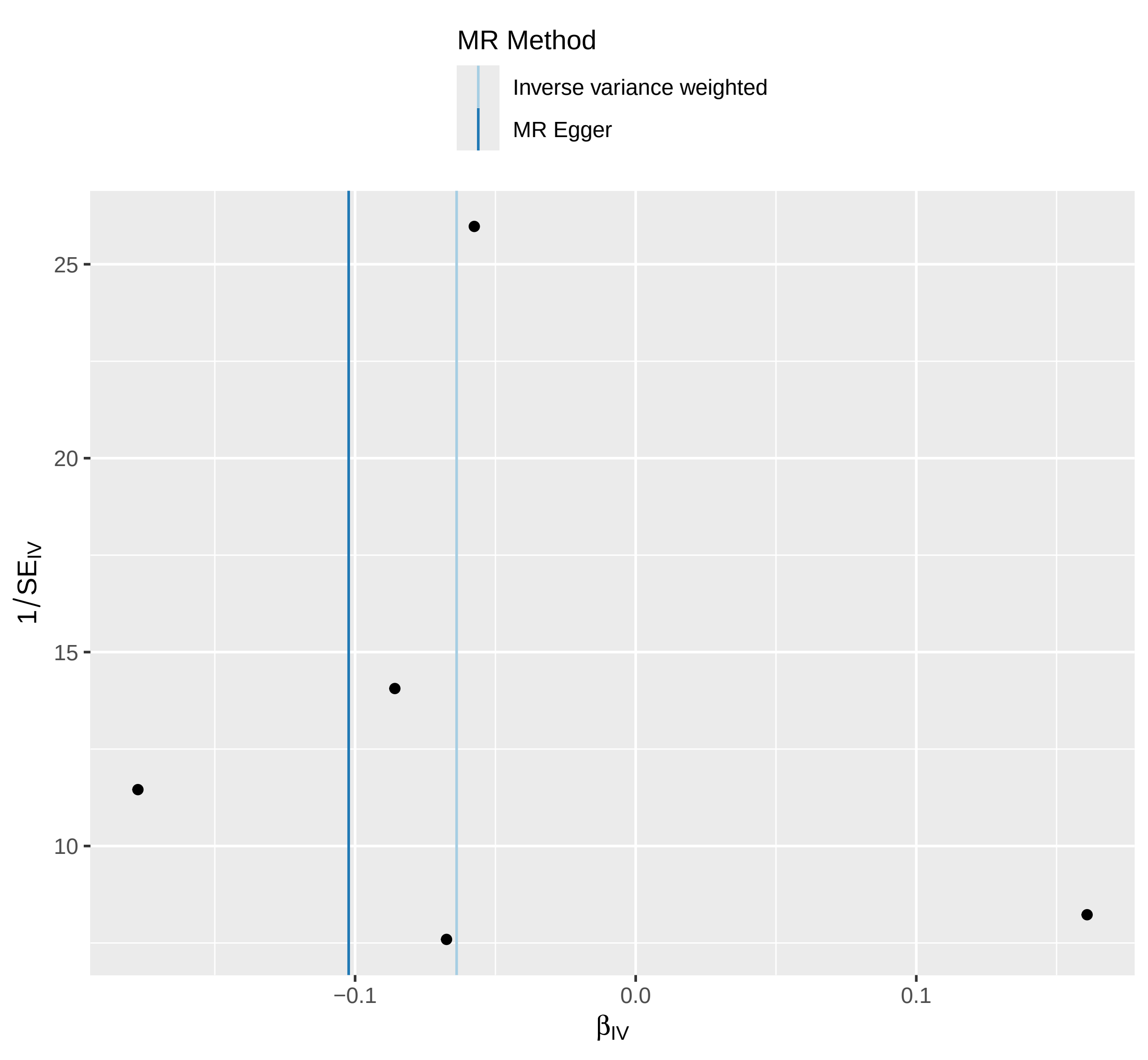
MR Method
Inverse variance weighted
MR Egger
25
20
15
10
1/SE_IV
−0.1
0.0
0.1
β_IV

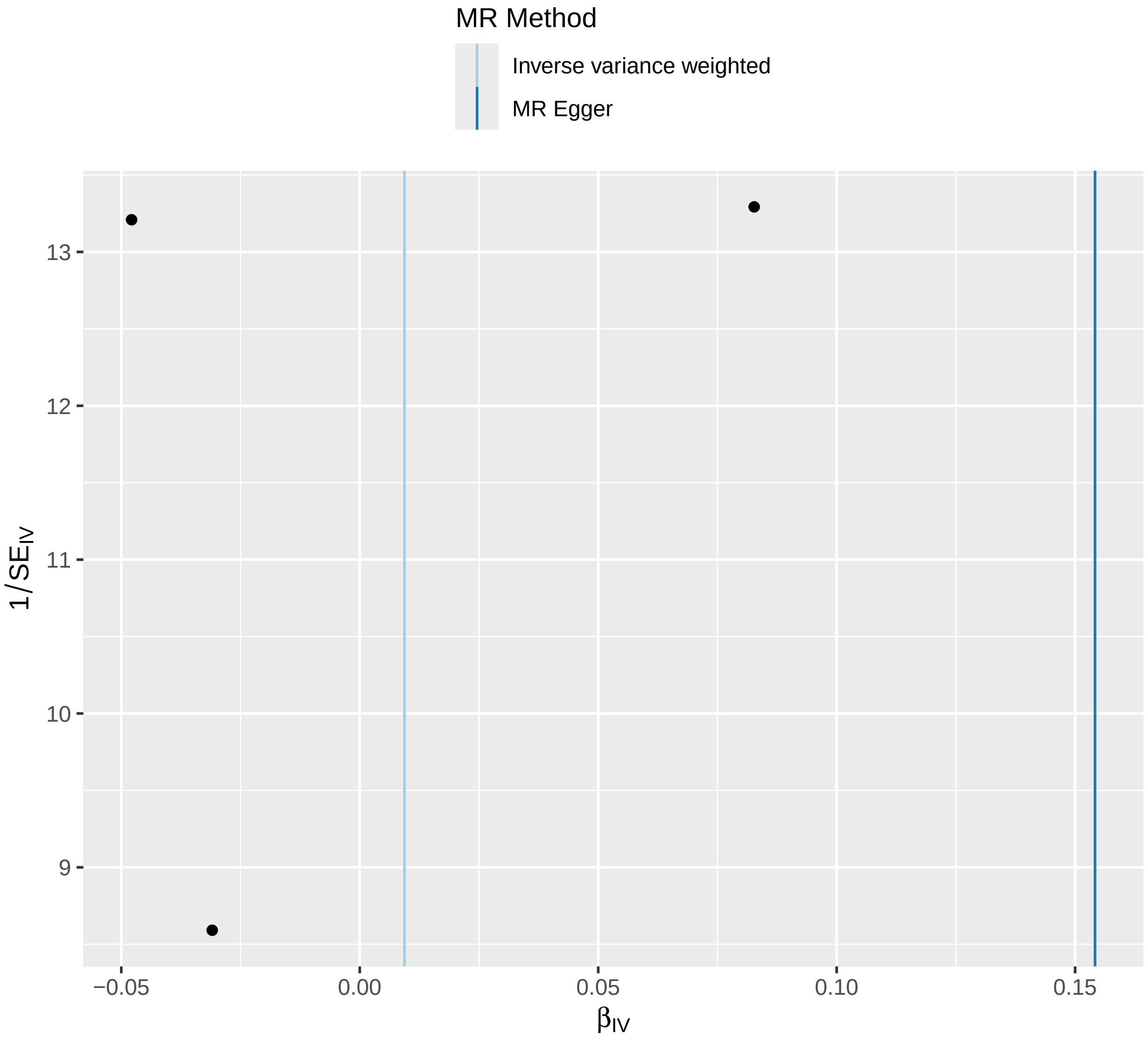

MR Method
Inverse variance weighted
MR Egger
13
12
11
10
9
1/SE$_{IV}$
−0.05
0.00
0.05
0.10
0.15
β_{IV}

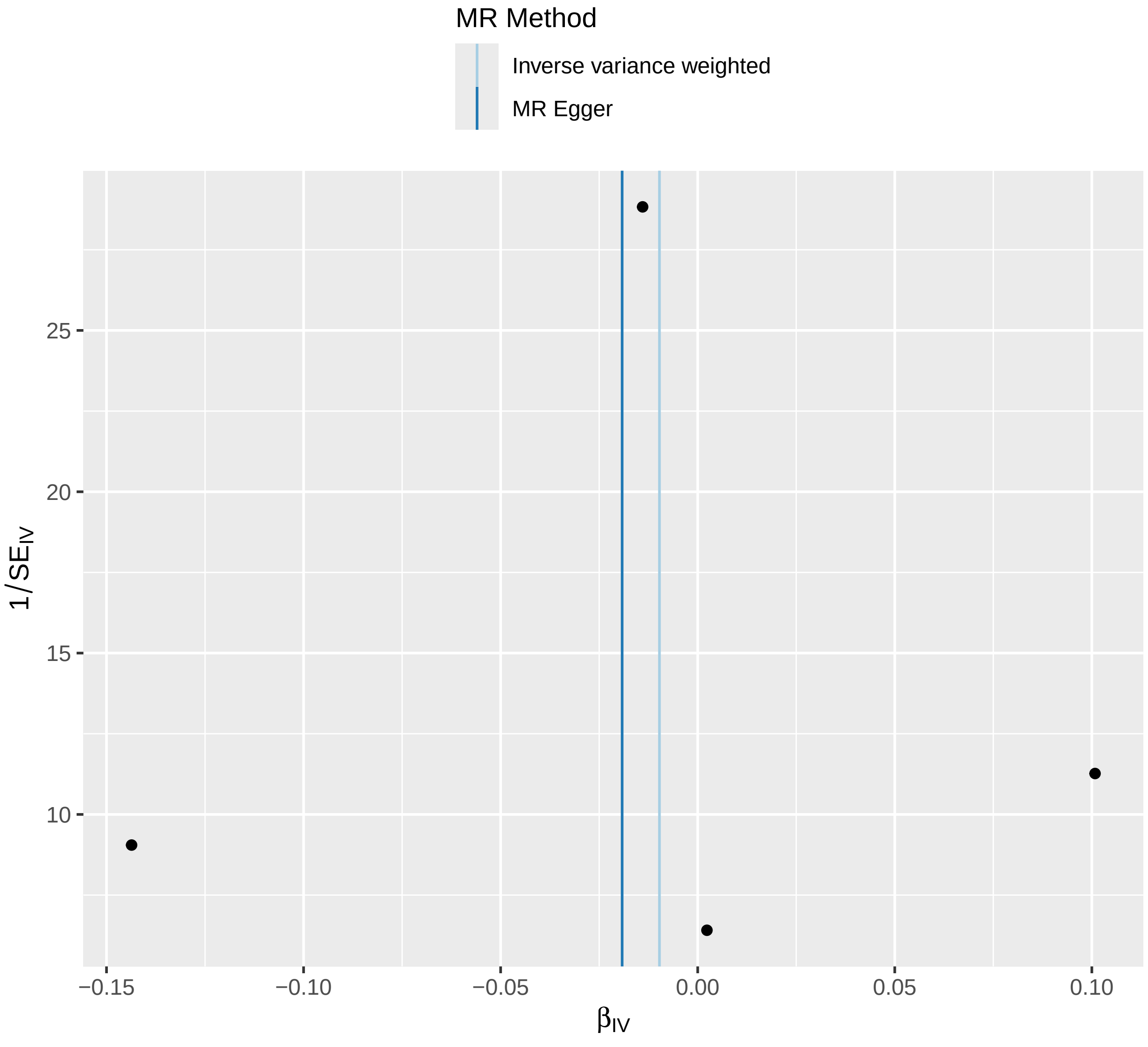

MR Method
Inverse variance weighted
MR Egger
1/SE_IV
25
20
15
10
−0.15
−0.10
−0.05
0.00
0.05
0.10
β_IV

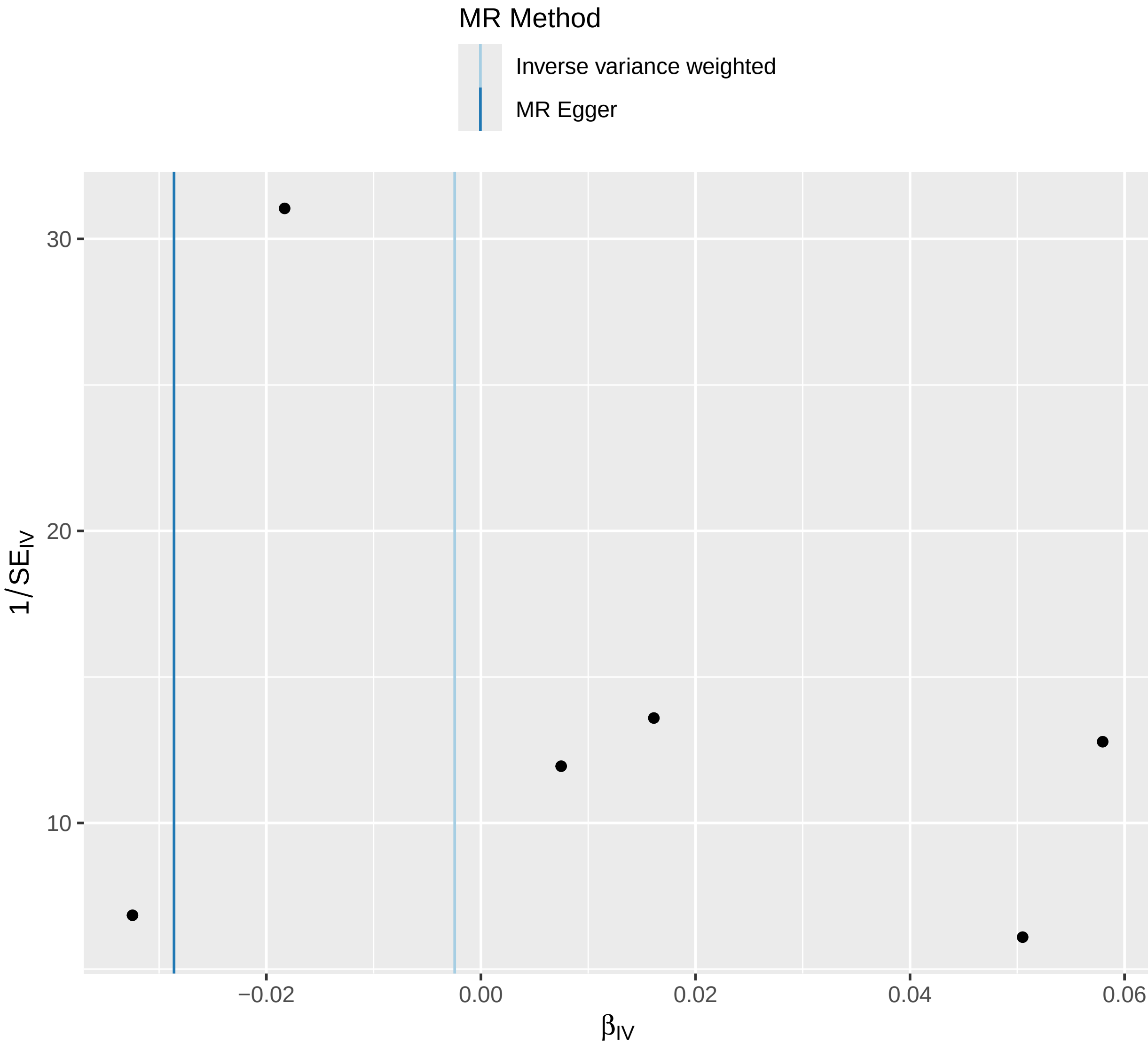
MR Method
Inverse variance weighted
MR Egger
30
20
10
1/SE_IV
−0.02
0.00
0.02
0.04
0.06
β_IV

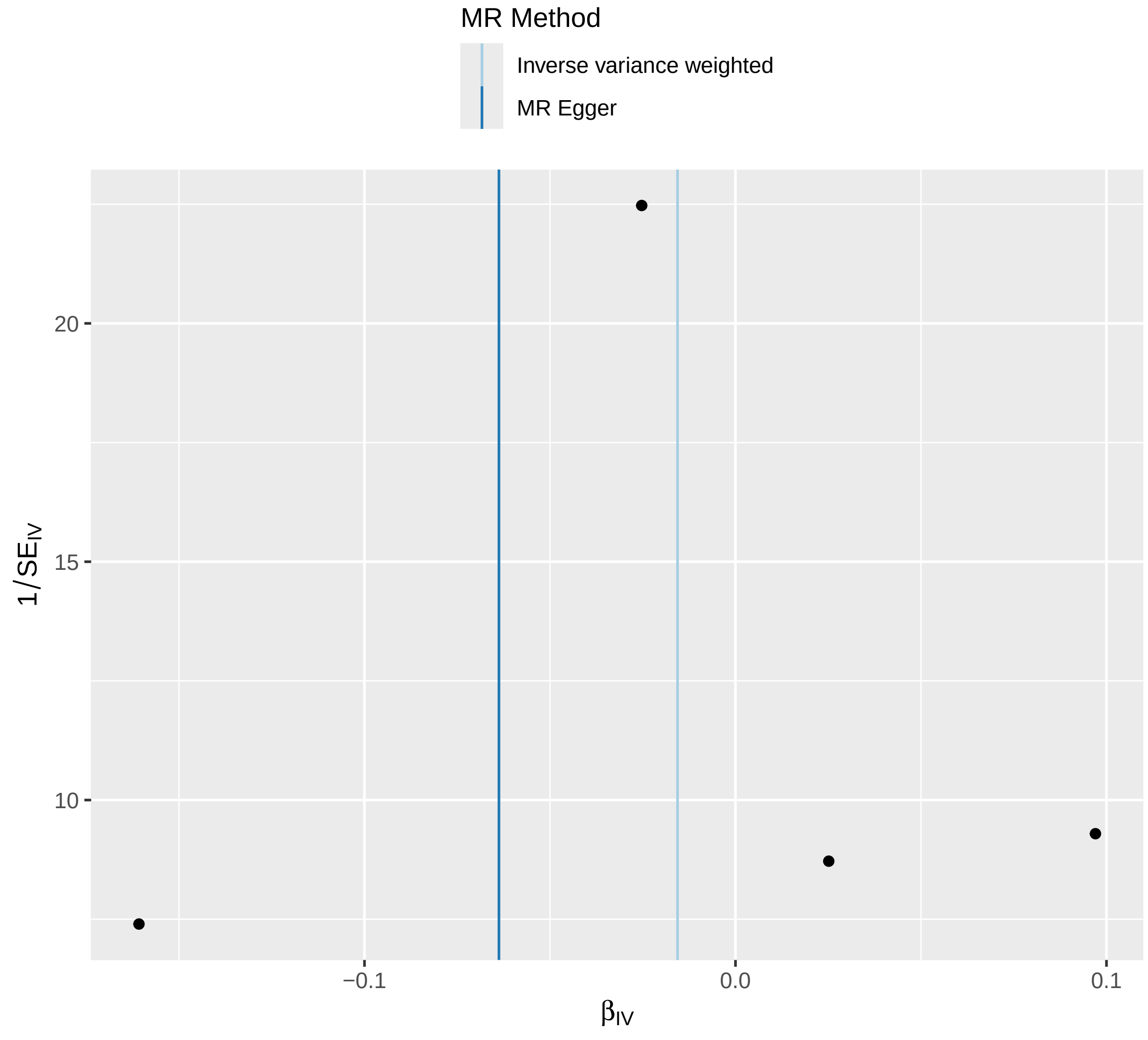

MR Method
Inverse variance weighted
MR Egger
20
15
10
1/SE_IV
−0.1
0.0
0.1
β_IV

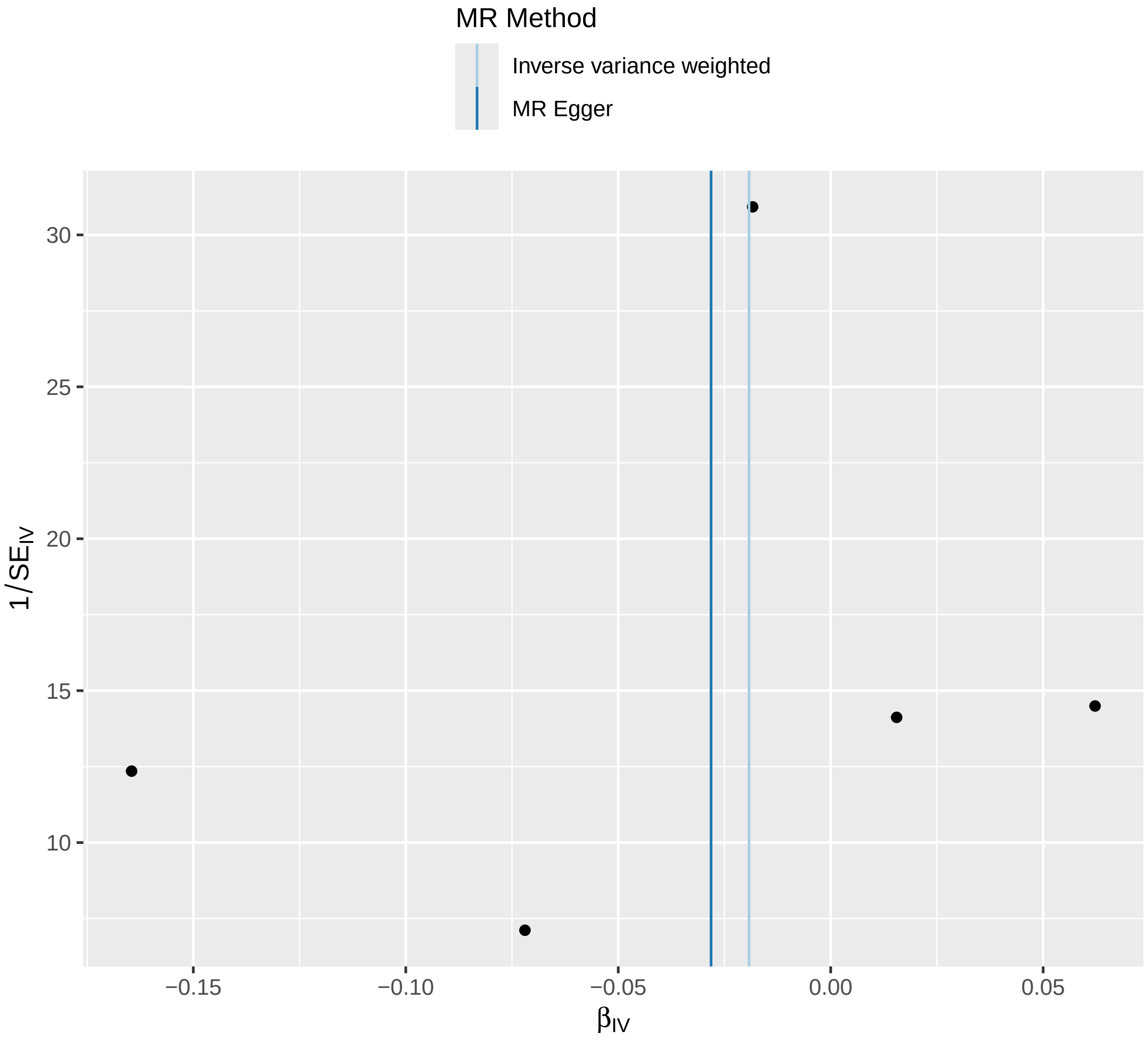
MR Method
Inverse variance weighted
MR Egger
30
25
20
15
10
$1/SE_{IV}$
−0.15
−0.10
−0.05
0.00
0.05
β_{IV}

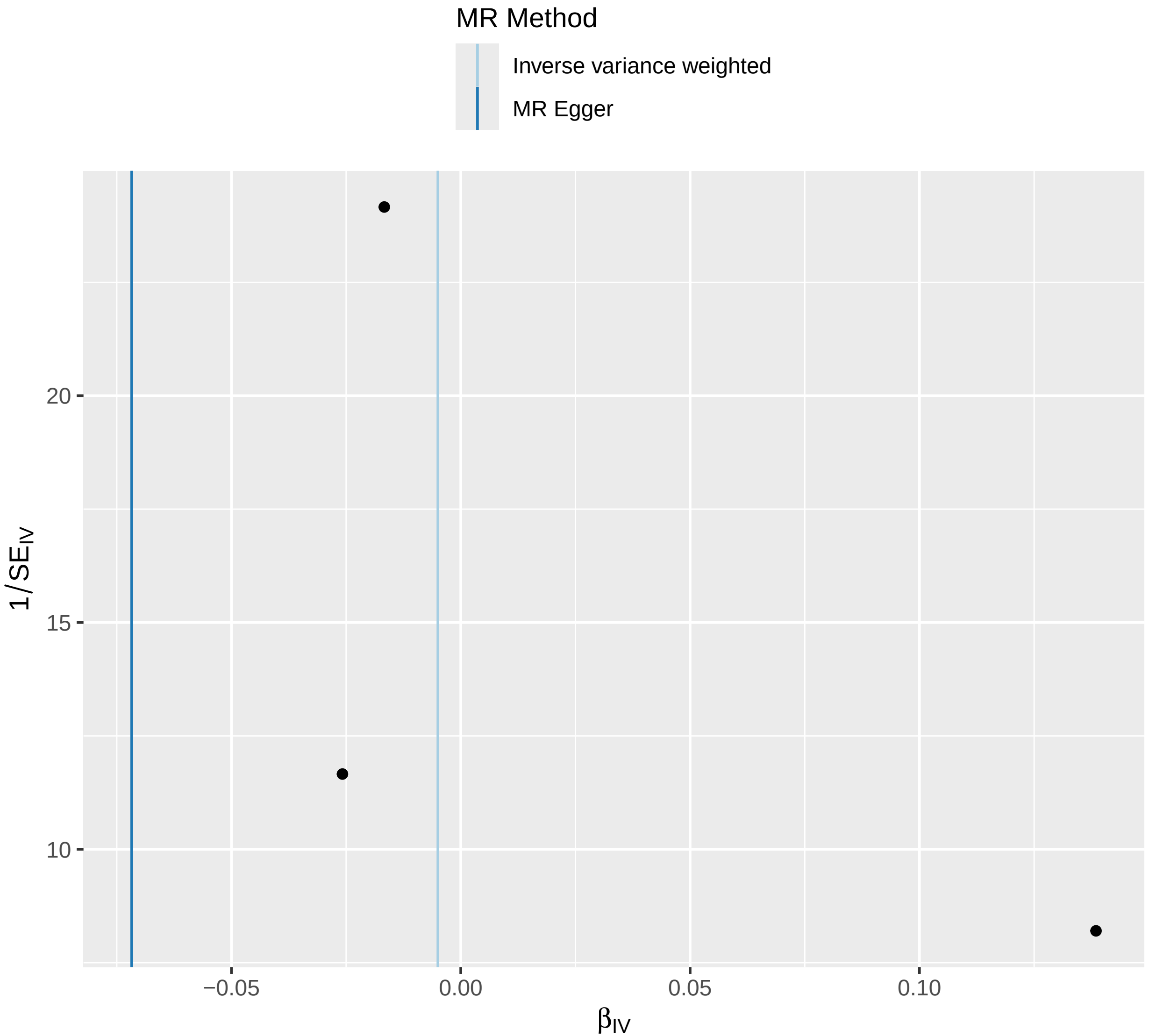
MR Method
Inverse variance weighted
MR Egger
20
15
10
1/SE_IV
−0.05
0.00
0.05
0.10
β_IV

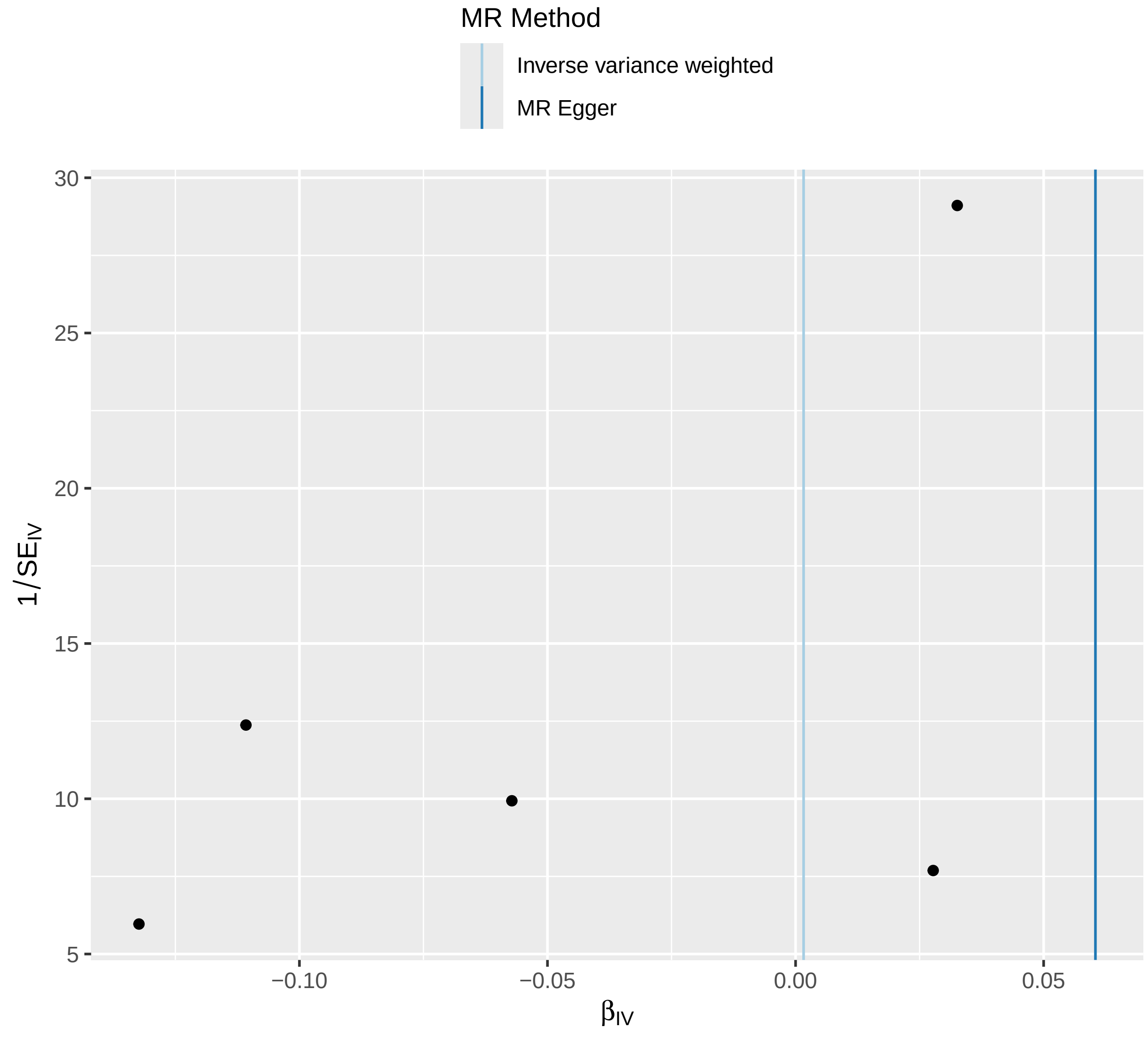
MR Method
Inverse variance weighted
MR Egger
30
25
20
15
10
5
1/SE_IV
−0.10
−0.05
0.00
0.05
β_IV

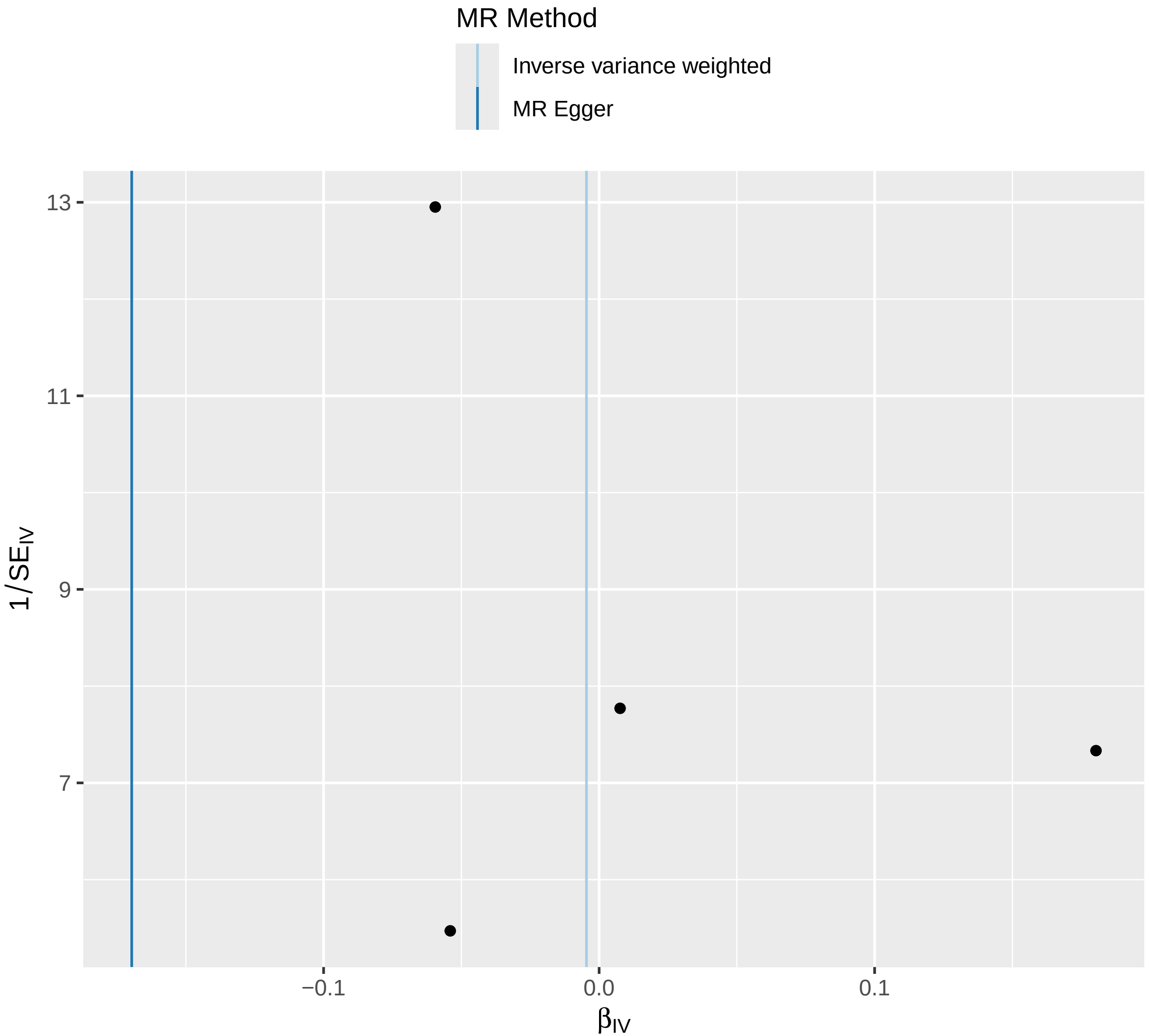
MR Method
Inverse variance weighted
MR Egger
13
11
9
7
1/SE_IV
−0.1
0.0
0.1
β_IV

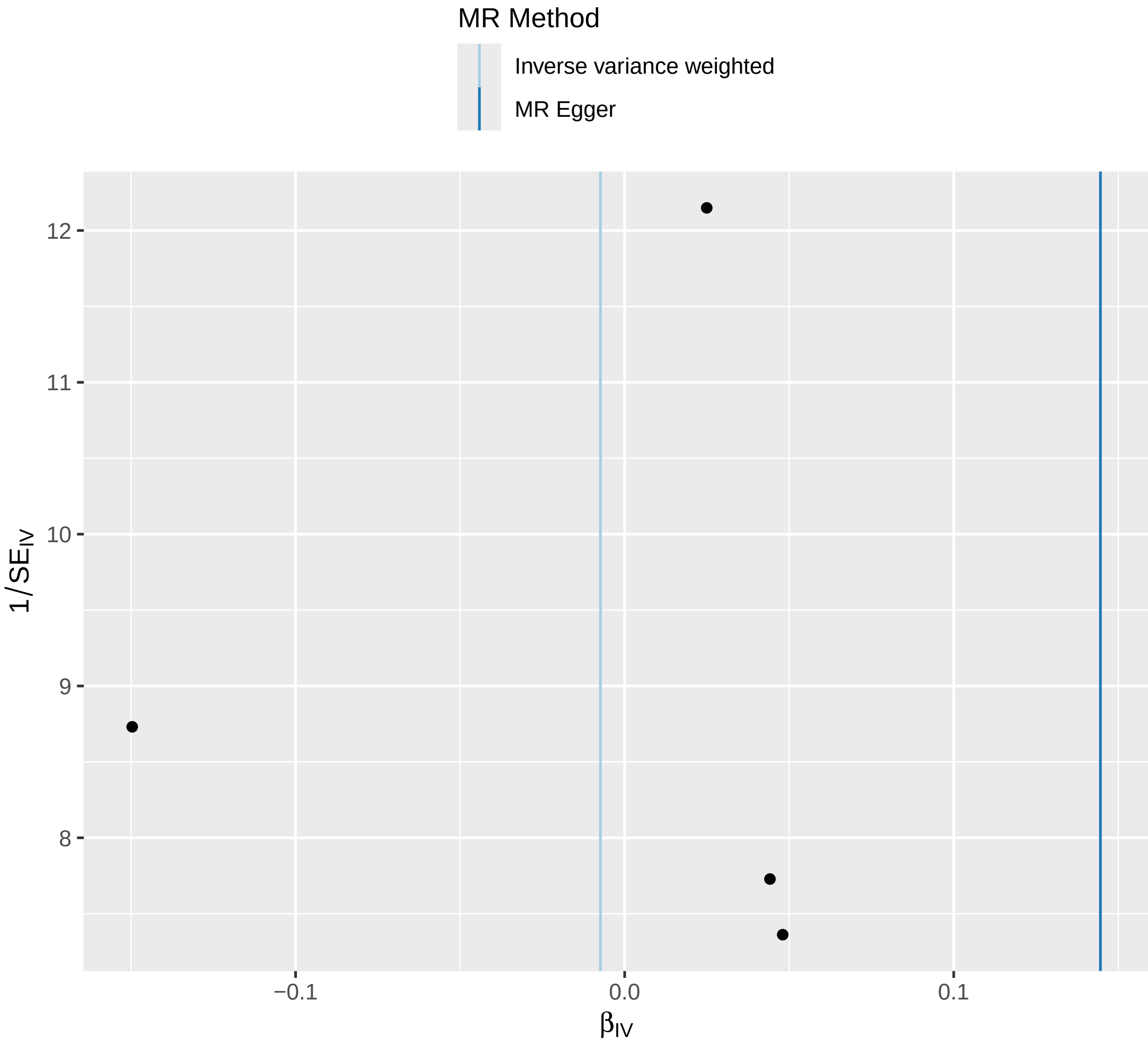
MR Method
Inverse variance weighted
MR Egger
1/SE_IV
12
11
10
9
8
−0.1
0.0
0.1
β_IV

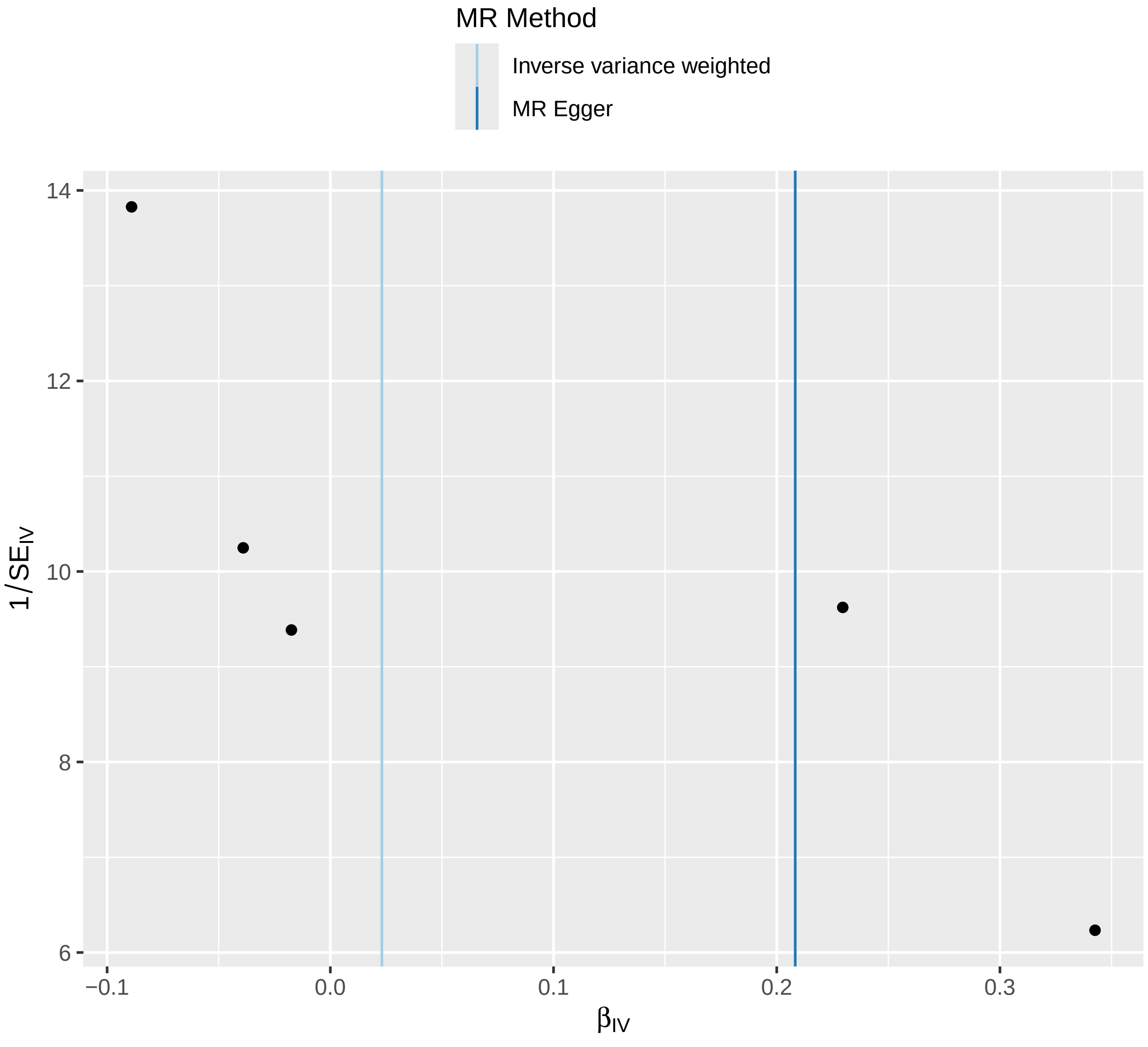
MR Method
Inverse variance weighted
MR Egger
14
12
10
8
6
1/SE_IV
−0.1
0.0
0.1
0.2
0.3
β_IV

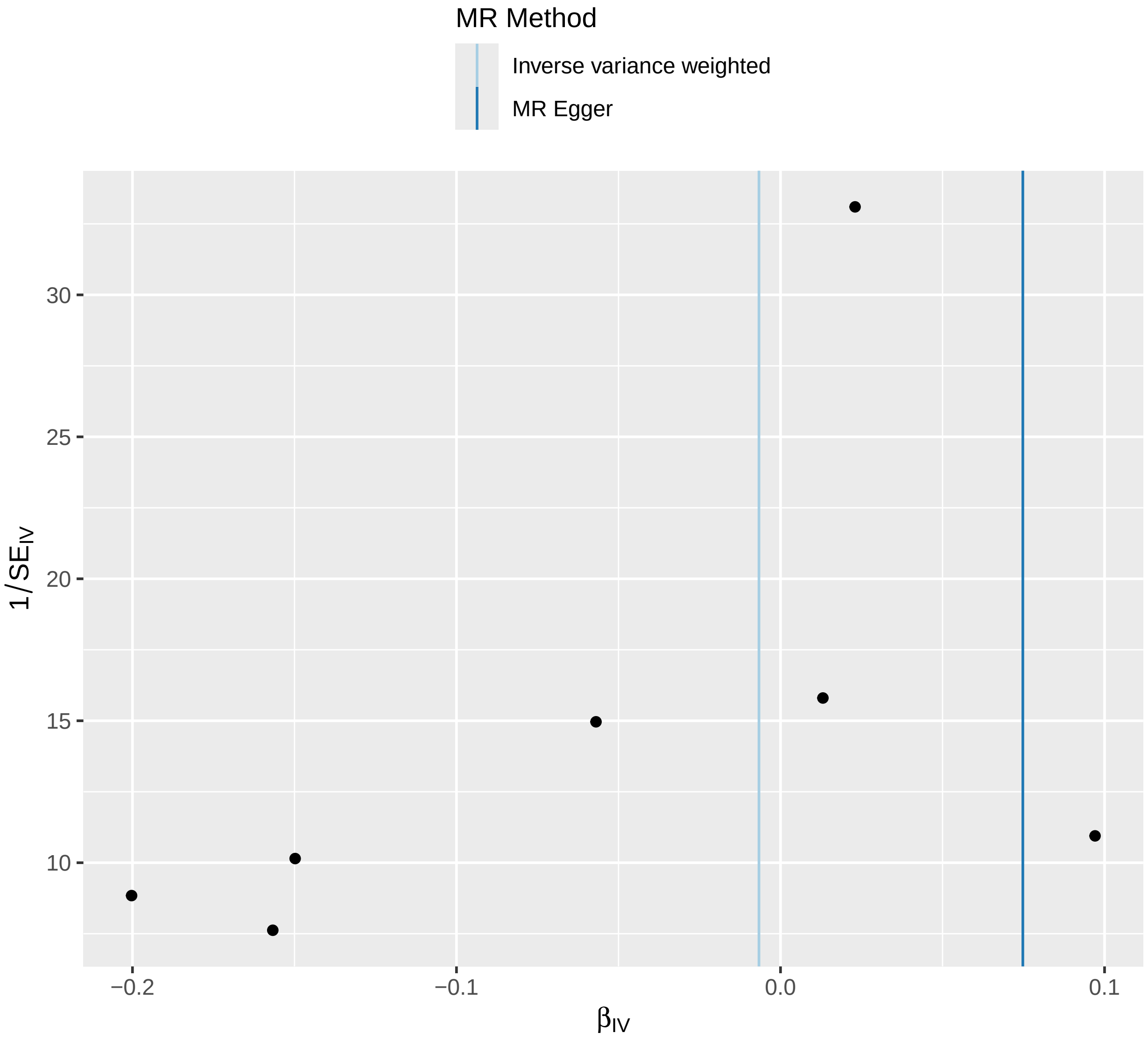

MR Method
Inverse variance weighted
MR Egger
30
25
20
15
10
$1/SE_{IV}$
−0.2
−0.1
0.0
0.1
β_{IV}

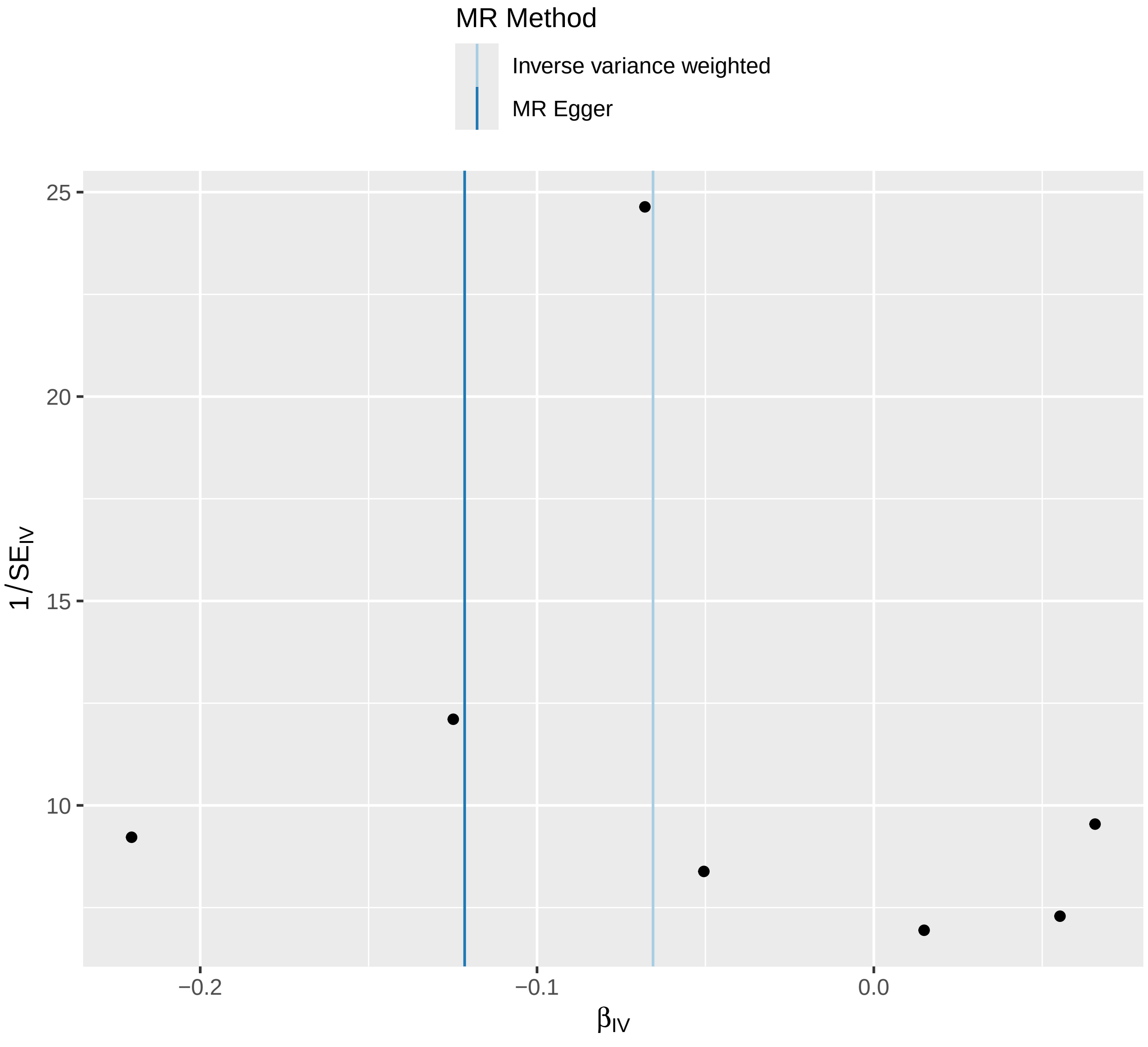

MR Method
Inverse variance weighted
MR Egger
25
20
15
10
1/SE_IV
−0.2
−0.1
0.0
β_IV

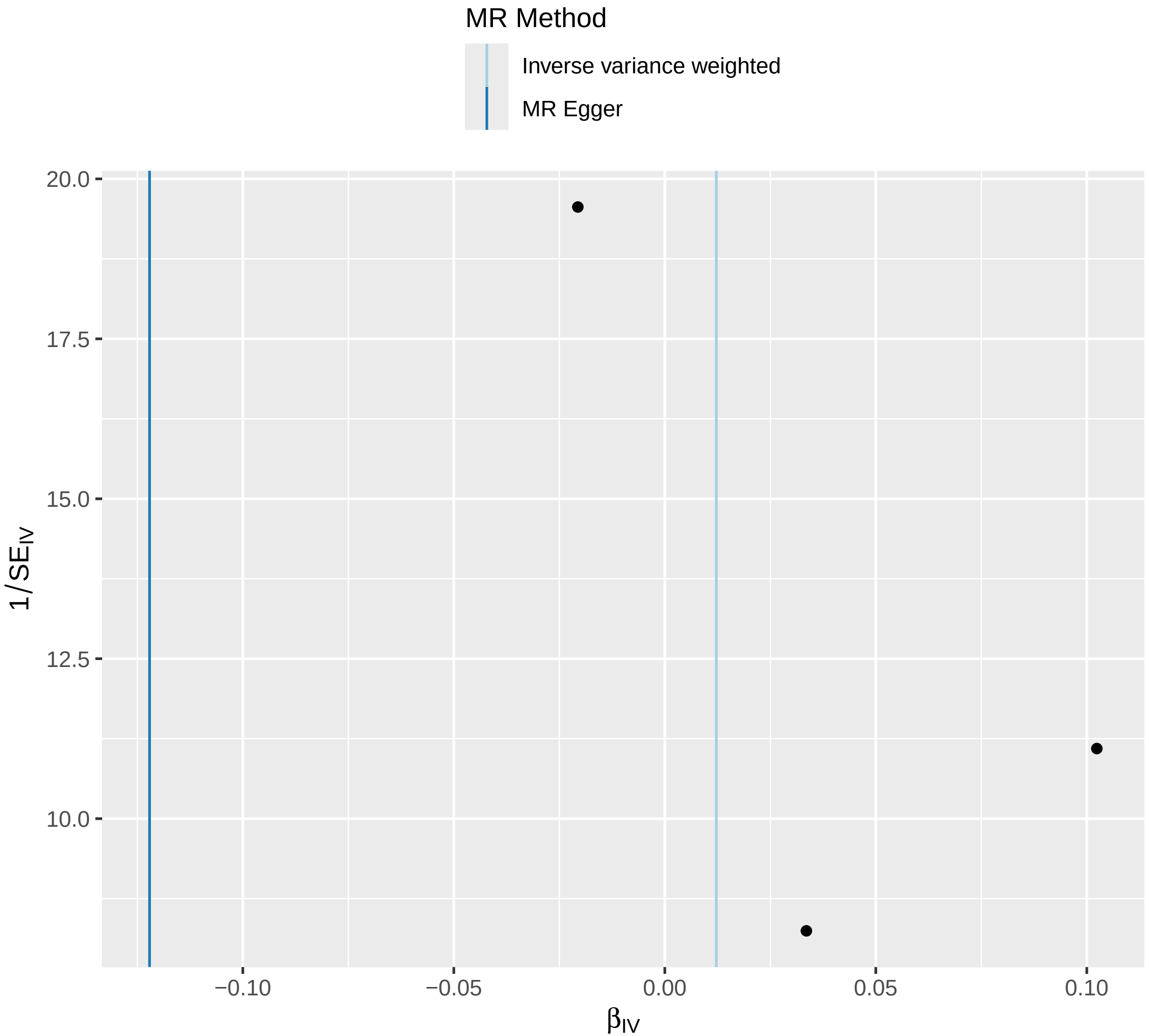
MR Method
Inverse variance weighted
MR Egger
20.0
17.5
15.0
12.5
10.0
1/SE_IV
−0.10
−0.05
0.00
0.05
0.10
β_IV

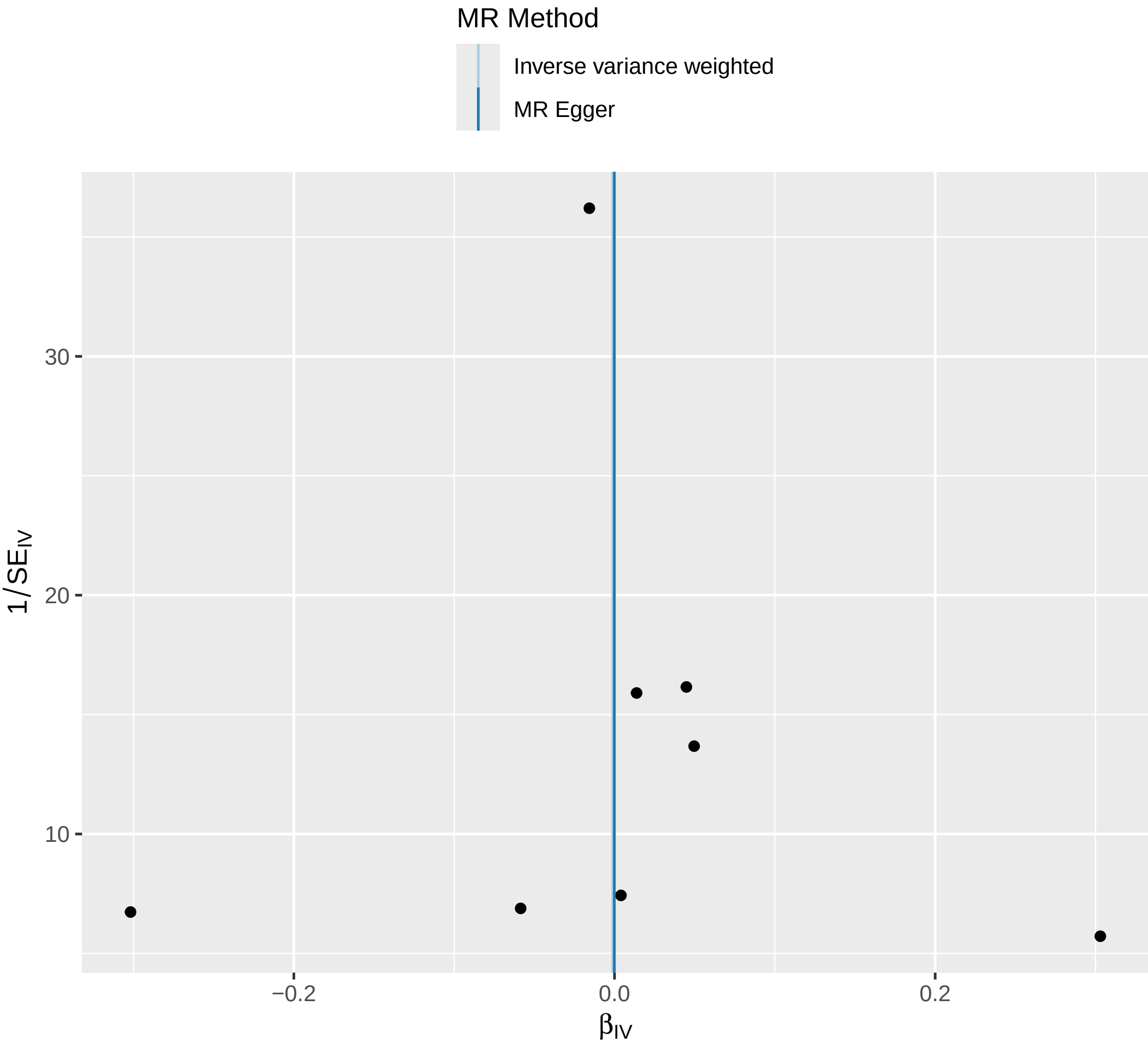
MR Method
Inverse variance weighted
MR Egger
30
20
10
1/SE$_{IV}$
−0.2
0.0
0.2
β$_{IV}$

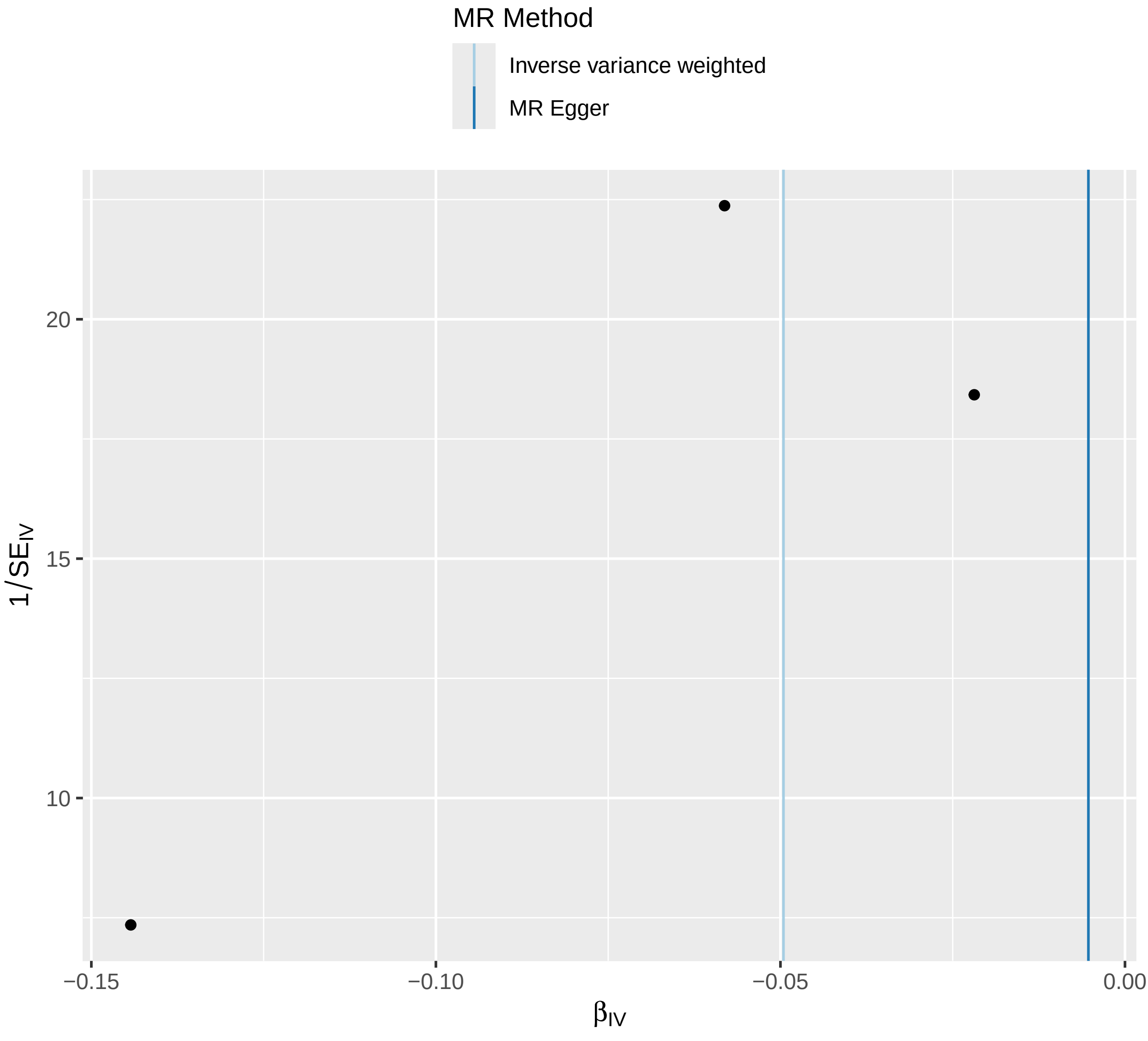
MR Method
Inverse variance weighted
MR Egger
20
15
10
1/SE_IV
−0.15
−0.10
−0.05
0.00
β_IV

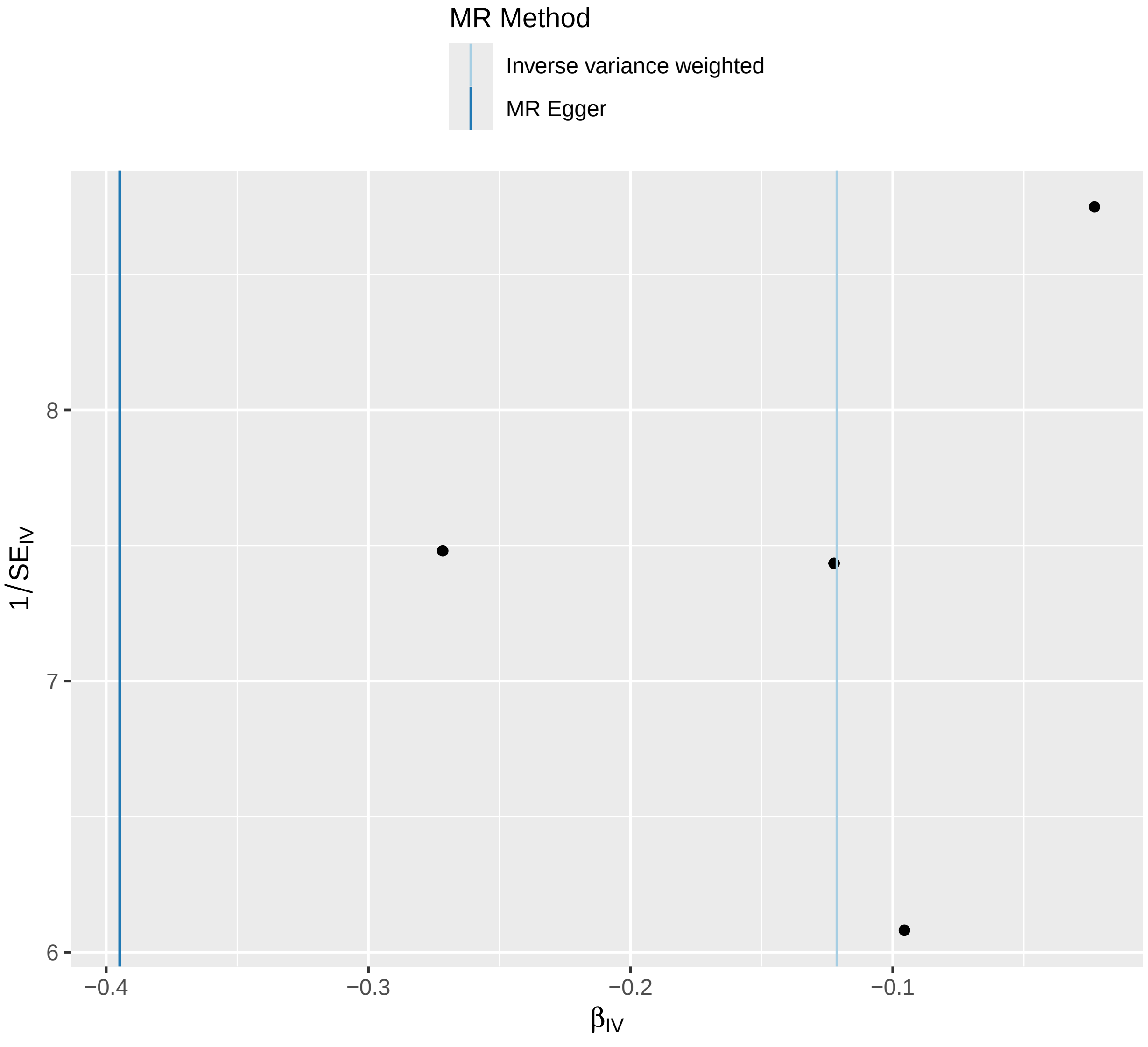

MR Method
Inverse variance weighted
MR Egger
8
7
6
−0.4
−0.3
−0.2
−0.1
$1/SE_{IV}$
β_{IV}

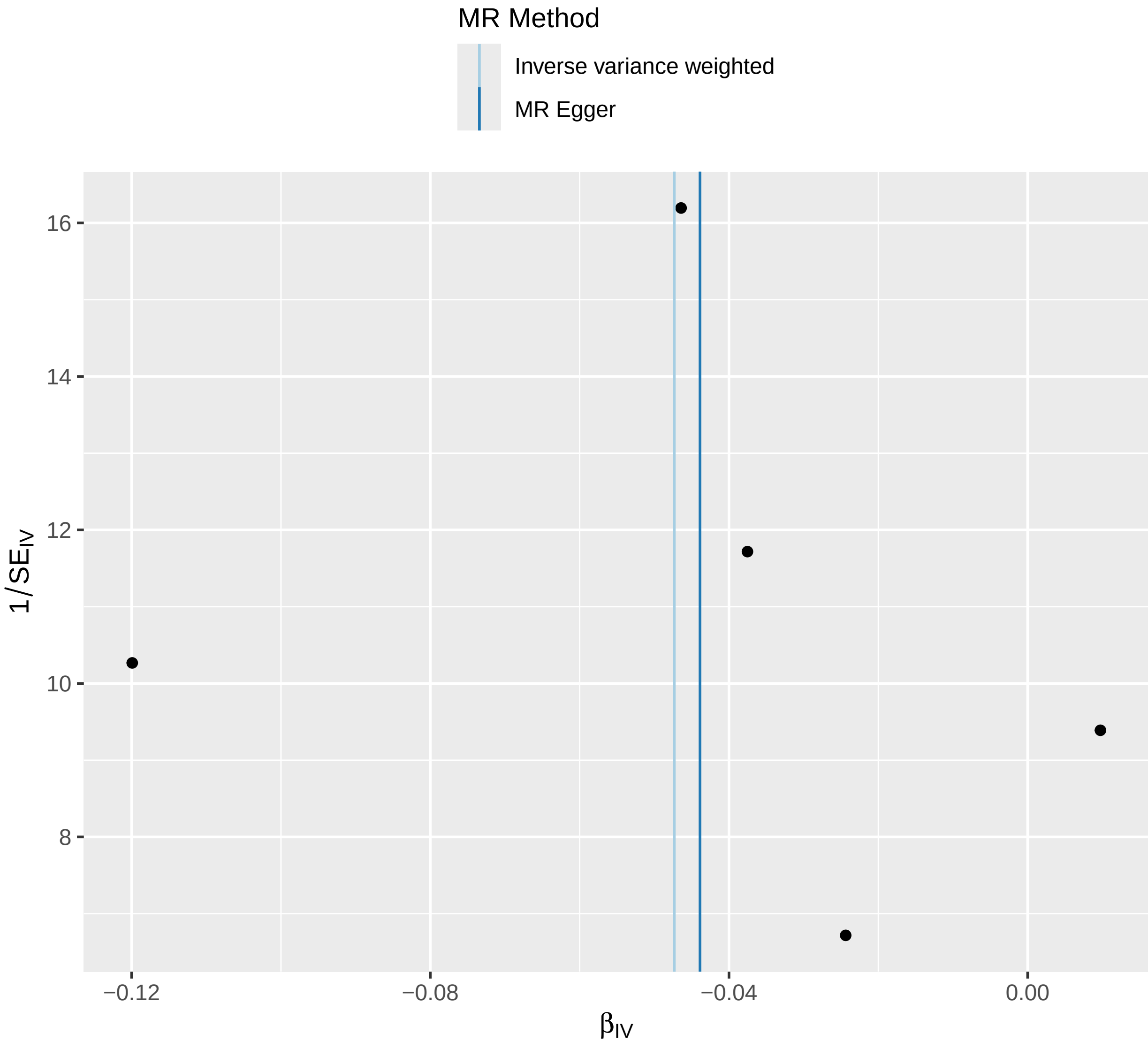
MR Method
Inverse variance weighted
MR Egger
16
14
12
10
8
1/SE_IV
−0.12
−0.08
−0.04
0.00
β_IV

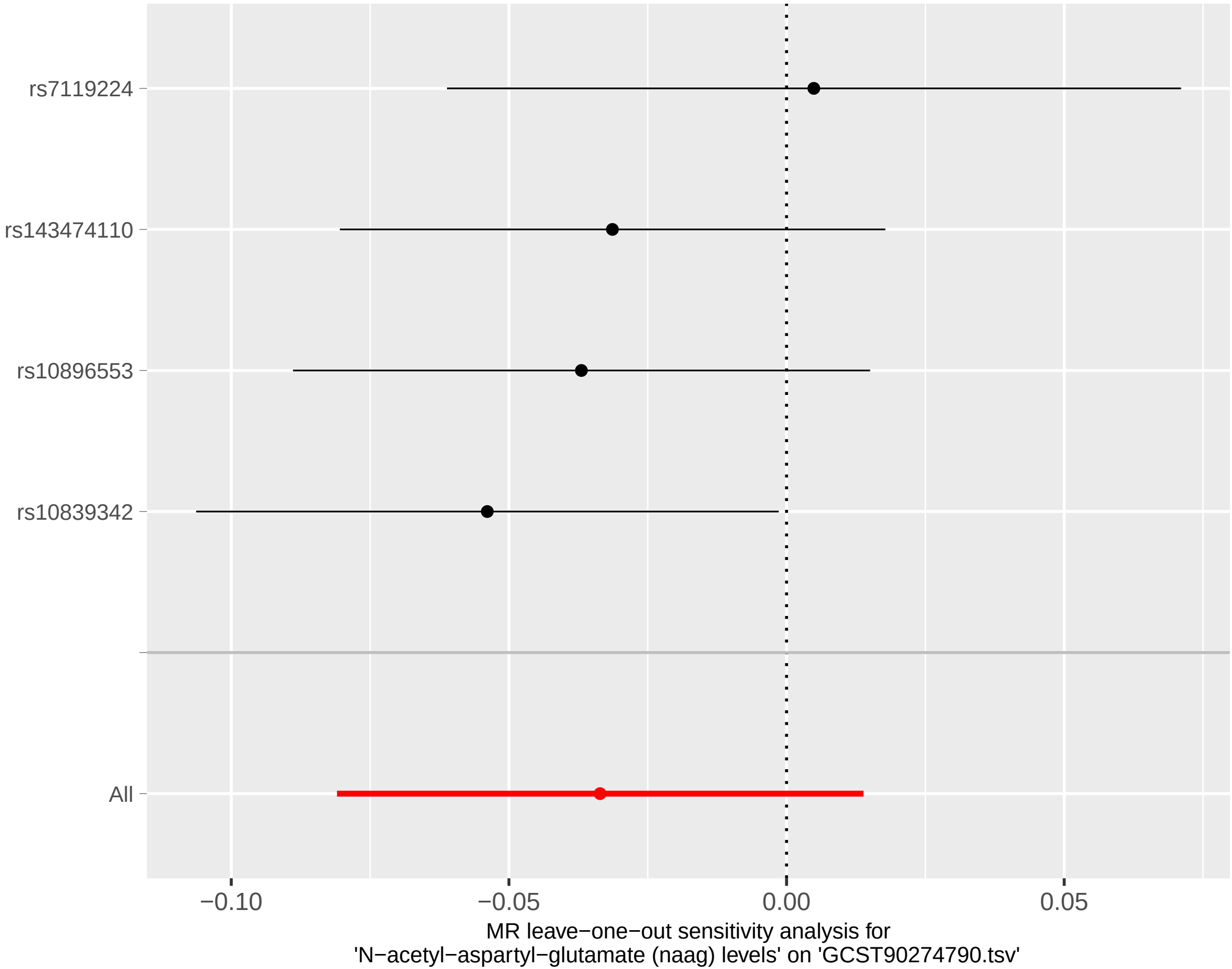
rs7119224
rs143474110
rs10896553
rs10839342
All
−0.10
−0.05
0.00
0.05
MR leave−one−out sensitivity analysis for
'N−acetyl−aspartyl−glutamate (naag) levels' on 'GCST90274790.tsv'

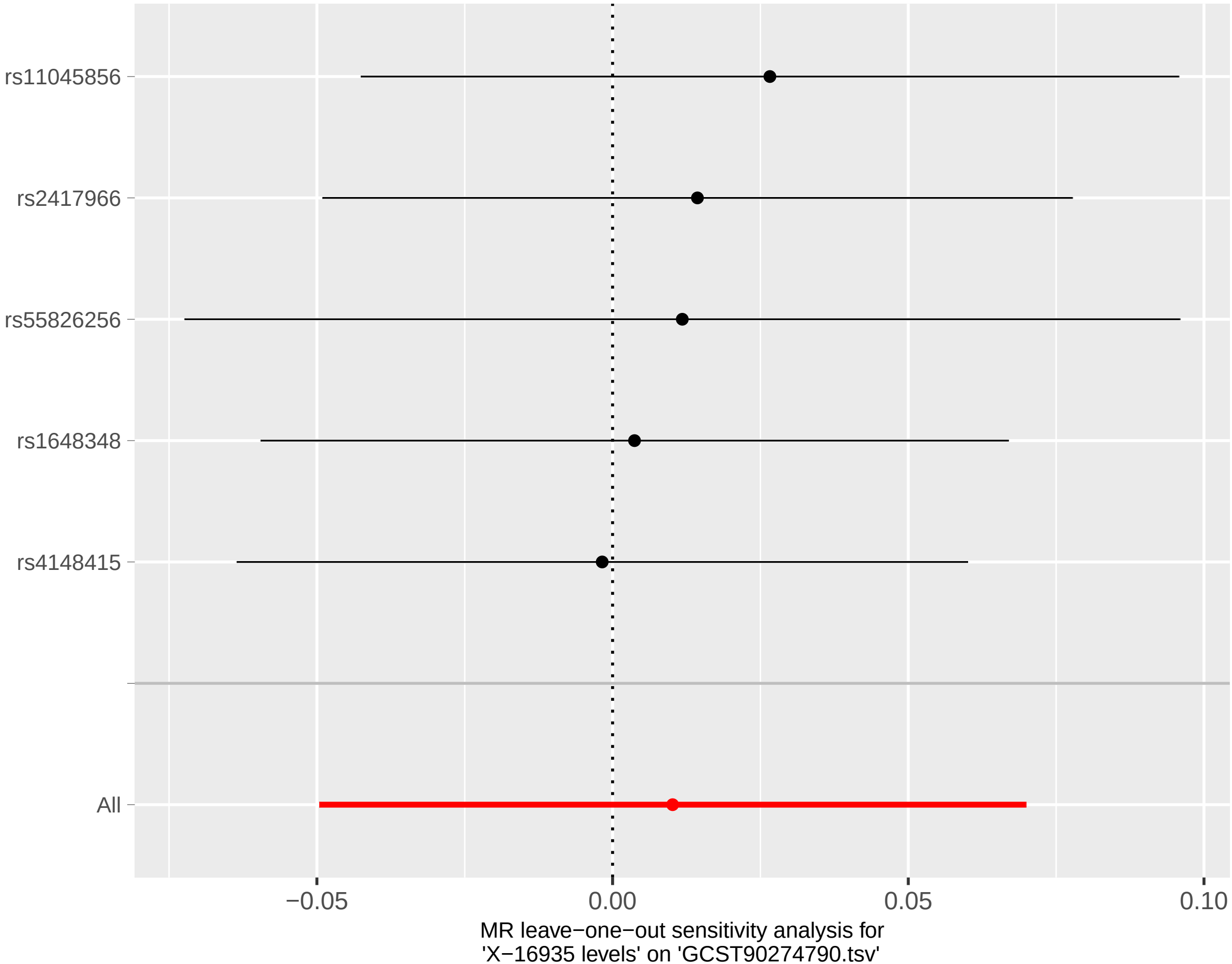
rs11045856
rs2417966
rs55826256
rs1648348
rs4148415
All
−0.05
0.00
0.05
0.10
MR leave−one−out sensitivity analysis for
'X−16935 levels' on 'GCST90274790.tsv'

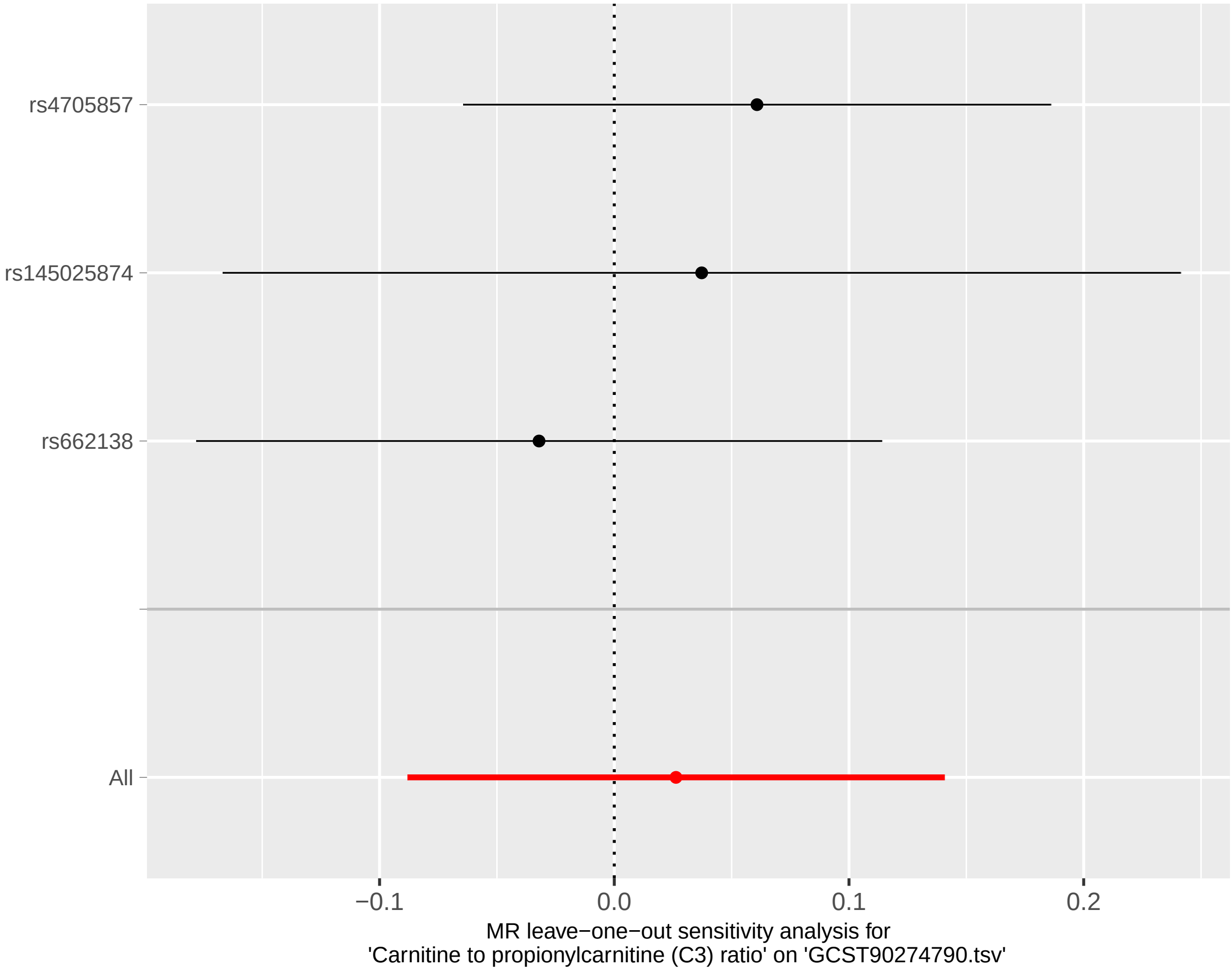
rs4705857
rs145025874
rs662138
All
−0.1
0.0
0.1
0.2
MR leave−one−out sensitivity analysis for
'Carnitine to propionylcarnitine (C3) ratio' on 'GCST90274790.tsv'

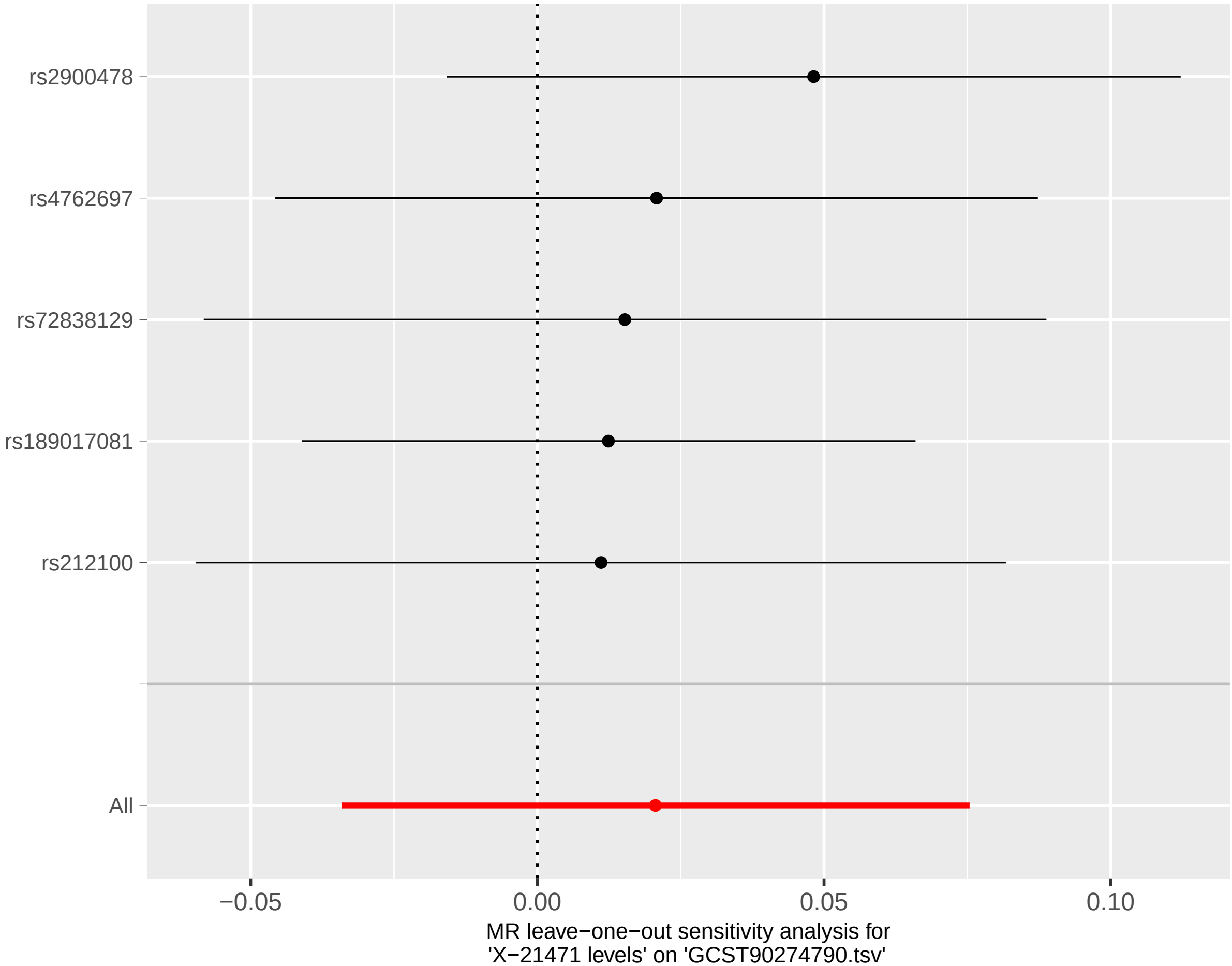
rs2900478
rs4762697
rs72838129
rs189017081
rs212100
All
−0.05
0.00
0.05
0.10
MR leave−one−out sensitivity analysis for
'X−21471 levels' on 'GCST90274790.tsv'

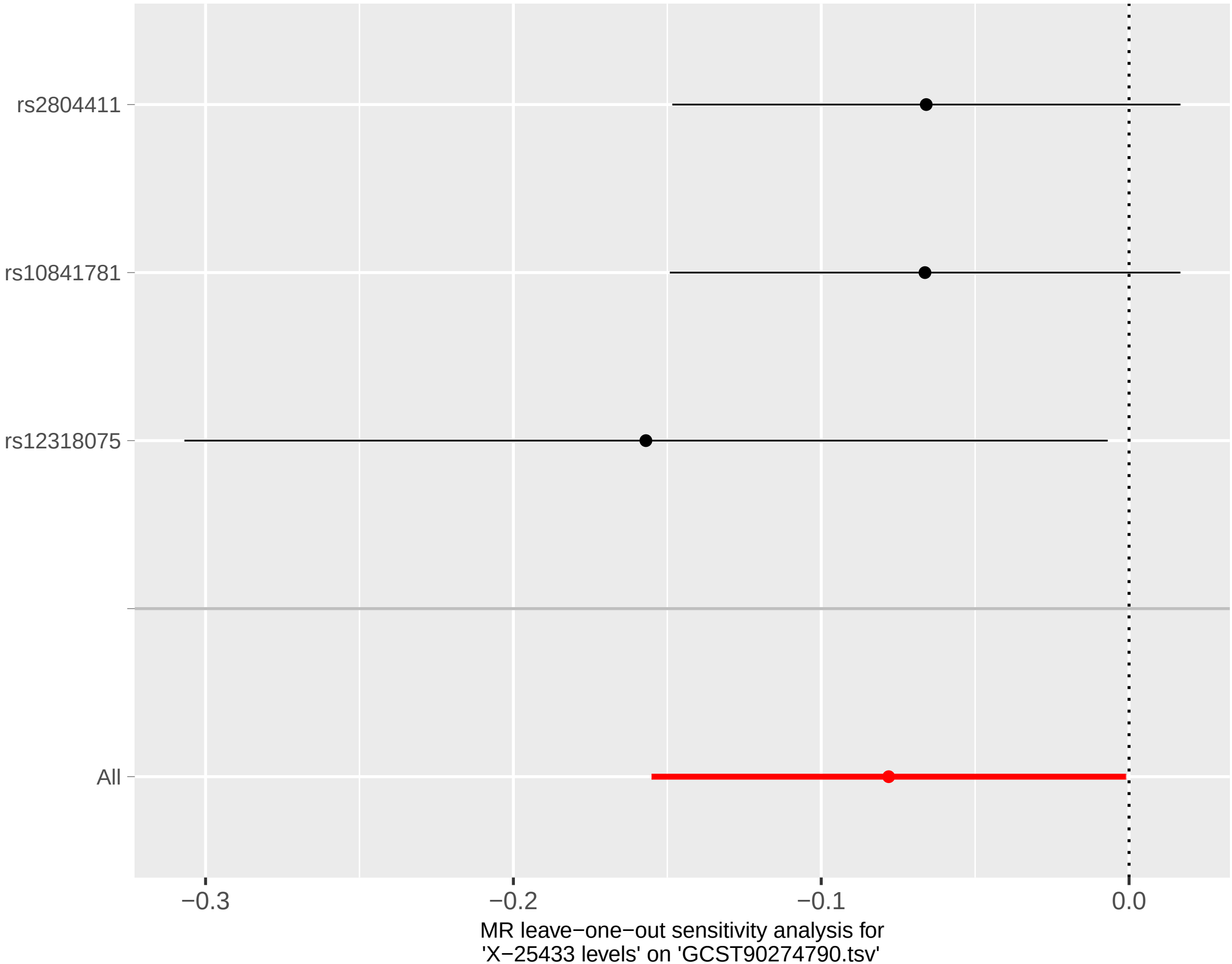
rs2804411
rs10841781
rs12318075
All
−0.3
−0.2
−0.1
0.0
MR leave−one−out sensitivity analysis for
'X−25433 levels' on 'GCST90274790.tsv'

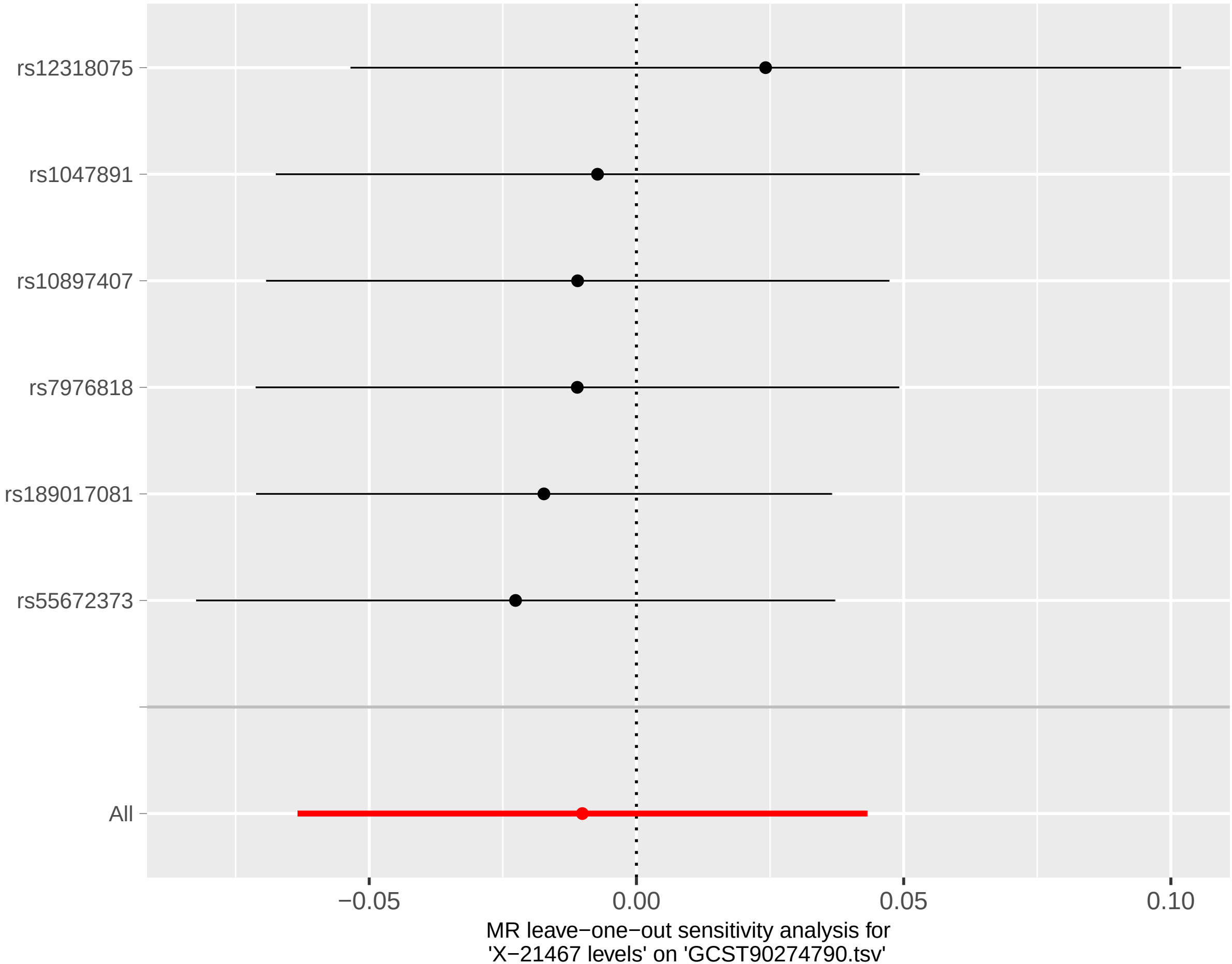
rs12318075
rs1047891
rs10897407
rs7976818
rs189017081
rs55672373
All
−0.05
0.00
0.05
0.10
MR leave−one−out sensitivity analysis for
'X−21467 levels' on 'GCST90274790.tsv'

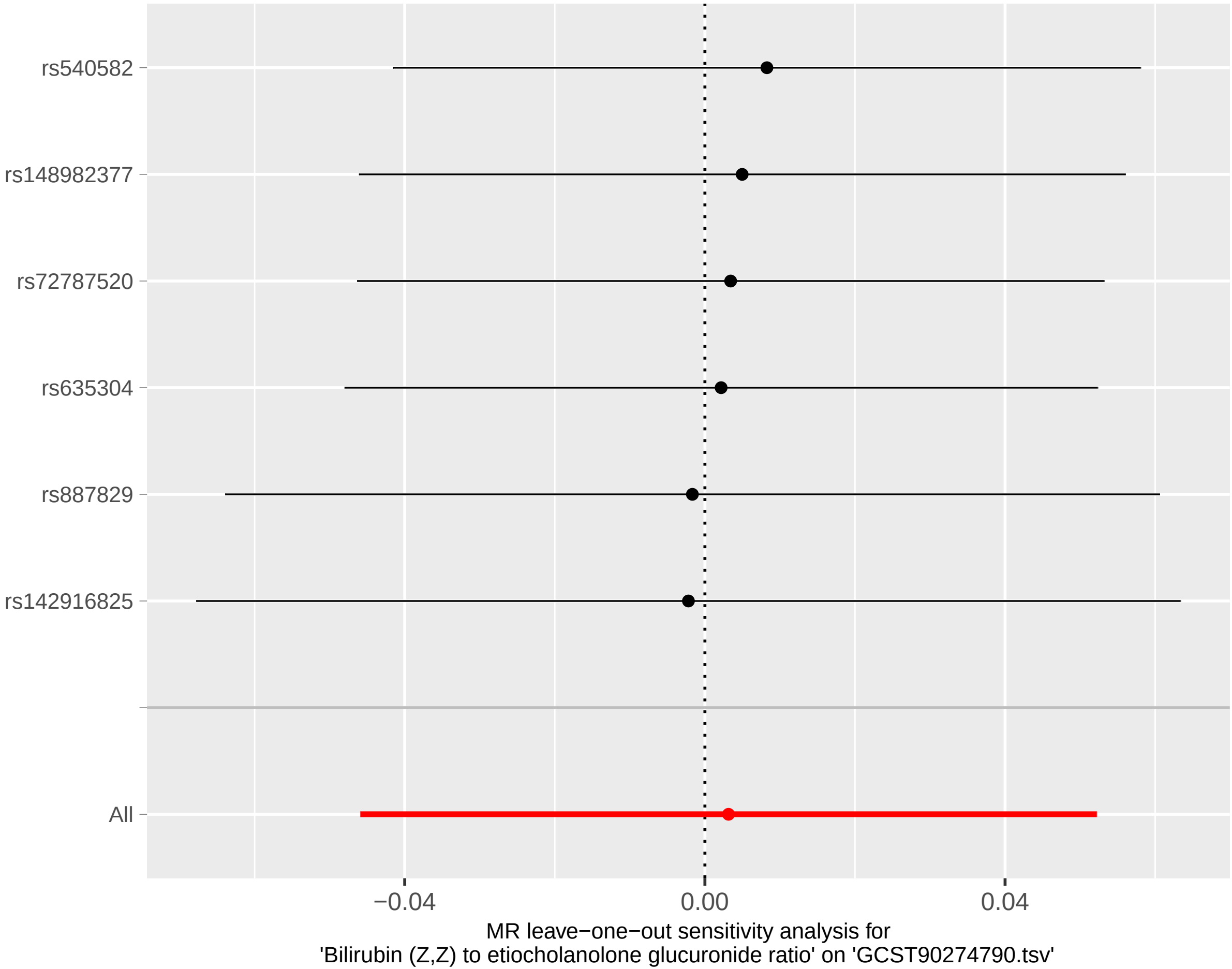
rs540582
rs148982377
rs72787520
rs635304
rs887829
rs142916825
All
−0.04
0.00
0.04
MR leave−one−out sensitivity analysis for
'Bilirubin (Z,Z) to etiocholanolone glucuronide ratio' on 'GCST90274790.tsv'

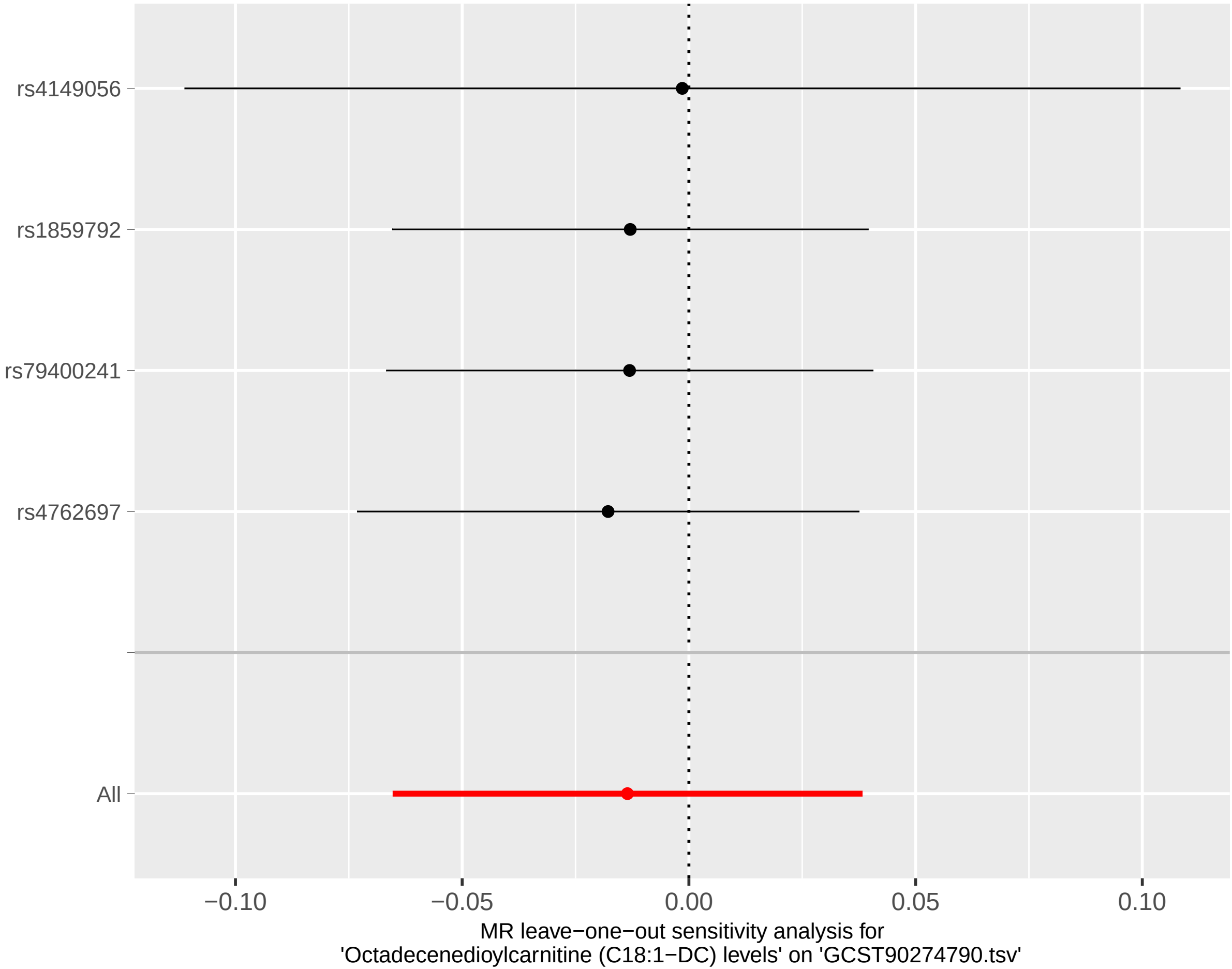
rs4149056
rs1859792
rs79400241
rs4762697
All
−0.10
−0.05
0.00
0.05
0.10
MR leave−one−out sensitivity analysis for
'Octadecenedioylcarnitine (C18:1−DC) levels' on 'GCST90274790.tsv'

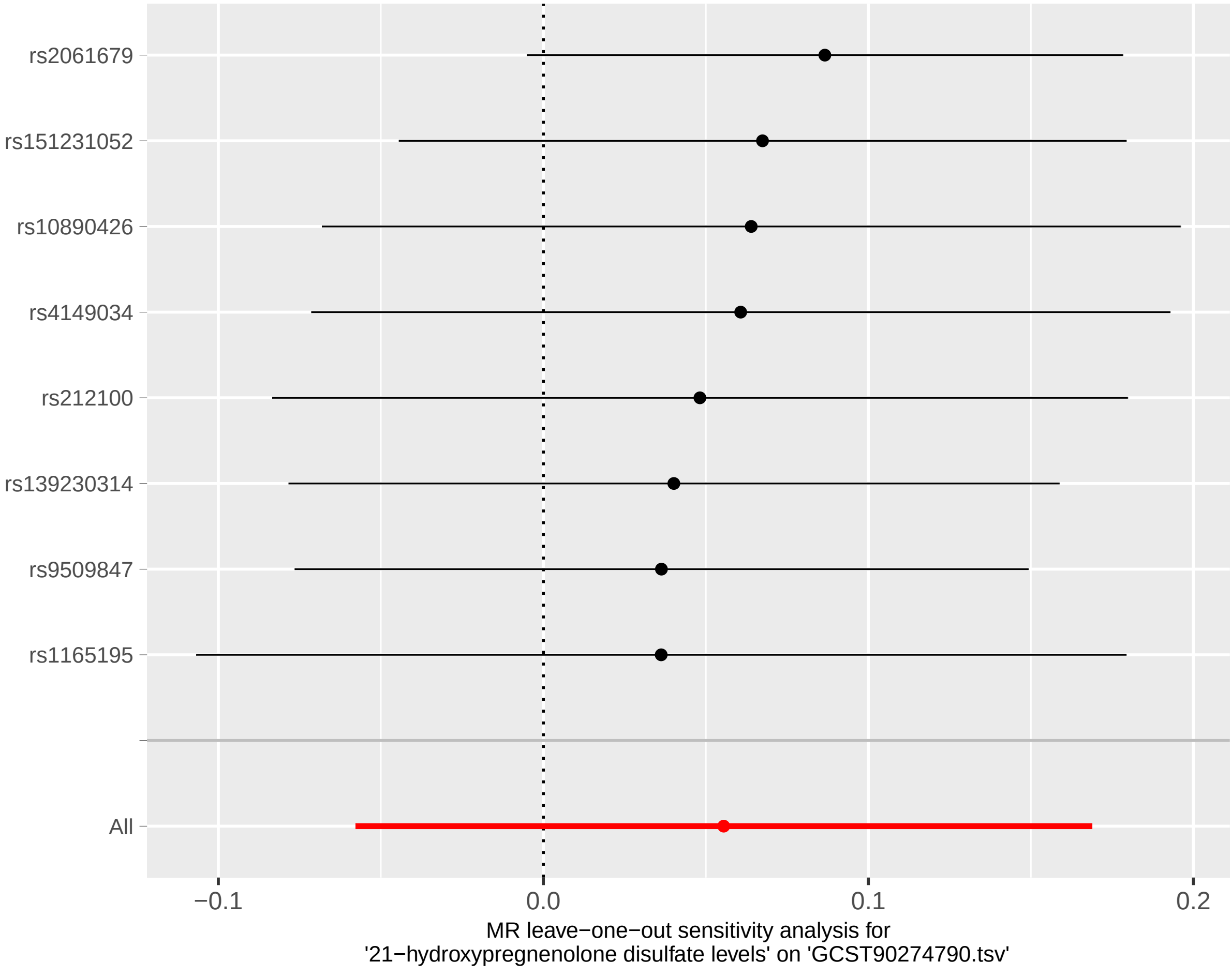
rs2061679
rs151231052
rs10890426
rs4149034
rs212100
rs139230314
rs9509847
rs1165195
All
−0.1
0.0
0.1
0.2
MR leave−one−out sensitivity analysis for
'21−hydroxypregnenolone disulfate levels' on 'GCST90274790.tsv'

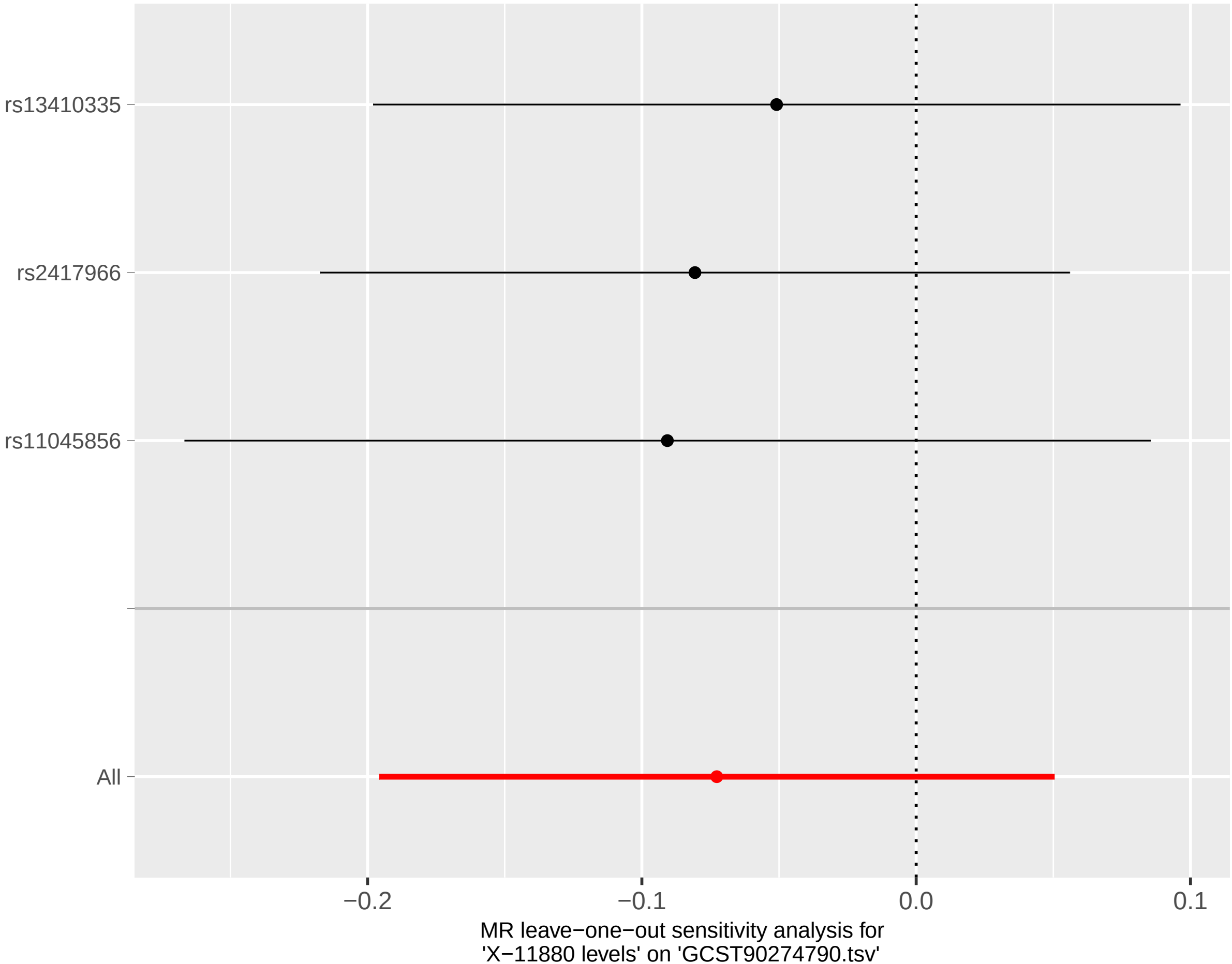
rs13410335
rs2417966
rs11045856
All
−0.2
−0.1
0.0
0.1
MR leave−one−out sensitivity analysis for
'X−11880 levels' on 'GCST90274790.tsv'

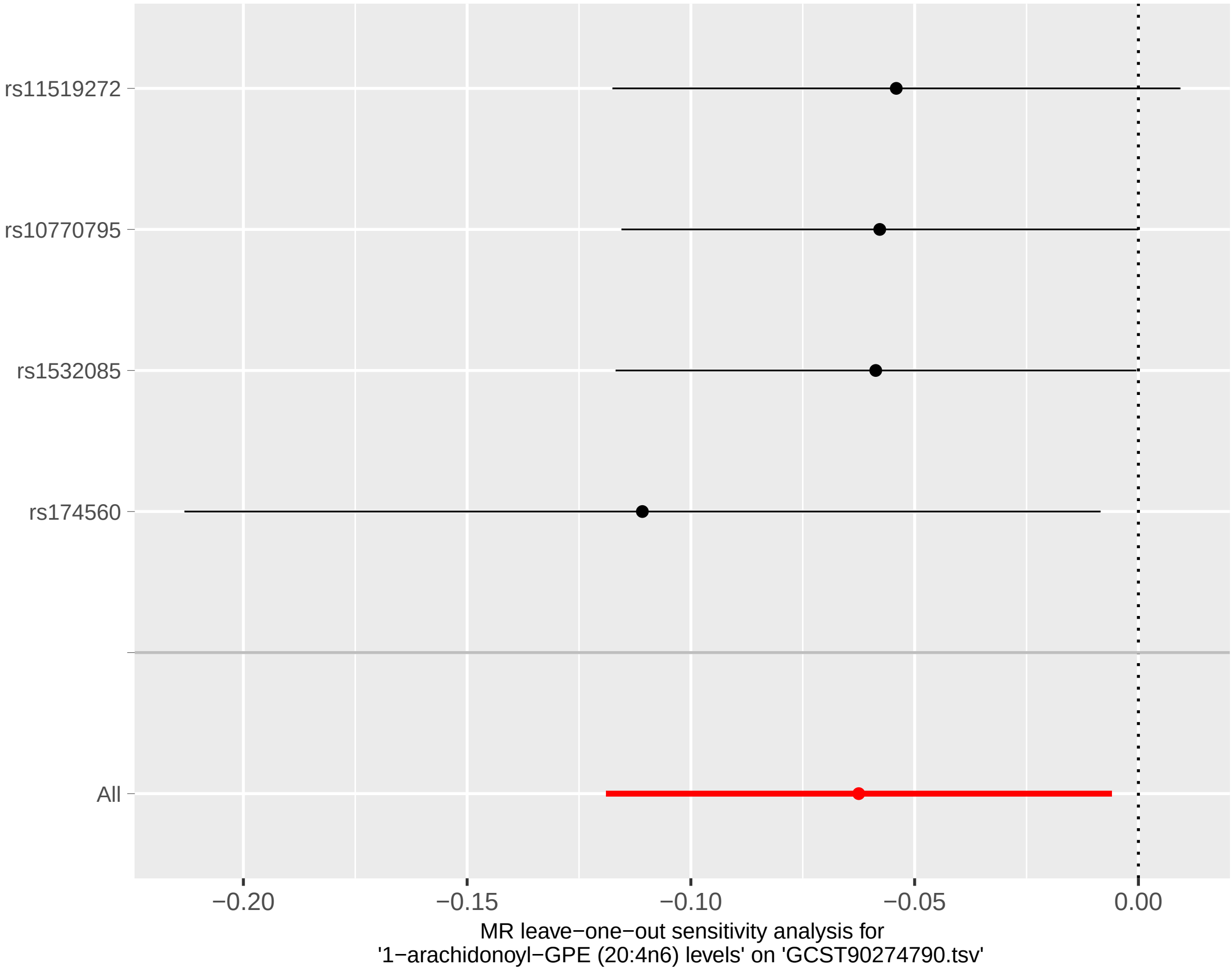
rs11519272
rs10770795
rs1532085
rs174560
All
−0.20
−0.15
−0.10
−0.05
0.00
MR leave−one−out sensitivity analysis for
'1−arachidonoyl−GPE (20:4n6) levels' on 'GCST90274790.tsv'

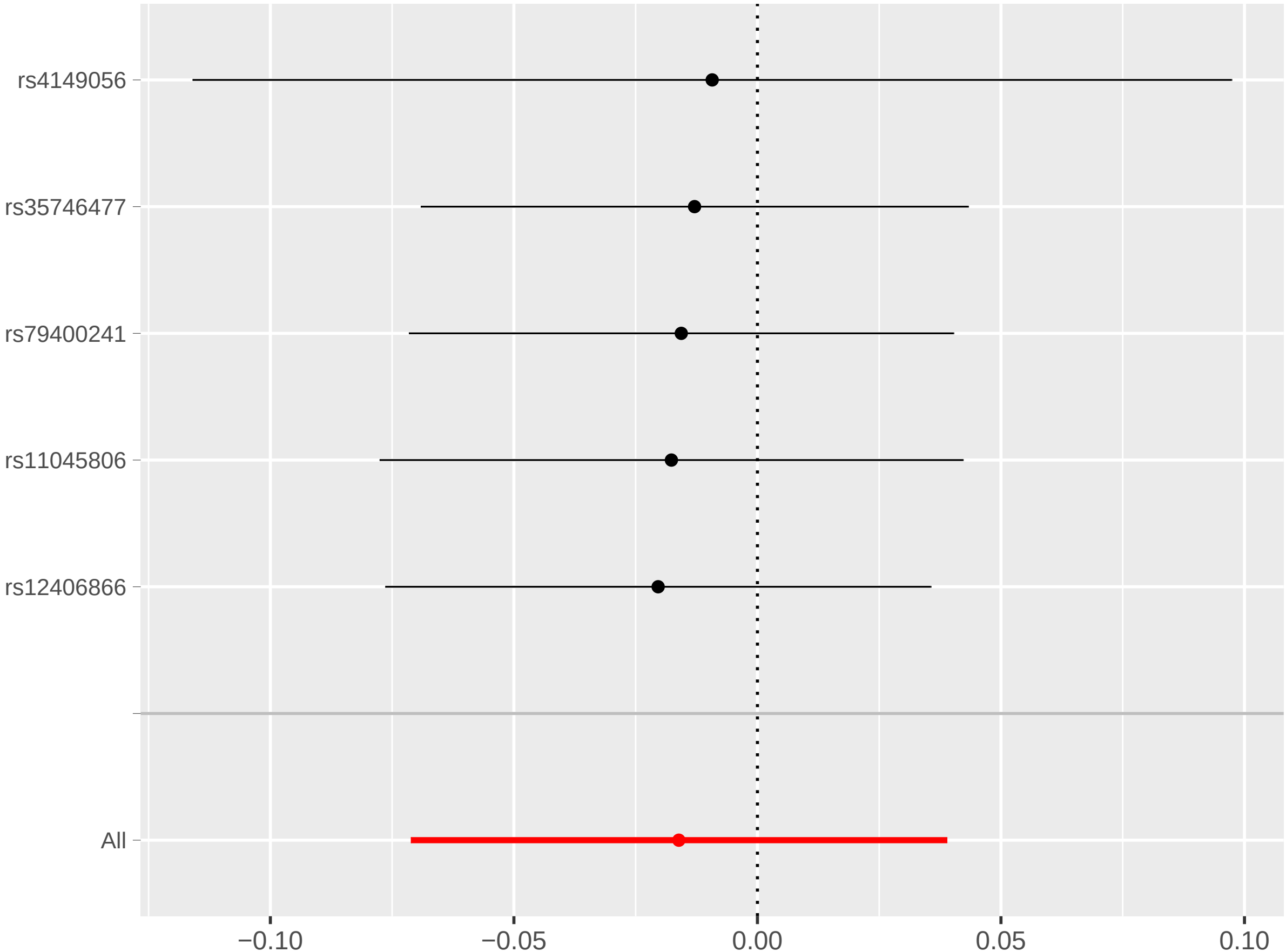

MR leave−one−out sensitivity analysis for
'Hexadecanedioate (C16−DC) levels' on 'GCST90274790.tsv'

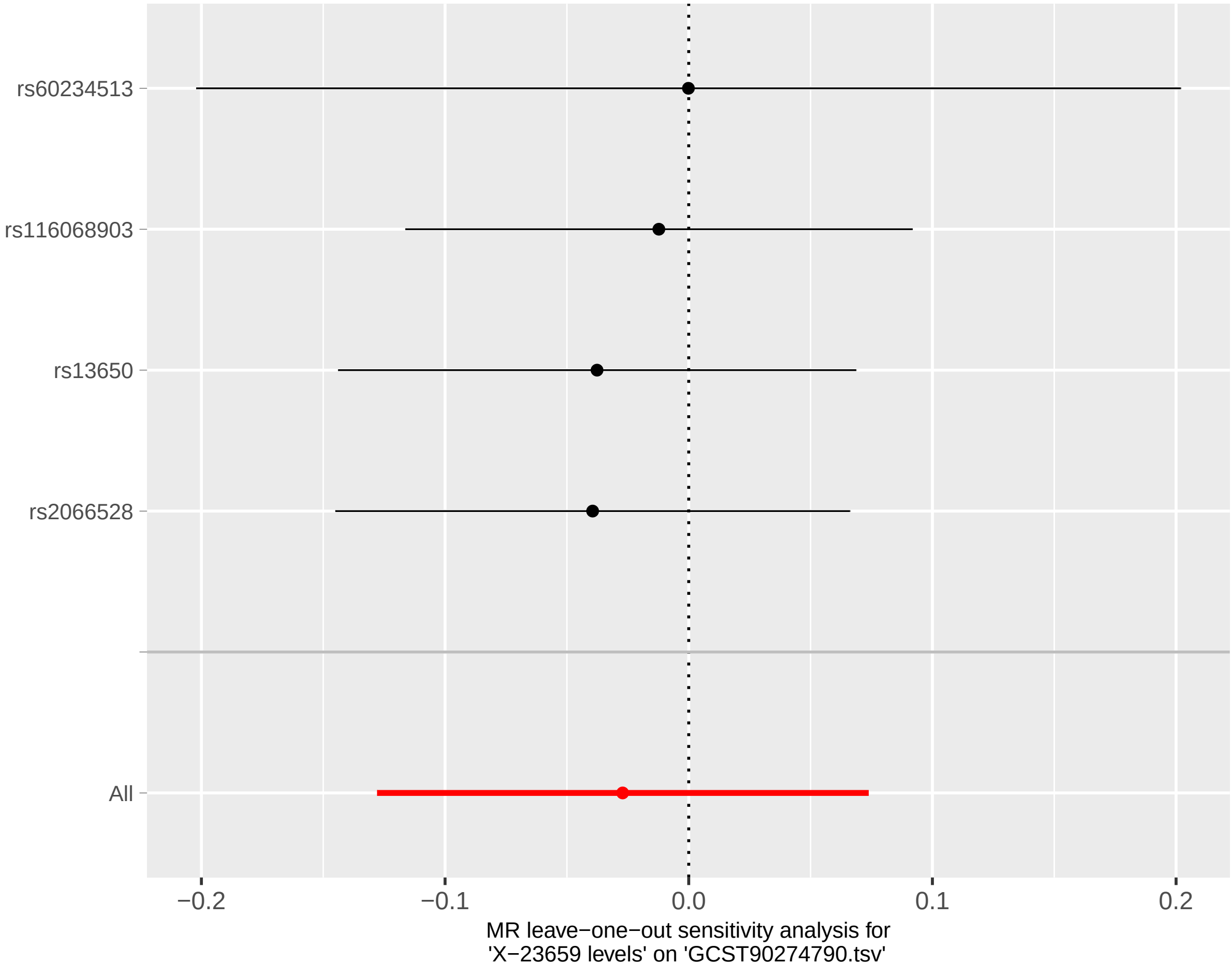
rs60234513
rs116068903
rs13650
rs2066528
All
−0.2
−0.1
0.0
0.1
0.2
MR leave−one−out sensitivity analysis for
'X−23659 levels' on 'GCST90274790.tsv'

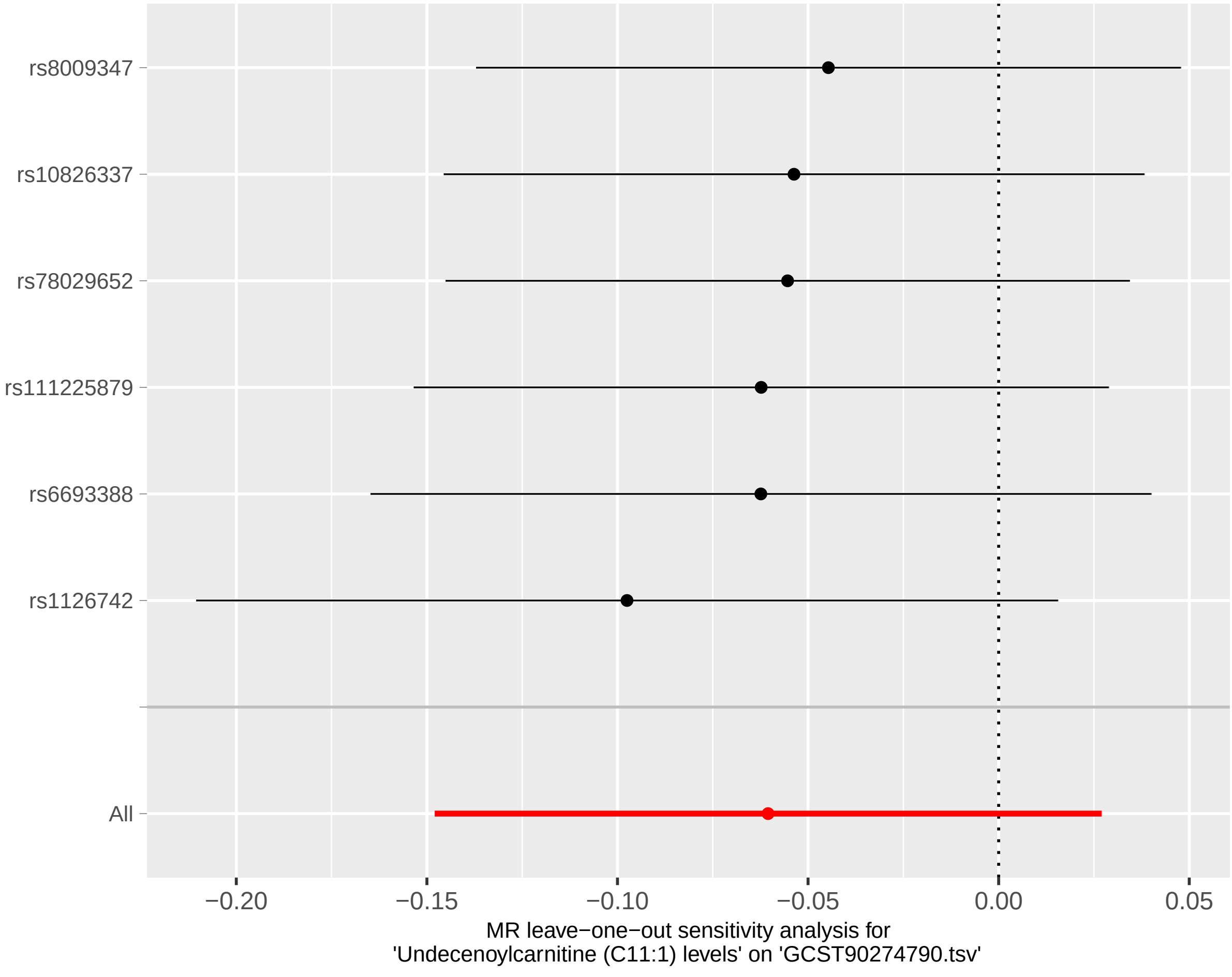
rs8009347
rs10826337
rs78029652
rs111225879
rs6693388
rs1126742
All
−0.20
−0.15
−0.10
−0.05
0.00
0.05
MR leave−one−out sensitivity analysis for
'Undecenoylcarnitine (C11:1) levels' on 'GCST90274790.tsv'

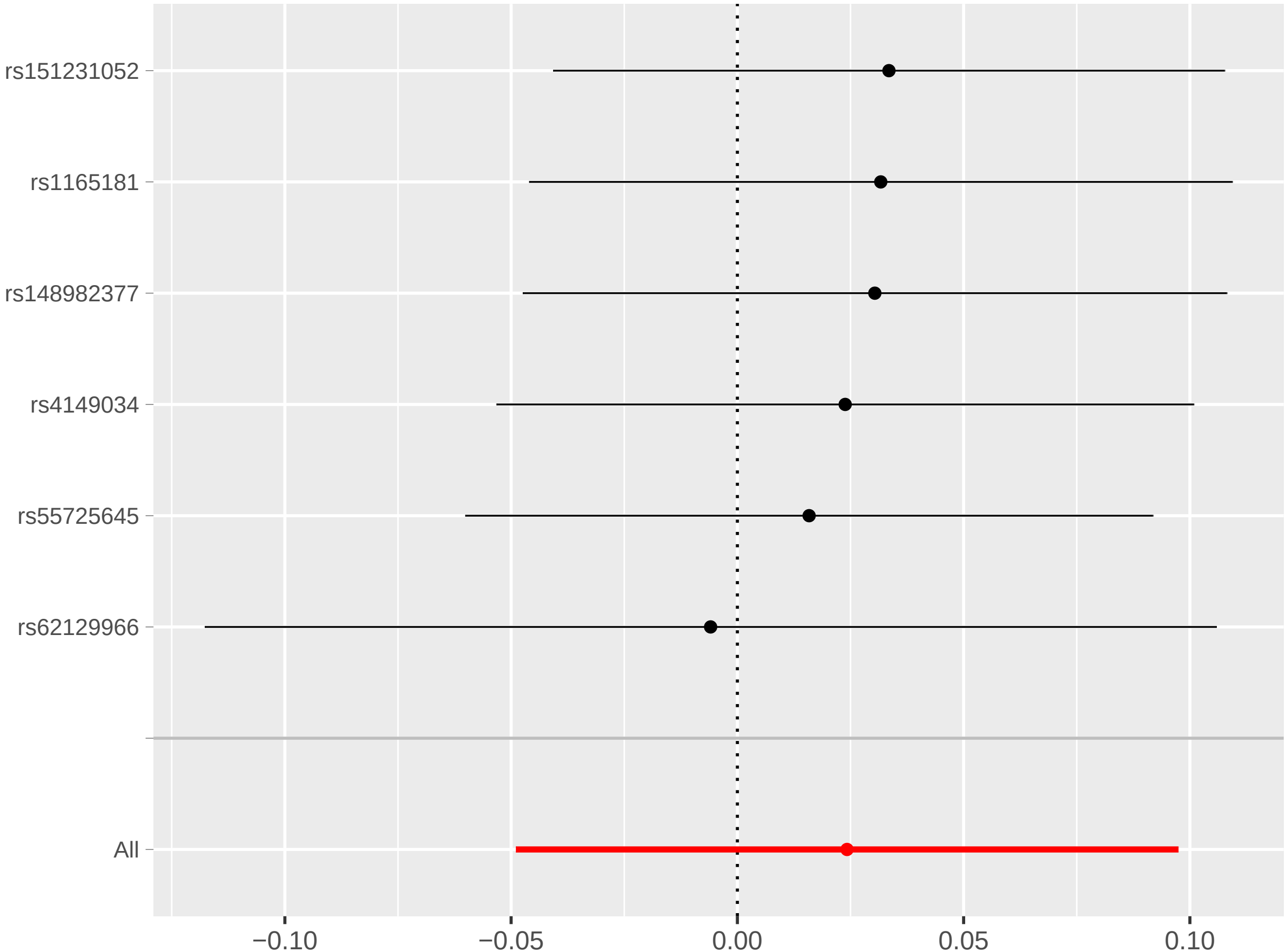

MR leave−one−out sensitivity analysis for
'X−21470 levels' on 'GCST90274790.tsv'

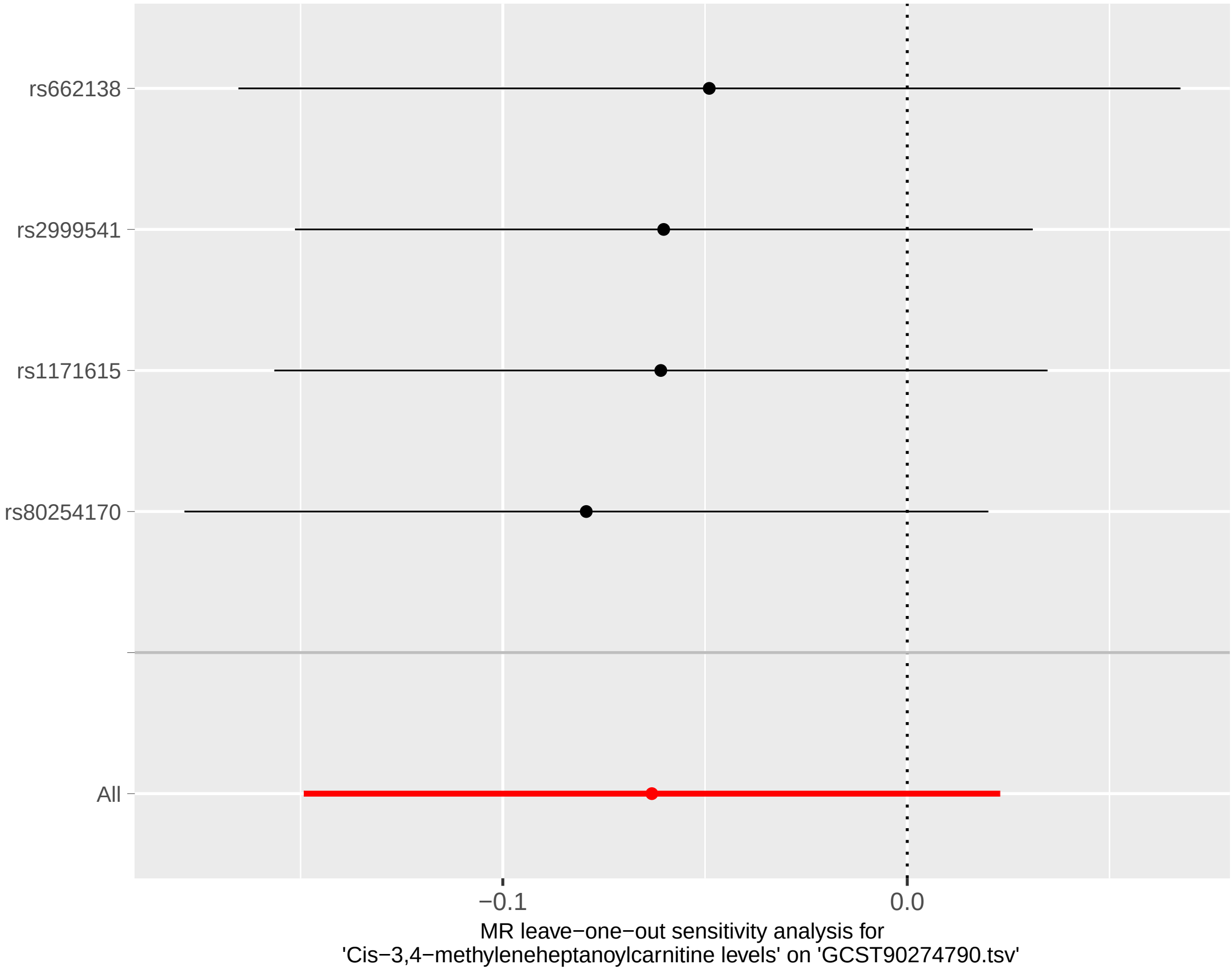

rs662138
rs2999541
rs1171615
rs80254170
All
−0.1
0.0
MR leave−one−out sensitivity analysis for
'Cis−3,4−methyleneheptanoylcarnitine levels' on 'GCST90274790.tsv'

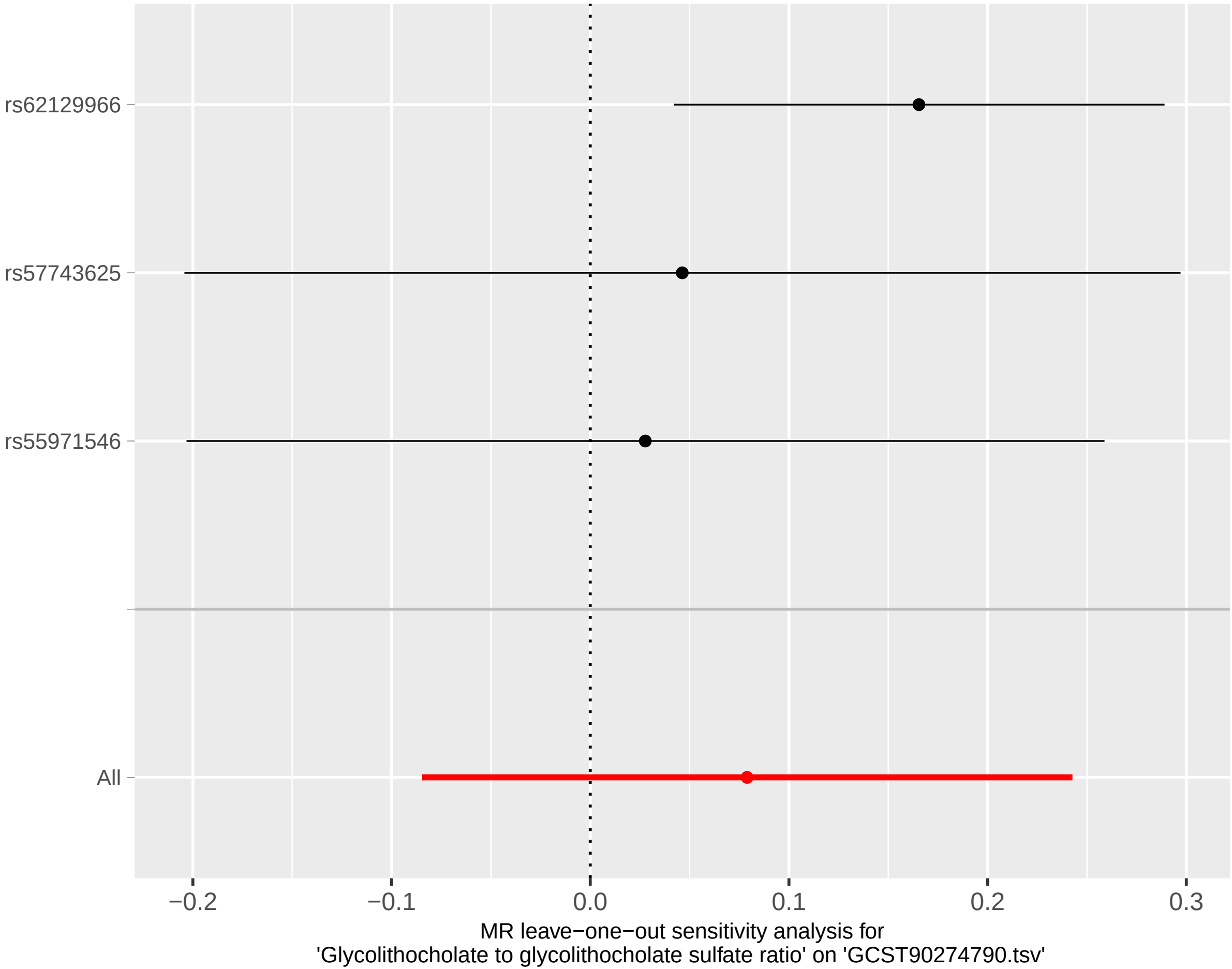
rs62129966
rs57743625
rs55971546
All
−0.2
−0.1
0.0
0.1
0.2
0.3
MR leave−one−out sensitivity analysis for
'Glycolithocholate to glycolithocholate sulfate ratio' on 'GCST90274790.tsv'

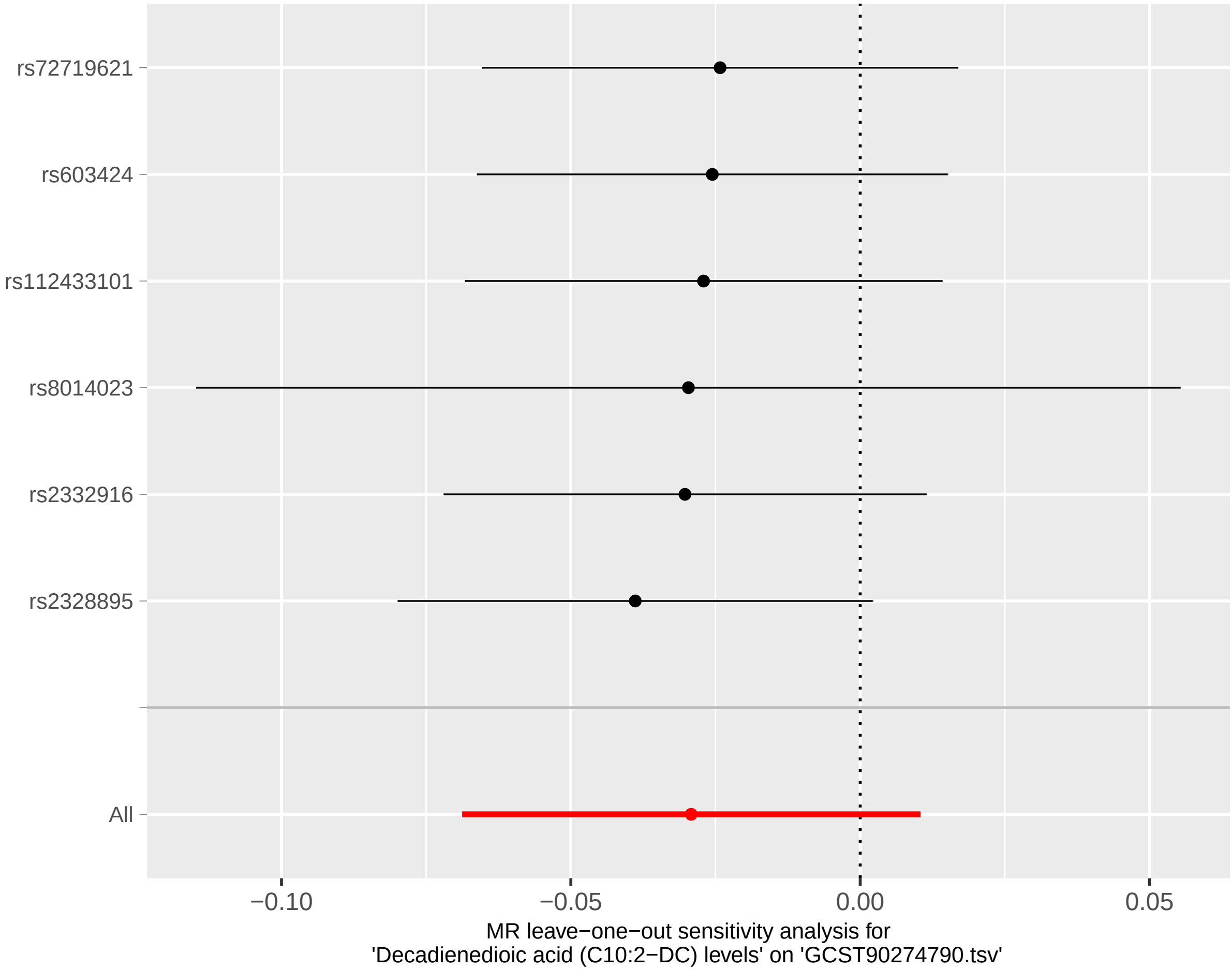

rs72719621
rs603424
rs112433101
rs8014023
rs2332916
rs2328895
All
−0.10
−0.05
0.00
0.05
MR leave−one−out sensitivity analysis for
'Decadienedioic acid (C10:2−DC) levels' on 'GCST90274790.tsv'

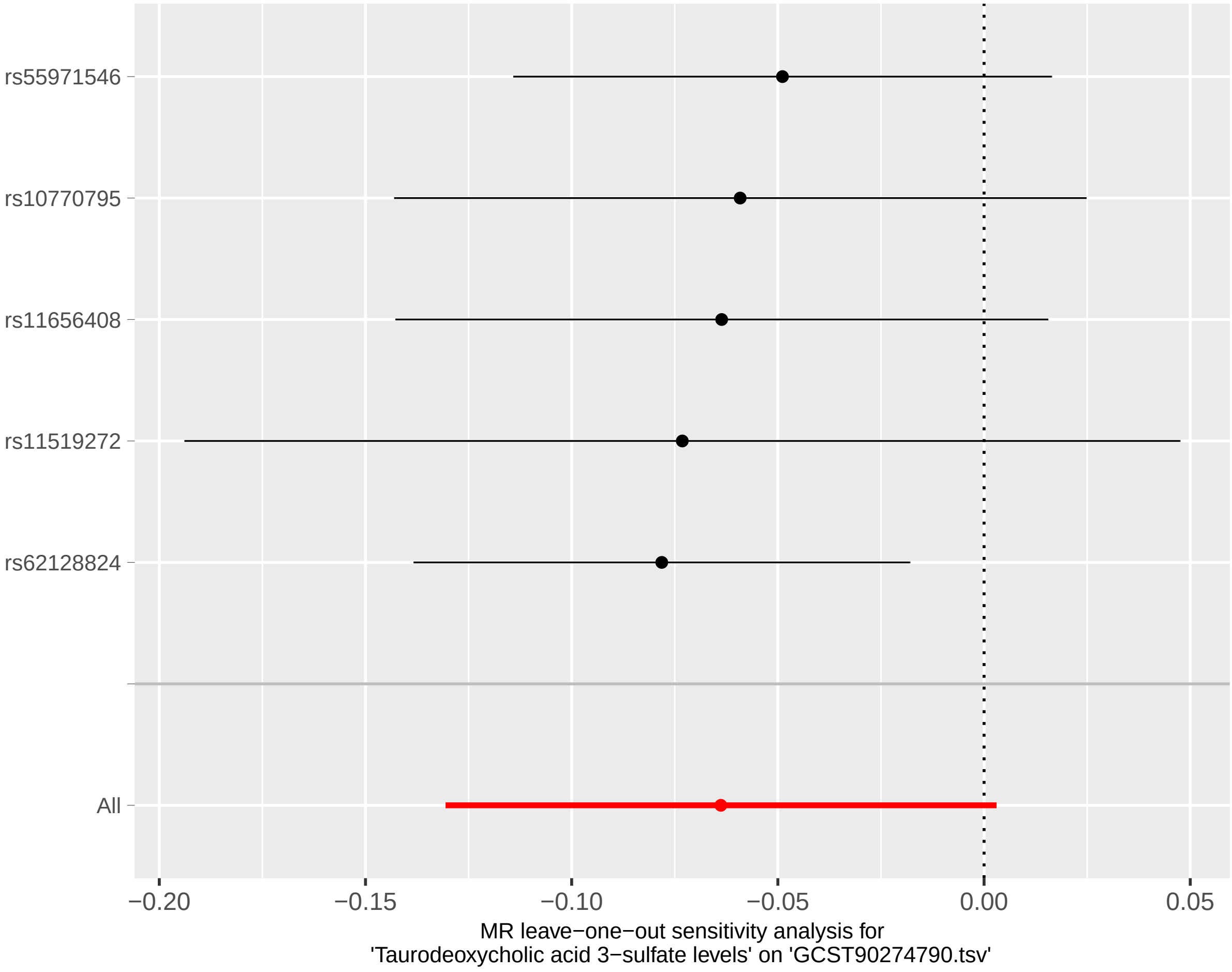

rs55971546
rs10770795
rs11656408
rs11519272
rs62128824
All
−0.20
−0.15
−0.10
−0.05
0.00
0.05
MR leave−one−out sensitivity analysis for
'Taurodeoxycholic acid 3−sulfate levels' on 'GCST90274790.tsv'

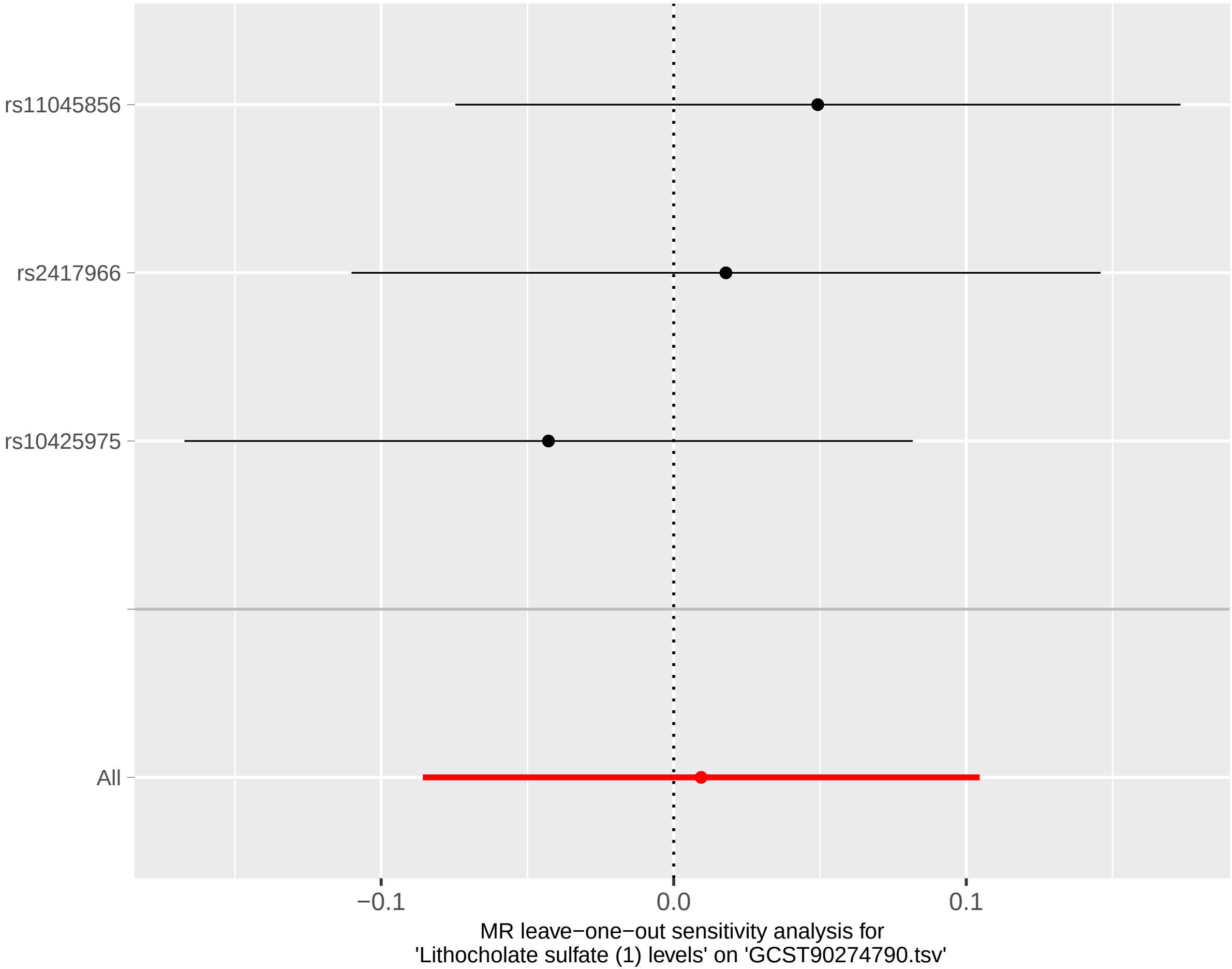
rs11045856
rs2417966
rs10425975
All
−0.1
0.0
0.1
MR leave−one−out sensitivity analysis for
'Lithocholate sulfate (1) levels' on 'GCST90274790.tsv'

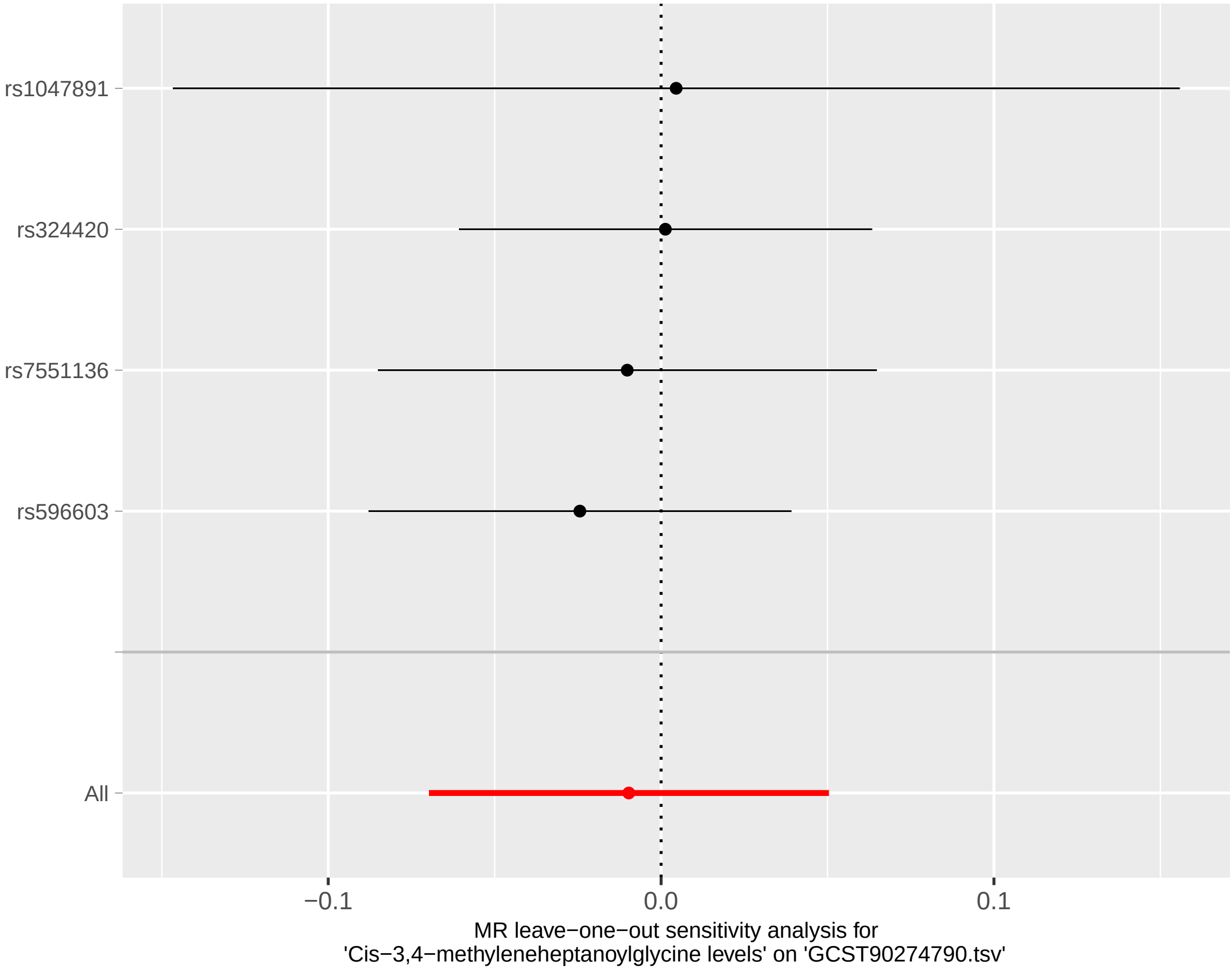
rs1047891
rs324420
rs7551136
rs596603
All
−0.1
0.0
0.1
MR leave−one−out sensitivity analysis for
'Cis−3,4−methyleneheptanoylglycine levels' on 'GCST90274790.tsv'

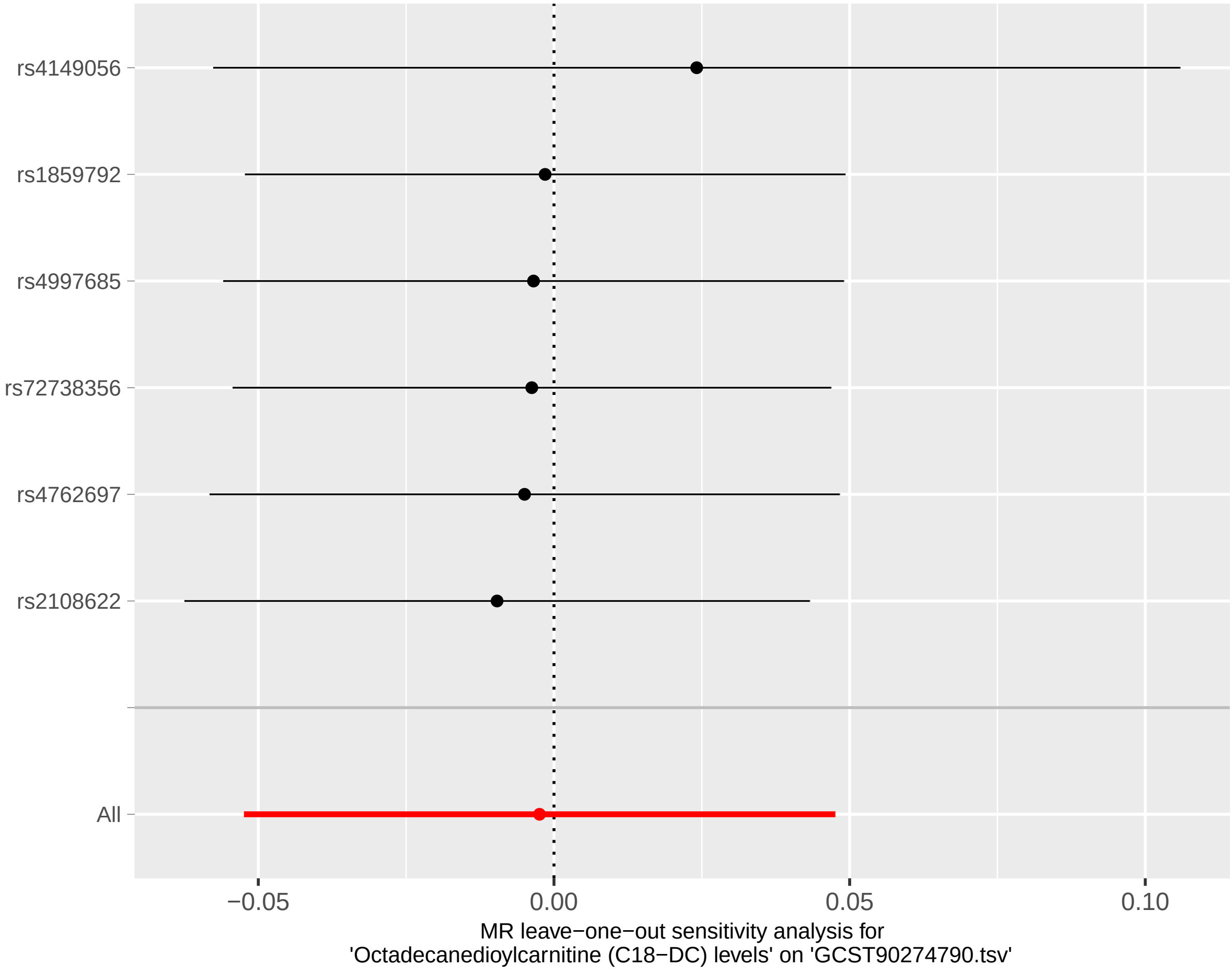
rs4149056
rs1859792
rs4997685
rs72738356
rs4762697
rs2108622
All
−0.05
0.00
0.05
0.10
MR leave−one−out sensitivity analysis for
'Octadecanedioylcarnitine (C18−DC) levels' on 'GCST90274790.tsv'

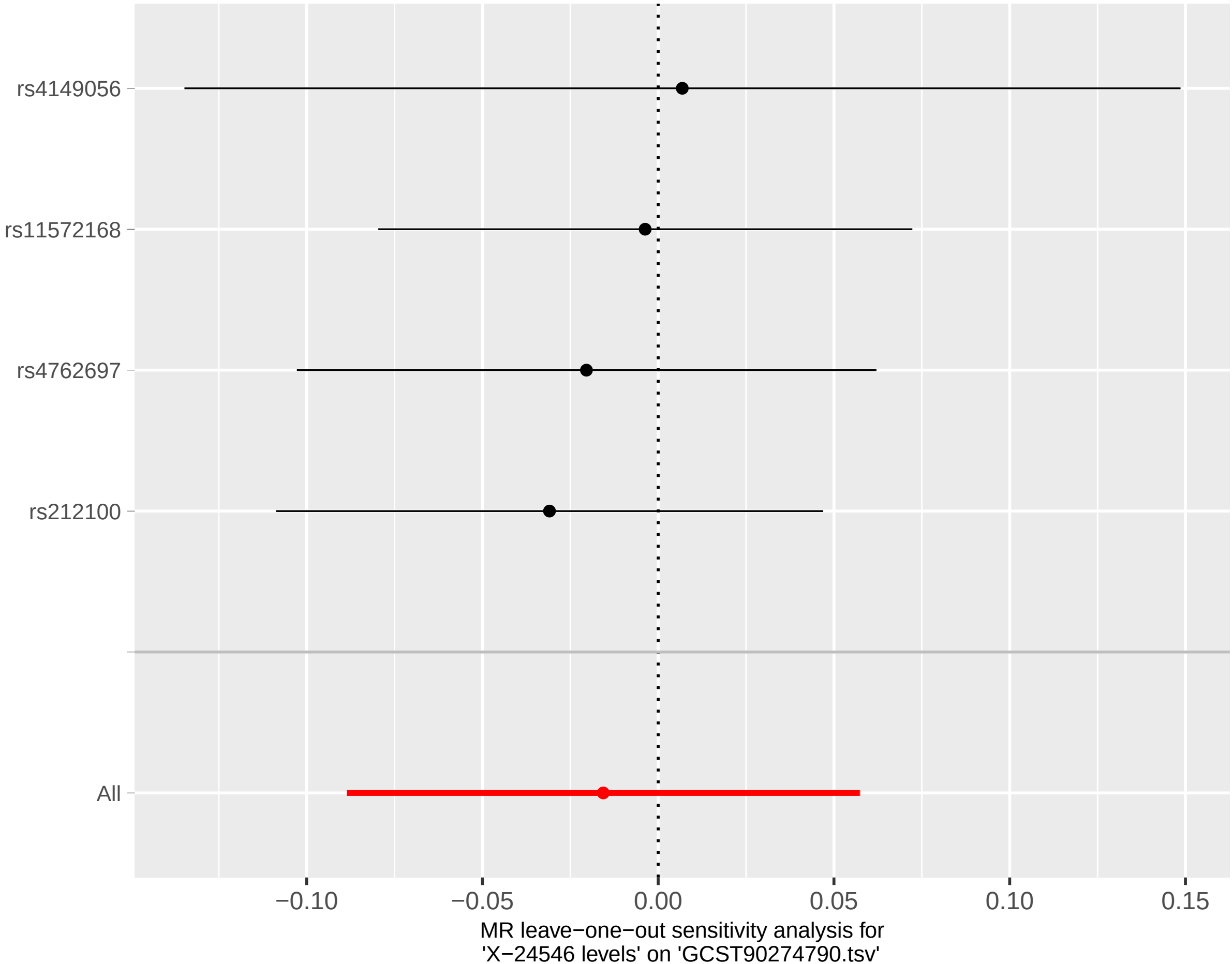
rs4149056
rs11572168
rs4762697
rs212100
All
−0.10
−0.05
0.00
0.05
0.10
0.15
MR leave−one−out sensitivity analysis for
'X−24546 levels' on 'GCST90274790.tsv'

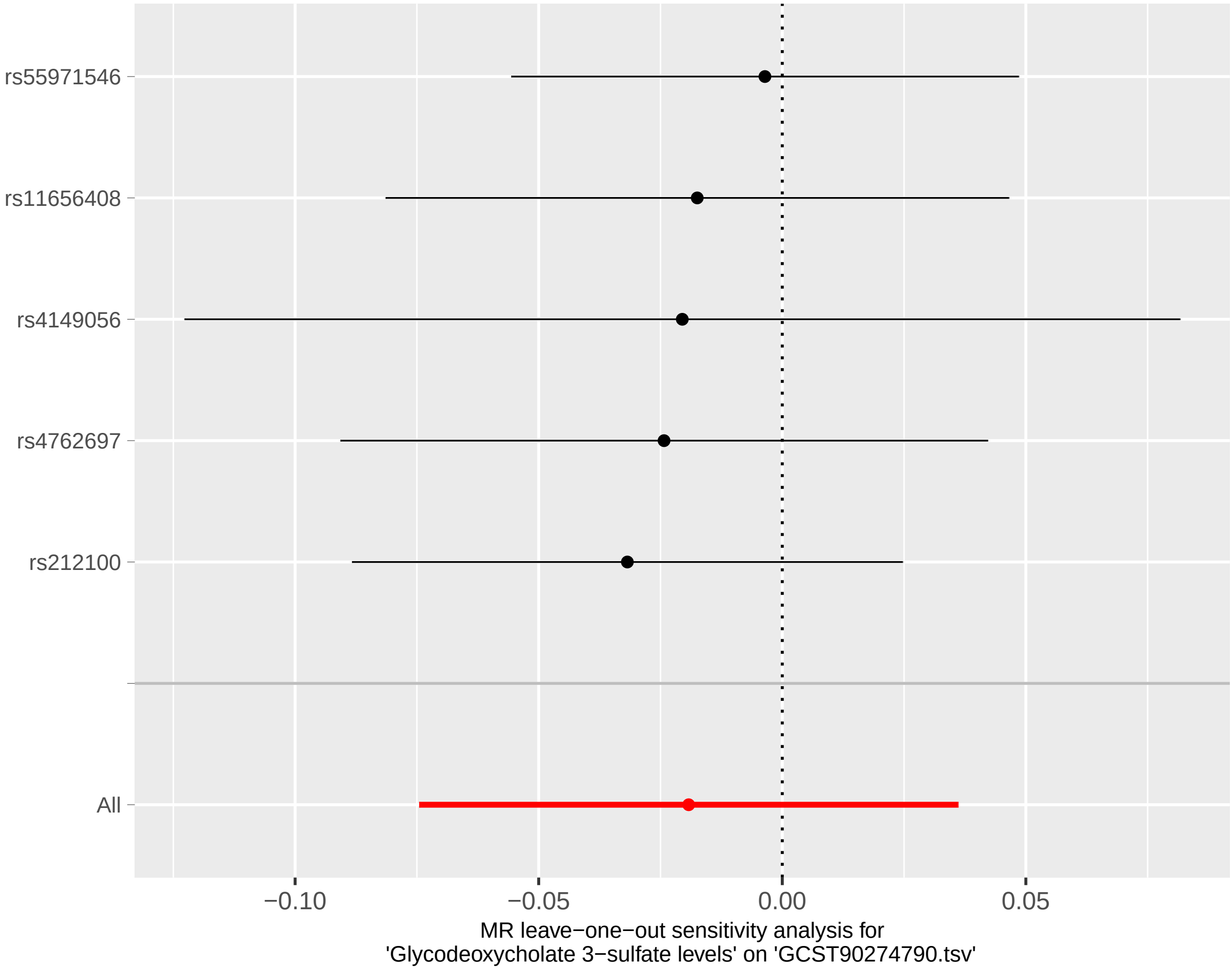
rs55971546
rs11656408
rs4149056
rs4762697
rs212100
All
−0.10
−0.05
0.00
0.05
MR leave−one−out sensitivity analysis for
'Glycodeoxycholate 3−sulfate levels' on 'GCST90274790.tsv'

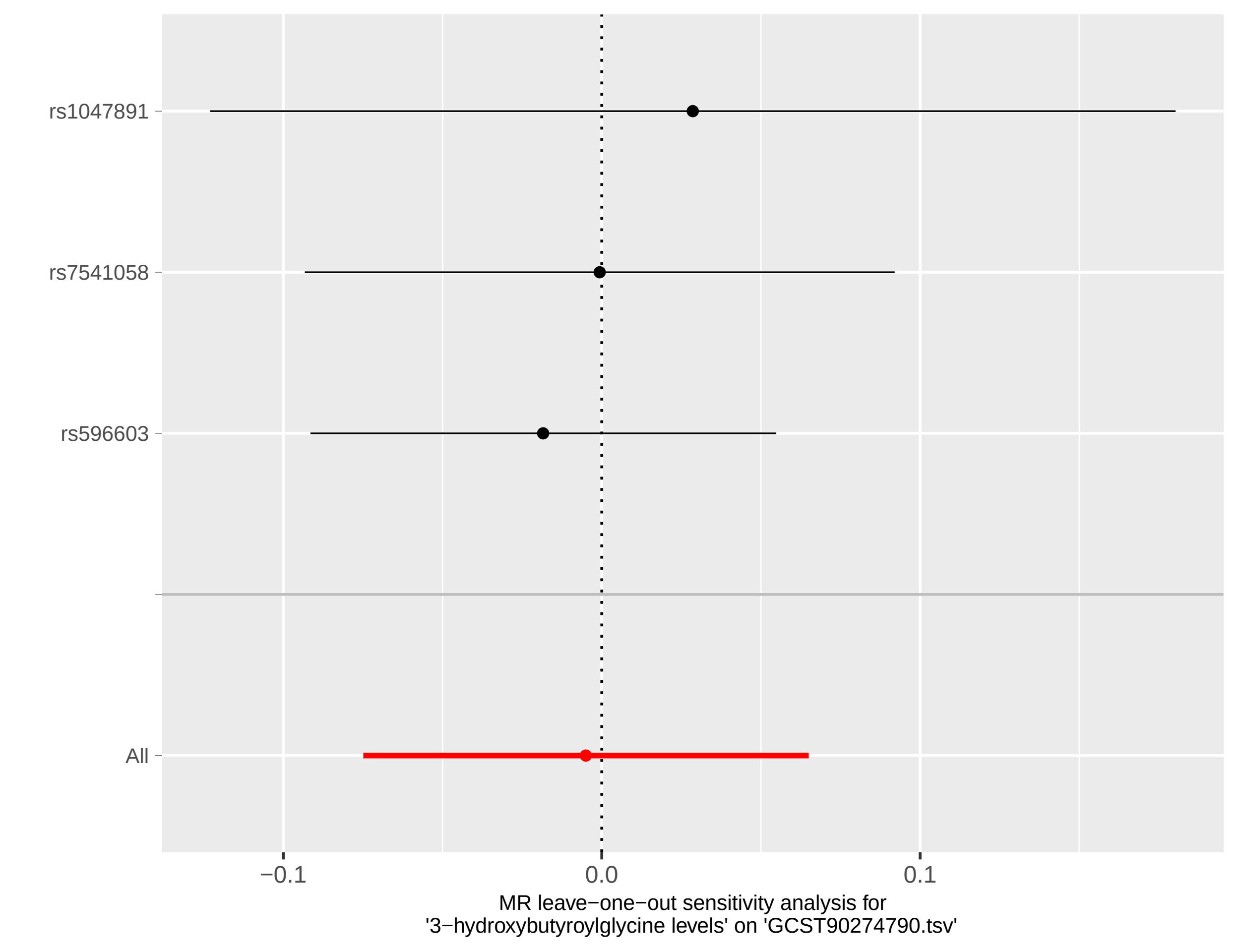
rs1047891
rs7541058
rs596603
All
−0.1
0.0
0.1
MR leave−one−out sensitivity analysis for
'3−hydroxybutyroylglycine levels' on 'GCST90274790.tsv'

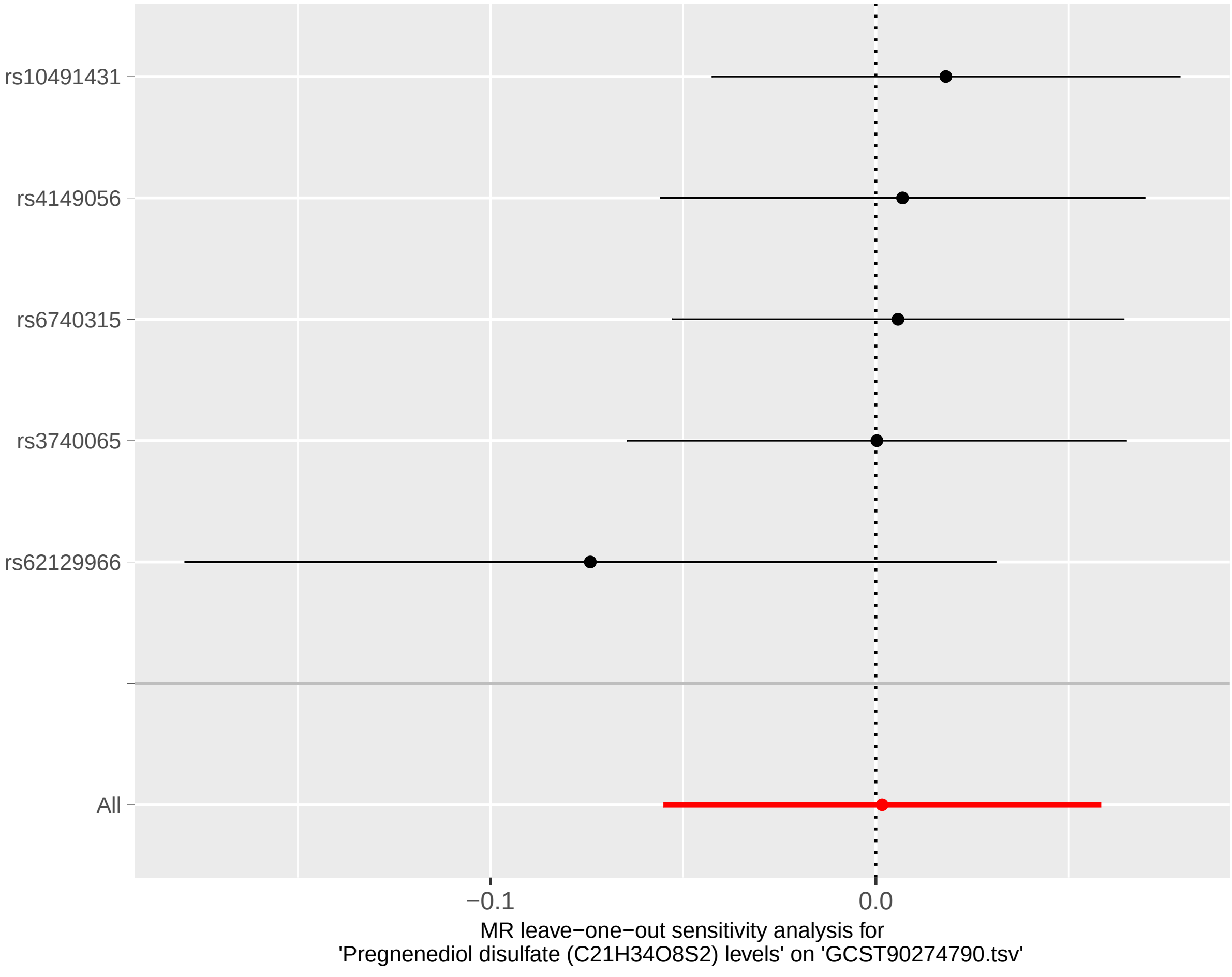
rs10491431
rs4149056
rs6740315
rs3740065
rs62129966
All
−0.1
0.0
MR leave−one−out sensitivity analysis for
'Pregnenediol disulfate (C21H34O8S2) levels' on 'GCST90274790.tsv'

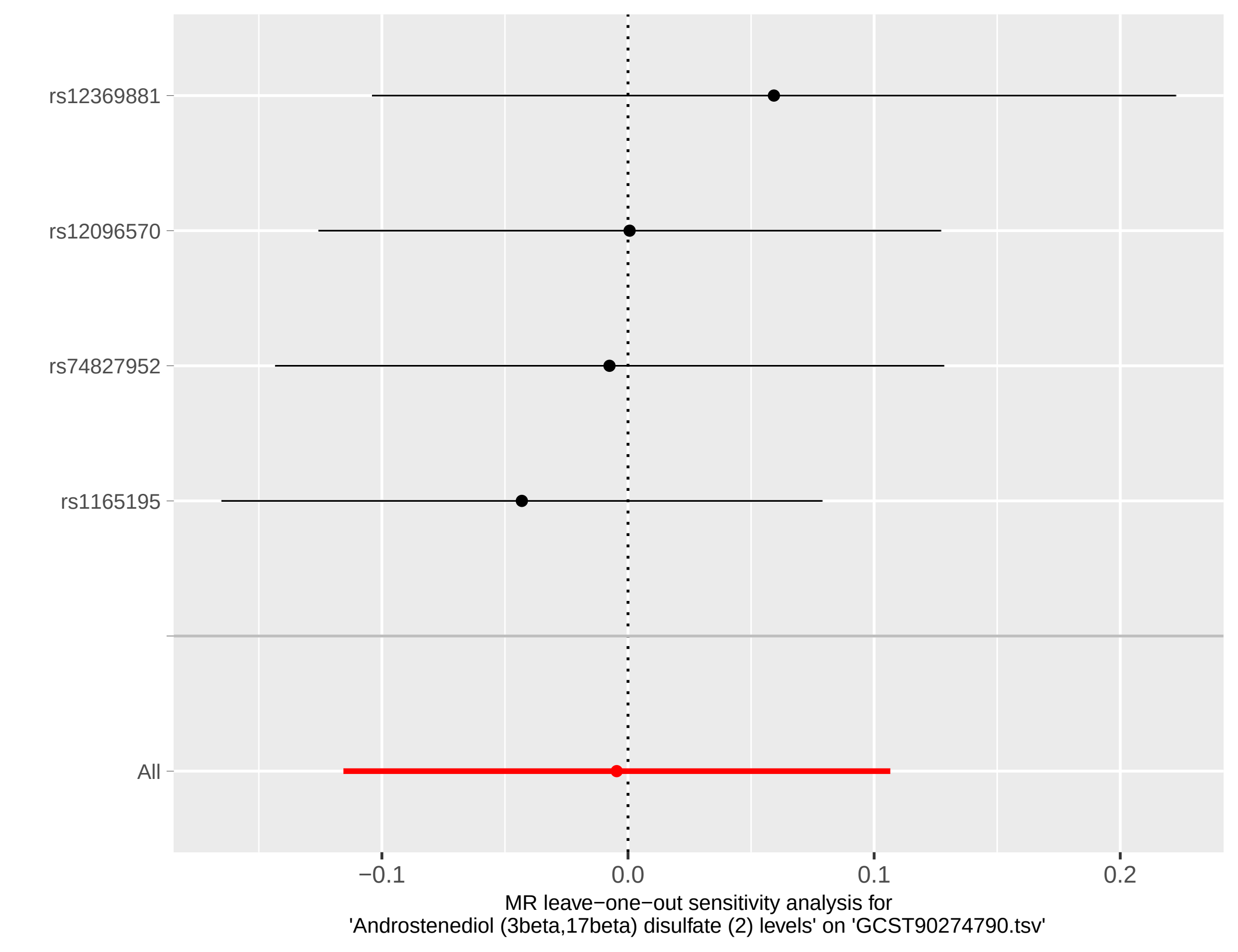
rs12369881
rs12096570
rs74827952
rs1165195
All
−0.1
0.0
0.1
0.2
MR leave−one−out sensitivity analysis for
'Androstenediol (3beta,17beta) disulfate (2) levels' on 'GCST90274790.tsv'

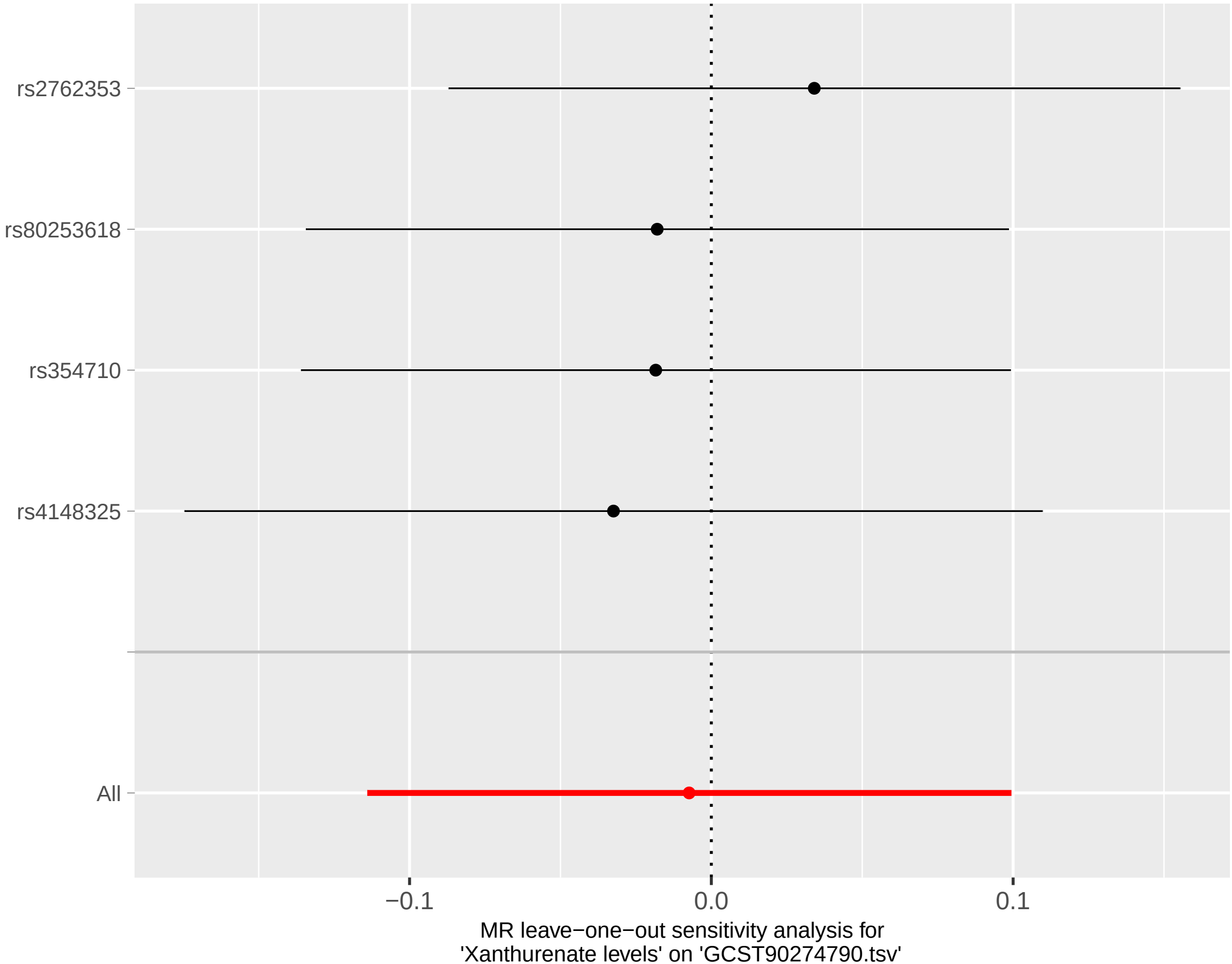

rs2762353
rs80253618
rs354710
rs4148325
All
−0.1
0.0
0.1
MR leave−one−out sensitivity analysis for
'Xanthurenate levels' on 'GCST90274790.tsv'

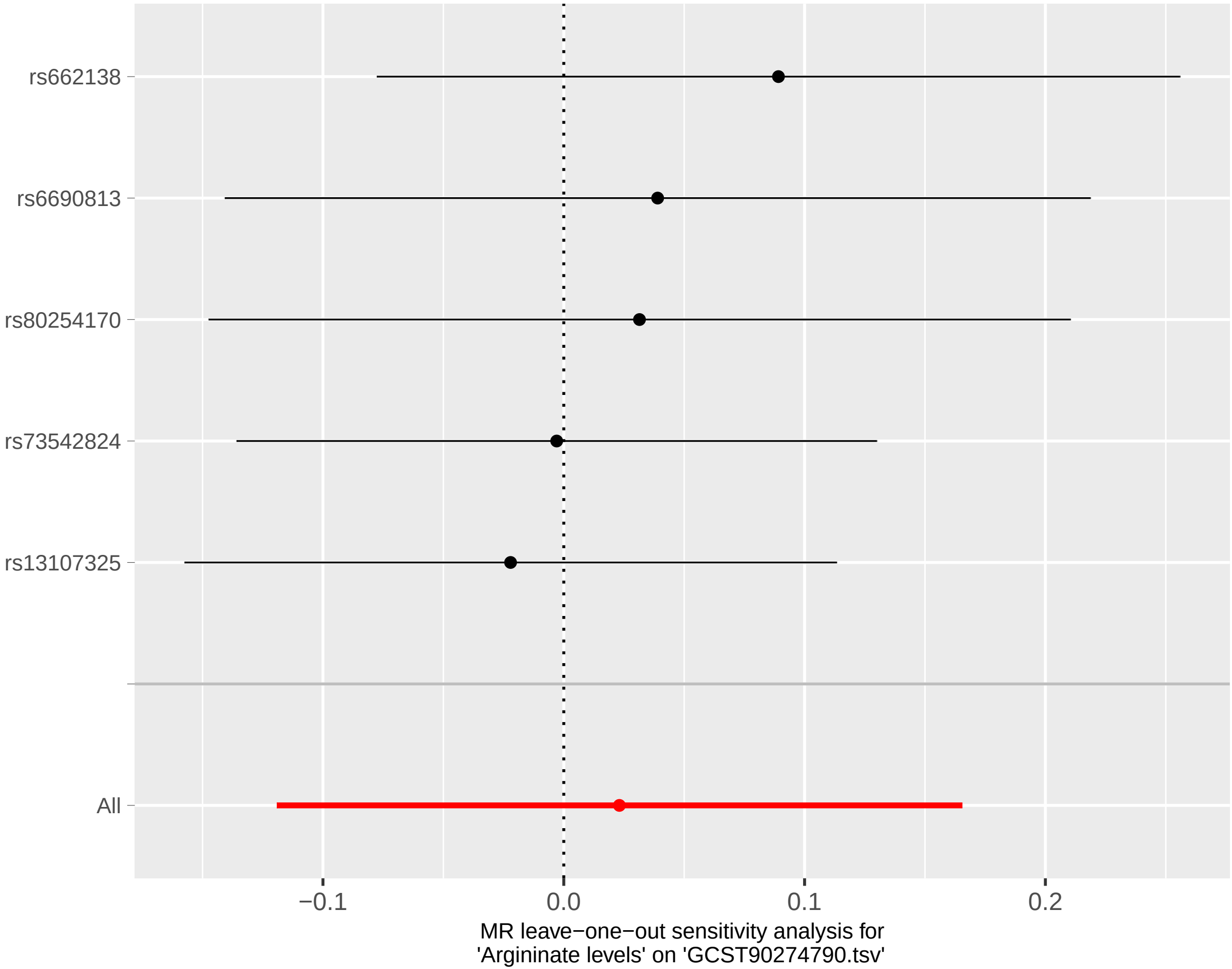

rs662138
rs6690813
rs80254170
rs73542824
rs13107325
All
−0.1
0.0
0.1
0.2
MR leave−one−out sensitivity analysis for
'Argininate levels' on 'GCST90274790.tsv'

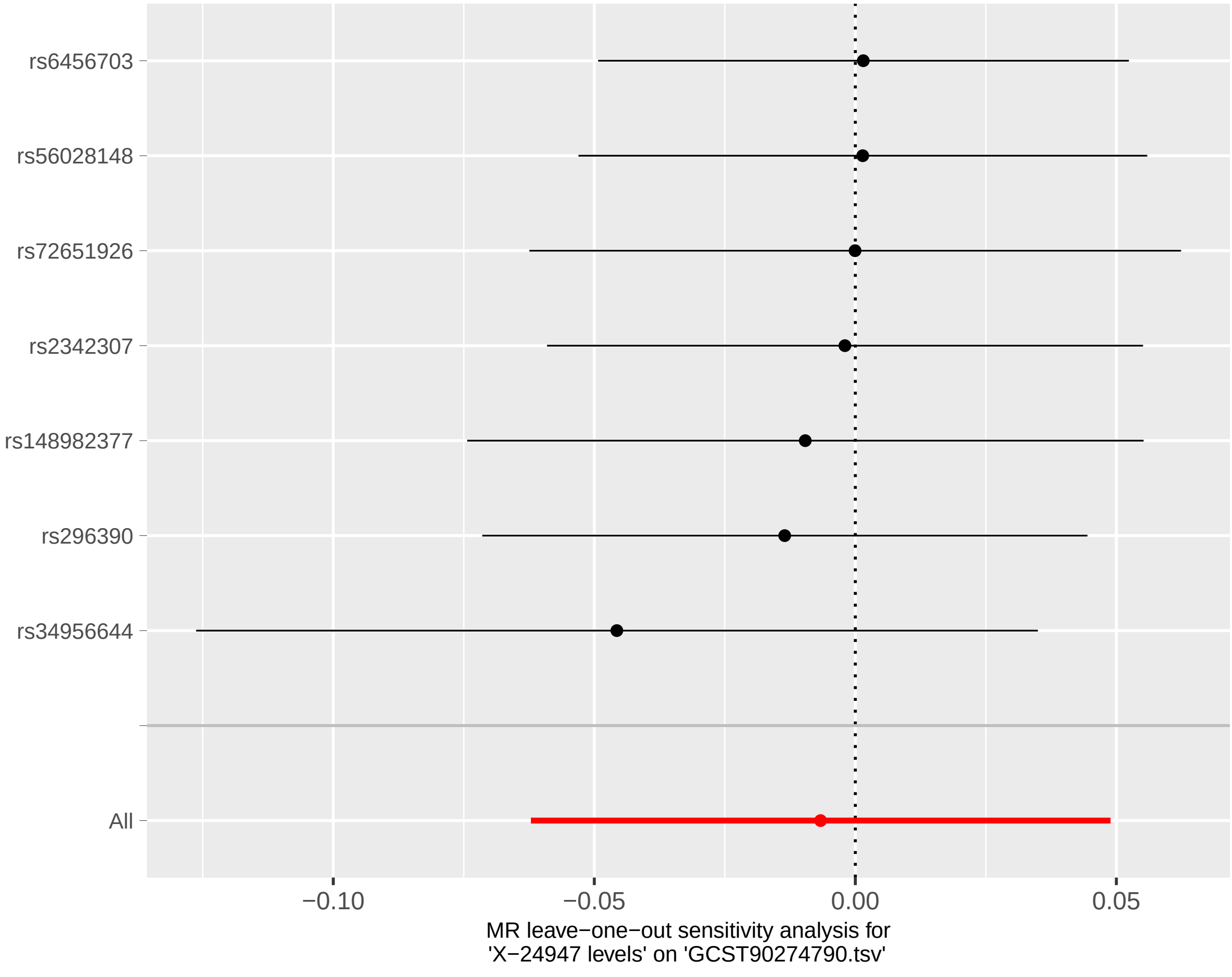
rs6456703
rs56028148
rs72651926
rs2342307
rs148982377
rs296390
rs34956644
All
−0.10
−0.05
0.00
0.05
MR leave−one−out sensitivity analysis for
'X−24947 levels' on 'GCST90274790.tsv'

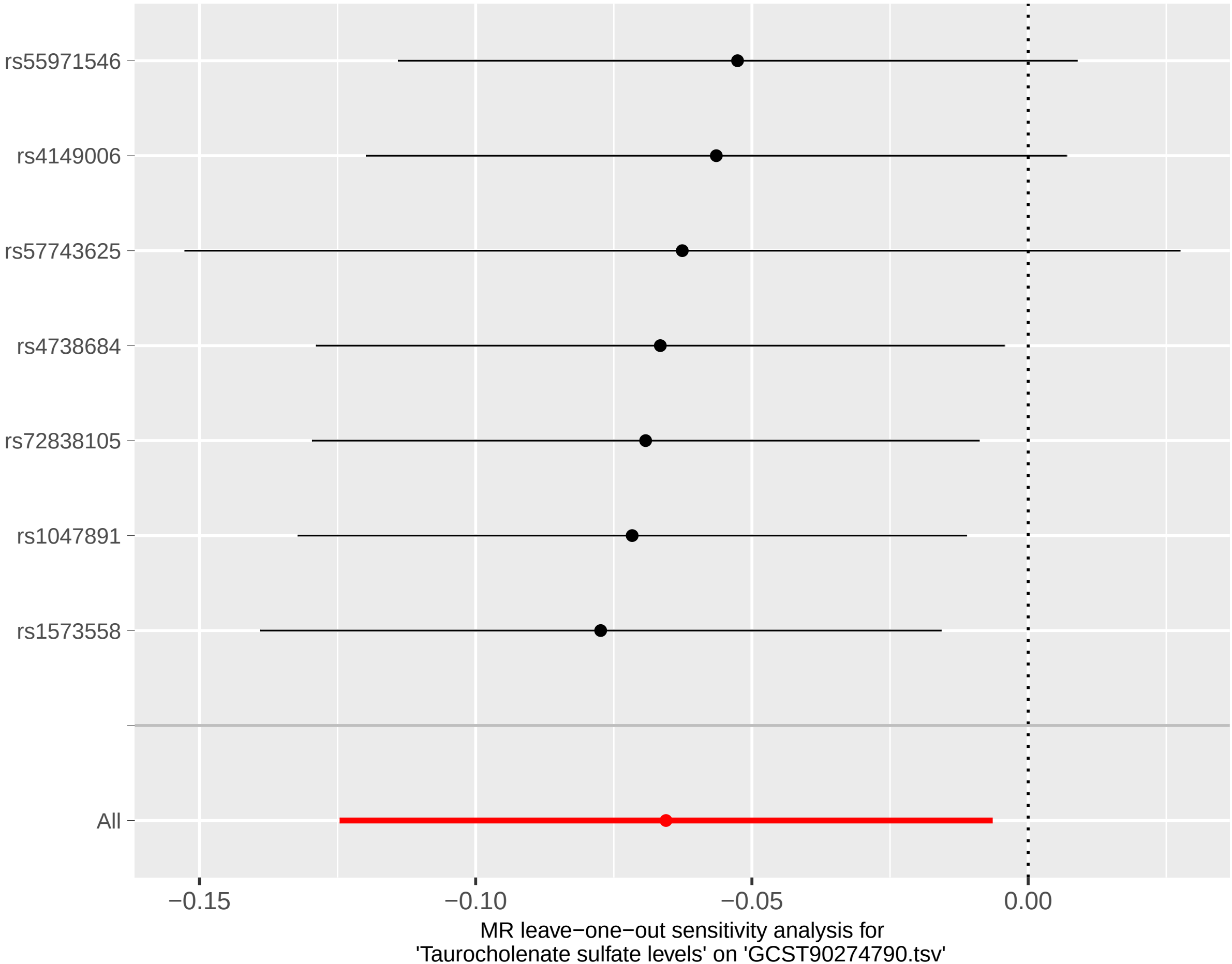
rs55971546
rs4149006
rs57743625
rs4738684
rs72838105
rs1047891
rs1573558
All
−0.15
−0.10
−0.05
0.00
MR leave−one−out sensitivity analysis for
'Taurocholenate sulfate levels' on 'GCST90274790.tsv'

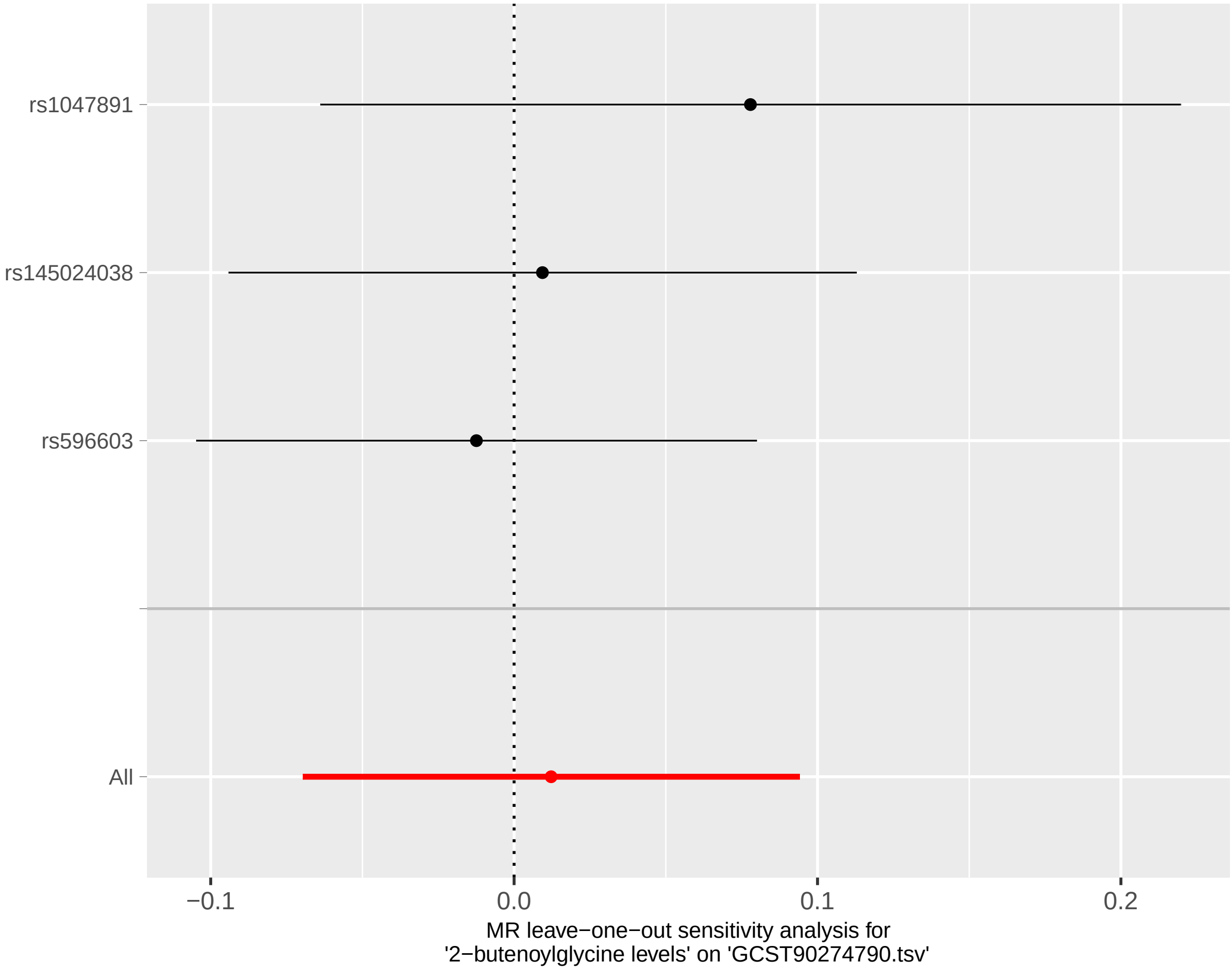
rs1047891
rs145024038
rs596603
All
−0.1
0.0
0.1
0.2
MR leave−one−out sensitivity analysis for
'2−butenoylglycine levels' on 'GCST90274790.tsv'

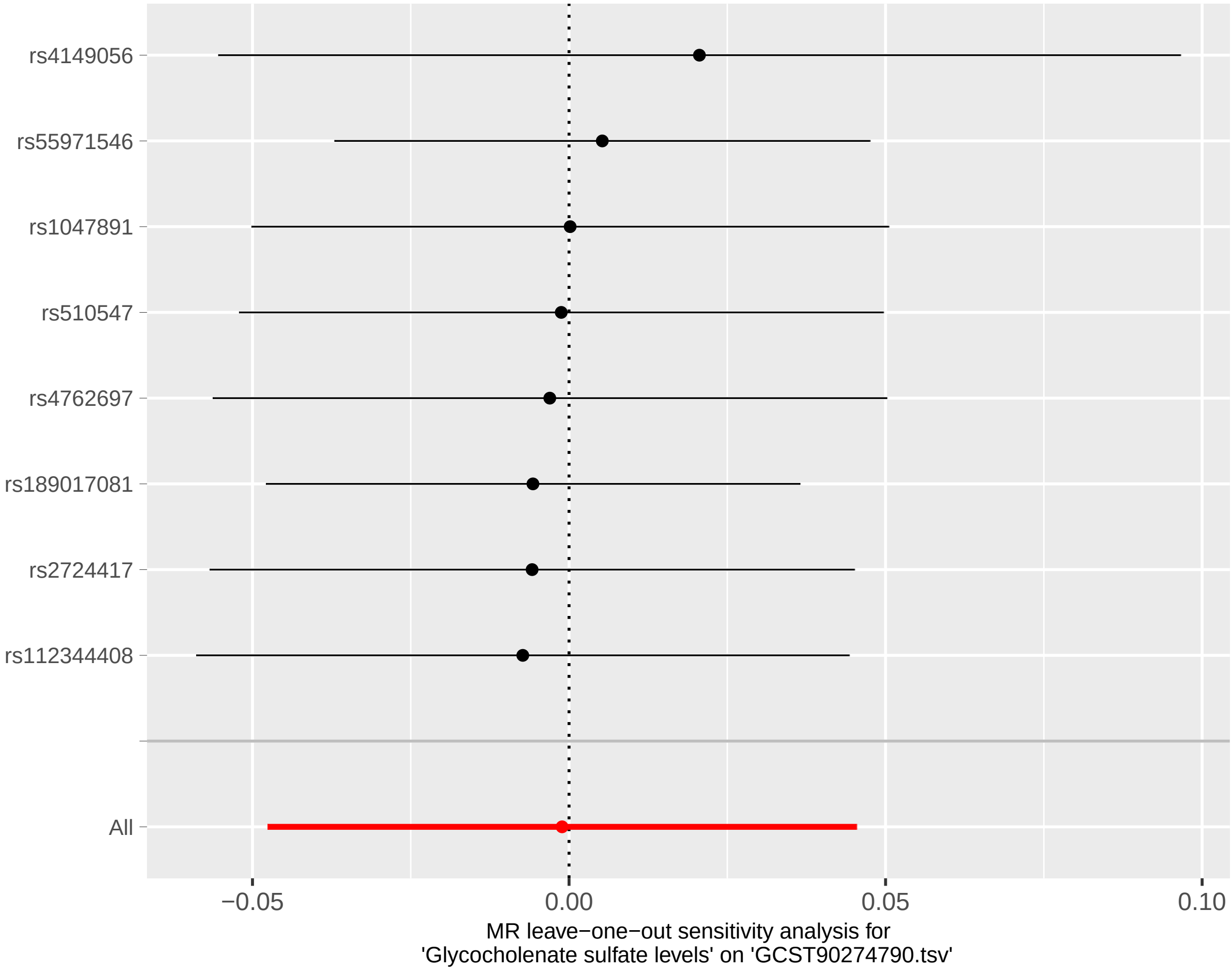

rs4149056
rs55971546
rs1047891
rs510547
rs4762697
rs189017081
rs2724417
rs112344408
All
−0.05
0.00
0.05
0.10
MR leave−one−out sensitivity analysis for
'Glycocholenate sulfate levels' on 'GCST90274790.tsv'

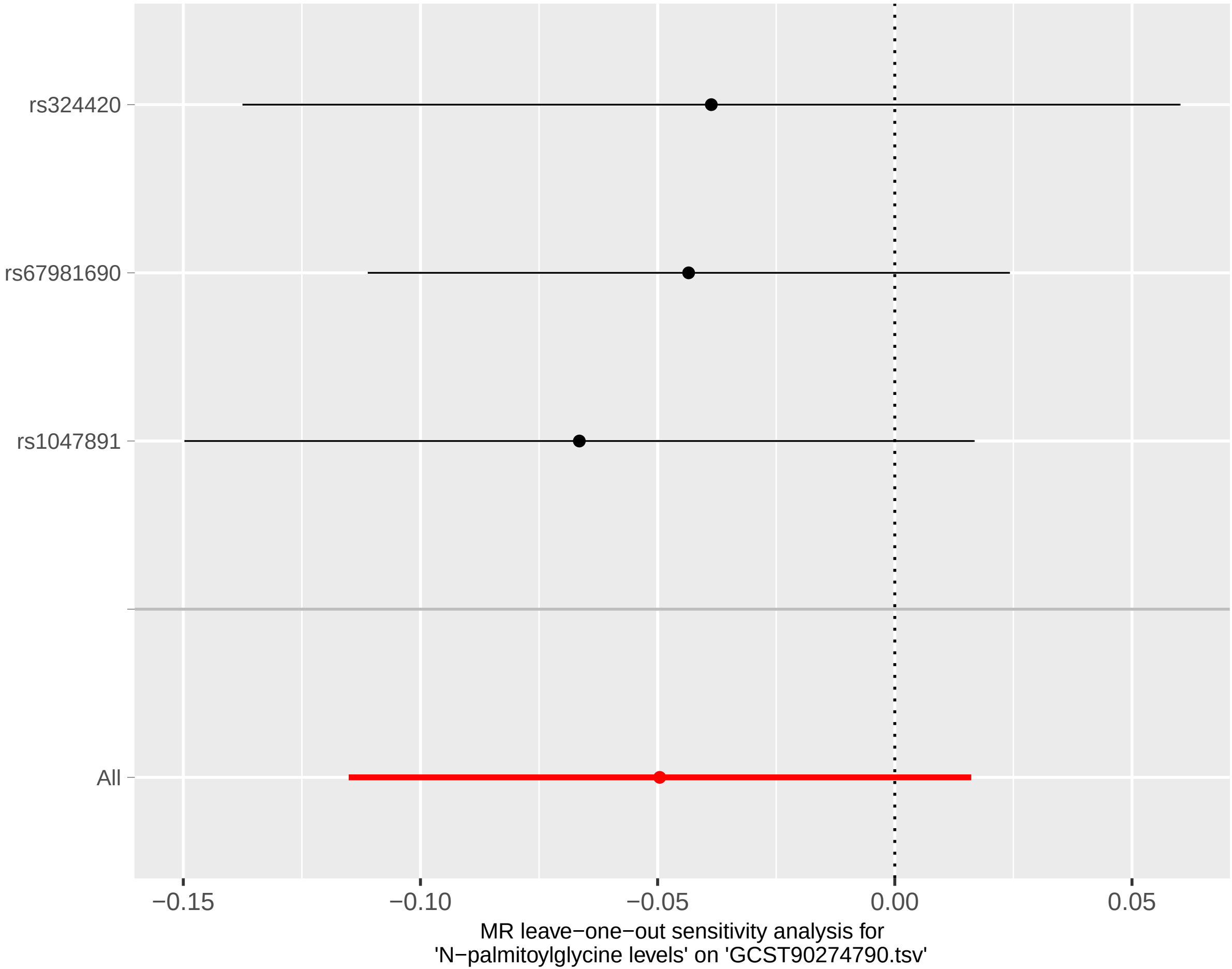
rs324420
rs67981690
rs1047891
All
−0.15
−0.10
−0.05
0.00
0.05
MR leave−one−out sensitivity analysis for
'N−palmitoylglycine levels' on 'GCST90274790.tsv'

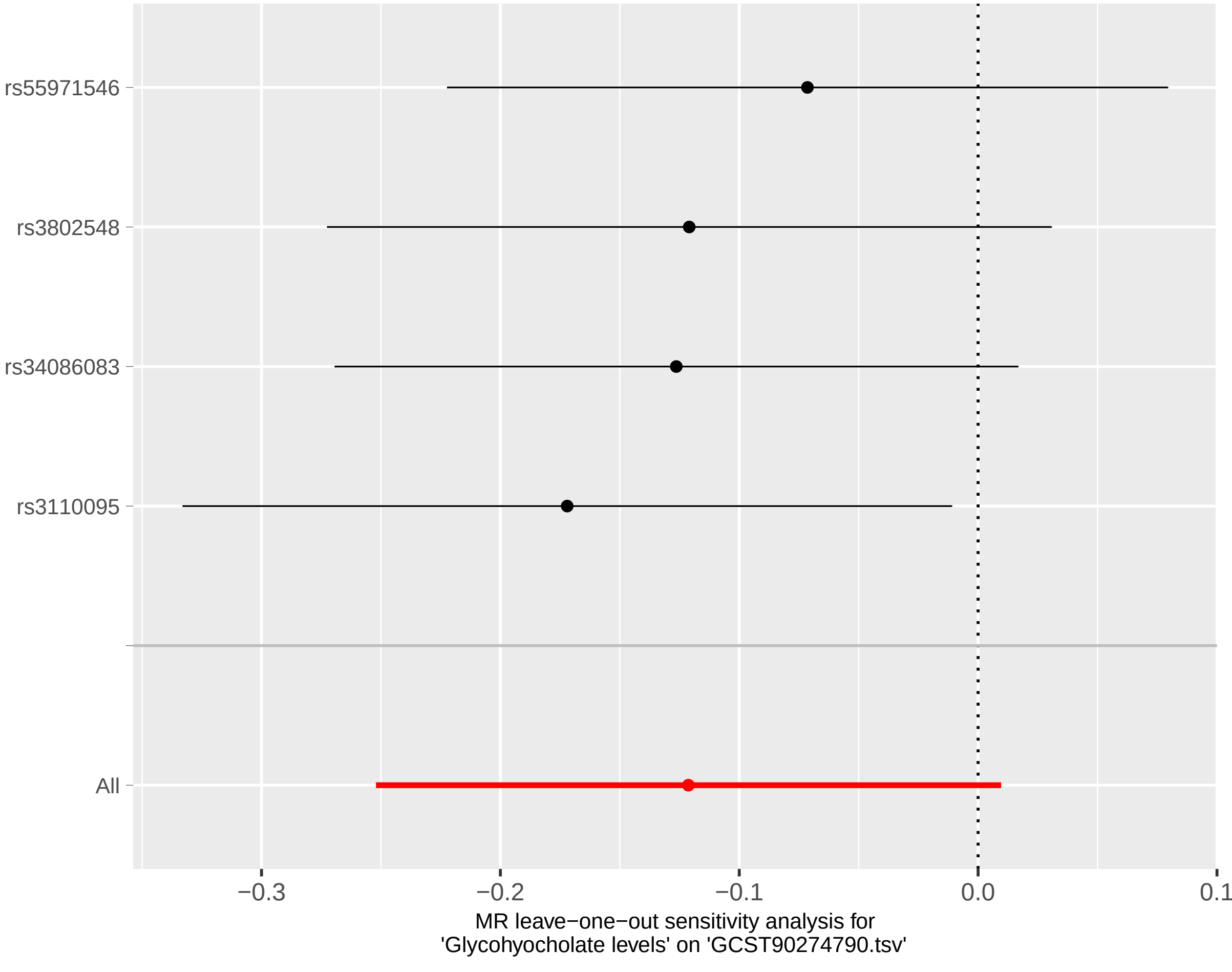
rs55971546
rs3802548
rs34086083
rs3110095
All
−0.3
−0.2
−0.1
0.0
0.1
MR leave−one−out sensitivity analysis for
'Glycohyocholate levels' on 'GCST90274790.tsv'

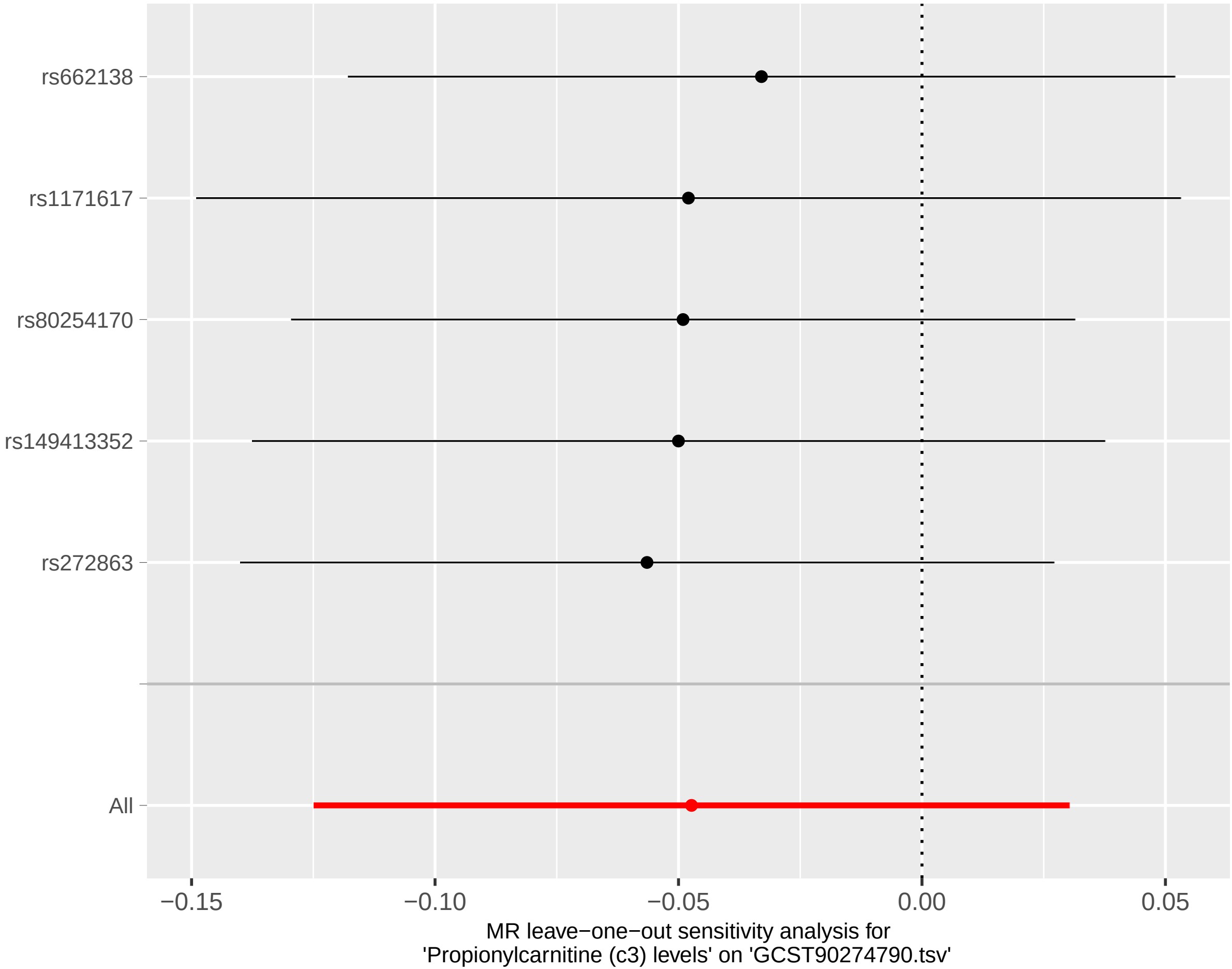
rs662138
rs1171617
rs80254170
rs149413352
rs272863
All
−0.15
−0.10
−0.05
0.00
0.05
MR leave−one−out sensitivity analysis for
'Propionylcarnitine (c3) levels' on 'GCST90274790.tsv'

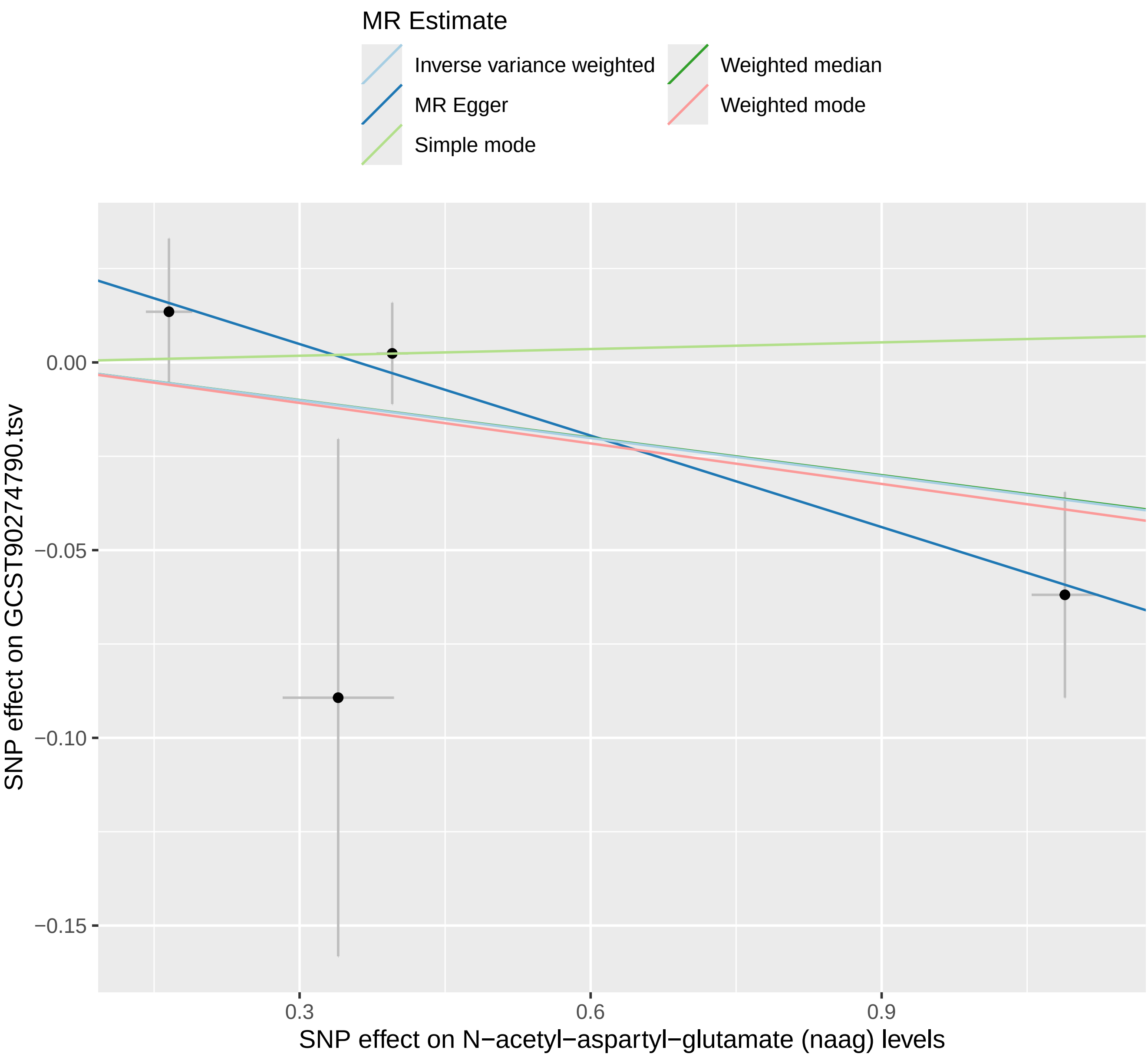
MR Estimate
Inverse variance weighted
MR Egger
Simple mode
Weighted median
Weighted mode
0.00
−0.05
−0.10
−0.15
0.3
0.6
0.9
SNP effect on GCST90274790.tsv
SNP effect on N−acetyl−aspartyl−glutamate (naag) levels

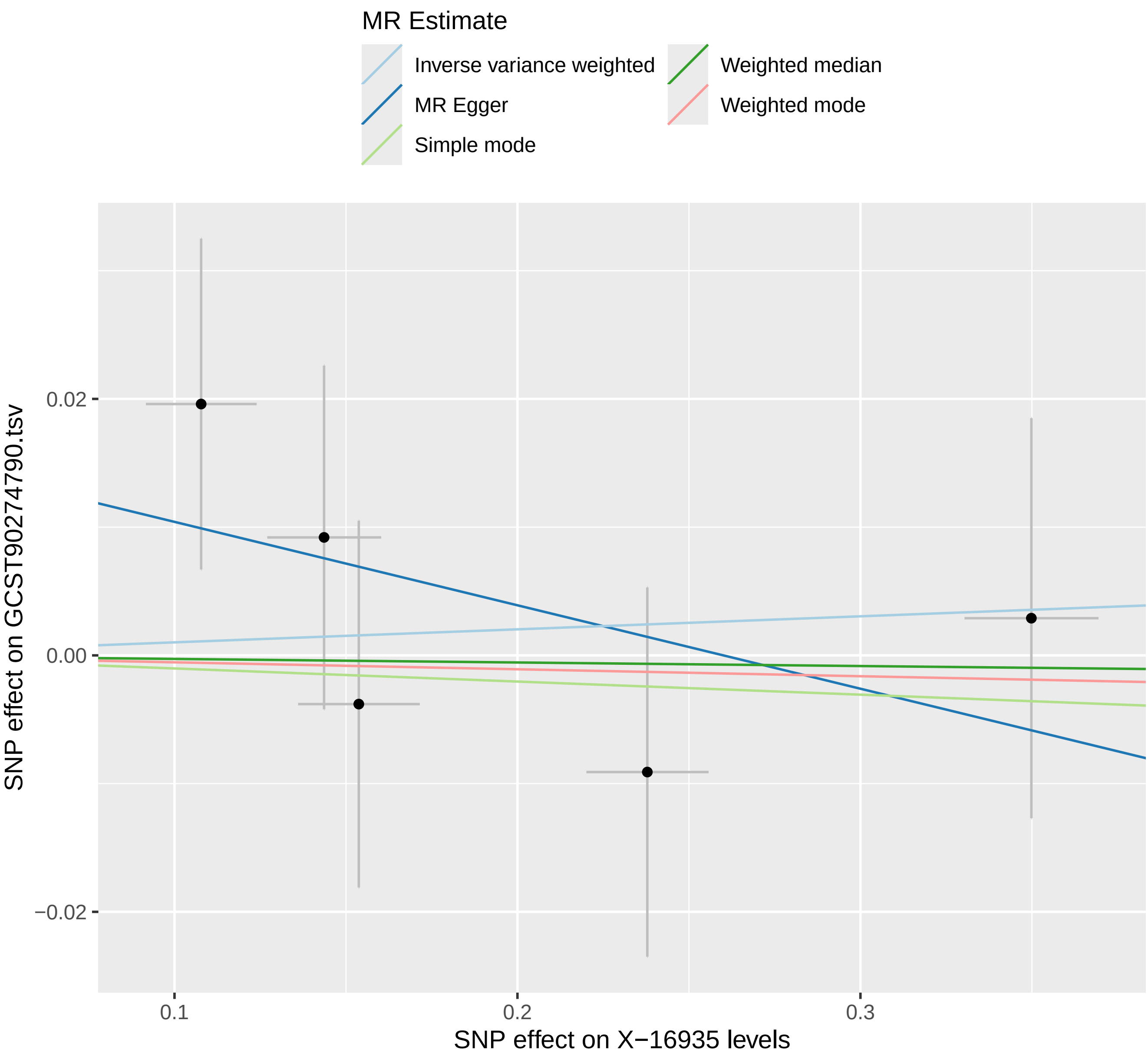
MR Estimate
Inverse variance weighted
MR Egger
Simple mode
Weighted median
Weighted mode
0.02
0.00
−0.02
SNP effect on GCST90274790.tsv
0.1
0.2
0.3
SNP effect on X−16935 levels

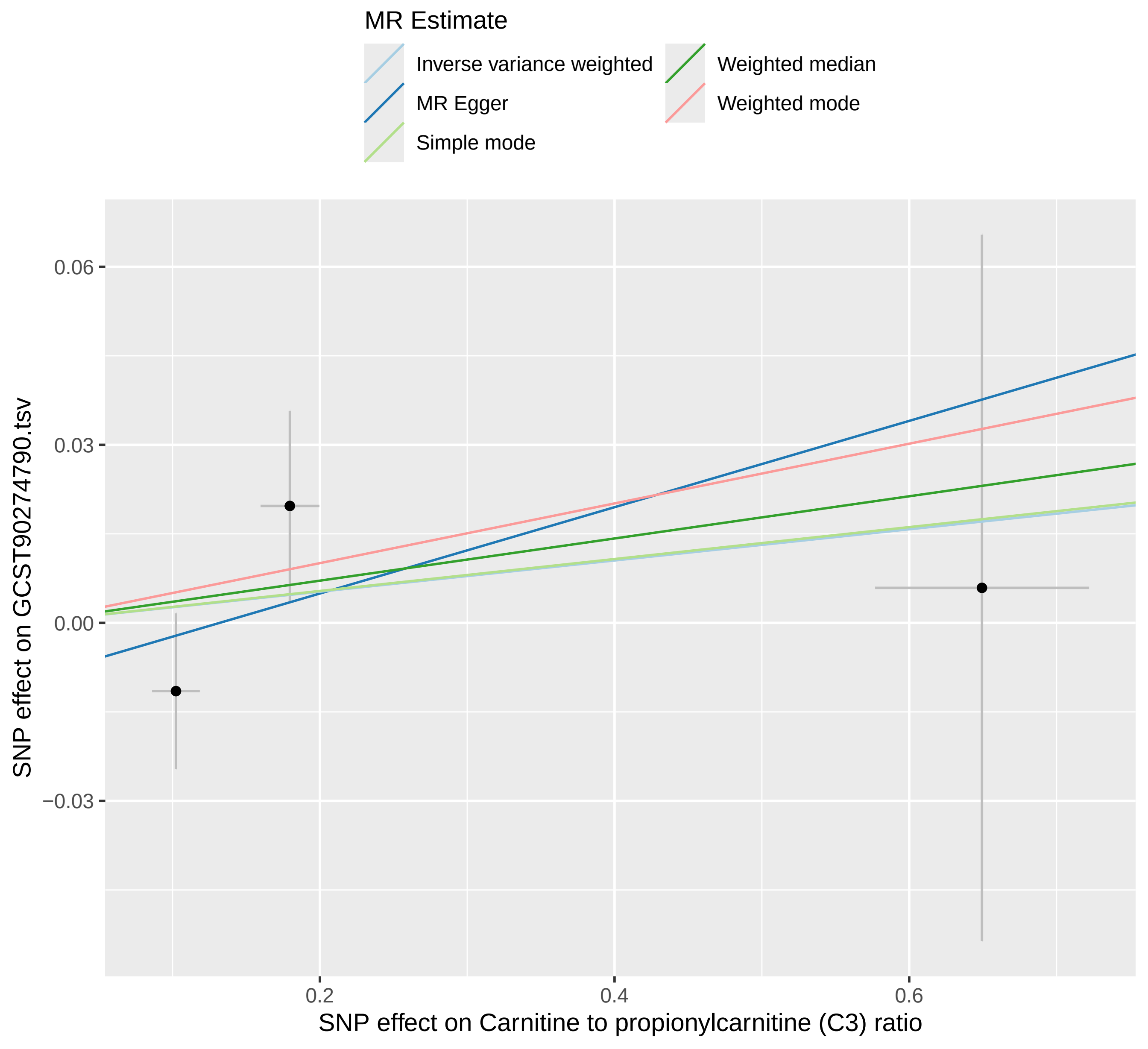

MR Estimate
Inverse variance weighted
MR Egger
Simple mode
Weighted median
Weighted mode
0.06
0.03
0.00
−0.03
SNP effect on GCST90274790.tsv
0.2
0.4
0.6
SNP effect on Carnitine to propionylcarnitine (C3) ratio

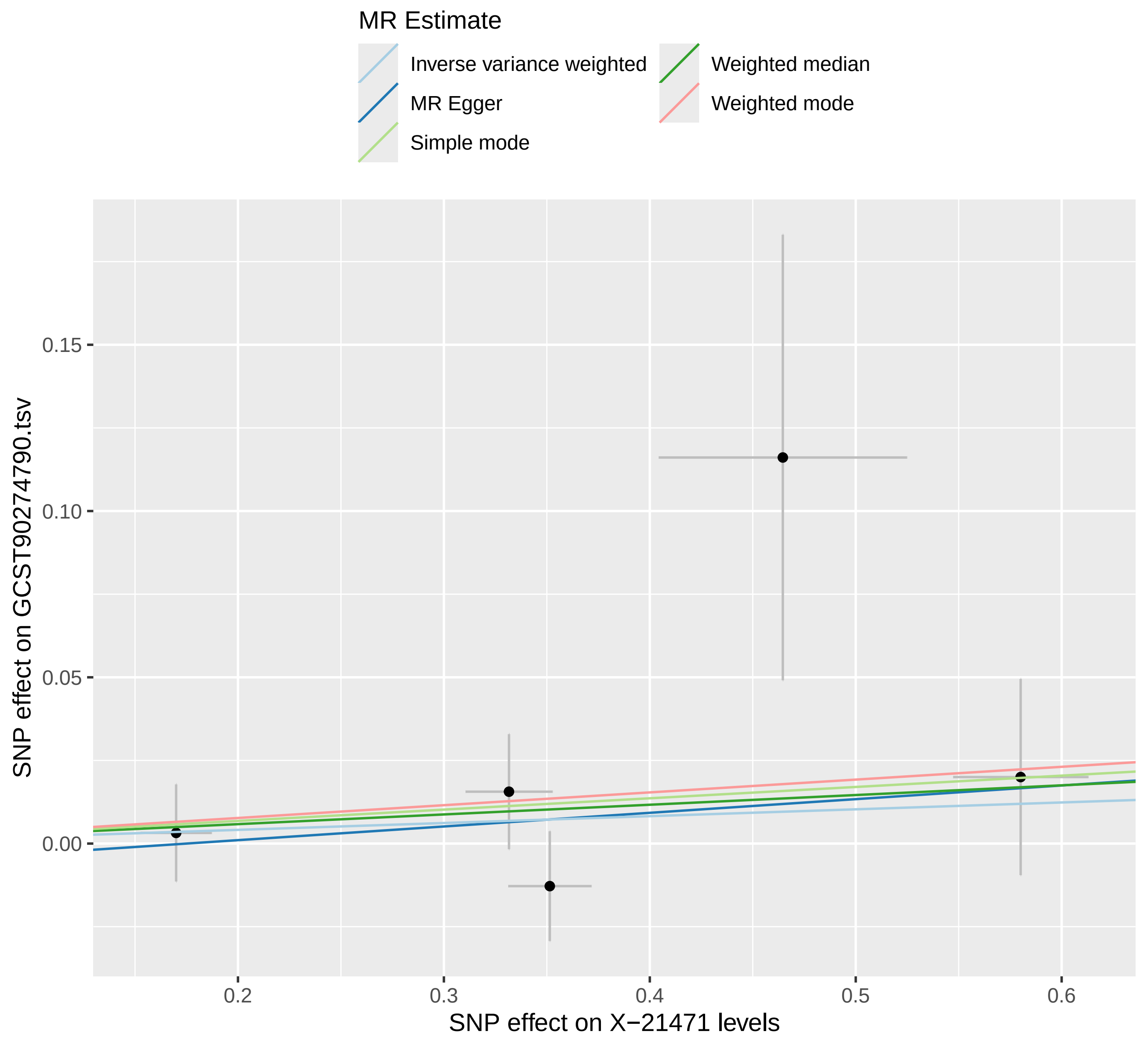

MR Estimate
Inverse variance weighted
MR Egger
Simple mode
Weighted median
Weighted mode
SNP effect on GCST90274790.tsv
0.15
0.10
0.05
0.00
0.2
0.3
0.4
0.5
0.6
SNP effect on X−21471 levels

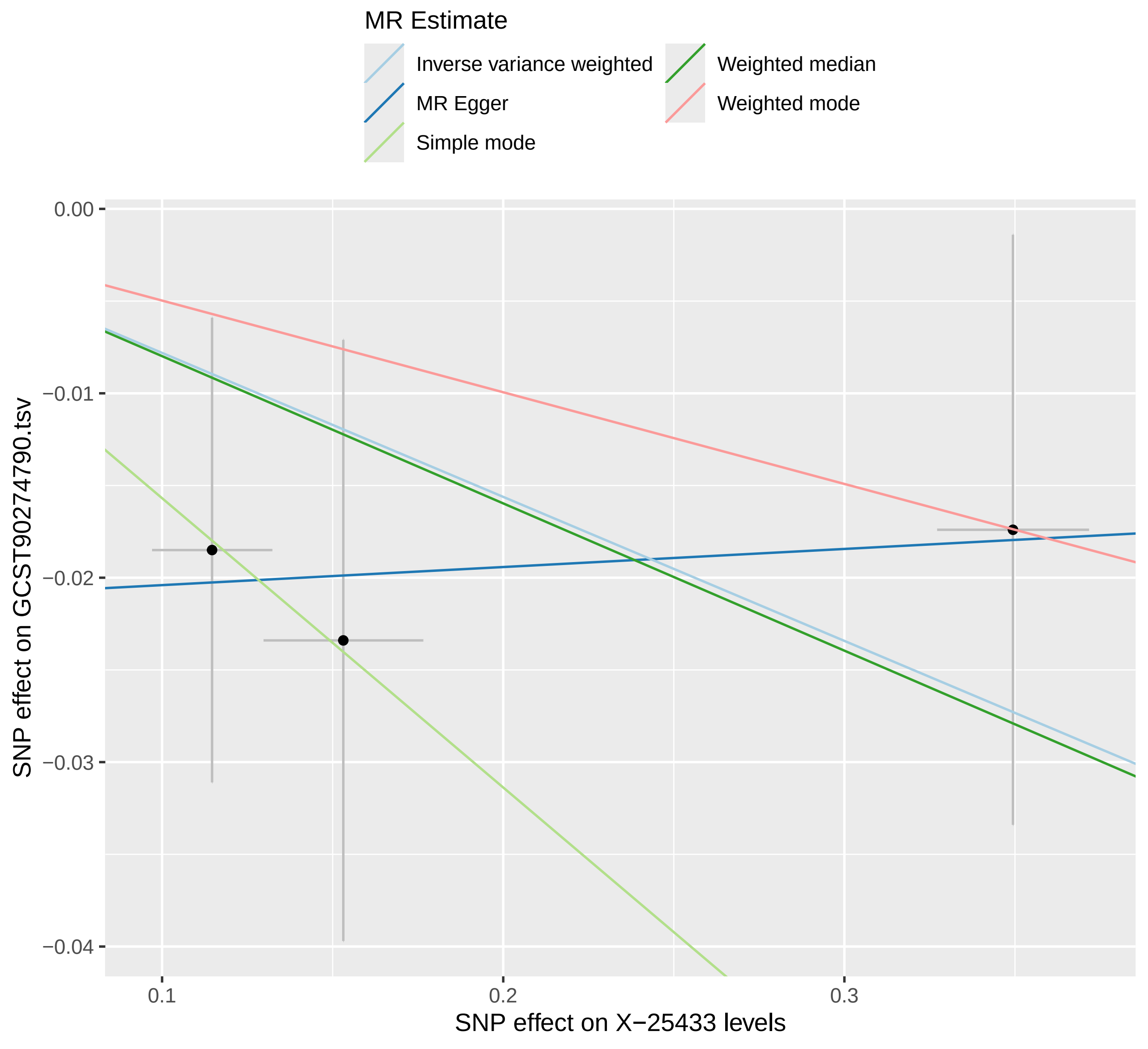

MR Estimate
Inverse variance weighted
MR Egger
Simple mode
Weighted median
Weighted mode
0.00
−0.01
−0.02
−0.03
−0.04
SNP effect on GCST90274790.tsv
0.1
0.2
0.3
SNP effect on X−25433 levels

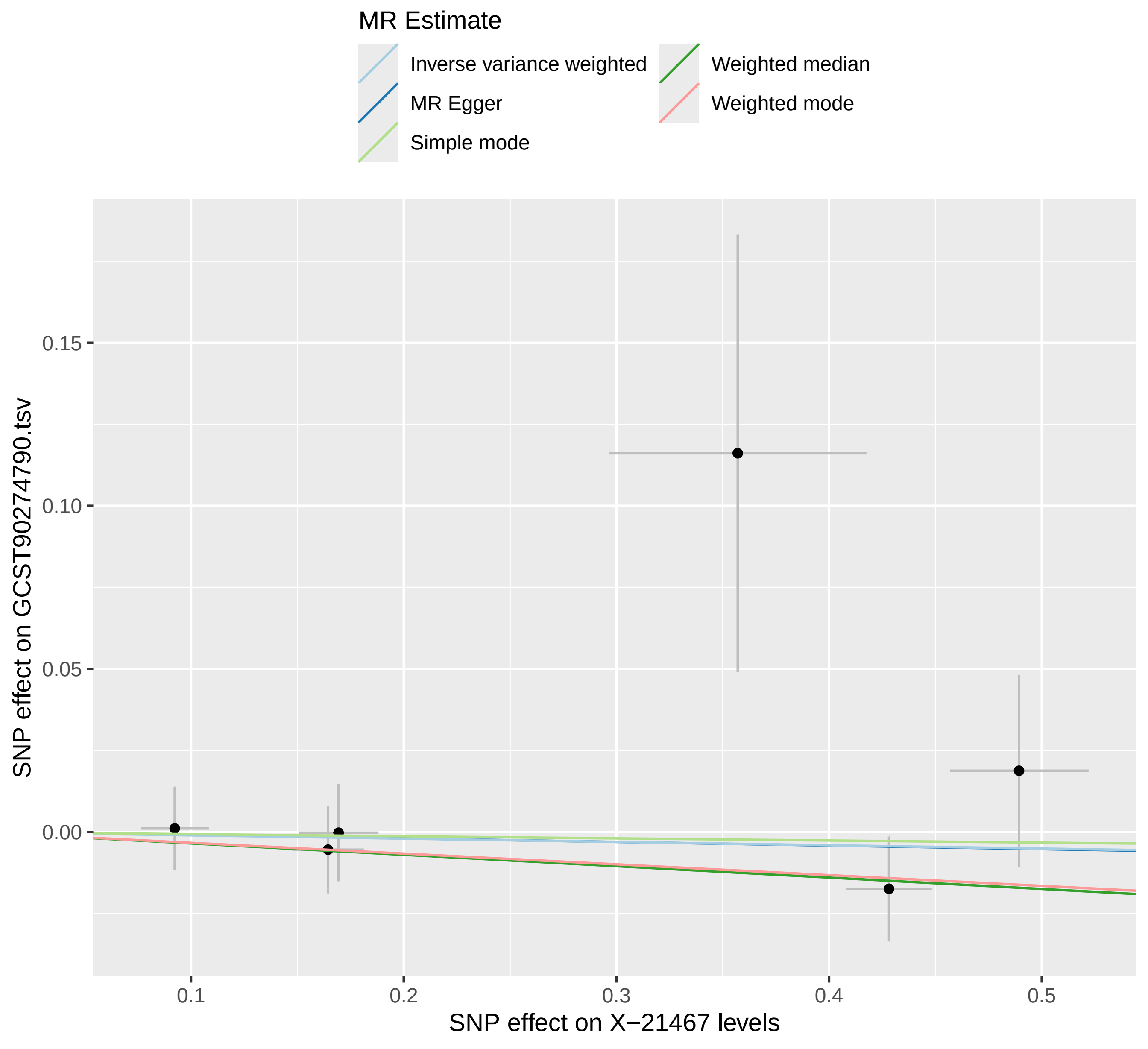

MR Estimate
Inverse variance weighted
MR Egger
Simple mode
Weighted median
Weighted mode
0.15
0.10
0.05
0.00
0.1
0.2
0.3
0.4
0.5
SNP effect on GCST90274790.tsv
SNP effect on X−21467 levels

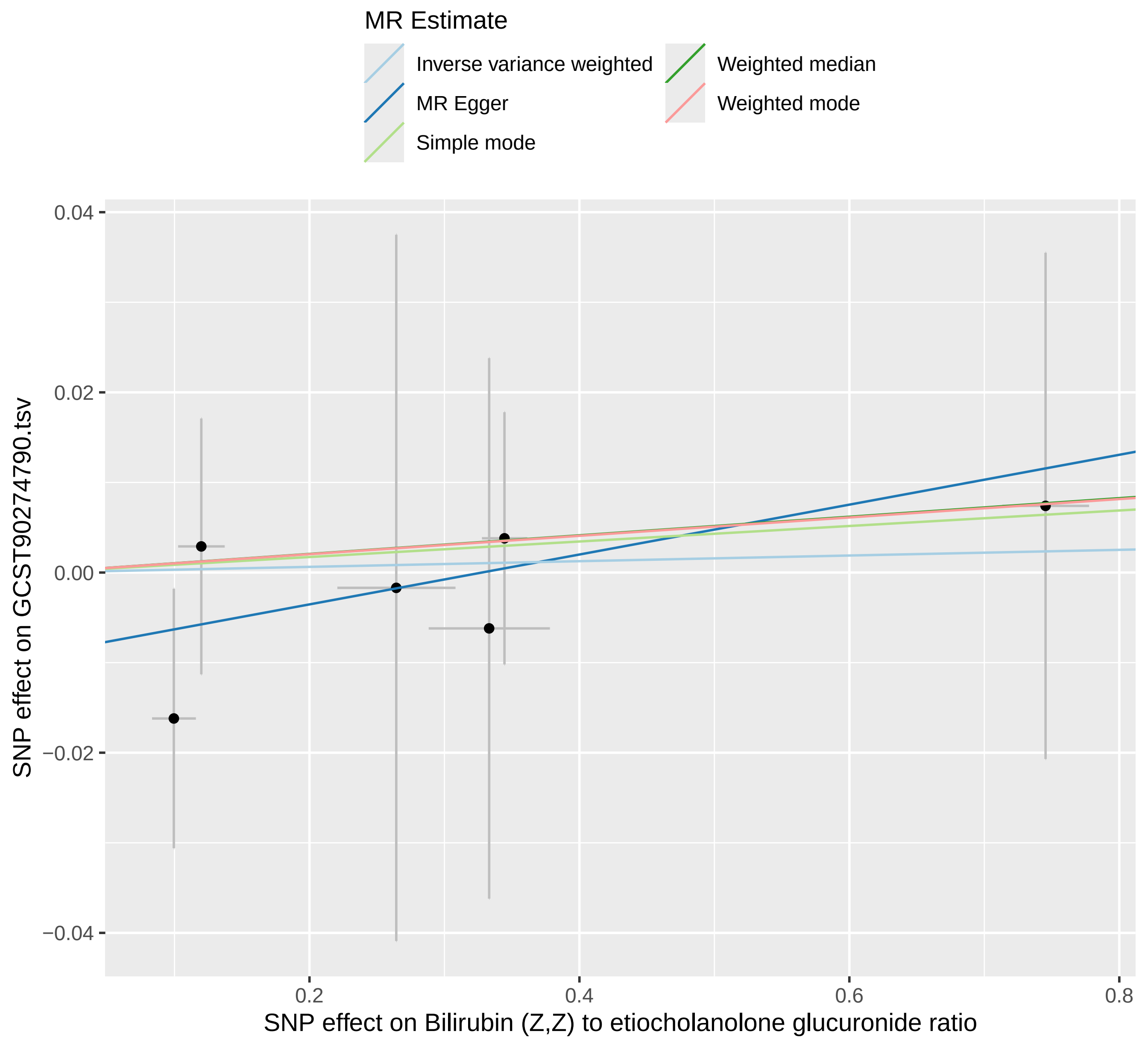
MR Estimate
Inverse variance weighted
MR Egger
Simple mode
Weighted median
Weighted mode
0.04
0.02
0.00
−0.02
−0.04
0.2
0.4
0.6
0.8
SNP effect on GCST90274790.tsv
SNP effect on Bilirubin (Z,Z) to etiocholanolone glucuronide ratio

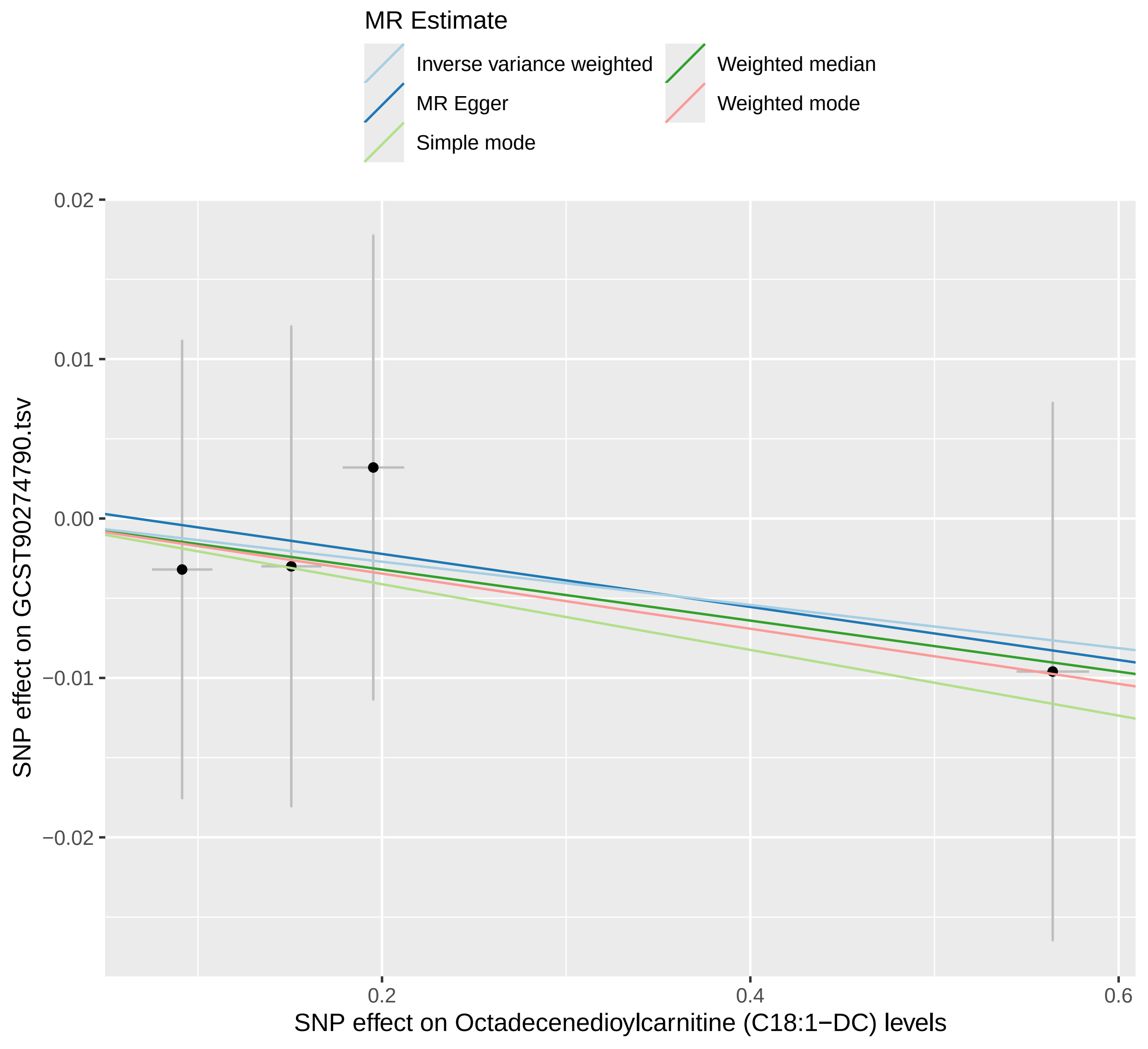
MR Estimate
Inverse variance weighted
MR Egger
Simple mode
Weighted median
Weighted mode
0.02
0.01
0.00
−0.01
−0.02
SNP effect on GCST90274790.tsv
0.2
0.4
0.6
SNP effect on Octadecenedioylcarnitine (C18:1−DC) levels

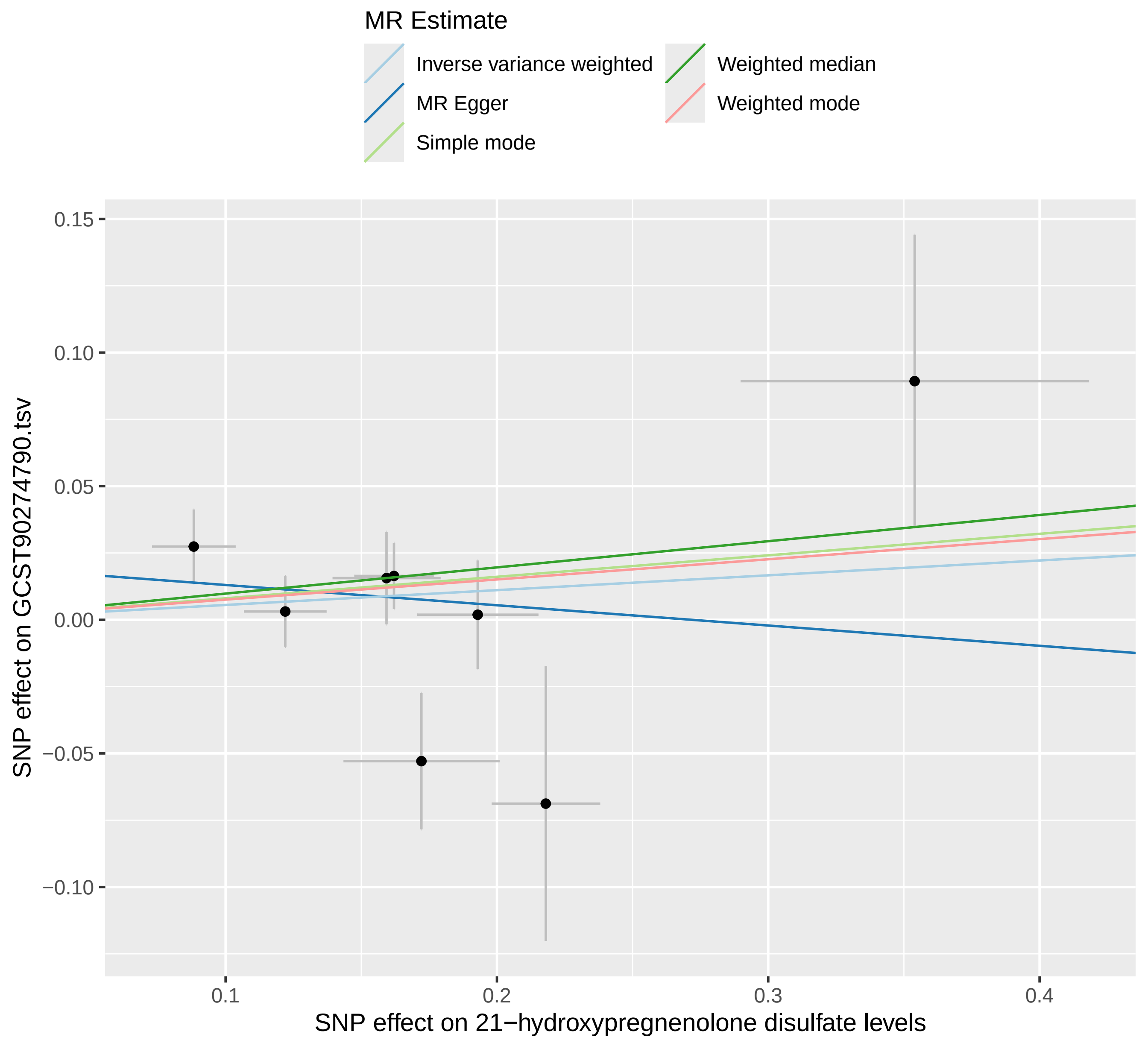

MR Estimate
Inverse variance weighted
MR Egger
Simple mode
Weighted median
Weighted mode
0.15
0.10
0.05
0.00
−0.05
−0.10
0.1
0.2
0.3
0.4
SNP effect on GCST90274790.tsv
SNP effect on 21−hydroxypregnenolone disulfate levels

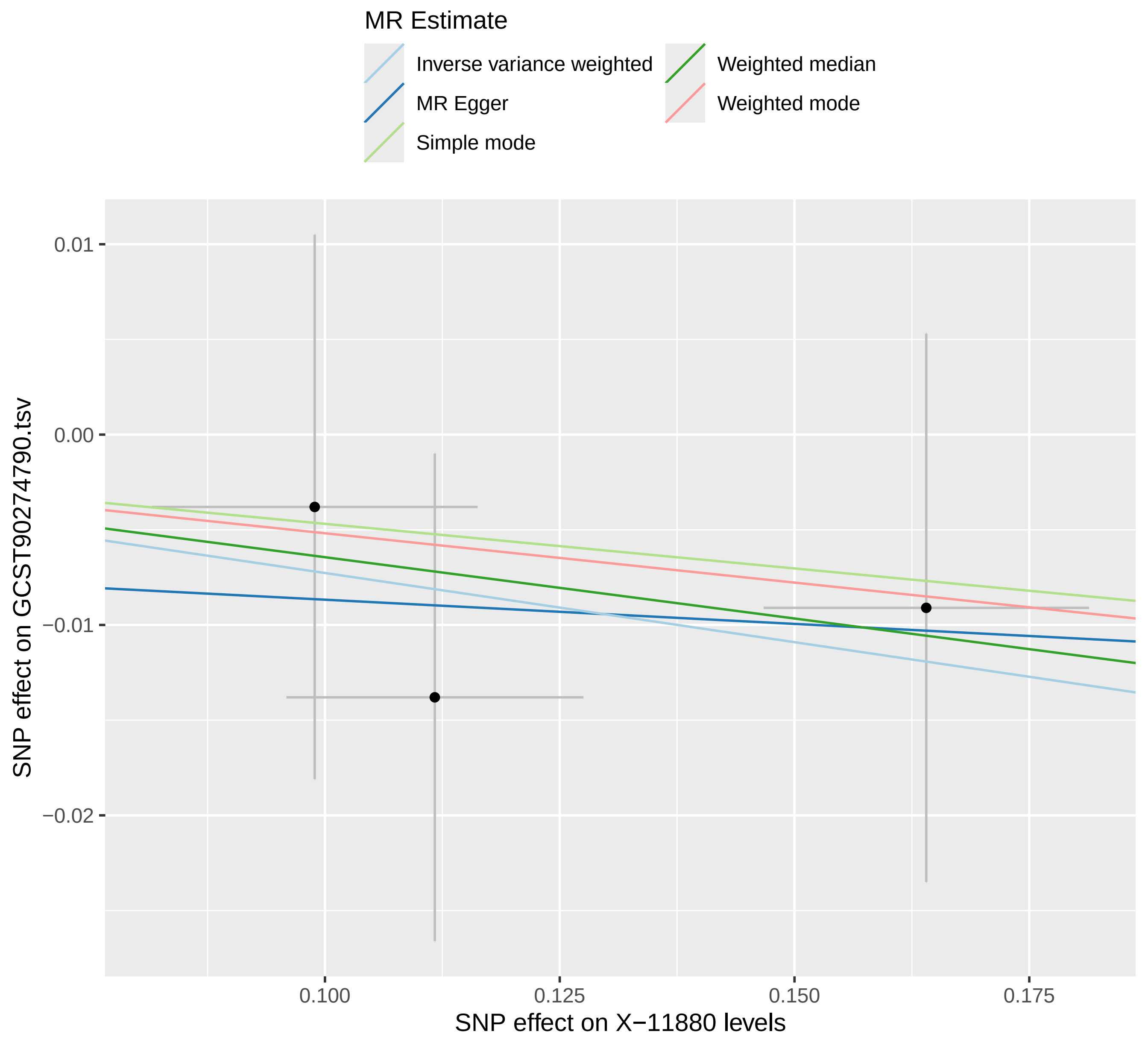

MR Estimate
Inverse variance weighted
MR Egger
Simple mode
Weighted median
Weighted mode
0.01
0.00
−0.01
−0.02
0.100
0.125
0.150
0.175
SNP effect on X−11880 levels
SNP effect on GCST90274790.tsv

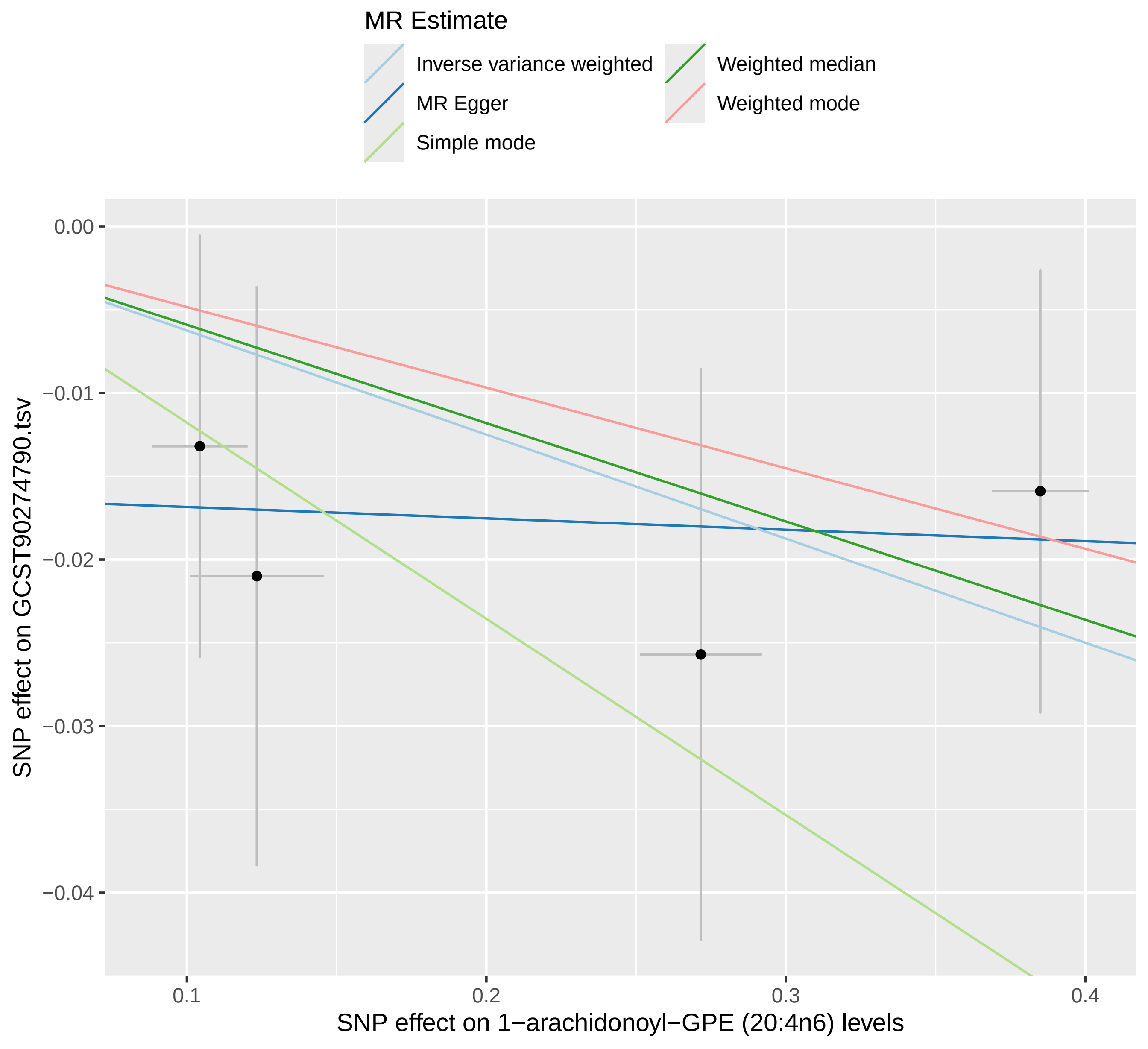
MR Estimate
Inverse variance weighted
MR Egger
Simple mode
Weighted median
Weighted mode
0.00
−0.01
−0.02
−0.03
−0.04
SNP effect on GCST90274790.tsv
0.1
0.2
0.3
0.4
SNP effect on 1−arachidonoyl−GPE (20:4n6) levels

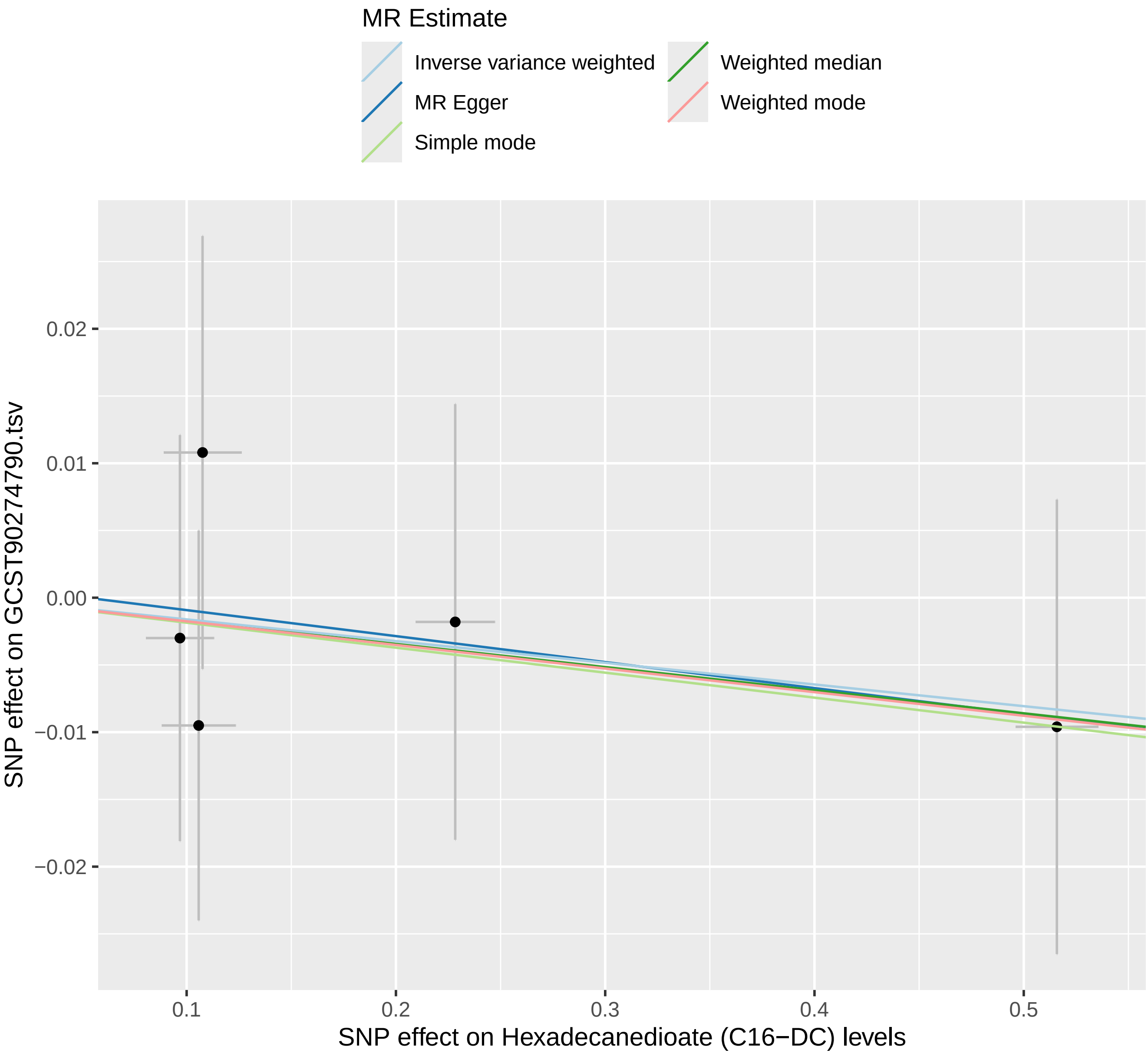
MR Estimate
Inverse variance weighted
MR Egger
Simple mode
Weighted median
Weighted mode
0.02
0.01
0.00
−0.01
−0.02
SNP effect on GCST90274790.tsv
0.1
0.2
0.3
0.4
0.5
SNP effect on Hexadecanedioate (C16−DC) levels

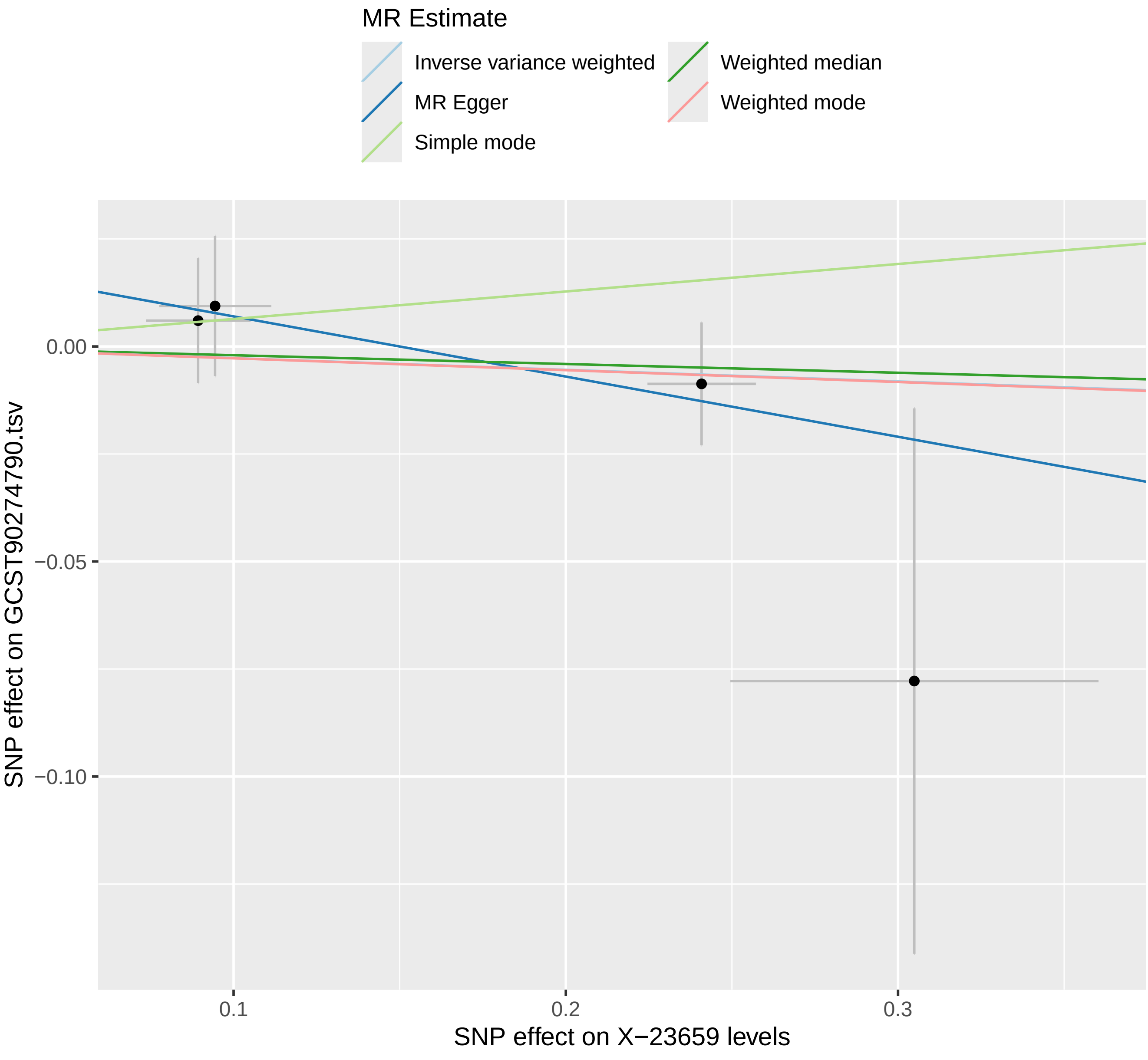
MR Estimate
Inverse variance weighted
MR Egger
Simple mode
Weighted median
Weighted mode
SNP effect on GCST90274790.tsv
0.00
−0.05
−0.10
0.1
0.2
0.3
SNP effect on X−23659 levels

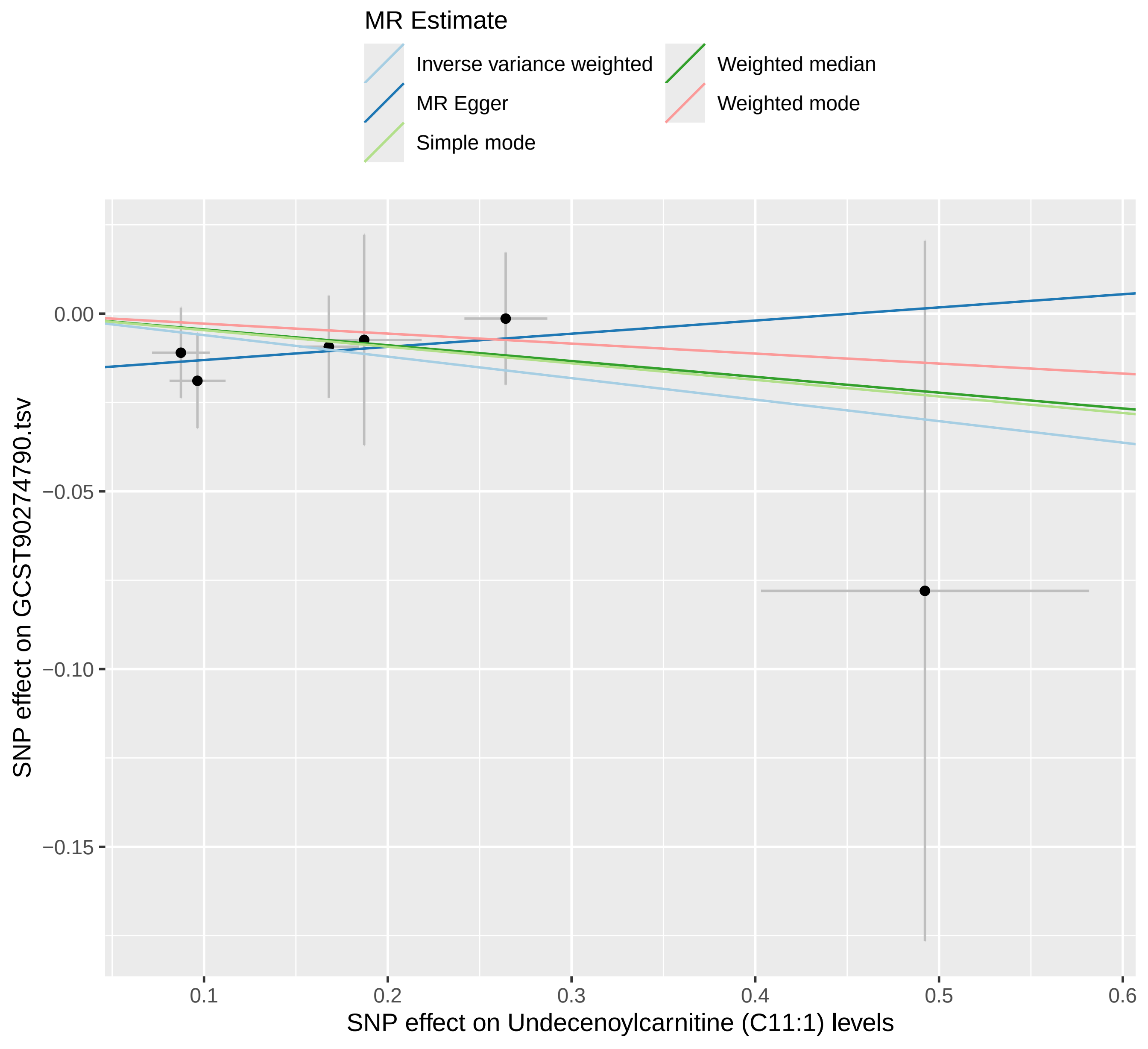
MR Estimate
Inverse variance weighted
MR Egger
Simple mode
Weighted median
Weighted mode
0.00
−0.05
−0.10
−0.15
0.1
0.2
0.3
0.4
0.5
0.6
SNP effect on GCST90274790.tsv
SNP effect on Undecenoylcarnitine (C11:1) levels

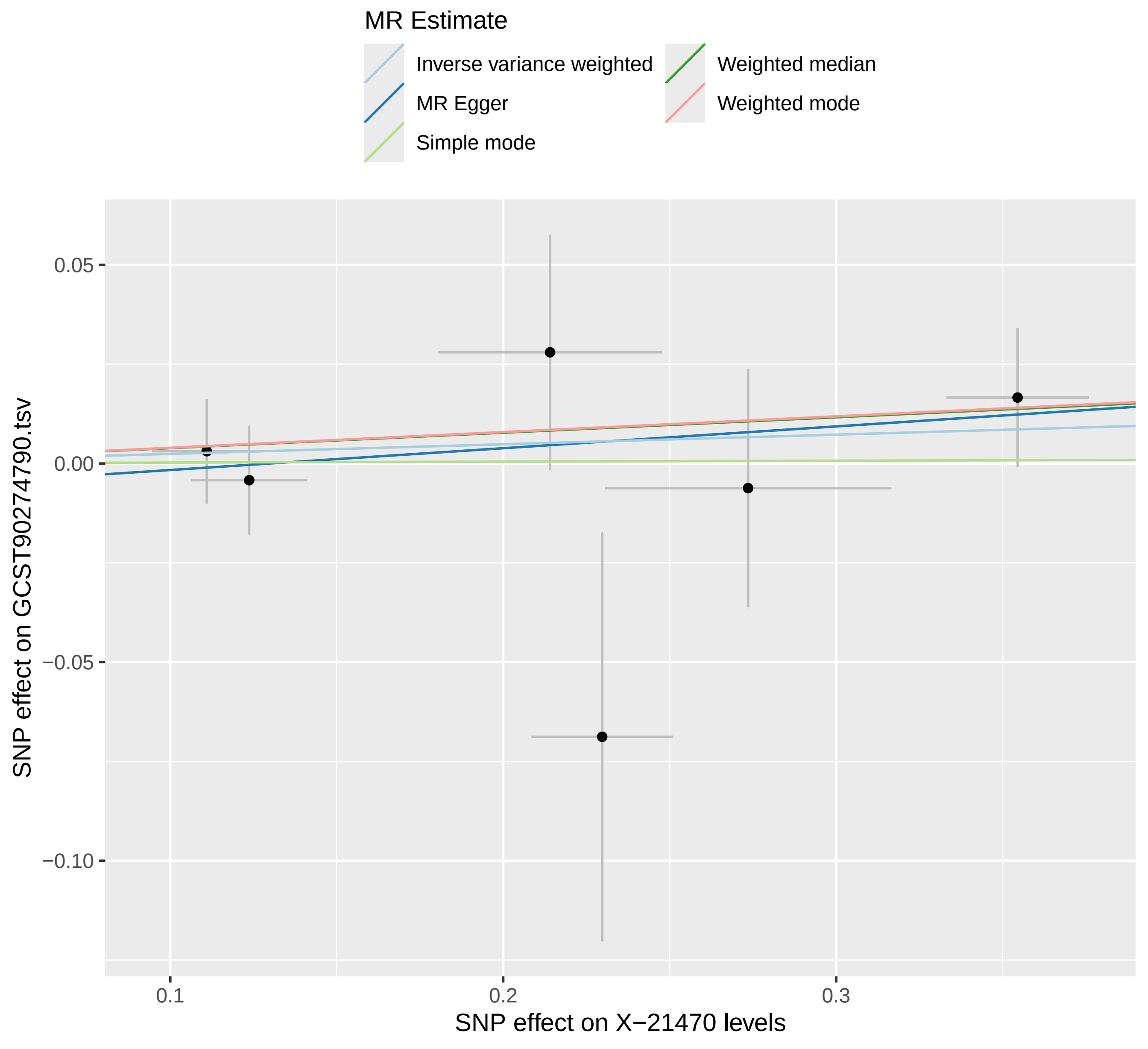
MR Estimate
Inverse variance weighted
MR Egger
Simple mode
Weighted median
Weighted mode
0.05
0.00
−0.05
−0.10
0.1
0.2
0.3
SNP effect on GCST90274790.tsv
SNP effect on X−21470 levels

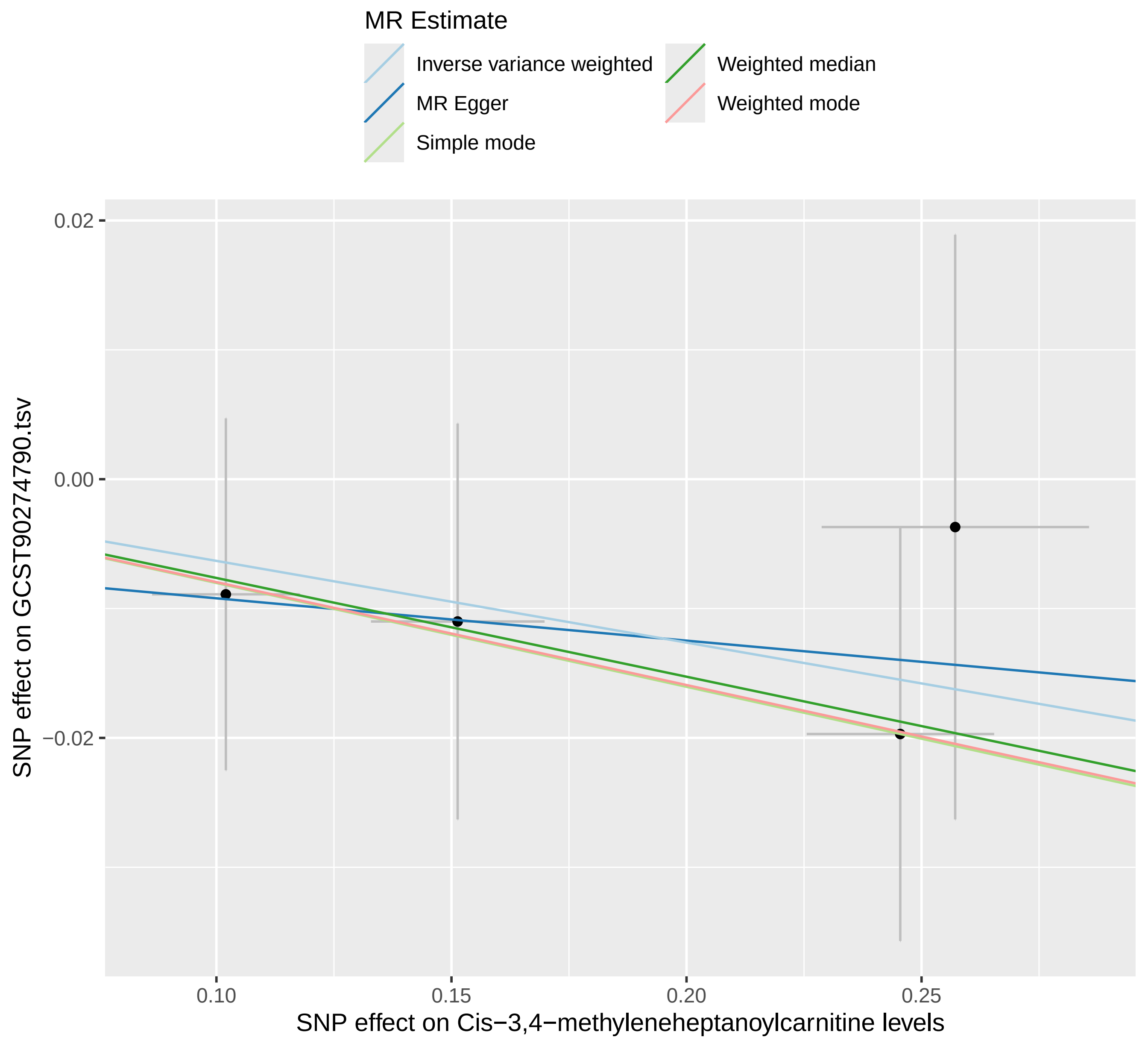
MR Estimate
Inverse variance weighted
MR Egger
Simple mode
Weighted median
Weighted mode
0.02
0.00
−0.02
0.10
0.15
0.20
0.25
SNP effect on GCST90274790.tsv
SNP effect on Cis−3,4−methyleneheptanoylcarnitine levels

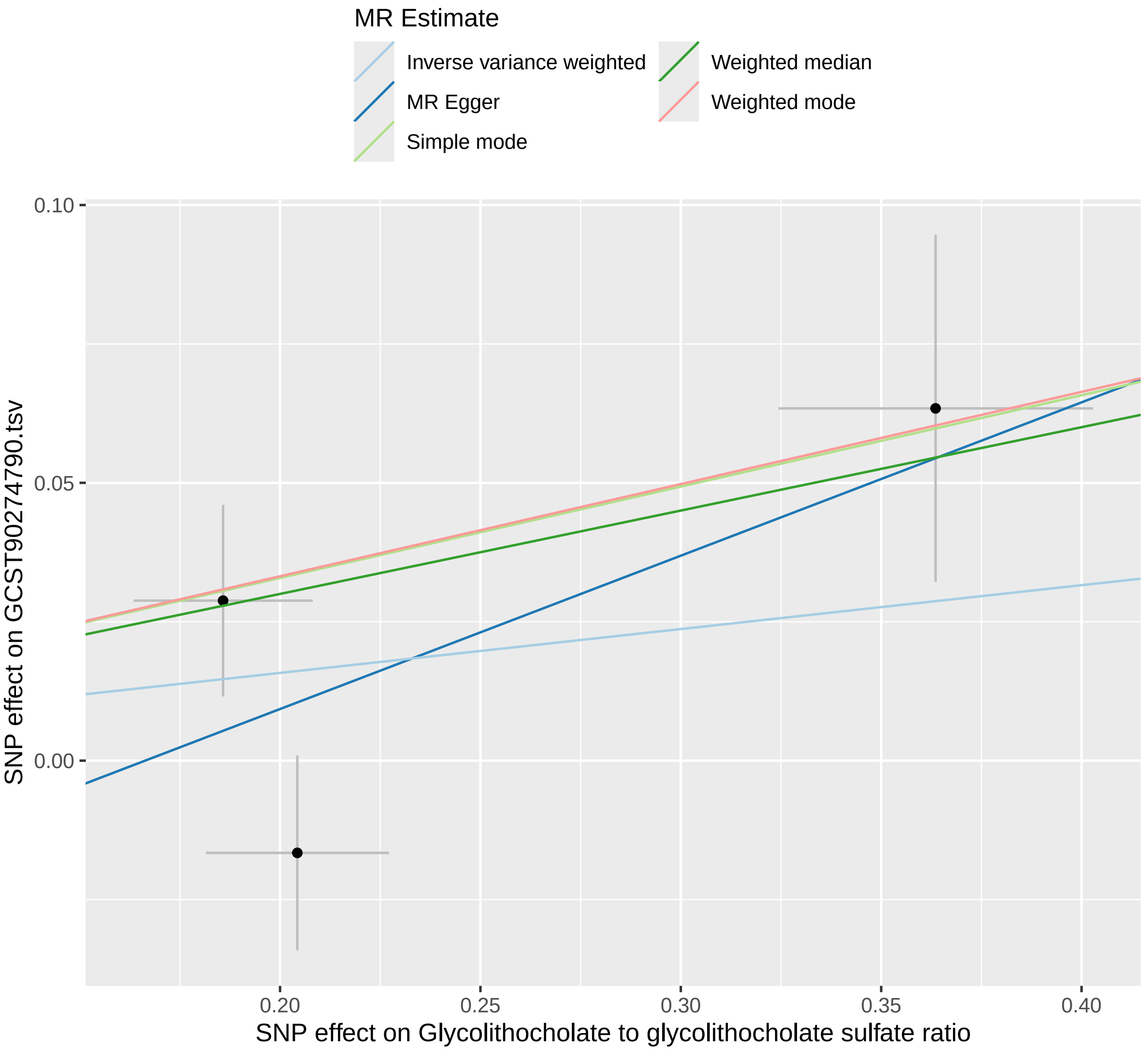
MR Estimate
Inverse variance weighted
MR Egger
Simple mode
Weighted median
Weighted mode
0.10
0.05
0.00
SNP effect on GCST90274790.tsv
0.20
0.25
0.30
0.35
0.40
SNP effect on Glycolithocholate to glycolithocholate sulfate ratio

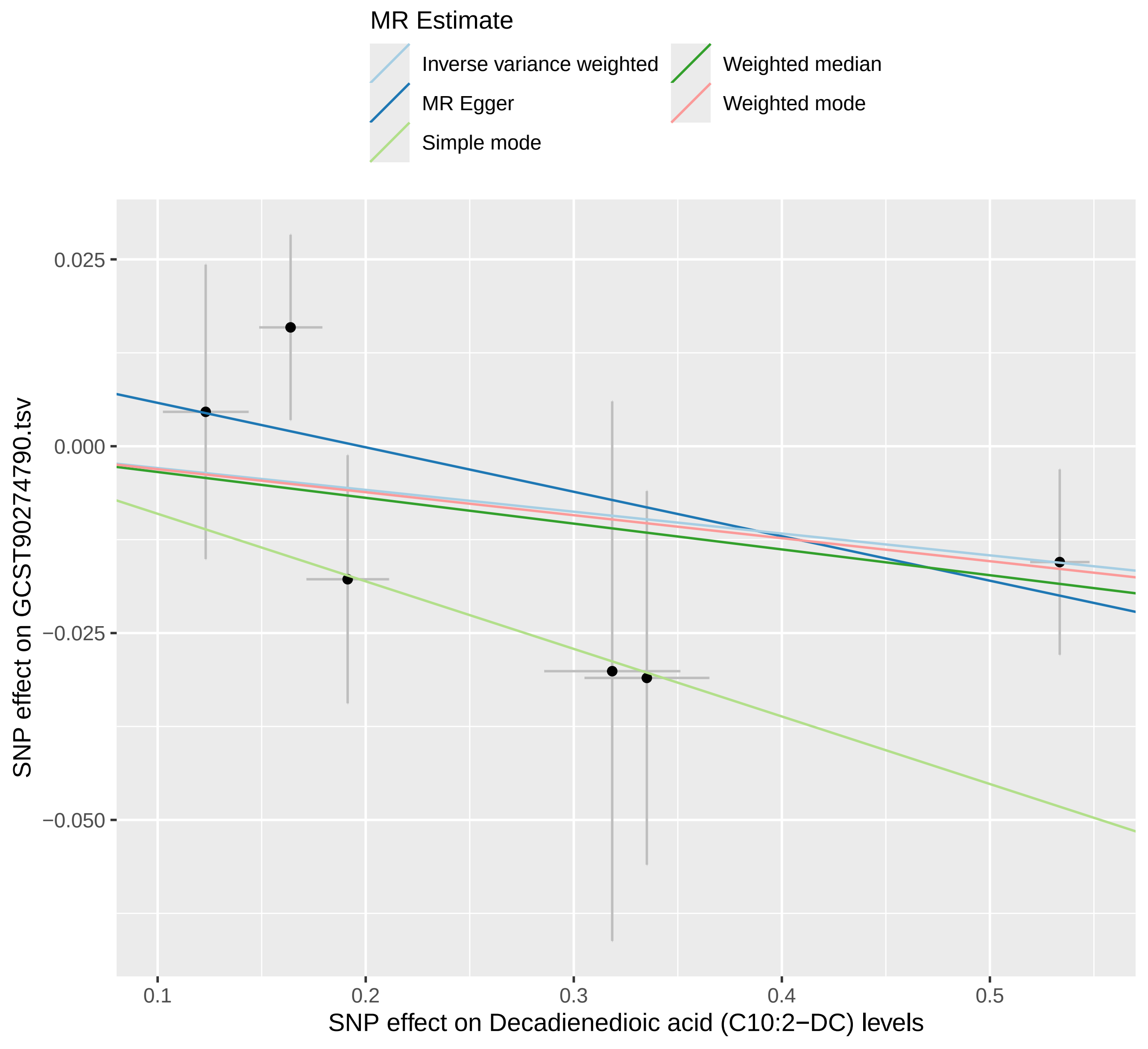
MR Estimate
Inverse variance weighted
MR Egger
Simple mode
Weighted median
Weighted mode
0.025
0.000
−0.025
−0.050
SNP effect on GCST90274790.tsv
0.1
0.2
0.3
0.4
0.5
SNP effect on Decadienedioic acid (C10:2−DC) levels

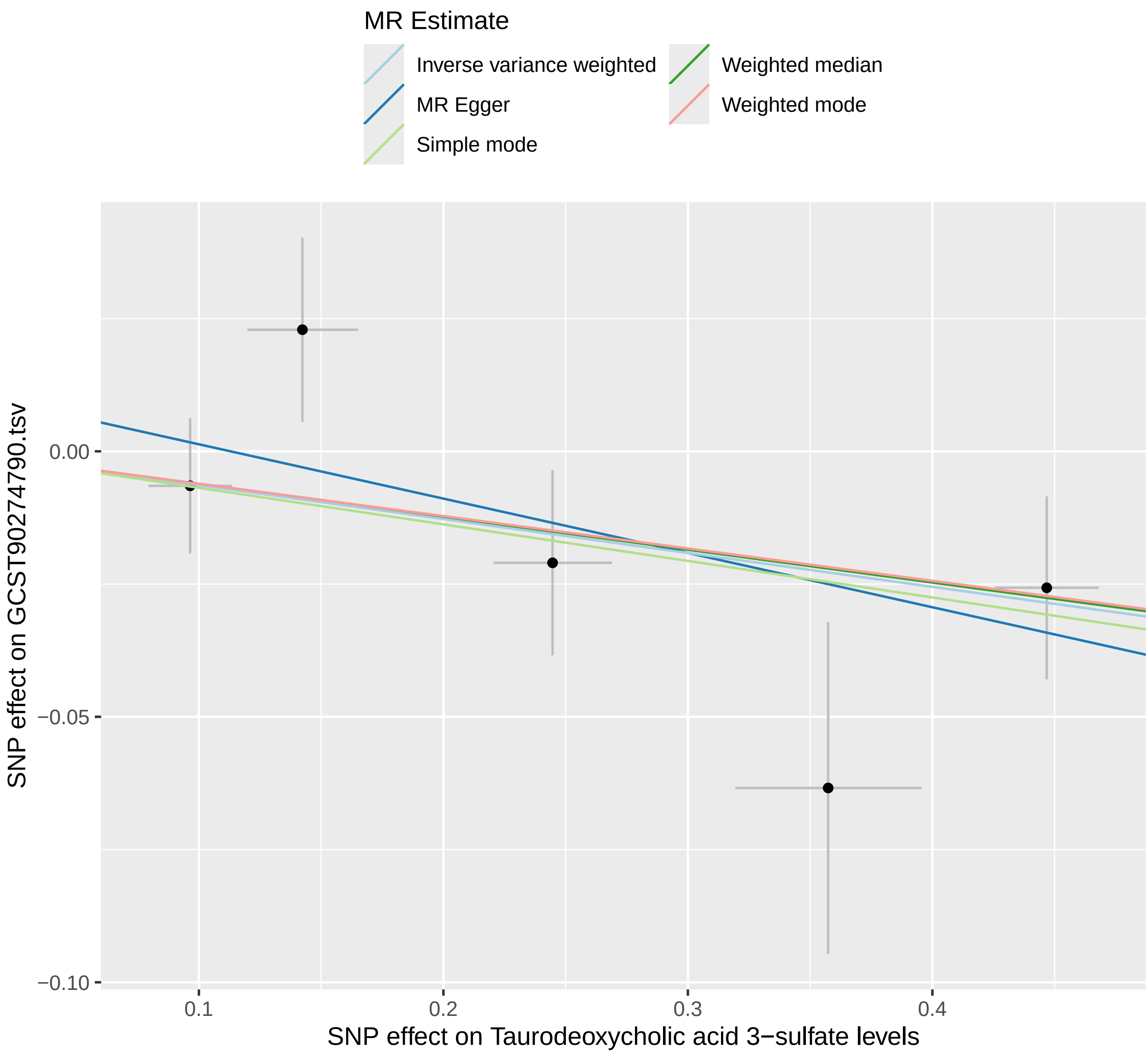
MR Estimate
Inverse variance weighted
MR Egger
Simple mode
Weighted median
Weighted mode
SNP effect on GCST90274790.tsv
0.00
−0.05
−0.10
0.1
0.2
0.3
0.4
SNP effect on Taurodeoxycholic acid 3−sulfate levels

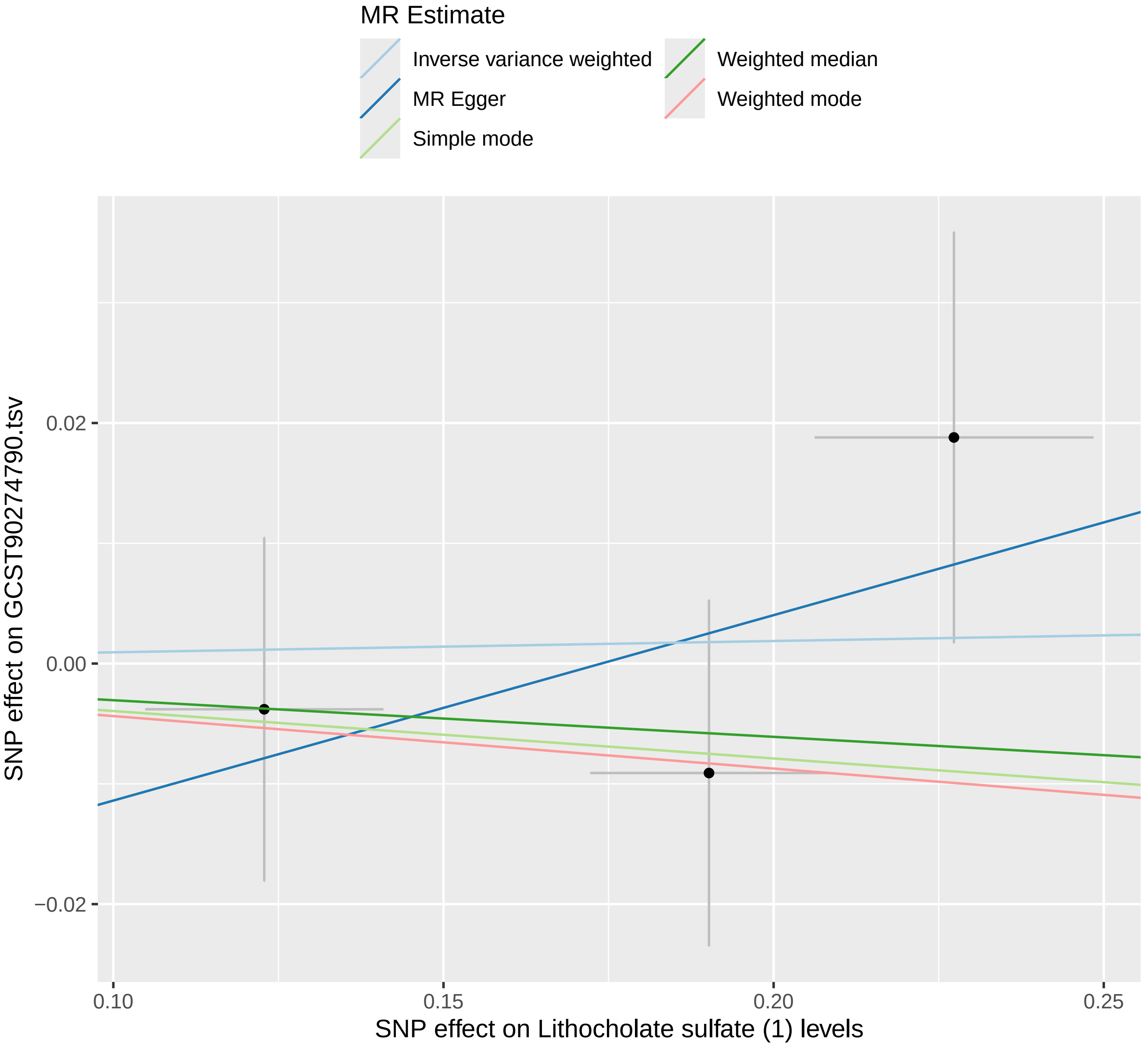
MR Estimate
Inverse variance weighted
MR Egger
Simple mode
Weighted median
Weighted mode
0.02
0.00
−0.02
SNP effect on GCST90274790.tsv
0.10
0.15
0.20
0.25
SNP effect on Lithocholate sulfate (1) levels

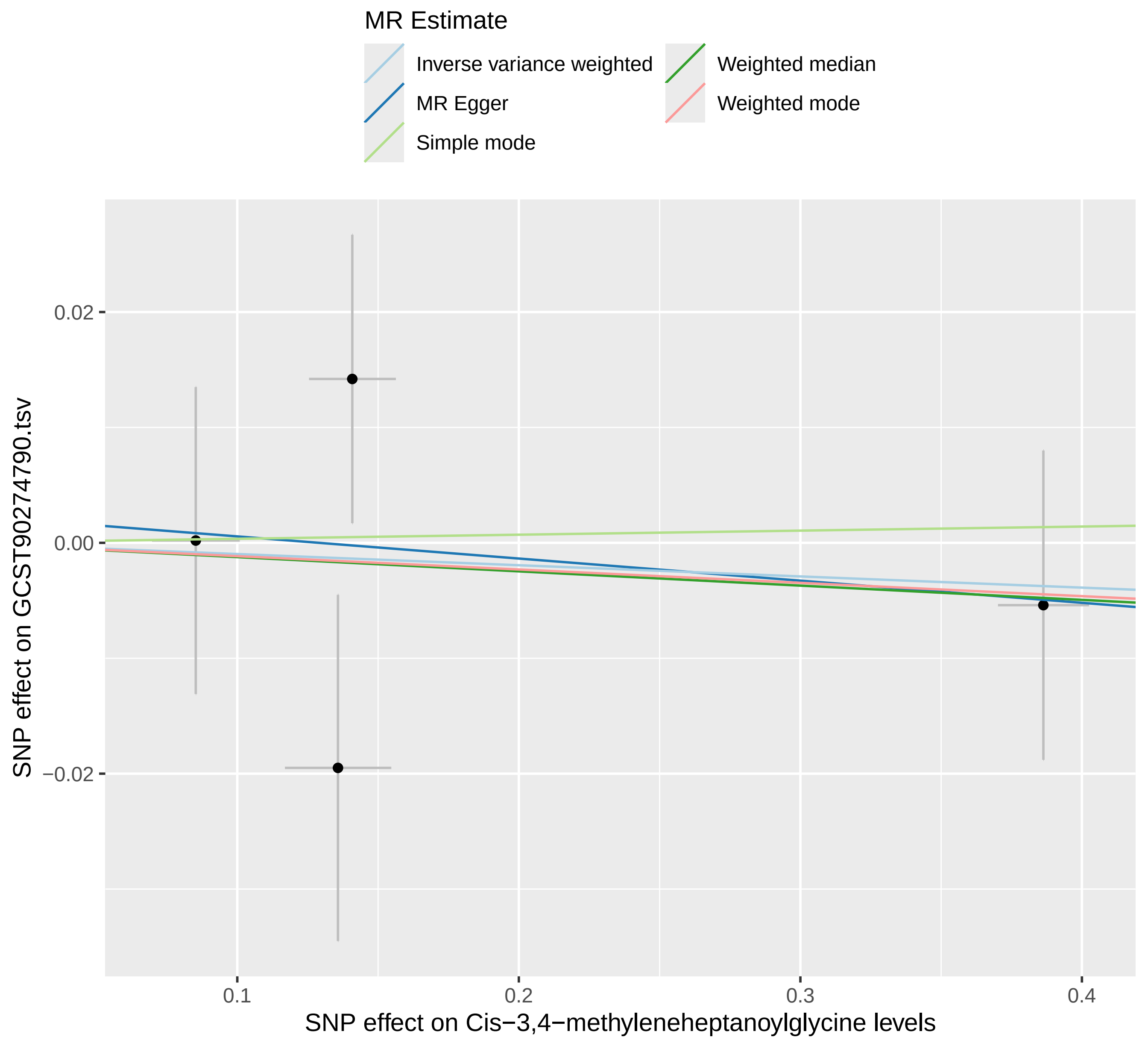
MR Estimate
Inverse variance weighted
MR Egger
Simple mode
Weighted median
Weighted mode
0.02
0.00
−0.02
0.1
0.2
0.3
0.4
SNP effect on GCST90274790.tsv
SNP effect on Cis−3,4−methyleneheptanoylglycine levels

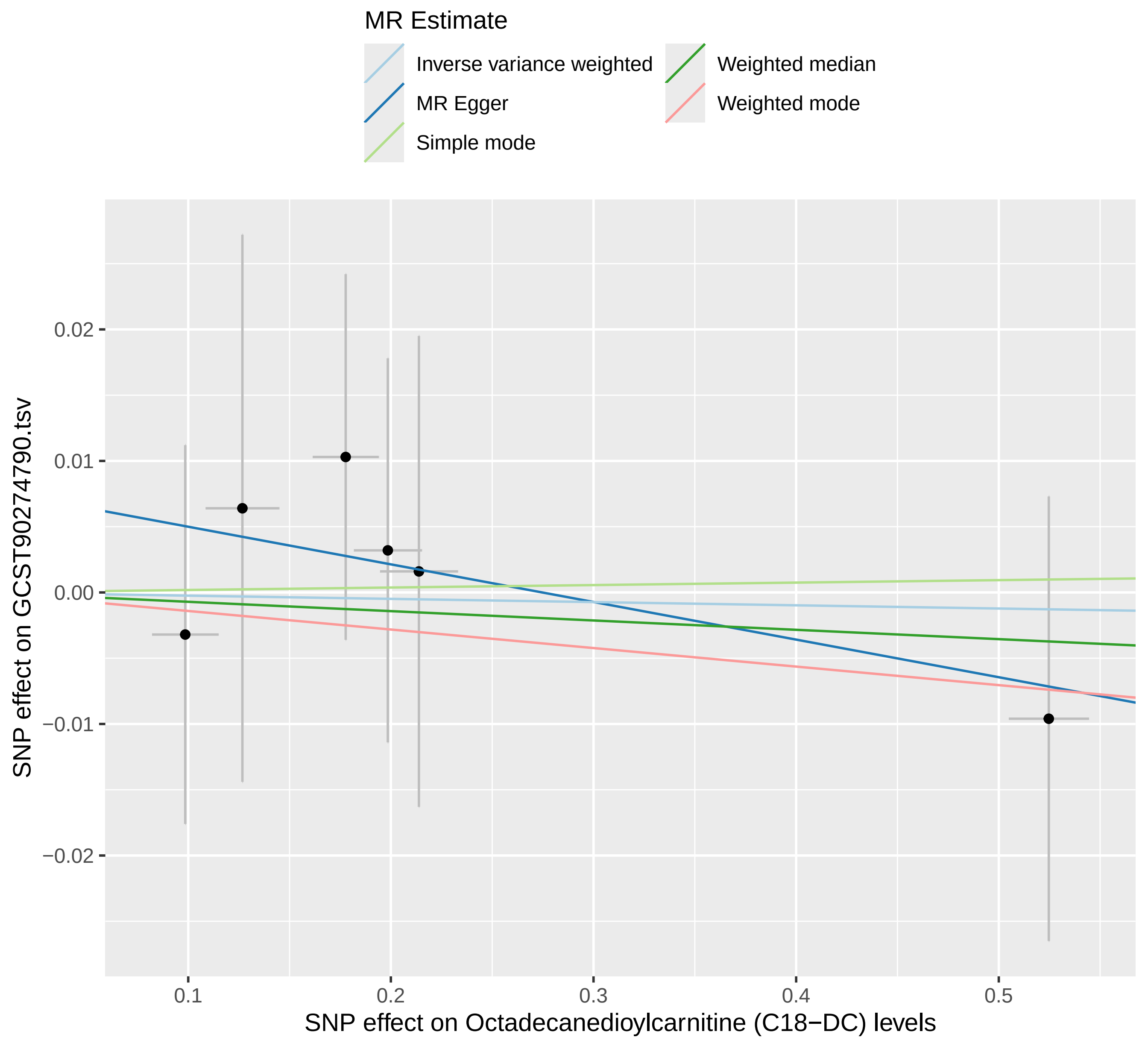
MR Estimate
Inverse variance weighted
MR Egger
Simple mode
Weighted median
Weighted mode
SNP effect on GCST90274790.tsv
0.02
0.01
0.00
−0.01
−0.02
0.1
0.2
0.3
0.4
0.5
SNP effect on Octadecanedioylcarnitine (C18−DC) levels

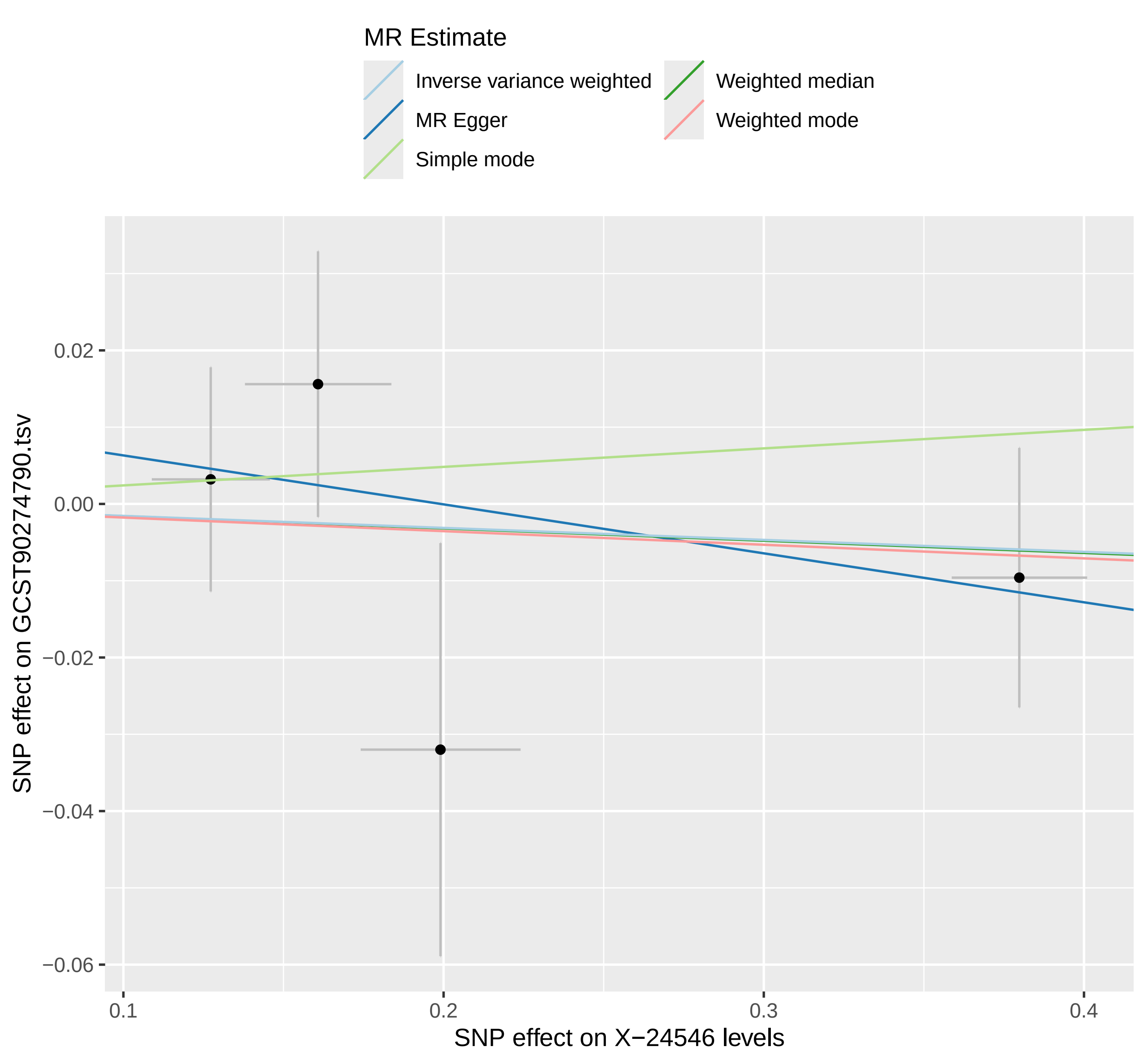

MR Estimate
Inverse variance weighted
MR Egger
Simple mode
Weighted median
Weighted mode
0.02
0.00
−0.02
−0.04
−0.06
0.1
0.2
0.3
0.4
SNP effect on GCST90274790.tsv
SNP effect on X−24546 levels

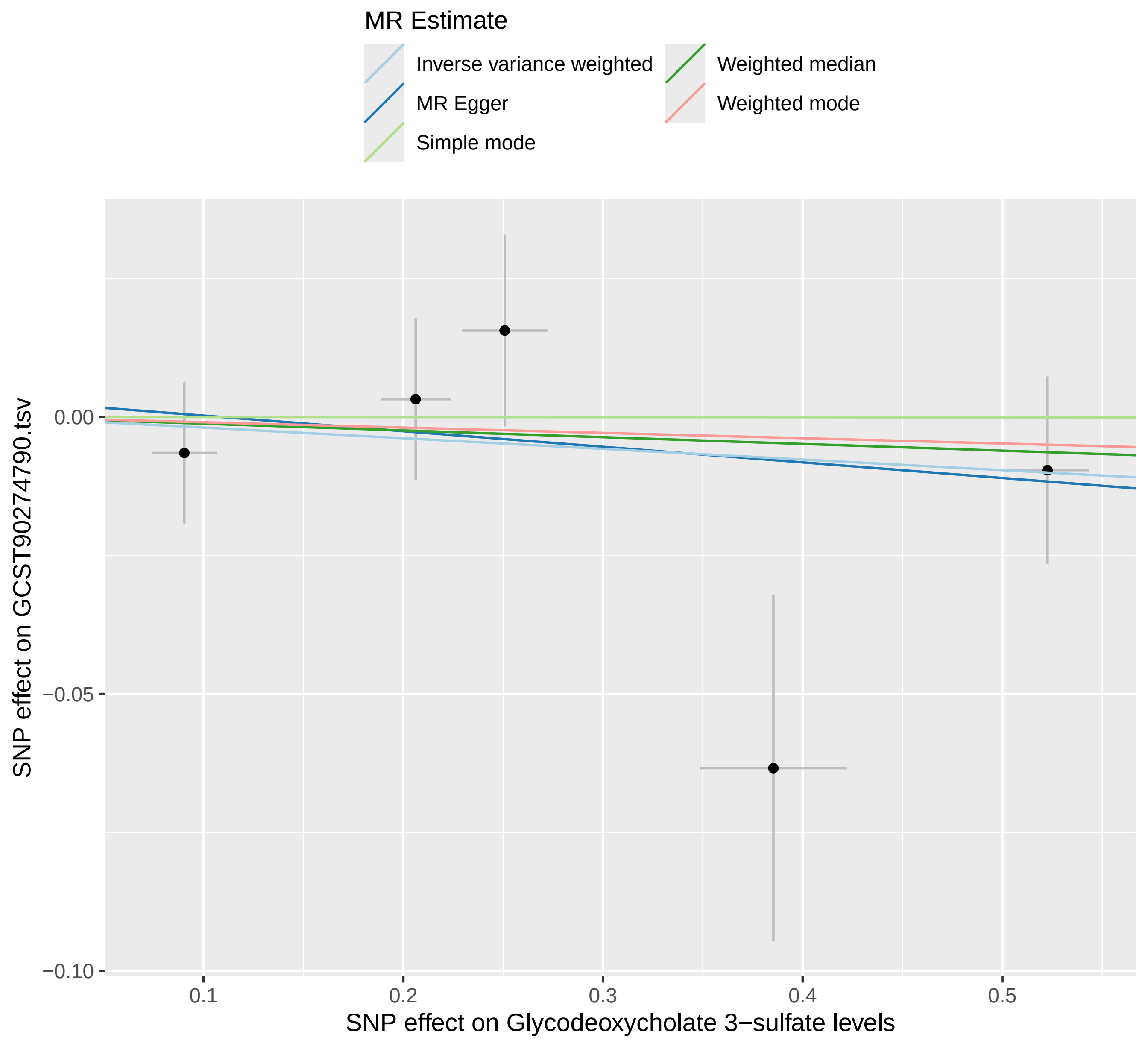
MR Estimate
Inverse variance weighted
MR Egger
Simple mode
Weighted median
Weighted mode
SNP effect on GCST90274790.tsv
0.00
−0.05
−0.10
0.1
0.2
0.3
0.4
0.5
SNP effect on Glycodeoxycholate 3−sulfate levels

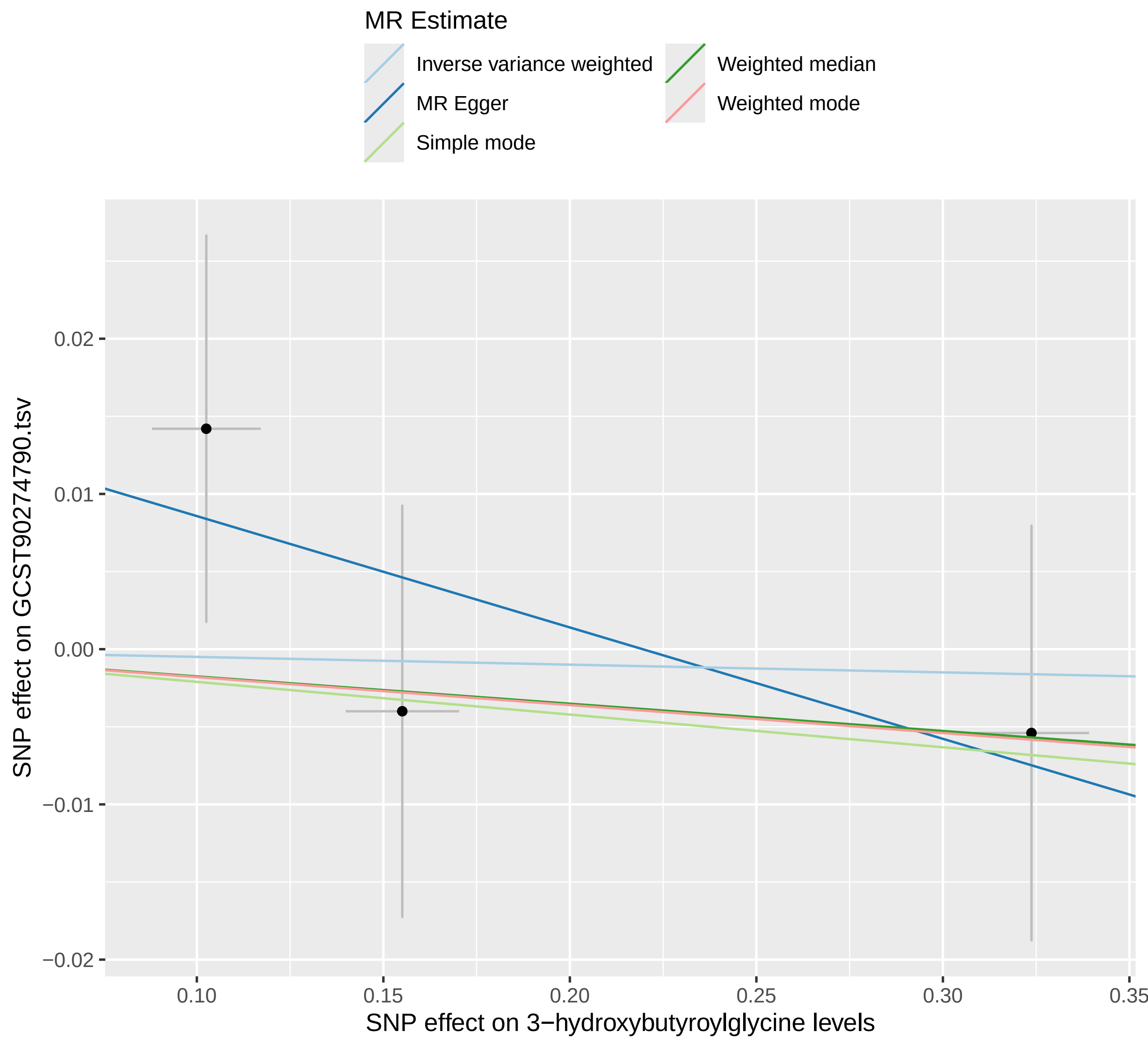
MR Estimate
Inverse variance weighted
MR Egger
Simple mode
Weighted median
Weighted mode
0.02
0.01
0.00
−0.01
−0.02
0.10
0.15
0.20
0.25
0.30
0.35
SNP effect on GCST90274790.tsv
SNP effect on 3−hydroxybutyroylglycine levels

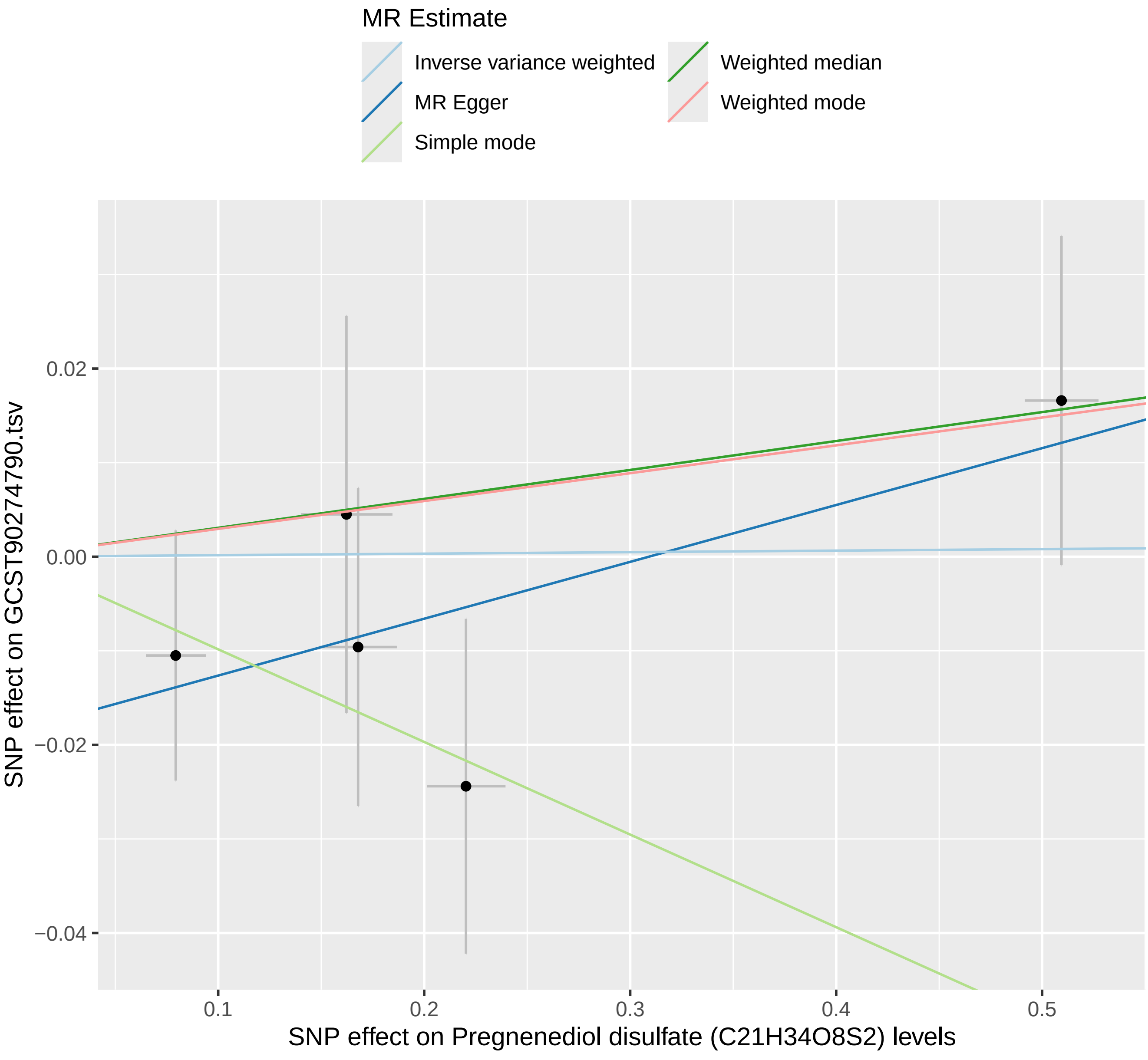
MR Estimate
Inverse variance weighted
MR Egger
Simple mode
Weighted median
Weighted mode
0.02
0.00
−0.02
−0.04
SNP effect on GCST90274790.tsv
0.1
0.2
0.3
0.4
0.5
SNP effect on Pregnenediol disulfate (C21H34O8S2) levels

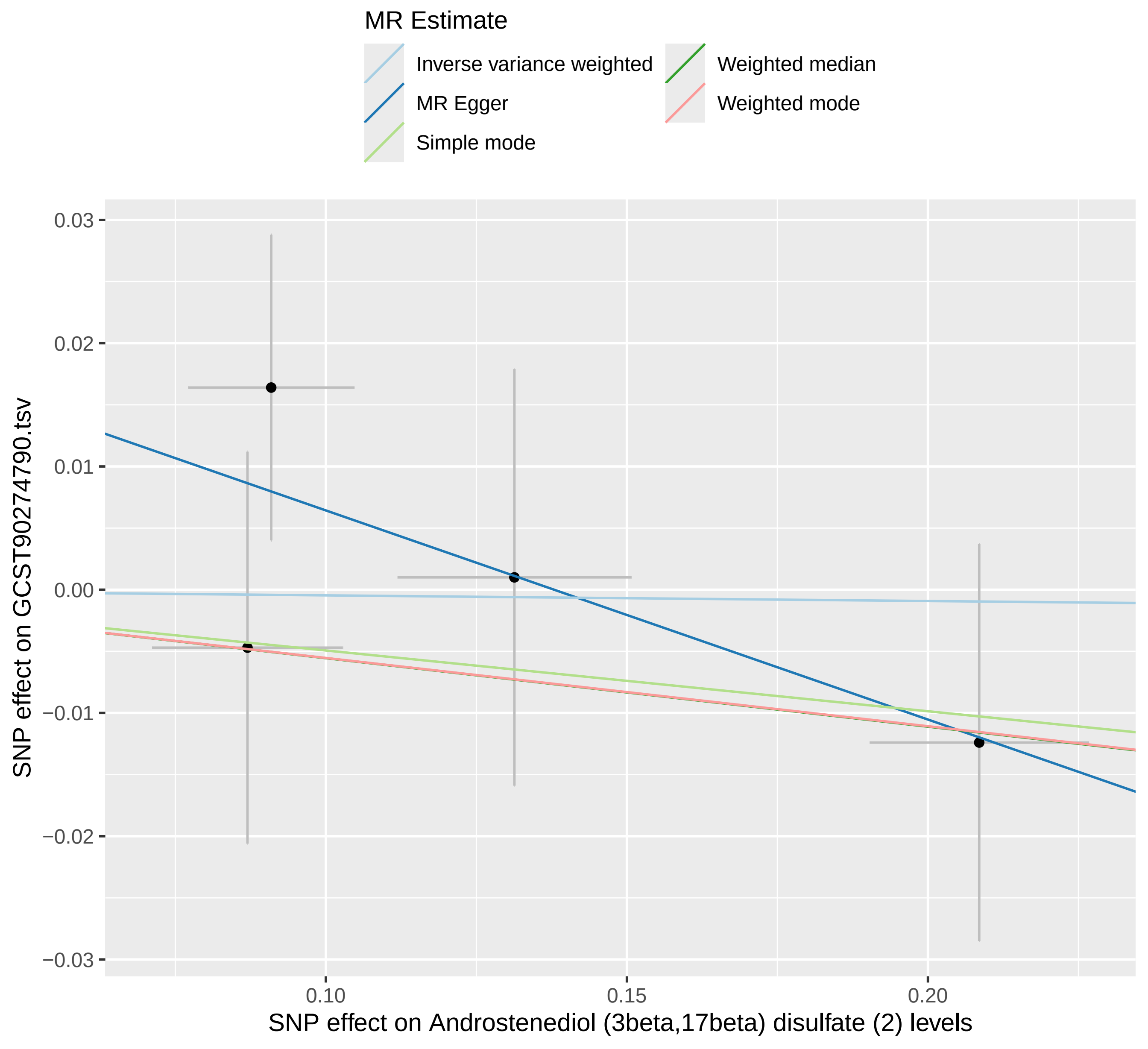
MR Estimate
Inverse variance weighted
MR Egger
Simple mode
Weighted median
Weighted mode
0.03
0.02
0.01
0.00
−0.01
−0.02
−0.03
0.10
0.15
0.20
SNP effect on GCST90274790.tsv
SNP effect on Androstenediol (3beta,17beta) disulfate (2) levels

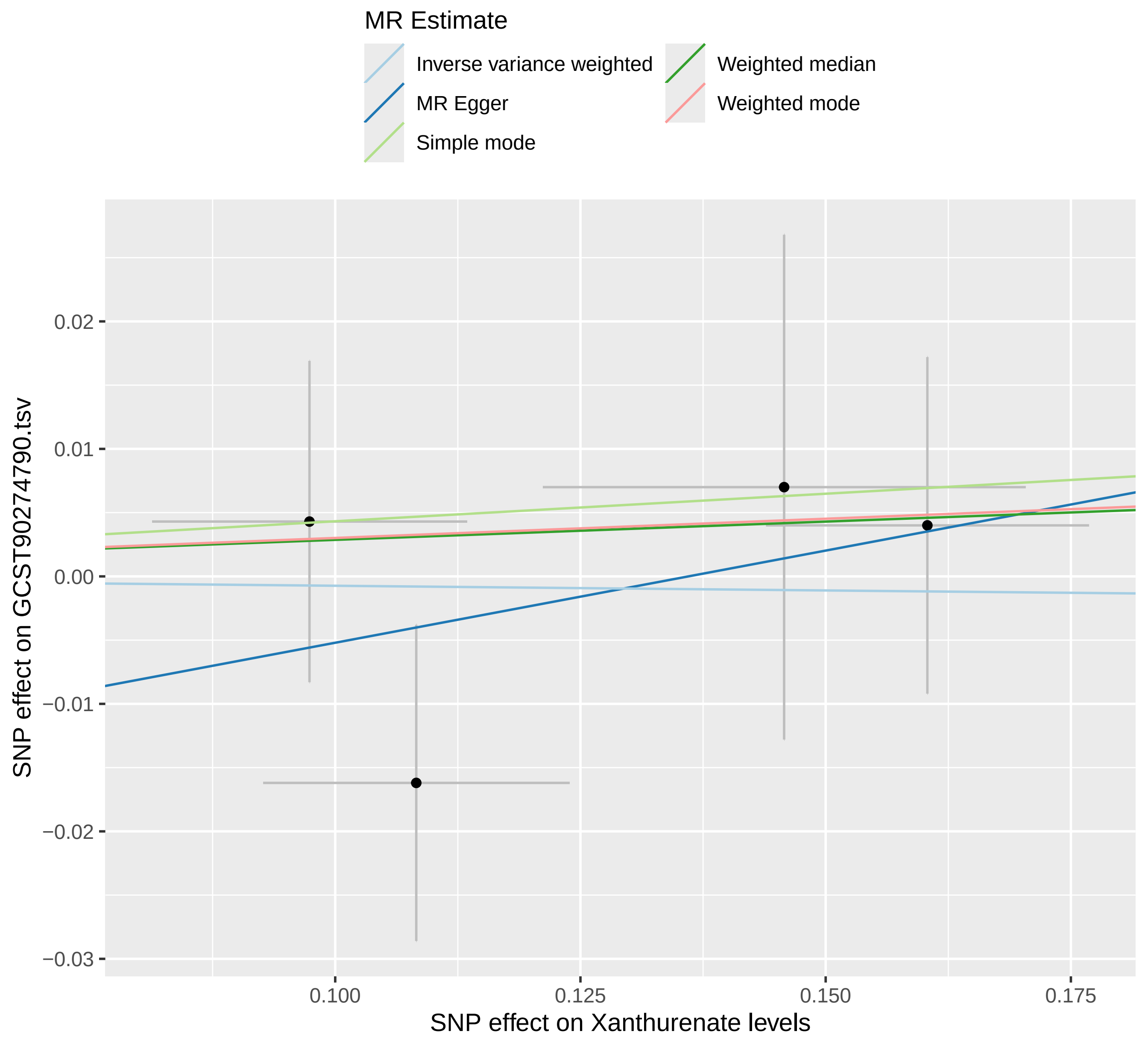

MR Estimate
Inverse variance weighted
MR Egger
Simple mode
Weighted median
Weighted mode
0.02
0.01
0.00
−0.01
−0.02
−0.03
0.100
0.125
0.150
0.175
SNP effect on GCST90274790.tsv
SNP effect on Xanthurenate levels

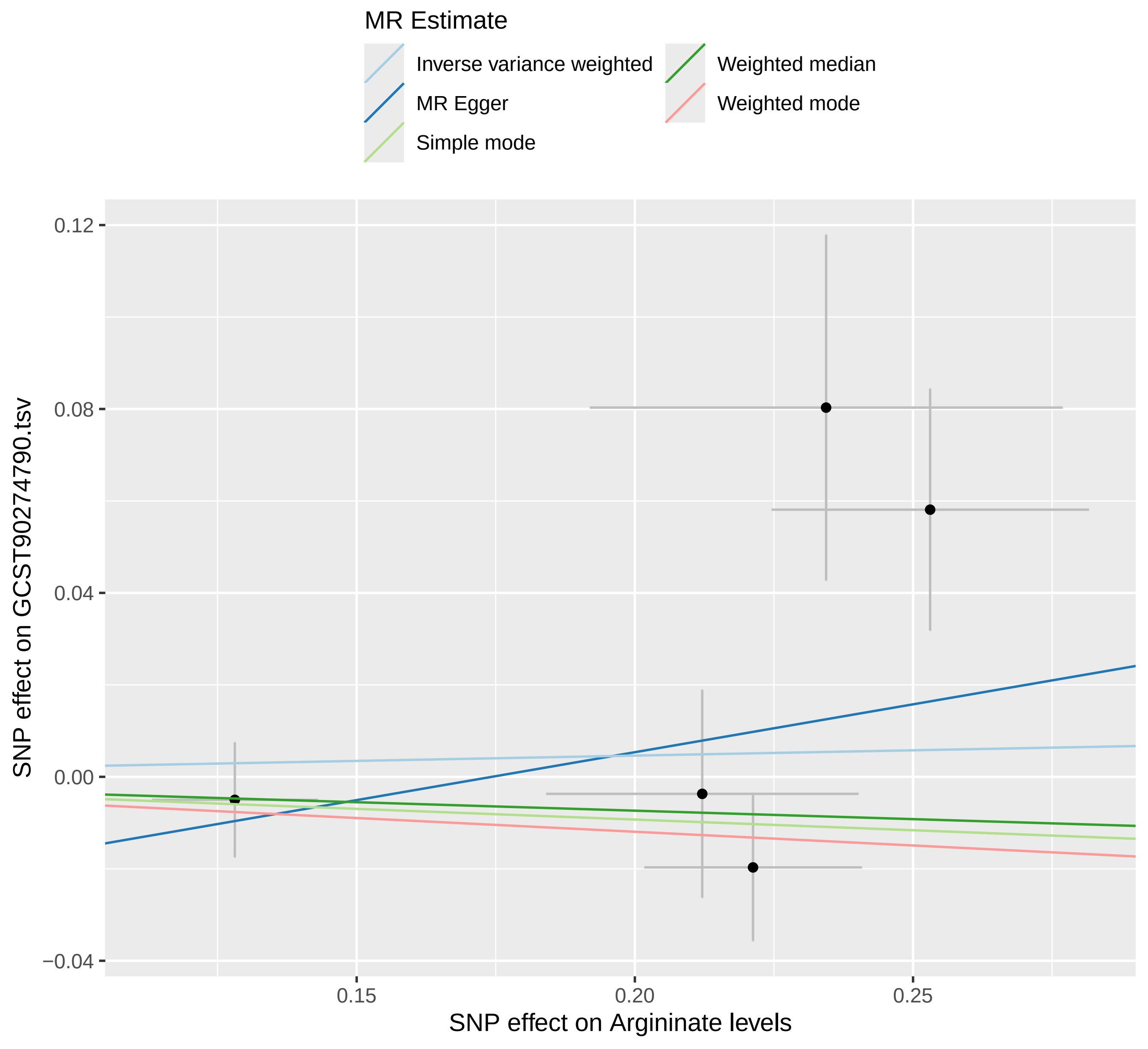

MR Estimate
Inverse variance weighted
MR Egger
Simple mode
Weighted median
Weighted mode
0.12
0.08
0.04
0.00
−0.04
0.15
0.20
0.25
SNP effect on GCST90274790.tsv
SNP effect on Argininate levels

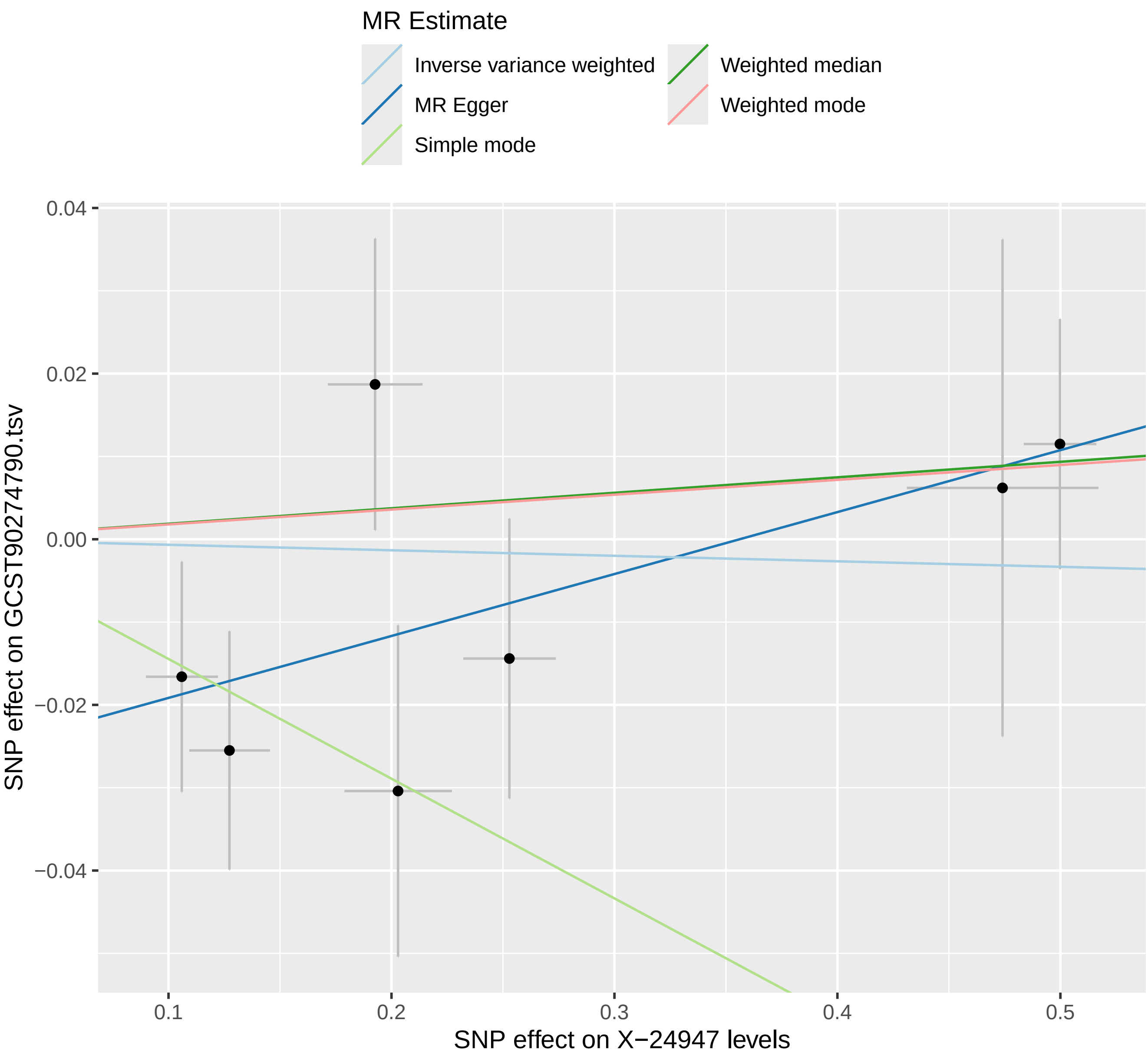
MR Estimate
Inverse variance weighted
MR Egger
Simple mode
Weighted median
Weighted mode
0.04
0.02
0.00
−0.02
−0.04
0.1
0.2
0.3
0.4
0.5
SNP effect on GCST90274790.tsv
SNP effect on X−24947 levels

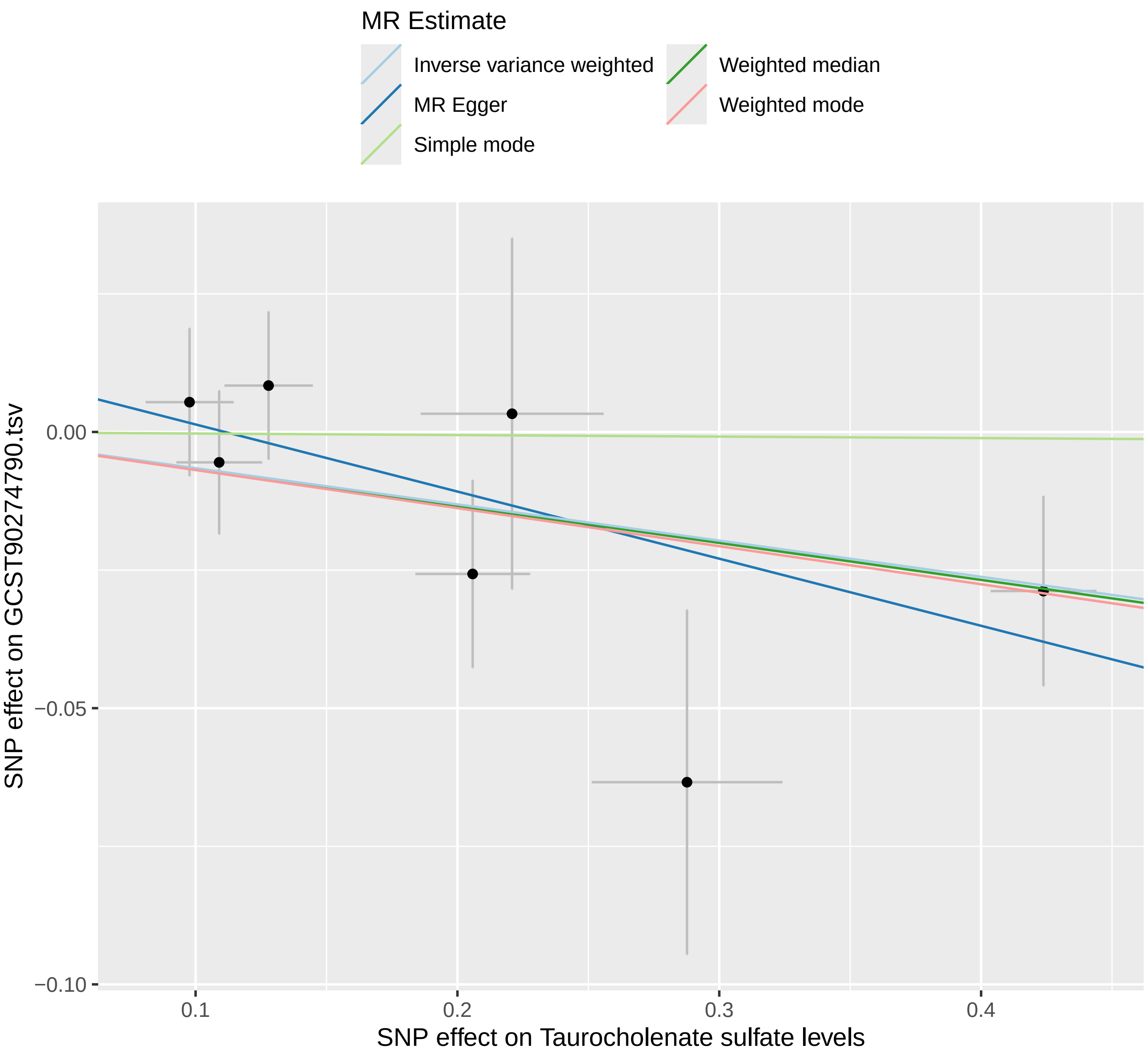
MR Estimate
Inverse variance weighted
MR Egger
Simple mode
Weighted median
Weighted mode
0.00
−0.05
−0.10
0.1
0.2
0.3
0.4
SNP effect on GCST90274790.tsv
SNP effect on Taurocholenate sulfate levels

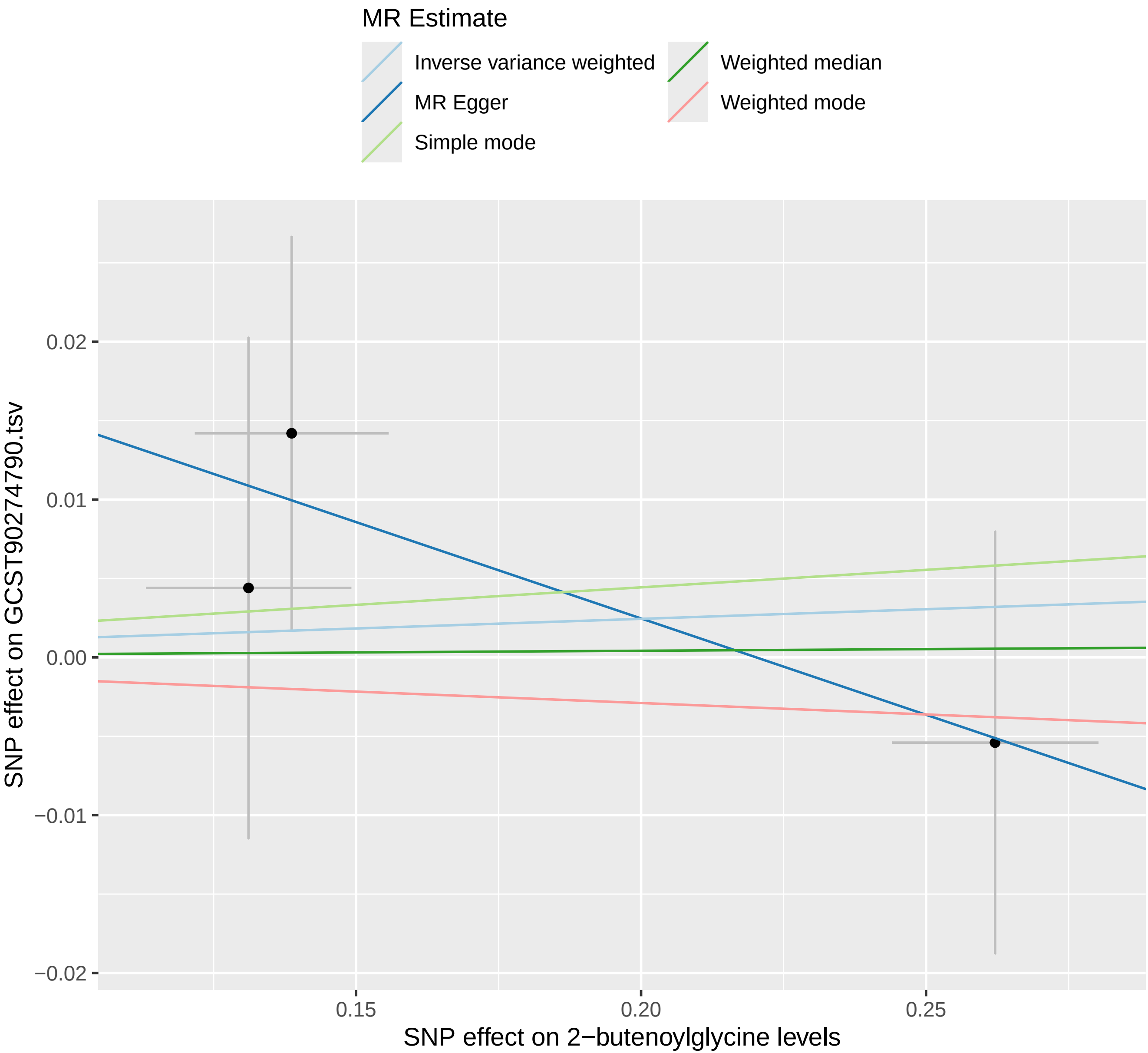
MR Estimate
Inverse variance weighted
MR Egger
Simple mode
Weighted median
Weighted mode
0.02
0.01
0.00
−0.01
−0.02
SNP effect on GCST90274790.tsv
0.15
0.20
0.25
SNP effect on 2−butenoylglycine levels

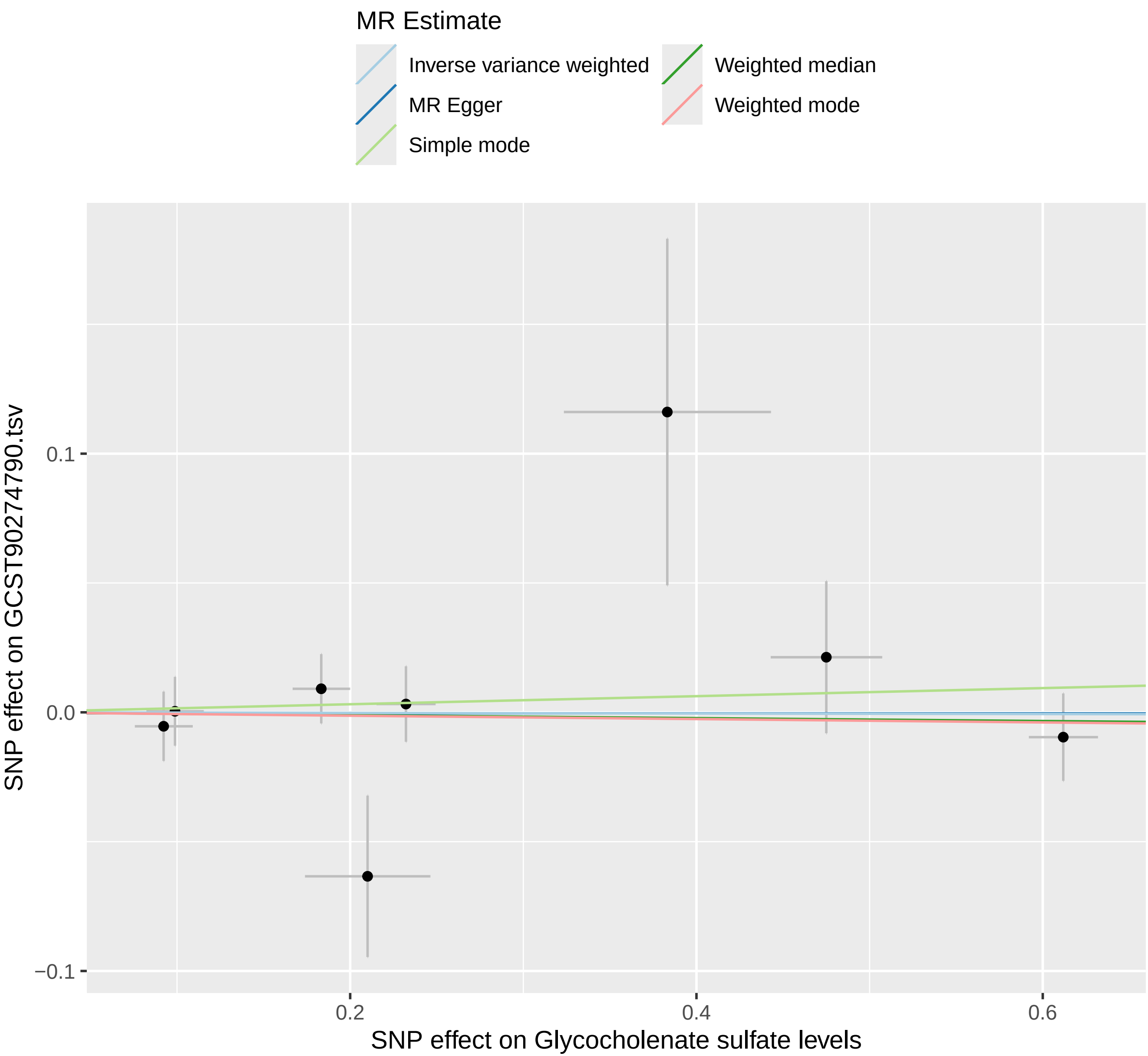
MR Estimate
Inverse variance weighted
MR Egger
Simple mode
Weighted median
Weighted mode
0.1
0.0
−0.1
0.2
0.4
0.6
SNP effect on GCST90274790.tsv
SNP effect on Glycocholenate sulfate levels

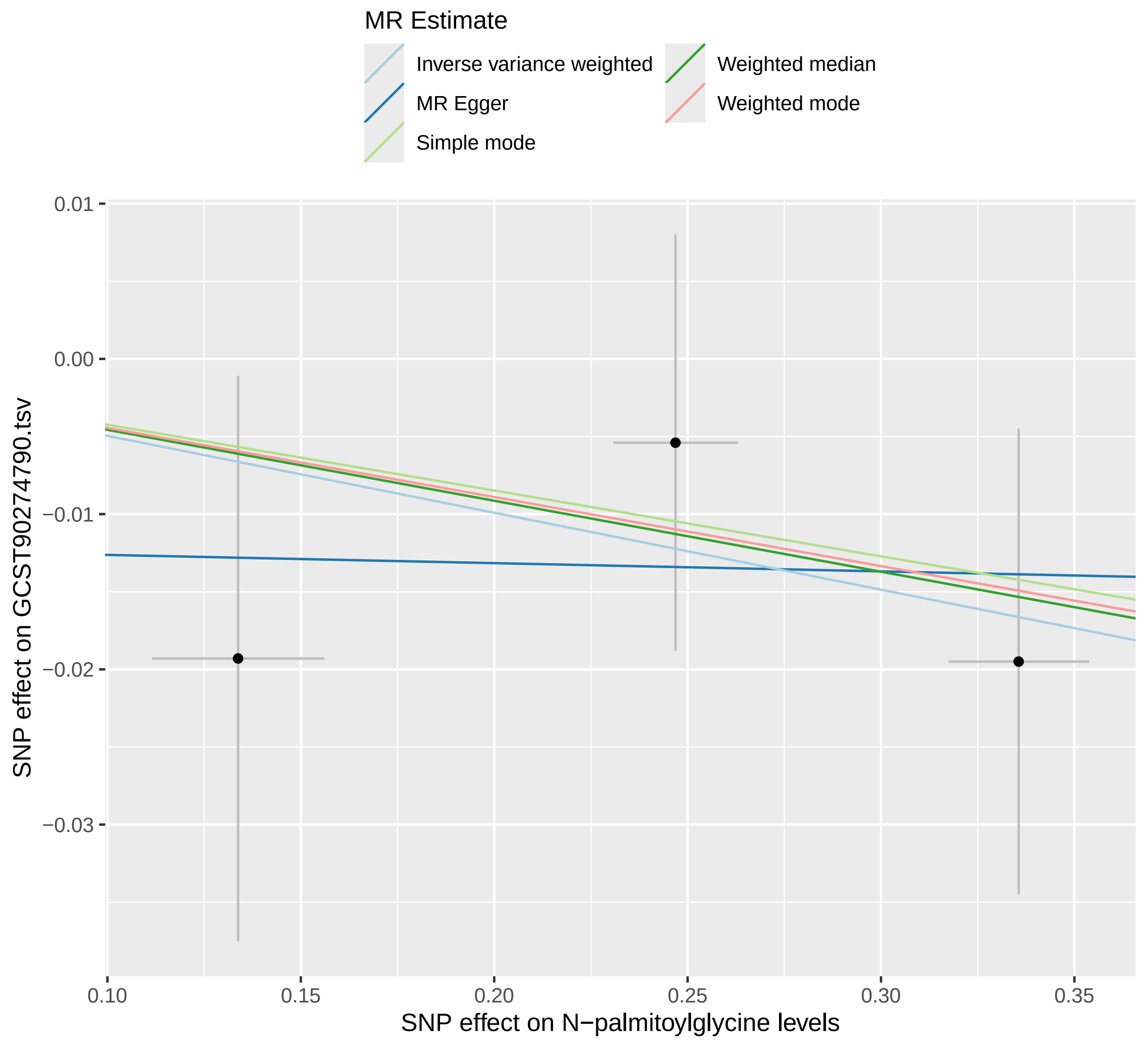
MR Estimate
Inverse variance weighted
MR Egger
Simple mode
Weighted median
Weighted mode
0.01
0.00
−0.01
−0.02
−0.03
SNP effect on GCST90274790.tsv
0.10
0.15
0.20
0.25
0.30
0.35
SNP effect on N−palmitoylglycine levels

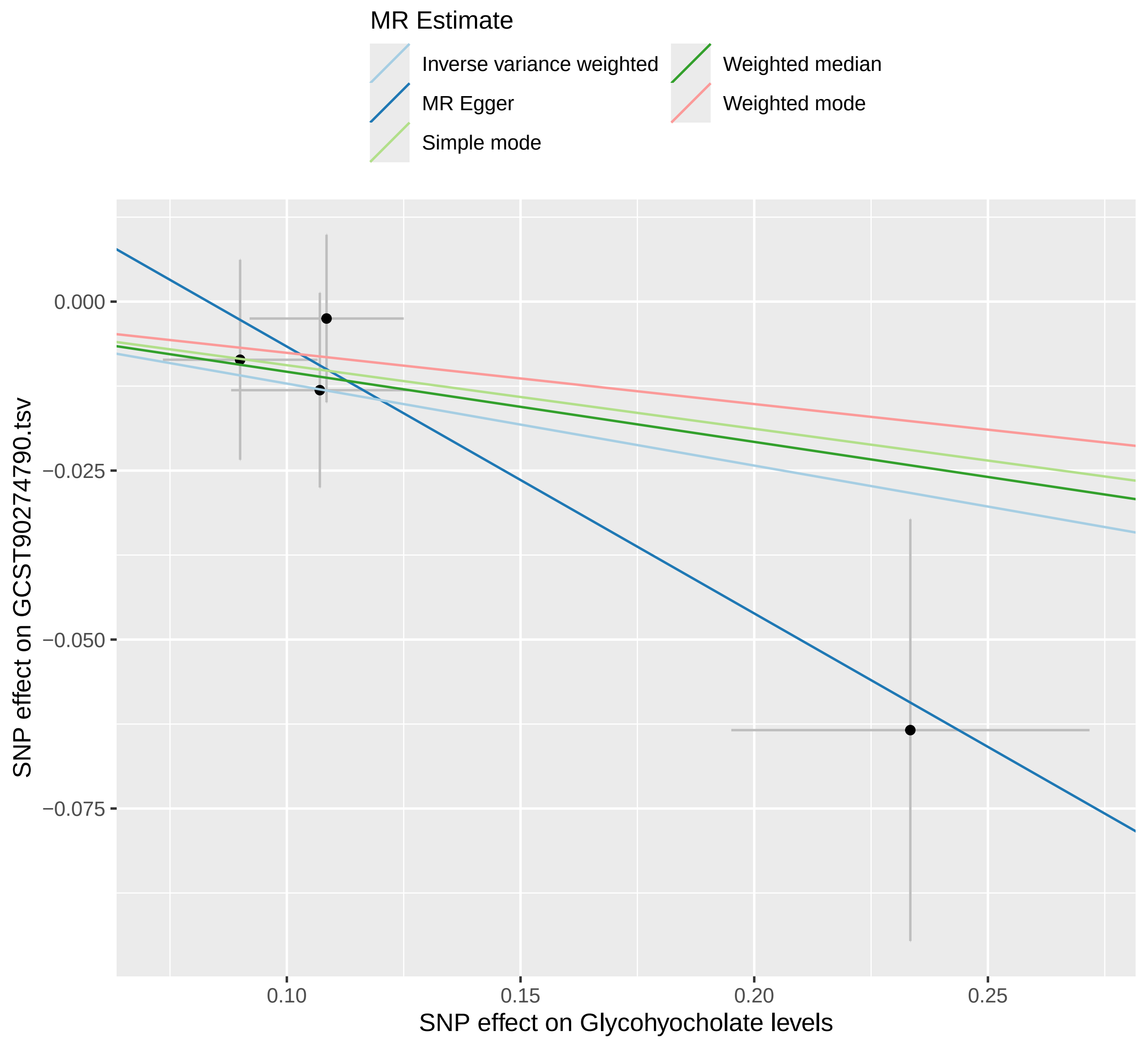

MR Estimate
Inverse variance weighted
MR Egger
Simple mode
Weighted median
Weighted mode
SNP effect on GCST90274790.tsv
0.000
−0.025
−0.050
−0.075
0.10
0.15
0.20
0.25
SNP effect on Glycohyocholate levels

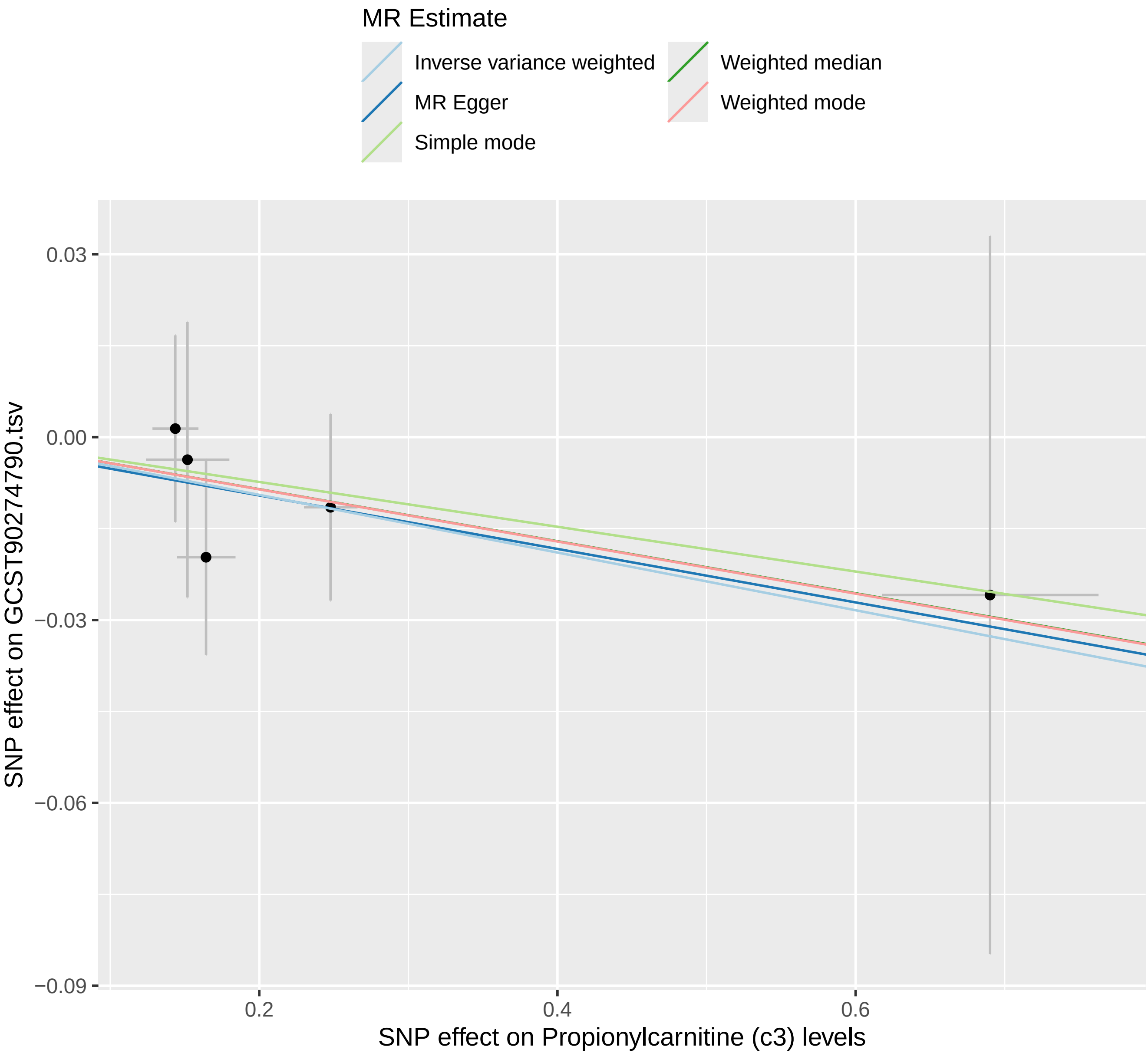
MR Estimate
Inverse variance weighted
MR Egger
Simple mode
Weighted median
Weighted mode
0.03
0.00
−0.03
−0.06
−0.09
0.2
0.4
0.6
SNP effect on GCST90274790.tsv
SNP effect on Propionylcarnitine (c3) levels
