# Supplementary material for: Uncovering metabolite–inflammatory protein interactions in gout pathogenesis: A 2-sample bidirectional Mendelian randomization study
Source: Medicine (Baltimore). 2026 Oct 30;105(44):e51276. doi: 10.1097/MD.0000000000051276 (PMC13727226; doi:10.1097/MD.0000000000051276)
Supplement: Supplementary file 4 [file medi-105-e51276-s010.pdf]

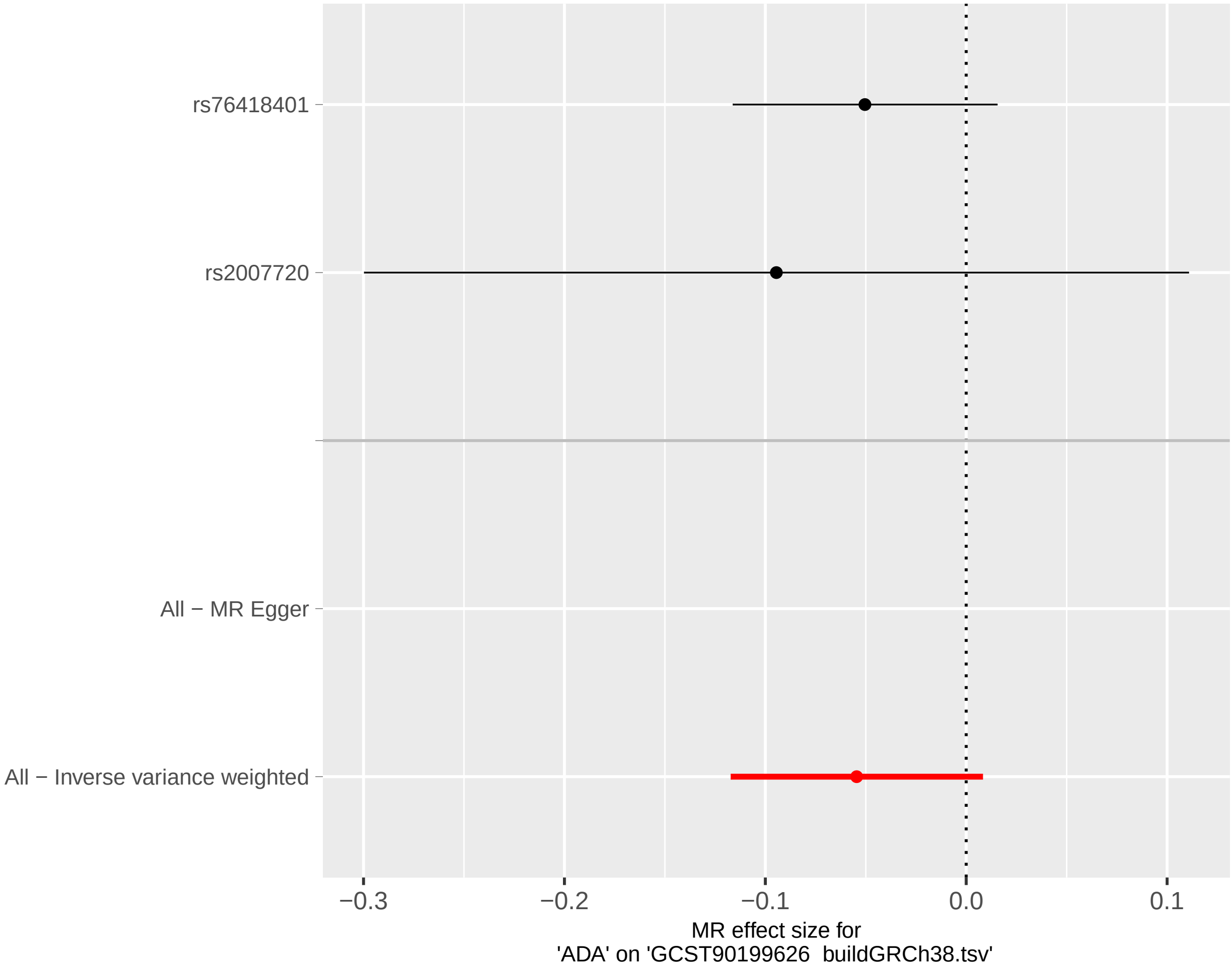
rs76418401
rs2007720
All − MR Egger
All − Inverse variance weighted
−0.3
−0.2
−0.1
0.0
0.1
MR effect size for
'ADA' on 'GCST90199626_buildGRCh38.tsv'

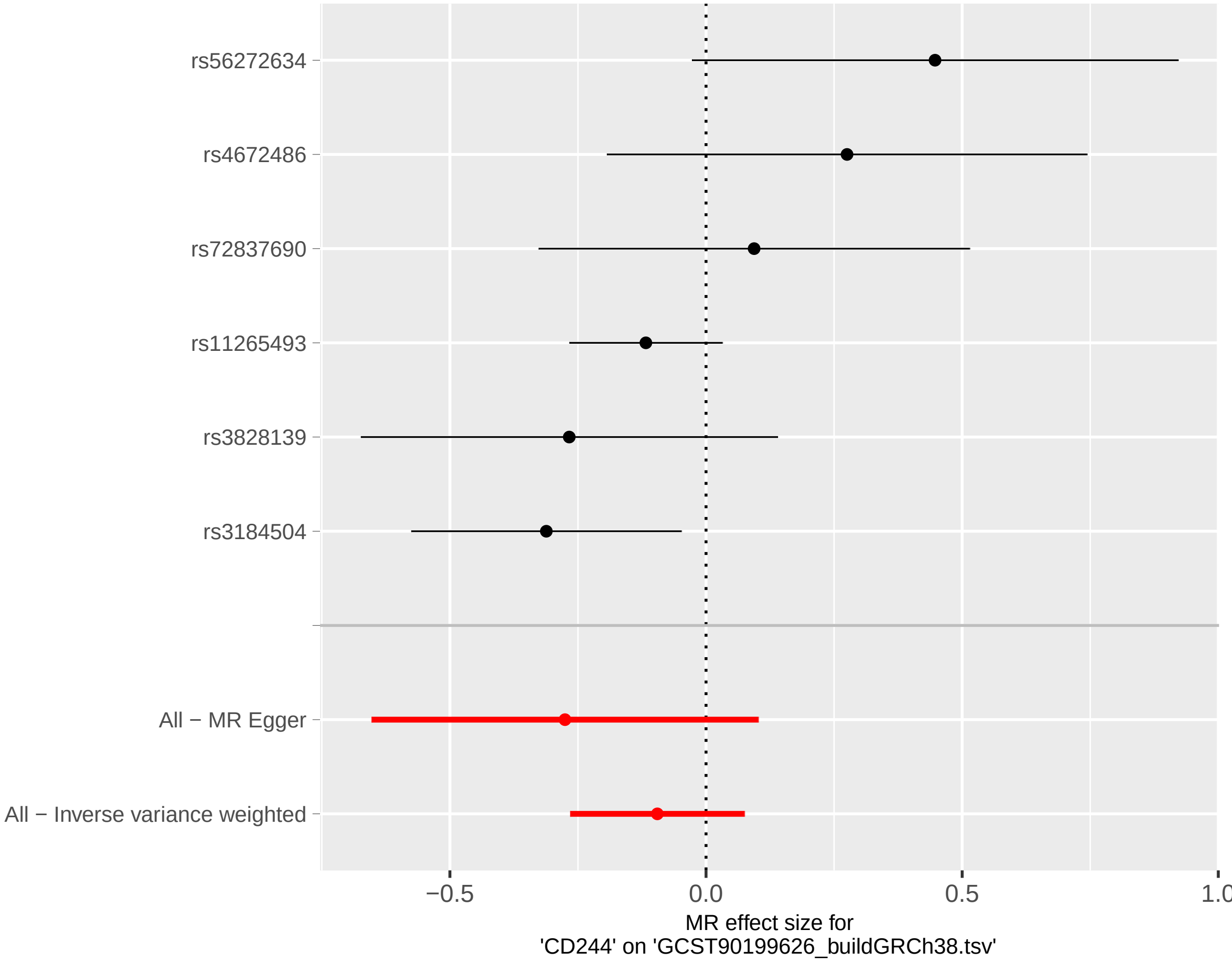

rs56272634
rs4672486
rs72837690
rs11265493
rs3828139
rs3184504
All − MR Egger
All − Inverse variance weighted
−0.5
0.0
0.5
1.0
MR effect size for
'CD244' on 'GCST90199626_buildGRCh38.tsv'

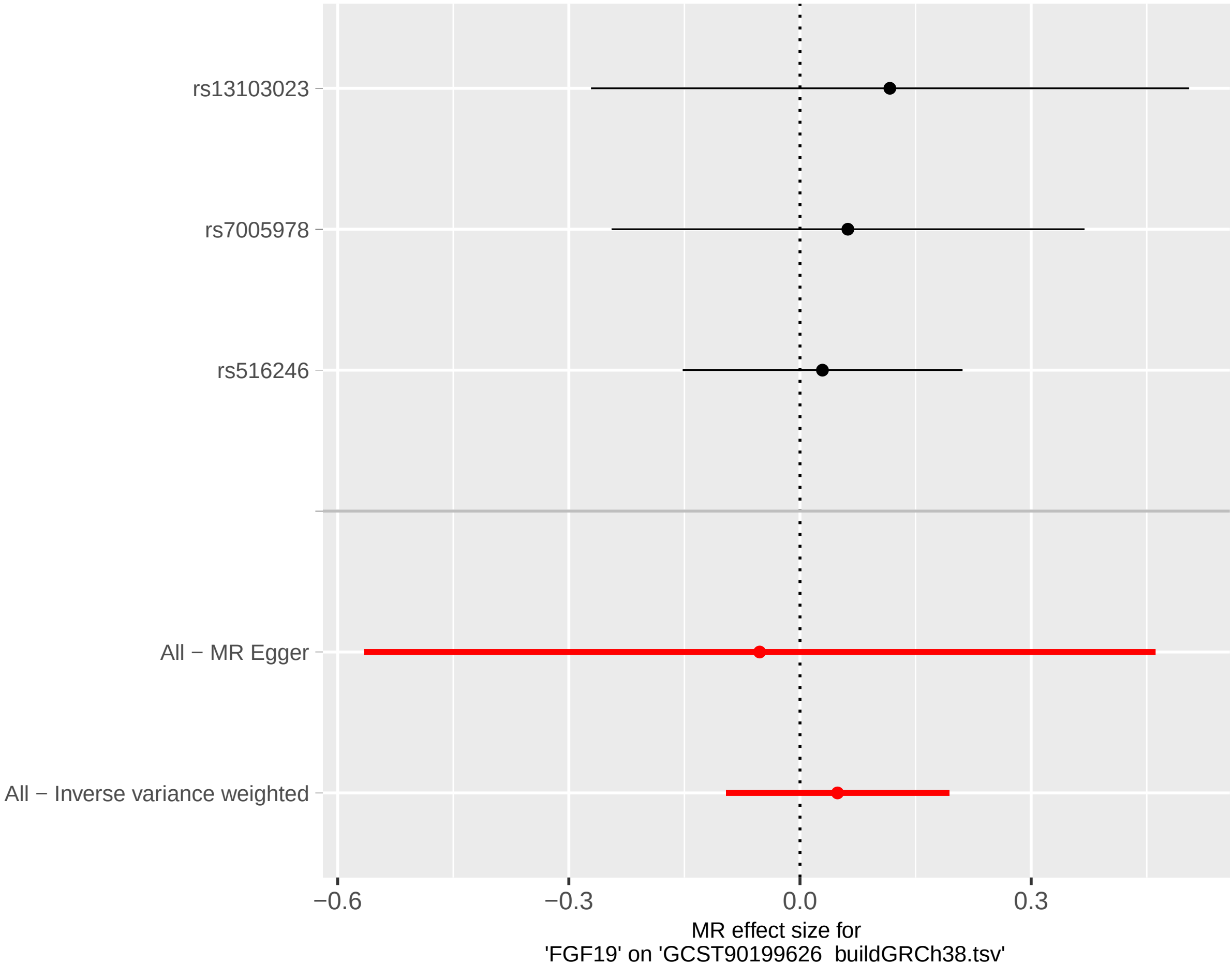

rs13103023
rs7005978
rs516246
All − MR Egger
All − Inverse variance weighted
−0.6
−0.3
0.0
0.3
MR effect size for
'FGF19' on 'GCST90199626_buildGRCh38.tsv'

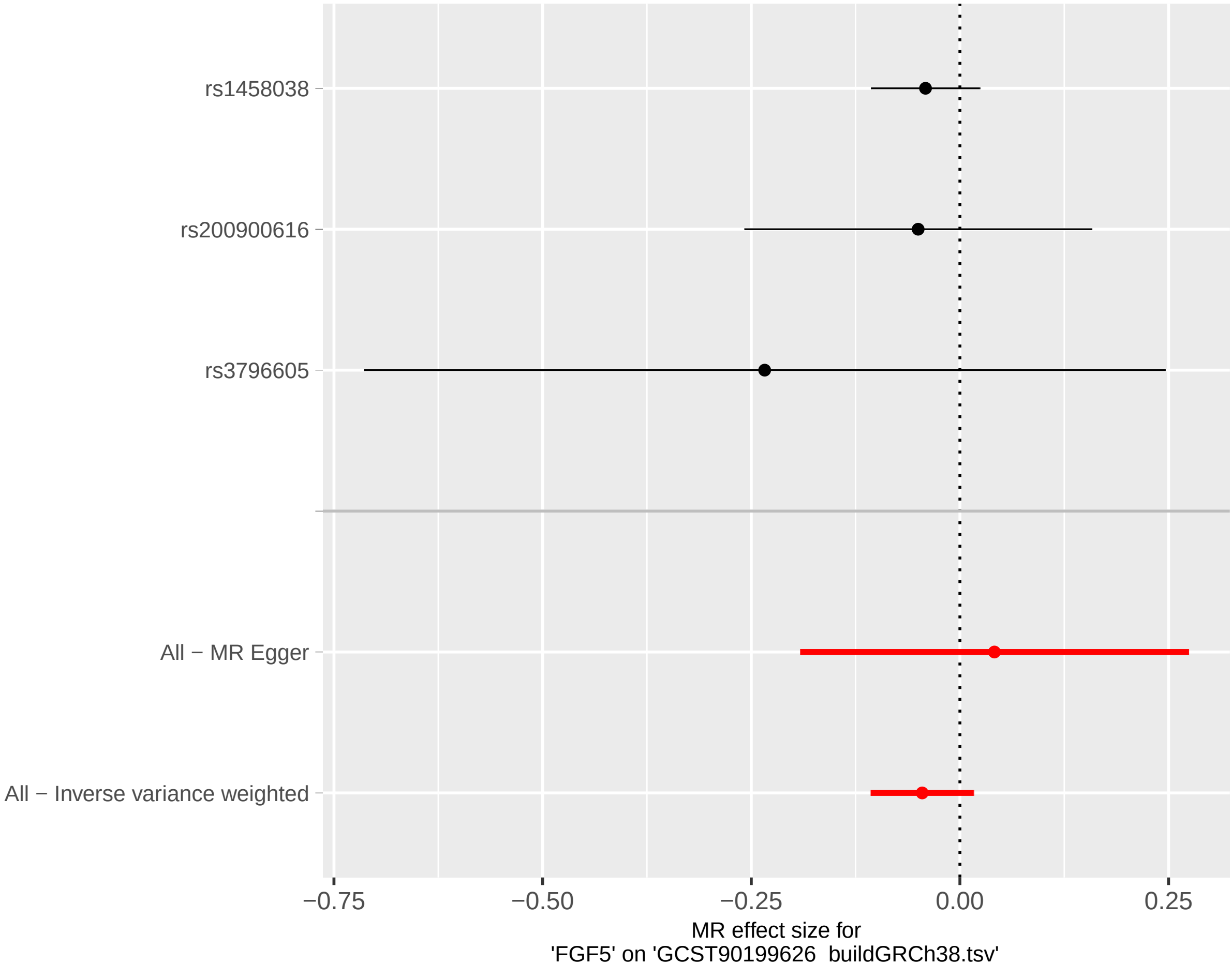
rs1458038
rs200900616
rs3796605
All − MR Egger
All − Inverse variance weighted
−0.75
−0.50
−0.25
0.00
0.25
MR effect size for
'FGF5' on 'GCST90199626_buildGRCh38.tsv'

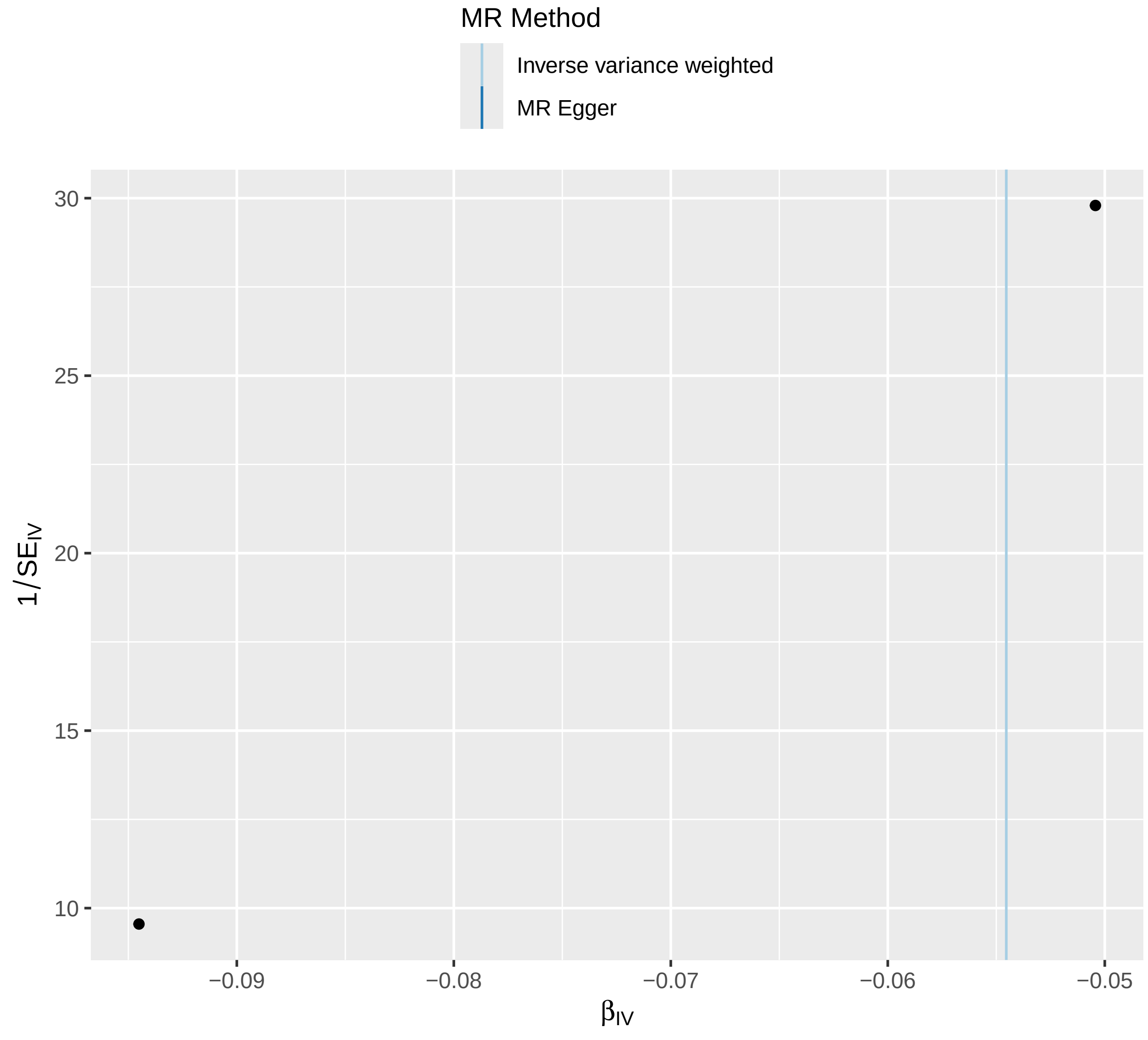
MR Method
Inverse variance weighted
MR Egger
1/SE_IV
30
25
20
15
10
−0.09
−0.08
−0.07
−0.06
−0.05
β_IV

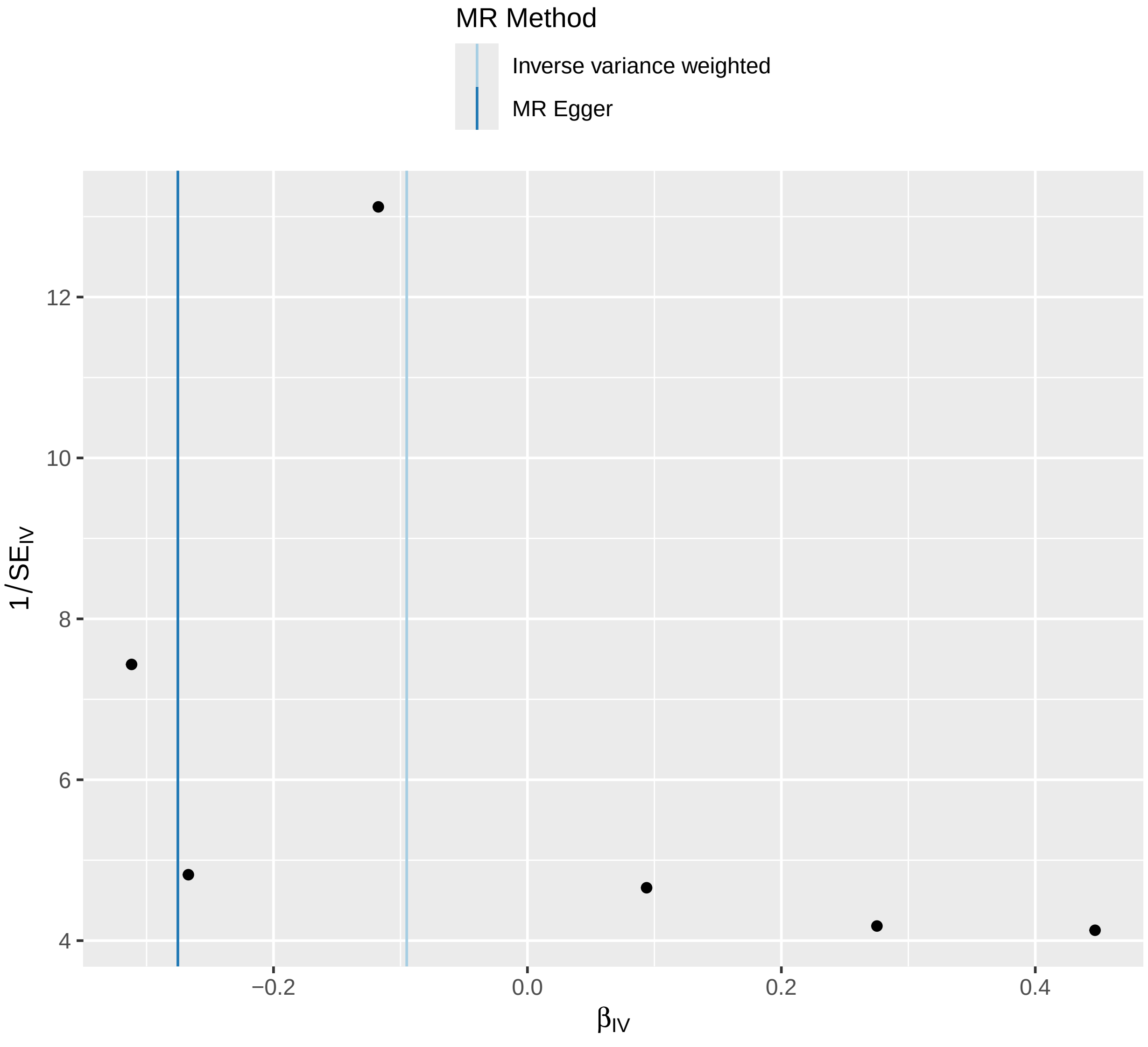

MR Method
Inverse variance weighted
MR Egger
12
10
8
6
4
1/SE_IV
−0.2
0.0
0.2
0.4
β_IV

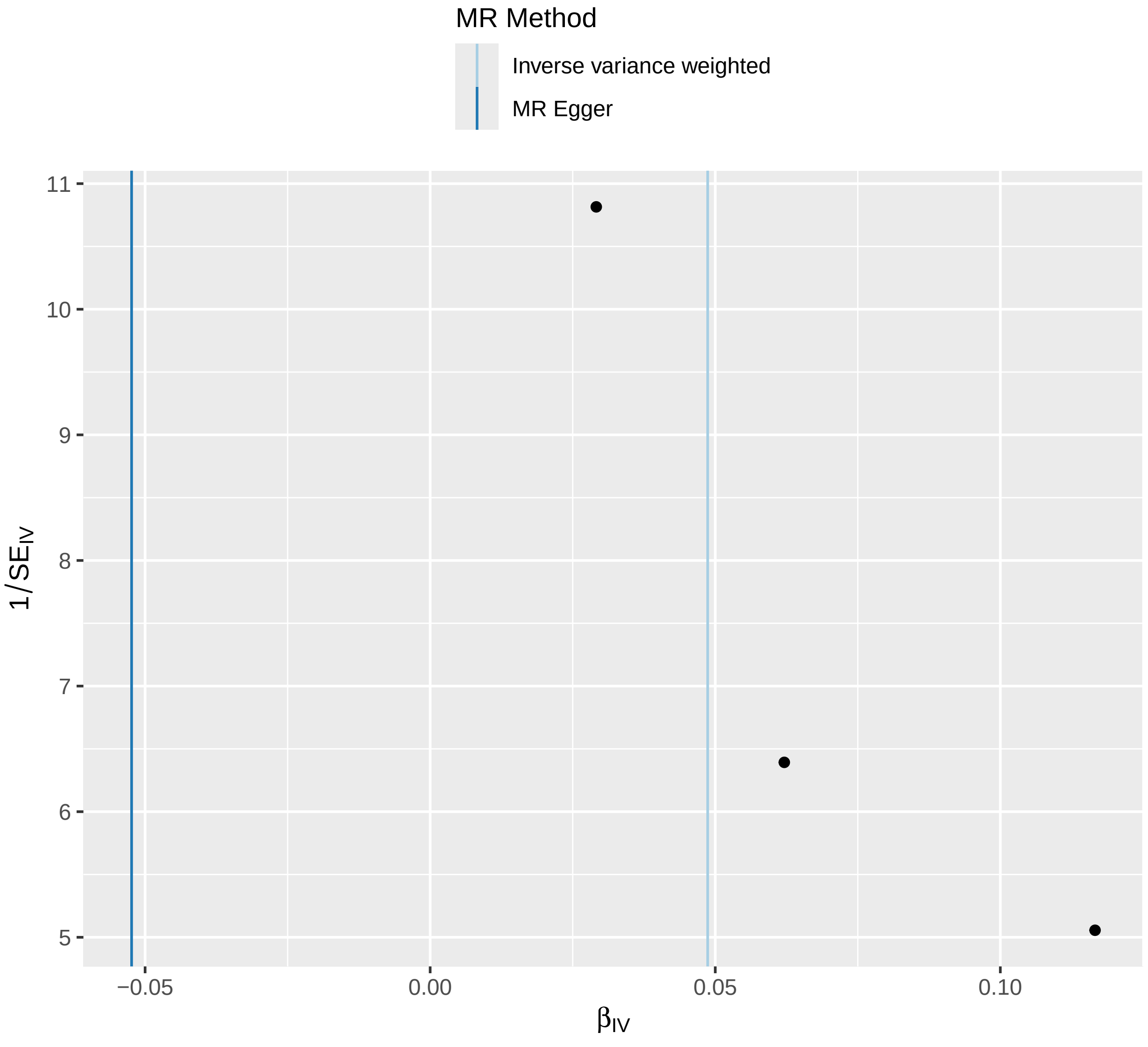
MR Method
Inverse variance weighted
MR Egger
11
10
9
8
7
6
5
1/SE_IV
−0.05
0.00
0.05
0.10
β_IV

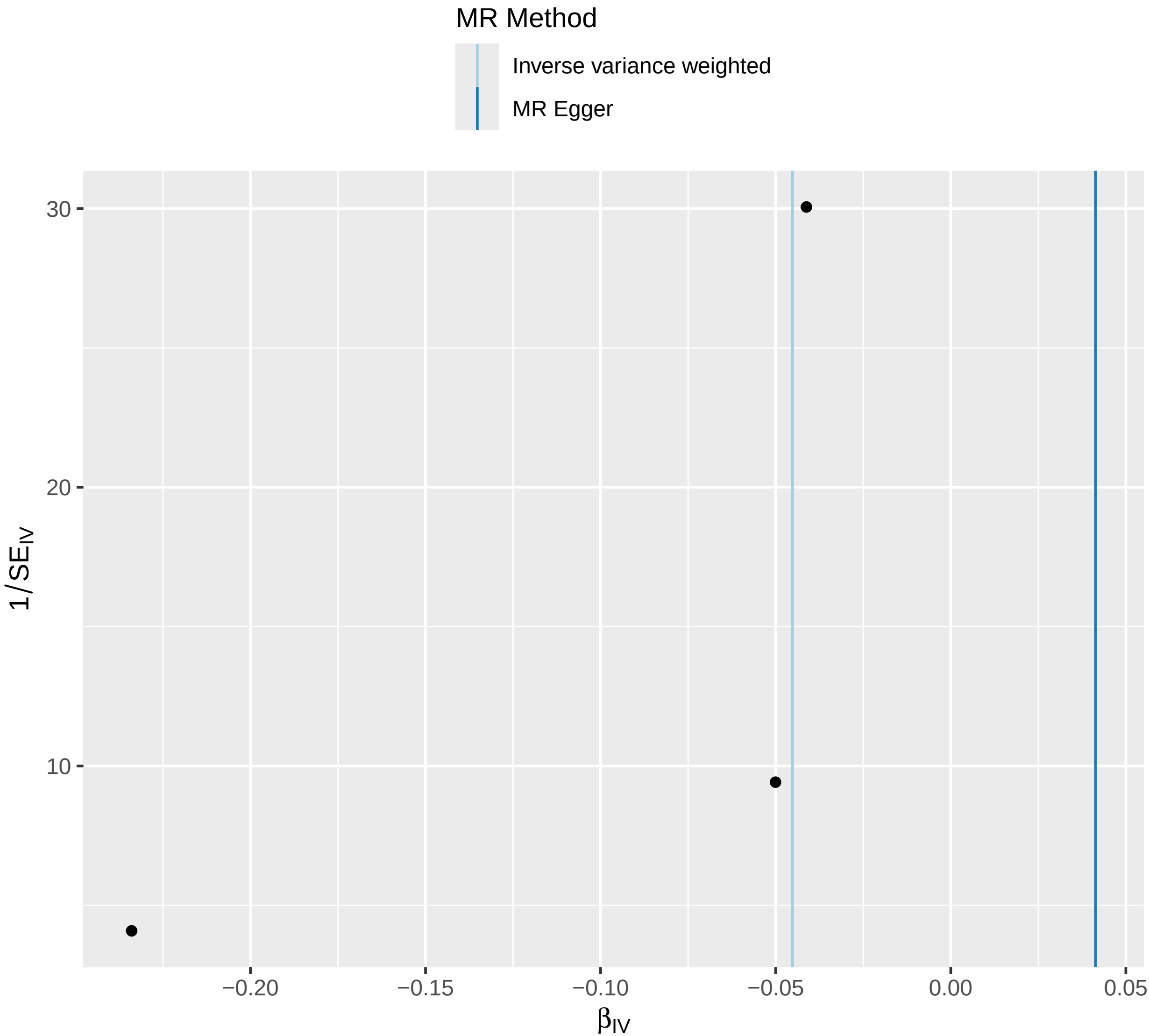
MR Method
Inverse variance weighted
MR Egger
30
20
10
$1/SE_{IV}$
−0.20
−0.15
−0.10
−0.05
0.00
0.05
β_{IV}

Insufficient number of SNPs

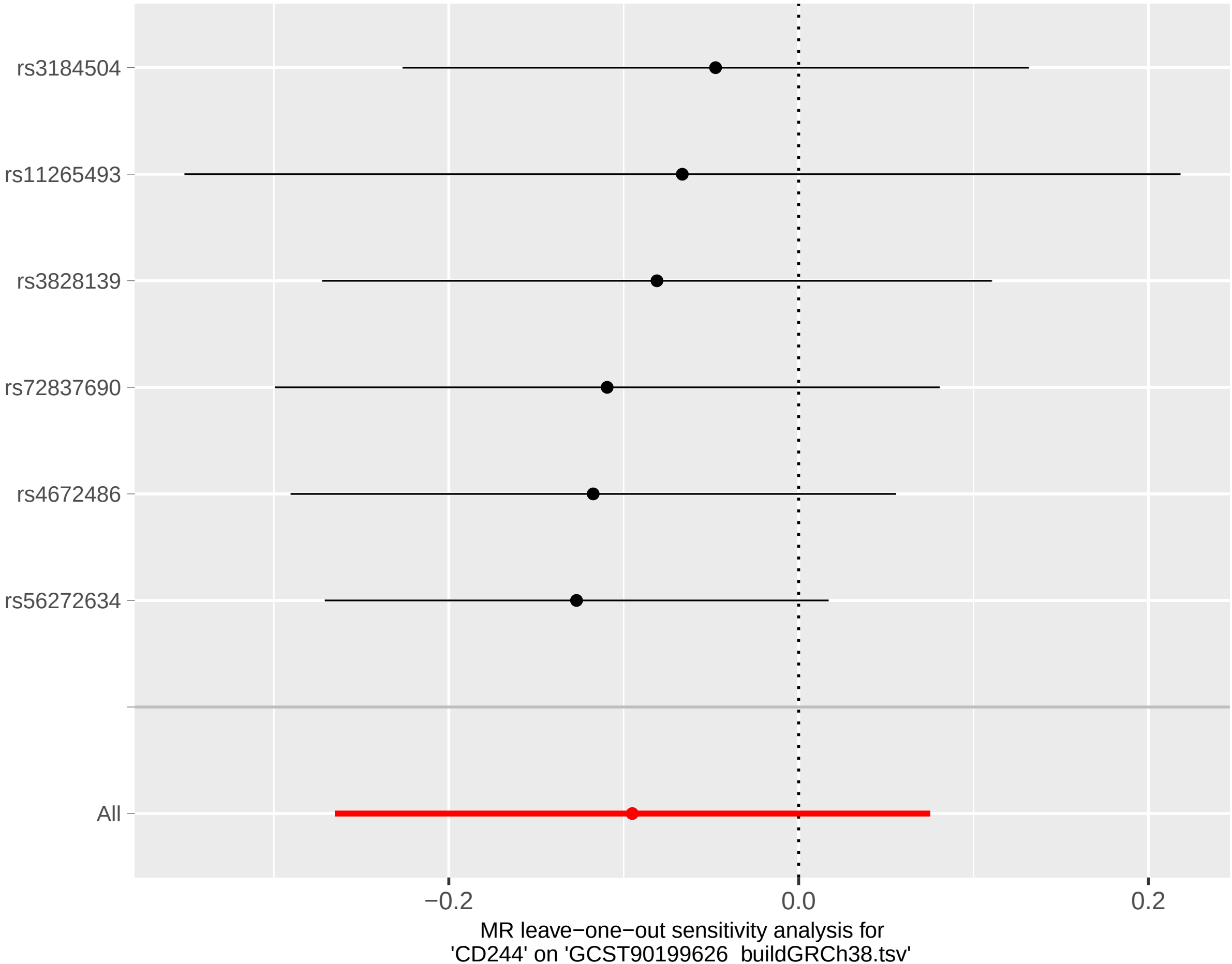
rs3184504
rs11265493
rs3828139
rs72837690
rs4672486
rs56272634
All
−0.2
0.0
0.2
MR leave−one−out sensitivity analysis for
'CD244' on 'GCST90199626_buildGRCh38.tsv'

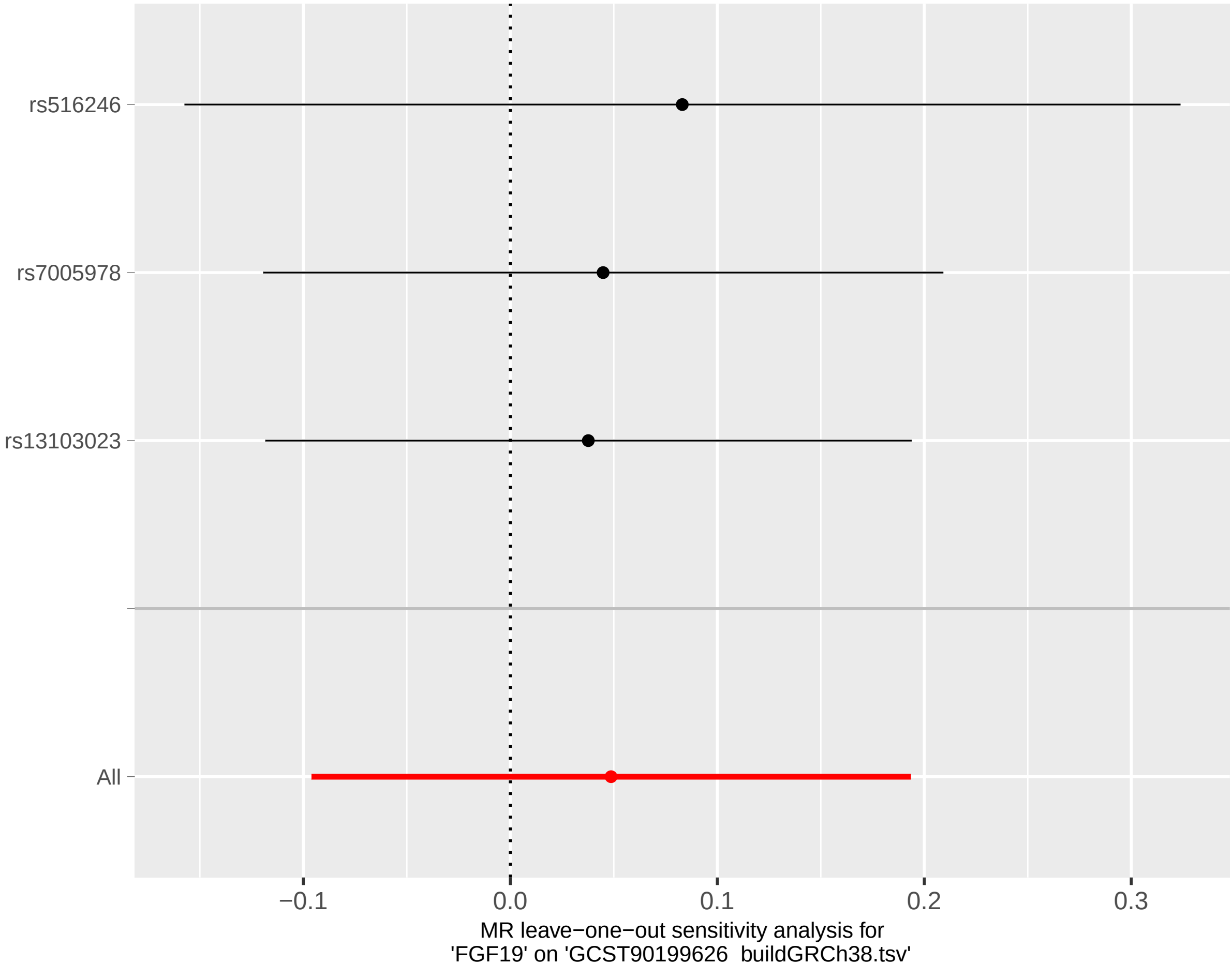
rs516246
rs7005978
rs13103023
All
−0.1
0.0
0.1
0.2
0.3
MR leave−one−out sensitivity analysis for
'FGF19' on 'GCST90199626_buildGRCh38.tsv'

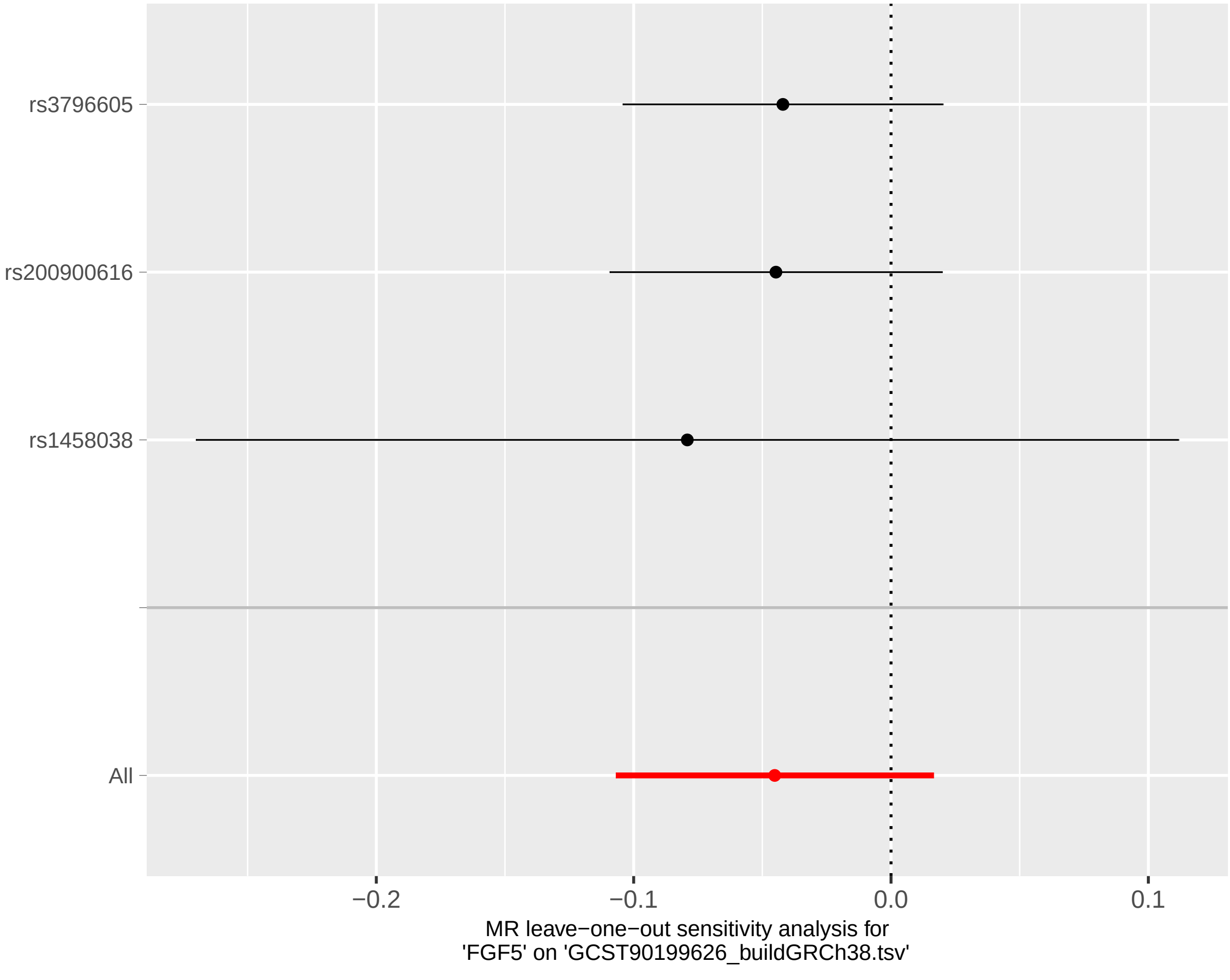
rs3796605
rs200900616
rs1458038
All
−0.2
−0.1
0.0
0.1
MR leave−one−out sensitivity analysis for
'FGF5' on 'GCST90199626_buildGRCh38.tsv'

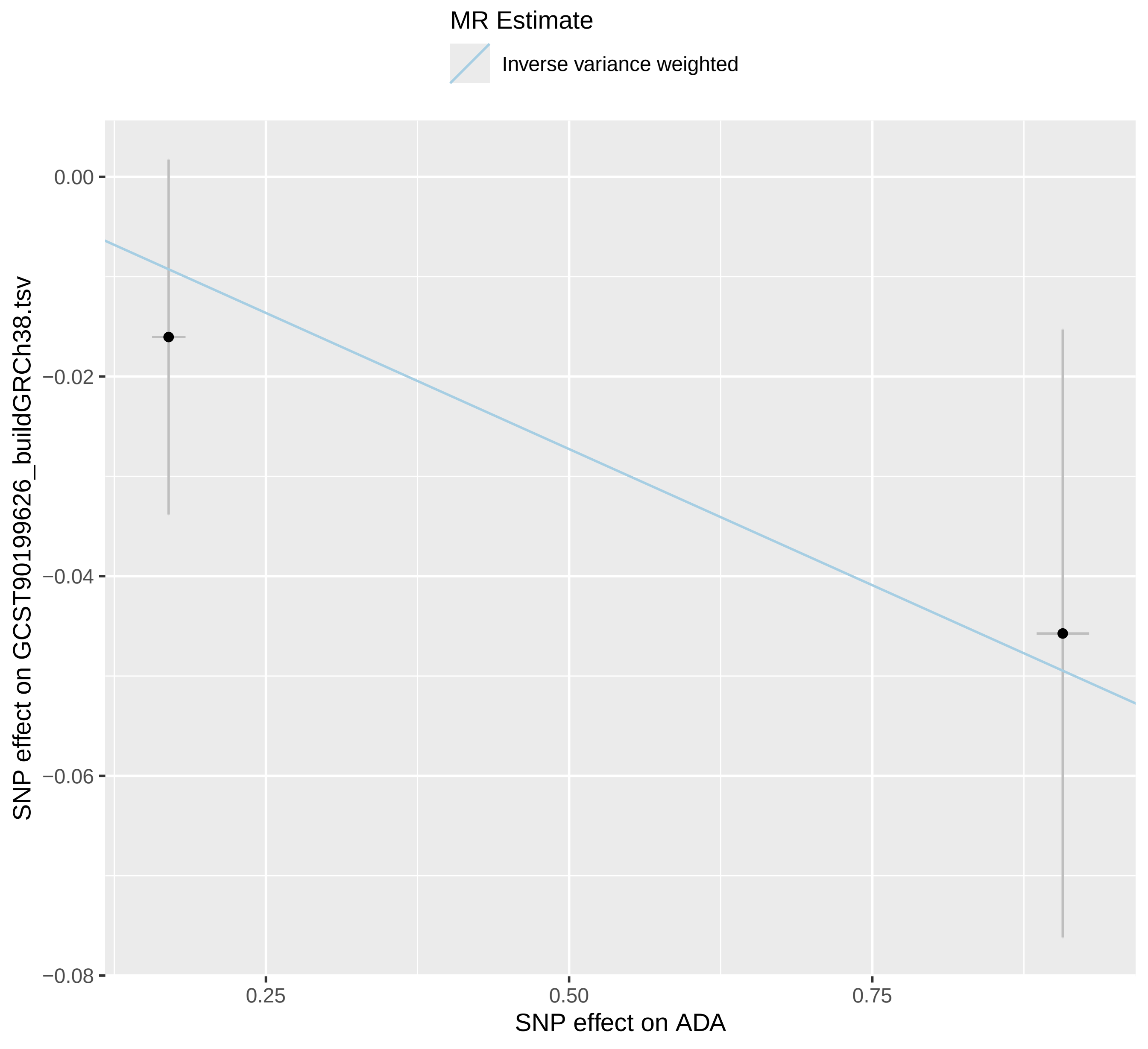
MR Estimate
Inverse variance weighted
0.00
−0.02
−0.04
−0.06
−0.08
SNP effect on GCST90199626_buildGRCh38.tsv
0.25
0.50
0.75
SNP effect on ADA

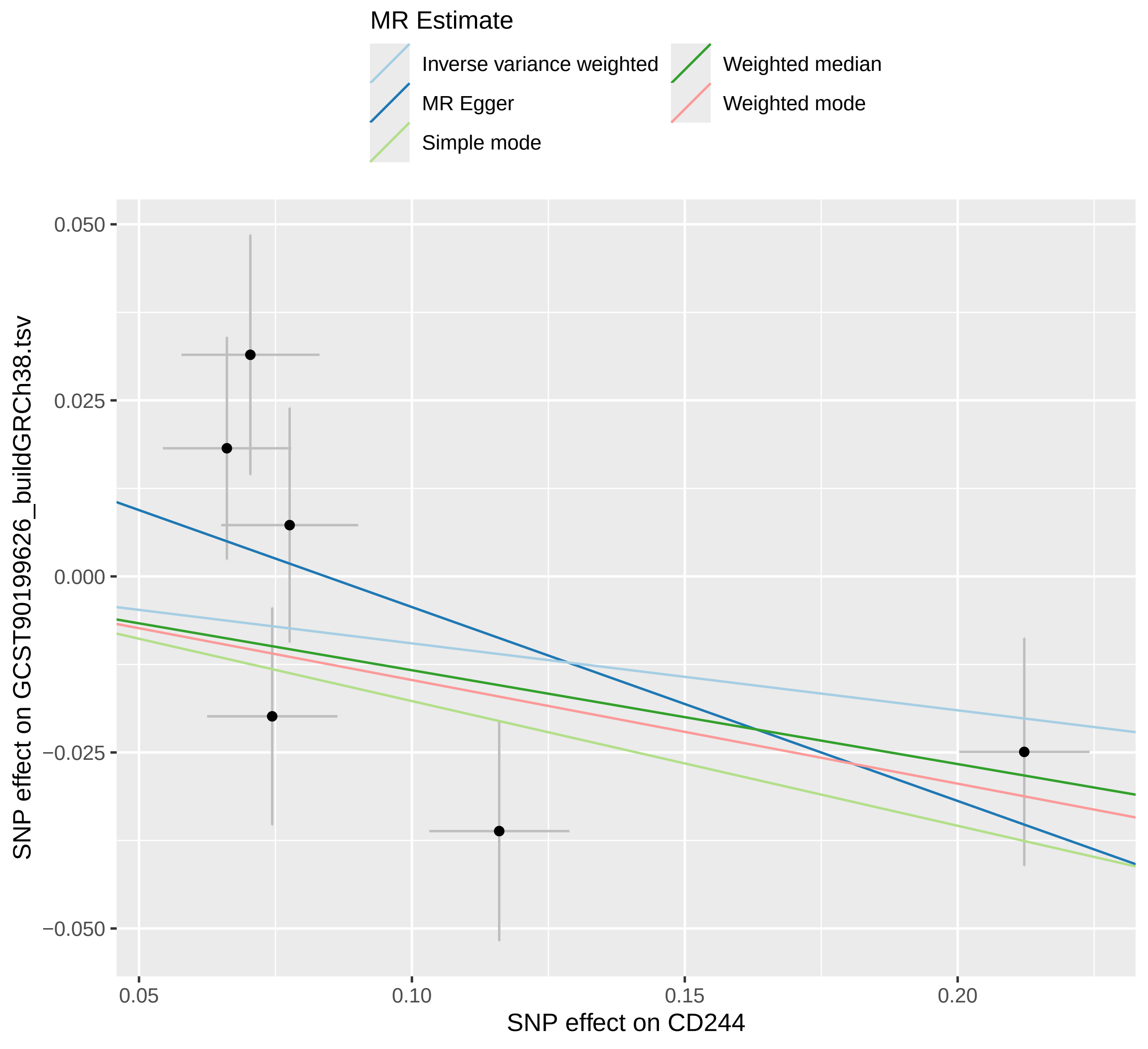
MR Estimate
Inverse variance weighted
MR Egger
Simple mode
Weighted median
Weighted mode
0.050
0.025
0.000
−0.025
−0.050
0.05
0.10
0.15
0.20
SNP effect on GCST90199626_buildGRCh38.tsv
SNP effect on CD244

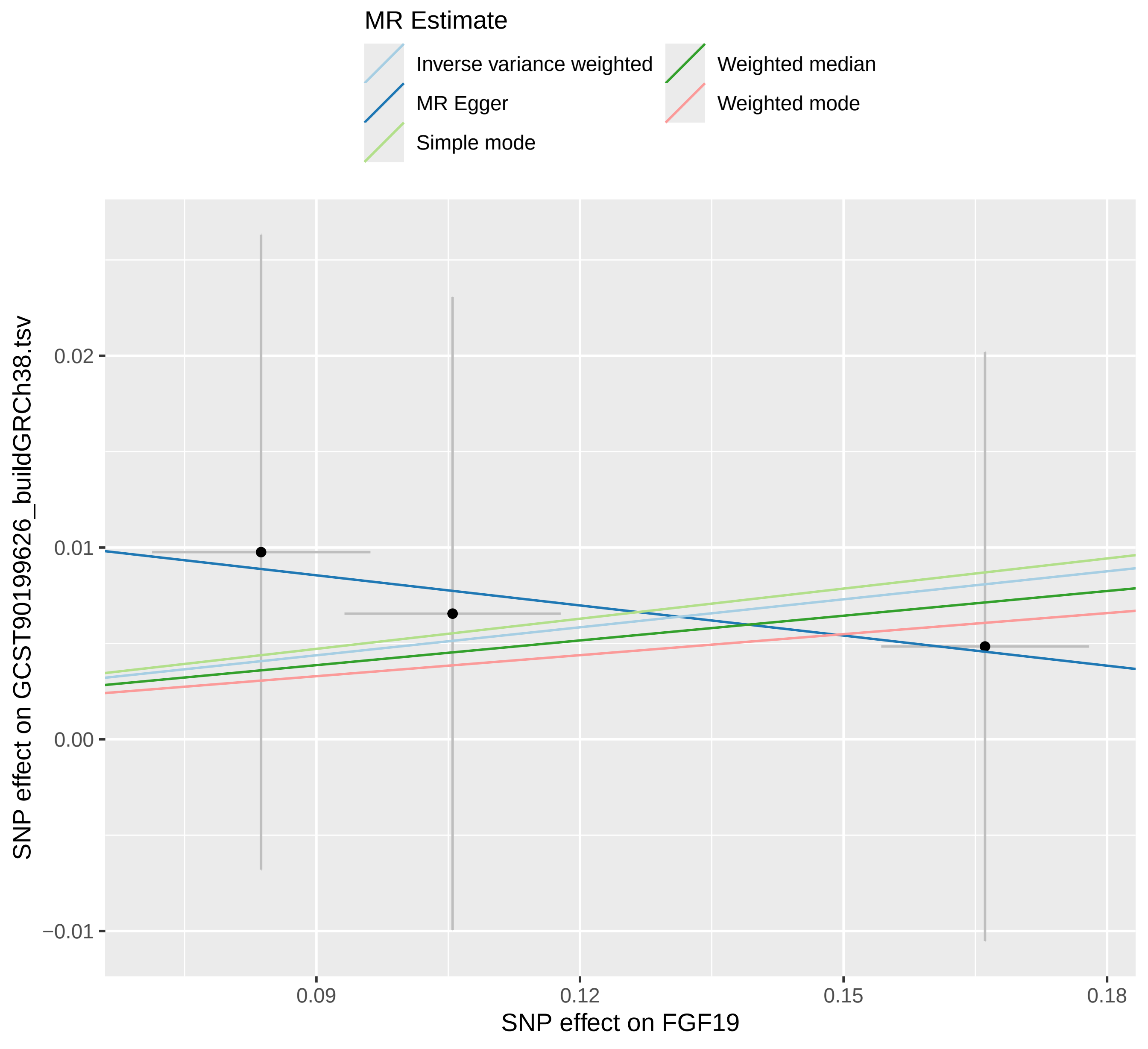

MR Estimate
Inverse variance weighted
MR Egger
Simple mode
Weighted median
Weighted mode
SNP effect on GCST90199626_buildGRCh38.tsv
0.02
0.01
0.00
−0.01
0.09
0.12
0.15
0.18
SNP effect on FGF19

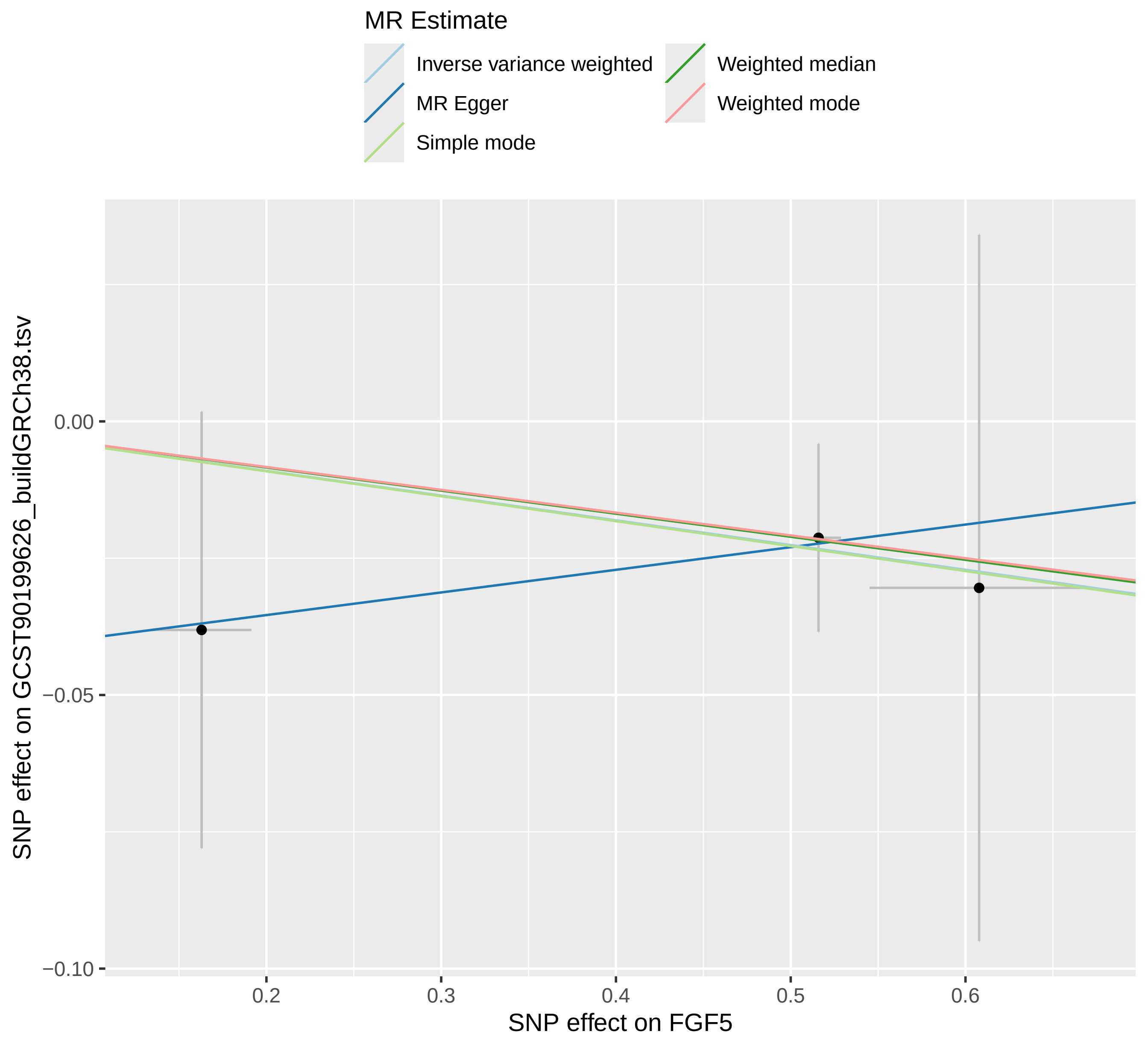

MR Estimate
Inverse variance weighted
MR Egger
Simple mode
Weighted median
Weighted mode
0.00
−0.05
−0.10
0.2
0.3
0.4
0.5
0.6
SNP effect on FGF5
SNP effect on GCST90199626_buildGRCh38.tsv

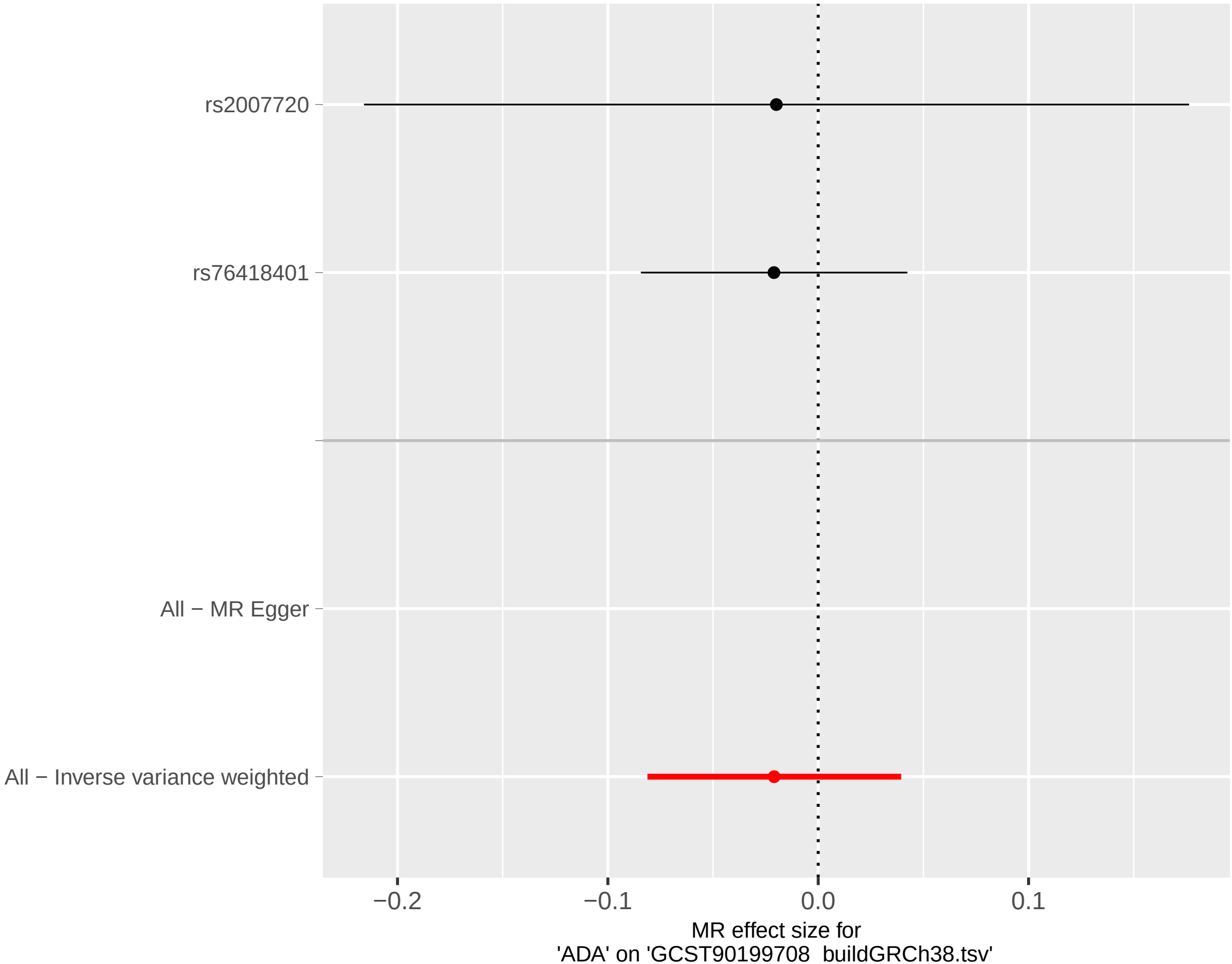
rs2007720
rs76418401
All − MR Egger
All − Inverse variance weighted
−0.2
−0.1
0.0
0.1
MR effect size for
'ADA' on 'GCST90199708_buildGRCh38.tsv'

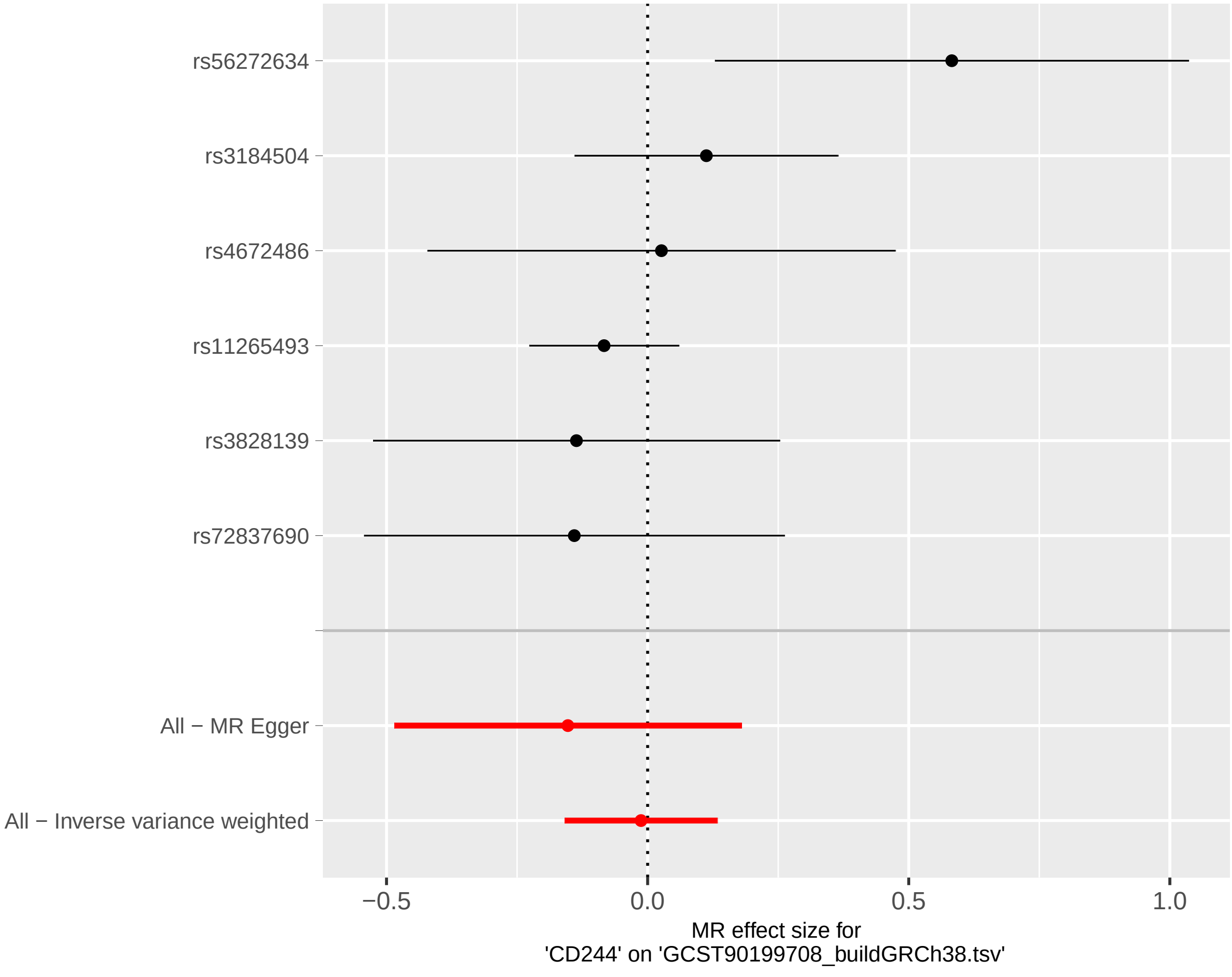
rs56272634
rs3184504
rs4672486
rs11265493
rs3828139
rs72837690
All − MR Egger
All − Inverse variance weighted
−0.5
0.0
0.5
1.0
MR effect size for
'CD244' on 'GCST90199708_buildGRCh38.tsv'

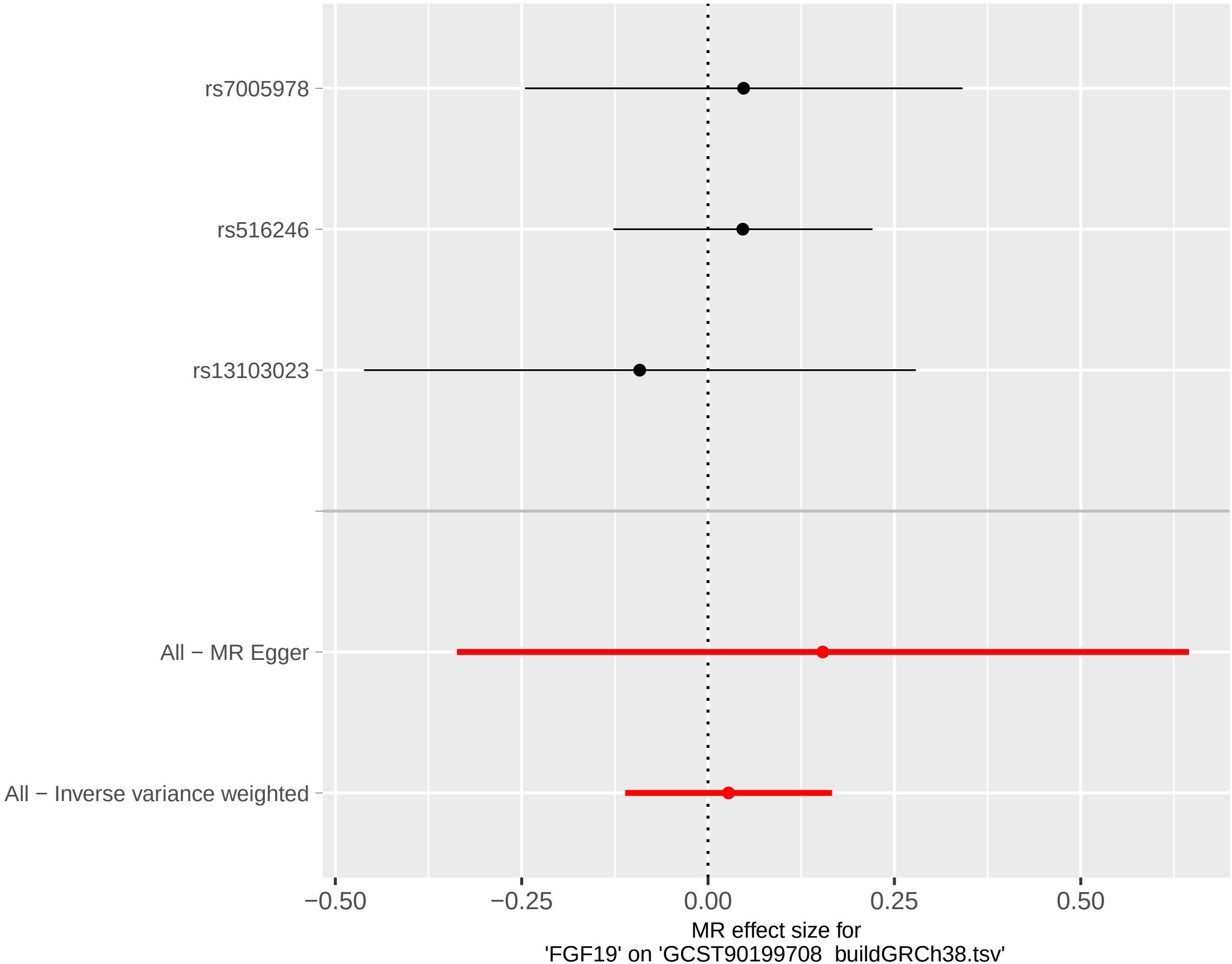
rs7005978
rs516246
rs13103023
All − MR Egger
All − Inverse variance weighted
−0.50
−0.25
0.00
0.25
0.50
MR effect size for
'FGF19' on 'GCST90199708_buildGRCh38.tsv'

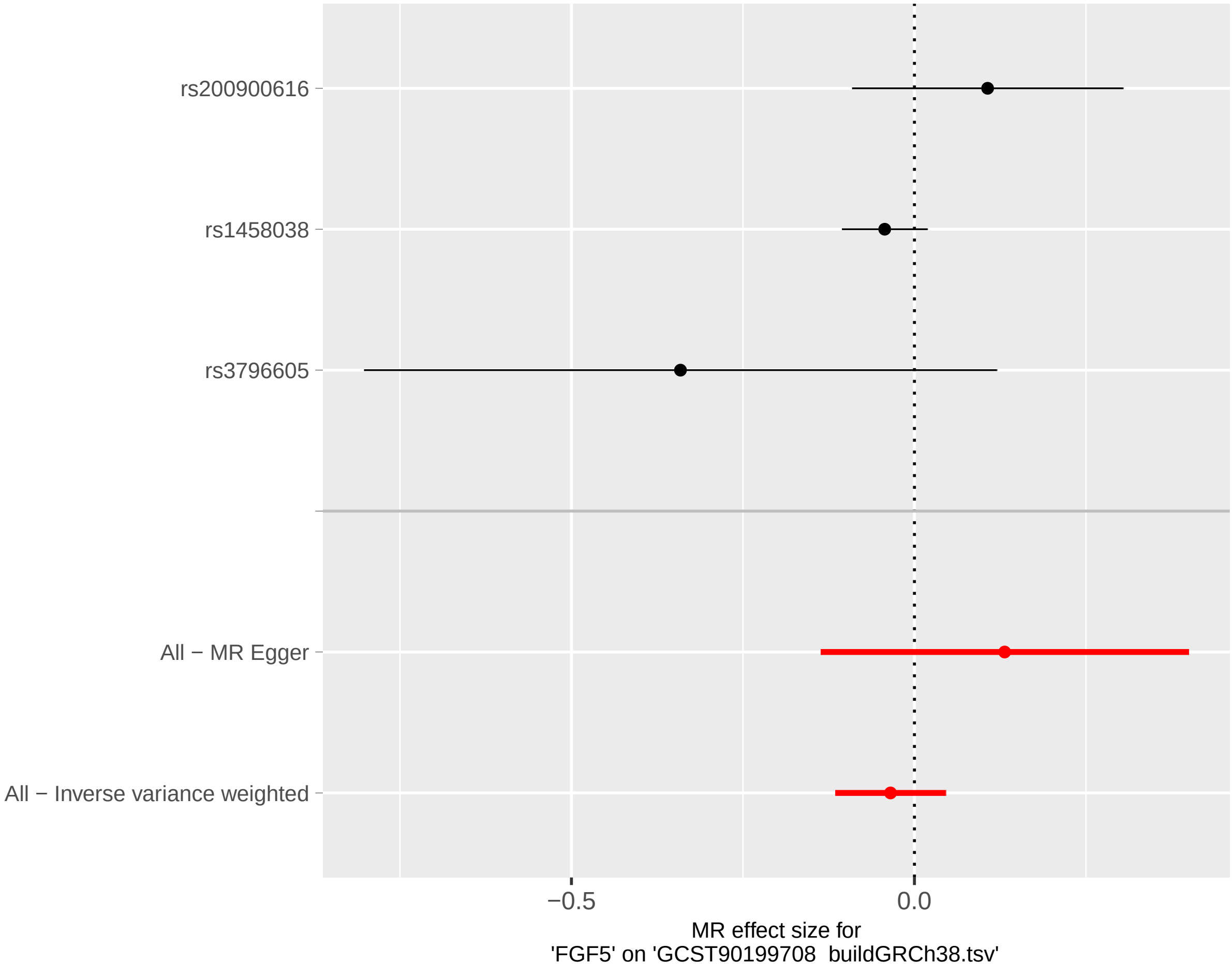
rs200900616
rs1458038
rs3796605
All − MR Egger
All − Inverse variance weighted
−0.5
0.0
MR effect size for
'FGF5' on 'GCST90199708_buildGRCh38.tsv'

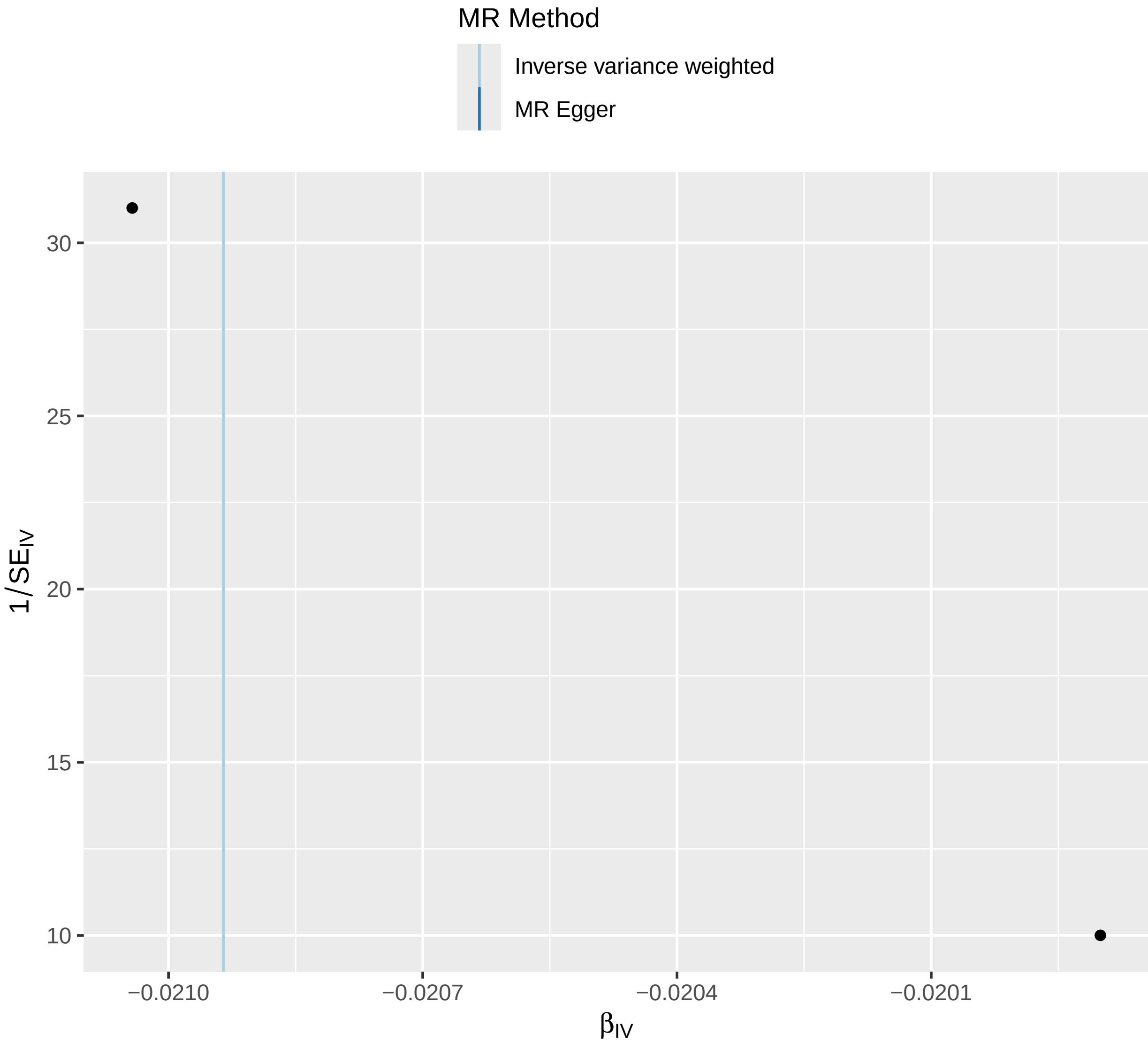
MR Method
Inverse variance weighted
MR Egger
30
25
20
15
10
1/SE$_{IV}$
−0.0210
−0.0207
−0.0204
−0.0201
β$_{IV}$

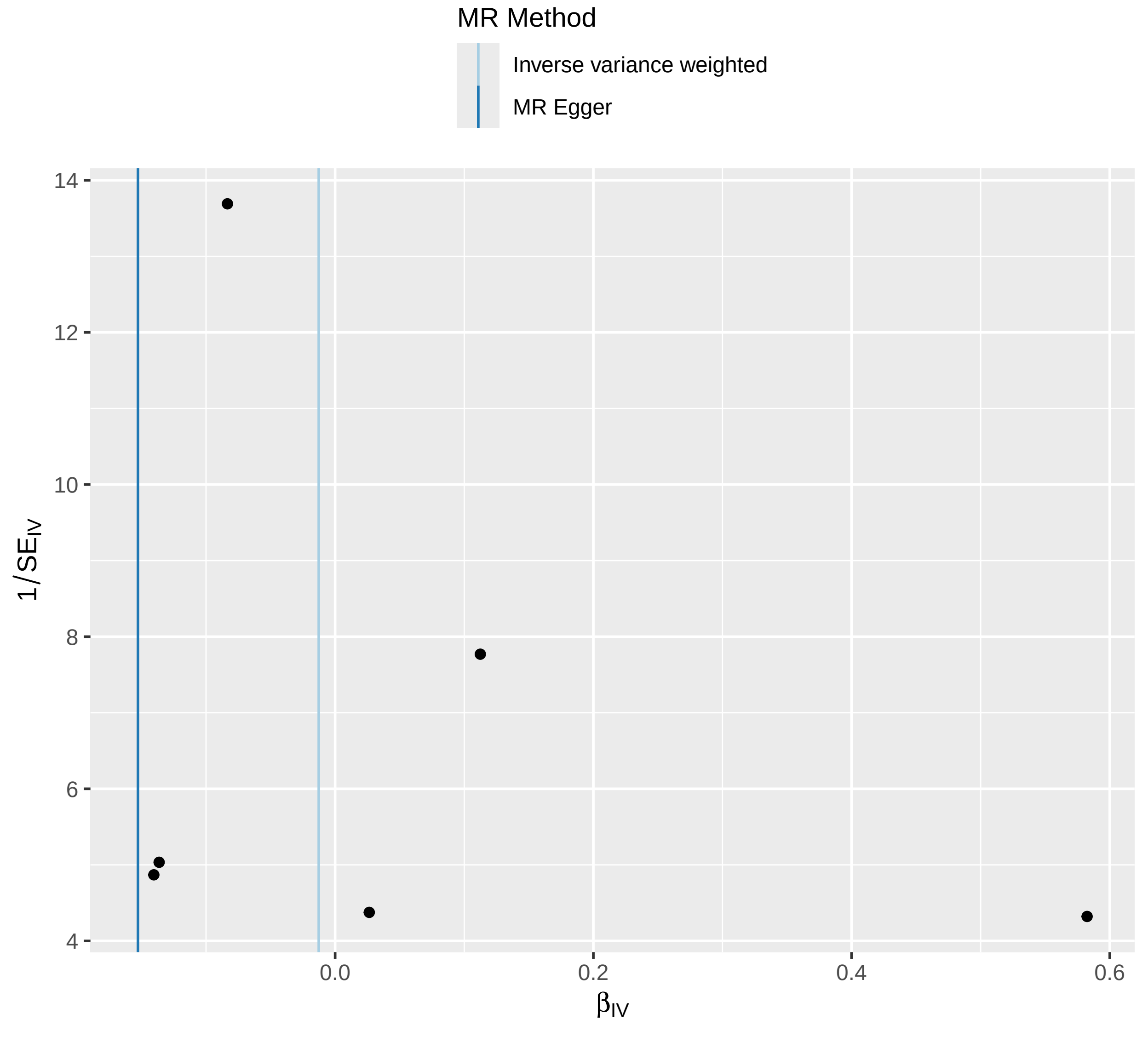

MR Method
Inverse variance weighted
MR Egger
14
12
10
8
6
4
1/SE_IV
0.0
0.2
0.4
0.6
β_IV

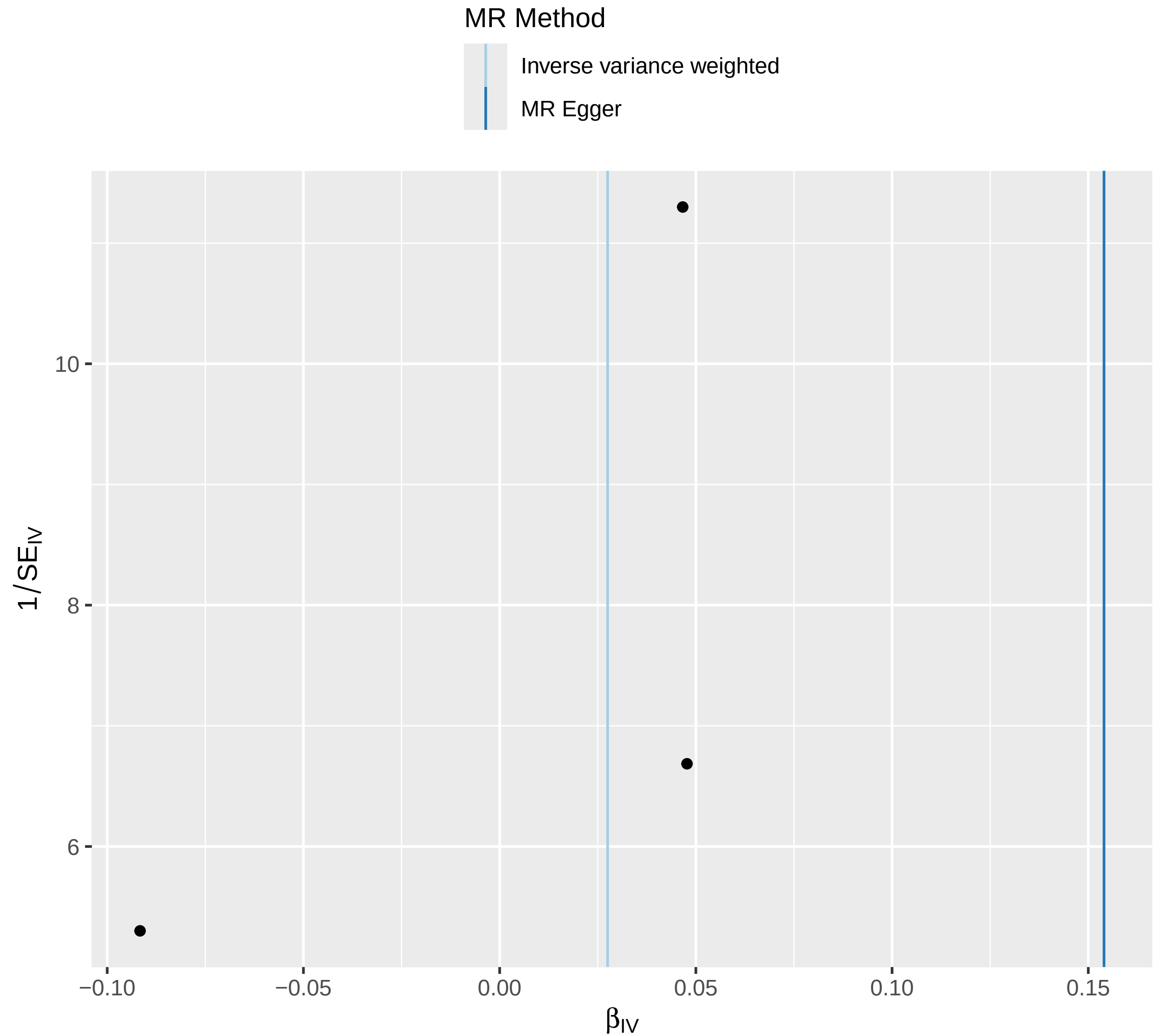
MR Method
Inverse variance weighted
MR Egger
1/SE_IV
10
8
6
−0.10
−0.05
0.00
0.05
0.10
0.15
β_IV

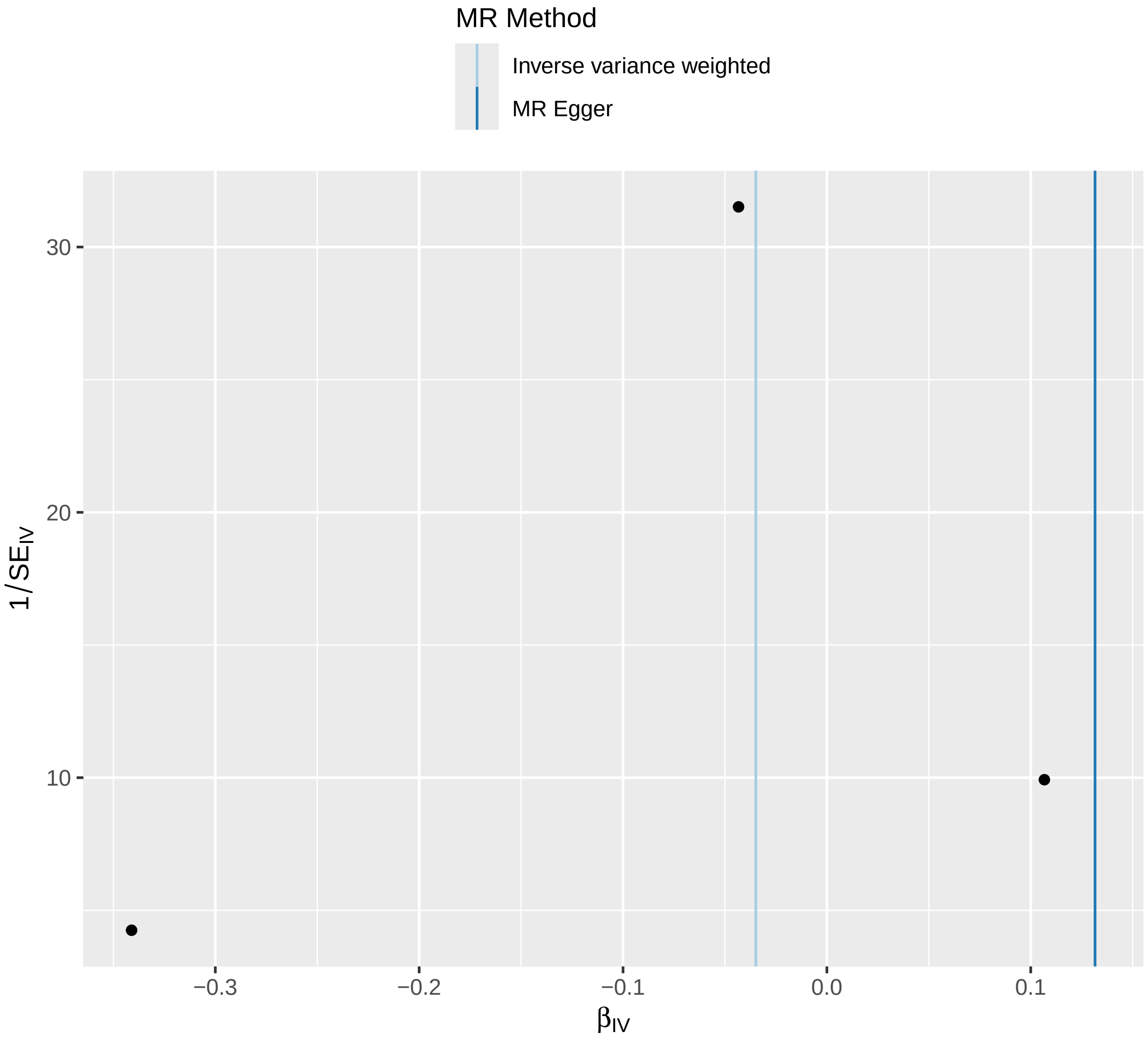
MR Method
Inverse variance weighted
MR Egger
30
20
10
1/SE_IV
−0.3
−0.2
−0.1
0.0
0.1
β_IV

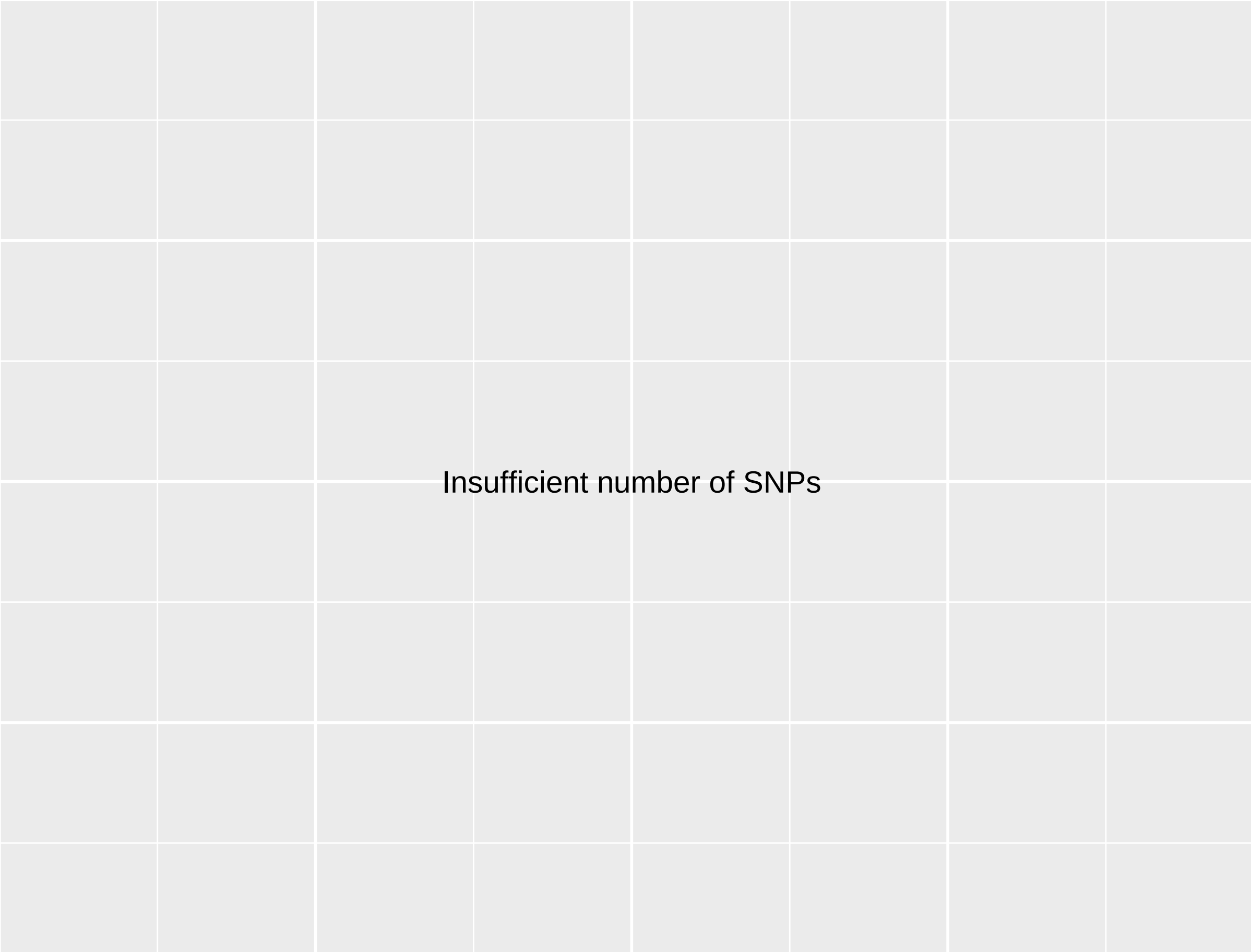
Insufficient number of SNPs

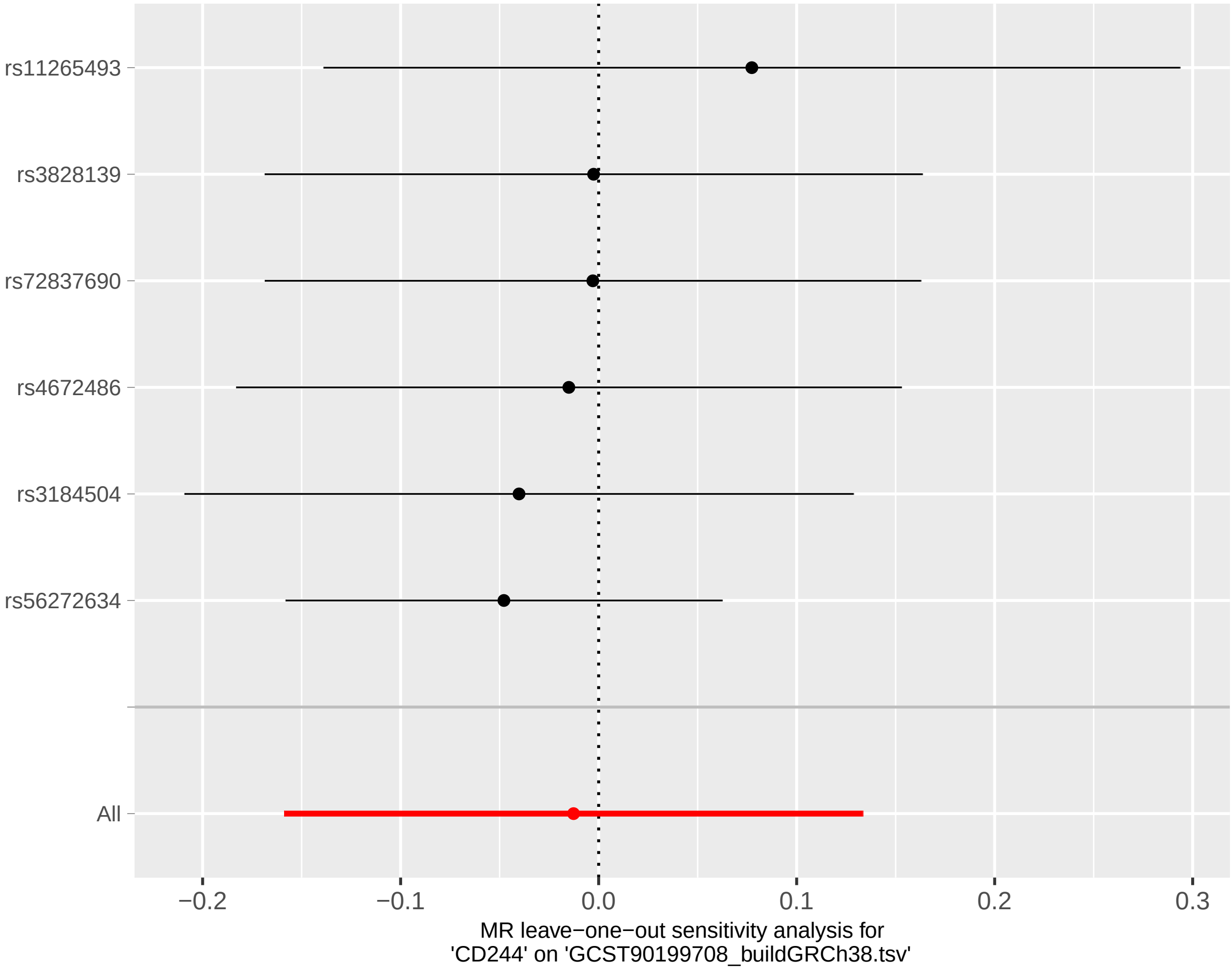
rs11265493
rs3828139
rs72837690
rs4672486
rs3184504
rs56272634
All
−0.2
−0.1
0.0
0.1
0.2
0.3
MR leave−one−out sensitivity analysis for
'CD244' on 'GCST90199708_buildGRCh38.tsv'

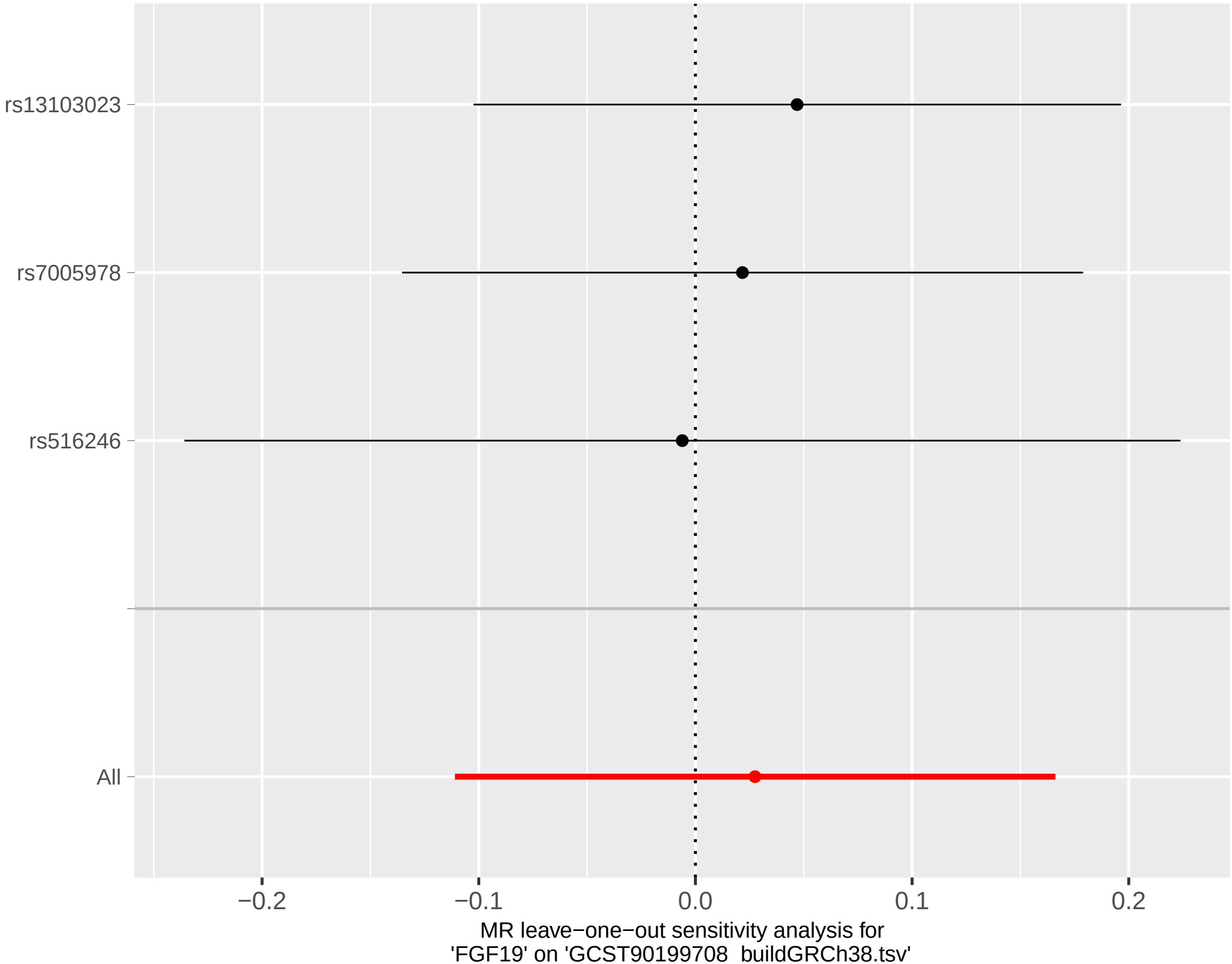
rs13103023
rs7005978
rs516246
All
−0.2
−0.1
0.0
0.1
0.2
MR leave−one−out sensitivity analysis for
'FGF19' on 'GCST90199708_buildGRCh38.tsv'

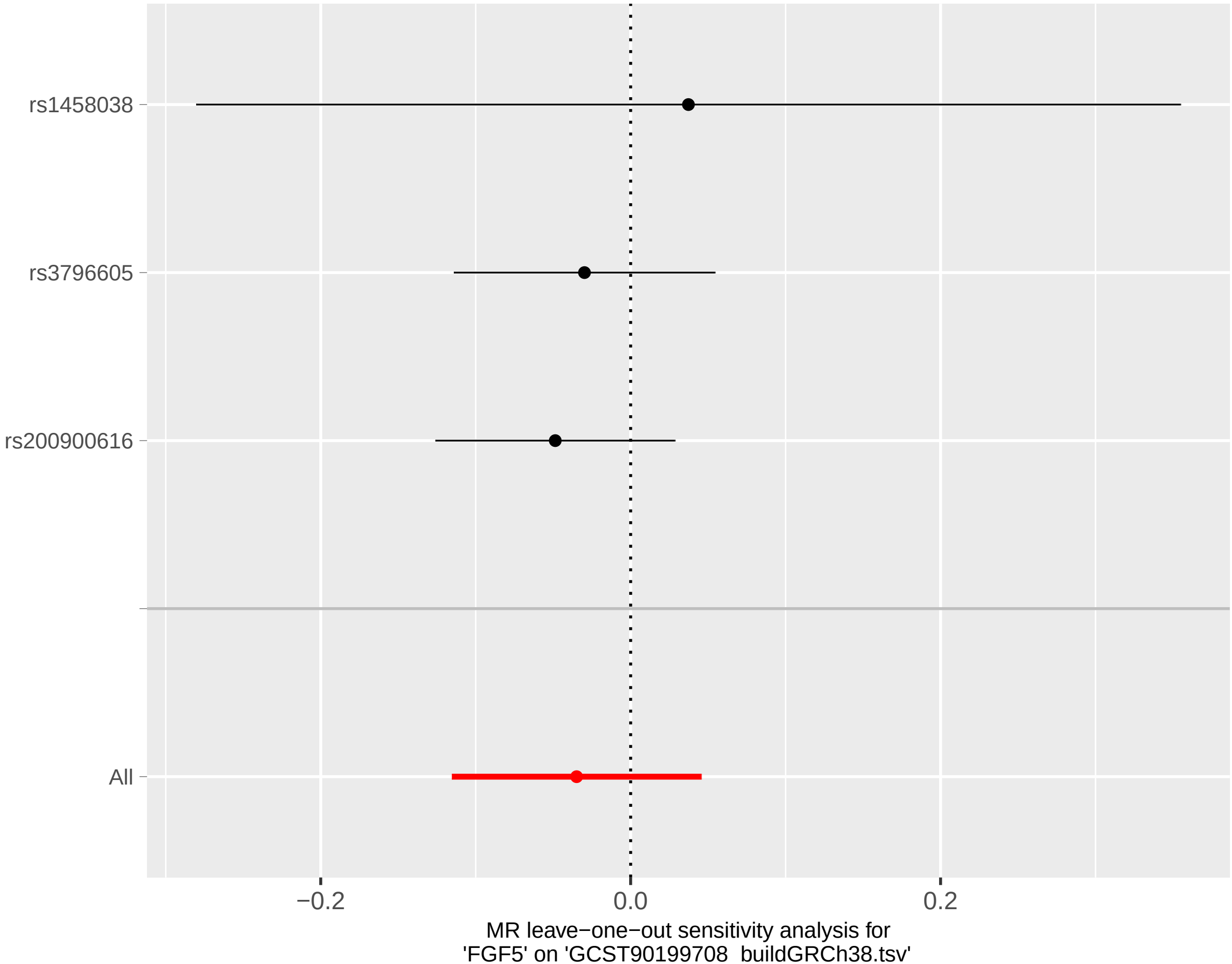
rs1458038
rs3796605
rs200900616
All
−0.2
0.0
0.2
MR leave−one−out sensitivity analysis for
'FGF5' on 'GCST90199708_buildGRCh38.tsv'

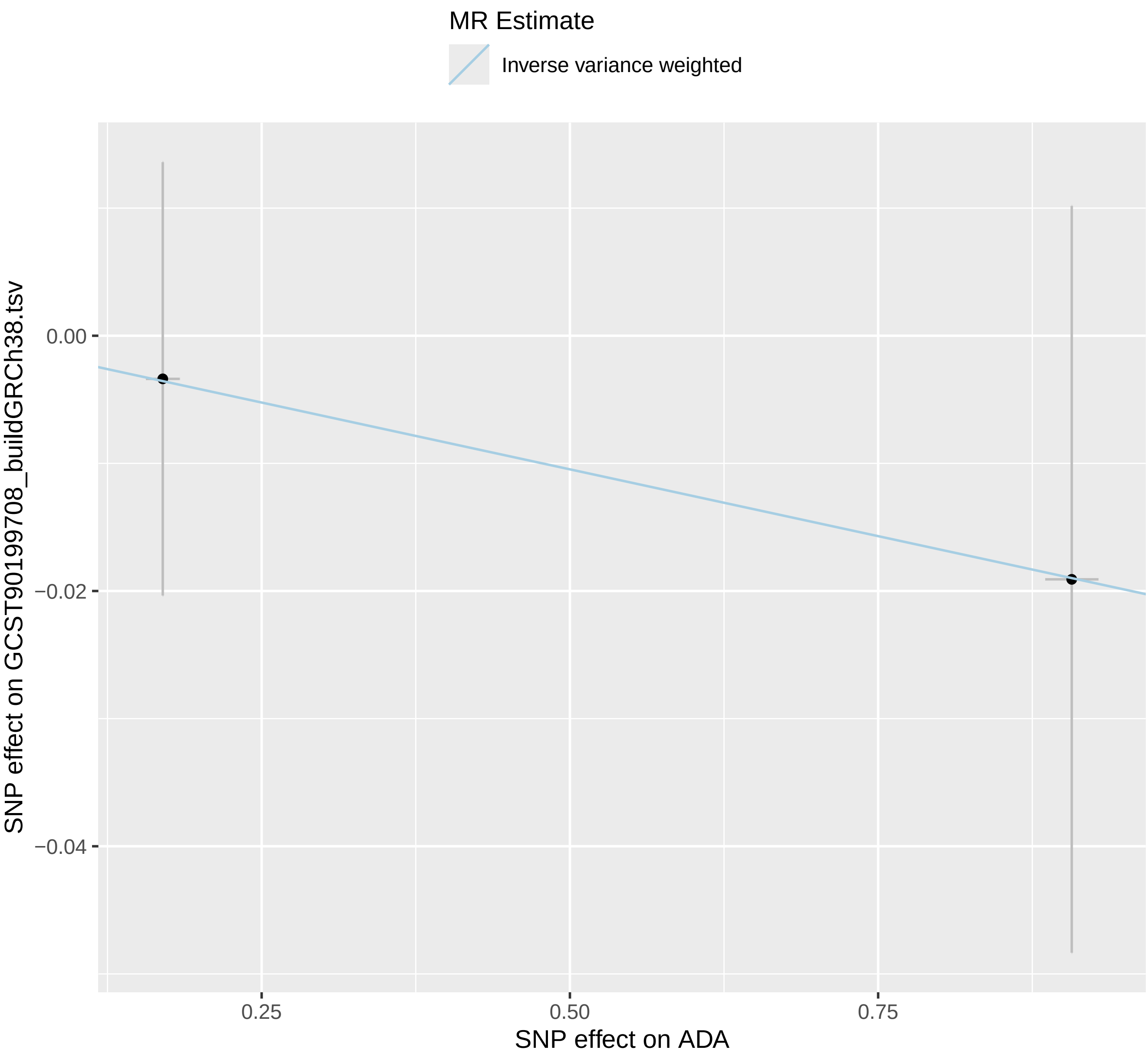
MR Estimate
Inverse variance weighted
SNP effect on GCST90199708_buildGRCh38.tsv
0.00
−0.02
−0.04
0.25
0.50
0.75
SNP effect on ADA

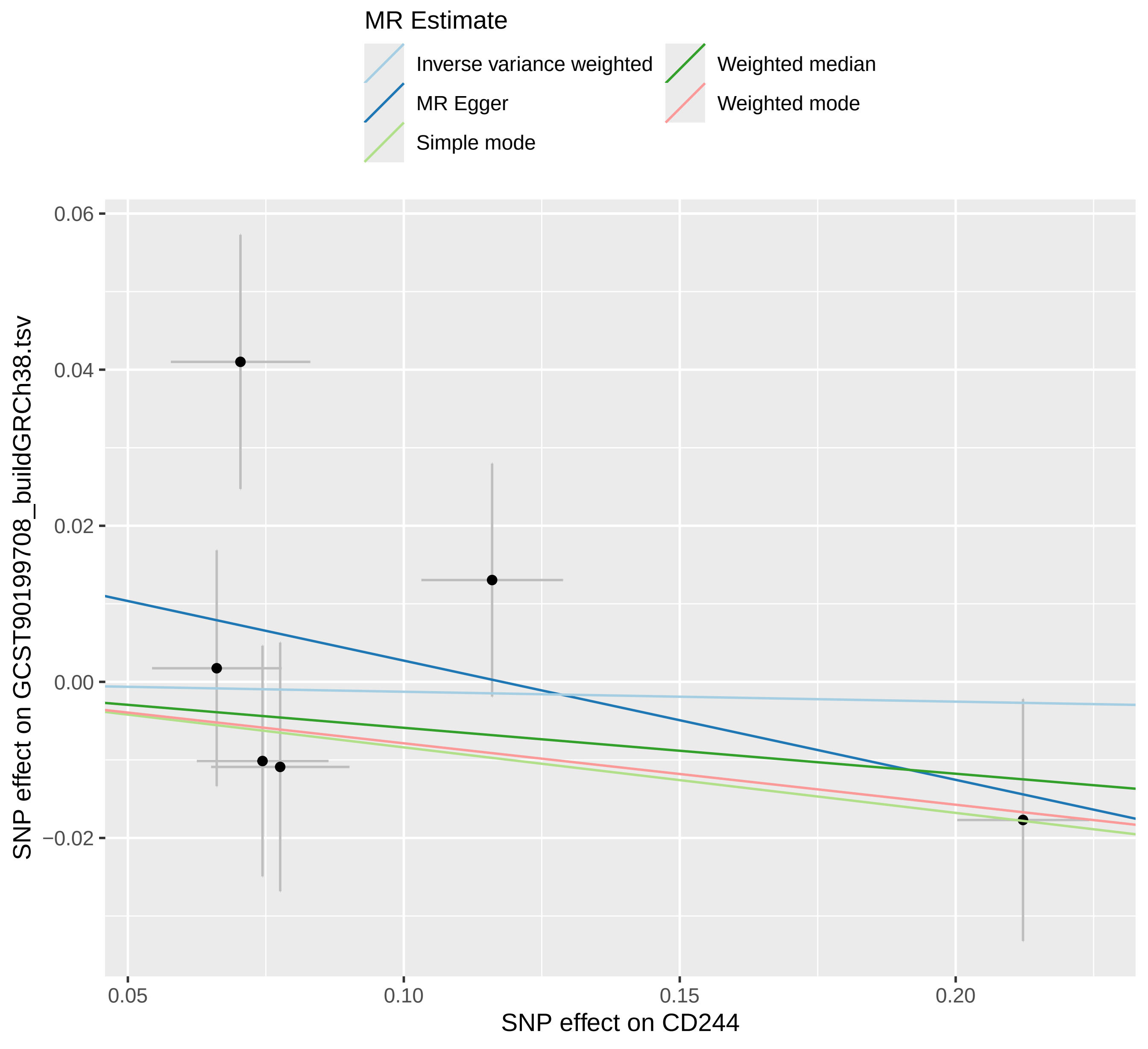
MR Estimate
Inverse variance weighted
MR Egger
Simple mode
Weighted median
Weighted mode
0.06
0.04
0.02
0.00
−0.02
SNP effect on GCST90199708_buildGRCh38.tsv
0.05
0.10
0.15
0.20
SNP effect on CD244

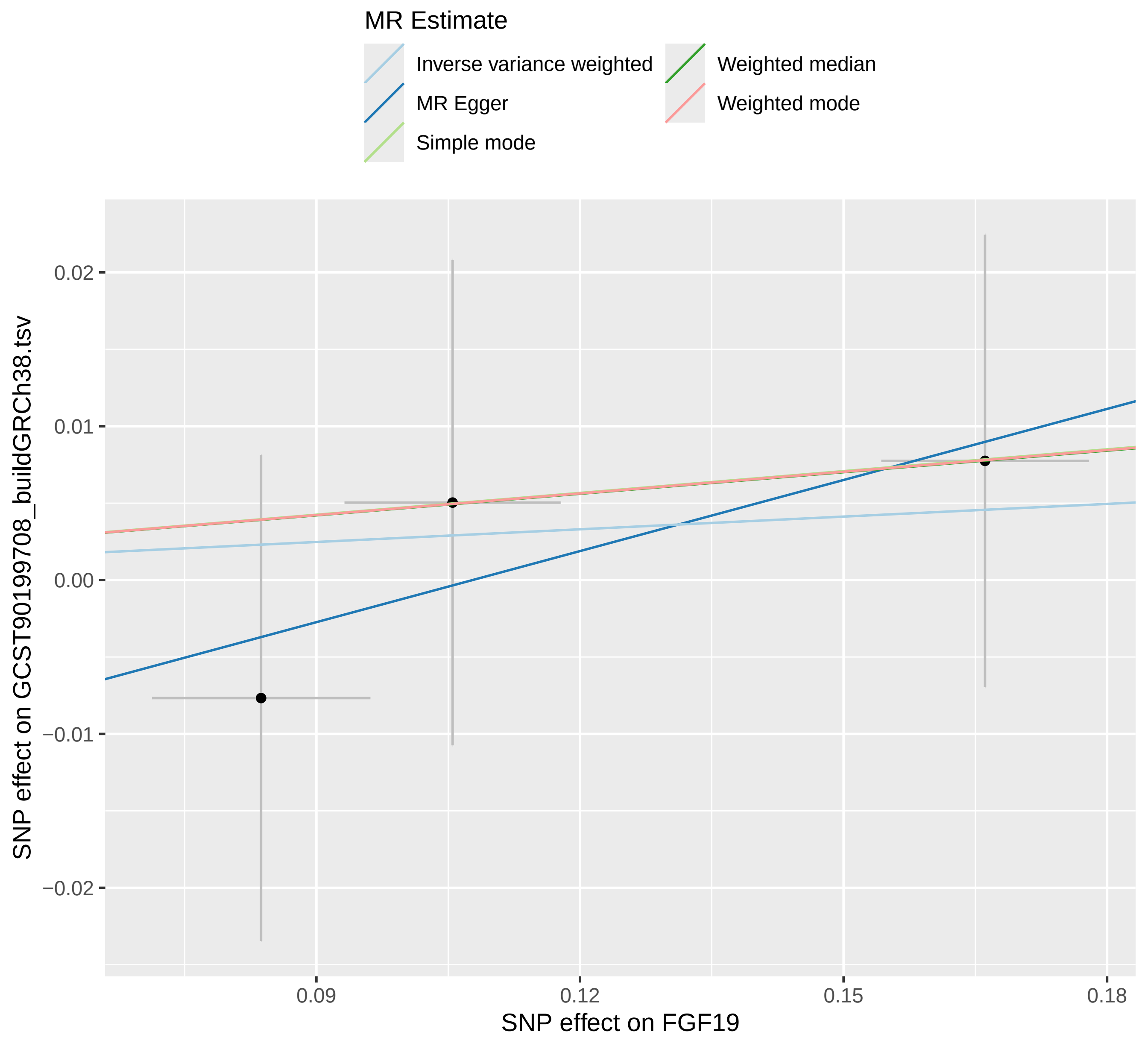

MR Estimate
Inverse variance weighted
MR Egger
Simple mode
Weighted median
Weighted mode
0.02
0.01
0.00
−0.01
−0.02
SNP effect on GCST90199708_buildGRCh38.tsv
0.09
0.12
0.15
0.18
SNP effect on FGF19

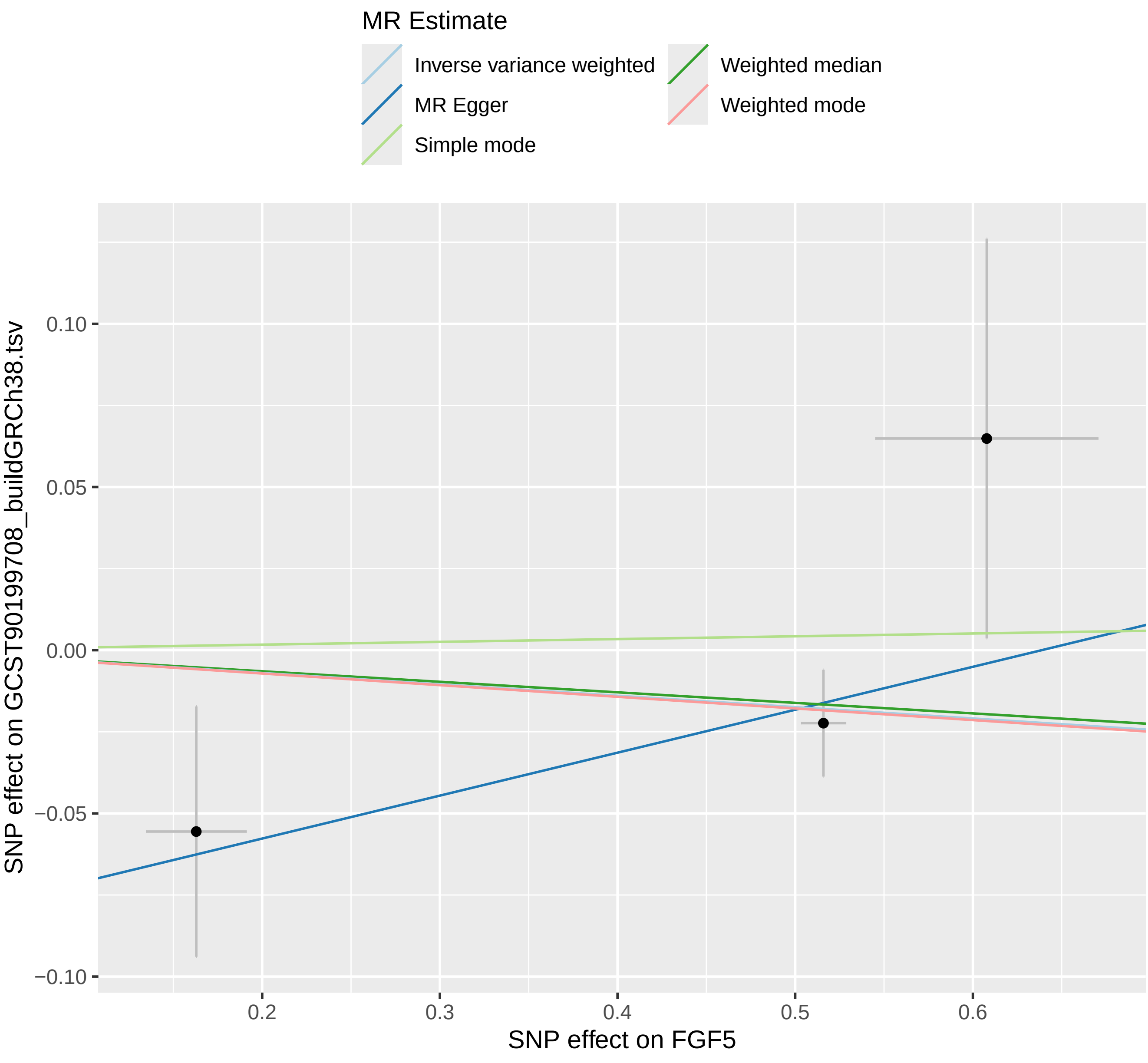
MR Estimate
Inverse variance weighted
MR Egger
Simple mode
Weighted median
Weighted mode
0.10
0.05
0.00
−0.05
−0.10
0.2
0.3
0.4
0.5
0.6
SNP effect on FGF5
SNP effect on GCST90199708_buildGRCh38.tsv

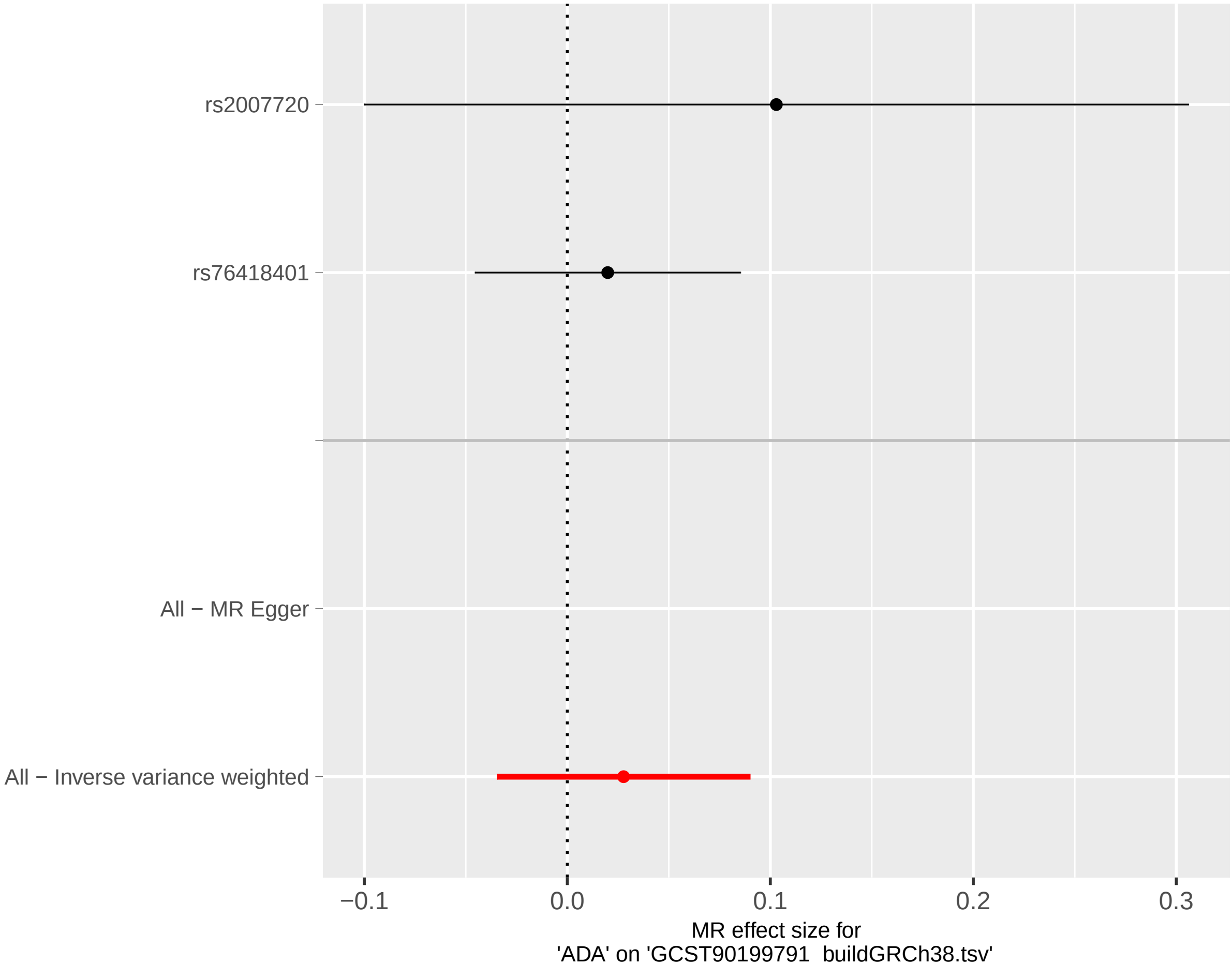
rs2007720
rs76418401
All − MR Egger
All − Inverse variance weighted
−0.1
0.0
0.1
0.2
0.3
MR effect size for
'ADA' on 'GCST90199791_buildGRCh38.tsv'

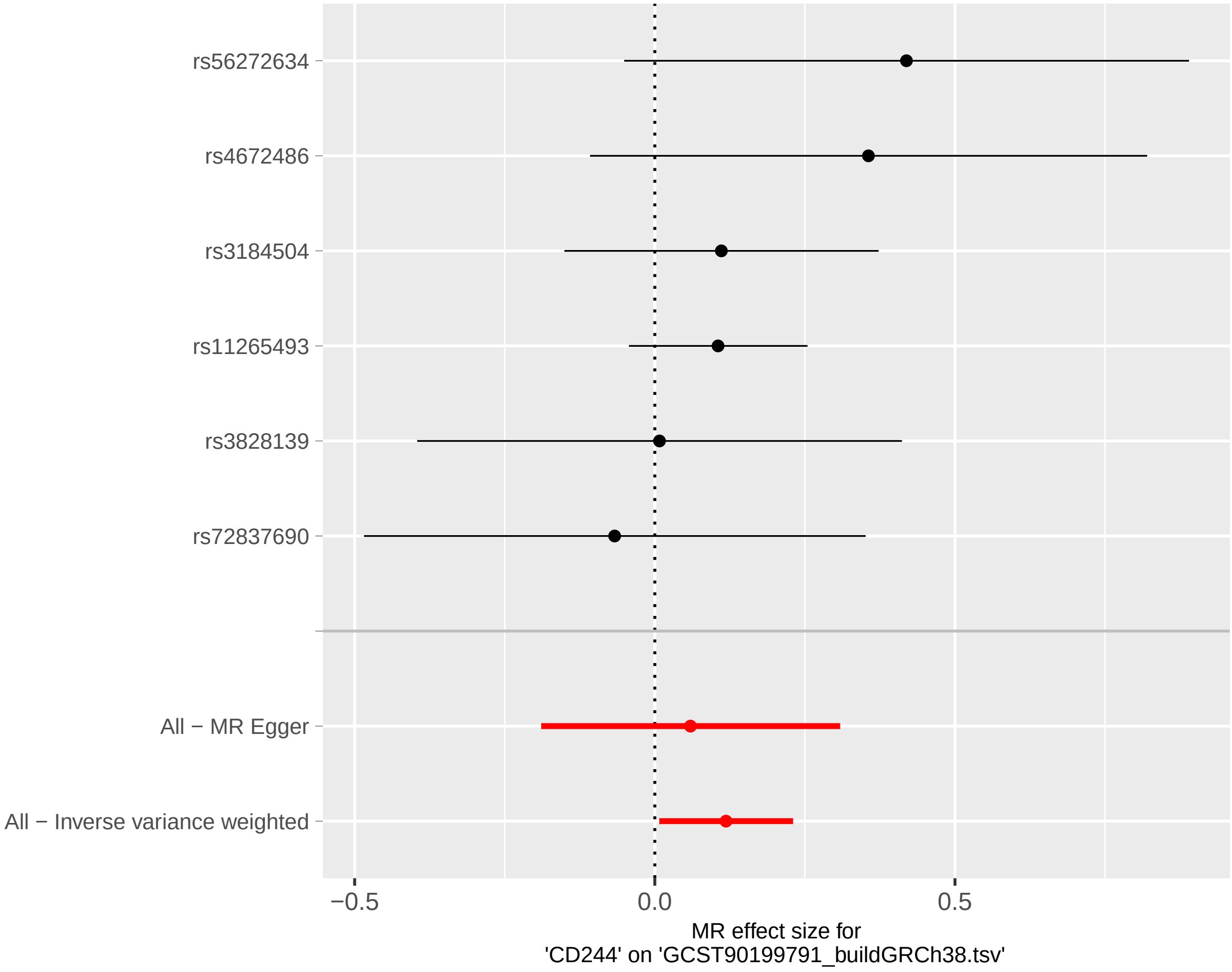
rs56272634
rs4672486
rs3184504
rs11265493
rs3828139
rs72837690
All − MR Egger
All − Inverse variance weighted
−0.5
0.0
0.5
MR effect size for
'CD244' on 'GCST90199791_buildGRCh38.tsv'

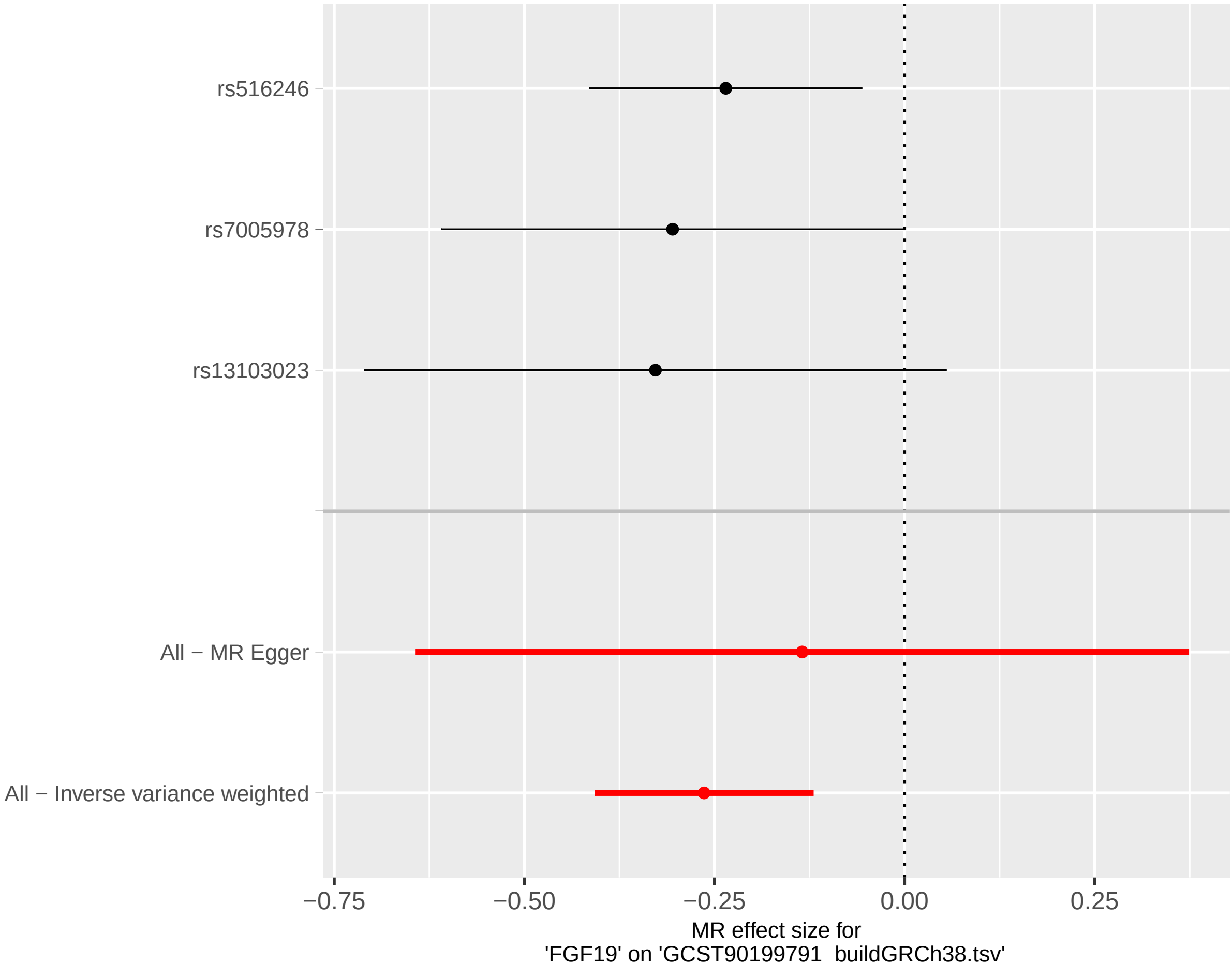

rs516246
rs7005978
rs13103023
All − MR Egger
All − Inverse variance weighted
−0.75
−0.50
−0.25
0.00
0.25
MR effect size for
'FGF19' on 'GCST90199791_buildGRCh38.tsv'

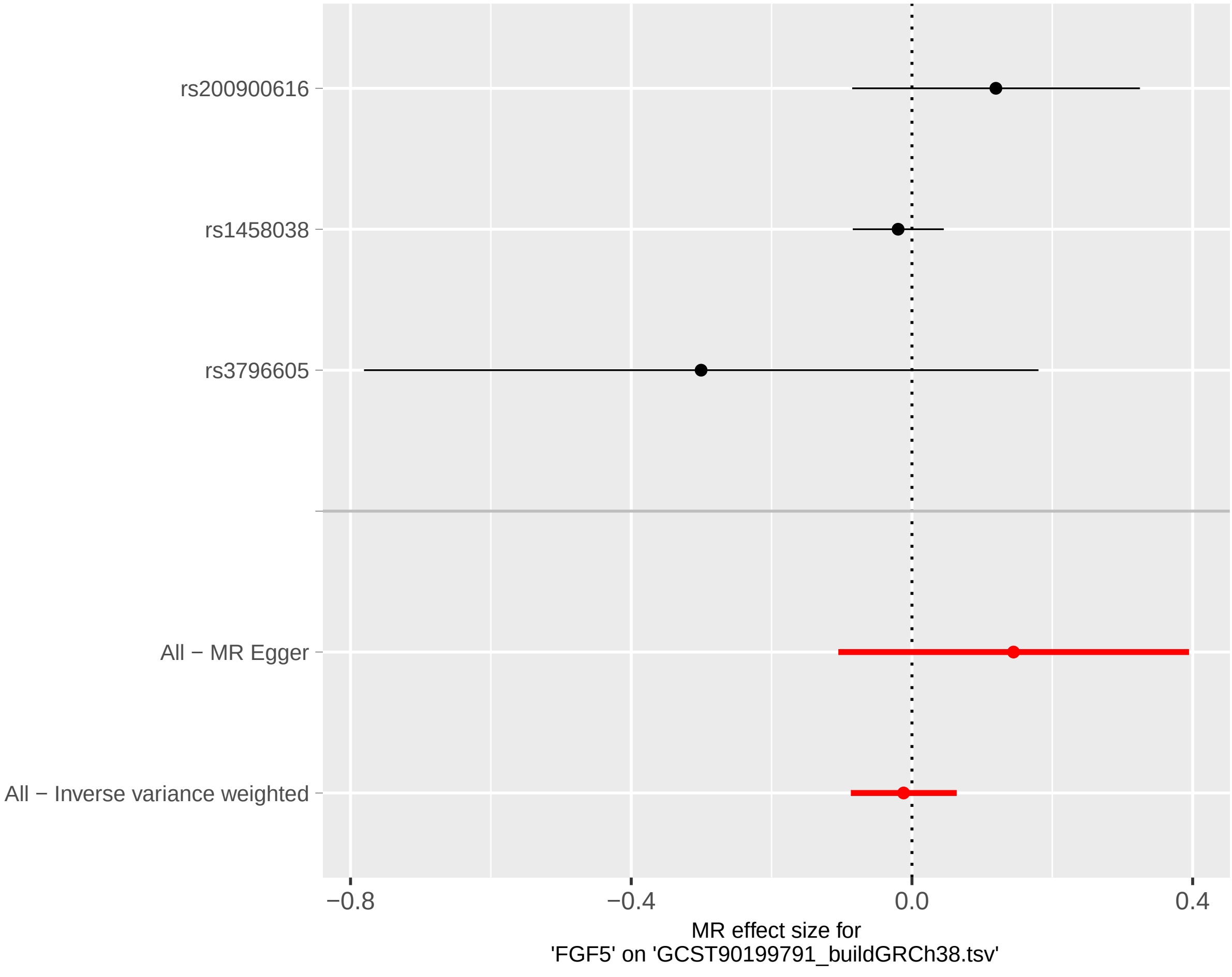
rs200900616
rs1458038
rs3796605
All − MR Egger
All − Inverse variance weighted
−0.8
−0.4
0.0
0.4
MR effect size for
'FGF5' on 'GCST90199791_buildGRCh38.tsv'

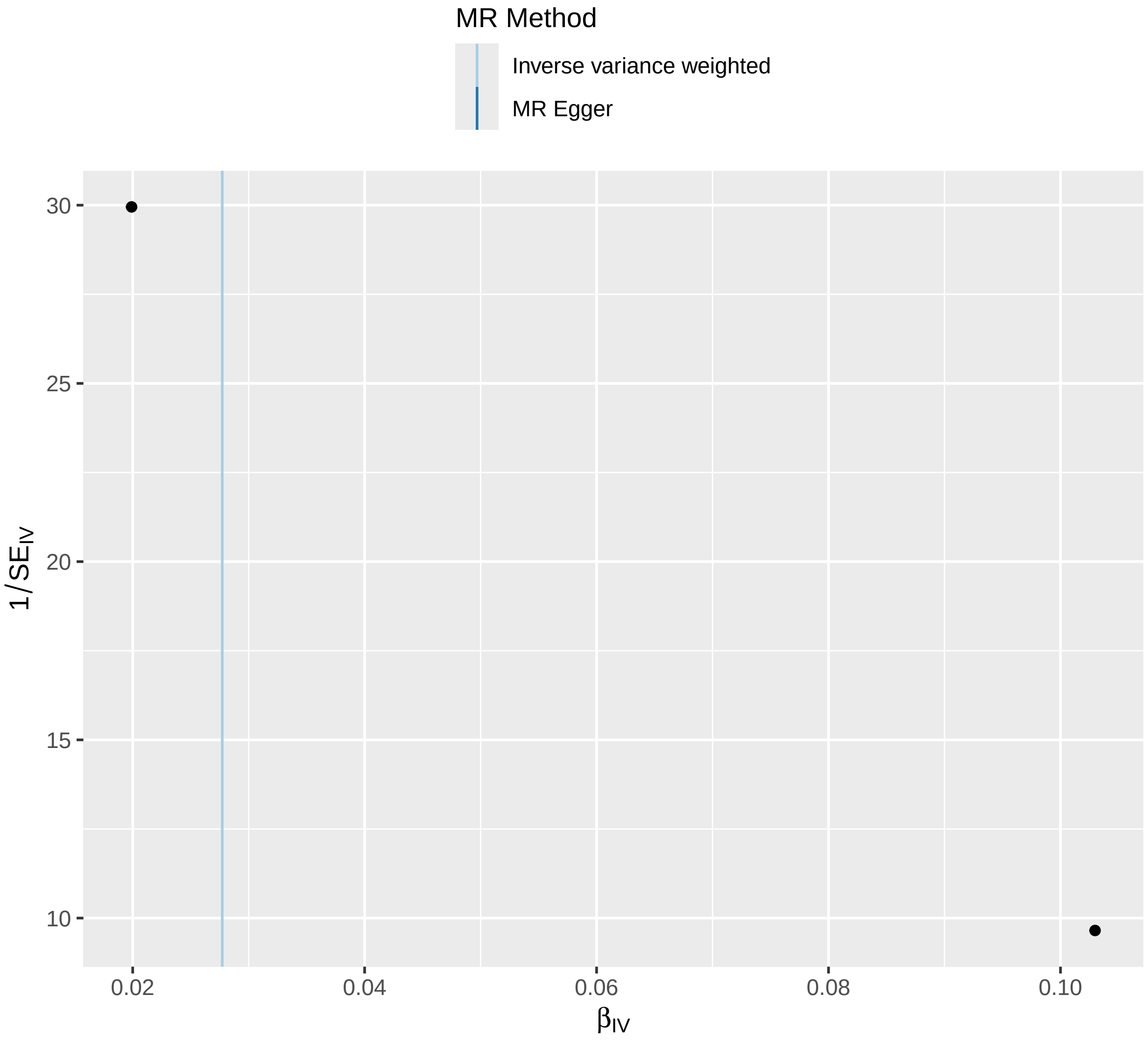

MR Method
Inverse variance weighted
MR Egger
30
25
20
15
10
0.02
0.04
0.06
0.08
0.10
1/SE_IV
β_IV

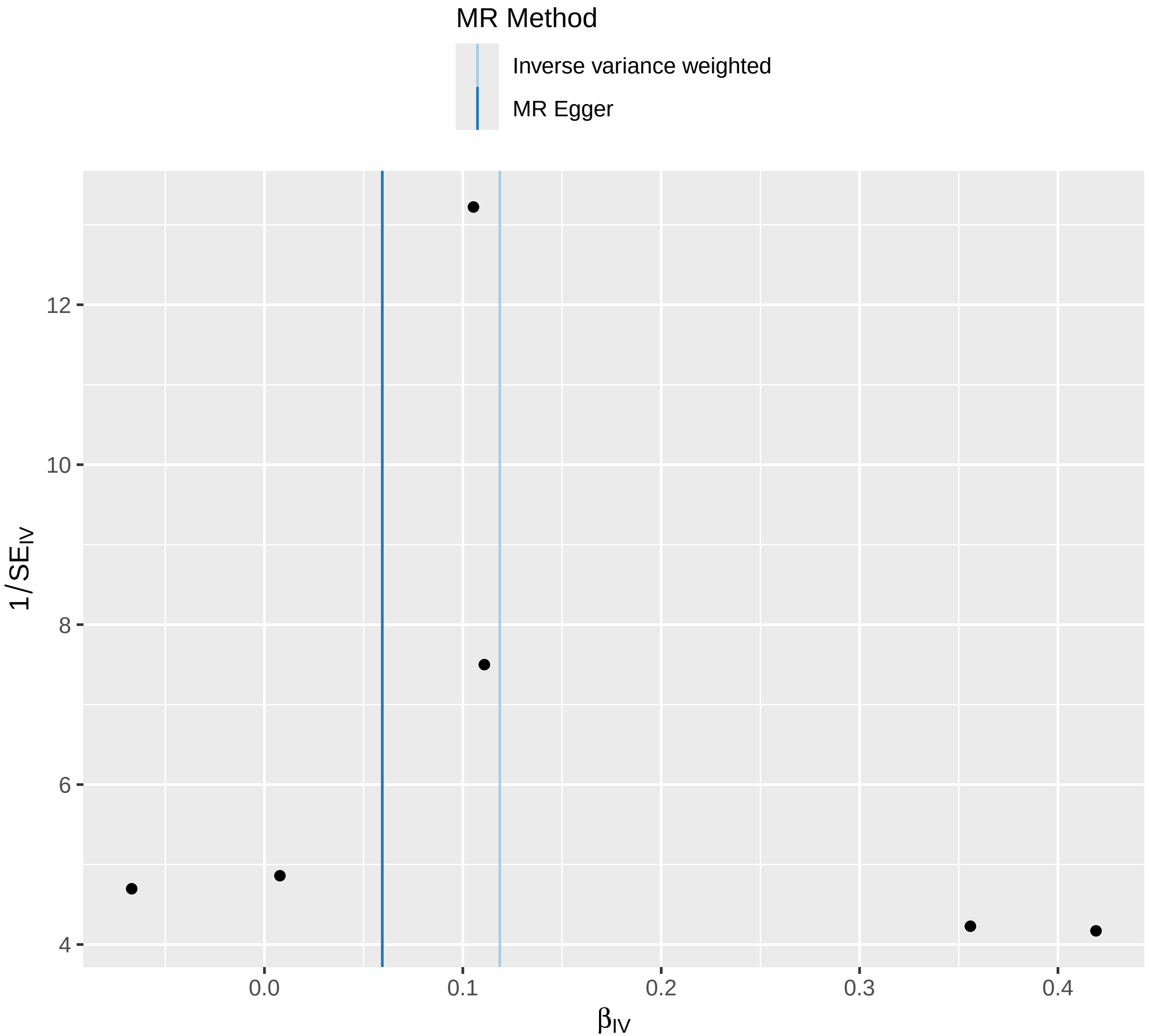

MR Method
Inverse variance weighted
MR Egger
12
10
8
6
4
1/SE_IV
0.0
0.1
0.2
0.3
0.4
β_IV

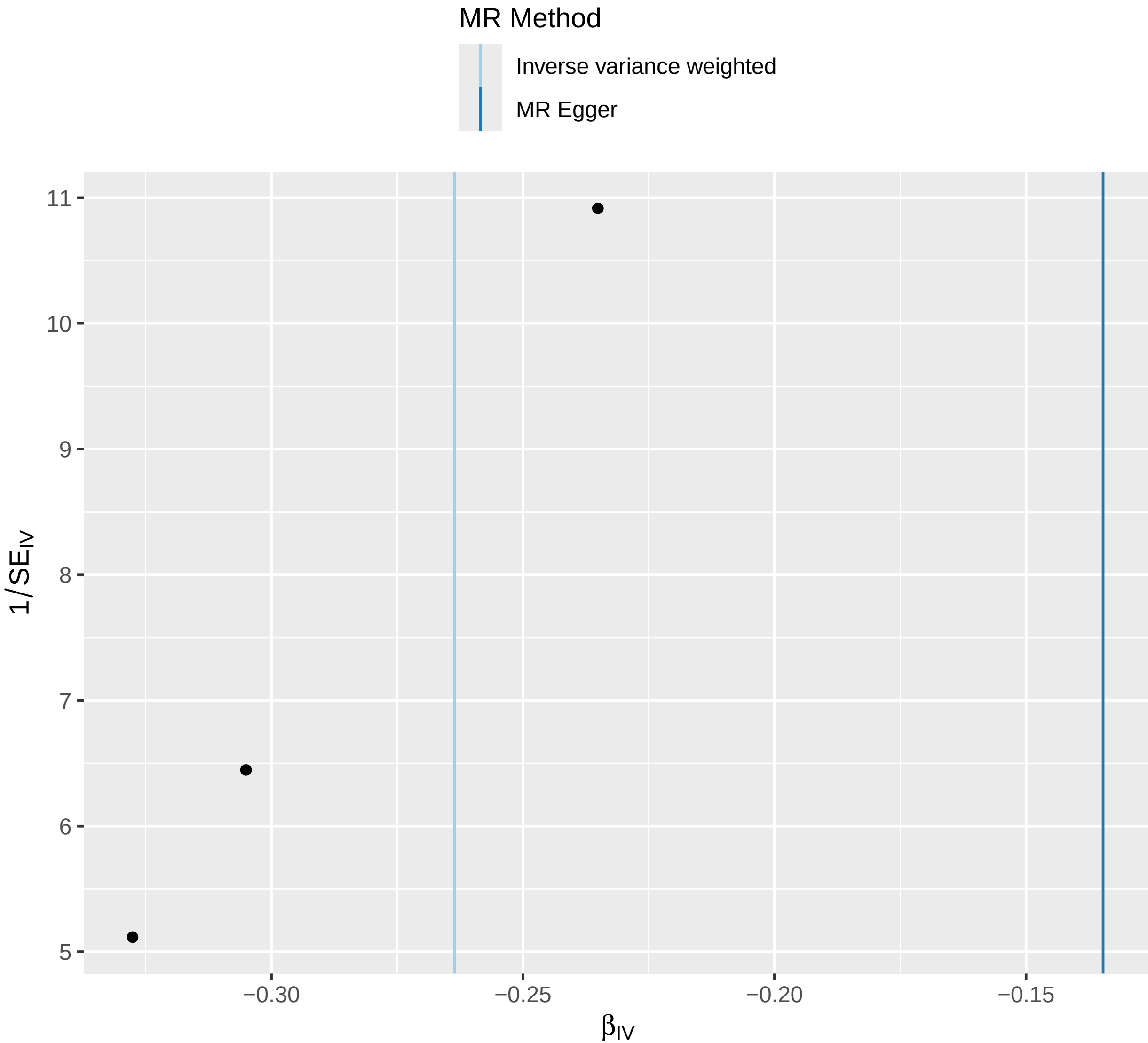
MR Method
Inverse variance weighted
MR Egger
11
10
9
8
7
6
5
1/SE$_{IV}$
−0.30
−0.25
−0.20
−0.15
β$_{IV}$

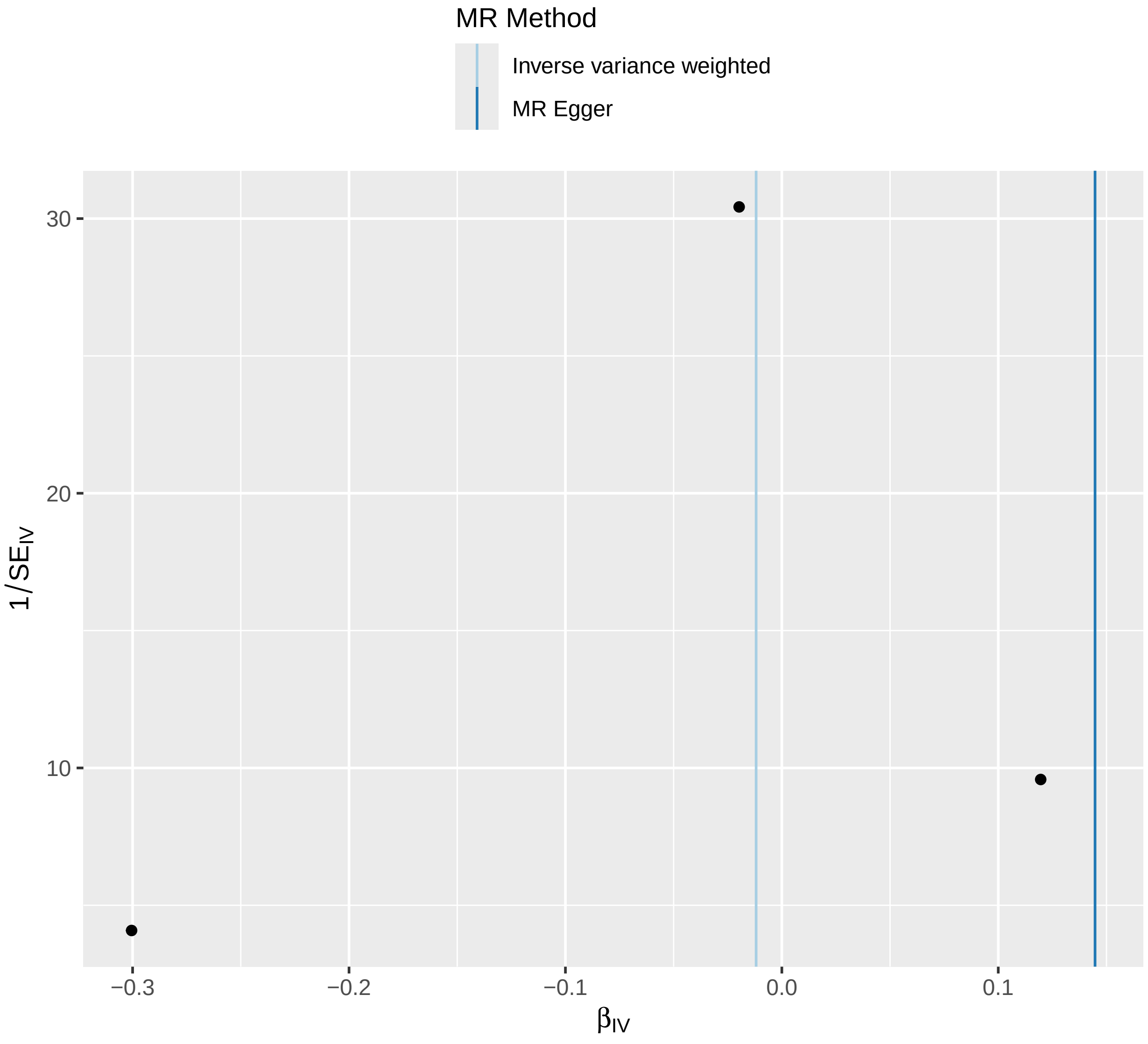
MR Method
Inverse variance weighted
MR Egger
30
20
10
1/SE_IV
−0.3
−0.2
−0.1
0.0
0.1
β_IV

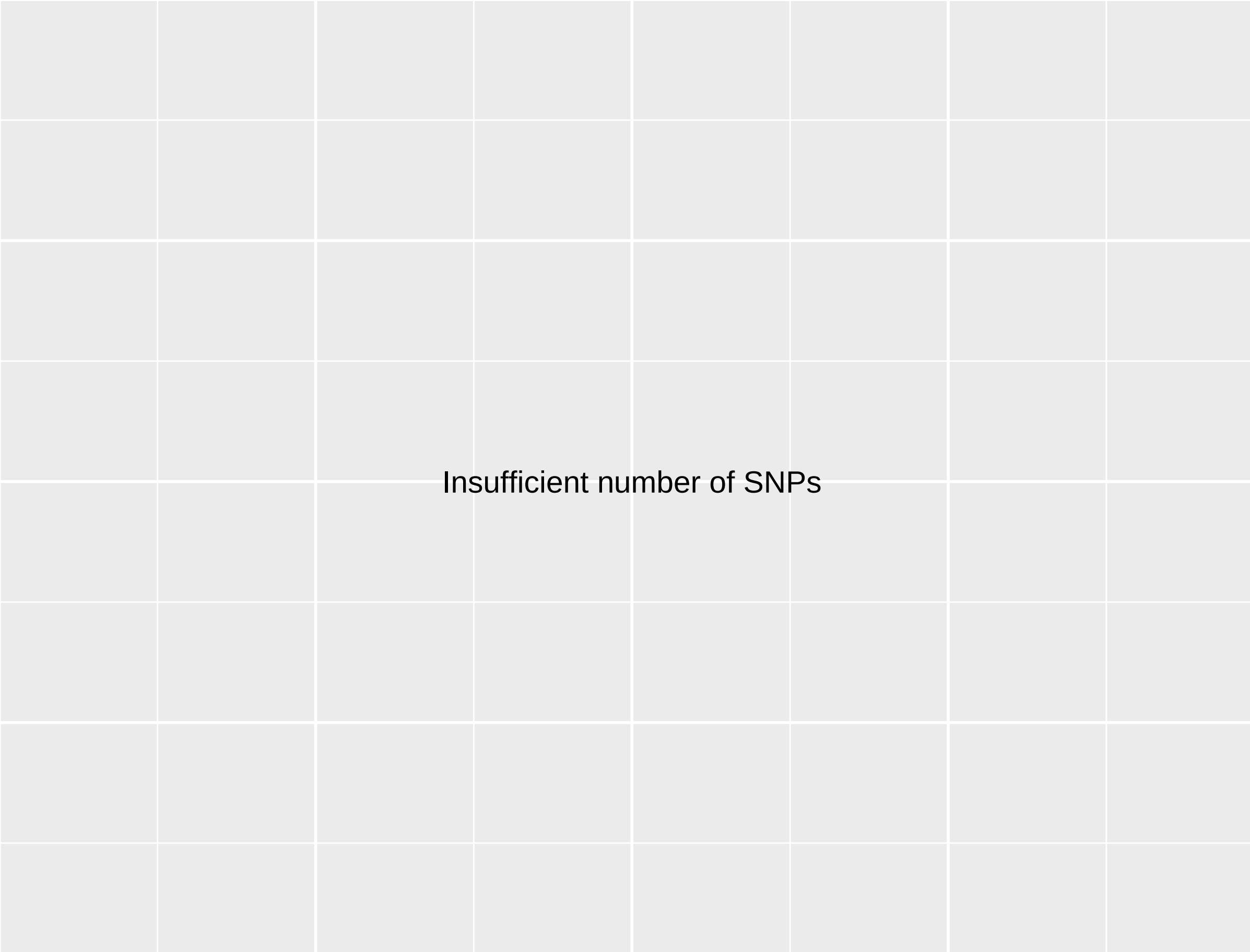
Insufficient number of SNPs

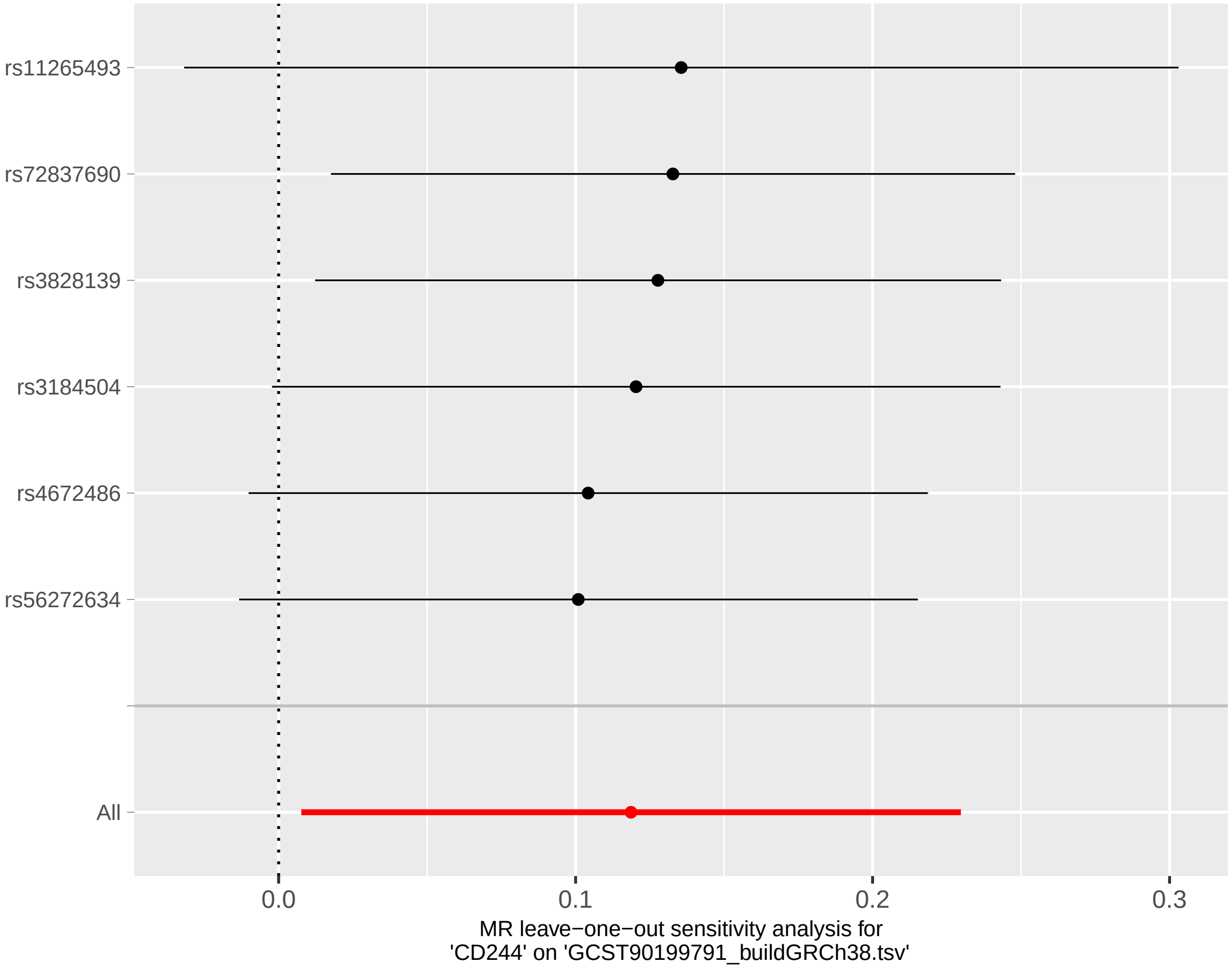
rs11265493
rs72837690
rs3828139
rs3184504
rs4672486
rs56272634
All
0.0
0.1
0.2
0.3
MR leave−one−out sensitivity analysis for
'CD244' on 'GCST90199791_buildGRCh38.tsv'

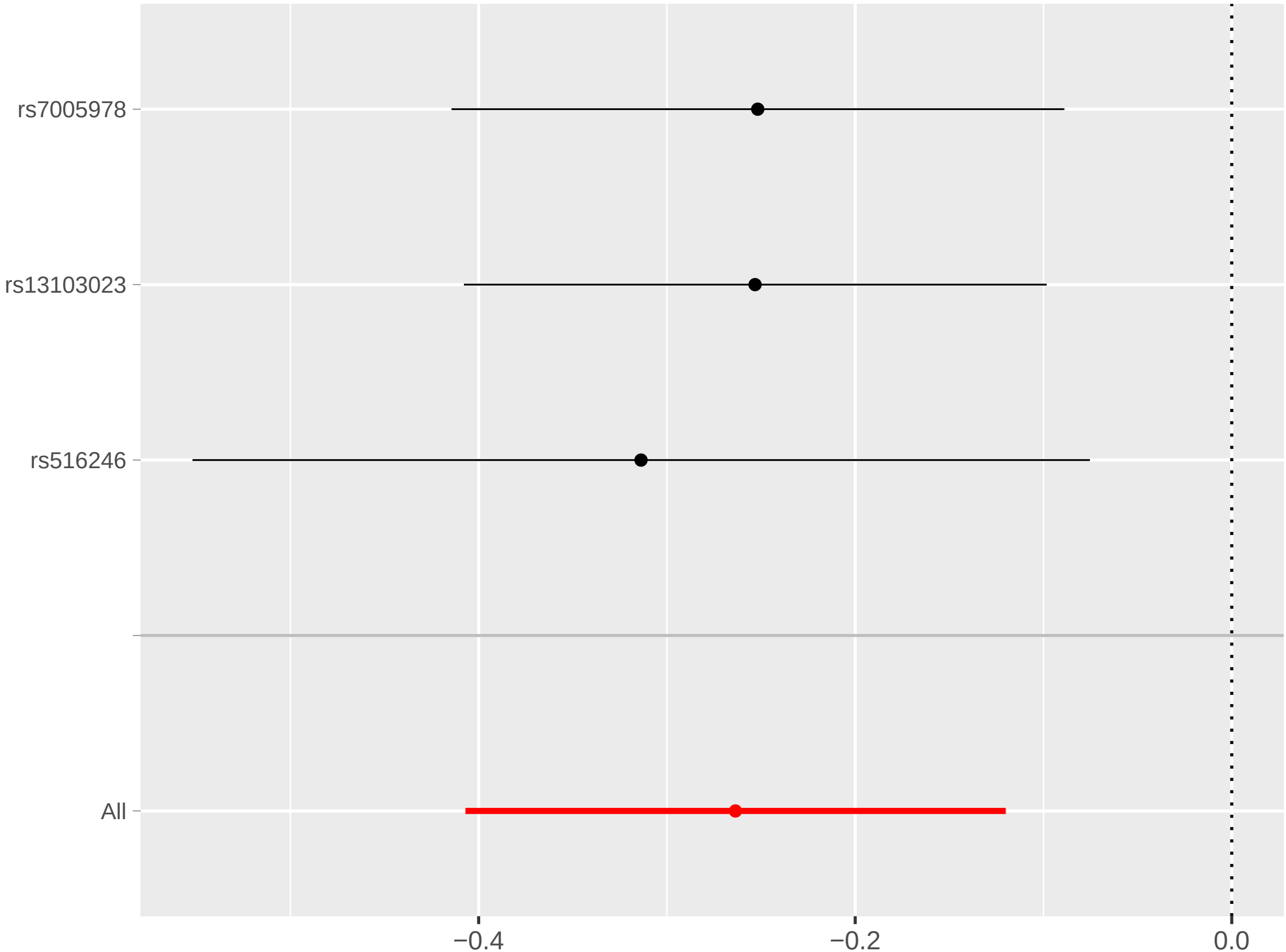

MR leave−one−out sensitivity analysis for
'FGF19' on 'GCST90199791_buildGRCh38.tsv'

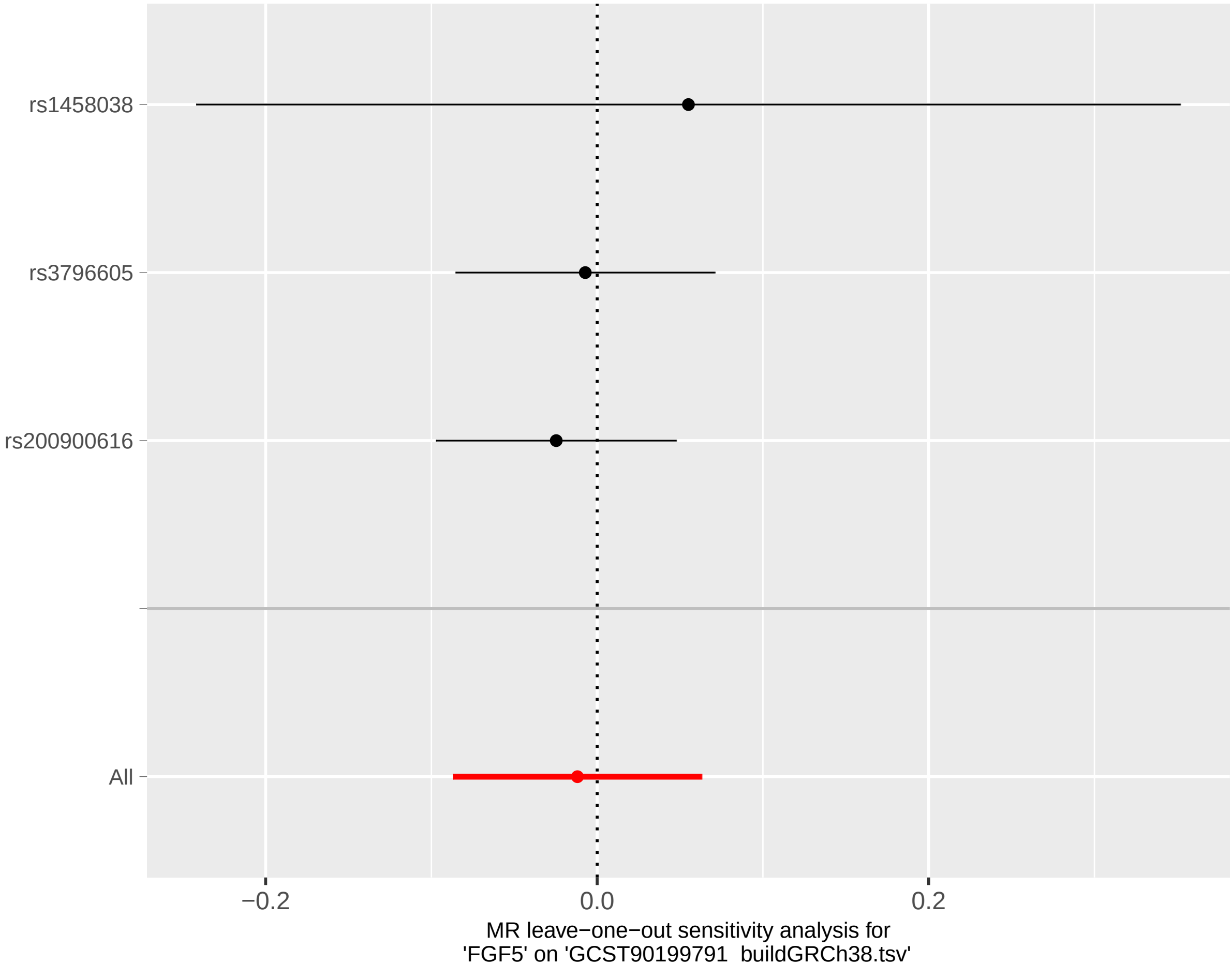
rs1458038
rs3796605
rs200900616
All
−0.2
0.0
0.2
MR leave−one−out sensitivity analysis for
'FGF5' on 'GCST90199791_buildGRCh38.tsv'

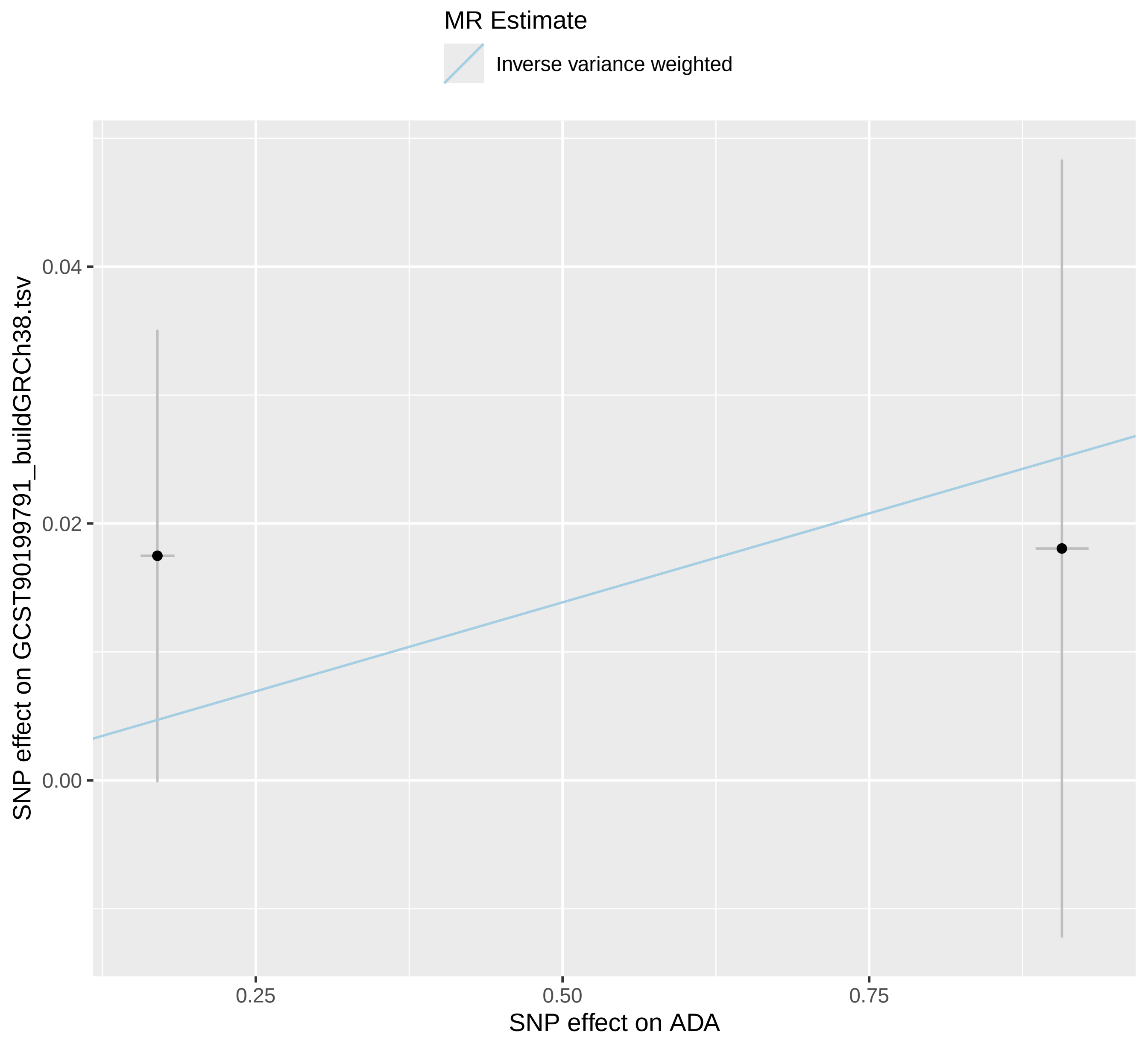
MR Estimate
Inverse variance weighted
0.04
0.02
0.00
0.25
0.50
0.75
SNP effect on ADA
SNP effect on GCST90199791_buildGRCh38.tsv

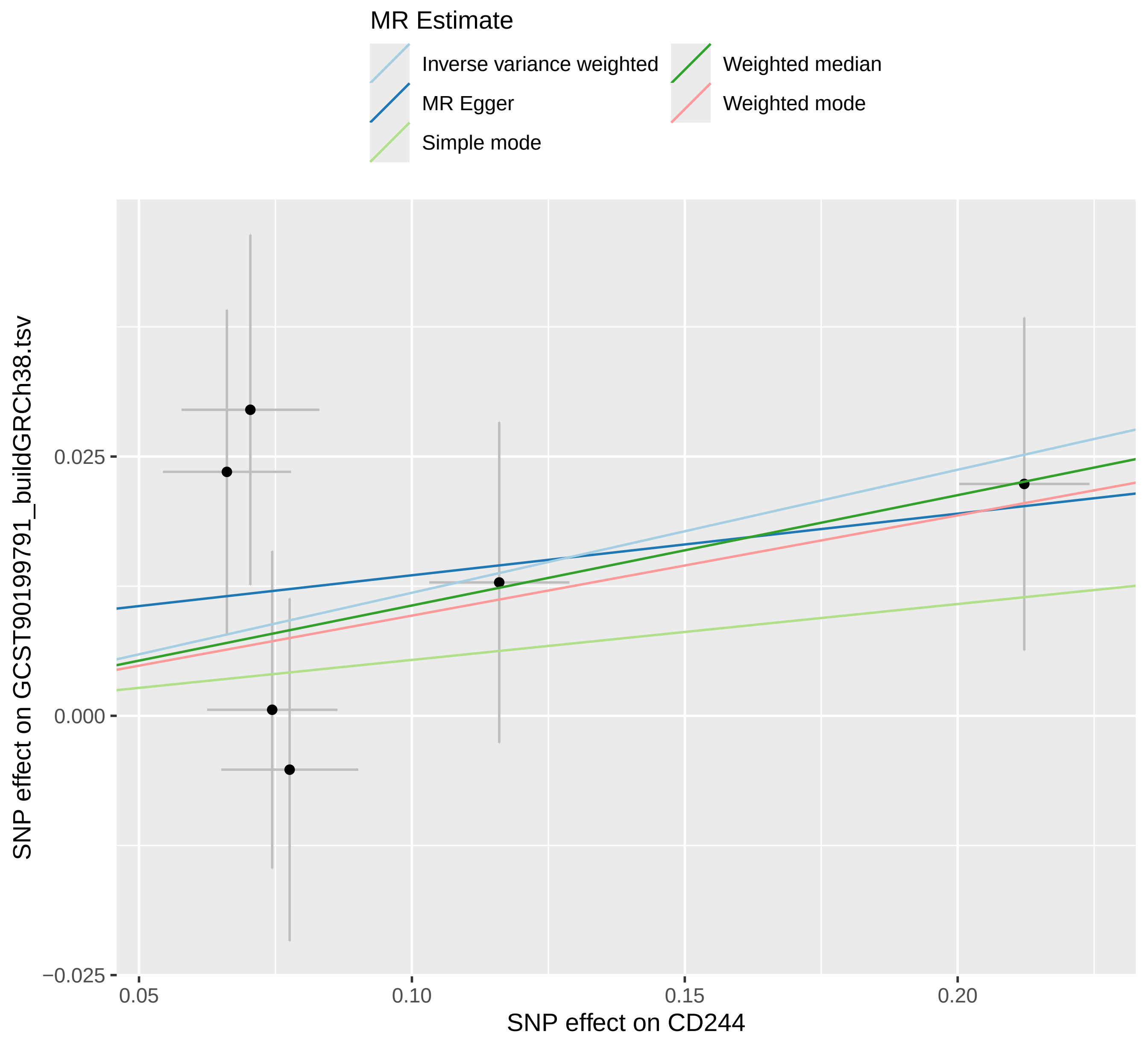
MR Estimate
Inverse variance weighted
MR Egger
Simple mode
Weighted median
Weighted mode
SNP effect on GCST90199791_buildGRCh38.tsv
0.025
0.000
−0.025
0.05
0.10
0.15
0.20
SNP effect on CD244

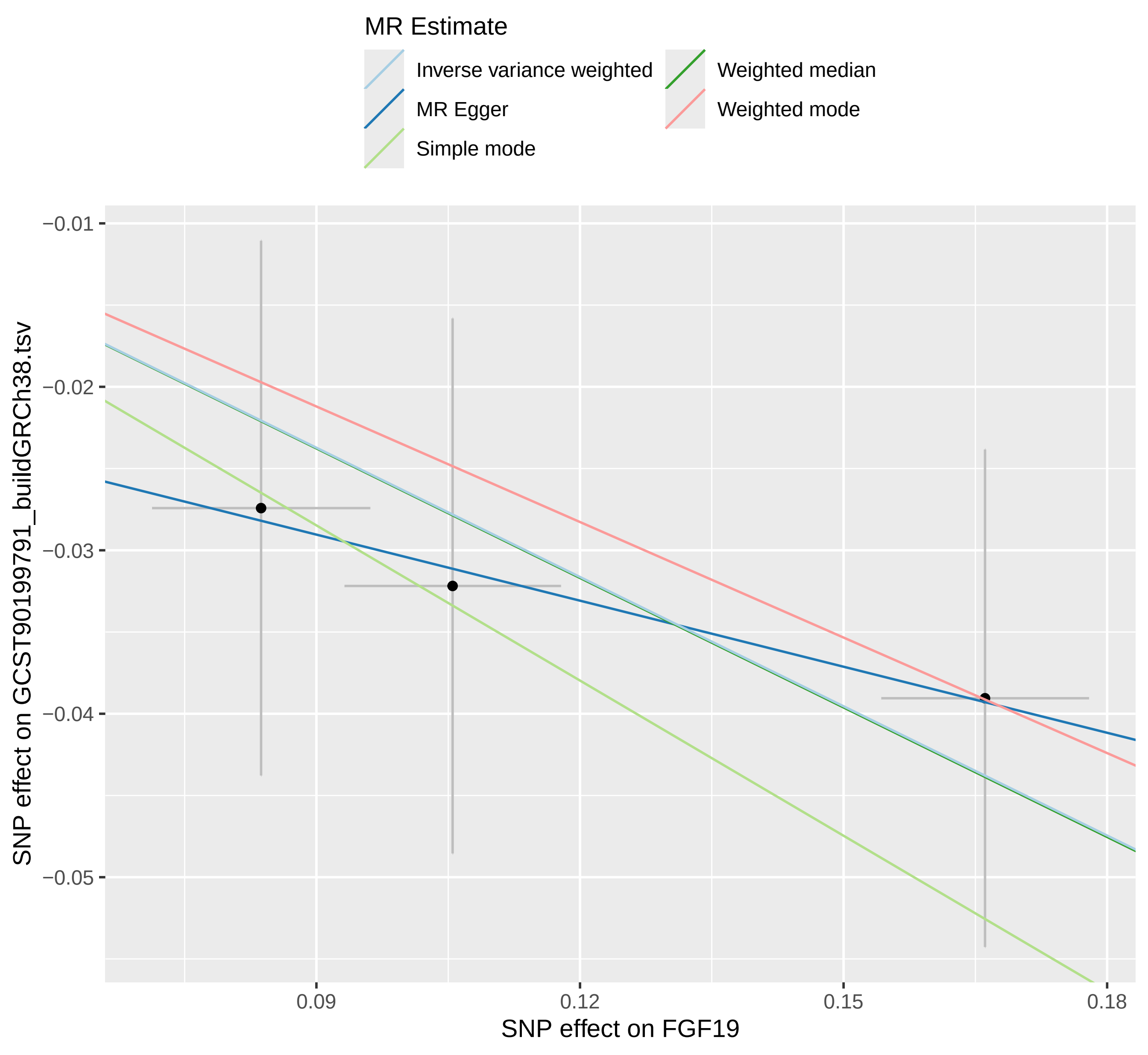

MR Estimate
Inverse variance weighted
MR Egger
Simple mode
Weighted median
Weighted mode
−0.01
−0.02
−0.03
−0.04
−0.05
0.09
0.12
0.15
0.18
SNP effect on GCST90199791_buildGRCh38.tsv
SNP effect on FGF19

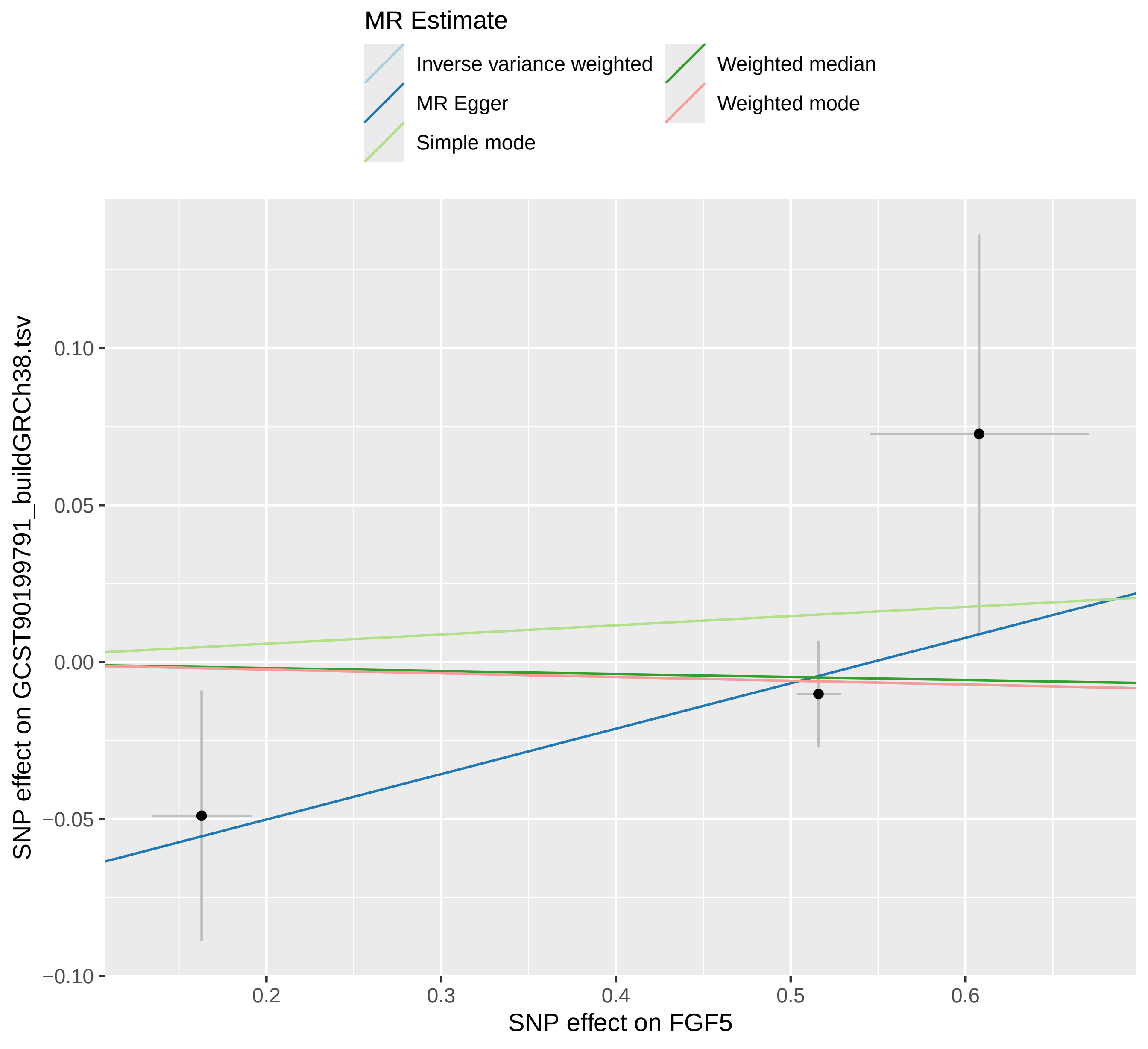

MR Estimate
Inverse variance weighted
MR Egger
Simple mode
Weighted median
Weighted mode
0.10
0.05
0.00
−0.05
−0.10
0.2
0.3
0.4
0.5
0.6
SNP effect on GCST90199791_buildGRCh38.tsv
SNP effect on FGF5

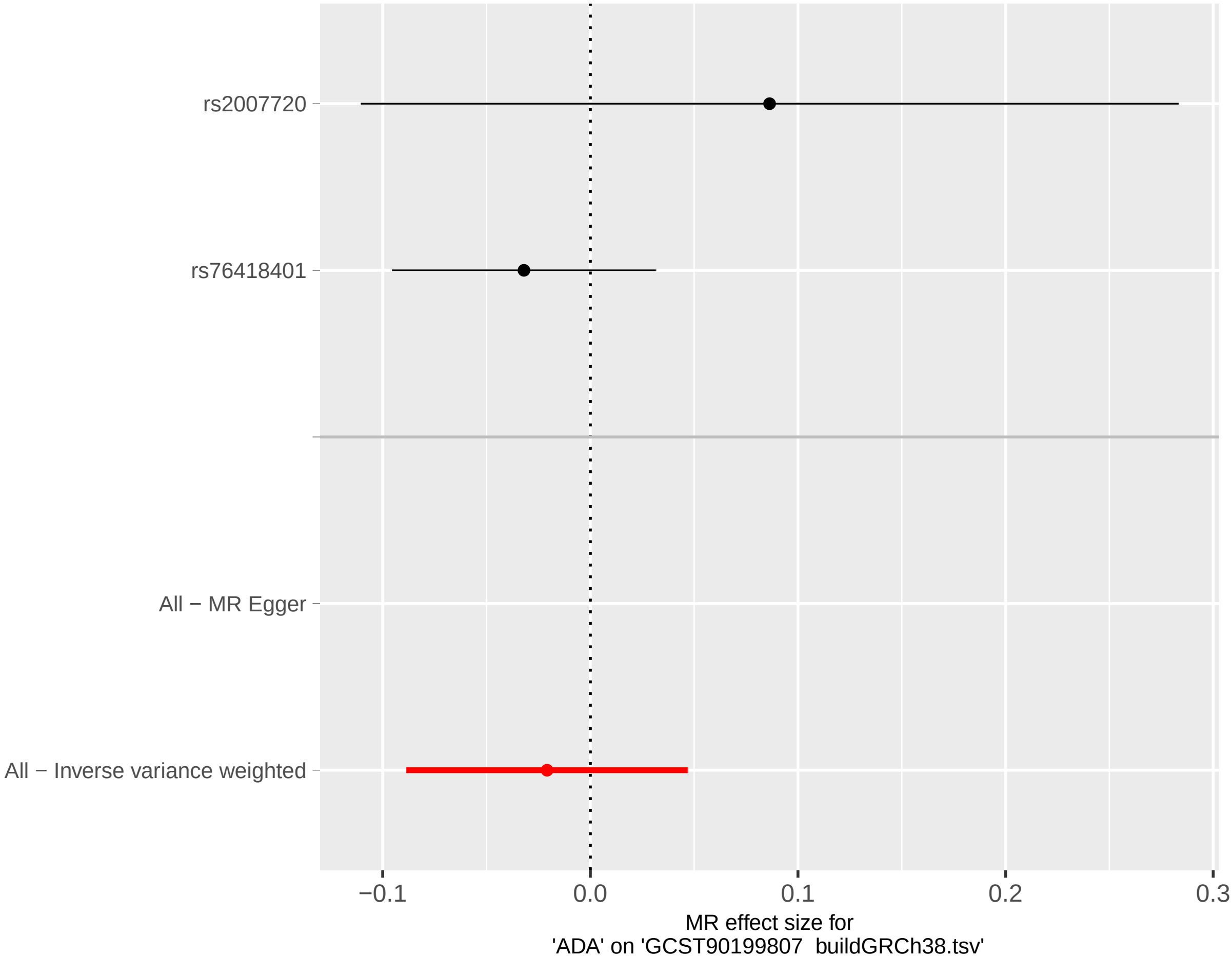
rs2007720
rs76418401
All − MR Egger
All − Inverse variance weighted
−0.1
0.0
0.1
0.2
0.3
MR effect size for
'ADA' on 'GCST90199807_buildGRCh38.tsv'

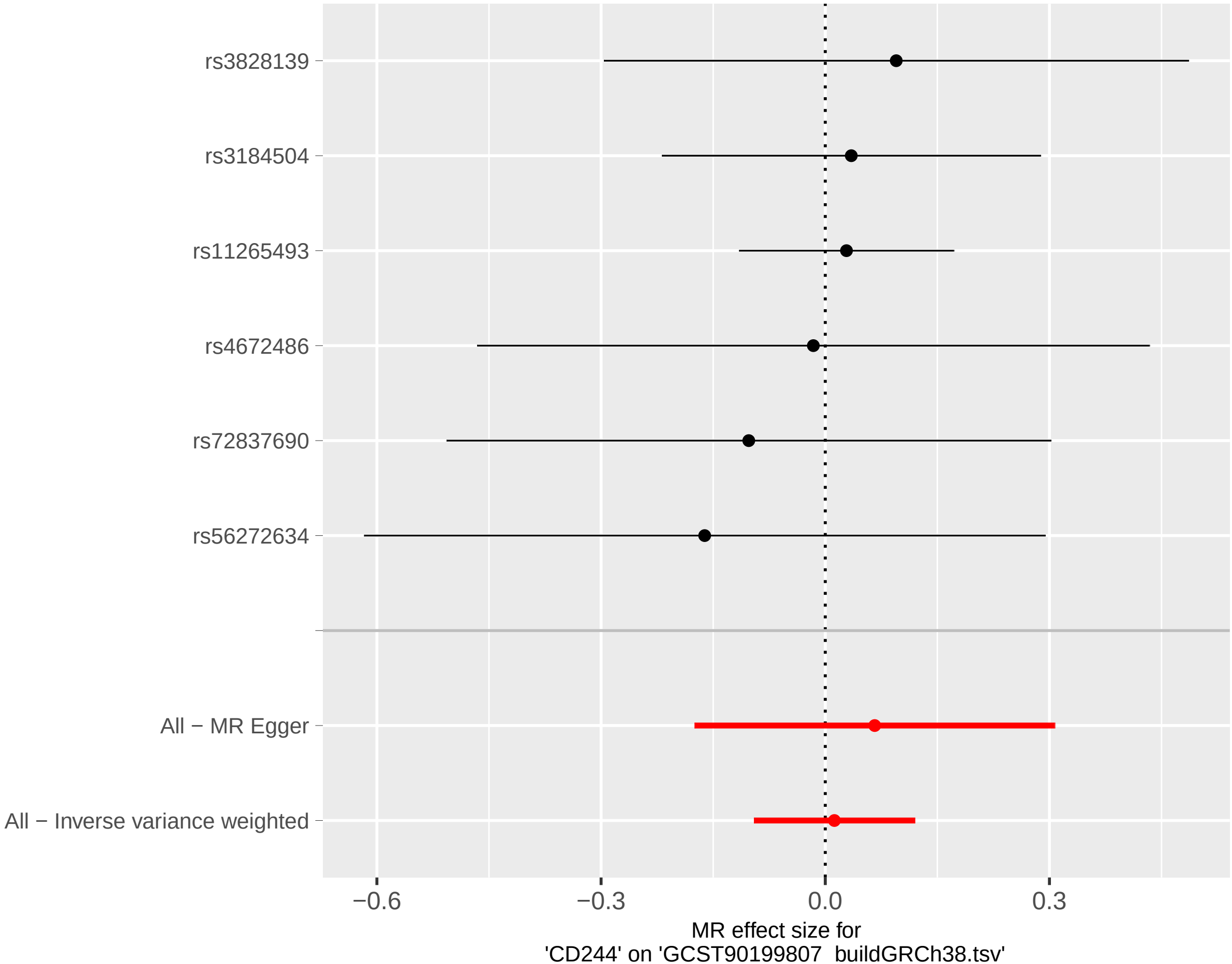
rs3828139
rs3184504
rs11265493
rs4672486
rs72837690
rs56272634
All − MR Egger
All − Inverse variance weighted
−0.6
−0.3
0.0
0.3
MR effect size for
'CD244' on 'GCST90199807_buildGRCh38.tsv'

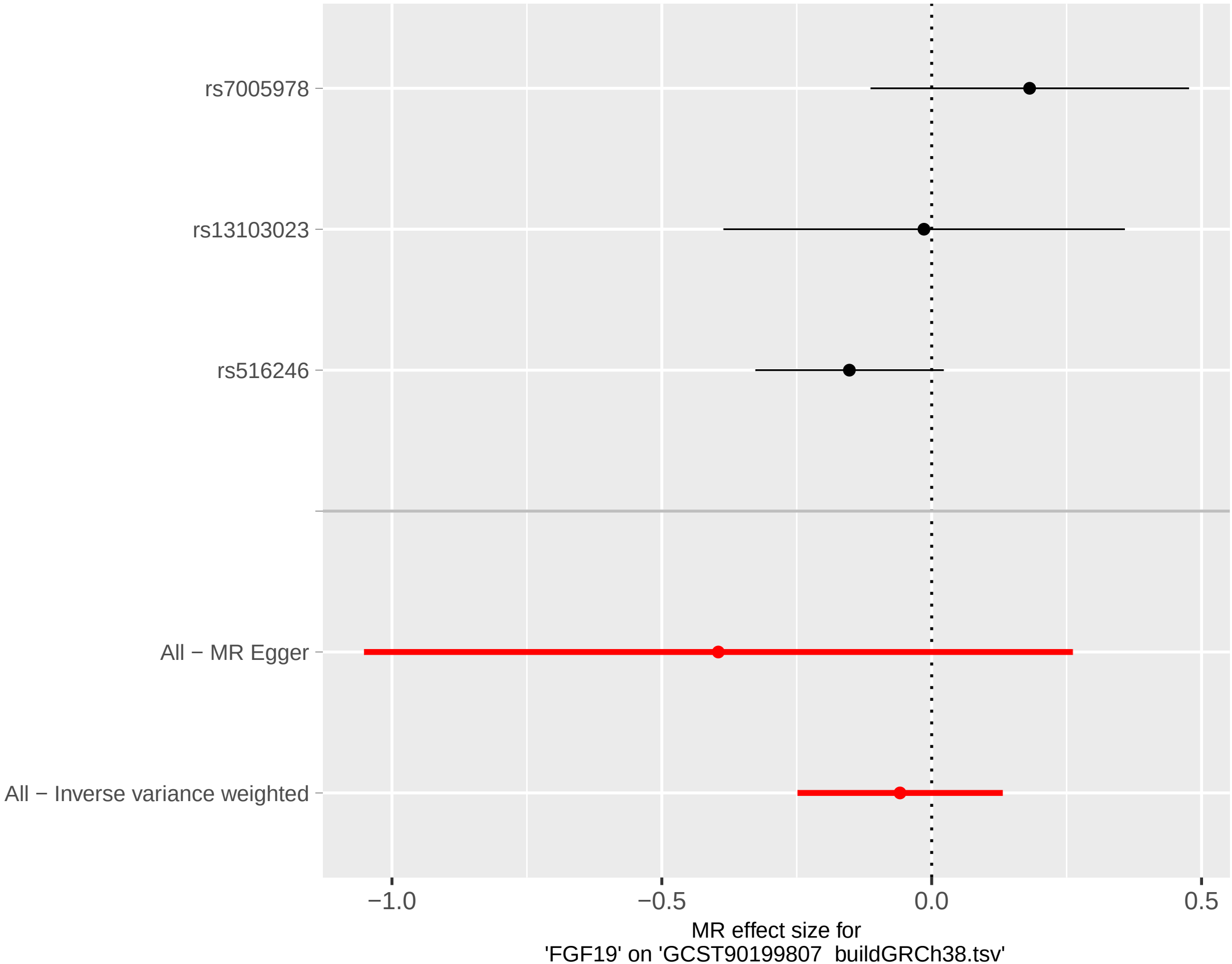
rs7005978
rs13103023
rs516246
All − MR Egger
All − Inverse variance weighted
−1.0
−0.5
0.0
0.5
MR effect size for
'FGF19' on 'GCST90199807_buildGRCh38.tsv'

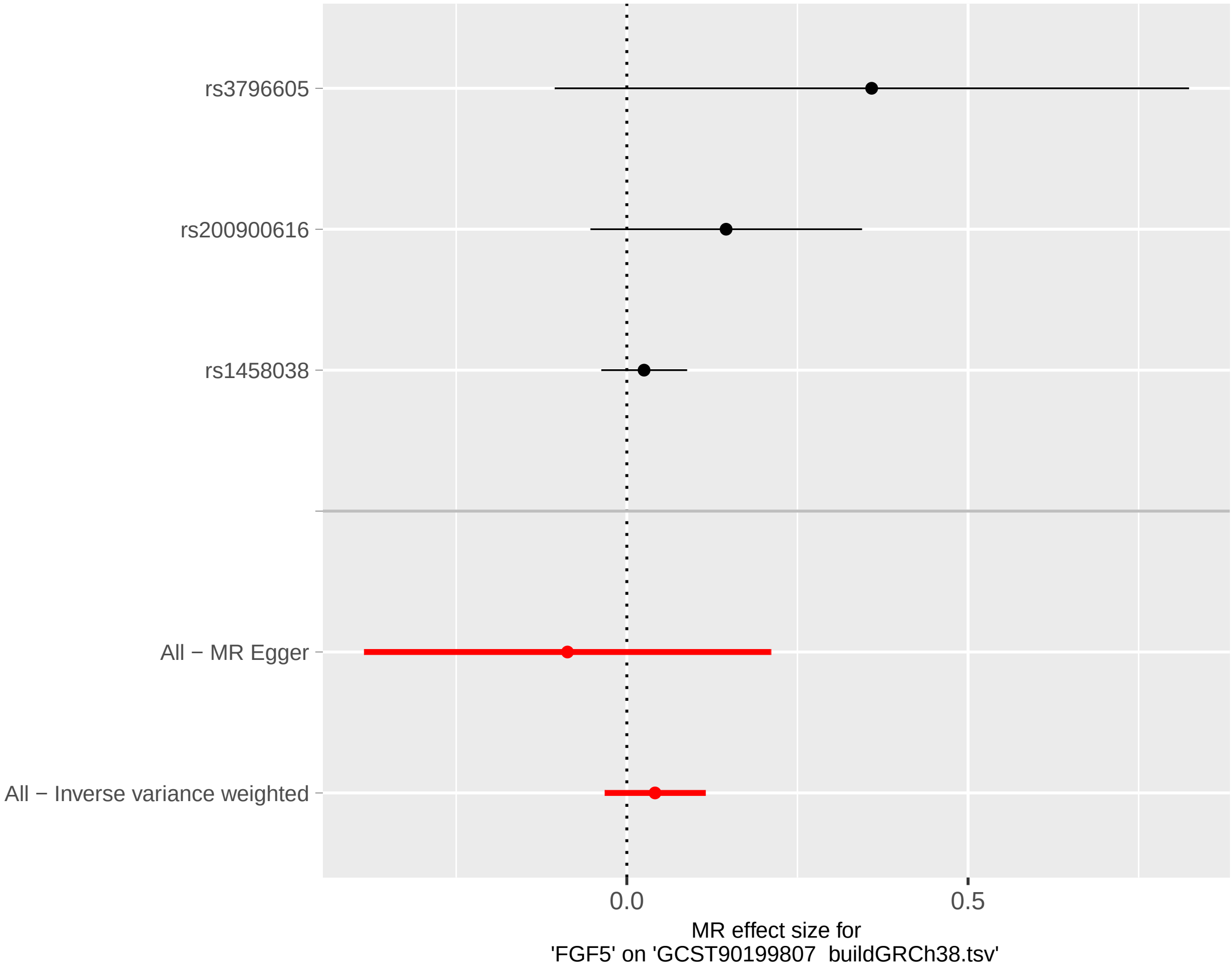

rs3796605
rs200900616
rs1458038
All − MR Egger
All − Inverse variance weighted
0.0
0.5
MR effect size for
'FGF5' on 'GCST90199807_buildGRCh38.tsv'

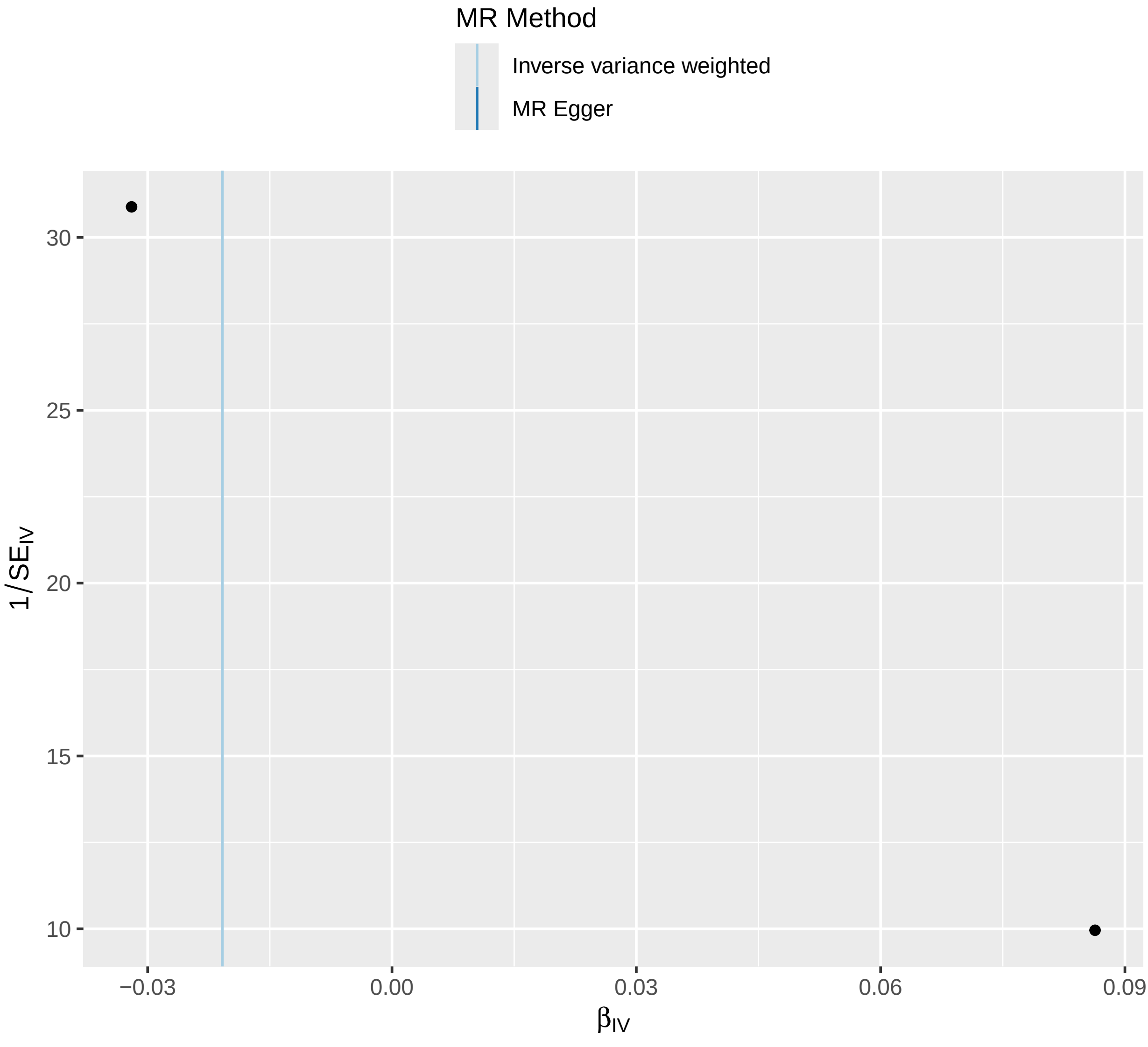
MR Method
Inverse variance weighted
MR Egger
30
25
20
15
10
−0.03
0.00
0.03
0.06
0.09
$1/SE_{IV}$
β_{IV}

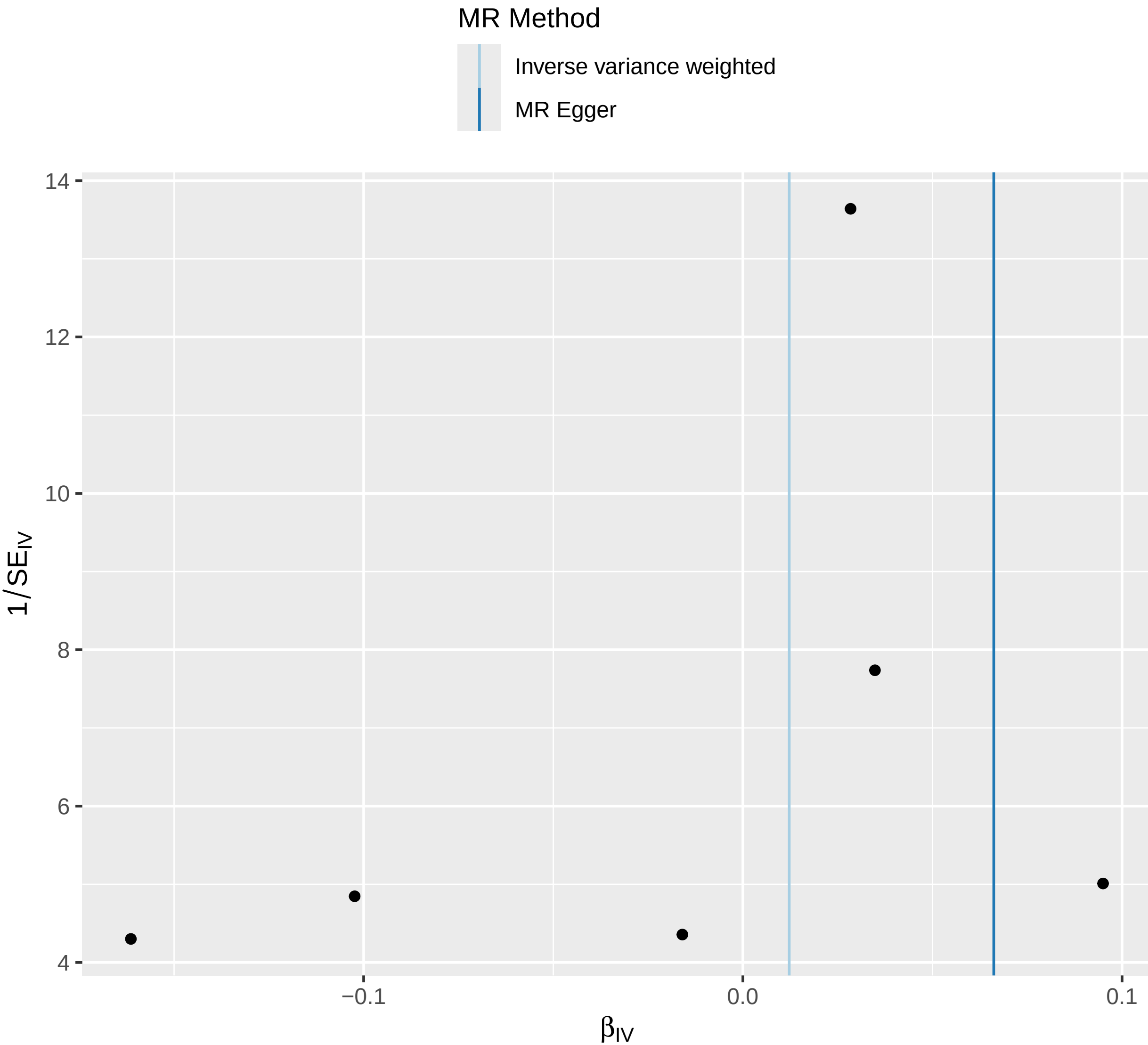
MR Method
Inverse variance weighted
MR Egger
14
12
10
8
6
4
1/SE_IV
−0.1
0.0
0.1
β_IV

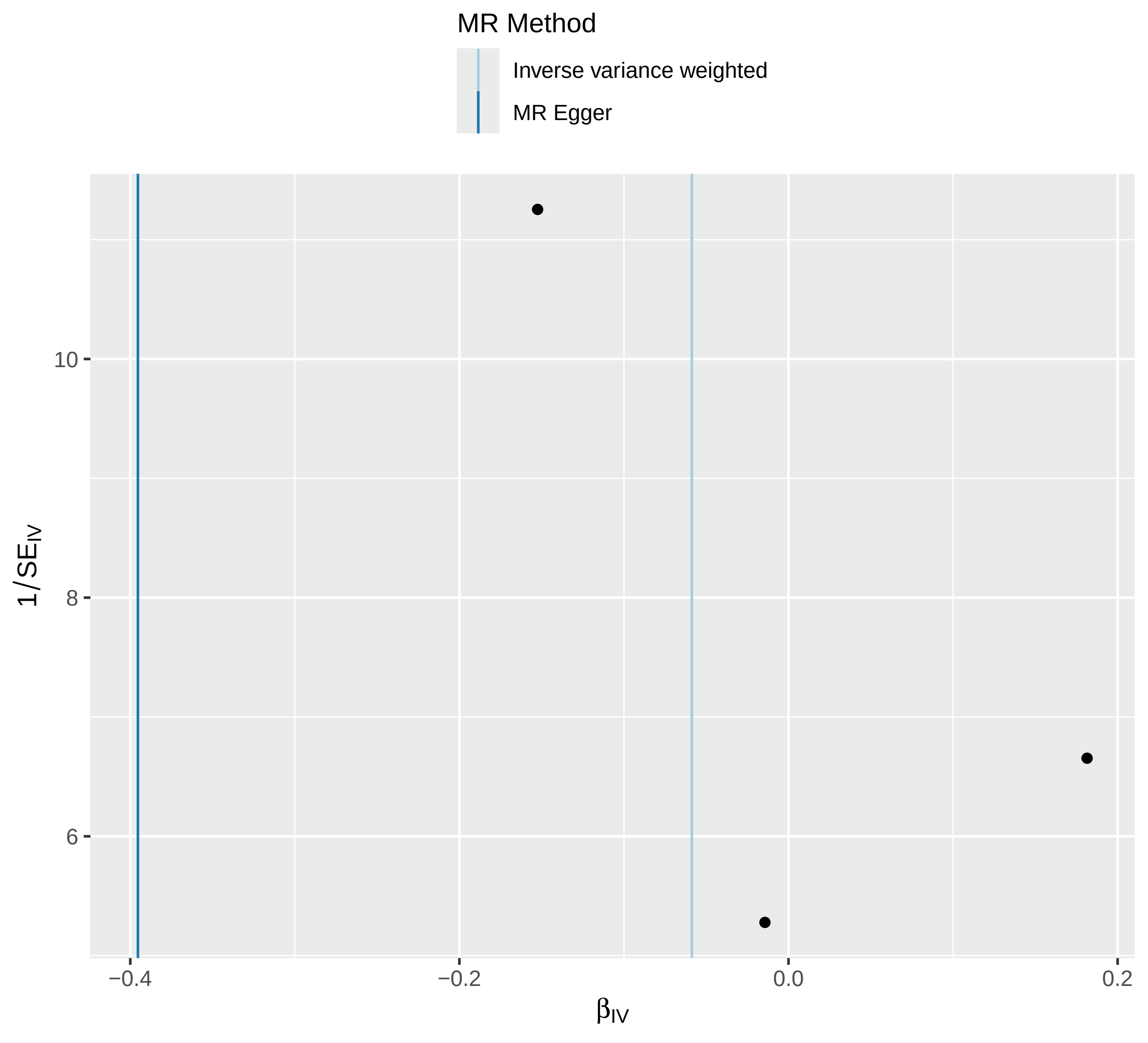

MR Method
Inverse variance weighted
MR Egger
10
8
6
1/SE$_{IV}$
−0.4
−0.2
0.0
0.2
β_{IV}

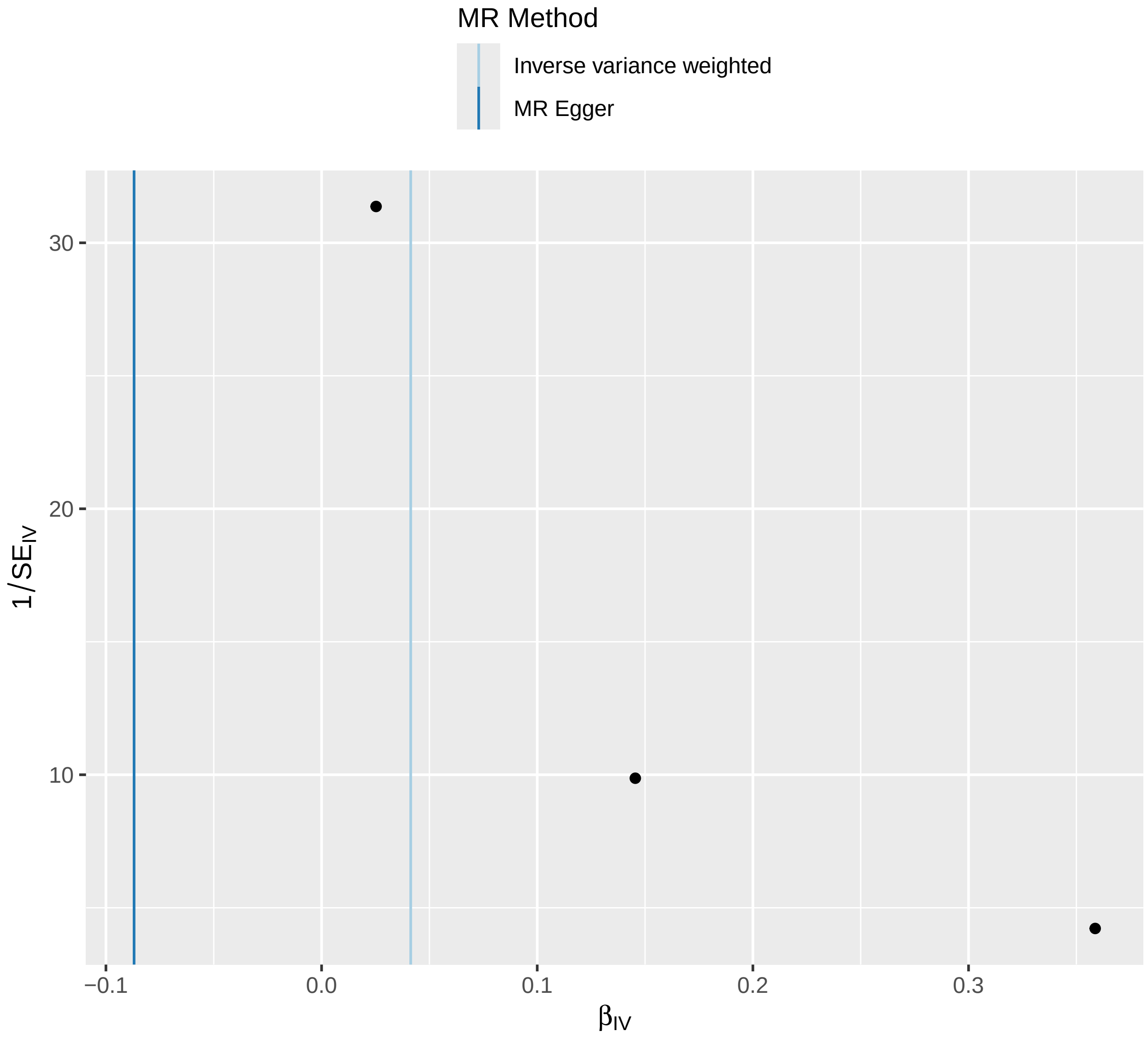
MR Method
Inverse variance weighted
MR Egger
30
20
10
1/SE_{IV}
−0.1
0.0
0.1
0.2
0.3
β_{IV}

Insufficient number of SNPs

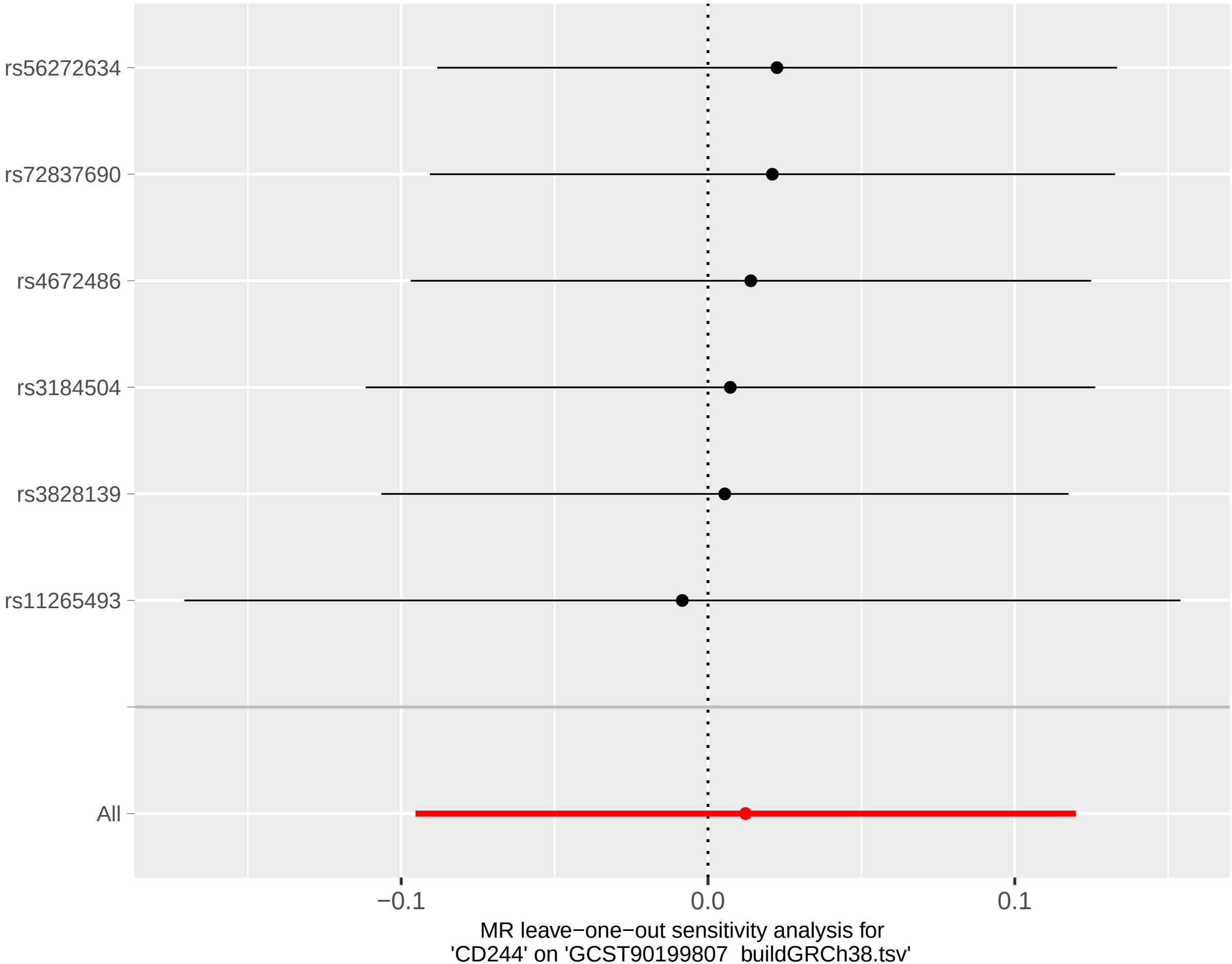
rs56272634
rs72837690
rs4672486
rs3184504
rs3828139
rs11265493
All
−0.1
0.0
0.1
MR leave−one−out sensitivity analysis for
'CD244' on 'GCST90199807_buildGRCh38.tsv'

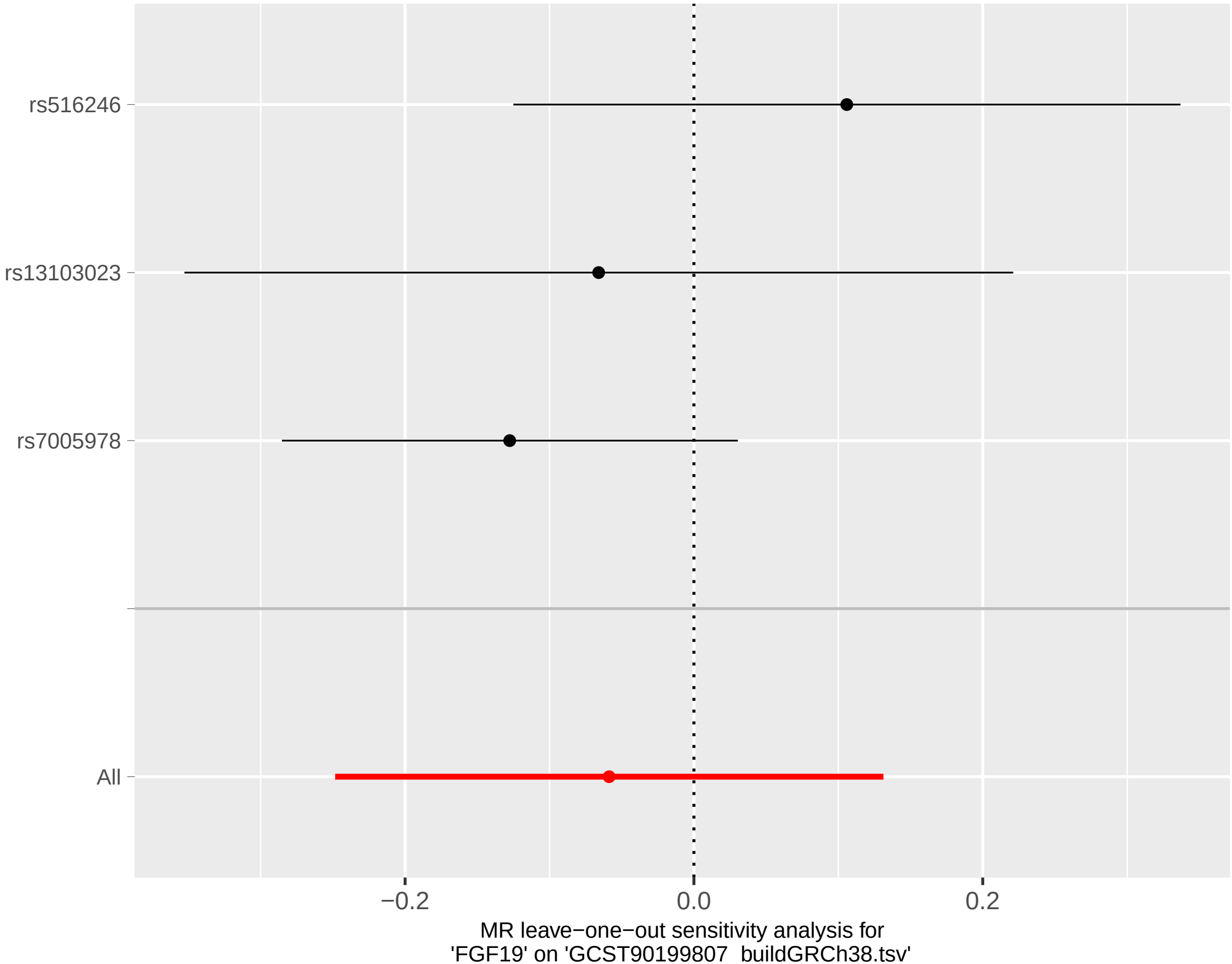
rs516246
rs13103023
rs7005978
All
−0.2
0.0
0.2
MR leave−one−out sensitivity analysis for
'FGF19' on 'GCST90199807_buildGRCh38.tsv'

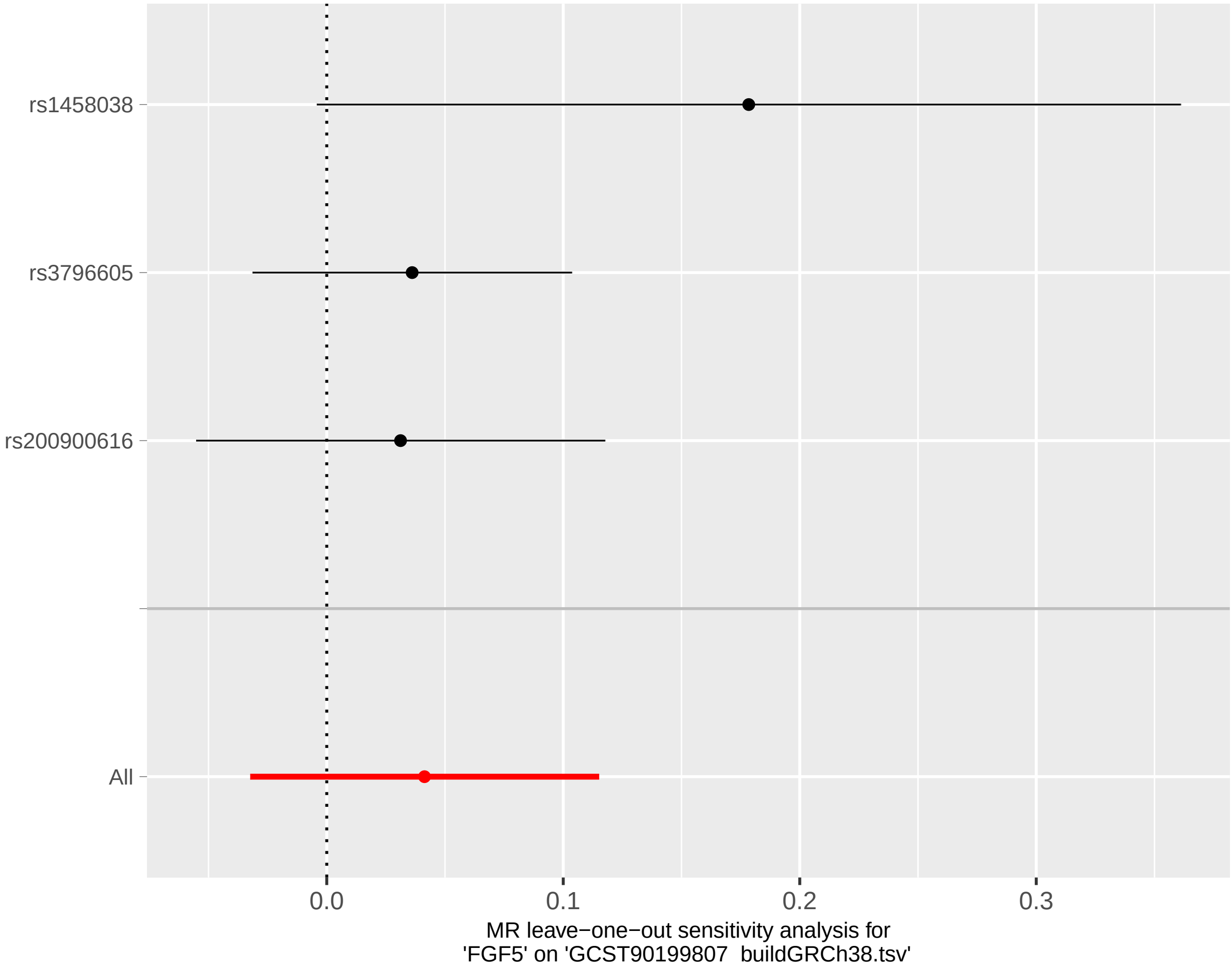
rs1458038
rs3796605
rs200900616
All
0.0
0.1
0.2
0.3
MR leave−one−out sensitivity analysis for
'FGF5' on 'GCST90199807_buildGRCh38.tsv'

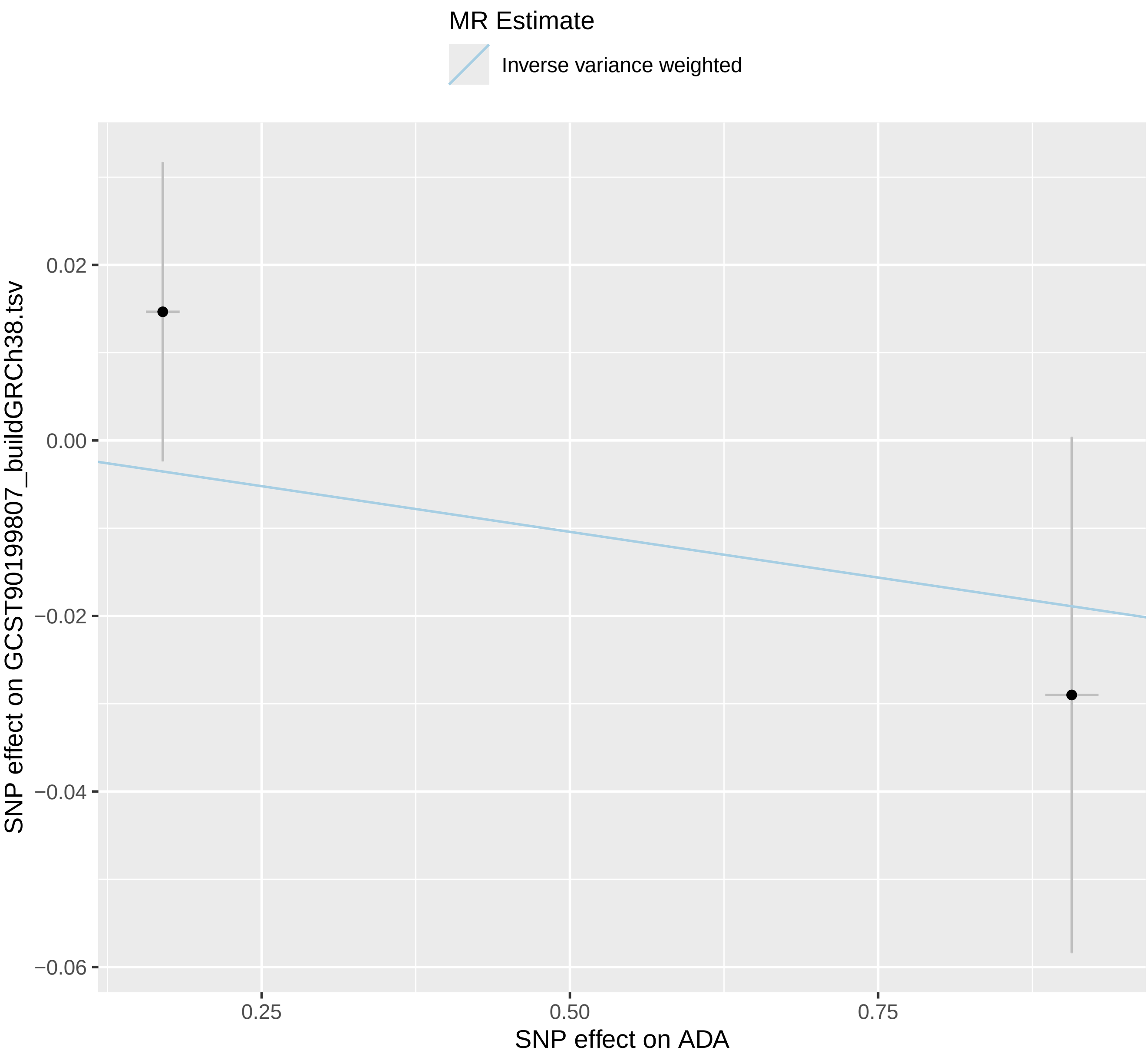
MR Estimate
Inverse variance weighted
0.02
0.00
−0.02
−0.04
−0.06
SNP effect on GCST90199807_buildGRCh38.tsv
0.25
0.50
0.75
SNP effect on ADA

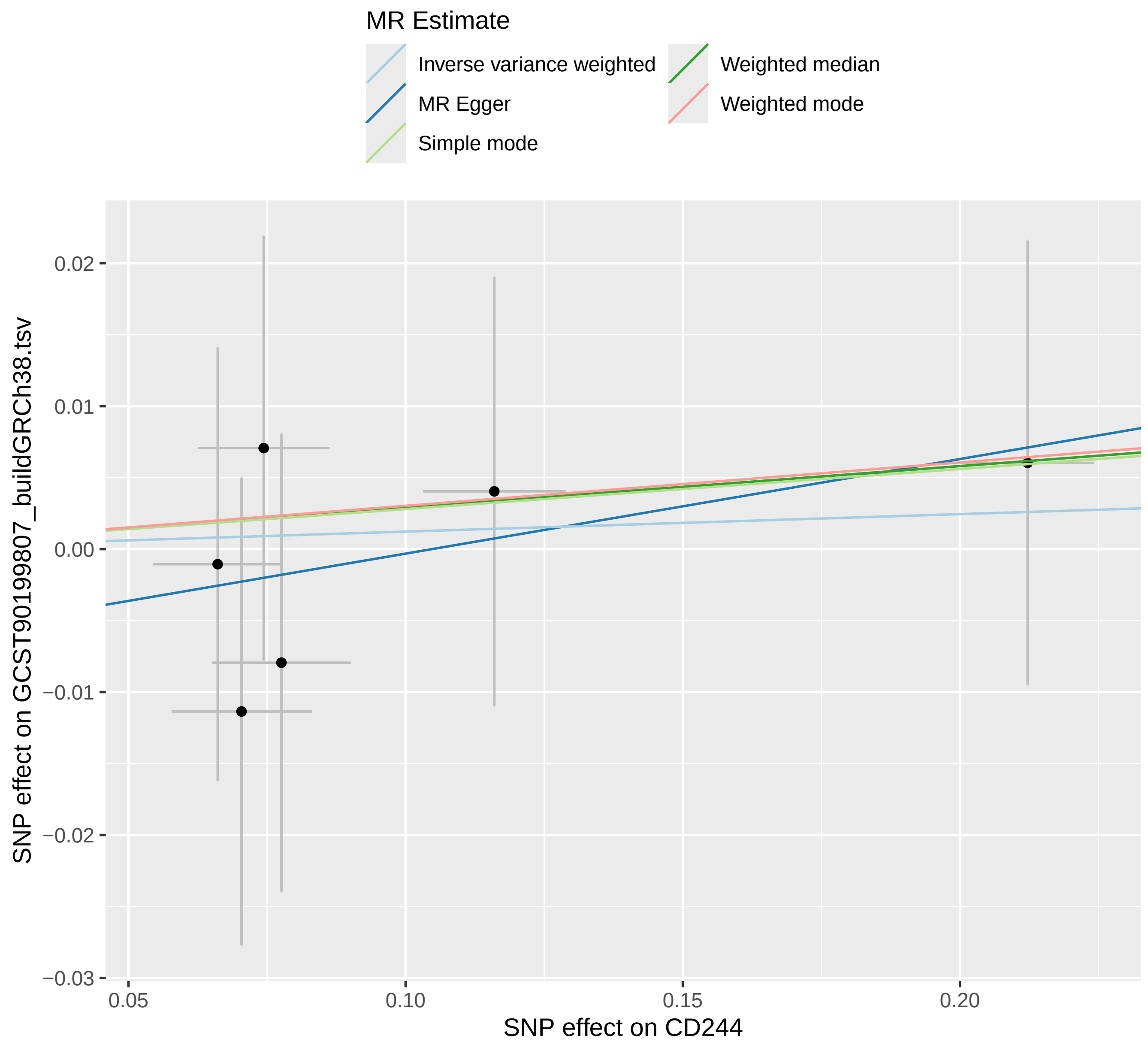
MR Estimate
Inverse variance weighted
MR Egger
Simple mode
Weighted median
Weighted mode
SNP effect on GCST90199807_buildGRCh38.tsv
SNP effect on CD244
0.02
0.01
0.00
−0.01
−0.02
−0.03
0.05
0.10
0.15
0.20

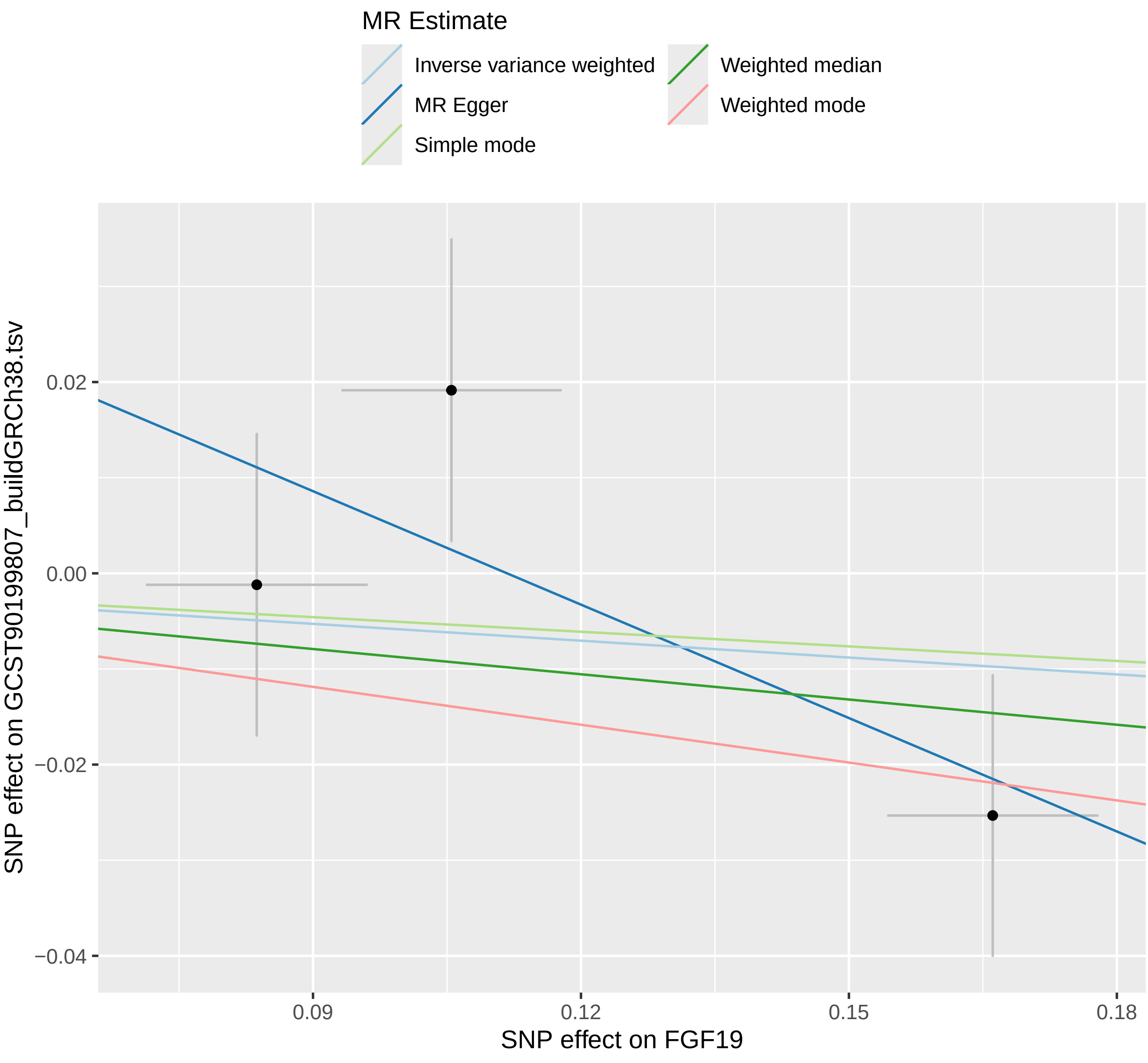
MR Estimate
Inverse variance weighted
MR Egger
Simple mode
Weighted median
Weighted mode
SNP effect on GCST90199807_buildGRCh38.tsv
0.02
0.00
−0.02
−0.04
0.09
0.12
0.15
0.18
SNP effect on FGF19

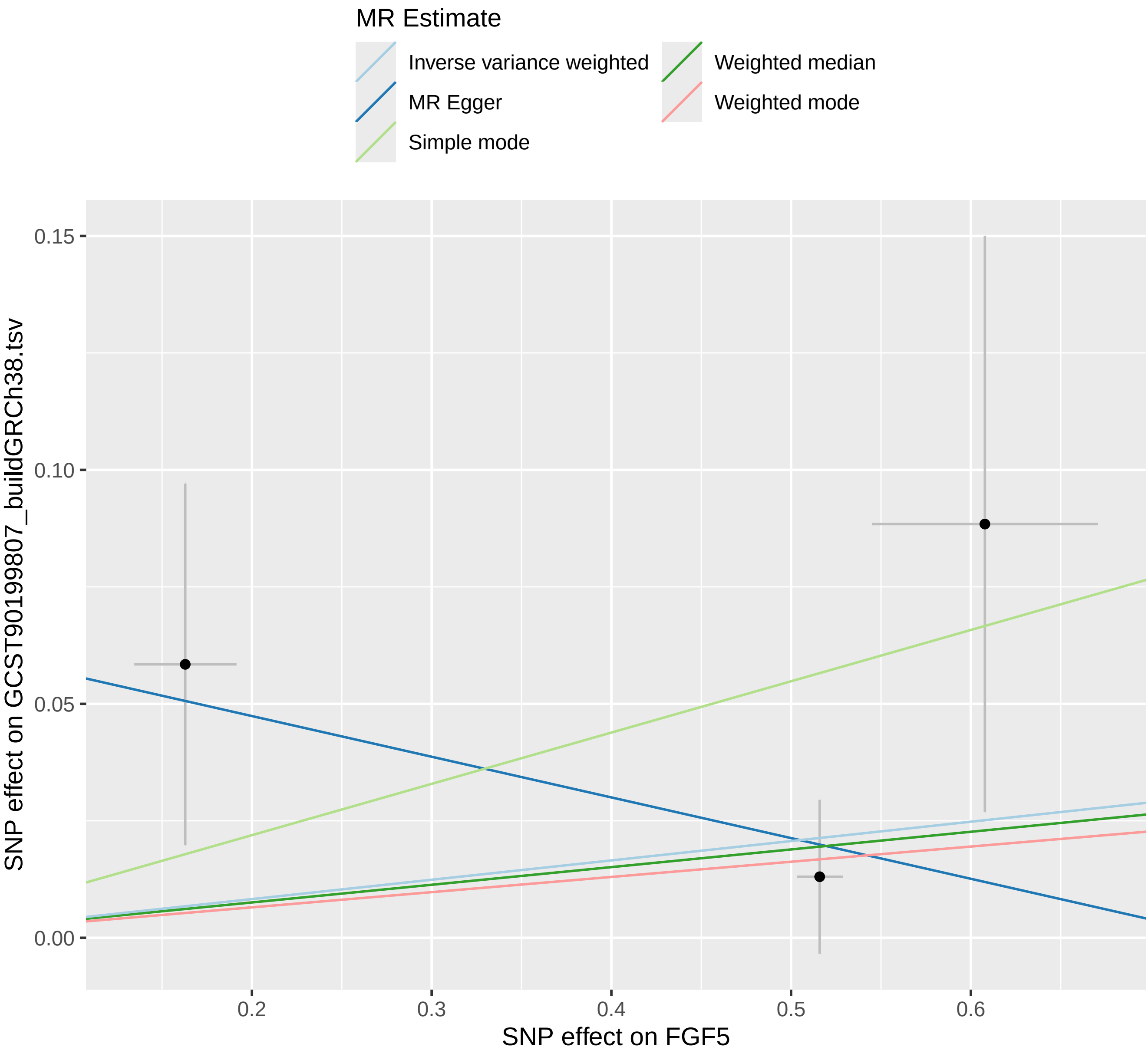

MR Estimate
Inverse variance weighted
MR Egger
Simple mode
Weighted median
Weighted mode
0.15
0.10
0.05
0.00
0.2
0.3
0.4
0.5
0.6
SNP effect on GCST90199807_buildGRCh38.tsv
SNP effect on FGF5

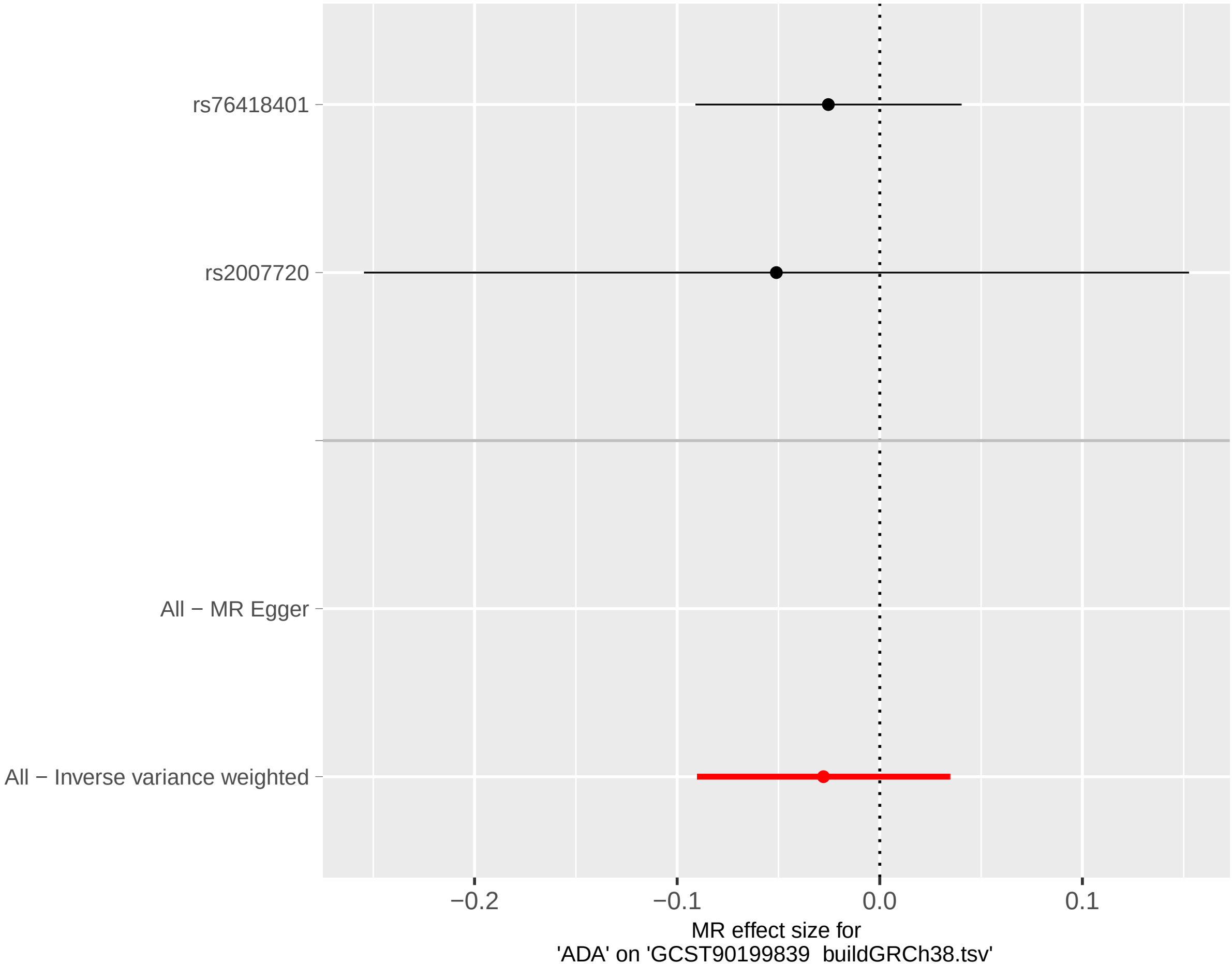
rs76418401
rs2007720
All − MR Egger
All − Inverse variance weighted
−0.2
−0.1
0.0
0.1
MR effect size for
'ADA' on 'GCST90199839_buildGRCh38.tsv'

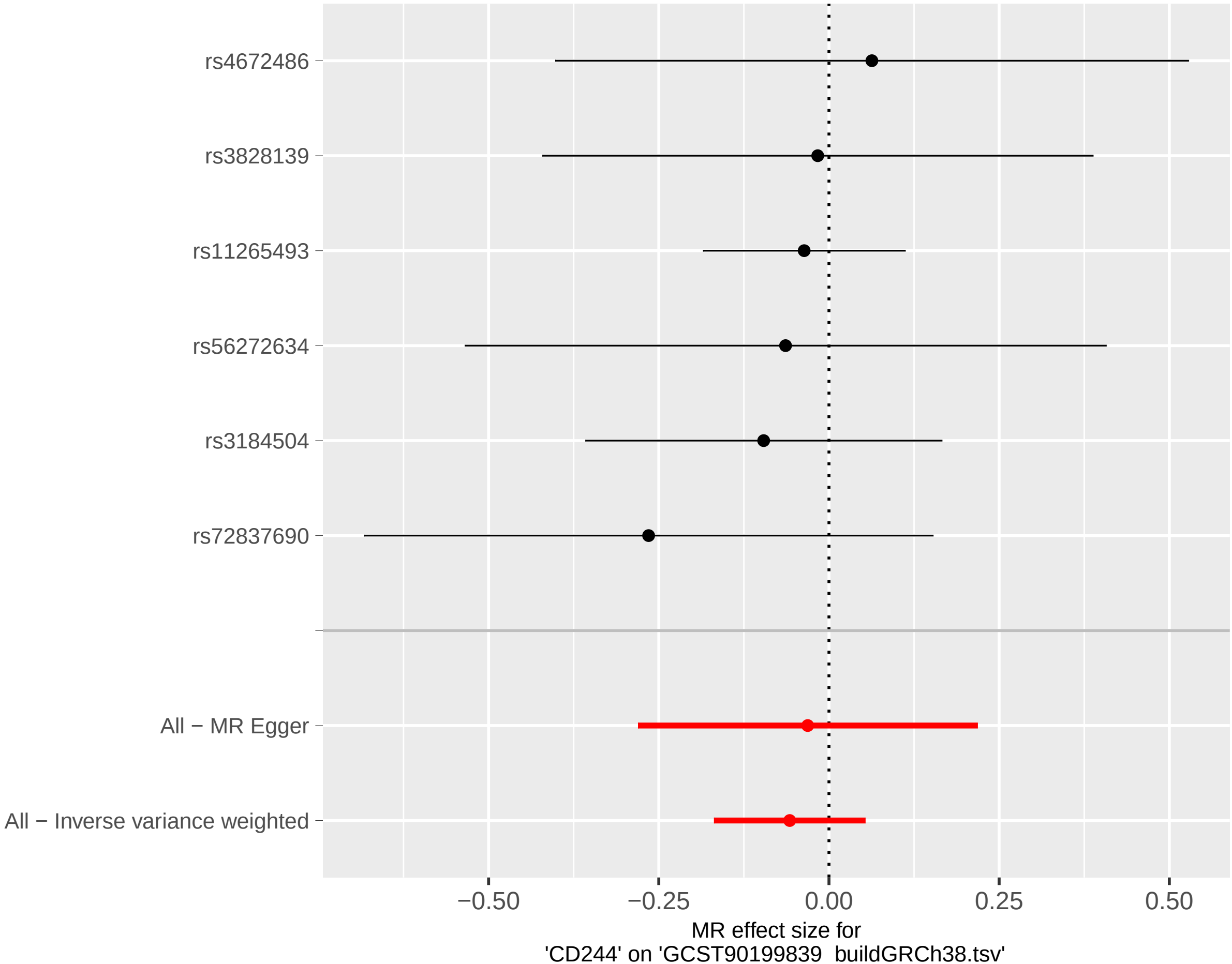
rs4672486
rs3828139
rs11265493
rs56272634
rs3184504
rs72837690
All − MR Egger
All − Inverse variance weighted
−0.50
−0.25
0.00
0.25
0.50
MR effect size for
'CD244' on 'GCST90199839_buildGRCh38.tsv'

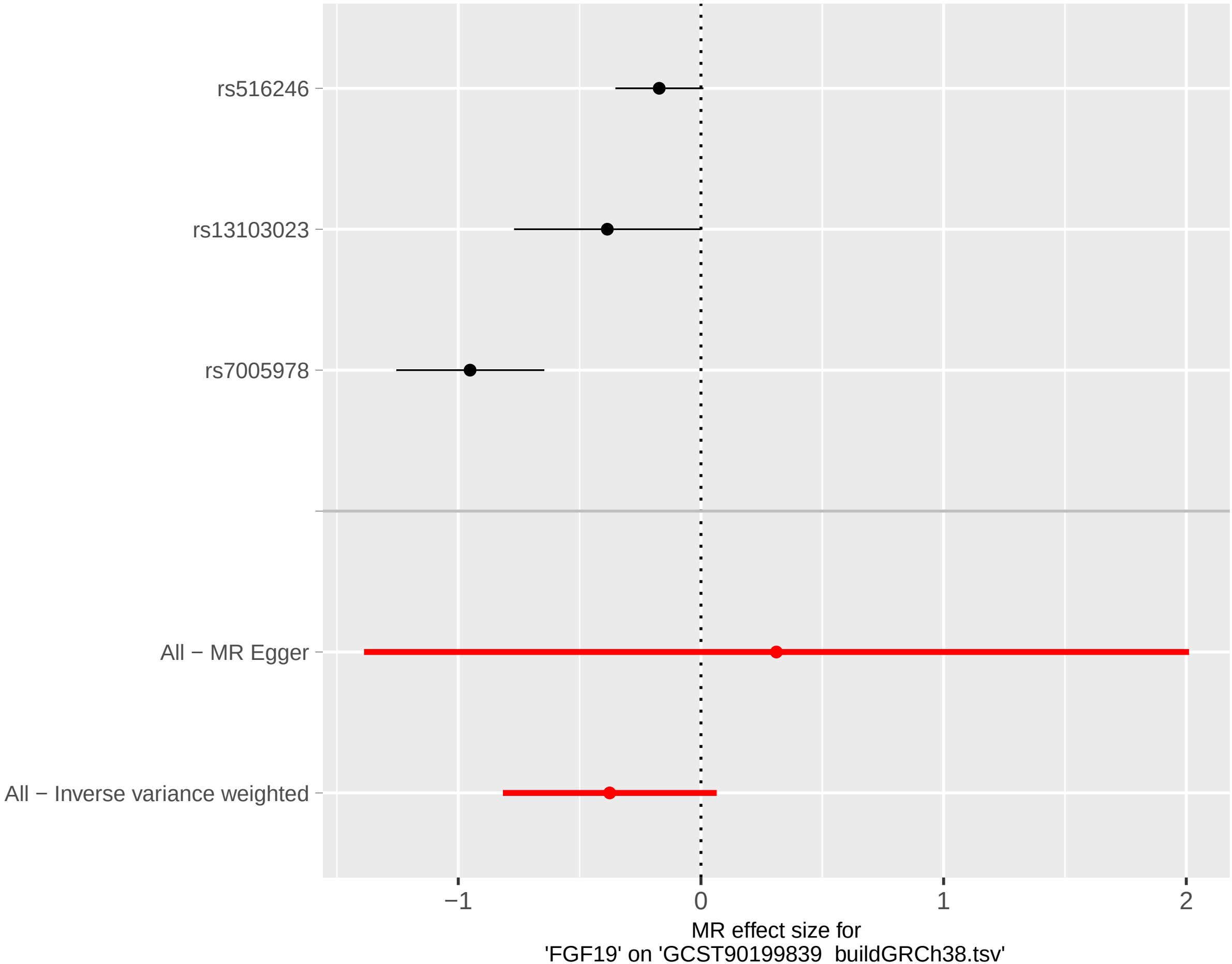
rs516246
rs13103023
rs7005978
All − MR Egger
All − Inverse variance weighted
−1
0
1
2
MR effect size for
'FGF19' on 'GCST90199839_buildGRCh38.tsv'

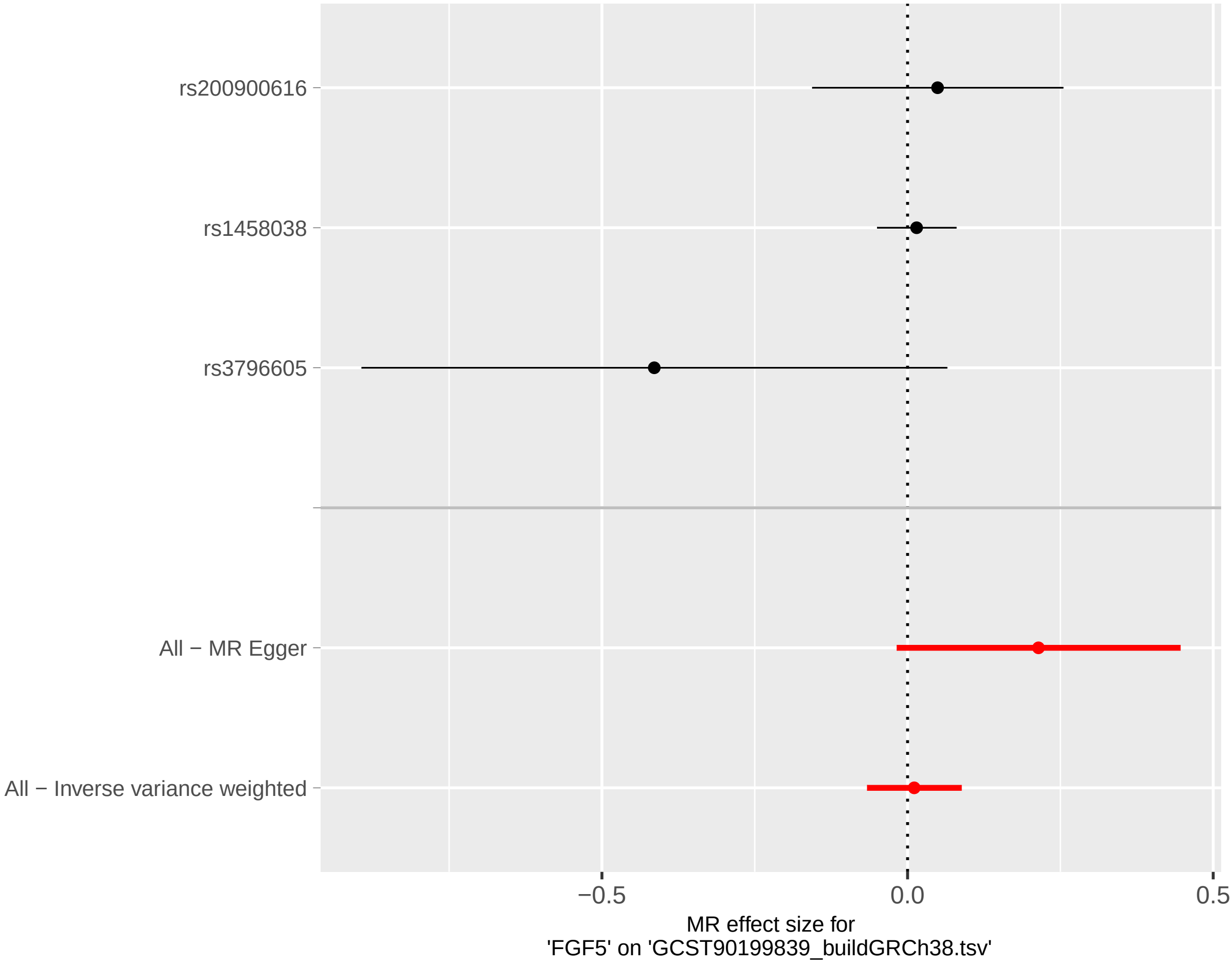
rs200900616
rs1458038
rs3796605
All − MR Egger
All − Inverse variance weighted
−0.5
0.0
0.5
MR effect size for
'FGF5' on 'GCST90199839_buildGRCh38.tsv'

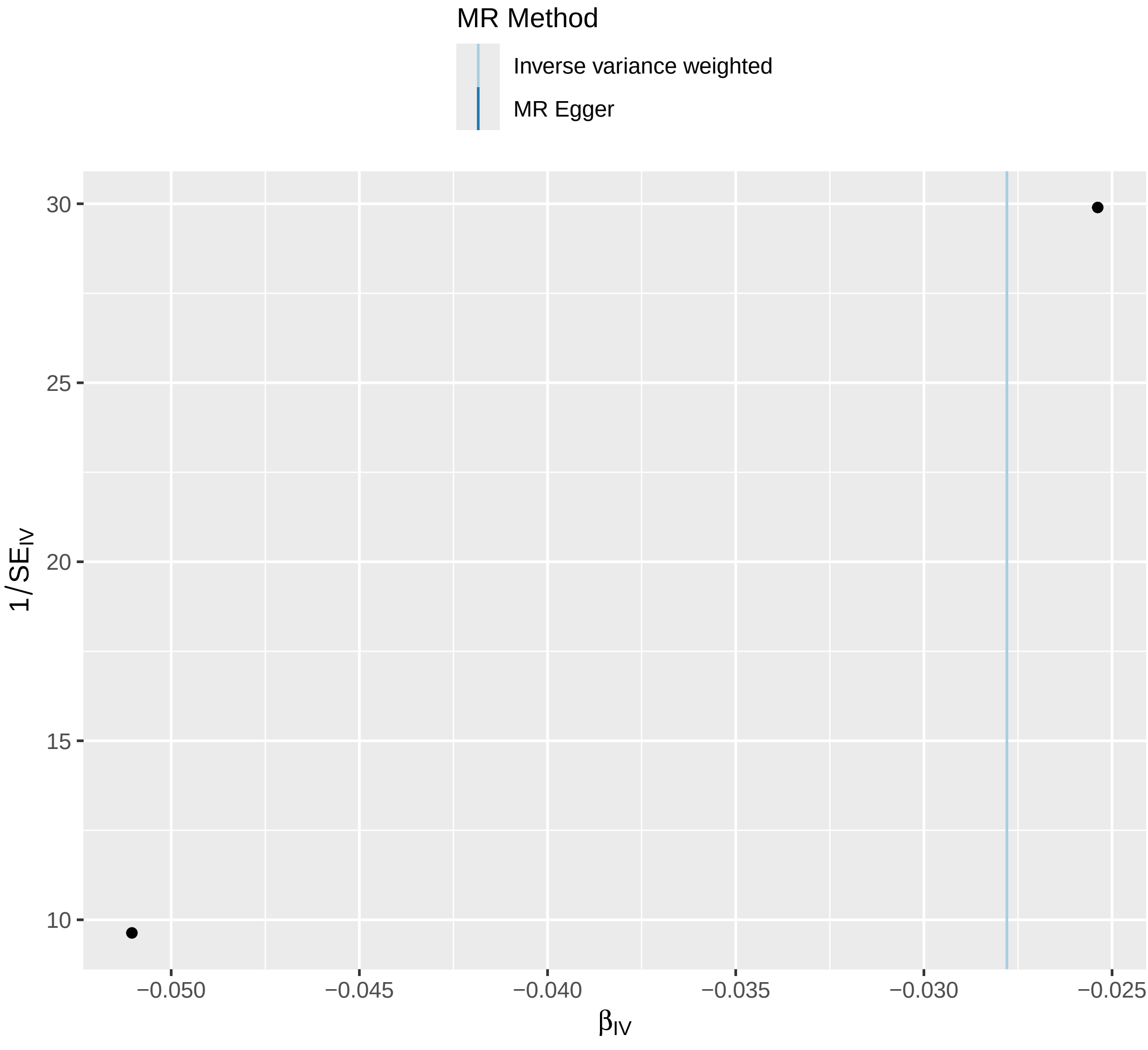
MR Method
Inverse variance weighted
MR Egger
30
25
20
15
10
1/SE$_{IV}$
−0.050
−0.045
−0.040
−0.035
−0.030
−0.025
β$_{IV}$

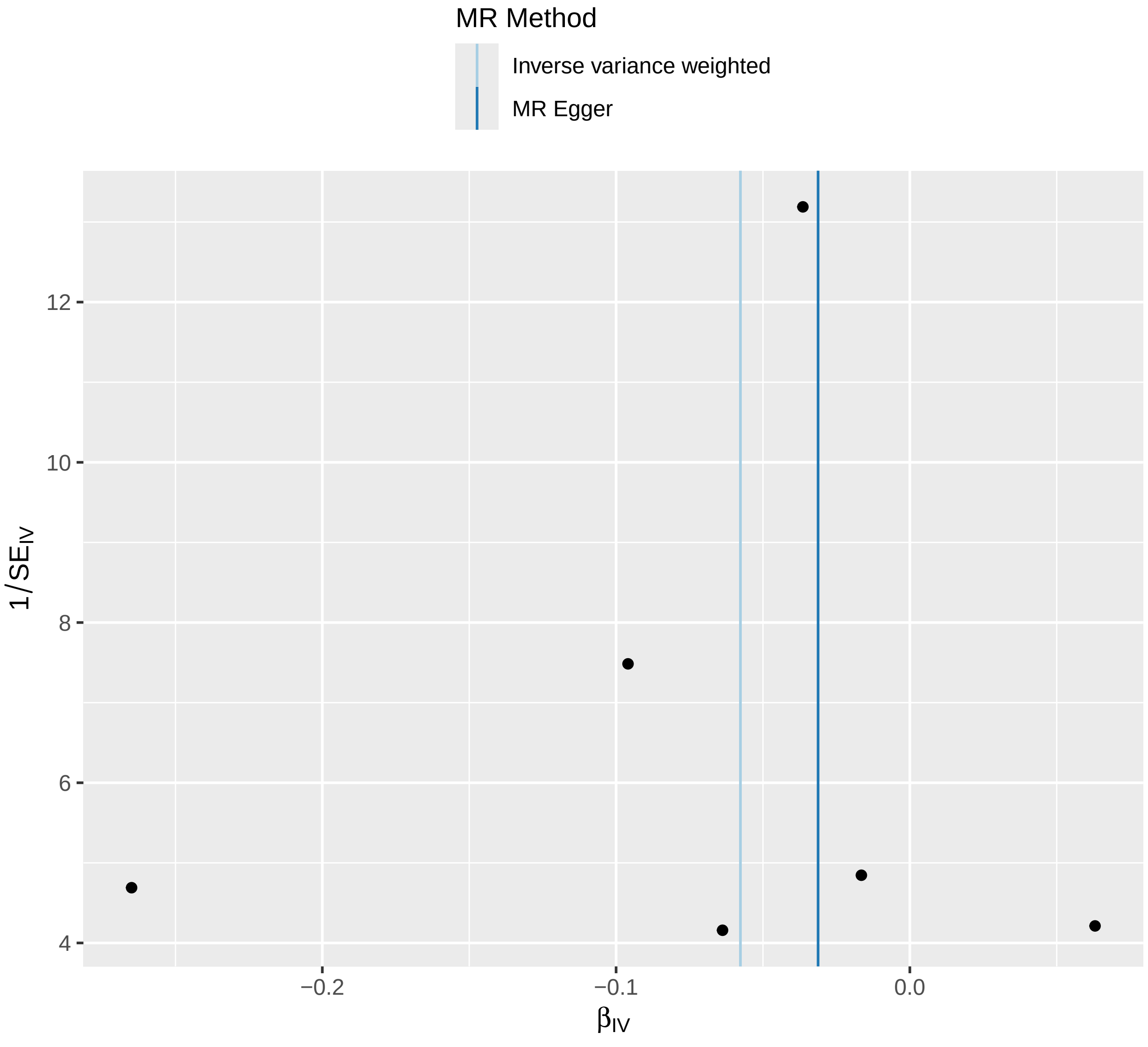
MR Method
Inverse variance weighted
MR Egger
12
10
8
6
4
1/SE_IV
−0.2
−0.1
0.0
β_IV

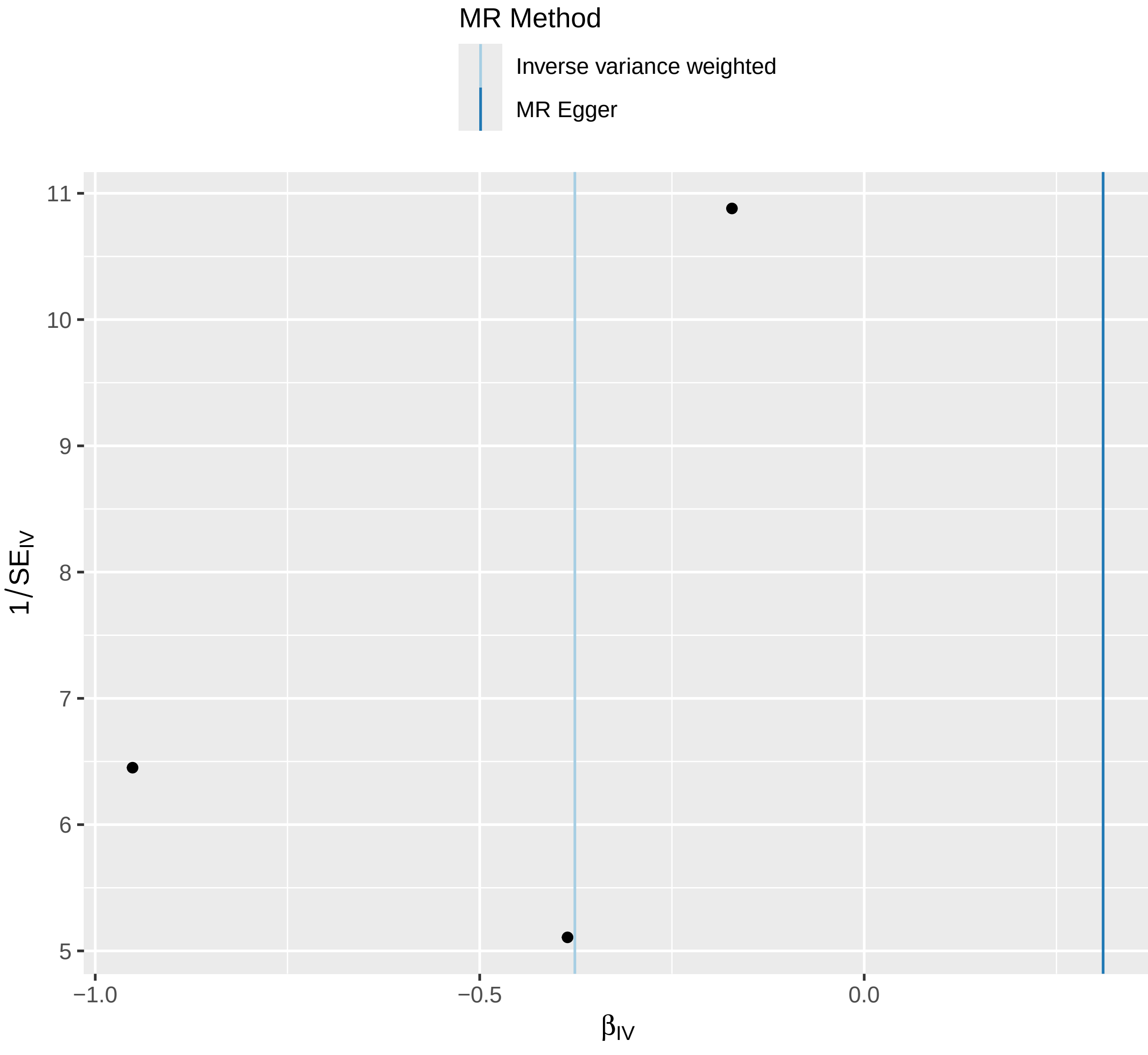
MR Method
Inverse variance weighted
MR Egger
1/SE_IV
11
10
9
8
7
6
5
−1.0
−0.5
0.0
β_IV

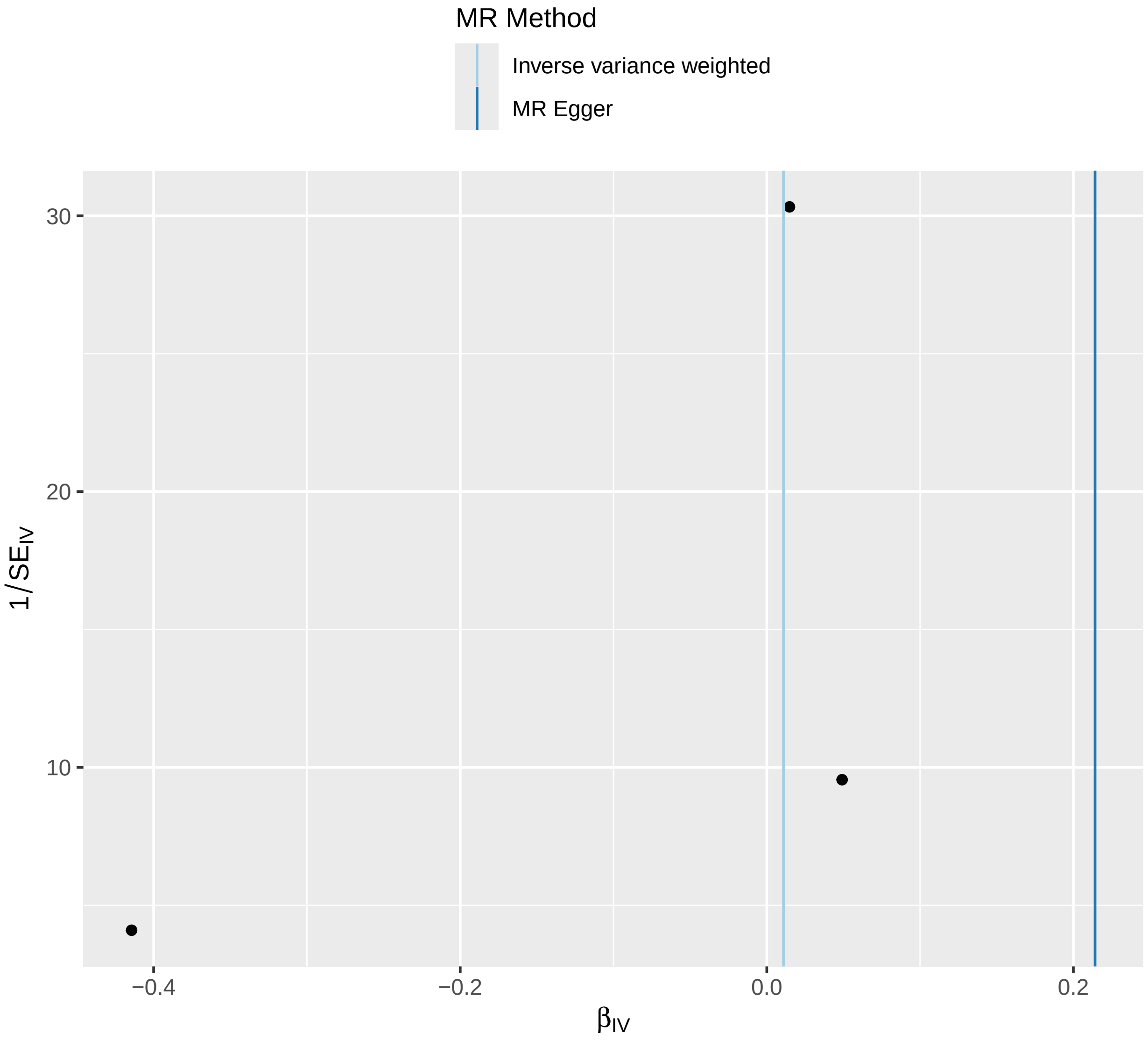
MR Method
Inverse variance weighted
MR Egger
30
20
10
1/SE$_{IV}$
−0.4
−0.2
0.0
0.2
β_{IV}

Insufficient number of SNPs

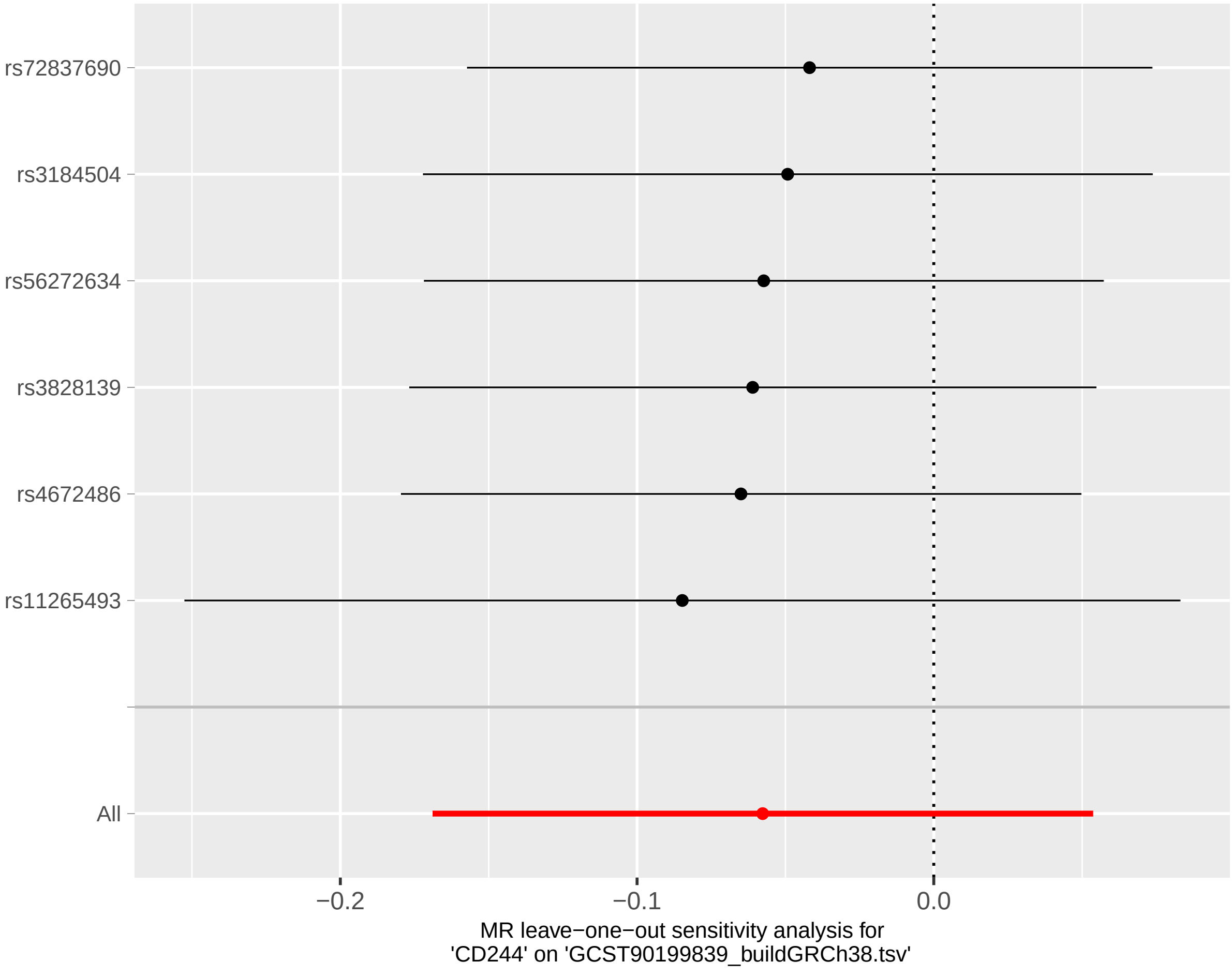

rs72837690
rs3184504
rs56272634
rs3828139
rs4672486
rs11265493
All
−0.2
−0.1
0.0
MR leave−one−out sensitivity analysis for
'CD244' on 'GCST90199839_buildGRCh38.tsv'

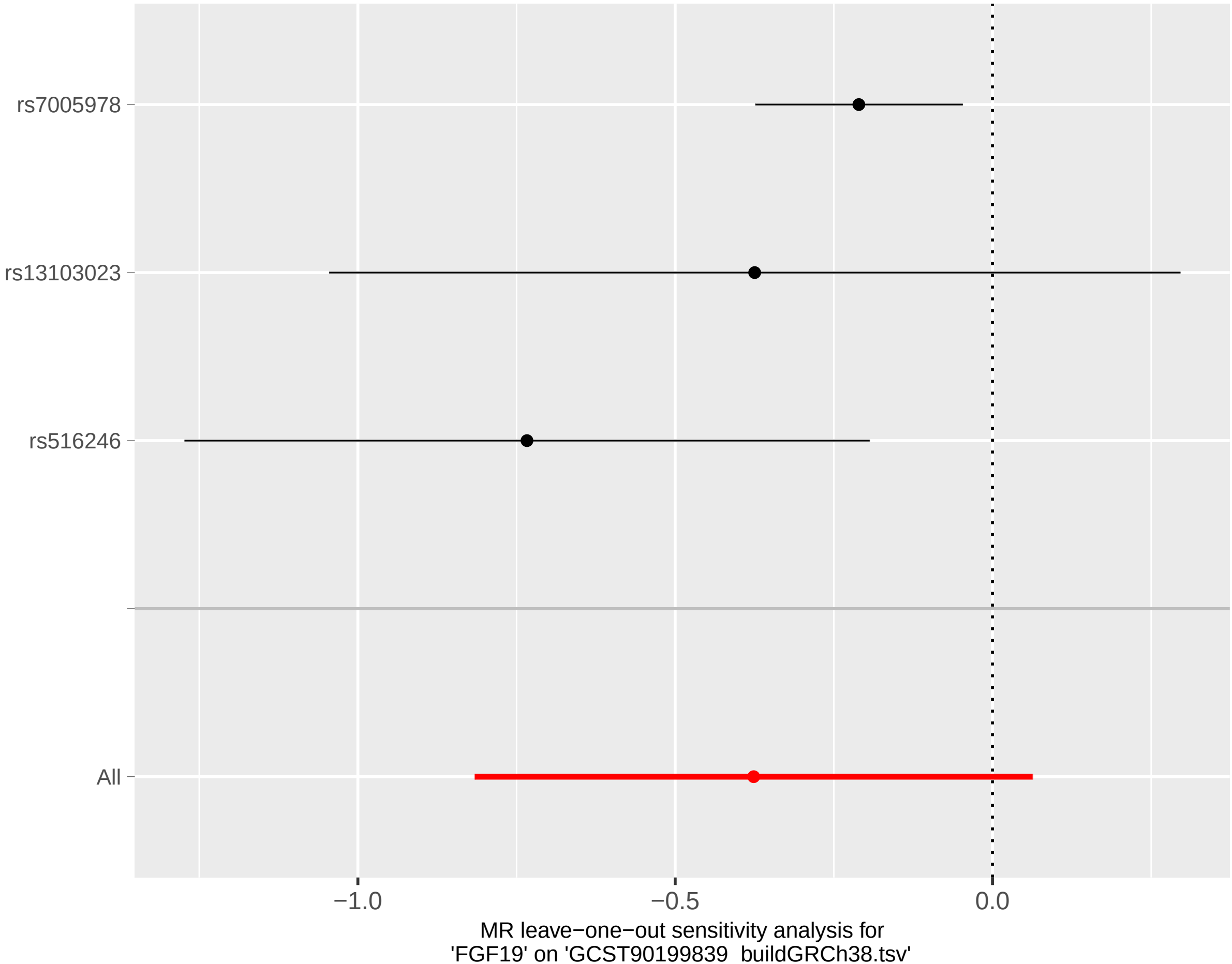
rs7005978
rs13103023
rs516246
All
−1.0
−0.5
0.0
MR leave−one−out sensitivity analysis for
'FGF19' on 'GCST90199839_buildGRCh38.tsv'

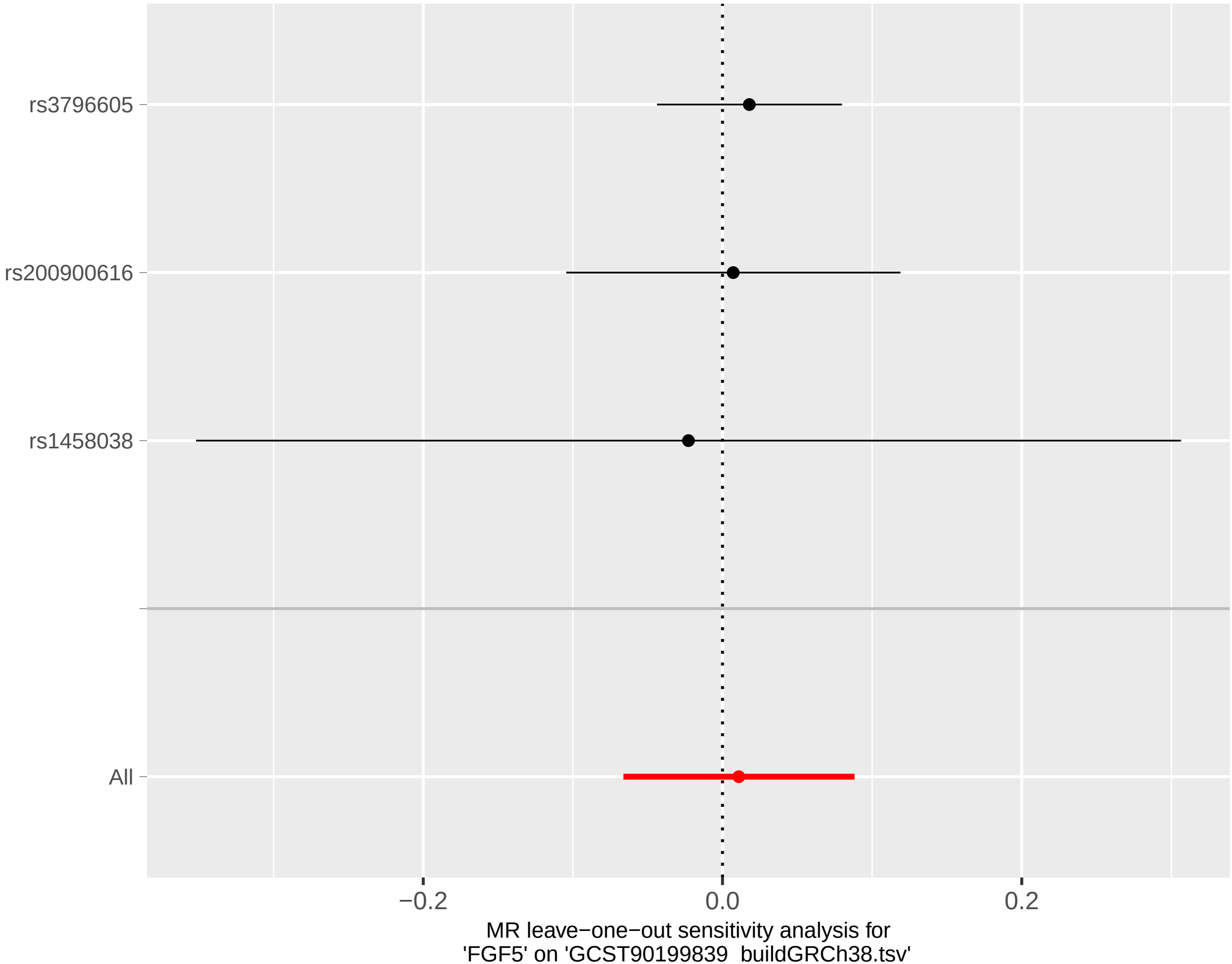
rs3796605
rs200900616
rs1458038
All
−0.2
0.0
0.2
MR leave−one−out sensitivity analysis for
'FGF5' on 'GCST90199839_buildGRCh38.tsv'

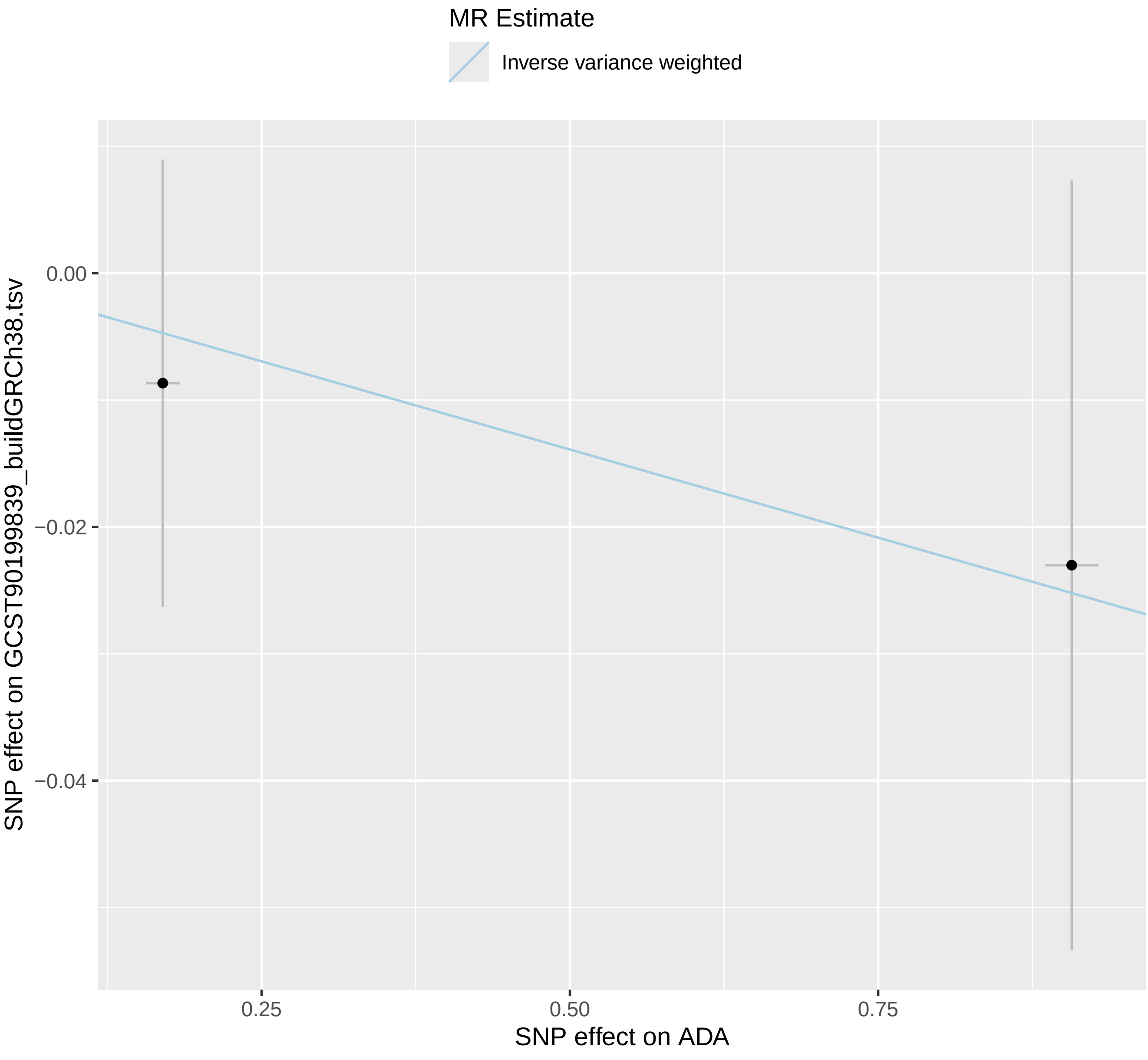
MR Estimate
Inverse variance weighted
0.00
−0.02
−0.04
0.25
0.50
0.75
SNP effect on ADA
SNP effect on GCST90199839_buildGRCh38.tsv

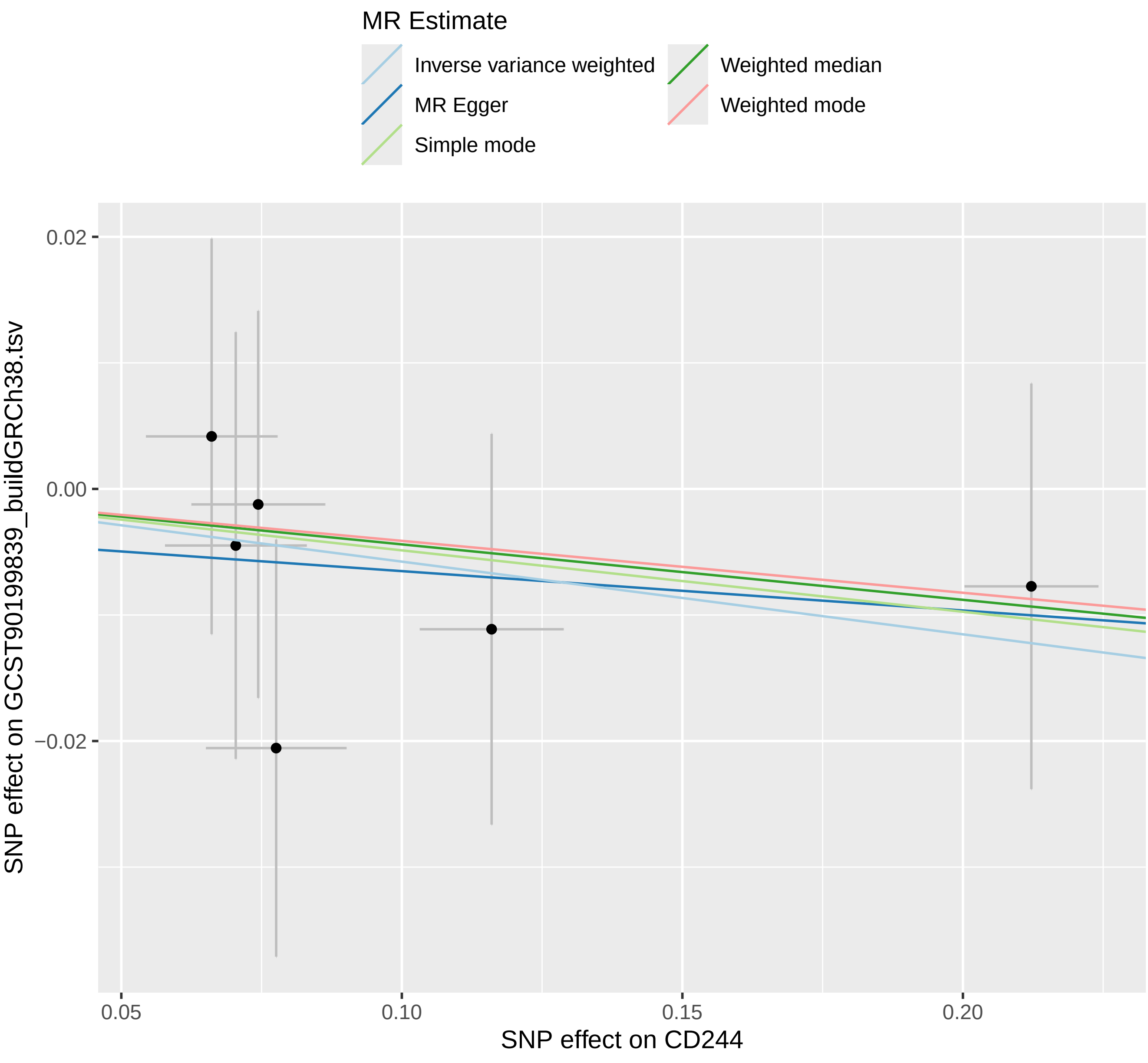
MR Estimate
Inverse variance weighted
MR Egger
Simple mode
Weighted median
Weighted mode
0.02
0.00
−0.02
0.05
0.10
0.15
0.20
SNP effect on GCST90199839_buildGRCh38.tsv
SNP effect on CD244

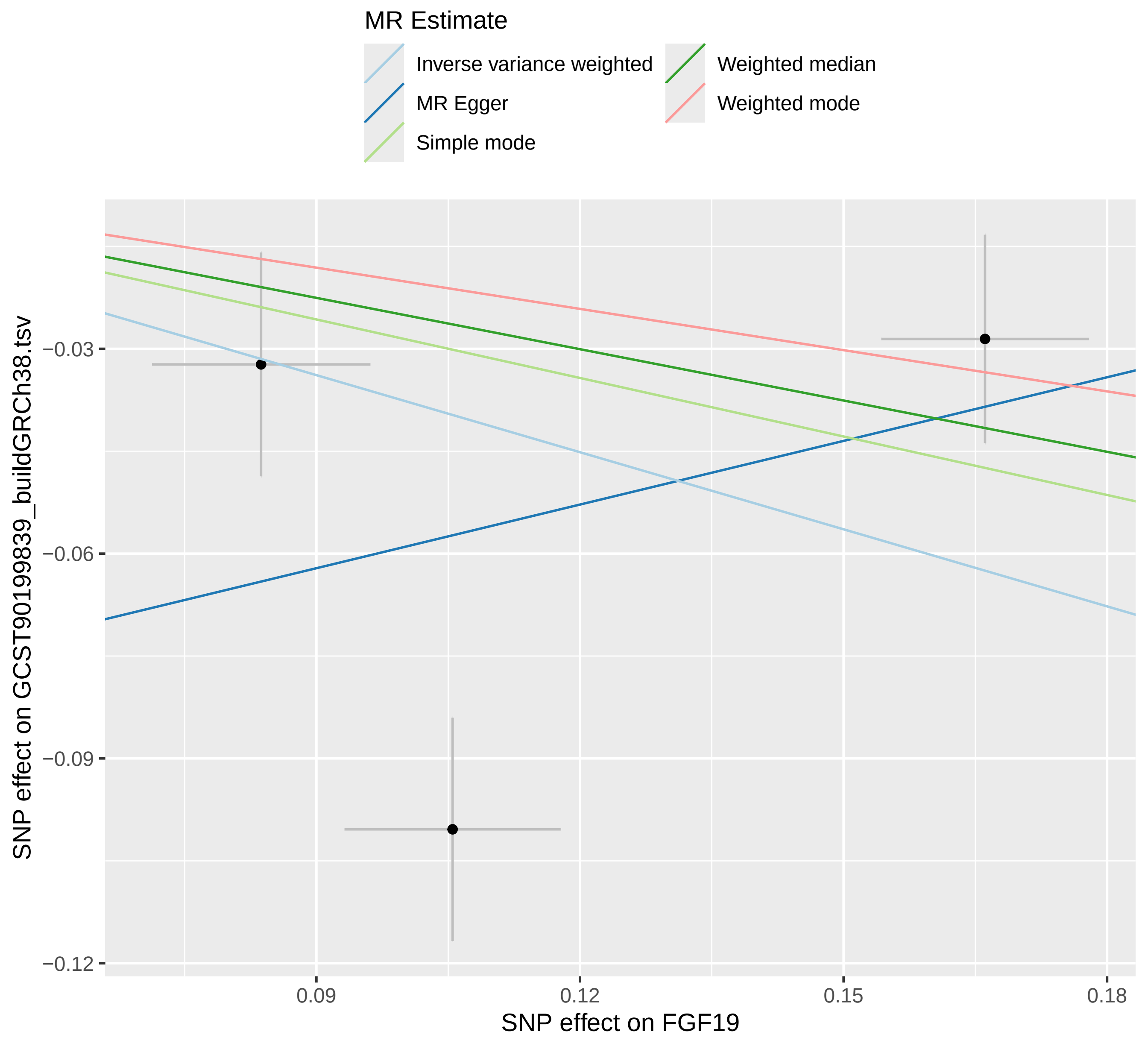
MR Estimate
Inverse variance weighted
MR Egger
Simple mode
Weighted median
Weighted mode
SNP effect on GCST90199839_buildGRCh38.tsv
−0.03
−0.06
−0.09
−0.12
0.09
0.12
0.15
0.18
SNP effect on FGF19

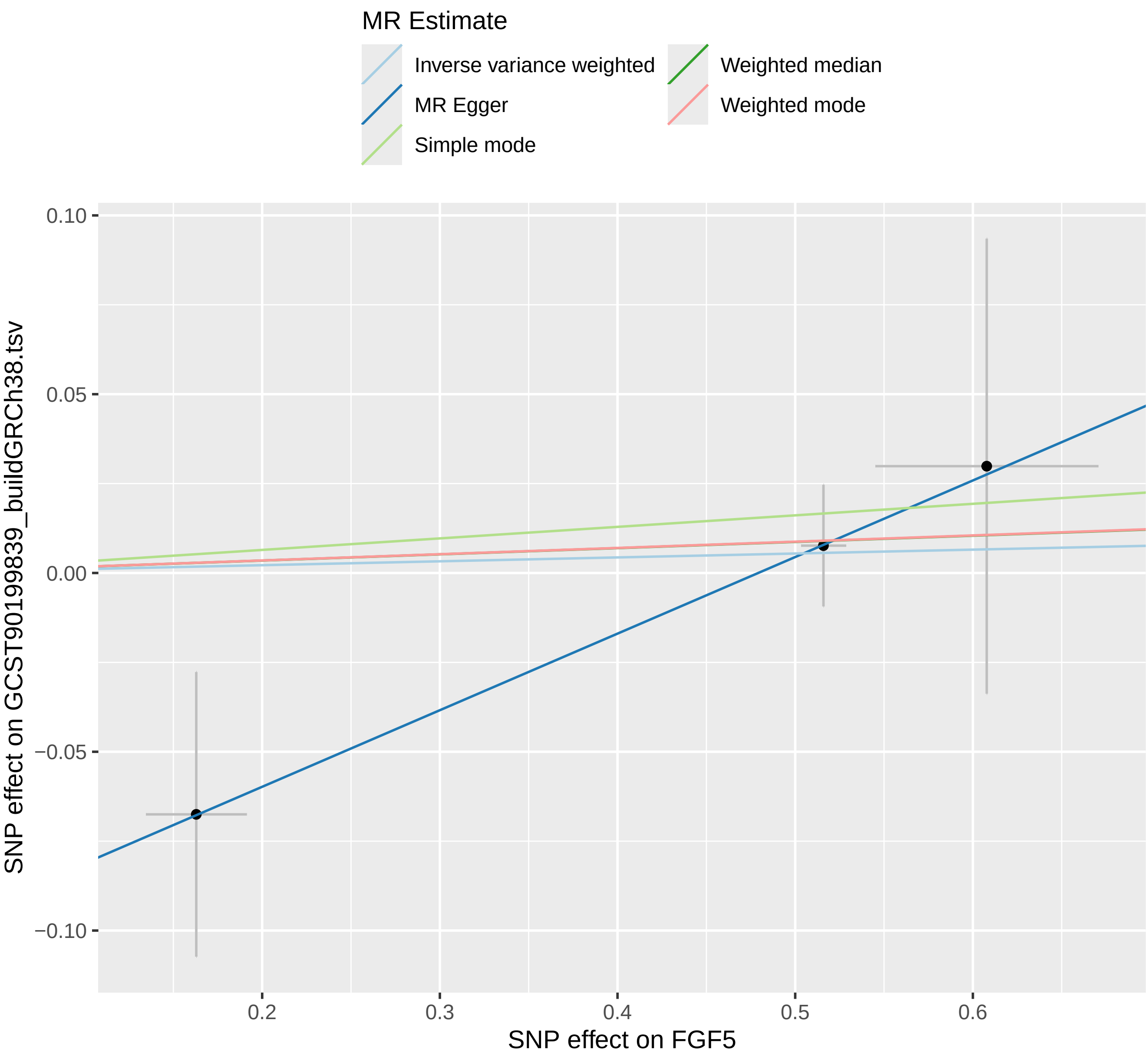
MR Estimate
Inverse variance weighted
MR Egger
Simple mode
Weighted median
Weighted mode
0.10
0.05
0.00
−0.05
−0.10
0.2
0.3
0.4
0.5
0.6
SNP effect on GCST90199839_buildGRCh38.tsv
SNP effect on FGF5

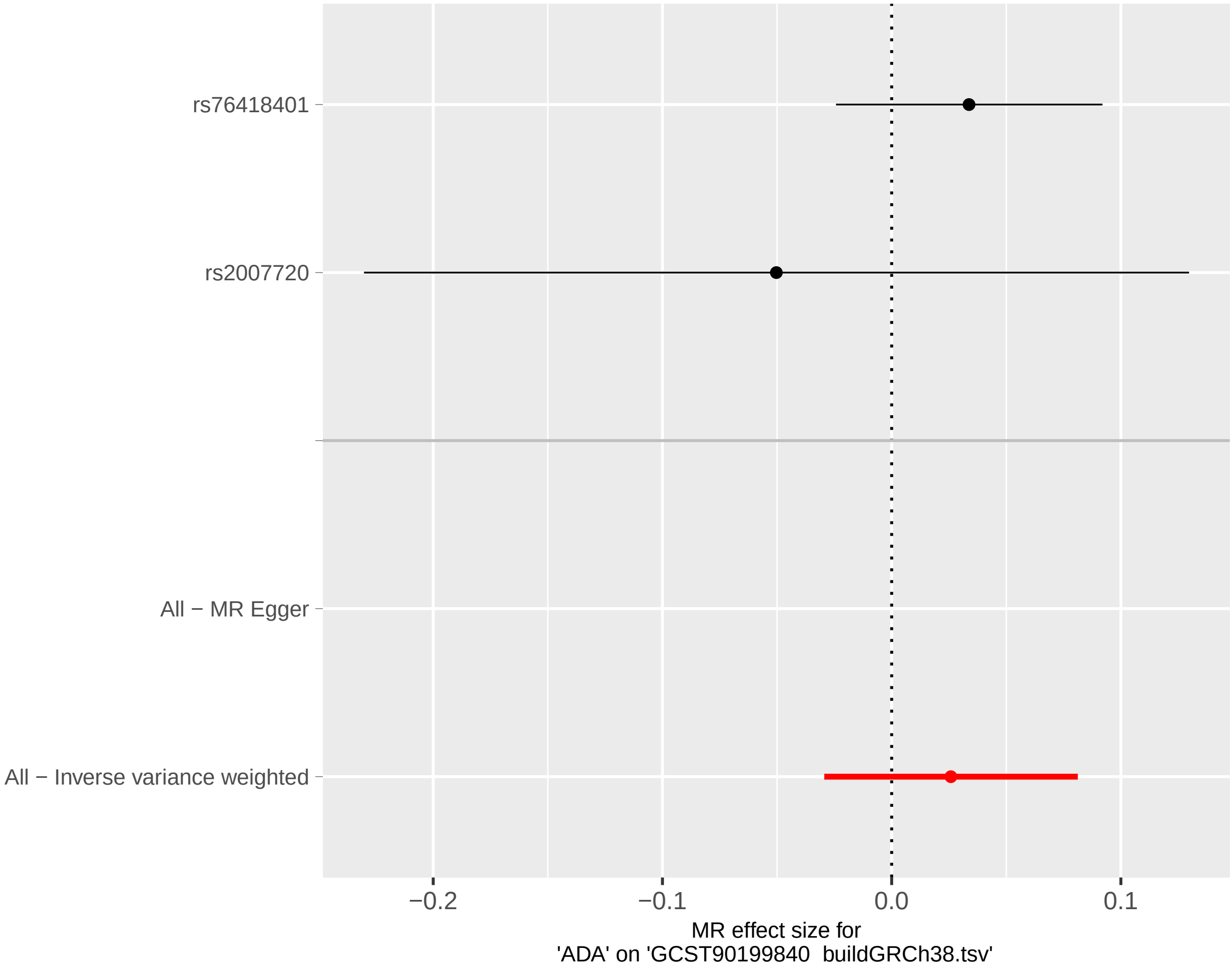
rs76418401
rs2007720
All − MR Egger
All − Inverse variance weighted
−0.2
−0.1
0.0
0.1
MR effect size for
'ADA' on 'GCST90199840_buildGRCh38.tsv'

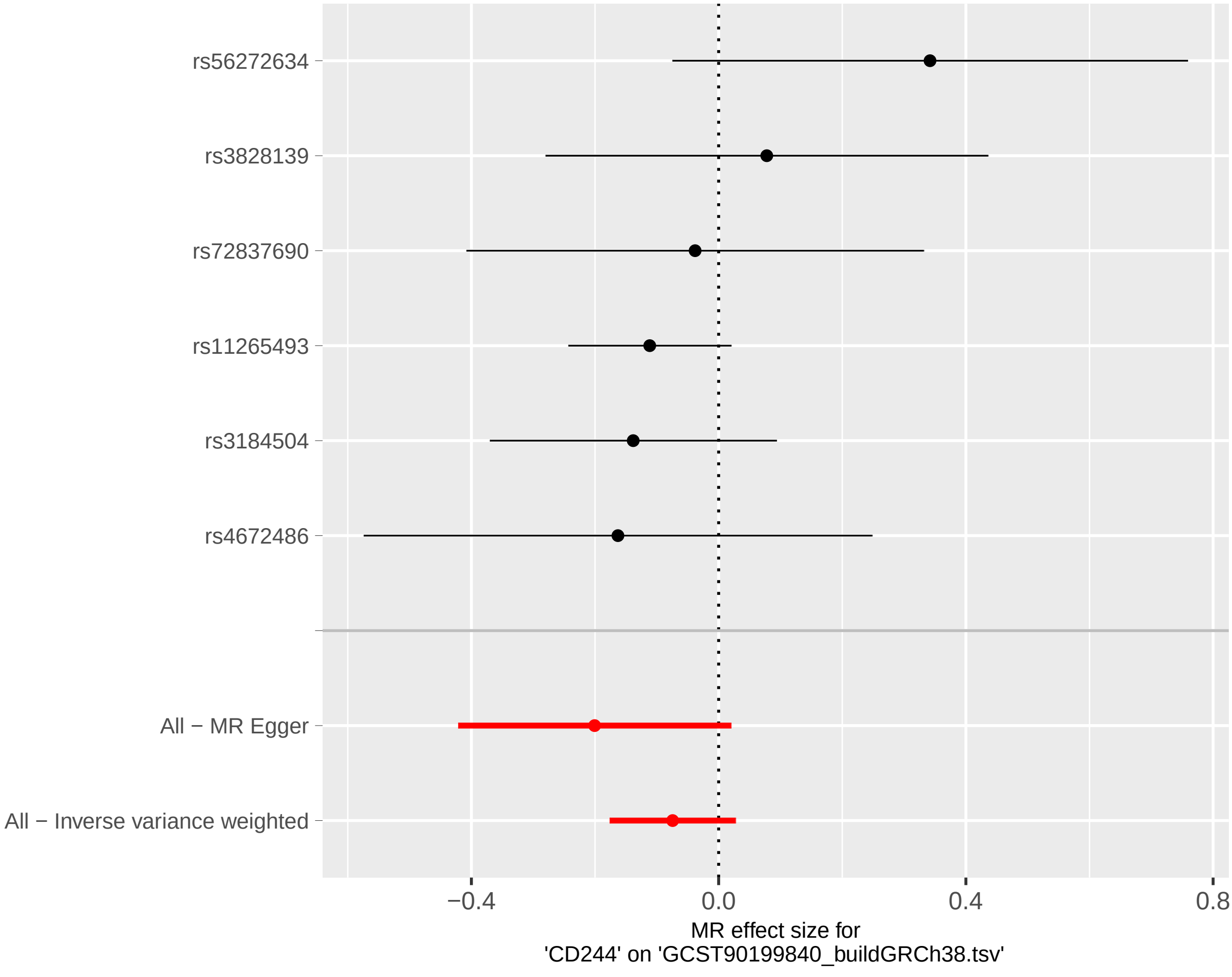
rs56272634
rs3828139
rs72837690
rs11265493
rs3184504
rs4672486
All − MR Egger
All − Inverse variance weighted
−0.4
0.0
0.4
0.8
MR effect size for
'CD244' on 'GCST90199840_buildGRCh38.tsv'

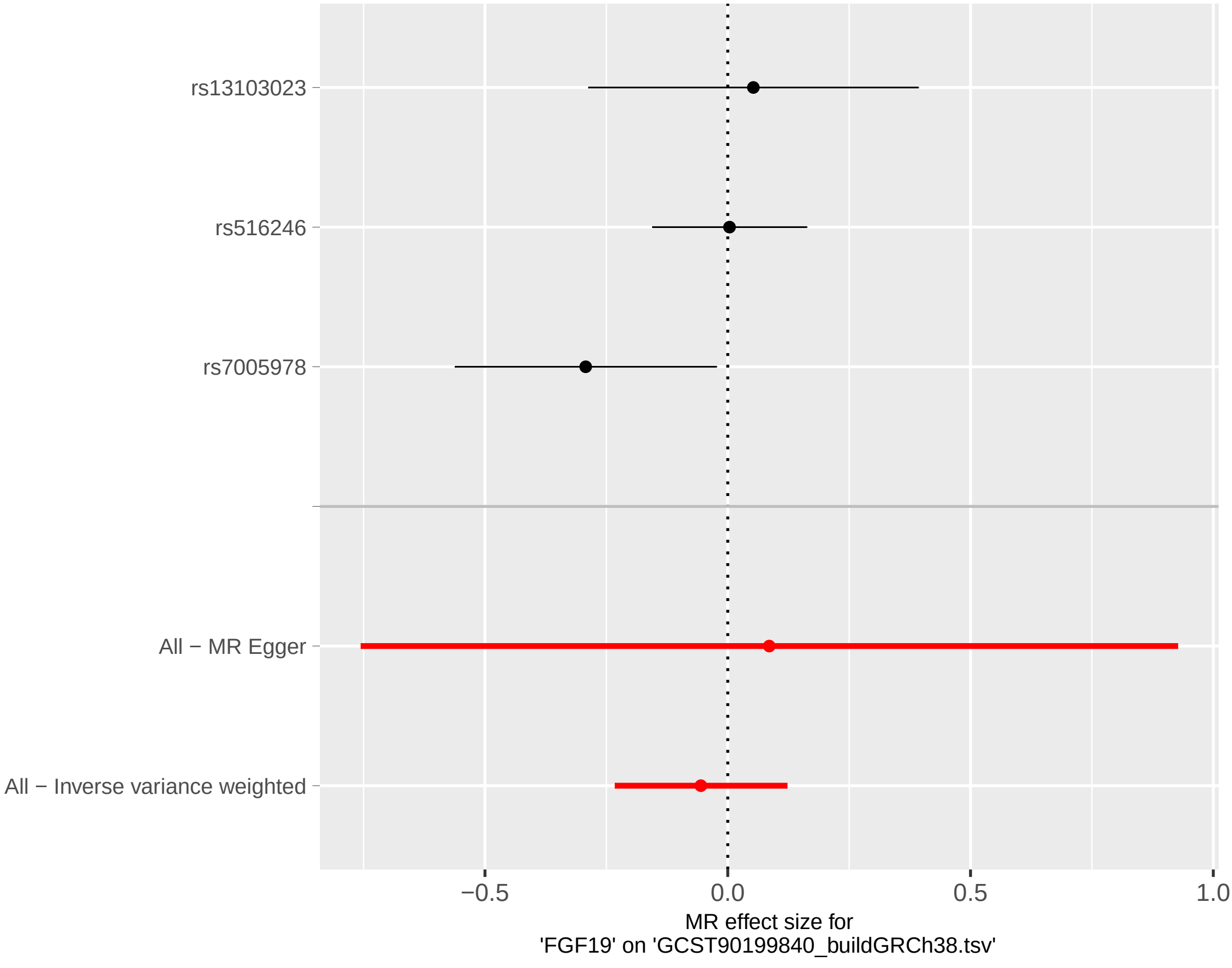
rs13103023
rs516246
rs7005978
All − MR Egger
All − Inverse variance weighted
−0.5
0.0
0.5
1.0
MR effect size for
'FGF19' on 'GCST90199840_buildGRCh38.tsv'

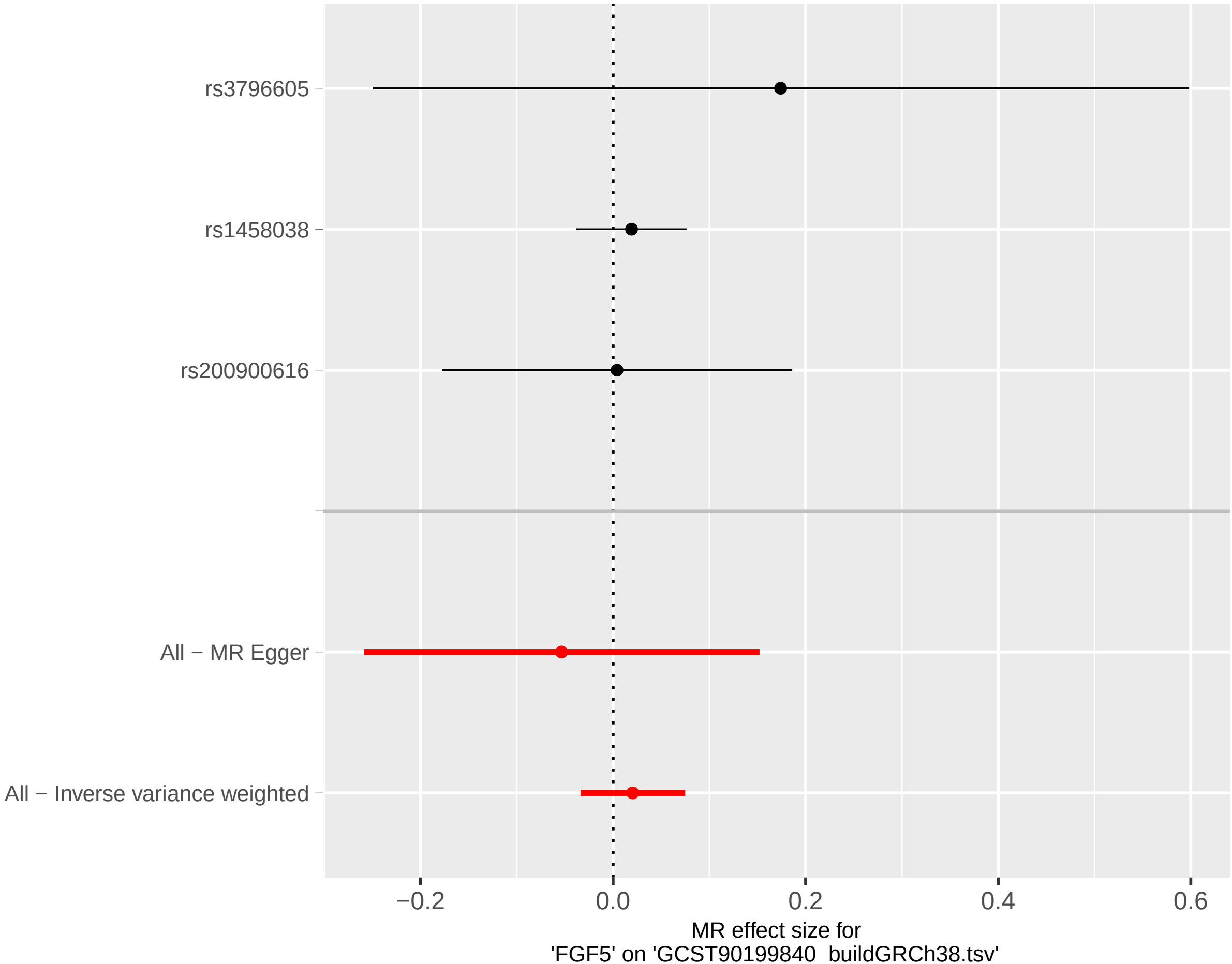

rs3796605
rs1458038
rs200900616
All − MR Egger
All − Inverse variance weighted
−0.2
0.0
0.2
0.4
0.6
MR effect size for
'FGF5' on 'GCST90199840_buildGRCh38.tsv'

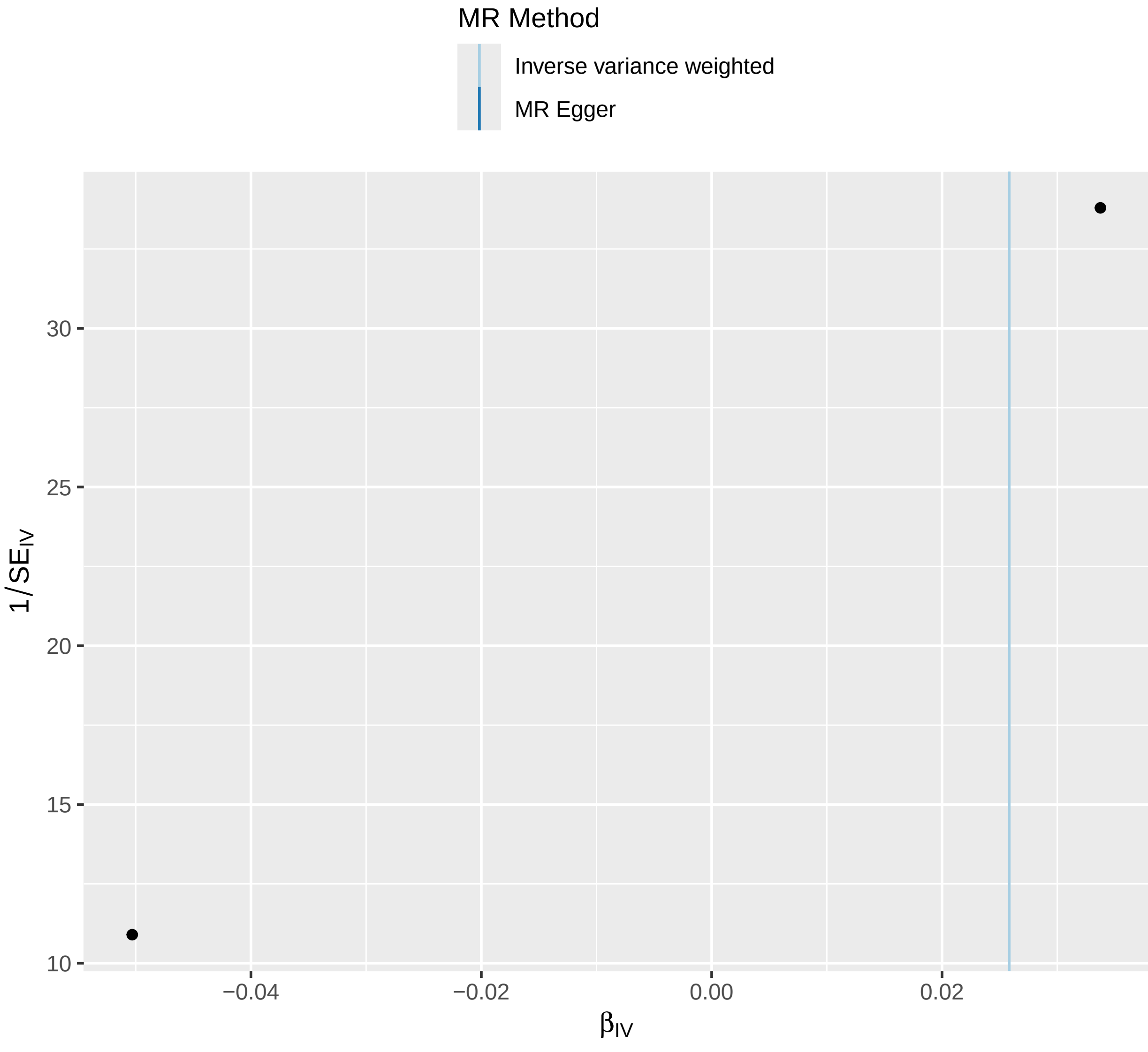
MR Method
Inverse variance weighted
MR Egger
30
25
20
15
10
1/SE_IV
−0.04
−0.02
0.00
0.02
β_IV

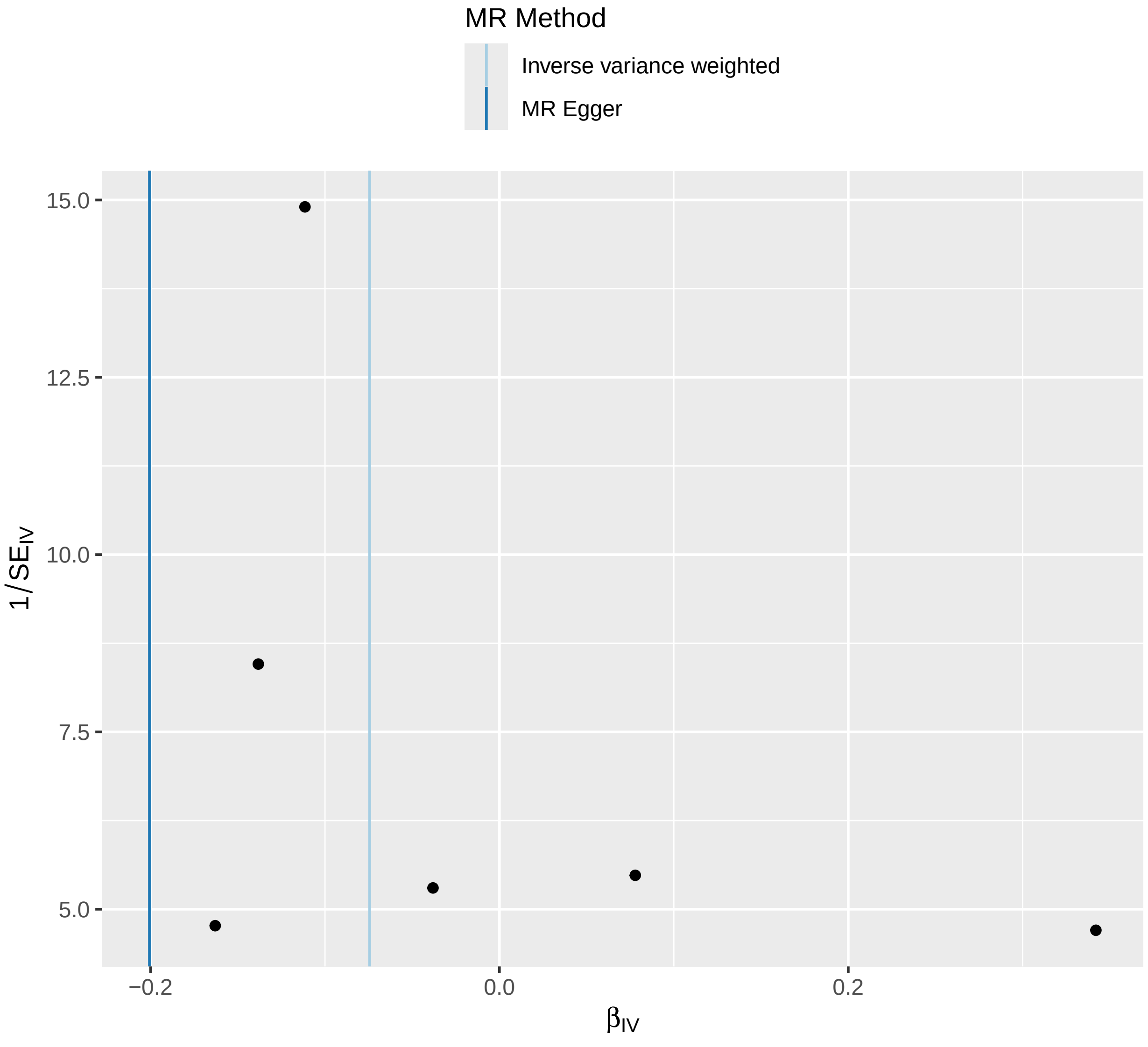
MR Method
Inverse variance weighted
MR Egger
15.0
12.5
10.0
7.5
5.0
1/SE_IV
−0.2
0.0
0.2
β_IV

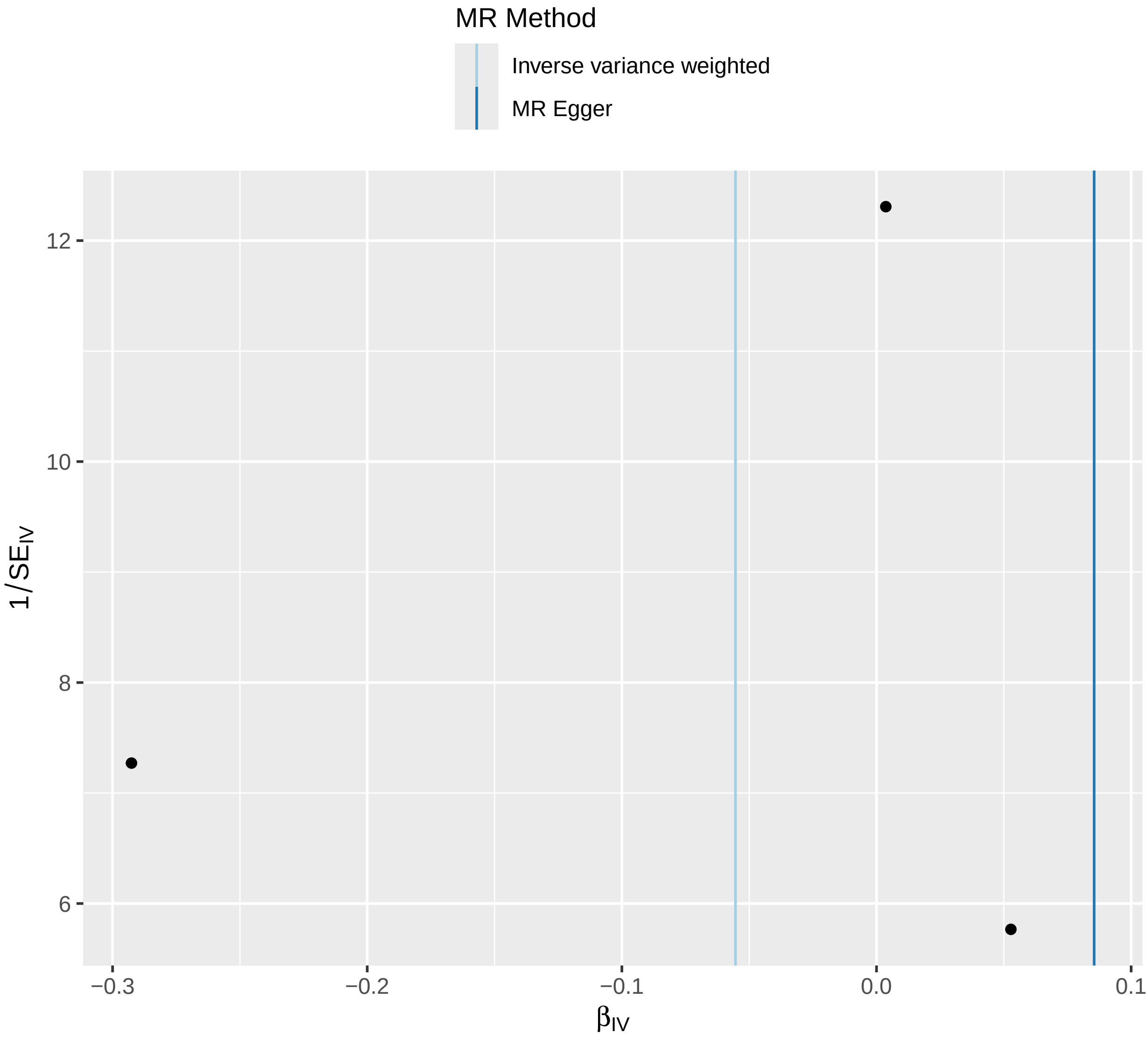
MR Method
Inverse variance weighted
MR Egger
12
10
8
6
1/SE$_{IV}$
−0.3
−0.2
−0.1
0.0
0.1
β$_{IV}$

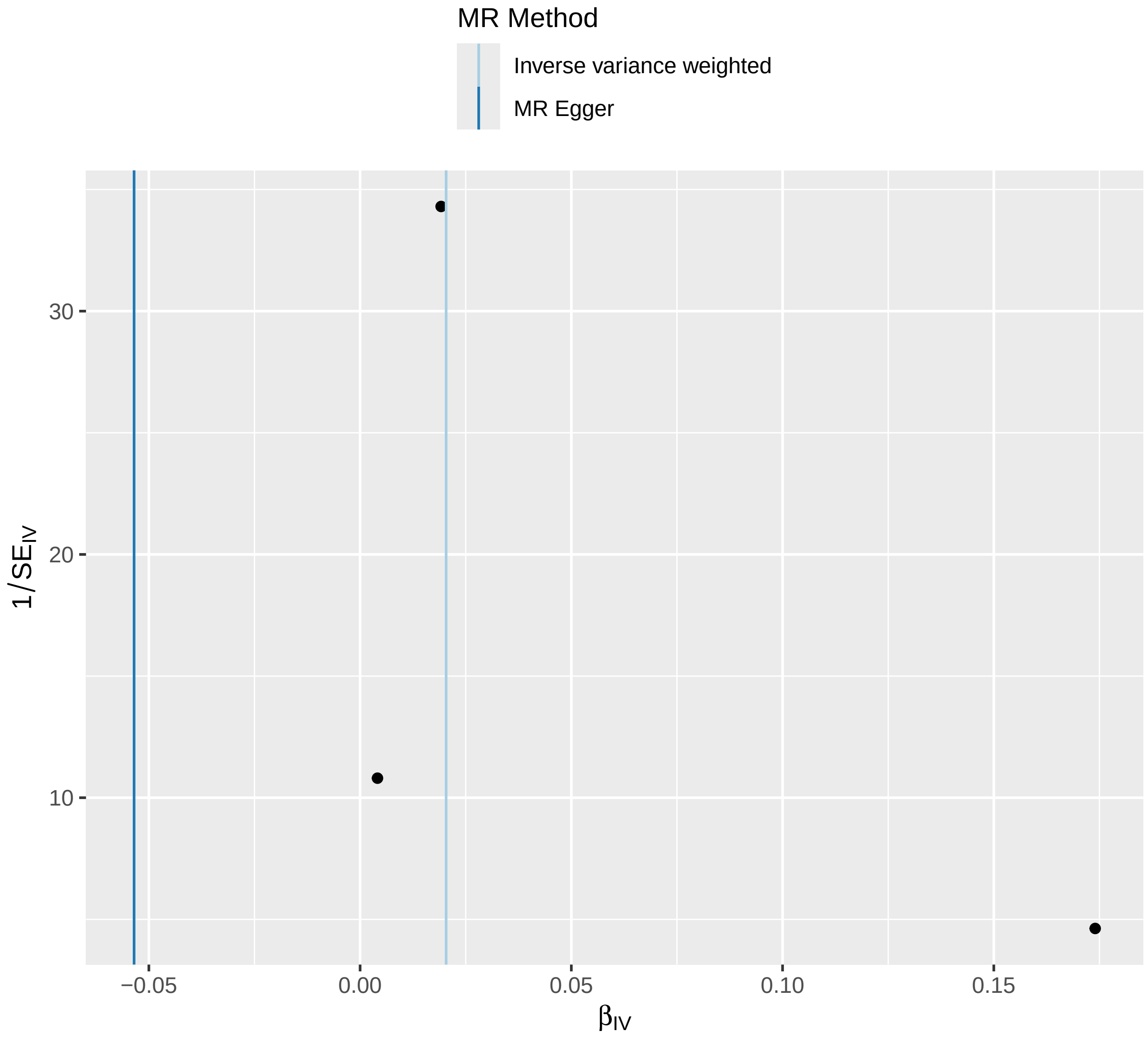

MR Method
Inverse variance weighted
MR Egger
30
20
10
$1/SE_{IV}$
−0.05
0.00
0.05
0.10
0.15
β_{IV}

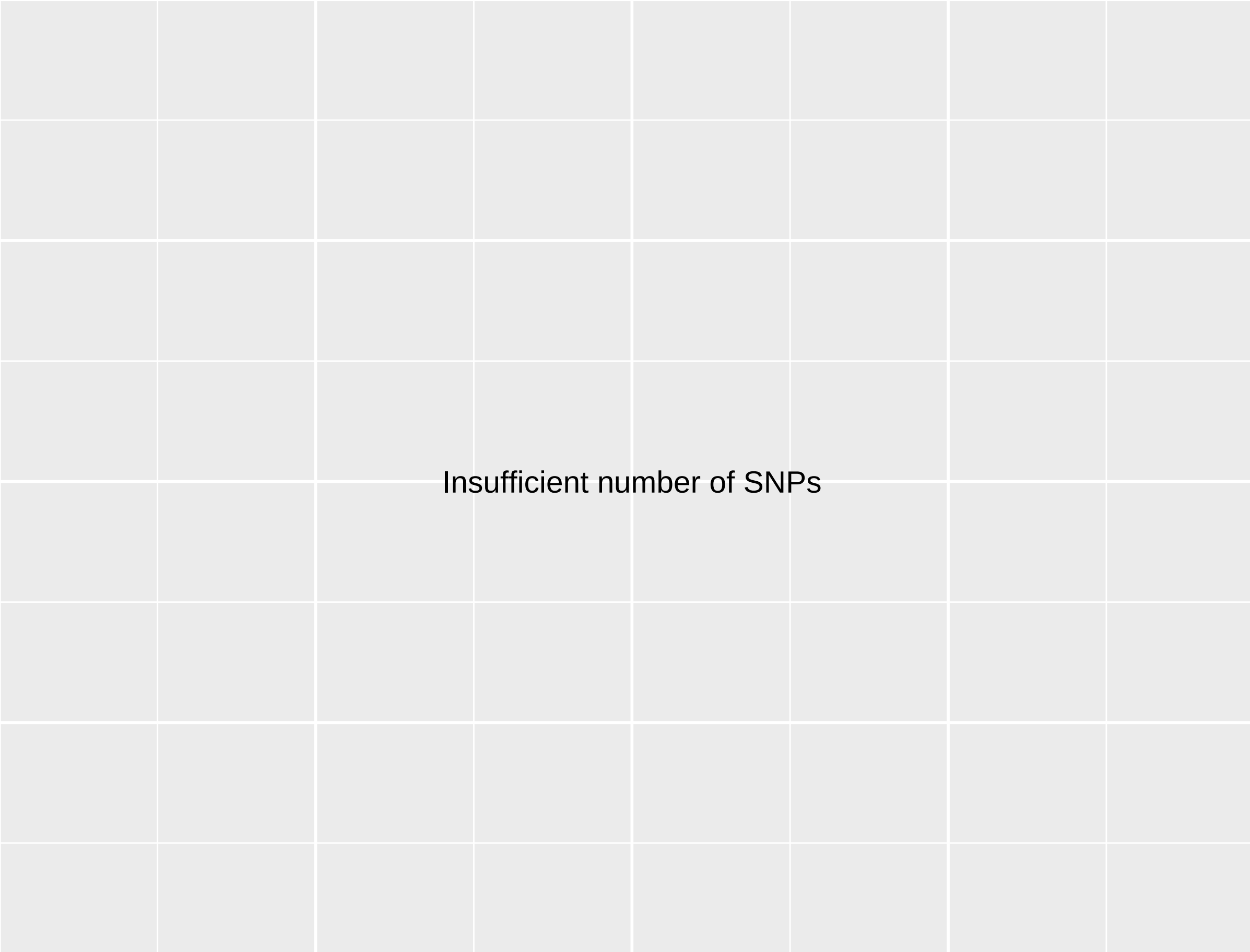
Insufficient number of SNPs

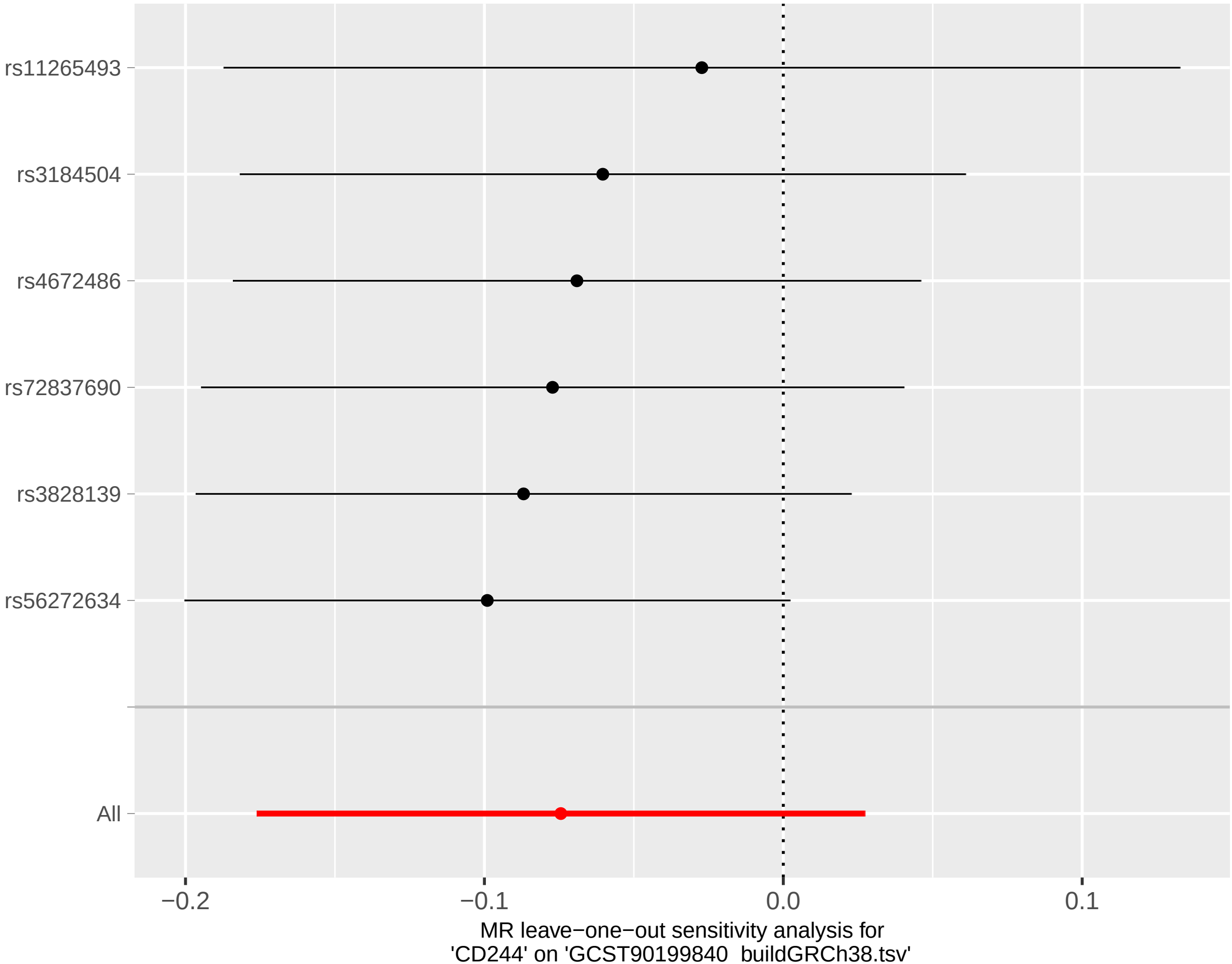

rs11265493
rs3184504
rs4672486
rs72837690
rs3828139
rs56272634
All
−0.2
−0.1
0.0
0.1
MR leave−one−out sensitivity analysis for
'CD244' on 'GCST90199840_buildGRCh38.tsv'

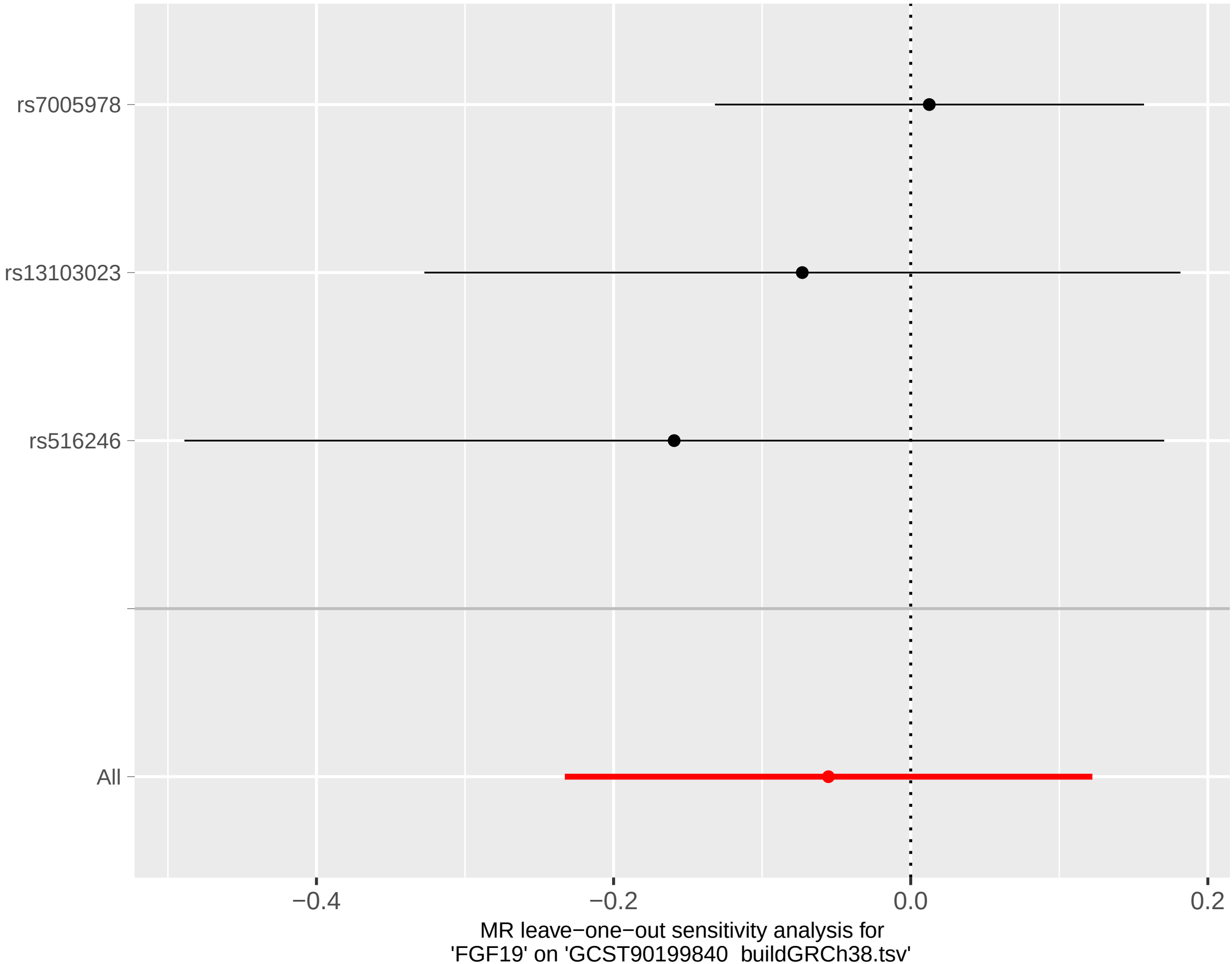
rs7005978
rs13103023
rs516246
All
−0.4
−0.2
0.0
0.2
MR leave−one−out sensitivity analysis for
'FGF19' on 'GCST90199840_buildGRCh38.tsv'

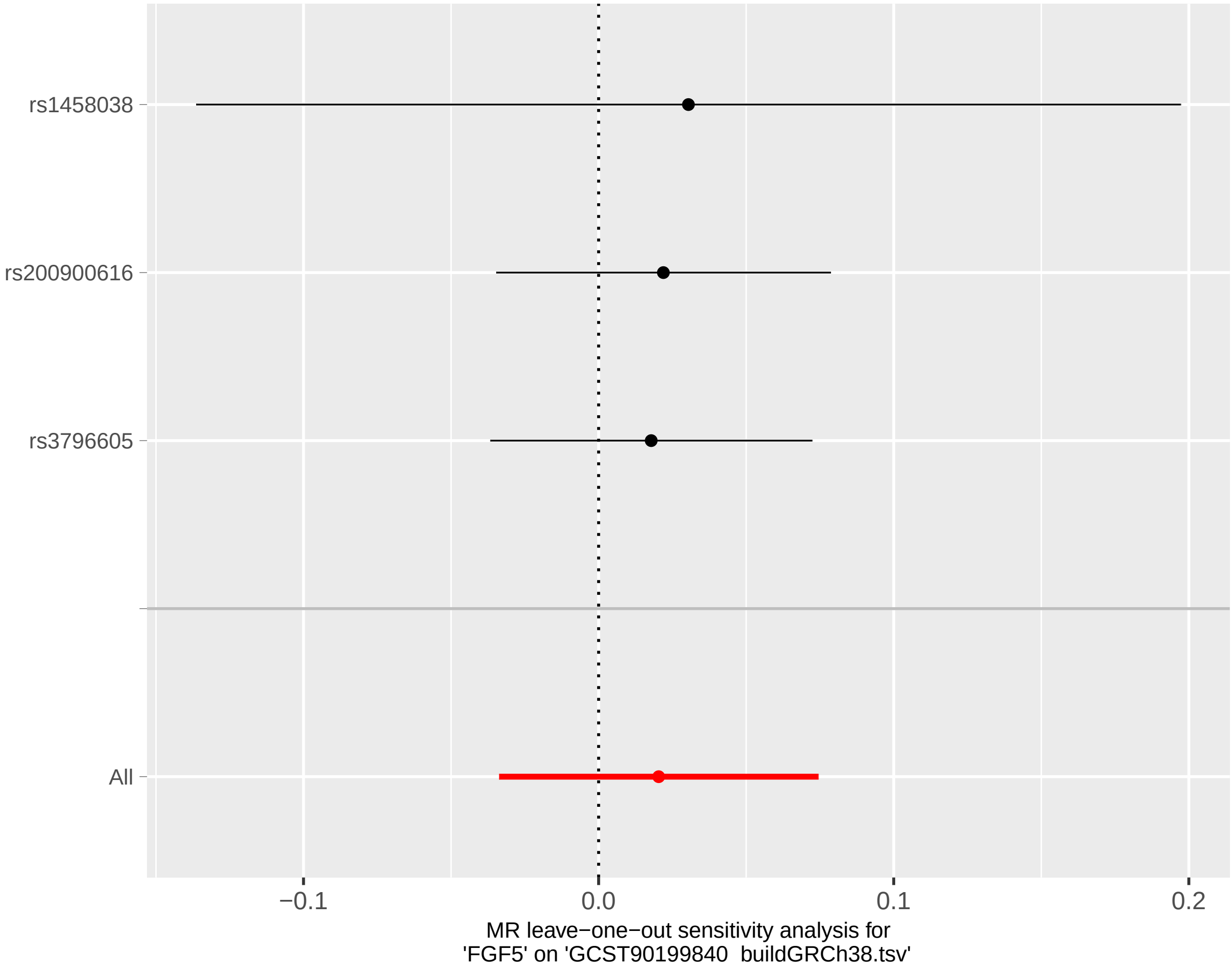
rs1458038
rs200900616
rs3796605
All
−0.1
0.0
0.1
0.2
MR leave−one−out sensitivity analysis for
'FGF5' on 'GCST90199840_buildGRCh38.tsv'

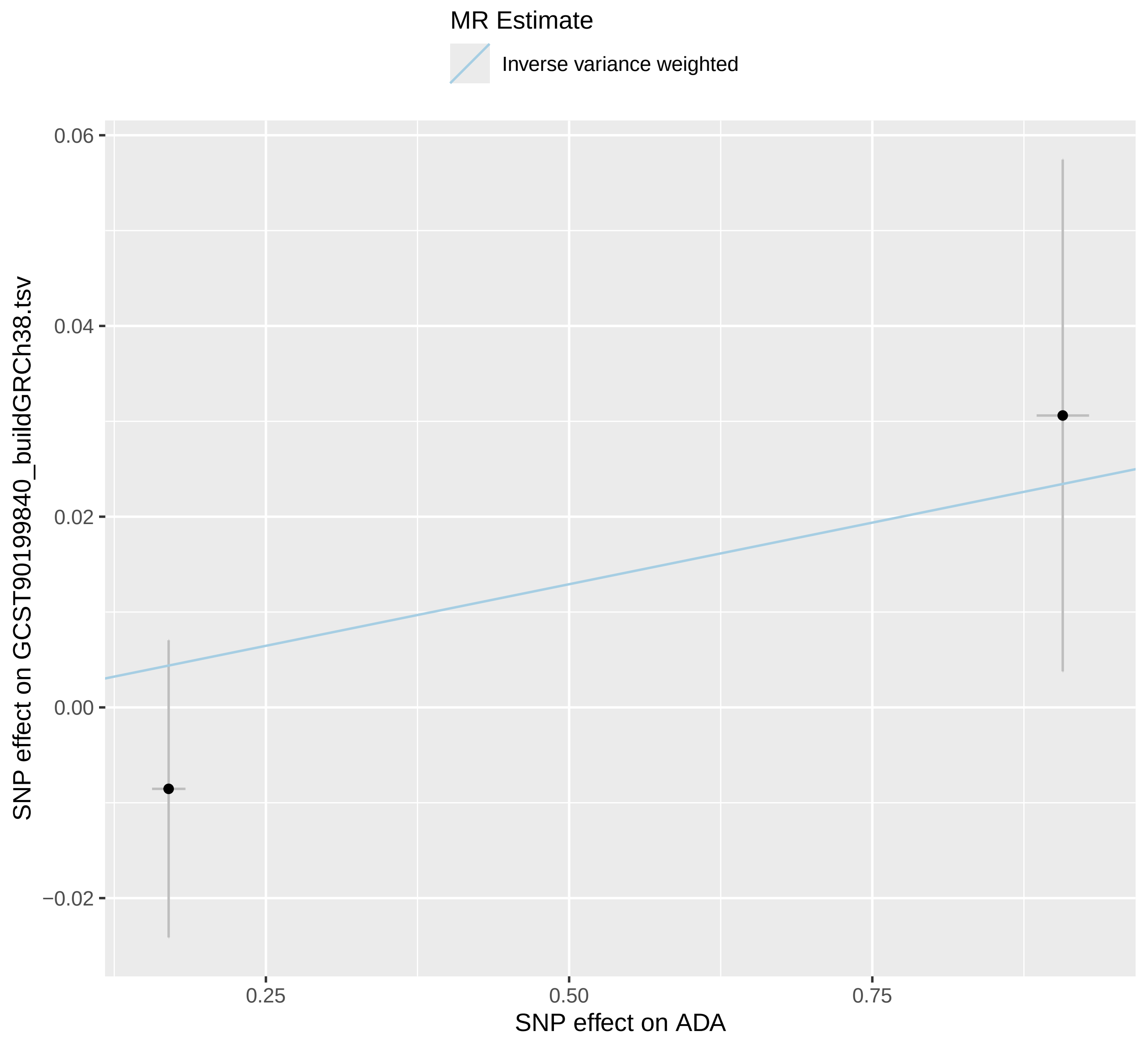

MR Estimate
Inverse variance weighted
0.06
0.04
0.02
0.00
−0.02
SNP effect on GCST90199840_buildGRCh38.tsv
0.25
0.50
0.75
SNP effect on ADA

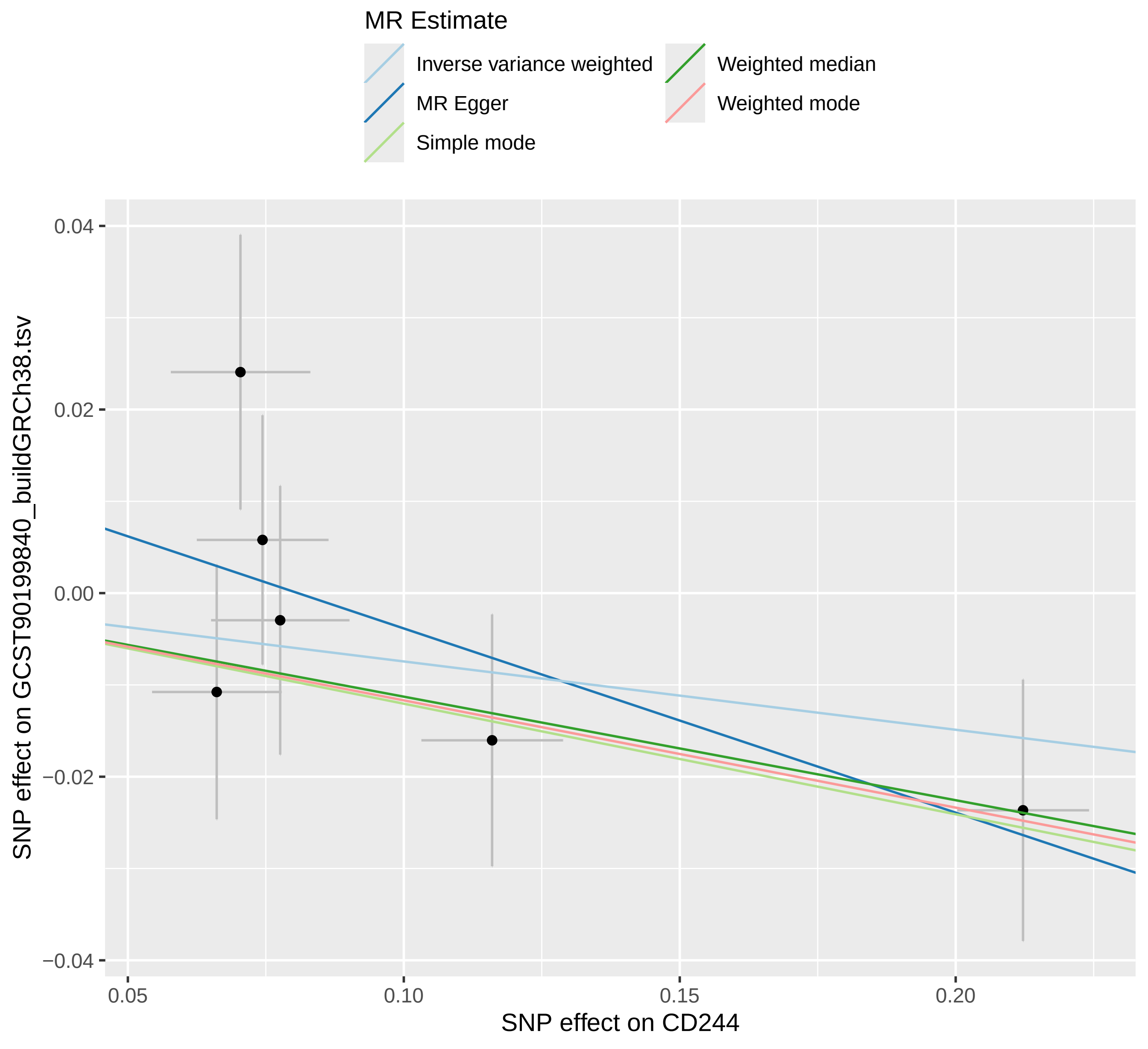

MR Estimate
Inverse variance weighted
MR Egger
Simple mode
Weighted median
Weighted mode
0.04
0.02
0.00
−0.02
−0.04
0.05
0.10
0.15
0.20
SNP effect on GCST90199840_buildGRCh38.tsv
SNP effect on CD244

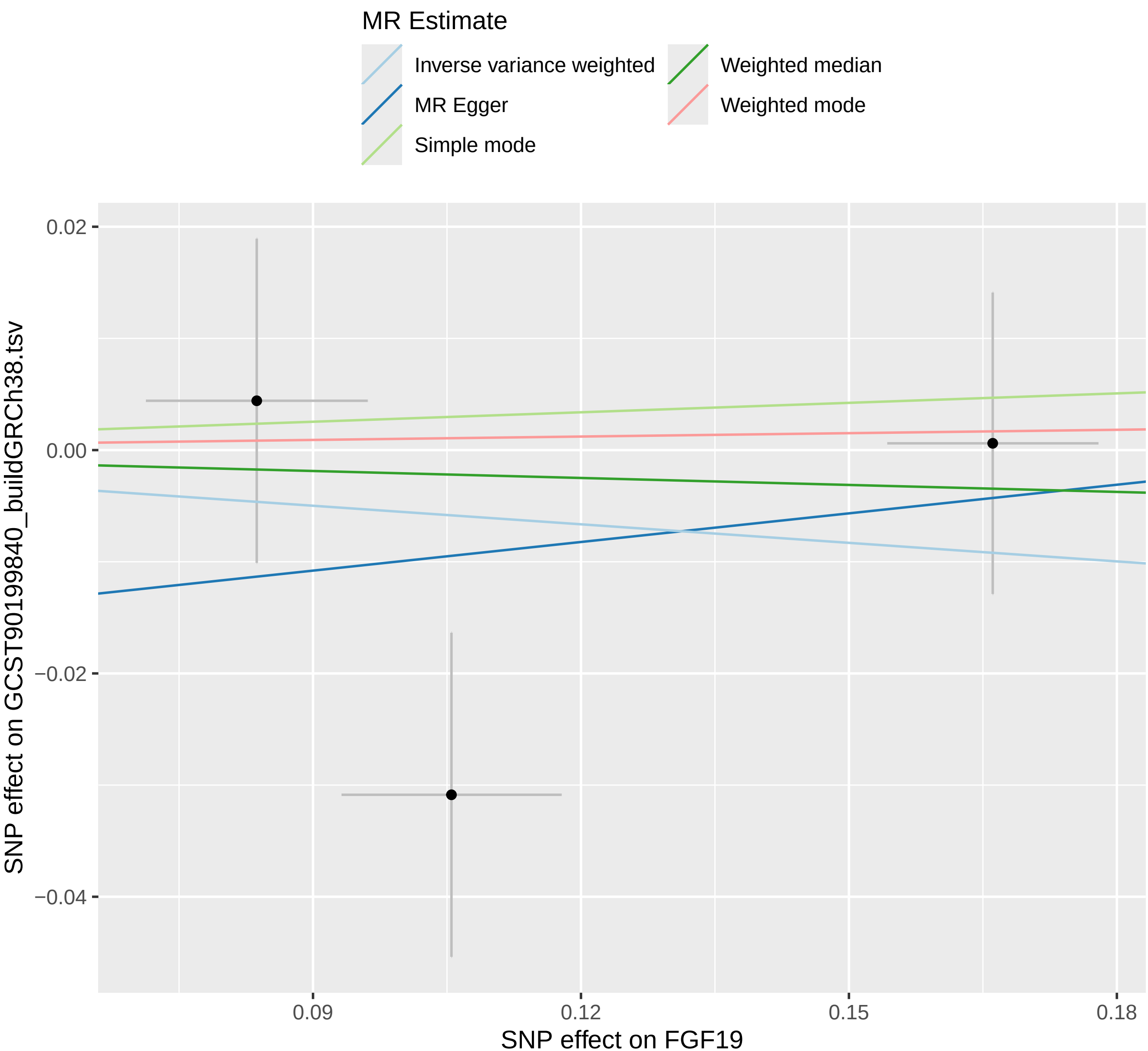
MR Estimate
Inverse variance weighted
MR Egger
Simple mode
Weighted median
Weighted mode
0.02
0.00
−0.02
−0.04
SNP effect on GCST90199840_buildGRCh38.tsv
0.09
0.12
0.15
0.18
SNP effect on FGF19

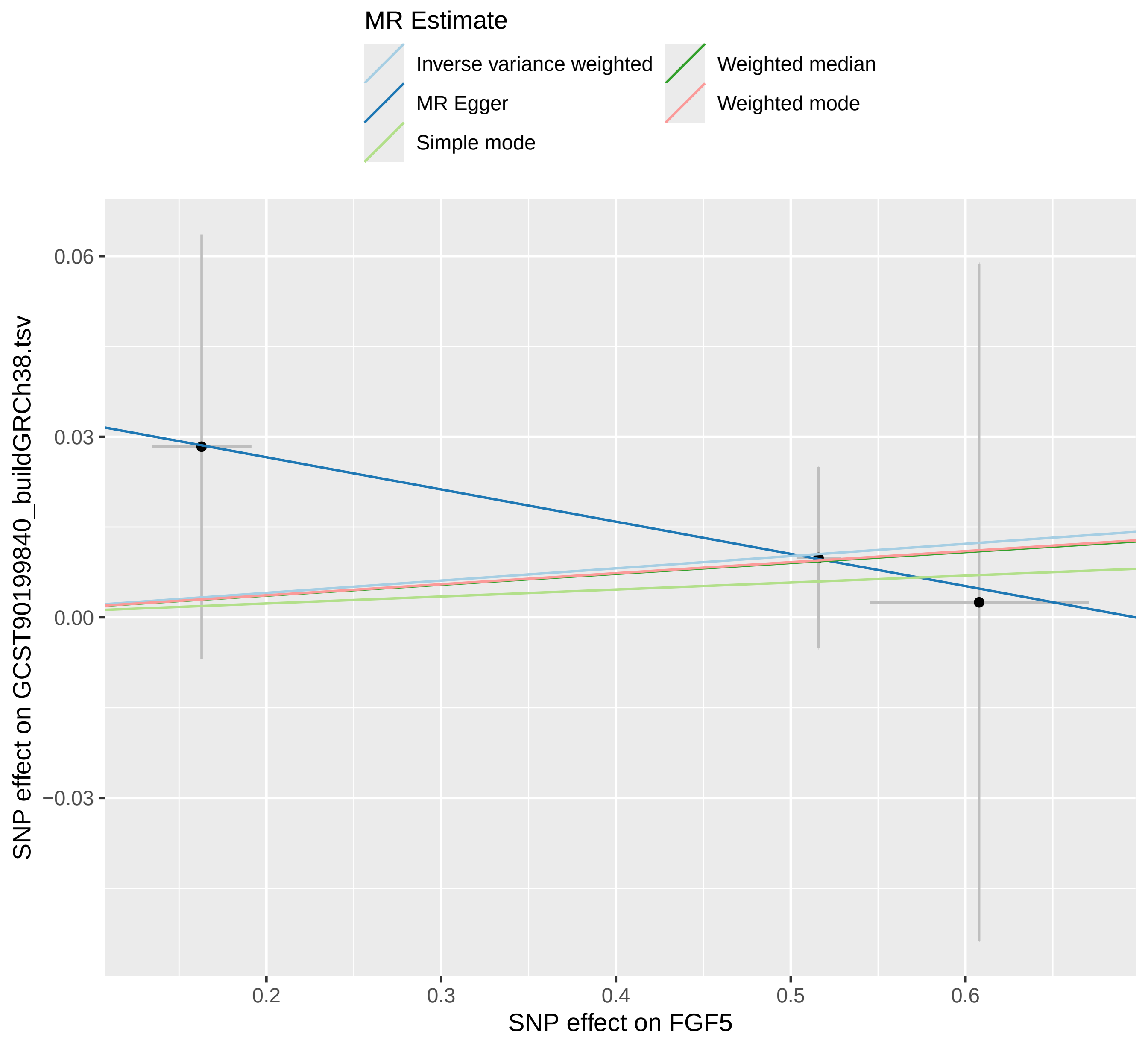

MR Estimate
Inverse variance weighted
MR Egger
Simple mode
Weighted median
Weighted mode
0.06
0.03
0.00
−0.03
SNP effect on GCST90199840_buildGRCh38.tsv
0.2
0.3
0.4
0.5
0.6
SNP effect on FGF5

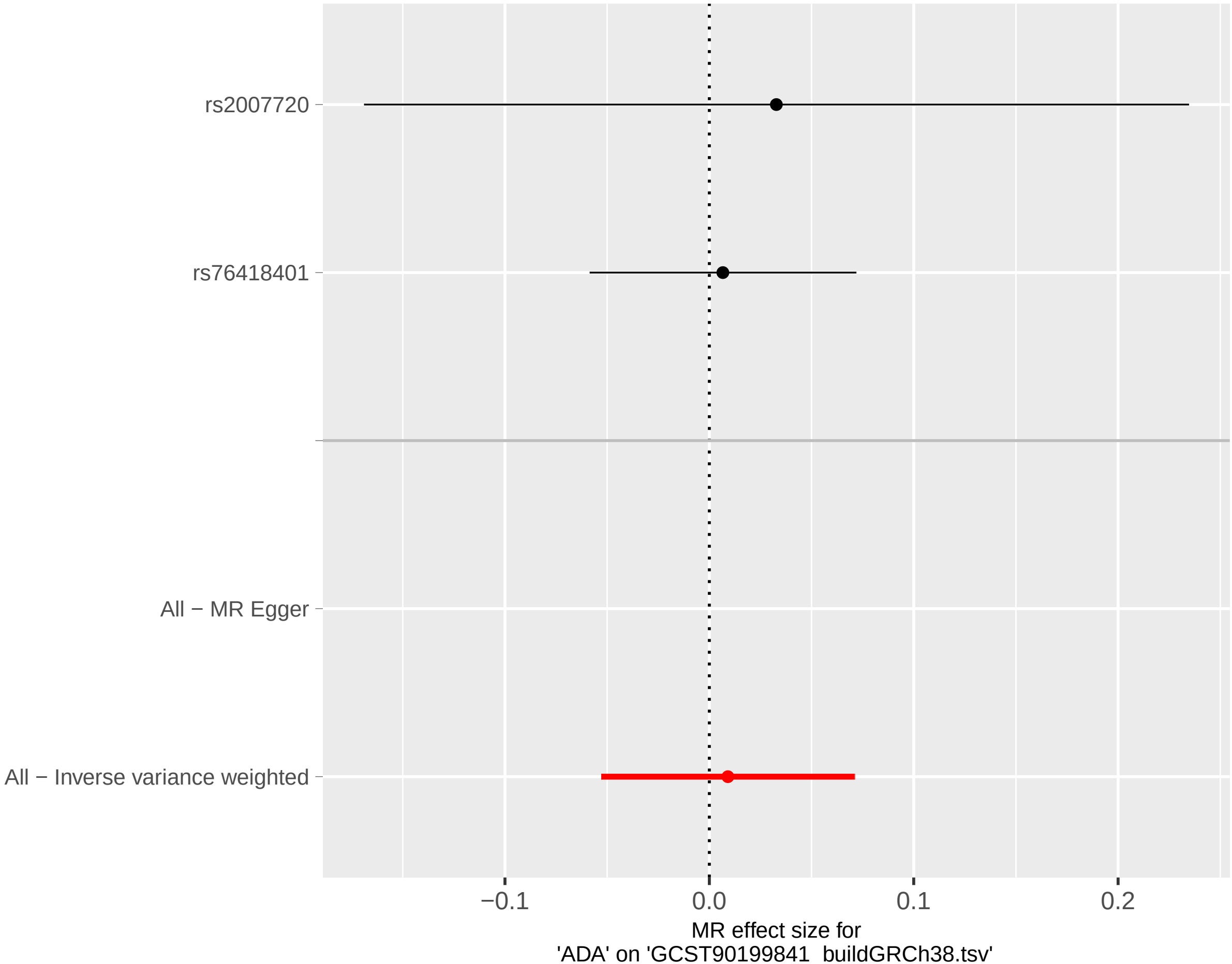
rs2007720
rs76418401
All − MR Egger
All − Inverse variance weighted
−0.1
0.0
0.1
0.2
MR effect size for
'ADA' on 'GCST90199841_buildGRCh38.tsv'

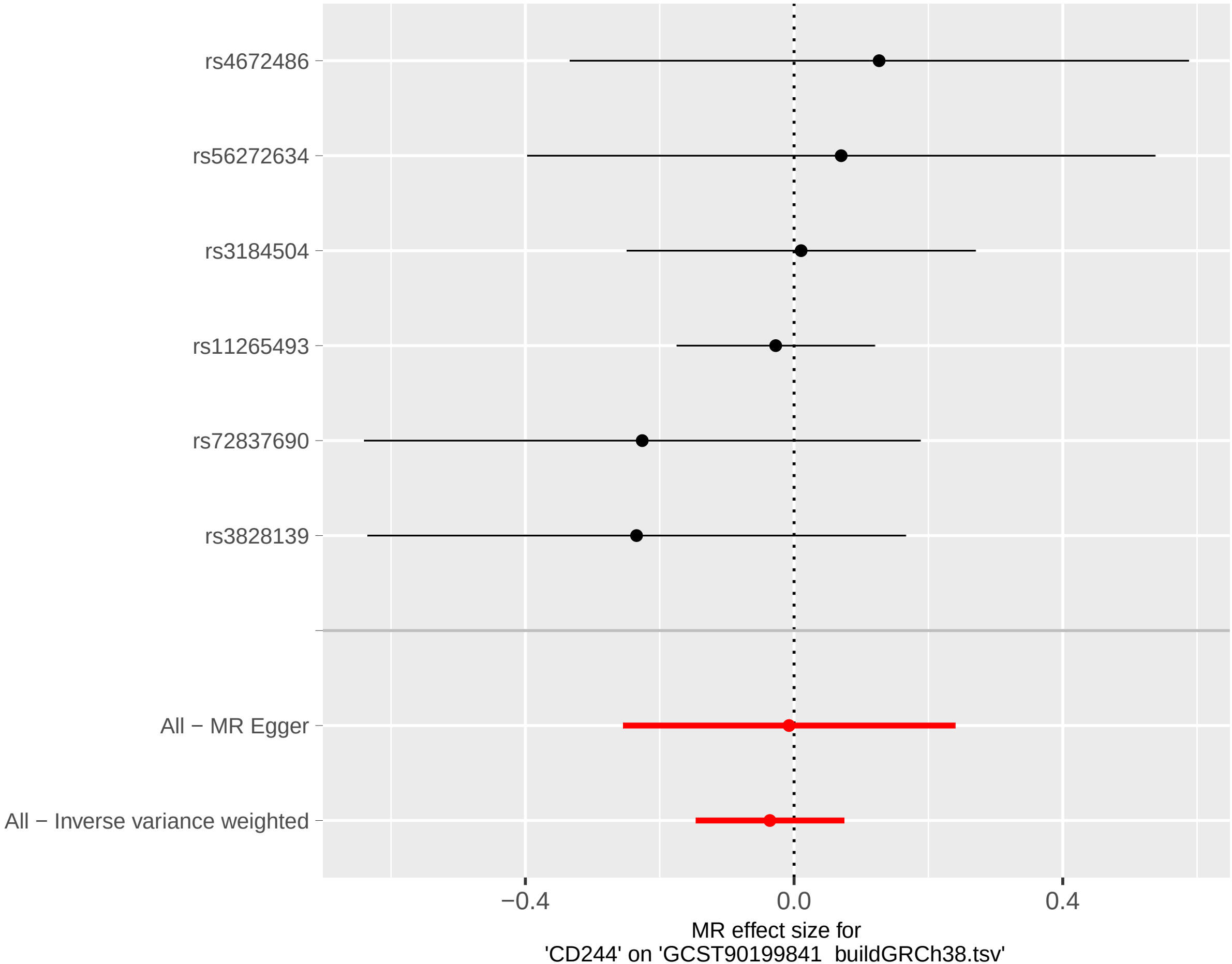
rs4672486
rs56272634
rs3184504
rs11265493
rs72837690
rs3828139
All − MR Egger
All − Inverse variance weighted
−0.4
0.0
0.4
MR effect size for
'CD244' on 'GCST90199841_buildGRCh38.tsv'

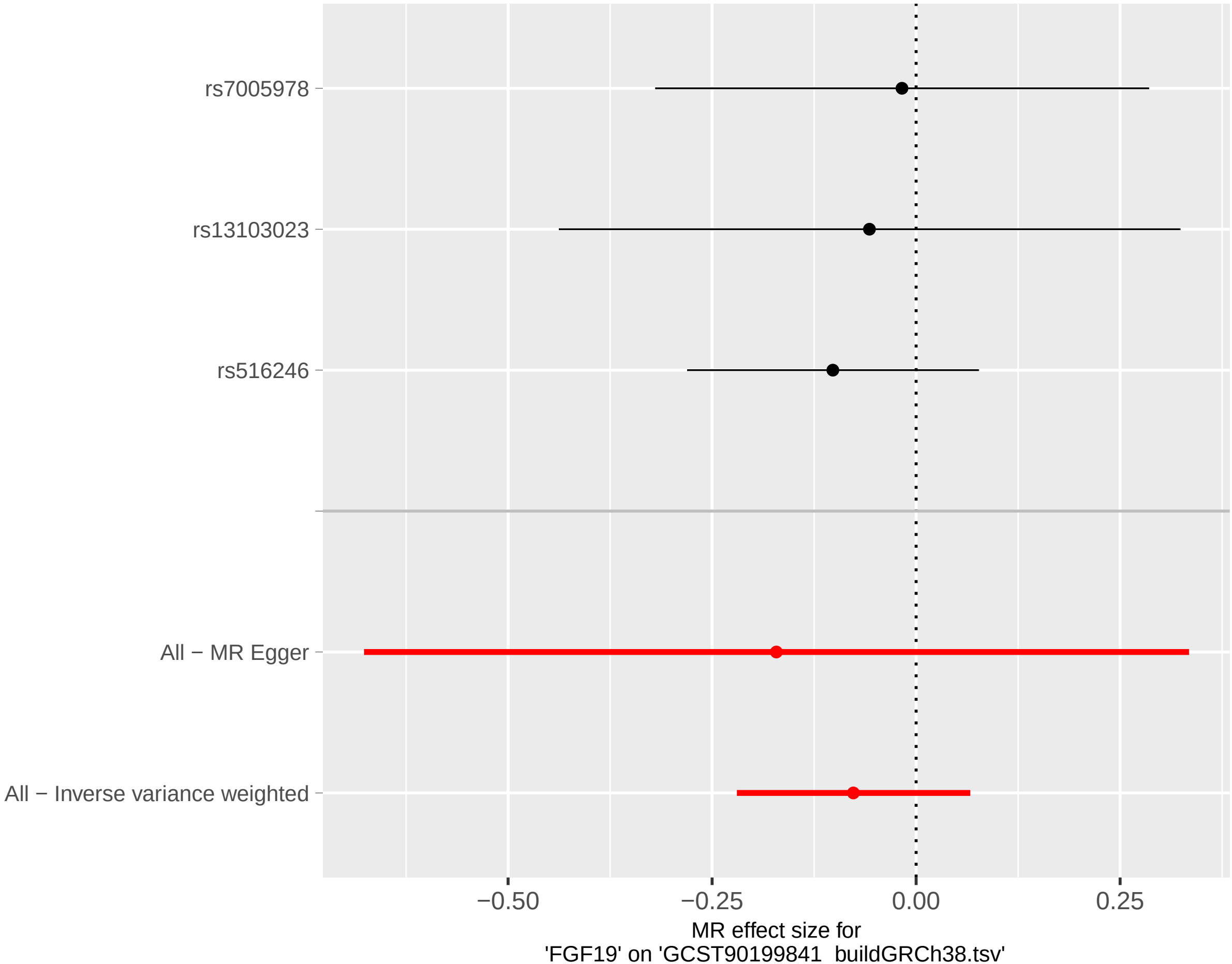

rs7005978
rs13103023
rs516246
All − MR Egger
All − Inverse variance weighted
−0.50
−0.25
0.00
0.25
MR effect size for
'FGF19' on 'GCST90199841_buildGRCh38.tsv'

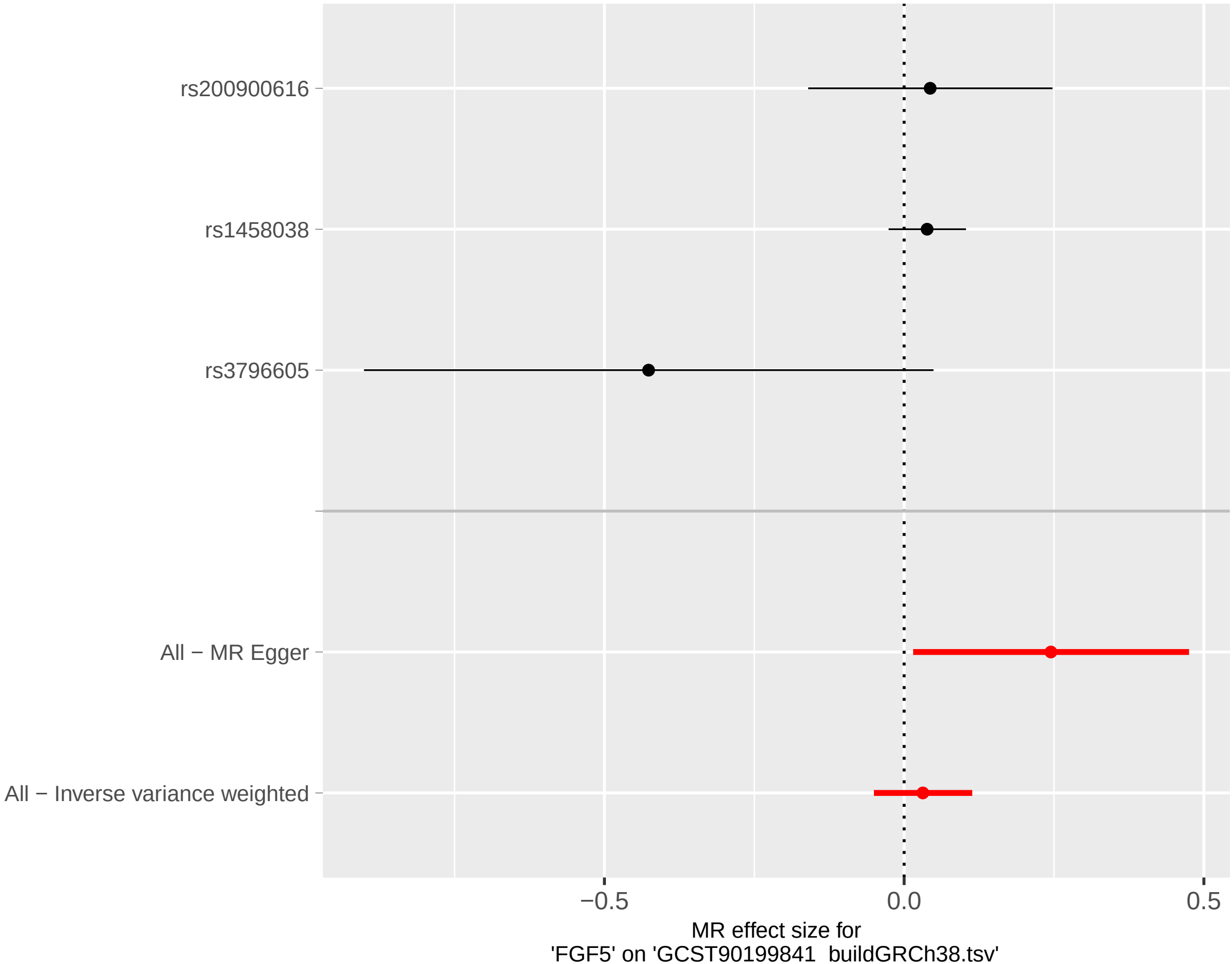
rs200900616
rs1458038
rs3796605
All − MR Egger
All − Inverse variance weighted
−0.5
0.0
0.5
MR effect size for
'FGF5' on 'GCST90199841_buildGRCh38.tsv'

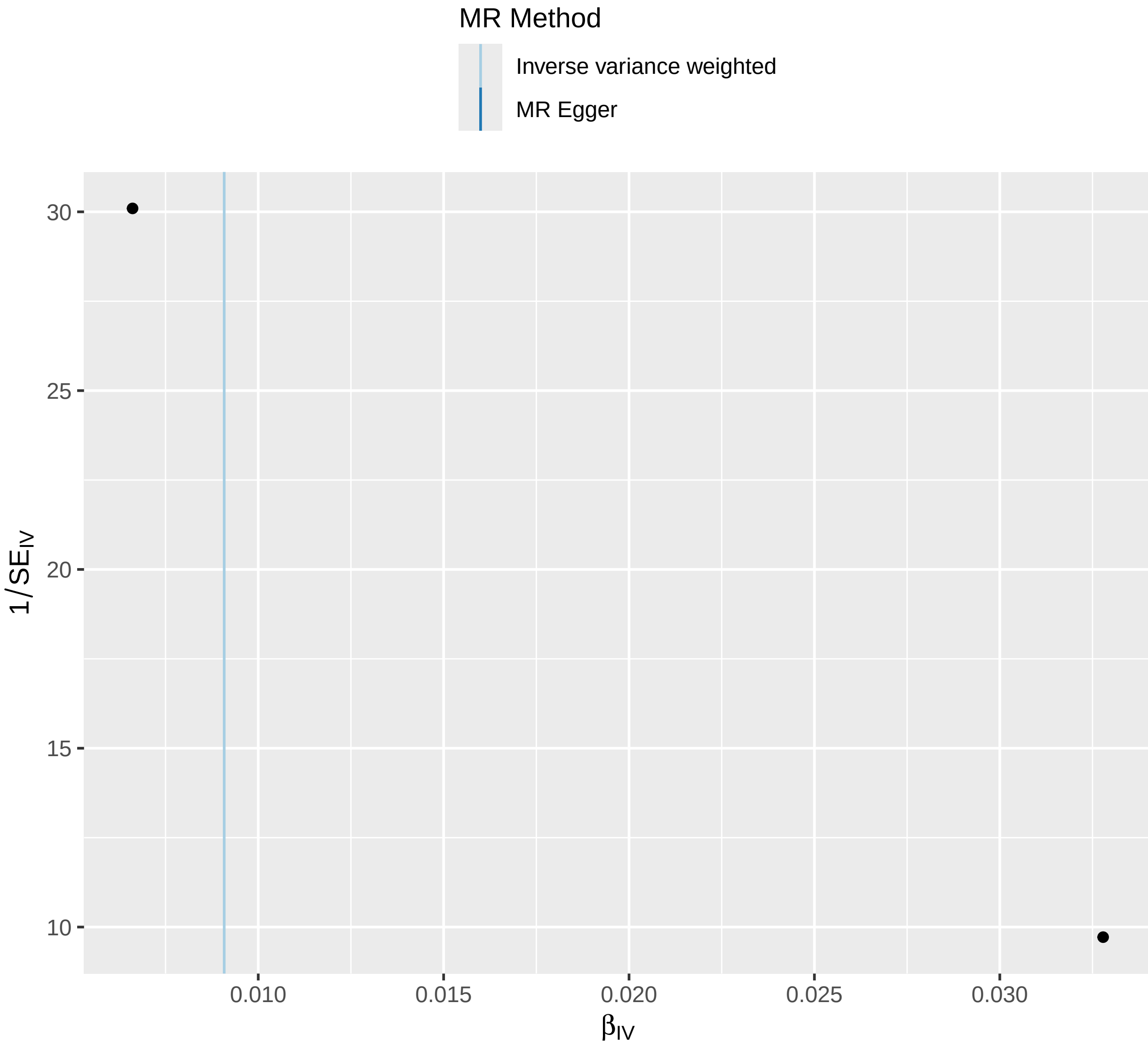

MR Method
Inverse variance weighted
MR Egger
30
25
20
15
10
1/SE_IV
0.010
0.015
0.020
0.025
0.030
β_IV

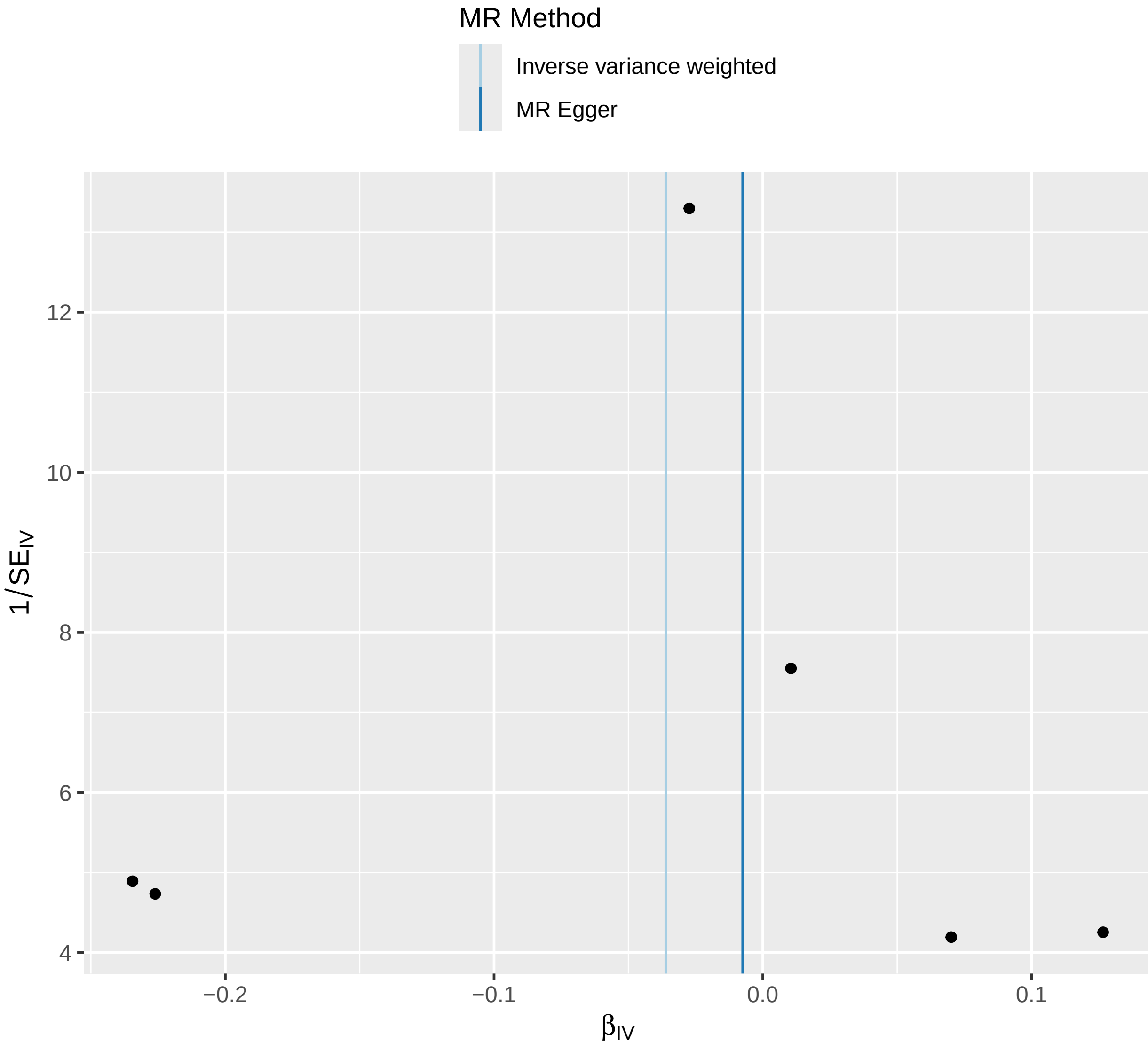
MR Method
Inverse variance weighted
MR Egger
12
10
8
6
4
1/SE$_{IV}$
−0.2
−0.1
0.0
0.1
β$_{IV}$

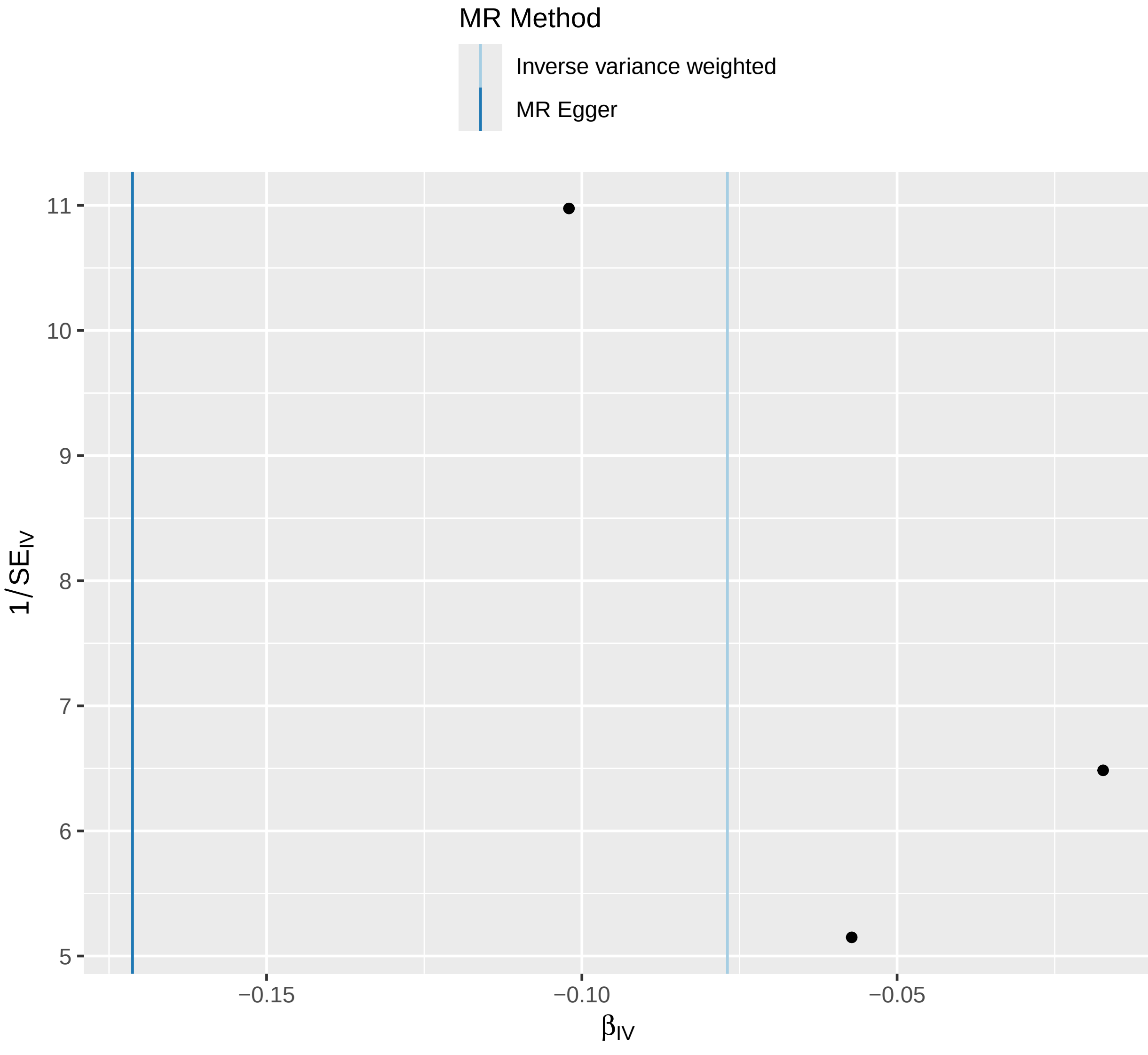

MR Method
Inverse variance weighted
MR Egger
11
10
9
8
7
6
5
$1/SE_{IV}$
−0.15
−0.10
−0.05
β_{IV}

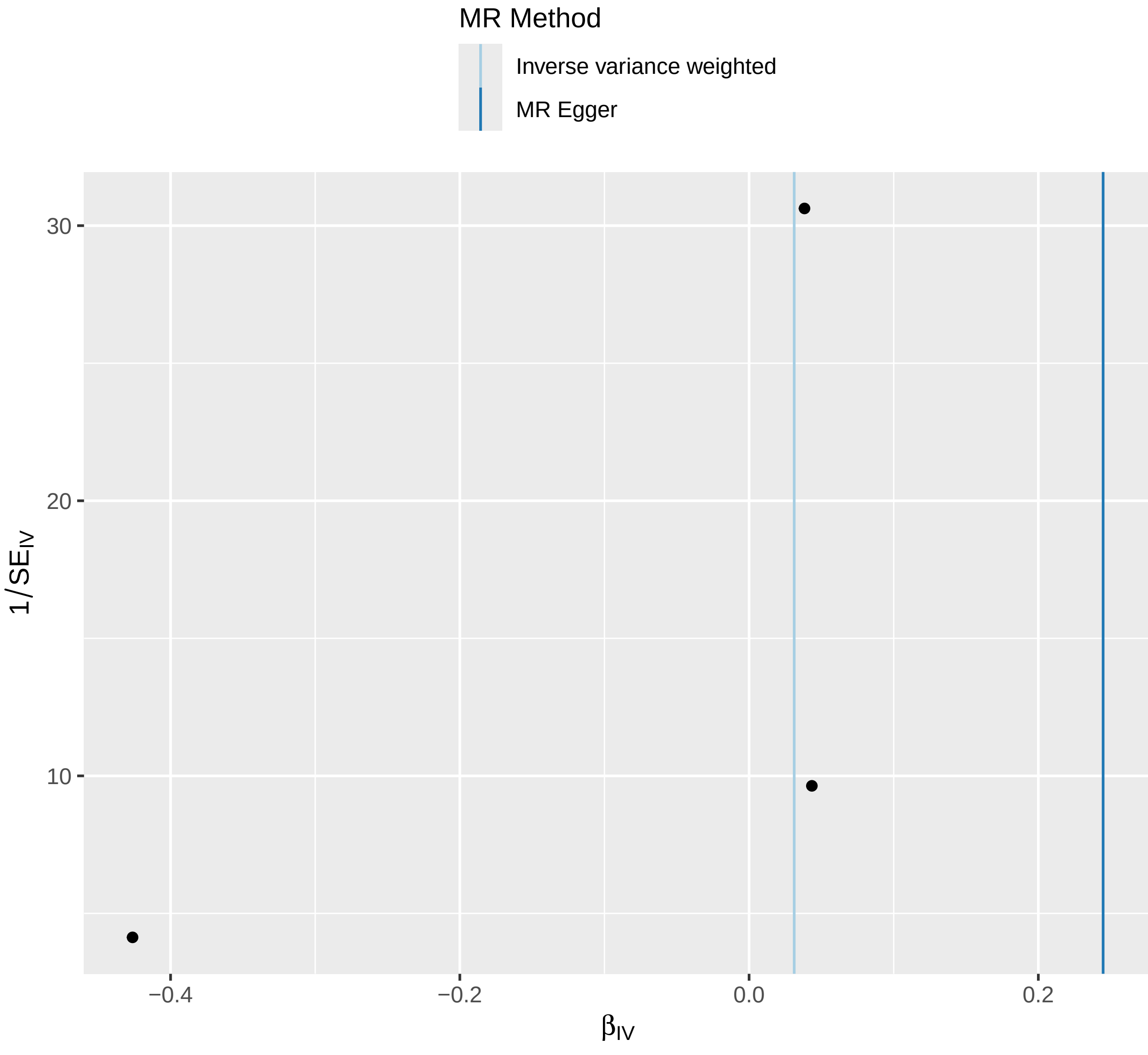
MR Method
Inverse variance weighted
MR Egger
30
20
10
1/SE_IV
−0.4
−0.2
0.0
0.2
β_IV

Insufficient number of SNPs

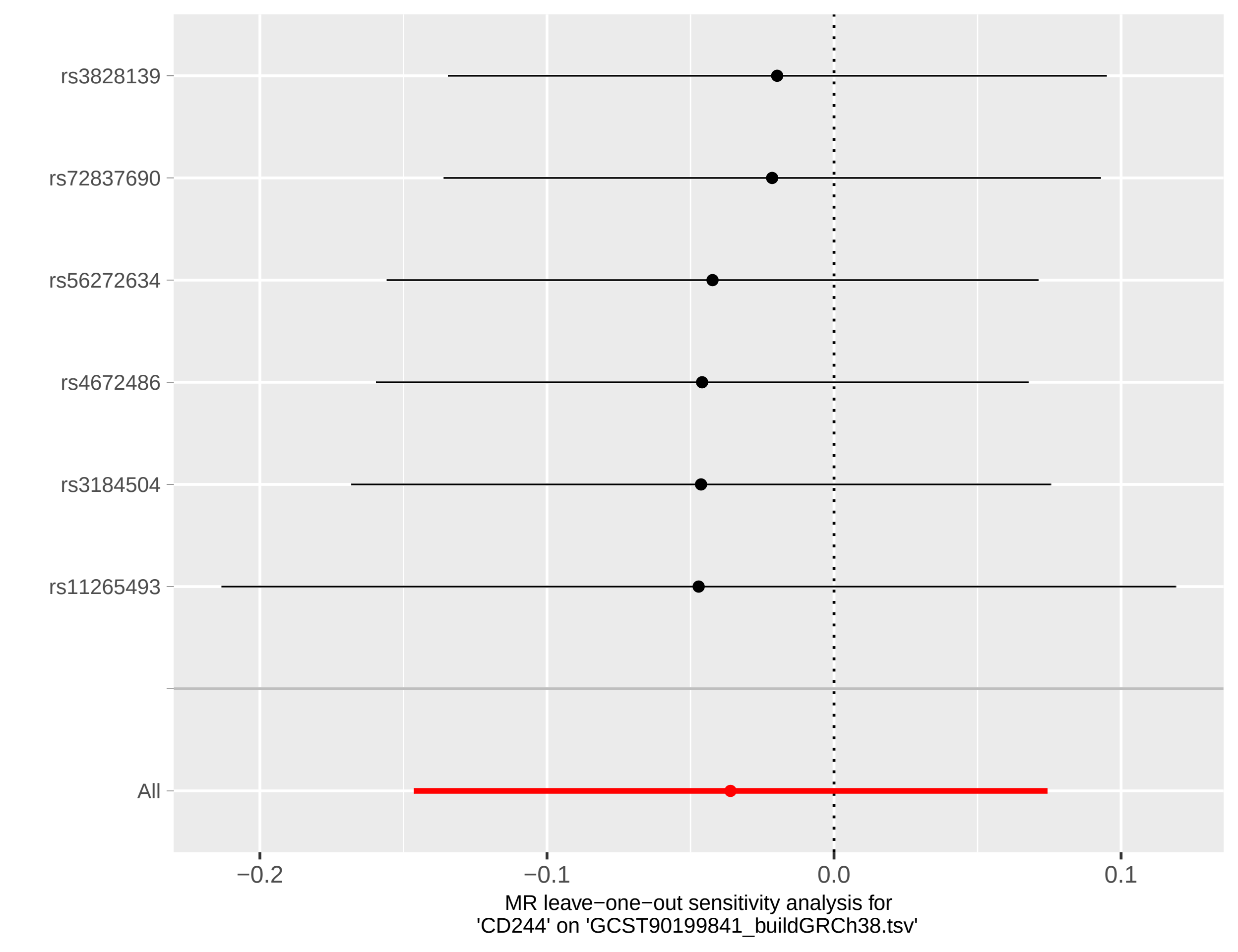
rs3828139
rs72837690
rs56272634
rs4672486
rs3184504
rs11265493
All
−0.2
−0.1
0.0
0.1
MR leave−one−out sensitivity analysis for
'CD244' on 'GCST90199841_buildGRCh38.tsv'

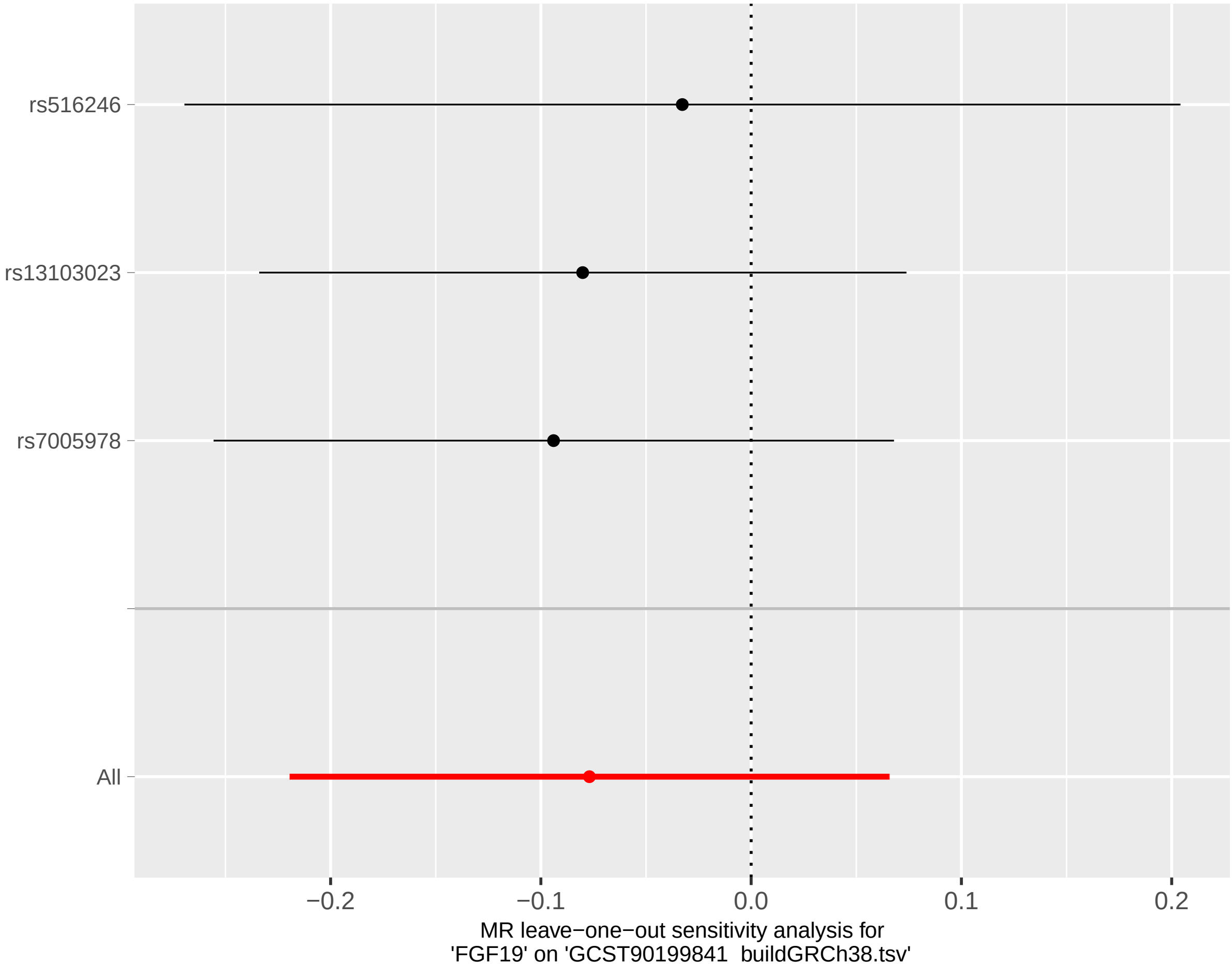
rs516246
rs13103023
rs7005978
All
−0.2
−0.1
0.0
0.1
0.2
MR leave−one−out sensitivity analysis for
'FGF19' on 'GCST90199841_buildGRCh38.tsv'

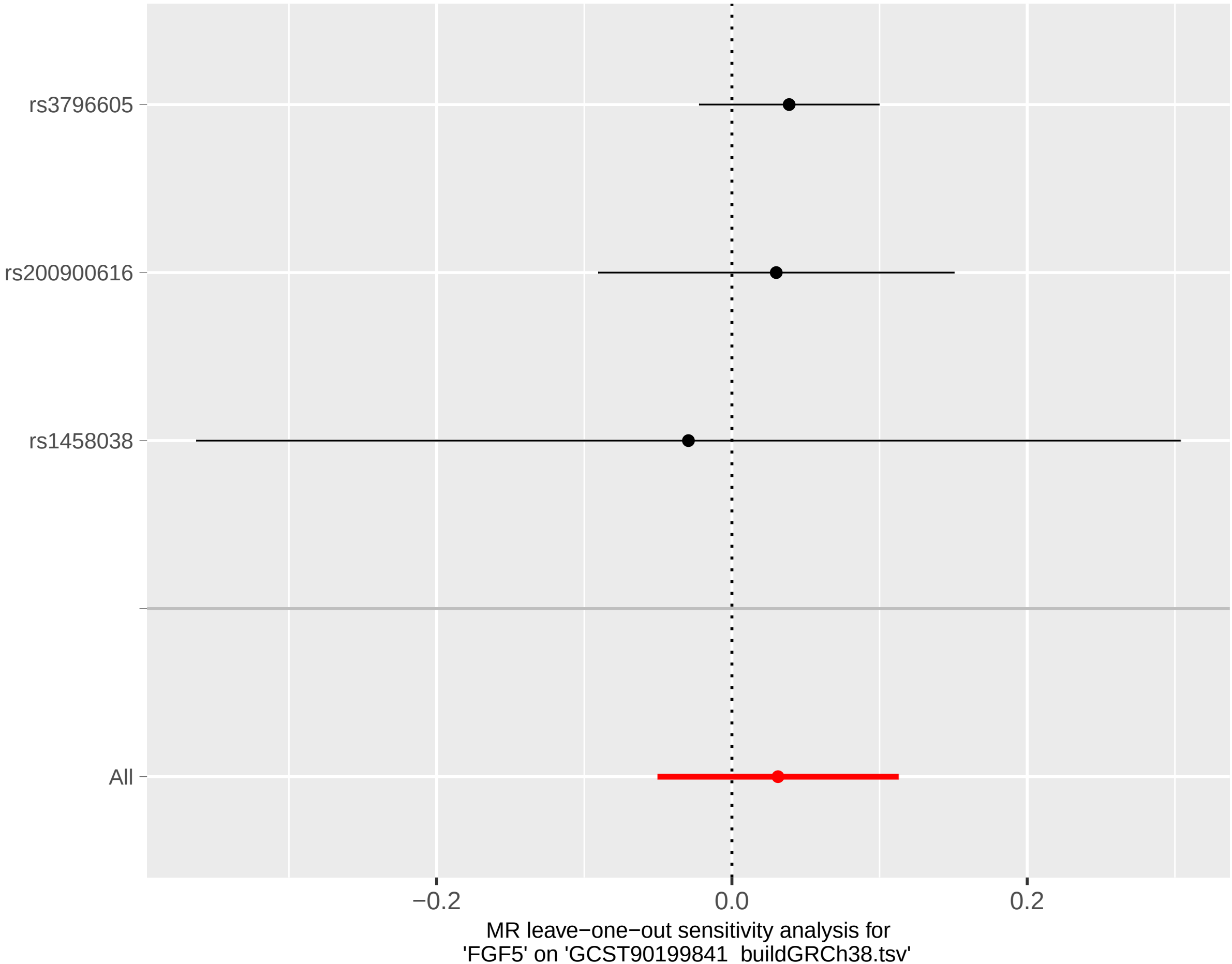

rs3796605
rs200900616
rs1458038
All
−0.2
0.0
0.2
MR leave−one−out sensitivity analysis for
'FGF5' on 'GCST90199841_buildGRCh38.tsv'

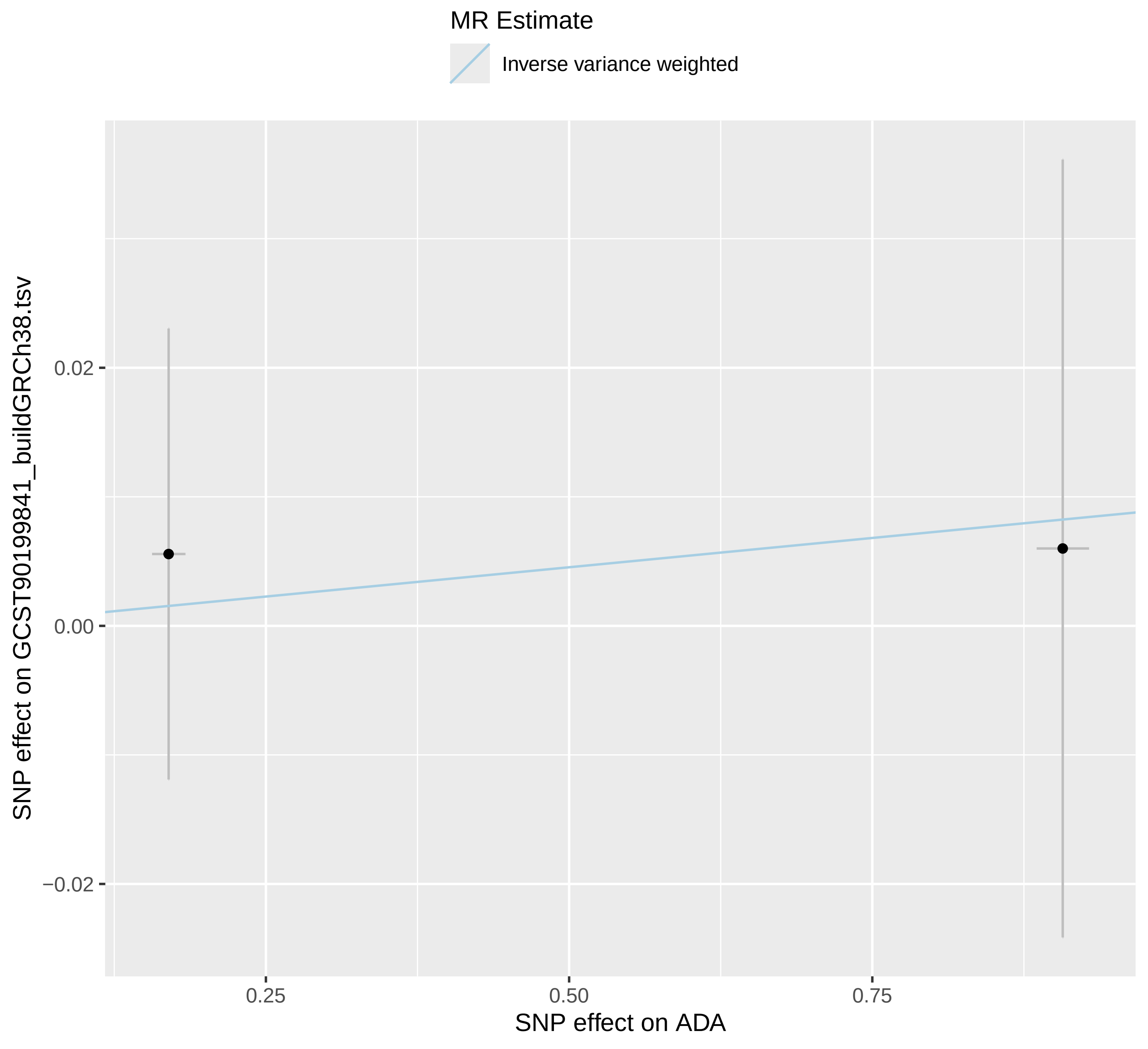
MR Estimate
Inverse variance weighted
SNP effect on GCST90199841_buildGRCh38.tsv
0.02
0.00
−0.02
0.25
0.50
0.75
SNP effect on ADA

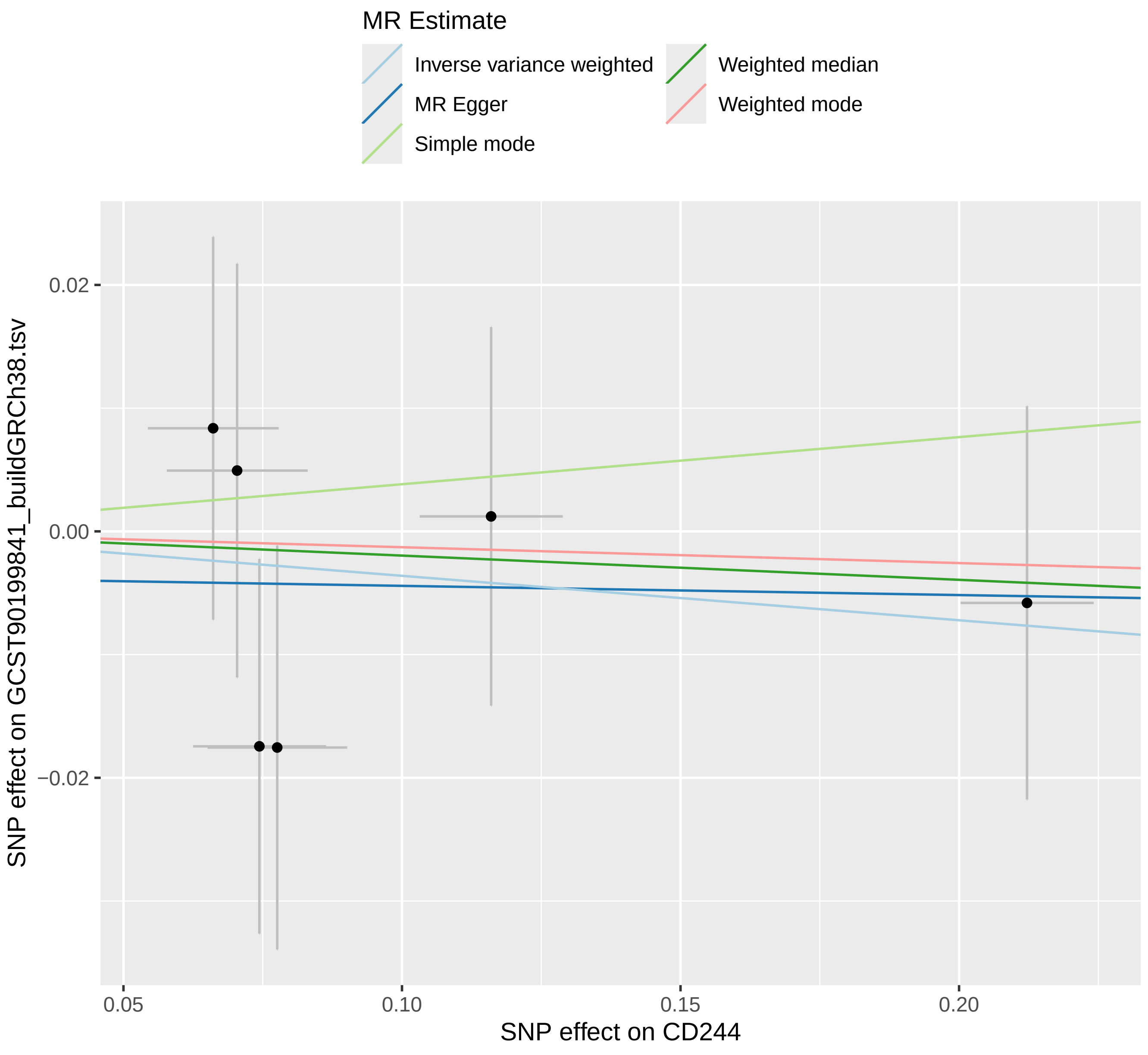
MR Estimate
Inverse variance weighted
MR Egger
Simple mode
Weighted median
Weighted mode
0.02
0.00
−0.02
0.05
0.10
0.15
0.20
SNP effect on GCST90199841_buildGRCh38.tsv
SNP effect on CD244

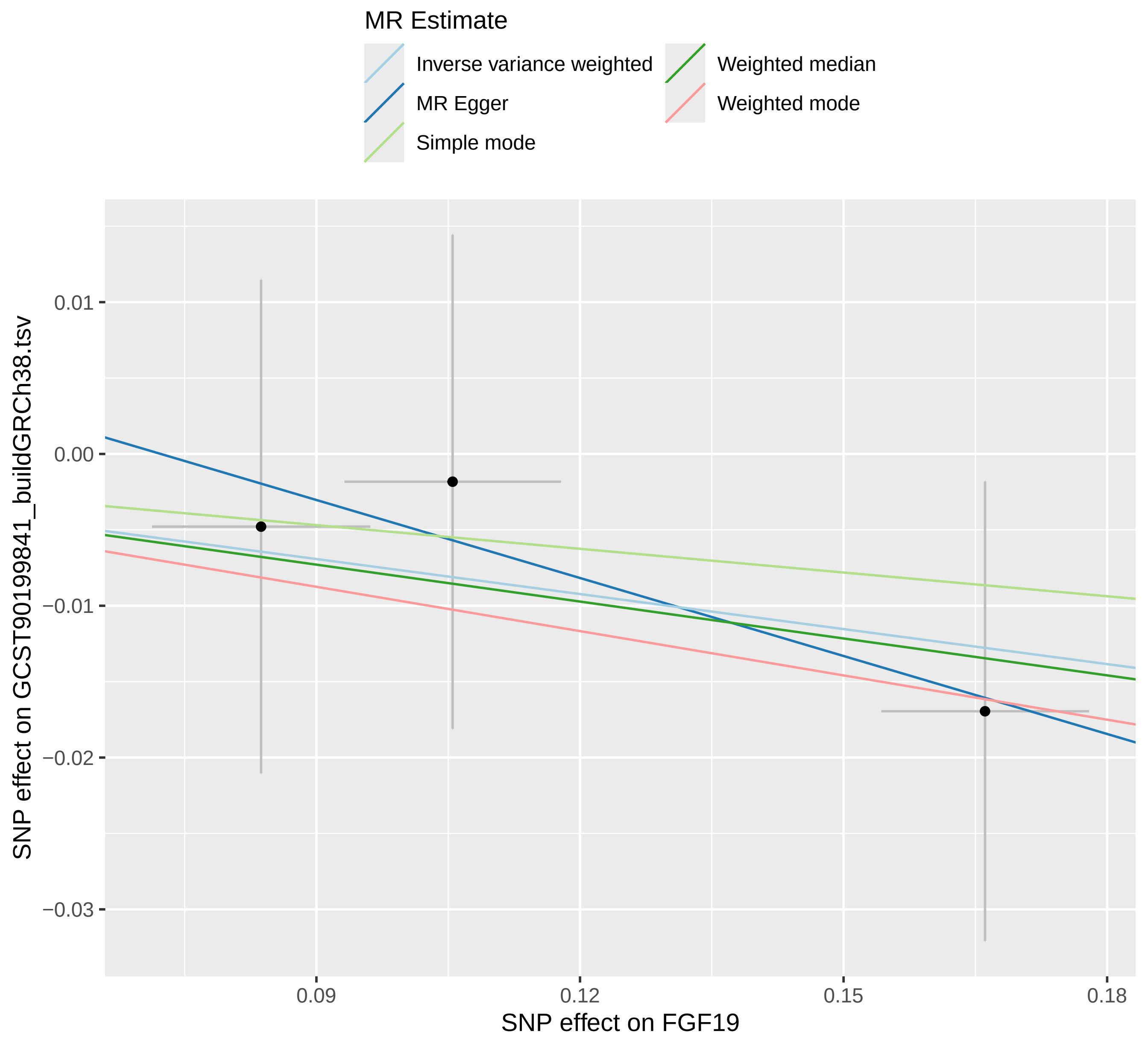
MR Estimate
Inverse variance weighted
MR Egger
Simple mode
Weighted median
Weighted mode
0.01
0.00
−0.01
−0.02
−0.03
SNP effect on GCST90199841_buildGRCh38.tsv
0.09
0.12
0.15
0.18
SNP effect on FGF19

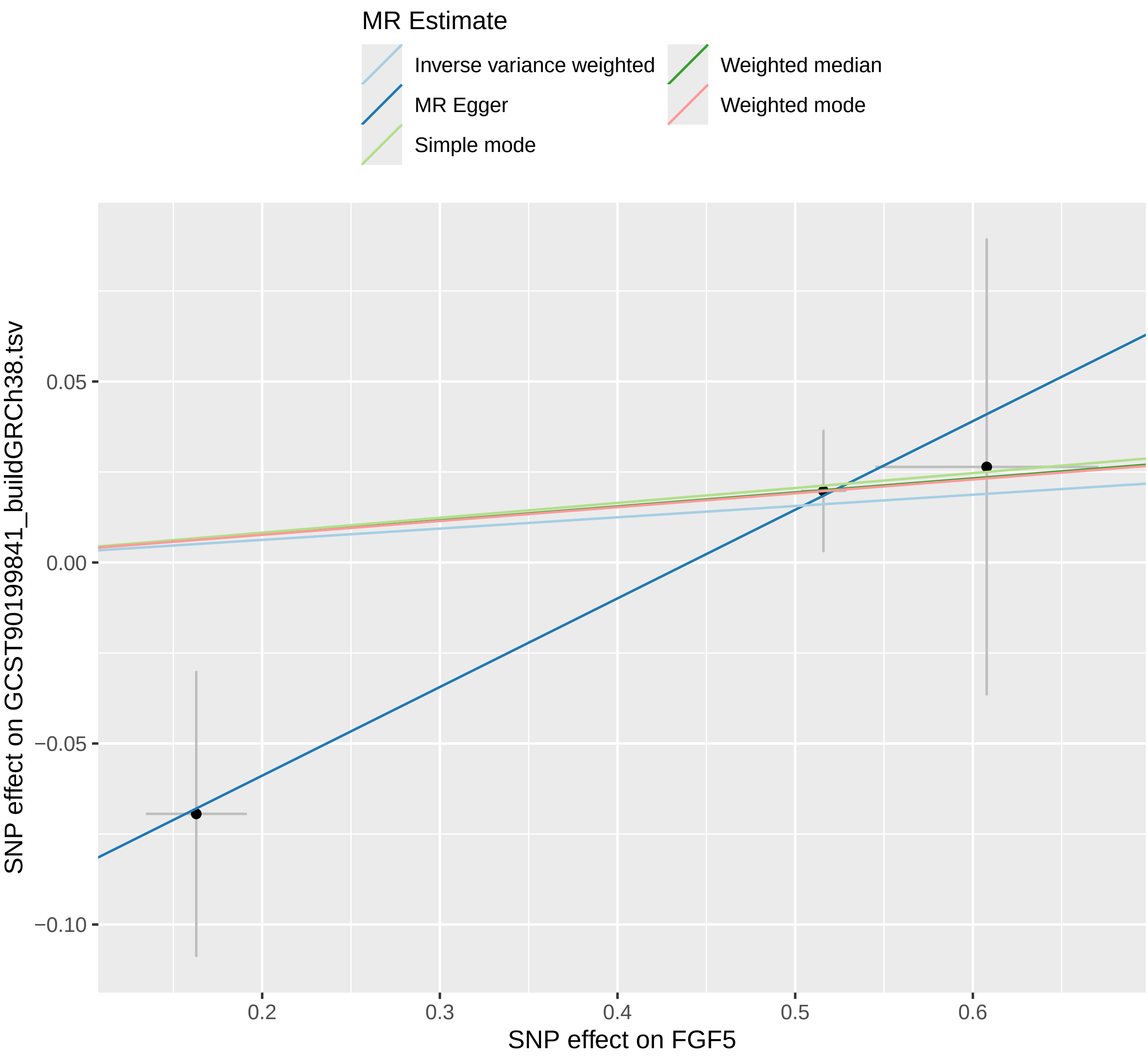
MR Estimate
Inverse variance weighted
MR Egger
Simple mode
Weighted median
Weighted mode
0.05
0.00
−0.05
−0.10
0.2
0.3
0.4
0.5
0.6
SNP effect on GCST90199841_buildGRCh38.tsv
SNP effect on FGF5

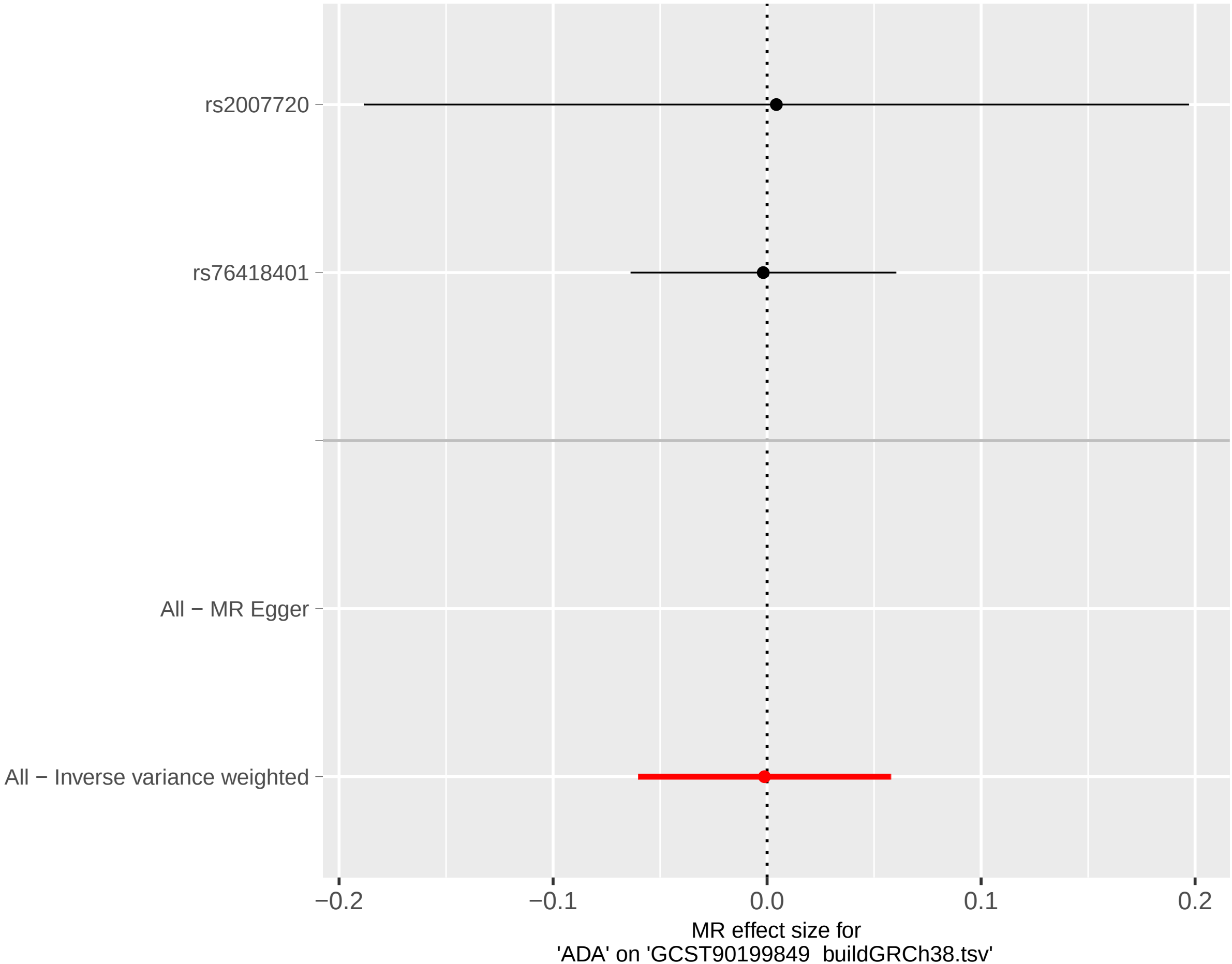

rs2007720
rs76418401
All − MR Egger
All − Inverse variance weighted
−0.2
−0.1
0.0
0.1
0.2
MR effect size for
'ADA' on 'GCST90199849_buildGRCh38.tsv'

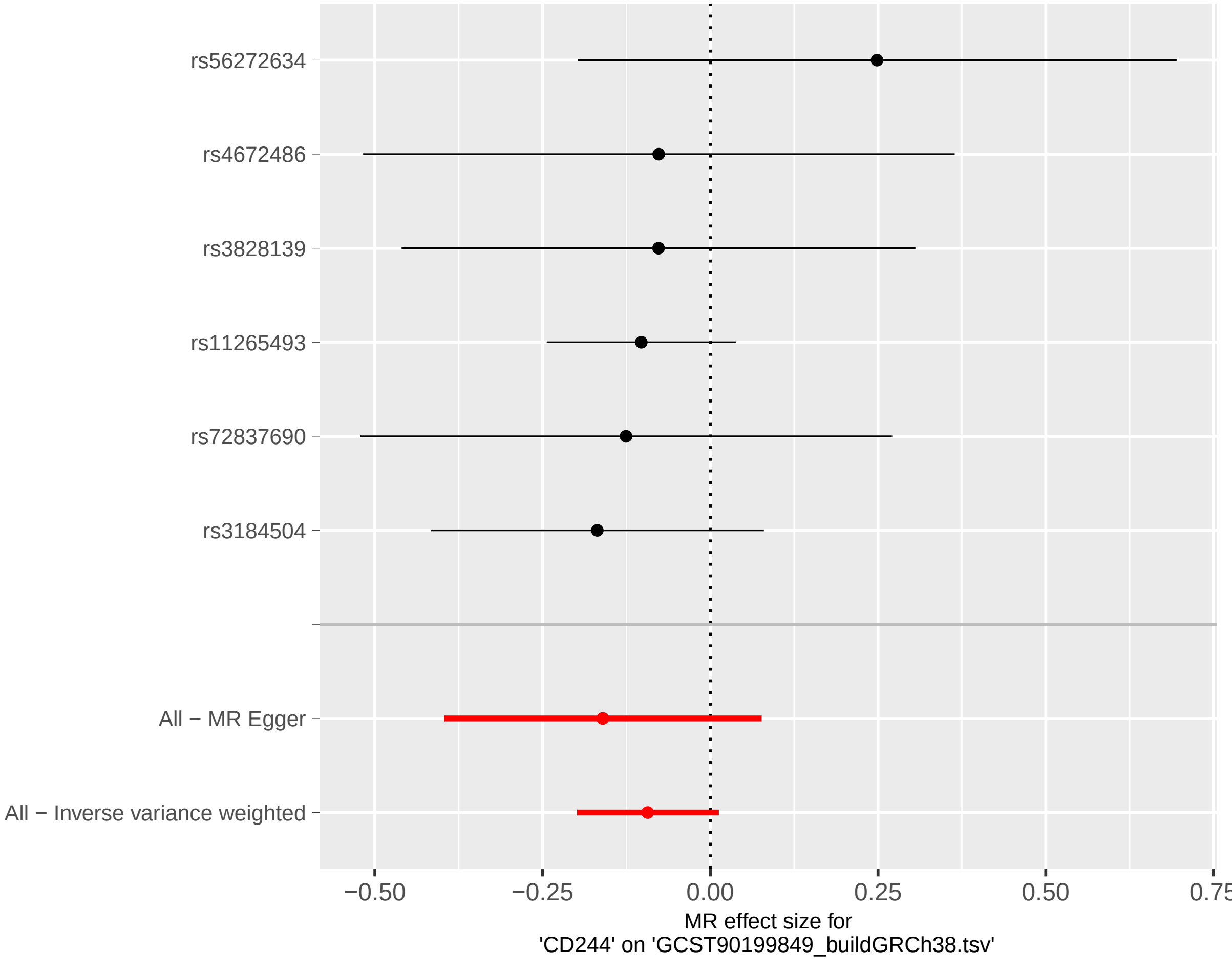
rs56272634
rs4672486
rs3828139
rs11265493
rs72837690
rs3184504
All − MR Egger
All − Inverse variance weighted
−0.50
−0.25
0.00
0.25
0.50
0.75
MR effect size for
'CD244' on 'GCST90199849_buildGRCh38.tsv'

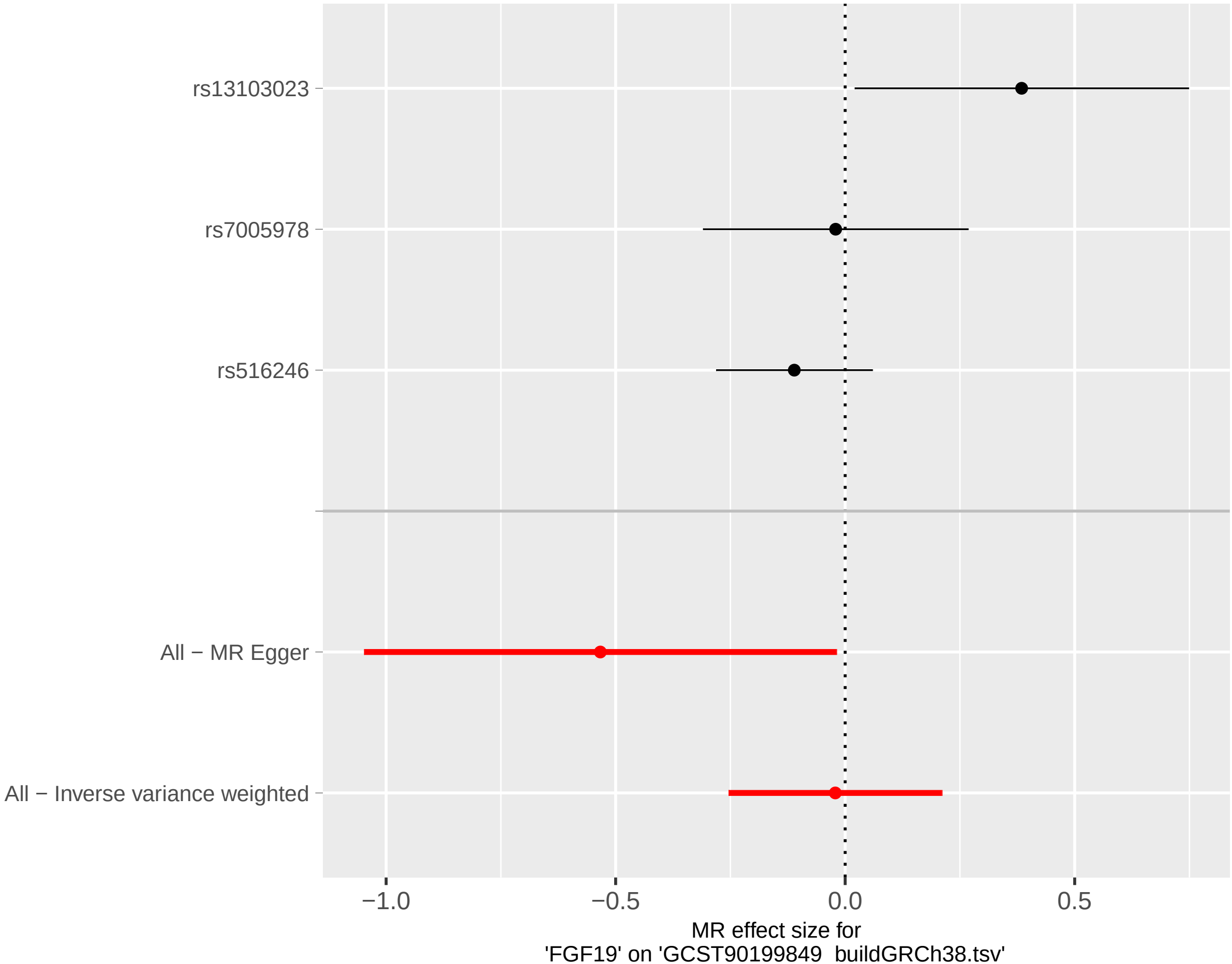

rs13103023
rs7005978
rs516246
All − MR Egger
All − Inverse variance weighted
−1.0
−0.5
0.0
0.5
MR effect size for
'FGF19' on 'GCST90199849_buildGRCh38.tsv'

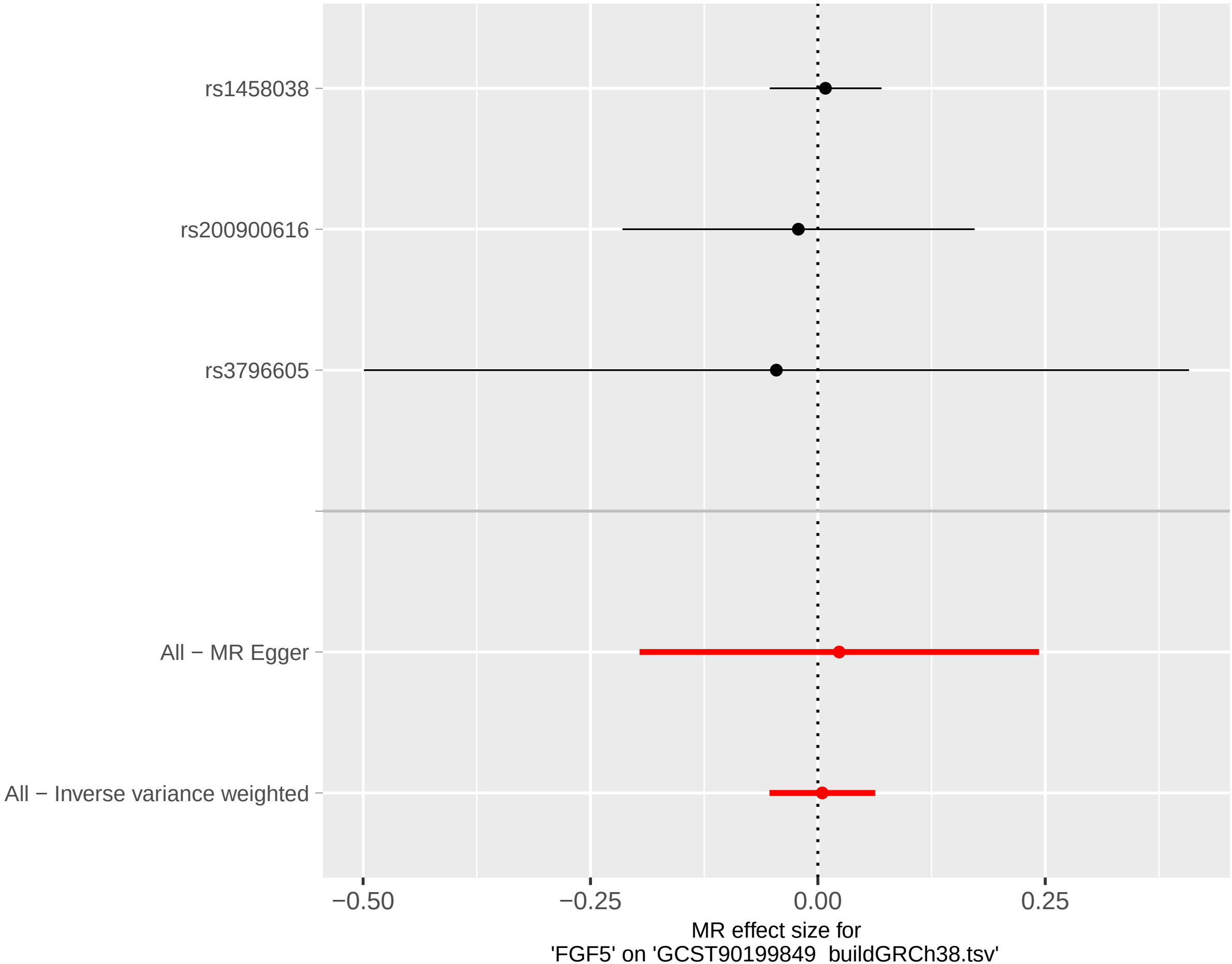
rs1458038
rs200900616
rs3796605
All − MR Egger
All − Inverse variance weighted
−0.50
−0.25
0.00
0.25
MR effect size for
'FGF5' on 'GCST90199849_buildGRCh38.tsv'

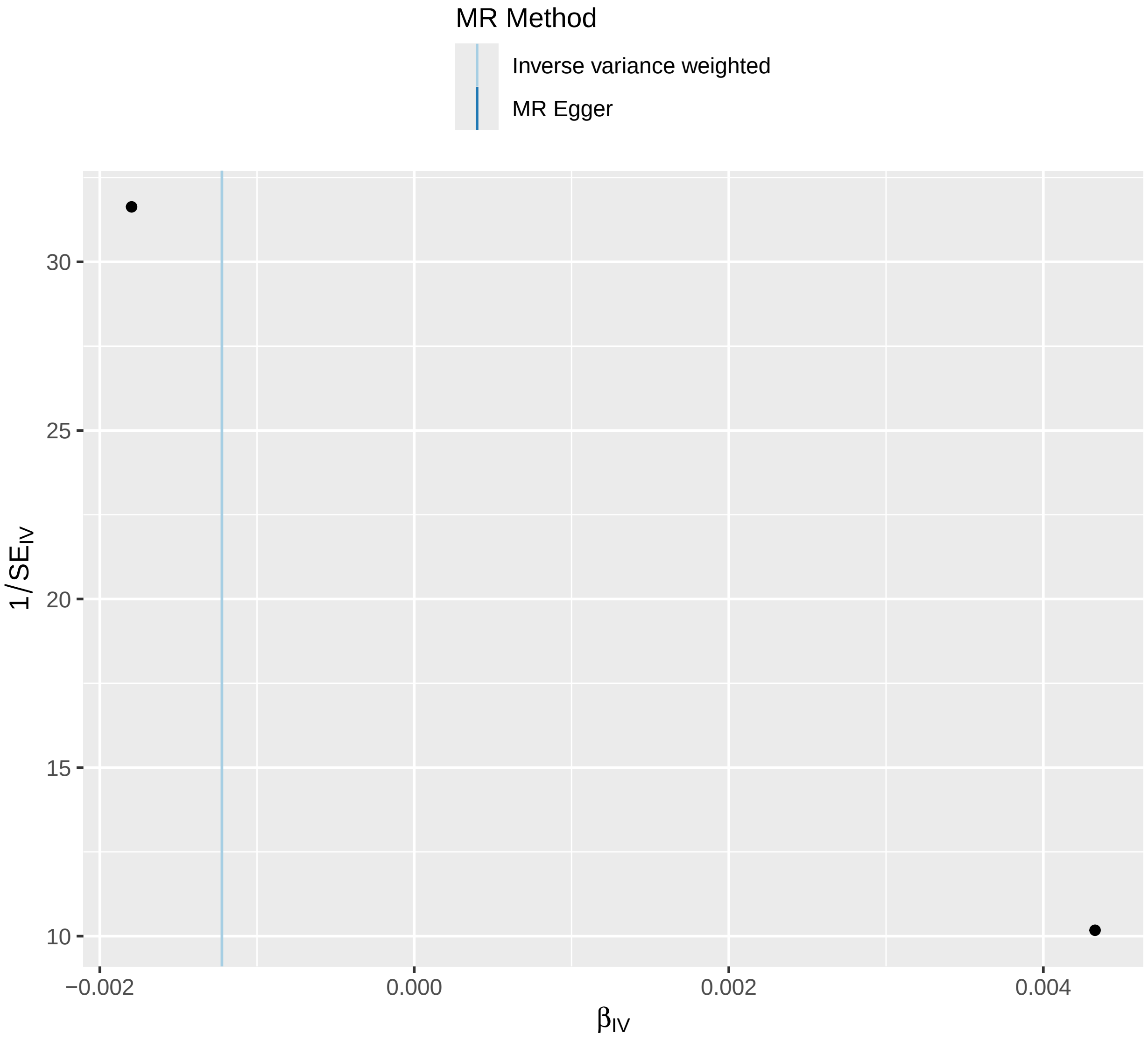
MR Method
Inverse variance weighted
MR Egger
1/SE_IV
30
25
20
15
10
−0.002
0.000
0.002
0.004
β_IV

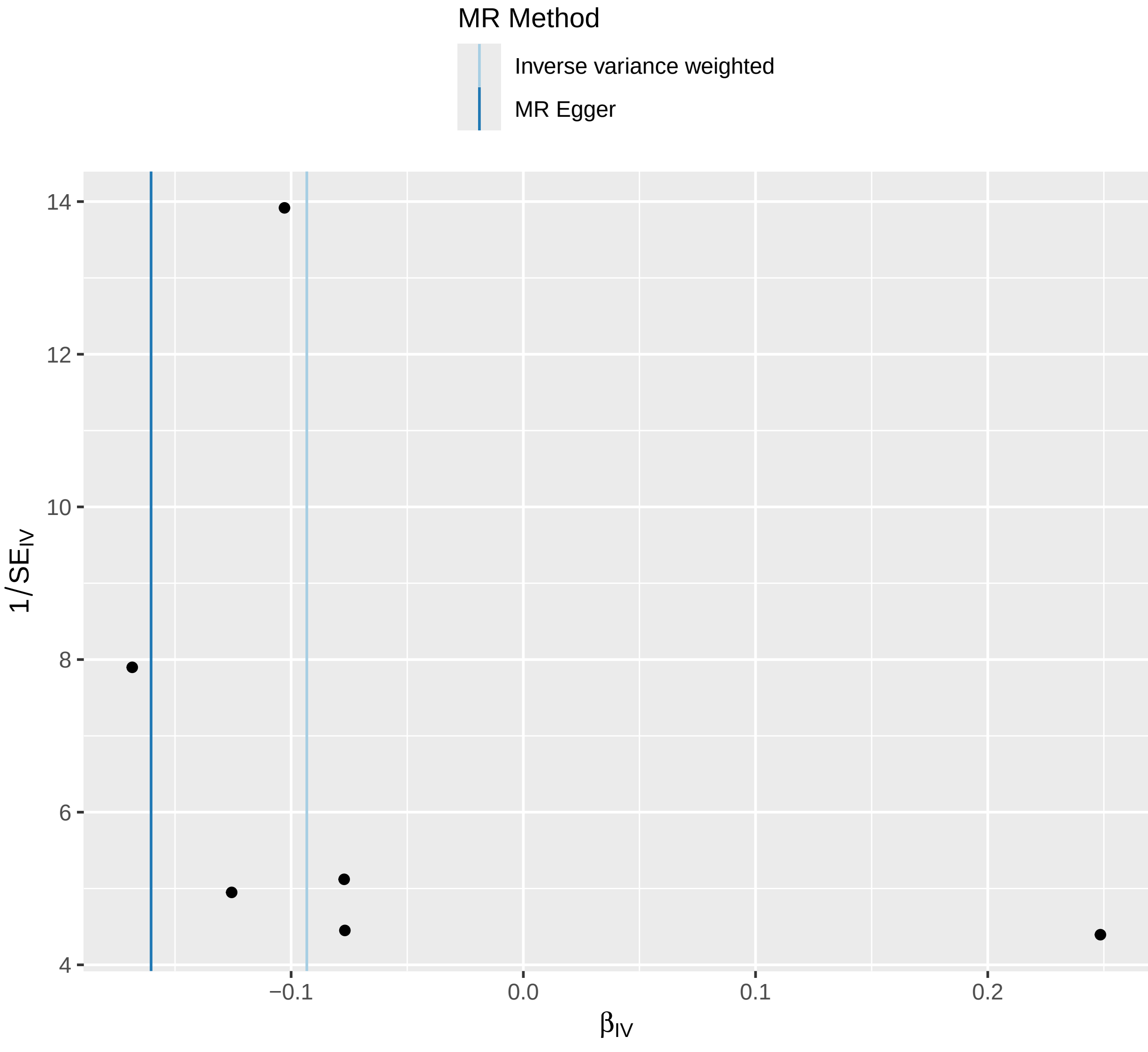
MR Method
Inverse variance weighted
MR Egger
14
12
10
8
6
4
$1/SE_{IV}$
−0.1
0.0
0.1
0.2
β_{IV}

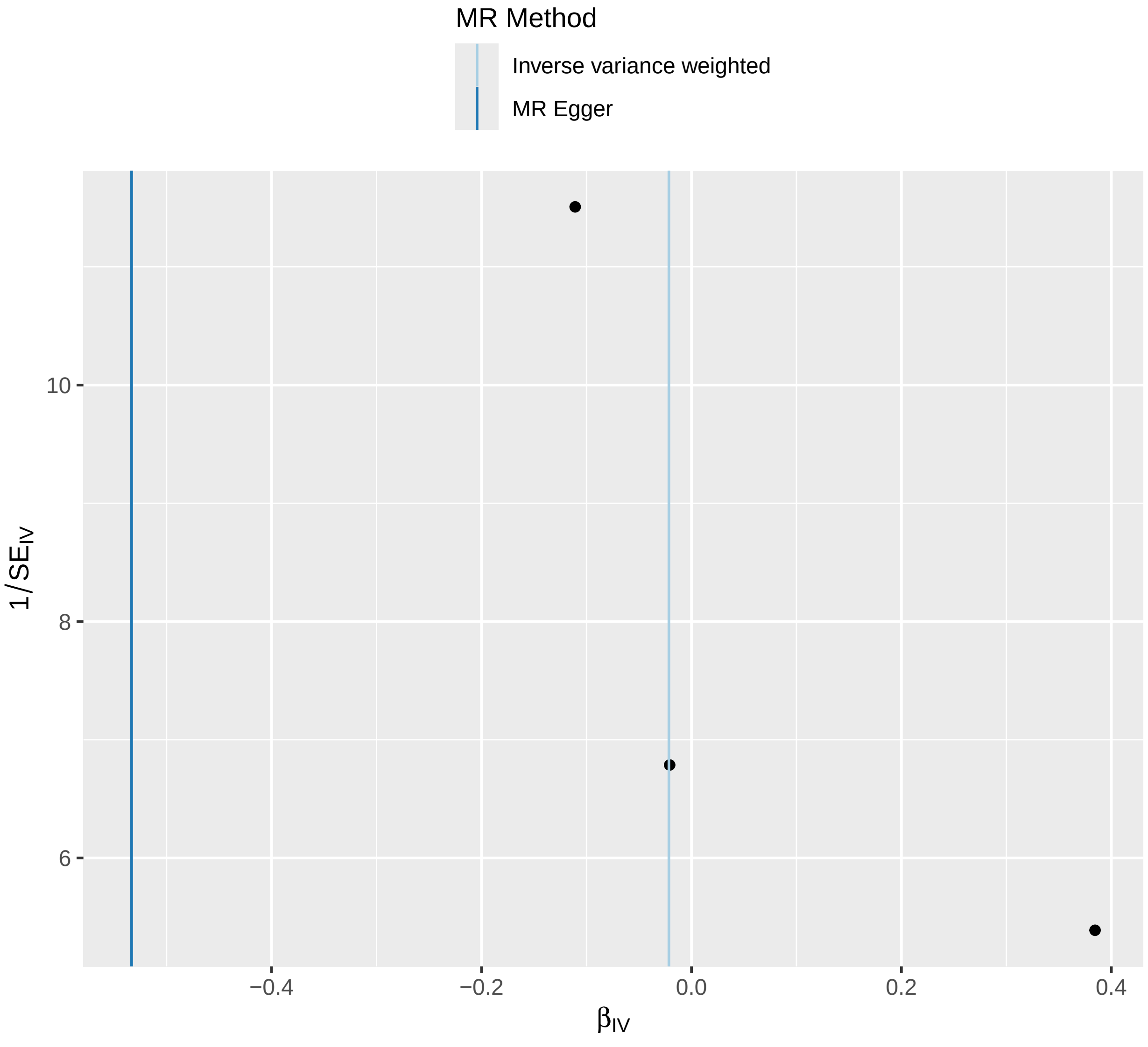

MR Method
Inverse variance weighted
MR Egger
10
8
6
1/SE_IV
−0.4
−0.2
0.0
0.2
0.4
β_IV

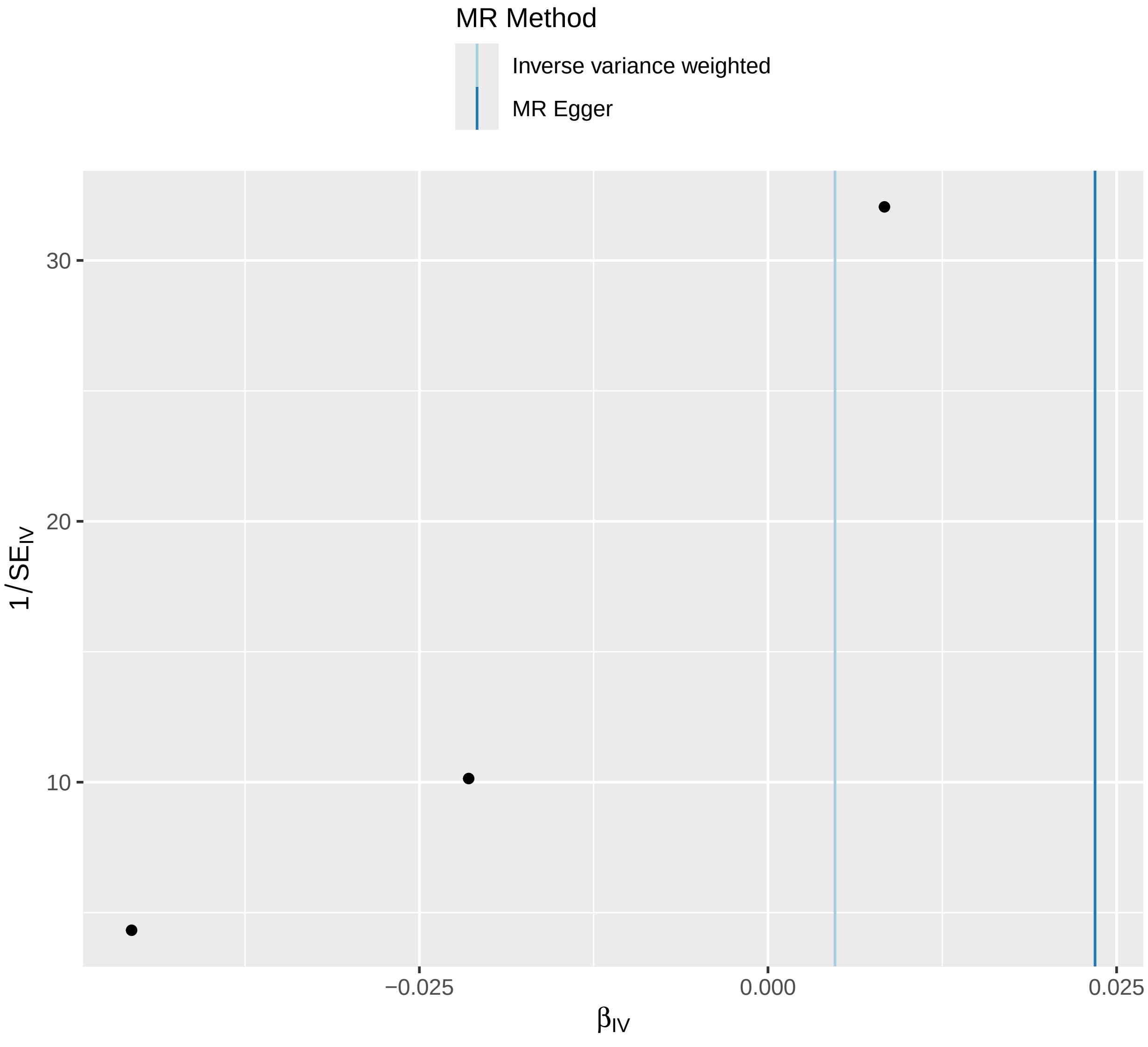
MR Method
Inverse variance weighted
MR Egger
30
20
10
1/SE$_{IV}$
−0.025
0.000
0.025
β$_{IV}$

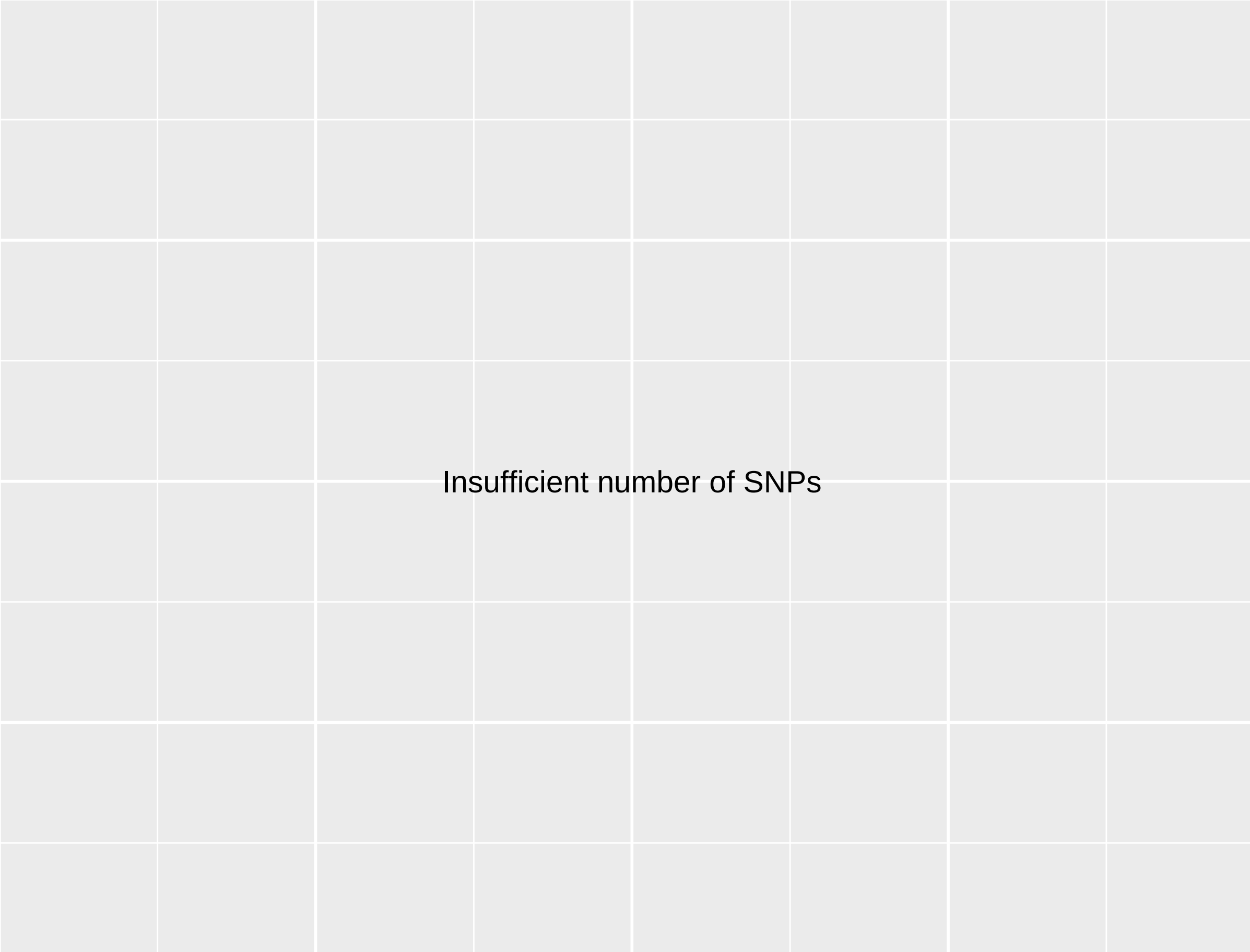
Insufficient number of SNPs

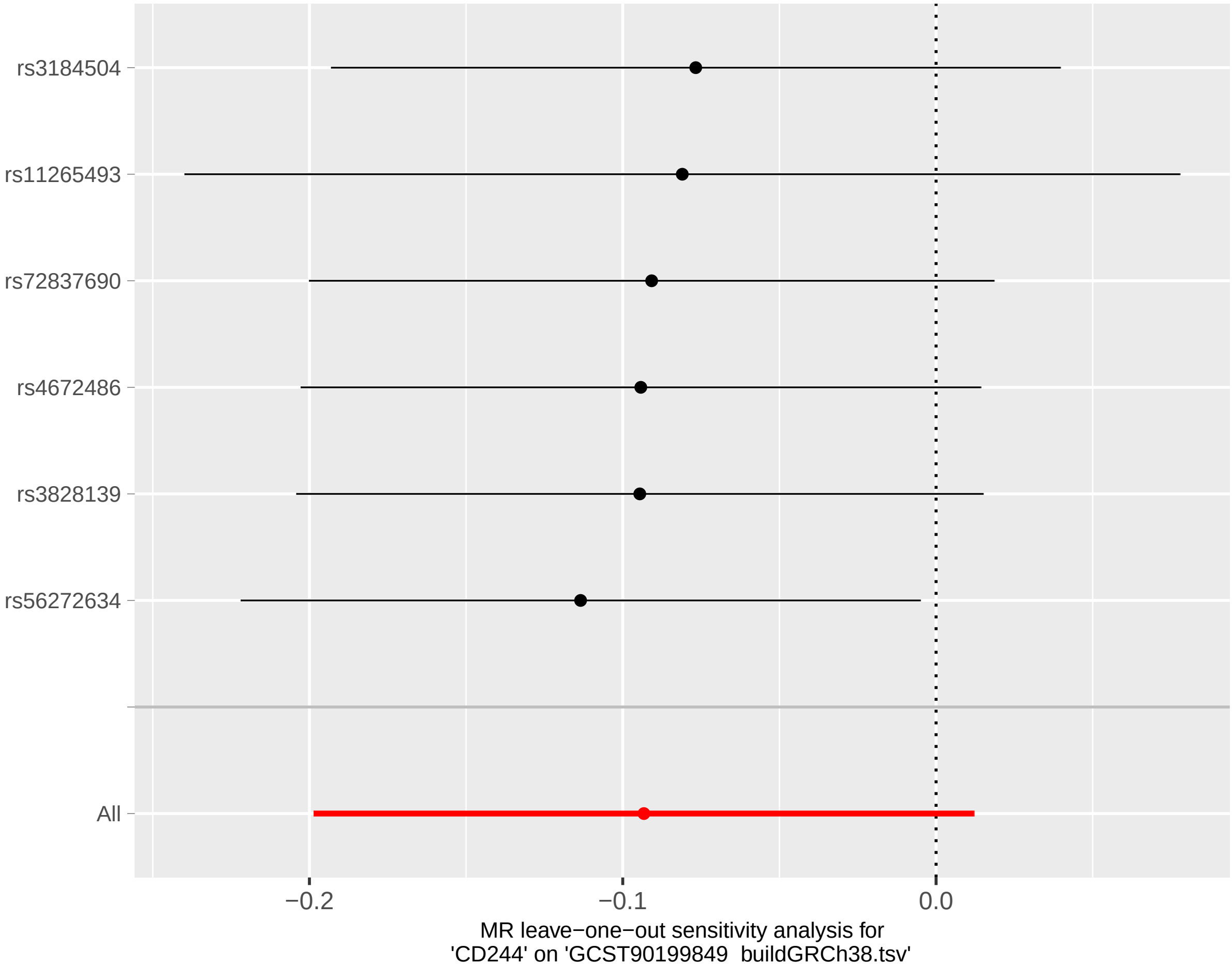
rs3184504
rs11265493
rs72837690
rs4672486
rs3828139
rs56272634
All
−0.2
−0.1
0.0
MR leave−one−out sensitivity analysis for
'CD244' on 'GCST90199849_buildGRCh38.tsv'

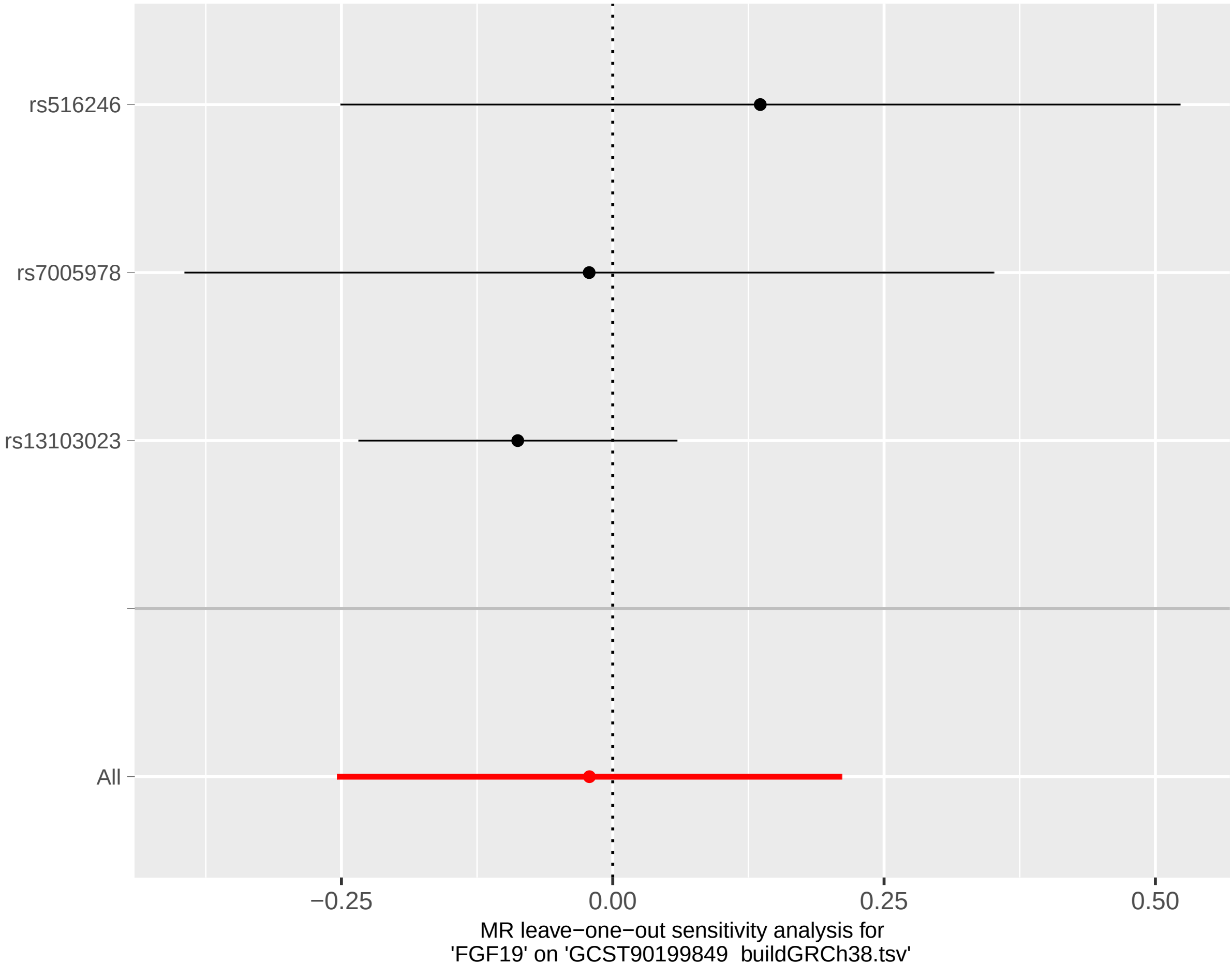

rs516246
rs7005978
rs13103023
All
−0.25
0.00
0.25
0.50
MR leave−one−out sensitivity analysis for
'FGF19' on 'GCST90199849_buildGRCh38.tsv'

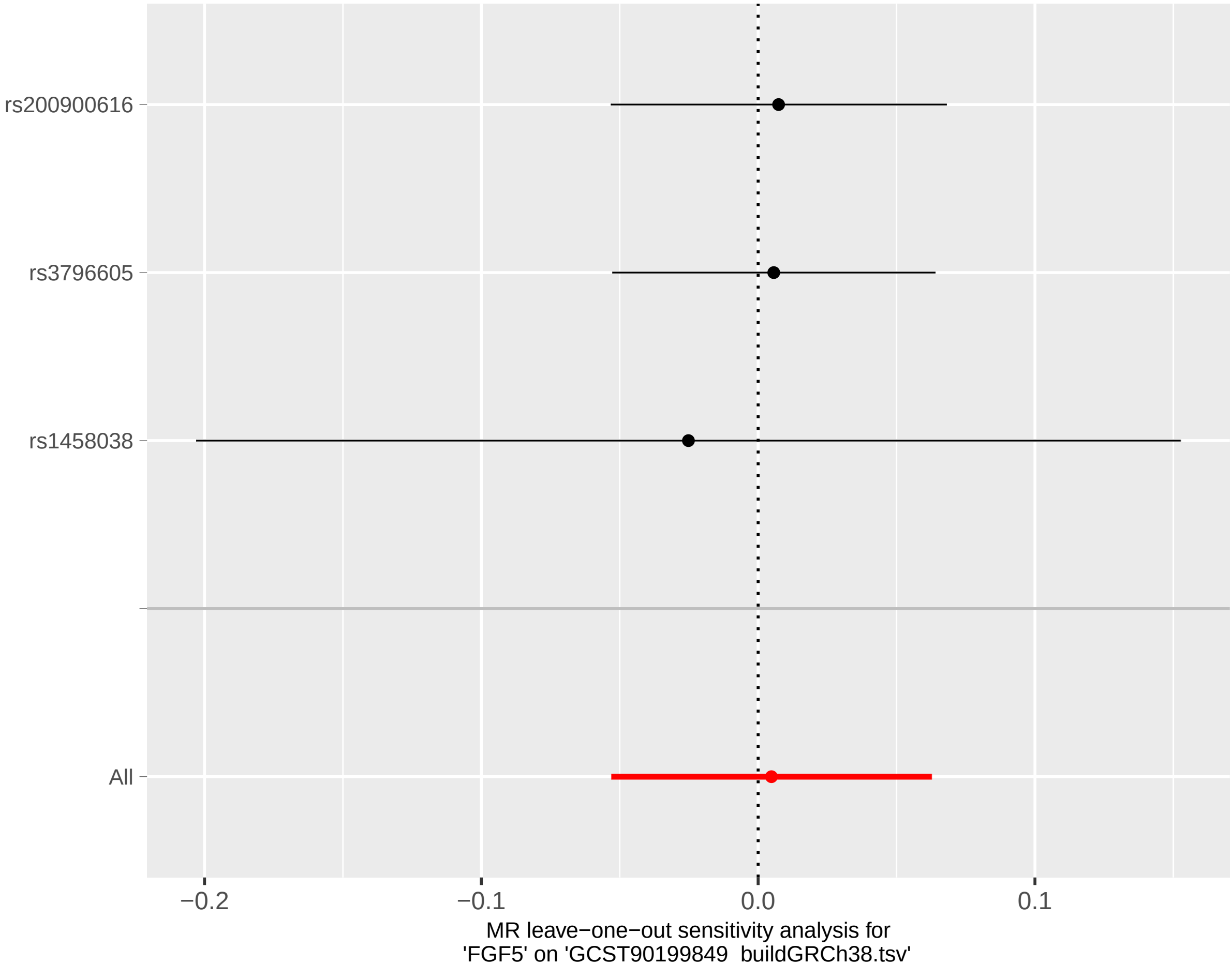
rs200900616
rs3796605
rs1458038
All
−0.2
−0.1
0.0
0.1
MR leave−one−out sensitivity analysis for
'FGF5' on 'GCST90199849_buildGRCh38.tsv'

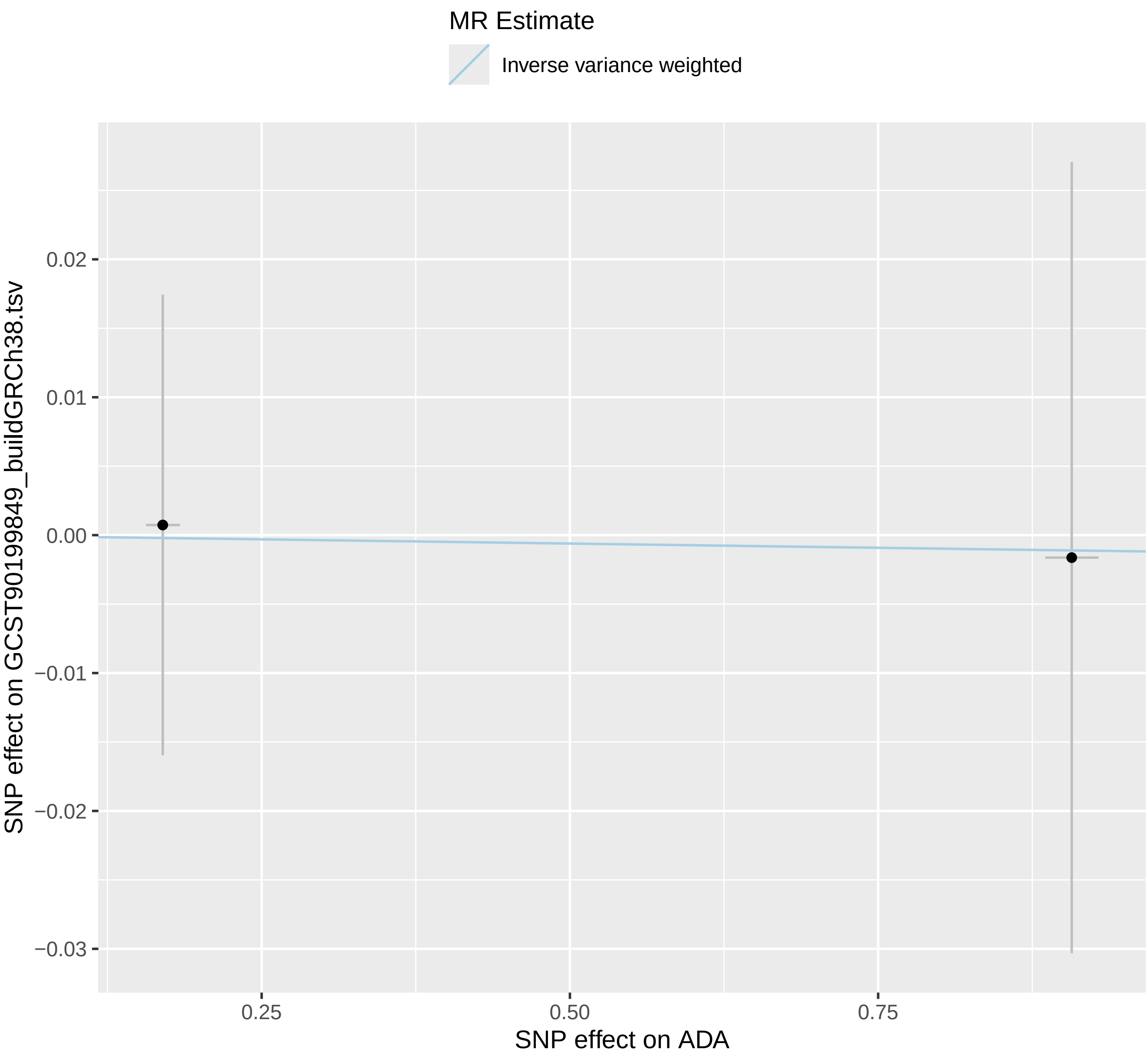
MR Estimate
Inverse variance weighted
SNP effect on GCST90199849_buildGRCh38.tsv
0.02
0.01
0.00
−0.01
−0.02
−0.03
0.25
0.50
0.75
SNP effect on ADA

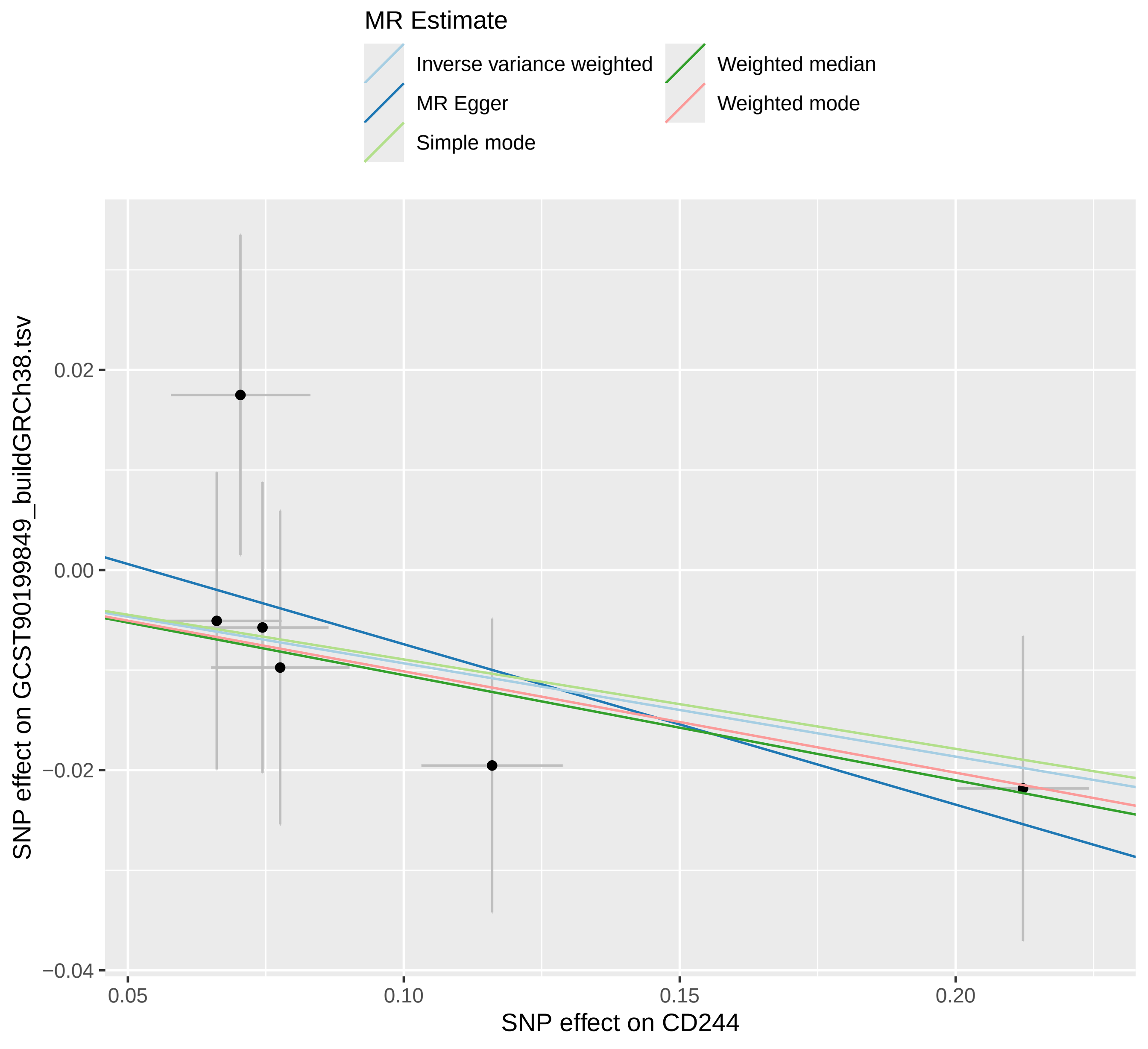
MR Estimate
Inverse variance weighted
MR Egger
Simple mode
Weighted median
Weighted mode
SNP effect on GCST90199849_buildGRCh38.tsv
0.02
0.00
−0.02
−0.04
0.05
0.10
0.15
0.20
SNP effect on CD244

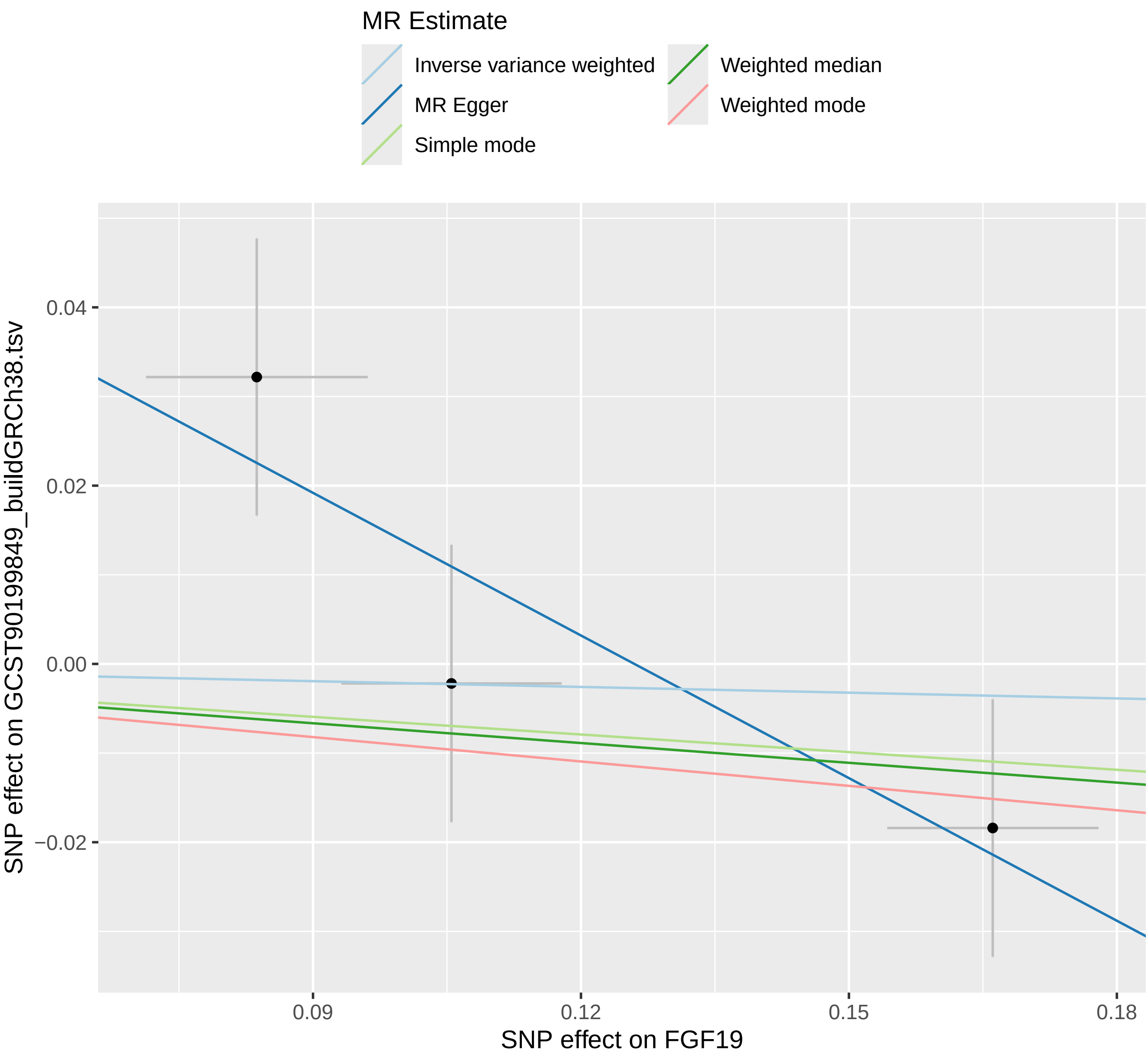
MR Estimate
Inverse variance weighted
MR Egger
Simple mode
Weighted median
Weighted mode
0.04
0.02
0.00
−0.02
0.09
0.12
0.15
0.18
SNP effect on GCST90199849_buildGRCh38.tsv
SNP effect on FGF19

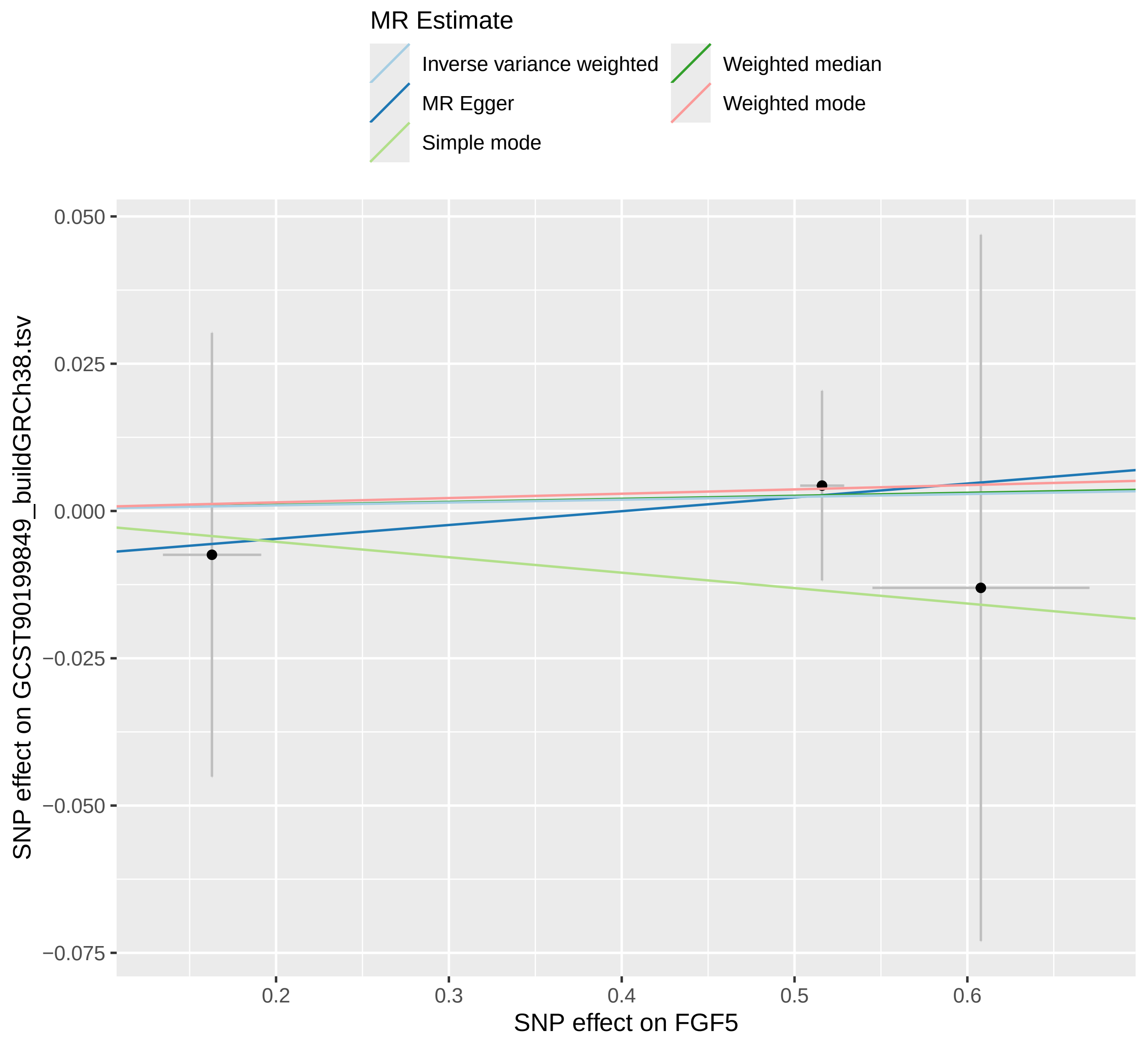
MR Estimate
Inverse variance weighted
MR Egger
Simple mode
Weighted median
Weighted mode
0.050
0.025
0.000
−0.025
−0.050
−0.075
SNP effect on GCST90199849_buildGRCh38.tsv
0.2
0.3
0.4
0.5
0.6
SNP effect on FGF5

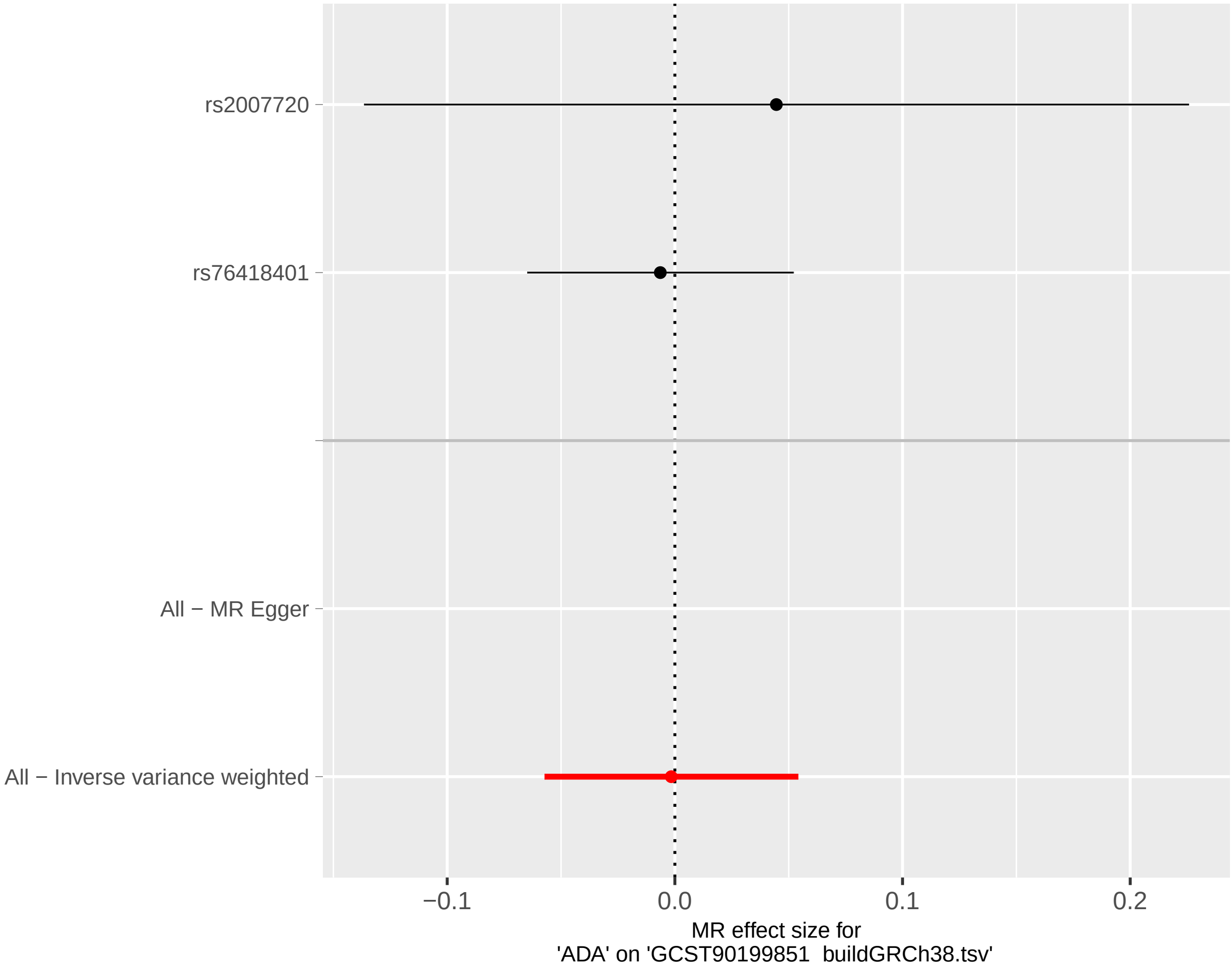
rs2007720
rs76418401
All − MR Egger
All − Inverse variance weighted
−0.1
0.0
0.1
0.2
MR effect size for
'ADA' on 'GCST90199851_buildGRCh38.tsv'

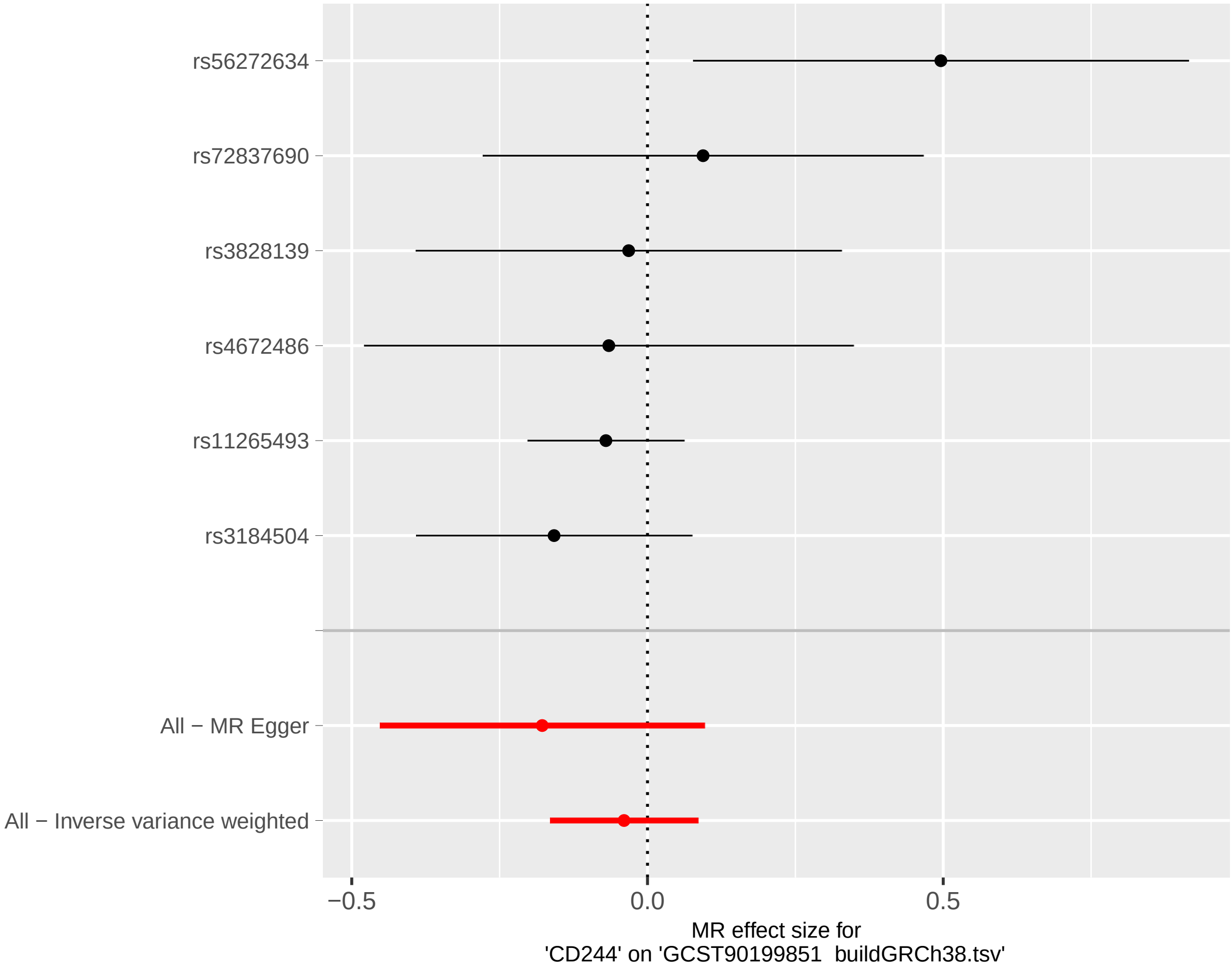
rs56272634
rs72837690
rs3828139
rs4672486
rs11265493
rs3184504
All − MR Egger
All − Inverse variance weighted
−0.5
0.0
0.5
MR effect size for
'CD244' on 'GCST90199851_buildGRCh38.tsv'

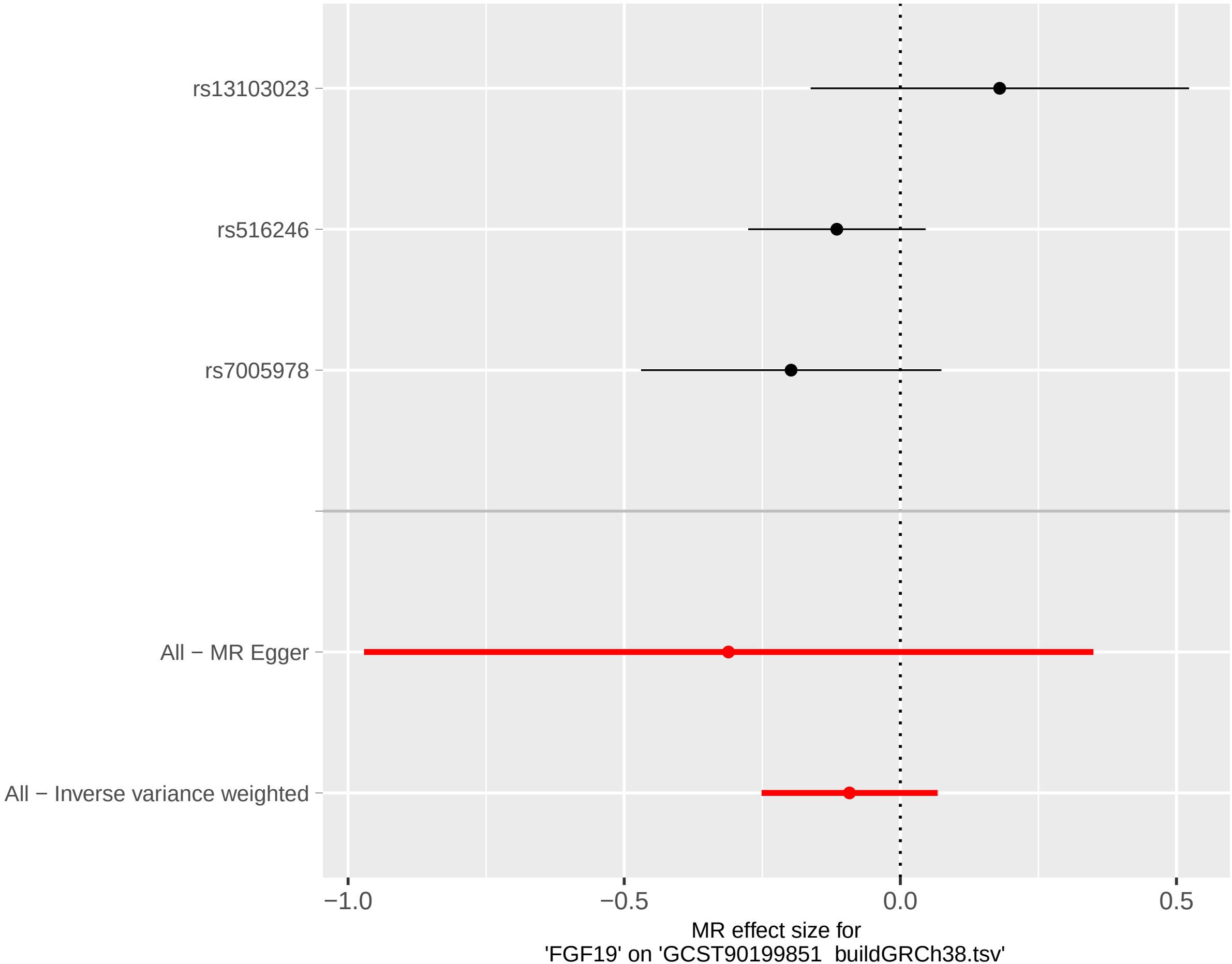
rs13103023
rs516246
rs7005978
All − MR Egger
All − Inverse variance weighted
−1.0
−0.5
0.0
0.5
MR effect size for
'FGF19' on 'GCST90199851_buildGRCh38.tsv'

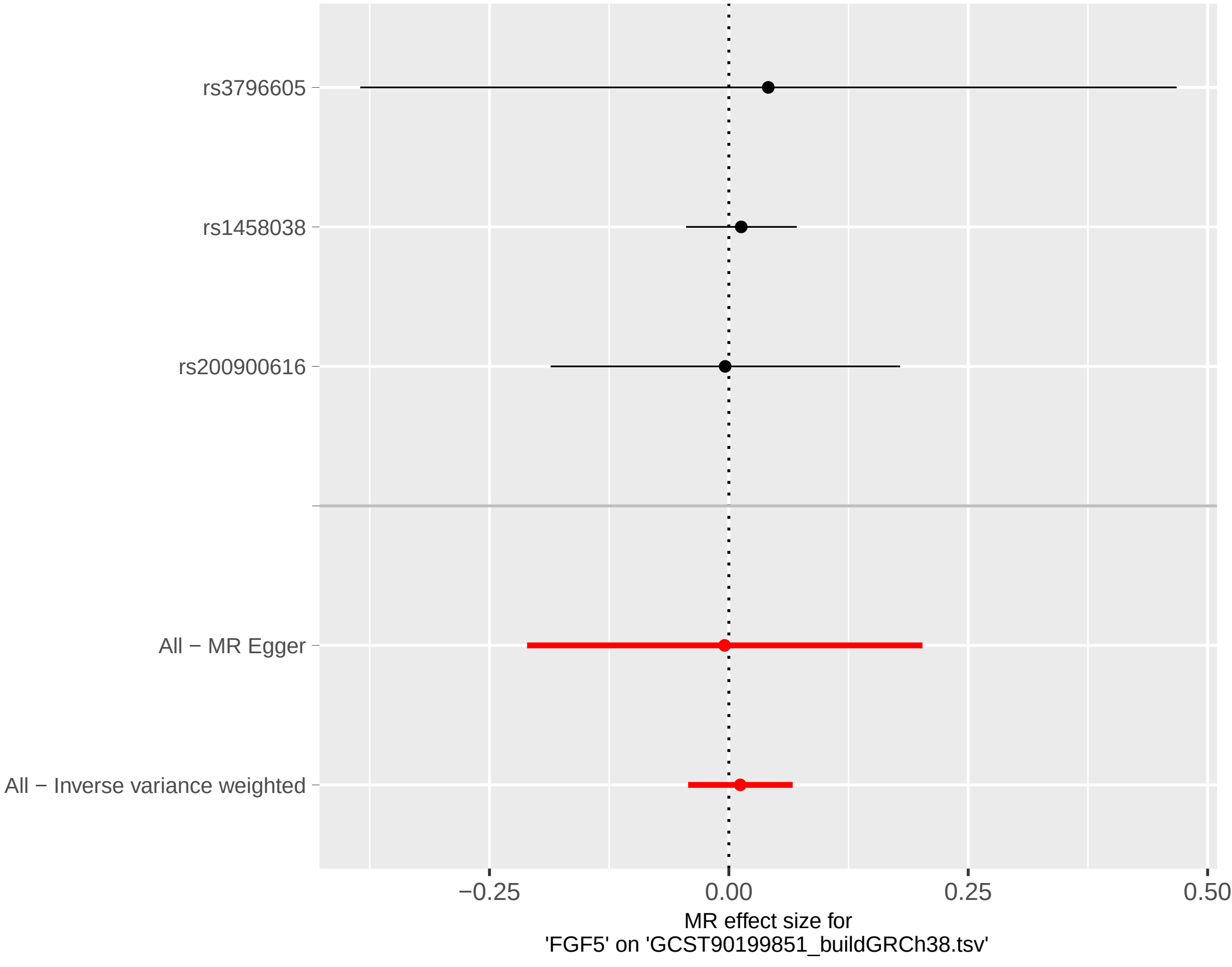
rs3796605
rs1458038
rs200900616
All − MR Egger
All − Inverse variance weighted
−0.25
0.00
0.25
0.50
MR effect size for
'FGF5' on 'GCST90199851_buildGRCh38.tsv'

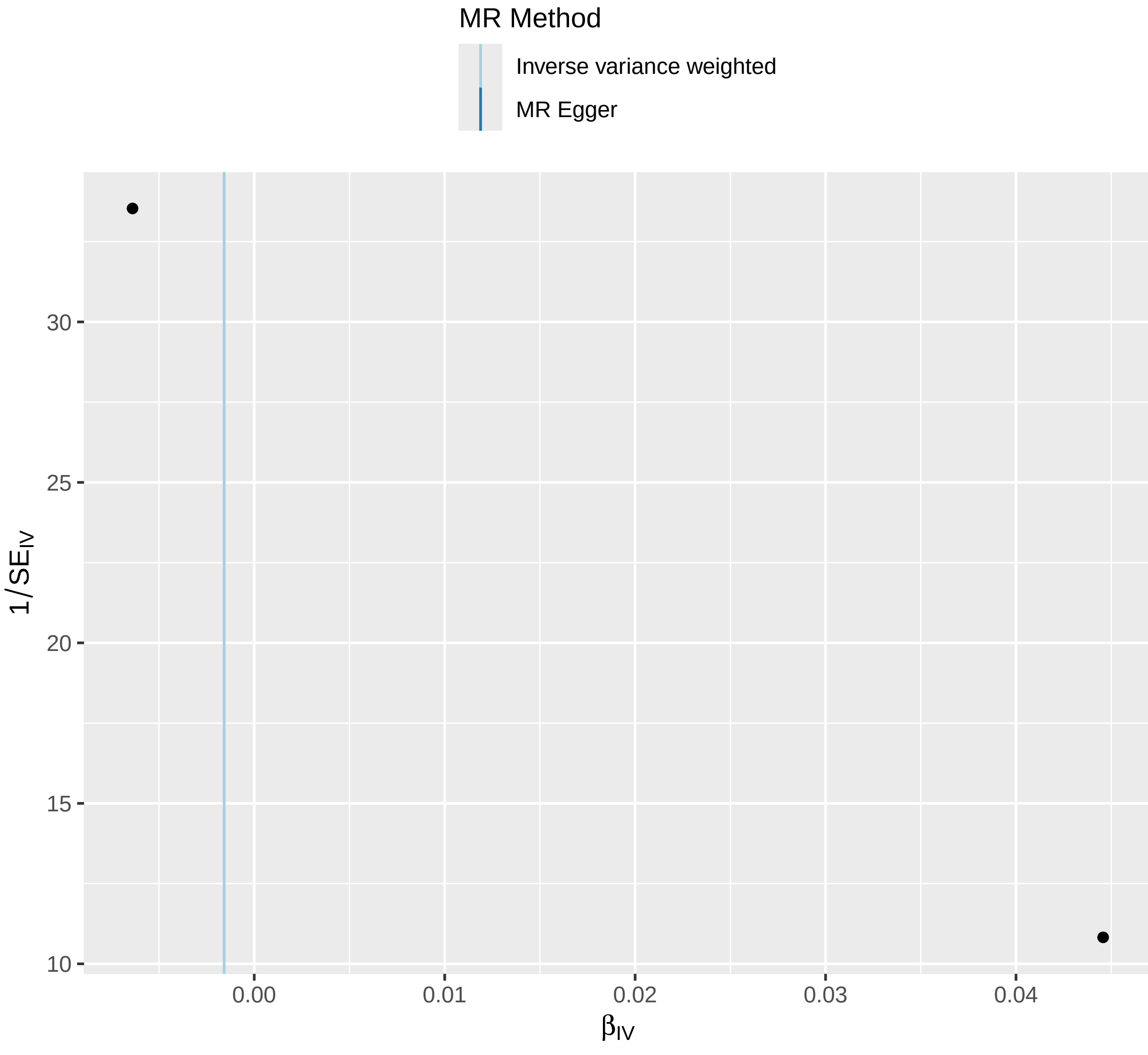
MR Method
Inverse variance weighted
MR Egger
30
25
20
15
10
0.00
0.01
0.02
0.03
0.04
1/SE_IV
β_IV

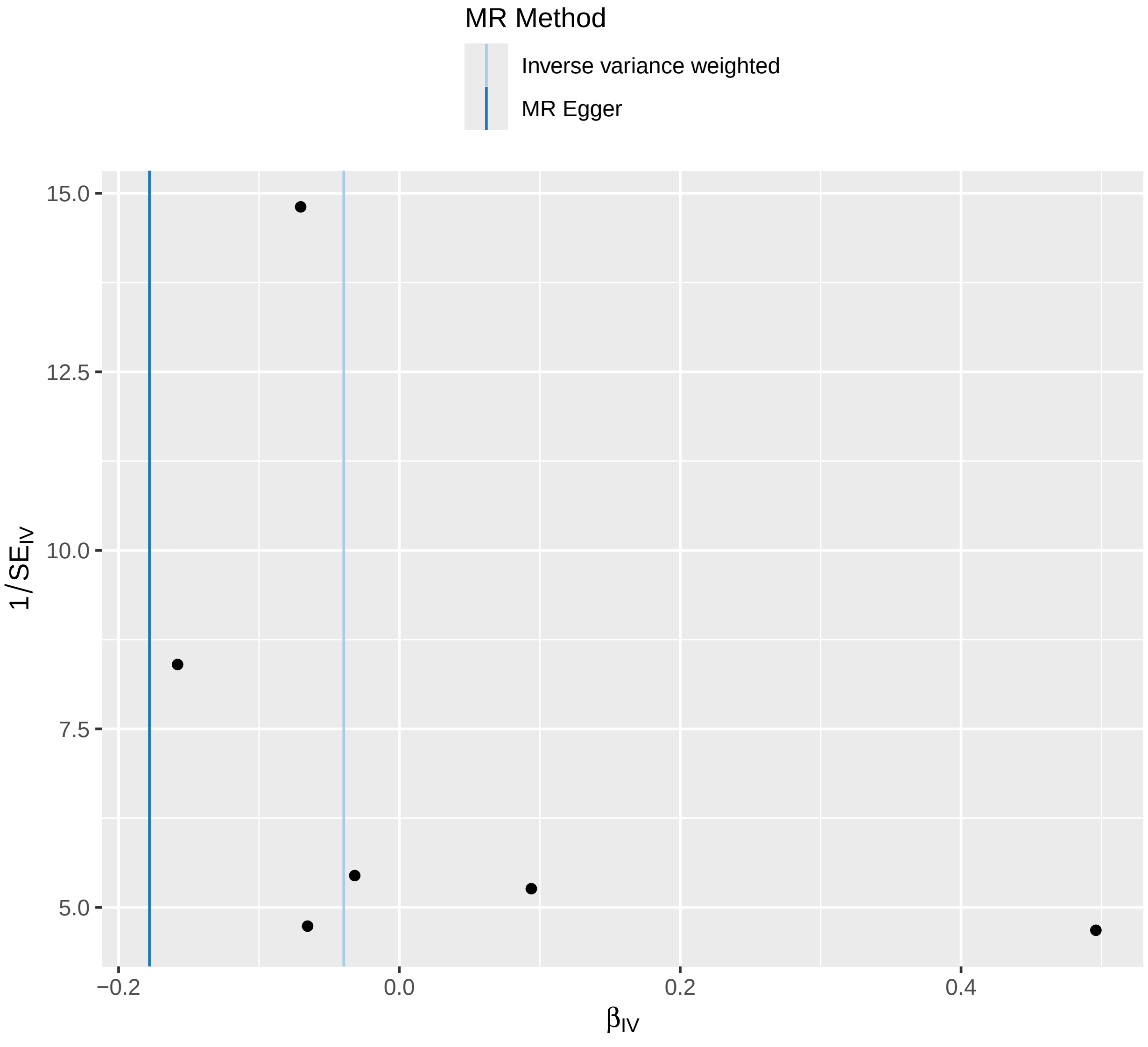
MR Method
Inverse variance weighted
MR Egger
15.0
12.5
10.0
7.5
5.0
1/SE_IV
−0.2
0.0
0.2
0.4
β_IV

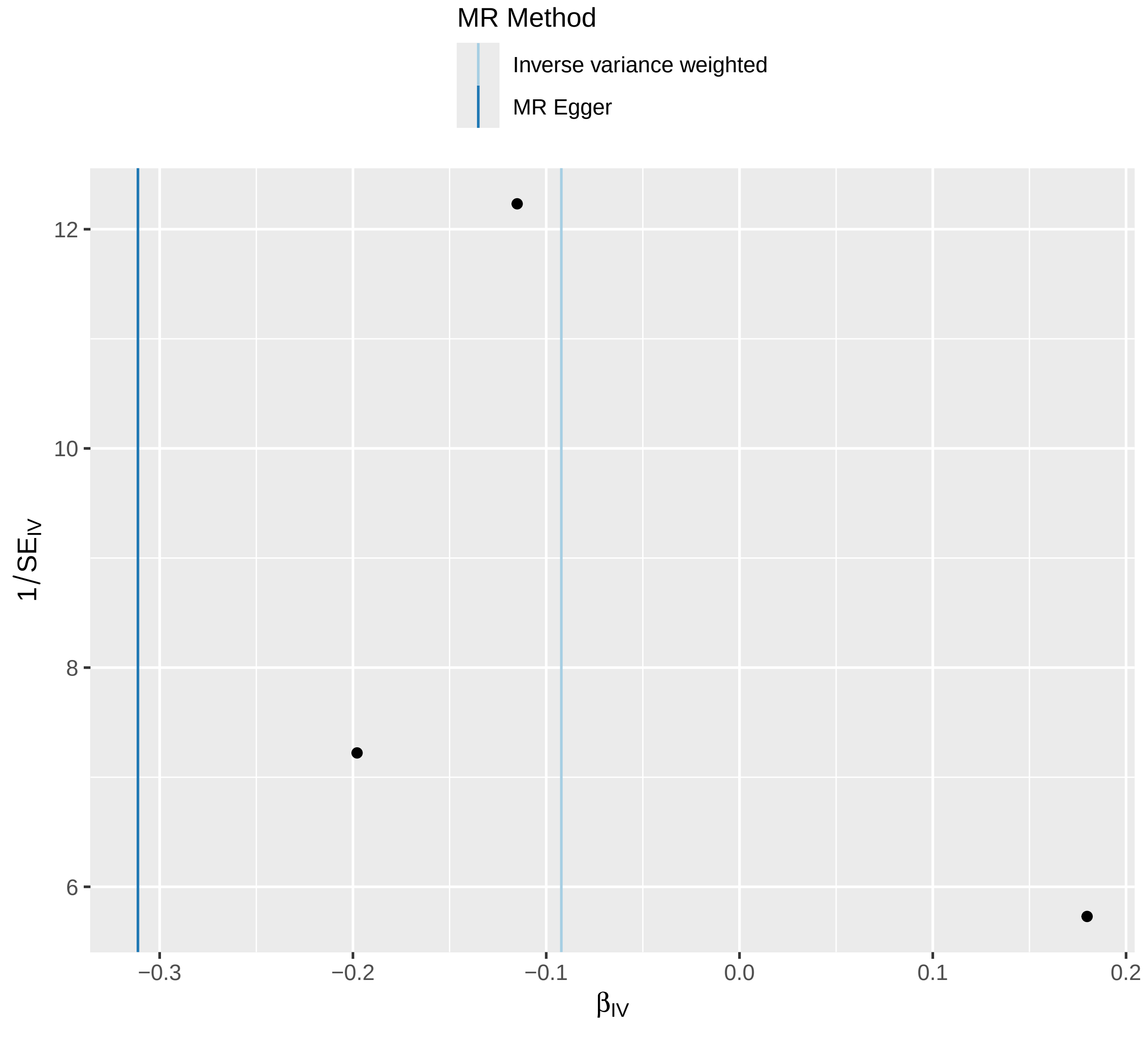

MR Method
Inverse variance weighted
MR Egger
12
10
8
6
1/SE_IV
−0.3
−0.2
−0.1
0.0
0.1
0.2
β_IV

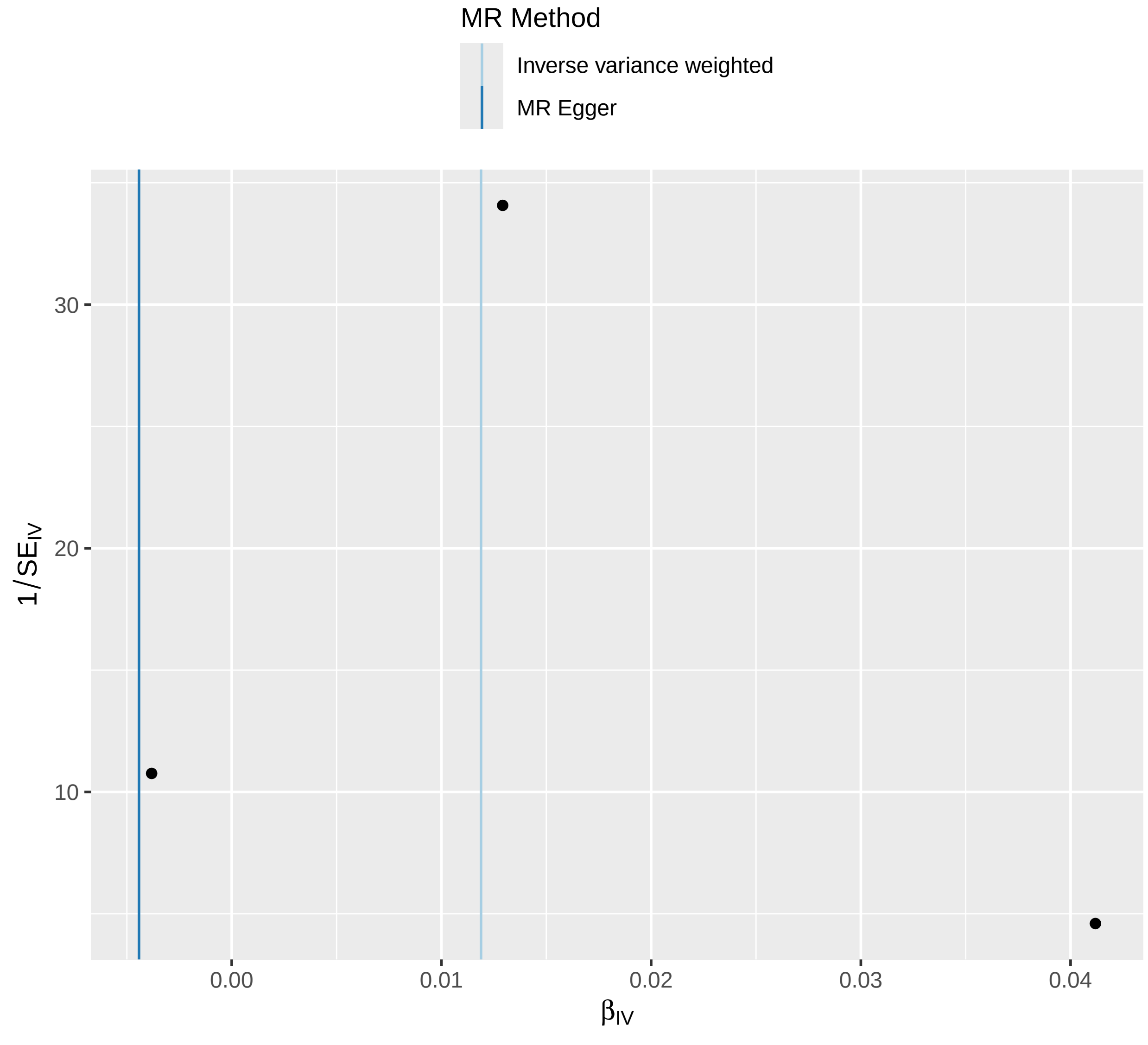

MR Method
Inverse variance weighted
MR Egger
30
20
10
1/SE_IV
0.00
0.01
0.02
0.03
0.04
β_IV

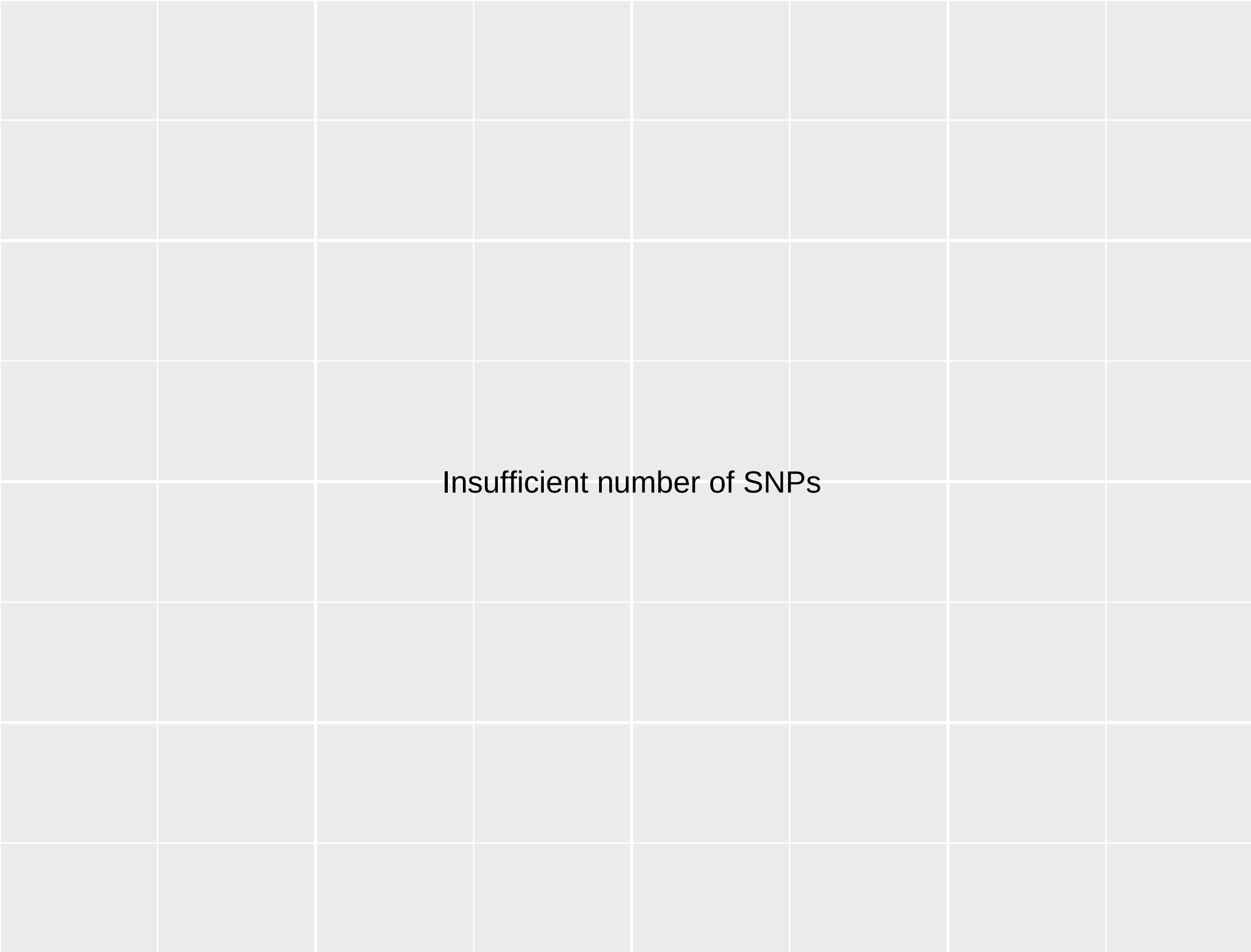
Insufficient number of SNPs

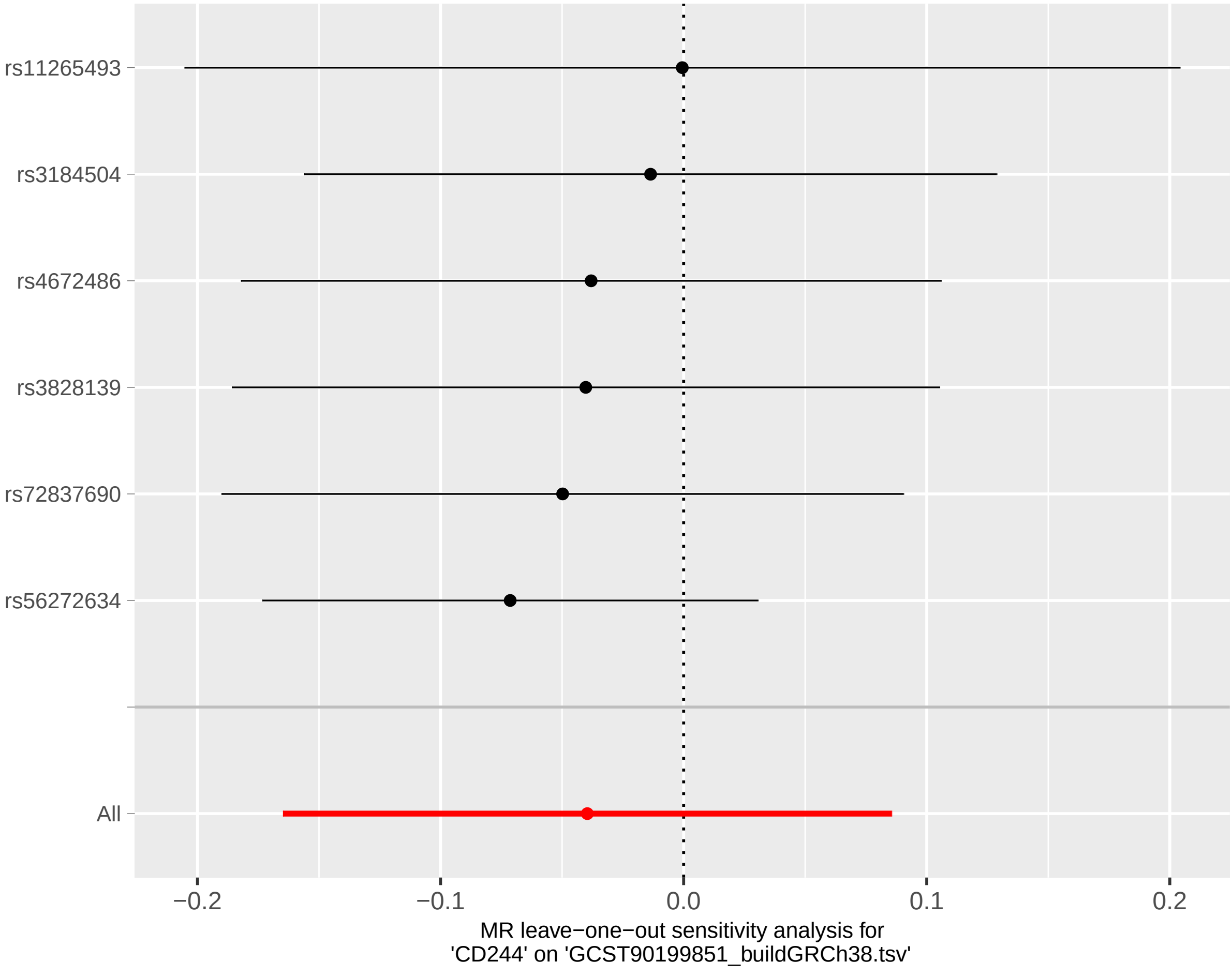
rs11265493
rs3184504
rs4672486
rs3828139
rs72837690
rs56272634
All
−0.2
−0.1
0.0
0.1
0.2
MR leave−one−out sensitivity analysis for
'CD244' on 'GCST90199851_buildGRCh38.tsv'

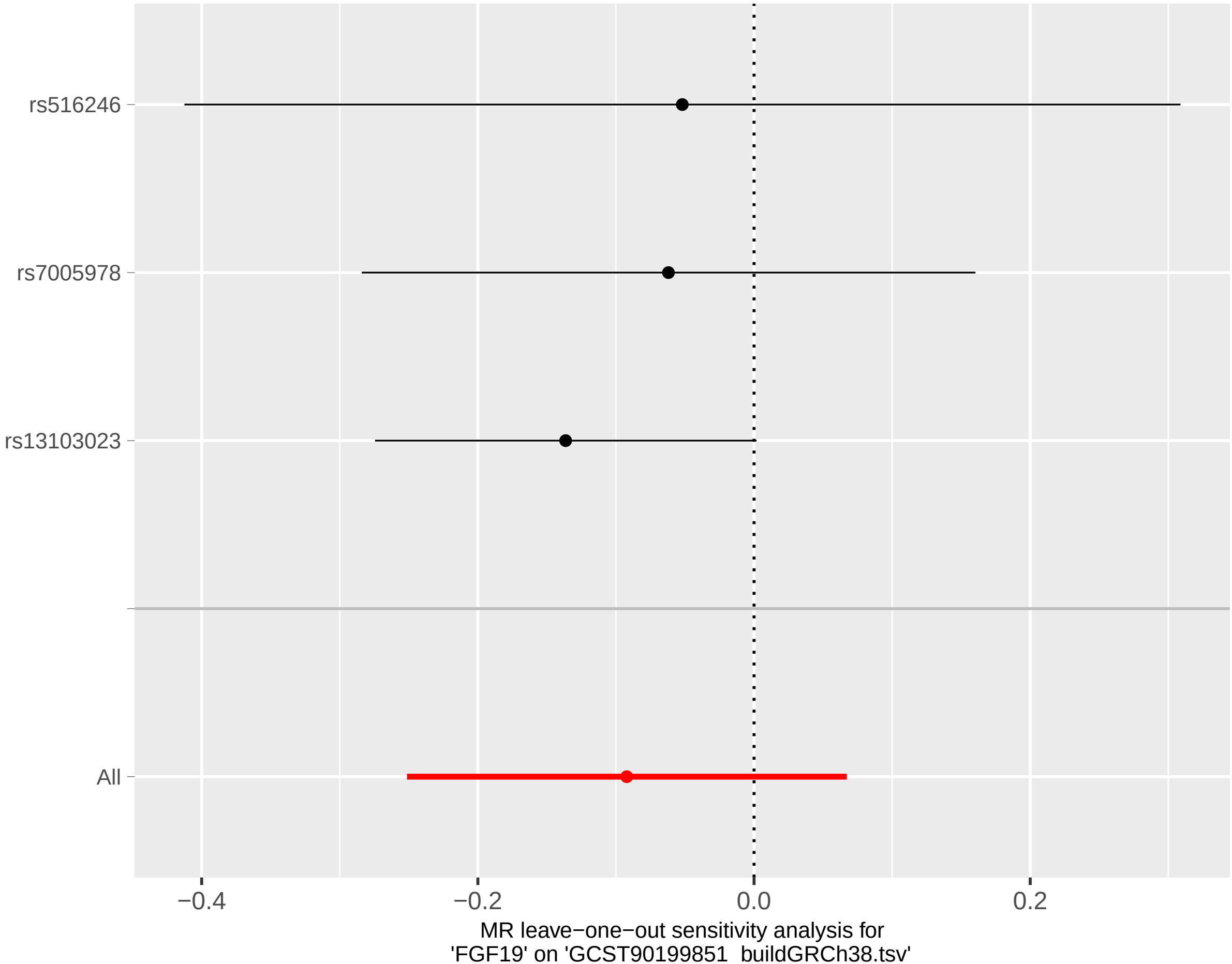
rs516246
rs7005978
rs13103023
All
−0.4
−0.2
0.0
0.2
MR leave−one−out sensitivity analysis for
'FGF19' on 'GCST90199851_buildGRCh38.tsv'

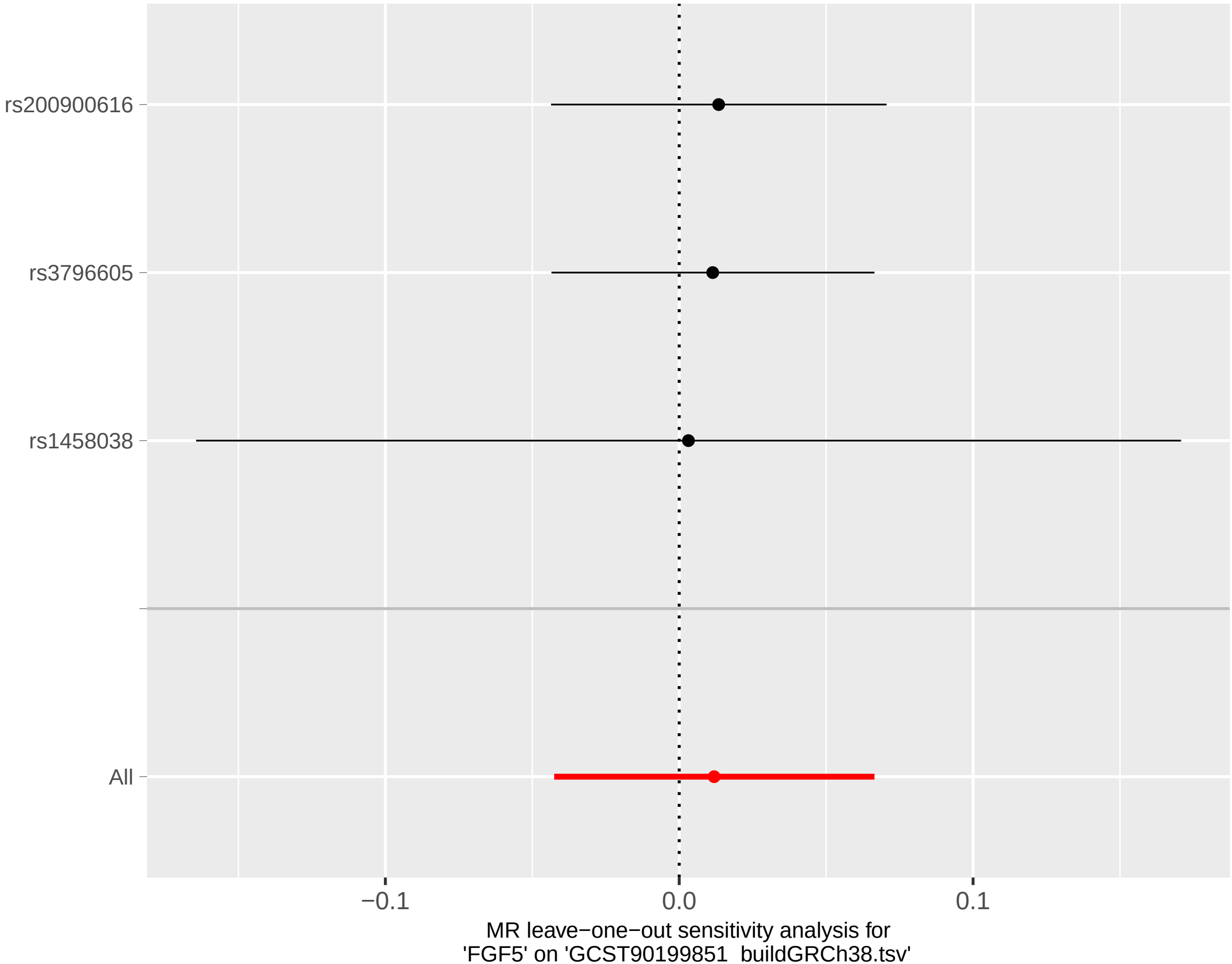
rs200900616
rs3796605
rs1458038
All
−0.1
0.0
0.1
MR leave−one−out sensitivity analysis for
'FGF5' on 'GCST90199851_buildGRCh38.tsv'

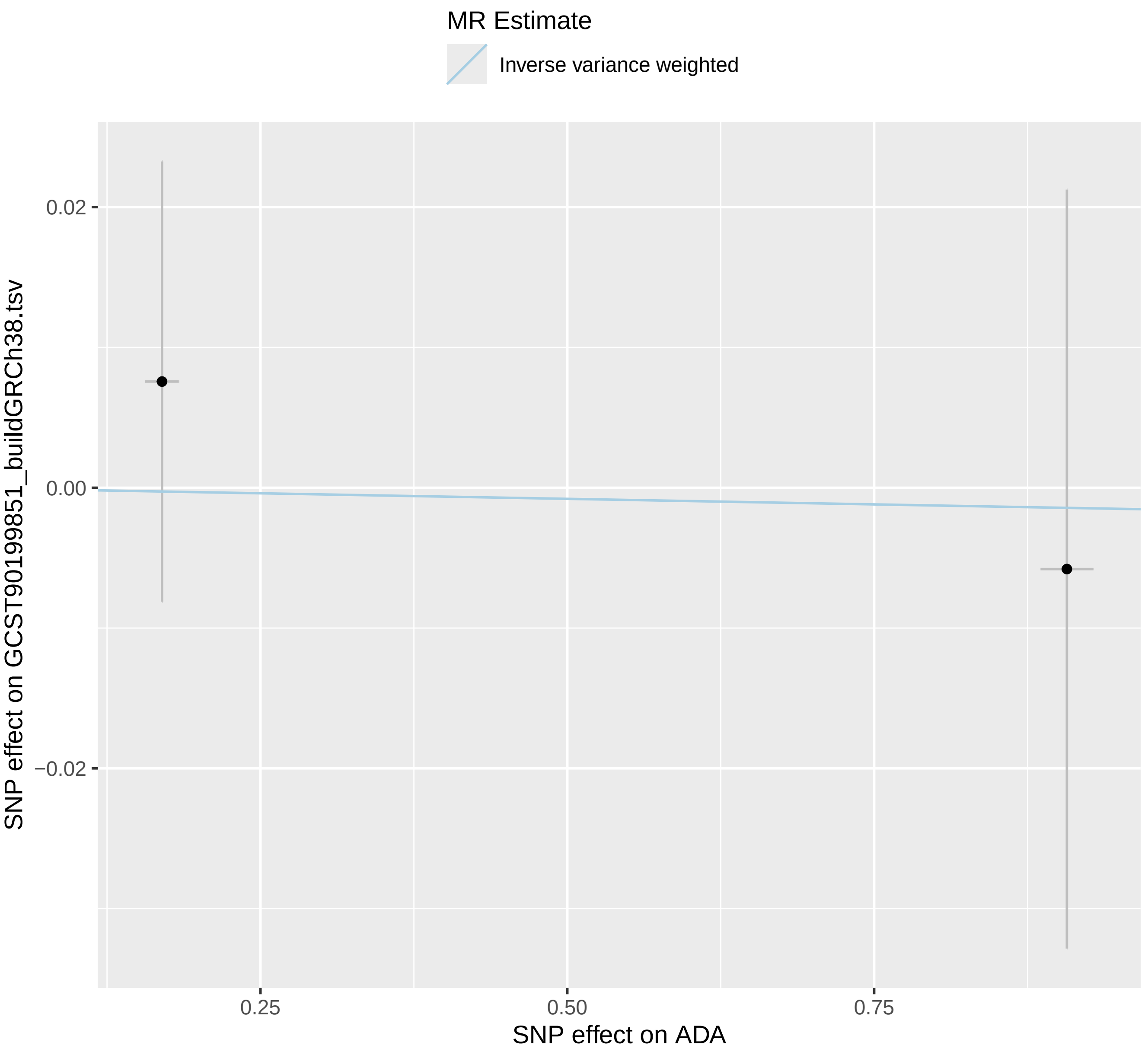
MR Estimate
Inverse variance weighted
0.02
0.00
−0.02
SNP effect on GCST90199851_buildGRCh38.tsv
0.25
0.50
0.75
SNP effect on ADA

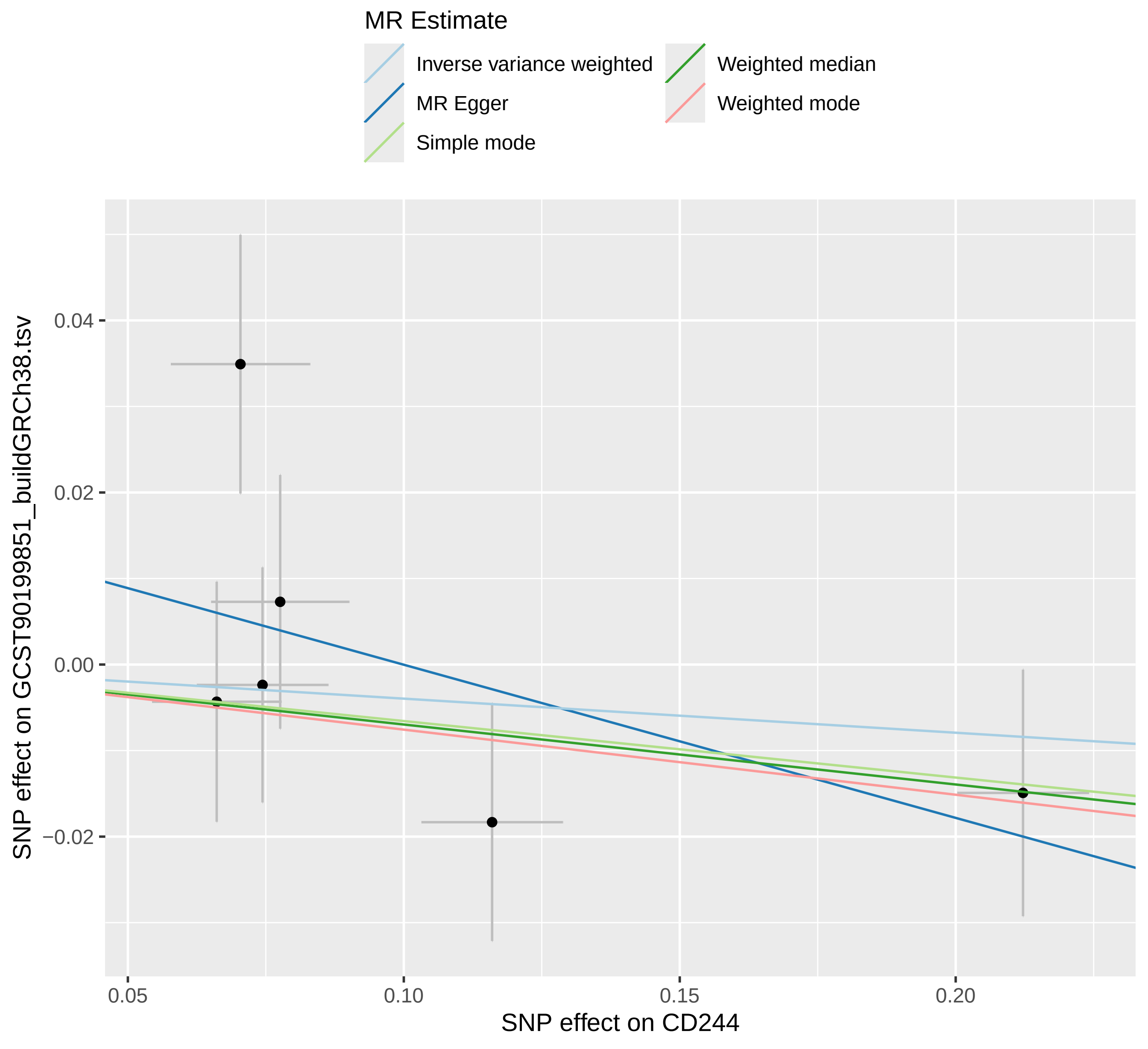

MR Estimate
Inverse variance weighted
MR Egger
Simple mode
Weighted median
Weighted mode
0.04
0.02
0.00
−0.02
0.05
0.10
0.15
0.20
SNP effect on GCST90199851_buildGRCh38.tsv
SNP effect on CD244

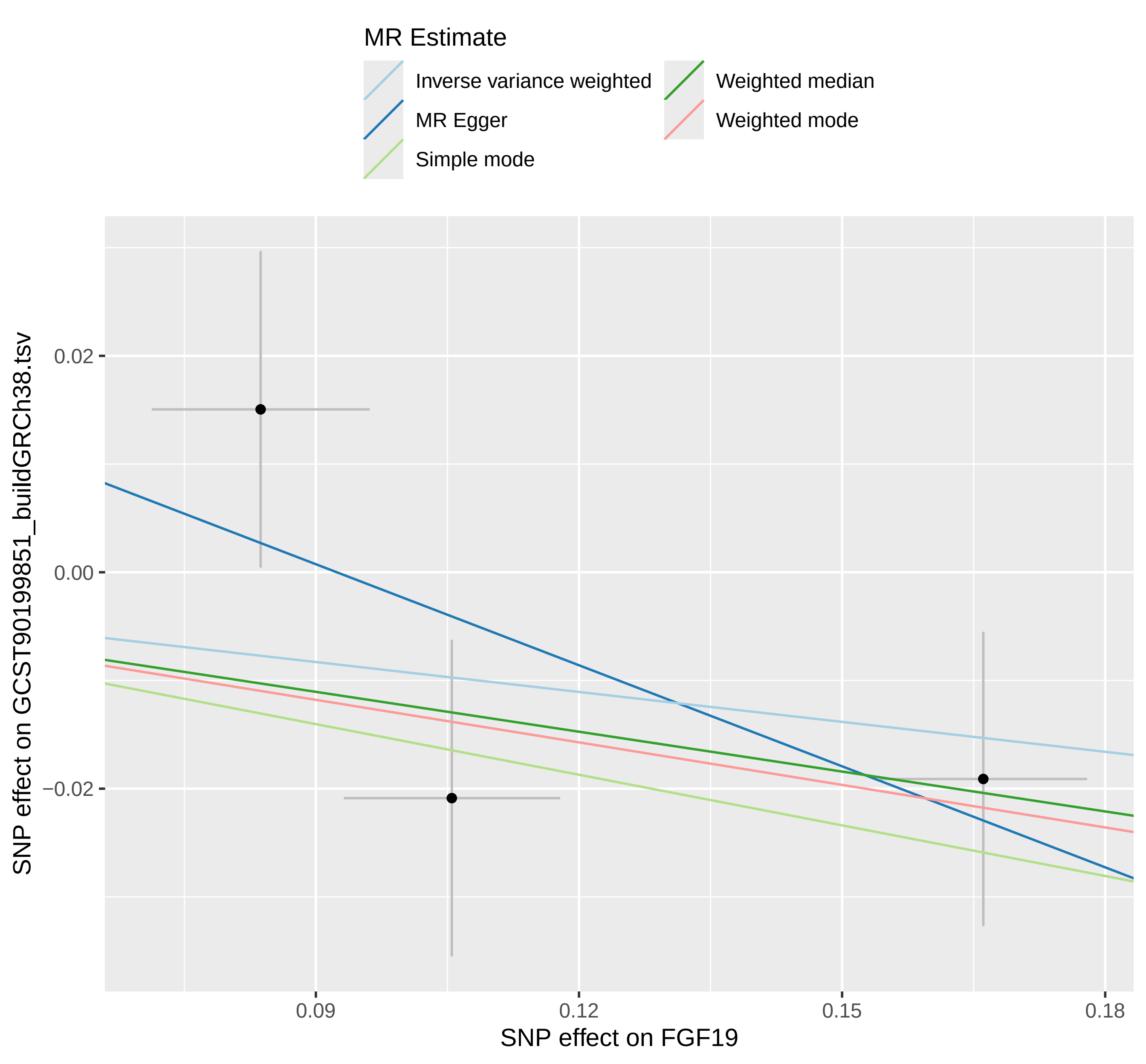
MR Estimate
Inverse variance weighted
MR Egger
Simple mode
Weighted median
Weighted mode
0.02
0.00
−0.02
0.09
0.12
0.15
0.18
SNP effect on GCST90199851_buildGRCh38.tsv
SNP effect on FGF19

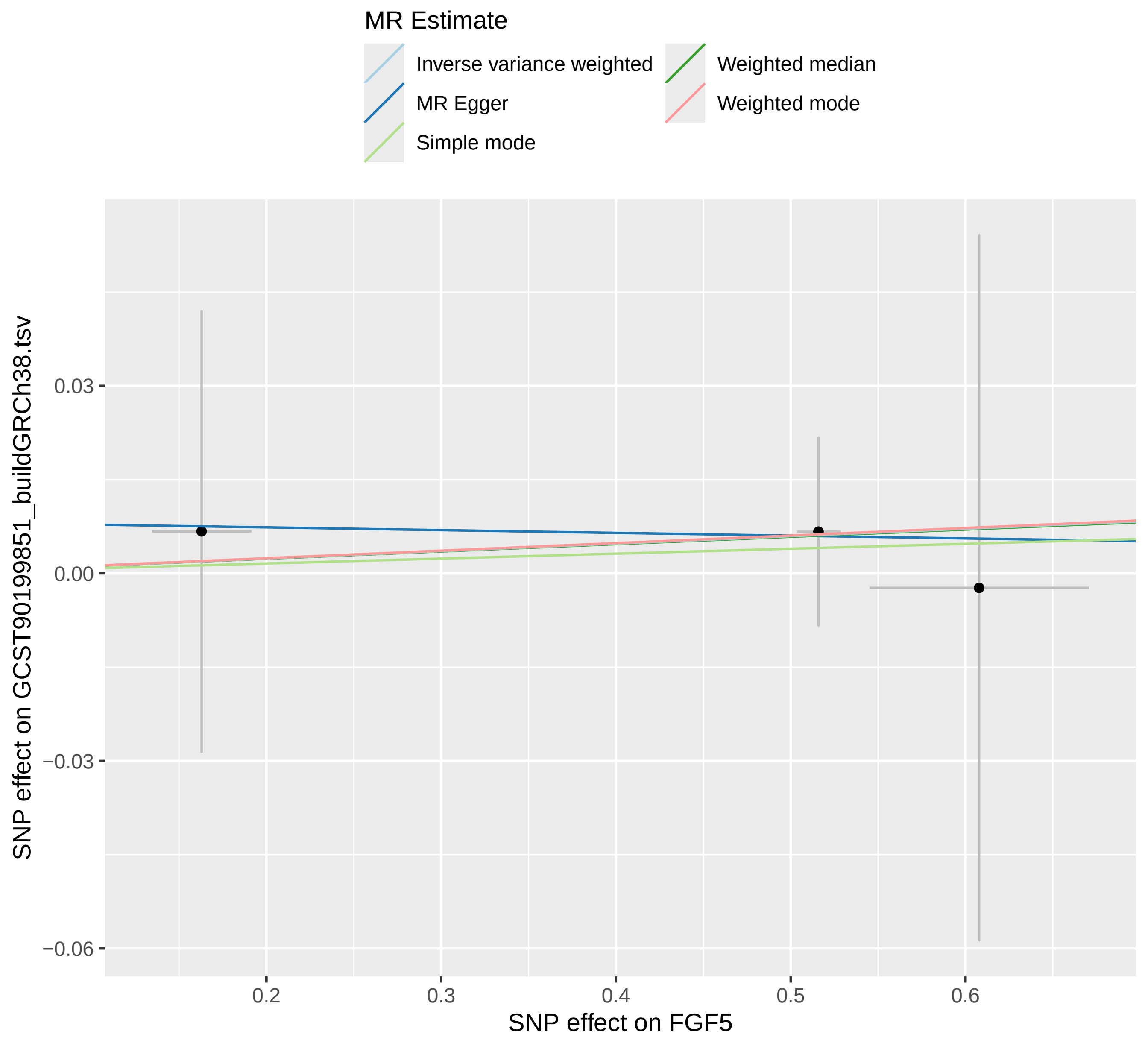

MR Estimate
Inverse variance weighted
MR Egger
Simple mode
Weighted median
Weighted mode
0.03
0.00
−0.03
−0.06
0.2
0.3
0.4
0.5
0.6
SNP effect on GCST90199851_buildGRCh38.tsv
SNP effect on FGF5

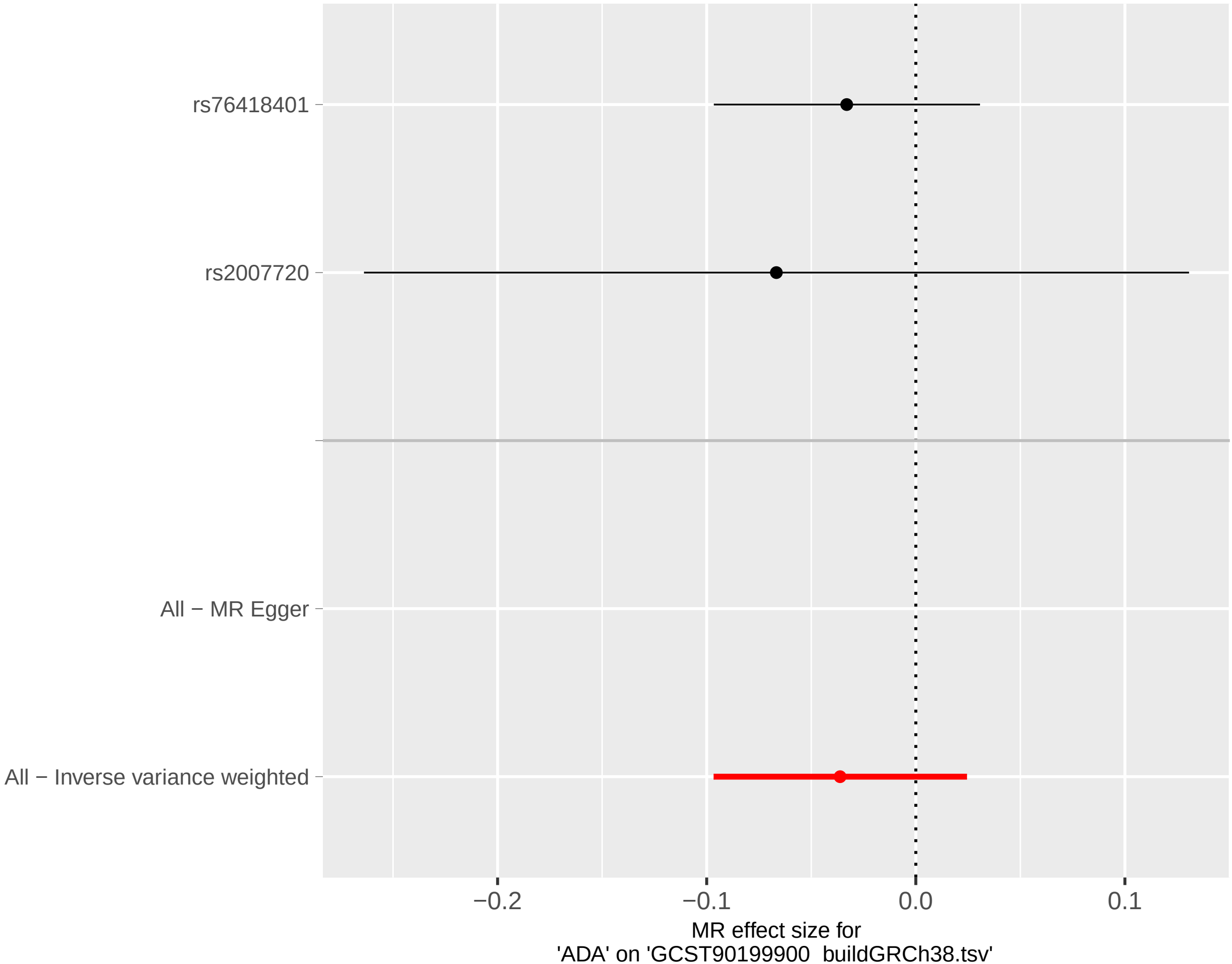

rs76418401
rs2007720
All − MR Egger
All − Inverse variance weighted
−0.2
−0.1
0.0
0.1
MR effect size for
'ADA' on 'GCST90199900_buildGRCh38.tsv'

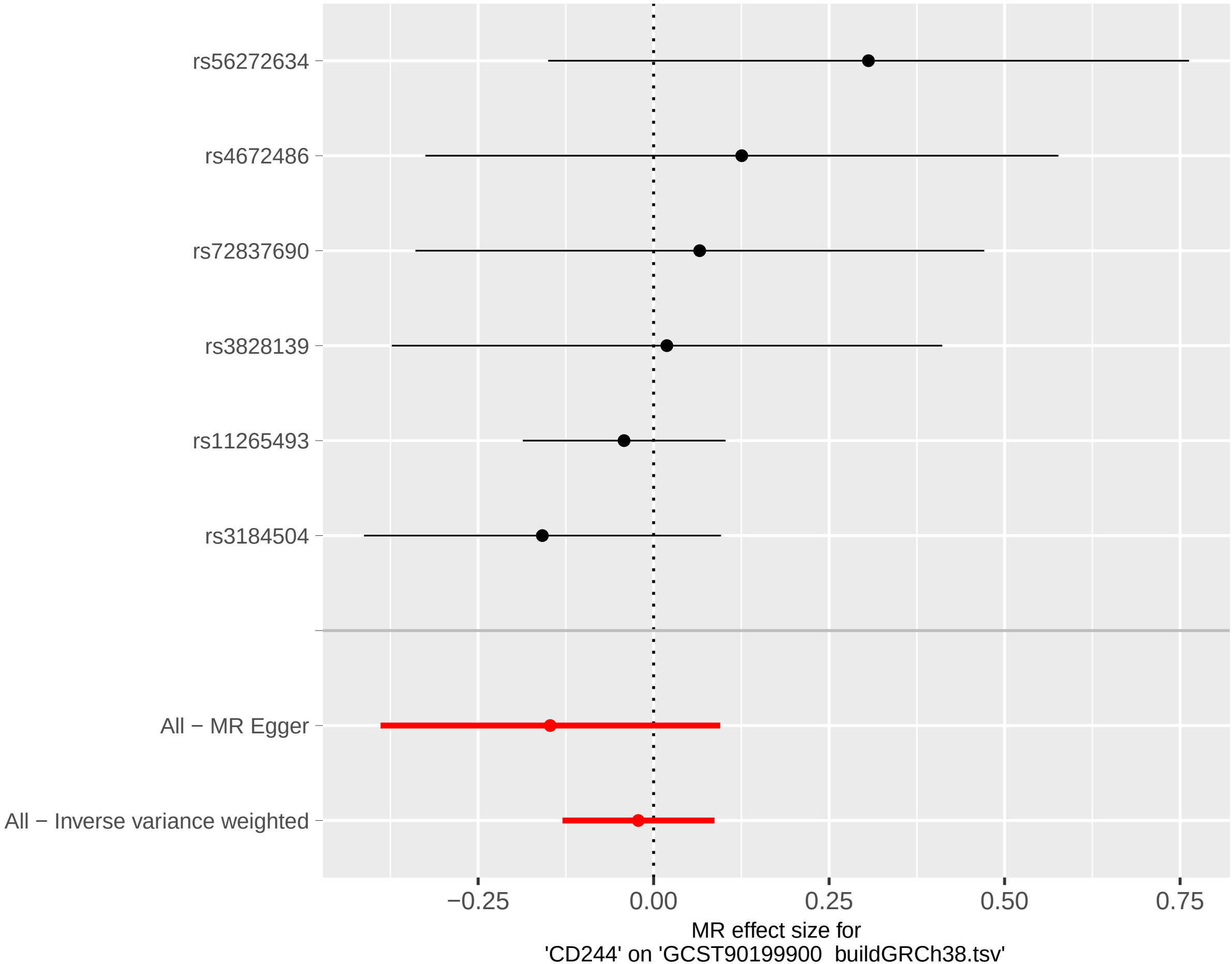
rs56272634
rs4672486
rs72837690
rs3828139
rs11265493
rs3184504
All − MR Egger
All − Inverse variance weighted
−0.25
0.00
0.25
0.50
0.75
MR effect size for
'CD244' on 'GCST90199900_buildGRCh38.tsv'

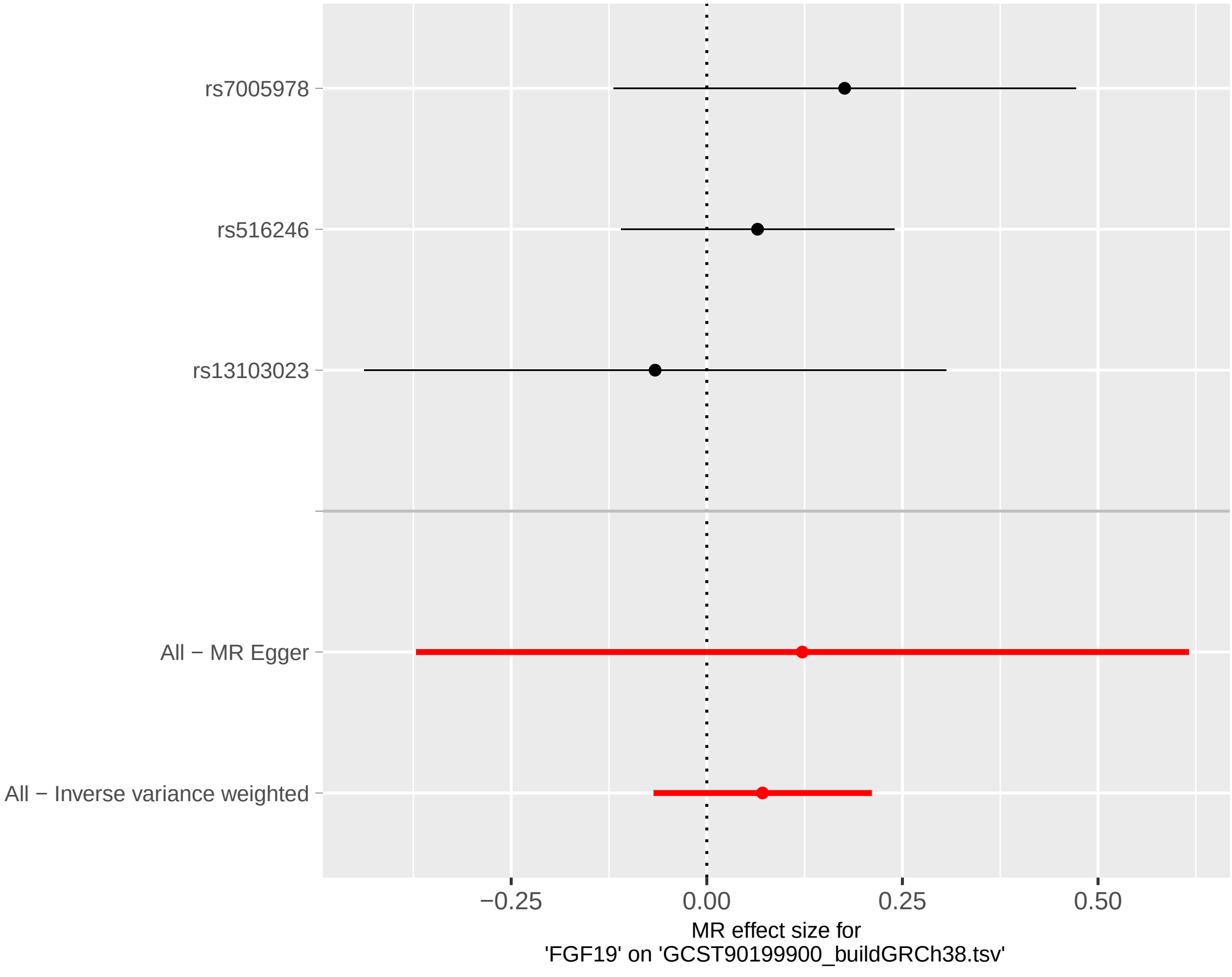
rs7005978
rs516246
rs13103023
All − MR Egger
All − Inverse variance weighted
−0.25
0.00
0.25
0.50
MR effect size for
'FGF19' on 'GCST90199900_buildGRCh38.tsv'

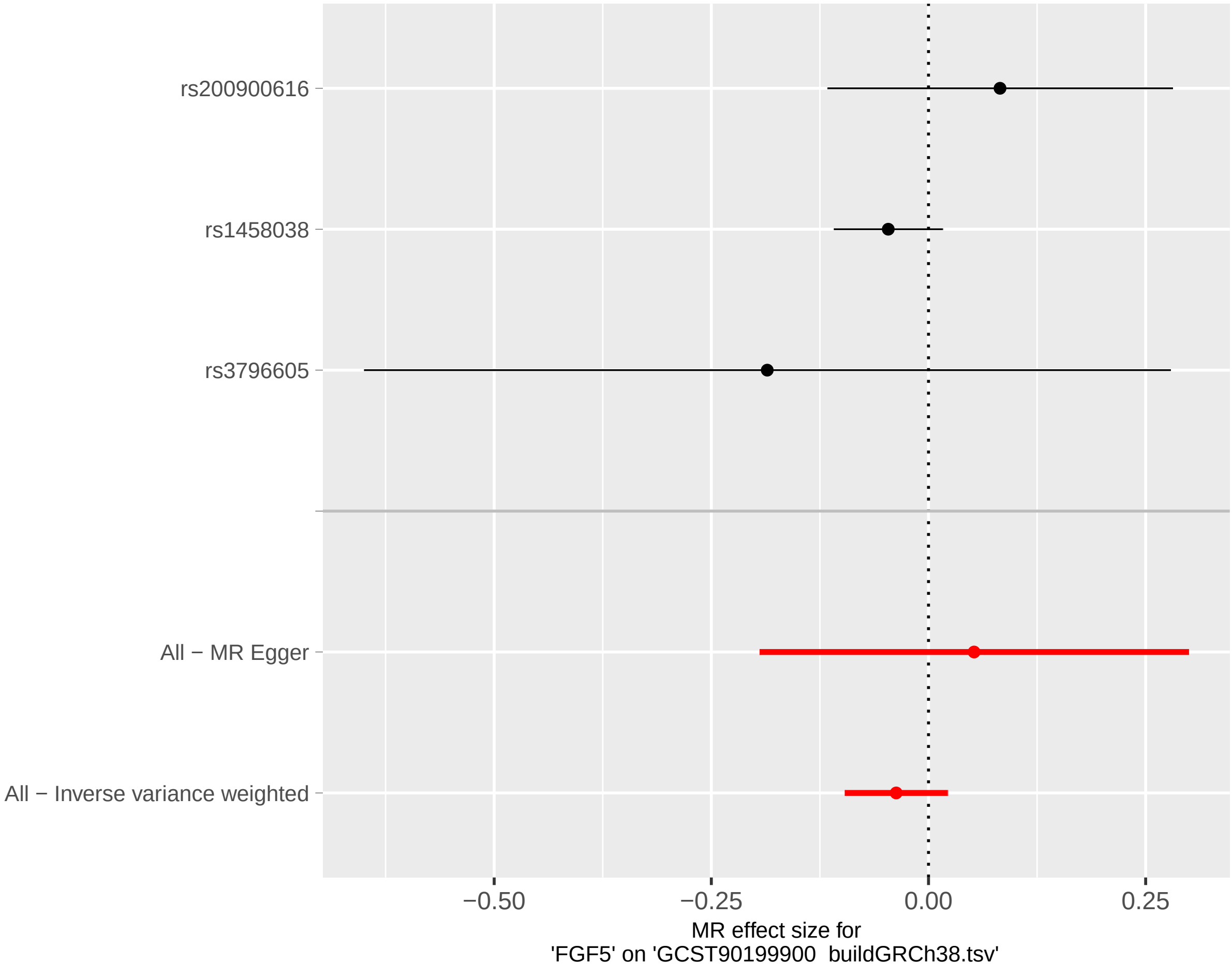
rs200900616
rs1458038
rs3796605
All − MR Egger
All − Inverse variance weighted
−0.50
−0.25
0.00
0.25
MR effect size for
'FGF5' on 'GCST90199900_buildGRCh38.tsv'

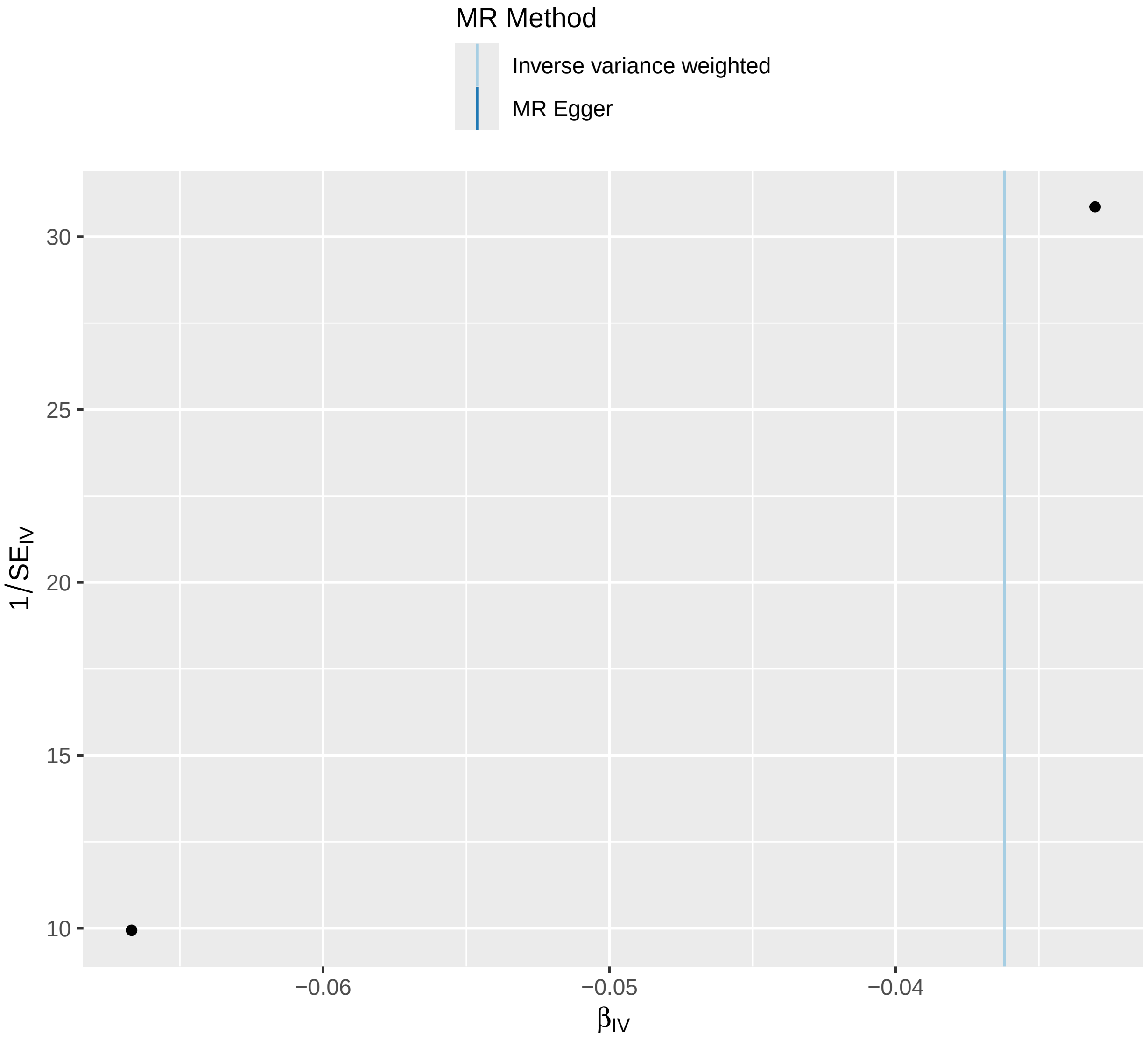
MR Method
Inverse variance weighted
MR Egger
30
25
20
15
10
1/SE_IV
−0.06
−0.05
−0.04
β_IV

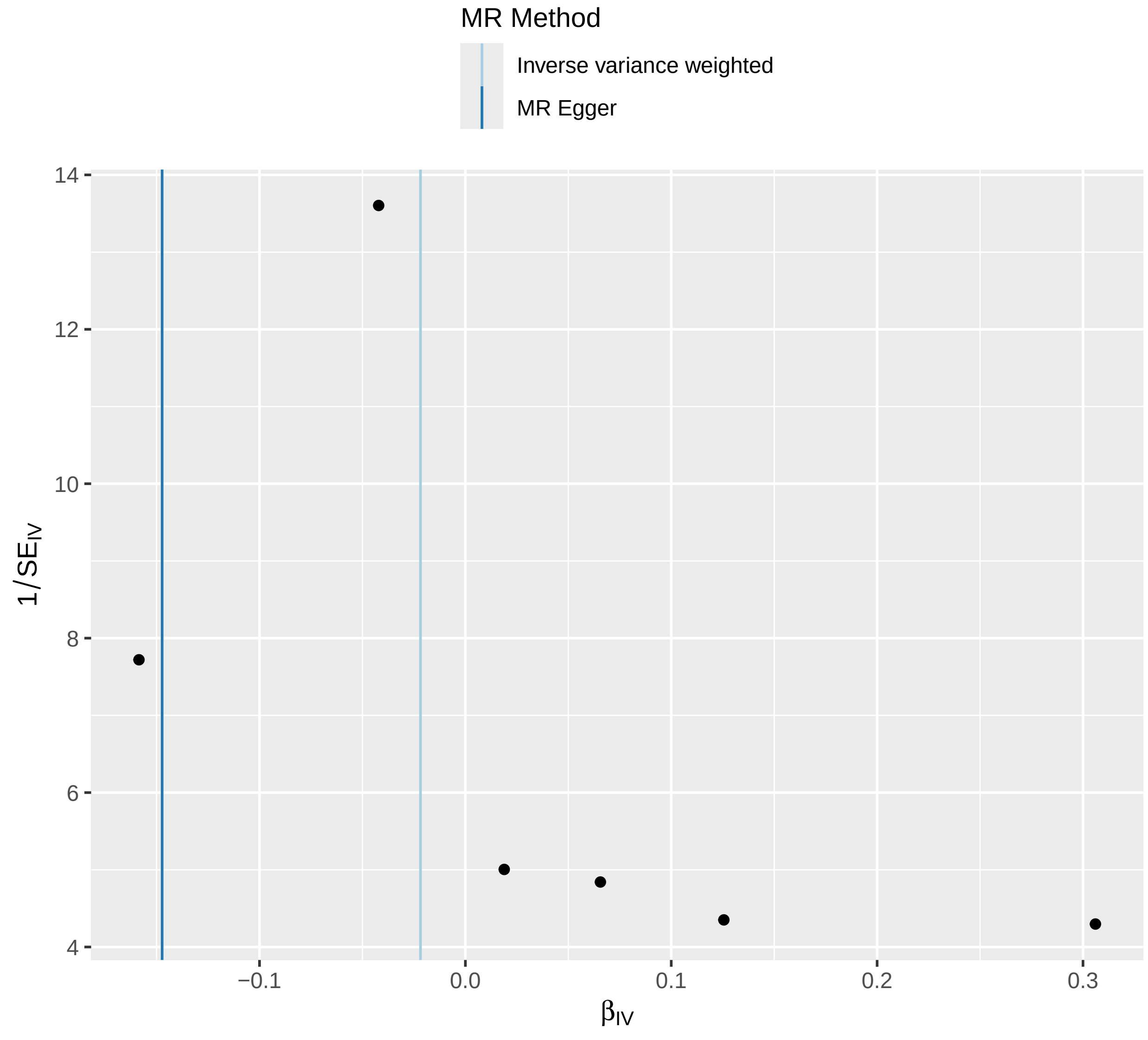

MR Method
Inverse variance weighted
MR Egger
14
12
10
8
6
4
1/SE$_{IV}$
−0.1
0.0
0.1
0.2
0.3
β$_{IV}$

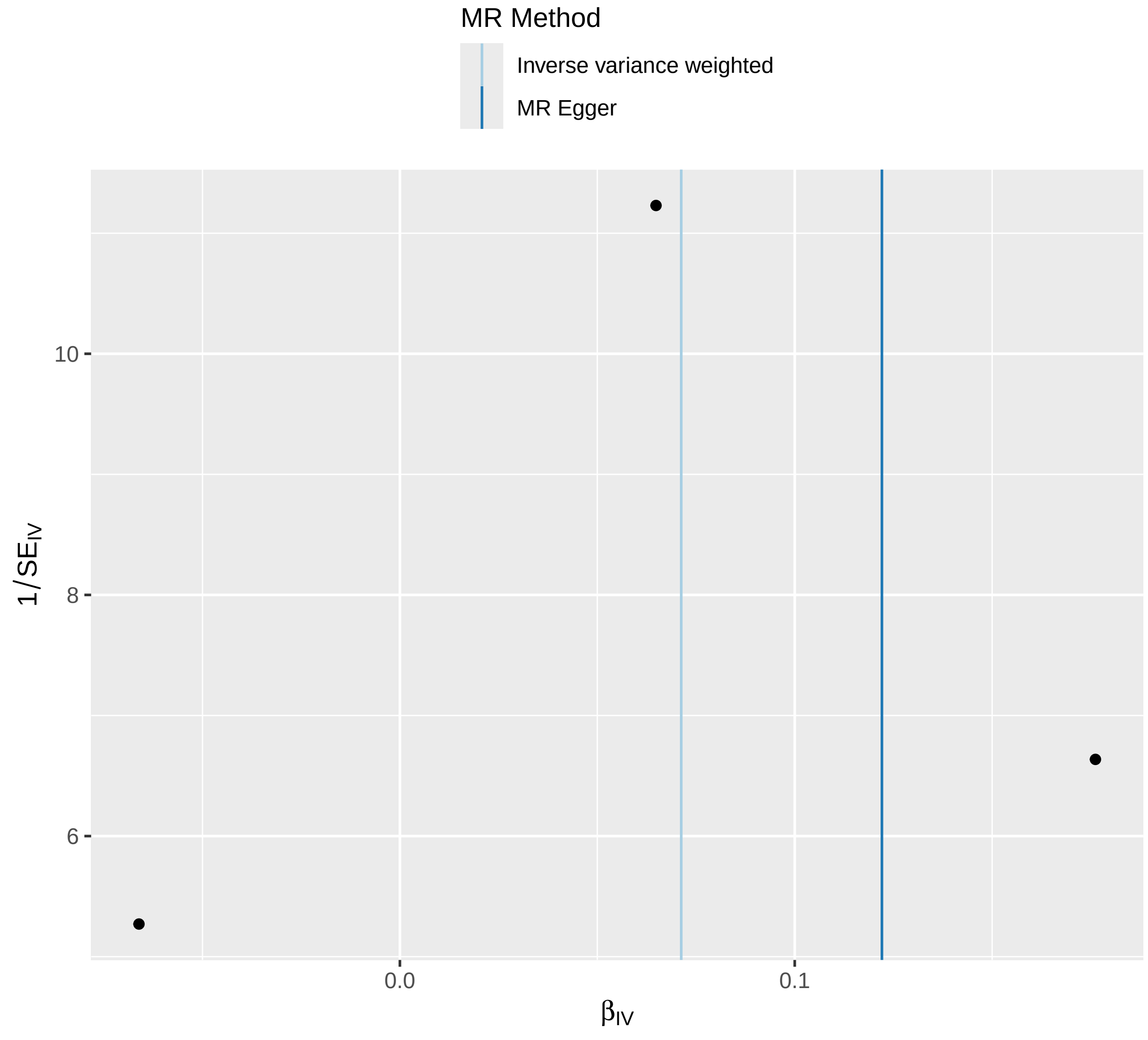

MR Method
Inverse variance weighted
MR Egger
10
8
6
1/SE_IV
0.0
0.1
β_IV

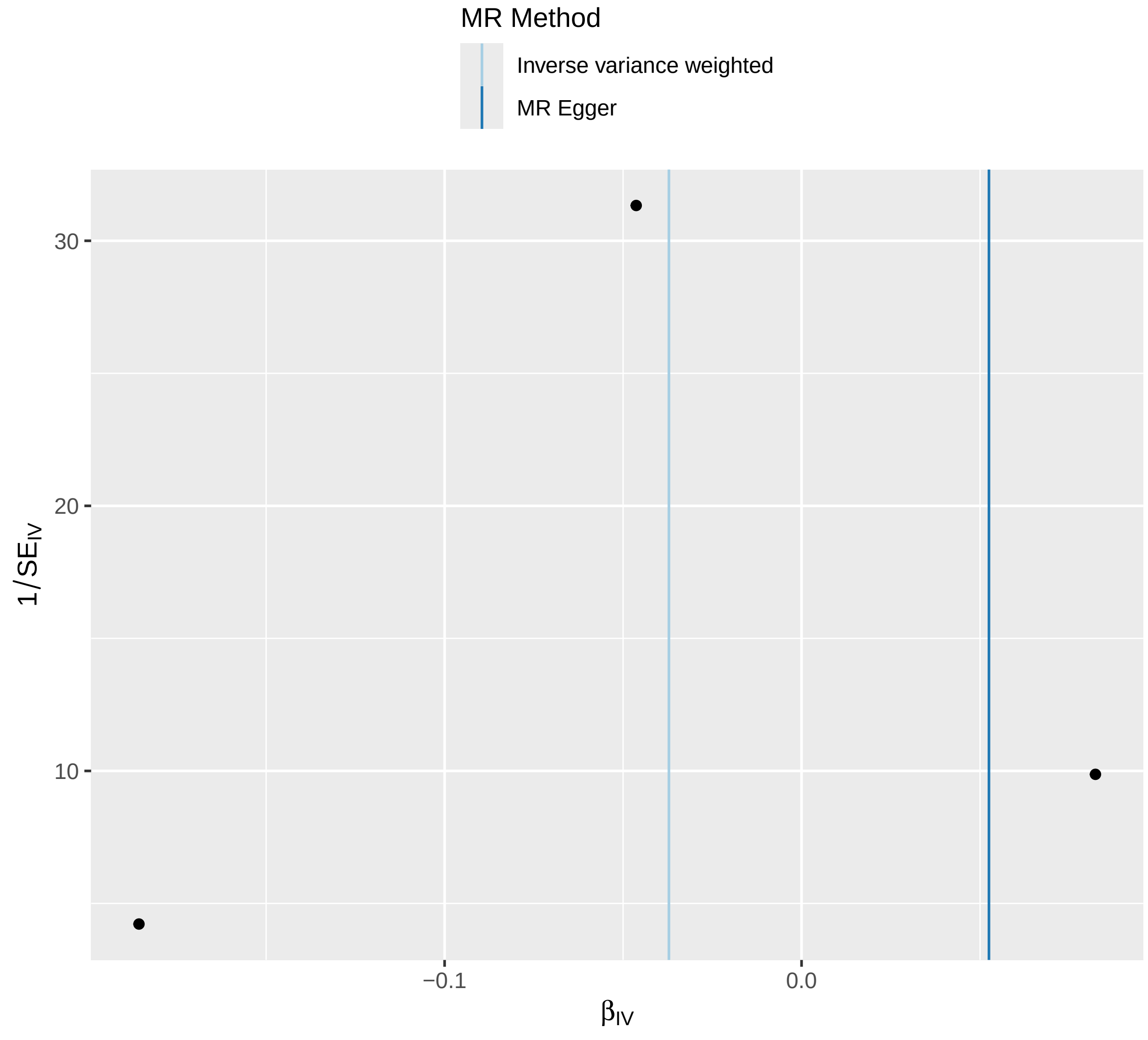
MR Method
Inverse variance weighted
MR Egger
30
20
10
1/SE_IV
−0.1
0.0
β_IV

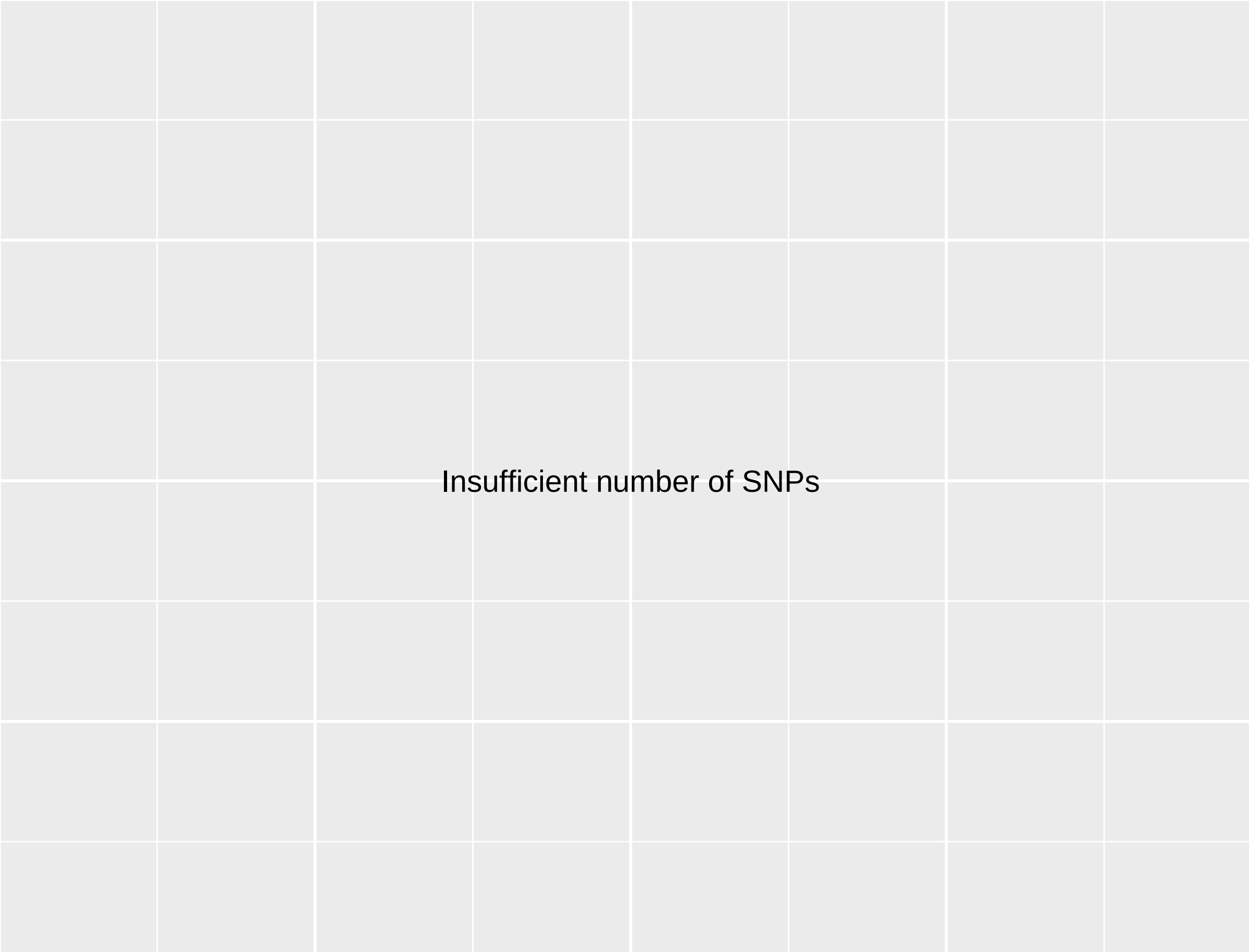
Insufficient number of SNPs

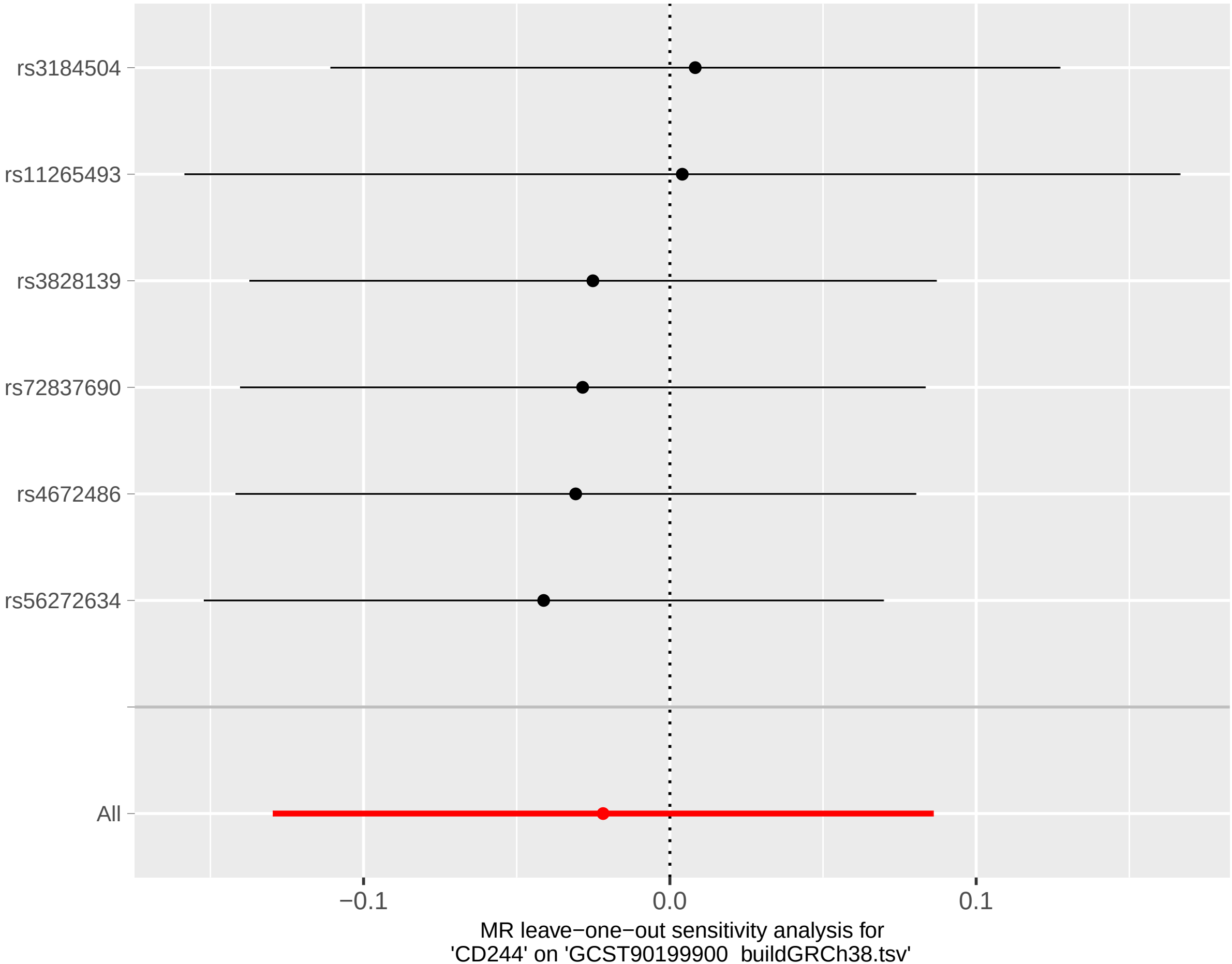
rs3184504
rs11265493
rs3828139
rs72837690
rs4672486
rs56272634
All
−0.1
0.0
0.1
MR leave−one−out sensitivity analysis for
'CD244' on 'GCST90199900_buildGRCh38.tsv'

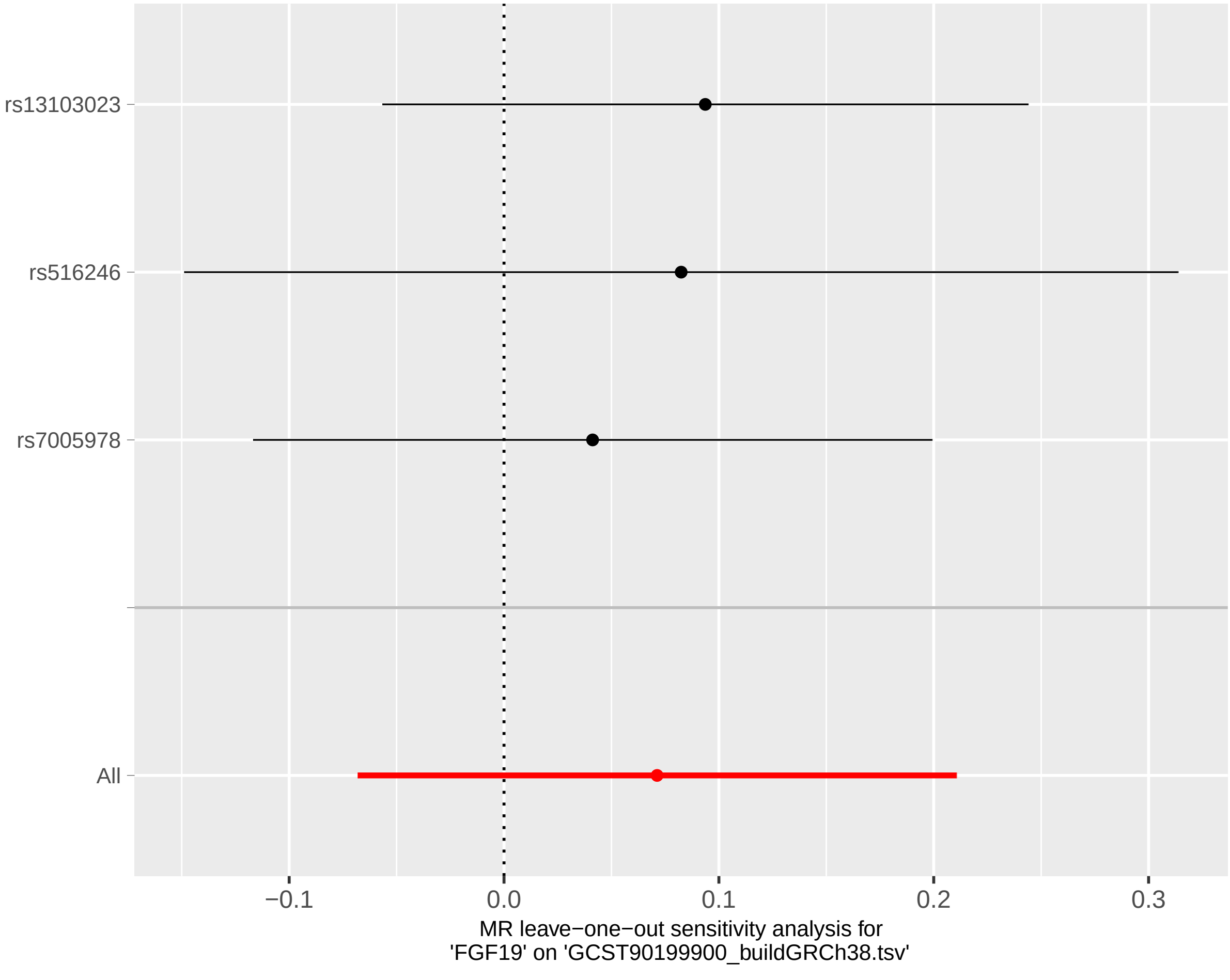
rs13103023
rs516246
rs7005978
All
−0.1
0.0
0.1
0.2
0.3
MR leave−one−out sensitivity analysis for
'FGF19' on 'GCST90199900_buildGRCh38.tsv'

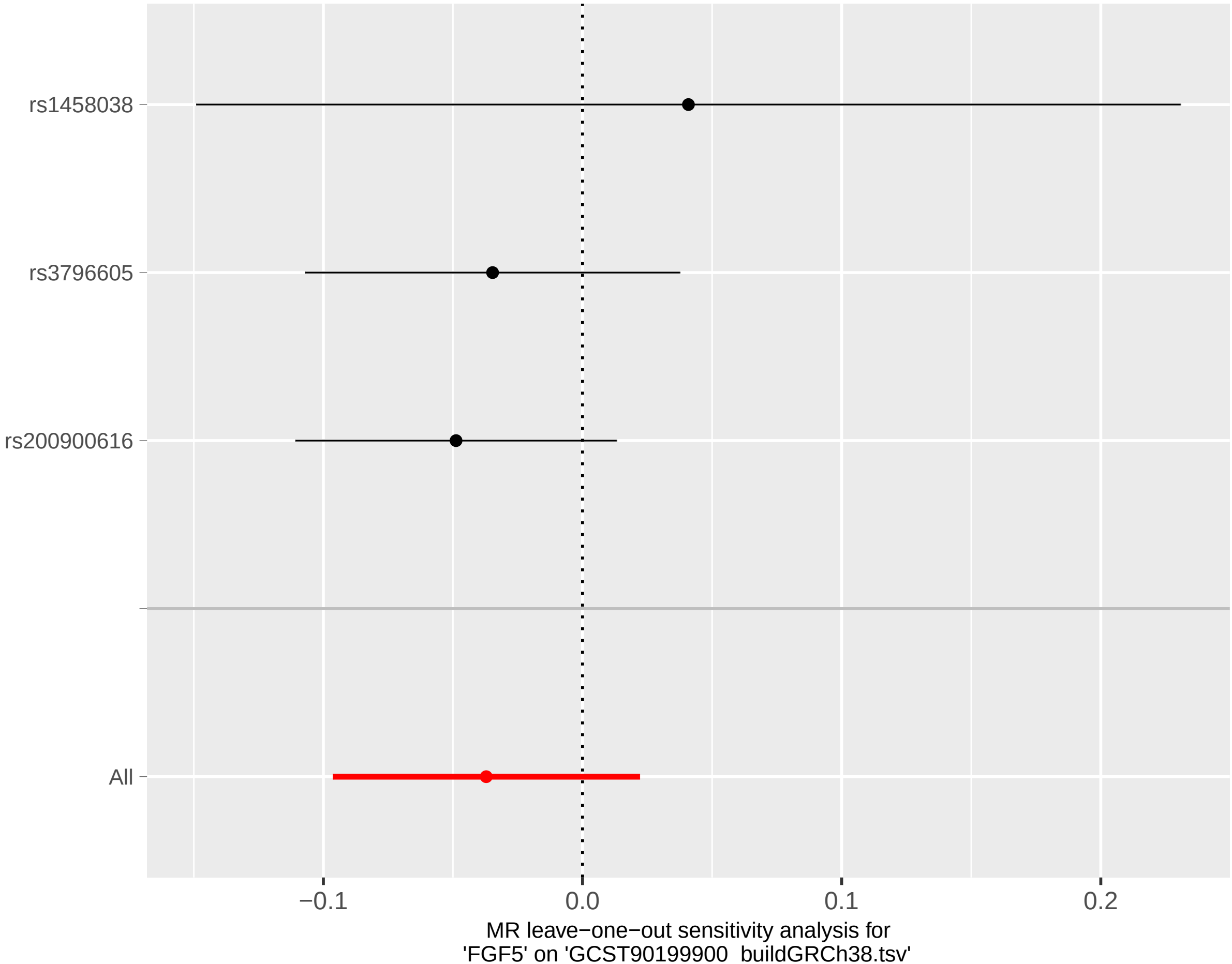

rs1458038
rs3796605
rs200900616
All
−0.1
0.0
0.1
0.2
MR leave−one−out sensitivity analysis for
'FGF5' on 'GCST90199900_buildGRCh38.tsv'

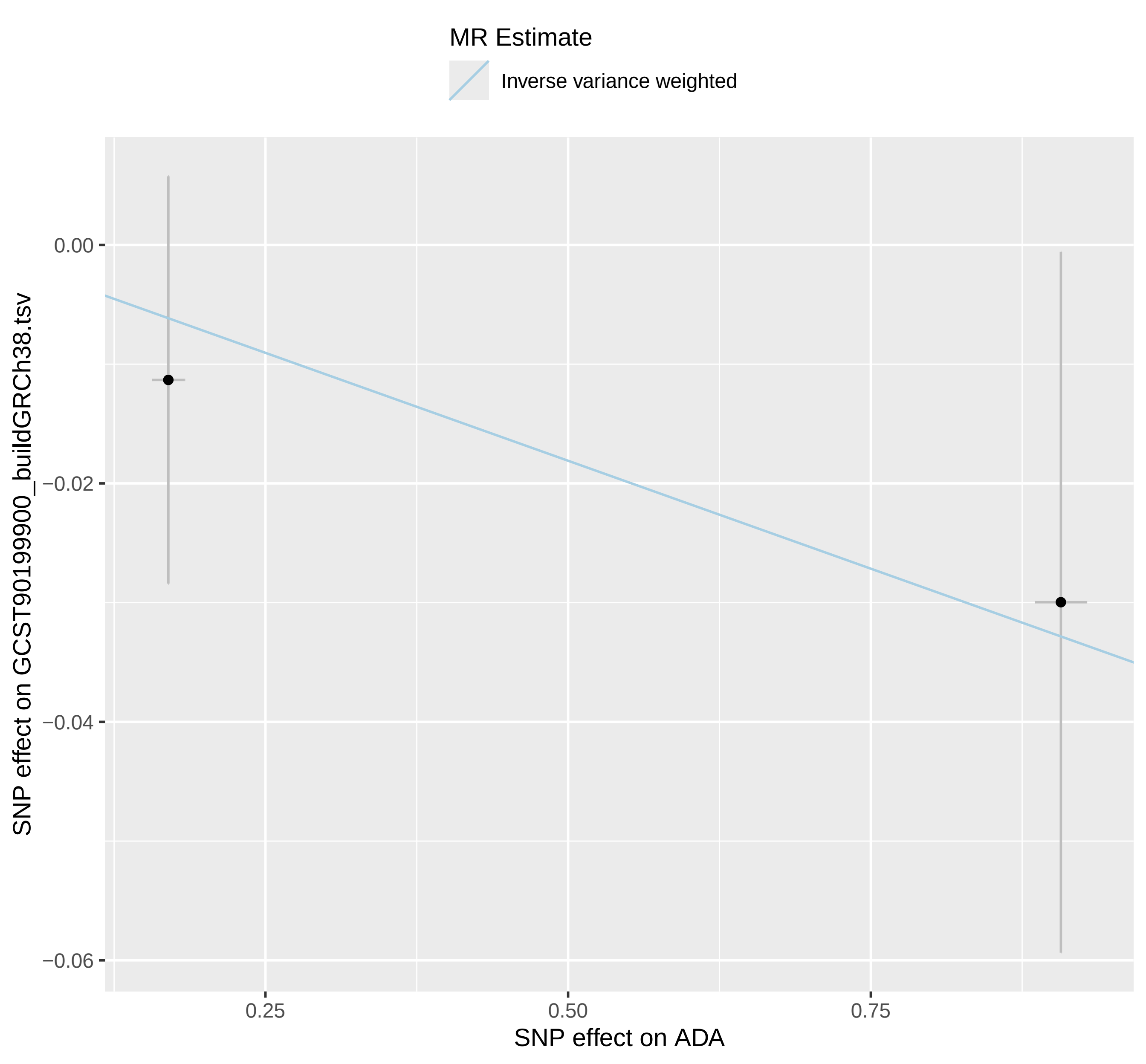
MR Estimate
Inverse variance weighted
SNP effect on GCST90199900_buildGRCh38.tsv
0.00
−0.02
−0.04
−0.06
0.25
0.50
0.75
SNP effect on ADA

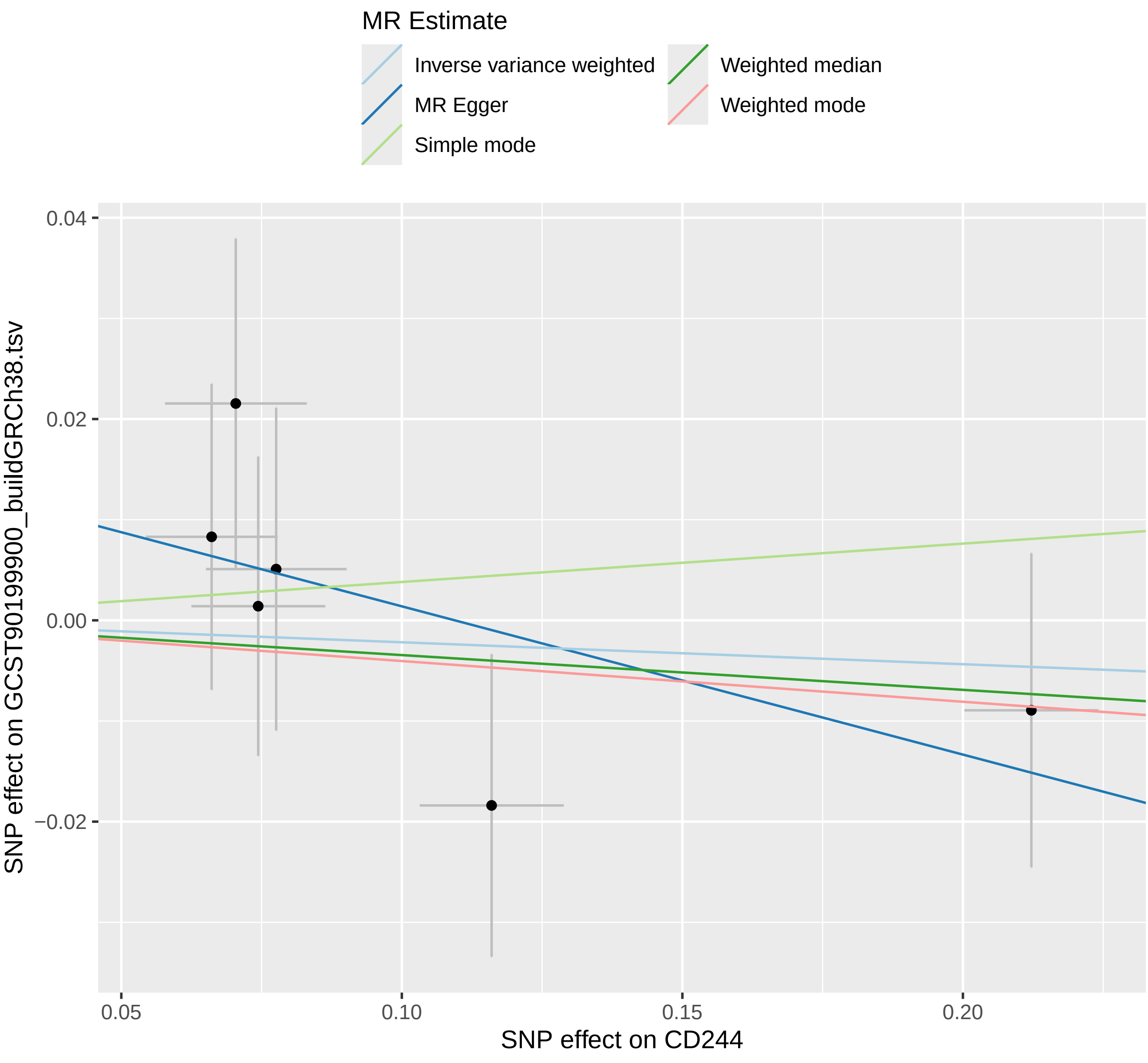
MR Estimate
Inverse variance weighted
MR Egger
Simple mode
Weighted median
Weighted mode
0.04
0.02
0.00
−0.02
0.05
0.10
0.15
0.20
SNP effect on GCST90199900_buildGRCh38.tsv
SNP effect on CD244

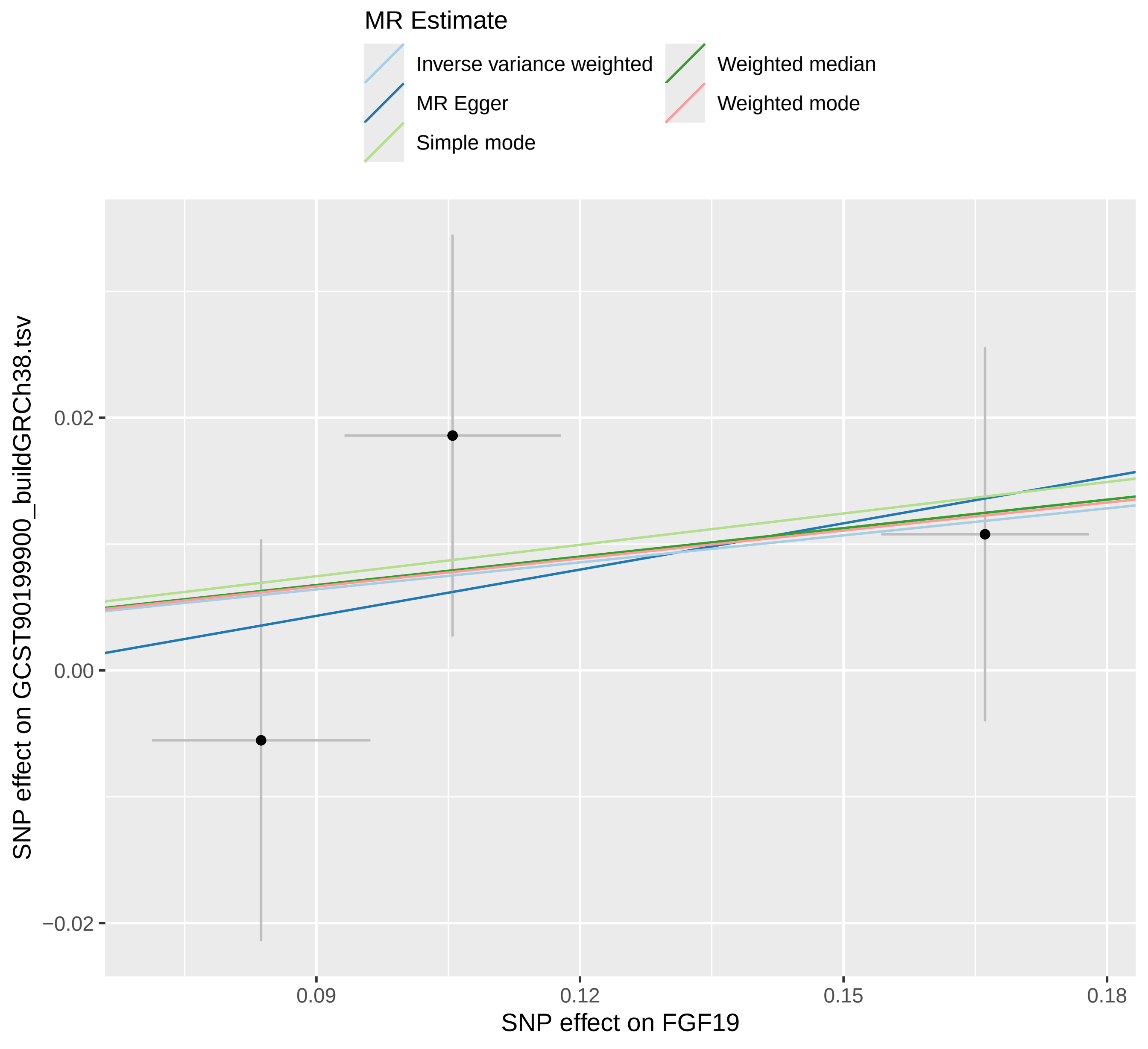
MR Estimate
Inverse variance weighted
MR Egger
Simple mode
Weighted median
Weighted mode
SNP effect on GCST90199900_buildGRCh38.tsv
0.02
0.00
−0.02
0.09
0.12
0.15
0.18
SNP effect on FGF19

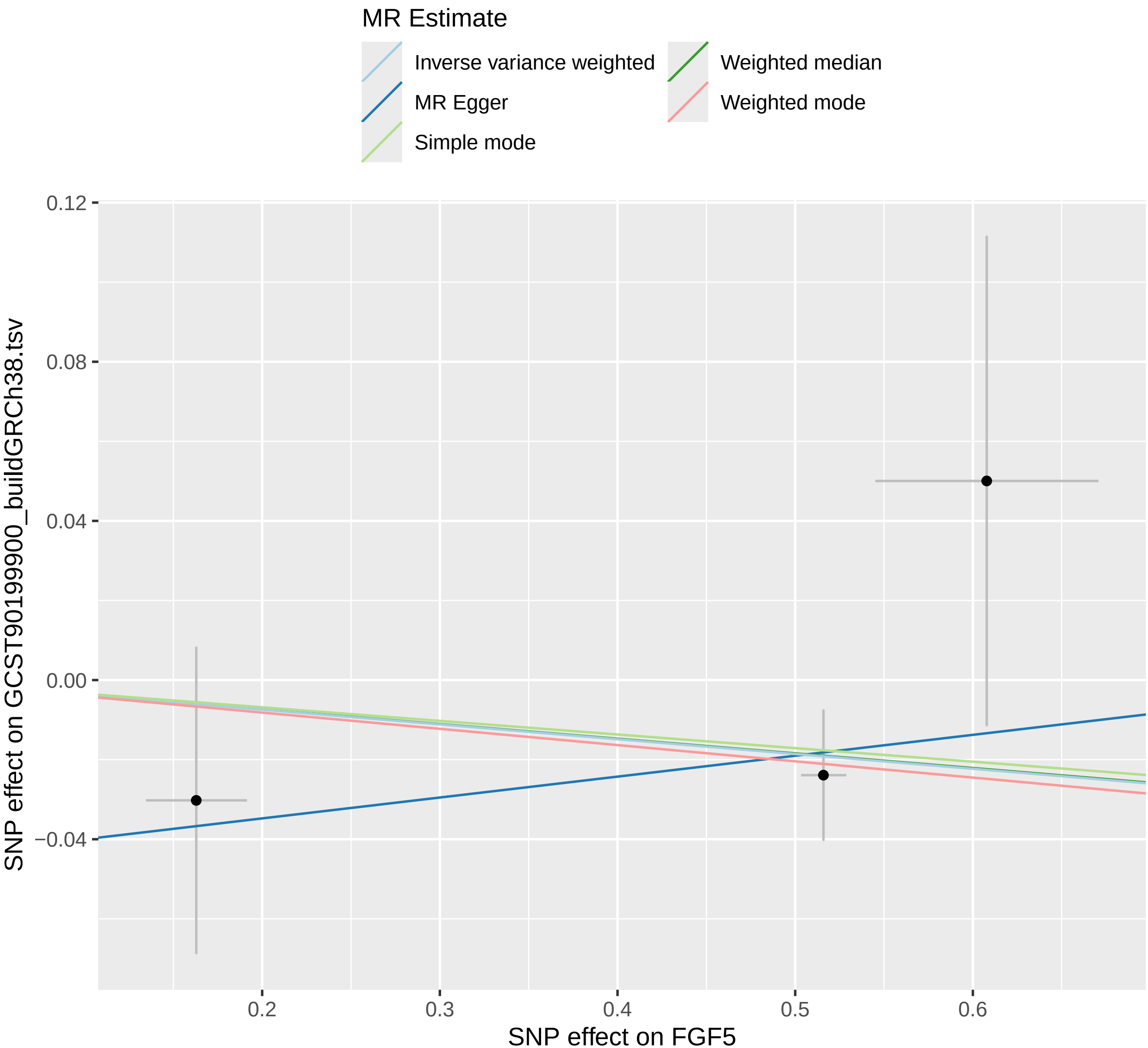
MR Estimate
Inverse variance weighted
MR Egger
Simple mode
Weighted median
Weighted mode
0.12
0.08
0.04
0.00
−0.04
0.2
0.3
0.4
0.5
0.6
SNP effect on GCST90199900_buildGRCh38.tsv
SNP effect on FGF5

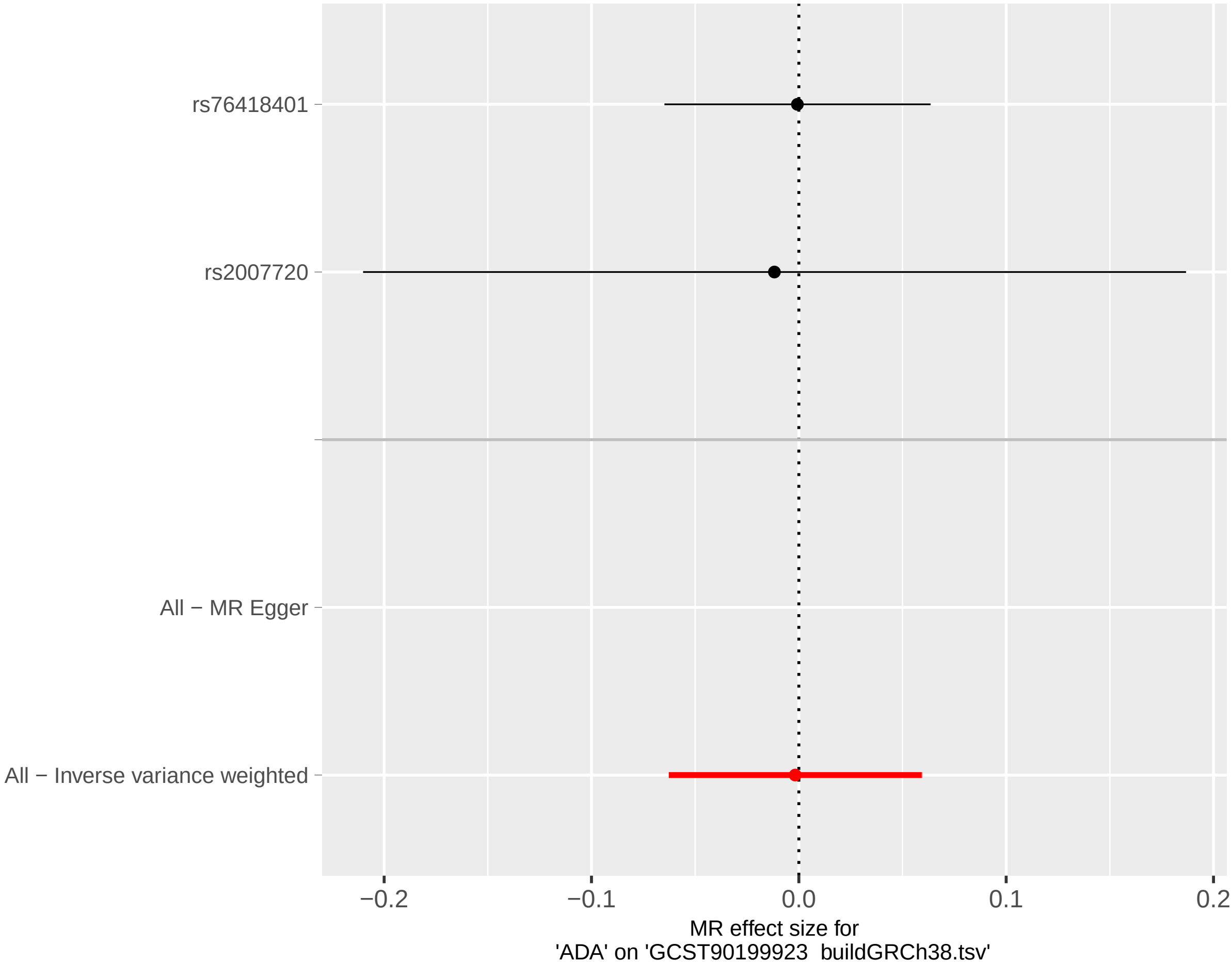
rs76418401
rs2007720
All − MR Egger
All − Inverse variance weighted
−0.2
−0.1
0.0
0.1
0.2
MR effect size for
'ADA' on 'GCST90199923_buildGRCh38.tsv'

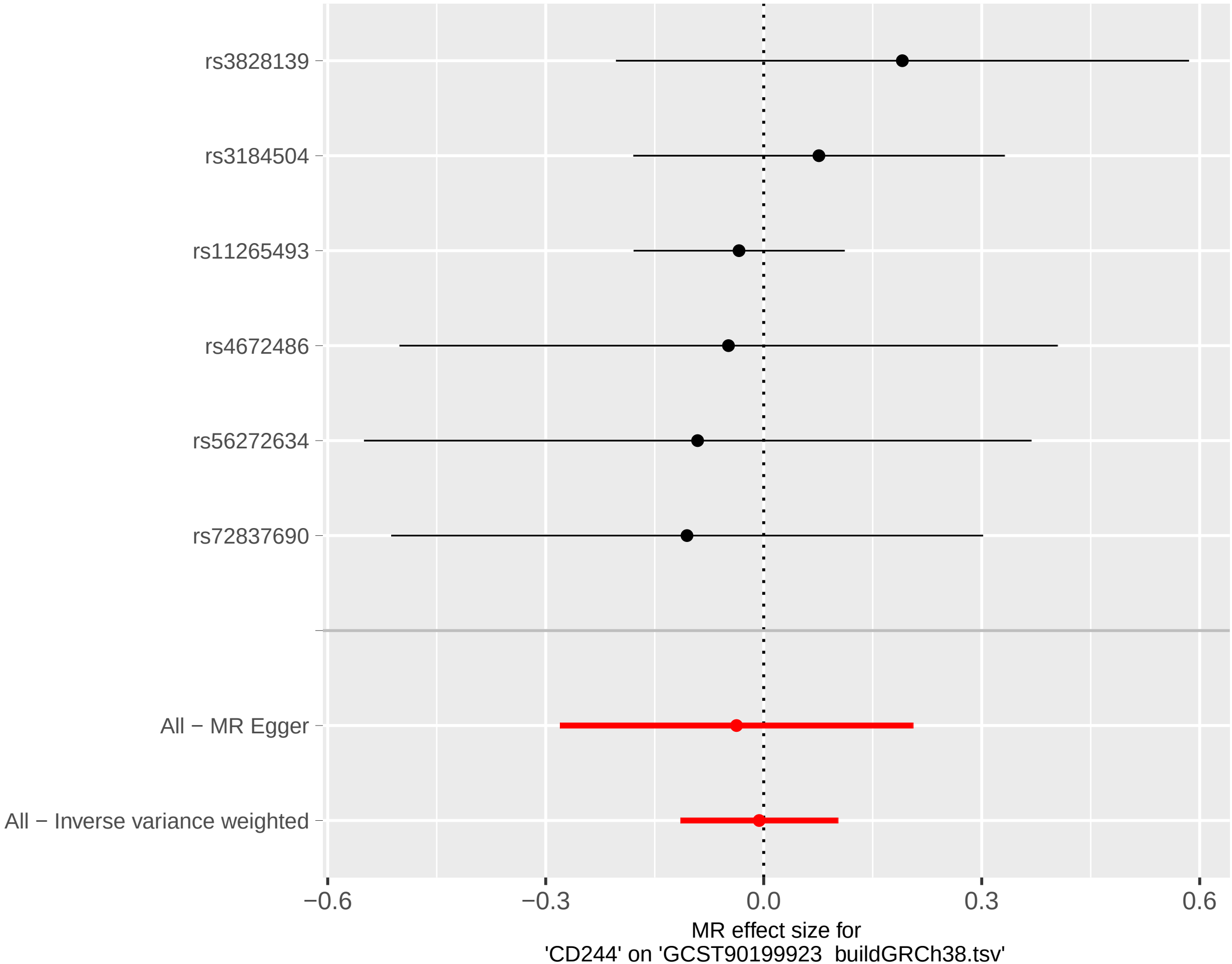

rs3828139
rs3184504
rs11265493
rs4672486
rs56272634
rs72837690
All − MR Egger
All − Inverse variance weighted
−0.6
−0.3
0.0
0.3
0.6
MR effect size for
'CD244' on 'GCST90199923_buildGRCh38.tsv'

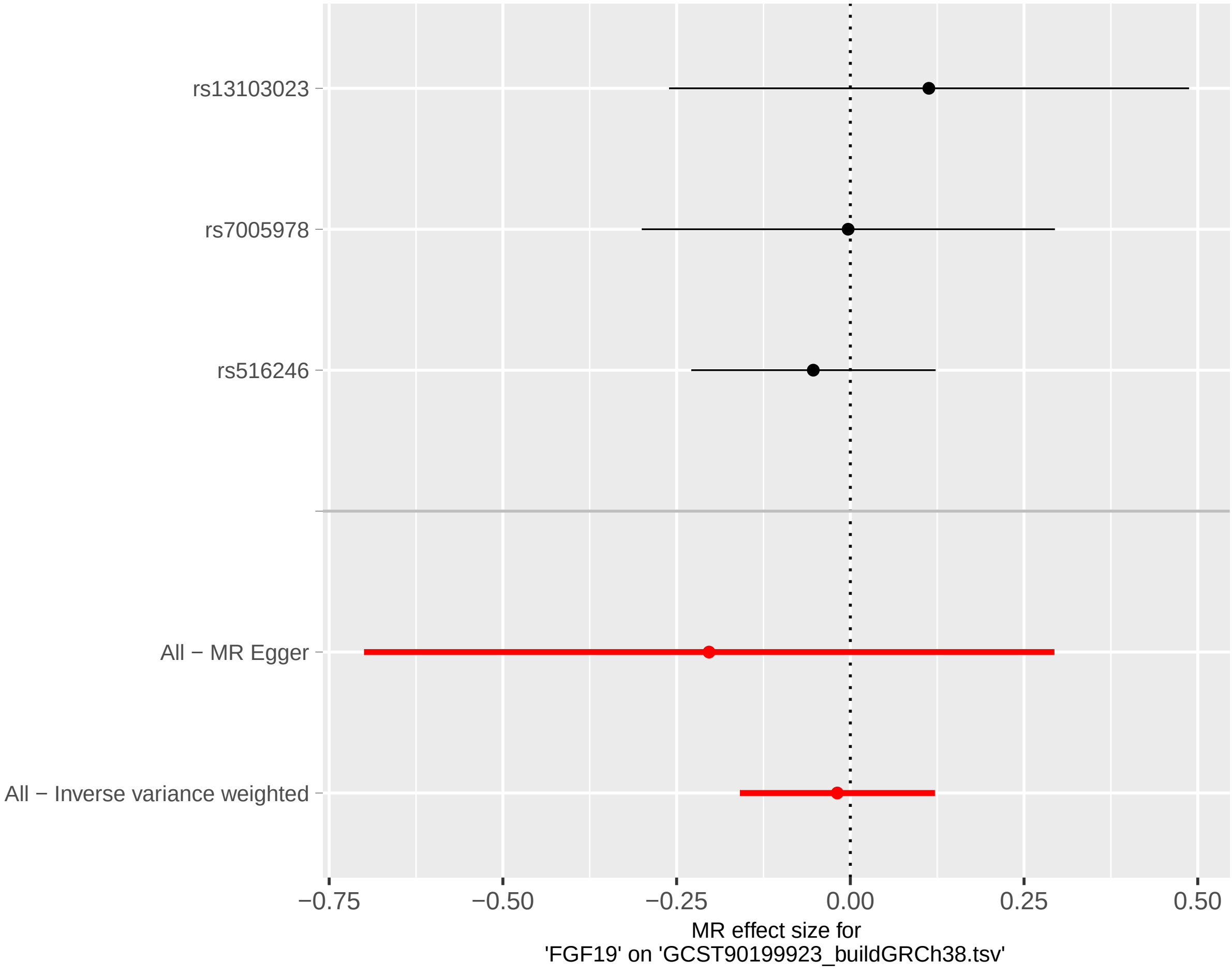
rs13103023
rs7005978
rs516246
All − MR Egger
All − Inverse variance weighted
−0.75
−0.50
−0.25
0.00
0.25
0.50
MR effect size for
'FGF19' on 'GCST90199923_buildGRCh38.tsv'

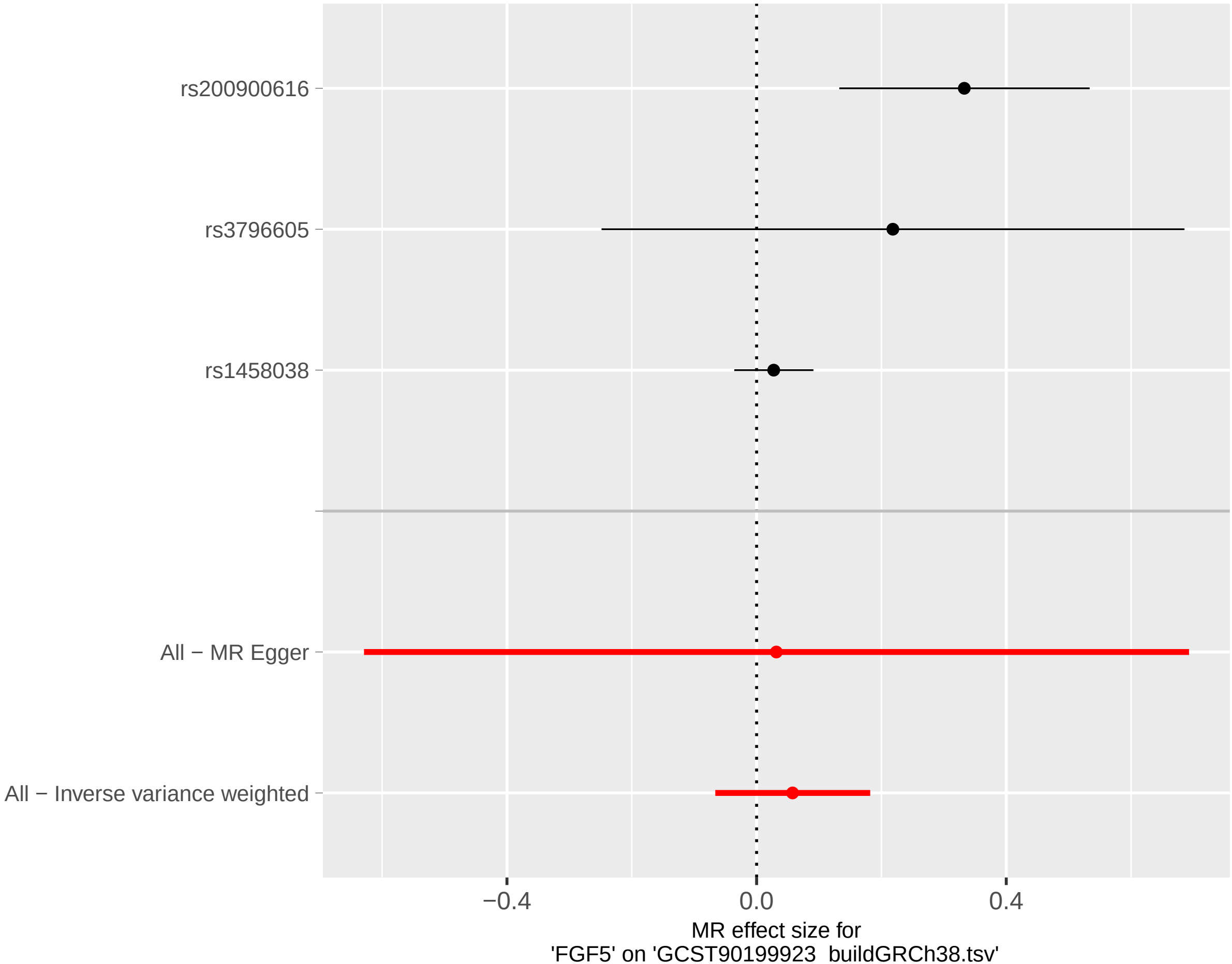
rs200900616
rs3796605
rs1458038
All − MR Egger
All − Inverse variance weighted
−0.4
0.0
0.4
MR effect size for
'FGF5' on 'GCST90199923_buildGRCh38.tsv'

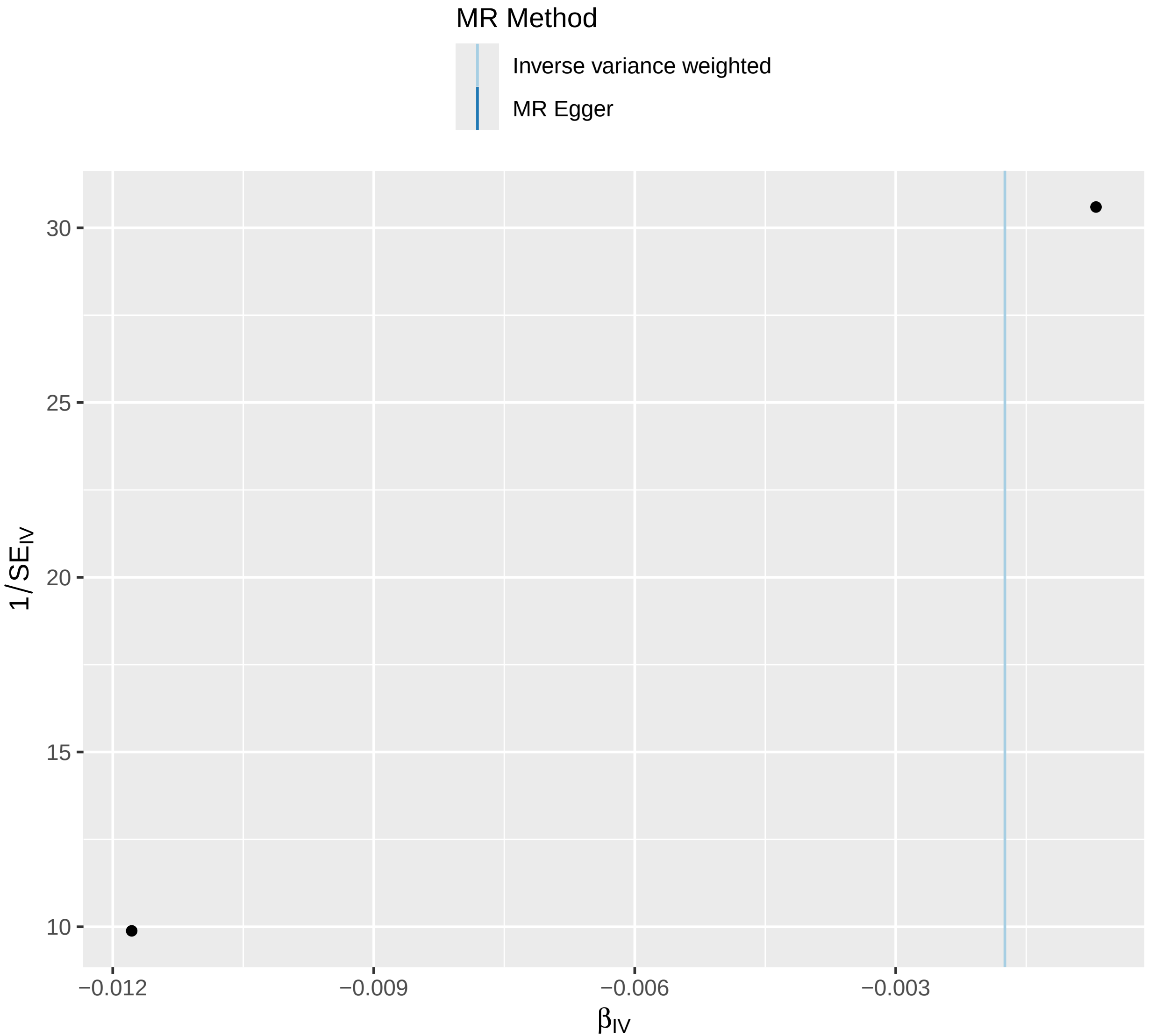

MR Method
Inverse variance weighted
MR Egger
30
25
20
15
10
1/SE$_{IV}$
−0.012
−0.009
−0.006
−0.003
β$_{IV}$

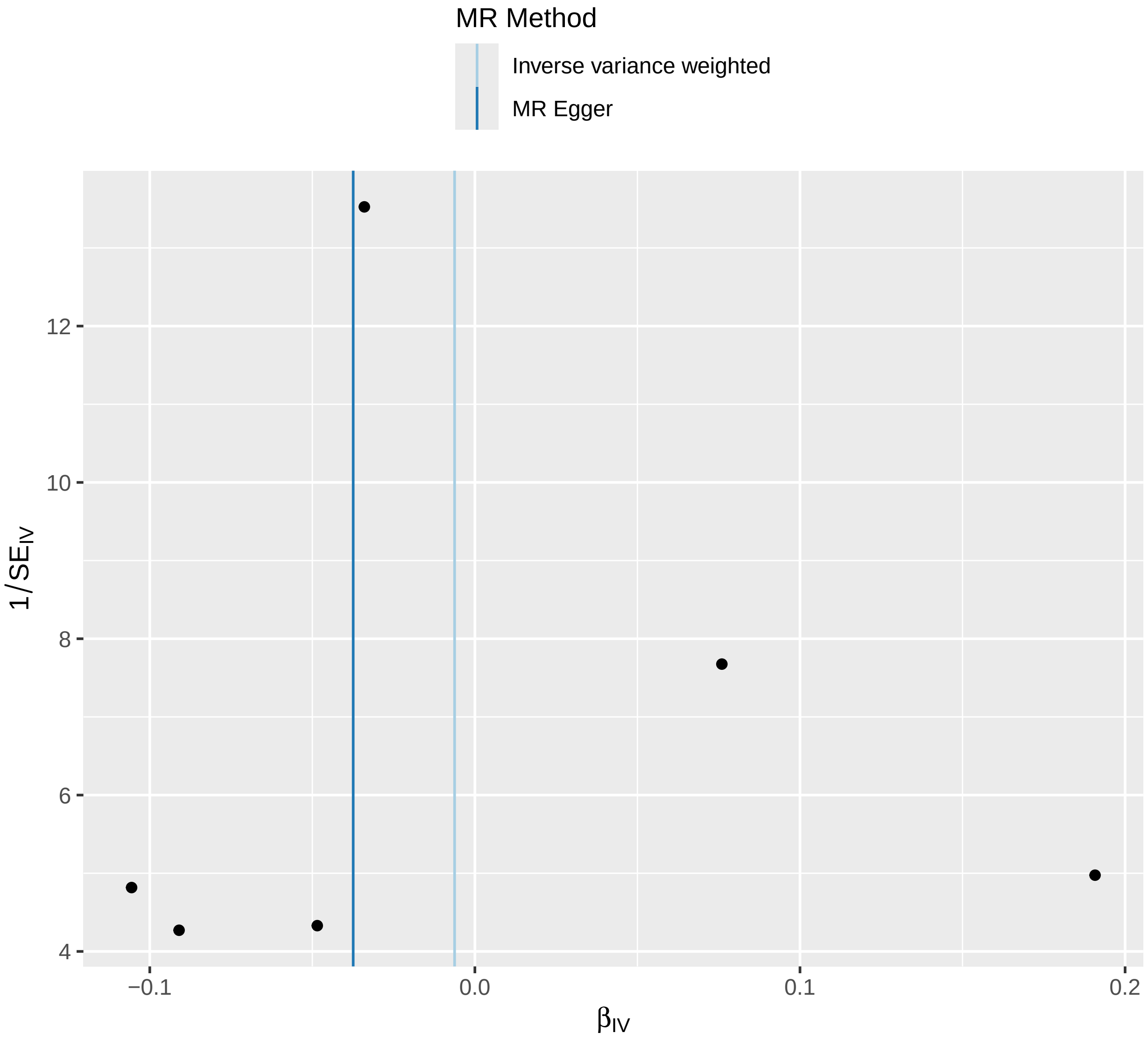
MR Method
Inverse variance weighted
MR Egger
12
10
8
6
4
$1/SE_{IV}$
−0.1
0.0
0.1
0.2
β_{IV}

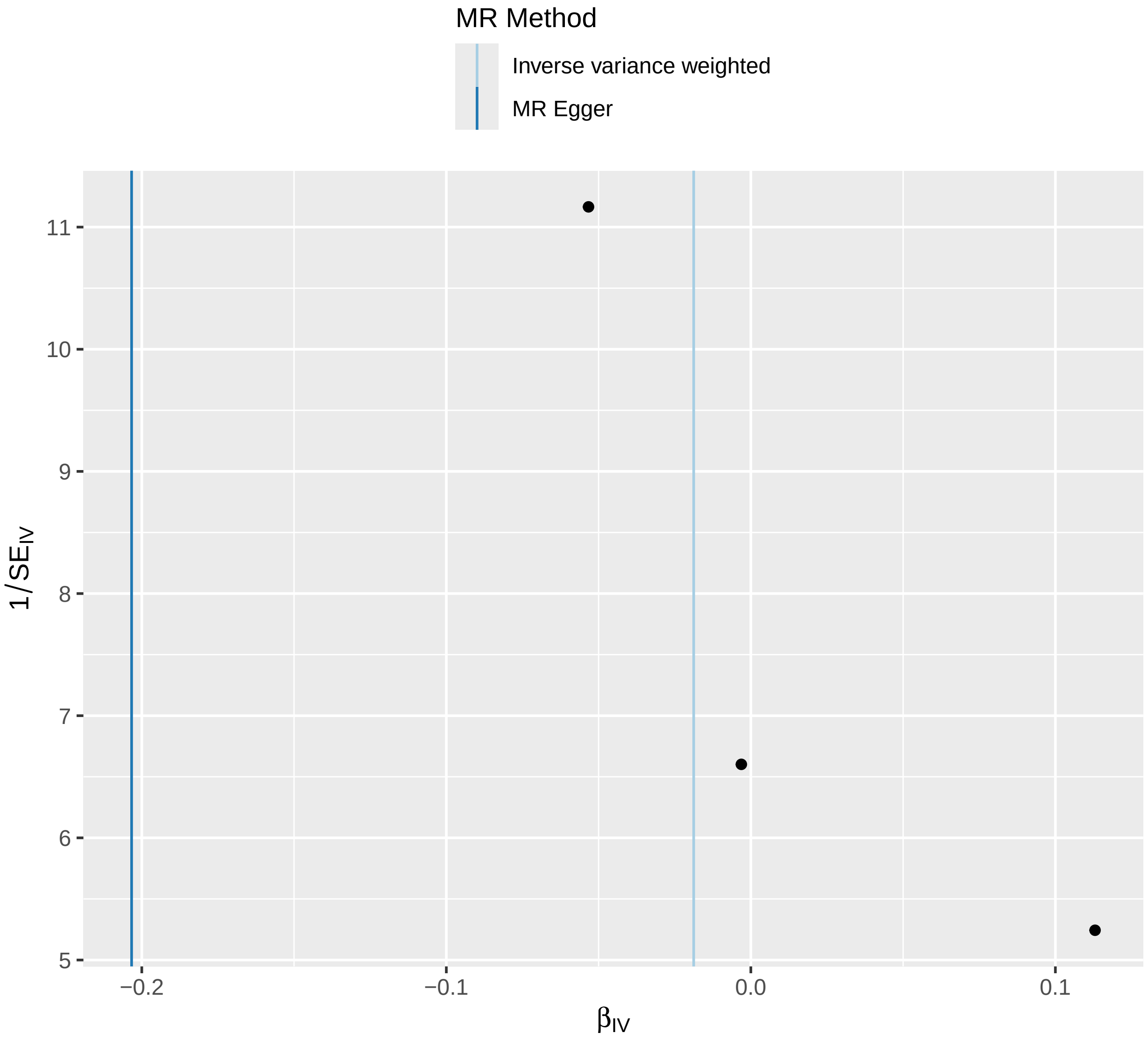
MR Method
Inverse variance weighted
MR Egger
11
10
9
8
7
6
5
1/SE_IV
−0.2
−0.1
0.0
0.1
β_IV

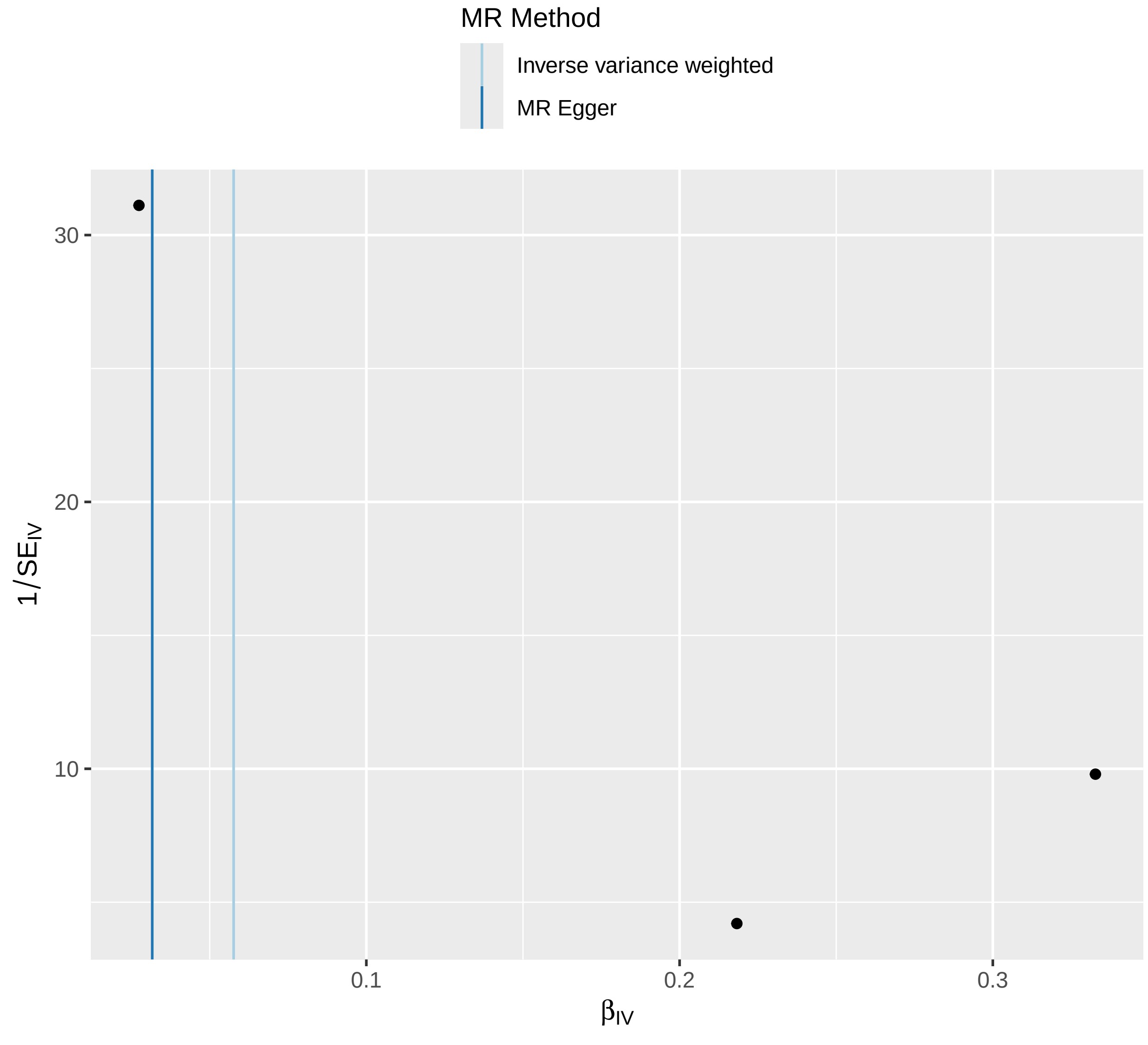
MR Method
Inverse variance weighted
MR Egger
30
20
10
0.1
0.2
0.3
1/SE_IV
β_IV

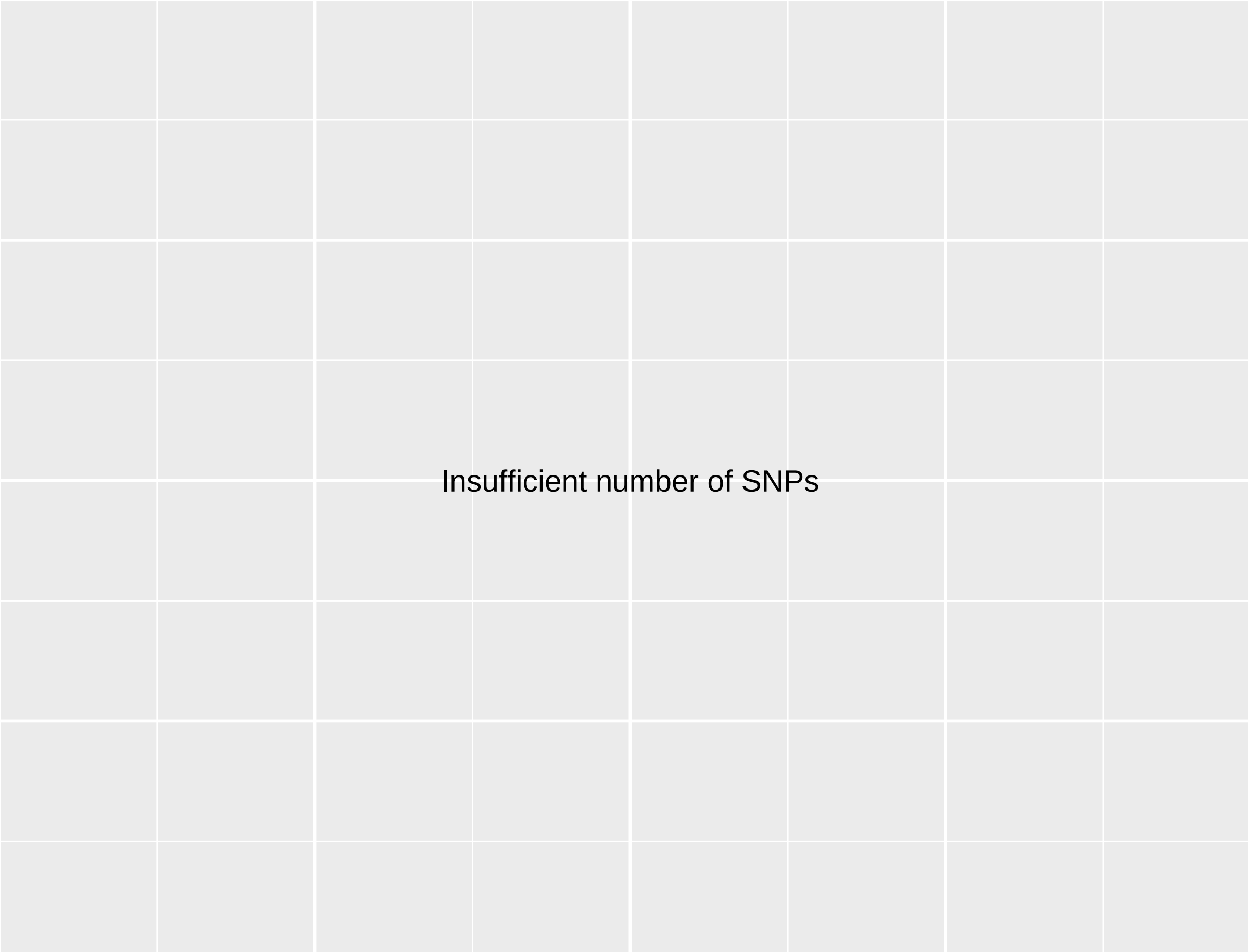
Insufficient number of SNPs

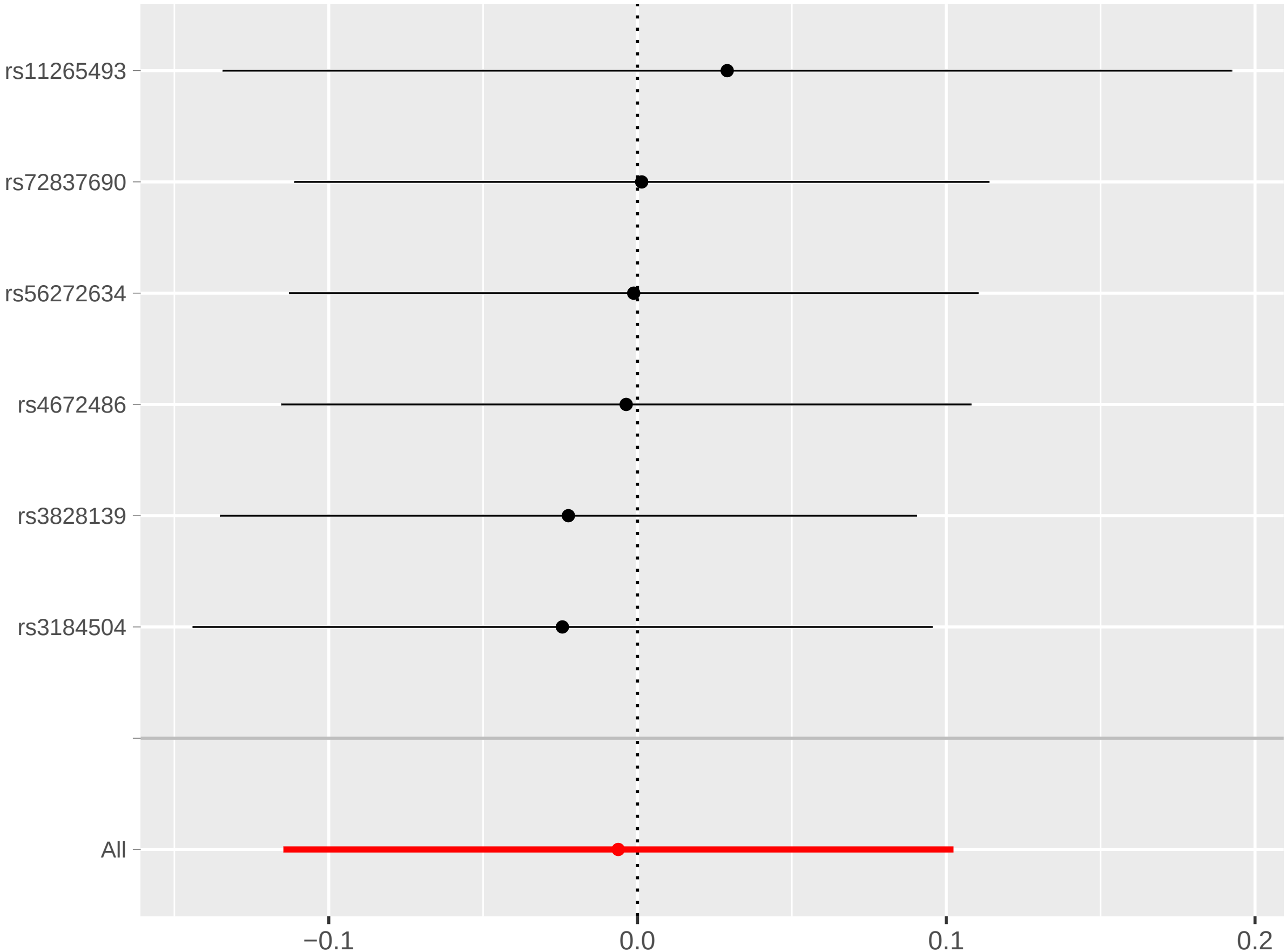

MR leave−one−out sensitivity analysis for
'CD244' on 'GCST90199923_buildGRCh38.tsv'

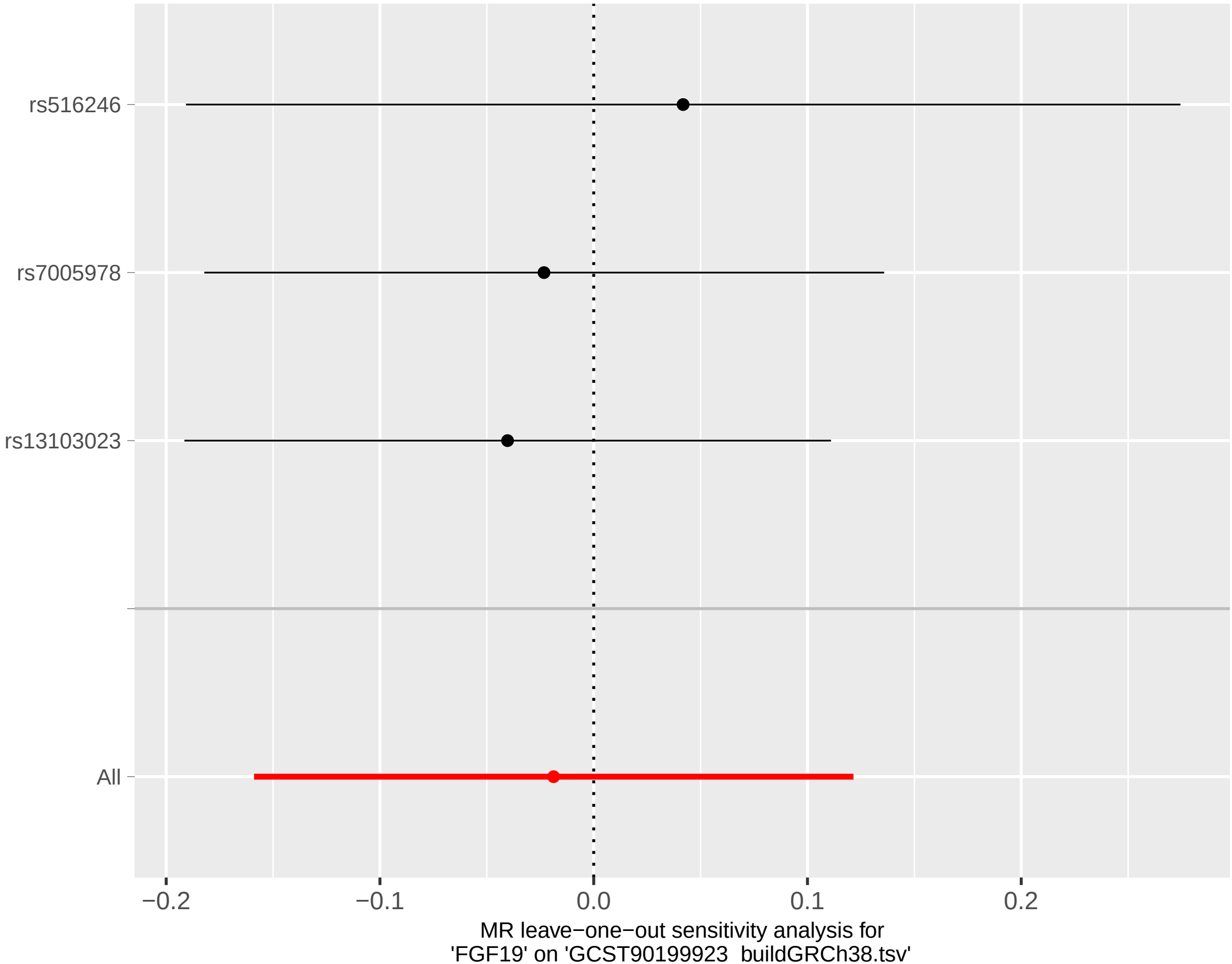
rs516246
rs7005978
rs13103023
All
−0.2
−0.1
0.0
0.1
0.2
MR leave−one−out sensitivity analysis for
'FGF19' on 'GCST90199923_buildGRCh38.tsv'

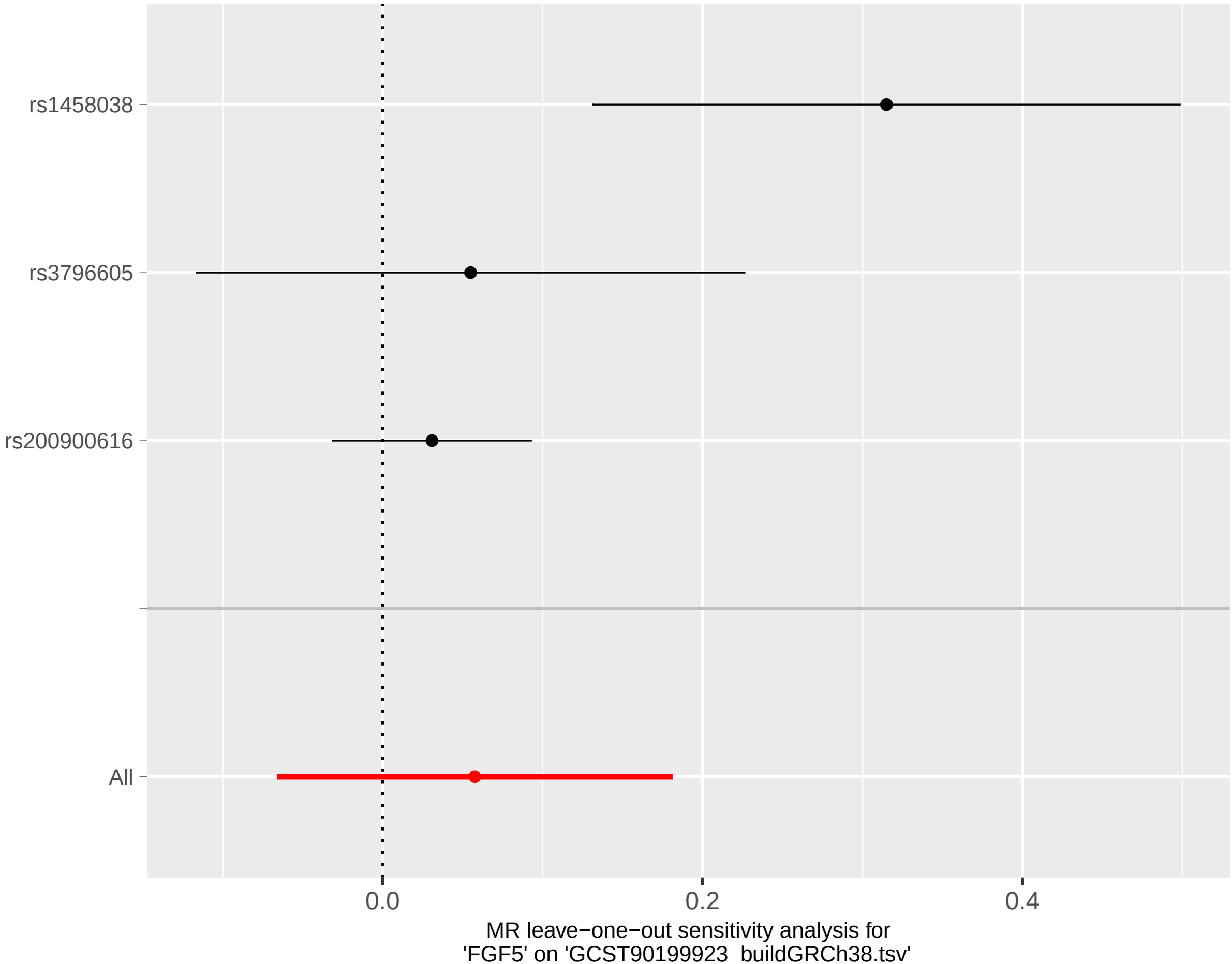
rs1458038
rs3796605
rs200900616
All
0.0
0.2
0.4
MR leave−one−out sensitivity analysis for
'FGF5' on 'GCST90199923_buildGRCh38.tsv'

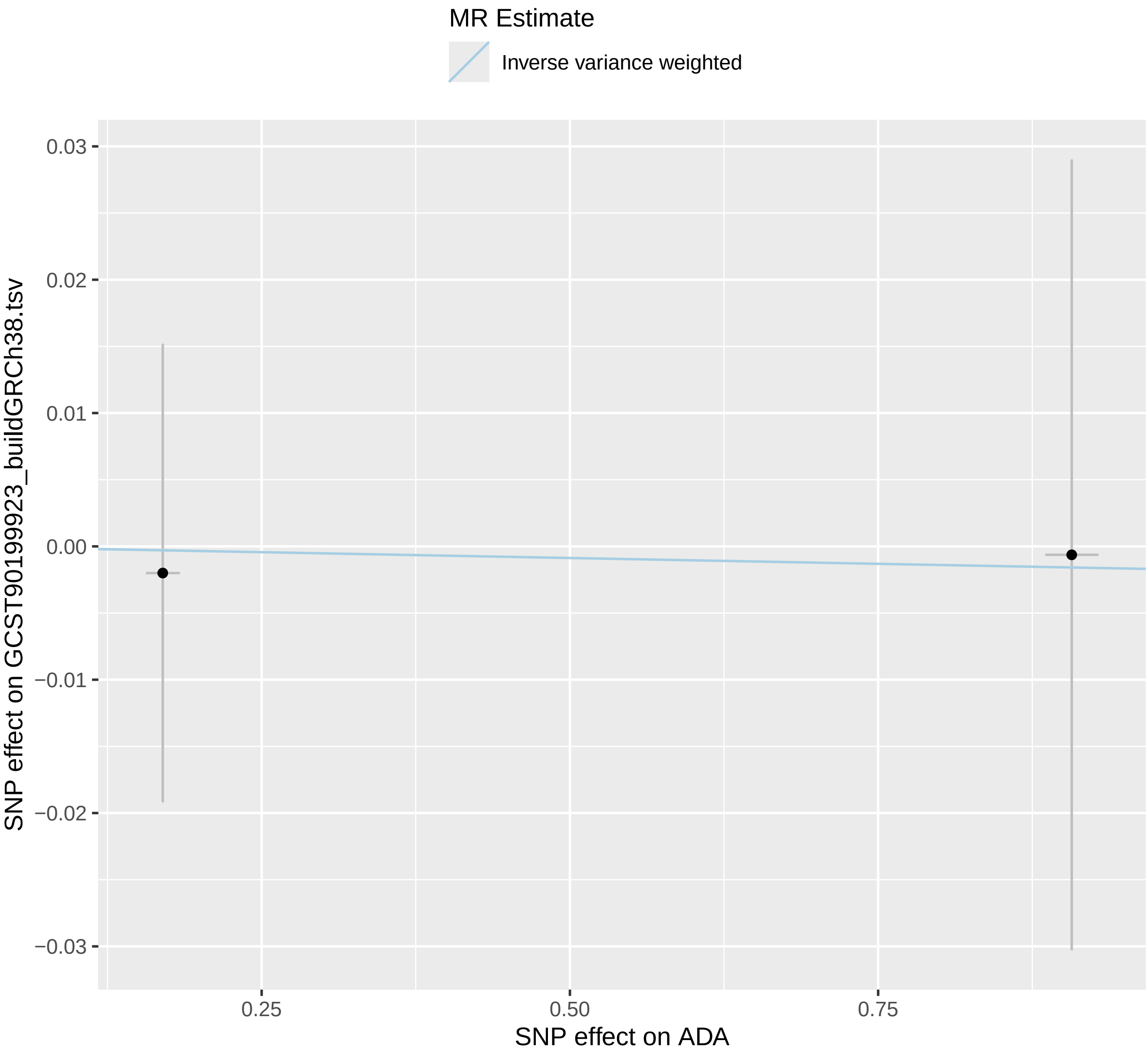
MR Estimate
Inverse variance weighted
0.03
0.02
0.01
0.00
−0.01
−0.02
−0.03
0.25
0.50
0.75
SNP effect on GCST90199923_buildGRCh38.tsv
SNP effect on ADA

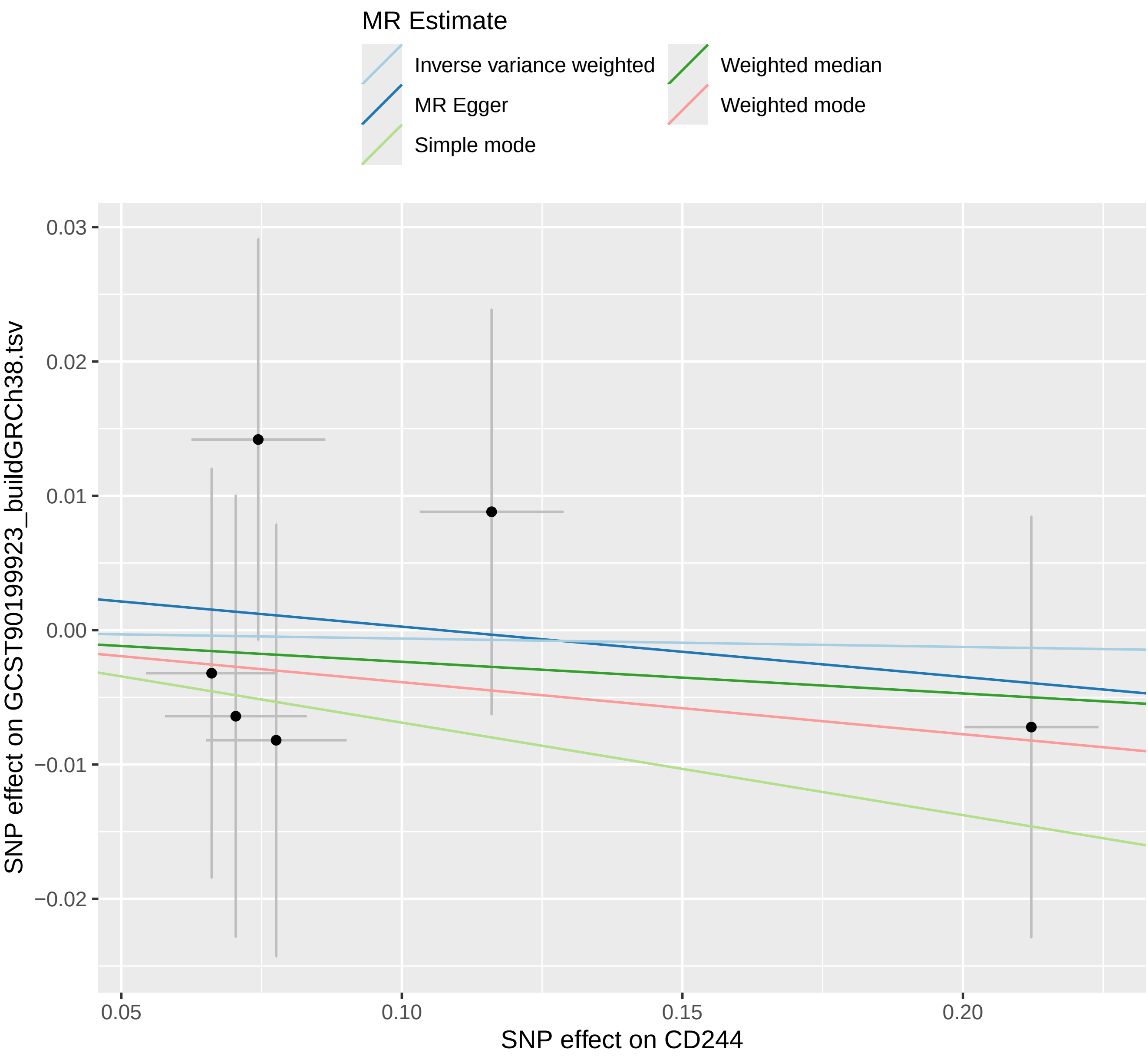
MR Estimate
Inverse variance weighted
MR Egger
Simple mode
Weighted median
Weighted mode
0.03
0.02
0.01
0.00
−0.01
−0.02
SNP effect on GCST90199923_buildGRCh38.tsv
0.05
0.10
0.15
0.20
SNP effect on CD244

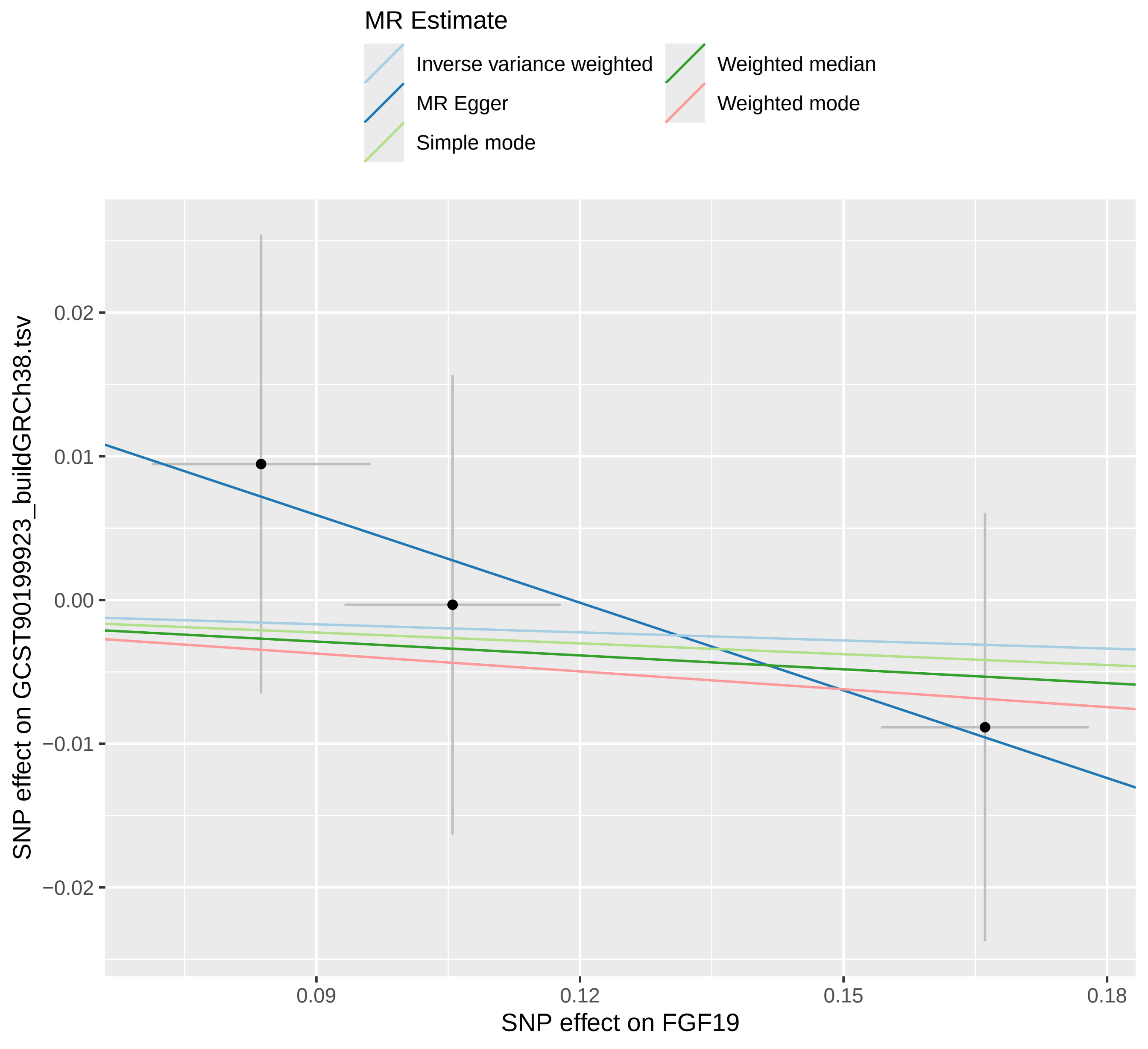
MR Estimate
Inverse variance weighted
MR Egger
Simple mode
Weighted median
Weighted mode
SNP effect on GCST90199923_buildGRCh38.tsv
0.02
0.01
0.00
−0.01
−0.02
0.09
0.12
0.15
0.18
SNP effect on FGF19

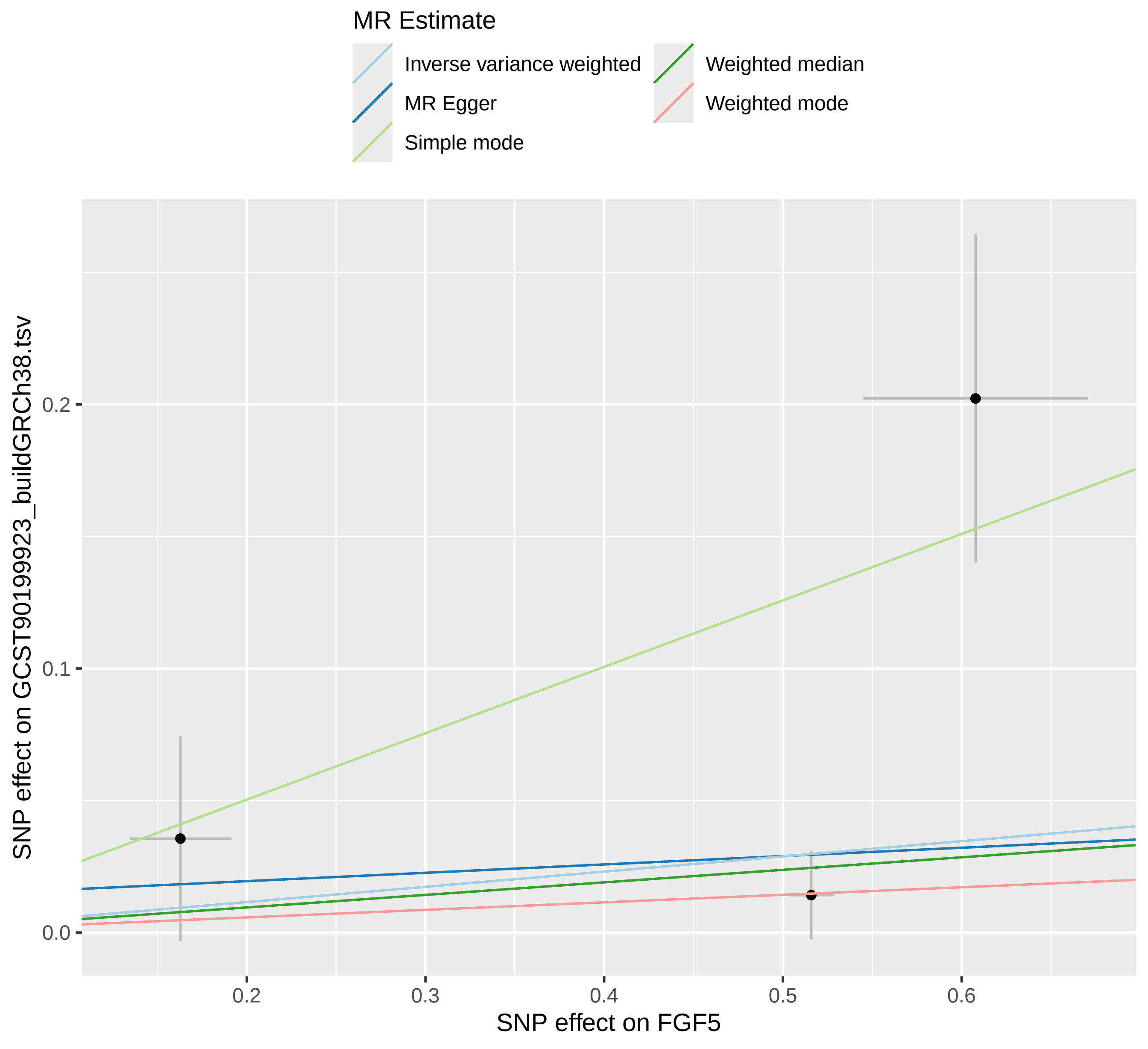
MR Estimate
Inverse variance weighted
MR Egger
Simple mode
Weighted median
Weighted mode
SNP effect on GCST90199923_buildGRCh38.tsv
0.0
0.1
0.2
0.2
0.3
0.4
0.5
0.6
SNP effect on FGF5

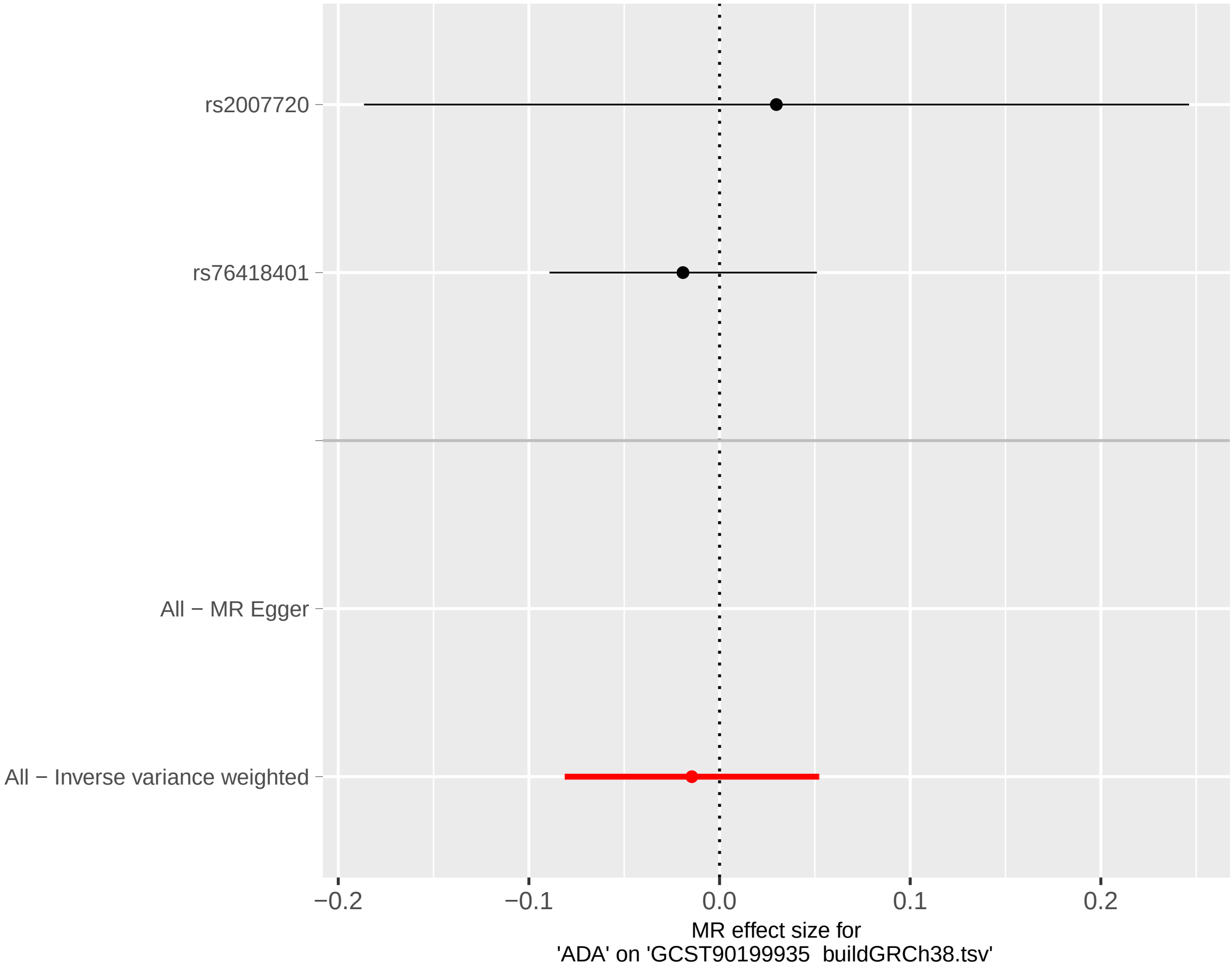

rs2007720
rs76418401
All − MR Egger
All − Inverse variance weighted
−0.2
−0.1
0.0
0.1
0.2
MR effect size for
'ADA' on 'GCST90199935_buildGRCh38.tsv'

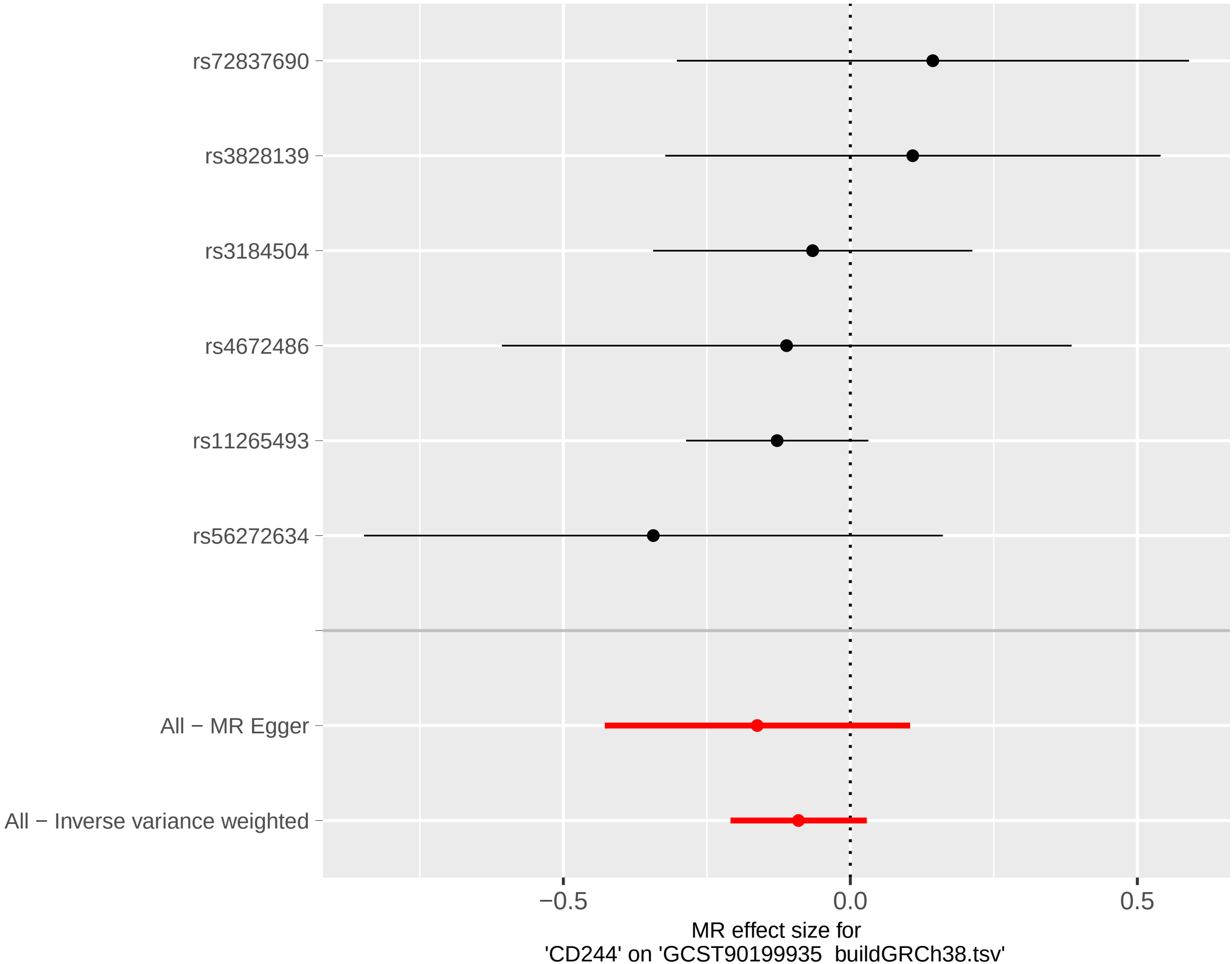
rs72837690
rs3828139
rs3184504
rs4672486
rs11265493
rs56272634
All − MR Egger
All − Inverse variance weighted
−0.5
0.0
0.5
MR effect size for
'CD244' on 'GCST90199935_buildGRCh38.tsv'

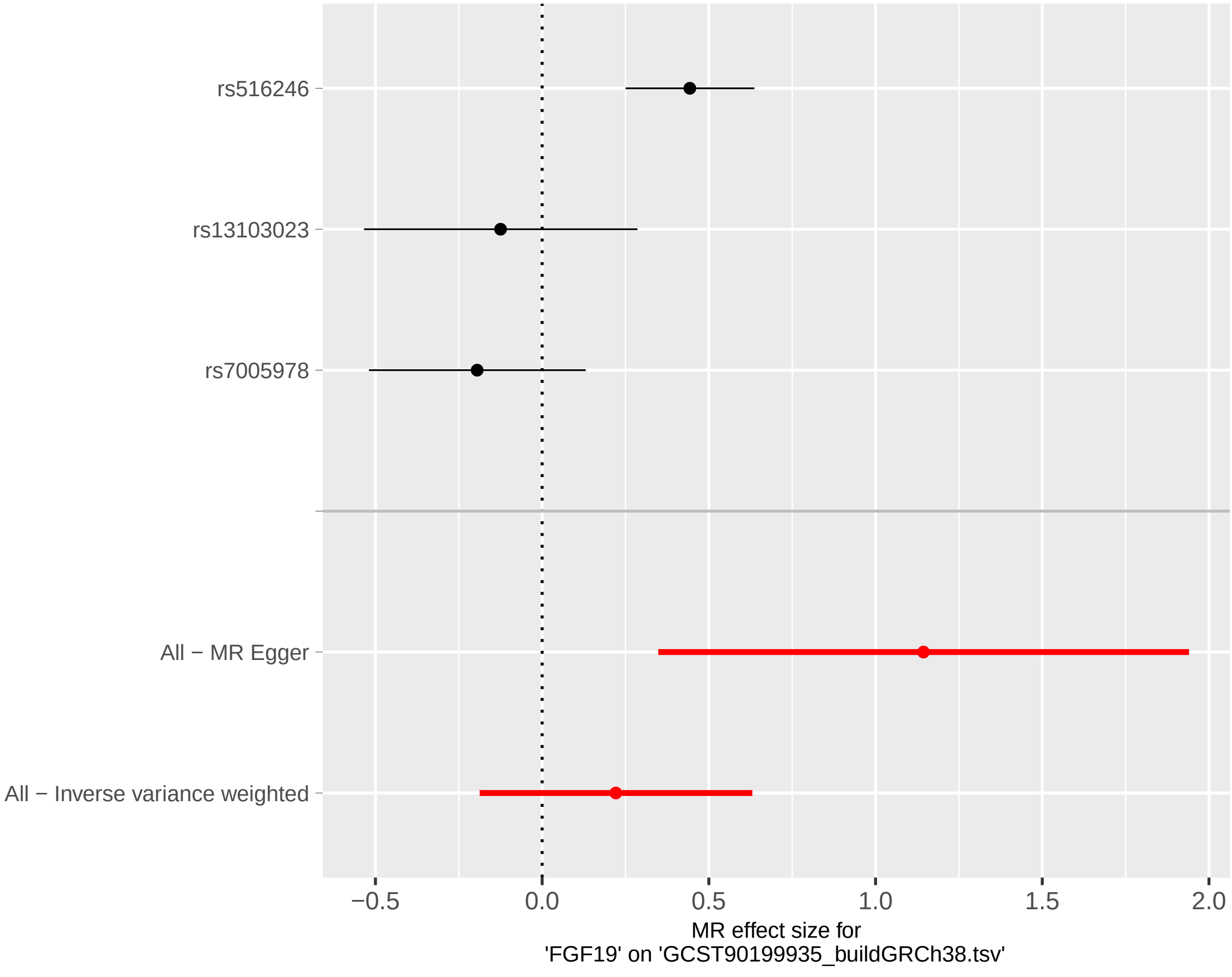
rs516246
rs13103023
rs7005978
All − MR Egger
All − Inverse variance weighted
−0.5
0.0
0.5
1.0
1.5
2.0
MR effect size for
'FGF19' on 'GCST90199935_buildGRCh38.tsv'

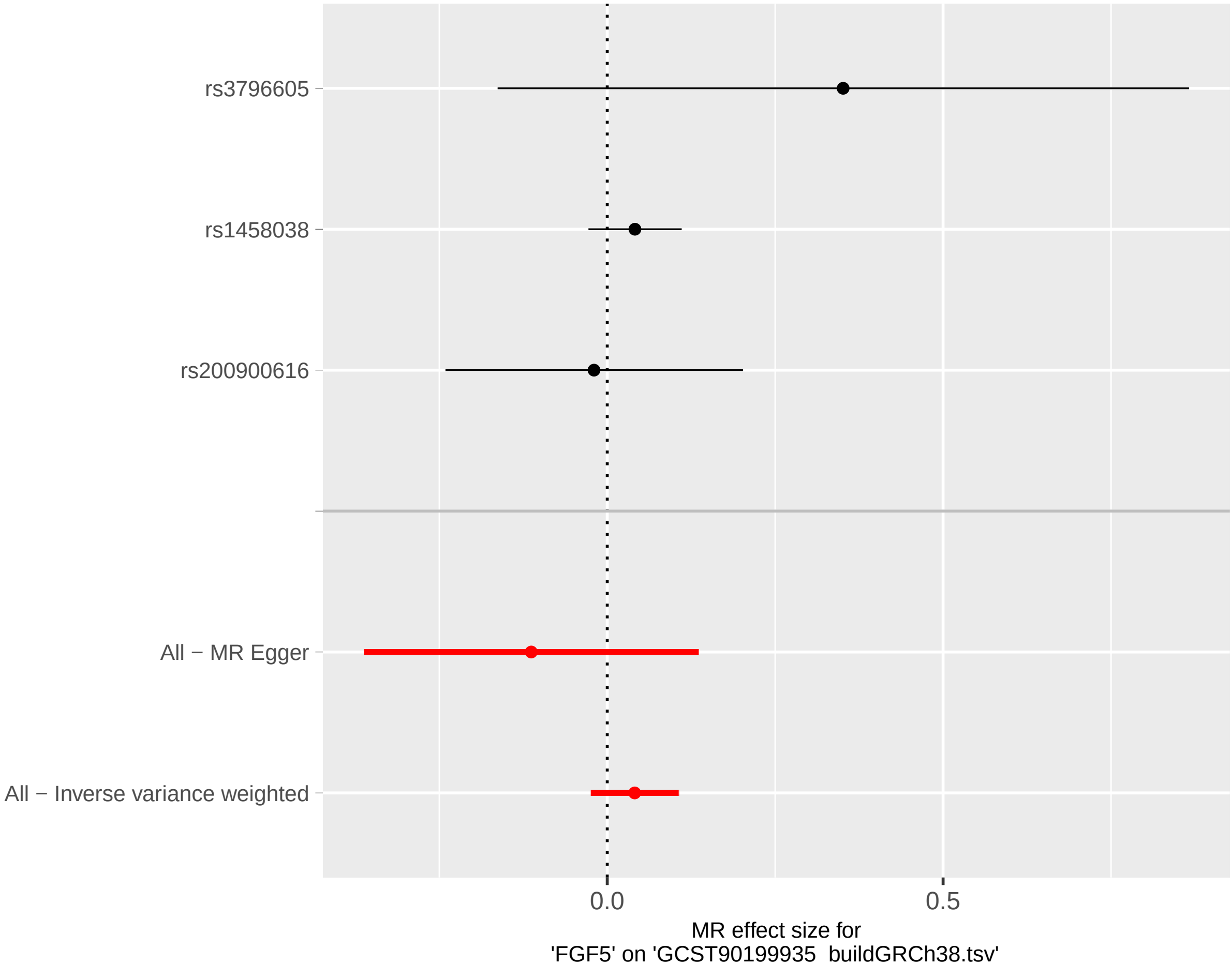

rs3796605
rs1458038
rs200900616
All − MR Egger
All − Inverse variance weighted
0.0
0.5
MR effect size for
'FGF5' on 'GCST90199935_buildGRCh38.tsv'

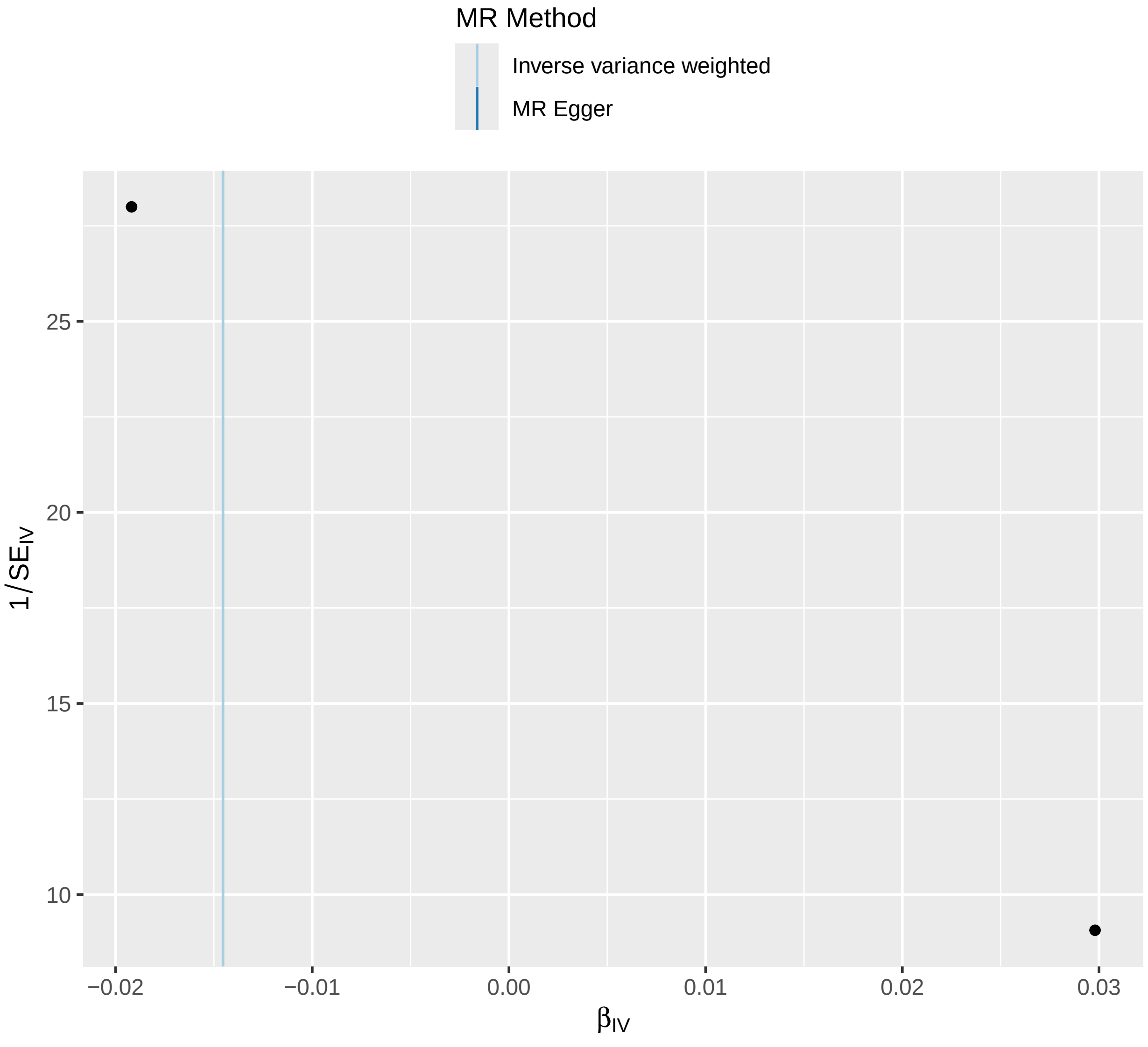
MR Method
Inverse variance weighted
MR Egger
25
20
15
10
1/SE$_{IV}$
−0.02
−0.01
0.00
0.01
0.02
0.03
β$_{IV}$

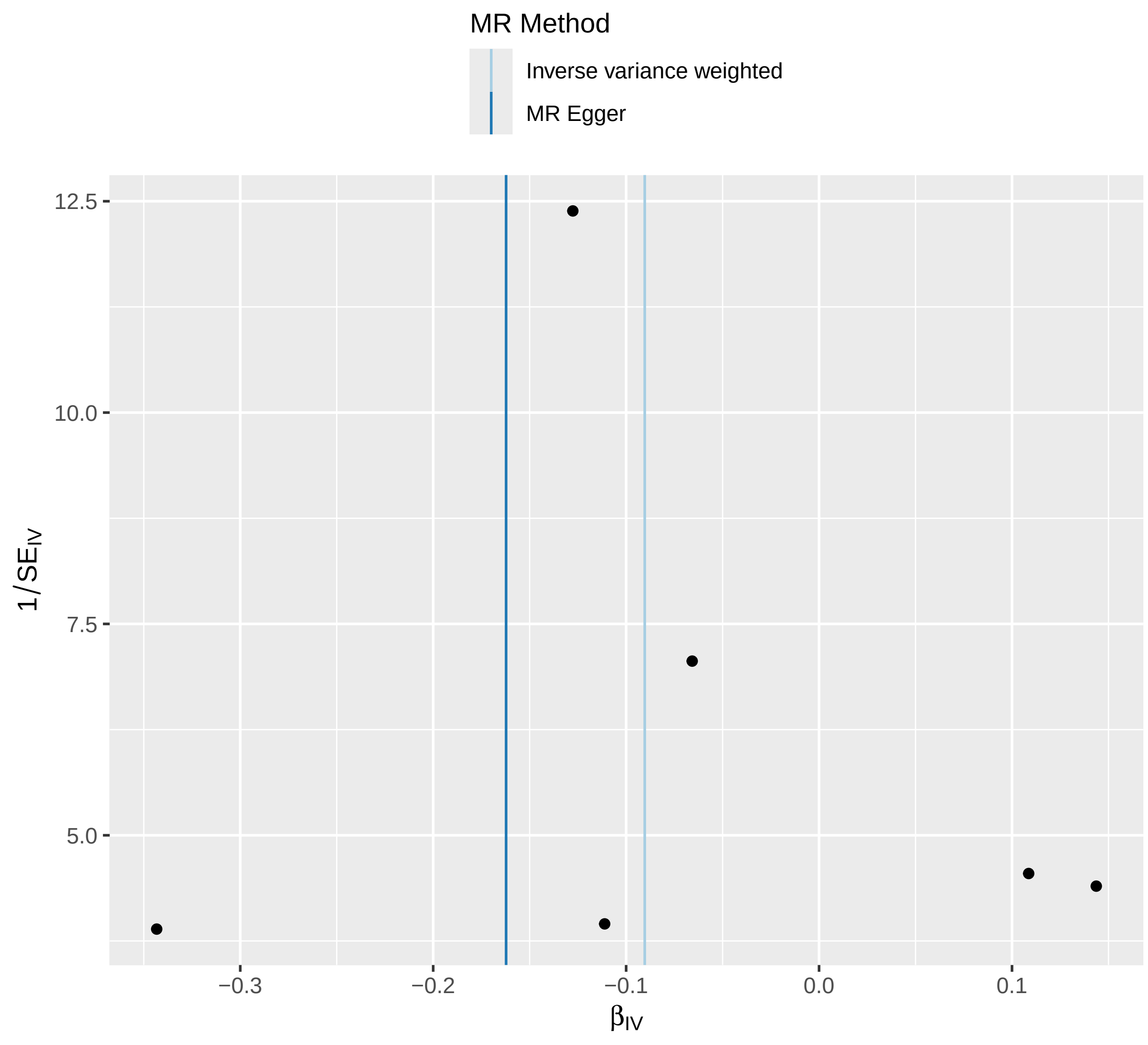

MR Method
Inverse variance weighted
MR Egger
12.5
10.0
7.5
5.0
1/SE_IV
−0.3
−0.2
−0.1
0.0
0.1
β_IV

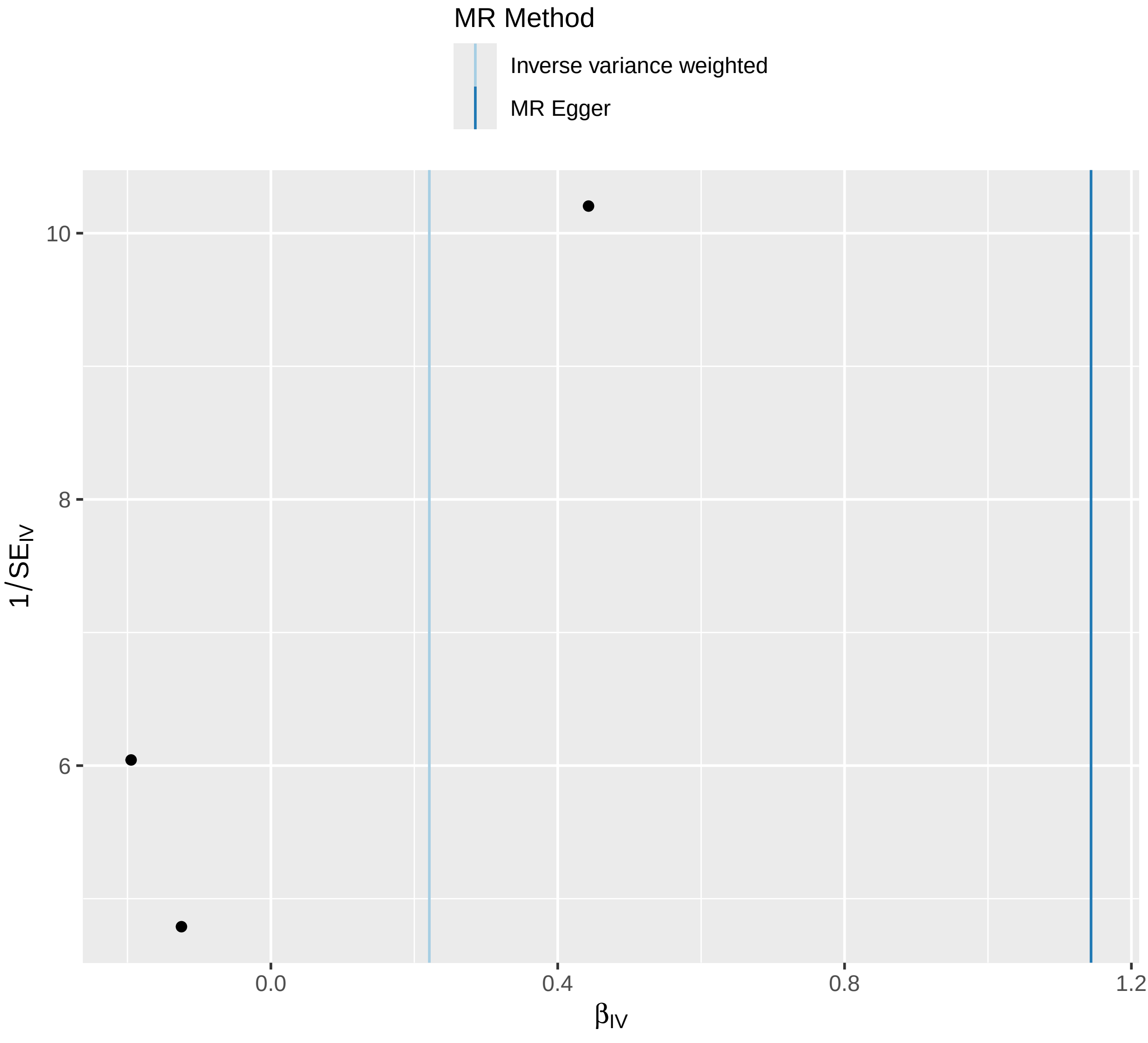
MR Method
Inverse variance weighted
MR Egger
10
8
6
1/SE_IV
0.0
0.4
0.8
1.2
β_IV

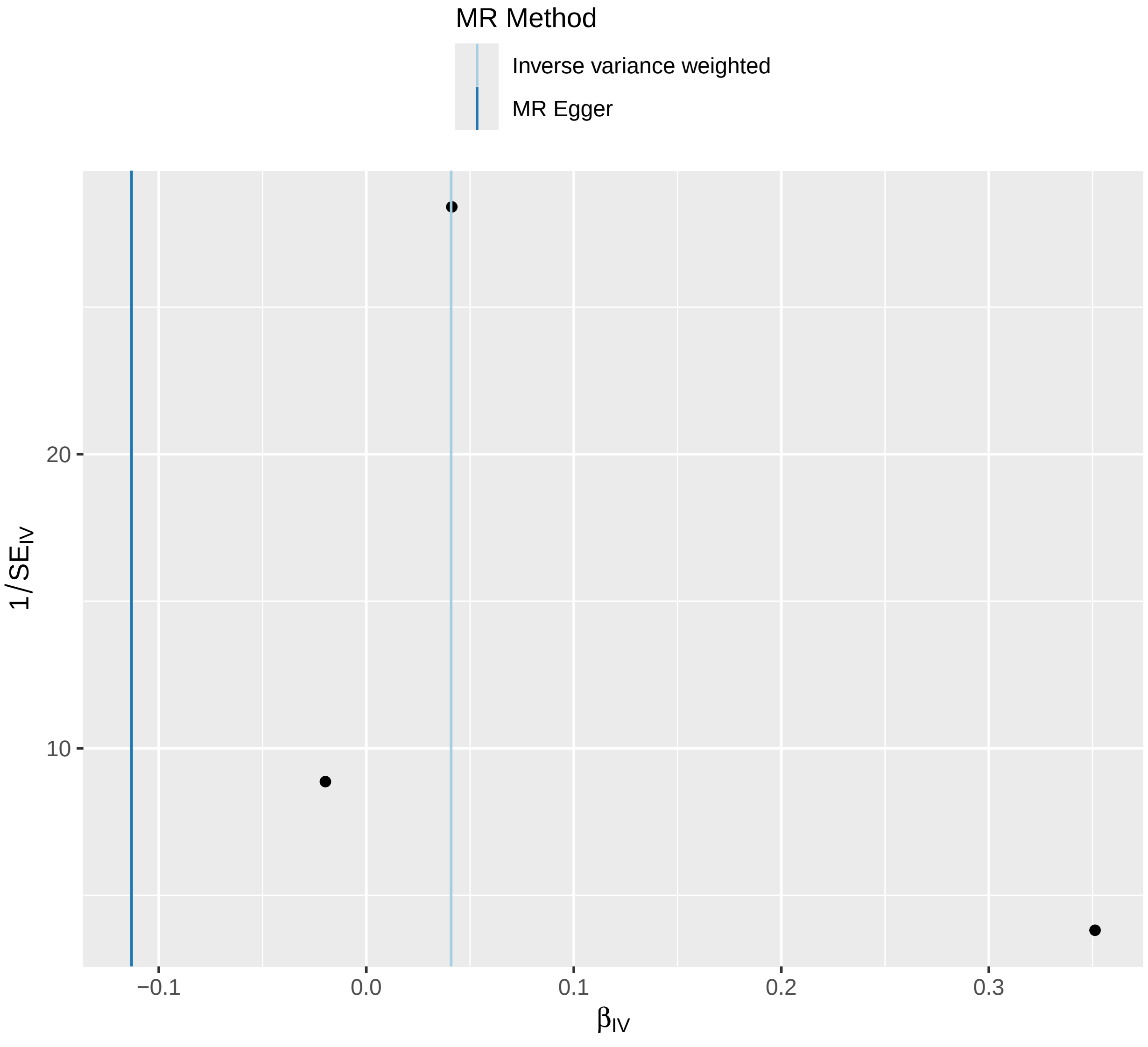

MR Method
Inverse variance weighted
MR Egger
20
10
1/SE_IV
−0.1
0.0
0.1
0.2
0.3
β_IV

Insufficient number of SNPs

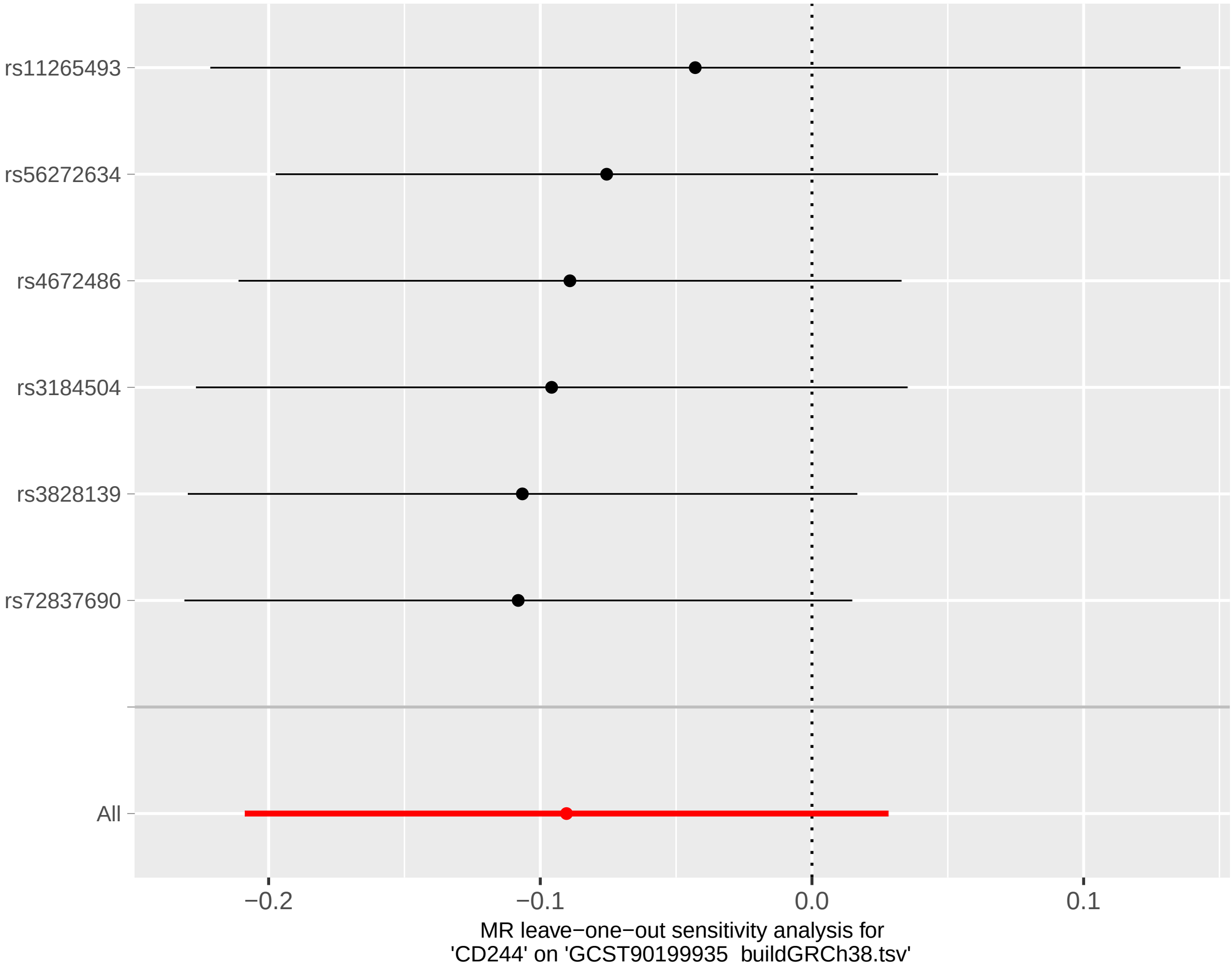
rs11265493
rs56272634
rs4672486
rs3184504
rs3828139
rs72837690
All
−0.2
−0.1
0.0
0.1
MR leave−one−out sensitivity analysis for
'CD244' on 'GCST90199935_buildGRCh38.tsv'

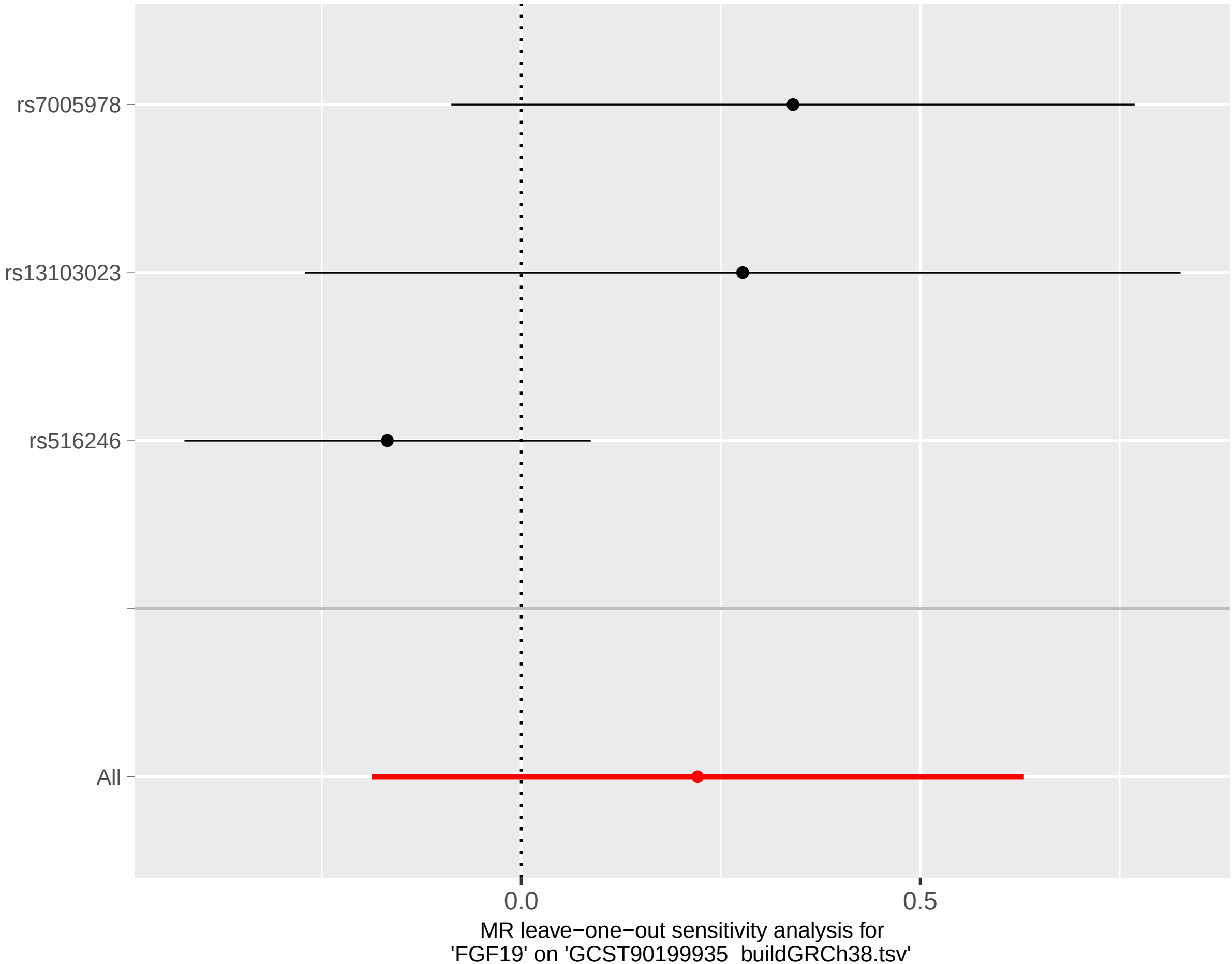
rs7005978
rs13103023
rs516246
All
0.0
0.5
MR leave−one−out sensitivity analysis for
'FGF19' on 'GCST90199935_buildGRCh38.tsv'

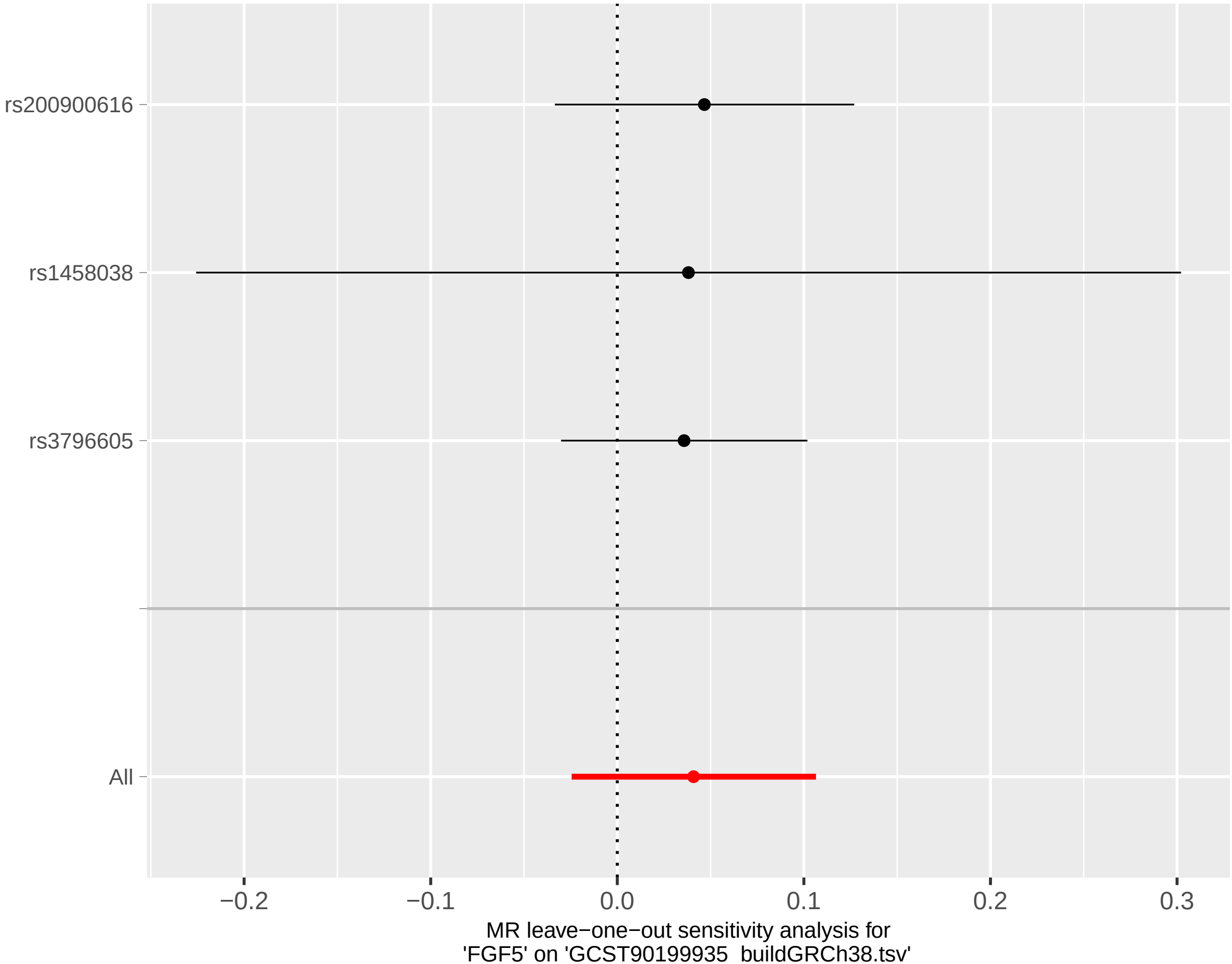

rs200900616
rs1458038
rs3796605
All
−0.2
−0.1
0.0
0.1
0.2
0.3
MR leave−one−out sensitivity analysis for
'FGF5' on 'GCST90199935_buildGRCh38.tsv'

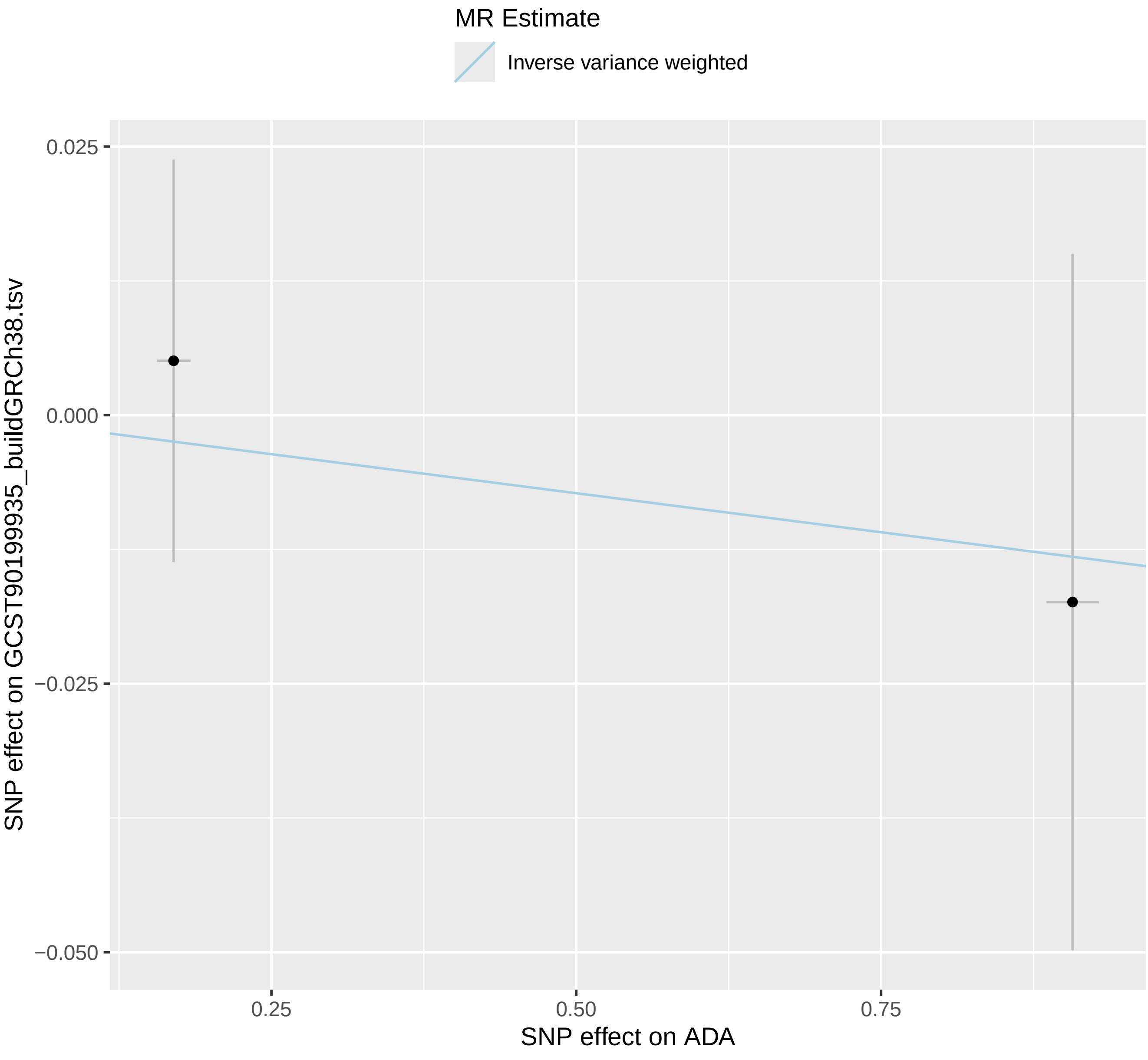
MR Estimate
Inverse variance weighted
0.025
0.000
−0.025
−0.050
0.25
0.50
0.75
SNP effect on ADA
SNP effect on GCST90199935_buildGRCh38.tsv

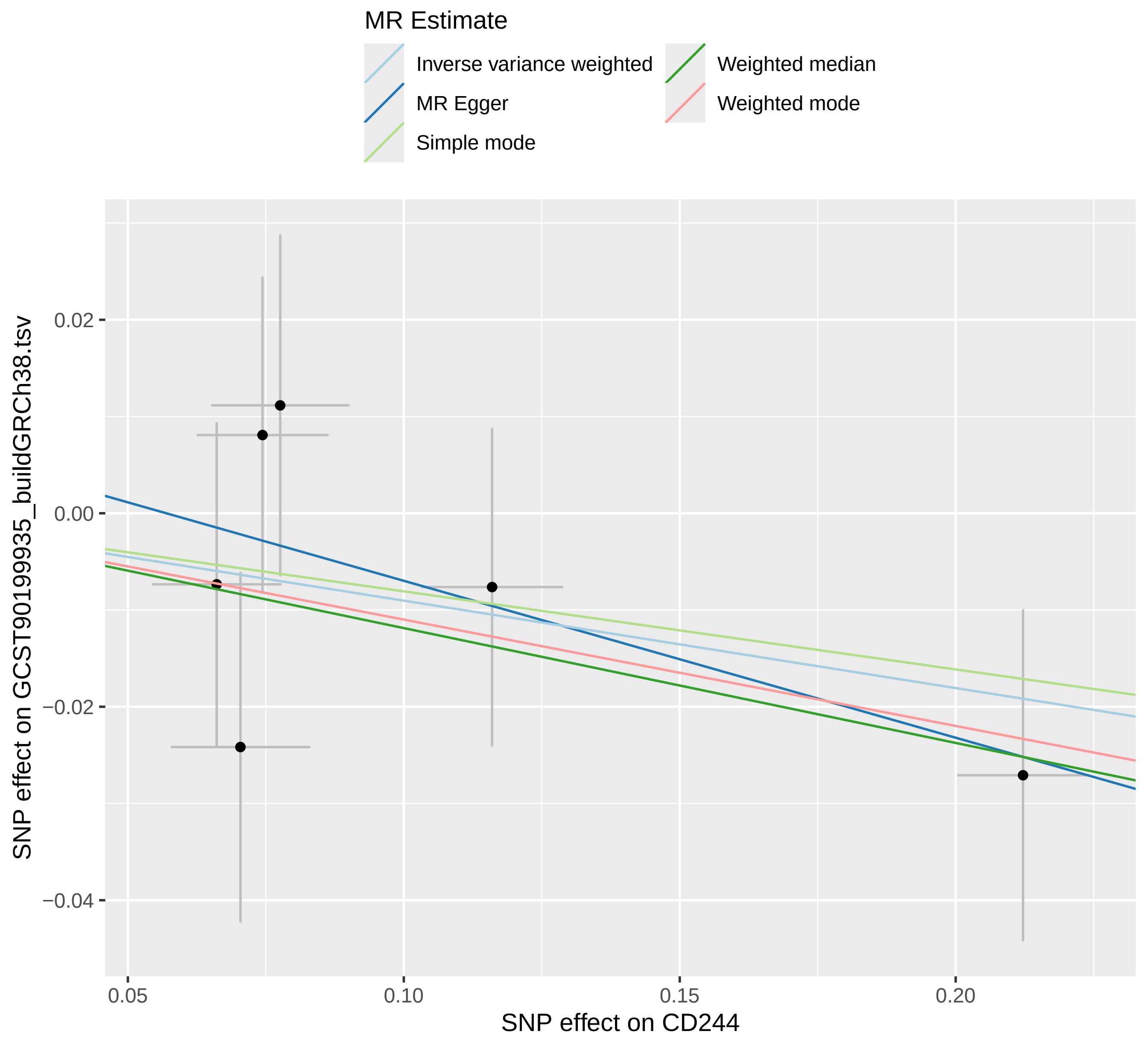
MR Estimate
Inverse variance weighted
MR Egger
Simple mode
Weighted median
Weighted mode
0.02
0.00
−0.02
−0.04
0.05
0.10
0.15
0.20
SNP effect on GCST90199935_buildGRCh38.tsv
SNP effect on CD244

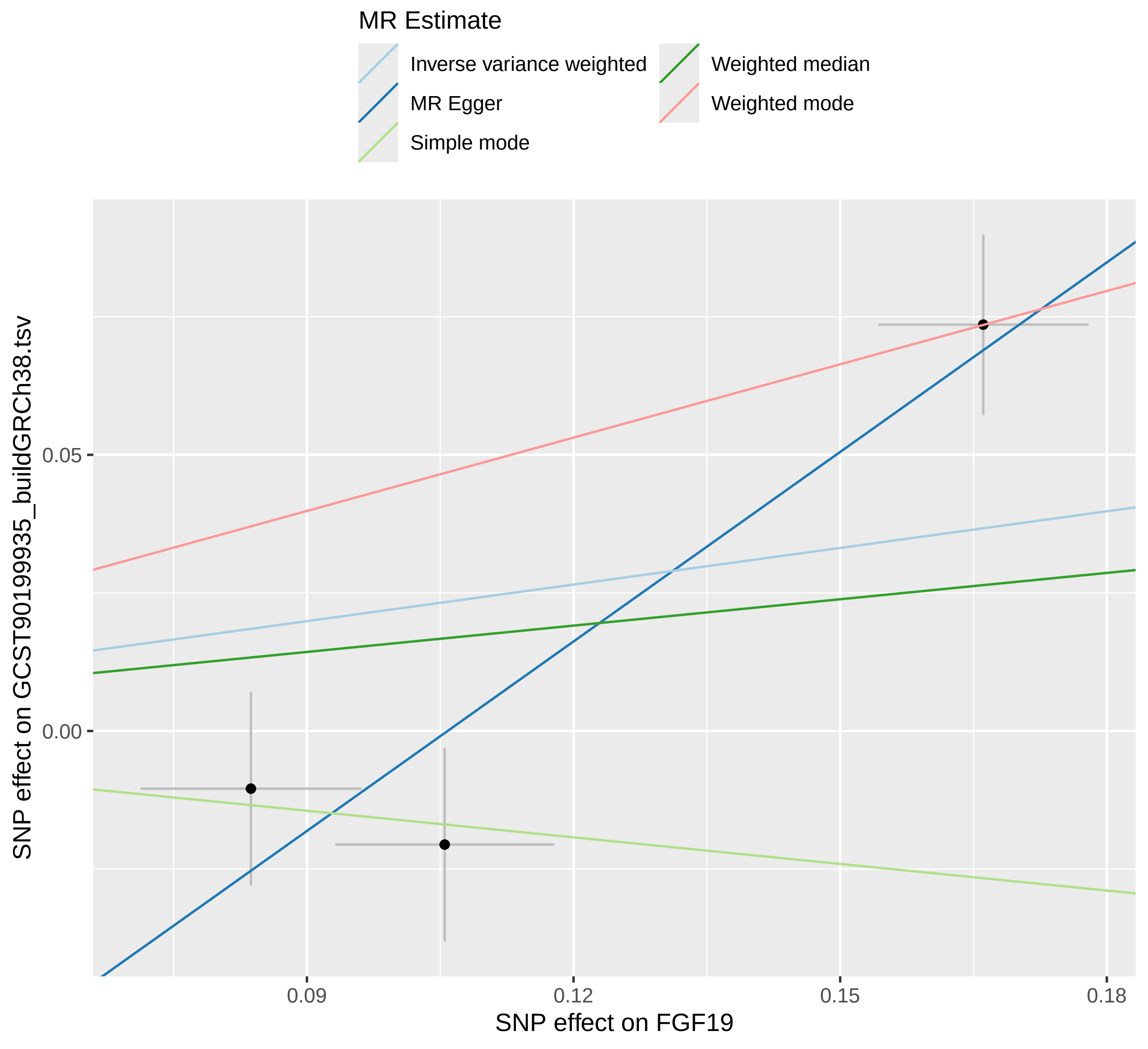
MR Estimate
Inverse variance weighted
MR Egger
Simple mode
Weighted median
Weighted mode
SNP effect on GCST90199935_buildGRCh38.tsv
0.05
0.00
0.09
0.12
0.15
0.18
SNP effect on FGF19

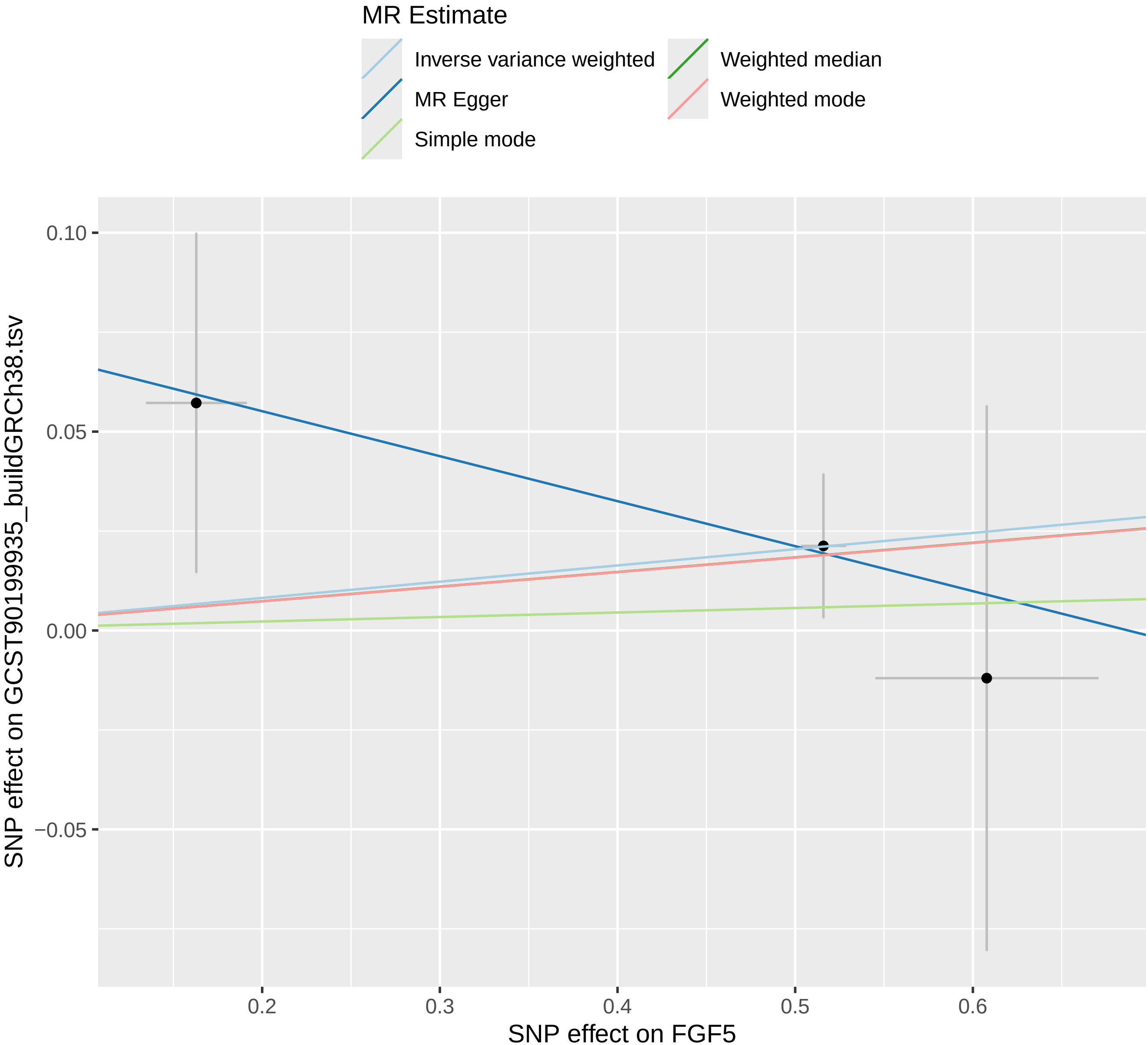

MR Estimate
Inverse variance weighted
MR Egger
Simple mode
Weighted median
Weighted mode
SNP effect on GCST90199935_buildGRCh38.tsv
0.10
0.05
0.00
−0.05
0.2
0.3
0.4
0.5
0.6
SNP effect on FGF5

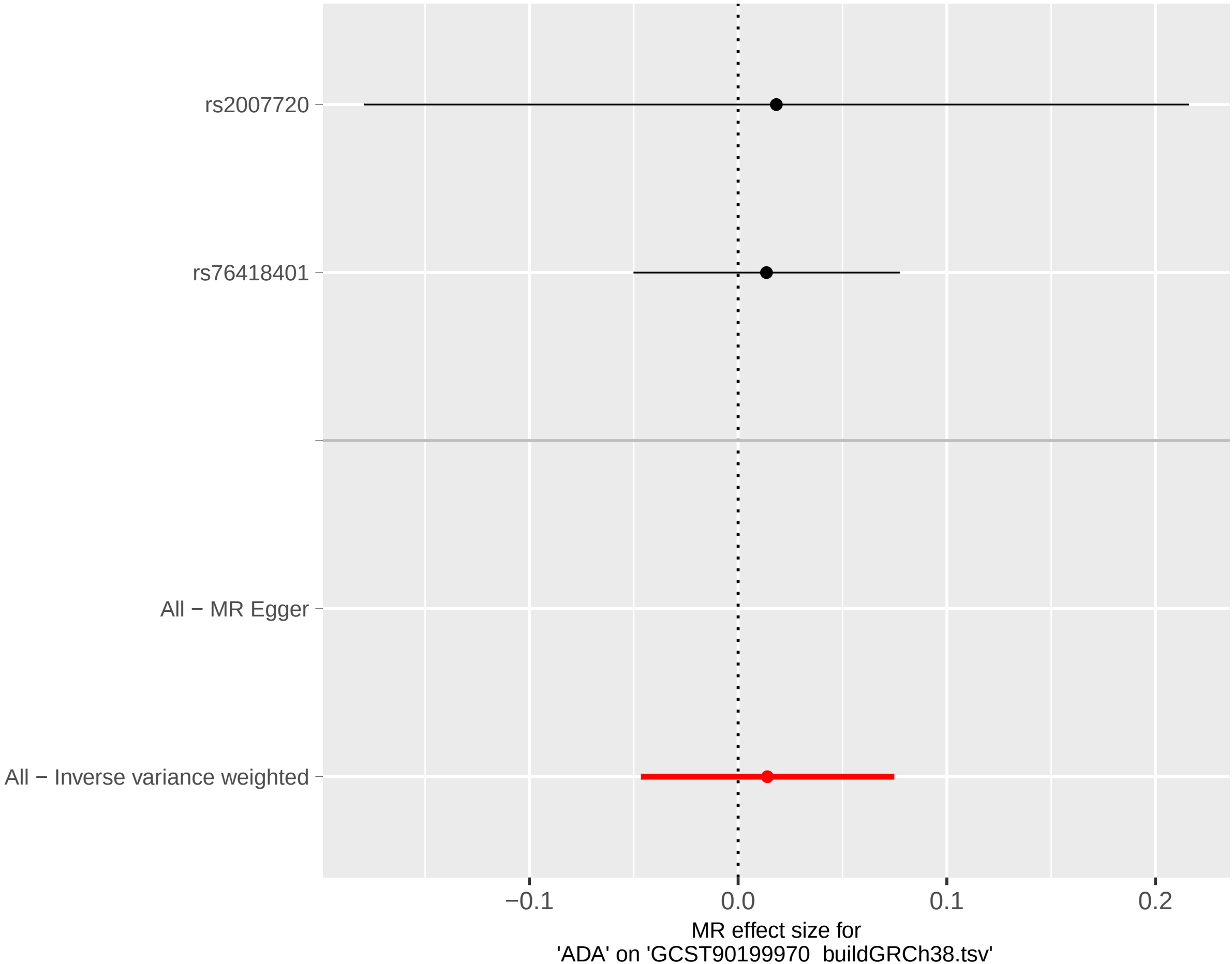
rs2007720
rs76418401
All − MR Egger
All − Inverse variance weighted
−0.1
0.0
0.1
0.2
MR effect size for
'ADA' on 'GCST90199970_buildGRCh38.tsv'

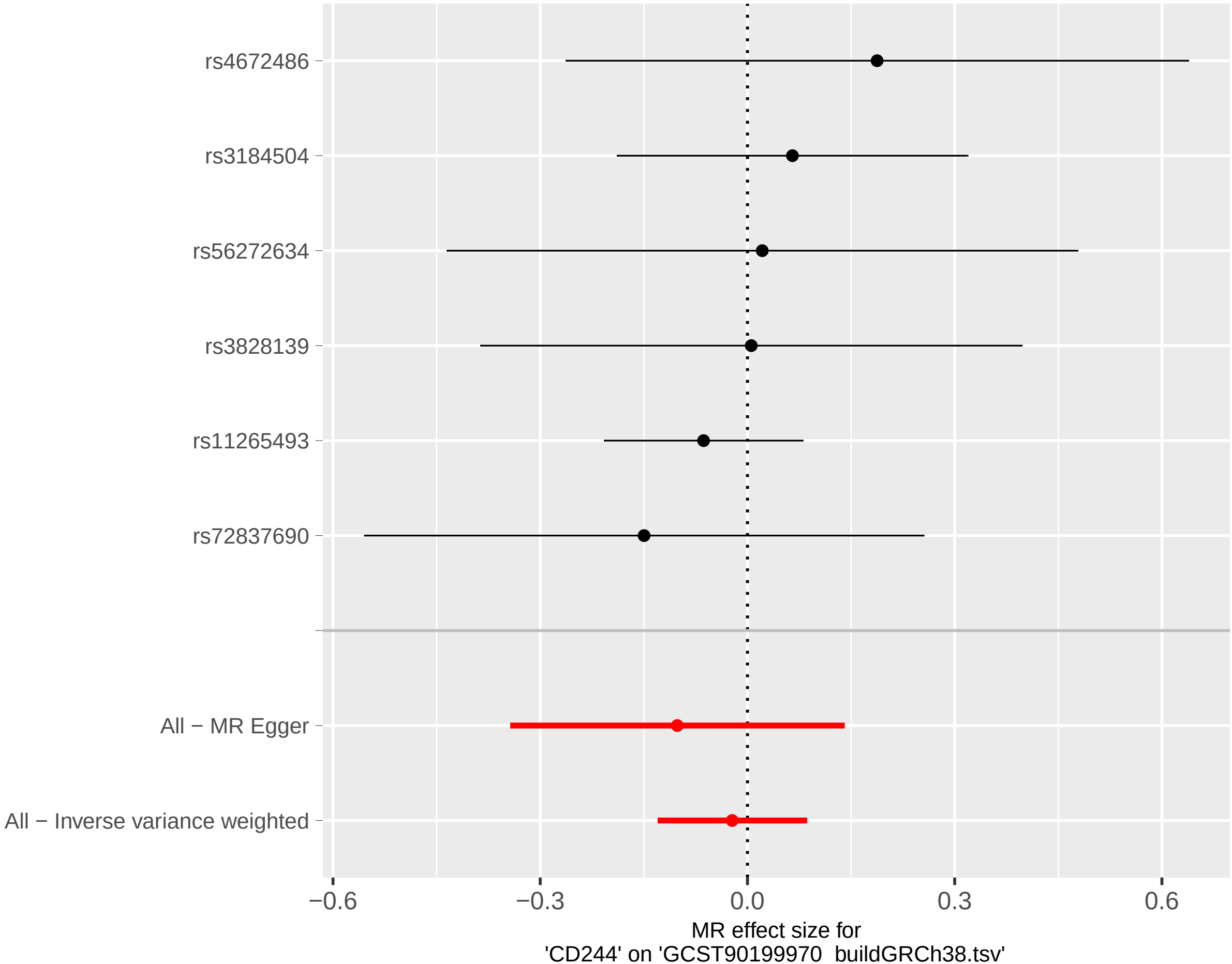
rs4672486
rs3184504
rs56272634
rs3828139
rs11265493
rs72837690
All − MR Egger
All − Inverse variance weighted
−0.6
−0.3
0.0
0.3
0.6
MR effect size for
'CD244' on 'GCST90199970_buildGRCh38.tsv'

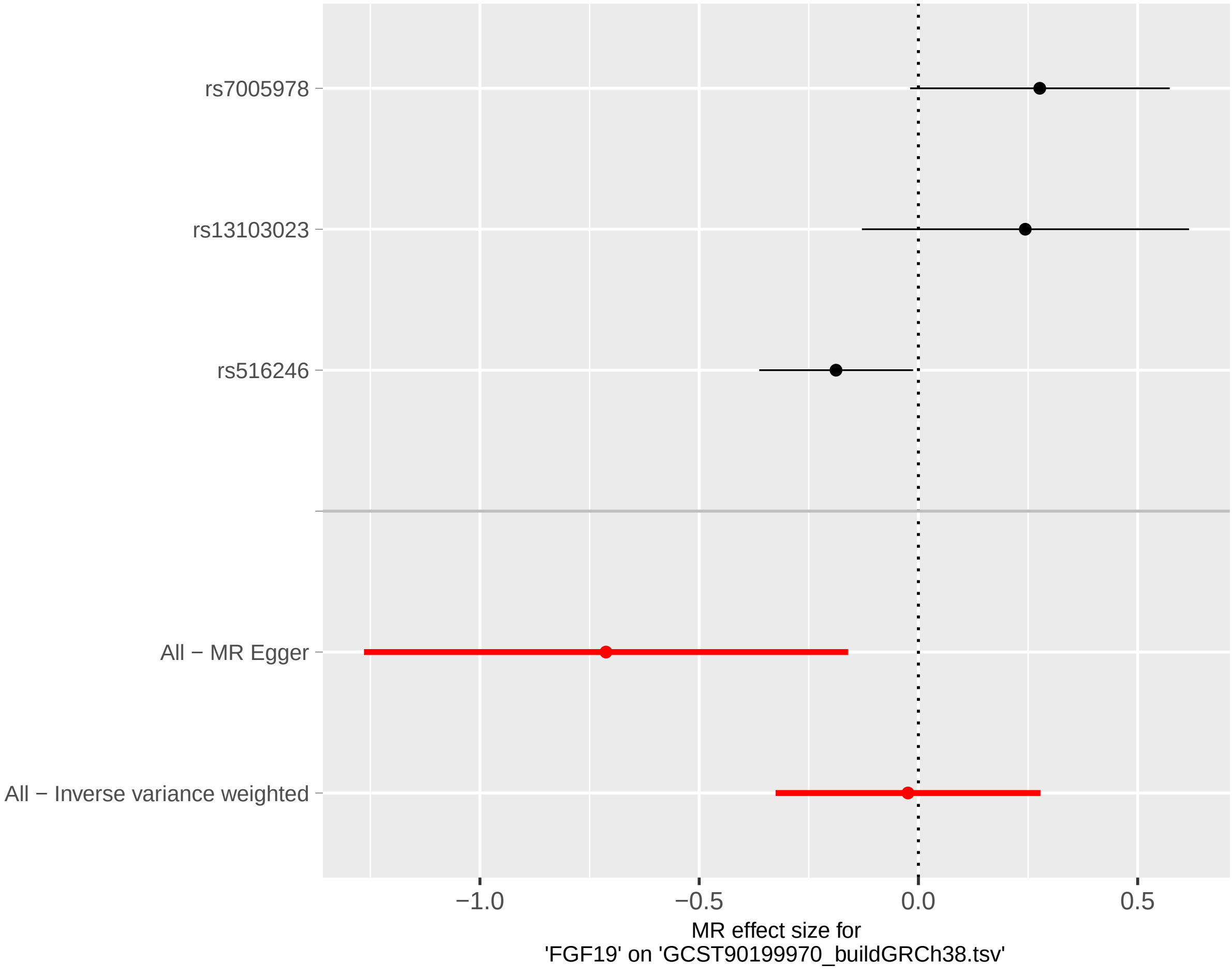
rs7005978
rs13103023
rs516246
All − MR Egger
All − Inverse variance weighted
−1.0
−0.5
0.0
0.5
MR effect size for
'FGF19' on 'GCST90199970_buildGRCh38.tsv'

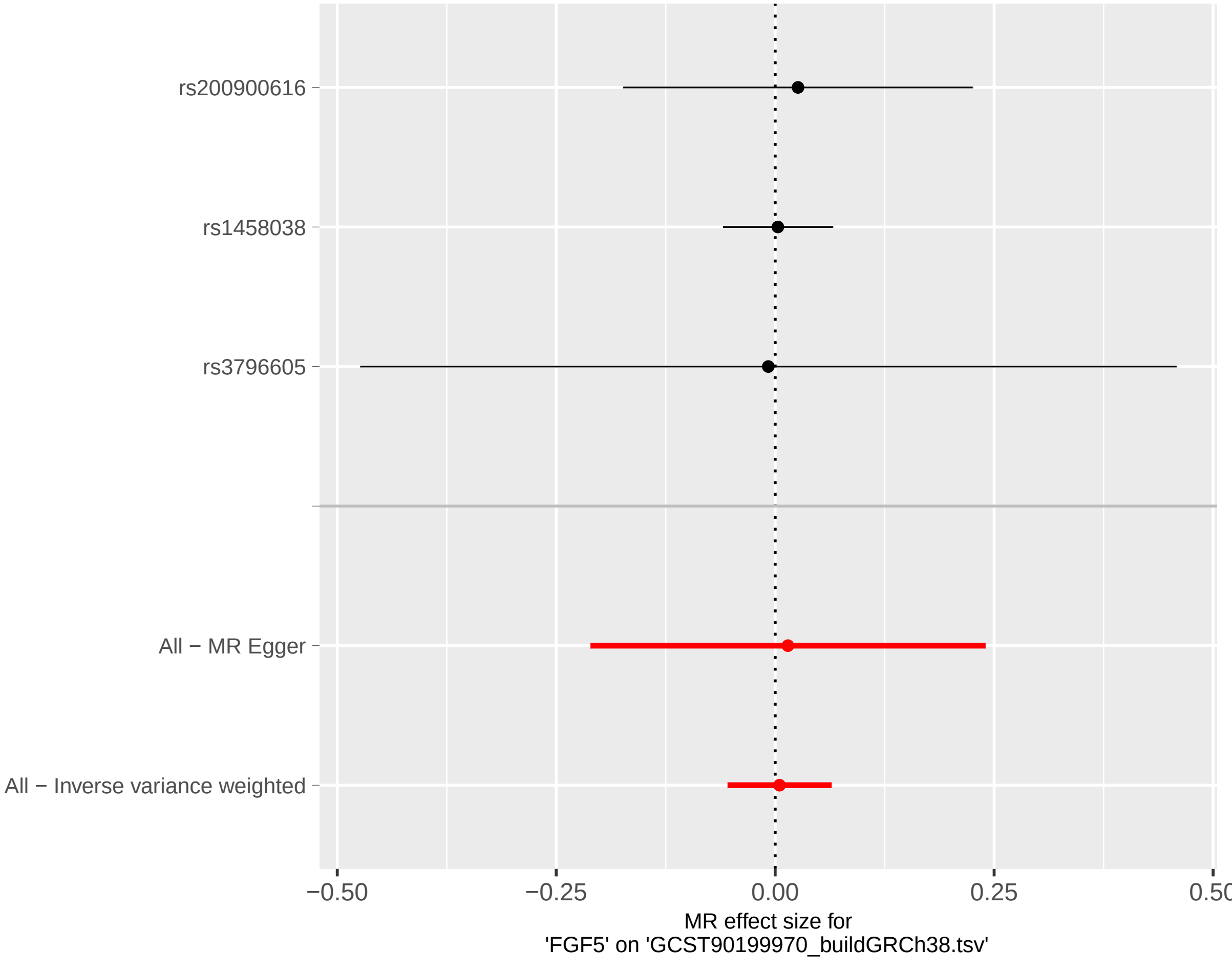
rs200900616
rs1458038
rs3796605
All − MR Egger
All − Inverse variance weighted
−0.50
−0.25
0.00
0.25
0.50
MR effect size for
'FGF5' on 'GCST90199970_buildGRCh38.tsv'

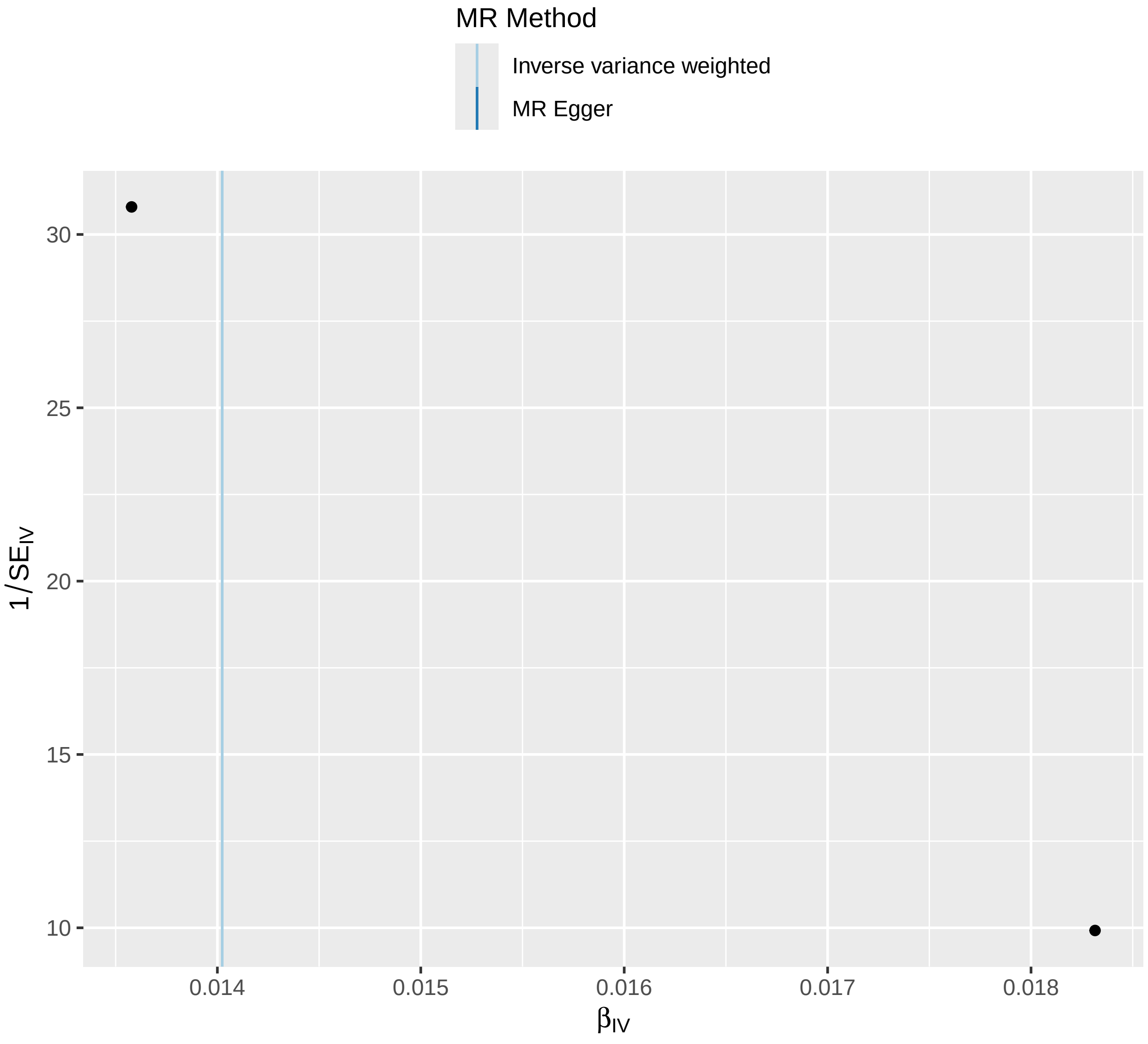
MR Method
Inverse variance weighted
MR Egger
$1/SE_{IV}$
30
25
20
15
10
0.014
0.015
0.016
0.017
0.018
β_{IV}

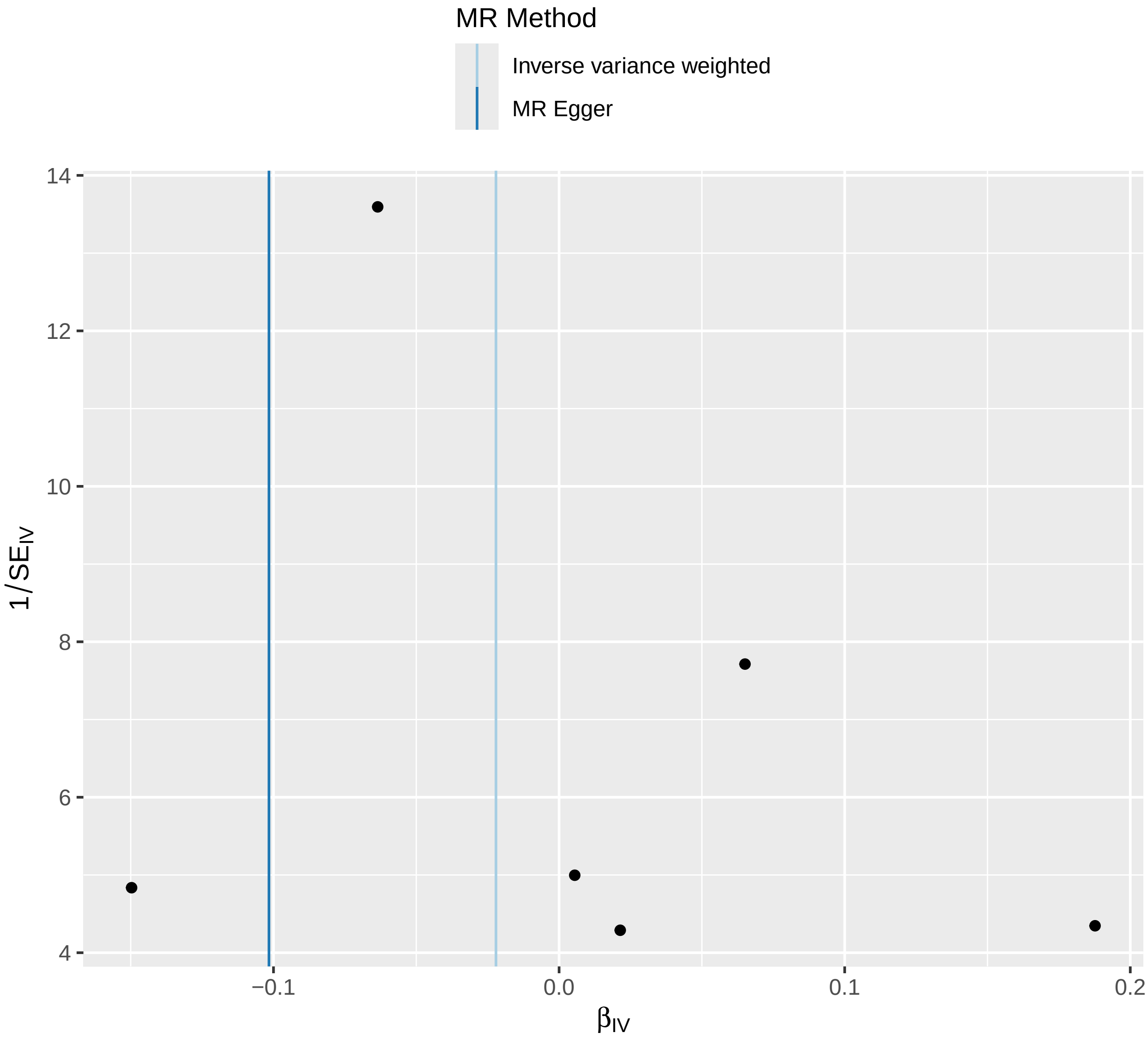

MR Method
Inverse variance weighted
MR Egger
14
12
10
8
6
4
1/SE_IV
−0.1
0.0
0.1
0.2
β_IV

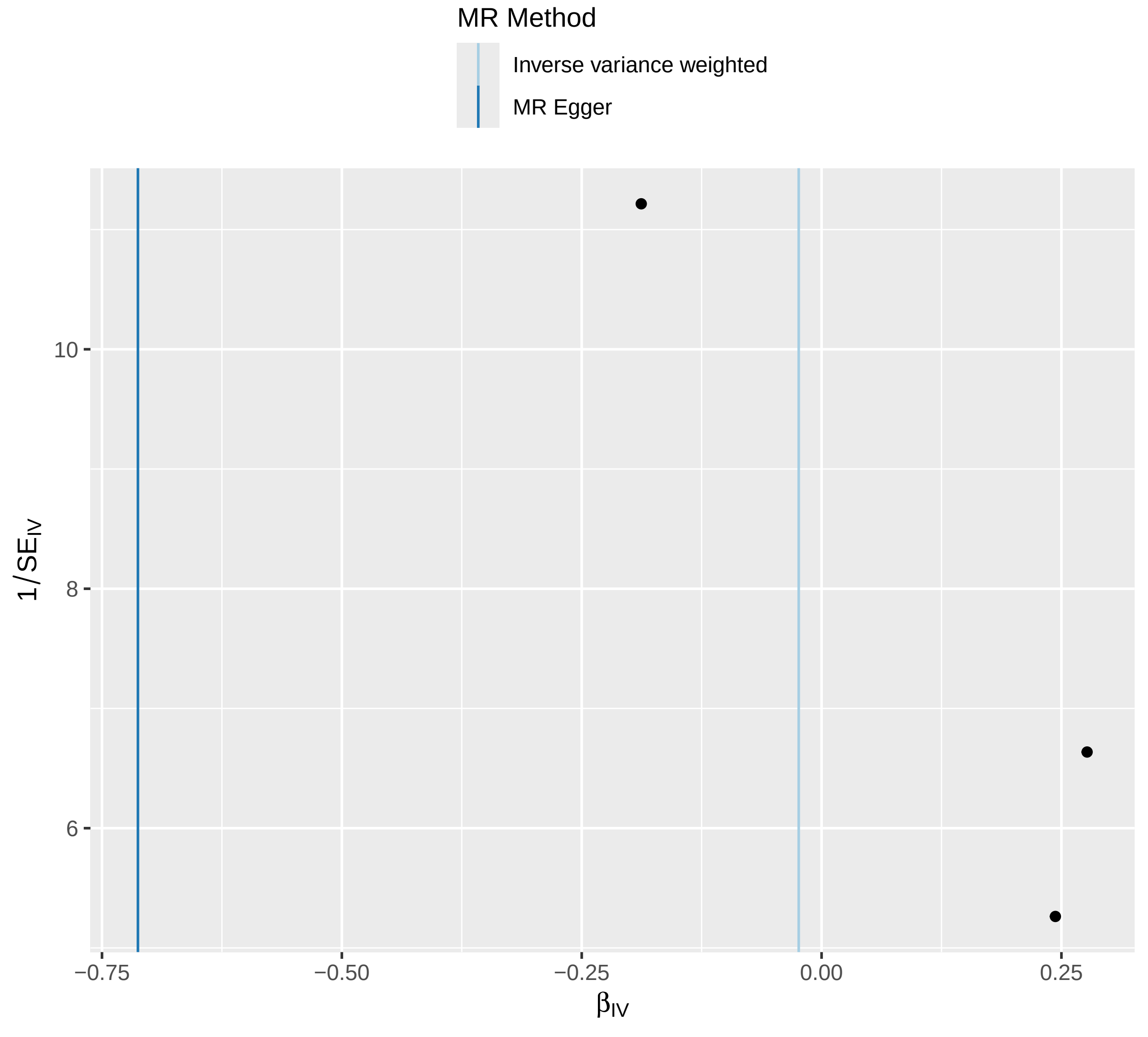
MR Method
Inverse variance weighted
MR Egger
1/SE_IV
10
8
6
−0.75
−0.50
−0.25
0.00
0.25
β_IV

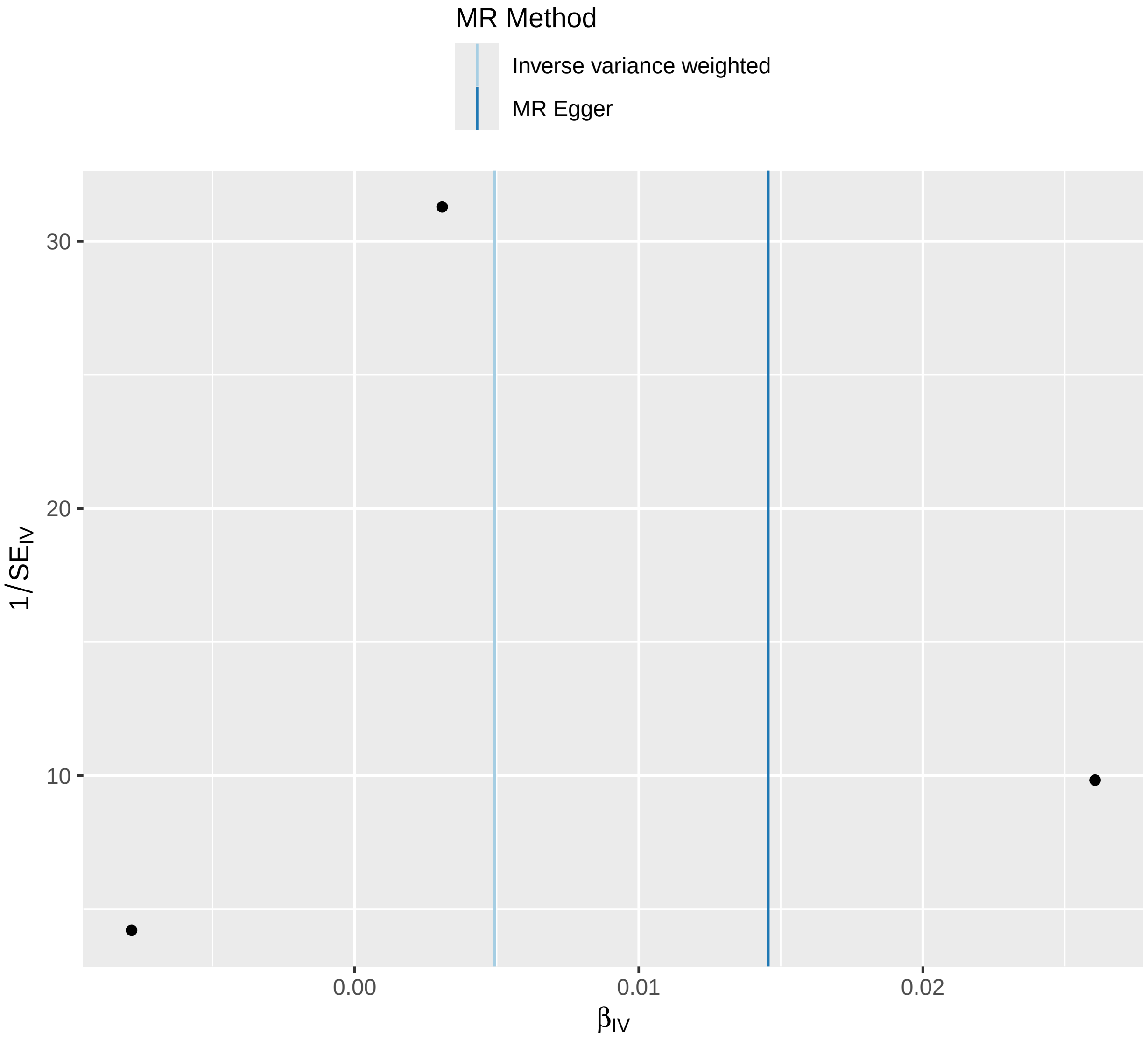
MR Method
Inverse variance weighted
MR Egger
30
20
10
1/SE_IV
0.00
0.01
0.02
β_IV

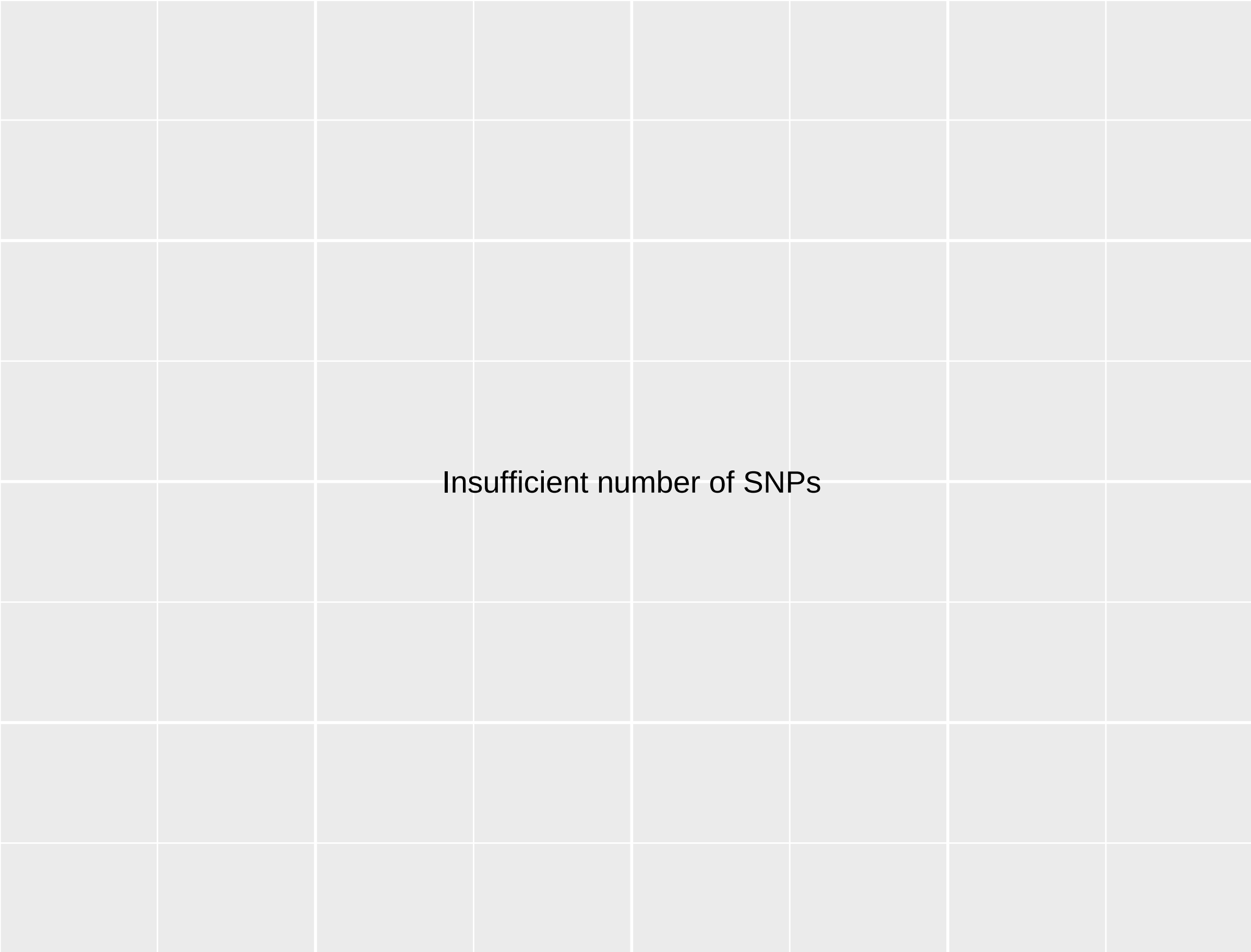
Insufficient number of SNPs

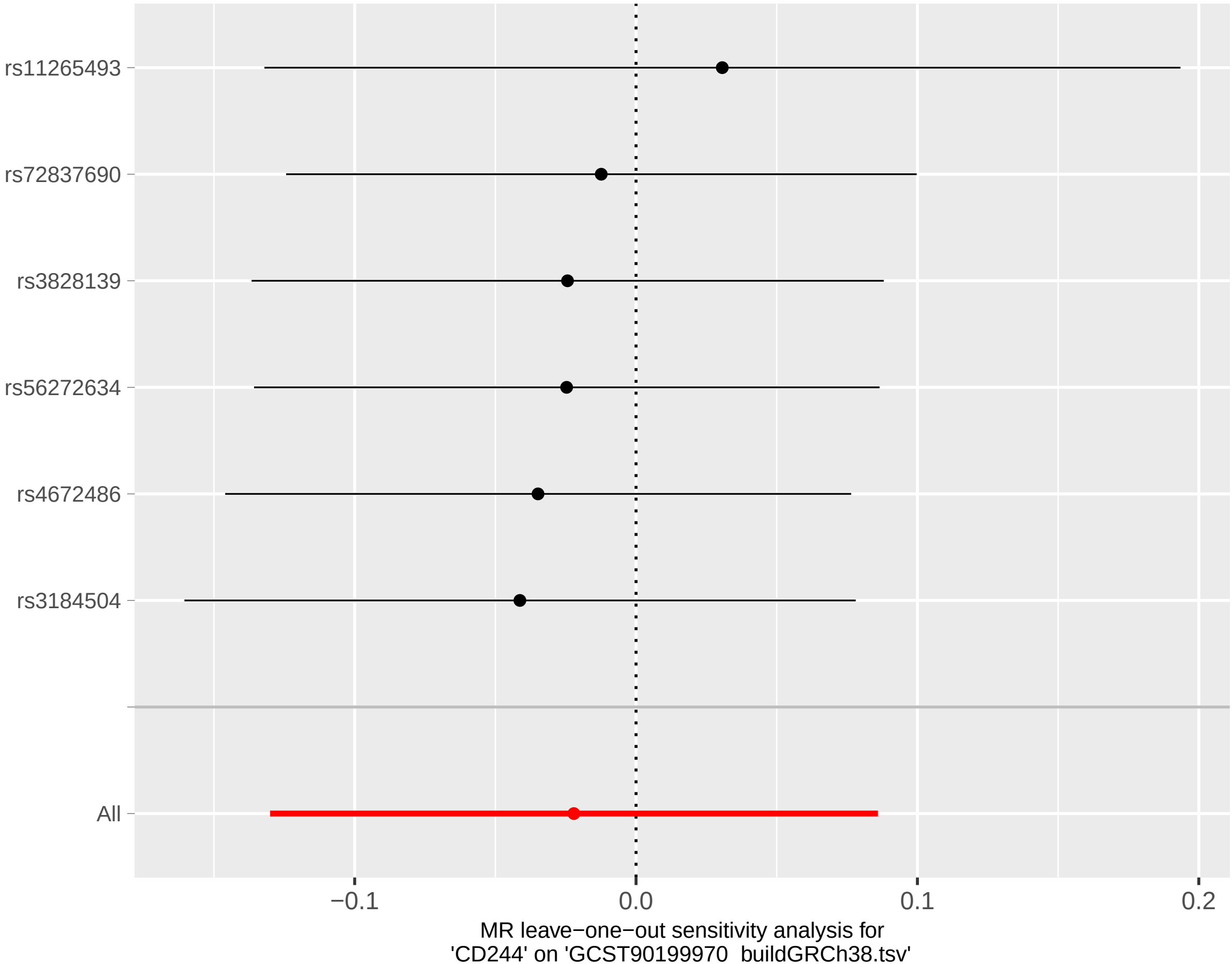

rs11265493
rs72837690
rs3828139
rs56272634
rs4672486
rs3184504
All
−0.1
0.0
0.1
0.2
MR leave−one−out sensitivity analysis for
'CD244' on 'GCST90199970_buildGRCh38.tsv'

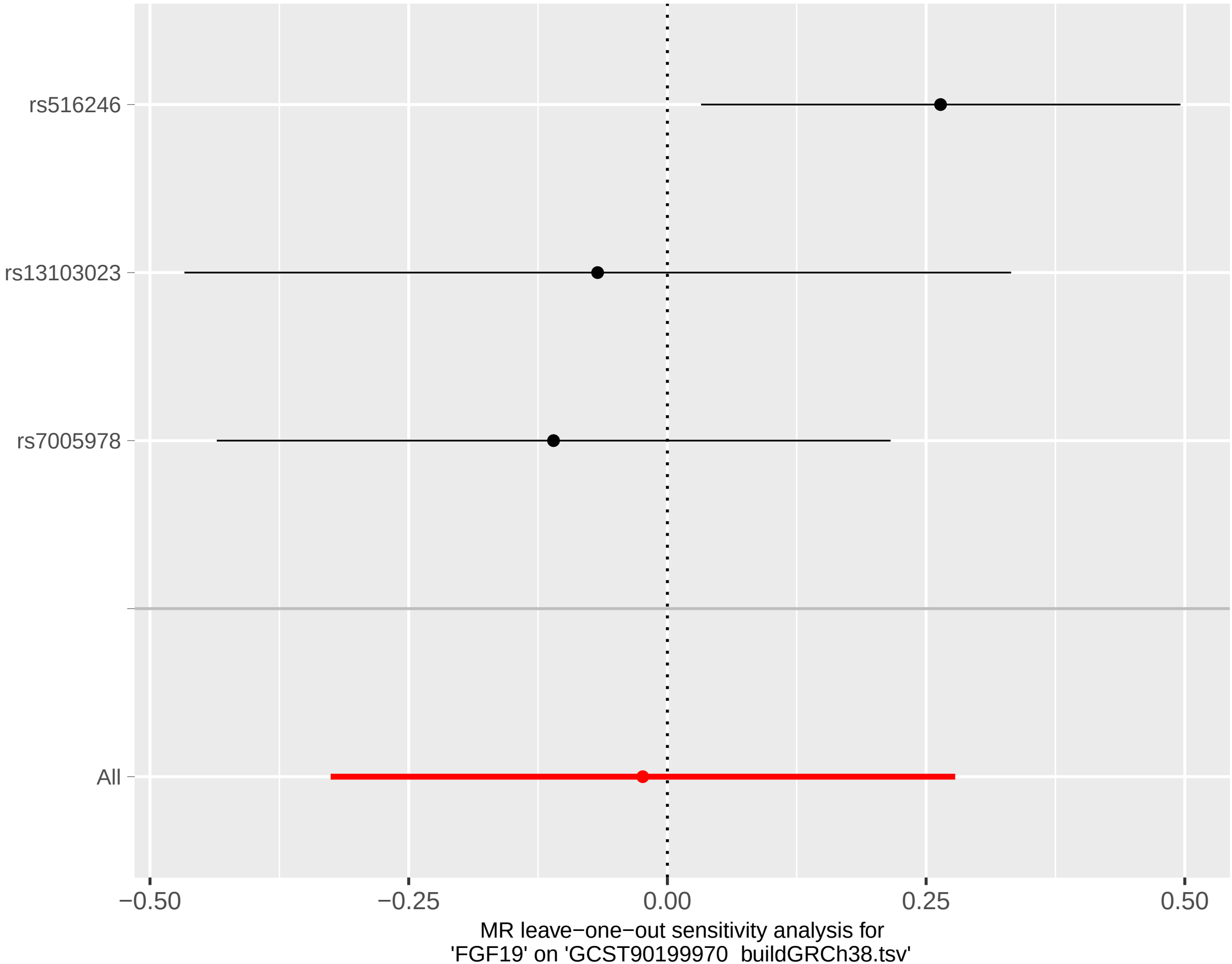
rs516246
rs13103023
rs7005978
All
−0.50
−0.25
0.00
0.25
0.50
MR leave−one−out sensitivity analysis for
'FGF19' on 'GCST90199970_buildGRCh38.tsv'

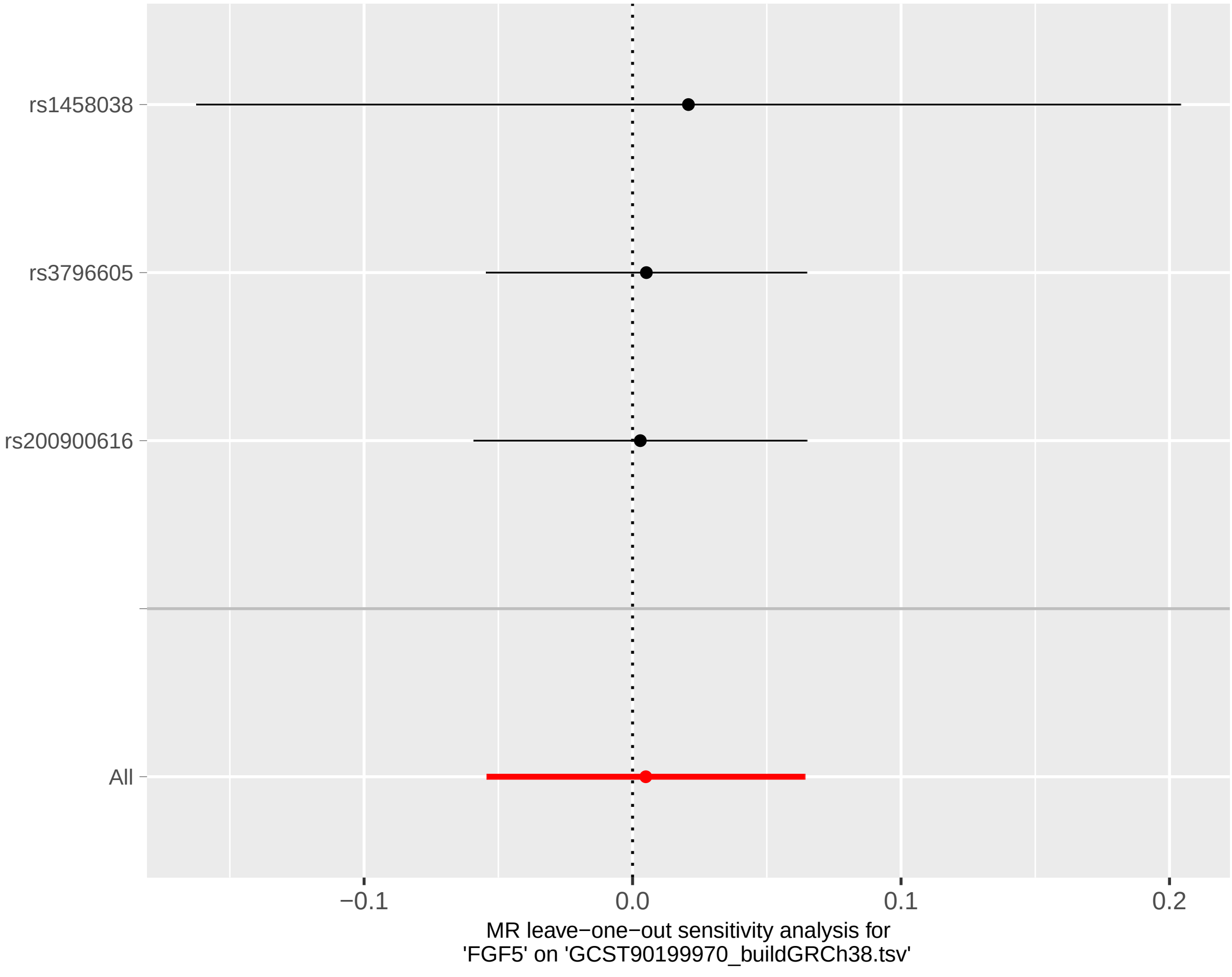
rs1458038
rs3796605
rs200900616
All
−0.1
0.0
0.1
0.2
MR leave−one−out sensitivity analysis for
'FGF5' on 'GCST90199970_buildGRCh38.tsv'

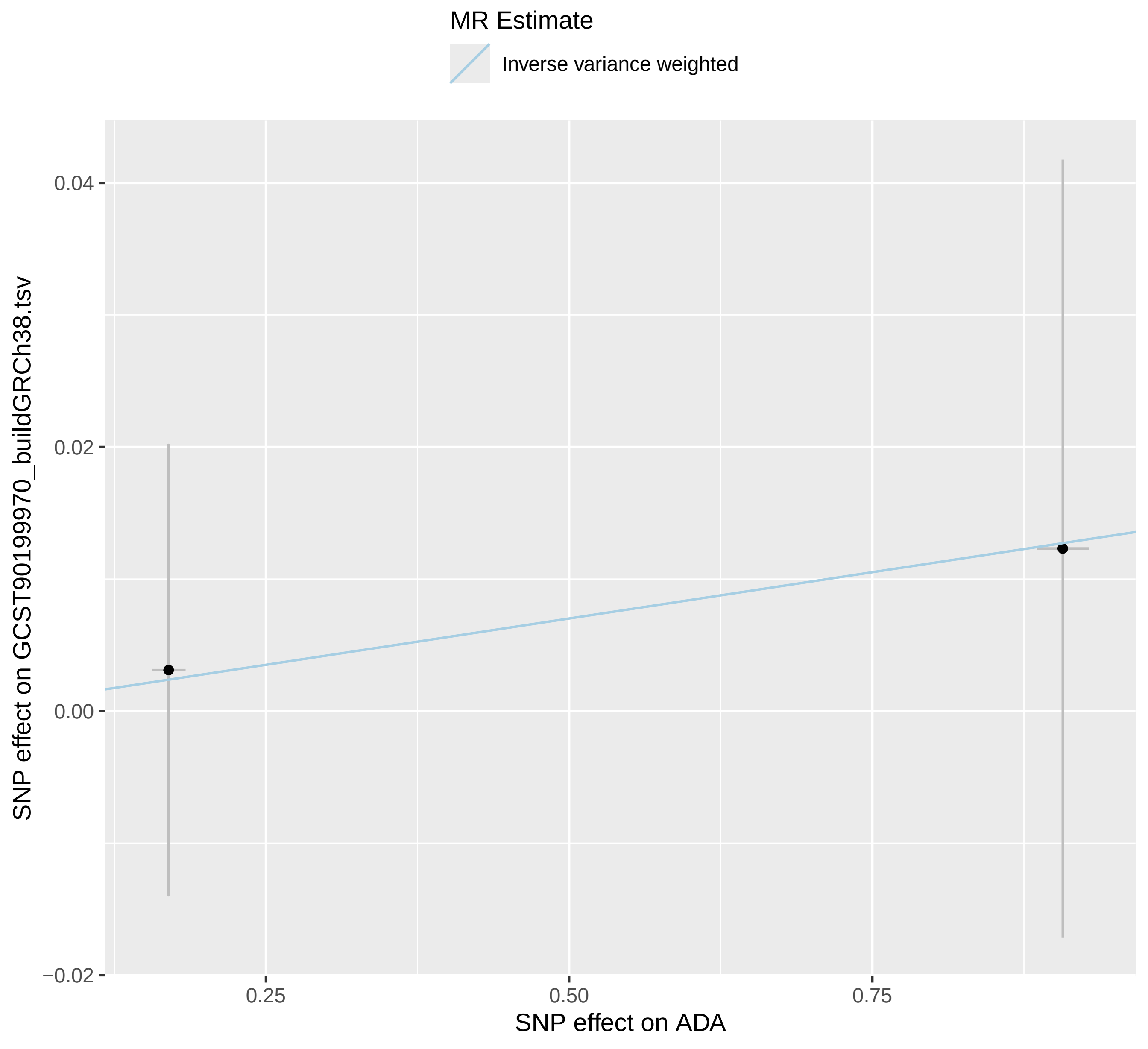
MR Estimate
Inverse variance weighted
0.04
0.02
0.00
−0.02
0.25
0.50
0.75
SNP effect on ADA
SNP effect on GCST90199970_buildGRCh38.tsv

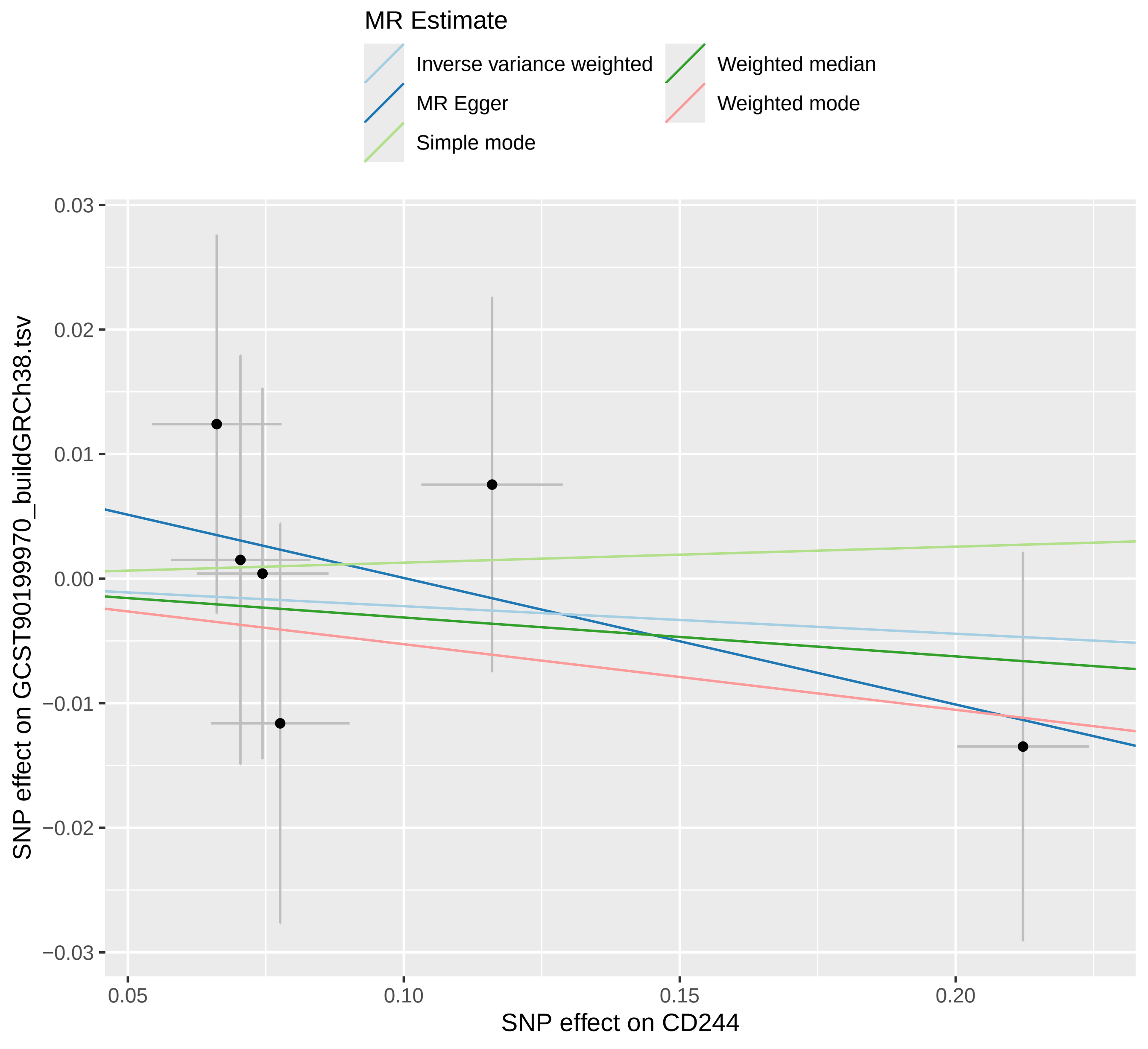
MR Estimate
Inverse variance weighted
MR Egger
Simple mode
Weighted median
Weighted mode
0.03
0.02
0.01
0.00
−0.01
−0.02
−0.03
SNP effect on GCST90199970_buildGRCh38.tsv
0.05
0.10
0.15
0.20
SNP effect on CD244

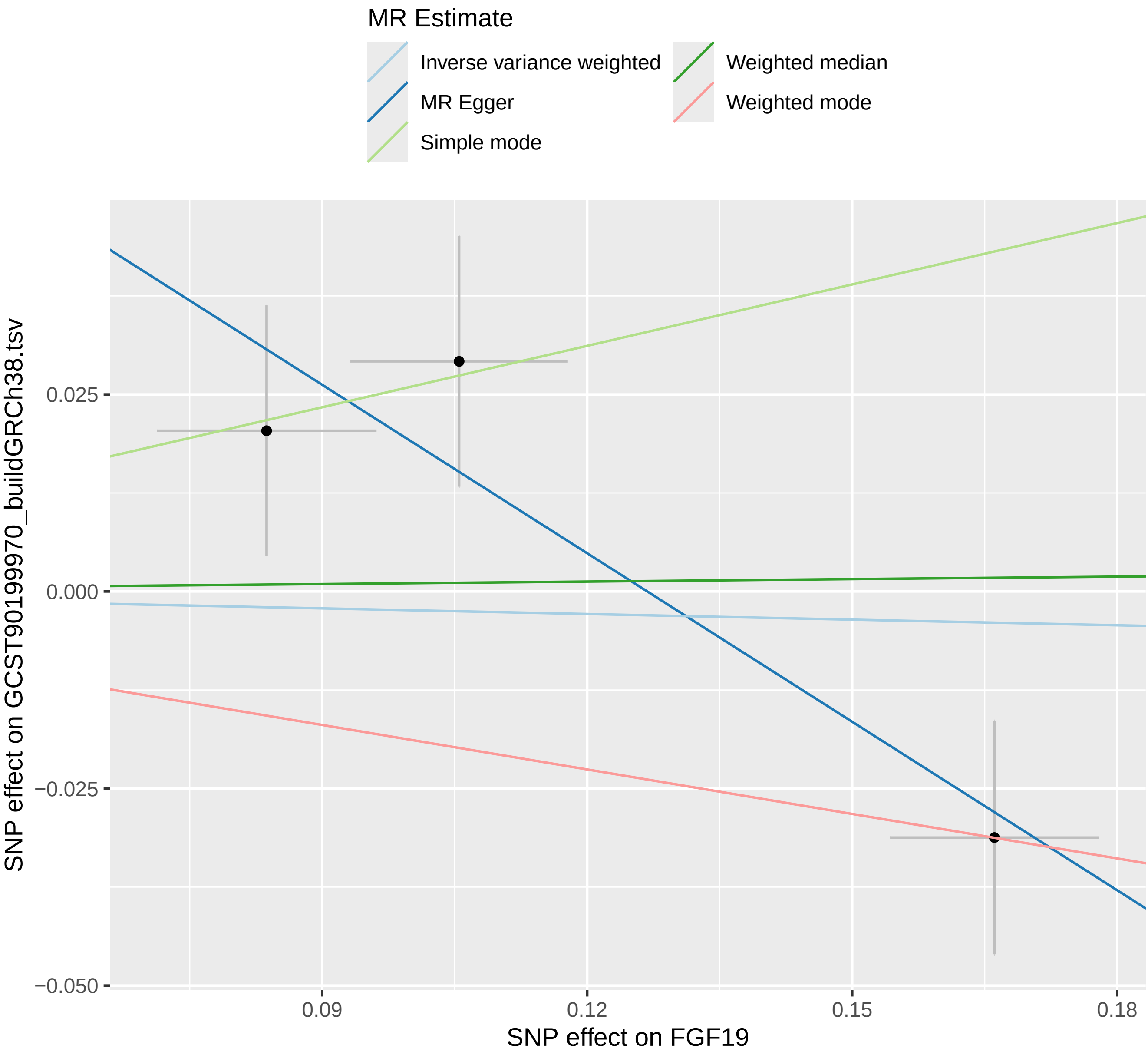

MR Estimate
Inverse variance weighted
MR Egger
Simple mode
Weighted median
Weighted mode
SNP effect on GCST90199970_buildGRCh38.tsv
0.025
0.000
−0.025
−0.050
0.09
0.12
0.15
0.18
SNP effect on FGF19

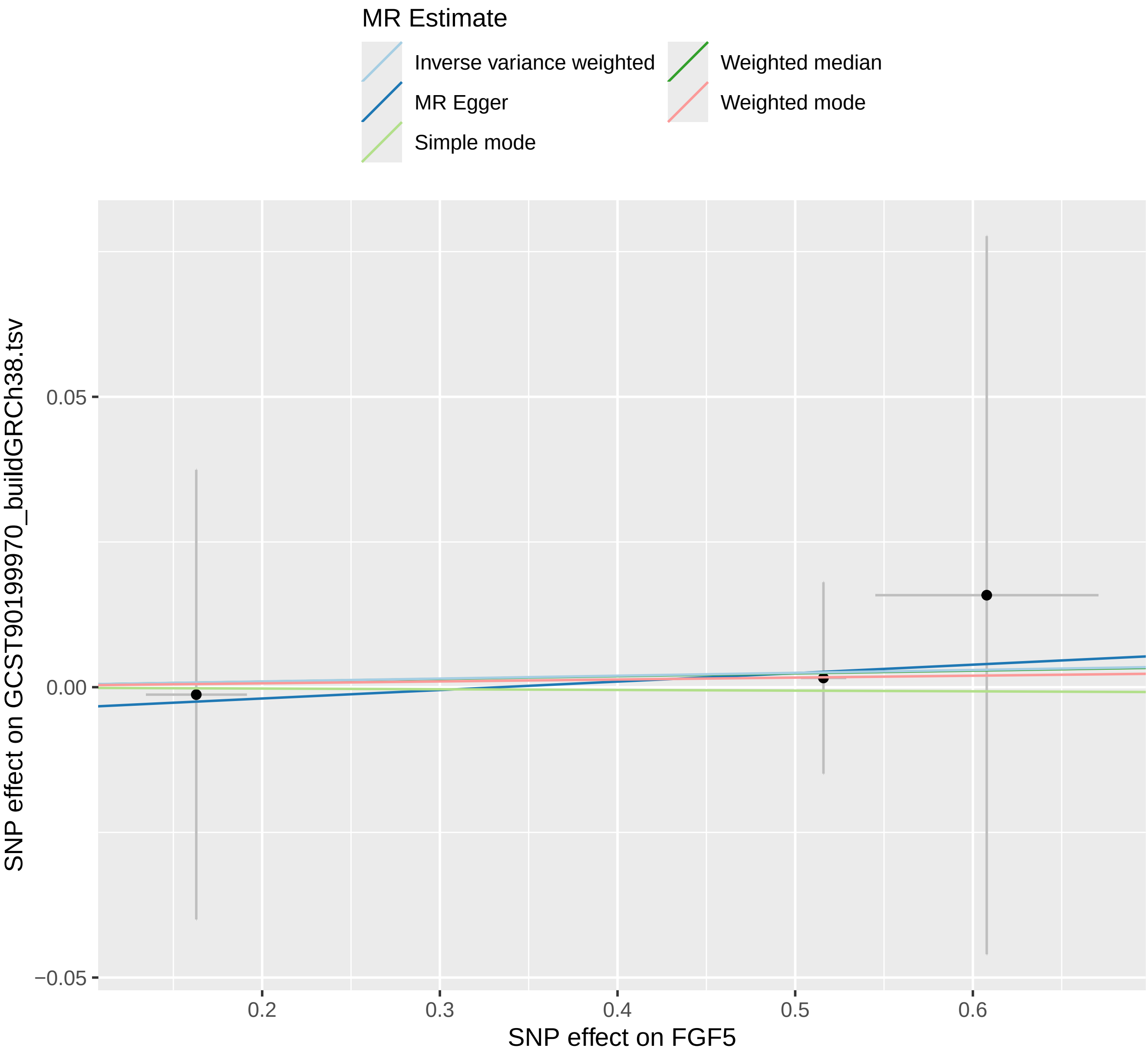

MR Estimate
Inverse variance weighted
MR Egger
Simple mode
Weighted median
Weighted mode
0.05
0.00
−0.05
0.2
0.3
0.4
0.5
0.6
SNP effect on GCST90199970_buildGRCh38.tsv
SNP effect on FGF5

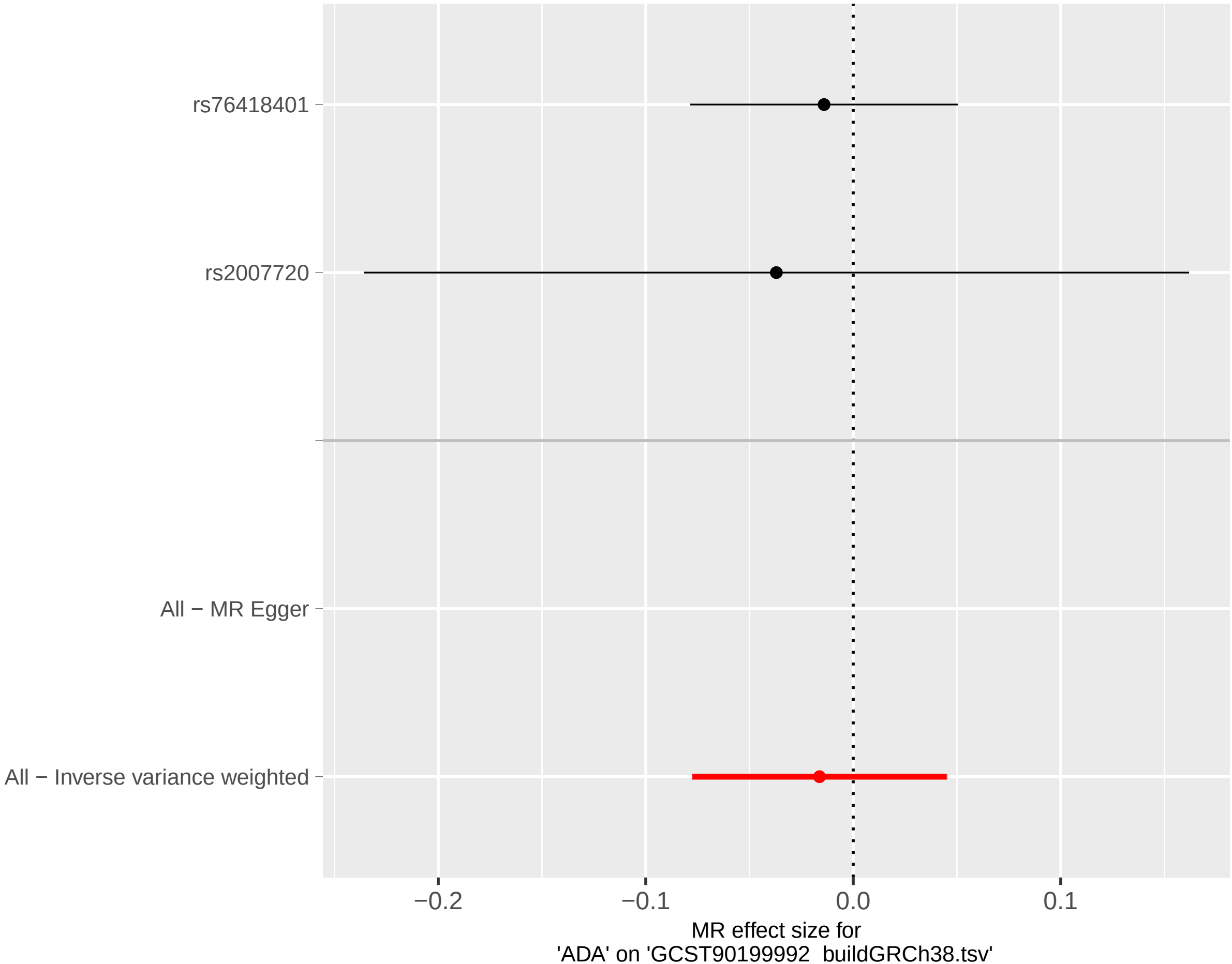
rs76418401
rs2007720
All − MR Egger
All − Inverse variance weighted
−0.2
−0.1
0.0
0.1
MR effect size for
'ADA' on 'GCST90199992_buildGRCh38.tsv'

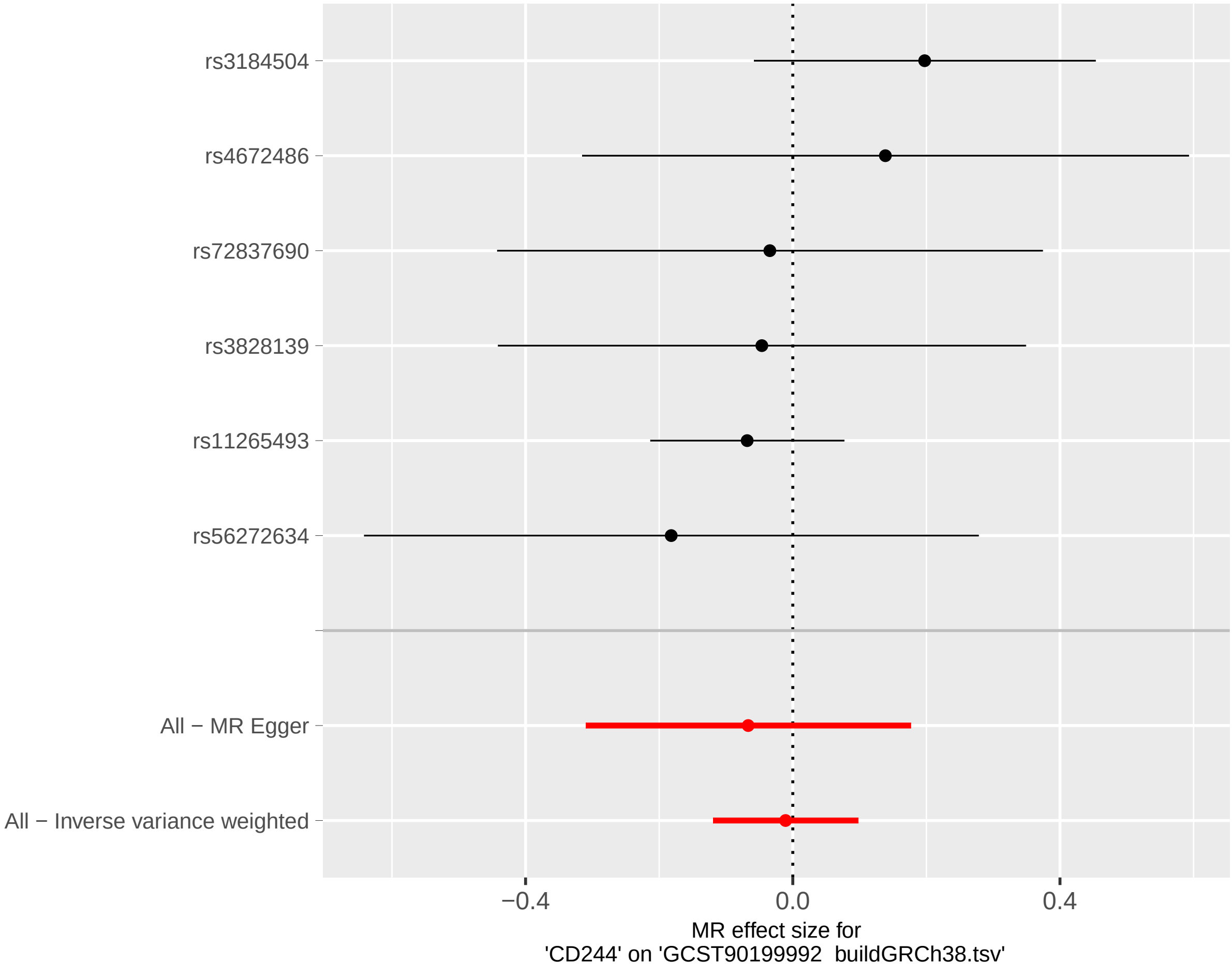
rs3184504
rs4672486
rs72837690
rs3828139
rs11265493
rs56272634
All − MR Egger
All − Inverse variance weighted
−0.4
0.0
0.4
MR effect size for
'CD244' on 'GCST90199992_buildGRCh38.tsv'

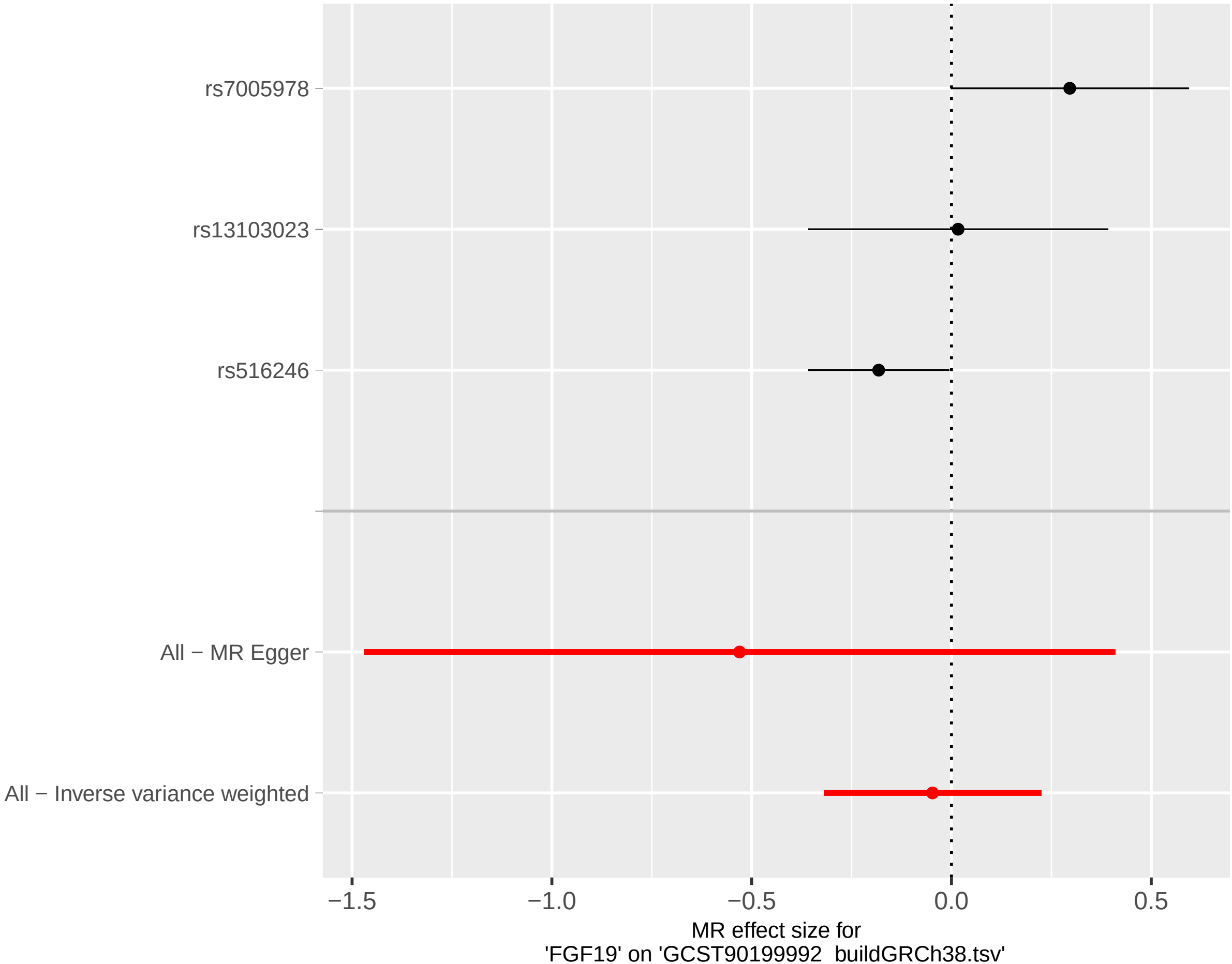
rs7005978
rs13103023
rs516246
All − MR Egger
All − Inverse variance weighted
−1.5
−1.0
−0.5
0.0
0.5
MR effect size for
'FGF19' on 'GCST90199992_buildGRCh38.tsv'

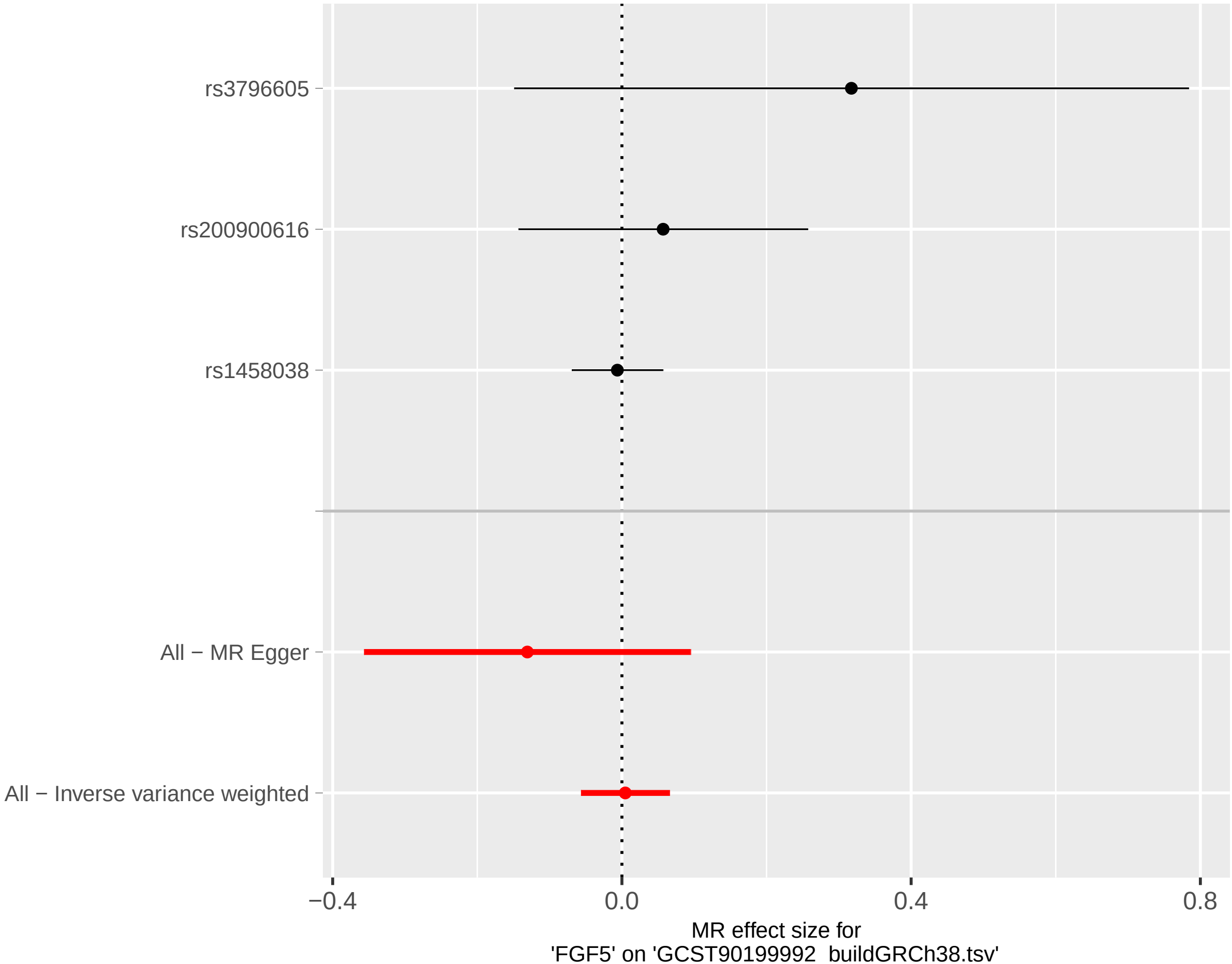
rs3796605
rs200900616
rs1458038
All − MR Egger
All − Inverse variance weighted
−0.4
0.0
0.4
0.8
MR effect size for
'FGF5' on 'GCST90199992_buildGRCh38.tsv'

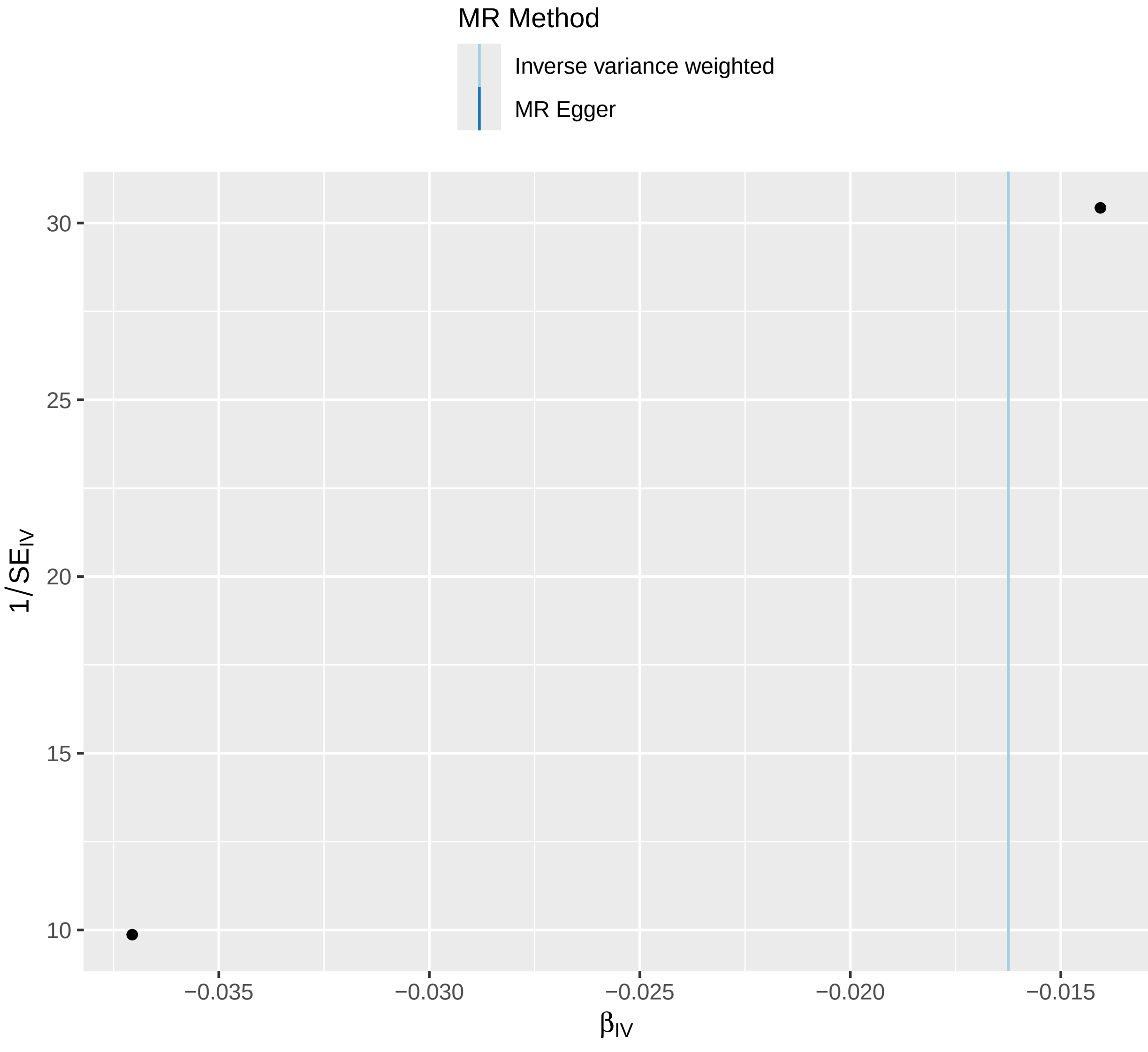
MR Method
Inverse variance weighted
MR Egger
30
25
20
15
10
1/SE_IV
−0.035
−0.030
−0.025
−0.020
−0.015
β_IV

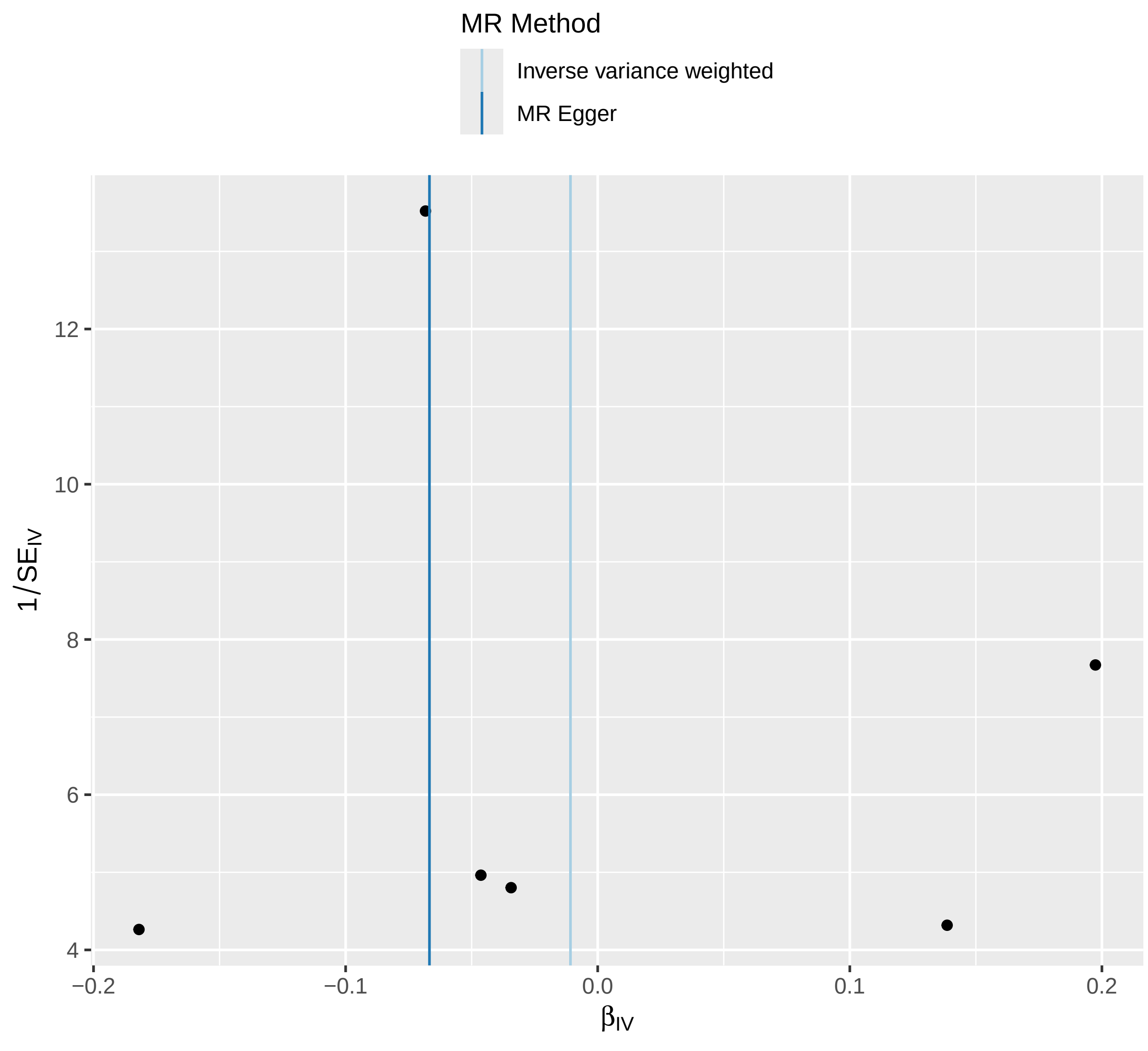

MR Method
Inverse variance weighted
MR Egger
12
10
8
6
4
1/SE_IV
−0.2
−0.1
0.0
0.1
0.2
β_IV

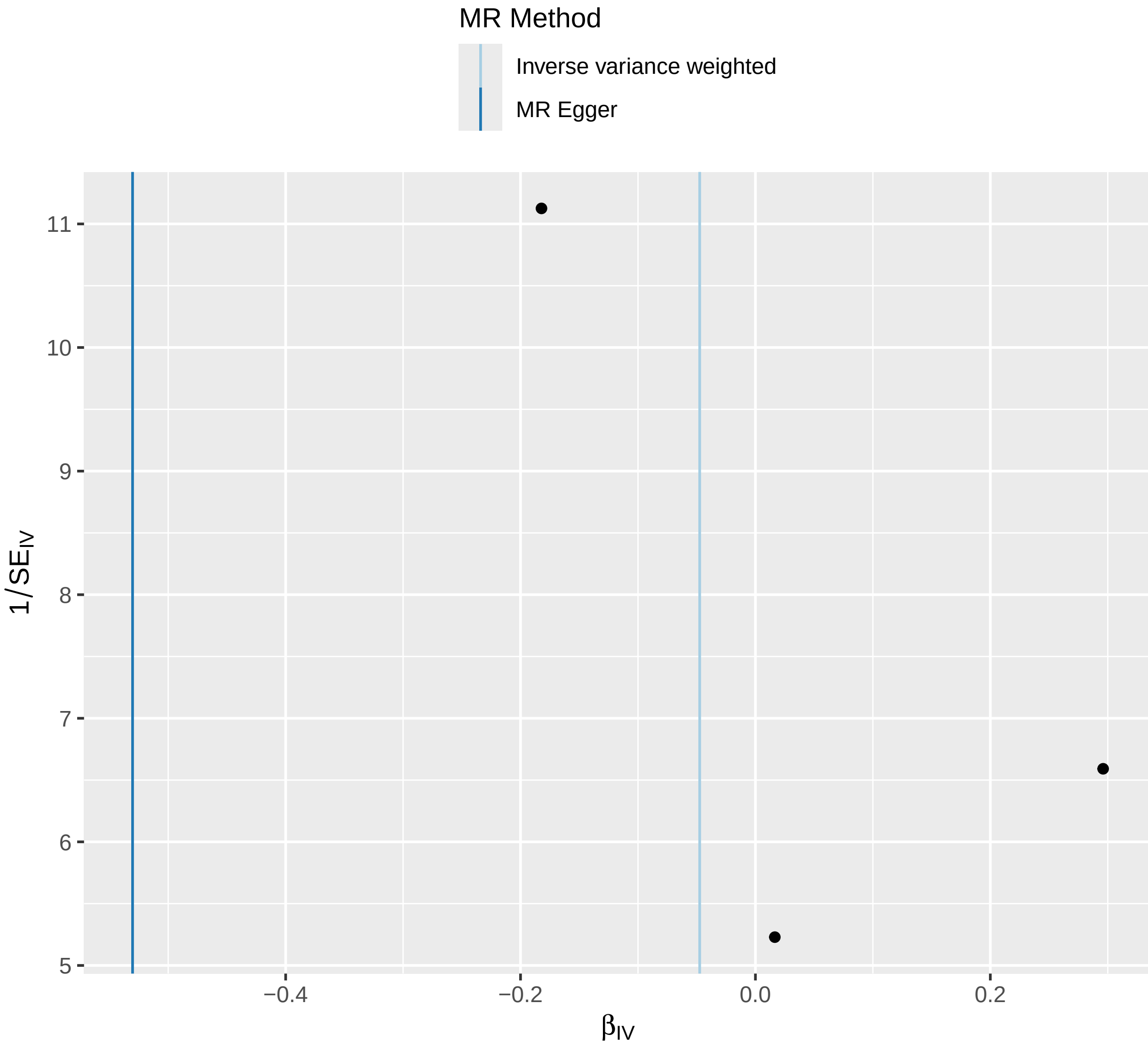
MR Method
Inverse variance weighted
MR Egger
11
10
9
8
7
6
5
1/SE$_{IV}$
−0.4
−0.2
0.0
0.2
β$_{IV}$

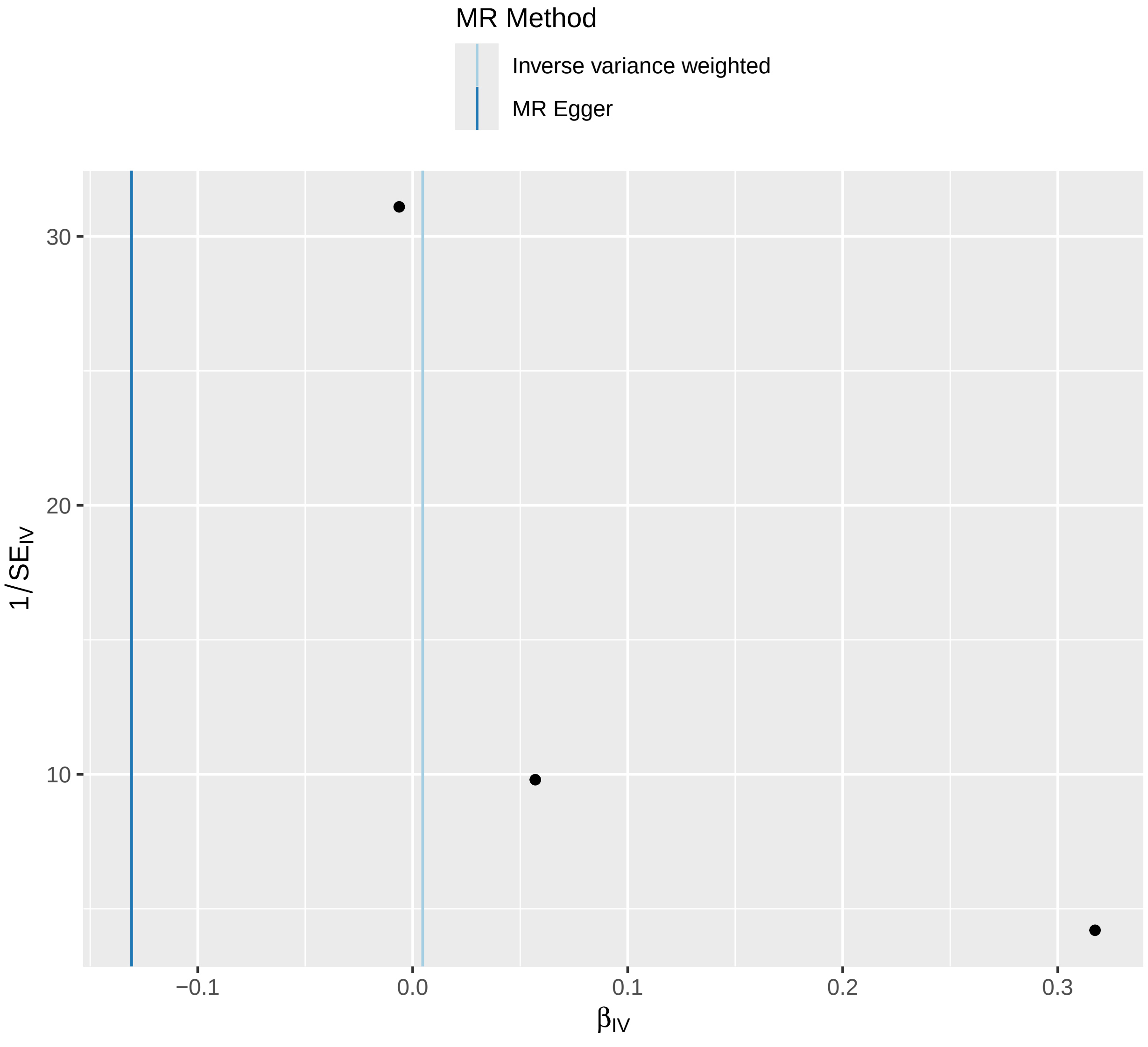
MR Method
Inverse variance weighted
MR Egger
30
20
10
1/SE_IV
−0.1
0.0
0.1
0.2
0.3
β_IV

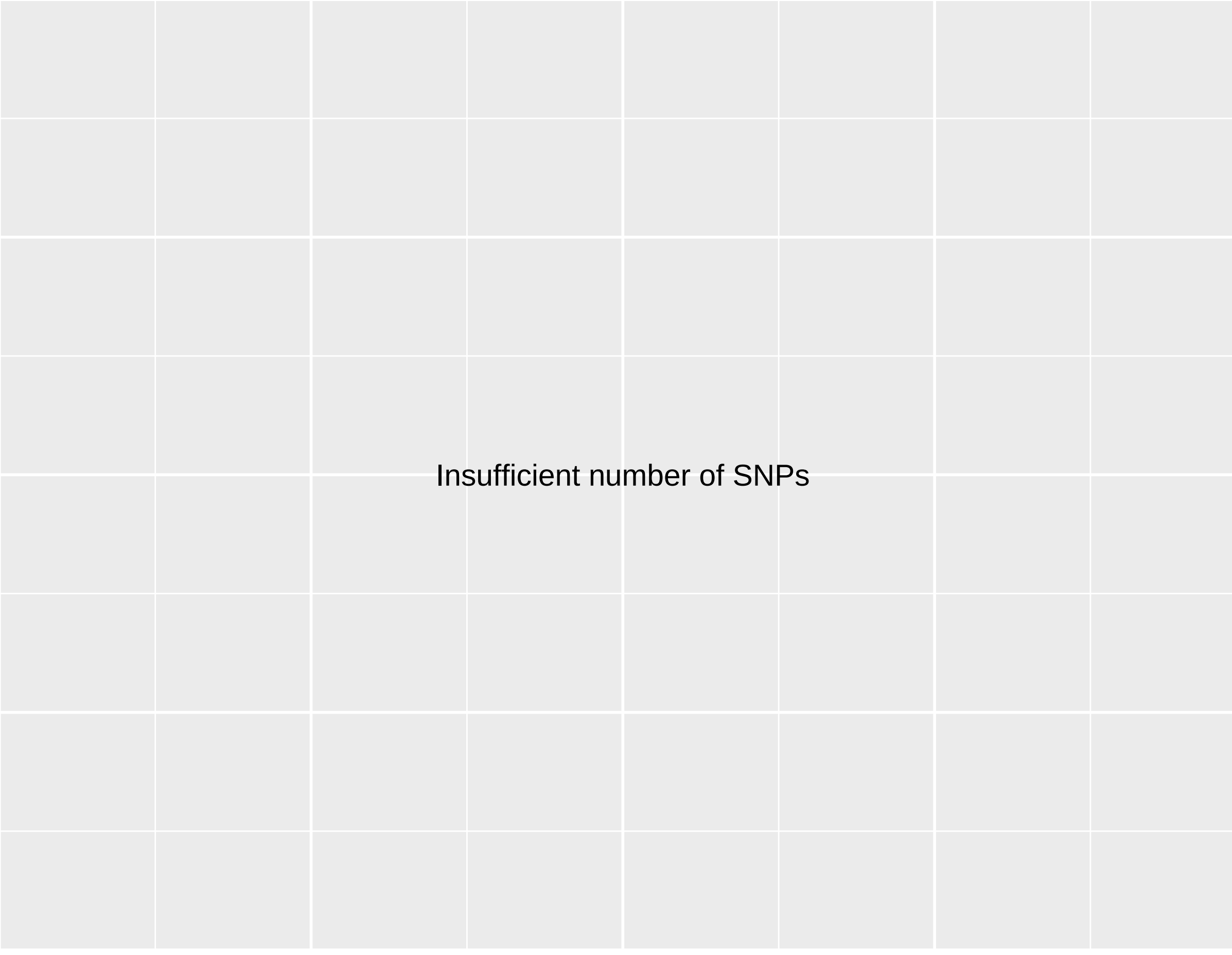
Insufficient number of SNPs

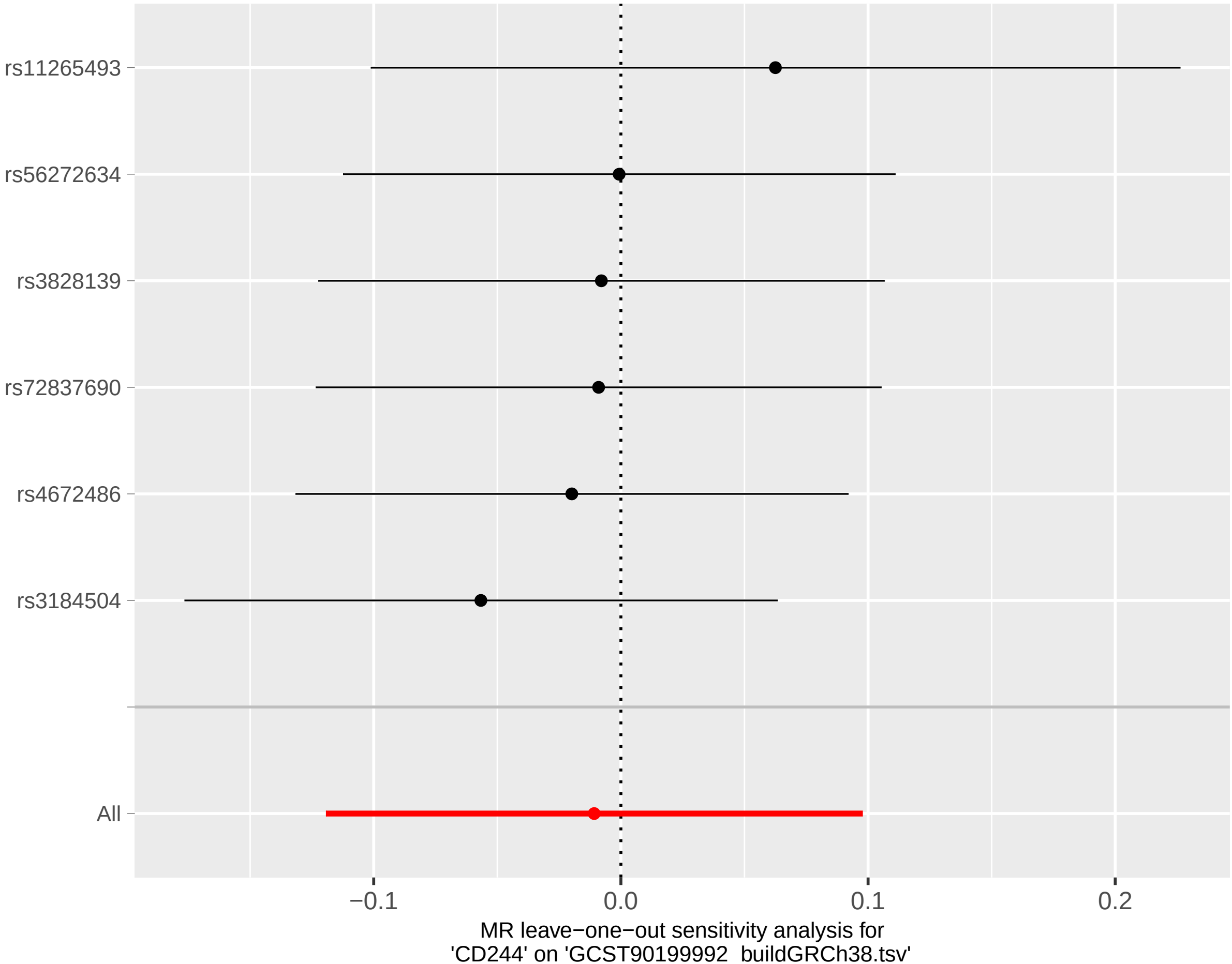
rs11265493
rs56272634
rs3828139
rs72837690
rs4672486
rs3184504
All
−0.1
0.0
0.1
0.2
MR leave−one−out sensitivity analysis for
'CD244' on 'GCST90199992_buildGRCh38.tsv'

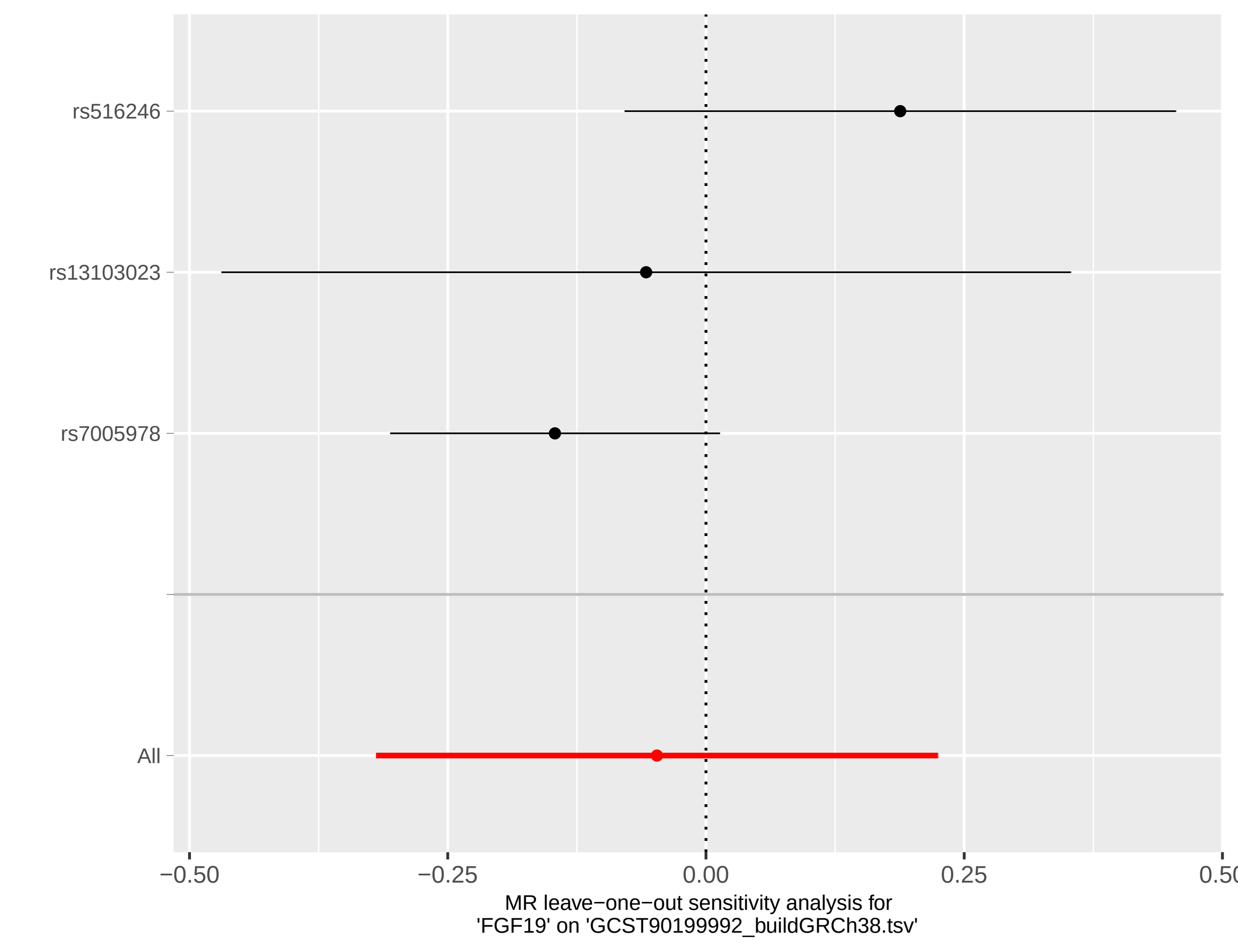

rs516246
rs13103023
rs7005978
All
−0.50
−0.25
0.00
0.25
0.50
MR leave−one−out sensitivity analysis for
'FGF19' on 'GCST90199992_buildGRCh38.tsv'

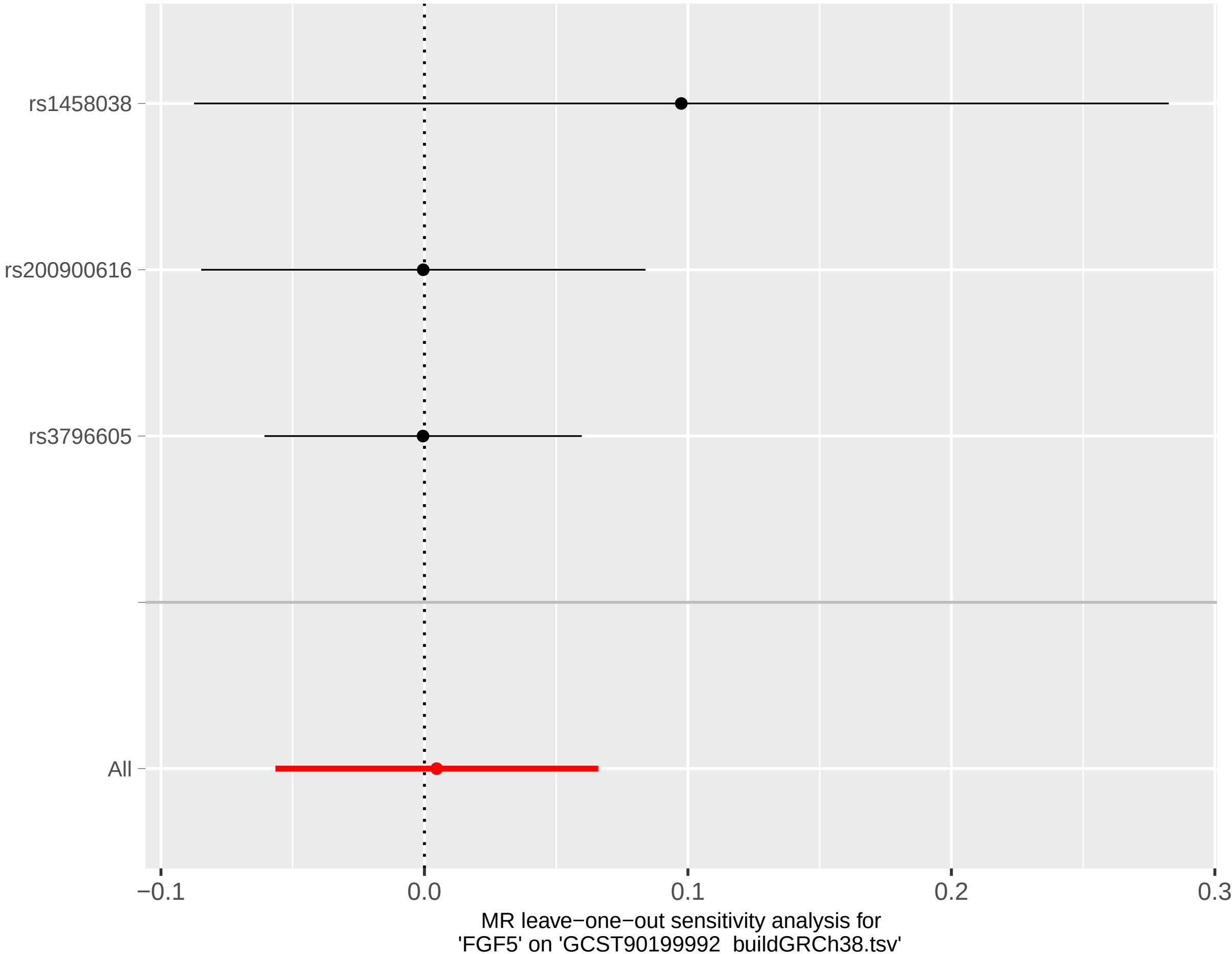
rs1458038
rs200900616
rs3796605
All
−0.1
0.0
0.1
0.2
0.3
MR leave−one−out sensitivity analysis for
'FGF5' on 'GCST90199992_buildGRCh38.tsv'

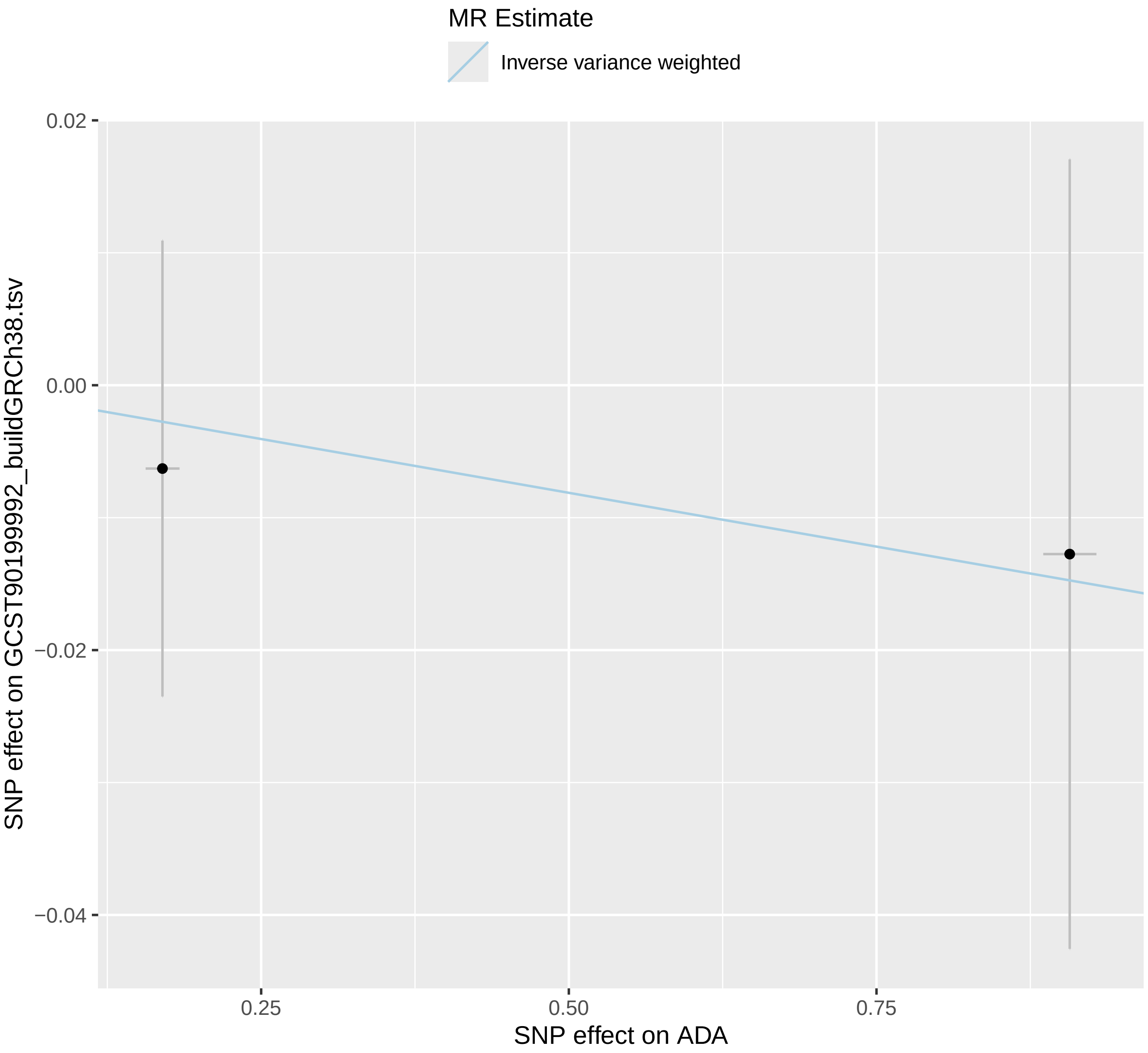

MR Estimate
Inverse variance weighted
0.02
0.00
−0.02
−0.04
SNP effect on GCST90199992_buildGRCh38.tsv
0.25
0.50
0.75
SNP effect on ADA

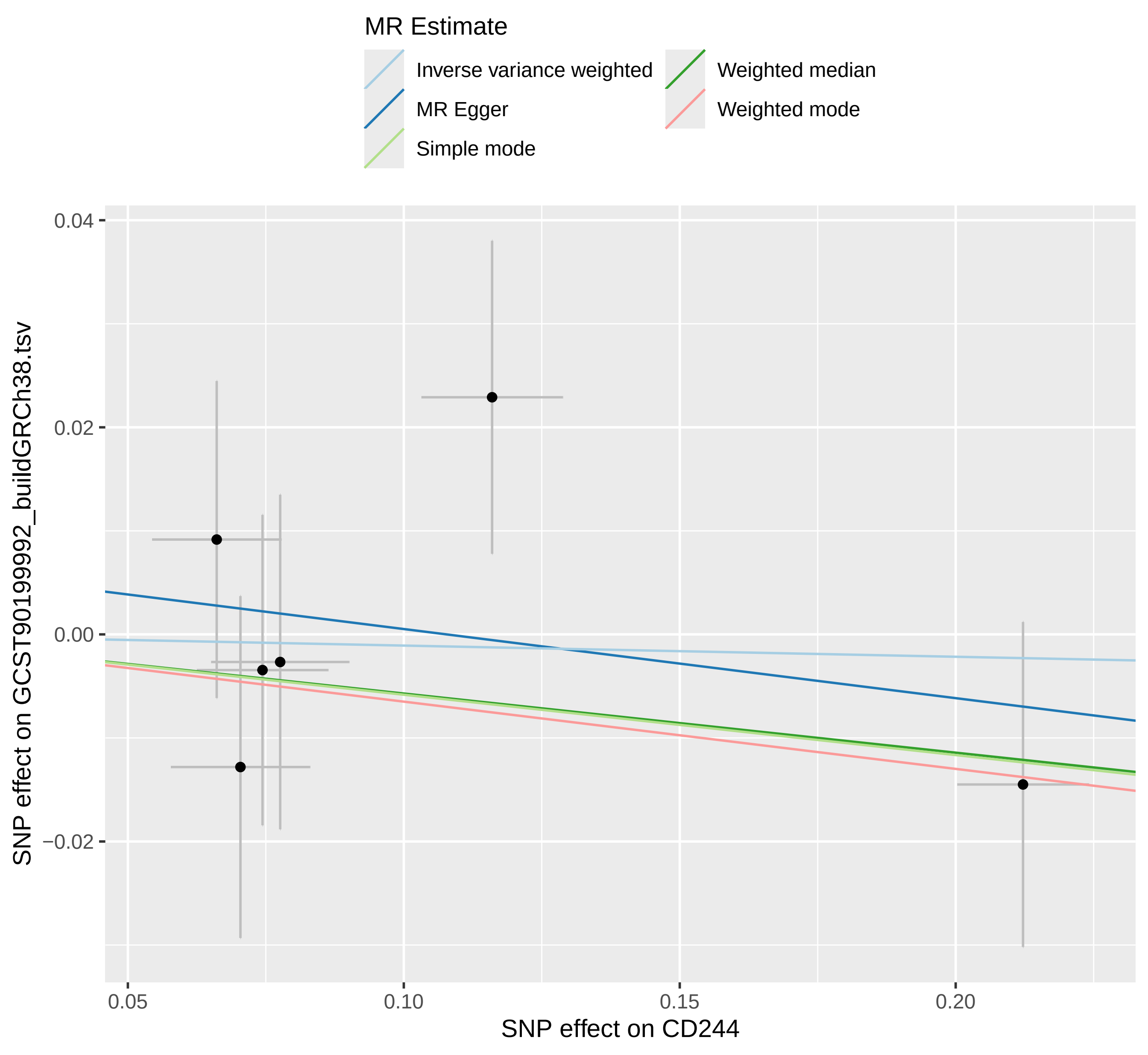

MR Estimate
Inverse variance weighted
MR Egger
Simple mode
Weighted median
Weighted mode
0.04
0.02
0.00
−0.02
0.05
0.10
0.15
0.20
SNP effect on GCST90199992_buildGRCh38.tsv
SNP effect on CD244

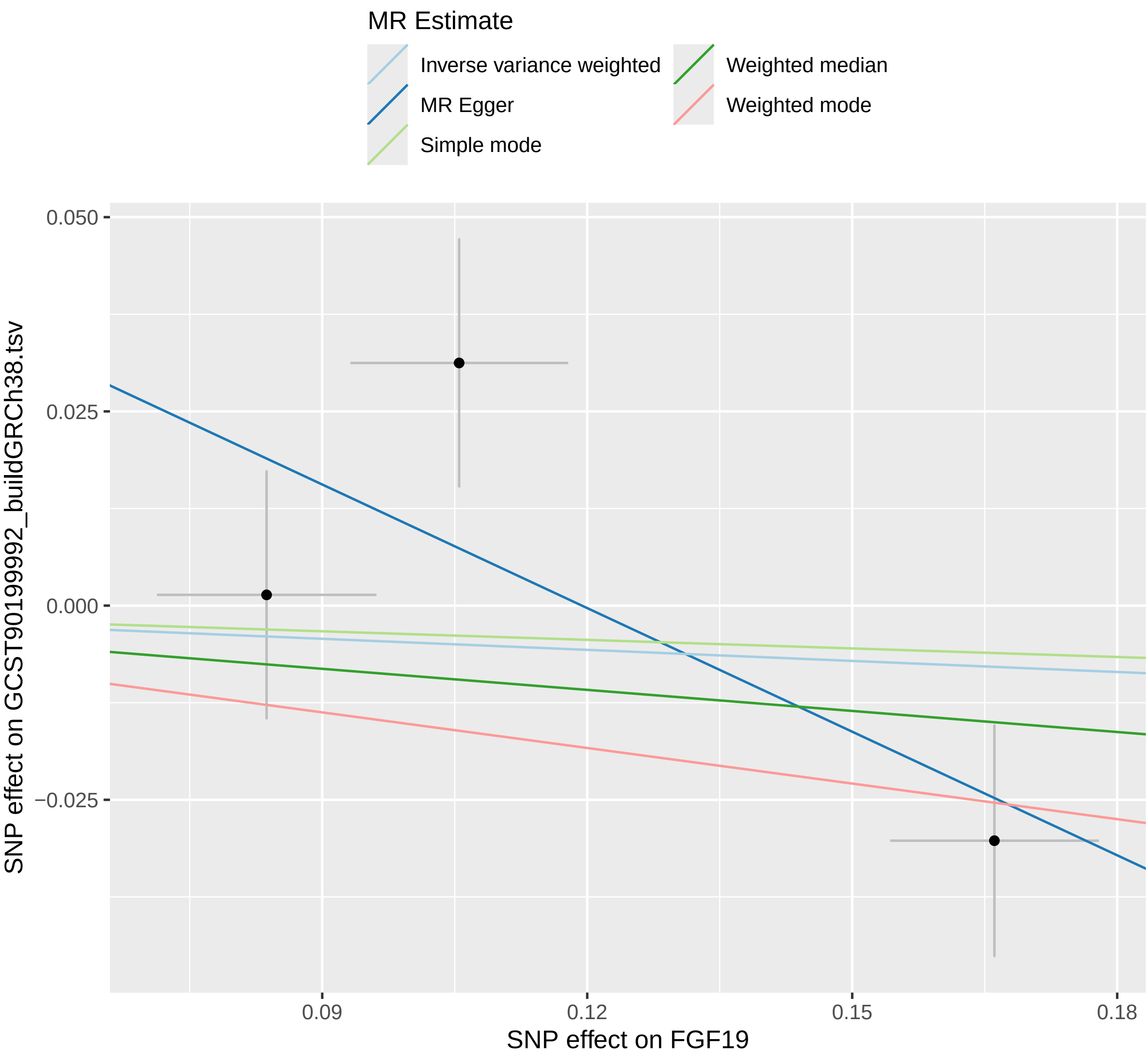
MR Estimate
Inverse variance weighted
MR Egger
Simple mode
Weighted median
Weighted mode
0.050
0.025
0.000
−0.025
0.09
0.12
0.15
0.18
SNP effect on GCST90199992_buildGRCh38.tsv
SNP effect on FGF19

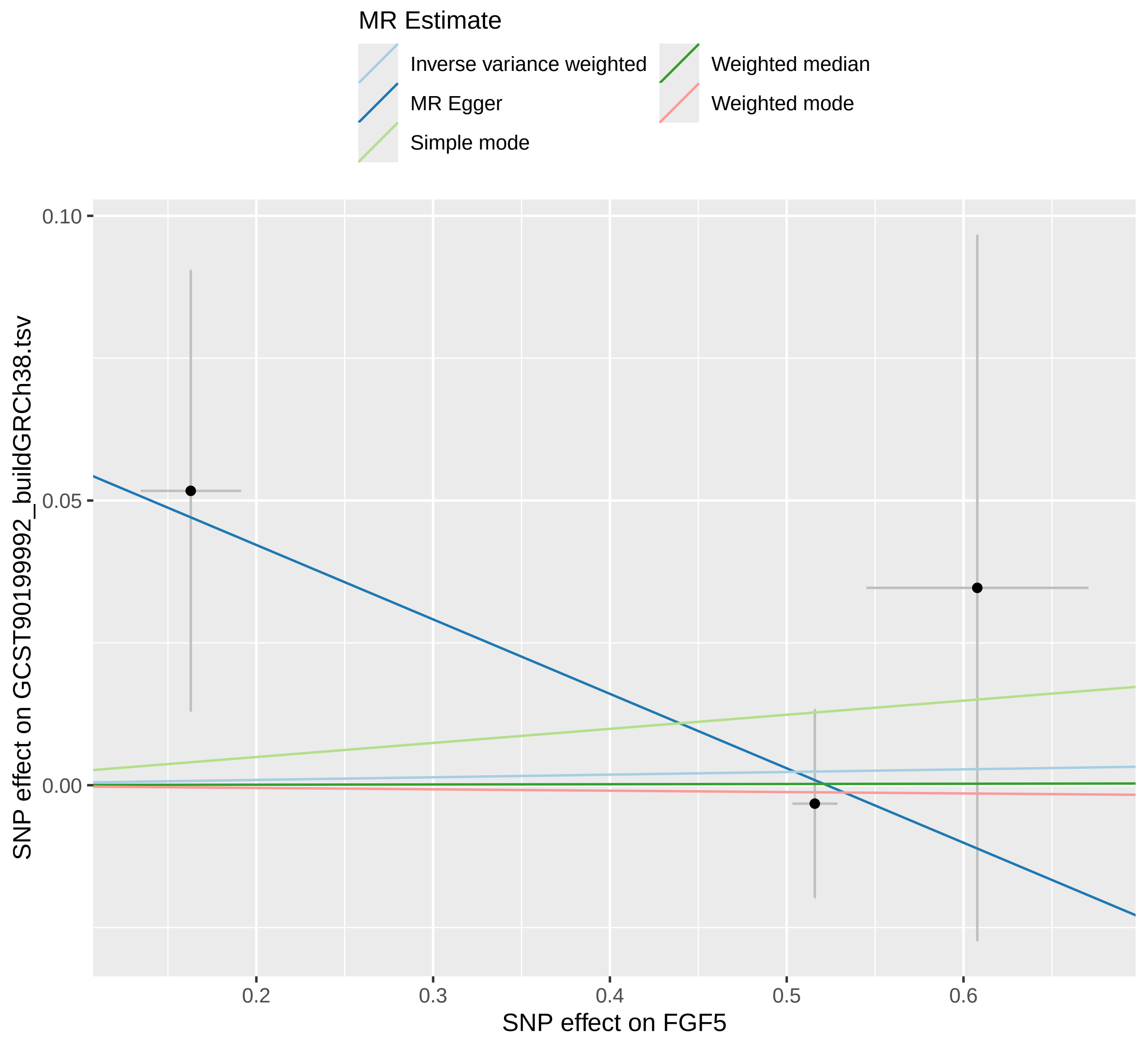

MR Estimate
Inverse variance weighted
MR Egger
Simple mode
Weighted median
Weighted mode
0.10
0.05
0.00
SNP effect on GCST90199992_buildGRCh38.tsv
0.2
0.3
0.4
0.5
0.6
SNP effect on FGF5

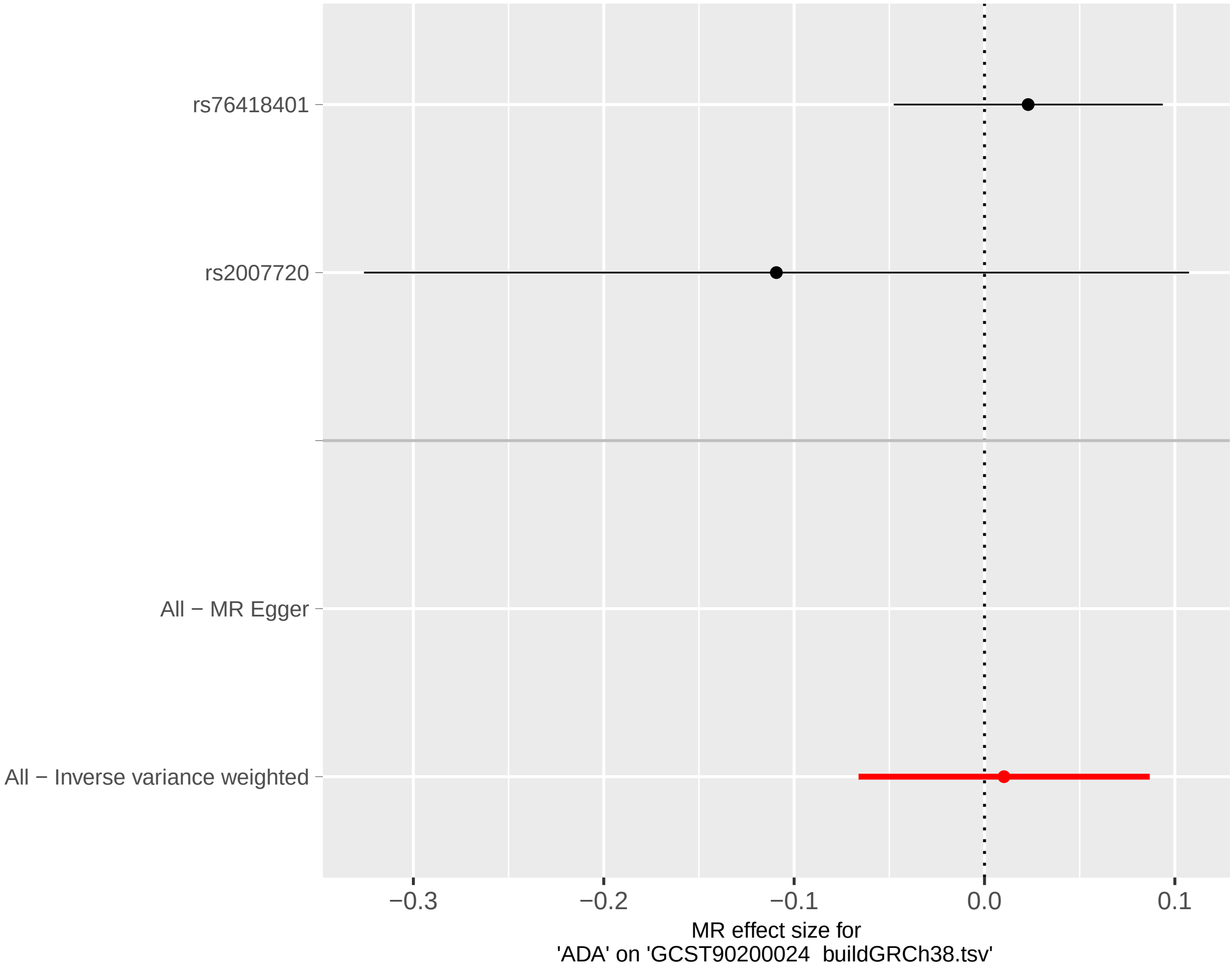
rs76418401
rs2007720
All − MR Egger
All − Inverse variance weighted
−0.3
−0.2
−0.1
0.0
0.1
MR effect size for
'ADA' on 'GCST90200024_buildGRCh38.tsv'

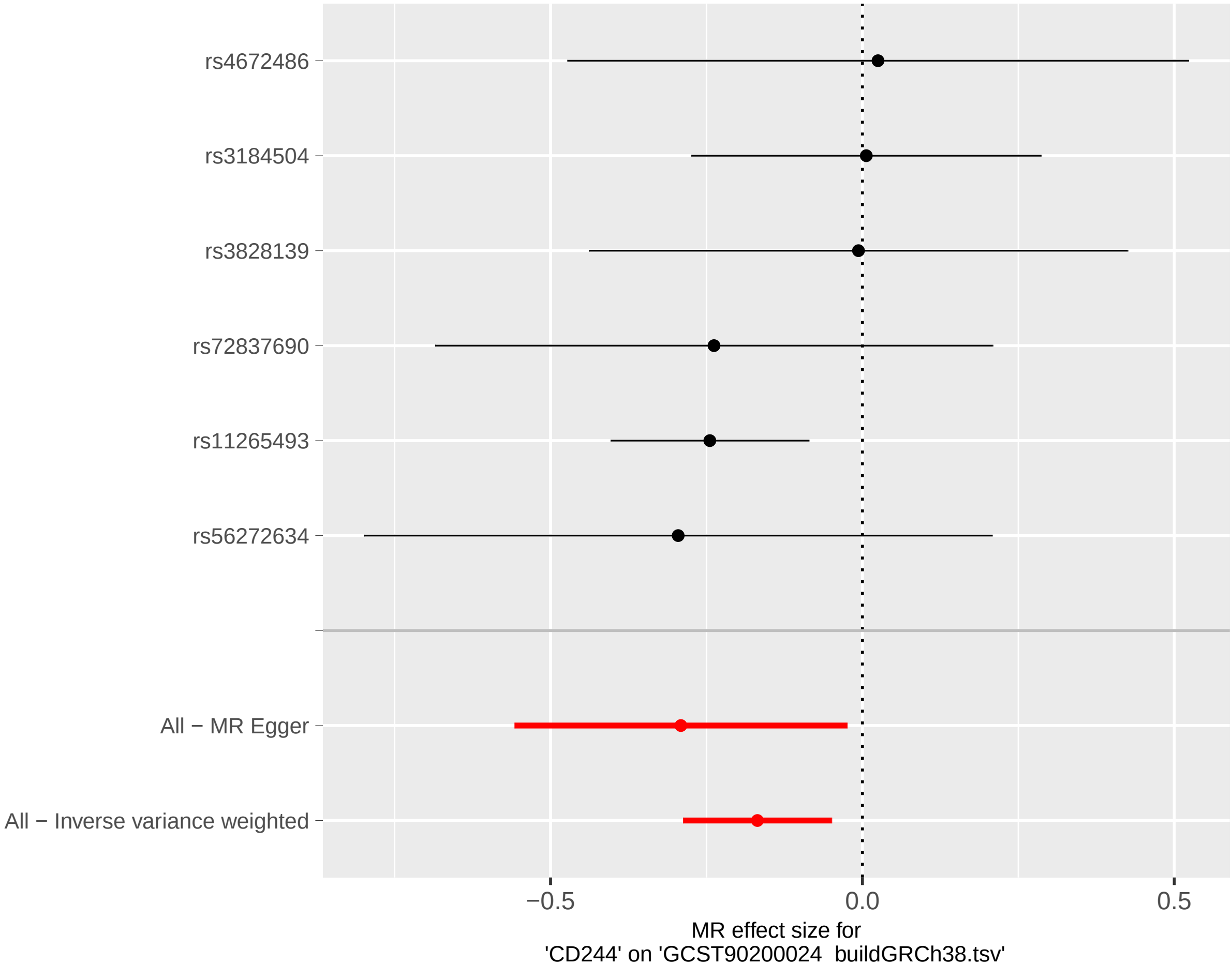

rs4672486
rs3184504
rs3828139
rs72837690
rs11265493
rs56272634
All − MR Egger
All − Inverse variance weighted
−0.5
0.0
0.5
MR effect size for
'CD244' on 'GCST90200024_buildGRCh38.tsv'

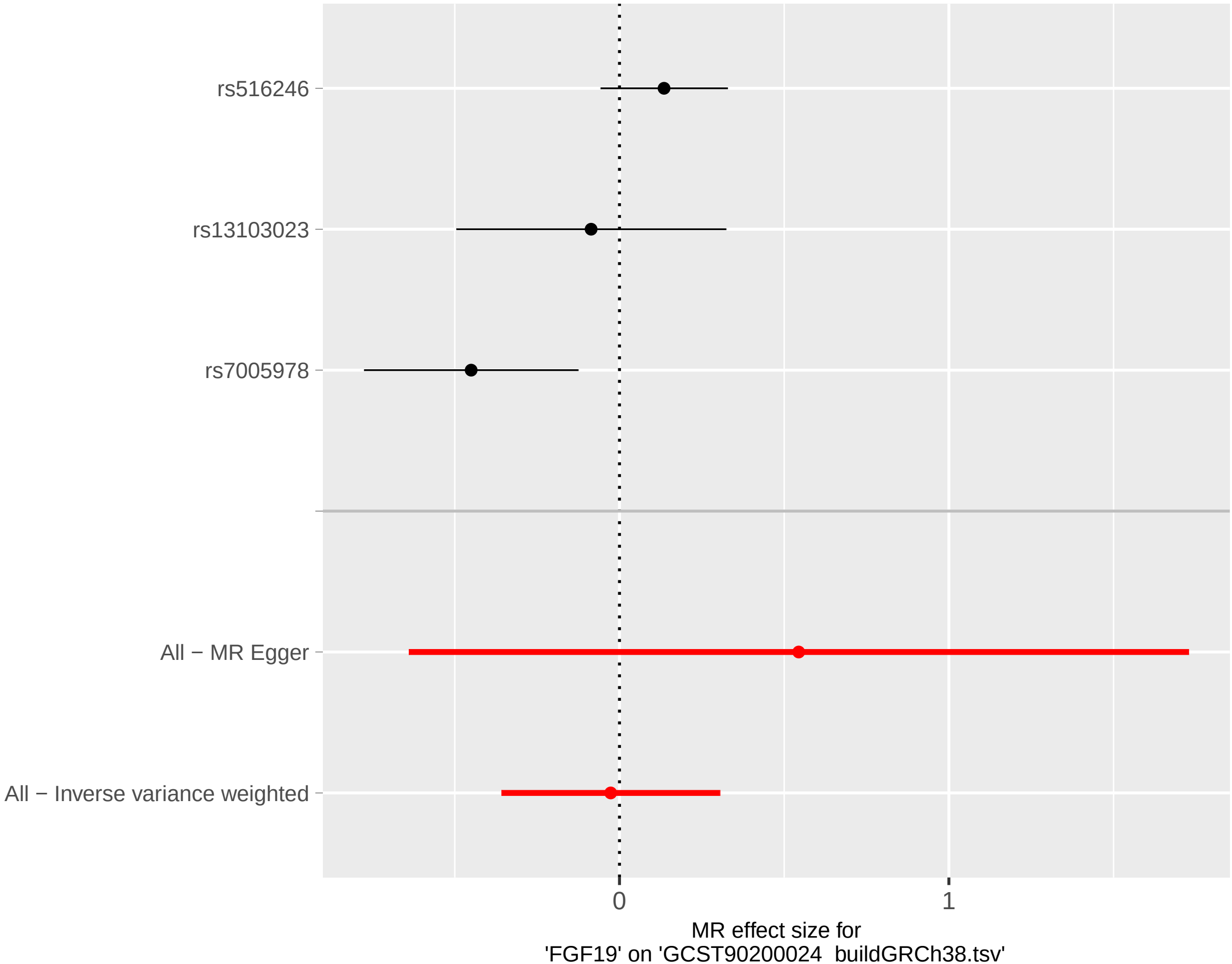
rs516246
rs13103023
rs7005978
All − MR Egger
All − Inverse variance weighted
0
1
MR effect size for
'FGF19' on 'GCST90200024_buildGRCh38.tsv'

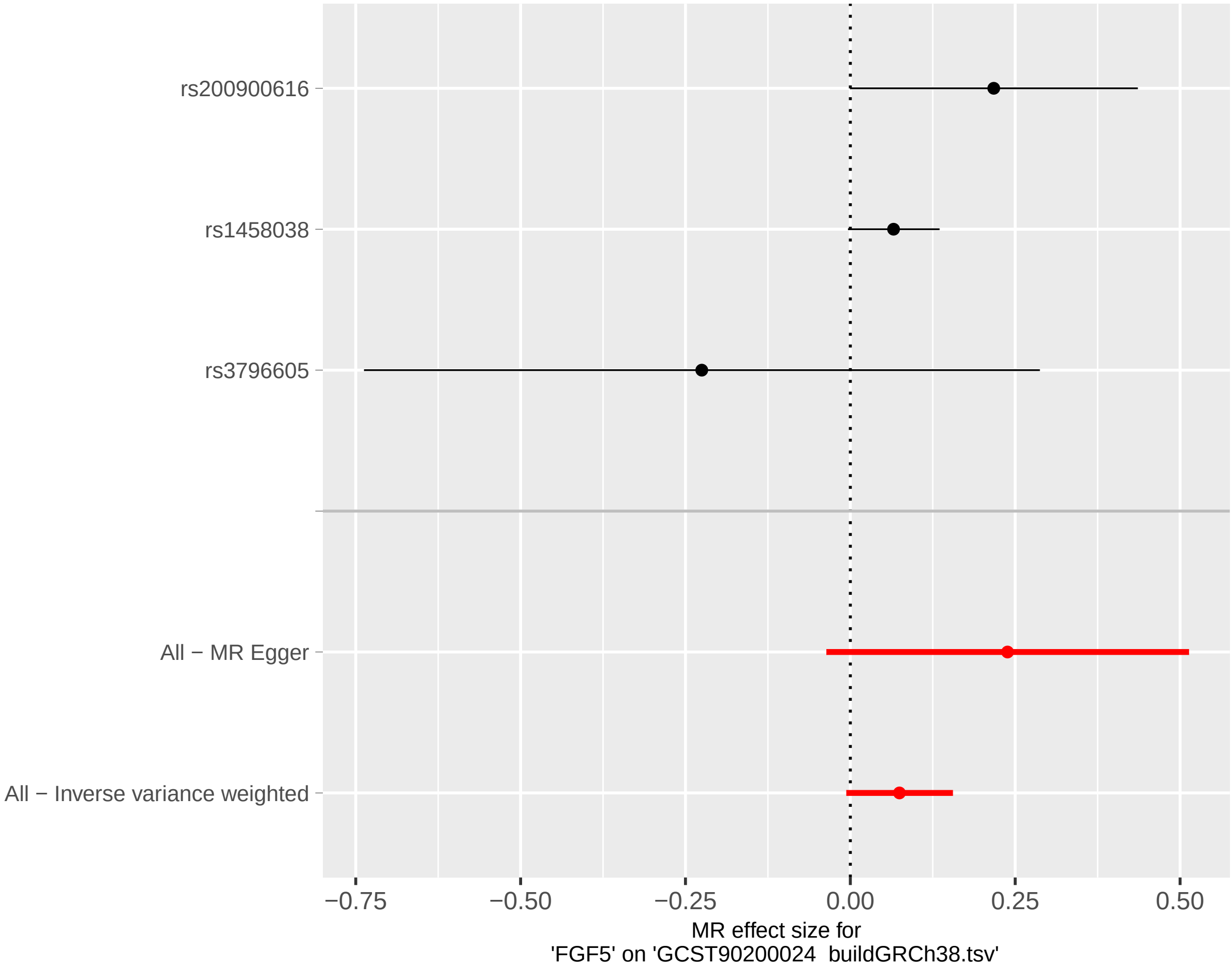
rs200900616
rs1458038
rs3796605
All − MR Egger
All − Inverse variance weighted
−0.75
−0.50
−0.25
0.00
0.25
0.50
MR effect size for
'FGF5' on 'GCST90200024_buildGRCh38.tsv'

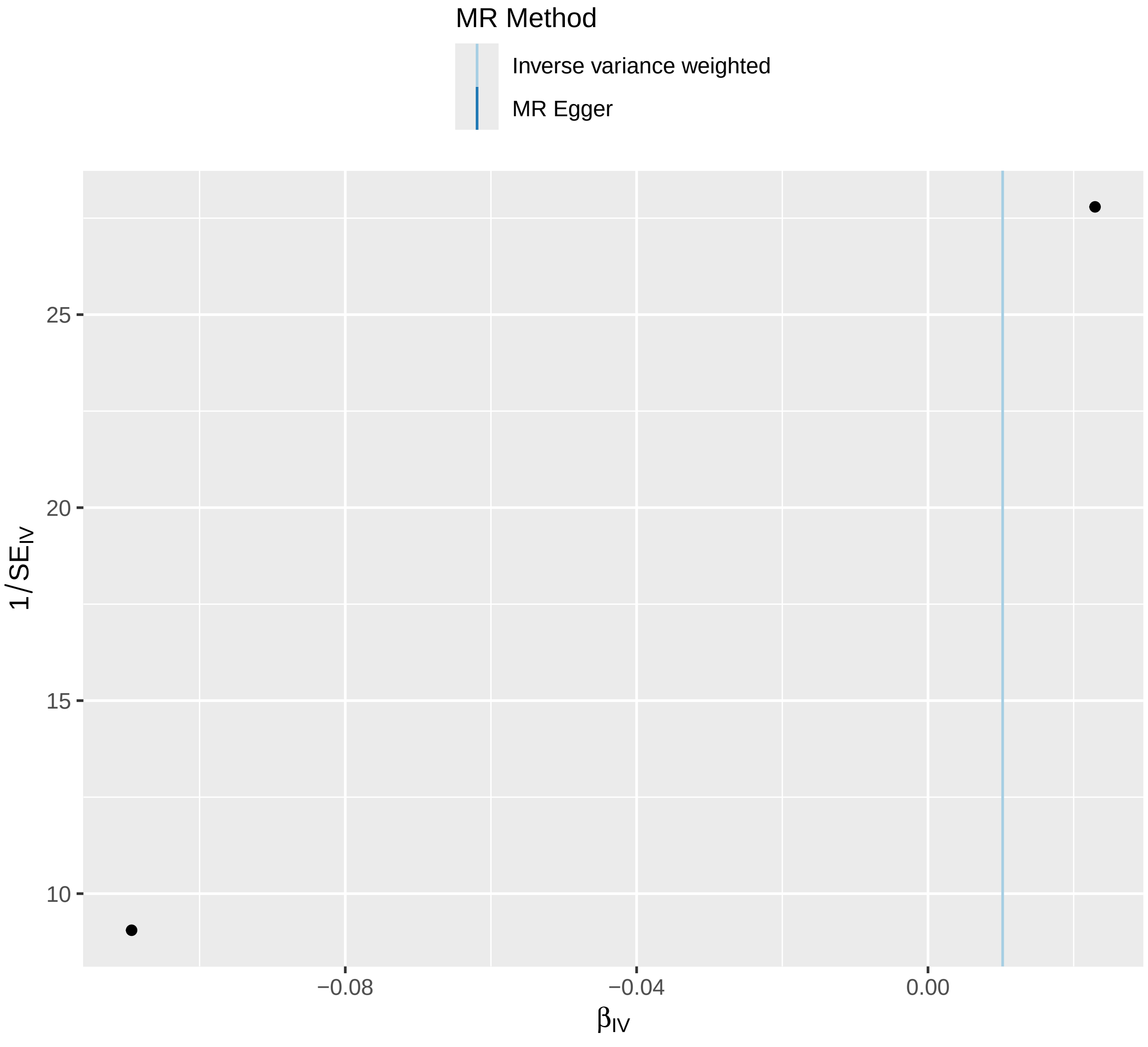
MR Method
Inverse variance weighted
MR Egger
1/SE$_{IV}$
25
20
15
10
−0.08
−0.04
0.00
β$_{IV}$

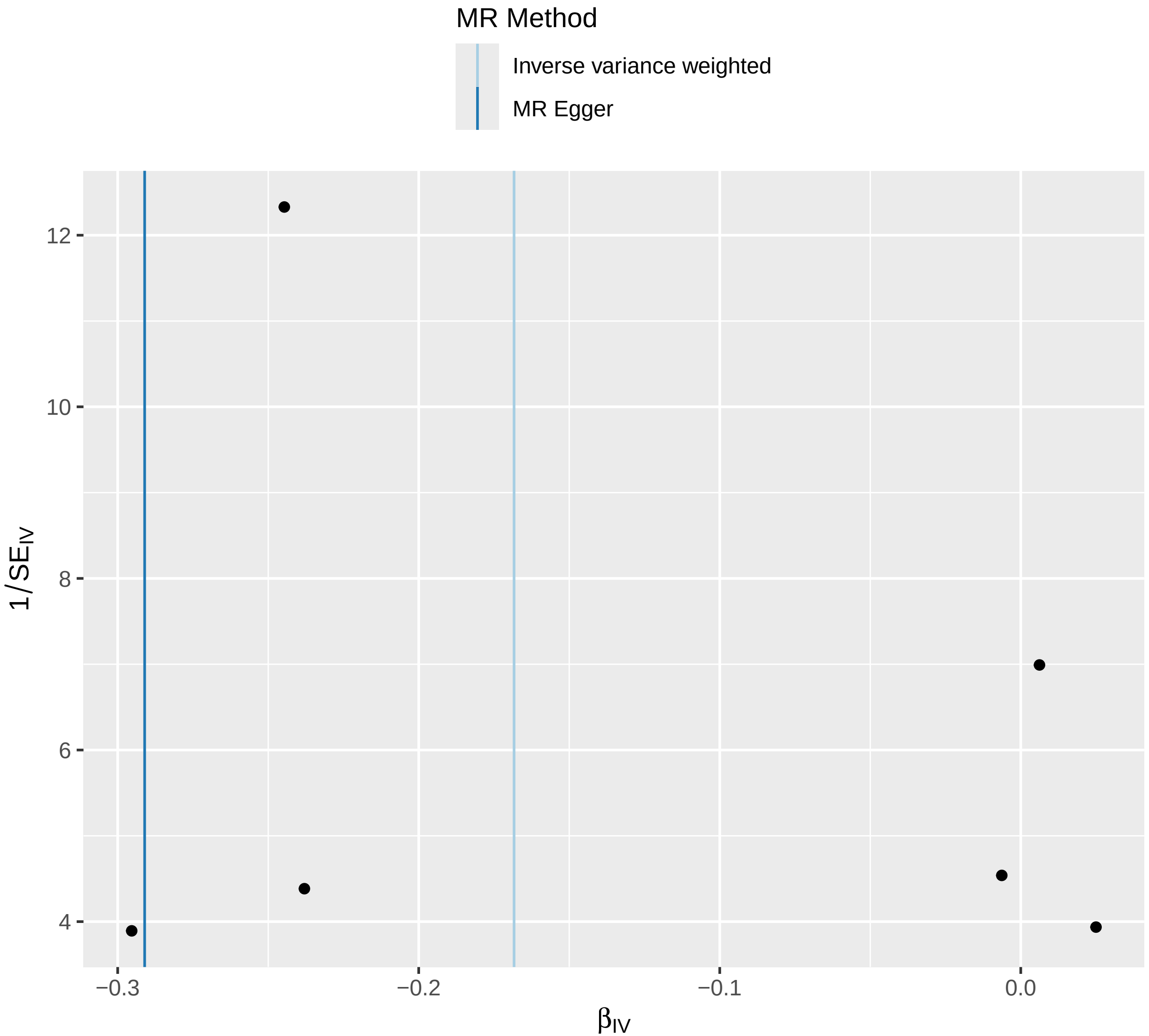

MR Method
Inverse variance weighted
MR Egger
12
10
8
6
4
1/SE_IV
−0.3
−0.2
−0.1
0.0
β_IV

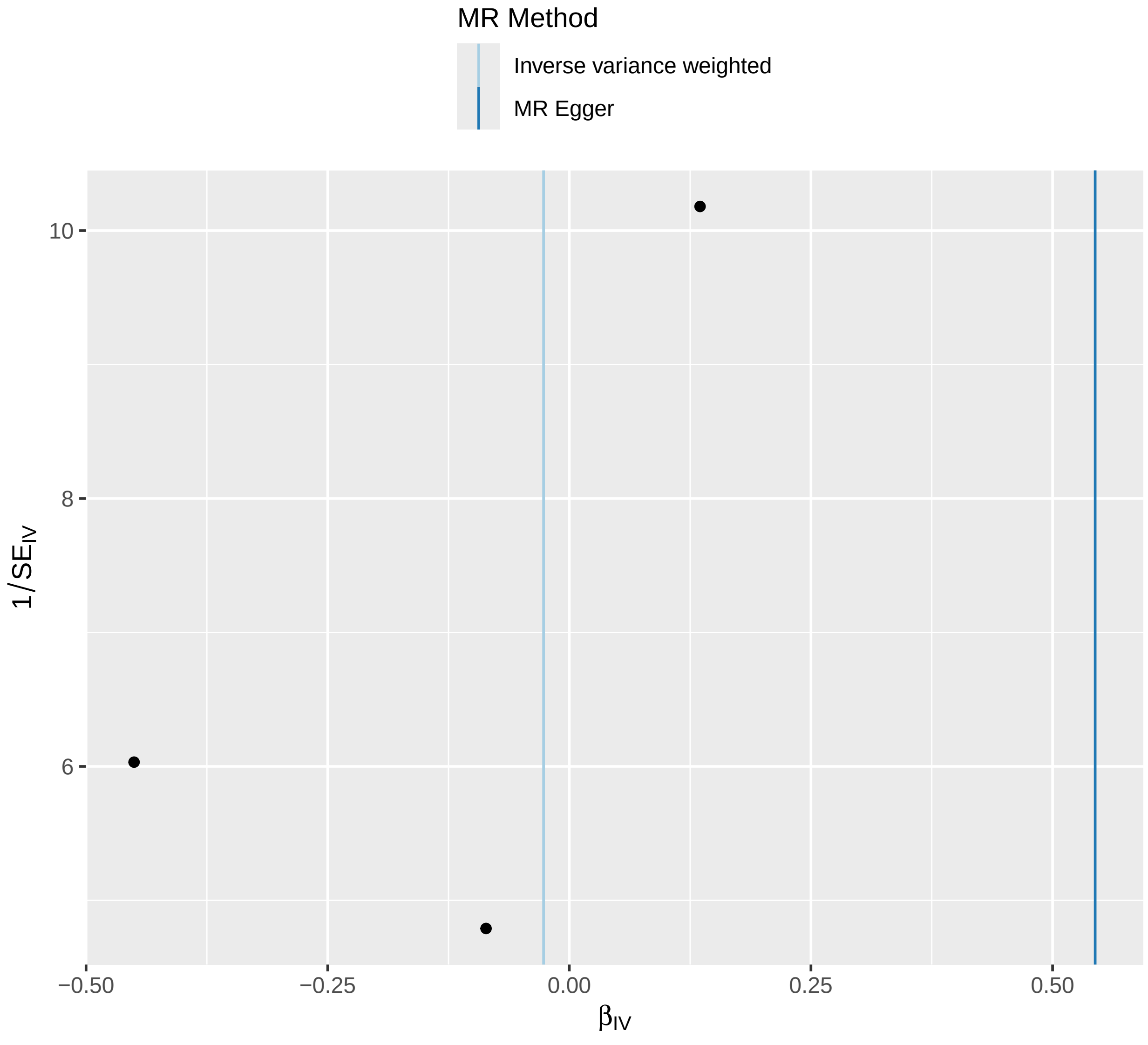
MR Method
Inverse variance weighted
MR Egger
10
8
6
1/SE_IV
−0.50
−0.25
0.00
0.25
0.50
β_IV

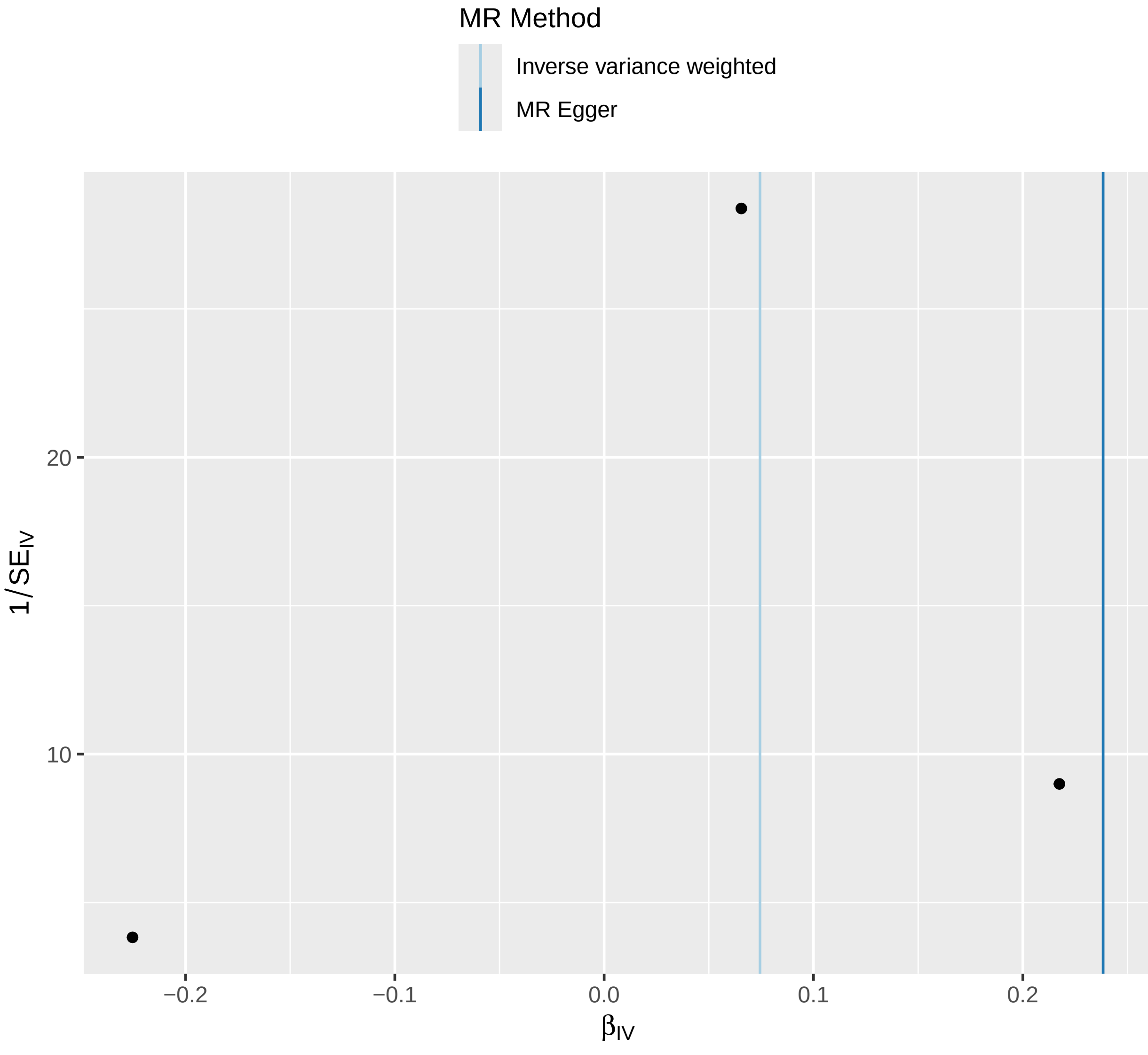
MR Method
Inverse variance weighted
MR Egger
20
10
1/SE$_{IV}$
−0.2
−0.1
0.0
0.1
0.2
β$_{IV}$

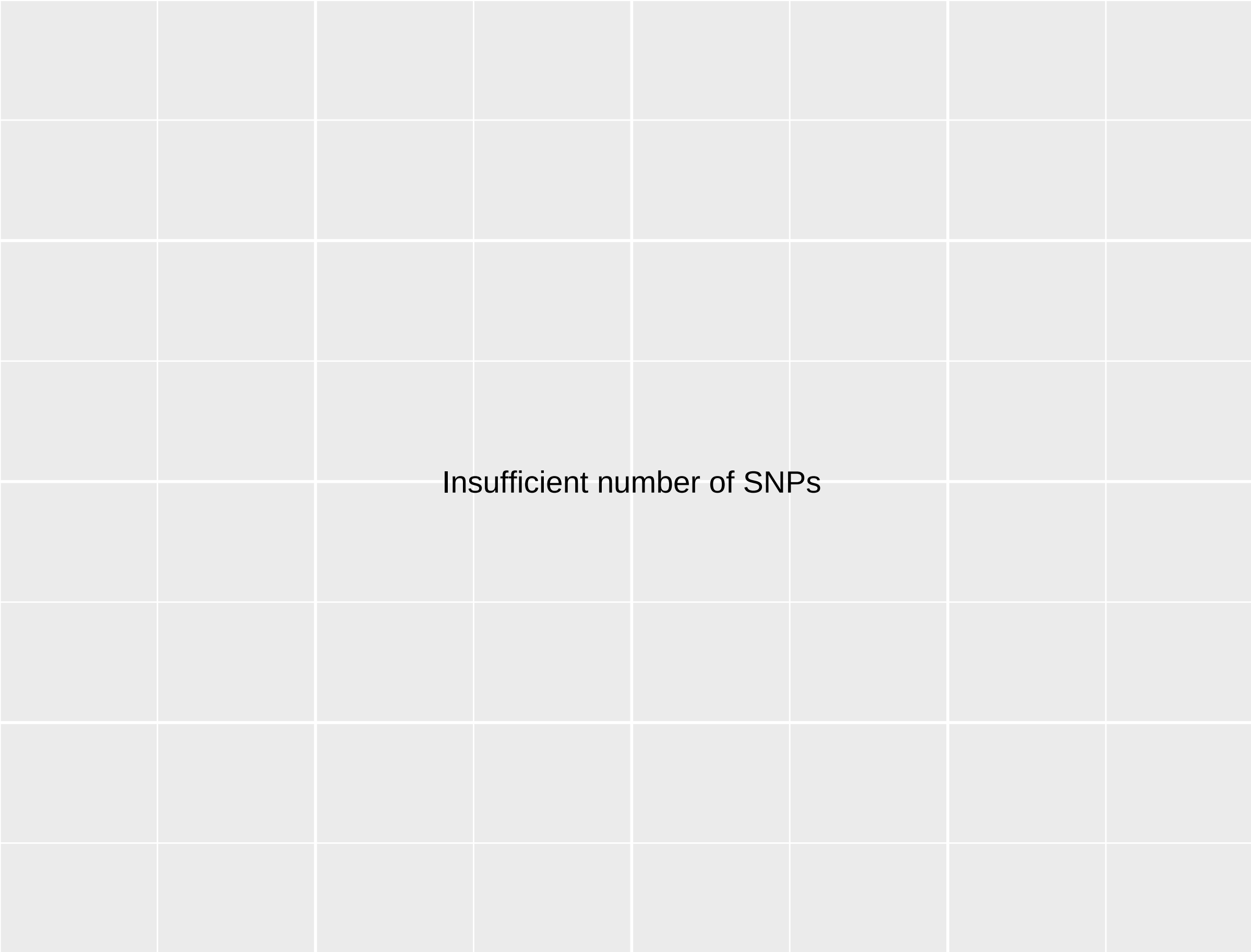
Insufficient number of SNPs

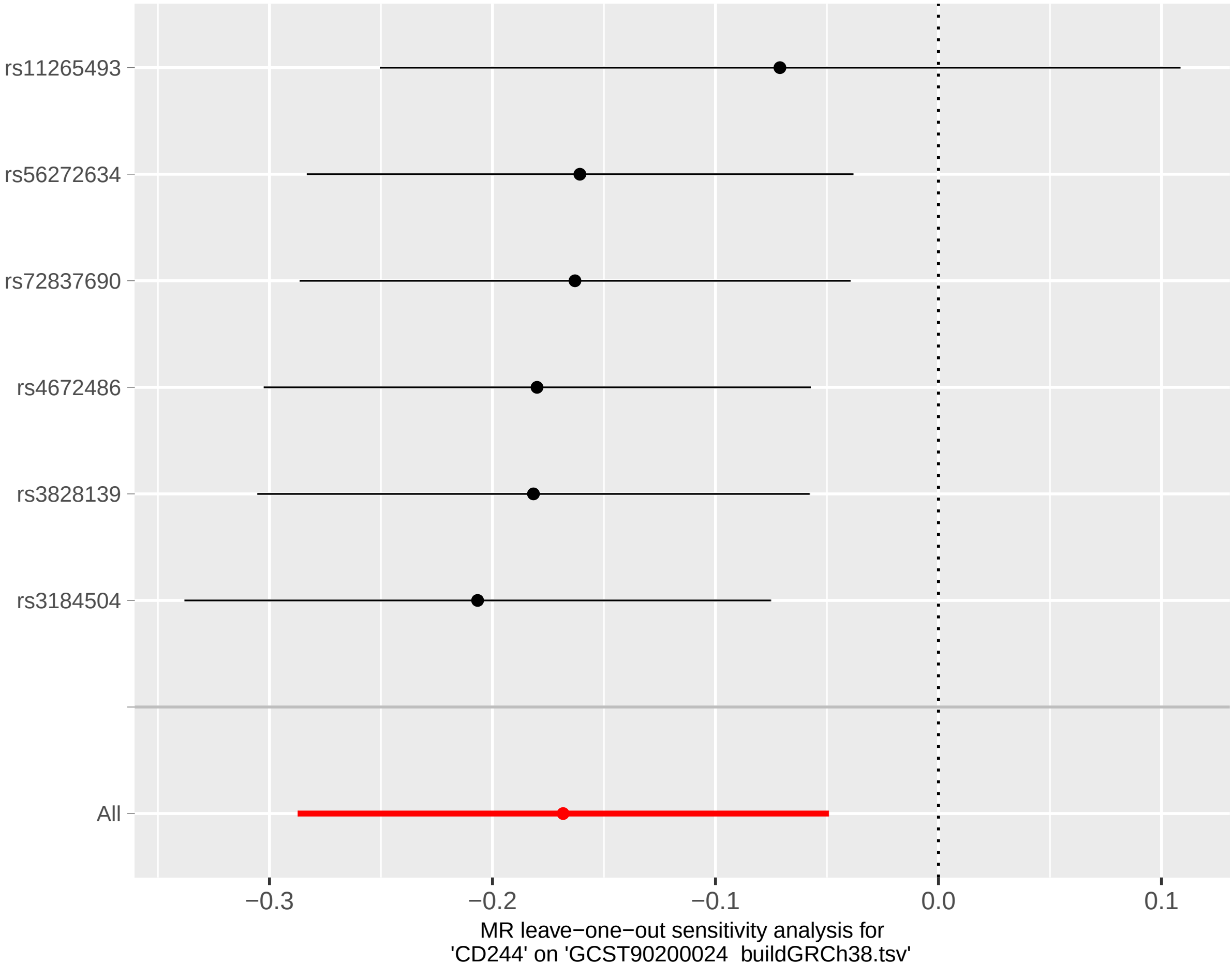
rs11265493
rs56272634
rs72837690
rs4672486
rs3828139
rs3184504
All
−0.3
−0.2
−0.1
0.0
0.1
MR leave−one−out sensitivity analysis for
'CD244' on 'GCST90200024_buildGRCh38.tsv'

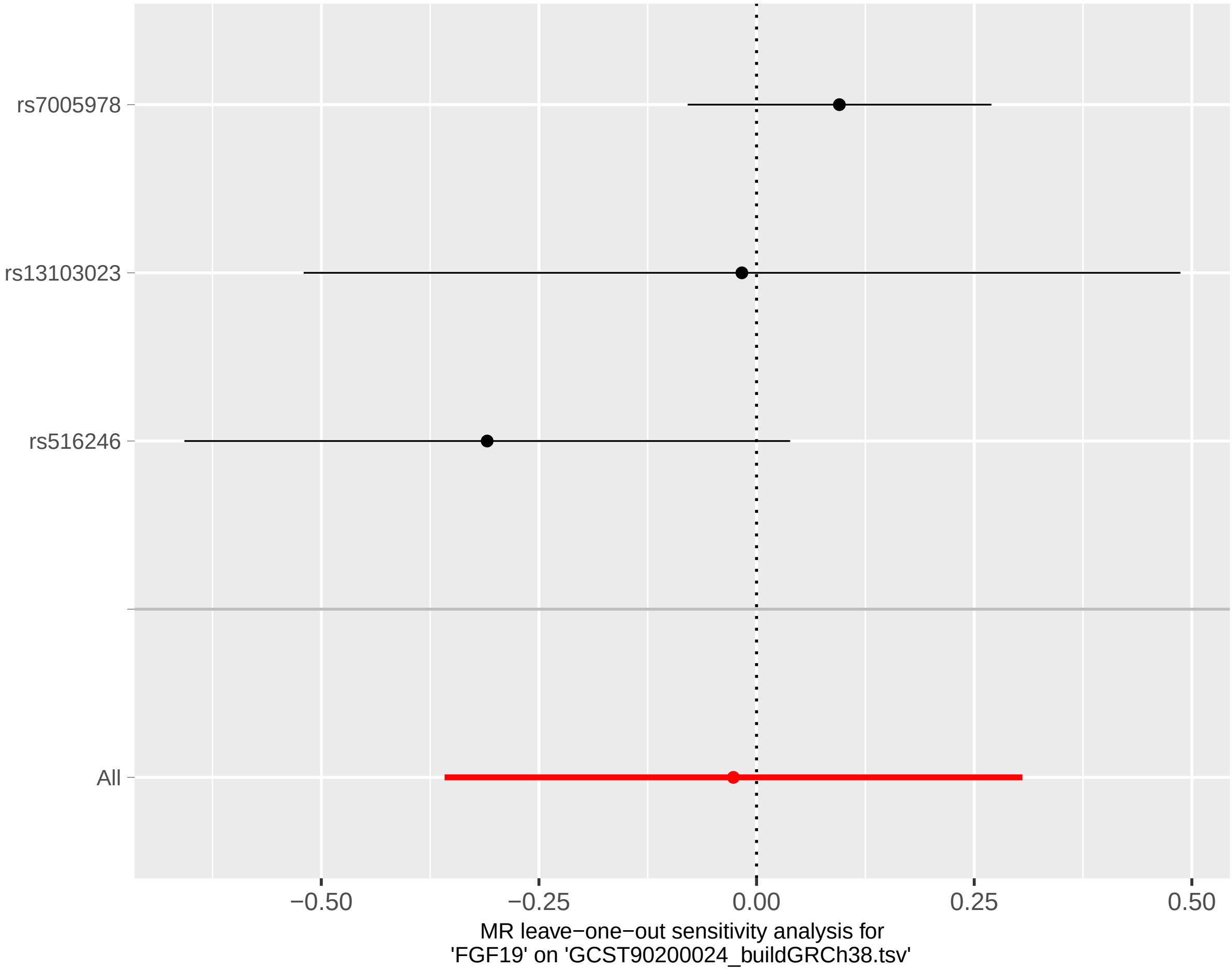
rs7005978
rs13103023
rs516246
All
−0.50
−0.25
0.00
0.25
0.50
MR leave−one−out sensitivity analysis for
'FGF19' on 'GCST90200024_buildGRCh38.tsv'

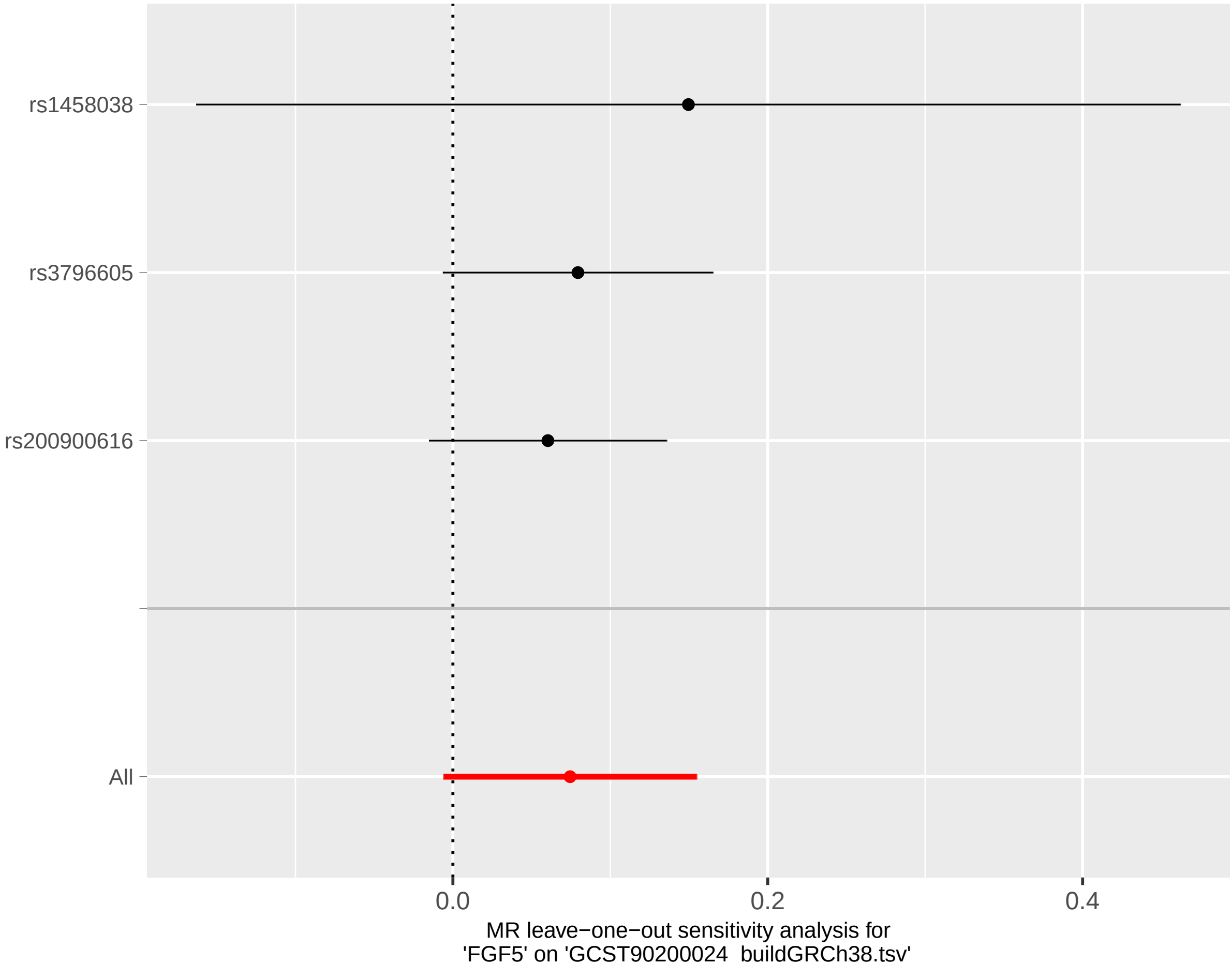
rs1458038
rs3796605
rs200900616
All
0.0
0.2
0.4
MR leave−one−out sensitivity analysis for
'FGF5' on 'GCST90200024_buildGRCh38.tsv'

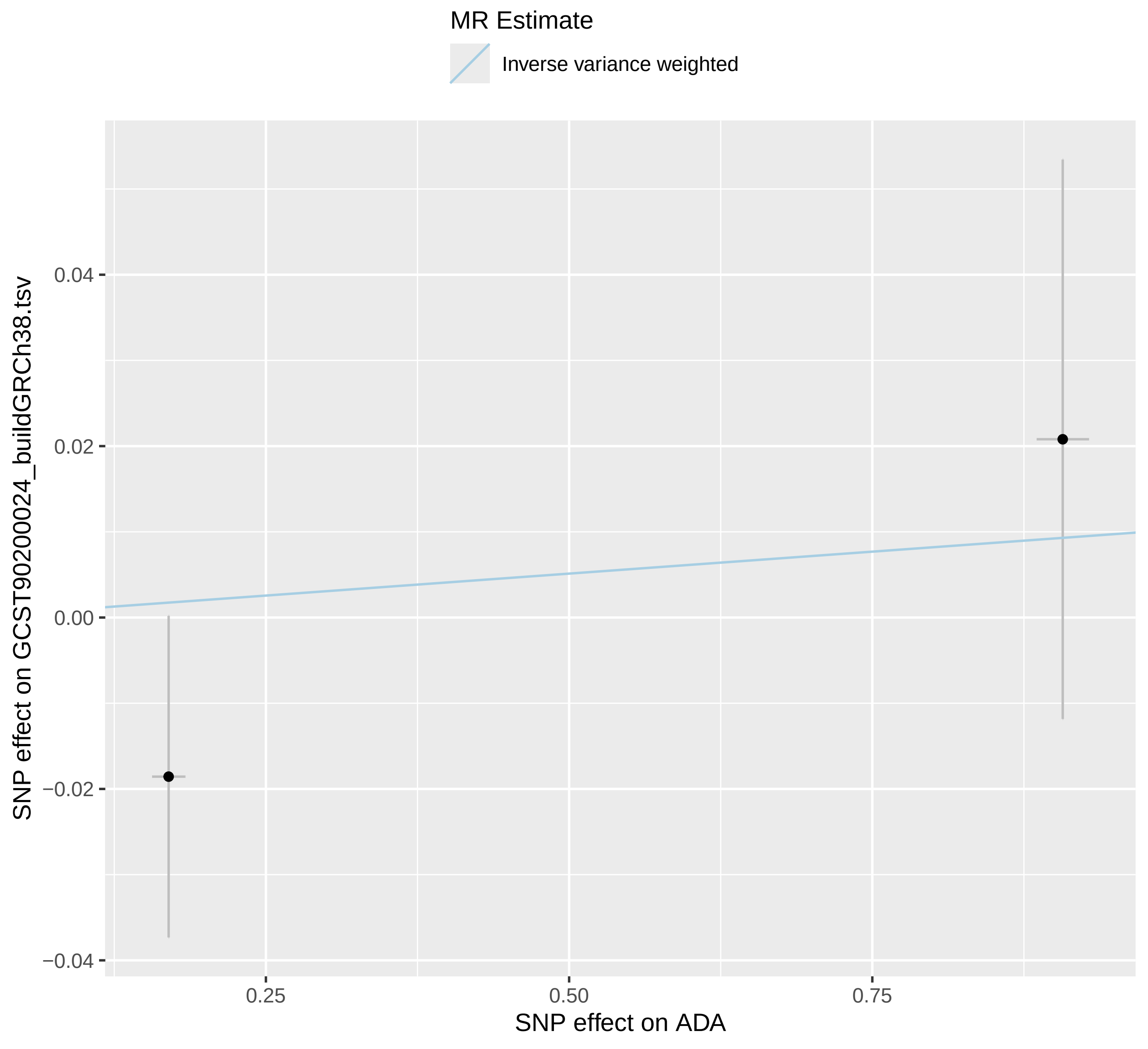

MR Estimate
Inverse variance weighted
0.04
0.02
0.00
−0.02
−0.04
SNP effect on GCST90200024_buildGRCh38.tsv
0.25
0.50
0.75
SNP effect on ADA

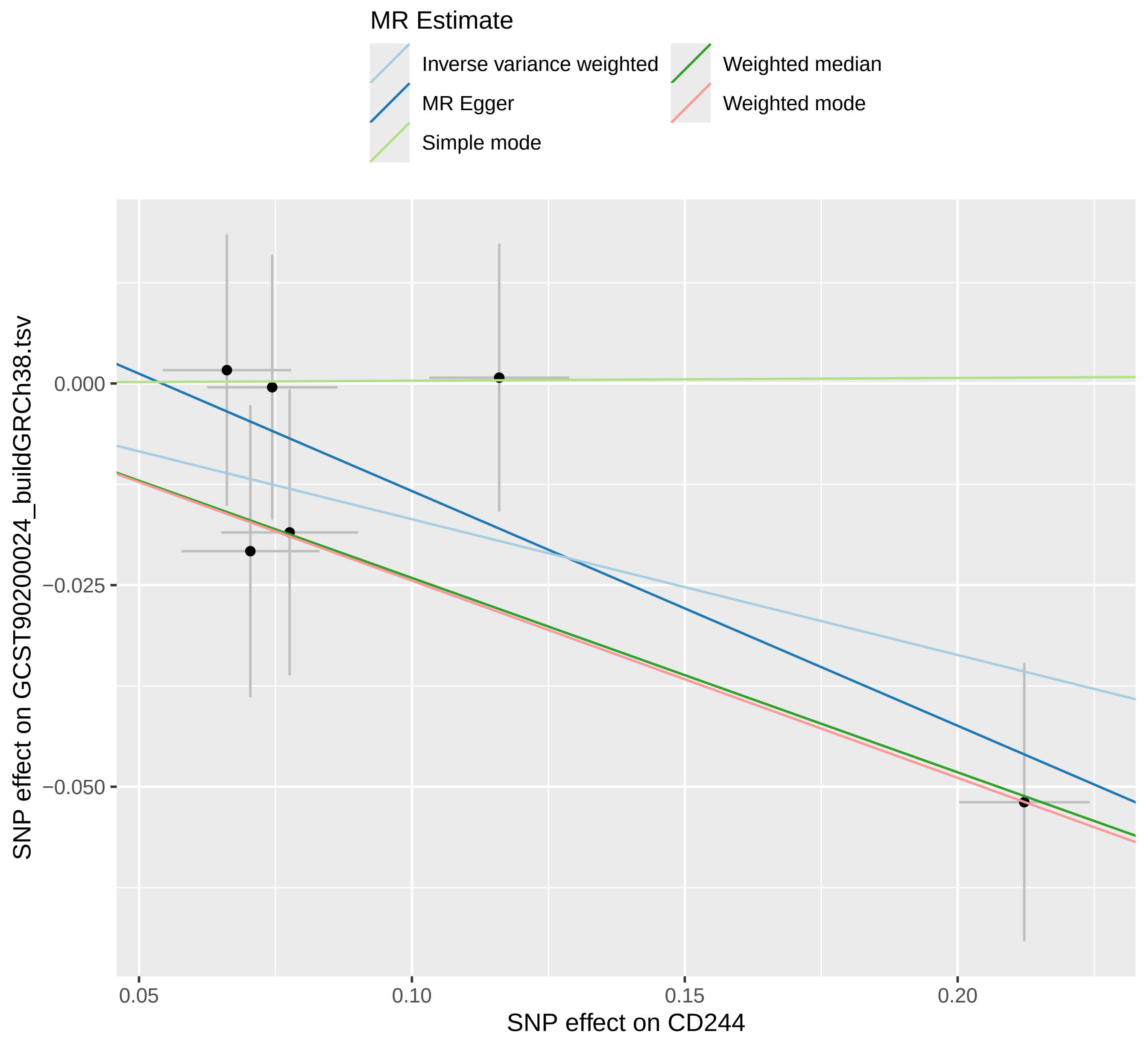
MR Estimate
Inverse variance weighted
MR Egger
Simple mode
Weighted median
Weighted mode
SNP effect on GCST90200024_buildGRCh38.tsv
0.000
−0.025
−0.050
0.05
0.10
0.15
0.20
SNP effect on CD244

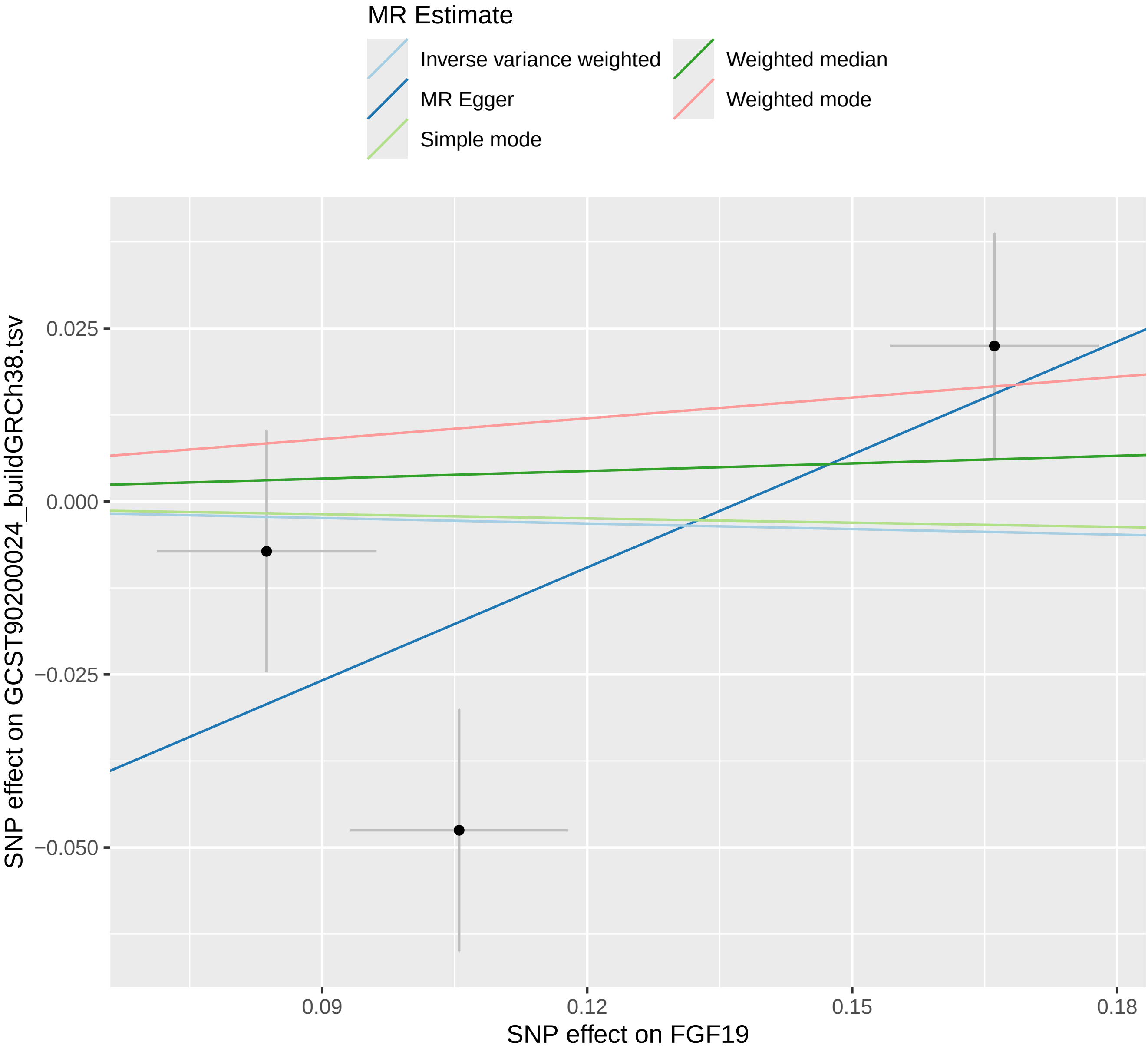
MR Estimate
Inverse variance weighted
MR Egger
Simple mode
Weighted median
Weighted mode
0.025
0.000
−0.025
−0.050
0.09
0.12
0.15
0.18
SNP effect on FGF19
SNP effect on GCST90200024_buildGRCh38.tsv

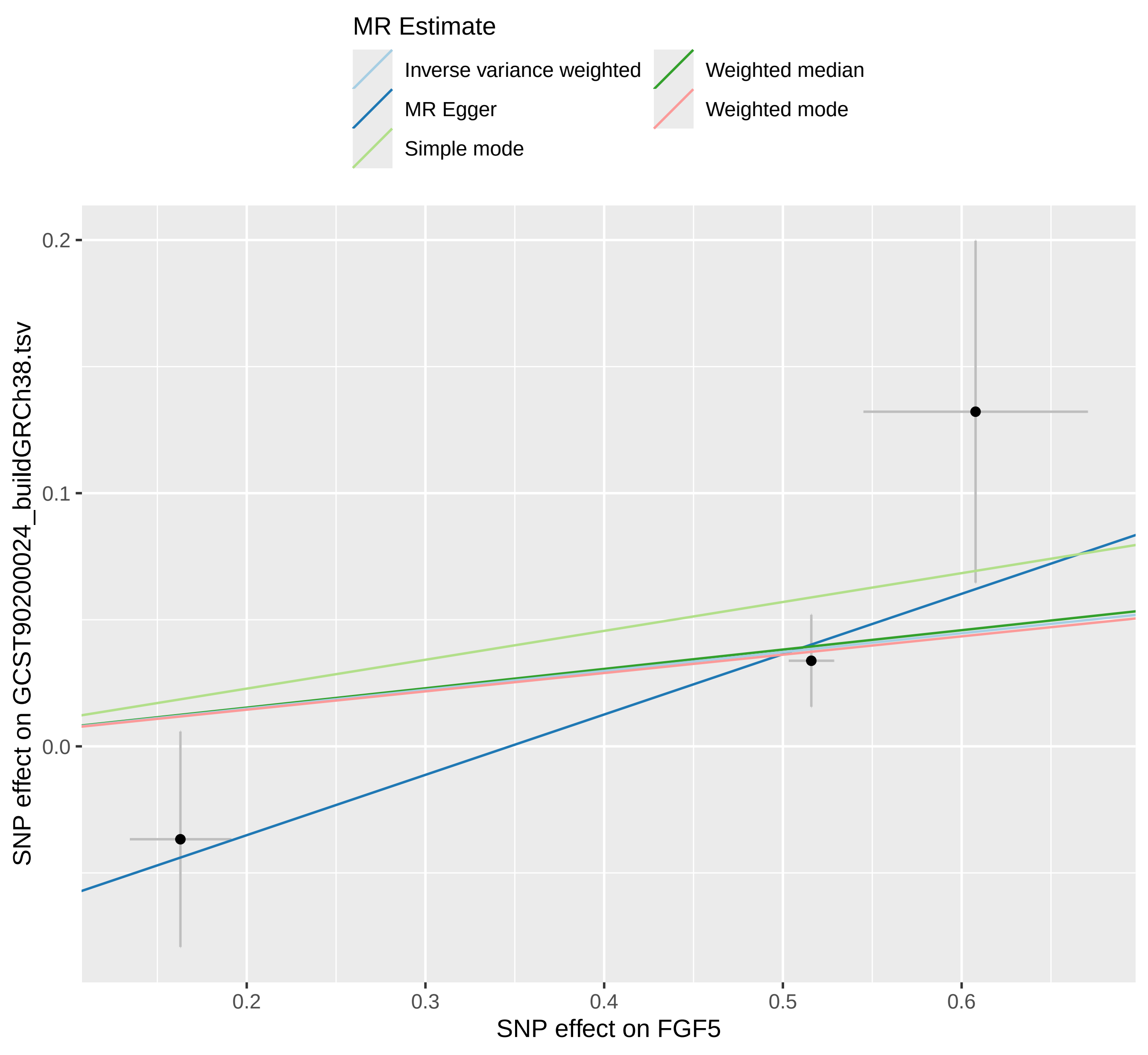
MR Estimate
Inverse variance weighted
MR Egger
Simple mode
Weighted median
Weighted mode
0.2
0.1
0.0
0.2
0.3
0.4
0.5
0.6
SNP effect on GCST90200024_buildGRCh38.tsv
SNP effect on FGF5

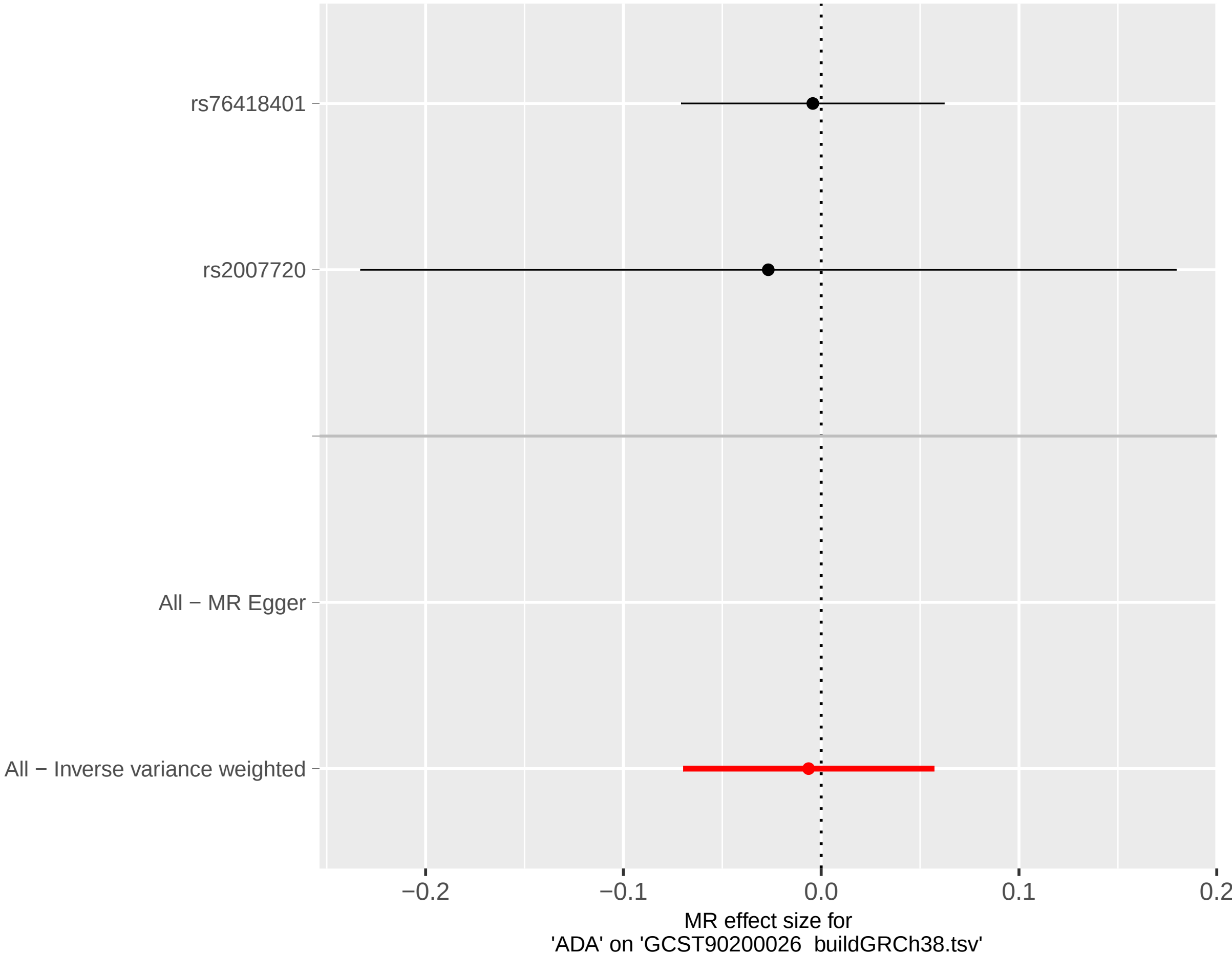
rs76418401
rs2007720
All − MR Egger
All − Inverse variance weighted
−0.2
−0.1
0.0
0.1
0.2
MR effect size for
'ADA' on 'GCST90200026_buildGRCh38.tsv'

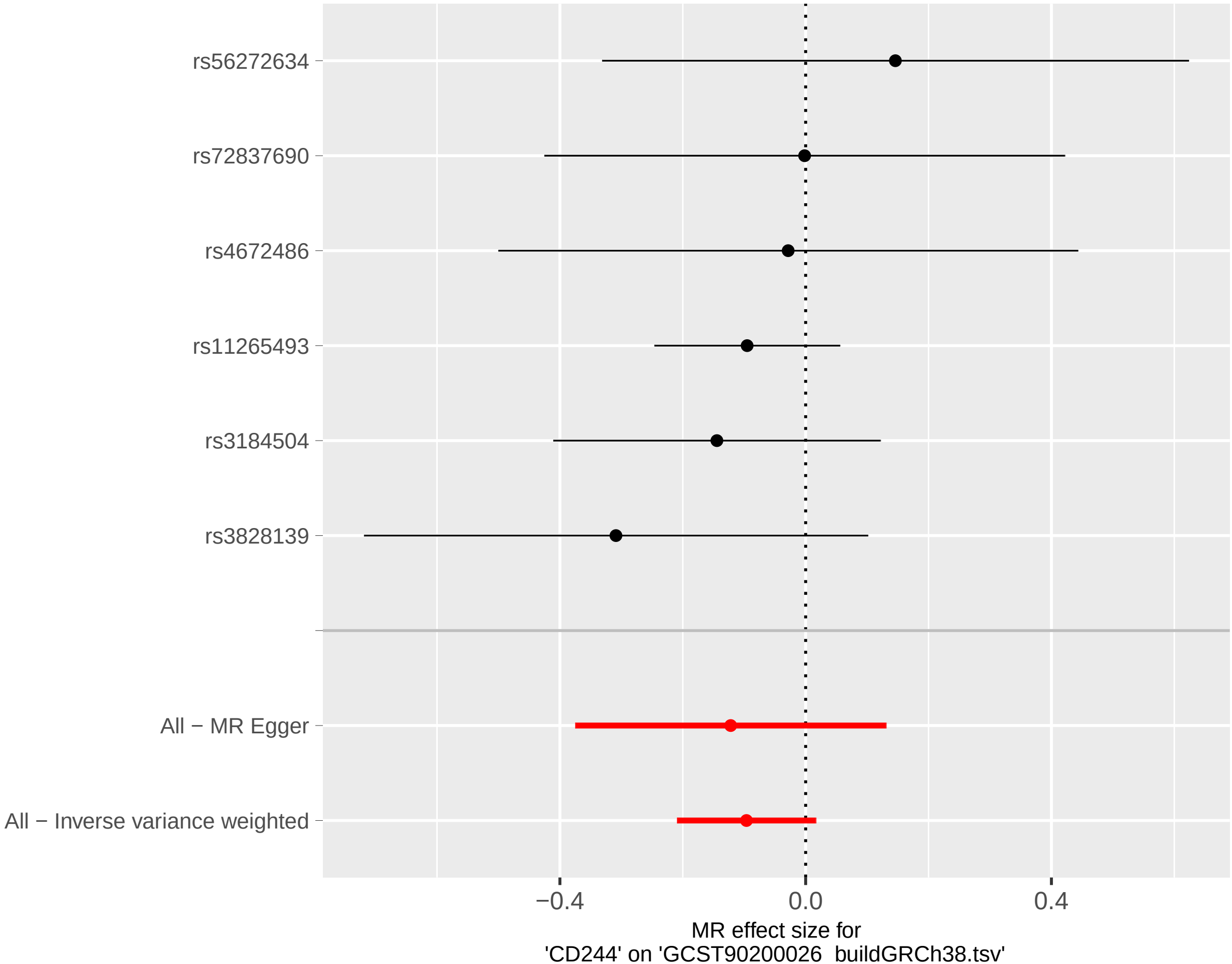
rs56272634
rs72837690
rs4672486
rs11265493
rs3184504
rs3828139
All − MR Egger
All − Inverse variance weighted
−0.4
0.0
0.4
MR effect size for
'CD244' on 'GCST90200026_buildGRCh38.tsv'

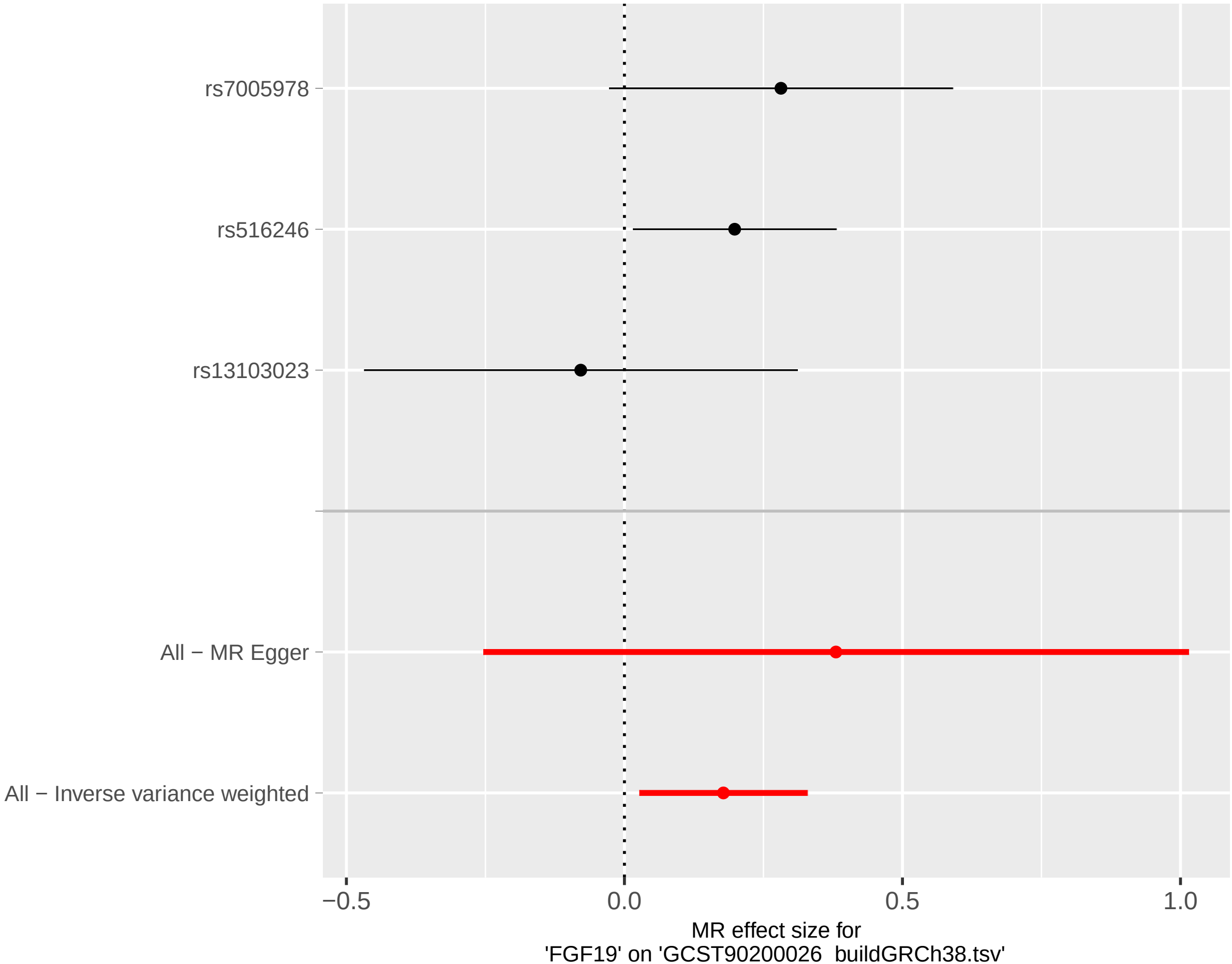
rs7005978
rs516246
rs13103023
All − MR Egger
All − Inverse variance weighted
−0.5
0.0
0.5
1.0
MR effect size for
'FGF19' on 'GCST90200026_buildGRCh38.tsv'

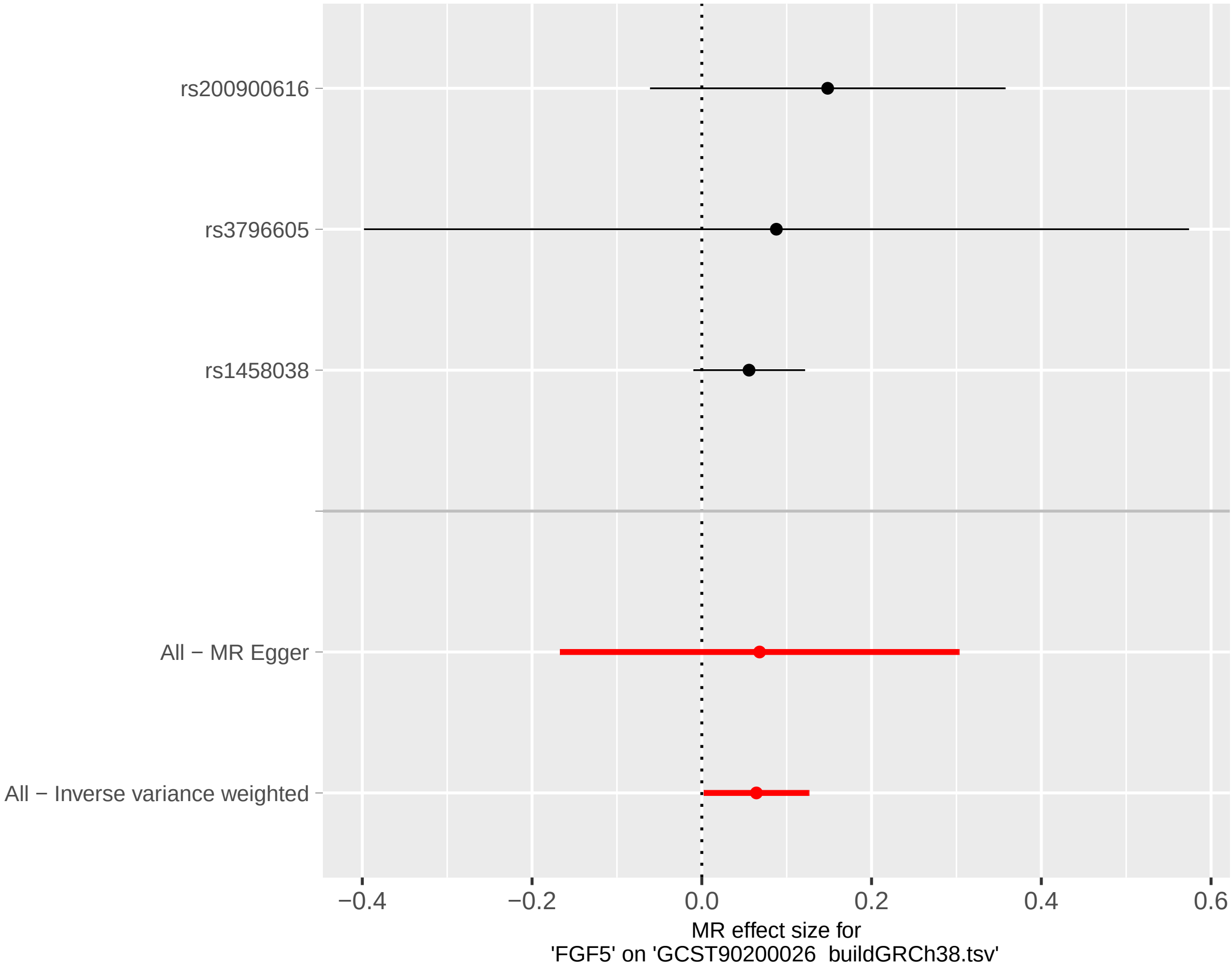

rs200900616
rs3796605
rs1458038
All − MR Egger
All − Inverse variance weighted
−0.4
−0.2
0.0
0.2
0.4
0.6
MR effect size for
'FGF5' on 'GCST90200026_buildGRCh38.tsv'

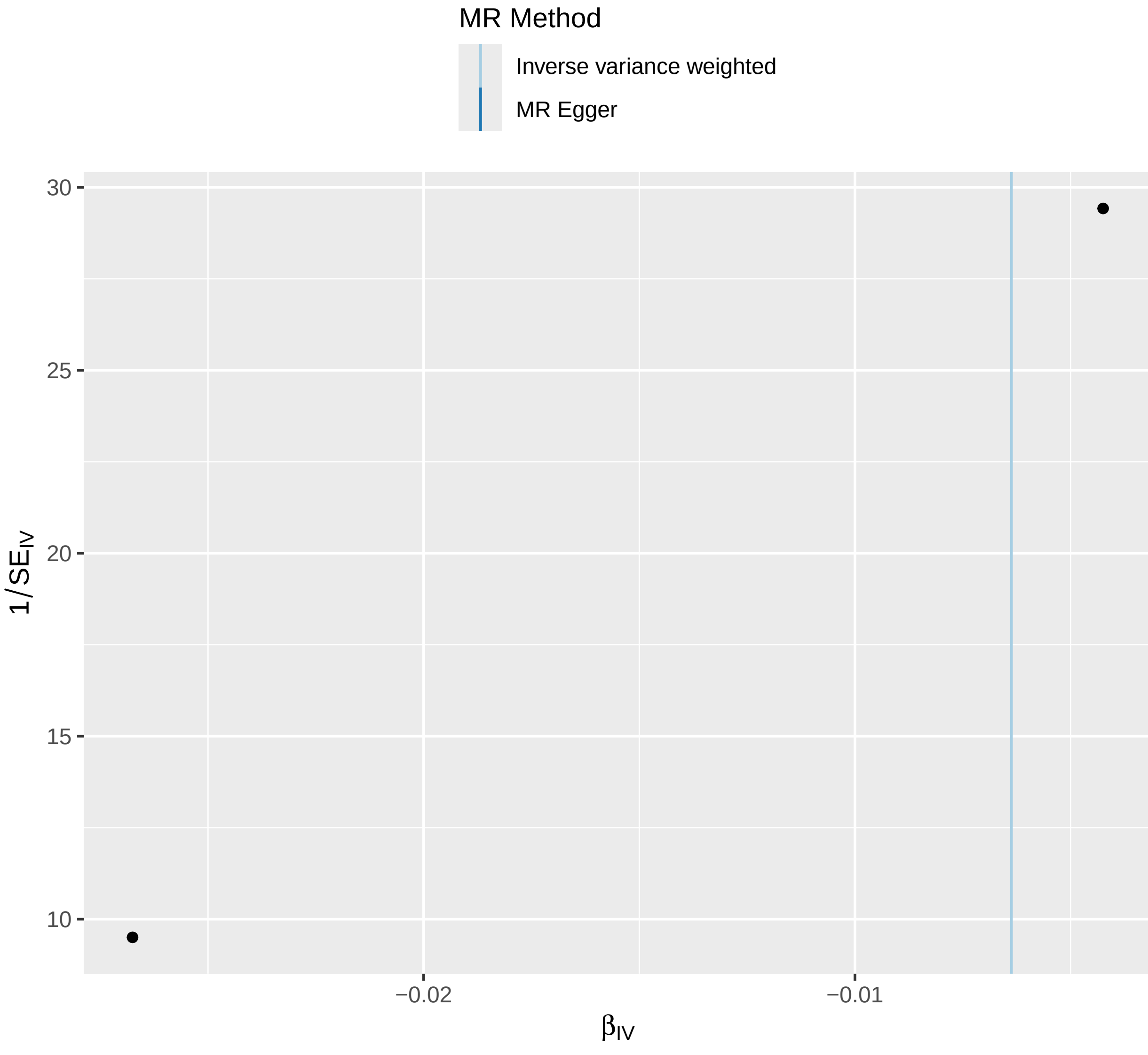
MR Method
Inverse variance weighted
MR Egger
30
25
20
15
10
1/SE_IV
−0.02
−0.01
β_IV

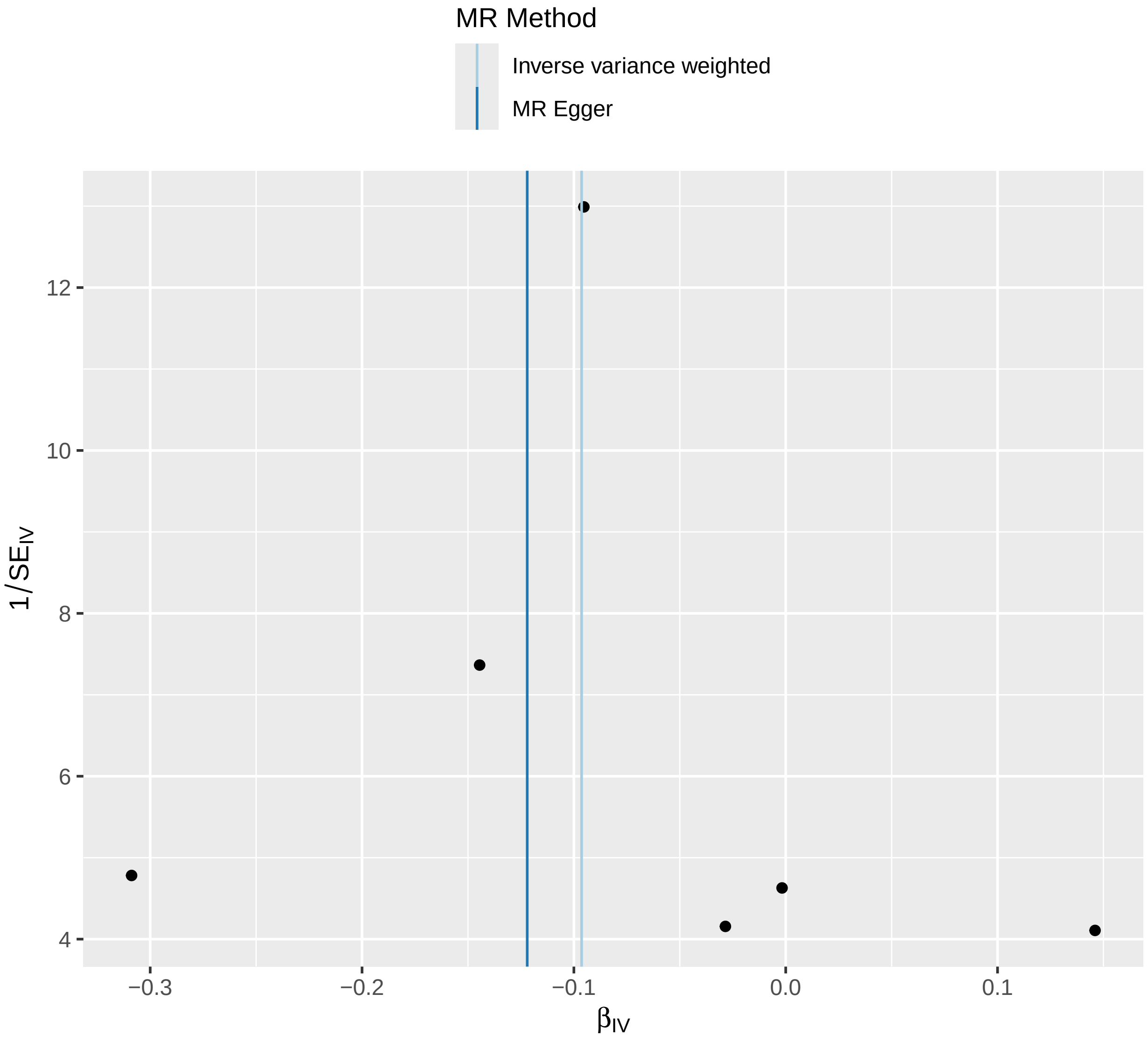
MR Method
Inverse variance weighted
MR Egger
12
10
8
6
4
1/SE_IV
−0.3
−0.2
−0.1
0.0
0.1
β_IV

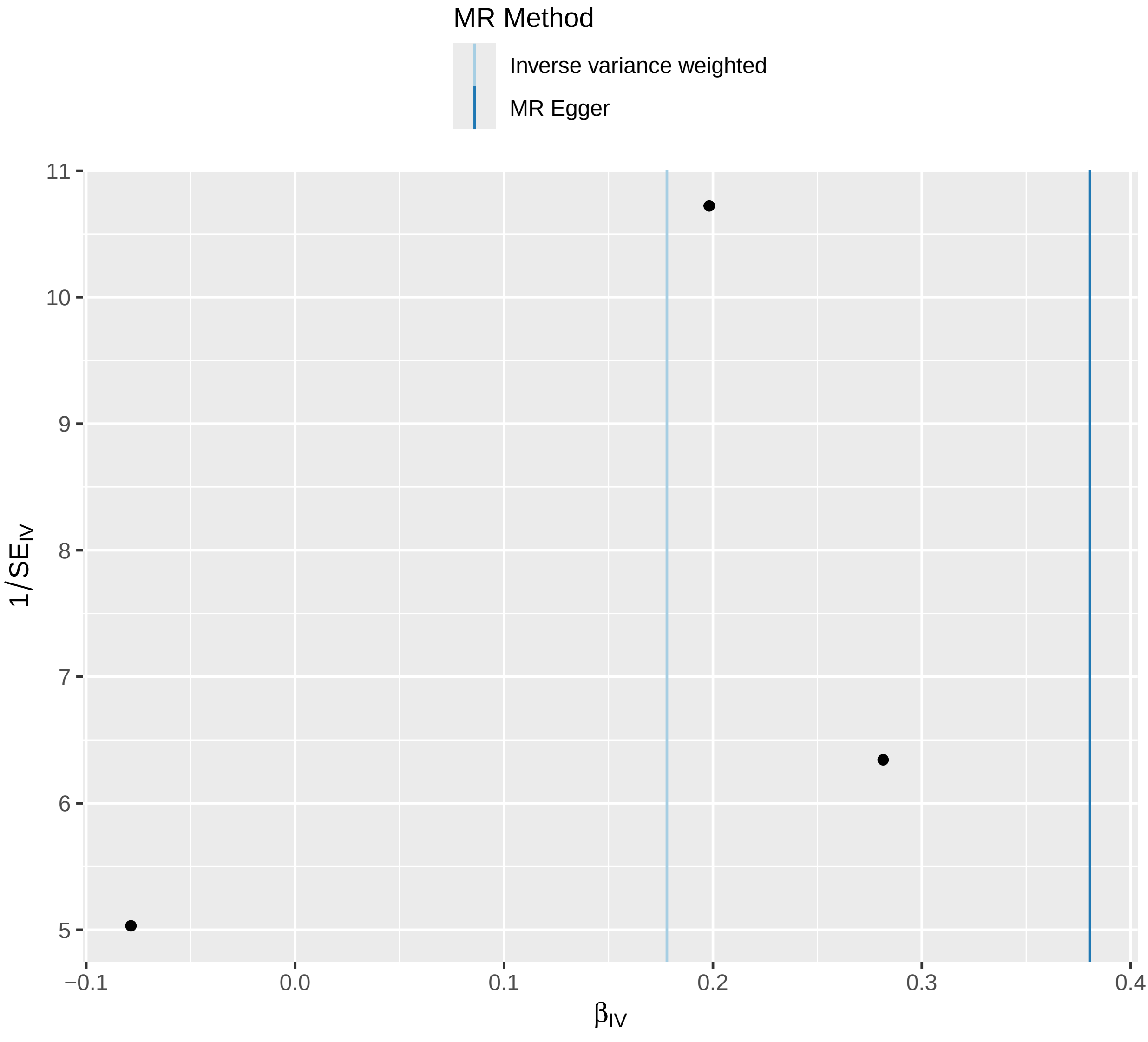

MR Method
Inverse variance weighted
MR Egger
11
10
9
8
7
6
5
1/SE$_{IV}$
−0.1
0.0
0.1
0.2
0.3
0.4
β$_{IV}$

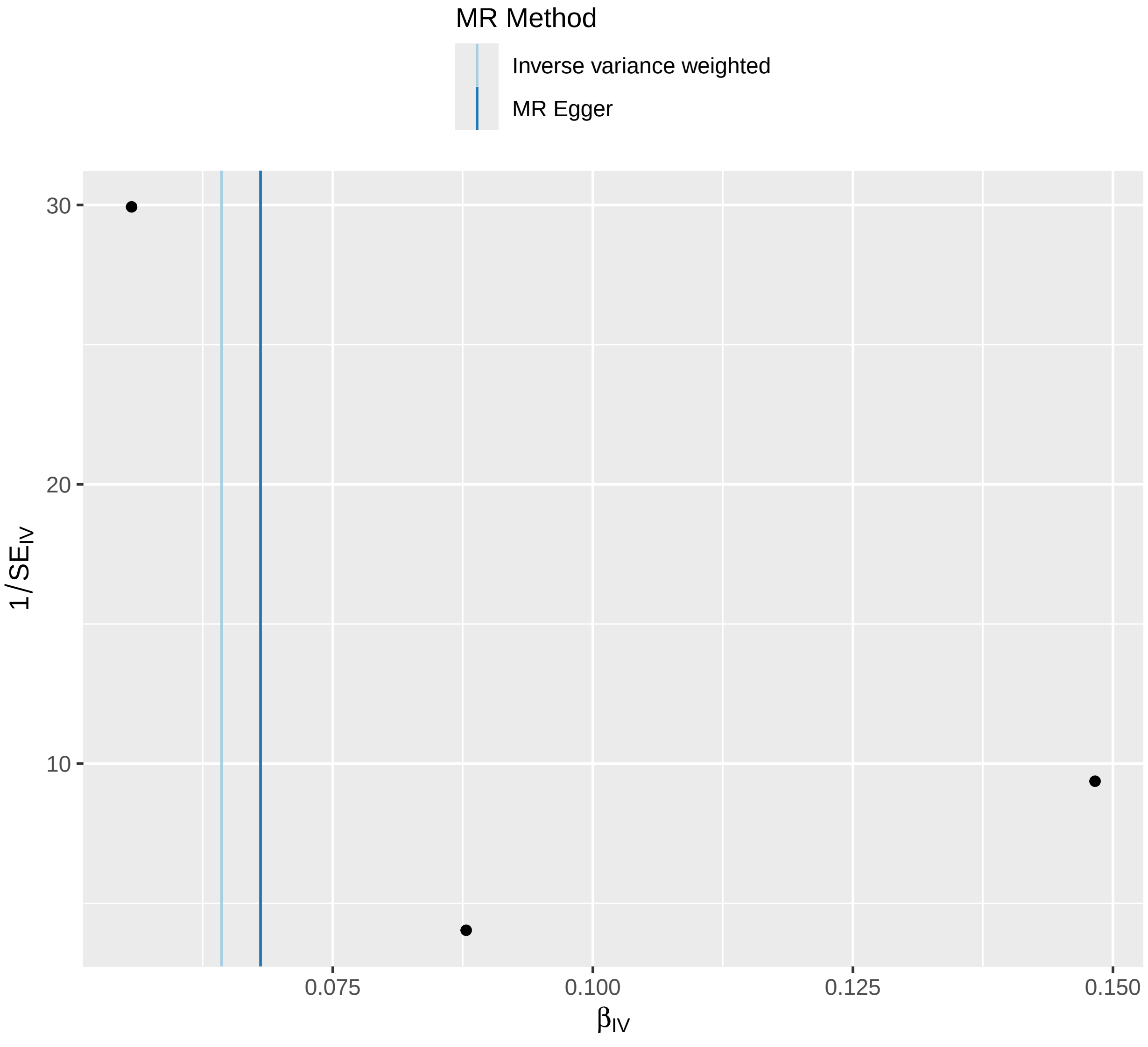
MR Method
Inverse variance weighted
MR Egger
30
20
10
1/SE_IV
0.075
0.100
0.125
0.150
β_IV

Insufficient number of SNPs

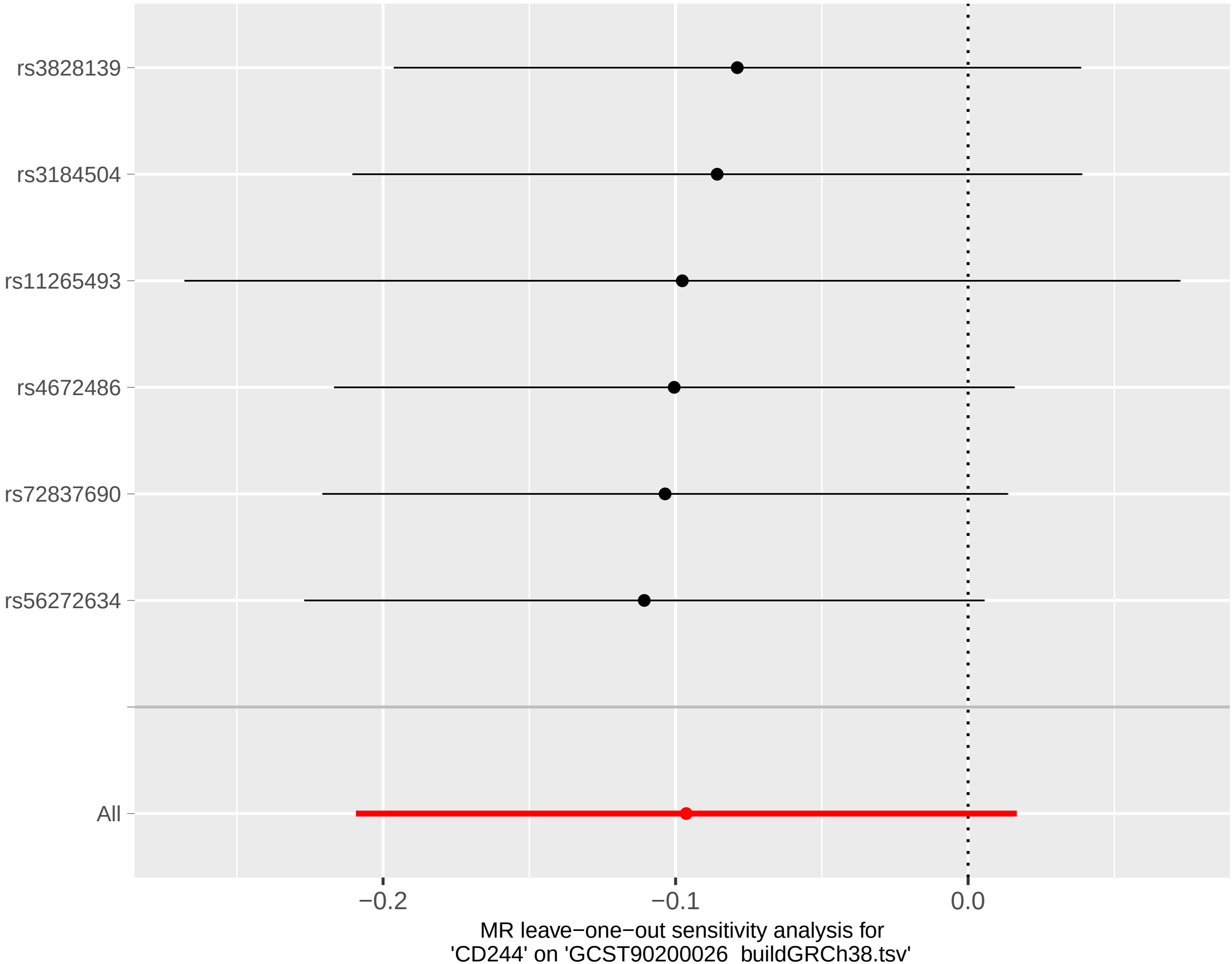

rs3828139
rs3184504
rs11265493
rs4672486
rs72837690
rs56272634
All
−0.2
−0.1
0.0
MR leave−one−out sensitivity analysis for
'CD244' on 'GCST90200026_buildGRCh38.tsv'

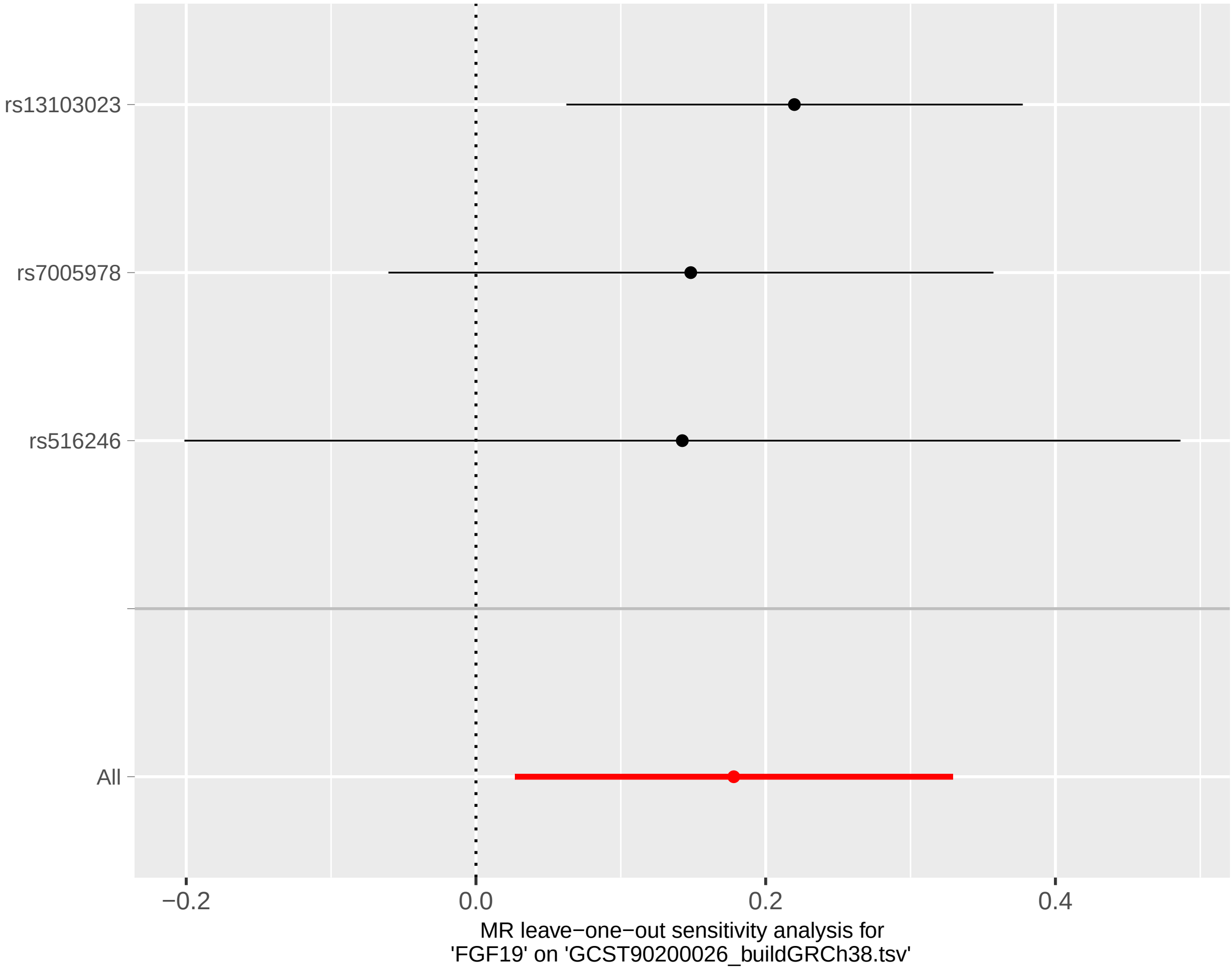
rs13103023
rs7005978
rs516246
All
−0.2
0.0
0.2
0.4
MR leave−one−out sensitivity analysis for
'FGF19' on 'GCST90200026_buildGRCh38.tsv'

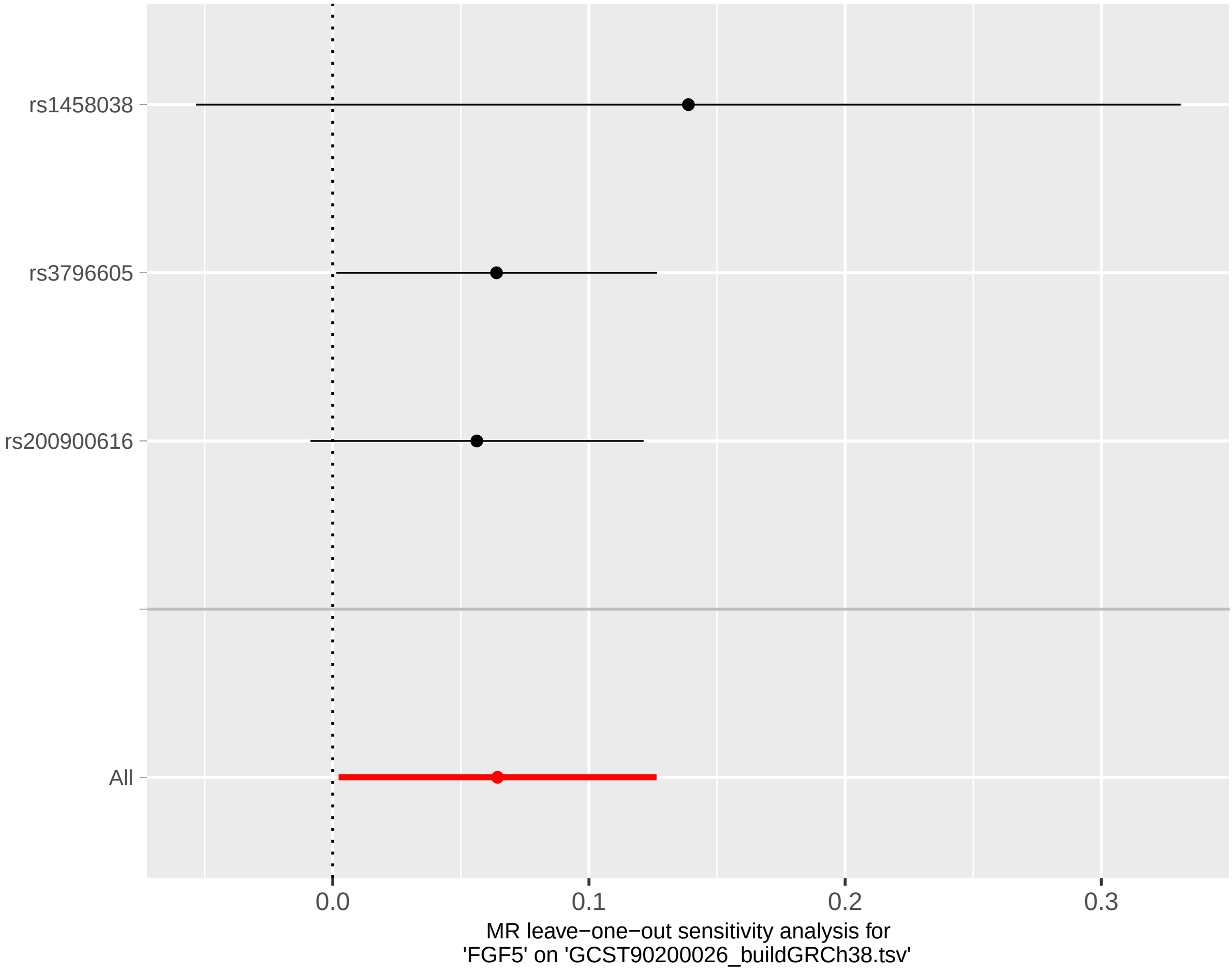

rs1458038
rs3796605
rs200900616
All
0.0
0.1
0.2
0.3
MR leave−one−out sensitivity analysis for
'FGF5' on 'GCST90200026_buildGRCh38.tsv'

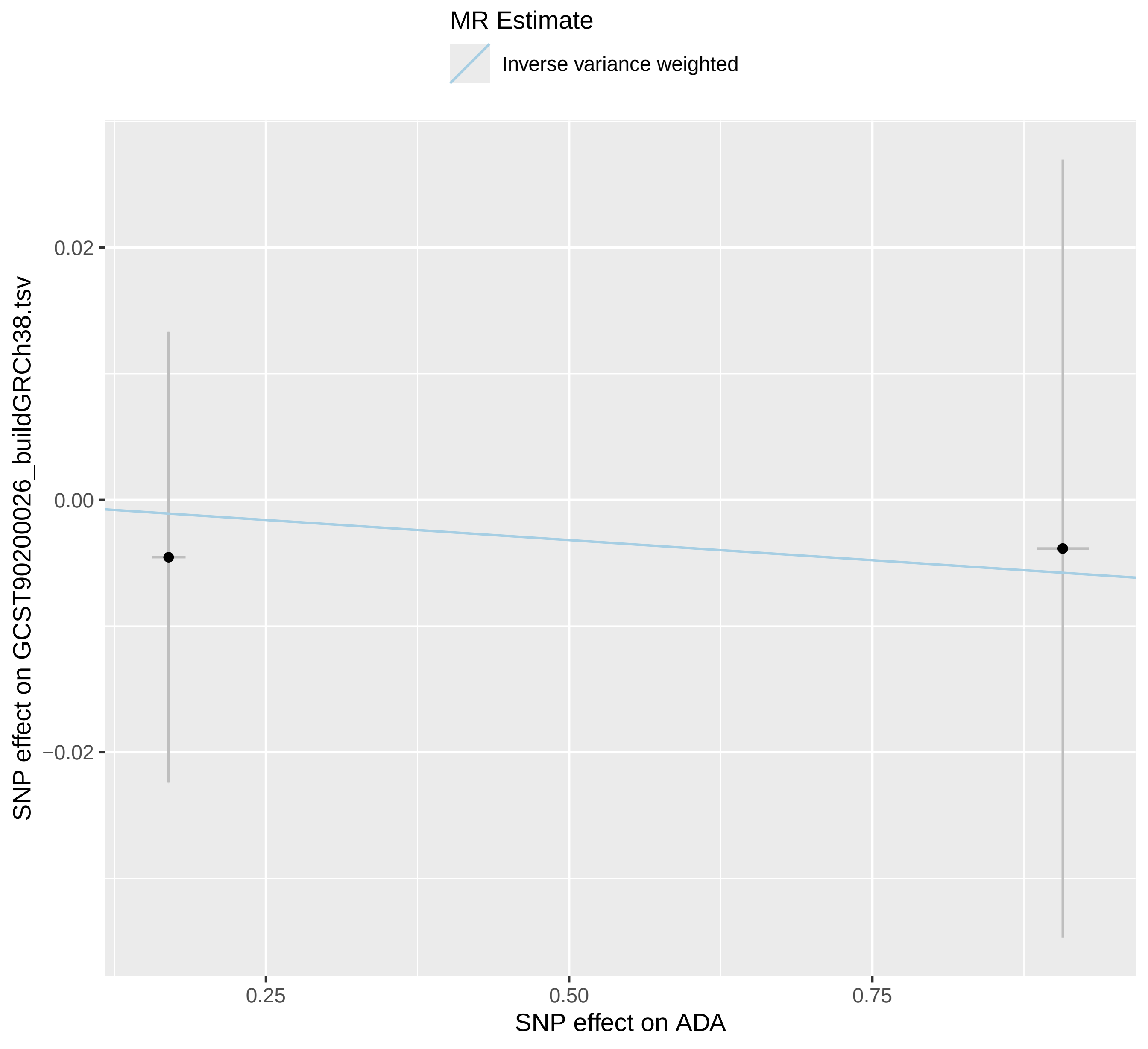
MR Estimate
Inverse variance weighted
0.02
0.00
−0.02
SNP effect on GCST90200026_buildGRCh38.tsv
0.25
0.50
0.75
SNP effect on ADA

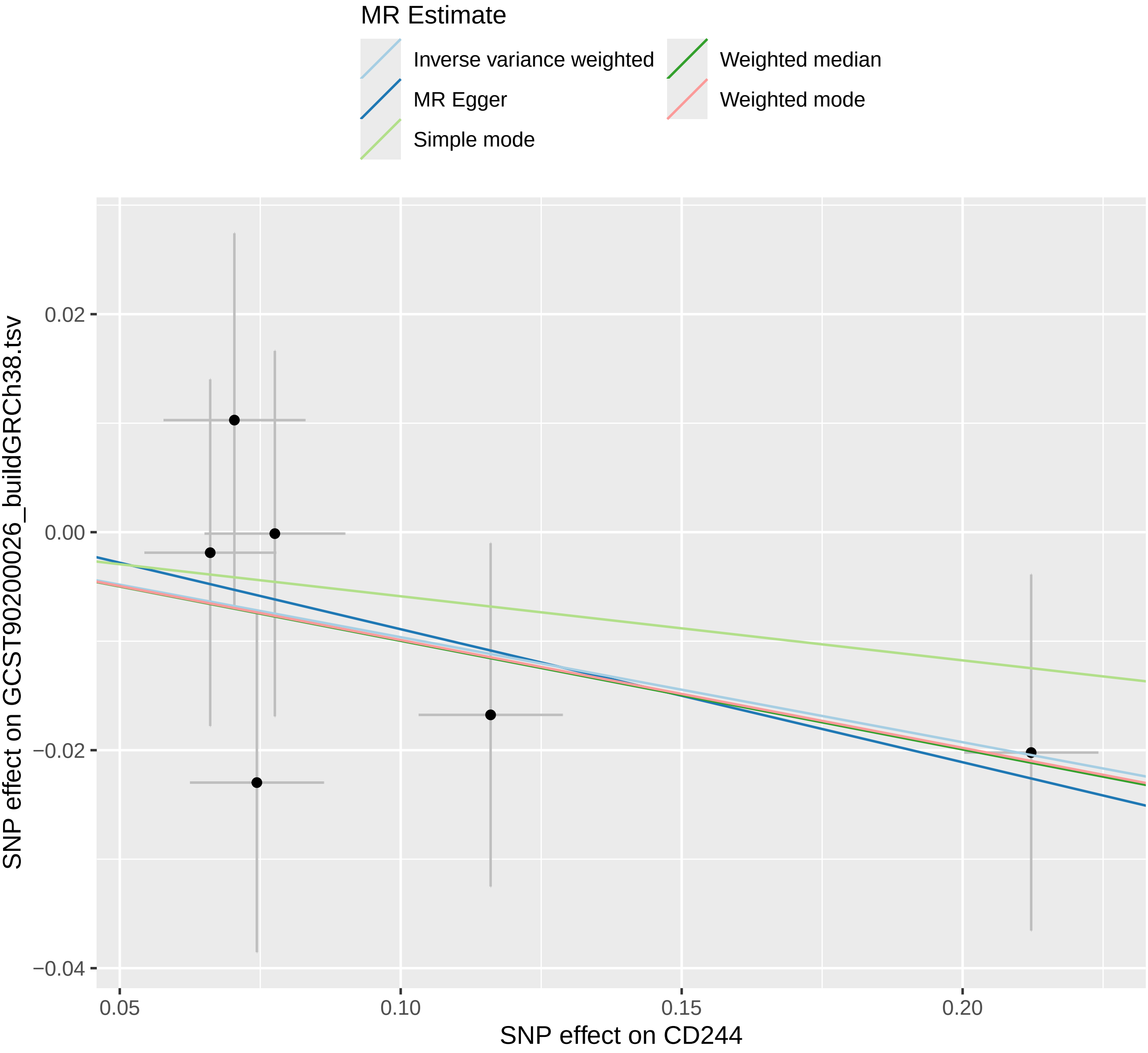
MR Estimate
Inverse variance weighted
MR Egger
Simple mode
Weighted median
Weighted mode
0.02
0.00
−0.02
−0.04
0.05
0.10
0.15
0.20
SNP effect on GCST90200026_buildGRCh38.tsv
SNP effect on CD244

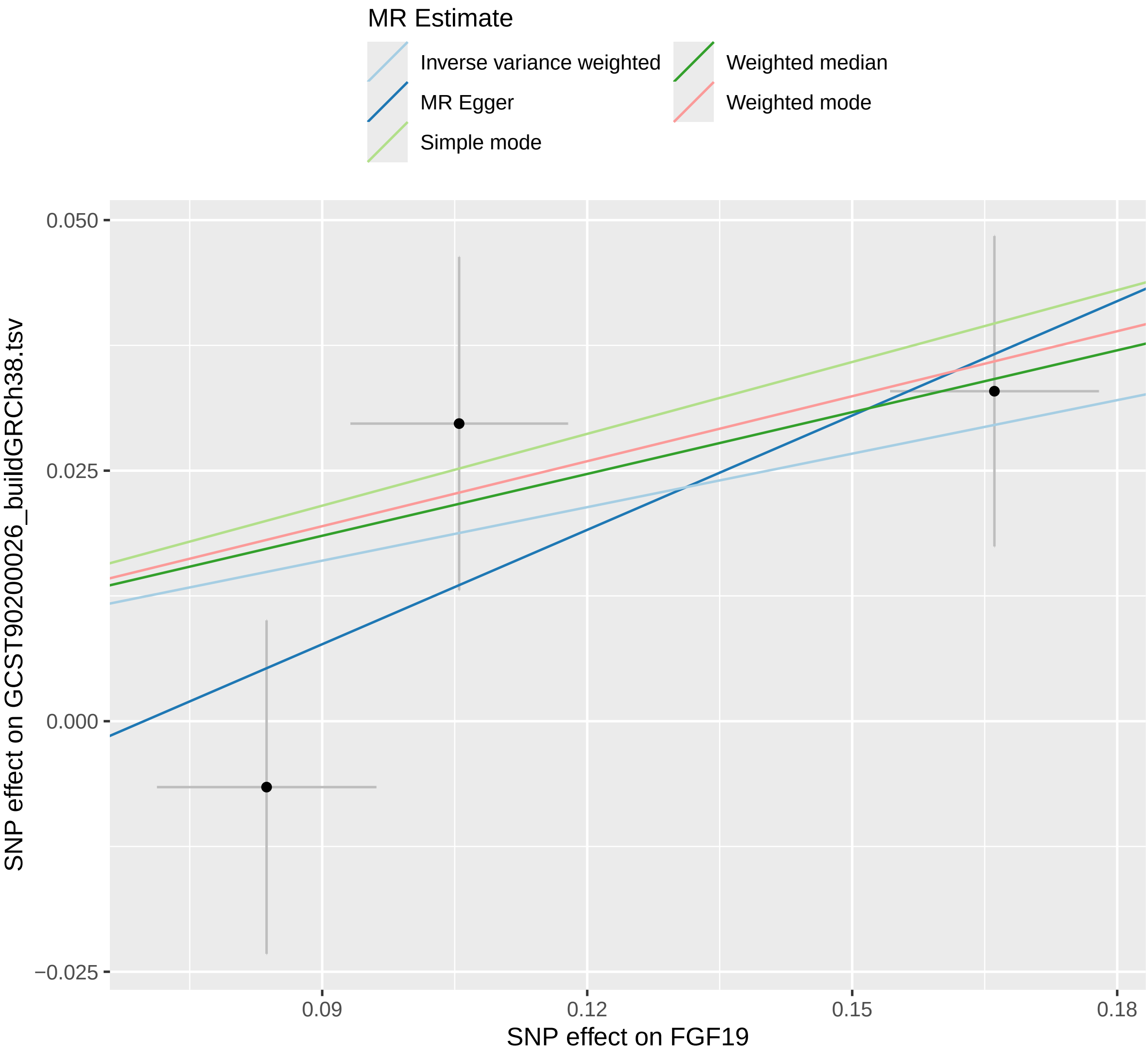

MR Estimate
Inverse variance weighted
MR Egger
Simple mode
Weighted median
Weighted mode
0.050
0.025
0.000
−0.025
0.09
0.12
0.15
0.18
SNP effect on GCST90200026_buildGRCh38.tsv
SNP effect on FGF19

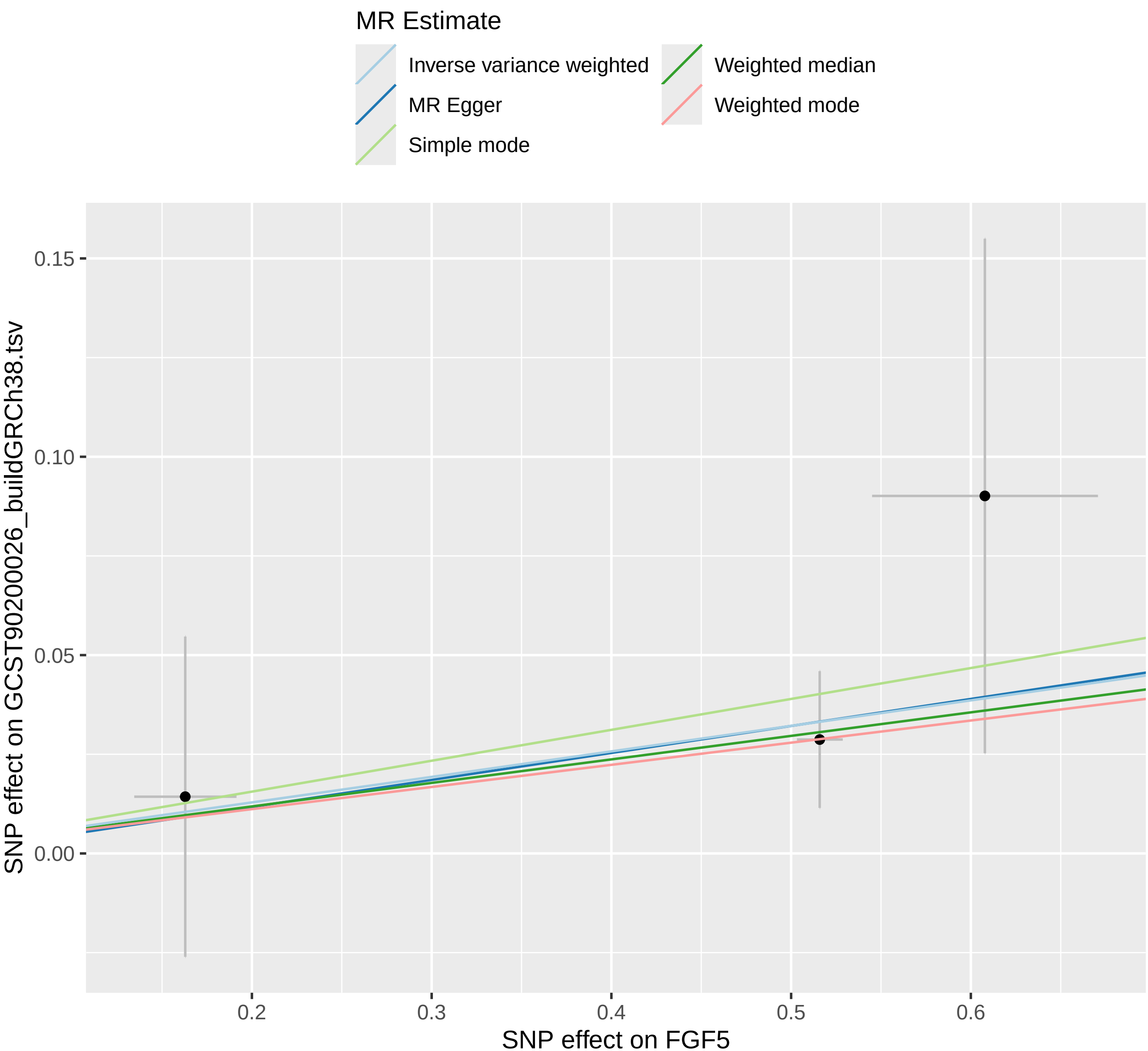
MR Estimate
Inverse variance weighted
MR Egger
Simple mode
Weighted median
Weighted mode
SNP effect on GCST90200026_buildGRCh38.tsv
0.15
0.10
0.05
0.00
0.2
0.3
0.4
0.5
0.6
SNP effect on FGF5

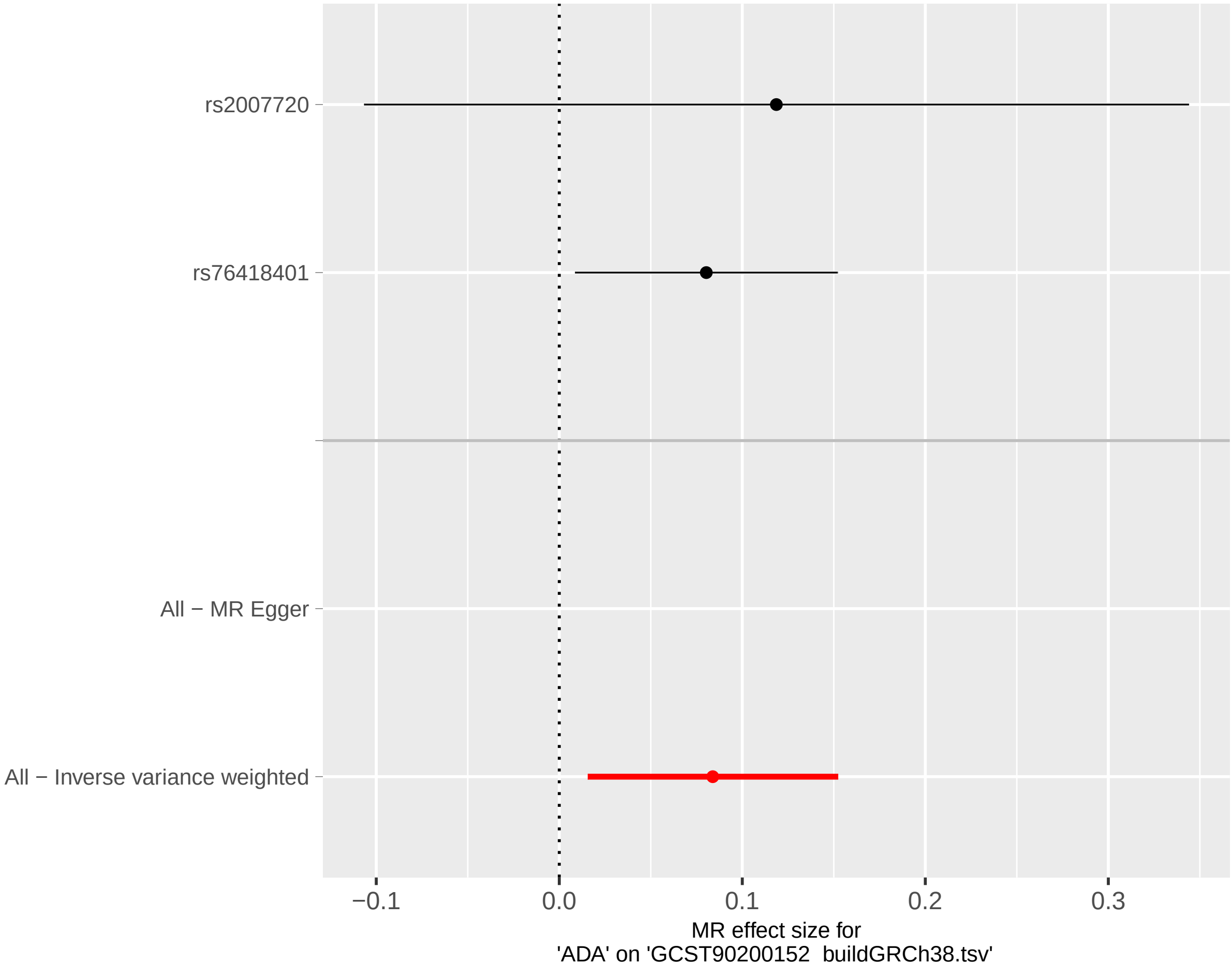
rs2007720
rs76418401
All − MR Egger
All − Inverse variance weighted
−0.1
0.0
0.1
0.2
0.3
MR effect size for
'ADA' on 'GCST90200152_buildGRCh38.tsv'

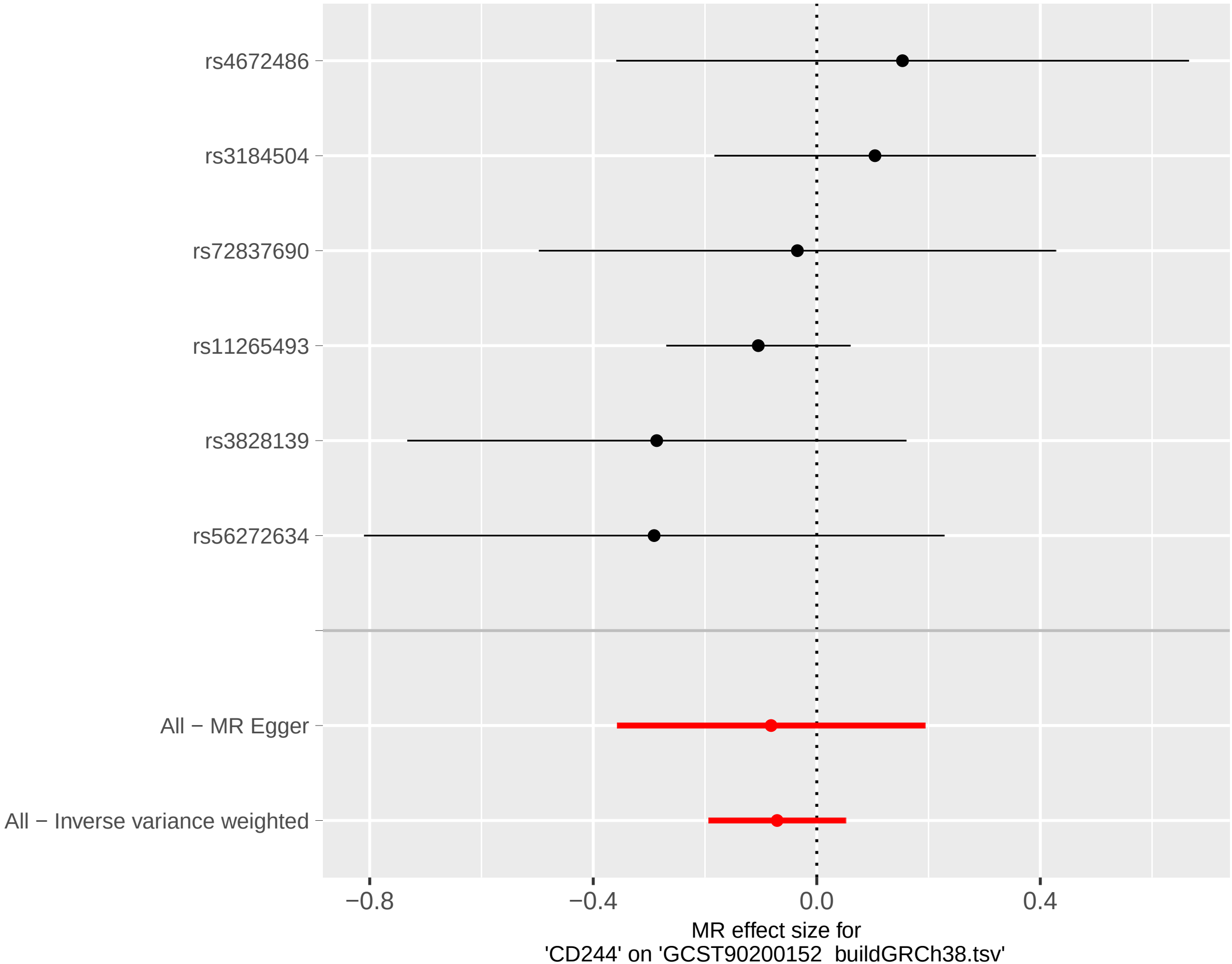
rs4672486
rs3184504
rs72837690
rs11265493
rs3828139
rs56272634
All − MR Egger
All − Inverse variance weighted
−0.8
−0.4
0.0
0.4
MR effect size for
'CD244' on 'GCST90200152_buildGRCh38.tsv'

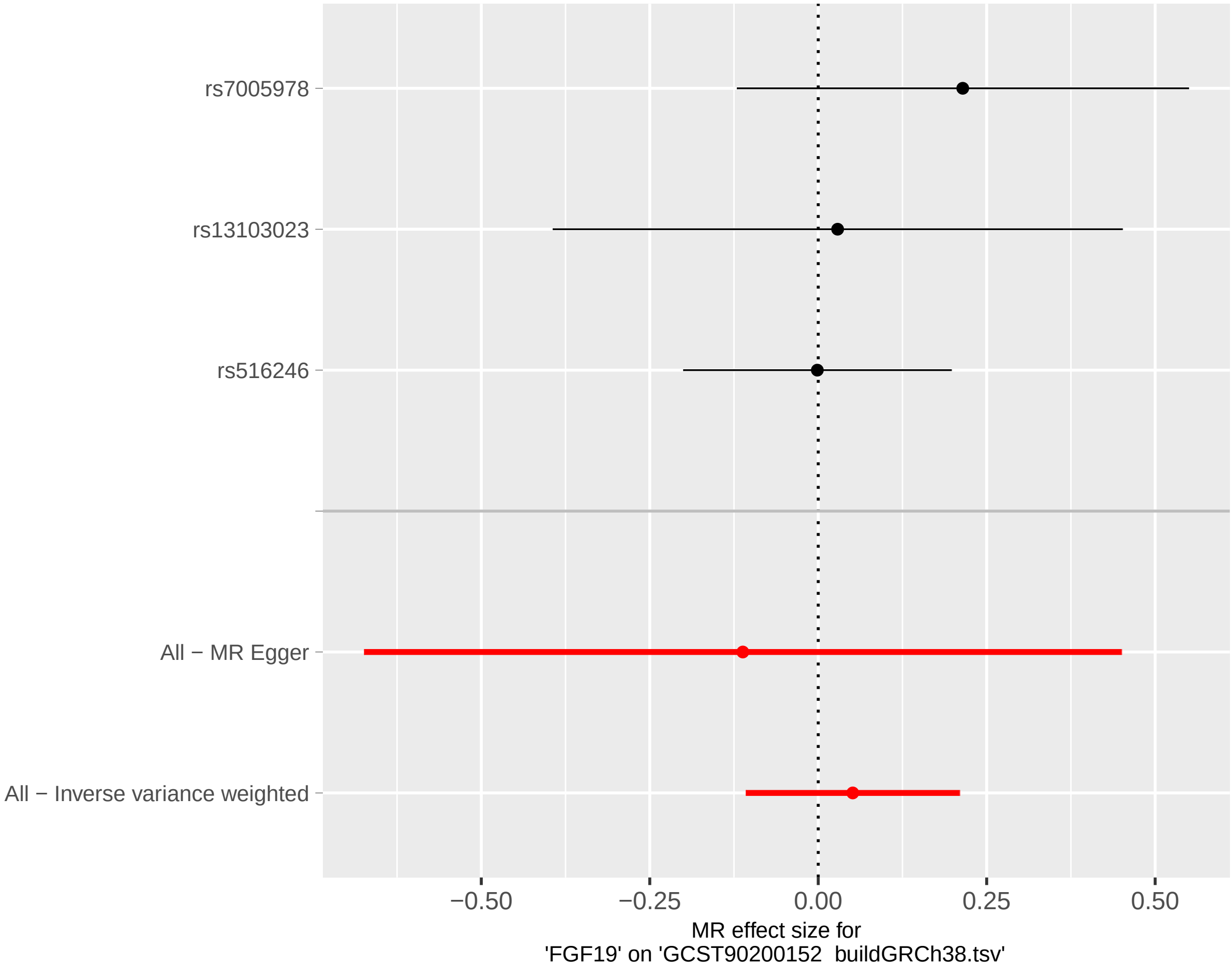
rs7005978
rs13103023
rs516246
All − MR Egger
All − Inverse variance weighted
−0.50
−0.25
0.00
0.25
0.50
MR effect size for
'FGF19' on 'GCST90200152_buildGRCh38.tsv'

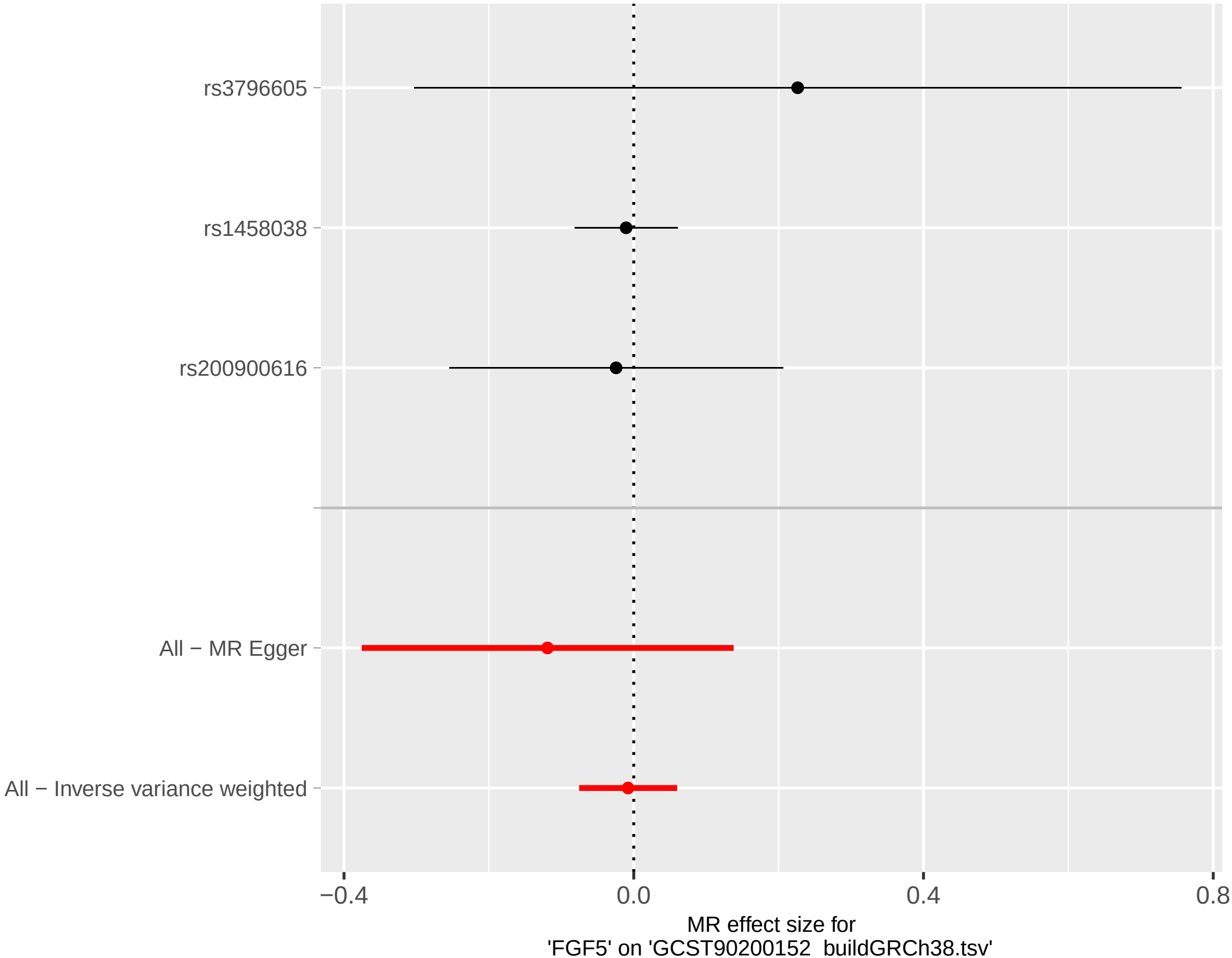
rs3796605
rs1458038
rs200900616
All − MR Egger
All − Inverse variance weighted
−0.4
0.0
0.4
0.8
MR effect size for
'FGF5' on 'GCST90200152_buildGRCh38.tsv'

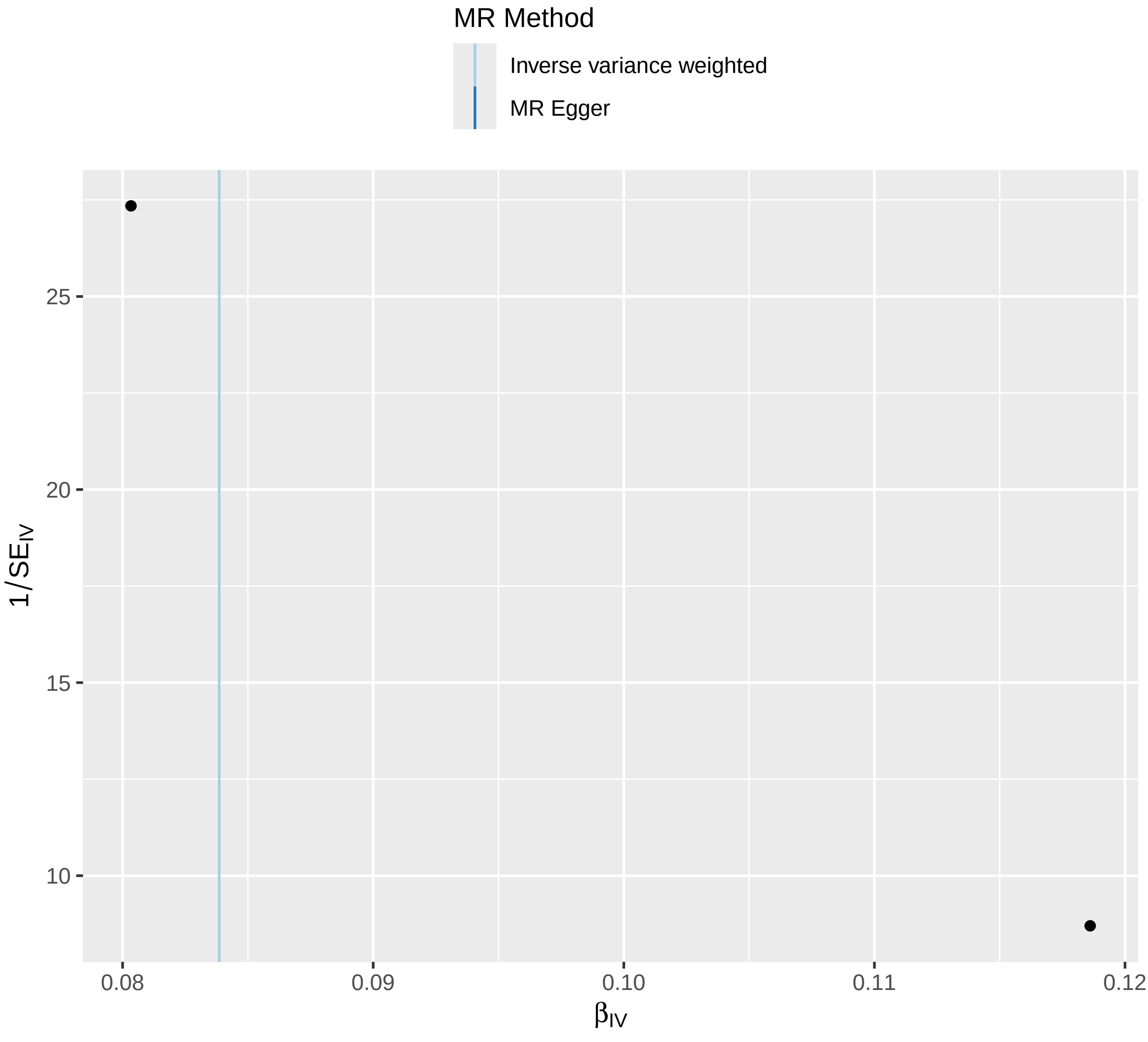

MR Method
Inverse variance weighted
MR Egger
25
20
15
10
1/SE$_{IV}$
0.08
0.09
0.10
0.11
0.12
β_{IV}

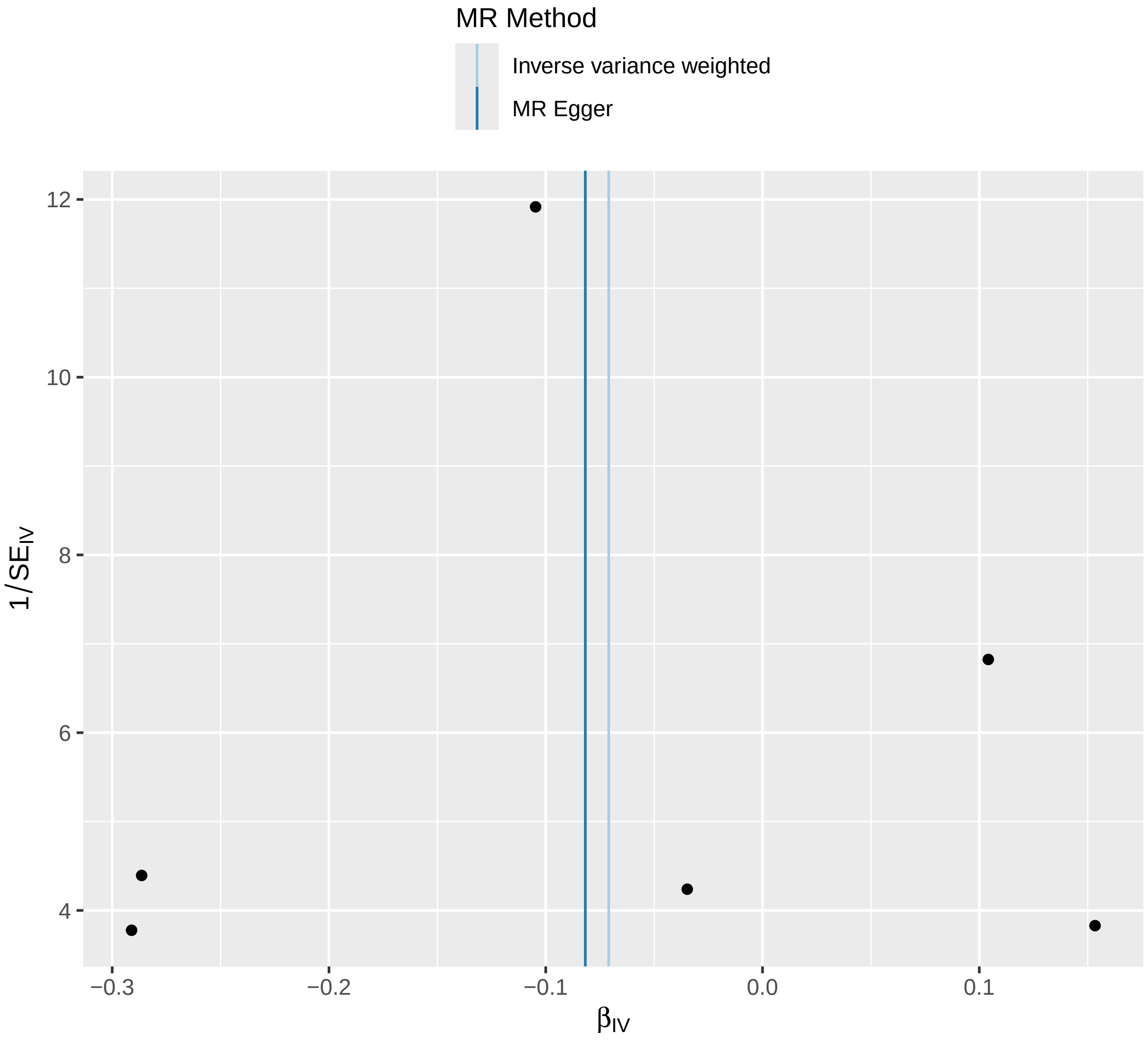

MR Method
Inverse variance weighted
MR Egger
12
10
8
6
4
1/SE_IV
−0.3
−0.2
−0.1
0.0
0.1
β_IV

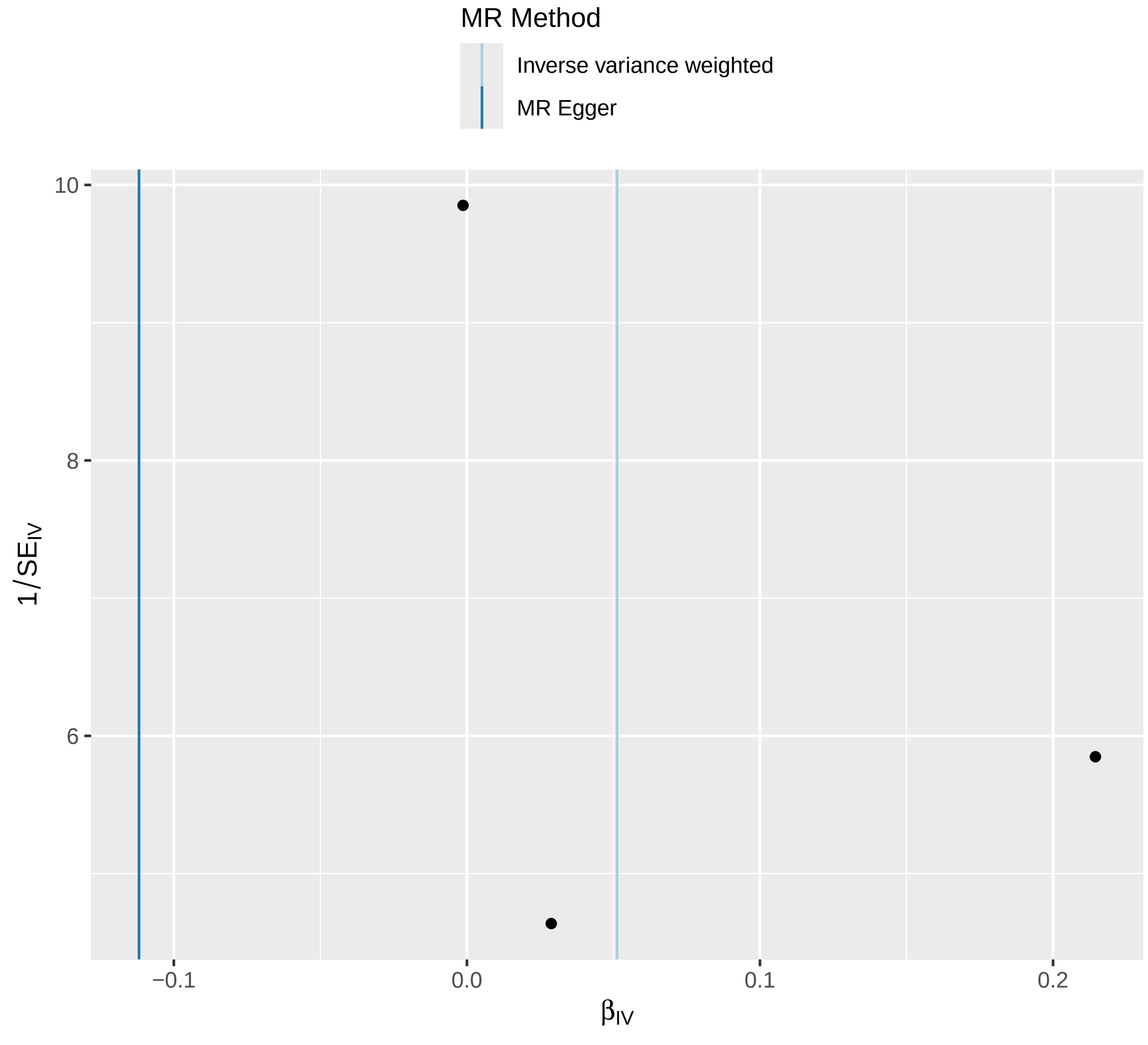
MR Method
Inverse variance weighted
MR Egger
10
8
6
1/SE_IV
−0.1
0.0
0.1
0.2
β_IV

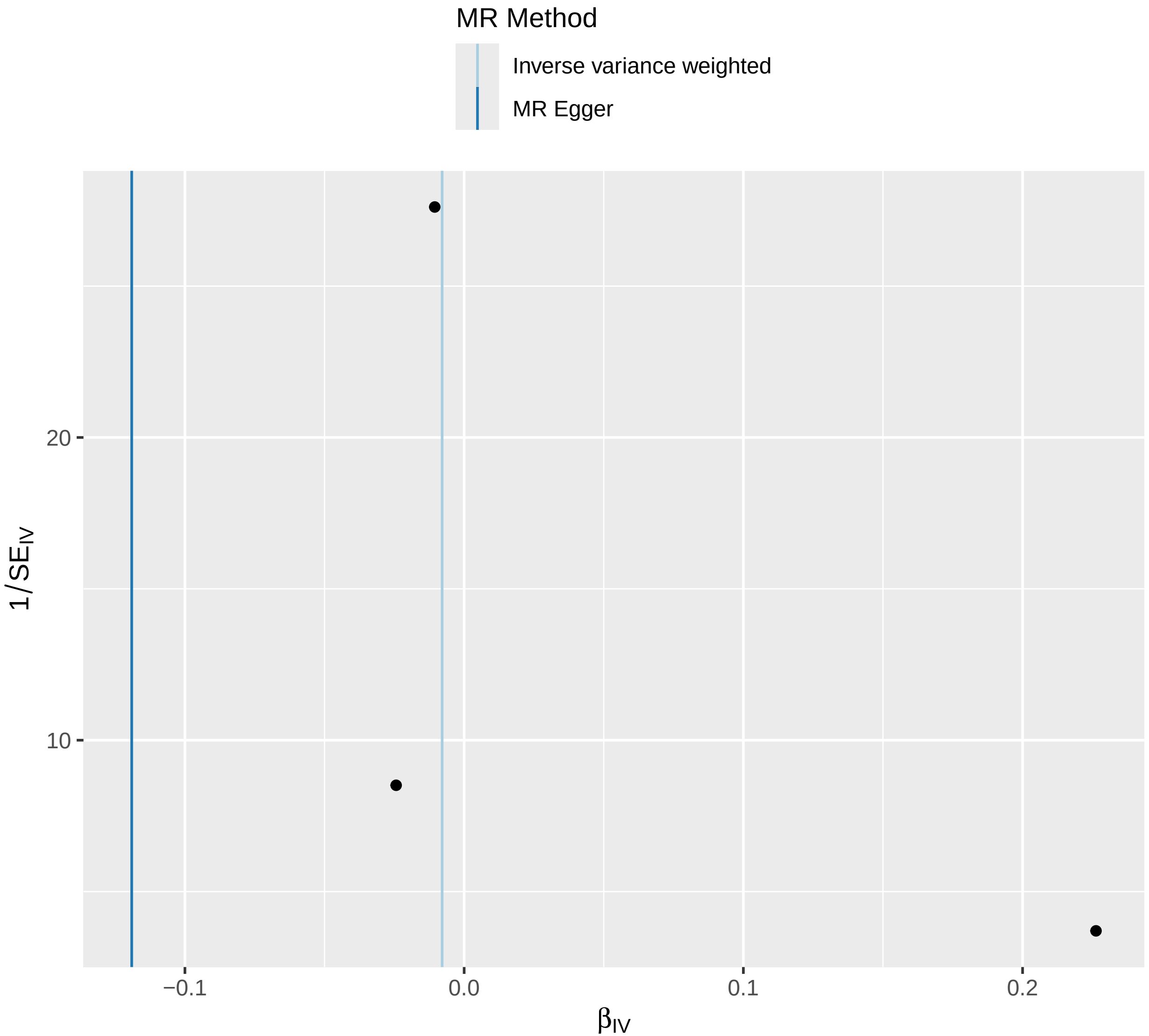

MR Method
Inverse variance weighted
MR Egger
20
10
$1/SE_{IV}$
−0.1
0.0
0.1
0.2
β_{IV}

Insufficient number of SNPs

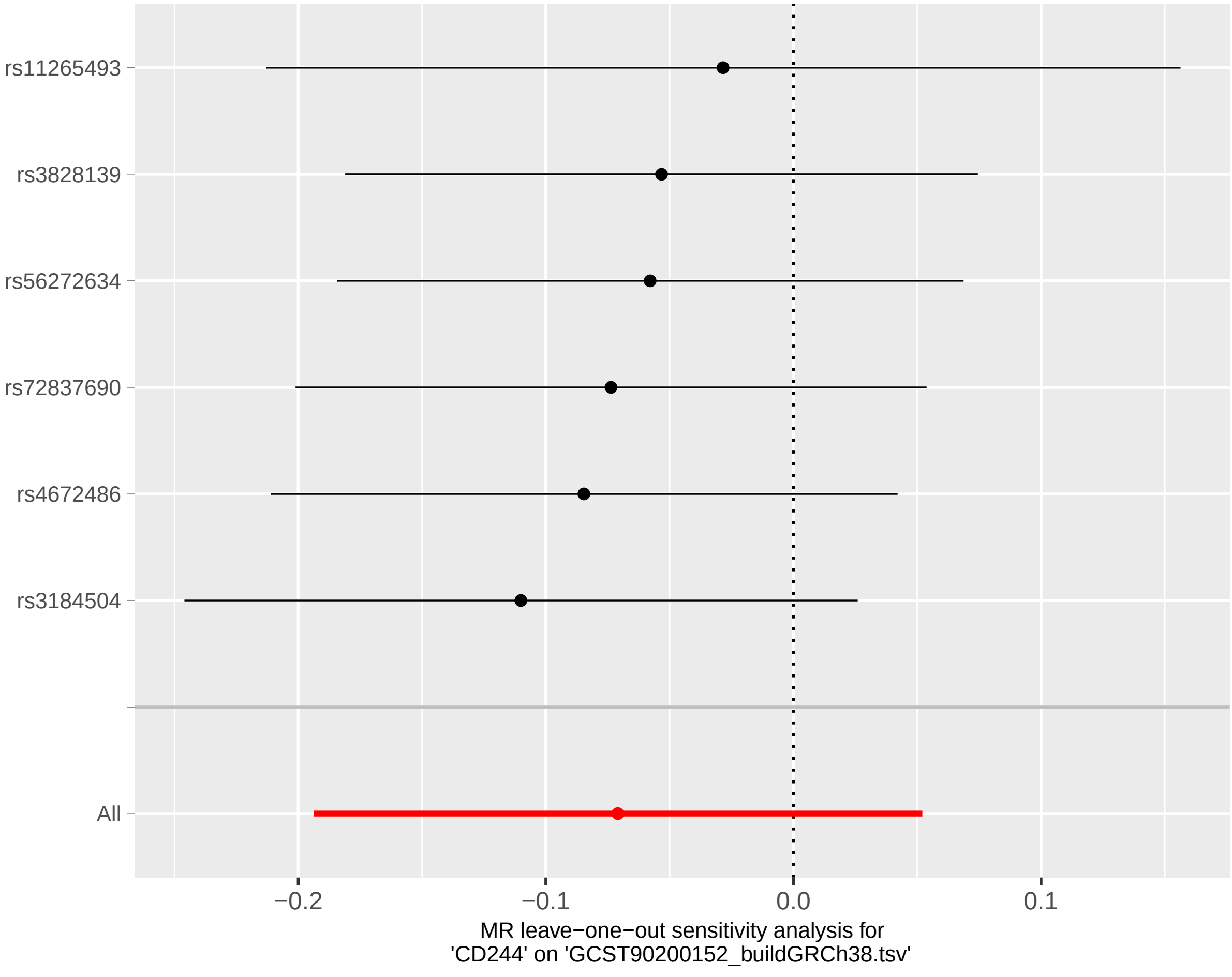

rs11265493
rs3828139
rs56272634
rs72837690
rs4672486
rs3184504
All
−0.2
−0.1
0.0
0.1
MR leave−one−out sensitivity analysis for
'CD244' on 'GCST90200152_buildGRCh38.tsv'

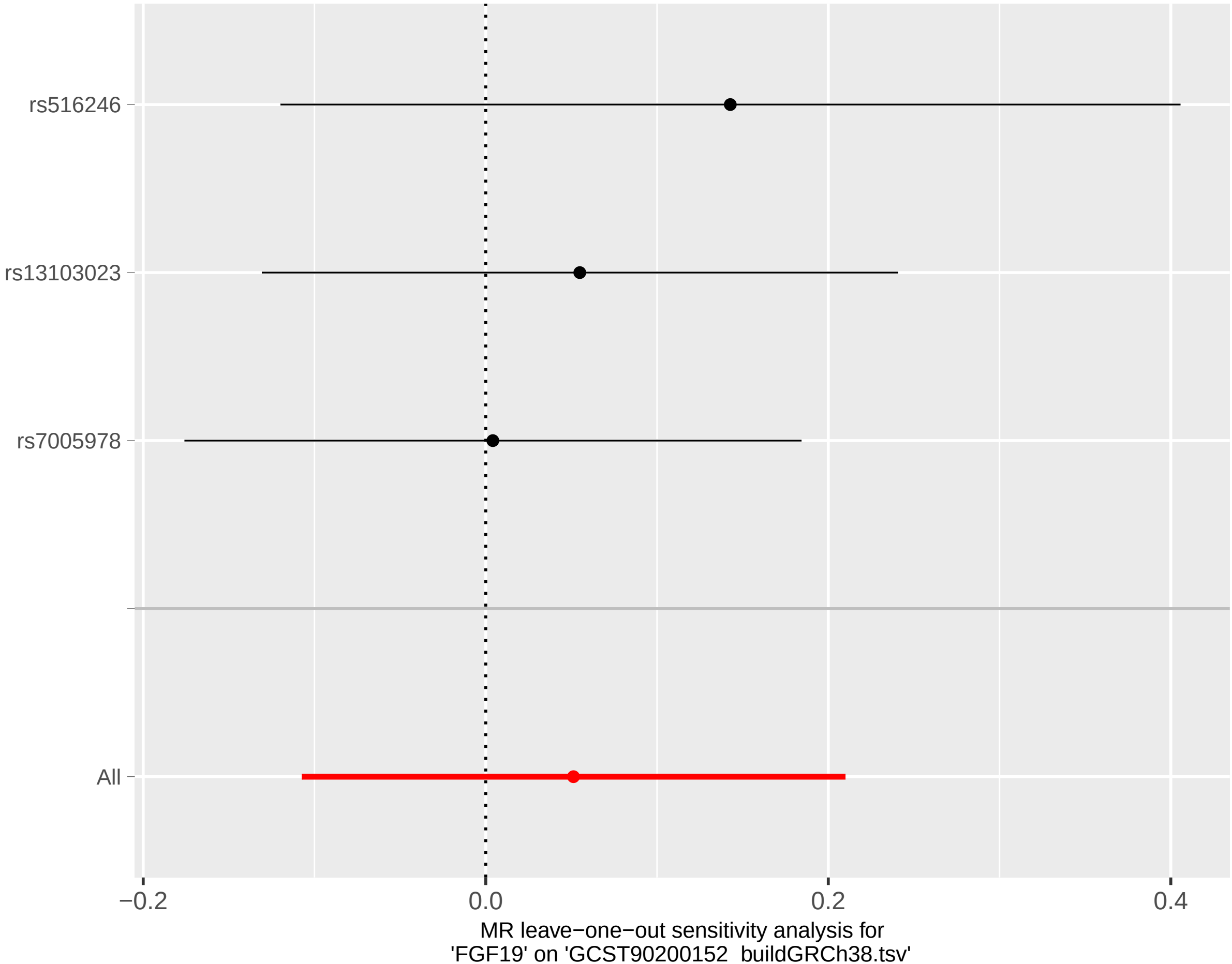
rs516246
rs13103023
rs7005978
All
−0.2
0.0
0.2
0.4
MR leave−one−out sensitivity analysis for
'FGF19' on 'GCST90200152_buildGRCh38.tsv'

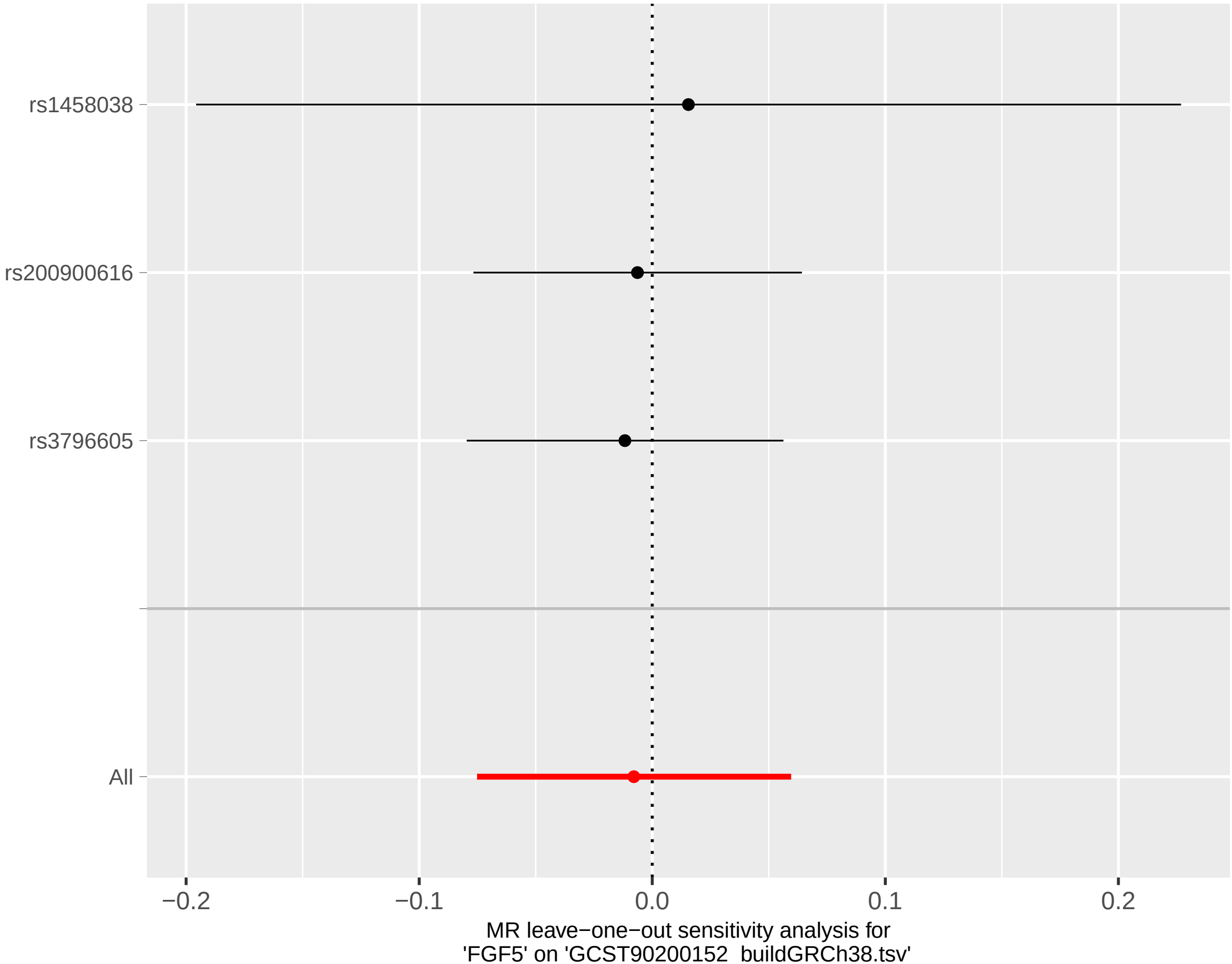
rs1458038
rs200900616
rs3796605
All
−0.2
−0.1
0.0
0.1
0.2
MR leave−one−out sensitivity analysis for
'FGF5' on 'GCST90200152_buildGRCh38.tsv'

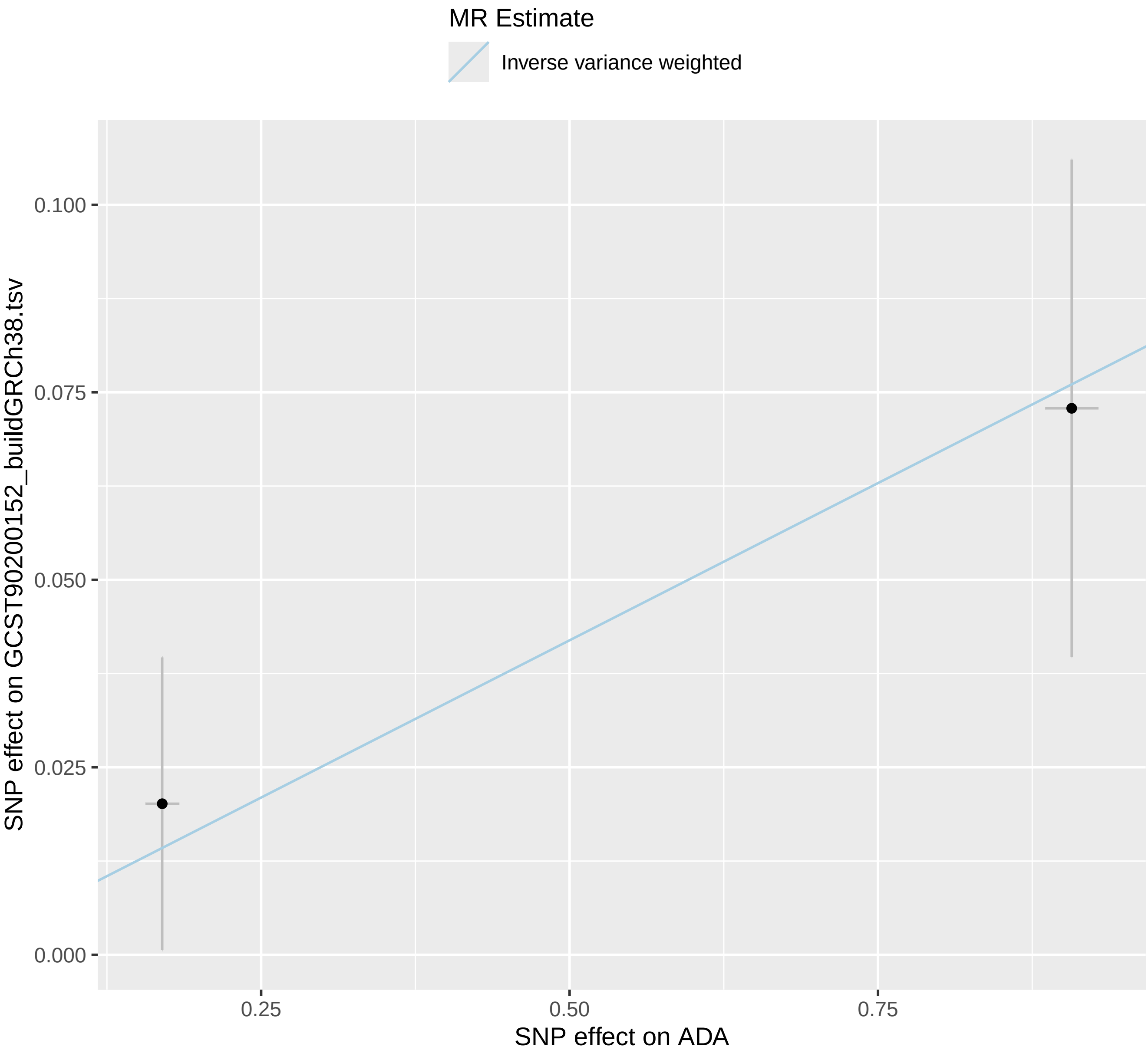
MR Estimate
Inverse variance weighted
0.100
0.075
0.050
0.025
0.000
0.25
0.50
0.75
SNP effect on ADA
SNP effect on GCST90200152_buildGRCh38.tsv

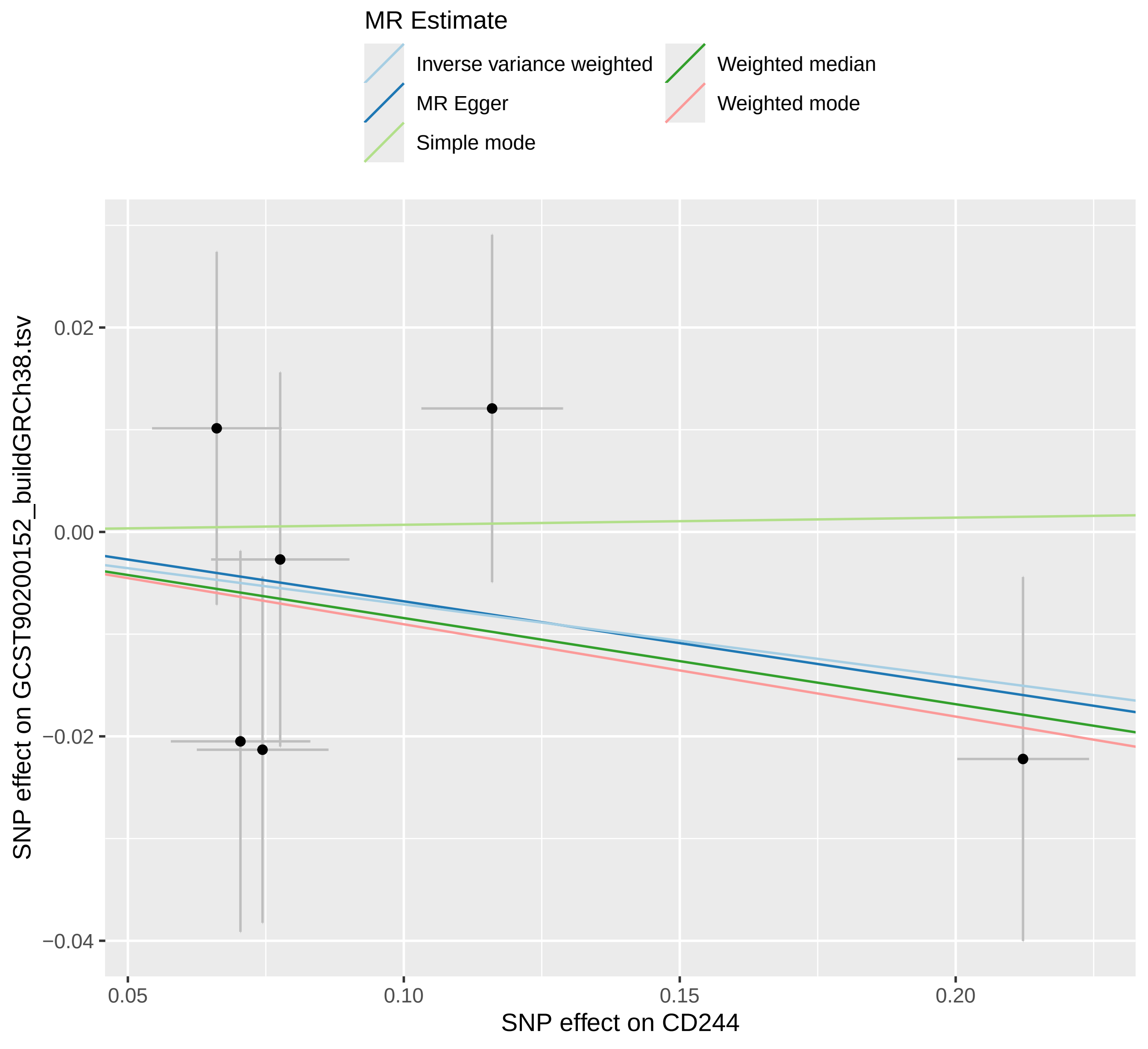
MR Estimate
Inverse variance weighted
MR Egger
Simple mode
Weighted median
Weighted mode
SNP effect on GCST90200152_buildGRCh38.tsv
0.02
0.00
−0.02
−0.04
0.05
0.10
0.15
0.20
SNP effect on CD244

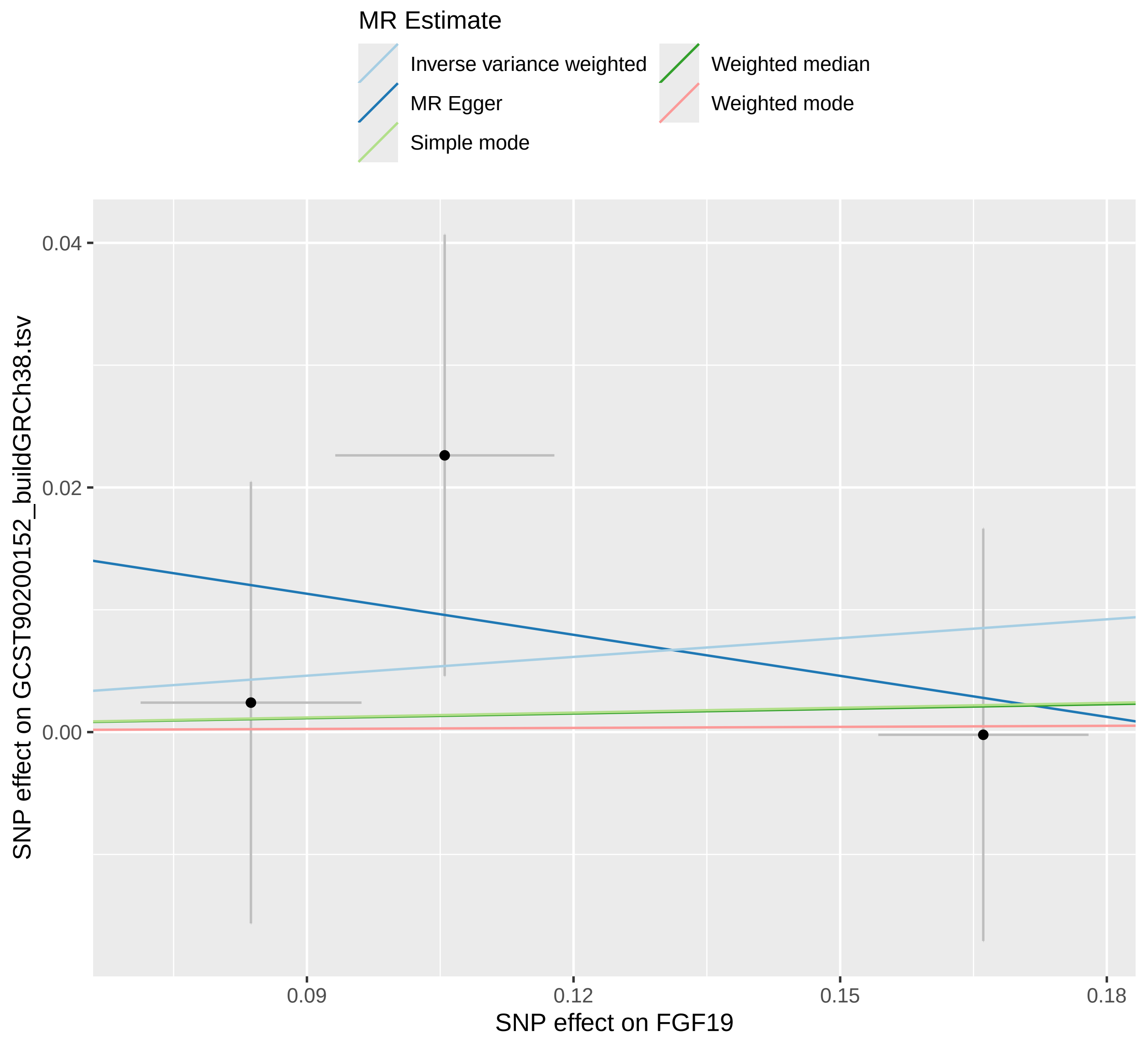

MR Estimate
Inverse variance weighted
MR Egger
Simple mode
Weighted median
Weighted mode
0.04
0.02
0.00
0.09
0.12
0.15
0.18
SNP effect on FGF19
SNP effect on GCST90200152_buildGRCh38.tsv

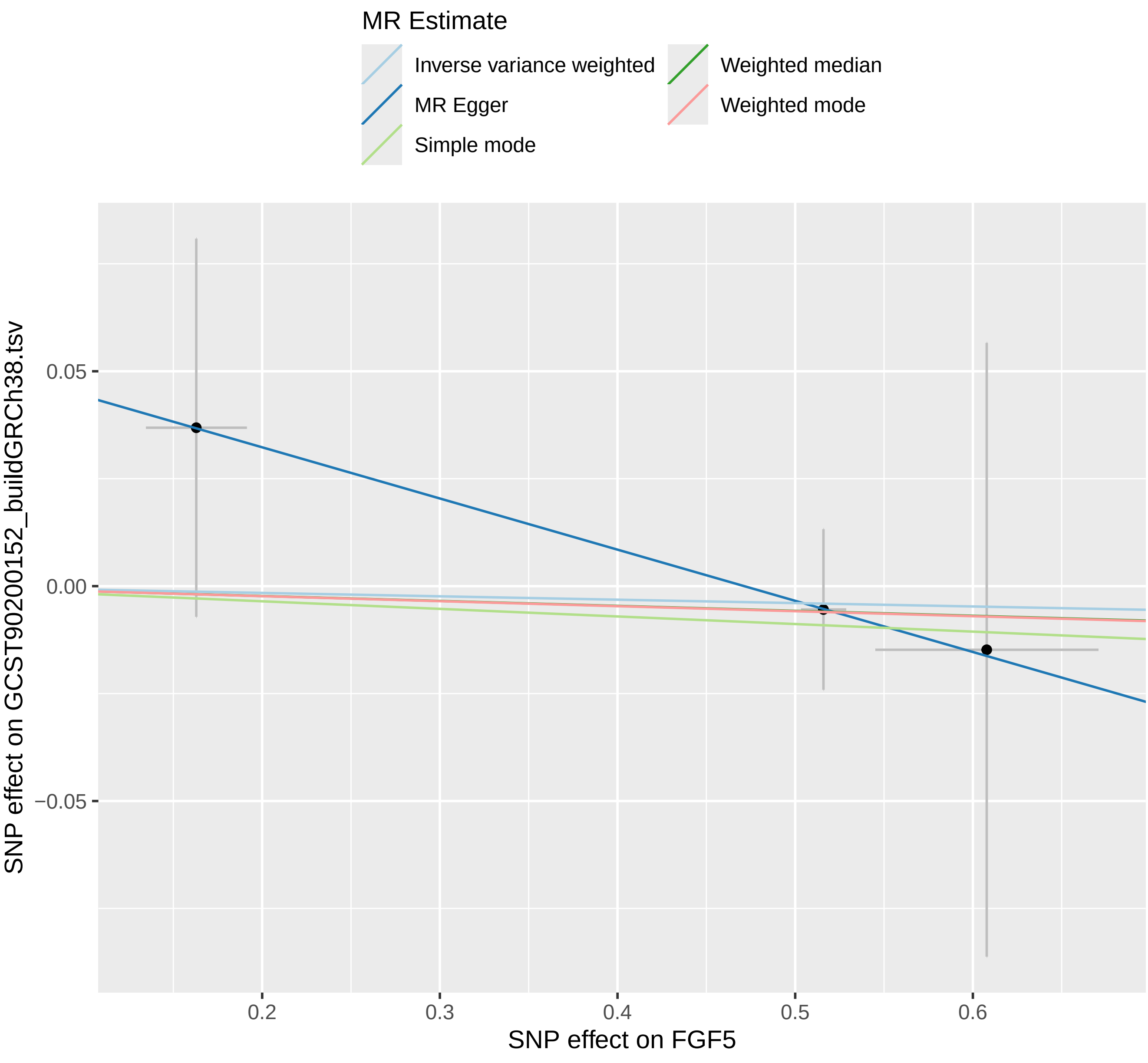
MR Estimate
Inverse variance weighted
MR Egger
Simple mode
Weighted median
Weighted mode
0.05
0.00
−0.05
0.2
0.3
0.4
0.5
0.6
SNP effect on GCST90200152_buildGRCh38.tsv
SNP effect on FGF5

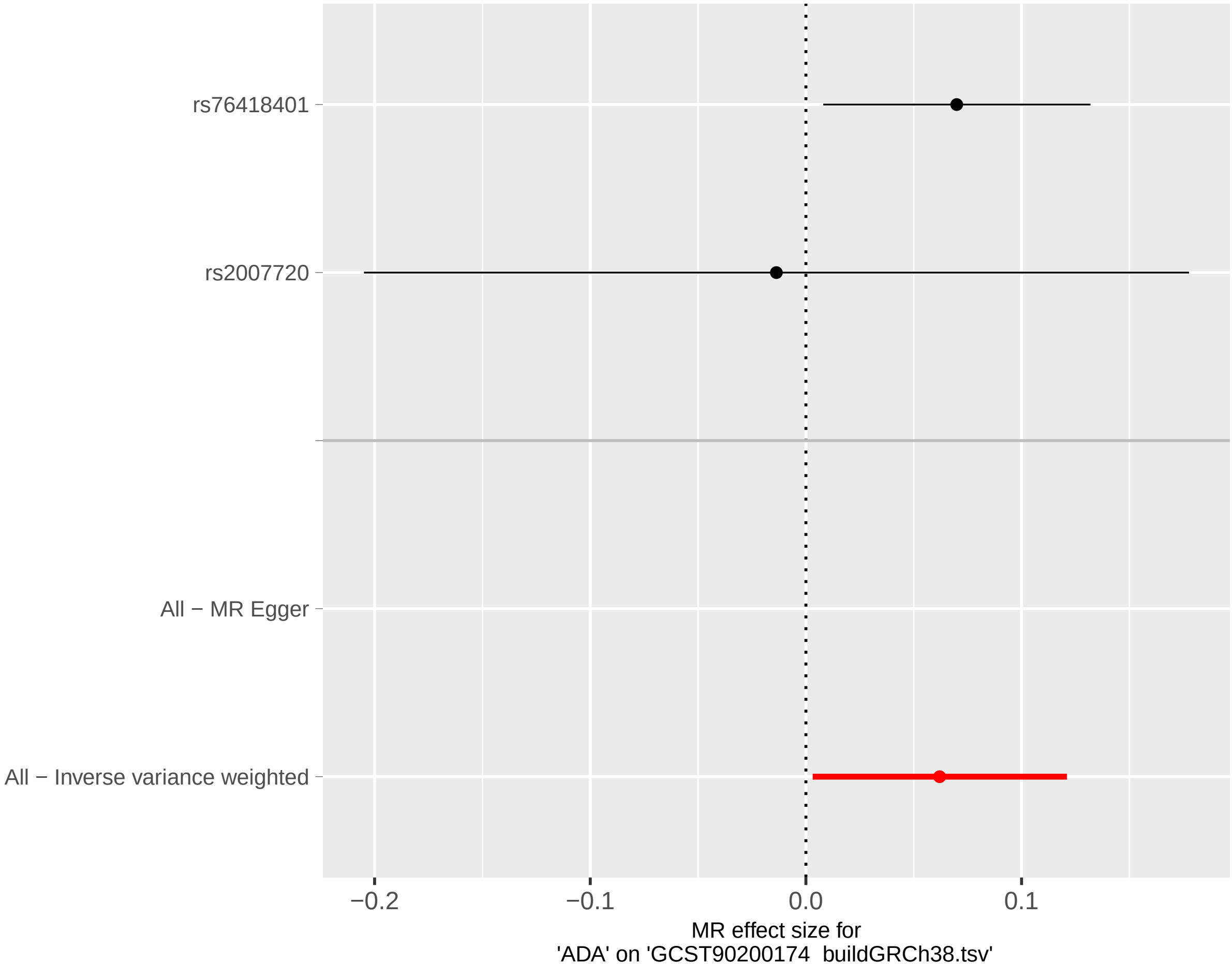
rs76418401
rs2007720
All − MR Egger
All − Inverse variance weighted
−0.2
−0.1
0.0
0.1
MR effect size for
'ADA' on 'GCST90200174_buildGRCh38.tsv'

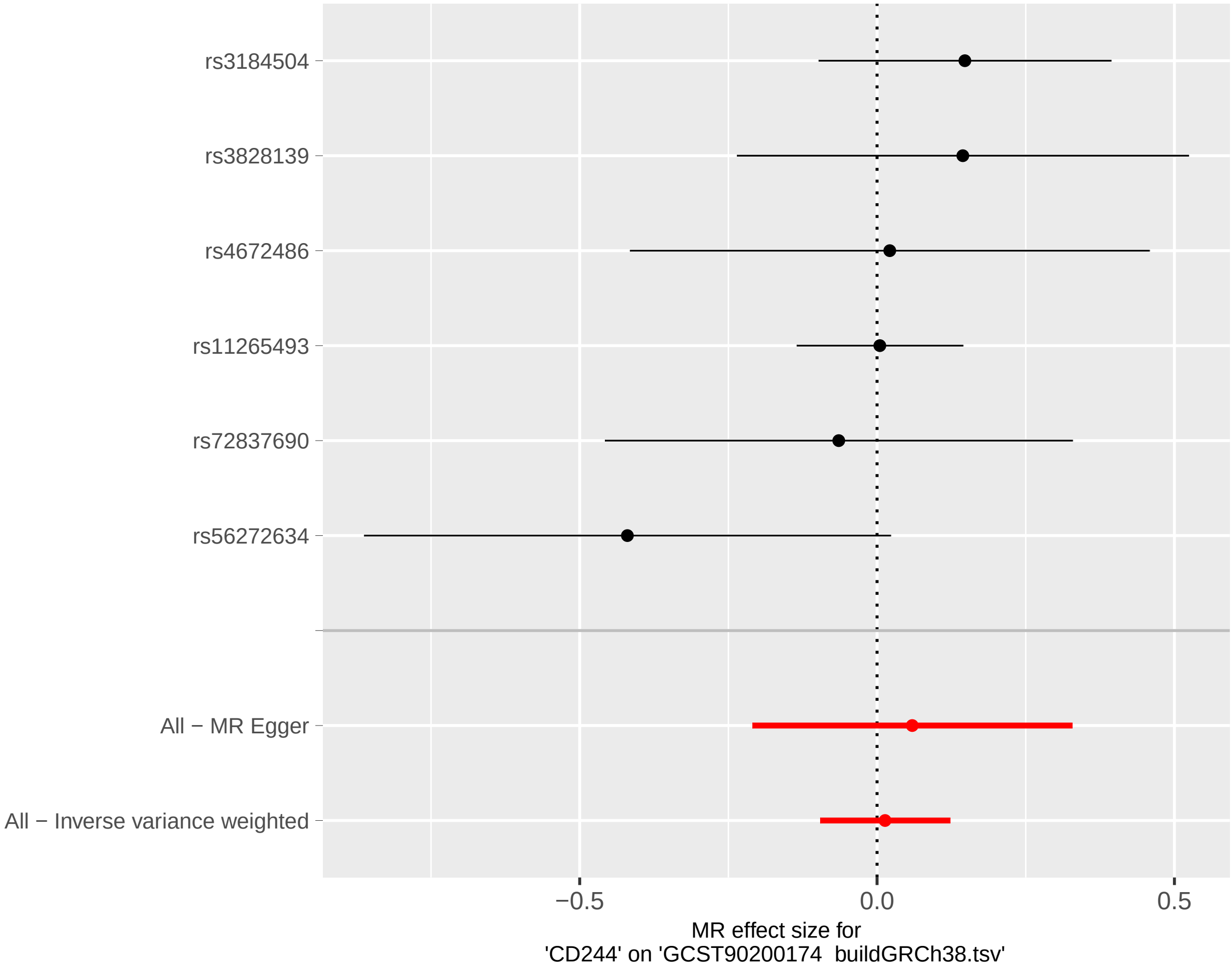
rs3184504
rs3828139
rs4672486
rs11265493
rs72837690
rs56272634
All − MR Egger
All − Inverse variance weighted
−0.5
0.0
0.5
MR effect size for
'CD244' on 'GCST90200174_buildGRCh38.tsv'

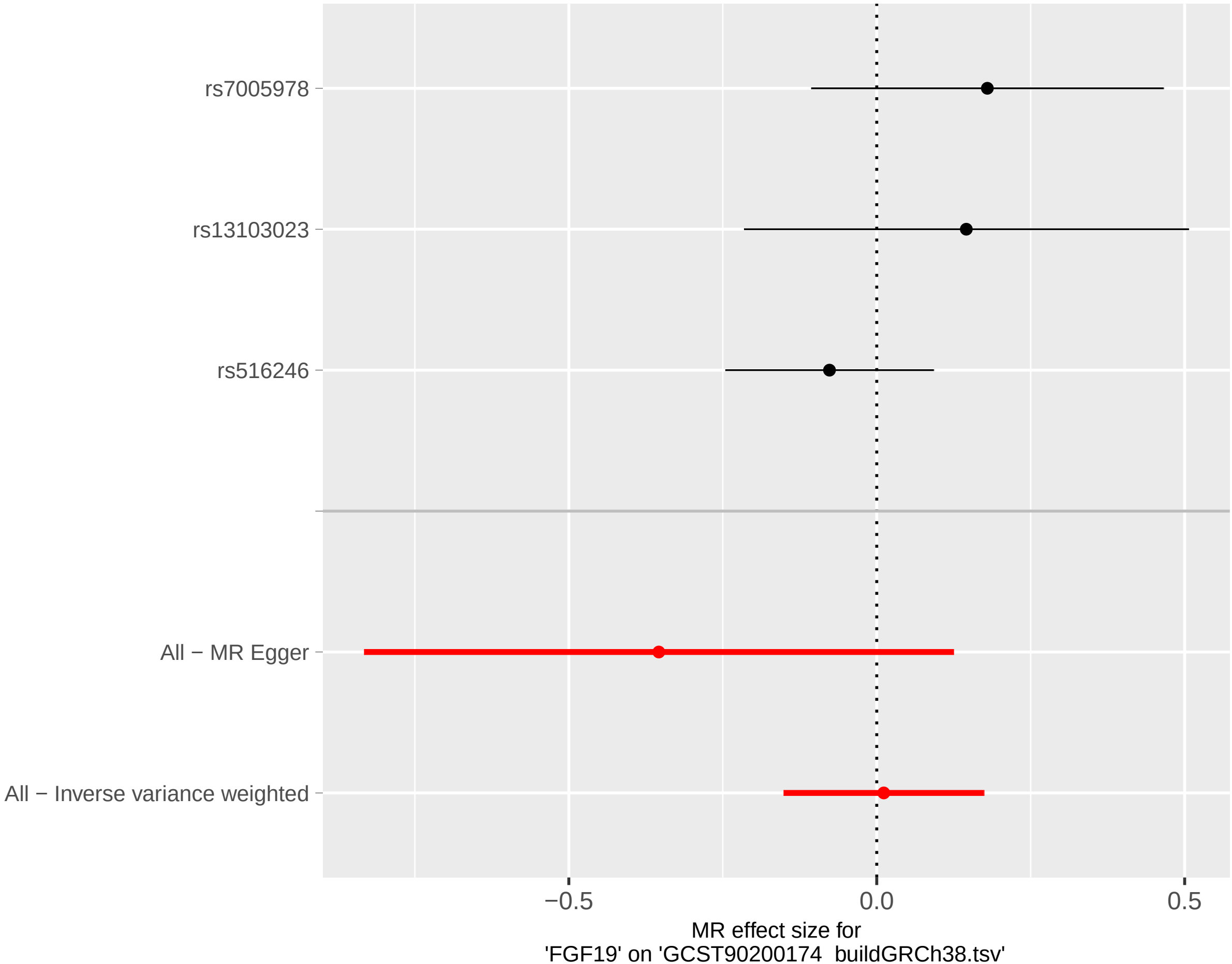

rs7005978
rs13103023
rs516246
All − MR Egger
All − Inverse variance weighted
−0.5
0.0
0.5
MR effect size for
'FGF19' on 'GCST90200174_buildGRCh38.tsv'

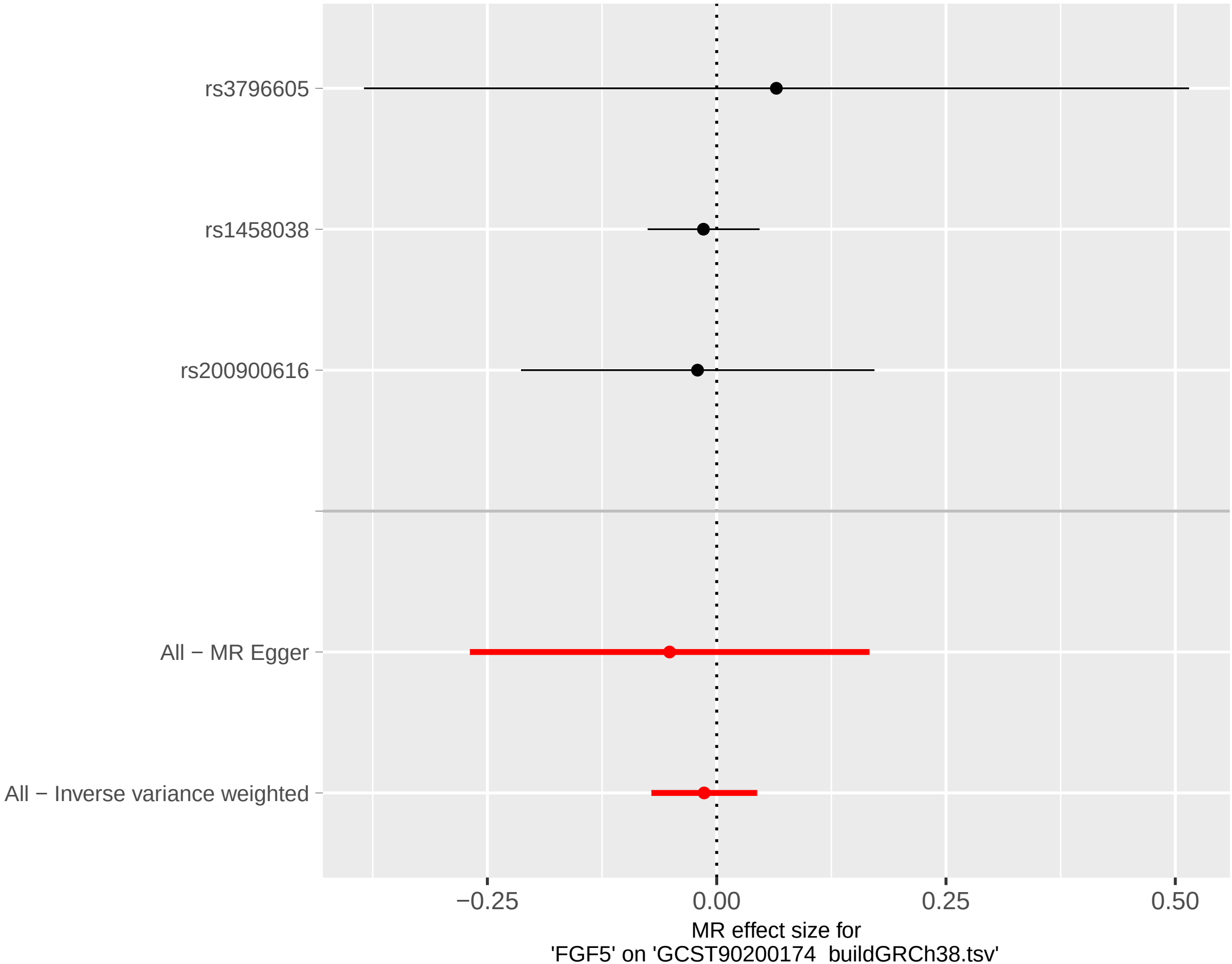

rs3796605
rs1458038
rs200900616
All − MR Egger
All − Inverse variance weighted
−0.25
0.00
0.25
0.50
MR effect size for
'FGF5' on 'GCST90200174_buildGRCh38.tsv'

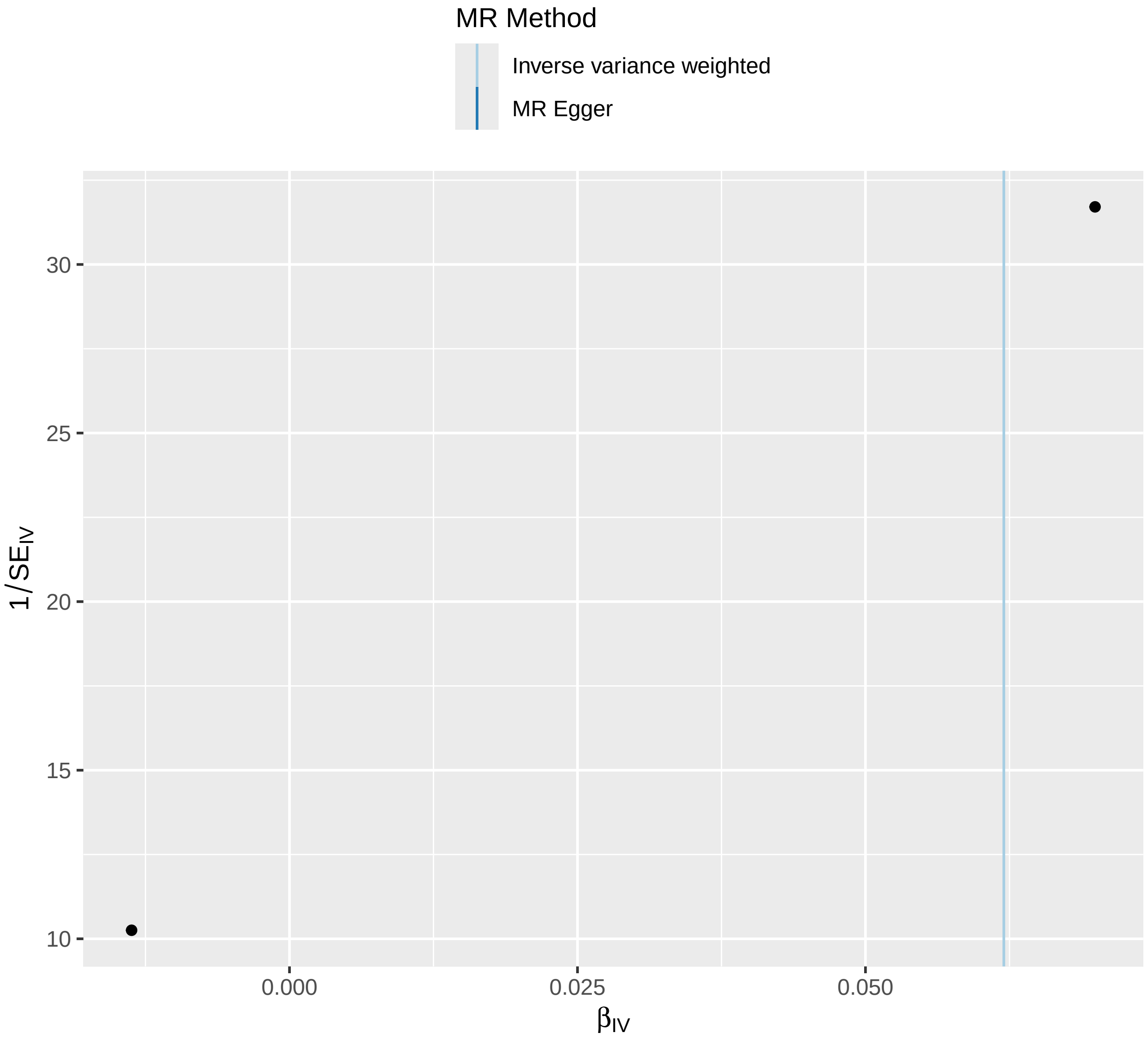
MR Method
Inverse variance weighted
MR Egger
30
25
20
15
10
0.000
0.025
0.050
1/SE_IV
β_IV

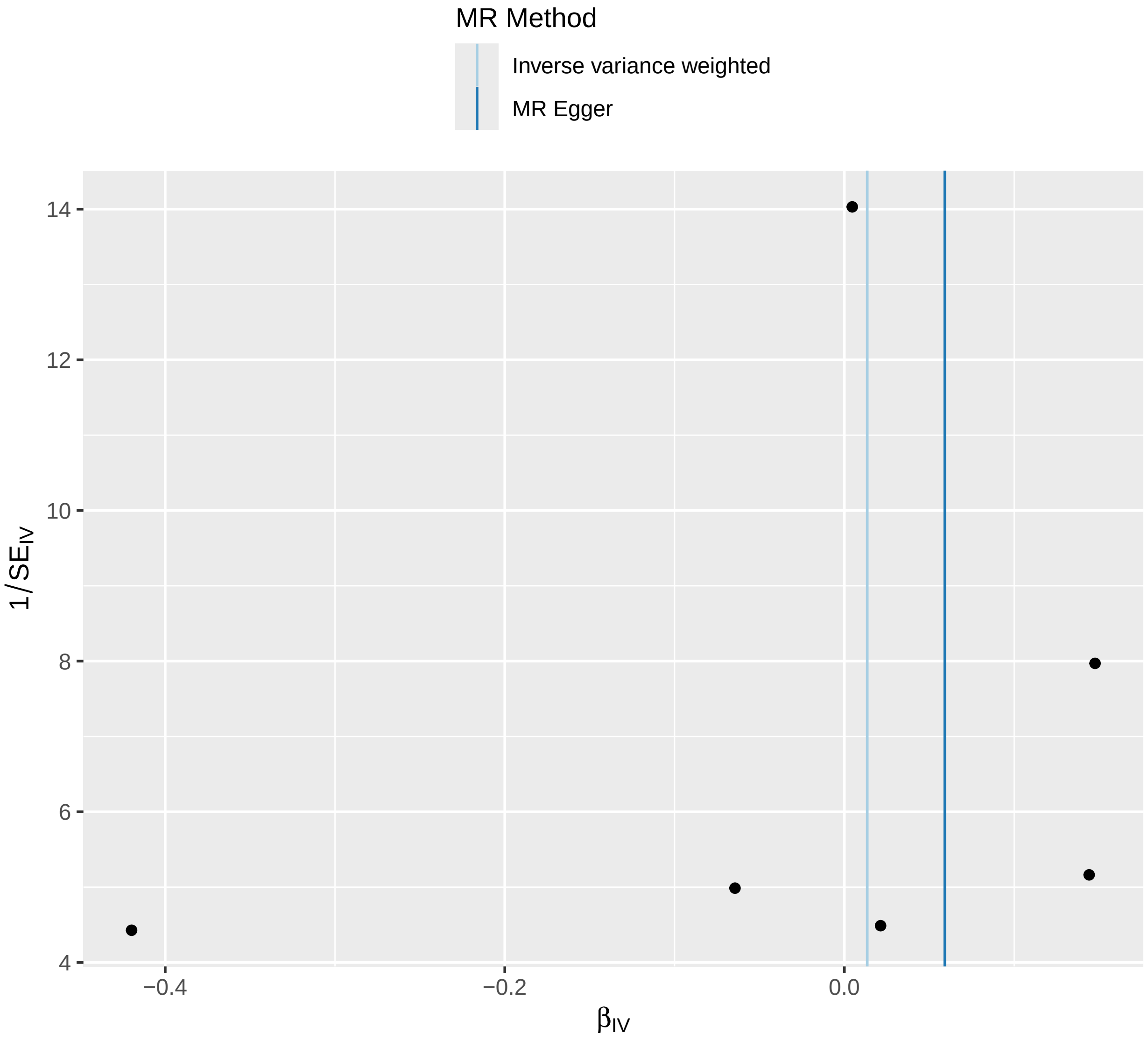

MR Method
Inverse variance weighted
MR Egger
14
12
10
8
6
4
$1/SE_{IV}$
−0.4
−0.2
0.0
β_{IV}

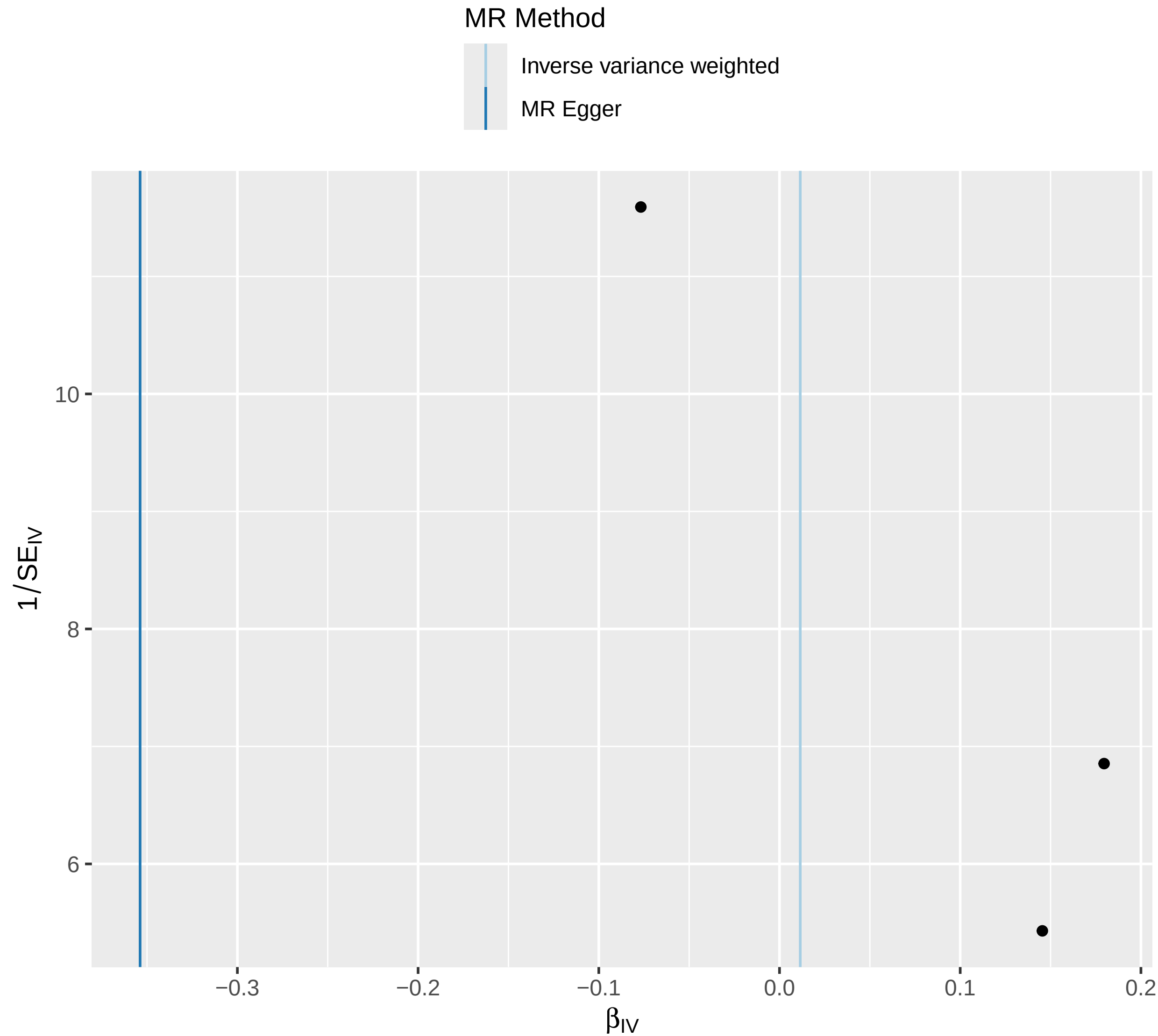
MR Method
Inverse variance weighted
MR Egger
1/SE_IV
10
8
6
−0.3
−0.2
−0.1
0.0
0.1
0.2
β_IV

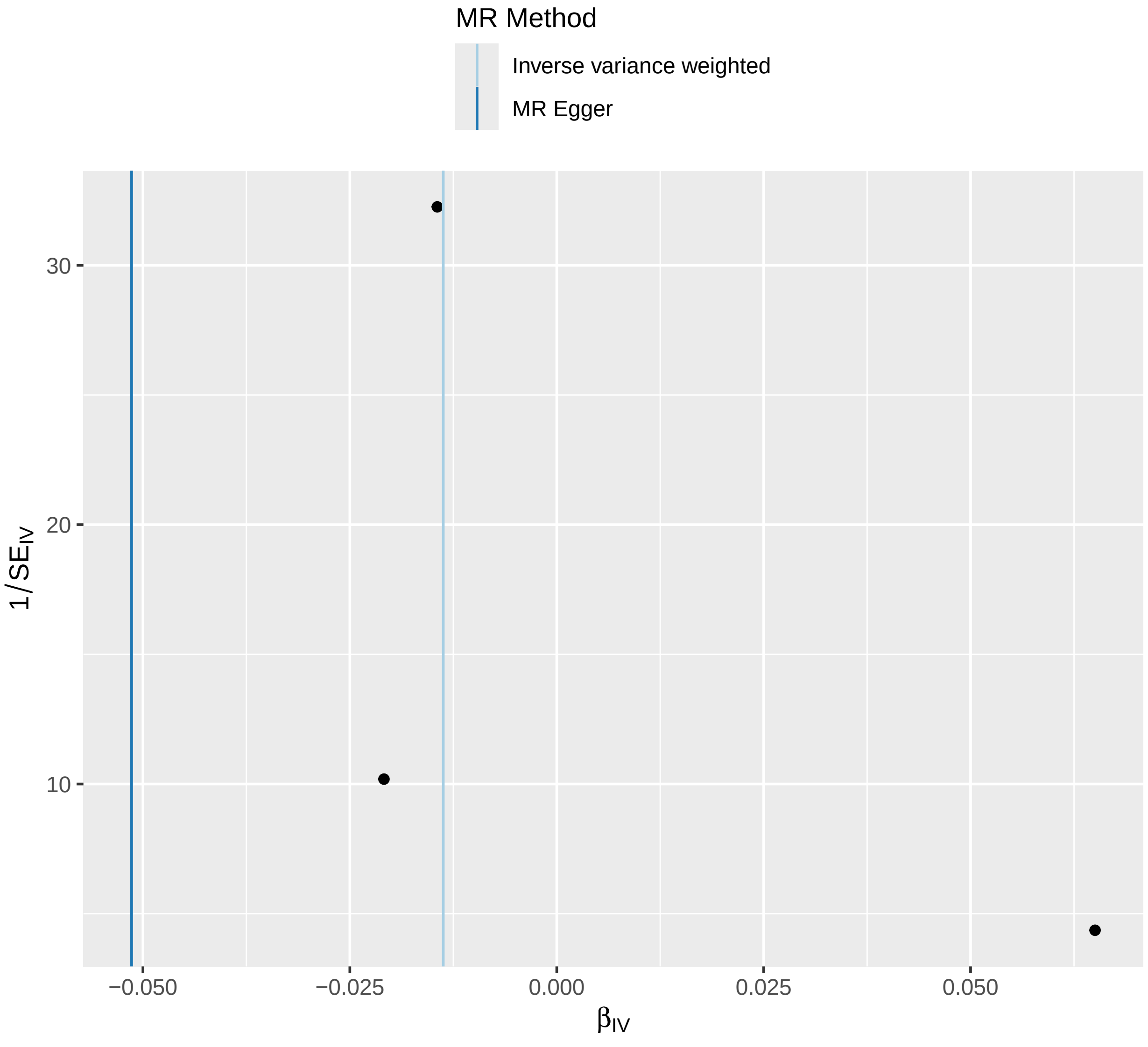

MR Method
Inverse variance weighted
MR Egger
30
20
10
1/SE$_{IV}$
−0.050
−0.025
0.000
0.025
0.050
β$_{IV}$

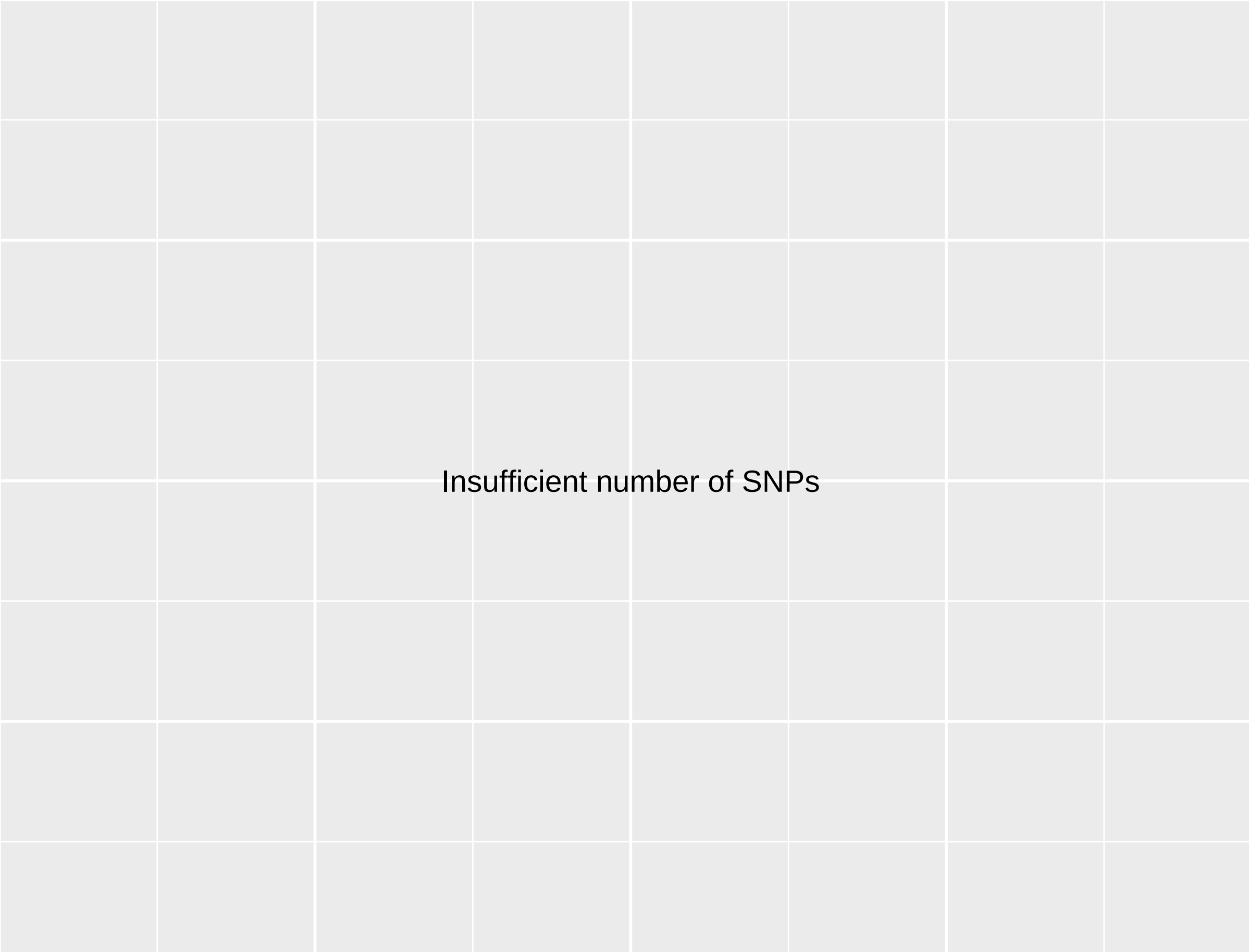
Insufficient number of SNPs

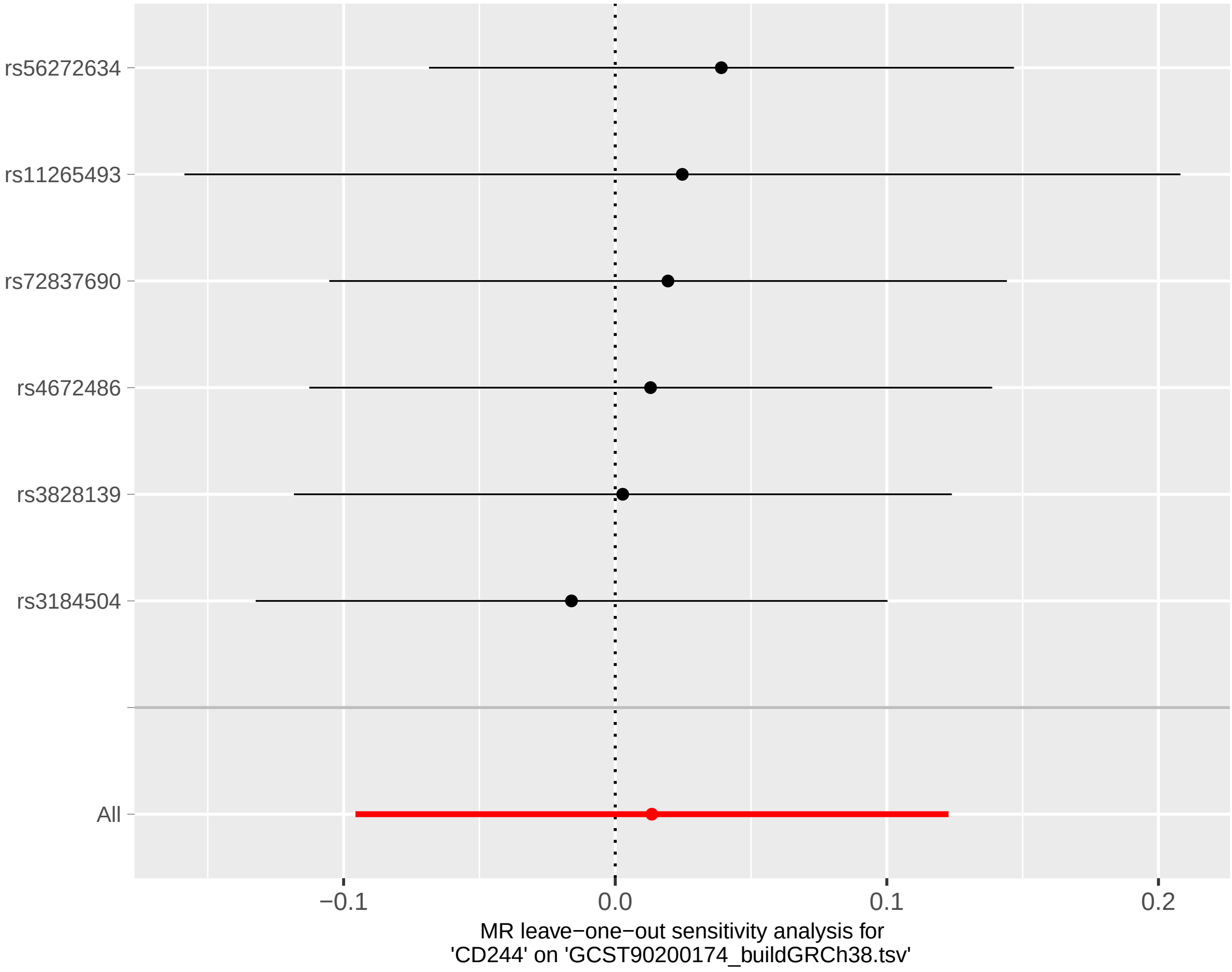
rs56272634
rs11265493
rs72837690
rs4672486
rs3828139
rs3184504
All
−0.1
0.0
0.1
0.2
MR leave−one−out sensitivity analysis for
'CD244' on 'GCST90200174_buildGRCh38.tsv'

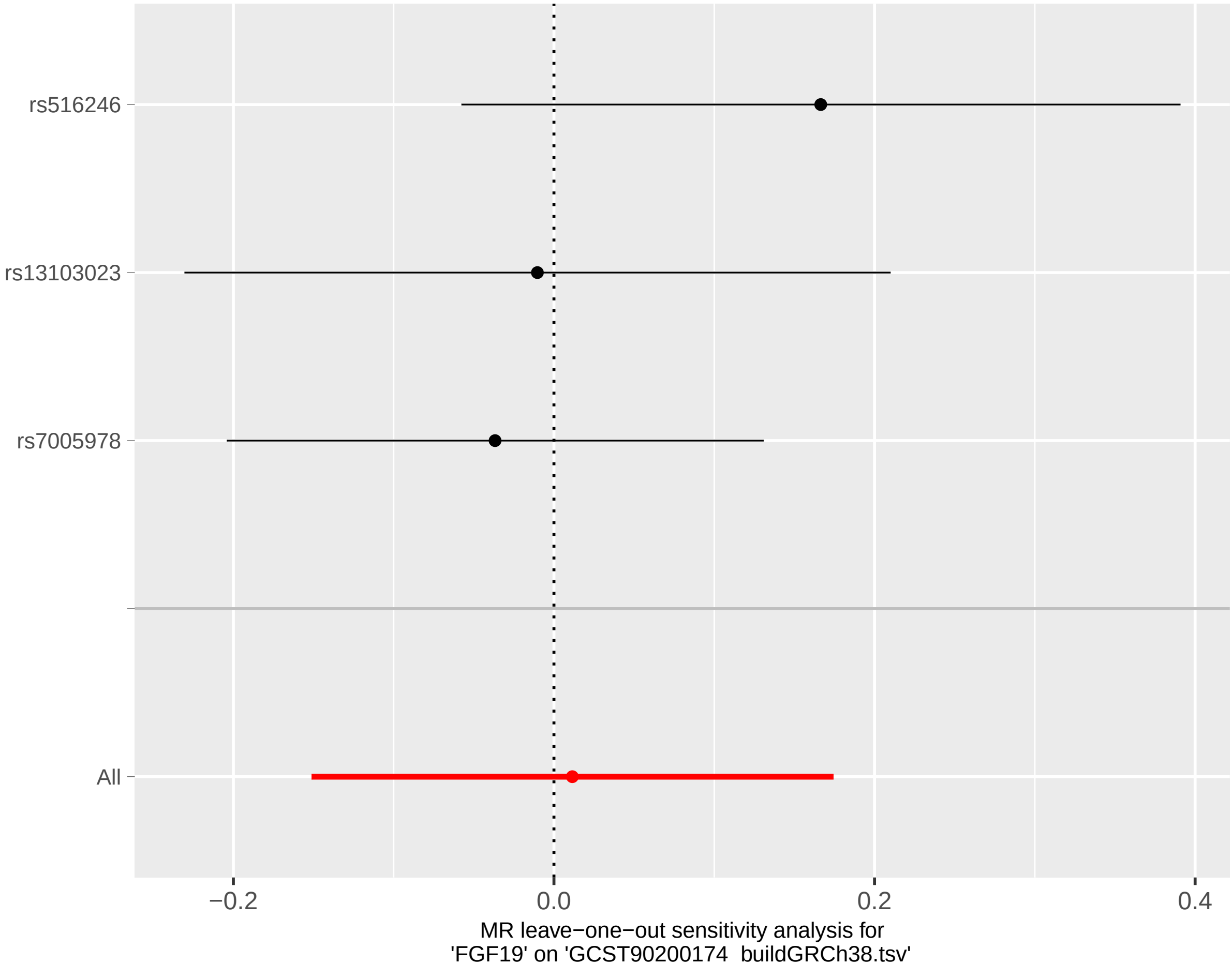
rs516246
rs13103023
rs7005978
All
−0.2
0.0
0.2
0.4
MR leave−one−out sensitivity analysis for
'FGF19' on 'GCST90200174_buildGRCh38.tsv'

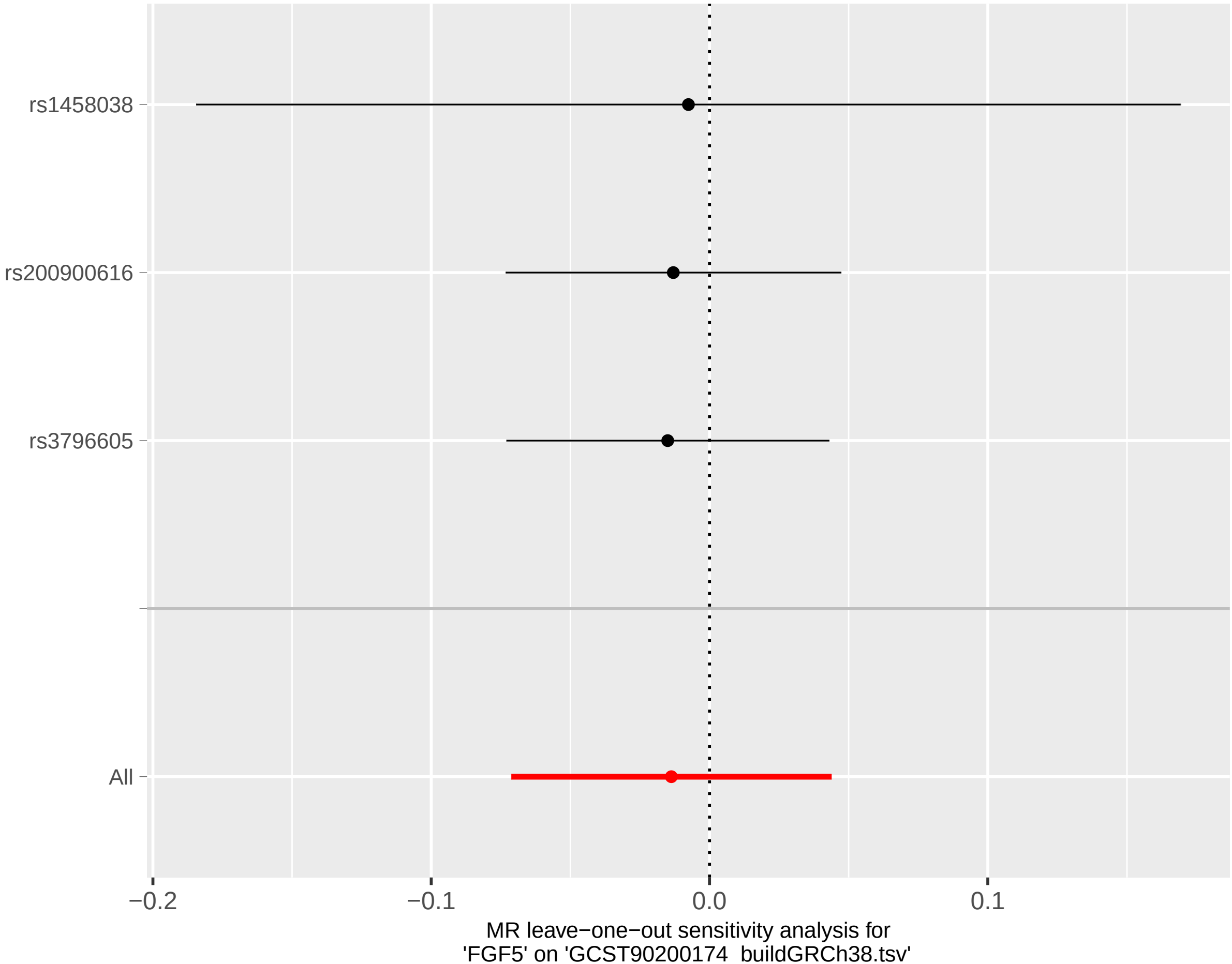
rs1458038
rs200900616
rs3796605
All
−0.2
−0.1
0.0
0.1
MR leave−one−out sensitivity analysis for
'FGF5' on 'GCST90200174_buildGRCh38.tsv'

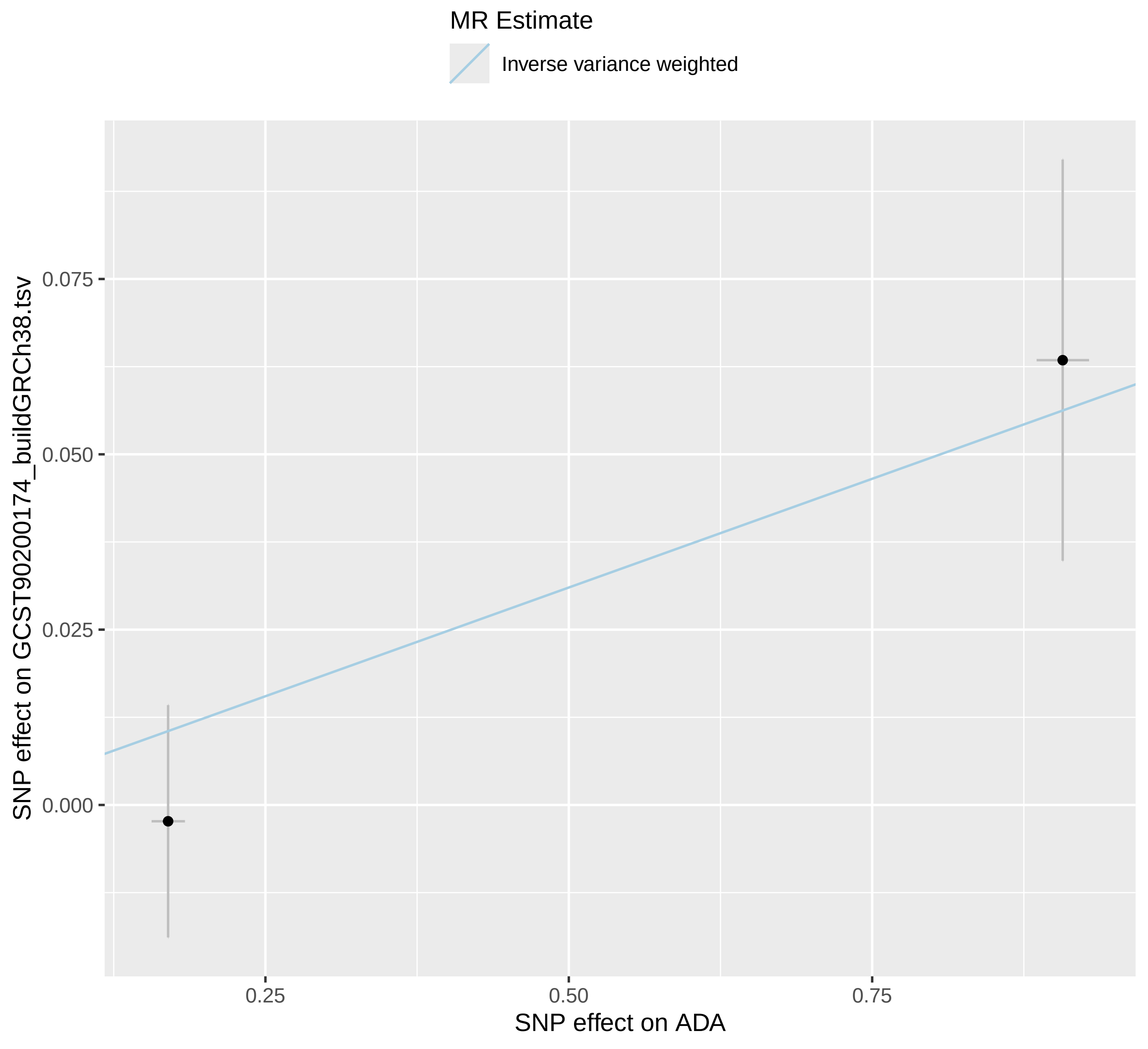

MR Estimate
Inverse variance weighted
0.075
0.050
0.025
0.000
SNP effect on GCST90200174_buildGRCh38.tsv
0.25
0.50
0.75
SNP effect on ADA

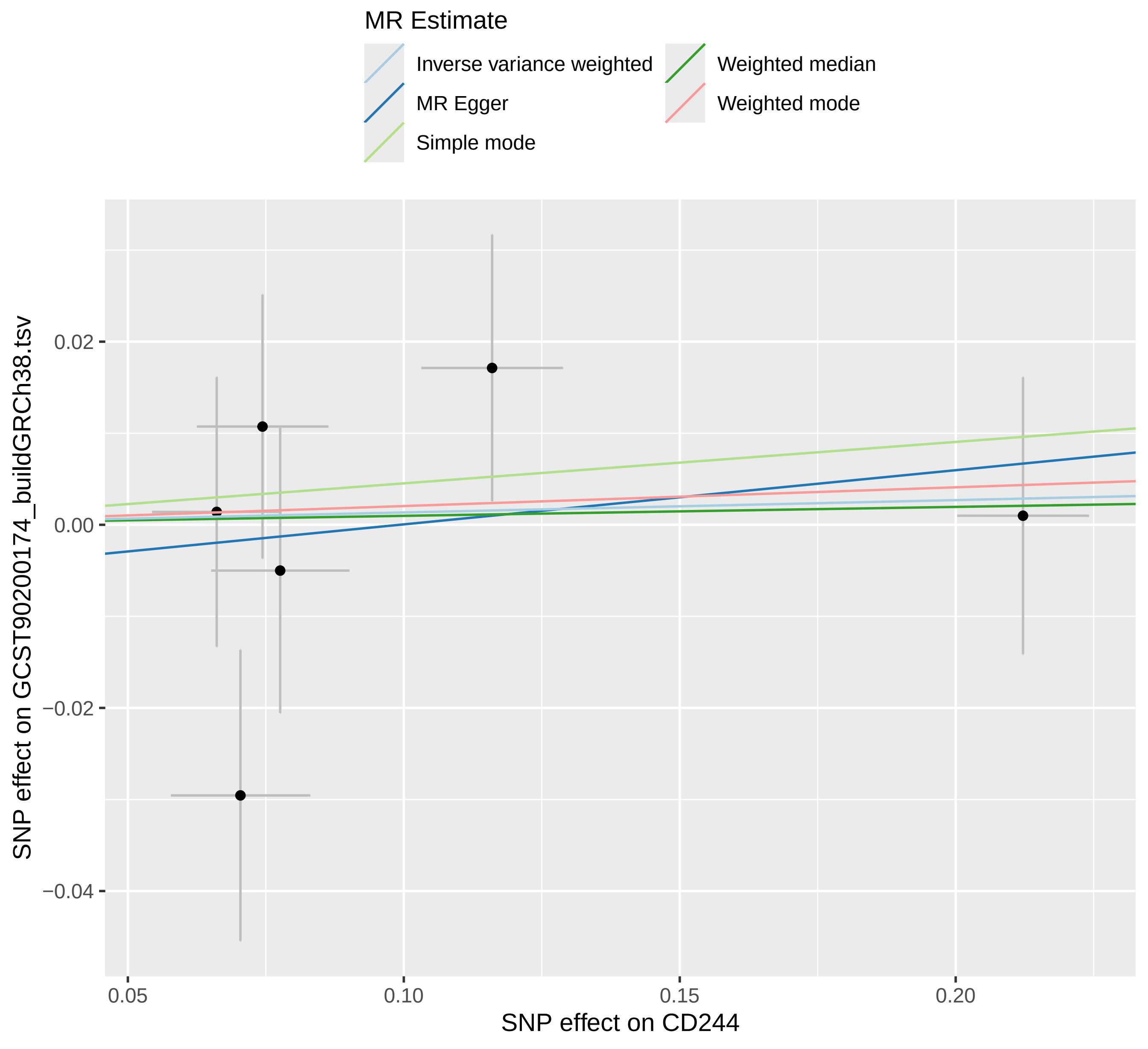
MR Estimate
Inverse variance weighted
MR Egger
Simple mode
Weighted median
Weighted mode
0.02
0.00
−0.02
−0.04
0.05
0.10
0.15
0.20
SNP effect on CD244
SNP effect on GCST90200174_buildGRCh38.tsv

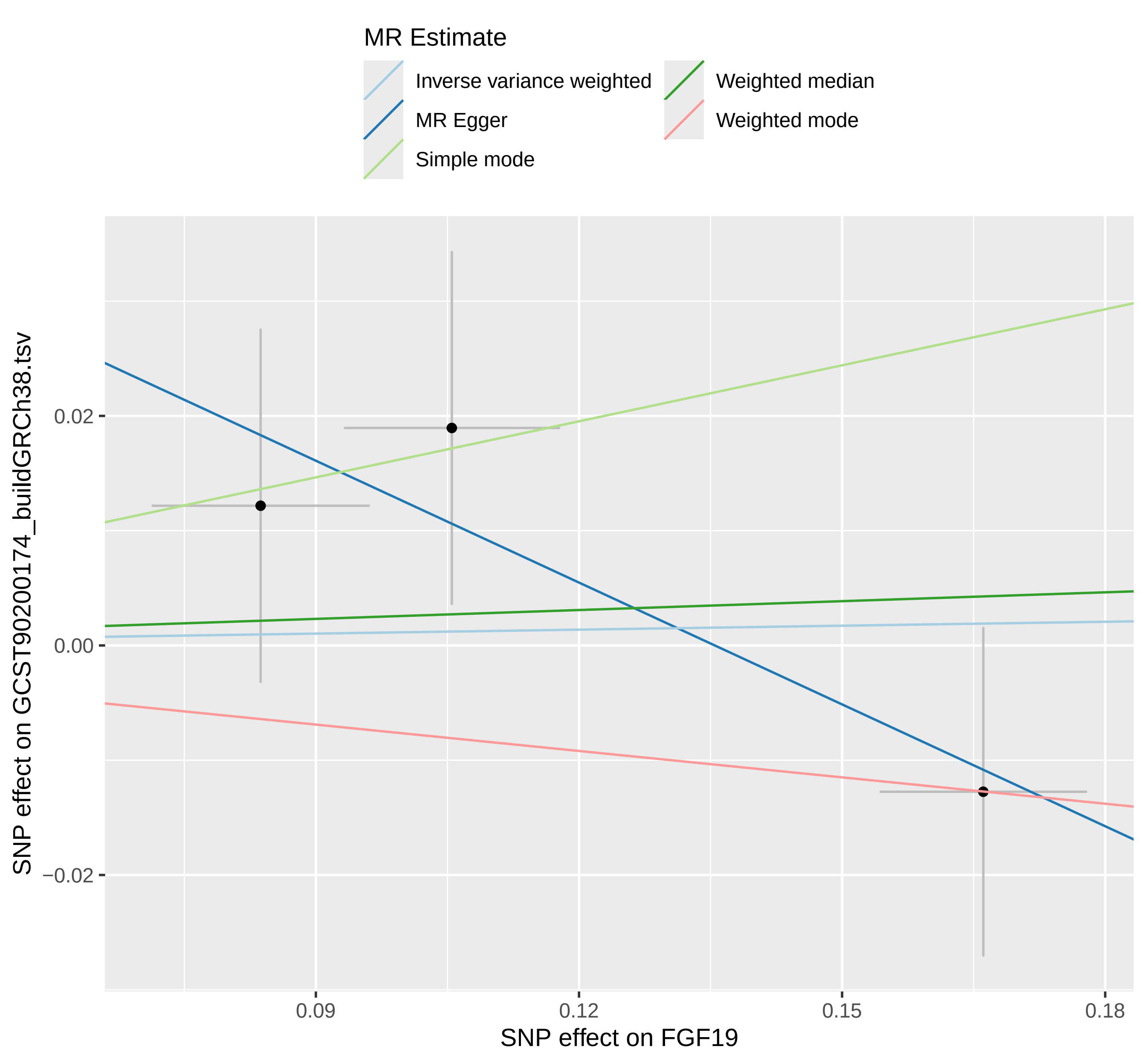
MR Estimate
Inverse variance weighted
MR Egger
Simple mode
Weighted median
Weighted mode
0.02
0.00
−0.02
0.09
0.12
0.15
0.18
SNP effect on GCST90200174_buildGRCh38.tsv
SNP effect on FGF19

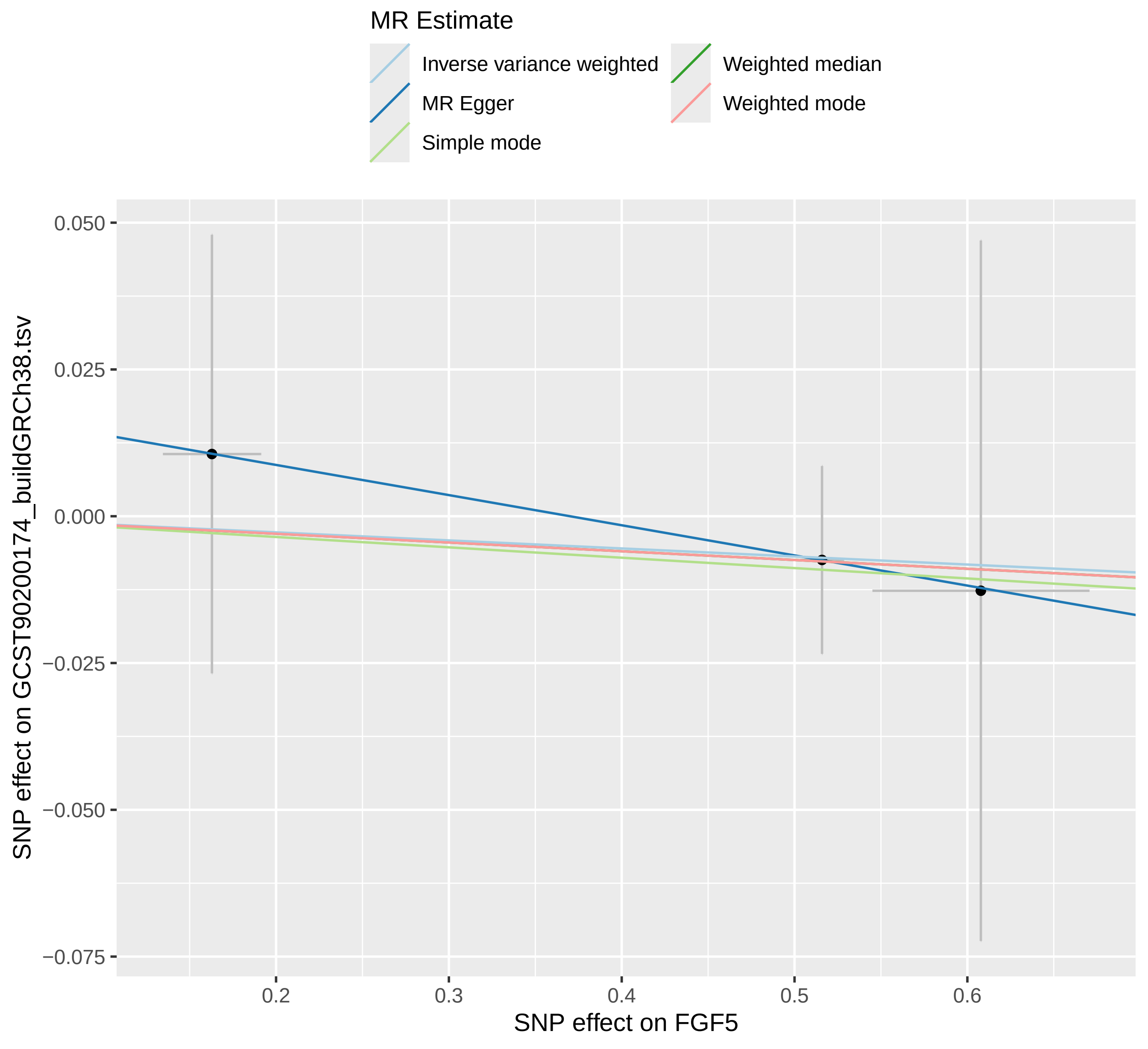
MR Estimate
Inverse variance weighted
MR Egger
Simple mode
Weighted median
Weighted mode
0.050
0.025
0.000
−0.025
−0.050
−0.075
0.2
0.3
0.4
0.5
0.6
SNP effect on GCST90200174_buildGRCh38.tsv
SNP effect on FGF5

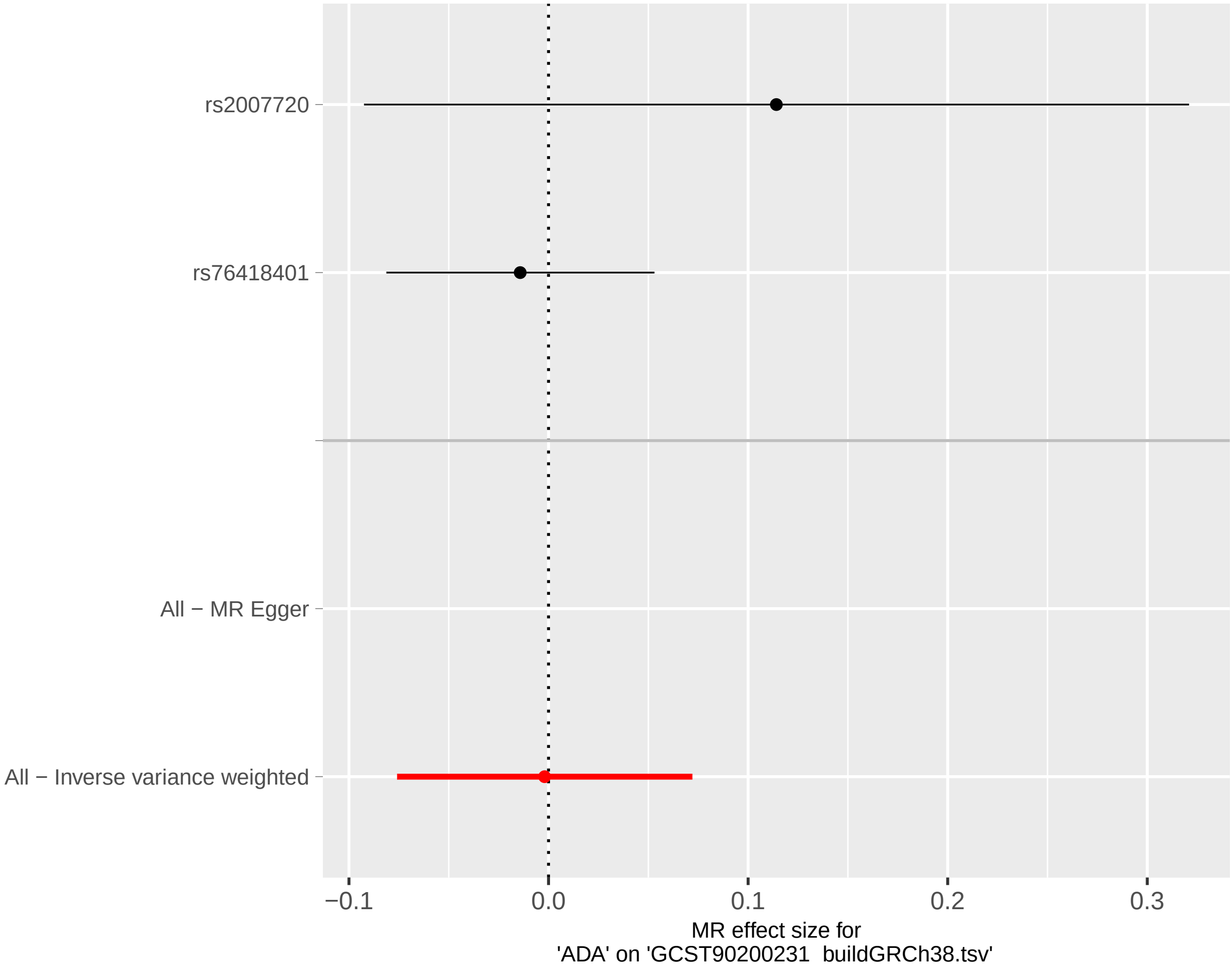
rs2007720
rs76418401
All − MR Egger
All − Inverse variance weighted
−0.1
0.0
0.1
0.2
0.3
MR effect size for
'ADA' on 'GCST90200231_buildGRCh38.tsv'

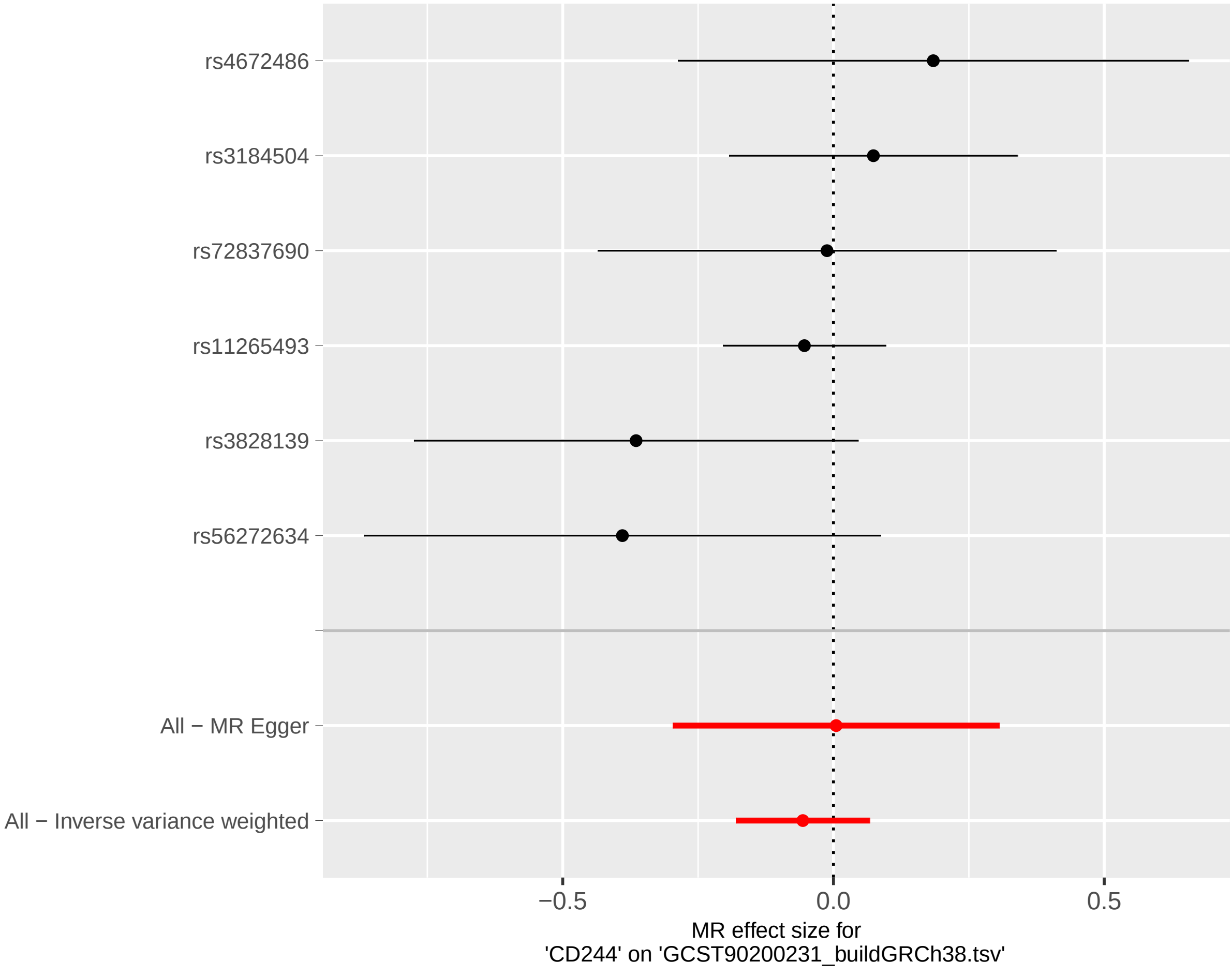
rs4672486
rs3184504
rs72837690
rs11265493
rs3828139
rs56272634
All − MR Egger
All − Inverse variance weighted
−0.5
0.0
0.5
MR effect size for
'CD244' on 'GCST90200231_buildGRCh38.tsv'

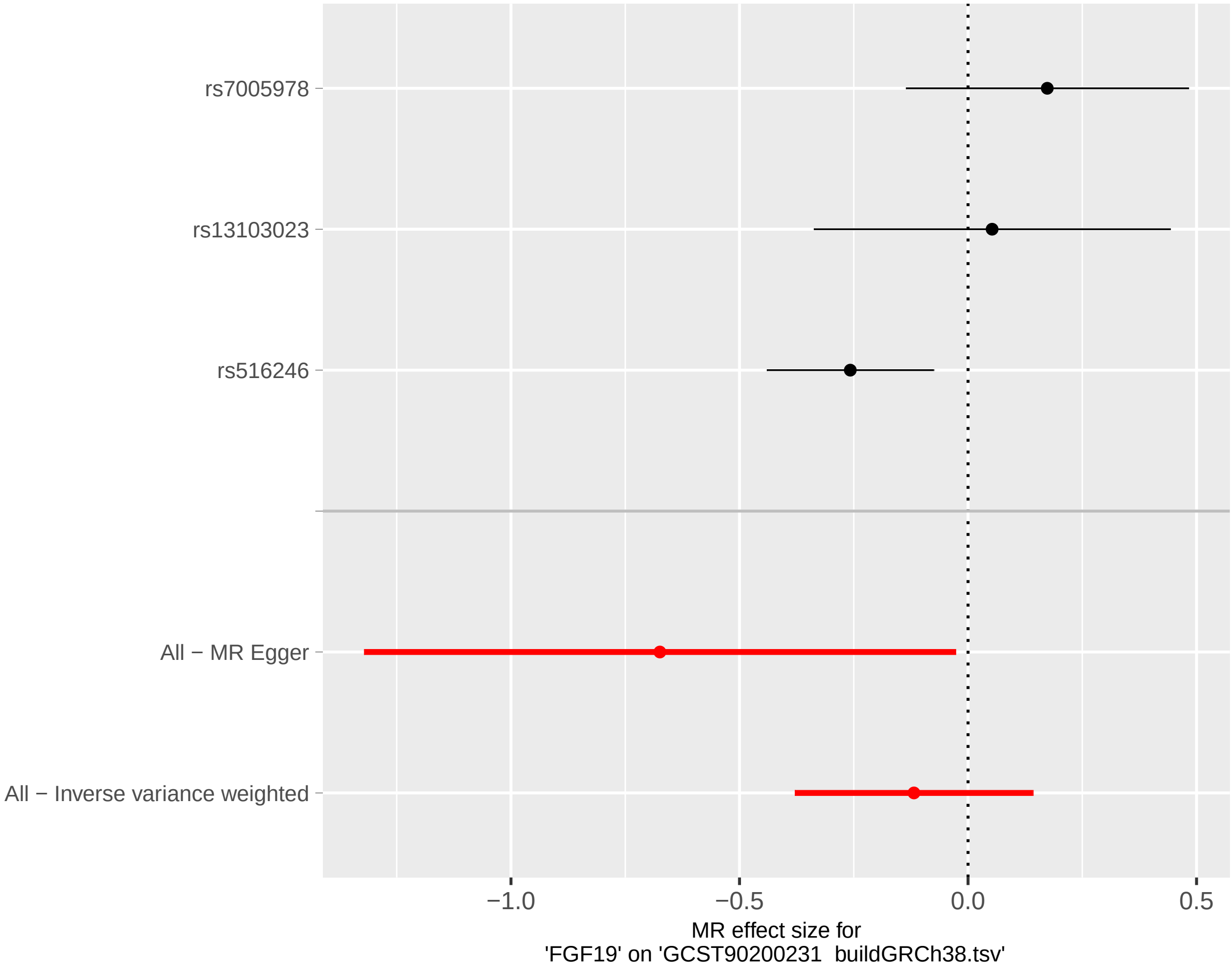
rs7005978
rs13103023
rs516246
All − MR Egger
All − Inverse variance weighted
−1.0
−0.5
0.0
0.5
MR effect size for
'FGF19' on 'GCST90200231_buildGRCh38.tsv'

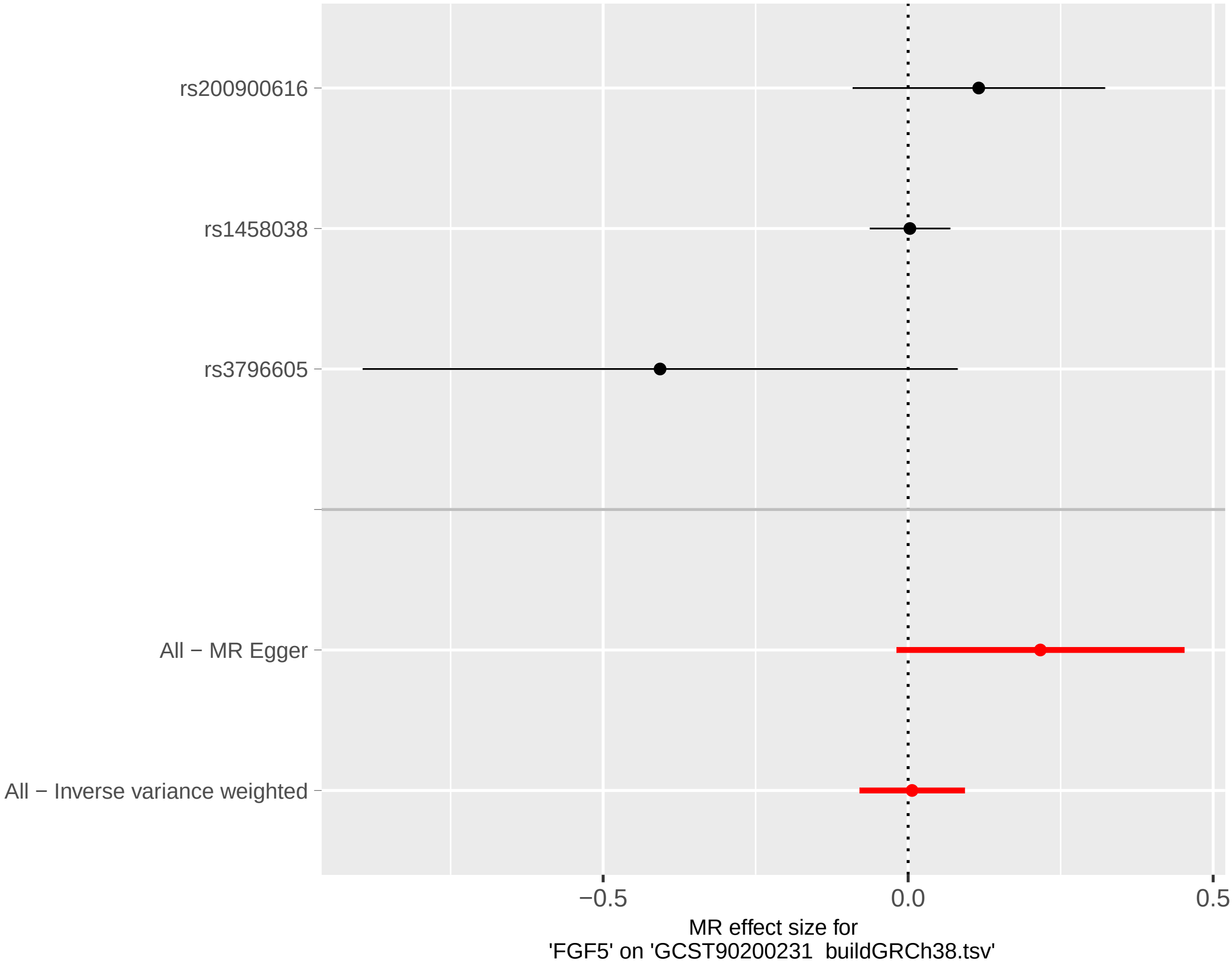

rs200900616
rs1458038
rs3796605
All − MR Egger
All − Inverse variance weighted
−0.5
0.0
0.5
MR effect size for
'FGF5' on 'GCST90200231_buildGRCh38.tsv'

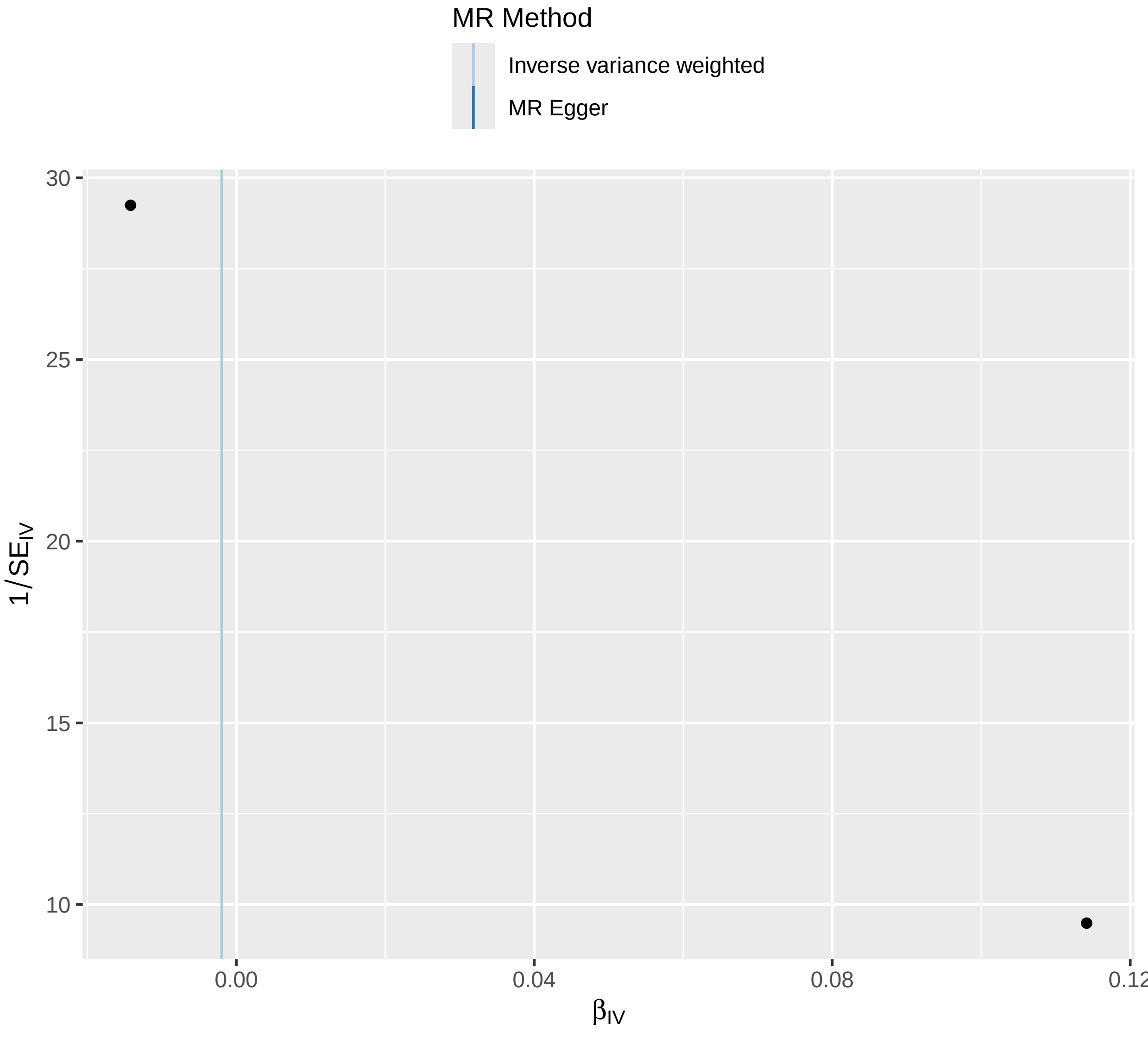
MR Method
Inverse variance weighted
MR Egger
30
25
20
15
10
$1/SE_{IV}$
0.00
0.04
0.08
0.12
β_{IV}

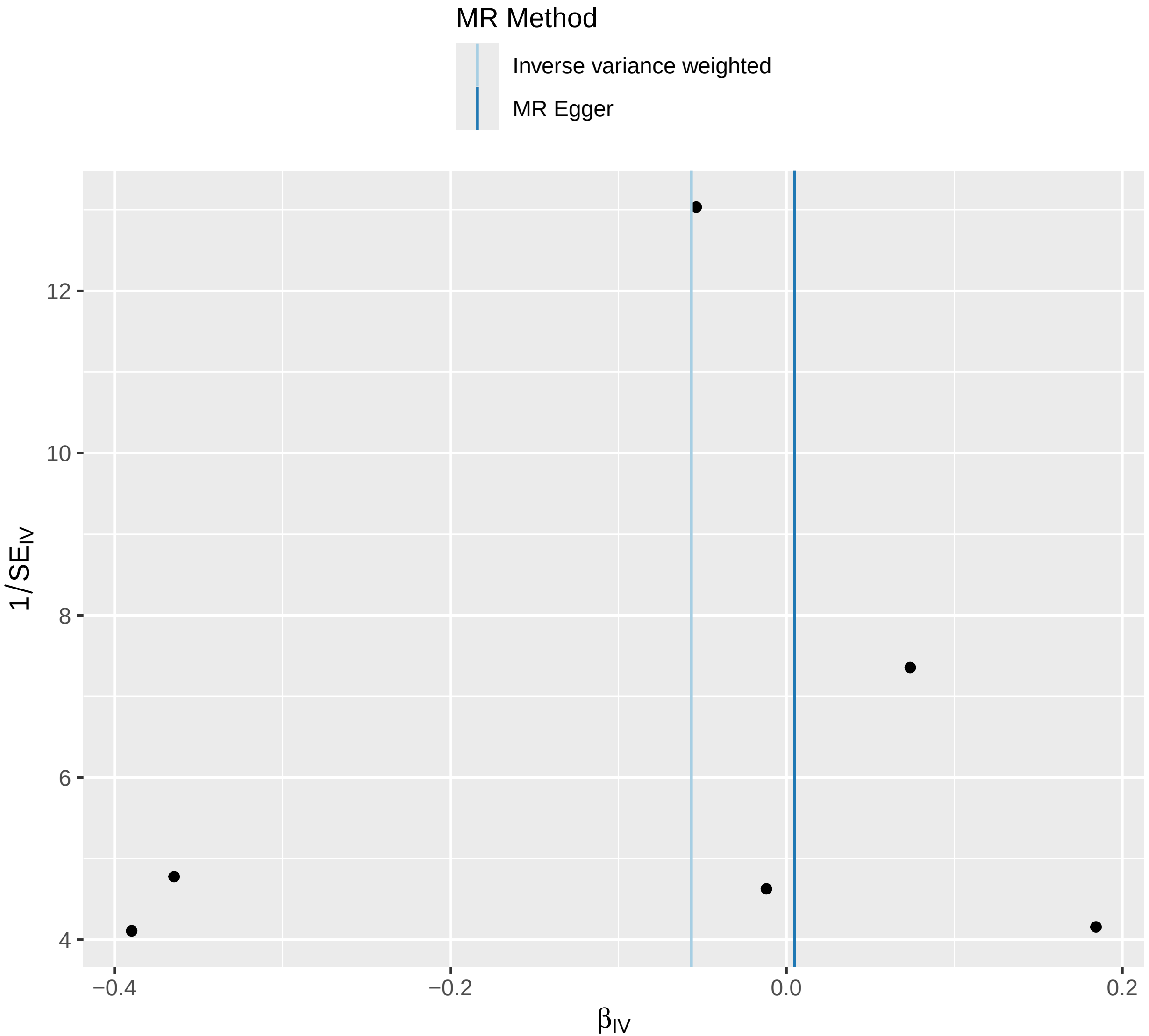
MR Method
Inverse variance weighted
MR Egger
12
10
8
6
4
1/SE_IV
−0.4
−0.2
0.0
0.2
β_IV

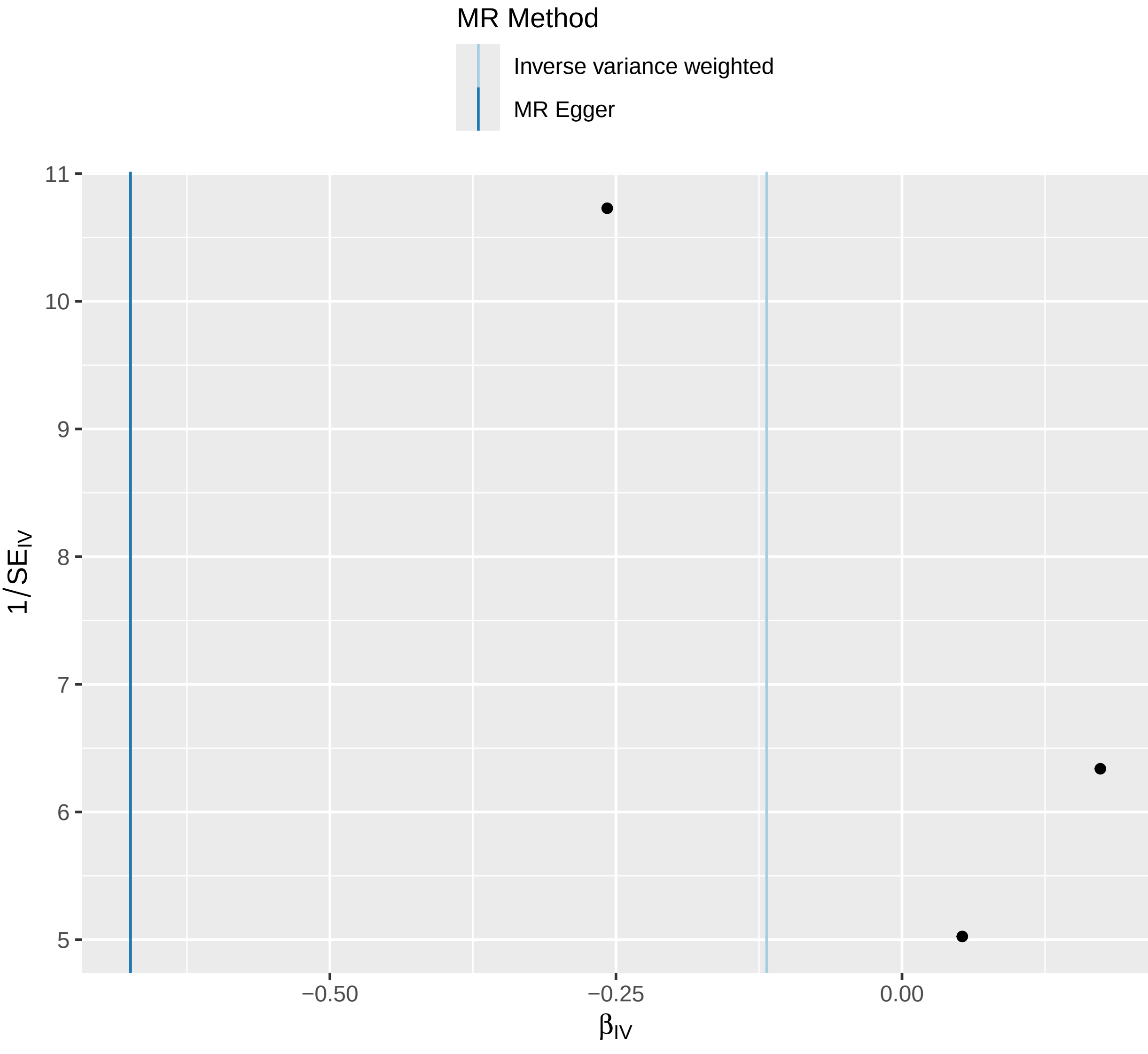

MR Method
Inverse variance weighted
MR Egger
11
10
9
8
7
6
5
1/SE_IV
−0.50
−0.25
0.00
β_IV

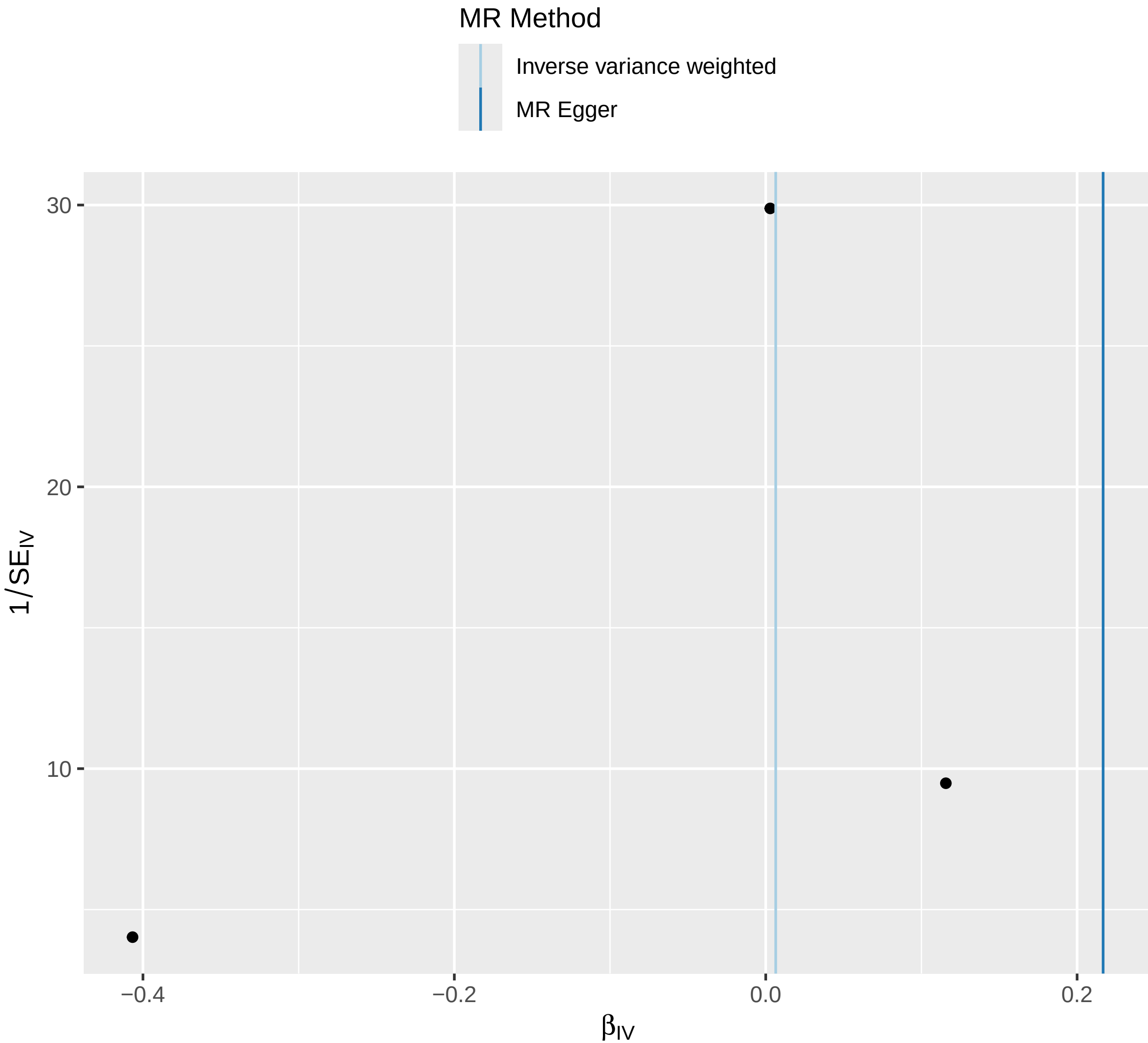

MR Method
Inverse variance weighted
MR Egger
30
20
10
1/SE_IV
−0.4
−0.2
0.0
0.2
β_IV

Insufficient number of SNPs

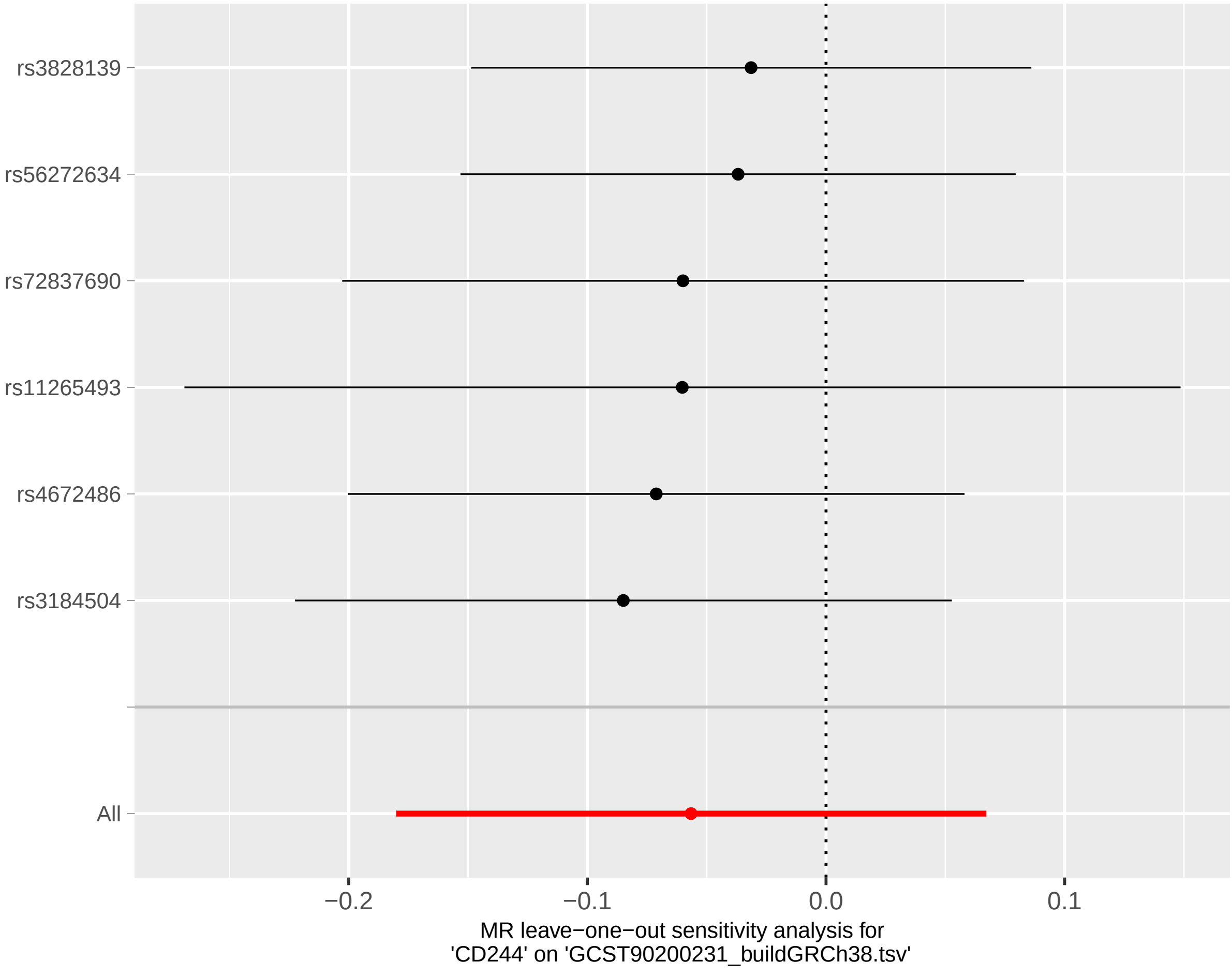
rs3828139
rs56272634
rs72837690
rs11265493
rs4672486
rs3184504
All
−0.2
−0.1
0.0
0.1
MR leave−one−out sensitivity analysis for
'CD244' on 'GCST90200231_buildGRCh38.tsv'

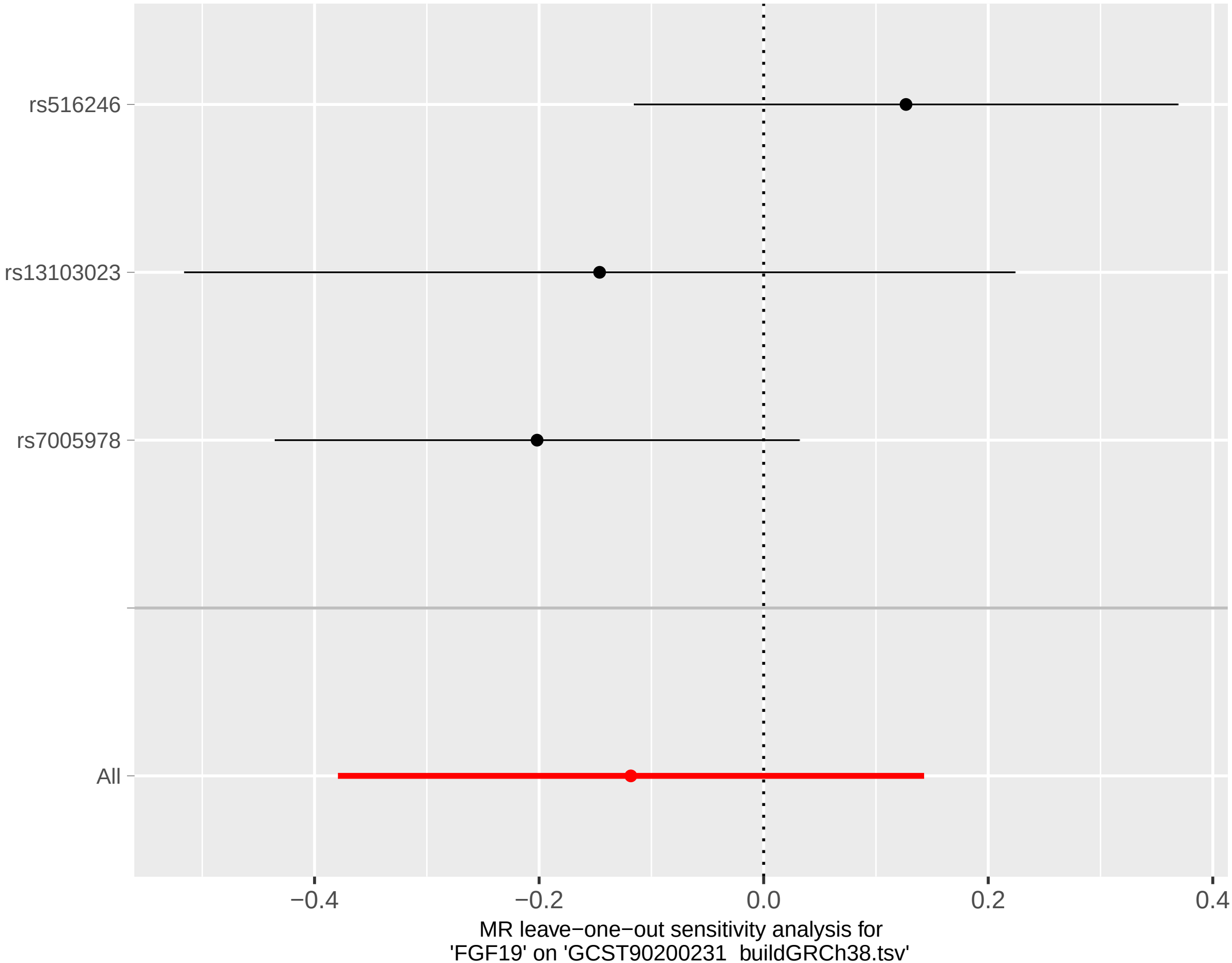
rs516246
rs13103023
rs7005978
All
−0.4
−0.2
0.0
0.2
0.4
MR leave−one−out sensitivity analysis for
'FGF19' on 'GCST90200231_buildGRCh38.tsv'

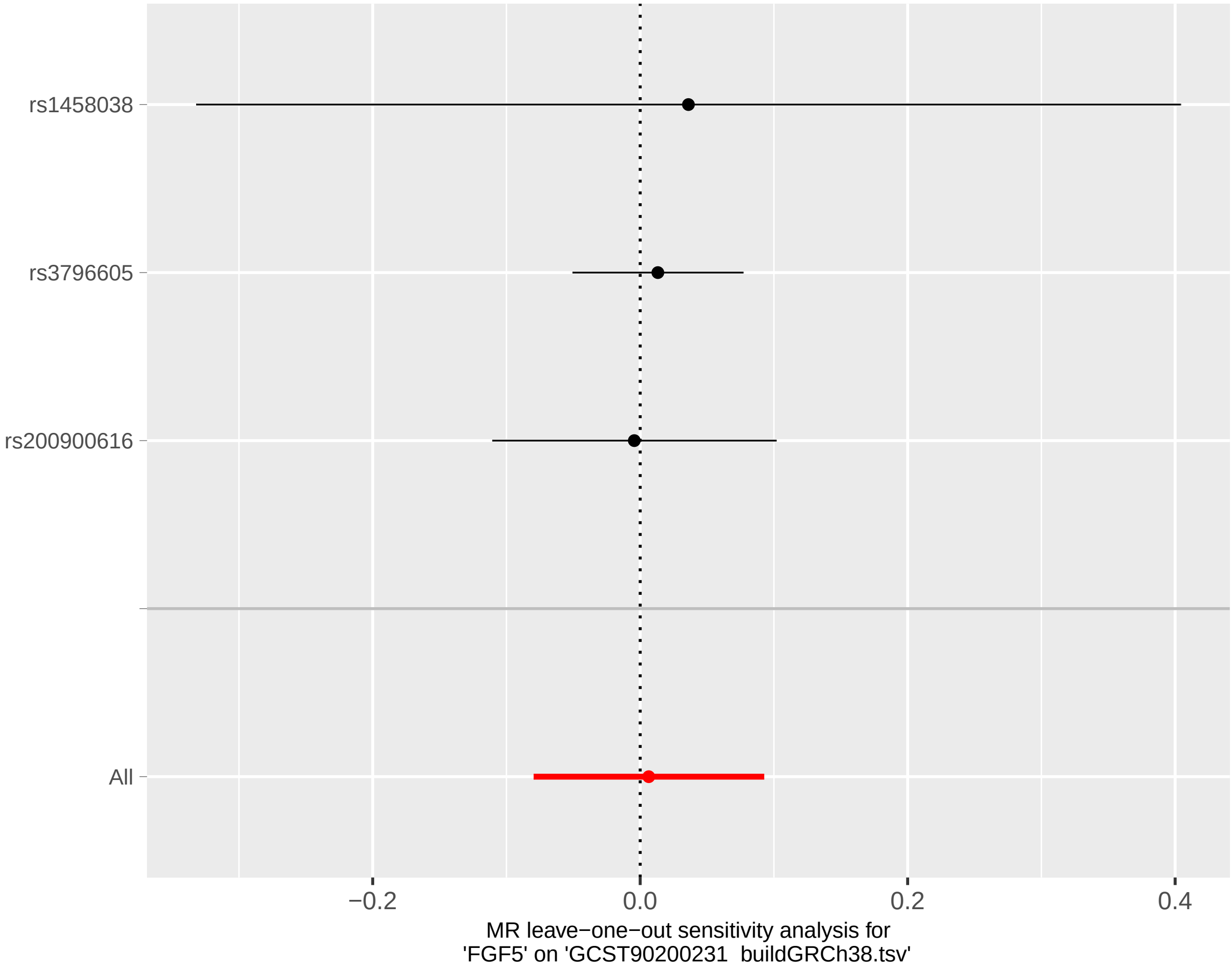
rs1458038
rs3796605
rs200900616
All
−0.2
0.0
0.2
0.4
MR leave−one−out sensitivity analysis for
'FGF5' on 'GCST90200231_buildGRCh38.tsv'

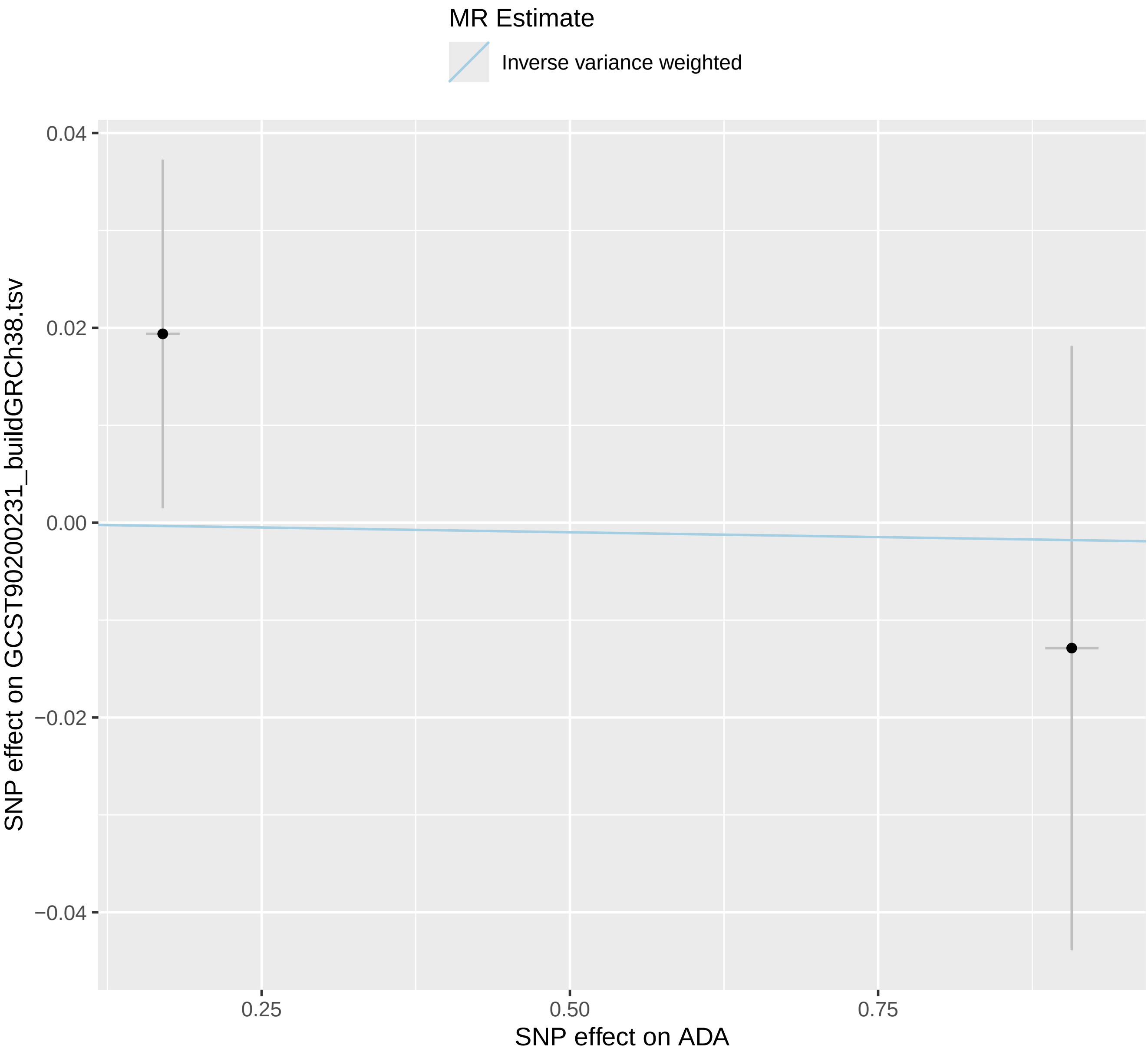

MR Estimate
Inverse variance weighted
SNP effect on GCST90200231_buildGRCh38.tsv
0.04
0.02
0.00
−0.02
−0.04
0.25
0.50
0.75
SNP effect on ADA

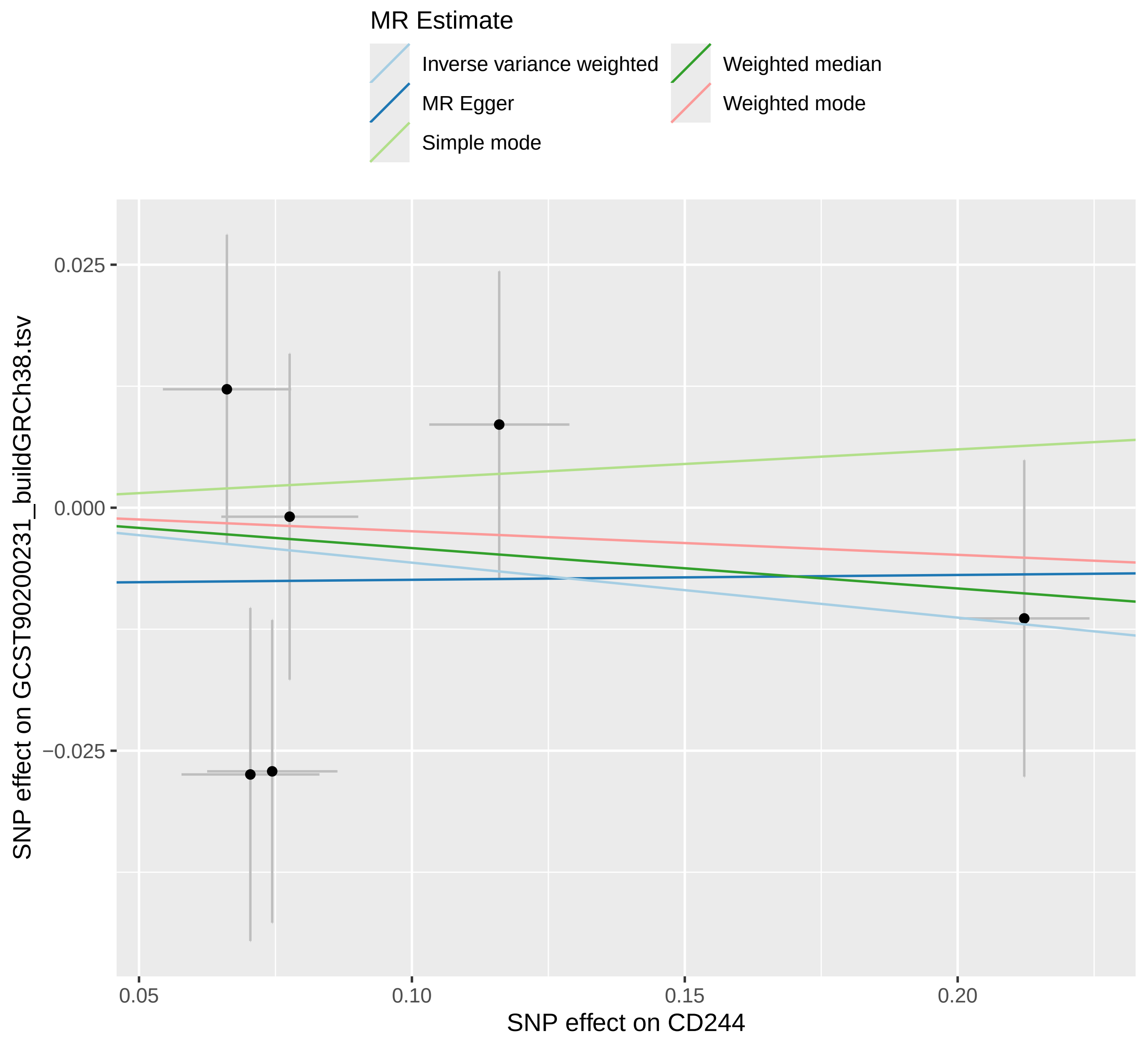

MR Estimate
Inverse variance weighted
MR Egger
Simple mode
Weighted median
Weighted mode
0.025
0.000
−0.025
SNP effect on GCST90200231_buildGRCh38.tsv
0.05
0.10
0.15
0.20
SNP effect on CD244

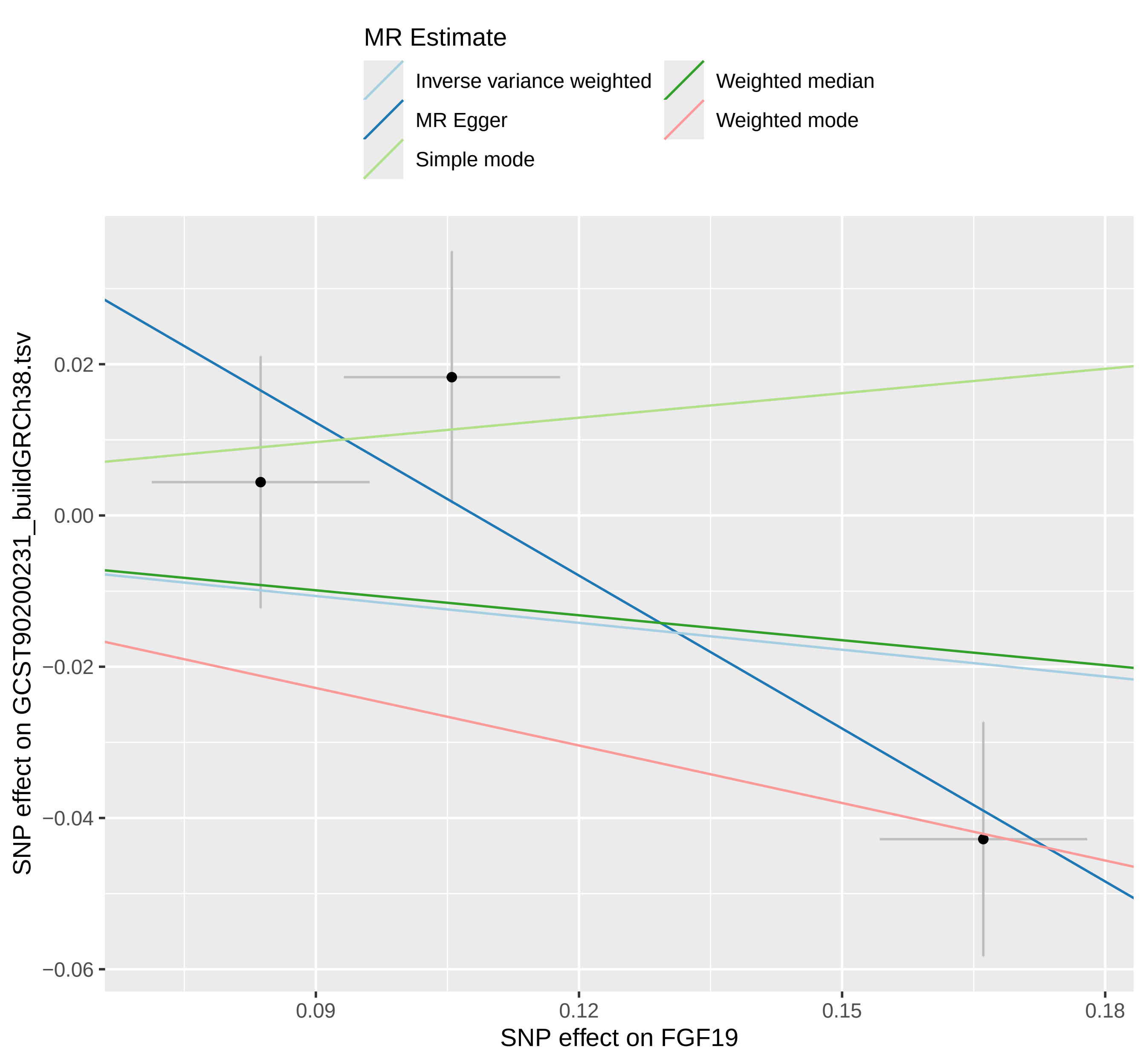
MR Estimate
Inverse variance weighted
MR Egger
Simple mode
Weighted median
Weighted mode
SNP effect on GCST90200231_buildGRCh38.tsv
0.02
0.00
−0.02
−0.04
−0.06
0.09
0.12
0.15
0.18
SNP effect on FGF19

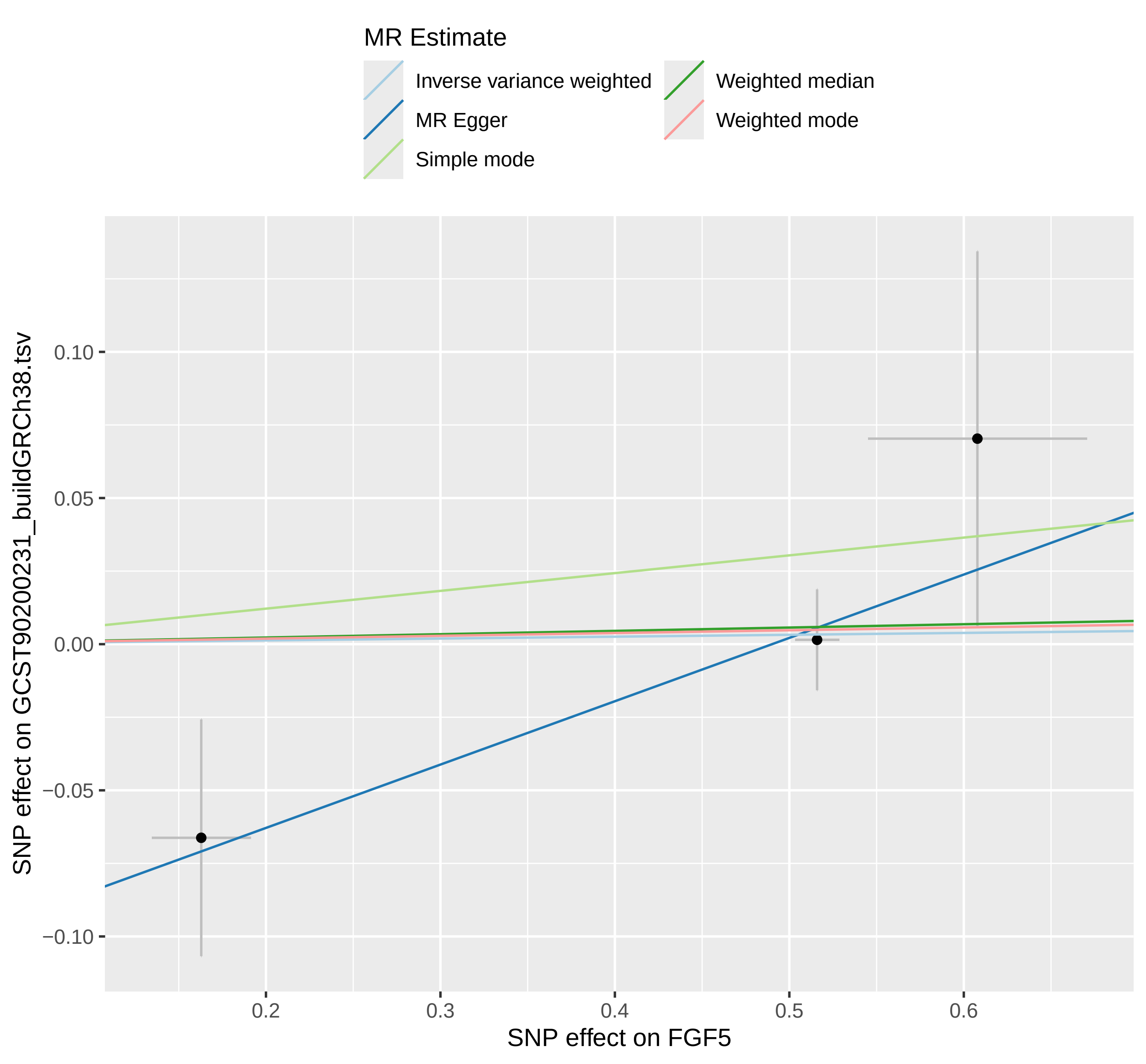

MR Estimate
Inverse variance weighted
MR Egger
Simple mode
Weighted median
Weighted mode
SNP effect on GCST90200231_buildGRCh38.tsv
0.10
0.05
0.00
−0.05
−0.10
0.2
0.3
0.4
0.5
0.6
SNP effect on FGF5

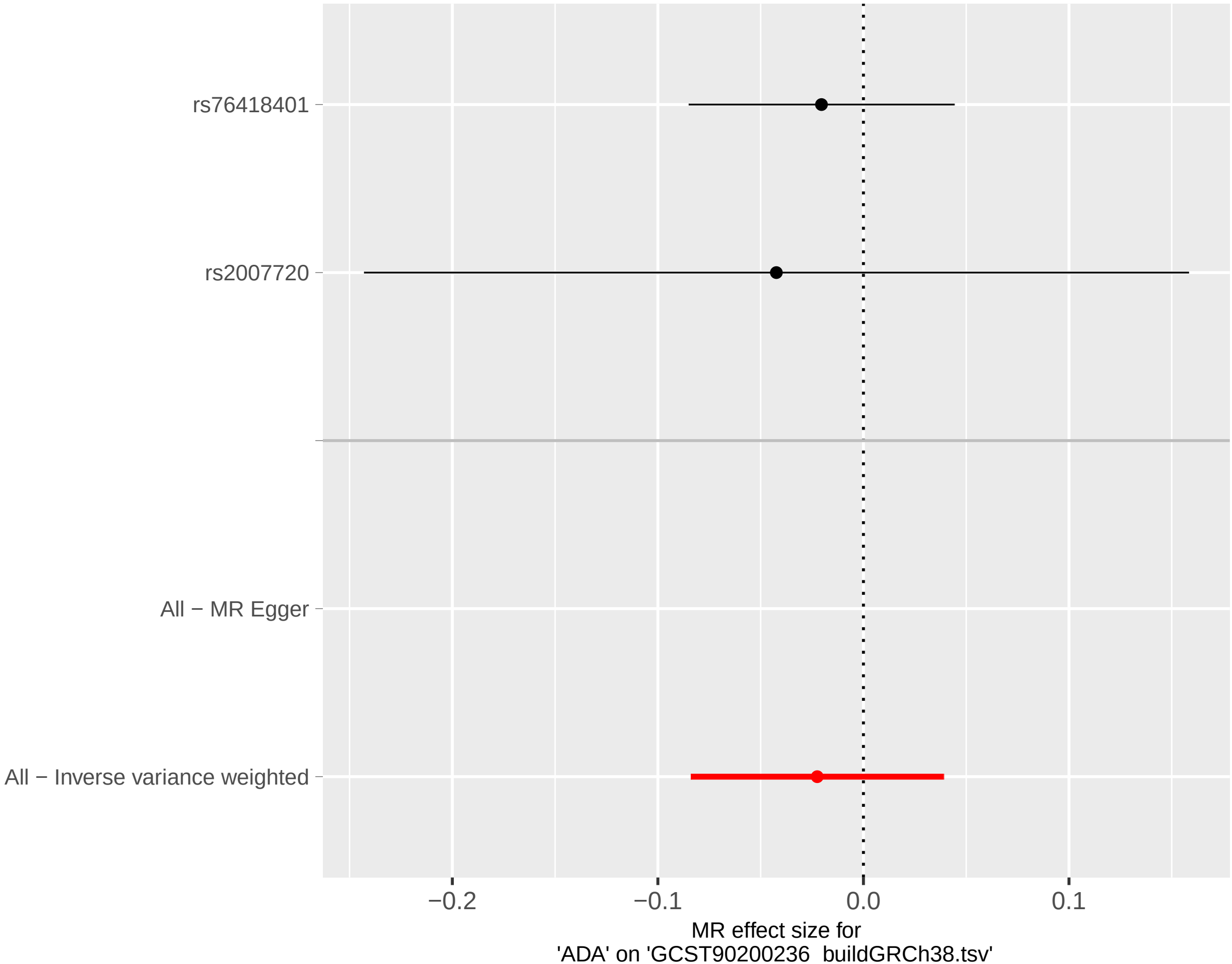

rs76418401
rs2007720
All − MR Egger
All − Inverse variance weighted
−0.2
−0.1
0.0
0.1
MR effect size for
'ADA' on 'GCST90200236_buildGRCh38.tsv'

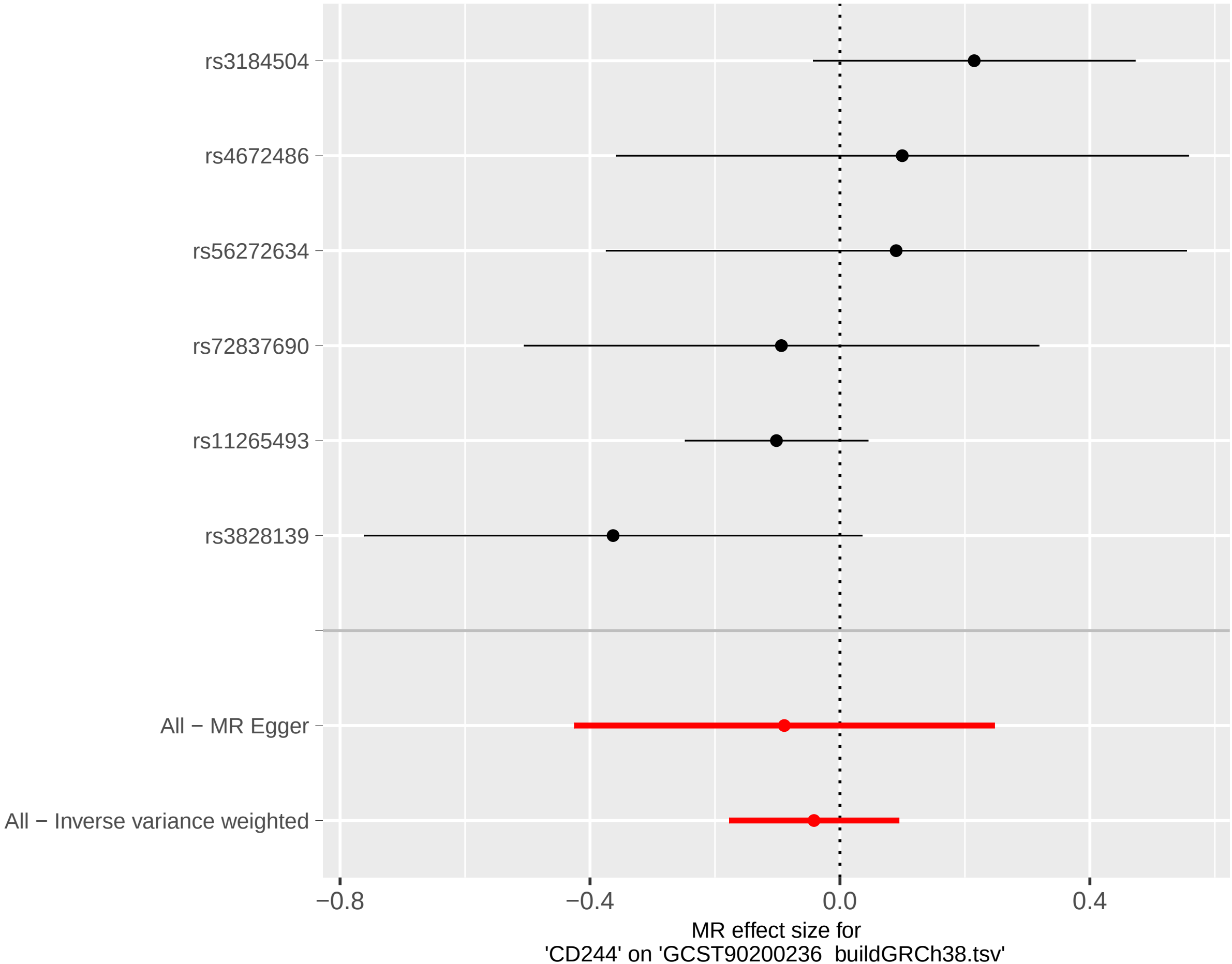

rs3184504
rs4672486
rs56272634
rs72837690
rs11265493
rs3828139
All − MR Egger
All − Inverse variance weighted
−0.8
−0.4
0.0
0.4
MR effect size for
'CD244' on 'GCST90200236_buildGRCh38.tsv'

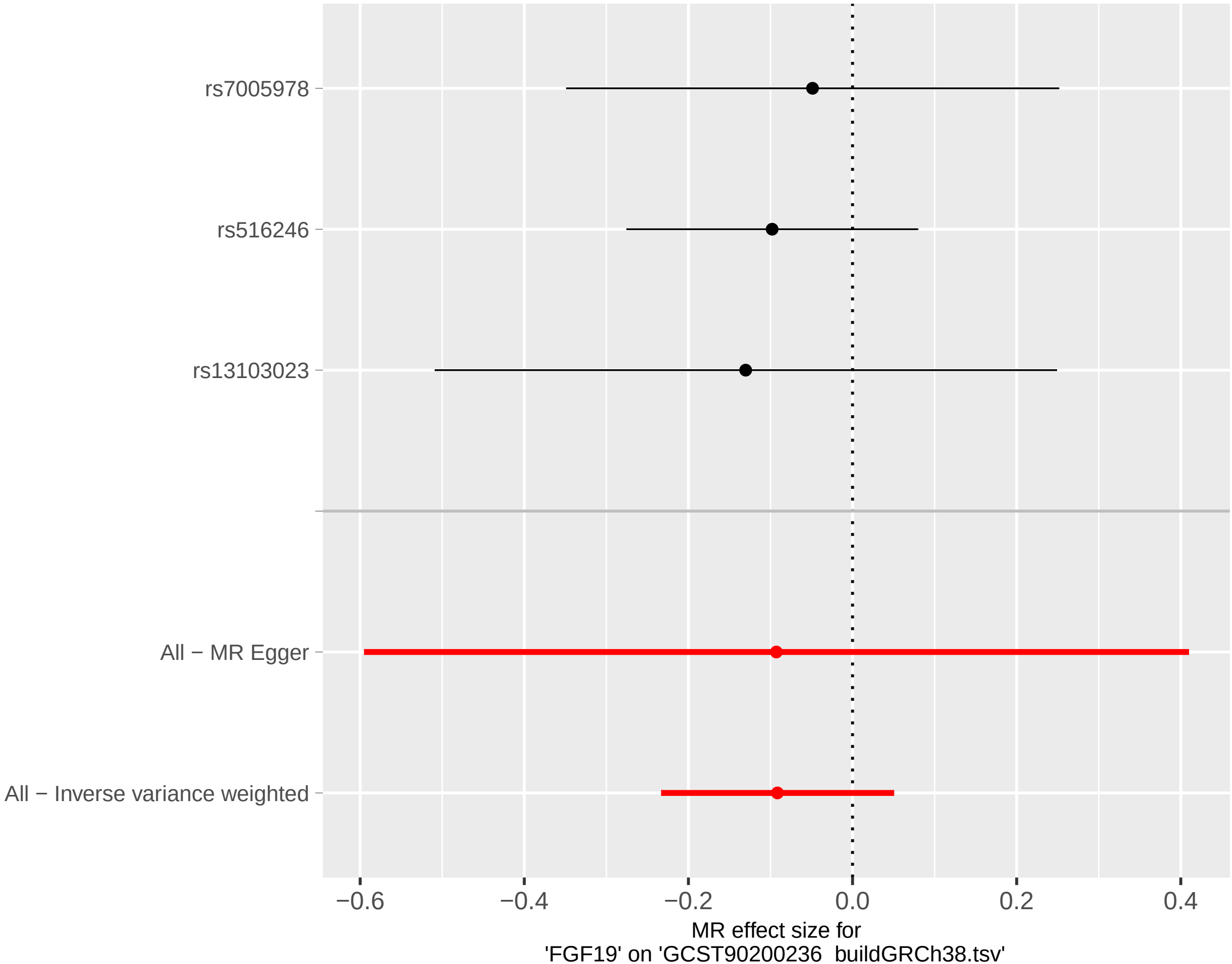
rs7005978
rs516246
rs13103023
All − MR Egger
All − Inverse variance weighted
−0.6
−0.4
−0.2
0.0
0.2
0.4
MR effect size for
'FGF19' on 'GCST90200236_buildGRCh38.tsv'

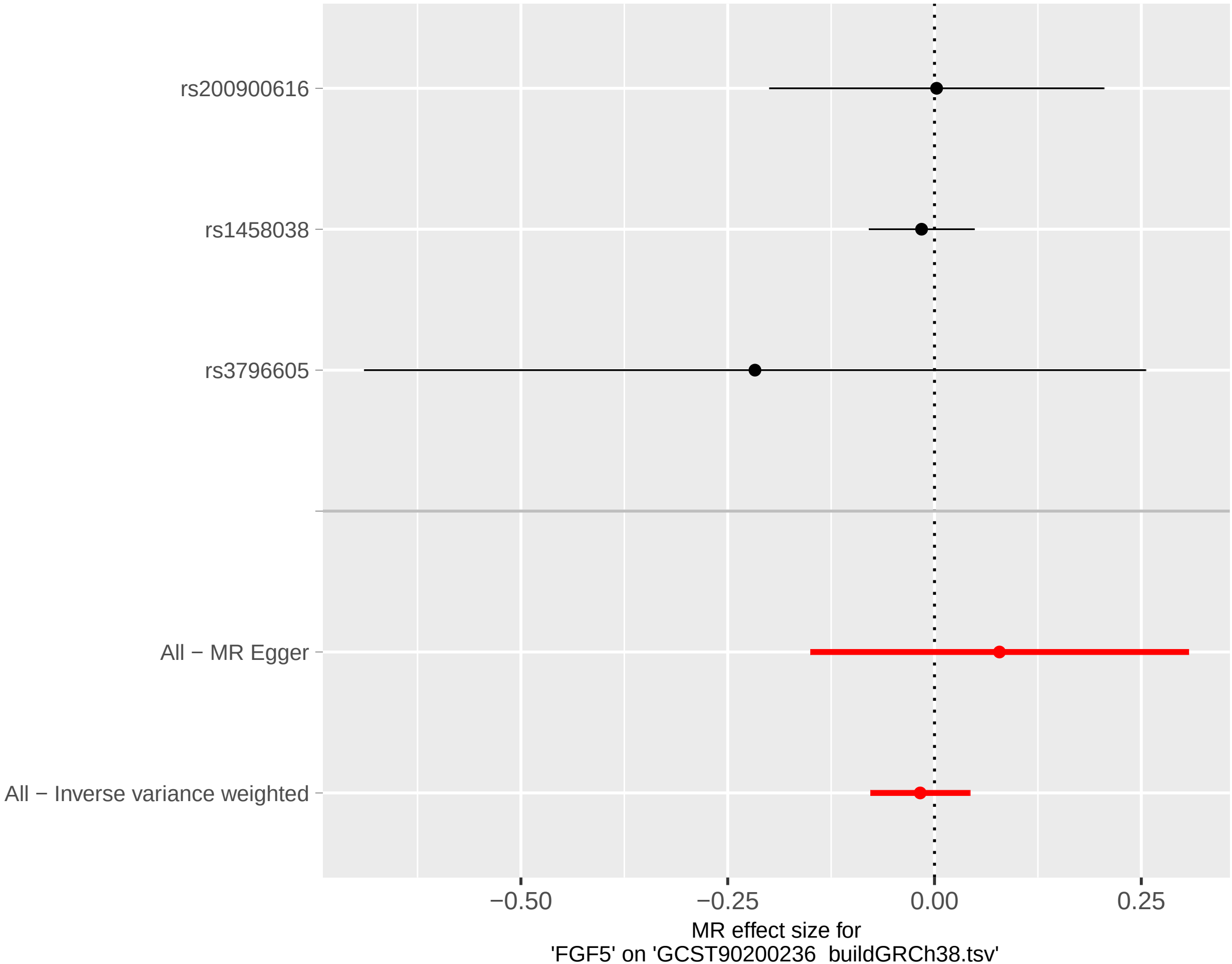

rs200900616
rs1458038
rs3796605
All − MR Egger
All − Inverse variance weighted
−0.50
−0.25
0.00
0.25
MR effect size for
'FGF5' on 'GCST90200236_buildGRCh38.tsv'

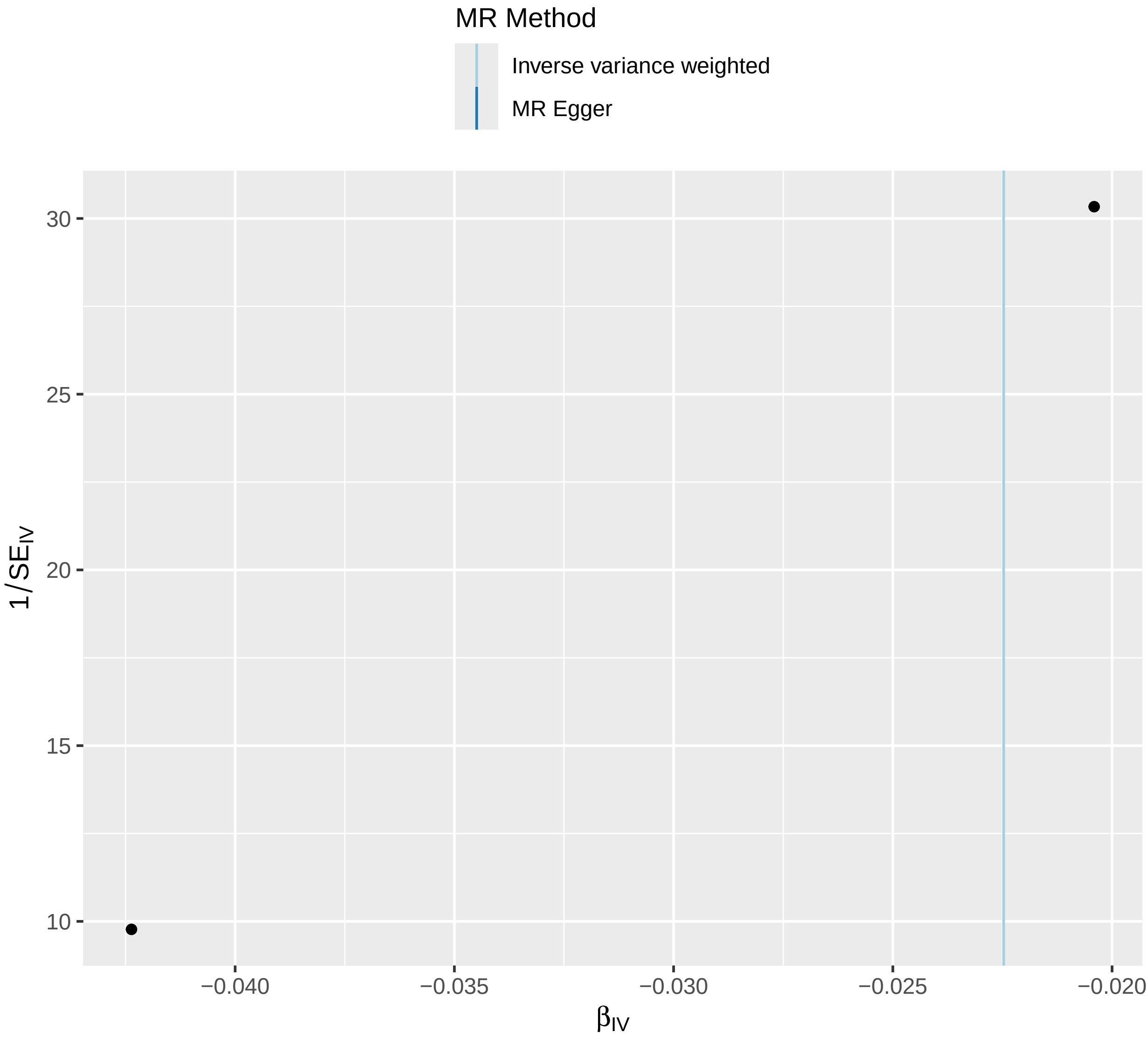
MR Method
Inverse variance weighted
MR Egger
30
25
20
15
10
1/SE$_{IV}$
−0.040
−0.035
−0.030
−0.025
−0.020
β$_{IV}$

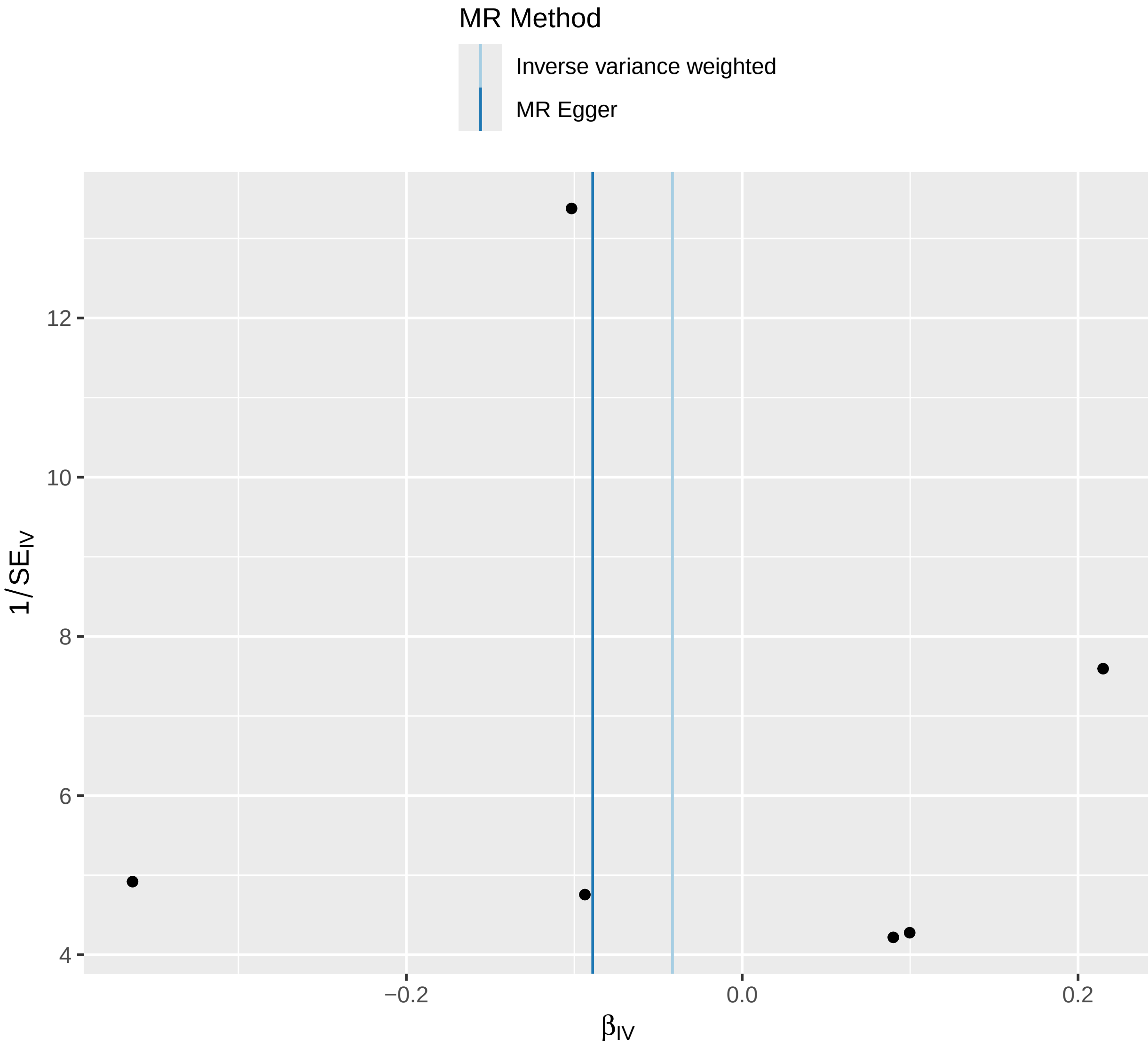

MR Method
Inverse variance weighted
MR Egger
12
10
8
6
4
1/SE_IV
−0.2
0.0
0.2
β_IV

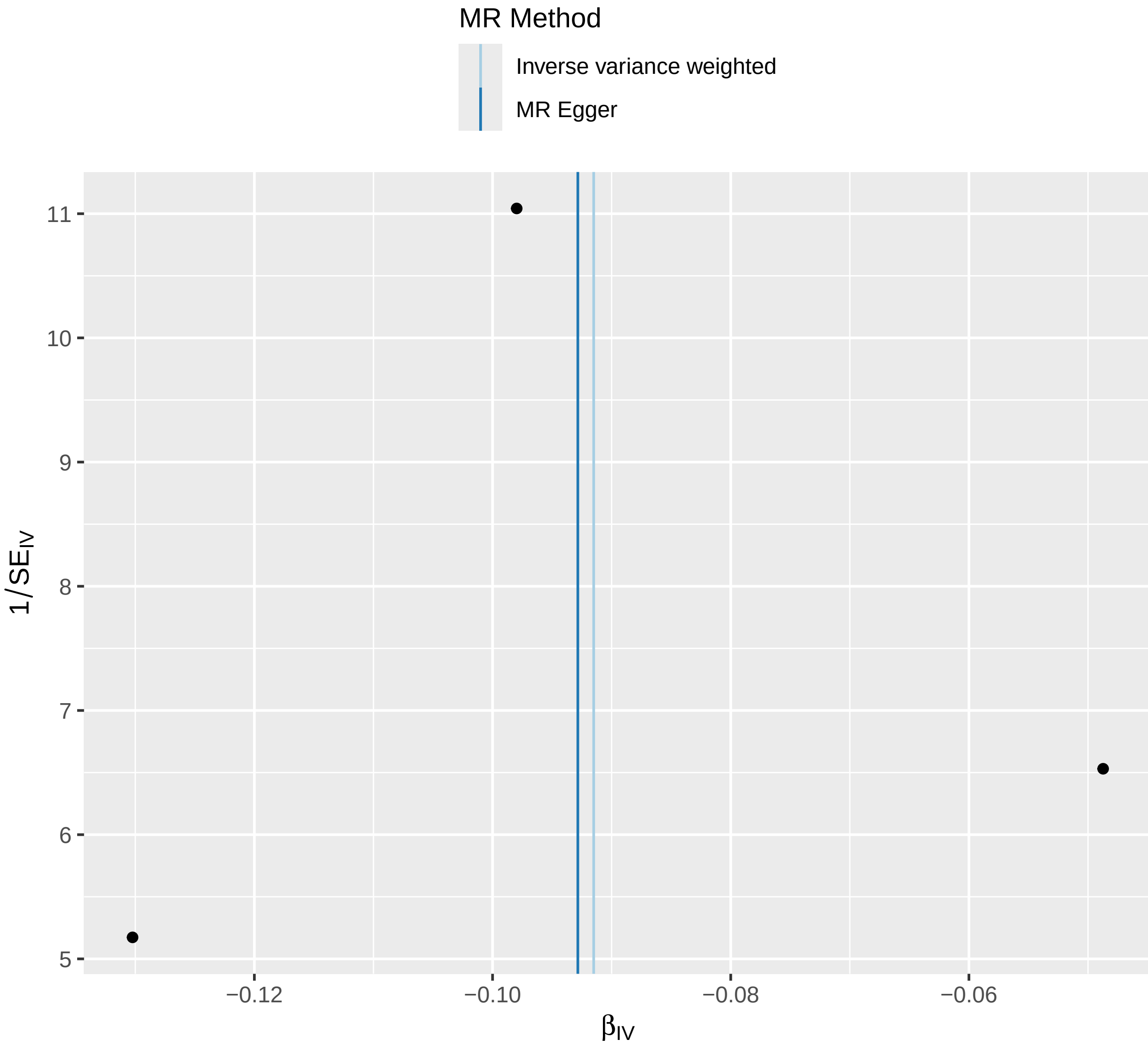

MR Method
Inverse variance weighted
MR Egger
11
10
9
8
7
6
5
1/SE_IV
−0.12
−0.10
−0.08
−0.06
β_IV

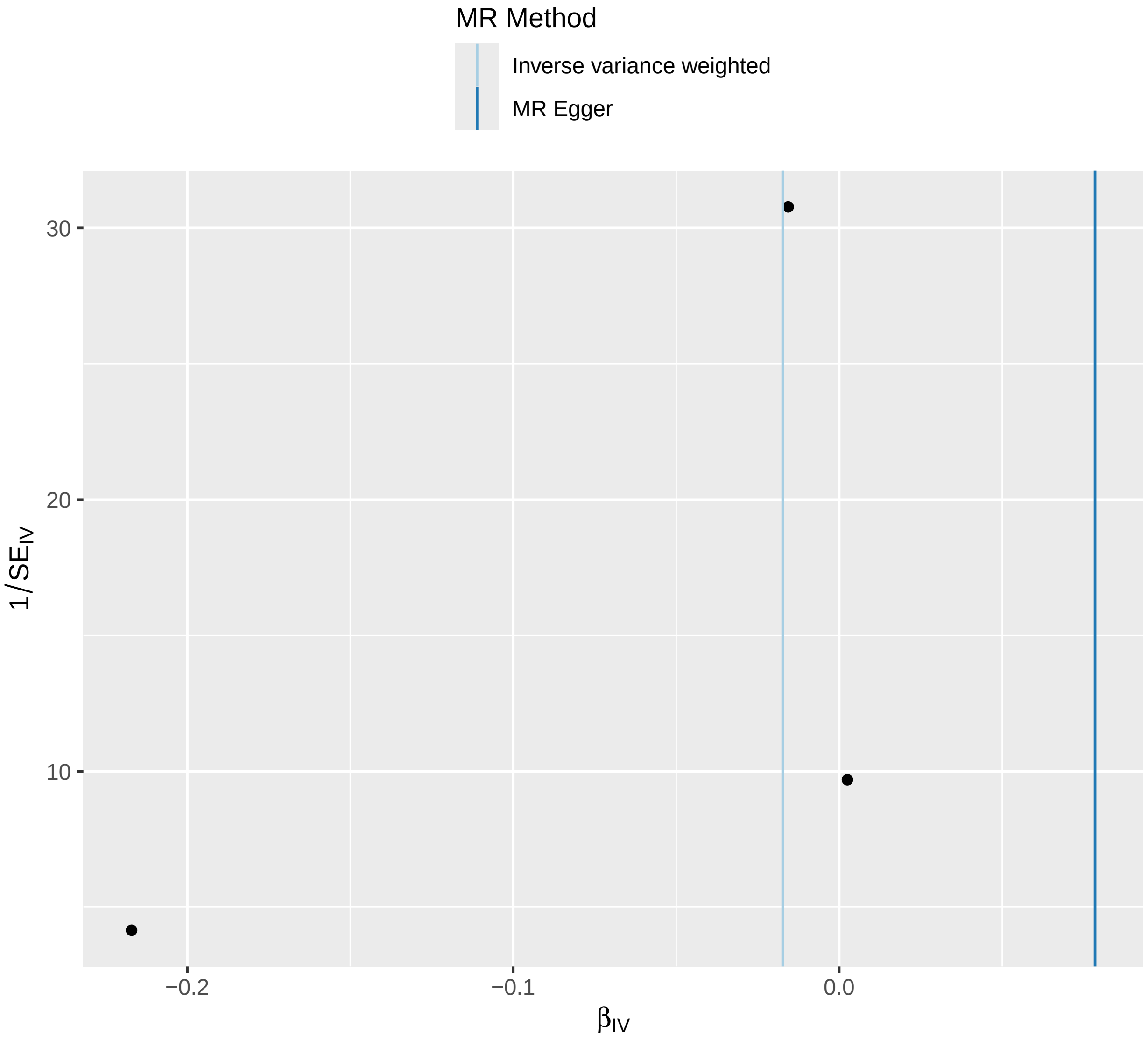
MR Method
Inverse variance weighted
MR Egger
30
20
10
1/SE_IV
−0.2
−0.1
0.0
β_IV

Insufficient number of SNPs

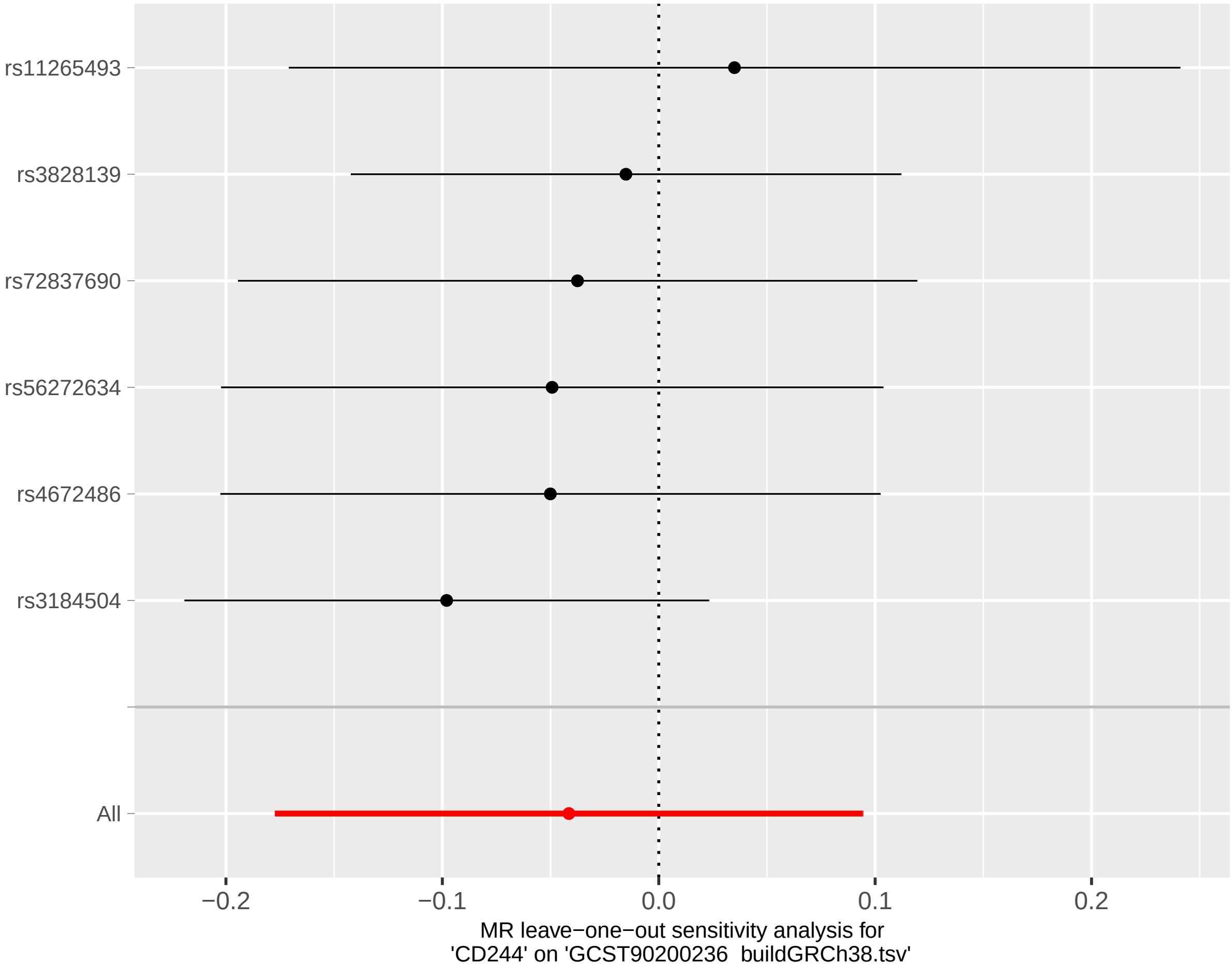

rs11265493
rs3828139
rs72837690
rs56272634
rs4672486
rs3184504
All
−0.2
−0.1
0.0
0.1
0.2
MR leave−one−out sensitivity analysis for
'CD244' on 'GCST90200236_buildGRCh38.tsv'

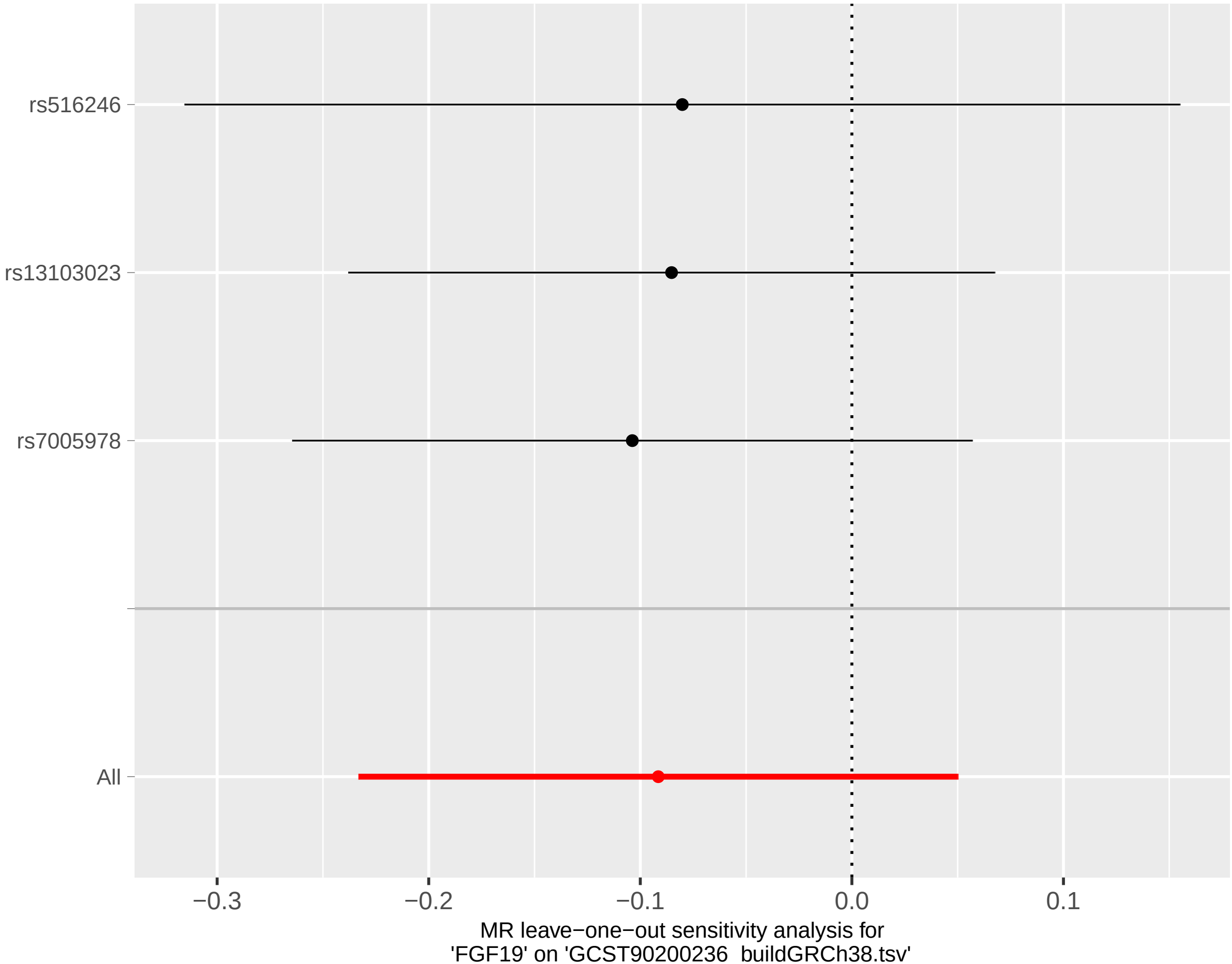
rs516246
rs13103023
rs7005978
All
−0.3
−0.2
−0.1
0.0
0.1
MR leave−one−out sensitivity analysis for
'FGF19' on 'GCST90200236_buildGRCh38.tsv'

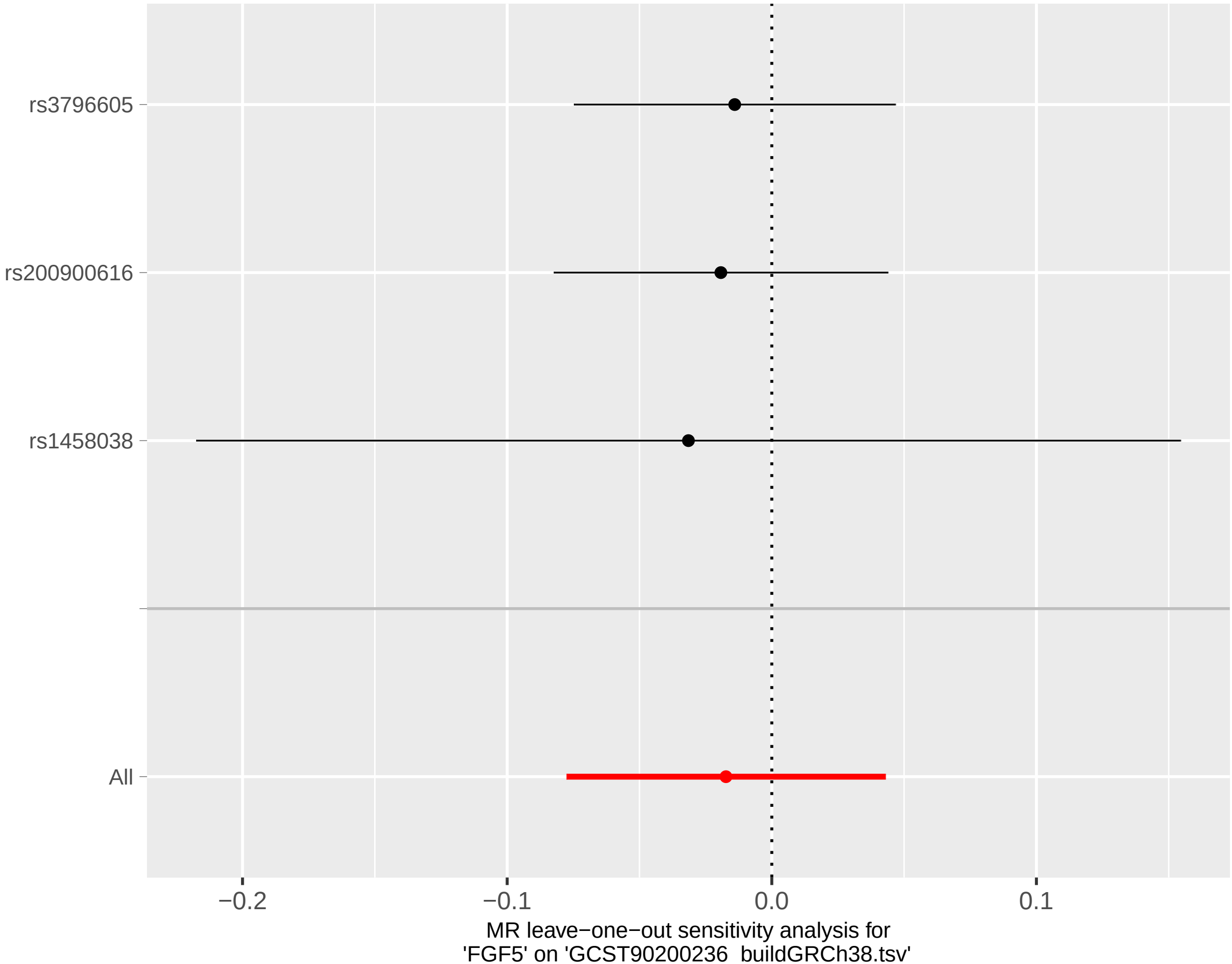
rs3796605
rs200900616
rs1458038
All
−0.2
−0.1
0.0
0.1
MR leave−one−out sensitivity analysis for
'FGF5' on 'GCST90200236_buildGRCh38.tsv'

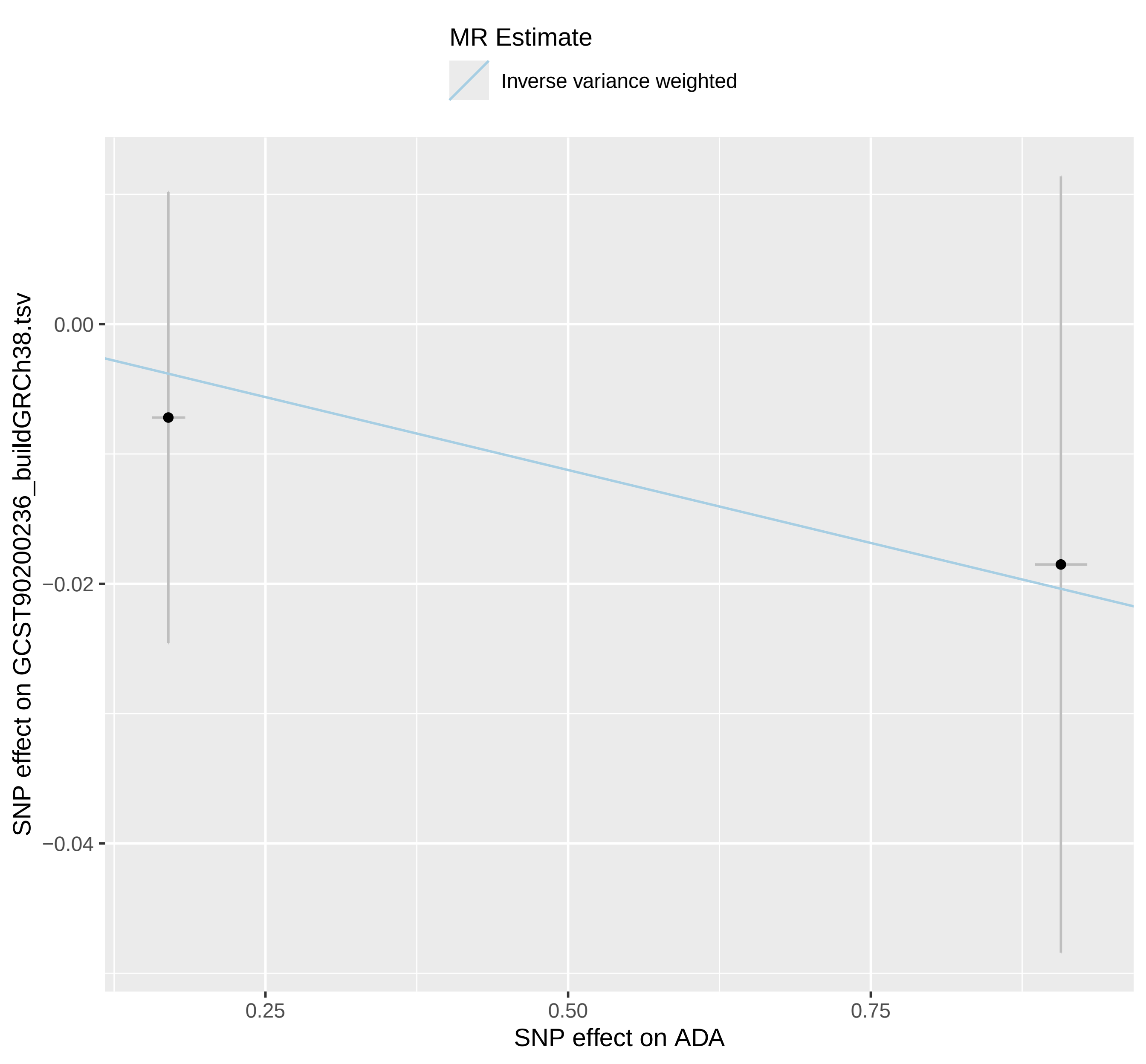
MR Estimate
Inverse variance weighted
SNP effect on GCST90200236_buildGRCh38.tsv
0.00
−0.02
−0.04
0.25
0.50
0.75
SNP effect on ADA

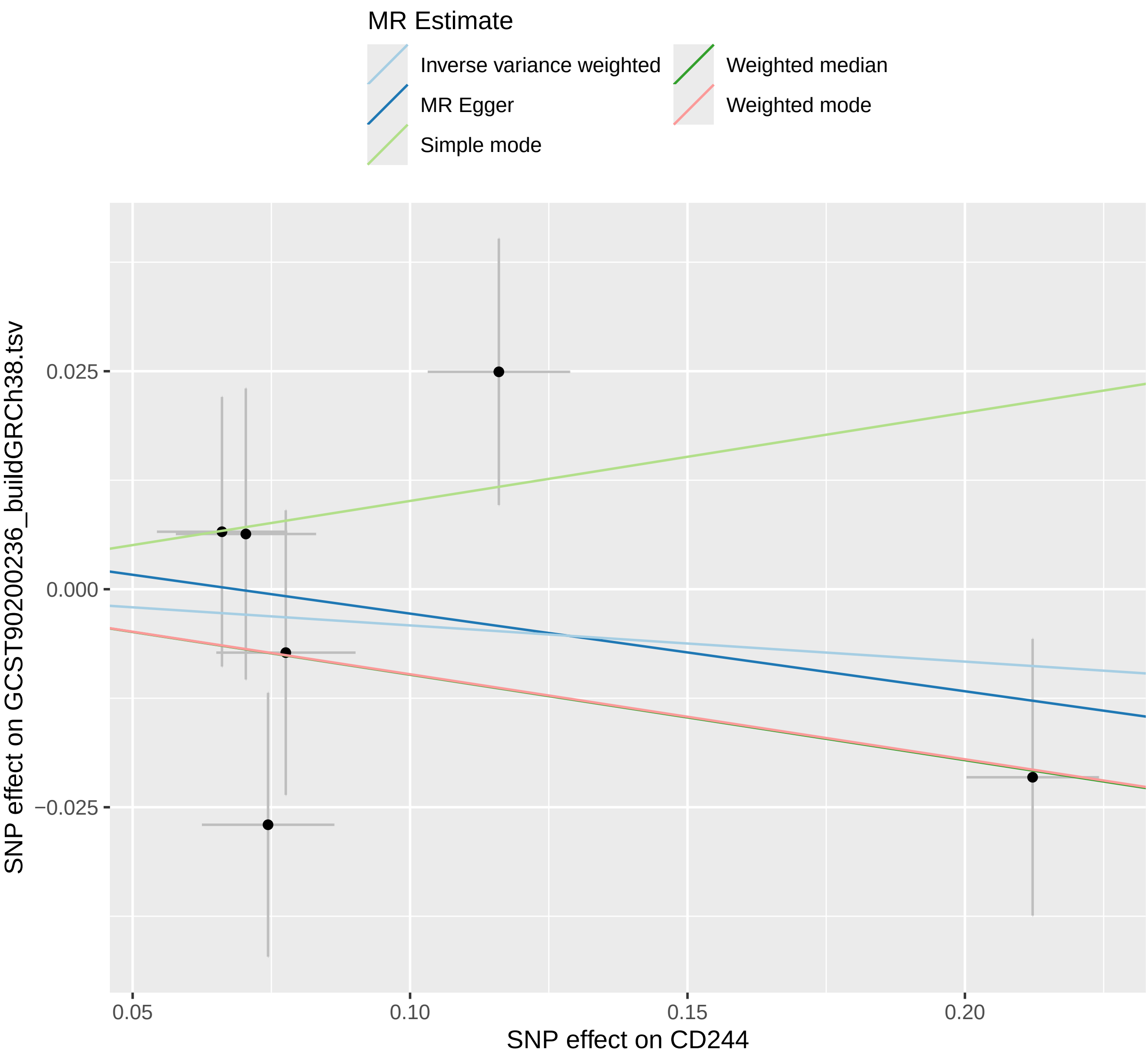
MR Estimate
Inverse variance weighted
MR Egger
Simple mode
Weighted median
Weighted mode
0.025
0.000
−0.025
0.05
0.10
0.15
0.20
SNP effect on GCST90200236_buildGRCh38.tsv
SNP effect on CD244

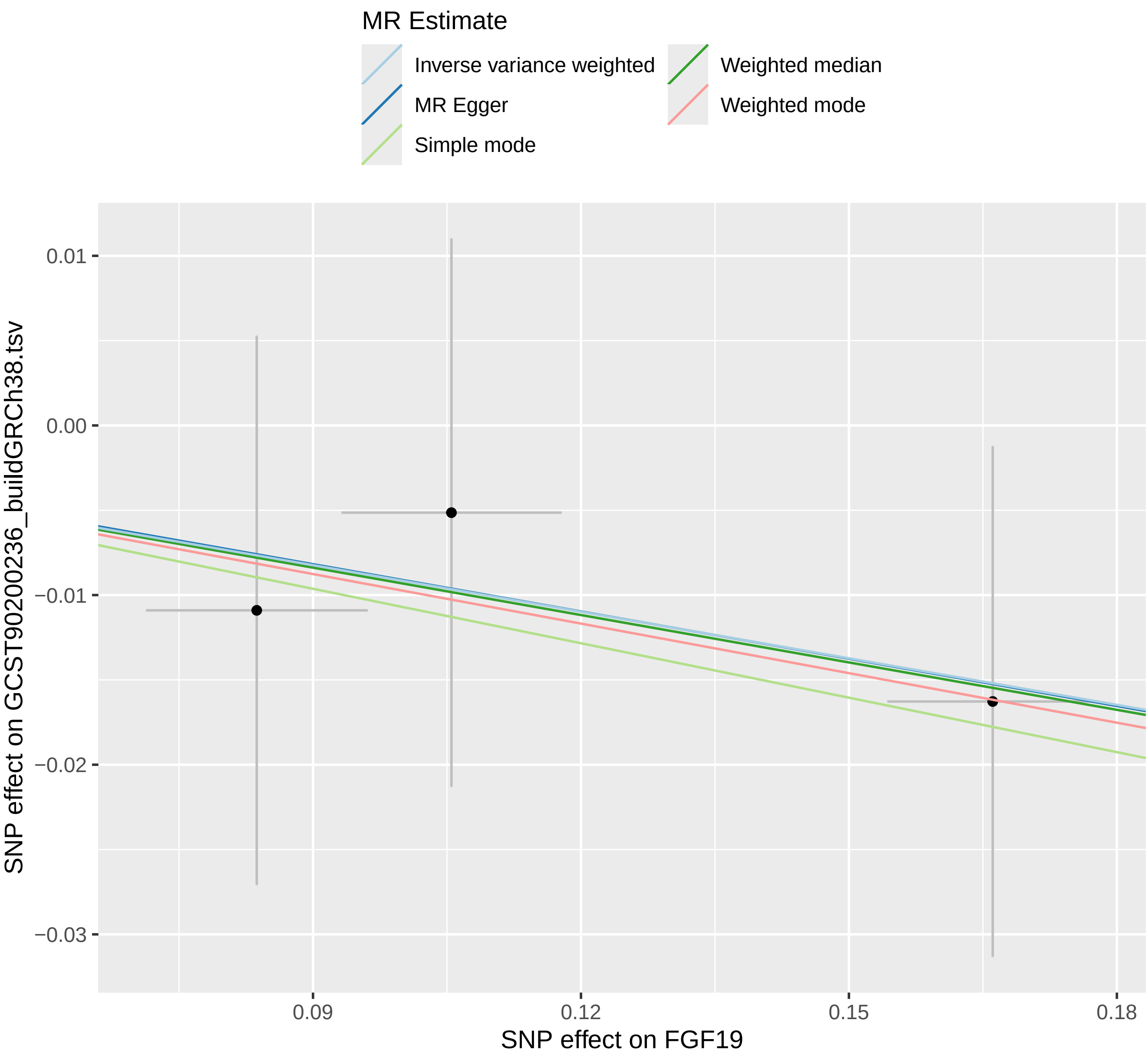
MR Estimate
Inverse variance weighted
MR Egger
Simple mode
Weighted median
Weighted mode
0.01
0.00
−0.01
−0.02
−0.03
SNP effect on GCST90200236_buildGRCh38.tsv
0.09
0.12
0.15
0.18
SNP effect on FGF19

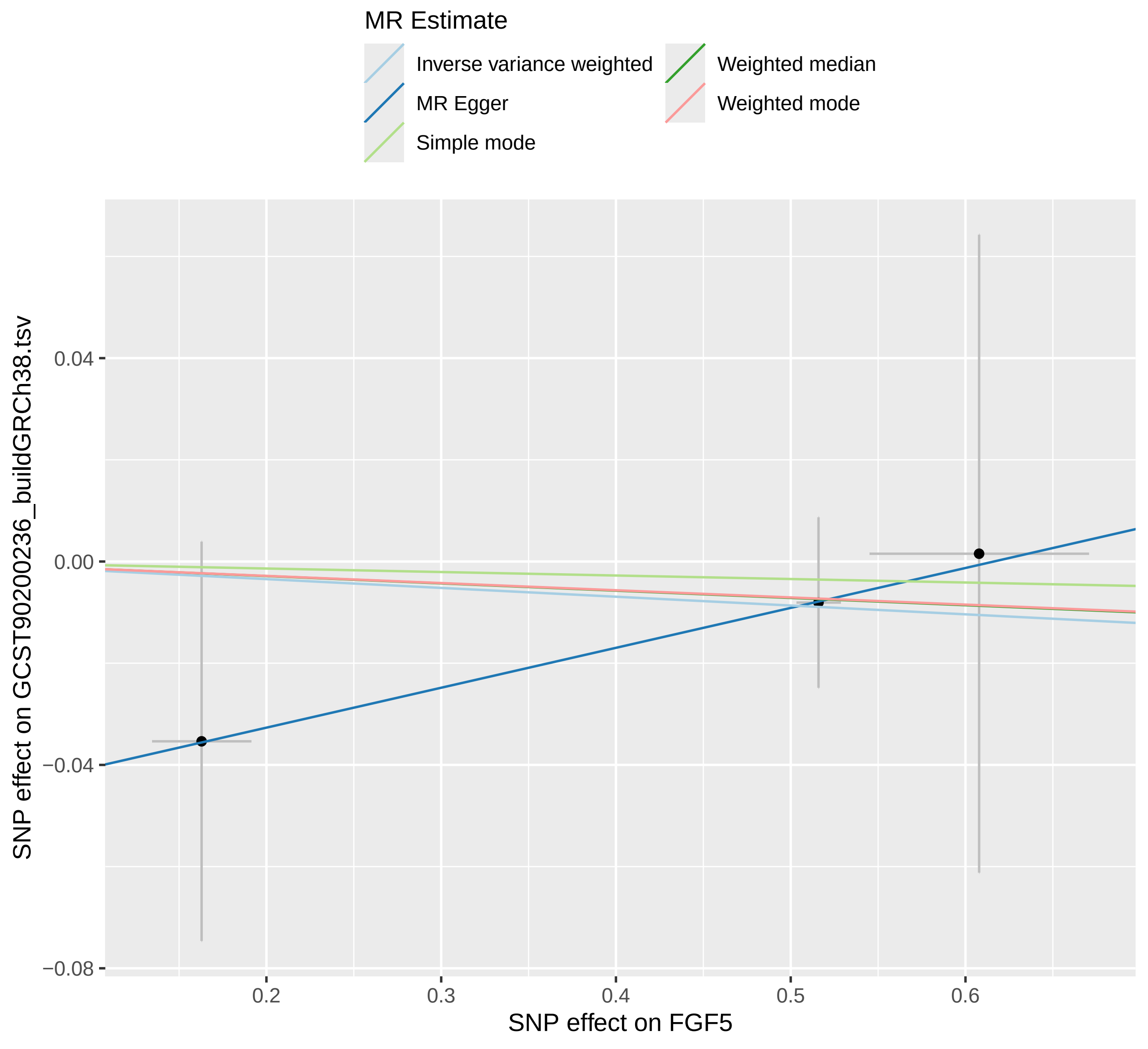

MR Estimate
Inverse variance weighted
MR Egger
Simple mode
Weighted median
Weighted mode
SNP effect on GCST90200236_buildGRCh38.tsv
0.04
0.00
−0.04
−0.08
0.2
0.3
0.4
0.5
0.6
SNP effect on FGF5

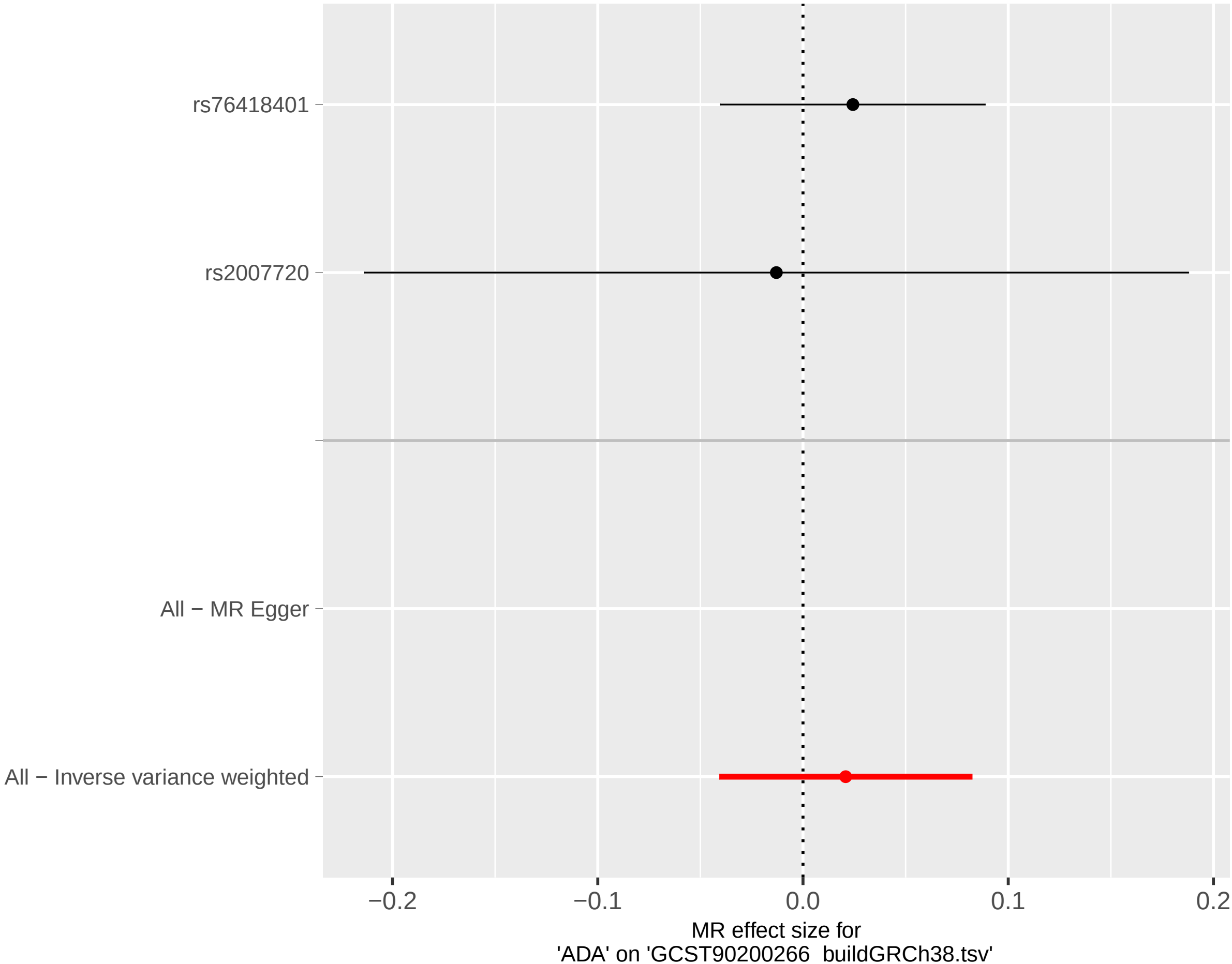
rs76418401
rs2007720
All − MR Egger
All − Inverse variance weighted
−0.2
−0.1
0.0
0.1
0.2
MR effect size for
'ADA' on 'GCST90200266_buildGRCh38.tsv'

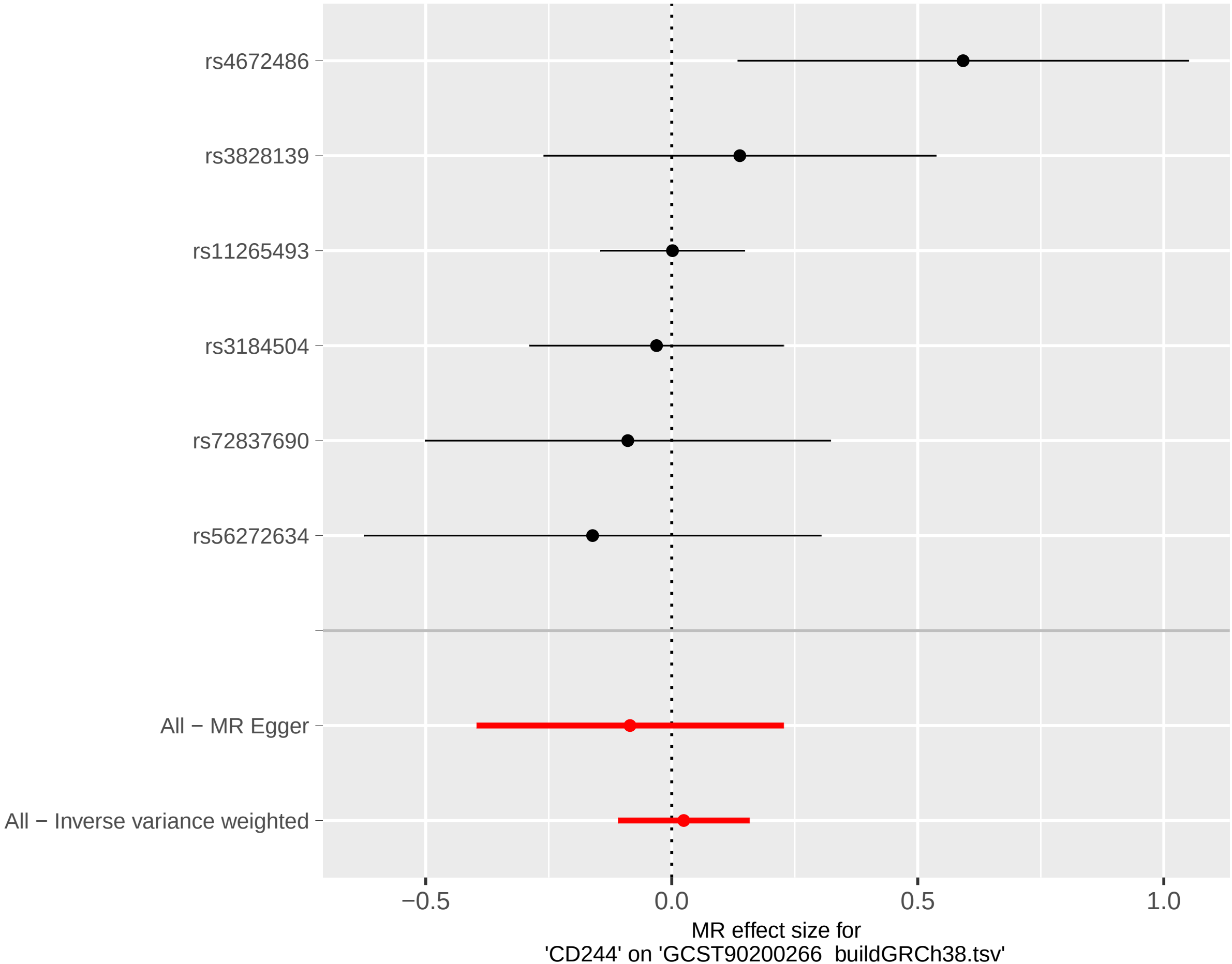
rs4672486
rs3828139
rs11265493
rs3184504
rs72837690
rs56272634
All − MR Egger
All − Inverse variance weighted
−0.5
0.0
0.5
1.0
MR effect size for
'CD244' on 'GCST90200266_buildGRCh38.tsv'

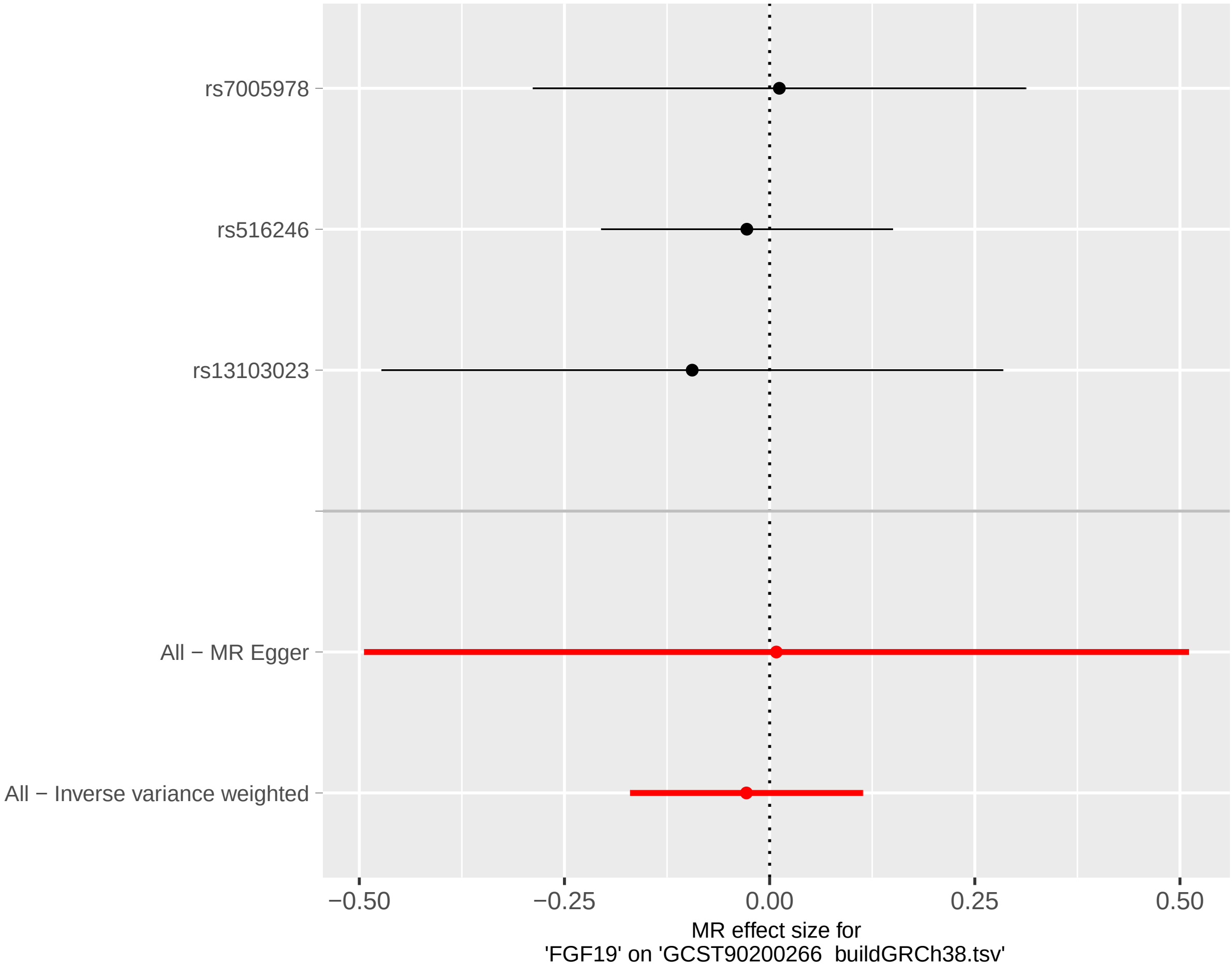
rs7005978
rs516246
rs13103023
All − MR Egger
All − Inverse variance weighted
−0.50
−0.25
0.00
0.25
0.50
MR effect size for
'FGF19' on 'GCST90200266_buildGRCh38.tsv'

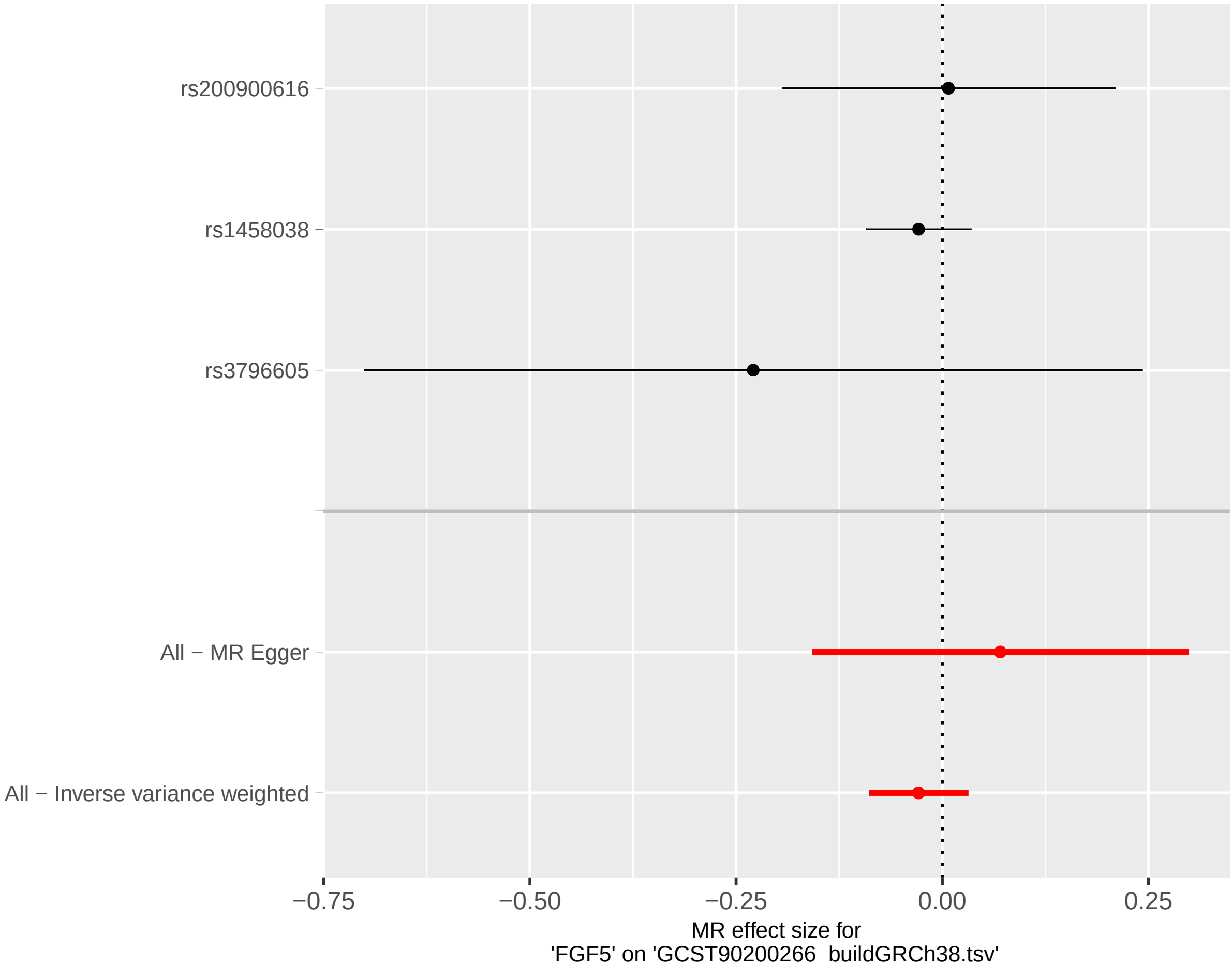

rs200900616
rs1458038
rs3796605
All − MR Egger
All − Inverse variance weighted
−0.75
−0.50
−0.25
0.00
0.25
MR effect size for
'FGF5' on 'GCST90200266_buildGRCh38.tsv'

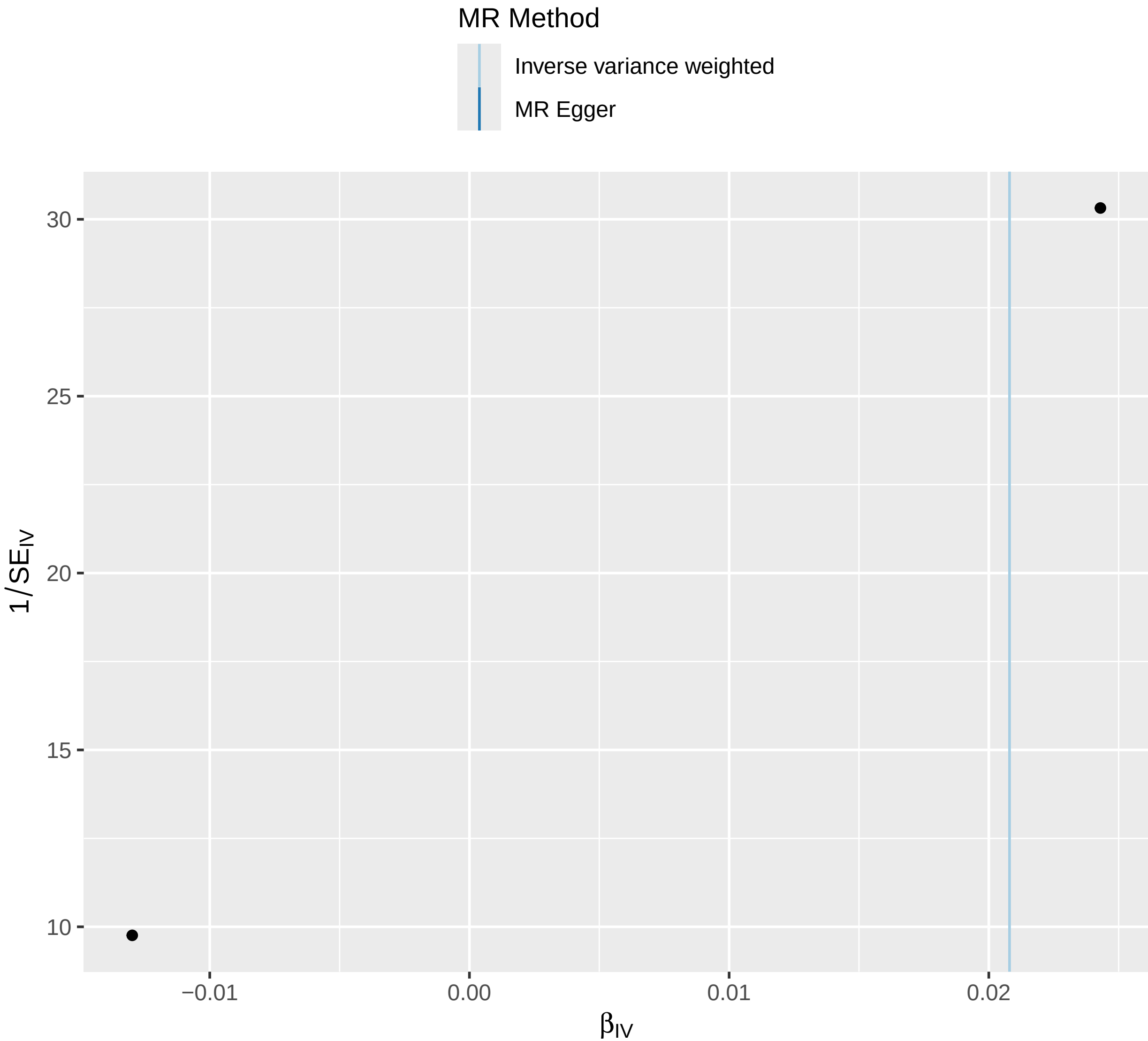
MR Method
Inverse variance weighted
MR Egger
30
25
20
15
10
1/SE$_{IV}$
−0.01
0.00
0.01
0.02
β$_{IV}$

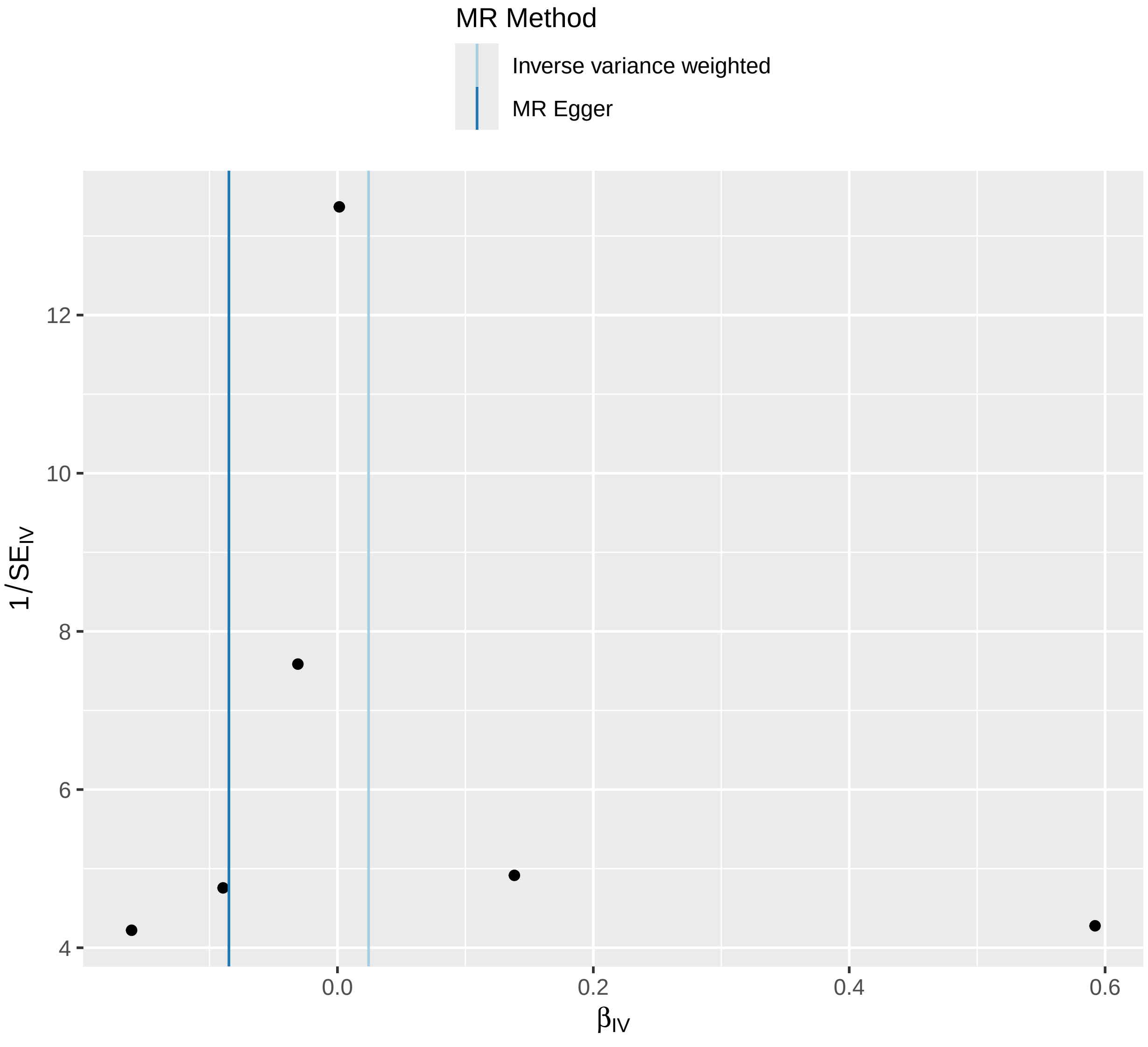
MR Method
Inverse variance weighted
MR Egger
12
10
8
6
4
0.0
0.2
0.4
0.6
$1/SE_{IV}$
β_{IV}

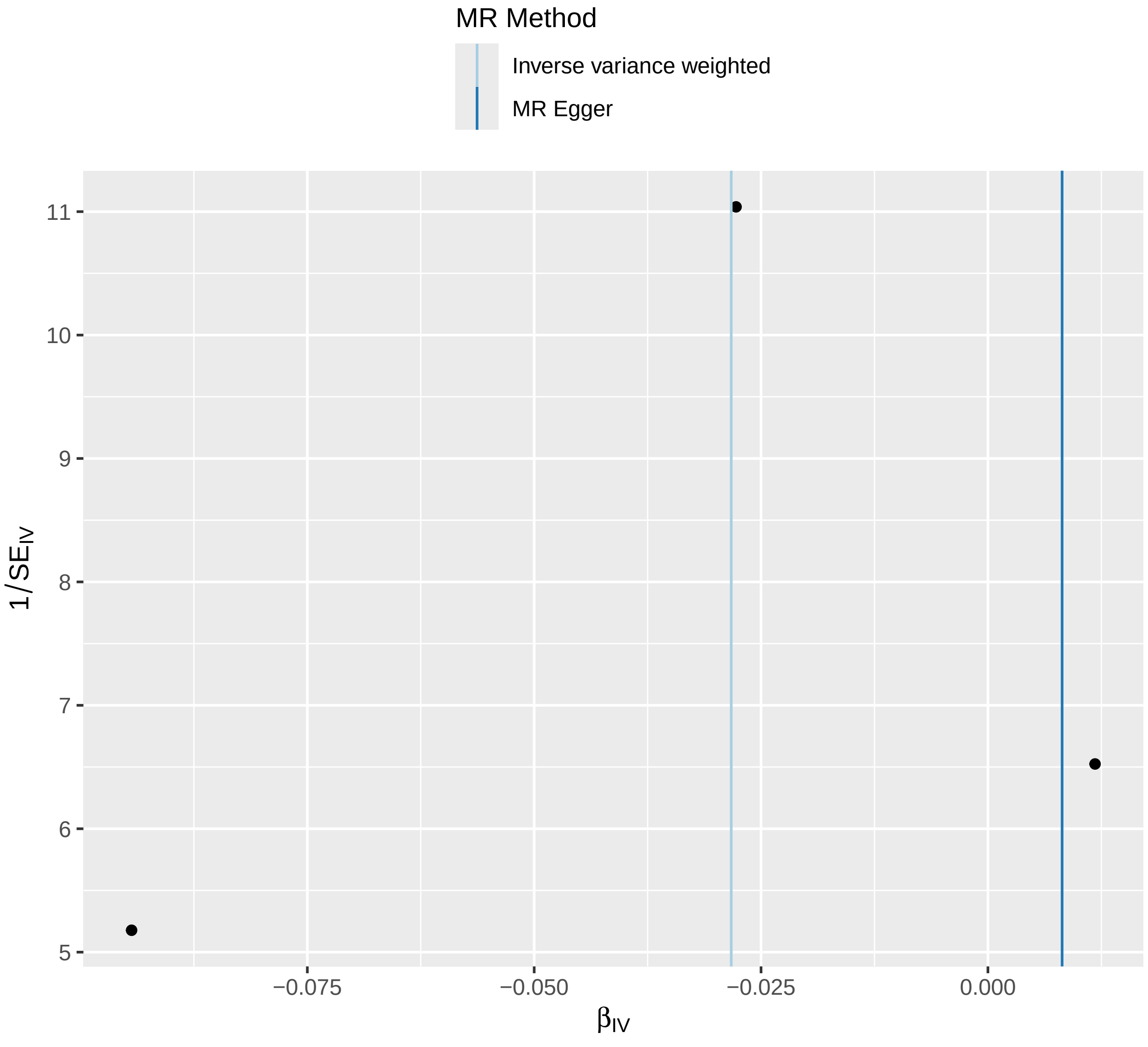

MR Method
Inverse variance weighted
MR Egger
11
10
9
8
7
6
5
1/SE_IV
−0.075
−0.050
−0.025
0.000
β_IV

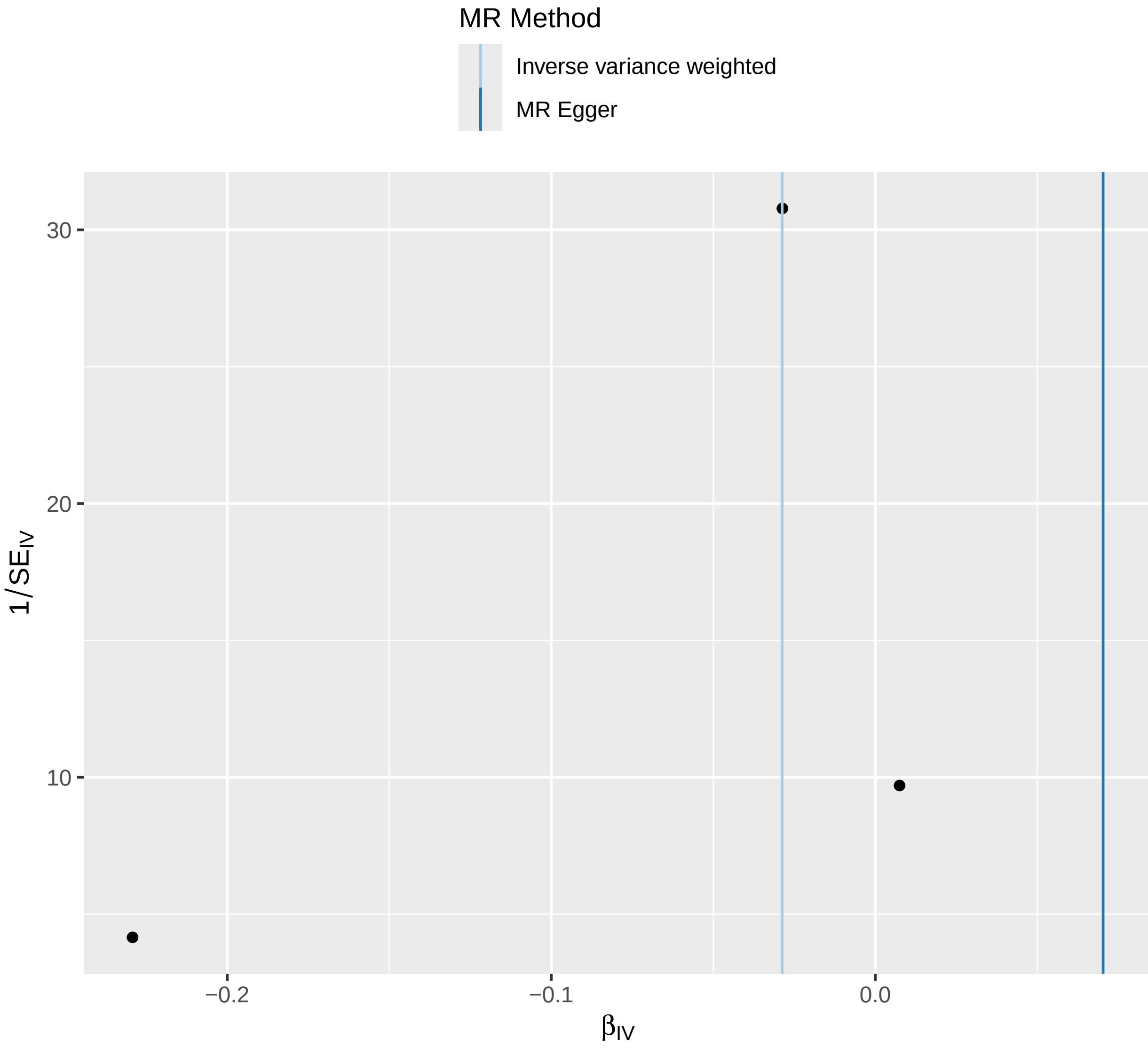
MR Method
Inverse variance weighted
MR Egger
30
20
10
1/SE
IV
−0.2
−0.1
0.0
β
IV

Insufficient number of SNPs

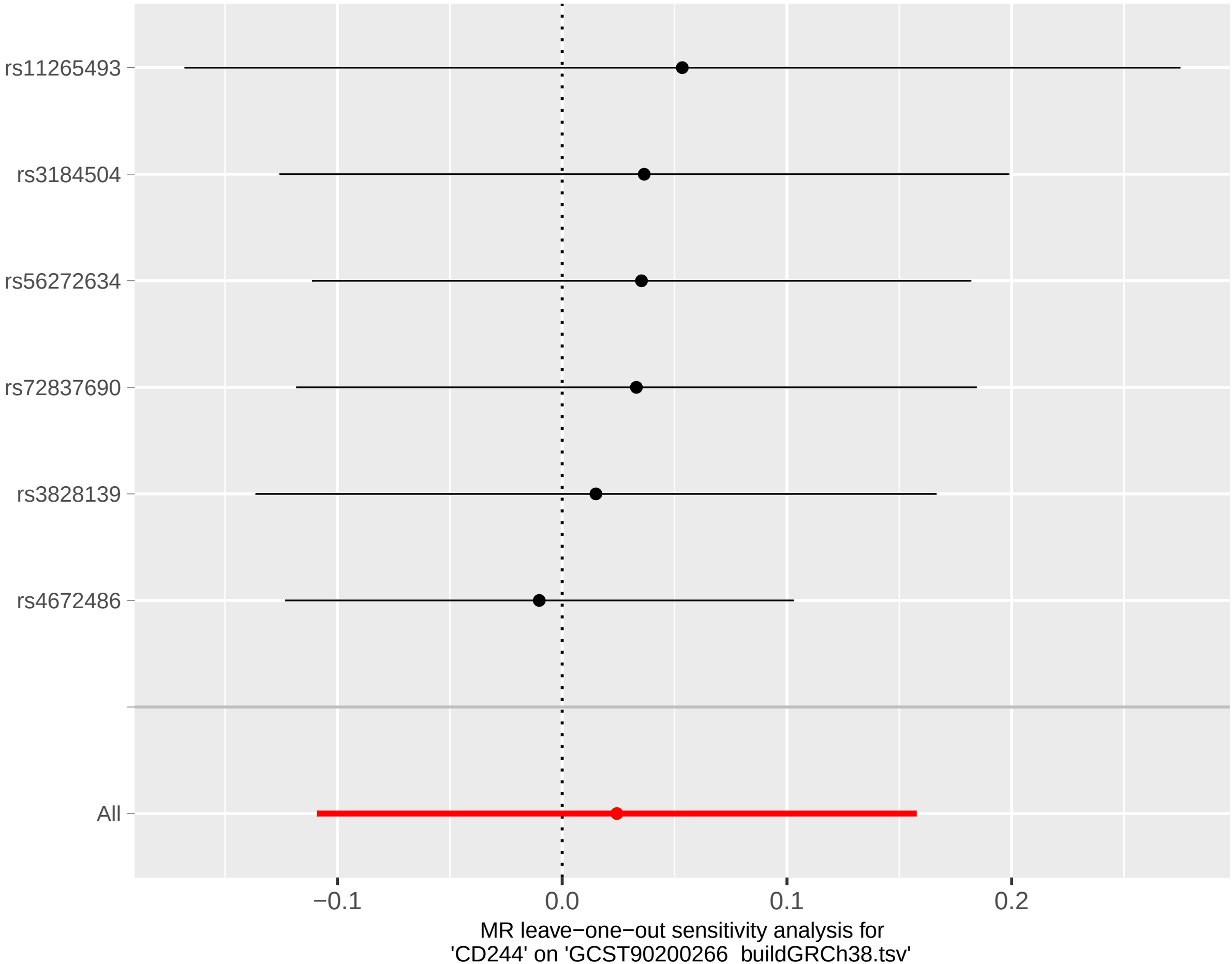
rs11265493
rs3184504
rs56272634
rs72837690
rs3828139
rs4672486
All
−0.1
0.0
0.1
0.2
MR leave−one−out sensitivity analysis for
'CD244' on 'GCST90200266_buildGRCh38.tsv'

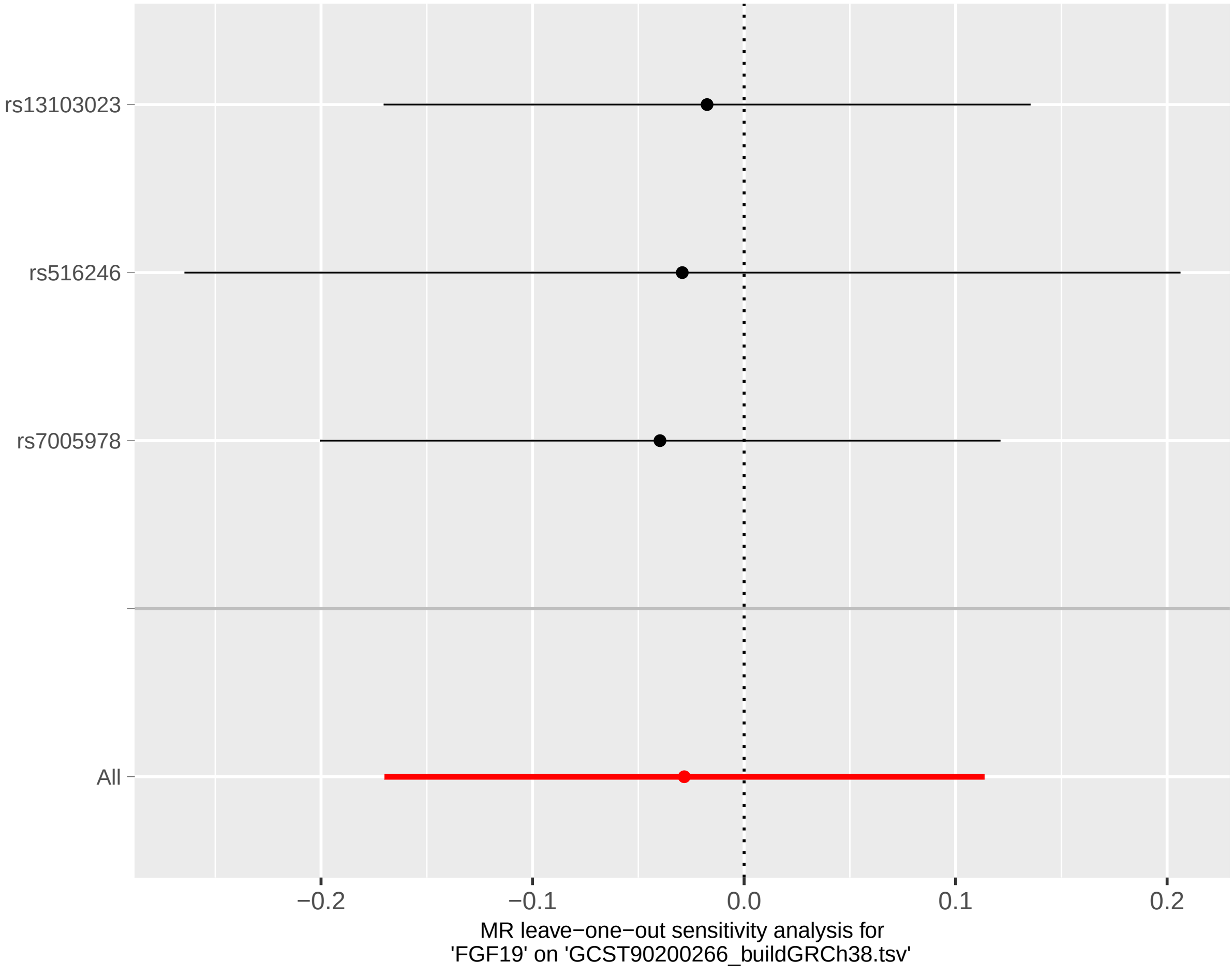
rs13103023
rs516246
rs7005978
All
−0.2
−0.1
0.0
0.1
0.2
MR leave−one−out sensitivity analysis for
'FGF19' on 'GCST90200266_buildGRCh38.tsv'

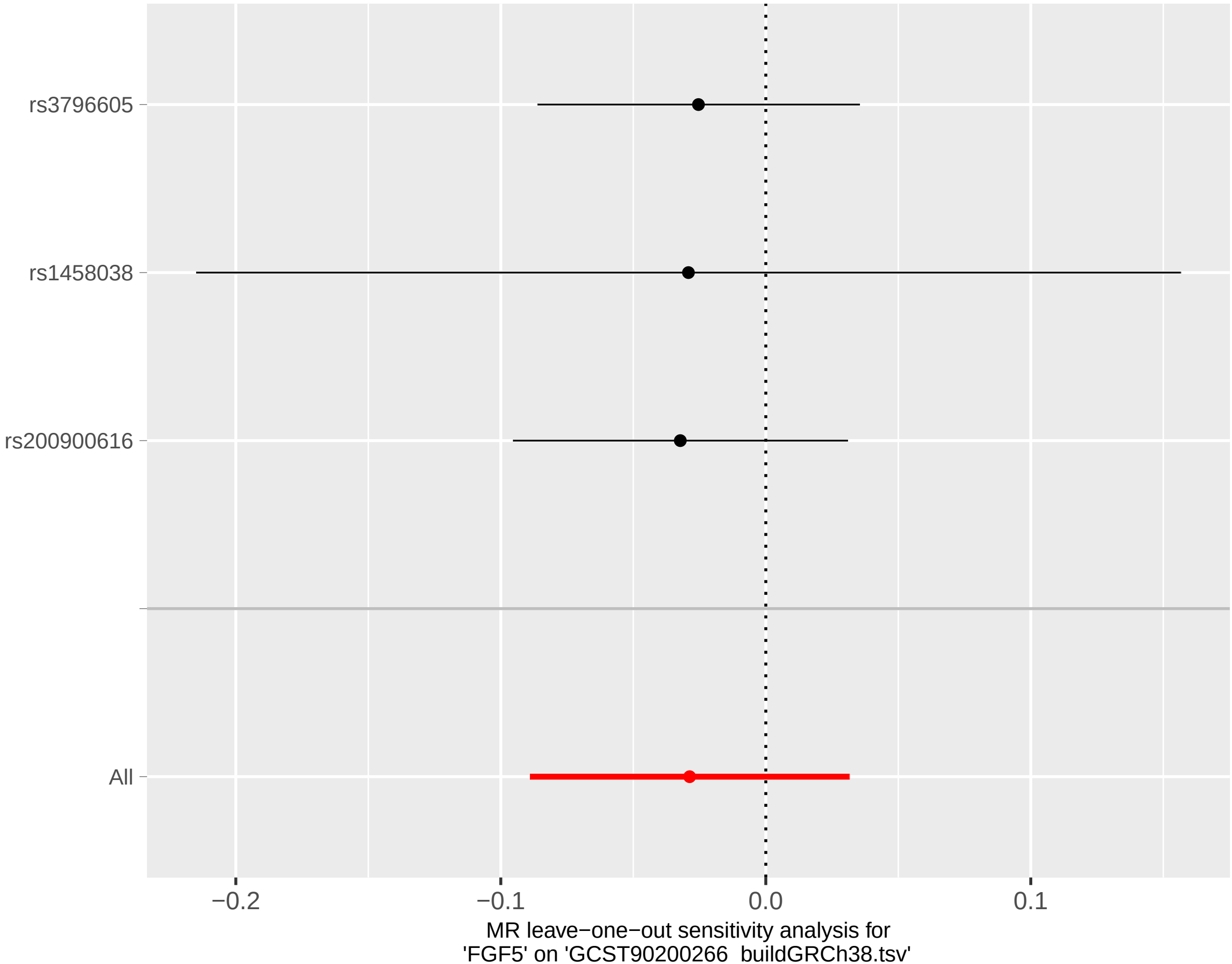
rs3796605
rs1458038
rs200900616
All
−0.2
−0.1
0.0
0.1
MR leave−one−out sensitivity analysis for
'FGF5' on 'GCST90200266_buildGRCh38.tsv'

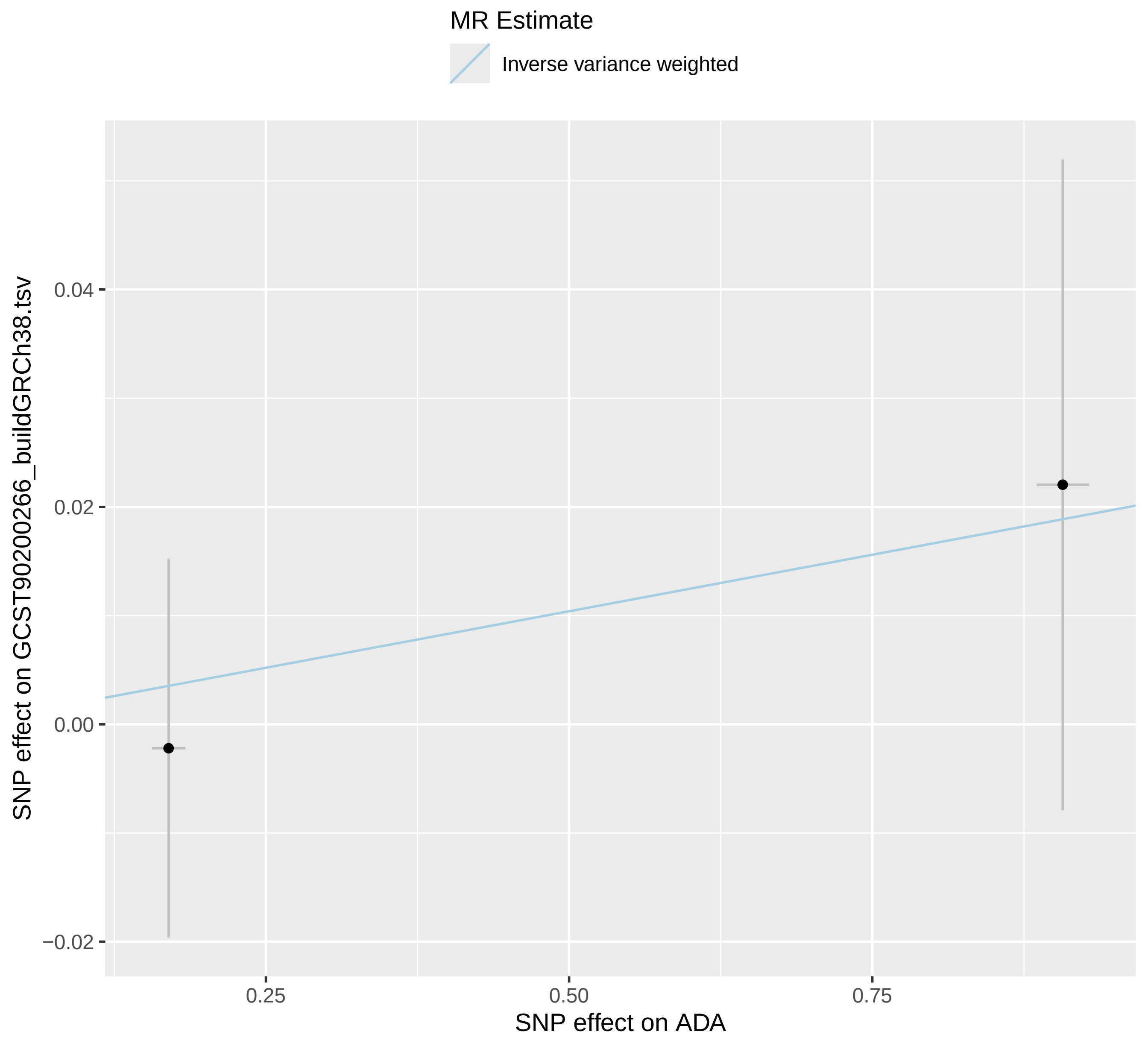

MR Estimate
Inverse variance weighted
SNP effect on GCST90200266_buildGRCh38.tsv
0.04
0.02
0.00
−0.02
0.25
0.50
0.75
SNP effect on ADA

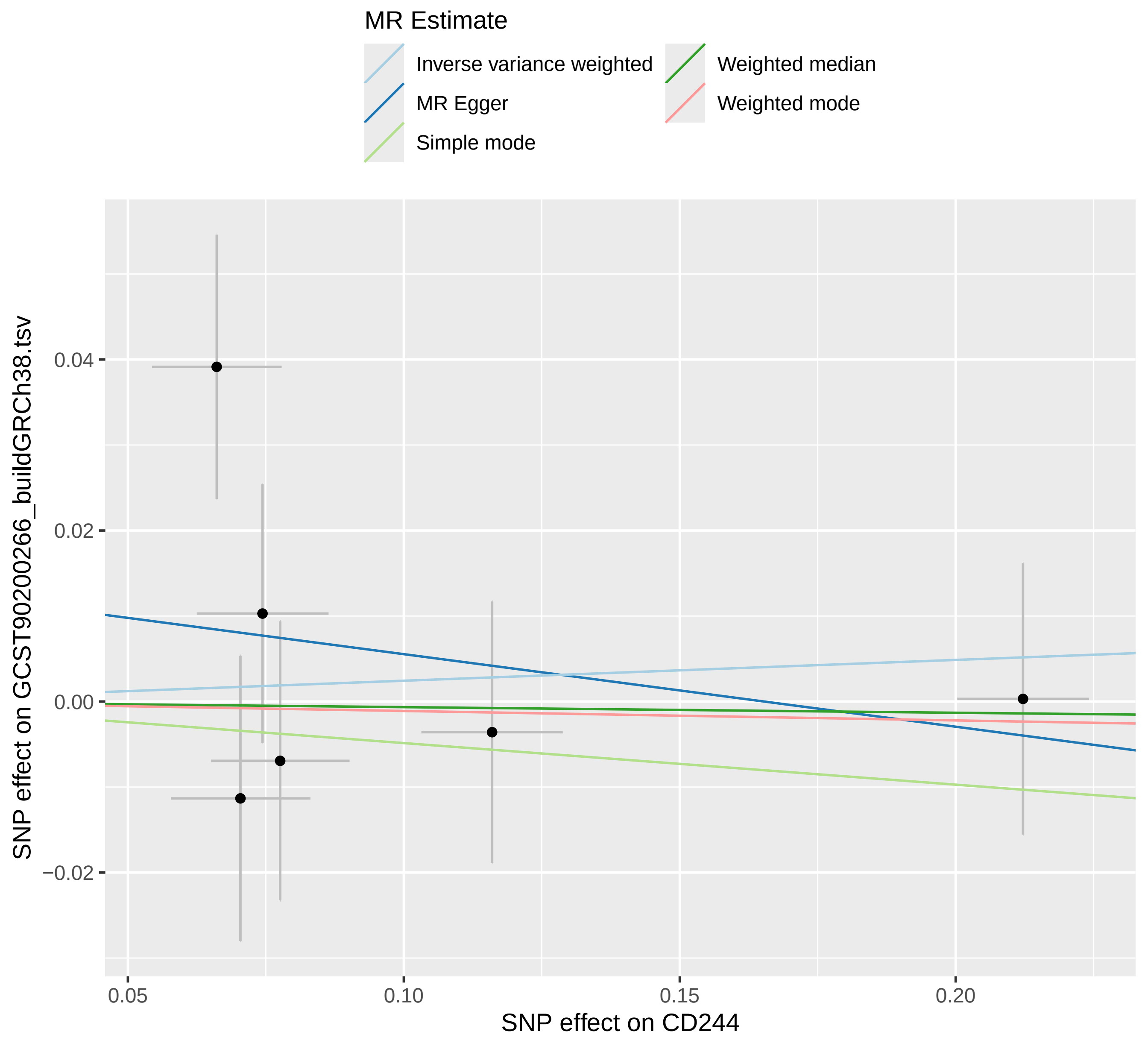

MR Estimate
Inverse variance weighted
MR Egger
Simple mode
Weighted median
Weighted mode
SNP effect on GCST90200266_buildGRCh38.tsv
0.04
0.02
0.00
−0.02
0.05
0.10
0.15
0.20
SNP effect on CD244

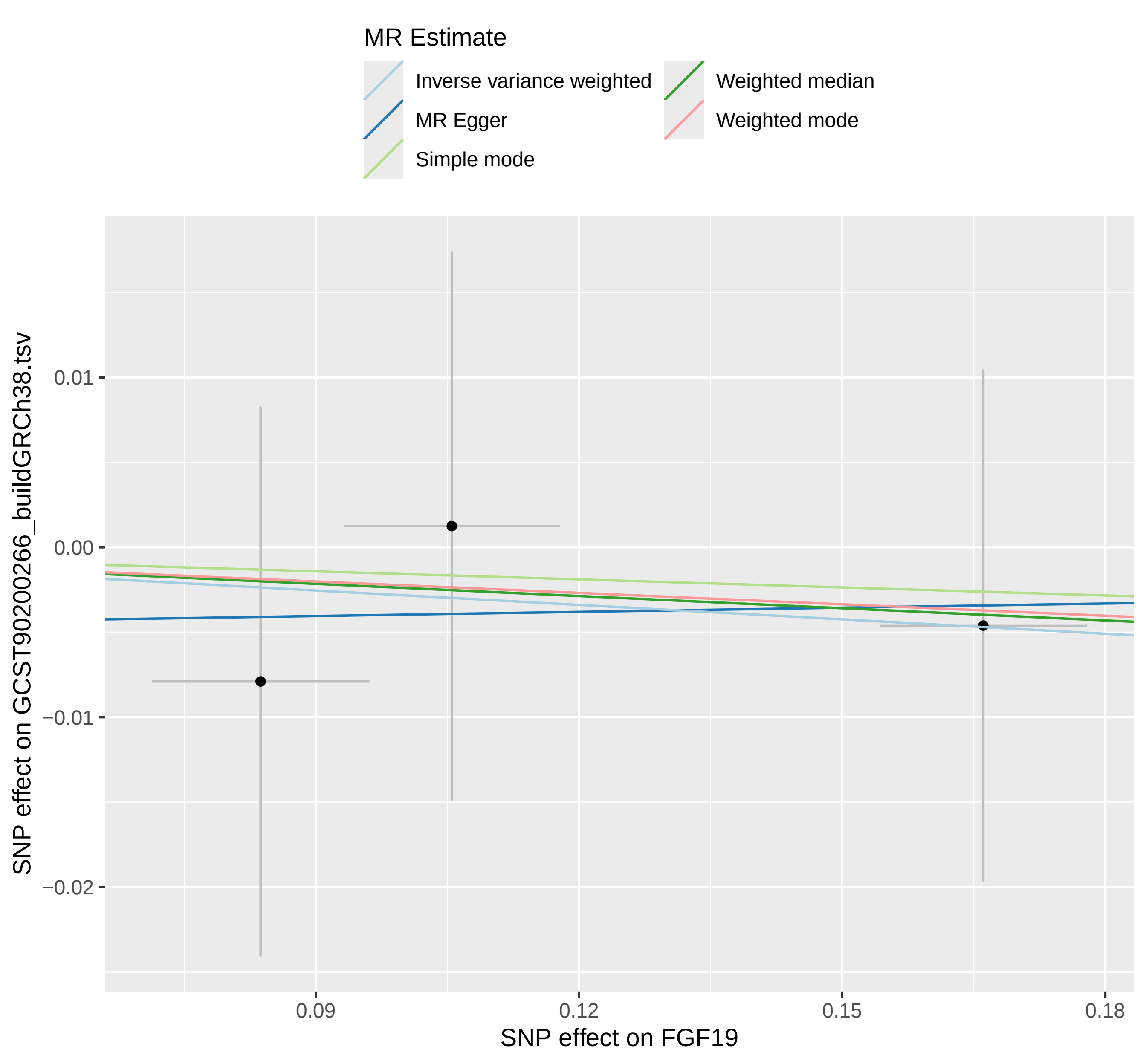

MR Estimate
Inverse variance weighted
MR Egger
Simple mode
Weighted median
Weighted mode
SNP effect on GCST90200266_buildGRCh38.tsv
0.01
0.00
−0.01
−0.02
0.09
0.12
0.15
0.18
SNP effect on FGF19

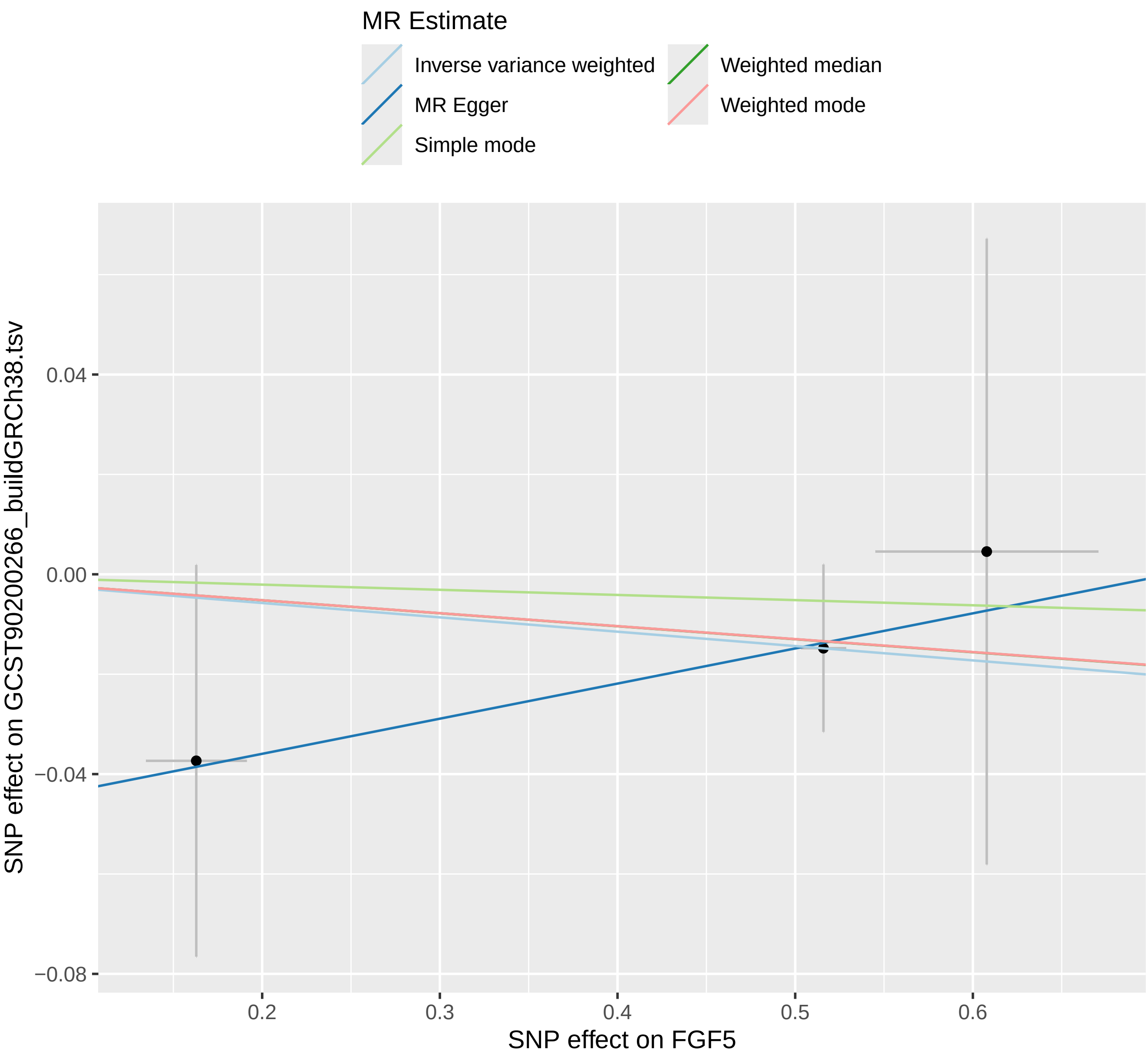

MR Estimate
Inverse variance weighted
MR Egger
Simple mode
Weighted median
Weighted mode
SNP effect on GCST90200266_buildGRCh38.tsv
0.04
0.00
−0.04
−0.08
0.2
0.3
0.4
0.5
0.6
SNP effect on FGF5

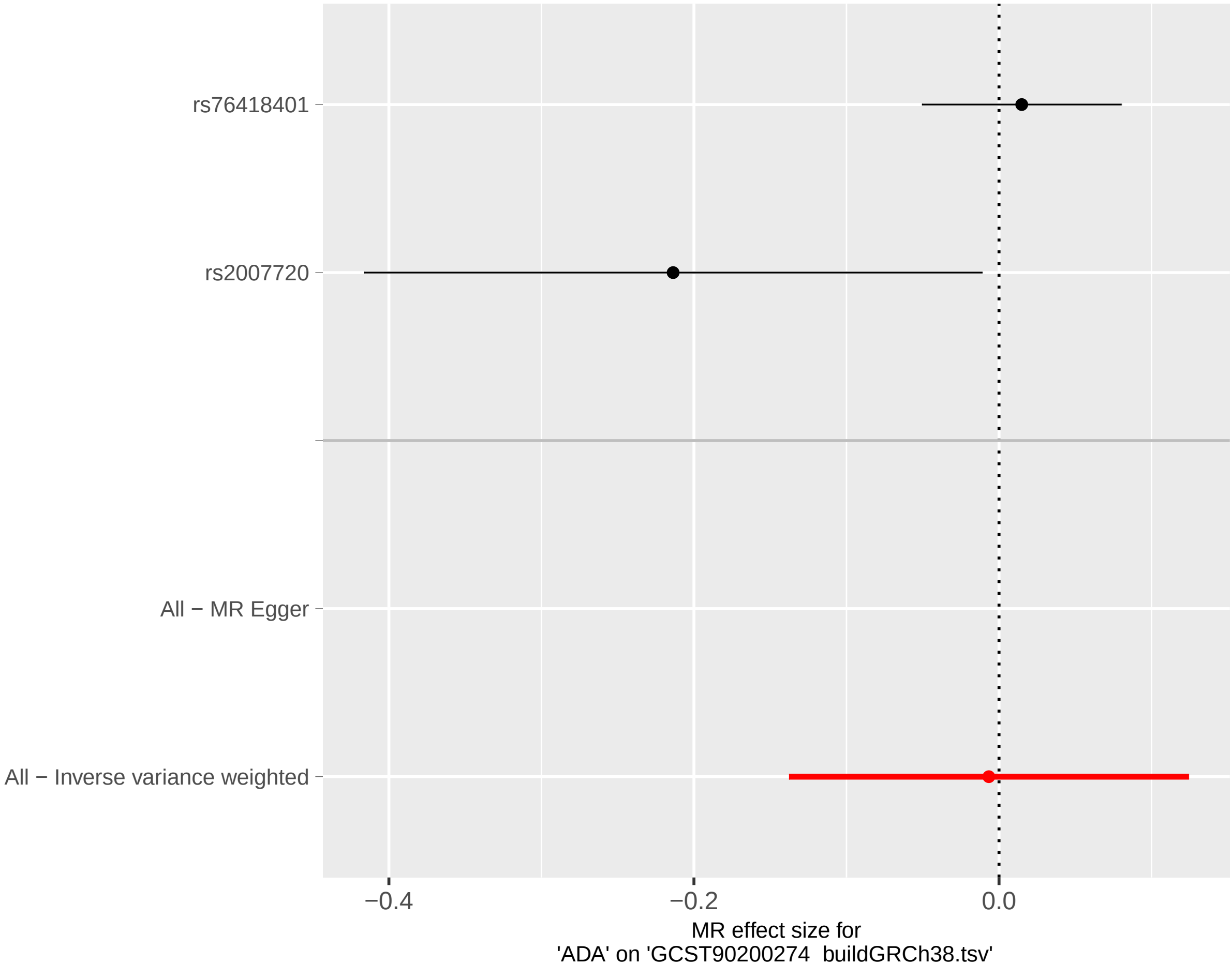

rs76418401
rs2007720
All − MR Egger
All − Inverse variance weighted
−0.4
−0.2
0.0
MR effect size for
'ADA' on 'GCST90200274_buildGRCh38.tsv'

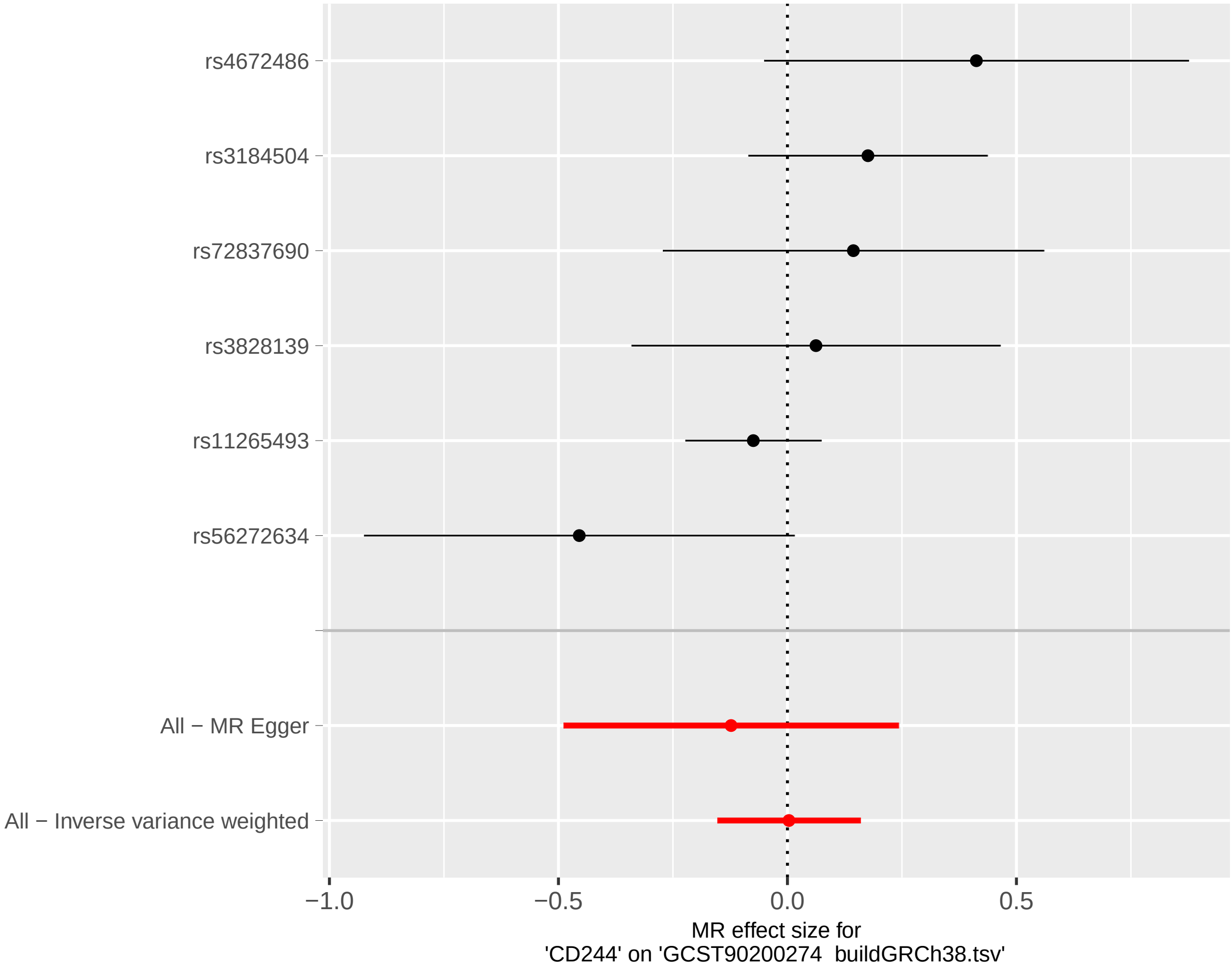

rs4672486
rs3184504
rs72837690
rs3828139
rs11265493
rs56272634
All − MR Egger
All − Inverse variance weighted
−1.0
−0.5
0.0
0.5
MR effect size for
'CD244' on 'GCST90200274_buildGRCh38.tsv'

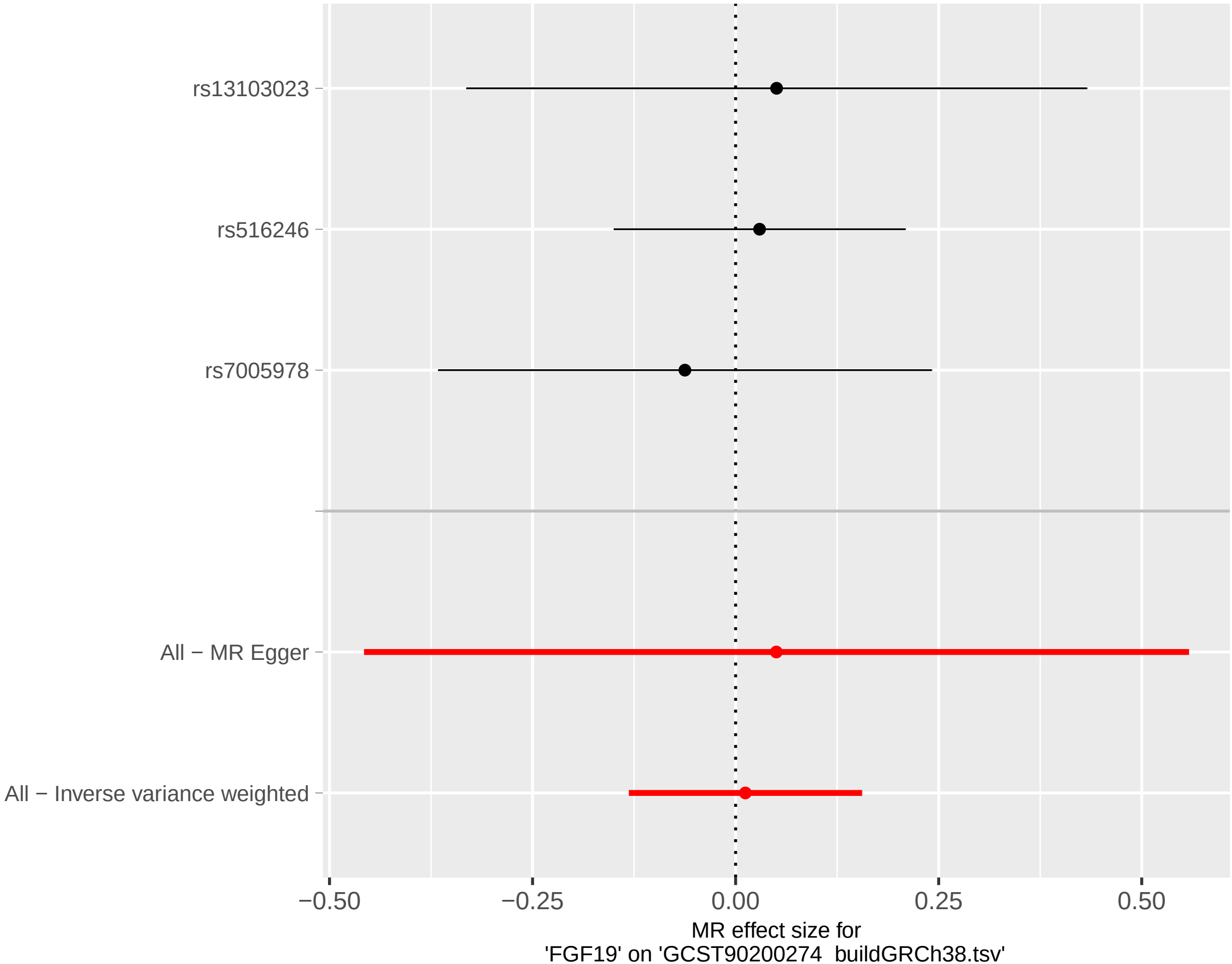

rs13103023
rs516246
rs7005978
All − MR Egger
All − Inverse variance weighted
−0.50
−0.25
0.00
0.25
0.50
MR effect size for
'FGF19' on 'GCST90200274_buildGRCh38.tsv'

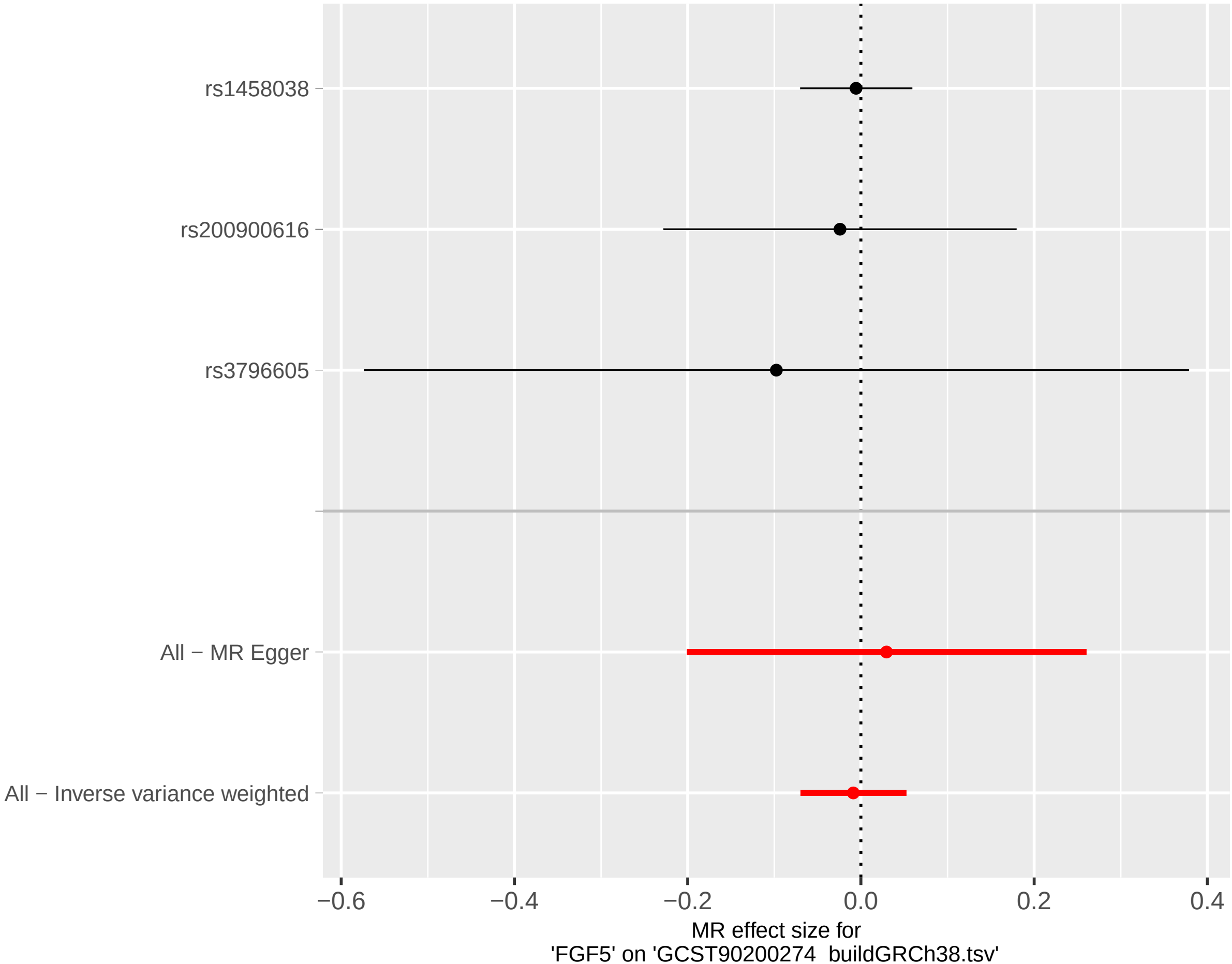
rs1458038
rs200900616
rs3796605
All − MR Egger
All − Inverse variance weighted
−0.6
−0.4
−0.2
0.0
0.2
0.4
MR effect size for
'FGF5' on 'GCST90200274_buildGRCh38.tsv'

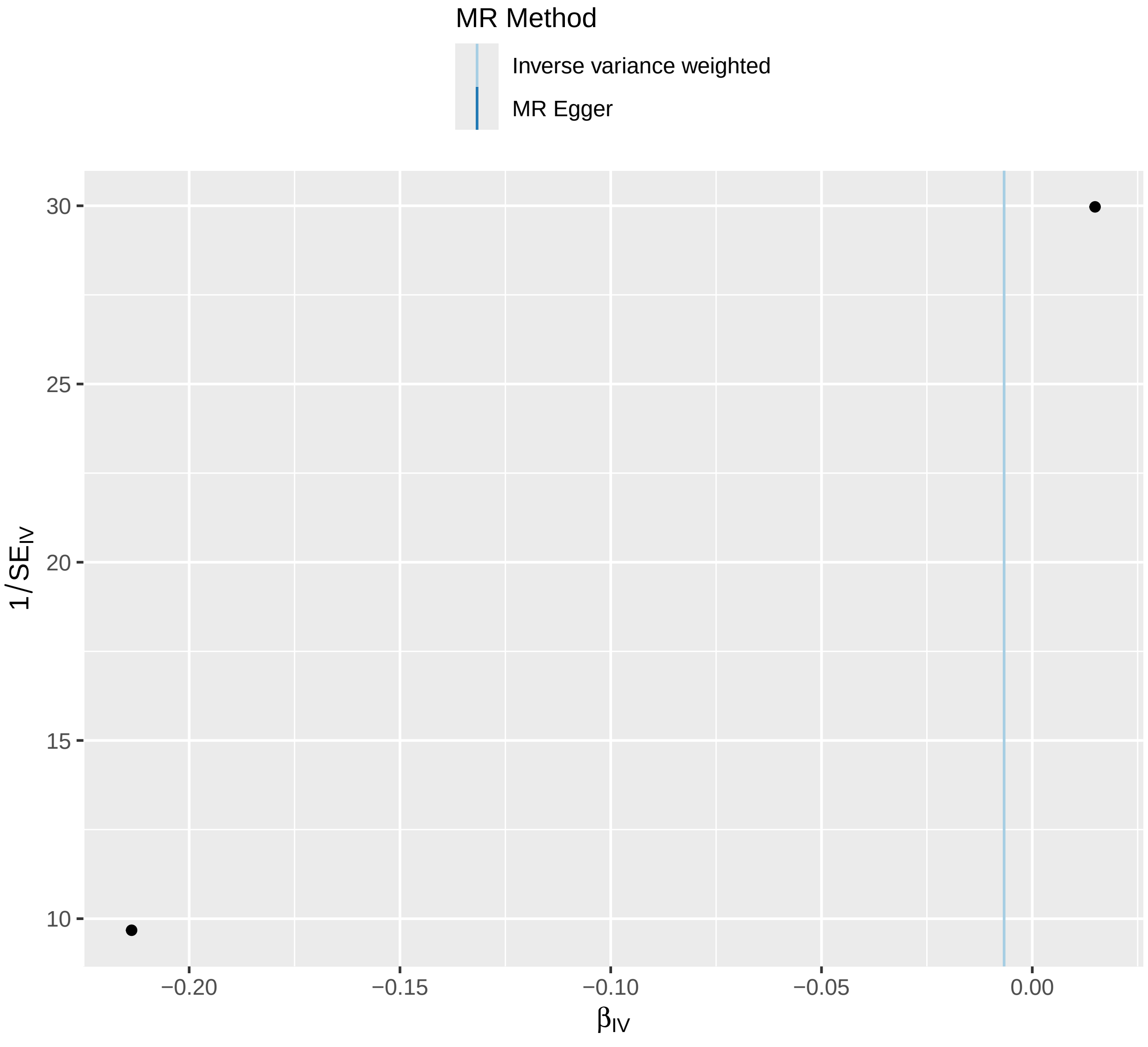

MR Method
Inverse variance weighted
MR Egger
30
25
20
15
10
1/SE_IV
−0.20
−0.15
−0.10
−0.05
0.00
β_IV

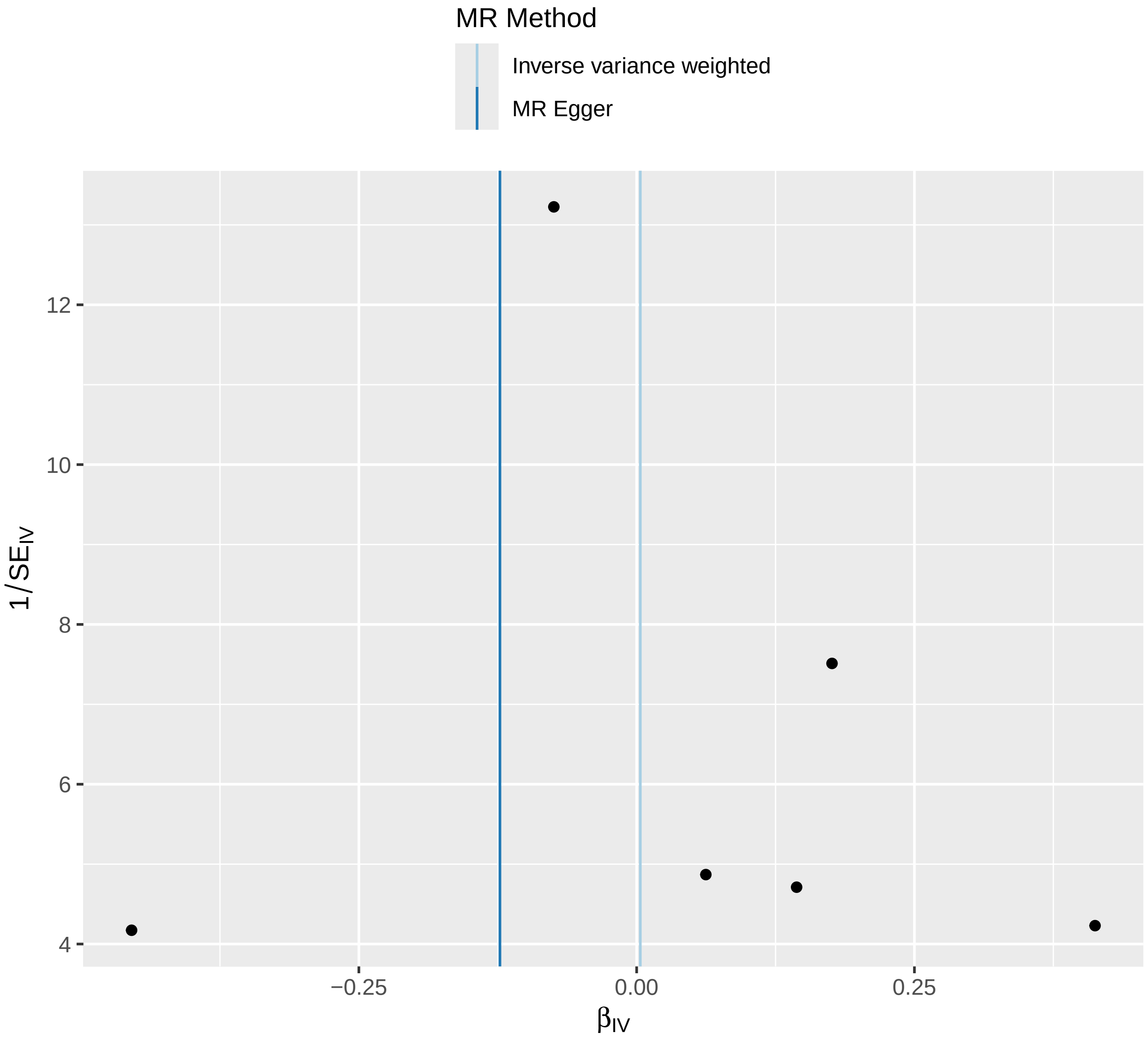
MR Method
Inverse variance weighted
MR Egger
12
10
8
6
4
1/SE_IV
−0.25
0.00
0.25
β_IV

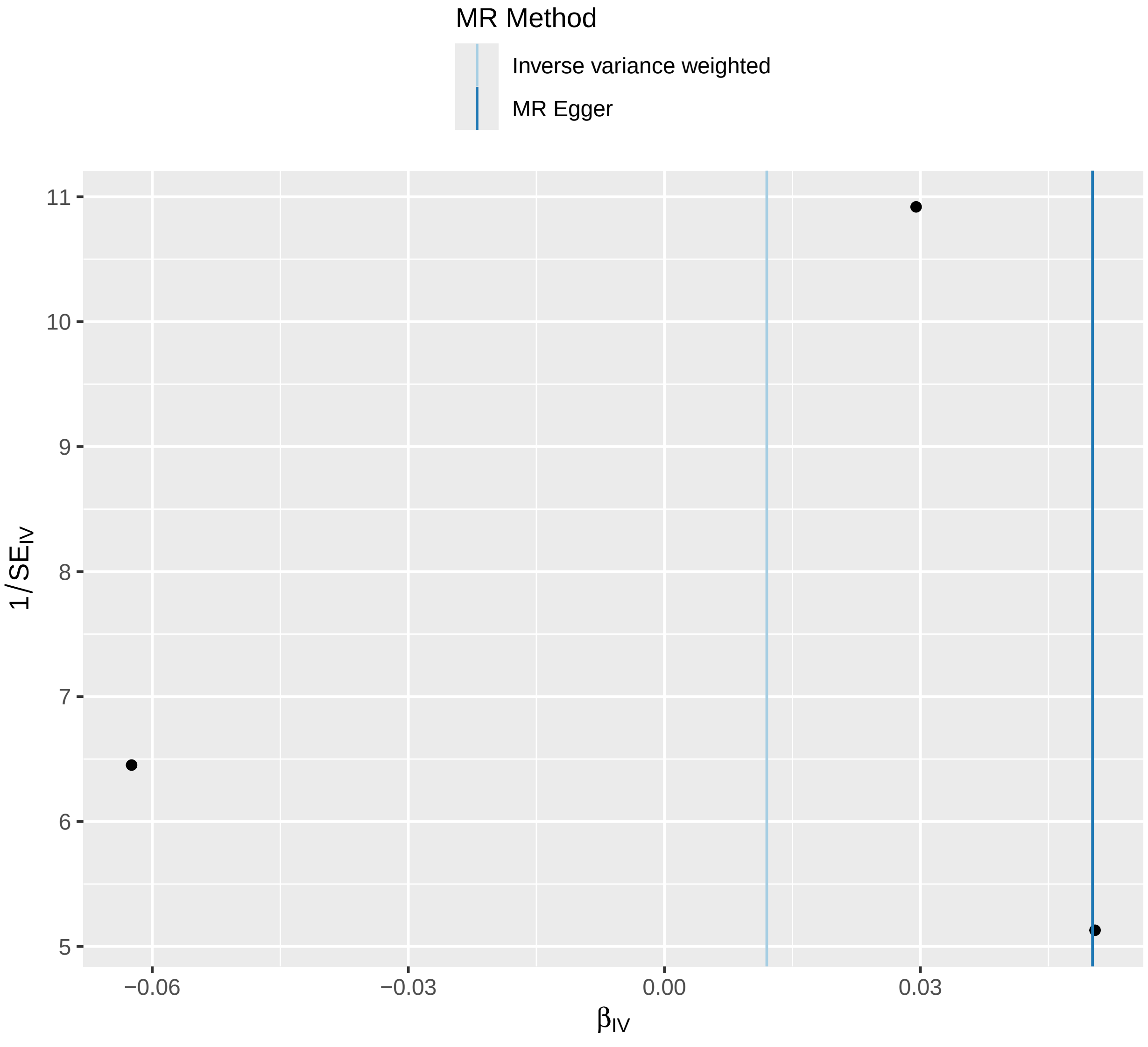
MR Method
Inverse variance weighted
MR Egger
11
10
9
8
7
6
5
1/SE_IV
−0.06
−0.03
0.00
0.03
β_IV

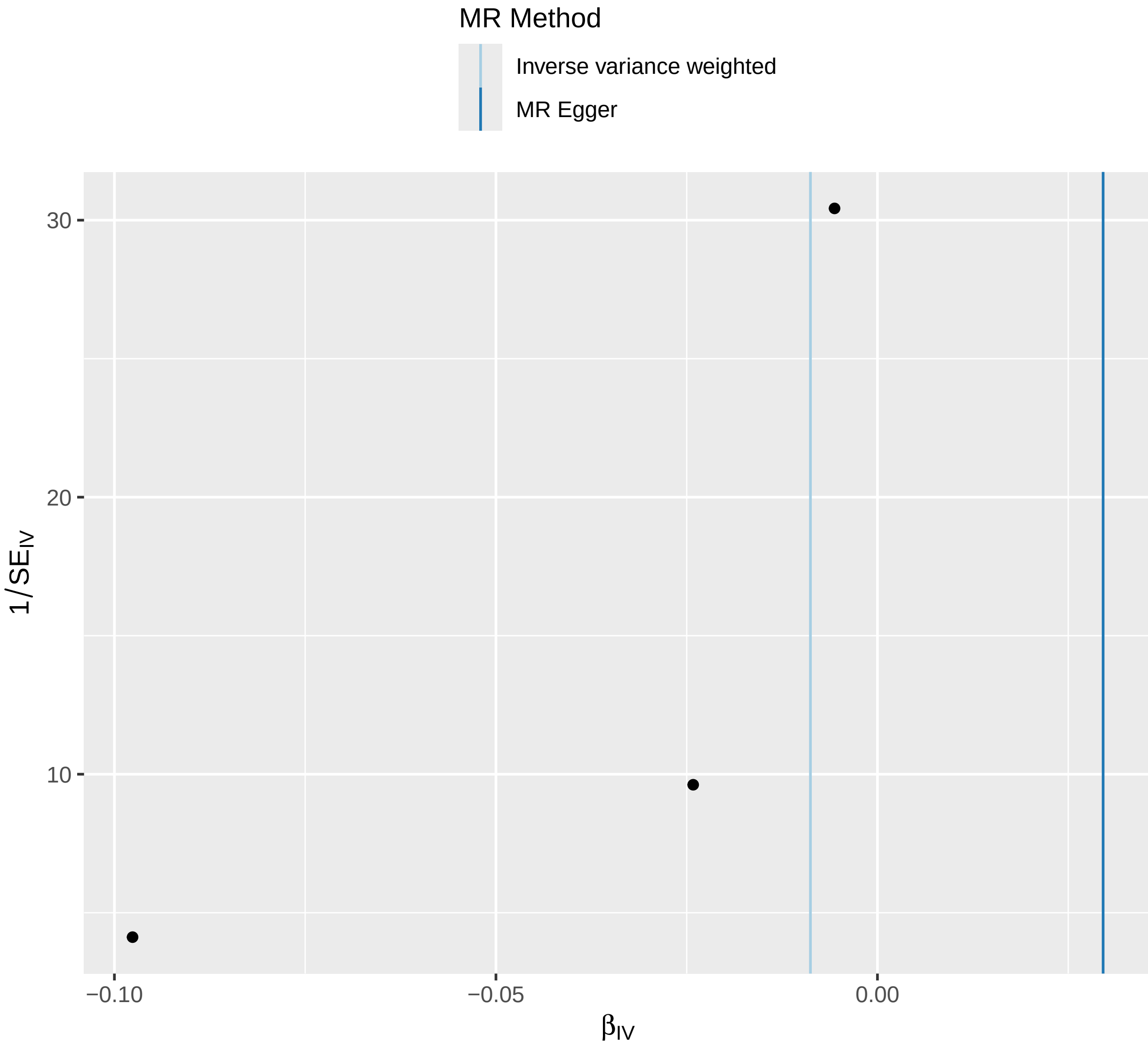
MR Method
Inverse variance weighted
MR Egger
30
20
10
$1/SE_{IV}$
−0.10
−0.05
0.00
β_{IV}

Insufficient number of SNPs

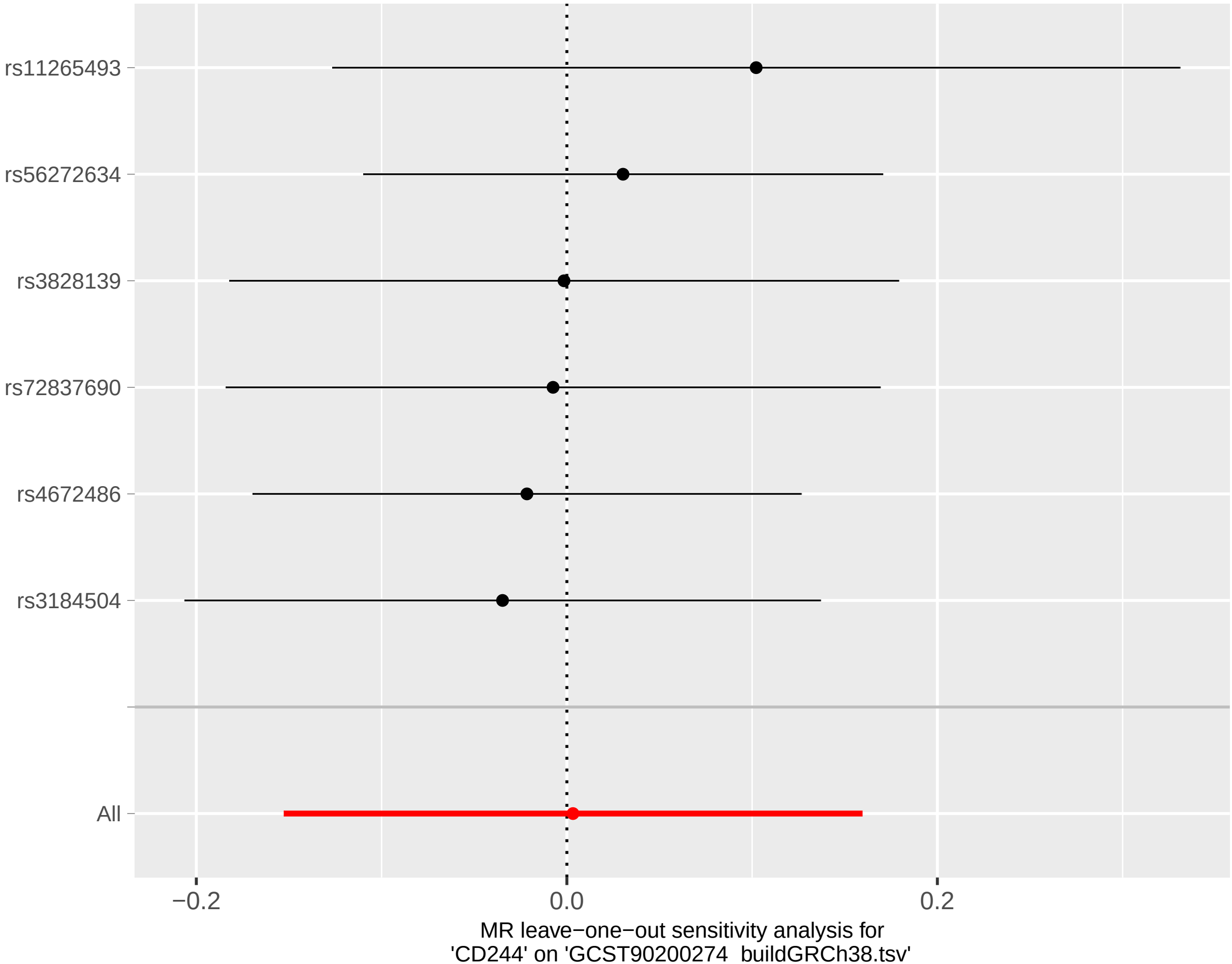
rs11265493
rs56272634
rs3828139
rs72837690
rs4672486
rs3184504
All
−0.2
0.0
0.2
MR leave−one−out sensitivity analysis for
'CD244' on 'GCST90200274_buildGRCh38.tsv'

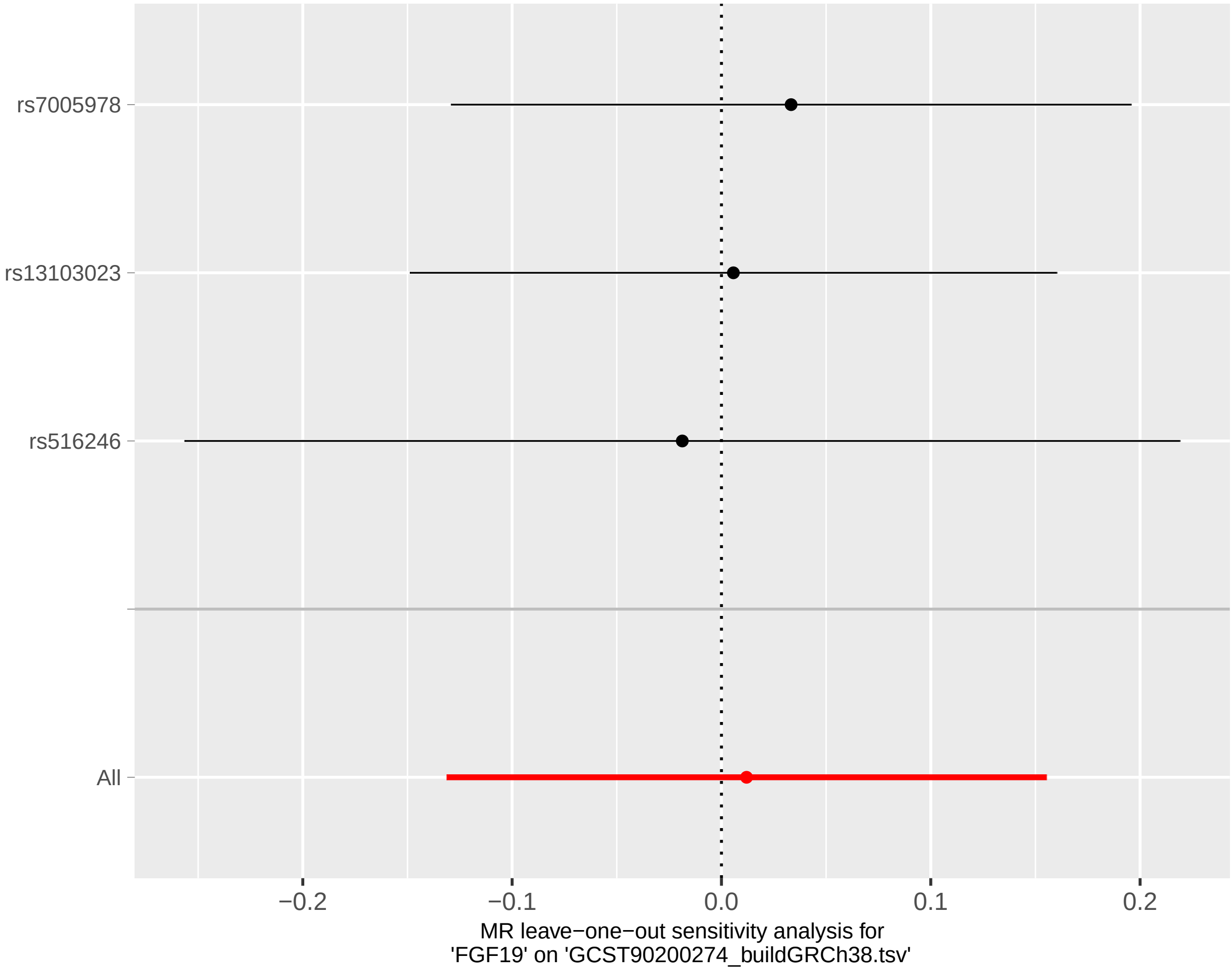

rs7005978
rs13103023
rs516246
All
−0.2
−0.1
0.0
0.1
0.2
MR leave−one−out sensitivity analysis for
'FGF19' on 'GCST90200274_buildGRCh38.tsv'

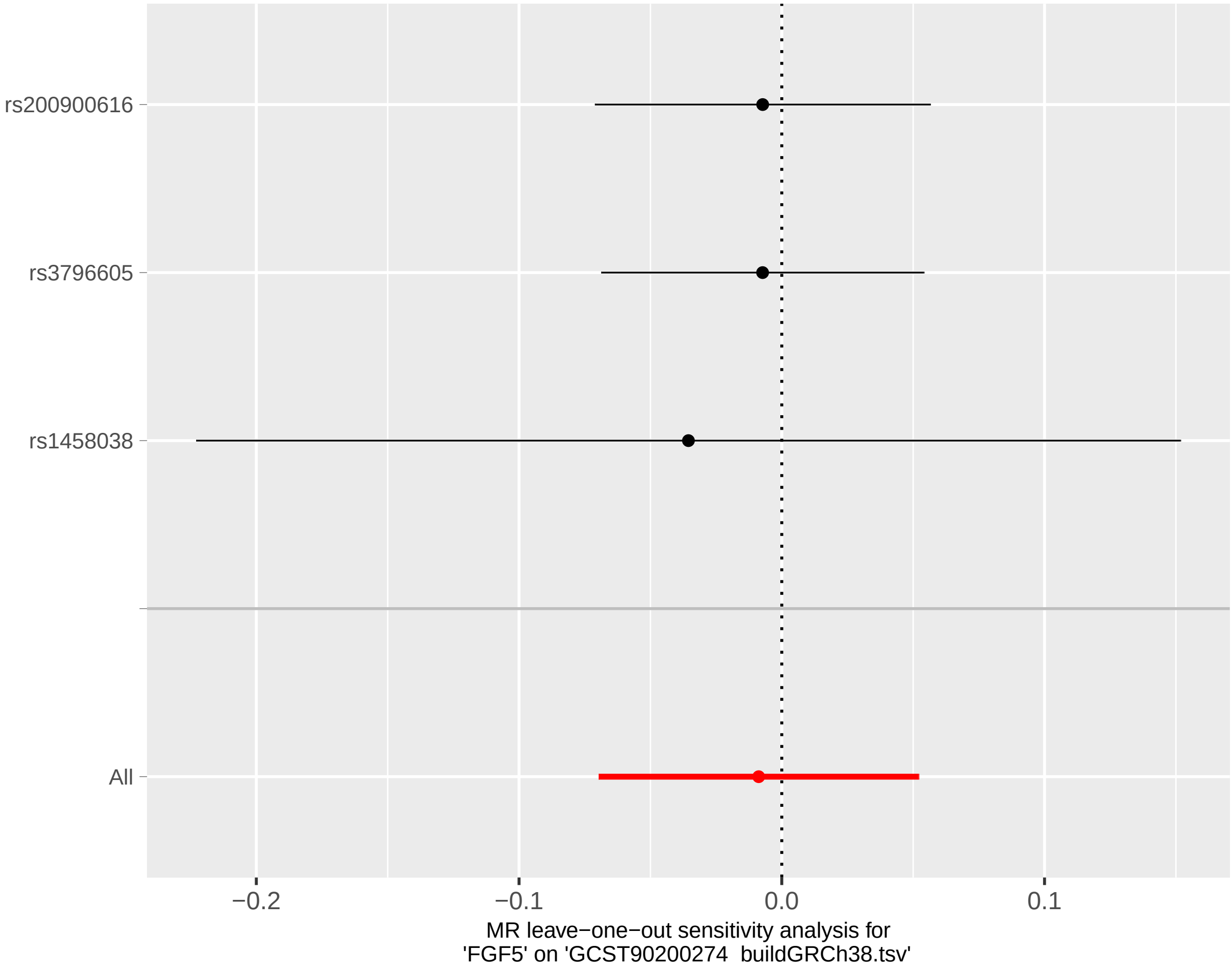
rs200900616
rs3796605
rs1458038
All
−0.2
−0.1
0.0
0.1
MR leave−one−out sensitivity analysis for
'FGF5' on 'GCST90200274_buildGRCh38.tsv'

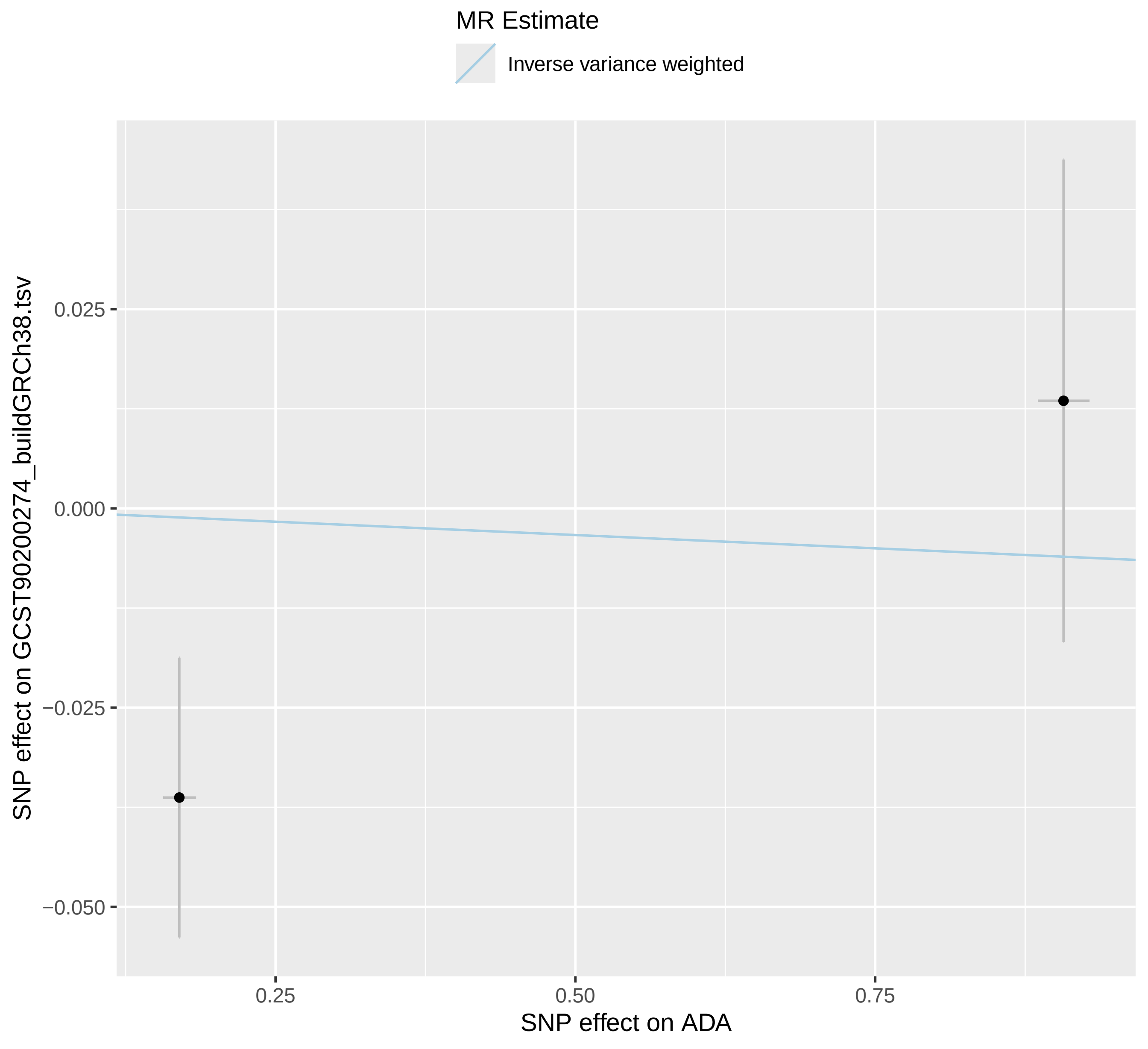
MR Estimate
Inverse variance weighted
SNP effect on GCST90200274_buildGRCh38.tsv
0.025
0.000
−0.025
−0.050
0.25
0.50
0.75
SNP effect on ADA

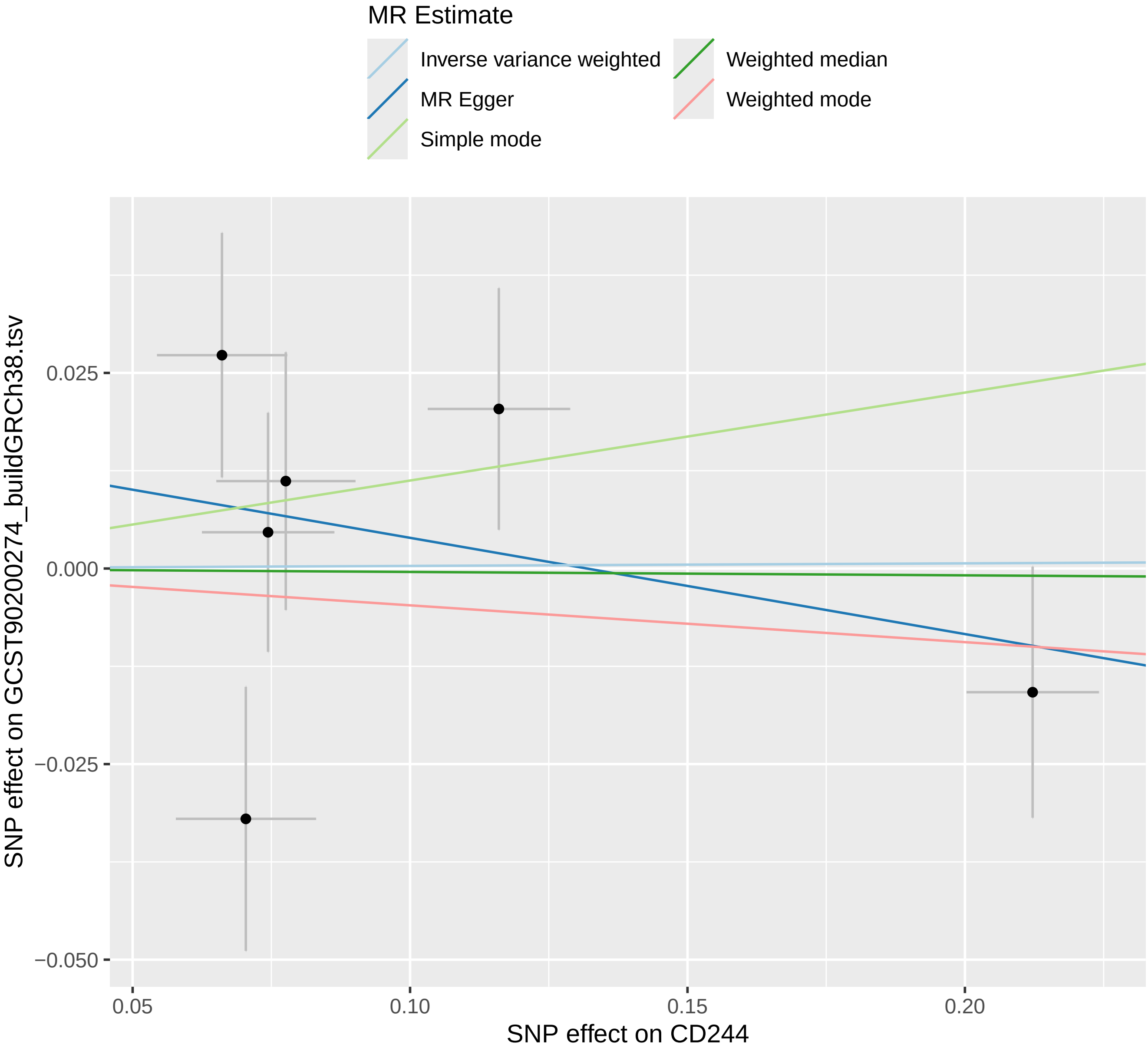
MR Estimate
Inverse variance weighted
MR Egger
Simple mode
Weighted median
Weighted mode
SNP effect on GCST90200274_buildGRCh38.tsv
0.025
0.000
−0.025
−0.050
0.05
0.10
0.15
0.20
SNP effect on CD244

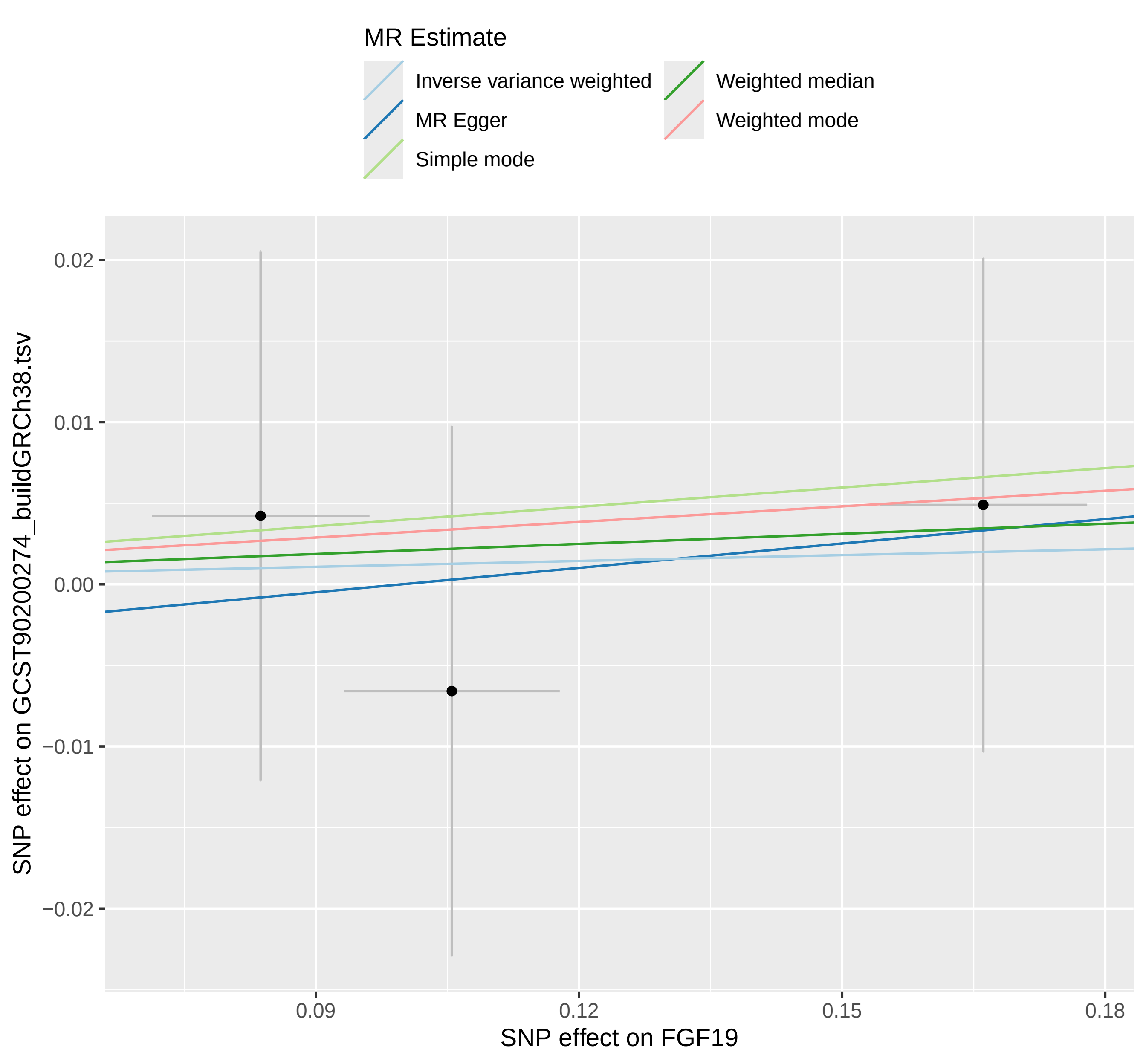

MR Estimate
Inverse variance weighted
MR Egger
Simple mode
Weighted median
Weighted mode
0.02
0.01
0.00
−0.01
−0.02
SNP effect on GCST90200274_buildGRCh38.tsv
0.09
0.12
0.15
0.18
SNP effect on FGF19

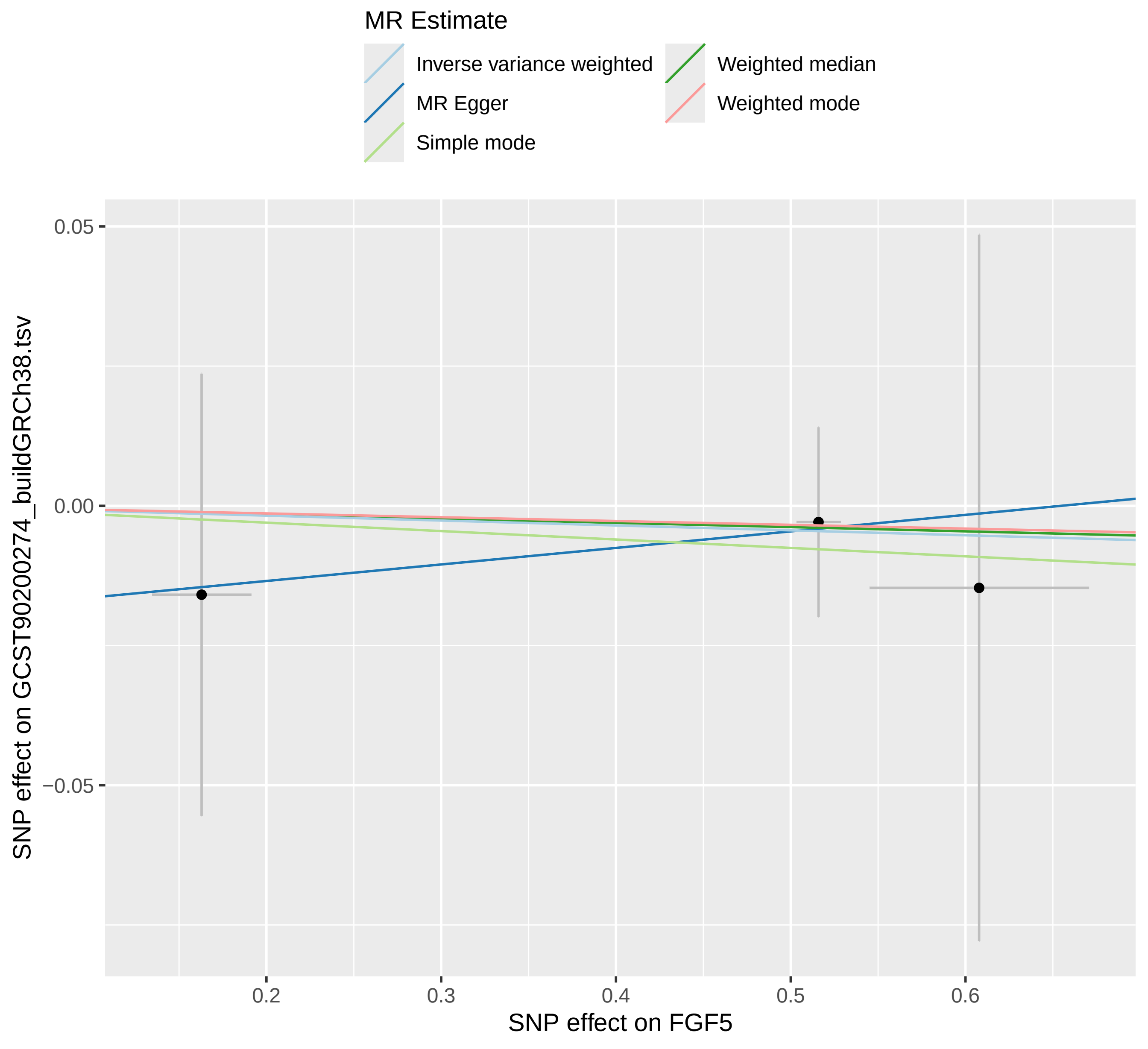

MR Estimate
Inverse variance weighted
MR Egger
Simple mode
Weighted median
Weighted mode
0.05
0.00
−0.05
SNP effect on GCST90200274_buildGRCh38.tsv
0.2
0.3
0.4
0.5
0.6
SNP effect on FGF5

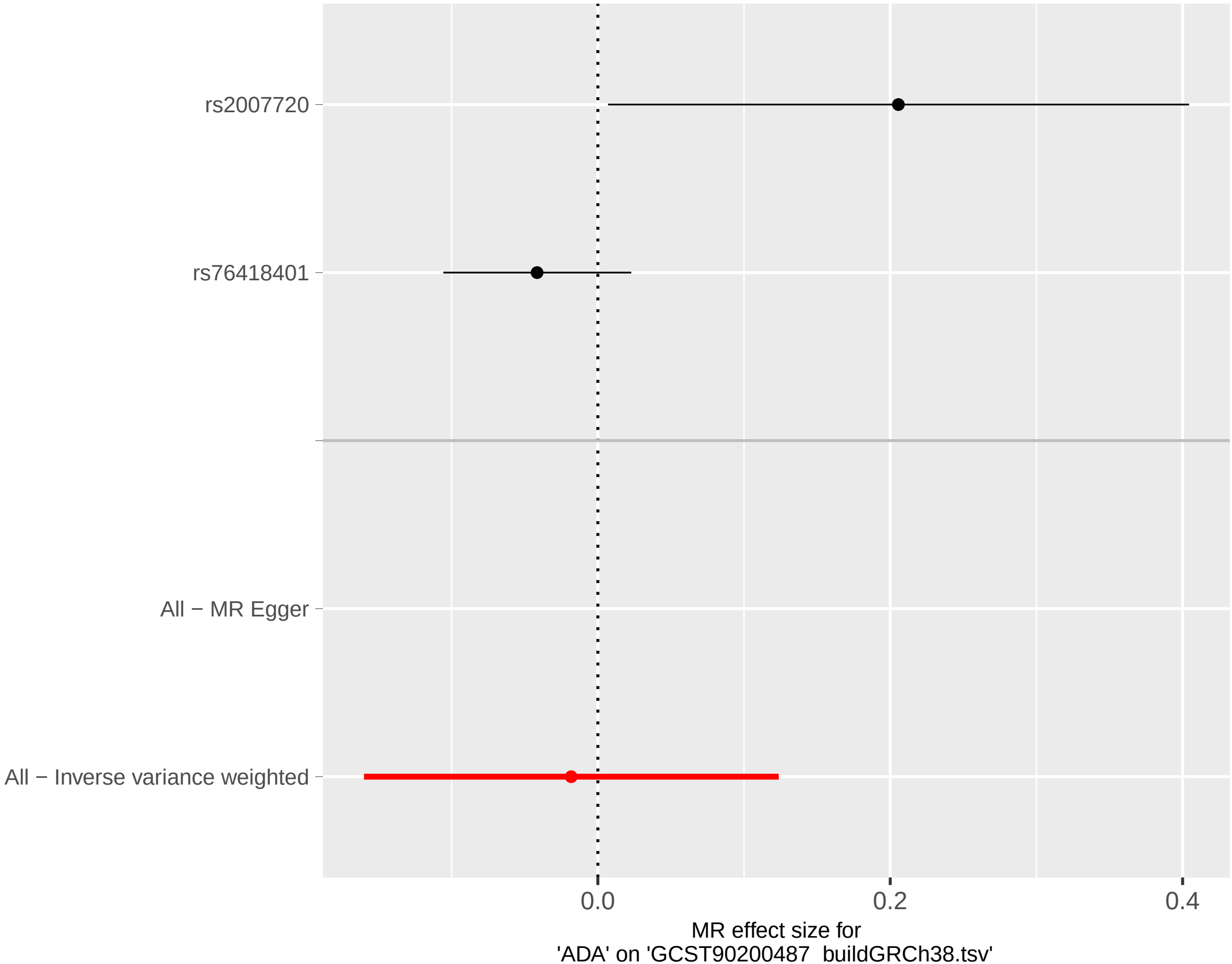

rs2007720
rs76418401
All − MR Egger
All − Inverse variance weighted
0.0
0.2
0.4
MR effect size for
'ADA' on 'GCST90200487_buildGRCh38.tsv'

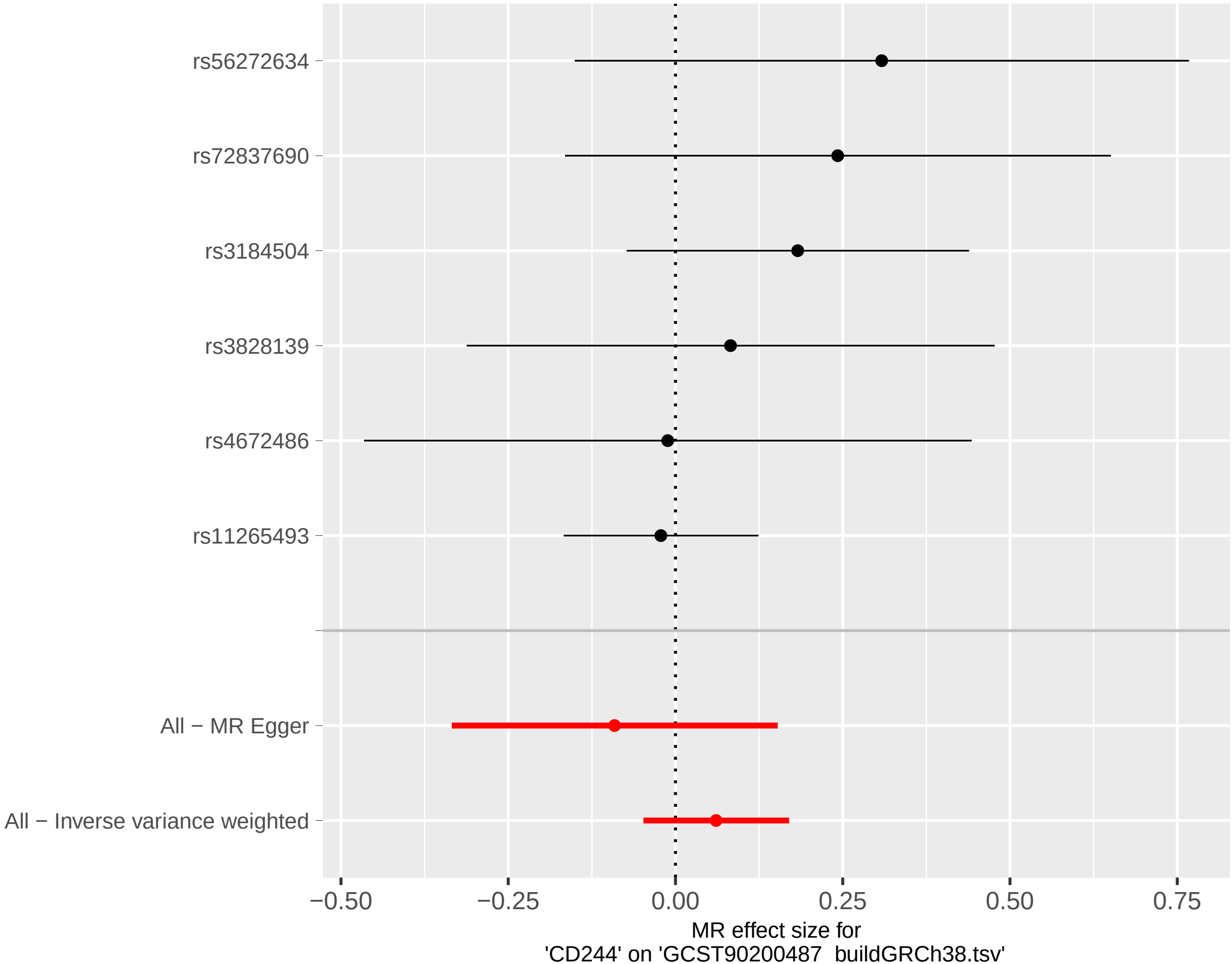
rs56272634
rs72837690
rs3184504
rs3828139
rs4672486
rs11265493
All − MR Egger
All − Inverse variance weighted
−0.50
−0.25
0.00
0.25
0.50
0.75
MR effect size for
'CD244' on 'GCST90200487_buildGRCh38.tsv'

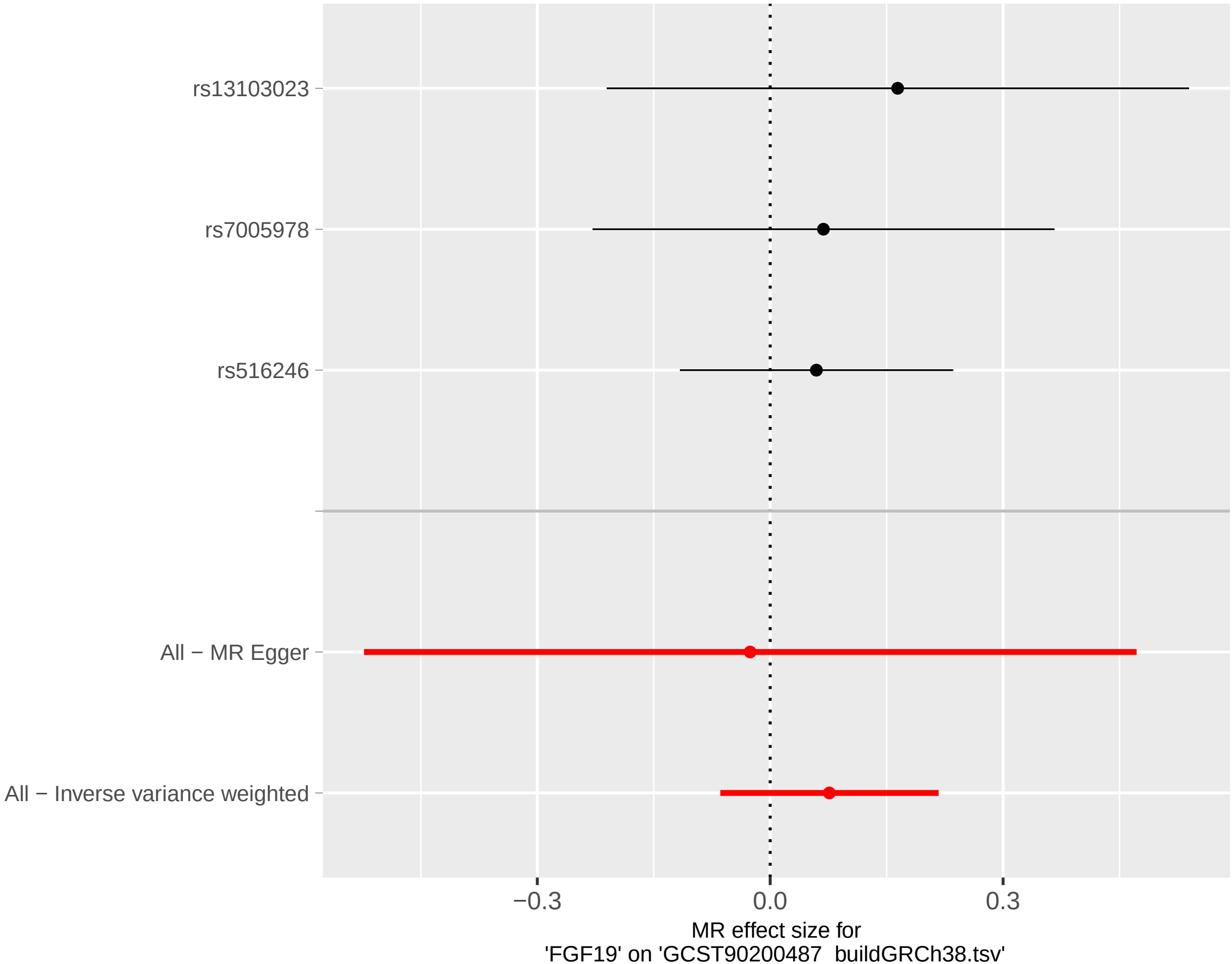
rs13103023
rs7005978
rs516246
All − MR Egger
All − Inverse variance weighted
−0.3
0.0
0.3
MR effect size for
'FGF19' on 'GCST90200487_buildGRCh38.tsv'

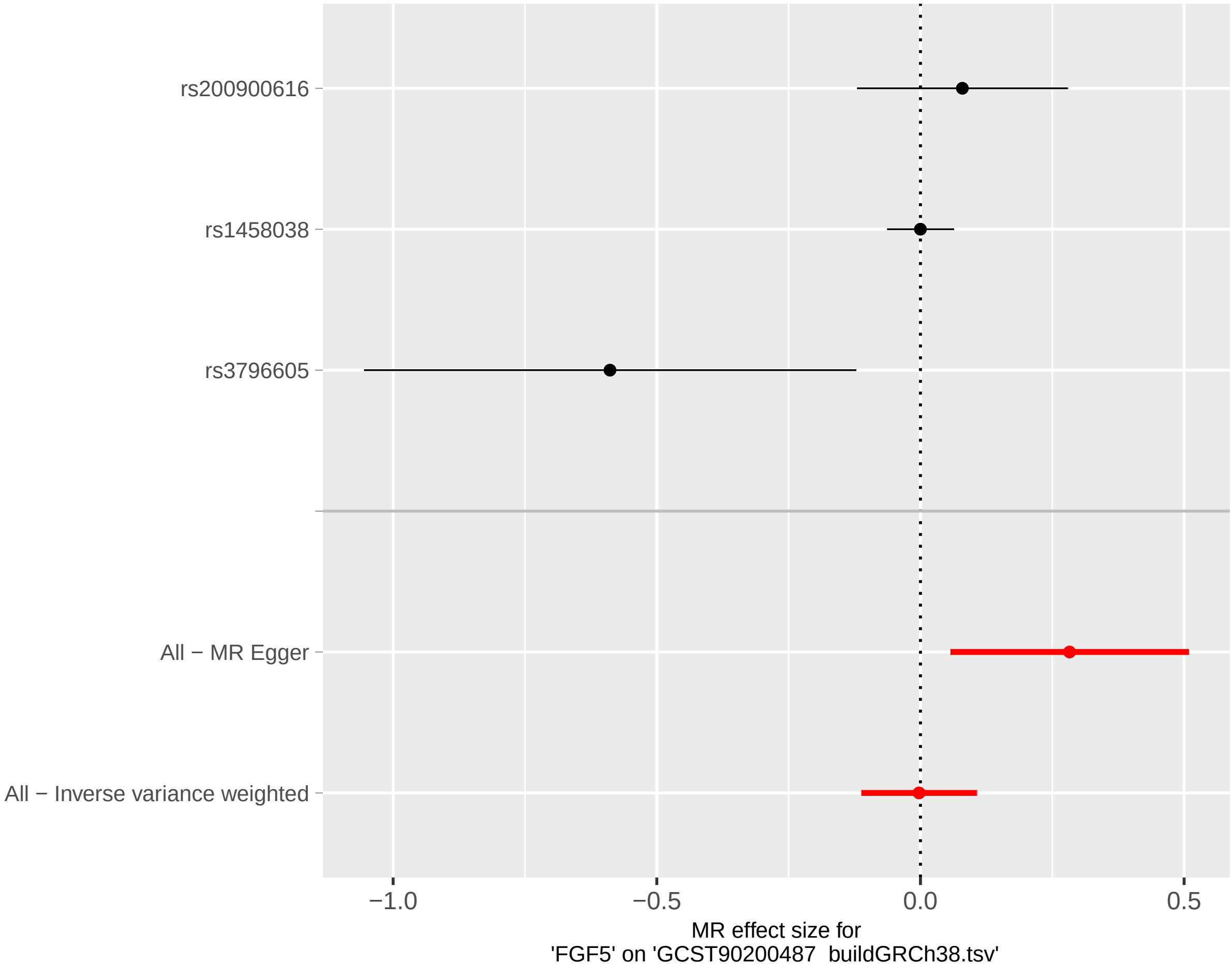

rs200900616
rs1458038
rs3796605
All − MR Egger
All − Inverse variance weighted
−1.0
−0.5
0.0
0.5
MR effect size for
'FGF5' on 'GCST90200487_buildGRCh38.tsv'

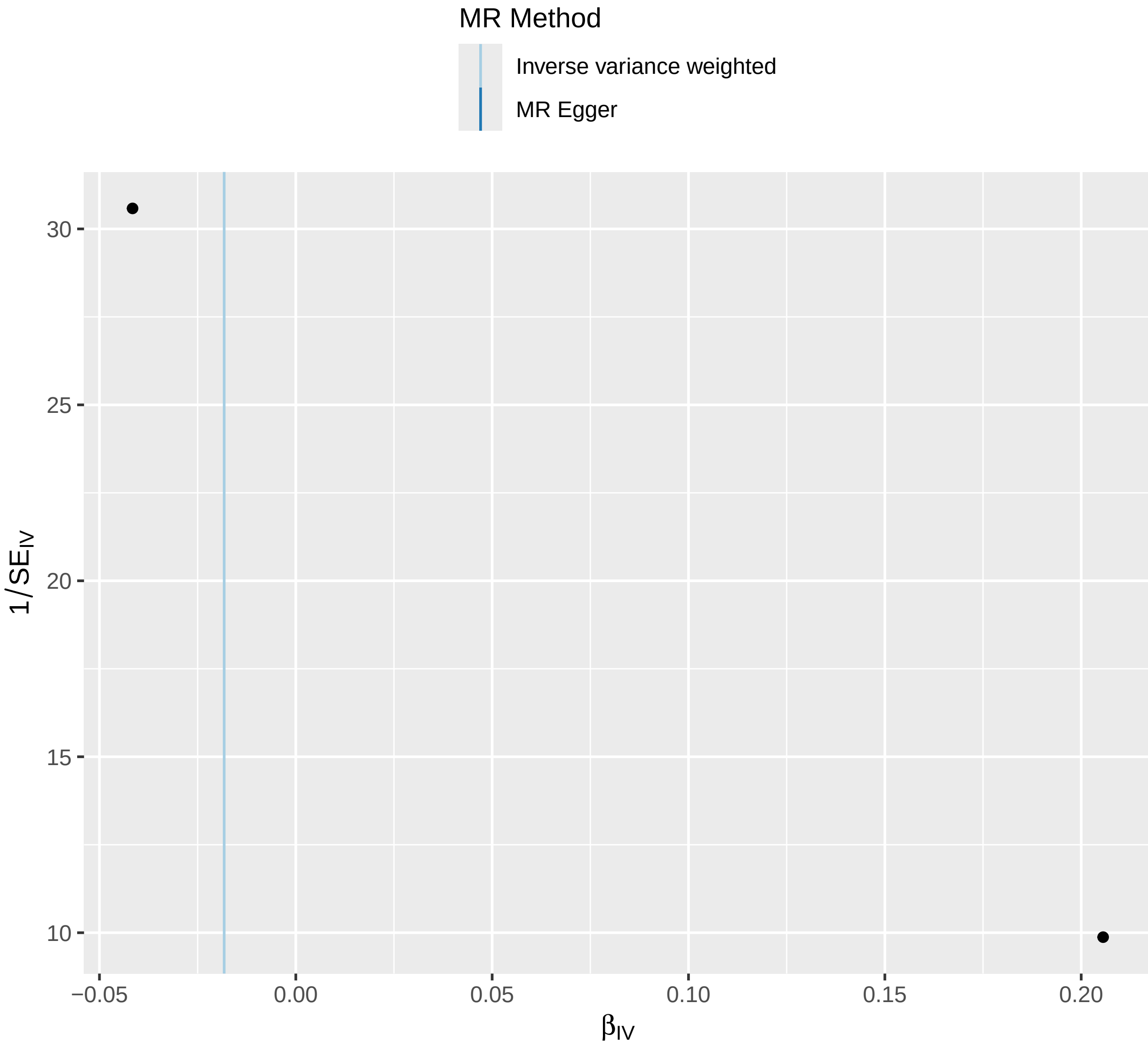
MR Method
Inverse variance weighted
MR Egger
30
25
20
15
10
$1/SE_{IV}$
−0.05
0.00
0.05
0.10
0.15
0.20
β_{IV}

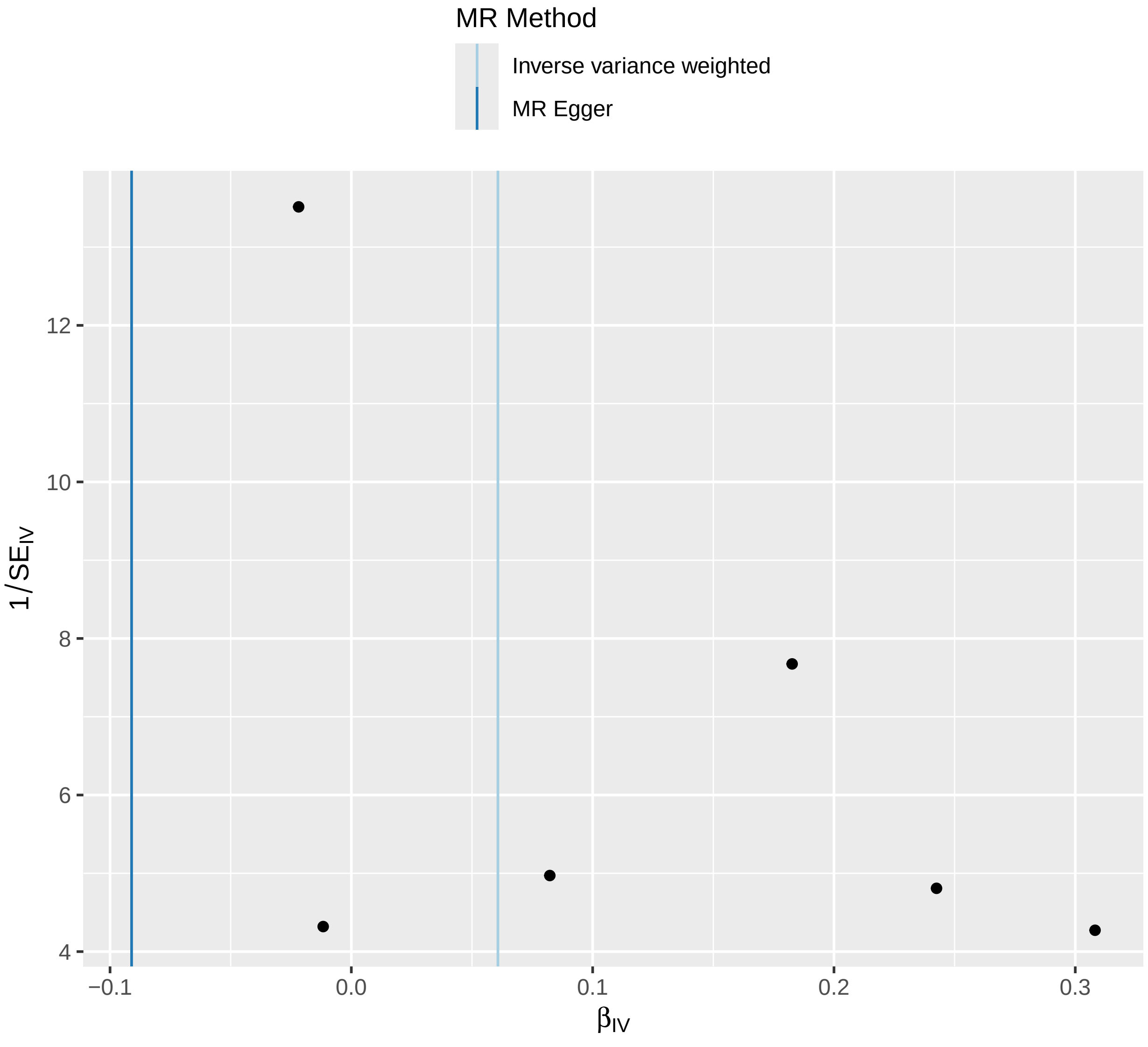
MR Method
Inverse variance weighted
MR Egger
12
10
8
6
4
−0.1
0.0
0.1
0.2
0.3
$1/SE_{IV}$
β_{IV}

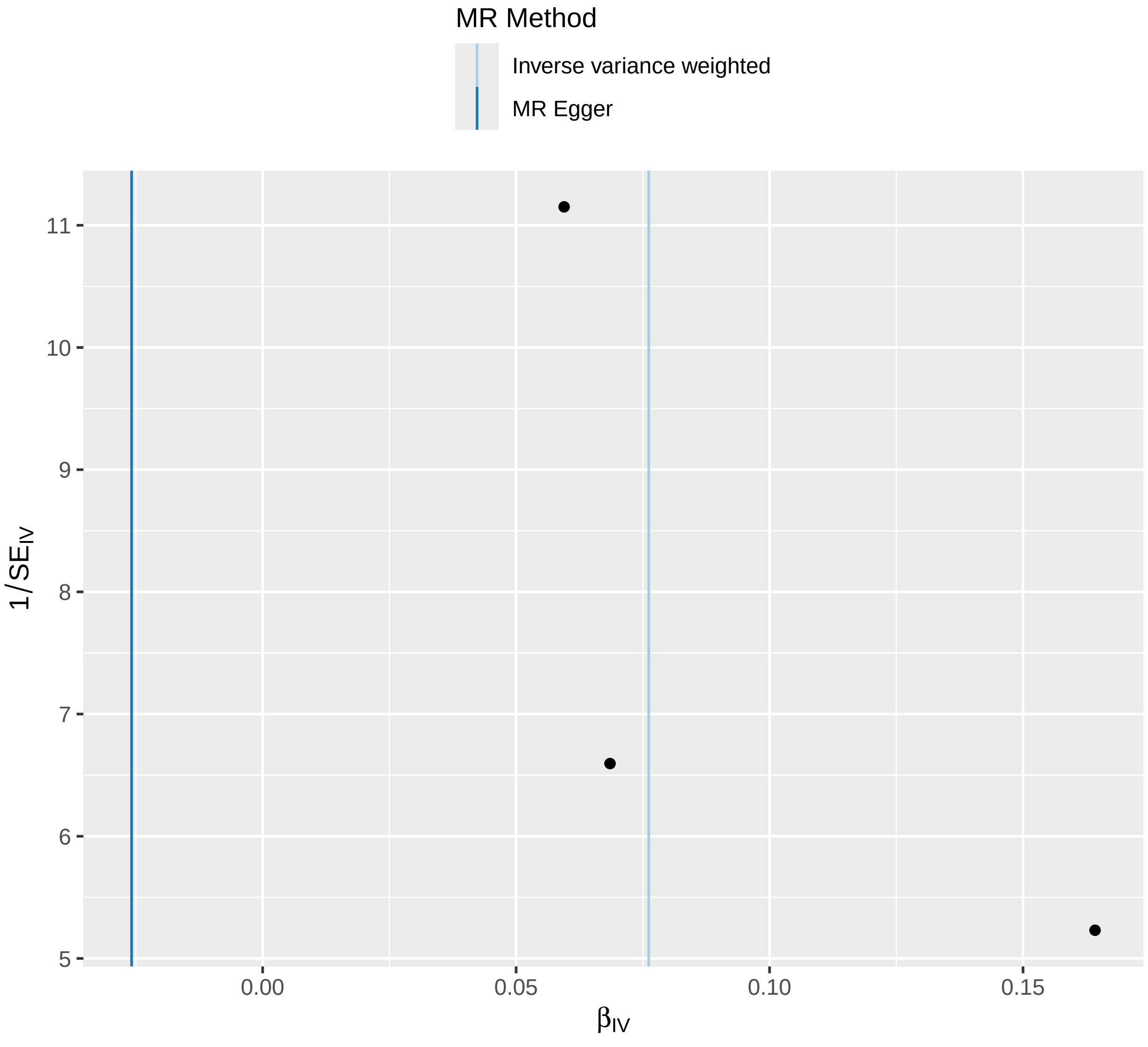
MR Method
Inverse variance weighted
MR Egger
1/SE_IV
β_IV
11
10
9
8
7
6
5
0.00
0.05
0.10
0.15

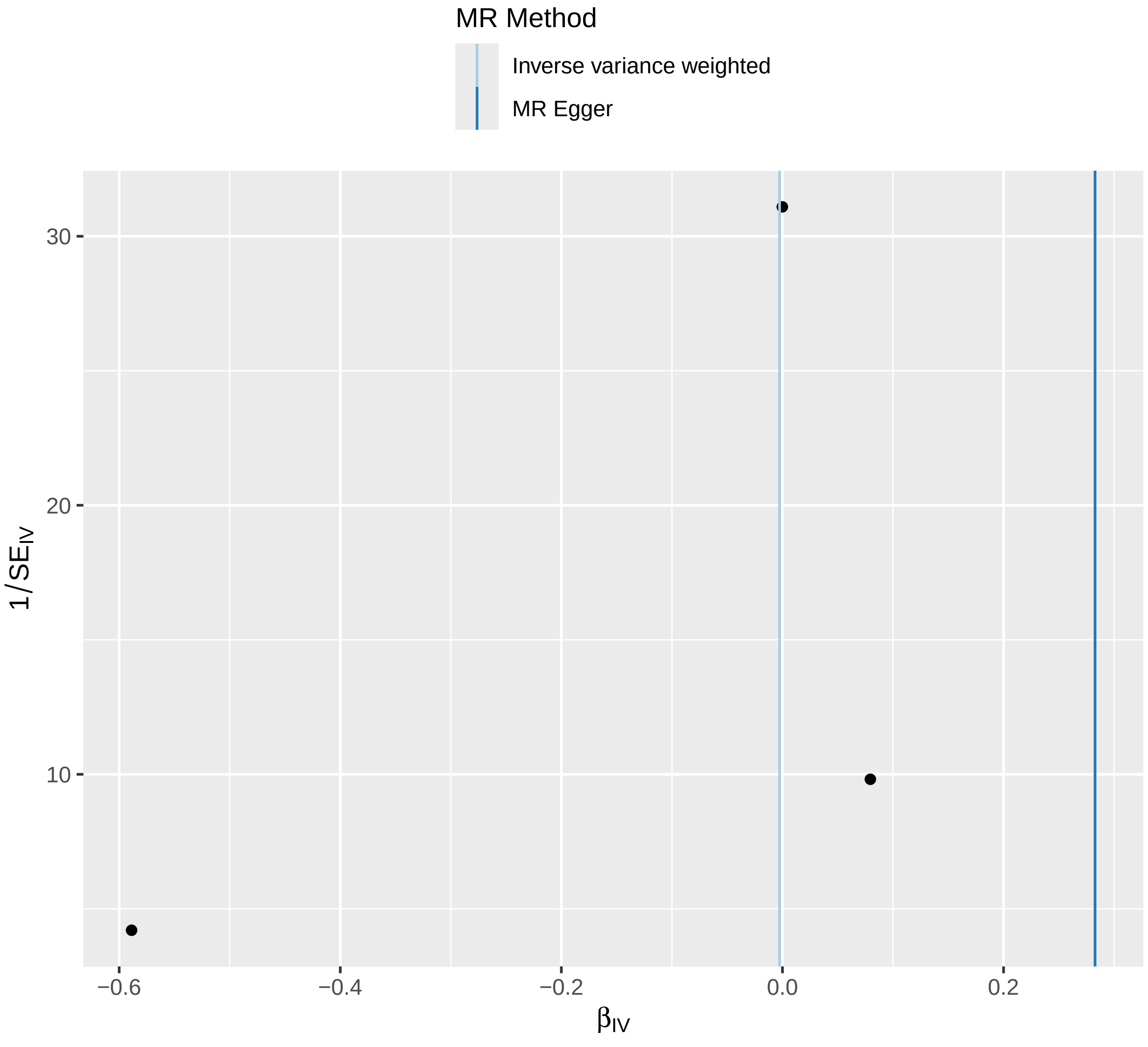
MR Method
Inverse variance weighted
MR Egger
30
20
10
1/SE_IV
−0.6
−0.4
−0.2
0.0
0.2
β_IV

Insufficient number of SNPs

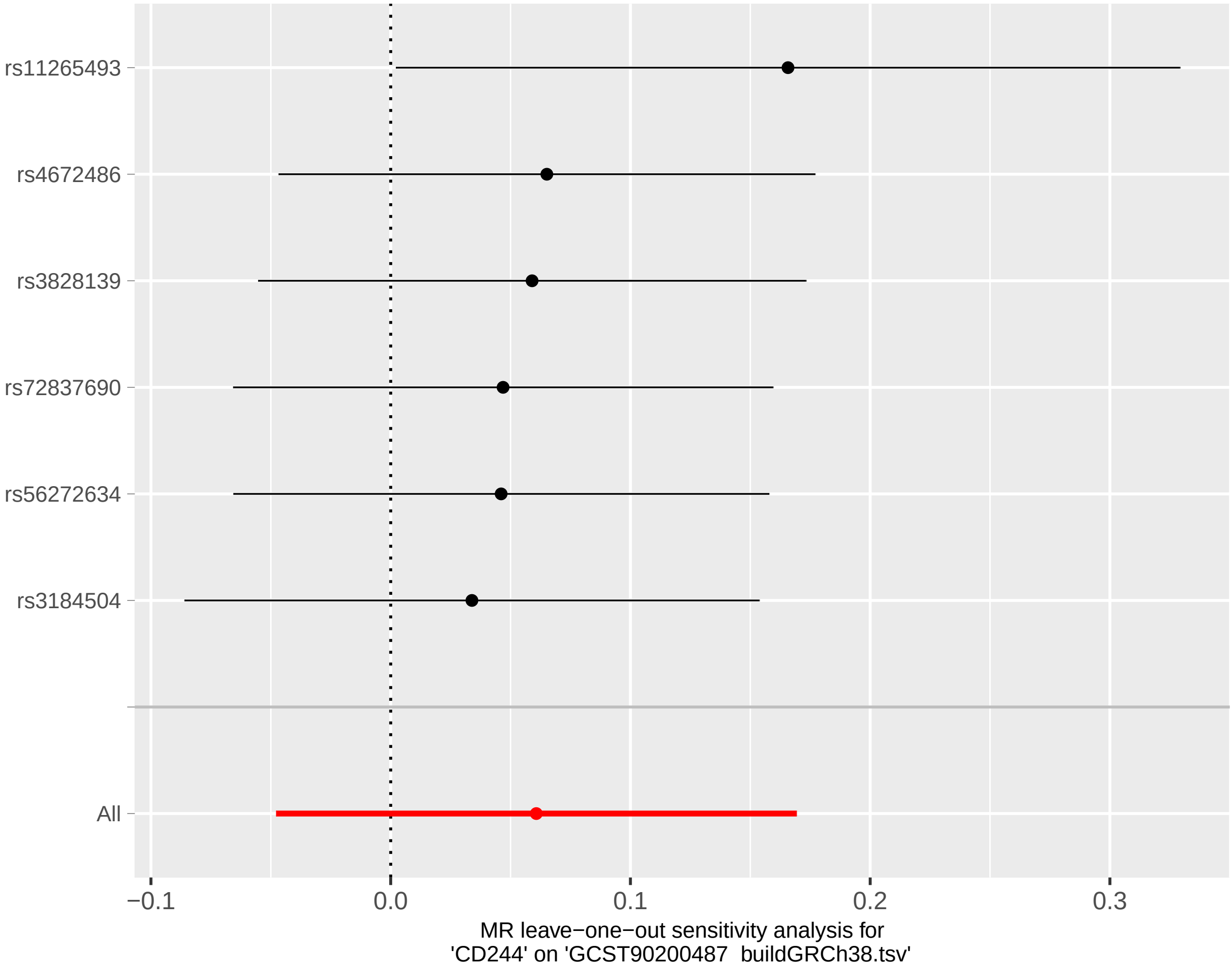
rs11265493
rs4672486
rs3828139
rs72837690
rs56272634
rs3184504
All
−0.1
0.0
0.1
0.2
0.3
MR leave−one−out sensitivity analysis for
'CD244' on 'GCST90200487_buildGRCh38.tsv'

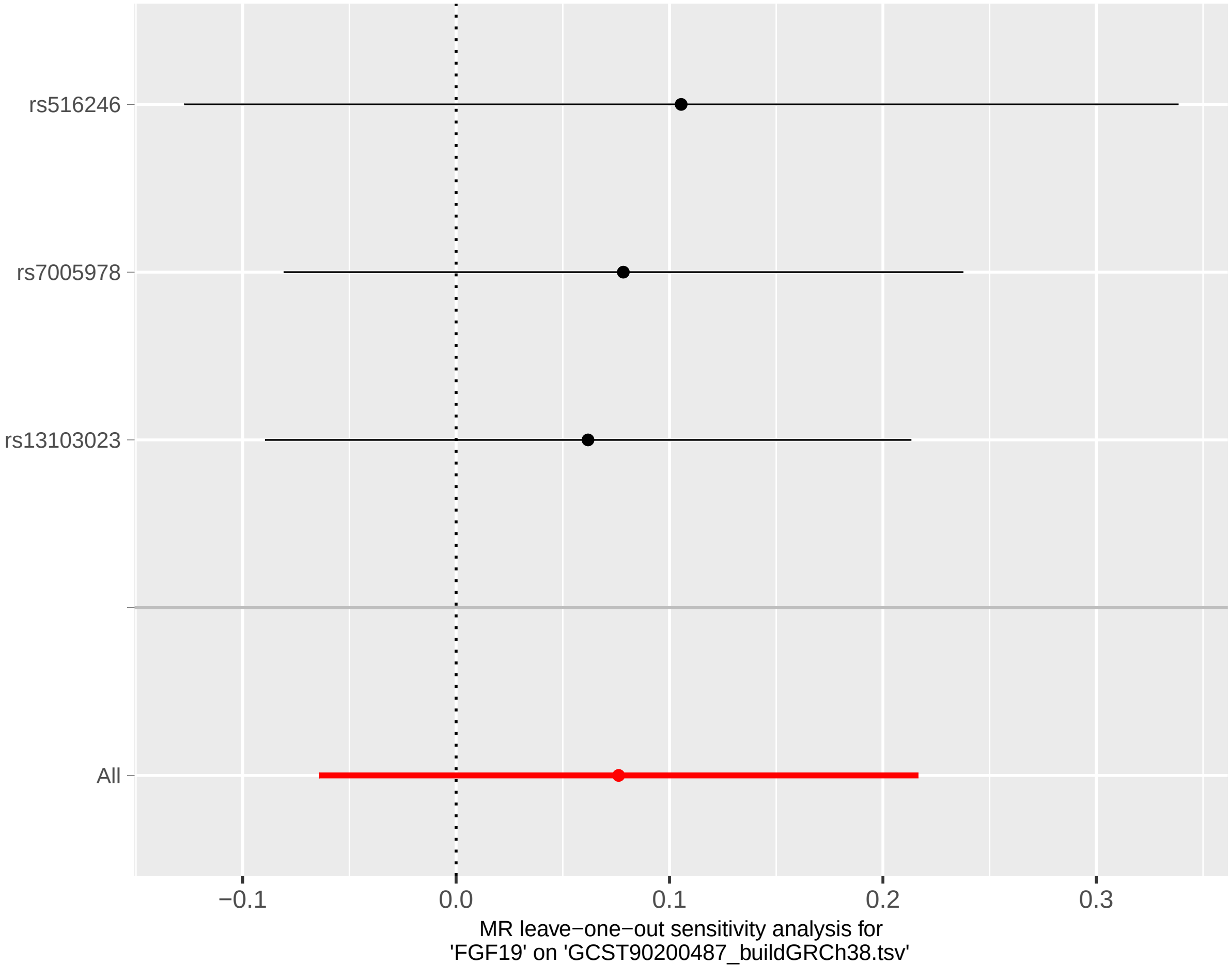
rs516246
rs7005978
rs13103023
All
−0.1
0.0
0.1
0.2
0.3
MR leave−one−out sensitivity analysis for
'FGF19' on 'GCST90200487_buildGRCh38.tsv'

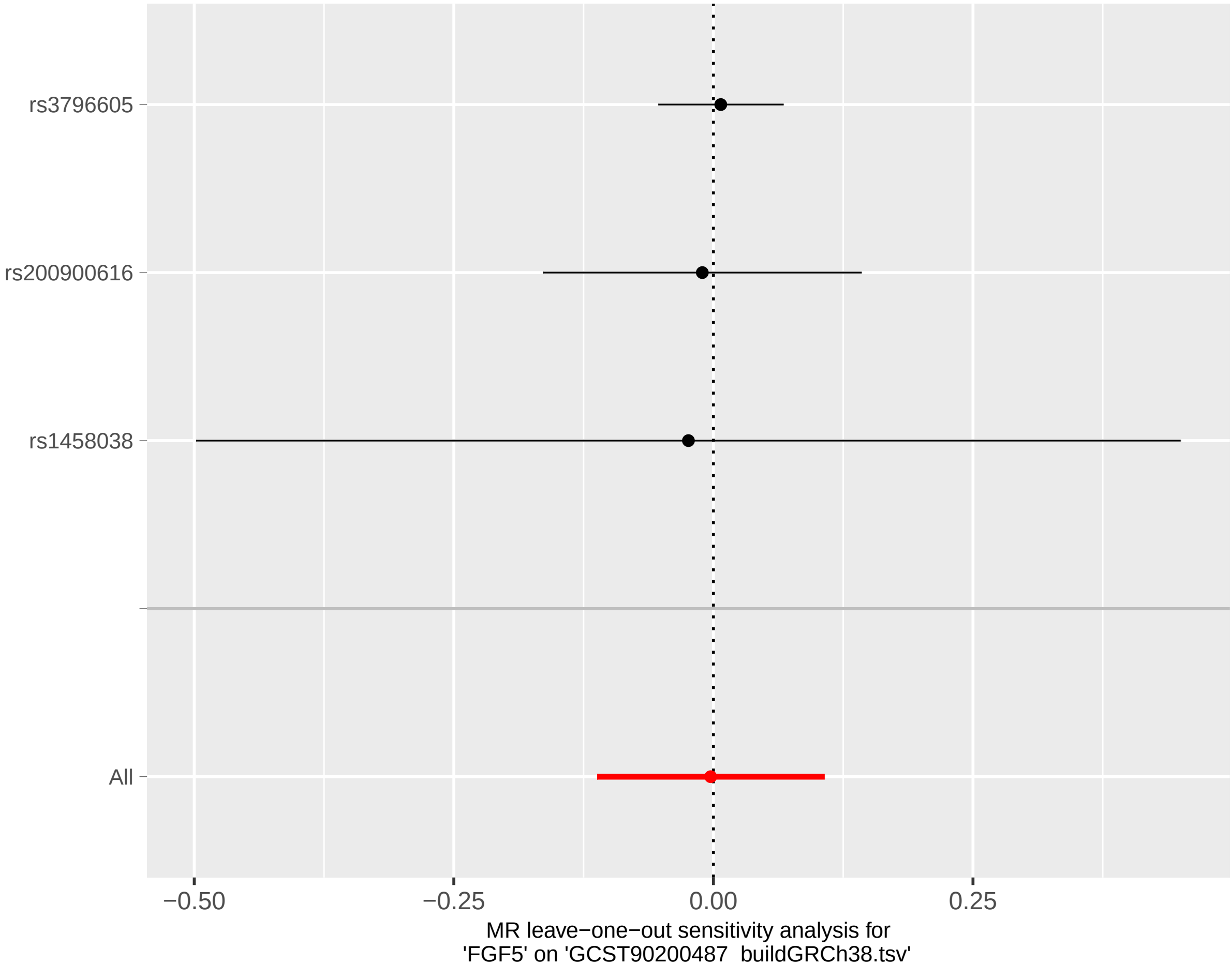
rs3796605
rs200900616
rs1458038
All
−0.50
−0.25
0.00
0.25
MR leave−one−out sensitivity analysis for
'FGF5' on 'GCST90200487_buildGRCh38.tsv'

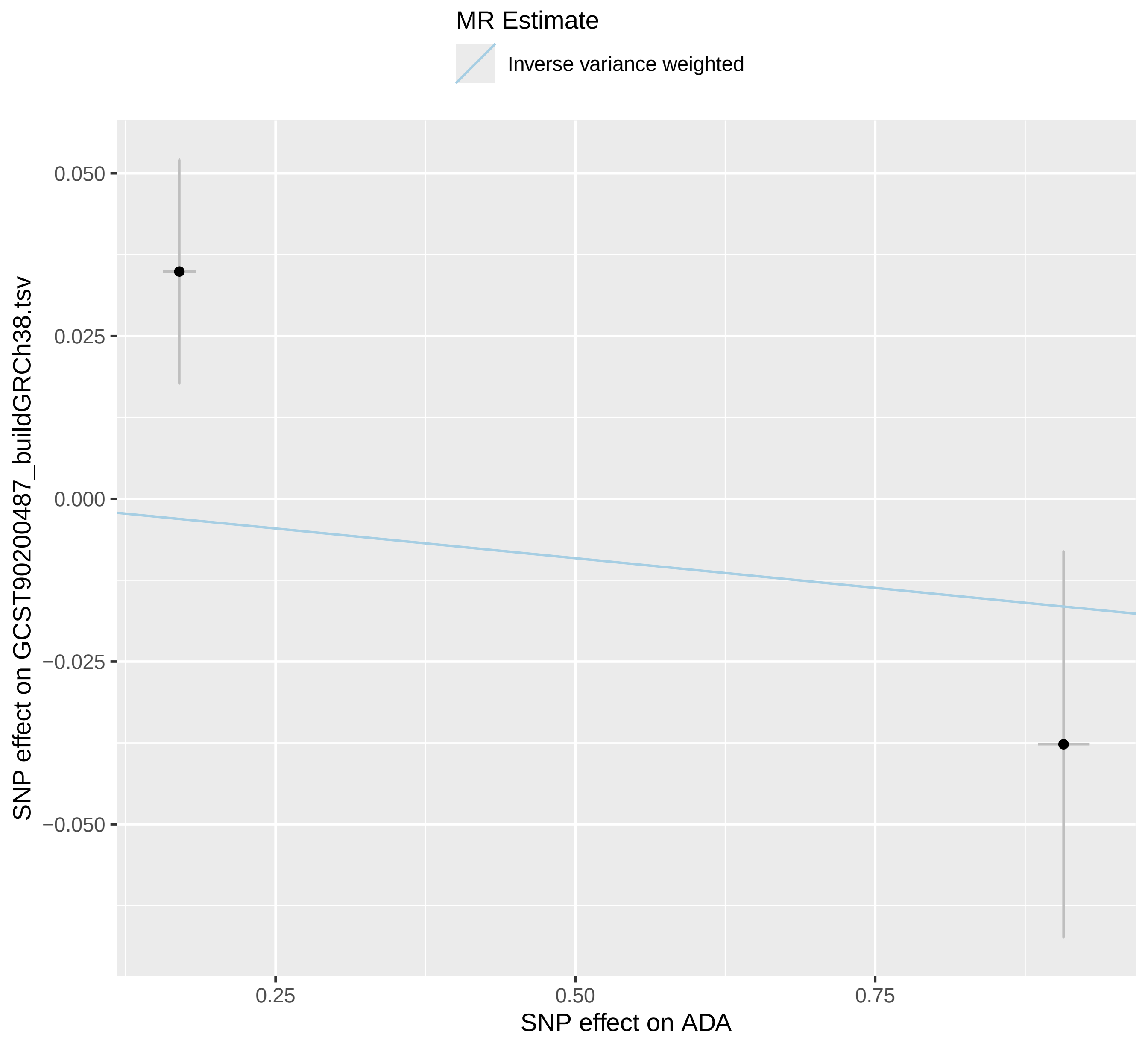
MR Estimate
Inverse variance weighted
0.050
0.025
0.000
−0.025
−0.050
SNP effect on GCST90200487_buildGRCh38.tsv
0.25
0.50
0.75
SNP effect on ADA

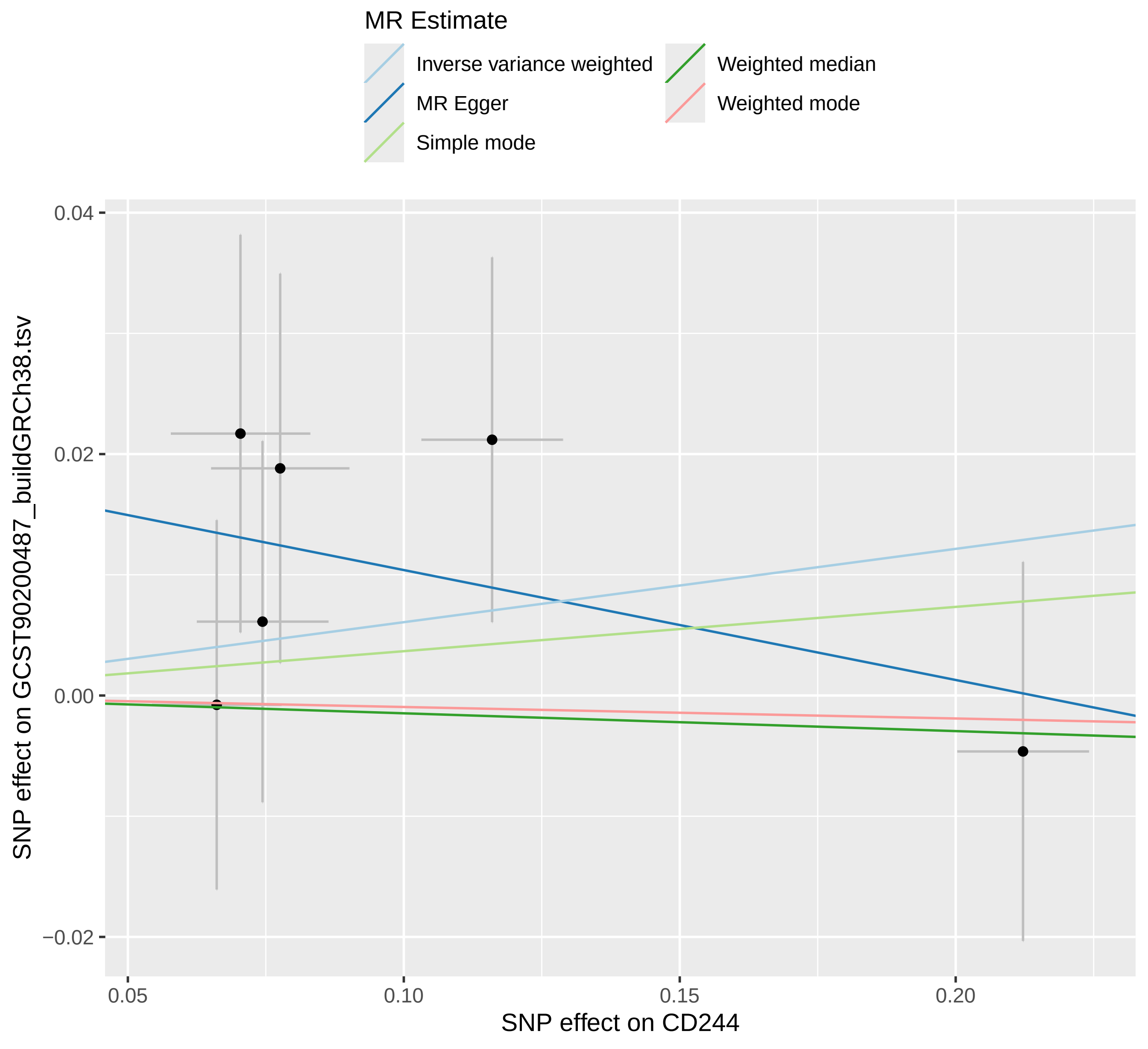
MR Estimate
Inverse variance weighted
MR Egger
Simple mode
Weighted median
Weighted mode
0.04
0.02
0.00
−0.02
SNP effect on GCST90200487_buildGRCh38.tsv
0.05
0.10
0.15
0.20
SNP effect on CD244

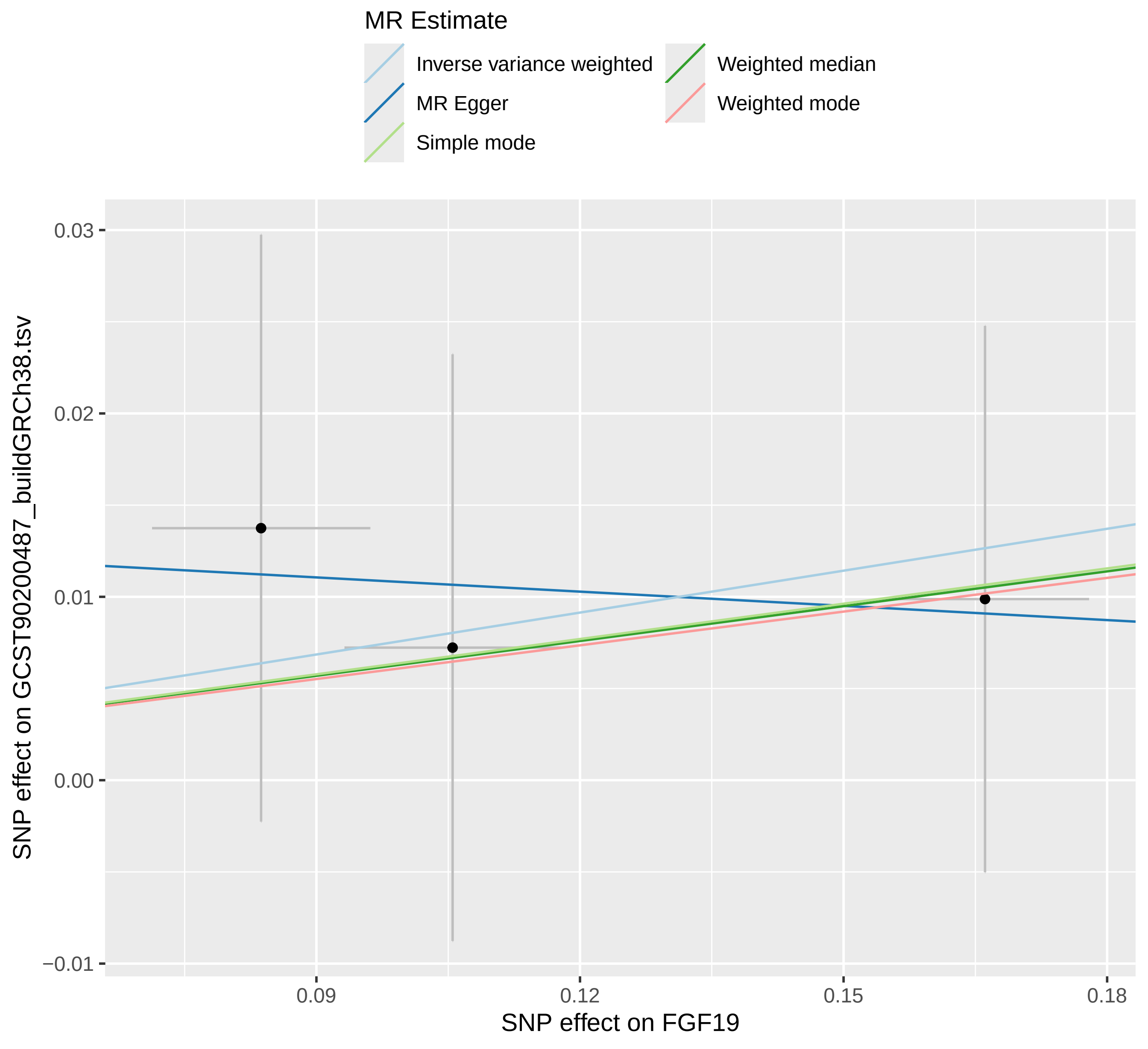

MR Estimate
Inverse variance weighted
MR Egger
Simple mode
Weighted median
Weighted mode
0.03
0.02
0.01
0.00
−0.01
0.09
0.12
0.15
0.18
SNP effect on GCST90200487_buildGRCh38.tsv
SNP effect on FGF19

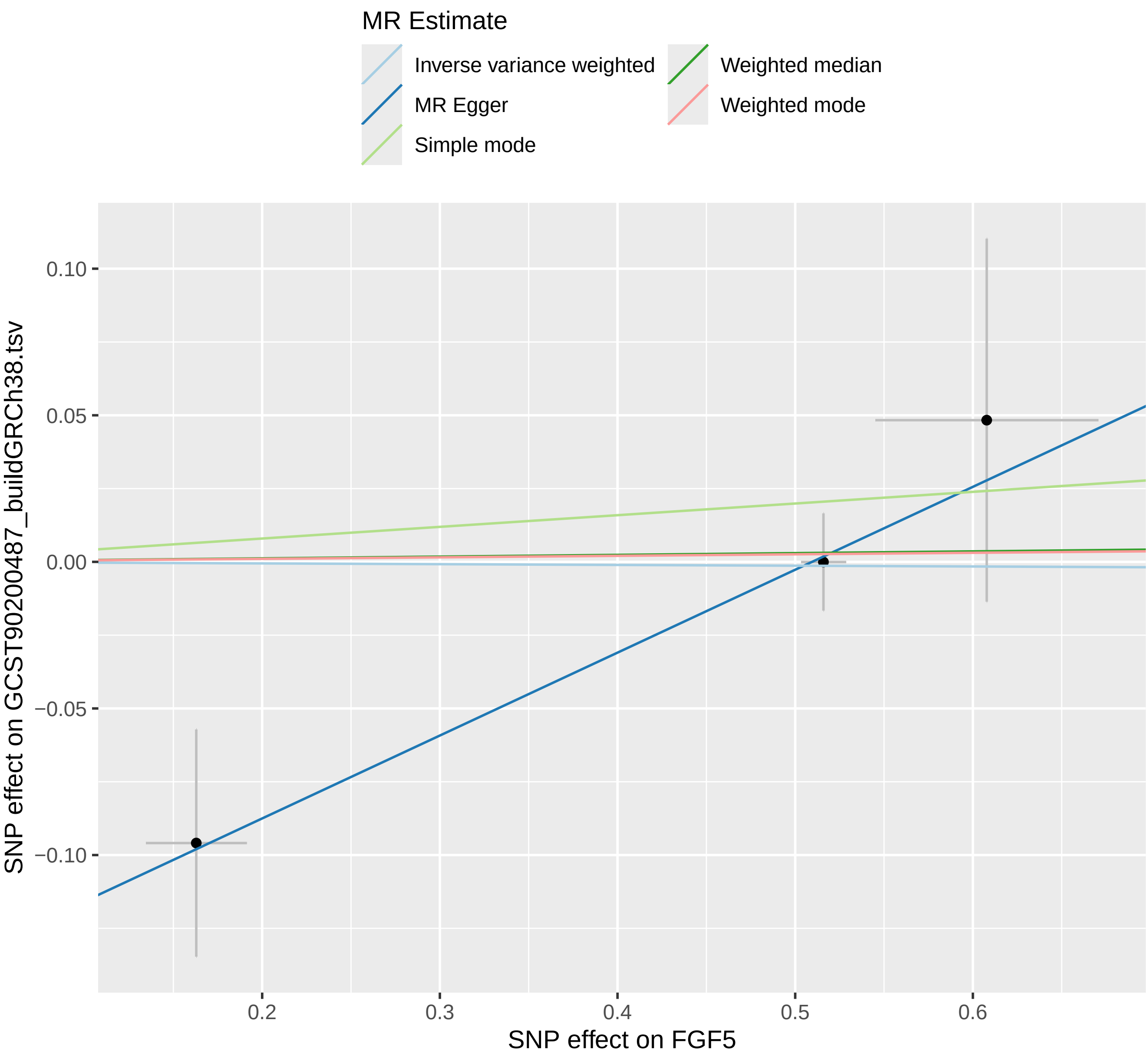

MR Estimate
Inverse variance weighted
MR Egger
Simple mode
Weighted median
Weighted mode
0.10
0.05
0.00
−0.05
−0.10
SNP effect on GCST90200487_buildGRCh38.tsv
0.2
0.3
0.4
0.5
0.6
SNP effect on FGF5

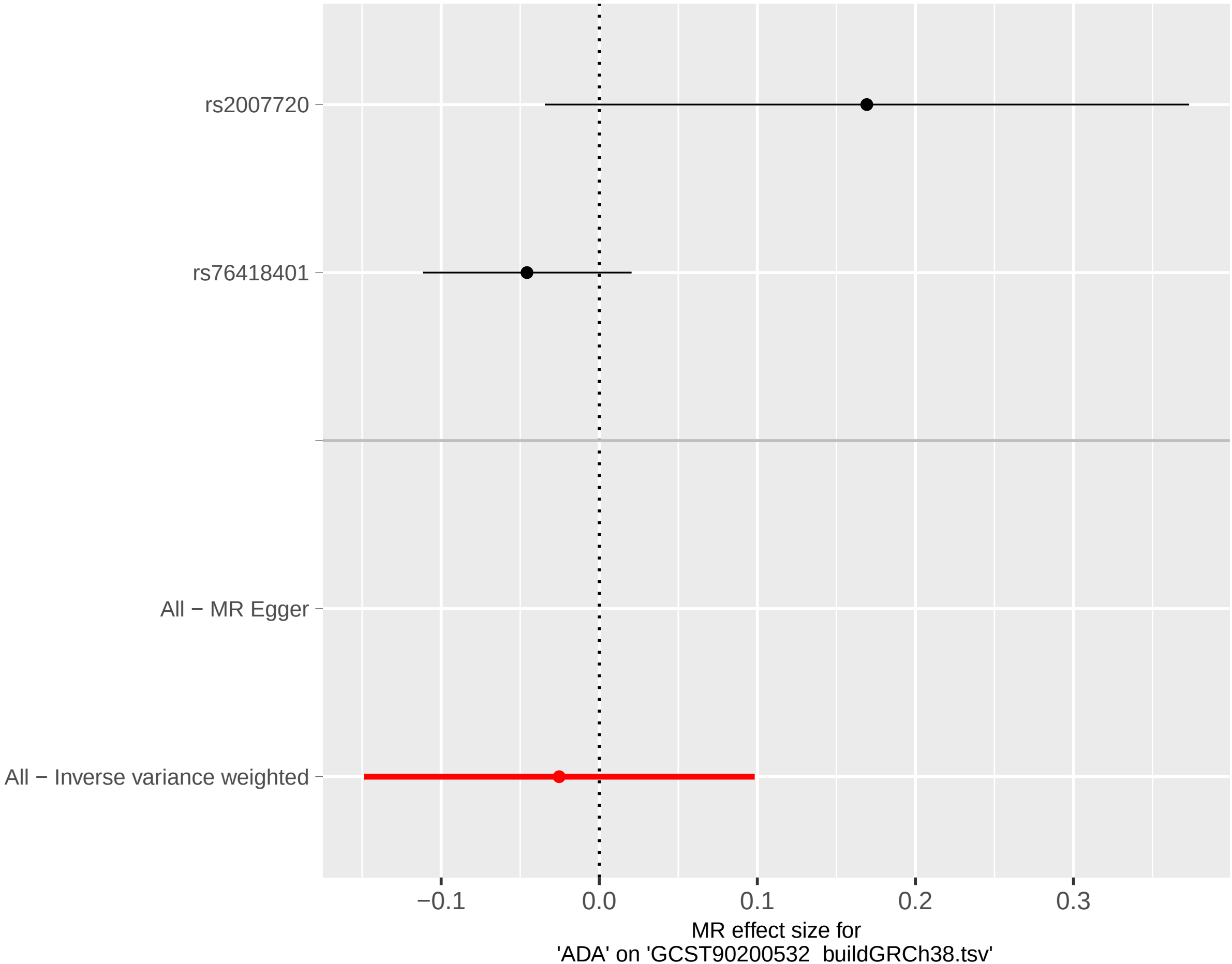
rs2007720
rs76418401
All − MR Egger
All − Inverse variance weighted
−0.1
0.0
0.1
0.2
0.3
MR effect size for
'ADA' on 'GCST90200532_buildGRCh38.tsv'

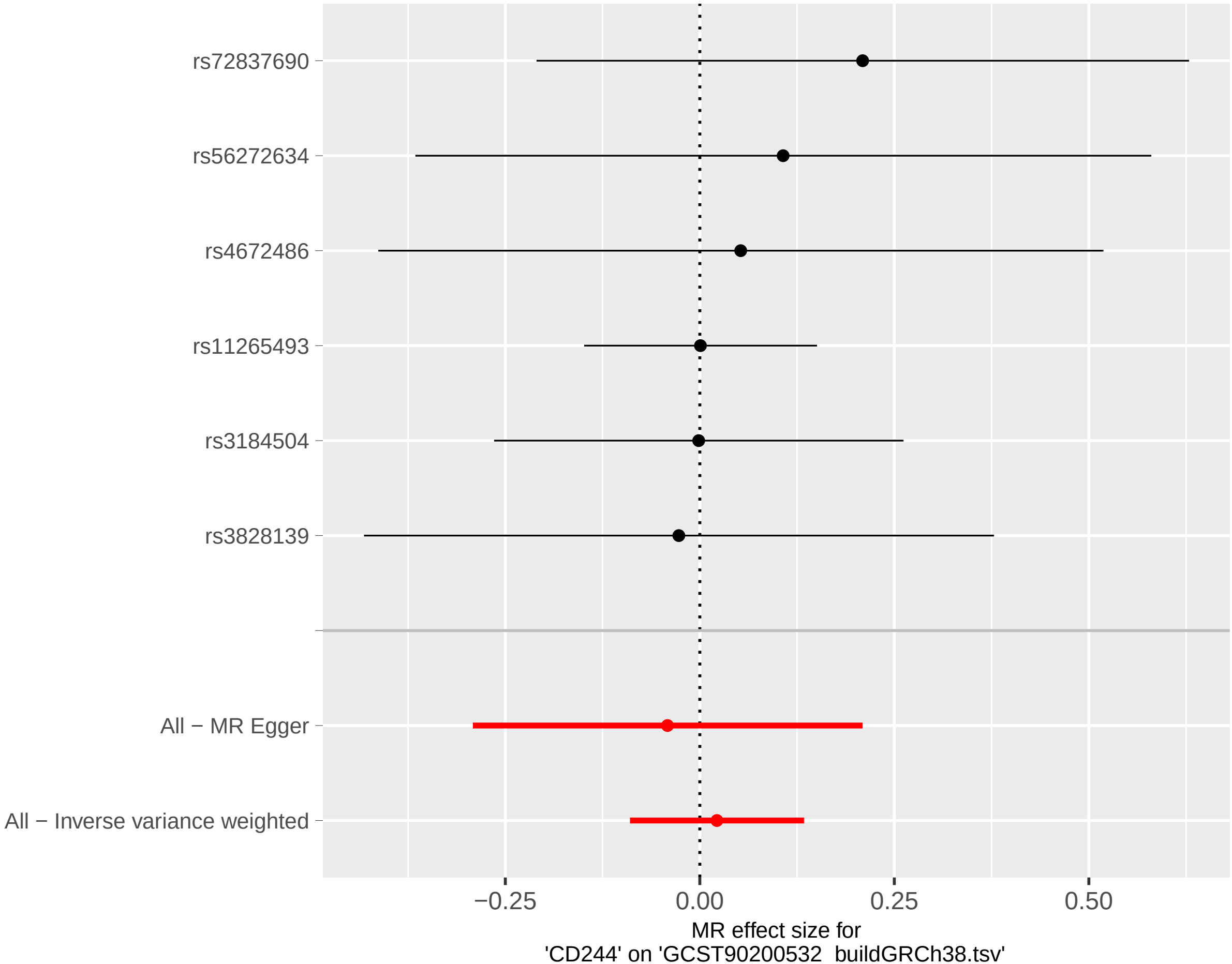
rs72837690
rs56272634
rs4672486
rs11265493
rs3184504
rs3828139
All − MR Egger
All − Inverse variance weighted
−0.25
0.00
0.25
0.50
MR effect size for
'CD244' on 'GCST90200532_buildGRCh38.tsv'

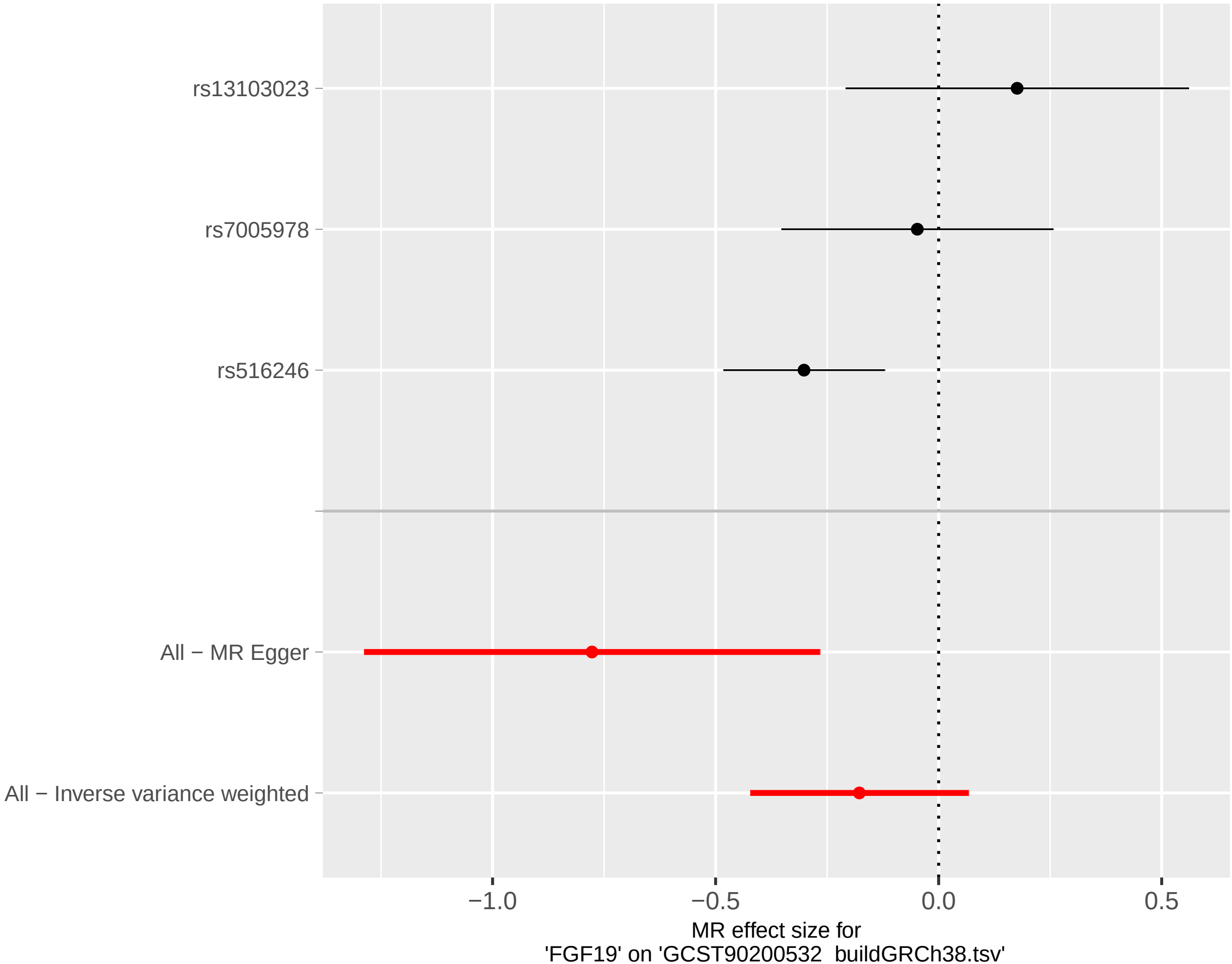
rs13103023
rs7005978
rs516246
All − MR Egger
All − Inverse variance weighted
−1.0
−0.5
0.0
0.5
MR effect size for
'FGF19' on 'GCST90200532_buildGRCh38.tsv'

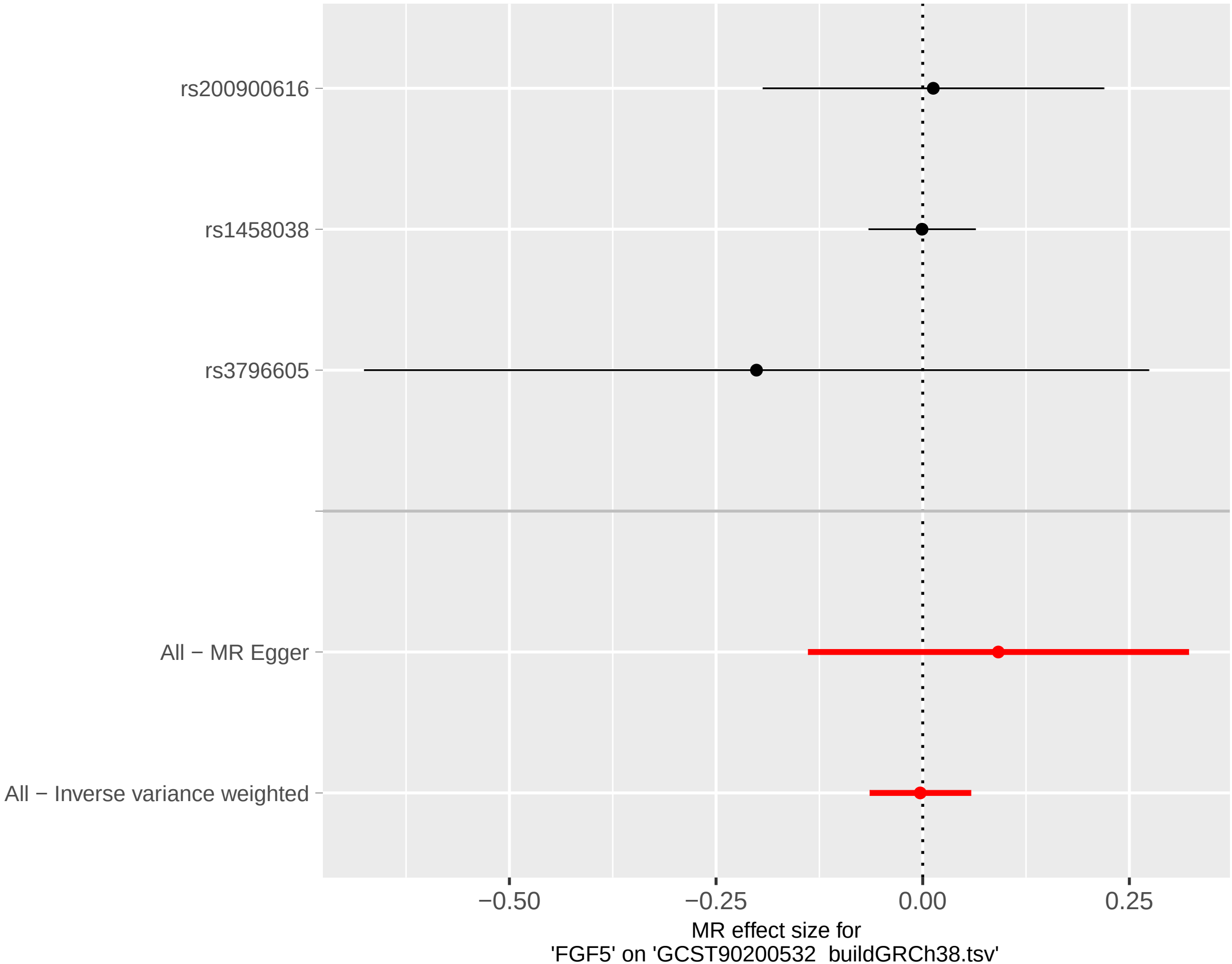

rs200900616
rs1458038
rs3796605
All − MR Egger
All − Inverse variance weighted
−0.50
−0.25
0.00
0.25
MR effect size for
'FGF5' on 'GCST90200532_buildGRCh38.tsv'

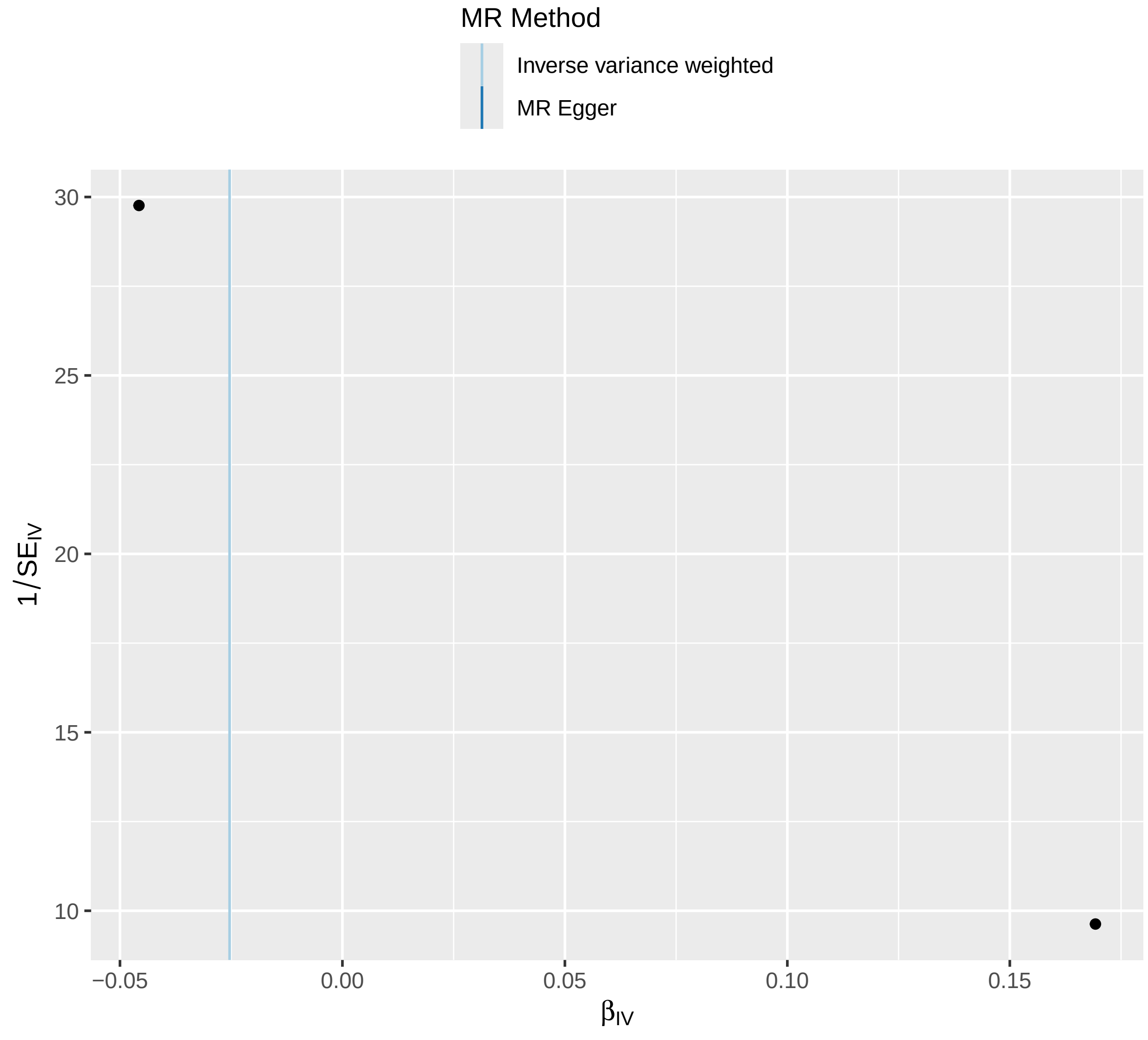

MR Method
Inverse variance weighted
MR Egger
30
25
20
15
10
1/SE_IV
−0.05
0.00
0.05
0.10
0.15
β_IV

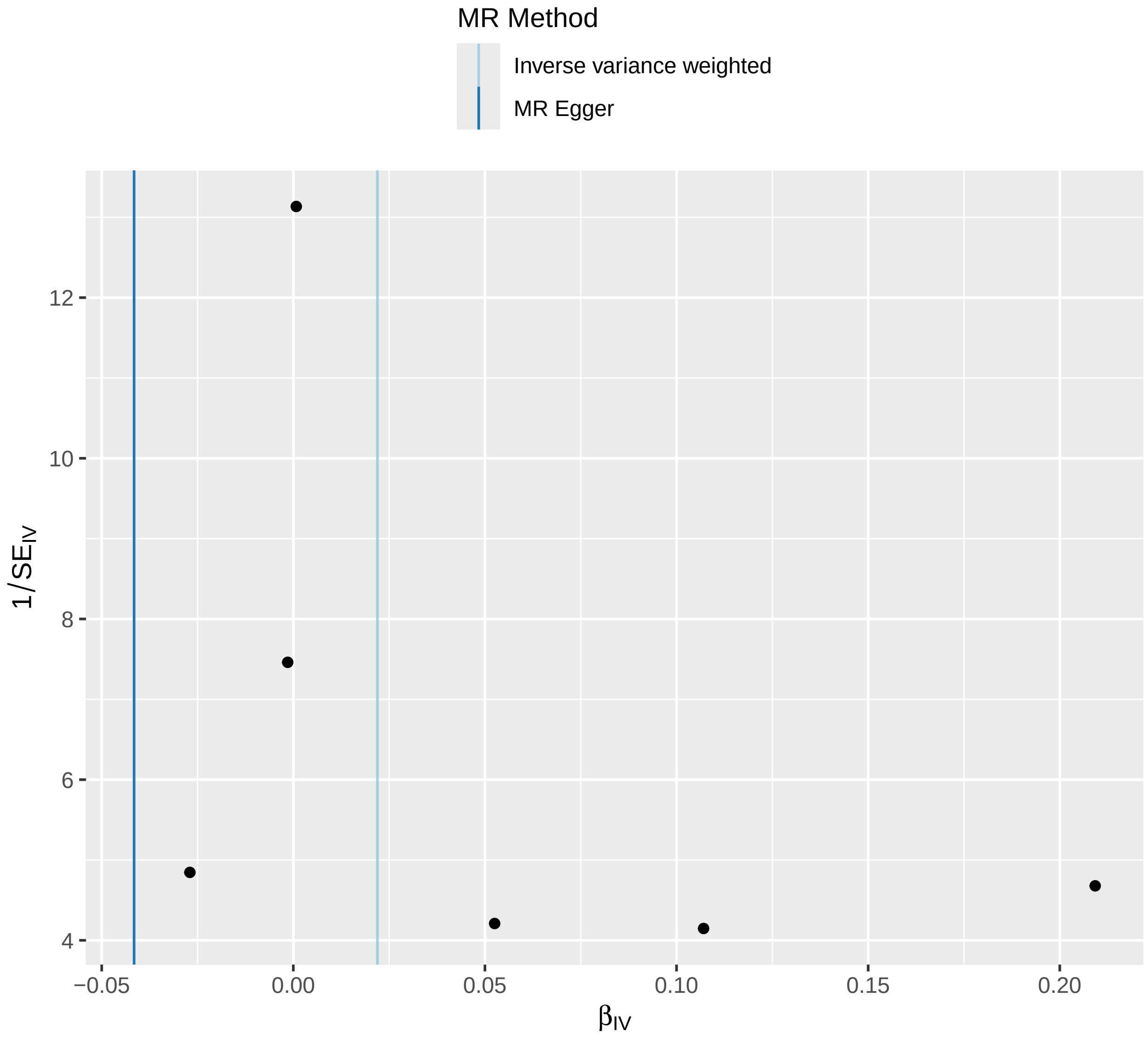

MR Method
Inverse variance weighted
MR Egger
12
10
8
6
4
1/SE$_{IV}$
−0.05
0.00
0.05
0.10
0.15
0.20
β$_{IV}$

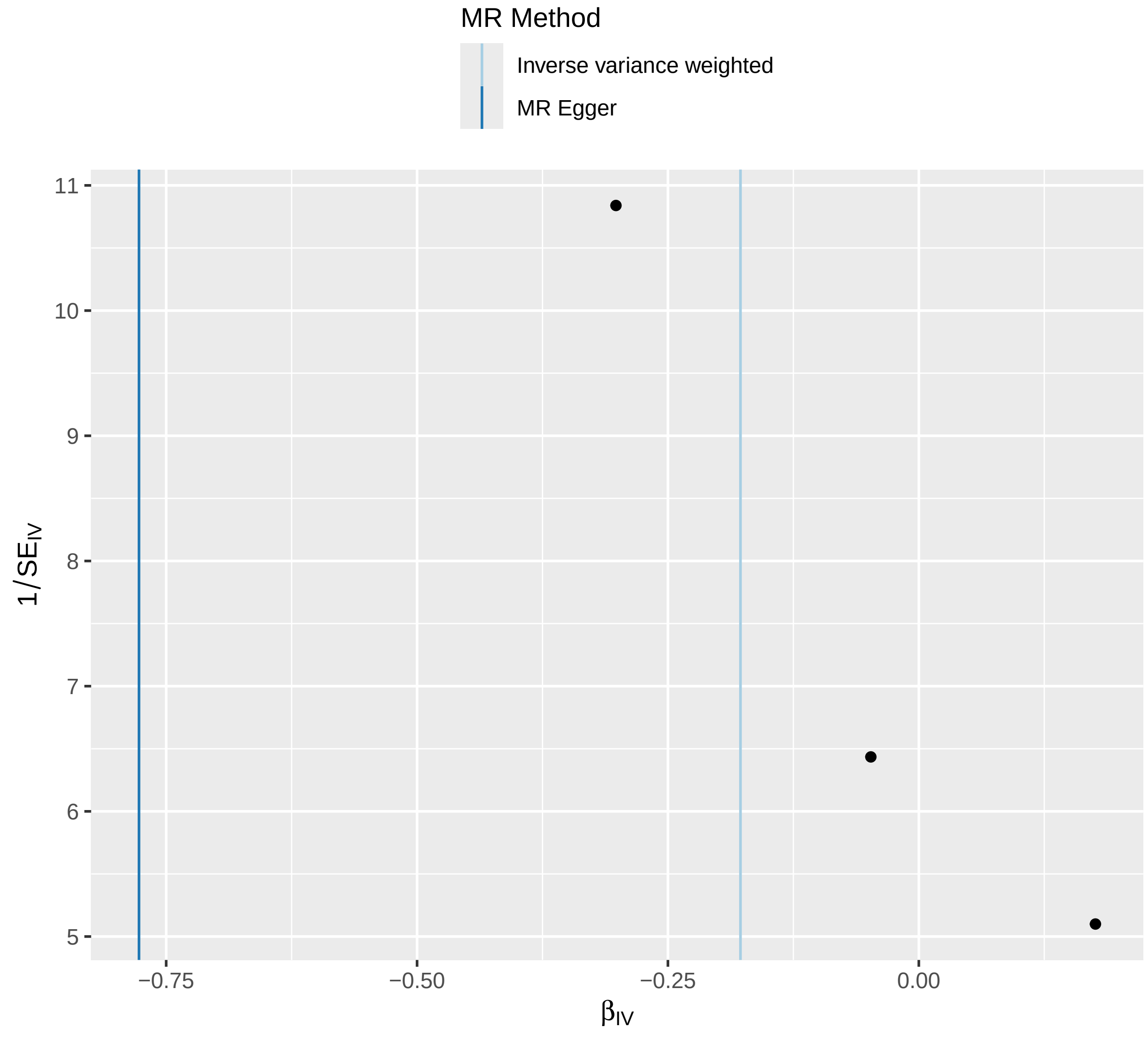
MR Method
Inverse variance weighted
MR Egger
1/SE_IV
β_IV
11
10
9
8
7
6
5
−0.75
−0.50
−0.25
0.00

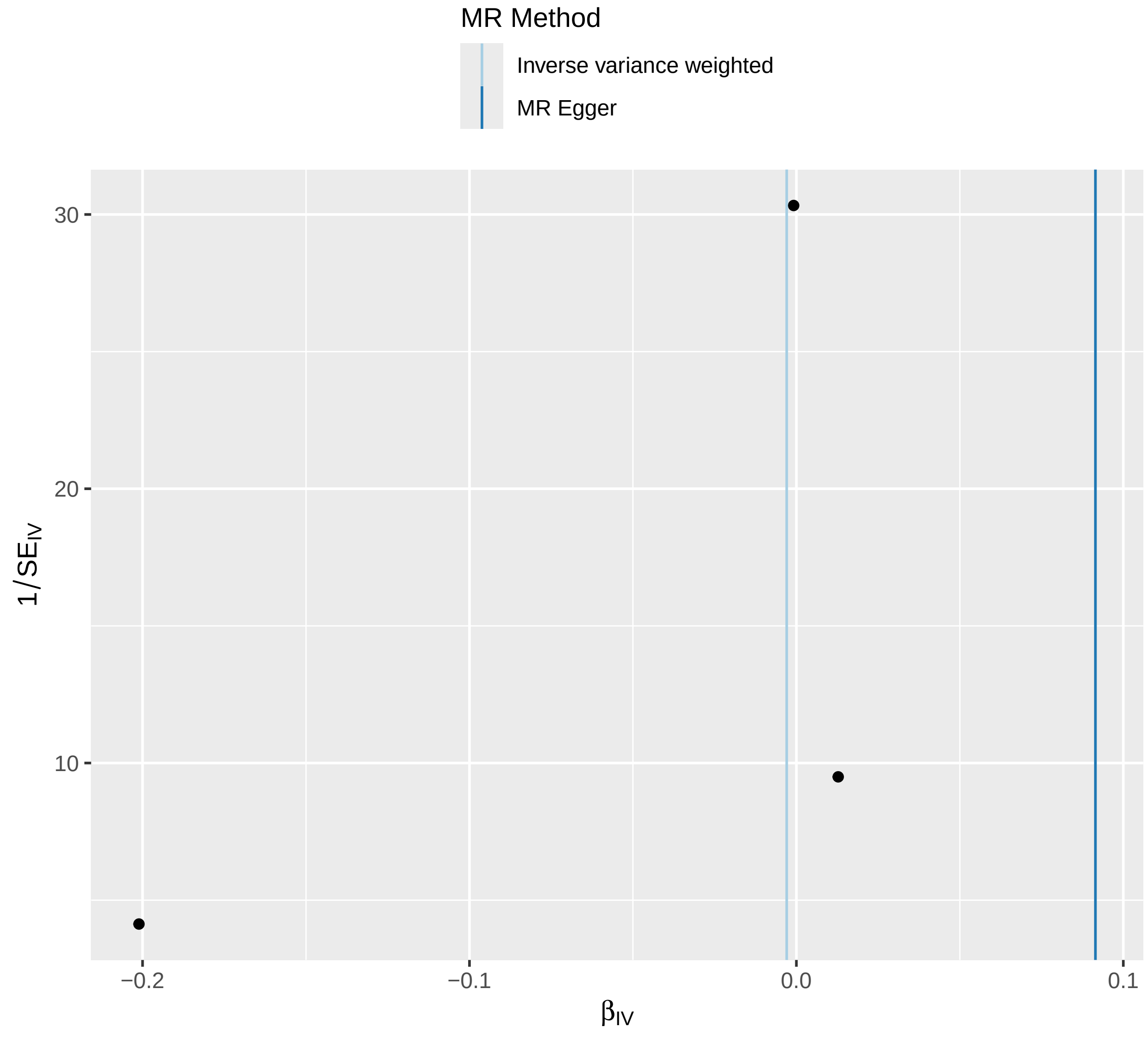
MR Method
Inverse variance weighted
MR Egger
30
20
10
1/SE_IV
−0.2
−0.1
0.0
0.1
β_IV

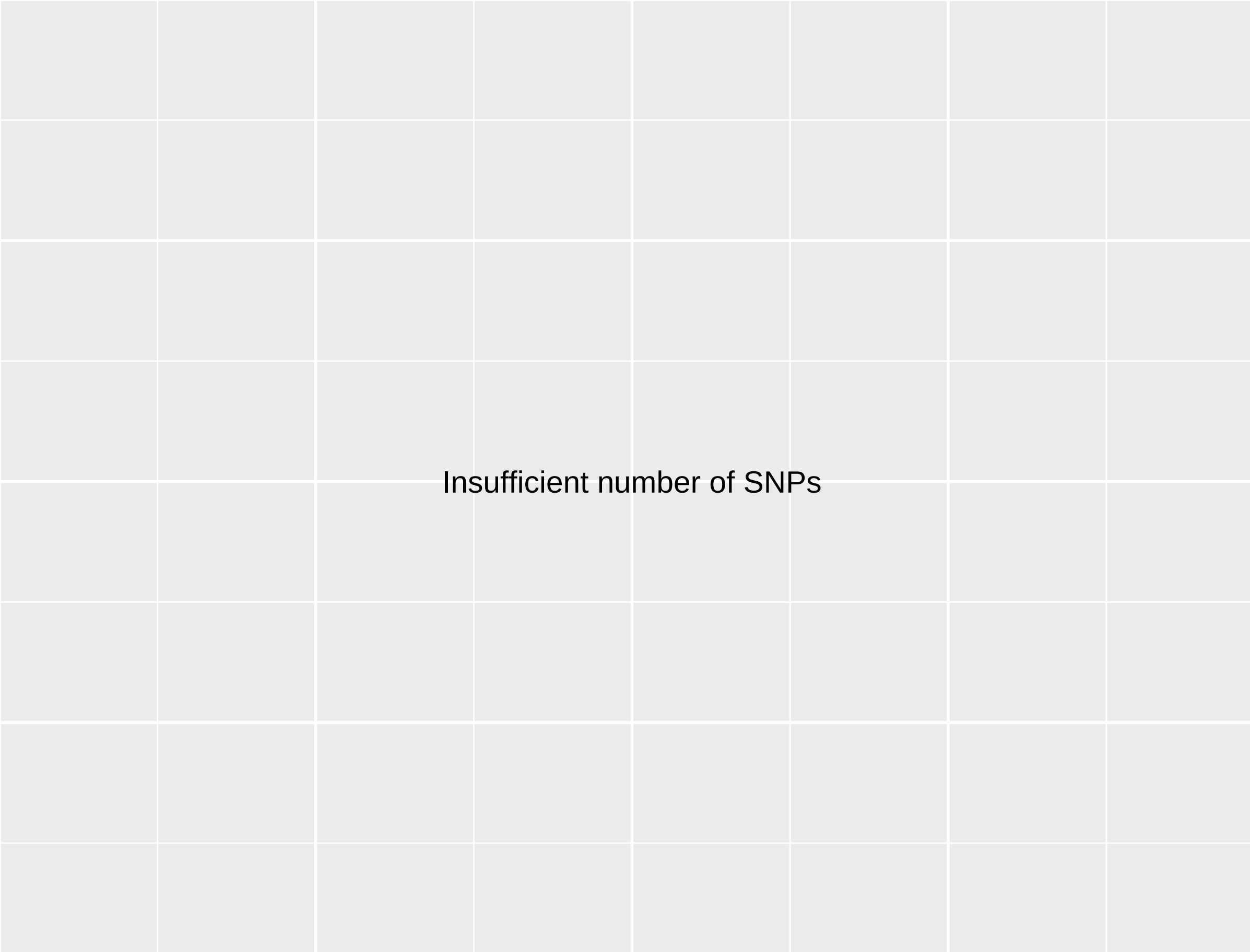
Insufficient number of SNPs

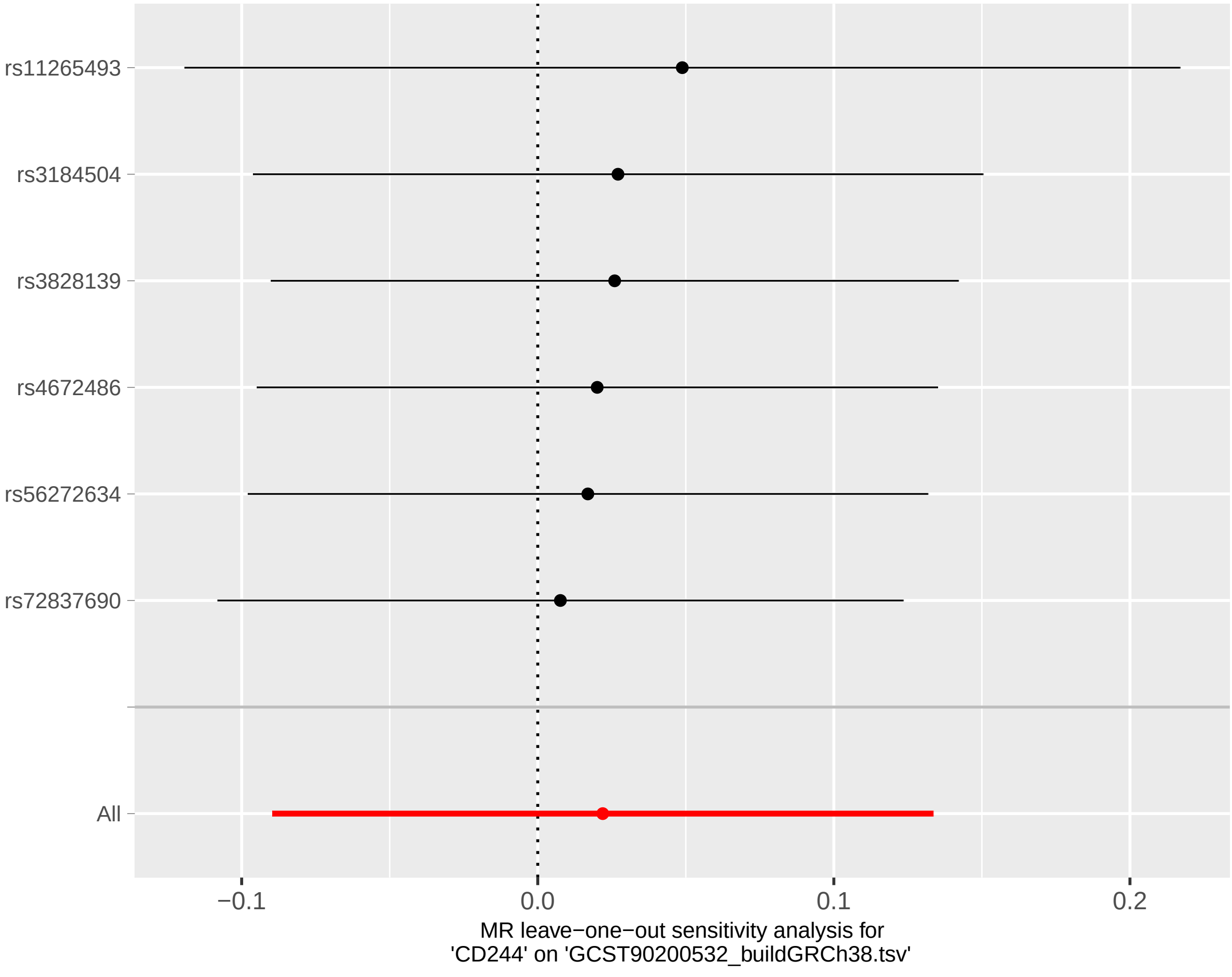
rs11265493
rs3184504
rs3828139
rs4672486
rs56272634
rs72837690
All
−0.1
0.0
0.1
0.2
MR leave−one−out sensitivity analysis for
'CD244' on 'GCST90200532_buildGRCh38.tsv'

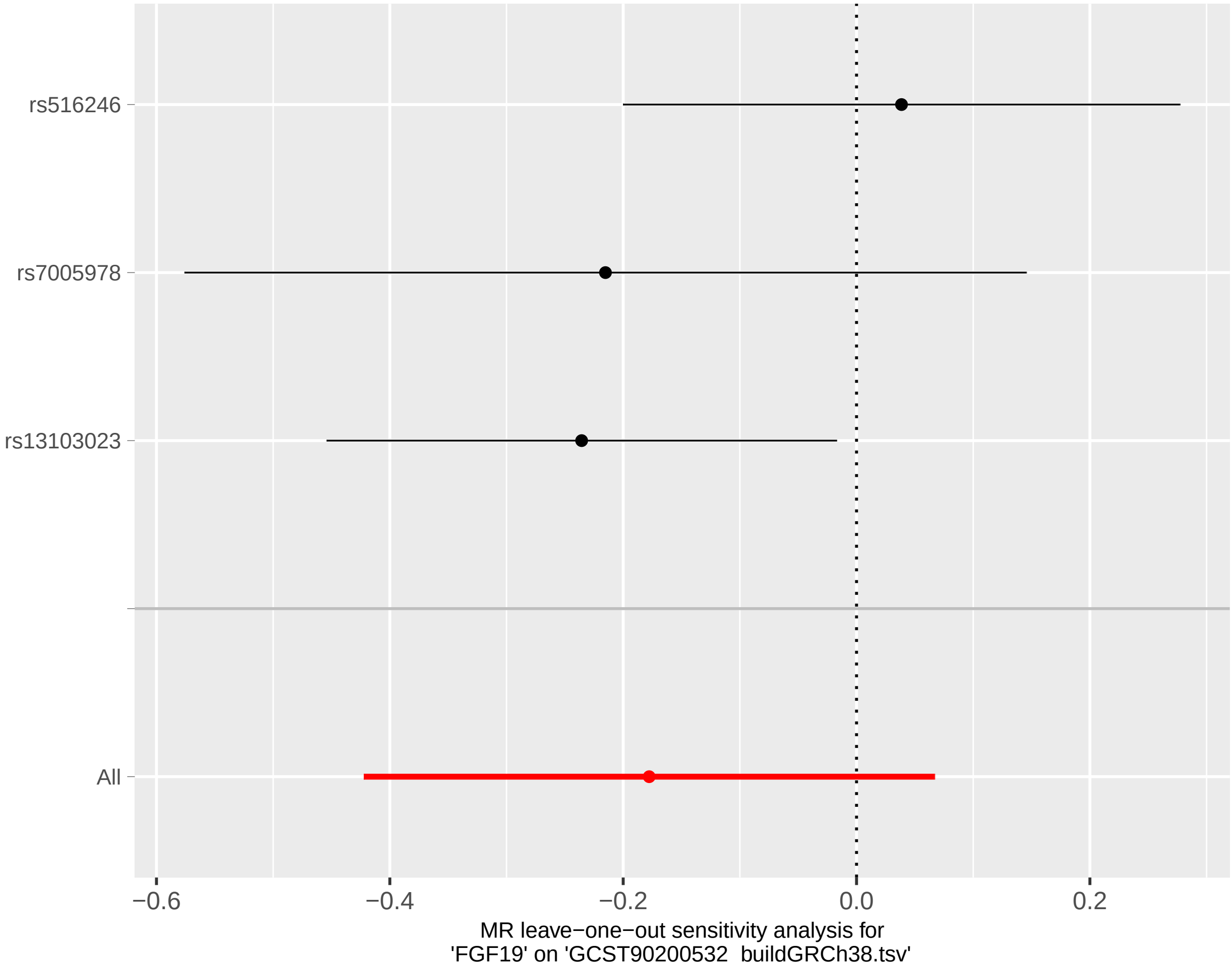

rs516246
rs7005978
rs13103023
All
−0.6
−0.4
−0.2
0.0
0.2
MR leave−one−out sensitivity analysis for
'FGF19' on 'GCST90200532_buildGRCh38.tsv'

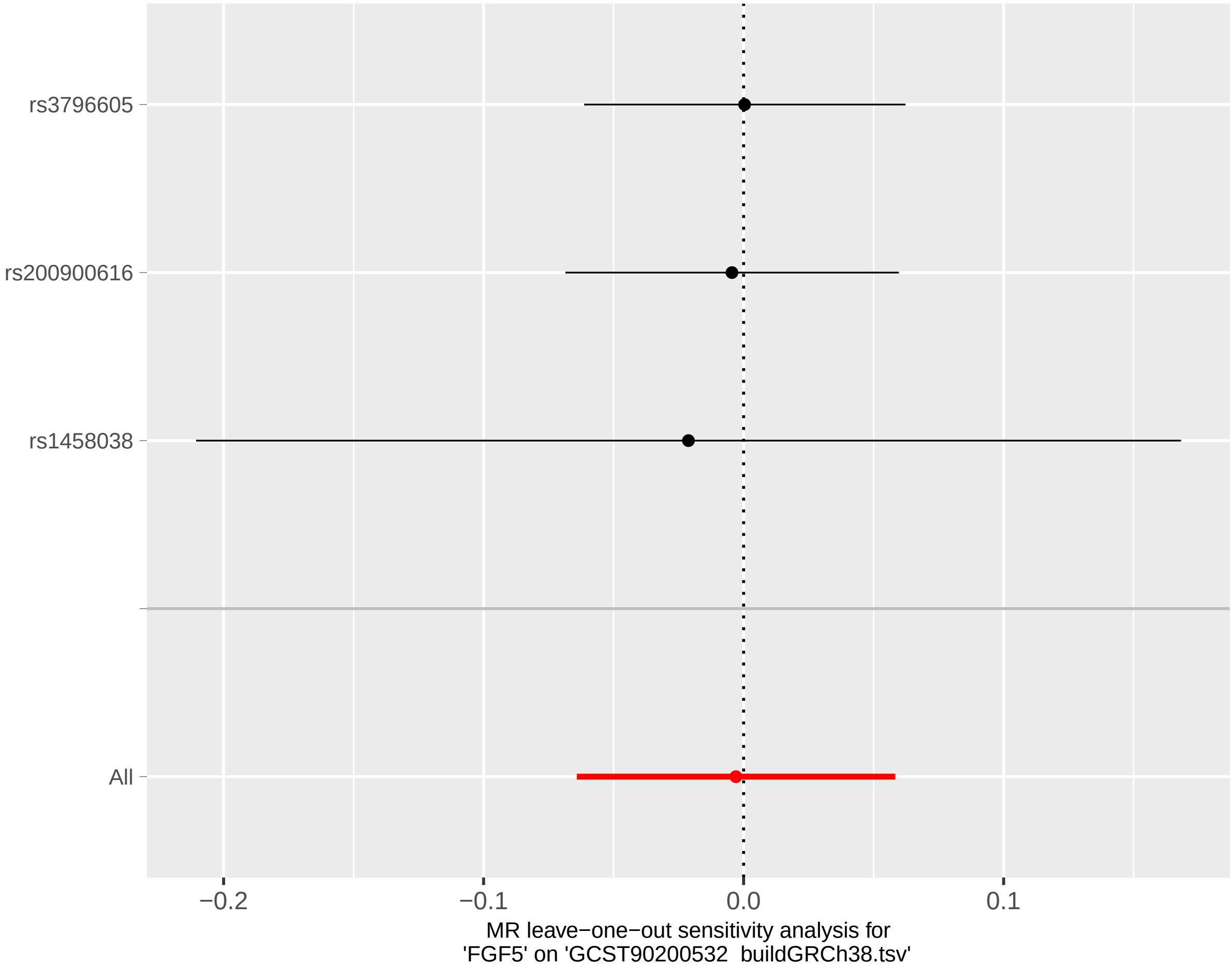
rs3796605
rs200900616
rs1458038
All
−0.2
−0.1
0.0
0.1
MR leave−one−out sensitivity analysis for
'FGF5' on 'GCST90200532_buildGRCh38.tsv'

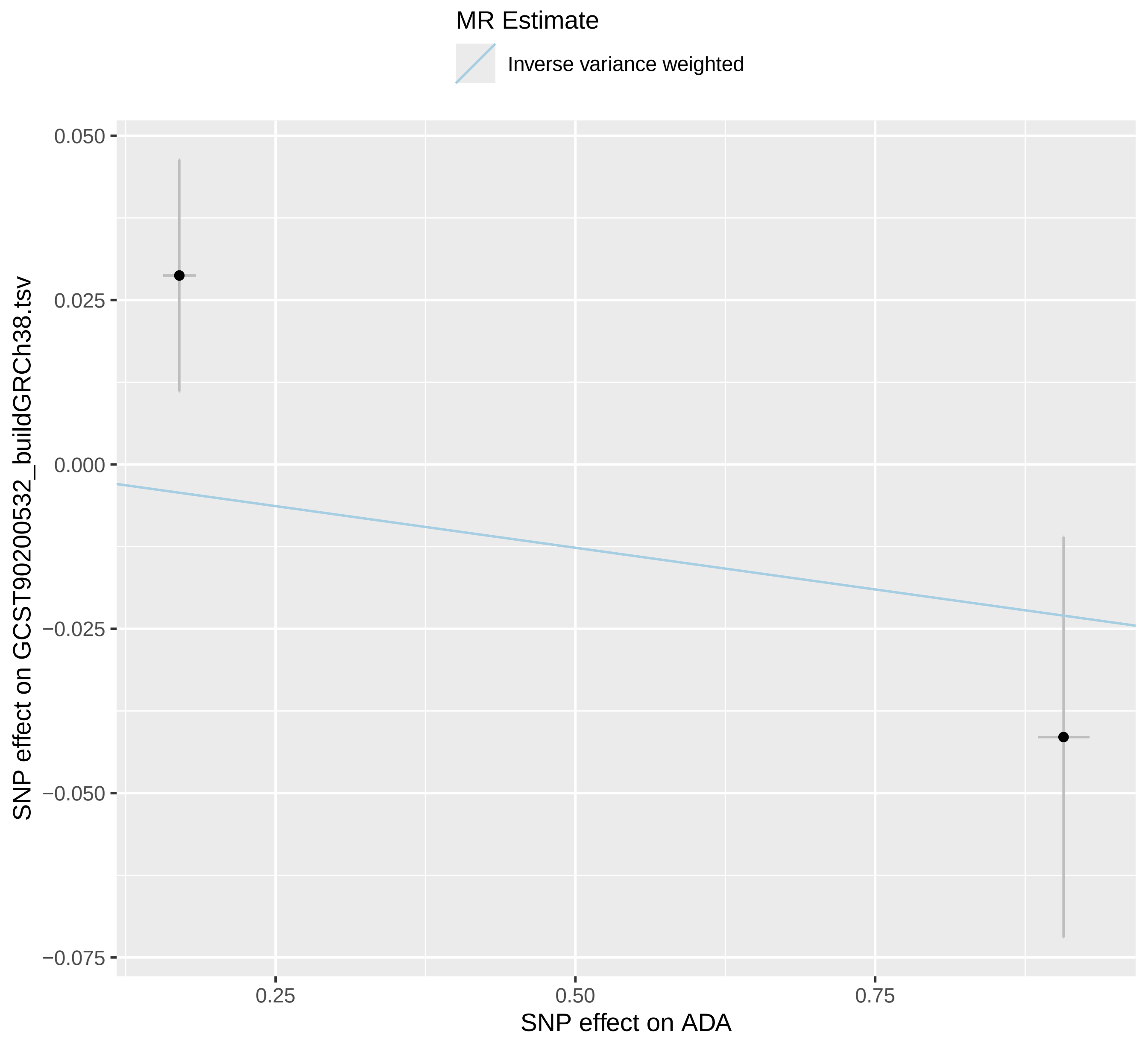
MR Estimate
Inverse variance weighted
0.050
0.025
0.000
−0.025
−0.050
−0.075
SNP effect on GCST90200532_buildGRCh38.tsv
0.25
0.50
0.75
SNP effect on ADA

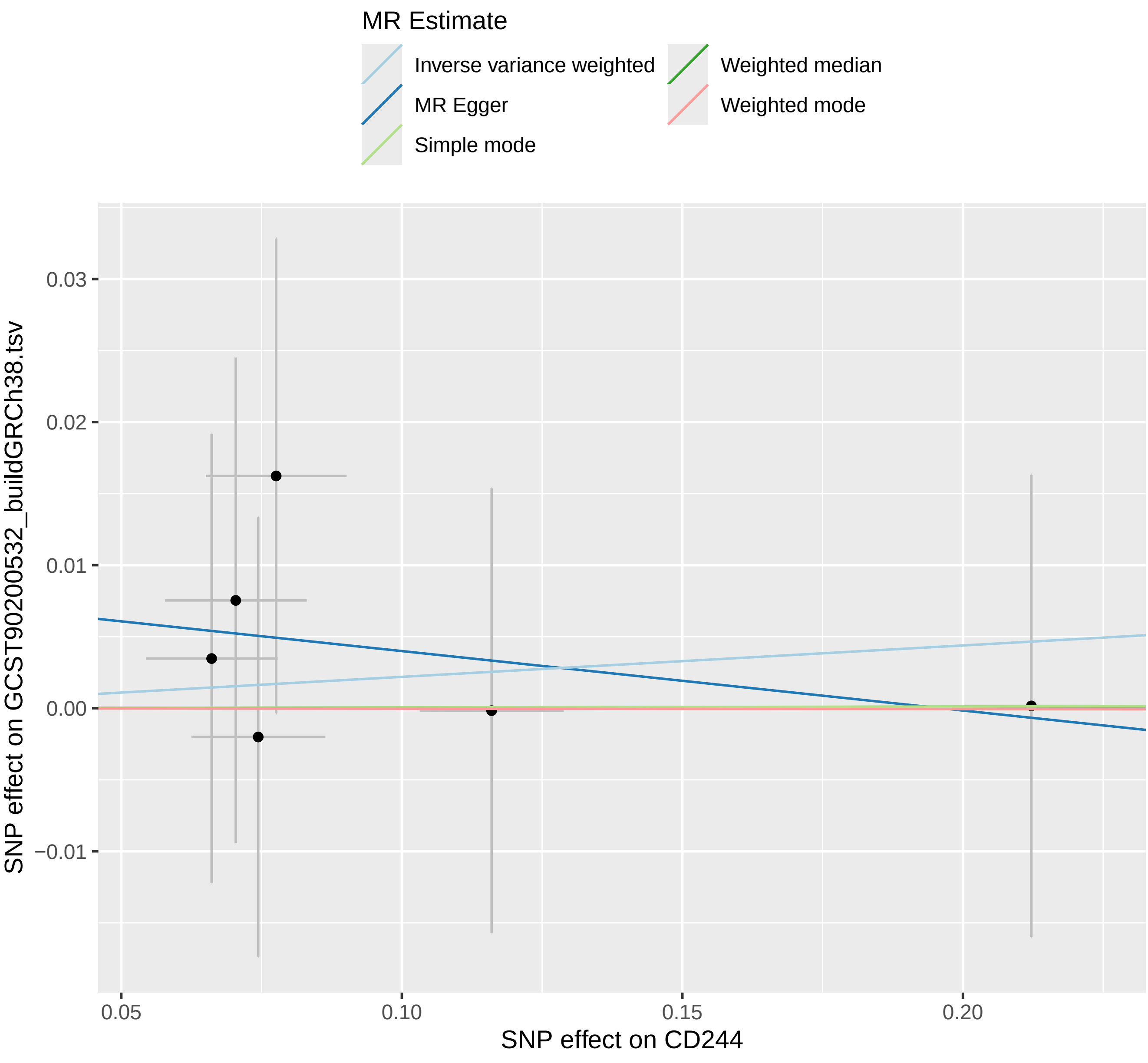
MR Estimate
Inverse variance weighted
MR Egger
Simple mode
Weighted median
Weighted mode
0.03
0.02
0.01
0.00
−0.01
SNP effect on GCST90200532_buildGRCh38.tsv
0.05
0.10
0.15
0.20
SNP effect on CD244

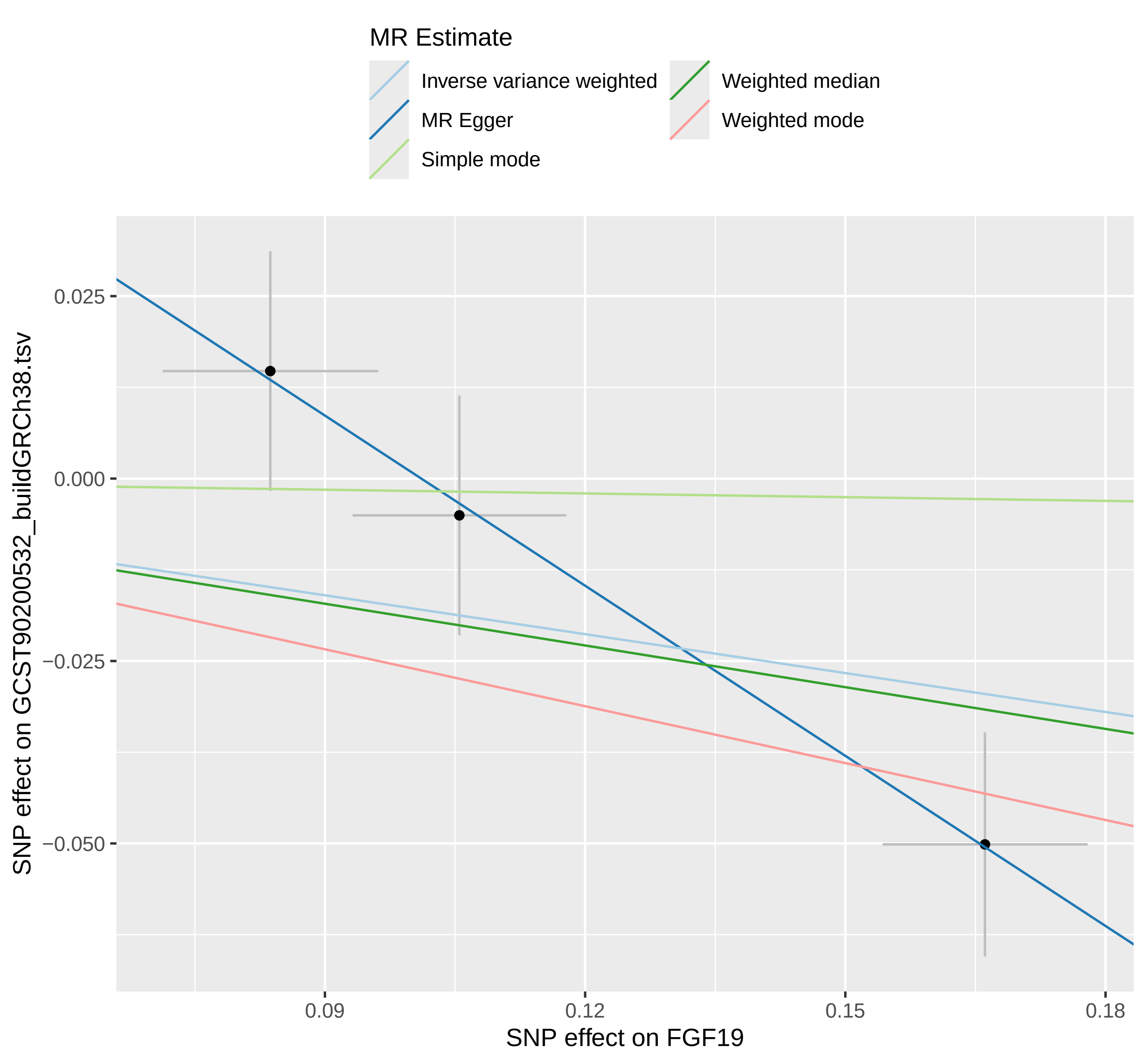
MR Estimate
Inverse variance weighted
MR Egger
Simple mode
Weighted median
Weighted mode
0.025
0.000
−0.025
−0.050
SNP effect on GCST90200532_buildGRCh38.tsv
0.09
0.12
0.15
0.18
SNP effect on FGF19

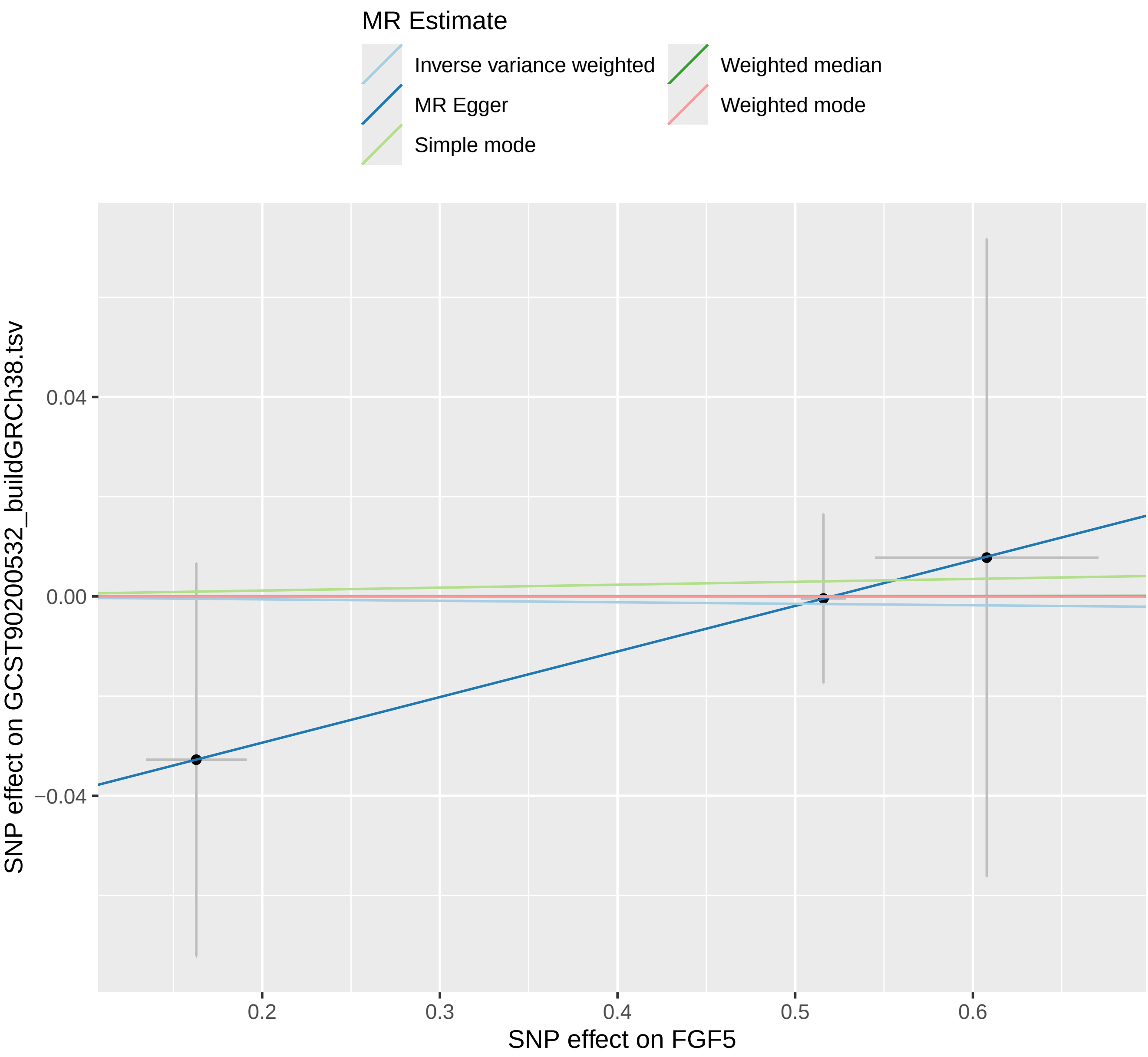
MR Estimate
Inverse variance weighted
MR Egger
Simple mode
Weighted median
Weighted mode
0.04
0.00
−0.04
0.2
0.3
0.4
0.5
0.6
SNP effect on GCST90200532_buildGRCh38.tsv
SNP effect on FGF5

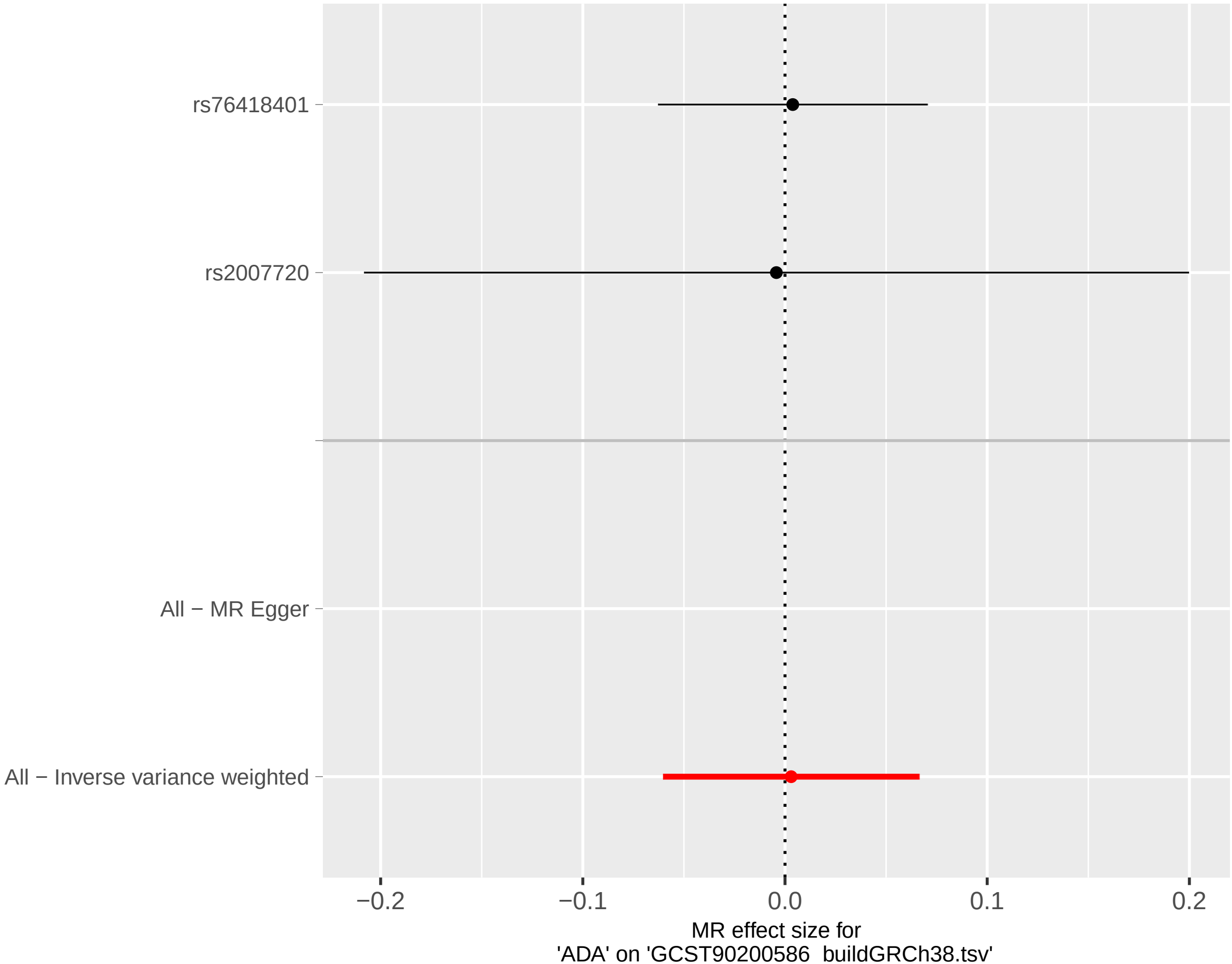
rs76418401
rs2007720
All − MR Egger
All − Inverse variance weighted
−0.2
−0.1
0.0
0.1
0.2
MR effect size for
'ADA' on 'GCST90200586_buildGRCh38.tsv'

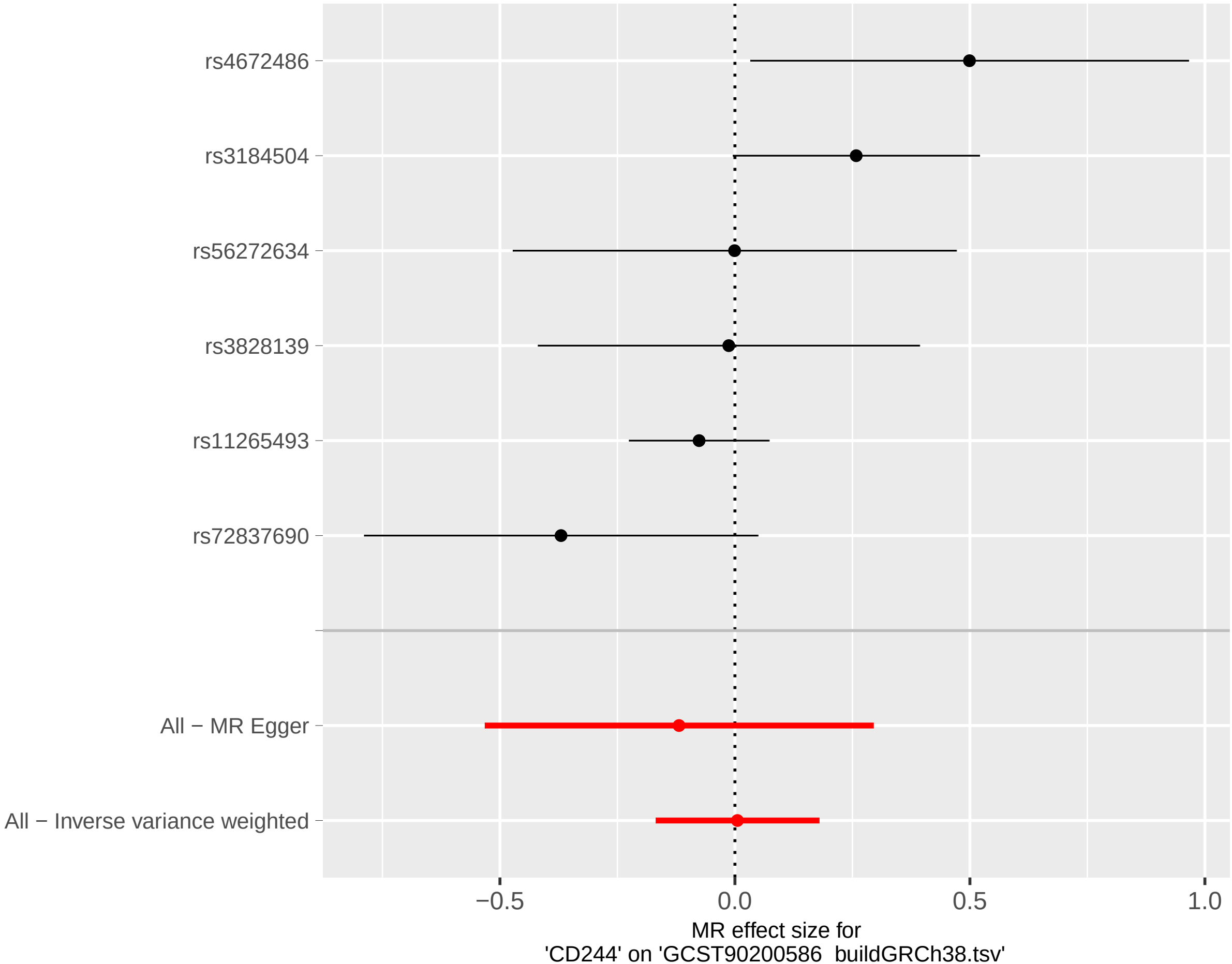

rs4672486
rs3184504
rs56272634
rs3828139
rs11265493
rs72837690
All − MR Egger
All − Inverse variance weighted
−0.5
0.0
0.5
1.0
MR effect size for
'CD244' on 'GCST90200586_buildGRCh38.tsv'

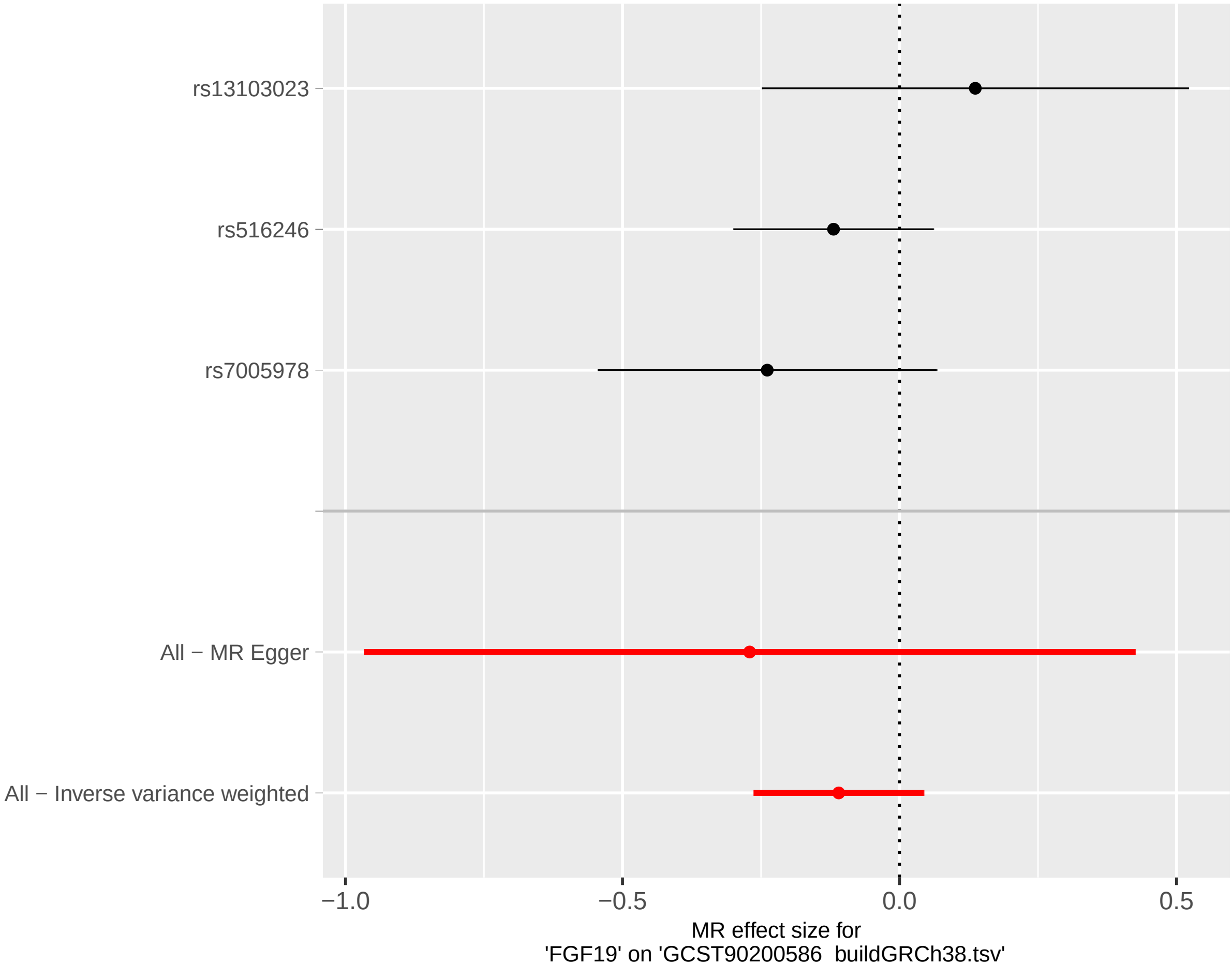
rs13103023
rs516246
rs7005978
All − MR Egger
All − Inverse variance weighted
−1.0
−0.5
0.0
0.5
MR effect size for
'FGF19' on 'GCST90200586_buildGRCh38.tsv'

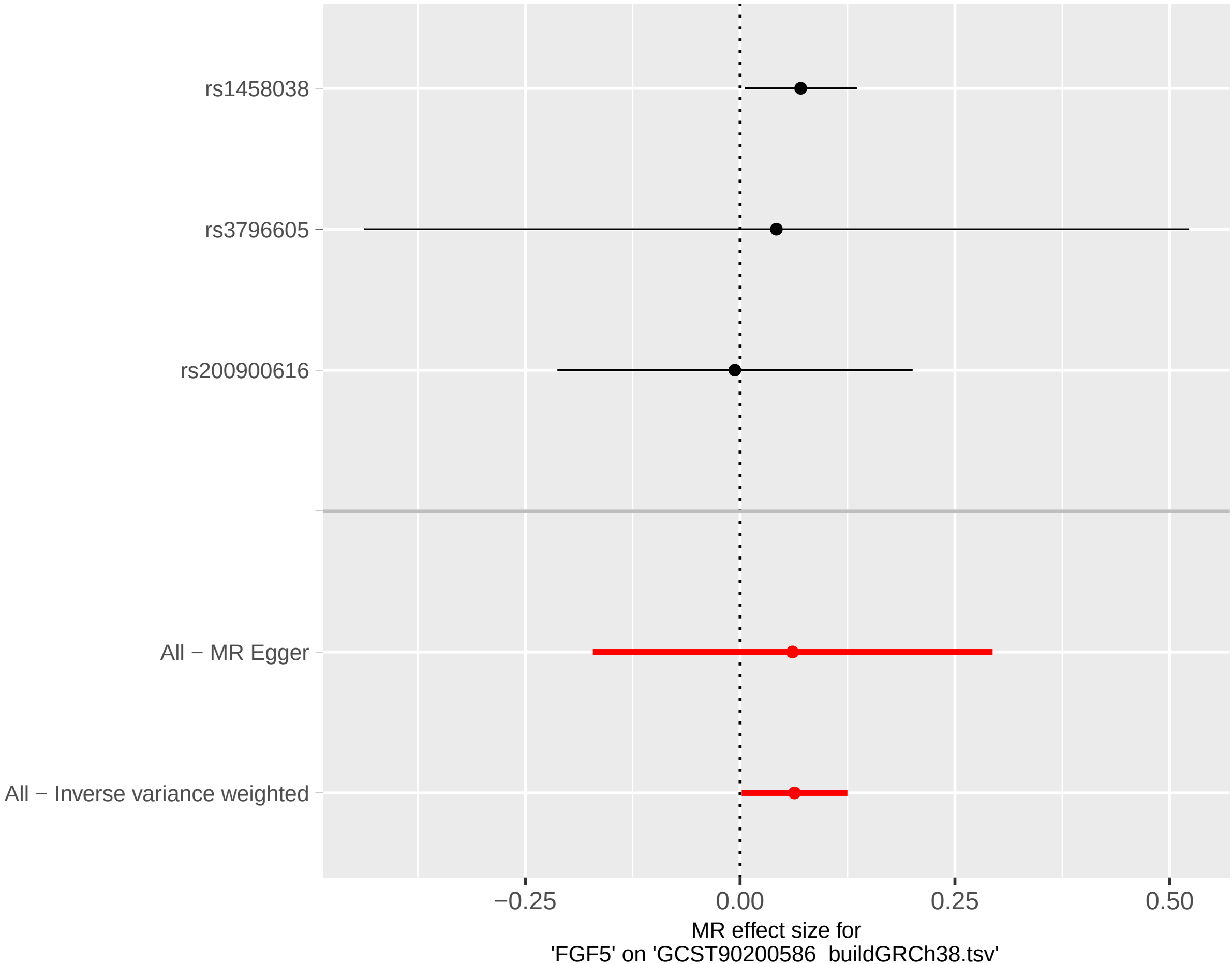
rs1458038
rs3796605
rs200900616
All − MR Egger
All − Inverse variance weighted
−0.25
0.00
0.25
0.50
MR effect size for
'FGF5' on 'GCST90200586_buildGRCh38.tsv'

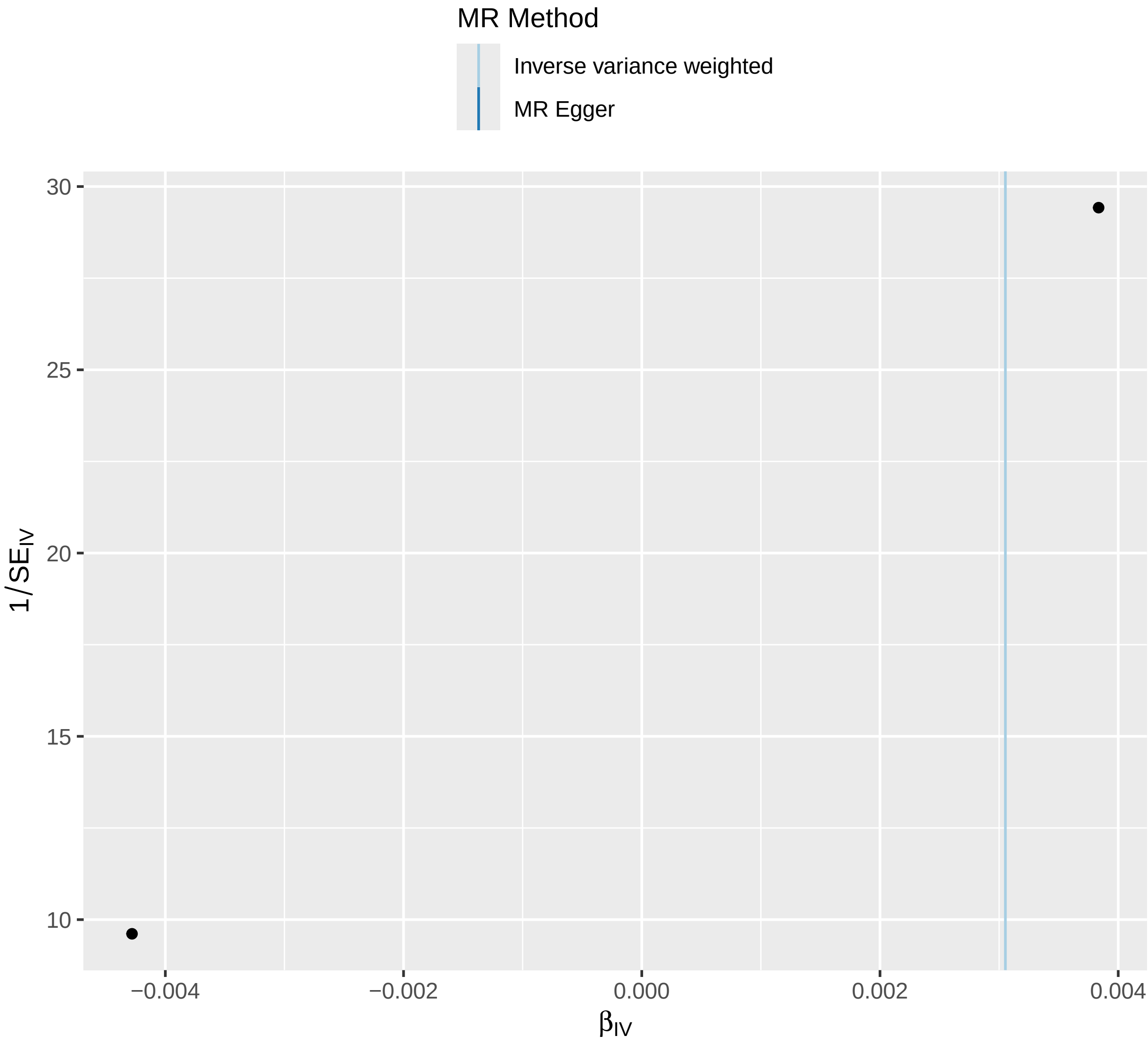

MR Method
Inverse variance weighted
MR Egger
30
25
20
15
10
1/SE_IV
−0.004
−0.002
0.000
0.002
0.004
β_IV

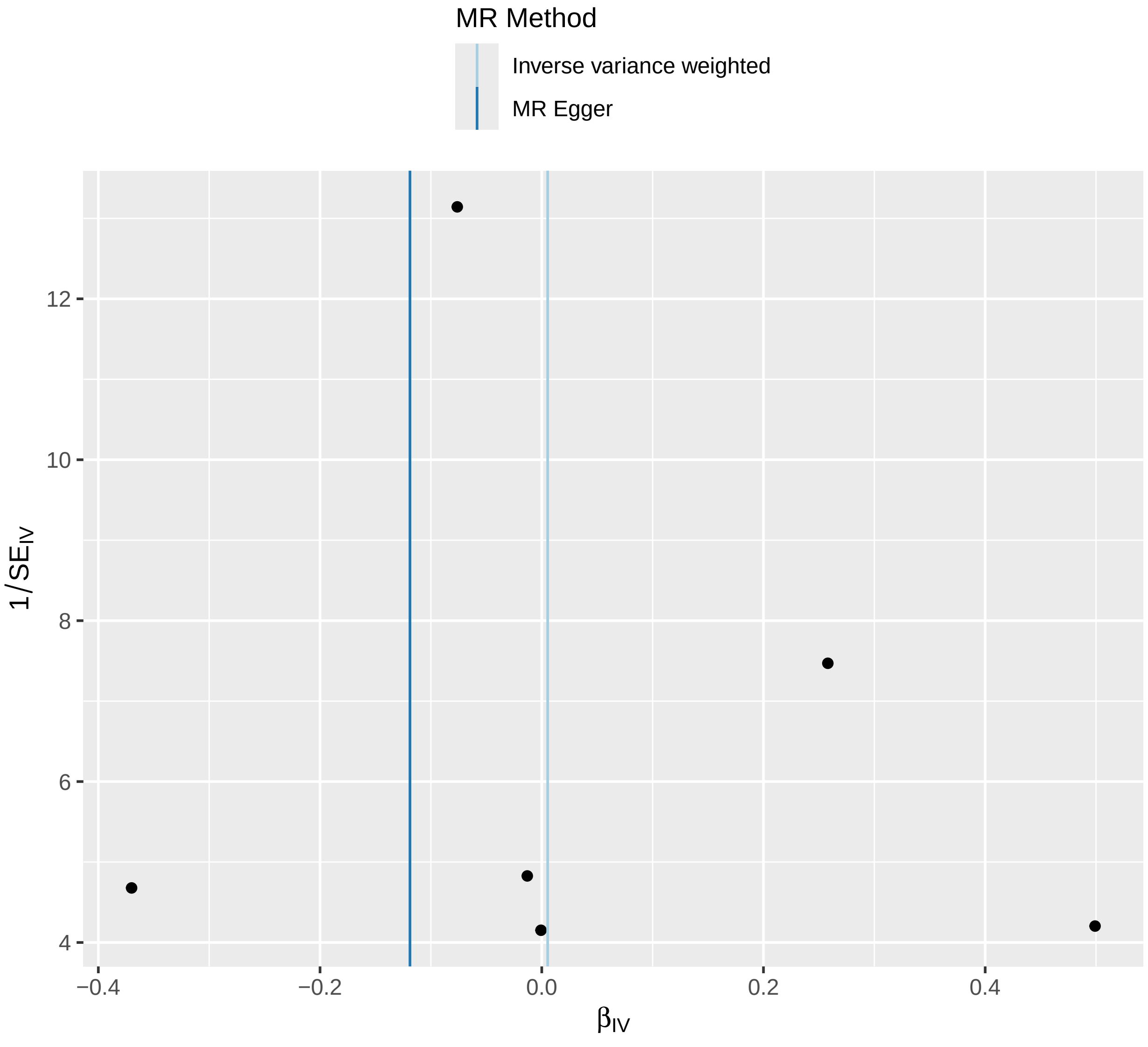

MR Method
Inverse variance weighted
MR Egger
12
10
8
6
4
1/SE_IV
−0.4
−0.2
0.0
0.2
0.4
β_IV

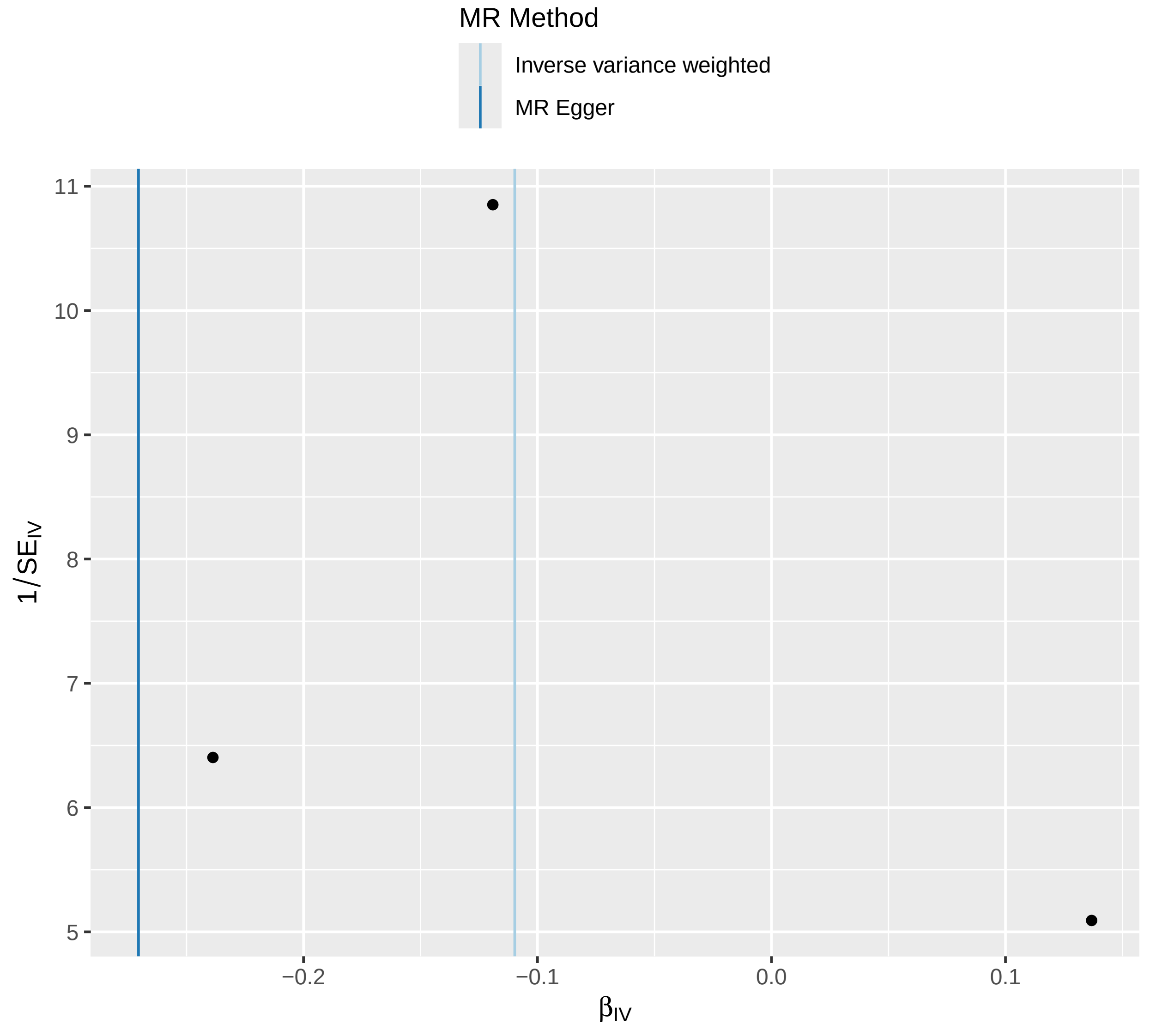
MR Method
Inverse variance weighted
MR Egger
1/SE$_{IV}$
11
10
9
8
7
6
5
−0.2
−0.1
0.0
0.1
β$_{IV}$

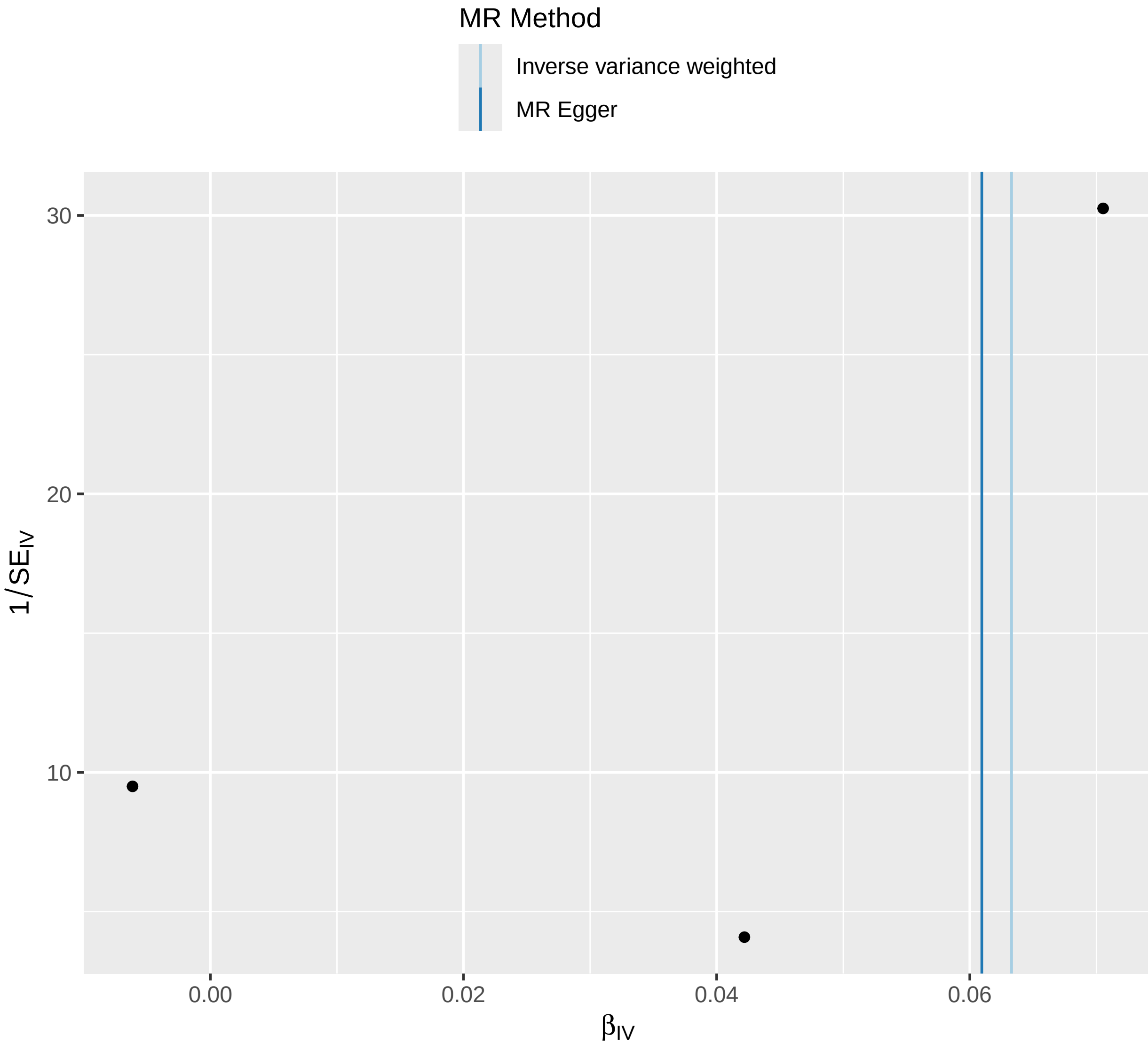

MR Method
Inverse variance weighted
MR Egger
30
20
10
1/SE_IV
0.00
0.02
0.04
0.06
β_IV

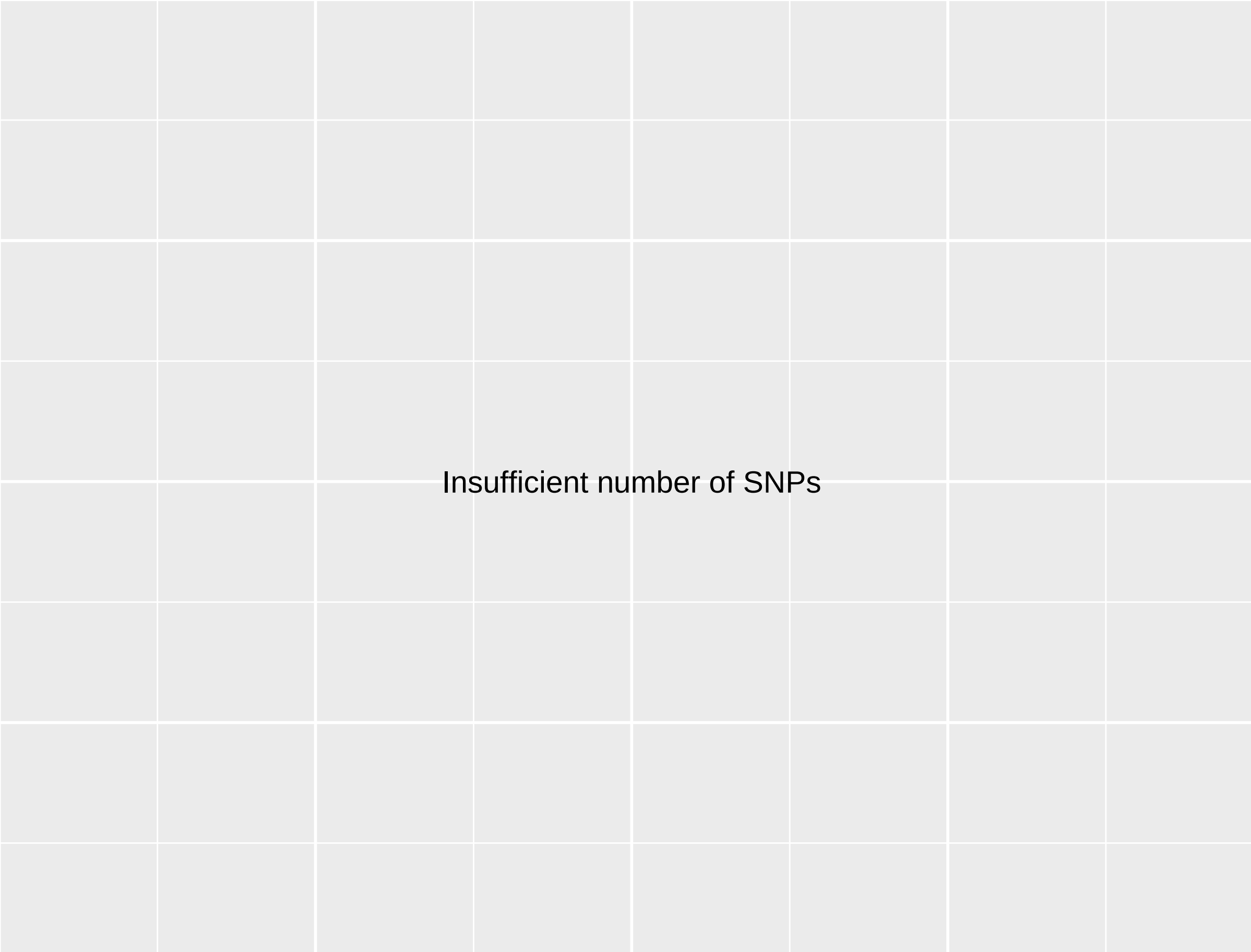
Insufficient number of SNPs

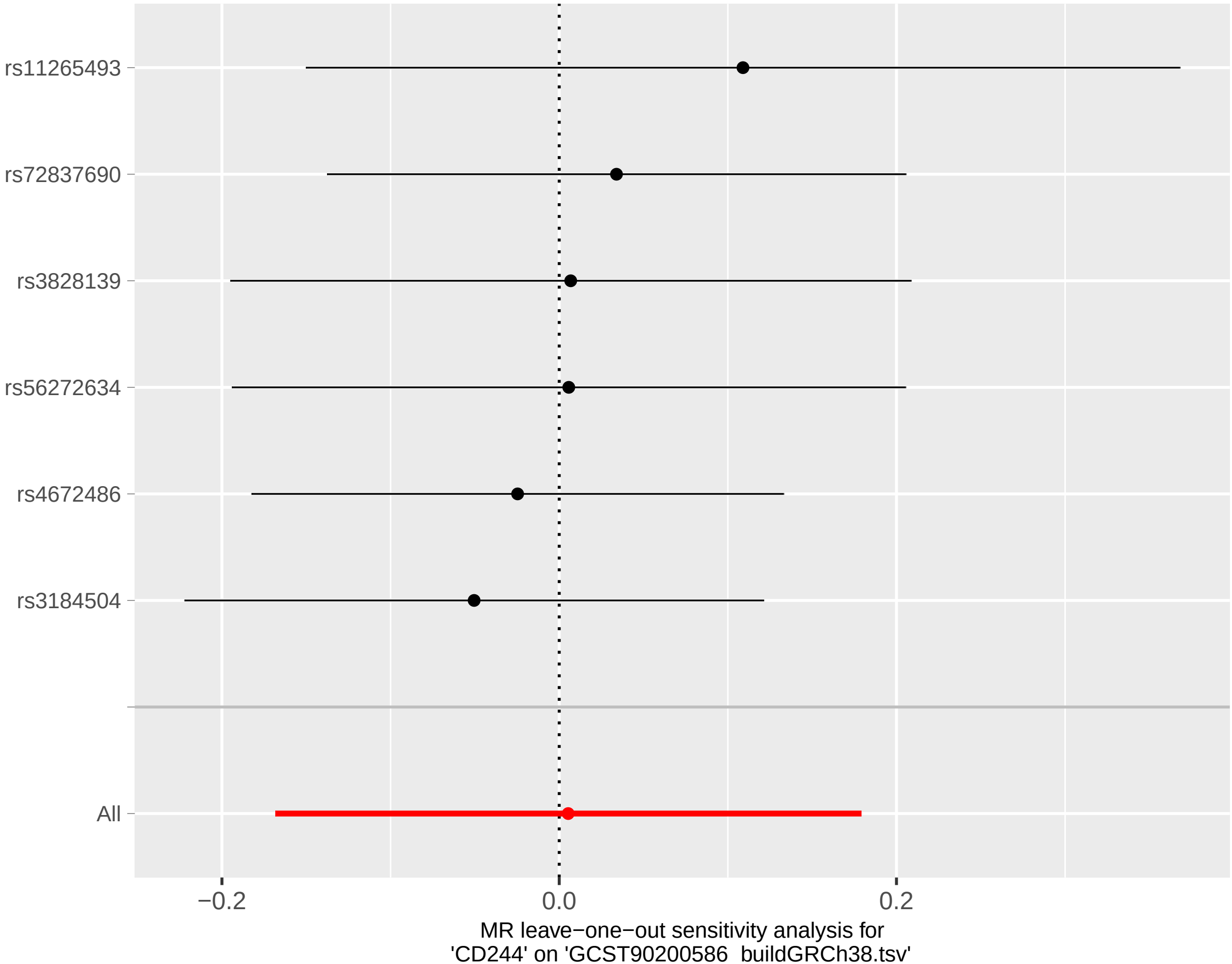
rs11265493
rs72837690
rs3828139
rs56272634
rs4672486
rs3184504
All
−0.2
0.0
0.2
MR leave−one−out sensitivity analysis for
'CD244' on 'GCST90200586_buildGRCh38.tsv'

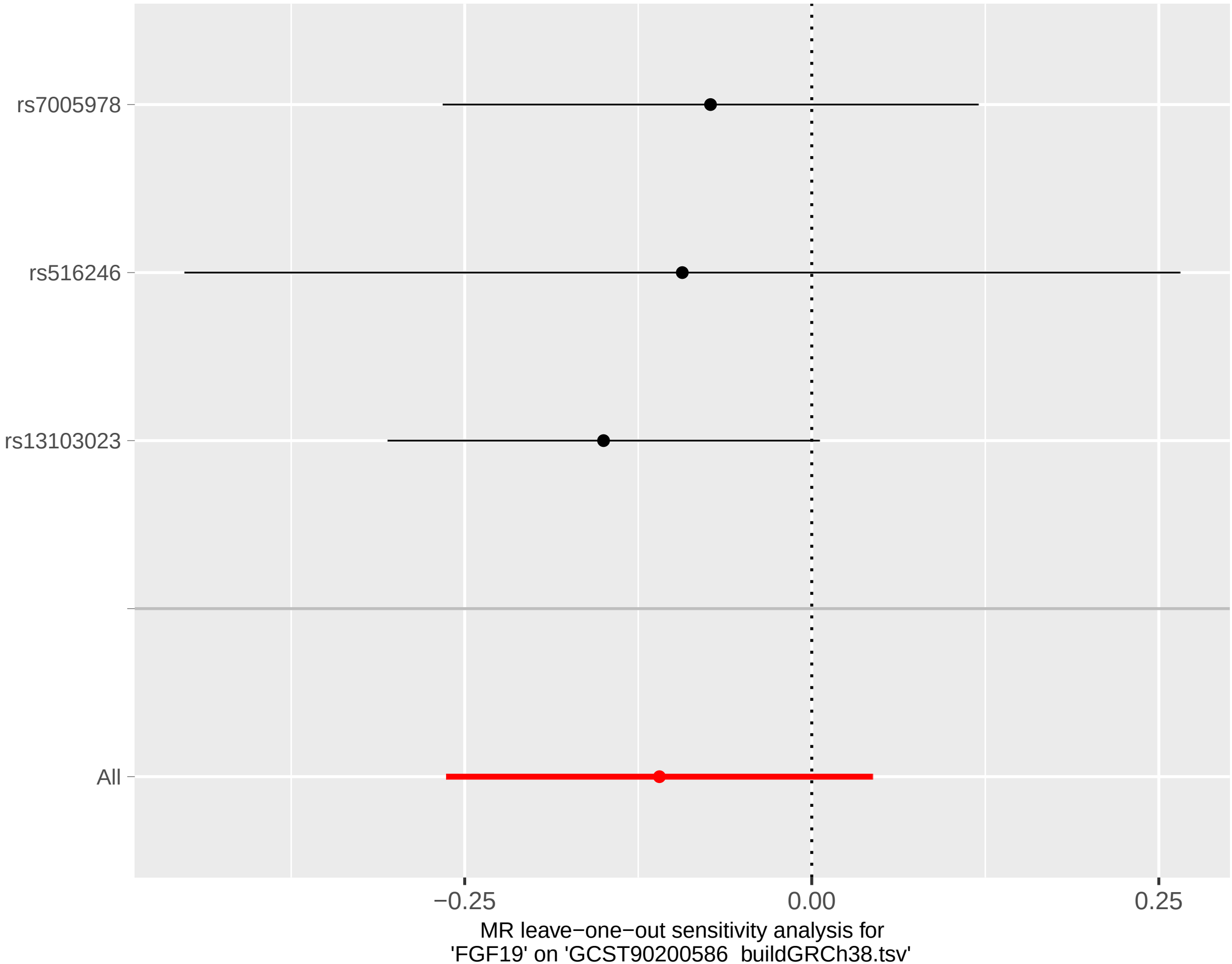
rs7005978
rs516246
rs13103023
All
−0.25
0.00
0.25
MR leave−one−out sensitivity analysis for
'FGF19' on 'GCST90200586_buildGRCh38.tsv'

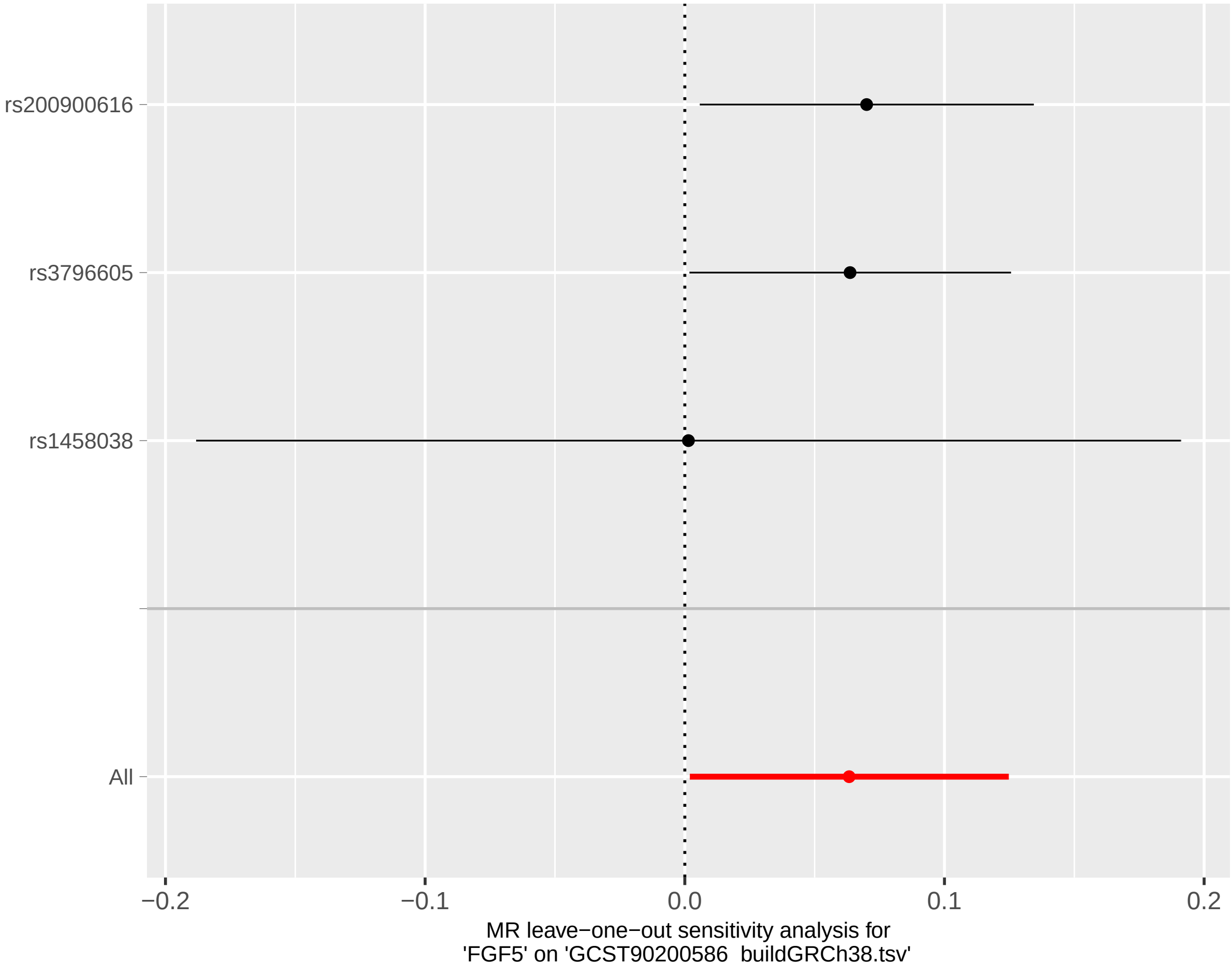
rs200900616
rs3796605
rs1458038
All
−0.2
−0.1
0.0
0.1
0.2
MR leave−one−out sensitivity analysis for
'FGF5' on 'GCST90200586_buildGRCh38.tsv'

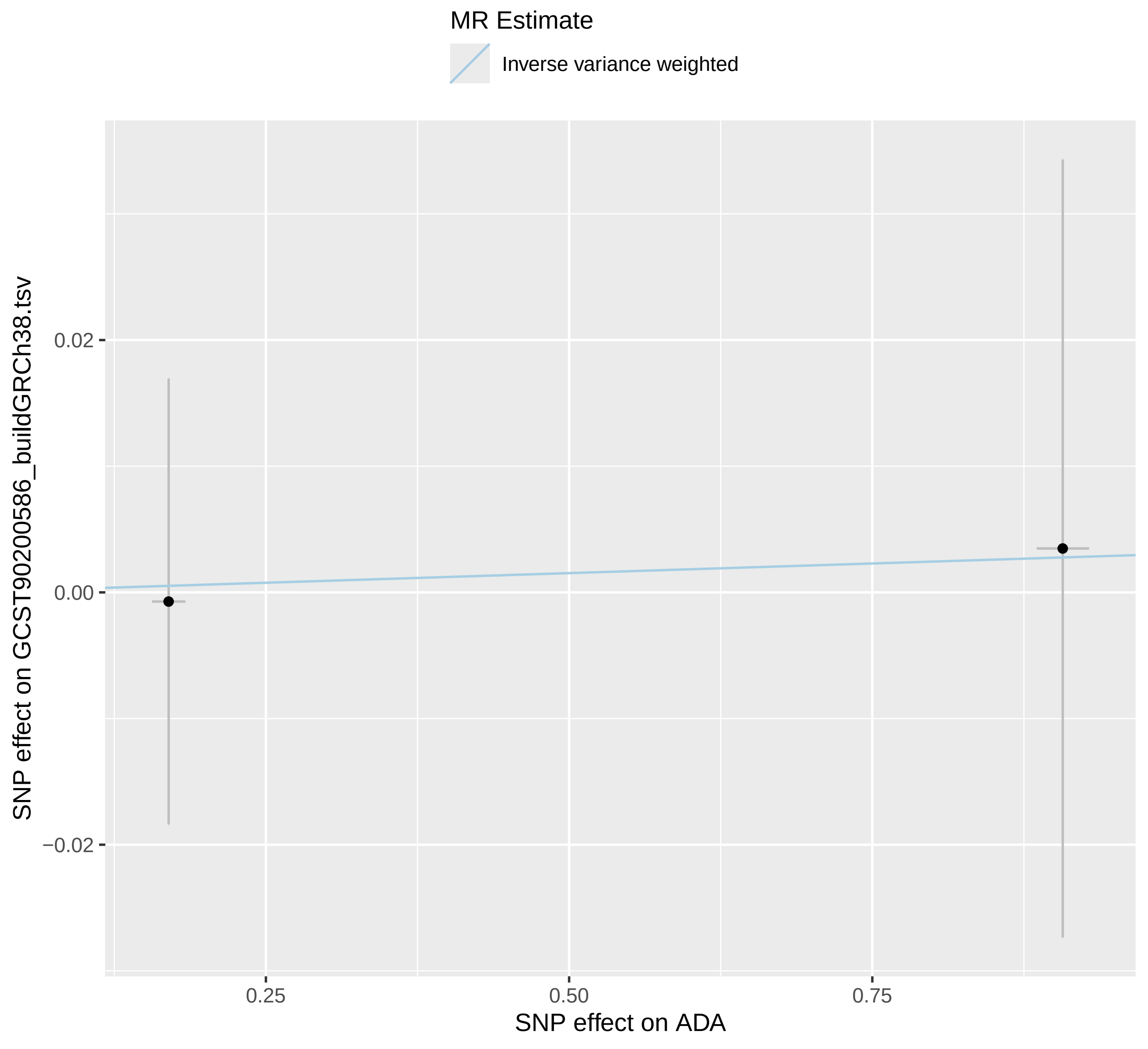

MR Estimate
Inverse variance weighted
SNP effect on GCST90200586_buildGRCh38.tsv
0.02
0.00
−0.02
0.25
0.50
0.75
SNP effect on ADA

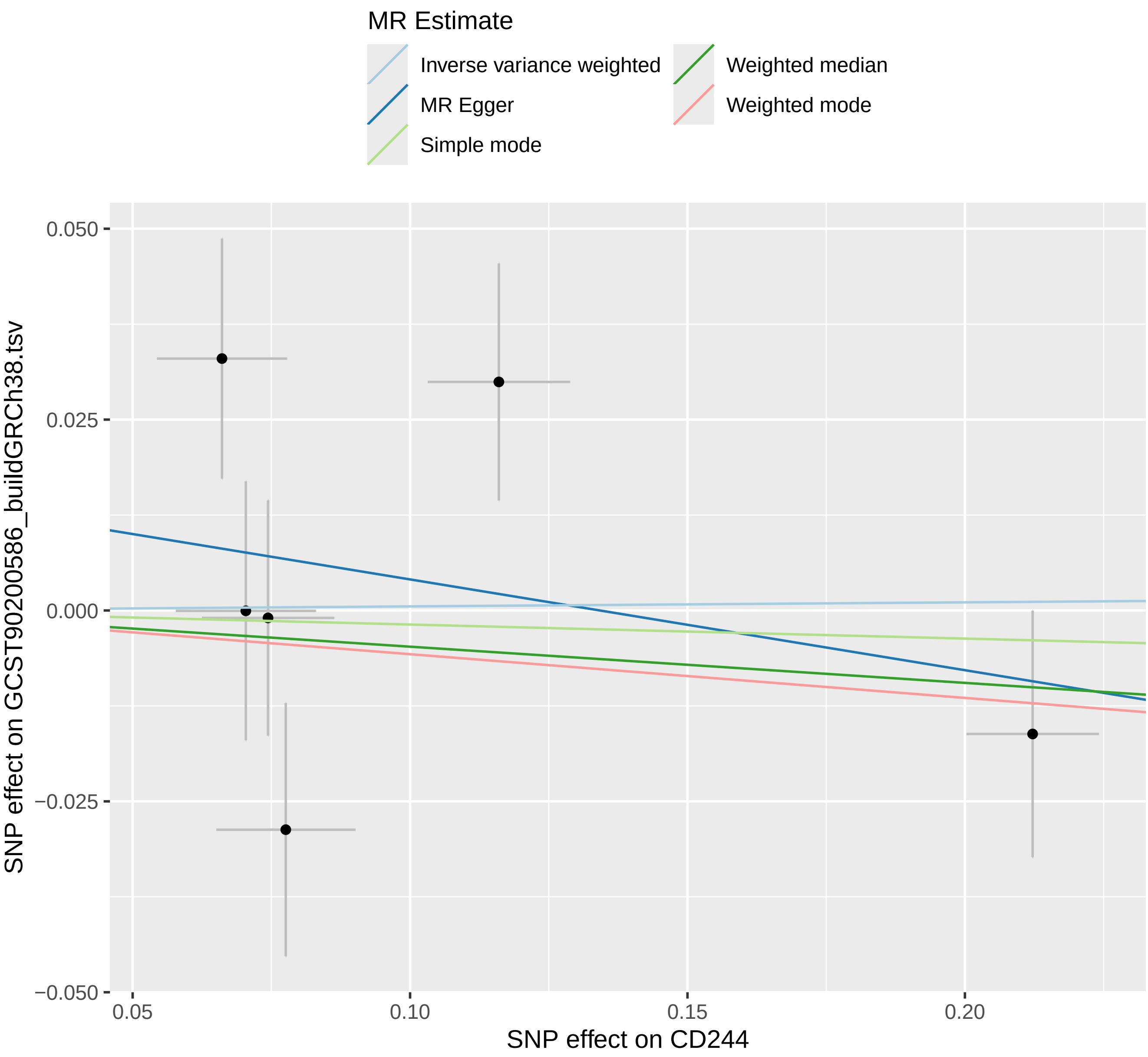

MR Estimate
Inverse variance weighted
MR Egger
Simple mode
Weighted median
Weighted mode
SNP effect on GCST90200586_buildGRCh38.tsv
0.050
0.025
0.000
−0.025
−0.050
0.05
0.10
0.15
0.20
SNP effect on CD244

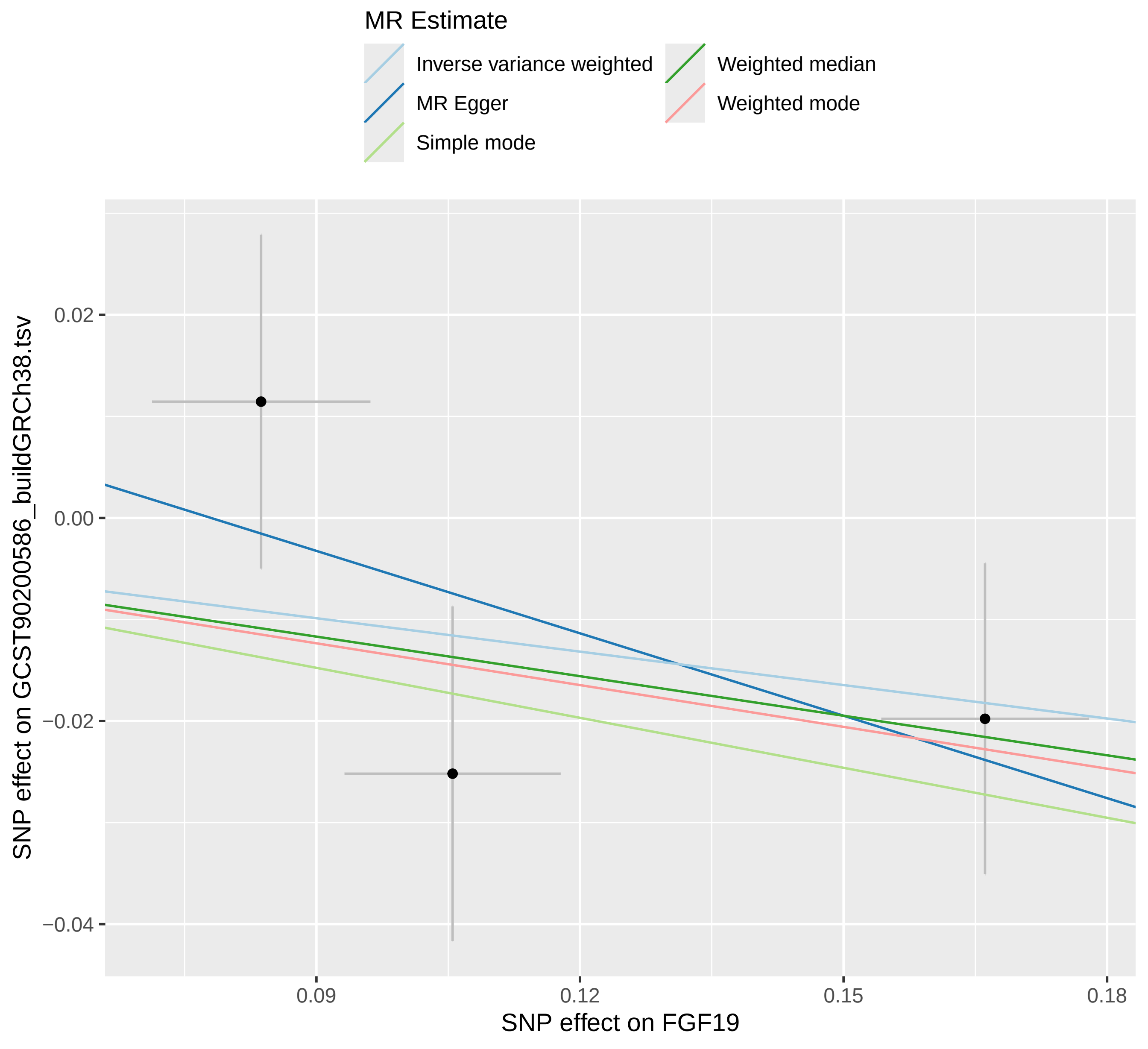

MR Estimate
Inverse variance weighted
MR Egger
Simple mode
Weighted median
Weighted mode
SNP effect on GCST90200586_buildGRCh38.tsv
0.02
0.00
−0.02
−0.04
0.09
0.12
0.15
0.18
SNP effect on FGF19

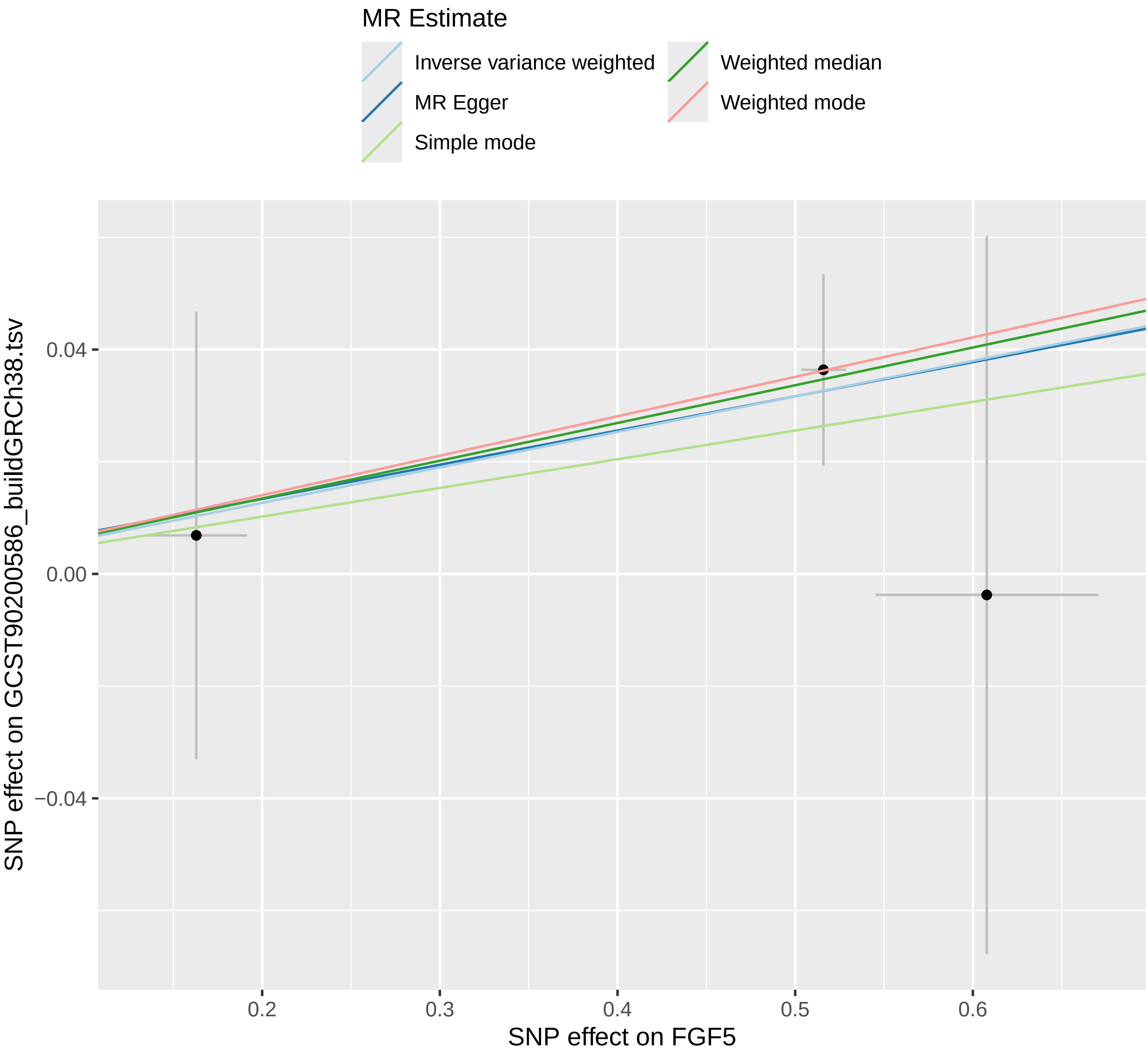

MR Estimate
Inverse variance weighted
MR Egger
Simple mode
Weighted median
Weighted mode
SNP effect on GCST90200586_buildGRCh38.tsv
0.04
0.00
−0.04
0.2
0.3
0.4
0.5
0.6
SNP effect on FGF5

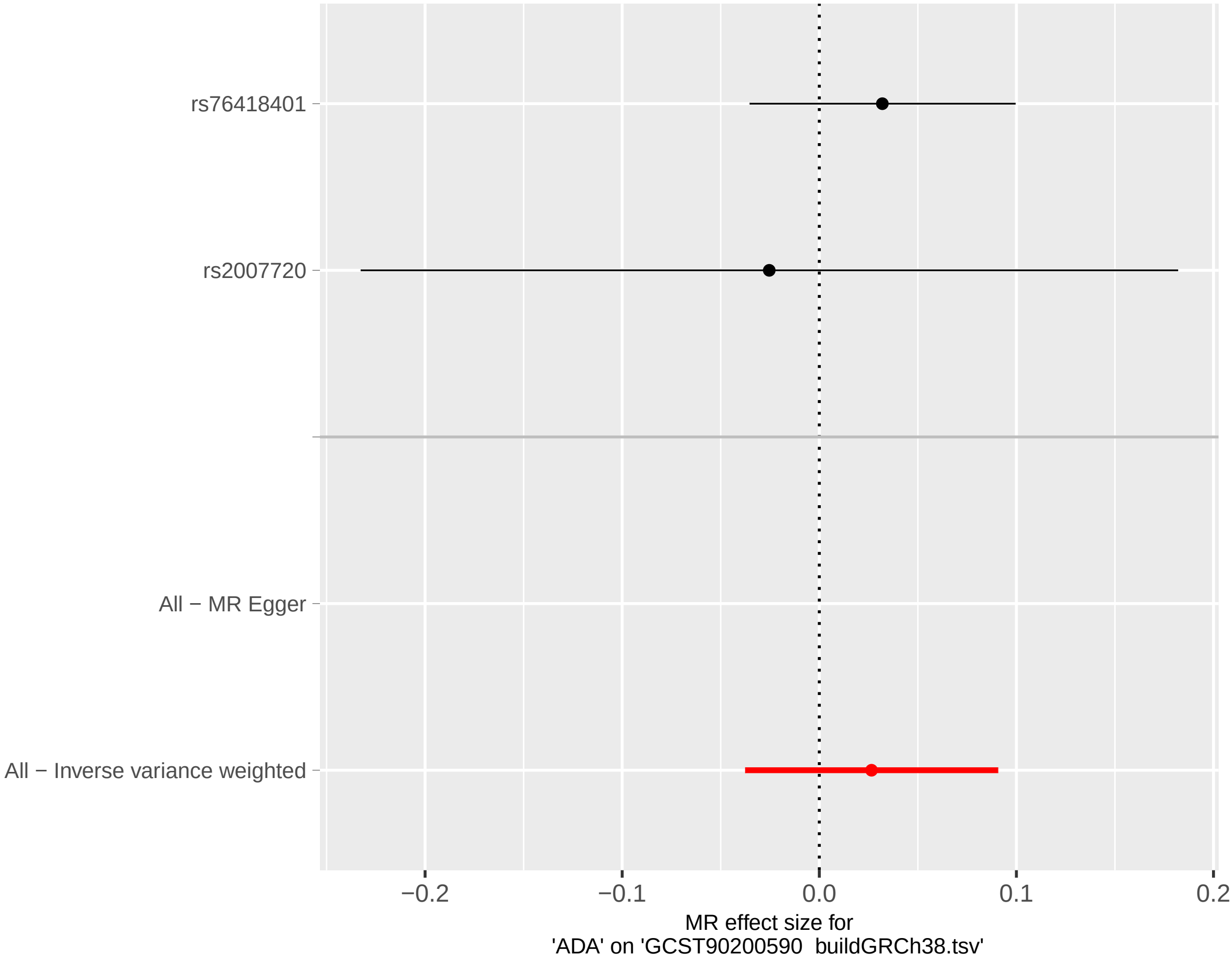
rs76418401
rs2007720
All − MR Egger
All − Inverse variance weighted
−0.2
−0.1
0.0
0.1
0.2
MR effect size for
'ADA' on 'GCST90200590_buildGRCh38.tsv'

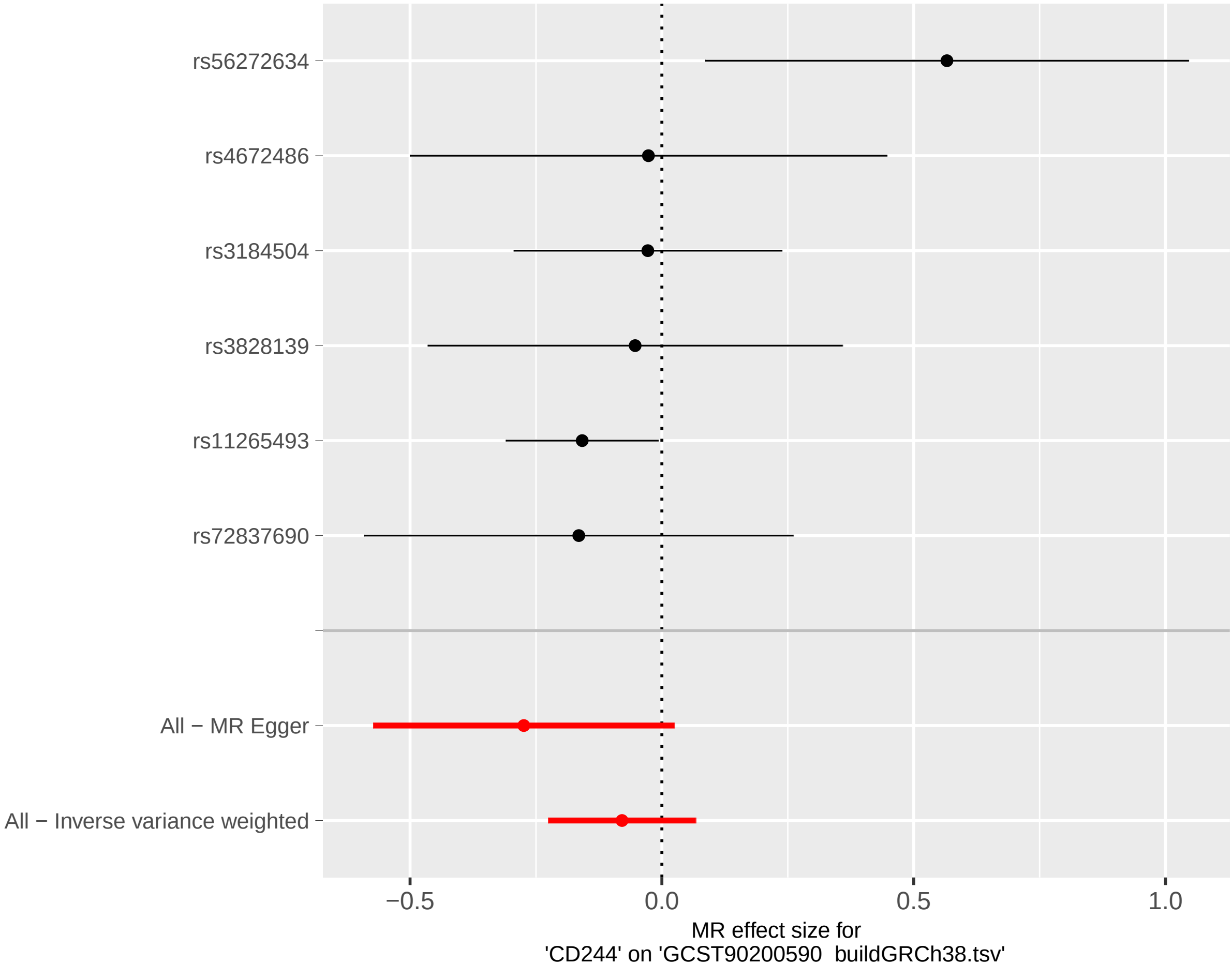
rs56272634
rs4672486
rs3184504
rs3828139
rs11265493
rs72837690
All − MR Egger
All − Inverse variance weighted
−0.5
0.0
0.5
1.0
MR effect size for
'CD244' on 'GCST90200590_buildGRCh38.tsv'

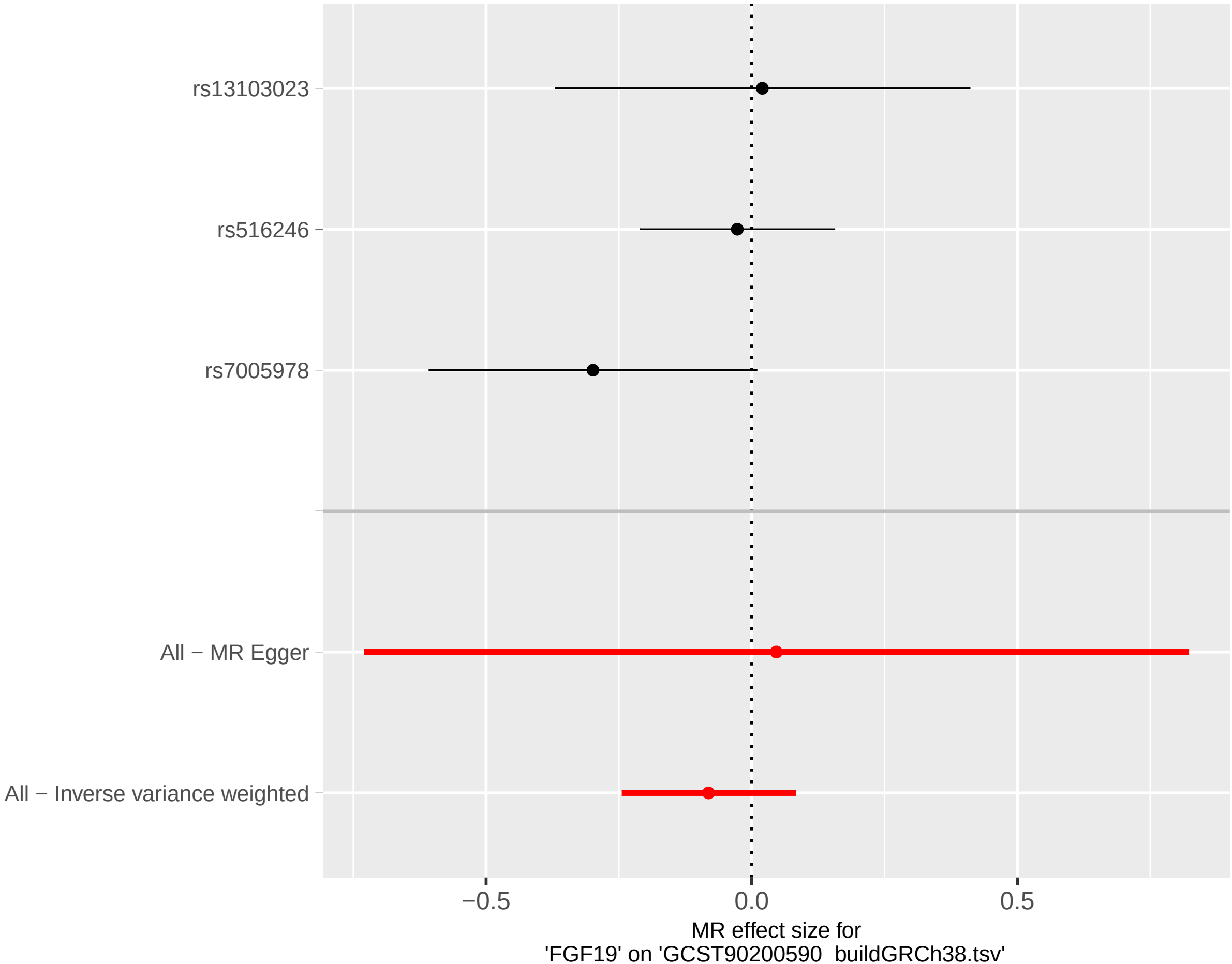

rs13103023
rs516246
rs7005978
All − MR Egger
All − Inverse variance weighted
−0.5
0.0
0.5
MR effect size for
'FGF19' on 'GCST90200590_buildGRCh38.tsv'

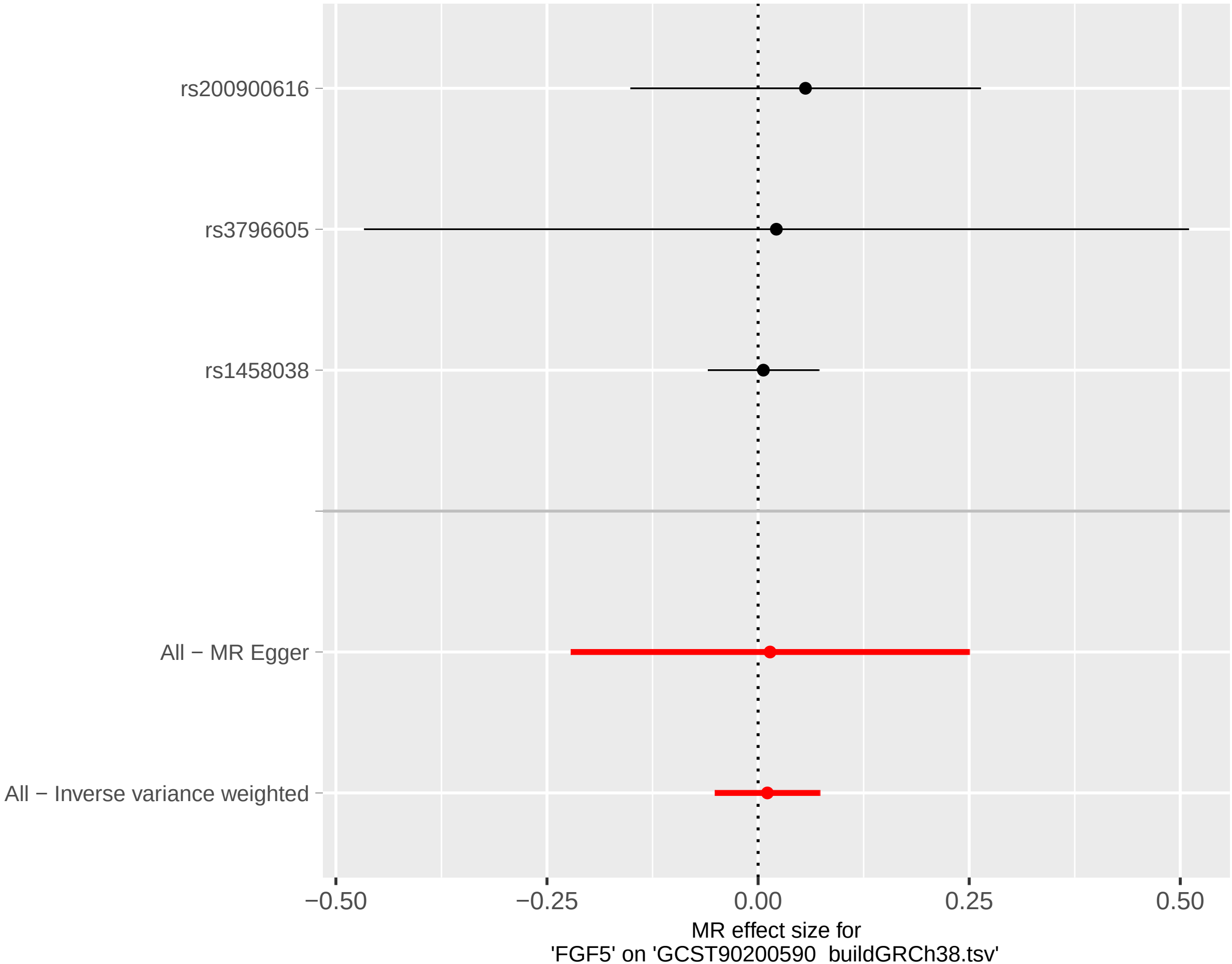
rs200900616
rs3796605
rs1458038
All − MR Egger
All − Inverse variance weighted
−0.50
−0.25
0.00
0.25
0.50
MR effect size for
'FGF5' on 'GCST90200590_buildGRCh38.tsv'

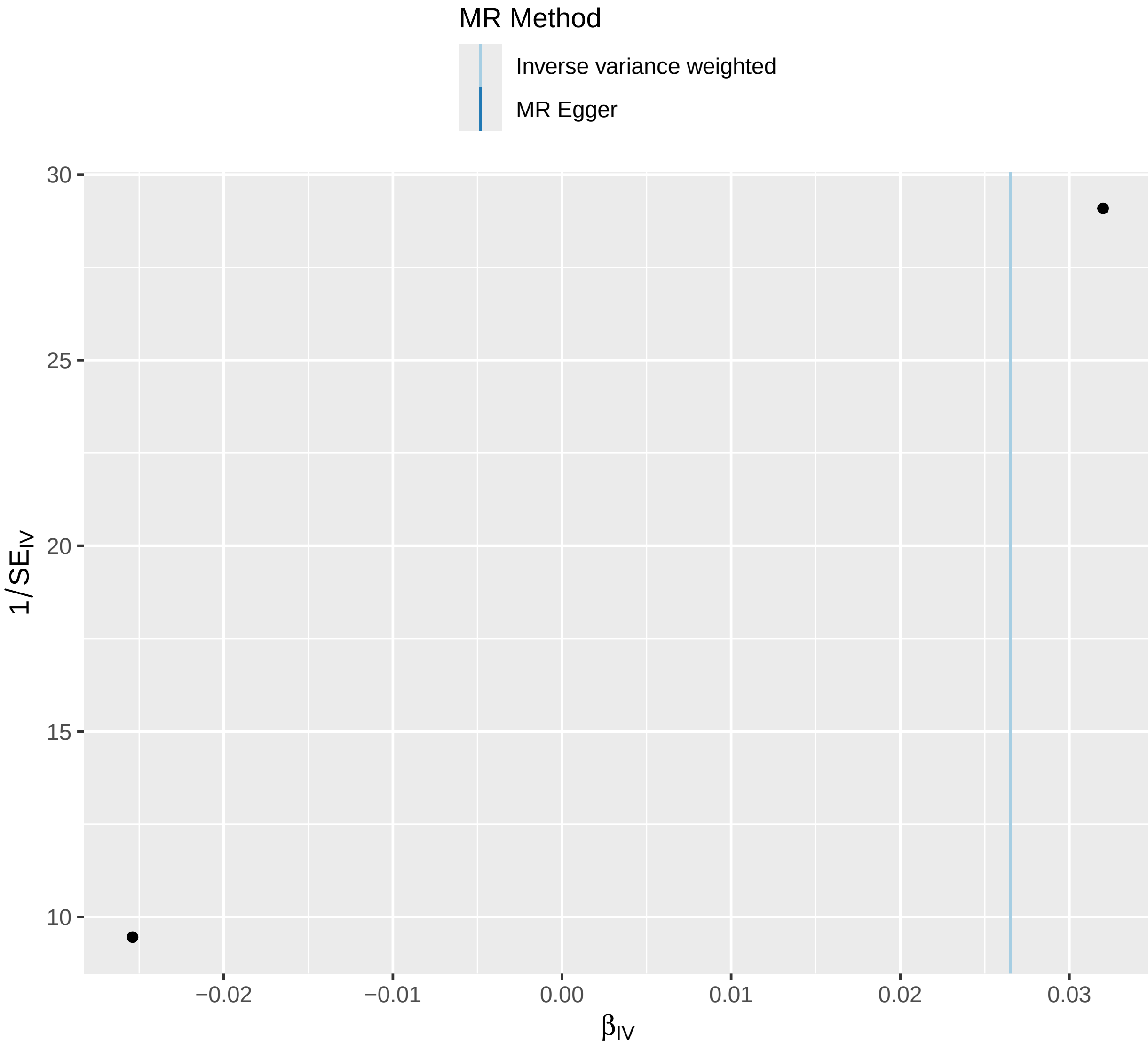
MR Method
Inverse variance weighted
MR Egger
30
25
20
15
10
1/SE_IV
−0.02
−0.01
0.00
0.01
0.02
0.03
β_IV

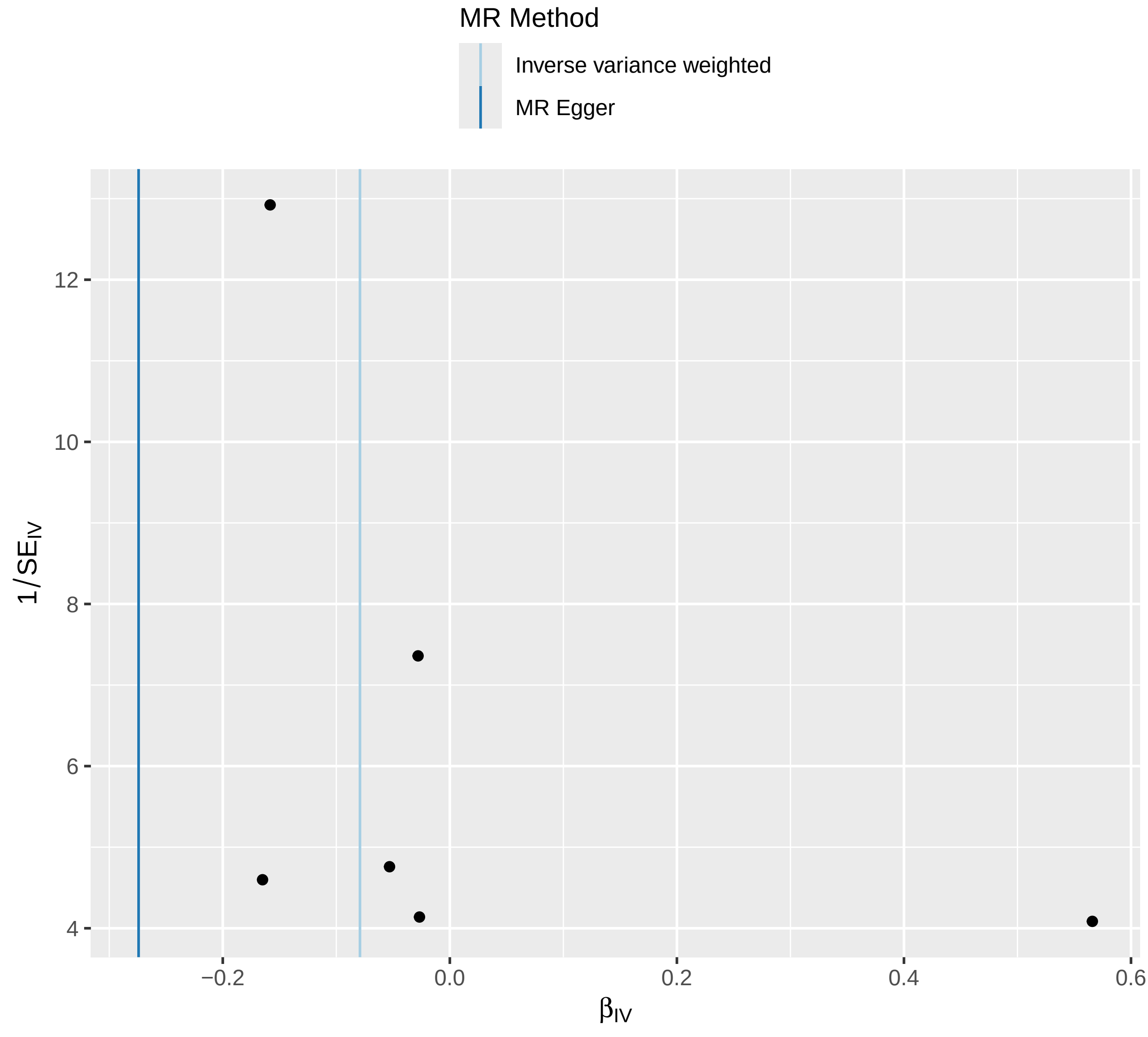

MR Method
Inverse variance weighted
MR Egger
12
10
8
6
4
1/SE_IV
−0.2
0.0
0.2
0.4
0.6
β_IV

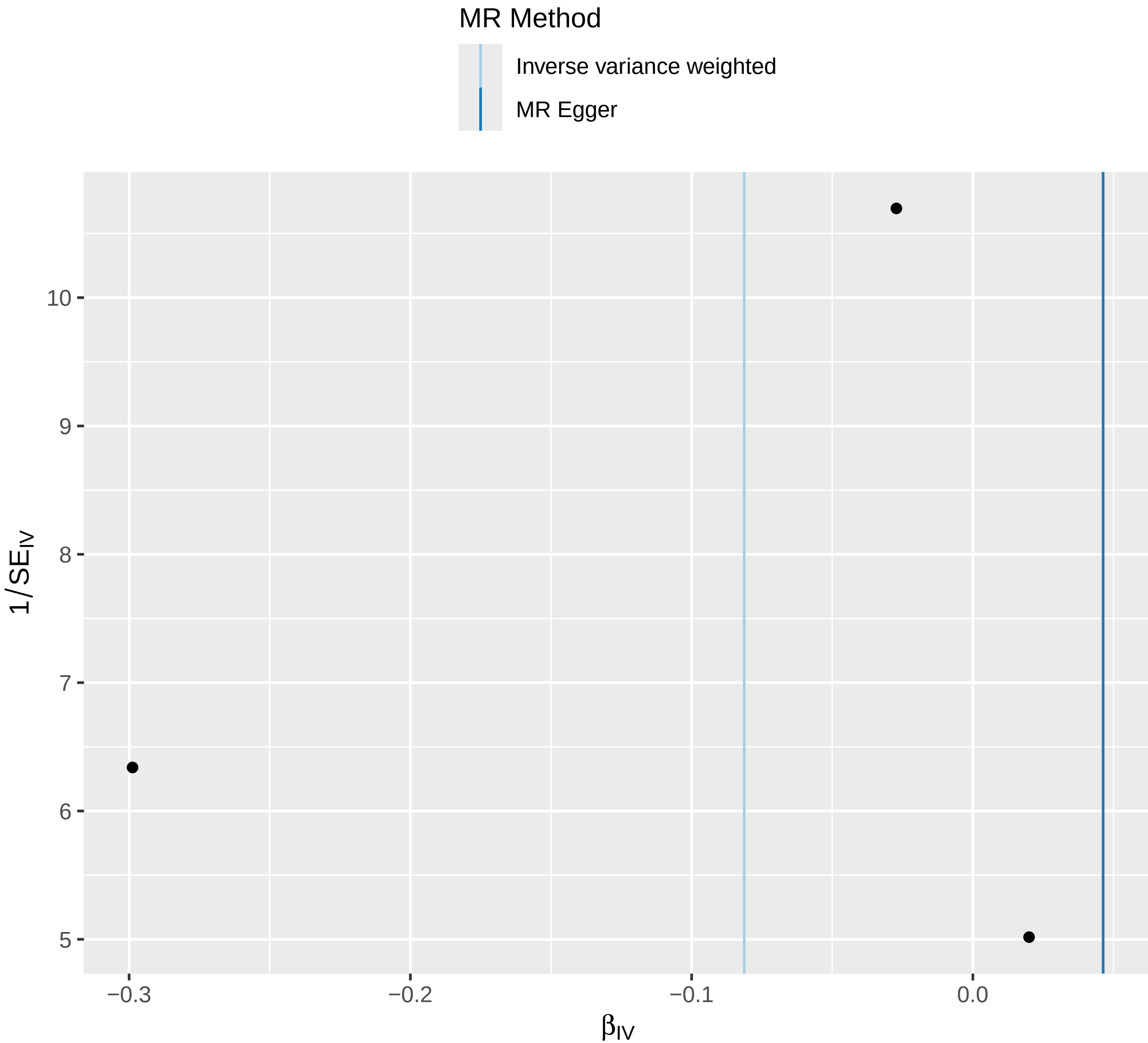

MR Method
Inverse variance weighted
MR Egger
10
9
8
7
6
5
1/SE_IV
−0.3
−0.2
−0.1
0.0
β_IV

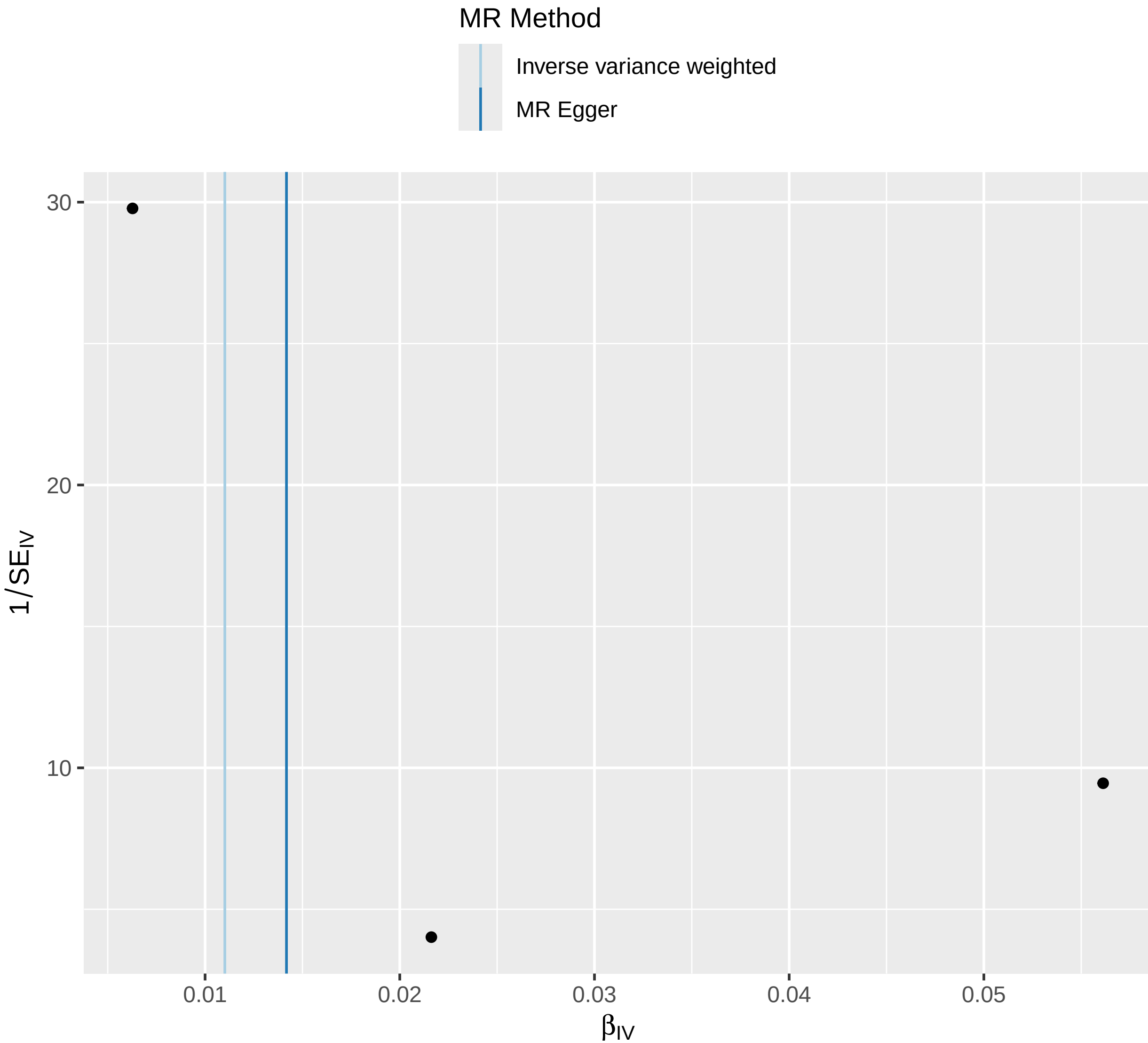
MR Method
Inverse variance weighted
MR Egger
30
20
10
1/SE_IV
0.01
0.02
0.03
0.04
0.05
β_IV

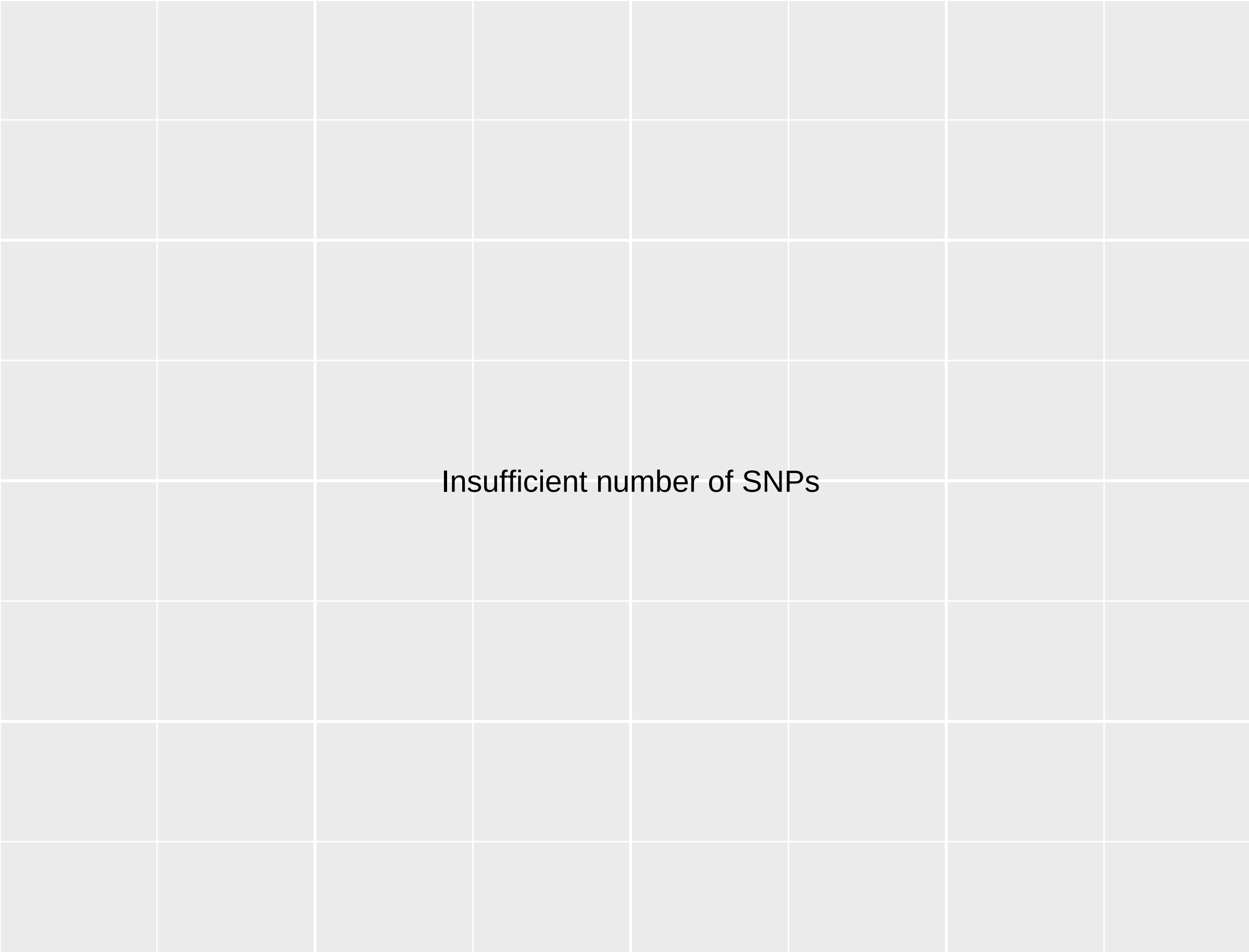
Insufficient number of SNPs

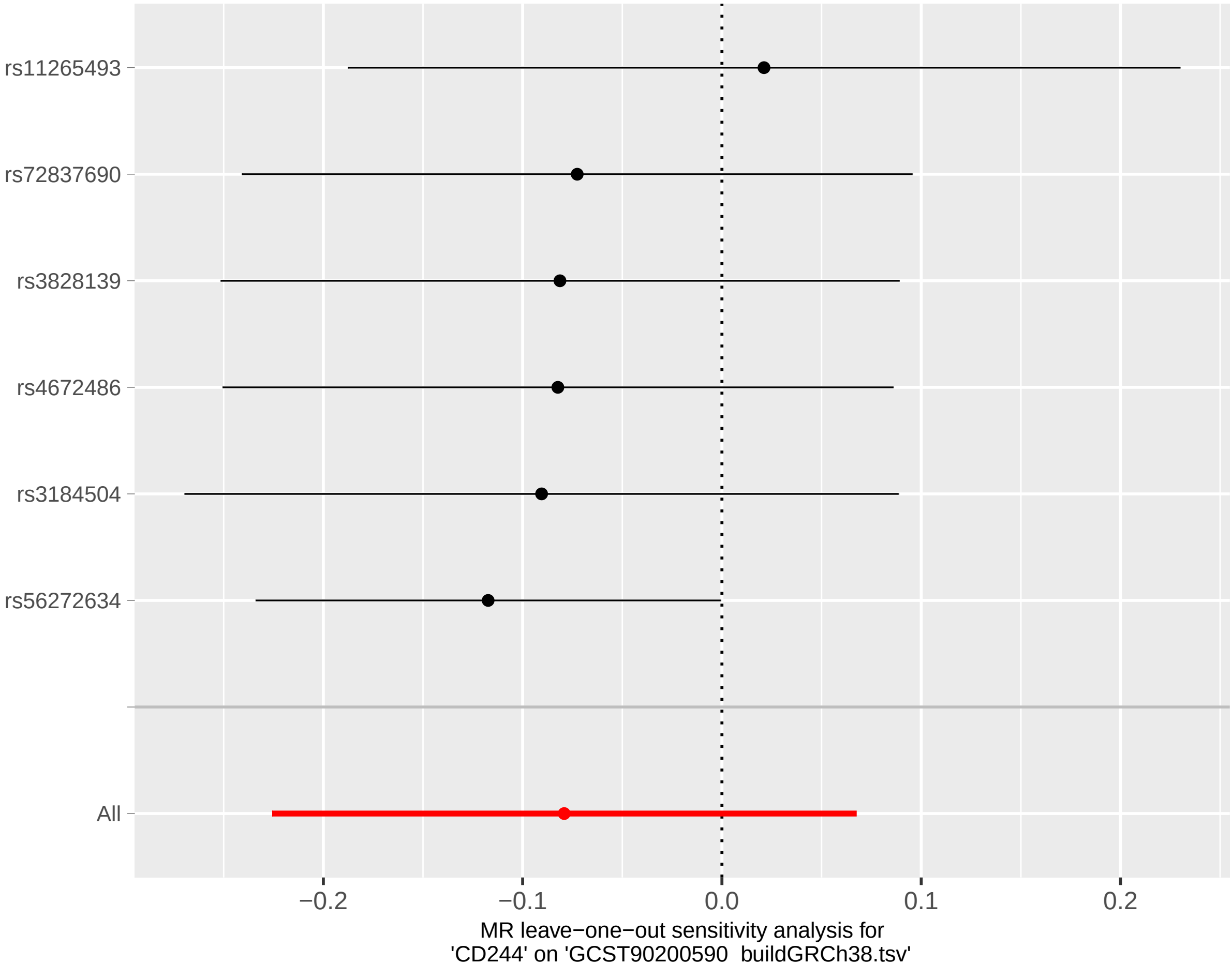
rs11265493
rs72837690
rs3828139
rs4672486
rs3184504
rs56272634
All
−0.2
−0.1
0.0
0.1
0.2
MR leave−one−out sensitivity analysis for
'CD244' on 'GCST90200590_buildGRCh38.tsv'

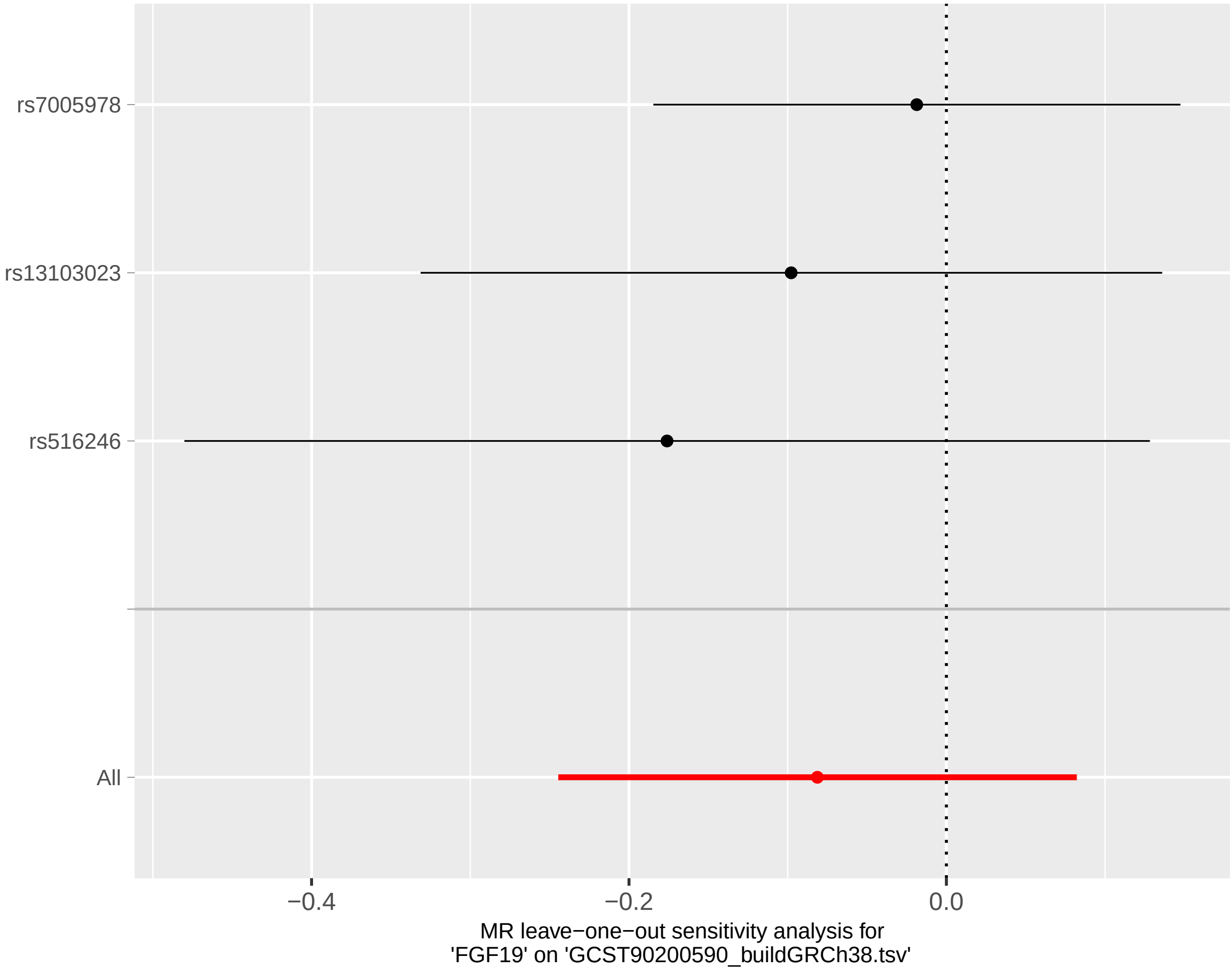
rs7005978
rs13103023
rs516246
All
−0.4
−0.2
0.0
MR leave−one−out sensitivity analysis for
'FGF19' on 'GCST90200590_buildGRCh38.tsv'

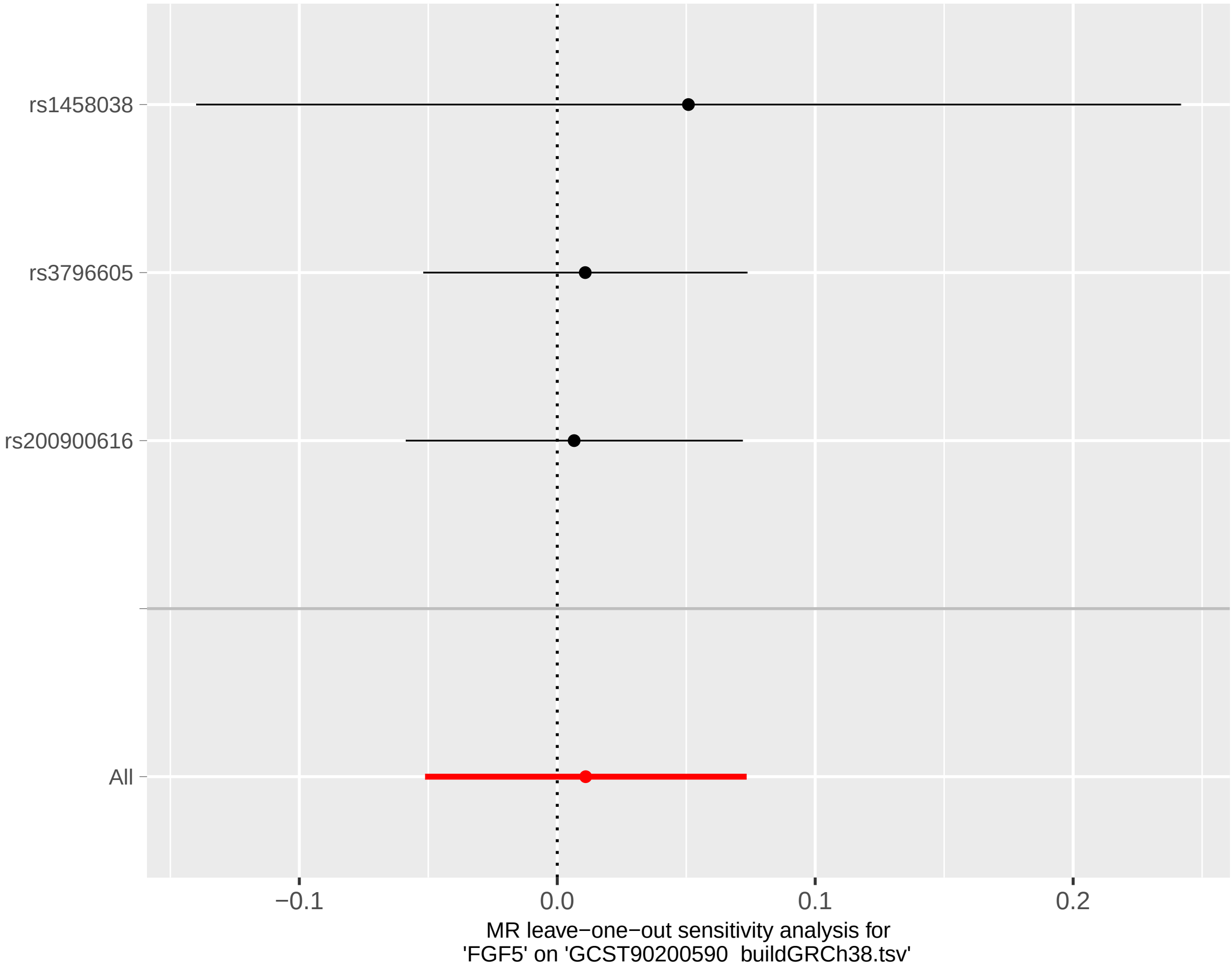

rs1458038
rs3796605
rs200900616
All
−0.1
0.0
0.1
0.2
MR leave−one−out sensitivity analysis for
'FGF5' on 'GCST90200590_buildGRCh38.tsv'

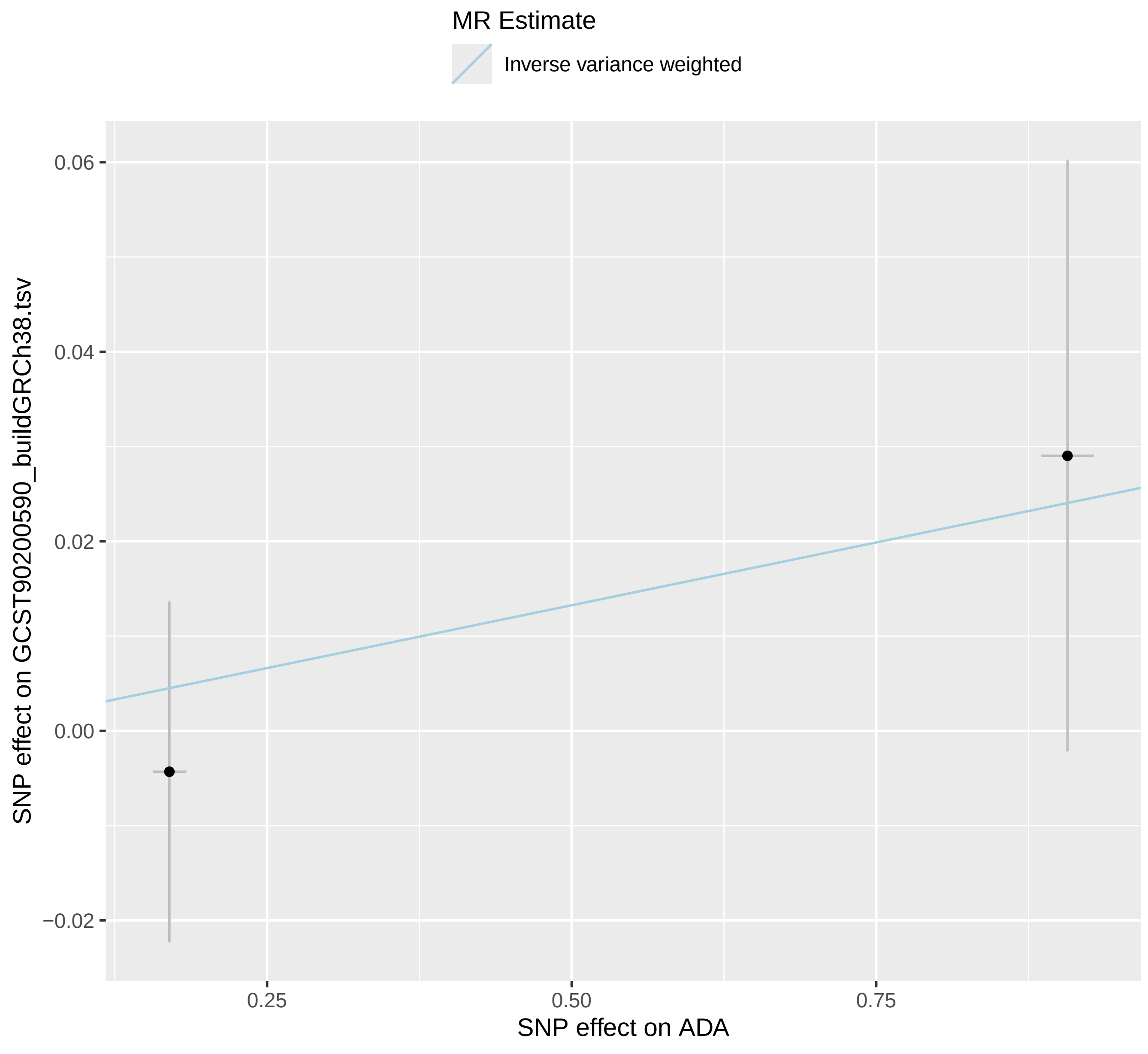
MR Estimate
Inverse variance weighted
SNP effect on GCST90200590_buildGRCh38.tsv
0.06
0.04
0.02
0.00
−0.02
0.25
0.50
0.75
SNP effect on ADA

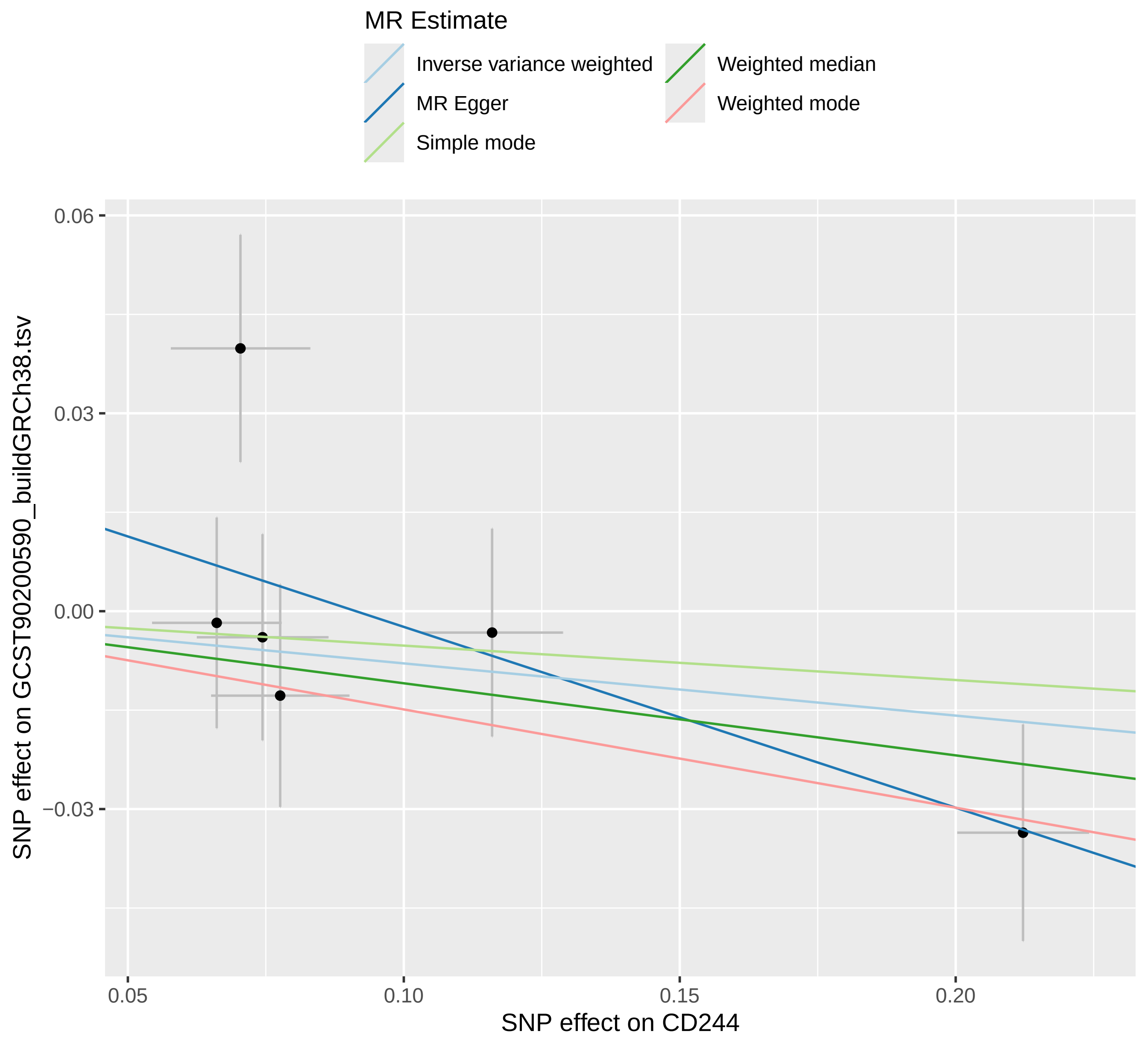

MR Estimate
Inverse variance weighted
MR Egger
Simple mode
Weighted median
Weighted mode
0.06
0.03
0.00
−0.03
0.05
0.10
0.15
0.20
SNP effect on GCST90200590_buildGRCh38.tsv
SNP effect on CD244

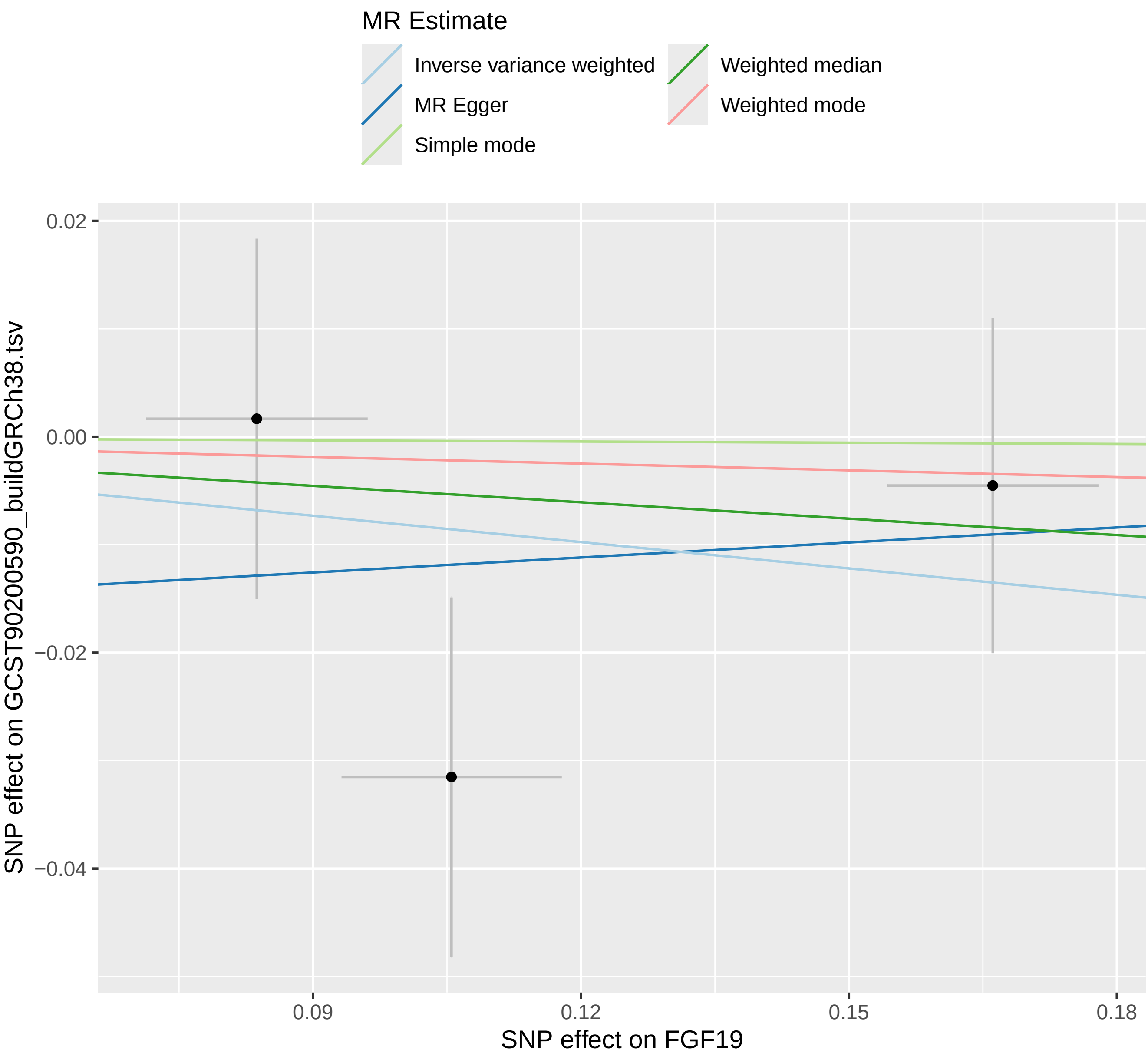
MR Estimate
Inverse variance weighted
MR Egger
Simple mode
Weighted median
Weighted mode
0.02
0.00
−0.02
−0.04
0.09
0.12
0.15
0.18
SNP effect on GCST90200590_buildGRCh38.tsv
SNP effect on FGF19

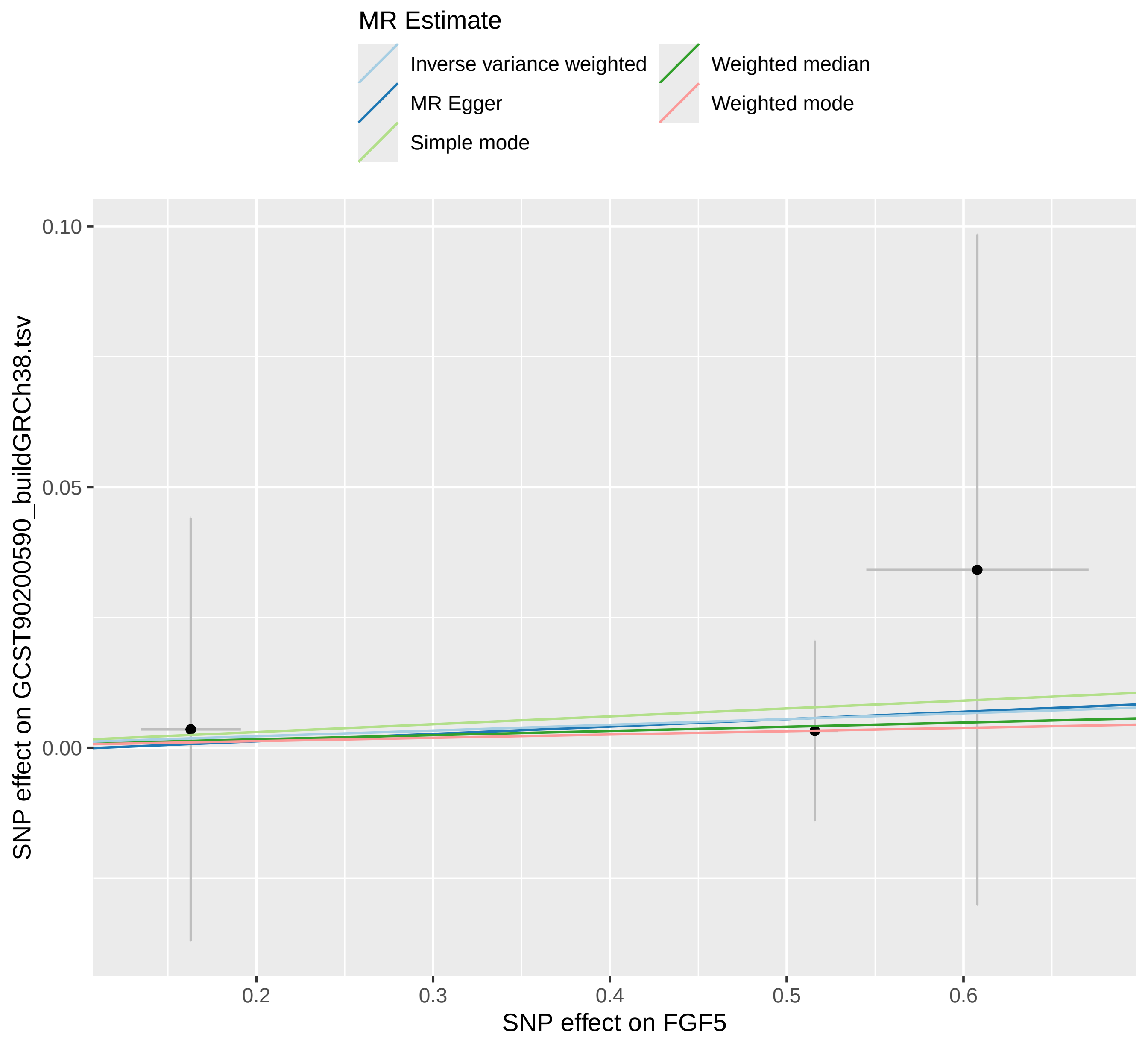

MR Estimate
Inverse variance weighted
MR Egger
Simple mode
Weighted median
Weighted mode
0.10
0.05
0.00
0.2
0.3
0.4
0.5
0.6
SNP effect on FGF5
SNP effect on GCST90200590_buildGRCh38.tsv

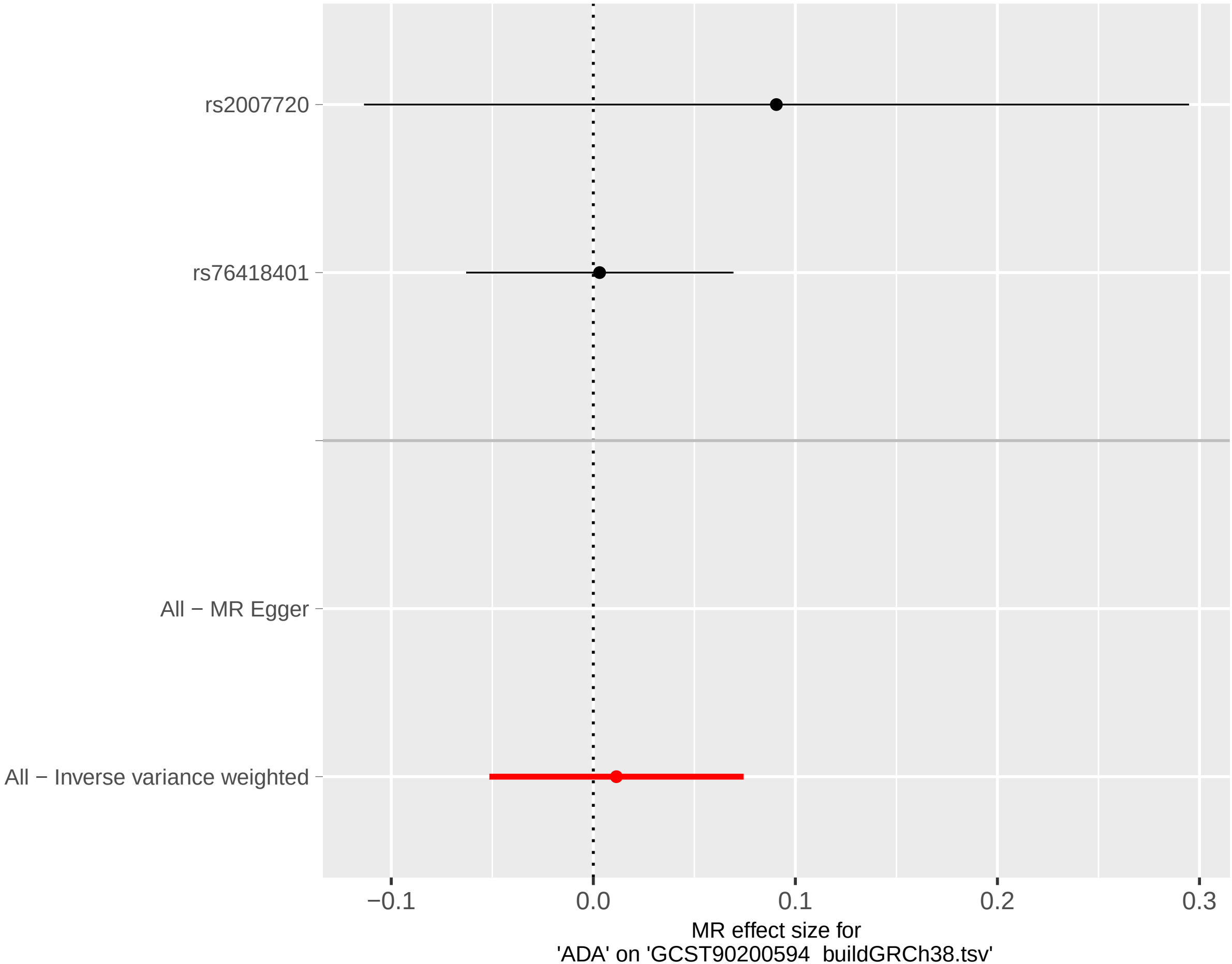

rs2007720
rs76418401
All − MR Egger
All − Inverse variance weighted
−0.1
0.0
0.1
0.2
0.3
MR effect size for
'ADA' on 'GCST90200594_buildGRCh38.tsv'

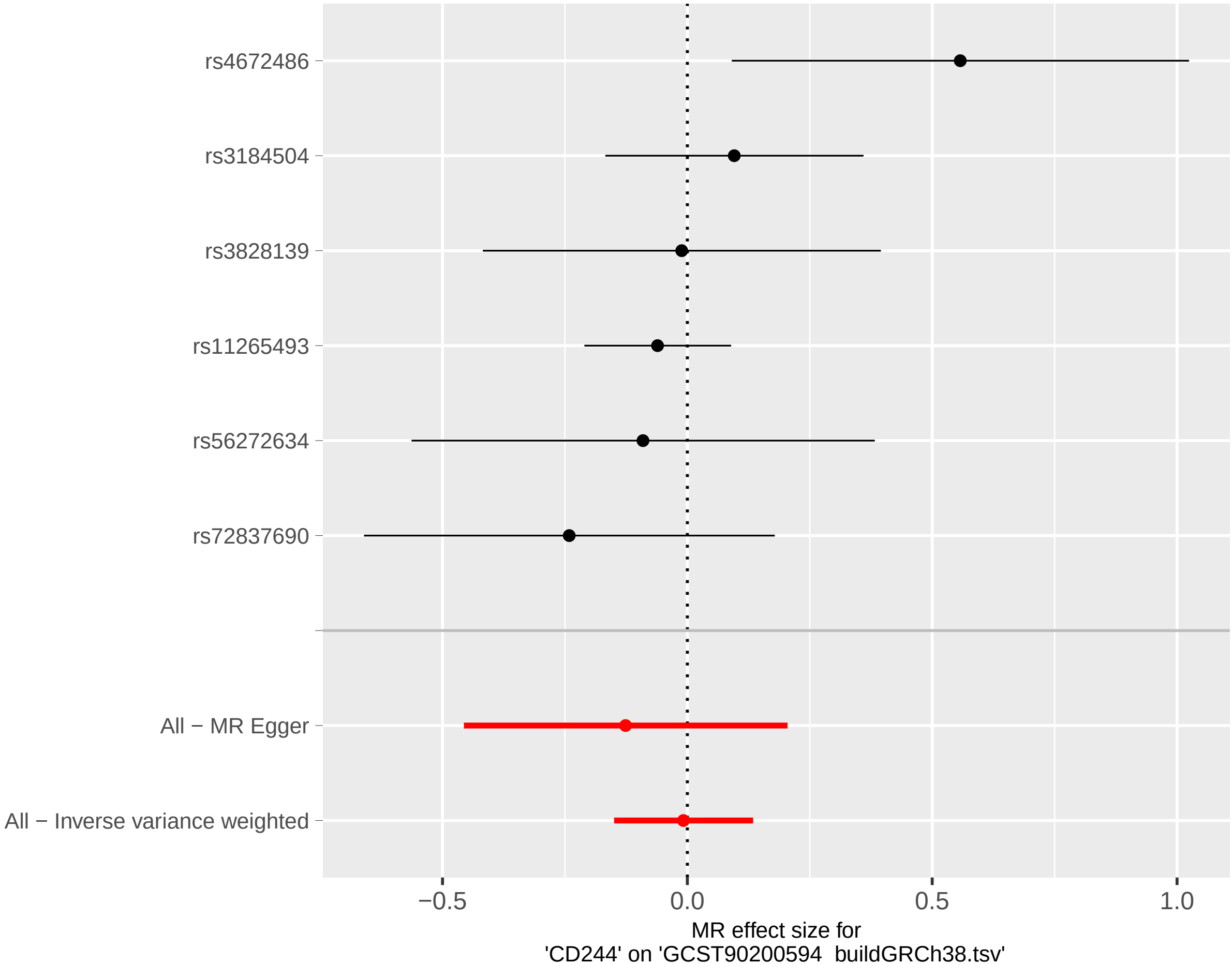
rs4672486
rs3184504
rs3828139
rs11265493
rs56272634
rs72837690
All − MR Egger
All − Inverse variance weighted
−0.5
0.0
0.5
1.0
MR effect size for
'CD244' on 'GCST90200594_buildGRCh38.tsv'

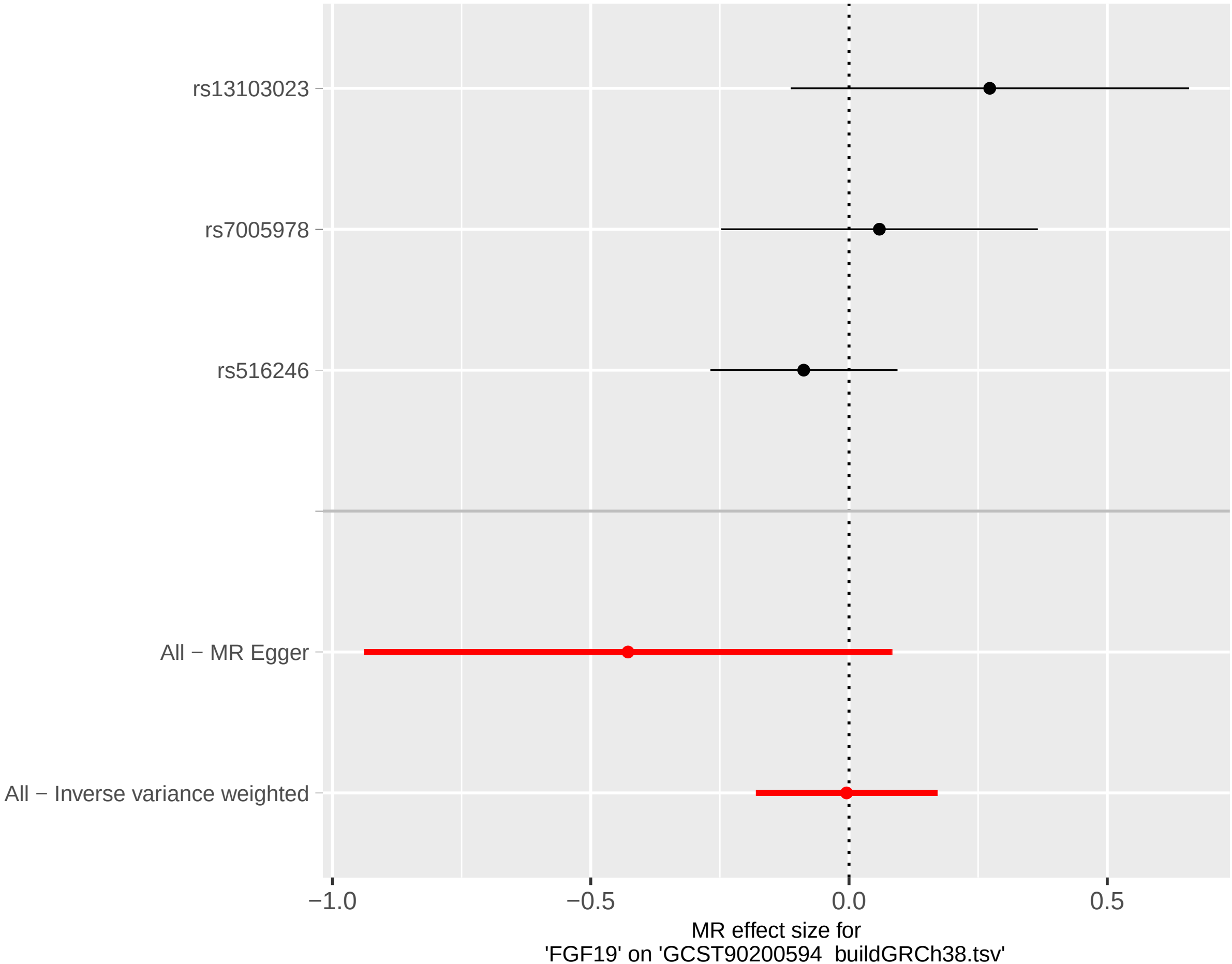
rs13103023
rs7005978
rs516246
All − MR Egger
All − Inverse variance weighted
−1.0
−0.5
0.0
0.5
MR effect size for
'FGF19' on 'GCST90200594_buildGRCh38.tsv'

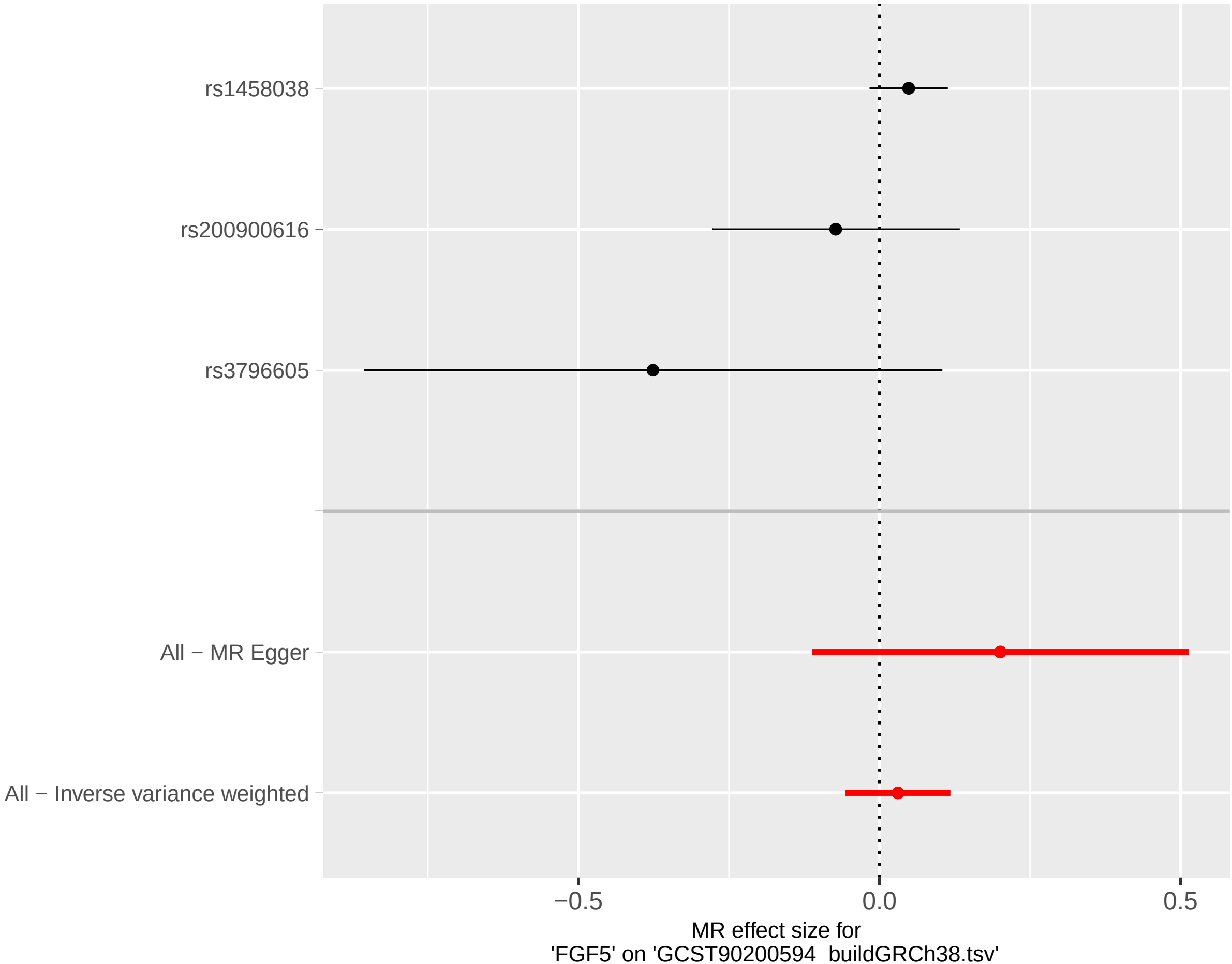
rs1458038
rs200900616
rs3796605
All − MR Egger
All − Inverse variance weighted
−0.5
0.0
0.5
MR effect size for
'FGF5' on 'GCST90200594_buildGRCh38.tsv'

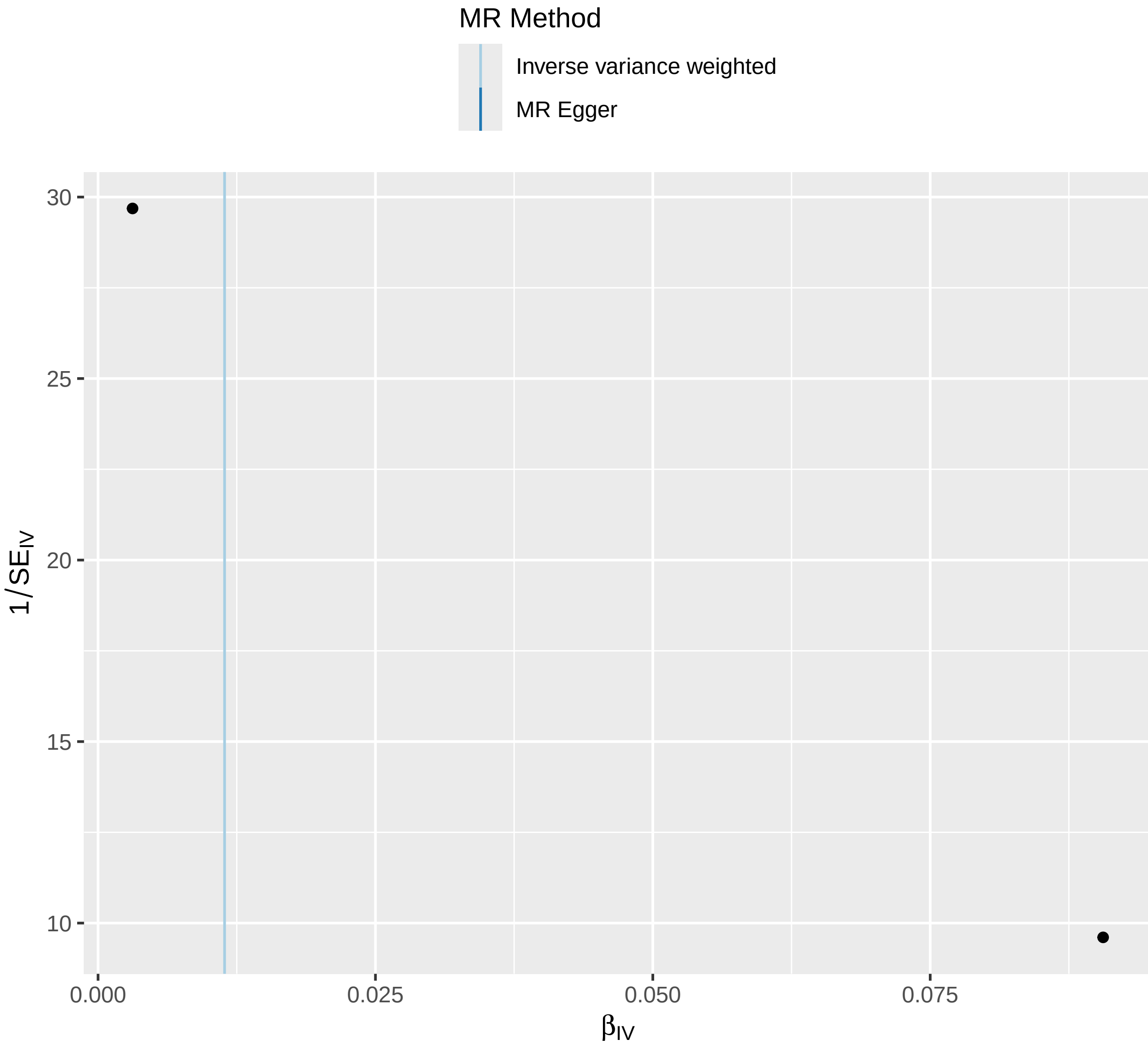

MR Method
Inverse variance weighted
MR Egger
30
25
20
15
10
1/SE_IV
0.000
0.025
0.050
0.075
β_IV

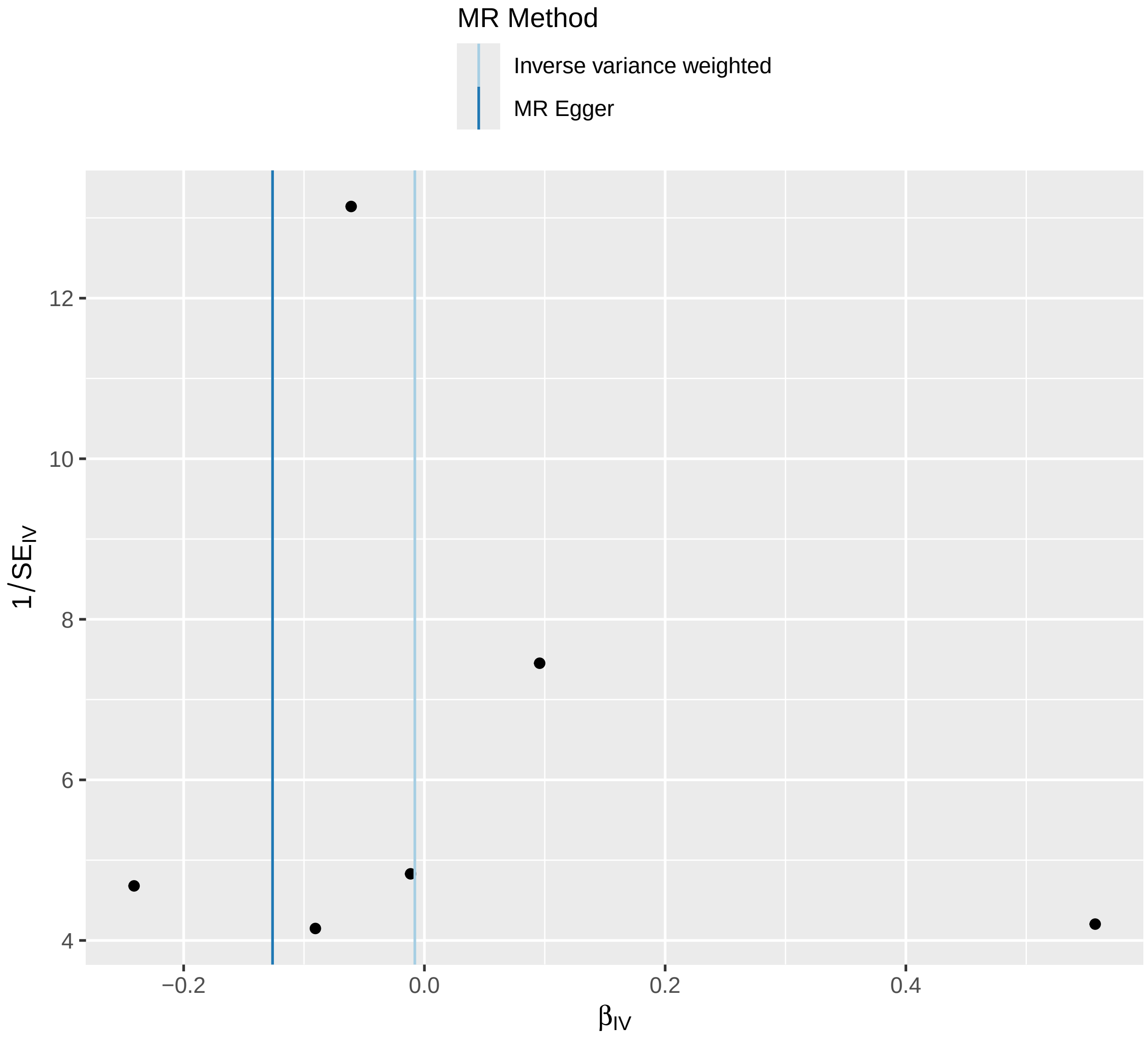

MR Method
Inverse variance weighted
MR Egger
12
10
8
6
4
1/SE_IV
−0.2
0.0
0.2
0.4
β_IV

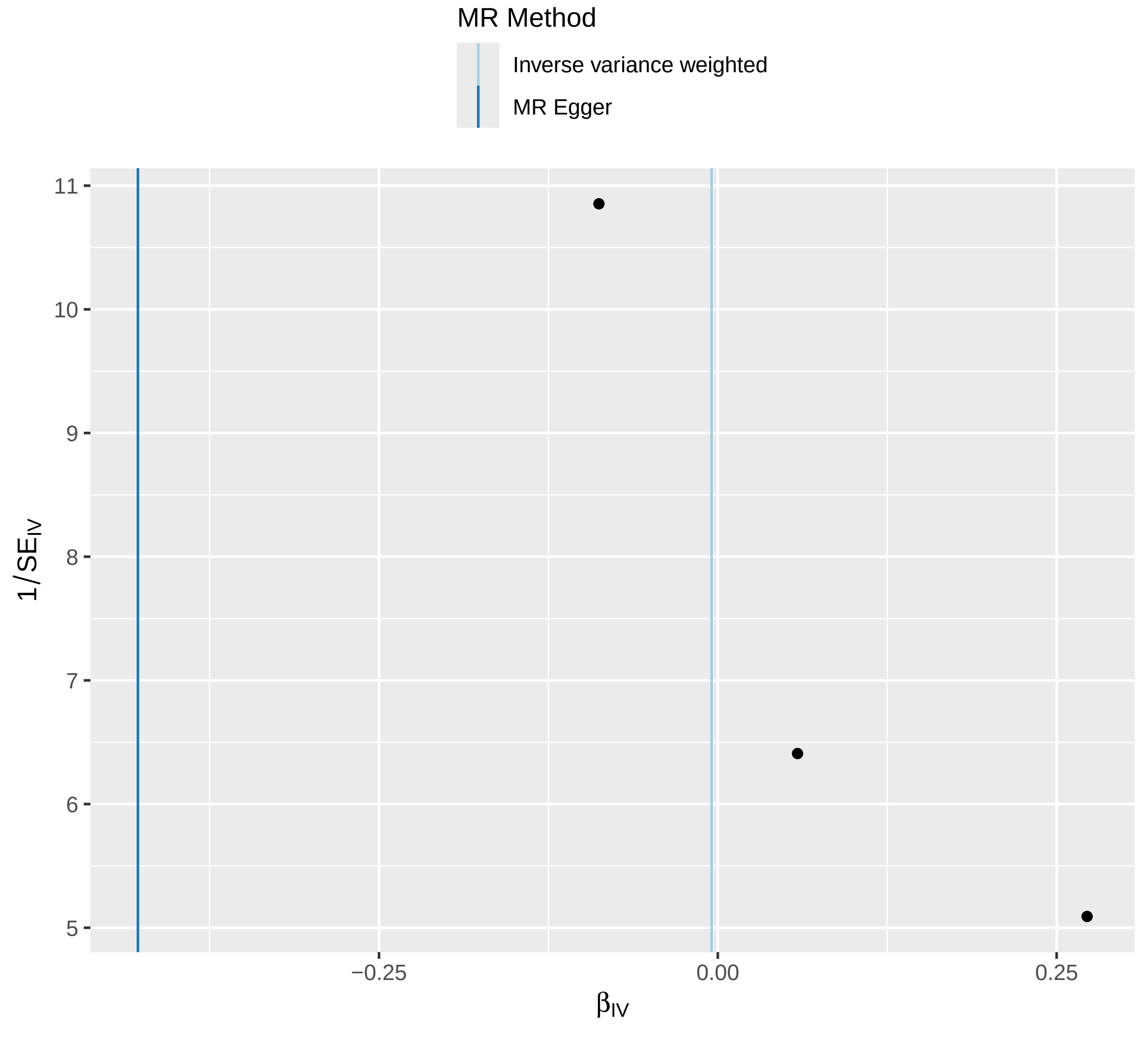

MR Method
Inverse variance weighted
MR Egger
11
10
9
8
7
6
5
1/SE_IV
−0.25
0.00
0.25
β_IV

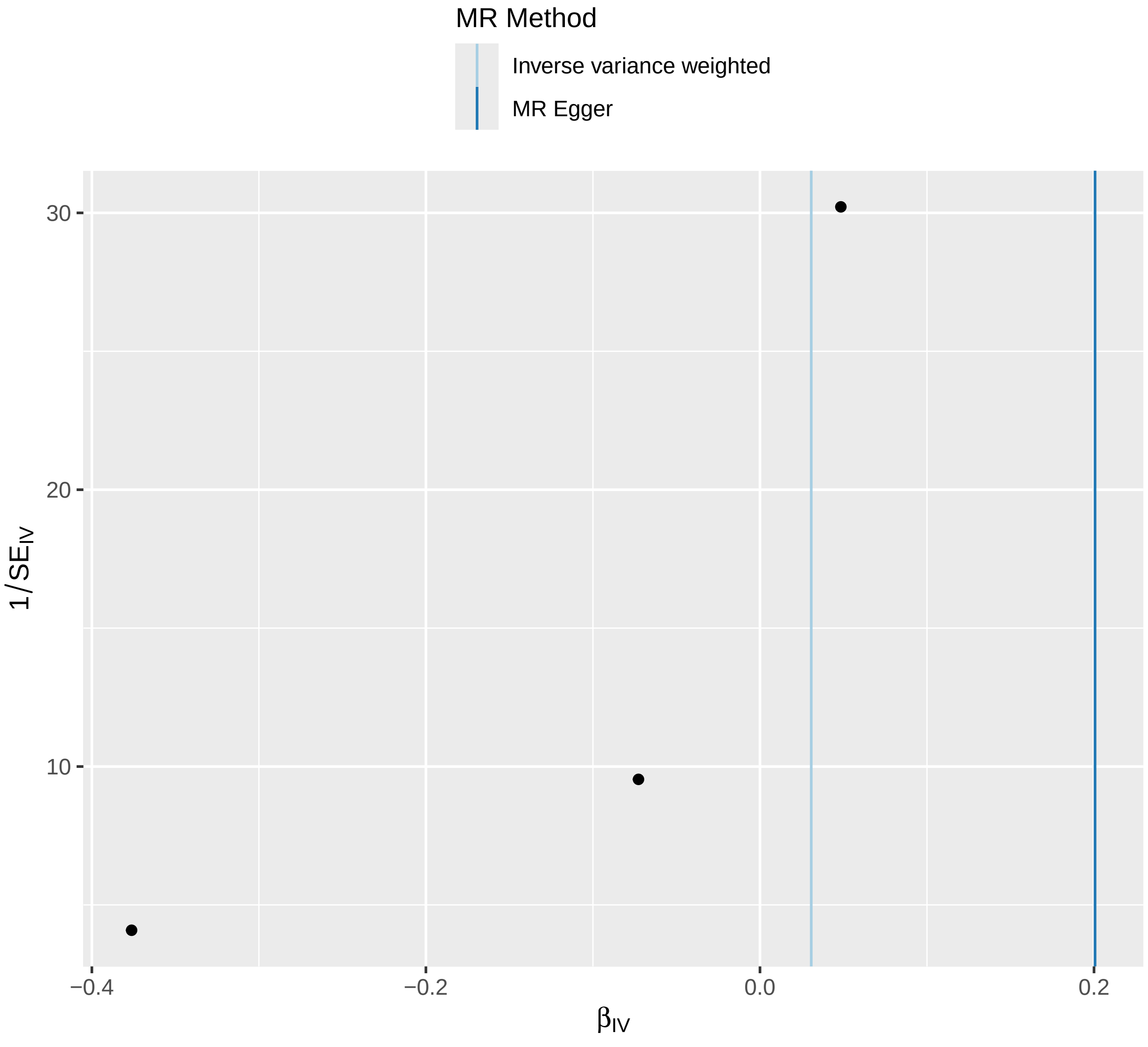
MR Method
Inverse variance weighted
MR Egger
30
20
10
1/SE_IV
−0.4
−0.2
0.0
0.2
β_IV

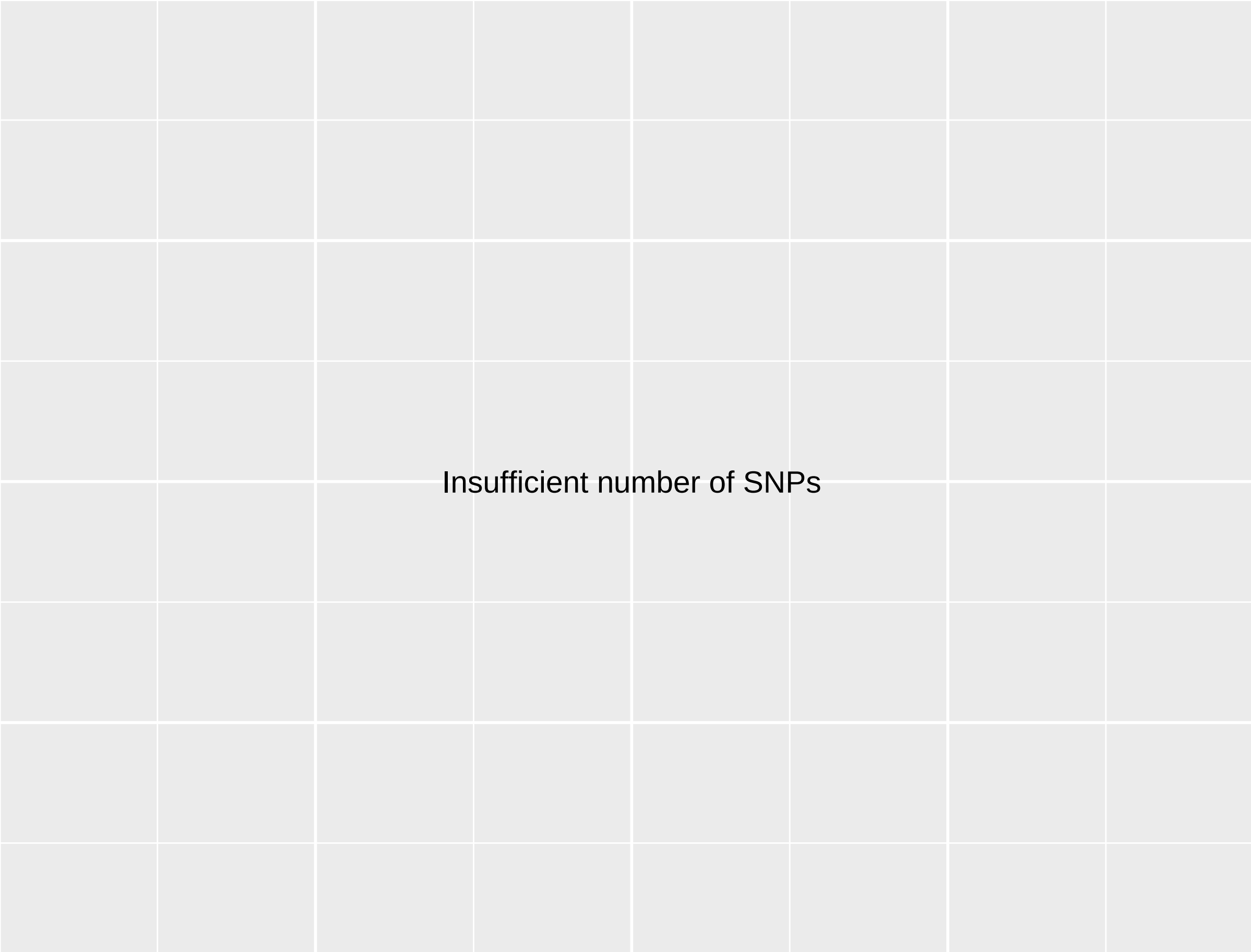
Insufficient number of SNPs

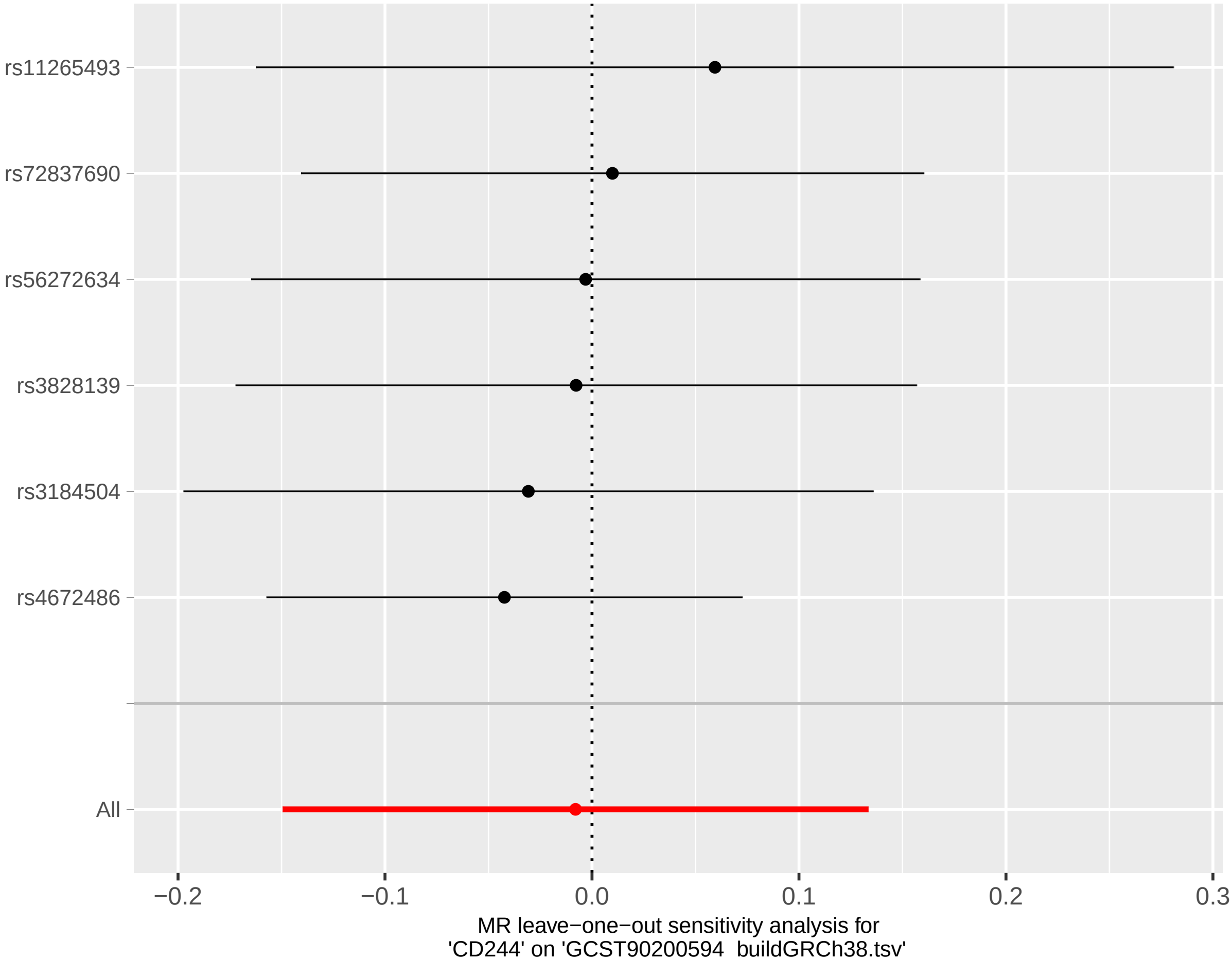
rs11265493
rs72837690
rs56272634
rs3828139
rs3184504
rs4672486
All
−0.2
−0.1
0.0
0.1
0.2
0.3
MR leave−one−out sensitivity analysis for
'CD244' on 'GCST90200594_buildGRCh38.tsv'

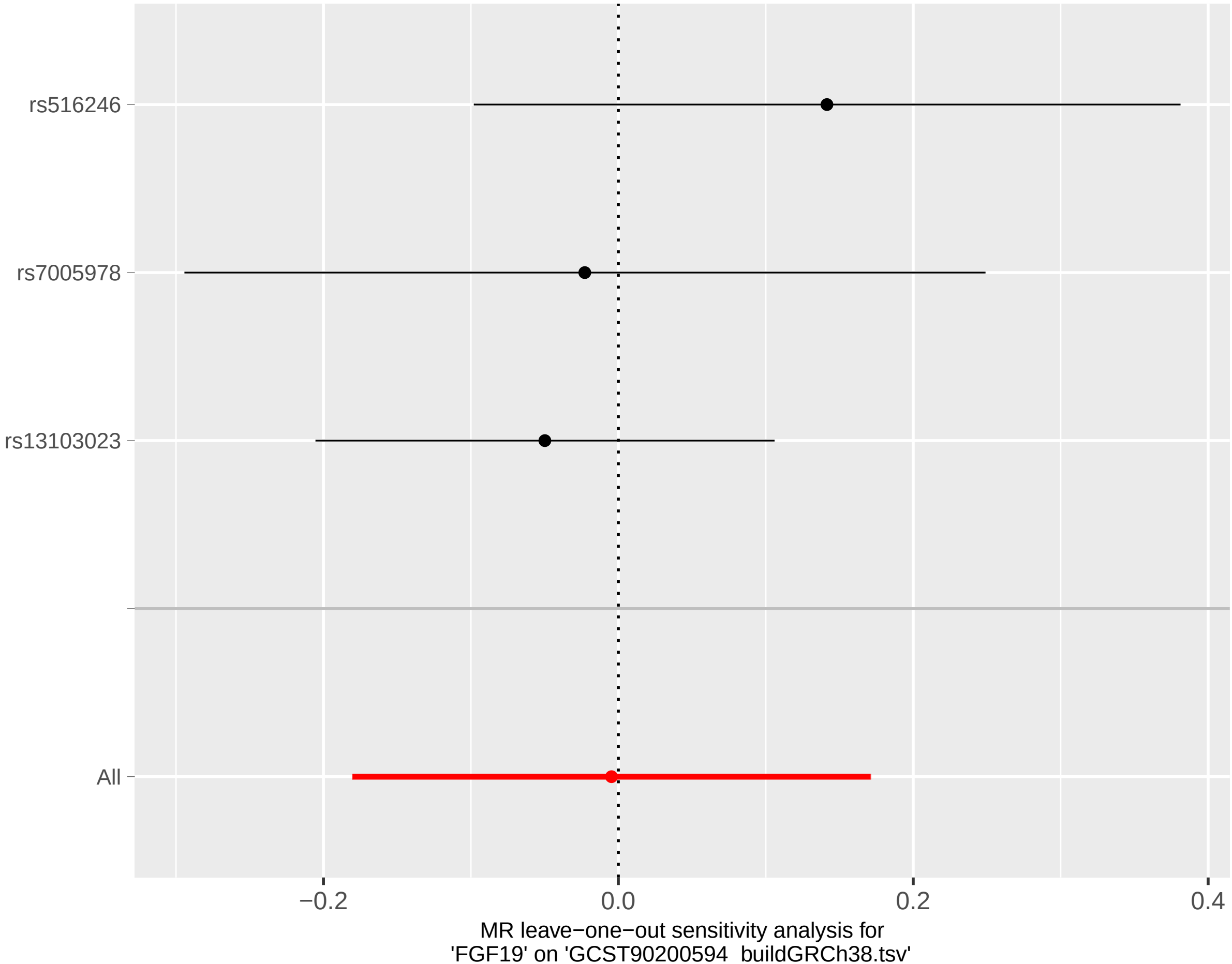
rs516246
rs7005978
rs13103023
All
−0.2
0.0
0.2
0.4
MR leave−one−out sensitivity analysis for
'FGF19' on 'GCST90200594_buildGRCh38.tsv'

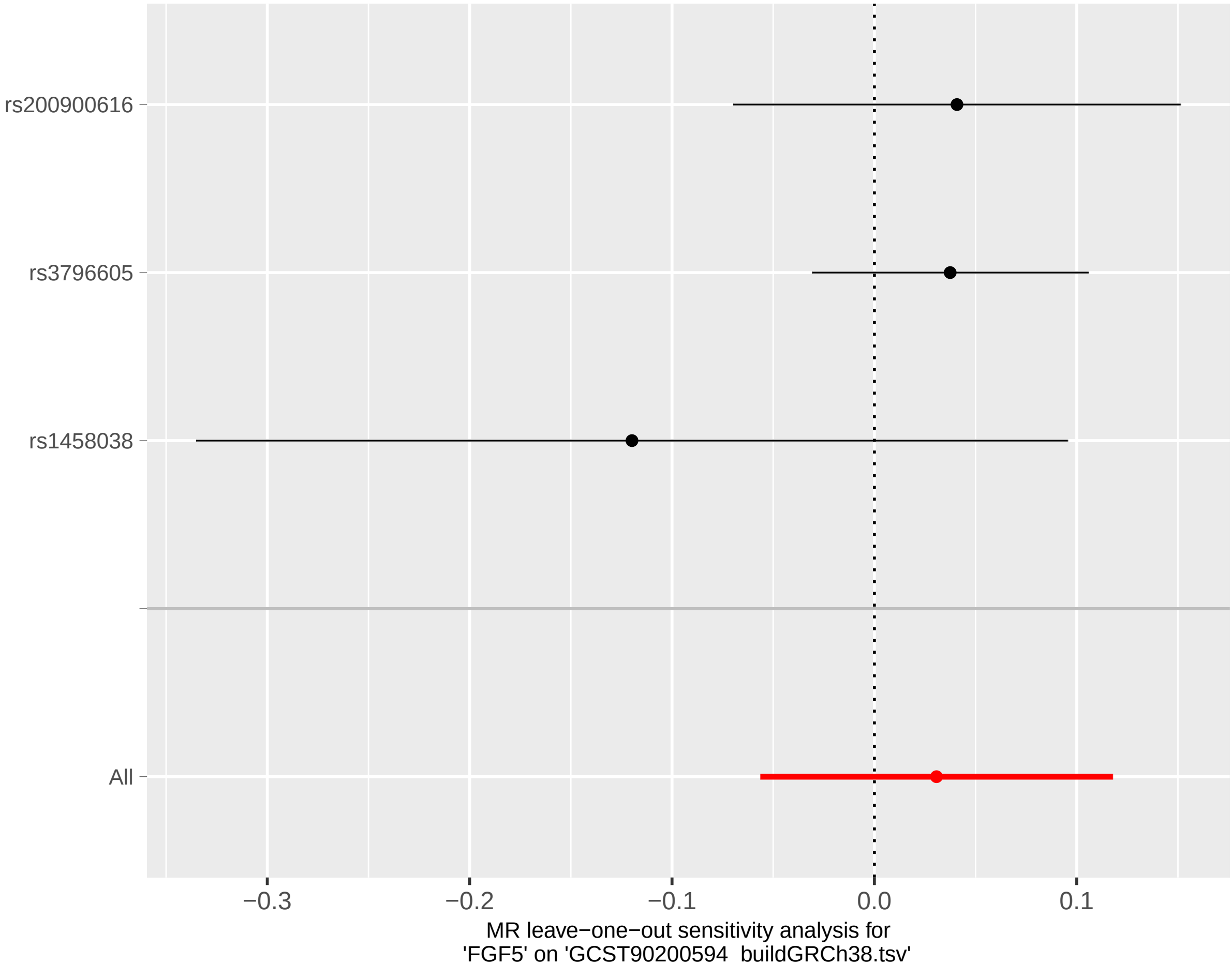
rs200900616
rs3796605
rs1458038
All
−0.3
−0.2
−0.1
0.0
0.1
MR leave−one−out sensitivity analysis for
'FGF5' on 'GCST90200594_buildGRCh38.tsv'

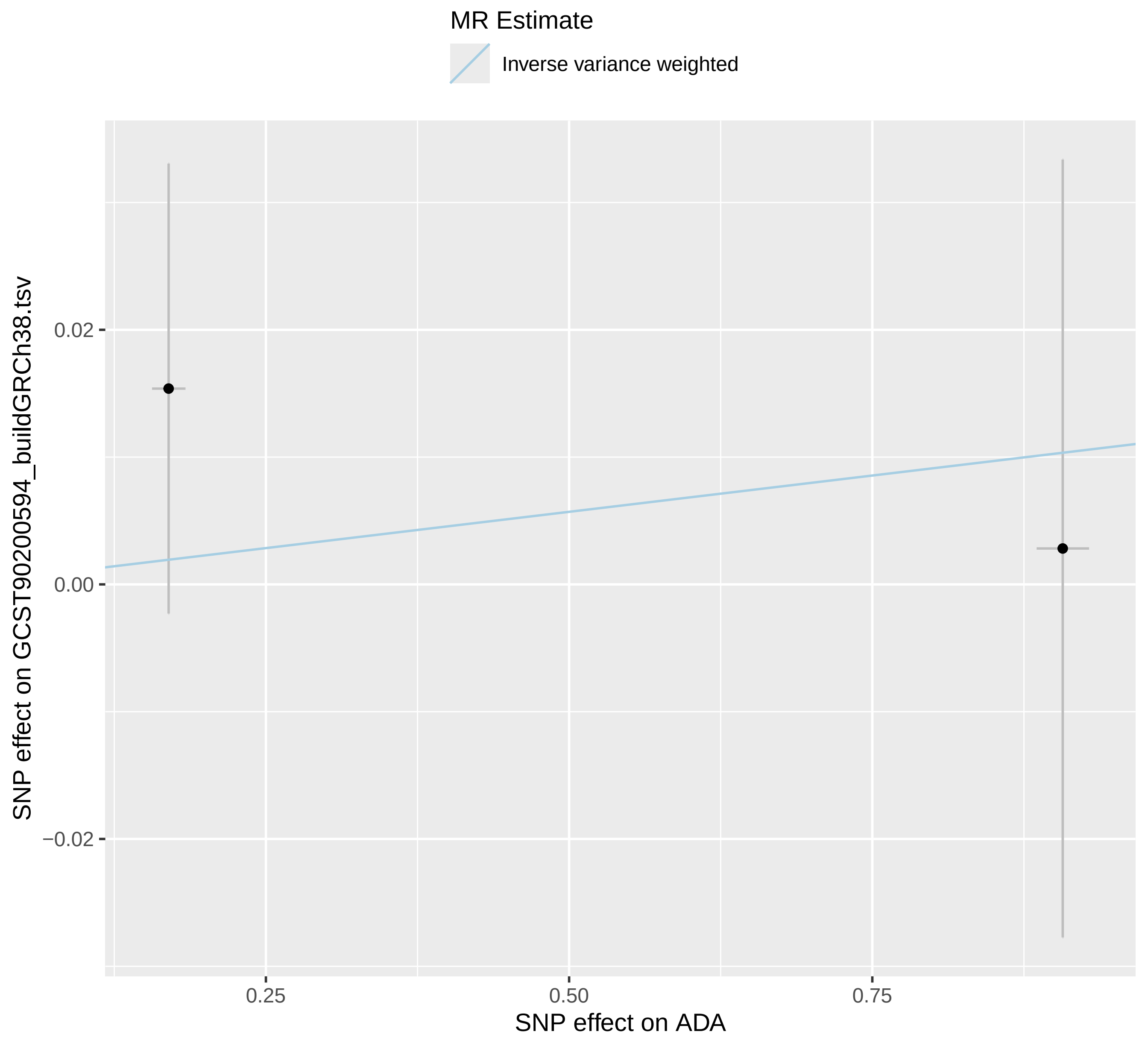
MR Estimate
Inverse variance weighted
SNP effect on GCST90200594_buildGRCh38.tsv
0.02
0.00
−0.02
0.25
0.50
0.75
SNP effect on ADA

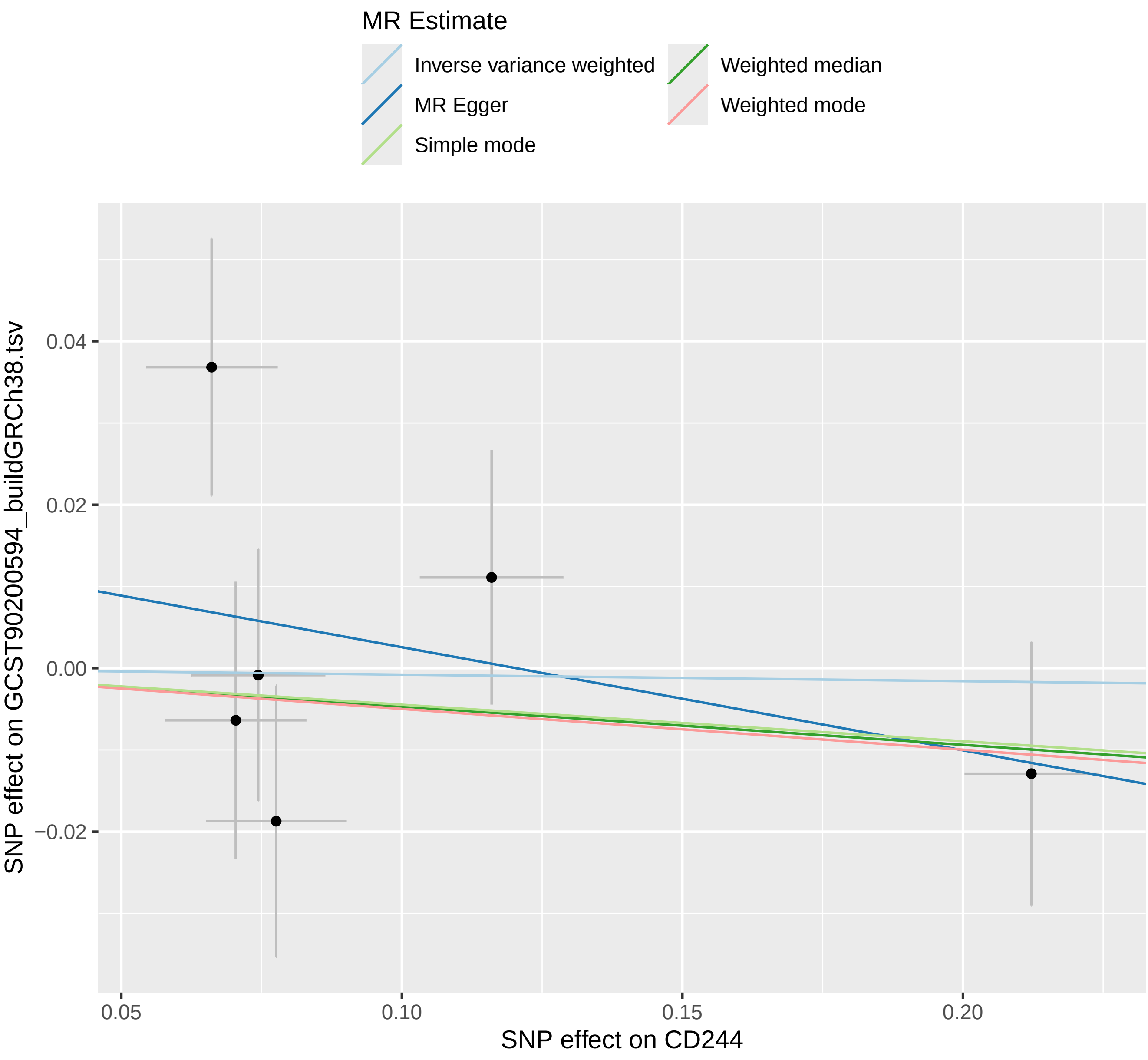
MR Estimate
Inverse variance weighted
MR Egger
Simple mode
Weighted median
Weighted mode
0.04
0.02
0.00
−0.02
SNP effect on GCST90200594_buildGRCh38.tsv
0.05
0.10
0.15
0.20
SNP effect on CD244

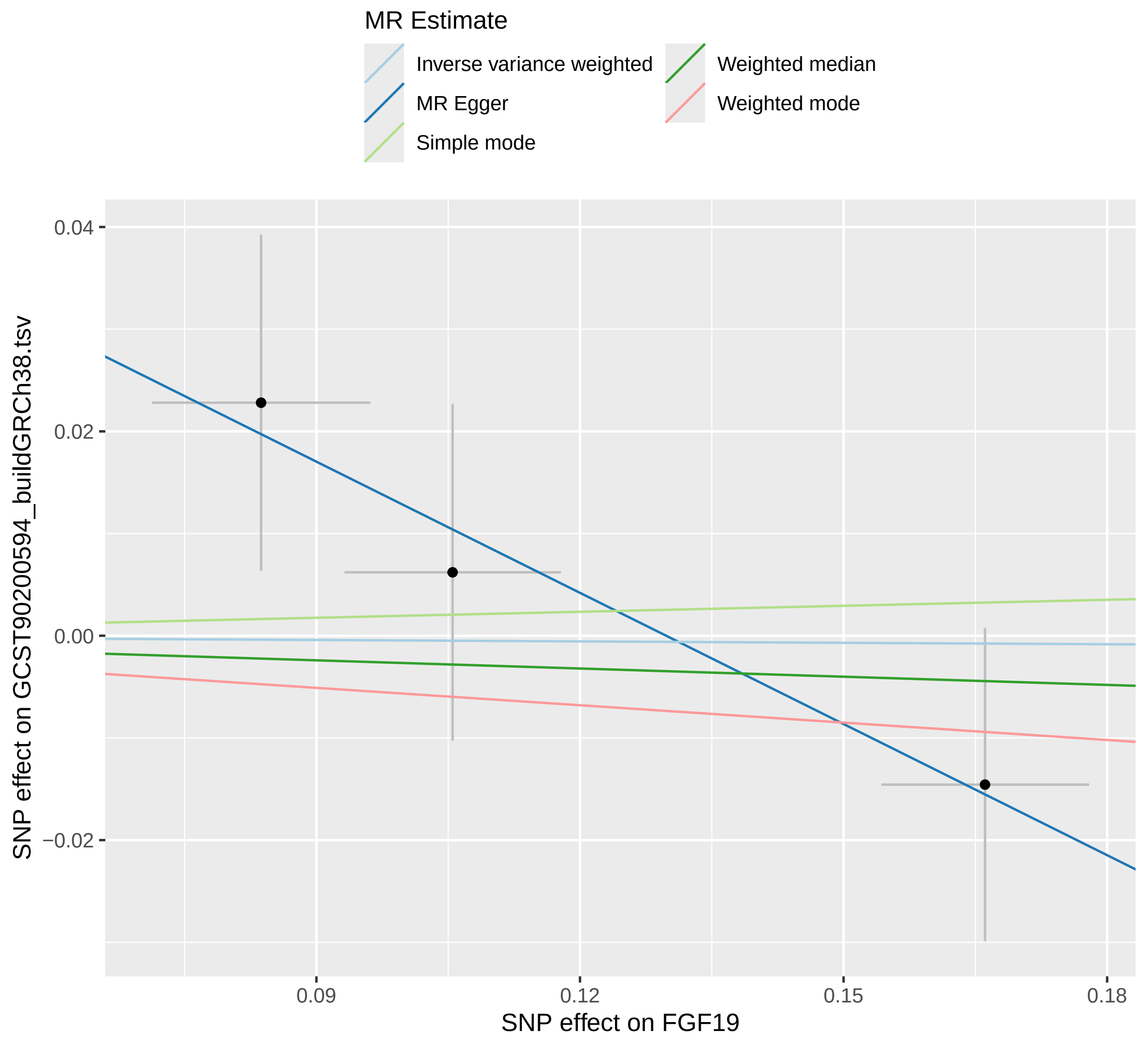
MR Estimate
Inverse variance weighted
MR Egger
Simple mode
Weighted median
Weighted mode
SNP effect on GCST90200594_buildGRCh38.tsv
0.04
0.02
0.00
−0.02
0.09
0.12
0.15
0.18
SNP effect on FGF19

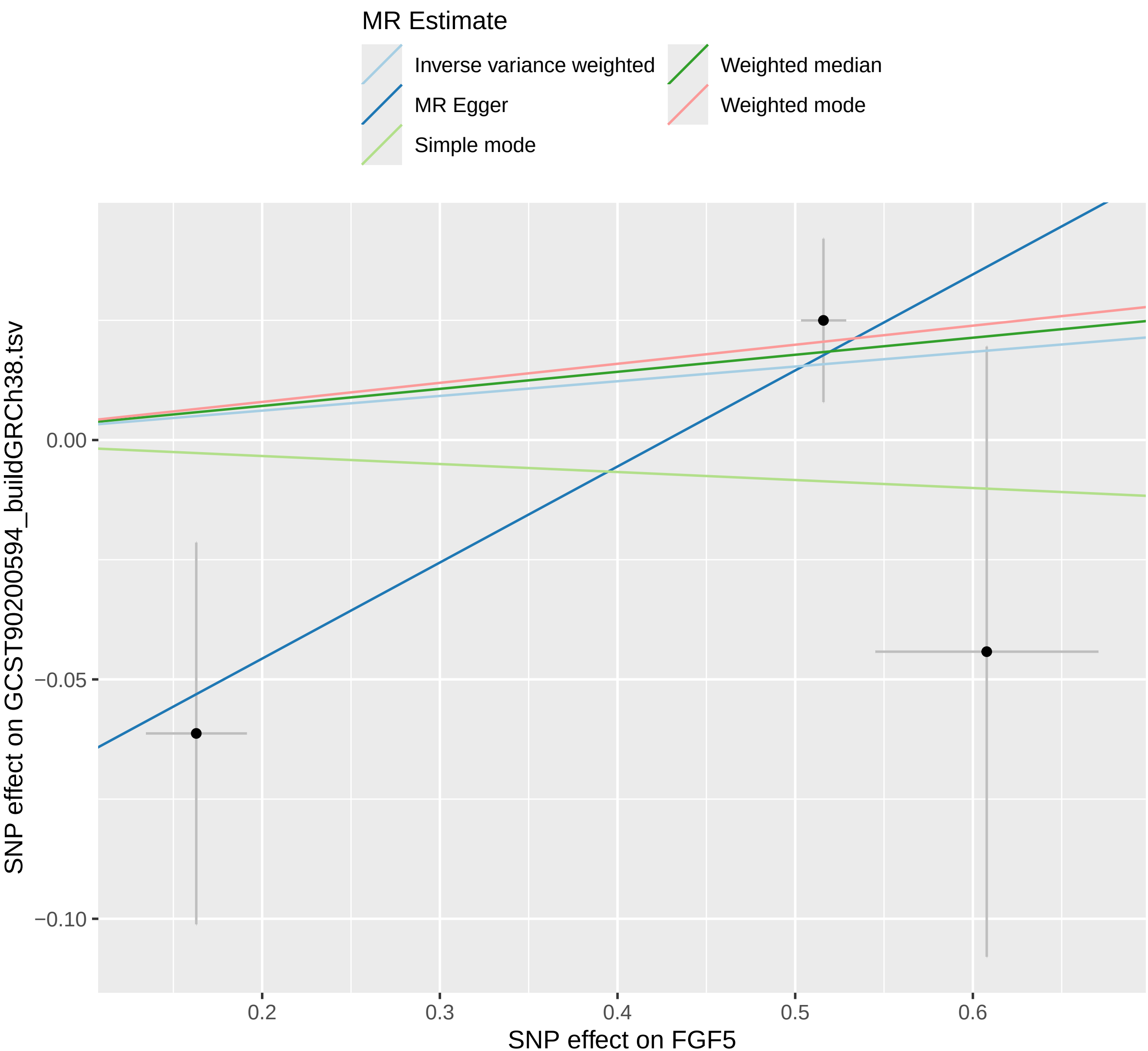

MR Estimate
Inverse variance weighted
MR Egger
Simple mode
Weighted median
Weighted mode
0.00
−0.05
−0.10
0.2
0.3
0.4
0.5
0.6
SNP effect on FGF5
SNP effect on GCST90200594_buildGRCh38.tsv

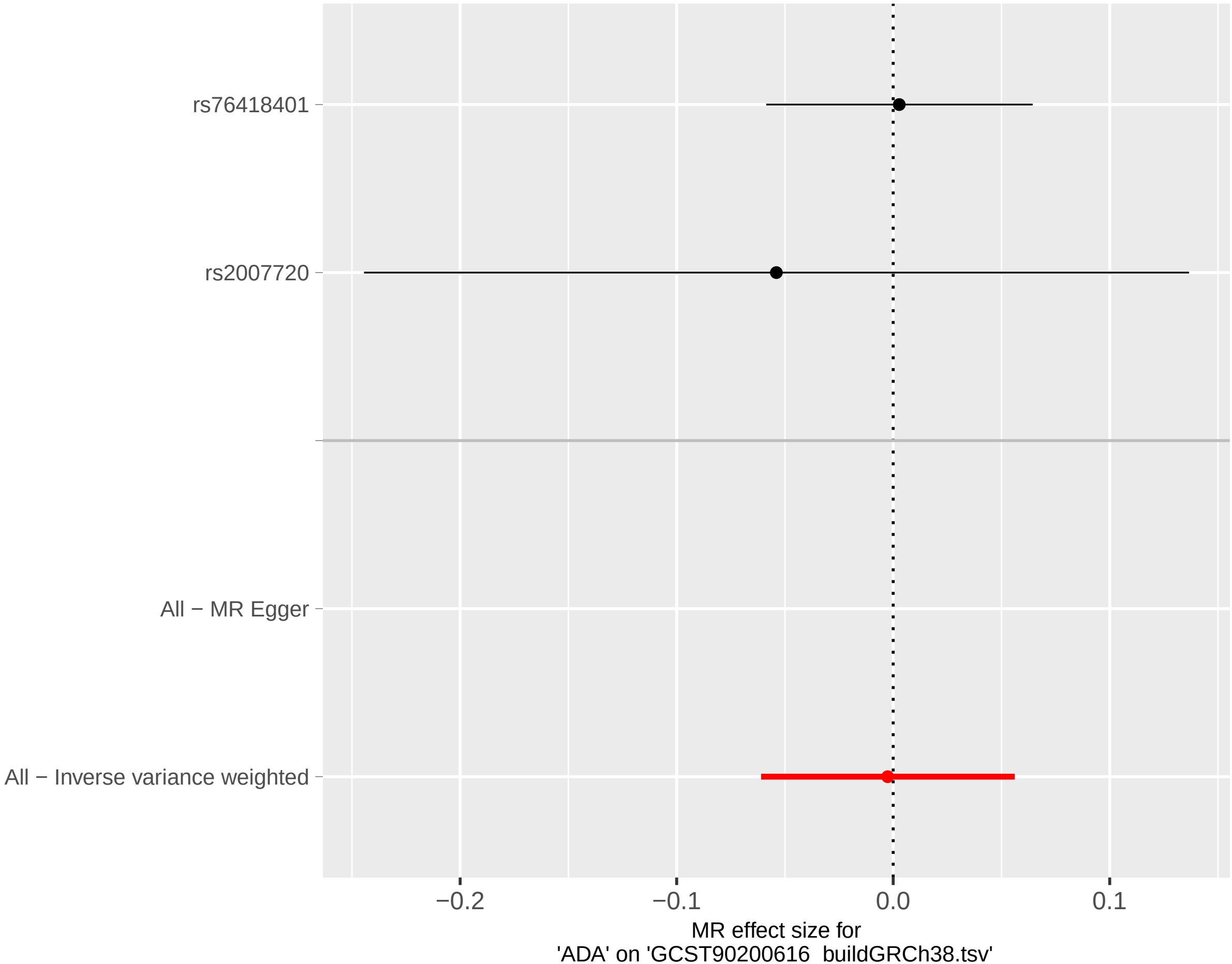

rs76418401
rs2007720
All − MR Egger
All − Inverse variance weighted
−0.2
−0.1
0.0
0.1
MR effect size for
'ADA' on 'GCST90200616_buildGRCh38.tsv'

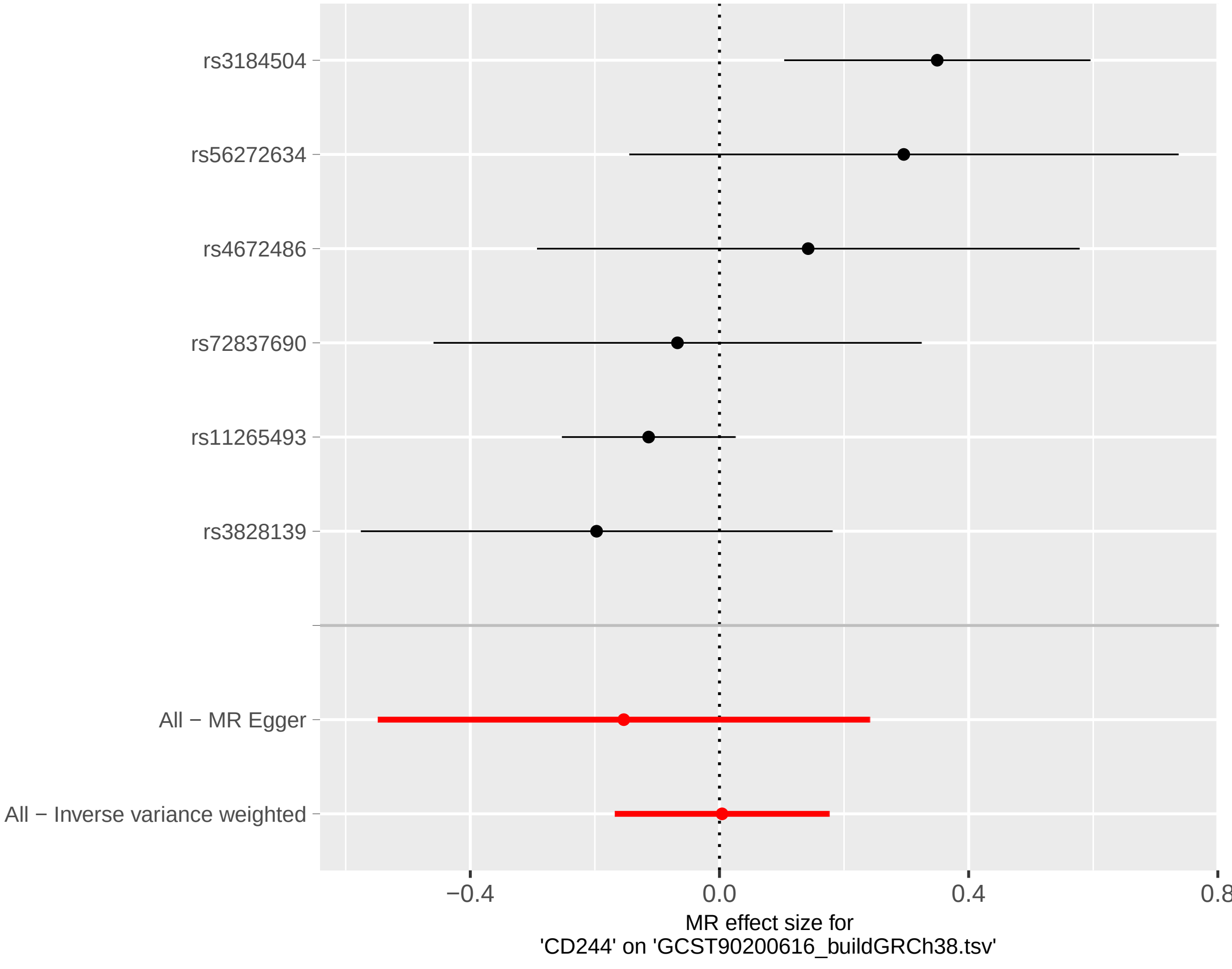
rs3184504
rs56272634
rs4672486
rs72837690
rs11265493
rs3828139
All − MR Egger
All − Inverse variance weighted
−0.4
0.0
0.4
0.8
MR effect size for
'CD244' on 'GCST90200616_buildGRCh38.tsv'

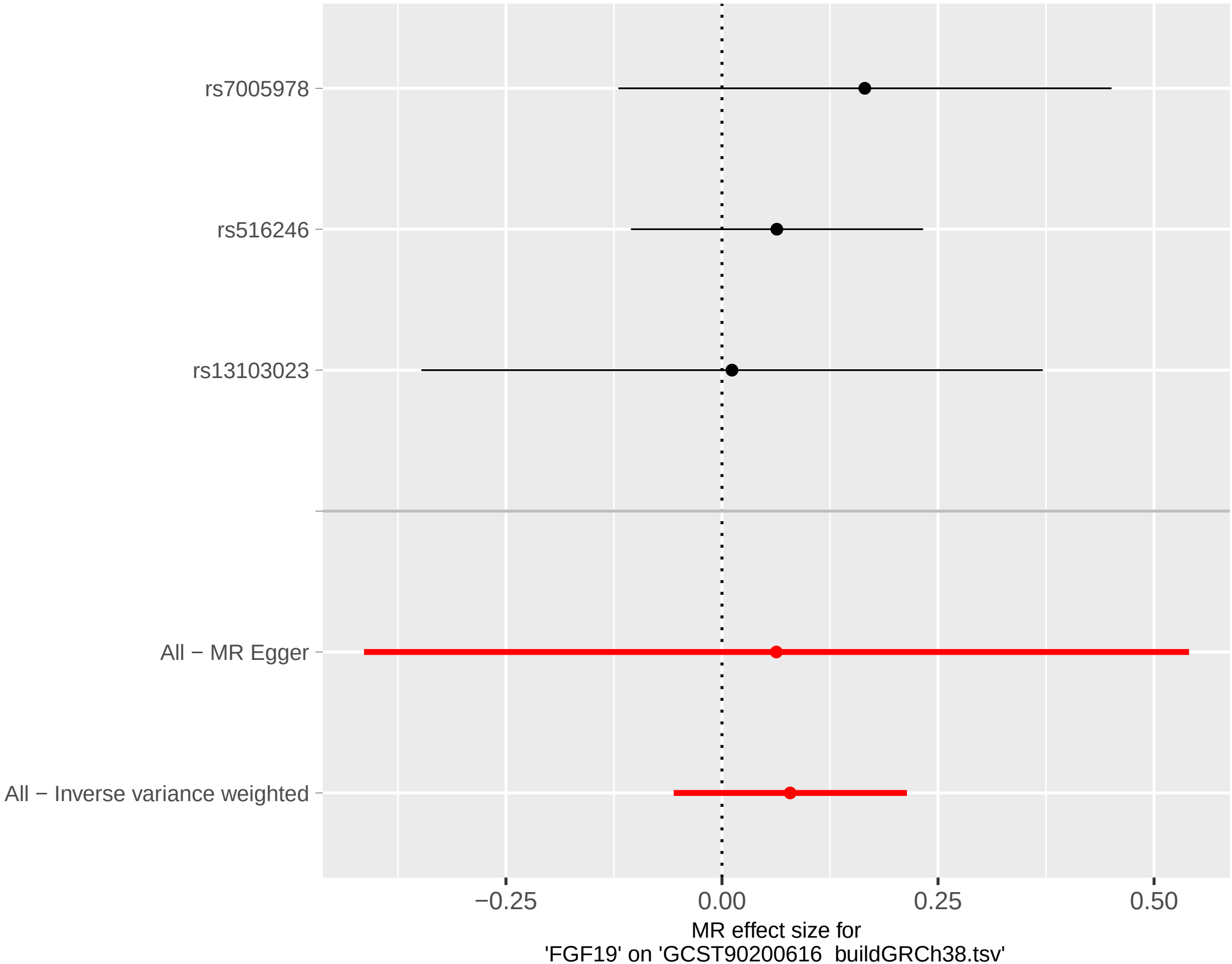
rs7005978
rs516246
rs13103023
All − MR Egger
All − Inverse variance weighted
−0.25
0.00
0.25
0.50
MR effect size for
'FGF19' on 'GCST90200616_buildGRCh38.tsv'

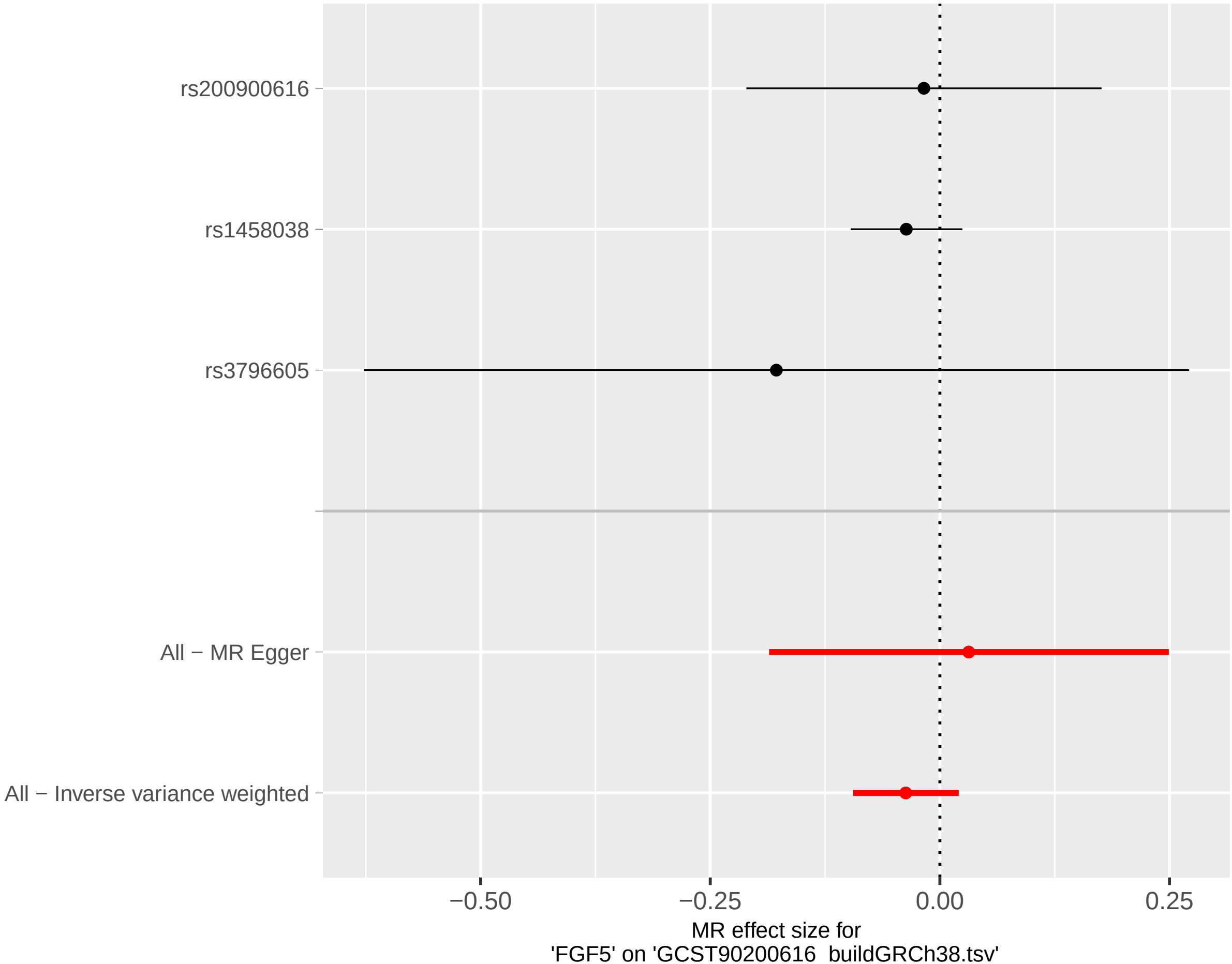
rs200900616
rs1458038
rs3796605
All − MR Egger
All − Inverse variance weighted
−0.50
−0.25
0.00
0.25
MR effect size for
'FGF5' on 'GCST90200616_buildGRCh38.tsv'

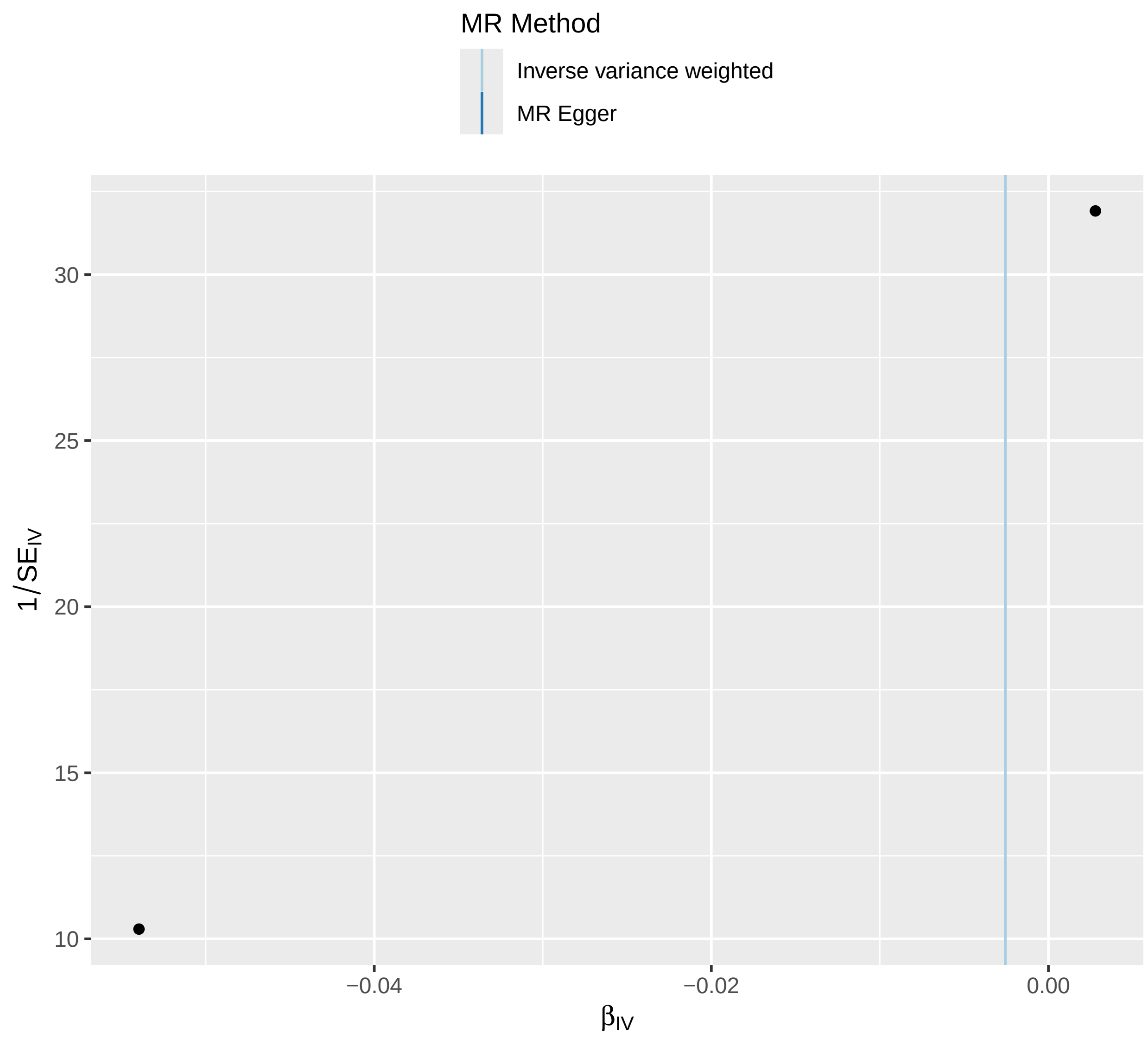
MR Method
Inverse variance weighted
MR Egger
30
25
20
15
10
1/SE_IV
−0.04
−0.02
0.00
β_IV

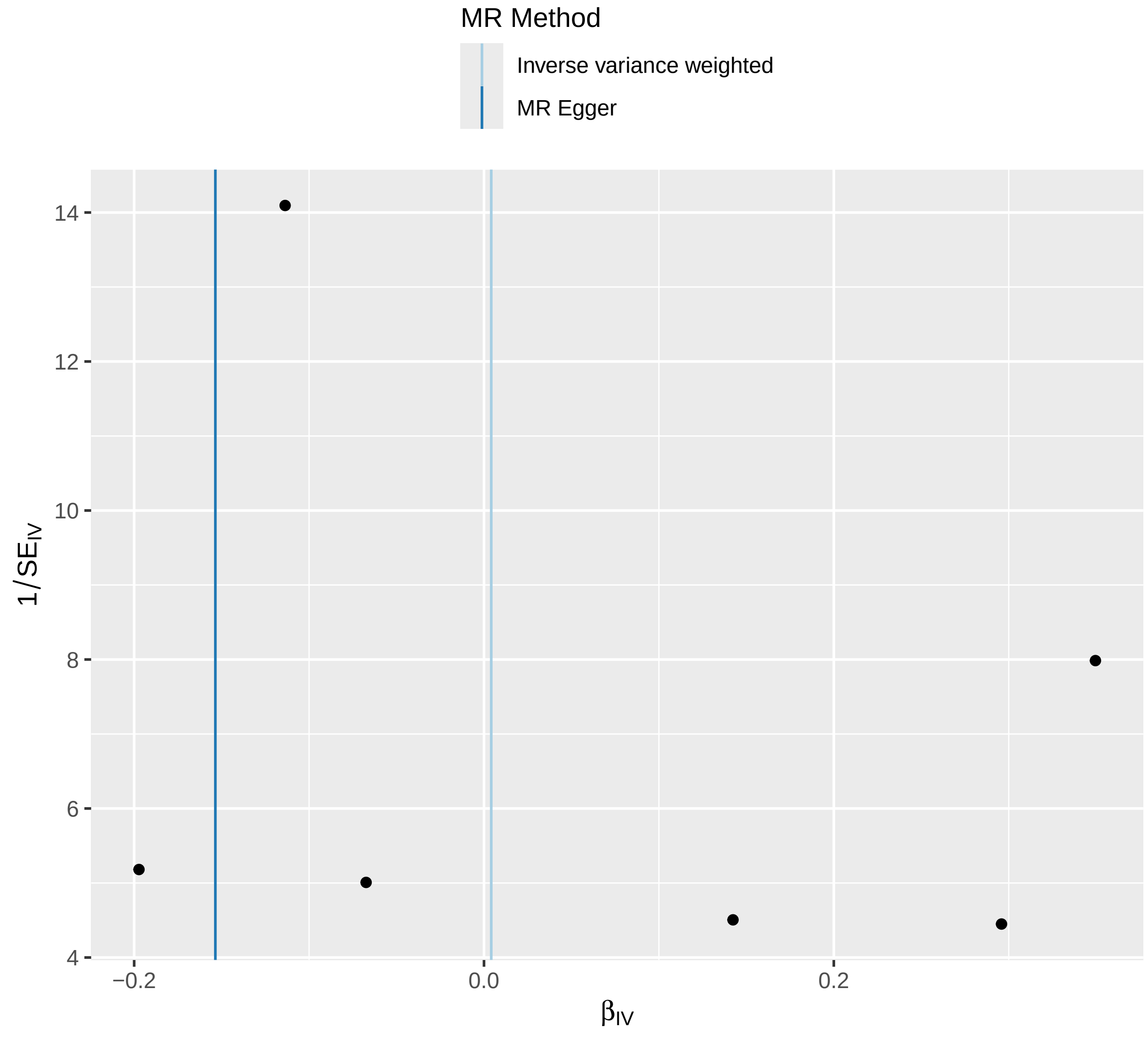
MR Method
Inverse variance weighted
MR Egger
14
12
10
8
6
4
1/SE$_{IV}$
−0.2
0.0
0.2
β_{IV}

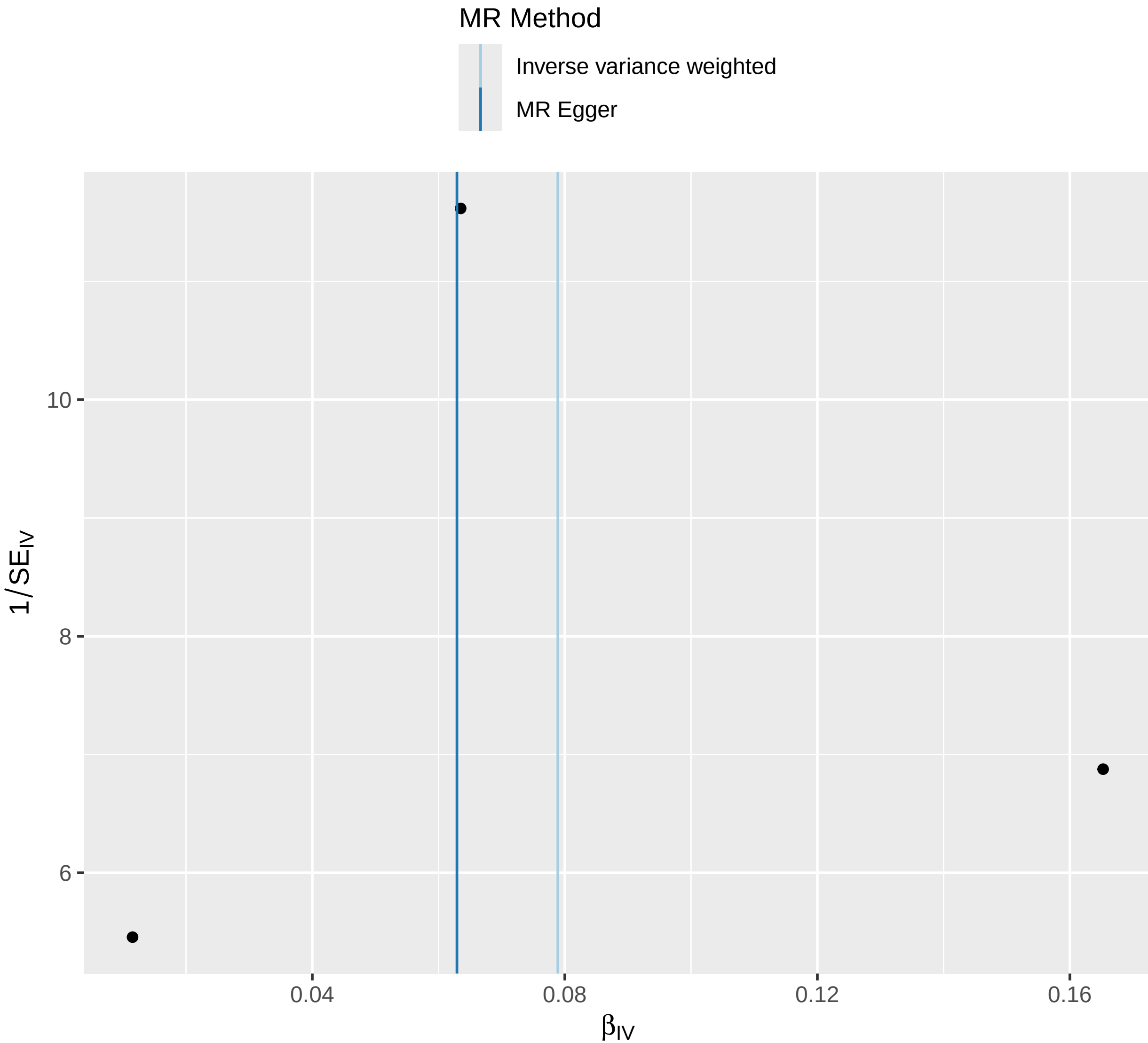
MR Method
Inverse variance weighted
MR Egger
10
8
6
1/SE_IV
0.04
0.08
0.12
0.16
β_IV

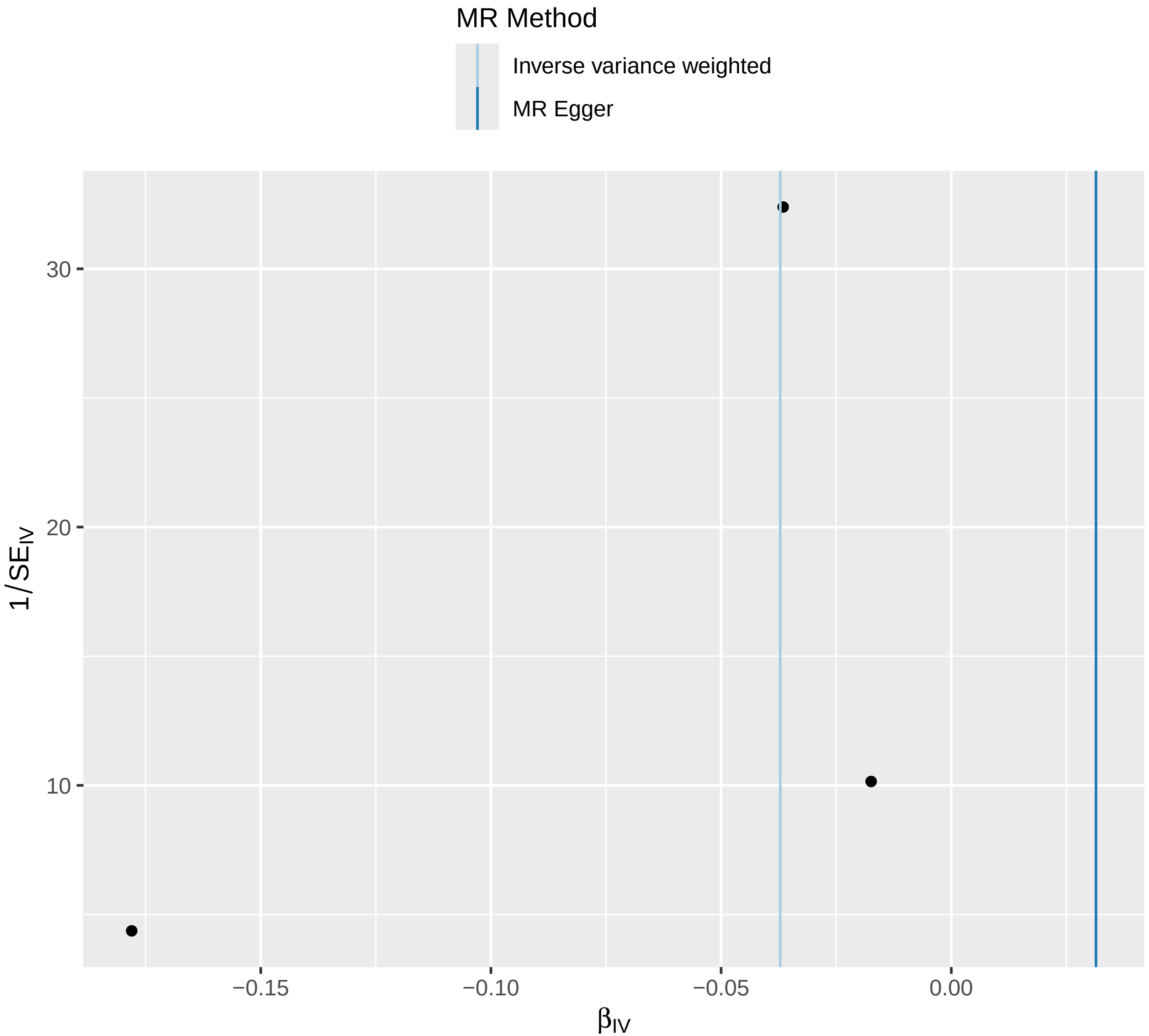
MR Method
Inverse variance weighted
MR Egger
30
20
10
1/SE$_{IV}$
−0.15
−0.10
−0.05
0.00
β$_{IV}$

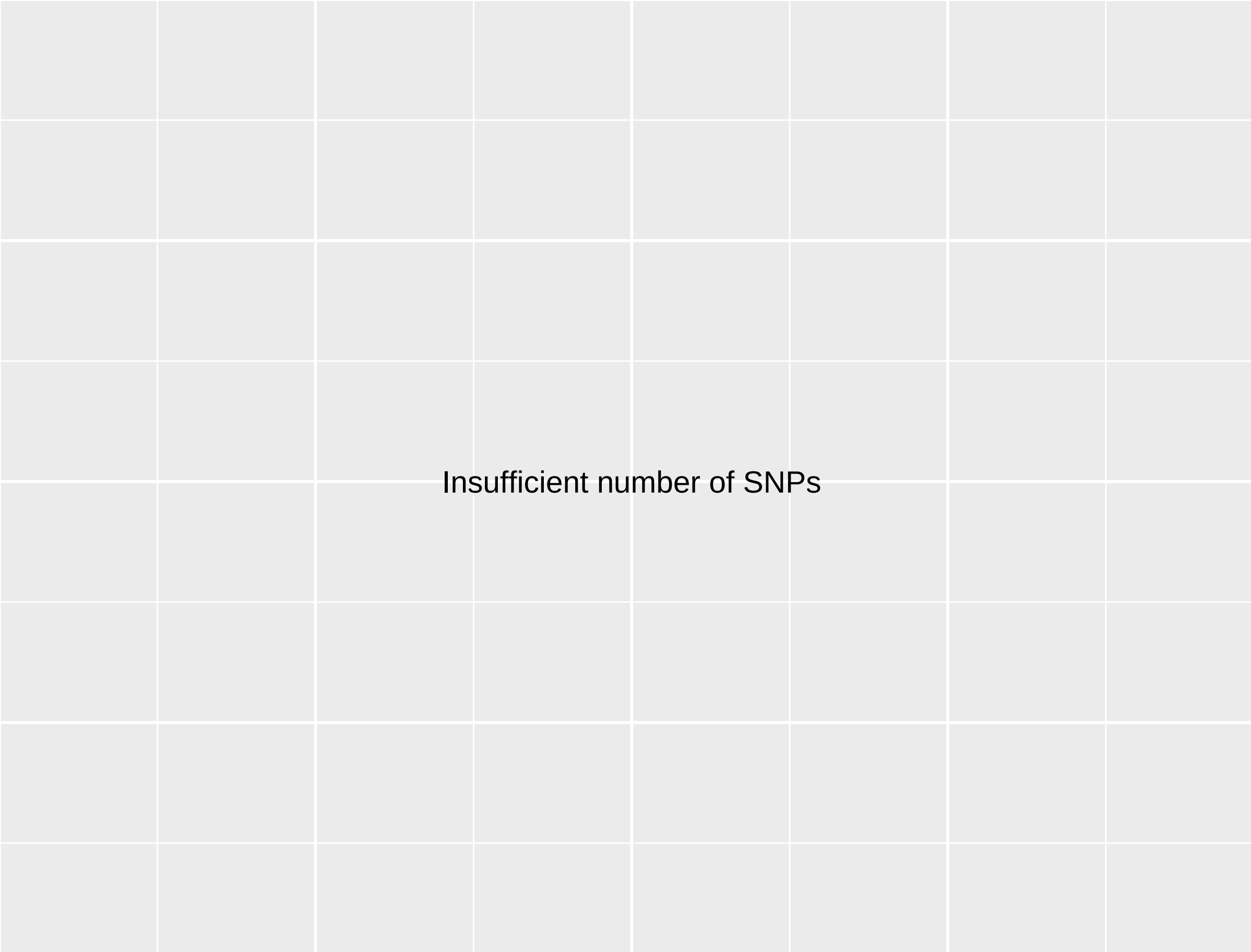
Insufficient number of SNPs

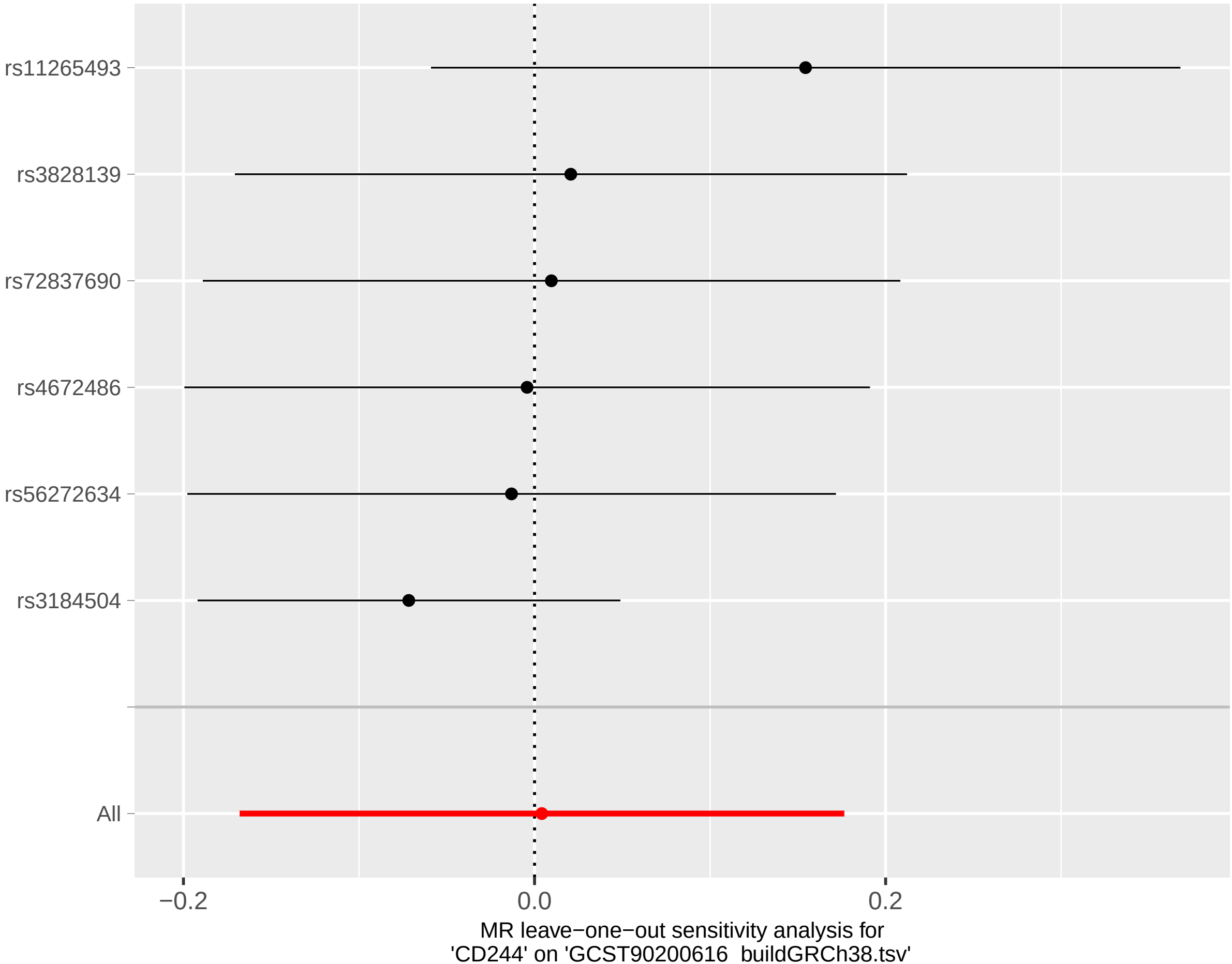
rs11265493
rs3828139
rs72837690
rs4672486
rs56272634
rs3184504
All
−0.2
0.0
0.2
MR leave−one−out sensitivity analysis for
'CD244' on 'GCST90200616_buildGRCh38.tsv'

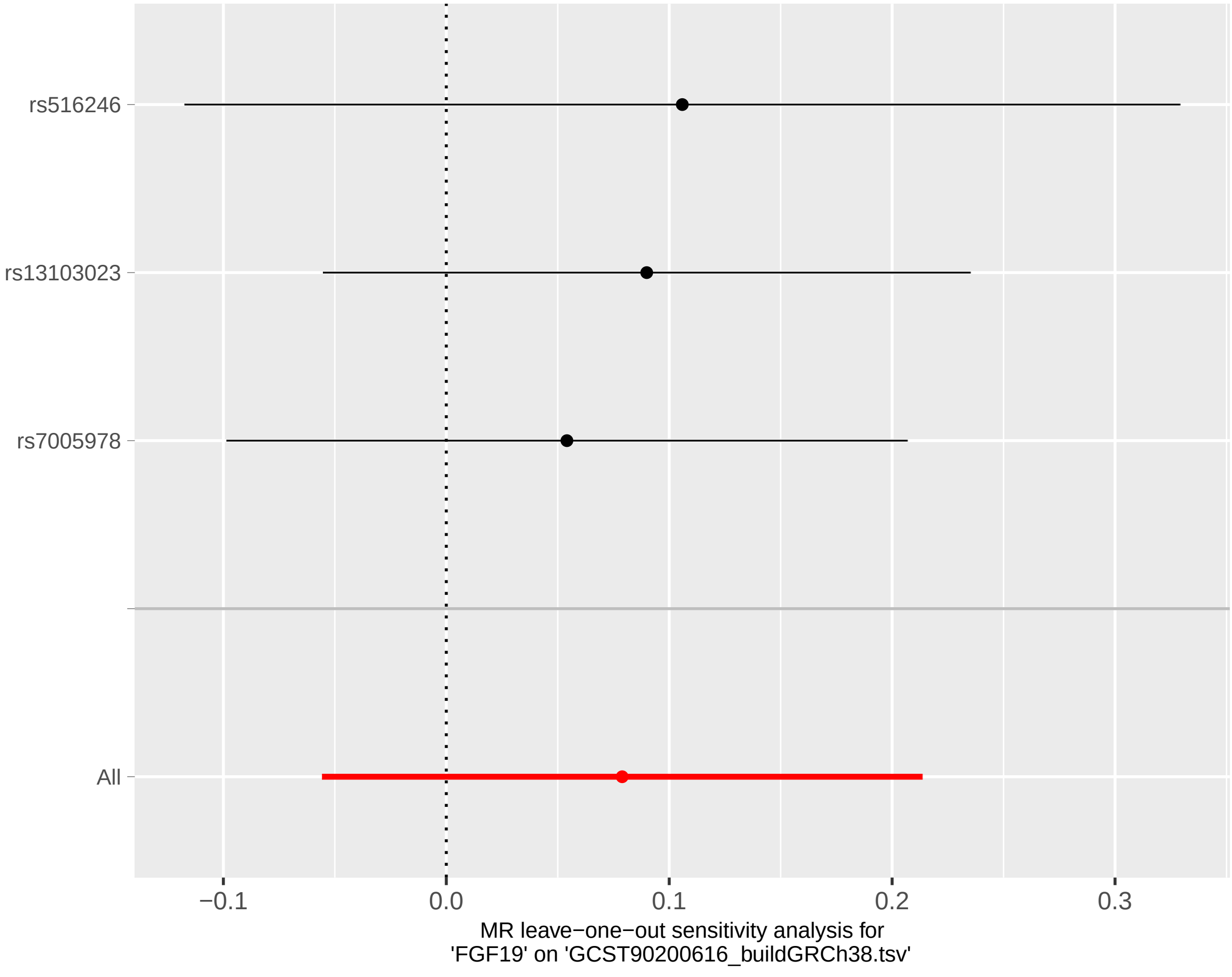
rs516246
rs13103023
rs7005978
All
−0.1
0.0
0.1
0.2
0.3
MR leave−one−out sensitivity analysis for
'FGF19' on 'GCST90200616_buildGRCh38.tsv'

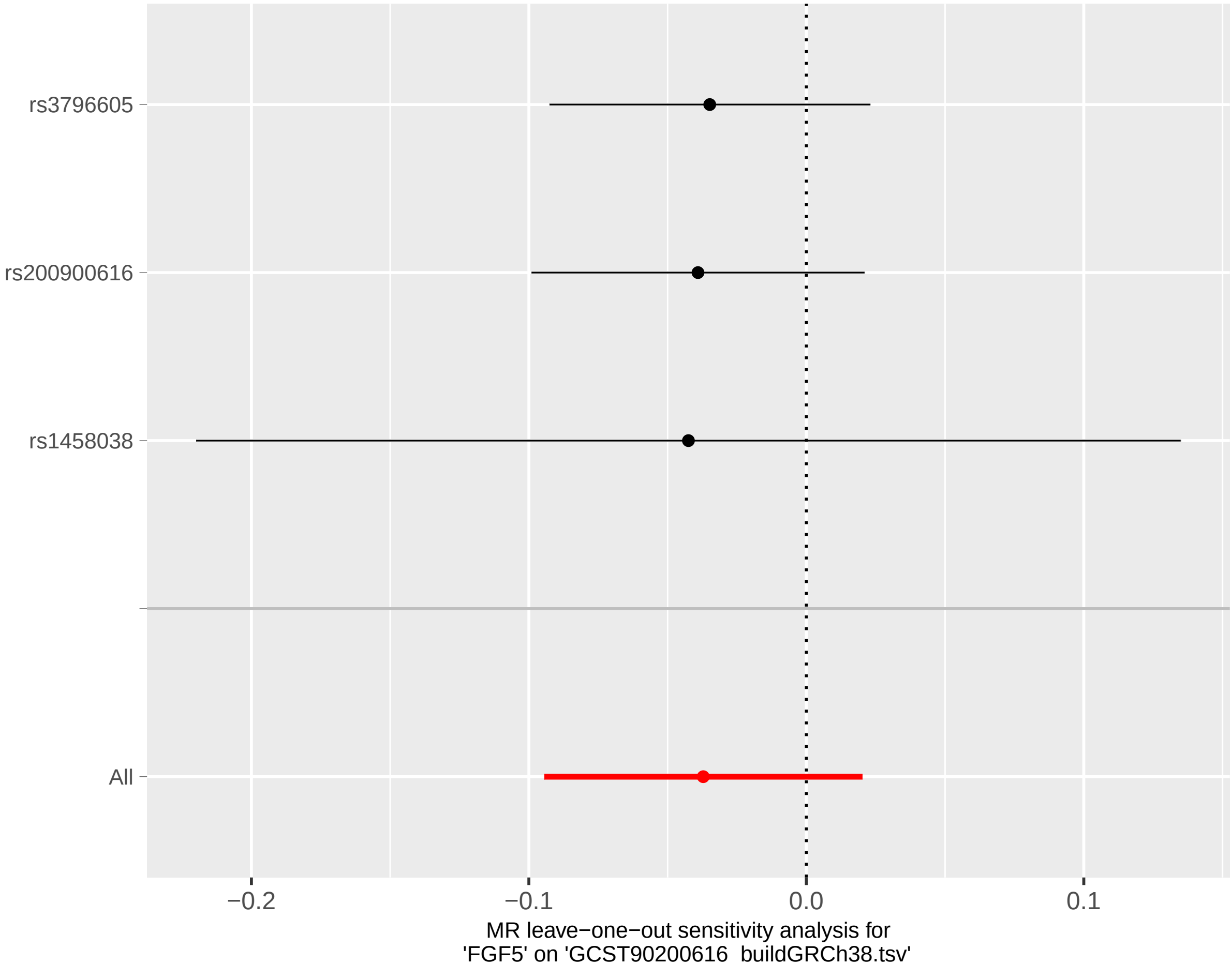
rs3796605
rs200900616
rs1458038
All
−0.2
−0.1
0.0
0.1
MR leave−one−out sensitivity analysis for
'FGF5' on 'GCST90200616_buildGRCh38.tsv'

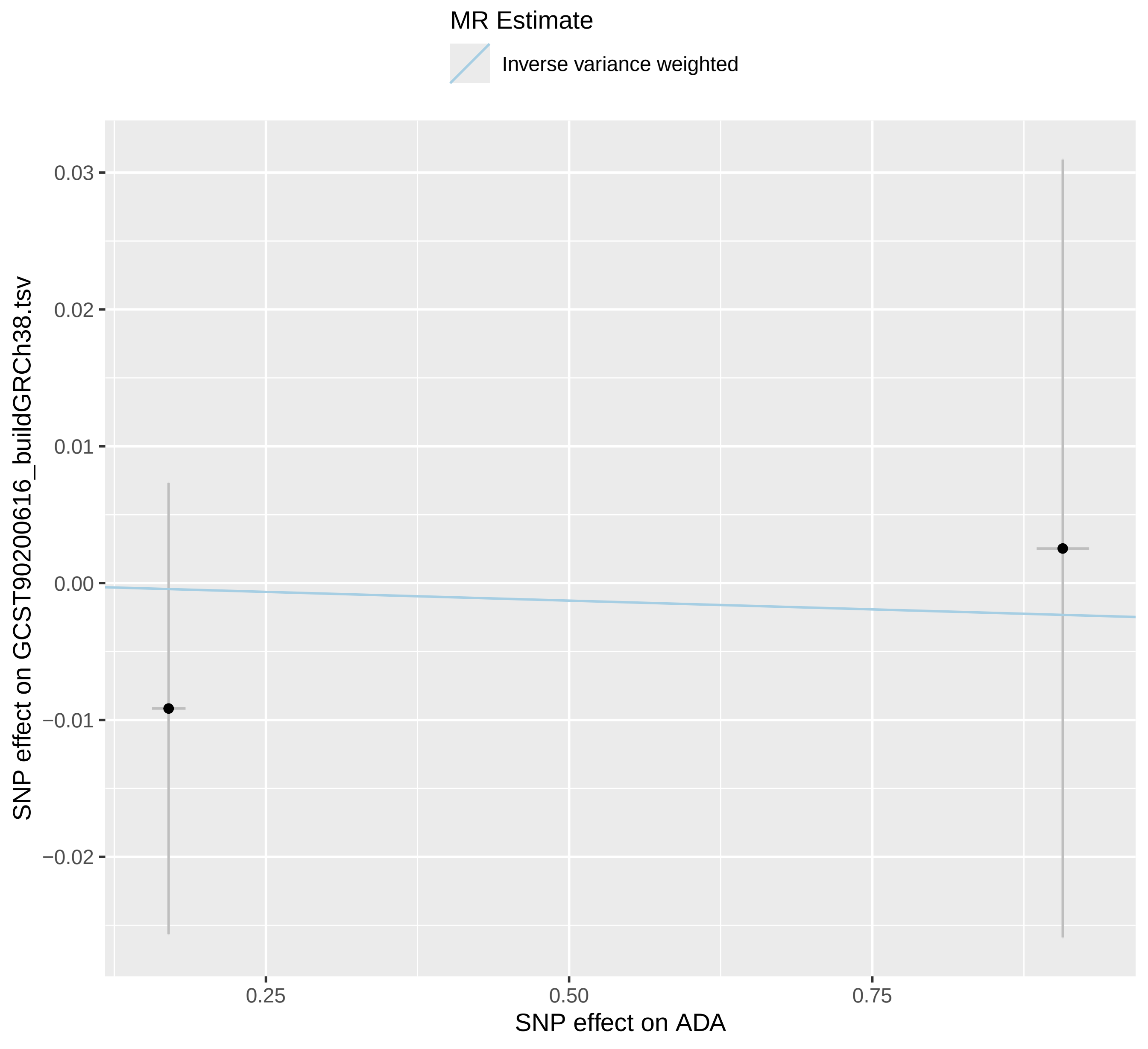

MR Estimate
Inverse variance weighted
0.03
0.02
0.01
0.00
−0.01
−0.02
0.25
0.50
0.75
SNP effect on ADA
SNP effect on GCST90200616_buildGRCh38.tsv

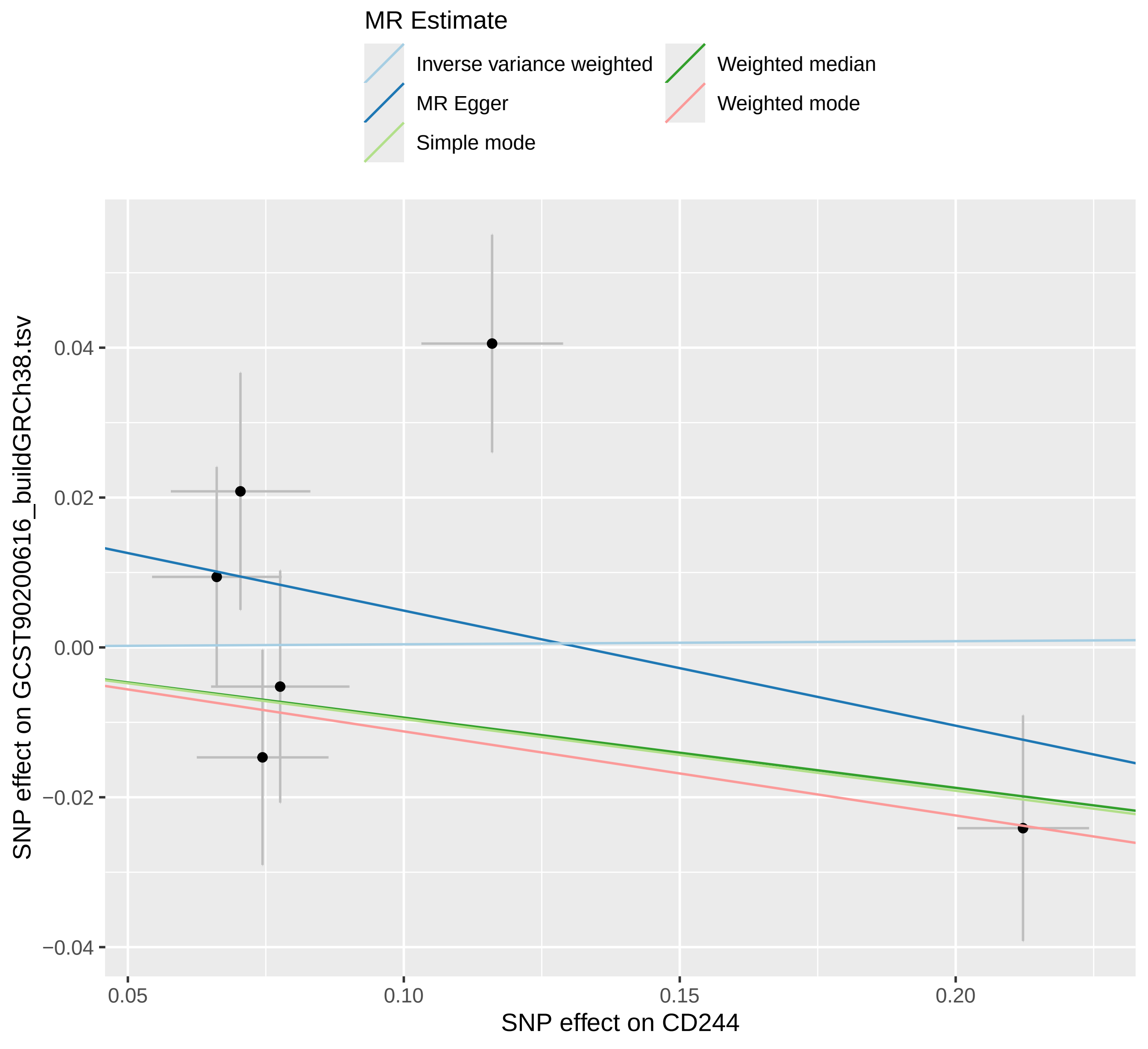
MR Estimate
Inverse variance weighted
MR Egger
Simple mode
Weighted median
Weighted mode
0.04
0.02
0.00
−0.02
−0.04
0.05
0.10
0.15
0.20
SNP effect on GCST90200616_buildGRCh38.tsv
SNP effect on CD244

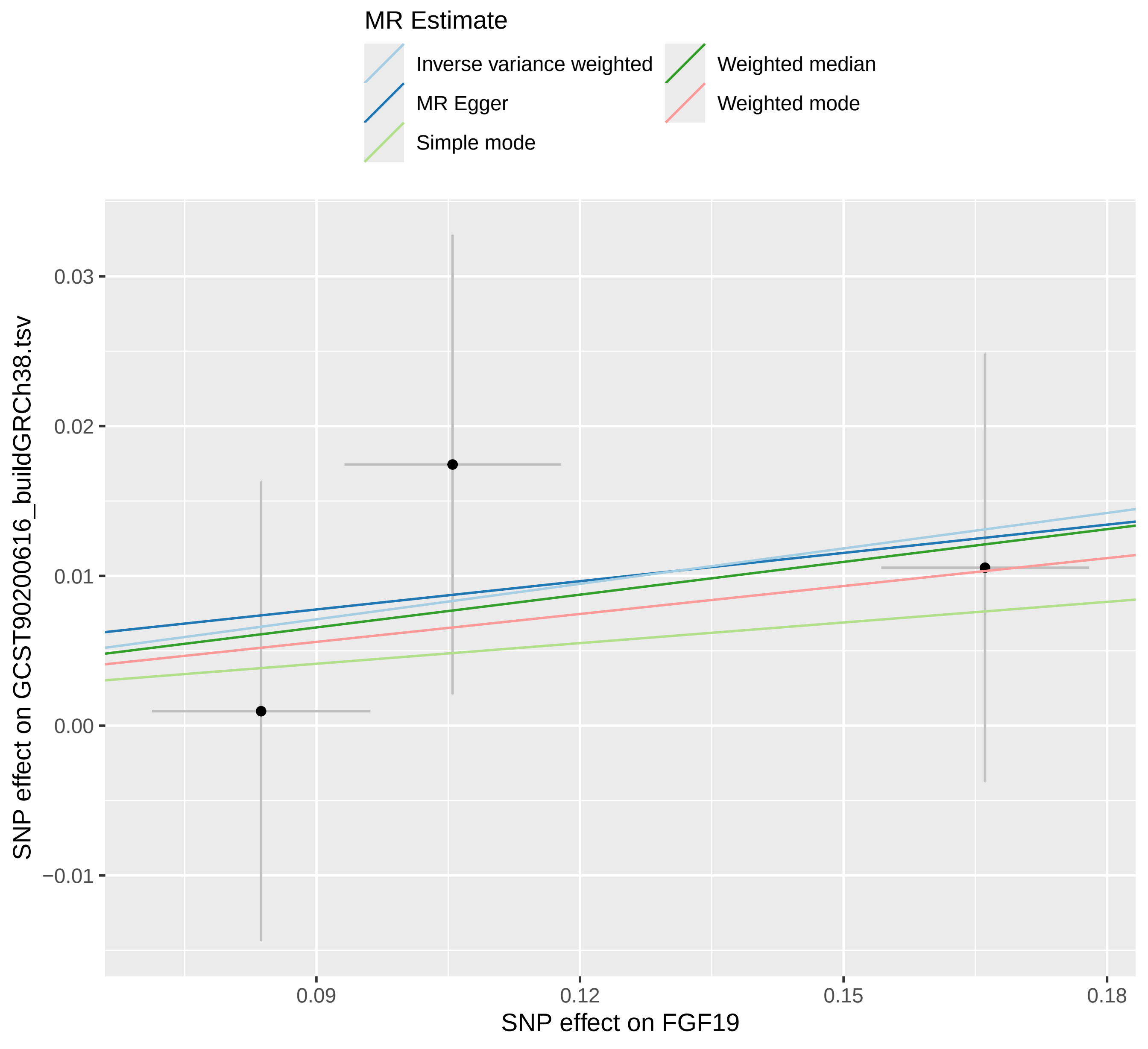
MR Estimate
Inverse variance weighted
MR Egger
Simple mode
Weighted median
Weighted mode
0.03
0.02
0.01
0.00
−0.01
SNP effect on GCST90200616_buildGRCh38.tsv
0.09
0.12
0.15
0.18
SNP effect on FGF19

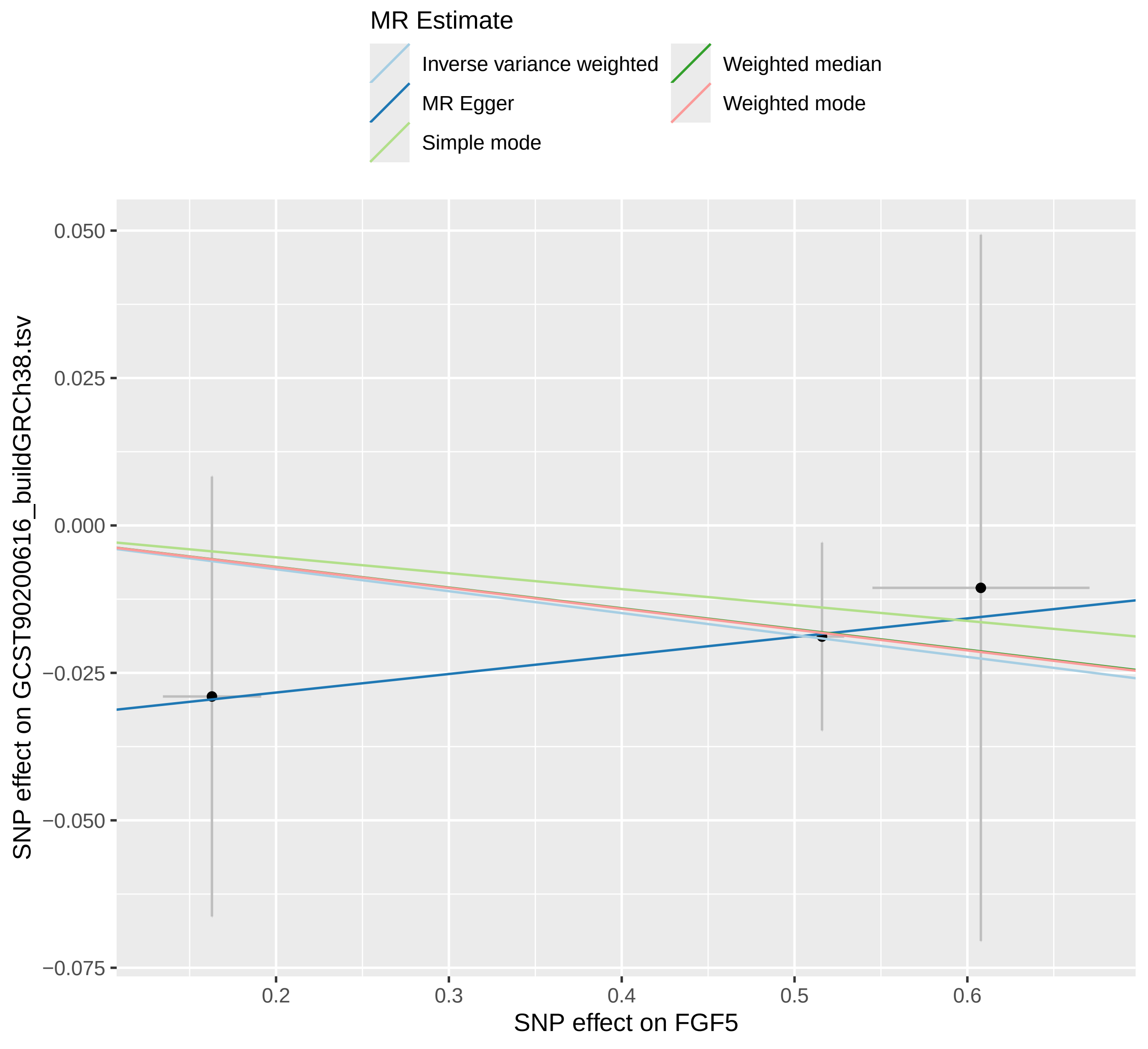
MR Estimate
Inverse variance weighted
MR Egger
Simple mode
Weighted median
Weighted mode
0.050
0.025
0.000
−0.025
−0.050
−0.075
SNP effect on GCST90200616_buildGRCh38.tsv
0.2
0.3
0.4
0.5
0.6
SNP effect on FGF5

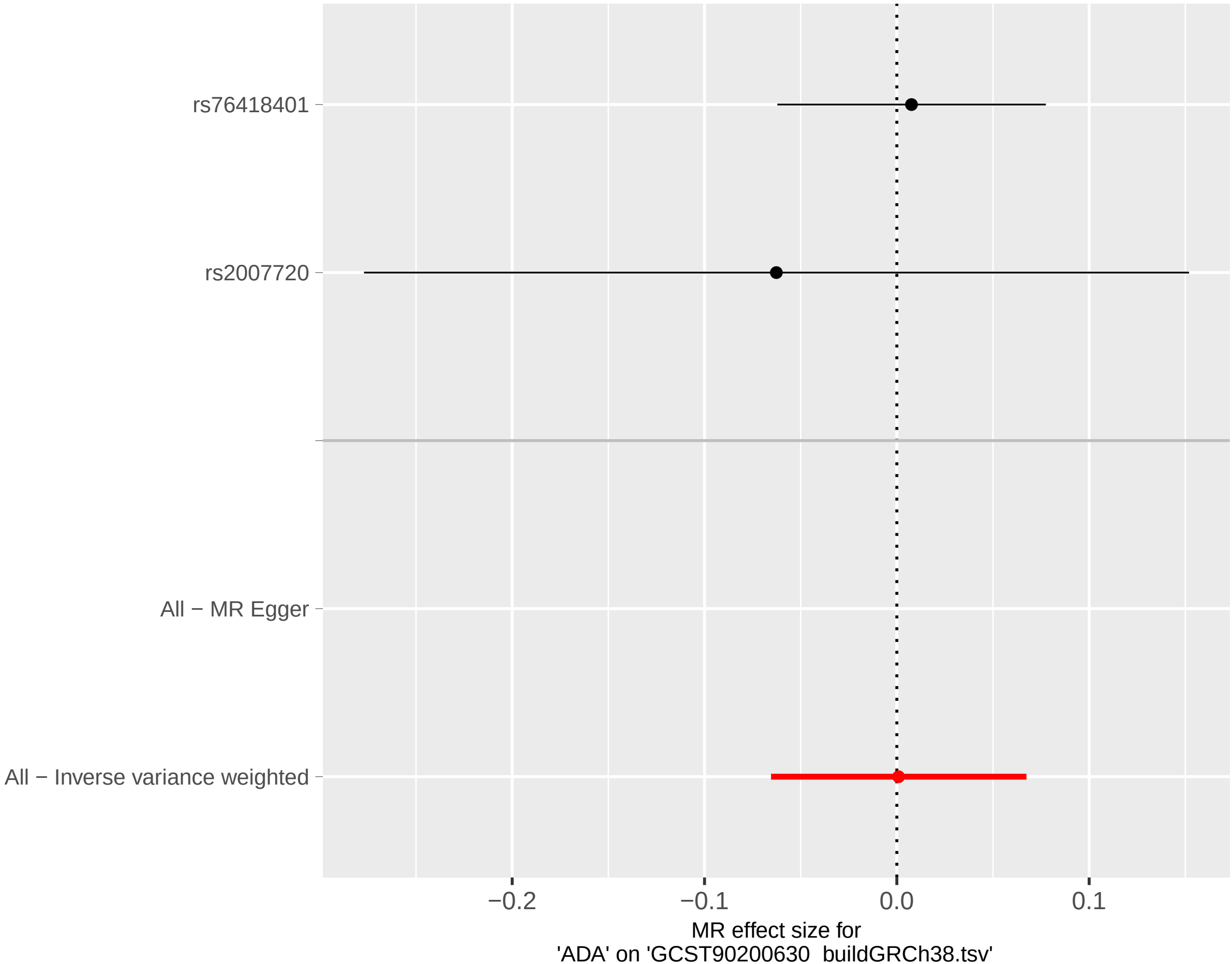
rs76418401
rs2007720
All − MR Egger
All − Inverse variance weighted
−0.2
−0.1
0.0
0.1
MR effect size for
'ADA' on 'GCST90200630_buildGRCh38.tsv'

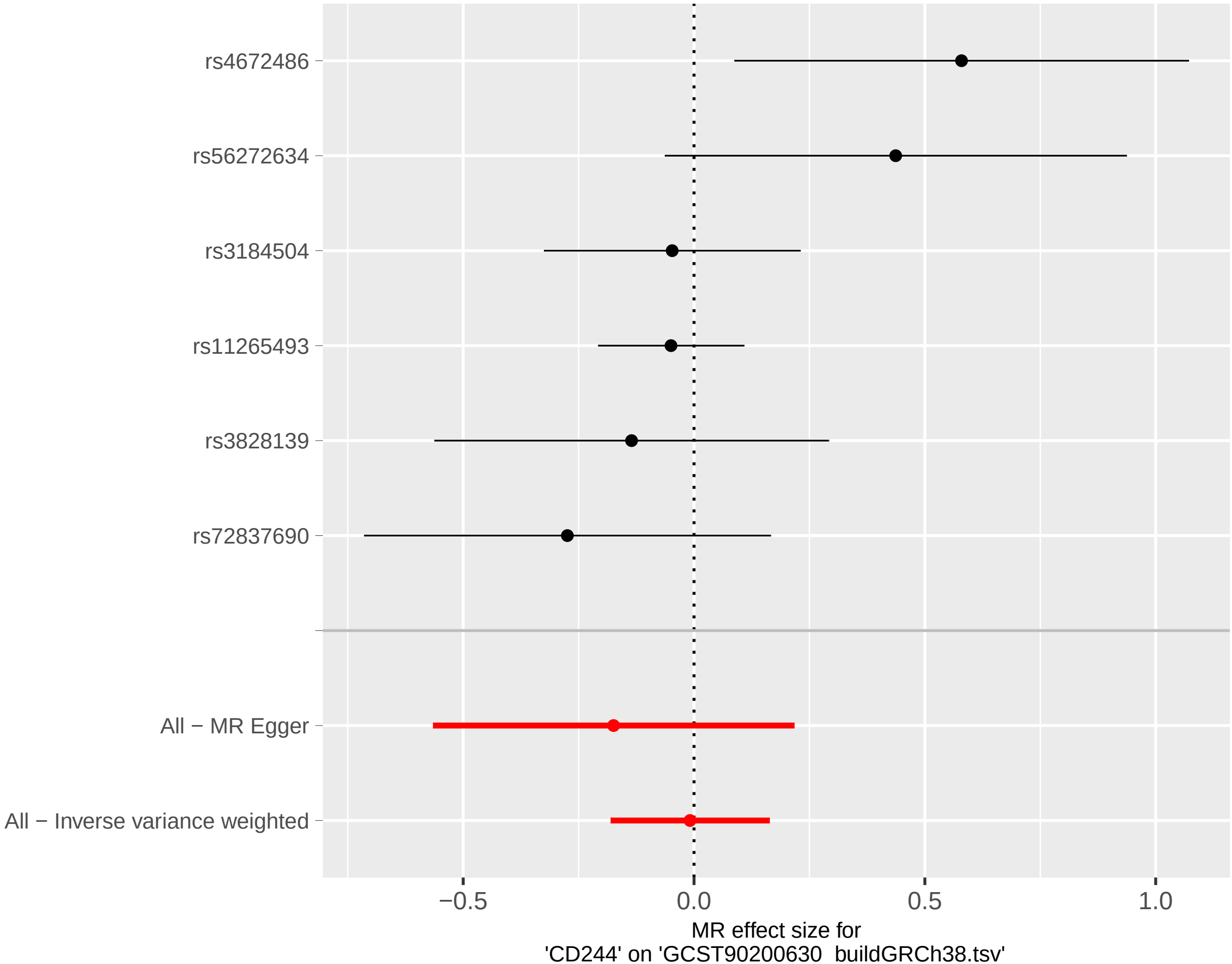
rs4672486
rs56272634
rs3184504
rs11265493
rs3828139
rs72837690
All − MR Egger
All − Inverse variance weighted
−0.5
0.0
0.5
1.0
MR effect size for
'CD244' on 'GCST90200630_buildGRCh38.tsv'

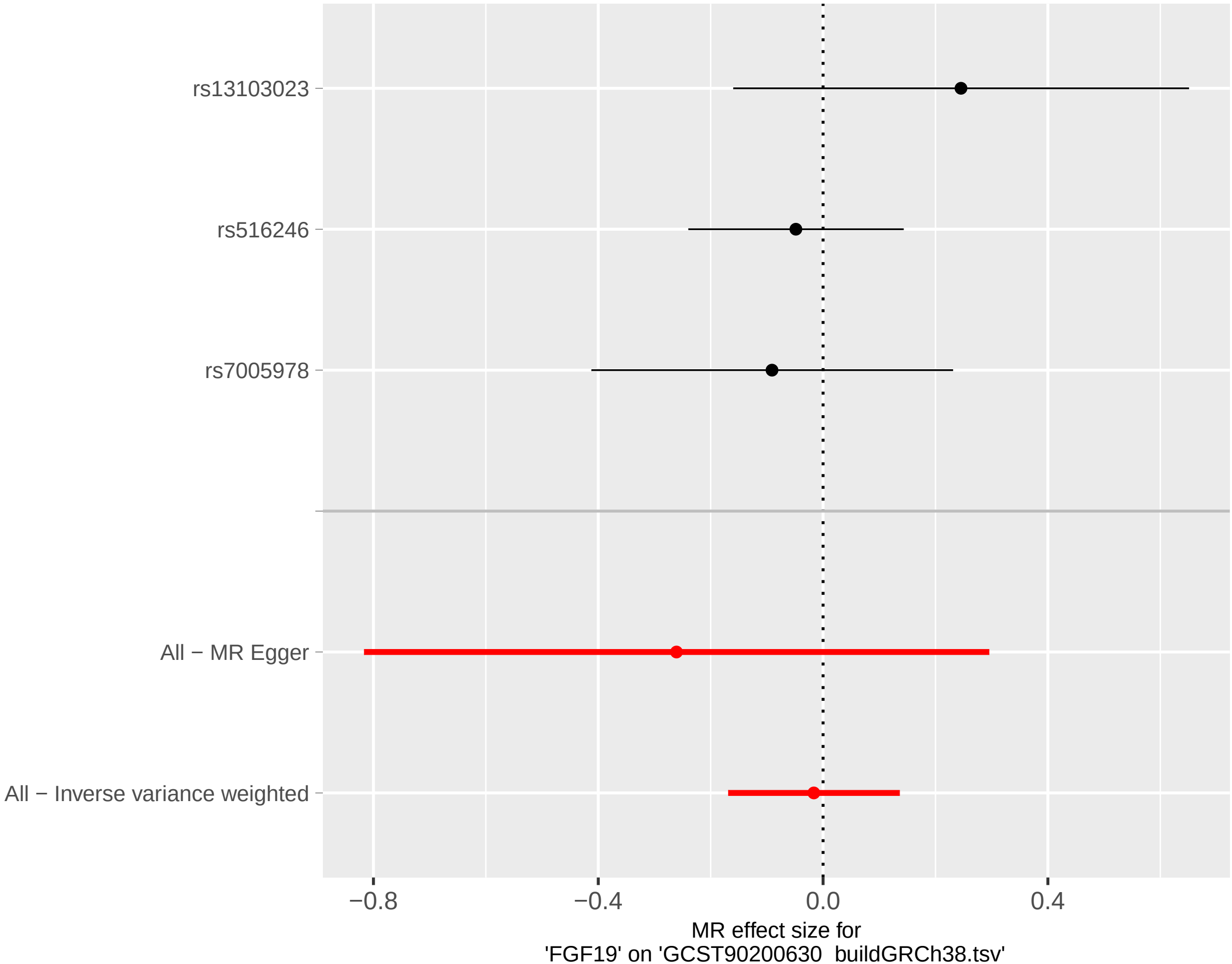
rs13103023
rs516246
rs7005978
All − MR Egger
All − Inverse variance weighted
−0.8
−0.4
0.0
0.4
MR effect size for
'FGF19' on 'GCST90200630_buildGRCh38.tsv'

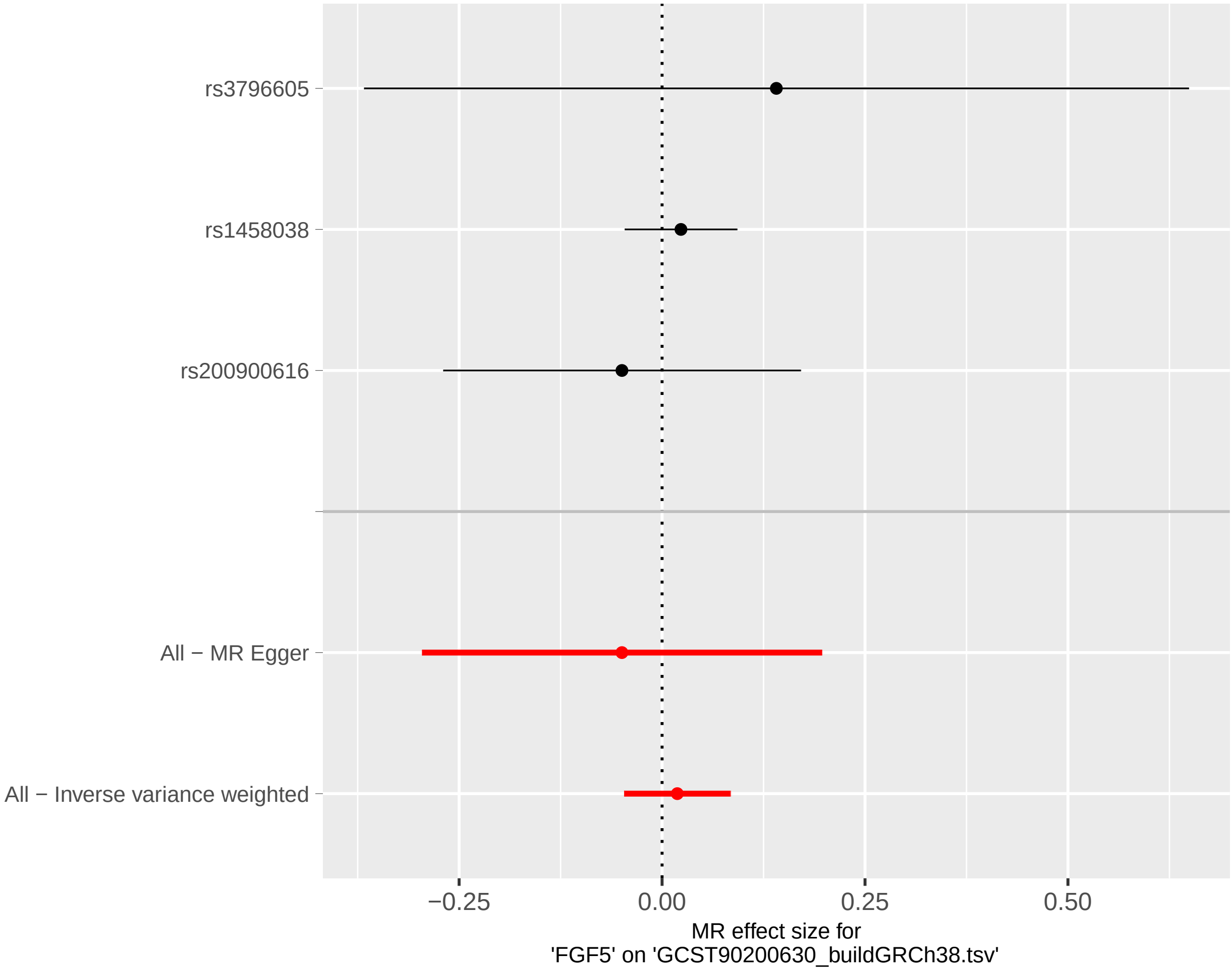
rs3796605
rs1458038
rs200900616
All − MR Egger
All − Inverse variance weighted
−0.25
0.00
0.25
0.50
MR effect size for
'FGF5' on 'GCST90200630_buildGRCh38.tsv'

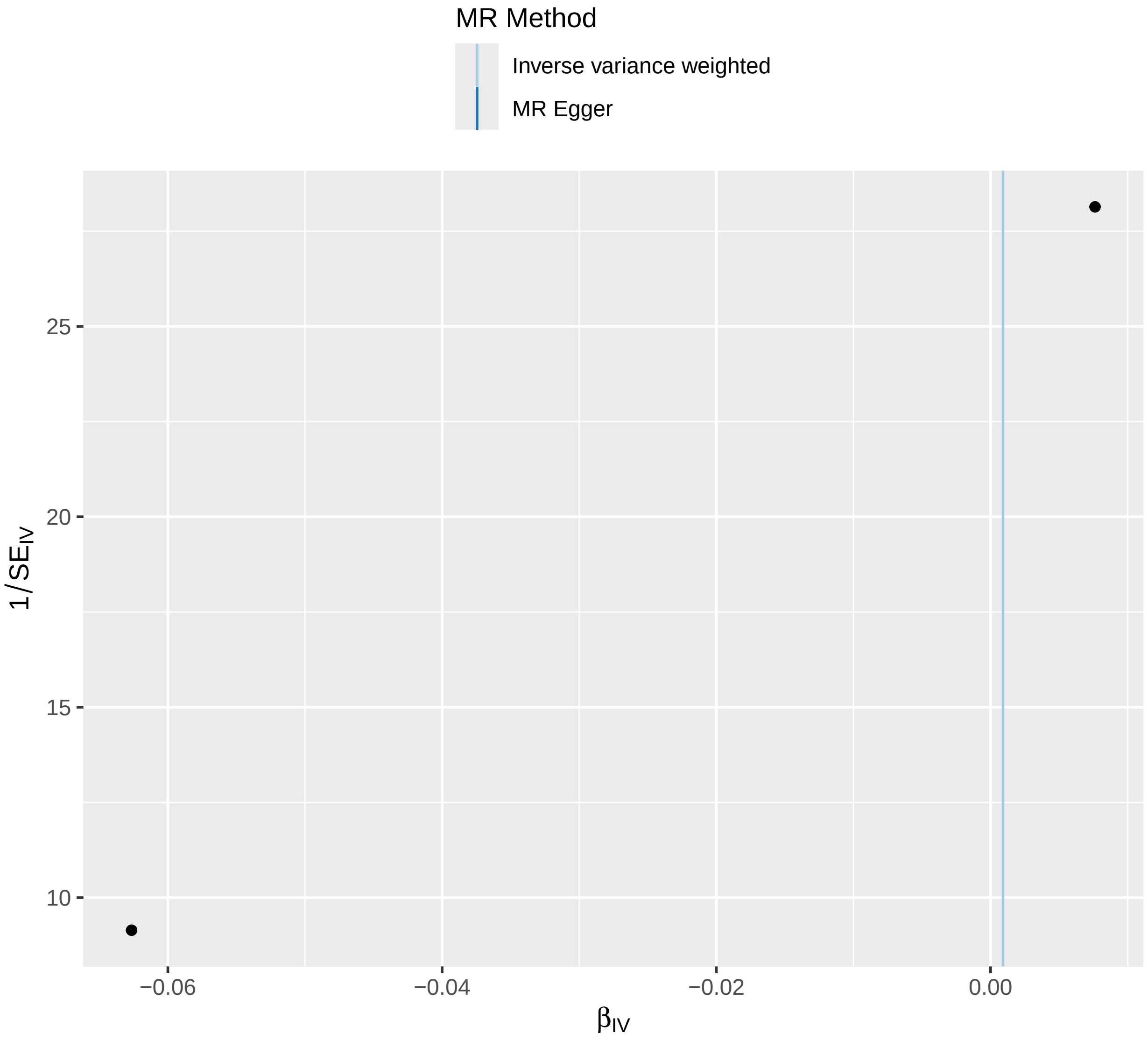

MR Method
Inverse variance weighted
MR Egger
25
20
15
10
1/SE_IV
−0.06
−0.04
−0.02
0.00
β_IV

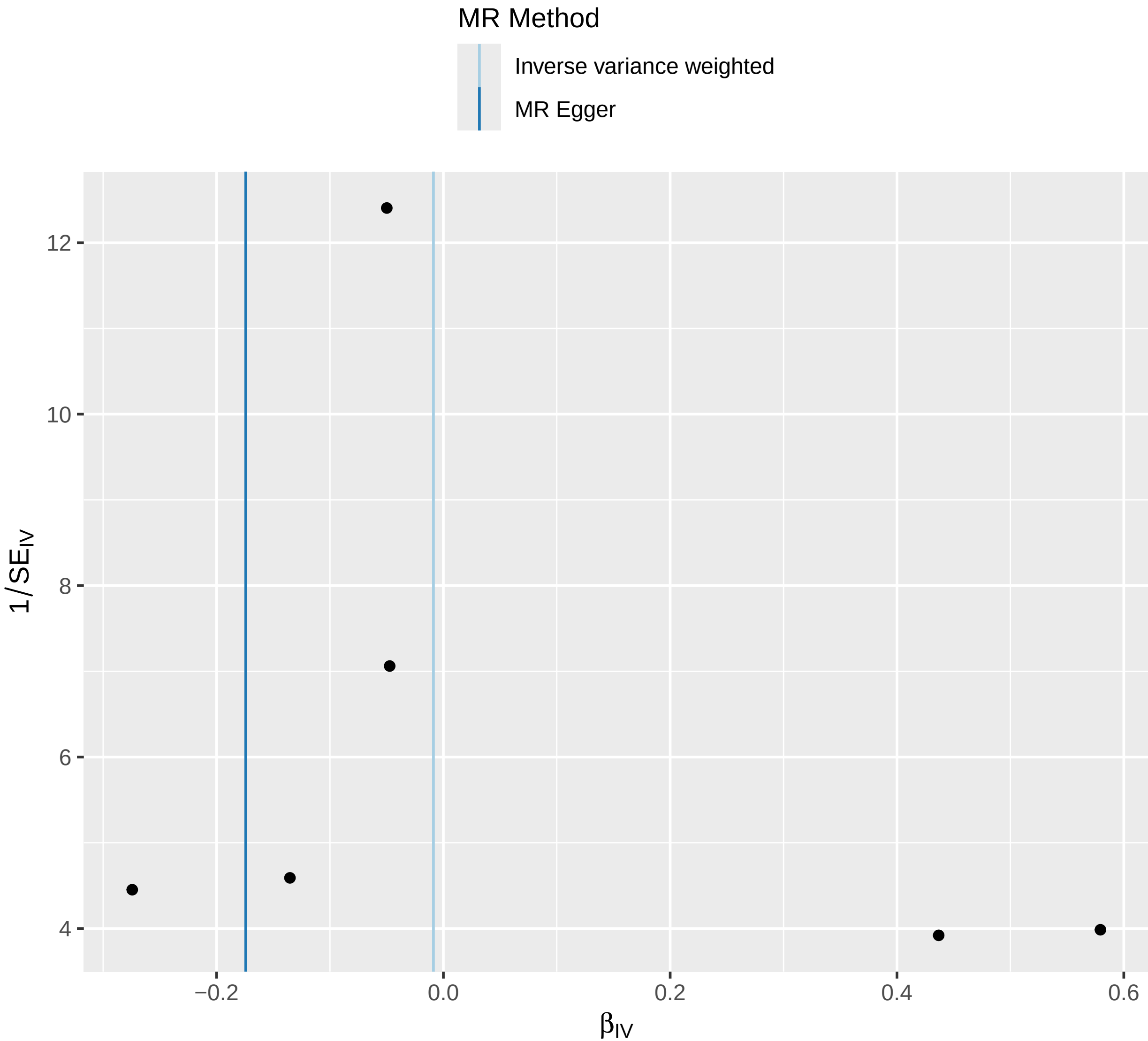

MR Method
Inverse variance weighted
MR Egger
12
10
8
6
4
$1/SE_{IV}$
−0.2
0.0
0.2
0.4
0.6
β_{IV}

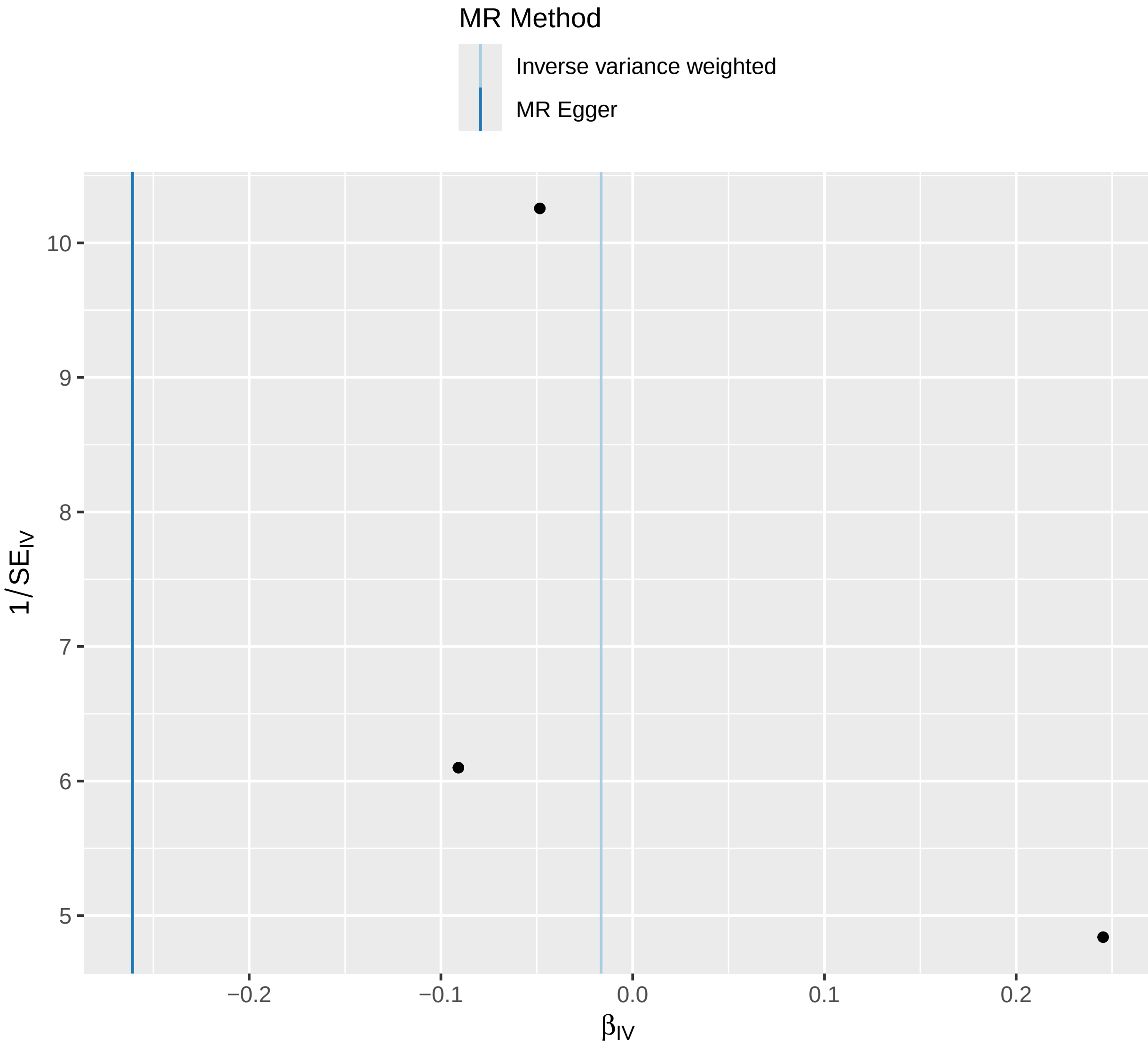
MR Method
Inverse variance weighted
MR Egger
10
9
8
7
6
5
$1/SE_{IV}$
−0.2
−0.1
0.0
0.1
0.2
β_{IV}

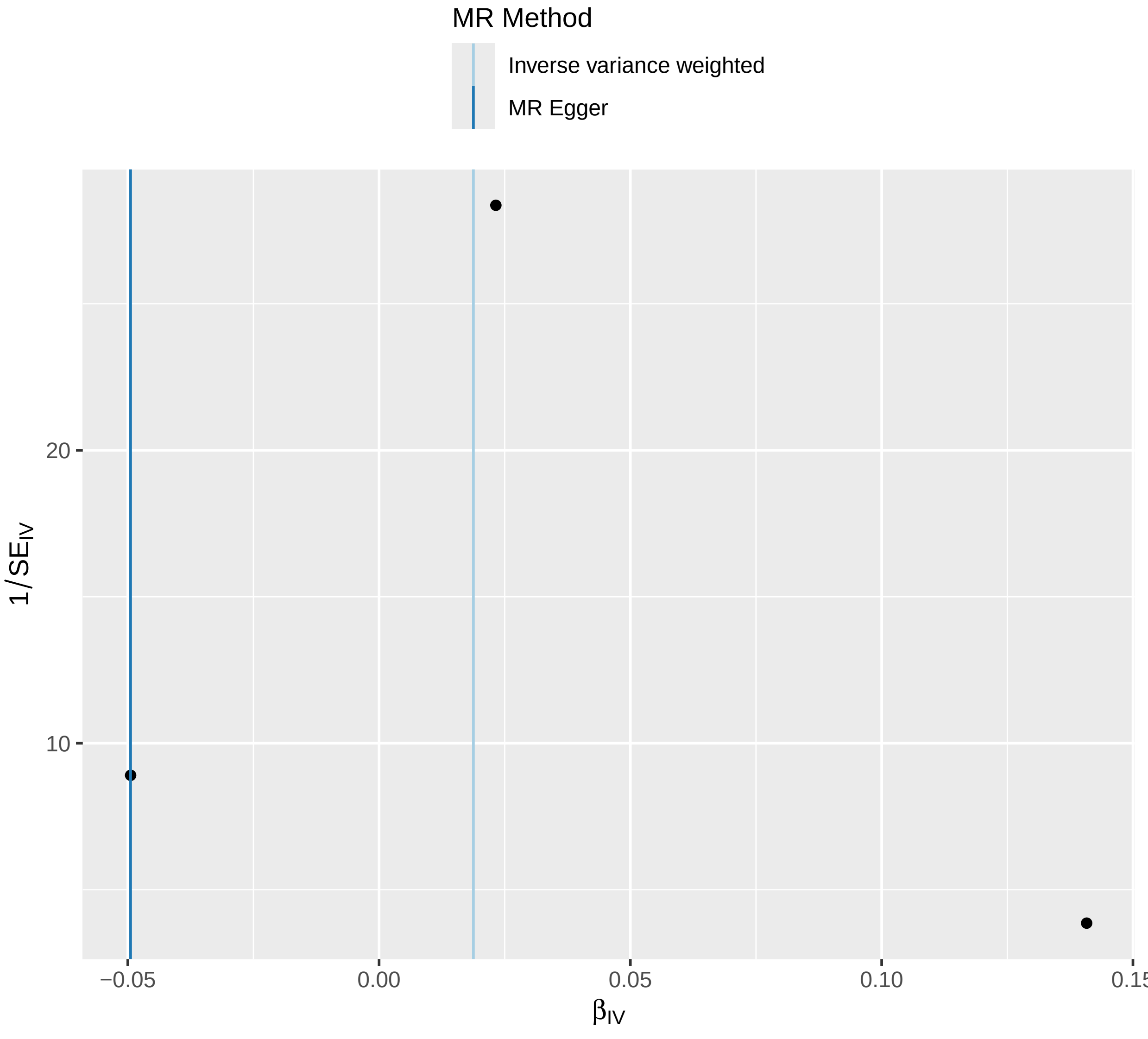
MR Method
Inverse variance weighted
MR Egger
20
10
1/SE$_{IV}$
−0.05
0.00
0.05
0.10
0.15
β$_{IV}$

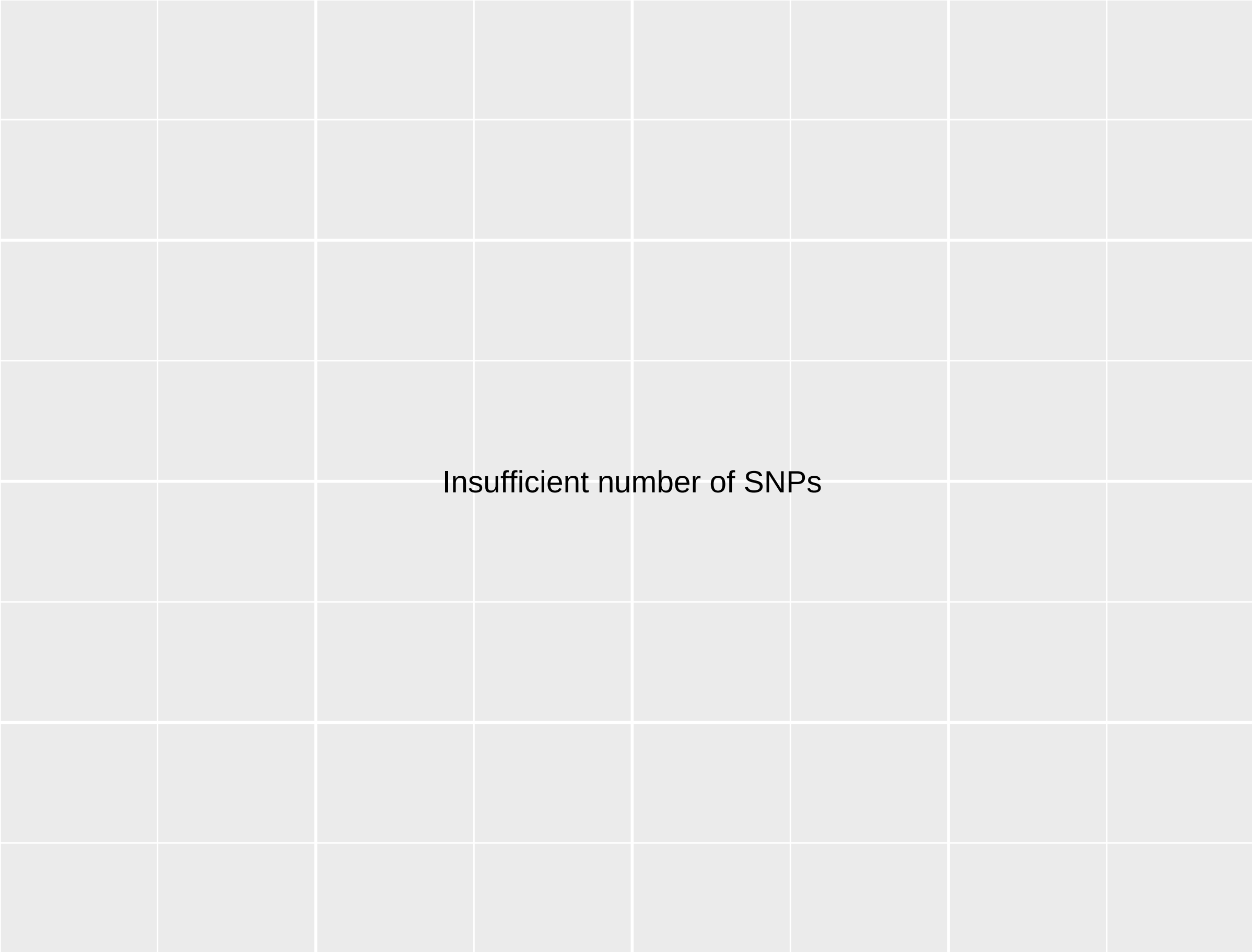
Insufficient number of SNPs

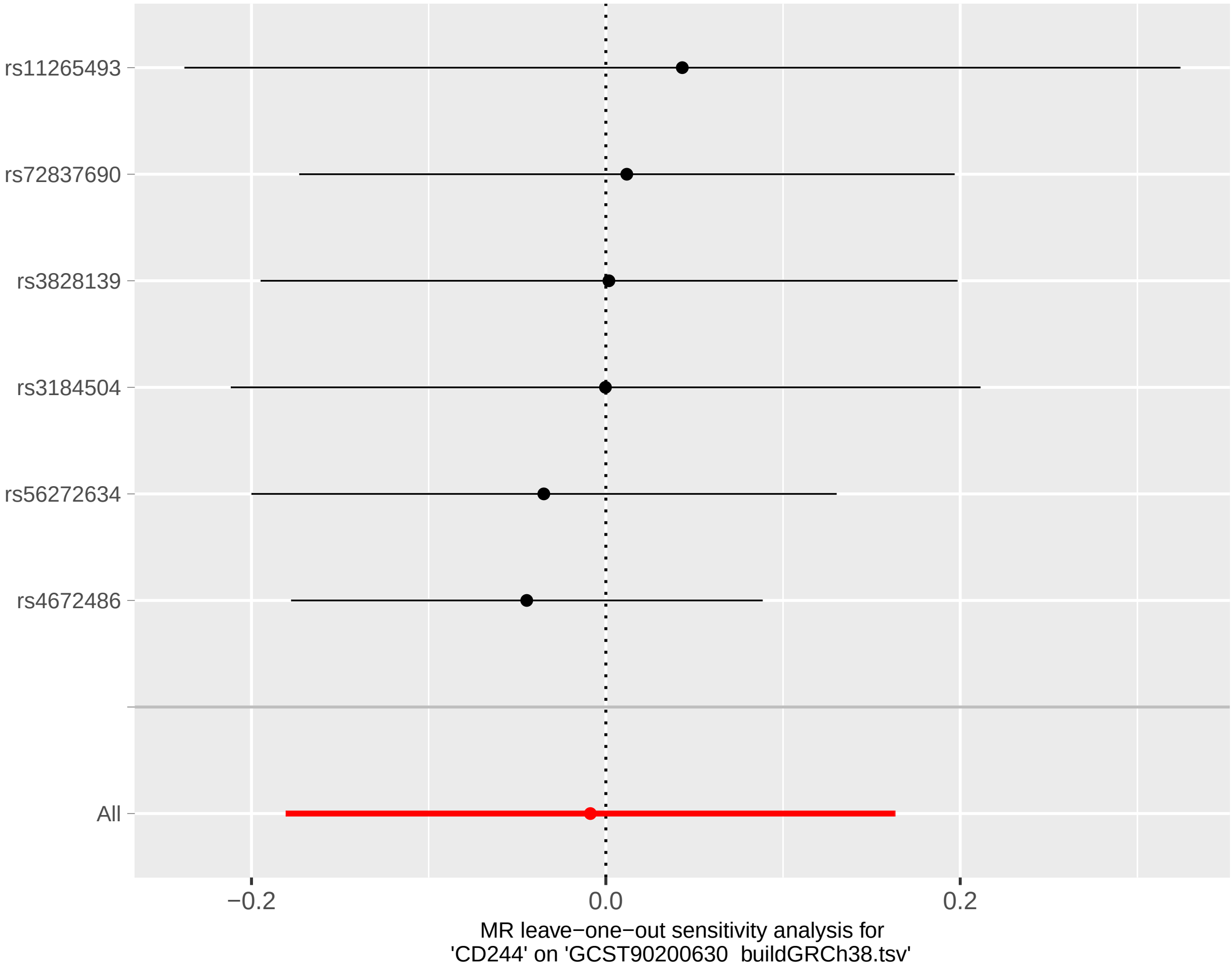

rs11265493
rs72837690
rs3828139
rs3184504
rs56272634
rs4672486
All
−0.2
0.0
0.2
MR leave−one−out sensitivity analysis for
'CD244' on 'GCST90200630_buildGRCh38.tsv'

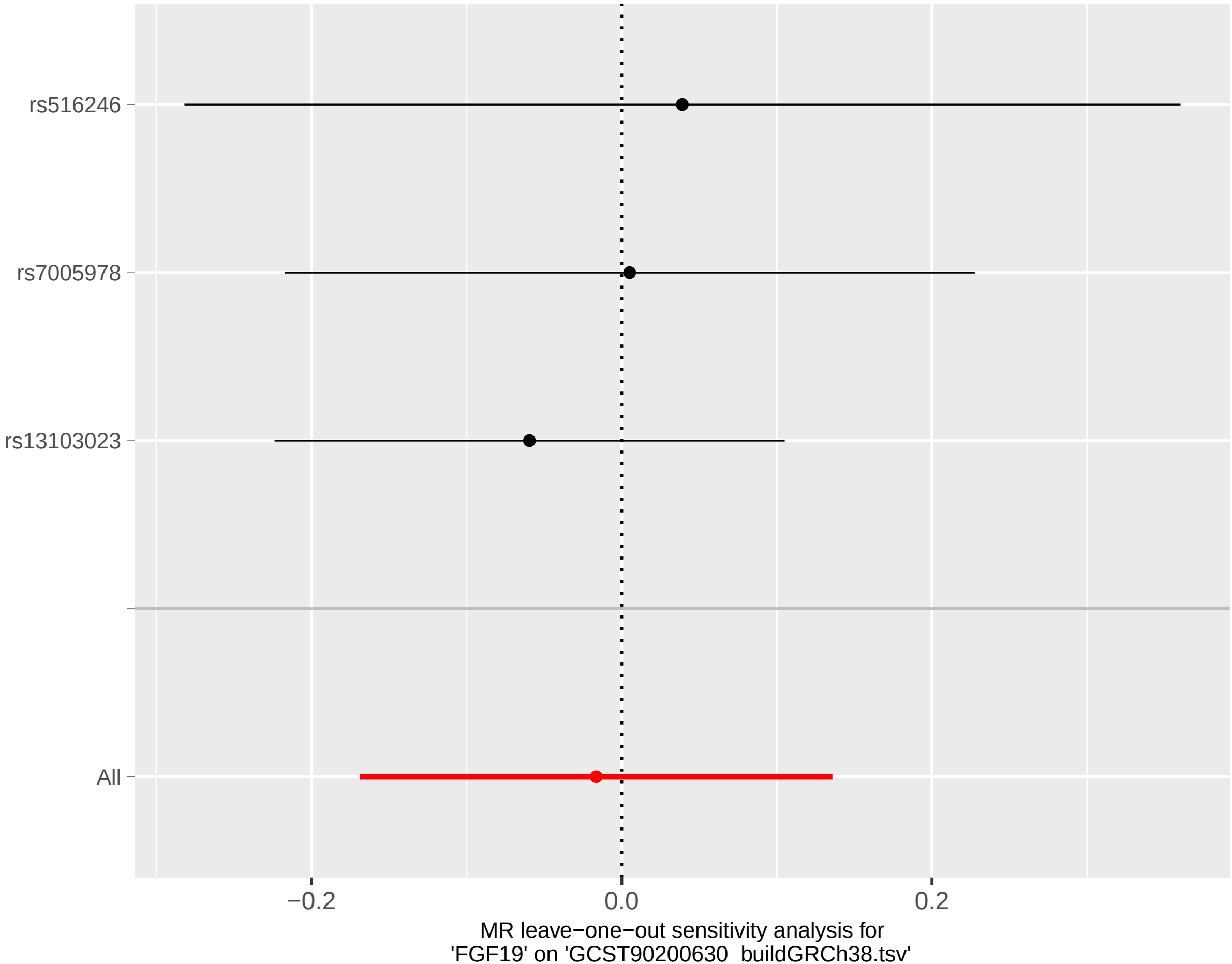
rs516246
rs7005978
rs13103023
All
−0.2
0.0
0.2
MR leave−one−out sensitivity analysis for
'FGF19' on 'GCST90200630_buildGRCh38.tsv'

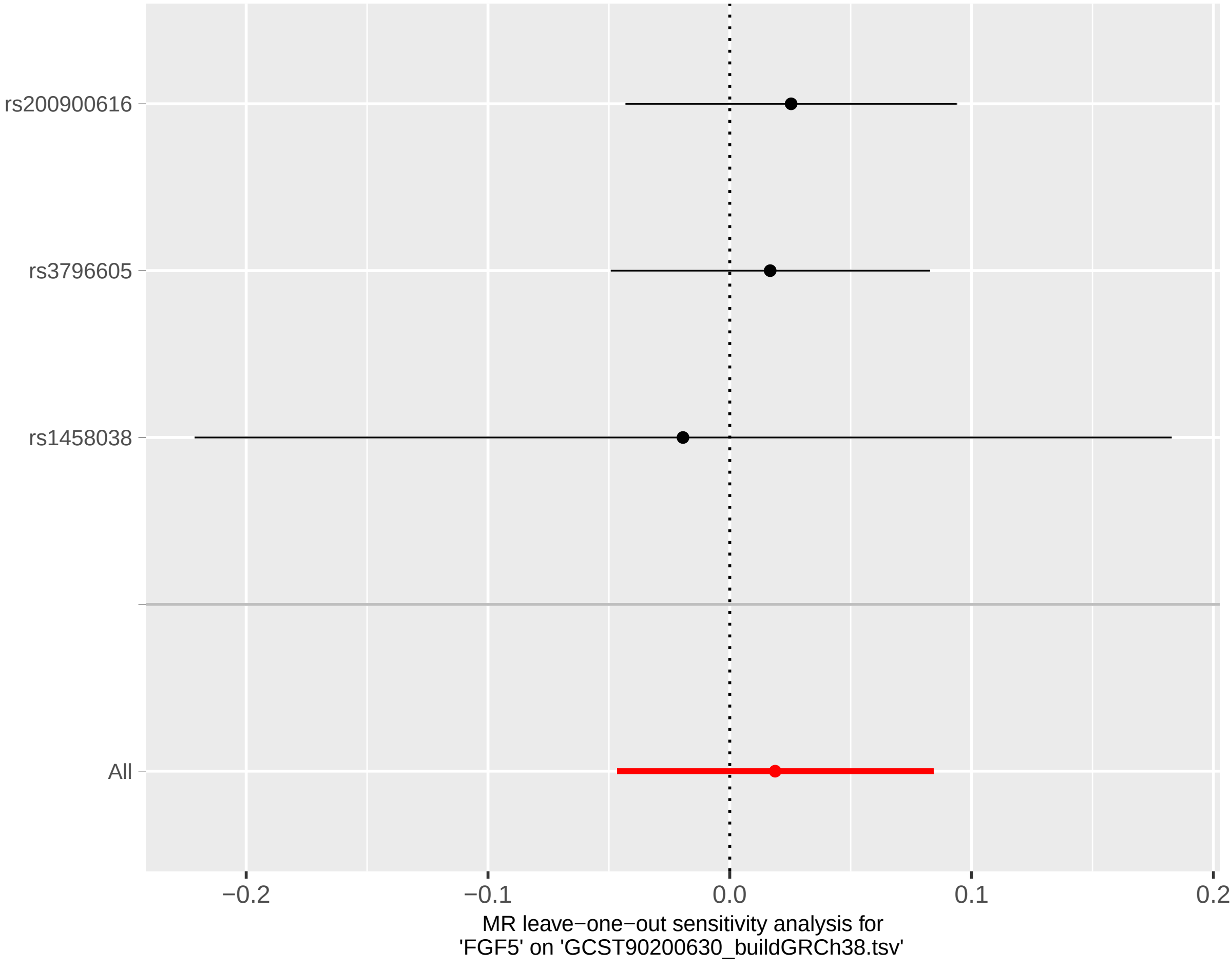
rs200900616
rs3796605
rs1458038
All
−0.2
−0.1
0.0
0.1
0.2
MR leave−one−out sensitivity analysis for
'FGF5' on 'GCST90200630_buildGRCh38.tsv'

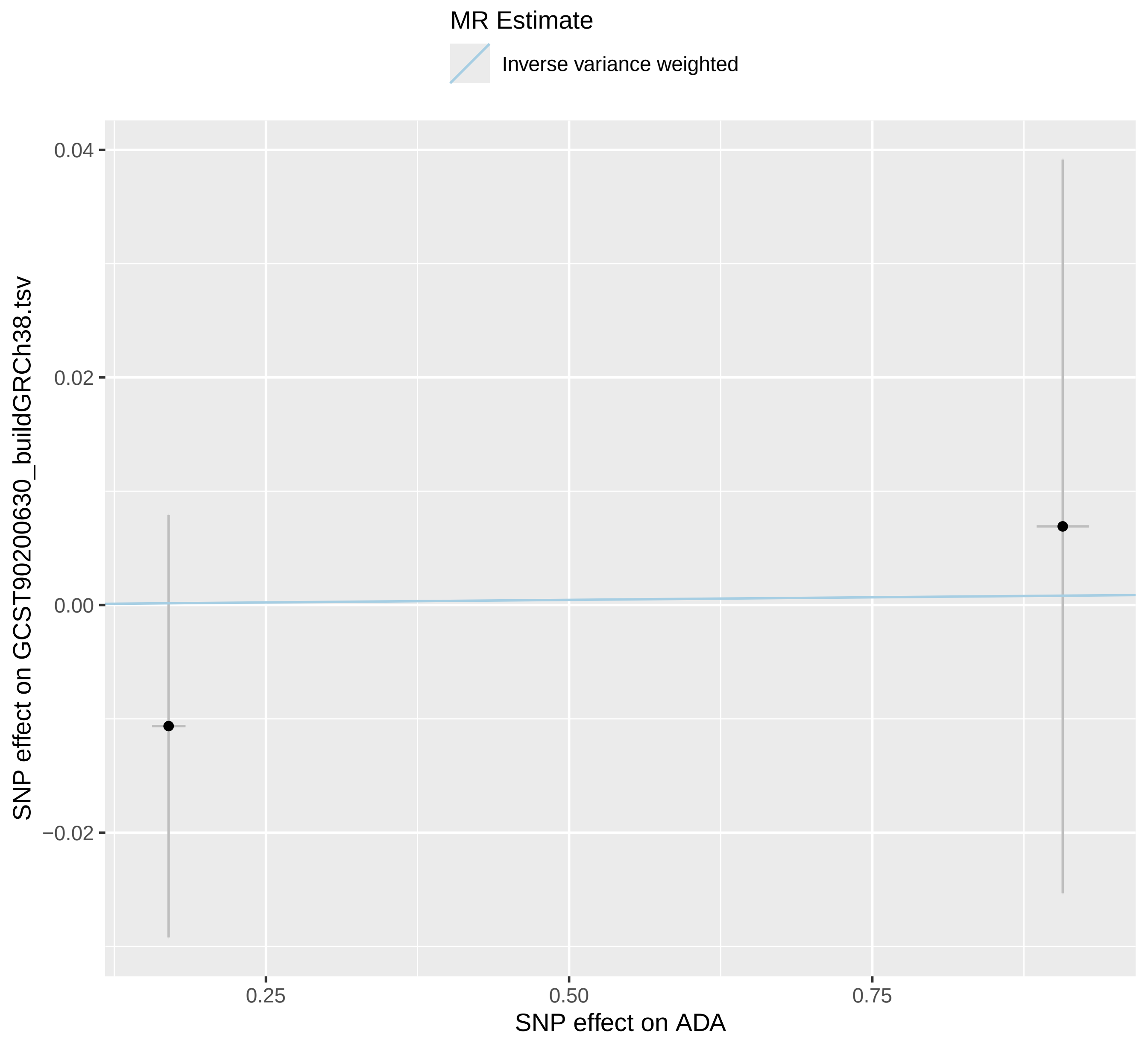
MR Estimate
Inverse variance weighted
0.04
0.02
0.00
−0.02
SNP effect on GCST90200630_buildGRCh38.tsv
0.25
0.50
0.75
SNP effect on ADA

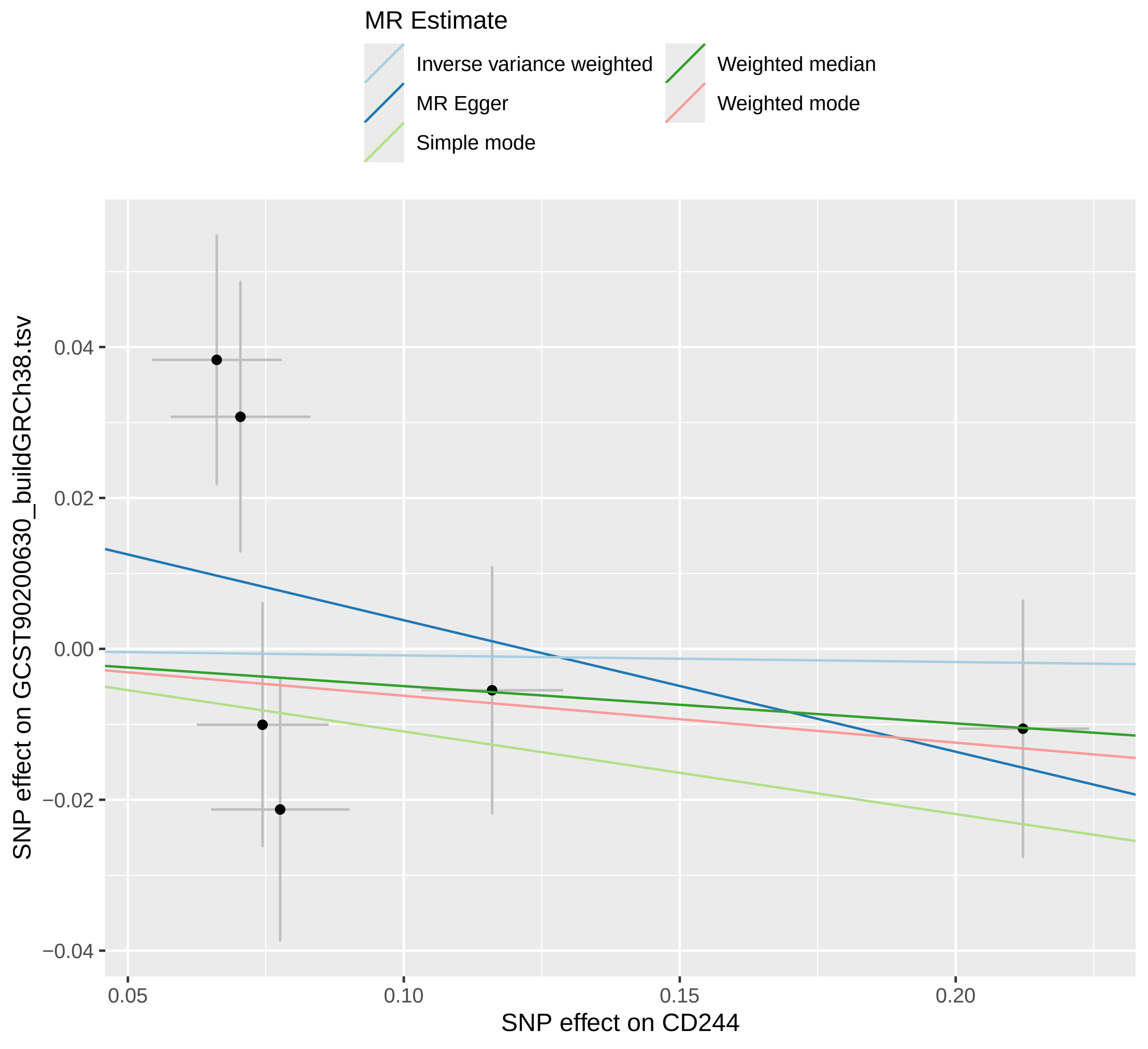

MR Estimate
Inverse variance weighted
MR Egger
Simple mode
Weighted median
Weighted mode
SNP effect on GCST90200630_buildGRCh38.tsv
0.04
0.02
0.00
−0.02
−0.04
0.05
0.10
0.15
0.20
SNP effect on CD244

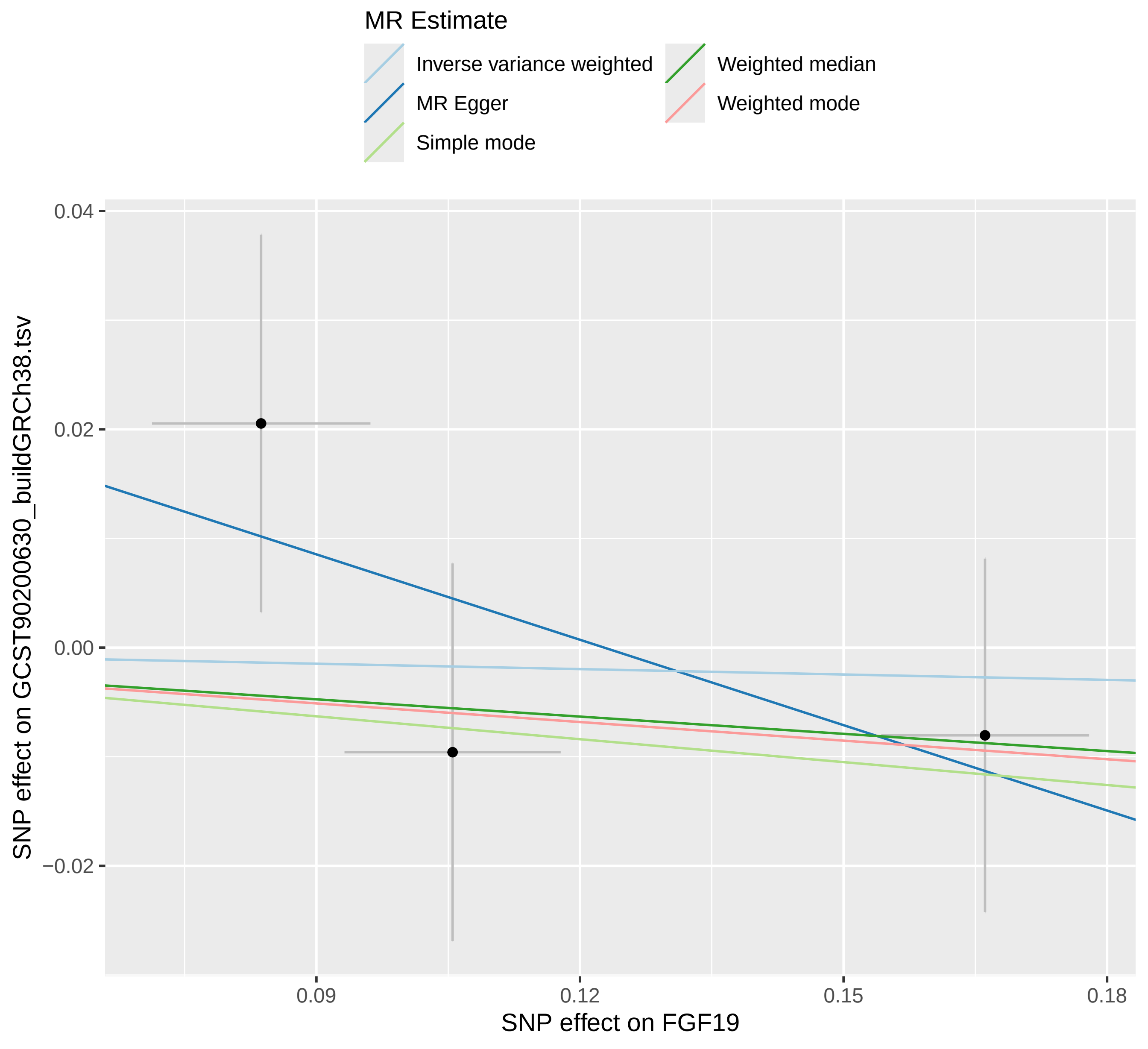
MR Estimate
Inverse variance weighted
MR Egger
Simple mode
Weighted median
Weighted mode
0.04
0.02
0.00
−0.02
SNP effect on GCST90200630_buildGRCh38.tsv
0.09
0.12
0.15
0.18
SNP effect on FGF19

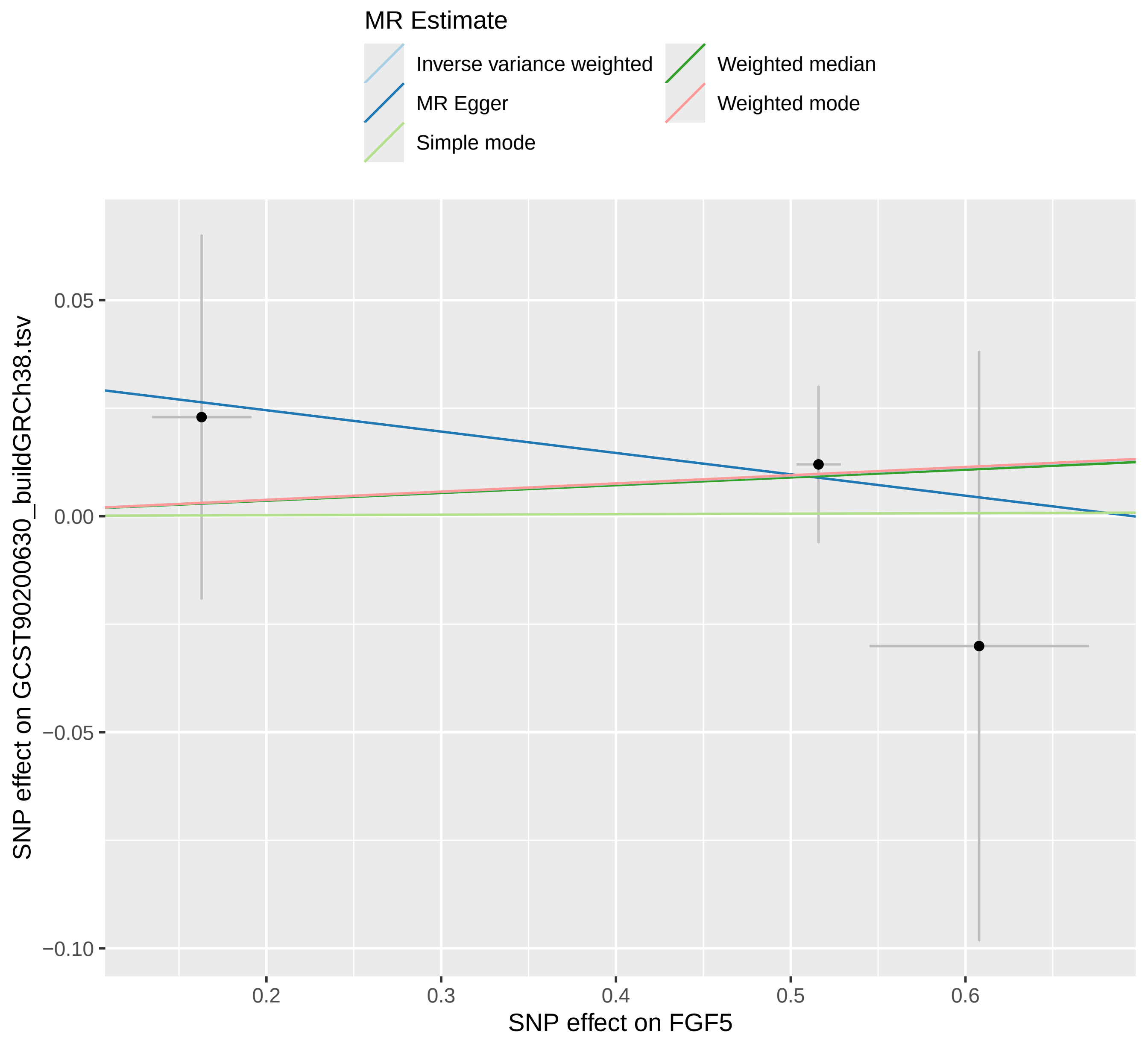
MR Estimate
Inverse variance weighted
MR Egger
Simple mode
Weighted median
Weighted mode
0.05
0.00
−0.05
−0.10
SNP effect on GCST90200630_buildGRCh38.tsv
0.2
0.3
0.4
0.5
0.6
SNP effect on FGF5

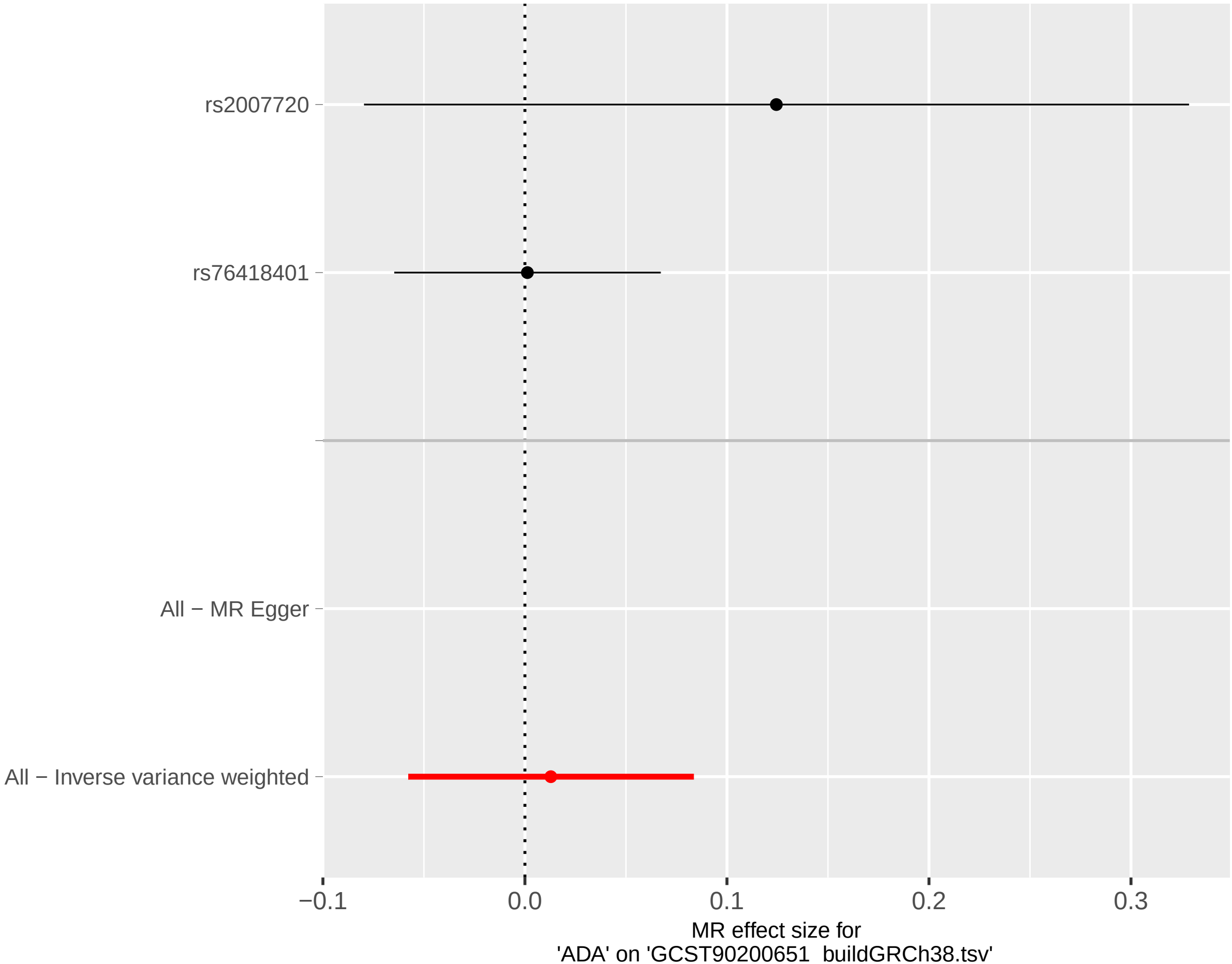
rs2007720
rs76418401
All − MR Egger
All − Inverse variance weighted
−0.1
0.0
0.1
0.2
0.3
MR effect size for
'ADA' on 'GCST90200651_buildGRCh38.tsv'

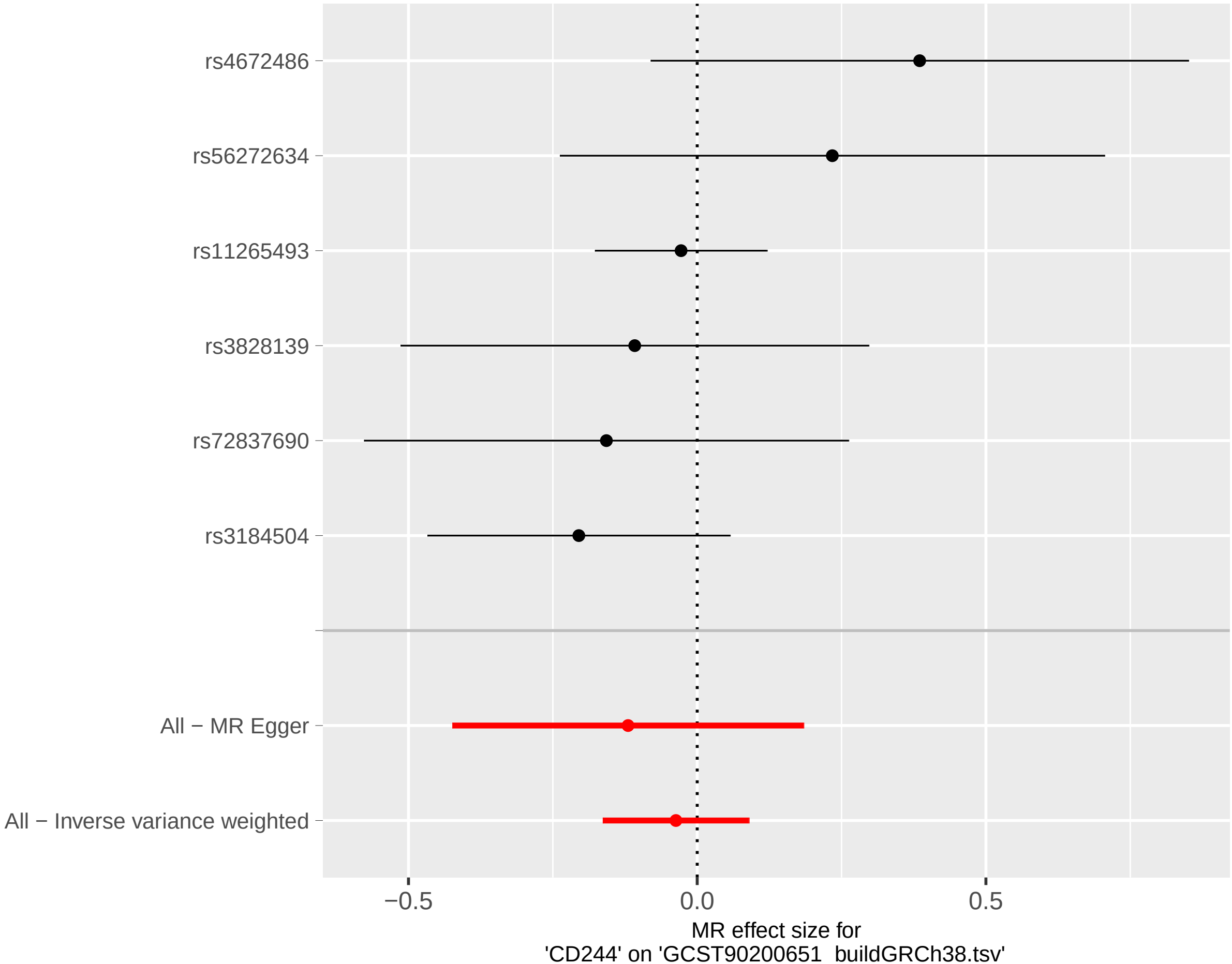
rs4672486
rs56272634
rs11265493
rs3828139
rs72837690
rs3184504
All − MR Egger
All − Inverse variance weighted
−0.5
0.0
0.5
MR effect size for
'CD244' on 'GCST90200651_buildGRCh38.tsv'

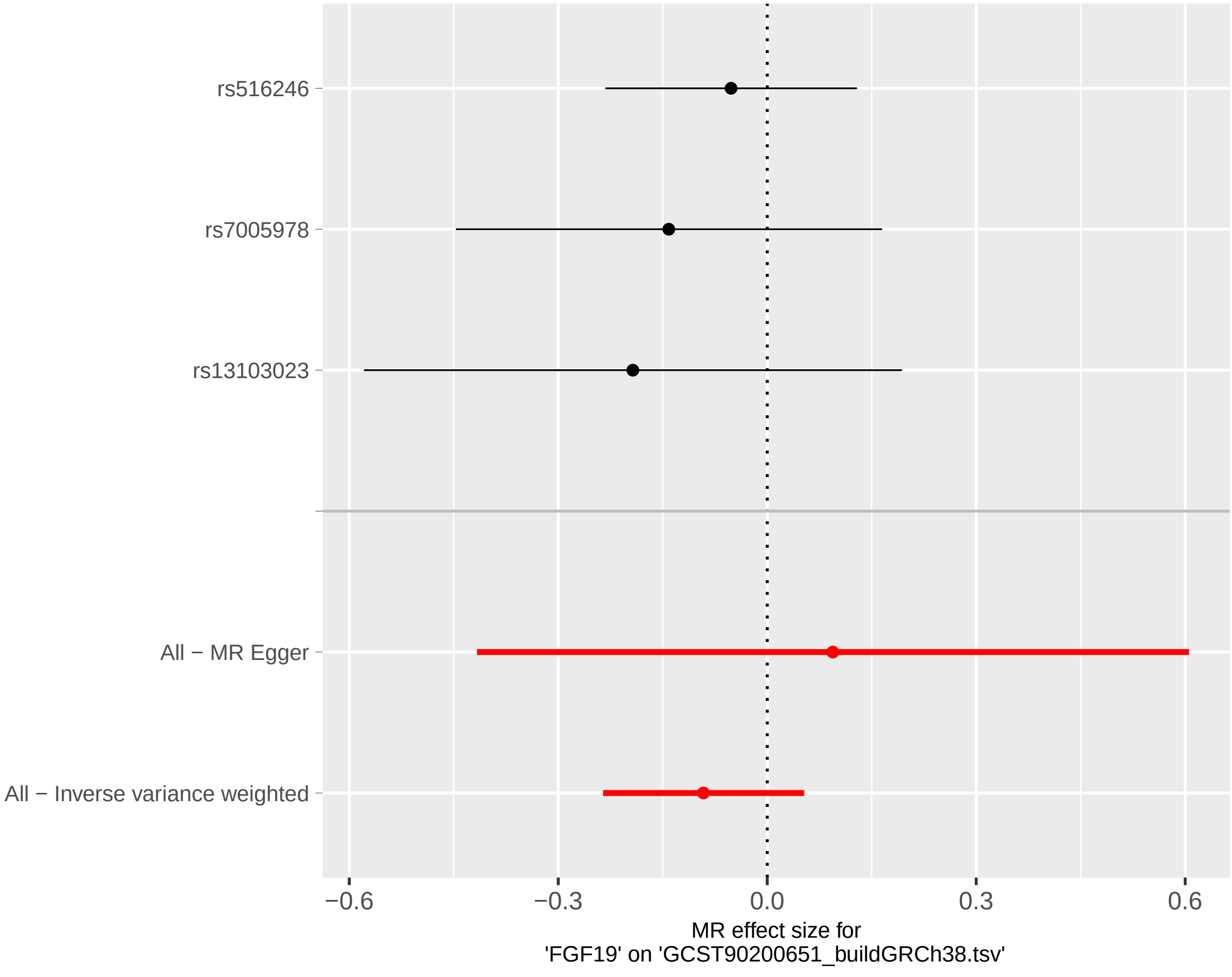
rs516246
rs7005978
rs13103023
All − MR Egger
All − Inverse variance weighted
−0.6
−0.3
0.0
0.3
0.6
MR effect size for
'FGF19' on 'GCST90200651_buildGRCh38.tsv'

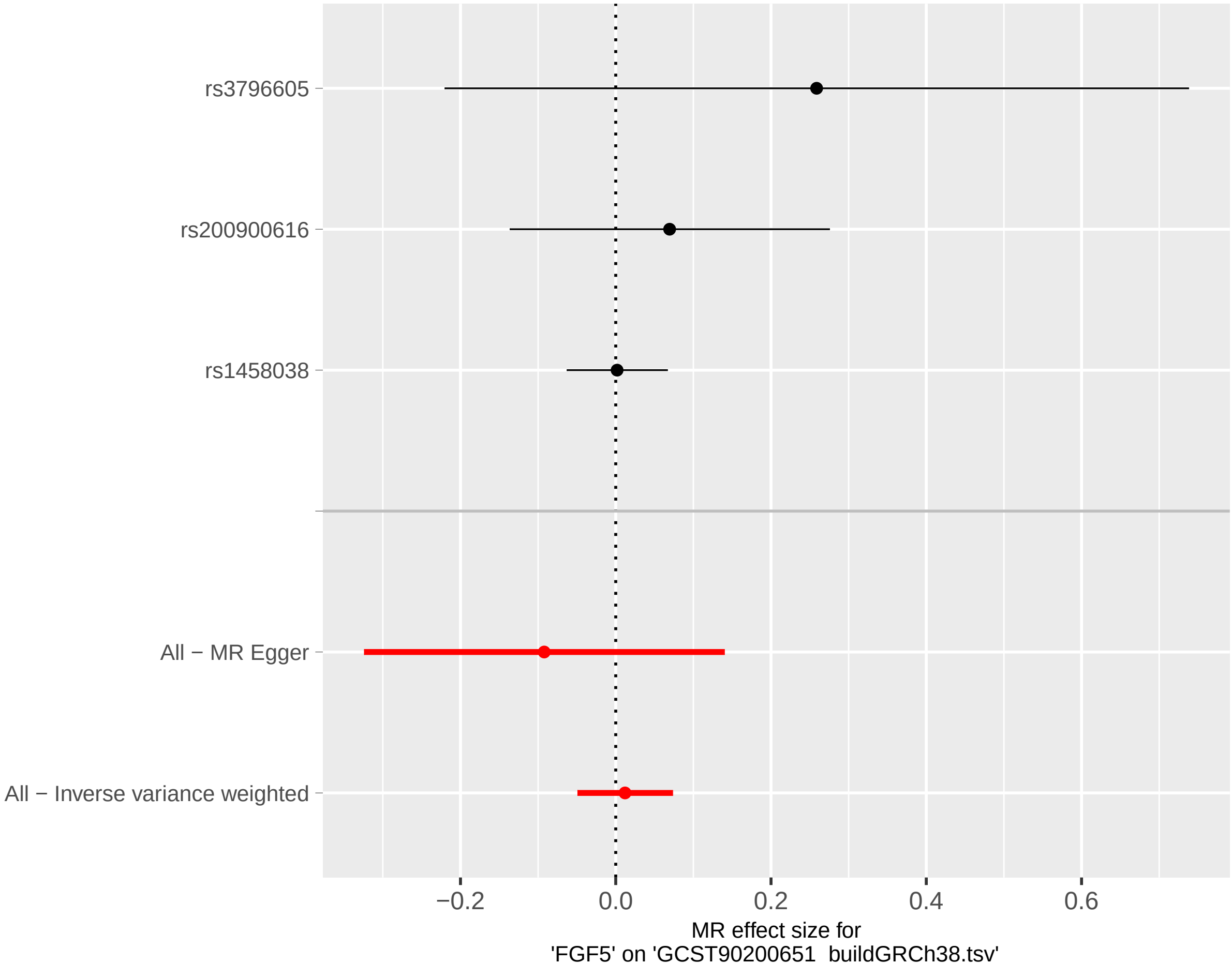
rs3796605
rs200900616
rs1458038
All − MR Egger
All − Inverse variance weighted
−0.2
0.0
0.2
0.4
0.6
MR effect size for
'FGF5' on 'GCST90200651_buildGRCh38.tsv'

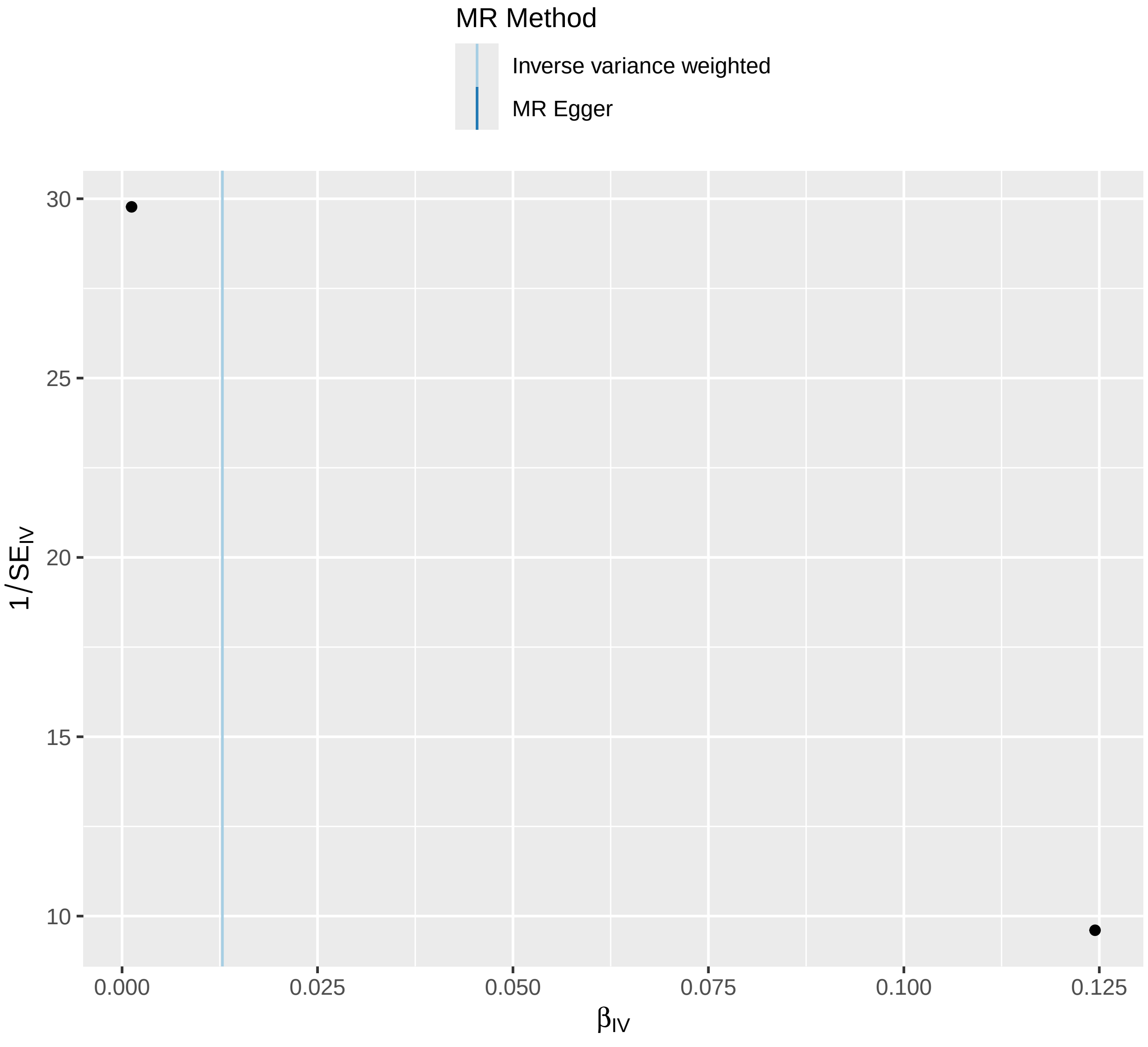
MR Method
Inverse variance weighted
MR Egger
30
25
20
15
10
1/SE_IV
0.000
0.025
0.050
0.075
0.100
0.125
β_IV

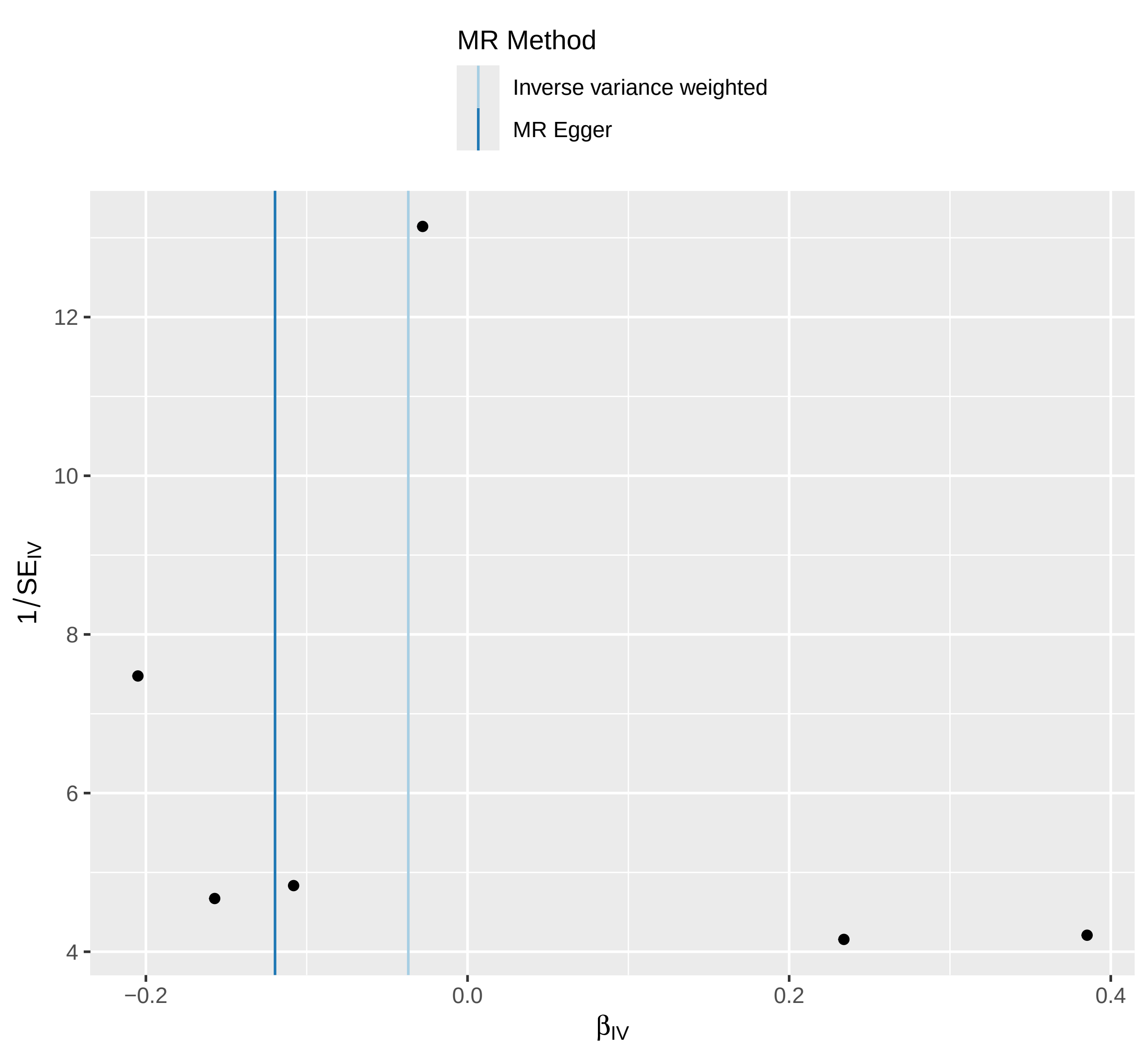
MR Method
Inverse variance weighted
MR Egger
1/SE_IV
β_IV
12
10
8
6
4
−0.2
0.0
0.2
0.4

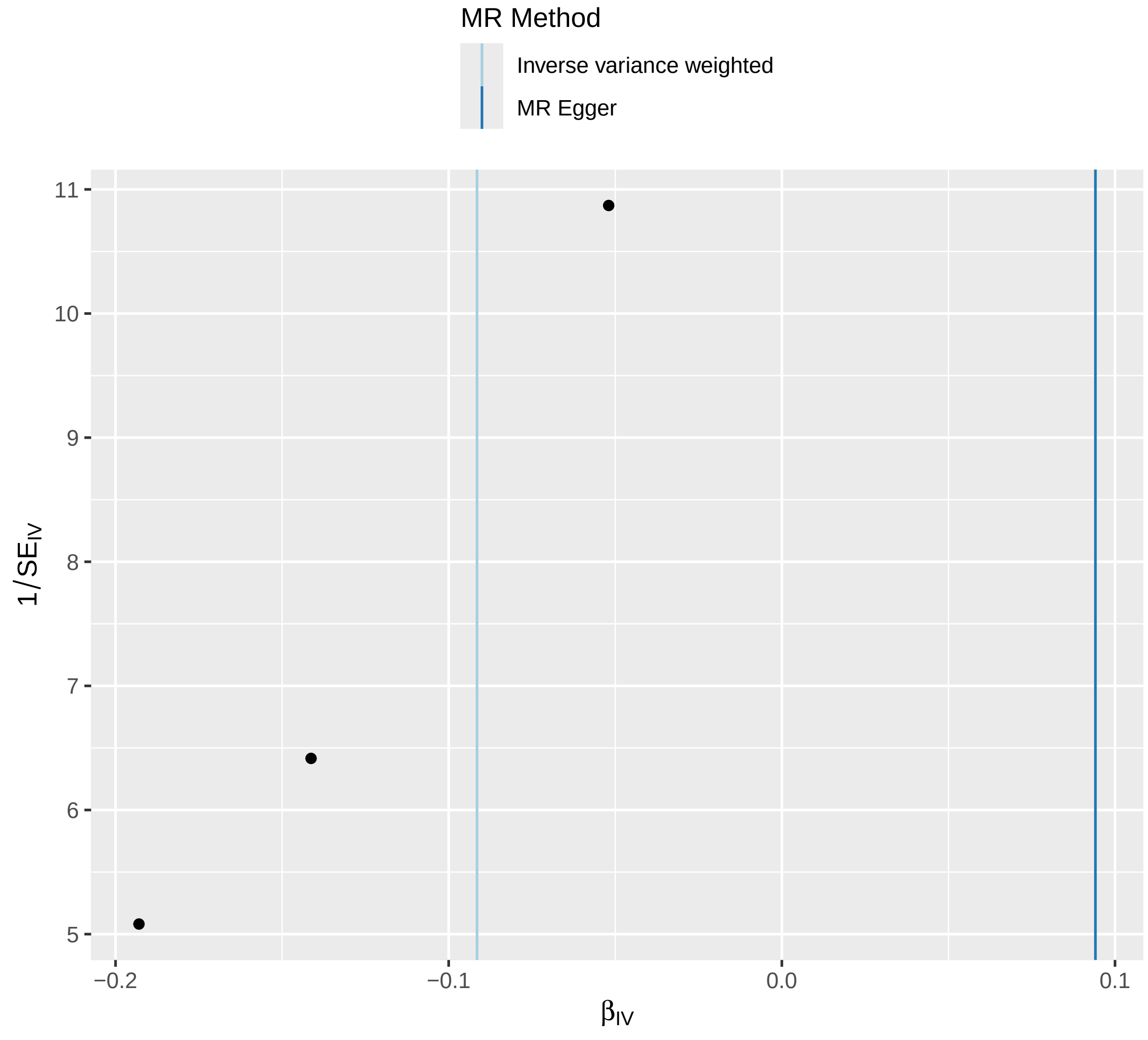
MR Method
Inverse variance weighted
MR Egger
11
10
9
8
7
6
5
1/SE_IV
−0.2
−0.1
0.0
0.1
β_IV

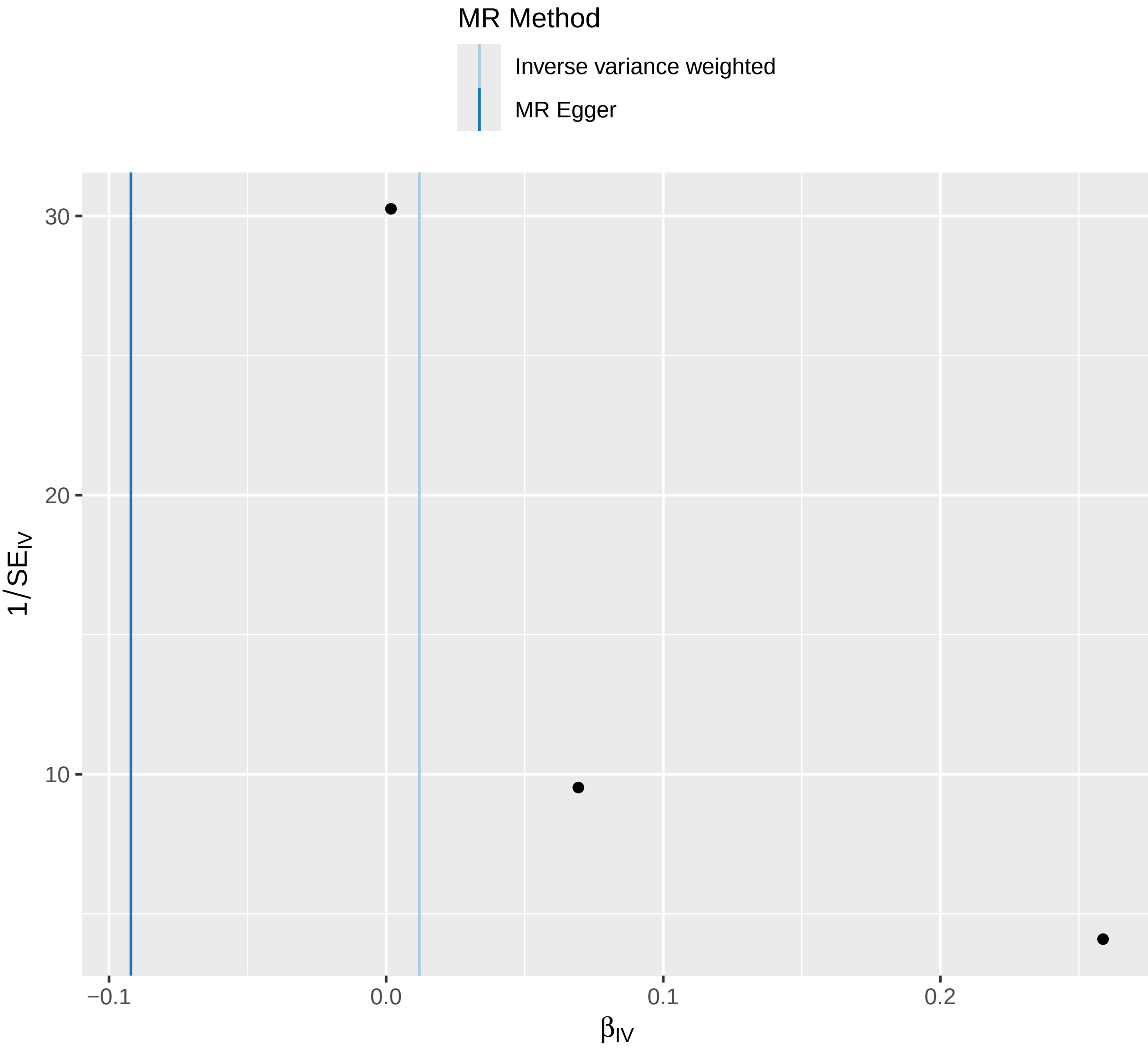
MR Method
Inverse variance weighted
MR Egger
30
20
10
1/SE$_{IV}$
−0.1
0.0
0.1
0.2
β$_{IV}$

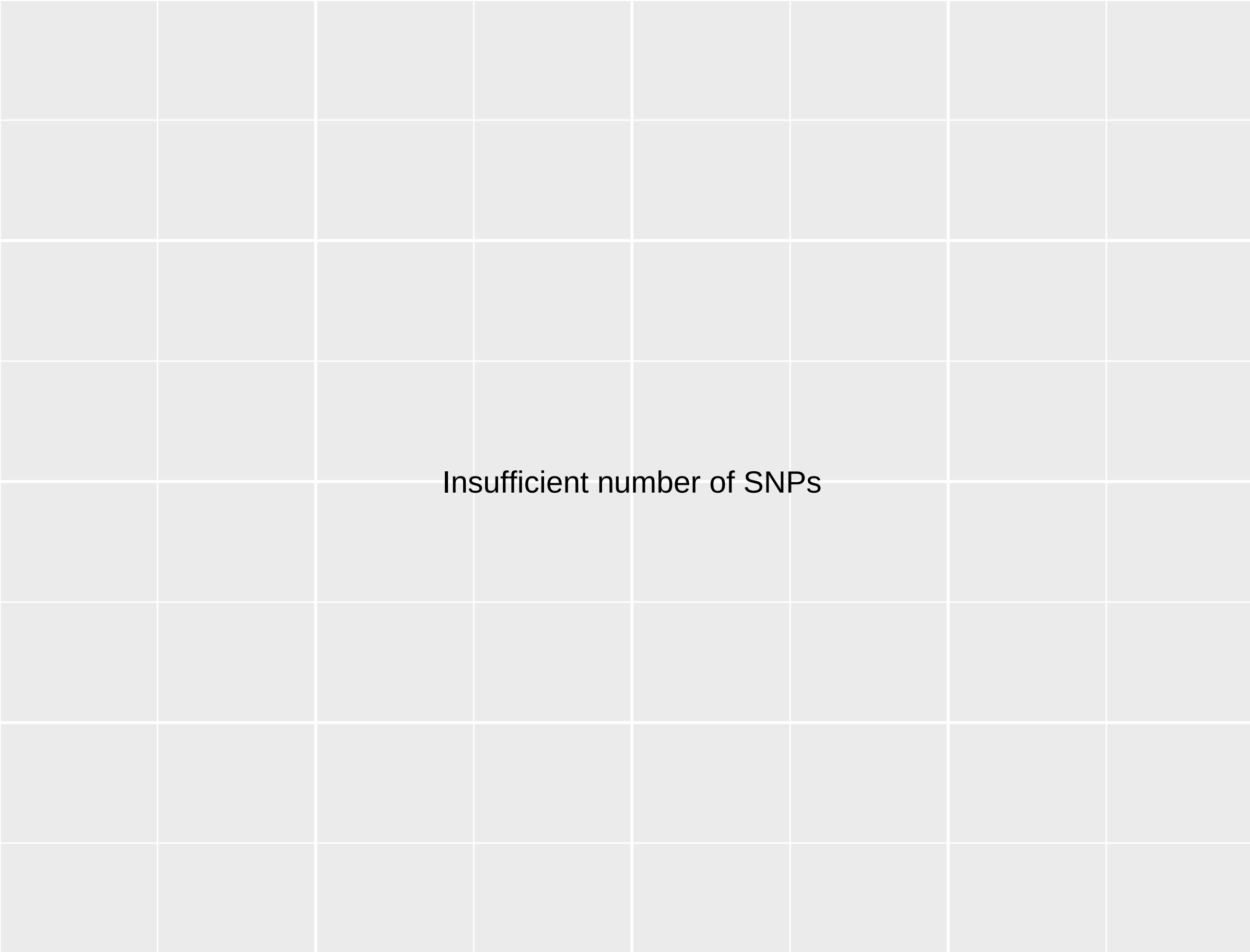
Insufficient number of SNPs

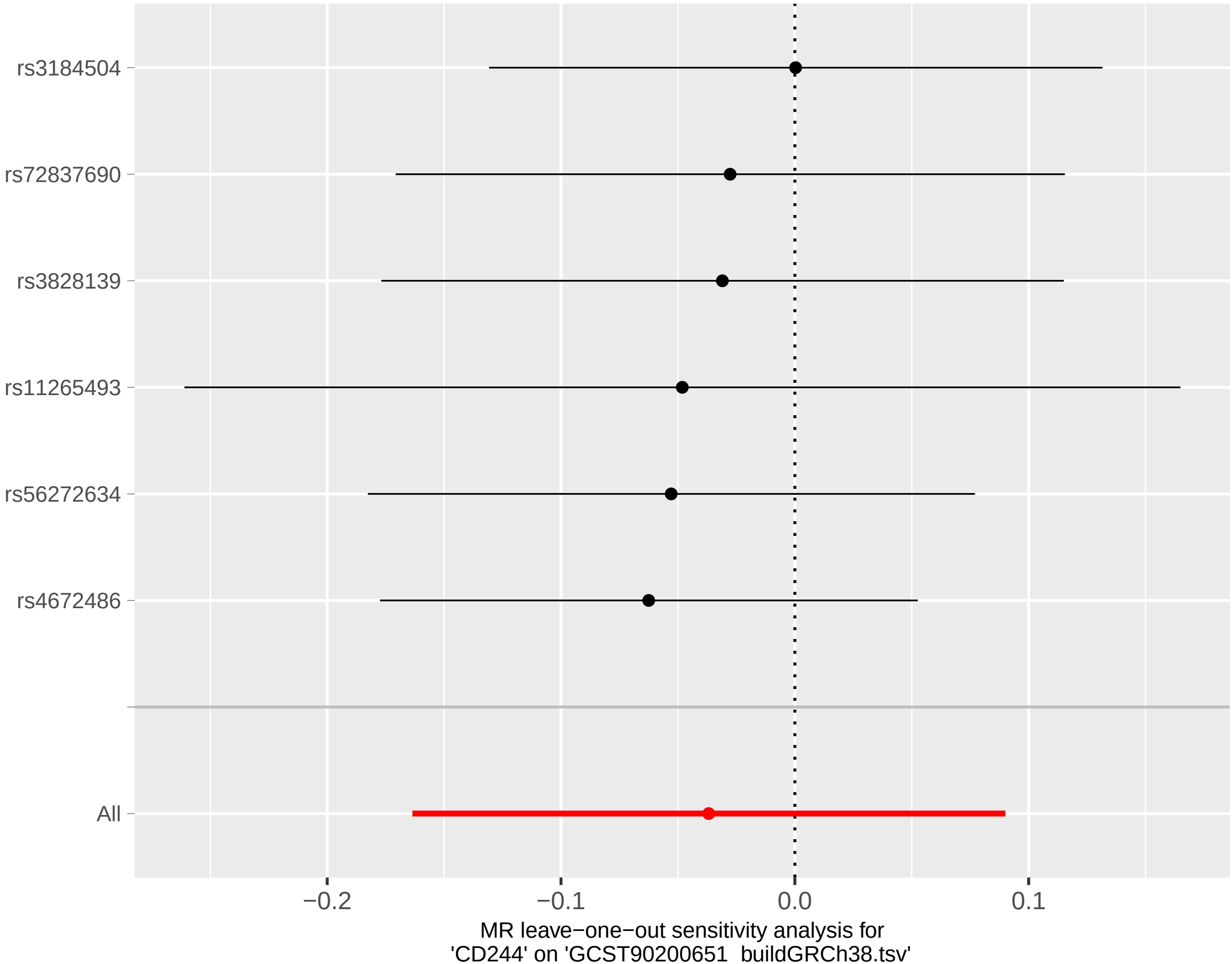
rs3184504
rs72837690
rs3828139
rs11265493
rs56272634
rs4672486
All
−0.2
−0.1
0.0
0.1
MR leave−one−out sensitivity analysis for
'CD244' on 'GCST90200651_buildGRCh38.tsv'

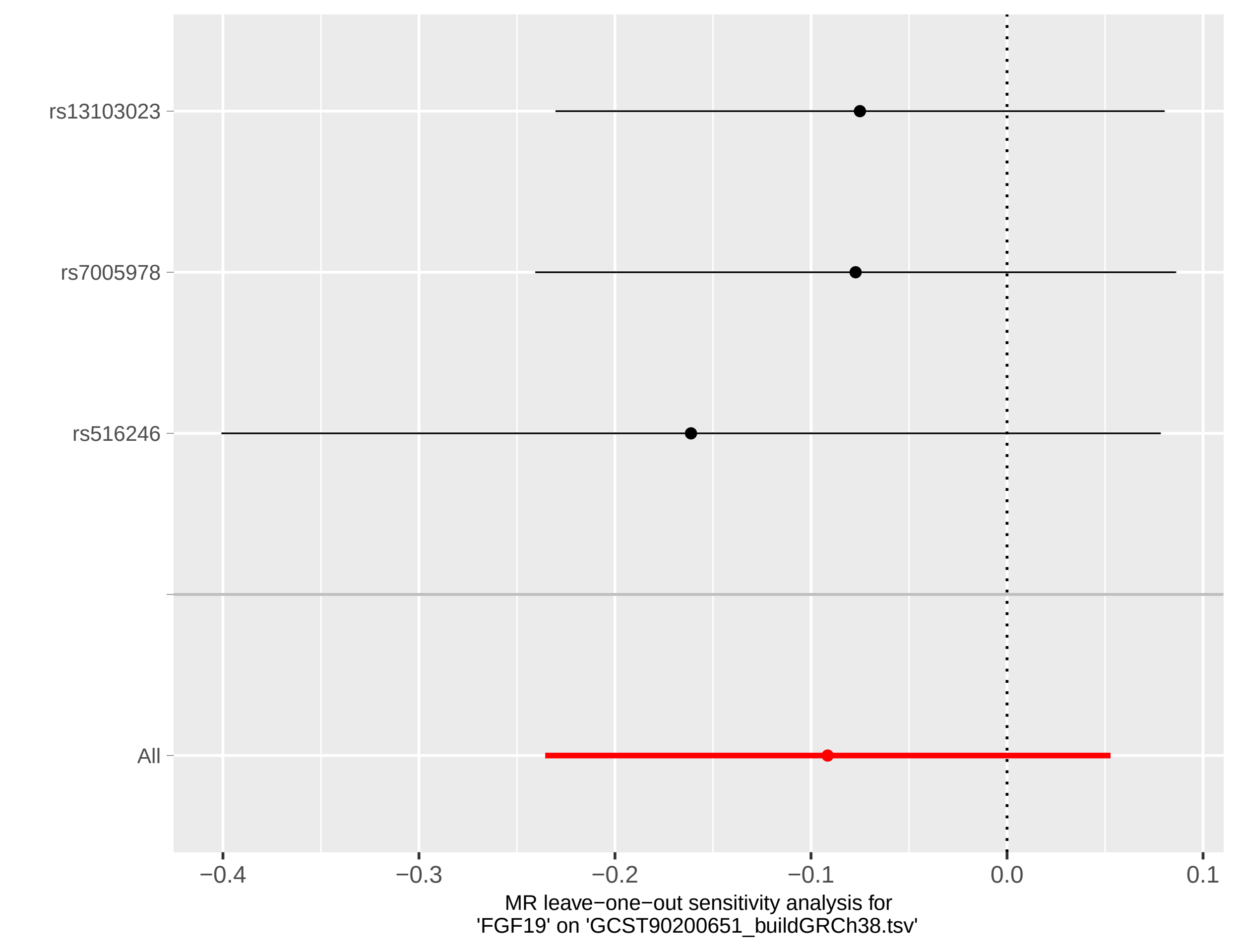
rs13103023
rs7005978
rs516246
All
−0.4
−0.3
−0.2
−0.1
0.0
0.1
MR leave−one−out sensitivity analysis for
'FGF19' on 'GCST90200651_buildGRCh38.tsv'

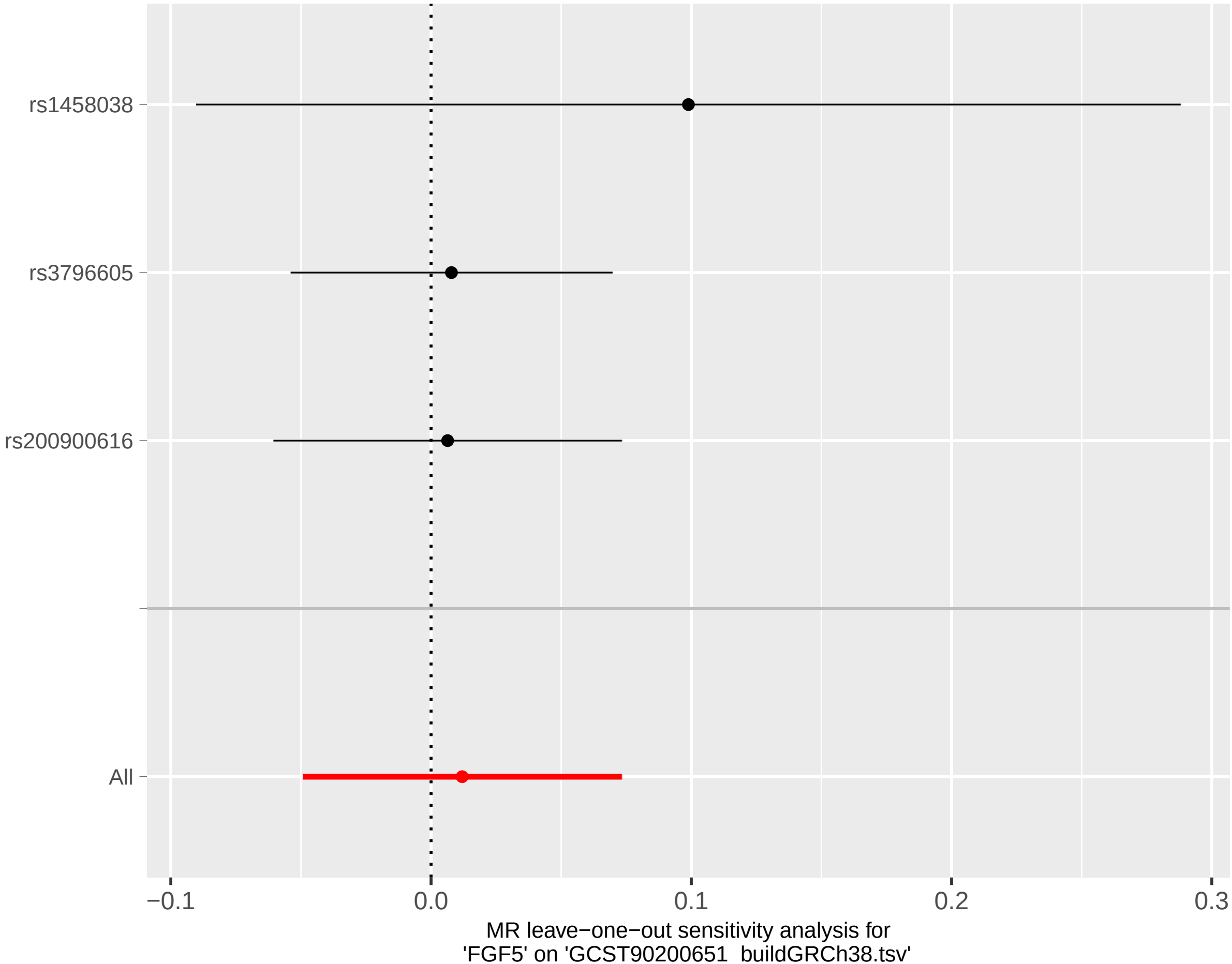
rs1458038
rs3796605
rs200900616
All
−0.1
0.0
0.1
0.2
0.3
MR leave−one−out sensitivity analysis for
'FGF5' on 'GCST90200651_buildGRCh38.tsv'

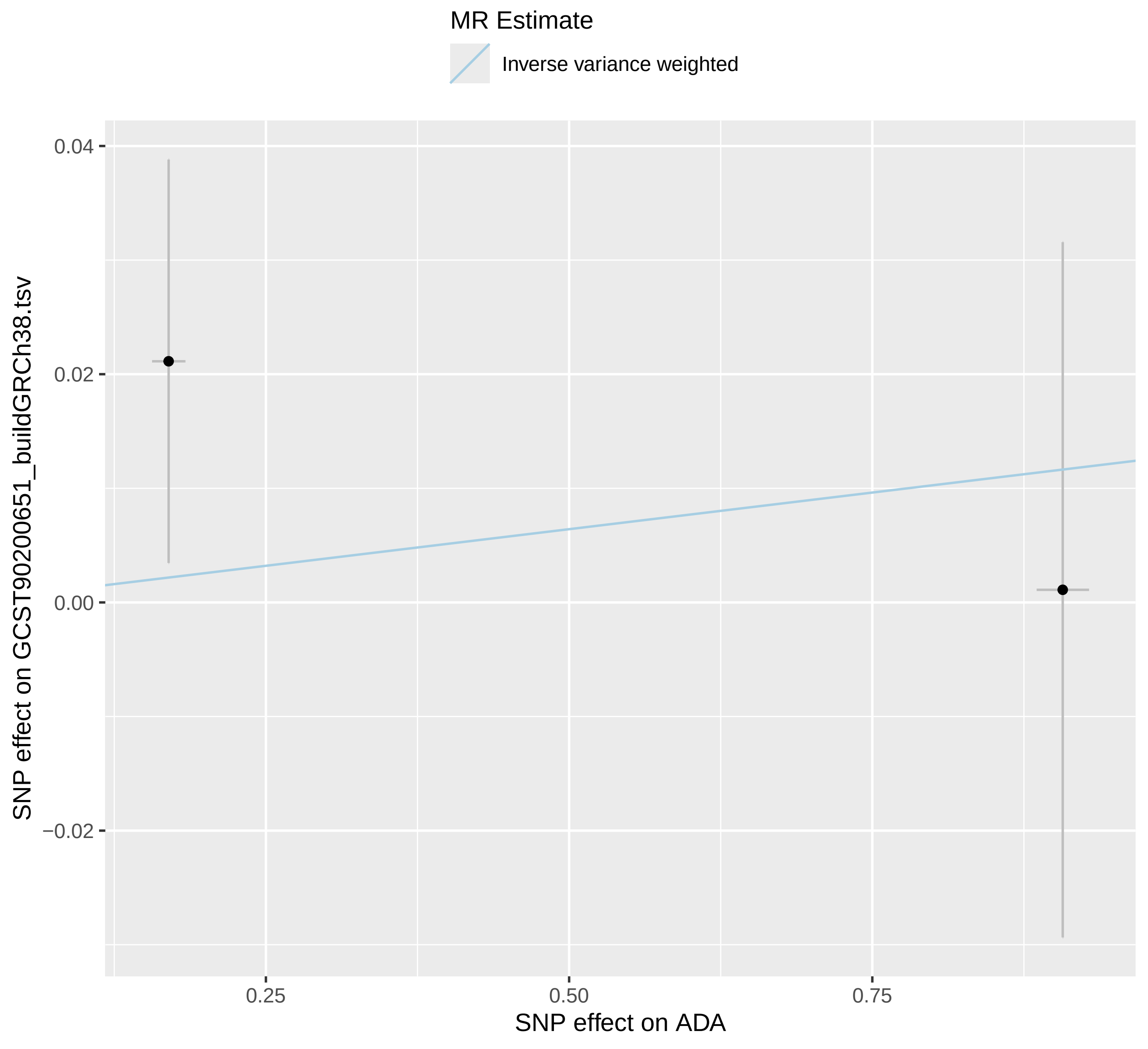
MR Estimate
Inverse variance weighted
0.04
0.02
0.00
−0.02
0.25
0.50
0.75
SNP effect on GCST90200651_buildGRCh38.tsv
SNP effect on ADA

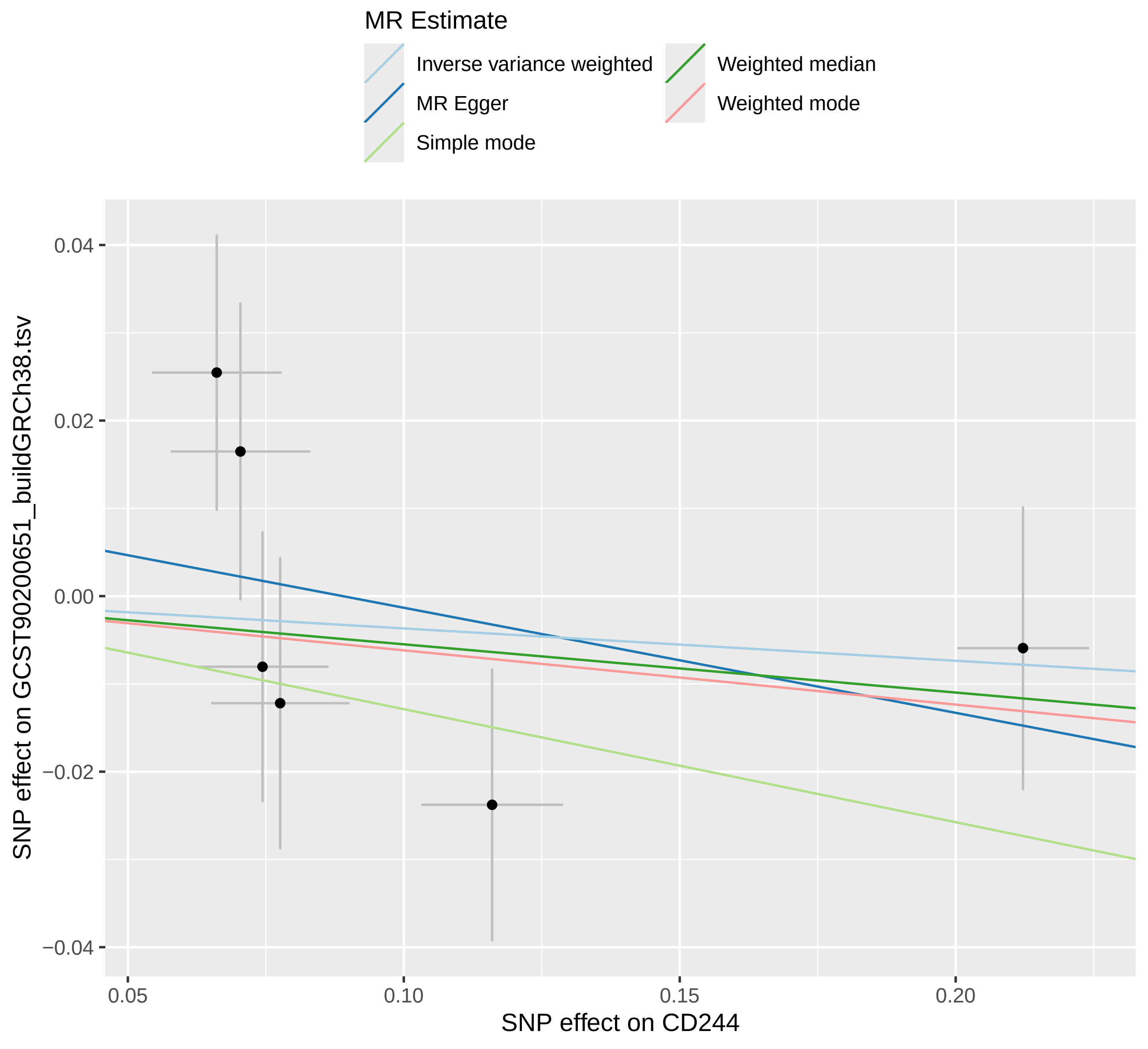

MR Estimate
Inverse variance weighted
MR Egger
Simple mode
Weighted median
Weighted mode
0.04
0.02
0.00
−0.02
−0.04
SNP effect on GCST90200651_buildGRCh38.tsv
0.05
0.10
0.15
0.20
SNP effect on CD244

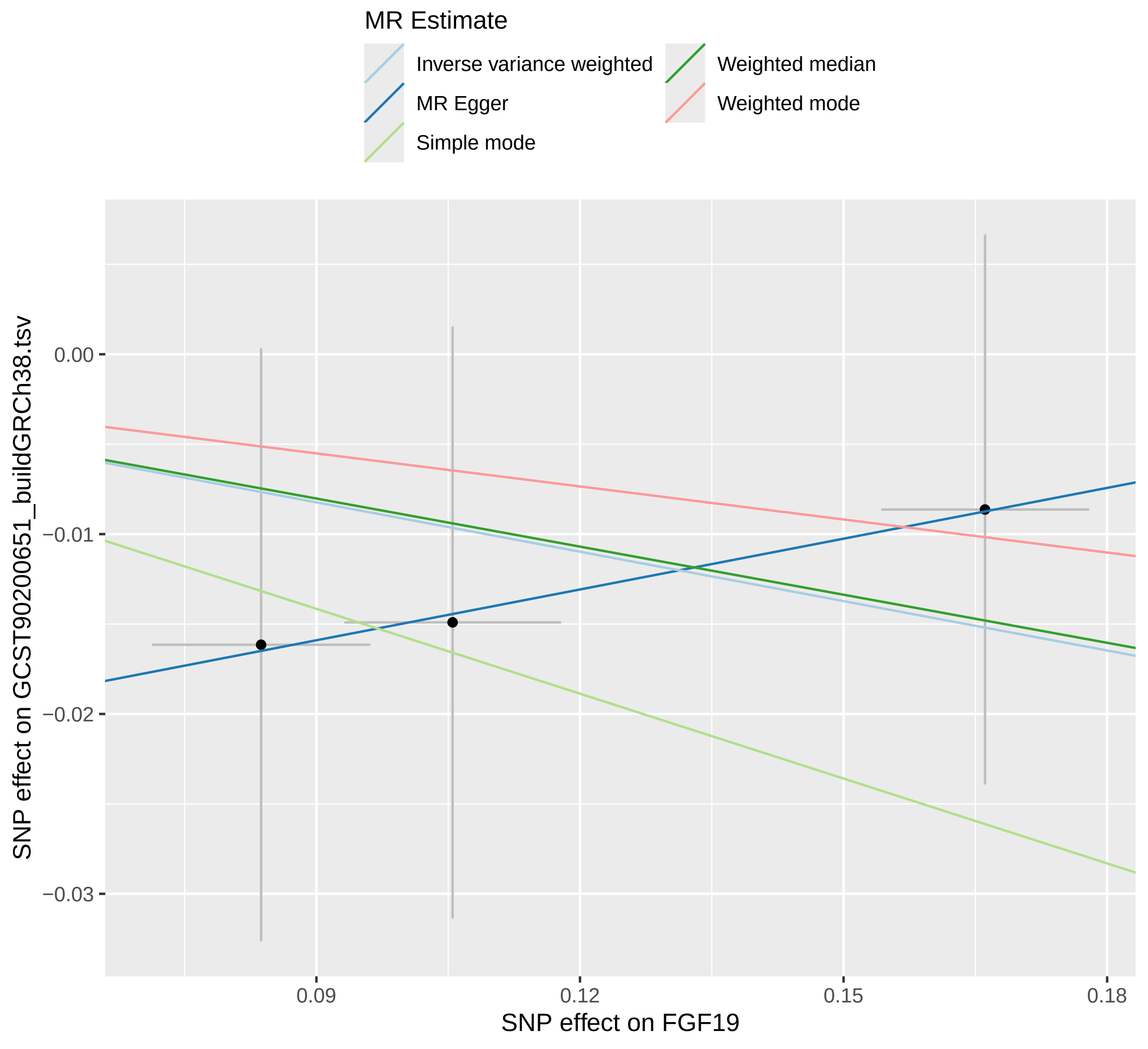
MR Estimate
Inverse variance weighted
MR Egger
Simple mode
Weighted median
Weighted mode
0.00
−0.01
−0.02
−0.03
0.09
0.12
0.15
0.18
SNP effect on FGF19
SNP effect on GCST90200651_buildGRCh38.tsv

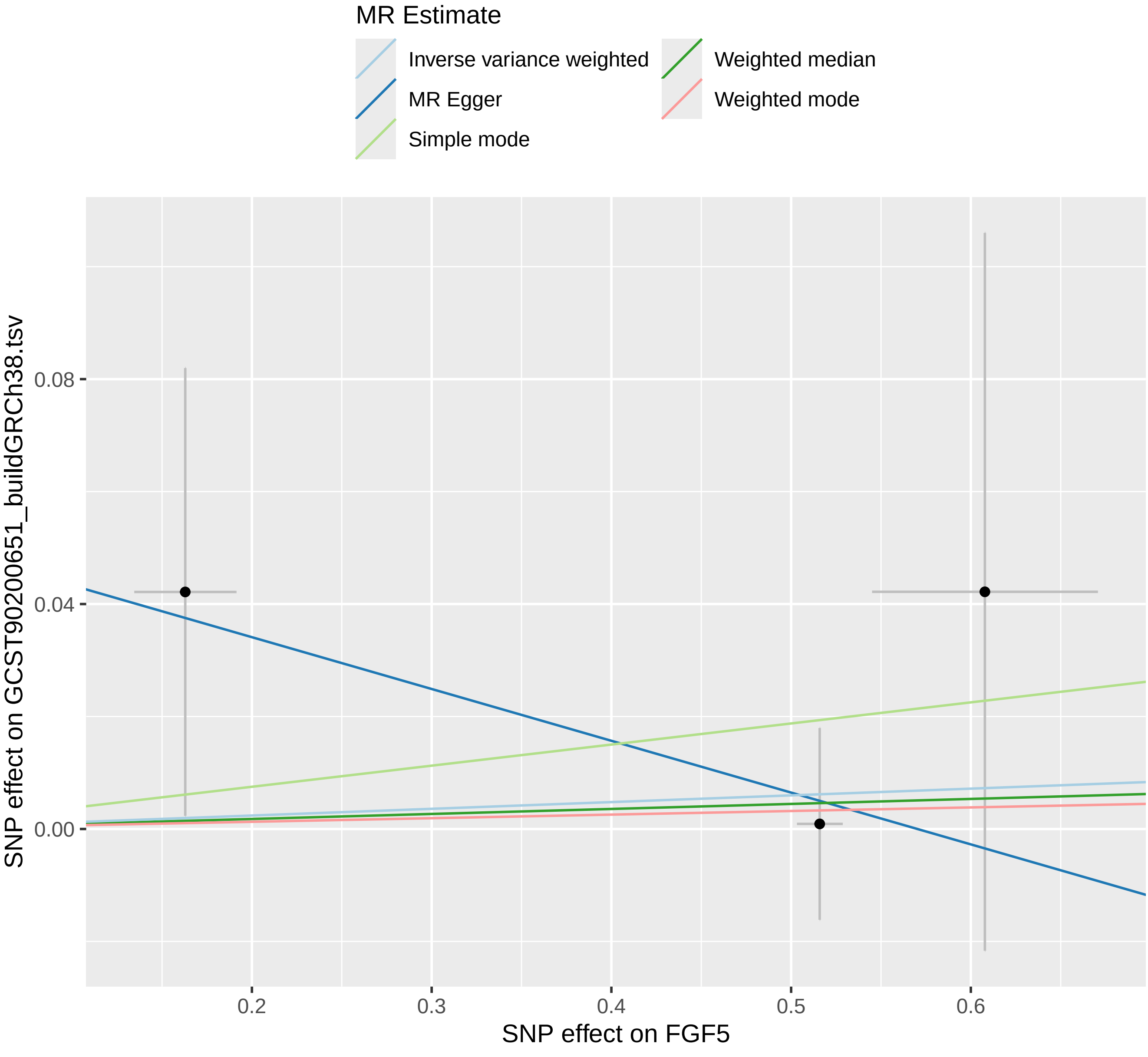
MR Estimate
Inverse variance weighted
MR Egger
Simple mode
Weighted median
Weighted mode
SNP effect on GCST90200651_buildGRCh38.tsv
0.08
0.04
0.00
0.2
0.3
0.4
0.5
0.6
SNP effect on FGF5

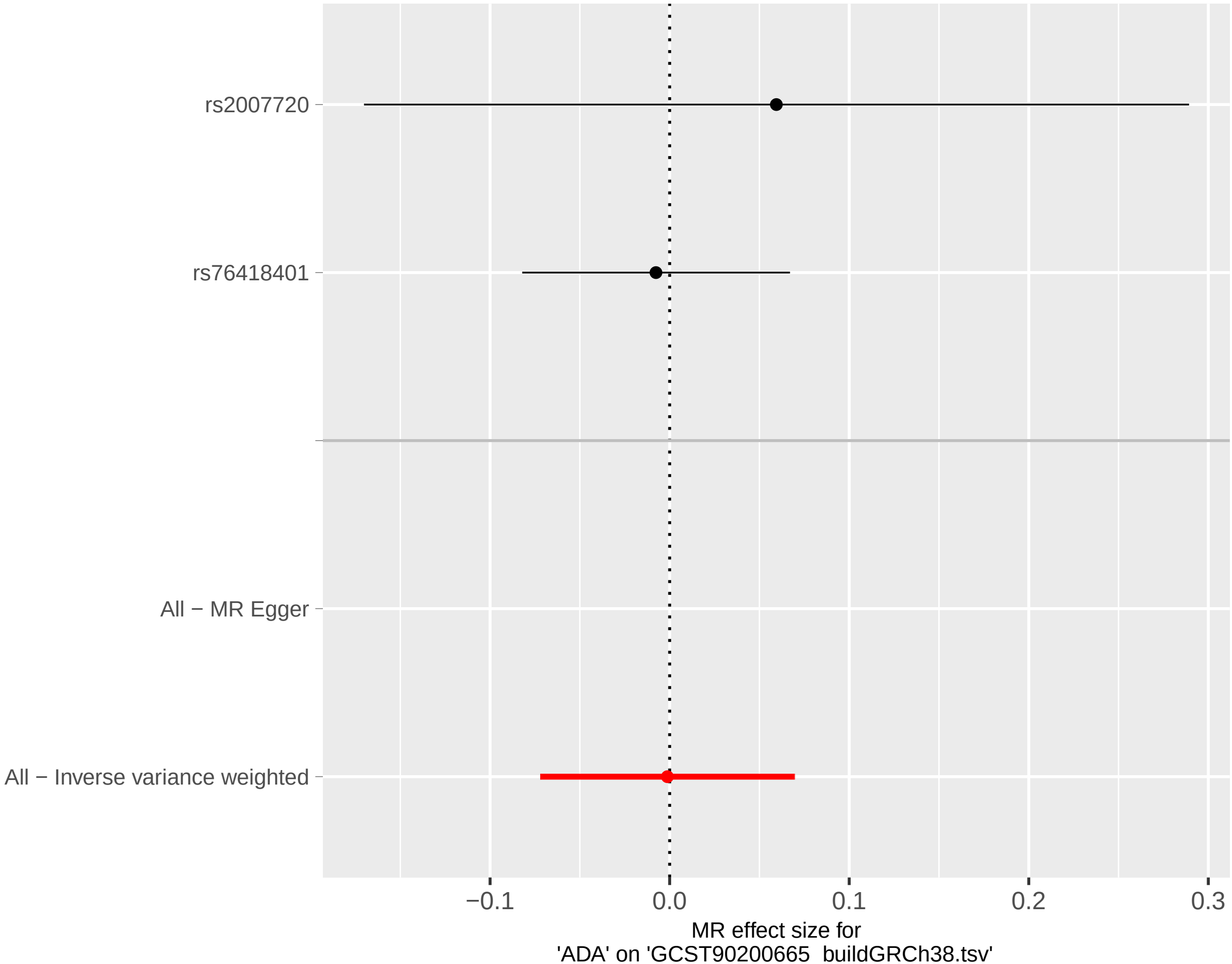
rs2007720
rs76418401
All − MR Egger
All − Inverse variance weighted
−0.1
0.0
0.1
0.2
0.3
MR effect size for
'ADA' on 'GCST90200665_buildGRCh38.tsv'

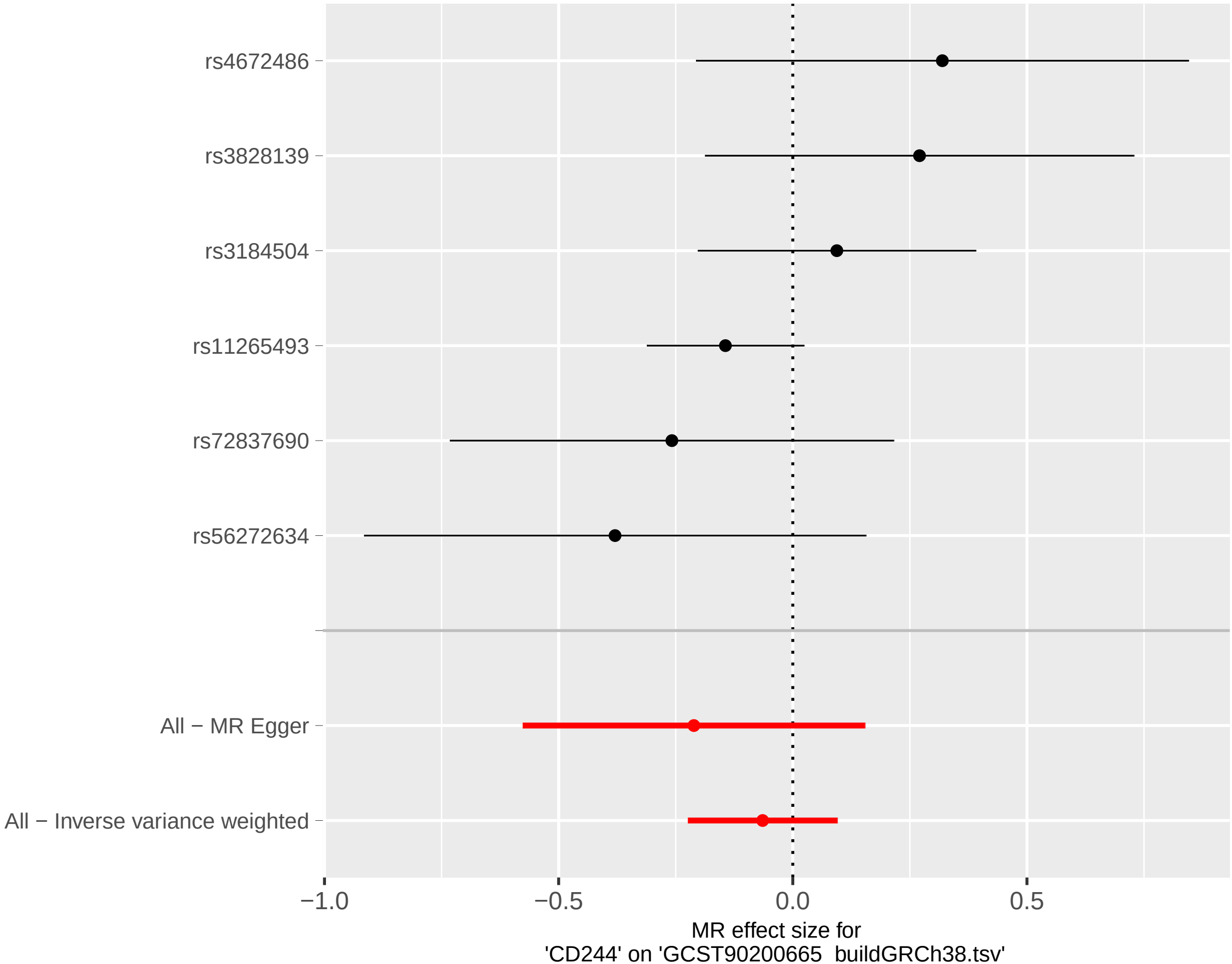

rs4672486
rs3828139
rs3184504
rs11265493
rs72837690
rs56272634
All − MR Egger
All − Inverse variance weighted
−1.0
−0.5
0.0
0.5
MR effect size for
'CD244' on 'GCST90200665_buildGRCh38.tsv'

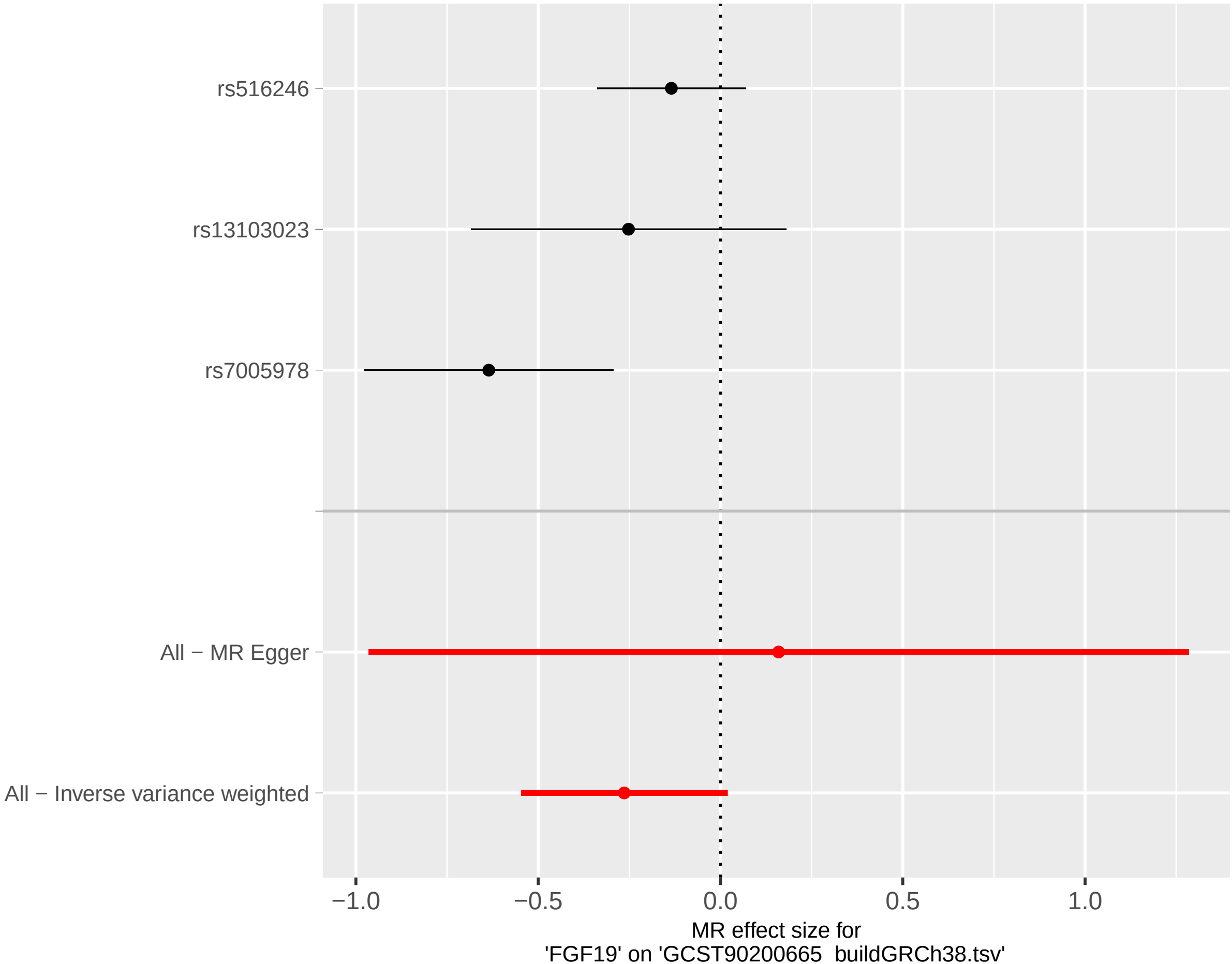

rs516246
rs13103023
rs7005978
All − MR Egger
All − Inverse variance weighted
−1.0
−0.5
0.0
0.5
1.0
MR effect size for
'FGF19' on 'GCST90200665_buildGRCh38.tsv'

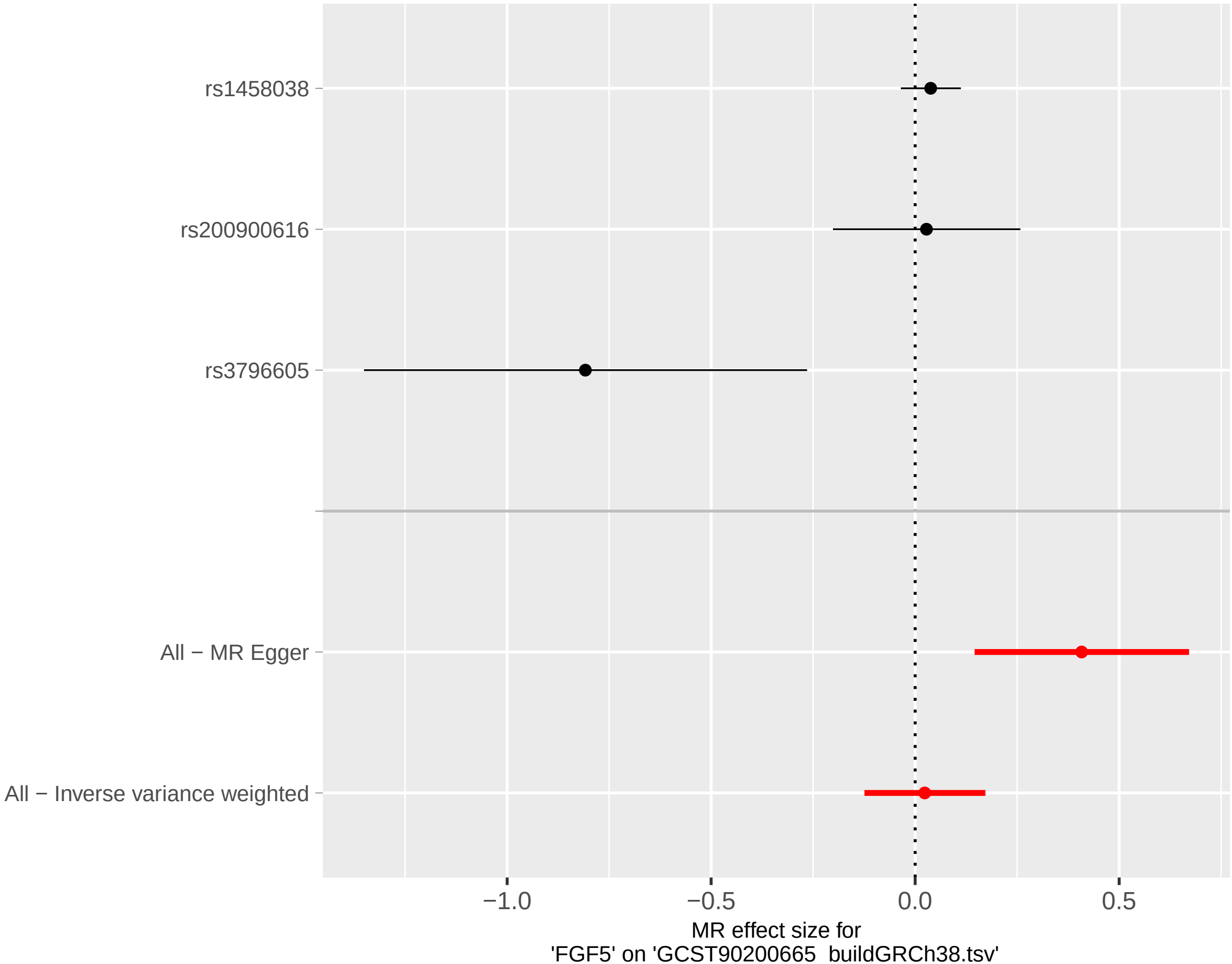
rs1458038
rs200900616
rs3796605
All − MR Egger
All − Inverse variance weighted
−1.0
−0.5
0.0
0.5
MR effect size for
'FGF5' on 'GCST90200665_buildGRCh38.tsv'

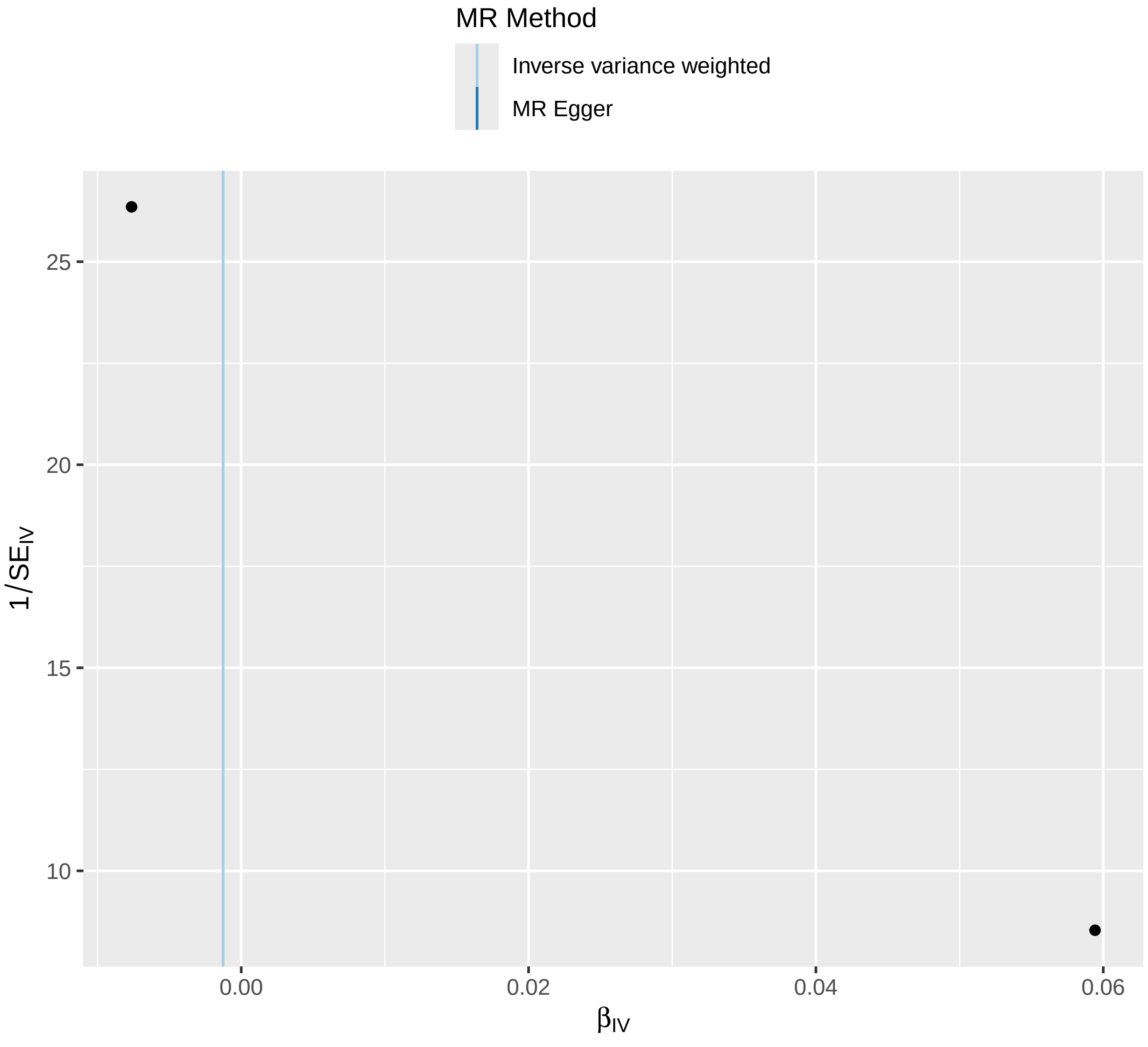
MR Method
Inverse variance weighted
MR Egger
25
20
15
10
1/SE_IV
0.00
0.02
0.04
0.06
β_IV

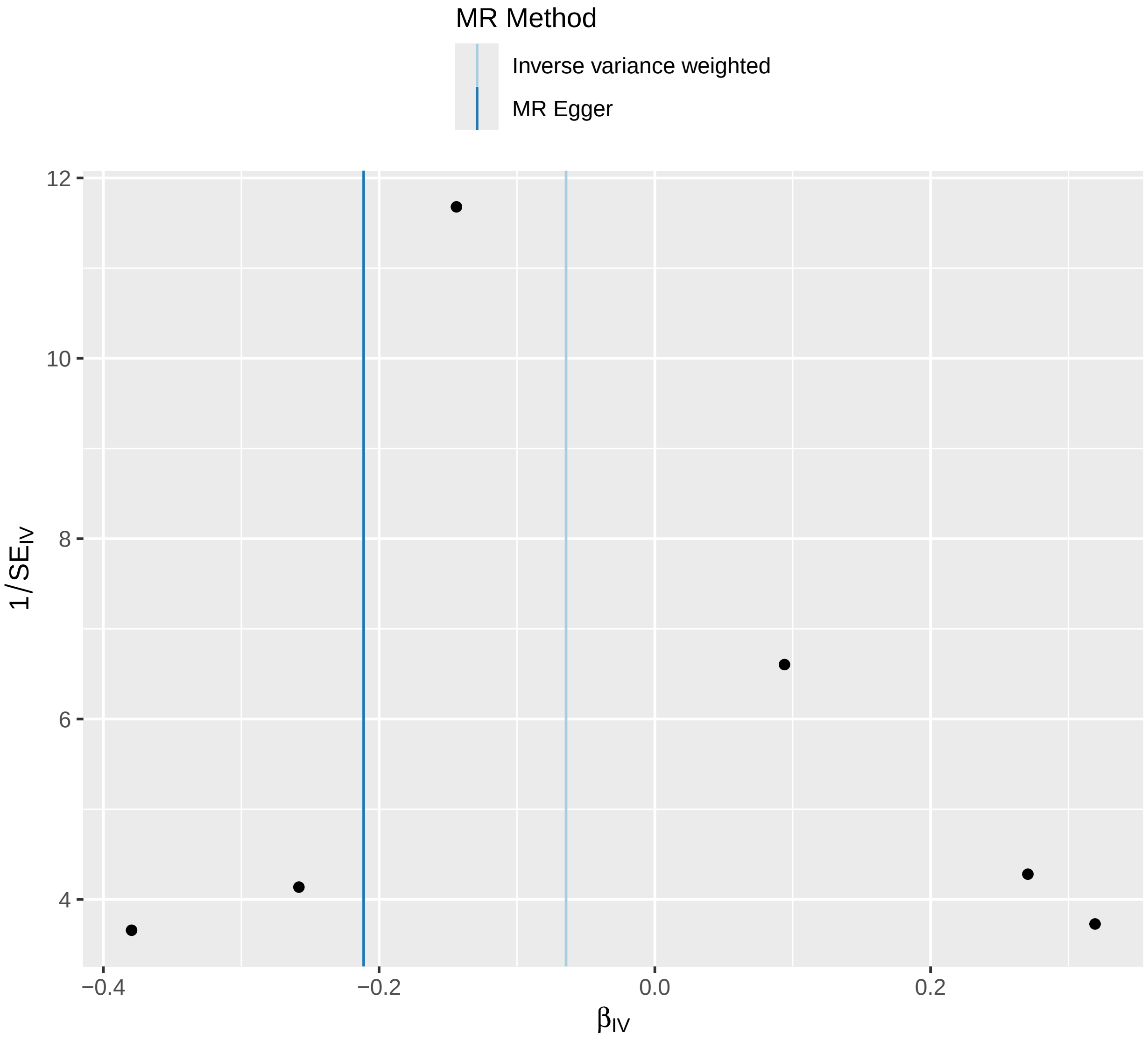

MR Method
Inverse variance weighted
MR Egger
12
10
8
6
4
1/SE_IV
−0.4
−0.2
0.0
0.2
β_IV

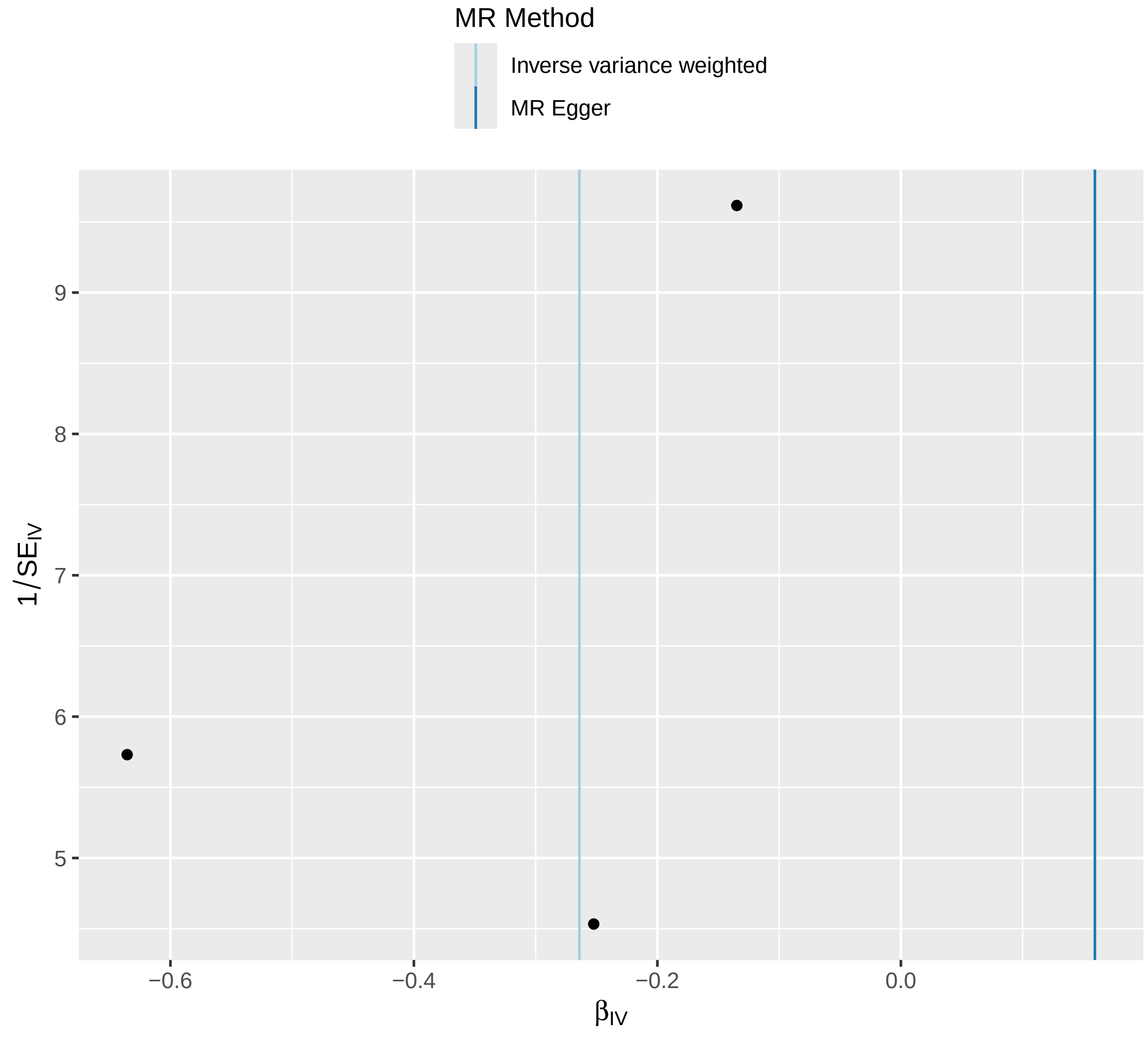
MR Method
Inverse variance weighted
MR Egger
1/SE_IV
9
8
7
6
5
−0.6
−0.4
−0.2
0.0
β_IV

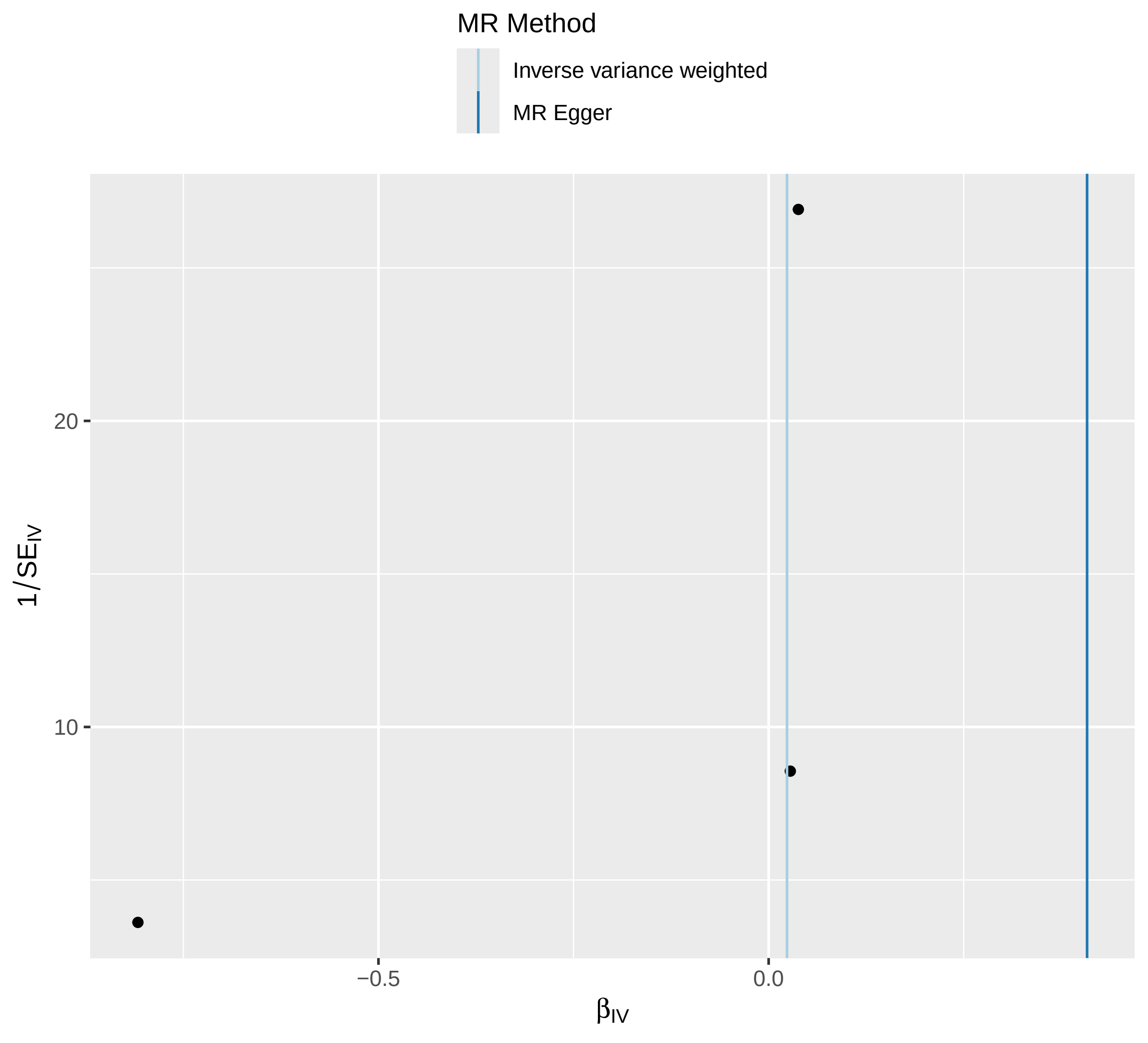

MR Method
Inverse variance weighted
MR Egger
20
10
1/SE_IV
−0.5
0.0
β_IV

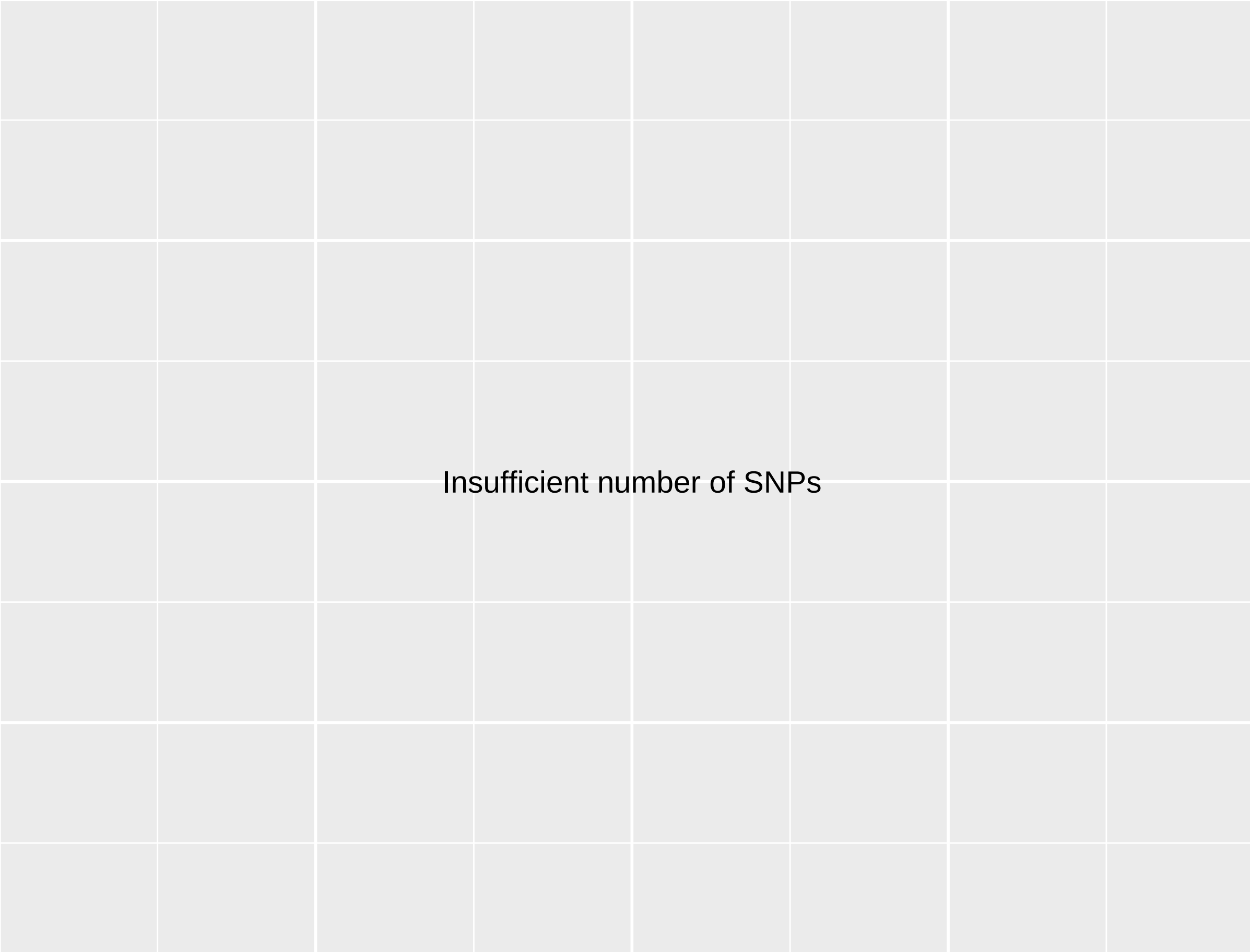
Insufficient number of SNPs

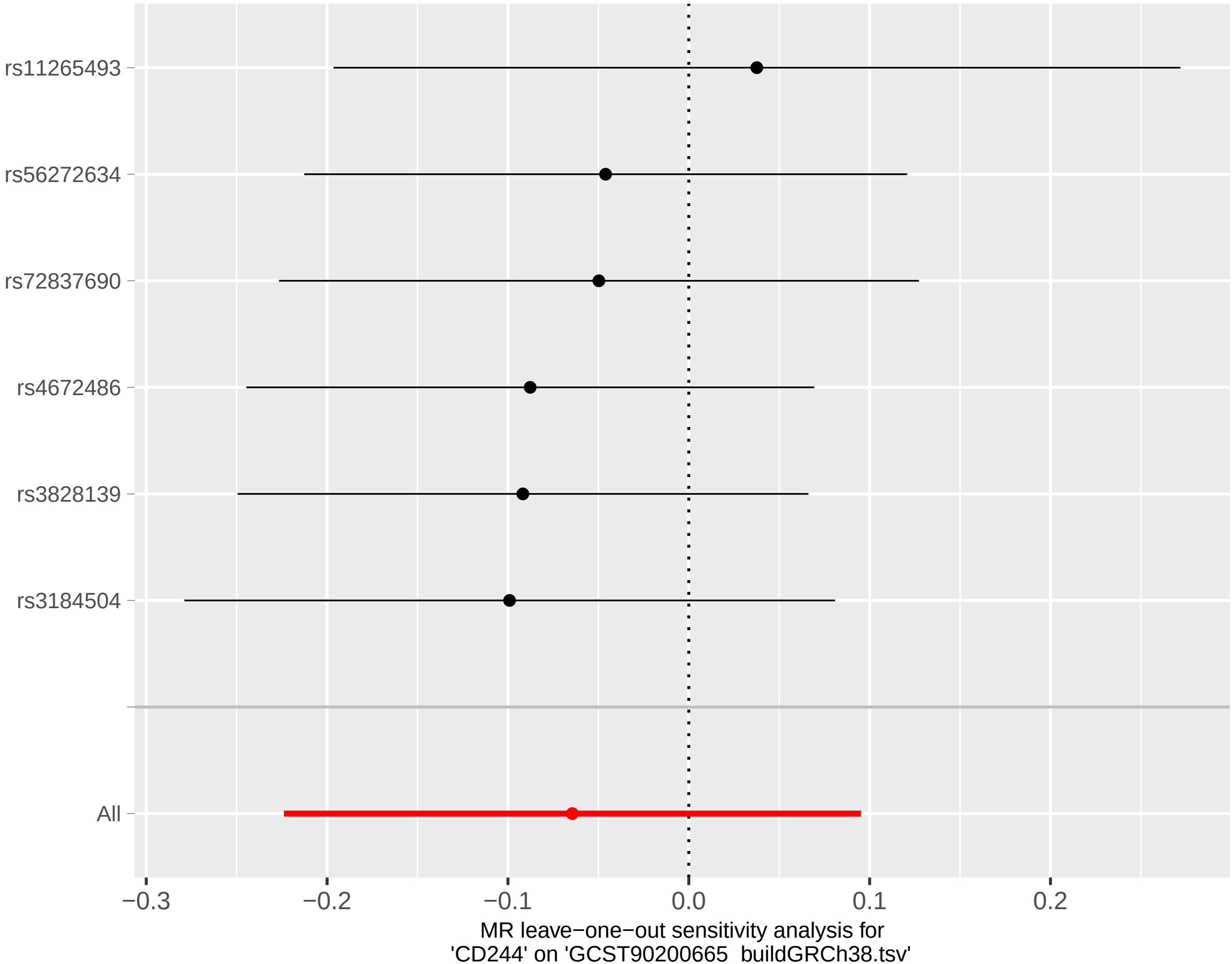
rs11265493
rs56272634
rs72837690
rs4672486
rs3828139
rs3184504
All
−0.3
−0.2
−0.1
0.0
0.1
0.2
MR leave−one−out sensitivity analysis for
'CD244' on 'GCST90200665_buildGRCh38.tsv'

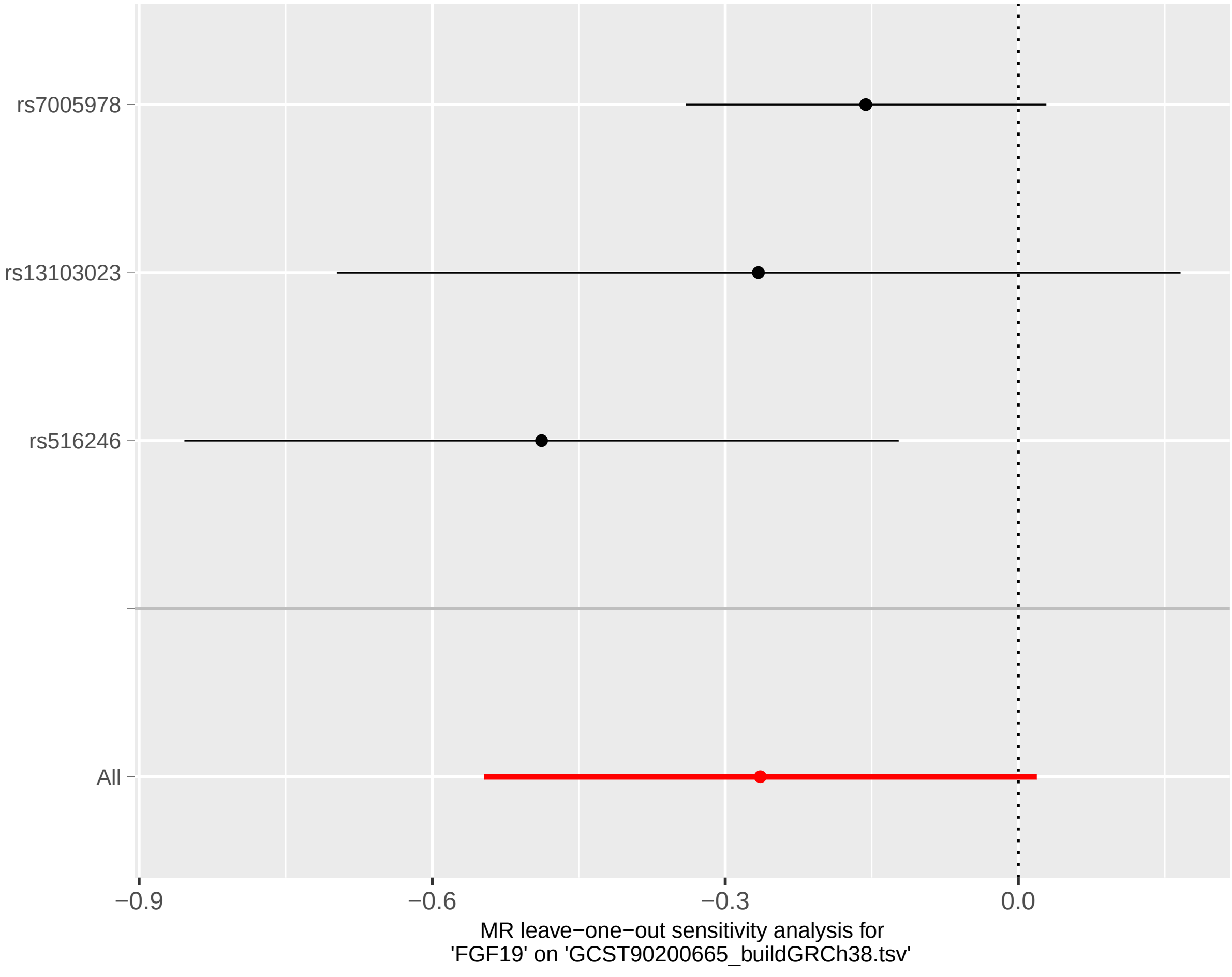
rs7005978
rs13103023
rs516246
All
−0.9
−0.6
−0.3
0.0
MR leave−one−out sensitivity analysis for
'FGF19' on 'GCST90200665_buildGRCh38.tsv'

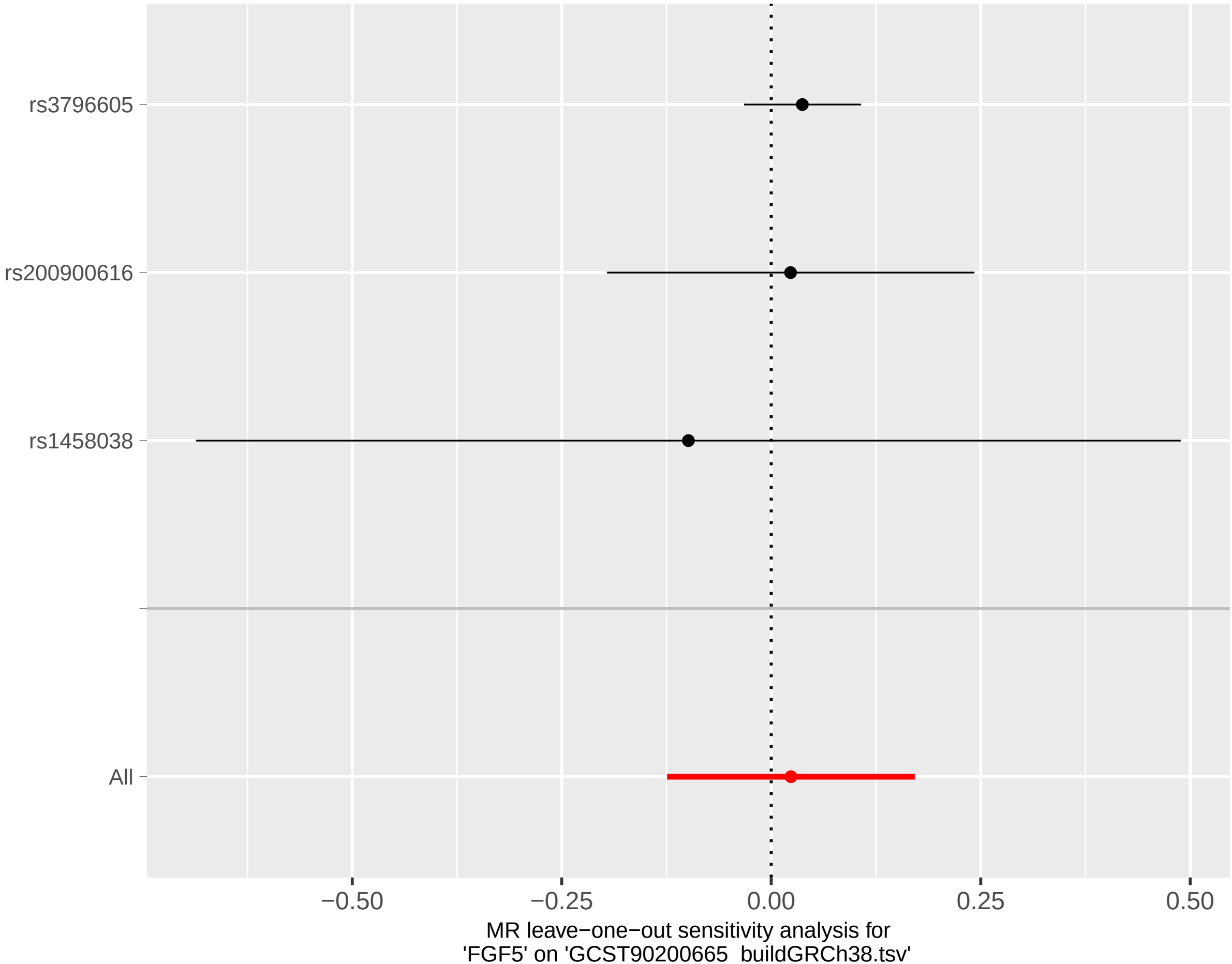
rs3796605
rs200900616
rs1458038
All
−0.50
−0.25
0.00
0.25
0.50
MR leave−one−out sensitivity analysis for
'FGF5' on 'GCST90200665_buildGRCh38.tsv'

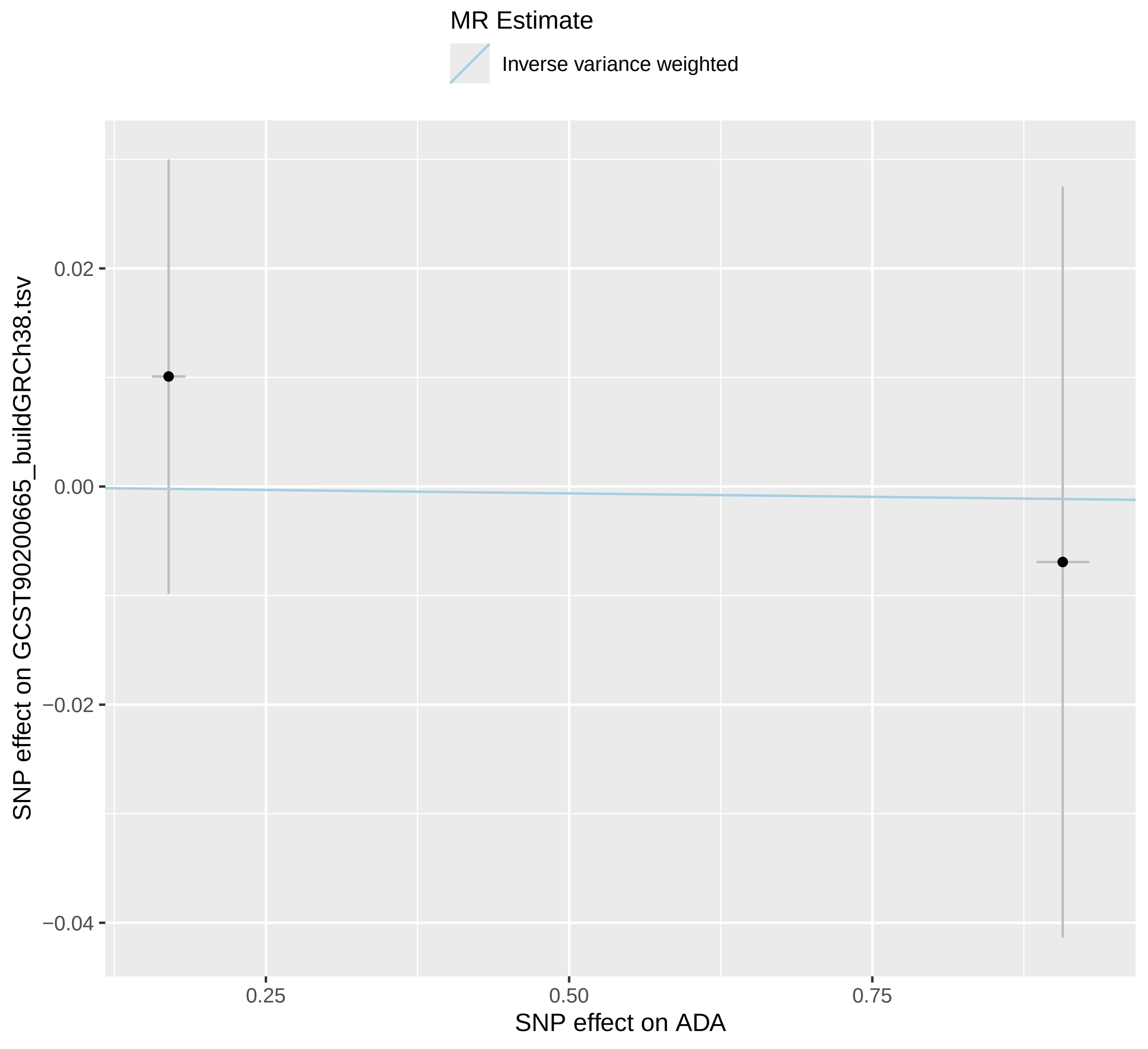
MR Estimate
Inverse variance weighted
0.02
0.00
−0.02
−0.04
0.25
0.50
0.75
SNP effect on ADA
SNP effect on GCST90200665_buildGRCh38.tsv

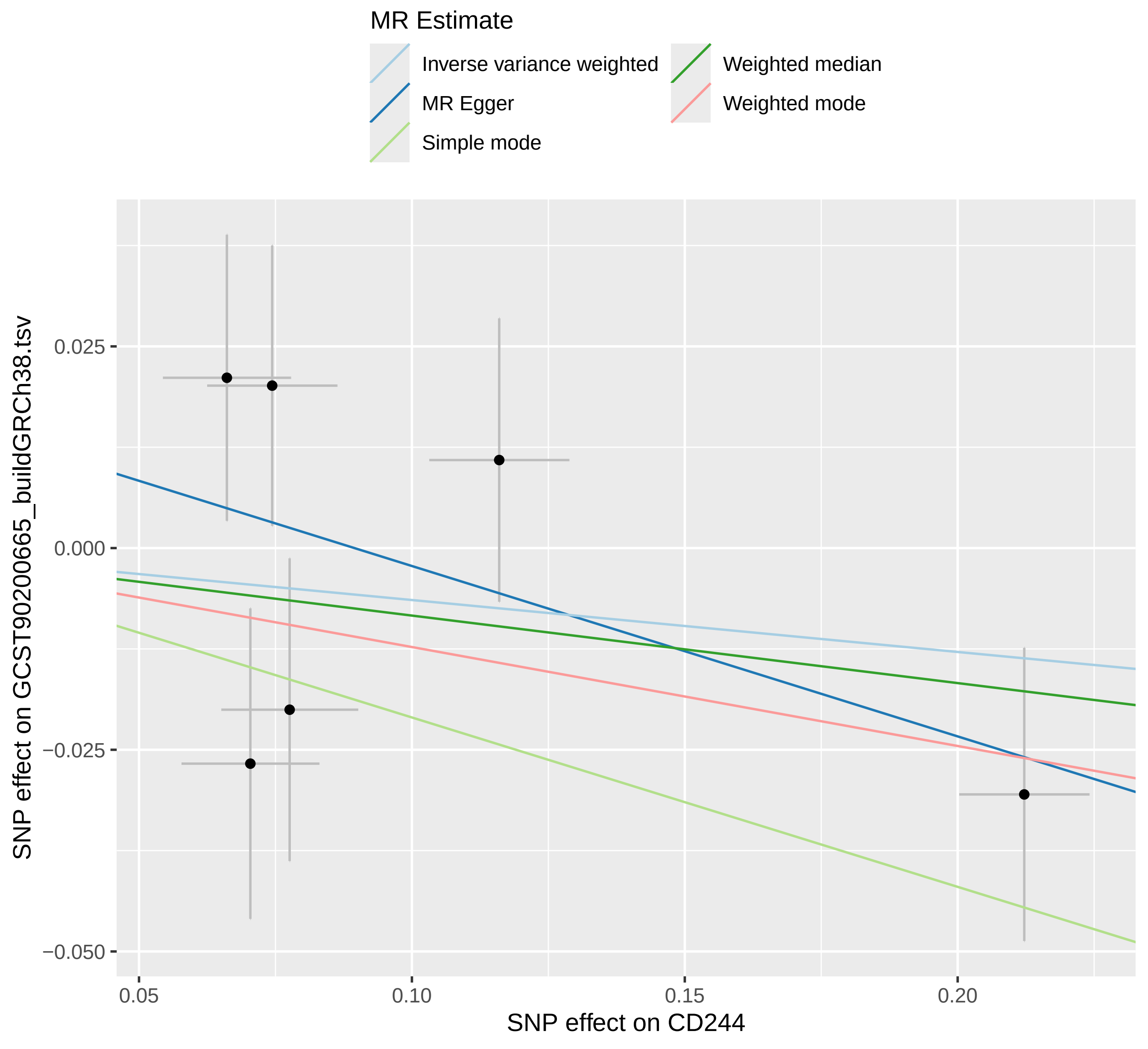

MR Estimate
Inverse variance weighted
MR Egger
Simple mode
Weighted median
Weighted mode
0.025
0.000
−0.025
−0.050
0.05
0.10
0.15
0.20
SNP effect on GCST90200665_buildGRCh38.tsv
SNP effect on CD244

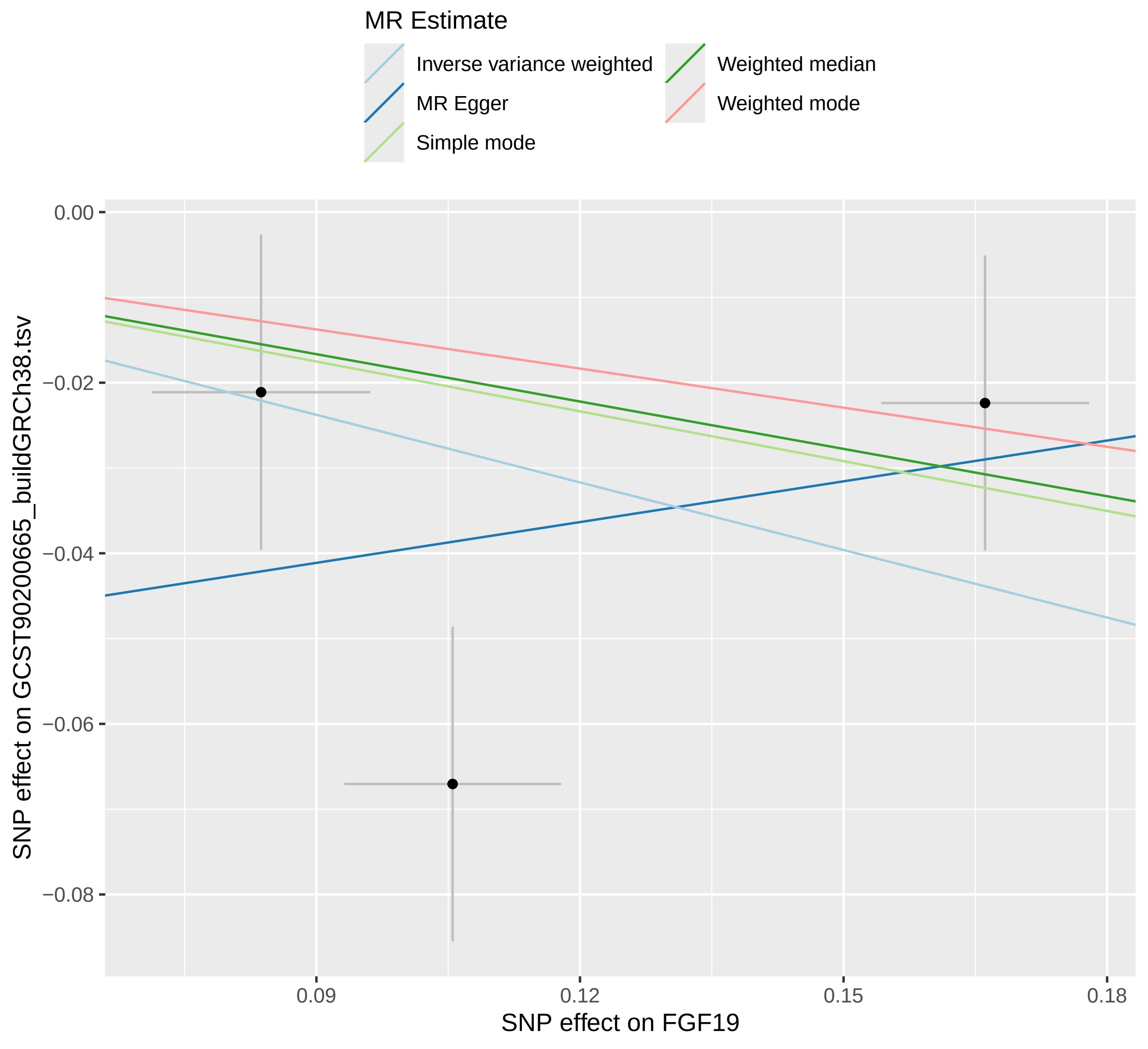
MR Estimate
Inverse variance weighted
MR Egger
Simple mode
Weighted median
Weighted mode
0.00
−0.02
−0.04
−0.06
−0.08
SNP effect on GCST90200665_buildGRCh38.tsv
0.09
0.12
0.15
0.18
SNP effect on FGF19

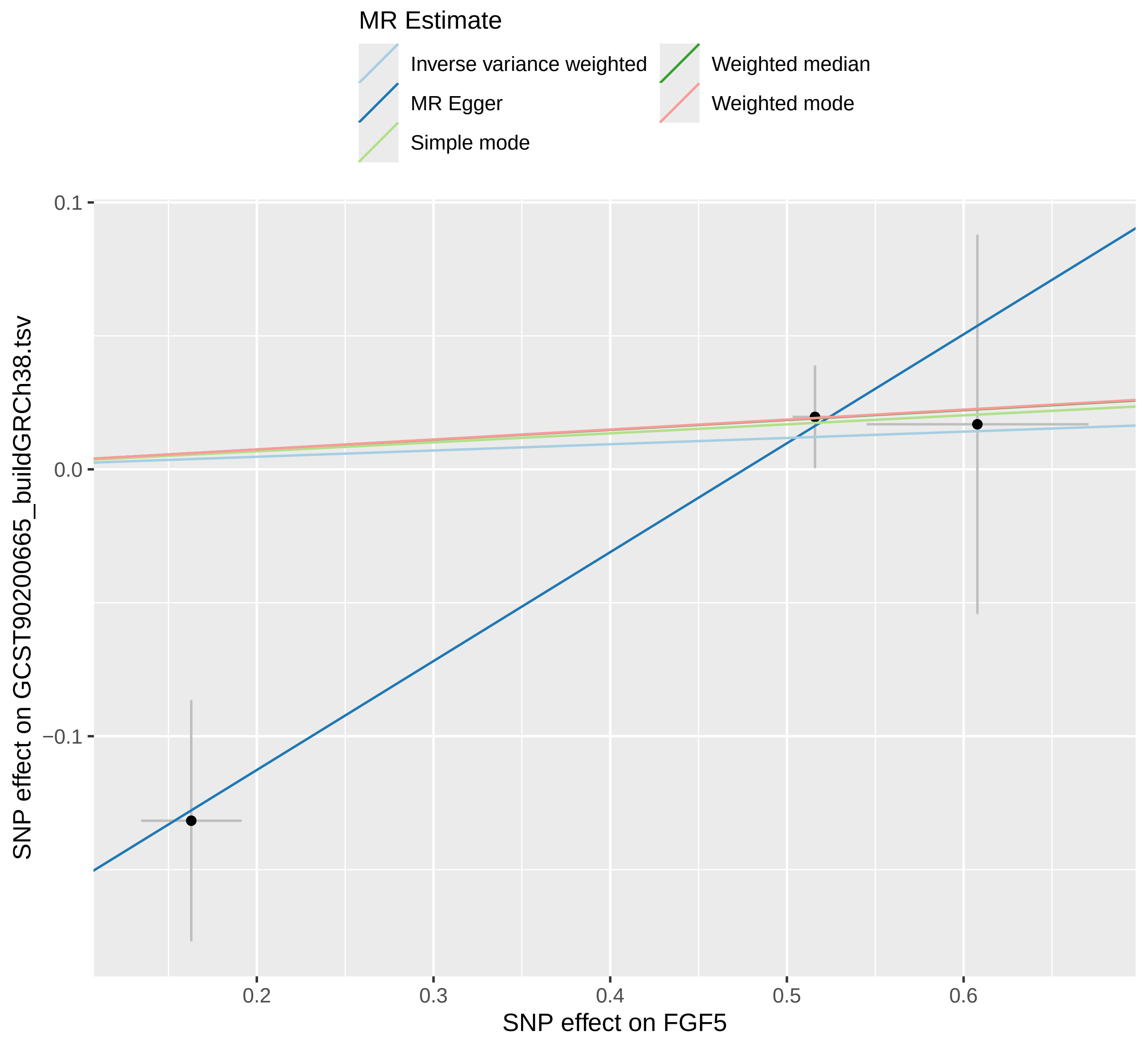

MR Estimate
Inverse variance weighted
MR Egger
Simple mode
Weighted median
Weighted mode
0.1
0.0
−0.1
0.2
0.3
0.4
0.5
0.6
SNP effect on GCST90200665_buildGRCh38.tsv
SNP effect on FGF5

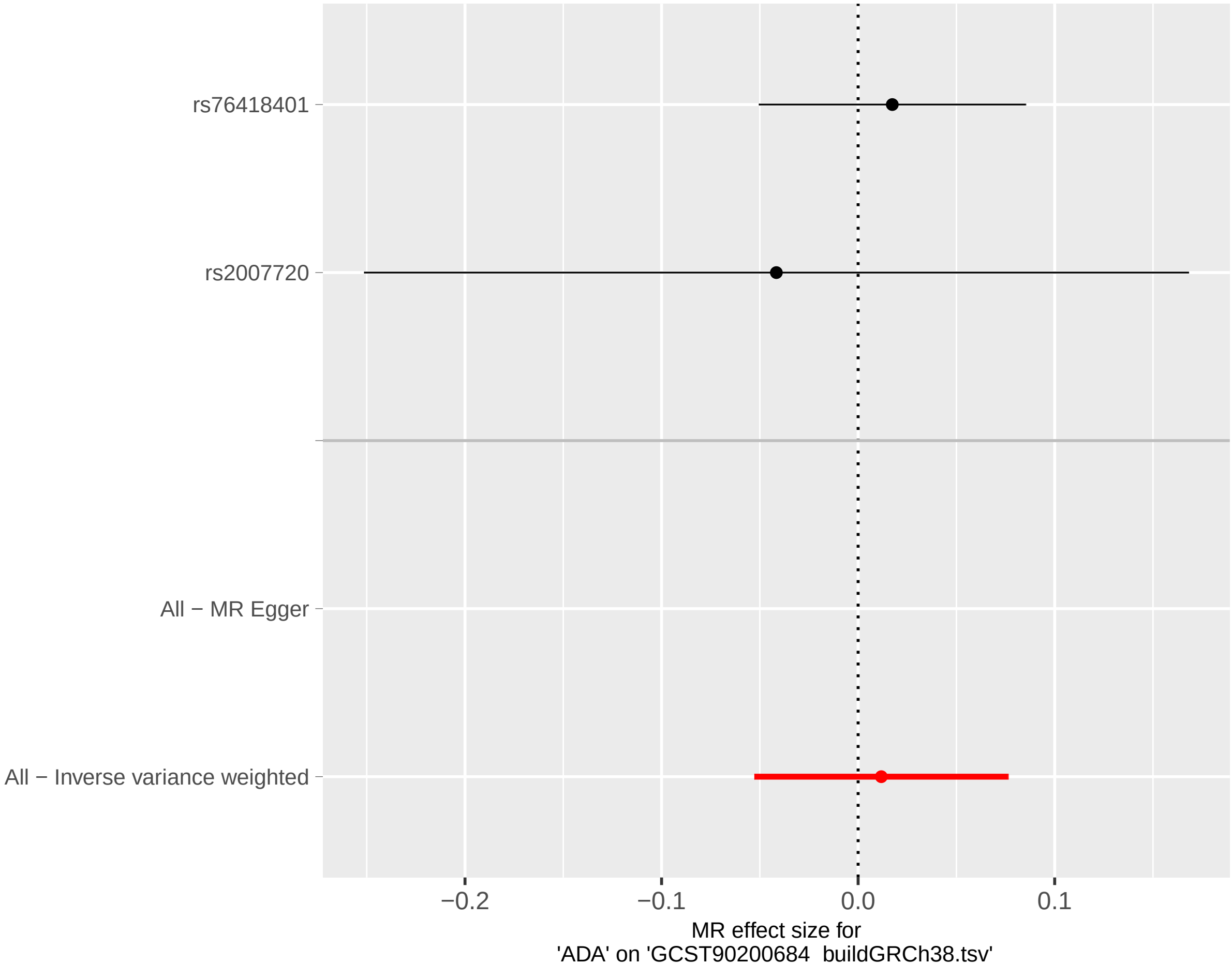

rs76418401
rs2007720
All − MR Egger
All − Inverse variance weighted
−0.2
−0.1
0.0
0.1
MR effect size for
'ADA' on 'GCST90200684_buildGRCh38.tsv'

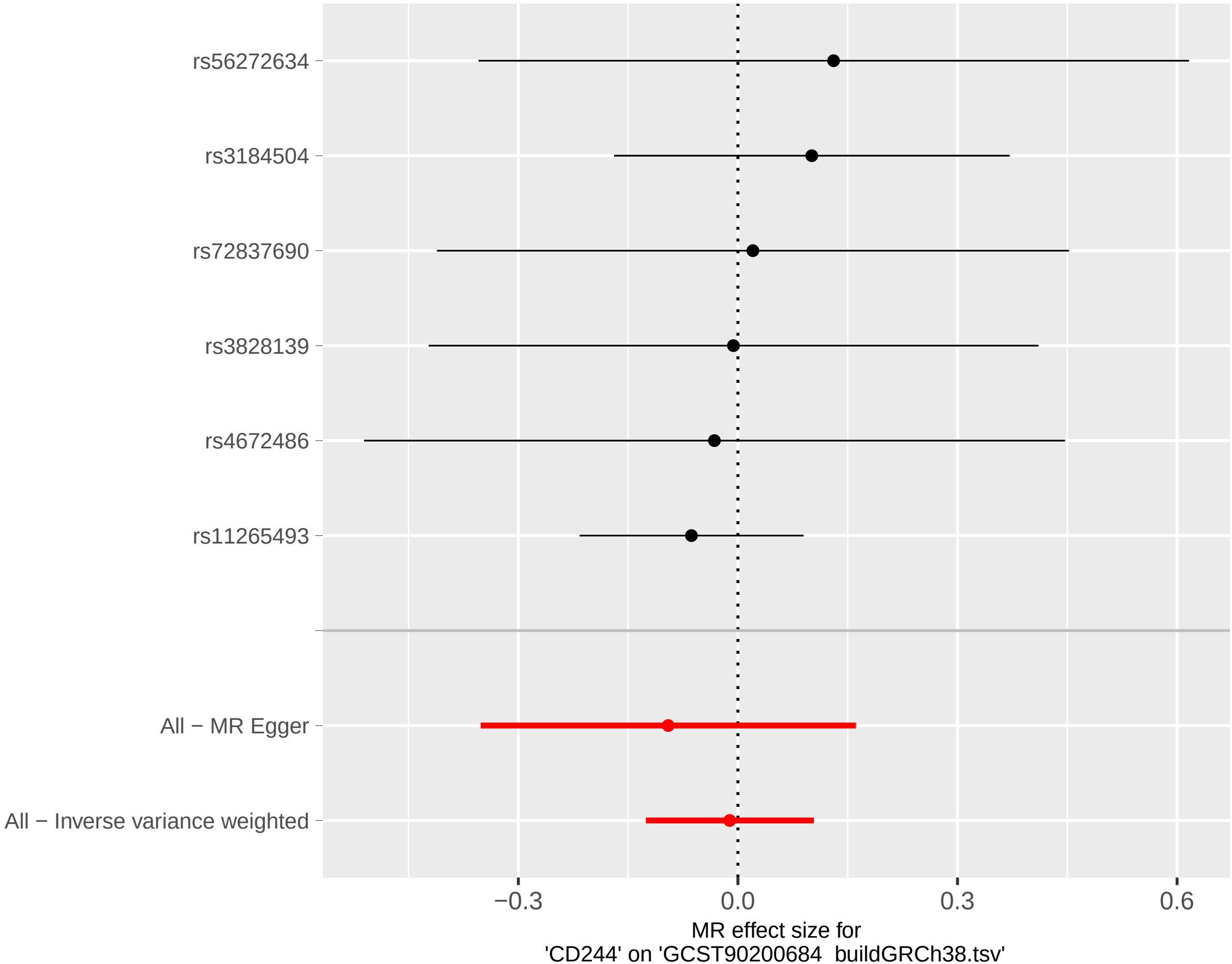
rs56272634
rs3184504
rs72837690
rs3828139
rs4672486
rs11265493
All − MR Egger
All − Inverse variance weighted
−0.3
0.0
0.3
0.6
MR effect size for
'CD244' on 'GCST90200684_buildGRCh38.tsv'

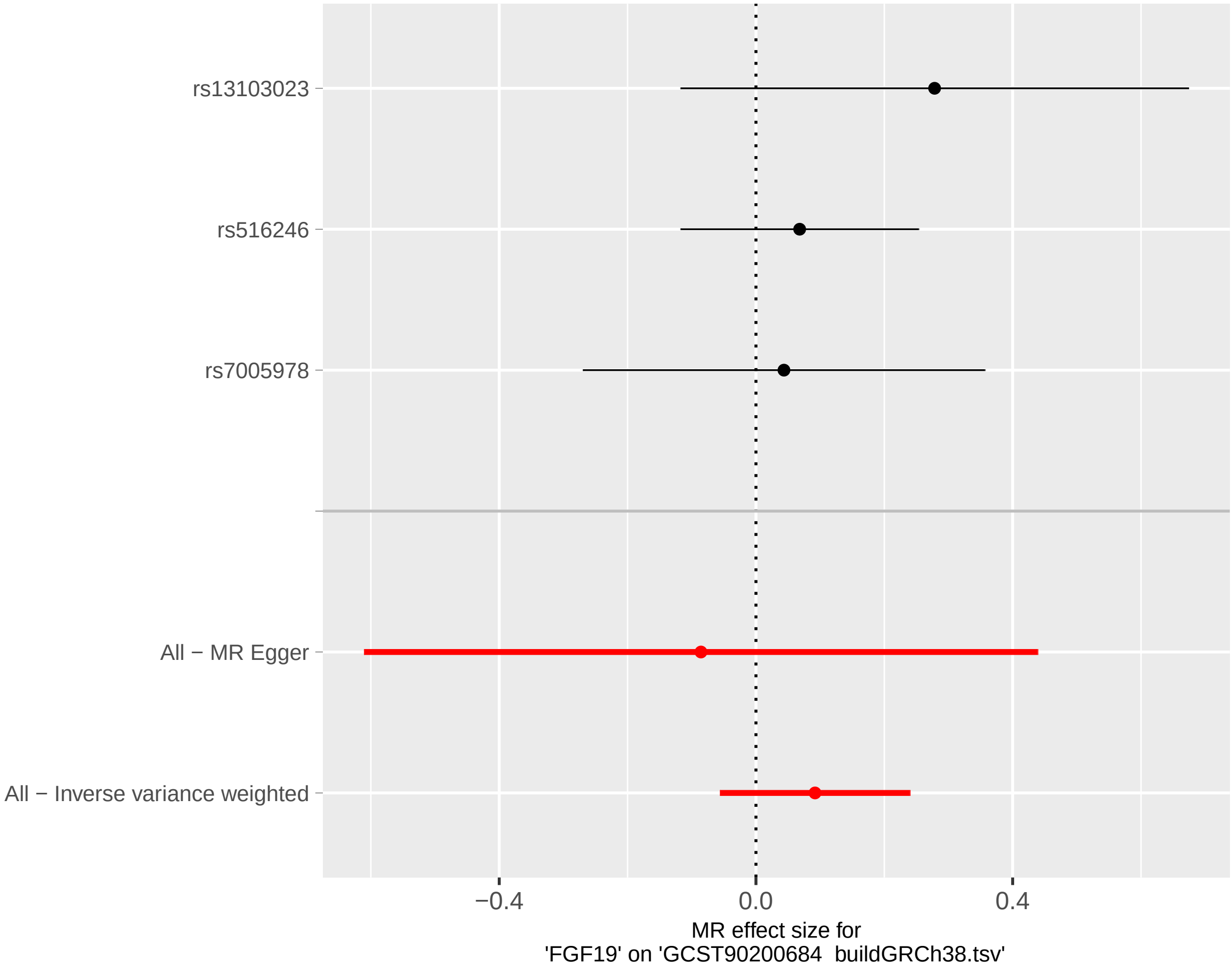
rs13103023
rs516246
rs7005978
All − MR Egger
All − Inverse variance weighted
−0.4
0.0
0.4
MR effect size for
'FGF19' on 'GCST90200684_buildGRCh38.tsv'

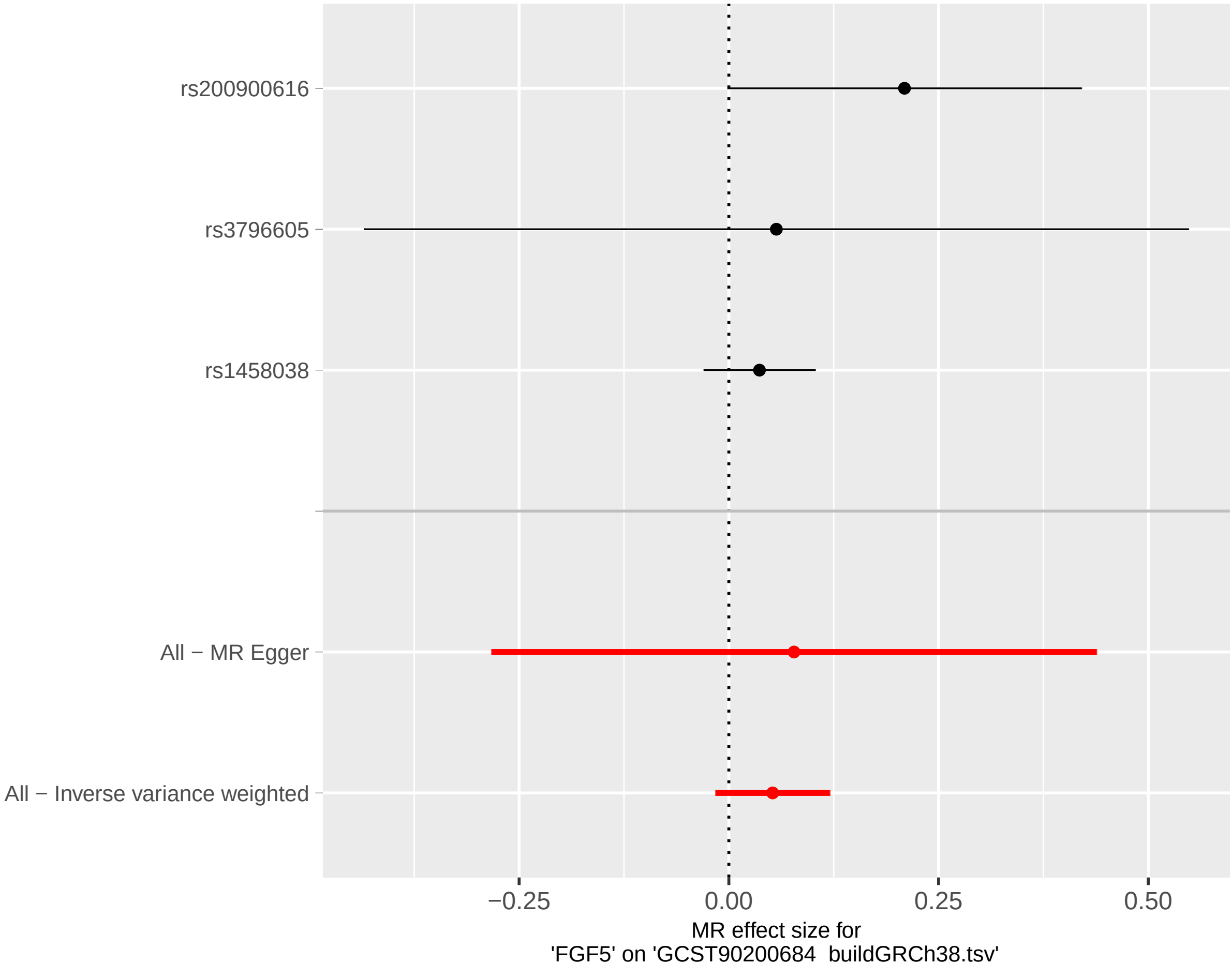

rs200900616
rs3796605
rs1458038
All − MR Egger
All − Inverse variance weighted
−0.25
0.00
0.25
0.50
MR effect size for
'FGF5' on 'GCST90200684_buildGRCh38.tsv'

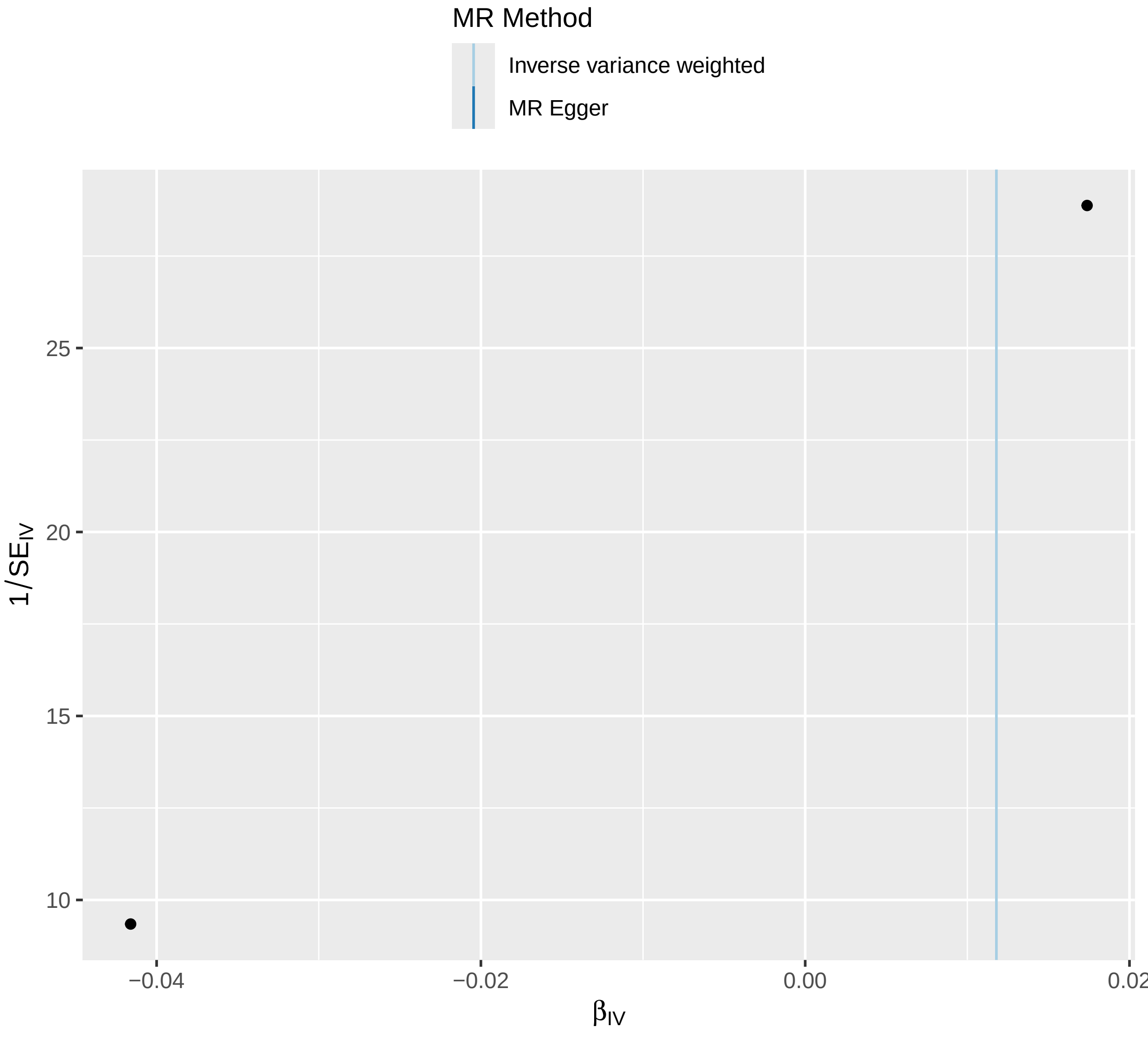

MR Method
Inverse variance weighted
MR Egger
25
20
15
10
1/SE_IV
−0.04
−0.02
0.00
0.02
β_IV

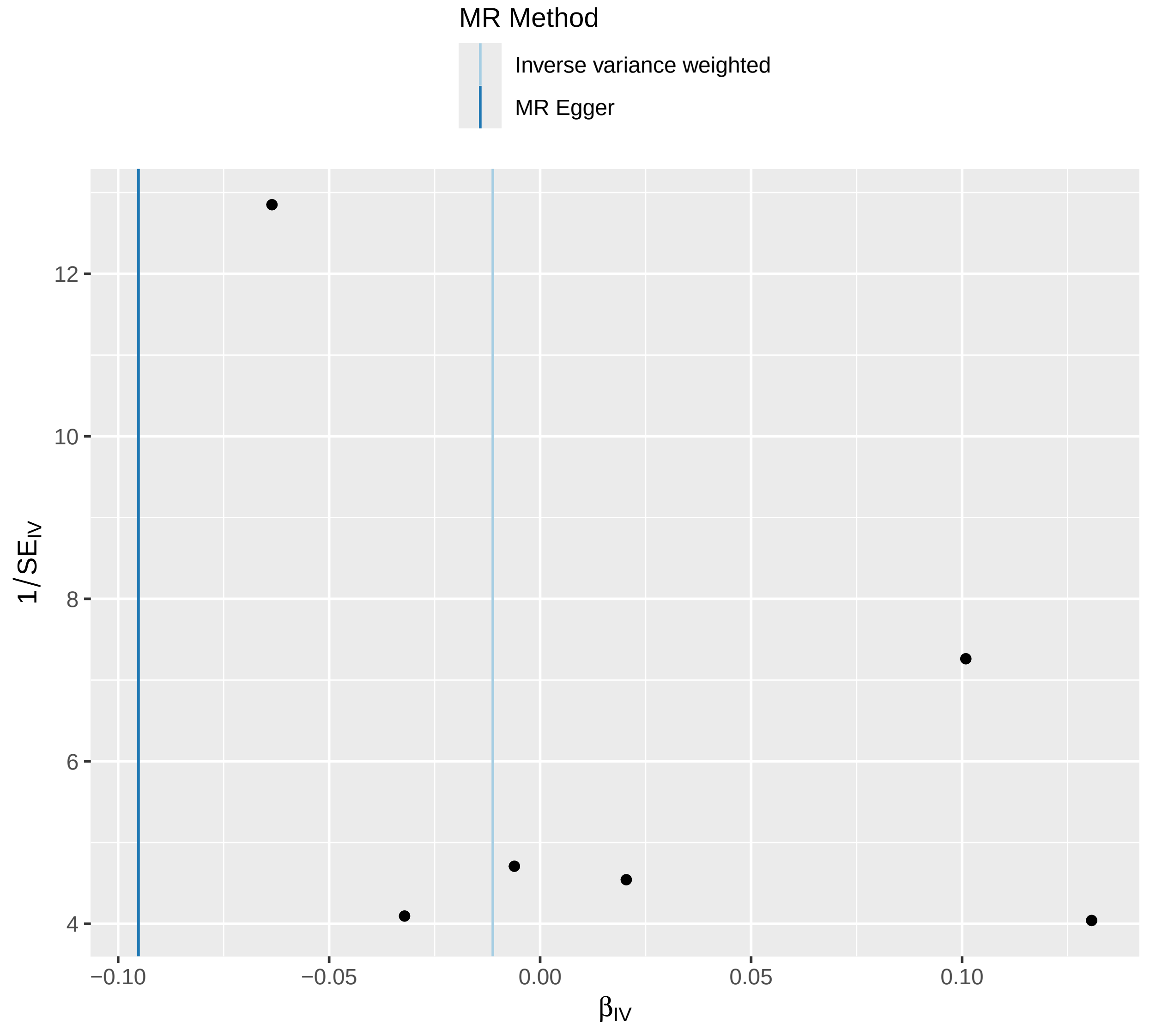
MR Method
Inverse variance weighted
MR Egger
12
10
8
6
4
1/SE_IV
−0.10
−0.05
0.00
0.05
0.10
β_IV

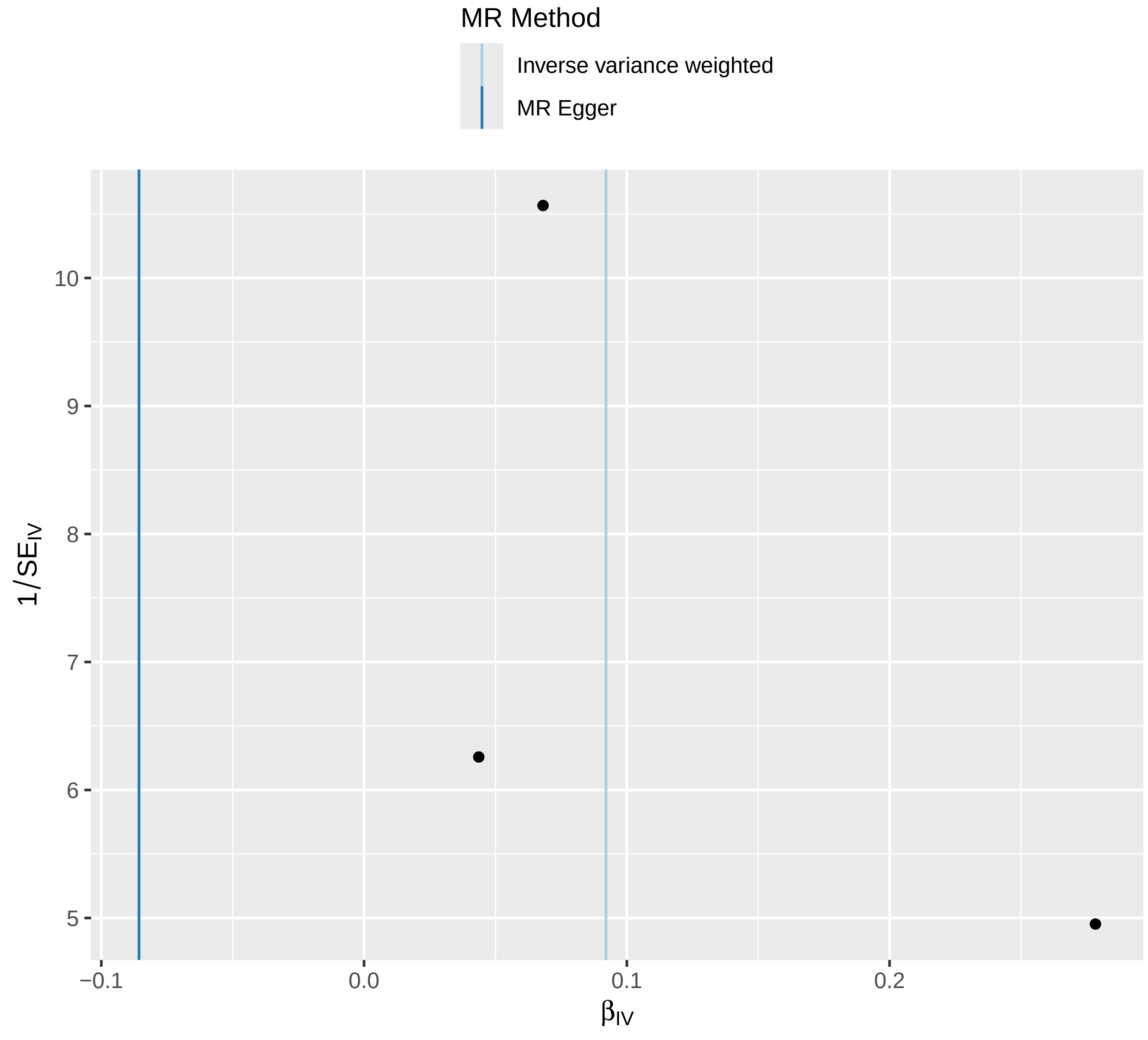
MR Method
Inverse variance weighted
MR Egger
10
9
8
7
6
5
1/SE$_{IV}$
−0.1
0.0
0.1
0.2
β$_{IV}$

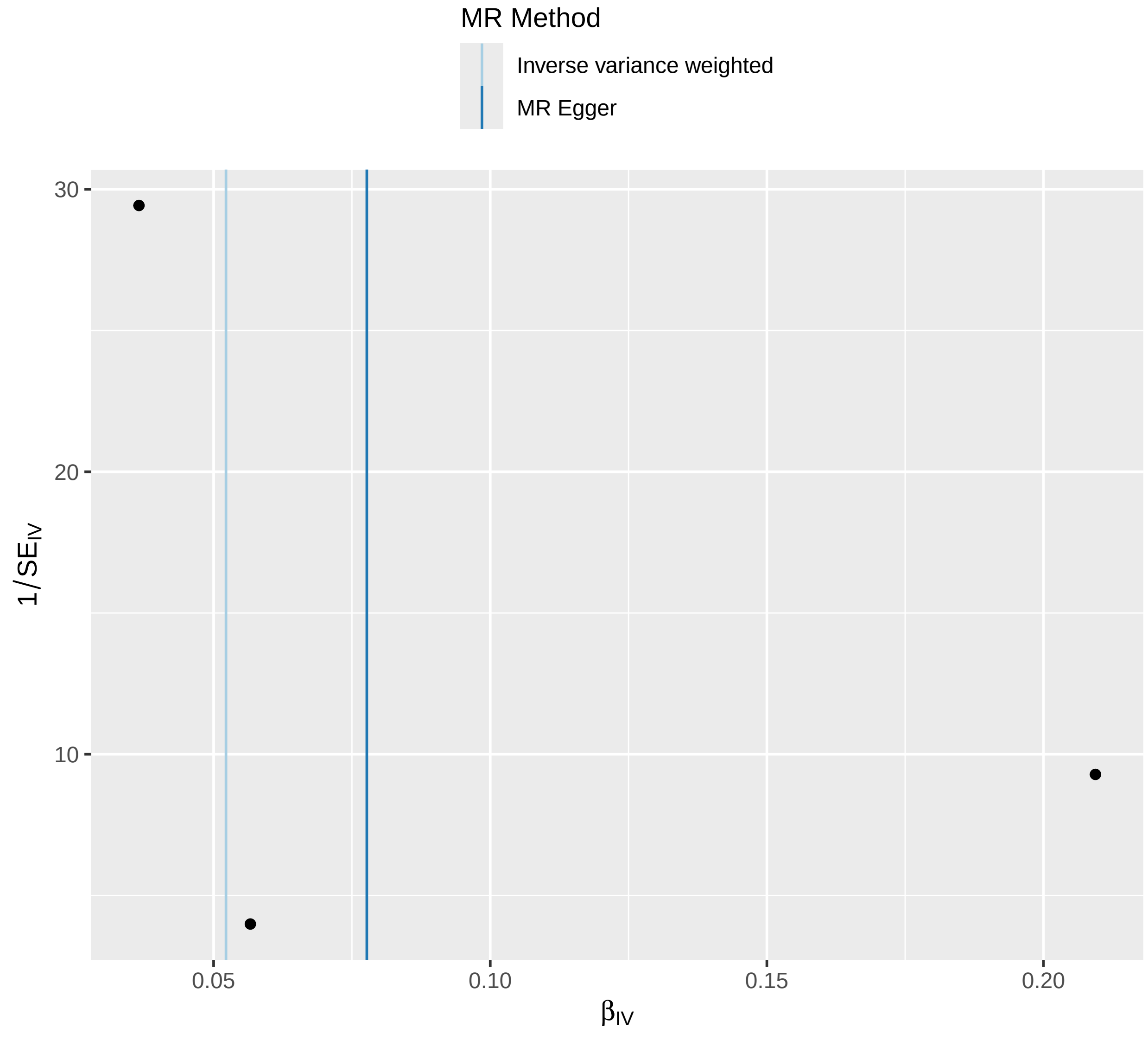
MR Method
Inverse variance weighted
MR Egger
30
20
10
0.05
0.10
0.15
0.20
1/SE_IV
β_IV

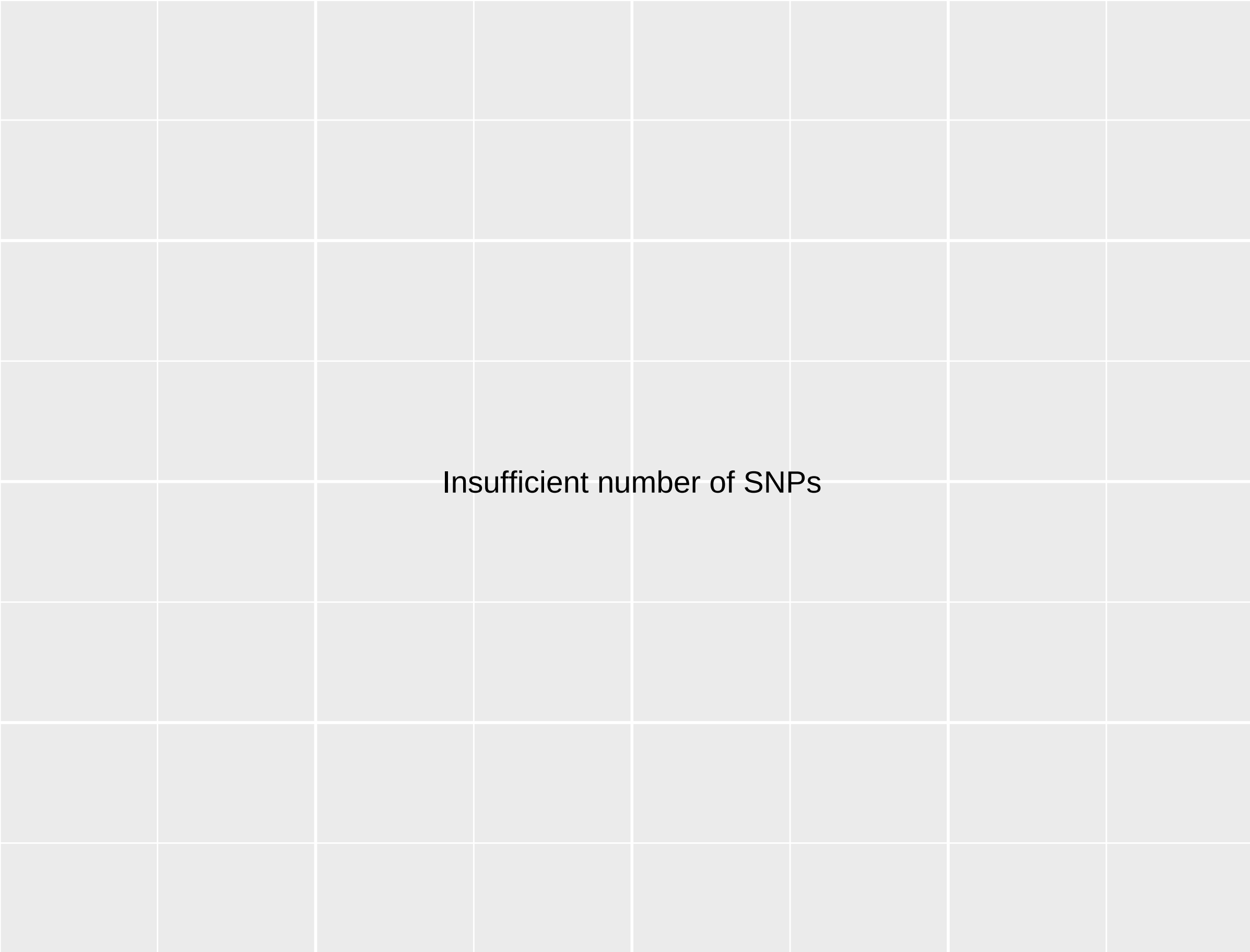
Insufficient number of SNPs

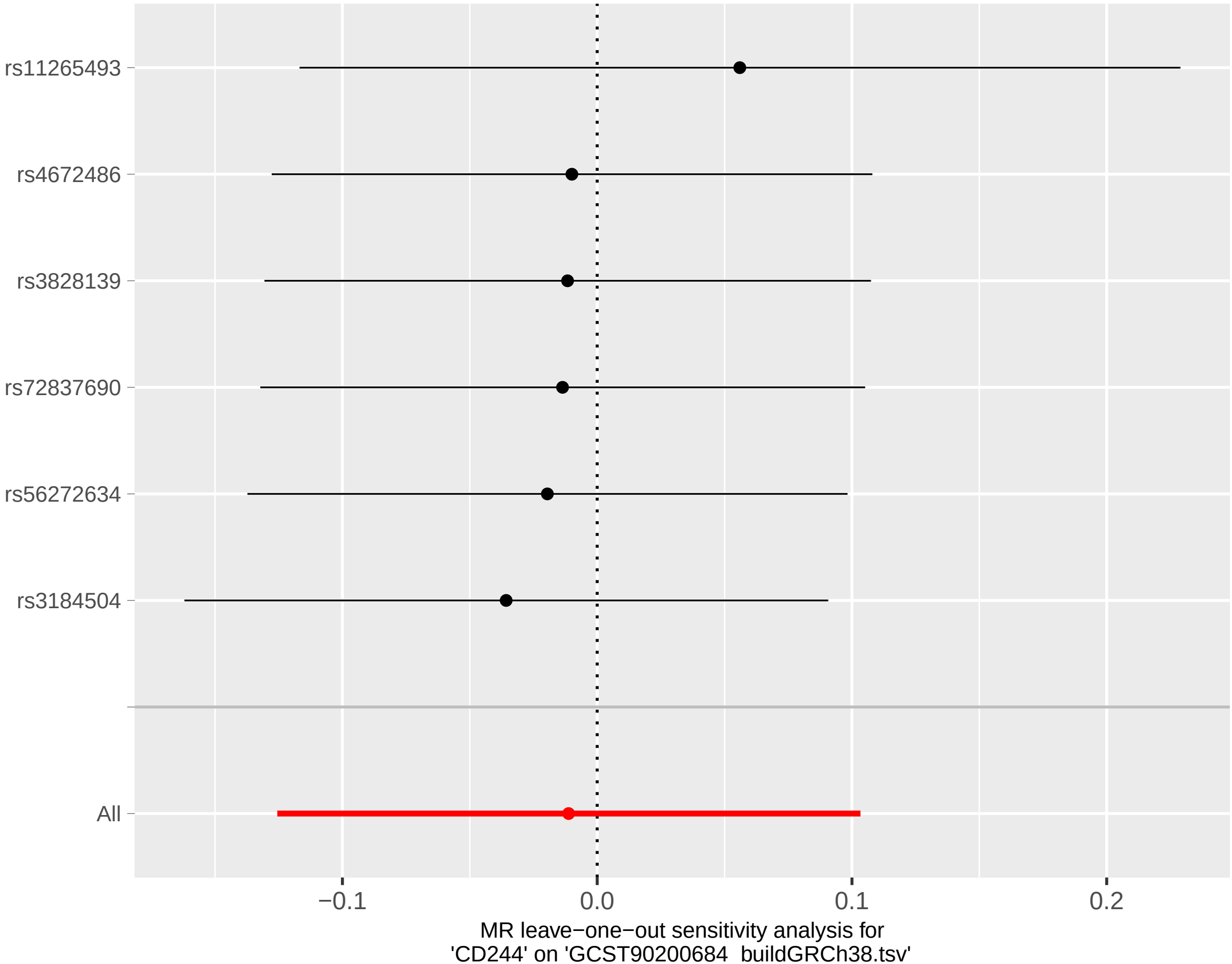
rs11265493
rs4672486
rs3828139
rs72837690
rs56272634
rs3184504
All
−0.1
0.0
0.1
0.2
MR leave−one−out sensitivity analysis for
'CD244' on 'GCST90200684_buildGRCh38.tsv'

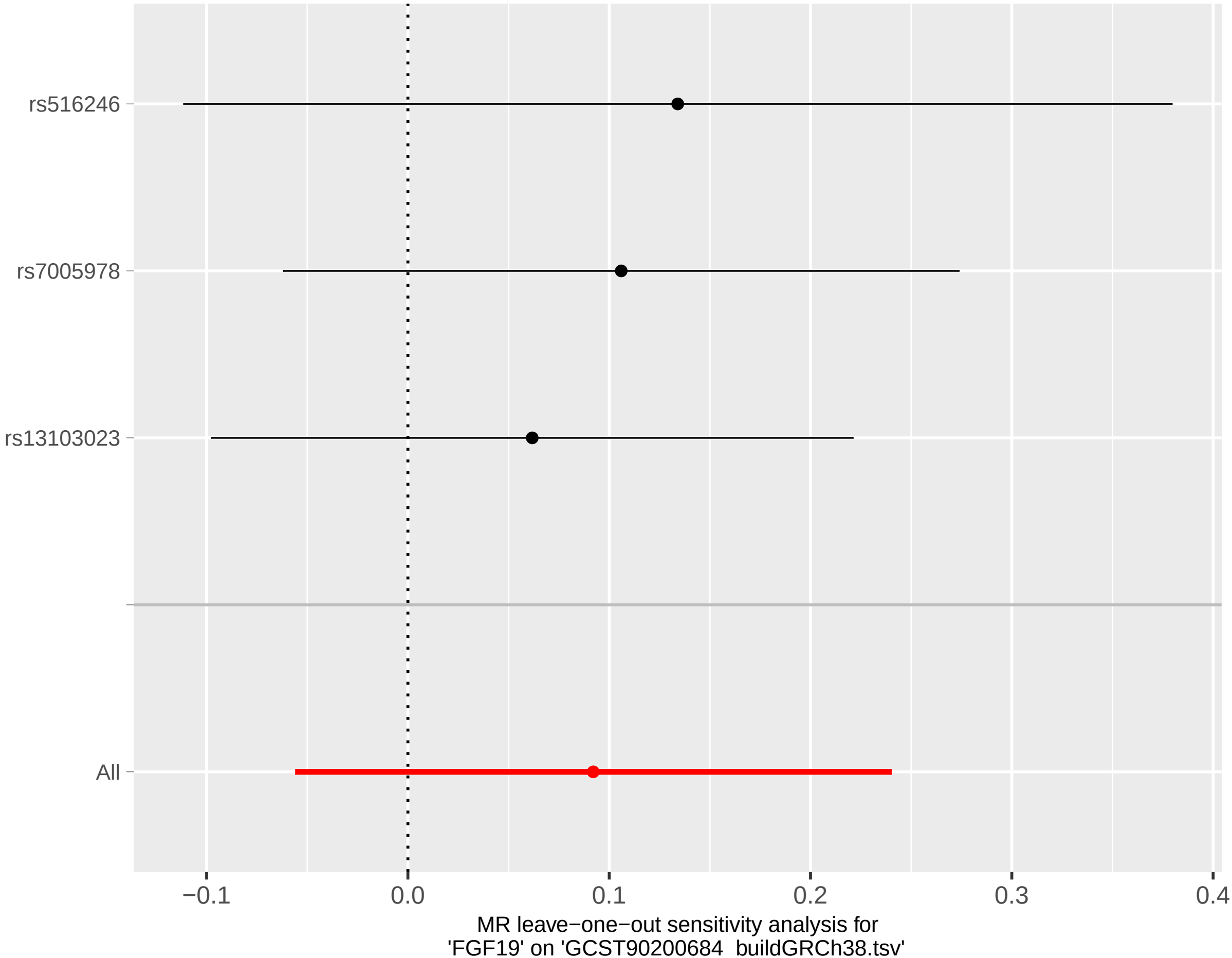
rs516246
rs7005978
rs13103023
All
−0.1
0.0
0.1
0.2
0.3
0.4
MR leave−one−out sensitivity analysis for
'FGF19' on 'GCST90200684_buildGRCh38.tsv'

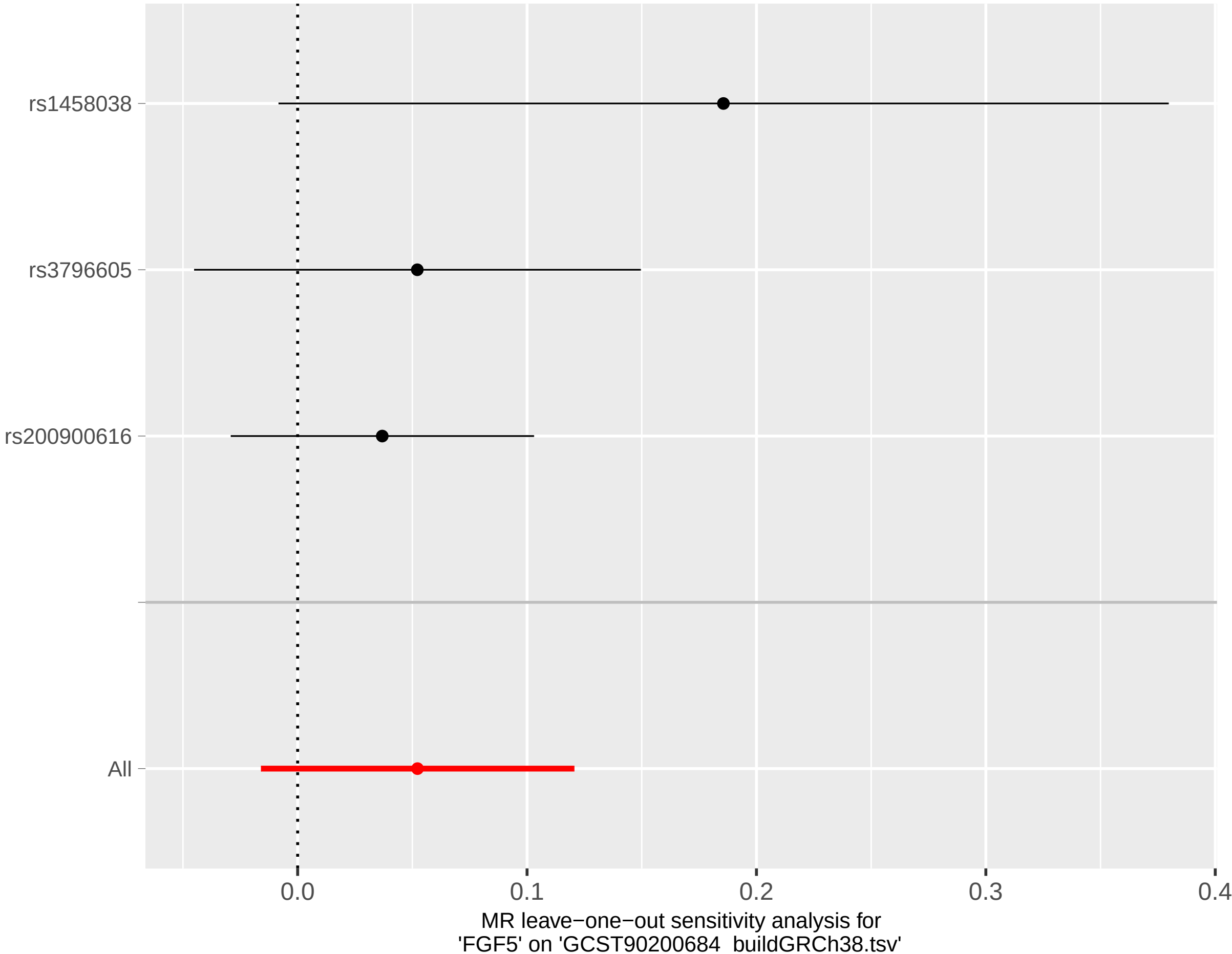
rs1458038
rs3796605
rs200900616
All
0.0
0.1
0.2
0.3
0.4
MR leave−one−out sensitivity analysis for
'FGF5' on 'GCST90200684_buildGRCh38.tsv'

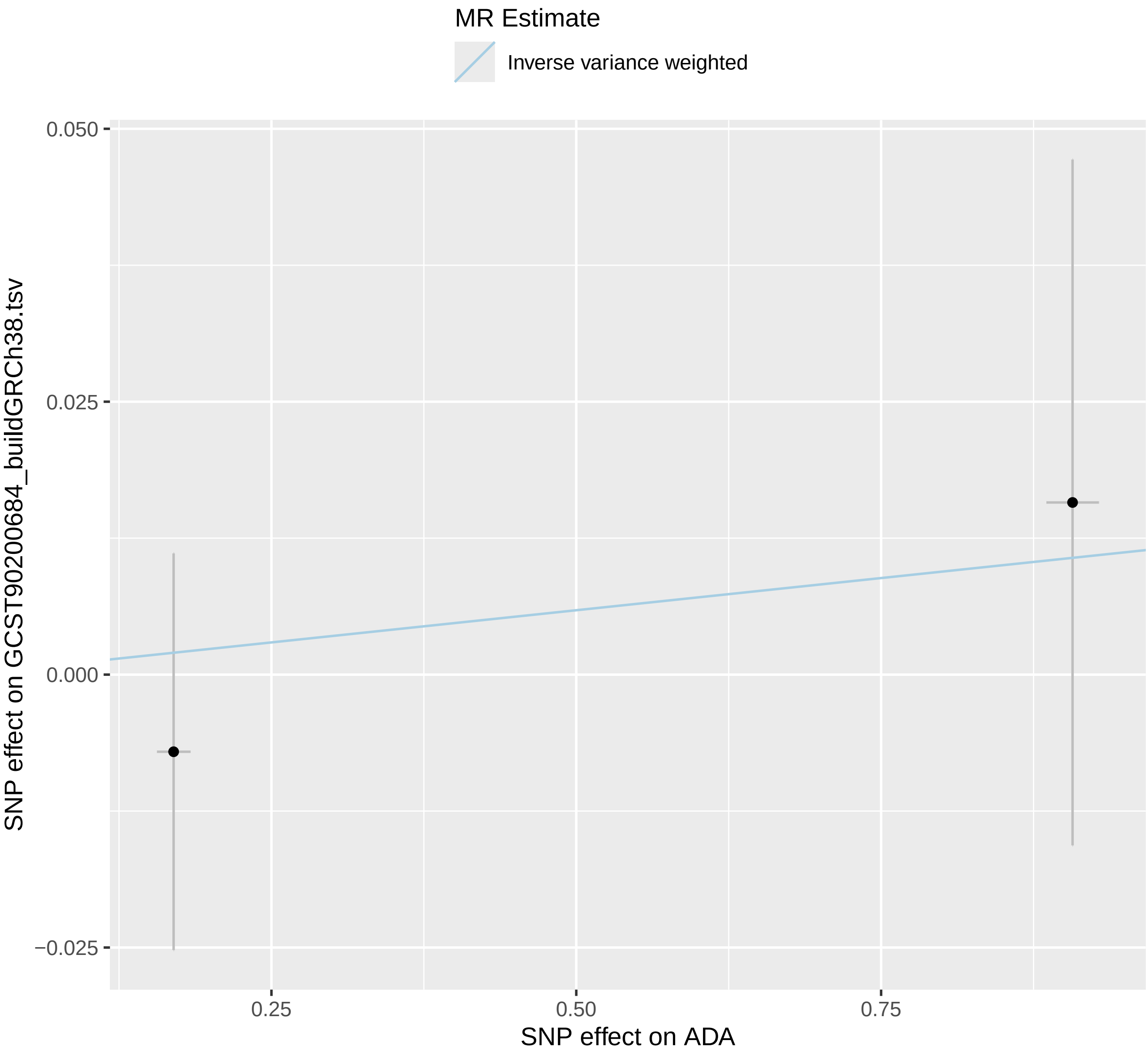

MR Estimate
Inverse variance weighted
0.050
0.025
0.000
−0.025
SNP effect on GCST90200684_buildGRCh38.tsv
0.25
0.50
0.75
SNP effect on ADA

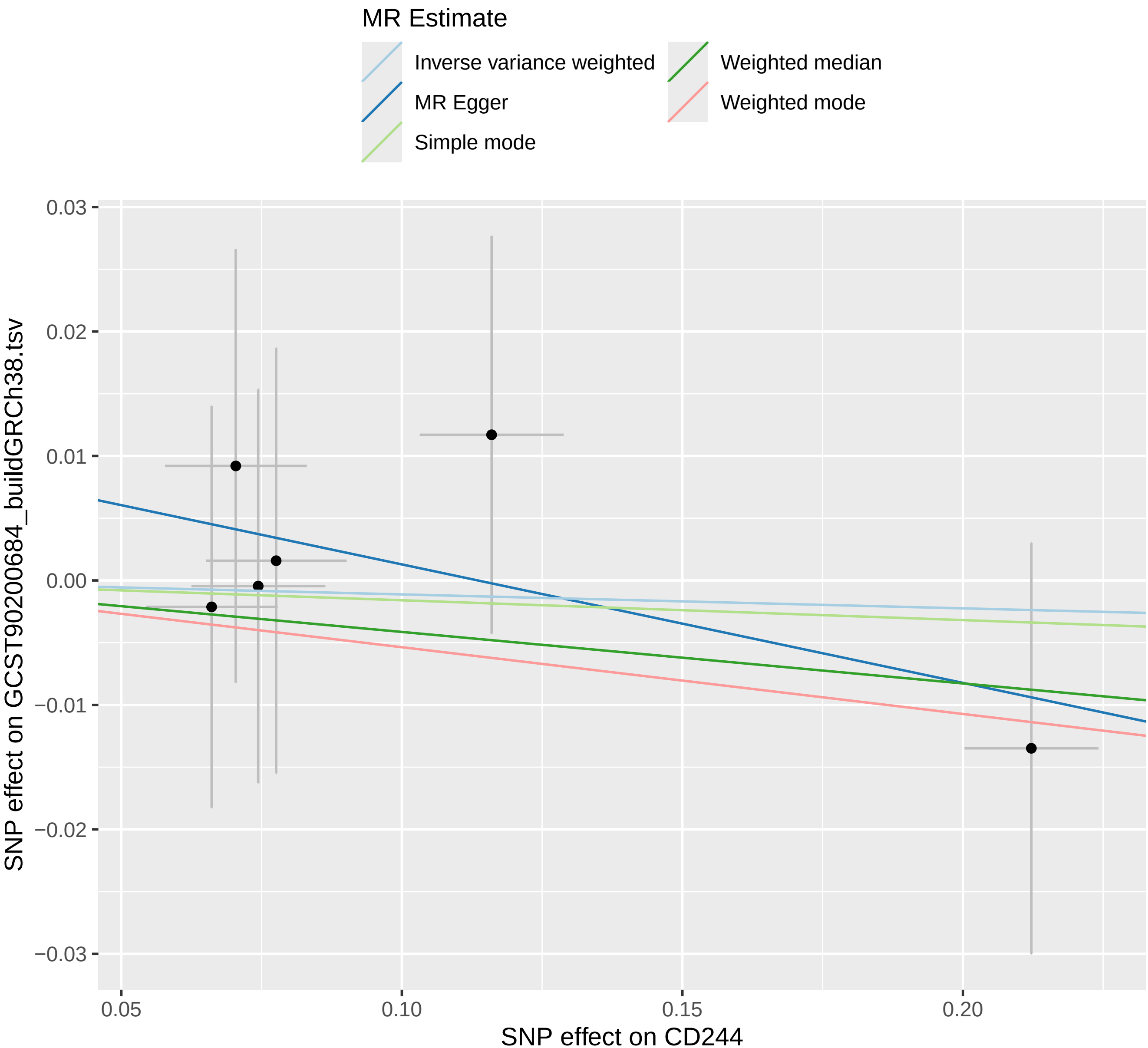
MR Estimate
Inverse variance weighted
MR Egger
Simple mode
Weighted median
Weighted mode
0.03
0.02
0.01
0.00
−0.01
−0.02
−0.03
0.05
0.10
0.15
0.20
SNP effect on CD244
SNP effect on GCST90200684_buildGRCh38.tsv

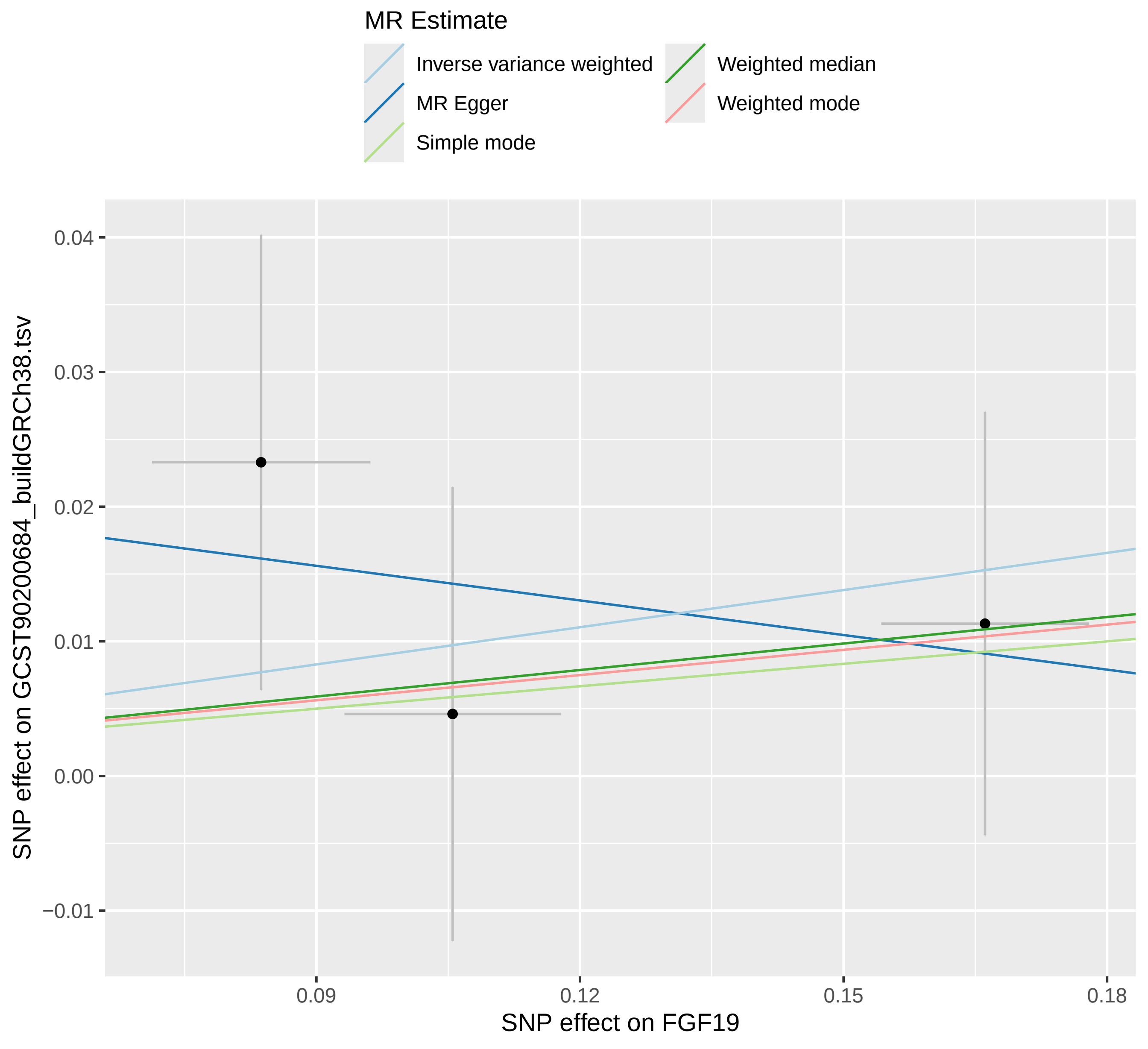
MR Estimate
Inverse variance weighted
MR Egger
Simple mode
Weighted median
Weighted mode
SNP effect on GCST90200684_buildGRCh38.tsv
0.04
0.03
0.02
0.01
0.00
−0.01
0.09
0.12
0.15
0.18
SNP effect on FGF19

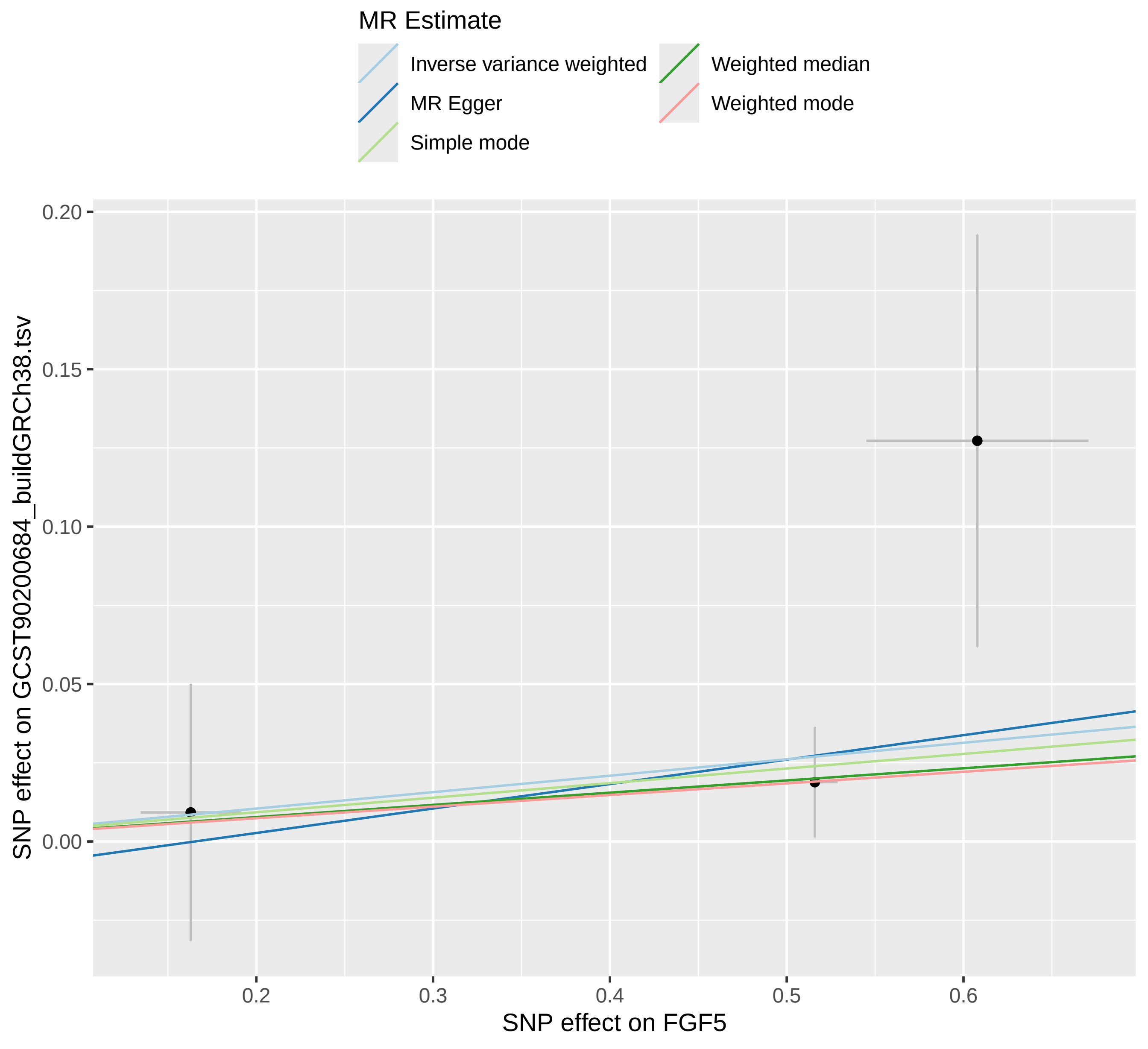
MR Estimate
Inverse variance weighted
MR Egger
Simple mode
Weighted median
Weighted mode
0.20
0.15
0.10
0.05
0.00
SNP effect on GCST90200684_buildGRCh38.tsv
0.2
0.3
0.4
0.5
0.6
SNP effect on FGF5

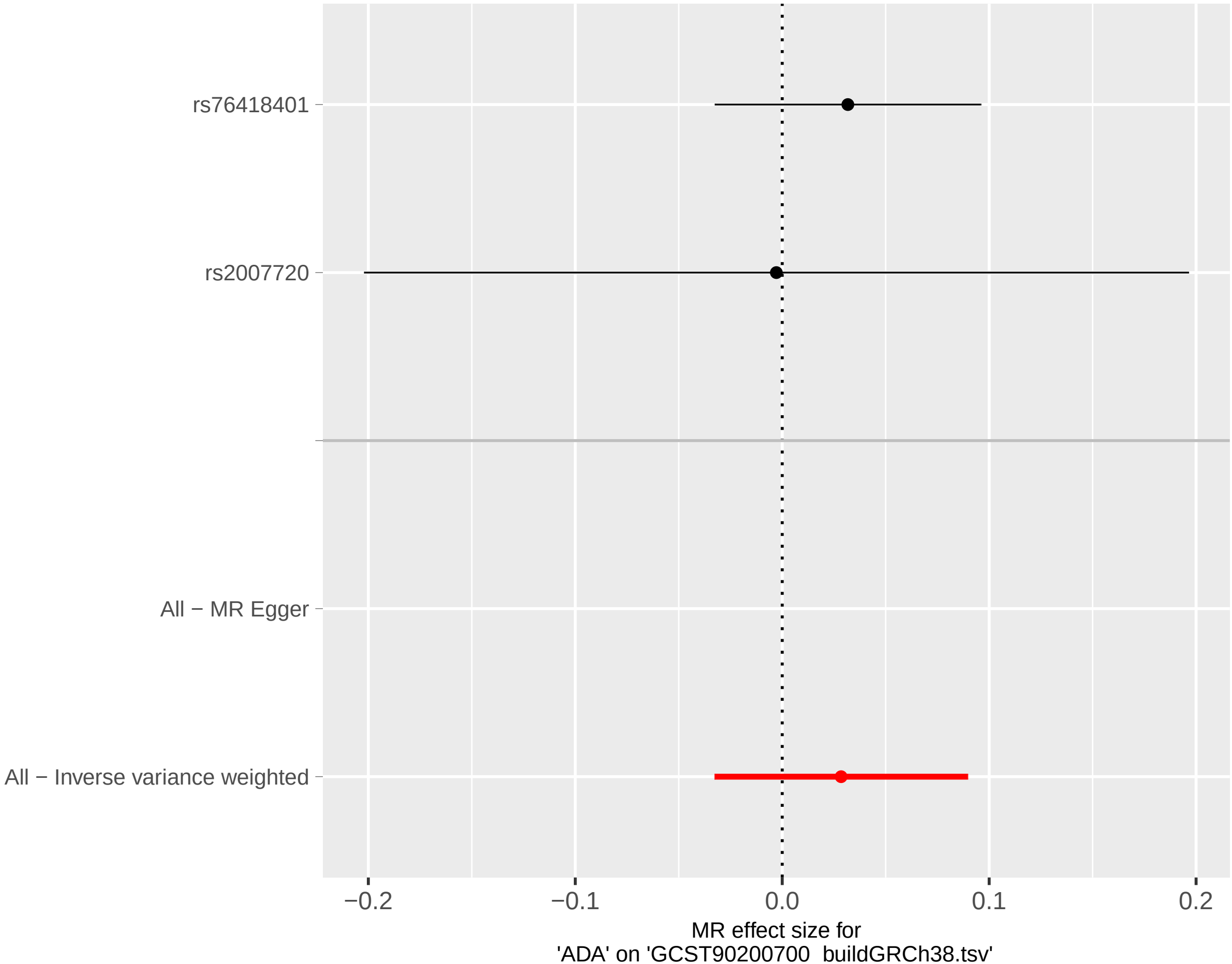
rs76418401
rs2007720
All − MR Egger
All − Inverse variance weighted
−0.2
−0.1
0.0
0.1
0.2
MR effect size for
'ADA' on 'GCST90200700_buildGRCh38.tsv'

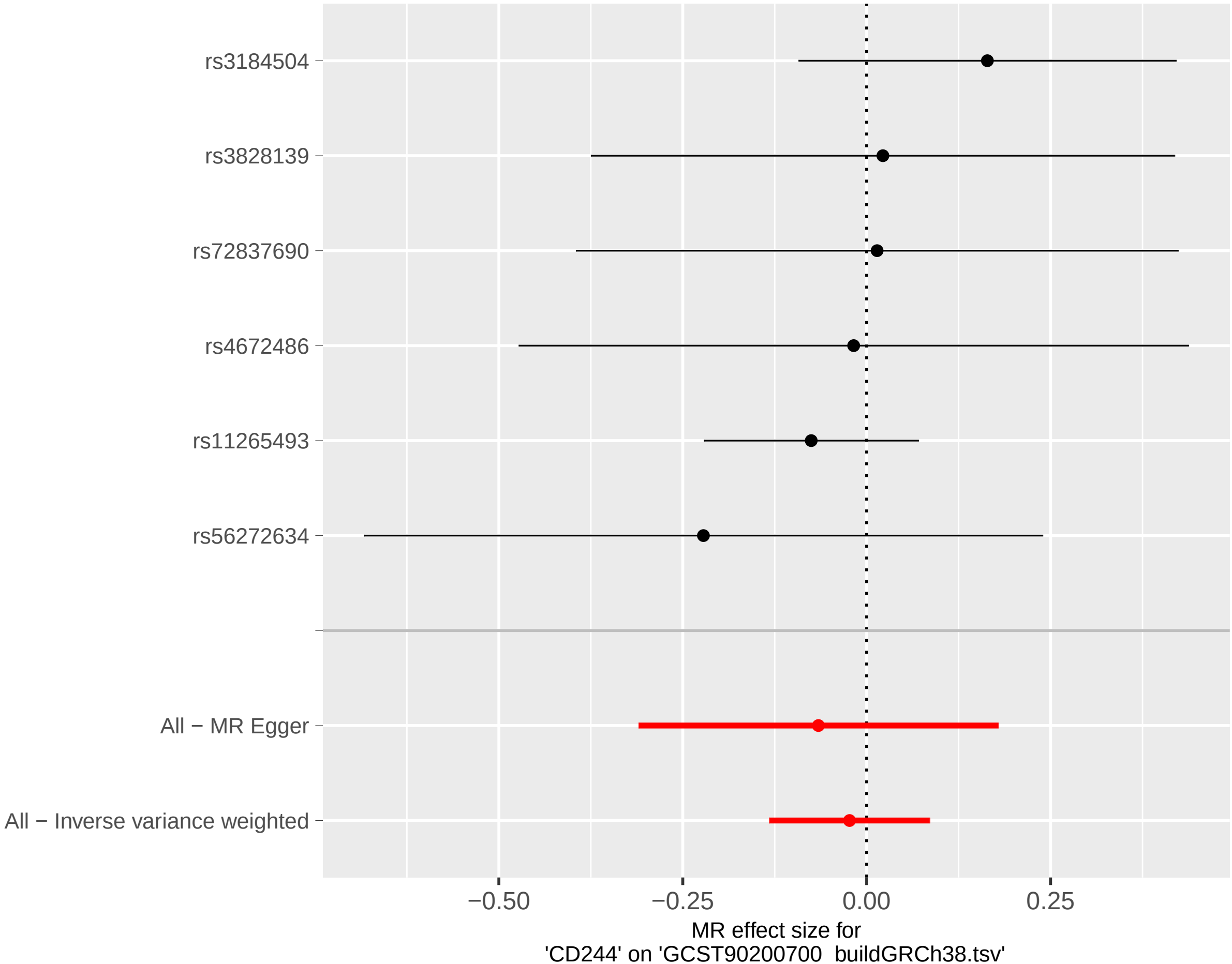

rs3184504
rs3828139
rs72837690
rs4672486
rs11265493
rs56272634
All − MR Egger
All − Inverse variance weighted
−0.50
−0.25
0.00
0.25
MR effect size for
'CD244' on 'GCST90200700_buildGRCh38.tsv'

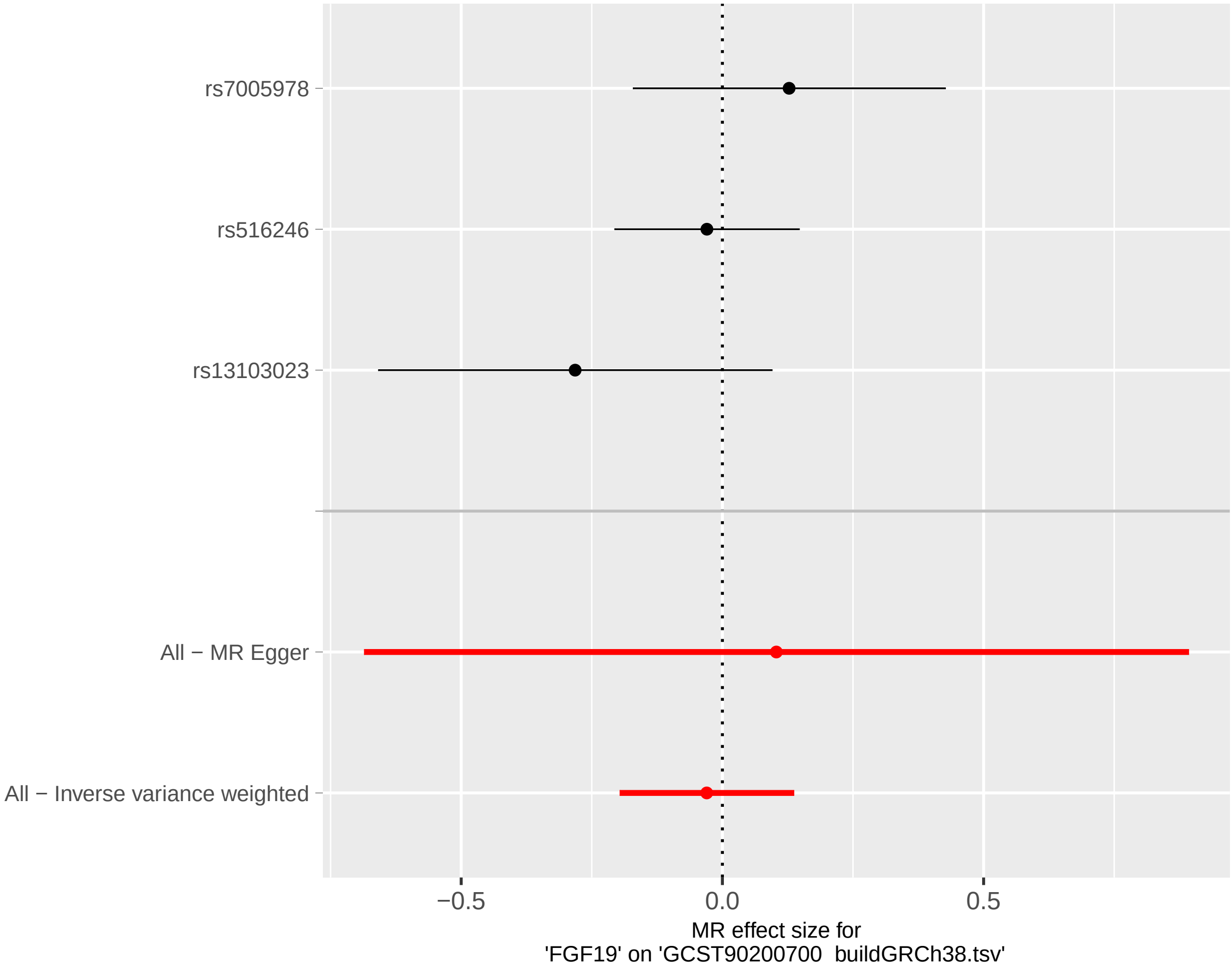
rs7005978
rs516246
rs13103023
All − MR Egger
All − Inverse variance weighted
−0.5
0.0
0.5
MR effect size for
'FGF19' on 'GCST90200700_buildGRCh38.tsv'

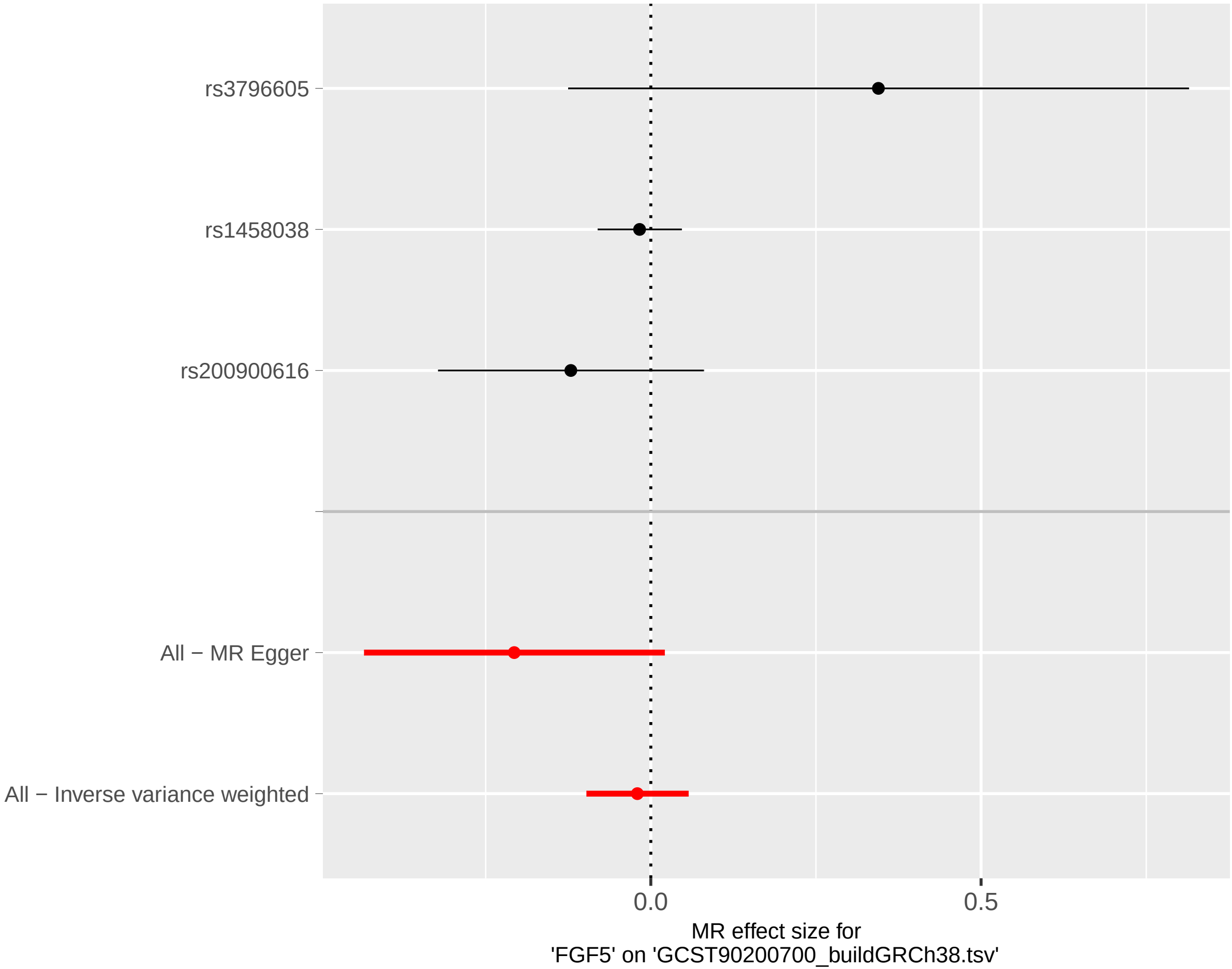

rs3796605
rs1458038
rs200900616
All − MR Egger
All − Inverse variance weighted
0.0
0.5
MR effect size for
'FGF5' on 'GCST90200700_buildGRCh38.tsv'

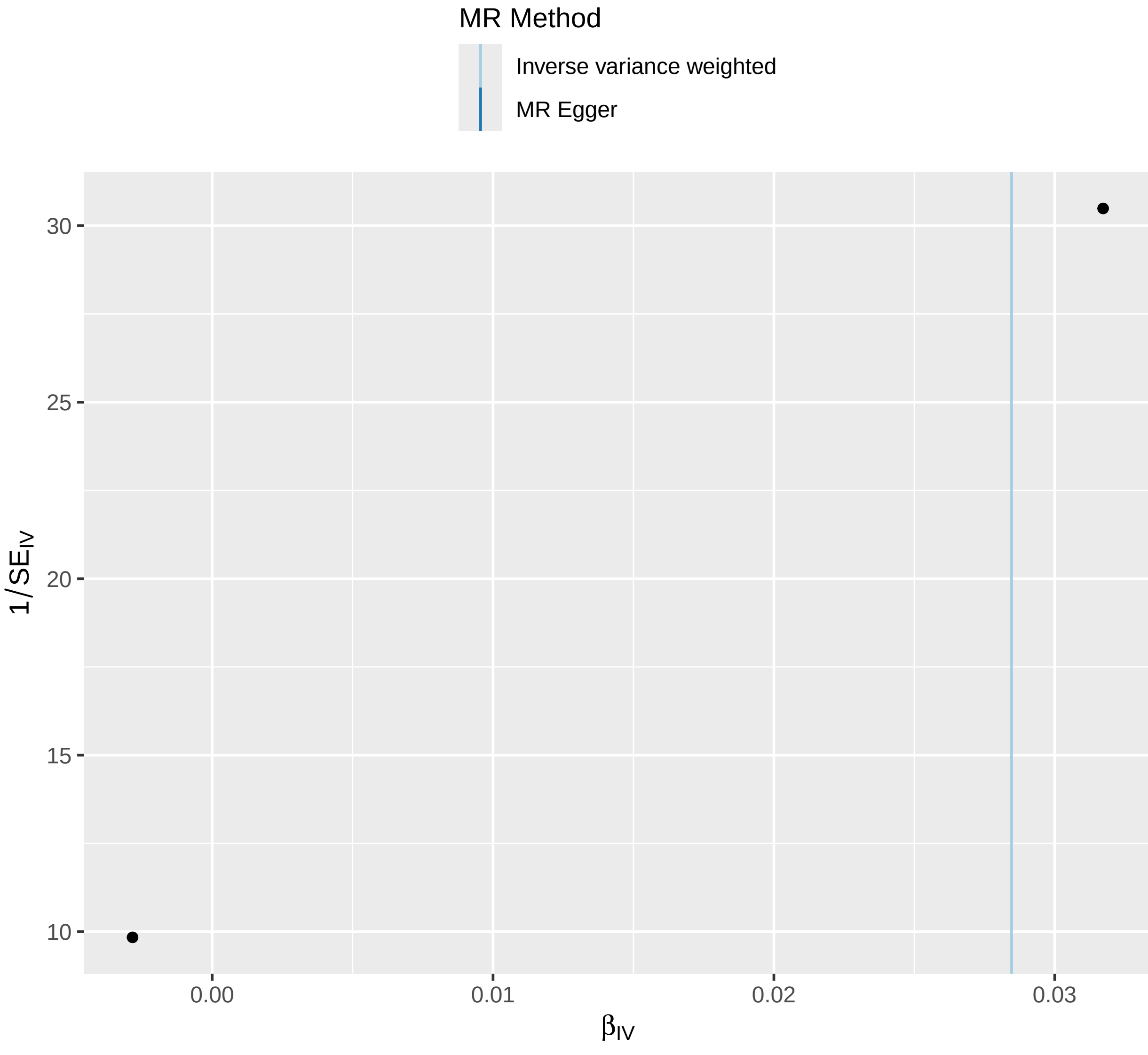

MR Method
Inverse variance weighted
MR Egger
30
25
20
15
10
1/SE_IV
0.00
0.01
0.02
0.03
β_IV

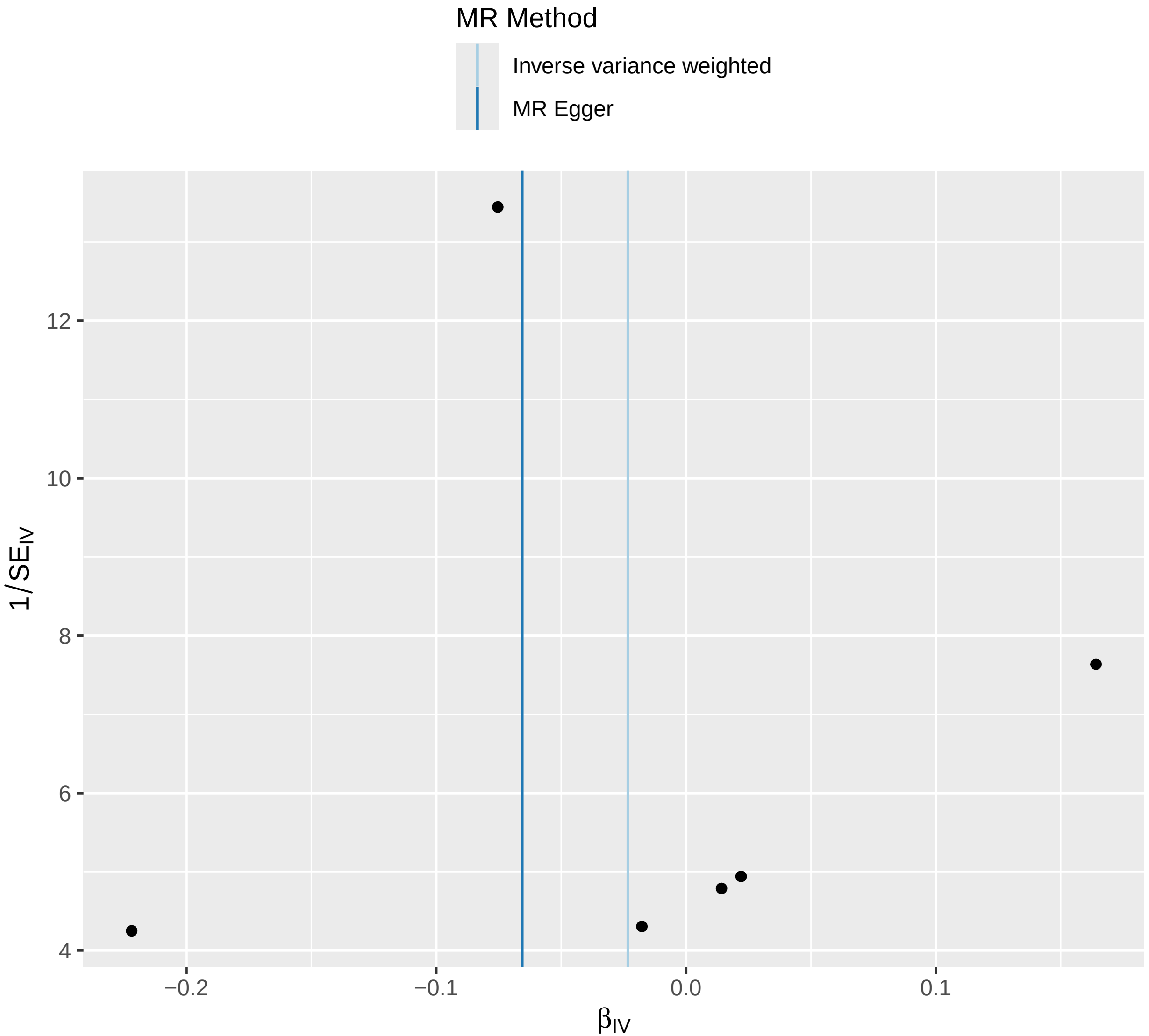

MR Method
Inverse variance weighted
MR Egger
12
10
8
6
4
1/SE_IV
−0.2
−0.1
0.0
0.1
β_IV

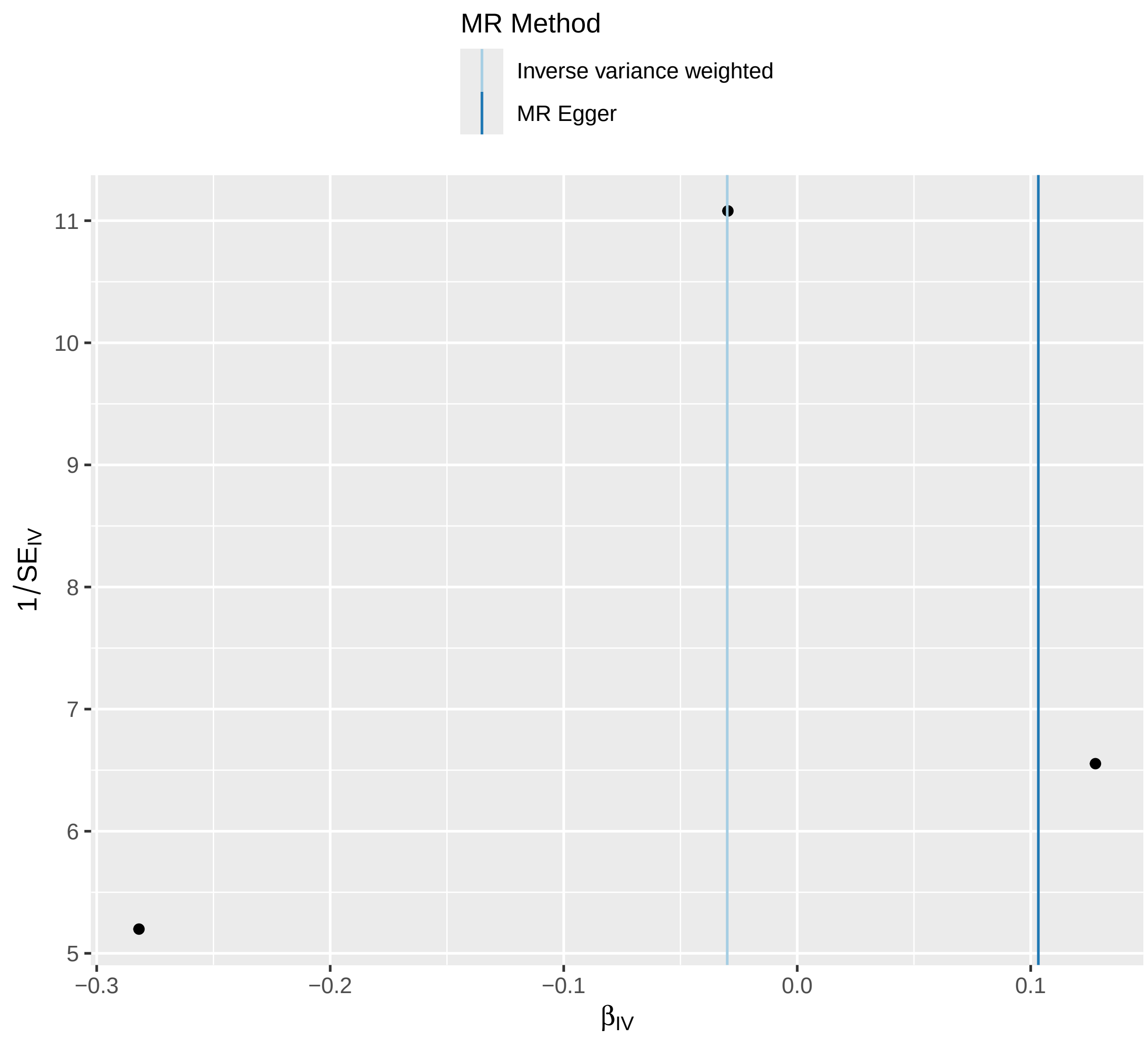

MR Method
Inverse variance weighted
MR Egger
11
10
9
8
7
6
5
$1/SE_{IV}$
−0.3
−0.2
−0.1
0.0
0.1
β_{IV}

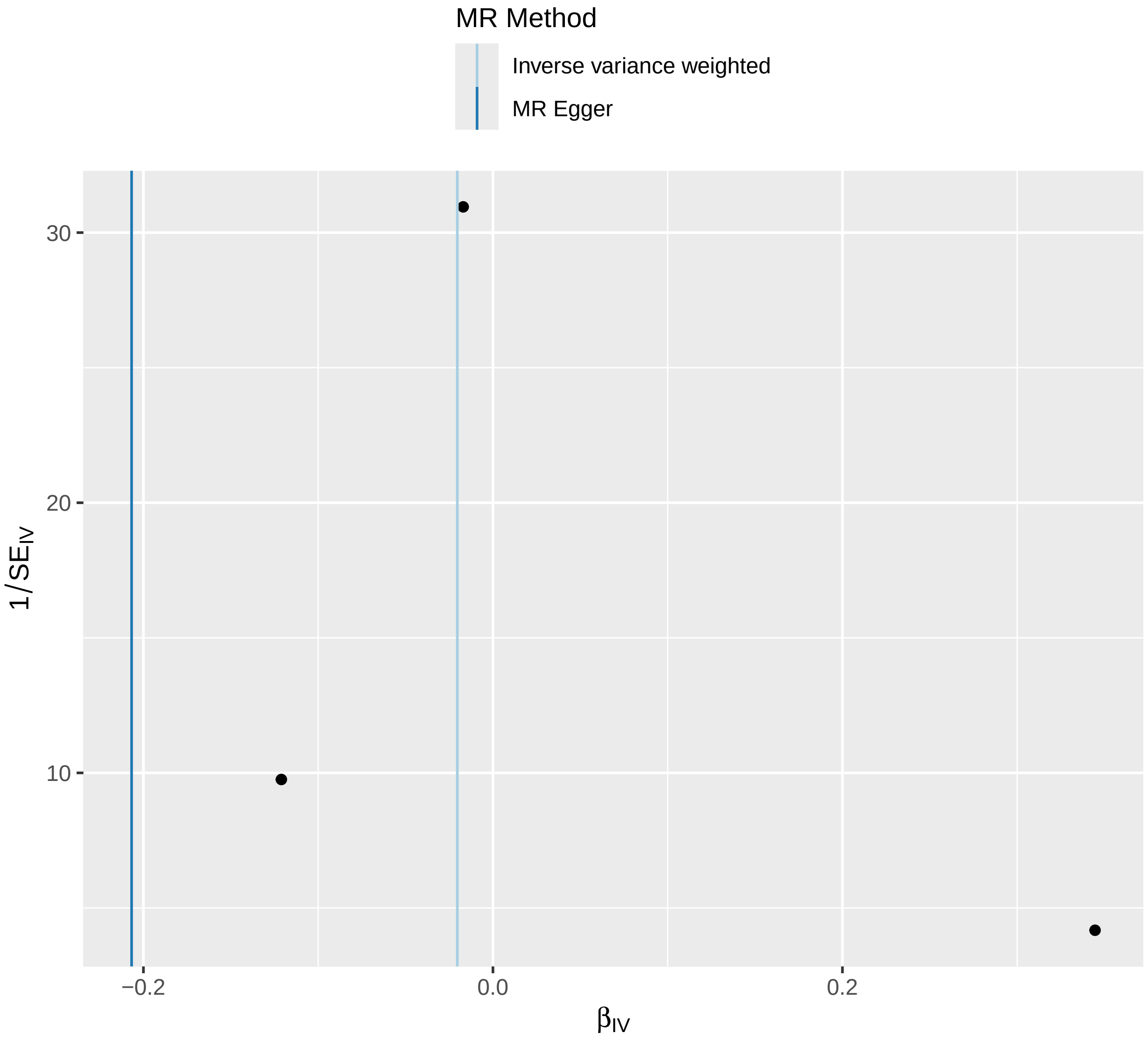

MR Method
Inverse variance weighted
MR Egger
30
20
10
1/SE_{IV}
−0.2
0.0
0.2
β_{IV}

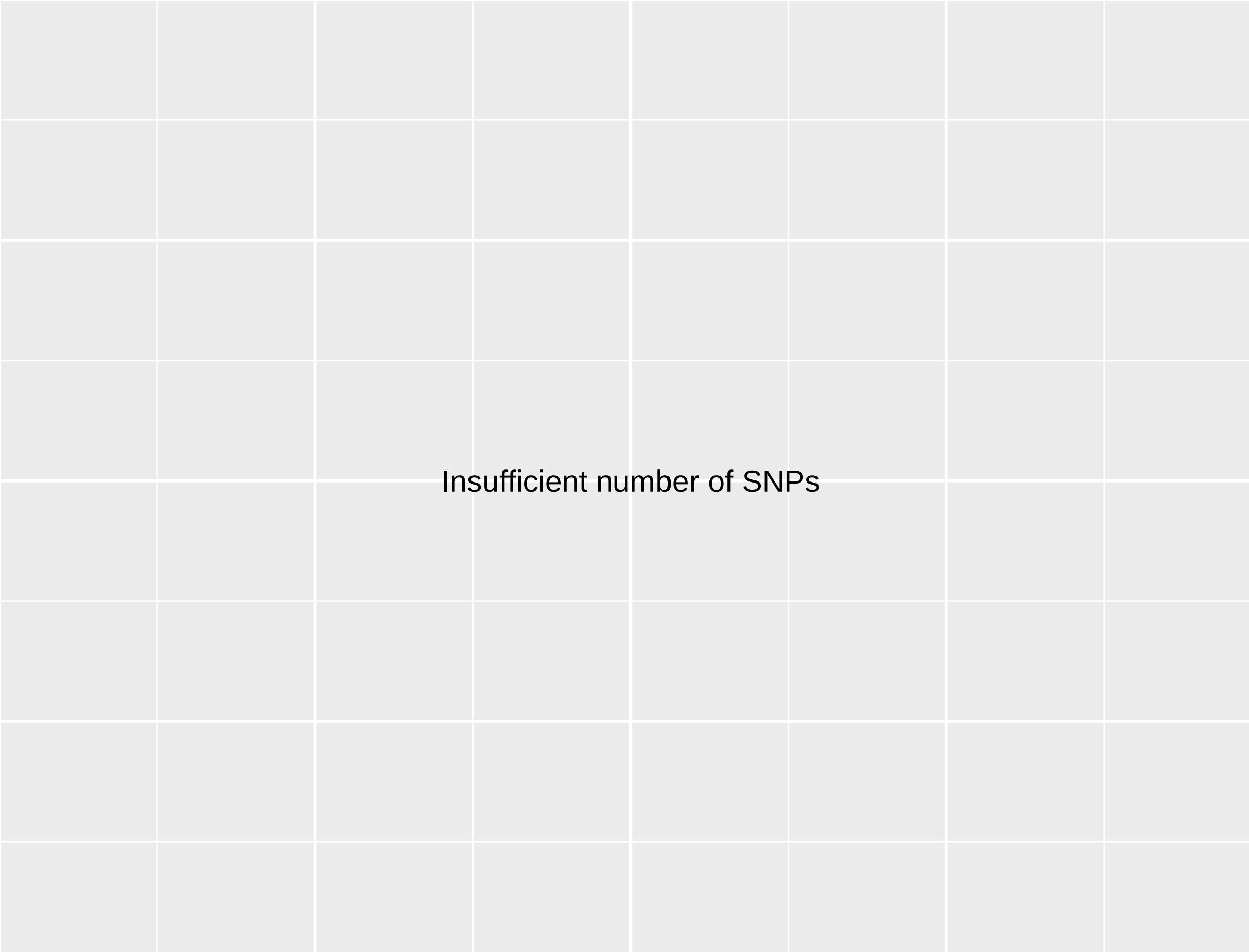
Insufficient number of SNPs

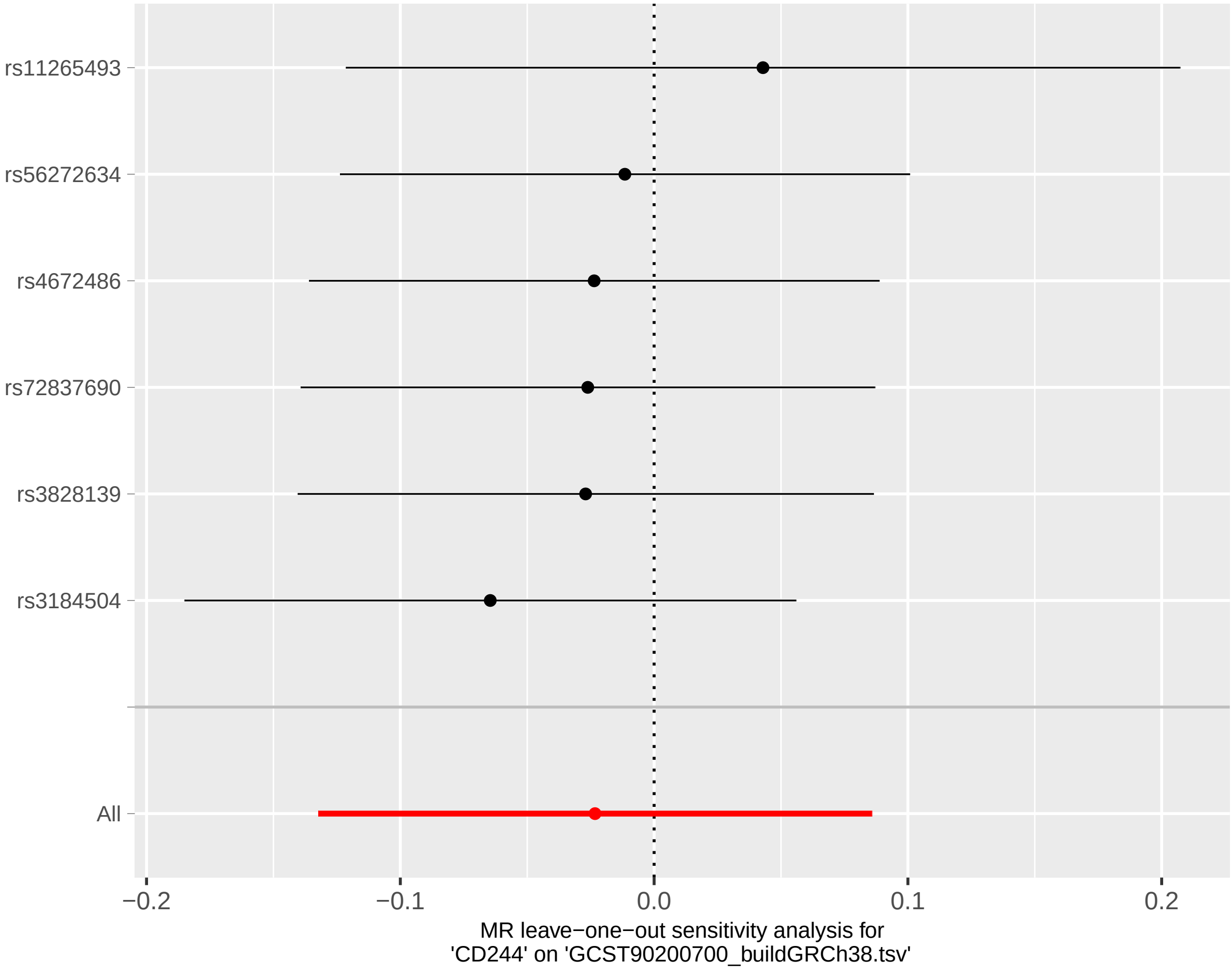

rs11265493
rs56272634
rs4672486
rs72837690
rs3828139
rs3184504
All
−0.2
−0.1
0.0
0.1
0.2
MR leave−one−out sensitivity analysis for
'CD244' on 'GCST90200700_buildGRCh38.tsv'

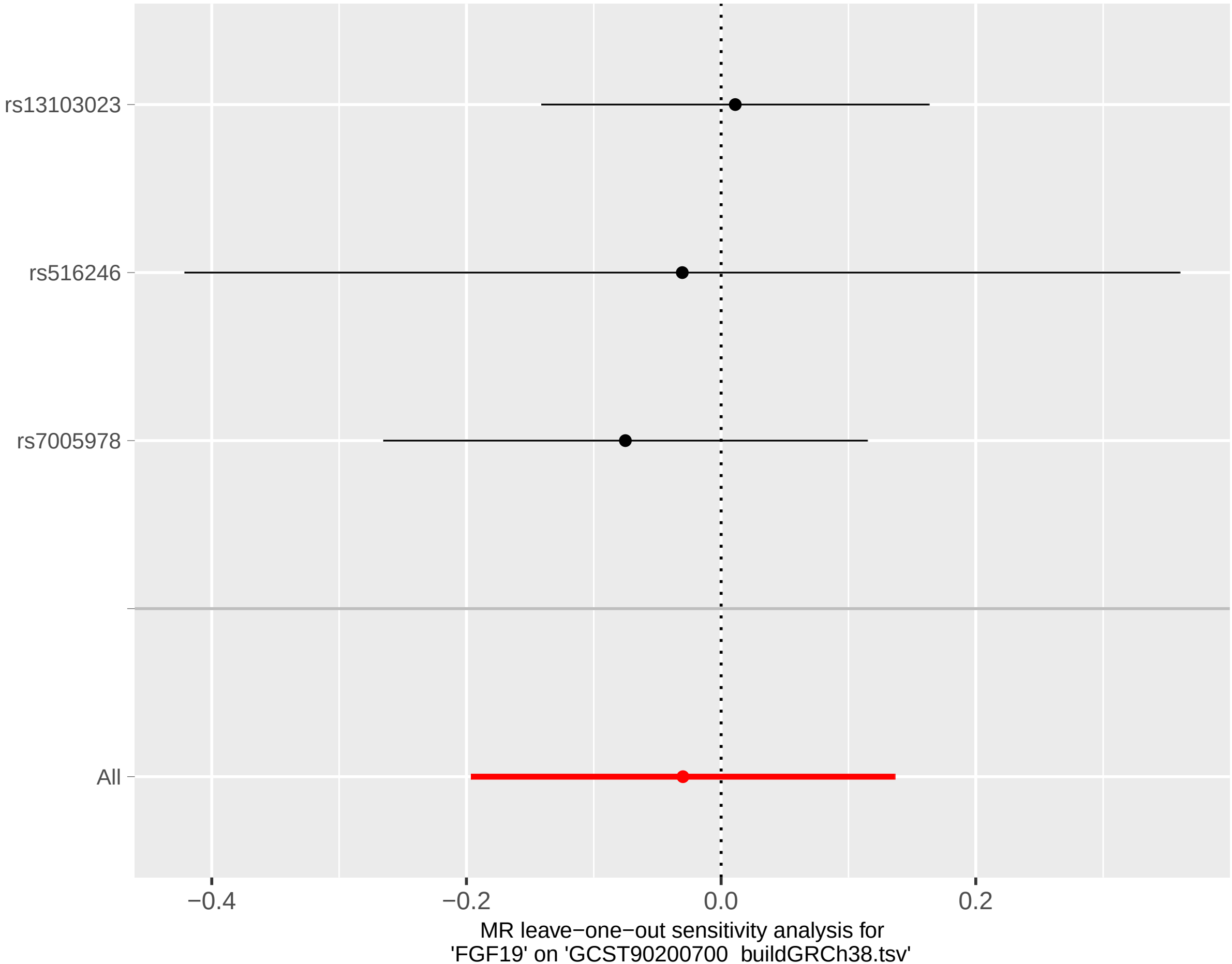
rs13103023
rs516246
rs7005978
All
−0.4
−0.2
0.0
0.2
MR leave−one−out sensitivity analysis for
'FGF19' on 'GCST90200700_buildGRCh38.tsv'

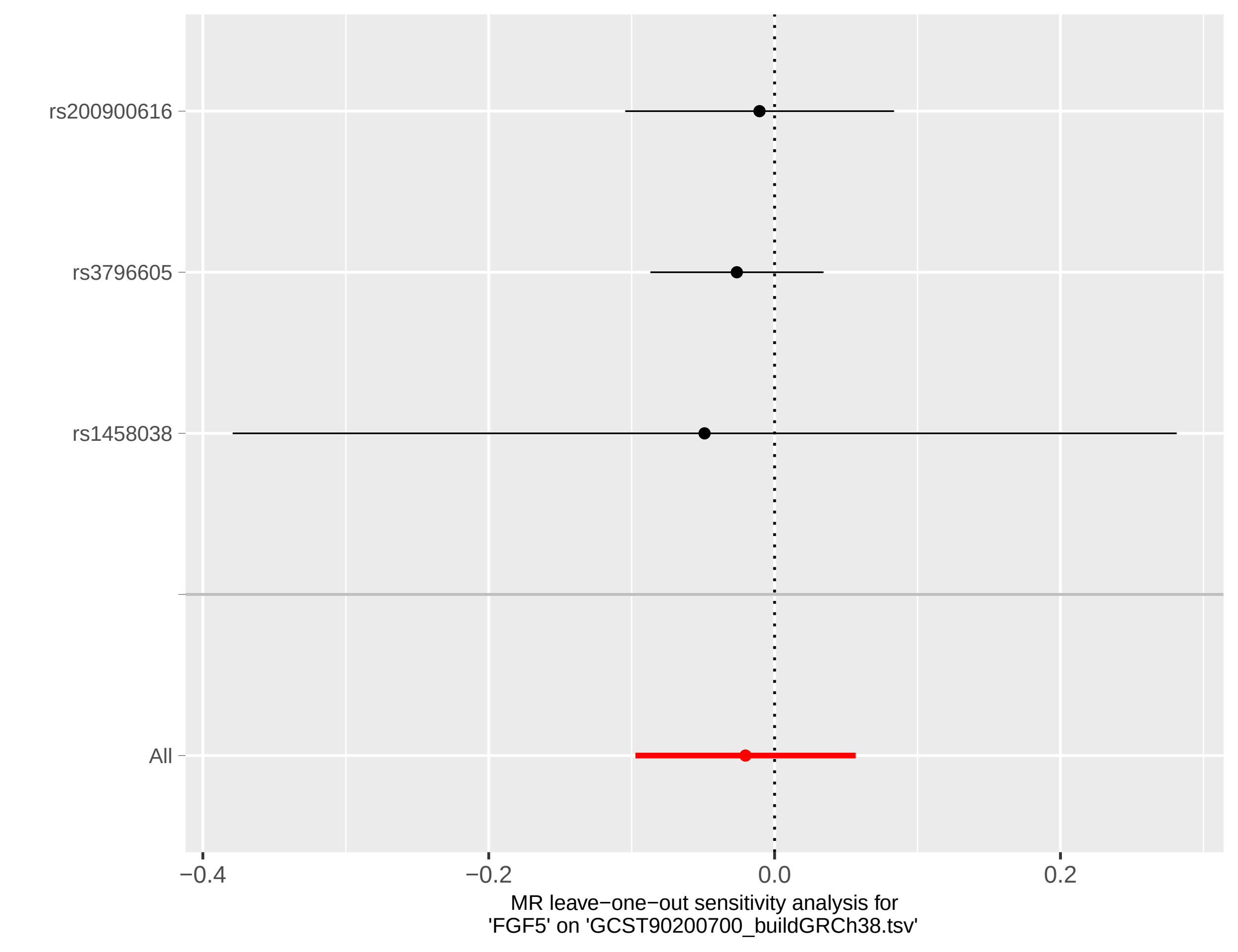
rs200900616
rs3796605
rs1458038
All
−0.4
−0.2
0.0
0.2
MR leave−one−out sensitivity analysis for
'FGF5' on 'GCST90200700_buildGRCh38.tsv'

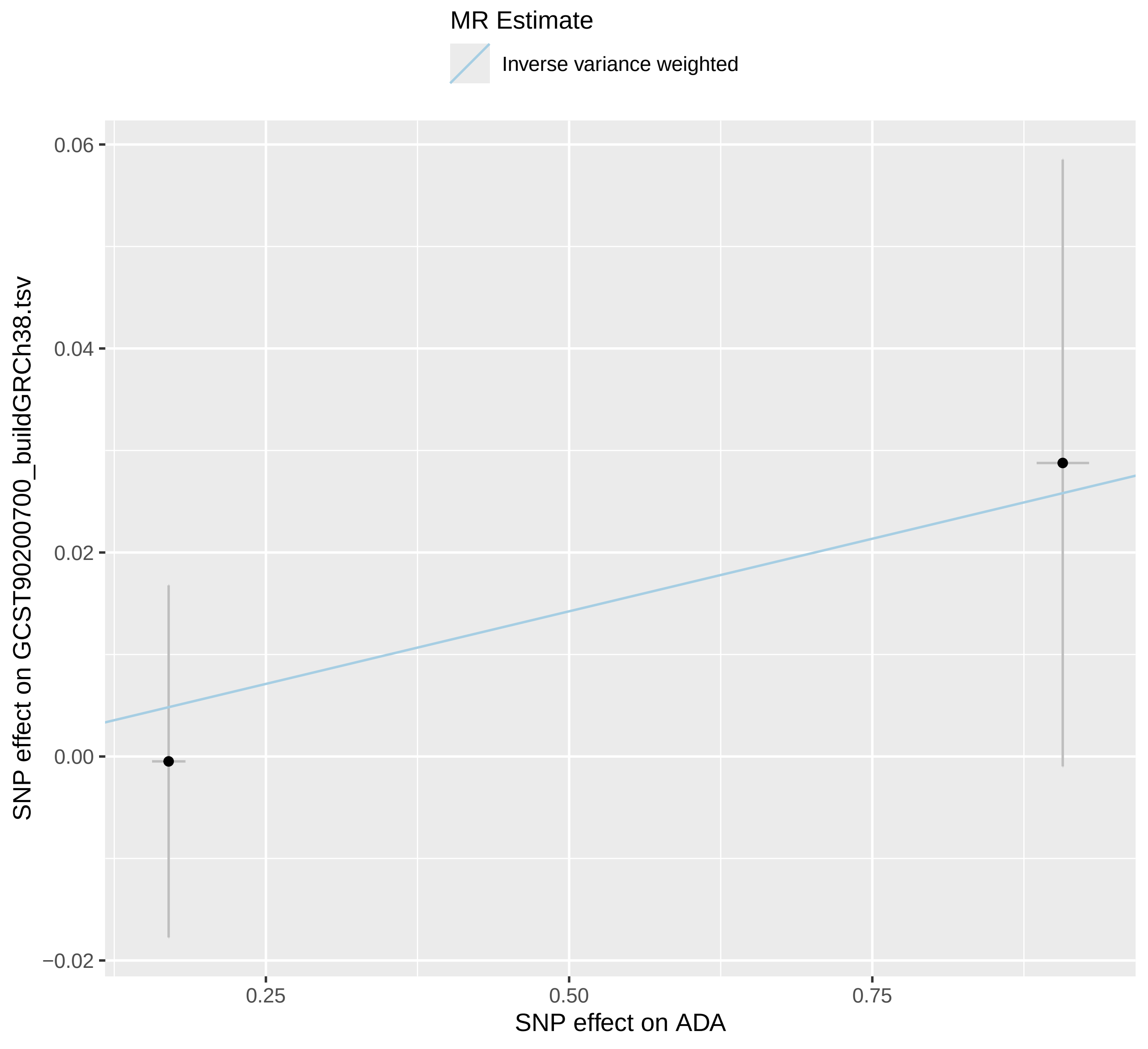

MR Estimate
Inverse variance weighted
SNP effect on GCST90200700_buildGRCh38.tsv
0.06
0.04
0.02
0.00
−0.02
0.25
0.50
0.75
SNP effect on ADA

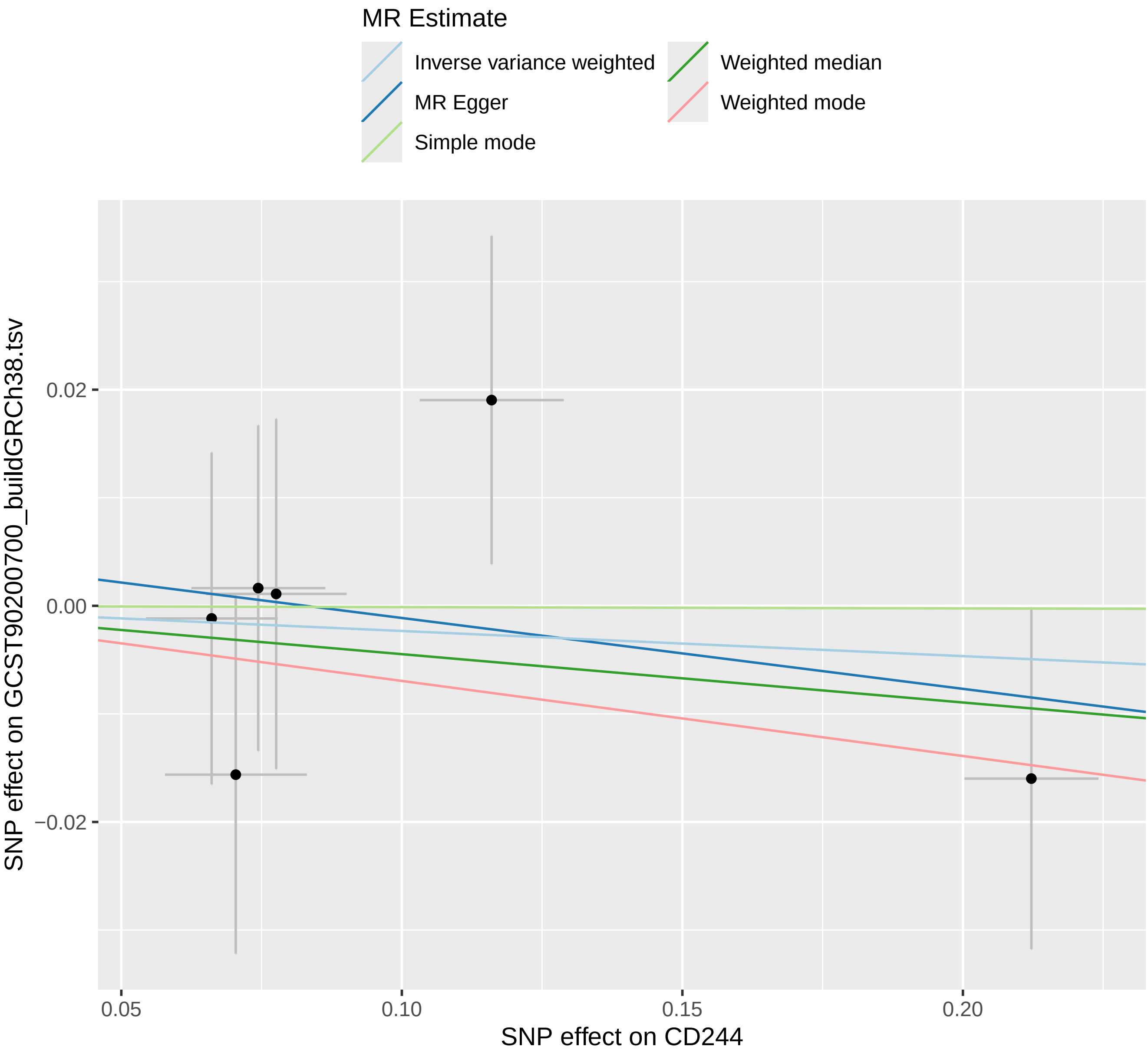
MR Estimate
Inverse variance weighted
MR Egger
Simple mode
Weighted median
Weighted mode
SNP effect on GCST90200700_buildGRCh38.tsv
0.02
0.00
−0.02
0.05
0.10
0.15
0.20
SNP effect on CD244

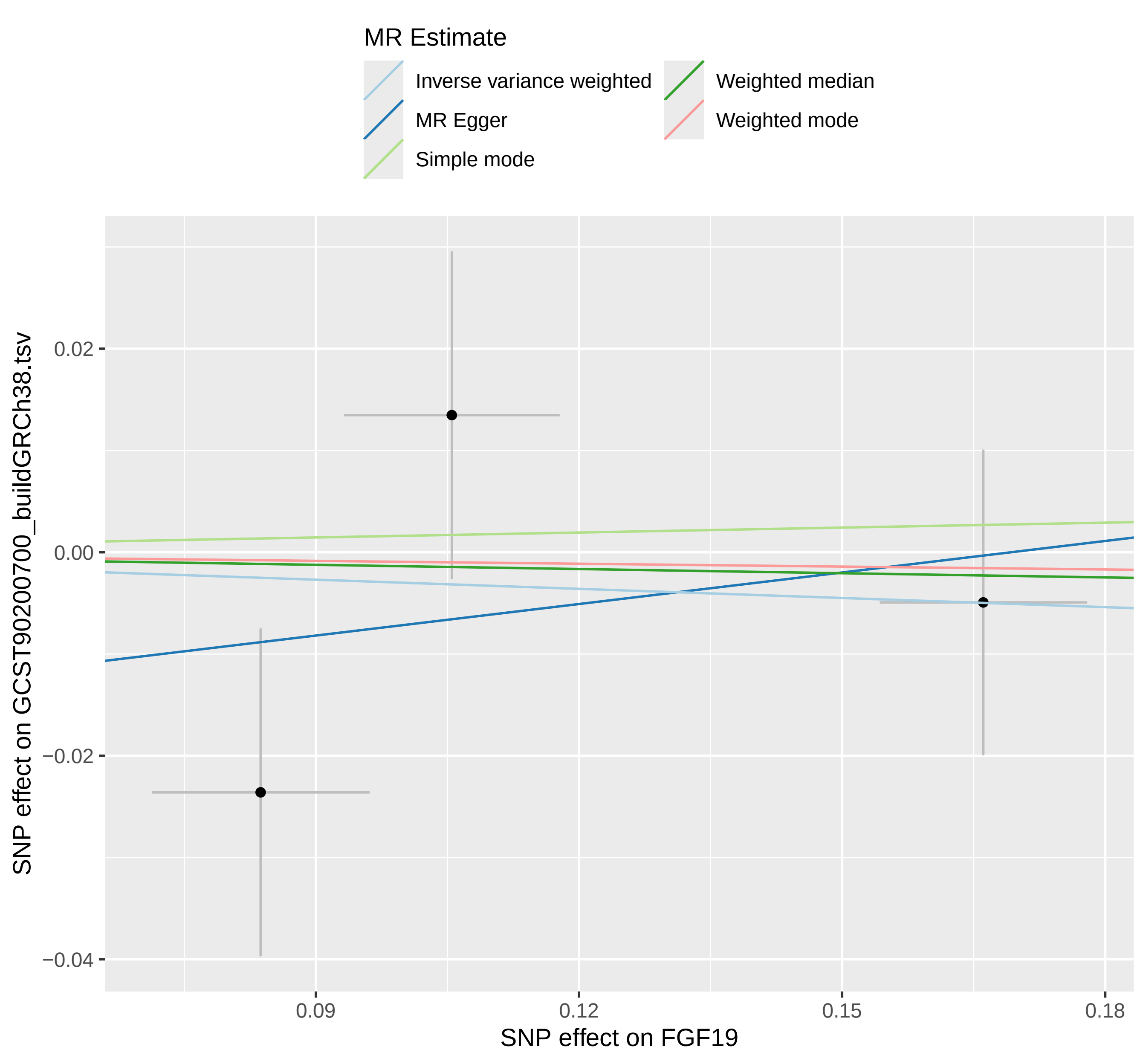

MR Estimate
Inverse variance weighted
MR Egger
Simple mode
Weighted median
Weighted mode
0.02
0.00
−0.02
−0.04
SNP effect on GCST90200700_buildGRCh38.tsv
0.09
0.12
0.15
0.18
SNP effect on FGF19

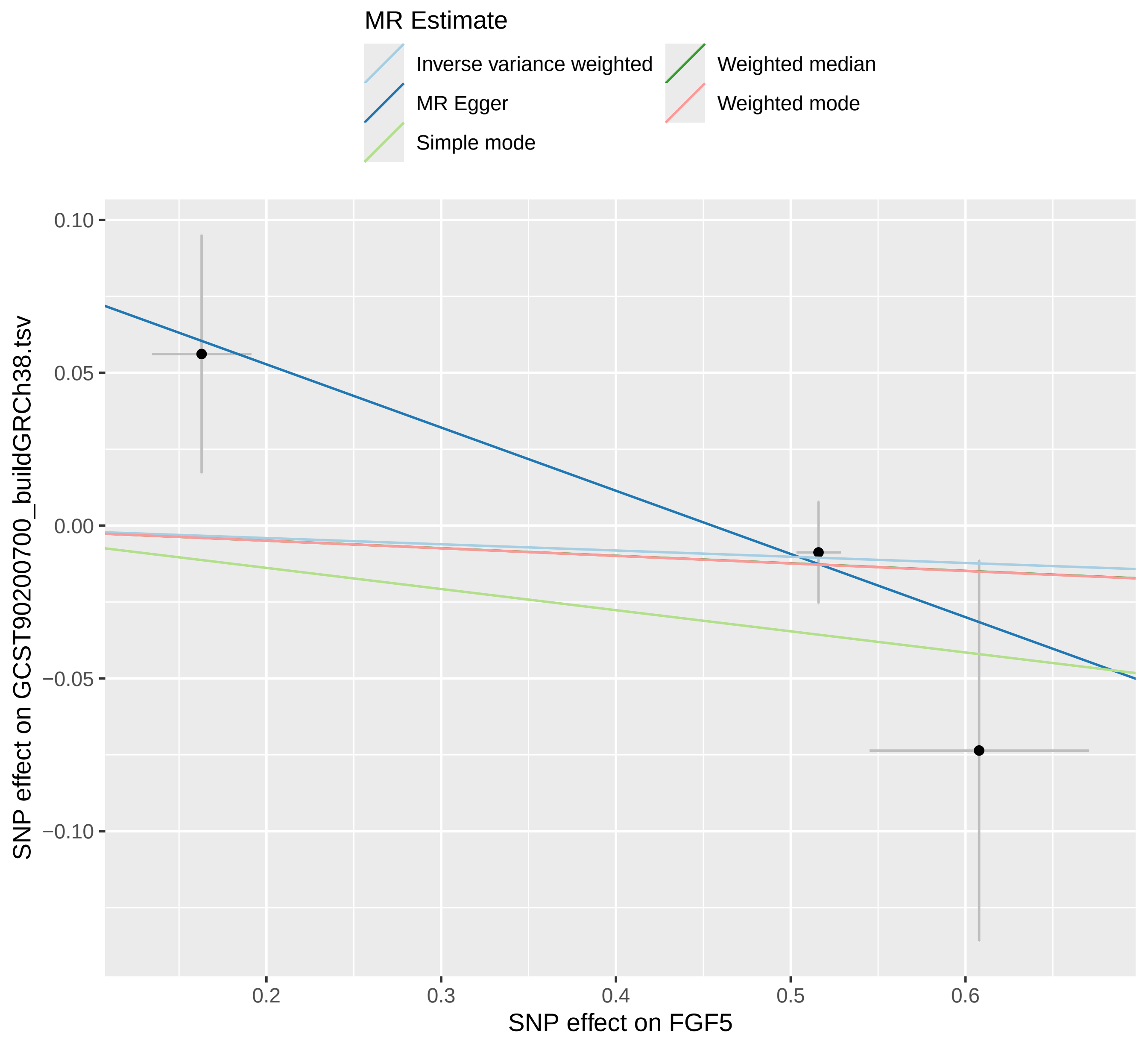

MR Estimate
Inverse variance weighted
MR Egger
Simple mode
Weighted median
Weighted mode
0.10
0.05
0.00
−0.05
−0.10
SNP effect on GCST90200700_buildGRCh38.tsv
0.2
0.3
0.4
0.5
0.6
SNP effect on FGF5

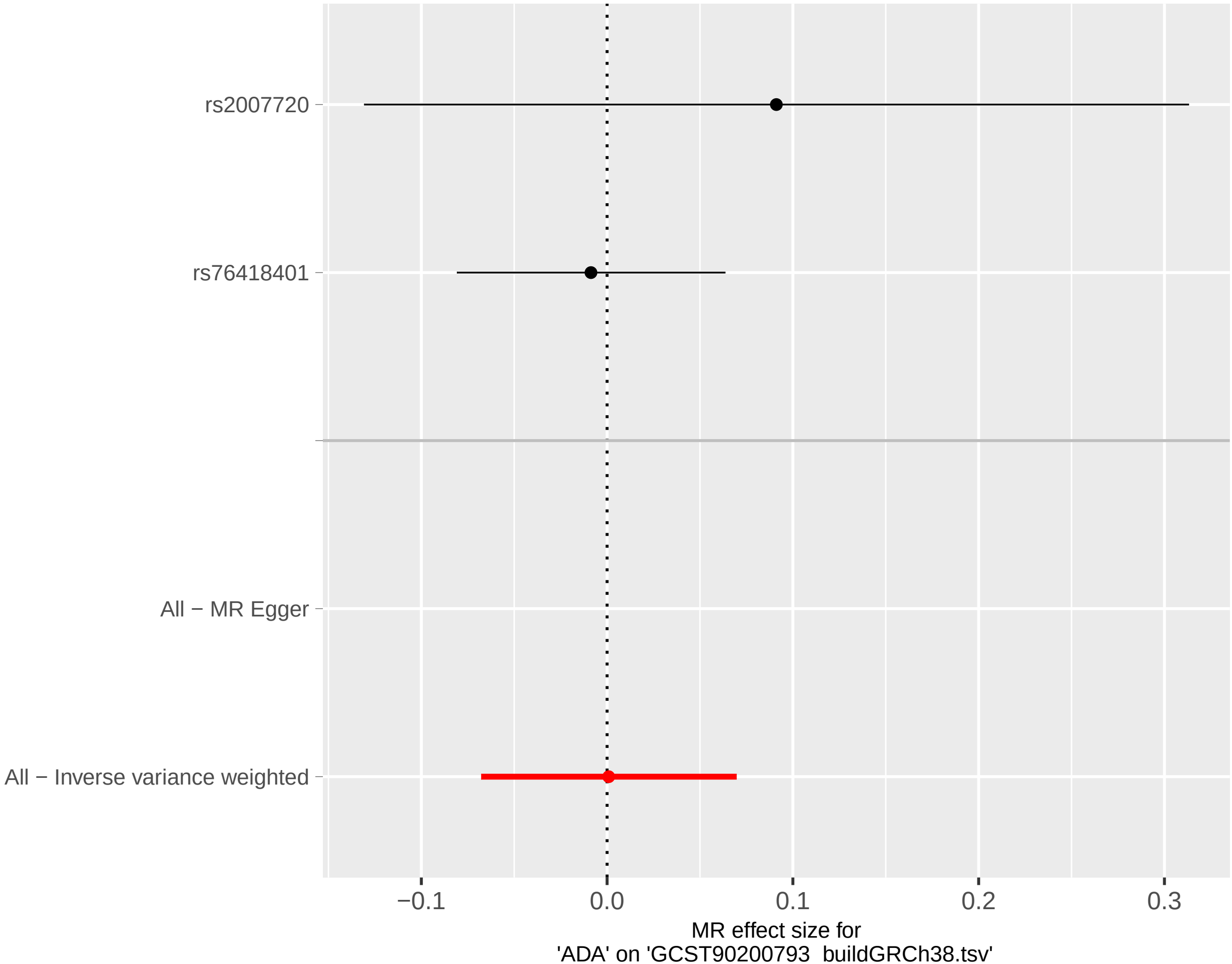
rs2007720
rs76418401
All − MR Egger
All − Inverse variance weighted
−0.1
0.0
0.1
0.2
0.3
MR effect size for
'ADA' on 'GCST90200793_buildGRCh38.tsv'

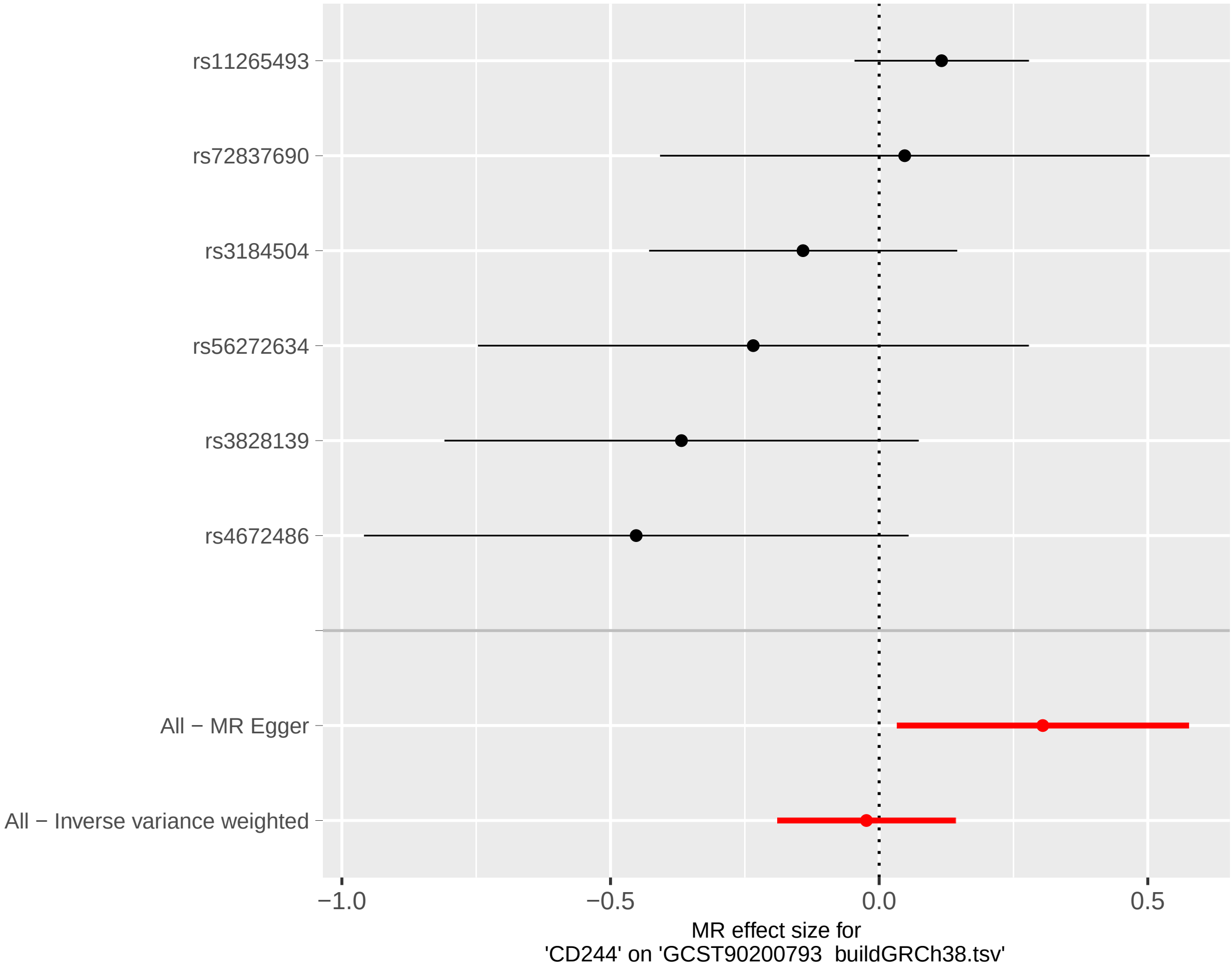
rs11265493
rs72837690
rs3184504
rs56272634
rs3828139
rs4672486
All − MR Egger
All − Inverse variance weighted
−1.0
−0.5
0.0
0.5
MR effect size for
'CD244' on 'GCST90200793_buildGRCh38.tsv'

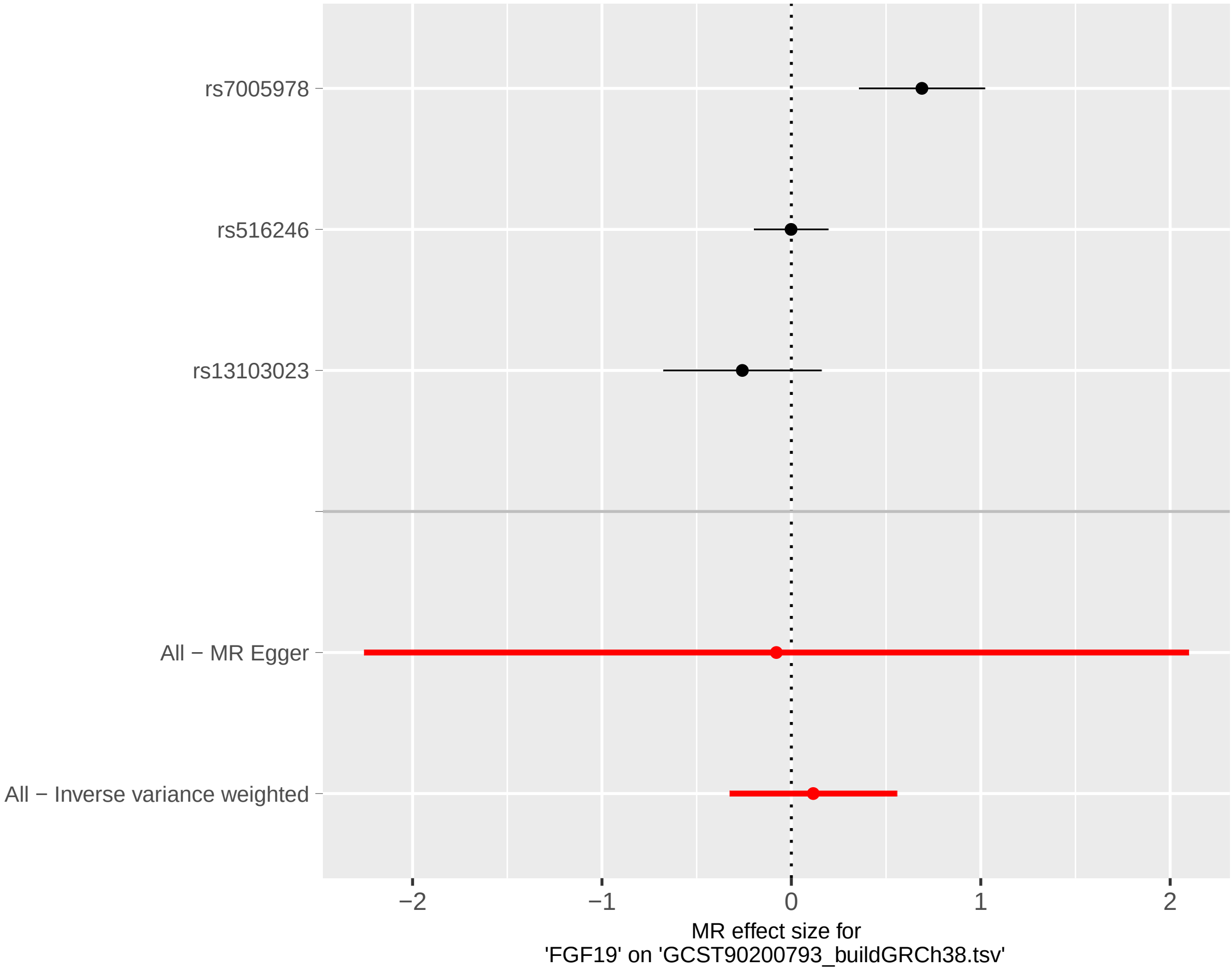

rs7005978
rs516246
rs13103023
All − MR Egger
All − Inverse variance weighted
−2
−1
0
1
2
MR effect size for
'FGF19' on 'GCST90200793_buildGRCh38.tsv'

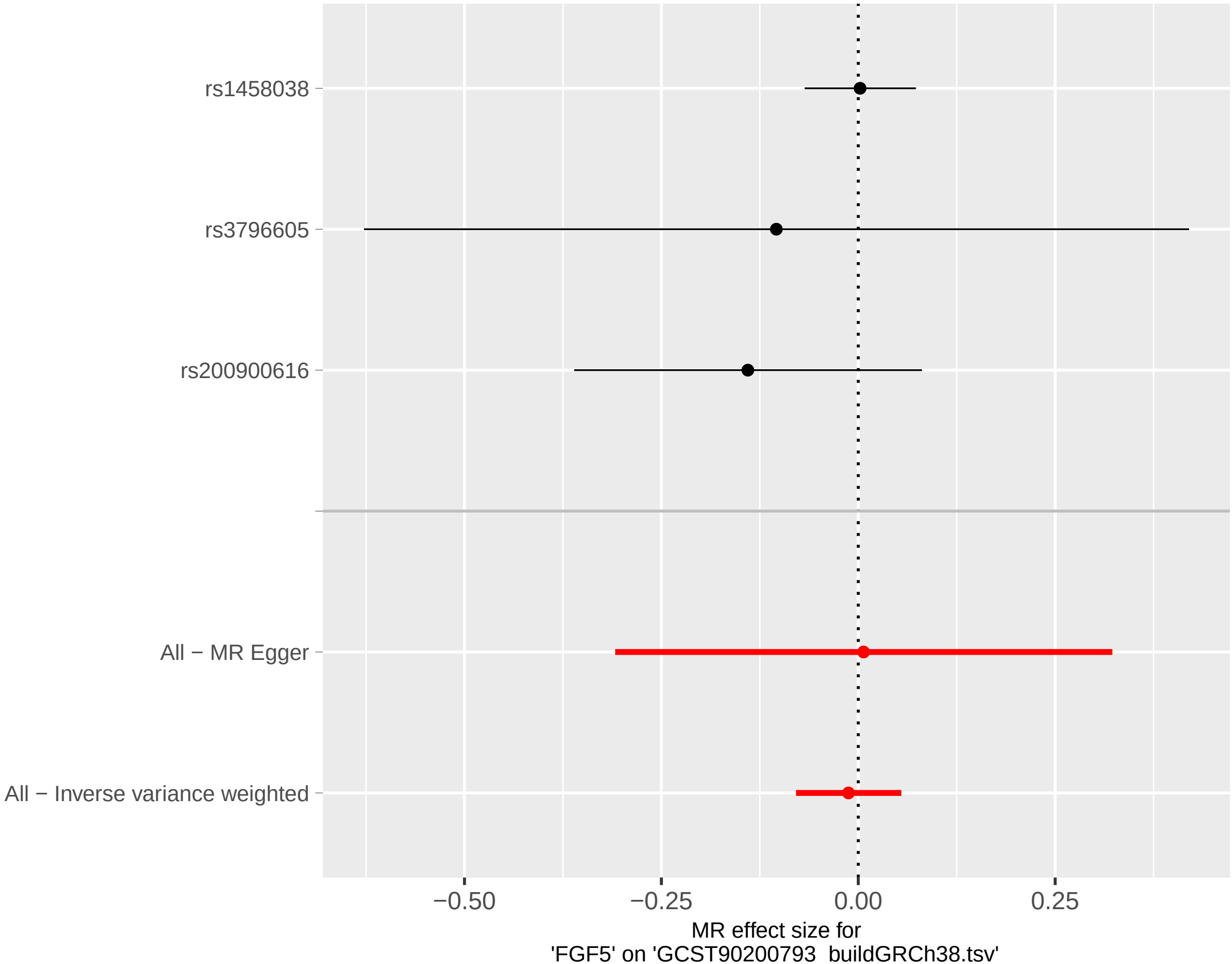
rs1458038
rs3796605
rs200900616
All − MR Egger
All − Inverse variance weighted
−0.50
−0.25
0.00
0.25
MR effect size for
'FGF5' on 'GCST90200793_buildGRCh38.tsv'

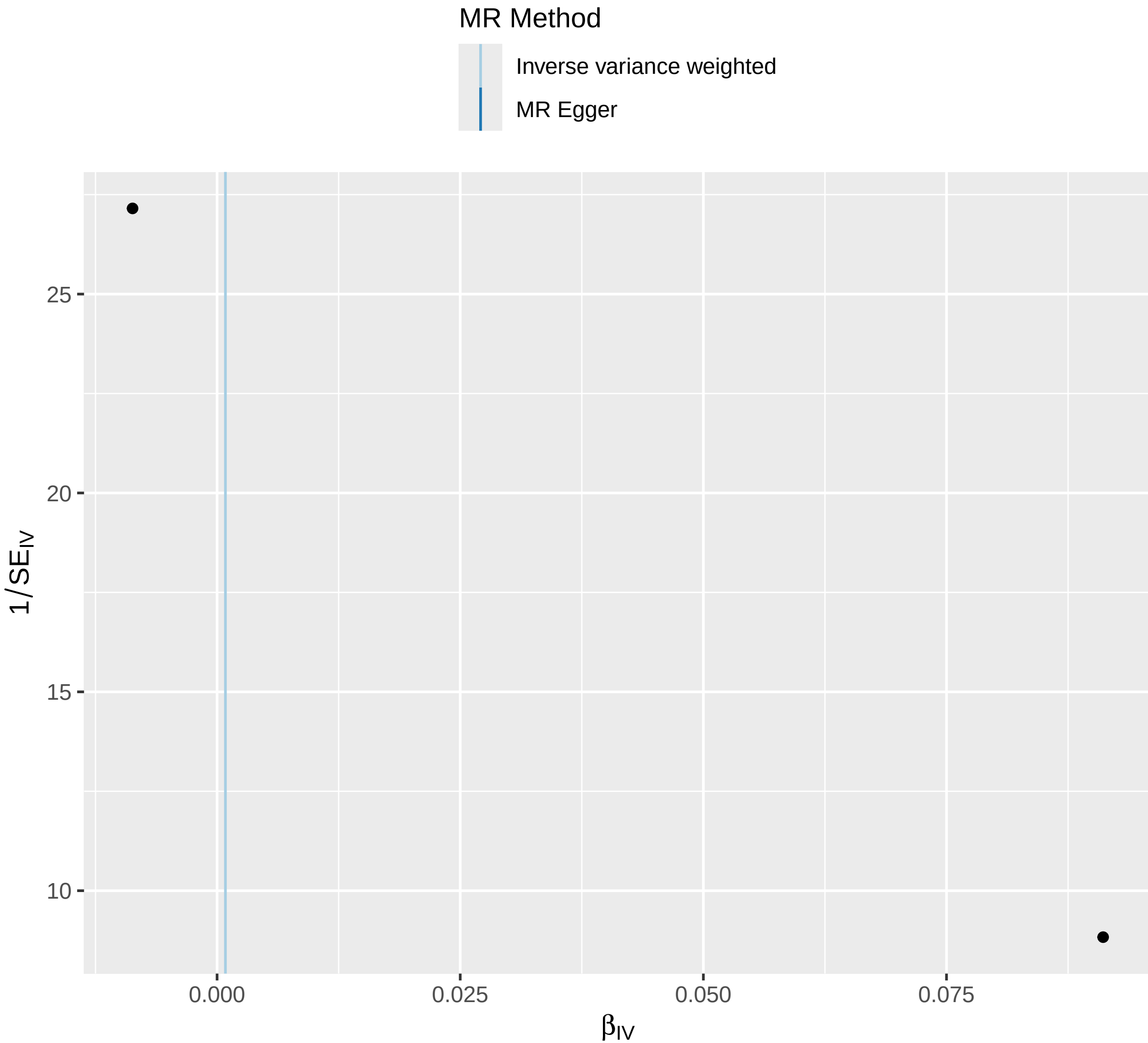

MR Method
Inverse variance weighted
MR Egger
25
20
15
10
$1/SE_{IV}$
0.000
0.025
0.050
0.075
β_{IV}

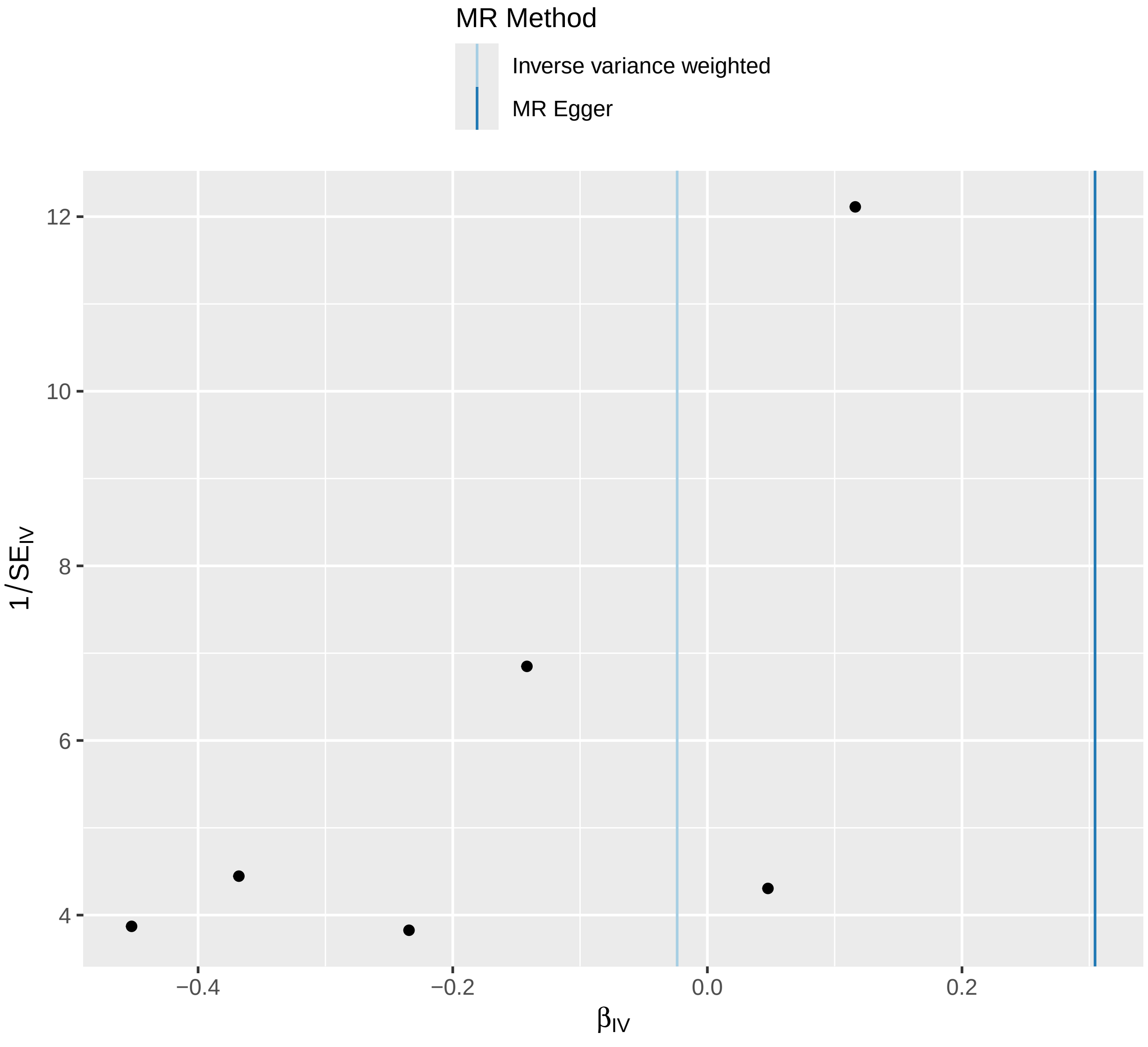

MR Method
Inverse variance weighted
MR Egger
12
10
8
6
4
1/SE_IV
−0.4
−0.2
0.0
0.2
β_IV

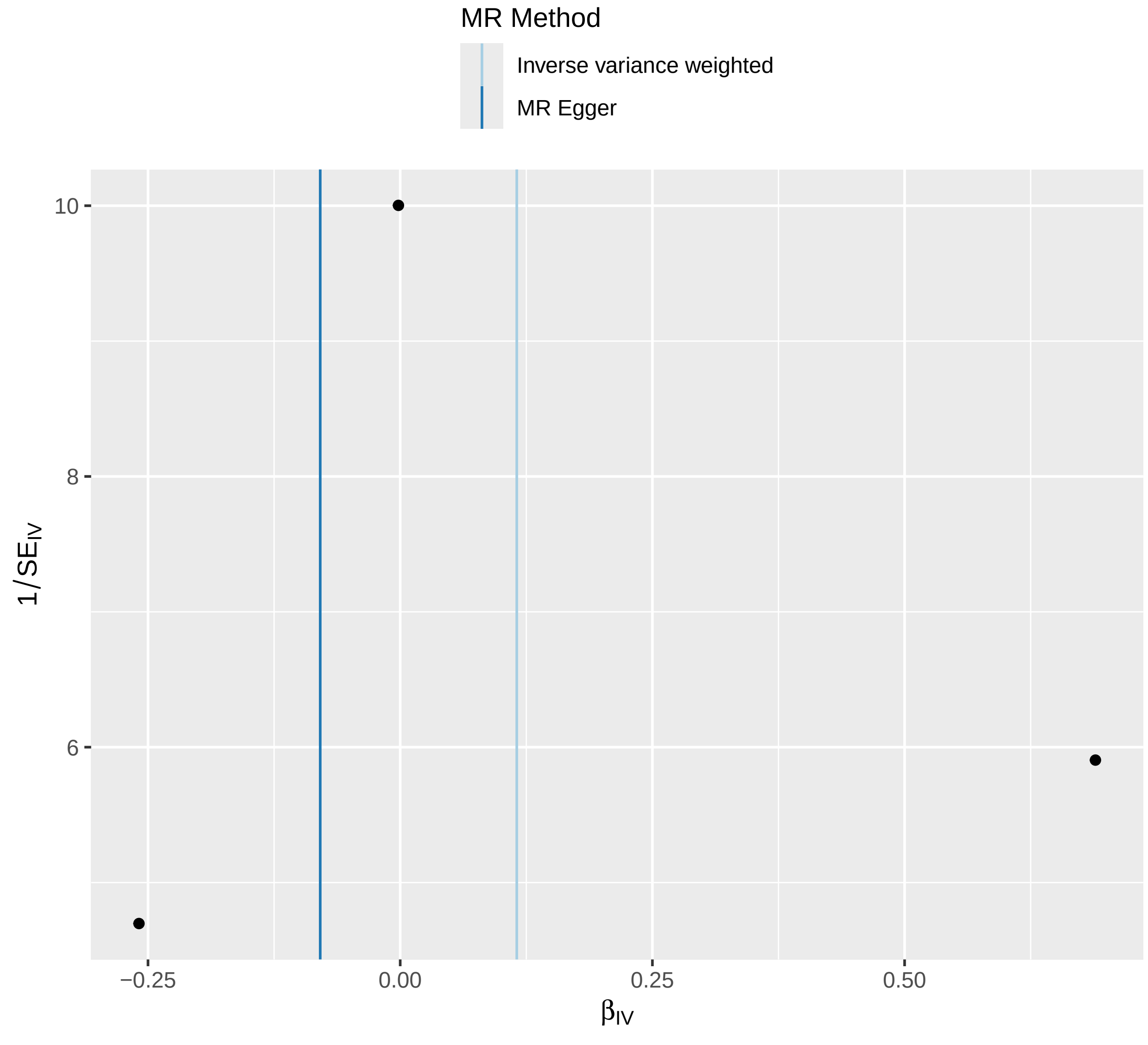
MR Method
Inverse variance weighted
MR Egger
10
8
6
1/SE_IV
−0.25
0.00
0.25
0.50
β_IV

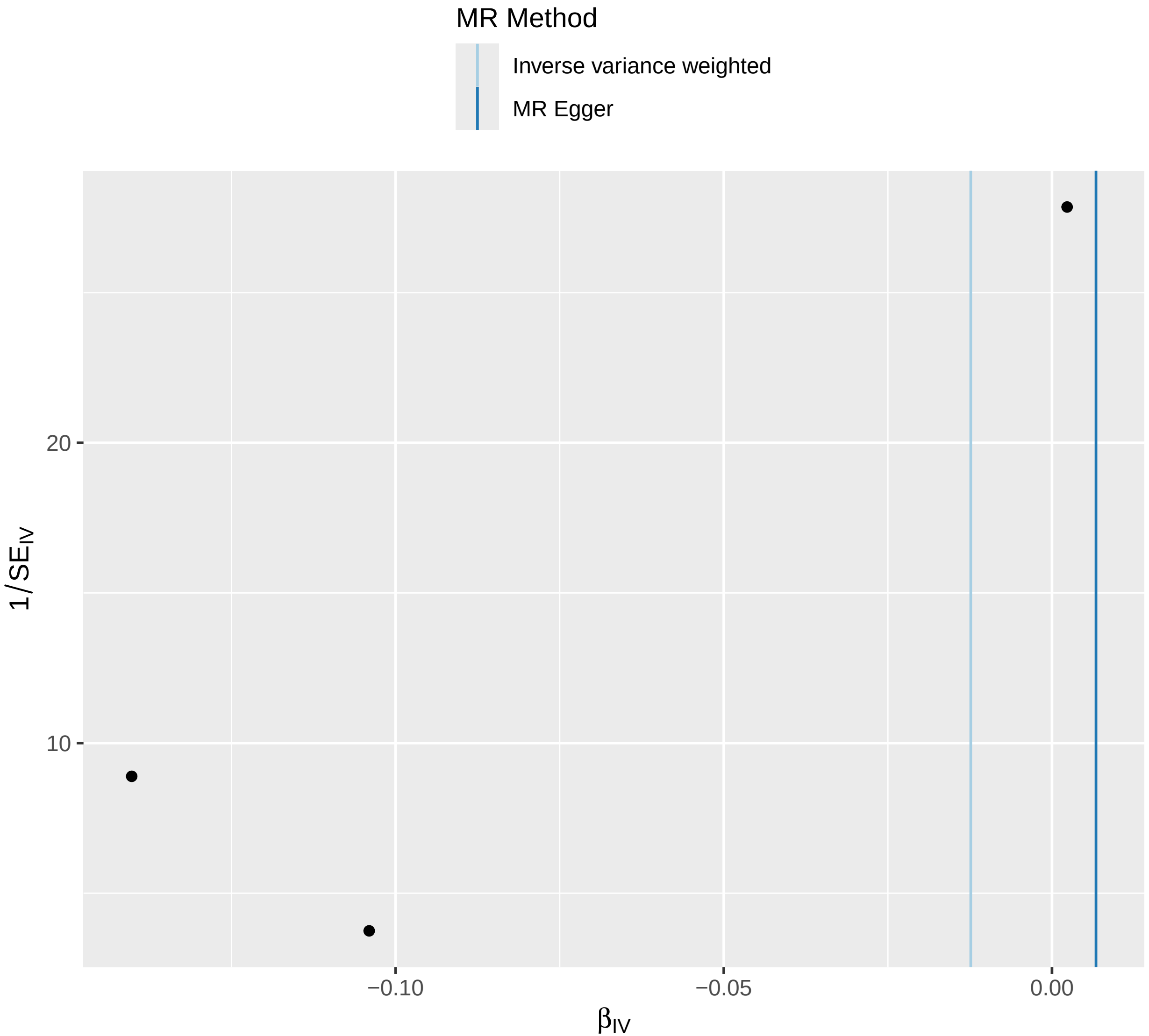
MR Method
Inverse variance weighted
MR Egger
20
10
1/SE_IV
−0.10
−0.05
0.00
β_IV

Insufficient number of SNPs

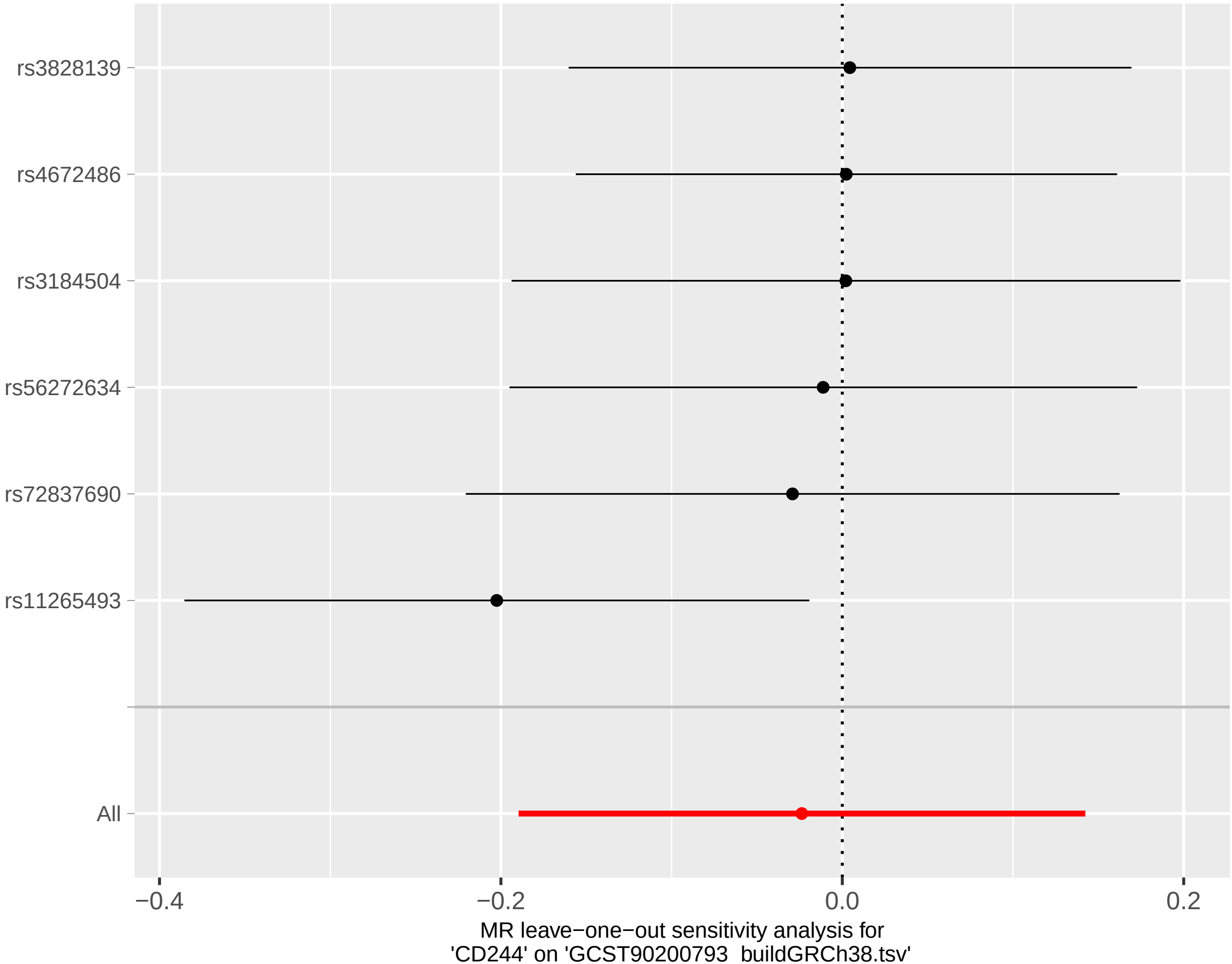

rs3828139
rs4672486
rs3184504
rs56272634
rs72837690
rs11265493
All
−0.4
−0.2
0.0
0.2
MR leave−one−out sensitivity analysis for
'CD244' on 'GCST90200793_buildGRCh38.tsv'

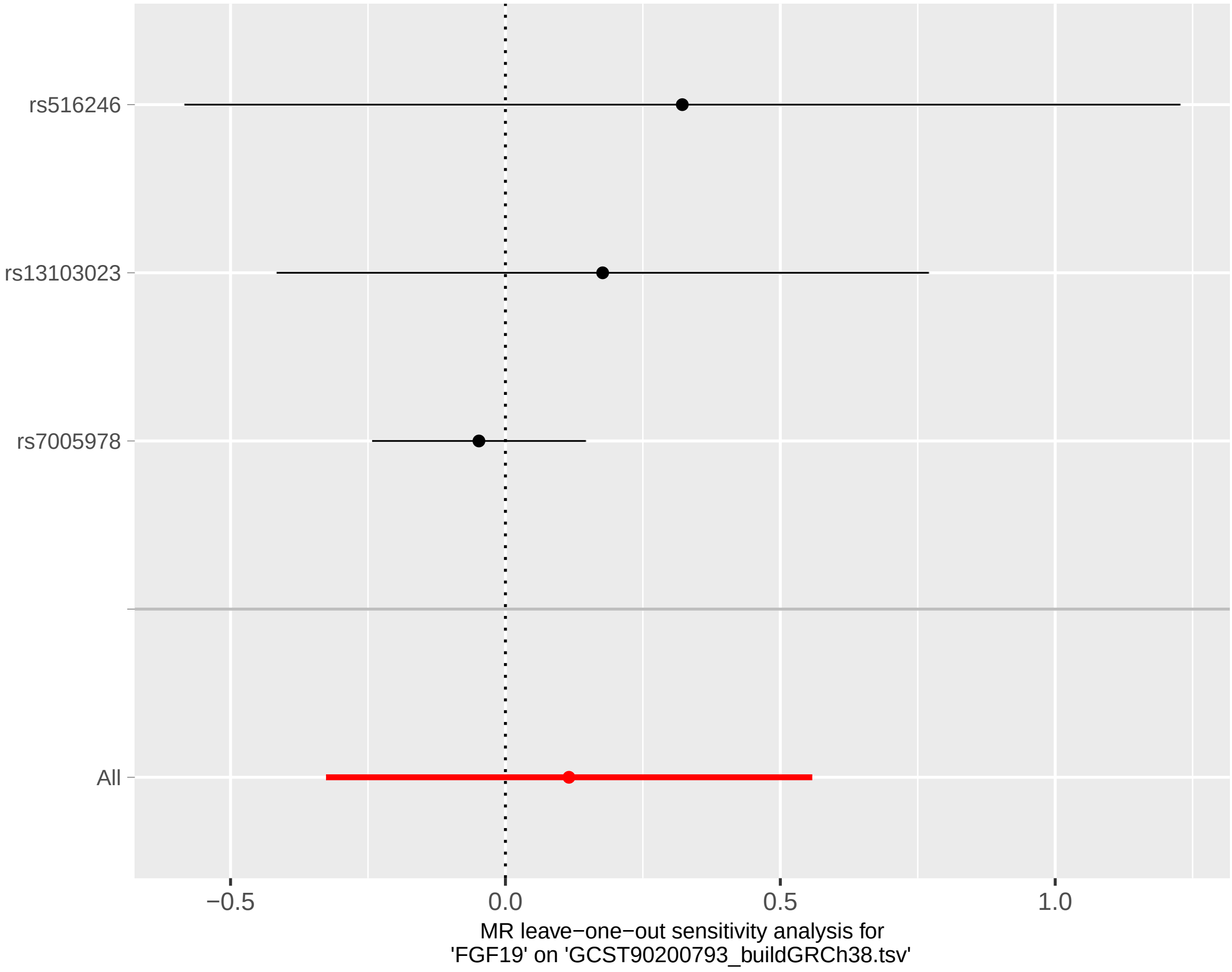

rs516246
rs13103023
rs7005978
All
−0.5
0.0
0.5
1.0
MR leave−one−out sensitivity analysis for
'FGF19' on 'GCST90200793_buildGRCh38.tsv'

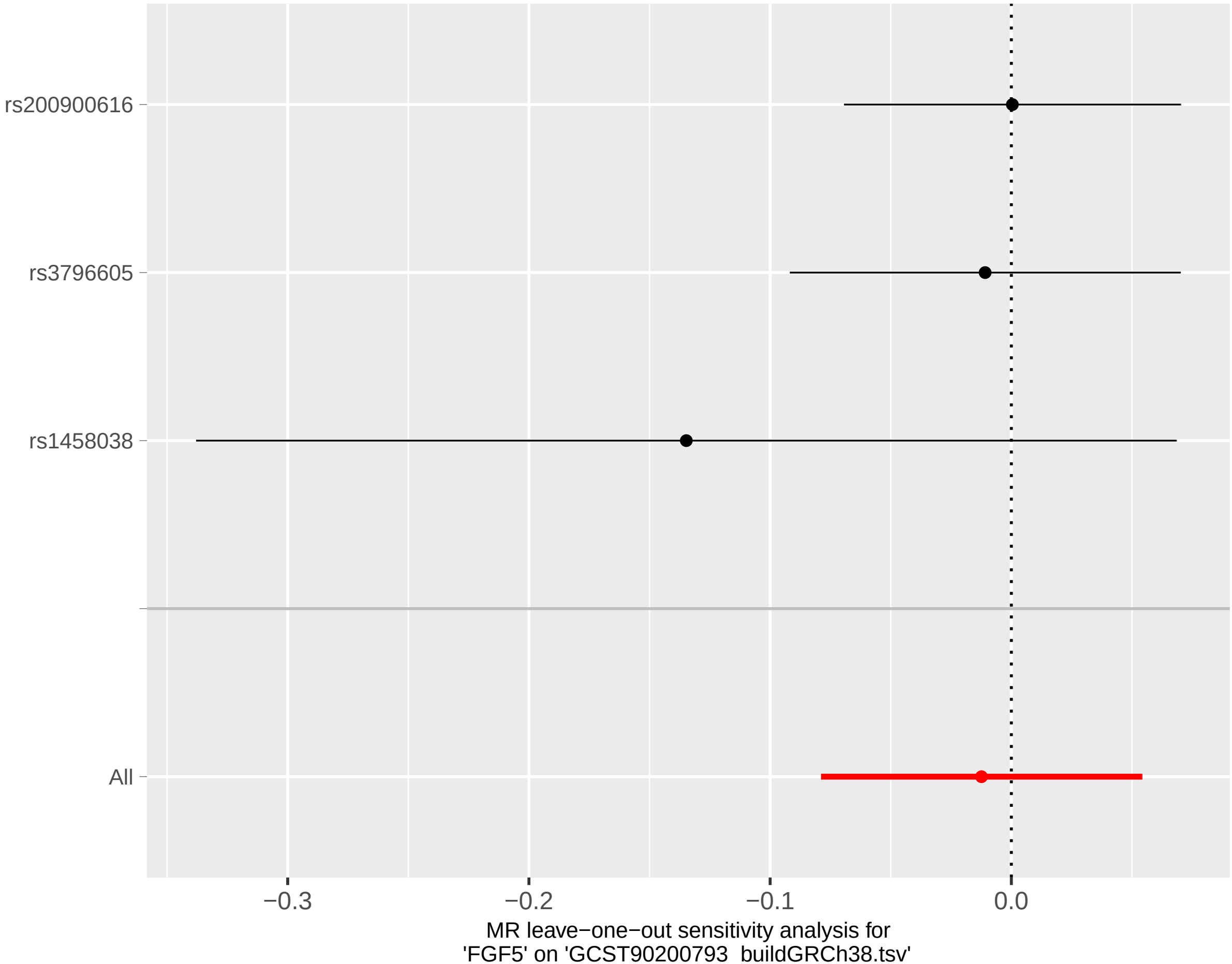
rs200900616
rs3796605
rs1458038
All
−0.3
−0.2
−0.1
0.0
MR leave−one−out sensitivity analysis for
'FGF5' on 'GCST90200793_buildGRCh38.tsv'

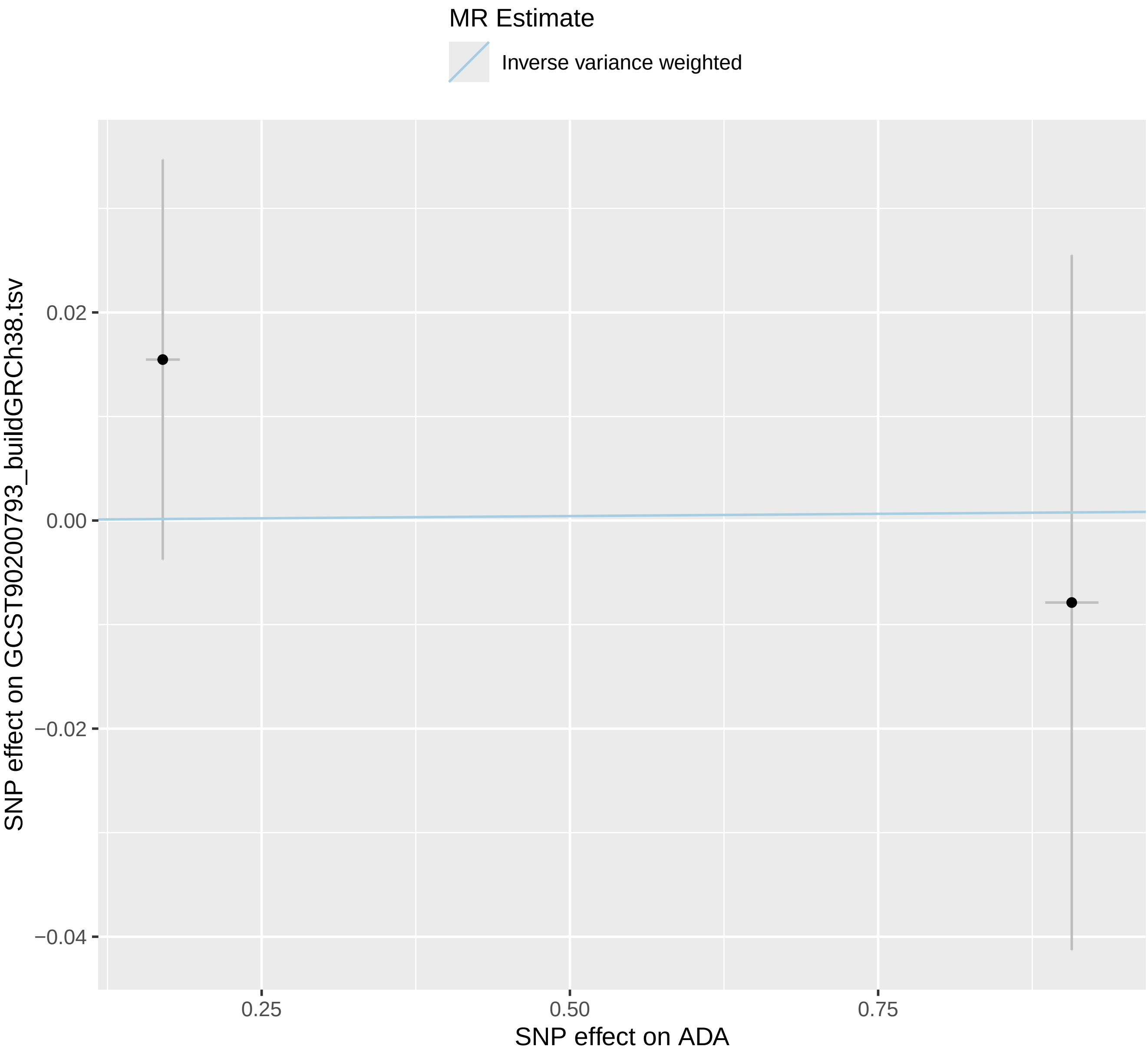

MR Estimate
Inverse variance weighted
SNP effect on GCST90200793_buildGRCh38.tsv
0.02
0.00
−0.02
−0.04
0.25
0.50
0.75
SNP effect on ADA

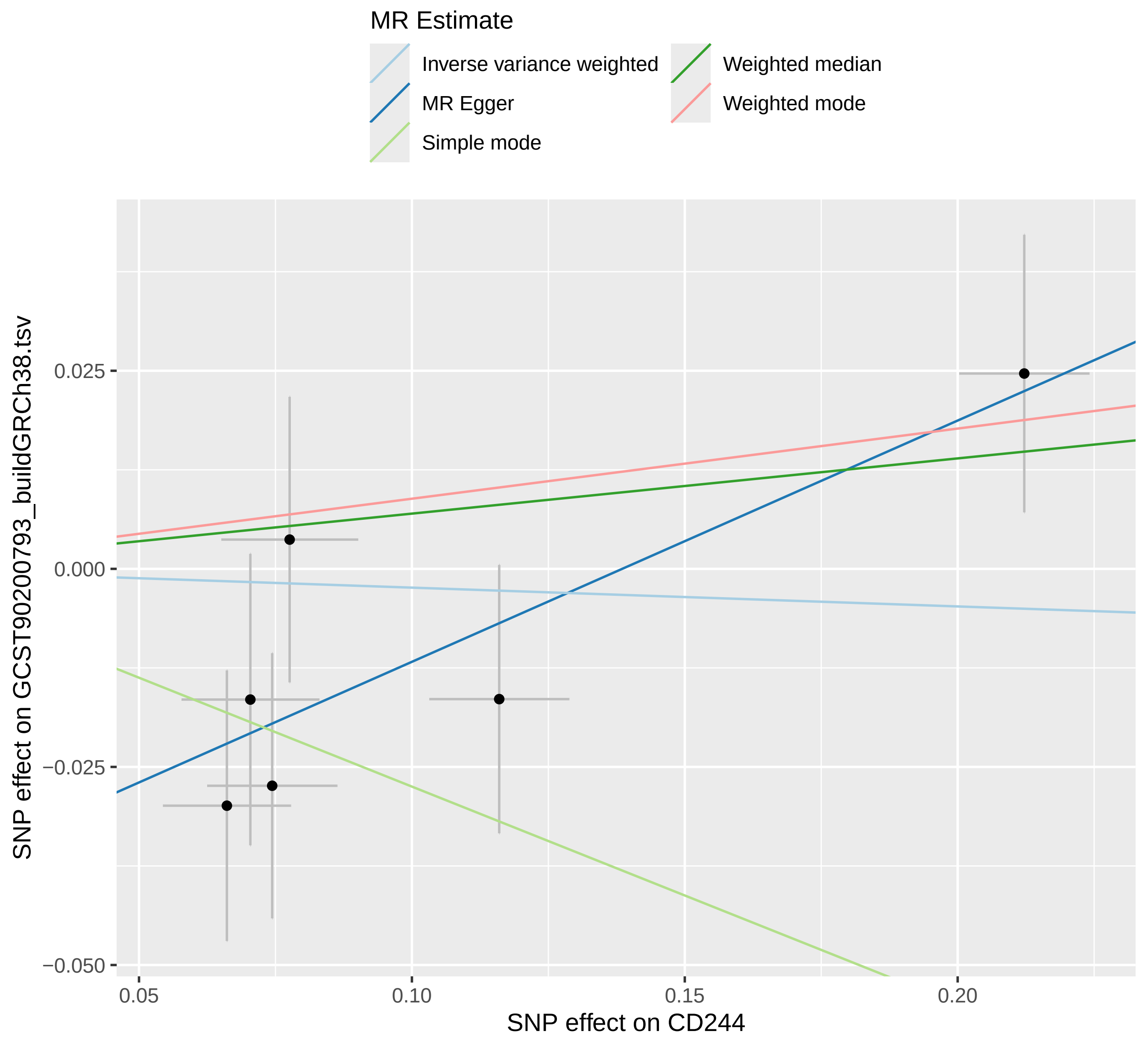
MR Estimate
Inverse variance weighted
MR Egger
Simple mode
Weighted median
Weighted mode
0.025
0.000
−0.025
−0.050
0.05
0.10
0.15
0.20
SNP effect on CD244
SNP effect on GCST90200793_buildGRCh38.tsv

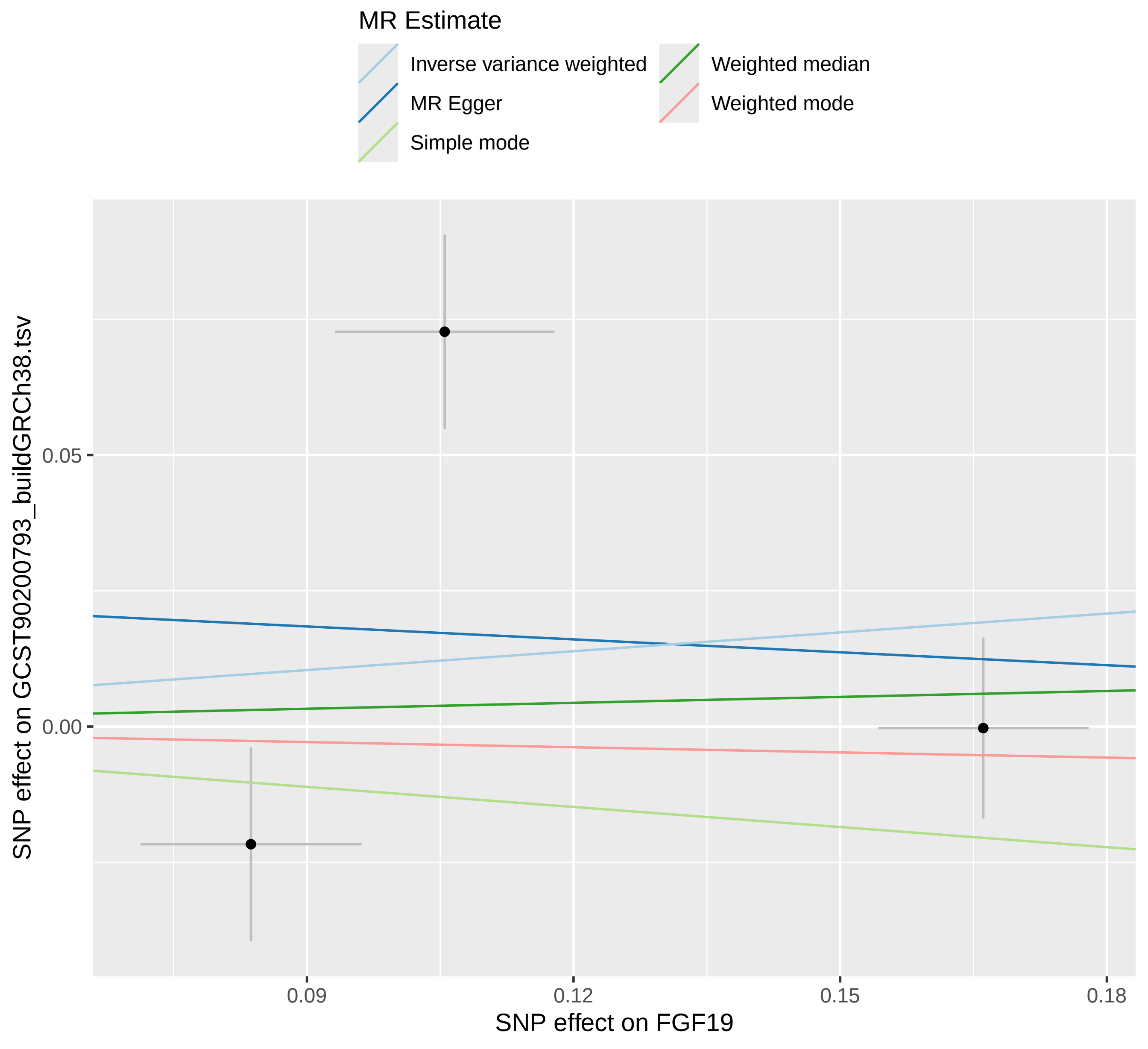
MR Estimate
Inverse variance weighted
MR Egger
Simple mode
Weighted median
Weighted mode
SNP effect on GCST90200793_buildGRCh38.tsv
0.05
0.00
0.09
0.12
0.15
0.18
SNP effect on FGF19

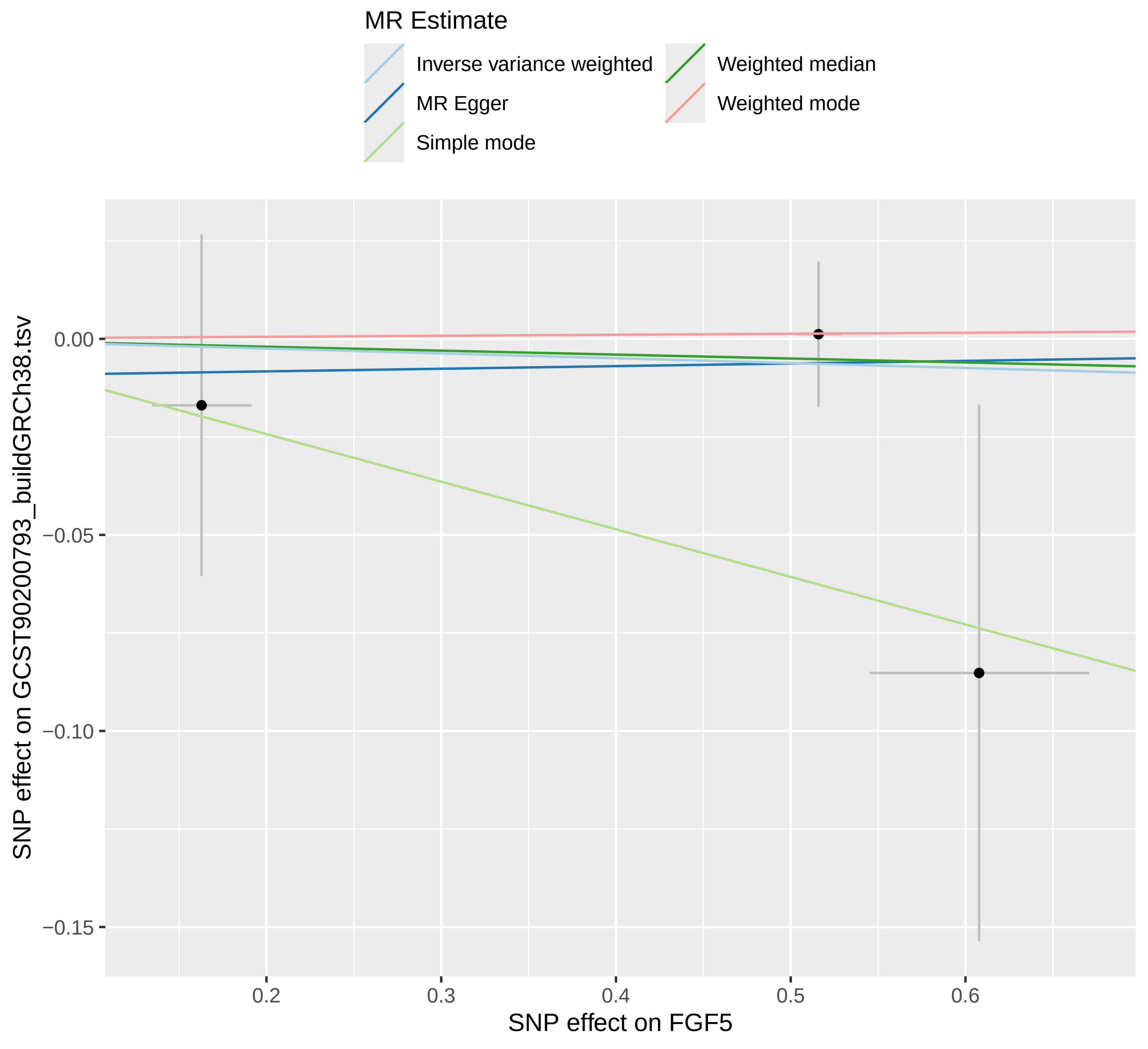
MR Estimate
Inverse variance weighted
MR Egger
Simple mode
Weighted median
Weighted mode
SNP effect on GCST90200793_buildGRCh38.tsv
0.00
−0.05
−0.10
−0.15
0.2
0.3
0.4
0.5
0.6
SNP effect on FGF5

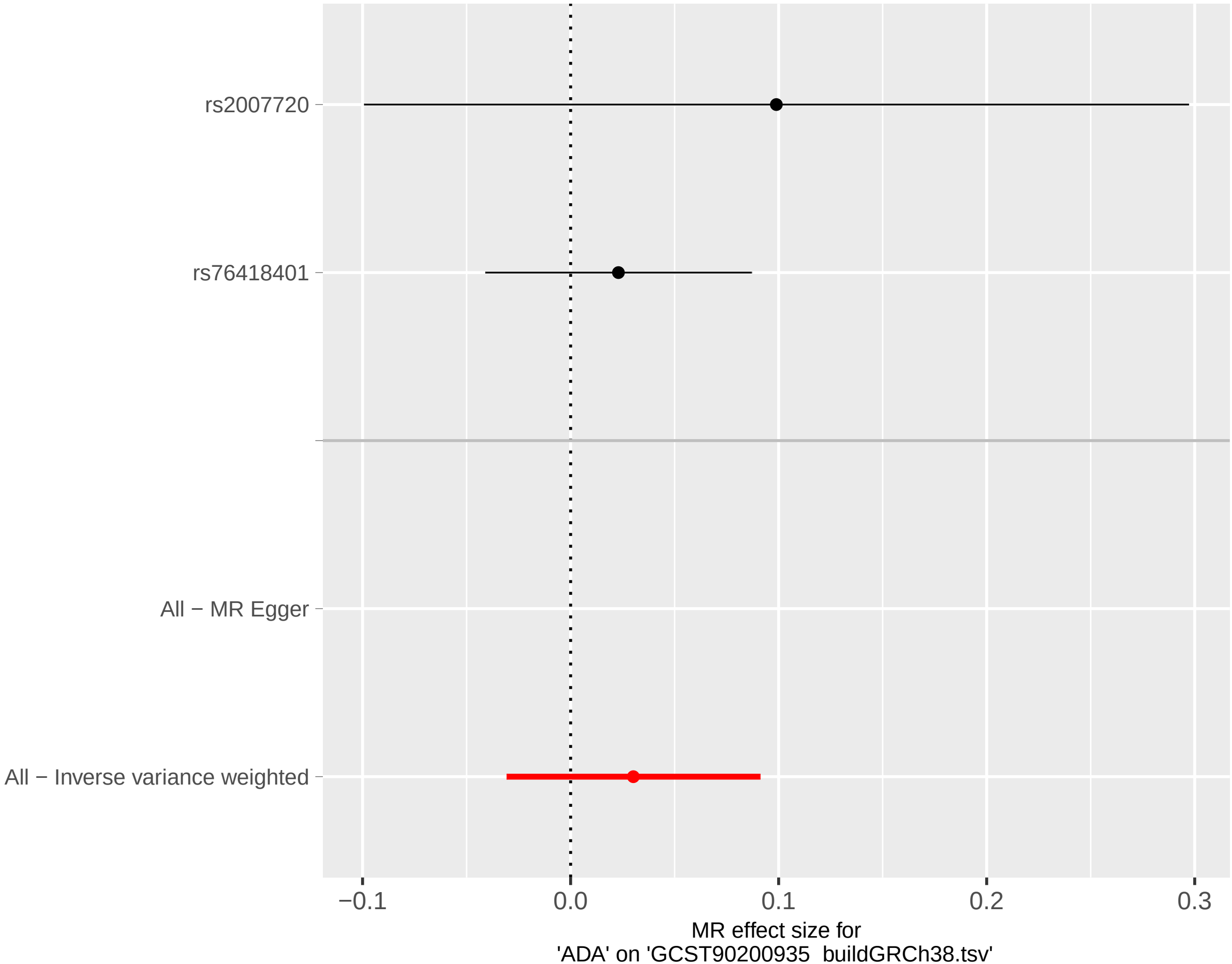
rs2007720
rs76418401
All − MR Egger
All − Inverse variance weighted
−0.1
0.0
0.1
0.2
0.3
MR effect size for
'ADA' on 'GCST90200935_buildGRCh38.tsv'

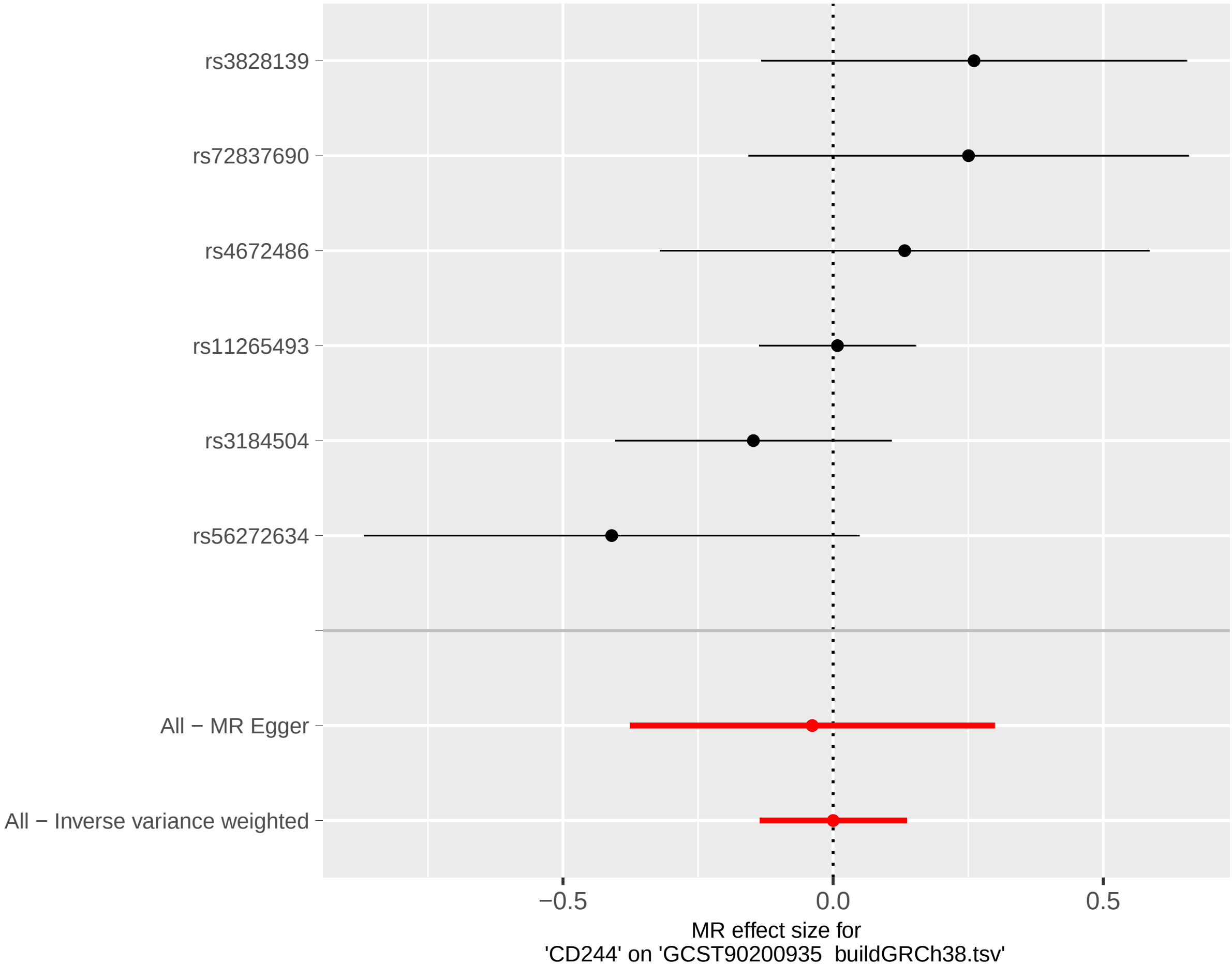
rs3828139
rs72837690
rs4672486
rs11265493
rs3184504
rs56272634
All − MR Egger
All − Inverse variance weighted
−0.5
0.0
0.5
MR effect size for
'CD244' on 'GCST90200935_buildGRCh38.tsv'

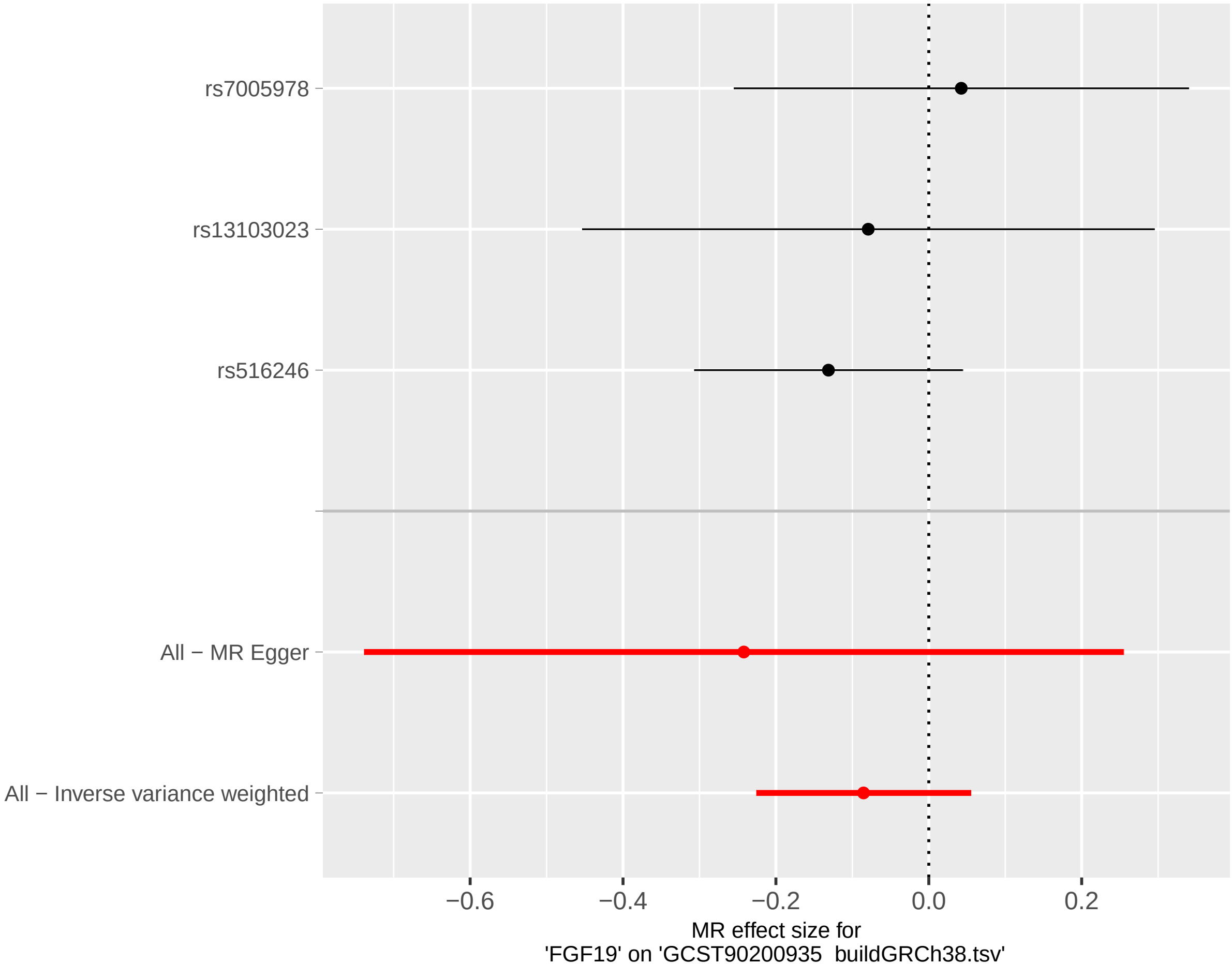
rs7005978
rs13103023
rs516246
All − MR Egger
All − Inverse variance weighted
−0.6
−0.4
−0.2
0.0
0.2
MR effect size for
'FGF19' on 'GCST90200935_buildGRCh38.tsv'

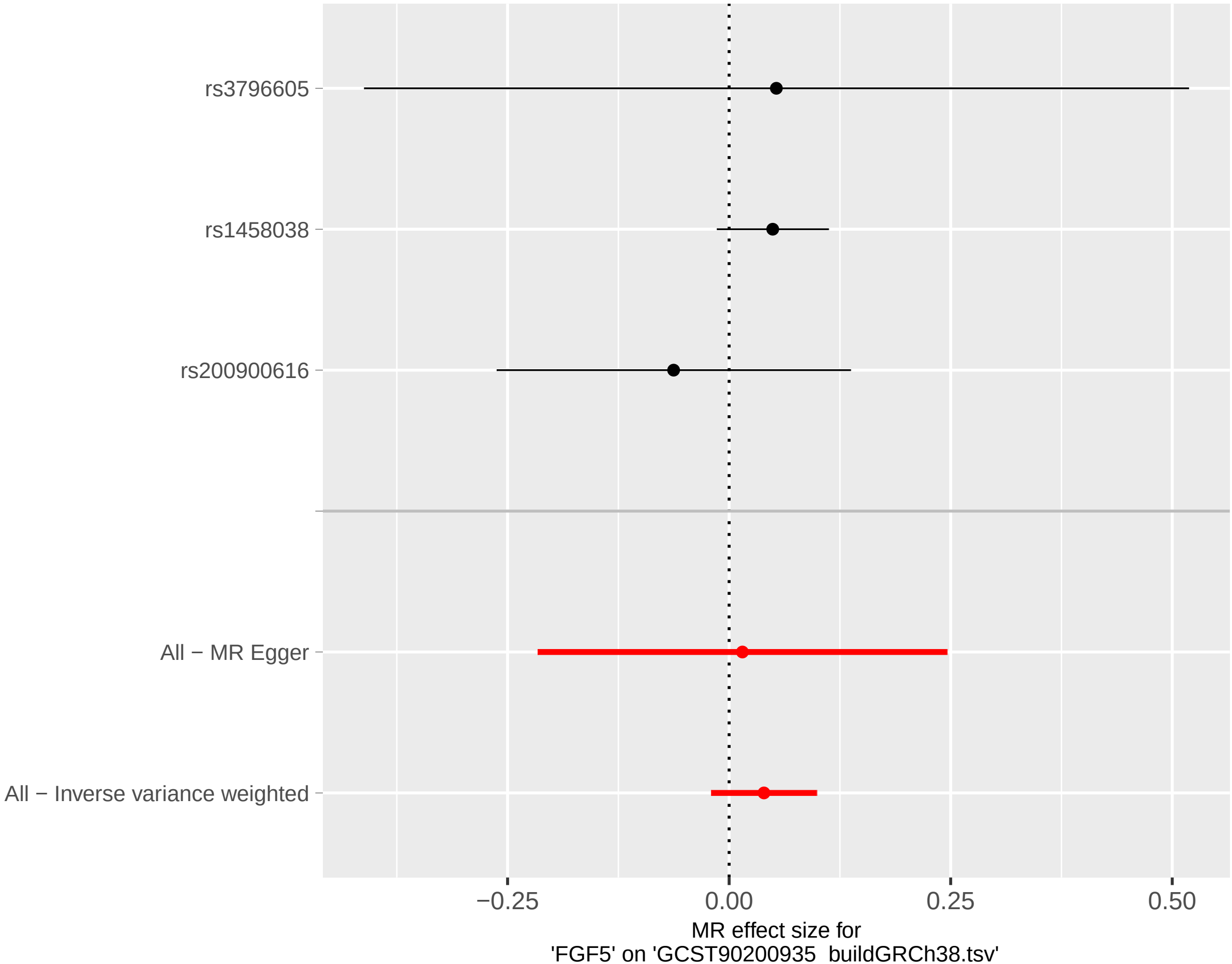

rs3796605
rs1458038
rs200900616
All − MR Egger
All − Inverse variance weighted
−0.25
0.00
0.25
0.50
MR effect size for
'FGF5' on 'GCST90200935_buildGRCh38.tsv'

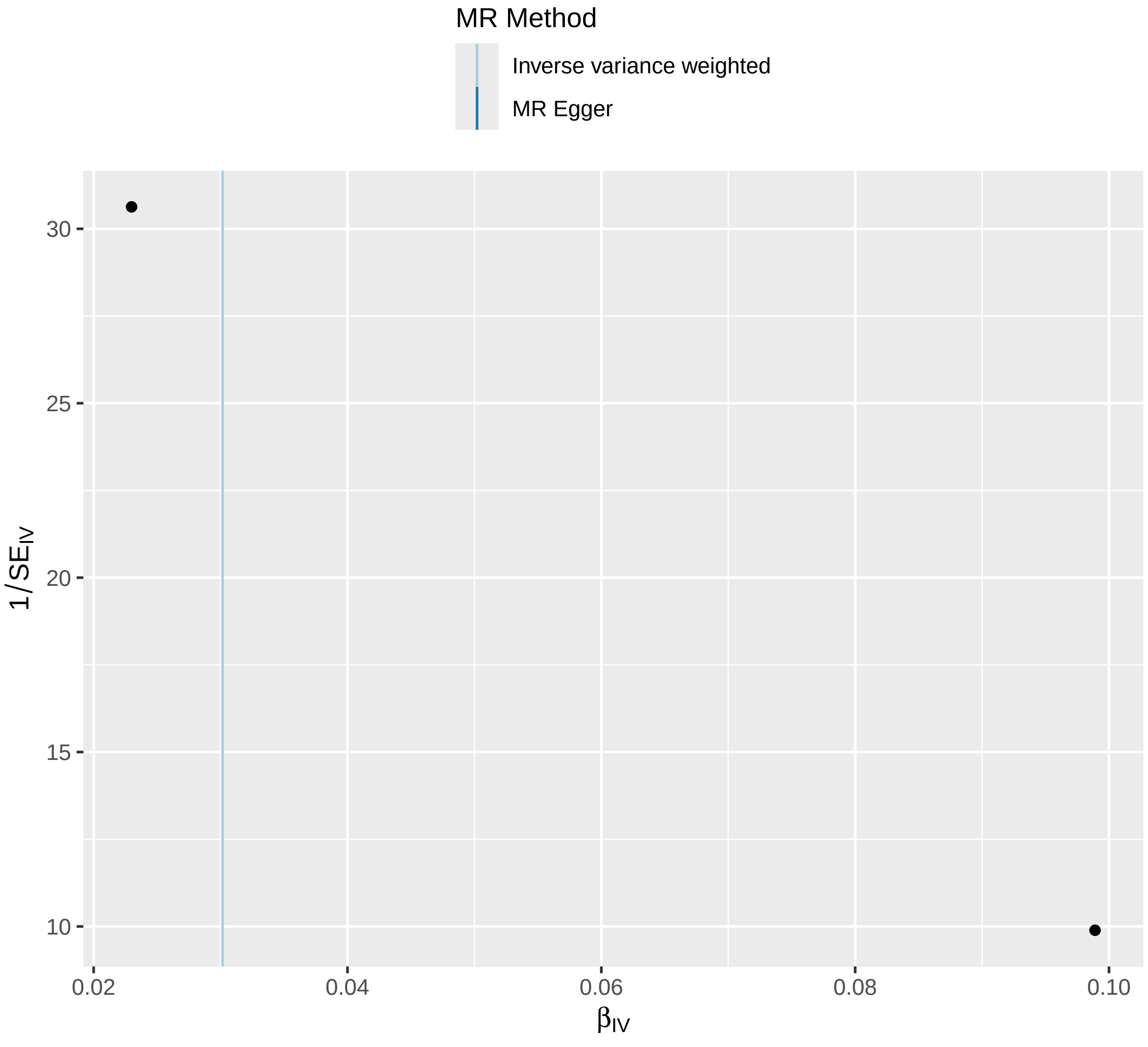

MR Method
Inverse variance weighted
MR Egger
1/SE_IV
30
25
20
15
10
0.02
0.04
0.06
0.08
0.10
β_IV

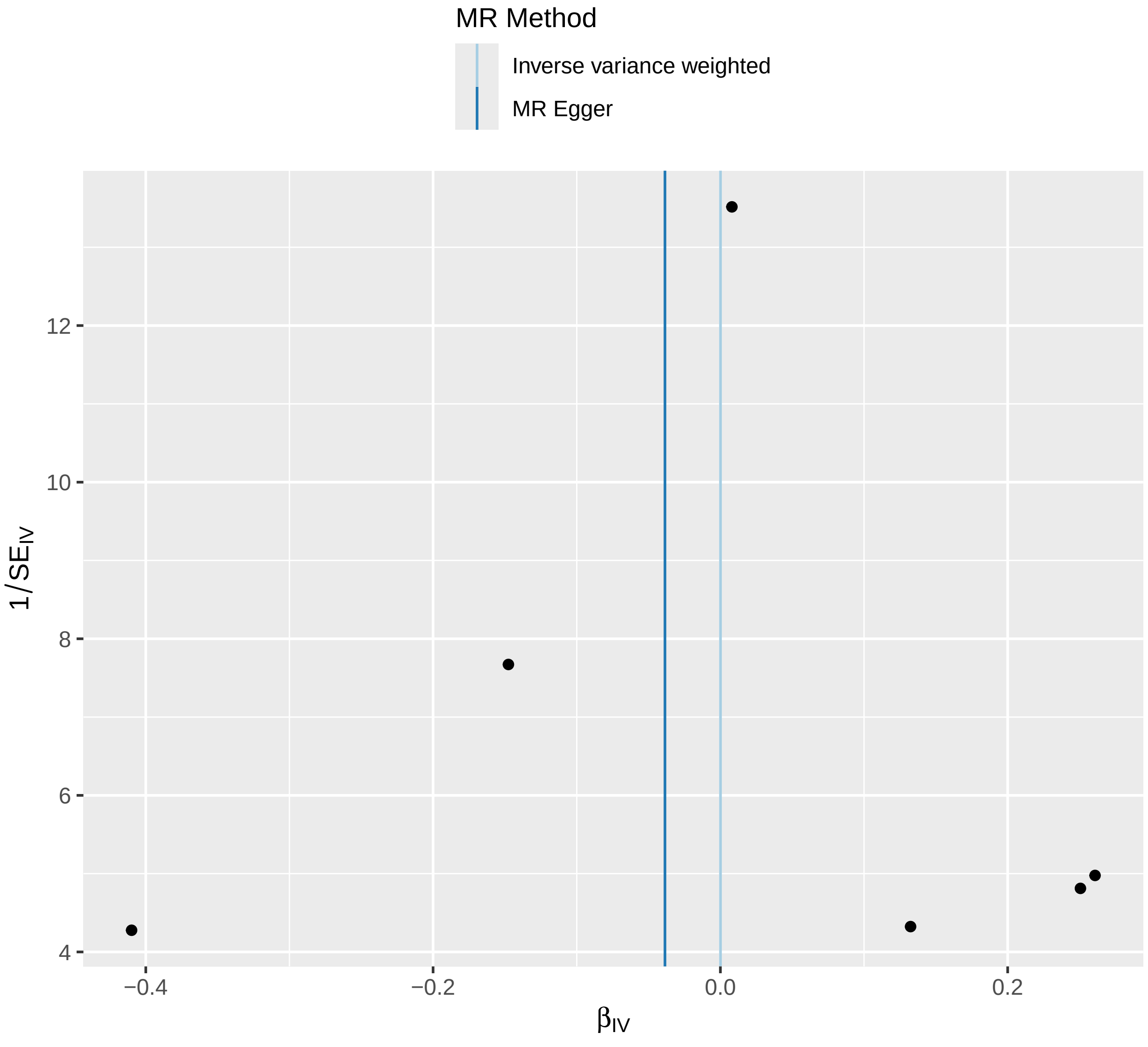

MR Method
Inverse variance weighted
MR Egger
12
10
8
6
4
1/SE$_{IV}$
−0.4
−0.2
0.0
0.2
β$_{IV}$

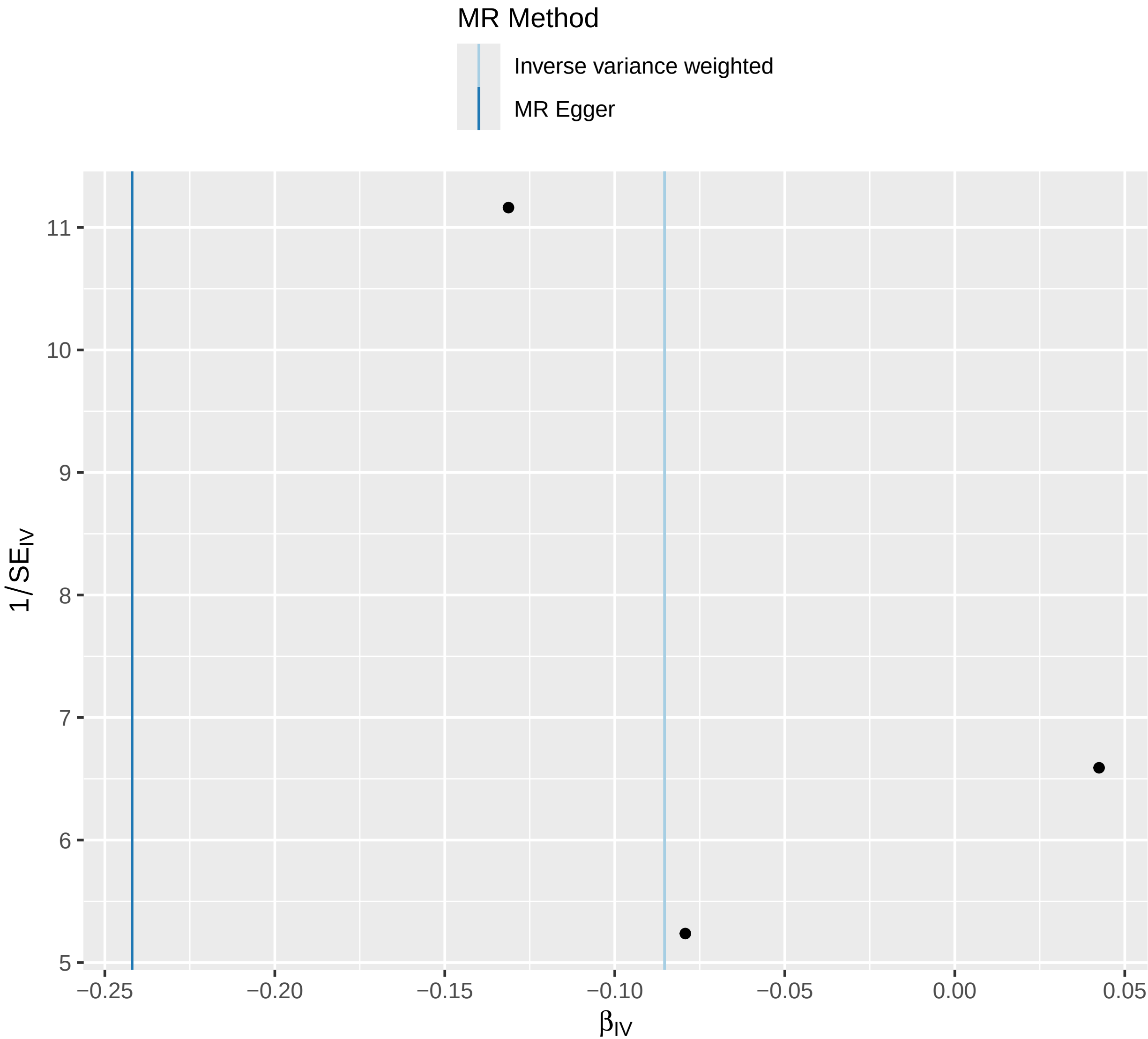
MR Method
Inverse variance weighted
MR Egger
11
10
9
8
7
6
5
1/SE_IV
−0.25
−0.20
−0.15
−0.10
−0.05
0.00
0.05
β_IV

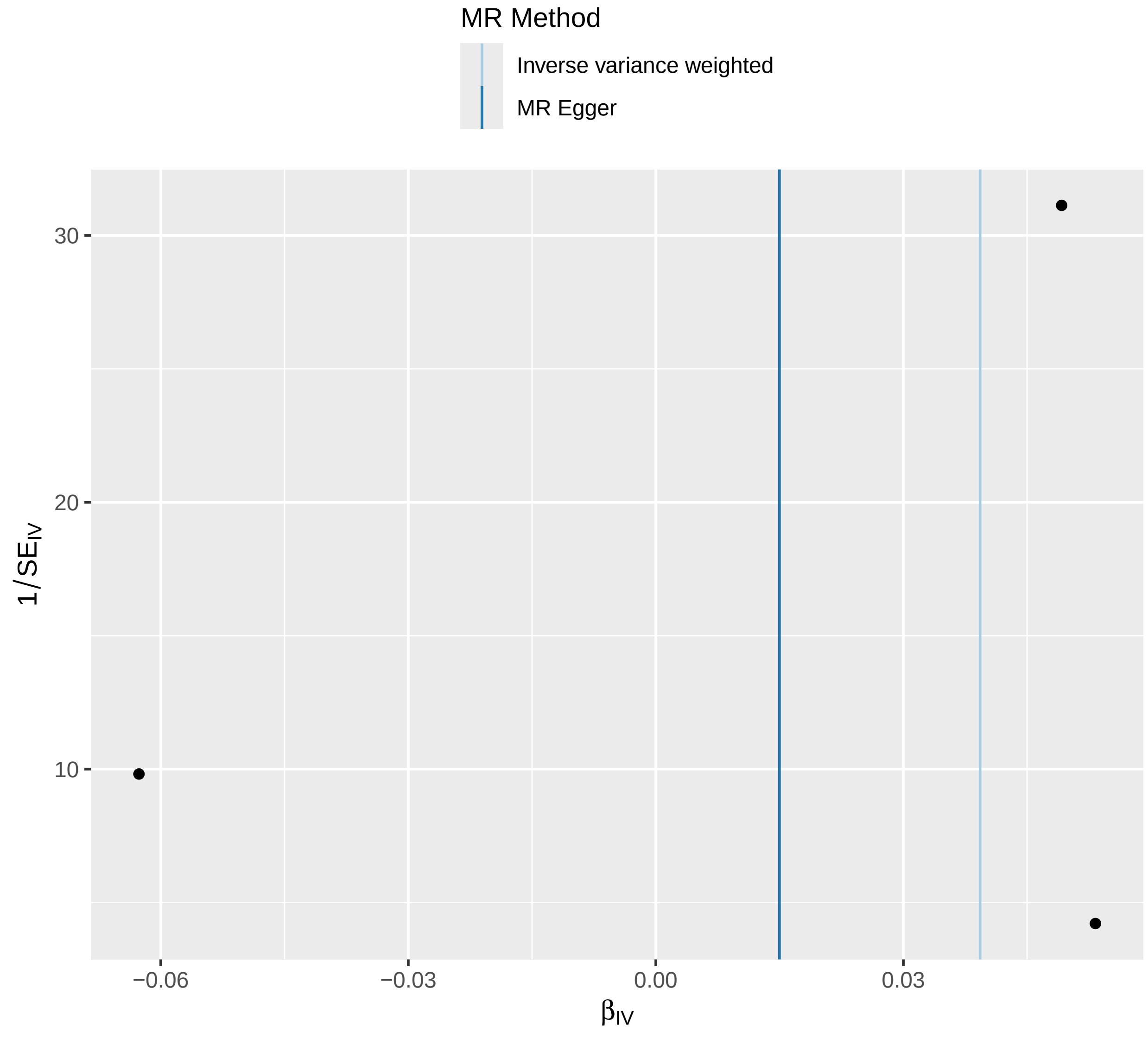
MR Method
Inverse variance weighted
MR Egger
30
20
10
1/SE_IV
−0.06
−0.03
0.00
0.03
β_IV

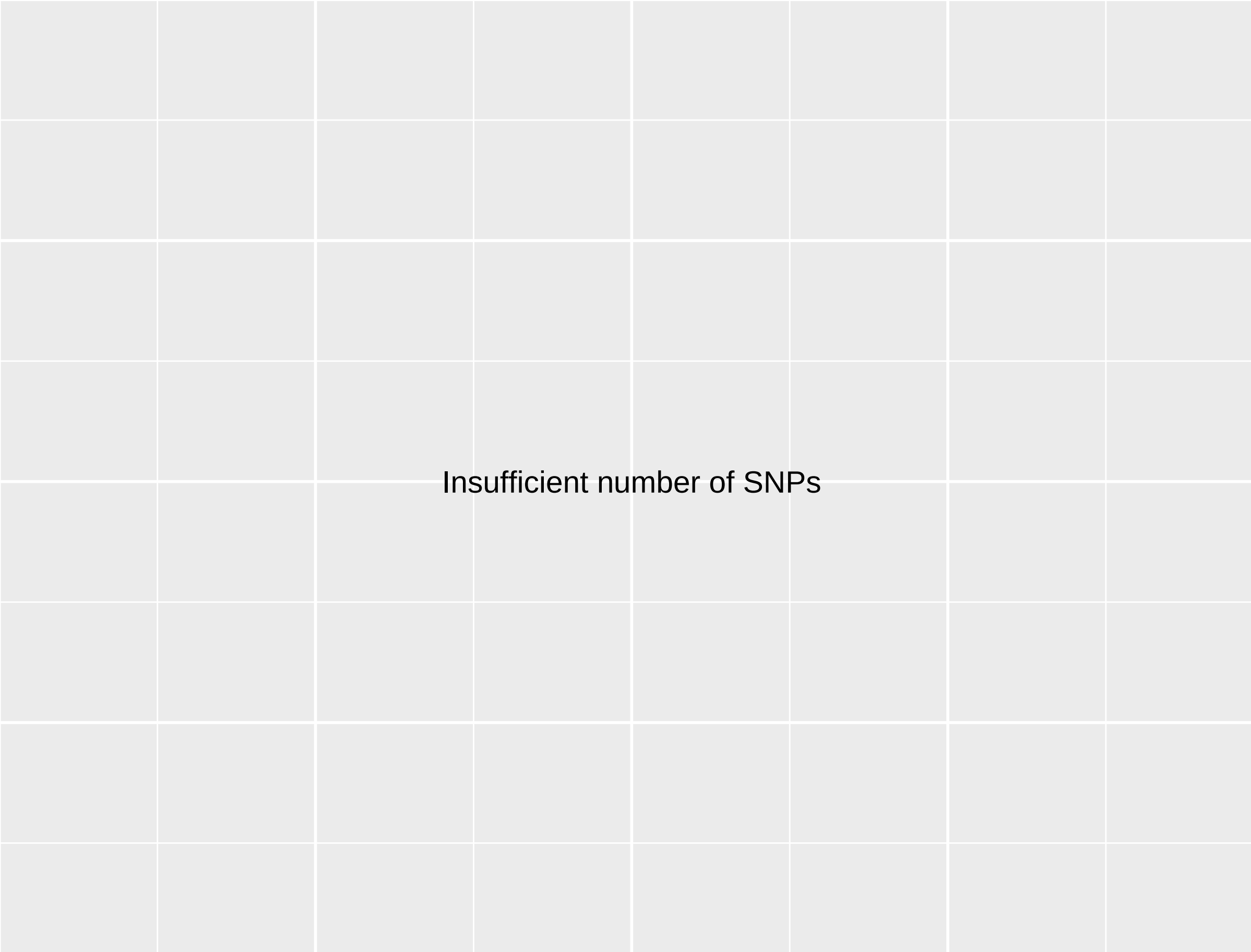
Insufficient number of SNPs

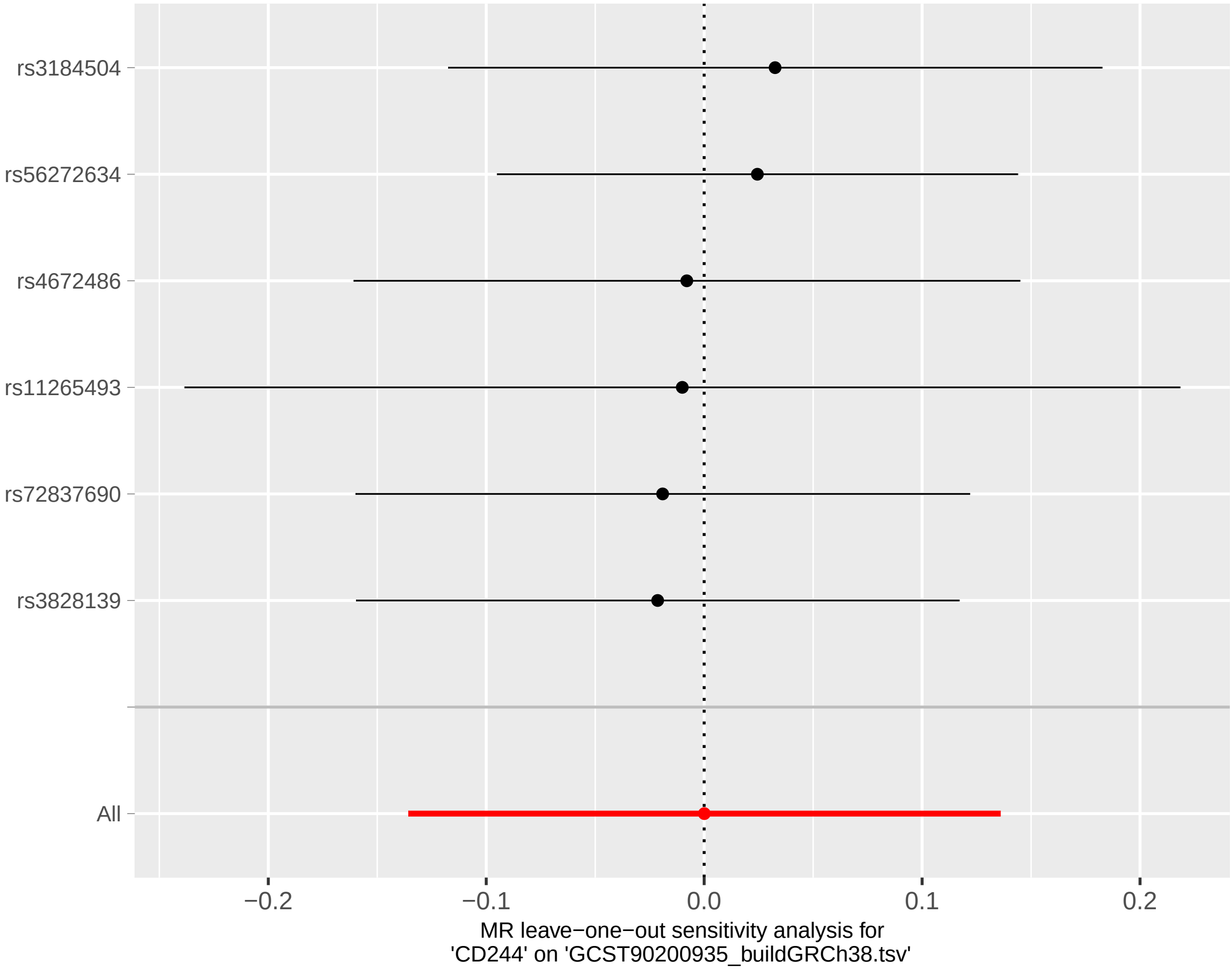
rs3184504
rs56272634
rs4672486
rs11265493
rs72837690
rs3828139
All
−0.2
−0.1
0.0
0.1
0.2
MR leave−one−out sensitivity analysis for
'CD244' on 'GCST90200935_buildGRCh38.tsv'

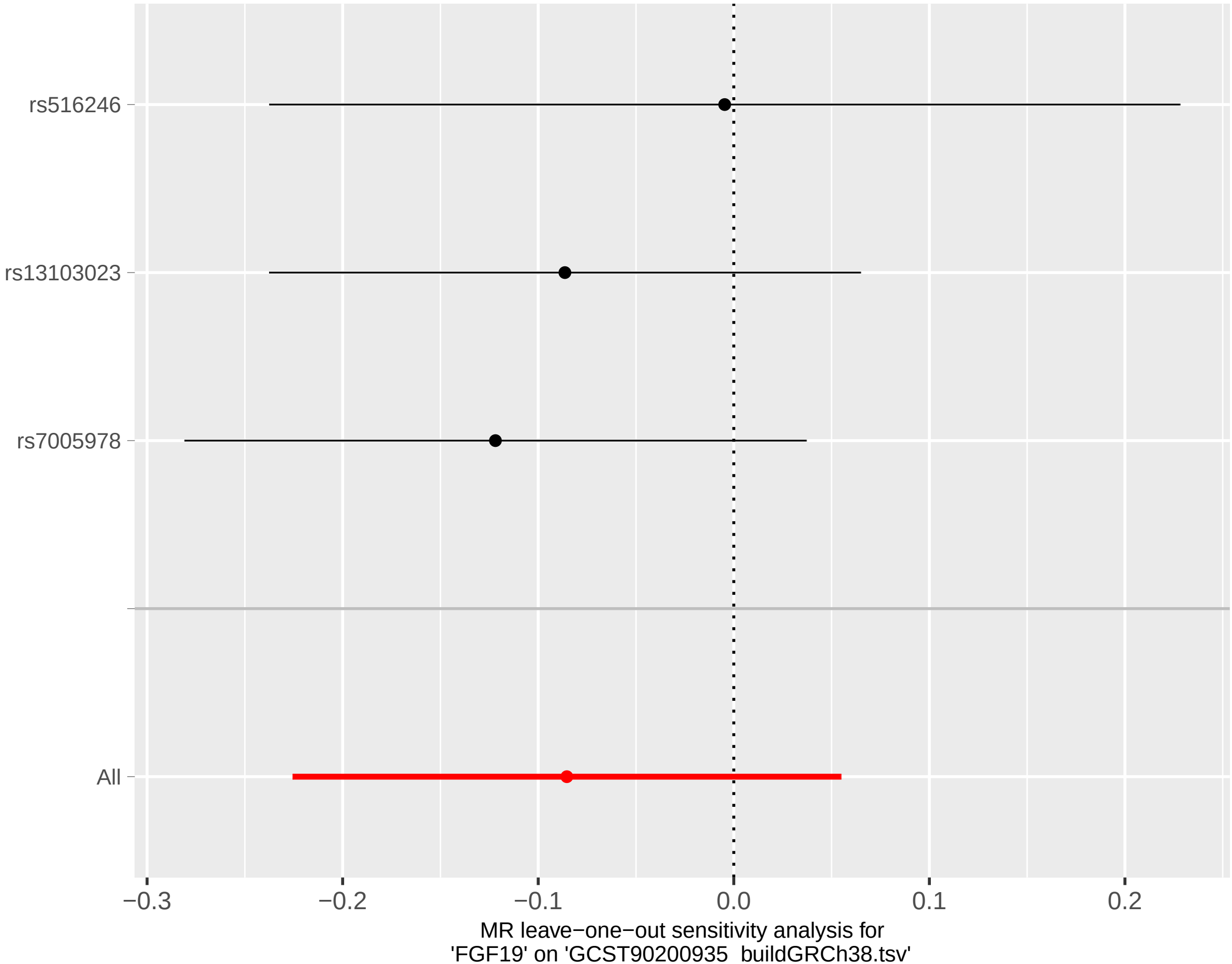
rs516246
rs13103023
rs7005978
All
−0.3
−0.2
−0.1
0.0
0.1
0.2
MR leave−one−out sensitivity analysis for
'FGF19' on 'GCST90200935_buildGRCh38.tsv'

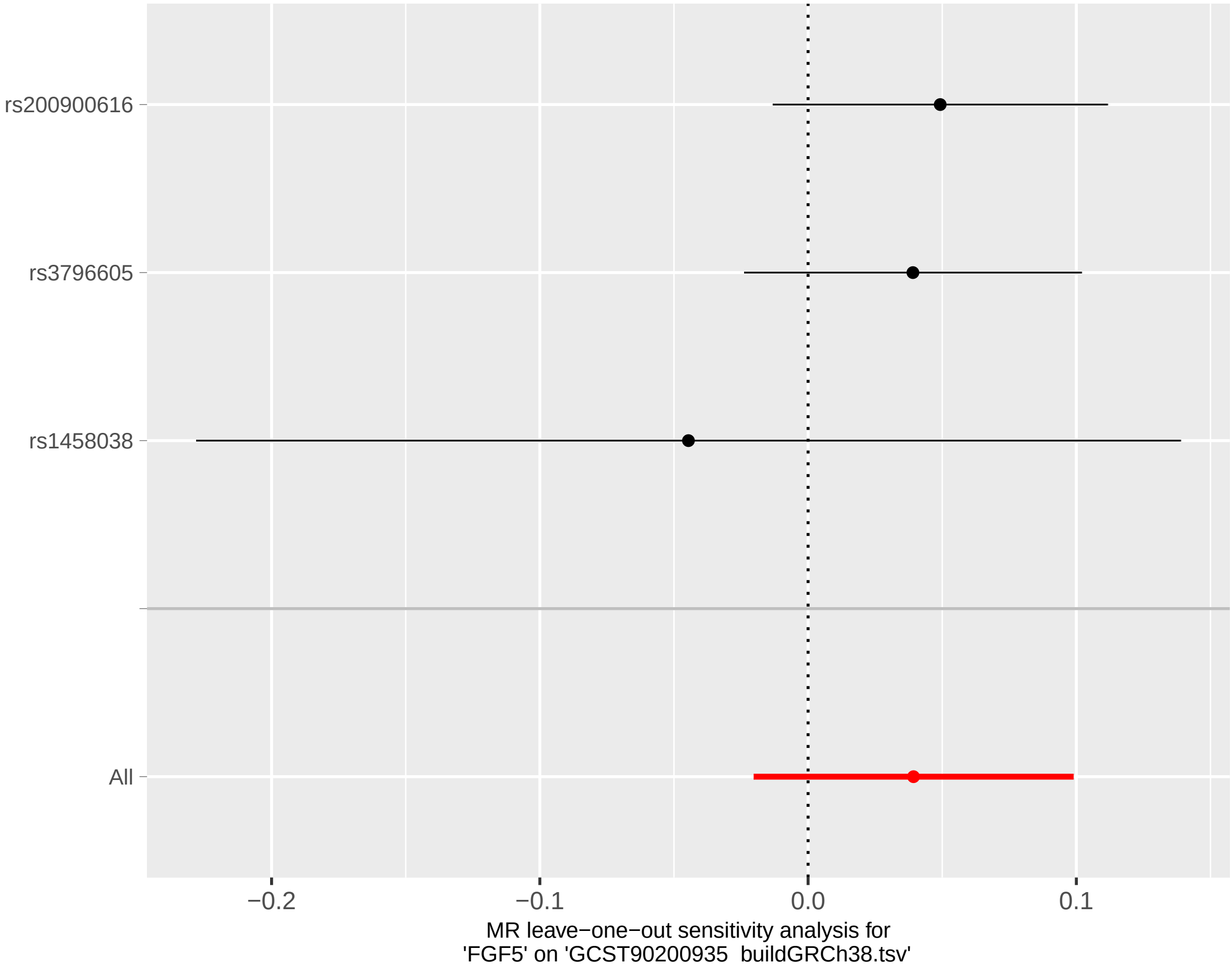
rs200900616
rs3796605
rs1458038
All
−0.2
−0.1
0.0
0.1
MR leave−one−out sensitivity analysis for
'FGF5' on 'GCST90200935_buildGRCh38.tsv'

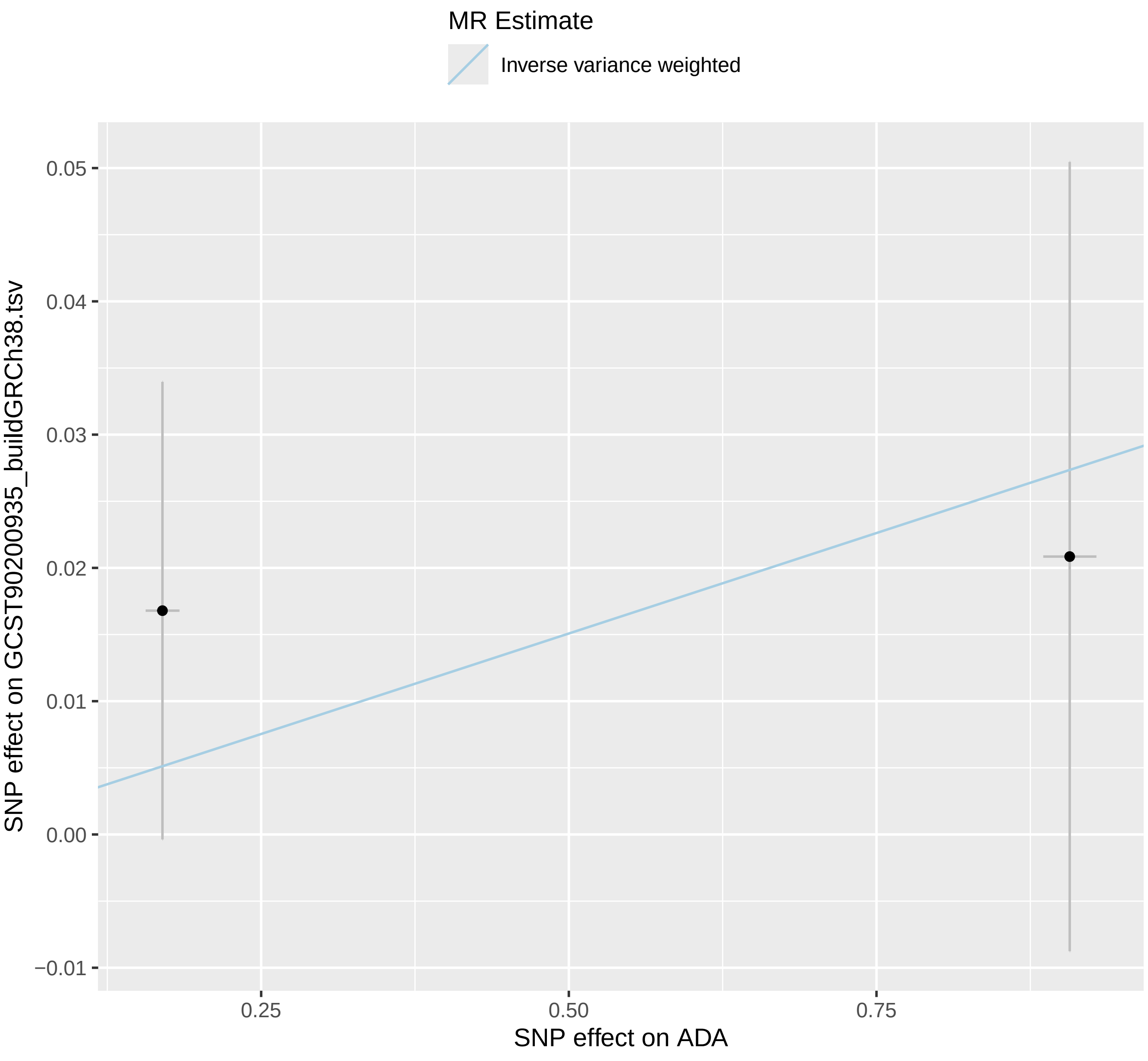

MR Estimate
Inverse variance weighted
0.05
0.04
0.03
0.02
0.01
0.00
−0.01
SNP effect on GCST90200935_buildGRCh38.tsv
0.25
0.50
0.75
SNP effect on ADA

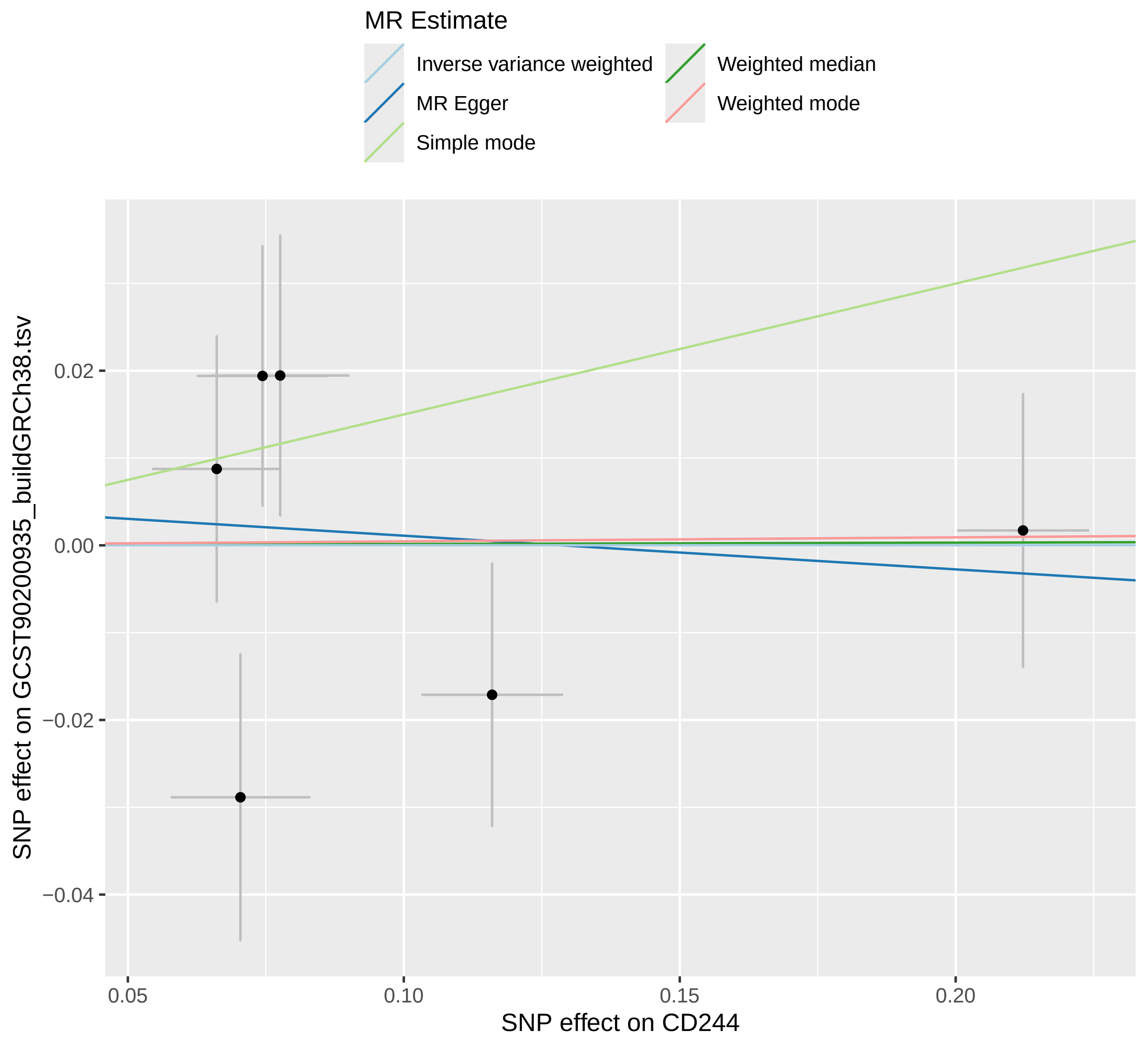

MR Estimate
Inverse variance weighted
MR Egger
Simple mode
Weighted median
Weighted mode
0.02
0.00
−0.02
−0.04
0.05
0.10
0.15
0.20
SNP effect on CD244
SNP effect on GCST90200935_buildGRCh38.tsv

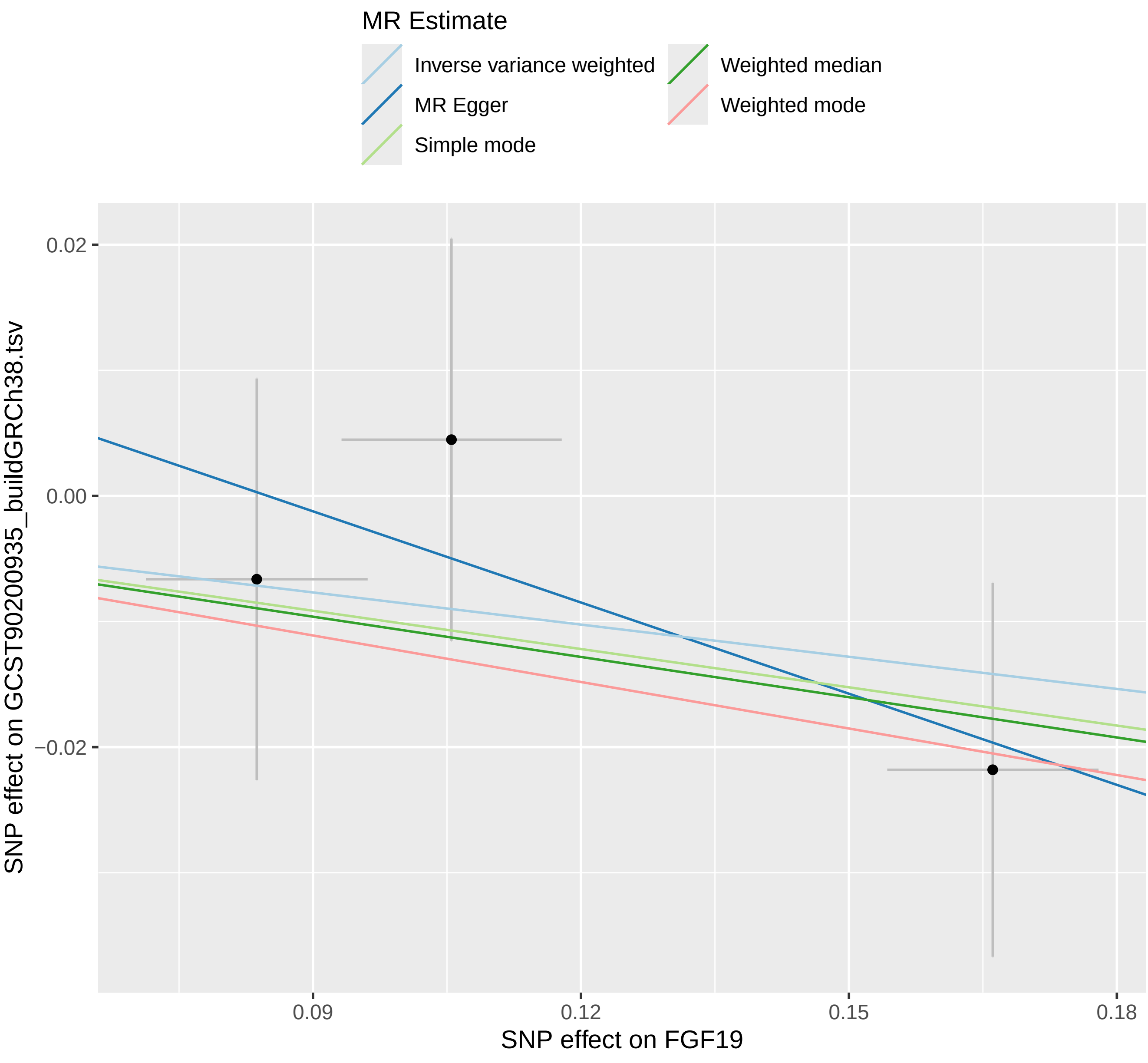
MR Estimate
Inverse variance weighted
MR Egger
Simple mode
Weighted median
Weighted mode
0.02
0.00
−0.02
SNP effect on GCST90200935_buildGRCh38.tsv
0.09
0.12
0.15
0.18
SNP effect on FGF19

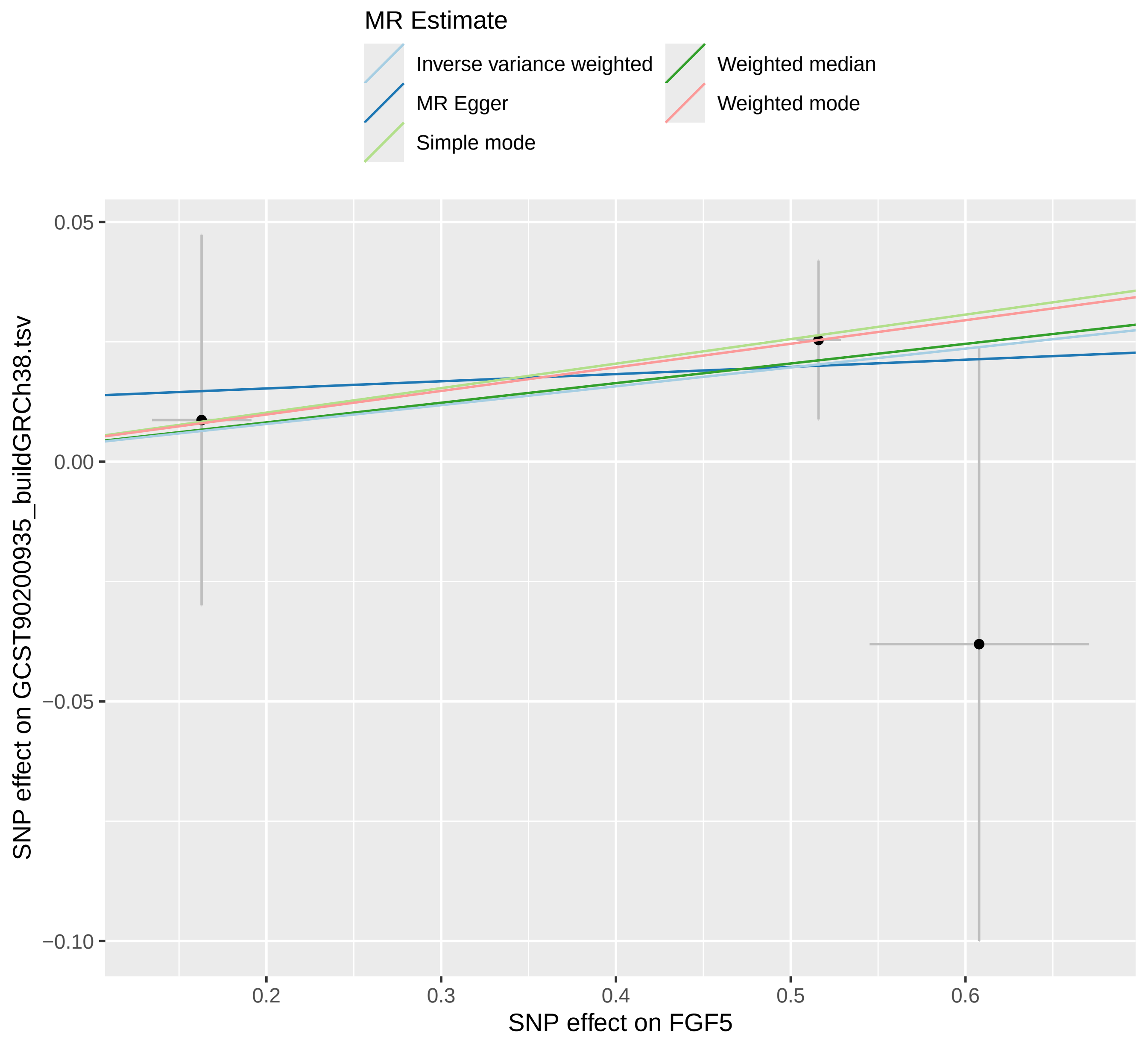

MR Estimate
Inverse variance weighted
MR Egger
Simple mode
Weighted median
Weighted mode
0.05
0.00
−0.05
−0.10
0.2
0.3
0.4
0.5
0.6
SNP effect on GCST90200935_buildGRCh38.tsv
SNP effect on FGF5

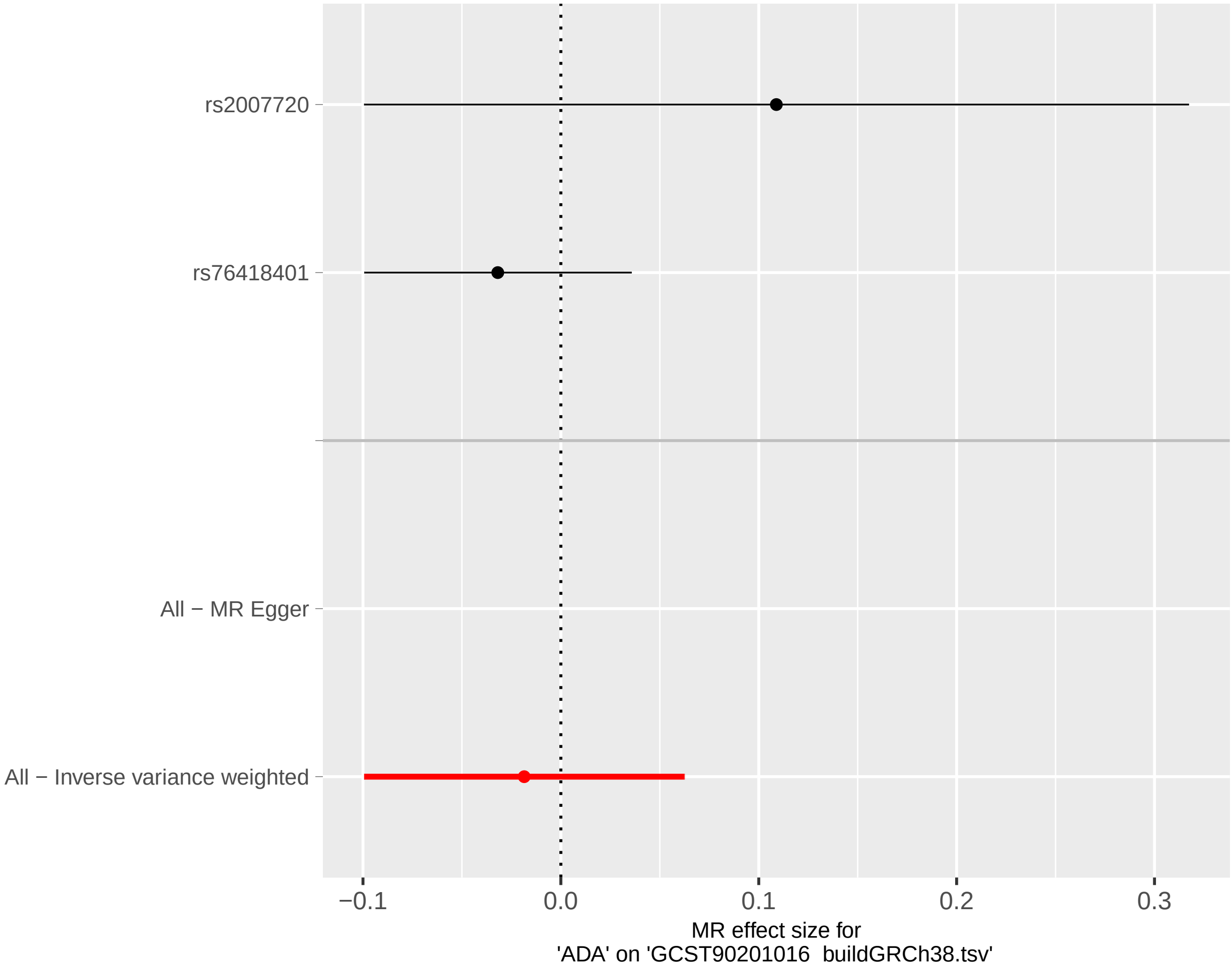
rs2007720
rs76418401
All − MR Egger
All − Inverse variance weighted
−0.1
0.0
0.1
0.2
0.3
MR effect size for
'ADA' on 'GCST90201016_buildGRCh38.tsv'

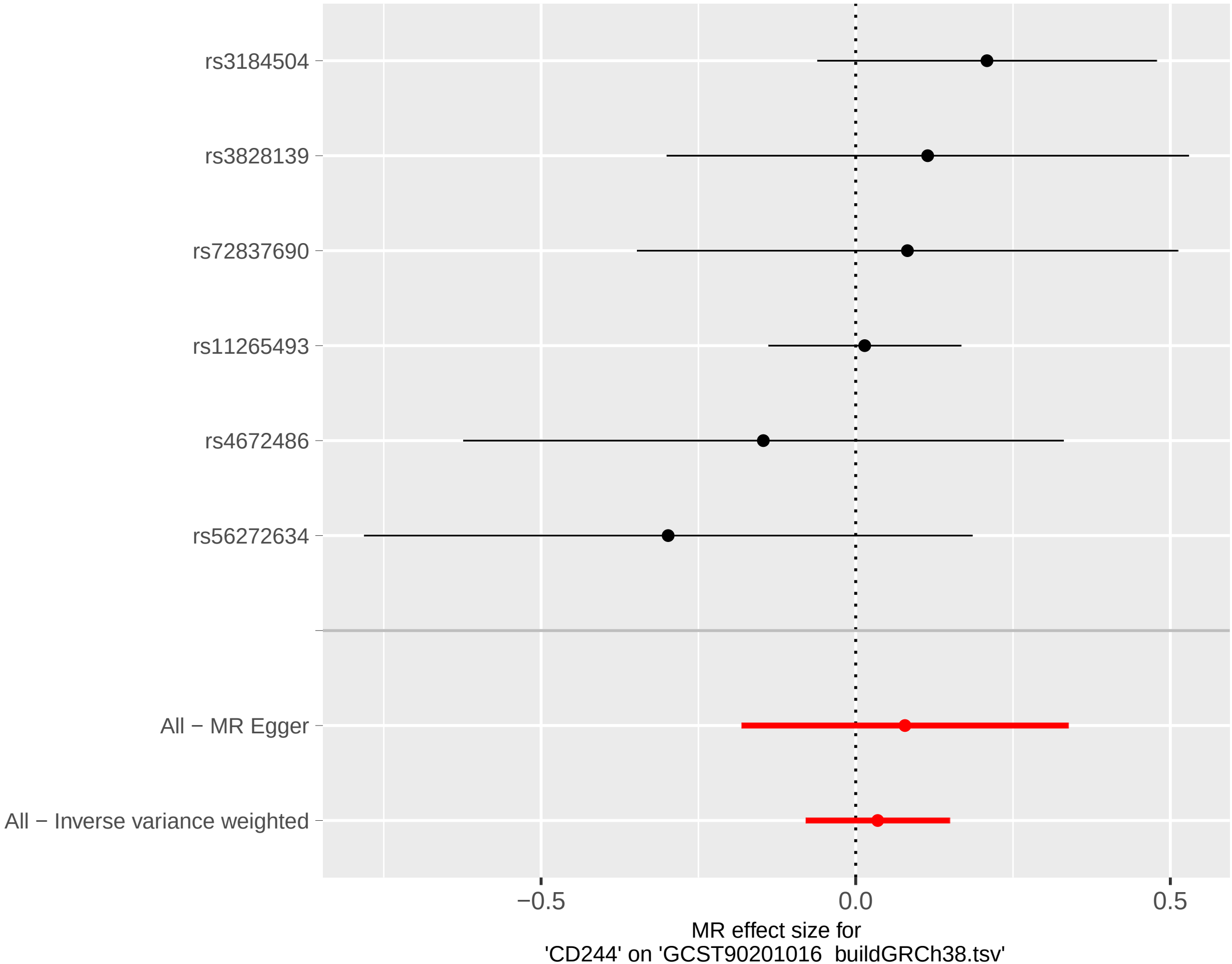
rs3184504
rs3828139
rs72837690
rs11265493
rs4672486
rs56272634
All − MR Egger
All − Inverse variance weighted
−0.5
0.0
0.5
MR effect size for
'CD244' on 'GCST90201016_buildGRCh38.tsv'

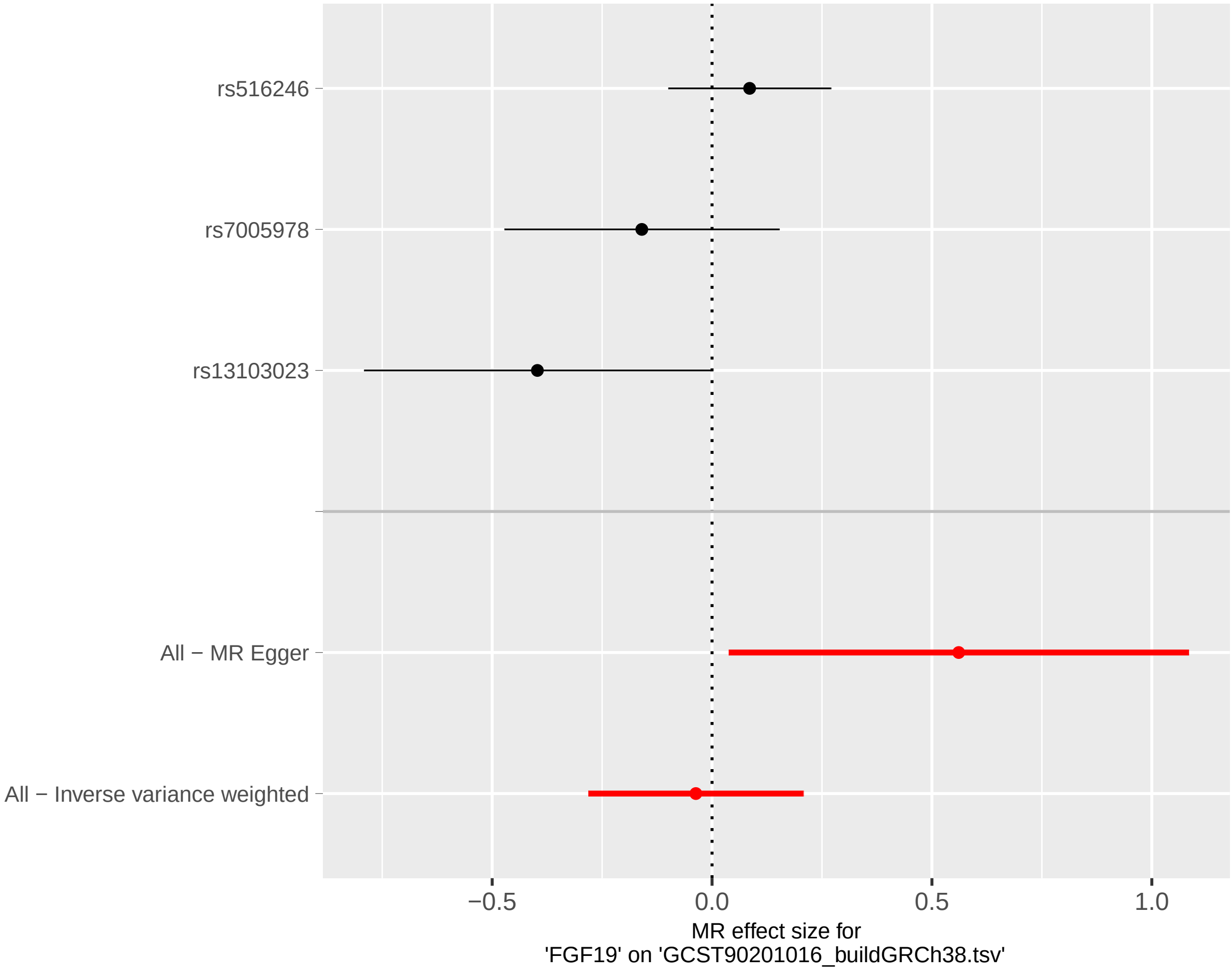
rs516246
rs7005978
rs13103023
All − MR Egger
All − Inverse variance weighted
−0.5
0.0
0.5
1.0
MR effect size for
'FGF19' on 'GCST90201016_buildGRCh38.tsv'

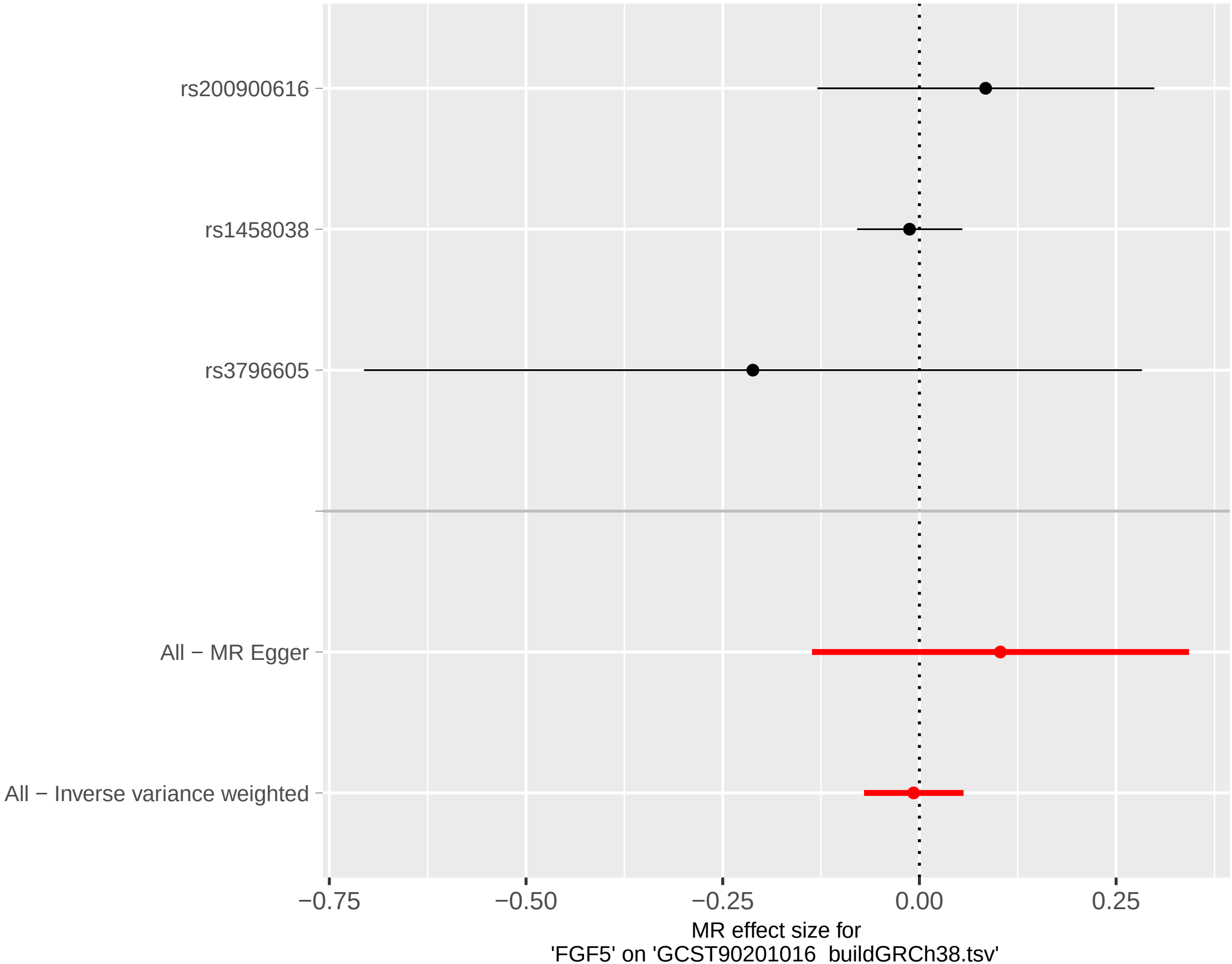
rs200900616
rs1458038
rs3796605
All − MR Egger
All − Inverse variance weighted
−0.75
−0.50
−0.25
0.00
0.25
MR effect size for
'FGF5' on 'GCST90201016_buildGRCh38.tsv'

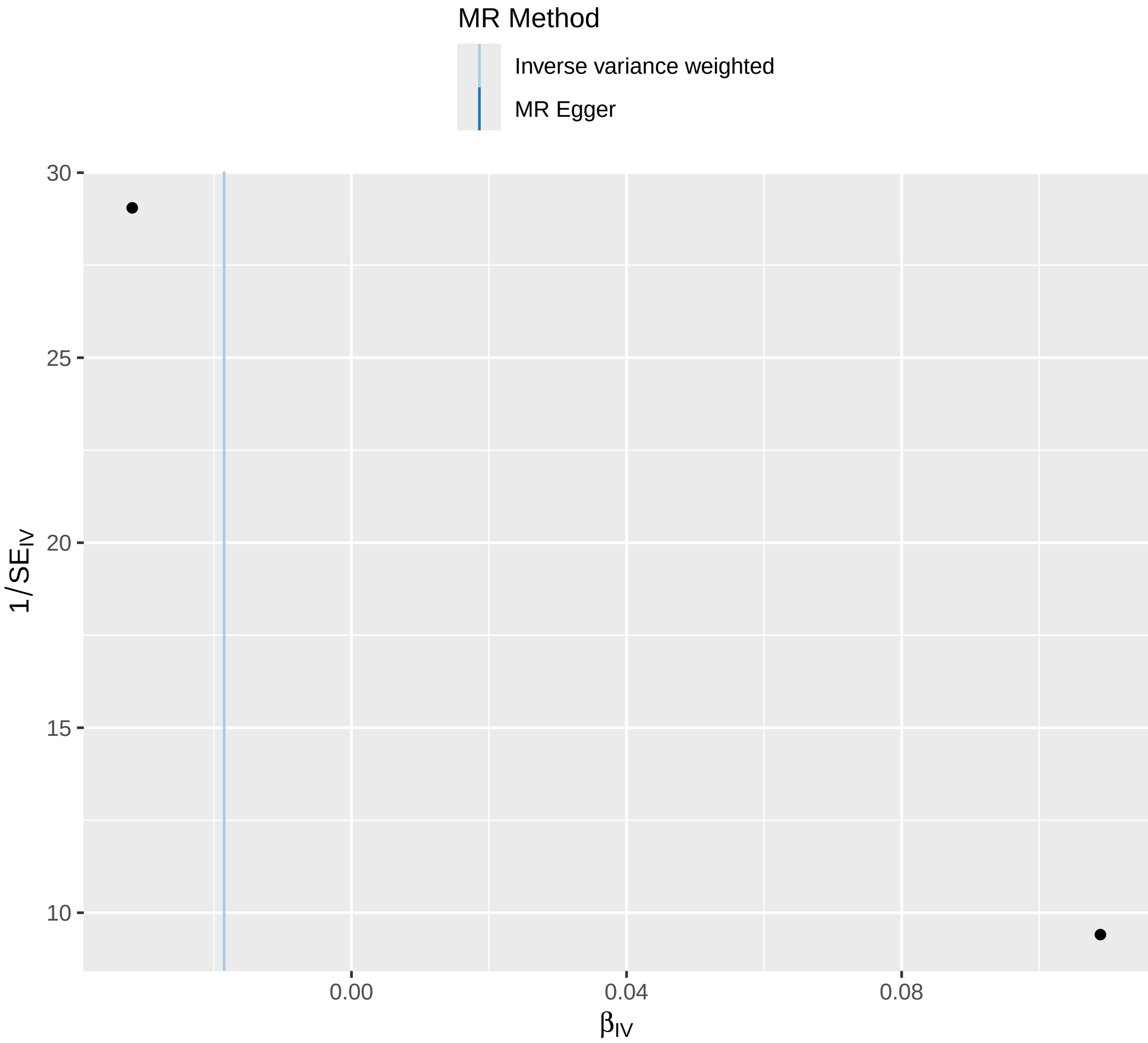

MR Method
Inverse variance weighted
MR Egger
30
25
20
15
10
1/SE$_{IV}$
0.00
0.04
0.08
β$_{IV}$

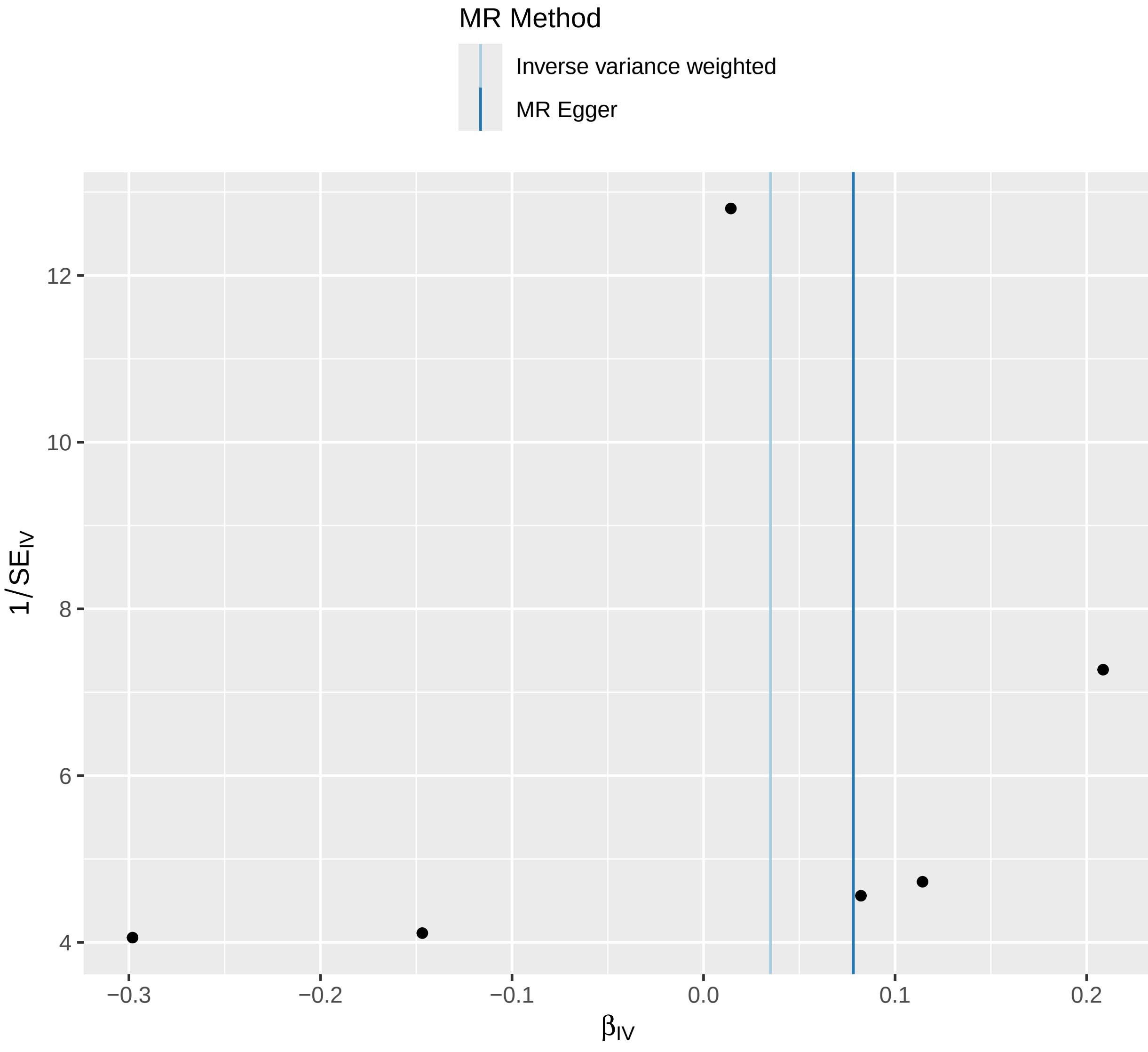
MR Method
Inverse variance weighted
MR Egger
1/SE_IV
12
10
8
6
4
−0.3
−0.2
−0.1
0.0
0.1
0.2
β_IV

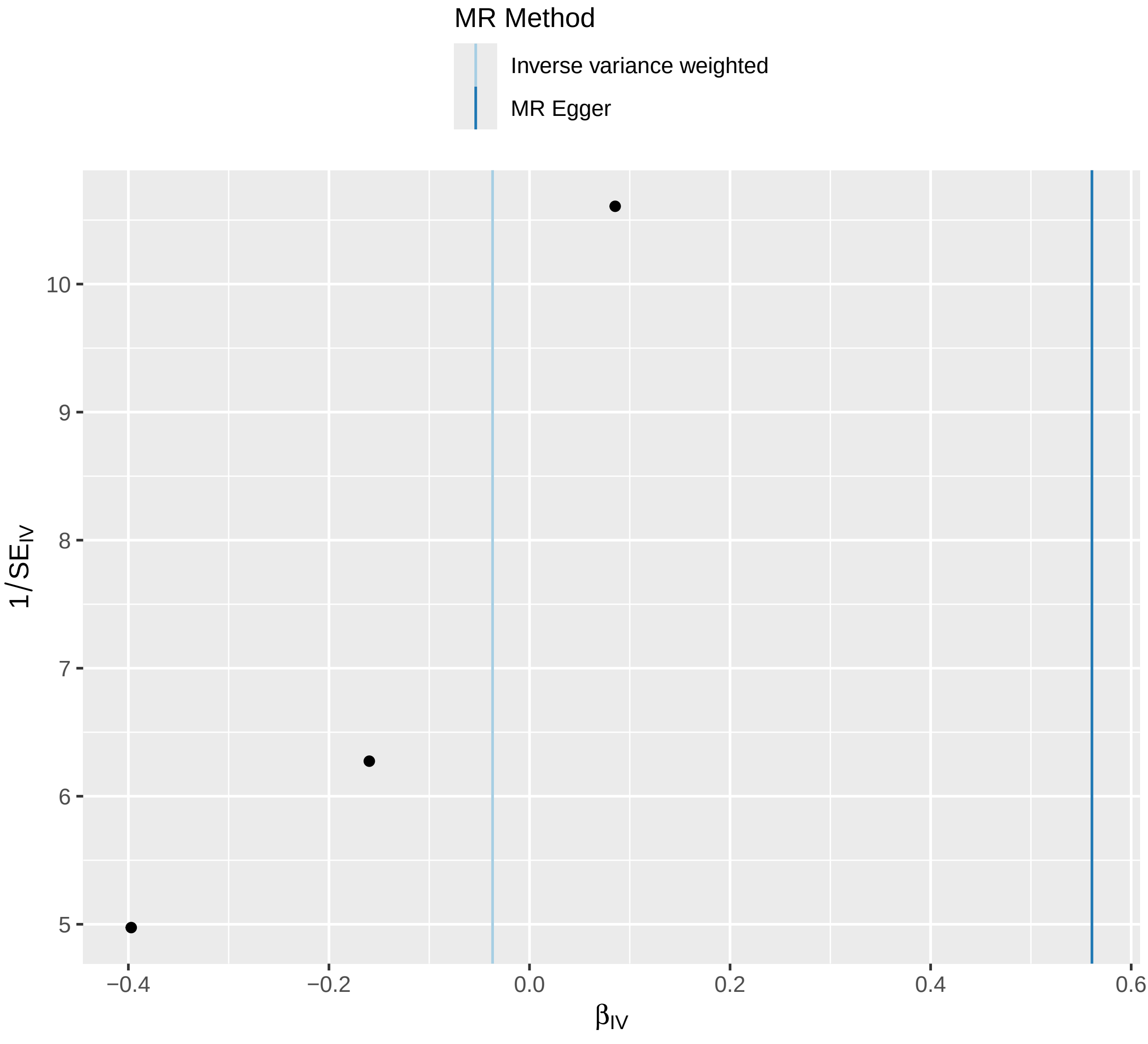
MR Method
Inverse variance weighted
MR Egger
10
9
8
7
6
5
$1/SE_{IV}$
−0.4
−0.2
0.0
0.2
0.4
0.6
β_{IV}

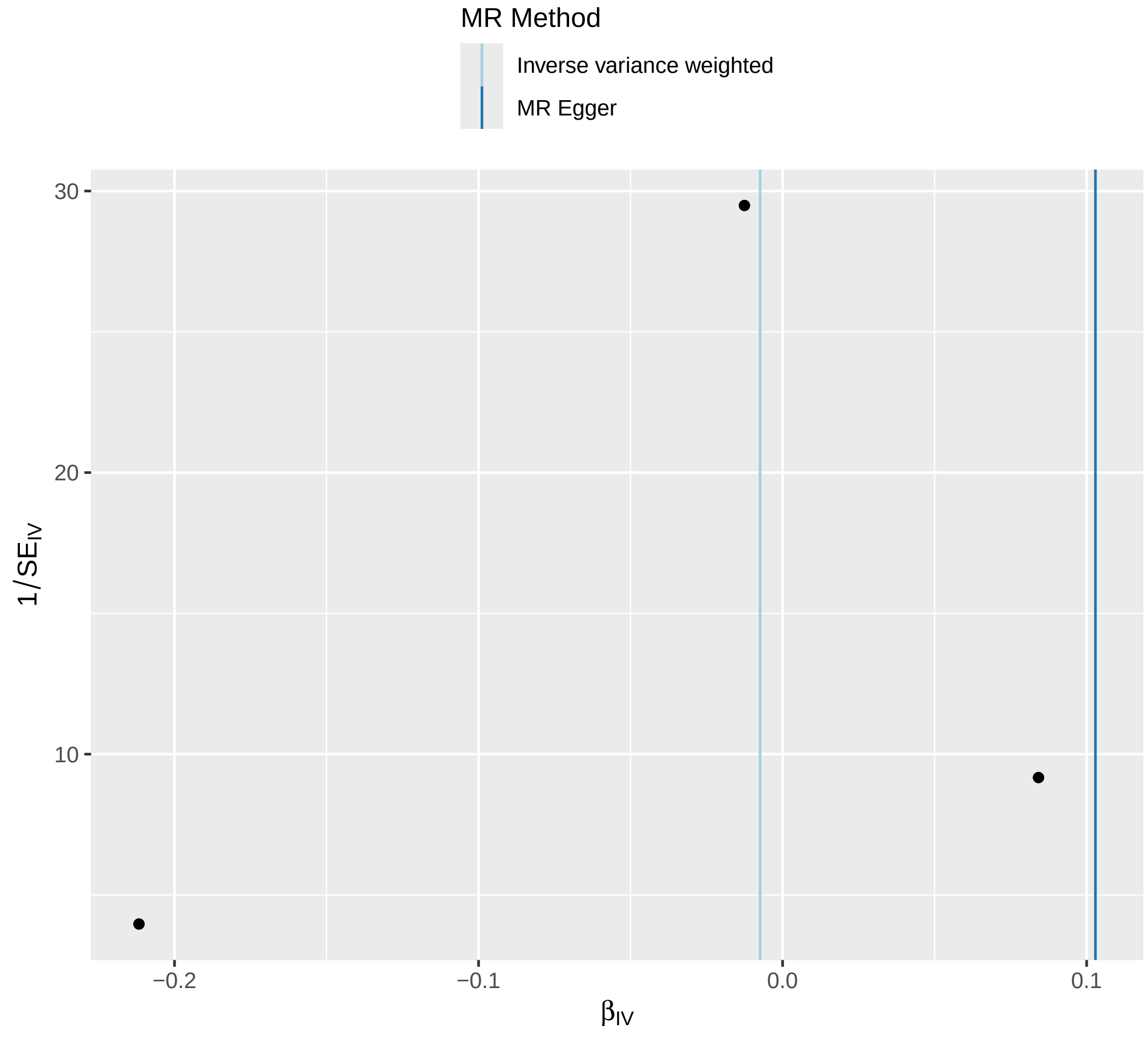

MR Method
Inverse variance weighted
MR Egger
30
20
10
1/SE_IV
−0.2
−0.1
0.0
0.1
β_IV

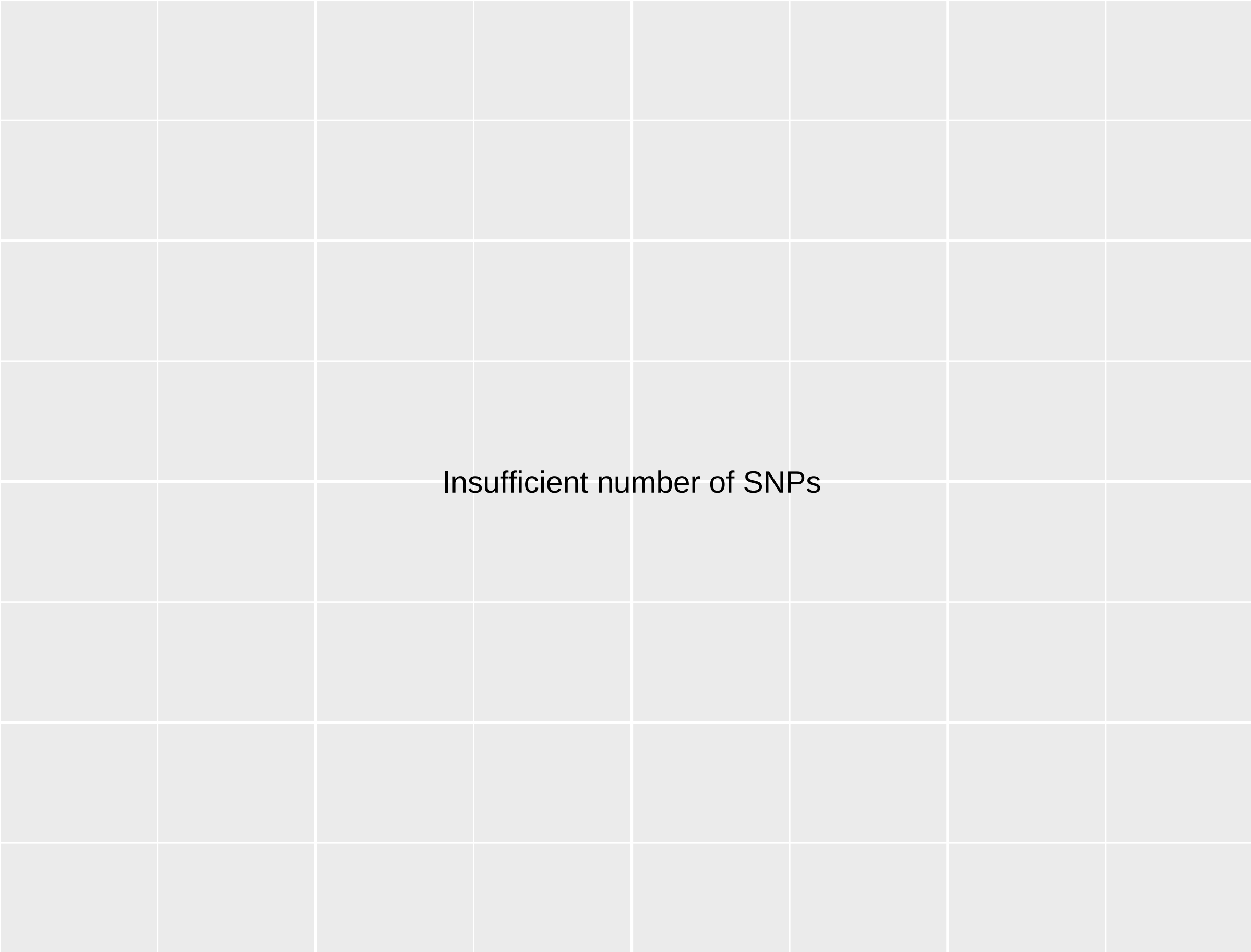
Insufficient number of SNPs

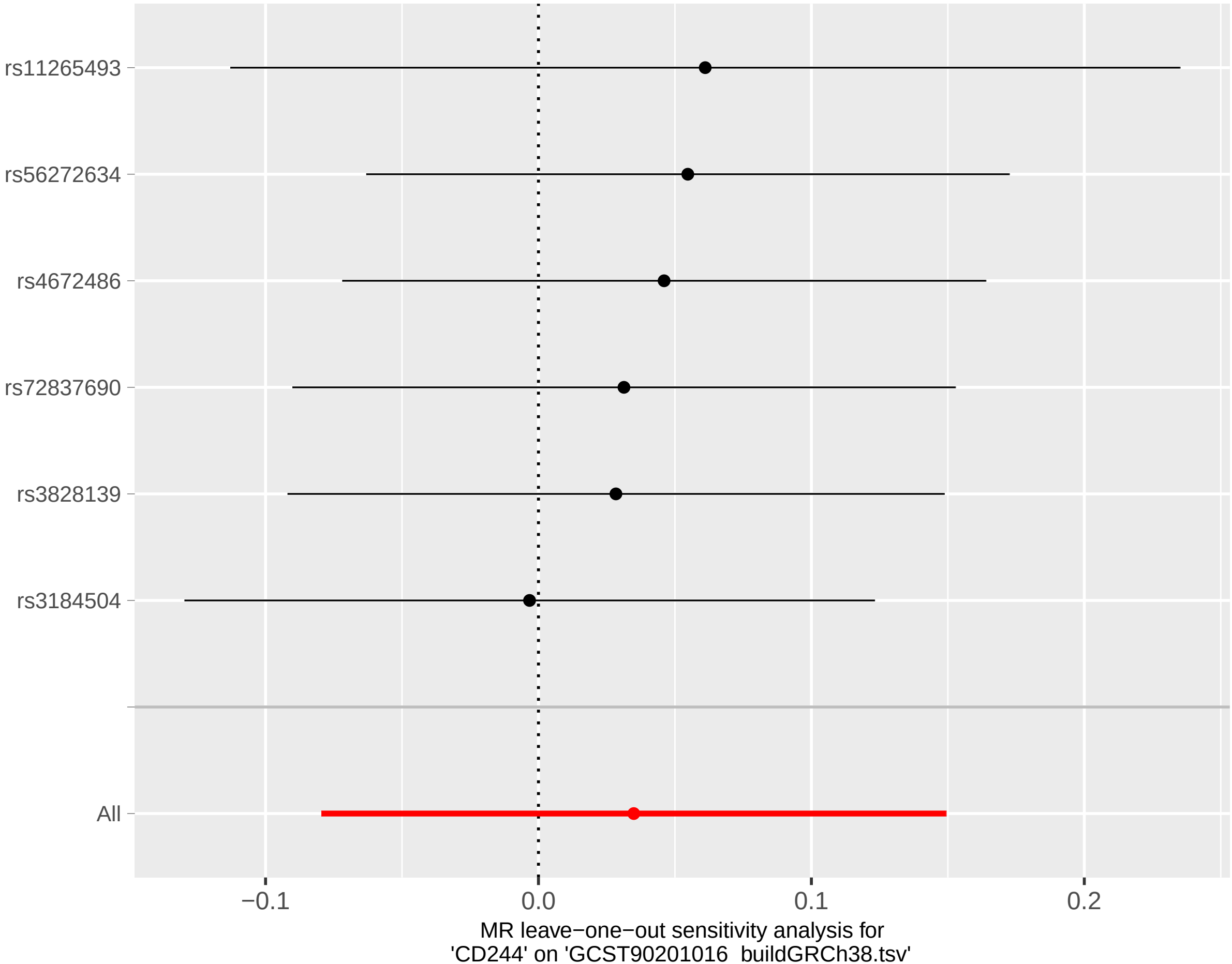
rs11265493
rs56272634
rs4672486
rs72837690
rs3828139
rs3184504
All
−0.1
0.0
0.1
0.2
MR leave−one−out sensitivity analysis for
'CD244' on 'GCST90201016_buildGRCh38.tsv'

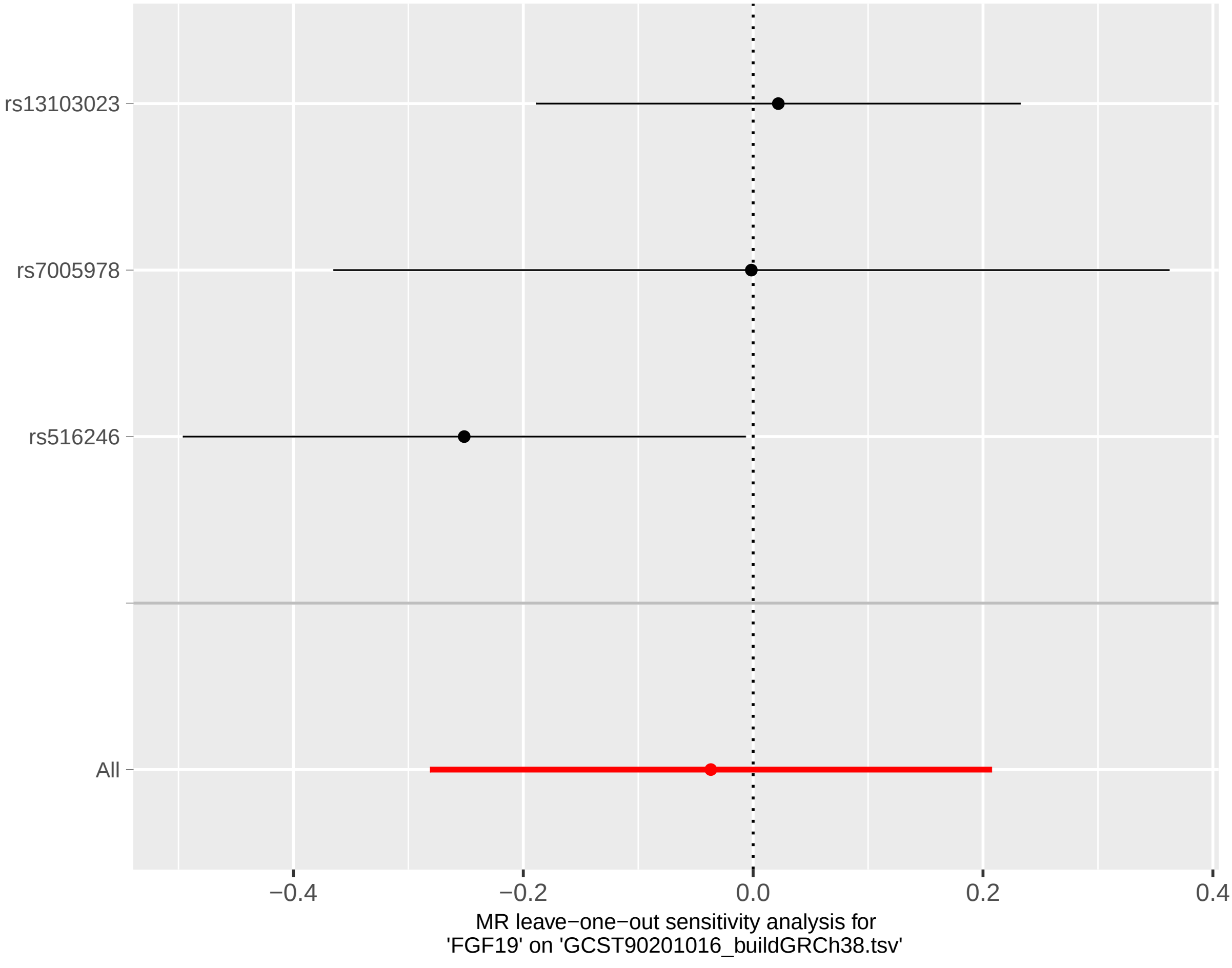
rs13103023
rs7005978
rs516246
All
−0.4
−0.2
0.0
0.2
0.4
MR leave−one−out sensitivity analysis for
'FGF19' on 'GCST90201016_buildGRCh38.tsv'

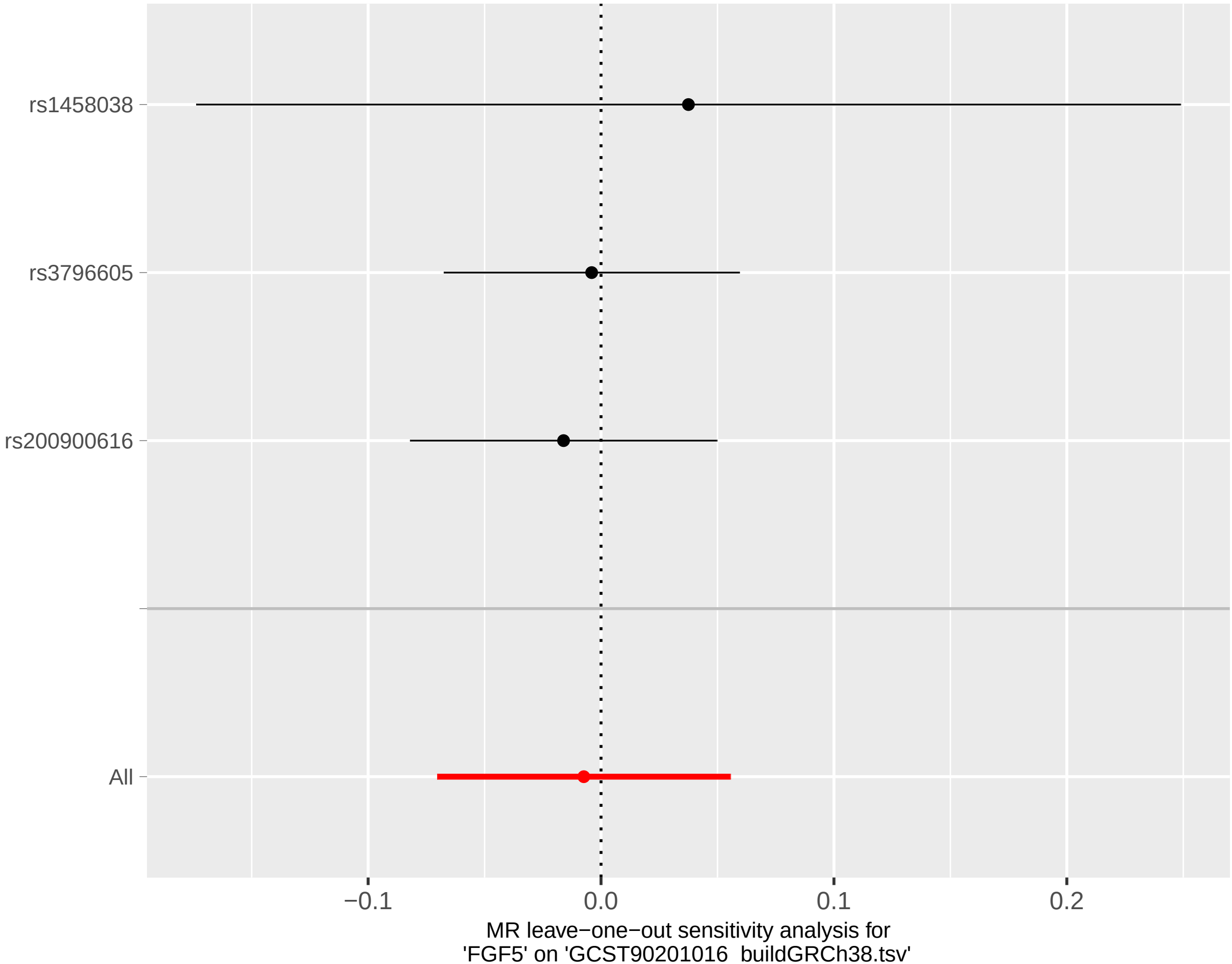
rs1458038
rs3796605
rs200900616
All
−0.1
0.0
0.1
0.2
MR leave−one−out sensitivity analysis for
'FGF5' on 'GCST90201016_buildGRCh38.tsv'

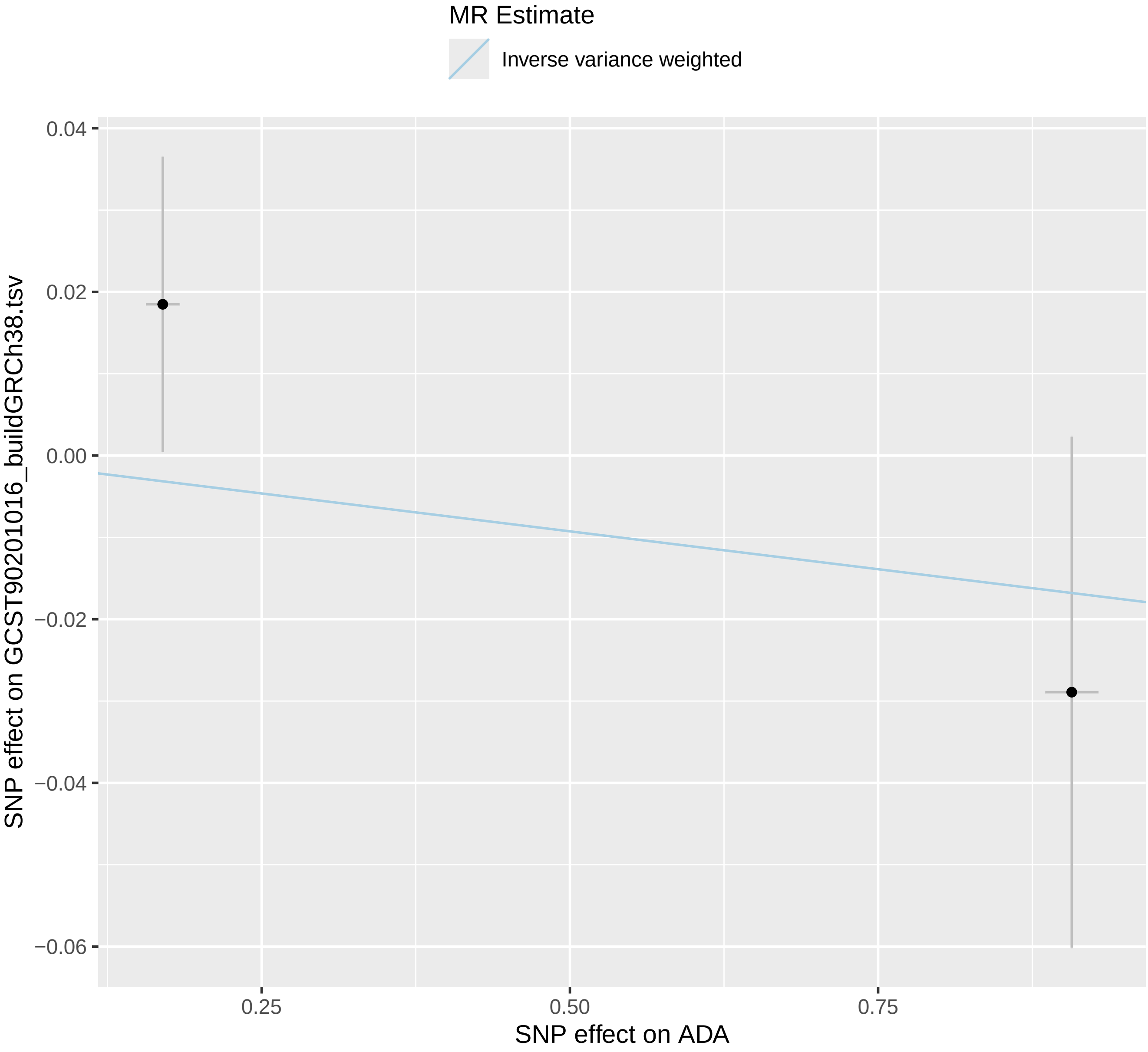
MR Estimate
Inverse variance weighted
0.04
0.02
0.00
−0.02
−0.04
−0.06
SNP effect on GCST90201016_buildGRCh38.tsv
0.25
0.50
0.75
SNP effect on ADA

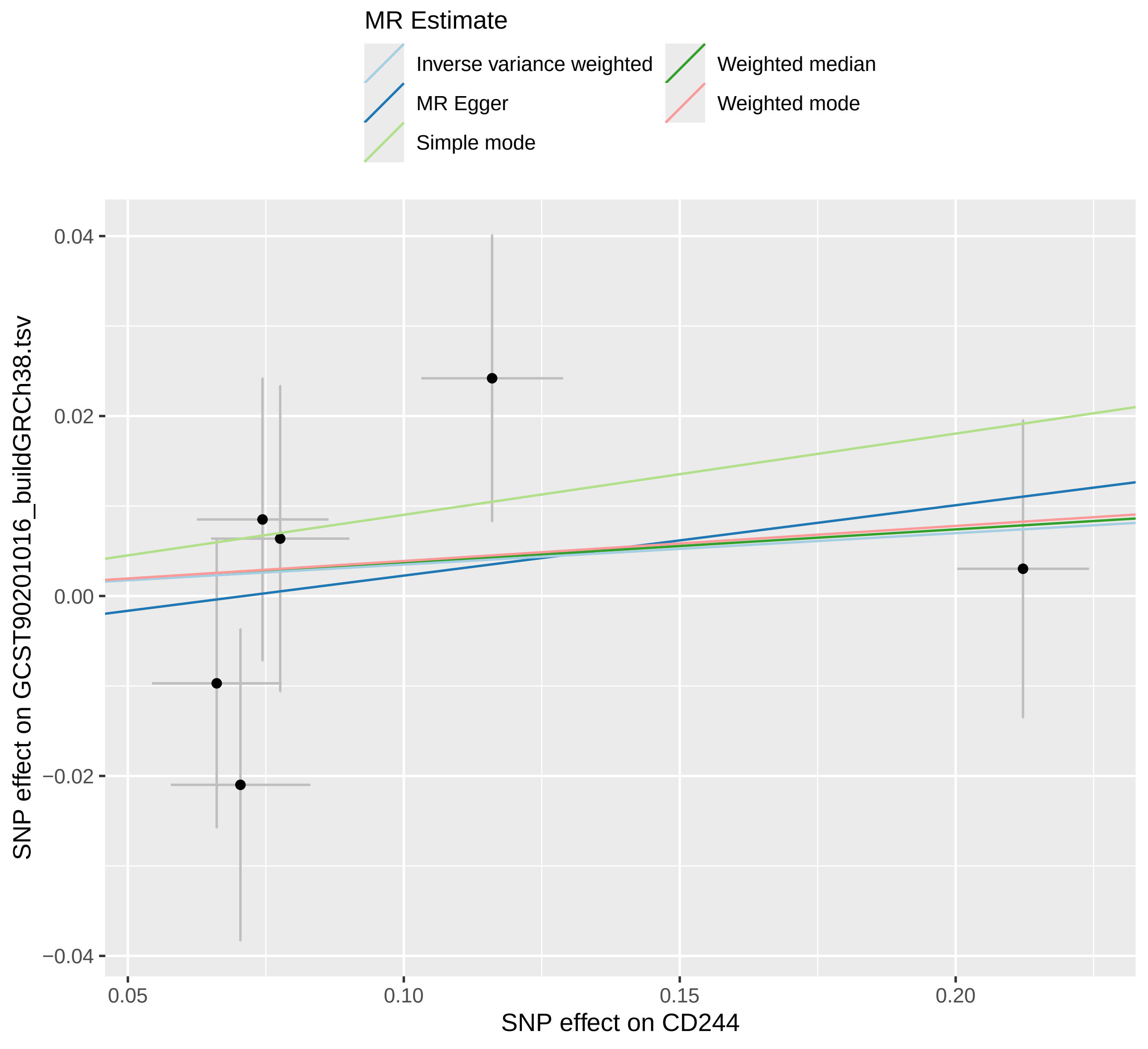

MR Estimate
Inverse variance weighted
MR Egger
Simple mode
Weighted median
Weighted mode
0.04
0.02
0.00
−0.02
−0.04
0.05
0.10
0.15
0.20
SNP effect on GCST90201016_buildGRCh38.tsv
SNP effect on CD244

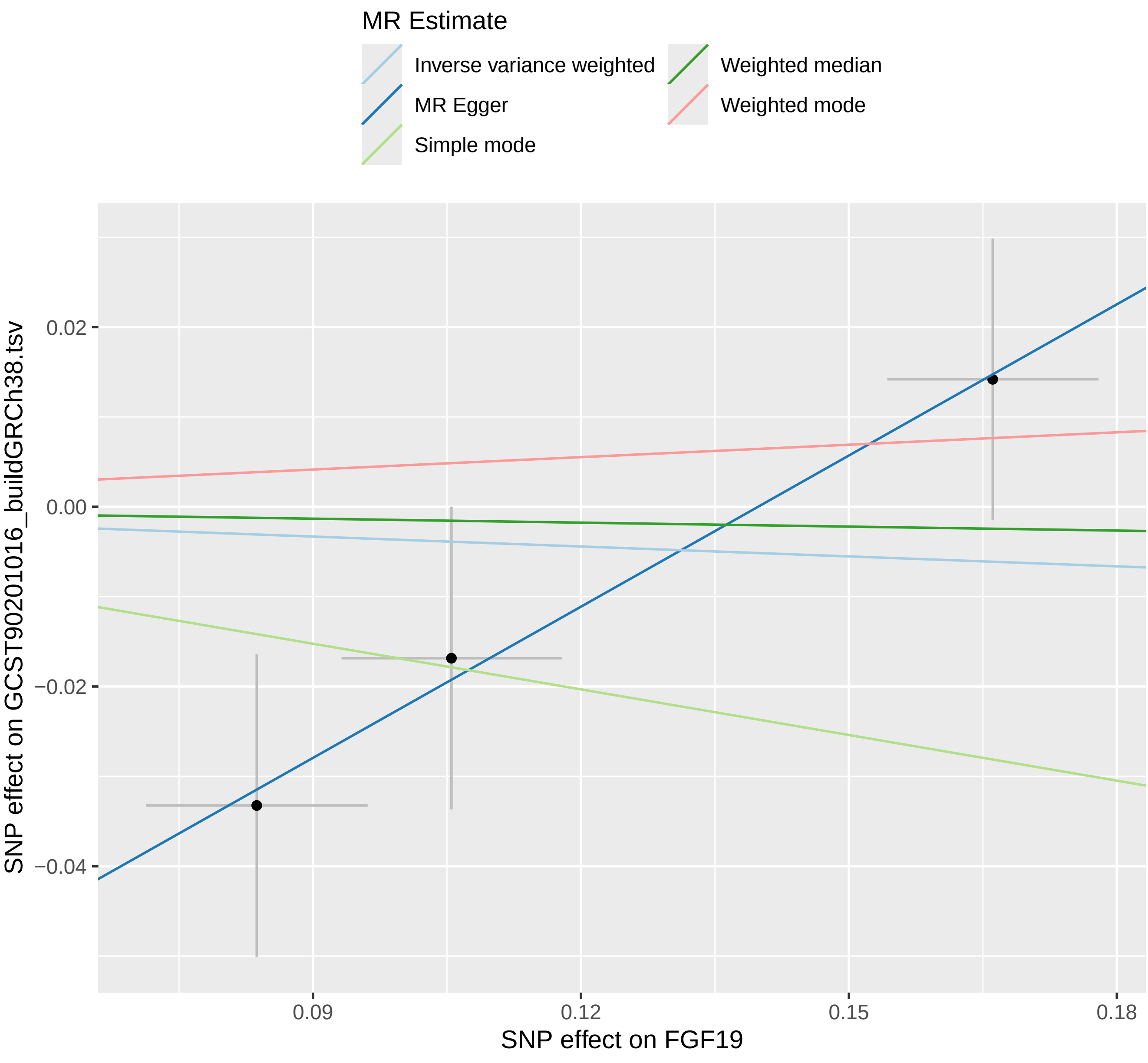

MR Estimate
Inverse variance weighted
MR Egger
Simple mode
Weighted median
Weighted mode
0.02
0.00
−0.02
−0.04
SNP effect on GCST90201016_buildGRCh38.tsv
0.09
0.12
0.15
0.18
SNP effect on FGF19

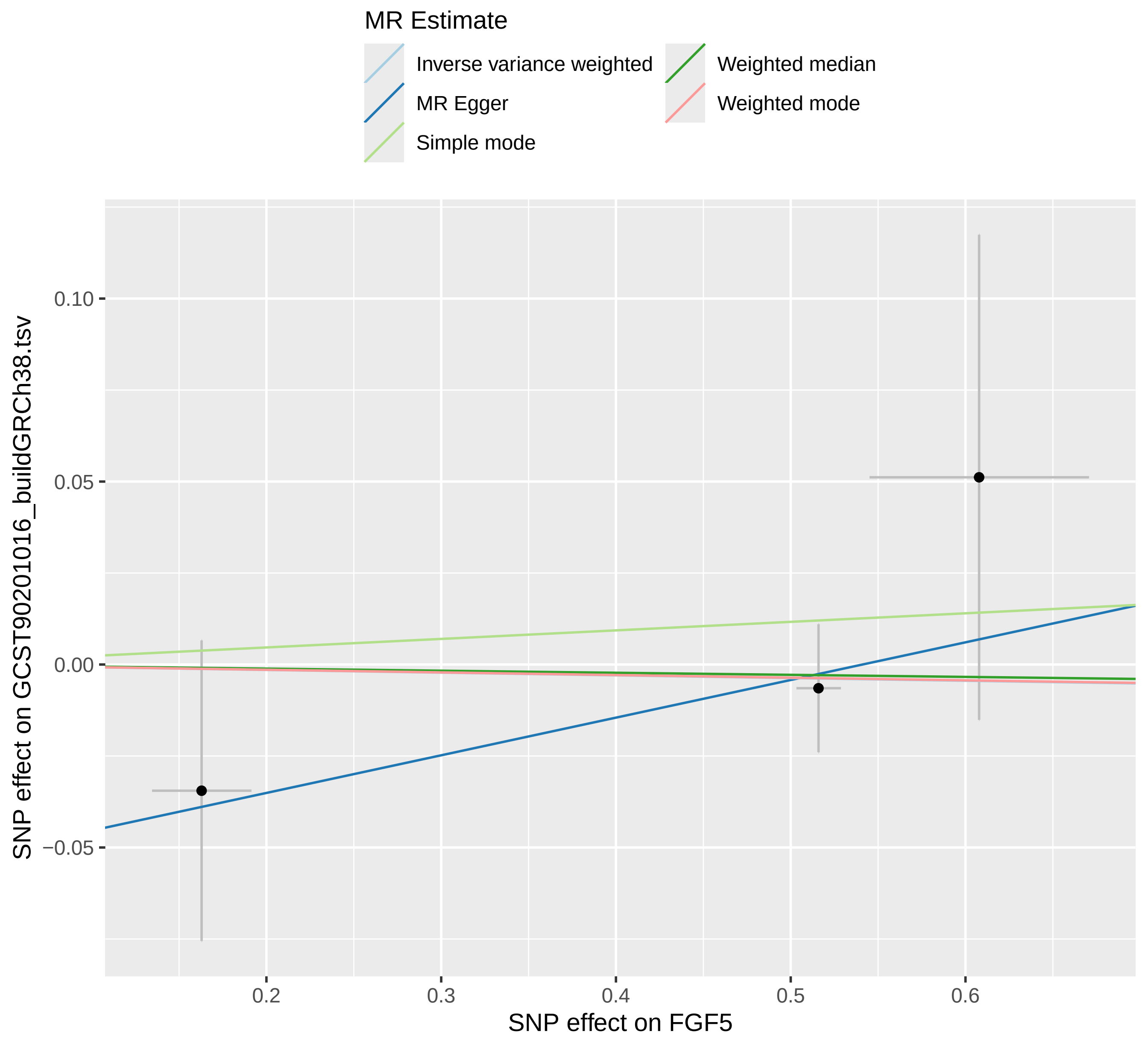
MR Estimate
Inverse variance weighted
MR Egger
Simple mode
Weighted median
Weighted mode
0.10
0.05
0.00
−0.05
SNP effect on GCST90201016_buildGRCh38.tsv
0.2
0.3
0.4
0.5
0.6
SNP effect on FGF5
