# Supplementary material for: Uncovering metabolite–inflammatory protein interactions in gout pathogenesis: A 2-sample bidirectional Mendelian randomization study
Source: Medicine (Baltimore). 2026 Oct 30;105(44):e51276. doi: 10.1097/MD.0000000000051276 (PMC13727226; doi:10.1097/MD.0000000000051276)
Supplement: Supplementary file 5 [file medi-105-e51276-s002.docx]

**1，Forward Mendelian Randomization Analysis：**

\# ============================================================

\# Forward Mendelian Randomization Analysis (Exposure → Outcome)

\# Exposure: Plasma metabolites (after MAF filtering)

\# Outcome: Gout (FinnGen R12 GOUT_STRICT)

\# ============================================================

\# Load required packages

library(VariantAnnotation)

library(gwasglue)

library(TwoSampleMR)

\# ============================================================

\# 1. Set file paths and parameters

\# ============================================================

exposureFile <- "血浆代谢物filtered_with_MAF.csv" # Exposure data (plasma metabolites after MAF filtering)

outcomeFile <- "finngen_R12_GOUT_STRICT.gz"	# Outcome data

outcomeName <- "GOUT"	# Name of the outcome shown in plots

\# ============================================================

\# 2. Read Exposure Data

\# ============================================================

exposure_dat <- read_exposure_data(

filename = exposureFile,

sep = ",",

snp_col = "SNP",

beta_col = "beta.exposure",

se_col = "se.exposure",

pval_col = "pval.exposure",

effect_allele_col = "effect_allele.exposure",

other_allele_col = "other_allele.exposure",

eaf_col = "eaf.exposure",

phenotype_col = "exposure",

id_col = "id.exposure",

samplesize_col = "samplesize.exposure",

chr_col = "chr.exposure",

pos_col = "pos.exposure",

clump = FALSE

)

\# ============================================================

\# 3. Read Outcome Data

\# ============================================================

outcomeData <- read_outcome_data(

snps = exposure_dat$SNP,

filename = outcomeFile,

sep = "\t",

snp_col = "rsids",

beta_col = "beta",

se_col = "sebeta",

effect_allele_col = "alt",

other_allele_col = "ref",

pval_col = "pval",

eaf_col = "af_alt"

)

\# Save outcome data

write.csv(outcomeData, file = "outcome.csv", row.names = FALSE)

\# Add outcome name and harmonize data

outcomeData$outcome <- outcomeName

dat <- harmonise_data(exposure_dat, outcomeData, action = 2)

\# Filter SNPs with very small p-values in outcome

dat <- dat[dat$pval.outcome > 4e-05, ]

\# Save instrumental variables

outTab <- dat[dat$mr_keep == "TRUE", ]

write.csv(outTab, file = "table.SNP.csv", row.names = FALSE)

\# ============================================================

\# 4. Main Mendelian Randomization Analysis

\# ============================================================

mrResult <- mr(dat)

\# Generate OR table

mrTab <- generate_odds_ratios(mrResult)

write.csv(mrTab, file = "table.MRresult.csv", row.names = FALSE)

\# Heterogeneity test

heterTab <- mr_heterogeneity(dat)

write.csv(heterTab, file = "table.heterogeneity.csv", row.names = FALSE)

\# Pleiotropy test

pleioTab <- mr_pleiotropy_test(dat)

write.csv(pleioTab, file = "table.pleiotropy.csv", row.names = FALSE)

\# ============================================================

\# 5. Visualization

\# ============================================================

\# Scatter plot

pdf(file = "pic.scatter_plot.pdf", width = 7.5, height = 7)

mr_scatter_plot(mrResult, dat)

dev.off()

\# Forest plot

res_single <- mr_singlesnp(dat)

pdf(file = "pic.forest.pdf", width = 7, height = 5.5)

mr_forest_plot(res_single)

dev.off()

\# Funnel plot

pdf(file = "pic.funnel_plot.pdf", width = 7, height = 6.5)

mr_funnel_plot(singlesnp_results = res_single)

dev.off()

\# Leave-one-out sensitivity analysis

pdf(file = "pic.leaveoneout.pdf", width = 7, height = 5.5)

mr_leaveoneout_plot(leaveoneout_results = mr_leaveoneout(dat))

dev.off()

\# ============================================================

\# 6. Filter Significant Results (IVW method)

\# ============================================================

pvalFilter <- 0.05

\# Read MR results

rt <- read.csv("table.MRresult.csv", header = TRUE, check.names = FALSE)

\# Extract IVW results and perform FDR correction

ivwRT <- rt[rt$method == "Inverse variance weighted", ]

ivwPval <- ivwRT$pval

ivwRT$fdr <- p.adjust(ivwPval, method = "fdr")

\# Keep exposures where all ORs are consistently >1 or <1

ivw <- data.frame()

for (protein in unique(ivwRT$exposure)) {

proteinData <- rt[rt$exposure == protein, ]

if (all(proteinData$or > 1) | all(proteinData$or < 1)) {

ivw <- rbind(ivw, ivwRT[ivwRT$exposure == protein, ])

}

}

\# Read pleiotropy results and filter (p > 0.05)

pleRT <- read.csv("table.pleiotropy.csv", header = TRUE, check.names = FALSE)

pleRT <- pleRT[pleRT$pval > 0.05, ]

proteinLists <- pleRT$exposure

\# Final filtering

outTab <- ivw[ivw$exposure %in% proteinLists, ]

outTab <- outTab[order(outTab$pval), ]

outTab <- outTab[!duplicated(outTab$exposure), ]

write.csv(outTab, file = "IVW.result.csv", row.names = FALSE)

\# Further filter by p-value and FDR

outTab <- outTab[(outTab$pval < pvalFilter) & (outTab$fdr < 1), ]

write.csv(outTab, file = "IVW.filter.csv", row.names = FALSE)

\# ============================================================

\# 7. Individual Analysis for Significant Exposures

\# ============================================================

library(TwoSampleMR)

exposureFile <- "血浆代谢物filtered_with_MAF.csv"

outcomeFile <- "outcome.csv"

sigGutFile	<- "IVW.filter.csv"

outcomeName <- "GOUT"

rt <- read.csv(exposureFile, header = TRUE, check.names = FALSE)

sigGut <- read.csv(sigGutFile, header = TRUE, check.names = FALSE)

for (i in sigGut$exposure) {

\# Create safe folder name

sanitized_name <- gsub("[^A-Za-z0-9_]", "_", i)

dir.create(sanitized_name, showWarnings = FALSE)

\# Subset exposure data

exposure_set <- rt[rt$exposure == i, ]

singleExposureFile <- file.path(sanitized_name, paste0(sanitized_name, ".exposure.csv"))

write.csv(exposure_set, file = singleExposureFile, row.names = FALSE)

\# Read exposure data

exposure_dat <- read_exposure_data(

filename = singleExposureFile,

sep = ",",

snp_col = "SNP",

beta_col = "beta.exposure",

se_col = "se.exposure",

pval_col = "pval.exposure",

effect_allele_col = "effect_allele.exposure",

other_allele_col = "other_allele.exposure",

eaf_col = "eaf.exposure",

phenotype_col = "exposure",

samplesize_col = "samplesize.exposure",

chr_col = "chr.exposure",

pos_col = "pos.exposure",

clump = FALSE

)

\# Read outcome data

outcome_data <- read_outcome_data(

snps = exposure_dat$SNP,

filename = outcomeFile,

sep = ",",

snp_col = "SNP",

beta_col = "beta.outcome",

se_col = "se.outcome",

effect_allele_col = "effect_allele.outcome",

other_allele_col = "other_allele.outcome",

pval_col = "pval.outcome",

eaf_col = "eaf.outcome"

)

outcome_data$outcome <- outcomeName

dat <- harmonise_data(exposure_dat, outcome_data, action = 2)

\# Save instrumental variables

outTab <- dat[dat$mr_keep == "TRUE", ]

write.csv(outTab, file = file.path(sanitized_name, paste0(sanitized_name, ".table.SNP.csv")), row.names = FALSE)

\# MR analysis

mrResult <- mr(dat)

mrTab <- generate_odds_ratios(mrResult)

write.csv(mrTab, file = file.path(sanitized_name, paste0(sanitized_name, ".table.MRresult.csv")), row.names = FALSE)

\# Heterogeneity test

heterTab <- mr_heterogeneity(dat)

write.csv(heterTab, file = file.path(sanitized_name, paste0(sanitized_name, ".table.heterogeneity.csv")), row.names = FALSE)

\# Pleiotropy test

pleioTab <- mr_pleiotropy_test(dat)

write.csv(pleioTab, file = file.path(sanitized_name, paste0(sanitized_name, ".table.pleiotropy.csv")), row.names = FALSE)

\# Visualization

\# Scatter plot

pdf(file = file.path(sanitized_name, paste0(sanitized_name, ".scatter_plot.pdf")), width = 7, height = 6.5)

p1 <- mr_scatter_plot(mrResult, dat)

print(p1)

dev.off()

\# Forest plot

res_single <- mr_singlesnp(dat)

pdf(file = file.path(sanitized_name, paste0(sanitized_name, ".forest.pdf")), width = 6.5, height = 5)

p2 <- mr_forest_plot(res_single)

print(p2)

dev.off()

\# Funnel plot

pdf(file = file.path(sanitized_name, paste0(sanitized_name, ".funnel_plot.pdf")), width = 6.5, height = 6)

p3 <- mr_funnel_plot(singlesnp_results = res_single)

print(p3)

dev.off()

\# Leave-one-out analysis

pdf(file = file.path(sanitized_name, paste0(sanitized_name, ".leaveoneout.pdf")), width = 6.5, height = 5)

p4 <- mr_leaveoneout_plot(leaveoneout_results = mr_leaveoneout(dat))

print(p4)

dev.off()

}

cat("Forward MR analysis completed successfully.\n")

**2.Exposure → Mediator Mendelian Randomization Analysis**

\# ============================================================

\# Exposure → Mediator Mendelian Randomization Analysis

\# Exposure: Plasma metabolites (positive SNPs after MAF filtering)

\# Mediators: Multiple outcomes (e.g., immune cells or gut microbiota)

\# ============================================================

\# Load required packages

library(VariantAnnotation)

library(gwasglue)

library(TwoSampleMR)

library(dplyr)

\# ============================================================

\# 1. Read Exposure Data

\# ============================================================

exposureFile <- "血浆代谢物阳性SNP_MAF.csv"	# Exposure data file (e.g., metabolites)

exposure_dat <- read_exposure_data(

filename = exposureFile,

sep = ",",

snp_col = "SNP",

beta_col = "beta.exposure",

se_col = "se.exposure",

pval_col = "pval.exposure",

effect_allele_col = "effect_allele.exposure",

other_allele_col = "other_allele.exposure",

eaf_col = "eaf.exposure",

phenotype_col = "exposure",

id_col = "id.exposure",

samplesize_col = "samplesize.exposure",

chr_col = "chr.exposure",

pos_col = "pos.exposure",

clump = FALSE

)

\# ============================================================

\# 2. Get all outcome files (e.g., immune cells or gut microbiota)

\# ============================================================

files <- list.files(pattern = "\\.h\\.tsv\\.gz$") # Get all .h.tsv.gz files in the directory

\# ============================================================

\# 3. Loop through each outcome file and perform MR analysis

\# ============================================================

for (file in files) {

outcomeFile <- file

outcomeName <- sub("\\.gz$", "", file)	# Remove .gz suffix to get clean name

\# Read outcome data

outcomeData <- read_outcome_data(

snps = exposure_dat$SNP,

filename = file,

sep = "\t",

snp_col = "hm_rsid",

beta_col = "hm_beta",

se_col = "standard_error",

effect_allele_col = "hm_effect_allele",

other_allele_col = "hm_other_allele",

pval_col = "p_value",

eaf_col = "hm_effect_allele_frequency"

)

\# Save outcome data as CSV

write.csv(outcomeData, file = paste0(outcomeName, "_outcome.csv"), row.names = FALSE)

\# Add outcome name

outcomeData$outcome <- outcomeName

\# Harmonize exposure and outcome data

dat <- harmonise_data(exposure_dat, outcomeData, action = 2)

\# Save instrumental variables

outTab <- dat[dat$mr_keep == "TRUE", ]

write.csv(outTab, file = paste0(outcomeName, "_table.SNP.csv"), row.names = FALSE)

\# ============================================================

\# 4. Mendelian Randomization Analysis

\# ============================================================

\# Main MR analysis

mrResult <- mr(dat)

\# Generate OR table and save

mrTab <- generate_odds_ratios(mrResult)

write.csv(mrTab, file = paste0(outcomeName, "_table.MRresult.csv"), row.names = FALSE)

\# Heterogeneity test

heterTab <- mr_heterogeneity(dat)

write.csv(heterTab, file = paste0(outcomeName, "_table.heterogeneity.csv"), row.names = FALSE)

\# Pleiotropy test

pleioTab <- mr_pleiotropy_test(dat)

write.csv(pleioTab, file = paste0(outcomeName, "_table.pleiotropy.csv"), row.names = FALSE)

\# ============================================================

\# 5. Visualization (saved as PDF)

\# ============================================================

\# Scatter plot

pdf(file = paste0(outcomeName, "_scatter_plot.pdf"), width = 7, height = 6.5)

p1 <- mr_scatter_plot(mrResult, dat)

print(p1)

dev.off()

\# Forest plot

res_single <- mr_singlesnp(dat)

pdf(file = paste0(outcomeName, "_forest.pdf"), width = 6.5, height = 5)

p2 <- mr_forest_plot(res_single)

print(p2)

dev.off()

\# Funnel plot

pdf(file = paste0(outcomeName, "_funnel_plot.pdf"), width = 6.5, height = 6)

p3 <- mr_funnel_plot(singlesnp_results = res_single)

print(p3)

dev.off()

\# Leave-one-out sensitivity analysis

pdf(file = paste0(outcomeName, "_leaveoneout.pdf"), width = 6.5, height = 5)

p4 <- mr_leaveoneout_plot(leaveoneout_results = mr_leaveoneout(dat))

print(p4)

dev.off()

}

\# ============================================================

\# 6. Combine Results from All Mediators

\# ============================================================

\# Combine all MR results

file_list <- list.files(pattern = "*.MRresult.csv")

combined_df <- file_list %>%

lapply(function(file) read.csv(file, fileEncoding = "UTF-8")) %>%

bind_rows()

write.csv(combined_df, "MRresult_combined_dataset.csv", row.names = FALSE)

\# Combine all heterogeneity results

file_list <- list.files(pattern = "*.heterogeneity.csv")

combined_df <- file_list %>%

lapply(function(file) read.csv(file, fileEncoding = "UTF-8")) %>%

bind_rows()

write.csv(combined_df, "heterogeneity_combined_dataset.csv", row.names = FALSE)

\# Combine all pleiotropy results

file_list <- list.files(pattern = "*.pleiotropy.csv")

combined_df <- file_list %>%

lapply(function(file) read.csv(file, fileEncoding = "UTF-8")) %>%

bind_rows()

write.csv(combined_df, "pleiotropy_combined_dataset.csv", row.names = FALSE)

cat("Exposure → Mediator MR analysis completed successfully.\n")

3，Reverse Mendelian Randomization Analysis (Outcome → Exposure)

\# ============================================================

\# Reverse Mendelian Randomization Analysis (Outcome → Exposure)

\# Outcome: Gout (FinnGen R12)

\# Exposure: Multiple traits (e.g., metabolites, immune cells, etc.)

\# ============================================================

\# Load required packages

library(VariantAnnotation)

library(gwasglue)

library(TwoSampleMR)

\# ============================================================

\# 1. Get all outcome files (.h.tsv.gz)

\# ============================================================

file_list <- list.files(pattern = ".gz$")

total_files <- length(file_list)

if (total_files > 0) {

for (i in seq_along(file_list)) {

file <- file_list[i]

\# Print progress

message(sprintf("Processing file %d/%d: %s", i, total_files, file))

\# Create clean output prefix (remove .tsv.gz)

output_prefix <- gsub("\\.tsv\\.gz$", "", file)

\# ============================================================

\# 2. Read Exposure Data (Gout as exposure in reverse MR)

\# ============================================================

exposure_dat <- read_exposure_data(

filename = "finngen_R12_GOUT_STRICT_clumped.F.csv", # Pre-clumped Gout data

sep = ",",

snp_col = "SNP",

beta_col = "beta.exposure",

se_col = "se.exposure",

pval_col = "pval.exposure",

effect_allele_col = "effect_allele.exposure",

other_allele_col = "other_allele.exposure",

eaf_col = "eaf.exposure",

phenotype_col = "exposure",

samplesize_col = "samplesize.exposure",

chr_col = "chr.exposure",

pos_col = "pos.exposure",

clump = FALSE

)

\# ============================================================

\# 3. Read Outcome Data (Current trait being tested)

\# ============================================================

outcome_data <- read_outcome_data(

snps = exposure_dat$SNP,

filename = file,

sep = "\t",

snp_col = "variant_id",

beta_col = "beta",

se_col = "standard_error",

effect_allele_col = "effect_allele",

other_allele_col = "other_allele",

pval_col = "p_value",

eaf_col = "effect_allele_frequency"

)

\# Set outcome name

outcome_data$outcome <- file

\# Harmonize exposure and outcome data

dat <- harmonise_data(exposure_dat, outcome_data, action = 2)

\# Save instrumental variables

outTab <- dat[dat$mr_keep == "TRUE", ]

write.csv(outTab, file = paste0(output_prefix, "_table.SNP.csv"), row.names = FALSE)

\# ============================================================

\# 4. Mendelian Randomization Analysis

\# ============================================================

\# Main MR analysis

mrResult <- mr(dat)

\# Generate OR table

mrTab <- generate_odds_ratios(mrResult)

write.csv(mrTab, file = paste0(output_prefix, "_table.MRresult.csv"), row.names = FALSE)

\# Heterogeneity test

heterTab <- mr_heterogeneity(dat)

write.csv(heterTab, file = paste0(output_prefix, "_table.heterogeneity.csv"), row.names = FALSE)

\# Pleiotropy test

pleioTab <- mr_pleiotropy_test(dat)

write.csv(pleioTab, file = paste0(output_prefix, "_table.pleiotropy.csv"), row.names = FALSE)

\# ============================================================

\# 5. Visualization

\# ============================================================

\# Scatter plot

pdf(file = paste0(output_prefix, "_scatter_plot.pdf"), width = 7, height = 6.5)

p1 <- mr_scatter_plot(mrResult, dat)

print(p1)

dev.off()

\# Forest plot

res_single <- mr_singlesnp(dat)

pdf(file = paste0(output_prefix, "_forest.pdf"), width = 6.5, height = 5)

p2 <- mr_forest_plot(res_single)

print(p2)

dev.off()

\# Funnel plot

pdf(file = paste0(output_prefix, "_funnel_plot.pdf"), width = 6.5, height = 6)

p3 <- mr_funnel_plot(singlesnp_results = res_single)

print(p3)

dev.off()

\# Leave-one-out sensitivity analysis

pdf(file = paste0(output_prefix, "_leaveoneout.pdf"), width = 6.5, height = 5)

p4 <- mr_leaveoneout_plot(leaveoneout_results = mr_leaveoneout(dat))

print(p4)

dev.off()

\# Print completion message

message(sprintf("Finished processing: %s (%d/%d)", file, i, total_files))

}

} else {

message("No .h.tsv.gz files found in the directory.")

}

message("All files have been processed.")

\# ============================================================

\# 6. Combine Results from All Reverse MR Analyses

\# ============================================================

\# Combine all MR results

mrresult_files <- list.files(pattern = "_table.MRresult.csv")

combined_mrresult <- do.call(rbind, lapply(mrresult_files, read.csv))

write.csv(combined_mrresult, "all_MRresults.csv", row.names = FALSE)

\# Combine all heterogeneity results

heter_files <- list.files(pattern = "_table.heterogeneity.csv")

combined_heter <- do.call(rbind, lapply(heter_files, function(x) {

df <- read.csv(x)

df$outcome <- gsub("_table.heterogeneity.csv", "", x)

return(df)

}))

write.csv(combined_heter, "all_heterogeneity_combined.csv", row.names = FALSE)

\# Combine all pleiotropy results

pleio_files <- list.files(pattern = "_table.pleiotropy.csv")

combined_pleio <- do.call(rbind, lapply(pleio_files, function(x) {

df <- read.csv(x)

df$outcome <- gsub("_table.pleiotropy.csv", "", x)

return(df)

}))

write.csv(combined_pleio, "all_pleiotropy_combined.csv", row.names = FALSE)

message("All results have been combined successfully.")

4，Mediation Analysis (Two-step MR)

\# ============================================================

\# Mediation Analysis (Two-step MR)

\# Step 1: Exposure → Mediator (b1)

\# Step 2: Mediator → Outcome (b2)

\# Step 3: Exposure → Outcome (total effect)

\# ============================================================

\# Load required package

library(openxlsx)

\# ============================================================

\# 1. Set input and output file paths

\# ============================================================

exp_to_med_file	<- "SM--IC.csv"	# b1: Exposure (e.g. metabolites) → Mediator (e.g. immune cells)

exp_to_disease_file <- "SM--IC.csv"	# ba: Exposure → Outcome (total effect)

med_to_disease_file <- "IC--AS.csv"	# b2: Mediator → Outcome

\# Extract disease name from filename (e.g. "Gout" from "IC--Gout.csv")

disease_name <- gsub(".*--(.*)\\.csv$", "\\1", med_to_disease_file)

\# Output files

output_file	<- paste0("SM--IC--AS--Mediation_All_Results_", disease_name, ".xlsx")

positive_output_file <- "SM--IC--AS--Positive_Mediation_Results.xlsx"

\# ============================================================

\# 2. Read input files

\# ============================================================

exp_to_med	<- read.csv(exp_to_med_file, header = TRUE, check.names = FALSE)

exp_to_disease <- read.csv(exp_to_disease_file, header = TRUE, check.names = FALSE)

med_to_disease <- read.csv(med_to_disease_file, header = TRUE, check.names = FALSE)

\# ============================================================

\# 3. Create workbooks

\# ============================================================

wb <- createWorkbook()

addWorksheet(wb, "Mediation")

wb_positive <- createWorkbook()

addWorksheet(wb_positive, "Positive_Results")

positive_row <- 1	# Row counter for positive results

current_row <- 1	# Row counter for main workbook

outTab	<- data.frame() # Store all calculated results

\# ============================================================

\# 4. Loop through each row in exp_to_med

\# ============================================================

for (i in 1:nrow(exp_to_med)) {

row_em <- exp_to_med[i, , drop = FALSE]

exp_name <- row_em$exposure

med_name <- row_em$outcome

\# Get corresponding row from exp_to_disease

row_ed <- exp_to_disease[exp_to_disease$exposure == exp_name, , drop = FALSE]

if (nrow(row_ed) == 0) next

row_ed <- row_ed[1, , drop = FALSE]

\# Get corresponding row from med_to_disease

row_md <- med_to_disease[med_to_disease$exposure == med_name, , drop = FALSE]

if (nrow(row_md) == 0) next

row_md <- row_md[1, , drop = FALSE]

\# Extract effect sizes

b1 <- row_em$b

se1 <- row_em$se

b2 <- row_md$b

se2 <- row_md$se

betaAll <- row_ed$b

\# Calculate mediated effect (product of coefficients)

beta12 <- b1 * b2

se12	<- sqrt((b1^2) * (se2^2) + (b2^2) * (se1^2))

ciLow <- beta12 - 1.96 * se12

ciHigh <- beta12 + 1.96 * se12

medEffect <- paste0(signif(beta12, digits = 3), " (",

signif(ciLow, digits = 3), ", ",

signif(ciHigh, digits = 3), ")")

\# Direct effect

betaDirect <- betaAll - beta12

\# Mediated proportion

if (abs(betaAll) < 1e-10) {

medRatioEffect <- "NA"

} else {

beta12Ratio <- beta12 / betaAll

ciLowRatio <- ciLow / betaAll

ciHighRatio <- ciHigh / betaAll

medRatioEffect <- paste0(signif(beta12Ratio * 100, digits = 3), "% (",

signif(ciLowRatio * 100, digits = 3), "%, ",

signif(ciHighRatio * 100, digits = 3), "%)")

}

\# P-value for mediated effect

zValue <- beta12 / se12

pvalue <- 2 * pnorm(abs(zValue), lower.tail = FALSE)

\# Create result row

new_row <- data.frame(

"Metabolite"	= exp_name,

"Immune cell"	= med_name,

"Outcome"	= row_ed$outcome,

"Mediated effect"	= medEffect,

"Mediated proportion" = medRatioEffect,

"BetaDirect"	= betaDirect,

"P.value"	= pvalue

)

outTab <- rbind(outTab, new_row)

\# ============================================================

\# 5. Write detailed steps to Excel

\# ============================================================

\# Step 1

writeData(wb, "Mediation", "Step 1 (Exposure → Mediator):", startRow = current_row, startCol = 1)

current_row <- current_row + 1

writeData(wb, "Mediation", row_em, startRow = current_row, startCol = 1, colNames = TRUE)

current_row <- current_row + nrow(row_em) + 2

\# Step 2

writeData(wb, "Mediation", "Step 2 (Exposure → Outcome):", startRow = current_row, startCol = 1)

current_row <- current_row + 1

writeData(wb, "Mediation", row_ed, startRow = current_row, startCol = 1, colNames = TRUE)

current_row <- current_row + nrow(row_ed) + 2

\# Step 3

writeData(wb, "Mediation", "Step 3 (Mediator → Outcome):", startRow = current_row, startCol = 1)

current_row <- current_row + 1

writeData(wb, "Mediation", row_md, startRow = current_row, startCol = 1, colNames = TRUE)

current_row <- current_row + nrow(row_md) + 2

\# Calculated results

writeData(wb, "Mediation", "Calculated Mediation Results:", startRow = current_row, startCol = 1)

current_row <- current_row + 1

calc_start_row <- current_row

writeData(wb, "Mediation", new_row, startRow = current_row, startCol = 1, colNames = TRUE)

\# Highlight if p < 0.05

if (!is.na(pvalue) && pvalue < 0.05) {

highlightStyle <- createStyle(fgFill = "yellow")

addStyle(wb, "Mediation", style = highlightStyle,

rows = calc_start_row:(calc_start_row + nrow(new_row)),

cols = 1:7, gridExpand = TRUE)

\# Also write to positive results sheet

writeData(wb_positive, "Positive_Results", new_row,

startRow = positive_row, startCol = 1, colNames = TRUE)

positive_row <- positive_row + nrow(new_row) + 1

}

current_row <- current_row + nrow(new_row) + 5

}

\# ============================================================

\# 6. Save Excel files

\# ============================================================

saveWorkbook(wb, output_file, overwrite = TRUE)

if (positive_row > 1) {

saveWorkbook(wb_positive, positive_output_file, overwrite = TRUE)

} else {

cat("No significant mediation results (p < 0.05) found. Positive results file was not created.\n")

}

\# Print number of significant results

count_positive <- sum(outTab$P.value < 0.05, na.rm = TRUE)

cat("Number of significant mediation results (p < 0.05):", count_positive, "\n")
